# Supplementary material for: Genomic diversity of 39 samples of Pyropia species grown in Japan
Source: PLoS One. 2021 Jun 9;16(6):e0252207. doi: 10.1371/journal.pone.0252207 (PMC8189503; doi:10.1371/journal.pone.0252207)
Supplement: S2 Fig — (PDF) [file pone.0252207.s002.pdf]

>Pyr_1

TATATGATAAATTAAGACATCTGTTATGTGAGAATTTCTAAAATATACAAAACTTTTTAT
TTAATATAGAAAAAGTAAATTAAAATAAGTTGAACATACTTTGCTCTAATCTAAACCTCT
TAATTGAGATTCTAATTAGAGACAAAAGTATTTACTTATTTTGTATCTATAGGAATAAAA
ATGAAACTTGCAGTTTATGGTAAAGGTGGTATAGGTAAATCTACAACTAGTTGCAATATT
TCTGTAGCACTTTCAAAAAGAGGTAAAAAAGTTCTACAAATTGGCTGCGACCCTAAACAT
GATAGTACATTTACGCTAACAGGTTTTTTAATCCCAACAATTATCGATACTCTTCAATCC
AAGGATTACCACTATGAGGACGTTTGGCCTGAAGATGTAATCTATAAGGGATATGGCGGT
GTAGACTGTGTTGAAGCTGGTGGACCGCCGGCTGGCGCTGGTTGCGGAGGCTACGTTGTA
GGTGAAACAGTTAAACTTTTAAAAGAATTAAATGCTTTTGATGAATACGATATTATCTTA
TTTGATGTTTTAGGTGATGTTGTATGTGGAGGTTTTGCAGCTCCATTAAATTATGCAGAC
TACTGCTTAATCATTACAGACAATGGATTTGATGCTTTATTTGCAGCTAATAGAATAGCA
GCTTCAGTACGAGAAAAAGCTAGAACGCACTCTCTGAGATTAGCTGGACTTGTTGGTAAT
AGAACAGATAAAAGAGATCTAATTGATAAATATATAGATTGTGTTCCAATGCCAGTATTA
GAAGTCTTGCCCTTGATTGAAGATATTAGAGTGTCCAGAGTAAAAGGTAAAACTTTATTT
GAAATGGCAGAAATTGATAAGGATTTAGCATATGTATGCGATTACTATTTGAATATTGCA
GATCAGTTGATTACAAGGCCAGAAGGTGTAGTTCCTAAAGAATCTCCGGATAGAGAATTA
TTTAGTCTTTTATCTGATTTTTACTTAAATCCTAAATCAAAGGTAGGACAAGAAAAAGTA
GATCAAGAAGAATTAGATTTGATGATAGTGTAAATAATATTTTCAGCATAATATAACAAG
GAATAAGATAATGTCTACAGCTCAATCAGATGCTCTTACTTTTGAATGTGAAACAGGTAA
TTATCATACTTTTTGTCCCATCAGTTGCGTTTCTTGGTTATATCAAAAAATTGAAGACAG
TTTTTTCTTAGTTATAGGGACTAAAACTTGTGGTTATTTCTTGCAAAATGCAATGGGAGT
AATGATTTTTGCTGAACCGAGATATGCTATGGCAGAATTAGAGGAAGGAGATATTTCAGC
TAAGCTAAATGATTATGGTGAACTCCGTAGATTATGCTTACAAATAAAAAAAGATAGAAA
TCCTAGTGTTATATTTTGGATTGGAACATGTACAACCGAAATTATAAAAATGGATCTGGA
AGGAATTGCTCCAAAATTGGAAGCAGAAATTCGTGTTCCTATAGTAGTTGCTAGAGCTAA
TGGTTTAGATTATGCCTTTACCCAAGGTGAAGATACAGTTCTAGCAGCTATGGCTCAAAG
GTGTCCATTAAATTTAAAGAATCAATCAGATAACACGAGTCTTAAAACTTCTCCTCATAT
TCCTTTAGTTTTATTTGGATCTCTTCCGGATCCAGTTGTAACCCAGTTAACTATGGAATT
AAAAAAACAAGGTATTTTTGTTTCTGGTTGGTTGCCATCAAAGAGGTATACTGAATTACC
GGTTATAAAAGAAGGATATTATGTTGCGGGAGTTAATCCATTTCTTAGTCGCACAGCTAC
TACACTAATGAGACGCCGAAAAACAAAACTAATTGGTGCACCATTTCCAATAGGTCCAGA
TGGTACTCGAGCCTGGATTGAAAAAATCTGTTCAGTAATGAATGTAAAGCCTATTGGATT
AGAAGATAGAGAAAAAGCAATTTGGGCTTCTTTAGAAGACTATATTTCTTTAATCCGAGG
TAAATCAGTTTTTTTTATGGGTGATAATTTACTAGAAGTATCTCTTGCAAGATTTTTAAC
TAGATGTGGAATGACTGTATATGAAATTGGTATTCCTTATATGGATAAACGCTATCAAGC

AGCAGAGCTAGCTTTATTAAAAGCTACATGTGATAAAATGAACGTTATGATGCCAACAAT

TGTAGAAAAACCAGATAATTATAATCAAGTAGACCGAATCCGTGATCTGAAACCCGATTT

AGTTATCACTGGTATGGCTCATGCAAATCCTTTAGAAGCCAGAGGTATTAATACAAAATG

GTCAGTAGAATTCACATTTGCACAAATTCATGGCTTTACCAATGCAAGAGATATTCTTGA

ACTCGTCACAAGACCATTACGGCGAAATCTTAGTTTGTCAGAATTAGGCTGGGATGTTTA

TAGCAAGCAAAGCTAGTATTCCTC----TTTATGACTTACATGGTATAAAAGCCTTGTAA

GTCTTTTATTTTATGTTAAAGAAGCATTTCTTGCTCTGCCATCACCAGCCCAAGCTTGTA

AACTTTCTACTTGTTCTTTGTCTGTAAATGCTAAAGGCACAAATTGTTTGATAGCTATTT

TAATATCTTCAGTACTAAATTCTCTCTCTTCACTAAACGCCGTATGCATACTTTCAATAA

TTGCTTGCTCAATTTCAGCGCCAGAAAATTTATTACATAATAAACTCAATTCATGAATAT

CATATTTTTGCCACGATCTTGGTCTAACTTTTGATAAATGTATCTTAAAAATTGATTCTC

TCTCTTGACGATTAGGTAAATCTAAGAAAAAAATTTCATCGAACCTGCCTTTCCTTAACA

TTTCGGAAGGTAAACTTTGAATTTTATTAGCTGTTGCGACAACAAATACTGGAGCTTTTT

TCTCTGATAGCCAGGTAATAAATGTACCAAATACTCTGGCACTAGTACCACTATCACCTT

GACTATGTAAACCAGAAAAAGCTTTATCTATTTCATCAATCCATAATATGCAAGGTGATA

ATCCTTCAGAGATATTTATCATTTCTCTCATTTTTGATTCCGATTCACCAACTAGTCCAC

CAAACAGTTTTCCCATGTCAAGACGTAGCAGAGGCAGCATCCAATCGCTTGCAATAGCTT

TCGCGGTTAAGGATTTACCCGTCCCCTGTATACCAACTAGCAATAAACCTTTGGGCGAAG

GAATACCGTAATTTAAACTTTGCTTCGAAAATGAGCGAGACCTTTTTTGTAGCCATTGTT

TTAATACATCTAAGCCACCAATATCCCTATTTACTTTACTATAAGGATAAAATTCTAATA

GATGCGTTTGATTAATAATTTGCCTTTTTTCTTCAATAATAATAGGTAAGCTCCGAGAGT

CTATCTGATTATATTGTGCAATAATTTTAGTAATCACTTTGCGGATTCTATCTATCGATA

ATCCTTGGCAGGATTTTGTTATATTATTAACGAGCTCTGAATTCAAATTAAGATTCAAAG

CTTTACTCAATCTTATGATCTCTTTTTTGATTTCTAGAAGACTAGGTAAGGGCAAATCTA

TTACTGTTATGATATCATTCAGAGCAAAAGGTATATTGATTTTGCAAGAAATAATAATAA

TGTGCTTAGATTGCGTTTTGATTATTTTAGCTAAATTGCGAAGTTTCCTAATTAATACTA

TTTCGTTTAGGAAAGAGTCAAAATCTTTTAAAAGAAAAAGATTTAAAGATTCATTATTCA

AGTTTTCAATAAATTCTAATGCTAATAAAGGATTTCTTTTTGCATAACCATTATCGCTAG

GATTGTTAGTGTATCCATCTACAAAGTCCCAGGAATATACTTGCTGACTGTTTGAACAGT

TTAAGCTGTGTTTTATAATATACTCTAGTCTATCTTCTTCTCTAGTATTTATTACAATAA

TAGGATATCGAGATTTTAAAAGTAATCGTAAATCTTGAGTGAAATTCATGTCAATATTTG

TTTCCATCAGACTTTTGTACTTCACCAATTGAAGAAAGACTAAATAAGATTATCTAATAT

TCCTTGGATATATTCAAAAGTCATCTCTCTATTTTCTAAATTATTGCTATTACTAATTTA

ACTTACCATGATTTTTTTTCTTCCTTTTCTTCGTCGACTATTTAATATAGAGCGTCCACA

TGGTGTTTTCATTCTTGCTCTGAAACCAGAAACTCTAATTTTTTTTCTTTTAGATCCTTG

TAGTGTTCTTTTAGTCATGATGTCTCCTCTGTTGTAAAAGATTAAATACTATTTTATCAT

CTTTTATATATCTTTATGATATAGATATGTATTAATATCAAAGTTAACAGCAATAATTTA
TATTAATCAAGAACTATTTATATAATATTAAACATTAAATATTAACTTTATGATATTGGC
TTTGCCCATTTTTTATTTAAGTATTTTGACCATCTTTTTGCTAATCTTAAACTGGTTAAT
TTTTCAGCAGCTAAAAACGATTTTGTTATTAGAATCTCAATTTAAGTATTTTGTTGATAA
AAGCCAAAATCGTAAATTAGAACCCGACGAAAGTTTTGCTTTTGCAAAAGTATGTGTAGC
TAAAAAATATTTTTCTAAAGCTATTATTGAAGGTCAATTAGCTTTAAAAAACTATCGAGA
CCTGAATATCTTAGATAATAATATAGTAATTGCTAATTTGTATAATATGCTAGGTTTTAT
TTATTTTGAAGCAGGCCAAACAAGTTTTGCAAAGAACTTTTATGAGCAAGCCTTACAAAT
AAACCCTAATTATGTTGTAGCTTTAAATAACCTGGCAAAAATTTATGAAGAGGTAAAAGA
TTTAAAAAAAGCCGAATCTCTATACGATAAAGTGTTAACTCTTAACTTAAATAATAAAAC
TGCTAATAGAAGGAAAGATTTTATAGCAAAAACTAAAAACATCTAATACTATTTGTAATC
GGGATAGCAGGATTTGAACCTGCGACATCCTGCTCCCAAAGCAGGCGCGCTACCAAACTG
CGCTATATCCCGTAAGACATAATTATTACTATATACCTTTTCTCTAATTATGTCTACTCT
TTCTGTATTTTATCTAGGATACCAAAACATGCCTTTAACTCCATCTGGATCTTTGATAAA
TCCTAATTTTTTATAAAAACTAACAACATCAGGTTCTGCGAATAGTGTAATAGTGCTAAT
TTCTGCCTGTCTCAATTGCTGTATTAATTGGTGTATGACTACTTTTCCTAAACCTAAACC
TTGGAAATCTGGATGAATAACTACGTCCCAGATAGTTGCATTAAATCCGTTGTCTGATGT
TGCTCTAGCAAATCCTACAAGTTTACTATTTGCATCTTTTTTTTGTATTAAAGAAATAAT
AATAGAACTATTTTTTAATGCGATCTTTACTTTTTTTAGAGGTCTTTTAACCCATCCGAC
TGAATCACAAAGCTGCTCCAATTCATATAAATTAATATTCTTATTGCTACTCAGATAGAT
ATCTTTAAATTCGATTTTATCGCAAGTTTTATCTAAAACAATAAGTTTTTTGAAATTTTT
TTCAGAGCTAGCATTGATATTGGAGTTTTGAAAAAAATTTTTCCAGAAGATCATAATGAT
TATGATATAATTAAATAAATATATATATTACAATATTGCTTTAACAAACAATATATTAAT
ATATGCTCAAATTTTTACATGAATCTATTAAGATAGAAGATTGTTAAAATATAGAGAAAT
AAAAATTTTTGTAAAATCTTTCAATAAAATGATATAAAGAGTGCTTTTTAAGACTAACAT
CTATTATTATAAATAAAGTCTCGAAATTGAGATGAAATAACACATTATTCTATGTCTAAT
GCCTTTTAAAGGAGTTATTTGTGAAAAAATCTATGTGTCTAACCTGTTTACTTGCTTTAC
TAATTATGAGCAACCCAATAATAGCTAACGCAGAAGTAGCTGGATTGGTGCCCTGCAAAG
ATTCTGCGGCATTCAATAAGCGTATGGTAAATAGTGTGAAAAAACTTCAAGCCAGATTAG
CTAAATATGATGCAGACACGCCACCAGCGTTAGCTTTAAATAAGCAAATAGAGAAAACTA
AAACTAGATTTGCAACTTATGGTCGAGCAGGTTTGCTGTGTGGTACTGATGGATTGCCAC
ATTTAATTTCTGATGGTCGATGGAGTAGAGCCGGGGACTTTGTTTTTCCAGGACTATTAT
TCTTGTATATTACAGGATGGATTGGCTGGGTAGGTAGAGGATATCTTTTATCTGTTGCTA
AGACTAGTAAGCCAACAGAAAAGGAAATCATTTTAGATGTGCCATTAGCTATCAAATTTA
TGTCATCCGGATTTGCATGGCCGCTAGCGGCTTGGCAAGAATTTAGTAGTGGACAATTAA
TTGCTTCCAATGACGATATTACTGTTTCACCCCGTTAGTAAAAAATTTATATGAATAATA

ATTTTACCAAATACTTATCAACAGCACCTGTAATTGGTGTATTGTGGATGACGTTTACAG
CCGGATTTATTATAGAATTGAACCGCTTTTTCCCAGATGTCTTATACTTTTACTTATAAA
TAAGATCGTATAAAAAACTACATAAATATAGAGAGATACTATTATTTGTGTAGTTTTTAA
TATATAACATAATTATTTATTTTTAAAAATGTTTATCTTTTCGATTATTTTAATATTAGA
TCTTACATAATAAAATAATATCAATATTATGAGCTTAGTAACCCAAATTATAGTTAATGC
CGATGATGAATTAAGATATCCTACAATTGGAGAATTGCAGTCAATTCAAGACTACTTAAC
TACAGGAAGCAATAGAATTAGAATTGCTACTATTATTAGAGATAAAGAAAAGGAGATTAT
ACAGAAAGCTAGTAAGCAAATTTTTCAGTTACATCCAGAATATATAGCTCCAGGTGGTAA
TGCAGCAGGTTCTAGGAAAAGATCGTTATGCTTACGTGATTATGGTTGGTATCTCAGACT
AATTACATACGGAGTATTAGCTGGGGATAAAGATTCTATAGAAACAATTGGTATTATAGG
AGTAAGAGAAATGTACAATTCTTTAGGTGTACCTATAATTGGAATGTTAGATGCAATCCA
GTGTTTAAAGGAAGCATCTTTAGAAATGCTTGGTCAAGATGATATTAGAATCATTTCTCC
TTATTTTGATTATATAATTCGTGGAATGTCATAAATCATAGCTTCAATTAATTAGTTGAA
TAAAAGCTTTTATAATGTTATAATTACAATAAGCCCGAGAGCATATAATTGTAAAAACTA
AATCTTGTCAGAACCGGAAGGTAGCAGCAATAATGTTTCAATACAAAAGTTATGATTTTC
GGGTGTTTATTGTTTTTATCTAATAGTCTCGACTTTTAATTAGTATGCTATGTATAAATC
TGTATATGACATGTAATCCATCAAACCTTCCAGCAAAAATAATTTAATAATTTGATATTA
ATACGTAAAACACCTATACTCTTTTATAAAATTTCTTTATATCTCATCTAAACAATGGAA
ACTTAACTTAATGGGTGTTCATATTTTATCAACAGGTTCGTCTGTCCCAAATTTTTCTGT
AGAGAATCAACAATTTGAAGACATGATCGAAACTTCCGACCATTGGATTTCAACAAGAAC
AGGAATAAAAAAAAGACATCTAGCTCCATCTTCTACTTCTTTAACTAAATTAGCGGCAGA
AGCTGCAAACAAAGCCTTATATGCAGCTAACTTAAAACCTACTGAGATCAGTTTAATTAT
TTTAGCTACGTCTACGCCTGATGATTTATTTGGTAGTGCTAGCCAGTTGCAAGCGGAAAT
AGGTGCAACAACATCAGTAGCTTTTGATATTACGGCTGCCTGCTCCGGTTTTATTGTTGC
TTTAGTAACAGCAGCTCAGTTCATTCAAACTGGTTCCTATGACAATATTTTAGTTGTTGG
AGCAGACACAATGTCTAGATGGATTAATTGGTCAGATAGAACTACCTGTATTTTATTTGG
TGATGGTGCTGGAGCAGTAGTATTGGGGCAAAGCCTCAAAAATAGTATTTTAGGCTTTAA
GTTATGTACAGACGGTCAGCTAAACAGTCATTTACAATTAATGAATAAACCTGTAAATAA
TCAAAAATTTGGTGGTACAGAAATTCCTCATGGAAACTATAATTCCATAACAATGAATGG
CAAGGAAGTGTACAAGTTCGCTGTATTTCAAGTTCCAACAGTAATTAGACAATGTTTGAA
TAATTTAAACATTTCAATAGATGAAGTTGATTGGTTTATATTGCATCAAGCAAACACTAG
AATCATAGAAGCAATTGCGAGCAGATTATCAGTACCTTTTTCTAAAATGATTACGAACTT
AGAGCATTATGGAAATACATCTGCAGCGTCAATCCCTTTAGCGTTAGATGAAGCTATTCA
ATCCAATAAAATTCAACCAGGTCAAATTATTGTTTTATCTGGTTTTGGAGCAGGCTTGAC
TTGGGGAGCAATTGTCTTGAAGTGGTGATTTATATATTGCGGATGACGAGACTCGAACTC
GTAAAGCTTTCGCTACACACCCCTCAAGCGTGCGTGTATACCAATTTCACCACATCCGCA

TTTTTTACCATGAATATTAAAAATTTATAATATTTATCTAAATATTCATATAATGTGTAT
ATATATATACATTCTTGTCAGAAACTCTCTTATATTTTATTTTAAGTGATAAAATTGAGT
TTAGTATAGATAGTAAAATTTTATTTTAAAATCATAAAAATAAATATATAAGGAAAATAA
AATATGACACCATCTTTATCAAGTTTTTTGAATAGTCTTATTCTTGGGGCAGTAATTGTA
GTCGTTCCCATAACTTTGGCTCTTTTATTTGTTAGTCAAAAAGACAGGACAATCCGGTCC
TAAAAATAACTTAGAAAGATATGGAGAAGCAATTTAAGATGAATAATGAAATATTTTTCA
TACTAATAAGCTCTCCATATATTGCTATACTTTAGAAGAATTTTTATTTTTTTATTTTGT
AACATCCTGTAAAGTTTATTCCGTTCTTTCTTTTTCCCATTTACTCTCTTATAATTAATG
ATACAAATGTCAGGTTAATAATAG-TTTTTTATTCTGTATCTTTCATTTTATATACGTAT
TATTATGTTCAATTAATAAAGAAAAGCTCATGTCTTTGTCTAATTGGCCTCTCAAAAAAG
AAAATTCTGAAGCATATAATATTAAGAATTCAAAACAAATCACAATTCCTGATGGTTTAT
GGATAAAATGCTTCGACTGTGGTCTATTAATGTATTCTAAAGTACTGAAGAGAAATTTAA
AAGTTTGCCCTCAATGTAGTTATCATTTTCAAGCTTCTAGTAACGAAAGAATTGATCAAT
TGATAGACCAAGGTAGTTGGCAACCAATGGATGTTCACTTGATCTCTACAGATCCATTAG
GCTTTAAAGACCAAAAGCTTTATAGTCAAAGGTTAAAAGATACTGCTTTCAAGACTGGCC
TGCAAGACGCAGTTCAAACAGGTACTGGGACTATGCAAGGTAAAAAAGTATGCTTAGGTA
TTATGGATTTTAGATTCATGGGGGGAAGCATGGGATCTGTTGTAGGCGAAAAACTAACAA
GACTGCTAGAAAAAGCAACTCAAGAAAAGTTGCCTGCAATTATACTTTGTGCATCAGGCG
GAGCTAGAATGCAAGAAGGTATGTTGAGCTTAATGCAAATGGCAAAAATTTCTTCTGCTC
TAGAAATGCATAAAAAAGAAAATCTACTATATATATCTGTTTTAACCTCTCCCACAACAG
GGGGTGTAACAGCTAGTTTTGCTATGCTTGGAGACTTAATTATTGCAGAGCCAAAAGCTC
TTATTGCATTTGCTGGTAGACGAGTTATAGAACAAACAATCAAAGAAGACTTGCCAGATA
ATTTTCAAAGTTCAGAATATTTATTTGAACATGGTTTCCTAGATTTAATTGTATCCAGAA
CTCAGCTTAGATCAAAGTTAATACAAATTTTGTCCTTACATAATCATAGTAAGTAATGAA
TTGAATATTGACTATAATATCAAAATGCTAGTAAAAATTAAGATATTAAGAAATTTTCAC
TAAAGATATTTGTAGACTAACTAGAATTACAATTACTATTGTAGAAAAATTTGTCTTAAT
GCCATTTTTTCAGTCTTTTAACAAATTGTTTTTATGACTTTAAATAATTGTTAGTTTAAT
ACAAATTTATAAAGAGAATACATACTAAGCTAAATATAGTAATATTATGTTTTCCTATAA
TACTGACCTATCAAATTTCGAGGAAATTTATGCTTAAAAGATCTTCTTGGCTTGCGGCTT
TATTGGGACTATTAACAGTAGTTTCTACAAGTACGCATACATATGCCATAGAGTTAGACG
AGGCAACAAGAACTGTTCCATTAGAATCTTCTGGCAGAACTGTAATTCTTACACCAGAAC
AAGTTAAAAGAGGCAAGCGATTATTTAATAATTCTTGTGCTATTTGCCATAATGGTGGTA
TCACGAAAACAAATCCGAATATTGGACTTGACCCAGAATCTTTAGGATTAGCTACGCCAC
AAAGAGATACCATTGAAGGACTAGTTGACTATATGAAAGATCCGACTAGTTATGATGGTG
CAGAGTCAATCGCAGAATTGCATCCAAGTATTAAAAGTGCTGAAATTTTTCCTAAAATGC
GCAATCTAACCGATGAAGACCTATTTACAATCGCAGGTCATATCTTACTTCAACCTAAAA

TTGTTTCTGAAAAGTGGGGCGGAGGAAAAATTTACTATTAGAACTTCAAAGACCTAAACC
TTGTGAATACTAGTTTATGTATTTGTTGCTTAAGTGTGATTATTTTGTTAGATCATGATA
TATATTAAAGATGGAGTTTGTTGACAGCAAGTTACTTTTTACCCGAACTTGTTTACACTA
TTCGTCTAGTTATTAAAAAGGAGACGTTAAATTGAAGAAGAAGCTTTCAGTTCTTTTCAC
TGTTTTTAGTTTTTTTGTAATAGGTTTCGCACAAATTGCTTTTGCTGCAGATCTAGATAA
TGGAGAAAAAGTTTTTTCTGCTAATTGTGCAGCATGTCATGCTGGCGGTAATAACGCCAT
TATGCCAGATAAAACCTTAAAAAAAGATGTACTTGAAGCTAATAGTATGAATACTATTGA
TGCTATTACTTATCAAGTACAAAATGGTAAAAATGCCATGCCTGCTTTCGGAGGTAGACT
GGTTGATGAAGATATTGAAGATGCAGCAAATTATGTATTATCTCAATCTGAAAAAGGTTG
GTAATTATACTTGATTTTATCCTGTATTAAAGAATAGACAATCTATTTAGTTGTTCATTA
GATTGTCTATTCTTTGTTTATGCTATTATATAAAGATATTTACACAATATTTTATGATGA
AAAGAATACCCGCAATTCTTGTACTAGAAGACGGTGCATATTATAAAGGATGGTCATTCC
AGCAAGATAAACAAGAGATTACTATTGGTGAAGTAGTTTTTAATACTGGAATGACAGGAT
ATCAAGAAATAATCACAGATCCTAGTTACTTCCATCAAATTGTCGCTTTTACCTACCCGG
AAATTGGGAATACAGGTATTAATAATCAAGATATTGAATCTCACAGTATTAGTATTAAAG
GACTTATTGCAAAAAATATTTGTAAAATTTCAAGCAGCTGGAGAGAGCAGCAATCTTTAG
TTAAGTATTTAAGTAGTAATAATATTCCTTTTATTTTCGGAATAGATACAAGGTCTTTAA
CCCAATACTTGCGTCAATTTGGTACAATGAACGGTTGTATCTCTACTGATAATTTAAATC
ATAGTTACTTAAAACAGAAAATTTGTGAGATTCCAAGTATGCAAGGTTTAGATTTAATCC
CGCATGTAACTACAAGAAATGTTTACCCCTGGGATGAAAAAAGTTTTCCGAATTGGTATT
TGACAGATAACATTAGAGTACACCGAGTTATTCAGTTGAAAGTTATTGTTATAGATTTTG
GAGTTAAACTAAATATACTCAGAAGACTAGCTACACTTGGATGTCAGATAACGGTTGTGC
CTGCCCACACTCCTTTAAAAGATATTTTGGCTTACCAGCCTGATGGTATATTACTCTCTA
ATGGTCCAGGAGATCCATCAGCAGTACATTACGGCATCCAGACAGTTACAAATTTACTAG
ATTACAATGTGCCTATATTTGGGATTTGTATGGGGCATCAAATTTTAAATTTAGCTCTTA
AAGCTAAAACTTTCAAACTTAAATTTGGCCATAGAGGTATTAACCATCCATCCGGATTGA
ACCAGCAAGTTGAAATAACTAGTCAAAATCATGGCTTTGCAGTTGAATTGACTTCGGTTT
TTGAATCTCCTGTAAGAGTGACTCATTTTAATCTAAATGACACTACTATTGCAGGAACTG
GACATAATCAAAGTCCTTATTTTTCTGTGCAATATCATCCAGAATCGAGCCCAGGCCCTC
ATGATGCTGATTATCTATTCGAAAGTTTTATAGAAATAATGACAAAGTCCAAGAATAAAG
TTAGTTAGTAATTTTCCCATGCTTTATGAGTGAATAAAGCGGATAGTACTTGTACTCCTC
GTAATTGATTAAAGAGAGGCAAATGTCCTCTGGGGCCATGAGAATTCCATTGAAATTCCT
CAGGATATCTGCAAGGAATTCTATCAACTTCCCAGCCGATTTTTTGCCAGAATTTTCCCC
AATCTTTTCCAACACTAAGCCATATTTGTCTTTGAACAAATAGCCCAAATTTACCTTTTG
AGTGTGTATGCCATAACTTATCAATAGTTTGTAAATCTTGAGCAGGTATTTTTTTTATAT
CTGTAAAATATAGCCAATTACGAGTTTGTGCATTTACACCAGCTAGTTGAATAAGCTTTT

GTTGAGTTAGCTGATCTGCTTTGAGCAAATCCCGATGAGTCAGTAACATTTGTAAATCTT
TGTAATTCATCTGTTGAGCAGAGCGCAATGGAACAATGCCATCGGGACATAAGTTAGAAG
CAAATTTCACTATTTCTTGATTTTTACTGTTAAGTAACTTTTCATAAATTAAACCGTCAA
CACAATTACTTTTATAGTTAGGCCCAGTAATCCTCTCAAAAAATAGATCTGCTAAATCTT
TTAACTCGATAGAGTCGTTGCTATTTATATTCTCAATAATTTCAAGTTGTTGCTTGACAT
TATTAGACTTAGTATTCTTATTCAATTCTAAAAGCTGGGCTCGGATTTGATTTGGCATCT
GATTTGTTTAATTAGTTTTAATAGTCATAAGGTCTTAGAGAGAATTAGATTGTTTTTTGA
AATTAGAAGAGCGCCGCAGTATTTCTAAAGAAGTTCGCATAATTTGATTCAATAAGTTTT
GTCCTGCATAAATTATAATTTGACCAATTAAAAAAATTGTGCTTTTTATTTTAGGAACAA
AAAAATCTTGTACCTCAAGCAGAAAAGTAATTATTATTTGCATTGGTGACAACATCTGTA
ATTCATTCGATCTGCAAGCATATATATACTGACAGTTTAAGCCTTCTTGATAAAATCCCC
ATACTTTGTAACGACTTTCGTAAATTGCTCGTGGTCGCTCAAGATAGTTTTTAATTATAG
TATTCCAAATTAGGTTATTCTTTAATTTTTCTAATTTTCTATCAGATAGAAATTTTACAT
TACAGAGATAACTTGCATTTGTCTCATTTACAGTATTAAAATTTTGAACTAACATATGAG
CAATAATGTTGCTTAGCTGAATTAAATAATTTTCAAGAAAAATTTCGACCTGTTTAACTG
GCATATTGTAAGTAAAGAGATTAAAAGTTTTATTCACAGTTTGAGATGACCCAAAAAGTA
GCTGAATAATTAAAATTTGTAGTAGCATTTTATAGTCGTTCAGTAGATAGTTCAAATCAT
GAATATCTTGGTAAATGTCAATACAGTCCAGTTCATATAACTTACAAAATCTTTTGATAC
ACCTATGGAATAAATCAATTAAAATATTTTCGGTCAAGTTTGTAATATCATTTACATCTA
AATTTGAATTATAAATTTCTAAAAGTATCTTTTCCAGTTCAGCTAATATGATTTTTAATA
AGTTTCTTTTTACTTCAGTGCGAAAAACATCTAAAATTAACACTTCCTGAGAGCAATTAG
TTAATCTCTTATTAATCTTTATAGAAGTTCTAACTAATAGCTCTGCTACCTCTTGATTGA
GTGTTGGTCCCTGAGAGCTTGGCCAATAATTATTCACGTTATACCATAGTAAGAAATATT
GAATTTAATGTTATACGATAACTTTTTAATATACTGAAAAGCAAGTTAATTACTCTATAG
CAACTAGTTTTTACAGATCTTAATAGTTATTTATCAAATAAAGTTATATGATAGTAGCGA
TTGAGCTAATTTTATTAAAATTTATAATAATGACAACTTATTACTTCGCCCTTGCGAGTC
AAAATTTTCTATTAGTACAAGAACCACTAGAAGAAGTTTTTAGAGAGAGAGTTAACTACT
ATCAGTCAAATAATAAAGCAATTGATTTTTGGCTAATACCAAACCCTTCTTTTCTAGAGA
AACCAGAAATGATTTCATTTAAAAACCTTGTACCTAAAGACGCTGTAGCTATAATCTCTA
CTAATCCAATATTTATTAATTGGTTAAAGCTAAGAATTGGCTATATCTGTATTGGACAAT
TTGAAGATAACCTACAACTTTCTGAAGAATCGTTAAATATTACTGTTTTAACAGACAAAA
TTTAACAATGCTTTTATTATGACTAGTGTTAAATTATTTTGGTTAAGTTATAATTATAGT
CTTTTAGTATTTAGACTCTTATAGTTTATCACTTAGTATTTTATGCTGAGATGGAGTGAC
AAATAGTGTAAGTATTTCTTGGCTAAACGTTTCAGTTGCCTTAGATTTATATCTATTTGG
ATTAACTATAATGGATAGCATTCGTTTTATAGTAACATTTTCAATTTGAGCCCAATGTAC
AATTCCAAGTTCCAATTCCTTAGCAATAGCTGAAACGGAAACAAAGGCAGCTCCTAGCCC

TGATTGCACAGCGTTTTTGATAGCTTCTATAGAATTCAATTCCATCTCTATTTTAAAGCG
ACTGCTGTCAATACCATGTTGACTGAGTACTTTATCTATCACTTTTCTAATTGTTGATTG
AGTATCTAATGCAATAAATCTAAGCCTGTATAAGTCTTCTTTTTGAATGTCTCCTAGTTT
AGAAAAAGGATGTGATTTGGGTAATATAAGTGCTAATTCGTCTTCCGCATAAGAAGTAAC
CTGCAAAACATCTTGCAATTCAGTAGGCACTTCTCCTCCAATAATTGCTAAATCAACTTG
ACCATTAGCTACGCTCCATGAAATAAGTCTAGTTGAATGTACTTGCAATTGAACAGCCAC
TTGTGGATATCTTTGCCTAAATAGTCCGATTAATCTTGGCATCAAATATGTCCCAGTTGT
CTGGCTAGCTCCAATAATTAATGTGCCACCTTGTAAGTTTTGTAAGTCGTCAAGAGCTCG
ACAAGTTTCTTCGCAGAGAGCTAAAATTCTGCCCCCGTATCGTAAAAGAAGACTTCCTGC
CTCAGTTAAAGTTGCCTTCTTATTACCTCTTTCAAATAGGGAAACATTTAATTGGCGCTC
TAAATTTTGAATTTGCAAACTAATAGCTGGCTGAGAAACATACAAGCTATTAGCTGCTTT
TTTAAAACTGCCCTCTTTGGCAATTGCTTTTAATATTCTTAACTGATCCAATGTAAATGG
AAGGTCTGTCATTAAAGAATTGTAGTATAATGTATATTATTTAGTATTGATCAATGTATG
CATTTGCCAACTAAAATATTTGTCATTACGCTTCATTATTTTCAAAATAATAAAAAGTAT
AATGTTAAATAAGCTGTATTAAAATTTAGTATCTGTTTGATATTAAATTTATACAAGAGA
TATCGTGAATTTAATTCTAACTATAAAATATAGGAGACAATATGGACTCTAGACTTTTAG
TTGTACTAATACCAGTTTTAGCAGCAGCATCTTGGGCAGTTTACAATATTGGTAGAGTTG
CACTACAGCAATTTAGAAAAATGACATCTTAGTTTTGCTTAGAATATAATTTTTAGACTT
TATGAGATAGAGGACTTTAAAATCCCTATCTCTATTGTATATCTTAATAAAAAAAGAAAG
CCCCCTTGTACTTACTGTTATTACACATTAATATTATTGGTGGTACCTAGATAATAAGTT
TAAGCTAACTTATTTTTTTATTTTATAATTAATTAAATTAATAACACAATTATGGCTGTTC
CAAAGAAAAGAACATCTAAAGCTAAAAAAAAATGCACGCAAAGCAAATTGGAAAAATCAAG
CAAAAACTGAAGCTCAAAGAGCTTTGTCTTTAGCAAAATCAGTATTAACTAGAAAATCCA
ATGGATTTATTTATAATCTAACTGAAGCATCAGATACTTTTAGCGATTAGAAGTGTTTTT
ACTACAAGTCTGAAAAAGTATTACGCAACAAGTTCTCAGTAATTTTTATCTATTAATCAT
TTGAAAATCATTCTATTTGATAATAAAATAAATAGCAGATTAAATAAAATCACATTAACA
AATTATGCAGCAAAGCTAGATCAAACTAGTGAAATTTGGTTGTTCAATTGTATTGAAAAT
ATTCAGCATATTTTTTTTAAAAGTCAATTAAAATCAAGTCATATTACTAAAATTTTTATT
TCTGGTACTAGCTTTGAATATACCGCAGGTCTACCAGGATTATTATCCAGCTTAACACTA
AGTGGTAGACTACATCCTATTAGTATATATAGTCCCCAGTCTCTCAAAAAGTATCTTGAA
GCATGTACCAAATATTCCCAAACTAATTTTTCTTTTCCTATTAATTTTCATAATTTACAA
TACGGAGGACAAGTTGTTAACCAATTTTATACAGTAATTTGTTTACCGTTGAGCAAAAAG
AGCCTGCTGTATGGATTTATTATTCTAAAAAAAGAAAAGCAGGGAGTATTTAATTTAGCA
CAAGCTAAAACCTTGAATATTCTTCAAGGACCTATATATGGAAAACTCAAAGAAAAGGAT
AATTTTTTAAGTCCAGATGGTTACTATCTATCCGGCCAAGACTTCTCTTCTAATACAATA
ATGGGACATAAAATATCGCTTCCGTTATTAGTCAGATATTCTAGAATTATTTCTGAGATG

CATTGGTTTTGCTCTTATCCTATTAGATTAAATACTTATTCGCATCAGCAAGGAGTAAAG
TGTTTACCACATAATGTTTTAACTGATATTATGAAATCCCAAATATATCAAGATAACAGT
TTTGTTGAATAATACTTATTCTTGATATATAATGGTCTCGGTAATCTAAATAAAATACTA
GAAATAATTTCTTAAATTTATTTGTCTATATTAAATTTACAATCATTTATGACATACGCA
ATTATTGAAGCAAGTGGCACGCAGCTTTGGATAGAAGAAGGCCGCTACTATGATTTAAAT
CATATACCTGTTGATCCAGGTCAGTCGATTATATTAGGAAAAGTCTTATTATTGAATAAA
AATGGGGAGGTTACTTTAGGACGCCCTTGTATAGAAGGGGTTACGATAAAGGCTACAGTA
ATGAGGCACTTGCGAGGAAAGAAGATAACTGTTTTCAAAATGAAACCAAAGAAGAAAATG
AGATTAAAAAAAGGTCATCGACAAGAATTGACTCGTTTAATGATCGATTCTATAACATCT
TAGATAAAATCTAGACTCATATTTTTAAAAACTTTTTAATATTTAACTAAAATAGATATA
ATGGCACATAAAAAAGGTAGTGGTAGTACAAGAAATGGCAGAGACTCTAATTCCAAGCGT
TTAGGTGTTAAAAAATATGGTGGAGAGCAAGTAACAGCAGGTAATATTTTAATCAGACAA
CGGGGAACTAAAGTTAAGCCTGGCCAAAATGTTGGAAAAGGAAAAGATGATACATTGTTT
TCTCTAATTGATGGTTTCGTGCTGTTTGAAAAGTCAAATCAAAAGCAAAAAACAATTAGT
GTTTATTCTGCTAAGAAATAGTTAAAACAATTGGTGCAAGATAACTTGAGTGTATTTATT
TCACTTACAGTGGCACCATATATTTGATTTATTAAGAAATGGAGTTCCTTAATGAACCAA
TGAATTTTATTAAAGATTTGCTGTGAAATTTAATGACAAACACTATTGTAATTTCCTGTC
TACACAATATGGCTGCTATTCTATATTGTGGTCAGATACAGAAACTAGTAGTAGCAAATG
CTCATTATCAAGTAAGTGATATCTACCTAGGTTGTGTTGATAAAATCTTCTCAGGAATAA
ATGCGGCATTTATTAACCTAGGAAAGAATGAGTACAGTGGTTTTATACATATCAGTGATA
CCGGTCCGCTTAAAAAGAAATATTATGTCAATAATATTACTAACATTTTAACAATACGGC
AAAAAATTTTAGTACAAATCATTAAAGAGCCAACTTTAAATAAAGGTCCAAGGCTCACTG
CCAATATTACATTATCAGGTCGATATATTGTATTAATGCCTTTTAGTCAATCAATCTGTA
TATCTCGAAAAATATATGATGAAGATGAGCGTCATTATTTGAAATCTTTAGCTATTTTAA
TTAAACCGGCAACAATGGGCTTGCTATTTAGACCTTCTGCTGTAGGTGTCGATGAGGAAA
TAATATTAAGCGAATTAAAAAATCTAAAAGAACAATGGAACTTTGTTCAAAAATCTGCAA
TTAATAGTTATTCACCTGTTCTTCTATATAAAGATGAAGATATTGTTAAAAAAGTAATCC
GAGATTTTTATAATAATAATACAAACAATATAGTAATTGATTCAAACCTGGGATTAAAAC
AATTAAATTATTATATCCACACTTGGCACTGTAATAACTCTAGCACAGTTCCTAAGATTA
AGCTTTATAGTAATAATCAATGTATACTAGATGCTTTTGGTATCAATCAGGCAATTTCCA
GAGCTCTCATTCCAAAAGTTGATCTTATACTTGGTGGCTATATGTTTATTGAAACTTTAG
AAGCTTTTACTATTATTGACGTTAATTCTGGATCTTTTAATAATTCCACTAGTGCACGAG
AAACAGTTTTAAAAACCAACTGTTCTGCAGCAACAGAAATAGCTTATCAGTTACAAATTA
GAAATATTACTGGTGTAATTATAATCGACTTCATTGATATGGAATCACAAAGAGATCAAT
TGCAATTATTAGAACACTTTAATAAAGAGCTATCACTTGATGATGCTAAACCACAAATTG
TACAGTTATCTGAATTAGGTTTAGTTGAATTGACTAGAAGAAGACAGGGCAAAAGTTTGT

ACGAGTTAATTAGTAGTGATTCTAATTACTTTTATTTTTTCACACAATCAGAGAGATCTC
AGTCTCTTAAGAGATTCGATGATAGACAGCAGAAACAACAGATTTTTAATAAATCTTGGC
TATCTGCAGAGATTAATACTATTAACAAGGTCTTTTTTCAAAAGTCAAATTTGTGCAGAC
CTGCTAACTTTTACCTAATTCGTAATCTCTATATAGTTAAAAGTAGTATTACTTATAAAC
AAAATTATTTATTAACTCACAGATCAAAACTTATTTATTCTAAAGAATACAGCAAAGTAT
TACCAAGCAGTTATTATCTAGCTAGTCTCAATAAGAATAGTAATCAAGAGTTCCTATCTT
AAGATTGTTATATCAACTGTTGAAAAAAAGAAGCTCTCTCTTATATAAGAGAGAGCTTCT
TTTATAGCTTACTAACTATCTAGTTAGTTAATTAAAAATTAACCGTTAACAGCTGGAGCT
GTTAGAGCTACTGGTAAAGATTCACCAGAAGCTAGATCTAGAGGGAAGTTGTGAGCGTTA
CGTTCGTGCATTACTTCCATACCTAGGTTAGCACGGTTGATGATATCAGCCCATGTGTTA
ATTACACGACCTTGGCTATCAACAACAGATTGGTTAAAGTTAAAACCATTCAAGTTGAAT
GCCATTGTGCTTACAGATAAAGCTGTTAGCCAGATACCAACTACAGGCCATAGACCTAAG
AAGAAATGTAGAGAACGAGAGTTGTTGAAACTAGCATATTGGAAGATTAAACGACCGAAG
TAGCCATGAGCTGCAACGATGTTATAAGTTTCTTCTTCTTGTCCGAATTTGTAACCATAG
TTAGCAGATTCGTTTTCGCTTGTTTCACGAATTAAGCTAGATGTAACTAGGGATCCGTGC
ATAGCACTGAACAGAGAACCACCAAACACACCAGCAACACCTAGTTGGTGGAATGGGTGC
ATTAAAATGTTGTGCTCAGCTTGGAATACAAGCATGAAGTTAAATGTTCCGGAGATACCT
AGAGGCATTCCATCAGAGAAACTACCTTGGCCAATTGGGTATACTAGGAATACTGCTGCT
GCTGCTGCTACTGGAGCAGTAAAAGCAACGGAAATCCATGGACGCATACCTAGGCGGTAG
CTTAGTTCCCACTCACGACCAATGTAGCAAGCTACGCCAGTTAGGAAATGAAGAACAACT
AATTGGTAAGGACCACCGTTGTATAGCCATTCGTCTAAAGAAGCAGCTTCCCAGATTGGG
TAGAAGTGAATACCGATAGCTGCAGAACTTGGAATAACAGCACCAGAAATGATGTTGTTT
CCGTATAGAAGGGAACCAGCAACTGGCTCACGAATTCCATCAATGTCTACTGGAGGTGCA
GCTACGAATGCAATGATGAATACAGATGTGGCAGTTAATAGAGTTGGAATCATTAATACA
CCAAACCAACCAATGTATAGGCGGTTTTCAGTACTAGTAATCCAAGAGCAGAAACGTTCC
CACAAGCTAGCGCTTTCGCGTCTTTGTAAAGTAGCAGTCATAATTTTTTATCAATTTTTA
AGGATTTAACAAAGATGCTTCCCAAACAATAAGTTGTCTGTTATGAAAATTATTACTTAA
GGCCTTTTCAAATGAGAACATTTTTAAAAAAAACATAGCAAAATCTAATCTCAGTAACTA
AAATTTTACTTGTTCATATATTGCTTAATAATTCAATAATAAATTTTTGCTTTTTGCAAA
ATTAACACTATTTTAGGGATATAATAATAATGCATATTAATGCTGCTTATTTTGATTTTA
ATAGTTTATATAATTTTGCCCTACTTAAATACGCATGTCACACTTTACAAAAATTCAGAC
GACCATACAAGATTTAAATCTGTTAAAGCATGCTTTAACAGATTTGGGTTTGATTTGGCA
AATGAATTCTAGTCATATAAAAGTTGGTGAAAATAATCAGCATAAGGTAGATATTTTAAT
TAAACAAGATAACTTGTCACATATTGGCTTTACTTGGAATGATAATAGATATCACTTAGT
TGCCGACTTACAACTTTGGGAACAACCCTGGTCTCTAGAAGTATTTTTGGATAAGTTATC
TCAGAAGTATGCTTACTACTCTATCATCGAAGAAACAAAGAAGCAAGGCTTTGAAAAAAT

GCAGCAGATTTCACAAAAAGATGGATCAATTAAGTTAATTGTGCAACGCTGGAATTATTA
ACTAACATACAAATGCGGGCGGAGAGACTTGAACTCTCACGAGATTATCTCACTAGAACC
TAAATCTAGCGCGTCTACCAATTCCACCACGCCCGCACTGTTGAGACAATATTATTCTAT
CATAATCTATACTAAAACACAAACTAATAGTATAAACTAGCTTCCAAGCTTAAAAGCGTC
TACAAATAGACCGTATGTTATACCAATTTTGAAGTTGTTGAATAAATTAATTTTTATATT
TAATTGCTTGTGGCTAGAGTAGACTATTTTACTAGTCAACTCAGTTGCTGCAACTATTAG
ACTGGCTGCAATAATACCCCAATCTCCTGTTTGTCCAGGAATCGTAGACAAACCTGTTGA
AATAAAAAATCCTAGCAATAAACTAATTAAGCCAGTTGTTAACTCGCTCAAAGAATAATA
AAGCTTATTATTTAAGTTTTTTATTAAGTAAGCAAAAAAAGTTGAAAGTCTAGTTTTAAT
CATAGTATTTAGTAACGGAGTTTTAAAAGGATGACAAGTATTAACAGTCATTAATATTGT
CGTATCCTTTTAAAACTCGTTAATACAAACAGCTGTTCTTGTTTTTAAAGGTCTTGTTCG
CTTAAAGCATTAATAAGATGCTGAAACGGCTCTTGTATAATATCTGTGCGTTTAATTGAT
TGCAAATCTAATTCTTCTTCTATGATTTCTTGTAATAACTGTATGCTTCTTACTGTTGGA
GCAACAGGTACAGATAGTGAATTGTAAGTATCTTTTAGCCCATTTAATACACGCTGATCG
AGAATATTTGTACTACCAGAAACCAAGGCATAACTAGCATACCGTAGGTAATATTCTATA
TCCCTTAGACAAGCTGCATATCTTCTTGTTGTATAAGAATTTCCTCCAGGCCTTAATAAT
TCAGGCTGTTCTTCGTACAACTGAGCTGCGGCCTCTTTTAAAATATTGGTAGCTTGATCA
TTAATAATTTCAGCTATTTTTATTCGATCTAGTCCACTCGAAAAGAAAGATTCTAGTTGT
CCGACAGCTGTCTTATCTAGATAACGACCAGTAAGATCGTAACGATTTAATATTGCTGTT
ATAGCATCTTGCATAAATTAATTCTCCTACTTGCTATCAGAGCGGTATTATAAATGACTA
ATATTGTAATATAATCATTAGTGTTTATGAAATTTATTTAAAACTTATTCTTTATTGCTT
AATGTGCAATTAAAAACTCGCATAGATTTTACACATATAGAGTATACGTAGTTTTTAATT
ATTTTTTTATAAAAACCAAGAAATAAAGCTTTTTTTAAGAGGTTCGTGAATTTGTTAATA
TAACATGAGCTTTCCTCCTAAATAAGTTTTGTAAAGTACAAAGTAAAAAAAACGATTAGTC
AATAATAGTAAACAATATTAATATGTATCAAAGTATAAATAAGCTTTTACTCCAGCATCA
AAAAAAATATTTGAGATATGTTGATACAACTGATTATTCAAGTGAAAACTTAGATTTAGA
CCAATTTAGCTATTTTATCATTACAATTAATAAAACTGATAAATGTATTCTTATAGAGCA
ATTTTGTTATAATGCTGAAGGCCATATTTACTCAATATTCTTCAAAGGTCCAACAGCTAA
ACATCTTTCTTCTATAATTTGTCATTTTCAACAATTAAATACAATAATATCTACAGCTCA
TGCCATATATTTAGGACGAGAGCTAATGAAGTCAGAATTAGCTCTCGTGCTAGATCAACA
ATATATTCAAGATTAAATTTTGTAAGTATTAATTATATTTATGCTAGTATAATATTAGTA
AGGGCATGTAACTCAGTGGATAGAGTATCAGATTCCGATTCTGATGGCCGTGGGTTCGAA
TCCCGCCCTGCCCGAGTACTCAGAGTACTATACTAAAACATCTTAATAGCTAGTTTGATA
ATAGTATGGGCATTGGGGCTGCAAGGTTTCTACATTATGAAAAGAAGAAAATATG-AAAA
AAACAAGCTCTCCAAAAGAGCTTTTAGTACAATCAATAAATGCAGAAAACAATATTGTTT
CTTTTTCTCGAAAATTAGCTTTTGCTTAAACACTGTTAATTTTTGGCATTCGACATGTTA

AACTCTTATTCATGGCGAATATTCTGTCAGAGTCGCTCTTAGTTTAAGAAAAGTCAGAAA
AAATATTTATCTTTACGTTTTTACCTATTTTTATTTAAATAAAGGTTAGTATTTTCAACT
TTTATAGTGGACGTGGGTTCGAGTCCCACCAGCTCCATCAATTTGATTTGTATAATTAGC
TAGTTTAACCTTTTTAATAACCAGATATATAATTGGAGGAAATATGGGTTTCATAACAAT
TACTAAGCCCGCTTTAAAGCAAATTGCAATCTTGAAAAATGATCATGAAAATGATGTACA
TCTAAGAATAGGTGTTAGACAAGGCGGATGTTCAGGTATGTCATACTCAATGAATTTTGA
ACATGTTGACAAATTAAAAGATACAGATGAACGGCTTCGTCTAGATAATTTTTCTGTTGT
CTGCGACCCTAAAAGTCTTCTTTATCTTTATGGATTATCATTAGATTTTAGCTCGGAATT
AATAGGAGGAGGATTTCAATTTTCTAACCCGAATGCCAGCCAAACTTGTGGCTGCGGTAA
ATCTTTTTCAGGCTGACCCTCGAACACGTTTTTTATATATTTTGTATTATATATATTATT
TTAGCAAGTCGTTTATTAGAGACAAGCAATGCTCTGTTAAAAACTAATTTCAAGAGTTTT
TATTGTATTATTTTATATATTTATTATCAACTACTTGAAAATAAATTAGCTAAGCAATCT
AATTTGTTATCAGTACCAAACTTTAAATGATTAACTTTTTATTGATATATGCTAATCTTT
ATCAGCCTAAACATATCTGTACATTACAATCTTTTGACATAAAAAGAGTCTTAGCATACC
TCTCAAAAATAAATTCAATATTTTATAAATACTCAACATGAATCTGTCACAACTTATCGT
TAATTCTTCTATGATGAAAATTCCTAATAACTTTTATTGCAATATAAAAGCTATTTCTTC
TGAGAATTTAAGTCACTTACAATTATCAAAAACAAATTTATTATCTCCTACAAAAATAAT
TGCTTTTGGACTACCTAAATTTGATTTTATTTCTACACAGATTACAAGTTCTATTAGAGA
AACAGTTGTTGTGTCTACTATTGATGATAATAATCTTTTTGCTGCCCCAACTAATGATTG
GATAAAAAAACACAAGGATAAAAATCATCATTCTTTGTTTTATAAAAAAGTTTTTAAAGC
TTTGCTAAACCATAAAATTAGAGTTGTTGCTGAGCCACTTCATAACTCCGAAATAAATAA
AGAAAAGAGCATAATGGCAAGTCAAAGATACATCTGGGGTAAAAGCTGGAAGCCTTCAAT
TATTTTATCTTGCTTAAAAAACAAAAAAAATAGCAAAATTTCTGATGCATCGAAAAAATT
TTTAACTGAACAGTTATCTGCATCACCAGTATTTGTGGTAAAAAATGGTTTTAATGAAAT
TATTTTAGGTCACCCATTATCTCGTGTTAAAAGAGGAAGAGTTAATAATCTGATGCATGC
GTTTTCTAATTTATTAAATCAGTCTAATGCAACTTATCCTATATCTACTGGTCTATTCTT
TTTTCATCCAGATGATGCATTTGAATTTAAAGACTTTATAATATCGGTCAATCCACTGGC
GGCCAAACATATGGAGATAAGCGTAGAGCCTGTTGGTCTACACTTTGCTTATAAAATGAA
TAGAAATATATCGTCAGATACTCAATTTTCGTTTATTCCAGATTTTAAAGAAGTAGGAGA
TTTATTATTCAAATATAGAAAAGGTAATCATTTAGTTTTTCATAAGAATCAGCATTATGG
CAAAGATTTTTTTCAGGGGCAACCAATATATATGATTCAGCCGATCACTTTTAAAGATCG
AGCCGGAAAGCTAAATATTATCAAATTTACGGGGCTAAATGACACAAGAGAAATTATTTT
TACTAATCTTGAAGCTGCTAATAGATCATGGGCACATTTTATAAAGAGGAATTCACAATT
AAAATCAATTAAAAATCCTACTCTATTAGTCTATAATTTAGAAAGTTTTTTAAAAGATCA
AGAACGATTGGATAATCAAGATTTAAATAAGTTTGTGGTGATCACTAATAAAGAATCTTA
TCTTGCCACAAAAGAGTTAATAGCTTTACCCGATTCTAATAGCTTTTCTAAGCACTTAAA

ATTAAATATAAAGCCTAAACTCTTTTTTGTGAAACTATGGGTAAGACGGTTATTTTCCAC
CTTAACTTATGAATAAATGCTATTGCCTTTATTTTCTCGAATAATATTCTATAACTAGTA
ATTCATTGAGTTGTAAAGCAACCCATTCCCTATCAATGACTCCATTGACTTTCCCTGATA
AATTGGATTTATTCAATTCTAAGTGGCTAGGAATATTAGCTAGTCCAGGGAAAGCTAAGT
AATTTTCTACTAGTTTTCGAGATGCTTCTTGATTTTTTACACTAATTGATTCTCCTGGTT
TACATTGATAACTACAGATAGATACTACTTGTCCATTAATACAAATATGACCATGGTTCA
CTAGTTGTCTAGCGGCAGGAATTGTAGGAGCCATACCAAGTCTAAAAACAGTATTGTCTA
GCCTCATCTCTAGTAGCTGTAACAAAATTTGACCTGTCGACCCTTGTAGTTTTTTAGCTG
CTTTTACGTACTTAAATAACTGTTTCTCACTTAATCCATAATTAAATCGCAACTTCTGTT
TTTCTTCTAATCTTACAGCATACTCAGAAGGTTTTCTGGACTTTTGTCCATGTTCTCCAG
GTGGATAAGATCTTTTAATCGCTTTTCTACTGAGTCCGGGTAAATCACCTAATCTACGGG
AAATGCGTACTCGTGGTCCTCTGTATCTAGACATATTATTTAAATTCTCCTAATGTAAAT
GATTAAGTAATGAGTAGCTATTTTTTAACACAAAAAGAGCATTAAAACAAAGCTTTTGCT
AAAAAATATTTACAAAAAATATAGGTTTGGACAAAATACATTACTATGCTTTTTAAGCGG
GTAGCGGGAATCGAACCCGCATCATTAGCTTGGAAGGCTAAGGTTTTACCACTAAACTAT
ACCCGCAATATTCAACAGATTGTCACTAACTCAATATAACATAGTTATGTTAAGTTAAGA
TAATCTTAAATTTAATAGAATTTTAAATTATTTATGCTCCTTCTAAAACCACATCAAGAA
AACGAGCAATTTCGGCAGCTTGTTCTTCAACTTCTGATAATAATAATGGTTGACCGACCC
TAGTTAATGGAATTACTCTTTTATCTTTTGTACATAAATAAATTTCACGTCTTGGATTTA
GCCCCTCTTTAATATCTATTTTTATTGATTTGATTTCTTTTATATTAAACTGAAGACAGA
TTTTTCTATTCTTTCCAGGGAAACCTAGCCGAAAAATTTTTACAATACCTTTATCTTTGT
TAAATTCATTATATCCAGCCCCTATATTCCAAATAATAGTTAGCCACAGAAACAAACTCA
GAAAGACTCCTATACTGCCATAGAATGTCATTACAATACCTTGTGGAATAAATACCAAAT
CGGTTGAATTTGTAAAAGGTAACAAATCAACTTGAAAATAACTTGATAGTCCAGCAAGAA
GAAAGCCCAAAGCCCCAATAAATATTATAGTAGCCCACCAGTAATTACTCAGTCTTCGTG
AACCTAAAATTAAATCTTTTCTAACTGAGTGTATAGATAATGTTTTTTTCATAATTTGTT
CTTTTAAAACAGTATACTAATGCAAAATGGAAAAATTTTGACAATTTTTATGCGCGCTTT
AGATTTTTTAAATAAGCTTGATTGCTTTTAGTCCTAAAAACAGTCCTGTTGCTAACCCAA
AAAAAGCAGCAAGAAATATTATATATCCTAGAAAAACTGACA---TATTATTATTTATTA
TTATTGTTACAATAGTGTTTACAAAAATAGTGAAAATCTATTACTACGGTATTATATTAC
GTTAAATGCCTTGCTGTCTACAGGAAAAAACTTTTTTCAGTTTTTAGCATTGTGTTTATA
TTAGAAATAATATTCTAGTAAATTTATTAAACTTCTGGAGAACAGAATCTTATGTCAGAA
TTTATCAAGCCTTATAACGATGATCCTTTTGTAGGCAATTTGTCCACGCCAGTTAGTACG
TCAAGTTTTAGTAAAGGACTTCTAGGAAATCTACCAGCTTACCGTCGAGGTTTATCTCCG
CTTCTTAGAGGATTAGAAATAGGAATGGCACATGGATACTTTTTAATTGGACCTTTTGAT
AAATTGGGCCCTTTACGAGGTACAGATGTAGCGTTACTAGCGGGATTTCTATCTTCGGTC

GGCCTCATTATTATTCTCACTACATGTTTATCCATGTATGGTAATGTATCTTTTACTAGA
GCAGATTCGAAAGATCCACTACAAACTTCTGAAGGCTGGGGACAATTCACTGCAGGATTT
CTAGTTGGAGCAGTAGGTGGTTCAGGATTTGCTTATTTATTGCTAGCTAATATACCTGTA
TTACAGACTGCAGGTCTTAGTTTATTCTCTTAAGCTAGTAAGGGGACTTGAACCCGTAAC
CTACTGATTACAAATCAGTTGCTCTACCAATTGAGCTATACTAGCATTCAGTTCATAATA
ACATATAAAGATATTTGTTGCAATCTGCTATTTTCAGATGTAACAAATATCTTTTTTATT
TAAACAGAACTGTTATGTTAACACGTATATTAATTAGAATCTGAATTATCTGACTCTGCA
GAAGATTCCTGATCACAGTTTAGATAATAACAAATTGTTTGATCTAGACAAGTTGCAGAC
CAACCAACAGGTGTTTCTCCTGATAATTCTTGCATATCTACAGAATTATAAAAATTATCA
TTTGGCTTATTACTGTTTGCTTGCATAAATATCACTCCCAAAACACTCTGAACTTAATGT
GAAGTAATTTAACTTCTCAATAATATATTAACACAAAATATTAAATATGGTGATTATTCT
TTAATTGTAAAGATGCTGCTAACTTTATAAAGTTTGAGTGAGAGTCTTGATAGCTTTTGT
TGAAATAAGCATTTTAACCCATTTATTTTGCCCGCTCGACCATACTTTTTTGTGTTGTAA
ATTAACTTTTTGTAGTTTTTTTGTTCGTTTATGAGAATGAGATACGGCATATCCATTATT
AGCTACTTTTCCCGTAAGCTGACACTTTTTTGACATAATTTTCTAACTTATATATTTATT
TAGAGTACTTGCTAAAGTTGACTTGGGCACAGCTCCAATCACTGTGTCTACTCTTTCTCC
TGCTTTAAAGATCATTAAAGTAGGTATACTTCTAATGCCATATTCAGCCGCAATAGTAGG
ATTATCGTCTGTGTTTATTTTTACTACCTTAATAGATGATTCATATTCTTCCGCAATTTC
ATCAACTACAGGAGAAACCATTCTACAAGGACCACACCATGGCGCCCAAAAATCTACGAG
TACAGGTAAGTTATTGTTAATAACTTCTTGTTTGAAAGAGGCATCTGTAACTTGAGATAC
TGACATATTCTTTAACCTTTAATATATGTTCCTTGCTAAGCAAATCTTATCACAAATTTC
AAGAAAAAATCTTCTATAGTGAATTACTATTAGTGTTGAATTATTTTTTTAAAAATTAGAC
ATTAAAAAAACTTATTACCTATATAAATAAAGTGGTGGCCGAGTGATATCTTGATAGTGA
TAATTTTTATATTAAGGTTGAACACTAGAAATAACTTTTCAAGCAAACTCTTATTTGAAA
AATTTCTCTGTGACCATAAAAACTTAATTATATTACAATATATTTTATTATTTGTAACTT
AGTTCTGATAATGGTATAAACAACGCAAAAGATACTGCCTTATAATCAAGGAGGAATACA
TGTCTCAATCCGTAGAATCACGGACTAGGATTAAAAGCGAACGTTACGAATCTGGAGTAA
TCCCTTACGCTAAAATGGGCTACTGGGATGCTGACTATGTGATTAAAGAAACAGATATTC
TAGCTCTTTTCAGAATCACTCCTCAACCAGGTGTTGACCCGATTGAAGCATCTGCTGCAA
TTGCAGGTGAATCTTCAACAGCTACTTGGACAGTTGTATGGACTGATTTATTAACAGCTT
GTGACTTATACAGAGCAAAAGCATATCGAGTAGATCCAGTTCCAAACGTGGCAGATCAAT
ATTTTGCTTACATAGCTTATGATATTGATTTGTTTGAAGAAGGTTCCATTGCGAACTTAA
CTGCTTCAATTATTGGTAACGTTTTTGGGTTTAAAGCTGTTAAAGCTCTTCGCTTGGAAG
ATATGCGTATGCCAGTAGCTTATCTAAAAACGTTCCAAGGTCCTGCAACTGGATTGATTG
TAGAACGTGAGCGTATGGATAAGTTCGGTAGACCTTTCTTAGGTGCTACAGTTAAACCTA
AACTAGGTTTATCTGGCAAAAACTACGGAAGAGTTGTATACGAAGGCCTGAAAGGCGGTC

TTGATTTCCTTAAAGATGATGAGAATATTAACTCACAACCATTTATGCGTTGGAGAGAAA
GATTTTTATATTCTATGGAAGGTGTAAATAAAGCATCGGCTTCTGCTGGCGAAATTAAAG
GTCATTACCTTAACGTAACAGCCGCGACAATGGAAGATATGTATGAGAGAGCCGAATTCT
CTAAAGAGGTTGGTAGTATCATTTGTATGATTGACCTTGTGATTGGTTATACTGCGATTC
AAAGTATGGCAATTTGGGCTCGTAAACATGACATGATTTTACATTTACATAGAGCTGGTA
ACTCAACTTACTCTCGTCAAAAAAATCATGGTATGAACTTCCGAGTTATTTGCAAATGGA
TGCGTATGGCTGGTGTTGACCATATTCACGCAGGTACAGTTGTAGGTAAGCTTGAAGGAG
ATCCTTTAATGATTAAAGGCTTCTACAATACTCTACTTGAAAGCGACACAGATATCAACC
TACCTCAAGGTCTGTTCTTTGCTCAAAATTGGGCTTCCCTACGTAAAGTTGTACCAGTAG
CATCTGGTGGTATTCATGCTGGTCAAATGCACCAACTTCTTGATTACTTAGGTGATGATG
TAGTTCTTCAGTTTGGTGGTGGTACAATTGGACATCCTGATGGTATCCAAGCAGGTGCAA
CTGCTAACAGAGTAGCACTAGAGTCCATGGTTATGGCAAGAAATGAAGGCCGTAACTATG
TAGCAGAAGGTCCACAAATCTTGAGGGACGCTGCTAAAACTTGTGGGCCTCTACAAACAG
CTTTAGATTTATGGAAAGATATTAGTTTTAACTACACTTCCACAGATACAGCTGATTTCG
TTGAGACTCCAACAGCAAACATCTAGTTTAATGACTACTTACTGATACTTTAAATAGTTA
ATTGTAAGTGAAATTAACTTATAACAATAAGGAGCATAGAATAGTGAGACTAACACAAGG
GACTTTTTCCTTCCTTCCAGATTTAACTGATGAGCAAATTAATAAACAGCTTGCTTATAT
CGTTTCTAAAGGCTTATCAGCAAACGTTGAGTATACTGACGATCCTCATCCAAGAAACTC
CTATTGGGAACTGTGGGGTTTACCTTTATTTGATGTAAAAGATGCTTCTGCTGTTATGTA
CGAAATTAGCTCATGCAGAAAAGCAAAACCTAATTATTATGTTAAAGTTAACGCTTTTGA
TAATACTAGAGGTATTGAAAGTTGTGTAATGTCTTTCATTGTAAATAGACCTGCTAATGA
ACCAGGATTCTTATTACAACGCCAAGACTTCGAAGGTAGAACTATGAAGTATAGTCTTCA
TAGCTATGCTACTGAAAAGCCTGAAGGAGCTAGGTATTAATATTAATTCTTAATTAATAT
TGGCTAATTATTCCCCTCTTAAAAATCATAATTAATTTAGTAATTGTGATTTTTAAGAGG
GATAGCTTCCCAAGTAAATTTTGACTAAATAGATTGAATAATTAAAAATACACAGAAATA
ATGCAATCACAGGATATAATTTCCAACGATACTCTTGTTAATTTACAAGAAGAATATGAT
AGAACACAAATCCAAGAAGTTTTAAATGAGTTAAATCAAGAACTTATAGGATTAGTGCCT
GTAAAGACCAGAATTCGCGAAATTGCTGCGCTATTATTGATTGACAGATTACGCAGAAAA
CTAGAACTAGTTTCTGGTAATCCAGGATTACACATGTCATTTACAGGTAGTCCAGGAACT
GGTAAAACTACAGTTGCTATGAAAATGGCTGATATTTTGCACAGACTTGGATATATAAAA
AAAGGGCATTTGTTGACAGTTACAAGAGATGATCTTGTAGGTCAATATATTGGACATACT
GCCCCTAAAACTAAGGAAGTTCTTAAACAAGCAATGGGAGGAGTTTTATTTATTGACGAA
GCTTACTATCTATATAAAGCAGATAATGAAAGAGACTATGGCTCTGAGGCAATTGAAATT
TTATTACAAGTAATGGAAAACCAAAGAAATGACTTAGTTGTTATCTTTGCTGGATATAAA
GATAGAATGGAAAAATTCTACGAATCCAACCCAGGACTCTCTTCTAGAGTAGCTAATCAT
GTAGACTTCCCAGATTATACTTCAGATGAATTATTACAAATAGCTAAAATGATGATAGAA

GAACAGCAGTACTGTTTTACAGAAGAAGCAGATAAAACTCTTTTAGAGTATACCGAGCGA
AGAATGAAACAGCCTTATTTTGCTAATGCAAGAAGTATTCGCAATGCTATTGACAGGGCT
AGAATGAGACAAGCCAATAGGATTTTTGCCAGTGGAGAAAAAGTATTAACAAAAGCTGAT
TTGGTAACGATTGAAGCAGAAGATATCTTGAAAAGTAGATTATTTTCATTACCTAATGCT
TAATATACACGTGATTTCATGAAACTATATATTATTAAAAAGTTTCATAATCAGTTGCAA
ATTTCTTGAAAAACCTTTATTATTATCTTAATGTAGGTGTGGCGGCATAGCCAAGTGGTA
AGGCAGAGGATTGCAAATCCTTCATCCCCCAGTTCAAATCTGGGTGCCGCCTAGTACTAA
AAAAGGGGGGTGTGGTGGAATGGTAGACACAACAGACTTAAAATCTGTTGATTTTTAGTA
ATCGTGAGGGTTCAAGTCCCTCCACCCCCATATATTGATTTATAAATAAAAAGAGATGTG
TATTTGTATTAATTGCAACCATATTACCAAATGTAATACCTATCACTTAATAGAATCTCA
ACATAAACAGCCTCATCTTACTAGAAGTCCATTGTTTATACCTAAATATCCTGTAGTTCA
TGTTAATATATCTAACAACTGTACCTATAATCAAATTGATTGGGATTTAGTGGAGTGTTT
ATCATTTGTAGAAAAACCGAATAGTTGGAATTTAGACGCTAATTAGTTACAAGAAAAATT
ATGTACCATAAGCAACAACTCTATTTTTTAGATACTCGGTATTAATTTTGGATGTTCTAA
CCAGTAAGATTTTACCCGTTCTTGCTATCTCTATAATGCCAAATTTAGTTAGTAATTGTT
CAATAGCAACAATCTTTCCCGGATCTCCAGTAACCTCTATAATTAAAAGATCTTCTGCAA
TGTCTACTATCTTAGCTCTAAAAATTCTTACAATTTCTAAAGCTTCTGTTCTAGTCTGAG
AATTGATCTGAATCTTAATTAACATTAGTTCTCTTTCAACTGAAGGAATATTTGTTACAT
CTTGAACGTTAAGGATATTTACTAACTTATATAATTGTTTCGTAAGTTGTTCAATAGTTC
TATTGTCTCCTTGAACTACCATTGTGATTCTAGAGACCCCAATTTGCTCTGCTGGTCCAA
CTGCTAAACTTGCGATATTAAAGCCTCTCCGGGCAAATAGACCAGATATTCTTGACAGTA
CTCCGGCTTCATCTTGAACTAAAACTGATAAGGTGTGTTTCATGAAAAATTAAATTTAAT
CTATTTGATCTATTAAGTATATTCTAAGAAAATTACAGAAAAAATACTAGTTAGTCTATA
ACTAAATTATCCAAACTAAACAAAATCCTACTATCAGATAATATATCTACCTTGTAATGC
TATACTATTAACTTAGTATTTAATGATATTCTAATTGCAAATAGAATGCTAATTTTTTTA
AATTTGTTAGGAACTTGTGCTAGAAAAATACAAAAACAGTAAAAAACTCTCTCGAGATTT
ACCTCAAATCAATGATCGCATTAGATTTCCAAAAGTCCGAGTAATTGATGACGAAGGTGA
ACAACTAGGTATTTTTGTGCCTGAAGAAGCTATACAATTAGCTGTCCAACAAGGTTTAGA
CTTAGTTGTTGTTAGTGATAAATCGGACCCGCCAGTATGCCGAATATTAGACTATGGTAA
ATATAAGTTTACACAAGAAAAAAGAGCTAGAGAAGCTAAGAAAAAGCAACATAACAGTAG
TATTAAAGAAGTAAAAATGCGATATAAGATAGAAGAGCATGATTATAAAGTTAGAATAAA
CCAGGCATCCAAATTTATTCAAGCAGGAGATAAAGTAAAAGCAACTATCACATTTCGGGG
GCGTGAAATCCAGCACTCTAATTTAGCTATAGATTTATTGAATAAGATGGCAAGCGATCT
AAATGCAATAGCTGAAATTCAGCAAGCTCCATCAAGAGATGGCAGAAATGTCATAATGCT
CTTATCTCCCAAAAAAGTTAGCTAAACTAATTTTTATTTAAATGCATCTGGCTGGATTCG
AACCAGCGACGTCTCTTTCGAAATGGCGGATTATGAGTCCGCTGCCTTCGGCCCCTCGGC

CACAGATGCATCACTTAAAGTTTATAACTCATAAAGCATATAAAAATCAATGTTTAGCTA
TTTTTAATAAAGTCTATAAAAGTCTATTATTCATTCATATTGTGATATGTCGCAACTGCT
GAAGGGGAAACTTGATTTAAGTATCGAAATATCCAGTATTTAAAAATTGTGTCGAGTATA
ACTGGAAAAGTTGAAATAAATAAAAAAATAAAATCTCGACTTTCCGGAAGTCCTAAATGT
CTTAAAATTACTTCAATAATAACTTCCCATCCATGAGGAGAATGAAAACCCACAAACATA
TCAGTAAAAAGAATGATTAAAAAAGCTTTAGCTGTATCACTAAGACCATAAATAATTTCA
TTTAAAAAAGATTTAACGACTGCAATTTGTCTTTGTCCAGTTATCATTAGTAATATAAAG
ACAAGTATTGATACAAGATCGGATAAAATATTTTTAACAGCATTTGCACTTTCGTTTGCA
TAATATTCTCCCAACTCTCTAGCCTTCAGCTGCACTCTTTTTTCAATGATTTCGTAAGAT
ATATCTTCCGAAGGATTCAGAAGAACTTCAAAGTGAATTTTCTCTTCAAATCTTTGTAAT
TCAGCAAAAGCTCTTTCCTCTTGAGAGGAATTTAAAAAAATTTTAGGCTGTTCTTGGTTC
CATAAATAATCAATACAAGGTCCAAATACAAAAAATTTTGAAGCCTGATTAACTAGTACT
GGAGAGATGAATAATAATAAAATATATTTTACGGAAGTAATAGTTTGGTGTCTAGATATT
CTAAACTCCTCTATAGCTTCAGATTCGCCATTAGGATCTAACTCTTTACGAAACTTTTCA
AAAGTATTAGTAATTGACCTAGGTATTGGCCCAACTTTTTCAAAGGCGAATTGATTATTT
CGTTTTAAATTCCAATATTTCATTTTTTATTGCTGCTAAAATTTCTATTAATCAATAATT
GATCACGTATTCACTATAAATATTAATTCTAGAACTAAACATATAAAACAATATTTATGA
AAAATAAAATACAATTAAAAATTAATTATGCATTAGTTCTATCTGCAGCTACAGCAGTTT
ATGGATATAAATCTTATCAGCCAACTATAAATACACTCTTAATCAAAGATGAAGAAAACT
GGCCTTTTTTCTCTAAAAGGCAGACTGCCTTTATTCCTATAATTGTATTAAAAAAAAATC
ATCAAAATCCCTGAACTCAGAACCTAGAAATAGTAAGTTATATCTTAATGGAATTGGAAG
TAGAAAATTTATCGACAGAATTGTCACTCTACTTGGATATTTAGAAAAACAGGCAAACTA
TACTGAATACTTGAACCAAATAAAAACAAGTGGTTATTTTTCTTTAGTCTACCTAGACAG
TGATATAATGTATAAGAAAAATATTAGTAACATTTTTTTACTACCTAATGATATTCTAAA
AAGAATCTATATTTGCAATAAGAAAAAAAAGCTTATACCGCATTCATTCTTAATAAATTT
ATTTCAGAAACAGATAGGCTATCCAAAAAGTTTTGCTAATTTAAATTGGGCCTTCTCTAA
AATTATAAAATGGTATTATGATAGAGGATACCAATGGTCTCTAGTTGAAGTTAAACAAGC
GTCTGATGCATCTTCTATTGTAATTGATATTCATGAAGGGGTAGTGAAGACAATTATAAC
AGAATACTACACTCTATCTTATAAAAGAGTCTCAGGTATCTTATGCGTAGAGTCAATAGA
GCAGTACCTTGGAGTAAGAGTGGGAGCTCCACTAAATATAATTGATTTACAAAAAAAAAT
TACTTACTTAAAAGATAATCAACTAGTTGGCGACATTATTTATAGTATTGAACGATCAAA
TAATAGCTCAATGAGTTTGGATATCAAATTTCAAATACAAGAATTAAAAGATAAAGAGAT
AATAGTGCTTGCAGAAAGTTCTTCTATTATCTCGCATGCATGTAATTTACTCAATCAATA
TAGAAATAGGCTAGTAGCTTCTAATATAGTTTCACTATCAACTAATAAATTACACGCATG
CTATTTTAATTATAAACTTGACTATCAATATAAGTATATAAACACCTCTAATCTTATAAA
ATTGTTAGCTTATTCTATTTCTTGTAGGAAGACACTACTTATTCATTATTTTAATTTACA

AACTTTAATAAGCTGTACAAAAAAAAATACTATTGGATTTCAGCTATATTTACGAAATCT
AAGTTTTGGGAAAGCTTTCTGTGTGCTTAGTATGAAATTTATCAAAAATGGACTTAATAT
AAAAATTTTATATATAAATCCTTCATTGATAGTTGATCAGAATTTTGTATTTCAATTTGC
TATACAAATCATTAAGCAATATCATACTGCTAAACCTCCAGCTTTATTTTTAACGAATCT
AGATCTTGAACAATATGTTGCTGAAAGTTTATTAATGTACCATTTTACATCTTGCTTCTC
AATATCTGAAAAAATATTATTATCTCGAATTATGCATACAGATTCTTTATTTTTCAACTC
TGAAAATTTTCATTTTGATGATAGAACTAACGCGATCAACAGTTATGATACTTTTAAACA
AAATACAAAAATATTTTATCAAGAGTTTCTTTCTTTATTATTAAGTTTACGTTATCAAAA
TTTTAATTATTTAGGTTGGCCTTTAAAAGGCCATTTTTTTGAAATAAAATCTTTATACTT
GGCTCCATTTCAAAAGAGTGATTTTTCTGATAGTCGTAAGACGCTATTCTTCCACAAAAT
GTCGTTAAAGCAAGTATCTAATTTTAATTTACCAGTATCTTTTAAAAGCCACCTTAACCA
TATTTTAGTTAGTACTATTAAATGTCAGTCAAATTTGAATATGAGAACTGTATCTTTGCT
ATTAATTGATTCACCTGCTGAATATCTGCTATACAAATCTATTCTTAATTTCTCTATTAA
AGTTAGGATGCAGTATTTCATACCTATGAGCAACAATATAAGATTATCTCTATTTTATAA
TTATTTAGATTGTTTTTTAATAAGATCATCTCAGAGTTGCATTCACATATGGCAAGATCT
AAGAACTCTTACACCAATTCAAAATTTTTGGTTAAAAAAATTTTCATATGGAGCTGGAAT
TCAGCTTAAATTACCAATTAAGCAGATGCCACCTCTTTCAATTGAATATACTGTAACTAG
TAGTCGCTACTTTTGTATTTACCTTCGTACTTATTATCAACGATAACTATTACTAAAATG
AAAAATTTAATTTCTTTTTTTGATCGCAGCAAAGGGAAATGGATTTCACAAAGAACAACT
TATGAATTATCTAATAAAAACATGAGTTCGGTACAGTCTCAGATGACAATGAAAATAGGC
AACTCATTGTCAGGATCTATAATACTTGCATCGTTAAATTGGGGGGACATTTATAGACAA
GTTGCCCATTACGCTAAAAATCACAGCCGGAGTGAATATGATAATAAGTTTAACTTGCAA
TTCGGTAATCAACTAAATAATCATAAATTATTAACGCTGTGCATAGTGACAGATCCTAGT
CTAATTAGTTTTAAAACTCGCTATGGAAGTACTACCATAGATGAAACATATTGGTTCGCA
ACAAATAATTTACGTCTAAGTACTAGTATTGTTAAACGATTCAACACTTGTGTGGCAGTA
TCTTTTTGTTCAGAAATTAAAGTTTAAACTCTATATAGAAGCAACTAATAGCCTTTAGGC
TATTAGTTGCTTCTATGTATCTAAATTTTGTATTAAAAATTATAATACTATATTATTCTA
AATCTTTACGATTGGGATTCCTGGAAGGATCATTAGAGAGGAACCCAAAGACAAACAAAG
AAATAAAGAAAATAACGGTAGTATATACAAAGATTTTCAAAGTAAACATATTTTCATCCT
TAAAAGCTATTAGTGCGATATTTACATATCTCCACATAATTTTATATCATGATATAAGTT
CCAGCACTATTTTATAAATAAATCAATATCTTTATTTTTTAGTAATGATTTATATTTAAA
TATGCGTGATGTACTAAATACTAAATCTTTTCCGACTTGTTTTGTCTTGTTTGTAAATAT
TTTGTTATTTCTATGGAGTTTACCTTCAATTATCACATAGTCTAACTTCTTTAGCTGTTT
AAAAGTATATGAAGATTTTTTGTTCCAAATAGTTAGATTGATTATAACTAATCGTTTTCT
TTTTAACAGTCTTGCTTTTAACTTAATTATTTGGCTTTTATTTTCACTAATTCTGATACT
CTTACAACTAAGAATTTGGACTAGAAGAGTACAGCTATTCATAATTTATAACAATTTTTT

AAAAAAATCTACTAAAATAGATGATTTTACAGAACTAAAATGATATTTCATTATCTTGTTG
ACCTTGCTCATAAGTAATATCTTTCAATACTTTTAATATTTTTCCAAAATACTCTTGCAG
GTATTTTTCTAAAGATATTACTTCCAACCTGTCAATTCCAAGAATACTGTAAACTTCATC
CATCGGAGCTGTAAATTGCTCTCCGCTACTTAAAATTTCGGCAAATGCTAAACGATCAGA
AATATTCCATGTCCATTGAAGAGTTTAGTAATTCTTCTTAAGGCTTTTAATAATCCAAT
CGGAATTTGTGAGATCTGTGTTTTTTGCCCAGATAATTTTTCACATAATTTAATAATTTC
AGCTGATGTCCAAGCGGTATTACCAACTAACGGCAGGGTCCTATTTTCTGTGGAAGGTAC
TCCAAGACTTTTTATAACTAACTTGGCTGCATCTTGAGTATCAATGTATGCAATGGGTGT
AGATTCTCCTGTAACCCAGACTGATTTCTTGTCTAAAATAGGAATGGCATATTGGTTGAT
TAAACCTTGAAAAAATCCTCCTAAAGAAAACACAGTATATTTCACATTGGACTTCTGAAG
GAAATCTACTACCTGAGACTTTAAGTTCATCAATGGAACATCTGGGTATTTCTCTGAATT
TAATATTGAAAAGAAAATAAATCTTTCAACTTTAGCCGCTTTAGCTGCTTCAATTAATGC
AATTTTCCCATCTAAATCTATTTTTTCTGCATTATAAGGATCAGTAGGACGAGATGTAGA
AGCATCTATGATTGCTGTTACCCCACAAAAAGATTGCAAAATACTTTCGGGTAATTTTAA
ATCACCATATATAAGTTCTGCTCCCCACTCTTTGAGAAAGGCAGACTTTCTTAAATTTCT
TACCATGCATTTTACATTGTAGCCTTCATCTAAAGCTCGTCTTACAATTTGACGTCCTAA
AGTTCCGGTTGCTCCAATTACTAAAAGAGTCATATTAGTTAAAAATAAGTATGTATAAAG
ACATTGATTTGGTTGACGGCAGAGAGTAAATTAGGTAGAGAGGGAATCGAACCCTCATGA
CCGAAGTCGTCACATTTTGAGTGTGATGCGTATACCAATTTCGCCATCTACCCTATTATA
GACCAACTACTACAAGTAATTTAGTCTTTAGCGTAAAAATAATACTAAGTTTCAATAGAT
ATGTCAACTTTTAAATTTATATTAATAAAAAAACGGAGTCTCTTATGGCCACTTTTGAAT
TAATTCCTTATAGATTGTATTTGAGTCAATCTAACACATGGATCCATAGAATAAAAGCAG
AAATTAAGATATATATAGTAGCGCTTTTATGGATTTCAATTTTTATTTTTTCTTACTTTA
AACTATGTATTATTGCTTTAAGCTTAATTGCCATAAGTTTTACTATAAGAAGTAAACAGA
ATATTATTCAAAAACATTTGTTACAAACGTTATTAATGACGTTCTTAACTACTGTTTTGT
CTTTTAGTGTGGCTATTAGTTATAAACAATATGCAGAACAAGAACAATCGCAGTATTTAT
CTGATTCCAAGAAATATAAAAATTCCAGCAGCTACTATTATATACAAATAGCGAACGATC
AAAGGATCAAAAGACAATACTTAATTACTACTTTAAAGCCTTCCTTGTACTTTTTTATTA
CTATATATTCTATTAAACTAGTTATGATAACAACCTCTCCAGAAGTTTTAGTAATTACTA
TTTATAGATCTAGGATAATAAATAAAATATTTAAAAATGAATTGCTATTTATCTTCCTTC
TTTCGTCACATATTGTTACTAGTATTATTAATAGAATCGATAAAGTGATTCAAGTAACTA
GCTTAAGAGGAAGCTTGAATTTATATAATTCATTGACAAGGCCTTTAATGTTTTCTTTGT
TAATATTTCAAGTCTTCTTTTTGGAGATTATTCGGGAGTCAAAAGAAATAGCTCAAGCTC
TTTATACTAGAAATCTCAATCAAGAAAACAATAATTTTTTGAAAATATATACAGTAAAAT
CTAACTTTAGTGATCGGCTCAATATAATTATTAGCACTTTGTACTTTATTATTTTAGCTC
TAGCGTAATAGAATCCACTTATTCTTCTGACTCTAGTAATACGAAGAGTAATAAAATTAG

TTAGTAGTTTAAATAGATAGAATTAAGATATTATATAAATAATTAAATGTTTCATTATGG
TCAATACTCAAAATCAAATTTCACAAACTTCAGAACTTGATTATATAGTTAATCAACCTT
ATAAATATGGCTTTAAAACTTCTGTTGAATCTGAGCAATTTCCTAGGGGAATAAGTGAAG
ATATTGTCCGCTTGATCTCCAAGAAAAAAGATGAGCCTGAATATCTATTGAATTTTAGGC
TTAAAGCATATAAAAAATGGAAAAAGATGAGTAGTCCGTCATGGGCTCATATTAAGCATC
CGAATATAGACTTTAATACGATTATTTATTACGCTGTTCCTAAATTAAAAAAAGAATTGA
AAAGTCTAGATGAGGTTGATCCAGAAATTCTTGACACTTTTAACAAGTTAGGTATATCTT
TAAATGAGCAAAAGCGAATTTCCAATGTAGCTGTTGATGCTGTTTTTGATAGTGTATCTA
TTGCAACTACTTTTAAGAAAGAACTGTCTGAAGCTGGTGTTATATTCTGTTCAATTTCTG
AAGCTATAAGGGATTATCCAGAATTAATAAAAAAATACTTAGGTACTGTTGTACCAGCTG
GTGATAATTATTTTGCTGCATTGAACTCTGCGGTATTTAGTGATGGCTCTTTTTGCTATA
TCCCTCCTAATACAGTTTGCCCTTTAGAATTATCAACTTACTTTCGTATTAACAACGAAG
AATCTGGGCAATTTGAAAGGACACTAATTATTGCTGATCGTGGCAGTAAAGTAAGTTATC
TTGAAGGTTGTACTGCTCCTCAATTTGACACAAATCAATTACATGCAGCGATTGTAGAAT
TAGTAGCTCTTGAAGGAGCCGAAATTAAATATTCTACAGTACAGAATTGGTATGCTGGTA
ACAAAGAAGGTAAAGGTGGTATATACAATTTTGTTACTAAACGAGGCTTATGCTCGGGTA
ACAATTCAAAAATTTCATGGACTCAAGTAGAAACTGGATCTGCAATTACCTGGAAATATC
CAAGCTGTATTTTAGCTGGTGAAAATTCTCAGGGAGAATTTTACTCTGTAGCTTTAACAA
ATAACTATCAAGAGGCTGATACAGGGACTAAAATGATCCATATTGGTAACAATACAAAAA
GTAGAATTATTTCTAAAGGTATTTCTGCGGGTAGATCGAAAAACAGTTATCGAGGCCTAG
TAAAAGTTGGCCCTCAGTCATTTAATTCTCGTAATTATTCTCAATGCGATTCTTTATTAA
TTGGTCAATCATCTCAAGCTAATACATTTCCTTATATTCAAGTACAGAATCCAACATCAA
AAGTAGAACATGAAGCATCCACTTCAAAAATTAGCGAGGACCAAATTTTTTATTTTTTAC
AAAGAGGGATCAATTTAGAAGAATCTATTGCTCTTATGATCAGTGGTTTTTGTAAAGATG
TCTTTAATGAATTGCCTATGGAATTTGCTACTGAAGCTGATCGTTTACTGAGTTTAAAAT
TAGAGGGAACTGTAGGATGAGCCAGACTATTTTAGAAATCAAAGATTTATATGCTTCTGT
TGGTGAAACAACAATTTTAAAAGGGGTAAACTTATCTATTCGAGCCGGCGAAATACATGC
GATTATGGGGCCTAACGGTTCAGGTAAAAGTACATTATCAAAAGTAATTGCAGGACATCC
AGCGTATTCACTAATAAGCGGAGATATTTTATTTTTTGGACAAAGTATTCTTGAAATGGA
GCCAGACGAGAGGGCAAAAGCAGGTATTTTTTTAGCTTTTCAGTATCCTGTTGAAATTCC
TGGAGTCAGTAATTCTGATTTCTTAAGAATTGCATTAAATGCTCGCAGAAAGTTTCAAGG
ATTATCGGAGTTTAGCCCCTTAGAATTTTTTCAACTAATAACAGAAAAAATAGATCTTGT
TGGCATGCAAGAAAGTTTTTTGACAAGAAATGTTAATGAAGGATTTTCTGGTGGAGAAAA
AAAACGTAACGAAATTCTTCAAATGGCTTTACTAGATAGCAAAATATCTATTTTAGATGA
AACAGATTCTGGACTAGATATTGATGCATTACGAGTAGTTGCAAAAGGAATTAACACTTT
AGCTAAATCAACAAATTCAATTATTTTGATTACTCACTATCAAAGATTATTAGATTATAT

TATTCCAGATTTTGTTCATATTATGAGTAACGGGCAAATTGTAAAAACTGGTAGTGTTAC
TCTTGCCCAAGATCTAGAAAAGCACGGATATGACTGGATTACGCAGACATAACTATTTAC
TACAATATTGAGACTAAAGAATATTCTTTAGTCTCAATATTTTTGCAAATGCAAAGTTAA
TTATTTAAGCTTTATCAAAACCTTGCTTAACATCTTCAATTGCTCTTTTTAGCAATTCTT
CACTAACTGGTTCTAATTTTTTAGTACTACGAACATTTTCACCAAATTCTGGTTTAGAAT
TCTTTAAATCTTCTCGAAGTTCTCTAATAAATTCAGCTACTTTTGAAACTTCAATATCAT
CTAGATAGCCATTAATTCCAGTGTAAATAATAGCAGTCTGTTCTTCAACTGGAATAGGAG
AATTTTGGGCTTGCTTCAAAATTTCTCGAAGACGTTGACCTCTTGCTAGTTGATTTTGGG
TTGCTTTATCTAGATCAGATGCAAACTGAGAAAAAGCTTCTAATTCAGCAAATTGTGCTA
ACTCTAGTTTTAACTTGCCTGCCACTTGTTTCATGGCTTTTATTTGCGCAGCAGAGCCTA
CTCTAGAGACTGAGATCCCAACATTAATAGCTGGTCTAATCCCTGAGTTAAATAAGTCAC
CAGATAGAAATATTTGTCCATCTGTAATTGAAATTACATTTGTTGGTATATATGCAGATA
CGTCCCCAGCTTGAGTTTCAATAATAGGGAGAGCTGTCATACTACCTCCACCTAATTCAG
CATTCAATTTAGCTGCTCTCTCTAGCAATCTAGAGTGTAAATAGAATACATCCCCAGGAT
AGGCTTCTCGTCCCGGAGGTCTTCTAAGCAAAAGAGACATCTGACGATAAGCTTGAGCTT
GCTTAGTTAAATCATCATAAATAACTAATGTTGCTTTACCTTTGTACATAAAATATTCAG
CTAAAGCCGCACCTGTATAAGGAGCAATATATTGTAAAGTCGCAGGACTATCTGCATTAG
CTGCAACTATAATAGTATAATCAAGAGCTCCTTTATCCTGTAGTGAAGATACTACTTGGG
CTACTGAAGATGCCTTTTGTCCTATCGCAACATATACACAAACAACATCTTGGCCTTTTT
GATTAATAATTGTGTCCAATGCAACAGCTGTTTTACCTGTTTGACGGTCACCAATAATTA
ATTCTCGCTGACCTCTACCAATTGGGATCATAGAGTCAATAGCAGTTATCCCTGTTTGCA
TAGGTTCACACACTGATTGCCTTCCAATTATACCAGGGGCCATTGACTCAATTAGCCTTG
TTCCATTACTTGCAGGTTCACCTTTATCATCTATTGGCCGAGCTAATGGATCAACAACTC
TACCTAAAAAAGCATCTCCGACCGGAATTTGAGCAATTCGACCAGTCCCTTTTACGGAAC
TGCCCTCTAAAATATCCCTACCATCTCCCATGAGTACTACACCAACGTTATCACTTTCTA
AATTTAAAGCAACCCCAATAGTTTTATCTTCAAATTCAAGTAGTTCACCAGCCATGACTT
CATCAAGTCCATATACTCGAGCGATACCATCACCAACTTGTAAAACTGTTCCTATATTGG
CTACTTCTACATCCTGATCATACTTTTCAATTTGTTGACGAATAATACTACTTATTTCAT
CAGGTCTAATATTTACCATATTTTTAATATTATTTTTATTAATTGGAAATTCTAAAGCGG
TAATTAGTCAGACTAATGCTATCTGTCTTTTTTACTTTATTTTAAGTTACTACAACATCA
AGGTGTGAAGCCATTTGTCTTAGCTGTCCTCTAATACTAGTATCAATGACTTTTGATCCA
ATCTGAATAGTAAAGCCACCAATTAGCTCGGGCTCGACTGAAATAATTAGTTTAACTTCT
TTAGCCTTAGTCATAACCTTGATTTTTTCTGTTAATAGAGTTTCCTGATCAGGGCTTAAA
GCAATAGAAGTATTAATATTTGCAATTGTTAAAGATTCCATTTGGTAAGCCAGTTCCAAA
TACTTACTAGCTATAGCATCTAACATTCCAATTCGCTTGCGATCAACTAAAATCATTAGA
AATGATAATGTATTTTCGTTAATTTGATCGCCAACAGTTGCAATAATTACTTCTTTTTTT

GCCTCTACAGTTTTTAATGGATTAGCTAAAAAAATTTTTAGCTTGTCAGATTGCGCTAAA
ATATTTTGTATTGACTGAATATCTTGGCTCACCTTTTCAGTAACTTTTTTAGTTTTAGCT
AAATCAAGAAGAGCTACTGCATAAGGCTGAGCTATTTTTATAACAACATTGTTACTGCTC
ATAGTTGATCTCCTAACTTAGCAATATTATTATCAATTATACGCAGTTGCATTTCGGAGC
TCATTTGATTTTCCAGCTGCAAAGTAACTCTTTTAAGAGCAAGAAATGTTATTTGTTGCT
GGATTTGCCTTCGGATCTGTTTTTCAGCTGTTTCTATATTTGATTTACCTGTTATGGCAA
GTCTTTCAATATCTAATTGGCCTTGAGCTAATATGGAACTTCTAACTTTTCCAGCTGTTA
GCTGAGCTTCTTTCTTAATTTGATCTATAATGATTTGAGTTTGAGCAAGTTGTTTTTCTG
ATTCTGACAATCTAGCACTTGCTTGCTCTAGTCTTTCTTCGGATTCTTGTATAGCTGCTA
AAACTTTTATCTGTCTTGCATTTAAACTTGATCCGAGAAATTGCTTCAAAACATAAATAA
GACCAAATAAAAGCAACAATATATTAATAACATTTGCTTCAAAAATATCAGAATTAAAGC
CGAATTTGTGTTCACTACTATGTTCTGATAAAATAGTAATTATTTGTGGTATTTTTACTA
TATTATTCATTTAAAATTCTTACTATTTTAATATTTTACTCTGGAATCATGATTTTCAAG
ATTGACTACTTAATAATTTAACTTTAATTTGATCGCTCAAAGTGTCAACCTGATTTTCTA
AAGTTTTAAGAGCTTCTTCTTTTTGAACATTTAACTGTTTCGATGCTTCAGCAATCAGTT
TCTCTGCATTCAATTGAGCTTTTTTTATATCTTCTGAGACTATATTTTGAGCTTCTTTTT
GAGAAGAGGCTATTTTTAATTGTGCATTGCGGCGAGCTTCAGATAGATCTTCTTCATATT
TTGCAGCTAGCTCATCTGCTTTAACAAGCATAGACGATGCAGTAGTCAATGTTGTTCGAA
TATATTCATCTCGTTCGTCTAGTACTTTAGTTACAGGTTTATAAAAAATAGTATTTAATA
AAACCATTAAAGTAAGAAACTGTAATGCCATTAAAGGCAAAGTACCATTGAAGTCAAATA
AGCCACCTTCAATTTCTTCGGCTAATAGAAATGGTAAATCAATCATTTTACTAATTATGT
TTATAGTTAATAATTTTGTTTCAATTAAGAAAAATAACTTTATAGTGCGCTTAGTCTCAT
CAGACTAAGCGCAAATGATTATTTTAACCAACGTATGGATTAGCGAACAATAAAGACAAC
GCAACAACTAGGCCGTAAATTGTTAAAGATTCCATAAATGCTAAACTTAGAAGAAGTGTA
CCCCGAATCTTTCCTTCTACTTCTGGCTGTCTAGCAATACCTTCGACAGCATTAGCTGCT
GCACTACCTTGACCAATGCCTGGGCCTATTGCAGCAAGACCTACAGCAAGACCGGCAGCG
ATAACGGATGCGGCTGAAACGATTGAATCCATAATGAATTTTAATTTTTTGTTATATGAG
TAGAAAATTGACAAATTTCGTATAATACTTAAAATCTATATAATTAAGTATAATAGATTC
TAATATTGTAATTATAGTATATTACTCTTCTCCATGACCTTCCATTGCTTCTCCAATATA
AGCTGCGGACAAAGTAGAAAAGATTAATGCCTGTATTGAACTCGCAAATAATCCGAGTAT
CATAACTGGTAATGGTATTAAAATTGGAATTAGCAAAGTAAATACAGAAACAACTAATTC
ATCTGCTAAGACGTTTCCAAATAATCGAAAACTTAAAGATAGTGGTTTCGTAAAATCTTC
TAGAATATTGATTGGTAGTAATACTGGAGTTGGCTGAATATATCTAGCAAAGTAGCCTAA
CCCTTTTTTACTTAAACCTGCATAAAAATAAGCTAAAGATGTAAGTAAAGACAGGGCCAC
AGTTGTATTAATGTCATTTGTTGGGGCAGCTAGCTCACCTTCAGGCAAGTGAATTAACTT
CCAAGGAATTAATGCACCTGCCCAGTTACAACCTAGAATAAATAGAAATAAAGTTGCAAT

GTAAGGCACCCAAGGGCGATATTCATGCTCACCAATCTGATTTTTTGCAATATCTTGCAG
AAATTCTAATATAAATTCCATAAAATTCTGAAATTTTTCAGGAATTCTTTGTAGATTTCT
AGTACCTAGAAATGATAAAGTTAAAAGTGTAGCAATTACTAACCAAGAGACAATGAAAAC
TTGCCCATGCAATTGTAAGCTACCTATTTTCCAATATAGATGTTTTCCAACTTCTACTGC
TGATAAACTATTATATGGGTTAAAATCTATGAGACTGTTTTGATACATAATAAATATTTT
TTTAAATAGTGCAAGAATCAAAAAATAAGTTTTTGAAGAATTAATATGCAATTAATTTAT
TCTTAAAATTAGACTATTAATAAATTAAAAGCAAAAATAAATTCTAATAGAGATAGCTAT
TTTTAGTAGTGATTATTTTTTGTAAAAAACAATTATTGCTACCTATAATGCTTTTACGTT
TAATTATATACCCATATATTAATTATTTAATATGATTATAGGTATTTTGCTACAAACTTT
ATTGTTTTATCCATTCTAAAGATTGTTATAGTTTATTTTTTATTTAAAATATCTGCGACT
TCATCTGCAAAATTAACTTTCAAATTTTCTTCTCCCCCTCCCAGAAGAAATCTTACAAAT
CTTCTAATTTTAATATTTTCACCTAGAACAGCAATATTTTGGTTTATTAAATCTTCAATA
CTAATATCTTGATTGCGAATAAACATTTGATCTAATAATGATAGCTCCTTAAGTCTCTTT
TTCATTCGTCCTTCAATGATTGTTTCGATTCTATCAACTGGTTTGTTTTTCAAGTCATCT
TTACCAGCTTCAACTCTTTGTTCCAGATTAATAATTTCACTAGGTATATCCTGTGTTGAT
ACGTATTCCACATTAGGCGATGCCGCTATTTGCATAGCAATATCTTTAGCTAACTTTTGG
AACTCTGGACGTCGTGCTACAAAATCTGTTTCACAGTTGACTTCAACTAATACACCTATT
CTTCCGCCAGTATGAATATAACTCTCTAGTAAACCTTCAATAGCGGTACGATTAGATTTC
TTGTTAGCTGAAGCCAGCCCTTTTTGTCTTAATGATTCTAAAGCTTTTTCTTCATTGCCA
TTATTAGCTTGTAAAGCTTTCTTGCAGTCCATCATACCTGCTCCAGTCTTATCACGTAAG
GCTTTTACGACTTGAGCAGAAATTTGTAGTGTCATATGCTTTCAAAAATTTTTAAGTATT
TAAATTAATTATTATAATTCACTTTGAGTAGGATGAGTTATTTCTTCTAAATCTGTTTGC
CCGTATTTACCATCATAAATTGCATCTGCTATTTTACCTACAATTAGTTTAATTGACCTA
ATGGCATCATCATTAGCTGGGATTGGAATATTAATAATTTCTGGACTACAATTAGTATCA
AGTATACAAATAGTTGGAATACCTAATTTTAAACACTCTTGTATAGCTGTTGTTTCTCTC
TTTTGATCTACAACTACAACAATATCAGGTAAACGAGTCATATTTTTAATACCATTTAGA
TGTTTACGAAGTTTATCTAATTCTCTTCGTAAAACAGCGGCTTCTTTTTTGGGTAACTGA
TCAATCATACCACTTTTATCTTGTTCTTCTAGCTGTCTCAGACGACTAACTCGAGACTTA
ATCGTTACCCAATTAGTTAACATTCCTCCAAGCCATCTCTGGTTAACATAATAAGAATCA
CAGCGTTGTGCTTCCTGTGCCACTATACCAGCGGCTTGTCTTTTTGTTCCTAGAAATAAA
ACTTTTTTACCATCCGACGAAGCCTGTTTTATAAGTTCACAAGCTTCTGTTAATAATTGT
GCAGTTTGGACTAGATCAATAATATGTATGCCATTTCTTTCTGTATAAATATATGGAAAC
ATTTTAGGATTCCATCTACGTGCTTGATGTCCAAAATGAACACCTGCTTCTAGTAATTCT
GCTAATGTAACAATAGCCATAAGATTATTAATATTTAAATAATGTAATTATTCGGGAAAT
CCCTTTGTTTAGAAACTGATTCAACTAACTTATTTGAATTTTAGTAAACTAATAATAAAT
TAACACACTTTTAAAGCAGATCAACAAAATAGATCCTGAAGAATTCATTCTACACTTTTA

TTGCTAAAATAATTACGCGCGGTTCTGTCGTCTAAAATGATATCATCTAAATCATCTCGT
ACAGATGAAATAGTACTTTCGTCATTAGTATTTGCAGTTAAATTCTTTTTCTCTAACGAT
GAGCCATTACAGTTATCATACATATTGAAGCCGGTACCAGCTGGTATAAGCCGACCTATA
ATAACATTTTCTTTCAGACCTCTTAACCAATCTAATTTACCAGATATAGCAGCTTCAGTT
AATACTTTTGTAGTTTCTTGAAAACTTGCTGCAGAAATAAAGCTTTCTGTATTTAAGGAA
GCTTGAGTAATACCTAATAGCACAGGCCTATATGATGCATTAATCTTATTTCTCAAGGTT
ATGGCTTTATTAGTCTGTTCAATCTTCTGTAACTCTACAAGCTCTCCAGGTAAATAGCCT
GTTTCTTCACCATTTTCTATTTTCACTTTAGAAGTCATCTGCCGTACTATTACTTCTACA
TGCTTATCAGAAATATTCACTCCTTGAGATTGATATACCAATTGTACTTCTTTAACAAGT
AGTAACTGAATCTCTTGAAAGCTTAATCGAGCAGCTTCATATAAGGCTAATCCACATTCA
ATATACAAATCAAAACTTGCATCGAGGATATCATGAGGATTTAACAATACGTCTGTTTTC
TTTCTTGCTTCAAGAATTTCTTCAATTCTTGGAAGCCCTTGTATAATGTCACCTGTTTTT
GCTCTATCAAAAACCAATATAGCTAATGTTTCTCCCCTTCTAATTAATGCATTGTTATCA
ACATGTAAAATAGCACCATTAGAAACCAAGTAAGGTCTAGCAATTCTTAATGTAACTGAT
CTAGAAGAAATTTCAATAATTTGTCCAGAGTCTAAAGAAGTCATATTTTCAGTAATAAAA
TCACCACAACGAATCCAATCACCCACACTTACTTTAACTGTTGATTTTCCTATATTAAAG
GTCTTTTTATCAGAAGATGTCGCAATCAAAATGCGCCTGCTAGTATTCTTTTCAGAATAA
ATCTCCTCAACAGTACCTTCACTCATTGCAATAATCTCAGTACTAGCAACAACTGTAAAT
GGCTTGATATATTCACCATTTTTTACAATAATTCTAGTTTGACTTTGTTGCTTCTCTGAT
TTATTTACATCAATACTTTTAATTGATAATGTTTCAAAAGTTGACAGCCTTAAGTCAAAA
TAATTATTGCCACTTTCTTTAGCAGAAAACTCAATTGAGGATATTAAATTATCATCTCGA
TGCTCAATTTCAGCCACAAGATTAGTTGTTACTAAATGAACTCCTTCTATAGATTTCACT
CTTTCTCCGTCCCGAAAAGGAGTTCTTTTAACTAGCTTTAGTTTAGTTTGACTTGGTTTT
TGTGAAACTAGATTTTCAATTAGACTGGATTTTGTTTCTGGTATTGAATAAACAATAACA
GGTCTAATAAGAATATAGGGCATTTGCTCGTTTTCTATATACTCCCAATAAACTAACTTA
TCTGTAGAAATATTATTATGAAGTCTTTCTCCAGGTCTTAAAAAACCTCTACTTTTATCA
TTATTACTATACACCTCACTAAGTTTATAGATAGATCCAGGCTTAATAATAATTTCTCGA
ACTATTCCATCCTTTTCGATTATTTCAACAATACCTGAGCTTTTAGAAAAAATATTTTTA
ACTAGCTCTGTTCCACTTTCTATAACATCTCCATTATGCACTAGTATTAGTGATGAATCT
TTATTAATTTCGTGAGTTTCTTCTGAAATCCATAAGATATATCCCGGACTTAAAATTTCG
TAGGCATCTTTATCTAAGCCTGTTTTCTTTTTTGATACATTTAAGTCTAAATATTTAATA
ATACCACCACTTGTCGTTTTATACGTATCAGATATTAGTTCTGCGACTGTGTAACCATCT
TGAATTTTTTGGTTTGGAATAGCTTTGAAAAGAAATTTTTGTTTTTTTTCTGTTTCTAAA
ATATAAGATTCTTTTTTGTGGATTACATCTGTATAAATATAACAACCTGGAATTACAATA
GATTCAGTAATAACTTGTATATTGGTAATACTGCTATTAGTATTCTGGTGTATCCTTACT
TCTCCAGCATAATGATTTATTAATTCTGTCTGTGCAAGTATAGTACCAGCTTTAGTTTTA

TCTTCTTTATGAACAATAACATTAGCAGAATCAGATATACTATAAACTTCACCTGAGAGC
ACCCAGATCAAGCCTCCAGTTTTAGTAATTCTTGTAGTATATTGATTATTATCTGTTTCT
TCAACTGTTAAATTGGAGAAACAGATTTTGCCAGAGAGATCTGAAACTACATGTTTTTGA
GCTCTTTCTGTCATGAGTCGATTTCTGCGGGGTGATTCAGCAATCACTTGATCCTTCGAT
ACTAATTCACCATCACATATTAAAAGAGTAGTACCTTTACTTAAGTTAATTATTGACTTC
TGATTTTTCTTAGATCTGATTGTAACTTGGGTTGGCTTTTTTGTTATTAATGCTTGCTCA
CCATGTCTGGTACGAACATCTGTATACGATTCAATATCTAGATCTATCAATTGACCATCT
ATAGGTGCGTAGATTTGTTCTGCTAATTCACCGGTAAATACTCCTCCAGTATGAAAGGTC
CTCATTGTCAATTGAGTGCCAGGCTCACCAATAGATTGAGCCGCAATAATACCAACAGCC
TCTCCTAAATCAACTAAGCGGCCATGTGCAAGATTCCATCCGTAACAATACTGACAAACA
GAACTTCTAGAGTTACATGTAACAGGAGATCTTACTAAAACTTTTTTGATACCAGCTTTA
GTTATCTCTTTTGCTAACTTAGGACTGACATCTTGGTTCGTATGAGCAATTAAACAATTT
GTCTCTGGATGGAATACATTTTCTGCTAATACTCGGCCAGATAAAGCTTGTTCTAGATTA
ATTAATATTTTCTGCGTATCTACTAAATCTTCTAGTATGATGCCTTTATTGGTCTTACAG
TCTACTTCTCTAATTATTACATCTTGAGAAACATCGACAAGTCGACGCGTCAAATAGCCG
GAATCTGCTGTTCTTAAAGCAGTATCAACTAATCCTTTTCTTGCTCCGTATGATGATATA
AAATAATCAGTAACAGTTAGTCCTTCTCTAAAATTACTCGATATTGGAAGATCAATAATT
TGTCCCTGTGGATCAGCCATAAGCCCTCTCATGCCCACTAACTGTCTTACTTGAGAAATA
TTACCTCTAGCACCTGAGAACGCCATCATATAAACAGCATTTAGAGGATCTGTCTCTTTA
AAGTATTTAATAACTTCTTGTTTCAAGGATTCACTCGCATTATTCCACGTATCAATAACT
TTTTGAAATCGTTCAACTGCTGTAATCTCACCTCGGCGATATTTGTTTTCAGTGGCCTTA
ATATCTTCAATCGTGCCTAATAAAAGACTTTGCTTACTAGGAGGAATTCTTAAATCTTCT
AAACTTAAAGAAATTCCTGCCTGGGTCGCATAATGAAATCCAAGATCTTTAAGCTTGTCG
GCCATATTGGCTGCTCGGGCTATACCATAATTACGAAAAGCCCAGACAATTAAATTTTTT
AGTTCGTTTTTATCAATAACCTTATTTGAAAAACTTGGCTGTGCAAGACTTCGTCTATTA
TCCACTAAATCTTCTCCATATTAAATGTTCGTTATTAACTATGCTACTAAAGACTCTTGA
ATAATTTTATTAAAGATTATACGGCCAGCTGTTGTTCTAATATATTGCACAATTCTCTGA
TTTTCAGCGTCTTCTTTTATGATATGGTTATTAAAAAATTTTGTTGTACTATTGTCAGAA
TGTTTTTCAATCTTAATCGGATCATGAGGTTGATCACCATCTACTAATCCATCAAAACGA
GCCCATATATAAGAATGTAAATCAATTTTCTTCTGTTCATAAGCCATAACTACATCTTCT
AAGCTTGCAAAATACTGATTAGATCCCTGCTGCTGAGATGGATTATTAGCTGTTAAATAA
TAGCAACCTAAAACCATGTCTTGGCTTGGCATAATAATTGGTTGACCTGTTGCTGGAGAG
AGAAAATTATGAGGGGCTAACATTAGCAATCTAGCTTCGGCTTGAGCCTCTAAAGACAAA
GGAACATGAACAGCCATTTGATCTCCGTCAAAATCAGCGTTAAACGCTGGGCATACTAAT
GGATGAAGCTTAATAGCTCTTCCTTCTACAAGAATAGGTTCAAATGCTTGAATACCTAAT
CTGTGTAGTGTGGGAGCTCTGTTCAATAGTACTGGGTGACCTTGAATAACTTCATTTAAG

ACATTCCAAATAGAAGATTCATTTTTTTGAATCATCTTTTTGGCAGCTTTAATATTGTTA
ACTAATCCCTGCAAAATTAGTCTGTGAATAACAAAAGGCTGAAATAACTCCAAAGCCATT
TCTCGTGGCAATCCACATTGATGTAGTTTTAAATGAGGACCTACAACAATTACAGATCTA
CCGGAATAATCTACTCTTTTACCTAGCAAGTTTTGTCTAAAACGACCTTGTTTTCCTTCT
ATAATATCTGACAAAGATTTTAATGGTCTATTATTTGCACCTACAACAGTTCTACCTCTA
CGACCATTATCCATGAGAGAGTCAACGGCCTCTTGCAGCATTCTTTTTTCATTTCTGATA
ATAATCTCCGGAGCTAAAATTGACTTTAGACGAGATAATCTATTATTTCTATTAATAATT
CGACGATAAAACTCATTCAAATCTGCCGTCGCAAATCTACCTCCATCTAATTGGACCATA
GGCCGTAAGTCTGGAGGTATCACAGGAATGACTGTGAATACCATCCAAGAAGGATCTGCA
CCTGTTGCTATGAAATTTTCAATCAATCTTAAGCGTTTCATTTTTTTATTGAATTTTAAT
GAAGGTGTCTTAAAACTTTTTGGTGGATTTGTAGCTTCAGACCTTAAGGTTTCAGCTATG
TGCTCTAAATCTAAATCTTTCAACAGTTTCTGAATAGCTTCTGCACCTATTCCTACTTCT
ACTTGGTTATTCTCATCTTGATTTTGGTAGATTTCCTCTTCAAGACTTTTCCATTCGTAA
CCTTCGAGCAGTTGCTTGTATTTTAAATTAATATCTGTATTAGATTGCGTAACTACATAA
GAATGAAAATAAACAATTTTTTCTACTTCTTTGACTTTTAAGTCTAATGCTAAAGCAATA
TAGCTTGTACTTCCTTTTAAGTACCAAACATGAGTTACTGGTGAGGCAAGTTCAATATAA
GCCATCCGATGTCTTCTCACGCGTGATTCTGTAACTTCTACGCCACATCGTTCACAAACT
ATACCTTTGTAGCGAAAACGTTTATATTTACCACAGTGACATTCCCAATCCTTAACTGGG
CCAAAAATTTTCTCACAGAATAAACCATCCATTTCTGGCTTTAAAGTTCTGTAATTAATT
GTTTCAGGCTTTGTAATTTCACCAACAATTTGTCCGTTGGGCAAACTTCTTTCGCCCCAC
TGTCGAATTTTTTCGGGAGAAGCTAAACTAATTTTTACGTAGTCAAAATATTGCTCAAAC
TTTGTCATAAATTTGAATACCTTAAAATCAAATAATTAGCCAATTTATTTACATGCTTCA
TATTGAAAAATGAATATCTTAATAAAGGAATTGTTCAAAATCGTCTACTGGAGGAGTATC
ATAATTAGAACGATCTACTCGGTTATCTTTAGAATCCGACATCAAGTCAACTTCAACTGT
CCGTCTTTGGCCATCTTCAAATAGTTTCAATTTATGTACTGCAATGTCTAAACCTAATGA
CTGCAGCTCTCGCATTAAGACTTTAAAAGATTCTGGAGTTCCAGGCTTCGGTATAGGCTT
CCCTTTAACTATTGCATTTAATGCTTCATTTCTAGCTTGCATATCATCCGACTTTACAGT
TAATAATTCTTGCAAAGTATATGCTGCACCAAAAGCTTCTAAAGCCCACACTTCCATTTC
ACCTAATCTTTGACCTCCATGCTGTGCTCTACCTCCTAATGGCTGCTGCGTAACTAGAGA
GTAAGGACCAGTGGATCTTGCATGGATTTTATCATCAACAAGATGAACAAGCTTAAGCAT
ATAAGCTCTACCAACTGTAACAGGATTATCAAAAGGCTCTCCTGTTCTACCATCAAAAAC
TTGCATTTTACCTGGATGCTGATCATTAAAAAGCCATTTATTGCTAGTAATCAATGATGC
TTCTTTTAATTTTCTATTTACAAGTGCTCTTGATGCTTCTGCTCCATACATTTCATCAAA
AGGTATTATTTTAAATCTCTTTCCTAAATAGCCACCTGCTAAGCCTAGTAGGCACTCAAA
TACTTGACCAACATTCATTCTAGAAGGTACACCTAAAGGATTTAAAACAATGTCCACTGG
TGTTCCATCTGATAAATAAGGCATATCTTGTTTAGGTAAGATCCTAGAAATGATACCTTT

ATTGCCGTGACGACCAGCCATTTTATCACCCACTTGGATTTTTCTCTTCTGCGCAACATA
GACGCGAATCATAGCATTTGTTCCGGGAGGCAGCTCATCTCCTTTCTGCCTAGTAAAAAC
CCTAACCTTAACTACTCTACCTTTTGCAGCATTAGGAAGCCGTAAAGATGTATCTCTCAC
ATCTCGAGCCTTTTCTCCAAAAATAGCTCGCAACAATTTACCTTCTGGTAGTTGATCAGC
TTCACCTTTAGGAGTAATTTTTCCTACTAAAATATCTCCAGCTTCAACCCAAGAACCACC
GACAACAATTCCATTTCTATCTAAATCCTTCAGAGAGTTATCGCTGACGTTAGGAATCTC
TCTAGTAATTTCTTCTGGTCCTAATTTAGTTTGACGGCATTCTACTTCATACTTTTCGAT
ATGGATAGAAGTATACAAGTCATCATAAACTAATCTTTCGCTAATTAAAAATGCATCTTC
GTAGTTATAACCTTCCCAAGGCATATAAGCAACTAGAATATTTCTCCCTAAAGCGATTTC
CCCTCCGTCTGTGGATGCGCCATCAGCTAAAGTTTGGCCAACAACTATTTTTTCTCCCAC
CCAAACAATTGGGCGCTGATTAATACAAGTATCTTGGTTAGACCGATAATATTTTTTTAA
GCGATAATGAACTGTTCTACCACTATTATCCTGAATGCCTATTTTATTAGCAGAAACATA
ATTTACATGACCAGATGTTCTGCTAATAACAACCATACCAGAGTCTCGAGCTATTTTAGT
CTCAAGACCAGTACCAATTATTGGCTTTTCTGGATATAATAATGGAACAGCTTGCCTTTG
CATATTAGATCCCATTAGAGCTCTATTCGCATCATCATGTTCTAAAAAAGGTATTAATGA
TGTTGCAGCTGAAATTACTTGAATAGGTGAAATTGCAATATAATCTACTTGGGTTGGGGT
TGTTGTAATAAACTCTTGACGATAACGTACAGGAATAATATCTCCTTCAATGTAATGTTG
CTTACTAACTTTAACATCTCCTGGAGCTACTCGGAAATCATCTTCCTCATCTGCTGTTAG
ATAGACAGGACTATTGTGATAAATCACTTGACCTTGGTTTACCGGATAAAATGGGGTTTC
TATGAAACCAAAAACATTGACCCGCGCACAGGTTGCTAGTGAACCAATTAAACCTGCGTT
CGGACCTTCCGGAGTTTCAATTGGGCAAATTCTACCATAGTGACTAGGATGTAAATCACG
AACTGCAAAACCTGCCCTGTCTTTATTAAAACCTCCTGGACCTAAGGCACTAATTCTTCT
CTTGTGAGTTAGTTCTGCAACTGGATTAGTTTGGTCCATAAATTGAGAAAGCTGACTAGA
TCCAAAGAATTCTCTCACTGACGCAATTAATGGCTTGGGATTAATTAGATTTGATAAACT
CAATGAATCTATATCACATATCATCATTCTCTCTCTAATAATGCGTTCTAAACGATTGAG
ACCTACTCTGAATTGGTTCTGCAGCAATTCTCCAACTGAACGAACTCTTCTATTACCAAG
ATGATCTATATCATCAAGATTACCTGAGTTTTTATCCTTAATATTAATTAGATAATCAAT
TGAGGATAAGATGTCTTGAGGAGACAATACTCGGAACGTTTTAGGTATATTGAGACCTAG
TTTTTTATTAATTTTATATCTACCAACTTCACCTAAGTCATATCTTTTAGGATCAAAAAA
ACGAGAGTAAAGCATCTGCTTAGCAACAGCAACTGTTGCTGGTTCATTAGGTCTAAGCTT
AGAATAAACTATGAGCAGGGCTTCCTCATCAGTTACTTCTTCAATATTATTTTTTCCAAT
TTCTTTAGCTAATTCTTTAACTGAATATATTTGGGATGCAGAAATAAGAAAAGCATATTT
ACTAAGTCCTTTTTCAATTTCATCTTTATTGAGTCCAATAGCTCGCAGAAAAATATAAGC
ATTTACTTTGTGGGTCTTATCTATTCTTATCCAAATTTCTCCCTTAGGATCAATTTCAAA
TTTTAACCAAGAACCTCTATTAGAAATTAGGCTTGCACTATAAATCTGCTTACCATTTTT
GTCTATCTCTTGTTTATAATAGATTCCTGGACTACGGATTATTTGGTTAATAATAACTCT

TTCTGTCCCAGATACAATAAAAGTTCCTCTATTTGTCATTATTGGCAAGTCTCCAATAAA
AACTAATCTCTTTTTGTACTTTTTATTTTTTATCTGCTTTTCAGCACTGAGCTGTAGATG
GGTTGATGATAAATCTAATGATTTAATATTTTTTTCTTGGTTTTTAGAAGGTAGATCAAT
ATCTTTTCTTGTTAACTTTGCAGGAACATATATTTGAGCACTATAAGTTTTATCCCTATT
TTTTGCCTGTCTGACACTATAACGAGGAAATTTTATCTTGTACTCATTACCAAATAATTG
TAATTCGAGTCGACTAGTAGGGTCTGATATCTTCGGAAAAAATTTCAAGAACTTCCGTCAA
ACCTTCTAATAAAAACCATTTAAAACTGGCCCTCTGAATTTCAACTAAATCTGGAAGAAG
TTTATTTTTTAAGCTTATACGTTGAACCATAGATCTCCTTTATAACAAATTTTGAAATCG
AAGAGTTTAATACTATTAATAGAGTACTGGTAGATAATGCATAAGCATAGTTTGCTACAT
TATTTCTTCTCTAGACATAACTAGAACATCATATTTTATTCAAAAAAAGATTTTTAAAAT
TTTTCTTCACACACACTATCTATAAGTACTTTGCTGCTAAAAGATAGTTCAAGCTATTAA
AAAAAACGAAATTAATACTTGTCTGTCTGTCAATTAATTGACATCAAGATAGACAAGTAT
TATTTATTTAATCAATTCTGTTTTTCTGAGCAAAAGACAATGCTCTCGCAAGTCTAGCTT
TTTTTCTAGCGCCTGTATTAGGGTGAAATGCCCCTTTTTTTACAGCTTTATCAATCTTAC
TATATACTTGAGAAATACTTGACTGCACATCATTGAAATTATCACTTTCTAAATTATCAA
TATTTAGAAGGCATCTTTTAGTTAAAGTTTTGACAACCGATTTATACTTACGGTTAATGA
GACGATTTCTTTCCGAAGTTTTAATGCGTTTAATCGCAGAAAGGTTCTTAGCCACAGTAA
ATCCAATAAAACTTTTATAAAATATTTTTAGAAACGAAAATACACAAATCTGGGATCTAT
TCAAAAAGGGAATATGCGGCGTATTATAACATTAAAGAAAAACAATAGGCAATAGTTCAA
CAAGAGCTCTTGTTATTACACAATAGAGATTGTCATTGCGAAAAACTTTGTAAGATAGTA
TTTAATTAAAATAAACTAACTAGACATATAAATATATATGAAAAAAATTGAAGCTATTAT
TAGACCTTTCAAGCTTAATGAAGTAAAACTTGCTCTAGTCAAGGAAGGTATTGGAGGAAT
GACGGTTATCAAAGTTTCTGGATTTGGGAGACAGAAGGGTCAAACTGAAAGATATAAAGG
ATCCGAATACTCTATTGATATCATTGATAAAATAAAAATTGAAATTATTATTAGCGATGA
TAAAGTAGAGAAAATTGTAGAGACGATTATTAAGGCTTCTAAAACAGGGGAAATTGGAGA
TGGAAAAATATTTATTAGTAGTATTGAAAGAGTAATAAGAATCAGGACTAATGACTTAAA
TTTTGAAGCTTTATAGTATTTTTACTTTTTTAGACTTGATACTTCTTCACTAAACATGAG
ATAATAAGCGTTTATTGTATACTTATAACTAAGGTGTAATACTAATGGCTAAAAGCAAAG
GTGCACGAATCGTAATAACTTTAGAGTGCTCTGATAAGGCTGGAGAGTTTGCTCAAAAAA
GGAAACCTGGCGTTTTTCGATATACAACTACTAAAAATAGACGAAATACACCAAGTAGAA
TTGAATTAAACAAGTTTTGTCCTAATTGTAATCAGCATTGTATCTTCAAAGAAATTAAAT
AGTTATTTATCTTATTAAAATAATCATATTATGGCTATATATAGAAAAAGAATATCTCCA
ATTAAGCCAACAGATGCTGTTGACTACAAAGATATTGACCTGCTAAGAAAATTTATTACA
GAACAAGGCAAAATACTGCCTAAAAGGTCAACTGGATTGACTTCGAAGCAGCAAAAAAAA
CTTACTAAAGCGATTAAACAAGCTAGAATCCTTTCTTTATTACCTTTTTTAAATAAAGAT
TAAATATATAGCCTAACTTCATATTTTTCCATCTAAATATTAAAAATTTCTATAGATTTG

TTTATTAAAATGATCTAACATACTGAATTACTAAGAAGATCTACTCATTACAAGTTTTGT
TCATTTTATAATTAATAATAGCATTATAATTTATTAAGCTAGATTC-----AGACATAAA
GGTAACCTCTTTTAATTGATTATATTATATAGTCTATTTAAAAGAGGAAATTTTTTTGCT
TCTAGCAATTCAAGATTCTTTAAGCTTTAACAAGGTATTTAAAACAAAGATTTTTGTTTA
GGTATCAGATCAAATGAATGAATCTTATCTCCCGACTGCCATAATTGAAATTCAGAGATG
AAAATTCCACATTCGTTGCCAGCTTGAATTTCTTCGACATCTTCTCTAACGCGCTTTAAA
GATTCAATTTTTCCTTGATAAATAACTTTTTCTTCTCGAATCACTTTAATCCAAGAGTTT
TTTAATAGCTTATTATTGATCACTCGACATCCTGCTATTTTTCTATTTGCTAACGAGAAT
ACTGTACTTACTTCTGCTTCTCCCACCGGTACTTCTGAATATTCAGGATCAAGTAAGTCT
TCCATCCTTCTTTTAATGTCTTCAATTAAGGCATAAATGATTTGATAGTTTTCTATTAAT
ATATTCGACTTTGCCGATGCTTGCTTTGTTCCTGGAGCAAAGTTGGTATTGAACCCAATG
AGAGTAGAATTTGTGGTTGAAGCTAGTTCAACATCTGTAGCAGTAATTTCTCCAGGCATG
ATTGAGACAACATTGAGTTGAACTTTACTCTGAGGAAATTGAGATAAAGAATCTAGGATT
GCTTCTGTAGAGCCTTGATTATCTGTTTTAATAATTAAAGAAATTTGCTTACTAATATCT
TTAGAATTAGTGTTTTTTAAAGTATCTAATGTAATTCGACTATTAAGTGCTCTTTGTTTC
TGTATAATTGATGAGTCTTTAGAGGTATTTTCAATCGCTTTAAGTTTGGCTTCTTTATCA
CTTTTAACTGCTAAAGCTATTTCTCCTGTAGCTGGTACAGATGACAATCCCCAAATTTCA
ACAACAGATGATGGTATTGCTAAATTAATTTTCTCTTTAGCGTTGTTAATTATAACTCGA
ATCTTTGCGTAAGCTGATCCAATAACCAAATTATCACTAATATTCAATGTACCATTTTGT
ATTAATAGAGTTGCTACTGGACCATGCGATTTATCTAAATGGGCTTCTATTATAATACCT
TGTGCAGGCTGTGTTGGATCAGCTTTCAAATCCTCTAATTCAGCTAACAAGGTAATAGTT
TCTAATAATTTATCAACATTTTGACCAGTAAGAGAACTAATAGGAATTATAGGCACTTGG
CCACCAAGTTTCTCCGACATGACATTGTATTTTAATAAATCTTGCTCAATAATATCTGTA
TTAGAACCAGCTTTATCTATCTTAGATATTGCTATGACAAAAGGGACATTCGCTTTTTGA
ATATGATTAATAGCCTCTATGGTTTGAGGTTTAACTCCGTCATCAGCAGCTATAATAATA
ATAGCGACATCTGTTAAATTAGCTCCCCTAGACCTCATACTTGTAAAAGCTTCATGGCCT
GGAGTATCCAAGAAAACAATTTTTTGTTTATTATCTTTCTTAATATACTCAACTTCATAA
GCTGCAATGGCTTGAGTAATTCCTCCAATTTCTTTGTTAGCATTATTAGATTTTCGAATA
TAATCTAATAGCGTTGTTTTGCCATGATCTACATGTCCCATAACTGTGACAATTGGAGGT
CTTTTAATATAATTGCCAGTTTCATAAAAAGCATTAGAGTTATCTAAATTACTAGAAAGT
CCATTATTATTTTCCTTTACATTTGATTCTACTGCTATACCAAAATTATCAGCCACTGAT
GATATTATTGAGGCGTCTATGGTTTGATTCATAGTAACTGATATTCTTTTAAGAAATAAA
TATTTTATAATATCTGTTTCTTGAACACAAATTAGTTTAGATAATTCTTGAATAGTTAGA
GGATTAGTAATACTAATAGACTCTGGCGGAGAATTAGATTGAATGCTTGCTGAATTTTGA
CTAGGAGTTACGGCTACTTTTTTCTGTCTTATAGATTTTTTATTACTAGTCAGTTTTTTT
ACAACTTCCACTTTTGGCTTAGGTGGACGCATTAAAGAGATAGCTAAATCTCCTGCAGTC

TGCGAAACATTAGAATTAGAGTCTCTAAAGTTGTCATCATCATCGTCAATATGTATTTTA
GTTTTTATTTTTTTGCGCTGTCTATTCTTATTTTTTTTACTATCTACTAAGTCGTGAATT
TTATTAAAATTTTTACTTTTTTTATCTAGTTTAGGTGGAGAACTTAATTCTAGATGCTGT
TCACCACCTGTATGTGATTCTGATTTATCAAGGTCTAAATTTAACAGATTATCATTATTG
ACTGACTCTAATCTAATTTTATAGATAATCTGAGGATTTTTCAAATCTACAATTGTTTCA
GAAGAATTGATATTTGAGCTAGATCTAGAGGATCTATTTTCAAAGTTTTGATTATTTAAA
AACATAAATATTGGTTTTATTATCTAATAGTAACTTTCTAATTTTAATACAGAGCTTAAT
ATAAACAAGAATCGAATATCTGTATAAATAATACAAATGTAGAGAATTAATCTATGTTGT
ATTAGATTTAATATAACACTAAAGTCCTTTGTTCGTTATACAAGTTTAAAACTTTTTATG
ATGATAAACACTAGATTCTAGTTTATTGGTATACAATCTATTTATCTAAATAATACTGTT
TTTGTCAGGGCTTTTAAATTATCTAATGTTATGTTCATTATGAAATTCTATTAAAATTTA
AAAACTCAACAATTAAGTAAGAATCGTTAATCACCTCCACGCCACTTATGCCACGATCTC
AAAAAAATGATAATTTTATTGACAAAACTTTTACAGTATTAGCTGATATTGTATTAAAAA
TACTTCCTACAAGTAAAGAGGAAAAAGAAGCTTTCTCTTACTATCGAGATGGAATGTCTG
CACAATCTGAAGGTGAGTATGCGGAAGCTTTAGAAAATTATTATGAGGCTTTAAAACTAG
AAGAAGATCCATATGATAGAAGCTATATTTTATATAATATAGGGCTTATTTATGCTAGTA
ATGGAGAGTATGTGAAAGCTTTAGAATACTACCATCAAGGATTAGAACTAAATTTTAAAT
TACCTCAGGCCCTTAATAATATAGCCGTGATATACCATTACCAAGGAGTTCAAGCAATTG
AGGATAAAGATACAGAGCTATCTAAATTAATGTTTGATAAGGCGGCTCAATATTGGCAGC
AGGCTATTAAGTTAGCTCCTGATAATTATATTGAAGCTCAAAATTGGCTGAAAACGACAG
GACGAATGAGGAATATACAAGGATATTAATATATTTAAGATATAATAAAGATATCAATTA
ATAAATTAAAATTAGTTAAAGAAACTGGTACTATATAGAGTACTTTACTATATATTATAA
TATTATACTAGTTGATCTCTATCTCTAAGACTTAGCTGAATAACCGAAATTAAATCTATT
GACTATATTCATGAATACAAATAAGCATAATGGTTAGTACAACAAAAAGTACCGGATCTG
TTACTCAAATTATCGGACCAGTTTTAGATATTGCATTTCCTAACGGACAGCTTCCGAAAG
TATTCAACGCACTCAAAGTACAAAGCTCAGAAGGAACTATTACTTGTGAAGTACAACAAC
TTTTGGGTGACAACAAGGTACGAGCTGTTTCTATGAGTTCCACTGAAGGACTACAAAGAG
GGGTAGAAGTTATTGATACTGGATCCCCTATATCTGTTCCTGTAGGTACAGATACTCTTG
GACGTATTTTTAATGTTTTAGGTGAACCTGTAGATAATTTGGGTCCCGTTGATTCTGAGA
GTACTTTACCTATCCATCGACCAGCACCTGCTTTTACTAAGCTAGAGACAAAACCAAATA
TTTTTGAAACAGGTATTAAAGTCGTCGATTTACTTGCTCCTTATAGAAGAGGTGGAAAA
TTGGTTTATTCGGAGGTGCTGGAGTAGGTAAAACTGTATTAATTATGGAACTAATTAATA
ACATCGCTAAAGCTCATGGTGGAGTATCTGTATTTGGAGGCGTTGGTGAAAGAACAAGAG
AAGGAAACGACCTATATATGGAAATGAAAGAGTCTAAAGTAATTGATGCAGATAATCTGA
AAGAATCTAAAGTAGCATTAGTATATGGTCAAATGAATGAACCTCCTGGAGCACGTATGC
GCGTTGGCTTAACTGCATTGACAATGGCAGAATACTTTAGAGATATTAACAAACAAGACG

TTCTATTGTTTATTGATAATATTTTTCGGTTTGTACAAGCTGGATCAGAAGTATCAGCTC
TACTAGGCCGTATGCCATCTGCTGTGGGTTACCAACCAACTCTAGCAACTGAAATGGGAG
CACTGCAAGAAAGAATTACTTCAACAACAGAAGGATCAATTACATCTATTCAAGCTGTAT
ATGTGCCGGCTGATGATTTAACAGACCCAGCTCCGGCCACTACATTTGCACATTTAGATG
CAACAACAGTACTTTCGAGGAACTTGGCGGCAAAAGGAATTTACCCTGCAGTGGATCCAC
TGGATTCAACATCAACAATGCTACAGCCTGGAATTGTTGGAACTGAGCATTATTCTACTG
CTCAAGAGGTAAAATCAACTTTACAAAGATATAAAGAACTGCAAGATATTATTGCTATTC
TTGGTCTTGATGAACTTTCAGAGGAAGACAGACAAACTGTATCAAGAGCAAGAAAAATTG
AAAGATTTTTATCTCAACCTTTTTTCGTGGCAGAAGTGTTTACTGGATCACCTGGGAAAT
ATGTATCTTTGGAAGATGCAATTAAAGGGTTTCAAATGATCTTAAAAGGCAATTTAGACG
ACTTGCCTGAGCAGGCATTTTATCTAGTAGGTGATATAGATGAAGCTATACAAAAAGCTG
ACAGCATGAAAGATTAATATAATTAACGATGACTTTAAATATAAGAATTATTGCTCCTGA
TCGGACTGTTTGGGATGCAGAAGCACAAGAAATTATTTTACCAAGTAGTACAGGGCAACT
TGGTATTTTAACAGGCCATGCACCTTTGCTTACAGCTTTAGATATTGGAGTTATGAGAGT
AAGAGTAGATAAAGAATGGATGCCGATTGTTTTGCTGGGCGGTTTTGCAGAAATAGAGAA
CAATCAATTAACTATTCTGGTTAATGGCGCAGAAGAAGCTAGTCAAATTGATTTATCAGA
AGCAGAAAAAAATTTAGACACTGCAACTCAACTCTTAAGTGATGCCTCGTCTAATAAAGA
AAAAATAGAAGCAACACAAAAAATACGAAAAGCTCGAGCTCGAGTACAAGCTGCAACAGC
AGCAACTTCGTAAAAGATTCCTCTTAAAACATAAAACTTTAGTAATTATTACTAAA----
---GTTTTATGTTTTATTTGTATGGTTATAGGCAGCTTAAATAAACTTAGCTTAGACCTG
AACAAATATAATCAAAATATAATCCCATCTCTTTACCTGCATCTGGTCCAACTAAACTAA
TAGTAACTTCTTTCATAGCTAAAATAGCTTGAATCGTTGCACCAATAGGAACACCTAGAG
AATTGTATGTTTCTTTTAGACCATTTAATACTCTTTCTTCAAGGATAGATGGATCTCCAG
CTAGCATTCCATAAGTAGCATAGCGTAAGTAGTAGTCTAAATCACGGATGCATGCAGCAT
AACGTCGAGTTGTATACATATTACCACCTGGGCGTGTAATATCCGAATAAAGTAGAGATT
TTGCTACGGATTCTTTAATAATTGTTGCTGCATTAGCTGCAATAGTAGCAGCAGCTCTGA
CTCGTAGCTCACCTGTTTGAAAATAGCCTCTTAATTTTTCAACAGAGCTATCATCTAAAT
ATTTACCTTGGACATCAGCTGCATTAATAACAGAAGTAATTGCGTCTTGCATAACTTTCA
CACATTCCTTAATTTTAAATATCTAATACTAGGGGTATATAACAAAATTACTAGATCTTT
TAGCGATATCAGTATTACTGCATAGCACCCAAAGTATAATCAAAGTAAAAACCAGCTTCT
GCTGAATCTTCACCTGCAAGCAGTGAGCAAGCTACACTTTTCATGCATTTTACACCTTCA
GCAACTCCTGAAATTGGTGTTCCTAAAGAATTATACATTTCTTTAACACCCACTAATCCA
ATTTCTTCAATAGGAGTTACATCGCCAGCAACTATTCCGTAAGTTACTAAGCGAAGATAA
TAGTCCAGATCTCGTAGACAAGTAGCAGTCATCTCTTCGCCATAAGCATTTCCACCAGGA
GAAACTACATCAGGTCTTTTTTGAAACAGTTGTTGGCCGCCTTGCTTTACAATACGCTCA
CGATTATCTGTTAAAATTTGAGCTATTCTTAAACGACGTTGTCCAGATAAAACAAAACTT

TTAATTCTATCTAATTCCCCAGGACTTAAATATCTTGCTTCTGCATCTGCATTTACAATT
GACTTTGTAACAATACTCATGGATAAAACTCCTGTAATGCTTT--AAAAAAAGCTTAATA
CTAATTATTTATGATATAACTCAGAAAGTAATGTACTTTTTGAGAATTTTCTAACCGAAA
CACGTTCATGATATATGAAGCAGGATAGATACTGAGGTAAGTACTAAAACGAGAAAGTTA
TATGTAGGAACTTCTGTTATTGATTTCCTAGTACTTTTTTAAAACTTGGTACCACAATTG
ATATATCCTGCTTAGTCAAACGATTATAGAGTGTTTCTGTATTAGGAAAGTTAGCTGCTG
GTAAAGTCGGAAATCTTCTGTATGGAACAGTATCTTTGCCGTATACCGCATTGTATTCAA
CACTTTCAACTAAATTACTTATAAATGCAGATAAGCCTTTTGAAGCTAAAATTTGATTAA
AGAATCTAATTTCTGCCTGATTATTAGGAGCTCTTCCTAAGATATGCTTAGTACCTAATT
CTATAACTTTTGTATTTGGATATGGCTGATAAAATTCTTTACCATATAGTTCAGATAGCG
CCAGTTTTTCTACTAGTTCTTTGACACAGATTTGTCTATTCAAGAAAGCTGACTTAATAT
CCAAGAATTCACCGCCGACACTAAAAGAGTTAAGATCTCTTTCGAAAATTTGACGATATG
CTGCTCTTAAAGCTTGTTCTAACATTTCTTTGTTACTATCACTATTCACTTCAAAGACAA
TAGATTGATCTCTTAAAGAAGTTACTCCTTGTGAAATACGAGACTGAATATCATTACTAC
TCCTCATTTCTTTGACAGTGCCTAATTCAACAAACCTAGCAGATTTACTAGCAATAACTT
TTTTGAATCTCTGGTCAATAATTCCTGGTCTTAAGCTTCTTAAAGCAACACCAGCTGGAG
TACTATATCTTTCATAAGGAACAGTATTATCTCCAAAAGTTTCTGTGTACTCTGAGCTAT
CAATTATTGTGTCTATTACTTGGTAATAGCCTTGCTTATATGCAATATCAAAATATTTAT
TAATTTCTTGCCGACCATATGTAGGGCGACCCAGTAAGCGGTTATGAATATATTCAATAG
CTTTACAAATATATAATGGCTCCCAATAAAGAGATCTAAATATACTTGATTTTGCTAACT
GTCTAACAAACTCGCGAACTGTAATTTGATTATCTTTTAGCTGACTTTCTATTGGCTTGA
GAATTAGTTTTTCTTCTTGATACACTTCTCTGCCAAAAACTCTTAAATAAGCAACTTTTG
TGACAACTTCTACTGAATTTTCAAAATTTTGGGATGAATCTGACTTTATAAGAGCGTTGG
ACAATTTAAAGATTTTTGGTCCCAGGGATCCTGCTGACTTAGGTCTTACCTGTGGATTAC
TAATCTGATTGTAGATTCCTGGCCCACGTCTAACTAATATCCTTCTTGTATCTTTACCAA
AAAGAGCTTGTCTTTTTCTTGGATCTTTATTTTCTTTAGGGAAAATAGCTCCAAATTGAA
TTGATAATGGATCATTACCTATTCCATATGGATGTTGATCAGGTAATGATTGCTTATAAT
CACTAAATAAAGTTATAAATTGAGGAACCTTACGAAAAGGCGCACTATAGGTGAGAAGAT
CTATTTGAGGCCCCCAATTACGGCACTCTTGAGGCTCTTCACCTAAATTTCTAAAGTAAG
GCACAGTCTCTTCACCGAAATAATCTGTATACTCAGAAGAATTTAAAAGTGCATTCACTA
AACCACTTAAACCAGTAGATGATAAAATAGCAAAATATTTTTGAAACTCTTCTAAAGAGC
TAGGACCTCTACCCAAGAAATGTCTAAATGCTAATTCTAATGCTCGACTATTGACAAAAG
GTTCATAAAATTGCTTTCTATAAATACTTGAAGTTCCTAACGAACGAATAAATTCCTTAA
TTGAAATTTGGCCATTTTTTACTTGCGACTCCAAATTCGATAAAGAGAGGTCATATGCTT
TAGCAATATCTCTTTCAAAAATTTGTCTATAACATGCTTTCACAACAATATTTTTCTCAT
CAGCAGATAAACTAGGCTTCATAACAAAACGAGGTGTTGACACTCCTGCCTGTACATAAG

TCTGAGGAAGACGCAAACCTTGTAAATCTCCAGATATTCTTTTTCTTAATTTATCAGTCA
AAGATGGAGCTTCAAATTCAGAAATCACAACATTAAAATACTCTTTAACTAAATCTTGAC
CTTTTATATCTTCCTCAAAGATTAATAATGCTGTCCGTCTCATCTCTCTAAGAGCCACAA
TTGCTGCTGCACTTGAGCAAGCGTTATCAATTAACTCTCTCAATCCTCGTATATTAACAG
ATAAAATATTAGGGTCGCCTGATACAATTGCATATGTCAAATACCGTAAAAACCAGTCTA
AATCTCGAAGAGATTTTCTCATTCTTGTAGTACCGTATCTCAAGACGTTTATAGGTTTAA
ACCCTGGCGGAGTAGCTCCCCCAGCATTAAATAAAGATCGGAAACTTTGGCCAAAATCTC
CTTGGATATTTCCTGATAACTCATTAATTTTATCTTGAGAACTTTGATCTCCTGCAATAA
TAACAGCTGCTTGCGGACGTTCTAAATAAGAAATTGCAGACCCACCTACGAATATTTTAT
CTGCTGCTCGTGCTACCAGAATATTTGCATTTTTAGTTAAAATATCTGCAACTTCCAGTC
TTTTTTGTCCAGAATTTAAAAAAGAAACTAATTGATTTAATTCACCAAGCTGTAAAAATC
GGTCTTGTTGCTCTGCCTGAGTAATAGTTAAAATTGAGGCAGTCCGATAAAGCTGGGGGC
GTGCTAATGGGCTTCCGCCACTTGCTTTGATACTCATTACTTTATTTATCTCCTTAACTG
ATATTGCTTGCTAGCGAATACAAATATAGCTTGATATTTCTAAATATTTAAAGATACTAT
GCAAGCACACTGTTTTCTATATTATTAGGTACTAGTACTTATAGTACAGAGAGTCTTATA
ACTAACTTCTATAAATACAAATTTTGTAATACTTCTATTAACATTTGTCAAAAAAAATATT
TAAGTGCTCACTCTTTTATATTAAAATTCAATTAGTGAGAGGTTGATGTGAAATAAAGTA
TACTAAGTAAGTTTTATAGATAGGAAAATTAAAAGATTTATTCTACAAACATTAATCACT
TGAATGTTTAGCAATTGTACAATTGTTATTGCTTTTAATTGGTTTAGACTAGTTTTATAT
TTTTTCTAACATAAACAATATTAATGAGCAATTTGTATGAATTTAAAGCCACAAAACAAC
CTATTAACTAACGAAAATATAGATCATGTAGATATACCCGAAAATGATATACCAATGTCA
ATTACGGAACATTTAGAAGAATTAAGACAACGGACTTTATTTGTGTTTTTATTCTTTTTG
TTTGCTACAACTATAAGTTTCACACAAATTAAGATCATTGTTGCAATACTGCAAGCTCCT
GCTGTTGGTATTAAGTTTTTACAACTGGCACCAGGAGAATATTTTTTTTCATCTATTAAG
GTTGCAATATATTGCGGGATTGTTGCAACAACTCCGTTTGCCGTTTATCAAGTTATATTA
TACATACTCCCAGGACTAACTGGAAAAGAAAGAAAAATTATTTTGCCTCTATTAATTAGT
TCTGTATTGCTTTTTATTACAGGTGGTATATTTGCTTACTTTGTTCTCGCACCAGCAGCT
TTAACATTTTTAATTAGCTATGGGTCTGATATTGTAGAACCATTATGGTCTTTCGAACAA
TACTTTGACTTTATTCTATTACTTTTACTTAGTACAGGATTAGCATTTGAGATACCAATC
ATACAATTATTGCTCGGTGTTTCAGGGACATTTTCTTCTAGTCAAATGATACGAGCTTGG
AGATATATTATTATTATAGCAACAATTGCTGGAGCTATTCTGACCCCTTCGACTGATCCC
GTTACACAATTAATAATGTCTTCAGCTGTTTTATTACTTTATTTTGGTGGAATTGTTATA
TTATTAGTCTTAAAAAAGTAGAGTAAATATTTTCTATTAATCCAATATATTTGTTATCTG
GGCAAATAATCTAT-AAAACAATTGCTAGATTTAAGTATCAGGCTTCTAAATATTTCAAG
TCTCTTTTTATCATTGAAATACAATCGATACATTGGCTTGTTTTTTCATCAATTATTTGT
GTTGCACACTTTTTAGTACTCTCTTTATTGTTCTTAGAATTATTTCTTTAATCTATTTAT

TAAATAGATATGAAGAGATTTAAGTATTGATTATGGTAATCTATATCAACGTAAAAAGTA
TTTTATTGTGAGTTTTATTTATATAAAGGCTTATTCATTATATAGTTATAACAGTAAGTC
TAATTAATAGTTTTCATGCTTAATTAAAGTAATAACTCTTAATTCTTTTGGCAGAAAAAT
CTGTCTTAATATTTTATCAAAAAAACAGAAAAATGAAGCTAAATAGTCTAATTAACTTAA
TTCAAAAGTCTATATATTCTTGTACACTTTTACTAATTATTTTAAATATTATTTGTGTCG
CACCTAATTCTAGTAATGCATTTCCAATTTATGCGCAACAAGCTTATGAAAGTCCAAGAG
AAGCAACTGGTAGGATAGTATGTGCCAATTGTCATCTTGCTCAAAAGCCTGTGGAGATAG
AAGCGCCTCAAGCAGTACTACCTAATACTGTTTTCGAGACTGTTGTGAAGATTCCATATG
ACAGCAATGCTAAACAGATTTTAGGTAATGGCAGTAAAGGAGGCTTAAATGTTGGAGCCG
TTGTAATATTACCTGAAGGATTTAAGTTAGCTCCTGTTAATAGATTATCTACAGAGTTAA
AAGAAAAGACTAGAAATCTTTACATTCAGCCGTACAGTGCTAAACAAGACAACATTTTAG
TAATTGGACCTATTTCTGGTGATAAAAATAGAGAAATAGTTTTCCCAATACTATCTCCCG
ATCCTGCAAAAGATAAAAAAGCTCATTTTTTCAAGTATCCAATATATGTTGGCGGAAATA
GAGGACGAGGCCAGATTTATCCAACGGGTGACAAAAGCAACAATAATATTGTCTCTGCTT
TAAGCAGTGGTAAAATTAATAAAATTGAATTACTAGACAAAGGTGGATTTATAATACATG
TGACTAACAGTAGCAATGTGGAGTCAACACAGAAGATTTCACCTGGTCTCGAACTTAGAG
TAAAAGAAGGGGATACAATTCAGCTTGATCAAGCTTTGAATAGTGATCCAAATGTAGGTG
GTTTTGGTCAGAACGAAACAGAAATAGTTTTACAGAGTCCAAATAGAATTAAGGGCATGA
TTGTTTTCTTTTTCGCTAGTGTTCTAGCTCAAATTTTCTTCGTATTAAAGAAAAAACAAT
TTGAAAAAGTTCAAGCAGCTGAAATGAATTTTTAAAGCAATATAAAACTGGCACTTACTA
TAAAGAAAGTATAAACACTTTATTCACAGGATGTACCAGTTTTTATGTTATGTAATAATC
GCTTTGACGGAAGTTATGATACTTTTAACAGAATCTACAATCTGACTAGGCTGAATTACT
GTAGCCTGCTCTAAGCTTCCATTATATGGTGTGGGTATATCCTGCGAAGATAGCCTCACG
ACAGGAGCATCTAATTCATCAAAAAGATACTCATTAATTTGTGCAATTAGCTCTGCTCCA
ATTCCGGCTGTTTTCATACACTCTTCTACAATTAAAACTTTGTGAGTTTTCTTTACTGAG
ATAGATATAGAGTCTATATCCAGAGGTTTTAAAGATATAAGATCTATAACTTCTGGATCA
TAACCTTCTTTTAATAAGGCCGGTAATGCTTGGATAACATGATGCCTCATTCTAGAATAG
GTTAAAATCGTAATATCTTTTCCTTTTCGTACAAATTCAACTTTATTGAGAGGTAAGAAA
TATTCTTCTTGAGGAATCTCTTCTTGTAAATTATAAAGTAGAACGTGCTCAAAGAATACA
ACTGGATTATTGTCTCGAATTGCAGATTTCAGTAATCCTTTTGCATTATAAGGAGTAGAA
CAAGCAACTATTTTTAAGCCGGGGATGGCTTGGAAATAAGCTTCCAGTCTCTGAGAATGC
TCTGCACCTAACTGCCTACCAACTCCTCCCGGTCCTCTAATAACTAAAGGCAATGTAAAA
TTACCTCCAGAAGTATAACGTAACATTCCTGCATTATTAGAAATTTGATTGAATGCTAAT
AATAAAAAACTCATGTTCATACCTTCAACAATTGGTCTTAGCCCTGTTATAGCTGCACCA
ATTGCCATACCAGTAAAGCTATTTTCTGCTATTGGTGTATCAAGAACTCTCAAATCCCCA
TATTTGCTATGCAAATCTTTAGTTACCTTATAAGATCCACCATAGTGACCAACATCTTCT

CCTATTACGCAGACAGTTGGATCTTTTGCCATTTCCTCATCTGTTGCCGCTCGTAAAGCG
TCAAACATAAAGATTTTACTCATTTTAATATTTGATTTTTATGATTTGACCACTGTTTTA
AAGTAACAGTTAAGACACCATATTCTATTTAGTTATCTGCAAAAAGATATCGTTTCAATT
CTGACATGTTAGGTTCTGGACTGGAAATAGCAAATTTCACAGCTTGTTCCAATTCGGTTT
TAACAGCATTCTGAATCTCATTAAGTTCACCTATATTAGCAATTTCATTATCTAGAATAT
ATTTTTTGAGTTTTTTGATAGGATCTCTTGCCACCCAAGCCTCTTTTTCTTGTCTTGATC
TTAGTTCATCAGGATCTGCGAGAGAGTGACCACGAAATCTATATGTTAATGCTTCTATTA
AGGTTGGACCATCACCTTGGCGAGCTCTTTGAACTGCTTGTTTTGCAGCTTGTCTTACAG
CTAGCACATCCATTCCATCAACTTCAATCCCAGGAAGCCCAAAAGCTTCTGCTTTTTTAT
GTATTTCAGGTATTGAAGAAGACCGGTGATGTGCCATACCTATAGCCCATTGATTATTTT
CAACAACAAATATAATAGGTAGTTTCCAGAGAACTGCCATATTCAGACATTCAAAAAATT
GCCCATTATTGGTAGTCCCATCACCAAAAAAGCAAGCCGTGACTCTTAAATCCTCTGTTT
CTTTAAGTACTTGCTGGCGGTAGATACTTTGAAAGGCTGCCCCTGTTGCAACCGGTATAC
CTTCCGCAATAAAAGCAAAGCCACCTAAAAAATTGTGAGGCGCAGAAAAAATATGCATCG
AACCTCCTCTGCCTTTACTACAACCAGTCTCTTTTCCAAATAACTCAGCCATCACATTTT
TAGATGGGACGCCTTTACTTAAAGCATGTACATGGTCTCGATAGGTACTGCAAACATAAT
CAGTTGGATTCAGAAGTTTAATTACACCTGTAGAAACAGCTTCTTGACCATTATAAAGAT
GAACAAAACCGAACATCTTTCCTTTATAATACATCTGAGCACACATATCTTCAAAATTTC
TGCCTAACAACATGTCTTCATATAAAACTAATAAATTACTCTTATTAAGATTAAGACCTG
TTGAATTATAGTTAGTCAGCGGCAATTGAACTTTCTTAGGATAACTCATAATTTGTAAAG
AAACCTCTTATTGTAAATAGGAATTTTGAAGAAACAGCTATCAATAGAAAACACACAGAT
AAAAACTTATAAATTATAATATGTTTTTTACTAAAGACTTTAATAATTATTTGAAACTAG
TTCAGTATATCAAATTAATTTTGCTTATACTACGGATGTCTGAAACTTATATAAAAAAGT
AGTAAAAAAGTTTATTAAAAGAATAGTTAAAAACAGATATAAATAGAAGTAAACAGAATT
ATTGAAAAAAAATGATTATTATTAATTAATACTTAATATTTCTAAATATTTTACAATTAA
TGGCTATGTCACCATCTTTATTTTATCCTGTTGAACAAGAACTATGTAGTCTTGAAAAAA
ATCTGAAAGCTGTTGCTGGGACTCGTCATCCAATTTTATATGCCGCAGCAAAGCATCTAT
TCGATGCTGGAGGAAAACGAGTTAGACCAGCTCTTGTATTTTTAGTGGCTAAAGCAACCT
CTGAGAAGCAAGATATAAATACTGGACAAAAAAGGCTAGCAGAAATTACTGAAATTATAC
ATACTGCTAGTTTGGTACATGATGATATTATTGACGAGTGCACAACACGTAGAGGAGTCA
AAACTGTACATAATTTATTCAATACTAAGATTGCTGTGCTAGCAGGAGATTTTTTATTTG
CACAGTCTTCTTGGTATTTAGCTAATATTGAAAATTTAGCCGTAGTCAAAGCTATTTCTA
AAGTCATCACCGACTTTGCAGAAGGAGAAATTAGGCAAGGCTTAGTTCATTTCGATCCCA
GTATTTCAATAGATGCTTACATTGAGAAATCATTTTACAAGACTGCTTCACTCATTGCTG
CTAGTTGTCGGGGTGCAGCTATGCTTAATGGTTCCAATCATCAAATAAATAATGATCTTT
ATCTTTACGGTAAACATATGGGATTAGCATTTCAAATTATGGACGATGTTCTAGATATAA

CTGGTTCTACTAAGAGCTTAGGGAAACCTGCTGGCGCTGATCTAATAAATGGAAATTTGA
CCTCTCCTCTCCTTTTTTCACTTACTCAAGAAGCAAGTTTAAATGATCTTATTGATAGGG
AGTTCTGTAATAGTACAGATATAGCCTCAACATTATTTCTTATAAAAAGAAGCGGGGGAA
TTACAAAAGCTAAAGATTTAGCTAAAGAACAGGTGCAGGCGGCACTTTCCTGCCTTCAGT
TTTTACCACAATCTACACCTGTATCTAGTTTAAAAGAATTAACACATTTCATAATCACAA
GATTGTCATAAAGGACTTGCTAAAATTAAAATGTTATTAACTAATTTTTTACTGTTTCTA
AAACAGTCTGAAAAGTTTGAATATCATTACTAGCTAGCTGCGCTAGCATTTTACGATTTA
ACGCAATATTCTCTTTTTTTAATGCACTAATAAAAGTACTATAATTCATGCCTTGATTGT
GAGCAGCAGCATTTATTCTAGTTATCCAAAGACGACGAAAATCTCTCTTTTTCCTTTTTC
GACCAACATAAGAATATCGAAGAGCTTTAAGGACTTGCTGCTTAGCTGTTCGAAATAAAC
ATTTATGTGCACCTTTAAAGCCTTTAGCTAGCTTAAAGATTTTAGCTCGTCTTTTTTTTG
CAACGTTACCTCTTTTAACTCTACTCATAAAATATTACTTAAATTTTAATTATTACCAGC
TTACTAAATTCTTATACTCTTTGATTATAAATAAGGTAATTTCATTGCAATGTTTTTAAT
ATCTTTTAAGTCAACCGAACAAGTAGAAGAAAGATGTCTTCTTTGTTTTGATGACTTTTT
TTGTAATAAATGACTTTTAGAAGCTTTATGTCGAAGAAATTTTCCGGATGAAGAGACTTT
AAATCTTTTTGCTATTGCTTTTGATGTTTTTAACTTAGGCATATTATAAACTTTTAATGA
TTAACAAAAGCATGAAAAGTCTGAGGATATCAATCTTAGACCTTTCAATACTTAGAATAA
TCTTAACACAATTTATTTTACTTACTACATAGGTTAACTGAAACAATTTATATATATAAT
ACTAATCAGTTAATCAACCATAGCTAAAGTGGATTTATATTTTGCTTTGAACTTTTGTAT
AGTATTAGCAATTAGCATTGTTATAATTTTAAGACTGCTTGAATTCAATTCTTCGAACAT
TTTCATATTTAATGTAAATGAAATATTGGCTTCTGAGACAATATTTTGAATTTGAACATC
AGACAAGGGAATCATATCTAATGCAGCTCTATATTGATCTTTAAATAATTTATCGTCTTT
TATTTGGTCAAAATCGTAAAATTTCGTTCCTCCTGAATCTGATAAATTCATAGCCCCTCT
AGCTATTTTTTTTAAAATTTGGCCGCCAGAAAGGTCACCCAAATAGCGCGTATAAGCATG
AGCAACTAATAATTCAGGTTGTTTATGTCCTATAGTGTGAATTCTATCAACATAAATTTT
TGTAGCAGGAGATGGTTCAATAAAATCTAACCAATCTGATCCATAATAATAGTTTAAATC
TTCAGATAGACTCGCTTTCCTATTAAGCTCTGTAAAATATATAGGTTTAATAGCTGGATG
ATTTTTATTAGAAAATAACTCTTCCTCTATTGCACAATAGACAAAGTATAGATTTGCAAC
CAATTTACGATATGACTTCTTATCTACAACTCCTCCCAAAAAAGATTTAACAAAACTAAC
ATTTTCTGCCATACTATGGGACTTAGTAGTACCTTCTCTTAGTTCATTCGCTAAAGTATT
AACCATAATATCAATATTCCATTTTTAAAGATAACAAATTAACAATAAATAGTTATCCGT
ATATTTATAATTGAGTTGCGCTAACTAGGCTGCTGCAAAATAATTTTTTGATTTTATTGG
ATCTGGATTCATTGTTTTGTCACCAGGCTTCCAATTAGCTGGACAAACTTCATCTGGATG
AGATTGTACATATTGAATTGCTTGTAAAACCCTCAAAGTTTCTTCTACACTCCTGCCAAA
CTCTAGATTATTGATTGTAGAGTACTGAATAATTCCTTTCGGGTCTATAATAAATAGTCC
TCTTAGAGCTACACCATCACTATTCAAAACATTATAAGCTGCACTGATCTCTTTTTTTAA

GTCTGATACTAATGGATATGAAAGATCTCCTAATCCACCTGATTCTCTGTCAGTCTGTAA
CCAAGCAAGATGAGAATACTCACTATCAACCGAGACTCCAAGAACTTCTGTATTAAGTTC
CGAAAAAGCATTATACTTATCGCTAAATGCTGTAATTTCTGTAGGGCACACAAATGTAAA
ATCTAAAGGATAAAAGAATAAGACAATATACTTATTCTTGAGATCAGATAATTTTAACGT
TTTAAATTCTTGGTCATAAACAGCTGTAGCTGAAAAATCAGGAGCTAGCTGACCTACTCG
AAGACAATTTGGTCCAGAAATCATTAATTTTTCCCAGAAGAATTGATATAATTTTGTTAT
TTAATAAATAATTATATAACAAAATTTAGTATATATTAAAATACTACAAGTCTTTTTTAA
ATTACTAAATGATATAATCAATACTATCTTTATAGCGGGGAATGGATTTGAACCATTGAC
CTTCGGGTTATGAGCCCGACGAGCTACCAGACTGCTCTACCCCGCGGATAACTAAAATCA
TATTAGTCTACATATATATATAAAAGCAAATGCATAATAGATTTTAGAAGTAAAAAAATA
GAAGAACTGGTCCACTGATCATTAAACTTAGAAACGACAAAGTATTTTTTATCTTATTTA
AAAGAGGGAAGACTTCATATAAACCAATAAATCTATCTTTTAAAAGAATTTCGGACGGCT
TTACCCAAATTTGACCGTCATACCAGCCTGATTCTTCATAAAATACTGTAGCACTCATTA
ATCGTTTTACTACGTATGACCAGCCTAAATATAATCTGATTAAAATAAATCCTGTCATTA
GGCTGGAAGTAATAAATTCTGAAAAGAAAAATTTCAAAGGTAGTTTAGTGATTGGAAAAA
TTGATAATAATATAGGACTAACTAATAAACAGTTAAGTAGTAGTGCAATAGTTATTTTTT
TATTATAAGATCTACGGCTTAAAGTTGGCCAGCAAAAGAACCAAGAATTTTTCAAAGAAG
TATATTCATGAACAGGTTGCTGCTCCTTTGGCACAGGACATTGAGTATTATATAAATTCA
TTGACATTAAAAAGTATTATAATAAAGACTTTGTTTATATTATCTAACATAGGTACGACA
TTAAATATTAACTAACTGCAATTATAGTAGTGAGTAGAAGAAATAAAACTAAAAATAGCC
AAGTAATTTTATTTAAAGTATTCTCAGTACTACGAGTATTACTAAAGAACTGATTCTGAG
CACCTACACTTCCTAAACCTTCGGATTTAGGATTGTGTATTAGAATAGTGAAGATTAATA
TTATTGTAGATGAGTACCAAAAAAATTTTAAAATTTGTTCCATTATGTAAAAGTTAATAA
AAATAGCACAAAGACAATCGTCTAAAAGATATTGATTGTCTTATCATATTAAAAAGTATT
TATTCACCCTGTACTTTTAAAAGTAAAAATCCTATTGCCAGCCCTACCAAAACTAGGACT
GAACTTAATACAGCACTTTCTACAATTTCTCCGACCATTTTATTTTTCCTTATTATTTTA
TTATTGTCATTCTTTTGACAAGATTATATTGAGCTAACTAAAATCCGTTTCTACCCCAAA
CTACCATTGCGATTGAAAATGTAAACATTGCCATTAGAGCTGCCCATCCCAAACTTAAAA
TATCCATAATTTATAATATTCCTAAAAATAAGATCTCTTTTACTTAAACTATAATATTCT
TAATATTAAAAATATCATTATATAATAAGCTAATTATAACAGAGTACTACTTAATTCGTA
TTTTTAGTGTATTTAAAGAAGATTGAATTTTTTCGAGGTACTGGATAAGTATTTCATCCT
AAGAACCATAGTCAACTTTTGATTTATTATAATGCTTAAATCTAAATATATATCACTCAA
GAATCATTAGTAATTATGATGTTTTTAATTATACGATTAACATATTTCAAAATAGAAGAA
TTCATAGCGACTAAGTTAATATGCTAATATAATTTATCAATAAAAAGGAAGTTAGTATTT
CAATTATGCAAACAACTATAAATAATGGGCAAACGTCTAGTAAAGAAACTTTACTAACAC
CTAGATTCTATACGACTGACTTTGAAGAGATGGCTAATATGGATATTTCCGGCAATCAAG

AAGATTTTTTGGCTATCCTCGAAGAATTTCGAGCTGACTATAATAGTGAACATTTTATTA
GAGACGAAGAGTTTAATCAATCTTGGTCTAATTTAGAACATAAAACTAAATCCTTATTTA
TTGAGTTTTTAGAAAGATCTTGTACCGCAGAATTTTCAGGTTTTTTACTATATAAGGAAT
TATCCAGAAAATTAAAAGACAGAAATCCGGTTATAGCTGAGTGCTTTTTATTAATGTCTA
GAGATGAAGCTAGGCATGCCGGTTTTTTAAATAAAGCTATCGGGGACTTCAATTTATCTT
TAGATTTAGGATTTTTAACGAAGAGTCGCAAGTATACTTTTTTCTCACCTAAATTTATTT
TTTATGCAACCTATCTTTCTGAAAAAATTGGATACTGGAGATACATAACTATTTACCGCC
ATCTTGAACAACATCCAGAACACCGTATTTATCCAATCTTTAGATTTTTTGAAAATTGGT
GTCAGGATGAAAATCGTCACGGAGATTTTTTTGCTGCTTTGCTCAAATCCCAACCTCATT
TTTTAAATGACTGGAAAGCAAAAATGTGGTGCAGATTTTTTCTATTAAGTGTATTTGCAA
CAATGTACTTAAACGACTTTCAAAGAATTGATTTTTATAATGCCATAGGCCTAGACTCTA
GACAGTATGATATGCAAGTAATACGAAAAACTAACGAAAGTGCCGCTAGAGTTTTCCCGG
TTGCTTTAGACGTGGACAATCCAAAATTTTTCAAATATTTAGATACTTGTGCATGTGATA
ATAGAGCTCTGATCGATATTGATAATAACAATTCTCCATTATTCATTAAATCTATCGTAA
AGATACCTCTATATTTTTCCTTATTTGCAAATTTACTAAAGATATATTTGATTAAGCCAA
TAGACTCAAAAACAGTATGGAATACAGTTCGATAGTTAAATACAAGGGAATGCTTTTTTC
TTATAAAAAGCTATCTAGTGATTAATATAAAATAAAAGCTTCCTTCTCGATGGAAGGAGC
TTTTATTTTGTCAAGAATATAATGGGATTATATAGATCTAGAACTAAGAACTTACTTTTT
CTTTTGCTTCATACATAACCTCTAAAGTAATTAGATTTTTAGAACAATCACGGGCAAATT
TCTCTGTATTTCTTTTTACTTTACCTCTAACAAACCCAGGAATTTTATTTAATTCTTTCT
GTGCTTCTTCTGACCAATTAATACTATCAACAGCAGAAATTCCTATACTTAATGCTTCTG
TTGTATCATGACCACCAAATATTTCTAGCAAATGATCTTCCATACCAAGTGTAAACGAAT
TATATACTAAATCAGCAATTTGATTTGTTCCCTCATAACCAAGAAATGGACGGTAACTTA
AAGGAAAGTTTTGTATATGCACTGGTGAAGAAATAACGCCACAAGGAATATTTAGACGCT
TACCAATATGTCTTTCCATCTGTGTTCCAAAAATTGCTGCAGGTTCAGTTTTAGCAATTA
AATCTCCAATTAAACCATGATCATCTGAAACGATTACTTCATCGCAGAATTCTTGAACTT
GATCTTTAAACCACTCTTCATCATATTTGCAATAAGTTCCACACCAGGCGACATGTATAC
CCATTTCCTGGTGTAAAATTCGAGTTATTGCTGCAGCATGAGTTGCATCTCCAAATACAA
TAGCCTTTTTACCTGTTAAATTCTGACAATCAATTGATCTTGAAAACCATGCAGATTGTG
AAATAAATCGGGTTTGCTCGTCTATGTATTTTTCGTAGTCTACAGCAGCTCCCAATGCAT
TTACTAATTGCTGTATTGATCGGATGCATGCAGCTGTTTGAACAATACCCATAGGTGTAA
TGTCAACATAAGGCATATTAAATTCTTTCTCTAAATATCGAGCTGTCATTAGTCCAGTTT
CTCTATATGGAATAAAATTAAACCATGCAGAGGGTAATTTTTTTAAATCTTGAACTGATG
CATTTTCTGGAATAATTTGGTTTATTTGGATATCTAGATCTTGAAATAACCTTTTTAATT
CTGCTATATCATGTTGATTATGAAATCCTAAGCTTACTGCTCCAATAATATTAACAGATG
GAGTTTTAGTCTTTTGAGTTAAAACTTGATTATTAGATTTAGCTTTTTCCATATAGAAAG

TGACAATTTGTTCTAATGTTCTATCACCTGCTTGCAGTTCATTTACTCTATAATGATTAA
CATCAGCTAATAAAACATCTGCTTCTGTTTCTATAGATGCTCTGCTAACAAAGTTTTGCA
AATCTTCTTGCAAAATACTCGAAGTACAAGTTGGTGTTAGAATAACTAAATCAGGGCTTT
CTTCTCTGTCTTTTCTAGTAATATTTTCTACAACTTTTTCTTGAGAACCACGGGCTAACA
CATGTCTATCTACAACACTTGCTGTAACAGGAGTAAAATCTCTGTCTCTTTCAAGCATTG
AACGCATTACATTAAAATAATCGTCTCCGAGAGGCGCATGCATAATTGCATGAACTTTTT
TAAAAGAACTGGCAATTCTTAAAGTTCCAATATGAGCAGGGCCTGCATACATCCAATAAG
CTAATTTCATATTAAATTTTCCTATGATTTATTTAGACATAGTAATACTTTTAAAGCTAA
CTAACAAACATTAGCCAAACATCTAATTTAACTCCTTGACAATTTTGTAATATTTTTTAA
ATGAAGCTTATCATTATAAATATTTAAAAAATATAATTCAGAATAGAAATAACTGTTAGA
TCAAGCTTGAAAGTAAAGATTTCTTGCAGTTATAGTTTTTCTTTAAGTAAACATGTAAAA
ATTGTACTATATATAAATGTACTATATATAAACTTTTTTTACTGAATATTGGTAATGCCA
GATACAATTAACTTAAATATGCCTTCCCCAACGTTTGGTGGAAGCACTGGCGGTTGGTTA
AGAGCCGCAGAAGTAGAAGAAAAGTATGCGATAACATGGACAGGCAAAAATGAAAGTAAG
TTTGAAATGCCAACTGGTGGTACCGCAACTATGCGAAATGGAGAAAACTTACTCTATTTA
GCTAAGAAAGAACAATGTTTAGCTTTAGGTACTCAACTAAAAAGTAAATTTAAAATATCT
GACTATAAGATTTACAGAGTTTTTCCTAACGGAGAAGTGCAATATTTGCATCCTAAAGAT
GGAGTCTTTCCAGAAAAAGTTAATACTGGGAGAGCTAGCGTCAACAGTGTTGATCATTCT
ATTGGACAAAATATTAATCCTGTAGATGTCAAATTCATGAATAAAGCAACTTACGATTAG
TCTCTCAAAAACCTAGACATAAAATAACTTTTATATAGTCTAGGTTTTTGATAATGTATA
CTGATAGTATGGAAGGGTGGCCGAGTGGTTGAAGGCGTCTGATTTGAAATCAGTTGAACT
ATCTGGTTCCGTGGGTTCGAATCCCACCCTTTCCGTTATAAAACTATAGTTGGCTTCTCA
AGGCAAGCTAGCTATACACTGATAGTATTAAGTTTAGACTTAATAAAGTCTACAACTTGT
GACAGGTTAGAAATTTTTTCGGCATCCTCATCTGGTATCTCTATACTAAATTTTTCTTCT
ATGGCCATTACCAGCTCAACTGTATCTAGAGAATCGGCTCCCAGGTCACTTGAAAAATTT
GCTTCCCTAGTCACTATTTTTTTTTCAATTCCTAGCTGTTCTGCTACAATATCTTGAACT
TTTTCAAAGATTTCATTATCTTGCATAATATATTATTTCCTCTAAGGTGTTATCAAAAAT
TTTTCGGCTTATATTATTATCCAACTCTAAAAAATTAGTAATTTTTAGTCGAGATTAAAA
TATTTATACCTATATGATATACTATTATTCTGGATAAATACGAAGTTTACGATATGGCTC
TTCTCCAAAACTACGAGCTCTCAACTGGTAGTTGTCAACTAGATTATGCTGCATTTTTCG
TATATAGGCTGATCGAGGTGTAAGCTGAACTATTGAACTTTCATTCAAGATAATAGTTTC
AATAGCCAATTTAGCTTCTTGAAGAGCCTGGATCTCATCAAAATTTTTATTTTTACAGAG
TTCTACCCAATTAAGACCAGATGAAGTATGAATGTTTAATATTTTTCTGAGTGCACGAGT
AATTTGTGGAACCGTACTATTCTGAATTGTATATATAATAATTTGTTTTGATTTTGCAAT
TTGTCTAAGTTTTGTATTTTGCTTTACTTGATTACGTAGCGCTAGAATAGCATCAGACTT
TTCTATCTCCTTGGTTAAAATAATTGGTAGATCTAGTGAAGAAATAACTGATGTAATATG

CTGCCAACTCAAAGAATAAGCATATAAGTACTGATGAGGTATTTCCAATTGCAAAGATTG
TTTATTAATATTAATAGGGGCATTGAAAGTAGTTGACAATAAAGATGTATCACGATTTTG
TGATTTATTCAAATCTAAGGACTTATTTCTTAATTCTCTATATTGTAAAACCGGAGGCGC
CTGCTGTGCGTTAATTGATTTTCGAGCATTAGAGATGTTTACAGGTAAGACTTCTATAGA
TTGCGATGGATAACATTTAATTAAAATTCTACCATTAGCTTGAATTTGTCTTTTTTGCAC
AAAAGGTTGATGGCCTTGCAAAATTTGATCTATTGTCTCTTTAACTTTATTATGAACTAT
CCAAACATTTCGCTCGTGTATTTCGATCGCAATCTGAAAAGCAGGCTCAGCTTTTCTTTC
TAGTATACTCTTTTGCGTACCTCTACGTTTAGCTTCATCGTCACCTAATGTTACATATTG
AATTCCACCAATTAGGTCCGCCAATGTTGGATTTTTAATTAAACTTTCTAAATGATTTCC
ATGCGCTGTACCTACTAATTGAACTCCTCTTTCTGCTATAGTTCGAGCAGCTAACGCTTC
TAATTCCGTGCCAATTTCATCTATAATAATAACCTCGGGCATGTGATTTTCAACAGCTTC
TATCATTACTTGATGTTGCAAATCGGGCCTTGCGACTTGCATTCTTCTAGCTCTACCAAT
AGCTGGATGAGGAATATCTCCGTCACCAGCTATTTCATTTGAAGTATCTATAATAACCAC
TCTTTTTTCCATTTCGTCAGCTAAAACACGGGCCATTTCTCGAACTGCGGTTGTTTTACC
AACTCCTGGTTTTCCTAAGAGTAAAATTGAATCACCTTGCTGTAGTAAATCTCGAATAAT
ACTAATTGTACCAAAGACTGCTCTGCCGACACGACAGGTTAAGCCAATAACACTACCTTC
TCTATTACGCAGTGAACTAATACGATGCAAGGTTTTTTCGATACCAGCTCGATTATCGCC
GCTAAAATTACCAACTTTTTTTACACAATAATCTAAGTCTTGCCAACTAATAGATCTTTG
AGATAAATATTCTGGGTTATCTGGAAATCTAGCTTCTGGACGACGACCTAAATCCATAAC
GACTTCTATTAAATTATTTCTATTAGGATGCTGTTGTAAAGGTTCTTTGACAAAATTTGG
CAAAATTTCTAGCAACTTATCTAAGTCATCTGCAATAAGCATGATTGAGTATATAATAAT
TAATTAATCAAATAGAAGATTCAAGATTCTAAAGATTACAATACATATTAAAGAATATTA
TGATTGGAATCAATTTTATACTTAAATTGAACAGGAAGTTATAGATGTAAAAGAACTGAT
TAAACTTTACGCAATAAATCGATTGGTCAAAATTAATAGTCGTGTTAACATGTTTATTGG
ACAAAAAGTTCGTATTAAATATAGCAAACAAAAAGTTGCTAGTGATATAGCAGATAAAGT
TGGTGAATTAGGAGTTATTAAGGGAATAAAGTTTATTAATAGTCAATGTGTTACTATTAT
TGTTGAGTTTGACAATCACACTAGGCTTTGGATGTTTAGAGAGGAATTAATCTGTCTAAA
TGAATTAAAAACATGAAACATAATTTATTTTATTTATTATACAAAATTTCTTATGAGTCG
TTAAAATAATAAATTTTAAAGGAGTACTTTTAATTGTGCAAATTACTATTAAAAAATTAC
AAGACTTACTTTCGTCTGTACAAAGAAAAAAGATCCAGACATTAAAACTAAAACAAGGTA
AGTTTGAACTTCTATTAAATAAGACCTATAAAAAAGTCAATCAAGAAATTATACCTTCGC
AGAAATCTGCTGTATTACAAAATAGTCCATCTACGATAATTAAATCAATAAATAATACAA
AAAAATCTTCTGTTGTCAATGAAGACCGTACAGAATATGCCACTATTGTTTCTCCAATGG
TTGGAACGTTTTATCATTCACCTGCTCCTGGTGAAAAAATTTTTGTACAAGTTGGCGATG
AGGTCAAATTCAATCAAACAGTCTGTATTATTGAAGCAATGAAGTTAATGAACGAAATCG
AAGCAGAAATTGAAGGCAAGATTATAGAAATTCTTGTTAAAGATGGTGATATAGTAGATT

GCGGGCAAGCCTTAATGAAGGTTGAAACATAATGCTAATTTTTCCATTTTACATATTGTT
AACAGAAATTATTTAGATAGAAAAATTAGCTTATACTATAGTTATGAAAACTTATTTCGC
AAAGGCACTAAAAACTAATATATAAAATTATTTACTTTATCTGCCAAAAATAGAAATAAG
CGTTTTGATTCCTTGTCTCAAGAGAGGAGAATCTCAATGGCAATTAGCTCAAAAGAGCAA
GAGACAAAGAAGGTAAAAATCTCGGTTGATAAAAATCCCGTAGATACTTCTTTCGAAAAG
TGGGCCCAACCAGGCCATTTTTCTCGTACACTAGCAAAAGGACCAAAAACTACTACTTGG
ATTTGGAATCTTCATGCTGATGCTCACGACTTCGATAGTCAAACCAGTTCTTTAGAAGAA
GTTTCACGTAAGATTTTCAGTGCACATTTTGGGCAGCTGTCTGTAATATTTTTATGGCTT
AGTGGAATGTATTTTCACGGAGCCCGCTTCTCTAATTATGTTGCTTGGTTAAGTAATCCA
ACAGGTATTAAGCCAAGTGCGCAGGTTGTTTGGCCTATAGTTGGGCAAGAAATTTTAAAT
GGCGATGTAGGTGGTGGCTTTCAAGGAGTACAAGTTACATCTGGATGGTTCCAACTGTGG
AGAGCATCAGGAATTACTACAGAATTTCAGCTTTACTGTACTGCTATTGGCGGATTAGCT
ATGGCTGCTTTAATGCTGTTTGCAGGATGGTTTCATTATCATAAAGCTGCTCCAAAGTTA
GAATGGTTTCAAAATGTTGAATCAATGATGAATCACCATTTAGCTGGGCTTTTAGGCTTA
GGCTGTTTAGGCTGGACAGGCCATCAAATCCATTTGTCTTTGCCTATTAATAAGCTACTA
GATTCTGGCGTGTCTCCGCAAGAAATTCCACTACCTCATGAGTTTTTAATTAATAGAGAG
CTTATGGCTCAGCTGTATCCAAGTTTTAGTAAAGGATTAGTTCCATTCTTTACTTTAAAT
TGGGCTGAATATTCCGACTTTTTAACTTTTAAAGGAGGTTTAAACCCTGTTACTGGAGGT
TTATGGCTAAGTGATACTGCTCATCATCATCTAGCATTAGCTGTTCTATTTCTTGCTGCA
GGTCATATGTATAGAACCAATTGGGGTATTGGACATAGCATGAAAGAAATTCTAGAAGCT
CACAAAGGACCTTTTACCGGCAACGGTCACGAAGGTCTATATGAAATTCTTACAACTTCT
TGGCATGCACAGCTTGCAATTAATTTAGCTATGATGGGATCTTTAAGCATCATTGTAGCA
CATCACATGTATGCAATGCCTCCTTATCCATATATTGCTACTGATTACCCAACTCAGTTA
TCGCTCTTCACTCATCATATGTGGATTGGAGGATTTTGTATTGTTGGAGCAGGAGCGCAT
GCTTCTATATTTATGGTAAGAGATTATAACCCTGCAGAAAATTATAACAATCTTTTAGAT
AGAGTCATTAGGCATCGAGATGCTATTGTTTCTCATCTAAATTGGGTATGTATATTTCTT
GGATTCCATTCATTTGGTTTATACATTCACAATGATACTATGCGGGCACTTGGAAGATCT
CAAGATATGTTCTCTGATACAGCTATACAGTTACAACCTATTTTTGCTCAATGGGTACAA
AGTATACACACTTTAGCTCCTGGAAATACAGCTCCAAATGCATTAGCAACAGCTAGTTAT
GCATTTGGAGGAGATATTGTTTCTGTTGGTAACAAAGTTGCAATGATGCCTATTTCTTTA
GGTACTGCAGATTTTTTAGTTCACCATATACATGCATTTACTATTCATGTAACTGTTTTA
ATTTTAGTTAAAGGGTTCCTTTTCTCAAGAAACTCTAGACTAATTCCTGACAAGGCCAAT
CTTGGCTTCAGGTTTCCATGTGATGGACCTGGTAGAGGTGGTACTTGCCAAGTGTCTGGC
TGGGATCATGTTTTTCTTGGCTTATTCTGGATGTACAATTCTCTGTCTGTAGCAATTTTT
CACTTTAGTTGGAAAATGCAATCAGATGTTTGGGGTAGTGTATCTCCGTCTGGAAATGTT
TCTCATATTACTGGCGGTAATTTTGCACAGAGTGCAATTACAATCAATGGATGGCTGAGA

GATTTCCTTTGGGCTCAAGCATCTCAAGTTATTCAATCATACGGTTCTGCGCTATCTGCA
TATGGATTAATTTTCTTAGCAGCACATTTTGTATGGGCATTCAGTTTGATGTTCTTATTT
AGTGGTAGAGGTTATTGGCAAGAGCTTATAGAATCAATCGTATGGGCGCACAATAAGATA
AAAGTTGCTCCTGCAATTCAACCAAGAGCTTTAAGTATTACTCAAGGTAGAGCAGTCGGT
GTTGCACACTACTTATTAGGTGGAATTGGTACAACTTGGGCATTCTTTTTAGCGAGAATT
ATTTCAGTAGGCTAATAGTGAAAATAGGATAAAAAAAACAATTATGGCAACAAAATTTCC
TAAGTTTAGCCAAGCTTTATCACAAGATCCTACAACTAGAAGGATTTGGTATGGTATTGC
TACGGCACATGACTTTGAAAGTCATGATGGAATGACAGAAGAAAATTTATATCAAAAGAT
ATTCGCTTCGCACTTTGGACATCTAGCAATTATCTTCTTATGGACATCTGGTAATTTATT
CCATGTAGCTTGGCAAGGCAACTTTGAACAGTGGGTATTAAATCCTTTGAAAGTTAAACC
AATTGCTCATGCAATTTGGGATCCGCATTTTGGACAACCTGCTTTGAAAGCTTTTAGTAA
AGGTGGCTCAGCTTATCCAGTAAATATAGCATATTCTGGCGTATACCACTGGTGGTATAC
TATTGGTATGAGAAGCAATCAAGACCTGTATTCTGGGGCTTTATTCTTACTAGTTTTATC
AGCTTTACTCTTATTTGGAGGGTGGCTACATCTACAACCAAAATTCAAGCCTGGTTTATC
ATGGTTTAAAAATAACGAATCAAGATTAAATCATCATTTATCTGGATTATTTGGGGTTAG
TTCTTTAGCCTGGACAGGTCATTTAGTACATGTTGCTATACCTGAGGCAAGAGGACAACA
TGTAGGATGGGATAATTTTACAACCGTATTACCTCATCCAGCCGGTTTACAGCCGTTTTT
CAGTGGTAATTGGAGTGTATATGCTCAAAATCCAGATACAGCTCAACATTTATTCGGAAC
TAATGAAGGTGCGGGTACAGCAATTCTGACATTTCTAGGAGGATTTCATCCTCAAAGTCA
GTCTTTGTGGCTAACTGATATGGCTCATCACCATTTGGCTATTGCAGTAGTATTCATTGT
TGCTGGACATATGTATAGGACTAATTGGGGAATTGGGCACAATCTAAAAGATATTTTAGA
TGCTCATAGACCACCTAGTGGTAGATTAGGAGCTGGACATAAAGGGCTATTTGATACTAT
TACTAATTCTTTACACATACAGTTGGGATTAGCATTGGCTTCCCTAGGTGTAATTACTTC
GTTGGTAGCTCAACATATGTATGCTATGCCTCCATATGCTTTCATGGCTAAAGATTTCAC
AACTCAAGCATCTTTGTACACACATCATCAATATATTGCTGGGTTTCTAATGGTTGGAGC
TTTTGCTCATGGGGCAATATTCTTTGTTCGAGACTATGACCCTGAACAGAATAAAGATAA
TGTTTTGGCTCGTATGCTAGAACATAAAGAAGCTATCATTTCTCATTTAAGTTGGGTAAC
TCTATTTTTAGGGTTTCATACATTAGGCCTTTATGTTCACAATGATACAATGATTGCTTT
TGGAACTCCTGAAAAACAAATTCTAATTGAGCCGGTATTTGCTCAATGGATTCAAGCCTC
TTCAGGGAAAGCACTTTATGGGTTTGATGTGTTACTATCATCCTCTACTAATATCGCAAC
ACAAGCTGGTAGCAATATTTGGCTGCCAGGCTGGTTAGAAGCGATTAATAGCGGAAAAAA
TTCATTGTTTTTAACAATTGGTCCTGGTGACTTCTTAGTTCATCATGCAATTGCATTGGG
ATTACATACTACTACGTTAATTTTAGTTAAAGGTGCTTTAGATGCAAGAGGCTCTAAACT
TATGCCGGACAAAAAAGACTTTGGATATAGTTTTCCTTGCGATGGACCTGGCAGAGGCGG
CACCTGTGATATATCTGCATGGGATGCGTTCTATTTAGCTGTATTTTGGATGCTAAATAC
AATAGGTTGGGTAACATTTTATTGGCATTGGAAACATATTACAATATGGCAAGGCAATGC

AACTCAATTCAATGAGTCTTCAACTTATCTAATGGGATGGTTTAGAGATTACTTATGGCT

AAATTCTTCTCCATTAATTAATGGTTATAATCCATATGGCATGAATAATTTATCAGTATG

GTCATGGATGTTCCTATTTGGACATTTAGTATGGGCAACAGGATTTATGTTCTTGATCTC

CTGGCGTGGCTATTGGCAAGAGTTAATTGAAACTCTAGCATGGGCGCATGAACGTACACC

TTTAGCAAACTTGATTCGTTGGAAAGATAAACCTGTTGCATTATCAATTGTACAGGCAAG

ATTAGTAGGTTTAGCACATTTTTCTGTAGGATATGTATTAACTTACGCAGCTTTTGTATT

AGCTTCAACAGCAGGCAAATTTGGTTAGACTAAATTCGAATTAGCTAAAAAAAAGTCAGT

CATAATAGTTTTATGACTGACTTTTTTAGATTTAAAACAAAAACTTACTAAAGTTTCACC

TCAATATCTACACCAGAAGGCAAATTTAACTTCATTAATGCATCTATCGTTTGAGAAGAA

GGTTGATGAATATCAATAATTCTTCTGTGAGATCTTATTTCAAAGTGTTCTCTTGAGTCT

TTATCTACATGTGGAGAACGTAAAACACAATAAATTCTTCTTTTTGTCGGTAAAGGAATT

GGCCCTACTGCAACGGCATTAGTTCTAGATGCGGTATCTAGTATTTTGTCACACGACGTA

TTAAGTATAATAGAATTATATGCTTTCAGTTTAATTCTGATTTTTGTCTGCTGAGTAATT

GTCATGTTAAAAAGATCTTATTATTTAAGAATTTTAGAGACAACACCTGCGCCTACGGTA

CGGCCACCTTCTCTAATAGCGAAACGCATACCTTGCTCAATTGCAATTGCATTAATTAAT

TCAGCAGTCATCTTAATTCTGTCACCAGGCATAACCATTTCTGCATCAGTACCATCATCA

GCAGTAAACTGATTAATAGTACCAGTTACATCAGTTGTTCTAACATAAAACTGAGGTCTA

TATCCTGGAAAAAATGGAGTATGTCTTCCGCCCTCCTCTTTAGTTAGAATATAAACTTCT

GCTTCAAATTGAGTATGAGGTGTAATTGTACCAGGTTTAGCTAATACCATACCTCTTTCA

ATATCTTTTTTCTGCACACCTCTTAAAAGAATTCCAATATTATCACCCGCTAGACCTTCT

TCTAACGTTTTTTGAAACATTTCTAATCCAGTAATAGTCGTTGTACGAGTTTCTCGTAAA

CCTACAATTTCAATTGTGTCACCAACTTTAATAATGCCTCTTTCAATTCTACCAGTGGCA

ACAGTTCCACGTCCTGTAATAGAAAAAACATCTTCTACAGCCATTAAGAAAGTTTTATCG

ACATCTCTCTCCGGCGTTGGAATATATGTGTCAACTGCTTCCATAAGTGAAAAAATCTTG

TCAACCCATTTATCTTCACCTTGCTTAATAGCTGGGTTTTTCGTCACAGCTTCTAATGCC

AATAAGGCAGAACCTGCAACAAAAGGAATATCATCTCCAGGAAAGTCGTATTGACTTAAT

AATTCCCTTCCTTCTAATTCTACTAATTCTAGTAGCTCTTCGTCATCTACTTGATCTTCC

TTATTTAGGAATACTACTAATGTAGGAACACCTACTTGTTTTGCTAATAAAATATGTTCA

CGAGTTTGTGGCATTGGACCGTCTGCCGCAGATACAACTAAAATAGCTCCATCCATCTGA

GCAGCACCCGTAATCATATTTTTTACGTAGTCGGCATGGCCTGGACAATCTACGTGAGCA

TAATGACGATTATCTGTTTCGTATTCAACATGAGCAGTATTAATAGTAATACCTCTAGCT

TTTTCTTCTGGAGCGGCATCAATTTCATCAAATTTTTTCGCTGCAGTAGACCCTAAAGTT

GATAAAGTCGCAGAGATCGCTGCTGTTAAAGTTGTCTTACCATGATCAACATGACCAATT

GTGCCAATATTGACATGAGGTTTTTTACGTTCAAATTTAGATCGAGCCATGCTTTTTGTT

TTCCTTATAGTAAAAGTTGCTTTATAGTTACTTTTAACGATAAATCTTGTGGATTGAAAG

ATTTATTATTTAATACAGACAAGATACAATATAACTAATCTAATATCGATAGTGAGCAAA

CGCTTTATTTGCTTCTGCCATTCTATGTGTATCCTCTCTCTTTCTAATAGAATTTCCTGT

TTCATTAGCTGCATCCATAATTTCATTAGCTAATTTCATAGACATACTTTTACCAGATCT

GTCTCTAGAGAATTTAGTAATCCATCTTAGTGCTAAATTCGTACCTCTATAAGCTCGTAC

TTCAATAGGAACTTGGTAAGTAGAACCACCAACTCTTCTTGCTTTTACTTCTACTAGAGG

AGTAATATTTCGGATGGCTTTTTCTAAAATATTTAAAGGATCTGATTCTGTTCTCTCTTT

AACGATATCTAAAGCCTGATATATAATTCTTTGAGATAAAGTTTTTTTACCACTTTTCAA

AATACGAACAGTTAACATACTTACGAGTCTGCTTTTATATAAAGGATCAGGTGATGCAAA

CCTTTTTTTAGCTGTATTACGACGAGACATAGTATTTAAAATTGGTTATTGTAATAATGT

TCTATTAGCAGGTATTTAAGATTTAGGCTTTTTTGTCCCATATTTAGATCGGCTTTTACG

ACGATCTTTTACTCCTGCAGCATCTAAAGTACCACGTACTACATGATATCGGACTCCAGG

CAAATCTTTAATTCGGCCGCCCCTAATCAGTACCACAGAATGCTCTTGAATATTATGACC

TACGCCAGGAATATAAGCTGTAACTTCAAATCCAGATGTTAATCTAACACGCGCTACTTT

TCTTAAAGCAGAGTTCGGTTTTTTAGGAGTTGTAGTATATACTCTTGTACAAACACCTCT

TCTTTGAGGGCAACTTTGAAGAGCTGGAGATTTTGTTTTTTTATGTATTTTTCGTCTTTC

GGATCTAACAAGTTGTTGAATTGTTGGCATAATAAAATTGATTTAAAGTCTTTAATAGAT

ACTATTTGAACTTGCTTTACATGAATTGGCTAGTTTTTATTTATCTTATACTTACGCATA

AATCTTTCTACTCTACCTTCTGTATCAATAATTCTCTGTGAACCAGTAAAGAATGGATGG

TTTCCTGACCAGATATCTACGTGTAGTTCTGGCTTTGTGGACCCAATCGTCATAATTAAC

TGGCCGTCACAGTAAACTTTTGCTTCTGGATACCAATTTGGATGTATATTATCTTTTGCC

ATTGTTTTTATAATTATATAAATGTATCAATAATAACTATTAACGCTTAGAAAATTGAGG

AGCCTTTCTGGCTTTCTTTAAACCATATTTTTTTCTCTCTTTTACTCTTGGATCTCTTGT

TAAATAGCCTTCAGACTTAAGTGTCGTTCTATTTTCTGGATTAATTGAGCACAATGCTCT

TGCAACACCTAAACGAATTGCATCAGCTTGCCCTGTCAAACCTCCTCCTCTAGCATTTAC

ATGGATATCATACTGGTTTAGTAGTCCTAAAACTTGTAATGGTGCATATGAAACTCTTAA

GTAATTAGGACTAAATTGAAGATAAGACTCTCCTGGTATACCGTTTATAATTAAATTACC

TGACCCTGGGACTAGTCTTACTTGTGCAACAGAACACTTCCGGCGACCTGTTCCAGAATA

GATTGCGCGAGTTTTAATTAATTCTGTCGACATAACATTCCTTGATAACTAAATAAATAT

ACATTCTTTATACTATATACTCTTGTGGCTTTTGCGCGACATGTGGGTGAATTGGGCCAG

AATACACTTTAAGCTTCGTAAATAGTTTTCTGCCTAATGGACCTTTAGGAAGCATACCTT

TAACCGATTTTTCAATAATTCTGTTAGGTAATCTTGTCTGAAGCTGATCAAATGTTTCAA

CTTTTAATCCGCCAGGCTGTCCAGAATGTCTTCTGTATAGTTTTTGATTCGTTTTATTTC

CACTTACAGATACGTGAGCAGAATTAATAACGATTACATAATCTCCAGTATCTAGATAGG

GTGTATAAGAAGGCTTGTTTTTACCTCTTAAGATATTAGAAATATGAGTAGATATTCTAC

CAAGTGTCTGATTTTTAGCGTCTATAACATACCAATGAGAATTAGTATTTAATGAGGGTG

ATTGCGTTTTATTCATGAAAAGATTAATACTTTTGGTTGACAATAAGAGAATATGTAAAT

GGTTTACATTCAATTTCTATACAAGTAGTATTTAAAAATATTATTAACAAAGAGAACTTT

TCTAACAGTTATTTAAATTGTTTGTCAGCAAGAAAATTTCTAGTTATCAATAACTTATAC
TATTTTTAATAATATAAAATATATTAATCACTTTTTTCTTTTGGCAAACTTATACCTAGT
TTTTTTTGCAAGGCATATATAACTTCTTCAGCTGATTTCTGGCCAAAATTTTTAATTTCT
AGCAGTTCTTCTTGAGAGTAATCTAGTAAATCTGCAATAGAATGGATTTGAGCTCGCTTT
AAGCAGTTATAAGCCCTAACAGATAACTGCAGTTCTTCTATTAAAACTTGACTAATTTTT
TTATCTTCTTTGCTACGATAATTATCTGCTGATTTAAAGTCTAAATTTCTTAGTGAACAA
AAAAGATTAGTTAAAACTGTAGCTCCTTGACTTATTGCTTCTTGAGGAGAGATGCTCCCA
TTTGTCCAGATTTGTATAATCAATCTATCTTTTATGCTGTTGCTACCAATACGGACTTCT
TCTACTTTATAATTAACTTTATTAACTGGCATAAAAACAGAGTCTACTTGCAAAAAAATCC
ACAGATAATTCATCTACAGCTTTTTCAGCTAAGCGATACCCGCAATTTTTTTCAATTTTA
AACTCCATTTCAAATATTGTATTATTGCAAATAGTTGCAATATACTGTCTAGGATCTACT
ACCTCTATATCAGAAGATAATTCAAATAGGCCGGCTGTAACTATAGCTGGCCCTTGAACT
CTAATTCGACCAATTTGAGATTCTTTGTTATAACTTTTAAATACTACTTCTTTTAGATTA
AGTAATATTTCTAACACATCTTCTCTTACCCCAGGAATTGTAGAAAACTCATGGTTCACT
CCAGCAATCCGTACAGCAACTATAGCAGTACCTTCAAGATCTGACAATATTGATCTTCTC
AAAGCATTACCTAATGTAATACCTTGTCCTTGATTTAATGGTTCTATTACAAAACTACCG
TACTGCCCACGCGCCCCATCTGTTCTTGACTCTATGCATTCAATTTGAAATTGAGCCACC
TAAGAAAGCTCCTTTAAAATAATCAGTGATTAGTAAGTCTCTCTAAAACAAGAAGGAATT
TATACTCGGCGTTTCTTAGGAGGGCGACATCCATTATGAGGTACAGGAGTAATATCTTTT
ATTAGAGTAATCTCTAATCCTGCAGCCTGCAAAGCTCTAATTGCTGTTTCTCGACCCGCT
CCTGGACCATTTACTAGAACTTCAGTTTGGCGCATACCTTGATCCATAGCTTGTCTAGCT
GCTTTTTCAGCTGCTGTTTGAGCTGCAAAAGGTGTTCCTTTTTTAGCTCCCTTAAATCCA
CTTGCACCAGAGGATGACCATGATAATGTTTCTCCTTTTAAATTAGTAATAGTAACAATT
GTATTATTGAATGTAGATTTAATATGTGTAATACCGTTAACTGCATTACGTTTAGTTTTT
CTTGCTCCGGATTTTTTTATTTGTCTAGCCATCGTGTCTCGTCTATTATATAGTTAATTA
AAAGATTATTTTCTTGGAGCTTTTTTCTTACCTGCTACTGTTTTTTTACCTCCTCTGCGT
GTTCTAGCATTAGTTCTAGTTCTTTGTCCTCTCAAAGGAAGACCAAGACGGTGTCTTCTA
CCTCTATAAGTACTAATTTCCATAAGTCTCTTAATGCTCATAGACTCAAAACGTTTGAGA
TCCCCTTCAATCTGATAATTAGACTCAAGAATTTCTCTTATACTGACAACTTGTTGATCA
TTTAAATCTTGACACTTAATATCAGCGTCTATGTTTGTTTTTTCTAATATTTCTTTCGAG
CGAGATAGTCCAATACCATAAATATATGTTAAAGCTATCTCTATTCTTTTGTTTCTTGGA
AGATCTACTCCAGCAATTCTGGCCACTTTGTCTTGTCTCCAATAAATAATATGTATTTTT
ATAACGGTTGTTTATAAGCTTAGCCTTGTCTCTGCTTATGTTTAGGATTAGTGCAAATTA
CCATCACCTTTCTGTGACGCCGAATTATTCTACATTTTTCACACATTTTTCGAACAGAAG
GACGAACTTTCATATTAACTCTATATACTAGGAATATTACTATATTAACTCTATTAATTA
TCGACATTAAAAATGTCAACAATATATTGTACTACTTTAACTGTTGTAGATTTTACTTCG

TCATACTATCATATTTTTTTGATATGACGTATGTCTGAATTTGCTTAGCTGTGTCTATTG
CGACTCCAACTAAAATAAGTAAAGACGTAGCCCCGAGACCTCTTAAGTTTTGGATCTGAG
TAACCTTTTCTATTATAAACGGAATCAGCGCTACTGTAAATAAAAACGAGGCTCCTAGGA
ATGTCAGTCTATTTAATATGACTTGTAAATAATCAATAGTCGCTTGACCTGGACGAATAT
TAGGAATGCTTGCACCCATTTTTTTTAAATTTATAGCAATATCTTCTGGATTCATTACTA
TTGATGTATAAAAATAGCTAAAGAAAAGGATCAAAGCACAATAAAGAAGAAGGTATAGTG
AACCATTAGGACAAAACAGGTATAGGATTTGGAGTAATGTTTTATTTTGAATAATTTGGG
TTAGATAAGATGGGAGGGCCATAGATGCAGATGCAAAAACAATAGGCATAACTCCACCTT
GATTTAATTTCAAGGGCAAGTAACTATTTGGATCCAAAATTGAAGATTTTCCTAGCTGTC
TTGCTGAAATAATTTTAATTCTTCTTGTTCCTTCTTGTACACAAATTGTAATTATTATCA
TTAATAAAAAGATCGCTATAAATAATCCGAACTTAAGACTTGCATTACTATAACTAGCAT
CAAAAAATGACTGTGTAAAATTCTTTGGTAGTCCTGACACAATGTTTTGAAAAATAAGTA
AAGAGGCTCCATTACCAATTCCCTTTTCCGTAATTAACTCTGATAACCACATAATAATCA
TAGAACCTGCTGTTAAAGCTAAAACAGACTCGCAGACAAATGCAAAATTCCAATTAAAAA
CATATGGTTTTACCCATATAGAGATTGCTCCAGATTGTAAAGTCGCCCAACCTAGAGCTA
AGTACCTTGTAATTTGAGTTATTTTTTGGCGGCCCAATTCACCTTCTTCCTTCTGTAATT
TTTCCAGATTGGGGACAATTTTCGTAAGTAGCTGCATTACAATTGAAGAGTTAATATAAG
GAACAATACCTAACGCAAAAATCCCTATTGTTGAAAAACCTCCTCCAGAAAAAATATTCA
GAAAATTTACTAAAGTATTTTTTTCTACACTTGCATAAAAGGCGTCATGATCTATACCTG
GAACAGGTATAAATATTCCTAAACGTGCTAAAACTAATAGAAAGAGAGTAAAGATAATAC
GATTTCTTAGATCACTTTTTTGGCTCATAAATAAATTTTAAATGAAAAGAGATACTTTTT
TATGTAAAAACCCTATTCCAAGACTTGTATAAGTTTAAAATAGGGAAGTAAAATATTATA
GTAATATTAATATAATATTCTATGTAGAATACAGGCTTTCTATTGGAACTCCTCGATCTT
TAGCTGCTTCACTAAAAGTTCTTAATTGAGTTAAGCCATTGAGAACAGCTCGCGCGTTAT
TTAATGTATTATTAGAACCTAATTGCTTAGCTAATATATTCTGTACACCCGACAATTCGA
GGACTGTCCTTACAGAACCTCCGGCAATTACACCTGAGCCAGGTGCAGAGGGCCTTAATA
TAACTTTCGCGGCACCAGAAATTCCGTTAATAGGATGAGGTATAGAATTCGATTTTGTCA
GTGGAACTGTAACTAGATGTTTTTTAGCATCTGTTACTCCTTTTTTTACTGCACCAATTA
CATCGCTTGCTTTTCCCACGCCGACACCAACTTGGCCTTGCTCATTACCAATAACAAGAA
TAACTCGAAAGCTTAGTTTTTTACCTCCTTTTACAACTTTAGTAACTCTTTTAACTTGTA
CGACTCTTTCTTCCCAGCCACTATCTTTATCTTTTCCTTTGCTCTGTTTTTTACGATTGG
CCATTTTAAGAATTATTCCTAATTAGTTAATTACGATTCTAGAAACCCATGCCTGCTTCC
TTAGCAGCTTCAGCCAAGGCCTTAACTCTTCCATGATATAACTTTCCTCCTCTATCGAAG
ACGACATTTTTAATGCCTTCTTTCATAGACTGTTCTGCTAACTGTTTACCAACTACGCGA
GAAGTATCACAGTTTGGTCTTATGTTATCAGATTCTTTATTATTTAAATTAACAGATGAT
GTAGCTACTAATGTAATACCTTGTGTGTCATCAATTATTTGTGCGTATATATGTTTATTA

GATCTAAATACACACAGACGAGGCCTACTTGAGGTTCCTTGAACTTTTTTCCGAACTCTT
TTATGCTTATGAATTCTAGTTTGTTTAGTGTTTAGTTTCATTATTATTTACCTTTTCCAG
CTTTCCCAGCTTTTCTTCTAACAAATTCACCTTGATATCTAATTCCTTTTCCTTTATAAG
GCTCAGGAGGCCTAATAGAACGAATAGTTGAAGCAACCTGACCGACAACTTCTTTATCTA
TGCCAGAGACAGTAATATTTGTGTTGTTTTCAACTTTAATTTCAATATTGGCAGGAGGTT
TAATTTTCACTACATGACTATAGCCAACACTTAAGATTAAATCTTGGTTATCAATTTGAG
AACGATAGCCTACACCTTGTATTTGCAGTTTTTTAAAAAATCCATTAGAAACACCTTCAA
TCATATTACTAATAAGAGTTCTGGATAGCCCATGTAACTGACTAGCCATTTTTGTTTGTC
CACTCGTCTTGACAGCAATTGTGTCGTTAAGTATTTCTAAATTAATACCAGCTGGTAAAG
TTCTGGATAGGGTACCTTTAGGTCCTGTCACGGTGATAGTCTGACCATCGAACTGAGTAC
TAAGATTTGTCGGCAATAAAATTATTTTTTTTCCAATACGAGACATATTCCACCTATAAA
ATAGTTACCAAATATAGCATAATATTTCACCACCAAGACCATCATGACGAGCTTGTCTAT
CTGTCATAACGCCTCTAGAAGTAGAAATAAGAGCGATGCCTAAACCTCCAAGAACTCTGG
GCAACTCTTTATGATTTGCGTAGACTCTTAGTCCGGGTTTACTAATTCTTTTCAGAGCAG
TAATAACTGGTTGACGATTTTTACCATTATACTTAAGAGAAATCATTAAATGAGTCTCTA
TACCTTCACCCATTTGTTCGAAATTTTGAACAAATCCTTCTTCTTTCAGTACTGTTGCCA
TATTGCATGTCATTTTCGTTGCTGGAACTTGCACAATTTGATGTCTTGCTAAGTTTGCGT
TACGAATACGTGTCAGCATATCGGCGATCGTATCGTTGACCACCTTGATCCTCCTTGCGA
TGAATTATTTGAAATATTTTCAAGATTCCCTGAAAGGCATCCCTAGCTTTTTTAACAAAG
CTAGACCTTCTTGATCTGTTTTAGCTGTAGTGACAATTGATATATCTAAACCACGAATTT
GATCAATATTATCATAGTCTATTTCTGGAAAGATCAACTGTTCACGTAAACCTAAATTGT
AATTACCTTTGCCGTCAAAACTTCTAGGACTAATTCCTCTGAAGTCTCTAATTCTTGGTA
ATGTTAAATTAATTAATTTCTCTAAAAAAGAATACATCTTGTCTTTTCTTAGATGCACAA
CAATTCCGATAGGAACCTCTTCTCGAATTTTAAATCCTGCAATAGATTTTTTAGCTTTTG
TGACAATTGGTTTTTGTCCAGTTATTAATGTCAATTCTTGAATACTACTTTCAAGAGCCT
TAGCATTCTGAGAAGCTTCACCTAAGCCACGGTTAATAGTAATTTTAGTAAACCTAGGAA
CTTCATGTACATTTTTGTACTGAAATTCATCTTTTAAAGATTGAGTAACAGTTGTTTTAT
ATTTTTCTTTTAATCCTATTGCCATTATATTTTCATCAATTATTTAATAAGTTCGCCAGT
CTTTTTCAGTTTTCGAATTTTTTGGCCTTTATCATTAATTATTACTGAGGATCGACTAGC
AATATTATTTTGTTCACTAAATAACATAACGTTAGAGGTATGTATTGGAGCTTCAAATTT
GATAATTTCTCCCGTTTCTCCTTCTTGTTGAGGTTTTTTATGCTTCACTTTGAGATTAAT
TCCTTTAACAATCACCTTATTTGTTTTATAAATAATTGCAATAATTTCACCTGTTTTTGT
TTTATCACTTCCAGAAATAACTTGAACTAAGTCCCCTTTTTTTAATTTAATTTTTGTATT
ATTTTTGGTTGTTTTAGAAGGACCTTTCATTATACTACCTCCGGTGCAAGAGAAACTATT
TTAGAAAAATTTTTATCTCTTAACTCTCGGGCTATAGGTCCGAAGACTCTTGTGCCACGT
GGATTATTATCTTGATTAATGATAACTGCCGCATTATCTCCAAATCTAATGCTCATACCG

TCAGTTCTCCGTAAAGCTTTACGAGTTCTTACTACAACGGCTCTGACAACATCAGATCGT
TTAACTGGCATATTGGGAGACGCATCTTTGACTACTCCAATAATAACATCTCCAATAGAT
GCATAAGAAGGATTACTAGTGCCTAGTACTCTAATACACATTATTTTTCTAGCACCACTA
TTATCTGCAACATTAAGATAGCTTTGAGTCTGTATCATACTTTTATTCTGTATAAGATTA
ATTATCAAAAGACTTAGATAAGATATTAACCATTGTCCAACACTTTGTGCGACTTAAAGG
TCGTGTCTCTTGTATTGTAACAATATCGCCGATTGTGCATTCATTATTTTCATCATGCGC
TTTATATTTCTTTGTCCGGATCATAGTTTTTGCGTACTTTCTATGAGAGATTCTATTTTC
TACAGCTACTACTATAGTTTTATTCATTTTATCGCTTACTACTTTACCTGTTGTTTCTTT
TAAAGGCATAGTCTTTATATTTTTTATATATAATTAAATTTTAAGATTATTACATGGTTG
ACTTAGTACGAGACTTTTCAACAGTTAAAAGTTGGGCTAATCTATGCTTAGAATGTTTAA
ATAGATGAGGCTGGAAATCTTGCCTTGTGGCTCTTTTTAGCCTTAAATCAAAAAGCTCTC
TTTTTATTACAAGGATTTCTTCAGCTAAAGAAGAAGAGTCCAGGTTTGTAACATCTGATA
TTTTAGGGAAAGTCATATTTTAGGATTCTGTTGTATTCCGAACTATAAATTTAGTCTTGA
TTGGTAATTTATAAGAAGCTAATTTCATAGCTTGTTGAGCAGTTTTTTGTGGTACACCTG
TAATTTCAAATAGAATATGTCCAGGCTTAATAACTGCAACCCAATATTCTGGAGCTCCTT
TACCTGACCCCATGCGAGTTTCAGCTGGACGGGCAGTGACTGGTTTATCTGGAAATACTC
TAATCCATAGTTTACCACCTCTTCTCACATATCTAGTAATAGTTCTTCGAGTAGCTTCTA
TTTGTCTAGAAGTTAACCAAACTGGCTCTGTCGCTTGTAATGCATAATCACCGAACGCAA
TTGTGTTACCTTTGCTAGCAGAACCTTTCATTCTACCTCTATGTTGTTTTCTAAATTTTG
TTTTCTTGGGGCTTAGCATAAAAATTAAAAAATAATAATAATTAAGAAGCTGTTGAATCA
GGAGTTTCCTGATTATAAGCAGATTCTAGACTTTTCGATTCTGGCAAAATTTCTCCTTTA
AAAAGCCAAACTTTTACTCCGAGCACACCATACGTAGTGTGAGCTTGGCGATGACAATAA
TCAATATCGGCTCTTAAAGTTTGTAAAGGTACACGACCTTCTCTAACCCATTCACTTCTA
GCTATCTCTGCACCATTAAGCCGACCAGATACTTGAATTTTAACACCCTGTGTATTTGCT
CTTTGAGCTCTTTGAACTGCTTGTCTGACAGCTCTACGAAAGGCCACTCTTTTTTCTAGC
TGTTGAGTAATAAATTCTGCTACTAAGGTTGCTTCAGAATCTGGATCTGCAATCTCTACA
ACATTTACTCTGAGTTGTTTGCTAGGGTCTAGGATTAAGGACAGCGATTTTCTTAAAGAC
TCAATTCCCGCTCCAGATTTTCCAAGTACGATTCCTGGTCGAGCTGTTGCTATGAGAATT
TCTACTTGATCAACTTTACGATTAATTTCAATTTTAGCAATACTAGCATTACTAAGTTTT
GAATGTATAAATGAGCGAATTTTATGATCTTCTTGTAAAAGAACTGGGTAGTCTTTAGAG
TTAGCAAACCATGAAGAACGATGTTTTTGGGTAATGCCTATGCGAAAGCCCAAAGGATGA
ATTTTTTGACCCACAGTATCCTTGATTATAAGTTAAAAGCTATAATGACTAATTGACGTT
AAAAGACTACAGTTCTGAAACACCTAATGTAATATGACAAGTTGGTTTATGTATTGGAAA
GGCTCTTCCTTGTGCTCTAGGCTGAAATCTTTTTAAAGTCGGGCCTTTGTCTGCAAAGGC
TTTACTAACAAACAATTGGTTTTTATTTAATCCATCATTATGCTCAGCATTCGCTGCAGC
AGATTCCAGGATTTGCTTTATATGAGAACAGACTCGGTATGGCATAAATTCCAGAATAAT

TAATGCTTCTTGGTATTTTCTACCTCTAATTTGGTCTAATACACGACGGACTTTATGCGG
AGAAAGACGAATGTATTTCCCTACTGCTTTAGTTTCTTTTACGTTTTTTGTAATACTCAT
AGTTATAGTTTAACGACGGGCTTTTCTGTCACCTTTTACGTGAGTGCGGAAAGTTCTTGT
TGGAACAAACTCTCCTAATTTATGTCCTACCATTTGGTCTGACACAAAGACAGGAAAATG
TTGTTTACCATTATAAACAGCTATTGTATGCCCTACCATATCGGGAATAATAGTAGATGC
TCTCGACCAAGTTTTTAGGACTTCTTTTTTTCCTGAAATATTTAATGCTTCTATACGTTT
AAGAAGACTAACATCAATAAAAGGGCCTTTATGTATAGATCTTGACATAATAATAAATCA
TTAGATTCTTAATAAAATAAATTCTATTTACGGCGACGTAAGACGTACGGGTTACTATAT
TTATTTGGATTTCGTGTTTTAACACCCAATGCAGGTTTACCCCAAGGTGTTACAGGACGA
GCTCGTCCAATTGGAGATTTGCCTTCACCACCACCATGTGGATGGTCTACAGGATTCATG
ACAACACCTCTAACAGTAGGTCTTTTACCTAACCAACGATTTCTACCAGCTTTACCAAGA
GTAATATTACTAGCATCAATATTACCAACTTGTCCAATAGTAGCGTAGCATTCTTTTCGA
ATCATTCGAACTTCGCTTGAAGGCAATTTTACAGTAACAAAAGTACCTTCTTTTGCTACT
ATTTGAGCATAAGTTCCTGCTGCACGAACAATCTGTCCTCCGCACGAAGGTCTTAATTCT
ATATTATGTACTGCTGTTCCTAAAGGAATACTAGATAAAGGCAAAGCATTTCCAACTTCG
ATAGGGGCAGTAGGACCAGAAAGAACCATAGATCCTACACTGAGAGATCGAGGGTGTAGA
ATATATCTTTTCTCACCATCAAGATAGTGTAATAATGCAATTCTTGCATTTCTATTCGGA
TCATATTCAATAGAAGCAACTTTAGCAACTATATTATGTCTATTTCTTTTAAAGTCAATT
AATCTGTACTGCTGCTTATGTCCACCACCTTTATGACGACAGGTAATGACACCTCTATTA
TTTCGACCTTTGCAGAAATGGTGTTTAACTATTAATGATTTTTCTGGCTTATCAGTAGTA
ATCTCTGAAAAGGTAGAAACCGTTCTATTTCTTGTCCCTGGTGTGTAGGCACGATATAAA
CGAATTGCCATATAAGAAGATTTGAGAAGAGTAAATGTTGAACTATGTGATATATTTAAG
TTTCTGGAAACAAATTAATAGAATCTTCTGAGGCAAGTGTTACAATTGCTTTCTTGTAAT
GTGGTCGCTTCCCTACGAATCTACCGATGCTCCTTTTTTTCTTAGGAGGATGACAAGTGT
TAACACCTGTAACTTGCACGTTGAAAATATACTGTATAGCAGCCTTAATATTAATTTTTG
TTGCTTTGGGATCAACGGCAAAACAATACTGATTCTCTTCTAATAATTTAGTTGTCTTAT
CAGTAATGATGGGATATTTAACCAGATCTAATAAACCTCTTGAATCAATGCTATCCATTA
TATACCTCTTGTATTTTAGATAAAGCATCAACTGTAATAATAATTTTATGTGCAGCTAAC
AAAGCCATAATATTTAGTGTATCAGCTGAAATAAGTTCTACGTTGTGTAGATTGCGAATA
GAAAGATAAACATTCGGATCTTTTTTGTCTACTATGACTAAAACTTTTTTATTTAAATCA
AGATTCCATCGATGTATAGCTTCCATAAATAGTTTAGTCTTGGGCTGATGAAAATAACTG
TTAAAATTTTCTACAATCAGTGTATTAACTGATTTGTTATTTAATGCTGTCCTTAATGCT
AATTGTCTTTCTTTTTTATTCATTTTTTTGGTAAAACTACGAGGTTTTGGCCCAAATATT
ACACCACCACCTCTCCATAGAGGTGAACGGATAGAACCTGCTCTTGCTCGACCAGTCCCT
TTTTGACGCCATGGTTTACGACCTCCACCTCGAACTTCACTTCTTGTTTTAGTATTAGCA
GAACCCTGGCGCTTTTCATTACTTTGCTTTACTAAAGCTCTATGGACTAAGTACATCCCA

GAATCTTGACTGACCTTAAGATTTAAATCAGCGTTGCCACTTACTTGACCTTCCCAATTA
TAGACTTGATAATTTAATTGCGTGTTAACTGTCATGAAATATATCGTATGAAAGAATATT
GCTACTTACTAATTTTTACCAAAGCGCCAGGTTTACCAGGAACAGCTCCCTTAACGATCA
ACAAATCATTCTCTGAGTTAATACTTACAATTTGCAAATTTTTTATTGTAACTTTTTTAT
TACCAAGTTGGCCAGCCATGTTTTTACCTGGATAAACTCTTCCAGGCGTTGTACCGGCTC
CAATAGAGCCAGGTTGCCTATGATTTTTCGAACCATGTGACATTGGGCCTCTACTAAAAT
GATGCCTCTTTTGGTAACCGGAGAAACCTTTACCTACACTTCTAGAAGAGACATTAATCT
TTTGGCCTACTTGAAATAGATCTGTAGATAGTATTTGGCTAACTTCGAAATCATCAGTAG
ATTTCATTTCGTATTCACGTAAATATTTTAGTGGGGGAGCCTGTGATTTTTTTAAGTGTC
CCAACAATGGTTTATTTAATTTTTGTTCAGCAACTTGCTTGTAACCGACTTGAATAGCAT
TGTAGCCATCAGTAGATACAGCTTTAATTTGAGTAATAACACATGGCCCAACTTGAATTA
CAGTAACTGGAATTGATAAACCAGCTTCATCAAAGAATTGGGTCATACCTACTTTAGTAC
CGAGTATACCAACAGACACTAGATTTCTCCTTTTTGAGAGATTTATACTATCATTGTACA
TAAAAATTCTACTTCATAGTTACTGAAGTAAATTAAACTAACAGATGCATGATATTATTC
ATGACAACATAAAGGTTAAAAACTTTACCTATTTAATCTAAAATAGAAACAGCTTTTTGT
CTAGTTACAGCGTATATAAATTAGAAGTATGCTTATTAAATTTTTTATAAACTACTTAGA
TTTAACTGATATTGATATCCGTAACTTACTTGCAGTAATATCAATCAACATGTATAAGTT
TAATTGCCTAAACTAGAACCAGCTATTATTAGAAATAATTCAAGTATAGATAAAAGCGTA
TTTGAACTTTTATATATTGACAAATATAATGGAATATATTGAGGTTCGGTAACTACACCT
TTTTATTTTTACATTTATTGTGCAGTATATATACATCTAATGCAGTAAACATAAGAATAT
AACTGTATAGCAAATGTAATTACACAAATTAGAGAGGTATTAATGGGCAAAGTCGTTGGA
ATTGATCTTGGAACAACGAATTCTGTAATTGCTGTTATGGAAGGAGGTAAACCTACCGTC
ATACCGAATGCAGAAGGTTTTAGAACTACAGCTTCTGTTGTTGCATATACTAAAAGTGGA
GATAAACTTGTAGGACAAATTGCCAGGAGGCAAGCTGTTATTAACCCAGAAAACACTTTC
TACTCTGTCAAAAGATTCATAGGACGTAAACAGAATGAAATTTCGCAAGAGATTCGGCAA
ACATCATATAATGTTAAAACTAGTGGATCAAGCATAAAAATTGCTTGCCCTGCACTGAAT
AAAGATTTTGCTCCAGAAGAAATTTCAGCTCAAGTACTGAGAAAACTTGTTGAAGATGCT
AGTACGTACTTAGGTGAGACTGTTACACAAGCAGTAATAACTGTACCTGCTTACTTTAAC
GATTCACAAAGACAAGCTACTAAAGACGCAGGTAAAATAGCAGGCTTAGATGTATTGAGA
ATTATTAATGAGCCTACCGCAGCTTCTTTGTCTTATGGACTAGACAAGCAAAATAATGAA
ACAATACTAGTATTTGACCTTGGTGGGGGCACATTTGATGTATCTGTATTAGAAGTTGGA
GATGGAGTATTTGAAGTACTCTCAACTTCTGGAGATACACATTTAGGCGGAGATGACTTT
GATCAGCAGATTGTAGAATGGCTAATCAAAGATTTTAAACAGAATGAAGGTATTGATCTT
GGTAAAGATAGACAAGCACTTCAGAGATTGACCGAGGCTGCAGAAAAAGCAAAGATAGAG
CTGTCAAATTTAACTCAAACAGAGATCAATCTTCCTTTTATTACTGCCACACAAGATGGT
CCAAAACATTTAGAAAAAACTGTAACTAGAGGGAAGTTTGAAGAACTTTGTTCAAATTTA

ATAGATAAATGTAGTATCCCTGTAAATAATGCTCTGAAAGATGCAAAACTAGAAGCTTCC
AGTATTGATGAAGTTGTTTTAGTTGGTGGATCTACAAGGATTCCAGCCATACAGCAAATG
GTTAAAAGATTAATTGGTAAAGATCCAAACCAAAGTGTCAATCCAGATGAAGTTGTTGCT
ATTGGTGCAGCCGTACAAGCTGGAGTTTTAGCAGGCGAAGTCAAAGATATTCTATTACTA
GATGTGACGCCGTTATCTTTAGGAGTGGAAACTCTGGGTGGCGTGATGACAAAGATTATA
CCAAGAAATACTACTATTCCTACAAAAAAATCAGAAGTATTTTCTACAGCTGTAGATAAT
CAACCAAATGTGGAAATTCAAGTACTTCAAGGCGAAAGAGAACTAACAAAAGACAATAAA
AGCTTAGGGACGTTCCGATTAGATGGCATTATGCCTGCACCTAGAGGAGTTCCTCAAATT
GAAGTTACCTTTGATATTGACGCTAACGGAATTTTATCTGTAAAAGCCAAAGAAAAGGCT
ACTGGTAAAGAGCAATCAATTACTATATCTGGAGCATCAACTTTACCTAAAGATGATGTT
GAAAGAATGGTAAAAGAAGCTGAAGAAAATTTCGATACAGACCAAAAAAGAAGAAAGAAT
ATTGACACAAAGAATCAAGCAGAGTCCCTATGTTACCAAGCTGAAAAGCAAGTTAAAGAG
TTTGAAGACAAAATTAGCCAAGATTTAAAAATAAAAATAGAAGAGCTAATTACAGAGCTT
AGATCTAGTCTAGAGAAAGAAGAATATGACAATATTGAATCTATTTCTCAGCAATTACAA
AATGCTCTGATGGACATTGGAAAAAATGCTGCTCAGACTGAAAGTAAAGATACAAAAGCG
AAGGATGACGACACTGTGATTGACACTGATTTCTCTGAAGCTAAGTAAAAGTAAGCGGGT
AACGCGATTCGAACGCGCGACATCAACCTTGGCAAGGTTGCGCTCTACCACTGAGCTATA
CCCGCATAGATTGTATTATTACAAAAATTAAGATATTTGTCAATCTGTTAATCTATACAT
CTAGACTATTAATTTTGGAATTACATATTAGCTAACTGTTGTAAATATAACAATTGCTAT
AATTTTTTAAAAGTACTTATATCATTATATAAGTACTTTTTATATTCAAAAATACAAATA
AGTATTATAGCTAAACTACTAAGGAATTAACTAAGCTAGTAACAATTACTAATCCTGTCC
ATAAGCCGGCTCCTGTATATATTAATCCTTTTGATTGCTCCCATTGGCCAGGGGATGCTA
AAACAACAGGTACGCCTACTACTAACACAGTCGATAATGCAATTAATAATAGCACTAATA
ATTGGATCGCAATAATCATTTAATATGTTCTCCTGAAAAATTCAAATGTTAATAATACTG
TATATAATACAATGAAGCTTTTGACCTTCAGCAATTTATCTATGAAGAAATAATTTAAAA
TTATTGTTTTCTAAATAAACCTTAAATTAAGTATTAAAATCTTTATTGTAAACAATAATA
CTATATTTTTTTAGTAGATAAAAAATTTAAAGAAAGTCTTTTTTAAAAAAAAATTAAGAT
AAGGTGATATGCTGTATAAAAAGCTAAGTATTATTAATTATTAGAGAAATTTAAAATAAG
AGGTAAAACATATGAACTCAGCCCTTTTTTTAGCAAAACTACCAGAAGCTTATGCCATTT
TTAAACCGATCATTGATATCTTACCCGTAATTCCTGTATTTTTTCTTCTTTTAGCTTTTG
TTTGGCAAGCAGCAATTGGTTTTAGATAACATTTAGGTTATAAAAAGAATAACAAATATA
CAGAGTGCCAATTTTTGAGCTCTAATCTTATATCTTACACAATAATAACTATTATGGTTG
AACCCTTACTATCAGGAATTGTTCTTGGACTTATTCCTATTACTTTATTCGGATTATTAG
TAGCAGCTTATTTACAATATCAACGGGGCAACCAATTAGGACTGTAAAGCCTAAATTATA
GATATTTATAAAAACTACAATAATTATAGTTTTTATAAATATCTACAAATAAGCTAATTT
TAAATACAGAAATAATACTATTGAAACTAGAGTGATAACAAAAAAAAATTTATGTAATAAC

AAGCTAATTGTTTACCAGCTTGACTTAGTAACTCCTGGTAAAAGACATTCATGAGACATT
TCTCGAAAAACATGGCGAGATAATCCAAAATCTCGATAATAACCTCTACTTCTACCTGTT
AACCAACATCTATTTCTCCCTCTAACAGCGGCGCTGTTTCTAGGCATTTCTTGGAGTTTT
TGTCTTAAATCCATTTTGTCAGAAAAGCTGCCAGCTTCTTTCAGTTGATCTTTAATAGCC
AGTCTTTTTAAATAATATTTTTTTGCAAGTTTCGATCTCTTAATCTCTCTTTGGATCATA
TTCTTTTTAGCCATGCTGTAAAACTCTCTTTTAAAATTATTTATTCAATATATTAGCTAA
AAAAGCTCCTGCCTGCAACTTTTATATATTTAAAAATAAATAGCATAAAGTAGTGAAATG
CAATTGATATTTAATCAACGATACCACTTTATGCTATTATCAAAAATTTATTTAGTAAAG
TTCTTGTTCAACATGAGTTGAAATAGTGCAATCAGATGAAGGATATGCAATACAGGTTAA
AACATAGCCAGCTAAAAGTTGATCATCATCTAAGAATGATTGATCAGCTTGATCTACAGT
TCCTTCTGTAACTTTACCTGCACAAGTTGAACAAGCTCCAGCTCTACAAGAGTAAGGAAG
TTCAATTCCTTCTTCTTCTGCTGCATCTAGAATATATGTATCCTCCGCACAATTAAATGT
GACATCAATTCCTTCTTCTTCACATAGTAAATGAATTTTATAATCAGCCATTTCTTCTTA
CTCCTTAATAAAGATACACTTGAAACTTAATAAAGCAAAATAATTTAAAGAACTTACCAA
AAAATTATATACTAGTTTCATTATGCATGATAGACTGGAAAAAAAGAAAATAGTATACTA
TTCTGAAAAATCAAGCTACATTTCCTACTAAATAGAACTATAGTAGGTCCAAAGTATATA
CATAAATTCATATAGTTAAAATTACTTTTATCATTTAAATAGCTGAACTTGATAGTATAG
ATTGATCATCAAGCTAGTCTTCTGCCAATAAATAATTAATTATGATTTCAATGCTTAATC
AACAAGTAGAACTTAAGCCTATTCTTAAATTTCAGATAACACAAATATATTCGCATTCTC
TTATTCAGGAAATTAAAGCCTTAATAACTAGACTTGTTTTGCAAGTTTGGAGAAGGCCGG
CTACATTGATGGCAGGTATTATCCAACCTTTATTATGGTTAATTCTATTTGGTGGGCTTT
TCTACAATGCTCCTATAAATTTGTTCACTATTAATACAAGCTATAATTGTTTTTTGAGCT
CTGGGATTATAATTTTTACCTCTTTTACTGGAGCTTTGAATTCAGGTCTTCCATTAATGT
TTGATAGAGAATTTGGATTTTTAAATAGATTATTAACGGCTCCTTTAGTATCGAGGACTT
CTATCATTTTATCTTCTGCTACTTTTATGACTTGTATTAGTTTAATACAAGTTGTATTTA
TAGTTACAGCTTCTCTTTTTATGGGAAACTCACCTCTAAATAGCGATAGTACTATGATTT
TTGGACTTATGATTCTATTAGTGACTGTAGGAGTTACAATGCTTAGTTTAGCTTTATCTT
TCACTCTGCCAGGTCATATTGAGCTATTAGCATTTATTTTAGTAGTTAACTTGCCCTTTT
TATTTTCTAGTACGGCTTTAGCTCCTTTATATTTCATGCCGCCATGGCTTCAGTTAATTG
CAAGTCTCAATCCATTGAGTTATGCAATAGAAGGTACAAGATACTTATATTCAAGCGTAA
ACTGGAATTTTACAGAGTGTGTGATTAAGATTAGCTGGGGAGATATTTGTTTAGGGCAAA
TTATTATTTTATTAATCGCTTTAGATATAATGGCAGCTTATCTTGTGTCTAATATATTAA
AAGCTAAACTTAATTAAAATTTTAATAAAAAATTTATTAAATTTATTTTACAAAGATTTT
TACTATATAATACTAATAGTAGTATGGATAATTGAAATAGAAATAGTTTTTCAAAACCAA
AGCTATTATCAACAATGATAAACATTTGTAAGAAAGTCAACAAAGTATGTTCTTATTCAT
AGGAGGCATGTAGTCAATGGGACTACCATGGTACCGTGTACACACGGTTGTTTTAAATGA

TCCTGGACGGTTAATTGCAGTCCACCTAATGCATACTGCACTTGTAGCGGGTTGGGCAGG
ATCTATGGCATTATACGAACTAGCTGTATTTGATCCTTCAGATCCAGTATTAAATCCAAT
GTGGCGACAAGGCATGTTTGTTATGCCATTTATGGCTAGACTGGGCGTAACAGATTCATG
GGGAGGATGGAGTATAACAGGAGAGAGCGTATCCAATCCTGGACTGTGGAGTTTTGAAGG
TGTAGCCTTAACTCATATAGTCCTTTCTGGCATGCTTTTTCTAGCTGCTATTTGGCATTG
GGTTTATTGGGATTTGGAATTATTTAGAGATCCACGAACTGGCGAGCCAGCCTTAGATTT
ACCGAAAATTTTTGGAATTCATTTATTGCTATCAAGTCTACTTTGTTTCGGATTTGGAGC
TTTCCATGTAACTGGACTTTTTGGACCAGGAATGTGGGTATCAGATGGGTACGGAGTAAC
CGGAAAGGTATTACCAGTAGCTCCAGCATGGGGACCAGAAGGATTCAACCCGTTTAATCC
TGGAGGGGTTGCATCTCACCACATTGCCGCAGGTACTGTAGGTATATTAGCTGGTGTTTT
CCATTTAACTGTTAGACCACCACAAAGACTGTATCGTGCTCTAAGAATGGGTAATATTGA
AACTGTATTATCAAGTAGTATCTCTGCTGTTTTTTTCTCAGCTTTTGTGACTTGTGGTAC
GATGTGGTACGGTTCTGCAACTACGCCTATTGAATTATTTGGTCCAACTAGATATCAGTG
GGATAGTGGATATTTTCAGCAAGAAATTGAGAAACGAGTAGAAAATGCTATTGCTGATGG
TGCTGCACCTAGCGAAGCATGGTCAAGAATTCCTGACAAGTTGGCATTCTATGACTATAT
TGGTAATAATCCAGCAAAAGGAGGATTATTCAGAGCGGGCCCTATGAATAAAGGCGACGG
AGTTGCTGAAGCATGGCTTGGACATCCTGTATTCCAAGATAAAGAAGGAAGAGAGCTTAG
TGTTCGCAGAATGCCTGCTTTTTTCGAAACTTTTCCTGTAATTTTAGTTGATAAAGATGG
TATTATACGAGCTGACATTCCATTTAGAAGAGCAGAGTCTAAGTATAGTATTGAACAAGT
AGGTGTAACAGCTAGTTTTTATGGTGGCAAATTAAATGGCCAAGTTTTCAACGATGCTCC
TAGCGTTAAAAAATATGCAAGGAAAGCTCAATTAGGTGAGGTATTTGAATTTGATCGAAC
TACATTAGAATCAGATGGAGTGTTTAGAAGTAGTCCTAGAGGCTGGTTTACATTCGGTCA
TGCTAATTTCGCTTTAATTTTCTTCTTTGGACATCTTTGGCATGGTTCAAGAACTATCTT
CCGGGATGTGTTTGCGGGAATCGGAGCTGAAGTTACTGAACAAGTTGAATTTGGAGCTTT
CCAAAAATTAGGAGATAGAAGTAGTAAAAAACAAGGAGCTGTATAAGACCAAAGTCAAAG
ATAGTCTTTATCAAATATTTCTAATATTAAAATAGTTAAGACAAAGTTTATTATCTAATA
GTTACTAATCAGGAGAATTTATGGAAGCCTTAGTCTATGTATTTTTACTAACAGGAACGC
TAATGGTTATATTCTTTGCGATCTTTTTTAGAGAACCTCCAAGAATAGCAAAGTAACTTG
ATTCCGTTTAAACTTATAATAATAGAACCACTTTCCAACTTATTAGTGGAAAGTGGTTTT
ATTGTAGATTACATTGTATTATTCTTCATGCTCTTCAAAAGGATCTCGGAGATCTTTTGA
AGCAGGACCAAATGCTGTGTATATCGAGTAACCTGTAATACCTAAAAGCAGACTCGAAAT
AAAAATACTAAGAACTGTTGCAGTTTCCATAATTTAAGCAGTATTAAGATGAACTTTTTC
TATAATGATATTATCAATATTAAAACATTTAATTATTAAAATTATCAAAATATACTATGG
CACTTAGAACTAGACTTGGAGAAATTTTAAGACCCTTAAATTCAGAGTATGGAAAAGTTG
CTCCAGGTTGGGGCACAACTCCTATTATGGGAATTTTCATGTTACTATTTTTCTTGTTTT
TATTAATCATTTTACAAATCTATAATTCTTCATTAGTATTAGAAAATGTAGATGTAGATT

GGGCTACTTTAGGTAGCTAACTAGATAAGTTCTTTGATTACACAATATTAAAGACAACAA
GCGATCGCTATTAGATTATAGTAGCGATCGCTTTAAAAGTATTTTACTATATATCCAGAG
AAAATTATTTACCTAAATTAATTAACTCGGTATCTGCAAAACTATTTGTGTTAACATTAT
TATAGTTAACTTTTTCAAACCTTACTAATACTGGATATTTGATACCACTTTTATCAATTG
CTGCAACAGTACCAATTTCCTGATACCAGTACGATTCTTTTCTCAATATTTTTACTTTTG
AGCCTCTTTCCATATTTTTAGTCCTCAGATAGATTATTAAAGATTATTATATTAAAAAAT
TAAAAAAAACTAAACAAAATATATAAAAATACTACCGACAATTGTAATATATAACTAATA
AAAGGAGAGAGAGGGATTCGAACCCTCGATAACAAAAGTTATGACAATTTTCGAAATTGT
TGCAATAAACCACTCTGCCATCTCTCCTGAATTAAAATACTGTTTTAAAAAATTAGACAA
GAATAACATAGAACTAAGGTAGTTGCCTAGAAAATTAAAACTATGTTAAATATAAATTAT
AATCTTTCTATAGATATATTTAAAAAATATGTACATAGAATCTATATCAGCTTACTTCAA
GATTTTTTAATCTTATACAATTTTTACTAAATAAATAAATTCTCTCATAATATGAAACTA
TCTTGGAAAACACTTCTTCTTTGGTCTTTACCAATATTTGTAATTGGTTTCTTTTTCTGG
CAAGGTTTTTTAGGTCCAACTACTACAGATGTTGGCAGTAATATCGCAAGTTCTAGAATG
ACATATGGACGATTTTTAGAATATTTAGATATGGGTTGGGTGAAACGGGTTGACCTCTAT
GAAAATAATCATACAGCAATTGTAGAAGCTGTTGGGCCAGAATTAGGAAACAGGGTTCAA
CGAATTCGAGTTGAACTGCCAGCAAGTGCGCCAGAATTAATTACAAAATTACGCAAAGCC
AATGTTGATCTAGATGCTCACCCCCCTAAAAGTACAAGTGCAGTATGGGGACTATTAGGC
AATTTACTATTTCCTTTACTATTAGTTGGCGGGTTAGCTTTCTTATTTAGAAGATCTAAT
AATGCTAGTGGTGGACCTGGTCAAGCAATGTCATTTGGCAAATCGAAAGCTTTATTTCAA
ATGGAGGCTAAAACGGGAGTAGTATTTAATGATGTAGCTGGAGTTGAAGAGGCAAAGGAA
GAATTTCAAGAAGTGGTAACATTTTTAAAACAGCCTGAATCATTTACTGCTGTTGGTGCA
AAAATACCAAAAGGCGTTTTATTAGTTGGACCTCCTGGCACAGGCAAAACATTACTAGCA
AAAGCTATTGCTGGCGAGGCTAGTGTTCCTTTTTTTAGTATCTCAGGCTCAGAATTTGTA
GAAATGTTTGTTGGTGTTGGCGCTTCTCGTGTGAGAGACCTATTCAAGAAAGCAAAAGAC
AATGCGCCTTGTATCGTTTTTATTGATGAAATTGATGCTGTTGGTAGACAACGAGGAACA
GGTGTTGGAGGTGGTAATGATGAAAGGGAACAAACATTAAATCAACTATTGACTGAAATG
GATGGCTTTGAAGGAAATACTGGTGTTATTGTAATTGCCGCTACTAACAGAGCTGATATT
TTAGATTCTGCATTATTAAGACCTGGAAGATTTGATAGACAAGTTTCTGTAGATGTACCA
GATTTTAAAGGCAGGTTAGCAATTCTTGAAGTTCATGCTAAAAATAAGAAAATGGAACCT
AAAGTATCTTTAGAAACCATTGCCAGAAGAACTCCCGGCTTTTCAGGAGCTGATTTAGCT
AACTTACTAAATGAGGCTGCTATCTTAACGGCTCGACGAAGAAAAAATGCAATGACTATG
TCTGAAATTGATACATCAATTGATCGAGTAGTAGCTGGGATGGAAGGCACTCCTTTAATT
GACAGTAAAAGCAAAAGATTAATTGCGTATCACGAAGTGGGTCACGCAATAATAGGCAGT
TTATTAGAGCATCATGATCCTGTGCAAAAAGTTACATTAATACCAAGAGGGCAAGCAAGA
GGCTTAACTTGGTTTACTCCTAGTGATGATCAAAGTCTAATATCAAGATCTCAAATACTA

GCCCGTATCGTAGGTGCTCTTGGTGGCAGAGCTGCAGAAGAAATCATTTTCGGTGACGCA
GAAGTTACTACTGGTGCAAGTAATGATTTACAGCAAGTAACATCAATGGCAAGACAAATG
GTCACTCGATTTGGAATGTCTAAAATTGGACCTTTATCTCTTGAAAGCCAAGGAGGAGAC
CCATTTTTAGGTAGAGGCATGGGAGGAGGCTCAGAATATTCAGATGAAGTTGCAACTAAT
ATTGATAAGCAAGTAAGGGAAATTGTCAGTGAATGCTATGCACAAGCTAAACACATTATT
ATAGATAATCGAGTAGTGATAGATAGATTAGTTGATTTACTAATTGAAAAAGAAACAATT
GAAGGCAATGAATTTAGAGACATCGTTAAGGAATACACTGCAATTCCTGAAAAAAATTAC
TACATATCACAATTTTAAATTAAACGGGACTGACGGGATTCGAACCCGCAACTTCCGCCG
TGACAGGGCGGTGCTCTAACCAGTTGAACTACAGTCCCAAAAAGATGAATACCCAGAGAT
AATCTCATAGTTAGCTTAAACTTGTCAAATTAGAATATTAAATATGAAGAGTATTTGTAA
TAATACTCTTCATATTTGATATAGTAGCTAAAATATTTCTTTTAGAAGGAGAGAGAGGGA
TTCGAACCCTCGGTACGAAGTTAATCGTACAGCAGATTAGCAATCTGCCGCTTTCGACCA
CTCAGCCACCTCTCCATGATATATATTATATATATATCAAATATACTTATATTTTAATAG
GTGTGCATAAATATTTATGGAGCTAAGCGGATTCGAACCGCTGACCCTCTCAATGCCATT
GAGATGCTCTACCAACTGAGCTATAACCCCTTTGGTAAGATATTAAGAAATGGCTCAAGC
GGGATTTGAACCTGCGACCTTGGGCTTATGAATCCCCTGCTCTAACCACTGAGCTACTGA
GCCATAGTATAAAATACTATTAAATATTATACCATAAATTATGGTCAAATATAAATCAAT
AAAAGAATAATTACTATCATTTACTACTTTTGTGCAAGTTGTAAATATTCGAATAAAATT
TCAAAATTAAAAATGTCTTTAACTTTTTGTTAGAATTATTGATTACGAAAATATTGATAT
TTAGAATTAACAATAAATTTTGTCTTTATTTGTAGACACAAAAATATTGGAAATTTTCAT
CAATTAATTAGTTAAACTATAACCGACGTACAAGAATTATTGAACTACGTTAAATTGTGA
TGCATTGGGCTTGATCAAAACTTCTCTAAAATCAATAGAATATAGTAATTCAAGTAAATC
AAATACTAACAAATGTGACAAACGCTTAAAAAAATAATACAATTAGAATCGTAAAAATTT
GGTTTTTGACTGTGTTAAATAAATTTGATTATCATAGATTTAATATTTAAAAGTGTTCTA
TTACTTGTATTGAGTAATTGTAATATTTAATATCTTCTCTTTTAGATAATAATCATAATA
TAAGCTAAAAAATAGTTTTTATTAAACAACTAGCATAGAGCCAATACCTTGATCAGTTAA
TATTTCTAGCAAGAGAGCATGATCTATTCTACCATCTAAAATATGCGCTGAAGCTACACC
CTGAGCTAAAGAACGAATACAACAATTCACTTTAGGAATCATGCCACCAGAAATTACTGC
TGTTTGAGTTAAATCTCTAGCTTCCTGTATATTCAGATGACTAATTAATGTTGAAGGATC
TGAAGAATTACGTAAAATGCCGGGCGTATCTGTGAGTAGAATTAGTTTTTCGGCATTAAG
AGCGGCTGCTATTTCTCCTGCGACAGTATCAGCATTAATATTATACGATTGGCCTTCCTT
GTCTGCTGCAACACTAGCTATAACAGGTATATAATTATTATTAATTAATATCTTCAGTAA
TTTAGTATCAATATTTTGTACTTCTCCAACAAAGCCAAGGTTTGCTTTTCCATTTGGTCT
CGGAGTAATAAGTAATCCATCTTTTCCCGACAAACCTACACTTTTACCACCTTGTTTATT
AATAGTTGCGACAAGATCTTTATTGACTCTTCCAACTAAAACCATTTCCACTATATCCAT
GGTAGGTTGATCTGTTACTCTAACGCCATCATCGAATTTTGGTAATATTTTGAGGCGATC

TAACCAAAAATTAATTTCTGGTCCTCCACCATGAACTAAAATGGGACGTAGTCCTATAAA
AGACAGAAAAACAAGATCACTAATCACTTGATCTTTTAGTTTCTGATTTTTCATAGCCGC
TCCCCCATATTTTATAACAATAATTCTGGAAGAAAATTGCTGAATATAAGGCAGAGCTTC
ACTTAAGACTTTTACTCTTTCTGAATTAGTCAACATAAATAAAGTAGGATTGTGATATAT
AAAATTATATACACAGCTTGTGTTAATTTAAAAGATACCAATGGGTACTTGAAAAAATAG
AAATATTACTAACAGTTAAAACTATAGGGATTATAATTATGAATAAATTTTGGGATAATG
TATTAAGATTTCCACGATTTTTAGTCAGCGTCATTTTAGGATTAATTTTGATAATTATTA
GTCCTTTTTTCGTGTTATTAAAAAAACCACTGACAAGTTTTTTTTTCATCATATCATTGG
CCGGATTAATCACAGTTTTGGCAATAATAATACAAAAAATGATAAATATCGAATGTTGTT
GAGAGAAATAAATAAATTTAATTAAAATTTTTGAACATATATAATATTATATTATCATTA
AATATGATAATTTATTTCCTTTTCTATAAAACCCATAAATATTAAATATTATTTGATAAA
TATTATGCCACTAAAACAAAGAGTTAGCTCTGAAAAGACCGGTGCCTTCGCACTTTTGGA
TAGTATTGTTAGGCACGGAGTTAAACATATATTTGGTTATCCAGGTGGAGCTATTCTTCC
TATTTATGATGAGCTTTATGCTTGGGAAGAAGCCTCTCTAATTAAACATATCCTTGTTCG
TCATGAGCAAGGCGCTGCTCATGCTGCAGATTCTTATTCTAGATCAACAGGAGAGGTTGG
AGTATGCTTCGCTACTTCTGGCCCAGGAGCAACTAATCTTGTCTCAGGTATAGCTACAGC
ACATATTGATTCTGTACCTATATTAGCTATAACAGGTCAAGTTGGGAGAGCTTTTATTGG
TACAGATGCTTTCCAAGAAGTAGATATTTTTGGGATTACACTTCCTATTGTAAAACATTC
ATATGTAGTTCGTGACCCTAGAGACATGTCTAGAATTGTTGCGGAAGCATTTTTTATTTG
TAAACACGGTAGACCAGGTCCAGTATTAATTGATGTTCCTAAAGATGTAGGATTAGAGAA
GTTTAATTATTTTTCTGTTGAGCCCGGAAAAGTTAATATTCCTGGCTGTAGGCCAATTAC
CAGCCTAAAGTCAAGACAAATCCTTATGGCAGCTAAAATGATACAGCAATCTAGCCAGCC
ATTGTTGTATATTGGTGGAGGAGCCATAATCTCTGATTCACATCAAATTATTAAAGAACT
TGTTGATTTTTATAAAATACCTGTTACTACTACTTTGATGGGGAAGGGGATTTTTAATGA
GGATAGCGACTATTGTCTAGGGATGTTAGGTATGCATGGTACTGCGTATGCTAATTTTGC
AGTTAGTGAGTGCGATCTTTTAATTGCTTTAGGAGCTAGATTTGATGATAGAGTTACTGG
AAAATTAGATGAATTTGCTTGTAATGCCCAAGTGATTCACGTAGATATTGATCCTGCTGA
AGTAGGAAAAAATAGGATTCCTCAAGTTGCTATTGTCGGTGACGTAGCAGAAGTTGTTAG
TGAAATATTGAATTTATTAAAGACTTCTTTCCCCCCTTATCCAGAGCAGATTATATCTTG
GCAAGAAAGAATTAATCGTTGGCGTCAACAGTATCCTTTACTGGTTCCTAGAAAATCAAC
AAGCATTTCACCTCAAGAGATTCTTGTTGCAACTAATAAATTAGCCCAAAATGCTTATTT
TACTACAGATGTTGGCCAGCATCAAATGTGGTCAGCTCAATTTCTGAAAGTAAAAGCTAA
GCATTGGCTTTCAAGTGCTGGATTAGGCACGATGGGTTATGGTTTACCTGCAGCAATTGG
CGCTCAAGTAGCACATCCAAATGACGTAGTCATTTGTATTAGTGGTGATTCTAGTTTTCA
AATGAATATGCAAGAGTTAGGAACTATCGCGCAATACCAGTTACCAGTTAAAATTATTAT
TATTAATAATCGATGGCAAGGGATGGTTAGACAATGGCAACAAGCTTTCTATGGTGAAAG

GTATTCACACTCAAGAATGACAGAAGGAGCACCTGATTTTCAAAAGCTTGCAGAAGCTTT
TGGTATTAAAGCTTTTACTATTAATAATAGGCAAAATATGCAATCTGCTTTACAAGTTGC
TATTGATTATCCTGGTCCAGTTTTATTAGATTGCCAAGTTACAGAAAATGAAAACTGTTA
TCCAATGGTTGCTCCTGGAAAAAGTAATGCACAAATGATAGGTATCGCTAAACCGCAGAG
AGGTACTGCTTCCAACTATATTAATAATAGTGTTTAAAGTAAATAGTTAGTATTTTTTAA
ACAAAGAATAAAGTACATAAATGTAATTTTTTTATACTATTAAAAACTATGTAAGAACAG
CCTTTGACGAGAATTGAACTCGTGACCTTCCCCTTACCAAGGGGATGCTCTACCTCTGAG
CCACAAAGGCTTTTTACTATATTGGGCCGGGTTGGATTTGAACCAACGTAGGCGAAGCCA
GCGGATTTACAGTCCGCCCCCATTAACCACTCGGGCACCGACCCTAATTAAACCTTATTC
AAGATATGACCTATAAGATATCATAACTATGTTGCTAAAAACAAGTACTTTGATAAATAA
AACTGAATAAGTAATGGACATAGCTGGACTTGAACCAGCGACTTTCACGATGTCGACGTG
ACACTCTAACCAACTGAGTTACATGTCCAAAACTTTAATTATTATTATAACACTGTTTAG
TGATTTTATAAGTATCTATTTCTAATTGAAGATTTGTTTTAATCGTCCGGCTTTACCGAG
CAGATCTCTTAGATAATACAGTTTAGCTCTACGTATTTTAGCTCTTCTAATCACTTTGAC
AAAAGTTATTCGCGGTGAATTGAGAAAAAATACTCTTTCAACTCCAATACCTTGAAATGA
GCATCTTAAAGTTAAACTAGTATTTAAGCTTTTTTTTCTTCTTCTCGCTAATACTACTCC
TTCACATAATTGTTCCCGAGTTTTACTACCTTCCTTAACCATTAGTCCAAGTTGTATTGT
ATCGCCAACTTTAATTTCAGGTACTTCTGTTTTGATAAAAGGTATCTCAACACTTTTCAT
TAGCTGACTTAGCTTTGTACTATTTGCTTTCATATATAGGATTAGTCTTGTGACATGTTT
TGAAATATTATACATTAAAAAAACAAATATGAAAAATTTTAGCGAATAAGCATACTAGTA
TTTATAAAACTCTTTTTATCTCCATTATACAGATCCATTATATTCAACTAGATTCTTTTA
TAGTTTCTTTGATAATCAGTACATCTAGATGATCAAATATTATTTTCAAACATGGCTTAT
GTATGAAAAAGTTCTATAAAAACCTAATGACTAAAATTAGGGCTTAAGTATTTCTTAGTT
TTGTGTTATAATTAGTTACTATCAAAGGTAGTGGCATAACACGAAGCATCAAAATGTTCG
AACGCTTTACTGAAAAAGCTATAAAAGTCATAATGCTAGCACAGGAAGAAGCTAGACGCT
TAGGACATAATTTTGTCGGAACTGAGCAGATACTATTAGGATTAGTTGGTGAAGGTACTG
GAATCGCAGCTCAAGTTTTGAAATCGATGAATGTGAATCTAAAAGATGCAAGAGTTGAAG
TCGAAAAAATTATAGGAAGAGGGTCTGGTTTTGTAGCGGTTGAAATTCCTTTCACTCCTC
GAGCAAAAAGAGTACTAGAATTATCTTTAGAGGAAGCACGTCAACTAGGCCATAACTATA
TTGGCACAGAACACTTGCTAATGGGCTTAGTCCGAGAAGGAGAAGGAGTTGCGGCAAGAG
TTTTAGAAAATCTGGCAGTCGATGTTTCTTCAATTAGAGCTGAAGTTATACAAATGCTCG
GAGAAAATGCGGAAGCCAATGTAAGTGGAAGCAATACTACGCAAGCTAGAAGTAAAACAC
CAACATTAGAAGAGTTCGGATCTAACTTAACTCAAATGGCTATGGAAGGTGGTTTAGATC
CTGTAGTCGGAAGACAAAAGGAAATAGAACGAGTTATTCAAATCTTAGGTAGAAGAACTA
AAAATAATCCTGTCTTAATTGGGGAGCCTGGTGTAGGTAAGACAGCGATTGCGGAAGGAT
TAGCTCAAAGAATTGCTAATAGAGATGTACCTTCTATTTTAGAAGATAAATTAGTTATTA

CTCTTGATGTAGGTCTATTAGTAGCCGGAACTAAATATAGAGGTGAATTTGAAGAAAGGC
TAAAACGTATTATGGATGAGATTAAATCAGCTGATAATGTAATATTAGTGATTGATGAAG
TTCATACATTGATTGGGGCTGGTGCTGCAGAAGGAGCAATAGATGCAGCTAATCTGCTTA
AGCCAGCTTTAGCAAGGGGAGAATTGCAATGTATAGGTGCAACAACTTTAGAAGAATATA
GAAAACATATAGAAAAAGATCCAGCATTAGAAAGAAGATTTCAACCAGTTGTAGTTGGAG
AGCCAAGTGTTGAAGAAACAATTGAAATTTTGTTTGGTCTTAGAGACCGTTATGAAAAGC
ACCATCAATTAACAATGTCAGATGGAGCTTTGGCTGCAGCTGCTAAATACGCTAATCAGT
ATATTTCTGACCGATTTTTGCCAGATAAAGCAATTGATTTAATTGATGAAGCTGGTTCTA
GAGTTCGTTTACTAAATTCTCAATTACCTCCTGCTGCCAGAGAATTAGATAAAGAGTTAA
GAGCTGTATTAAAAACAAAAGATGAAGCTATTAGAGCTCAAAAATATGAAACAGCAGAGC
AGTATAGAGCAAGAGAAATGGAAATTAAAGCTCAAATTGCAGCAATTGCTCAAAGTAAAA
AGAATGAGCCTGATTTAAATTTAGAAGATCCTGTTGTTACAGAAGATGATATTGCTGAAA
TTGTTGCTGCATGGACTGGTATACCAGTAACTAAGCTTACTAAAAGTGAGTCAGAAAAAT
TAATGCACATGGAAGAAACTTTGCATGGACGTATTATTGGTCAAGACGAAGCGGTTGTAG
CTGTCTCTAGAGCGATAAGACGCGCAAGAGTAGGTCTAAAAAATCCTAACAGACCAATTG
CAAGCTTTATTTTTTCCGGACCGACGGGTGTAGGAAAAACAGAATTAACAAAAGCTTTGG
CTTCTTATTTCTTTGGTTCAGAAGCTTCTATGATACGGCTAGATATGTCAGAATACATGG
AAAGACACACTGTATCTAAACTAATTGGTTCTCCTCCAGGATATGTGGGTTATAGTGAAG
GTGGTTATCTAACAGAAGCGGTAAGAAAAAAACCATATACTGTCATCTTATTTGACGAAA
TTGAAAAAGCTCATCCGGATATTTTTAATCTACTTCTTCAAATTTTAGAAGATGGCCGAC
TAACAGATGCAAAAGGTAGAACTATTGATTTTAAGAATACTCTTTTAATTATGACTTCTA
ATATTGGATCTAAAGTTATTGAAAAAGGAGGAGGTAGTTTAGGCTTTGAATTATCAGAAG
ATCAAACAGAATCCCAATATACTAGAGTACGATCTTTAGTAAATGAAGAACTGAAACAAT
ACTTTAGACCAGAGTTTCTAAATCGATTAGATGAAATTATTGTATTTCGTCAGCTTACTA
AAGATGAAGTTAGAGAAATTGCAGAATTAATGCTTAATGAAGTCTTTGCGAGAATTAAGC
AGCAAGATATTCAATTAAATGTAACAGAACGATTTAAGCAACGATTAGTAGAAGAAGGAT
ATAATCCAAGCTATGGAGCTAGACCACTTAGACGAGCAGTAATGAGGCTTTTAGAAGATA
GTCTGGCTGAAGAAGTTTTATCTGGTAAAATTAAAGCTGGTGATAGTGCAGTAGTAGATG
TTACTAATGAAGGAGAAGTTACAGTTTTATTAGGTGAAAAATTAGAACTGTTAACATAAA
AAACAATTTATTAATTAAAGCATTGAAGAGGTTATATTCAATGCTATGAAAAAAATTAAT
AAGATGGGTTGCTATAATTGACAACCTCTGATACTCTTTATTTGTATATAAAGCCGGGAT
AGCTCAGTTGGTAGAGCAGTGGATTGAAAATCCTCGTGTCACCAGTTCAAATCTGGTTCT
TGGCATTTTAAGCATCAGAAAATTTTGCTATCTGAGCATTAAATTTTAATTGAAATGTTC
CTGTCGGACCATTCCTGTGTTTTGCTATTATAACTTCTGTGAAGTCTCTACTTCTAGTTT
CTTGTGTATAGTAACTTTCTCTATATAGCATTATTACTAAATCTGCATCTTGTTCTATAG
AATTATGAACAATAAAGTTGTTTGAAATAAAATTGCATAAAGGTTTACATTCTAAATCAA

ACACTATTTGTAAACTTGTAACGTTTATTTTTTGTATACTTTCAAAAGTCAAAGATGAAA
AAGAATTAAAAACATTTTTTTGTTCTTCAAAGTTTTTTATTTGAATAGCAATCATGTCAT
TTTGATTAATTTTATCACATCTTTTCCATCCTTGTGTTGTGAGTAGTTTATGGTTACTTG
TTAATTGTATATATTTCCCAGCTTCGGTAATAATTTTATATACAGTTTTTTTCCCTGTTT
TCGAAATACTACATTTAATTGCTAGAAATAACTGTTGTTTTTGCGCACTAAAGTTAATGA
CTTCAATCTTTTTATAATAGCAATTAAATAATATTTGAAGTTGATTATATAATGATGTCT
GAAGATAATTGAATTTGGATATACACCCACTTTCTCTTAAATCGGATAACAAAGGCCTTT
TGTCACTCCGAGTTTCAAGGTTTCTATTTAGTTGAGATAATGCCAAGATAGGCAAGCTTA
AATCTTTTGCTAATATTTTAAGTGATCGAGTAATTAATGATAGTTCTTGAGATCTATTGT
CAGATTGTCTACTCTCCTGCAGCAATTGAAGATAATCTATAATAATTAATTCTATATTTT
TTCCTTGTAATTTAAAGAATTTTACTTTTGTCTTTATAGTGTCAGTAGAAATCTTAGCGC
TATCATCAATATAAAGATTAAGGTTAGCAAGAGTTTTACTTTTTTGAATAACATATTGCC
ATTCATCATTATTAAGCTGACCAGATTGAATTTTTTGGCCGTTTAAATTACACTCTTGAG
CTAAAATTCTTCTTAGTAGTTGCTCTGTAGACATCTCTAAGCTAAATAAAATAACATAAG
ATCTTTTCTGATTAATTACATATCTAGTTATATTAATAGCAAAAGCAGTTTTCCCCATTG
AAGGTCTTCCTGCAAGAATAATTAAATCTGACTTTTGAAACCCTTGTGTTATAGAGTCGA
GTTCTGTAAAACCAGAAAGTATACTACTATTTATACTAATCTTTTTCCTTGTATCCAAAT
GGACTAATAGGTTTGCAAGAATTGTAGCTAATGTTTGCGTATCTTTTTCGTCAAGTATTT
CATAAGCTTTTGTTAATTGACTAACAATAGAAGTTATGTTTTTTTGTTCAATAAGTTGTT
TTGAGCAGCTAATTAAACATAATGAATCTCCACATGCTAAAAGTAATCTTTTAATGTAGT
TATCTATAACGACTGCTGAATATTCGCTTATAGTATCAGATAGGGGAGCTTGTTCTATTA
GACTAAAAACTTCATTTAGCTCATTCAAATCTTGAATCATTTTTTCATTTTTTAAGTTAA
TGAAAAAATTTCTTACGCTTTTTGTCTTTGCATGGTTAACATTTTCGAGTGCTGCTCTAT
AAAGTAATGAAGTTGATGTAAAATAAAAAAAATCAGGAGAGATTTTTTCTACTGATTTTA
GTAATGATATCGATTGCTGAGTTAATATTATACTAATTAATATTTTTTCAGCTAAAATAT
TATGAGGAGGTAGATATTTATAAATACTTATTACTTCCTCCTTGTAACATTACTCTTGTG
GTATTAGTTAACTATTGATTTAATTTGATTCTGGAAGAACTTGCAATTTAATATTTGCAG
TTACTTGATGTAATAGCTTAATTTCTACATCGTAAATGCCAATTGTTTTCACGTCTGGTA
AAGAGATACTTTGTTTTTCAATGTCTATGTTAGTAGTGTTTTTAATAATTTGTGAAATTT
CTTTTTCTGTGACACTACCAAAAATATTGTGGCCATCTCCTGTTTTTTTACTAACGCTAA
ACCTTTGTATTTCTTCTAGAAGTTGCTTGACTTTTTTAGCATTTTCTTTAGCTGTTTTTA
ATTTTTCTTCTCTTATAGCTGCATAAAATTTTTGTTGTTTTAAAATACCATTTGTGGCAA
CTGCCGCCATTTTATTCGGGATTAAAAAATTTCTTGCATAACCAGAAGCTACTTTGATAA
CGTCATTACTCTTGCCAAGTTTTTGAATATTCTCTTTTAGCACAACATTTATAACTTTTT
TACTCATCTTAATTCTAAAAAATCTGTATTATCCTATTATACCTGTTGTATACAGATTTT
AATAGTAAGTATTTATTTGTTGATGTAATATTCTTGTAAAAAAAGGTAATTTTTTATTTT

TTTCTGATATAATCACTTTCACAATAATTCATTAGGAGAGGTGGCCGAGTGGTTGAAGGC
GCAGCATTGGAAATGCTGTTTAGGAGCAATCTTAACGAGGGTTCGAATCCCTCTCTCTCC
GTACAAAAAATATAAAAAGTTTTATAGAGTCCAATCTAAATCTCCAATAATATTCTTCCA
AGCATTTAATCCACCCCGAACTCTAACAATATTTAGTTTTTGAGCTATTAAAAACTTAGA
AGCAAATATTGATCTAGAATCTAAGCTACAGTAAACAAAACAAATTTTATCTTGTAAATT
GATATGAGAGTAATTCATTCCTTTTATTTTTTGTAAAGGTAGATTTAATGAATGTATTAA
ATGACTTTTATGATATTCTTCATGATTTCTTACATCTATTAAAATATATTGTGGATTCCT
ACTAATTAAAAACTTTTGTAATTGAATAACACTAATTTCTCGTACAAATGTACTAGATTT
ACTGCCATAATATTTGTTCCAATGCTTTTTTGTGGATAATACAAATTTAGTATTTATAAT
CTTAAATTTGTTAAATGATGAAGTTAATGAGTTGTACGTTAATATAGTTCCACTTAATAT
AGACTTATAACCCAGAACGATTTTGACAGCTTCAGTTGCTTGAAGTGTACCAACAATACC
CGGTAATAGACCTAAAACTCCGGAATTACTACAAGTATCTCTTGCGCTCTCTTTATTTTC
AGTTTCACTGTAGAAGTCTCGATAAACAGGTCCACCTTGATAATTAAATACACTAACTTG
TCCTTCAAATTGAAAAATGGCACCATAAATATGTATTTTATTTAACTCTAGGCAAGTATC
ATTCAAGAGATATCGAGTATCGAAGTTATCAGATCCATCTATAATAATATCATATTGTCT
AATAATGTCGATTGAATTCTCATAGCTTAGTCTTGTTTTGAACACAGTTACTATACACTG
TGGATTTAAATCTAATATTTTTTTTTAGCTATTTCTACTTTTGAATATCCTATATCATGC
ATCGTGTATAAAATTTGCCTCTGTAAATTAGAGAGATCAATTATGTCATCGTCTATAATT
CCAATGCTGCCAATTCCTGCAGCAGCAAGGTAAATGATTCCTGGAGATCCTAGCCCACCA
GCCCCAATGAATAAAACTTTTGCTTCTTTTAATCTTTCTTGTCCTTCTAATTGAATTTGT
GGCAATACTAAATGCTTAGAATATCTTGTATATTCTTCTAGTGAGTATTTTGTATTTTCT
GTTTTAAAATTTAGCATAAAAAATTTATAGTTTATTTAATAGCACTTAAAAGCTAGCTGAT
AAGTTTATTAAAATGACTTTTGTAAACTTGGTTCTTCTCTATCTAAAATAGCTCCTTGTG
TAGGACAGACCTGAACACAAATACTACAATCAATACAGGCAGAAAAGTCAATCCAATACC
AATTAGTGTTATTATTGTTTTTTCCCTTTCCTTGATGAATACAAGCTACGGGGCAAGCTT
CCACGCATTCAGAAACTCCAATACATTTTTCTGTTACAATTGTATGAGACATATTATTTA
ATTAATAAAAGATAAGCTATTTACATCTAGTAGTTTTGTTGTATACTAAATTTACATATG
CTATCCAGGCCAATATTTAAGGTAGTCTATTTCTACAGTTTACTAATTTGATATGCGCCT
TTAGTTCAGTTGGTAGAACGCAGGTCTCCAAAACCTGATGTCGAGGGTTCAAGTCCTTCA
GGGCGCGTTTAGTTTTATCGTAATTCAATAATATCATTTTATCTTTTTGCAAACATCAAA
ATGATATGATATAAAAGATCATATTTTATACATTGAATATTCTATGTACTAGTACTGTTT
TTTAATATGTTATTATTGATAAGTTAATTATACTAATTCTATTTTTGTAATAAGTAAAAA
CAGTTCTAATTAAGTATGCACGTATTTAATAGTCTTTTGTATATATAACTTGAAAAAATT
TAAACTATGGCTAAAAAAGTTACTGGAATTGTTAAACTAGCATTAAATGCAGGTAAAGCT
ACTCCTGCGCCACCTGTCGGACCTGCTTTAGGACAGCACGGCGTGAATATTGTTATGTTC
TGCAAAGAGTACAATGCACGTACTGCTGACAAGTCAGGACTAGTAATCCCTGTAGAGATC

TCAATATATGAAGATAGAAGTTTTACTTTTATACTTAAAACTCCTCCTGCTTCTGTACTA
ATCGCTAAAGCTGCTGGACTAAATAAAGGTTCGGGTGAACCAAATACCAAAAAAGTAGGC
AGCATAACAAATAAACAGTTAGAATCTATTGCAGAAACAAAACTTCCCGATTTGAACACT
AACAATATACCTCAAGCTATGAAAATAGTTGGAGGTACGGCTAAAAATATGGGAATTCTA
ATTAAAGATTAAAAGTTTCAATTATATTTACTATTTTTATGAAAAAATTTTCACGTCGAC
TTACAACATTAAAATCAAAAGTTGAGCCTAAACTTTACACTATTAATGAAGCAGTATCTA
TATTAAAAGCAACGTCAAATGCTAAATTTAAGGAAACTGCAGAAGCTCATATTGCTCTAG
GTTTAAATCCTAAATATGCAGACCAGCAACTCAGAGCAACAGTTATATTACCGAAAGGTA
CAGGTAAATTGATAAAAGTAGCAGTTATTGCTAAGGGAGAAAAATTAACAGAAGCAATTA
GTGCGGGAGCTGATGTCAGTGGCTCCGAAGAACTAATTGATGAAATACTGAAAGGTAGAT
TAGATTTTGATAAGCTGATAGCAACACCAGATGTTATGCCTTTAATAGCAAAGCTGGGAC
GAGTATTAGGCCCTCGAGGGTTAATGCCTTCTCCTAAAGCAGGCACGGTAACATTGGACG
TAGCAAAAGCCGTGAATGAATTTAAAGGGGGTAAAGTTGAATACAGAGTTGATAGAACAG
GTATAATTCATGTACCTTTTGGAAAATCTAGTTTTTCACAAGAAGATCTAGTTTTGAACC
TCCAAACAATTAAAGAATCGATTGATAGAAACAAGCCTTCTGGAGCAAAAGGGAAGTACT
GGAAAACTTTTTTCTTATCTAGCACCATGGGGCCATCTATTCAAATTGACATCACTAGCC
TTCTATGAAAAATTTTTGTATAATTATAGAACTTGAATAATCTATTATTTCAAATTATAA
TTTGCTAGCATTATTAACGTAACAACTTGCTTACTATATCTTATCTGCATTAAAAACTTT
TTATATATCTTATTAATTATGAGTACAAAGGTTGAAAATATCTTGGAAGAGCTAAAATCT
TTAAACCTTCTAGAGGCTGCTGAACTAGTTAAACAAATAGAAGAAACGTTTGATGTTGAT
GCATCTGCGGCTTCAGGAGGTATGATGATGGCCACACCAACTTCAGCACCAGCGTCTGCT
GAGGTTGAAGAGAAGACAGAATTTGATGTTGTCCTAGAGGAAGTCCCAGCACCTAAAAAG
ATTTCTGTATTAAAAGTTGTTCGCTCTCTGACTGGTTTGGGTTTAAAAGAAGCAAAAGAT
CTTGTTGAATCTACTCCAAAAGTCTTAAAAGAAGGTGCTTCTAAAGATGATGCAGAAACT
ATGAAAAAACAGCTAGAAGATGCTGGCGCAACAGTTATTGTTAAGTAATGAAGAAGCGCT
TCTGTTTAAGTATAAACAGAAGTGCCTCTTTATTTATTATAAGCAATTATACTAGAAAAG
TCCAAGTGTAATTGCATTGTTGATAGGCATAGTTGCACCAATACCTAGCCAAATACTTAC
AACGGTACCAATTAAGAAAACAGTGGTTGCAATTGGTCTTCTAAATGGATTTTGAAATTT
GTTAACATTTTCAATGAATGGAACAGTAAGTAAACCAGCTGGTACAGCAGCCATACTTAG
AACACCTAATAGTTTATTAGGAATCACTCTTAATAGATTAAATGTTGGAAAGAAGTACCA
TTCTGGTAAAATCTCTAATGGAGTCGCAAATGGATTAGACTTTTCACCTATAGAAGATGG
TTCTAAAATTGCGAGTCCAATACTGCAGGCAAAAGTGCCAATAATTACAACTGGAAAGAC
GTATAATAGATCATTTGGCCAAGCTGGCTCTCCGTAATAATTATGGCCCATTCCTTTTGC
TAATTTAGCTCTCAGCTTCGGATCTGTTAAATCTGGTTTTTTAAGAATTGACATATTGTT
TTTGTGTTTAATATAAAGATTAGTCTAATTACTGTTTTAAAGTGTAATTTTCAACTATTA
TAATGGTCCAGAAATTCCTTGTTTACGTATCATTAAAAAATGCATAAGCATGAAAACGGC

AGTTAGTAGCGGAAGTACAAAAGTGTGTAAACTATAGAACCTAGTTAAAGTTCCTTGGCC

TACACTAACTCCTCCTCTTAACAATTCTACTATACTCGCTCCGACAACTGGAACAGCATC

AGGGACACCTGTAACAATTTTTACAGCCCAGTATCCGATTTGATCCCATGGCAAAGAATA

ACCTGTTACCCCAAAAGAAACAGTAAGCACTCCTAAAATTACACCTGTTACCCATGTCAA

TTCTCTAGGTTTTTTGAAACCTCCTGTTAAATAAACACGGAATACATGTAGAATCATCAT

CAAGACCATCATGCTCGCTGACCATCTATGAATTGATCTAATAAGCCATCCAAAGTTTAC

ATCAGTCATAATGTACTCTACTGATGTAAAAGCTTCTGCAACTGTAGGTCTATAATAGAA

TGTCATTGCAAATCCAGTTGCAACTTGAATTAGAAAAGATACAAATACAATACCCCCTAA

GCAATAAAAAATATTAACATGAGGTGGTACATACTTACTAGAAATATCGTCAGCAATTGC

TTGAATTTCTAATCTCTCTTCAAACCAGTCATAAATTTTACTCATAAAATAGCTTCAAAA

AGCTCTTCTAATATATTTTGCGTTTAAGCTACTATTAATTCAATAGTTATTGAACTTATA

TTTTTTTATAATCAATACTTATTAACTATTATAACATCTTTTTATTGATTTAAAATTCTC

TGACTTAGTAAAACAGGGCTATGTACTATAATTCTTTTTTTTTGTATAGAGATTAATTTC

GTTTTTAGTAAATTAGCTAAAATTCTTGTTACACTGACTCTATTACTACCTATAATCTGT

GCTAAAATTTTATGTGTAATAGTAAAGTTAAGCATAATACCATTATTAACTTGAGTTCCA

TTTTGCTCAGCTAATAATAATAATAAGCTCGCCAGTCTACTGGTTATACTTTTATGTGAA

ATGATTTCTATAAAATGATAAGCTTTTGCCGAGCAAAATACTAAATGATTTACAAAAAAT

AAATTGAATGGAGCGCAGTTACTACATGCTTTTATAATAGTAGTATAGTTAATACAGGCA

ACTTCTGTTTTATCTATTGCTTCTGCTTCGTAGTAAAAATTATCGTCGACCAATTCTATT

TGTCCAAATGTATCTCCTGTAGTTAATAAGTTAAGAGTTACTTTATGTGTATTTCTCAGG

ACTTTTGTAATAATTAAAGATCCTATCAATATAATGTATAATCTAGAGCTGCTATTAAAC

AAAAGAGTATCATTTTTCTTAAGTGAAAAAATTTGATAATTAATTTTATTACGATTAAAA

AATAGTAGCCATGGATTGCAAGAAAAAAAATCACCTGAACTATTTTCAGAGAATATTGGA

ATACAATTTTCTTGATTTAAACAATTTTTAGTCATAATTACTACTTTATAAAAAAAATATT

CTAATAATTAAGAACTGAAATTAAATGTCATATAAATTGATGTTAGTTGAGAATGACATT

GTATTATCAAAGGCCATTCAAGAATACTTAATAGATCAAGGCTTCAATGTTTATATTGCC

AATAATGGATTAGAAGCACTGAATCTAGCTTATCAATATAATTTCGACTTGATAATTTCT

GATATAATGATGCCGCTAGTTAATGGCTATGAACTATTAGCAAAACTTAAAAAAAACAAA

GCATTATCCAAAATACCTGTTATCTTTTTGACAGCTAAAGGAATGACTAAAGACAGAATA

AAAGGCTACGACATGGGGTGCTATGGTTATTTATCTAAACCATTTGATCCTAATGAGTTG

CTTTCAATTATTAACAACTTAATCGCTAGAGATGTCTTAAAAGAAGCTTCTTTACAAAAC

TCGGCAACATCAAACCAGCAATTAAATCATAAAATACGTTTGACACCTAGAGAAAAAAGT

ATTTTAGATCTTGTTGTTGATGGACTTACTAATAAAGAAATATCTACTATACTGAATACT

AGTGTTAGAAATGTAGAAAAATATGTTAGTCGACTCTTACACAAAACTAATATGAAGAAT

AGGACTTTGCTAGTCAAGTATTCTATAAATAATAATCTATTAAATAATGAGATCAATGAA

AGGGCGAATGACGGGACTCGAACCCGCGAATGATGGAGCCACAACCCATTGCCTTAACCC

CTTGGCCACACTCGCCATATTACATACACCACTATAGCTTGTTTTTTGATATATCGTCTA
GTTTTCGAGTTATATATTTATCTAAAACAATATTTACTTTGAATTTTTATGATACATAGC
AATATTAGTATTCAAATTAATGGGGAACCATTTAATTGCTCAAAGCCCATCTCATTGCAA
TTTTTATTAAATTATCTTGATTTTAATTCCGAGCGTGTAGCAGTCGAGCTAAATGACATT
CTTTTACCAGAGACTCTGTTTCACTCAACTTATTTGAATGATCAAGATAAGCTAGAGGTT
ATTACCATTGTAGGTGGTGGATAATCTATTTGTACATACACAGTTTTCTATCTCAAATGT
TTCATAGATAGAGATAATCTTCCTTGTTTTTTATCTACATGAATAATAACAGCTTTAATA
GTGTCACCTATTTTAAATTGTGATGATATCTGCTCTAATTCTTTGATATTAATCTCAGAA
ATATGGACAAGACCTTTGAGATTTCCTACCTTTATAAATAATCCGTAAGGTGTAATCTGA
TTTATTATGCCTTCAATAATATTACCAACAATTAAATTAGATGATGCTTGAGATATTAGA
GCTCTTCTATGACTCAGTATTAAATTATTAGATTTCTCTTCGACATTGAGTAATTTTAGC
TTAATAAATTTATTATTAAATTGCTCACTTTTTTGAAAATTACCGAGATGAGAATTAGGT
ACAAATCCAGATATACCTTCAAGGTTAATTATCATTCCTCCCTTATTGAATCCTTTTATC
ATTACATTAAGTAAAGAGTCTTCAGCTAATAGTTGCCTAATTCTTTTCCATGCTCTTATA
TATTCAAGGCGACGAATTGATAATATTAATTGTTTCGACTGAATATTATAATCTAATAAA
AAAAATTCTCTTGTGTCATTAATGTTTAAAGAAGTGAAATTATTTAAATCTTGATTACTA
GATACTTCTTGAATAGGCAAGTATGCAGATATTGGTGTTCCTATATCTACTAATACCCCA
TTTAATTCAAAGCTAAATATAGTTCCAGCTACAATATCTCCAAGATTCAAATCATACTTG
TATTTTTGTAAAACAGCTGCAAAATTTCTATGAGTAAATCCTTCATTATTTTTTGTCATA
TTGAATAGTATGAATTAAAGAGTAACTTTGTACAATATATTAAGGTAAATAAATGTTTGT
CGAATTGTATGAAAAAATTTTAATTTGTTCGGTTGTAATTAAGTATATAAACATATTAAA
ATATCTAGGAGACATGAAAAGTAGACGTAGATAGCTGCAGATTTAATACTATATAATCTG
AGGAAAGTCCGGGCTCCACAAATACAATTTATGCTGGAGAAATCCCAGTGTAGGTAACTG
CGAGGATAGTACCACAGAAAAAAACCGCCAGAATAAGTTATATTAGCTGGTAAGGGTGCA
AAGGCAAGTTAAAAGCTTACCAAAAGTACTGCAAAGTATTTGTTAGGTAAACCCCTAAAA
TGGAGCAAAGCTACTAAACAAATATTTGTGTATATTTTATCTAGTTTAGTTGAAAATACT
GCATGAAGTTATTAGTAACAATAACTCGAGAGGAATAGCTATCCTTTACAATATATCTCG
ATTATATTGAAAGAACAGAACCCGGCTTATGTAGTACTTTTTATGTCTTGTATTATTATG
TCTATTGTCGAATCTTTAAATTTAACTGTTTGACAATTTTTTTTTGTAGCTGATGTTGTT
TATAATCTATTTCTGAATTGGTTAAAGTTTTTTCATTAGATCGATATGTAAATCTTAGAC
CTATACTTTTTTTCTCTTTTCCAATTGATTCATCTATATATTGATCAAATAATGTTGTAC
TTTCTAAATCTTTATCATAAAATTGATCTAATAATTTTAATAAGTATTTAATTTCCATAT
TCTTAGGTATTATTAAAGAGAGATCTCTTATGATCGATGGATACTTAGAGTATGGTTGAA
TCTGATAACTTAAATAATTGAATTCATTATGACAATCTTCTAAAATGTTTAAGTTAACTT
CAAGAAAAAAAAGTTTTGTATTGAGTCCTAATTCACTATATGTTGCTTGATTAAGTTCAC
TGAATATGCCAATAATAATATTGTTATAGATTAGTGTGGCAAATTTTCCTTTTTGAATGA

ACTTTATTCTACCATCGACTATATCTTTTTTTGTCCACTCAATTTGTCTATTAATCTTTT
GAAAAAAATTTTCTACGATCCCTTTAGCTTCATACCAATTTAAAGAATGCGCAGGGTGTG
ACCATGTAGATCGGATATCTAGATTACCACCCAATATGATAGCTAGATTAGTAGTTTCTA
CAATTTTGTTTTGATCATTATGAAATACTGTTCCTATCTCAAAGCTATCTATAGTTTGGT
TGCTTTGTTTAATATTATAAAGATTTGATTCAATTAATCCTTCCAGCAAACTGCCGCGTA
AATTAGAATAATCTTGAATAAGAGGATTGTTTAAAGCTATATTTCCTTTGGACTTTATTA
AAGAATAATGAACTAATTCAGTTAATCCTAGATTTCTTAAAATACTTCTACTTTTGTCTA
TGAATTTTCTCTTTGTGGATAACCTTTTGTTGAATTGAATATTTGGTACTGAGCTCCTGA
ATTTATGATAACCATAAATTCTAGCAATTTCTTCAATAACATCTATTTCTCGAAAAACAT
CTTCTCGTCTATAATTTGGAACAGTAACTTCTATATTTTCCTTATTTTGATAAATAAGAT
CAAAATTTAAAGAGTGAAGAATATTTTGAATTTCTTCAAAGAATAAAAAACGTGTTTGAC
CATTATACATAATTGGTCCTAAAATGTCATGAACTTTTTTAATTGATAAATTAATATTAA
GAGCATGATCAGTTATTTTTTCTCGACAGAAAGTATCTCTAATATTTCCGCATGTTAAAT
CTGTGATTAAAGCAAGAGCTTCAAAATGTGCATTTTTCCAATTATCTACATTTAGCCCAC
GTTCTTGTCTAATTGAACTTTCTGTTCGAATATTAAGAACTCGAGAAGACTTTCTAACTA
CAGATTGTTTAAATATAGCAGACTCTACAAGTATCGATTTTGTATTATAATCTGTATCAA
AATCAGAGTTGCTTCCAATACCAGCTATACTAGTTACATTTGTATTTACTTGAGTAACTA
AAATATTTTTGTTTAACTCAATATTTTCATTGTTTAACTTAATCTGATCATTACTTCCAA
GAGGAAAATTGCTTCGAATTGTTAGACTATTTTTATGATTCATATTATTAATTTTATTCA
AATCAATAATATTAATAGGCTGTCCCCATTTCAACATAATATAATTGCTTATATCTGTTA
ACAAATTTCTGTGTGTAAACCCAGAAGAGAGCAACCGATTTTTTAGCCAATCTGGAGAAT
CTTTAACTCTTATTTCGTCTATAATGGCAGCAAAATAATTGTCACAATTTAATAAATCGT
GATTACTGATTATAGTCTCCTTTTTAGAGAAATATCTATTATTGGGTTGATGTATAGCTG
ATAACATTGAGGACCCTGTTAAAGCCGAGACTTCTCTTGATAGACCAATCATGCTTAACA
CGTCTGACCTGTTAGCTGTTGATGATATATCTAATATATGATCAATCTCACCTCCAATTA
TAATAGATTCAATAGCTTCAACTTCAAAACCTGCTTGTGTTAGTTGATTAGTTAAATTGT
CTATATCTATTGTTTTGATATTACCAAGTTCTTTCAACCAATTTAAAGAAACTTTCATAA
TTAATTGTCATCTATCTTATAGAAGTATTTATTTTACTTATGATTAAAAATCAATTTAAA
AAATTCATCAAATTAATATAAGTTATTTGATGAATTTGCTTATACAATATATTATTGTGA
TATCTTACTTTTATATGGCAGTTATTAAGAACTAGCTTTTGTAAAAGTTAGCCCATTATC
TTGGGCATATCTTTCCATAAATCTCATGAATCTATCCCAATCATCAGGACTTTTAATAAC
ATGTACAGCTTCTATTGCTTGTGGTTTACCATTAATAAACTTGGCATTAACATCTCTTGT
AATAATTTGTCCCTCTGTGTCCATCAGGTACATACCTGTGATTTCACCTTTGTCTGCCAT
ACTAGCATCTAAAATTTTGGGATTTGTAAACCGAAAAGTTGCTGTTCCTGTACTACCATC
TCGAGACCTTGTTAAACGTACATCTGGTACAACTTCTTCATTGATACCTTGAATAAATTG
AATTGTTGCCATATCAATCTTCTTTATTTGTATATAATTTATATTGGATAAAACTGATTT

CTAGTTCTTGAATGCTTGTTGAGAAACTGTTTTAAAAGCTTATAGTATCAGTAGATCAAA
TTTTATGTATTATATAAAACTGTAACTTTTCTTTTTTGATCTGTTCAAAGAACTAACTTA
TTATAAGCTAATCATACGAATATTAGCCATATAATGTTCGTAAGTGAAATTAAGGGGTTG
TAGCTCAGAAGGATAGAGCAAGCGCCTCCTAAGCGCTAGGTCAGCGGTTCAAGTCCGCTC
AACCCTGTTAACGAATTAAGTAGTATTATTTAACTTGTTCTGCAAATCAGTATTGATCTA
AGTTAAAGTACTTGACAGCTAAAACAAAAGAATCTGAGAAATATTTTCATTTAAAGAACG
AACAATTATCTTAACTCTAAATATCTGATAATTAGTAAAAAATTCTCTTTTTAAAACACA
AGATGATGAAGATATTTAAACTAATAATTGAAATTAAGTTTTTGAAGTTATAGAAGCCAA
TATAATACTTTCTATTAACAGATGCTTGCTTCTATTACTATCTGTTTACAAGAAATTATG
AGTATCAAAATAATTTTAGCTCCACTGACTACTAATAAATATTTATTTTAGTCTAGACAA
GATGAATCTTCATGGTATAACAGAGCTATTCTTTGTGTCATACTTATATAAAATTAGGAT
ACTGTTTTCATTACCATAACTGGGGTGGGAGGATTCGAACCTGCGAATGGCGGAGTCAAA
GTCCGCTGCCTTACCACTTGGCTACACCCCAACATGTGAACATTATAATAGCAGTCGTAT
ACCCTGCCATGTCAATAGACTATTGTCTTTATTTAATAATTTTTGCTGCTCTTGTAGAAT
AAAATCTTTTTTTTCAAATAAGATACGCTATGTTTAAGTTGCACGACTGTTATGTTTTCT
TGCTTTTGACTGAACAGCCACTTGATAGTAATTATTCCTTCTGCAATTTCGTTACTTCCT
ATAATAATACAAGCTACAGCTCTTTTTTTATTTGCTTGCTTAATCTGCTTACTAAAATTA
CTTGAGCTAACATCGATTTCTATTTTAAAAAACTGCTGATGTAAAAAACGCATTATTTGC
ATACCAGTTTCGTTAGCTTTTGTACCTTGTGTAGCAATATAAAAATCGATAGATTTATTA
GGCAAGTCTATATTATCTTTTGCAATCAGTAATAGGCGTTCTAATCCTATTGCACATCCA
ACAGCTGGTGTTGCTTTGCCACCTAATTGGTGAACTAAGCTGTCATATCTTCCACCACCA
CATATAGTATCTTGCCCTTTTGATGTCAATGTCTTAATTTCGAAAGCAGTATCATTATAA
TAATCTAATCCTCGAACTAACTTATTATTTATATTGTAGGGAATATTAAGTAATTTTAGA
TAATTGCAAACGTCACCAAAGTGTTGTTGTGATTCCAAACTTAAAAAATCAGAAATTTTT
GGAGCCTCTGTCAGTATTTTTTGTGTATTAGAATCTTTTGAATCTAAAATTCTAATTGGA
TTACTAGTTAATCTTTTTTGTGAGTCAGTGTCTAAATCATCATGGTACTTTGTAAGATAA
TCTCGTAATTTAACTTGATAAATACTACGATCCTCTACTTTTCCAATTGAATTCAGATCA
AGTTTCAAGTTGTGCAGATTGAGATTGTTAAATATGCTCATAGCTAAATGTATAACTTCA
CTATCTGCTCTTGCATCTAAGCTACCAATAAACTCAATGCCAAGTTGGTGAAATTGTCGT
TGTCGTCCACTTTGTGGTCTTTCGTACCTGAACATTGGACCGCTATACCATAATCTTTGT
AAACTATGGTGGTAACTCATTTTATTTTCAATAAAAGATCTCACAATACCGGCAGTACCT
TCTGGCCTTAAGGTAATATCTCTATTGCTGCGATCATGAAACCGATACATTTCTTTATTT
ACAATATCAGTATCTTCACCAATACCCCTATCATATAATTCACTATTTTCAAAAATAGGT
GTTCTAATTTCTTGGTAATTTGCACATTCTAATAATTTGGAAATTTTATTATGTATAAAC
TGCCAATATTGAATTTCATCGGGAAGAATATCTTTTGTCCCTCTAATAGCCTGAATTTTT
GCCATGATAATTTTTTGATTACCTTCTTAATATGTCTGCATTAAATAGCAGTTGTAGACT

GTTATTCAGTCATGTATTATACATTAGAACATACTGATGTAATTTATAAAGTACGGGCAA

GGAGGGATTCGAACCCCCGACACCATGGTTCGTAGCCATGTGCTCTAATCCACTGAGCTA

CAAGCCCACTTAATAAGTACCTTAACTATATCATTGTTCACTAATGTATACAACTATATT

CTTTTTATCTTACATGTGAAATTTTTGTAAAAGTAAAAAATTATGTTTGTTTTTTACAAT

TGGAACTATTCATATATAATGCTTAATTTATTATAACTTTGTAAAAATATCTATAAGTTT

AATTGTATGATTCTATTATATTGGTAATCTTGTTATAATTTTGCGCTCTTATCTTTCAAT

TGGCTAGCATCTAATTAACTTATATTTCAAATAAAAAAATCTTATCTAAAATGAGTAAAC

AAATTCTATATCAGGATGATGCCAGAAAAGCATTAGAAAAAGGCATGGATATTTTAACAG

AAGCTGTTTCTGTGACTTTAGGACCAAAAGGAAGAAATGTTGTCTTAGAAAAAAAATTCG

GTGCCCCTCAAATTATTAATGATGGTGTAACGATTGCAAAGGAAATTAGTTTAGAAAATC

ATATTGAAAATACCGGAGTCGCACTGATTAGACAAGCAGCATCTAAAACAAATGATGTAG

CTGGTGATGGTACAACAACAGCTACAGTACTGGCTTCAGCAATTGTTAAGCAAGGAATGA

GAAATGTTGCAGCAGGTTCGAATCCGATGGCTATTAAAAAAGGTATTGAAAAAGCAACAA

ATTTTGTTGTTAGTAAAATAGCTGAGTATGCTAAGCCAGTAGAAGATACAAAAGCTATTA

TACAAGTTGCTTCTTTGTCATCAGGAAATGATATAGAGGTAGGTAAAATGATAGCGAATG

CTATAGAAAAAGTTGGCAGAGAAGGAGTTATTTCTTTAGAAGAAGGGAAATCAACTAATA

CGATTCTTGAGATCACAGAAGGAATGCAGTTTGAAAAAGGCTTTATTTCTCCTTATTTCG

TTACAGATACAGAACGAATGGAAGTTCTTCAAGAAAATCCTTTTATTTTATTTACAGACA

AAAAAATTACTTTGGTGCAGCAAGAACTTGTGCCATTGCTTGAGCAAATTGCAAAAACAT

CTCGGCCTTTATTAATAATAGCTGAAGACATCGAGAAAGAAGCATTAGCCACAATTGTAG

TTAATAAATTAAGAGGGATTTTGAATGTTGTAGCGGTTAGAGCTCCTGGATTCGGTGATA

GAAGAAAATCTTTACTTGAAGATATGAGTATCTTAACGAACGGACAAGTAATTACTGAAG

ATGCAGGTTTATCACTTGATACAGTTCAATTAGATATGTTAGGAAAAGCCCGAAGAGTTA

TTGTTACTAAAGACTCGACAACAATAATTGCAGATGGTCATGAAATTAAAGTTAAATCAA

GATGTGAGCAAATTAAGCGGCAAATAGAGACGAGTGACTCTTTATATGAAAGAGAAAAAT

TGCAAGAACGATTAGCAAAGCTTTCTGGAGGAGTTGCCGTCATTAAAGTTGGTGCAGCTA

CAGAAACAGAGATGAAAGATAAAAAACTAAGACTAGAAGATGCAATTAATGCAACAAAAG

CAGCAATTGAAGAAGGTATTGTACCAGGAGGAGGAGCTACTAATGTCCATATCTCTAGTG

AATTATTTACATGGGCTAAAAACAATTTAGTTGAAGATGAATTAATTGGTGCTTTAATAG

TTGAACGAGCTGTGACCTATCCGTTAAGACGAATAGCTTTTAATGCAGGTGATAATGGAG

CAGTAATAGTGGAAAAAGTTAAAAGTCACGATTTTCATATAGGCTATGATGCAGCAAACG

GTAATATTGTAAATATGTACGATAGAGGTATTATAGATCCAGCGAAAGTAGCTAGATCAG

CTTTGCAAAACGCAGCTTCTATTGCAGCAATGGTTTTAACTACTGAATGTATTGTAGTTG

ATAAAGTCGATGATTGAGCTATAAGATAATAGATAATCTCAAACAAAGAATAATTTCTAA

TTATAGAAATTATTCTTTGTTTCAATTAATCTAATTAATTGATCAAATGTATAATTTTTA

TAATAAAGCCAAAGTTTAAACACGTCTTCTTTTTCAACTATTAAGTAGAGAAGATATAAG

CCCGTAATACCTAAATTATCAATTAAAATATTATTCTCATATATAGATTTTTCTAAAGAT
CTAAAAAATGACATTTTTGAAAAATAATACCGAAATCTTCTCTTATAGTTATTTGCTAAA
TCATTACTGTCTAAAGATTGATTTCCAATTGAATTCAGAACCTTTAACAGAATACTTTTT
AAATCTTTATCTGACTTTATTTGAGAATCAATAATAATTTGATTCAATTGTATGTCAAAA
CTAAGATTAAAAATATTACTTGTTAAAGATATTTGATTTTGAATATCTAGAGATTTTATA
GCCAAGAAGATTAAACTTTTATTGTTCATTGTAAAATATAAGACAATTGATGATGTATTC
TTTAAATACTAAATTTATCTTCTTGTGCTTCTATCGTTTTTTGTAAAATAATTAATGTTT
TAATTTTTTAGTTTTAGTTACTACCCGAAAAAATAAATTCTGGAAATCTAGCTTGAGCTT
TAGCTAGAGAGTGTTTTTTCCCTTCTTCTTGCCAGTAATTGATAACTTCTGTTGTTTTGT
TTAATAACTCAATTCTGTCTCTTTCAGCAATCCACGGCTTGGATTCTAGTTCTGCTTTTA
ATTCTTCCCAAGCATCATTCCTTGGCCAGAAAAAAATAAGAAGTAAGTGGACTAGTGCCAT
TTCCAACAATCTGATCAATAGCAATAGCAATATTATTTTCTAACCAAAGTACTTTTAGTG
TAAATTTTGACAAGTTTTTATCTCCATTATTAATATTAAGTATTGGTACAGTAGAATACT
GTACTAGTTATTTTTTTCAGCAACAATTTGAGCTTTGTTTAAAATGTTTTTTACCGTTTT
AGTAGCTTGTGCACCTTGAGATAATCGTTGCAAAATTTTTGAAATGTTAACACGAGTTTC
ATTATTTATAGGATTATAAAATCCTAATTCTTCTATAGCTTTACCATCGCGTTTATTTCT
GCTATCCATTACAACAATTCTATAGCTTGGTTGTTTTTTTCTTCCGTATCGCTTTAACCT
TAACTTGACCATATTAGTCACCTCGCTTATTTTTTATATCTTTATTAAATTATATCTTAC
GACATTATGCAATAATTCAAACAATTAAGCTGATTCAAGTCTAACATTATTAAATATTAC
CATTAAACATTGTAGAAAAATAATACCTAACATAGGTGACATGTCCATTCCAAACATGGG
AGGAATGCTTCCTCTGAATAGTTTTAAGTATGGATCAGTAATTCTATTTAATGAACAGAA
TGGTTCATTGTACCAATTTACAGTTGGAAACCATGCCAATGATAATTTAAGTAAAATTAA
AATTAAATAAATCTCAGAAAAATTAGCTATCGATCCAAGCAATAAATTTAATGTACCAGG
AAGAGTGTTCATACGTATTTTATAATTATTATATACTTCGTTATACTAATAGTTTGATTA
TATATTATAATCGATGTAAATAAATCAGCTAAACAAAAAACTTATCCTTAAAATCATCTT
CTCTTGAGCACTTTTCTTTATCTACATTAGTATTTACTTTTGTCGTATAAATTTCCAGTT
TAGACCAAATCTGTCCTAACTGATTTAATTGAGATGTTCCACATTCTAAACAGGCTATTT
TTATATTGCTCAAAACAGCACCTTTTTTTATAAAATCGTTCAATAATGGCGTAATAGTTG
CTTCATCCAAAAATAAATCTAAGACAAGTATTTTTGTAAATGAATTTAATTGATCTGGGA
CATTGTTAATAATATCATTATAATTAACTAAAACTATATTAGCTGTAGGAAGTAAAGATC
GTGCTCCTTCCGCTAAGATAAAGCCATAAGGCATAATAATTACAATAATATATTTATAAT
GACTGCTAATAAGACTTATAGTGGTATTAGCTTTTAATTCGATTGTTTCTGTTACTAGCC
ACTCTCTCATTATTTCGTAAGTAATCCATTTGCCTAGTTCTGAGCAAGCAGTTCTTAAAA
TCGTACCTGGATTATTGTCGTTTTTGAGAATCCCTGACCAATGTTGTATTAATGGATGGG
ATGCTATATTAATTTGGAGTTGCATGTTTTGTTAAATATATTTAACATTTAATTTGTTGA
TTGAATCTTCAATTCCTATTTGTTGAGTATTATTAAAATAGCATATATATTAAAGAAAAT

AATTATAAAAAATGAAAAAAAGTCTTTGGCTGTGGGGTTTTACAGATAGTGCTGAAACTT

GGAATGGTAGATTTGCAATGATTGGTTTTATATCAGTTATTTTTATCGAAGTAGTTACAG

GACAAGGATTATTATATTTAATAGGTATGATGTCATAATAAAAATCCAAAAAAGTTCTTG

TAATTTTTTATAAGAACTTTTTTGGATTTTTATAAATCTAATCTAGCGGTCTCATAGATA

GAACAGGTTCATTCTCTCTATTGATACCTTTTTCAAATCCAGCAGCAGCAGCTCTTGCTC

TGCCTGCATGCCAAAGATGTCCAATAAATAGGAAGAATCCTAGGAAGAAATGAGATGTTG

TTAACCAAGATCTAGGAGATACATAGTTAACTGAATTAATTTCTGTAGCTACACCACCAA

CGGAATTCAAAGAACCTAGTGGTGCGTGAGTCATATATTCTGCGGCACGCCTTTCCTGCC

ACGGTTGAATATCGTTCTTGATTTTATTTAAATCAAGACCATTTGGTCCTCTAAGAGGCT

CAACCCAAGGAGCTCGCAAGTCCCAAAATCTCATGGTTTCACCACCAAAAATAATTTCTC

CACTAGGAGATCTCATTAGGTATTTACCTAATCCAGTAGGTCCTTGAGAAGATGCAACAT

TCGCACCTAGTCTTTGATCTCTAACTAAGAAAGTAAAAGCTTGAGCTTGTGAAGCTTCAG

GACCAGTTGGTCCATAGAATTCACTAGGATAAGCTGTGTTGTTATACCAAACAAAATTAG

AAGCCGTAAGCCCCATGATTGATAAAGCACCTAGACTATAAGATAAGTAAGCCTCACCAG

ACCATACAAAAGCTCTTCTAGCCCAAGCAAAAGGTTTTGTTAGTATATGCCAGATTCCGC

CAGCAATACAAATAATGCCAATCCAGACATGACCACCTACAAGATCTTCCATGTTATTTA

CACTTACGATCCATCCATCACCGCCAAACGGAGACTTTAAGACATAACCAAAGATAACTA

GGGGATTAAGTGTAGGATTGCTAACAAATCTAACATCTCCACCACCTGGAGCCCAAGTGT

CGTATACCCCACCAATAAATAAAGCTTTAATAACTAACAGAAAAGCGCCAATGCCTAATA

ACACTAAGTGTATACCAAGTATTGTTGTCATTTTGTTTTTATCCCGCCAATCGTATCCGA

AGAAAGGAAATGATTCTTCAAGAGTATCAGGGCCAATCAGAGAATGATAAAGACCTCCAA

AGCCAAGAACAGCTGAAGAAATTAAATGCACTACGCCTACTACAAAGTATGGGTATGTAT

TGAAAATCTCGCCGCCTGGACCAACACCCCAGCCTAGTGTAGCTAAGTGCGGAATTAGAA

TAAACCCTTGTTCGTACAAAGGCTTCTCGGGAACAAAATGTGCTACCTCAAAAAGAGTCA

TGGCACCAGTCCAAAAGACCATTATACCTGCATGAGCAACATGAGCACCAAGCAATTTGC

CAGAAACGTTAATTAAGCGTGCATTGCCAGACCACCAGGCAAACCCGGTAGACTCAATGT

CTCTACCGCCAACACCAACAGTTGTATTAAAGGGCGTTTCCACGTGGTAAAACCTCCTCA

GGGAATATAAAGTTTTCATGAGGTTGATCTTGAGCAGCCATCCAAGAGCGAATACCTTCG

TTCAATAGGATATTTTTAGTATAAAAAGTTTCAAACTCAGGATCTTCCGCAGCTCTCAAC

TCTTGAGAAACAAAATCGTAAGCTCTTAAATTTAGAGCTAGTCCAACAATTCCAAATGCG

CTTGTCCATAATCCAGTTACTGGTACAAATAACATGAAAAAATGTAGCCAACGTTTATTA

GAAAAAGCAACACCAAAAATCTGTGACCAGAATCTATTAGCTGTTACCATCGAATAAGTT

TCTTCAGATTGTGTTGGAGTAAATGCACGAAAAGTATCTGCAGCATCACCATCTTCGAAT

AAGGTATTCTGTACAGTTGCACCGTGAATAGCACATAGTAAAGCACCACCTAAGATTCCT

GCAACGCCCATCATATGGAACGGATTCAGGGTCCAGTTATGAAATCCTTGCAAAAATAGC

AAAAATCTAAAAATTGCAGCAACTCCAAGGCTTGGGGCAAAGAACCAGCTCGCTTGGCCT

AGAGGGTACATTAGAAAGACGGAAACGAATACTGCAATTGGTCCAGAAAATGCAATAGCG
TTGTATGGTCTAAGACCAACTAGCCTAGCAATTTCAAATTGTCTTAAACAGAATCCAATT
AGTCCAAATGATCCATGTAAAGCTATAAATGCCCACAGACCACCAATTTGGCACCAGCGA
GTAAAATCTCCTTGAGCTTCAGGTCCCCAAAGGAAAAGAAGTGAATGTCCCATGCTGTTT
GCTGGGGTAGAAACAGCCGCAGTTAAAAAATTGCATCCTTCTAGATATGAACTGGCTAGC
CCATGAGTATACCAAGAAGTAACAAAAGTAGTCCCAGTTAGCCAGCCACCTACAGCAAGG
TAAGCGCAAGGAAACAGAAGTAGTCCAGACCAACCTACGAATACAAATCGGTCTCTTTTT
AGCCAATCGTCTACAAGATCAAAACCACCACGAGTTTTTTCTTGTCCAATTGCTATGGTC
ATAATTTTAATCTCCAGAGCAAATTTAATAAGTAATTTGTTTAAATAAACACTTTAGATA
TACTCTAAAATTTTACTTTTCTTTACGGTCAATATAATTATAAGTCAAAAGTAACCAAAA
TTCACGCTTTTAATAACATTTTTTTAGCTCATTAAGTTAATTTTTTAATATATAAACAGT
AATAGAGTTATTGTTTAACTTACAATACTCACAATTTATTCATTTCTATAATCCTTTGAA
AAAGATATCCTGTTCCTCGTGCTGTTAAAATCAAGTCGGGATTACTTGGATCATCTTCTA
GTTTAGCTCTCAATCGTGAAATATGAACATCAACAACTCTCGTGTCAACATGCCGTTCGG
GAGTATAGCCCCAAACTTCTTGCAAGATAGAAGCTCTAGAAAAAGGTTCTCCAGCCTTAC
TAACTAAAAGCTCGAGAAGACTGAATTCCATACCTGTTAATCGAACTCTTTCATTATTTT
TATAAACTTGTCTTTTGTTAGTATCTATTTTTAAAAATCCAATACTAATAATCCCAGAGT
TTGGGACTCCAAGACTAGTGGTGATTTTATCAGCTCTTCGAAGAACAGAACGAATGCGAG
CTTCTAGCTCCTTAGGAGAAAATGGTTTAACGACATAGTCATCAGCACCTATTTCTAATC
CGGTAATTCTATCGCAGACTTCACCTAACGCTGTTAGCATTATAATAGGAACATCTGATT
CTTTTCGAAGTTCTTGACAAACACCGTAACCATCTAGTTTAGGCATCATAACATCTAAAA
CAACAAGGCTAGGATACTCTTTTCTAAACACGAGTAAAGCTTCTTCTCCATTTGAAGCAG
TGATAACTTCATAACCTATTATTGTTAGTCTTGTTTCTAAAATTCTTCTTATGCTGGCCT
CATCGTCAACAACGAGAATCTTTTCTTTCTGGTTCTCCAATTTCTTATTTACTCCTGTAA
CTCACCGATCATGATTACCATAACCTTAAATTTTTAGCGCTATGAGTATCTTTATTCACT
TTAGAATTTAGTTATTATAAATTTTGCAAGTCTAGTTTACATTAATTAGTACCAGTCAAT
ATTTTTGTTTTAAGTTATGACTATTTTAATCCATAAGTTGACTCTAGTTTGCAGAATTCA
ATATATACATATTTTCTACCCAAGTAAATTGTATTCTATTTTCTAAATAAAAAAGGGGTT
GACAAACCAGTTGATAGAAAGTATTCTACATTACTATAAGTTAATTCCAACAAAGAAGTT
TTAAATCTTCTTTCGGAGTAATGAATACTTTAAT
>SRR9587917
TATATGATAAATTAAGACGTCTGTTATGTGAGAATTTCTAAAATATACAAAACTTTTTAT
TTAATATAGAAAAAGTAAATTAAAATAAGTTGAACATACTTTGCTCTAATCTAAACCTCT
TAATTGAGATTCTAATTAGAGACAAAAGTATTTACTTATTTTGTATCTATAGGAATAAAA
ATGAAACTTGCAGTTTATGGTAAAGGTGGTATAGGTAAATCTACAACTAGTTGCAATATT
TCTGTAGCACTTTCAAAAAGAGGTAAAAAAGTTCTACAAATTGGCTGCGACCCTAAACAT

GATAGTACATTTACGCTAACAGGTTTTTTAATCCCAACAATTATCGATACTCTTCAATCC
AAGGATTACCACTATGAGGACGTTTGGCCTGAAGATGTAATCTATAAGGGATATGGCGGT
GTAGACTGTGTTGAAGCTGGTGGACCGCCGGCTGGCGCTGGTTGCGGAGGCTACGTTGTA
GGTGAAACAGTTAAACTTTTAAAAGAATTAAATGCTTTTGATGAATACGATATTATCTTA
TTTGATGTTTTAGGTGATGTTGTATGTGGAGGTTTTGCAGCTCCATTAAATTATGCAGAC
TACTGCTTAATCATTACAGACAATGGATTTGATGCTTTATTTGCAGCTAATAGAATAGCA
GCTTCAGTACGAGAAAAAGCTAGAACGCACTCTCTGAGATTAGCTGGACTTGTTGGTAAT
AGAACAGATAAAAGAGATCTAATTGATAAATATATAGATTGTGTTCCAATGCCAGTATTA
GAAGTCTTGCCCTTGATTGAAGATATTAGAGTGTCCAGAGTAAAAGGTAAAACTTTATTT
GAAATGGCAGAAATTGATAAGGATTTAGCATATGTATGCGATTACTATTTGAATATTGCA
GATCAGTTGATTACAAGGCCAGAAGGTGTAGTTCCTAAAGAATCTCCGGATAGAGAATTA
TTTAGTCTTTTATCTGATTTTTACTTAAATCCTAAATCAAAGGTAGGACAAGAAAAAGTA
GATCAAGAAGAATTAGATTTGATGATAGTGTAAATAATATTTTCAGCATAATATAACAAG
GAATAAGATAATGTCTACAGCTCAATCAGATGCTCTTACTTTTGAATGTGAAACAGGTAA
TTATCATACTTTTTGTCCCATCAGTTGCGTTTCTTGGTTATATCAAAAAATTGAAGACAG
TTTTTTCTTAGTTATAGGGACTAAAACTTGTGGTTATTTCTTGCAAAATGCAATGGGAGT
AATGATTTTTGCTGAACCGAGATATGCCATGGCAGAATTAGAGGAAGGAGATATTTCAGC
TAAGCTAAATGATTATGGTGAACTCCGTAGATTATGCTTACAAATAAAAAAAGATAGAAA
TCCTAGTGTTATATTTTGGATTGGAACATGTACAACCGAAATTATAAAAATGGATCTGGA
AGGAATTGCTCCAAAATTGGAAGCAGAAATTCGTGTTCCTATAGTAGTTGCTAGAGCTAA
TGGTTTAGATTATGCCTTTACCCAAGGTGAAGATACAGTTCTAGCAGCTATGGCTCAAAG
GTGTCCATTAAATTTAAAGAATCAATCAGATAACACGAGTCTTAAAACTTCTCCTCATAT
TCCTTTAGTTTTATTTGGATCTCTTCCGGATCCAGTTGTAACCCAGTTAACTATGGAATT
AAAAAAACAAGGTATTTTTGTTTCTGGTTGGTTGCCATCAAAGAGGTATACTGAATTACC
GGTTATAAAAGAAGGATATTATGTTGCGGGAGTTAATCCATTTCTTAGTCGCACAGCTAC
TACACTAATGAGACGCCGAAAAACAAAACTAATTGGTGCACCATTTCCAATAGGTCCAGA
TGGTACTCGAGCCTGGATTGAAAAAATCTGTTCAGTAATGAATGTAAAGCCTATTGGATT
AGAAGATAGAGAAAAAGCAATTTGGGCTTCTTTAGAAGACTATATTTCTTTAATCCGAGG
TAAATCAGTTTTTTTTATGGGTGATAATTTACTAGAAGTATCTCTTGCAAGATTTTTAAC
TAGATGTGGAATGACTGTATATGAAATTGGTATTCCTTATATGGATAAACGCTATCAAGC
AGCAGAGCTAGCTTTATTAAAAGCTACATGTGATAAAATGAACGTTATGATGCCAACAAT
TGTAGAAAAACCAGATAATTATAATCAAGTAGACCGAATCCGTGATCTGAAACCCGATTT
AGTTATCACTGGTATGGCTCATGCAAACCCTTTAGAAGCCAGAGGTATTAATACAAAATG
GTCAGTAGAATTCACATTTGCACAAATTCATGGCTTTACCAATGCAAGAGATATTCTTGA
ACTCGTCACAAGACCATTACGGCGAAATCTTAGTTTGTCAGAATTAGGCTGGGATGTTTA
TAGCAAGCAAAGCTAGTATTCCTCTTTATTTATGACTTACATGGTATAAAAGCCTTGTAA

GTCTTTTATTTTATGTTAAAGAAGCATTTCTTACTCTGCCATCACCAGCCCAAGCTTGTA
AACTTTCTACTTGTTCTTTGTCTGTAAATGCTAAAGGCACAAACTGTTTGATAGCTATTT
TAATATCTTCAGTACTAAATTCTCTCTCTTCACTAAACGCCGTATGCATACTTTCAATAA
TTGCTTGCTCAATTTCAGCGCCAGAAAATTTATTACATAATAAACTCAATTCATGAATAT
CATATTCTTGCCACGATCTTGGTCTAACTTTTGATAAATGTATCTTAAAAATTGACTCTC
TCTCTTGACGATTAGGTAAATCTAAGAAAAAAATTTCATCGAACCTGCCTTTCCTTAACA
TTTCGGAAGGTAAACTTTGAATTTTATTAGCTGTTGCGACAACAAATACTGGAGCTTTTT
TCTCTGATAGCCAGGTAATAAATGTACCAAATACTCTGGCACTAGTACCACTATCACCTT
GACTATGTAAACCAGAAAAAGCTTTATCTATTTCATCAATCCATAATATGCAAGGCGATA
ATCCTTCAGAGATATTTATCATTTCTCTCATTTTTGATTCCGATTCACCAACTAGTCCAC
CAAACAGTTTTCCCATGTCAAGACGTAGCAGAGGCAGCATCCAATCGCTTGCAATAGCTT
TCGCGGTTAAGGATTTACCCGTCCCCTGTATACCAACTAGCAATAAACCTTTGGGCGAAG
GAATACCGTAATTTAAACTTTGCTTCGAAAATGAGCGAGACCTTTTTTGTAGCCATTGTT
TTAATACATCTAAGCCACCAATATCCCTATTTACTTTACTATAAGGATAAAATTCTAATA
GATGCGTTTGATTAATAATTTGTCTTTTTTCTTCAATAATAATAGGTAAGCTCCGAGAGT
CTATCTGATTATATTGTGCAATAATTTTAGTAATCACTTTGCGGATTCTATCTATCGATA
ATCCTTGGCAGGATTTTGTTATATTATTAACGAGCTCTGAATTCAAATTAAGATTCAAAG
CTTTACTCAATCTTATGATCTCTTTTTTGATTTCTAGAAGACTAGGTAAGGGCAAATCTA
TTACCGTTATGATATCATTCAGAGCAAAAGGTATATTGATTTTGCAAGAAATAATAATAA
TGTGCTTAGATTGCGTTTTGATTATTTTAGCTAAATTGCGAAGTTTCCTAATTAATACTA
TTTCGTTTAGGAAAGAGTCAAAATCTTTTAAAAGAAAAAGATTTAAAGATTCATTATTCA
AGTTTTCAATAAATTCTAATGCTAATAAAGGATTTCTTTTTGCATAACCATTATCGCTAG
GATTGTTAGTGTATCCATCTACAAAGTCCCAGGAATATACTTGTTGACTGTTTGAACAGT
TTAAGCTGTGTTTTATAATATACTCTAGTCTATCTTCTTCTCTAGTATTTATTACAATAA
TAGGATATCGAGATTTTAAAAGTAATCGTAAATCTTGAGTGAAATTCATGTCAATATTTG
TTTCCATCAGACTTTTGTACTTCACCAATTGAAGAAAGACTAAATAAGATTATCTAATAT
TCCTTGGATATATTCAAAAGTCATCTCTCTATTTTCTAAATTATTGCTATTACTAATTTA
ACTTACCATGATTTTTTTTCTTCCTTTTCTTCGTCGACTATTTAATATAGAGCGTCCACA
TGGTGTTTTCATTCTTGCTCTGAAACCAGAAACTCTAATTTTTTTTCTTTTAGATCCTTG
TAGTGTTCTTTTAGTCATGATGTTTCCTCTGTTGTAAAAGATTAAATACTATTTTATCAT
CTTTTATATATCTTTATGATATAGATATGTATTAATATCAAAGTTAACAGCAATAATTTA
TATTAATCAAGAATTATTTATATAATATTAAACATTAAATATTAACTTTATGATATTGGC
TTTGCCCATTTTTTATTTAAGTATTTTGACCATCTTTTTGCTAATCTTAAACTGGTTAAT
TTTTCAGCAGCTAAAAACGATTTTGTTATTAGAATCTCAATTTAAGTATTTTGTTGATAA
AAGCCAAAATCGTAAATTAGAACCCGAAGAAAGTTTTGCTTTTGCAAAAGTATGTGTAGC
TAAAAAATATTTTTCTAAAGCTATTATTGAAGGTCAATTAGCTTTAAAAAACTATCGAGA

CCTGAATATCTTAGATAATAATATAGTAATTGCTAATTTGTATAATATGCTAGGTTTTAT
TTATTTTGAAGCAGGCCAAACAAGTTTTGCAAAGAACTTTTATGAGCAAGCCTTACAAAT
AAACCCTAATTATGTTGTAGCTTTAAATAACCTGGCAAAAATTTACGAAGAGGTAAAAGA
TTTAAAAAAAGCCGAATCTCTATACGATAAAGTGTTAACTCTTAACTTAAATAATAAAAC
TGCTAATAGAAGGAAAGATTTTATAGCAAAAACTAAAAACATCTAATACTATTTGTAATC
GGGATAGCAGGATTTGAACCTGCGACATCCTGCTCCCAAAGCAGGCGCGCTACCAAACTG
CGCTATATCCCGTAAGACATAATTATTACTATATACCTTTTCTCTAATTATGTCTACTCT
TTCTGTATTTTATCTAGGATACCAAAACATGCCTTTAACTCCATCTGGATCTTTGATAAA
TCCTAATTTTTTATAAAAACTAACAACATCAGGTTCTGCGAATAGTGTAATAGTGCTAAT
TTCTGCCTGTCTCAACTGCTGTATTAATTGATGTATGATTACTTTTCCTAAACCTAAACC
TTGGAAATCTGGATGAATAACTACGTCCCAGATAGTTGCATTAAATCCGTTGTCTGATGT
TGCTCTAGCAAATCCTACAAGTTTACTATTTGCATCTTTTTTTTGTATTAAAGAAATAAT
AATAGAACTATTTTTTTAATGCGATCTTTACTTTTTTTAGAGGTCTTTTAACCCATCCGAC
TGAATCACAAAGCTGCTCCAATTCGTATAAATTAATATTCTTATTGCTACTCAGATAGAT
ATCTTTAAATTCGATTTTATCGCAAGTTTTATCTAAAACAATAAGTTTTTTGAAATTTTT
TTCAGAGCTAGCATTGATATCGGAGTTTTGAAAAAAATTTTTCCAGAAGATCATAATGAT
TATGATATAATTAAATAAATATATATATTACAATATTGCTTTAACAAACAATATATTAAT
ATATGCTCAAATTTTTACATGAATCTATTAAGATAGAAGATTGTTAAAATATAGAGAAAT
AAAAATTTTTGTAAAATCTTTCAATAAAATGATATAAAGAGTGCTTTTTAAGACTAACAT
CTATTATTATAAATAAAGTCTCGAAATTGAGATGAAATAACACATTATTCTATGTCTAAT
GCCTTTTAAAGGAGTTATTTGTGAAAAAATCTATGTGTCTAACCTGTTTACTTGCTTTAC
TAATTATGAGCAACCCAATAATAGCTAACGCAGAAGTAGCTGGATTGGTGCCCTGCAAAG
ATTCTGCGGCATTCAATAAGCGTATGGTAAATAGTGTGAAAAAACTTCAAGCCAGATTAG
CTAAATATGATGCAGACACGCCACCAGCGTTAGCTTTAAATAAGCAAATAGAGAAAACTA
AAACTAGATTTGCAACTTATGGTCGAGCAGGTTTGCTGTGTGGTACTGATGGATTGCCAC
ATTTAATTTCTGATGGTCGATGGAGTAGAGCCGGGGACTTTGTTTTTCCAGGACTATTAT
TCTTGTATATTACAGGATGGATTGGCTGGGTAGGTAGAGGATATCTTTTATCTGTTGCTA
AGACTAGTAAGCCAACAGAAAAGGAAATCATTTTAGATGTGCCATTAGCTATCAAATTTA
TGTCATCCGGATTTGCATGGCCGCTAGCGGCTTGGCAAGAATTTAGTAGTGGACAATTAA
TTGCTTCCAATGACGATATTACTGTTTCACCCCGTTAGTAAAAAATTTATATGAATAATA
ATTTTACCAAATACTTATCAACAGCACCTGTAATTGGTGTATTGTGGATGACGTTTACAG
CCGGATTTATTATAGAATTGAACCGCTTTTTCCCAGATGTCTTATACTTTTACTTATAAA
TAAGATCGTATAAAAAACTACATAAATATAGAGAAATACTATTATTTGTGTAGTTTTTAA
TATATAACATAATTATTTATTTTTAAAAATGTTTATCTTTTCGATTATTTTAATATTAGA
TCTTACATAATAAAATAATATCAATATTATGAGCTTAGTAACCCAAATTATAGTTAATGC
CGATGATGAATTAAGATATCCTACAATTGGAGAATTGCAGTCAATTCAAGACTACTTAAC

TACAGGAAGCAATAGAATTAGAATTGCTACTATTATTAGAGATAAAGAAAAGGAGATTAT
ACAGAAAGCTAGTAAGCAAATTTTTCAGTTACATCCAGAATATATAGCTCCAGGTGGTAA
TGCAGCAGGTTCTAGGAAAAGATCGTTATGCTTACGTGATTATGGTTGGTATCTCAGACT
AATTACATACGGAGTATTAGCTGGGGATAAAGATTCTATAGAAACAATTGGTATTATAGG
AGTAAGAGAAATGTACAATTCTTTAGGTGTACCTATAATTGGAATGTTAGATGCAATCCA
GTGTTTAAAGGAAGCATCTTTAGAAATGCTTGGTCAAGATGATATTAGAATCATTTCTCC
TTATTTTGATTATATAATTCGTGGAATGTCATAAATCATAGCTTCAATTAATTAGTTGAA
TAAAAGCTTTTATAATGTTATAATTACAATAAGCCCGAAAGCATATAATTGTAAAAACTA
AATCTTGTCAGAACCGGAAGGTAGCAGCAATAATGTTTCAATACAAAAGTTATGATTTTC
GGGTGTTTATTGTTTTTATCTAATAGTCTCGACTTTTAATTAGTATGCTATGTATAAATC
TGTATATGACATGTAATCCATCAAACCTTCCAGCAAAAATAATTTAATAATTTGATATTA
ATACGTAAAACACCTATACTCTTTTATAAAATTTCTTTATATCTCATCTAAACAATGGAA
ACTTAACTTAATGGGTGTTCATATTTTATCAACAGGTTCGTCTGTCCCAAATTTTTCTGT
AGAGAATCAACAATTTGAAGATATGATCGAAACTTCCGACCATTGGATTTCAACAAGAAC
AGGAATAAAAAAAAGACATCTAGCTCCATCTTCTACTTCTTTAACTAAATTAGCGGCAGA
AGCTGCAAACAAAGCCTTATATGCAGCTAACTTAAAACCTACTGAGATCAGTTTAATTAT
TTTAGCTACGTCTACGCCTGATGATTTATTTGGTAGTGCTAGCCAGTTGCAAGCAGAAAT
AGGTGCAACAACATCGGTAGCTTTTGATATTACGGCTGCCTGCTCCGGTTTTATTGTTGC
TTTAGTAACAGCAGCTCAGTTCATTCAAACTGGTTCCTATGACAATATTTTAGTTGTTGG
AGCAGACACAATGTCTAGATGGATTAATTGGTCAGATAGAACTACCTGTATTTTATTTGG
TGATGGTGCTGGAGCAGTAGTATTGGGGCAAAGCCTCAAAAATAGTATTTTAGGCTTTAA
GTTATGTACAGATGGTCAGCTAAACAGTCATTTACAATTAATGAATAAACCTGTAAATAA
TCAAAAATTTGGTGTTACAGAAATTCCTCATGGAAACTATAATTCCATAACAATGAATGG
CAAGGAAGTGTACAAGTTCGCTGTATTTCAAGTTCCAACAGTAATTAGACAATGTTTGAA
TAATTTAAACATTTCAATAGATGAAGTTGATTGGTTTATATTGCATCAAGCAAACACTAG
AATCATAGAAGCAATTGCGAGCAGATTATCAGTACCTTTTTCTAAAATGATTACGAACTT
AGAGCATTATGGAAATACATCTGCAGCGTCAATCCCTTTAGCGTTAGATGAAGCTATTCA
ATCCAATAAAATTCAACCAGGCCAAATTATTGTTTTATCTGGTTTTGGAGCAGGCTTGAC
TTGGGGAGCAATTGTCTTGAAGTGGTGATTTATATATTGCGGATGACGAGACTCGAACTC
GTAAAGCTTTCGCTACACACCCCTCAAGCGTGCGTGTATACCAATTTCACCACATCCGCA
TTTTTTACCATGAATATTAAAAATTTATAATATTTATCTAAATATTCATATAATGTGTAT
ATATATATACATTCTTGTCAGAAACTCTCTTATATTTTATTTTAAGTGATAAAATTGAGT
TTAGTATAGATAGTAAAATTTTATTTTAAAATCATAAAAATAAATATATAAGGAAAATAA
AATATGACACCATCTTTATCAAGTTTTTTGAATAGTCTTATTCTTGGGGCAGTAATTGTA
GTCGTTCCCATAACTTTGGCTCTTTTATTTGTTAGTCAAAAAGACAGGACAATCCGGTCC
TAAAAATAACTTAGAAAGATATGGAGAAGCAATTTAAAATGAATAATAAAATATTTTTCA

TACTAATAAGCTCTCCATATATTGCTATACTTTAGAAGAATTTTTATTTTTTTATTTTGT
AACATCCTGTAAAGTTTATTCCGTTCTTTCTTTTTCCCATTTACTCTCTTATAATTAATG
ATACAAATGTCAGGTTAATAATAGTTTTTTTATTCTGTATCTTTCATTTTATATACGTAT
TATTATGTTCAATTAATAAAGAAAAGCTCATGTCTTTGTCTAATTGGCCTCTCAAAAAAG
AAAATTCTGAAGCATATAATATTAAGAATTCAAAACAAATCACAATTCCTGATGGTTTAT
GGATAAAATGCTTCGACTGTGGTCTATTAATGTATTCTAAAGTACTGAAGAGAAATTTAA
AAGTTTGCCCTCAATGTAGTTATCATTTTCAAGCTTCTAGTAACGAAAGAATTGATCAAT
TGATAGACCAAGGTAGTTGGCAACCAATGGATGTTCACTTGATCTCTACAGATCCATTAG
GCTTTAAAGACCAAAAGCTTTATAGTCAAAGGTTAAAAGATACTGCTTTCAAGACTGGCC
TGCAAGACGCAGTTCAAACAGGTACTGGGACTATGCAAGGTAAAAAAGTATGCTTAGGTA
TTATGGATTTTAGATTCATGGGGGGAAGCATGGGATCTGTTGTAGGCGAAAAACTAACAA
GACTGCTAGAAAAAGCAACTCAAGAAAAGTTGCCTGCAATTATACTTTGTGCATCAGGCG
GAGCTAGAATGCAAGAAGGTATGTTGAGCTTAATGCAAATGGCAAAAATTTCTTCTGCTC
TAGAAATGCATAAAAAAGAAAATCTACTATATATATCTGTTTTAACCTCTCCCACAACAG
GGGGTGTAACAGCTAGTTTTGCTATGCTTGGAGACTTAATTATTGCAGAGCCAAAAGCTC
TTATTGCATTTGCTGGTAGACGAGTTATAGAACAAACAATCAAAGAAGACTTGCCAGATA
ATTTTCAAAGTTCAGAATATTTATTTGAACATGGTTTCCTAGATTTAATTGTATCCAGAA
CTCAGCTTAGATCAAAGTTAATACAAATTTTGTCCTTACATAATCATAGTAAGTAATGAA
TTGAATATTGACTATAATATCAAAATGCTAGTAAAAATTAAGATATTAAGAAATTTTCAC
TAAAGATATTTGTAGACTAACTAGAATTACAATTACTATTGTAGAAAAATTTGTCTTAAT
GCCATTTTTTCAGTCTTTTAACAAATTGTTTTTATGACTTTAAATAATTGTTAGTTTAAT
ACAAATTTATAAAGAGAATACATACTAAGCTAAATATAGTAATATTATGTTTTCCTATAA
TACTGACCTATCAAATTTCGAGGAAATTTATGCTTAAAAGATCTTCTTGGCTTGCGGCTT
TATTGGGACTATTAACAGTAGTTTCTACAAGTACGCATACATATGCCATAGAGTTAGACG
AGGCAACAAGAACTGTTCCATTAGAATCTTCTGGCAGAACTGTAATTCTTACACCAGAAC
AAGTTAAAAGAGGCAAGCGATTATTTAATAATTCTTGTGCTATTTGCCATAATGGTGGTA
TCACGAAAACAAATCCGAATATTGGACTTGACCCAGAATCTTTAGGATTAGCTACGCCAC
AAAGAGATACCATTGAAGGACTAGTTGACTATATGAAAGATCCGACTAGTTATGATGGTG
CAGAGTCAATCGCAGAATTGCATCCAAGTATTAAAAGTGCTGAAATTTTTCCTAAAATGC
GCAATCTAACCGATGAAGACCTATTTACAATCGCAGGTCATATCTTACTTCAACCTAAAA
TTGTTTCTGAAAAGTGGGGCGGAGGAAAAATTTACTATTAGAACTTCAAAGACCTAAACC
TTGTGAATACTAGTTTATGTATTTGTTGCTTAAGTGTGATTATTTTGTTAGATCATGATA
TATATTAAAGATGGAGTTTGTTGACAGCAAGTTACTTTTTACCCGAACTTGTTTACACTA
TTCGTCTAGTTATTAAAAAGGAGACGTTAAATTGAAGAAGAAGCTTTCAGTTCTTTTCAC
TGTTTTTAGTTTTTTTGTAATAGGTTTCGCACAAATTGCTTTTGCTGCAGATCTAGATAA
TGGAGAAAAAGTTTTTTCTGCTAATTGTGCAGCATGTCATGCTGGCGGTAATAACGCCAT

TATGCCAGATAAAACCTTAAAAAAAGATGTACTTGAAGCTAATAGTATGAATACTATTGA
TGCTATTACTTATCAAGTACAAAATGGTAAAAATGCCATGCCTGCTTTCGGAGGTAGACT
GGTTGATGAAGATATTGAAGATGCAGCAAATTATGTATTATCTCAATCTGAAAAAGGTTG
GTAATTATACTTGATTTTATCCTGTATTAAAGAATAGACAATCTATTTAGTTGTTCATTA
GATTGTCTATTCTTTGTTTATGCTATTATATAAAGATATTTACACAATATTTTATGATGA
AAAGAATACCCGCAATTCTTGTACTAGAAGACGGTGCGTATTATAAAGGATGGTCATTCC
AGCAAGATAAACAAGAGATTACTATTGGTGAAGTAGTTTTTAATACTGGAATGACAGGAT
ATCAAGAAATAATCACAGATCCTAGTTACTTCCATCAAATTGTCGCTTTTACCTACCCGG
AAATTGGGAATACAGGTATTAATAATCAAGATATTGAATCTCACAGTATTAGTATTAAAG
GACTTATTGCAAAAAATATTTGTAAAATTTCAAGCAGCTGGAGAGAGCAGCAATCTTTAG
TTAAGTATTTAAGTAGTAATAATATTCCTTTTATTTTCGGAATAGATACAAGGTCTTTAA
CCCAATACTTGCGTCAATTTGGTACAATGAACGGTTGTATCTCTACTGATAATTTAAATC
ATAGTTACTTAAAACAGAAAATTTGTGAGATTCCAAGTATGCAAGGTTTAGATTTAATCC
CGCATGTAACTACAAGAAATGTTTACCCCTGGGATGAAAAAAGTTTTCCAAATTGGTATT
TGACAGATAACATTAGAGTGCACCGAGTTATTCAGTTGAAAGTTATTGTTATAGATTTTG
GAGTTAAACTAAATATACTCAGAAGACTAGCTACACTTGGATGTCAGATAACGGTTGTGC
CTGCCCACACTCCTTTAAAAGATATTTTGGCTTACCAGCCTGATGGTATATTACTCTCTA
ATGGTCCAGGAGATCCATCAGCAGTACATTACGGCATCCAGACAGTTACAAAATTACTAG
ATTACAATGTGCCTATATTTGGGATTTGTATGGGGCATCAAATTTTAAATTTAGCTCTTA
AAGCTAAAACTTTCAAACTTAAATTTGGTCATAGAGGTATTAACCATCCATCCGGATTGA
ACCAGCAAGTTGAAATAACTAGTCAAAATCATGGCTTTGCAGTTGAATTGACTTCGGTTT
TTGAATCTCCTGTAAGAGTGACTCATTTTAATCTAAATGACACTACTATTGCAGGAACTG
GACATAATCAAAGTCCTTATTTTTCTGTGCAATATCATCCAGAATCGAGCCCAGGCCCTC
ATGATGCTGATTATCTATTCGAAAATTTTATAGAAATAATGACAAAGTCCAAGAATAAAG
TTAGTTAGTAATTTTCCCATGCTTTATGAGTGAATAAAGCGGATAGTACTTGTACTCCTC
GTAATTGATTAAAGAGAGGCAAATGTCCTCTGGGGCCATGAGAATTCCATTGAAATTCCT
CAGGATATCTGCAAGGAATTCTATCAACTTCCCAGCCGATTTTTTGCCAGAATTTTCCCC
AATCTTTTCCAACACTAAGCCATATTTGTCTTTGAACAAATAGCCCAAATTTACCTTTTG
AGTGTGTATGCCATAACTTATCAATAGTTTGTAAATCTTGAGCAGGTATTTTTTTTATAT
CTGTAAAATATAGCCAATTACGAGTTTGTGCATTTACACCAGCTAGTTGAATAAGCTTTT
GTTGAGTTAGCTGATCTGCTTTGAGCAAATCCCGATGAGTCAGTAACATTTGTAAATCTT
TGTAATTCATCTGTTGAGCAGAGCGCAATGGAACAATGCCATCGGGACATAAGTTAGAAG
CAAATTTCACTATTTCTTGATTTTTACTGTTAAGTAACTTTTCATAAATTAAACCGTCAA
CACAATTACTTTTATAGTTAGGCCCAGTAATCCTCTCAAAAAATAGATCTGCTAAATCTT
TTAACTCGATAGAGTCTTTGCTATTTATATTCTCAATAATTTCAAGTTGTTGCTTGACAT
TATTAGACTTAGTATTCTTATTCAATTCTAAAAGCTGGGCTCGGATTTGATTTGGCATCT

GATTTGTTTAATTAGTTTTAATAGTCATAAGGTCTTAGAGAGAATTAGATTGTTTTTTGA
AATTAGAAGAGCGCCGCAGTATTTCTAAAGAAGTTCGCATAATTTGATTCAATAAGTTTT
GTCCTGCATAAATTATAATTTGACCAATTAAAAAAATTGTGCTTTTTATTTTAGGAACAA
AAAAATCTTGTACCTCAAGCAGAAAAGTAATTATTATTTGCATTGGTGACAACATCTGTA
ATTCATTCGATCTGCAAGCATATATATACTGACAGTTTAAGCCTTCTTGATAAAATCCCC
ATACTTTGTAACGACTTTCGTAAATTGCTCGTGGTCGCTCAAGATAGTTTTTAATTATAG
TATTCCAAATTAGGTTATTCTTTAATTTTTCTAATTTTCTATCAGATAGAAATTTTACAT
TACAGAGATAACTTGCATTTGTCTCATTTACAGTATTAAAATTTTGAACTAACATATGAG
CAATAATGTTGCTTAGCTGAATTAAATAATTTTCAAGAAAAATTTCGACCTGCTTAACTG
GCATATTGTAAGTAAAGAGATTAAAAGTTTTATTCACAGTTTGAGATGACCCAAAAAGTA
GCTGAATAATTAAAATTTGTAGTAGCATCTTATAGTCGTTCAGTAGATAGTTCAAATCAT
GAATATCTTGGTAAATGTCAATACAGTCCAGTTCATATAACTTACAAAATCTTTTGATAC
ATCTATGGAATAAATCAATTAAAATATTTTCGGTCAAGTTTGTAATATCATTTACATCTA
AATTTGAATTATAAATTTCTAAAAGTATCTTTTCCAGTTCAGCTAATATGATTTTTAATA
AGTTTCTTTTTACTTCAGTGCGAAAAACATCTAAAATTAACACTTCCTGAGAGCAATTAG
TTAATCTCTTATTAATCTTTATAGAAGTTCTAACTAATAGCTCTGCTACCTCTTGATTGA
GTGTTGGTCCCTGAGAGCTTGGCCAATAATTATTCACGTTATACCATAGTAAGAAATATT
GAATTTAATGTTATACGATAACTTTTTAATATACTGAAAAGCAAGTTAATTACTCTATAG
CAACTAGTTTTTACAGATCTTAATAGTTATTTATCAAATAAAGTTATATGATAGTAGCGA
TTGAGCTAATTTTATTAAAATTTATAATAATGACAACTTATTACTTCGCCCTTGCGAGTC
AAAATTTTCTATTAGTACAAGAACCACTAGAAGAAGTTTTTAGAGAGAGAGTTAACTACT
ATCAGTCAAATAATAAAGCAATTGATTTTTGGCTAATACCAAACCCTTCTTTTCTAGAGA
AACCAGAAATGATTTCATTTAAAAACCTTGTACCTAAAGACGCTGTAGCTATAATCTCTA
CTAATCCAATATTTATTAATTGGTTAAAGCTAAGAATCGGCTATATCTGTATTGGGCAAT
TTGAAGATAACCTACAACTTTCTGAAGAATCGTTAAATATTACTGTTTTAACAGACAAAA
TTTAACAATGCTTTTATTATGACTAGTGTTAAATTATTTTGGTTAAGTTATAATTATAGT
CTTTTAGTATTTAGACTCTTATAGTTTATCACTTAGTATTTTATGCTGAGATGGAGTGAC
AAATAGTGTAAGTATTTCTTGGCTAAACGTTTCAGTTGCCTTAGATTTATATCTATTTGG
ATTAACTATAATGGATAGCATTCGTTTTATAGTAACATTTTCAATTTGAGCCCAATGTAC
AATTCCAAGTTCCAATTCCTTAGCAATAGCTGAAACGGAAACAAAGGCAGCTCCTAGCCC
TGATTGCACAGCGTTTTTGATAGCTTCTATAGAATTCAATTCCATCTCTATTTTAAAGCG
ACTGCTGTCAATACCATGTTGACTGAGTACTTTATCTATGACTTTTCTAATTGTTGATTG
AGTATCTAACGCAATAAATCTAAGCCTGTATAAGTCTTCTTTTTGAATGTCTCCTAGTTT
AGAAAAAGGATGTGATTTGGGTAATATAAGTGCTAATTCGTCTTCCGCATAAGAAGTAAC
CTGCAAAACATCTTGCAATTCAGTAGGCACTTCTCCTCCAATAATTGCTAAATCAACTTG
ACCATTAGCTACGCTCCATGAAATAAGTCTAGTTGAATGTACTTGCAATTGAACAGCCAC

TTGTGGATATCTTTGCCTAAATAGTCCGATTAATCTTGGCATCAAATATGTCCCAGTTGT
CTGGCTAGCTCCAATAATTAATGTGCCACCTTGTAAGTTTTGTAAGTCGTCAAGAGCTCG
ACAAGTTTCTTCGCAGAGAGCTAAAATTCTGCCCCCATATCGTAAAAGAAGACTTCCTGC
CTCAGTTAAAGTTGCCTTCTTATTACCTCTTTCAAATAGGGAAACATTCAATTGGCGCTC
TAAATTTTGAATTTGCAAACTAATAGCTGGCTGAGAAACATACAAGCTATTAGCTGCTTT
TTTAAAACTGCCCTCTTTGGCAATTGCTTTTAATATTCTTAACTGATCCAATGTAAATGG
AAGGTCTGTCATTAAAGAATTGTAGTATAATGTATATTATTTAGTATTGATCAATGTATG
CATTTGCCAACTAAAATATTTGTCATTACGCTTCATTATTTTCAAAATAATAAAAAGTAT
AATGTTAAATAAGCTGTATTAAAATTTAGTATCTGTTTGATATTAGATTTATACAAGAGA
TATCGTGAATTTAATTCTAACTATAAAATATAGGAGACAATATGGACTCTAGACTTTTAG
TTGTACTAATACCAGTTTTAGCAGCAGCATCTTGGGCAGTTTACAATATTGGTAGAGTTG
CACTACAGCAATTTAGAAAAATGACATCTTAGTTTTGCTTAGAATATAATTTTTAGACTT
TATGAGATAGAGGACTTTAAAATCCCTATCTCTATTGTATATCTTAATAAAAAAAGAAAG
CCCCCTTGTACTTACTGTTATTACACATTAATATTATTGGTGGTACCTAGATAATAAGTT
TAAGCTAACTTATTTTTTATTTTATAATTAATTAAATTAATAACACAATTATGGCTGTTC
CAAAGAAAAGAACATCTAAAGCTAAAAAAAATGCACGCAAAGCAAATTGGAAAAATCAAG
CAAAAACTGAAGCTCAAAGAGCTTTGTCTTTAGCAAAATCAGTATTAACTAGAAAATCCA
ATGGATTTATTTATAATCTAACTGAAGCATCAGATACTTTTAGCGATTAGAAGTGTTTTT
ACTACAAGTCTGAAAAAGTATTACGCAACAAGTTCTCAGTAATTTTTATCTATTAATCAT
TTGAAAATCATTCTATTTGATAATAAAATAAATAGCAGATTAAATAAAATCACATTAACA
AATTATGCAGCAAAGCTAGATCAAACTAGTGAAATTTGGTTGTTCAATTGTATTGAAAAT
ATTCAGCATATTTTTTTTAAAAGTCAATTAAAATCAAGTCATATTACTAAAATTTTTATT
TCTGGTACTAGCTTTGAATATACCGCAGGTCTACCAGGATTATTATCCAGCTTAACACTA
AGTGGTAGACTACATCCTATTAGTATATATAGTCCCCAGTCTCTCAAAAAGTATCTTGAA
GCATGTACCAAATATTCCCAAACTAATTTTTCTTTTCCTATTAATTTTCATAATTTACAA
TACGGAGGACAAGTTGTTAACCAATTTTATACAGTAATTTGTTTACCGTTGAGCAAAAAG
AGCCTGCTGTATGGATTTATTATTCTAAAAAAAGAAAAGCAGGGAGTATTTAATTTAGCA
CAAGCTAAAACCTTGAATATTCTTCAAGGACCTATATATGGAAAACTCAAAGAAAAGGAT
AATTTTTTAAGTCCAGATGGTTACTATCTATCCGGCCAAGACTTCTCTTCTAATACAATA
ATGGGACATAAAATATCGCTTCCGTTATTAGTCAGATATTCTAGAATTATTTCTGAGATG
CATTGGTTTTGCTCTTATCCTATTAGATTAAATACTTATTCGCATCAGCAAGGAGCAAAG
TGTTTACCACATAATGTTTTAACTGATATTATGAAATCCCAAATATATCAAGATAACAGT
TTTGTTGAATAATACTTATTCTTGATATATAATGGTCTCGGTAATCTAAATAAAATACTA
GAAATAATTTCTTAAATTTATTTGTCTATATTAAATTTACAATCATTTATGACATACGCA
ATTATTGAAGCAAGTGGCACGCAGCTTTGGATAGAAGAAGGCCGCTACTATGATTTAAAT
CATATACCTGTTGATCCAGGTCAGTCGATTATATTAGGAAAAGTCTTATTATTGAATAAA

AATGGGGAGGTTACTTTAGGCCGCCCTTGTATAGAAGGGGTTACGATAAAGGCTACAGTA
ATGAGGCACTTGCGAGGAAAGAAGATAACTGTTTTCAAAATGAAACCAAAGAAGAAAATG
AGATTAAAAAAAGGTCATCGACAAGAATTGACTCGTTTAATGATCGATTCTATAACATCT
TAGATAAAATCTAGACTCATATTTTTAAAAACTTTTTAATATTTAACTAAAATAGATATA
ATGGCACATAAAAAAGGTAGTGGTAGTACAAGAAATGGCAGAGACTCTAATTCCAAGCGT
TTAGGTGTTAAAAAATATGGTGGAGAGCAAGTAACAGCAGGTAATATTTTAATCAGACAA
CGGGGAACTAAAGTTAAGCCTGGCCAAAATGTTGGAAAAGGAAAAGATGATACATTGTTT
TCTCTAATTGATGGTTTCGTGCTGTTTGAAAAGTCAAATCAAAAGCAAAAAACAATTAGT
GTTTATTCTGCTAAGAAATAGTTAAAACAATTGGTGCAAGATAACTTGAGTGTATTTATT
TCACTTACAGTGGCACCATATATTTGATTTATTAAGAAATGGAGTTCCTTAATGAACCAA
TGAATTTTATTAAAGATTTGCTGTGAAATTTAATGACAAACACTATTGTAATTTCCTGTC
TACACAATATGGCTGCTATTCTATATTGTGGTCAGATACAGAAACTAGTAGTAGCAAATG
CTCATTATCAAGTAAGTGATATCTACCTAGGTTGTGTTGATAAAATCTTCTCAGGAATAA
ATGCGGCATTTATTAACCTAGGAAAGAATGAGTACAGTGGTTTTATACATATCAGTGATA
CCGGTCCGCTTAAAAAGAAATATTATGTCAATAATATTACTAACATTTTAACAATACGGC
AAAAAATTTTAGTACAAATTATTAAAGAGCCAACTTTAAATAAAGGTCCAAGGCTCACTG
CCAATATTACATTATCAGGTCGATATATTGTATTAATGCCTTTTAGTCAATCAATCTGTA
TATCTCGAAAAATATATGATGAAGATGAGCGTCATTATTTGAAGTCTTTAGCTATTTTAA
TTAAACCGGCAACAATGGGCTTGCTATTTAGACCTTCTGCTGTAGGTGTCGATGAGGAAA
TAATATTAAGCGAATTAAAAAATCTAAAAGAACAATGGAACTTTGTTCAAAAATCTGCAA
TTAATAGTTATTCACCTGTTCTTCTATATAAAGATGAAGATATTGTTAAAAAGGTAATCC
GAGATTTTTATAATAATAATACAAACAATATAGTAATTGATTCAAACCTGGGATTAAAAC
AATTAAATTATTATATCCACACTTGGCACTGTAATAACTCTAGCACAGTTCCTAAGATTA
AGCTTTATAGTAATAATCAATGTATACTAGATGCTTTTGGTATCAATCAGGCAATTTCCA
GAGCTCTCATTCCAAAAGTTGATCTTATACTTGGTGGCTATATGTTTATTGAAACTTTAG
AAGCTTTTACTATTATTGACGTTAATTCTGGATCTTTTAATAATTCCACTAGTGCACGAG
AAACAGTTTTAAAAACCAACTGTTCTGCAGCAACAGAAATAGCTTATCAGTTACAAATTA
GAAATATTACTGGTGTAATTATAATCGACTTCATTGATATGGAATCACAAAGAGATCAAT
TGCAATTATTAGAACACTTTAATAAAGAGCTATCACTTGATGATGCTAAACCACAAATTG
TACAGTTATCTGAATTAGGTTTAGTTGAATTGACTAGAAGAAGACAGGGCAAAAGTTTGT
ACGAGTTAATTAGTAGTGATTCTAATTACTTTTATTTTTTCACACAATCAGAGAGATCTC
AGTCTCTTAAGAGATTCGATGATAGACAGCAGAAACAACAGATTTTTAATAAATCTTGGC
TATCTGCAGAGATTAATACTATTAACAAGGTCTTTTTTCAAAAGTCCAATTTGTGCAGAC
CTGCTAACTTTTACCTAATTCGTAATCTCTATATAGTTAAAAGTAGTATTACTTATAAAC
AAAATTATTTATTAACTCACAGATCAAAACTTATTTATTCTAAAGAATACAGCAAAGTAT
TACCAAGCAGTTATTATCTAGCTAGTCTCAATAAGAATAGTAATCAAGAGTTCCTACCTT

AAGATTGTTATATCAACTGTTGAAAAAAAGAAGCTCTCTCTTATATAAGAGAGAGCTTCT
TTTGTAGCTTACTAACTATCTAGTTAGTTAATTAAAAATTAACCGTTAACAGCTGGAGCT
GTTAGAGCTACTGGTAAAGATTCACCAGAAGCTAGATCTAGAGGGAAGTTGTGAGCGTTA
CGTTCGTGCATTACTTCCATACCTAGGTTAGCACGGTTGATGATATCAGCCCATGTGTTA
ATTACACGACCTTGGCTATCAACAACAGATTGGTTAAAGTTAAAACCATTCAAGTTGAAT
GCCATTGTGCTTACAGATAAAGCTGTTAGCCAGATACCAACTACAGGCCATAGACCTAAG
AAGAAATGTAGAGAACGAGAGTTGTTGAAACTAGCATATTGGAAGATTAAACGACCGAAG
TAGCCATGAGCTGCAACGATGTTATAAGTTTCTTCTTCTTGTCCGAATTTGTAACCATAG
TTAGCAGATTCGTTTTCGCTTGTTTCACGAATTAAGCTAGATGTAACTAGGGATCCGTGC
ATAGCACTGAACAGAGAACCACCAAACACACCAGCAACACCTAGTTGGTGGAATGGGTGC
ATTAAAATGTTGTGCTCAGCTTGGAATACAAGCATGAAGTTAAATGTTCCGGAGATACCT
AGAGGCATTCCATCAGAGAAACTACCTTGGCCAATTGGGTATACTAGGAATACTGCTGCT
GCTGCTGCTACTGGAGCAGTAAAAGCAACGGAAATCCATGGACGCATACCTAGGCGGTAG
CTTAGTTCCCACTCACGACCAATGTAGCAAGCTACGCCAGTTAGGAAATGAAGAACAACT
AATTGGTAAGGACCACCGTTGTATAGCCATTCGTCTAAAGAAGCAGCTTCCCAGATTGGG
TAGAAGTGAATACCGATAGCTGCAGAACTTGGAATAACAGCACCAGAAATGATGTTGTTT
CCGTATAGAAGGGAACCAGCAACTGGCTCACGAATTCCATCAATGTCTACTGGAGGTGCA
GCTACGAATGCAATGATGAATACAGATGTGGCAGTTAATAGAGTTGGAATCATTAATACA
CCAAACCAACCAATGTATAGGCGGTTTTCAGTACTAGTAATCCAAGAGCAGAAACGTTCC
CACAAGCTAGCGCTTTCGCGTCTTTGTAAAGTAGCAGTCATAATTTTTTATCAATTTTTA
AGGATTTAACAAAGATGCTTCCCAAACAATAAGTTGTCTGTTATGGAAATTATTACTTAA
GGCCTTTTCAAATGAGAACATTTTTAAAAAAAACATAGTAAAATCTAATCTCAGTAACTA
AAATTTTACTTGTTCATATATTGCTTAATAATTCAATAATAAATTTTTGCTTTTTGCAAA
ATTAACACTATTTTAGGGATATAATAATAATGCATATTAATGCTGCTTATTTTGATTTTA
ATAGTTTATATAATTTTGCCCTACTTAAATACGCATGTCACACTTTACAAAAATTCAGAC
GACCATACAAGATTTAAATCTGTTAAAGCATGCTTTAACAGATTTGGGTTTGATTTGGCA
AATGAATTCTAGTCATATAAAAGTTGGTGAAAATAATCAGCACAAGGTAGATATTTTAAT
TAAACAAGATAATTTGTCACATATTGGCTTTACTTGGAATGATAATAGATATCACTTAGT
TGCCGACTTACAACTTTGGGAACAACCCTGGTCTCTAGAAGTATTTTTGGATAAGTTATC
TCAGAAGTATGCTTACTACTCTATCATCGAAGAAACAAAGAAGCAAGGCTTTGAAAAAAT
GCAGCAGATTTCACAAAAAGATGGATCAATTAAGTTAATTGTGCAACGCTGGAATTATTA
ACTAACATACAAATGCGGGCGGAGAGACTTGAACTCTCACGAGATTATCTCACTAGAACC
TAAATCTAGCGCGTCTACCAATTCCACCACGCCCGCACTGTTGAGACAATATTACTCTAT
CATAATCTATACTAAAACACAAACTAATAGTATAAACTAGCTTCCAAGCTTAAAAGCGTC
TACAAATAGACCGTATGTTATACCAATTTTGAAGTTGTTGAATAAATTAATTTTTATATT
TAATTGCTTGTGGCTAGAGTAGACTATTTTACTAGTCAACTCAGTTGCTGCAACTATTAG

ACTGGCTGCAATAATACCCCAATCTCCTGTTTGTCCAGGAATCGTAGACAAACCTGTTGA
AATAAAAAATCCTAGCAATAAACTAATTAAGCCAGTTGTTAACTCGCTCAAAGAATAATA
AAGCTTATTATTTAAGTTTTTTATTAAGTAAGCAAAAAAAGTTGAAAGTCTAGTTTTAAT
CATAGTATTTAGTAACGGAGTTTTAAAAGGATGACAAGTATTAACAGTCATTAATATTGT
CGTATCCTTTTAAAACTCGTTAATACAAACAGCTGTTCTTGTTTTTAAAGGTCTTGTTCG
CTTAAAGCATTAATAAGATGCTGAAACGGCTCTTGTATAATATCTGTGCGTTTAATTGAT
TGCAAATCTAATTCTTCTTCTATGATTTCTTGTAATAACTGTATGCTTCTTACTGTTGGA
GCAACAGGTACAGATAGTGAATTGTAAGTATCTTTTAGCCCATTTAATACACGCTGATCG
AGAATATTTGTACTACCAGAAACCAAGGCATAACTAGCATACCGTAGGTAATATTCTATA
TCCCTCAGACAAGCTGCATATCTTCTTGTTGTATAAGAATTTCCTCCAGGCCTTAATAAT
TCAGGCTGTTCTTCGTACAACTGAGCTGCGGCCTCTTTTAAAATATTGGTAGCTTGATCA
TTAATAATTTCAGCTATTTTTATTCGATCTAGTCCACTCGAAAAGAAAGATTCTAGTTGT
CCGACAGCTGTCTTATCTAGATAACGACCAGTAAGATCGTAACGATTTAATATTGCTGTT
ATAGCATCTTGCATAAATTAATTCTCCTACTTGCTATCAGAGCGGTATTATAAATGACTA
ATATTGTAATATAATCATTAGTGTTTATGAAATTTATTTAAAACTTATTCTTTATTGCTT
AATGTGCAATTAAAAACTCGCATAGATTTTACACATATAGAGTATACGTAGTTTTTAATT
ATTTTTTTATAAAAACCAAGAAATAAAGCTTTTTTTAAGAGGTTCGTGAATTTGTTAATA
TAACATGAGCTTTCCTCCTAAATAAGTTTTGTAAAGTACAAAGTAAAAAAACGATTAGTC
AATAATAGTAAACAATATTAATATGTATCAAAGTATAAATAAGCTTTTACTCCAGCATCA
AAAAAAATATTTGAGATATGTTGATACAACTGATTATTCAAGTGAAAACTTAGATTTAGA
CCAATTTAGCTATTTTATCATTACAATTAATAAAACTGATAAATGTATTCTTATAGAGCA
ATTTTGTTATAATGCTGAAGGCCATATTTACTCAATATTCTTCAAAGGTCCAACAGCTAA
ACATCTTTCTTCTATAATTTGTCATTTTCAACAATTAAATACAATAATATCTACAGCTCA
TGCCATATATTTAGGACGAGAGCTAATGAAGTCAGAATTAGCTCTCGTGCTAGATCAACA
ATATATTCAAGATTAAATTTTGTAAGTATTAATTATATTTATGCTAGTATAATATTAGTA
AGGGCATGTAACTCAGTGGATAGAGTATCAGATTCCGATTCTGATGGCCGTGGGTTCGAA
TCCCGCCCTGCCCGAGTATTCAGAGTACTATACTAAAACATCTTAATAGCTAGTTTGATA
ATAGTATGGGCATTGGGGCTGCAAGGTTTCTACATTATGAAAAGAAGAAAATATGAAAAA
AAACAAGCTCTCCAAAAGAGCTTTTAGTACAATCAATAAATGCAGAAAACAATATTGTTT
CTTTTTCTCGAAAATTAGCTCTTGCTTAAACACTGTTAATTTTTGGCATTCGACATGTTA
AACTCTTATTCATGGCGAATATTCTGTCAGAGTCGCTCTTAGTTTAAGAAAAGTCAGAAA
AAATATTTATCTTTACGTTTTTACCTATTTTTATTTAAATAAAGGTTAGTATTTTCAACT
TTTATAGTGGACGTGGGTTCGAGTCCCACCAGCTCCATCAATTTGATTTGTATAATTAGC
TAGTTTAACCTTTTTAATAACCAGATATATAATTGGAGGAAATATGGGTTTCATAACAAT
TACTAAGCCCGCTTTAAAGCAAATTGCAATCTTGAAAAATGATCATGAAAATGATGTACA
TCTAAGAATAGGTGTTAGACAAGGCGGATGTTCAGGTATGTCATACTCAATGAATTTTGA

ACATGTTGACAAATTAAAAGATACAGATGAACGGCTTCGTCTAGATAATTTTTCTGTTGT
CTGCGACCCTAAAAGTCTTCTTTATCTTTATGGATTATCATTAGATTTTAGCTCGGAATT
AATAGGAGGAGGATTTCAATTTTCTAACCCGAATGCCAGCCAAACTTGTGGCTGCGGTAA
ATCTTTTTCAGGCTGACCCTCTAACACGTTTTTATATATTTTGTATTATATATATTATT
TTAGCAAGTTGTTTATTAGAGACAAGCAATGCTCTGTTAAAAACTAATTTCAAGAGTTTT
TATTGTATTATTTTATATATTTATTATCAACTACTTGAAAATAAATTAGCTAAGCAATCT
AATTTGTTATCAGTACCAAACTTTAAATGATTAACTTTTTATTGATATATGCTAATCTTT
ATCAGCCTAAACATATCTGTACATTACAATCTTTTGACATAAAAAGAGTCTTAACATACC
TCTCAAAAATAAATTCAATATTTTATAAATACTCAACATGAATCTGTCACAACTTATCGT
TAATTCTTCTATGATGAAAATTCCTAATAACTTTTATTGCAATATAAAAGCTATTTCTTC
TGAGAATTTAAGTCACTTACAATTATCAAAAACAAATTTATTATCTCCTACAAAAATAAT
TGCTTTTGGACTACCTAAATTTGATTTTATTTCTACACAGATTACAAGTTCTATTAGAGA
AACAGTTGTTGTGTCTACTATTGATGATAATAATCTTTTTGCTGCCCCAACTAATGATTG
GATAAAAAAACACAAGGATAAAAATCATCATTCTTTGTTTTATAAAAAAGTTTTTAAAGC
TTTGCTAAACCATAAAATTAGAGTTGTTGCTGAGCCATTTCATAACTCCGAAATAAATAA
AGAAAAGAGCATAATGGCAAGTCAAAGATACATCTGGGGTAAAAGCTGGAAGCCTTCAAT
TATTTTATCTTGCTTAAAAAACAAAAAAAATAGCAAAATTTCTGATGCATCGAAAAAATT
TTTAACTGAACAGTTATCTGCATCACCAGTATTTGTAGTAAAAAATGGTTTTAATGAAAT
TATTTTAGGTCACCCATTATCTCGTGTTAAAAGAGGAGGAGTTAATAATCTGATGCATGC
GTTTTCTAATTTATTAAATCAGTCTAATGCAACTTATCCTATATCTACTGGTCTATTCTT
TTTTCATCCAGATGATGCATTTGAATTTAAAGACTTTATAATATCGGTCAATCCACTGGC
GGCCAAACATATGGAGATAAGCGTAGAGCCTGTTGGTCTACACTTTGCTTATAAAATGAA
TAGAAATATATCGTCAGATACTCAATTTTCGTTTATTCCAGATTTTAAAGAAGTAGGAGA
TTTATTATTCAAATATAGAAAAGGTAATCATTTAGTTTTTCATAAGAATCAGCATTATGG
CAAAGATTTTTTTCAGGGGCAACCAATATATATGATTCAGCCGATCACTTTTAAAGATCG
AGCCGGAAAGCTAAATATTATCAAATTTACGGGGCTAAATGACACAAGAGAAATTATTTT
TACTAATCTTGAAGCTGCTAATAGATCATGGGCACATTTTATAAAGAGGAATTCACAATT
AAAATCAATTAAAAATCCTACTCTATTAGTCTATAATTTAGAAAGTTTTTTAAAAGATCA
AGAACGATTGGATAATCAAGATTTAAATAAGTTTGTGGTGATCACTAATAAAGAATCTTA
TCTTGCCACAAAAGAGTTAATAGCTTTACCCGATTCTAATAGCTTTTCTAAGCACTTAAA
ATTAAATATAAAGCCTAAACTCTTTTTTGTGAAACTATGGGTAAGACGGTTATTTTCCAC
CTTAACTTATGAATAAATGCTATTGCCTTTATTTTCTCGAATAATATTCTATAACTAGTA
ATTCATTGAGTTGTAAAGCAACCCATTCCCTATCAATGACTCCATTGACTTTCCCTGATA
AATTGGATTTATTCAATTCTAAGTGGCTAGGAATATTAGCTAGTCCAGGGAAAGCTAAGT
AATTTTCTACTAGTTTTCGAGATGCTTCTTGATTTTTTACACTAATTGATTCTCCTGGTT
TACATTGATAACTACAGATAGATACTACTTGTCCATTAATACAAATATGACCATGGTTCA

CTAGTTGTCTAGCGGCAGGAATTGTAGGAGCCATACCAAGTCTAAAAACAGTATTGTCTA
GCCTCATCTCTAGTAGCTGTAACAAAATTTGACCTGTCGACCCTTGTAGTTTTTTAGCTG
CTTTTACGTACTTAAATAACTGTTTCTCACTTAATCCATAATTAAATCGCAACTTCTGTT
TTTCTTCTAATCTTACAGCATACTCAGAAGGTTTTCTGGACTTTTGTCCATGTTCTCCAG
GTGGATAAGATCTTTTAATCGCTTTTCTACTGAGTCCGGGTAAATCACCTAATCTACGGG
AAATGCGTACTCGTGGCCCTCTGTATCTAGACATATTATTTAAATTCTCCTAATGTAAAT
GATTAAGTAATGAGTAGCTATTTTTTAACACAAAAAGAGCATTAAAACAAAGCTTTTGCT
AAAAAATATTTACAAAAAATATAGATTTGGACAAAATACATTACTATGCTTTTTAAGCGG
GTAGCGGGAATCGAACCCGCATCATTAGCTTGGAAGGCTAAGGTTTTACCACTAAACTAT
ACCCGCAATATTCAACAGATTGTCACTAACTCAATATAACATAGTTATGTTAAGTTAAGA
TAATCTTAAATTTAATAGAATTTTAAATTATTTATGCTCCTTCTAAAACCACATCAAGAA
AACGAGCAATTTCGGCAGCTTGTTCTTCAACTTCTGATAATAATAATGGTTGACCGACCC
TAGTCAATGGAATTACTCTTTTATCTTTTGTACATAAATAAATTTCACGTCTTGGATTTA
GCCCCTCTTTAATATCTATTTTTATTGATTTGATTTCTTTTATATTAAACTGAAGACAGA
TTTTTCTATTCTTTCCAGGGAAACCTAGCCGAAAAATTTTTACGATACCTTTATCTTTGT
TAAATTCATTATATCCAGCCCCTATATTCCAAATAATAGTTAGCCACAGAAACAAACTCA
GAAAGACTCCTATACTGCCATAGAATGTCATTACAATACCTTGTGGAATAAATACCAAAT
CGGTTGAATTTGTAAAAGGTAACAAATCAACTTGAAAATAACTTGATAGTCCAGCAAGAA
GAAAGCCCAAAGCCCCAATAAATATTATAGTAGCCCACCAGTAATTACTCAGTCTTCGTG
AACCTAAAATTAAATCTTTTCTAACTGAGTGTATAGATAATGTTTTTTTCATAATTTGTT
CTTTTAAAACAGTATACTAATGCAAAATGGAAAAATTTTGACAATTTTTATGCGCGCTTT
AGATTTTTTAAATAAGCTTGATTGCTTTTAGTCCTAAAAACAGTCCTGTTGCTAACCCAA
AAAAAGCAGCAAGAAATATTATATATCCTAGAAAAACTGACATATTATTATTATTTATTA
TTATTGTTACAATAGTGTTTACAAAAATAGTGAAAATCTATTACTACGGTATTATATTAC
GTTAAATGCCTTGCTGTCTACAGGAAAAAACTTTTTTCAGTTTTTAGCATTGTGTTTATA
TTAGAAATAATATTCTAGTAAATTTATTAAACTTCTGGAGAACAGAATCTTATGTCAGAA
TTTATCAAGCCTTATAACGATGATCCTTTTGTAGGCAATTTGTCCACGCCAGTTAGTACG
TCAAGTTTTAGTAAAGGACTTCTAGGAAATCTACCAGCTTACCGTCGAGGTTTATCTCCG
CTTCTTAGAGGATTAGAAATAGGAATGGCACATGGATACTTTTTAATTGGACCTTTTGAT
AAATTGGGCCCTTTACGAGGTACAGATGTAGCGTTACTAGCGGGATTTCTATCTTCGGTC
GGCCTCATTATTATTCTCACTACATGTTTATCCATGTATGGTAATGTATCTTTTACTAGA
GCAGATTCGAAAGATCCACTACAAACTTCTGAAGGCTGGGGACAATTCACTGCAGGATTT
CTAGTTGGAGCAGTAGGTGGTTCAGGATTTGCTTATTTATTGCTAGCTAATATACCTGTA
TTACAGACTGCAGGTCTTAGTTTATTCTCTTAAGCTAGTAAGGGGACTTGAACCCGTAAC
CTACTGATTACAAATCAGTTGCTCTACCAATTGAGCTATACTAGCATTCAGTTCACAATA
ACACATAAAGATATTTGTTGCAATCTGCTATTTTCAGATGTAACAAATATCTTTTTTATT

TAAACAGAACTGTTATGTTAACACGTATATTAATTAGAATCTGAATTATCTGACTCTGCA
GAAGATTCCTGATCACAGTTTAGATAATAACAAATTGTTTGATCTAGACAAGTTGCAGAC
CAACCAACAGGTGTTTCTCCTGATAATTCTTGCATATCTACAGAATTATAAAAATTATCA
TTTGGCTTATTACTGTTTGCTTGCATAAATATCACTCCCAAAACACTCTGAACTTAATGT
GAAGTAATTTAACTTCTCAATAATATATTAACACAAAATATTAAATATGGTGATTATTCT
TTAATTGTAAAGATGCTGCTAACTTTATAAAGTTTGAGTGAGAGTCTTGATAGCTTTTGT
TGAAATAAGCATTTTAACCCATTTATTTTGCCCTCTCGACCATACTTTTTTGTGTTGTAA
ATTAACTTTTTGTAGTTTTTTTGTTCGTTTATGAGAATGAGATACGGCATATCCATTATT
AGCTACTTTTCCCGTAAGCTGACACTTTTTTGACATAATTTTCTAACTTATATATTTATT
TAGAGTACTTGCTAAAGTTGACTTGGGCACAGCTCCAATCACTGTGTCTACTCTTTCTCC
TGCTTTAAAGATCATTAAAGTAGGTATACTTCTAATGCCATATTCAGCTGCAATAGTAGG
ATTATCGTCTGTGTTTATTTTTACTACCTTAATAGATGATTCATATTCTTCCGCAATTTC
ATCAACTACAGGAGAAACCATTCTACAAGGACCACACCATGGCGCCCAAAAATCTACGAG
TACAGGTAAGTTATTGTTAATAACTTCTTGTTTGAAAGAGGCATCTGTAACTTGAGATAC
TGACATATTCTTTAACCTTTAATATATGTTCCTTGCTAAGCAAATCTTATCACAAATTTC
AAGAAAAATCTTCTATAGTGAATTACTATTAGTGTTGAATTATTTTTTTAAAAATTAGAC
ATTAAAAAAACTTATTACCTATATAAATAAAGTGGTGGCCGAGTGATATCTTGATAGTGA
TAATTTTTATATTAAGGTTGAACACTAGAAATAACTTTTCAAGCAAACTCTTGTTTAAAA
AATTTCTCTGTGACCATAAAAACTTAATTATATTACAATATATTTTATTATTTGTAACTT
AGTTCTGATAATGGTATAAACAACGCAAAAGATACTGCCTTATAATCAAGGAGGAATACA
TGTCTCAATCCGTAGAATCACGGACTAGGATTAAAAGCGAACGTTACGAATCTGGAGTAA
TCCCCTACGCTAAAATGGGCTACTGGGATGCTGACTATGTGATTAAAGAAACAGATATTC
TAGCTCTTTTCAGAATCACTCCTCAACCAGGTGTTGACCCGATTGAAGCATCTGCTGCAA
TTGCAGGTGAATCTTCAACAGCTACTTGGACAGTTGTATGGACTGATTTATTAACAGCTT
GTGACTTATACAGAGCAAAAGCATATCGAGTAGATCCAGTTCCAAACGTGGCAGATCAAT
ATTTTGCTTACATAGCTTATGATATTGATTTGTTTGAAGAAGGTTCCATTGCGAACTTAA
CTGCTTCAATTATTGGTAACGTTTTTGGGTTTAAAGCTGTTAAAGCTCTTCGCTTGGAAG
ATATGCGTATGCCAGTAGCTTATCTAAAAACGTTCCAAGGTCCTGCAACTGGATTGATTG
TAGAACGTGAGCGTATGGATAAGTTCGGTAGACCTTTCTTAGGTGCTACAGTTAAACCTA
AACTAGGTTTATCTGGCAAAAACTACGGAAGAGTTGTATACGAAGGCCTGAAAGGCGGTC
TTGATTTCCTTAAAGATGATGAGAATATTAACTCACAACCATTTATGCGTTGGAGAGAAA
GATTTTTATATTCTATGGAAGGTGTAAATAAAGCATCGGCTTCTGCTGGCGAAATTAAAG
GTCATTACCTTAACGTAACAGCCGCGACAATGGAAGATATGTATGAGAGAGCCGAATTCT
CTAAAGAGGTTGGTAGTATCATTTGTATGATTGACCTTGTGATTGGTTATACTGCGATTC
AAAGTATGGCAATTTGGGCTCGTAAACATGACATGATTTTACATTTACATAGAGCTGGTA
ACTCAACTTACTCTCGTCAAAAAAATCATGGTATGAACTTCCGAGTTATTTGCAAATGGA

TGCGTATGGCTGGTGTTGACCATATTCACGCAGGTACAGTTGTAGGTAAGCTTGAAGGAG
ATCCTTTAATGATTAAAGGCTTCTACAATACTCTACTTGAAAGCGACACAGATATCAACC
TACCTCAAGGTCTGTTCTTTGCTCAAAATTGGGCTTCCCTACGTAAAGTTGTACCAGTAG
CATCTGGTGGTATTCATGCTGGTCAAATGCACCAACTTCTTGATTACTTAGGTGATGATG
TAGTTCTTCAGTTTGGTGGTGGTACAATTGGACATCCTGATGGTATCCAAGCAGGTGCAA
CTGCTAACAGAGTAGCACTAGAGTCCATGGTTATGGCAAGAAATGAAGGCCGTAACTATG
TAGCAGAAGGTCCACAAATCTTGAGGGACGCTGCTAAAACTTGTGGGCCTCTACAAACAG
CTTTAGATTTATGGAAAGATATTAGTTTCAACTACACTTCCACAGATACAGCTGATTTCG
TTGAGACTCCAACAGCAAACATCTAGTTTAATGACTACTTACTGATACTTTAAATAGTCA
ATTGTAAGTGGAATTAACTTATAACAATAAGGAGCATAGAATAGTGAGACTAACACAAGG
GACTTTTTCCTTCCTTCCAGATTTAACTGATGAGCAAATTAATAAACAGCTTGCTTATAT
CGTTTCTAAAGGCTTATCAGCAAACGTTGAGTATACTGACGATCCTCATCCAAGAAACTC
CTATTGGGAACTGTGGGGTTTACCTTTATTTGATGTAAAAGATGCTTCTGCTGTTATGTA
CGAAATTAGCTCATGCAGAAAAGCAAAACCTAATTATTATGTTAAAGTTAACGCTTTTGA
TAATACTAGAGGTATTGAAAGTTGTGTAATGTCTTTCATTGTAAATAGACCTGCTAATGA
ACCAGGATTCTTATTACAACGCCAAGACTTCGAAGGTAGAACTATGAAGTATAGTCTTCA
TAGCTATGCTACTGAAAAGCCTGAAGGAGCTAGGTATTAATATTAATTAAGAATTAATAT
TGGCTAATTATTACCCTCTTAAAAATCATAATTAATTTAGTAATTGTGATTTTTAAGAGG
GATAGCTTCCCAAGTAAATTTTGACTAAATAGATTGAATAATTAAAAATACACAGAAATA
ATGCAATCACAGGATATAATTTCCAACGATACTCTTGTTAATTTACAAGAAGAATATGAT
AGAACACAAATCCAAGAAGTTTTAAATGAGTTAAATCAAGAACTTATAGGATTAGTGCCT
GTAAAGACCAGAATTCGCGAAATTGCTGCGCTATTATTGATTGACAGATTACGCAGAAAA
CTAGAACTAGTTTCTGGTAATCCAGGATTACACATGTCATTTACAGGTAGTCCAGGAACT
GGTAAAACTACAGTTGCTATGAAAATGGCTGATATTTTGCACAGACTTGGATATATAAAA
AAAGGGCATTTGTTGACAGTTACAAGAGATGATCTTGTAGGTCAATATATTGGACATACT
GCCCCTAAAACTAAGGAAGTTCTTAAACAAGCAATGGGAGGAGTTTTATTTATTGACGAA
GCTTACTATCTATATAAAGCAGATAATGAAAGAGACTATGGCTCTGAGGCAATTGAAATT
TTATTACAAGTAATGGAAAACCAAAGAAATGACTTAGTTGTTATCTTTGCTGGATATAAA
GATAGAATGGAAAAATTCTACGAATCCAACCCAGGACTCTCTTCTAGAGTAGCTAATCAT
GTAGACTTCCCAGATTATACTTCAGATGAATTATTACAAATAGCTAAAATGATGATAGAA
GAACAGCAGTACTGTTTTACAGAAGAAGCAGATAAAACTCTTTTAGAGTATACCGAGCGA
AGAATGAAACAGCCTTATTTTGCTAATGCAAGAAGTATTCGCAATGCTATTGACAGGGCT
AGAATGAGACAAGCCAATAGGATTTTTGCCAGTGGAGAAAAAGTATTAACAAAAGCTGAT
TTGGTAACGATTGAAGCAGAAGATATCTTGAAAAGTAGATTATTTTCATTACCTAATGCT
TAATATACACGTGATTTCATGAAACTATATATTATTAAAAAGTTTCATAATCAGTTGCAA
ATTTCTTGAAAAACCTTTATTATTATCTTAATGCAGGTGTGGCGGCATAGCCAAGTGGTA

AGGCAGAGGATTGCAAATCCTTCATCCCCCAGTTCAAATCTGGGTGCCGCCTAGTACTAA
AAAAGGGGGGTGTGGTGGAATGGTAGACACAACAGACTTAAAATCTGTTGATTTTTAGTA
ATCGTGAGGGTTCAAGTCCCTCCACCCCCATATATTGATTTATAAATAAAAAGAGATGTG
TATTTGTATTAATTGCAACCATATTACCAAATGTAATACCTATCACTTAATAGAATCTCA
ACATAAACAGCCTCATCTTACTAGAAGTCCATTGTTTATACCTAAATATCCTGTAGTTCA
TGTTAATATATCTAACAACTGTACCTATAATCAAATTGATTGGGATTTAGTGGAGTGTTT
ATCATTTGTAGAAAAACCGAATAGTTGGAATTTAGACGCTAATTAGTTACAAGAAAAATT
ATGTACCATAAGCAACAACTCTATTTTTTAGATACTCGGTATTAATTTTGGATGTTCTAA
CCAGTAAGATTTTACCCGTTCTTGCTATCTCTATAATGCCAAATTTAGTTAGTAATTGTT
CAATAGCAACAATCTTTCCCGGATCTCCAGTAACCTCTATAATTAAAAGATCTTCTGCAA
TGTCTACTATCTTAGCTCTAAAAATTCTTACAATTTCTAAAGCTTCTGTTCTAGTCTGAG
AATTGATCTGAATCTTAATTAACATTAGTTCTCTTTCAACTGAAGGAATATTTGTTACAT
CTTGAACGTTAAGGATATTTACTAACTTATATAATTGTTTCGTAAGTTGTTCAATAGTTC
TATTGTCTCCTTGAACTACCATTGTGATTCTAGAGACCCCAATTTGCTCTGCTGGTCCAA
CTGCTAAACTTGCGATATTAAACCCTCTCCGGGCAAATAGACCAGATATTCTTGACAGTA
CTCCGGCTTCATCTTGAACTAAAACTGATAAGGTGTGTTTCATGAAAAATTAAATTTAAT
CTATTTGATCTGTTAAGTATATTCTAAGAAAATTACAGAAAAAATACTAGTTAGTCTATA
ACTAAATTATCCAAACTAAACAAAATCCTACTATCAGATAATATATCTACCTTGTAATGC
TATACTATTAACTTAGTATTTAATGATATTCTAATTGCAAATAGAATGCTAATTTTTTAA
AATTTGTTAGGAACTTGTGCTAGAAAAATACAAAAACAGTAAAAAAACTCTCTCGAGATTT
ACCTCAAATCAATGATCGCATTAGATTTCCAAAAGTCCGAGTAATTGATGACGAAGGTGA
ACAACTAGGTATTTTTGTGCCTGAAGAAGCTATACAATTAGCTGTCCAACAAGGTTTAGA
CTTAGTTGTTGTTAGTGATAAATCGGACCCGCCAGTATGCCGAATATTAGACTATGGTAA
ATATAAGTTTACACAAGAAAAAAGAGCTAGAGAAGCTAAGAAAAAGCAACATAACAGTAG
TATTAAAGAAGTAAAAATGCGATATAAGATAGAAGAGCATGATTATAAAGTTAGAATAAA
CCAGGCATCCAAATTTATTCAAGCAGGAGATAAAGTAAAAGCAACTATCACATTTCGGGG
GCGTGAAATCCAGCACTCTAATTTAGCTATAGATTTATTGAATAAAATGGCAAGCGATCT
AAATGCAATAGCTGAAATTCAGCAAGCTCCATCAAGAGATGGCAGAAATGTCATAATGCT
CTTATCTCCCAAAAAAGTTAGCTAAACTAATTTTTATTTAAATGCATCTGGCTGGATTCG
AACCAGCGACGTCTCTTTCGAAATGGCGGATTATGAGTCCGCTGCCTTCGGCCCCTCGGC
CACAGATGCATCACTTAAAGTTTATACCTCATAAAGCATATAAAAATCAATGTTTAGCTA
TTTTTAATAAAGTCTATAAGAGTCTATTATTCATTCATATTGTGATATGTCGCAACTGCT
GAAGGGGAAACTTGATTTAAGTATCGAAATATCCAGTATTTAAAAATTGTGTCGAGTATA
ACTGGAAAAGTTGAAATAAATAAAAAAATAAAATCTCGACTTTCCGGAAGTCCTAAATGT
CTTAAAATTACTTCAATAATAACTTCCCATCCATGAGGAGAATGAAAACCCACAAACATA
TCAGTAAAAAGAATGATTAAAAAAGCTTTAGCTGTATCACTAAGACCATAAATAATTTCA

TTTAAAAAAAGATTTAACGACTGCAATTTGTCTTTGTCCAGTTATCATTAGTAATATAAAG
ACAAGTATTGATACAAGATCGGATAAAATATTTTTAACAGCATTTGCACTTTCATTTGCA
TAATATTCTCCCAACTCTCTAGCCTTCAGCTGCACTCTTTTTTCAATGATTTCGTAAGAT
ATATCTTCCGAAGGATTCAGAAGAACTTCAAAGTGAATTTTCTCTTCAAATCTTTGTAAT
TCAGCAAAAGCTCTTTCCTCTTGAGAGGAATTTAAAAAAATTTTAGGCTGTTCTTGGTTC
CATAAATAATCAATACAAGGTCCAAATACAAAAAATTTTGAAGCCTGATTAACTAGTACT
GGAGAGATGAATAATAATAAAATATATTTTACGGAAGTAATAGTTTGGTGTCTAGATATT
CTAAACTCCTCTATAGCTTCAGATTCGCCATTAGGATCTAACTCTTTACGAAACTTTTCA
AAAGTATTAGTAATTGACCTAGGTATTGGCCCAACTTTTTCAAAGGCGAATTGATTATTT
CGTTTTAAATTCCAATATTTCATTTTTTATTGCTGCTAAAATCTCTATTAATCAATAATT
GATCACGTATTCACTATAAATATTAATTCTAGAACTAAACATATAAAACAATATTTATGA
AAAATAAAATACAATTAAAAATTAATTATGCATTAGTTCTATCTGCAGCTACAGCAGTTT
ATGGATATAAATCTTATCAGCCAACTATAAATACACTCTTAATCAAAGATGAAGAAAACT
GGCCTTTTTTCTCTAAAAGGCAGACTGCCTTTATTCCTATAATTGTATTAAAAAAAAATC
ATCAAAATCCCTGAACTCAGAACCTAGAAATAGTAAGTTATATCTTAATGGAATTGGAAG
TAGAAAATTTATCGACAGAATTGTCACTCTACTTGGATATTTAGAAAAACAGGCAAACTA
TACTGAATACTTGAACCAAATAAAAACAAGTGGTTATTTTTCTTTAGTCTACCTAGACAG
TGATATAATGTATAAGAAAAATATTAGTAACATTTTTTTACTACCTAATGATATTCTAAA
AAGAATCTATATTTGCAATAAGAAAAAAAAGCTTATACCGCATTCATTCTTAATAAATTT
ATTTCAGAAACAGATAGGCTATCCAAAAAGTTTTGCTAATTTAAATTGGGCCTTCTCCAA
AATTATAAAATGGTATTATGATAGAGGATACCAATGGTCTCTAGTTGAAGTTAAACAAGC
GTCTGATGCATCTTCTATTGTAATTGATATTCATGAAGGGGTAGTGAAGACAATTATAAC
AGAATACTACACTCTATCTTATAAAAGAGTCTCAGGTATCTTATGCGTAGAGTCAATAGA
GCAGTACCTTGGAGTAAGAGTGGGAGCTCCACTAAATATAATTGATTTACAAAAAAAAAT
TACTTACTTAAAAGATAATCAACTAGTTGGCGACATTATTTATAGTATTGAACGATCAAA
TAATAGCTCAATGAGTTTGGATATCAAATTTCAAATACAAGAATTAAAAGATAAAGAGAT
AATAGTGCTTGCAAAAAGTTCTTCTATTATCTCGCATGCATGTAATTTACTTAATCAATA
TAGAAATAGGCTAGTAGCTTCTAATATAGTTTCACTATCAACTAATAAATTACACGCATG
CTATTTTAATTATAAACTTGACTATCAATATAAGTATATAAACACGTCTAATCTTATAAA
ATTGTTAGCTTATTCTATTTCTTGTAGGAAGACACTACTTATTCATTATTTTAATTTACA
AACTTTAATAAGCTATACAAAAAAAAATACTATTGGATTTCAGCTATATTTACGAAATCT
AAGTTTTGGGAAAGCTTTCTGTGTGCTTAGTATGAAATTTATCAAAAATGGACTTAATAT
AAAAATTTTATATATAAATCCTTCATTGATAGTTGATCAGAATTTTGTATTTCAATTTGC
TATACAAATCATTAAGCAATATCATACTGCTAAACCTCCAGCTTTATTTTTAACGAATCT
AGATCTTGAACAATATGTTGCTGAAAGTTTATTAATGTACCATTTTACATCTTGCTTCTC
AATATCTGAAAAAATATTATTATCTCGAATTATGCATACAGATTCTTTATTTTTCAACTC

TGAAAATTTTCATTTTGATGATAGAACTAACGCGATCAACAGTTATGATACTTTTAAACA
AAATACAAAAATATTTTATCAAGAGTTTCTTTCTTTATTATTAAGTTTACGTTATCAAAA
TTTTAATTATTTAGGTTGGCCTTTAAAAGGCCATTTTTTTGAAATAAAATCTTTATACTT
GGCTCCATTTCAAAAGAGTGATTTTTCTGATAGTCGTAAGACCCTATTCTTCCACAAAAT
GTCGTTAAAGCAAGTATCTAATTTTAATTTACCAATATCTTTTAAAAGCCACCTTAACCA
TATTTTAGTTAGTACTATTAAATGTCAGTCAAATTTGAATATGAGAACTGTATCTTTGCT
ATTAATTGATTCACCTGCTGAATATATGCTATACAAATCTATTCTTAATTTCTCTATTAA
AGTTAGGATGCAGTATTTCATACCTATGAGCAACAATATAAGATTATCTCTATTTTATAA
TTATTTAGATTGTTTTTTAATAAGATCATCTCAGAGTCGCATTCATATATGGCAAGATCT
AAGAACTCTTACACCAATTCAAAATTTTTGGTTAAAAAAATTTTCATATGGAGCTGGAAT
TCAGCTTAAATTACCAATTAAGCAGATGCCACCTCTTTCAATTGAATATACTGTAACTAG
TAGTCGCTACTTTTGTATTTACCTTCGTACTTATTATCAACGATAACTATTACTAAAATG
AAAAATTTAATTTCTTTTTTTGATCGCAGCAAAGGGAAATGGATTTCACAAAGAACAACT
TATGAATTATCTAATAAAAACATGAGTTCGATACAGTCTCAGATGACAATGAAAATAGGC
AACTCATTGTCAGGATCTATAATACTTGCATCGTTAAATTGGGGGGACATTTATAGACAA
GTTGCCCATTACGCTAAAAATCACAGCCGGAGTGAATATGATAATAAGTTTAACTTGCAA
TTCAGTAATCAACTAAATAATCATAAATTATTAACGCTGTGCATAGTGACAGATCCTAGT
CTAATTAGTTTTAAAACTCGCTATGGAAGTACTACCATAGATGAAACATATTGGTTCGCA
ACAAATAATTTACGTCTAAGTACTAGTATTGTTAAACGATTCAACACTTGTGTGGCAGTA
TCTTTTTGTTCAGAAATTAAAGTTTAAACTCTATATAGAAGCAACTAATAGCCTAAAGGC
TATTAGTTGCTTCTATATATCTAAATTTTGTATTAAAAATTATAATACTATATTATTCTA
AGTCTTTACGATTGGGATTCCTGGAAGGATCATTAGAGAGGAACCCAAAGACAAACAAAG
AAATAAAGAAAATAACGGTAGTATATACAAAGATTTTCAAAGTAAACATATTTTCATCCT
TAAAAGCTATTAGTGCGATATTTACATATCTCCACATAATTTTATATCATGATATAAGTT
CCAGCACTATTTTATAAATAAATCAATATCTTTATTTTTTAGTAATGATTTATATTTAAA
TATGCGTGATGTACTAAATACTAAATCTTTTCCGACTTGTTTTGTCTTGTTTGTAAATAT
TTTGTTATTTCTATGGAGTTTACCTTCAATTATCACATAGTCTAACTTCTTTAGCTGTTT
AAAAGTATATGAAGATTTTTTGTTCCAAATAGTTAGATTGATTATAACTAATTGTTTTCT
TTTTAACAGTCTTGCTTTTAACTTAATTATTTGGCTTTTATTTTCACTAATTCTGATACT
CTTACAACTAAGAATTTGGACTAGAAGAGTACAGCTATTCATAATTTATAACAATTTTTT
AAAAAATCTACTAAAATAGATGATTTTACAGAACTAAAATGATATTTCATTATCTTGTTG
ACCTTGCTCATAAGTAATATCTTTCAATACTTTTAATATTTTTCCAAAATACTCTTGCAG
GTATTTCTCTAAAGATATTACTTCCAACCTGTCAATTCCAAGAATACTGTAAACTTCATC
CATCGGAGCTGTAAATTGCTCTCCGCTACTTAAAATTTCGGCAAATGCTAAACGATCAGA
AATATTCCATGTCCATTGAAGAGTTTTAGTAATTCTTCTTAAGGCTTTTAATAATCCAAT
CGGAATTTGTGAGATCTGTGTTTTTTGCCCAGATAATTTTTCACATAATTTAATAATTTC

AGCTGATGTCCAAGCGGTATTACCAACTAACGGCAGGGTCCTATTTTCTGTGGAAGGTAC
TCCAAGACTTTTTATAACTAACTTTGCTGCATCTTGAGTATCAATGTATGCAATGGGTGT
AGATTCTCCTGTAACCCAGACTGATTTTTTGTCTAAAATAGGAATGGCATATTGGTTGAT
TAAACCTTGAAAAAATCCTCCTAAAGAAAACACAGTATATTTCACATTGGACTTCTGAAG
GAAATCTACTACCTGAGACTTTAAGTTCATCAATGGAACATCTGGGTATTTCTCTGAATT
TAATATTGAAAAGAAAATAAATCTTTCAACTTTAGCCGCTTTAGCTGCTTCAATTAATGC
AATTTTCCCATCTAAATCTATTTTTTCTGCATTGTAAGGATCAGTAGGACGAGATGTAGA
AGCATCTATGATTGCTGTTACCCCACAAAAAGATTGCAAAATACTTTCGGGTAATTTTAA
ATCACCATATATAAGTTCTGCTCCCCACTCTTTGAGAAAGGCAGACTTTCTTAAATTTCT
TACCATGCATTTTACATTGTAGCCTTCATCTAAAGCTCGTCTTACAATTTGACGTCCTAA
AGTTCCGGTTGCTCCAATTACTAAAAGAGTCATATTAGTTAAAAATAAGTATGTATAAAG
ACATTGATTTGGTTGACGGCAGAGAGTAAATTAGGTAGAGAGGGAATCGAACCCTCATGA
CCGAAGTCGTCACATTTTGAGTGTGATGCGTATACCAATTTCGCCATCTACCCTATTATA
GACCAACTACTACAAGTAATTTAGTCTTTAGCGTAAAAATAATACTAAGTTTCAATGGAT
ATGTCAACTTTTAAATTTATATTAATAAAAAAACGGAGTCTCTTATGGCCACTTTTGAAT
TAATTCCTTATAGATTGTATTTGAGTCAATCTAACACATGGATCCATAGAATAAAAGCAG
AAATTAAGATATATATAGTAACGCTTTTATGGATTTCAATTTTTATTTTTTCTTACTTTA
AACTATGTATTATTGCTTTAAGCTTAATTGCCATAAGTTTTACTATAAGAAGTAAACAGA
ATATTATCCAAAAACATTTGTTACAAACGTTATTAATAACGTTCTTAACTACTGTTTTGT
CTTTTAGTGTGGCTATTAGTTATAAACAATATGCAGAACAAGAACAATCGCAGTATTTAT
CTGATTCCAAGAAATATAAAAATTCCAGCAGCTACTATTATATACAAATAGCTAACGATC
AAAGGATCAAAAGACAATACTTAATTACTACTTTAAAGCCTTCCTTGTACTTTTTTATTA
CTATATATTCTATTAAACTAGTTATGATAACAACTTCTCCAGAAGTTTTAGTAATTACTA
TTTATAGATCTAGGATAATAAATAAAATATTTAAAAATGAATTGCTATTTATCTTCCTTC
TTTCGTCACATATTGTTACTAGTATTATTAATAGAATCGATAAAGTAATTCAAGTAACTA
GCTTAAGAGGAAGCTTGAATTTATATAATTCATTGACAAGGCCTTTAATGTTTTCTTTGT
TAATATTTCAAGTCTTCTTTTTGGAGATTATTCGGGAGTCAAAAGAAATAGCTCAAGCTC
TTTATACTAGAAATCTCAATCAAGAAAACAATAATTTTTTGAAAATATATACAGTAAAAT
CTAACTTTAGTGATCGGCTCAATATAATTATTAGCACTTTGTACTTTATTATTTTAGCTC
TAGCGTAATAGAATCCACTTATTCTTCTGACTCTAGTAATACGAAGAGTAATAAAATTAG
TTAGTAGTTTAAATAGATAGAATTAAGATATTATATAAATAATTAAATGTTTCATTATGG
TTAATACTCAAAATCAAATTTCACAAACTTCAGAACTTGATTATATAGTTAATCAACCTT
ATAAATATGGCTTTAAAACTTCTGTTGAATCTGAGCAATTTCCTAGGGGAATAAGTGAAG
ATATTGTCCGCTTGATCTCCAAGAAAAAAGATGAGCCTGAATATCTATTGAATTTTAGGC
TTAAAGCATATAAAAAATGGAAAAAGATGAGTAGTCCGTCATGGGCTCATATTAAGCATC
CGAATATAGACTTTAATACGATTATTTATTACGCTGTTCCTAAATTAAAAAAAGAATTGA

AAAGTCTAGATGAGGTTGATCCAGAAATTCTTGACACTTTTAACAAGTTAGGTATATCTT

TAAATGAGCAAAAGCGAATTTCCAATGTAGCCGTTGATGCTGTTTTTGATAGTGTATCTA

TTGCAACTACTTTTAAGAAAGAACTGTCTGAAGCTGGTGTTATATTCTGTTCAATTTCTG

AAGCTATAAGGGATTATCCAGAATTAATAAAAAAATACTTAGGTACTGTTGTACCAGCTG

GTGATAATTATTTTGCTGCATTGAACTCTGCGGTATTTAGTGATGGCTCTTTTTGCTATA

TCCCTCCTAATACAGTTTGCCCTTTAGAATTATCAACTTACTTTCGTATTAACAACGAAG

AATCTGGGCAATTTGAAAGGACACTAATTATTGCTGATCGTGGCAGTAAAGTAAGTTATC

TTGAAGGTTGTACTGCTCCTCAATTTGACACAAATCAATTACATGCAGCGATTGTAGAAT

TAGTAGCTCTTGAAGGAGCCGAAATTAAATATTCTACAGTACAGAATTGGTATGCTGGTA

ACAAAGAAGGTAAAGGTGGTATATACAATTTTGTTACTAAACGAGGCTTATGCTCGGGTA

ACAATTCAAAAATTTCATGGACTCAAGTAGAAACTGGATCTGCAATTACCTGGAAATATC

CAAGCTGTATTTTAGCTGGTGAAAATTCTCAGGGAGAATTTTACTCTGTAGCTTTAACAA

ATAACTATCAAGAGGCTGATACAGGGACTAAAATGATCCATATTGGTAACAATACAAAAA

GTAGAATTATTTCTAAAGGTATTTCTGCGGGTAGATCGAAAAACAGTTATCGAGGCCTAG

TAAAAGTTGGCCCTCAGTCATTTAATTCTCGTAATTATTCTCAATGCGATTCTTTATTAA

TTGGTCAATCATCTCAAGCTAATACATTTCCCTATATTCAAGTACAGAATCCAACATCAA

AAGTAGAACATGAAGCATCCACTTCAAAAATTAGCGAGGACCAAATTTTTTATTTTTTAC

AAAGAGGGATCAATTTAGAAGAATCTATTGCTCTTATGATCAGTGGTTTTTGTAAAGATG

TCTTTAATGAATTGCCTATGGAATTTGCTACTGAAGCTGATCGTTTACTGAGTTTAAAAT

TAGAGGGAACTGTAGGATGAGCCAGACTATTTTAGAAATCAAAGATTTATATGCTTCTGT

TGGTGAAACAACAATTTTAAAAGGGGTAAACTTATCTATTCGAGCCGGCGAAATACATGC

GATTATGGGGCCTAACGGTTCAGGTAAAAGTACATTATCAAAAGTAATTGCAGGACATCC

AGCGTATTCACTAATAAGCGGAGACATTTTATTTTTTGGACAAAGTATTCTTGAAATGGA

GCCAGACGAGAGGGCAAAAGCAGGTATTTTTTTAGCTTTTCAGTATCCTGTTGAAATTCC

TGGAGTCAGTAATTCTGATTTCTTAAGAATTGCATTAAATGCTCGCAGAAAGTTTCAAGG

ATTATCGGAGTTTAGCCCCTTAGAATTTTTTCAACTAATAACAGAAAAAATAGATCTTGT

TGGCATGCAAGAAAGTTTTTTGACAAGAAATGTTAATGAAGGATTTTCTGGTGGAGAAAA

AAAACGTAACGAAATTCTTCAAATGGCTTTACTAGATAGCAAAATATCTATTTTAGATGA

AACAGATTCTGGACTAGATATTGATGCATTACGAGTAGTTGCAAAAGGAATTAACACTTT

AGCTAAATCAACAAATTCAATTATTTTGATTACTCACTATCAAAGATTATTAGATTATAT

TATTCCAGATTTTGTTCATATTATGAGTAACGGACAAATTGTAAAAACTGGTAGTGTTAC

TCTTGCCCAAGATCTAGAAAAGCACGGATATGACTGGATTACGCAGACATAACTATTTAC

TACAATATTGAGACTAAAGAATATTCTTTAGTCTCAATATTTTTGCAAATGCAAAGTTAA

TTATTTAAGCTTTATCAAAACCTTGCTTAACATCTTCAATTGCTCTTTTTAGCAATTCTT

CACTAACTGGTTCTAATTTTTTAGTACTACGAACATTTTCACCAAATTCTGGTTTAGAAT

TCTTTAAATCTTCTCGAAGTTCTCTAATAAATTCAGCTACTTTTGAAACTTCAATATCAT

CTAGATAGCCATTAATGCCAGTGTAAATAATAGCAGTCTGTTCTTCAACTGGAATAGGAG
AATTTTGAGCTTGTTTCAAAATTTCTCGAAGACGTTGACCTCTTGCTAGTTGATTTTGGG
TTGCTTTATCTAGATCAGATGCAAACTGAGAAAAAGCTTCTAATTCAGCAAATTGTGCTA
ACTCTAGTTTTAACTTGCCTGCCACTTGTTTCATGGCTTTTATTTGCGCAGCAGAGCCTA
CTCTAGAGACTGAGATCCCAACATTAATAGCTGGTCTAATCCCTGAGTTAAATAAGTCAC
CAGATAGAAATATTTGTCCATCTGTAATTGAAATTACATTTGTTGGTATATATGCAGATA
CGTCCCCAGCTTGAGTTTCAATAATAGGGAGAGCTGTCATACTACCTCCACCTAATTCAG
CATTCAATTTAGCTGCTCTCTCTAGCAATCTAGAGTGTAAATAGAATACATCCCCAGGAT
AGGCTTCTCGTCCCGGAGGTCTTCTAAGCAAAAGAGACATCTGACGATAAGCTTGAGCTT
GCTTAGTTAAATCATCATAAATAACTAATGTTGCTTTACCTTTGTACATAAAATATTCAG
CTAAAGCCGCACCTGTATAAGGAGCAATATATTGTAAAGTCGCAGGACTATCTGCATTAG
CTGCAACTATAATAGTATAATCAAGAGCTCCTTTATCCTGTAGTGAAGATACTACTTGGG
CTACTGAAGATGCCTTTTGTCCTATCGCAACATATACACAAACAACATCTTGGCCTTTTT
GATTAATAATTGTGTCCAATGCAACAGCTGTTTTACCTGTTTGACGGTCACCAATAATTA
ATTCTCGCTGACCTCTACCAATTGGGATCATAGAGTCAATAGCAGTTATCCCTGTTTGCA
TAGGTTCACAAACTGATTGCCTTCCAATTATACCAGGGGCCATTGACTCAATTAGCCTTG
TTCCATTACTTGCAGGTTCACCTTTATCATCTATTGGCCGAGCTAATGGATCAACAACTC
TACCTAAAAAAGCATCTCCGACCGGAATTTGAGCAATTCGACCAGTCCCTTTTACGGAAC
TGCCCTCTAAAATATCCCTACCATCTCCCATGAGTACTACACCAACGTTATCACTTTCTA
AATTTAAAGCAACCCCAATAGTTTTATCTTCAAATTCAAGTAGTTCACCAGCCATGACTT
CATCAAGTCCATATACTCGAGCGATACCATCACCAACTTGTAAAACTGTTCCTATATTGG
CTACTTCTACATCCTGATCATACTTTTCAATTTGTTGACGAATAATACTACTTATTTCAT
CAGGTCTAATATTTACCATATTTTTAATATTATTTTTATTAATTGGAAATTCTAAAGCGG
TAATTAGTCAGACTAATGCTATCTGTCTTTTTTACTTTATTTTAAGTTACTACAACATCA
AGGTGTGAAGCCATTTGTCTTAGCTGTCCTCTAATACTAGTATCAATGACTTTTGATCCA
ATCTGAATAGTAAAGCCACCAATTAGCTCGGGCTCGACTGAAATAATTAGTTTAACTTCT
TTAGCCTTAGTCATAACCTTGATTTTTTCTGTTAATAGAGTTTCCTGATCAGGGCTTAAA
GCAATAGAAGTATTAATATTTGCAATTGTTAAAGATTCCATTTGGTAAGCCAGTTCCAAA
TACTTACTAGCTATAGCATCTAACATTCCAATTCGCTTGCGATCAACTAAAATCATTAGA
AATGATAATGTATTTTCGTTAATTTGATCGCCAACAGTTGCAATAATTACTTCTTTTTTT
GCCTCTACAGTTTTTAATGGATTAGCTAAAAAAATTTTTAGCTTGTCAGATTGCGCTAAA
ATATTTTGTATTGACTGAATATCTTGGCTCACCTTTTCAGTAACTTTTTTAGTTTTAGCT
AAATCAAGAAGAGCTACTGCATAAGGCTGAGCTATTTTTATAACAACATTGTTACTGCTC
ATAGTTGATCTCCTAACTTAGCAATATTATTATCAATTATACGCAGTTGCATTTCGGAGC
TCATTTGATTTTCCAGCTGCAAAGTAACTCTTTTAAGAGCAAGAAATGTTATTTGTTGCT
GGATTTGCCTTCGGATCTGTTTTTCAGCTGTTTCTATATTTGATTTACCTGTTATGGCAA

GTCTTTCAATATCTAATTGGCCTTGAGCTAATATGGAACTTCTAACTTTTCCAGCTGTTA
GCTGAGCTTCTTTCTTAATTTGATCTATAATGATTTGAGTTTGAGCAAGTTGTTTTTCTG
ATTCTGACAATCTAGCACTTGCTTGCTCTAGTCTTTCTTCGGATTCTTGTATAGCTGCTA
AAACTTTTATCTGTCTTGCATTTAAACTTGATCCGAGAAATTGCTTCAAAACATAAATAA
GACCAAATAAAAGCAACAATATATTAATAACATTTGCTTCAAAAATATCAGAATTAAAGC
CGAATTTGTGTTCACTACTATGTTCTGATAAAATAGTAATTATTTGTGGTATTTTTACTA
TATTATTCATTTAAAATTCTTACTATTTTAATATTTTACTCTGGAATCATGATTTTCAAG
ATTGACTACTTAATAATTTAACTTTAATTTGATCGCTCAAAGTGTCAACCTGATTTTCTA
AAGTTTTAAGAGCTTCTTCTTTTTGAACATTTAACTGTTTCGATGCTTCAGCAATCAGTT
TCTCTGCATTCAATTGAGCTTTTTTTATATCTTCTGAGACTATATTTTGAGCTTCTTTTT
GAGAAGAGGCTATTTTTAATTGTGCATTGCGGCGAGCTTCAGATAAATCTTCTTCATATT
TTGCAGCTAGCTCATCTGCTTTAACAAGCATAGACGATGCAGTAGTCAATGTTGTTCGAA
TATATTCATCTCGTTCGTCTAGTACTTTAGTTACAGGTTTATAAAAAAATAGTATTTAATA
AAACCATTAAAGTAAGAAACTGTAATGCCATTAAAGGCAAAGTACCATTGAAGTCAAATA
AGCCACCTTCAATTTCTTCGGCTAATAGAAATGGTAAATCAATCATTTTACTAATTATGT
TTATAGTTAATAATTTTGTTTCAATTAAGAAAAATAACTTTATAGTGCGCTTAGTCTCAT
CAGACTAAGCGCAAATGATTATTTTAACCAACGTATGGATTAGCGAACAATAAAGACAAC
GCAACAACTAGGCCGTAAATTGTTAAAGATTCCATAAATGCTAAACTTAGAAGAAGTGTA
CCCCGAATCTTTCCTTCTACTTCTGGCTGTCTAGCAATACCTTCGACAGCATTAGCTGCT
GCACTACCTTGACCAATGCCTGGGCCTATTGCAGCAAGACCTACAGCAAGACCGGCAGCG
ATAACGGATGCGGCTGAAACGATTGAATCCATAATGAATTTTAATTTTTTGTTATATGAG
TAGAAAATTGACAAATTTCGTATAATACTTAAAATCTATATAATTAAGTATAATAGATTC
TAATATTGTAATTATAGTATATTACTCTTCTCCATGACCTTCCATTGCTTCTCCAATATA
AGCTGCGGACAAAGTAGAAAAGATTAATGCCTGTATTGAACTCGCAAATAATCCGAGTAT
CATAACTGGTAATGGTATTAAAATTGGAATTAGCAAAGTAAATACAGAAACAACTAATTC
ATCTGCTAAGACGTTTCCAAATAATCGAAAACTTAAAGATAGTGGTTTCGTAAAATCTTC
TAGAATATTGATTGGGAGTAATACTGGAGTTGGCTGAATATATCTAGCAAAGTAGCCTAA
CCCTTTTTTACTTAAACCTGCATAAAAATAAGCTAAAGATGTAAGTAAAGACAGGGCCAC
AGTTGTATTAATGTCATTTGTTGGGGCAGCTAGCTCACCTTCAGGCAAGTGAATTAACTT
CCAAGGAATTAATGCACCTGCCCAGTTACAACCTAGAATAAATAGAAATAAAGTTGCAAT
GTAAGGCACCCAAGGGCGATATTCATGCTCACCAATCTGATTTTTTGCAATATCTTGCAG
AAATTCTAATATAAATTCCATAAAATTCTGGAATTTTTCAGGAATTCTTTGTAGATTTCT
AGTACCTAGAAATGATAAAGTTAAAAGTGTAGCAATTACTAACCAAGAGACAATGAAAAC
TTGCCCATGCAATTGTAAGCTACCTATTTTCCAATATAGATGTTTTCCAACTTCTACTGC
TGATAAACTATTATATGGGTTAAAATCTATGAGACTGTTTTGATACATAATAAATATTTT
TTTAAATAGTGCAAGAATCAAAAAAATAAGTTTTTGAAGAATTAATATGCAATTAATTTAT

TCTTAAAATTAGACTATTAATAAATTAAAAGCAAAAATAAATTCTAATAGAGATAGCTAT
TTTTAGTAGTGATTATTTTTTGTAAAAAACAATTATTGCTACCTATAATGCTTTTACGTT
TAATTATATACCCATATATTAATTATTTAATATGATTATAGGTATTTTGCTACAAACTTT
ATTGTTTTATCCATTCTAAAGATTGTTATAGTTTATTTTTTATTTAAAATATCTGCGACT
TCATCTGCAAAATTAACTTTCAAATTTTCTTCTCCCCCTCCCAGAAGAAATCTTACAAAT
CTTCTAATTTTAATATTTTCACCTAGAACAGCAATATTTTGGTTTATTAAATCTTCAATA
CTAATATCTTGATTGCGGATAAACATTTGATCTAATAATGAGAGCTCCTTAAGTCTCTTT
TTCATTCGTCCTTCAATGATTGTTTCGATTCTATCAACTGGTTTGTTTTTCAAGTCATCT
TTACCAGCTTCAACTCTTTGTTCCAGATTAATAATTTCACTAGGTATATCCTGTGTTGAT
ACGTATTCCACATTAGGCGATGCCGCTATTTGCATAGCAATATCTTTAGCTAACTTTTGG
AACTCTGGACGTCGTGCTACAAAATCTGTTTCACAGTTGACTTCAACTAATACACCTATT
CTTCCGCCAGTATGAATATAACTCTCTAGTAAACCTTCAATAGCGGTACGATTAGATTTC
TTGTTAGCTGAAGCCAGCCCTTTTTGTCTTAATGATTCTAAAGCTTTTTCTTCATTGCCA
TTATTAGCTTGTAAAGCTTTCTTGCAGTCCATCATACCTGCTCCAGTCTTATCACGTAAG
GCTTTTACGACTTGAGCAGAAATTTGTAGTGTCATATGCTTTCAAAAATTTTTAAGTATT
TAAATTAATTATTATAATTCACTTTGAGTAGGATGAGTTATTTCTTCTAAATCTGTTTGC
CCGTATTTACCATCATAAATTGCATCTGCTATTTTACCTACAATTAGTTTAATTGACCTA
ATGGCATCATCATTAGCTGGGATTGGAATATTAATAATTTCTGGACTACAATTAGTATCA
AGTATACAAATAGTTGGAATACCTAATTTTAAACACTCTTGTATAGCTGTTGTTTCTCTC
TTTTGATCTACAACTACAACAATATCAGGTAAACGAGTCATATTTTTAATACCATTTAGA
TGTTTACGAAGTTTATCTAATTCTCTTCGTAAAACAGCAGCTTCTTTTTTGGGTAACTGA
TCAATCATACCACTTTTATCTTGTTCTTCTAGCTGTCTCAGACGATTAACTCGAGACTTA
ATCGTTACCCAATTAGTTAACATTCCTCCAAGCCATCTCTGGTTAACATAATAAGAATCA
CAGCGTTGTGCTTCCTGTGCCACTATACCAGCGGCTTGTCTTTTTGTTCCTAGAAATAAA
ACTTTTTTACCATCCGACGAAGCCTGTTTTATAAATTCACAAGCTTCTGTTAATAATTGT
GCAGTTTGGACTAGATCAATAATATGTATGCCATTTCTTTCTGTATAAATATATGGAAAC
ATTTTAGGATTCCATCTACGTGCTTGATGTCCAAAATGAACACCTGCTTCTAGTAATTCT
GCTAATGTAACAATAGCCATAAGATTATTAATATTTAAATAATGTAATTATTCGGGAAAT
CCCTTTGTTTAGAAACTGATTCAACTAACTTATTTGAATTTTAGTAAACTAATAATAAAT
TAACACACTTTTAAAGCAGATCAACAAAATAGATCCTGAAGAATTCATTCTACACTTTTA
TTGCTAAAATAATTACGCGCGGTTCTGTCGTCTAAAATGATATCATCTAAATCATCTCGT
ACAGATGAAATAGTACTTTCGTCATTAGTATTTGCAGTTAAATTCTTTTTCTCTAACGAT
GAGCCATTACAGTTATCATACATATTGAAGCCGGTACCAGCCGGTATAAGCCGACCTATA
ATAACATTTTCTTTCAGACCTCTTAACCAATCTAATTTACCAGATATAGCAGCTTCAGTT
AATACTTTTGTAGTTTCTTGAAAACTTGCTGCAGAAATAAAGCTTTCTGTATTTAAGGAA
GCTTGAGTAATACCTAATAGCACAGGCCTATATGATGCATTAATCTTATTTCTCAAGGTT

ATGGCTTTATTAGTCTGTTCAATCTTCTGTAACTCTACAAGCTCTCCAGGTAAATAGCCT
GTTTCTTCACCATTTTCTATTTTCACTTTAGAAGTCATCTGCCGTACTATTACTTCTACA
TGCTTATCAGAAATATTCACTCCTTGAGATTGATATACCAATTGTACTTCTTTAACAAGT
AGTAACTGAATCTCTTGAAAGCTTAATCGAGCAGCTTCATATAAGGCTAATCCACATTCA
ATATACAAATCAAAACTTGCATCGAGGATATCATGAGGATTTAACAATACGTCTGTTTTC
TTTCTTGCTTCAAGAATTTCTTCAATTCTTGGAAGCCCTTGTATAATGTCACCTGTTTTT
GCTCTATCAAAAACCAATATAGCTAATGTTTCTCCCCTTCTAATTAATGCATTGTTATCA
ACATGTAAAATAGCACCATTAGAAACCAAGTAAGGTCTAGCAATTCTTAATGTAACTGAT
CTAGAAGAAATTTCAATAATTTGTCCAGAGTCTAAAGAAGTCATATTTTCAGTAATAAAA
TCACCACAACGAATCCAATCACCCACACTTACTTTAACTATTGATTTTCCTATATTAAAG
GTCTTTTTATCAGAAGATGTCGCAATCAAAATGCGCCTGCTAGTATTCTTTTCAGAATAA
ATCTCCTCAACAGTACCTTCACTCATTGCAATAATCTCAGTACTAGCAACAACTGTAAAT
GGCTTGATATATTCACCATTTTTTACAATAATTCTAGTTTGACTTTGTTGCTTCTCTGAT
TTATTTACATCAATACTTTTAATTGATAATGTTTCAAAAGTTGACAGCCTTAAGTCAAAA
TAATTATTGCCACTTTCTTTAGCAGAAAACTCAATTGAGGATATTAAATTATCATCTCGA
TGCTCAATTTCAGCCACAAGATTAGTTGTTACTAAATGAACTCCTTCTATAGATTTCACT
CTTTCTCCGTCCCGAAAAGGAGTTCTTTTAACTAGCTTTAGTTTAGTTTGACTTGGTTTT
TGTGAAACTAGATTTTCAATTAGACTGGATTTTGTTTCTGGTATTGAATAAACAATAACA
GGTCTAATAAGAATATAGGGCATTTGCTCGTTTTCTATATACTCCCAATAAACTAACTTA
TCTGTAGAAATATTATTATGAAGTCTTTCTCCAGGTCTTAAAAAACCTCTACTTTTATCA
TTATTACTATACACCTCACTAAGTTTATAGATAGATCCAGGCTTAATAATAATTTCTCGA
ACTATTCCATCCTTTTCGATTATTTCAACAATACCTGAGCTTTTAGAAAAAATATTTTTA
ACTAGCTCTGTTCCACTTTCTATAACATCTCCATTATGCACTAGTAGTAGTGATGAATCT
TTATTAATTTCGTGAGTTTCTTCTGAAATCCATAAGATATATCCCGGACTTAAAATTTCG
TAGGCATCTTTATCTAAGCCTGTTTTCTTTTTTGATACATTTAAGTCTAAATATTTAATA
ATACCACCACTTGTCGTTTTATACGTATCAGATATTAGTTCTGCGACTGTGTAACCATCT
TGAATTTTTTGGTTTGGAATAGCTTTGAAAAGAAATTTTTGTTTTTTTTCTGTTTCTAAA
ATATAAGATTCTTTTTTGTGGATTACATCTGTATAAATATAACAACCTGGAATTACAATA
GATTCAGTAATAACTTGTATATTGGTAATACTGCTATTAGTATTCTGGTGTATCCTTACT
TCTCCAGCATAATGATTTATTAATTCTGTCTGTGCAAGTATCGTACCAGCTTTAGTTTTA
TCTTCTTTATGAACAATAACATTAGCAGAATCAGATATACTATAAACTTCACCTGAGAGC
ACCCAGATCAAGCCTCCAGTTTTAGTAATTCTTGTAGTATATTGATTATTATCTGTTTCT
TCAACTGTTAAGTTGGAGAAACAGATTTTGCCAGAGAGATCTGAGACTACATGTTTTTGA
GCTCTTTCTGTCATGAGTCGATTTCTGCGGGGTGATTCAGCAATCACTTGATCCTTCGAT
ACTAATTCACCATCACATATTAAAAGAGTAGTACCTTTACTTAAGTTAATTATTGACTTC
TGATTTTTCTTAGATCTGATTGTAACTTGGGTTGGCTTTTTTGTTATTAATGCTTGCTCA

CCATGTCTGGTACGAACATCTGTATACGATTCAATATCTAGATCTATCAATTGACCATCT
ATAGGTGCGTAGATTTGTTCTGCTAATTCACCGGTAAATACTCCTCCAGTATGAAAGGTC
CTCATTGTCAATTGAGTGCCAGGCTCACCAATAGATTGAGCCGCAATAATACCAACAGCC
TCTCCTAAATCAACTAAGCGGCCATGTGCAAGATTCCATCCGTAACAATACTGACAAACA
GAACTTCTAGAGTTACATGTAACAGGAGATCTTACTAAAACTTTTTTGATACCAGCTTTA
GTTATCTCTTTTGCTAACTTAGGACTGACATCTTGGTTCGTATGAGCAATTAAACAATTT
GTCTCTGGATGGAATACATTTTCTGCTAATACTCGGCCAGATAAAGCTTGTTCTAGATTA
ATTAATATTTTCTGCGTATCTACTAAATCTTCTAGTATGATGCCTTTATTGGTCTTACAG
TCTACTTCTCTAATTATTACATCTTGAGAAACATCGACAAGTCGACGCGTCAAATAGCCG
GAATCTGCTGTTCTTAAAGCAGTATCAACTAATCCTTTTCTTGCTCCGTATGATGATATA
AAATAATCAGTAACAGTTAGTCCTTCTCTAAAATTACTCGATATTGGAAGATCAATAATT
TGTCCCTGTGGATCAGCCATAAGCCCTCTCATGCCCACTAACTGTCTTACTTGAGAAATA
TTACCTCTAGCACCTGAGAACGCCATCATATAAACAGCATTTAGAGGATCTGTCTCTTTA
AAGTATTTAATAACTTCTTGTTTCAAGGATTCACTCGCATTATTCCACGTATCAATAACT
TTTTGAAATCGTTCAACTGCTGTAATCTCACCTCGGCGATATTTGTTTTCAGTGGCCTTA
ATATCTTCAATCGTGCCTAATAAAAGACTTTGCTTACTAGGAGGAATTCTTAAATCTTCT
AAACTTAAAGAAATTCCTGCCTGGGTCGCATAATGAAATCCAAGATCTTTAAGCTTGTCG
GCCATATTGGCTGCTCGGGCTATACCATAATTACGAAAAGCCCAGACAATTAAATTTTTT
AGTTCGTTTTTATCAATAACCTTATTTGAAAAACTTGGCTGTGCAAGACTTCGTCTATTA
TCCACTAAATCTTCTCCATATTAAATGTTCGTTATTAACTATGCTACTAAAGACTCTTGA
ATAATTTTATTAAAGATTATACGGCCAGCTGTTGTTCTAATATATTGCACAATTCTCTGA
TTTTCAGCGTCTTCTTTTATGATATGGTTATTAAAAAATTTGTTGTACTATTGTCAGAA
TGTTTTTCAATCTTAATCGGATCATGAGGTTGATCACCATCTACTAATCCATCAAAACGA
GCCCATATATAAGAATGTAAATCAATTTTCTTCTGTTCATAAGCCATAACTACATCTTCT
AAGCTTGCAAAATACTGATTAGATCCCTGCTGCTGAGATGGATTATTAGCTGTTAAATAA
TAGCAACCTAAAACCATGTCTTGGCTTGGCATAATAATTGGTTGACCTGTTGCTGGAGAG
AGAAAATTATGAGGGGCTAACATTAGCAATCTAGCTTCGGCTTGAGCCTCTAAAGACAAA
GGAACATGAACAGCCATTTGATCTCCGTCAAAATCAGCGTTAAACGCTGGGCATACTAAT
GGATGAAGCTTAATAGCTCTTCCTTCTACAAGAATAGGTTCAAATGCTTGAATACCTAAT
CTGTGTAGTGTGGGAGCTCTGTTCAATAGTACTGGGTGACCTTGAATAACTTCATTTAAG
ACATTCCAAATAGAAGATTCATTTTTTTGAATCATCTTTTTGGCAGCTTTAATATTGTTA
ACTAATCCCTGCAAAATTAGTCTGTGAATAACAAAAGGCTGAAATAACTCCAAAGCCATT
TCTCGTGGCAATCCACATTGATGTAGTTTTAAATGAGGACCTACAACAATTACAGATCTA
CCGGAATAATCTACTCTTTTACCTAGCAAGTTTTGTCTAAAACGACCTTGTTTTCCTTCT
ATAATATCTGACAAAGATTTTAATGGTCTATTATTTGCACCTACAACAGTTCTACCTCTA
CGACCATTATCCATGAGAGAGTCAACGGCCTCTTGCAGCATTCTTTTTTCATTTCTGATA

ATAATCTCCGGAGCTAAAATTGACTTTAGACGAGATAATCTATTATTTCTATTAATAATT
CGACGATAAAACTCATTCAAATCTGCCGTCGCAAATCTACCTCCATCTAATTGGACCATA
GGCCGTAAGTCTGGAGGTATCACAGGAATGACTGTGAATACCATCCAAGAAGGATCTGCA
CCTGTTGCTATGAAATTTTCAATCAATCTTAAGCGTTTCATTTTTTTATTGAATTTTAAT
GAAGGTGTCTTAAAACTTTTTGGTGGATTTGTAGCTTCAGACCTTAAGGTTTCAGCTATG
TGCTCTAAATCTAAATCTTTCAACAGTTTCTGAATAGCTTCTGCACCTATTCCTACTTCT
ACTTGGTTATTCTCATCTTGATTTTGGTAGATTTCCTCTTCAAGACTTTTCCATTCGTAA
CCTTCGAGAAGTTGCTTGTATTTTAAATTAATATCTGTATTAGATTGCGTAACTACATAA
GAATGAAAATAAACAATTTTTTCTACTTCTTTGACTTTTAAGTCTAATGCTAAAGCAATA
TAGCTTGTACTTCCTTTTAAGTACCAAACATGAGTTACTGGTGAGGCAAGTTCAATATAA
GCCATCCGATGTCTTCTCACGCGTGATTCTGTAACTTCTACGCCACATCGTTCACAAACT
ATACCTTTGTAGCGAAAACGTTTATATTTACCACAGTGACATTCCCAATCCTTAACTGGG
CCAAAAATTTTCTCACAGAATAAACCATCCATTTCTGGCTTTAAAGTTCTGTAATTAATT
GTTTCAGGCTTTGTAATTTCACCAACAATTTGTCCGTTGGGCAAACTTCTTTCGCCCCAC
TGTCGAATTTTTTCGGGAGAAGCTAAACTAATTTTTACGTAGTCAAAATATTGCTCAAAC
TTTGTCATAAATTTGAATACCTTAAAATCAAATAATTAGCCAATTTATTTACATGCTTCA
TATTGAAAAATGAATATCTTAATAAAGGAATTGTTCAAAATCGTCTACTGGAGGAGTATC
ATAATTAGAACGATCTACTCGGTTATCTTTAGAATCCGACATCAAGTCAACTTCAACTGT
CCGTCTTTGGCCATCTTCAAATAGTTTCAATTTATGTACTGCAATGTCTAAACCTAATGA
CTGCAGCTCTCGCATTAAGACTTTAAAAGATTCTGGAGTTCCAGGCTTCGGTATAGGCTT
CCCTTTAACTATTGCATTTAATGCTTCATTTCTAGCTTGCATATCATCCGACTTTACAGT
TAATAATTCTTGCAAAGTATATGCTGCACCAAAAGCTTCTAAAGCCCACACTTCCATTTC
ACCTAATCTTTGACCTCCATGCTGTGCTCTACCTCCTAATGGCTGCTGCGTAACTAGAGA
GTAAGGACCAGTGGATCTTGCATGGATTTTATCATCAACAAGATGAACAAGCTTAAGCAT
ATAAGCTCTACCAACTGTAACAGGATTATCAAAAGGCTCTCCTGTTCTACCATCAAAAAC
TTGCATTTTACCTGGATGCTGATCATTAAAAAGCCATTTATTGCTAGTAATCAATGATGC
TTCTTTTAATTTTCTATTTACAAGTGCTCTTGATGCTTCTGCTCCATACATTTCATCAAA
AGGTATTATTTTAAATCTCTTTCCTAAATAGCCACCTGCTAAGCCTAGTAGGCACTCAAA
TACTTGACCAACATTCATTCTAGAAGGTACACCTAAAGGATTTAAAACAATGTCCACTGG
TGTTCCATCTGATAAATAAGGCATATCTTGTTTAGGTAAGATCCTAGAAATGATACCTTT
ATTGCCGTGACGACCAGCCATTTTATCACCCACTTGGATTTTTCTCTTCTGCGCAACATA
GACGCGAATCATAGCATTTGTTCCGGGAGGCAGCTCATCTCCTTTCTGCCTAGTAAAAAC
CCTAACCTTAACTACTCTACCTTTTGCAGCATTAGGAAGCCGTAAAGATGTATCTCTCAC
ATCTCGAGCCTTTTCTCCAAAAATAGCTCGCAACAATTTACCTTCTGGTAGTTGATCAGC
TTCACCTTTAGGAGTAATTTTTCCTACTAAAATATCTCCAGCTTCAACCCAAGAACCACC
GACAACAATTCCATTTCTATCTAAATCCTTCAGAGAGTTATCGCTGACGTTAGGAATCTC

TCTAGTAATTTCTTCTGGTCCTAATTTAGTTTGACGGCATTCTACTTCATACTTTTCGAT
ATGGATAGAAGTATACAAGTCATCATAAACTAATCTTTCGCTAATTAAAAATGCATCTTC
GTAGTTATAACCTTCCCAAGGCATATAAGCAACTAGAATATTTCTCCCTAAAGCGATTTC
CCCTCCGTCTGTGGATGCGCCATCAGCTAAAGTTTGGCCAACAACTATTTTTTCTCCTAC
CCAAACAATTGGGCGCTGATTAATACAAGTATCTTGGTTAGACCGATAATATTTTTTTAA
GCGATAATGAACTGTTCTACCACTATTATCCTGAATGCCTATTTTATTAGCAGAAACATA
ATTTACATGACCAGATGTTCTGCTAATAACAACCATACCAGAGTCTCGAGCTATTTTAGT
CTCAAGACCAGTACCAATTATTGGCTTTTCTGGATATAATAATGGAACAGCTTGCCTTTG
CATATTAGATCCCATTAGAGCTCTATTCGCATCATCATGTTCCAAAAAAGGTATTAATGA
TGTTGCAGCTGAAATTACTTGAATAGGTGAAATTGCAATATAATCTACTTGGGTTGGGGT
TGTTGTAATAAACTCTTGACGATAACGTACAGGAATAATATCTCCTTCAATGTAATGTTG
TTTACTAACTTTAACATCTCCTGGAGCTACTCGGAAATCATCTTCCTCATCTGCTGTTAG
ATAGACAGGACTATTGTGATAAATCACTTGACCTTGGTTTACCGGATAAAATGGGGTTTC
TATGAAACCAAAAACATTGACCCGCGCACAGGTTGCTAGTGAACCAATTAAACCTGCGTT
CGGACCTTCCGGAGTTTCAATTGGGCAAATTCTACCATAGTGACTAGGATGTAAATCACG
AACTGCAAAACCTGCCCTGTCTTTATTAAAACCTCCTGGACCTAAGGCACTAATTCTTCT
CTTGTGAGTTAGTTCTGCAACTGGATTAGTTTGGTCCATAAATTGAGAAAGCTGACTAGA
TCCAAAGAATTCTCTCACTGACGCAATTAATGGCTTGGGATTAATTAGATTTGATAAACT
CAATGAATCTATATCACATATCATCATTCTCTCTCTAATAATGCGTTCTAAACGATTGAG
ACCTACTCTGAATTGGTTCTGCAGCAATTCTCCAACTGAACGAACTCTTCTATTACCAAG
ATGATCTATATCATCAAGATTACCTGAGTTTTTATCCTTAATATTAATTAGATAATCAAT
TGAGGATAAGATGTCTTGAGGAGACAATACTCGGAACGTTTTAGGTATATTGAGACCTAG
TTTTTTATTAATTTTATATCTACCAACTTCACCCAAGTCATATCTTTTAGGATCAAAAAA
ACGAGAGTAAAGCATCTGCTTAGCAACAGCAACTGTTGCTGGTTCATTAGGTCTAAGCTT
AGAATAAACTATGAGCAGGGCTTCCTCATCAGTTACTTCTTCAATATTATTTTTTCCAAT
TTCTTTAGCTAATTCTTTAACTGAATATATTTGGGATGCAGAAATAAGAAAAGCATATTT
ACTAAGTCCTTTTTCAATTTCATCTTTATTGAGTCCAATAGCTCGCAGAAAAATATAAGC
ATTTACTTTGTGGGTCTTATCTATTCTTATCCAAATTTCTCCCTTAGGATCAATTTCAAA
TTTTAACCAAGAACCTCTATTAGAAATTAGGCTTGCACTATAAATCTGCTTACCATTTTT
GTCTATCTCTTGTTTATAATAGATTCCTGGACTACGGATTATTTGGTTAATAATAACTCT
TTCCGTCCCAGATACAATAAAAGTTCCTCTATTTGTCATTATTGGCAAGTCTCCAATAAA
AATTAATCTCTTTTTGTATTTTTTATTTTTTATCTGCTTTTCAGCACTGAGCTGTAGATG
GGTTGATGATAAATCTAATGATTTAATATTTTTTTCCTGGTTTTTAGAAGGTAGATCAAT
ATCTTTTCTTGTTAACTTTGCAGGAACATATATTTGAGCACTATAAGTTTTATCCCTATT
TTTTGCCTGTCTGACACTATAACGAGGAAATTTTATCTTGTACTCATTACCAAATAATTG
TAATTCGAGTCGACTAGTAGGGTCTGATATCTTCGGAAAAATTTCAAGAACTTCCGTCAA

ACCTTCTAATAAAAACCATTTAAAACTGGCCCTCTGAATTTCAACTAAATCTGGAAGAAG
TTTATTTTTTAAGCTTATACGTTGAACCATAGATCTCCTTTATAACAAATTTTGAAATCG
AAGAGTTTAATACTATTAATAGAGTACTGGTAGATAATGCATAAGCATAGTTTGCTACAT
TATTTCTTCTCTAGACATAACTAGAACATCATATTTTATTCAAAAAAAGATTTTTAAAAT
TTTTCTTCACACACACTATCTATAAGTACTTTGCTGCTAAAAGATAGTTCAAGCTATTAA
AAAAAACGAAATTAATACTTGTCTGTCTGTCAATTAATTGACATCAAGATAGACAAGTAT
TATTTATTTAATCAATTCTGTTTTTCTGAGCAAAAGACAATGCTCTCGCAAGTCTAGCTT
TTTTTCTAGCGCCTGTATTAGGGTGAAAGGCCCCTTTTTTTACAGCTTTATCAATCTTAC
TATATACTTGAGAAATACTTGACTGCACATCATTGAAATTATCACTTTCTAAATTATCAA
TATTTAGAAGGCATCTTTTAGTTAAAGTTTTGACAACCGATTTATACTTACGGTTAATGA
GACGATTTCTTTCCGAAGTTTTAATGCGTTTAATCGCAGAAAGGTTCTTAGCCACAGTAA
ATCCAATAAAACTTTTATAAAATATTTTTAGAAACGAAAATACACAAATCTGGGATCTAT
TCAAAAAGGGAATATGCGGCGTATTATAACATTAAAGAAAAACAATAGGCAATAGTTCAC
CAAGAGCTCTTATTATTACACAATAGAGATTGTCATTGCGAAAAACTTTGTAAGATAGTA
TTTAATTAAAATAAACTAACTAGACATATAAATATATATGAAAAAAATTGAAGCTATTAT
TAGACCTTTCAAGCTTAATGAAGTAAAACTTGCTCTAGTCAAGGAAGGTATTGGAGGAAT
GACGGTTATCAAAGTTTCTGGATTTGGGAGACAGAAGGGTCAAACTGAAAGATATAAAGG
ATCCGAATACTCTATTGATATCATTGATAAAATAAAAATTGAAATTATTATTAGCGATGA
TAAAGTAGAGAAAATTGTAGAGACGATTATTAAGGCTTCTAAAACAGGGGAAATTGGAGA
TGGAAAAATATTTATTAGTAGTATTGAAAGAGTAATAAGAATCAGGACTAATGACTTAAA
TTTTGAAGCTTTATAGTATTTTTACTTTTTTAGACTTGATACTTCTTCACTAAACATGAG
ATAATAAGCGTTTATTGTATACTTATAACTAAGGTGTAATACTAATGGCTAAAAGCAAAG
GTGCACGAATCGTAATAACTTTAGAGTGCTCTGATAAAGCTGGAGAGTTTGCTCAAAAAA
GGAAACCTGGCGTTTTTCGATATACAACTACTAAAAATAGACGAAATACACCAAGTAGAA
TTGAATTAAACAAGTTTTGTCCTAATTGTAATCAGCATTGTATCTTCAAAGAAATTAAAT
AGTTATTTATCTTATTAAAATAATCATATTATGGCTATATACAGAAAAAGAATATCTCCA
ATTAAGCCAACAGATGCTGTTGACTACAAAGATATTGACTTGCTAAGAAAATTTATTACA
GAACAAGGCAAAATACTGCCTAAAAGGTCAACTGGATTGACTTCGAAGCAGCAAAAAAAA
CTTACTAAAGCGATTAAACAAGCTAGAATCCTTTCTTTATTACCTTTTTTAAATAAAGAT
TAAATATATAGCCTAACTTCATATTTTTCCATCTAAATATTAAAAATTTCTATAGATTTG
TTTATTAAAATGATCTAACATACTGAATTACTAAGAAGATCTACTCATTACAAGTTTTGT
TCATTTTATAATTAATAATAGCATCATAATTTATTAAGCTAGATTCAGACAAGACATAAA
GGTAACCTCTTTTAATTGATTATATTATATAGTCTATTTAAAAGAGGAAATTTTTTTGCT
TCTAGCAATTCAAGATTCTTTAAGCTTTAACAAGGTATTTAAAACAAAGATTTTTGTTTA
GGTATCAGATCAAATGAATGAATCTTATCTCCCGACTGCCATAATTGAAATTCAGAGATG
AAAATTCCACATTCGTTGCCAGCTTGAATTTCTTCGACATCTTCTCTAACGCGCTTTAAA

GATTCAATTTTTCCTTGATAAATAACTTTTTCTTCTCGAATCACTTTAATCCAAGAGTTT
TTTAATAGCTTATTATTGATCACTCGACATCCTGCTATTTTTCTATTTGCTAACGAGAAT
ACTGTACTTACTTCTGCTTCTCCCACCGGTACTTCTGAATATTCAGGATCAAGTAAGTCT
TCCATCCTTCTTTTAATGTCTTCAATTAAGGCATAAATGATTTGATAGTTTTCTATTAAT
ATATTCGACTTTGCCGATGCTTGCTTTGTTCCTGGAGCAAAGTTGGTATTGAACCCAATG
AGGGTAGAATTTGTGGTTGAAGCTAGTTCAACATCTGTAGCAGTAATTTCTCCAGGCATG
ATTGAGACAACATTGAGTTGAACTTTACTCTGAGGAAATTGAGATAAAGAATCTAGGATT
GCTTCTGTAGAGCCTTGATTATCTGTTTTAATAATTAAAGAAATTTGCTTACTAATATCT
TTAGAATTAGTGTTTTTTAAAGTATCTAATGTAATTCGACTATTAAGTGCTCTTTGTTTC
TGTATAATTGATGAGTCTTTAGAGGTATTTTCAATCGCTTTAAGTTTGGCTTCTTTATCA
CTTTTAACTGCTAAAGCTATTTCTCCTGTAGCTGGTACAGATGACAATCCCCAAATTTCA
ACAACAGATGATGGTATTGCTAAATTAATTTTCTCTTTAGCGTTGTTAATTATAACTCGA
ATCTTTGCGTAAGCTGATCCAATAACCAAATTATCACTAATATTCAATGTACCATTTTGT
ATTAATAGAGTTGCTACTGGACCATGCGATTTATCTAAATGGGCTTCTATTATAATACCT
TGTGCAGGCTGTGTTGGATCAGCTTTCAAATCCTCTAATTCAGCTAACAAGGTAATAGTT
TCTAATAATTTATCAACATTTTGACCAGTAAGAGAACTAATAGGAATTATAGGCACTTGG
CCACCAAGTTTCTCCGACATGACATTGTATTTTAATAAATCTTGCTCAATAATATCTGTA
TTAAAACCAGCTTTATCTATCTTAGATATTGCTATGACAAAAGGGACATTCGCTTTTTGA
ATATGATTAATAGCCTCTATGGTTTGAGGTTTAACTCCGTCATCAGCAGCTATAATAATA
ATAGCGACATCTGTTAAATTAGCTCCCCTAGACCTCATACTTGTAAAAGCTTCATGGCCT
GGAGTATCCAAGAAAACAATTTTTTGTTTATTATCTTTCTTAATATACTCAACTTCATAA
GCTGCAATTGCTTGAGTAATTCCTCCAATTTCTTTGTTAGCATTATTAGATTTTCGAATA
TAATCTAATAGCGTTGTTTTGCCATGATCTACATGTCCCATCACTGTGACAATTGGAGGT
CTTTTAATATAATTGCCAGTTTCATAAAGAGCATTAGAGTGATCTAAATTACTAGAAAGT
CCATTATTATTTTCCTTTACATTTGATTCTACTGCTATACCAAAATTATCAGCCACTGAT
GATATTATTGAGGCGTCTATGGTTTGATTCATAGTAACTGATATTCTTTTAAGAAATAAA
TATTTTATAATATCTGTTTCTTGAACACAAATTAGTTTAGATAATTCTTGAATAGTTAGA
GGATTAGTAATACTAATAGACTCTGGCGGAGAATTAGATTGAATGCTTGCTGAATTTTGA
CTAGGAGTTACTGCTACTTTTTTCTGTCTTATAGATTTTTTATTACTAGTCAGTTTTTTT
ACAACTTCCACTTTTGGCTTAGGTGGACGCATTAAAGAGATAGCTAAATCTCCTGCAGTC
TGCGAAACATTAGAATTAGAGTCTCTAAAGTTGTCATCATCATCGTCAATATGTATTTTA
GTTTTTATTTTTTTGCGCTGTCTATTCTTATTTTTTTTACTATCTACTAAGTCGTGAATT
TTATTAAAATTTTTACTTTTTTTATCTAGTTTAGGTGGAGAACTTAATTCTAGATGCTGT
TCACCACCTGTATGTGATTCTGATTTATCAAGGTCTAAATTTAACAGATTATCATTATTG
ACTGACTCTAATCTAATTTTATAGATAATCTGAGGATTTTTCAAATCTACAATTGTTTCA
GAAGAATTGATATTTGAGCTAGATCTAGAGGATCTATTTTCAAAGTTTTGATTATTTAAA

AACATAAATATTGGTTTTATTATCTAATAGTAACTTTCTAATTTTAATACAGAGCTTAAT
ATAAACAAGAATCGAATATCTGTATAAATAATACAAATGTAGAGAATTAATCTATGTTGT
ATTAGATTTAATATAACACTAAAGTCCTTTGTTCGTTATACAAGTTTAAAACTTTTTATG
ATGATAAACACTAGATTCTAGTTTATTGGTATACAATCTATTTATCTAAATAATACTGTT
TTTGTCAGGGCTTTTAAATTATCTAATGTTATGTTCATTATGAAATTCTATTAAAATTTA
AAAACTCAACAATTAAGTAAGAATCGTTAATCACCTCCACGCCACTTATGCCACGATCTC
AAAAAAATGATAATTTTATTGACAAAACTTTTACAGTATTAGCTGATATTGTATTAAAAA
TACTTCCTACAAGTAAAGAGGAAAAAGAAGCTTTCTCTTACTATCGAGATGGAATGTCTG
CACAATCTGAAGGTGAGTATGCGGAAGCTTTAGAAAATTATTATGAGGCTTTAAAACTAG
AGGAAGATCCATATGATAGAAGCTATATTTTATATAATATAGGGCTTATTTATGCTAGTA
ATGGTGAGTATGTAAAAGCTTTAGAATACTACCATCAAGGATTAGAACTAAATTTTAAAT
TACCTCAGGCCCTTAATAATATAGCCGTGATATACCATTACCAAGGAGTTCAAGCAATTG
AGGATAAAGATACAGAGCTATCTAAATTAATGTTTGATAAGGCGGCTCAATATTGGCAGC
AGGCTATTAAGTTAGCTCCTGATAATTATATTGAAGCTCAAAATTGGCTGAAAACGACAG
GACGAATGAGGAATATACAAGGATATTAATATATTTAAGATATAATAAAGATATCAATTA
ATAAATTAAAATTAGTTAAAGAAACTGGTACTATATAGAGTACTTTACTATATATTATAA
TATTATACTAGTTGA------TCTCTAAGACTTAGCTGAATAAACGAAATTAAATCTATT
GACTATATTCATGAATACAAATAAGCATAATGGTTAGTACAACAAAAAGTACCGGATCTG
TTACTCAAATTATTGGACCAGTTTTAGATATTGCATTTCCTAACGGACAGCTTCCGAAAG
TATTCAACGCACTCAAAGTACAAAGCTCAGAAGGAACTATTACTTGTGAAGTACAACAAC
TTTTGGGTGACAACAAGGTACGAGCTGTTTCTATGAGTTCCACTGAAGGACTACAAAGAG
GGGTAGAAGTTATTGATACTGGATCCCCTATATCTGTTCCTGTAGGTACAGATACTCTTG
GACGTATTTTTAATGTTTTAGGTGAACCTGTAGATAATTTGGGTCCCGTTGATTCTGAGA
GTACTTTACCTATCCATCGACCAGCACCTGCTTTTACTAAGTTAGAGACAAAACCAAATA
TTTTTGAAACAGGTATTAAAGTCGTCGATTTACTTGCTCCTTATAGAAGAGGTGGGAAAA
TTGGTTTATTCGGAGGTGCTGGAGTAGGTAAAACTGTATTAATTATGGAACTAATTAATA
ACATCGCTAAAGCTCATGGTGGAGTATCTGTATTTGGAGGCGTTGGTGAAAGAACAAGAG
AAGGAAACGACCTCTATATGGAAATGAAAGAGTCTAAAGTAATTGATGCAGATAATCTGA
AAGAATCTAAAGTAGCATTAGTATATGGTCAAATGAATGAACCTCCTGGAGCACGTATGC
GCGTTGGCTTAACTGCATTGACAATGGCAGAATACTTTAGAGATATTAACAAACAAGACG
TTCTATTGTTTATTGATAATATTTTTCGGTTTGTACAAGCTGGATCAGAAGTATCAGCTC
TACTAGGCCGTATGCCATCTGCTGTGGGTTACCAACCAACTCTAGCAACTGAAATGGGAG
CACTTCAAGAAAGAATTACTTCAACAACAGAAGGATCAATTACATCTATTCAAGCTGTAT
ATGTGCCGGCTGATGATTTAACAGACCCAGCTCCGGCCACTACATTTGCACATTTAGATG
CAACAACAGTACTTTCGAGGAACTTGGCGGCAAAAGGAATTTACCCTGCAGTGGATCCAC
TGGATTCAACATCAACAATGCTACAGCCTGGAATTGTTGGAACTGAGCATTATTCTACTG

CTCAAGAGGTAAAATCAACTTTACAAAGATATAAAGAACTGCAAGATATTATTGCTATTC
TTGGTCTTGATGAACTTTCAGAGGAAGACAGACAAACTGTATCAAGAGCAAGAAAAATTG
AAAGATTTTTATCTCAACCTTTTTTCGTGGCAGAAGTGTTTACTGGATCACCTGGGAAAT
ATGTATCTTTGGAAGATGCAATTAAAGGGTTTCAAATGATCTTAAAAGGCAATTTAGACG
ACTTGCCTGAGCAGGCATTTTATCTAGTAGGTGATATAGATGAAGCTATACAAAAAGCTG
ACAGCATGAAAGATTAATATAATTAATGATGACTTTAAATATAAGAATTATTGCTCCTGA
TCGGACTGTTTGGGATGCAGAAGCACAAGAAATTATTTTACCAAGTAGTACAGGGCAACT
TGGTATTTTAACAGGCCATGCACCTTTGCTTACAGCTTTAGATATTGGAGTTATGAGAGT
AAGAGTAGATAAAGAATGGATGCCGATTGTTTTGCTGGGCGGTTTTGCAGAAATAGAGAA
CAATCAATTAACTATTCTGGTTAATGGCGCAGAAGAAGCTAGTCAAATTGATTTATCAGA
AGCAGAAAAAAATTTAGACACTGCAACTCAACTCTTAAGTGATGCCTCGTCTAATAAAGA
AAAAATAGAAGCAACACAAAAAATACGAAAAGCTCGAGCTCGAGTACAAGCTGCAACAGC
AGCAACTTCGTAAAAGATTCCTCTTAAAACATAAAACTTTAGTAATGATTACTAAA----
---GTTTTATGTTTTATTTGTATGGTTATAGGCAGCTTAAATAAACTTAGCTTAGACCTG
AACAAATATAATCAAAATATAATCCCATCTCTTTACCTGCATCTGGTCCAACTAAACTAA
TAGTAACTTCTTTCATAGCTAAAATAGCTTGAATCGTTGCACCAATAGGAACACCTAGAG
AATTGTATGTTTCTTTTAGACCATTTAATACTCTTTCTTCAAGGATAGATGGATCTCCAG
CTAGCATTCCATAAGTAGCATAGCGTAAGTAGTAGTCTAAATCACGGATGCATGCAGCAT
AACGTCGAGTTGTATACATATTACCACCTGGGCGTGTAATATCCGAATAAAGTAGAGATT
TTGCTACGGATTCTTTAATAATTGTTGCTGCATTAGCTGCAATAGTAGCAGCAGCTCTGA
CTCGTAGCTCACCTGTTTGAAAATAGCCTCTTAATTTTTCAACAGAGCTATCATCTAAAT
ATTTACCTTGGACATCAGCTGCATTAATAACAGAAGTAATTGCGTCTTGCATAACTTTCA
CACATTCCTTAATTTTAAATATCTAATACTAGGGGTATATAACAAAATTACTAGATCTTT
TAGCAATATCAGTATTACTGCATAGCACCCAAAGTATAATCAAAGTAAAAACCAGCTTCT
GCTGAATCTTCACCTGCAAGCAGTGAGCAAGCTACACTTTTCATGCATTTTACACCTTCA
GCAACTCCTGAAATTGGTGTTCCTAAAGAATTATACATTTCTTTAACACCCACTAATCCA
ATTTCTTCAATAGGAGTTACATCGCCAGCAACTATTCCGTAAGTTACTAAGCGAAGATAA
TAGTCCAGATCTCGTAGACAAGTAGCAGTCATCTCTTCGCCATAAGCATTTCCACCAGGA
GAAACTACATCAGGTCTTTTTTGAAACAGTTGTTGGCCGCCTTGCTTTACAATACGCTCA
CGATTATCTGTTAAAATTTGAGCTATTCTTAAACGACGTTGTCCAGATAAAACAAAACTT
TTAATTCTATCTAATTCCCCAGGACTTAAATATCTTGCTTCTGCATCTGCATTTACAATT
GACTTTGTAACAATACTCATGGATAAAACTCCTGTAATGCTTTAAAAAAAAAGCTTAATA
CTAATTATTTATGATATAACTCAGAAAGTAATGTACTTTTTGAGAATTTTATAACCGAAA
CACGTTCATGATATATGAAGCAGGATAGATACTGAGGTAAGTACTAAAACGAGAAAGTTA
TATGTAGGAACTTCTGCTATTGATTTCCTAGTACTTTTTTAAAACTTGGTACCACAATTG
ATATATCCTGCTTAGTCAAACGATTATAGAGTGTTTCTGTATTAGGAAAGTTAGCTGCTG

GTAAAGTCGGAAATCTTCTGTATGGAACAGTATCTTTGCCATATACCGCATTGTATTCAA
CACTTTCAACTAAATTACTTATAAATGCAGATAAGCCTTTTGAAGCTAAAATTTGATTAA
AGAATCTAATTTCTGCCTGATTATTAGGAGCTCTTCCTAAGATATGCTTAGTACCTAATT
CTATAACTTTTGTATTTGGATATGGCTGATAAAATTCTTTACCATATAGTTCAGATAGCG
CCAGTTTTTCTACTAGTTCTTTGACACAGATTTGTCTATTCAAGAAAGCTGACTTAATAT
CCAAGAATTCACCGCCGACACTAAAAGAGTTAAGATCTCTTTCGAAAATTTGACGATATG
CTGCTCTTAAAGCTTGTTCTAACATTTCTTTGTTACTATCACTATTGACTTCAAAGACAA
TAGATTGATCTCTTAAAGAAGTTACTCCCTGTGAAATACGAGACTGAATATCATTACTAC
TCCTCATTTCTTTGACAGTGCCTAATTCAACAAACCTAGCAGATTTACTAGCAATAACTT
TTTTGAATCTCTGGTCAATAATTCCTGGTCTTAAGCTTCTTAAAGCAACACCAGCTGGAG
TACTATATCTTTCATAAGGAACAGTATTATCTCCAAAAGTTTCTGTGTACTCCGAGCTAT
CAATTATTGTGTCTATTACTTGGTAATAGCCTTGCTTATATGCAATATCAAAATATTTAT
TAATTTCTTGCCGACCATATGTAGGGCGACCCAGTAAGCGGTTATGAATATATTCAATAG
CTTTACAAATATATAATGGCTCCCAATAAAGAGATCTAAATATACTTGATTTTGCTAACT
GTCTAACAAACTCGCGAACTGTAATTTGATTATCTTTTAGCTGACTTTCTATTGGCTTGA
GAATTAGTTTTTCTTCTTGATACACTTCTCTGCCAAAAACTCTTAAATAAGCAACTTTTG
TGACAACTTCTACTGAATTTTCAAAATTTTGGGATGAATCTGACTTTATAAGAGCGTTGG
ACAATTTAAAGATTTTTGGTCCCAGGGATCCTGCTGACTTAGGTCTTACCTGTGGATTAC
TAATCTGATTGTAGATTCCTGGCCCACGTCTAACTAATATCCTTCTTGTATCTTTACCAA
AAAGAGCTTGTCTTTTTCTTGGATCTTTATTTTCTTTAGGGAAAATAGCTCCAAATTGAA
TTGATAATGGATCATTACCTATTCCATATGGATGTTGATCAGGTAATGATTGCTTATAAT
CACTAAATAAAGTTATAAATTGAGGAACCTTACGAAAAGGCGCACTATAGGTGAAAAGAT
CTATTTGAGGCCCCCAATTACGGCACTCTTGAGGCTCTTCACCTAAATTTCTAAAGTAAG
GCACAGTCTCTTCACCGAAATAATCTGTATACTCAGAAGAATTTAAAAGTGCATTCACTA
AACCACTTAAACCAGTAGATGATAAAATAGCAAAATATTTTTGAAACTCTTCTAAAGAGC
TAGGACCTCTACCCAAGAAATGTCTAAATGCTAATTCTAATGCTCGACTATTGACAAAAG
GTTCATAAAATTGCTTTCTATAAATACTTGAAGTTCCTAACGAACGAATGAATTCCTTAA
TTGAAATTTGGCCATTTTTTACTTGCGACTCCAAATTCGATAAAGAGAGGTCATATGCTT
TAGCAATATCTCTTTCAAAAATTTGTCTATAACATGCTTTCACAACAATATTTTTCTCAT
CAGCAGATAAACTAGGCTTCATAACAAAACGAGGTGTTGACACTCCTGCCTGTACATAAG
TTTGAGGAAGACGCAAACCTTGTAAATCTCCAGATATTCTTTTTCTTAATTTATCAGTCA
AAGATGGAGCTTCAAATTCAGAAATCACAACATTAAAATACTCTTTAACTAAATCTTGAC
CTTTTATATCTTCCTCAAAGATTAATAATGCTGTCCGTCTCATCTCTCTAAGAGCCACAA
TTGCTGCTGCACTTGAGCAAGCGTTATCAATTAACTCTCTCAATCCTCGTATATTAACAG
ATAAAATATTAGGGTCGCCTGATACAATTGCATATGTCAAATACCGTAAAAACCAGTCTA
AATCTCGAAGAGATTTTCTCATTCTTGTAGTACCGTATCTCAAGACGTTTATAGGTTTAA

ACCCTGGCGGAGTAGCTCCCCCAGCATTAAATAAAGATCGGAAACTTTGGCCAAAATCTC

CTTGGATATTTCCTGATAACTCATTAATTTTATCTTGAGAACTTTGATCTCCTGCAATAA

TAACAGCTGCTTGCGGACGTTCTAAATAAGAAATTGCAGACCCACCTACGAATATTTTAT

CTGCTGCTCGTGCTACCAGAATATTTGCATTTTTAGTTAAAATATCTGCAACTTCCAGTC

TTTTTTGTCCAGAATTTAAAAAAGAAACTAATTGATTTAATTCACCAAGCTGTAAAAATC

GGTCTTGTTGCTCTGCCTGAGTAATAGTTAAAATTGAGGCAGTCCGATAAAGCTGGGGGC

GTGCTAATGGGCTTCCGCCACTTGCTTTGATACTCATTACTTTATTTATCTCCTTAACTG

ATATTGCTTGCTAGCGAATACAAATATAGCTTGATATTTCTAAATATTTAAAGATACTAT

GCAAGCACACTGTTTTCTATATTATTAGGTACTAGTACTTATAGTACAGAGAGTCTTATA

ACTAACTTCTATAAATACAAATTTTGTAATACTTCTATTAACATTTGTCAAAAAAATATT

TAAGTGCTCACTCTTTTATATTAAAATTCAATTAGTGAGAGATTGATGTGAAATAAAGTA

TACTAAGTAAGTTTTATAGATAGGAAAATTAAAAGATTTATTCTACAAACATTAATCACT

TGAATGTTTAGCAATTGTACAATTGTTATTGCTTTTAATTGGTTTAGACTAGTTTTATAT

TTTTTCTAACATAAACAATATTAATGAGCAATTTGTATGAATTTAAAGCCACAAAACAAC

CTATTAACTAACGAAAATATAAATCATGTAGATATACCCGAAAATGATATACCAATGTCA

ATTACGGAACATTTAGAAGAATTAAGACAACGGACTTTATTTGTGTTTTTATTCTTTTTG

TTTGCTACAACTATAAGTTTCACACAAATTAAGATCATTGTTGCAATACTGCAAGCTCCT

GCTGTTGGTATTAAGTTTTTACAACTGGCACCAGGAGAATATTTTTTTTCATCTATTAAG

GTTGCAATATATTGCGGGATTGTTGCAACAACTCCGTTTGCCGTTTATCAAGTTATATTA

TACATACTCCCAGGACTAACTGGAAAAGAAAGAAAAATTATTTTGCCTCTATTAATTAGT

TCTGTATTGCTTTTTATTACAGGTGGTATATTTGCTTACTTTGTTCTCGCACCAGCAGCT

TTAACATTTTTAATTAGCTATGGGTCTGATATTGTAGAACCATTATGGTCTTTCGAACAA

TACTTTGACTTTATTCTATTACTTTTACTTAGTACAGGATTAGCATTTGAGATACCAATC

ATACAATTATTGCTCGGTGTTTCAGGGACATTTTCTTCTAGTCAAATGATACGAGCTTGG

AGATATATTATTATTATAGCAACAATTGCTGGAGCTATTCTGACCCCTTCGACTGATCCC

GTTACACAATTAATAATGTCTTCAGCTGTTTTATTACTTTATTTTGGTGGAATTGTTATA

TTATTAGTCTTAAAAAAGTAGAGTAAATATTTTCTATTAATCCAATATATTTGTTATCTG

GGCAAATAATCTATAAAAACAATTGCTAGATTTAAGTATCAGGCTTCTAAATATTTCAAG

TCTCTTTTTATCATTTAAATACAATCGATACATTGGCTTGTTTTTTCATCAATTATTTGT

GTTGCACACTTTTTAGCACTCTCTTTATTGTTCTTAGAATTATTTCTTTAATCTATTTAT

TAAATAGATATGAAGAGATTTAAGTATTGATTATGGTAATCTATATCAACGTAAAAAGTA

TTTTATTGTGAGTTTTATTTATATAAAGGCTTATTCATTATATAGTTATAACAGTAAGTC

TAATTAATAGTTTTCATGCTTAATTAAAGTAATAACTCTTAATTCTTTTGGCAGAAAAAT

CTGTCTTAATATTTTATCAAAAAAACAGAAAAATGAAGCTAAATAGTCTAATTAACTTAA

TTCAAAAGTCTATATATTCTTGTACACTTTTACTAATTATTTTAAATATTATTTGTGTCG

CACCTAATTCTAGTAATGCATTTCCAATTTATGCGCAACAAGCTTATGAAAGTCCAAGAG

AAGCAACTGGTAGGATAGTATGTGCCAATTGTCATCTTGCTCAAAAGCCTGTGGAGATAG
AAGCGCCTCAAGCAGTACTACCTAATACTGTTTTCGAGACTGTTGTGAAGATTCCATATG
ACAGCAATGCTAAACAGATTTTAGGTAATGGCAGTAAAGGAGGCTTAAATGTTGGAGCCG
TTGTAATATTACCTGAAGGATTTAAGTTAGCTCCTGTTAATAGATTATCTACAGAGTTAA
AAGAAAAGACTAGAAATCTTTACATTCAGCCGTACAGTGCTAAACAAGACAACATTTTAG
TAATTGGACCTATTTCTGGTGATAAAAATAGAGAAATAGTTTTCCCAATACTATCTCCCG
ATCCTGCAAAAGATAAAAAAGCTCATTTTTTCAAGTATCCAATATATGTTGGCGGAAATA
GAGGACGAGGCCAGATTTATCCAACGGGTGACAAAAGCAACAATAATATTGTCTCTGCTT
TAAGCAGTGGTAAAATTAATAAAATTGAATTACTAGACAAAGGTGGATTTATAATACATG
TGACTAACAGTAGCAATGTAGAGTCAACACAGAAGATTTCACCTGGTCTCGAACTTAGAG
TAAAAGAAGGGGATACAATTCAGCTTGATCAAGCTTTGAATAGTGATCCAAATGTAGGTG
GTTTTGGTCAGAACGAAACAGAAATAGTTTTACAGAGTCCAAATAGAATTAAGGGCATGA
TTGTTTTCTTTTTCGCTAGTGTTCTAGCTCAAATTTTCTTCGTATTAAAGAAAAAACAAT
TTGAAAAAGTTCAAGCAGCTGAAATGAATTTTTAAAGCAATATAAAACTGGCACTTACTA
TAAAGAAAGTATAAACACTTTATTCACAGGATGTACCAGTTTTTATGTTATGTAATAATC
GCTTTGACGGAAGTTATGATACTTTTAACAGAATCTACAATCTGACTAGGCTGAATTACT
GTAGCCTGCTCTAAGCTTCCATTATATGGTGTGGGTATATCCTGCGAAGATAGCCTCACG
ACAGGAGCATCTAATTCATCAAAAAGGTACTCATTAATTTGTGCAATTAGCTCTGCTCCA
ATTCCGGCTGTTTTCATACACTCTTCTACAATTAAAACTTTGTGAGTTTTCTTTACTGAG
ATAGATATAGAGTCTATATCCAGAGGTTTTAAAGATATAAGATCTATAACTTCTGGATCA
TAACCTTCTTTTAATAAGGCCGGTAATGCTTGGATAACATGATGCCTCATTCTAGAATAG
GTTAAAATCGTAATATCTTTTCCTTTTCGTACAAATTCAACTTTATTGAGAGGTAAGAAA
TATTCTTCTTGAGGAATCTCTTCTTGTAAATTATAAAGTAGAACGTGCTCAAAGAATACA
ACTGGATTATTGTCTCGAATTGCAGATTTCAGTAATCCTTTTGCATTATAAGGAGTAGAA
CAAGCAACTATTTTTAAGCCGGGGATGGCTTGGAAATAAGCTTCCAGTCTCTGAGAATGC
TCTGCACCTAACTGCCTACCAACTCCTCCCGGTCCTCTAATAACTAAAGGCAATGTAAAA
TTACCTCCAGAAGTATAACGTAACATTCCTGCATTATTAGAAATTTGATTGAATGCTAAT
AATAAAAAACTCATGTTCATACCTTCAACAATTGGTCTTAGCCCTGTTATAGCTGCACCA
ATTGCCATACCAGTAAAGCTATTTTCTGCTATTGGTGTATCAAGAACTCTTAAATCCCCA
TATTTGCTATGCAAATCTTTAGTTACCTTATAAGATCCACCATAGTGACCAACATCTTCT
CCTATTACGCAGACAGTTGGATCTTTTGCCATTTCCTCATCTGTTGCCGCTCGTAAAGCG
TCAAACATAAAGATTTTACTCATTTTAATATTTGATTTTTATGATTTGACCACTGTTTTA
AAGTAACAGTTAAGACACCATATTCTATTTAGTTATCTGCAAAAAGATATCGTTTCAATT
CTGACATGTTAGGTTCTGGACTGGAAATAGCAAATTTCACAGCTTGTTCCAATTCGGTTT
TAACAGCATTCTGAATCTCATTAAGTTCACCTATATTAGCAATTTCATTATCTAGAATAT
ATTTTTTGAGTTTTTTGATAGGATCTCTTGCCACCCAAGCCTCTTTTTCTTGTCTTGATC

TTAGTTCATCAGGATCTGCGAGAGAGTGACCACGAAATCTATATGTTAATGCTTCTATTA
AGGTTGGACCATCACCTTGGCGAGCTCTTTGAACTGCTTGTTTTGCAGCTTGTCTTACAG
CTAGCACATCCATTCCATCAACTTCAATCCCAGGAAGCCCAAAAGCTTCTGCTTTTTTAT
GTATTTCAGGTATTGAAGAAGACCGGTGATGTGCCATACCTATAGCCCACTGATTATTTT
CAACAACAAATATAATAGGTAGTTTCCAGAGAACTGCCATATTCAGACATTCAAAAAATT
GCCCATTATTGGTAGTCCCATCACCAAAAAAGCAAGCCGTGACTCTTAAATCCTCTGTTT
CTTTAAGTACTTGCTGGCGGTAGATACTTTGAAAGGCTGCCCCTGTTGCAACCGGTATAC
CTTCCGCAATAAAAGCAAAGCCACCTAAAAAATTGTGAGGCGCAGAAAAAAATATGCATCG
AACCTCCTCTGCCTTTACTACAACCAGTCTCTTTTCCAAATAACTCAGCCATCACATTTT
TAGATGGGACGCCTTTACTTAAAGCATGTACATGGTCTCGATAGGTACTGCAAACATAAT
CAGTTGGATTCAGAAGTTTAATTACACCTGTAGAAACAGCTTCTTGACCATTATAAAGAT
GAACAAAACCAAACATCTTTCCTTTATAATACATCTGAGCACACATATCTTCAAAATTTC
TGCCTAACAACATGTCTTCATATAAAACTAATAAATTACTCTTATTAAGATTAAGACCTG
TTGAATTATAGTTAGTCAGCGGCAATTGAACTTTCTTAGGATAACTCATAATTTGTAAAG
AAACCTCTTATTGTAAATAGGAATTTTGAAGAAACAGCTATCAATAGAAAACACACAGAT
AAAAACTTATAAATTATAATATGTTTTTTACTAAAGACTTTAATAATTATTTGAAACTAG
TTCAGTATATCAAATTAATTTTGCTTATACTACGGATGTCTAAAACTTATATAAAAAAGT
AGTAAAAAAGTTTATTAAAAGAATAGTTAAAAACAGATATAAATAGAAGTAAACAGAATT
ATTGAAAAAAAATGATTATTATTAATTAATACTTAATATTTCTAAATATTTTACAATTAA
TGGCTATGTCACCATCTTTATTTTATCCTGTTGAACAAGAACTATGTAGTCTTGAAAAAA
ATCTGAAAGCTGTTGCTGGGACTCGTCATCCAATTTTATATGCCGCAGCAAAGCATCTAT
TCGATGCTGGAGGAAAACGAGTTAGACCAGCTCTTGTATTTTTAGTGGCTAAAGCAACCT
CTGAGAAGCAAGATATAAATACTGGACAAAAAAGGCTAGCAGAAATTACTGAAATTATAC
ATACTGCTAGTTTGGTACATGATGATATTATTGACGAGTGCACAACACGTAGAGGAGTCA
AAACTGTACATAATTTATTCAATACTAAGATTGCTGTGCTAGCAGGAGATTTTTTATTTG
CACAGTCTTCTTGGTATTTAGCTAATATTGAAAATTTAGCCGTAGTCAAAGCTATTTCTA
AAGTCATCACCGACTTTGCAGAAGGAGAAATTAGGCAAGGCTTAGTTCATTTCGATCCCA
GTATTTCAATAGATGCTTACATTGAGAAATCATTTTACAAGACTGCTTCACTCATTGCTG
CTAGTTGTCGGGGTGCAGCTATGCTTAATGGTTCCAATCATCAAATAAATAATGATCTTT
ATCTTTACGGTAAACATATGGGATTAGCATTTCAAATTATGGACGATGTTCTAGATATAA
CTGGTTCTACTAAGAGCTTAGGAAAACCTGCTGGCGCTGATCTAATAAATGGAAATTTGA
CCTCTCCTCTCCTTTTTTCACTTACTCAAGAAGCAAGTTTAAATGATCTTATTGATAGGG
AGTTCTGTAATAGTACAGATATAGCCTCAACATTATTTCTTATAAAAAGAAGCGGGGGAA
TTACAAAAGCTAAAGATTTAGCTAAAGAACAGGTGCAGGCGGCACTTTCCTGCCTTCAGT
TTTTACCACAATCTACACCTGTATCTAGTTTAAAAGAATTAACACATTTCATAATCACAA
GATTGTCATAAAGGACTTGCTAAAATTAAAATGTTATTAACTAATTTTTTACTGTTTCTA

AAACAGTCTGAAAAGTTTGAATATCATTACTAGCTAGCTGCGCTAGCATTTTACGATTTA
ACGCAATATTCTCTTTTTTTAATGCACTAATAAAAGTACTATAATTCATGCCTTGATTGT
GAGCAGCAGCATTTATTCTAGTTATCCAAAGACGACGAAAATCTCTCTTTTTCCTTTTTC
GACCAACATAAGAATATCGAAGAGCTTTAAGGACTTGCTGCTTAGCTGTTCGAAATAAAC
ATTTATGTGCACCTTTAAAGCCTTTAGCTAGCTTAAAGATTTTAGCTCGTCTTTTTTTTG
CAACGTTACCTCTTTTAACTCTACTCATAAAATATTACTTAAATTTTAATTATTACCAGC
TTACTAAATTCTTATACTCTTTGATTATAAATAAGGTAATTTCATTGCAATGTTTTTAAT
ATCTTTTAAGTCAACCGAACAAGTAGAAGAAAGATGTCTTCTTTGTTTTGATGACTTTTT
TTGTAATAAATGACTTTTAGAAGCTTTATGTCGAAGAAATTTTCCGGATGAAGAGACTTT
AAATCTTTTTGCTATTGCTTTTGATGTTTTTAACTTAGGCATATTATAAACTTTTAATGA
TTAACAAAAGCATGAAAAGTCTGAGGATATCAATCTTAGACCTTTCAATACTTAGAATAA
TCTTAACACAATTTATTTTACTTACTACATAGGTTAACTGAAACAATTTATATATATAAT
ACTAATCAGTTAATCAACCATAGCTAAAGTGGATTTATATTTTGCTTTGAACTTTTGTAT
AGTATTAGCAATTAGCATTGTTATAATTTTAAGACTGCTTGAATTCAATTCTTCGAACAT
TTTCATATTTAATGTAAATGAAATATTGGCTTCTGAGACAATATTTTGAATTTGAACATC
AGACAAGGGAATCATATCTAATGCAGCTCTATATTGATCTTTAAATAATTTATCGTCTTT
TATTTGGTCAAAATCGTAAAATTTCGTTCCTCCTGAATCTGATAAATTCATAGCCCCTCT
AGCTATTTTTTTTAAAATTTGGCCACCAGAAAGGTCACCCAAATAGCGCGTATAAGCATG
AGCAACTAGTAATTCAGGTTGTTTATGTCCTATAGTGTGAATTCTATCAACATAAATTTT
TGTAGCAGGAGATGGTTCAATAAAATCTAACCAATCTGATCCATAATAATAGTTTAAATC
TTCAGATAGACTCGCTTTCCTATTAAGCTCTGTAAAATATATAGGTTTAATAGCTGGATG
ATTTTTATTAGAAAATAACTCTTCCTCTATTGCACAATAGACAAAGTATAGATTTGCAAC
CAATTTACGATATGACTTCTTATCTACAACTCCTCCCAAAAAAGATTTAACAAAACTAAC
ATTTTCTGCCATACTATGGGACTTAGTAGTACCTTCTCTTAGTTCATTCGCTAAAGTATT
AACCATAATATCAATATTCCATTTTTAAAGATAACAAATTAACAATAAATAGTTATCCGT
ATATTTATAATTGAGTTGCGCTAACTAGGCTGCTGCAAAATAATTTTTTGATTTTATTGG
ATCTGGATTCATTGTTTTGTCACCAGGCTTCCAATTAGCTGGACAAACTTCATCTGGATG
AGATTGTACATATTGAATTGCTTGTAAAACCCTCAAAGTTTCTTCTACACTCCTGCCAAA
CTCTAGATTATTGATTGTAGAGTACTGAATAATTCCTTTCGGGTCTATAATAAATAGTCC
TCTTAGAGCTACACCATCACTATTCAAAACATTATAAGCTGCACTGATCTCTTTTTTTAA
GTCTGATACTAATGGATATGAAAGATCTCCTAATCCACCTGATTCTCTGTCAGTCTGTAA
CCAAGCAAGATGAGAATACTCACTATCAACCGAGACTCCAAGAACTTCTGTATTAAGTTC
CGAAAAAGCATTATACTTATCGCTAAATGCTGTAATTTCTGTAGGGCACACAAATGTAAA
ATCTAAAGGATAAAAGAATAAGACAATATACTTATTCTTGAGATCAGATAATTTTAACGT
TTTAAATTCTTGGTCATAAACAGCTGTAGCTGAAAAATCAGGAGCTAGCTGACCTACTCG
AAGACAATTTGGTCCAGAAATCATTAATTTTTCCCAGAAGAATTGATATAATTTTGTTAT

TTAATAAATAATTATATAACAAAATTTAGTATATATTAAAATACTACCAGTCTTTTTTAA
ATTACTAAATGATATAATCAATACTATCTTTATAGCGGGGAATGGATTTGAACCATTGAC
CTTCGGGTTATGAGCCCGACGAGCTACCAGACTGCTCTACCCCGCGGATAACTAAAATCA
TATTAGTCTACATATATATATAAAAGCAAATGCATAATAGATTTAGAAGTAAAAAAATA
GAAGAACTGGTCCACTGATCATTAAACTTAGAAACGACAAAGTATTTTTTATCTTATTTA
AAAGAGGAAAGACTTCATATAAACCAATAAATCTATCTTTTAAAAGAATTTCGGACGGCT
TTACCCAAATTTGACCGTCATACCAGCCTGATTCTTCATAAAATACTGTAGCACTCATTA
ATCGTTTTACTACGTATGACCAGCCTAAATATAATCTGATTAAAATAAATCCTGTCATTA
GGCTGGAAGTAATAAATTCTGAAAAGAAAAATTTCAAAGGTAGTTTAGTGATTGGAAAAA
TTGATAATAATATAGGACTAACTAATAAACAGTTAAGTAGTAGTGCAATAGTTATTTTTT
TGTTATAAGATCTATGGCTTAAAGTTGGCCAGCAAAAGAACCAAGAATTTTTCAAAGAAG
TATATTCATGAACAGGTTGCTGCTCCTTTGGCACAGGACATTGAGTATTATATAAATTCA
TTGACATTAAAAAGTATTATAATAAAGACTTTGTTTATATTATCTAACATAGGTACGACA
TTAAATATTAACTAACTGCAATTATAGTAGTGAGTAGAAGAAATAAAACTAAAAATAGCC
AAGTAATTTTATTTAAAGTATTCTCAGTACTACGAGTATTACTAAAGAACTGATTCTGAG
CACCTACACTTCCTAAACCTTCGGATTTAGGATTGTGTATTAGAATAGTGAAGATTAATA
TTATTGTAGATGAGTACCAAAAAAATTTTAAAATTTGTTCCATTATGTAAAAGTTAATAA
AAATAGCACAAAGACAATCGTCTAAAAGATATTGATTGTCTTATCATATTAAAAAGTATT
TATTCACCCTGTACTTTTAAAAGTAAAAATCCTATTGCCAGCCCTACCAAAACTAGGACT
GAACTTAATACAGCACTTTCTACAATTTCTCCGACCATTTTATTTTTCCTTATTATTTTA
TTATTGTCATTCTTTTGACAAGATTATATTGAGCTAACTAAAATCCGTTTCTACCCCAAA
CTACCATTGCAATTGAAAATGTAAACATTGCCATTAGAGCTGCCCACCCCAAACTTAAAA
TATCCATAATTTATAATATTCCTAAAAATAAGATCTCTTTTACTTAAACTATAATATTCT
TAATATTAAAAATATCATTATATAATAAGCTAATTATAACAGAGTACTACTTAATTCGTA
TTTTTAGTGTATTTAAAGAAGATTGAATTTTTTCGAGGTACTGGATAAGTATTTCATCCT
AAGAACCATATTCAACTTTTGATTTATTATAATGCTTAAATCTAAATATATATCACTCAA
GAATCATTAGTAATTATGATGTTTTTAATTATACGATTAACATATTTCAAAATAGAAGAA
TTCATAGCGACTAAGTTAATATGCTAATATAATTTATCAATAAAAAGGAAGTTAGTATTT
CAATTATGCAAACAACTATAAATAATGGGCAAACGTCTAGTAAAGAAACTTTACTAACAC
CTAGATTCTACACGACTGACTTTGAAGAGATGGCTAATATGGATATTTCCGGCAATCAAG
AAGATTTTTTGGCTATCCTCGAAGAATTTCGAGCTGACTATAATAGTGAACATTTTATTA
GAGATGAAGAGTTTAATCAATCTTGGTCTAATTTAGAACATAAAACTAAATCCTTATTTA
TTGAGTTTTTAGAAAGATCTTGTACCGCAGAATTTTCAGGTTTTTTACTATATAAGGAAT
TATCCAGAAAATTAAAAGACAGAAATCCGGTTATAGCTGAGTGCTTTTTATTAATGTCTA
GAGATGAAGCTAGGCATGCCGGTTTTTTAAATAAAGCTATCGGGGACTTCAATTTATCTT
TAGATTTAGGATTTTTAACGAAGAGTCGCAAGTATACTTTTTTCTCACCTAAATTTATTT

TTTATGCAACCTATCTTTCTGAAAAAATTGGATACTGGAGATACATAACTATTTACCGCC
ATCTTGAACAACATCCAGAACACCGTATTTATCCAATCTTTAGATTTTTTGAAAATTGGT
GTCAGGATGAAAATCGTCACGGAGATTTTTTTGCTGCTTTGCTCAAATCCCAACCTCATT
TTTTAAATGACTGGAAAGCAAAAATGTGGTGCAGATTTTTTCTATTAAGTGTATTTGCAA
CAATGTACTTAAACGACTTTCAAAGAATTGATTTTTATAATGCCATAGGCCTAGACTCTA
GACAGTATGATATGCAAGTAATACGAAAAACTAACGAAAGTGCCGCTAGAGTTTTCCCGG
TTGCTTTAGACGTGGACAATCCAAAATTTTTCAAATATTTAGATACTTGTGCATGTGATA
ATAGAGCTCTGATCGATATTGATAATAACAATTCTCCATTATTCATTAAATCTATCGTAA
AGATACCTCTATATTTTTCCTTATTTGCAAATTTACTAAAGATATATTTGATTAAGCCAA
TAGACTCAAAAACAGTATGGAATACAGTTCGATAGTTAAATACAAGGGAATGCTTTTTTC
TTATAAAAAGCTATCTAGTGATTAATATAAAATAAAAGCTTCCTTCTCGATGGAAGGAGC
TTTTATTTTGTCAAGAATATAATGGGATTATATAGATCTAGAACTAAGAACTTACTTTTT
CTTTTGCTTCATACATAACCTCTAAAGTAATTAGATTTTTAGAACAATCACGGGCAAATT
TCTCTGTATTTCTTTTTACTTTACCTCTAACAAACCCAGGAATTTTATTTAATTCTTTCT
GTGCTTCTTCTGACCAATTAATACTATCAACAGCAGAAATTCCTATACTTAATGCTTCTG
TTGTATCATGACCACCAAATATTTCTAGCAAATGATCTTCCATACCAAGTGTAAACGAAT
TATATACTAAATCAGCAATTTGATTTGTTCCCTCATAACCAAGAAATGGACGGTAACTTA
AAGGAAAGTTTTGTATATGCACTGGTGAAGAAATAACGCCACAAGGAATATTTAGACGCT
TACCAATATGTCTTTCCATCTGTGTTCCAAAAATTGCTGCAGGTTCAGTTTTAGCAATTA
AATCTCCAATTAAACCATGATCATCTGAAACGATTACTTCATCGCAGAATTCTTGAACTT
GATCTTTAAACCACTCTTCATCATATTTGCAATAAGTTCCACACCAGGCGACATGTATAC
CCATTTCCTGGTGTAAAATTCGAGTTATTGCTGCAGCATGAGTTGCATCTCCAAATACAA
TAGCCTTTTTACCTGTTAAATTCTGACAATCAATTGATCTTGAAAACCATGCAGATTGTG
AAATAAATCGGGTTTGCTCGTCTATGTATTTTTCGTAGTCTACAGCAGCTCCCAATGCAT
TTACTAATTGCTGTATTGATCGGATGCATGCAGCTGTTTGAACAATACCCATAGGTGTAA
TGTCAACATAAGGCATATTAAATTCTTTCTCTAAATATCGAGCTGTCATTAGTCCAGTTT
CTCTATATGGAATAAAATTAAACCATGCAGAGGGTAATTTTTTTAAATCTTGAACTGATG
CATTTTCTGGAATAATTTGGTTTATTTGGATATCTAGATCTTGAAATAACCTTTTTAATT
CTGCTATATCATGTTGATTATGAAATCCTAAGCTTACTGCTCCAATAATATTAACAGATG
GAGTTTTAGTCTTTTGAGTTAAAACTTGATTATTAGATTTAGCTTTTTCCATATAGAAAG
TGACAATTTGTTCTAACGTTCTATCACCTGCTTGCAGTTCATTTACTCTATAATGATTAA
CATCAGCTAATAAAACATCTGCTTCTGTTTCTATAGATGCTCTGCTAACAAAGTTTTGCA
AATCTTCTTGCAAAATACTCGAAGTACAAGTTGGTGTTAGAATAACTAAATCAGGGCTTT
CTTCTCTGTCTTTTCTAGTAATATTTTCTACAACTTTTTCTTGAGAACCACGGGCTAACA
CATGTCTATCTACAACACTTGCTGTAACAGGAGTAAAATCTCTGTCTCTTTCAAGCATTG
AACGCATTACATTAAAATAATCGTCTCCAAGAGGCGCATGCATAATTGCATGAACTTTTT

TAAAAGAACTCGCAATTCTTAAAGTTCCAATATGAGCAGGGCCTGCATACATCCAATAAG
CTAATTTCATATTAAATTTTCCTATGATTTATTTAGACATAGTAATACTTTTAAAGCTAA
CTAACAAACATTAGCCAAACATCTAATTTAACTCCTTGACAATTTTGTAATATTTTTTAA
ATGAAGCTTATCATTATAAATATTTAAAAAATATAATTCAGAATAGAAATAACTGTTAGA
TCAAGCTTGAAAGTAAAGATTTCTTGCAGTTATAGTTTTTCTTTAAGTAAACATGTAAAA
ATTGTACTATATATAAATGTACTATATATAAACTTTTTTTACTGAATATTGGTAATGCCA
GATACAATTAACTTAAATATGCCTTCCCCAACGTTTGGTGGAAGCACTGGCGGTTGGTTA
AGAGCCGCAGAAGTAGAAGAAAAGTATGCGATAACATGGACAGGCAAAAATGAAAGTAAG
TTTGAAATGCCAACTGGTGGTACCGCAACTATGCGAAATGGAGAAAACTTACTCTATTTA
GCTAAGAAAGAACAGTGTTTAGCTTTAGGTACTCAACTAAAAAGTAAATTTAAAATATCT
GACTATAAGATTTACAGAGTTTTTCCTAACGGAGAAGTGCAATATTTGCATCCTAAAGAT
GGAGTCTTTCCAGAAAAAGTTAATACTGGGAGAGCTAGCGTCAACAGTGTTGATCATTCT
ATTGGACAAAATATTAATCCTGTAGATGTCAAATTCATGAATAAAGCAACTTACGATTAG
TCTCTCAAAAACCTAGACATAAAATAACTTTTATATAGTCTAGGTTTTTGATAATGTATA
CTGATAGTATGGAAGGGTGGCCGAGTGGTTGAAGGCGTCTGATTTGAAATCAGTTGAACT
ATCTGGTTCCGTGGGTTCGAATCCCACCCTTTCCGTTATAAAACTATAGTTGGCTTCTCA
AGGCAAGCTAGCTATACACTGATAGTATTAAGTTTAGACTTAATAAAGTCTACAACTTGT
GACAGGTTAGAAATTTTTTCGGCATCCTCATCTGGTATCTCTATACTAAATTTTTCTTCT
ATGGCCATTACCAGCTCAACTGTATCTAGAGAATCGGCTCCCAGGTCACTTGAAAAATTT
GCTTCCCTAGTCACTATTTTTTTTTCAATTCCTAGCTGTTCTGCTACAATATCTTGAACT
TTTTCAAAGATTTCATTATCTTGCATAATATATTATTTCCTCTAAGGTGTTATCAAAAAT
TTTTCGGCTTATATTATTATCCAACTCTAAAAAATTAGTAATTTTTAGTCGAGATTAAAA
TATTTATACCTATATGATATACTATTATTCTGGATAAATACGAAGTTTACGATATGGCTC
TTCTCCAAAACTACGAGCTCTCAACTGGTAGTTGTCAACTAGATTATGCTGCATTTTTCG
TATATAGGCTGATCGAGGTGTAAGCTGAACTATTGAACTTTCATTCAAGATAATAGTTTC
AATAGCCAATTTAGCTTCTTGAAGAGCCTGGATCTCATCAAAATTTTTATTTTTACAGAG
TTCTACCCAATTAAGACCAGATGAAGTATGAATGTTTAATATTTTTCTGAGTGCACGAGT
AATTTGTGGAACCGTACTATTCTGAATTGTATATATAATAATTTGTTTTGATTTTGCAAT
TTGTCTAAGTTTTGTATTTTGCTTTACTTGATTACGTAGCGCTAGAATAGCATCAGACTT
TTCTATCTCCTTGGTTAAAATAATTGGTAGATCTAGTGAAGAAATAACTGATGTAATATG
CTGCCAACTCAAAGAATAAGCATATAAGTACTGATGAGGTATTTCCAATTGCAAAGATTG
TTTATTAATATTAATAGGGGCATTGAAAGTAGTTGACAATAAAGATGTATCACGATTTTG
TGATTTATTCAAATCTAAGGACTTATTTCTTAATTCTCTATATTGTAAAACCGGAGGCGC
CTGCTGTGCGTTAATTGATTTACGAGCATTAGAGATGTTTACAGGTAAGACTTCTATAGA
TTGCGATGGATAACATTTAATTAAAATTCTACCATTAGCTTGAATTTGTCTTTTTTGCAC
AAAAGGTTGATGGCCTTGCAAAATTTGATCTATTGTCTCTTTAACTTTATTATGAACTAT

CCAAACATTTCGCTCGTGTATTTCGATCGCAATCTGAAAAGCAGGCTCAGCTTTTCTTTC
TAGTATACTCTTTTGCGTACCTCTACGTTTAGCTTCATCGTCACCTAATGTTACATATTG
AATTCCACCAATTAGGTCCGCCAATGTTGGATTTTTAATTAAACTTTCTAAATGATTTCC
ATGCGCTGTACCTACTAATTGAACTCCTCTTTCTGCTATAGTTCGAGCAGCTAACGCTTC
TAATTCCGTGCCAATTTCATCTATAATAATAACCTCGGGCATGTGATTTTCAACAGCTTC
TATCATTACTTGATGTTGCAAATCGGGCCTTGCGACTTGCATTCTTCTAGCTCTACCAAT
AGCTGGATGAGGAATATCTCCGTCACCAGCTATTTCATTTGAAGTATCTATAATAACTAC
TCTTTTTTCCATTTCGTCAGCTAAAACACGGGCCATTTCTCGAACTGCGGTTGTTTTACC
AACTCCTGGTTTTCCTAAGAGTAAAATTGAATCACCTTGCTGTAGTAAATCTCGAATAAT
ACTAATTGTACCAAAGACTGCTCTGCCGACACGACAGGTTAAGCCAATAACACTACCTTC
TCTATTACGCAGTGAACTAATACGATGCAAGGTTTTTTCGATACCAGCTCGATTATCGCC
GCTAAAATTACCAACTTTTTTTACACAATAATCTAAGTCTTGCCAACTAATAGATCTTTG
AGATAAATATTCTGGGTTATCTGGAAATCTAGCTTCTGGACGACGACCTAAATCCATAAC
GACTTCTATTAAATTATTTCTATTAGGATGCTGTTGTAAAGGTTCTTTGACAAAATTTGG
CAAAATTTCTAGCAACTTATCTAAGTCATCTGCAATAAGCATGATTGAGTATATAATAAT
TAATTAATCAAATAGAAGATTCAAGATTCTAAAGATTACAATACATATTAAAGAATATTA
TGATTGGAATCAATTTTATACTTAAATTGAACAGGAAGTTATAGATGTAAAAGAACTGAT
TAAACTTTACGCAATAAATCGATTGGTCAAAATTAATAGTCGTGTTAACATGTTTATTGG
ACAAAAAGTTCGTATTAAATATAGCAAACAAAAAGTTGCTAGTGATATAGCAGATAAAGT
TGGTGAATTAGGAGTTATTAAGGGAATAAAGTTTATTAATAGTCAATGTGTTACTATTAT
TGTTGAGTTTGACAATCACACTAGGCTTTGGATGTTTAGAGAGGAATTAATCTGTCTAAA
TGAATTAAAAACATGAAACATAATTTATTTTATTTATTATACAAAATTTCTTATGAGTCG
TTAAAATAATAAATTTTAAAGGAGTACTTTTAATTGTGCAAATTACTATTAAAAAAATTAC
AAGACTTACTTTCGTCTGTACAAAGAAAAAAGATCCAGACATTAAAACTAAAACAAGGTA
AGTTTGAACTTCTATTAAATAAGACCTATAAAAAAGTCAATCAAGAAATTATACCTTCGC
AGAAATCTGCTGTATTACAAAATAGTCCATCTACGATAATTAAATCAATAAATAATACAA
AAAAATCTTCTGTTGTTAATGAAGACCGTACAGAATATGCCACTATTGTTTCTCCAATGG
TTGGAACGTTTTATCATTCACCTGCTCCTGGTGAAAAAATTTTTGTACAAGTTGGCGATG
AGGTCAAATTCAATCAAACAGTCTGTATTATTGAAGCAATGAAGTTAATGAACGAAATCG
AGGCAGAAATTGAAGGCAAGATTATAGAAATTCTTGTTAAAGATGGTGATATAGTAGATT
GCGGGCAAGCCTTAATGAAGGTTGAAACATAATGCTAATTTTTCCATTTTACATATTGTT
AACAGAAATTATTTAGATAGAAAAATTAGCTTATACTATAGTTATGAAAACTTATTTCGC
AAAGGCACTAAAAACTAATATATAAAATTATTTACTTTATCTGCCAAAAATAGAAATAAG
CGTTTTGATTCCTTGTCTCAAGAGAGGAGAATCTCAATGGCAATTAGCTCAAAAGAGCAA
GAGACAAAGAAGGTAAAAATCTCGGTTGATAAAAATCCCGTAGATACTTCTTTCGAAAAG
TGGGCCCAACCAGGCCATTTTTCTCGTACACTAGCAAAAGGACCAAAAACTACTACTTGG

ATTTGGAATCTTCATGCTGATGCTCACGACTTCGATAGTCAAACCAGTTCTTTAGAAGAA
GTTTCACGTAAGATTTTCAGTGCACATTTTGGGCAGCTGTCTGTAATATTTTTATGGCTT
AGTGGAATGTATTTTCACGGAGCCCGCTTCTCTAACTATGTTGCTTGGTTAAGTAATCCA
ACAGGTATTAAGCCAAGTGCGCAGGTTGTTTGGCCTATAGTTGGGCAAGAAATTTTAAAT
GGCGATGTAGGTGGTGGCTTTCAAGGAGTACAAGTTACATCTGGATGGTTCCAACTGTGG
AGAGCATCAGGAATTACTACAGAATTTCAGCTTTACTGTACTGCTATTGGCGGATTAGCT
ATGGCTGCTTTAATGCTGTTTGCAGGATGGTTTCATTATCATAAAGCTGCTCCAAAGTTA
GAATGGTTTCAAAATGTTGAATCAATGATGAATCACCATTTAGCTGGGCTTTTAGGCTTA
GGCTGTTTAGGCTGGGCAGGCCATCAAATCCATTTGTCTTTGCCTATTAATAAGCTACTA
GATTCTGGCGTGTCTCCGCAAGAAATTCCACTACCTCATGAGTTTTTAATTAATAGAGAG
CTTATGGCTCAGCTGTATCCAAGTTTTAGTAAAGGATTAGTTCCATTCTTTACTTTAAAT
TGGGCTGAATATTCCGACTTTTTAACTTTTAAAGGAGGTTTAAACCCTGTTACTGGAGGT
TTATGGCTAAGTGATACTGCTCATCATCATCTAGCATTAGCTGTTCTATTTCTTGCTGCA
GGTCATATGTATAGAACCAATTGGGGTATTGGACATAGCATGAAAGAAATTCTAGAAGCT
CACAAAGGACCTTTTACCGGCAACGGTCACGAAGGTCTATATGAAATTCTTACAACTTCT
TGGCATGCACAGCTTGCAATTAATTTAGCTATGATGGGATCTTTAAGCATCATTGTAGCA
CATCACATGTATGCAATGCCTCCTTATCCATATATTGCTACTGATTACCCGACTCAGTTA
TCGCTCTTCACTCATCATATGTGGATTGGAGGATTTTGTATTGTTGGAGCAGGAGCGCAT
GCTTCTATATTTATGGTAAGGGATTATAATCCTGCAGAAAATTATAACAATCTTTTAGAT
AGAGTCATTAGGCATCGAGATGCTATTGTTTCTCATCTAAATTGGGTATGTATATTTCTT
GGATTCCATTCATTTGGTTTATACATTCACAATGATACTATGCGGGCACTTGGAAGATCT
CAAGATATGTTCTCTGATACAGCTATACAGTTACAACCTATTTTTGCTCAATGGGTACAA
AGTATACACACTTTAGCTCCTGGAAATACAGCTCCAAATGCATTAGCAACAGCTAGTTAT
GCATTTGGAGGAGATATTGTTTCTGTTGGTAACAAAGTTGCAATGATGCCTATTTCTTTA
GGTACTGCAGATTTTTTAGTTCACCATATACATGCATTTACTATTCATGTAACTGTTTTA
ATTTTAGTTAAAGGGTTCCTTTTCTCAAGAAACTCTAGACTAATTCCTGACAAGGCCAAT
CTTGGCTTCAGGTTTCCATGTGATGGACCTGGTAGAGGTGGTACTTGCCAAGTGTCTGGC
TGGGATCATGTTTTTCTTGGCTTATTCTGGATGTACAATTCTCTGTCTGTAGCAATTTTT
CACTTTAGTTGGAAAATGCAATCAGATGTTTGGGGTAGTGTATCTCCGTCTGGAAATGTT
TCTCATATTACTGGCGGTAATTTTGCACAGAGTGCAATTACAATCAATGGATGGCTGAGA
GATTTCCTTTGGGCTCAAGCATCTCAAGTTATTCAATCATACGGTTCTGCGCTATCTGCA
TATGGATTAATTTTCTTAGCAGCACATTTTGTATGGGCATTCAGTTTGATGTTCTTATTT
AGTGGTAGAGGTTATTGGCAAGAGCTTATAGAATCAATCGTATGGGCGCATAATAAGATA
AAAGTTGCTCCTGCAATTCAACCAAGAGCTTTAAGTATTACTCAAGGTAGAGCAGTCGGT
GTTGCACACTACTTATTAGGTGGAATTGGTACAACTTGGGCATTCTTTTTAGCGAGAATT
ATTTCAGTAGGCTAATAGTGAAAATAGGATAAAAAAAACAATTATGGCAACAAAATTTCC

TAAGTTTAGCCAAGCTTTATCACAAGATCCTACAACTAGAAGGATTTGGTATGGTATTGC
TACGGCACATGACTTTGAAAGTCATGATGGAATGACAGAAGAAAATTTATATCAAAAGAT
ATTCGCTTCGCACTTTGGACATCTAGCAATTATCTTCTTATGGACATCTGGTAATTTATT
CCATGTAGCTTGGCAAGGCAACTTTGAACAGTGGGTATTAAATCCTTTGAAAGTTAAACC
AATTGCTCATGCAATTTGGGATCCGCATTTTGGACAACCTGCTTTGAAAGCTTTTAGTAA
AGGTGGCTCAGCTTATCCAGTAAATATAGCATATTCTGGCGTATATCACTGGTGGTATAC
TATTGGTATGAGAAGCAATCAAGACCTGTATTCTGGGGCTTTATTCTTACTAGTTTTATC
AGCTTTACTCTTATTTGGAGGGTGGCTACATCTACAACCAAAATTCAAGCCTGGTTTATC
ATGGTTTAAAAATAACGAATCAAGATTAAATCATCATTTATCTGGATTATTTGGGGTTAG
TTCTTTAGCCTGGACAGGTCATTTAGTACATGTTGCTATACCTGAGGCAAGAGGACAACA
TGTAGGATGGGATAATTTTACAACCGTATTACCTCATCCAGCCGGTTTACAGCCGTTTTT
CAGTGGTAATTGGAGTGTATATGCTCAAAATCCAGATACAGCTCAACATTTATTCGGAAC
TAATGAAGGTGCGGGTACAGCAATTCTGACATTTCTAGGAGGATTTCATCCTCAAAGTCA
GTCTTTGTGGCTAACTGATATGGCTCATCACCATTTGGCTATTGCAGTAGTATTCATTGT
TGCTGGACATATGTATAGGACTAATTGGGGAATTGGGCACAATCTAAAAGATATTTTAGA
TGCTCATAGACCACCTAGTGGTAGATTAGGAGCTGGACATAAAGGGCTATTTGATACTAT
TACTAATTCTTTACACATACAGTTGGGATTAGCATTGGCTTCCCTAGGTGTAATTACTTC
GTTGGTAGCTCAACATATGTATGCTATGCCTCCATATGCTTTCATGGCTAAAGATTTCAC
AACTCAAGCATCTTTGTACACACATCATCAATATATTGCTGGGTTTCTAATGGTTGGAGC
TTTTGCTCATGGGGCAATATTCTTTGTTCGAGACTATGACCCTGAACAGAATAAAGATAA
TGTTTTAGCTCGTATGCTAGAACATAAAGAAGCTATCATTTCTCATTTAAGTTGGGTAAC
TCTATTTTTAGGGTTTCATACATTAGGCCTTTATGTTCACAATGACACAATGATTGCTTT
TGGAACTCCTGAAAAACAAATTCTAATTGAGCCGGTATTTGCTCAATGGATTCAAGCCTC
TTCAGGGAAAGCACTTTATGGGTTTGATGTGTTACTATCATCCTCTACTAATATCGCAAC
ACAAGCTGGTAGCAATATTTGGCTGCCAGGCTGGTTAGAAGCGATTAATAGCGGAAAAAA
TTCATTGTTTTTAACAATTGGTCCTGGTGACTTCTTAGTTCATCATGCAATTGCATTGGG
ATTACATACTACTACGTTAATTTTAGTTAAAGGTGCTTTAGATGCAAGAGGCTCTAAACT
TATGCCGGACAAAAAAGACTTTGGATATAGTTTTCCTTGCGATGGACCTGGCAGAGGCGG
CACCTGTGATATATCTGCATGGGATGCGTTCTATTTAGCTGTATTTTGGATGCTAAATAC
AATAGGTTGGGTAACATTTTATTGGCATTGGAAACATATTACAATATGGCAAGGCAATGC
AACTCAATTCAATGAGTCTTCAACTTATCTAATGGGATGGTTTAGAGATTACTTATGGCT
AAATTCTTCTCCATTAATTAATGGTTATAATCCATATGGCATGAATAATTTATCAGTATG
GTCATGGATGTTCCTATTTGGACATTTAGTATGGGCAACAGGATTTATGTTCTTGATCTC
CTGGCGTGGCTATTGGCAAGAGTTAATTGAAACTCTAGCATGGGCGCATGAACGTACACC
TTTAGCAAACTTGATTCGTTGGAAAGATAAACCTGTTGCATTATCAATTGTACAGGCAAG
ATTAGTAGGTTTAGCACATTTTTCTGTAGGATATGTATTAACTTACGCAGCTTTTGTATT

AGCTTCAACAGCAGGCAAATTTGGTTAGACTAAATTCGAATTAGCT-AAAAAAAGTCAGT
CATAATAGTTTTATGACTGACTTTTTTAGATTTAAAACAAAAACTTACTAAAGTTTCACC
TCAATATCTACACCAGAAGGCAAATTTAACTTCATTAATGCATCTATCGTTTGAGAAGAA
GGTTGATGAATATCAATAATTCTTCTGTGAGATCTTATTTCAAAGTGTTCTCTTGAGTCT
TTATCTACATGTGGAGAACGTAAAACACAATAAATTCTTCTTTTTGTCGGTAAAGGAATT
GGCCCTACTGCAACGGCATTAGTTCTAGATGCGGTATCTAGTATTTTGTCACACGACGTA
TTAAGTATAATAGAATTATATGCTTTCAGTTTAATTCTGATTTTTGTCTGCTGAGTAATT
GTCATGTTAAAAAGATCTTATTATTTAAGAATTTTAGAGACAACACCTGCGCCTACGGTA
CGGCCACCTTCTCTAATAGCGAAACGCATACCTTGCTCAATTGCAATTGCATTAATTAAT
TCAGCAGTCATCTTAATTCTGTCACCAGGCATAACCATTTCTGCATCAGTACCATCATCA
GCAGTAAACTGATTAATAGTACCAGTTACATCAGTTGTTCTAACATAAAACTGAGGTCTA
TATCCTGGAAAAAATGGAGTATGTCTTCCGCCCTCCTCTTTAGTTAAAATATAAACTTCT
GCTTCAAATTGAGTATGAGGTGTAATTGTACCAGGTTTAGCTAATACCATACCTCTTTCA
ATATCTTTTTTCTGCACACCTCTTAAAAGAATTCCAATATTATCACCCGCTAGACCTTCT
TCTAACGTTTTTTGAAACATTTCTAATCCAGTAATAGTCGTTGTACGAGTTTCTCGTAAA
CCTACAATTTCAATTGTGTCACCAACTTTAATAATGCCTCTTTCAATTCTACCAGTGGCA
ACAGTTCCACGTCCTGTAATAGAAAAAACATCTTCTACAGCCATTAAGAAAGTTTTATCG
ACATCTCTCTCCGGCGTTGGAATATATGTGTCAACTGCTTCCATAAGTGAAAAAATCTTG
TCAACCCATTTATCTTCACCTTGCTTAGTAGCTGGGTTTTTCGTCACAGCTTCTAATGCC
AATAAGGCAGAACCTGCAACAAAAGGAATATCATCTCCAGGAAAGTCGTATTGACTTAAT
AATTCCCTTCCTTCTAATTCTACTAATTCTAGTAGCTCTTCGTCATCTACTTGATCTTCC
TTATTTAGGAATACTACTAATGTAGGAACACCTACTTGTTTTGCTAATAAAATATGTTCA
CGAGTTTGTGGCATTGGACCGTCTGCCGCAGATACAACTAAAATAGCTCCATCCATCTGA
GCAGCACCCGTAATCATATTTTTTACGTAGTCGGCATGGCCTGGACAATCTACGTGAGCA
TAATGACGATTATCTGTTTCGTATTCAACATGAGCAGTATTAATAGTAATACCTCTAGCT
TTTTCTTCTGGAGCGGCATCAATTTCATCAAATTTTTTCGCTGCAGTAGACCCTAAAGTT
GATAAAGTCGCGGAGATCGCTGCTGTTAAAGTTGTCTTACCATGATCAACATGACCAATT
GTGCCAATATTGACATGAGGTTTTTTACGTTCAAATTTAGATCGAGCCATGCTTTTTGTT
TTCCTTATAGTAAAAGTTGCTTTATAGTTACTTTTAACGATAAATCTTGTGAATTGAAAG
ATTTATTATTTAATACAGACAAGATACAATATAACTAATCTAATATCGATAGTGAGCAAA
CGCTTTATTTGCTTCTGCCATTCTATGTGTATCCTCTCTCTTTCTAATAGAATTTCCTGT
TTCATTAGCTGCATCCATAATTTCATTAGCTAATTTCATAGACATACTTTTACCAGATCT
GTCTCTAGAGAATTTAGTAATCCATCTTAGTGCTAAATTCGTACCTCTATAAGCTCGTAC
TTCAATAGGAACTTGGTAAGTAGAACCACCAACTCTTCTTGCTTTTACTTCTACTAGAGG
AGTAATATTTCGGATGGCTTTTTCTAAAATATTTAAAGGATCTGATTCTGTTCTCTCTTT
AACGATATCTAAAGCCTGATATATAATTCTTTGAGATAAAGTTTTTTTACCACTTTTCAA

AATACGAACAGTTAACATACTTACGAGTCTGCTTTTATATAAAGGATCAGGTGATGCAAA
CCTTTTTTTAGCTGTATTACGACGAGACATAGTATTTAAAATTGGTTATTGTAATAATGT
TCTATTAGCAGGTATTTAAGATTTAGGCTTTTTTGTCCCATATTTAGATCGGCTTTTACG
ACGATCTTTTACTCCTGCAGCATCTAAAGTACCACGTACTACATGATATCGGACTCCAGG
CAAATCTTTAATTCGGCCGCCCCTAATCAGTACCACAGAATGCTCTTGAATATTATGACC
TACGCCAGGAATATAAGCTGTAACTTCAAATCCAGATGTTAATCTAACGCGCGCTACTTT
TCTTAAAGCAGAGTTCGGTTTTTTAGGAGTTGTAGTATATACTCTTGTACAAACACCTCT
TCTTTGAGGGCAACTTTGAAGAGCTGGAGATTTTGTTTTTTTATGTATTTTTCGTCTTTC
GGATCTAACAAGTTGTTGAATTGTTGGCATAATAAAATTGATTTAAAGTCTTTAATAGAT
ACTATTTGAACCTGCTTTACATGAATTGGCTAGTTTTTATTTATCTTATACTTACGCATA
AATCTTTCTACTCTACCTTCTGTATCAATAATTCTCTGTGAACCAGTAAAGAATGGATGG
TTTCCTGACCAGATATCTACGTGTAGTTCTGGCTTTGTGGACCCAATCGTCATAATTAAC
TGGCCGTCACAGTAAACTTTTGCTTCTGGATACCAATTTGGATGTATATTATCTTTTGCC
ATTGTTTTTATAATTATATAAATGTATCAATAATAACTATTAACGCTTAGAAAATTGAGG
AGCCTTTCTTGCTTTCTTTAAACCATATTTTTTTCTCTCTTTTACTCTTGGATCTCTTGT
TAAATAGCCTTCAGACTTGAGTGTCGTTCTATTTTCTGGATTAATTGAGCACAATGCTCT
TGCAACACCTAAACGAATTGCATCAGCTTGCCCTGTCAAACCTCCTCCTCTAGCATTTAC
ATGGATATCATACTGGTTTAGTAGTCCTAAAACTTGTAATGGTGCATATGAAACTCTTAA
GTAATTAGGACTAAATTGAAGATAAGACTCTCCTGGTATACCGTTTATAATTAAATTACC
TGACCCTGGGACTAGTCTTACTTGTGCAACAGAACACTTCCGGCGACCTGTTCCAGAATA
GATTGCGCGAGTTTTAATTAATTCTGTCGACATAACATTCCTTGATAACTAAATAAATAT
ACATTCTTTATACTATATACTCTTGTGGCTTTTGCGCGACATGTGGGTGAATTGGGCCAG
AATACACTTTAAGCTTCGTAAATAGTTTTCTGCCTAATGGACCTTTAGGAAGCATACCTT
TAACCGATTTTTCAATAATTCTGTTAGGTAATCTTGTCTGAAGCTGATCAAATGTTTCAA
CTTTTAATCCGCCAGGCTGTCCAGAATGTCTTCTGTATAGTTTTTGATTCGTTTTATTTC
CACTTACAGATACGTGAGCAGAATTAATAACGATTACATAATCTCCAGTATCTAGATAGG
GTGTATAAGAAGGCTTGTTTTTACCTCTTAAGATATTAGAAATATGAGTAGATATTCTAC
CAAGTGTCTGATTTTTAGCGTCTATAACATACCAATGAGAATTAGTATTTAATGAGGGTG
ATTGCGTTTTATTCATGAAAAGATTAATACTTTTGGTTGACAATAAGAGAATATGTAAAT
GGTTTACATTCAATTTCTATACAAGTAGTATTTAAAAATATTATTAACAAAGAGAACTTT
TCTAACAGTTATTTAAATTGTTTGTCAGCAAGAAAATTTCTAGTTATCAATAACTTATAC
TATTTTTAATAATATAAAATATATTAATCACTTTTTTCTTTTGGCAAACTTATACCTAGT
TTTTTTTGCAAGGCATATATAACTTCTTCAGCTGATTTCTGGCCAAAATTTTTAATTTCT
AGCAGTTCTTCTTGAGAGTAATCTAGTAAATCTGCAATAGAATGGATTTGAGCTCGCTTT
AAGCAGTTATAAGCCCTAACAGATAACTGCAGTTCTTCTATTAAAACTTGACTAATTTTT
TTATCTTCTTTGCTACGATAATTATCTGCTGATTTAAAGTCTAAATTTCTTAGTGAACAA

AAAAGATTAGTTAAAACTGTAGCTCCTTGACTTATTGCTTCTTGGGGAGATATGCTCCCA
TTTGTCCAGATTTGTATAATCAATCTATCTTTTATGCTGTTGCTACCAATACGGACTTCT
TCTACTTTATAATTAACTTTATTAACTGGCATAAAAACAGAGTCTACTTGCAAAAAATCC
ACAGATAATTCATCTACAGCTTTTTCAGCTAAGCGATACCCGCAATTTTTTTCAATTTTA
AACTCCATTTCAAATATTGTGTTATTGCAAATAGTTGCAATATACTGTCTAGGATCTACT
ACCTCTATATCAGAAGATAATTCAAATAGGCCGGCTGTAACTATAGCTGGCCCTTGAACT
CTAATTCGACCAATTTGAGATTCTTTGTTATAACTTTTAAATACTACTTCTTTTAGATTA
AGTAATATTTCTAACACATCTTCTCTTACCCCAGGAATTGTGGAAAACTCATGGTTCACT
CCAGCAATCCGTACAGCAACTATAGCAGTACCTTCAAGATCTGACAATATTGATCTTCTC
AAAGCATTACCTAATGTAATACCTTGTCCTTGATTTAATGGTTCTATTACAAAACTACCG
TACTGCCCACGCGCCCCATCTGTTCTTGACTCTATGCATTCAATTTGAAATTGAGCCACC
TAAGAAAGCTCCTTTAAAATAATCAGTGATTAGTAAGTCTCTCTAAAACAAGAAGGAATT
TATACTCGGCGTTTCTTAGGAGGGCGACATCCATTATGAGGTACAGGAGTAATATCTTTT
ATTAGAGTAATCTCTAATCCTGCAGCCTGCAAAGCTCTAATTGCTGTTTCTCGACCCGCT
CCTGGACCATTTACTAGAACTTCAGTTTGGCGCATACCTTGATCCATAGCTTGTCTAGCT
GCTTTTTCAGCTGCTGTTTGAGCTGCAAAAGGTGTTCCTTTTTTAGCTCCCTTAAATCCA
CTTGCACCAGAGGATGACCATGATAATGTTTCTCCTTTTAAATTAGTAATAGTAACAATT
GTATTATTGAATGTAGATTTAATATGTGTAATACCGTTAACTGCATTACGTTTAGTTTTT
CTTGCTCCGGATTTTTTTATTTGTCTAGCCATCGTGTCTCGTCTATTATATAGTTAATTA
AAAGATTATTTTCTTGGAGCTTTTTTCTTACCTGCTACTGTTTTTTTACCTCCTCTGCGT
GTTCTAGCATTAGTTCTAGTTCTTTGTCCTCTCAAAGGAAGACCAAGACGGTGTCTTCTA
CCTCTATAAGTACTAATTTCCATAAGTCTCTTAATGCTCATAGACTCAAAACGTTTGAGA
TCCCCTTCAATCTGATAATTAGACTCAAGAATTTCTCTTATACTGACAACTTGTTGATCA
TTTAAATCTTGACACTTAATATCAGCGTCTATGTTTGTTTTTTCTAATATTTCTTTCGAG
CGAGATAGTCCAATACCATAAATATATGTTAAAGCTATCTCTATTCTTTTGTTTCTTGGA
AGATCTACTCCAGCAATTCTGGCCACTTTGTCTTGTCTCCAATAAATAATATGTATTTTT
ATAACGGTTATTTATAAGCTTAGCCTTGTCTCTGCTTATGTTTAGGATTAGTGCAAATTA
CCATCACCTTTCTGTGACGCCGAATTATTCTACATTTTTCACACATTTTTCGAACAGAAG
GACGAACTTTCATATTAACTCTATATACTAGGAATATTACTATATTAACTCTATTAATTA
TCGACATTAAAAATGTCAACAATATATTGTATTACTTTAACTGTTGTAGATTTTACTTCG
TCATACTATCATATTTTTTTGATATGACGTATGTCTGAATTTGCTTAGCTGTGTCTATTG
CGACTCCAACTAAAATAAGTAAAGACGTAGCCCCGAGACCTCTTAAGTTTTGGATCTGAG
TAACCTTTTCTATTATAAACGGAATCAGCGCTACTGTAAATAAAAACGAGGCTCCTAGGA
ATGTCAGTCTATTTAATATGACTTGTAAATAATCAATAGTCGCTTGACCTGGACGAATAT
TAGGAATGCTTGCACCCATTTTTTTTAAATTTATAGCAATATCTTCTGGATTCATTACTA
TTGATGTATAAAAATAGCTAAAGAAAAGGATCAAAGCACAATAAAGAAGAAGGTATAGTG

AACCATTAGGACAAAACAGGTATAGGATTTGGAGTAATGTTTTATTTTGAATAATTTGGG
TTAGATAAGATGGGAGGGCCATAGATGCAGATGCAAAAACAATAGGCATAACTCCACCTT
GATTTAATTTCAAGGGCAAGTAACTATTTGGATCCAAAATTGAAGATTTTCCTAGCTGTC
TTGCTGAAATAATTTTAATTCTTCTTGTTCCTTCTTGTACACAAATTGTAATTATTATCA
TTAATAAAAAGATCGCTATAAATAATCCGAACTTAAGACTTGCATTACTATAACTAGCAT
CAAAAAATGACTGTGTAAAATTCTTTGGTAGTCCTGACACAATGTTTTGAAAAATAAGTA
AAGAGGCTCCATTACCAATTCCCTTTTCCGTAATTAACTCTGATAACCACATAATAATCA
TAGAGCCTGCTGTTAAAGCTAAAACAGACTCGCAGACAAATGCAAAATTCCAATTAAAAA
CATATGGTTTTACCCATATAGAGATTGCTCCAGATTGTAAAGTCGCCCAACCTAGAGCTA
AGTACCTTGTAATTTGAGTTATTTTTTGGCGGCCCAATTCACCTTCTTCCTTCTGTAATT
TTTCCAGATTGGGGACAATTTTCGTAAGTAGCTGCATTACAATTGAAGAGTTAATATAAG
GAACAATACCTAACGCAAAAATCCCTATTGTTGAAAAACCTCCTCCAGAAAAAATATTCA
GAAAATTTACTAAAGTATTTTTTTCTACACTTGCATAAAAGGCGTCATGATCTATACCTG
GAACAGGTATAAATATTCCTAAACGTGCTAAAACTAATAGAAAGAGAGTAAAGATAATAC
GATTTCTTAGATCACTTTTTTTGGCTCATAAATAAATTTTAAATGAAAAGAGATACTTTTT
TATGTAAAAACCCTATTCCAAGACTTGTATAAGTTTAAAATAGGGAAGTAAAATATTATA
GTAATATTAATATAATATTCTATGTAGAATACAGGCTTTCTATTGGAACTCCTCGATCTT
TAGCTGCTTCACTAAAAGTTCTTAATTGAGTTAAGCCATTGAGAACAGCTCGCGCGTTAT
TTAATGTATTATTAGAACCTAATTGCTTAGCTAATATATTCTGTACACCCGACAATTCTA
GGACTGTCCTTACAGAACCTCCGGCAATTACACCTGAGCCAGGTGCAGAGGGCCTTAATA
TAACTTTCGCGGCACCAGAAATTCCGTTAATAGGATGAGGTATAGAATTCGATTTTGTCA
GTGGAACTGTAACTAGATGTTTTTTAGCATCTGTTACTCCTTTTTTTACTGCACCAATTA
CATCGCTTGCTTTTCCCACGCCGACACCAACTTGGCCTTGCTCATTACCAATAACAAGAA
TAACTCGAAAGCTTAGTTTTTTACCTCCTTTTACAACTTTAGTAACTCTTTTAACTTGTA
CGACTCTTTCTTCCCAGCCACTATCTTTATCTTTTCCTTTGCTCTGTTTTTTACGATTGG
CCATTTTAAGAATTATTCCTAATTAGTTAATTACGATTCTAGAAACCCATGCCTGCTTCC
TTAGCAGCTTCAGCCAAGGCCTTAACTCTTCCATGATATAACTTTCCTCCTCTATCGAAG
ACGACATTTTTAATGCCTTCTTTCATAGACTGTTCTGCTAACTGTTTACCAACTACGCGA
GAAGTATCACAGTTTGGTCTTATGTTATCAGATTCTTTATTATTTAAATTAACAGATGAT
GTAGCTACTAATGTAATACCTTGTGTGTCATCAATTATTTGTGCGTATATATGTTTATTA
GATCTAAATACACACAGACGAGGCCTACTTGAGGTTCCTTGAACTTTTTTCCGAACTCTT
TTATGCTTATGAATTCTAGTTTGTTTAGTGTTTAGTTTCATTATTATTTACCTTTTCCAG
CTTTCCCAGCTTTTCTTCTAACAAATTCACCTTGATATCTAATTCCTTTTCCTTTATAAG
GCTCAGGAGGCCTAATAGAACGAATAGTTGAAGCAACCTGACCGACAACTTCTTTATCTA
TGCCAGAGACAGTAATATTTGTGTTGTTTTCAACTTTAATTTCAATATTGGCAGGAGGTT
TAATTTTCACTACATGACTATAGCCAACACTTAAGATTAAATCTTGGTTATCAATTTGAG

AACGATAGCCTACACCTTGTATTTGCAGTTTTTTAAAAAATCCATTAGAAACACCTTCAA
TCATATTACTAATAAGAGTTCTGGATAGCCCATGTAACTGACTAGCCATTTTTGTTTGTC
CACTCGTCTTGACAGCAATTGTGTCGTTAAGTATTTCTAAATTAATACCAGCTGGTAAAG
TTCTGGATAGGGTACCTTTAGGTCCTGTCACGGTGATAGTCTGACCATCGAACTGAGTAC
TAAGATTTGTCGGCAATAAAATTATTTTTTTTCCAATACGAGACATATTCCACCTATAAA
ATAGTTACCAAATATAGCATAATATTTCACCACCAAGACCATCATGACGAGCTTGTCTAT
CTGTCATAACGCCTCTAGAAGTAGAAATAAGAGCGATGCCTAAACCTCCAAGAACTCTGG
GCAACTCTTTATGATTTGCGTAGACTCTTAGTCCGGGTTTACTAATTCTTTTCAGAGCAG
TAATAACTGGTTGACGATTTTTACCATTATACTTAAGAGAAATCATTAAATGAGTCTCTA
TACCTTCACCCATTTGTTCGAAATTTTGAACAAATCCTTCTTCTTTTAGTACTGTTGCCA
TATTGCATGTCATTTTCGTTGCTGGAACTTGCACAATTTGATGTCTTGCTAAGTTTGCGT
TACGAATACGTGTCAGCATATCGGCGATCGTATCGTTGACCACCTTGATCCTCCTTGCGA
TGAATTATTTGAAATATTTTCAAGATTCCCTGAAAGGCATCCCTAGCTTTTTTAACAAAG
CTAGACCTTCTTGATCTGTTTTAGCTGTAGTGACAATTGATATATCTAGACCACGAATTT
GATCAATATTATCATAGTCTATTTCTGGAAAGATCAACTGTTCACGTAAACCTAAATTGT
AATTACCTTTGCCATCAAAACTTCTAGGACTAATTCCTCTAAAGTCTCTAATTCTTGGTA
ATGTTAAATTAATTAATTTCTCTAAAAAAGAATACATCTTGTCTTTTCTTAGATGCACAA
CAATTCCGATAGGAACCTCTTCTCGAATTTTAAATCCTGCAATAGATTTTTTAGCTTTTG
TGACAATTGGTTTTTGTCCAGTTATTAATGTTAATTCTTGAATACTACTTTCAAGAGCCT
TAGCATTCTGAGAAGCTTCACCTAAGCCACGGTTAATAGTAATTTTAGTAAACCTAGGAA
CTTCATGTACATTTTTGTACTGAAATTCATCTTTTAAAGATTGAGTAACAGTTGTTTTAT
ATTTTTCTTTTAATCCTATTGCCATTATATTTTCATCAATTATTTAATAAGTTCGCCAGT
CTTTTTCAGTTTTCGAATTTTTTGGCCTTTATCATTAATTATTACTGAGGATCGACTAGC
AATATTATTTTGTTCACTAAATAACATAACGTTAGAGGTATGTATTGGAGCTTCAAATTT
GATAATTTCTCCCGTTTCTCCTTCTTGCTGAGGTTTTTTATGCTTCACTTTGAGATTAAT
TCCTTTAACAATCACCTTATTTGTTTTATAAATGATTGCAATAATTTCACCTGTTTTTGT
TTTATCACTTCCAGAAATAACTTGAACTAAGTCCCCTTTTTTTAATTTAATTTTTGTATT
ATTTTTGGTTGTTTTAGAAGGACCTTTCATTATACTACCTCCGGTGCAAGAGAAACTATT
TTAGAAAAATTTTTATCTCTTAACTCTCGGGCTATAGGTCCGAAGACTCTTGTGCCACGT
GGATTATTATCTTGATTAATGATAACTGCCGCATTATCTCCAAATCTAATGCTCATACCG
TCAGTTCTCCGTAAAGCTTTACGAGTTCTTACTACAACAGCTCTGACAACATCAGATCGT
TTAACTGGCATATTGGGAGACGCATCTTTGACTACTCCAATAATAACATCTCCAATAGAT
GCATAAGAAGGATTACTAGTGCCTAATACTCTAATACACATTATTTTTCTAGCACCACTA
TTATCTGCAACATTAAGATAGCTTTGAGTCTGTATCATACTTTTATTCTGTATAAGATTA
ATTATCAAAAGACTTAGATAAGATATTAACCATTGTCCAACACTTTGTGCGACTTAAAGG
TCGTGTCTCTTGTATTGTAACAATATCGCCGATTGTGCATTCATTATTTTCATCATGCGC

TTTATATTTCTTTGTCCGGATCATAGTTTTTGCGTACTTTCTATGAGAGATTCTATTTTC

TACAGCTACTACTATAGTTTTATTCATTTTATCGCTTACTACTTTACCTGTTGTTTCTTT

TAAAGGCATAGTCTTTATATTTTTTATATATAATTAAATTTTAAGATTATTACATGGTTG

ACTTAGTACGAGACTTTTCAACAGTTAAAAGTTGGGCTAATCTATGCTTAGAATGTTTAA

ATAGATGAGGCTGGAAATCTTGCCTTGTGGCTCTTTTTAGCCTTAAATCAAAAAGCTCTC

TTTTTATTACAAGGATTTCTTCAGCTAAAGAAGAAGAGTCCAGGTTTGTAACATCTGATA

TTTTAGGGAAAGTCATATTTTAGGATTCTGTTGTATTCCGAACTATAAATTTAGTCTTGA

TTGGTAATTTATAAGAAGCTAATTTCATAGCTTGTTGAGCAGTTTTTTGTGGTACACCTG

TAATTTCAAATAGAATATGTCCAGGCTTAATAACTGCAACCCAATATTCTGGAGCTCCTT

TACCTGACCCCATGCGAGTTTCAGCTGGACGGGCAGTGACTGGTTTATCTGGAAATACTC

TAATCCATAGTTTACCACCTCTTCTCACATATCTAGTAATAGTTCTTCGAGTAGCTTCTA

TTTGTCTAGAAGTTAACCAAACTGGCTCTGTCGCTTGTAATGCATAATCACCGAACGCAA

TTGTGTTACCTTTGCTAGCAGAACCTTTCATTCTACCTCTATGTTGTTTTCTAAATTTTG

TTTTCTTGGGGCTTAGCATAAAAATTAAAAAATAATAATAATTAAGAAGCTGTTGAATCA

GGAGTTTCCTGATTATAAGCAGATTCTAGACTTTTCGATTCTGGCAAAATTTCTCCTTTA

AAAAGCCAAACTTTTACTCCGAGCACACCATACGTAGTGTGAGCTTGGCGATGACAATAA

TCAATATCGGCTCTTAAAGTTTGTAAAGGTACACGACCTTCTCTAACCCATTCACTTCTA

GCTATCTCTGCACCATTAAGCCGACCAGATACTTGAATTTTAACACCCTGTGTATTTGCT

CTTTGAGCTCTTTGAACTGCTTGTCTGACAGCTCTACGAAAGGCCACTCTTTTTTCTAGC

TGTTGAGTAATAAATTCTGCTACTAAGGTTGCTTCAGAATCTGGATCTGCAATCTCTACA

ACATTTACTCTGAGTTGTTTGCTAGGGTCTAGGATTAAGGACAGCGATTTTCTTAAAGAC

TCAATTCCCGCTCCAGATTTTCCAAGTACGATTCCTGGTCGAGCTGTTGCTATGAGAATT

TCTACTTGATCAACTTTACGATTAATTTCAATTTTAGCAATACTAGCATTACTAAGTTTT

GAATGTATAAATGAGCGAATTTTATGATCTTCTTGTAAAAGAACTGGGTAGTCTTTAGAG

TTAGCAAACCATGAAGAACGATGTTTTTGGGTAATGCCTATGCGAAAGCCCAAAGGATGA

ATTTTTTGACCCACAGTATCCTTGATTATAAGTTAAAAGCTATAATGACTAATTGACGTT

AAAAGACTACAGTTCTGAAACACCTAATGTAATATGACAAGTTGGTTTATGTATTGGAAA

GGCTCTTCCTTGTGCTCTAGGCTGAAATCTTTTTAAAGTCGGGCCTTTGTCTGCAAAGGC

TTTACTAACAAACAATTGGTTTTTATTTAATCCATCATTATGCTCAGCATTCGCTGCAGC

AGATTCCAGGATTTGCTTTATATGAGAACAGACTCGGTATGGCATAAATTCCAGAATAAT

TAATGCTTCTTGGTATTTTCTACCTCTAATTTGGTCTAATACACGACGGACTTTATGCGG

AGAAAGACGAATGTATTTCCCTACTGCTTTAGTTTCTTTTACGTTTTTTGTAATACTCAT

AGTTATAGTTTAACGACGGGCTTTTCTGTCACCTTTTACGTGAGTGCGGAAAGTTCTTGT

TGGAACAAACTCTCCTAATTTATGTCCTACCATTTGGTCTGACACAAAGACAGGAAAATG

TTGTTTACCATTATAAACAGCTATTGTATGCCCTACCATATCGGGAATAATAGTAGATGC

TCTCGACCAAGTTTTTAGGACTTCTTTTTTTCCTGAAATATTTAATGCTTCTATACGTTT

AAGAAGACTAACATCAATAAAAGGGCCTTTATGTATAGATCTTGACATAATAATAAATCA
TTAGATTCTTAATAAAATAAATTCTATTTACGGCGACGTAAGACGTACGGGTTACTATAT
TTATTTGGATTTCGTGTTTTAACACCCAATGCAGGTTTACCCCAAGGTGTTACAGGACGA
GCTCGTCCAATTGGAGATTTGCCTTCACCACCACCATGTGGATGGTCTACAGGATTCATG
ACAACACCTCTAACAGTAGGTCTTTTACCTAACCAACGATTTCTACCAGCTTTACCAAGA
GTAATATTACTAGCGTCAATATTACCAACTTGTCCAATAGTAGCGTAGCATTCTTTTCGA
ATCATTCGAACTTCGCTTGAAGGCAATTTTACAGTAACAAAAGTACCTTCTTTTGCTACT
ATTTGAGCATAAGTTCCTGCTGCACGAACAATCTGTCCTCCGCACGAAGGTCTTAATTCT
ATATTATGTACTGCTGTTCCTAAAGGAATACTAGATAAAGGCAAAGCATTTCCAACTTCG
ATAGGGGCAGTAGGACCAGAAAGAACCATAGATCCTACACTGAGAGATCGAGGGTGTAGA
ATATATCTTTTCTCACCATCAAGATAGTGTAATAATGCAATTCTTGCATTTCTATTCGGA
TCATATTCAATAGAAGCAACTTTAGCAACTATATTATGTCTATTTCTTTTAAAGTCAATT
AATCTGTACTGCTGCTTATGTCCACCACCTTTATGACGACAGGTAATGACACCTCTATTA
TTTCGACCTTTGCAGAAATGGTGTTTAACTATTAATGATTTTTCTGGCTTATCAGTAGTA
ATCTCTGAAAAGGTAGAAACCGTTCTATTTCTTGTCCCTGGTGTGTAGGCACGATATAAA
CGAATTGCCATATAAGAAGATTTGAGAAGAGTAAATGTTGAACTATGTGATATATTTAAG
TTTCTGGAAACAAATTAATAGAATCTTCTGAGGCAAGTGTTACAATTGCTTTCTTGTAAT
GTGGTCGCTTCCCTACGAATCTACCGATGCTCCTTTTTTTCTTAGGAGGATGACAAGTGT
TAACACCTGTAACTTGCACGTTGAAAATATACTGTATAGCAGCCTTAATATTAATTTTTG
TTGCTTTGGGATCAACGGCAAAACAATACTGATTTTCTTCTAATAATTTAGTTGTCTTAT
CAGTAATGATGGGATATTTAACCAGATCTAATAAACCTCTTGAATCAATGCTATCCATTA
TATACCTCTTGTATTTTAGATAAAGCATCAACTGTAATAATAATTTTATGTGCAGCTAAC
AAAGCCATAATATTTAGTGTATCAGCTGAAATAAGTTCTACGTTGTGTAGATTGCGAATA
GAAAGATAAACATTCGGATCTTTTTTGTCTACGATGACTAAAACTTTTTTATTTAAATCA
AGATTCCATCGATGTATAGCTTCCATAAATAGTTTAGTCTTGGGCTGATGAAAATAACTG
TTAAAATTTTCTACAATCAGTGTATTAACTGATTTGTTATTTAATGCTGTCCTTAATGCT
AATTGTCTTTCTTTTTTATTCATTTTTTTGGTAAAACTACGAGGTTTTGGCCCAAATATT
ACACCACCACCTCTCCATAGAGGTGAACGGATAGAACCTGCTCTTGCTCGACCAGTCCCT
TTTTGACGCCATGGTTTACGACCTCCACCTCGAACTTCACTTCTTGTTTTAGTATTAGCA
GAACCCTGGCGCTTTTCATTACTTTGCTTTACTAAAGCTCTATGGACTAAGTACATCCCA
GAATCTTGACTGACCTTAAGATTTAAATCAGCGTTGCCACTTACTTGACCTTCCCAATTA
TAGACTTGATAATTTAATTGCGTGTTAACTGTCATGAAATATATCGTATGAAAGAATATT
GCTACTTACTAATTTTTACTAAAGCGCCAGGTTTACCAGGAACAGCTCCCTTAACGATCA
ACAAATCATTCTCTGAGTTAATACTTACAATTTGCAAATTTTTTATTGTAACTTTTTTAT
TACCAAGTTGGCCAGCCATGTTTTTACCTGGATAAACTCTTCCAGGCGTTGTACCGGCTC
CAATAGAGCCAGGTTGCCTATGATTTTTCGAACCATGTGACATTGGGCCTCTACTAAAAT

GATGCCTCTTTTGGTAACCGGAGAAACCTTTACCTACACTTCTAGAAGAGACATTAATCT
TTTGGCCTACTTGAAATAGATCTGTAGATAGTATTTGGCTAACTTCGAAATCATCAGTAG
ATTTCATTTCGTATTCACGTAAATATTTTAGTGGGGGAGCCTGTGATTTTTTTAAGTGTC
CCAACAATGGTTTATTTAATTTTTGTTCAGCAACTTGCTTGTAACCGACTTGAATAGCAT
TGTAGCCATCAGTAGATACAGCTTTAATTTGAGTAATAACACATGGCCCAACTTGAATTA
CAGTAACTGGAATTGATAAACCAGCTTCATCAAAGAATTGGGTCATACCTACTTTAGTAC
CGAGTATACCAACAGACACTAGATTTCTCCTTTTTGAGAGATTTATACTATCATTGTACA
TAAAAATTCTACTTCATAGTTACTGAAGTAAATTAAACTAACAGATGCATGATATTATTC
ATGACAACATAAAGGTTAAAAACTTTACCTATTTAATCTAAAATAGAAACAGCTTTTTGT
CTAGTTACAGCGTATATAAATTAGAAGTATGCTTATTAAATTTTTTATAAACTACTTAGA
TTTAACTGATATTGATATCCGTAACTTACTTGCAGTAATATCAATCAACATGTATAAGTT
TAATTGCCTAAACTAGAACCAGCTATTATTAGAAATAATTCAAGTATAGATAAAAGCGTA
TTTGAACTTTTATATATTGACAAATATAATAGAATATATTGAGGTTCGGTAACTACACCT
TTTTATTTTTACATTTATTGTGCAGTATATATACATCTAATGCAGTAAACATAAGAATAT
AACTGTATAGCAAATGTAATTACACAAATTAGAGAGGTATTAATGGGCAAAGTCGTTGGA
ATTGATCTTGGAACAACGAATTCTGTAATTGCTGTTATGGAAGGAGGTAAACCTACCGTC
ATACCGAATGCAGAAGGTTTTAGAACTACAGCTTCTGTTGTTGCATATACTAAAAGTGGA
GATAAACTTGTAGGACAAATTGCCAGGAGGCAAGCTGTTATTAACCCAGAAAACACTTTC
TACTCTGTCAAAAGATTCATAGGACGTAAACAGAATGAAATTTCGCAAGAGATTCGGCAA
ACATCATATAATGTTAAAACTAGTGGATCAAGCATAAAAATTGCTTGCCCTGCACTGAAT
AAAGATTTTGCTCCAGAAGAAATTTCAGCTCAAGTACTGAGAAAACTTGTTGAAGATGCT
AGTACGTACTTAGGTGAGACTGTTACACAAGCAGTAATAACTGTACCGGCTTACTTTAAC
GATTCACAAAGACAAGCTACTAAAGACGCAGGTAAAATAGCAGGCTTAGATGTATTGAGA
ATTATTAATGAGCCTACCGCAGCTTCTTTGTCTTATGGACTAGACAAACAAAATAATGAA
ACAATACTAGTATTTGACCTTGGTGGGGGCACATTTGATGTATCTGTATTAGAAGTTGGA
GATGGAGTATTTGAAGTACTCTCAACTTCTGGAGATACACATTTAGGCGGAGATGACTTT
GATCAGCAGATTGTAGAATGGCTAATCAAAGATTTTAAACAGAATGAAGGTATTGATCTT
GGTAAAGATAGACAAGCACTTCAGAGATTGACCGAGGCTGCAGAAAAAGCAAAGATAGAA
CTGTCAAATTTAACTCAAACAGAGATCAATCTTCCTTTTATTACTGCCACACAAGATGGT
CCAAAACATTTAGAAAAAACTGTAACTAGAGGGAAGTTTGAAGAACTTTGTTCAAATTTA
ATAGATAAATGTAGTATCCCTGTAAATAATGCTCTGAAAGATGCAAAACTAGAAGCTTCC
AGTATTGATGAAGTTGTTTTAGTTGGTGGATCTACAAGGATTCCAGCCATACAGCAAATG
GTTAAAAGATTAATTGGTAAAGATCCAAACCAAAGTGTCAATCCAGATGAAGTTGTTGCT
ATTGGTGCAGCCGTACAAGCTGGAGTTTTAGCAGGCGAAGTCAAAGATATTCTATTACTA
GATGTGACGCCGTTATCTTTAGGAGTGGAAACTTTGGGTGGCGTGATGACAAAGATTATA
CCAAGAAATACTACTATTCCTACAAAAAAATCAGAAGTATTTTCTACAGCTGTAGATAAT

CAACCAAATGTGGAAATTCAAGTACTTCAAGGCGAAAGAGAACTAACAAAAGACAATAAA
AGCTTAGGGACGTTCCGATTAGATGGCATTATGCCTGCACCTAGAGGAGTTCCTCAAATT
GAAGTTACCTTTGATATTGACGCTAACGGAATTTTATCTGTAAAAGCCAAAGAAAAGGCT
ACTGGTAAAGAGCAATCAATTACTATATCTGGAGCATCAACTTTACCTAAAGATGATGTT
GAAAGAATGGTAAAAGAAGCTGAAGAAAATTTCGATACAGACCAAAAAAGAAGAAAGAAT
ATTGACACAAAGAATCAAGCAGAGTCCCTATGTTACCAAGCTGAAAAGCAAGTTAAAGAG
TTTGAAGACAAAATTAGCCAAGATTTAAAAATAAAAATAGAGGAGCTAATTACAGAGCTT
AGATCTAGTCTAGAGAAAGAAGAATATGACAATATTGAATCCATTTCTCAGCAATTACAA
AATGCTCTGATGGACATTGGAAAAAATGCTGCTCAGACTGAAAGTAAAGATACAAAAGCG
AAGGATGACGACACTGTGATTGACACTGATTTCTCTGAAGCTAAGTAAAAGTAAGCGGGT
AACGCGATTCGAACGCGCGACATCAACCTTGGCAAGGTTGCGCTCTACCACTGAGCTATA
CCCGCATAGATTGTATTATTACAAAAATTAAGATATTTGTCAATCTGTTAATCTATACAT
CTAGACTATTAATTTTGGAATTACATATTAGCTAACTGTTGTAAATATAACAATTGCTAT
AATTTTTTAAAAGTACTTATATCATTATATAAGTACTTTTTATATTCAAAAATACAAATA
AGTATTATAGCTAAACTACTAAGGAGTTAACTAAGCTAGTAACAATTACTAATCCTGTCC
ATAAGCCGGCTCCTGTATATATTAATCCTTTTGATTGCTCCCATTGGCCAGGGGATGCTA
AAACAACAGGTACGCCTACTACTAACACAGTCGATAATGCAATTAATAATAGCACTAATA
ATTGGATCGCAATAATCATTTAATATGTTCTCCTGAAAAATTCAAATGTTAATAATACTG
TATATAATATAATGAAGCTTTTGACCTTCAGCAATTTATCTATGAAGAAATAATTTAAAA
TTATTGTTTTCTAAATAAACCTTAAATTAAGTATTAAAATCTTTATTGTAAACAATAATA
CTATATTTTTTTAGTAGATAAAAAATTTAAAGAAAGTCTTTTTTAAAAAAAAAATTAAGAT
AAGGTGATATGCTGTATAAAAAGCTAAGTATTATTAATTATTAGAGAAATTTAAAATAAG
AGGTAAAACATATGAACTCAGCCCTTTTTTTAGCAAAACTACCAGAAGCTTATGCCATTT
TTAAACCGATCATTGATATCTTACCCGTAATTCCTGTATTTTTTCTTCTTTTAGCTTTTG
TTTGGCAAGCAGCAATTGGTTTTAGATAACATTTAGGTTATAAAAAGAATAACAAATATA
CAGAGTGCCAATTTTTGAGCTCTAATCTTATATCTTACACAATAATAACTATTATGGTTG
AACCCTTACTATCAGGAATTGTTCTTGGACTTATTCCTATTACTTTATTCGGATTATTAG
TAGCAGCTTATTTACAATATCAACGGGGCAACCAATTAGGACTGTAAAGCCTAAATTATA
GATATTTATAAAAACTACAATAATTATAGTTTTTATAAATATCTACAAATAAGCTAATTT
TAAATACAGAAATAATACTATTGAAACTAGAGTGATAACAAAAAAAATTTATGTAATAAC
AAGCTAATTGTTTACCAGCTCGACTTAGTAACTCCTGGTAAAAGACATTCATGAGACATT
TCTCGAAAAACATGGCGAGATAATCCAAAATCTCGATAATAACCTCTACTTCTACCTGTT
AACCAACATCTATTTCTCCCTCTAACAGCGGCGCTGTTTCTAGGCATTTCTTGGAGTTTT
TGTCTTAAATCCATTTTGTCAGAAAAGCTGCCAGCTTCTTTCAGTTGATCTTTAATAGCC
AGTCTTTTTAAATAATATTTTTTTGCAAGTTTCGATCTCTTAATCTCTCTTTGGATCATA
TTCTTTTTAGCCATGCTGTAAAACTCTCTTTTAAAATTATTTATTCAATATATTAGCTAA

AAAAGCTCCTGCCTGCAACTTTTATATATTTAAAAATAAATAGCATAAAGTAGTGAAATG
CAATTGATATTTAATCAACGATACCACTTTATGCTATTATCAAAAATTTATTTAGTAAAG
TTCTTGTTCAACATGAGTTGAAATAGTGCAATCAGATGAAGGATATGCAATACAGGTTAA
AACATAGCCAGCTAAAAGTTGATCATCATCTAAGAATGATTGATCAGCTTGATCTACAGT
TCCTTCTGTAACTTTACCTGCACAAGTTGAACAAGCTCCAGCTCTACAAGAGTAAGGAAG
TTCAATTCCTTCTTCTTCTGCTGCATCTAGAATATATGTATCCTCTGCACAATTAAATGT
GACATCAATTCCTTCTTCTTCACATAGTAAATGAATTTTATAATCAGCCATTTCTTCTTA
CTCCATAATAAAGATACACTTGAAACTTAATAAAGCAAAATAATTTAAAGAACTTACCAA
AAAATTATATACTAGTTTCATTATGCATGATAGACTGGAAAAAAAGAAAATAGTATACTA
TTCTGAAAAATCAAGCTACATTTCCTACTAAATAGAACTATAGTAGGTCCAAAGTATATA
CATAAATTCATATAGTTAAAATTACTTTTATCATTTAAATAGCTGAACTTGATAGTATAG
ATTGATCATCAAGCTAGTCTTCTGCCAATAAATAATTAATTATGATTTCAATGCTTAATC
AACAAGTAGAACTTAAGCCTATTCTTAAATTTCAGATAACACAAATATATTCGCATTCTC
TTATTCAGGAAATTAAAGCCTTAATAACTAGACTTGTTTTGCAAGTTTGGAGAAGGCCAG
CTACATTGATGGCAGGTATTATCCAACCTTTATTATGGTTAATTCTATTTGGTGGGCTTT
TCTACAATGCTCCTATAAATTTGTTCACTATTAATACAAGCTATAATTGTTTTTTGAGCT
CTGGGATTATAATTTTCACCTCTTTTACTGGAGCTTTGAATTCAGGTCTTCCATTAATGT
TTGATAGAGAATTTGGATTTTTAAATAGATTATTAACGGCTCCCTTAGTATCGAGGACTT
CTATCATTTTATCTTCTGCTACTTTTATGACTTGTATTAGTTTAATACAAGTTGTATTTA
TAGTTACAGCTTCTCTTTTTATGGGAAACCCACCTCTAAATAGCGATAGTACTATGATTT
TTGGACTTATGATTCTATTAGTGACTGTAGGAGTTACAATGCTTAGTTTAGCTTTATCTT
TCACTCTGCCAGGTCATATTGAGCTATTAGCATTTATTTTAGTAGTTAACTTGCCCTTTT
TATTTTCTAGTACGGCTTTAGCTCCTTTATATTTCATGCCGCCATGGCTTCAGTTAATTG
CAAGTCTCAATCCATTGAGTTATGCAATAGAAGGTACAAGATACTTATATTCAAGCGTAA
ACTGGAATTTTACAGAGTGTGTGATTAAGATTAGCTGGGGAGATATTTGTTTAGGGCAAA
TTATTATTTTATTAATCGCTTTAGATATAATGGCAGCTTATCTTGTGTCTAATATATTAA
AAGCTAAACTTAATTAAAATTTTAATAAAAAATTTATTAAATTTATTTTACAAAGATTTT
TACTATATAATACTAATAGTAGTATGGATAATTGAAATAGAAATAGTTTTTCAAAACCAA
AGCTATTATCAACAATGATAAACATTTGTAAGAAAGTCAACAAAGTATGTTCTTATTCAT
AGGAGGCATGTAGTCAATGGGACTACCATGGTACCGTGTACACACGGTTGTTTTAAATGA
TCCTGGACGGTTAATTGCAGTCCACCTAATGCATACTGCACTTGTAGCGGGTTGGGCAGG
ATCTATGGCATTATACGAACTAGCTGTATTTGATCCTTCAGATCCAGTATTAAATCCAAT
GTGGCGACAAGGCATGTTTGTTATGCCATTTATGGCTAGACTGGGCGTAACAGATTCATG
GGGAGGATGGAGTATAACAGGAGAGAGCGTATCCAATCCTGGACTGTGGAGTTTTGAAGG
TGTAGCCTTAACTCATATAGTCCTTTCTGGCATGCTTTTTCTAGCTGCTATTTGGCATTG
GGTTTATTGGGATTTGGAATTATTTAGAGATCCACGAACTGGCGAGCCAGCCTTAGATTT

ACCGAAAATTTTTGGAATTCATTTATTGCTATCAAGTCTACTTTGTTTCGGATTTGGAGC

TTTCCATGTAACTGGACTTTTTGGACCAGGAATGTGGGTATCAGATGGGTACGGAGTAAC

CGGAAAGGTATTACCAGTAGCTCCAGCATGGGGACCAGAAGGATTCAACCCGTTTAATCC

TGGAGGAGTTGCATCTCACCACATTGCCGCAGGTACTGTAGGTATATTAGCTGGTGTTTT

CCATTTAACTGTTAGACCACCACAAAGACTGTATCGTGCTCTAAGAATGGGTAATATTGA

AACTGTATTATCAAGTAGTATCTCTGCTGTTTTTTTCTCAGCTTTTGTGACTTGTGGTAC

GATGTGGTACGGCTCTGCAACTACGCCTATTGAATTATTTGGTCCAACTAGATATCAGTG

GGATAGTGGATATTTTCAGCAAGAAATTGAGAAACGAGTAGAAAATGCTATTGCTGATGG

TGCTGCACCTAGCGAAGCATGGTCAAGAATTCCTGACAAGTTGGCATTCTATGACTATAT

TGGTAATAATCCAGCAAAAGGAGGATTATTCAGAGCGGGCCCTATGAATAAAGGCGACGG

AGTTGCTGAAGCATGGCTTGGACATCCTGTATTCCAAGATAAAGAAGGAAGAGAGCTTAG

TGTTCGCAGAATGCCTGCTTTTTTCGAAACTTTTCCTGTAATTTTAGTTGATAAAGATGG

TATTATACGAGCTGACATTCCATTTAGAAGAGCAGAGTCTAAGTATAGTATTGAACAAGT

AGGTGTAACAGCTAGTTTTTATGGTGGCAAATTAAATGGCCAAGTTTTCAACGATGCTCC

TAGCGTTAAAAAATATGCAAGGAAAGCTCAATTAGGTGAGGTATTTGAATTTGATCGAAC

TACATTAGAATCAGATGGAGTGTTTAGAAGTAGTCCTAGAGGCTGGTTTACATTCGGTCA

TGCTAATTTCGCTTTAATTTTCTTCTTTGGACATCTTTGGCATGGTTCAAGAACTATCTT

CCGGGATGTGTTTGCGGGAATCGGAGCTGAAGTTACTGAACAAGTTGAATTTGGAGCTTT

CCAAAAATTAGGAGATAGAAGTAGTAAAAAACAAGGAGCTGTATAAGACCAAAGTCAAAG

ATAGTCTTTATCAAATATTTCTAATATTAAAATAGTTAAGACAAAGTTTATTATCTAATA

GTTACTAATCAGGAGAATTTATGGAAGCCTTAGTCTATGTATTTTTACTAACAGGAACGC

TAATGGTTATATTCTTTGCGATCTTTTTTAGAGAACCTCCAAGAATAGCAAAGTAACTTG

ATTCCGTTTAAACTTATAATAATAGAACCACTTTCCAACTTATTAGTGGAAAGTGGTTTT

ATTGTAGATTACATTGTATTATTCTTCATGCTCTTCAAAAGGATCTCGGAGATCTTTTGA

AGCAGGACCAAATGCTGTGTATATCGAGTAACCTGTAATACCTAAAAGCAGACTCGAAAT

AAAAATACTAAGAACTGTTGCAGTTTCCATAATTTAAGCAGTATTAAGATGAACTTTTTC

TATAATGATATTATCAATATTAAAACATTTAATTATTAAAATTATCAAAATATACTATGG

CACTTAGAACTAGACTTGGAGAAATTTTAAGACCTTTAAATTCAGAGTATGGAAAAGTTG

CTCCAGGTTGGGGCACAACTCCTATTATGGGAATTTTCATGTTACTATTTTTCTTGTTTT

TATTAATCATTTTACAAATCTATAATTCTTCATTAGTATTAGAAAATGTAGATGTAGATT

GGGCTACTTTAGGTAGCTAACTAGAGAAGTTCTTTGATTACACAATATTAAAGACAACAA

GCGATCGCTATTAGATTATAGTAGCGATCGCTTTAAAAGTATTTTACTATATATCCAGAG

AAAATTATTTACCTAAATTAATTAACTCGGTATCTGCAAAACTATTTGTGTTAACATTAT

TATAGTTAACTTTTTCAAACCTTACTAATACTGGATATTTGATACCACTTTTATCAATTG

CTGCAACAGTACCAATTTCCTGATACCAGTACGATTCTTTTCTCAATATTTTTACTTTTG

AGCCTCTTTCCATATTTTTAGTCCTCAGATAGATTATTAAAGATTATTATATTAAAAAAT

T-TAAAAAACTAAACAAAATATATAAAAATACTACCGACAATTGTAATATATAACTAATA
AAAGGAGAGAGAGGGATTCGAACCCTCGATAACAAAAGTTATGACAATTTTCGAAATTGT
TGCAATAAACCACTCTGCCATCTCTCCTGAATTAAAATACTGTTTTAAAAAATTAGACAA
GAATAACATAGAACTAAGGTAGTTGCCTAGAAAATTAAAACTATGTTAAATATAAATTAT
AATCTTTCTATAGATATATTTAAAAAATATGTACATAGAATCTATATCAGCTTATTTCAA
GATTTTTTAATCTTATACAATTTTTACTAAATAAATAAATTCTCTCATAATATGAAACTA
TCTTGGAAAACACTTCTTCTTTGGTCTTTACCAATATTTGTAATTGGTTTCTTTTTCTGG
CAAGGTTTTTTAGGTCCAACTACTACAGATGTTGGCAGTAATATCGCAAGTTCTAGAATG
ACATATGGACGATTTTTAGAATATTTAGATATGGGTTGGGTGAAACGGGTTGACCTCTAT
GAAAATAATCATACAGCAATTGTAGAAGCTGTTGGGCCAGAATTAGGAAATAGGGTTCAA
CGAATTCGAGTTGAACTGCCAGCAAGTGCGCCAGAATTAATTACAAAATTACGCAAAGCC
AATGTTGATCTAGATGCTCACCCCCCTAAAAGTACAAGTGCAGTATGGGGACTATTAGGC
AATTTACTATTTCCTTTACTATTAGTTGGCGGGTTAGCTTTCTTATTTAGAAGATCTAAT
AATGCTAGTGGTGGACCTGGTCAAGCAATGTCATTTGGCAAATCGAAAGCTTTATTTCAA
ATGGAGGCTAAAACGGGAGTAGTATTTAATGATGTAGCTGGAGTTGAAGAGGCAAAGGAA
GAATTTCAAGAAGTGGTAACATTTTTAAAACAGCCTGAATCATTTACTGCTGTTGGTGCA
AAAATACCAAAAGGCGTTTTATTAGTTGGACCTCCTGGCACAGGCAAAACATTACTAGCA
AAAGCTATTGCTGGCGAGGCTAGTGTTCCTTTTTTTAGTATCTCAGGCTCAGAATTTGTA
GAAATGTTTGTTGGTGTTGGCGCTTCTCGTGTGAGAGACCTATTCAAGAAAGCAAAAGAC
AATGCGCCTTGTATCGTTTTTATTGATGAAATTGATGCTGTTGGTAGACAACGAGGAACA
GGTGTTGGAGGTGGTAATGATGAAAGGGAACAAACATTAAATCAACTATTGACTGAAATG
GATGGCTTTGAAGGAAATACTGGTGTTATTGTAATTGCCGCTACTAACAGAGCTGATATT
TTAGATTCTGCATTATTAAGACCTGGAAGATTTGATAGACAAGTTTCTGTAGATGTACCA
GATTTTAAAGGCAGGTTAGCAATTCTTGAAGTTCATGCTAAAAATAAGAAAATGGAACCT
AAAGTATCTTTAGAAACCATTGCCAGAAGAACTCCCGGCTTTTCAGGAGCTGATTTAGCT
AACTTACTAAATGAGGCTGCTATCTTAACGGCTCGACGAAGAAAAAATGCAATGACTATG
TCTGAAATTGATACATCAATTGATCGAGTAGTAGCCGGGATGGAAGGCACTCCTTTAATT
GACAGTAAAAGCAAAAGATTAATTGCGTATCACGAAGTGGGTCACGCAATAATAGGCAGT
TTATTAGAGCATCATGATCCTGTGCAAAAAGTTACATTAATACCAAGAGGGCAAGCAAGA
GGCTTAACTTGGTTTACTCCTAGTGATGATCAAAGTCTAATATCAAGATCTCAAATACTA
GCCCGTATCGTAGGTGCTCTTGGTGGCAGAGCTGCAGAAGAAATCATTTTCGGTGACGCA
GAAGTTACTACTGGTGCAAGTAATGATTTACAGCAAGTAACATCAATGGCAAGACAAATG
GTCACTCGATTTGGAATGTCTAAAATTGGACCTTTATCTCTTGAAAGCCAAGGAGGAGAC
CCATTTTTAGGTAGAGGCATGGGAGGAGGCTCAGAATATTCAGATGAAGTTGCAACTAAT
ATTGATAAGCAAGTAAGGGAAATTGTCAGTGAATGCTATGCACAAGCTAAACACATTATT
ATAGATAATCGAGTAGTGATAGATAGATTAGTTGATTTACTAATTGAAAAAGAAACAATT

GAAGGCAATGAATTTAGAGACATCGTTAAGGAATACACTGCAATTCCTGAAAAAAATTAC
TACATATCACAATTTTAAATTAAACGGGACTGACGGGATTCGAACCCGCAACTTCCGCCG
TGACAGGGCGGTGCTCTAACCAGTTGAACTACAGTCCCAAAAAGATGAATACCCAGAGAT
AATCTCATAGTTAGCTTAAACTTGTCAAATTAGAATATTAAATATGAAGAGTATTTGTAA
TAATACTCTTCATATTTAATATAGTAGCTAAAATATTTCTTTTAGAAGGAGAGAGAGGGA
TTCGAACCCTCGGTACGAAGTTAATCGTACAGCAGATTAGCAATCTGCCGCTTTCGACCA
CTCAGCCACCTCTCCATGATATATATTATATATATATCAAATATACTTATATTTTAATAG
GTGTGCATAAATATTTATGGAGCTAAGCGGATTCGAACCGCTGACCCTCTCAATGCCATT
GAGATGCTCTACCAACTGAGCTATAACCCCTTTGGTAAGATATTAAGAAATGGCTCAAGC
GGGATTTGAACCTGCGACCTTGGGCTTATGAATCCCCTGCTCTAACCACTGAGCTACTGA
GCCATAGTATAAAATATTATTAAGTATTATACCATAAATTATGGTCAAATATAAATCAAT
AAAAGAATAATTACTATCATTTACTACTTTTGTGCAAGTTGTAAATATTCGAATAAAATT
TCAAAATTAAAAATGTCTTTAACTTTTTGTTAGAATTATTGATTACGAAAGTATTGATAT
TTAGAATTAACAATAAATTTTGTCTTTATTTGTAGACACAAAAATATTGGAAATTTTCAT
CAATTAATTAGTTAAACTATAACCGACATACAAGAATTATTGAACTACGTTAAATTGTGA
TGCATTGGGCTTGATCAAAACTTCTCTAAAATCAATAGAATATAGTAATTCAAGTAGATC
AAATACTAACAAATGTGACAAACGCTTAAAAAAATAATACAATTAGAATCGTAAAAATTT
GGTTTTTGACTGTGTTAAATAAATTTGATTATCATAGATTTAATATTTAAAAGTGTTCTA
TTACTTGTATTGAGTAATTGTAATATTTAATATCTTCTTTTTTAGATAACAATCATAATA
TAAGCTAAAAAATAGTTTTTATTAAACAACTAGCATAGAGCCAATACCTTGATCAGTTAA
TATTTCTAGCAAGAGAGCATGATCTATTCTACCATCTAAAATATGCGCTGAAGCTACACC
CTGAGCCAAAGAACGAATACAACAATTCACTTTAGGAATCATGCCACCAGAAATTACTGC
TGTTTGAGTTAAATCTCTAGCTTCCTGTATATTCAGATGACTAATTAATGTTGAAGGATC
TGAAGAATTACGTAAAATGCCGGGCGTATCTGTGAGTAGAATTAGTTTTTCGGCATTAAG
AGCGGCTGCTATTTCTCCTGCGACAGTATCAGCATTAATATTATACGATTGGCCTTCCTT
GTCTGCTGCAACACTAGCTATAACAGGTATATAATTATTATTAATTAATATCTTCAGTAA
TTTAGTATCAATATTTTGTACTTCTCCAACAAAACCAAGGTTTGCTTTTCCATTTGGTCT
CGGAGTAATAAGTAATCCATCTTTTCCCGACAAACCTACACTTTTACCACCTTGTTTATT
AATAGTTGCGACAAGATCTTTATTGACTCTTCCAACTAAAACCATTTCCACTATATCCAT
GGTAGGTTGATCTGTTACTCTAACGCCATCATCGAATTTTGGTAATATTTTGAGGCGATC
TAACCAAAAATTAATTTCTGGTCCTCCACCATGAACTAAAATGGGACGTAGTCCTATAAA
AGACAGAAAAACAAGATCACTAATCACTTGATCTTTTAGTTTCTGATTTTTCATAGCCGC
TCCCCCATATTTTATAACAATAATTCTGGAAGAAAATTGCTGAATATAAGGCAGAGCTTC
ACTTAAGACTTTTACTCTTTCTGAATTAGTCAACATAAATAAAGTAGGATTGTGATATAT
AAAATTATATACACAGCTTGTGTTAATTTAAAAGATACCAATGGGTACTTGAAAAAATAG
AAATATTACTAACAGTTAAAACTATAGGGATTATAATTATGAATAAATTTTGGGATAATG

TATTAAGATTTCCTCGATTTTTAGTCAGCGTCATTTTAGGATTAATTTTGATAATTATTA
GTCCTTTTTTCGTGTTATTAAAAAAACCACTGACAAGTTTTTTTTTCATCATATCATTGG
CCGGATTAATCACAGTTTTGGCAATAATAATACAAAAAATGATAAATATCGAATGTTGTT
GAGAGAAATAAATAAATTTAATTAAAATTTTTGAACATATATAATATTATATTATCATTA
GATATGATAATTTATTTCCTTTTCTATAAAACCCATAAATATTAAATATTATTTGATAAA
TATTATGCCACTAAAACAAAGAGTTAGCTCTGAAAAGACCGGTGCCTTCGCACTTTTGGA
TAGTATTGTTAGGCACGGAGTTAAACATATATTTGGTTATCCAGGTGGAGCTATTCTTCC
TATTTATGATGAGCTTTATGCTTGGGAAGAAGCCTCTCTAATTAAACATATCCTTGTTCG
TCATGAGCAAGGCGCTGCTCATGCTGCAGATTCTTATTCTAGATCAACAGGAGAGGTTGG
AGTATGCTTCGCTACTTCTGGCCCAGGAGCAACTAATCTTGTCTCAGGTATAGCTACAGC
ACATATTGATTCTGTACCTATATTAGCTATAACAGGTCAAGTTGGGAGAGCTTTTATTGG
TACAGATGCTTTCCAAGAAGTAGATATTTTTGGGATTACACTTCCTATTGTAAAACATTC
ATATGTAGTTCGTGACCCTAGAGACATGTCTAGAATTGTTGCGGAAGCATTTTTTATTTG
TAAACACGGTAGACCAGGTCCAGTATTAATTGATGTTCCTAAAGATGTAGGATTAGAGAA
GTTTAATTATTTTTCTGTTGAGCCCGGAAAAGTTAATATTCCTGGCTGTAGGCCAATTAC
CAGCCTAAAGTCAAGACAAATCCTTATGGCAGCTAAAATGATACAGCAATCTAGCCAGCC
ATTGTTGTATATTGGTGGAGGAGCCATAATCTCTGATTCACATCAAATTATTAAAGAACT
TGTTGATTTTTATAAAATACCTGTTACTACTACTTTGATGGGGAAGGGGATTTTTAATGA
GGATAGCGATTATTGTCTAGGGATGTTAGGTATGCATGGTACTGCGTATGCTAATTTTGC
AGTTAGTGAGTGCGATCTTTTAATTGCTTTAGGAGCTAGATTTGATGATAGAGTTACTGG
AAAATTAGATGAATTTGCTTGTAATGCACAAGTGATTCACGTAGACATTGATCCTGCTGA
AGTAGGAAAAAATAGGATTCCTCAAGTTGCTATTGTCGGTGACGTAGCAGAAGTTGTTAG
TGAAATATTGAATTTATTAAAGACTTCTTTCCCCCCTTATCCAGAGCAGATTATATCTTG
GCAAGAAAGAATTAATCGTTGGCGTCAACAGTATCCTTTACTGGTTCCTAGAAAATCAAC
AAGCATTTCACCTCAAGAGATTCTTGTTGCAACTAATAAATTAGCCCAAAATGCTTATTT
TACTACAGATGTTGGCCAGCATCAAATGTGGTCAGCTCAATTTCTGAAAGTAAAAGCTAA
GCATTGGCTTTCAAGTGCTGGATTAGGCACGATGGGTTATGGTTTACCTGCAGCAATTGG
CGCTCAAGTAGCACATCCAAATGACGTAGTCATTTGTATTAGTGGTGATTCTAGTTTTCA
AATGAATATGCAAGAGTTAGGAACTATCGCGCAATACCAGTTACCAGTTAAAATTATTAT
TATTAATAATCGATGGCAAGGGATGGTTAGACAGTGGCAACAAGCTTTCTATGGTGAAAG
GTATTCACACTCAAGAATGACAGAAGGAGCACCTGATTTTCAAAAGCTTGCAGAAGCTTT
TGGTATTAAAGCTTTTACTATTAATAATAGGCAAAATATGCAATCTGCTTTACAAGTTGC
TATTGATTATCCTGGTCCAGTTTTATTAGATTGCCAAGTTACAGAAAATGAAAACTGTTA
TCCAATGGTTGCTCCTGGAAAAAGTAATGCACAAATGATAGGTATCGCTAAACCGCAGAG
AGGTACTGCTTCCAACTATATTAATAATAGTGTTTGAAGTAAATAGTTAGTATTTTTTAA
ACAAAGAATAAAGTACATAAATGTATTTTTTTTATACTATTAAAAACTATGTAAGAACAG

CCTTTGACGAGAATTGAACTCGTGACCTTCCCCTTACCAAGGGGATGCTCTACCTCTGAG
CCACAAAGGCTTTTTACTATATTGGGCCGGGTTGGATTTGAACCAACGTAGGCGAAGCCA
GCGGATTTACAGTCCGCCCCCATTAACCACTCGGGCACCGACCCTAATTAAACCTTATTC
AAGATATGACCTATAAGATATCATAACTATGTTGCTAAAAACAAGTACTTTGATAAATAA
AACTGAATAAGTAATGGACATAGCTGGACTTGAACCAGCGACTTTCACGATGTCGACGTG
ACACTCTAACCAACTGAGTTACATGTCCAAAACTTTAATTATCATTATAACACTGTTTAG
TGATTTTATAAGTATCTATTTCTAATTGAAGATTTGTTTTAATCGTCCGGCTTTACCGAG
CAGATCTCTTAGATAATACAGTTTAGCTCTACGTATTTTAGCTCTTCTAATCACTTTGAC
AAAAGTTATTCGCGGTGAATTGAGAAAAAAATACTCTTTCAACTCCAATACCTTGAAATGA
GCATCTTAAAGTTAAACTAGTATTTAAGCTTTTTTTTCTTCTTCTTGCTAATACTACTCC
TTCACATAATTGTTCCCGAGTTTTACTACCTTCCTTAACCATTAGTCCAAGTTGTATTGT
ATCGCCAACTTTAATTTCAGGTACTTCTGTTTTGATAAAAGGTATCTCAACACTTTTCAT
TAGCTGACTTAGCTTTGTACTATTTGCTTTCATATATAGGATTAGTCTTGTGACATGTTT
TGAAATATTATACATTAAAAAAACAAATATGAAAAATTTTAGCGAATAAGCATATTAGTA
TTTATAAAACTCTTTTTATCTCCATTATACAGATCCATTATATTCAACTAGATTCTTTCA
TAGTTTCTTTGATAATCAGTACATCTAGATGATCAAATATTATTTTCAAACATGGCTTAT
GTATGAAAAAGTTCTATAAAAACCTAATGACTAAAATTAGGGCTTAAGTATTTCTTAGTT
TTGTGTTATAATTAGTTACTATCAAAGGTAGTGGCATAACACGAAGCATCAAAATGTTCG
AACGCTTTACTGAAAAAGCTATAAAAGTCATAATGCTAGCACAGGAAGAAGCTAGACGCT
TAGGACATAATTTTGTCGGAACTGAGCAGATACTATTAGGATTAGTTGGTGAAGGTACTG
GAATCGCAGCTCAAGTTTTGAAATCGATGAATGTGAATCTAAAAGATGCAAGAGTTGAAG
TCGAAAAAATTATAGGAAGAGGGTCTGGTTTTGTAGCGGTTGAAATTCCTTTCACTCCTC
GAGCAAAAAGAGTACTAGAATTATCTTTAGAGGAAGCACGTCAACTAGGCCATAACTATA
TTGGCACAGAACACTTGCTAATGGGCTTAGTCCGAGAAGGAGAAGGAGTTGCGGCAAGAG
TTTTAGAAAATTTGGCAGTCGATGTTTCTTCAATTAGAGCTGAAGTTATACAAATGCTCG
GAGAAAATGCGGAAGCCAATGTAAGTGGAAGCAATACTACGCAAGCTAGAAGTAAAACAC
CAACATTAGAAGAGTTCGGATCTAACTTAACTCAAATGGCTATGGAAGGTGGTTTAGATC
CTGTAGTCGGAAGACAAAAGGAAATAGAACGAGTTATTCAAATCTTAGGTAGAAGAACTA
AAAATAATCCTGTCTTAATTGGGGAGCCTGGTGTAGGTAAGACAGCGATTGCGGAAGGAT
TAGCTCAAAGAATTGCTAATAGAGATGTACCTTCTATTTTAGAAGATAAATTAGTTATTA
CTCTTGATGTCGGTCTATTAGTAGCCGGAACTAAATATAGAGGTGAATTTGAAGAAAGAC
TAAAACGTATTATGGATGAGATTAAATCAGCTGATAATGTAATATTAGTGATTGATGAAG
TTCATACATTGATTGGGGCTGGTGCTGCAGAAGGAGCAATAGATGCAGCTAATCTGCTTA
AGCCAGCTTTAGCAAGGGGAGAATTGCAATGTATAGGTGCAACAACTTTAGAAGAATATA
GAAAACATATAGAAAAAGATCCAGCATTAGAAAGAAGATTTCAACCAGTTGTAGTTGGAG
AGCCAAGTGTTGAAGAAACAATTGAAATTTTGTTTGGTCTTAGAGACCGTTATGAAAAGC

ACCATCAATTAACAATGTCAGATGGAGCTTTGGCTGCAGCTGCTAAATACGCTAATCAGT
ATATTTCTGACCGATTTTTGCCAGATAAAGCAATTGATTTAATTGATGAAGCTGGTTCTA
GAGTCCGTTTACTAAATTCTCAATTACCTCCTGCTGCCAGAGAATTAGATAAAGAGTTAA
GAGCTGTATTAAAAACAAAAGATGAAGCTATTAGAGCTCAAAAATATGAAACAGCAGAGC
AGTATAGAGCAAGAGAAATGGAAATTAAAGCTCAAATTGCAGCAATTGCTCAAAGTAAAA
AGAATGAGCCTGATTTAAATTTAGAAGATCCTGTTGTTACAGAAGATGATATTGCTGAAA
TTGTTGCTGCATGGACTGGTATACCAGTAACTAAGCTTACTAAAAGTGAGTCAGAAAAAT
TAATGCACATGGAAGAAACTTTGCATGGACGTATTATTGGTCAAGACGAAGCGGTTGTAG
CTGTCTCTAGAGCGATAAGACGCGCAAGAGTAGGTCTAAAAAATCCTAACAGACCAATTG
CAAGCTTTATTTTTTCCGGACCGACGGGTGTAGGAAAAACAGAATTAACAAAAGCTTTGG
CTTCTTATTTCTTTGGTTCAGAAGCTTCTATGATACGGCTAGATATGTCAGAATACATGG
AAAGACACACTGTATCTAAACTAATTGGTTCTCCTCCAGGATATGTGGGTTATAGTGAAG
GTGGTTATCTAACAGAAGCGGTAAGAAAAAAACCATATACTGTCATCTTATTTGACGAAA
TTGAAAAAGCTCATCCGGATATTTTTAATCTACTTCTTCAAATTTTAGAAGATGGCCGAC
TAACAGATGCAAAAGGCAGAACTATTGATTTTAAGAATACTCTTTTAATTATGACTTCTA
ATATTGGATCTAAAGTTATTGAAAAAGGAGGAGGTAGTTTAGGCTTTGAATTATCAGAAG
ATCAAACAGAATCCCAATATACTAGAGTACGATCTTTAGTAAATGAAGAACTGAAACAAT
ACTTTAGACCAGAGTTTCTAAATCGATTAGATGAAATTATTGTATTTCGTCAGCTTACTA
AAGATGAAGTTAGAGAAATTGCAGAATTAATGCTTAATGAAGTCTTTGCGAGAATTAAGC
AGCAAGATATTCAATTAAATGTAACAGAACGATTTAAGCAACGATTAGTAGAAGAAGGAT
ATAATCCAAGCTATGGAGCTAGACCACTTAGACGAGCAGTAATGAGGCTTTTAGAAGATA
GTCTGGCTGAAGAAGTTTTATCTGGTAAAATTAAAGCTGGTGATAGTGCAGTAGTAGATG
TTACTAATGAAGGAGAAGTTACAGTTTTATTAGGTGAAAAATTAGAACTGTTAACATAAA
AAACAATTTATTAATTAAAGCATTGAAGAGGTTATATTCAATGCTATGAAAAAAATTAAT
AAGATGGGTTGCTATAATTGACAACCTCTGATATTCTTTATTTGTATATAAAGCCGGGAT
AGCTCAGTTGGTAGAGCAGTGGATTGAAAATCCTCGTGTCACCAGTTCAAATCTGGTTCT
TGGCATTTTAAGCATCAGAAAATTTTGCTATCTGAGCATTAAATTTTAATTGAAATGTTC
CTGTGGGACCATTCCTGTGTTTTGCTATTATAACTTCTGTGAAGTCTCTACTTCTAGTTT
CTTGTGTATAGTAACTTTCTCTATATAGCATTATTACTAAATCTGCATCTTGTTCTATAG
AATTATGAACAATAAAGTTATTTGAAATAAAATTGCATAAAGGTTTACATTCTAAATCAA
ACACTATTTGTAAACTTGTAACGTTTATTTTTTGTATACTTTCAAAAGTCAAAGATGAAA
AAGAATTAAAAACATTTTTTTGTTCTTCAAAGTTTTTTATTTGAATAGCAATCATGTCAT
TTTGATTAATTTTATCACATCTTTTCCATCCTTGTGTTGTGAGTAGTTTATGGTTACTTG
TTAATTGTATATATTTCCCAGCTTCGGTAATAATTTTATATACAGTTTTTTTCCCTGTTT
TCGAAATACTACATTTAATTGCTAGAAATAACTGTCGTTTTTGCGCACTAAAGTTAATGA
CTTCAATCTTTTTATAATAGCAATTAAATAATATTTGAGGTTGATTATATAATGATGTCT

GAAGATAATTGAATTTGGATATACACCCACTTTCTCTTAAATCGGATAACAAAGGCCTTT
TGTCACTCCGAGTTTCAAGGTTTCTATTTAGTTGAGATAATGCCAAGATAGGCAAGCTTA
AATCTTTTGCTAATATTTTAAGTGATCGAGTAATTAATGATAGTTCTTGAGATCTATTGT
CAGATTGTCTACTCTCCTGCAGTAATTGAAGATAATCTATAATAATTAATTCTATATTTT
TTCCTTGTAATTTAAAGAATTTTACTTTTGTCTTTATAGTGTCAGTAGAAATCTTAGCGC
TATCATCAATATAAAGATTAAGGTTAGCAAGAGTTTTACTTTTTTGAATAACATATTGCC
ATTCATCATTATTAAGCTGACCAGATTGAATTTTTTGGCCGTTTAAATTACACTCTTGAG
CTAAAATTCTTCTTAGTAGTTGCTCTGTAGACATCTCTAAGCTAAATAAAATAACATAAG
ATCTTTTCTGATTAATTACATATCTAGTTATATTAATAGCAAAAGCAGTTTTCCCCATTG
AAGGTCTTCCTGCAAGAATAATTAAATCTGACTTTTGAAACCCTTGTGTTATAGAGTCGA
GTTCTGTAAAACCAGAAAGTATACTACTATTTATACTAATCTTTTTCCTTGTATCCAAAT
GGACTAATAGTCTTGCAAGAATTGTAGCTAATGTTTGCGTATCTTTTTCGTCAAGTATTT
CATAAGCTTTTGTTAATTGACTAACAATAGAAGTTATGTTTTTTTGTTCAATAAGTTGTT
TTGAGTAGCTAATTAAACATAATGAATCTCCACATGCTAAAAGTAATCTTTTAATGTAGT
TATCTATAACGACTGCTGAATATTCGCTTATAGTATCAGATAGGGGAGCTTGTTCTATTA
GACTAAAAACTTCATTTAGCTCATTCAAGTCTTGAATCATTTTTTCATTTTTTAAGTTAA
TGAAAAAATTTCTTACGCTTTTTGTCTTTGCATGGTTAACATTTTCGAGTGCTGCTCTAT
AAAGTAATGAAGTTGATGTAAAATAAAAAAAAATCAGGAGAGATTTTTTCTACTGATTTTA
GTAATGATATCGATTGCTGAGTTAATATTATACTAATTAATATTTTTTCAGCTAAAATAT
TATGAGGAGGTAGATATTTATAAATACTTATTACTTCCTCCTTGTAACATTACTCTTGTG
GTATTAGTTAACTATTGATTTAATTTGATTCTGGAAGAACTTGCAATTTAATATTTGCAG
TTACTTGATGTAATAGCTTAATTTCTACATCGTAAATGCCAATTGTTTTCACGTCTGGTA
AAGAGATACTTTGTTTTTCAATGTCTATGTTGGTAGTGTTTTTAATAATTTGTGAAATTT
CTTTTTCTGTGACACTACCAAAAATATTGTGGCCATCTCCTGTTTTTTTACTAACGCTAA
ACCTTTGTATTTCTTCTAGAAGTTGCTTGACTTTTTTAGCATTTTCTTTAGCTGTTTTTA
ATTTTTCTTCTCTTATAGCTGCATAAAATTTTTGTTGTTTTAAAATACCATTTGTGGCAA
CTGCCGCCATTTTATTCGGGATTAAAAAATTTCTTGCATAACCAGAAGCTACTTTGATAA
CGTCATTACTCTTGCCAAGTTTTTGAATATTCTCTTTTAGCACAACATTTATAACTTTTT
TACTCATCTTAATTCTAAAAAATCTGTATTATCCTATTATACCTGTTGTATACAGATTTT
AATAGGAAGTATTTATTTGTTGATATAATATTCTTGTAAAAAAAGGTAATTTTTTATTTT
TTTCTGATATAATCACTTTCACAATAATTCATTAGGAGAGGTGGCCGAGTGGTTGAAGGC
GCAGCATTGGAAATGCTGTTTAGGAGCAATCTTAACGAGGGTTCGAATCCCTCTCTCTCC
GTACAAAAAATATAAAAAGTTTTATAGAGTCCAATCTAAATCTCCAATAATATTCTTCCA
AGCATTTAATCCACCCCGAACTCTAACAATATTTAGTTTTTGAGCTATTAAAAACTCAGA
AGCAAATATTGATCTAGAATCTAAGCTACAGTAAACAAAACAAATTTTATCTTGTAAATT
GATATGAGAGTAATTCATTCCTTTTATTTTTTGTAAAGGTAGATTTAATGAATATATTAA

ATGACTTTTATGATATTCTTCATGATTTCTTACATCTATTAAAATATATTGTGGATTCCT
ACTAATTAAAAACTTTTGTAATTGAATAACACTAATTTCTCGTACAAATGTATTAGATTT
ACTGCCATAATATTTGTTCCAATGCTTTTTTGTGGATAATACAAATTTAGTATTTATAAT
CTTAAATTTGTTAAATGATGAAGTTAATGAGTTGTACGTTAATATAGTTCCACTTAATAT
AGACTTATAACCCAGAACGATTTTGACAGCTTCAGTTGCTTGAAGTGTACCAACAATACC
CGGTAAGAGACCTAAAACTCCGGAATTACTACAAGTATCTCTTGCGCTCTCTTTATTTTC
AGTTTCACTGTAGAAGTCTCGATAAACAGGTCCACCTTGATAATTAAATACACTAACTTG
TCCTTCAAATTGAAAAATGGCACCATAAATATGTATTTTATTTAACTCTAGGCAAGTATC
ATTCAAGAGATATCGAGTATCGAAGTTATCAGATCCATCTATAATAATATCATATTGTCT
AATAATGTCGATTGAATTCTCATAGCTTAGTCTTGTTTTGAACACAGTTACTATACACTG
TGGATTTAAATCTAATATTTTTTTTTAGCTATTTCTACTTTTGAATATCCTATATCATGC
ATCGTGTATAAAATTTGCCTCTGTAAATTAGAGAGATCAATTATGTCATCGTCTATAATT
CCAATGCTGCCAATTCCTGCAGCAGCAAGGTAAATGATTCCTGGAGATCCTAGCCCACCA
GCACCAATGAATAAAACTTTTGCTTCTTTTAATCTTTCTTGTCCTTCTAATTGAATTTGT
GGCAATACTAAATGCTTAGAATATCTTGTATATTCTTCTAGTGAGTATTTTGTATTTTCT
GTTTTAAAATTTAGCATAAAAATTTATAGTTTATTTAATAGCACTTAAAAGCTAGCTGAT
AAGTTTATTAAAATGACTTTTGTAAACTTGGTTCTTCTCTATCTAAAATAGCTCCTTGTG
TAGGACAGACCTGAACACAAATACTACAATCAATACAGGCAGAAAAGTCAATCCAATACC
AATTAGTGTTATTATTGTTTTTCCCCTTTCCTTGATGAATACAAGCTACGGGGCAAGCTT
CCACGCATTCAGCAACTCCAATACATTTTTCTGTTACAATTGTATGAGACATATTATTTA
ATTAATAAAAGATAAGCTATTTACATCTAGTAGTTTTGTTGTATACTAAATTTACATATG
CTATCCAGGCCAATATTTAAGGTAGTCTATTTCTACAGTTTACTAATTTGATATGCGCCT
TTAGTTCAGTTGGTAGAACGCAGGTCTCCAAAACCTGATGTCGAGGGTTCAAGTCCTTCA
GGGCGCGTTTAGTTTTATCGTAATTCAATAATATCATTTTATCTTTTTGCAAACATCAAA
ATGATATGATATAAAAGATCATATTTTATACATTGAATATTCTATGTACTAGTACTGTTT
TTTAATATGTTATTATTGATAAGTTAATTATACTAATTCTATTTTTGTAATAAGTAAAAA
CAGTTCTAATTAAGTATGCACGTATTTAATAGTCTTTTGTATATATAACTTGAAAAAATT
TAAACTATGGCTAAAAAAGTTACTGGAATTGTTAAACTAGCATTAAATGCAGGTAAAGCT
ACTCCTGCGCCACCTGTCGGACCTGCTTTAGGACAGCACGGCGTGAATATTGTTATGTTC
TGCAAAGAGTACAATGCACGTACTGCTGACAAGTCAGGACTAGTAATCCCTGTAGAGATC
TCAATATATGAAGATAGAAGTTTTACTTTTATACTTAAAACTCCTCCTGCTTCTGTACTA
ATCGCTAAAGCTGCTGGACTAAATAAAGGTTCGGGTGAACCAAATACCAAAAAAGTAGGC
AGCATAACAAATAAACAGTTAGAATCTATTGCAGAAACAAAACTTCCCGATTTGAACACT
AACAATATACCTCAAGCTATGAAAATAGTTGGAGGTACGGCTAAAAATATGGGAATTCTA
ATTAAAGATTAAAAGTTTCAATTATATTTACTATTTTTATGAAAAAATTTTCACGTCGAC
TTACAACATTAAAATCAAAAGTTGAGCCTAAACTTTACACTATTAATGAAGCAGTATCTA

TATTAAAAGCAACGTCAAATGCTAAATTTAAGGAAACTGCAGAAGCTCATATTGCTCTAG
GTTTAAATCCTAAATATGCAGACCAGCAACTCAGAGCAACAGTTATATTACCGAAAGGTA
CAGGTAAATTGATAAAAGTAGCAGTTATTGCTAAGGGAGAAAAATTAACAGAAGCAATTA
GTGCGGGAGCTGATGTTAGTGGCTCCGAAGAACTAATTGATGAAATACTGAAAGGTAGAT
TAGATTTTGATAAGCTGATAGCAACACCAGATGTTATGCCTTTAATAGCAAAGCTGGGAC
GAGTATTAGGCCCTCGAGGGTTAATGCCTTCTCCTAAAGCAGGCACGGTAACATTGGACG
TAGCAAAAGCCGTGAATGAATTTAAAGGGGGTAAAGTTGAATACAGAGTTGATAGAACAG
GTATAATTCATGTACCTTTTGGAAAATCTAGTTTTTCACAAGAAGATCTAGTTTTGAACC
TCCAAACAATTAAAGAATCGATTGATAGAAACAAGCCTTCTGGAGCAAAAGGGAAGTACT
GGAAAACTTTTTTCTTATCTAGCACCATGGGGCCATCTATTCAAATTGACATCACTAGCC
TTCTATGAAAAATTTTTGTATAATTATAGAACTTGAATAATCTATTATTTCAAATTATAA
TTTGCTAGCATTATTAACGTAACAACTTGCTTACTATATCTTATCTGCATTAAAAACTTT
TTATATATCTTATTAATTATGAGTACAAAGGTTGAAAATATCTTGGAAGAGCTAAAATCT
TTAAACCTTCTAGAGGCTGCTGAACTAGTTAAACAAATAGAAGAAACGTTTGATGTTGAT
GCATCTGCGGCTTCAGGAGGTATGATGATGGCCGCACCAACTTCAGCACCAGCGTCTGCT
GAGGTTGAAGAGAAGACAGAATTTGATGTTGTCCTAGAGGAAGTCCCAGCACCTAAAAAG
ATTTCTGTATTAAAAGCTGTTCGCTCTCTGACTGGTTTGGGTTTAAAAGAAGCAAAAGAT
CTTGTTGAATCTACGCCAAAAGTCTTAAAAGAAGGTGCTTCTAAAGATGATGCAGAAACT
ATGAAAAAACAGCTAGAAGATGCTGGCGCAACAGTTATTGTTAAGTAATGAAGAAGCGCT
TCTGTTTAAGTATAAACAGAAGTGCCTCTTTATTTATTATAAGCAATTATACTAGAAAAG
TCCAAGTGTAATTGCATTGTTGATAGGCATAGTTGCACCAATACCTAGCCAAATACTTAC
AACGGTACCAATTAAGAAAACAGTGGTTGCAATTGGTCTTCTAAATGGATTTTGAAATTT
GTTAACATTTTCAATGAATGGAACAGTAAGTAAACCAGCTGGTACAGCAGCCATACTTAG
AACACCTAATAGTTTATTAGGAATCACTCTTAATAGATTAAATGTTGGAAAGAAGTACCA
TTCTGGTAAAATCTCTAATGGAGTCGCAAATGGATTAGACTTTTCACCTATAGAAGATGG
TTCTAAAATTGCGAGTCCAATACTGCAGGCAAAAGTGCCAATAATTACAACTGGAAAGAC
GTATAATAGATCATTTGGCCAAGCTGGCTCTCCGTAATAATTATGGCCCATTCCTTTTGC
TAATTTAGCTCTCAGCTTCGGATCTGTTAAATCTGGTTTTTTAAGAATTGACATATTGTT
TTTGTGTTTAATATAAAGATTAGTCTAATTACTGTTTTAAAGTGTAATTTTCAACTATTA
TAATGGTCCAGAAATTCCTTGTTTACGTATCATTAAAAAATGCATAAGCATGAAAACGGC
AGTTAGTAGCGGAAGTACAAAAGTGTGTAAACTATAGAACCTAGTTAAAGTTCCTTGGCC
TACACTGACTCCTCCTCTTAACAATTCTACTATACTCGCTCCGACAACTGGAACAGCATC
AGGGACACCTGTAACAATTTTTACAGCCCAGTATCCGATTTGATCCCATGGCAAAGAATA
ACCTGTTACCCCAAAAGAAACAGTAAGCACTCCTAAAATTACACCTGTTACCCATGTCAA
TTCTCTAGGTTTTTTGAAACCTCCTGTTAAATAAACACGGAATACATGTAGAATCATCAT
CAAGACCATCATGCTCGCTGACCATCTATGAATTGATCTAATAAGCCATCCAAAGTTTAC

ATCAGTCATAATGTACTCTACTGATGTAAAAGCTTCTGCAACTGTAGGTCTATAATAGAA
TGTCATTGCAAATCCAGTTGCAACTTGAATTAGAAAAGATACAAATACAATACCCCCTAA
GCAATAAAAAAATATTAACATGAGGTGGTACATACTTACTAGAAATATCGTCAGCAATTGC
TTGAATTTCTAATCTCTCTTCAAACCAGTCATAAATTTTACTCATAAAATAGCTTC-AAA
AGCTCTTCTAATATATTTTGCGTTTAAGCTACTATTAATTCAATAGTTATTGAACTTATA
TTTTTTTATAATCAATACTTATTAACTATTATAACATCTTTTTATTGATTTAAAATTCTC
TGACTTAGTAAAACAGGGCTATGTACTATAATTCTTTTTTTTTGTATAGAGATTAATTTC
GTTTTTAGTAAATTAGCTAAAATTCTTGTTACACTGACTCTATTACTACCTATAATCTGT
GCTAAAATTTTATGTGTAATAGTAAAGTTAAGCATAATACCATTATTAACTTGAGTTCCA
TTTTGCTCAGCTAATAATAATAATAAGCTCGCCAGTCTACTGGTTATACTTTTATGTGAA
ATGATTTCTATAAAATGATAAGCTTTTGCCGAGCAAAATACTAAATGATTTACAAAAAAT
AAATTGAATGGAGCGCAGTTACTACACGCTTTTATAATAGTAGTATAGTTAATACAGGCA
ACTTCTGTTTTATCTATTGCTTCTGCTTCGTAGTAAAAATTATCGTCGACCAATTCTATT
TGTCCAAATGTATCTCCTGTAGTTAATAAGTTAAGAGTTACTTTATGTGTATTTCTCAGG
ACTTTTGTAATAATTAAAGATCCTATCAATATAATGTATAATCTAGAGCTGCTATTAAAC
AAAAGAGTATCATTTTTCTTAAGTGAAAAAATTTGATAATTAATTTTATTACGATTAAAA
AATAGTAGCCATGGATTGCAAGAAAAAAAATCACCTGAACTATTTTCAGAGAATATTGGA
ATACAATTTTCTTGATTTAAACAATTTTTAGTCATAATTACTACTTTATAAAAAAATATT
CTAATAATTAAGAACTGAAATTAAATGTCATATAAATTGATGTTAGTTGAGAATGACATT
GTATTATCAAAGGCCATTCAAGAATACTTAATAGATCAAGGCTTCAATGTTTATATTGCC
AATAATGGATTAGAAGCACTGAATCTAGCTTATCAATATAATTTCGACTTGATAATTTCT
GATATAATGATGCCGCTAGTTAATGGCTATGAACTATTAGCAAAACTTAAAAAAAACAAA
GCATTATCCAAAATACCTGTTATCTTTTTGACAGCTAAAGGAATGACTAAAGACAGAATA
AAAGGCTACGACATGGGGTGCTATGGTTATTTATCTAAACCATTTGATCCTAATGAGTTG
CTTTCAATTATTAACAACTTAATCGCTAGAGATGTCTTAAAAGAAGCTTCTTTACAAAAC
TTGGCAACATCAAACCAGCAATTAAATCATAAAATACGTTTGACACCTAGAGAAAAAAGT
ATTTTAGATCTTGTTGTTGATGGACTTACTAATAAAGAAATATCTACTATACTGAATACT
AGTGTTAGAAATGTAGAAAAATATGTTAGTCGACTCTTACACAAAACTAATATGAAGAAT
AGGACTTTGCTAGTCAAGTATTCTATAAATAATAATCTATTAAATAATGAGATCAATGAA
AGGGCGAATGACGGGACTCGAACCCGCGAATGATGGAGCCACAACCCATTGCCTTAACCC
CTTGGCCACACTCGCCATATTACACATACCACTATAGCTTGTTTTTTGATATATCGTCTA
GTTTTCGAGTTATATATTTATCTAAAACAATATTTACTTTGAATTTTTATGATACATAGC
AATATTAGTATTCAAATTAATGGGGAACCATTTAATTGCTCAAAGCCCATCTCATTGCAA
TTTTTATTAAATTATCTTGATTTTAATTCCGAGCGTGTAGCAGTCGAGCTAAATGACATT
CTGTTACCAGAGACTCTGTTTCACTCAACTTATTTGAATGATCAAGATAAGCTAGAGGTT
ATTACCATTGTAGGTGGTGGATAATCTATTTGTACATACACAGTTCTCTATCTCAAATGT

TTCATAGATAGAGATAATCTTCCTTGTTTTTTATCTACATGAATAATAACAGCTTTAATA
GTGTCACCTATTTTAAATTGTGATGATATCTGCTCTAATTCTTTGATATTAATCTCAGAA
ATATGGACAAGACCTTTGAGATTTCCTACCTTTATAAATAATCCGTAAGGTGTAATCTGA
TTTATTATGCCTTCAATAATATTACCAACAATTAAATTAGATGATGCTTGAGATATTAGA
GCTCTTCTATGACTCAGTATTAAATTATTAGATTTCTCTTCGACATTGAGTAATTTTAGC
TTAATAAATTTATTATTAAATTGCTCACTTTTTTGAAAATTACCGAGATGAGAATTAGGT
ACAAATCCAGATATACCTTCAAGGTTAATTATCATTCCTCCCTTATTGAATCCTTTTATC
ATTACATTAAGTAAAGAGTCTTCAGCTAATAGTTGCCTAATTCTTTTCCATGCTCTTATA
TATTCAAGGCGACGAATTGATAATATTAATTGTTTCGACTGAATATTATAATCTAATAAA
AAAAATTCTCTTGTGTCATTAATGTTTAAAGAAGTGAAATTATTTAAATCTTGATTACTA
GATACTTCTTGAATAGGCAAGTATGCAGATATTGGTGTTCCTATATCTACTAATACCCCA
TTTAATTCAAAGCTAAATATAGTTCCAGCTACAATATCTCCAAGATTCAAATCATACTTG
TATTTTTGTAAAACAGCTGCAAAATTTCTATGAGTAAATCCTTCATTATTTTTTGTCATA
TTGAATAGTATGAATTAAAGAGTAACTTTGTACAATATATTAAGGTAAATAAATGTTTGT
CGAATTGTATGAAAAAATTTTAATTTGTTCGGTTGTAATTAAGTATATAAACATATTAAA
ATATCTAGGAGACATGAAAAGTAGACGTAGATAGCTGCAGATTTAATACTATATAATCTG
AGGAAAGTCCGGGCTCCACAAATACAATTTATGCTGGAGAAATCCCAGTGTAGGTAACTG
CGAGGATAGTACCACAGAAAAAAACCGCCAGAATAAGTTATATTAGCTGGTAAGGGTGCA
AAGGCAAGTTAAAAGCTTACCAAAAGTACTGCAAAGTATTTGTTAGGTAAACCCCTAAAA
TGGAGCAAAGCTACTAAACAAATATTTGTGTATATTTTATCTAGTTTAGTTGAAAATACT
GCATGAAGTTATTAGTAACAATAACTCGAGAGGAATAGCTATCCTTTACAATATATCTCG
ATTATATTGAAAGAACAGAACCCGGCTTATGTAGTACTTTTTATGTCTTGTATTATTATG
TCTATTGTCGAATCTTTAAATTTAACTGTTTGACAATTTTTTTTTGTAGCTGATGTTGTT
TATAATCTATTTCTGAATTGGTTAAAGTTTTTTCATTAGATCGATATGTAAATCTTAGAC
CTATACTTTTTTTCTCTTTTCCAATTGATTCATCTATATATTGATCAAATAATGTTGTAC
TTTCTAAATCTTTATCATAAAATTGATCCAATAATTTTAATAAGTATTTAATTTCCATAT
TCTTAGGTATTATTAAAGAGAGATCTCTTATGATCGATGGATACTTAGAGTATGGTTGAA
TCTGATAACTTAAATAATTGAATTCATTATGACAATCTTCTAAAATGTTTAAGTTAACTT
CAAGAAAAAAAAGTTTTGTGTTGAGTCCTAATTCACTATATGTTGCTTGATTAAGTTCAC
TGAATATGCCAATAATAATATTGTTATAGATTAGTGTGGCAAATTTTCCTTTTTGAATGA
ACTTTATTCTACCATCGACTATATCTTTTTTTGTCCACTCAATTTGTCTATTAATCTTTT
GAAAAAAATTTTCTACGATCCCTTTAGCTTCATACCAATTTAAAGAATGCGCAGGGTGTG
ACCATGTAGATCGGATATCTAAATTACCACCCAATATGATAGCTAGATTATTAGTTTCTA
CAATTTTGTTTTGATCATTATGAAATACTGTTCCTATCTCAAAGCTATCTATAGTTTGGT
TGCTTTGTTTAATATTATAAAGATTTGATTCAATTAATCCTTCCAGCAAACTGCCGCGTA
AATTAGAATAATCTTGAATAAGAGGATTGTTTAAAGCTATATTTCCTTTGGACTTTATTA

AAGAATAATGAACTAATTCAGTTAATCCTAGATTTCTTAAAATACTTCTACTTTTATCTA

TGAATTGTCTCTTTGTGGATAACTTTTTGTTGAATTGAATATTTGGTACTGAGCTCCTGA

ATTTATGATAACCATAAATTCTAGCAATTTCTTCAATAACATCTATTTCTCGAAAAACAT

CTTCTCGTCTATAATTTGGAACAGTAACTTCTATATTTTCCTTATTTTGATAAATAAGAT

CAAAATTTAAAGAGTGAAGAATATTTTGAATTTCTTCAAAGAATAAAAAACGTGTTTGAC

CATTATACATAATTGGTCCTAAAATGTCATGAACTTTTTTAATTGATAAATTAATATTAA

GAGCATGATCATTTATTTTTTCTCGACAGAAAGTCTCTCTAATATTTCCGCCTGTTAGAT

CTGTGATTAAAGCAAGAGCTTCAAAATGTGCATTTTTCCAATTATCTACATTTAGCCCAC

GTTCTTGTCTAATTGAACTTTCTGTTCGAATATTAAGAACTCGAGAAGATTTTCTAACTA

CAGATTGTTTAAATATAGCAGACTCTACAAGTATCGATTTTGTATTATAATCTGTATCAA

AATCAGAGTTGCTTCCAATACCAGCTATACTAGTTACATTTGTATTTACTTGAGTAACTA

AAATATTTTTGTTTAACTCAATATTTTCATTGTTTAACTTAATCTGATCATTACTTCCAA

GAGGAAAATTGCTTCGAATTGTTAGACTATTTTTATGATTCATATTATTAATTTTATTCA

AATCAACAATATTAATAGGCTGTCCCCATTTCAACATAATATAATTGCTTATATCTGTTA

ACAAATTTCTGTGTGTAAACCCAGAAGAGAGCAACCGATTTTTTAGCCAATCTGGAGAAT

CTTTAACTCTTATTTCGTCTATAATGGCAGCAAAATAATTGTCACAATTTAATAAATCGT

GATTACTGATTATAGTCTCCTTTTTAGAGAAATATCTATTATTGGGTTGATGTATAGCTG

ATAACATTGAGGACCCTGTTAAAGCCGAGACTTCTCTTGATAGACCAATCATGCTTAACA

CGTCTGACCTGTTAGCTGTTGATGATATATCTAATATATGATCAATCTCACTTCCAATTA

TAATAGATTCAATAGCTTCAACTTCAAAACCTGCTTGTGTTAGTTGATTAGTTAAATTGT

CTATATCTATTGTTTTGATATTACCAAGTTCTTTCAACCAATTTAAAGAAACTTTCATAA

TTAATTGTCATCTATCTTATAGAAGTATTTATTTTACTTATGATTAAAAATCAATTTAAA

AAATTCATCAAATTAATATAAGTTATTTGATGAATTTGCTTATACAATATATTATTGTGA

TATCTTACTTTTATATGGCAGTTATTAAGAACTAGCTTTTGTAAAAGTTAGCCCATTATC

TTGGGCATATCTTTCCATAAATCTCATGAATCTATCCCAATCATCAGGACTTTTAATAAC

ATGTACAGCTTCTATTGCTTGTGGTTTACCATTAATAAACTTGGCATTAACATCTCTTGT

AATAATTTGTCCCTCTGTGTCCATCAGGTACATACCTGTGATTTCACCTTTGTCTGCCAT

ACTAGCATCTAAAATTTTGGGATTTGTAAACCGAAAAGTTGCTGTTCCTGTACTACCATC

TCGAGACCTTGTTAAACGTACATCTGGTACAACTTCTTCATTGATACCTTGAATAAATTG

AATTGTTGCCATATCAATCTTCTTTATTTGTATATAATTTATATTGGATAAAACTGATTT

CTAGTTCTTGAATGCTTGTTGAGAAACTGTTTTAAAAGCTTATAGTAGCAGTAGATCAAA

TTTTATGTATTATATAAAACTGTAACTTTTCTTTTTTGATCTGTTCAAAGAACTAACTTA

TTATAAGCTAATCATACGAATATTAGCCATATAATGTTCGTAAGTGAAATTAAGGGGTTG

TAGCTCAGAAGGATAGAGCAAGCGCCTCCTAAGCGCTAGGTCAGCGGTTCAAGTCCGCTC

AACCCTGTTAACGAATTAAGTAGTATTATTTAACTTGTTCTGCAAATCAGTATTGATCTA

AGTTAAAGTACTTGACAGCTAAAACAAAAGAATCTGAGAAATATTTTCATTTAAAGAACG

AACAATTATCTTAACTCTAAATATCTGATAATTATTAAAAAATTCTCTTTTTAAAACACA
AGATGATGAAGATATTTAAACTAATAATTGAAATTAAGTTTTTGAAGTTATAGAAGCCAA
TATAATACTTTCTATTAACAGATGCTTGCTTCTATTACTATCTGTTTACAAGAAATTATG
AGTATCAAAATAATTTTAGCTCTACTGACTACTAATAAATATTTATTTTAGTCTAGACAA
GATGAATCTTCATGGTATAACAGAGCTATTCTTTGTGTCATACTTATATAAAATTAGGAT
ACTGTTTTCATTATTATAACTGGGGTGGGAGGATTCGAACCTGCGAATGGCGGAGTCAAA
GTCCGCTGCCTTACCACTTGGCTACACCCCAACATGTGAACATTATAATAGCAGTCGTAT
ACCCTGCCATGTCAATAGACTATTGTCTTTATTTAATAATTTTTGCTGCTCTTGTAGAAT
AAAATCTTTTTTTTCAAATAAGATACGCTATGTTTAAGTTGCACGACTGTTATGTTTTCT
TGCTTTTGACTGAACAGCCACTTGATAGTAATTATTCCTTCTGCAATTTCGTTACTTCCT
ATAATAATACAAGCTACAGCTCTTTTTTTATTTGCTTGCTTAATCTGCTTACTAAAATTA
CTTGAGCTAACATCGATTTCTATTTTAAAAAACTGCTGATGTAAAAAACGCATTATTTGC
ATACCAGTTTCGTTAGCTTTTGTACCTTGTGTAGCAATATAAAAATCGATAGATTGATTA
GGCAAGTCTATATTATCTTTTGCAATCAGTAATAGGCGTTCTAATCCTATTGCACATCCA
ACAGCTGGTGTTGCTTTGCCACCTAATTGGTGAACTAAGCTGTCATATCTTCCACCACCA
CATATAGTATCTTGCCCTTTTGATGTCAATGTCTTAATTTCGAAAGCAGTATCATTATAA
TAATCTAATCCTCGAACTAACTTATTATTTATATTGTAGGGAATATTAAGTAATTTTAGA
TAATTGCAAACGTCACCAAAGTGTTTTTGTGATTCCAAACTTAAAAAATCAGAAATTTTT
GGAGCCTCTGTCAGTATTTTTTGTGTATTAGAATCTTTTGAATCTAAAATTCTAATTGGA
TTACTAGTTAATCTTTTTTGTGAGTCAGTGTCTAAATCATCATGGTACTTTGTAAGATAA
TCTCGTAATTTAACTTGATAAATACTACGATCCTCTACTTTTCCAATTGAATTCAGATCA
AGTTTCAAGTTGTGCAGATTGAGATTGTTAAATATGCTCATAGCTAAATGTATAACTTCA
CTATCTGCTCTTGCATCTAAGCTACCAATAAACTCAATGCCAAGTTGGTGAAATTGTCGT
TGTCGTCCACTTTGTGGTCTTTCGTACCTGAACATTGGACCGCTATACCATAATCTTTGT
AAACTATGGTGGTAACTCATTTTATTTTCAATAAAAGATCTCACAATACCGGCAGTACCT
TCTGGCCTTAAGGTAATATCTCTATTGCTGCGATCATGAAACCGATACATTTCTTTATTT
ACAATATCAGTATCTTCACCAATACCCCTATCATATAATTCACTATTTTCAAAAATAGGT
GTTCTAATTTCTTGGTAATTTGCACATTCTAATAATTTGGAAATTTTATTATGTATAAAC
TGCCAATATTGAATTTCATCGGGAAGAATATCTTTTGTCCCTCTAATAGCCTGAATTTTT
GCCATGATAATTTTTTGATTACCTTCTTAATATGTCTGCATTAAATAGCAGTTGTAGACT
GTTATTCAGTCATGTATTATACATTAGAACATACTGATGTAATTTATAAAGTACGGGCAA
GGAGGGATTCGAACCCCCGACACCATGGTTCGTAGCCATGTGCTCTAATCCACTGAGCTA
CAAGCCCACTTAATAAGTACCTTAACTATATCATTGTTCACTAATGTATACAACTATATT
CTTTTTATCTTACATGTGAAATTTTTGTAAAAGTAAAAAATTATGTTTGTTTTTTACAAT
TGGAGCTATTCATATATAATGCTTAATTTATTATAACTTTGTAAAAATATCTATAAGTTT
AATTGTATGATTCTATTATATTGGTAATCTTGTTATAATTTTGCGCTCTTATTTTTCAAT

TGGCTAGCATTTAATTAACTTATATTTCAAATAAAAAAATCTTATCTAAAATGAGTAAAC
AAATTCTATATCAGGATGATGCCAGAAAAGCATTAGAAAAAGGCATGGATATTTTAACAG
AAGCTGTTTCTGTGACTTTAGGACCAAAAGGAAGAAATGTTGTCTTAGAAAAAAAATTCG
GTGCCCCTCAAATTATTAATGATGGTGTAACGATTGCAAAGGAAATTAGTTTAGAAAATC
ATATTGAAAATACCGGAGTCGCACTGATTAGACAAGCAGCATCTAAAACAAATGATGTAG
CTGGTGATGGTACAACAACAGCTACAGTACTGGCTTCAGCAATTGTTAAGCAAGGAATGA
GAAATGTTGCAGCAGGTTCGAATCCGATGGCTATTAAAAAAGGTATTGAAAAAGCAACAA
ATTTTGTTGTTAGTAAAATAGCTGAGTATGCTAAGCCAGTAGAAGATACAAAAGCTATTA
TACAAGTTGCTTCTTTGTCATCAGGAAATGATATAGAGGTAGGTAAAATGATAGCGAATG
CTATAGAAAAAGTTGGCAGAGAAGGAGTTATTTCTTTAGAAGAAGGGAAATCAACTAATA
CGATTCTTGAGATCACAGAAGGAATGCAGTTTGAAAAAGGCTTTATTTCTCCTTATTTCG
TTACAGATACAGAACGAATGGAAGTTCTTCAAGAAAATCCTTTTATTTTATTTACAGACA
AAAAAATTACTTTGGTGCAGCAAGAACTTGTGCCATTGCTTGAGCAAATTGCAAAAACAT
CTCGGCCTTTATTAATAATAGCTGAAGACATCGAGAAAGAAGCATTAGCCACAATTGTAG
TTAATAAATTAAGAGGGATTTTGAATGTTGTAGCGGTTAGAGCTCCTGGATTCGGTGATA
GAAGAAAATCTTTACTTGAAGATATGAGTATCTTAACGAACGGACAAGTAATTACTGAAG
ATGCAGGTTTATCACTTGATACAGTTCAATTAGATATGTTAGGAAAAGCCCGAAGAGTTA
TTGTTACTAAAGACTCGACAACAATAATTGCAGATGGTCATGAAATTAAAGTTAAATCAA
GATGTGAGCAAATTAAGCGGCAAATAGAGACGAGTGACTCTTTATATGAAAGAGAAAAAT
TGCAAGAACGATTAGCAAAGCTTTCTGGAGGAGTTGCCGTCATTAAAGTTGGTGCAGCTA
CAGAAACAGAGATGAAAGATAAAAAACTAAGACTAGAAGATGCAATTAATGCAACAAAAG
CAGCAATTGAAGAAGGTATTGTACCAGGAGGAGGAGCTACTAATGTCCATATCTCTAGTG
AATTATTTACATGGGCTAAAAACAATTTAGTTGAAGATGAATTAATTGGTGCTTTAATAG
TTGAACGAGCTGTGACCTATCCGTTAAGACGAATAGCTTTTAATGCAGGTGATAATGGAG
CAGTAATAGTGGAAAAAGTTAAAAGTCACGATTTTCATATAGGCTATGATGCAGCAAACG
GTAATATTGTAAATATGTACGATAGAGGTATTATAGATCCAGCGAAAGTAGCTAGATCAG
CTTTGCAAAACGCAGCTTCTATTGCAGCAATGGTTTTAACTACTGAATGTATTGTAGTTG
ATAAAGTCGATGATTGAGCTATAAGATAATAGATAATCTCAAACAAAGAATAATTTCTAA
TTATAGAAATTATTCTTTGTTTCAATTAATCTAATTAATTGATCAAATGTATAATTTTTA
TAATAAAGCCAAAGTTTAAACACGTCTTCTTTTTCAACTATTAAGTAGAGAAGATATAAG
CCCGTAATACCTAAATTATCAATTAAAATATTATTTTCATATATAGATTTTTCTAAAGAT
CTAAAAAATGACATTTTTGAAAAATAATACCGAAATCTTCTCTTATAGTTATTTGCTAAA
TCATTACTGTCTAAAGATTGATTTCCAATTGAATTCAGAACCTTTAACAGAATACTTTTT
AAATCTTTATCTGACTTTATTTGAGAATCAATAATAATTTGATTCAATTGTATGTCAAAA
CTAAGATTAAAAATATTACTTGTTAAAGATATTTGATTTTGAATATCTAGAGATTTTATA
GCCAAGAAGATTAAACTTTTATTGTTCATTGTAAAATATAAGACAATTGATGATGTATTC

TTTAAATACTAAATTTATCTTCTTGTGCTTCTATCATTTTTTGTAAAATAATTAATGTTT
TAATTTTTTAGTTTTAGTTACTACCCGAAAAAATAAATTCTGGAAATCTAGCTTGAGCTT
TAGCTAGAGAGTGTTTTTTCCCTTCTTCTTGCCAGTAATTGATAACTTCTGTTGTTTTGT
TTAATAACTCAATTCTGTCTCTTTCAGCAATCCACGGCTTGGATTCTAGTTCTGCTTTTA
ATTCTTCCCAAGCATCATTCCTTGGCCAGAAAAAATAAGAAGTAAGTGGACTAGTGCCAT
TTCCAACAATCTGATCAATAGCAATAGCAATATTATTTTCTAACCAAAGTACTTTTAGTG
TAAATTTTGACAAGTTTTTATCTCCATTATTAATATTAAGTATTGGTACAGTAGAATACT
GTACTAGTTATTTTTTTCAGCAACAATTTGAGCTTTGTTTAAAATGTTTTTTACCGTTTT
AGTAGCTTGTGCACCTTGAGATAATCGTTGCAAAATTTTTGAAATGTTAACACGAGTTTC
ATTATTTATAGGATTATAAAATCCTAATTCTTCTATAGCTTTACCATCGCGTTTATTTCT
GCTATCCATTACAACAATTCTATAGCTTGGTTGTTTTTTTCTTCCGTATCGCTTTAACCT
TAACTTGACCATATTAGTCACCTCGCTTATTTTTTATATCTTTATTAAATTATATCTTAC
GACATTATGCAATAATTCAAACAATTAAGCTGATTCAAGTCTAACATTATTAAATATTAC
CATTAAACATTGTAGAAAAATAATACCTAACATAGGTGACATGTCCATTCCAAACATGGG
AGGAATGCTTCCTCTGAATAGTTTTAAGTATGGATCAGTAATTCTATTTAATGAACAGAA
TGGTTCATTGTACCAATTTACAGTTGGAAACCATGCCAATGATAATTTAAGTAAAATTAA
AATTAAATAAATCTCAGAAAAATTAGCTATAGATCCAAGCAATAAATTTAATGTACCAGG
AAGAGTGTTCATACGTATTTTATAATTATTATATACTTCGTTATACTAATAGTTTGATTA
TATATTATAATCGATGTAAATAAATCAGCTAAACAAAAAACTTATCCTTAAAATCATCTT
CTCTTGAGCATTTTTTTTTATCTACATTAGTATTTACTTTTGTCGTATAAATTTCCAGTT
TAGACCAAATATGTCCTAACTGATTTAATTGAGATGTTCCACATTCTAAACAGGCTATTT
TTATATTGCTCAAAACAGCACCTTTTTTTATAAAATCGTTCAATAATGGCGTAATAGTTG
CTTCATCCAAAAATAAATCTAAGACAAGTATTTTTGTAAATGAATTTAATTGATCTGGGA
CATTGTTAATAATATCATTATAATTAACTAAAACTATATTAGCTGTAGGAAGTAAAGATC
GTGCTCCTTCCGCTAAGATAAAGCCATAAGGCATAATAATTACAATAATATATTTATAAT
GACTGCTAATAAGACTTATAGTGGTATTAGCTTTTAATTCGATTGTTTCTGTTACTAGCC
ACTCTCTCATTATTTCGTAAGTAATCCATTTGCCTAGTTCTGAGCAAGCAGTTCTTAAAA
TCGTACCTGGATTATTGTCGTTTTCGAGAATCCCTGACCAATGTTGTATTAATGGATGGG
ATGCTATATTAATTTGGAGTTGCATGTTTTGTTAAATATATTTAACATTTAATTTGTTGA
TTGAATCTTCAATTCCTATTTGTTGAGTATTATTAAAATAGCATATATATTAAAGAAAAT
AATTATAAAAAATGAAAAAAAGTCTTTGGCTGTGGGGTTTTACAGATAGTGCTGAAACTT
GGAATGGTAGATTTGCAATGATTGGTTTTATATCAGTTATTTTTATCGAAGTAGTTACAG
GACAAGGATTATTATATTTAATAGGTATGATGTCATAATAAAAATCCAAAAAAGTTCTTG
TAATTTTTTATAAGAACTTTTTTGGATTTTTATAAATCTAATCTAGCGGTCTCATAGATA
GAACAGGTTCATTCTCTCTATTGATACCTTTTTCAAATCCAGCAGCAGCAGCTCTTGCTC
TGCCTGCATGCCAAAGATGTCCAATAAATAGGAAGAATCCTAGGAAGAAATGAGATGTTG

TTAACCAAGATCTAGGAGATACATAGTTAACTGAATTAATTTCTGTAGCTACACCACCAA
CGGAATTCAAAGAACCTAGTGGTGCGTGAGTCATATATTCTGCGGCACGCCTTTCCTGCC
ACGGTTGAATATCGTTCTTGATTTTATTTAAATCAAGACCATTTGGTCCTCTAAGAGGCT
CAACCCAAGGAGCTCGCAAGTCCCAAAATCTCATGGTTTCACCACCAAAAATAATTTCTC
CACTAGGAGATCTCATTAGGTATTTACCTAATCCAGTAGGTCCTTGAGAAGATGCAACAT
TCGCACCTAGTCTTTGATCTCTAACTAAGAAAGTAAAAGCTTGAGCTTGTGAAGCTTCAG
GACCAGTTGGTCCATAGAATTCACTAGGATAAGCTGTGTTGTTATACCAAACAAAATTAG
AAGCCGTAAGCCCCATGATTGATAAAGCACCTAGACTATAAGATAAGTAAGCCTCACCAG
ACCATACAAAAGCTCTTCTAGCCCAAGCAAAAGGTTTTGTTAGTATATGCCAGATTCCGC
CAGCAATACAGATAATGCCAATCCAGACATGACCACCTACAAGATCTTCCATGTTATTTA
CACTTACGATCCATCCATCACCGCCAAACGGAGACTTTAAGACATAACCAAAGATAACTA
GGGGATTAAGTGTAGGATTGCTAACAAATCTAACATCTCCACCACCTGGAGCCCAAGTGT
CGTATACCCCACCAATAAATAAAGCTTTAATAACTAACAGAAAAGCGCCAATGCCTAATA
ACACTAAGTGTATACCAAGTATTGTTGTCATTTTGTTTTTATCCCGCCAATCGTATCCGA
AGAAAGGAAATGATTCTTCAAGAGTATCAGGGCCAATCAGAGAATGATAAAGACCTCCAA
AGCCAAGAACAGCTGAAGAAATTAAATGCACTACGCCTACTACAAAGTATGGGTATGTAT
TGAAAATCTCGCCGCCTGGACCAACACCCCAGCCTAGTGTAGCTAAGTGCGGAATTAGAA
TAAACCCTTGTTCGTACAAAGGCTTCTCGGGAACAAAGTGTGCTACCTCAAAAAGAGTCA
TGGCACCAGTCCAAAAGACCATTATACCTGCATGAGCAACATGAGCACCAAGCAATTTGC
CAGAAACGTTAATTAAGCGTGCATTGCCAGACCACCAGGCAAACCCGGTAGACTCAATGT
CTCTACCGCCAACACCAACAGTTGTATTAAAGGGCGTTTCCACGTGGTAAAACCTCCTCA
GGGAATATAAAGTTTTCATGAGGTTGATCTTGAGCAGCCATCCAAGAGCGAATACCTTCG
TTCAATAGGATATTTTTAGTATAAAAAGTTTCAAACTCAGGATCTTCCGCAGCTCTCAAC
TCTTGAGAAACAAAATCGTAAGCTCTTAAATTTAGAGCTAGTCCAACAATTCCAAATGCG
CTTGTCCATAATCCAGTTACTGGTACAAATAACATGAAAAAATGTAGCCAACGTTTATTA
GAGAAAGCAACACCAAAAATCTGTGACCAGAATCTATTAGCTGTTACCATCGAATAAGTT
TCTTCAGATTGTGTTGGAGTAAATGCACGAAAAGTATCTGCAGCATCACCATCTTCGAAT
AAGGTATTCTGTACAGTTGCACCGTGAATAGCACATAGTAAAGCACCACCTAAGATTCCT
GCAACGCCCATCATATGGAACGGATTCAGAGTCCAGTTATGAAATCCTTGCAAAAATAGC
AAAAATCTAAAAATTGCAGCAACTCCAAGGCTTGGGGCAAAGAACCAGCTCGCTTGGCCT
AGAGGGTACATTAGAAAGACGGAAACGAATACTGCAATTGGTCCAGAAAATGCAATAGCG
TTGTATGGTCTAAGACCAACTAGCCTAGCAATTTCAAATTGTCTTAAACAGAATCCAATT
AGTCCAAATGATCCATGTAAAGCTATAAATGCCCACAGACCACCAATTTGGCACCAGCGA
GTAAAATCTCCTTGAGCTTCAGGTCCCCAAAGGAAAAGAAGTGAATGTCCCATGCTGTTT
GCTGGGGTAGAAACAGCCGCAGTTAAAAAATTGCATCCTTCTAGATATGAACTGGCTAGC
CCATGAGTATACCAAGAAGTAACAAAAGTAGTCCCAGTTAGCCAGCCACCTACAGCAAGG

TAAGCGCAAGGAAACAGAAGTAGTCCAGACCAACCTACGAATACAAATCGGTCTCTTTTT
AGCCAATCGTCTACAAGATCAAAACCACCACGAGTTTTTTCTTGTCCAATTGCTATGGTC
ATAATTTTAATCTCCAGAGCAAATTTAATAAGTAATTTGTTTAAATAAACACTTTAGATA
TACTCTAAAATTTTACTTTTCTTTACGGTCAATATAATTATAAGTCAAAATTAACCAAAA
TTCACGCTTTTAATAACATTTTTTTAGCTCATTAAGTTAATTTTTTAATATATAAACAGT
AATAGAGTTATTGTTTAACTTACAATACTCATAATTTATTCATTTCTATAATCCTTTGAA
AAAGATATCCTGTTCCTCGTGCTGTTAAAATCAAGTCGGGATTACTTGGATCATCTTCTA
GTTTAGCTCTCAATCGTGAAATATGAACATCAACAACTCTCGTGTCAACATGCCGTTCGG
GAGTATAGCCCCAAACTTCTTGCAAGATAGAAGCTCTAGAAAAAGGTTCTCCAGCCTTAC
TAACTAAAAGCTCGAGAAGACTGAATTCCATACCTGTTAATCGAACTCTTTCATTATTTT
TATAAACTTGTCTTTTGTTAGTATCTATTTTTAGAAATCCAATACTAATAATCCCAGAGT
TTGGGACTCCAAGACTAGTGGTGATTTTATCAGCTCTTCGAAGAACAGAACGAATGCGAG
CTTCTAGCTCCTTAGGAGAAAATGGTTTAACGACATAGTCATCAGCACCTATTTCTAATC
CGGTAATTCTATCGCAGACTTCACCTAACGCTGTTAGCATTATGATAGGAACATCTGATT
CTTTTCGAAGTTCTTGACAAACACCGTAACCATCTAGTTTAGGCATCATAACATCTAAAA
CAACAAGGCTAGGATACTCTTTTCTAAACACGAGTAAAGCTTCTTCTCCATTTGAAGCAG
TGATAACTTCATAACCTATTATTGTTAGTCTTGTTTCTAAAATTCTTCTTATGCTGGCCT
CATCGTCAACAACGAGAATCTTTTCTTTCTGGTTCTCCAATTTCTTATTTACTCCTATAA
CTCACCGATCATGATTACTATAACCTTAAATTTTTAGCGCTATGAGTATCTTTATTCACT
TTAGAATTTAGTTATTATAAATTTTGCAAGTCTAGTTTACATTAATTAGTACCAGTCAAT
ATTTTTGTTTTAAGTTATGACTATTTTAATCCATAAGTTGACTCTAGTTTGCAGAATTCA
ATATATACATATTTTCTAGCCAAGTAAATTGTATTCTATTTTCTAAATAAAAAAGGGGTT
GACAAACCAGTTGATAGAAAGTATTCTACATTACTATAAGTTAATTCCAACAAAGAAGTT
TTAAATCTTCTTTCGGAGTAATGAATACTTTAAT
>SRR9587921
TATATGATAAATTAAGACGTCTGTTATGTGAGAATTTCTAAAATATACAAAACTTTTTAT
TTAATATAGAAAAAGTAAATTAAAATAAGTTGAACATACTTTGCTCTAATCTAAACCTCT
TAATTGAGATTCTAATTAGAGACAAAAGTATTTACTTATTTTGTATCTATAGGAATAAAA
ATGAAACTTGCAGTTTATGGTAAAGGTGGTATAGGTAAATCTACAACTAGTTGCAATATT
TCTGTAGCACTTTCAAAAAGAGGTAAAAAAGTTCTACAAATTGGCTGCGACCCTAAACAT
GATAGTACATTTACGCTAACAGGTTTTTTAATCCCAACAATTATCGATACTCTTCAATCC
AAGGATTACCACTATGAGGACGTTTGGCCTGAAGATGTAATCTATAAGGGATATGGCGGT
GTAGACTGTGTTGAAGCTGGTGGACCGCCGGCTGGCGCTGGTTGCGGAGGCTACGTTGTA
GGTGAAACAGTTAAACTTTTAAAAGAATTAAATGCTTTTGATGAATACGATATTATCTTA
TTTGATGTTTTAGGTGATGTTGTATGTGGAGGTTTTGCAGCTCCATTAAATTATGCAGAC
TACTGCTTAATCATTACAGACAATGGATTTGATGCTTTATTTGCAGCTAATAGAATAGCA

GCTTCAGTACGAGAAAAAGCTAGAACGCACTCTCTGAGATTAGCTGGACTTGTTGGTAAT
AGAACAGATAAAAGAGATCTAATTGATAAATATATAGATTGTGTTCCAATGCCAGTATTA
GAAGTCTTGCCCTTGATTGAAGATATTAGAGTGTCCAGAGTAAAAGGTAAAACTTTATTT
GAAATGGCAGAAATTGATAAGGATTTAGCATATGTATGCGATTACTATTTGAATATTGCA
GATCAGTTGATTACAAGGCCAGAAGGTGTAGTTCCTAAAGAATCTCCGGATAGAGAATTA
TTTAGTCTTTTATCTGATTTTTACTTAAATCCTAAATCAAAGGTAGGACAAGAAAAAGTA
GATCAAGAAGAATTAGATTTGATGATAGTGTAAATAATATTTTCAGCATAATATAACAAG
GAATAAGATAATGTCTACAGCTCAATCAGATGCTCTTACTTTTGAATGTGAAACAGGTAA
TTATCATACTTTTTGTCCCATCAGTTGCGTTTCTTGGTTATATCAAAAAATTGAAGACAG
TTTTTTCTTAGTTATAGGGACTAAAACTTGTGGTTATTTCTTGCAAAATGCAATGGGAGT
AATGATTTTTGCTGAACCGAGATATGCCATGGCAGAATTAGAGGAAGGAGATATTTCAGC
TAAGCTAAATGATTATGGTGAACTCCGTAGATTATGCTTACAAATAAAAAAAGATAGAAA
TCCTAGTGTTATATTTTGGATTGGAACATGTACAACCGAAATTATAAAAATGGATCTGGA
AGGAATTGCTCCAAAATTGGAAGCAGAAATTCGTGTTCCTATAGTAGTTGCTAGAGCTAA
TGGTTTAGATTATGCCTTTACCCAAGGTGAAGATACAGTTCTAGCAGCTATGGCTCAAAG
GTGTCCATTAAATTTAAAGAATCAATCAGATAACACGAGTCTTAAAACTTCTCCTCATAT
TCCTTTAGTTTTATTTGGATCTCTTCCGGATCCAGTTGTAACCCAGTTAACTATGGAATT
AAAAAAACAAGGTATTTTTGTTTCTGGTTGGTTGCCATCAAAGAGGTATACTGAATTACC
GGTTATAAAAGAAGGATATTATGTTGCGGGAGTTAATCCATTTCTTAGTCGCACAGCTAC
TACACTAATGAGACGCCGAAAAACAAAACTAATTGGTGCACCATTTCCAATAGGTCCAGA
TGGTACTCGAGCCTGGATTGAAAAAATCTGTTCAGTAATGAATGTAAAGCCTATTGGATT
AGAAGATAGAGAAAAAGCAATTTGGGCTTCTTTAGAAGACTATATTTCTTTAATCCGAGG
TAAATCAGTTTTTTTTATGGGTGATAATTTACTAGAAGTATCTCTTGCAAGATTTTTAAC
TAGATGTGGAATGACTGTATATGAAATTGGTATTCCTTATATGGATAAACGCTATCAAGC
AGCAGAGCTAGCTTTATTAAAAGCTACATGTGATAAAATGAACGTTATGATGCCAACAAT
TGTAGAAAAACCAGATAATTATAATCAAGTAGACCGAATCCGTGATCTGAAACCCGATTT
AGTTATCACTGGTATGGCTCATGCAAACCCTTTAGAAGCCAGAGGTATTAATACAAAATG
GTCAGTAGAATTCACATTTGCACAAATTCATGGCTTTACCAATGCAAGAGATATTCTTGA
ACTCGTCACAAGACCATTACGGCGAAATCTTAGTTTGTCAGAATTAGGCTGGGATGTTTA
TAGCAAGCAAAGCTAGTATTCCTC----TTTATGACTTACATGGTATAAAAGCCTTGTAA
GTCTTTTATTTTATGTTAAAGAAGCATTTCTTACTCTGCCATCACCAGCCCAAGCTTGTA
AACTTTCTACTTGTTCTTTGTCTGTAAATGCTAAAGGCACAAACTGTTTGATAGCTATTT
TAATATCTTCAGTACTAAATTCTCTCTCTTCACTAAACGCCGTATGCATACTTTCAATAA
TTGCTTGCTCAATTTCAGCGCCAGAAAATTTATTACATAATAAACTCAATTCATGAATAT
CATATTCTTGCCACGATCTTGGTCTAACTTTTGATAAATGTATCTTAAAAATTGACTCTC
TCTCTTGACGATTAGGTAAATCTAAGAAAAAAATTTCATCGAACCTGCCTTTCCTTAACA

TTTCGGAAGGTAAACTTTGAATTTTATTAGCTGTTGCGACAACAAATACTGGAGCTTTTT
TCTCTGATAGCCAGGTAATAAATGTACCAAATACTCTGGCACTAGTACCACTATCACCTT
GACTATGTAAACCAGAAAAAGCTTTATCTATTTCATCAATCCATAATATGCAAGGCGATA
ATCCTTCAGAGATATTTATCATTTCTCTCATTTTTGATTCCGATTCACCAACTAGTCCAC
CAAACAGTTTTCCCATGTCAAGACGTAGCAGAGGCAGCATCCAATCGCTTGCAATAGCTT
TCGCGGTTAAGGATTTACCCGTCCCCTGTATACCAACTAGCAATAAACCTTTGGGCGAAG
GAATACCGTAATTTAAACTTTGCTTCGAAAATGAGCGAGACCTTTTTTGTAGCCATTGTT
TTAATACATCTAAGCCACCAATATCCCTATTTACTTTACTATAAGGATAAAATTCTAATA
GATGCGTTTGATTAATAATTTGTCTTTTTTCTTCAATAATAATAGGTAAGCTCCGAGAGT
CTATCTGATTATATTGTGCAATAATTTTAGTAATCACTTTGCGGATTCTATCTATCGATA
ATCCTTGGCAGGATTTTGTTATATTATTAACGAGCTCTGAATTCAAATTAAGATTCAAAG
CTTTACTCAATCTTATGATCTCTTTTTTGATTTCTAGAAGACTAGGTAAGGGCAAATCTA
TTACCGTTATGATATCATTCAGAGCAAAAGGTATATTGATTTTGCAAGAAATAATAATAA
TGTGCTTAGATTGCGTTTTGATTATTTTAGCTAAATTGCGAAGTTTCCTAATTAATACTA
TTTCGTTTAGGAAAGAGTCAAAATCTTTTAAAAGAAAAAGATTTAAAGATTCATTATTCA
AGTTTTCAATAAATTCTAATGCTAATAAAGGATTTCTTTTTGCATAACCATTATCGCTAG
GATTGTTAGTGTATCCATCTACAAAGTCCCAGGAATATACTTGTTGACTGTTTGAACAGT
TTAAGCTGTGTTTTATAATATACTCTAGTCTATCTTCTTCTCTAGTATTTATTACAATAA
TAGGATATCGAGATTTTAAAAGTAATCGTAAATCTTGAGTGAAATTCATGTCAATATTTG
TTTCCATCAGACTTTTGTACTTCACCAATTGAAGAAAGACTAAATAAGATTATCTAATAT
TCCTTGGATATATTCAAAAGTCATCTCTCTATTTTCTAAATTATTGCTATTACTAATTTA
ACTTACCATGATTTTTTTTCTTCCTTTTCTTCGTCGACTATTTAATATAGAGCGTCCACA
TGGTGTTTTCATTCTTGCTCTGAAACCAGAAACTCTAATTTTTTTTCTTTTAGATCCTTG
TAGTGTTCTTTTAGTCATGATGTTTCCTCTGTTGTAAAAGATTAAATACTATTTTATCAT
CTTTTATATATCTTTATGATATAGATATGTATTAATATCAAAGTTAACAGCAATAATTTA
TATTAATCAAGAATTATTTATATAATATTAAACATTAAATATTAACTTTATGATATTGGC
TTTGCCCATTTTTTATTTAAGTATTTTGACCATCTTTTTGCTAATCTTAAACTGGTTAAT
TTTTCAGCAGCTAAAAACGATTTTGTTATTAGAATCTCAATTTAAGTATTTTGTTGATAA
AAGCCAAAATCGTAAATTAGAACCCGAAGAAAGTTTTGCTTTTGCAAAAGTATGTGTAGC
TAAAAAATATTTTTCTAAAGCTATTATTGAAGGTCAATTAGCTTTAAAAAACTATCGAGA
CCTGAATATCTTAGATAATAATATAGTAATTGCTAATTTGTATAATATGCTAGGTTTTAT
TTATTTTGAAGCAGGCCAAACAAGTTTTGCAAAGAACTTTTATGAGCAAGCCTTACAAAT
AAACCCTAATTATGTTGTAGCTTTAAATAACCTGGCAAAAATTTACGAAGAGGTAAAAGA
TTTAAAAAAAGCCGAATCTCTATACGATAAAGTGTTAACTCTTAACTTAAATAATAAAAC
TGCTAATAGAAGGAAAGATTTTATAGCAAAAACTAAAAACATCTAATACTATTTGTAATC
GGGATAGCAGGATTTGAACCTGCGACATCCTGCTCCCAAAGCAGGCGCGCTACCAAACTG

CGCTATATCCCGTAAGACATAATTATTACTATATACCTTTTCTCTAATTATGTCTACTCT
TTCTGTATTTTATCTAGGATACCAAAACATGCCTTTAACTCCATCTGGATCTTTGATAAA
TCCTAATTTTTTATAAAAACTAACAACATCAGGTTCTGCGAATAGTGTAATAGTGCTAAT
TTCTGCCTGTCTCAACTGCTGTATTAATTGATGTATGATTACTTTTCCTAAACCTAAACC
TTGGAAATCTGGATGAATAACTACGTCCCAGATAGTTGCATTAAATCCGTTGTCTGATGT
TGCTCTAGCAAATCCTACAAGTTTACTATTTGCATCTTTTTTTTGTATTAAAGAAATAAT
AATAGAACTATTTTTTAATGCGATCTTTACTTTTTTTAGAGGTCTTTTAACCCATCCGAC
TGAATCACAAAGCTGCTCCAATTCGTATAAATTAATATTCTTATTGCTACTCAGATAGAT
ATCTTTAAATTCGATTTTATCGCAAGTTTTATCTAAAACAATAAGTTTTTTGAAATTTTT
TTCAGAGCTAGCATTGATATCGGAGTTTTGAAAAAAATTTTTCCAGAAGATCATAATGAT
TATGATATAATTAAATAAATATATATATTACAATATTGCTTTAACAAACAATATATTAAT
ATATGCTCAAATTTTTACATGAATCTATTAAGATAGAAGATTGTTAAAATATAGAGAAAT
AAAAATTTTTGTAAAATCTTTCAATAAAATGATATAAAGAGTGCTTTTTAAGACTAACAT
CTATTATTATAAATAAAGTCTCGAAATTGAGATGAAATAACACATTATTCTATGTCTAAT
GCCTTTTAAAGGAGTTATTTGTGAAAAAATCTATGTGTCTAACCTGTTTACTTGCTTTAC
TAATTATGAGCAACCCAATAATAGCTAACGCAGAAGTAGCTGGATTGGTGCCCTGCAAAG
ATTCTGCGGCATTCAATAAGCGTATGGTAAATAGTGTGAAAAAACTTCAAGCCAGATTAG
CTAAATATGATGCAGACACGCCACCAGCGTTAGCTTTAAATAAGCAAATAGAGAAAACTA
AAACTAGATTTGCAACTTATGGTCGAGCAGGTTTGCTGTGTGGTACTGATGGATTGCCAC
ATTTAATTTCTGATGGTCGATGGAGTAGAGCCGGGGACTTTGTTTTTCCAGGACTATTAT
TCTTGTATATTACAGGATGGATTGGCTGGGTAGGTAGAGGATATCTTTTATCTGTTGCTA
AGACTAGTAAGCCAACAGAAAAGGAAATCATTTTAGATGTGCCATTAGCTATCAAATTTA
TGTCATCCGGATTTGCATGGCCGCTAGCGGCTTGGCAAGAATTTAGTAGTGGACAATTAA
TTGCTTCCAATGACGATATTACTGTTTCACCCCGTTAGTAAAAAATTTATATGAATAATA
ATTTTACCAAATACTTATCAACAGCACCTGTAATTGGTGTATTGTGGATGACGTTTACAG
CCGGATTTATTATAGAATTGAACCGCTTTTTCCCAGATGTCTTATACTTTTACTTATAAA
TAAGATCGTATAAAAAACTACATAAATATAGAGAAATACTATTATTTGTGTAGTTTTTAA
TATATAACATAATTATTTATTTTTAAAAATGTTTATCTTTTCGATTATTTTAATATTAGA
TCTTACATAATAAAATAATATCAATATTATGAGCTTAGTAACCCAAATTATAGTTAATGC
CGATGATGAATTAAGATATCCTACAATTGGAGAATTGCAGTCAATTCAAGACTACTTAAC
TACAGGAAGCAATAGAATTAGAATTGCTACTATTATTAGAGATAAAGAAAAGGAGATTAT
ACAGAAAGCTAGTAAGCAAATTTTTCAGTTACATCCAGAATATATAGCTCCAGGTGGTAA
TGCAGCAGGTTCTAGGAAAAGATCGTTATGCTTACGTGATTATGGTTGGTATCTCAGACT
AATTACATACGGAGTATTAGCTGGGGATAAAGATTCTATAGAAACAATTGGTATTATAGG
AGTAAGAGAAATGTACAATTCTTTAGGTGTACCTATAATTGGAATGTTAGATGCAATCCA
GTGTTTAAAGGAAGCATCTTTAGAAATGCTTGGTCAAGATGATATTAGAATCATTTCTCC

TTATTTTGATTATATAATTCGTGGAATGTCATAAATCATAGCTTCAATTAATTAGTTGAA
TAAAAGCTTTTATAATGTTATAATTACAATAAGCCCGAAAGCATATAATTGTAAAAACTA
AATCTTGTCAGAACCGGAAGGTAGCAGCAATAATGTTTCAATACAAAAGTTATGATTTTC
GGGTGTTTATTGTTTTTATCTAATAGTCTCGACTTTTAATTAGTATGCTATGTATAAATC
TGTATATGACATGTAATCCATCAAACCTTCCAGCAAAAATAATTTAATAATTTGATATTA
ATACGTAAAACACCTATACTCTTTTATAAAATTTCTTTATATCTCATCTAAACAATGGAA
ACTTAACTTAATGGGTGTTCATATTTTATCAACAGGTTCGTCTGTCCCAAATTTTTCTGT
AGAGAATCAACAATTTGAAGATATGATCGAAACTTCCGACCATTGGATTTCAACAAGAAC
AGGAATAAAAAAAAGACATCTAGCTCCATCTTCTACTTCTTTAACTAAATTAGCGGCAGA
AGCTGCAAACAAAGCCTTATATGCAGCTAACTTAAAACCTACTGAGATCAGTTTAATTAT
TTTAGCTACGTCTACGCCTGATGATTTATTTGGTAGTGCTAGCCAGTTGCAAGCAGAAAT
AGGTGCAACAACATCGGTAGCTTTTGATATTACGGCTGCCTGCTCCGGTTTTATTGTTGC
TTTAGTAACAGCAGCTCAGTTCATTCAAACTGGTTCCTATGACAATATTTTAGTTGTTGG
AGCAGACACAATGTCTAGATGGATTAATTGGTCAGATAGAACTACCTGTATTTTATTTGG
TGATGGTGCTGGAGCAGTAGTATTGGGGCAAAGCCTCAAAAATAGTATTTTAGGCTTTAA
GTTATGTACAGATGGTCAGCTAAACAGTCATTTACAATTAATGAATAAACCTGTAAATAA
TCAAAAATTTGGTGTTACAGAAATTCCTCATGGAAACTATAATTCCATAACAATGAATGG
CAAGGAAGTGTACAAGTTCGCTGTATTTCAAGTTCCAACAGTAATTAGACAATGTTTGAA
TAATTTAAACATTTCAATAGATGAAGTTGATTGGTTTATATTGCATCAAGCAAACACTAG
AATCATAGAAGCAATTGCGAGCAGATTATCAGTACCTTTTTCTAAAATGATTACGAACTT
AGAGCATTATGGAAATACATCTGCAGCGTCAATCCCTTTAGCGTTAGATGAAGCTATTCA
ATCCAATAAAATTCAACCAGGCCAAATTATTGTTTTATCTGGTTTTGGAGCAGGCTTGAC
TTGGGGAGCAATTGTCTTGAAGTGGTGATTTATATATTGCGGATGACGAGACTCGAACTC
GTAAAGCTTTCGCTACACACCCCTCAAGCGTGCGTGTATACCAATTTCACCACATCCGCA
TTTTTTACCATGAATATTAAAAATTTATAATATTTATCTAAATATTCATATAATGTGTAT
ATATATATACATTCTTGTCAGAAACTCTCTTATATTTTATTTTAAGTGATAAAATTGAGT
TTAGTATAGATAGTAAAATTTTATTTTAAAATCATAAAAATAAATATATAAGGAAAATAA
AATATGACACCATCTTTATCAAGTTTTTTGAATAGTCTTATTCTTGGGGCAGTAATTGTA
GTCGTTCCCATAACTTTGGCTCTTTTATTTGTTAGTCAAAAAGACAGGACAATCCGGTCC
TAAAAATAACTTAGAAAGATATGGAGAAGCAATTTAAAATGAATAATAAAATATTTTTCA
TACTAATAAGCTCTCCATATATTGCTATACTTTAGAAGAATTTTTATTTTTTTATTTTGT
AACATCCTGTAAAGTTTATTCCGTTCTTTCTTTTTCCCATTTACTCTCTTATAATTAATG
ATACAAATGTCAGGTTAATAATAGTTTTTTTATTCTGTATCTTTCATTTTATATACGTAT
TATTATGTTCAATTAATAAAGAAAAGCTCATGTCTTTGTCTAATTGGCCTCTCAAAAAAG
AAAATTCTGAAGCATATAATATTAAGAATTCAAAACAAATCACAATTCCTGATGGTTTAT
GGATAAAATGCTTCGACTGTGGTCTATTAATGTATTCTAAAGTACTGAAGAGAAATTTAA

AAGTTTGCCCTCAATGTAGTTATCATTTTCAAGCTTCTAGTAACGAAAGAATTGATCAAT
TGATAGACCAAGGTAGTTGGCAACCAATGGATGTTCACTTGATCTCTACAGATCCATTAG
GCTTTAAAGACCAAAAGCTTTATAGTCAAAGGTTAAAAGATACTGCTTTCAAGACTGGCC
TGCAAGACGCAGTTCAAACAGGTACTGGGACTATGCAAGGTAAAAAAGTATGCTTAGGTA
TTATGGATTTTAGATTCATGGGGGGAAGCATGGGATCTGTTGTAGGCGAAAAACTAACAA
GACTGCTAGAAAAAGCAACTCAAGAAAAGTTGCCTGCAATTATACTTTGTGCATCAGGCG
GAGCTAGAATGCAAGAAGGTATGTTGAGCTTAATGCAAATGGCAAAAATTTCTTCTGCTC
TAGAAATGCATAAAAAAGAAAATCTACTATATATATCTGTTTTAACCTCTCCCACAACAG
GGGGTGTAACAGCTAGTTTTGCTATGCTTGGAGACTTAATTATTGCAGAGCCAAAAGCTC
TTATTGCATTTGCTGGTAGACGAGTTATAGAACAAACAATCAAAGAAGACTTGCCAGATA
ATTTTCAAAGTTCAGAATATTTATTTGAACATGGTTTCCTAGATTTAATTGTATCCAGAA
CTCAGCTTAGATCAAAGTTAATACAAATTTTGTCCTTACATAATCATAGTAAGTAATGAA
TTGAATATTGACTATAATATCAAAATGCTAGTAAAAATTAAGATATTAAGAAATTTTCAC
TAAAGATATTTGTAGACTAACTAGAATTACAATTACTATTGTAGAAAAATTTGTCTTAAT
GCCATTTTTTCAGTCTTTTAACAAATTGTTTTTATGACTTTAAATAATTGTTAGTTTAAT
ACAAATTTATAAAGAGAATACATACTAAGCTAAATATAGTAATATTATGTTTTCCTATAA
TACTGACCTATCAAATTTCGAGGAAATTTATGCTTAAAAGATCTTCTTGGCTTGCGGCTT
TATTGGGACTATTAACAGTAGTTTCTACAAGTACGCATACATATGCCATAGAGTTAGACG
AGGCAACAAGAACTGTTCCATTAGAATCTTCTGGCAGAACTGTAATTCTTACACCAGAAC
AAGTTAAAAGAGGCAAGCGATTATTTAATAATTCTTGTGCTATTTGCCATAATGGTGGTA
TCACGAAAACAAATCCGAATATTGGACTTGACCCAGAATCTTTAGGATTAGCTACGCCAC
AAAGAGATACCATTGAAGGACTAGTTGACTATATGAAAGATCCGACTAGTTATGATGGTG
CAGAGTCAATCGCAGAATTGCATCCAAGTATTAAAAGTGCTGAAATTTTTCCTAAAATGC
GCAATCTAACCGATGAAGACCTATTTACAATCGCAGGTCATATCTTACTTCAACCTAAAA
TTGTTTCTGAAAAGTGGGGCGGAGGAAAAATTTACTATTAGAACTTCAAAGACCTAAACC
TTGTGAATACTAGTTTATGTATTTGTTGCTTAAGTGTGATTATTTTGTTAGATCATGATA
TATATTAAAGATGGAGTTTGTTGACAGCAAGTTACTTTTTACCCGAACTTGTTTACACTA
TTCGTCTAGTTATTAAAAAGGAGACGTTAAATTGAAGAAGAAGCTTTCAGTTCTTTTCAC
TGTTTTTAGTTTTTTTGTAATAGGTTTCGCACAAATTGCTTTTGCTGCAGATCTAGATAA
TGGAGAAAAAGTTTTTTCTGCTAATTGTGCAGCATGTCATGCTGGCGGTAATAACGCCAT
TATGCCAGATAAAACCTTAAAAAAAGATGTACTTGAAGCTAATAGTATGAATACTATTGA
TGCTATTACTTATCAAGTACAAAATGGTAAAAATGCCATGCCTGCTTTCGGAGGTAGACT
GGTTGATGAAGATATTGAAGATGCAGCAAATTATGTATTATCTCAATCTGAAAAAGGTTG
GTAATTATACTTGATTTTATCCTGTATTAAAGAATAGACAATCTATTTAGTTGTTCATTA
GATTGTCTATTCTTTGTTTATGCTATTATATAAAGATATTTACACAATATTTTATGATGA
AAAGAATACCCGCAATTCTTGTACTAGAAGACGGTGCGTATTATAAAGGATGGTCATTCC

AGCAAGATAAACAAGAGATTACTATTGGTGAAGTAGTTTTTAATACTGGAATGACAGGAT
ATCAAGAAATAATCACAGATCCTAGTTACTTCCATCAAATTGTCGCTTTTACCTACCCGG
AAATTGGGAATACAGGTATTAATAATCAAGATATTGAATCTCACAGTATTAGTATTAAAG
GACTTATTGCAAAAAATATTTGTAAAATTTCAAGCAGCTGGAGAGAGCAGCAATCTTTAG
TTACGTATTTAAGTAGTAATAATATTCCTTTTATTTTCGGAATAGATACAAGGTCTTTAA
CCCAATACTTGCGTCAATTTGGTACAATGAACGGTTGTATCTCTACTGATAATTTAAATC
ATAGTTACTTAAAACAGAAAATTTGTGAGATTCCAAGTATGCAAGGTTTAGATTTAATCC
CGCATGTAACTACAAGAAATGTTTACCCCTGGGATGAAAAAAGTTTTCCAAATTGGTATT
TGACAGATAACATTAGAGTGCACCGAGTTATTCAGTTGAAAGTTATTGTTATAGATTTTG
GAGTTAAACTAAATATACTCAGAAGACTAGCTACACTTGGATGTCAGATAACGGTTGTGC
CTGCCCACACTCCTTTAAAAGATATTTTGGCTTACCAGCCTGATGGTATATTACTCTCTA
ATGGTCCAGGAGATCCATCAGCAGTACATTACGGCATCCAGACAGTTACAAAATTACTAG
ATTACAATGTGCCTATATTTGGGATTTGTATGGGGCATCAAATTTTAAATTTAGCTCTTA
AAGCTAAAACTTTCAAACTTAAATTTGGTCATAGAGGTATTAACCATCCATCCGGATTGA
ACCAGCAAGTTGAAATAACTAGTCAAAATCATGGCTTTGCAGTTGAATTGACTTCGGTTT
TTGAATCTCCTGTAAGAGTGACTCATTTTAATCTAAATGACACTACTATTGCAGGAACTG
GACATAATCAAAGTCCTTATTTTTCTGTGCAATATCATCCAGAATCGAGCCCAGGCCCTC
ATGATGCTGATTATCTATTCGAAAATTTTATAGAAATAATGACAAAGTCCAAGAATAAAG
TTAGTTAGTAATTTTCCCATGCTTTATGAGTGAATAAAGCGGATAGTACTTGTACTCCTC
GTAATTGATTAAAGAGAGGCAAATGTCCTCTGGGGCCATGAGAATTCCATTGAAATTCCT
CAGGATATCTGCAAGGAATTCTATCAACTTCCCAGCCGATTTTTTGCCAGAATTTTCCCC
AATCTTTTCCAACACTAAGCCATATTTGTCTTTGAACAAATAGCCCAAATTTACCTTTTG
AGTGTGTATGCCATAACTTATCAATAGTTTGTAAATCTTGAGCAGGTATTTTTTTTATAT
CTGTAAAATATAGCCAATTACGAGTTTGTGCATTTACACCAGCTAGTTGAATAAGCTTTT
GTTGAGTTAGCTGATCTGCTTTGAGCAAATCCCGATGAGTCAGTAACATTTGTAAATCTT
TGTAATTCATCTGTTGAGCAGAGCGCAATGGAACAATGCCATCGGGACATAAGTTAGAAG
CAAATTTCACTATTTCTTGATTTTTACTGTTAAGTAACTTTTCATAAATTAAACCGTCAA
CACAATTACTTTTATAGTTAGGCCCAGTAATCCTCTCAAAAAATAGATCTGCTAAATCTT
TTAACTCGATAGAGTCTTTGCTATTTATATTCTCAATAATTTCAAGTTGTTGCTTGACAT
TATTAGACTTAGTATTCTTATTCAATTCTAAAAGCTGGGCTCGGATTTGATTTGGCATCT
GATTTGTTTAATTAGTTTTAATAGTCATAAGGTCTTAGAGAGAATTAGATTGTTTTTTGA
AATTAGAAGAGCGCCGCAGTATTTCTAAAGAAGTTCGCATAATTTGATTCAATAAGTTTT
GTCCTGCATAAATTATAATTTGACCAATTAAAAAAATTGTGCTTTTTATTTTAGGAACAA
AAAAATCTTGTACCTCAAGCAGAAAAGTAATTATTATTTGCATTGGTGACAACATCTGTA
ATTCATTCGATCTGCAAGCATATATATACTGACAGTTTAAGCCTTCTTGATAAAATCCCC
ATACTTTGTAACGACTTTCGTAAATTGCTCGTGGTCGCTCAAGATAGTTTTTAATTATAG

TATTCCAAATTAGGTTATTCTTTAATTTTTCTAATTTTCTATCAGATAGAAATTTTACAT
TACAGAGATAACTTGCATTTGTCTCATTTACAGTATTAAAATTTTGAACTAACATATGAG
CAATAATGTTGCTTAGCTGAATTAAATAATTTTCAAGAAAAATTTCGACCTGCTTAACTG
GCATATTGTAAGTAAAGAGATTAAAAGTTTTATTCACAGTTTGAGATGACCCAAAAAGTA
GCTGAATAATTAAAATTTGTAGTAGCATCTTATAGTCGTTCAGTAGATAGTTCAAATCAT
GAATATCTTGGTAAATGTCAATACAGTCCAGTTCATATAACTTACAAAATCTTTTGATAC
ATCTATGGAATAAATCAATTAAAATATTTTCGGTCAAGTTTGTAATATCATTTACATCTA
AATTTGAATTATAAATTTCTAAAAGTATCTTTTCCAGTTCAGCTAATATGATTTTTAATA
AGTTTCTTTTTACTTCAGTGCGAAAAACATCTAAAATTAACACTTCCTGAGAGCAATTAG
TTAATCTCTTATTAATCTTTATAGAAGTTCTAACTAATAGCTCTGCTACCTCTTGATTGA
GTGTTGGTCCCTGAGAGCTTGGCCAATAATTATTCACGTTATACCATAGTAAGAAATATT
GAATTTAATGTTATACGATAACTTTTTAATATACTGAAAAGCAAGTTAATTACTCTATAG
CAACTAGTTTTTACAGATCTTAATAGTTATTTATCAAATAAAGTTATATGATAGTAGCGA
TTGAGCTAATTTTATTAAAATTTATAATAATGACAACTTATTACTTCGCCCTTGCGAGTC
AAAATTTTCTATTAGTACAAGAACCACTAGAAGAAGTTTTTAGAGAGAGAGTTAACTACT
ATCAGTCAAATAATAAAGCAATTGATTTTTGGCTAATACCAAACCCTTCTTTTCTAGAGA
AACCAGAAATGATTTCATTTAAAAACCTTGTACCTAAAGACGCTGTAGCTATAATCTCTA
CTAATCCAATATTTATTAATTGGTTAAAGCTAAGAATCGGCTATATCTGTATTGGGCAAT
TTGAAGATAACCTACAACTTTCTGAAGAATCGTTAAATATTACTGTTTTAACAGACAAAA
TTTAACAATGCTTTTATTATGACTAGTGTTAAATTATTTTGGTTAAGTTATAATTATAGT
CTTTTAGTATTTAGACTCTTATAGTTTATCACTTAGTATTTTATGCTGAGATGGAGTGAC
AAATAGTGTAAGTATTTCTTGGCTAAACGTTTCAGTTGCCTTAGATTTATATCTATTTGG
ATTAACTATAATGGATAGCATTCGTTTTATAGTAACATTTTCAATTTGAGCCCAATGTAC
AATTCCAAGTTCCAATTCCTTAGCAATAGCTGAAACGGAAACAAAGGCAGCTCCTAGCCC
TGATTGCACAGCGTTTTTGATAGCTTCTATAGAATTCAATTCCATCTCTATTTTAAAGCG
ACTGCTGTCAATACCATGTTGACTGAGTACTTTATCTATGACTTTTCTAATTGTTGATTG
AGTATCTAACGCAATAAATCTAAGCCTGTATAAGTCTTCTTTTTGAATGTCTCCTAGTTT
AGAAAAAGGATGTGATTTGGGTAATATAAGTGCTAATTCGTCTTCCGCATAAGAAGTAAC
CTGCAAAACATCTTGCAATTCAGTAGGCACTTCTCCTCCAATAATTGCTAAATCAACTTG
ACCATTAGCTACGCTCCATGAAATAAGTCTAGTTGAATGTACTTGCAATTGAACAGCCAC
TTGTGGATATCTTTGCCTAAATAGTCCGATTAATCTTGGCATCAAATATGTCCCAGTTGT
CTGGCTAGCTCCAATAATTAATGTGCCACCTTGTAAGTTTTGTAAGTCGTCAAGAGCTCG
ACAAGTTTCTTCGCAGAGAGCTAAAATTCTGCCCCCATATCGTAAAAGAAGACTTCCTGC
CTCAGTTAAAGTTGCCTTCTTATTACCTCTTTCAAATAGGGAAACATTCAATTGGCGCTC
TAAATTTTGAATTTGCAAACTAATAGCTGGCTGAGAAACATACAAGCTATTAGCTGCTTT
TTTAAAACTGCCCTCTTTGGCAATTGCTTTTAATATTCTTAACTGATCCAATGTAAATGG

AAGGTCTGTCATTAAAGAATTGTAGTATAATGTATATTATTTAGTATTGATCAATGTATG
CATTTGCCAACTAAAATATTTGTCATTACGCTTCATTATTTTCAAAATAATAAAAAGTAT
AATGTTAAATAAGCTGTATTAAAATTTAGTATCTGTTTGATATTAGATTTATACAAGAGA
TATCGTGAATTTAATTCTAACTATAAAATATAGGAGACAATATGGACTCTAGACTTTTAG
TTGTACTAATACCAGTTTTAGCAGCAGCATCTTGGGCAGTTTACAATATTGGTAGAGTTG
CACTACAGCAATTTAGAAAAATGACATCTTAGTTTTGCTTAGAATATAATTTTTAGACTT
TATGAGATAGAGGACTTTAAAATCCCTATCTCTATTGTATATCTTAATAAAAAAAGAAAG
CCCCCTTGTACTTACTGTTATTACACATTAATATTATTGGTGGTACCTAGATAATAAGTT
TAAGCTAACTTATTTTTTATTTTATAATTAATTAAATTAATAACACAATTATGGCTGTTC
CAAAGAAAAGAACATCTAAAGCTAAAAAAAATGCACGCAAAGCAAATTGGAAAAATCAAG
CAAAAACTGAAGCTCAAAGAGCTTTGTCTTTAGCAAAATCAGTATTAACTAGAAAATCCA
ATGGATTTATTTATAATCTAACTGAAGCATCAGATACTTTTAGCGATTAGAAGTGTTTTT
ACTACAAGTCTGAAAAAGTATTACGCAACAAGTTCTCAGTAATTTTTATCTAGTAATCAT
TTGAAAATCATTCTATTTGATAATAAAATAAATAGCAGATTAAATAAAATCACATTAACA
AATTATGCAGCAAAGCTAGATCAAACTAGTGAAATTTGGTTGTTCAATTGTATTGAAAAT
ATTCAGCATATTTTTTTTAAAAGTCAATTAAAATCAAGTCATATTACTAAAATTTTTATT
TCTGGTACTAGCTTTGAATATACCGCAGGTCTACCAGGATTATTATCCAGCTTAACACTA
AGTGGTAGACTACATCCTATTAGTATATATAGTCCCCAGTCTCTCAAAAAGTATCTTGAA
GCATGTACCAAATATTCCCAAACTAATTTTTCTTTTCCTATTAATTTTCATAATTTACAA
TACGGAGGACAAGTTGTTAACCAATTTTATACAGTAATTTGTTTACCGTTGAGCAAAAAG
AGCCTGCTGTATGGATTTATTATTCTAAAAAAAGAAAAGCAGGGAGTATTTAATTTAGCA
CAAGCTAAAACCTTGAATATTCTTCAAGGACCTATATATGGAAAACTCAAAGAAAAGGAT
AATTTTTTAAGTCCAGATGGTTACTATCTATCCGGCCAAGACTTCTCTTCTAATACAATA
ATGGGACATAAAATATCGCTTCCGTTATTAGTCAGATATTCTAGAATTATTTCTGAGATG
CATTGGTTTTGCTCTTATCCTATTAGATTAAATACTTATTCGCATCAGCAAGGAGCAAAG
TGTTTACCACATAATGTTTTAACTGATATTATGAAATCCCAAATATATCAAGATAACAGT
TTTGTTGAATAATACTTATTCTTGATATATAATGGTCTCGGTAATCTAAATAAAATACTA
GAAATAATTTCTTAAATTTATTTGTCTATATTAAATTTACAATCATTTATGACATACGCA
ATTATTGAAGCAAGTGGCACGCAGCTTTGGATAGAAGAAGGCCGCTACTATGATTTAAAT
CATATACCTGTTGATCCAGGTCAGTCGATTATATTAGGAAAAGTCTTATTATTGAATAAA
AATGGGGAGGTTACTTTAGGCCGCCCTTGTATAGAAGGGGTTACGATAAAGGCTACAGTA
ATGAGGCACTTGCGAGGAAAGAAGATAACTGTTTTCAAAATGAAACCAAAGAAGAAAATG
AGATTAAAAAAAGGTCATCGACAAGAATTGACTCGTTTAATGATCGATTCTATAACATCT
TAGATAAAATCTAGACTCATATTTTTAAAAACTTTTTAATATTTAACTAAAATAGATATA
ATGGCACATAAAAAAGGTAGTGGTAGTACAAGAAATGGCAGAGACTCTAATTCCAAGCGT
TTAGGTGTTAAAAAATATGGTGGAGAGCAAGTAACAGCAGGTAATATTTTAATCAGACAA

CGGGGAACTAAAGTTAAGCCTGGCCAAAATGTTGGAAAAGGAAAAGATGATACATTGTTT
TCTCTAATTGATGGTTTCGTGCTGTTTGAAAAGTCAAATCAAAAGCAAAAAACAATTAGT
GTTTATTCTGCTAAGAAATAGTTAAAACAATTGGTGCAAGATAACTTGAGTGTATTTATT
TCACTTACAGTGGCACCATATATTTGATTTATTAAGAAATGGAGTTCCTTAATGAACCAA
TGAATTTTATTAAAGATTTGCTGTGAAATTTAATGACAAACACTATTGTAATTTCCTGTC
TACACAATATGGCTGCTATTCTATATTGTGGTCAGATACAGAAACTAGTAGTAGCAAATG
CTCATTATCAAGTAAGTGATATCTACCTAGGTTGTGTTGATAAAATCTTCTCAGGAATAA
ATGCGGCATTTATTAACCTAGGAAAGAATGAGTACAGTGGTTTTATACATATCAGTGATA
CCGGTCCGCTTAAAAAGAAATATTATGTCAATAATATTACTAACATTTTAACAATACGGC
AAAAAATTTTAGTACAAATTATTAAAGAGCCAACTTTAAATAAAGGTCCAAGGCTCACTG
CCAATATTACATTATCAGGTCGATATATTGTATTAATGCCTTTTAGTCAATCAATCTGTA
TATCTCGAAAAATATATGATGAAGATGAGCGTCATTATTTGAAGTCTTTAGCTATTTTAA
TTAAACCGGCAACAATGGGCTTGCTATTTAGACCTTCTGCTGTAGGTGTCGATGAGGAAA
TAATATTAAGCGAATTAAAAAATCTAAAAGAACAATGGAACTTTGTTCAAAAATCTGCAA
TTAATAGTTATTCACCTGTTCTTCTATATAAAGATGAAGATATTGTTAAAAAGGTAATCC
GAGATTTTTATAATAATAATACAAACAATATAGTAATTGATTCAAACCTGGGATTAAAAC
AATTAAATTATTATATCCACACTTGGCACTGTAATAACTCTAGCACAGTTCCTAAGATTA
AGCTTTATAGTAATAATCAATGTATACTAGATGCTTTTGGTATCAATCAGGCAATTTCCA
GAGCTCTCATTCCAAAAGTTGATCTTATACTTGGTGGCTATATGTTTATTGAAACTTTAG
AAGCTTTTACTATTATTGACGTTAATTCTGGATCTTTTAATAATTCCACTAGTGCACGAG
AAACAGTTTTAAAAACCAACTGTTCTGCAGCAACAGAAATAGCTTATCAGTTACAAATTA
GAAATATTACTGGTGTAATTATAATCGACTTCATTGATATGGAATCACAAAGAGATCAAT
TGCAATTATTAGAACACTTTAATAAAGAGCTATCACTTGATGATGCTAAACCACAAATTG
TACAGTTATCTGAATTAGGTTTAGTTGAATTGACTAGAAGAAGACAGGGCAAAAGTTTGT
ACGAGTTAATTAGTAGTGATTCTAATTACTTTTATTTTTTCACACAATCAGAGAGATCTC
AGTCTCTTAAGAGATTCGATGATAGACAGCAGAAACAACAGATTTTTAATAAATCTTGGC
TATCTGCAGAGATTAATACTATTAACAAGGTCTTTTTTCAAAAGTCAAATTTGTGCAGAC
CTGCTAACTTTTACCTAATTCGTAATCTCTATATAGTTAAAAGTAGTATTACTTATAAAC
AAAATTATTTATTAACTCACAGATCAAAACTTATTTATTCTAAAGAATACAGCAAAGTAT
TACCAAGCAGTTATTATCTAGCTAGTCTCAATAAGAATAGTAATCAAGAGTTCCTACCTT
AAGATTGTTATATCAACTGTTGAAAAAAAGAAGCTCTCTCTTATATAAGAGAGAGCTTCT
TTTGTAGCTTACTAACTATCTAGTTAGTTAATTAAAAATTAACCGTTAACAGCTGGAGCT
GTTAGAGCTACTGGTAAAGATTCACCAGAAGCTAGATCTAGAGGGAAGTTGTGAGCGTTA
CGTTCGTGCATTACTTCCATACCTAGGTTAGCACGGTTGATGATATCAGCCCATGTGTTA
ATTACACGACCTTGGCTATCAACAACAGATTGGTTAAAGTTAAAACCATTCAAGTTGAAT
GCCATTGTGCTTACAGATAAAGCTGTTAGCCAGATACCAACTACAGGCCATAGACCTAAG

AAGAAATGTAGAGAACGAGAGTTGTTGAAACTAGCATATTGGAAGATTAAACGACCGAAG
TAGCCATGAGCTGCAACGATGTTATAAGTTTCTTCTTCTTGTCCGAATTTGTAACCATAG
TTAGCAGATTCGTTTTCGCTTGTTTCACGAATTAAGCTAGATGTAACTAGGGATCCGTGC
ATAGCACTGAACAGAGAACCACCAAACACACCAGCAACACCTAGTTGGTGGAATGGGTGC
ATTAAAATGTTGTGCTCAGCTTGGAATACAAGCATGAAGTTAAATGTTCCGGAGATACCT
AGAGGCATTCCATCAGAGAAACTACCTTGGCCAATTGGGTATACTAGGAATACTGCTGCT
GCTGCTGCTACTGGAGCAGTAAAAGCAACGGAAATCCATGGACGCATACCTAGGCGGTAG
CTTAGTTCCCACTCACGACCAATGTAGCAAGCTACGCCAGTTAGGAAATGAAGAACAACT
AATTGGTAAGGACCACCGTTGTATAGCCATTCGTCTAAAGAAGCAGCTTCCCAGATTGGG
TAGAAGTGAATACCGATAGCTGCAGAACTTGGAATAACAGCACCAGAAATGATGTTGTTT
CCGTATAGAAGGGAACCAGCAACTGGCTCACGAATTCCATCAATGTCTACTGGAGGTGCA
GCTACGAATGCAATGATGAATACAGATGTGGCAGTTAATAGAGTTGGAATCATTAATACA
CCAAACCAACCAATGTATAGGCGGTTTTCAGTACTAGTAATCCAAGAGCAGAAACGTTCC
CACAAGCTAGCGCTTTCGCGTCTTTGTAAAGTAGCAGTCATAATTTTTTATCAATTTTTA
AGGATTTAACAAAGATGCTTCCCAAACAATAAGTTGTCTGTTATGGAAATTATTACTTAA
GGCCTTTTCAAATGAGAACATTTTTAAAAAAAACATAGCAAAATCTAATCTCAGTAACTA
AAATTTTACTTGTTCATATATTGCTTAATAATTCAATAATAAATTTTTGCTTTTTGCAAA
ATTAACACTATTTTAGGGATATAATAATAATGCATATTAATGCTGCTTATTTTGATTTTA
ATAGTTTATATAATTTTGCCCTACTTAAATACGCATGTCACACTTTACAAAAATTCAGAC
GACCATACAAGATTTAAATCTGTTAAAGCATGCTTTAACAGATTTGGGTTTGATTTGGCA
AATGAATTCTAGTCATATAAAAGTTGGTGAAAATAATCAGCACAAGGTAGATATTTTAAT
TAAACAAGATAATTTGTCACATATTGGCTTTACTTGGAATGATAATAGATATCACTTAGT
TGCCGACTTACAACTTTGGGAACAACCCTGGTCTCTAGAAGTATTTTTGGATAAGTTATC
TCAGAAGTATGCTTACTACTCTATCATCGAAGAAACAAAGAAGCAAGGCTTTGAAAAAAT
GCAGCAGATTTCACAAAAAGATGGATCAATTAAGTTAATTGTGCAACGCTGGAATTATTA
ACTAACATACAAATGCGGGCGGAGAGACTTGAACTCTCACGAGATTATCTCACTAGAACC
TAAATCTAGCGCGTCTACCAATTCCACCACGCCCGCACTGTTGAGACAATATTACTCTAT
CATAATCTATACTAAAACACAAACTAATAGTATAAACTAGCTTCCAAGCTTAAAAGCGTC
TACAAATAGACCGTATGTTATACCAATTTTGAAGTTGTTGAATAAATTAATTTTTATATT
TAATTGCTTGTGGCTAGAGTAGACTATTTTACTAGTCAACTCAGTTGCTGCAACTATTAG
ACTGGCTGCAATAATACCCCAATCTCCTGTTTGTCCAGGAATCGTAGACAAACCTGTTGA
AATAAAAAAATCCTAGCAATAAACTAATTAAGCCAGTTGTTAACTCGCTCAAAGAATAATA
AAGCTTATTATTTAAGTTTTTTATTAAGTAAGCAAAAAAAGTTGAAAGTCTAGTTTTAAT
CATAGTATTTAGTAACGGAGTTTTAAAAGGATGACAAGTATTAACAGTCATTAATATTGT
CGTATCCTTTTAAAACTCGTTAATACAAACAGCTGTTCTTGTTTTTAAAGGTCTTGTTCG
CTTAAAGCATTAATAAGATGCTGAAACGGCTCTTGTATAATATCTGCGCGTTTAATTGAT

TGCAAATCTAATTCTTCTTCTATGATTTCTTGTAATAACTGTATGCTTCTTACTGTTGGA
GCAACAGGTACAGATAGTGAATTGTAAGTATCTTTTAGCCCATTTAATACACGCTGATCG
AGAATATTTGTACTACCAGAAACCAAGGCATAACTAGCATACCGTAGGTAATATTCTATA
TCCCTTAGACAAGCTGCATATCTTCTTGTTGTATAAGAATTTCCTCCAGGCCTTAATAAT
TCAGGCTGTTCTTCGTACAACTGAGCTGCGGCCTCTTTTAAAATATTGGTAGCTTGATCA
TTAATAATTTCAGCTATTTTTATTCGATCTAGTCCACTCGAAAAGAAAGATTCTAGTTGT
CCGACAGCTGTCTTATCTAGATAACGACCAGTAAGATCGTAACGATTTAATATTGCTGTT
ATAGCATCTTGCATAAATTAATTCTCCTACTTGCTATCAGAGCGGTATTATAAATGACTA
ATATTGTAATATAATCATTAGTGTTTATGAAATTTATTTAAAACTTATTCTTTATTGCTT
AATGTGCAATTAAAAACTCGCATAGATTTTACACATATAGAGTATACGTAGTTTTTAATT
ATTTTTTTATAAAAACCAAGAAATAAAGCTTTTTTTAAGAGGTTCGTGAATTTGTTAATA
TAACATGAGCTTTCCTCCTAAATAAGTTTTGTAAAGTACAAAGTAAAAAAACGATTAGTC
AATAATAGTAAACAATATTAATATGTATCAAAGTATAAATAAGCTTTTACTCCAGCATCA
AAAAAAATATTTGAGATATGTTGATACAACTGATTATTCAAGTGAAAACTTAGATTTAGA
CCAATTTAGCTATTTTATCATTACAATTAATAAAACTGATAAATGTATTCTTATAGAGCA
ATTTTGTTATAATGCTGAAGGCCATATTTACTCAATATTCTTCAAAGGTCCAACAGCTAA
ACATCTTTCTTCTATAATTTGTCATTTTCAACAATTAAATACAATAATATCTACAGCTCA
TGCCATATATTTAGGACGAGAGCTAATGAAGTCAGAATTAGCTCTCGTGCTAGATCAACA
ATATATTCAAGATTAAATTTTGTAAGTATTAATTATATTTATGCTAGTATAATATTAGTA
AGGGCATGTAACTCAGTGGATAGAGTATCAGATTCCGATTCTGATGGCCGTGGGTTCGAA
TCCCGCCCTGCCCGAGTATTCAGAGTACTATACTAAAACATCTTAATAGCTAGTTTGATA
ATAGTATGGGCATTGGGGCTGCAAGGTTTCTACATTATGAAAAGAAGAAAATATGAAAAA
AAACAAGCTCTCCAAAAGAGCTTTTAGTACAATCAATAAATGCAGAAAACAATATTGTTT
CTTTTTCTCGGAAATTAGCTCTTGCTTAAACACTGTTAATTTTTGGCATTCGACATGTTA
AACTCTTATTCATGGCGAATATTCTGTCAGAGTCGCTCTTAGTTTAAGAAAAGTCAGAAA
AAATATTTATCTTTACGTTTTTACCTATTTTTATTTAAATAAAGGTTAGTATTTTCAACT
TTTATAGTGGACGTGGGTTCGAGTCCCACCAGCTCCATCAATTTGATTTGTATAATTAGC
TAGTTTAACCTTTTTAATAACCAGATATATAATTGGAGGAAATATGGGTTTCATAACAAT
TACTAAGCCCGCTTTAAAGCAAATTGCAATCTTGAAAAATGATCATGAAAATGATGTACA
TCTAAGAATAGGTGTTAGACAAGGCGGATGTTCAGGTATGTCATACTCAATGAATTTTGA
ACATGTTGACAAATTAAAAGATACAGATGAACGGCTTCGTCTAGATAATTTTTCTGTTGT
CTGCGACCCTAAAAGTCTTCTTTATCTTTATGGATTATCATTAGATTTTAGCTCGGAATT
AATAGGAGGAGGATTTCAATTTTCTAACCCGAATGCCAGCCAAACTTGTGGCTGCGGTAA
ATCTTTTTCAGGCTGACCCTCTAACACGTTTTTTATATATTTTGTATTATATATATTATT
TTAGCAAGTTGTTTATTAGAGACAAGCAATGCTCTGTTAAAAACTAATTTCAAGAGTTTT
TATTGTATTATTTTATATATTTATTATCAACTACTTGAAAATAAATTAGCTAAGCAATCT

AATTTGTTATCAGTACCAAACTTTAAATGATTAACTTTTTATTGATATATGCTAATCTTT
ATCAGCCTAAACATATCTGTACATTACAATCTTTTGACATAAAAAGAGTCTTAACATACC
TCTCAAAAATAAATTCAATATTTTATAAATACTCAACATGAATCTGTCACAACTTATCGT
TAATTCTTCTATGATGAAAATTCCTAATAACTTTTATTGCAATATAAAAGCTATTTCTTC
TGAGAATTTAAGTCACTTACAATTATCAAAAACAAATTTATTATCTCCTACAAAAATAAT
TGCTTTTGGACTACCTAAATTTGATTTTATTTCTACACAGATTACAAGTTCTATTAGAGA
AACAGTTGTTGTGTCTACTATTGATGATAATAATCTTTTTGCTGCCCCAACTAATGATTG
GATAAAAAAACACAAGGATAAAAATCATCATTCTTTGTTTTATAAAAAAGTTTTTAAAGC
TTTGCTAAACCATAAAATTAGAGTTGTTGCTGAGCCATTTCATAACTCCGAAATAAATAA
AGAAAAGAGCATAATGGCAAGTCAAAGATACATCTGGGGTAAAAGCTGGAAGCCTTCAAT
TATTTTATCTTGCTTAAAAAACAAAAAAAATAGCAAAATTTCTGATGCATCGAAAAAATT
TTTAACTGAACAGTTATCTGCATCACCAGTATTTGTAGTAAAAAATGGTTTTAATGAAAT
TATTTTAGGTCACCCATTATCTCGTGTTAAAAGAGGAGGAGTTAATAATCTGATGCATGC
GTTTTCTAATTTATTAAATCAGTCTAATGCAACTTATCCTATATCTACTGGTCTATTCTT
TTTTCATCCAGATGATGCATTTGAATTTAAAGACTTTATAATATCGGTCAATCCACTGGC
GGCCAAACATATGGAGATAAGCGTAGAGCCTGTTGGTCTACACTTTGCTTATAAAATGAA
TAGAAATATATCGTCAGATACTCAATTTTCGTTTATTCCAGATTTTAAAGAAGTAGGAGA
TTTATTATTCAAATATAGAAAAGGTAATCATTTAGTTTTTCATAAGAATCAGCATTATGG
CAAAGATTTTTTTCAGGGGCAACCAATATATATGATTCAGCCGATCACTTTTAAAGATCG
AGCCGGAAAGCTAAATATTATCAAATTTACGGGGCTAAATGACACAAGAGAAATTATTTT
TACTAATCTTGAAGCTGCTAATAGATCATGGGCACATTTTATAAAGAGGAATTCACAATT
AAAATCAATTAAAAATCCTACTCTATTAGTCTATAATTTAGAAAGTTTTTTAAAAGATCA
AGAACGATTGGATAATCAAGATTTAAATAAGTTTGTGGTGATCACTAATAAAGAATCTTA
TCTTGCCACAAAAGAGTTAATAGCTTTACCCGATTCTAATAGCTTTTCTAAGCACTTAAA
ATTAAATATAAAGCCTAAACTCTTTTTTGTGAAACTATGGGTAAGACGGTTATTTTCCAC
CTTAACTTATGAATAAATGCTATTGCCTTTATTTTCTCGAATAATATTCTATAACTAGTA
ATTCATTGAGTTGTAAAGCAACCCATTCCCTATCAATGACTCCATTGACTTTCCCTGATA
AATTGGATTTATTCAATTCTAAGTGGCTAGGAATATTAGCTAGTCCAGGGAAAGCTAAGT
AATTTTCTACTAGTTTTCGAGATGCTTCTTGATTTTTTACACTAATTGATTCTCCTGGTT
TACATTGATAACTACAGATAGATACTACTTGTCCATTAATACAAATATGACCATGGTTCA
CTAGTTGTCTAGCGGCAGGAATTGTAGGAGCCATACCAAGTCTAAAAACAGTATTGTCTA
GCCTCATCTCTAGTAGCTGTAACAAAATTTGACCTGTCGACCCTTGTAGTTTTTTAGCTG
CTTTTACGTACTTAAATAACTGTTTCTCACTTAATCCATAATTAAATCGCAACTTCTGTT
TTTCTTCTAATCTTACAGCATACTCAGAAGGTTTTCTGGACTTTTGTCCATGTTCTCCAG
GTGGATAAGATCTTTTAATCGCTTTTCTACTGAGTCCGGGTAAATCACCTAATCTACGGG
AAATGCGTACTCGTGGCCCTCTGTATCTAGACATATTATTTAAATTCTCCTAATGTAAAT

GATTAAGTAATGAGTAGCTATTTTTTAACACAAAAAGAGCATTAAAACAAAGCTTTTGCT

AAAAAATATTTACAAAAAATATAGATTTGGACAAAATACATTACTATGCTTTTTAAGCGG

GTAGCGGGAATCGAACCCGCATCATTAGCTTGGAAGGCTAAGGTTTTACCACTAAACTAT

ACCCGCAATATTCAACAGATTGTCACTAACTCAATATAACATAGTTATGTTAAGTTAAGA

TAATCTTAAATTTAATAGAATTTTAAATTATTTATGCTCCTTCTAAAACCACATCAAGAA

AACGAGCAATTTCGGCAGCTTGTTCTTCAACTTCTGATAATAATAATGGTTGACCGACCC

TAGTCAATGGAATTACTCTTTTATCTTTTGTACATAAATAAATTTCACGTCTTGGATTTA

GCCCCTCTTTAATATCTATTTTTATTGATTTGATTTCTTTTATATTAAACTGAAGACAGA

TTTTTCTATTCTTTCCAGGGAAACCTAGCCGAAAAATTTTTACGATACCTTTATCTTTGT

TAAATTCATTATATCCAGCCCCTATATTCCAAATAATAGTTAGCCACAGAAACAAACTCA

GAAAGACTCCTATACTGCCATAGAATGTCATTACAATACCTTGTGGAATAAATACCAAAT

CGGTTGAATTTGTAAAAGGTAACAAATCAACTTGAAAATAACTTGATAGTCCAGCAAGAA

GAAAGCCCAAAGCCCCAATAAATATTATAGTAGCCCACCAGTAATTACTCAGTCTTCGTG

AACCTAAAATTAAATCTTTTCTAACTGAGTGTATAGATAATGTTTTTTTCATAATTTGTT

CTTTTAAAACAGTATACTAATGCAAAATGGAAAAATTTTGACAATTTTTATGCGCGCTTT

AGATTTTTTAAATAAGCTTGATTGCTTTTAGTCCTAAAAACAGTCCTGTTGCTAACCCAA

AAAAAGCAGCAAGAAATATTATATATCCTAGAAAAACTGACATATTATTATTATTTATTA

TTATTGTTACAATAGTGTTTACAAAAATAGTGAAAATCTATTACTACGGTATTATATTAC

GTTAAATGCCTTGCTGTCTACAGGAAAAAACTTTTTTCAGTTTTTAGCATTGTGTTTATA

TTAGAAATAATATTCTAGTAAATTTATTAAACTTCTGGAGAACAGAATCTTATGTCAGAA

TTTATCAAGCCTTATAACGATGATCCTTTTGTAGGCAATTTGTCCACGCCAGTTAGTACG

TCAAGTTTTAGTAAAGGACTTCTAGGAAATCTACCAGCTTACCGTCGAGGTTTATCTCCG

CTTCTTAGAGGATTAGAAATAGGAATGGCACATGGATACTTTTTAATTGGACCTTTTGAT

AAATTGGGCCCTTTACGAGGTACAGATGTAGCGTTACTAGCGGGATTTCTATCTTCGGTC

GGCCTCATTATTATTCTCACTACATGTTTATCCATGTATGGTAATGTATCTTTTACTAGA

GCAGATTCGAAAGATCCACTACAAACTTCTGAAGGCTGGGGACAATTCACTGCAGGATTT

CTAGTTGGAGCAGTAGGTGGTTCAGGATTTGCTTATTTATTGCTAGCTAATATACCTGTA

TTACAGACTGCAGGTCTTAGTTTATTCTCTTAAGCTAGTAAGGGGACTTGAACCCGTAAC

CTACTGATTACAAATCAGTTGCTCTACCAATTGAGCTATACTAGCATTCAGTTCACAATA

ACACATAAAGATATTTGTTGCAATCTGCTATTTTCAGATGTAACAAATATCTTTTTTATT

TAAACAGAACTGTTATGTTAACACGTATATTAATTAGAATCTGAATTATCTGACTCTGCA

GAAGATTCCTGATCACAGTTTAGATAATAACAAATTGTTTGATCTAGACAAGTTGCAGAC

CAACCAACAGGTGTTTCTCCTGATAATTCTTGCATATCTACAGAATTATAAAAATTATCA

TTTGCCTTATTACTGTTTGCTTGCATAAATATCACTCCCAAAACACTCTGAACTTAATGT

GAAGTAATTTAACTTCTCAATAATATATTAACACAAAATATTAAATATGGTGATTATTCT

TTAATTGTAAAGATGCTGCTAACTTTATAAAGTTTGAGTGAGAGTCTTGATAGCTTTTGT

TGAAATAAGCATTTTAACCCATTTATTTTGCCCTCTCGACCATACTTTTTTGTGTTGTAA
ATTAACTTTTTGTAGTTTTTTTGTTCGTTTATGAGAATGAGATACGGCATATCCATTATT
AGCTACTTTTCCCGTAAGCTGACACTTTTTTGACATAATTTTCTAACTTATATATTTATT
TAGAGTACTTGCTAAAGTTGACTTGGGCACAGCTCCAATCACTGTGTCTACTCTTTCTCC
TGCTTTAAAGATCATTAAAGTAGGTATACTTCTAATGCCATATTCAGCTGCAATAGTAGG
ATTATCGTCTGTGTTTATTTTTACTACCTTAATAGATGATTCATATTCTTCCGCAATTTC
ATCAACTACAGGAGAAACCATTCTACAAGGACCACACCATGGCGCCCAAAAATCTACGAG
TACAGGTAAGTTATTGTTAATAACTTCTTGTTTGAAAGAGGCATCTGTAACTTGAGATAC
TGACATATTCTTTAACCTTTAATATATGTTCCTTGCTAAGCAAATCTTATCACAAATTTC
AAGAAAAATCTTCTATAGTGAATTACTATTAGTGTTGAATTATTTTTTTAAAAATTAGAC
ATTAAAAAAACTTATTACCTATATAAATAAAGTGGTGGCCGAGTGATATCTTGATAGTGA
TAATTTTTATATTAAGGTTGAACACTAGAAATAACTTTTCAAGCAAACTCTTGTTTAAAA
AATTTCTCTGTGACCATAAAAACTTAATTATATTACAATATATTTTATTATTTGTAACTT
AGTTCTGATAATGGTATAAACAACGCAAAAGATACTGCCTTATAATCAAGGAGGAATACA
TGTCTCAATCCGTAGAATCACGGACTAGGATTAAAAGCGAACGTTACGAATCTGGAGTAA
TCCCCTACGCTAAAATGGGCTACTGGGATGCTGACTATGTGATTAAAGAAACAGATATTC
TAGCTCTTTTCAGAATCACTCCTCAACCAGGTGTTGACCCGATTGAAGCATCTGCTGCAA
TTGCAGGTGAATCTTCAACAGCTACTTGGACAGTTGTATGGACTGATTTATTAACAGCTT
GTGACTTATACAGAGCAAAAGCATATCGAGTAGATCCAGTTCCAAACGTGGCAGATCAAT
ATTTTGCTTACATAGCTTATGATATTGATTTGTTTGAAGAAGGTTCCATTGCGAACTTAA
CTGCTTCAATTATTGGTAACGTTTTTGGGTTTAAAGCTGTTAAAGCTCTTCGCTTGGAAG
ATATGCGTATGCCAGTAGCTTATCTAAAAACGTTCCAAGGTCCTGCAACTGGATTGATTG
TAGAACGTGAGCGTATGGATAAGTTCGGTAGACCTTTCTTAGGTGCTACAGTTAAACCTA
AACTAGGTTTATCTGGCAAAAACTACGGAAGAGTTGTATACGAAGGCCTGAAAGGCGGTC
TTGATTTCCTTAAAGATGATGAGAATATTAACTCACAACCATTTATGCGTTGGAGAGAAA
GATTTTTATATTCTATGGAAGGTGTAAATAAAGCATCGGCTTCTGCTGGCGAAATTAAAG
GTCATTACCTTAACGTAACAGCCGCGACAATGGAAGATATGTATGAGAGAGCCGAATTCT
CTAAAGAGGTTGGTAGTATCATTTGTATGATTGACCTTGTGATTGGTTATACTGCGATTC
AAAGTATGGCAATTTGGGCTCGTAAACATGACATGATTTTACATTTACATAGAGCTGGTA
ACTCAACTTACTCTCGTCAAAAAAATCATGGTATGAACTTCCGAGTTATTTGCAAATGGA
TGCGTATGGCTGGTGTTGACCATATTCACGCAGGTACAGTTGTAGGTAAGCTTGAAGGAG
ATCCTTTAATGATTAAAGGCTTCTACAATACTCTACTTGAAAGCGACACAGATATCAACC
TACCTCAAGGTCTGTTCTTTGCTCAAAATTGGGCTTCCCTACGTAAAGTTGTACCAGTAG
CATCTGGTGGTATTCATGCTGGTCAAATGCACCAACTTCTTGATTACTTAGGTGATGATG
TAGTTCTTCAGTTTGGTGGTGGTACAATTGGACATCCTGATGGTATCCAAGCAGGTGCAA
CTGCTAACAGAGTAGCACTAGAGTCCATGGTTATGGCAAGAAATGAAGGCCGTAACTATG

TAGCAGAAGGTCCACAAATCTTGAGGGACGCTGCTAAAACTTGTGGGCCTCTACAAACAG
CTTTAGATTTATGGAAAGATATTAGTTTCAACTACACTTCCACAGATACAGCTGATTTCG
TTGAGACTCCAACAGCAAACATCTAGTTTAATGACTACTTACTGATACTTTAAATAGTCA
ATTGTAAGTGGAATTAACTTATAACAATAAGGAGCATAGAATAGTGAGACTAACACAAGG
GACTTTTTCCTTCCTTCCAGATTTAACTGATGAGCAAATTAATAAACAGCTTGCTTATAT
CGTTTCTAAAGGCTTATCAGCAAACGTTGAGTATACTGACGATCCTCATCCAAGAAACTC
CTATTGGGAACTGTGGGGTTTACCTTTATTTGATGTAAAAGATGCTTCTGCTGTTATGTA
CGAAATTAGCTCATGCAGAAAAGCAAAACCTAATTATTATGTTAAAGTTAACGCTTTTGA
TAATACTAGAGGTATTGAAAGTTGTGTAATGTCTTTCATTGTAAATAGACCTGCTAATGA
ACCAGGATTCTTATTACAACGCCAAGACTTCGAAGGTAGAACTATGAAGTATAGTCTTCA
TAGCTATGCTACTGAAAAGCCTGAAGGAGCTAGGTATTAATATTAATTAAGAATTAATAT
TGGCTAATTATTACCCTCTTAAAAATCATAATTAATTTAGTAATTGTGATTTTTAAGAGG
GATAGCTTCCCAAGTAAATTTTGACTAAATAGATTGAATAATTAAAAATACACAGAAATA
ATGCAATCACAGGATATAATTTCCAACGATACTCTTGTTAATTTACAAGAAGAATATGAT
AGAACACAAATCCAAGAAGTTTTAAATGAGTTAAATCAAGAACTTATAGGATTAGTGCCT
GTAAAGACCAGAATTCGCGAAATTGCTGCGCTATTATTGATTGACAGATTACGCAGAAAA
CTAGAACTAGTTTCTGGTAATCCAGGATTACACATGTCATTTACAGGTAGTCCAGGAACT
GGTAAAACTACAGTTGCTATGAAAATGGCTGATATTTTGCACAGACTTGGATATATAAAA
AAAGGGCATTTGTTGACAGTTACAAGAGATGATCTTGTAGGTCAATATATTGGACATACT
GCCCCTAAAACTAAGGAAGTTCTTAAACAAGCAATGGGAGGAGTTTTATTTATTGACGAA
GCTTACTATCTATATAAAGCAGATAATGAAAGAGACTATGGCTCTGAGGCAATTGAAATT
TTATTACAAGTAATGGAAAACCAAAGAAATGACTTAGTTGTTATCTTTGCTGGATATAAA
GATAGAATGGAAAAATTCTACGAATCCAACCCAGGACTCTCTTCTAGAGTAGCTAATCAT
GTAGACTTCCCAGATTATACTTCAGATGAATTATTACAAATAGCTAAAATGATGATAGAA
GAACAGCAGTACTGTTTTACAGAAGAAGCAGATAAAACTCTTTTAGAGTATACCGAGCGA
AGAATGAAACAGCCTTATTTTGCTAATGCAAGAAGTATTCGCAATGCTATTGACAGGGCT
AGAATGAGACAAGCCAATAGGATTTTTGCCAGTGGAGAAAAAGTATTAACAAAAGCTGAT
TTGGTAACGATTGAAGCAGAAGATATCTTGAAAAGTAGATTATTTTCATTACCTAATGCT
TAATATACACGTGATTTCATGAAACTATATATTATTAAAAAGTTTCATAATCAGTTGCAA
ATTTCTTGAAAAACCTTTATTATTATCTTAATGCAGGTGTGGCGGCATAGCCAAGTGGTA
AGGCAGAGGATTGCAAATCCTTCATCCCCCAGTTCAAATCTGGGTGCCGCCTAGTACTAA
AAAAGGGGGGTGTGGTGGAATGGTAGACACAACAGACTTAAAATCTGTTGATTTTTAGTA
ATCGTGAGGGTTCAAGTCCCTCCACCCCCATATATTGATTTATAAATAAAAAGAGATGTG
TATTTGTATTAATTGCAACCATATTACCAAATGTAATACCTATCACTTAATAGAATCTCA
ACATAAACAGCCTCATCTTACTAGAAGTCCATTGTTTATACCTAAATATCCTGTAGTTCA
TGTTAATATATCTAACAACTGTACCTATAATCAAATTGATTGGGATTTAGTGGAGTGTTT

ATCATTTGTAGAAAAACCGAATAGTTGGAATTTAGACGCTAATTAGTTACAAGAAAAATT
ATGTACCATAAGCAACAACTCTATTTTTTAGATACTCGGTATTAATTTTGGATGTTCTAA
CCAGTAAGATTTTACCCGTTCTTGCTATCTCTATAATGCCAAATTTAGTTAGTAATTGTT
CAATAGCAACAATCTTTCCCGGATCTCCAGTAACCTCTATAATTAAAAGATCTTCTGCAA
TGTCTACTATCTTAGCTCTAAAAATTCTTACAATTTCTAAAGCTTCTGTTCTAGTCTGAG
AATTGATCTGAATCTTAATTAACATTAGTTCTCTTTCAACTGAAGGAATATTTGTTACAT
CTTGAACGTTAAGGATATTTACTAACTTATATAATTGTTTCGTAAGTTGTTCAATAGTTC
TATTGTCTCCTTGAACTACCATTGTGATTCTAGAGACCCCAATTTGCTCTGCTGGTCCAA
CTGCTAAACTTGCGATATTAAACCCTCTCCGGGCAAATAGACCAGATATTCTTGACAGTA
CTCCGGCTTCATCTTGAACTAAAACTGATAAGGTGTGTTTCATGAAAAATTAAATTTAAT
CTATTTGATCTGTTAAGTATATTCTAAGAAAATTACAGAAAAAATACTAGTTAGTCTATA
ACTAAATTATCCAAACTAAACAAAATCCTACTATCAGATAATATATCTACCTTGTAATGC
TATACTATTAACTTAGTATTTAATGATATTCTAATTGCAAATAGAATGCTAATTTTTTAA
AATTTGTTAGGAACTTGTGCTAGAAAAATACAAAAACAGTAAAAAACTCTCTCGAGATTT
ACCTCAAATCAATGATCGCATTAGATTTCCAAAAGTCCGAGTAATTGATGACGAAGGTGA
ACAACTAGGTATTTTTGTGCCTGAAGAAGCTATACAATTAGCTGTCCAACAAGGTTTAGA
CTTAGTTGTTGTTAGTGATAAATCGGACCCGCCAGTATGCCGAATATTAGACTATGGTAA
ATATAAGTTTACACAAGAAAAAAGAGCTAGAGAAGCTAAGAAAAAGCAACATAACAGTAG
TATTAAAGAAGTAAAAATGCGATATAAGATAGAAGAGCATGATTATAAAGTTAGAATAAA
CCAGGCATCCAAATTTATTCAAGCAGGAGATAAAGTAAAAGCAACTATCACATTTCGGGG
GCGTGAAATCCAGCACTCTAATTTAGCTATAGATTTATTGAATAAAATGGCAAGCGATCT
AAATGCAATAGCTGAAATTCAGCAAGCTCCATCAAGAGATGGCAGAAATGTCATAATGCT
CTTATCTCCCAAAAAAGTTAGCTAAACTAATTTTTATTTAAATGCATCTGGCTGGATTCG
AACCAGCGACGTCTCTTTCGAAATGGCGGATTATGAGTCCGCTGCCTTCGGCCCCTCGGC
CACAGATGCATCACTTAAAGTTTATACCTCATAAAGCATATAAAAATCAATGTTTAGCTA
TTTTTAATAAAGTCTATAAGAGTCTATTATTCATTCATATTGTGATATGTCGCAACTGCT
GAAGGGGAAACTTGATTTAAGTATCGAAATATCCAGTATTTAAAAATTGTGTCGAGTATA
ACTGGAAAAGTTGAAATAAATAAAAAAATAAAATCTCGACTTTCCGGAAGTCCTAAATGT
CTTAAAATTACTTCAATAATAACTTCCCATCCATGAGGAGAATGAAAACCCACAAACATA
TCAGTAAAAAGAATGATTAAAAAAGCTTTAGCTGTATCACTAAGACCATAAATAATTTCA
TTTAAAAAAGATTTAACGACTGCAATTTGTCTTTGTCCAGTTATCATTAGTAATATAAAG
ACAAGTATTGATACAAGATCGGATAAAATATTTTTAACAGCATTTGCACTTTCATTTGCA
TAATATTCTCCCAACTCTCTAGCCTTCAGCTGCACTCTTTTTTCAATGATTTCGTAAGAT
ATATCTTCCGAAGGATTCAGAAGAACTTCAAAGTGAATTTTCTCTTCAAATCTTTGTAAT
TCAGCAAAAGCTCTTTCCTCTTGAGAGGAATTTAAAAAAATTTTAGGCTGTTCTTGGTTC
CATAAATAATCAATACAAGGTCCAAATACAAAAAATTTTGAAGCCTGATTAACTAGTACT

GGAGAGATGAATAATAATAAAATATATTTTACGGAAGTAATAGTTTGGTGTCTAGATATT
CTAAACTCCTCTATAGCTTCAGATTCGCCATTAGGATCTAACTCTTTACGAAACTTTTCA
AAAGTATTAGTAATTGACCTAGGTATTGGCCCAACTTTTTCAAAGGCGAATTGATTATTT
CGTTTTAAATTCCAATATTTCATTTTTTATTGCTGCTAAAATCTCTATTAATCAATAATT
GATCACGTATTCACTATAAATATTAATTCTAGAACTAAACATATAAAACAATATTTATGA
AAAATAAAATACAATTAAAAATTAATTATGCATTAGTTCTATCTGCAGCTACAGCAGTTT
ATGGATATAAATCTTATCAGCCAACTATAAATACACTCTTAATCAAAGATGAAGAAAACT
GGCCTTTTTTCTCTAAAAGGCAGACTGCCTTTATTCCTATAATTGTATTAAAAAAAAATC
ATCAAAATCCCTGAACTCAGAACCTAGAAATAGTAAGTTATATCTTAATGGAATTGGAAG
TAGAAAATTTATCGACAGAATTGTCACTCTACTTGGATATTTAGAAAAACAGGCAAACTA
TACTGAATACTTGAACCAAATAAAAACAAGTGGTTATTTTTCTTTAGTCTACCTAGACAG
TGATATAATGTATAAGAAAAATATTAGTAACATTTTTTACTACCTAATGATATTCTAAA
AAGAATCTATATTTGCAATAAGAAAAAAAAGCTTATACCGCATTCATTCTTAATAAATTT
ATTTCAGAAACAGATAGGCTATCCAAAAAGTTTTGCTAATTTAAATTGGGCCTTCTCCAA
AATTATAAAATGGTATTATGATAGAGGATACCAATGGTCTCTAGTTGAAGTTAAACAAGC
GTCTGATGCATCTTCTATTGTAATTGATATTCATGAAGGGGTAGTGAAGACAATTATAAC
AGAATACTACACTCTATCTTATAAAAGAGTCTCAGGTATCTTATGCGTAGAGTCAATAGA
GCAGTACCTTGGAGTAAGAGTGGGAGCTCCACTAAATATAATTGATTTACAAAAAAAAAT
TACTTACTTAAAAGATAATCAACTAGTTGGCGACATTATTTATAGTATTGAACGATCAAA
TAATAGCTCAATGAGTTTGGATATCAAATTTCAAATACAAGAATTAAAAGATAAAGAGAT
AATAGTGCTTGCAAAAAGTTCTTCTATTATCTCGCATGCATGTAATTTACTTAATCAATA
TAGAAATAGGCTAGTAGCTTCTAATATAGTTTCACTATCAACTAATAAATTACACGCATG
CTATTTTAATTATAAACTTGACTATCAATATAAGTATATAAACACGTCTAATCTTATAAA
ATTGTTAGCTTATTCTATTTCTTGTAGGAAGACACTACTTATTCATTATTTTAATTTACA
AACTTTAATAAGCTATACAAAAAAAAAATACTATTGGATTTCAGCTATATTTACGAAATCT
AAGTTTTGGGAAAGCTTTCTGTGTGCTTAGTATGAAATTTATCAAAAATGGACTTAATAT
AAAAATTTTATATATAAATCCTTCATTGATAGTTGATCAGAATTTTGTATTTCAATTTGC
TATACAAATCATTAAGCAATATCATACTGCTAAACCTCCAGCTTTATTTTTAACGAATCT
AGATCTTGAACAATATGTTGCTGAAAGTTTATTAATGTACCATTTTACATCTTGCTTCTC
AATATCTGAAAAAATATTATTATCTCGAATTATGCATACAGATTCTTTATTTTTCAACTC
TGAAAATTTTCATTTTGATGATAGAACTAACGCGATCAACAGTTATGATACTTTTAAACA
AAATACAAAAATATTTTATCAAGAGTTTCTTTCTTTATTATTAAGTTTACGTTATCAAAA
TTTTAATTATTTAGGTTGGCCTTTAAAAGGCCATTTTTTTGAAATAAAATCTTTATACTT
GGCTCCATTTCAAAAGAGTGATTTTTCTGATAGTCGTAAGACCCTATTCTTCCACAAAAT
GTCGTTAAAGCAAGTATCTAATTTTAATTTACCAATATCTTTTAAAAGCCACCTTAACCA
TATTTTAGTTAGTACTATTAAATGTCAGTCAAATTTGAATATGAGAACTGTATCTTTGCT

ATTAATTGATTCACCTGCTGAATATATGCTATACAAATCTATTCTTAATTTCTCTATTAA
AGTTAGGATGCAGTATTTCATACCTATGAGCAACAATATAAGATTATCTCTATTTTATAA
TTATTTAGATTGTTTTTTAATAAGATCATCTCAGAGTCGCATTCATATATGGCAAGATCT
AAGAACTCTTACACCAATTCAAAATTTTTGGTTAAAAAAATTTTCATATGGAGCTGGAAT
TCAGCTTAAATTACCAATTAAGCAGATGCCACCTCTTTCAATTGAATATACTGTAACTAG
TAGTCGCTACTTTTGTATTTACCTTCGTACTTATTATCAACGATAACTATTACTAAAATG
AAAAATTTAATTTCTTTTTTTGATCGCAGCAAAGGGAAATGGATTTCACAAAGAACAACT
TATGAATTATCTAATAAAAACATGAGTTCGATACAGTCTCAGATGACAATGAAAATAGGC
AACTCATTGTCAGGATCTATAATACTTGCATCGTTAAATTGGGGGACATTTATAGACAA
GTTGCCCATTACGCTAAAAATCACAGCCGGAGTGAATATGATAATAAGTTTAACTTGCAA
TTCAGTAATCAACTAAATAATCATAAATTATTAACGCTGTGCATAGTGACAGATCCTAGT
CTAATTAGTTTTAAAACTCGCTATGGAAGTACTACCATAGATGAAACATATTGGTTCGCA
ACAAATAATTTACGTCTAAGTACTAGTATTGTTAAACGATTCAACACTTGTGTGGCAGTA
TCTTTTTGTTCAGAAATTAAAGTTTAAACTCTATATAGAAGCAACTAATAGCCTAAAGGC
TATTAGTTGCTTCTATATATCTAAATTTTGTATTAAAAATTATAATACTATATTATTCTA
AGTCTTTACGATTGGGATTCCTGGAAGGATCATTAGAGAGGAACCCAAAGACAAACAAAG
AAATAAAGAAAATAACGGTAGTATATACAAAGATTTTCAAAGTAAACATATTTTCATCCT
TAAAAGCTATTAGTGCGATATTTACATATCTCCACATAATTTTATATCATGATATAAGTT
CCAGCACTATTTTATAAATAAATCAATATCTTTATTTTTTAGTAATGATTTATATTTAAA
TATGCGTGATGTACTAAATACTAAATCTTTTCCGACTTGTTTTGTCTTGTTTGTAAATAT
TTTGTTATTTCTATGGAGTTTACCTTCAATTATCACATAGTCTAACTTCTTTAGCTGTTT
AAAAGTATATGAAGATTTTTTGTTCCAAATAGTTAGATTGATTATAACTAATTGTTTTCT
TTTTAACAGTCTTGCTTTTAACTTAATTATTTGGCTTTTATTTTCACTAATTCTGATACT
CTTACAACTAAGAATTTGGACTAGAAGAGTACAGCTATTCATAATTTATAACAATTTTTT
AAAAAATCTACTAAAATAGATGATTTTACAGAACTAAAATGATATTTCATTATCTTGTTG
ACCTTGCTCATAAGTAATATCTTTCAATACTTTTAATATTTTTCCAAAATACTCTTGCAG
GTATTTCTCTAAAGATATTACTTCCAACCTGTCAATTCCAAGAATACTGTAAACTTCATC
CATCGGAGCTGTAAATTGCTCTCCGCTACTTAAAATTTCGGCAAATGCTAAACGATCAGA
AATATTCCATGTCCATTGAAGAGTTTTAGTAATTCTTCTTAAGGCTTTTAATAATCCAAT
CGGAATTTGTGAGATCTGTGTTTTTTGCCCAGATAATTTTTCACATAATTTAATAATTTC
AGCTGATGTCCAAGCGGTATTACCAACTAACGGCAGGGTCCTATTTTCTGTGGAAGGTAC
TCCAAGACTTTTTATAACTAACTTTGCTGCATCTTGAGTATCAATGTATGCAATGGGTGT
AGATTCTCCTGTAACCCAGACTGATTTTTTGTCTAAAATAGGAATGGCATATTGGTTGAT
TAAACCTTGAAAAAATCCTCCTAAAGAAAACACAGTATATTTCACATTGGACTTCTGAAG
GAAATCTACTACCTGAGACTTTAAGTTCATCAATGGAACATCTGGGTATTTCTCTGAATT
TAATATTGAAAAGAAAATAAATCTTTCAACTTTAGCCGCTTTAGCTGCTTCAATTAATGC

AATTTTCCCATCTAAATCTATTTTTTCTGCATTGTAAGGATCAGTAGGACGAGATGTAGA

AGCATCTATGATTGCTGTTACCCCACAAAAAGATTGCAAAATACTTTCGGGTAATTTTAA

ATCACCATATATAAGTTCTGCTCCCCACTCTTTGAGAAAGGCAGACTTTCTTAAATTTCT

TACCATGCATTTTACATTGTAGCCTTCATCTAAAGCTCGTCTTACAATTTGACGTCCTAA

AGTTCCGGTTGCTCCAATTACTAAAAGAGTCATATTAGTTAAAAATAAGTATGTATAAAG

ACATTGATTTGGTTGACGGCAGAGAGTAAATTAGGTAGAGAGGGAATCGAACCCTCATGA

CCGAAGTCGTCACATTTTGAGTGTGATGCGTATACCAATTTCGCCATCTACCCTATTATA

GACCAACTACTACAAGTAATTTAGTCTTTAGCGTAAAAATAATACTAAGTTTCAATGGAT

ATGTCAACTTTTAAATTTATATTAATAAAAAAACGGAGTCTCTTATGGCCACTTTTGAAT

TAATTCCTTATAGATTGTATTTGAGTCAATCTAACACATGGATCCATAGAATAAAAGCAG

AAATTAAGATATATATAGTAACGCTTTTATGGATTTCAATTTTTATTTTTTCTTACTTTA

AACTATGTATTATTGCTTTAAGCTTAATTGCCATAAGTTTTACTATAAGAAGTAAACAGA

ATATTATCCAAAAACATTTGTTACAAACTTTATTAATAACGTTCTTAACTACTGTTTTGT

CTTTTAGTGTGGCTATTAGTTATAAACAATATGCAGAACAAGAACAATCGCAGTATTTAT

CTGATTCCAAGAAATATAAAAATTCCAGCAGCTACTATTATATACAAATAGCTAACGATC

AAAGGATCAAAAGACAATACTTAATTACTACTTTAAAGCCTTCCTTGTACTTTTTTATTA

CTATATATTCTATTAAACTAGTTATGATAACAACTTCTCCAGAAGTTTTAGTAATTACTA

TTTATAGATCTAGGATAATAAATAAAATATTTAAAAATGAATTGCTATTTATCTTCCTTC

TTTCGTCACATATTGTTACTAGTATTATTAATAGAATCGATAAAGTGATTCAAGTAACTA

GCTTAAGAGGAAGCTTGAATTTATATAATTCATTGACAAGGCCTTTAATGTTTTCTTTGT

TAATATTTCAAGTCTTCTTTTTGGAGATTATTCGGGAGTCAAAAGAAATAGCTCAAGCTC

TTTATACTAGAAATCTCAATCAAGAAAACAATAATTTTTTGAAAATATATACAGTAAAAT

CTAACTTTAGTGATCGGCTCAATATAATTATTAGCACTTTGTACTTTATTATTTTAGCTC

TAGCGTAATAGAATCCACTTATTCTTCTGACTCTAGTAATACGAAGAGTAATAAAATTAG

TTAGTAGTTTAAATAGATAGAATTAAGATATTATATAAATAATTAAATGTTTCATTATGG

TTAATACTCAAAATCAAATTTCACAAACTTCAGAACTTGATTATATAGTTAATCAACCTT

ATAAATATGGCTTTAAAACTTCTGTTGAATCTGAGCAATTTCCTAGGGGAATAAGTGAAG

ATATTGTCCGCTTGATCTCCAAGAAAAAAGATGAGCCTGAATATCTATTGAATTTTAGGC

TTAAAGCATATAAAAAATGGAAAAAGATGAGTAGTCCGTCATGGGCTCATATTAAGCATC

CGAATATAGACTTTAATACGATTATTTATTACGCTGTTCCTAAATTAAAAAAAGAATTGA

AAAGTCTAGATGAGGTTGATCCAGAAATTCTTGACACTTTTAACAAGTTAGGTATATCTT

TAAATGAGCAAAAGCGAATTTCCAATGTAGCCGTTGATGCTGTTTTTGATAGTGTATCTA

TTGCAACTACTTTTAAGAAAGAACTGTCTGAAGCTGGTGTTATATTCTGTTCAATTTCTG

AAGCTATAAGGGATTATCCAGAATTAATAAAAAAATACTTAGGTACTGTTGTACCAGCTG

GTGATAATTATTTTGCTGCATTGAACTCTGCGGTATTTAGTGATGGCTCTTTTTGCTATA

TCCCTCCTAATACAGTTTGCCCTTTAGAATTATCAACTTACTTTCGTATTAACAACGAAG

AATCTGGGCAATTTGAAAGGACACTAATTATTGCTGATCGTGGCAGTAAAGTAAGTTATC
TTGAAGGTTGTACTGCTCCTCAATTTGACACAAATCAATTACATGCAGCGATTGTAGAAT
TAGTAGCTCTTGAAGGAGCCGAAATTAAATATTCTACAGTACAGAATTGGTATGCTGGTA
ACAAAGAAGGTAAAGGTGGTATATACAATTTTGTTACTAAACGAGGCTTATGCTCGGGTA
ACAATTCAAAAATTTCATGGACTCAAGTAGAAACTGGATCTGCAATTACCTGGAAATATC
CAAGCTGTATTTTAGCTGGTGAAAATTCTCAGGGAGAATTTTACTCTGTAGCTTTAACAA
ATAACTATCAAGAGGCTGATACAGGGACTAAAATGATCCATATTGGTAACAATACAAAAA
GTAGAATTATTTCTAAAGGTATTTCTGCGGGTAGATCGAAAAACAGTTATCGAGGCCTAG
TAAAAGTTGGCCCTCAGTCATTTAATTCTCGTAATTATTCTCAATGCGATTCTTTATTAA
TTGGTCAATCATCTCAAGCTAATACATTTCCCTATATTCAAGTACAGAATCCAACATCAA
AAGTAGAACATGAAGCATCCACTTCAAAAATTAGCGAGGACCAAATTTTTTATTTTTTAC
AAAGAGGGATCAATTTAGAAGAATCTATTGCTCTTATGATCAGTGGTTTTTGTAAAGATG
TCTTTAATGAATTGCCTATGGAATTTGCTACTGAAGCTGATCGTTTACTGAGTTTAAAAT
TAGAGGGAACTGTAGGATGAGCCAGACTATTTTAGAAATCAAAGATTTATATGCTTCTGT
TGGTGAAACAACAATTTTAAAAGGGGTAAACTTATCTATTCGAGCCGGCGAAATACATGC
GATTATGGGGCCTAACGGTTCAGGTAAAAGTACATTATCAAAAGTAATTGCAGGACATCC
AGCGTATTCACTAATAAGCGGAGACATTTTATTTTTTGGACAAAGTATTCTTGAAATGGA
GCCAGACGAGAGGGCAAAAGCAGGTATTTTTTTAGCTTTTCAGTATCCTGTTGAAATTCC
TGGAGTCAGTAATTCTGATTTCTTAAGAATTGCATTAAATGCTCGCAGAAAGTTTCAAGG
ATTATCGGAGTTTAGCCCCTTAGAATTTTTTCAACTAATAACAGAAAAAATAGATCTTGT
TGGCATGCAAGAAAGTTTTTTGACAAGAAATGTTAATGAAGGATTTTCTGGTGGAGAAAA
AAAACGTAACGAAATTCTTCAAATGGCTTTACTAGATAGCAAAATATCTATTTTAGATGA
AACAGATTCTGGACTAGATATTGATGCATTACGAGTAGTTGCAAAAGGAATTAACACTTT
AGCTAAATCAACAAATTCAATTATTTTGATTACTCACTATCAAAGATTATTAGATTATAT
TATTCCAGATTTTGTTCATATTATGAGTAACGGACAAATTGTAAAAACTGGTAGTGTTAC
TCTTGCCCAAGATCTAGAAAAGCACGGATATGACTGGATTACGCAGACATAACTATTTAC
TACAATATTGAGACTAAAGAATATTCTTTAGTCTCAATATTTTTGCAAATGCAAAGTTAA
TTATTTAAGCTTTATCAAAACCTTGCTTAACATCTTCAATTGCTCTTTTTAGCAATTCTT
CACTAACTGGTTCTAATTTTTTAGTACTACGAACATTTTCACCAAATTCTGGTTTAGAAT
TCTTTAAATCTTCTCGAAGTTCTCTAATAAATTCAGCTACTTTTGAAACTTCAATATCAT
CTAGATAGCCATTAATGCCAGTGTAAATAATAGCAGTCTGTTCTTCAACTGGAATAGGAG
AATTTTGAGCTTGTTTCAAAATTTCTCGAAGACGTTGACCTCTTGCTAGTTGATTTTGGG
TTGCTTTATCTAGATCAGATGCAAACTGAGAAAAAGCTTCTAATTCAGCAAATTGTGCTA
ACTCTAGTTTTAACTTGCCTGCCACTTGTTTCATGGCTTTTATTTGCGCAGCAGAGCCTA
CTCTAGAGACTGAGATCCCAACATTAATAGCTGGTCTAATCCCTGAGTTAAATAAGTCAC
CAGATAGAAATATTTGTCCATCTGTAATTGAAATTACATTTGTTGGTATATATGCAGATA

CGTCCCCAGCTTGAGTTTCAATAATAGGGAGAGCTGTCATACTACCTCCACCTAATTCAG
CATTCAATTTAGCTGCTCTCTCTAGCAATCTAGAGTGTAAATAGAATACATCCCCAGGAT
AGGCTTCTCGTCCCGGAGGTCTTCTAAGCAAAAGAGACATCTGACGATAAGCTTGAGCTT
GCTTAGTTAAATCATCATAAATAACTAATGTTGCTTTACCTTTGTACATAAAATATTCAG
CTAAAGCCGCACCTGTATAAGGAGCAATATATTGTAAAGTCGCAGGACTATCTGCATTAG
CTGCAACTATAATAGTATAATCAAGAGCTCCTTTATCCTGTAGTGAAGATACTACTTGGG
CTACTGAAGATGCCTTTTGTCCTATCGCAACATATACACAAACAACATCTTGGCCTTTTT
GATTAATAATTGTGTCCAATGCAACAGCTGTTTTACCTGTTTGACGGTCACCAATAATTA
ATTCTCGCTGACCTCTACCAATTGGGATCATAGAGTCAATAGCAGTTATCCCTGTTTGCA
TAGGTTCACAAACTGATTGCCTTCCAATTATACCAGGGGCCATTGACTCAATTAGCCTTG
TTCCATTACTTGCAGGTTCACCTTTATCATCTATTGGCCGAGCTAATGGATCAACAACTC
TACCTAAAAAAGCATCTCCGACCGGAATTTGAGCAATTCGACCAGTCCCTTTTACGGAAC
TGCCCTCTAAAATATCCCTACCATCTCCCATGAGTACTACACCAACGTTATCACTTTCTA
AATTTAAAGCAACCCCAATAGTTTTATCTTCAAATTCAAGTAGTTCACCAGCCATGACTT
CATCAAGTCCATATACTCGAGCGATACCATCACCAACTTGTAAAACTGTTCCTATATTGG
CTACTTCTACATCCTGATCATACTTTTCAATTTGTTGACGAATAATACTACTTATTTCAT
CAGGTCTAATATTTACCATATTTTTAATATTATTTTTATTAATTGGAAATTCTAAAGCGG
TAATTAGTCAGACTAATGCTATCTGTCTTTTTTACTTTATTTTAAGTTACTACAACATCA
AGGTGTGAAGCCATTTGTCTTAGCTGTCCTCTAATACTAGTATCAATGACTTTTGATCCA
ATCTGAATAGTAAAGCCACCAATTAGCTCGGGCTCGACTGAAATAATTAGTTTAACTTCT
TTAGCCTTAGTCATAACCTTGATTTTTTCTGTTAATAGAGTTTCCTGATCAGGGCTTAAA
GCAATAGAAGTATTAATATTTGCAATTGTTAAAGATTCCATTTGGTAAGCCAGTTCCAAA
TACTTACTAGCTATAGCATCTAACATTCCAATTCGCTTGCGATCAACTAAAATCATTAGA
AATGATAATGTATTTTCGTTAATTTGATCGCCAACAGTTGCAATAATTACTTCTTTTTTT
GCCTCTACAGTTTTTAATGGATTAGCTAAAAAAATTTTTAGCTTGTCAGATTGCGCTAAA
ATATTTTGTATTGACTGAATATCTTGGCTCACCTTTTCAGTAACTTTTTTAGTTTTAGCT
AAATCAAGAAGAGCTACTGCATAAGGCTGAGCTATTTTTATAACAACATTGTTACTGCTC
ATAGTTGATCTCCTAACTTAGCAATATTATTATCAATTATACGCAGTTGCATTTCGGAGC
TCATTTGATTTTCCAGCTGCAAAGTAACTCTTTTAAGAGCAAGAAATGTTATTTGTTGCT
GGATTTGCCTTCGGATCTGTTTTTCAGCTGTTTCTATATTTGATTTACCTGTTATGGCAA
GTCTTTCAATATCTAATTGGCCTTGAGCTAATATGGAACTTCTAACTTTTCCAGCTGTTA
GCTGAGCTTCTTTCTTAATTTGATCTATAATGATTTGAGTTTGAGCAAGTTGTTTTTCTG
ATTCTGACAATCTAGCACTTGCTTGCTCTAGTCTTTCTTCGGATTCTTGTATAGCTGCTA
AAACTTTTATCTGTCTTGCATTTAAACTTGATCCGAGAAATTGCTTCAAAACATAAATAA
GACCAAATAAAAGCAACAATATATTAATAACATTTGCTTCAAAAATATCAGAATTAAAGC
CGAATTTGTGTTCACTACTATGTTCTGATAAAATAGTAATTATTTGTGGTATTTTTACTA

TATTATTCATTTAAAATTCTTACTATTTTAATATTTTACTCTGGAATCATGATTTTCAAG
ATTGACTACTTAATAATTTAACTTTAATTTGATCGCTCAAAGTGTCAACCTGATTTTCTA
AAGTTTTAAGAGCTTCTTCTTTTTGAACATTTAACTGTTTCGATGCTTCAGCAATCAGTT
TCTCTGCATTCAATTGAGCTTTTTTATATCTTCTGAGACTATATTTGAGCTTCTTTTT
GAGAAGAGGCTATTTTTAATTGTGCATTGCGGCGAGCTTCAGATAAATCTTCTTCATATT
TTGCAGCTAGCTCATCTGCTTTAACAAGCATAGACGATGCAGTAGTCAATGTTGTTCGAA
TATATTCATCTCGTTCGTCTAGTACTTTAGTTACAGGTTTATAAAAAATAGTATTTAATA
AAACCATTAAAGTAAGAAACTGTAATGCCATTAAAGGCAAAGTACCATTGAAGTCAAATA
AGCCACCTTCAATTTCTTCGGCTAATAGAAATGGTAAATCAATCATTTTACTAATTATGT
TTATAGTTAATAATTTTGTTTCAATTAAGAAAAATAACTTTATAGTGCGCTTAGTCTCAT
CAGACTAAGCGCAAATGATTATTTTAACCAACGTATGGATTAGCGAACAATAAAGACAAC
GCAACAACTAGGCCGTAAATTGTTAAAGATTCCATAAATGCTAAACTTAGAAGAAGTGTA
CCCCGAATCTTTCCTTCTACTTCTGGCTGTCTAGCAATACCTTCGACAGCATTAGCTGCT
GCACTACCTTGACCAATGCCTGGGCCTATTGCAGCAAGACCTACAGCAAGACCGGCAGCG
ATAACGGATGCGGCTGAAACGATTGAATCCATAATGAATTTTAATTTTTTGTTATATGAG
TAGAAAATTGACAAATTTCGTATAATACTTAAAATCTATATAATTAAGTATAATAGATTC
TAATATTGTAATTATAGTATATTACTCTTCTCCATGACCTTCCATTGCTTCTCCAATATA
AGCTGCGGACAAAGTAGAAAAGATTAATGCCTGTATTGAACTCGCAAATAATCCGAGTAT
CATAACTGGTAATGGTATTAAAATTGGAATTAGCAAAGTAAATACAGAAACAACTAATTC
ATCTGCTAAGACGTTTCCAAATAATCGAAAACTTAAAGATAGTGGTTTCGTAAAATCTTC
TAGAATATTGATTGGGAGTAATACTGGAGTTGGCTGAATATATCTAGCAAAGTAGCCTAA
CCCTTTTTTACTTAAACCTGCATAAAAATAAGCTAAAGATGTAAGTAAAGACAGGGCCAC
AGTTGTATTAATGTCATTTGTTGGGGCAGCTAGCTCACCTTCAGGCAAGTGAATTAACTT
CCAAGGAATTAATGCACCTGCCCAGTTACAACCTAGAATAAATAGAAATAAAGTTGCAAT
GTAAGGCACCCAAGGGCGATATTCATGCTCACCAATCTGATTTTTTGCAATATCTTGCAG
AAATTCTAATATAAATTCCATAAAATTCTGGAATTTTTCAGGAATTCTTTGTAGATTTCT
AGTACCTAGAAATGATAAAGTTAAAAGTGTAGCAATTACTAACCAAGAGACAATGAAAAC
TTGCCCATGCAATTGTAAGCTACCTATTTTCCAATATAGATGTTTTCCAACTTCTACTGC
TGATAAACTATTATATGGGTTAAAATCTATGAGACTGTTTTGATACATAATAAATATTTT
TTTAAATAGTGCAAGAATCAAAAAAATAAGTTTTTGAAGAATTAATATGCAATTAATTTAT
TCTTAAAATTAGACTATTAATAAATTAAAAGCAAAAATAAATTCTAATAGAGATAGCTAT
TTTTAGTAGTGATTATTTTTTGTAAAAAACAATTATTGCTACCTATAATGCTTTTACGTT
TAATTATATACCCATATATTAATTATTTAATATGATTATAGGTATTTTGCTACAAACTTT
ATTGTTTTATCCATTCTAAAGATTGTTATAGTTTATTTTTTATTTAAAATATCTGCGACT
TCATCTGCAAAATTAACTTTCAAATTTTCTTCTCCCCCTCCCAGAAGAAATCTTACAAAT
CTTCTAATTTTAATATTTTCACCTAGAACAGCAATATTTTGGTTTATTAAATCTTCAATA

CTAATATCTTGATTGCGGATAAACATTTGATCTAATAATGAGAGCTCCTTAAGTCTCTTT
TTCATTCGTCCTTCAATGATTGTTTCGATTCTATCAACTGGTTTGTTTTTCAAGTCATCT
TTACCAGCTTCAACTCTTTGTTCCAGATTAATAATTTCACTAGGTATATCCTGTGTTGAT
ACGTATTCCACATTAGGCGATGCCGCTATTTGCATAGCAATATCTTTAGCTAACTTTTGG
AACTCTGGACGTCGTGCTACAAAATCTGTTTCACAGTTGACTTCAACTAATACACCTATT
CTTCCGCCAGTATGAATATAACTCTCTAGTAAACCTTCAATAGCGGTACGATTAGATTTC
TTGTTAGCTGAAGCCAGCCCTTTTTGTCTTAATGATTCTAAAGCTTTTTCTTCATTGCCA
TTATTAGCTTGTAAAGCTTTCTTGCAGTCCATCATACCTGCTCCAGTCTTATCACGTAAG
GCTTTTACGACTTGAGCAGAAATTTGTAGTGTCATATGCTTTCAAAAATTTTTAAGTATT
TAAATTAATTATTATAATTCACTTTGAGTAGGATGAGTTATTTCTTCTAAATCTGTTTGC
CCGTATTTACCATCATAAATTGCATCTGCTATTTTACCTACAATTAGTTTAATTGACCTA
ATGGCATCATCATTAGCTGGGATTGGAATATTAATAATTTCTGGACTACAATTAGTATCA
AGTATACAAATAGTTGGAATACCTAATTTTAAACACTCTTGTATAGCTGTTGTTTCTCTC
TTTTGATCTACAACTACAACAATATCAGGTAAACGAGTCATATTTTTAATACCATTTAGA
TGTTTACGAAGTTTATCTAATTCTCTTCGTAAAACAGCAGCTTCTTTTTTGGGTAACTGA
TCAATCATACCACTTTTATCTTGTTCTTCTAGCTGTCTCAGACGATTAACTCGAGACTTA
ATCGTTACCCAATTAGTTAACATTCCTCCAAGCCATCTCTGGTTAACATAATAAGAATCA
CAGCGTTGTGCTTCCTGTGCCACTATACCAGCGGCTTGTCTTTTTGTTCCTAGAAATAAA
ACTTTTTTACCATCCGACGAAGCCTGTTTTATAAATTCACAAGCTTCTGTTAATAATTGT
GCAGTTTGGACTAGATCAATAATATGTATGCCATTTCTTTCTGTATAAATATATGGAAAC
ATTTTAGGATTCCATCTACGCGCTTGATGTCCAAAATGAACACCTGCTTCTAGTAATTCT
GCTAATGTAACAATAGCCATAAGATTATTAATATTTAAATAATGTAATTATTCGGGAAAT
CCCTTTGTTTAGAAACTGATTCAACTAACTTATTTGAATTTTAGTAAACTAATAATAAAT
TAACACACTTTTAAAGCAGATCAACAAAATAGATCCTGAAGAATTCATTCTACACTTTTA
TTGCTAAAATAATTACGCGCGGTTCTGTCGTCTAAAATGATATCATCTAAATCATCTCGT
ACAGATGAAATAGTACTTTCGTCATTAGTATTTGCAGTTAAATTCTTTTTCTCTAACGAT
GAGCCATTACAGTTATCATACATATTGAAGCCGGTACCAGCCGGTATAAGCCGACCTATA
ATAACATTTTCTTTCAGACCTCTTAACCAATCTAATTTACCAGATATAGCAGCTTCAGTT
AATACTTTTGTAGTTTCTTGAAAACTTGCTGCAGAAATAAAGCTTTCTGTATTTAAGGAA
GCTTGAGTAATACCTAATAGCACAGGCCTATATGATGCATTAATCTTATTTCTCAAGGTT
ATGGCTTTATTAGTCTGTTCAATCTTCTGTAACTCTACAAGCTCTCCAGGTAAATAGCCT
GTTTCTTCACCATTTTCTATTTTCACTTTAGAAGTCATCTGCCGTACTATTACTTCTACA
TGCTTATCAGAAATATTCACTCCTTGAGATTGATATACCAATTGTACTTCTTTAACAAGT
AGTAACTGAATCTCTTGAAAGCTTAATCGAGCAGCTTCATATAAGGCTAATCCACATTCA
ATATACAAATCAAAACTTGCATCGAGGATATCATGAGGATTTAACAATACGTCTGTTTTC
TTTCTTGCTTCAAGAATTTCTTCAATTCTTGGAAGCCCTTGTATAATGTCACCTGTTTTT

GCTCTATCAAAAACCAATATAGCTAATGTTTCTCCCCTTCTAATTAATGCATTGTTATCA
ACATGTAAAATAGCACCATTAGAAACCAAGTAAGGTCTAGCAATTCTTAATGTAACTGAT
CTAGAAGAAATTTCAATAATTTGTCCAGAGTCTAAAGAAGTCATATTTTCAGTAATAAAA
TCACCACAACGAATCCAATCACCCACACTTACTTTAACTATTGATTTTCCTATATTAAAG
GTCTTTTTATCAGAAGATGTCGCAATCAAAATGCGCCTGCTAGTATTCTTTTCAGAATAA
ATCTCCTCAACAGTACCTTCACTCATTGCAATAATCTCAGTACTAGCAACAACTGTAAAT
GGCTTGATATATTCACCATTTTTTACAATAATTCTAGTTTGACTTTGTTGCTTCTCTGAT
TTATTTACATCAATACTTTTAATTGATAATGTTTCAAAAGTTGACAGCCTTAAGTCAAAA
TAATTATTGCCACTTTCTTTAGCAGAAAACTCAATTGAGGATATTAAATTATCATCTCGA
TGCTCAATTTCAGCCACAAGATTAGTTGTTACTAAATGAACTCCTTCTATAGATTTCACT
CTTTCTCCGTCCCGAAAAGGAGTTCTTTTAACTAGCTTTAGTTTAGTTTGACTTGGTTTT
TGTGAAACTAGATTTTCAATTAGACTGGATTTTGTTTCTGGTATTGAATAAACAATAACA
GGTCTAATAAGAATATAGGGCATTTGCTCGTTTTCTATATACTCCCAATAAACTAACTTA
TCTGTAGAAATATTATTATGAAGTCTTTCTCCAGGTCTTAAAAAACCTCTACTTTTATCA
TTATTACTATACACCTCACTAAGTTTATAGATAGATCCAGGCTTAATAATAATTTCTCGA
ACTATTCCATCCTTTTCGATTATTTCAACAATACCTGAGCTTTTAGAAAAAATATTTTTA
ACTAGCTCTGTTCCACTTTCTATAACATCTCCATTATGCACTAGTAGTAGTGATGAATCT
TTATTAATTTCGTGAGTTTCTTCTGAAATCCATAAGATATATCCCGGACTTAAAATTTCG
TAGGCATCTTTATCTAAGCCTGTTTTCTTTTTTGATACATTTAAGTCTAAATATTTAATA
ATACCACCACTTGTCGTTTTATACGTATCAGATATTAGTTCTGCGACTGTGTAACCATCT
TGAATTTTTTGGTTTGGAATAGCTTTGAAAAGAAATTTTTGTTTTTTTTCTGTTTCTAAA
ATATAAGATTCTTTTTTGTGGATTACATCTGTATAAATATAACAACCTGGAATTACAATA
GATTCAGTAATAACTTGTATATTGGTAATACTGCTATTAGTATTCTGGTGTATCCTTACT
TCTCCAGCATAATGATTTATTAATTCTGTCTGTGCAAGTATCGTACCAGCTTTAGTTTTA
TCTTCTTTATGAACAATAACATTAGCAGAATCAGATATACTATAAACTTCACCTGAGAGC
ACCCAGATCAAGCCTCCAGTTTTAGTAATTCTTGTAGTATATTGATTATTATCTGTTTCT
TCAACTGTTAAGTTGGAGAAACAGATTTTGCCAGAGAGATCTGAGACTACATGTTTTTGA
GCTCTTTCTGTCATGAGTCGATTTCTGCGGGGTGATTCAGCAATCACTTGATCCTTCGAT
ACTAATTCACCATCACATATTAAAAGAGTAGTACCTTTACTTAAGTTAATTATTGACTTC
TGATTTTTCTTAGATCTGATTGTAACTTGGGTTGGCTTTTTTGTTATTAATGCTTGCTCA
CCATGTCTGGTACGAACATCTGTATACGATTCAATATCTAGATCTATCAATTGACCATCT
ATAGGTGCGTAGATTTGTTCTGCTAATTCACCGGTAAATACTCCTCCAGTATGAAAGGTC
CTCATTGTCAATTGAGTGCCAGGCTCACCAATAGATTGAGCCGCAATAATACCAACAGCC
TCTCCTAAATCAACTAAGCGGCCATGTGCAAGATTCCATCCGTAACAATACTGACAAACA
GAACTTCTAGAGTTACATGTAACAGGAGATCTTACTAAAACTTTTTTGATACCAGCTTTA
GTTATCTCTTTTGCTAACTTAGGACTGACATCTTGGTTCGTATGAGCAATTAAACAATTT

GTCTCTGGATGGAATACATTTTCTGCTAATACTCGGCCAGATAAAGCTTGTTCTAGATTA
ATTAATATTTTCTGCGTATCTACTAAATCTTCTAGTATGATGCCTTTATTGGTCTTACAG
TCTACTTCTCTAATTATTACATCTTGAGAAACATCGACAAGTCGACGCGTCAAATAGCCG
GAATCTGCTGTTCTTAAAGCAGTATCAACTAATCCTTTTCTTGCTCCGTATGATGATATA
AAATAATCAGTAACAGTTAGTCCTTCTCTAAAATTACTCGATATTGGAAGATCAATAATT
TGTCCCTGTGGATCAGCCATAAGCCCTCTCATGCCCACTAACTGTCTTACTTGAGAAATA
TTACCTCTAGCACCTGAGAACGCCATCATATAAACAGCATTTAGAGGATCTGTCTCTTTA
AAGTATTTAATAACTTCTTGTTTCAAGGATTCACTCGCATTATTCCACGTATCAATAACT
TTTTGAAATCGTTCAACTGCTGTAATCTCACCTCGGCGATATTTGTTTTCAGTGGCCTTA
ATATCTTCAATCGTGCCTAATAAAAGACTTTGCTTACTAGGAGGAATTCTTAAATCTTCT
AAACTTAAAGAAATTCCTGCCTGGGTCGCATAATGAAATCCAAGATCTTTAAGCTTGTCG
GCCATATTGGCTGCTCGGGCTATACCATAATTACGAAAAGCCCAGACAATTAAATTTTTT
AGTTCGTTTTTATCAATAACCTTATTTGAAAAACTTGGCTGTGCAAGACTTCGTCTATTA
TCCACTAAATCTTCTCCATATTAAATGTTCGTTATTAACTATGCTACTAAAGACTCTTGA
ATAATTTTATTAAAGATTATACGGCCAGCTGTTGTTCTAATATATTGCACAATTCTCTGA
TTTTCAGCGTCTTCTTTTATGATATGGTTATTAAAAAATTTTGTTGTACTATTGTCAGAA
TGTTTTTCAATCTTAATCGGATCATGAGGTTGATCACCATCTACTAATCCATCAAAACGA
GCCCATATATAAGAATGTAAATCAATTTTCTTCTGTTCATAAGCCATAACTACATCTTCT
AAGCTTGCAAAATACTGATTAGATCCCTGCTGCTGAGATGGATTATTAGCTGTTAAATAA
TAGCAACCTAAAACCATGTCTTGGCTTGGCATAATAATTGGTTGACCTGTTGCTGGAGAG
AGAAAATTATGAGGGGCTAACATTAGCAATCTAGCTTCGGCTTGAGCCTCTAAAGACAAA
GGAACATGAACAGCCATTTGATCTCCGTCAAAATCAGCGTTAAACGCTGGGCATACTAAT
GGATGAAGCTTAATAGCTCTTCCTTCTACAAGAATAGGTTCAAATGCTTGAATACCTAAT
CTGTGTAGTGTGGGAGCTCTGTTCAATAGTACTGGGTGACCTTGAATAACTTCATTTAAG
ACATTCCAAATAGAAGATTCATTTTTTTGAATCATCTTTTTGGCAGCTTTAATATTGTTA
ACTAATCCCTGCAAAATTAGTCTGTGAATAACAAAAGGCTGAAATAACTCCAAAGCCATT
TCTCGTGGCAATCCACATTGATGTAGTTTTAAATGAGGACCTACAACAATTACAGATCTA
CCGGAATAATCTACTCTTTTACCTAGCAAGTTTTGTCTAAAACGACCTTGTTTTCCTTCT
ATAATATCTGACAAAGATTTTAATGGTCTATTATTTGCACCTACAACAGTTCTACCTCTA
CGACCATTATCCATGAGAGAGTCAACGGCCTCTTGCAGCATTCTTTTTTCATTTCTGATA
ATAATCTCCGGAGCTAAAATTGACTTTAGACGAGATAATCTATTATTTCTATTAATAATT
CGACGATAAAACTCATTCAAATCTGCCGTCGCAAATCTACCTCCATCTAATTGGACCATA
GGCCGTAAGTCTGGAGGTATCACAGGAATGACTGTGAATACCATCCAAGAAGGATCTGCA
CCTGTTGCTATGAAATTTTCAATCAATCTTAAGCGTTTCATTTTTTTATTGAATTTTAAT
GAAGGTGTCTTAAAACTTTTTGGTGGATTTGTAGCTTCAGACCTTAAGGTTTCAGCTATG
TGCTCTAAATCTAAATCTTTCAACAGTTTCTGAATAGCTTCTGCACCTATTCCTACTTCT

ACTTGGTTATTCTCATCTTGATTTTGGTAGATTTCCTCTTCAAGACTTTTCCATTCGTAA
CCTTCGAGAAGTTGCTTGTATTTTAAATTAATATCTGTATTAGATTGCGTAACTACATAA
GAATGAAAATAAACAATTTTTTCTACTTCTTTGACTTTTAAGTCTAATGCTAAAGCAATA
TAGCTTGTACTTCCTTTTAAGTACCAAACATGAGTTACTGGTGAGGCAAGTTCAATATAA
GCCATCCGATGTCTTCTCACGCGTGATTCTGTAACTTCTACGCCACATCGTTCACAAACT
ATACCTTTGTAGCGAAAACGTTTATATTTACCACAGTGACATTCCCAATCCTTAACTGGG
CCAAAAATTTTCTCACAGAATAAACCATCCATTTCTGGCTTTAAAGTTCTGTAATTAATT
GTTTCAGGCTTTGTAATTTCACCAACAATTTGTCCGTTGGGCAAACTTCTTTCGCCCCAC
TGTCGAATTTTTTCGGGAGAAGCTAAACTAATTTTTACGTAGTCAAAATATTGCTCAAAC
TTTGTCATAAATTTGAATACCTTAAAATCAAATAATTAGCCAATTTATTTACATGCTTCA
TATTGAAAAATGAATATCTTAATAAAGGAATTGTTCAAAATCGTCTACTGGAGGAGTATC
ATAATTAGAACGATCTACTCGGTTATCTTTAGAATCCGACATCAAGTCAACTTCAACTGT
CCGTCTTTGGCCATCTTCAAATAGTTTCAATTTATGTACTGCAATGTCTAAACCTAATGA
CTGCAGCTCTCGCATTAAGACTTTAAAAGATTCTGGAGTTCCAGGCTTCGGTATAGGCTT
CCCTTTAACTATTGCATTTAATGCTTCATTTCTAGCTTGCATATCATCCGACTTTACAGT
TAATAATTCTTGCAAAGTATATGCTGCACCAAAAGCTTCTAAAGCCCACACTTCCATTTC
ACCTAATCTTTGACCTCCATGCTGTGCTCTACCTCCTAATGGCTGCTGCGTAACTAGAGA
GTAAGGACCAGTGGATCTTGCATGGATTTTATCATCAACAAGATGAACAAGCTTAAGCAT
ATAAGCTCTACCAACTGTAACAGGATTATCAAAAGGCTCTCCTGTTCTACCATCAAAAAC
TTGCATTTTACCTGGATGCTGATCATTAAAAAGCCATTTATTGCTAGTAATCAATGATGC
TTCTTTTAATTTTCTATTTACAAGTGCTCTTGATGCTTCTGCTCCATACATTTCATCAAA
AGGTATTATTTTAAATCTCTTTCCTAAATAGCCACCTGCTAAGCCTAGTAGGCACTCAAA
TACTTGACCAACATTCATTCTAGAAGGTACACCTAAAGGATTTAAAACAATGTCCACTGG
TGTTCCATCTGATAAATAAGGCATATCTTGTTTAGGTAAGATCCTAGAAATGATACCTTT
ATTGCCGTGACGACCAGCCATTTTATCACCCACTTGGATTTTTCTCTTCTGCGCAACATA
GACGCGAATCATAGCATTTGTTCCGGGAGGCAGCTCATCTCCTTTCTGCCTAGTAAAAAC
CCTAACCTTAACTACTCTACCTTTTGCAGCATTAGGAAGCCGTAAAGATGTATCTCTCAC
ATCTCGAGCCTTTTCTCCAAAAATAGCTCGCAACAATTTACCTTCTGGTAGTTGATCAGC
TTCACCTTTAGGAGTAATTTTTCCTACTAAAATATCTCCAGCTTCAACCCAAGAACCACC
GACAACAATTCCATTTCTATCTAAATCCTTCAGAGAGTTATCGCTGACGTTAGGAATCTC
TCTAGTAATTTCTTCTGGTCCTAATTTAGTTTGACGGCATTCTACTTCATACTTTTCGAT
ATGGATAGAAGTATACAAGTCATCATAAACTAATCTTTCGCTAATTAAAAATGCATCTTC
GTAGTTATAACCTTCCCAAGGCATATAAGCAACTAGAATATTTCTCCCTAAAGCGATTTC
CCCTCCGTCTGTGGATGCGCCATCAGCTAAAGTTTGGCCAACAACTATTTTTTCTCCTAC
CCAAACAATTGGGCGCTGATTAATACAAGTATCTTGGTTAGACCGATAATATTTTTTTAA
GCGATAATGAACTGTTCTACCACTATTATCCTGAATGCCTATTTTATTAGCAGAAACATA

ATTTACATGACCAGATGTTCTGCTAATAACAACCATACCAGAGTCTCGAGCTATTTTAGT
CTCAAGACCAGTACCAATTATTGGCTTTTCTGGATATAATAATGGAACAGCTTGCCTTTG
CATATTAGATCCCATTAGAGCTCTATTCGCATCATCATGTTCCAAAAAAGGTATTAATGA
TGTTGCAGCTGAAATTACTTGAATAGGTGAAATTGCAATATAATCTACTTGGGTTGGGGT
TGTTGTAATAAACTCTTGACGATAACGTACAGGAATAATATCTCCTTCAATGTAATGTTG
TTTACTAACTTTAACATCTCCTGGAGCTACTCGGAAATCATCTTCCTCATCTGCTGTTAG
ATAGACAGGACTATTGTGATAAATCACTTGACCTTGGTTTACCGGATAAAATGGGGTTTC
TATGAAACCAAAAACATTGACCCGCGCACAGGTTGCTAGTGAACCAATTAAACCTGCGTT
CGGACCTTCCGGAGTTTCAATTGGGCAAATTCTACCATAGTGACTAGGATGTAAATCACG
AACTGCAAAACCTGCCCTGTCTTTATTAAAACCTCCTGGACCTAAGGCACTAATTCTTCT
CTTGTGAGTTAGTTCTGCAACTGGATTAGTTTGGTCCATAAATTGAGAAAGCTGACTAGA
TCCAAAGAATTCTCTCACTGACGCAATTAATGGCTTGGGATTAATTAGATTTGATAAACT
CAATGAATCTATATCACATATCATCATTCTCTCTCTAATAATGCGTTCTAAACGATTGAG
ACCTACTCTGAATTGGTTCTGCAGCAATTCTCCAACTGAACGAACTCTTCTATTACCAAG
ATGATCTATATCATCAAGATTACCTGAGTTTTTATCCTTAATATTAATTAGATAATCAAT
TGAGGATAAGATGTCTTGAGGAGACAATACTCGGAACGTTTTAGGTATATTGAGACCTAG
TTTTTTATTAATTTTATATCTACCAACTTCACCCAAGTCATATCTTTTAGGATCAAAAAA
ACGAGAGTAAAGCATCTGCTTAGCAACAGCAACTGTTGCTGGTTCATTAGGTCTAAGCTT
AGAATAAACTATGAGCAGGGCTTCCTCATCAGTTACTTCTTCAATATTATTTTTTCCAAT
TTCTTTAGCTAATTCTTTAACTGAATATATTTGGGATGCAGAAATAAGAAAAGCATATTT
ACTAAGTCCTTTTTCAATTTCATCTTTATTGAGTCCAATAGCTCGCAGAAAAATATAAGC
ATTTACTTTGTGGGTCTTATCTATTCTTATCCAAATTTCTCCCTTAGGATCAATTTCAAA
TTTTAACCAAGAACCTCTATTAGAAATTAGGCTTGCACTATAAATCTGCTTACCATTTTT
GTCTATCTCTTGTTTATAATAGATTCCTGGACTACGGATTATTTGGTTAATAATAACTCT
TTCCGTCCCAGATACAATAAAAGTTCCTCTATTTGTCATTATTGGCAAGTCTCCAATAAA
AATTAATCTCTTTTTGTATTTTTTATTTTTTATCTGCTTTTCAGCACTGAGCTGTAGATG
GGTTGATGATAAATCTAATGATTTAATATTTTTTTCCTGGTTTTTAGAAGGTAGATCAAT
ATCTTTTCTTGTTAACTTTGCAGGAACATATATTTGAGCACTATAAGTTTTATCCCTATT
TTTTGCCTGTCTGACACTATAACGAGGAAATTTTATCTTGTACTCATTACCAAATAATTG
TAATTCGAGTCGACTAGTAGGGTCTGATATCTTCGGAAAAATTTCAAGAACTTCCGTCAA
ACCTTCTAATAAAAACCATTTAAAACTGGCCCTCTGAATTTCAACTAAATCTGGAAGAAG
TTTATTTTTTAAGCTTATACGTTGAACCATAGATCTCCTTTATAACAAATTTTGAAATCG
AAGAGTTTAATACTATTAATAGAGTACTGGTAGATAATGCATAAGCATAGTTTGCTACAT
TATTTCTTCTCTAGACATAACTAGAACATCATATTTTATTCAAAAAAAGATTTTTAAAAT
TTTTCTTCACACACACTATCTATAAGTACTTTGCTGCTAAAAGATAGTTCAAGCTATTAA
AAAAAACGAAATTAATACTTGTCTGTCTGTCAATTAATTGACATCAAGATAGACAAGTAT

TATTTATTTAATCAATTCTGTTTTTCTGAGCAAAAGACAATGCTCTCGCGAGTCTAGCTT
TTTTTCTAGCGCCTGTATTAGGGTGAAAGGCCCCTTTTTTTACAGCTTTATCAATCTTAC
TATATACTTGAGAAATACTTGACTGCACATCATTGAAATTATCACTTTCTAAATTATCAA
TATTTAGAAGACATCTTTTAGTTAAAGTTTTGACAACCGATTTATACTTACGGTTAATGA
GACGATTTCTTTCCGAAGTTTTAATGCGTTTAATCGCAGAAAGGTTCTTAGCCACAGTAA
ATCCAATAAAACTTTTATAAAATATTTTTAGAAACGAAAATACACAAATCTGGGATCTAT
TCAAAAAGGGAATATGCGGCGTATTATAACATTAAAGAAAAACAATAGGCAATAGTTCAC
CAAGAGCTCTTATTATTACACAATAGAGATTGTCATTGCGAAAAACTTTGTAAGATAGTA
TTTAATTAAAATAAACTAACTAGACATATAAATATATATGAAAAAAATTGAAGCTATTAT
TAGACCTTTCAAGCTTAATGAAGTAAAACTTGCTCTAGTCAAGGAAGGTATTGGAGGAAT
GACGGTTATCAAAGTTTCTGGATTTGGGAGACAGAAGGGTCAAACTGAAAGATATAAAGG
ATCCGAATACTCTATTGATATCATTGATAAAATAAAAATTGAAATTATTATTAGCGATGA
TAAAGTAGAGAAAATTGTAGAGACGATTATTAAGGCTTCTAAAACAGGGGAAATTGGAGA
TGGAAAAATATTTATTAGTAGTATTGAAAGAGTAATAAGAATCAGGACTAATGACTTAAA
TTTTGAAGCTTTATAGTATTTTTACTTTTTTAGACTTGATACTTCTTCACTAAACATGAG
ATAATAAGCGTTTATTGTATACTTATAACTAAGGTGTAATACTAATGGCTAAAAGCAAAG
GTGCACGAATCGTAATAACTTTAGAGTGCTCTGATAAAGCTGGAGAGTTTGCTCAAAAAA
GGAAACCTGGCGTTTTTCGATATACAACTACTAAAAATAGACGAAATACACCAAGTAGAA
TTGAATTAAACAAGTTTTGTCCTAATTGTAATCAGCATTGTATCTTCAAAGAAATTAAAT
AGTTATTTATCTTATTAAAATAATCATATTATGGCTATATACAGAAAAAGAATATCTCCA
ATTAAGCCAACAGATGCTGTTGACTACAAAGATATTGACTTGCTAAGAAAATTTATTACA
GAACAAGGCAAAATACTGCCTAAAAGGTCAACTGGATTGACTTCGAAGCAGCAAAAAAAA
CTTACTAAAGCGATTAAACAAGCTAGAATCCTTTCTTTATTACCTTTTTTAAATAAAGAT
TAAATATATAGCCTAACTTCATATTTTTCCATCTAAATATTAAAAATTTCTATAGATTTG
TTTATTAAAATGATCTAACATACTGAATTACTAAGAAGATCTACTCATTACAAGTTTTGT
TCATTTTATAATTAATAATAGCATCATAATTTATTAAGCTAGATTCAGACAAGACATAAA
GGTAACCTCTTTTAATTGATTATATTATATAGTCTATTTAAAAGAGGAAATTTTTTTGCT
TCTAGCAATTCAAGATTCTTTAAGCTTTAACAAGGTATTTAAAACAAAGATTTTTGTTTA
GGTATCAGATCAAATGAATGAATCTTATCTCCCGACTGCCATAATTGAAATTCAGAGATG
AAAATTCCACATTCGTTGCCAGCTTGAATTTCTTCGACATCTTCTCTAACGCGCTTTAAA
GATTCAATTTTTCCTTGATAAATAACTTTTTCTTCTCGAATCACTTTAATCCAAGAGTTT
TTTAATAGCTTATTATTGATCACTCGACATCCTGCTATTTTTCTATTTGCTAACGAGAAT
ACTGTACTTACTTCTGCTTCTCCCACCGGTACTTCTGAATATTCAGGATCAAGTAAGTCT
TCCATCCTTCTTTTAATGTCTTCAATTAAGGCATAAATGATTTGATAGTTTTCTATTAAT
ATATTCGACTTTGCCGATGCTTGCTTTGTTCCTGGAGCAAAGTTGGTATTGAACCCAATG
AGGGTAGAATTTGTGGTTGAAGCTAGTTCAACATCTGTAGCAGTAATTTCTCCAGGCATG

ATTGAGACAACATTGAGTTGAACTTTACTCTGAGGAAATTGAGATAAAGAATCTAGGATT
GCTTCTGTAGAGCCTTGATTATCTGTTTTAATAATTAAAGAAATTTGCTTACTAATATCT
TTAGAATTAGTGTTTTTTAAAGTATCTAATGTAATTCGACTATTAAGTGCTCTTTGTTTC
TGTATAATTGATGAGTCTTTAGAGGTATTTTCAATCGCTTTAAGTTTGGCTTCTTTATCA
CTTTTAACTGCTAAAGCTATTTCTCCTGTAGCTGGTACAGATGACAATCCCCAAATTTCA
ACAACAGATGATGGTATTGCTAAATTAATTTTCTCTTTAGCGTTGTTAATTATAACTCGA
ATCTTTGCGTAAGCTGATCCAATAACCAAATTATCACTAATATTCAATGTACCATTTTGT
ATTAATAGAGTTGCTACTGGACCATGCGATTTATCTAAATGGGCTTCTATTATAATACCT
TGTGCAGGCTGTGTTGGATCAGCTTTCAAATCCTCTAATTCAGCTAACAAGGTAATAGTT
TCTAATAATTTATCAACATTTTGACCAGTAAGAGAACTAATAGGAATTATAGGCACTTGG
CCACCAAGTTTCTCCGACATGACATTGTATTTTAATAAATCTTGCTCAATAATATCTGTA
TTAGAACCAGCTTTATCTATCTTAGATATTGCTATGACAAAAGGGACATTCGCTTTTTGA
ATATGATTAATAGCCTCTATGGTTTGAGGTTTAACTCCGTCATCAGCAGCTATAATAATA
ATAGCGACATCTGTTAAATTAGCTCCCCTAGACCTCATACTTGTAAAAGCTTCATGGCCT
GGAGTATCCAAGAAAACAATTTTTTGTTTATTATCTTTCTTAATATACTCAACTTCATAA
GCTGCAATTGCTTGAGTAATTCCTCCAATTTCTTTGTTAGCATTATTAGATTTTCGAATA
TAATCTAATAGCGTTGTTTTGCCATGATCTACATGTCCCATCACTGTGACAATTGGAGGT
CTTTTAATATAATTGCCAGTTTCATAAAGAGCATTAGAGTGATCTAAATTACTAGAAAGT
CCATTATTATTTTCCTTTACATTTGATTCTACTGCTATACCAAAATTATCAGCCACTGAT
GATATTATTGAGGCGTCTATGGTTTGATTCATAGTAACTGATATTCTTTTAAGAAATAAA
TATTTTATAATATCTGTTTCTTGAACACAAATTAGTTTAGATAATTCTTGAATAGTTAGA
GGATTAGTAATACTAATAGACTCTGGCGGAGAATTAGATTGAATGCTTGCTGAATTTTGA
CTAGGAGTTACTGCTACTTTTTTCTGTCTTATAGATTTTTTATTACTAGTCAGTTTTTTT
ACAACTTCCACTTTTGGCTTAGGTGGACGCATTAAAGAGATAGCTAAATCTCCTGCAGTC
TGCGAAACATTAGAATTAGAGTCTCTAAAGTTGTCATCATCATCGTCAATATGTATTTTA
GTTTTTATTTTTTTGCGCTGTCTATTCTTATTTTTTTTACTATCTACTAAGTCGTGAATT
TTATTAAAATTTTTACTTTTTTTATCTAGTTTAGGTGGAGAACTTAATTCTAGATGCTGT
TCACCACCTGTATGTGATTCTGATTTATCAAGGTCTAAATTTAACAGATTATCATTATTG
ACTGACTCTAATCTAATTTTATAGATAATCTGAGGATTTTTCAAATCTACAATTGTTTCA
GAAGAATTGATATTTGAGCTAGATCTAGAGGATCTATTTTCAAAGTTTTGATTATTTAAA
AACATAAATATTGGTTTTATTATCTAATAGTAACTTTCTAATTTTAATACAGAGCTTAAT
ATAAACAAGAATCGAATATCTGTATAAATAATACAAATGTAGAGAATTAATCTATGTTGT
ATTAGATTTAATATAACACTAAAGTCCTTTGTTCGTTATACAAGTTTAAAACTTTTTATG
ATGATAAACACTAGATTCTAGTTTATTGGTATACAATCTATTTATCTAAATAATACTGTT
TTTGTCAGGGCTTTTAAATTATCTAATGTTATGTTCATTATGAAATTCTATTAAAATTTA
AAAACTCAACAATTAAGTAAGAATCGTTAATCACCTCCACGACACTTATGCCACGATCTC

AAAAAAATGATAATTTTATTGACAAAACTTTTACAGTATTAGCTGATATTGTATTAAAAA
TACTTCCTACAAGTAAAGAGGAAAAAGAAGCTTTCTCTTACTATCGAGATGGAATGTCTG
CACAATCTGAAGGTGAGTATGCGGAAGCTTTAGAAAATTATTATGAGGCTTTAAAACTAG
AGGAAGATCCATATGATAGAAGCTATATTTTATATAATATAGGGCTTATTTATGCTAGTA
ATGGTGAGTATGTAAAAGCTTTAGAATACTACCATCAAGGATTAGAACTAAATTTTAAAT
TACCTCAGGCCCTTAATAATATAGCCGTGATATACCATTACCAAGGAGTTCAAGCAATTG
AGGATAAAGATACAGAGCTATCTAAATTAATGTTTGATAAGGCGGCTCAATATTGGCAGC
AGGCTATTAAGTTAGCTCCTGATAATTATATTGAAGCTCAAAATTGGCTGAAAACGACAG
GACGAATGAGGAATATACAAGGATATTAATATATTTAAGATATAATAAAGATATCAATTA
ATAAATTAAAATTAGTTAAAGAAACTGGTACTATATAGAGTACTTTACTATATATTATAA
TATTATACTAGTTGA------TCTCTAAGACTTAGCTGAATAAACGAAATTAAATCTATT
GACTATATTCATGAATACAAATAAGCATAATGGTTAGTACAACAAAAAGTACCGGATCTG
TTACTCAAATTATTGGACCAGTTTTAGATATTGCATTTCCTAACGGACAGCTTCCGAAAG
TATTCAACGCACTCAAAGTACAAAGCTCAGAAGGAACTATTACTTGTGAAGTACAACAAC
TTTTGGGTGACAACAAGGTACGAGCTGTTTCTATGAGTTCCACTGAAGGACTACAAAGAG
GGGTAGAAGTTATTGATACTGGATCCCCTATATCTGTTCCTGTAGGTACAGATACTCTTG
GACGTATTTTTAATGTTTTAGGTGAACCTGTAGATAATTTGGGTCCCGTTGATTCTGAGA
GTACTTTACCTATCCATCGACCAGCACCTGCTTTTACTAAGTTAGAGACAAAACCAAATA
TTTTTGAAACAGGTATTAAAGTCGTCGATTTACTTGCTCCTTATAGAAGAGGTGGGAAAA
TTGGTTTATTCGGAGGTGCTGGAGTAGGTAAAACTGTATTAATTATGGAACTAATTAATA
ACATCGCTAAAGCTCATGGTGGAGTATCTGTATTTGGAGGCGTTGGTGAAAGAACAAGAG
AAGGAAACGACCTATATATGGAAATGAAAGAGTCTAAAGTAATTGATGCAGATAATCTGA
AAGAATCTAAAGTAGCATTAGTATATGGTCAAATGAATGAACCTCCTGGAGCACGTATGC
GCGTTGGCTTAACTGCATTGACAATGGCAGAATACTTTAGAGATATTAACAAACAAGACG
TTCTATTGTTTATTGATAATATTTTTCGGTTTGTACAAGCTGGATCAGAAGTATCAGCTC
TACTAGGCCGTATGCCATCTGCTGTGGGTTACCAACCAACTCTAGCAACTGAAATGGGAG
CACTTCAAGAAAGAATTACTTCAACAACAGAAGGATCAATTACATCTATTCAAGCTGTAT
ATGTGCCGGCTGATGATTTAACAGACCCAGCTCCGGCCACTACATTTGCACATTTAGATG
CAACAACAGTACTTTCGAGGAACTTGGCGGCAAAAGGAATTTACCCTGCAGTGGATCCAC
TGGATTCAACATCAACAATGCTACAGCCTGGAATTGTTGGAACTGAGCATTATTCTACTG
CTCAAGAGGTAAAATCAACTTTACAAAGATATAAAGAACTGCAAGATATTATTGCTATTC
TTGGTCTTGATGAACTTTCAGAGGAAGACAGACAAACTGTATCAAGAGCAAGAAAAATTG
AAAGATTTTTATCTCAACCTTTTTTCGTGGCAGAAGTGTTTACTGGATCACCTGGGAAAT
ATGTATCTTTGGAAGATGCAATTAAAGGGTTTCAAATGATCTTAAAAGGCAATTTAGACG
ACTTGCCTGAGCAGGCATTTTATCTAGTAGGTGATATAGATGAAGCTATACAAAAAGCTG
ACAGCATGAAAGATTAATATAATTAATGATGACTTTAAATATAAGAATTATTGCTCCTGA

TCGGACTGTTTGGGATGCAGAAGCACAAGAAATTATTTTACCAAGTAGTACAGGGCAACT
TGGTATTTTAACAGGCCATGCACCTTTGCTTACAGCTTTAGATATTGGAGTTATGAGAGT
AAGAGTAGATAAAGAATGGATGCCGATTGTTTTGCTGGGCGGTTTTGCAGAAATAGAGAA
CAATCAATTAACTATTCTGGTTAATGGCGCAGAAGAAGCTAGTCAAATTGATTTATCAGA
AGCAGAAAAAAATTTAGACACTGCAACTCAACTCTTAAGTGATGCCTCGTCTAATAAAGA
AAAAATAGAAGCAACACAAAAAATACGAAAAGCTCGAGCTCGAGTACAAGCTGCAACAGC
AGCAACTTCGTAAAAGATTCCTCTTAAAACATAAAACTTTAGTAATGATTACTAAA----
---GTTTTATGTTTTATTTGTATGGTTATAGGCAGCTTAAATAAACTTAGCTTAGACCTG
AACAAATATAATCAAAATATAATCCCATCTCTTTACCTGCATCTGGTCCAACTAAACTAA
TAGTAACTTCTTTCATAGCTAAAATAGCTTGAATCGTTGCACCAATAGGAACACCTAGAG
AATTGTATGTTTCTTTTAGACCATTTAATACTCTTTCTTCAAGGATAGATGGATCTCCAG
CTAGCATTCCATAAGTAGCATAGCGTAAGTAGTAGTCTAAATCACGGATGCATGCAGCAT
AACGTCGAGTTGTATACATATTACCACCTGGGCGTGTAATATCCGAATAAAGTAGAGATT
TTGCTACGGATTCTTTAATAATTGTTGCTGCATTAGCTGCAATAGTAGCAGCAGCTCTGA
CTCGTAGCTCACCTGTTTGAAAATAGCCTCTTAATTTTTCAACAGAGCTATCATCTAAAT
ATTTACCTTGGACATCAGCTGCATTAATAACAGAAGTAATTGCGTCTTGCATAACTTTCA
CACATTCCTTAATTTTAAATATCTAATACTAGGGGTATATAACAAAATTACTAGATCTTT
TAGCAATATCAGTATTACTGCATAGCACCCAAAGTATAATCAAAGTAAAAACCAGCTTCT
GCTGAATCTTCACCTGCAAGCAGTGAGCAAGCTACACTTTTCATGCATTTTACACCTTCA
GCAACTCCTGAAATTGGTGTTCCTAAAGAATTATACATTTCTTTAACACCCACTAATCCA
ATTTCTTCAATAGGAGTTACATCGCCAGCAACTATTCCGTAAGTTACTAAGCGAAGATAA
TAGTCCAGATCTCGTAGACAAGTAGCAGTCATCTCTTCGCCATAAGCATTTCCACCAGGA
GAAACTACATCAGGTCTTTTTTGAAACAGTTGTTGGCCGCCTTGCTTTACAATACGCTCA
CGATTATCTGTTAAAATTTGAGCTATTCTTAAACGACGTTGTCCAGATAAAACAAAACTT
TTAATTCTATCTAATTCCCCAGGACTTAAATATCTTGCTTCTGCATCTGCATTTACAATT
GACTTTGTAACAATACTCATGGATAAAACTCCTGTAATGCTTT-AAAAAAAAGCTTAATA
CTAATTATTTATGATATAACTCAGAAAGTAATGTACTTTTTGAGAATTTTATAACCGAAA
CACGTTCATGATATATGAAGCAGGATAGATACTGAGGTAAGTACTAAAACGAGAAAGTTA
TATGTAGGAACTTCTGCTATTGATTTCCTAGTACTTTTTTAAAACTTGGTACCACAATTG
ATATATCCTGCTTAGTCAAACGATTATAGAGTGTTTCTGTATTAGGAAAGTTAGCTGCTG
GTAAAGTCGGAAATCTTCTGTATGGAACAGTATCTTTGCCATATACCGCATTGTATTCAA
CACTTTCAACTAAATTACTTATAAATGCAGATAAGCCTTTTGAAGCTAAAATTTGATTAA
AGAATCTAATTTCTGCCTGATTATTAGGAGCTCTTCCTAAGATATGCTTAGTACCTAATT
CTATAACTTTTGTATTTGGATATGGCTGATAAAATTCTTTACCATATAGTTCAGATAGCG
CCAGTTTTTCTACTAGTTCTTTGACACAGATTTGTCTATTCAAGAAAGCTGACTTAATAT
CCAAGAATTCACCGCCGACACTAAAAGAGTTAAGATCTCTTTCGAAAATTTGACGATATG

CTGCTCTTAAAGCTTGTTCTAACATTTCTTTGTTACTATCACTATTGACTTCAAAGACAA
TAGATTGATCTCTTAAAGAAGTTACTCCCTGTGAAATACGAGACTGAATATCATTACTAC
TCCTCATTTCTTTGACAGTGCCTAATTCAACAAACCTAGCAGATTTACTAGCAATAACTT
TTTTGAATCTCTGGTCAATAATTCCTGGTCTTAAGCTTCTTAAAGCAACACCAGCTGGAG
TACTATATCTTTCATAAGGAACAGTATTATCTCCAAAAGTTTCTGTGTACTCCGAGCTAT
CAATTATTGTGTCTATTACTTGGTAATAGCCTTGCTTATATGCAATATCAAAATATTTAT
TAATTTCTTGCCGACCATATGTAGGGCGACCCAGTAAGCGGTTATGAATATATTCAATAG
CTTTACAAATATATAATGGCTCCCAATAAAGAGATCTAAATATACTTGATTTTGCTAACT
GTCTAACAAACTCGCGAACTGTAATTTGATTATCTTTTAGCTGACTTTCTATTGGCTTGA
GAATTAGTTTTTCTTCTTGATACACTTCTCTGCCAAAAACTCTTAAATAAGCAACTTTTG
TGACAACTTCTACTGAATTTTCAAAATTTTGGGATGAATCTGACTTTATAAGAGCGTTGG
ACAATTTAAAGATTTTTGGTCCCAGGGATCCTGCTGACTTAGGTCTTACCTGTGGATTAC
TAATCTGATTGTAGATTCCTGGCCCACGTCTAACTAATATCCTTCTTGTATCTTTACCAA
AAAGAGCTTGTCTTTTTCTTGGATCTTTATTTTCTTTAGGGAAAATAGCTCCAAATTGAA
TTGATAATGGATCATTACCTATTCCATATGGATGTTGATCAGGTAATGATTGCTTATAAT
CACTAAATAAAGTTATAAATTGAGGAACCTTACGAAAAGGCGCACTATAGGTGAAAAGAT
CTATTTGAGGCCCCCAATTACGGCACTCTTGAGGCTCTTCACCTAAATTTCTAAAGTAAG
GCACAGTCTCTTCACCGAAATAATCTGTATACTCAGAAGAATTTAAAAGTGCATTCACTA
AACCACTTAAACCAGTAGATGATAAAATAGCAAAATATTTTTGAAACTCTTCTAAAGAGC
TAGGACCTCTACCCAAGAAATGTCTAAATGCTAATTCTAATGCTCGACTATTGACAAAAG
GTTCATAAAATTGCTTTCTATAAATACTTGAAGTTCCTAACGAACGAATGAATTCCTTGA
TTGAAATTTGGCCATTTTTTACTTGCGACTCCAAATTCGATAAAGAGAGGTCATATGCTT
TAGCAATATCTCTTTCAAAAATTTGTCTATAACATGCTTTCACAACAATATTTTTCTCAT
CAGCAGATAAACTAGGCTTCATAACAAAACGAGGTGTTGACACTCCTGCCTGTACATAAG
TTTGAGGAAGACGCAAACCTTGTAAATCTCCAGATATTCTTTTTCTTAATTTATCAGTCA
AAGATGGAGCTTCAAATTCAGAAATCACAACATTAAAATACTCTTTAACTAAATCTTGAC
CTTTTATATCTTCCTCAAAGATTAATAATGCTGTCCGTCTCATCTCTCTAAGAGCCACAA
TTGCTGCTGCACTTGAGCAAGCGTTATCAATTAACTCTCTCAATCCTCGTATATTAACAG
ATAAAATATTAGGGTCGCCTGATACAATTGCATATGTCAAATACCGTAAAAACCAGTCTA
AATCTCGAAGAGATTTTCTCATTCTTGTAGTACCGTATCTCAAGACGTTTATAGGTTTAA
ACCCTGGCGGAGTAGCTCCCCCAGCATTAAATAAAGATCGGAAACTTTGGCCAAAATCTC
CTTGGATATTTCCTGATAACTCATTAATTTTATCTTGAGAACTTTGATCTCCTGCAATAA
TAACAGCTGCTTGCGGACGTTCTAAATAAGAAATTGCAGACCCACCTACGAATATTTTAT
CTGCTGCTCGTGCTACCAGAATATTTGCATTTTTAGTTAAAATATCTGCAACTTCCAGTC
TTTTTTGTCCAGAATTTAAAAAAGAAACTAATTGATTTAATTCACCAAGCTGTAAAAATC
GGTCTTGTTGCTCTGCCTGAGTAATAGTTAAAATTGAGGCAGTCCGATAAAGCTGGGGGC

GTGCTAATGGGCTTCCGCCACTTGCTTTGATACTCATTACTTTATTTATCTCCTTAACTG
ATATTGCTTGCTAGCGAATACAAATATAGCTTGATATTTCTAAATATTTAAAGATACTAT
GCAAGCACACTGTTTTCTATATTATTAGGTACTAGTACTTATAGTACAGAGAGTCTTATA
ACTAACTTCTATAAATACAAATTTTGTAATACTTCTATTAACATTTGTCAAAAAAATATT
TAAGTGCTCACTCTTTTATATTAAAATTCAATTAGTGAGAGATTGATGTGAAATAAAGTA
TACTAAGTAAGTTTTATAGATAGGAAAATTAAAAGATTTATTCTACAAACATTAATCACT
TGAATGTTTAGCAATTGTACAATTGTTATTGCTTTTAATTGGTTTAGACTAGTTTTATAT
TTTTTCTAACATAAACAATATTAATGAGCAATTTGTATGAATTTAAAGCCACAAAACAAC
CTATTAACTAACGAAAATATAAATCATGTAGATATACCCGAAAATGATATACCAATGTCA
ATTACGGAACATTTAGAAGAATTAAGACAACGGACTTTATTTGTGTTTTTATTCTTTTTG
TTTGCTACAACTATAAGTTTCACACAAATTAAGATCATTGTTGCAATACTGCAAGCTCCT
GCTGTTGGTATTAAGTTTTTACAACTGGCACCAGGAGAATATTTTTTTTCATCTATTAAG
GTTGCAATATATTGCGGGATTGTTGCAACAACTCCGTTTGCCGTTTATCAAGTTATATTA
TATATACTCCCAGGACTAACTGGAAAAGAAAGAAAAATTATTTTGCCTCTATTAATTAGT
TCTGTATTGCTTTTTATTACAGGTGGTATATTTGCTTACTTTGTTCTCGCACCAGCAGCT
TTAACATTTTTAATTAGCTATGGGTCTGATATTGTAGAACCATTATGGTCTTTCGAACAA
TACTTTGACTTTATTCTATTACTTTTACTTAGTACAGGATTAGCATTTGAGATACCAATC
ATACAATTATTGCTCGGTGTTTCAGGGACATTTTCTTCTAGTCAAATGATACGAGCTTGG
AGATATATTATTATTATAGCAACAATTGCTGGAGCTATTCTGACCCCTTCGACTGATCCC
GTTACACAATTAATAATGTCTTCAGCTGTTTTATTACTTTATTTTGGTGGAATTGTTATA
TTATTAGTCTTAAAAAAGTAGAGTAAATATTTTCTATTAATCCAATATATTTGTTATCTG
GGCAAATAATCTATAAAAACAATTGCTAGATTTAAGTATCAGGCTTCTAAATATTTCAAG
TCTCTTTTTATCATTTAAATACAATCGATACATTGGCTTGTTTTTTCATCAATTATTTGT
GTTGCACACTTTTTAGCACTCTCTTTATTGTTCTTAGAATTATTTCTTTAATCTATTTAT
TAAATAGATATGAAGAGATTTAAGTATTGATTATGGTAATCTATATCAACGTAAAAAGTA
TTTTATTGTGAGTTTTATTTATATAAAGGCTTATTCATTATATAGTTATAACAGTAAGTC
TAATTAATAGTTTTCATGCTTAATTAAAGTAATAACTCTTAATTCTTTTGGCAGAAAAAT
CTGTCTTAATATTTTATCAAAAAAACAGAAAAATGAAGCTAAATAGTCTAATTAACTTAA
TTCAAAAGTCTATATATTCTTGTACACTTTTACTAATTATTTTAAATATTATTTGTGTCG
CACCTAATTCTAGTAATGCATTTCCAATTTATGCGCAACAAGCTTATGAAAGTCCAAGAG
AAGCAACTGGTAGGATAGTATGTGCCAATTGTCATCTTGCTCAAAAGCCTGTGGAGATAG
AAGCGCCTCAAGCAGTACTACCTAATACTGTTTTCGAGACTGTTGTGAAGATTCCATATG
ACAGCAATGCTAAACAGATTTTAGGTAATGGCAGTAAAGGAGGCTTAAATGTTGGAGCCG
TTGTAATATTACCTGAAGGATTTAAGTTAGCTCCTGTTAATAGATTATCTACAGAGTTAA
AAGAAAAGACTAGAAATCTTTACATTCAGCCGTACAGTGCTAAACAAGACAACATTTTAG
TAATTGGACCTATTTCTGGTGATAAAAATAGAGAAATAGTTTTCCCAATACTATCTCCCG

ATCCTGCAAAAGATAAAAAAGCTCATTTTTTCAAGTATCCAATATATGTTGGCGGAAATA
GAGGACGAGGCCAGATTTATCCAACGGGTGACAAAAGCAACAATAATATTGTCTCTGCTT
TAAGCAGTGGTAAAATTAATAAAATTGAATTACTAGACAAAGGTGGATTTATAATACATG
TGACTAACAGTAGCAATGTAGAGTCAACACAGAAGATTTCACCTGGTCTCGAACTTAGAG
TAAAAGAAGGGGATACAATTCAGCTTGATCAAGCTTTGAATAGTGATCCAAATGTAGGTG
GTTTTGGTCAGAACGAAACAGAAATAGTTTTACAGAGTCCAAATAGAATTAAGGGCATGA
TTGTTTTCTTTTTCGCTAGTGTTCTAGCTCAAATTTTCTTCGTATTAAAGAAAAAACAAT
TTGAAAAAGTTCAAGCAGCTGAAATGAATTTTTAAAGCAATATAAAACTGGCACTTACTA
TAAAGAAAGTATAAACACTTTATTCACAGGATGTACCAGTTTTTATGTTATGTAATAATC
GCTTTGACGGAAGTTATGATACTTTTAACAGAATCTACAATCTGACTAGGCTGAATTACT
GTAGCCTGCTCTAAGCTTCCATTATATGGTGTGGGTATATCCTGCGAAGATAGCCTCACG
ACAGGAGCATCTAATTCATCAAAAAGGTACTCATTAATTTGTGCAATTAGCTCTGCTCCA
ATTCCGGCTGTTTTCATACACTCTTCTACAATTAAAACTTTGTGAGTTTTCTTTACTGAG
ATAGATATAGAGTCTATATCCAGAGGTTTTAAAGATATAAGATCTATAACTTCTGGATCA
TAACCTTCTTTTAATAAGGCCGGTAATGCTTGGATAACATGATGCCTCATTCTAGAATAG
GTTAAAATCGTAATATCTTTTCCTTTTCGTACAAATTCAACTTTATTGAGAGGTAAGAAA
TATTCTTCTTGAGGAATCTCTTCTTGTAAATTATAAAGTAGAACGTGCTCAAAGAATACA
ACTGGATTATTGTCTCGAATTGCAGATTTCAGTAATCCTTTTGCATTATAAGGAGTAGAA
CAAGCAACTATTTTTAAGCCGGGGATGGCTTGGAAATAAGCTTCCAGTCTCTGAGAATGC
TCTGCACCTAACTGCCTACCAACTCCTCCCGGTCCTCTAATAACTAAAGGCAATGTAAAA
TTACCTCCAGAAGTATAACGTAACATTCCTGCATTATTAGAAATTTGATTGAATGCTAAT
AATAAAAAACTCATGTTCATACCTTCAACAATTGGTCTTAGCCCTGTTATAGCTGCACCA
ATTGCCATACCAGTAAAGCTATTTTCTGCTATTGGTGTATCAAGAACTCTTAAATCCCCA
TATTTGCTATGCAAATCTTTAGTTACCTTATAAGATCCACCATAGTGACCAACATCTTCT
CCTATTACGCAGACAGTTGGATCTTTTGCCATTTCCTCATCTGTTGCCGCTCGTAAAGCG
TCAAACATAAAGATTTTACTCATTTTAATATTTGATTTTTATGATTTGACCACTGTTTTA
AAGTAACAGTTAAGACACCATATTCTATTTAGTTATCTGCAAAAAGATATCGTTTCAATT
CTGACATGTTAGGTTCTGGACTGGAAATAGCAAATTTCACAGCTTGTTCCAATTCGGTTT
TAACAGCATTCTGAATCTCATTAAGTTCACCTATATTAGCAATTTCATTATCTAGAATAT
ATTTTTTGAGTTTTTTGATAGGATCTCTTGCCACCCAAGCCTCTTTTTCTTGTCTTGATC
TTAGTTCATCAGGATCTGCGAGAGAGTGACCACGAAATCTATATGTTAATGCTTCTATTA
AGGTTGGACCATCACCTTGGCGAGCTCTTTGAACTGCTTGTTTTGCAGCTTGTCTTACAG
CTAGCACATCCATTCCATCAACTTCAATCCCAGGAAGCCCAAAAGCTTCTGCTTTTTTAT
GTATTTCAGGTATTGAAGAAGACCGGTGATGTGCCATACCTATAGCCCACTGATTATTTT
CAACAACAAATATAATAGGTAGTTTCCAGAGAACTGCCATATTCAGACATTCAAAAAATT
GCCCATTATTGGTAGTCCCATCACCAAAAAAGCAAGCCGTGACTCTTAAATCCTCTGTTT

CTTTAAGTACTTGCTGGCGGTAGATACTTTGAAAGGCTGCCCCTGTTGCAACCGGTATAC
CTTCCGCAATAAAAGCAAAGCCACCTAAAAAATTGTGAGGCGCAGAAAAAATATGCATCG
AACCTCCTCTGCCTTTACTACAACCAGTCTCTTTTCCAAATAACTCAGCCATCACATTTT
TAGATGGGACGCCTTTACTTAAAGCATGTACATGGTCTCGATAGGTACTGCAAACATAAT
CAGTTGGATTCAGAAGTTTAATTACACCTGTAGAAACAGCTTCTTGACCATTATAAAGAT
GAACAAAACCAAACATCTTTCCTTTATAATACATCTGAGCACACATATCTTCAAAATTTC
TGCCTAACAACATGTCTTCATATAAAACTAATAAATTACTCTTATTAAGATTAAGACCTG
TTGAATTATAGTTAGTCAGCGGCAATTGAACTTTCTTAGGATAACTCATAATTTGTAAAG
AAACCTCTTATTGTAAATAGGAATTTTGAAGAAACAGCTATCAATAGAAAACACACAGAT
AAAAACTTATAAATTATAATATGTTTTTTACTAAAGACTTTAATAATTATTTGAAACTAG
TTCAGTATATCAAATTAATTTTGCTTATACTACGGATGTCTAAAACTTATATAAAAAAGT
AGTAAAAAAGTTTATTAAAAGAATAGTTAAAAACAGATATAAATAGAAGTAAACAGAATT
ATTGAAAAAAAATGATTATTATTAATTAATACTTAATATTTCTAAATATTTTACAATTAA
TGGCTATGTCACCATCTTTATTTTATCCTGTTGAACAAGAACTATGTAGTCTTGAAAAAA
ATCTGAAAGCTGTTGCTGGGACTCGTCATCCAATTTTATATGCCGCAGCAAAGCATCTAT
TCGATGCTGGAGGAAAACGAGTTAGACCAGCTCTTGTATTTTTAGTGGCTAAAGCAACCT
CTGAGAAGCAAGATATAAATACTGGACAAAAAAGGCTAGCAGAAATTACTGAAATTATAC
ATACTGCTAGTTTGGTACATGATGATATTATTGACGAGTGCACAACACGTAGAGGAGTCA
AAACTGTACATAATTTATTCAATACTAAGATTGCTGTGCTAGCAGGAGATTTTTTATTTG
CACAGTCTTCTTGGTATTTAGCTAATATTGAAAATTTAGCCGTAGTCAAAGCTATTTCTA
AAGTCATCACCGACTTTGCAGAAGGAGAAATTAGGCAAGGCTTAGTTCATTTCGATCCCA
GTATTTCAATAGATGCTTACATTGAGAAATCATTTTACAAGACTGCTTCACTCATTGCTG
CTAGTTGTCGGGGTGCAGCTATGCTTAATGGTTCCAATCATCAAATAAATAATGATCTTT
ATCTTTACGGTAAACATATGGGATTAGCATTTCAAATTATGGACGATGTTCTAGATATAA
CTGGTTCTACTAAGAGCTTAGGAAAACCTGCTGGCGCTGATCTAATAAATGGAAATTTGA
CCTCTCCTCTCCTTTTTTCACTTACTCAAGAAGCAAGTTTAAATGATCTTATTGATAGGG
AGTTCTGTAATAGTACAGATATAGCCTCAACATTATTTCTTATAAAAAGAAGCGGGGGAA
TTACAAAAGCTAAAGATTTAGCTAAAGAACAGGTGCAGGCGGCACTTTCCTGCCTTCAGT
TTTTACCACAATCTACACCTGTATCTAGTTTAAAAGAATTAACACATTTCATAATCACAA
GATTGTCATAAAGGACTTGCTAAAATTAAAATGTTATTAACTAATTTTTTACTGTTTCTA
AAACAGTCTGAAAAGTTTGAATATCATTACTAGCTAGCTGCGCTAGCATTTTACGATTTA
ACGCAATATTCTCTTTTTTTAATGCACTAATAAAAGTACTATAATTCATGCCTTGATTGT
GAGCAGCAGCATTTATTCTAGTTATCCAAAGACGACGAAAATCTCTCTTTTTCCTTTTTC
GACCAACATAAGAATATCGAAGAGCTTTAAGGACTTGCTGCTTAGCTGTTCGAAATAAAC
ATTTATGTGCACCTTTAAAGCCTTTAGCTAGCTTAAAGATTTTAGCTCGTCTTTTTTTTG
CAACGTTACCTCTTTTAACTCTACTCATAAAATATTACTTAAATTTTAATTATTACCAGC

TTACTAAATTCTTATACTCTTTGATTATAAATAAGGTAATTTCATTGCAATGTTTTTAAT
ATCTTTTAAGTCAACCGAACAAGTAGAAGAAAGATGTCTTCTTTGTTTTGATGACTTTTT
TTGTAATAAATGACTTTTAGAAGCTTTATGTCGAAGAAATTTTCCGGATGAAGAGACTTT
AAATCTTTTTGCTATTGCTTTTGATGTTTTTAACTTAGGCATATTATAAACTTTTAATGA
TTAACAAAAGCATGAAAAGTCTGAGGATATCAATCTTAGACCTTTCAATACTTAGAATAA
TCTTAACACAATTTATTTTACTTACTACATAGGTTAACTGAAACAATTTATATATATAAT
ACTAATCAGTTAATCAACCATAGCTAAAGTGGATTTATATTTTGCTTTGAACTTTTGTAT
AGTATTAGCAATTAGCATTGTTATAATTTTAAGACTGCTTGAATTCAATTCTTCGAACAT
TTTCATATTTAATGTAAATGAAATATTGGCTTCTGAGACAATATTTTGAATTTGAACATC
AGACAAGGGAATCATATCTAATGCAGCTCTATATTGATCTTTAAATAATTTATCGTCTTT
TATTTGGTCAAAATCGTAAAATTTCGTTCCTCCTGAATCTGATAAATTCATAGCCCCTCT
AGCTATTTTTTTTAAAATTTGGCCACCAGAAAGGTCACCCAAATAGCGCGTATAAGCATG
AGCAACTAGTAATTCAGGTTGTTTATGTCCTATAGTGTGAATTCTATCAACATAAATTTT
TGTAGCAGGAGATGGTTCAATAAAATCTAACCAATCTGATCCATAATAATAGTTTAAATC
TTCAGATAGACTCGCTTTCCTATTAAGCTCTGTAAAATATATAGGTTTAATAGCTGGATG
ATTTTTATTAGAAAATAACTCTTCCTCTATTGCACAATAGACAAAGTATAGATTTGCAAC
CAATTTACGATATGACTTCTTATCTACAACTCCTCCCAAAAAAGATTTAACAAAACTAAC
ATTTTCTGCCATACTATGGGACTTAGTAGTACCTTCTCTTAGTTCATTCGCTAAAGTATT
AACCATAATATCAATATTCCATTTTTAAAGATAACAAATTAACAATAAATAGTTATCCGT
ATATTTATAATTGAGTTGCGCTAACTAGGCTGCTGCAAAATAATTTTTTGATTTTATTGG
ATCTGGATTCATTGTTTTGTCACCAGGCTTCCAATTAGCTGGACAAACTTCATCTGGATG
AGATTGTACATATTGAATTGCTTGTAAAACCCTCAAAGTTTCTTCTACACTCCTGCCAAA
CTCTAGATTATTGATTGTAGAGTACTGAATAATTCCTTTCGGGTCTATAATAAATAGTCC
TCTTAGAGCTACACCATCACTATTCAAAACATTATAAGCTGCACTGATCTCTTTTTTTAA
GTCTGATACTAATGGATATGAAAGATCTCCTAATCCACCTGATTCTCTGTCAGTCTGTAA
CCAAGCAAGATGAGAATACTCACTATCAACCGAGACTCCAAGAACTTCTGTATTAAGTTC
CGAAAAAGCATTATACTTATCGCTAAATGCTGTAATTTCTGTAGGGCACACAAATGTAAA
ATCTAAAGGATAAAAGAATAAGACAATATACTTATTCTTGAGATCAGATAATTTTAACGT
TTTAAATTCTTGGTCATAAACAGCTGTAGCTGAAAAATCAGGAGCTAGCTGACCTACTCG
AAGACAATTTGGTCCAGAAATCATTAATTTTTCCCAGAAGAATTGATATAATTTTGTTAT
TTAATAAATAATTATATAACAAAATTTAGTATATATTAAAATACTACCAGTCTTTTTTAA
ATTACTAAATGATATAATCAATACTATCTTTATAGCGGGGAATGGATTTGAACCATTGAC
CTTCGGGTTATGAGCCCGACGAGCTACCAGACTGCTCTACCCCGCGGATAACTAAAATCA
TATTAGTCTACATATATATATAAAAGCAAATGCATAATAGATTTTAGAAGTAAAAAAATA
GAAGAACTGGTCCACTGATCATTAAACTTAGAAACGACAAAGTATTTTTTATCTTATTTA
AAAGAGGAAAGACTTCATATAAACCAATAAATCTATCTTTTAAAAGAATTTCGGACGGCT

TTACCCAAATTTGACCGTCATACCAGCCTGATTCTTCATAAAATACTGTAGCACTCATTA

ATCGTTTTACTACGTATGACCAGCCTAAATATAATCTGATTAAAATAAATCCTGTCATTA

GGCTGGAAGTAATAAATTCTGAAAAGAAAAATTTCAAAGGTAGTTTAGTGATTGGAAAAA

TTGATAATAATATAGGACTAACTAATAAACAGTTAAGTAGTAGTGCAATAGTTATTTTTT

TGTTATAAGATCTATGGCTTAAAGTTGGCCAGCAAAAGAACCAAGAATTTTTCAAAGAAG

TATATTCATGAACAGGTTGCTGCTCCTTTGGCACAGGACATTGAGTATTATATAAATTCA

TTGACATTAAAAAGTATTATAATAAAGACTTTGTTTATATTATCTAACATAGGTACGACA

TTAAATATTAACTAACTGCAATTATAGTAGTGAGTAGAAGAAATAAAACTAAAAATAGCC

AAGTAATTTTATTTAAAGTATTCTCAGTACTACGAGTATTACTAAAGAACTGATTCTGAG

CACCTACACTTCCTAAACCTTCGGATTTAGGATTGTGTATTAGAATAGTGAAGATTAATA

TTATTGTAGATGAGTACCAAAAAAATTTTAAAATTTGTTCCATTATGTAAAAGTTAATAA

AAATAGCACAAAGACAATCGTCTAAAAGATATTGATTGTCTTATCATATTAAAAAGTATT

TATTCACCCTGTACTTTTAAAAGTAAAAATCCTATTGCCAGCCCTACCAAAACTAGGACT

GAACTTAATACAGCACTTTCTACAATTTCTCCGACCATTTTATTTTTCCTTATTATTTTA

TTATTGTCATTCTTTTGACAAGATTATATTGAGCTAACTAAAATCCGTTTCTACCCCAAA

CTACCATTGCAATTGAAAATGTAAACATTGCCATTAGAGCTGCCCACCCCAAACTTAAAA

TATCCATAATTTATAATATTCCTAAAAATAAGATCTCTTTTACTTAAACTATAATATTCT

TAATATTAAAAATATCATTATATAATAAGCTAATTATAACAGAGTACTACTTAATTCGTA

TTTTTAGTGTATTTAAAGAAGATTGAATTTTTTCGAGGTACTGGATAAGTATTTCATCCT

AAGAACCATATTCAACTTTTGATTTATTATAATGCTTAAATCTAAATATATATCACTCAA

GAATCATTAGTAATTATGATGTTTTTAATTATACGATTAACATATTTCAAAATAGAAGAA

TTCATAGCGACTAAGTTAATATGCTAATATAATTTATCAATAAAAAGGAAGTTAGTATTT

CAATTATGCAAACAACTATAAATAATGGGCAAACGTCTAGTAAAGAAACTTTACTAACAC

CTAGATTCTACACGACTGACTTTGAAGAGATGGCTAATATGGATATTTCCGGCAATCAAG

AAGATTTTTTGGCTATCCTCGAAGAATTTCGAGCTGACTATAATAGTGAACATTTTATTA

GAGATGAAGAGTTTAATCAATCTTGGTCTAATTTAGAACATAAAACTAAATCCTTATTTA

TTGAGTTTTTAGAAAGATCTTGTACCGCAGAATTTTCAGGTTTTTTACTATATAAGGAAT

TATCCAGAAAATTAAAAGACAGAAATCCGGTTATAGCTGAGTGCTTTTTATTAATGTCTA

GAGATGAAGCTAGGCATGCCGGTTTTTTAAATAAAGCTATCGGGGACTTCAATTTATCTT

TAGATTTAGGATTTTTAACGAAGAGTCGCAAGTATACTTTTTTCTCACCTAAATTTATTT

TTTATGCAACCTATCTTTCTGAAAAAATTGGATACTGGAGATACATAACTATTTACCGCC

ATCTTGAACAACATCCAGAACACCGTATTTATCCAATCTTTAGATTTTTTGAAAATTGGT

GTCAGGATGAAAATCGTCACGGAGATTTTTTTGCTGCTTTGCTCAAATCCCAACCTCATT

TTTTAAATGACTGGAAAGCAAAAATGTGGTGCAGATTTTTTCTATTAAGTGTATTTGCAA

CAATGTACTTAAACGACTTTCAAAGAATTGATTTTTATAATGCCATAGGCCTAGACTCTA

GACAGTATGATATGCAAGTAATACGAAAAACTAACGAAAGTGCCGCTAGAGTTTTCCCGG

TTGCTTTAGACGTGGACAATCCAAAATTTTTCAAATATTTAGATACTTGTGCATGTGATA
ATAGAGCTCTGATCGATATTGATAATAACAATTCTCCATTATTCATTAAATCTATCGTAA
AGATACCTCTATATTTTTCCTTATTTGCAAATTTACTAAAGATATATTTGATTAAGCCAA
TAGACTCAAAAACAGTATGGAATACAGTTCGATAGTTAAATACAAGGGAATGCTTTTTTC
TTATAAAAAGCTATCTAGTGATTAATATAAAATAAAAGCTTCCTTCTCGATGGAAGGAGC
TTTTATTTTGTCAAGAATATAATGGGATTATATAGATCTAGAACTAAGAACTTACTTTTT
CTTTTGCTTCATACATAACCTCTAAAGTAATTAGATTTTTAGAACAATCACGGGCAAATT
TCTCTGTATTTCTTTTTACTTTACCTCTAACAAACCCAGGAATTTTATTTAATTCTTTCT
GTGCTTCTTCTGACCAATTAATACTATCAACAGCAGAAATTCCTATACTTAATGCTTCTG
TTGTATCATGACCACCAAATATTTCTAGCAAATGATCTTCCATACCAAGTGTAAACGAAT
TATATACTAAATCAGCAATTTGATTTGTTCCCTCATAACCAAGAAATGGACGGTAACTTA
AAGGAAAGTTTTGTATATGCACTGGTGAAGAAATAACGCCACAAGGAATATTTAGACGCT
TACCAATATGTCTTTCCATCTGTGTTCCAAAAATTGCTGCAGGTTCAGTTTTAGCAATTA
AATCTCCAATTAAACCATGATCATCTGAAACGATTACTTCATCGCAGAATTCTTGAACTT
GATCTTTAAACCACTCTTCATCATATTTGCAATAAGTTCCACACCAGGCGACATGTATAC
CCATTTCCTGGTGTAAAATTCGAGTTATTGCTGCAGCATGAGTTGCATCTCCAAATACAA
TAGCCTTTTTACCTGTTAAATTCTGACAATCAATTGATCTTGAAAACCATGCAGATTGTG
AAATAAATCGGGTTTGCTCGTCTATGTATTTTTCGTAGTCTACAGCAGCTCCCAATGCAT
TTACTAATTGCTGTATTGATCGGATGCATGCAGCTGTTTGAACAATACCCATAGGTGTAA
TGTCAACATAAGGCATATTAAATTCTTTCTCTAAATATCGAGCTGTCATTAGTCCAGTTT
CTCTATATGGAATAAAATTAAACCATGCAGAGGGTAATTTTTTTAAATCTTGAACTGATG
CATTTTCTGGAATAATTTGGTTTATTTGGATATCTAGATCTTGAAATAACCTTTTTAATT
CTGCTATATCATGTTGATTATGAAATCCTAAGCTTACTGCTCCAATAATATTAACAGATG
GAGTTTTAGTCTTTTGAGTTAAAACTTGATTATTAGATTTAGCTTTTTCCATATAGAAAG
TGACAATTTGTTCTAACGTTCTATCACCTGCTTGCAGTTCATTTACTCTATAATGATTAA
CATCAGCTAATAAAACATCTGCTTCTGTTTCTATAGATGCTCTGCTAACAAAGTTTTGCA
AATCTTCTTGCAAAATACTCGAAGTACAAGTTGGTGTTAGAATAACTAAATCAGGGCTTT
CTTCTCTGTCTTTTCTAGTAATATTTTCTACAACTTTTTCTTGAGAACCACGGGCTAACA
CATGTCTATCTACAACACTTGCTGTAACAGGAGTAAAATCTCTGTCTCTTTCAAGCATTG
AACGCATTACATTAAAATAATCGTCTCCAAGAGGCGCATGCATAATTGCATGAACTTTTT
TAAAAGAACTCGCAATTCTTAAAGTTCCAATATGAGCAGGGCCTGCATACATCCAATAAG
CTAATTTCATATTAAATTTTCCTATGATTTATTTAGACATAGTAATACTTTTAAAGCTAA
CTAACAAACATTAGCCAAACATCTAATTTAACTCCTTGACAATTTTGTAATATTTTTTAA
ATGAAGCTTATCATTATAAATATTTAAAAAATATAATTCAGAATAGAAATAACTGTTAGA
TCAAGCTTGAAAGTAAAGATTTCTTGCAGTTATAGTTTTTCTTTAAGTAAACATGTAAAA
ATTGTACTATATATAAATGTACTATATATAAACTTTTTTTACTGAATATTGGTAATGCCA

GATACAATTAACTTAAATATGCCTTCCCCAACGTTTGGTGGAAGCACTGGCGGTTGGTTA
AGAGCCGCAGAAGTAGAAGAAAAGTATGCGATAACATGGACAGGCAAAAATGAAAGTAAG
TTTGAAATGCCAACTGGTGGTACCGCAACTATGAGAAATGGAGAAAACTTACTCTATTTA
GCTAAGAAAGAACAGTGTTTAGCTTTAGGTACTCAACTAAAAAGTAAATTTAAAATATCT
GACTATAAGATTTACAGAGTTTTTCCTAACGGAGAAGTGCAATATTTGCATCCTAAAGAT
GGAGTCTTTCCAGAAAAAGTTAATACTGGGAGAGCTAGCGTCAACAGTGTTGATCATTCT
ATTGGACAAAATATTAATCCTGTAGATGTCAAATTCATGAATAAAGCAACTTACGATTAG
TCTCTCAAAAACCTAGACATAAAATAACTTTTATATAGTCTAGGTTTTTGATAATGTATA
CTGATAGTATGGAAGGGTGGCCGAGTGGTTGAAGGCGTCTGATTTGAAATCAGTTGAACT
ATCTGGTTCCGTGGGTTCGAATCCCACCCTTTCCGTTATAAAACTATAGTTGGCTTCTCA
AGGCAAGCTAGCTATACACTGATAGTATTAAGTTTAGACTTAATAAAGTCTACAACTTGT
GACAGGTTAGAAATTTTTTCGGCATCCTCATCTGGTATCTCTATACTAAATTTTTCTTCT
ATGGCCATTACCAGCTCAACTGTATCTAGAGAATCGGCTCCCAGGTCACTTGAAAAATTT
GCTTCCCTAGTCACTATTTTTTTTTCAATTCCTAGCTGTTCTGCTACAATATCTTGAACT
TTTTCAAAGATTTCATTATCTTGCATAATATATTATTTCCTCTAAGGTGTTATCAAAAAT
TTTTCGGCTTATATTATTATCCAACTCTAAAAAATTAGTAATTTTTAGTCGAGATTAAAA
TATTTATACCTATATGATATACTATTATTCTGGATAAATACGAAGTTTACGATATGGCTC
TTCTCCAAAACTACGAGCTCTCAACTGGTAGTTGTCAACTAGATTATGCTGCATTTTTCG
TATATAGGCTGATCGAGGTGTAAGCTGAACTATTGAACTTTCATTCAAGATAATAGTTTC
AATAGCCAATTTAGCTTCTTGAAGAGCCTGGATCTCATCAAAATTTTTATTTTTACAGAG
TTCTACCCAATTAAGACCAGATGAAGTATGAATGTTTAATATTTTTCTGAGTGCACGAGT
AATTTGTGGAACCGTACTATTCTGAATTGTATATATAATAATTTGTTTTGATTTTGCAAT
TTGTCTAAGTTTTGTATTTTGCTTTACTTGATTACGTAGCGCTAGAATAGCATCAGACTT
TTCTATCTCCTTGGTTAAAATAATTGGTAGATCTAGTGAAGAAATAACTGATGTAATATG
CTGCCAACTCAAAGAATAAGCATATAAGTACTGATGAGGTATTTCCAATTGCAAAGATTG
TTTATTAATATTAATAGGGGCATTGAAAGTAGTTGACAATAAAGATGTATCACGATTTTG
TGATTTATTCAAATCTAAGGACTTATTTCTTAATTCTCTATATTGTAAAACCGGAGGCGC
CTGCTGTGCGTTAATTGATTTACGAGCATTAGAGATGTTTACAGGTAAGACTTCTATAGA
TTGCGATGGATAACATTTAATTAAAATTCTACCATTAGCTTGAATTTGTCTTTTTTGCAC
AAAAGGTTGATGGCCTTGCAAAATTTGATCTATTGTCTCTTTAACTTTATTATGAACTAT
CCAAACATTTCGCTCGTGTATTTCGATTGCAATCTGAAAAGCAGGCTCAGCTTTTCTTTC
TAGTATACTCTTTTGCGTACCTCTACGTTTAGCTTCATCGTCACCTAATGTTACATATTG
AATTCCACCAATTAGGTCCGCCAATGTTGGATTTTTAATTAAACTTTCTAAATGATTTCC
ATGCGCTGTACCTACTAATTGAACTCCTCTTTCTGCTATAGTTCGAGCAGCTAACGCTTC
TAATTCCGTGCCAATTTCATCTATAATAATAACCTCGGGCATGTGATTTTCAACAGCTTC
TATCATTACTTGATGTTGCAAATCGGGCCTTGCGACTTGCATTCTTCTAGCTCTACCAAT

AGCTGGATGAGGAATATCTCCGTCACCAGCTATTTCATTTGAAGTATCTATAATAACTAC
TCTTTTTTCCATTTCGTCAGCTAAAACACGGGCCATTTCTCGAACTGCGGTTGTTTTACC
AACTCCTGGTTTTCCTAAGAGTAAAATTGAATCACCTTGCTGTAGTAAATCTCGAATAAT
ACTAATTGTACCAAAGACTGCTCTGCCGACACGACAGGTTAAGCCAATAACACTACCTTC
TCTATTACGCAGTGAACTAATACGATGCAAGGTTTTTTCGATACCAGCTCGATTATCGCC
GCTAAAATTACCAACTTTTTTTACACAATAATCTAAGTCTTGCCAACTAATAGATCTTTG
AGATAAATATTCTGGGTTATCTGGAAATCTAGCTTCTGGACGACGACCTAAATCCATAAC
GACTTCTATTAAATTATTTCTATTAGGATGCTGTTGTAAAGGTTCTTTGACAAAATTTGG
CAAAATTTCTAGCAACTTATCTAAGTCATCTGCAATAAGCATGATTGAGTATATAATAAT
TAATTAATCAAATAGAAGATTCAAGATTCTAAAGATTACAATACATATTAAAGAATATTA
TGATTGGAATCAATTTTATACTTAAATTGAACAGGAAGTTATAGATGTAAAAGAACTGAT
TAAACTTTACGCAATAAATCGATTGGTCAAAATTAATAGTCGTGTTAACATGTTTATTGG
ACAAAAAGTTCGTATTAAATATAGCAAACAAAAAGTTGCTAGTGATATAGCAGATAAAGT
TGGTGAATTAGGAGTTATTAAGGGAATAAAGTTTATTAATAGTCAATGTGTTACTATTAT
TGTTGAGTTTGACAATCACACTAGGCTTTGGATGTTTAGAGAGGAATTAATCTGTCTAAA
TGAATTAAAAACATGAAACATAATTTATTTTATTTATTATACAAAATTTCTTATGAGTCG
TTAAAATAATAAATTTTAAAGGAGTACTTTTAATTGTGCAAATTACTATTAAAAAATTAC
AAGACTTACTTTCGTCTGTACAAAGAAAAAAGATCCAGACATTAAAACTAAAACAAGGTA
AGTTTGAACTTCTATTAAATAAGACCTATAAAAAAGTCAATCAAGAAATTATACCTTCGC
AGAAATCTGCTGTATTACAAAATAGTCCATCTACGATAATTAAATCAATAAATAATACAA
AAAAATCTTCTGTTGTTAATGAAGACCGTACAGAATATGCCACTATTGTTTCTCCAATGG
TTGGAACGTTTTATCATTCACCTGCTCCTGGTGAAAAAATTTTTGTACAAGTTGGCGATG
AGGTCAAATTCAATCAAACAGTCTGTATTATTGAAGCAATGAAGTTAATGAACGAAATCG
AGGCAGAAATTGAAGGCAAGATTATAGAAATTCTTGTTAAAGATGGTGATATAGTAGATT
GCGGGCAAGCCTTAATGAAGGTTGAAACATAATGCTAATTTTTCCATTTTACATATTGTT
AACAGAAATTATTTAGATAGAAAAATTAGCTTATACTATAGTTATGAAAACTTATTTCGC
AAAGGCACTAAAAACTAATATATAAAATTATTTACTTTATCTGCCAAAAATAGAAATAAG
CGTTTTGATTCCTTGTCTCAAGAGAGGAGAATCTCAATGGCAATTAGCTCAAAAGAGCAA
GAGACAAAGAAGGTAAAAATCTCGGTTGATAAAAATCCCGTAGATACTTCTTTCGAAAAG
TGGGCCCAACCAGGCCATTTTTCTCGTACACTAGCAAAAGGACCAAAAACTACTACTTGG
ATTTGGAATCTTCATGCTGATGCTCACGACTTCGATAGTCAAACCAGTTCTTTAGAAGAA
GTTTCACGTAAGATTTTCAGTGCACATTTTGGGCAGCTGTCTGTAATATTTTTATGGCTT
AGTGGAATGTATTTTCACGGAGCCCGCTTCTCTAACTATGTTGCTTGGTTAAGTAATCCA
ACAGGTATTAAGCCAAGTGCGCAGGTTGTTTGGCCTATAGTTGGGCAAGAAATTTTAAAT
GGCGATGTAGGTGGTGGCTTTCAAGGAGTACAAGTTACATCTGGATGGTTCCAACTGTGG
AGAGCATCAGGAATTACTACAGAATTTCAGCTTTACTGTACTGCTATTGGCGGATTAGCT

ATGGCTGCTTTAATGCTGTTTGCAGGATGGTTTCATTATCATAAAGCTGCTCCAAAGTTA
GAATGGTTTCAAAATGTTGAATCAATGATGAATCACCATTTAGCTGGGCTTTTAGGCTTA
GGCTGTTTAGGCTGGGCAGGCCATCAAATCCATTTGTCTTTGCCTATTAATAAGCTACTA
GATTCTGGCGTGTCTCCGCAAGAAATTCCACTACCTCATGAGTTTTTAATTAATAGAGAG
CTTATGGCTCAGCTGTATCCAAGTTTTAGTAAAGGATTAGTTCCATTCTTTACTTTAAAT
TGGGCTGAATATTCCGACTTTTTAACTTTTAAAGGAGGTTTAAACCCTGTTACTGGAGGT
TTATGGCTAAGTGATACTGCTCATCATCATCTAGCATTAGCTGTTCTATTTCTTGCTGCA
GGTCATATGTATAGAACCAATTGGGGTATTGGACATAGCATGAAAGAAATTCTAGAAGCT
CACAAAGGACCTTTTACCGGCAACGGTCACGAAGGTCTATATGAAATTCTTACAACTTCT
TGGCATGCACAGCTTGCAATTAATTTAGCTATGATGGGATCTTTAAGCATCATTGTAGCA
CATCACATGTATGCAATGCCTCCTTATCCATATATTGCTACTGATTACCCGACTCAGTTA
TCGCTCTTCACTCATCATATGTGGATTGGAGGATTTTGTATTGTTGGAGCAGGAGCGCAT
GCTTCTATATTTATGGTAAGGGATTATAATCCTGCAGAAAATTATAACAATCTTTTAGAT
AGAGTCATTAGGCATCGAGATGCTATTGTTTCTCATCTAAATTGGGTATGTATATTTCTT
GGATTCCATTCATTTGGTTTATACATTCACAATGATACTATGCGGGCACTTGGAAGATCT
CAAGATATGTTCTCTGATACAGCTATACAGTTACAACCTATTTTTGCTCAATGGGTACAA
AGTATACACACTTTAGCTCCTGGAAATACAGCTCCAAATGCATTAGCAACAGCTAGTTAT
GCATTTGGAGGAGATATTGTTTCTGTTGGTAACAAAGTTGCAATGATGCCTATTTCTTTA
GGTACTGCAGATTTTTTAGTTCACCATATACATGCATTTACTATTCATGTAACTGTTTTA
ATTTTAGTTAAAGGGTTCCTTTTCTCAAGAAACTCTAGACTAATTCCTGACAAGGCCAAT
CTTGGCTTCAGGTTTCCATGTGATGGACCTGGTAGAGGTGGTACTTGCCAAGTGTCTGGC
TGGGATCATGTTTTTCTTGGCTTATTCTGGATGTACAATTCTCTGTCTGTAGCAATTTTT
CACTTTAGTTGGAAAATGCAATCAGATGTTTGGGGTAGTGTATCTCCGTCTGGAAATGTT
TCTCATATTACTGGCGGTAATTTTGCACAGAGTGCAATTACAATCAATGGATGGCTGAGA
GATTTCCTTTGGGCTCAAGCATCTCAAGTTATTCAATCATACGGTTCTGCGCTATCTGCA
TATGGATTAATTTTCTTAGCAGCACATTTTGTATGGGCATTCAGTTTGATGTTCTTATTT
AGTGGTAGAGGTTATTGGCAAGAGCTTATAGAATCAATCGTATGGGCGCATAATAAGATA
AAAGTTGCTCCTGCAATTCAACCAAGAGCTTTAAGTATTACTCAAGGTAGAGCAGTCGGT
GTTGCACACTACTTATTAGGTGGAATTGGTACAACTTGGGCATTCTTTTTAGCGAGAATT
ATTTCAGTAGGCTAATAGTGAAAATAGGATAAAAAAAACAATTATGGCAACAAAATTTCC
TAAGTTTAGCCAAGCTTTATCACAAGATCCTACAACTAGAAGGATTTGGTATGGTATTGC
TACGGCACATGACTTTGAAAGTCATGATGGAATGACAGAAGAAAATTTATATCAAAAGAT
ATTCGCTTCGCACTTTGGACATCTAGCAATTATCTTCTTATGGACATCTGGTAATTTATT
CCATGTAGCTTGGCAAGGCAACTTTGAACAGTGGGTATTAAATCCTTTGAAAGTTAAACC
AATTGCTCATGCAATTTGGGATCCGCATTTTGGACAACCTGCTTTGAAAGCTTTTAGTAA
AGGTGGCTCAGCTTATCCAGTAAATATAGCATATTCTGGCGTATATCACTGGTGGTATAC

TATTGGTATGAGAAGCAATCAAGACCTGTATTCTGGGGCTTTATTCTTACTAGTTTTATC
AGCTTTACTCTTATTTGGAGGGTGGCTACATCTACAACCAAAATTCAAGCCTGGTTTATC
ATGGTTTAAAAATAACGAATCAAGATTAAATCATCATTTATCTGGATTATTTGGGGTTAG
TTCTTTAGCCTGGACAGGTCATTTAGTACATGTTGCTATACCTGAGGCAAGAGGACAACA
TGTAGGATGGGATAATTTTACAACCGTATTACCTCATCCAGCCGGTTTACAGCCGTTTTT
CAGTGGTAATTGGAGTGTATATGCTCAAAATCCAGATACAGCTCAACATTTATTCGGAAC
TAATGAAGGTGCGGGTACAGCAATTCTGACATTTCTAGGAGGATTTCATCCTCAAAGTCA
GTCTTTGTGGCTAACTGATATGGCTCATCACCATTTGGCTATTGCAGTAGTATTCATTGT
TGCTGGACATATGTATAGGACTAATTGGGGAATTGGGCACAATCTAAAAGATATTTTAGA
TGCTCATAGACCACCTAGTGGTAGATTAGGAGCTGGACATAAAGGGCTATTTGATACTAT
TACTAATTCTTTACACATACAGTTGGGATTAGCATTGGCTTCCCTAGGTGTAATTACTTC
GTTGGTAGCTCAACATATGTATGCTATGCCTCCATATGCTTTCATGGCTAAAGATTTCAC
AACTCAAGCATCTTTGTACACACATCATCAATATATTGCTGGGTTTCTAATGGTTGGAGC
TTTTGCTCATGGGGCAATATTCTTTGTTCGAGACTATGACCCTGAACAGAATAAAGATAA
TGTTTTAGCTCGTATGCTAGAACATAAAGAAGCTATCATTTCTCATTTAAGTTGGGTAAC
TCTATTTTTAGGGTTTCATACATTAGGCCTTTATGTTCACAATGACACAATGATTGCTTT
TGGAACTCCTGAAAAACAAATTCTAATTGAGCCGGTATTTGCTCAATGGATTCAAGCCTC
TTCAGGGAAAGCACTTTATGGGTTTGATGTGTTACTATCATCCTCTACTAATATCGCAAC
ACAAGCTGGTAGCAATATTTGGCTGCCAGGCTGGTTAGAAGCGATTAATAGCGGAAAAAA
TTCATTGTTTTTAACAATTGGTCCTGGTGACTTCTTAGTTCATCATGCAATTGCATTGGG
ATTACATACTACTACGTTAATTTTAGTTAAAGGTGCTTTAGATGCAAGAGGCTCTAAACT
TATGCCGGACAAAAAAGACTTTGGATATAGTTTTCCTTGCGATGGACCTGGCAGAGGCGG
CACCTGTGATATATCTGCATGGGATGCGTTCTATTTAGCTGTATTTTGGATGCTAAATAC
AATAGGTTGGGTAACATTTTATTGGCATTGGAAACATATTACAATATGGCAAGGCAATGC
AACTCAATTCAATGAGTCTTCAACTTATCTAATGGGATGGTTTAGAGATTACTTATGGCT
AAATTCTTCTCCATTAATTAATGGTTATAATCCATATGGCATGAATAATTTATCAGTATG
GTCATGGATGTTCCTATTTGGACATTTAGTATGGGCAACAGGATTTATGTTCTTGATCTC
CTGGCGTGGCTATTGGCAAGAGTTAATTGAAACTCTAGCATGGGCGCATGAACGTACGCC
TTTAGCAAACTTGATTCGTTGGAAAGATAAACCTGTTGCATTATCAATTGTACAGGCAAG
ATTAGTAGGTTTAGCACATTTTTCTGTAGGATATGTATTAACTTACGCAGCTTTTGTATT
AGCTTCAACAGCAGGCAAATTTGGTTAGACTAAATTCGAATTAGCT-AAAAAAAGTCAGT
CATAATAGTTTTATGACTGACTTTTTTAGATTTAAAACAAAAACTTACTAAAGTTTCACC
TCAATATCTACACCAGAAGGCAAATTTAACTTCATTAATGCATCTATCGTTTGAGAAGAA
GGTTGATGAATATCAATAATTCTTCTGTGAGATCTTATTTCAAAGTGTTCTCTTGAGTCT
TTATCTACATGTGGAGAACGTAAAACACAATAAATTCTTCTTTTTGTCGGTAAAGGAATT
GGCCCTACTGCAACGGCATTAGTTCTAGATGCGGTATCTAGTATTTTGTCACACGACGTA

TTAAGTATAATAGAATTATATGCTTTCAGTTTAATTCTGATTTTTGTCTGCTGAGTAATT
GTCATGTTAAAAAGATCTTATTATTTAAGAATTTTAGAGACAACACCTGCGCCTACGGTA
CGGCCACCTTCTCTAATAGCGAAACGCATACCTTGCTCAATTGCAATTGCATTAATTAAT
TCAGCAGTCATCTTAATTCTGTCACCAGGCATAACCATTTCTGCATCAGTACCATCATCA
GCAGTAAACTGATTAATAGTACCAGTTACATCAGTTGTTCTAACATAAAACTGAGGTCTA
TATCCTGGAAAAAATGGAGTATGTCTTCCGCCCTCCTCTTTAGTTAAAATATAAACTTCT
GCTTCAAATTGAGTATGAGGTGTAATTGTACCAGGTTTAGCTAATACCATACCTCTTTCA
ATATCTTTTTTCTGCACACCTCTTAAAAGAATTCCAATATTATCACCCGCTAGACCTTCT
TCTAACGTTTTTTGAAACATTTCTAATCCAGTAATAGTCGTTGTACGAGTTTCTCGTAAA
CCTACAATTTCAATTGTGTCACCAACTTTAATAATGCCTCTTTCAATTCTACCAGTGGCA
ACAGTTCCACGTCCTGTAATAGAAAAAACATCTTCTACAGCCATTAAGAAAGTTTTATCG
ACATCTCTCTCCGGCGTTGGAATATATGTGTCAACTGCTTCCATAAGTGAAAAAATCTTG
TCAACCCATTTATCTTCACCTTGCTTAGTAGCTGGGTTTTTCGTCACAGCTTCTAATGCC
AATAAGGCAGAACCTGCAACAAAAGGAATATCATCTCCAGGAAAGTCGTATTGACTTAAT
AATTCCCTTCCTTCTAATTCTACTAATTCTAGTAGCTCTTCGTCATCTACTTGATCTTCC
TTATTTAGGAATACTACTAATGTAGGAACACCTACTTGTTTTGCTAATAAAATATGTTCA
CGAGTTTGTGGCATTGGACCGTCTGCCGCAGATACAACTAAAATAGCTCCATCCATCTGA
GCAGCACCCGTAATCATATTTTTTACGTAGTCGGCATGGCCTGGACAATCTACGTGAGCA
TAATGACGATTATCTGTTTCGTATTCAACATGAGCAGTATTAATAGTAATACCTCTAGCT
TTTTCTTCTGGAGCGGCATCAATTTCATCAAATTTTTTCGCTGCAGTAGACCCTAAAGTT
GATAAAGTCGCGGAGATCGCTGCTGTTAAAGTTGTCTTACCATGATCAACATGACCAATT
GTGCCAATATTGACATGAGGTTTTTTACGTTCAAATTTAGATCGAGCCATGCTTTTTGTT
TTCCTTATAGTAAAAGTTGCTTTATAGTTACTTTTAACGATAAATCTTGTGAATTGAAAG
ATTTATTATTTAATACAGACAAGATACAATATAACTAATCTAATATCGATAGTGAGCAAA
CGCTTTATTTGCTTCTGCCATTCTATGTGTATCCTCTCTCTTTCTAATAGAATTTCCTGT
TTCATTAGCTGCATCCATAATTTCATTAGCTAATTTCATAGACATACTTTTACCAGATCT
GTCTCTAGAGAATTTAGTAATCCATCTTAGTGCTAAATTCGTACCTCTATAAGCTCGTAC
TTCAATAGGAACTTGGTAAGTAGAACCACCAACTCTTCTTGCTTTTACTTCTACTAGAGG
AGTAATATTTCGGATGGCTTTTTCTAAAATATTTAAAGGATCTGATTCTGTTCTCTCTTT
AACGATATCTAAAGCCTGATATATAATTCTTTGAGATAAAGTTTTTTTACCACTTTTCAA
AATACGAACAGTTAACATACTTACGAGTCTGCTTTTATATAAAGGATCAGGTGATGCAAA
CCTTTTTTTAGCTGTATTACGACGAGACATAGTATTTAAAATTGGTTATTGTAATAATGT
TCTATTAGCAGGTATTTAAGATTTAGGCTTTTTTGTCCCATATTTAGATCGGCTTTTACG
ACGATCTTTTACTCCTGCAGCATCTAAAGTACCACGTACTACATGATATCGGACTCCAGG
CAAATCTTTAATTCGGCCGCCCCTAATCAGTACCACAGAATGCTCTTGAATATTATGACC
TACGCCAGGAATATAAGCTGTAACTTCAAATCCAGATGTTAATCTAACGCGCGCTACTTT

TCTTAAAGCAGAGTTCGGTTTTTTAGGAGTTGTAGTATATACTCTTGTACAAACACCTCT
TCTTTGAGGGCAACTTTGAAGAGCTGGAGATTTTGTTTTTTTATGTATTTTTCGTCTTTC
GGATCTAACAAGTTGTTGAATTGTTGGCATAATAAAATTGATTTAAAGTCTTTAATAGAT
ACTATTTGAACCTGCTTTACATGAATTGGCTAGTTTTTATTTATCTTATACTTACGCATA
AATCTTTCTACTCTACCTTCTGTATCAATAATTCTCTGTGAACCAGTAAAGAATGGATGG
TTTCCTGACCAGATATCTACGTGTAGTTCTGGCTTTGTGGACCCAATCGTCATAATTAAC
TGGCCGTCACAGTAAACTTTTGCTTCTGGATACCAATTTGGATGTATATTATCTTTTGCC
ATTGTTTTTATAATTATATAAATGTATCAATAATAACTATTAACGCTTAGAAAATTGAGG
AGCCTTTCTTGCTTTCTTTAAACCATATTTTTTTCTCTCTTTTACTCTTGGATCTCTTGT
TAAATAGCCTTCAGACTTGAGTGTCGTTCTATTTTCTGGATTAATTGAGCACAATGCTCT
TGCAACACCTAAACGAATTGCATCAGCTTGCCCTGTCAAACCTCCTCCTCTAGCATTTAC
ATGGATATCATACTGGTTTAGTAGTCCTAAAACTTGTAATGGTGCATATGAAACTCTTAA
GTAATTAGGACTAAATTGAAGATAAGACTCTCCTGGTATACCGTTTATAATTAAATTACC
TGACCCTGGGACTAGTCTTACTTGTGCAACAGAACACTTCCGGCGACCTGTTCCAGAATA
GATTGCGCGAGTTTTAATTAATTCTGTCGACATAACATTCCTTGATAACTAAATAAATAT
ACATTCTTTATACTATATACTCTTGTGGCTTTTGCGCGACATGTGGGTGAATTGGGCCAG
AATACACTTTAAGCTTCGTAAATAGTTTTCTGCCTAATGGACCTTTAGGAAGCATACCTT
TAACCGATTTTTCAATAATTCTGTTAGGTAATCTTGTCTGAAGCTGATCAAATGTTTCAA
CTTTTAATCCGCCAGGCTGTCCAGAATGTCTTCTGTATAGTTTTTGATTCGTTTTATTTC
CACTTACAGATACGTGAGCAGAATTAATAACGATTACATAATCTCCAGTATCTAGATAGG
GTGTATAAGAAGGCTTGTTTTTACCTCTTAAGATATTAGAAATATGAGTAGATATTCTAC
CAAGTGTCTGATTTTTAGCGTCTATAACATACCAATGAGAATTAGTATTTAATGAGGGTG
ATTGCGTTTTATTCATGAAAAGATTAATACTTTTGGTTGACAATAAGAGAATATGTAAAT
GGTTTACATTCAATTTCTATACAAGTAGTATTTAAAAATATTATTAACAAAGAGAACTTT
TCTAACAGTTATTTAAATTGTTTGTCAGCAAGAAAATTTCTAGTTATCAATAACTTATAC
TATTTTTAATAATATAAAATATATTAATCACTTTTTTCTTTTGGCAAACTTATACCTAGT
TTTTTTTGCAAGGCATATATAACTTCTTCAGCTGATTTCTGGCCAAAATTTTTAATTTCT
AGCAGTTCTTCTTGAGAGTAATCTAGTAAATCTGCAATAGAATGGATTTGAGCTCGCTTT
AAGCAGTTATAAGCCCTAACAGATAACTGCAGTTCTTCTATTAAAACTTGACTAATTTTT
TTATCTTCTTTGCTACGATAATTATCTGCTGATTTAAAGTCTAAATTTCTTAGTGAACAA
AAAAGATTAGTTAAAACTGTAGCTCCTTGACTTATTGCTTCTTGGGGAGATATGCTCCCA
TTTGTCCAGATTTGTATAATCAATCTATCTTTTATGCTGTTGCTACCAATACGGACTTCT
TCTACTTTATAATTAACTTTATTAACTGGCATAAAAACAGAGTCTACTTGCAAAAAATCC
ACAGATAATTCATCTACAGCTTTTTCAGCTAAGCGATACCCGCAATTTTTTTCAATTTTA
AACTCCATTTCAAATATTGTGTTATTGCAAATAGTTGCAATATACTGTCTAGGATCTACT
ACCTCTATATCAGAAGATAATTCAAATAGGCCGGCTGTAACTATAGCTGGCCCTTGAACT

CTAATTCGACCAATTTGAGATTCTTTGTTATAACTTTTAAATACTACTTCTTTTAGATTA
AGTAATATTTCTAACACATCTTCTCTTACCCCAGGAATTGTGGAAAACTCATGGTTCACT
CCAGCAATCCGTACAGCAACTATAGCAGTACCTTCAAGATCTGACAATATTGATCTTCTC
AAAGCATTACCTAATGTAATACCTTGTCCTTGATTTAATGGTTCTATTACAAAACTACCG
TACTGCCCACGCGCCCCATCTGTTCTTGACTCTATGCATTCAATTTGAAATTGAGCCACC
TAAGAAAGCTCCTTTAAAATAATCAGTGATTAGTAAGTCTCTCTAAAACAAGAAGGAATT
TATACTCGGCGTTTCTTAGGAGGGCGACATCCATTATGAGGTACAGGAGTAATATCTTTT
ATTAGAGTAATCTCTAATCCTGCAGCCTGCAAAGCTCTAATTGCTGTTTCTCGACCCGCT
CCTGGACCATTTACTAGAACTTCAGTTTGGCGCATACCTTGATCCATAGCTTGTCTAGCT
GCTTTTTCAGCTGCTGTTTGAGCTGCAAAAGGTGTTCCTTTTTTAGCTCCCTTAAATCCA
CTTGCACCAGAGGATGACCATGATAATGTTTCTCCTTTTAAATTAGTAATAGTAACAATT
GTATTATTGAATGTAGATTTAATATGTGTAATACCGTTAACTGCATTACGTTTAGTTTTT
CTTGCTCCGGATTTTTTTATTTGTCTAGCCATCGTGTCTCGTCTATTATATAGTTAATTA
AAAGATTATTTTCTTGGAGCTTTTTTCTTACCTGCTACTGTTTTTTTACCTCCTCTGCGT
GTTCTAGCATTAGTTCTAGTTCTTTGTCCTCTCAAAGGAAGACCAAGACGGTGTCTTCTA
CCTCTATAAGTACTAATTTCCATAAGTCTCTTAATGCTCATAGACTCAAAACGTTTGAGA
TCCCCTTCAATCTGATAATTAGACTCAAGAATTTCTCTTATACTGACAACTTGTTGATCA
TTTAAATCTTGACACTTAATATCAGCGTCTATGTTTGTTTTTTCTAATATTTCTTTCGAG
CGAGATAGTCCAATACCATAAATATATGTTAAAGCTATCTCTATTCTTTTGTTTCTTGGA
AGATCTACTCCAGCAATTCTGGCCACTTTGTCTTGTCTCCAATAAATAATATGTATTTTT
ATAACGGTTATTTATAAGCTTAGCCTTGTCTCTGCTTATGTTTAGGATTAGTGCAAATTA
CCATCACCTTTCTGTGACGCCGAATTATTCTACATTTTTCACACATTTTTCGAACAGAAG
GACGAACTTTCATATTAACTCTATATACTAGGAATATTACTATATTAACTCTATTAATTA
TCGACATTAAAAATGTCAACAATATATTGTATTACTTTAACTGTTGTAGATTTTACTTCG
TCATACTATCATATTTTTTTGATATGACGTATGTCTGAATTTGCTTAGCTGTGTCTATTG
CGACTCCAACTAAAATAAGTAAAGACGTAGCCCCGAGACCTCTTAAGTTTTGGATCTGAG
TAACCTTTTCTATTATAAACGGAATCAGCGCTACTGTAAATAAAAACGAGGCTCCTAGGA
ATGTCAGTCTATTTAATATGACTTGTAAATAATCAATAGTCGCTTGACCTGGACGAATAT
TAGGAATGCTTGCACCCATTTTTTTTAAATTTATAGCAATATCTTCTGGATTCATTACTA
TTGATGTATAAAAATAGCTAAAGAAAAGGATCAAAGCACAATAAAGAAGAAGGTATAGTG
AACCATTAGGACAAAACAGGTATAGGATTTGGAGTAATGTTTTATTTTGAATAATTTGGG
TTAGATAAGATGGGAGGGCCATAGATGCAGATGCAAAAACAATAGGCATAACTCCACCTT
GATTTAATTTCAAGGGCAAGTAACTATTTGGATCCAAAATTGAAGATTTTCCTAGCTGTC
TTGCTGAAATAATTTTAATTCTTCTTGTTCCTTCTTGTACACAAATTGTAATTATTATCA
TTAATAAAAAGATCGCTATAAATAATCCGAACTTAAGACTTGCATTACTATAACTAGCAT
CAAAAAATGACTGTGTAAAATTCTTTGGTAGTCCTGACACAATGTTTTGAAAAATAAGTA

AAGAGGCTCCATTACCAATTCCCTTTTCCGTAATTAACTCTGATAACCACATAATAATCA
TAGAGCCTGCTGTTAAAGCTAAAACAGACTCGCAGACAAATGCAAAATTCCAATTAAAAA
CATATGGTTTTACCCATATAGAGATTGCTCCAGATTGTAAAGTCGCCCAACCTAGAGCTA
AGTACCTTGTAATTTGAGTTATTTTTTGGCGGCCCAATTCACCTTCTTCCTTCTGTAATT
TTTCCAGATTGGGGACAATTTTCGTAAGTAGCTGCATTACAATTGAAGAGTTAATATAAG
GAACAATACCTAACGCAAAAATCCCTATTGTTGAAAAACCTCCTCCAGAAAAAATATTCA
GAAAATTTACTAAAGTATTTTTTTCTACACTTGCATAAAAGGCGTCATGATCTATACCTG
GAACAGGTATAAATATTCCTAAACGTGCTAAAACTAATAGAAAGAGAGTAAAGATAATAC
GATTTCTTAGATCACTTTTTTGGCTCATAAATAAATTTTAAATGAAAAGAGATACTTTTT
TATGTAAAAACCCTATTCCAAGACTTGTATAAGTTTAAAATAGGGAAGTAAAATATTATA
GTAATATTAATATAATATTCTATGTAGAATACAGGCTTTCTATTGGAACTCCTCGATCTT
TAGCTGCTTCACTAAAAGTTCTTAATTGAGTTAAGCCATTGAGAACAGCTCGCGCGTTAT
TTAATGTATTATTAGAACCTAATTGCTTAGCTAATATATTCTGTACACCCGACAATTCTA
GGACTGTCCTTACAGAACCTCCGGCAATTACACCTGAGCCAGGTGCAGAGGGCCTTAATA
TAACTTTCGCGGCACCAGAAATTCCGTTAATAGGATGAGGTATAGAATTCGATTTTGTCA
GTGGAACTGTAACTAGATGTTTTTTAGCATCTGTTACTCCTTTTTTTACTGCACCAATTA
CATCGCTTGCTTTTCCCACGCCGACACCAACTTGGCCTTGCTCATTACCAATAACAAGAA
TAACTCGAAAGCTTAGTTTTTTACCTCCTTTTACAACTTTAGTAACTCTTTTAACTTGTA
CGACTCTTTCTTCCCAGCCACTATCTTTATCTTTTCCTTTGCTCTGTTTTTTACGATTGG
CCATTTTAAGAATTATTCCTAATTAGTTAATTACGATTCTAGAAACCCATGCCTGCTTCC
TTAGCAGCTTCAGCCAAGGCCTTAACTCTTCCATGATATAACTTTCCTCCTCTATCGAAG
ACGACATTTTTAATGCCTTCTTTCATAGACTGTTCTGCTAACTGTTTACCAACTACGCGA
GAAGTATCACAGTTTGGTCTTATGTTATCAGATTCTTTATTATTTAAATTAACAGATGAT
GTAGCTACTAATGTAATACCTTGTGTGTCATCAATTATTTGTGCGTATATATGTTTATTA
GATCTAAATACACACAGACGAGGCCTACTTGAGGTTCCTTGAACTTTTTTCCGAACTCTT
TTATGCTTATGAATTCTAGTTTGTTTAGTGTTTAGTTTCATTATTATTTACCTTTTCCAG
CTTTCCCAGCTTTTCTTCTAACAAATTCACCTTGATATCTAATTCCTTTTCCTTTATAAG
GCTCAGGAGGCCTAATAGAACGAATAGTTGAAGCAACCTGACCGACAACTTCTTTATCTA
TGCCAGAGACAGTAATATTTGTGTTGTTTTCAACTTTAATTTCAATATTGGCAGGAGGTT
TAATTTTCACTACATGACTATAGCCAACACTTAAGATTAAATCTTGGTTATCAATTTGAG
AACGATAGCCTACACCTTGTATTTGCAGTTTTTTAAAAAATCCATTAGAAACACCTTCAA
TCATATTACTAATAAGAGTTCTGGATAGCCCATGTAACTGACTAGCCATTTTTGTTTGTC
CACTCGTCTTGACAGCAATTGTGTCGTTAAGTATTTCTAAATTAATACCAGCTGGTAAAG
TTCTGGATAGGGTACCTTTAGGTCCTGTCACGGTGATAGTCTGACCATCGAACTGAGTAC
TAAGATTTGTCGGCAATAAAATTATTTTTTTTCCAATACGAGACATATTCCACCTATAAA
ATAGTTACCAAATATAGCATAATATTTCACCACCAAGACCATCATGACGAGCTTGTCTAT

CTGTCATAACGCCTCTAGAAGTAGAAATAAGAGCGATGCCTAAACCTCCAAGAACTCTGG
GCAACTCTTTATGATTTGCGTAGACTCTTAGTCCGGGTTTACTAATTCTTTTCAGAGCAG
TAATAACTGGTTGACGATTTTTACCATTATACTTAAGAGAAATCATTAAATGAGTCTCTA
TACCTTCACCCATTTGTTCGAAATTTTGAACAAATCCTTCTTCTTTTAGTACTGTTGCCA
TATTGCATGTCATTTTCGTTGCTGGAACTTGCACAATTTGATGTCTTGCTAAGTTTGCGT
TACGAATACGTGTCAGCATATCGGCGATCGTATCGTTGACCACCTTGATCCTCCTTGCGA
TGAATTATTTGAAATATTTTCAAGATTCCCTGAAAGGCATCCCTAGCTTTTTTAACAAAG
CTAGACCTTCTTGATCTGTTTTAGCTGTAGTGACAATTGATATATCTAGACCACGAATTT
GATCAATATTATCATAGTCTATTTCTGGAAAGATCAACTGTTCACGTAAACCTAAATTGT
AATTACCTTTGCCATCAAAACTTCTAGGACTAATTCCTCTAAAGTCTCTAATTCTTGGTA
ATGTTAAATTAATTAATTTCTCTAAAAAAGAATACATCTTGTCTTTTCTTAGATGCACAA
CAATTCCGATAGGAACCTCTTCTCGAATTTTAAATCCTGCAATAGATTTTTTAGCTTTTG
TGACAATTGGTTTTTGTCCAGTTATTAATGTTAATTCTTGAATACTACTTTCAAGAGCCT
TAGCATTCTGAGAAGCTTCACCTAAGCCACGGTTAATAGTAATTTTAGTAAACCTAGGAA
CTTCATGTACATTTTTGTACTGAAATTCATCTTTTAAAGATTGAGTAACAGTTGTTTTAT
ATTTTTCTTTTAATCCTATTGCCATTATATTTTCATCAATTATTTAATAAGTTCGCCAGT
CTTTTTCAGTTTTCGAATTTTTTGGCCTTTATCATTAATTATTACTGAGGATCGACTAGC
AATATTATTTTGTTCACTAAATAACATAACGTTAGAGGTATGTATTGGAGCTTCAAATTT
GATAATTTCTCCCGTTTCTCCTTCTTGCTGAGGTTTTTTATGCTTCACTTTGAGATTAAT
TCCTTTAACAATCACCTTATTTGTTTTATAAATGATTGCAATAATTTCACCTGTTTTTGT
TTTATCACTTCCAGAAATAACTTGAACTAAGTCCCCTTTTTTTAATTTAATTTTTGTATT
ATTTTTGGTTGTTTTAGAAGGACCTTTCATTATACTACCTCCGGTGCAAGAGAAACTATT
TTAGAAAAATTTTTATCTCTTAACTCTCGGGCTATAGGTCCGAAGACTCTTGTGCCACGT
GGATTATTATCTTGATTAATGATAACTGCCGCATTATCTCCAAATCTAATGCTCATACCG
TCAGTTCTCCGTAAAGCTTTACGAGTTCTTACTACAACAGCTCTGACAACATCAGATCGT
TTAACTGGCATATTGGGAGACGCATCTTTGACTACTCCAATAATAACATCTCCAATAGAT
GCATAAGAAGGATTACTAGTGCCTAATACTCTAATACACATTATTTTTCTAGCACCACTA
TTATCTGCAACATTAAGATAGCTTTGAGTCTGTATCATACTTTTATTCTGTATAAGATTA
ATTATCAAAAGACTTAGATAAGATATTAACCATTGTCCAACACTTTGTGCGACTTAAAGG
TCGTGTCTCTTGTATTGTAACAATATCGCCGATTGTGCATTCATTATTTTCATCATGCGC
TTTATATTTCTTTGTCCGGATCATAGTTTTTGCGTACTTTCTATGAGAGATTCTATTTTC
TACAGCTACTACTATAGTTTTATTCATTTTATCGCTTACTACTTTACCTGTTGTTTCTTT
TAAAGGCATAGTCTTTATATTTTTTATATATAATTAAATTTTAAGATTATTACATGGTTG
ACTTAGTACGAGACTTTTCAACAGTTAAAAGTTGGGCTAATCTATGCTTAGAATGTTTAA
ATAGATGAGGCTGGAAATCTTGCCTTGTGGCTCTTTTTAGCCTTAAATCAAAAAGCTCTC
TTTTTATTACAAGGATTTCTTCAGCTAAAGAAGAAGAGTCCAGGTTTGTAACATCTGATA

TTTTAGGGAAAGTCATATTTTAGGATTCTGTTGTATTCCGAACTATAAATTTAGTCTTGA
TTGGTAATTTATAAGAAGCTAATTTCATAGCTTGTTGAGCAGTTTTTTGTGGTACACCTG
TAATTTCAAATAGAATATGTCCAGGCTTAATAACTGCAACCCAATATTCTGGAGCTCCTT
TACCTGACCCCATGCGAGTTTCAGCTGGACGGGCAGTGACTGGTTTATCTGGAAATACTC
TAATCCATAGTTTACCACCTCTTCTCACATATCTAGTAATAGTTCTTCGAGTAGCTTCTA
TTTGTCTAGAAGTTAACCAAACTGGCTCTGTCGCTTGTAATGCATAATCACCGAACGCAA
TTGTGTTACCTTTGCTAGCAGAACCTTTCATTCTACCTCTATGTTGTTTTCTAAATTTTG
TTTTCTTGGGGCTTAGCATAAAAATTAAAAAATAATAATAATTAAGAAGCTGTTGAATCA
GGAGTTTCCTGATTATAAGCAGATTCTAGACTTTTCGATTCTGGCAAAATTTCTCCTTTA
AAAAGCCAAACTTTTACTCCGAGCACACCATACGTAGTGTGAGCTTGGCGATGACAATAA
TCAATATCGGCTCTTAAAGTTTGTAAAGGTACACGACCTTCTCTAACCCATTCACTTCTA
GCTATCTCTGCACCATTAAGCCGACCAGATACTTGAATTTTAACACCCTGTGTATTTGCT
CTTTGAGCTCTTTGAACTGCTTGTCTGACAGCTCTACGAAAGGCCACTCTTTTTTCTAGC
TGTTGAGTAATAAATTCTGCTACTAAGGTTGCTTCAGAATCTGGATCTGCAATCTCTACA
ACATTTACTCTGAGTTGTTTGCTAGGGTCTAGGATTAAGGACAGCGATTTTCTTAAAGAC
TCAATTCCCGCTCCAGATTTTCCAAGTACGATTCCTGGTCGAGCTGTTGCTATGAGAATT
TCTACTTGATCAACTTTACGATTAATTTCAATTTTAGCAATACTAGCATTACTAAGTTTT
GAATGTATAAATGAGCGAATTTTATGATCTTCTTGTAAAAGAACTGGGTAGTCTTTAGAG
TTAGCAAACCATGAAGAACGATGTTTTTGGGTAATGCCTATGCGAAAGCCCAAAGGATGA
ATTTTTTGACCCACAGTATCCTTGATTATAAGTTAAAAGCTATAATGACTAATTGACGTT
AAAAGACTACAGTTCTGAAACACCTAATGTAATATGACAAGTTGGTTTATGTATTGGAAA
GGCTCTTCCTTGTGCTCTAGGCTGAAATCTTTTTAAAGTCGGGCCTTTGTCTGCAAAGGC
TTTACTAACAAACAATTGGTTTTTATTTAATCCATCATTATGCTCAGCATTCGCTGCAGC
AGATTCCAGGATTTGCTTTATATGAGAACAAACTCGGTATGGCATAAATTCCAGAATAAT
TAATGCTTCTTGGTATTTTCTACCTCTAATTTGGTCTAATACACGACGGACTTTATGCGG
AGAAAGACGAATGTATTTCCCTACTGCTTTAGTTTCTTTTACGTTTTTTGTAATACTCAT
AGTTATAGTTTAACGACGGGCTTTTCTGTCACCTTTTACGTGAGTGCGGAAAGTTCTTGT
TGGAACAAACTCTCCTAATTTATGTCCTACCATTTGGTCTGACACAAAGACAGGAAAATG
TTGTTTACCATTATAAACAGCTATTGTATGCCCTACCATATCGGGAATAATAGTAGATGC
TCTCGACCAAGTTTTTAGGACTTCTTTTTTTCCTGAAATATTTAATGCTTCTATACGTTT
AAGAAGACTAACATCAATAAAAGGGCCTTTATGTATAGATCTTGACATAATAATAAATCA
TTAGATTCTTAATAAAATAAATTCTATTTACGGCGACGTAAGACGTACGGGTTACTATAT
TTATTTGGATTTCGTGTTTTAACACCCAATGCAGGTTTACCCCAAGGTGTTACAGGACGA
GCTCGTCCAATTGGAGATTTGCCTTCACCACCACCATGTGGATGGTCTACAGGATTCATG
ACAACACCTCTAACAGTAGGTCTTTTACCTAACCAACGATTTCTACCAGCTTTACCAAGA
GTAATATTACTAGCGTCAATATTACCAACTTGTCCAATAGTAGCGTAGCATTCTTTTCGA

ATCATTCGAACTTCGCTTGAAGGCAATTTTACAGTAACAAAAGTACCTTCTTTTGCTACT
ATTTGAGCATAAGTTCCTGCTGCACGAACAATCTGTCCTCCGCACGAAGGTCTTAATTCT
ATATTATGTACTGCTGTTCCTAAAGGAATACTAGATAAAGGCAAAGCATTTCCAACTTCG
ATAGGGGCAGTAGGACCAGAAAGAACCATAGATCCTACACTGAGAGATCGAGGGTGTAGA
ATATATCTTTTCTCACCATCAAGATAGTGTAATAATGCAATTCTTGCATTTCTATTCGGA
TCATATTCAATAGAAGCAACTTTAGCAACTATATTATGTCTATTTCTTTTAAAGTCAATT
AATCTGTACTGCTGCTTATGTCCACCACCTTTATGACGACAGGTAATGACACCTCTATTA
TTTCGACCTTTGCAGAAATGGTGTTTAACTATTAATGATTTTTCTGGCTTATCAGTAGTA
ATCTCTGAAAAGGTAGAAACCGTTCTATTTCTTGTCCCTGGTGTGTAGGCACGATATAAA
CGAATTGCCATATAAGAAGATTTGAGAAGAGTAAATGTTGAACTATGTGATATATTTAAG
TTTCTGGAAACAAATTAATAGAATCTTCTGAGGCAAGTGTTACAATTGCTTTCTTGTAAT
GTGGTCGCTTCCCTACGAATCTACCGATGCTCCTTTTTTTCTTAGGAGGATGACAAGTGT
TAACACCTGTAACTTGCACGTTGAAAATATACTGTATAGCAGCCTTAATATTAATTTTTG
TTGCTTTGGGATCAACGGCAAAACAATACTGATTTTCTTCTAATAATTTAGTTGTCTTAT
CAGTAATGATGGGATATTTAACCAGATCTAATAAACCTCTTGAATCAATGCTATCCATTA
TATACCTCTTGTATTTTAGATAAAGCATCAACTGTAATAATAATTTTATGTGCAGCTAAC
AAAGCCATAATATTTAGTGTATCAGCTGAAATAAGTTCTACGTTGTGTAGATTGCGAATA
GAAAGATAAACATTCGGATCTTTTTTGTCTACGATGACTAAAACTTTTTTATTTAAATCA
AGATTCCATCGATGTATAGCTTCCATAAATAGTTTAGTCTTGGGCTGATGAAAATAACTG
TTAAAATTTTCTACAATCAGTGTATTAACTGATTTGTTATTTAATGCTGTCCTTAATGCT
AATTGTCTTTCTTTTTTATTCATTTTTTTGGTAAAACTACGAGGTTTTGGCCCAAATATT
ACACCACCACCTCTCCATAGAGGTGAACGGATAGAACCTGCTCTTGCTCGACCAGTCCCT
TTTTGACGCCATGGTTTACGACCTCCACCTCGAACTTCACTTCTTGTTTTAGTATTAGCA
GAACCCTGGCGCTTTTCATTACTTTGCTTTACTAAAGCTCTATGGACTAAGTACATCCCA
GAATCTTGACTGACCTTAAGATTTAAATCAGCGTTGCCACTTACTTGACCTTCCCAATTA
TAGACTTGATAATTTAATTGCGTGTTAACTGTCATGAAATATATCGTATGAAAGAATATT
GCTACTTACTAATTTTTACTAAAGCGCCAGGTTTACCAGGAACAGCTCCCTTAACGATCA
ACAAATCATTCTCTGAGTTAATACTTACAATTTGCAAATTTTTTATTGTAACTTTTTTAT
TACCAAGTTGGCCAGCCATGTTTTTACCTGGATAAACTCTTCCAGGCGTTGTACCGGCTC
CAATAGAGCCAGGTTGCCTATGATTTTTCGAACCATGTGACATTGGGCCTCTACTAAAAT
GATGCCTCTTTTGGTAACCGGAGAAACCTTTACCTACACTTCTAGAAGAGACATTAATCT
TTTGGCCTACTTGAAATAGATCTGTAGATAGTATTTGGCTAACTTCGAAATCATCAGTAG
ATTTCATTTCGTATTCACGTAAATATTTTAGTGGGGGAGCCTGTGATTTTTTTAAGTGTC
CCAACAATGGTTTATTTAATTTTTGTTCAGCAACTTGCTTGTAACCGACTTGAATAGCAT
TGTAGCCATCAGTAGATACAGCTTTAATTTGAGTAATAACACATGGCCCAACTTGAATTA
CAGTAACTGGAATTGATAAACCAGCTTCATCAAAGAATTGGGTCATACCTACTTTAGTAC

CGAGTATACCAACAGACACTAGATTTCTCCTTTTTGAGAGATTTATACTATCATTGTACA
TAAAAATTCTACTTCATAGTTACTGAAGTAAATTAAACTAACAGATGCATGATATTATTC
ATGACAACATAAAGGTTAAAAACTTTACCTATTTAATCTAAAATAGAAACAGCTTTTTGT
CTAGTTACAGCGTATATAAATTAGAAGTATGCTTATTAAATTTTTTATAAACTACTTAGA
TTTAACTGATATTGATATCCGTAACTTACTTGCAGTAATATCAATCAACATGTATAAGTT
TAATTGCCTAAACTAGAACCAGCTATTATTAGAAATAATTCAAGTATAGATAAAAGCGTA
TTTGAACTTTTATATATTGACAAATATAATAGAATATATTGAGGTTCGGTAACTACACCT
TTTTATTTTTACATTTATTGTGCAGTATATGTACATCTAATGCAGTAAACATAAGAATAT
AACTGTATAGCAAATGTAATTACACAAATTAGAGAGGTATTAATGGGCAAAGTCGTTGGA
ATTGATCTTGGAACAACGAATTCTGTAATTGCTGTTATGGAAGGAGGTAAACCTACCGTC
ATACCGAATGCAGAAGGTTTTAGAACTACAGCTTCTGTTGTTGCATATACTAAAAGTGGA
GATAAACTTGTAGGACAAATTGCCAGGAGGCAAGCTGTTATTAACCCAGAAAACACTTTC
TACTCTGTCAAAAGATTCATAGGACGTAAACAGAATGAAATTTCGCAAGAGATTCGGCAA
ACATCATATAATGTTAAAACTAGTGGATCAAGCATAAAAATTGCTTGCCCTGCACTGAAT
AAAGATTTTGCTCCAGAAGAAATTTCAGCTCAAGTACTGAGAAAACTTGTTGAAGATGCT
AGTACGTACTTAGGTGAGACTGTTACACAAGCAGTAATAACTGTACCGGCTTACTTTAAC
GATTCACAAAGACAAGCTACTAAAGACGCAGGTAAAATAGCAGGCTTAGATGTATTGAGA
ATTATTAATGAGCCTACCGCAGCTTCTTTGTCTTATGGACTAGACAAACAAAATAATGAA
ACAATACTAGTATTTGACCTTGGTGGGGGCACATTTGATGTATCTGTATTAGAAGTTGGA
GATGGAGTATTTGAAGTACTCTCAACTTCTGGAGATACACATTTAGGCGGAGATGACTTT
GATCAGCAGATTGTAGAATGGCTAATCAAAGATTTTAAACAGAATGAAGGTATTGATCTT
GGTAAAGATAGACAAGCACTTCAGAGATTGACCGAGGCTGCAGAAAAAGCAAAGATAGAA
CTGTCAAATTTAACTCAAACAGAGATCAATCTTCCTTTTATTACTGCCACACAAGATGGT
CCAAAACATTTAGAAAAAACTGTAACTAGAGGAAAGTTTGAAGAACTTTGTTCAAATTTA
ATAGATAAATGTAGTATCCCTGTAAATAATGCTCTGAAAGATGCAAAACTAGAAGCTTCC
AGTATTGATGAAGTTGTTTTAGTTGGTGGATCTACAAGGATTCCAGCCATACAGCAAATG
GTTAAAAGATTAATTGGTAAAGATCCAAACCAAAGTGTCAATCCAGATGAAGTTGTTGCT
ATTGGTGCAGCCGTACAAGCTGGAGTTTTAGCAGGCGAAGTCAAAGATATTCTATTACTA
GATGTGACGCCGTTATCTTTAGGAGTGGAAACTTTGGGTGGCGTGATGACAAAGATTATA
CCAAGAAATACTACTATTCCTACAAAAAAATCAGAAGTATTTTCTACAGCTGTAGATAAT
CAACCAAATGTGGAAATTCAAGTACTTCAAGGCGAAAGAGAACTAACAAAAGACAATAAA
AGCTTAGGGACGTTCCGATTAGATGGCATTATGCCTGCACCTAGAGGAGTTCCTCAAATT
GAAGTTACCTTTGATATTGACGCTAACGGAATTTTATCTGTAAAAGCCAAAGAAAAGGCT
ACTGGTAAAGAGCAATCAATTACTATATCTGGAGCATCAACTTTACCTAAAGATGATGTT
GAAAGAATGGTAAAAGAAGCTGAAGAAAATTTCGATACAGACCAAAAAAGAAGAAAGAAT
ATTGACACAAAGAATCAAGCAGAGTCCCTATGTTACCAAGCTGAAAAGCAAGTTAAAGAG

TTTGAAGACAAAATTAGCCAAGATTTAAAAATAAAAATAGAGGAGCTAATTACAGAGCTT
AGATCTAGTCTAGAGAAAGAAGAATATGACAATATTGAATCCATTTCTCAGCAATTACAA
AATGCTCTGATGGACATTGGAAAAAATGCTGCTCAGACTGAAAGTAAAGATACAAAAGCG
AAGGATGACGACACTGTGATTGACACTGATTTCTCTGAAGCTAAGTAAAAGTAAGCGGGT
AACGCGATTCGAACGCGCGACATCAACCTTGGCAAGGTTGCGCTCTACCACTGAGCTATA
CCCGCATAGATTGTATTATTACAAAAATTAAGATATTTGTCAATCTGTTAATCTATACAT
CTAGACTATTAATTTTGGAATTACATATTAGCTAACTGTTGTAAATATAACAATTGCTAT
AATTTTTTAAAAGTACTTATATCATTATATAAGTACTTTTTATATTCAAAAATACAAATA
AGTATTATAGCTAAACTACTAAGGAGTTAACTAAGCTAGTAACAATTACTAATCCTGTCC
ATAAGCCGGCTCCTGTATATATTAATCCTTTTGATTGCTCCCATTGGCCAGGGGATGCTA
AAACAACAGGTACGCCTACTACTAACACAGTCGATAATGCAATTAATAATAGCACTAATA
ATTGGATCGCAATAATCATTTAATATGTTCTCCTGAAAAATTCAAATGTTAATAATACTG
TATATAATATAATGAAGCTTTTGACCTTCAGCAATTTATCTATGAAGAAATAATTTAAAA
TTATTGTTTTCTAAATAAACCTTAAATTAAGTATTAAAATCTTTATTGTAAACAATAATA
CTATATTTTTTTAGTAGATAAAAAATTTAAAGAAAGTCTTTTTTAAAAAAAAATTAAGAT
AAGGTGATATGCTGTATAAAAAGCTAAGTATTATTAATTATTAGAGAAATTTAAAATAAG
AGGTAAAACATATGAACTCAGCCCTTTTTTTAGCAAAACTACCAGAAGCTTATGCCATTT
TTAAACCGATCATTGATATCTTACCCGTAATTCCTGTATTTTTTCTTCTTTTAGCTTTTG
TTTGGCAAGCAGCAATTGGTTTTAGATAACATTTAGGTTATAAAAAGAATAACAAATATA
CAGAGTGCCAATTTTTGAGCTCTAATCTTATATCTTACACAATAATAACTATTATGGTTG
AACCCTTACTATCAGGAATTGTTCTTGGACTTATTCCTATTACTTTATTCGGATTATTAG
TAGCAGCTTATTTACAATATCAACGGGGCAACCAATTAGGACTGTAAAGCCTAAATTATA
GATATTTATAAAAACTACAATAATTATAGTTTTTATAAATATCTACAAATAAGCTAATTT
TAAATACAGAAATAATACTATTGAAACTAGAGTGATAACAAAAAAAATTTATGTAATAAC
AAGCTAATTGTTTACCAGCTCGACTTAGTAACTCCTGGTAAAAGACATTCATGAGACATT
TCTCGAAAAACATGGCGAGATAATCCAAAATCTCGATAATAACCTCTACTTCTACCTGTT
AACCAACATCTATTTCTCCCTCTAACAGCGGCGCTGTTTCTAGGCATTTCTTGGAGTTTT
TGTCTTAAATCCATTTTGTCAGAAAAGCTGCCAGCTTCTTTCAGTTGATCTTTAATAGCC
AGTCTTTTTAAATAATATTTTTTTGCAAGTTTCGATCTCTTAATCTCTCTTTGGATCATA
TTCTTTTTAGCCATGCTGTAAAACTCTCTTTTAAAATTATTTATTCAATATATTAGCTAA
AAAAGCTCCTGCCTGCAACTTTTATATATTTAAAAATAAATAGCATAAAGTAGTGAAATG
CAATTGATGTTTAATCAACGATACCACTTTATGCTATTATCAAAAATTTATTTAGTAAAG
TTCTTGTTCAACATGAGTTGAAATAGTGCAATCAGATGAAGGATATGCAATACAGGTTAA
AACATAGCCAGCTAAAAGTTGATCATCATCTAAGAATGATTGATCAGCTTGATCTACAGT
TCCTTCTGTAACTTTACCTGCACAAGTTGAACAAGCTCCAGCTCTACAAGAGTAAGGAAG
TTCAATTCCTTCTTCTTCTGCTGCATCTAGAATATATGTATCCTCTGCACAATTAAATGT

GACATCAATTCCTTCTTCTTCACATAGTAAATGAATTTTATAATCAGCCATTTCTTCTTA
CTCCATAATAAAGATACACTTGAAACTTAATAAAGCAAAATAATTTAAAGAACTTACCAA
AAAATTATATACTAGTTTCATTATGCATGATAGACTGGAAAAAAAGAAAATAGTATACTA
TTCTGAAAAATCAAGCTACATTTCCTACTAAATAGAACTATAGTAGGTCCAAAGTATATA
CATAAATTCATATAGTTAAAATTACTTTTATCATTTAAATAGCTGAACTTGATAGTATAG
ATTGATCATCAAGCTAGTCTTCTGCCAATAAATAATTAATTATGATTTCAATGCTTAATC
AACAAGTAGAACTTAAGCCTATTCTTAAATTTCAGATAACACAAATATATTCGCATTCTC
TTATTCAGGAAATTAAAGCCTTAATAACTAGACTTGTTTTGCAAGTTTGGAGAAGGCCAG
CTACATTGATGGCAGGTATTATCCAACCTTTATTATGGTTAATTCTATTTGGTGGGCTTT
TCTACAATGCTCCTATAAATTTGTTCACTATTAATACAAGCTATAATTGTTTTTTGAGCT
CTGGGATTATAATTTTCACCTCTTTTACTGGAGCTTTGAATTCAGGTCTTCCATTAATGT
TTGATAGAGAATTTGGATTTTTAAATAGATTATTAACGGCTCCCTTAGTATCGAGGACTT
CTATCATTTTATCTTCTGCTACTTTTATGACTTGTATTAGTTTAATACAAGTTGTATTTA
TAGTTACAGCTTCTCTTTTTATGGGAAACCCACCTCTAAATAGCGATAGTACTATGATTT
TTGGACTTATGATTCTATTAGTGACTGTAGGAGTTACAATGCTTAGTTTAGCTTTATCTT
TCACTCTGCCAGGTCATATTGAGCTATTAGCATTTATTTTAGTAGTTAACTTGCCCTTTT
TATTTTCTAGTACGGCTTTAGCTCCTTTATATTTCATGCCGCCATGGCTTCAGTTAATTG
CAAGTCTCAATCCATTGAGTTATGCAATAGAAGGTACAAGATACTTATATTCAAGCGTAA
ACTGGAATTTTACAGAGTGTGTGATTAAGATTAGCTGGGGAGATATTTGTTTAGGGCAAA
TTATTATTTTATTAATCGCTTTAGATATAATGGCAGCTTATCTTGTGTCTAATATATTAA
AAGCTAAACTTAATTAAAATTTTAATAAAAAATTTATTAAATTTATTTTACAAAGATTTT
TACTATATAATACTAATAGTAGTATGGATAATTGAAATAGAAATAGTTTTTCAAAACCAA
AGCTATTATCAACAATGATAAACATTTGTAAGAAAGTCAACAAAGTATGTTCTTATTCAT
AGGAGGCATGTAGTCAATGGGACTACCATGGTACCGTGTACACACGGTTGTTTTAAATGA
TCCTGGACGGTTAATTGCAGTCCACCTAATGCATACTGCACTTGTAGCGGGTTGGGCAGG
ATCTATGGCATTATACGAACTAGCTGTATTTGATCCTTCAGATCCAGTATTAAATCCAAT
GTGGCGACAAGGCATGTTTGTTATGCCATTTATGGCTAGACTGGGCGTAACAGATTCATG
GGGAGGATGGAGTATAACAGGAGAGAGCGTATCCAATCCTGGACTGTGGAGTTTTGAAGG
TGTAGCCTTAACTCATATAGTCCTTTCTGGCATGCTTTTTCTAGCTGCTATTTGGCATTG
GGTTTATTGGGATTTGGAATTATTTAGAGATCCACGAACTGGCGAGCCAGCCTTAGATTT
ACCGAAAATTTTTGGAATTCATTTATTGCTATCAAGTCTACTTTGTTTCGGATTTGGAGC
TTTCCATGTAACTGGACTTTTTGGACCAGGAATGTGGGTATCAGATGGGTACGGAGTAAC
CGGAAAGGTATTACCAGTAGCTCCAGCATGGGGACCAGAAGGATTCAACCCGTTTAATCC
TGGAGGAGTTGCATCTCACCACATTGCCGCAGGTACTGTAGGTATATTAGCTGGTGTTTT
CCATTTAACTGTTAGACCACCACAAAGACTGTATCGTGCTCTAAGAATGGGTAATATTGA
AACTGTATTATCAAGTAGTATCTCTGCTGTTTTTTTCTCAGCTTTTGTGACTTGTGGTAC

GATGTGGTACGGCTCTGCAACTACGCCTATTGAATTATTTGGTCCAACTAGATATCAGTG
GGATAGTGGATATTTTCAGCAAGAAATTGAGAAACGAGTAGAAAATGCTATTGCTGATGG
TGCTGCACCTAGCGAAGCATGGTCAAGAATTCCTGACAAGTTGGCATTCTATGACTATAT
TGGTAATAATCCAGCAAAAGGAGGATTATTCAGAGCGGGCCCTATGAATAAAGGCGACGG
AGTTGCTGAAGCATGGCTTGGACATCCTGTATTCCAAGATAAAGAAGGAAGAGAGCTTAG
TGTTCGCAGAATGCCTGCTTTTTTCGAAACTTTTCCTGTAATTTTAGTTGATAAAGATGG
TATTATACGAGCTGACATTCCATTTAGAAGAGCAGAGTCTAAGTATAGTATTGAACAAGT
AGGTGTAACAGCTAGTTTTTATGGTGGCAAATTAAATGGCCAAGTTTTCAACGATGCTCC
TAGCGTTAAAAAATATGCAAGGAAAGCTCAATTAGGTGAGGTATTTGAATTTGATCGAAC
TACATTAGAATCAGATGGAGTGTTTAGAAGTAGTCCTAGAGGCTGGTTTACATTCGGTCA
TGCTAATTTCGCTTTAATTTTCTTCTTTGGACATCTTTGGCATGGTTCAAGAACTATCTT
CCGGGATGTGTTTGCGGGAATCGGAGCTGAAGTTACTGAACAAGTTGAATTTGGAGCTTT
CCAAAAATTAGGAGATAGAAGTAGTAAAAAACAAGGAGCTGTATAAGACCAAAGTCAAAG
ATAGTCTTTATCAAATATTTCTAATATTAAAATAGTTAAGACAAAGTTTATTATCTAATA
GTTACTAATCAGGAGAATTTATGGAAGCCTTAGTCTATGTATTTTTACTAACAGGAACGC
TAATGGTTATATTCTTTGCGATCTTTTTTAGAGAACCTCCAAGAATAGCAAAGTAACTTG
ATTCCGTTTAAACTTATAATAATAGAACCACTTTCCAACTTATTAGTGGAAAGTGGTTTT
ATTGTAGATTACATTGTATTATTCTTCATGCTCTTCAAAAGGATCTCGGAGATCTTTTGA
AGCAGGACCAAATGCTGTGTATATCGAGTAACCTGTAATACCTAAAAGCAGACTCGAAAT
AAAAATACTAAGAACTGTTGCAGTTTCCATAATTTAAGCAGTATTAAGATGAACTTTTTC
TATAATGATATTATCAATATTAAAACATTTAATTATTAAAATTATCAAAATATACTATGG
CACTTAGAACTAGACTTGGAGAAATTTTAAGACCTTTAAATTCAGAGTATGGAAAAGTTG
CTCCAGGTTGGGGCACAACTCCTATTATGGGAATTTTCATGTTACTATTTTTCTTGTTTT
TATTAATCATTTTACAAATCTATAATTCTTCATTAGTATTAGAAAATGTAGATGTAGATT
GGGCTACTTTAGGTAGCTAACTAGAGAAGTTCTTTGATTACACAATATTAAAGACAACAA
GCGATCGCTATTAGATTATAGTAGCGATCGCTTTAAAAGTATTTTACTATATATCCAGAG
AAAATTATTTACCTAAATTAATTAACTCGGTATCTGCAAAACTATTTGTGTTAACATTAT
TATAGTTAACTTTTTCAAACCTTACTAATACTGGATATTTGATACCACTTTTATCAATTG
CTGCAACAGTACCAATTTCCTGATACCAGTACGATTCTTTTCTCAATATTTTTACTTTTG
AGCCTCTTTCCATATTTTTAGTCCTCAGATAGATTATTAAAGATTATTATATTAAAAAAT
T-TAAAAAACTAAACAAAATATATAAAAATACTACCGACAATTGTAATATATAACTAATA
AAAGGAGAGAGAGGGATTCGAACCCTCGATAACAAAAGTTATGACAATTTTCGAAATTGT
TGCAATAAACCACTCTGCCATCTCTCCTGAATTAAAATACTGTTTTAAAAAATTAGACAA
GAATAACATAGAACTAAGGTAGTTGCCTAGAAAATTAAAACTATGTTAAATATAAATTAT
AATCTTTCTATAGATATATTTAAAAAATATGTACATAGAATCTATATCAGCTTATTTCAA
GATTTTTTAATCTTATACAATTTTTACTAAATAAATAAATTCTCTCATAATATGAAACTA

TCTTGGAAAACACTTCTTCTTTGGTCTTTACCAATATTTGTAATTGGTTTCTTTTTCTGG
CAAGGTTTTTTAGGTCCAACTACTACAGATGTTGGCAGTAATATCGCAAGTTCTAGAATG
ACATATGGACGATTTTTAGAATATTTAGATATGGGTTGGGTGAAACGGGTTGACCTCTAT
GAAAATAATCATACAGCAATTGTAGAAGCTGTTGGGCCAGAATTAGGAAATAGGGTTCAA
CGAATTCGAGTTGAACTGCCAGCAAGTGCGCCAGAATTAATTACAAAATTACGCAAAGCC
AATGTTGATCTAGATGCTCACCCCCCTAAAAGTACAAGTGCAGTATGGGGACTATTAGGC
AATTTACTATTTCCTTTACTATTAGTTGGCGGGTTAGCTTTCTTATTTAGAAGATCTAAT
AATGCTAGTGGTGGACCTGGTCAAGCAATGTCATTTGGCAAATCGAAAGCTTTATTTCAA
ATGGAGGCTAAAACGGGAGTAGTATTTAATGATGTAGCTGGAGTTGAAGAGGCAAAGGAA
GAATTTCAAGAAGTGGTAACATTTTTAAAACAGCCTGAATCATTTACTGCTGTTGGTGCA
AAAATACCAAAAGGCGTTTTATTAGTTGGACCTCCTGGCACAGGCAAAACATTACTAGCA
AAAGCTATTGCTGGCGAGGCTAGTGTTCCTTTTTTTAGTATCTCAGGCTCAGAATTTGTA
GAAATGTTTGTTGGTGTTGGCGCTTCTCGTGTGAGAGACCTATTCAAGAAAGCAAAAGAC
AATGCGCCTTGTATCGTTTTTATTGATGAAATTGATGCTGTTGGTAGACAACGAGGAACA
GGTGTTGGAGGTGGTAATGATGAAAGGGAACAAACATTAAATCAACTATTGACTGAAATG
GATGGCTTTGAAGGAAATACTGGTGTTATTGTAATTGCCGCTACTAACAGAGCTGATATT
TTAGATTCTGCATTATTAAGACCTGGAAGATTTGATAGACAAGTTTCTGTAGATGTACCA
GATTTTAAAGGCAGGTTAGCAATTCTTGAAGTTCATGCTAAAAATAAGAAAATGGAACCT
AAAGTATCTTTAGAAACCATTGCCAGAAGAACTCCCGGCTTTTCAGGAGCTGATTTAGCT
AACTTACTAAATGAGGCTGCTATCTTAACGGCTCGACGAAGAAAAAATGCAATGACTATG
TCTGAAATTGATACATCAATTGATCGAGTAGTAGCCGGGATGGAAGGCACTCCTTTAATT
GACAGTAAAAGCAAAAGATTAATTGCGTATCACGAAGTGGGTCACGCAATAATAGGCAGT
TTATTAGAGCATCATGATCCTGTGCAAAAAGTTACATTAATACCAAGAGGGCAAGCAAGA
GGCTTAACTTGGTTTACTCCTAGTGATGATCAAAGTCTAATATCAAGATCTCAAATACTA
GCCCGTATCGTAGGTGCTCTTGGTGGCAGAGCTGCAGAAGAAATCATTTTCGGTGACGCA
GAAGTTACTACTGGTGCAAGTAATGATTTACAGCAAGTAACATCAATGGCAAGACAAATG
GTCACTCGATTTGGAATGTCTAAAATTGGACCTTTATCTCTTGAAAGCCAAGGAGGAGAC
CCATTTTTAGGTAGAGGCATGGGAGGAGGCTCAGAATATTCAGATGAAGTTGCAACTAAT
ATTGATAAGCAAGTAAGGGAAATTGTCAGTGAATGCTATGCACAAGCTAAACACATTATT
ATAGATAATCGAGTAGTGATAGATAGATTAGTTGATTTACTAATTGAAAAAGAAACAATT
GAAGGCAATGAATTTAGAGACATCGTTAAGGAATACACTGCAATTCCTGAAAAAAATTAC
TACATATCACAATTTTAAATTAAACGGGACTGACGGGATTCGAACCCGCAACTTCCGCCG
TGACAGGGCGGTGCTCTAACCAGTTGAACTACAGTCCCAAAAAGATGAATACCCAGAGAT
AATCTCATAGTTAGCTTAAACTTGTCAAATTAGAATATTAAATATGAAGAGTATTTGTAA
TAATACTCTTCATATTTAATATAGTAGCTAAAATATTTCTTTTAGAAGGAGAGAGAGGGA
TTCGAACCCTCGGTACGAAGTTAATCGTACAGCAGATTAGCAATCTGCCGCTTTCGACCA

CTCAGCCACCTCTCCATGATATATATTATATATATATCAAATATACTTATATTTTAATAG
GTGTGCATAAATATTTATGGAGCTAAGCGGATTCGAACCGCTGACCCTCTCAATGCCATT
GAGATGCTCTACCAACTGAGCTATAACCCCTTTGGTAAGATATTAAGAAATGGCTCAAGC
GGGATTTGAACCTGCGACCTTGGGCTTATGAATCCCCTGCTCTAACCACTGAGCTACTGA
GCCATAGTATAAAATATTATTAAGTATTATACCATAAATTATGGTCAAATATAAATCAAT
AAAAGAATAATTACTATCATTTACTACTTTTGTGCAAGTTGTAAATATTCGAATAAAATT
TCAAAATTAAAAATGTCTTTAACTTTTTGTTAGAATTATTGATTACGAAAGTATTGATAT
TTAGAATTAACAATAAATTTTGTCTTTATTTGTAGACACAAAAATATTGGAAATTTTCAT
CAATTAATTAGTTAAACTATAACCGACATACAAGAATTATTGAACTACGTTAAATTGTGA
TGCATTGGGCTTGATCAAAACTTCTCTAAAATCAATAGAATATAGTAATTCAAGTAGATC
AAATACTAACAAATGTGACAAACGCTTAAAAAAATAATACAATTAGAATCGTAAAAATTT
GGTTTTTGACTGTGTTAAATAAATTTGATTATCATAGATTTAATATTTAAAAGTGTTCTA
TTACTTGTATTGAGTAATTGTAATATTTAATATCTTCTTTTTTAGATAACAATCATAATA
TAAGCTAAAAAATAGTTTTTATTAAACAACTAGCATAGAGCCAATACCTTGATCAGTTAA
TATTTCTAGCAAGAGAGCATGATCTATTCTACCATCTAAAATATGCGCTGAAGCTACACC
CTGAGCCAAAGAACGAATACAACAATTCACTTTAGGAATCATGCCACCAGAAATTACTGC
TGTTTGAGTTAAATCTCTAGCTTCCTGTATATTCAGATGACTAATTAATGTTGAAGGATC
TGAAGAATTACGTAAAATGCCGGGCGTATCTGTGAGTAGAATTAGTTTTTCGGCATTAAG
AGCGGCTGCTATTTCTCCTGCGACAGTATCAGCATTAATATTATACGATTGGCCTTCCTT
GTCTGCTGCAACACTAGCTATAACAGGTATATAATTATTATTAATTAATATCTTCAGTAA
TTTAGTATCAATATTTTGTACTTCTCCAACAAAACCAAGGTTTGCTTTTCCATTTGGTCT
CGGAGTAATAAGTAATCCATCTTTTCCCGACAAACCTACACTTTTACCACCTTGTTTATT
AATAGTTGCGACAAGATCTTTATTGACTCTTCCAACTAAAACCATTTCCACTATATCCAT
GGTAGGTTGATCTGTTACTCTAACGCCATCATCGAATTTTGGTAATATTTTGAGGCGATC
TAACCAAAAATTAATTTCTGGTCCTCCACCATGAACTAAAATGGGACGTAGTCCTATAAA
AGACAGAAAAACAAGATCACTAATCACTTGATCTTTTAGTTTCTGATTTTTCATAGCCGC
TCCCCCATATTTTATAACAATAATTCTGGAAGAAAATTGCTGAATATAAGGCAGAGCTTC
ACTTAAGACTTTTACTCTTTCTGAATTAGTCAACATAAATAAAGTAGGATTGTGATATAT
AAAATTATATACACAGCTTGTGTTAATTTAAAAGATACCAATGGGTACTTGAAAAAATAG
AAATATTACTAACAGTTAAAACTATAGGGATTATAATTATGAATAAATTTTGGGATAATG
TATTAAGATTTCCTCGATTTTTAGTCAGCGTCATTTTAGGATTAATTTTGATAATTATTA
GTCCTTTTTTCGTGTTATTAAAAAAACCACTGACAAGTTTTTTTTTCATCATATCATTGG
CCGGATTAATCACAGTTTTGGCAATAATAATACAAAAAATGATAAATATCGAATGTTGTT
GAGAGAAATAAATAAATTTAATTAAAATTTTTGAACATATATAATATTATATTATCATTA
GATATGATAATTTATTTCCTTTTCTATAAAACCCATAAATATTAAATATTATTTGATAAA
TATTATGCCACTAAAACAAAGAGTTAGCTCTGAAAAGACCGGTGCCTTCGCACTTTTGGA

TAGTATTGTTAGGCACGGAGTTAAACATATATTTGGTTATCCAGGTGGAGCTATTCTTCC
TATTTATGATGAGCTTTATGCTTGGGAAGAAGCCTCTCTAATTAAACATATCCTTGTTCG
TCATGAGCAAGGCGCTGCTCATGCTGCAGATTCTTATTCTAGATCAACAGGAGAGGTTGG
AGTATGCTTCGCTACTTCTGGCCCAGGAGCAACTAATCTTGTCTCAGGTATAGCTACAGC
ACATATTGATTCTGTACCTATATTAGCTATAACAGGTCAAGTTGGGAGAGCTTTTATTGG
TACAGATGCTTTCCAAGAAGTAGATATTTTTGGGATTACACTTCCTATTGTAAAACATTC
ATATGTAGTTCGTGACCCTAGAGACATGTCTAGAATTGTTGCGGAAGCATTTTTTATTTG
TAAACACGGTAGACCAGGTCCAGTATTAATTGATGTTCCTAAAGATGTAGGATTAGAGAA
GTTTAATTATTTTTCTGTTGAGCCCGGAAAAGTTAATATTCCTGGCTGTAGGCCAATTAC
CAGCCTAAAGTCAAGACAAATCCTTATGGCAGCTAAAATGATACAGCAATCTAGCCAGCC
ATTGTTGTATATTGGTGGAGGAGCCATAATCTCTGATTCACATCAAATTATTAAAGAACT
TGTTGATTTTTATAAAATACCTGTTACTACTACTTTGATGGGGAAGGGGATTTTTAATGA
GGATAGCGATTATTGTCTAGGGATGTTAGGTATGCATGGTACTGCGTATGCTAATTTTGC
AGTTAGTGAGTGCGATCTTTTAATTGCTTTAGGAGCTAGATTTGATGATAGAGTTACTGG
AAAATTAGATGAATTTGCTTGTAATGCCCAAGTGATTCACGTAGACATTGATCCTGCTGA
AGTAGGAAAAAATAGGATTCCTCAAGTTGCTATTGTCGGTGACGTAGCAGAAGTTGTTAG
TGAAATATTGAATTTATTAAAGACTTCTTTCCCCCCTTATCCAGAGCAGATTATATCTTG
GCAAGAAAGAATTAATCGTTGGCGTCAACAGTATCCTTTACTGGTTCCTAGAAAATCAAC
AAGCATTTCACCTCAAGAGATTCTTGTTGCAACTAATAAATTAGCCCAAAATGCTTATTT
TACTACAGATGTTGGCCAGCATCAAATGTGGTCAGCTCAATTTCTGAAAGTAAAAGCTAA
GCATTGGCTTTCAAGTGCTGGATTAGGCACGATGGGTTATGGTTTACCTGCAGCAATTGG
CGCTCAAGTAGCACATCCAAATGACGTAGTCATTTGTATTAGTGGTGATTCTAGTTTTCA
AATGAATATGCAAGAGTTAGGAACTATCGCGCAATACCAGTTACCAGTTAAAATTATTAT
TATTAATAATCGATGGCAAGGGATGGTTAGACAGTGGCAACAAGCTTTCTATGGTGAAAG
GTATTCACACTCAAGAATGACAGAAGGAGCACCTGATTTTCAAAAGCTTGCAGAAGCTTT
TGGTATTAAAGCTTTTACTATTAATAATAGGCAAAATATGCAATCTGCTTTACAAGTTGC
TATTGATTATCCTGGTCCAGTTTTATTAGATTGCCAAGTTACAGAAAATGAAAACTGTTA
TCCAATGGTTGCTCCTGGAAAAAGTAATGCACAAATGATAGGTATCGCTAAACCGCAGAG
AGGTACTGCTTCCAACTATATTAATAATAGTGTTTGAAGTAAATAGTTAGTATTTTTTAA
ACAAAGAATAAAGTACATAAATGTATTTTTTTTATACTATTAAAAACTATGTAAGAACAG
CCTTTGACGAGAATTGAACTCGTGACCTTCCCCTTACCAAGGGGATGCTCTACCTCTGAG
CCACAAAGGCTTTTTACTATATTGGGCCGGGTTGGATTTGAACCAACGTAGGCGAAGCCA
GCGGATTTACAGTCCGCCCCCATTAACCACTCGGGCACCGACCCTAATTAAACCTTATTC
AAGATATGACCTATAAGATATCATAACTATGTTGCTAAAAACAAGTACTTTGATAAATAA
AACTGAATAAGTAATGGACATAGCTGGACTTGAACCAGCGACTTTCACGATGTCGACGTG
ACACTCTAACCAACTGAGTTACATGTCCAAAACTTTAATTATCATTATAACACTGTTTAG

TGATTTTATAAGTATCTATTTCTAATTGAAGATTTGTTTTAATCGTCCGGCTTTACCGAG

CAGATCTCTTAGATAATACAGTTTAGCTCTACGTATTTTAGCTCTTCTAATCACTTTGAC

AAAAGTTATTCGCGGTGAATTGAGAAAAAATACTCTTTCAACTCCAATACCTTGAAATGA

GCATCTTAAAGTTAAACTAGTATTTAAGCTTTTTTTTCTTCTTCTTGCTAATACTACTCC

TTCACATAATTGTTCCCGAGTTTTACTACCTTCCTTAACCATTAGTCCAAGTTGTATTGT

ATCGCCAACTTTAATTTCAGGTACTTCTGTTTTGATAAAAGGTATCTCAACACTTTTCAT

TAGCTGACTTAGCTTTGTACTATTTGCTTTCATATATAGGATTAGTCTTGTGACATGTTT

TGAAATATTATACATTAAAAAAACAAATATGAAAAATTTTAGCGAATAAGCATATTAGTA

TTTATAAAACTCTTTTTATCTCCATTATACAGATCCATTATATTCAACTAGATTCTTTCA

TAGTTTCTTTGATAATCAGTACATCTAGATGATCAAATATTATTTTCAAACATGGCTTAT

GTATGAAAAAGTTCTATAAAAACCTAATGACTAAAATTAGGGCTTAAGTATTTCTTAGTT

TTGTGTTATAATTAGTTACTATCAAAGGTAGTGGCATAACACGAAGCATCAAAATGTTCG

AACGCTTTACTGAAAAAGCTATAAAAGTCATAATGCTAGCACAGGAAGAAGCTAGACGCT

TAGGACATAATTTTGTCGGAACTGAGCAGATACTATTAGGATTAGTTGGTGAAGGTACTG

GAATCGCAGCTCAAGTTTTGAAATCGATGAATGTGAATCTAAAAGATGCAAGAGTTGAAG

TCGAAAAAATTATAGGAAGAGGGTCTGGTTTTGTAGCGGTTGAAATTCCTTTCACTCCTC

GAGCAAAAAGAGTACTAGAATTATCTTTAGAGGAAGCACGTCAACTAGGCCATAACTATA

TTGGCACAGAACACTTGCTAATGGGCTTAGTCCGAGAAGGAGAAGGAGTTGCGGCAAGAG

TTTTAGAAAATTTGGCAGTCGATGTTTCTTCAATTAGAGCTGAAGTTATACAAATGCTCG

GAGAAAATGCGGAAGCCAATGTAAGTGGAAGCAATACTACGCAAGCTAGAAGTAAAACAC

CAACATTAGAAGAGTTCGGATCTAACTTAACTCAAATGGCTATGGAAGGTGGTTTAGATC

CTGTAGTCGGAAGACAAAAGGAAATAGAACGAGTTATTCAAATCTTAGGTAGAAGAACTA

AAAATAATCCTGTCTTAATTGGGGAGCCTGGTGTAGGTAAGACAGCGATTGCGGAAGGAT

TAGCTCAAAGAATTGCTAATAGAGATGTACCTTCTATTTTAGAAGATAAATTAGTTATTA

CTCTTGATGTCGGTCTATTAGTAGCCGGAACTAAATATAGAGGTGAATTTGAAGAAAGAC

TAAAACGTATTATGGATGAGATTAAATCAGCTGATAATGTAATATTAGTGATTGATGAAG

TTCATACATTGATTGGGGCTGGTGCTGCAGAAGGAGCAATAGATGCAGCTAATCTGCTTA

AGCCAGCTTTAGCAAGGGGAGAATTGCAATGTATAGGTGCAACAACTTTAGAAGAATATA

GAAAACATATAGAAAAAGATCCAGCATTAGAAAGAAGATTTCAACCAGTTGTAGTTGGAG

AGCCAAGTGTTGAAGAAACAATTGAAATTTTGTTTGGTCTTAGAGACCGTTATGAAAAGC

ACCATCAATTAACAATGTCAGATGGAGCTTTGGCTGCAGCTGCTAAATACGCTAATCAGT

ATATTTCTGACCGATTTTTGCCAGATAAAGCAATTGATTTAATTGATGAAGCTGGTTCTA

GAGTCCGTTTACTAAATTCTCAATTACCTCCTGCTGCCAGAGAATTAGATAAAGAGTTAA

GAGCTGTATTAAAAACAAAAGATGAAGCTATTAGAGCTCAAAAATATGAAACAGCAGAGC

AGTATAGAGCAAGAGAAATGGAAATTAAAGCTCAAATTGCAGCAATTGCTCAAAGTAAAA

AGAATGAGCCTGATTTAAATTTAGAAGATCCTGTTGTTACAGAAGATGATATTGCTGAAA

TTGTTGCTGCATGGACTGGTATACCAGTAACTAAGCTTACTAAAAGTGAGTCAGAAAAAT
TAATGCACATGGAAGAAACTTTGCATGGACGTATTATTGGTCAAGACGAAGCGGTTGTAG
CTGTCTCTAGAGCGATAAGACGCGCAAGAGTAGGTCTAAAAAATCCTAACAGACCAATTG
CAAGCTTTATTTTTTCCGGACCGACGGGTGTAGGAAAAACAGAATTAACAAAAGCTTTGG
CTTCTTATTTCTTTGGTTCAGAAGCTTCTATGATACGGCTAGATATGTCAGAATACATGG
AAAGACACACTGTATCTAAACTAATTGGTTCTCCTCCAGGATATGTGGGTTATAGTGAAG
GTGGTTATCTAACAGAAGCGGTAAGAAAAAAACCATATACTGTCATCTTATTTGACGAAA
TTGAAAAAGCTCATCCGGATATTTTTAATCTACTTCTTCAAATTTTAGAAGATGGCCGAC
TAACAGATGCAAAAGGCAGAACTATTGATTTTAAGAATACTCTTTTAATTATGACTTCTA
ATATTGGATCTAAAGTTATTGAAAAAGGAGGAGGTAGTTTAGGCTTTGAATTATCAGAAG
ATCAAACAGAATCCCAATATACTAGAGTACGATCTTTAGTAAATGAAGAACTGAAACAAT
ACTTTAGACCAGAGTTTCTAAATCGATTAGATGAAATTATTGTATTTCGTCAGCTTACTA
AAGATGAAGTTAGAGAAATTGCAGAATTAATGCTTAATGAAGTCTTTGCGAGAATTAAGC
AGCAAGATATTCAATTAAATGTAACAGAACGATTTAAGCAACGATTAGTAGAAGAAGGAT
ATAATCCAAGCTATGGAGCTAGACCACTTAGACGAGCAGTAATGAGGCTTTTAGAAGATA
GTCTGGCTGAAGAAGTTTTATCTGGTAAAATTAAAGCTGGTGATAGTGCAGTAGTAGATG
TTACTAATGAAGGAGAAGTTACAGTTTTATTAGGTGAAAAATTAGAACTGTTAACATAAA
AAACAATTTATTAATTAAAGCATTGAAGAGGTTATATTCAATGCTATGAAAAAAATTAAT
AAGATGGGTTGCTATAATTGACAACCTCTGATATTCTTTATTTGTATATAAAGCCGGGAT
AGCTCAGTTGGTAGAGCAGTGGATTGAAAATCCTCGTGTCACCAGTTCAAATCTGGTTCT
TGGCATTTTAAGCATCAGAAAATTTTGCTATCTGAGCATTAAATTTTAATTGAAATGTTC
CTGTGGGACCATTCCTGTGTTTTGCTATTATAACTTCTGTGAAGTCTCTACTTCTAGTTT
CTTGTGTATAGTAACTTTCTCTATATAGCATTATTACTAAATCTGCATCTTGTTCTATAG
AATTATGAACAATAAAGTTATTTGAAATAAAATTGCATAAAGGTTTACATTCTAAATCAA
ACACTATTTGTAAACTTGTAACGTTTATTTTTTGTATACTTTCAAAAGTCAAAGATGAAA
AAGAATTAAAAACATTTTTTTGTTCTTCAAAGTTTTTTATTTGAATAGCAATCATGTCAT
TTTGATTAATTTTATCACATCTTTTCCATCCTTGTGTTGTGAGTAGTTTATGGTTACTTG
TTAATTGTATATATTTCCCAGCTTCGGTAATAATTTTATATACAGTTTTTTTCCCTGTTT
TCGAAATACTACATTTAATTGCTAGAAATAACTGTCGTTTTTGCGCACTAAAGTTAATGA
CTTCAATCTTTTTATAATAGCAATTAAATAATATTTGAGGTTGATTATATAATGATGTCT
GAAGATAATTGAATTTGGATATACACCCACTTTCTCTTAAATCGGATAACAAAGGCCTTT
TGTCACTCCGAGTTTCAAGGTTTCTATTTAGTTGAGATAATGCCAAGATAGGCAAGCTTA
AATCTTTTGCTAATATTTTAAGTGATCGAGTAATTAATGATAGTTCTTGAGATCTATTGT
CAGATTGTCTACTCTCCTGCAGTAATTGAAGATAATCTATAATAATTAATTCTATATTTT
TTCCTTGTAATTTAAAGAATTTTACTTTTGTCTTTATAGTGTCAGTAGAAATCTTAGCGC
TATCATCAATATAAAGATTAAGGTTAGCAAGAGTTTTACTTTTTTGAATAACATATTGCC

ATTCATCATTATTAAGCTGACCAGATTGAATTTTTTGGCCGTTTAAATTACACTCTTGAG
CTAAAATTCTTCTTAGTAGTTGCTCTGTAGACATCTCTAAGCTAAATAAAATAACATAAG
ATCTTTTCTGATTAATTACATATCTAGTTATATTAATAGCAAAAGCAGTTTTCCCCATTG
AAGGTCTTCCTGCAAGAATAATTAAATCTGACTTTTGAAACCCTTGTGTTATAGAGTCGA
GTTCTGTAAAACCAGAAAGTATACTACTATTTATACTAATCTTTTTCCTTGTATCCAAAT
GGACTAATAGTCTTGCAAGAATTGTAGCTAATGTTTGCGTATCTTTTTCGTCAAGTATTT
CATAAGCTTTTGTTAATTGACTAACAATAGAAGTTATGTTTTTTTGTTCAATAAGTTGTT
TTGAGTAGCTAATTAAACATAATGAATCTCCACATGCTAAAAGTAATCTTTTAATGTAGT
TATCTATAACGACTGCTGAATATTCGCTTATAGTATCAGATAGGGGAGCTTGTTCTATTA
GACTAAAAACTTCATTTAGCTCATTCAAGTCTTGAATCATTTTTTCATTTTTTAAGTTAA
TGAAAAAATTTCTTACGCTTTTTGTCTTTGCATGGTTAACATTTTCGAGTGCTGCTCTAT
AAAGTAATGAAGTTGATGTAAAATAAAAAAAATCAGGAGAGATTTTTTCTACTGATTTTA
GTAATGATATCGATTGCTGAGTTAATATTATACTAATTAATATTTTTTCAGCTAAAATAT
TATGAGGAGGTAGATATTTATAAATACTTATTACTTCCTCCTTGTAACATTACTCTTGTG
GTATTAGTTAACTATTGATTTAATTTGATTCTGGAAGAACTTGCAATTTAATATTTGCAG
TTACTTGATGTAATAGCTTAATTTCTACATCGTAAATGCCAATTGTTTTCACGTCTGGTA
AAGAGATACTTTGTTTTTCAATGTCTATGTTGGTAGTGTTTTTAATAATTTGTGAAATTT
CTTTTTCTGTGACACTACCAAAAATATTGTGGCCATCTCCTGTTTTTTTACTAACGCTAA
ACCTTTGTATTTCTTCTAGAAGTTGCTTGACTTTTTTAGCATTTTCTTTAGCTGTTTTTA
ATTTTTCTTCTCTTATAGCTGCATAAAATTTTTGTTGTTTTAAAATACCATTTGTGGCAA
CTGCCGCCATTTTATTCGGGATTAAAAAATTTCTTGCATAACCAGAAGCTACTTTGATAA
CGTCATTACTCTTGCCAAGTTTTTGAATATTCTCTTTTAGCACAACATTTATAACTTTTT
TACTCATCTTAATTCTAAAAAATCTGTATTATCCTATTATACCTGTTGTATACAGATTTT
AATAGGAAGTATTTATTTGTTGATATAATATTCTTGTAAAAAAAGGTAATTTTTTATTTT
TTTCTGATATAATCACTTTCACAATAATTCATTAGGAGAGGTGGCCGAGTGGTTGAAGGC
GCAGCATTGGAAATGCTGTTTAGGAGCAATCTTAACGAGGGTTCGAATCCCTCTCTCTCC
GTACAAAAAATATAAAAAGTTTTATAGAGTCCAATCTAAATCTCCAATAATATTCTTCCA
AGCATTTAATCCACCCCGAACTCTAACAATATTTAGTTTTTGAGCTATTAAAAACTCAGA
AGCAAATATTGATCTAGAATCTAAGCTACAGTAAACAAAACAAATTTTATCTTGTAAATT
GATATGAGAGTAATTCATTCCTTTTATTTTTTGTAAAGGTAGATTTAATGAATATATTAA
ATGACTTTTATGATATTCTTCATGATTTCTTACATCTATTAAAATATATTGTGGATTCCT
ACTAATTAAAAACTTTTGTAATTGAATAACACTAATTTCTCGTACAAATGTATTAGATTT
ACTGCCATAATATTTGTTCCAATGCTTTTTTGTGGATAATACAAATTTAGTATTTATAAT
CTTAAATTTGTTAAATGATGAAGTTAATGAGTTGTACGTTAATATAGTTCCACTTAATAT
AGACTTATAACCCAGAACGATTTTGACAGCTTCAGTTGCTTGAAGTGTACCAACAATACC
CGGTAAGAGACCTAAAACTCCGGAATTACTACAAGTATCTCTTGCGCTCTCTTTATTTTC

AGTTTCACTGTAGAAGTCTCGATAAACAGGTCCACCTTGATAATTAAATACACTAACTTG
TCCTTCAAATTGAAAAATGGCACCATAAATATGTATTTTATTTAACTCTAGGCAAGTATC
ATTCAAGAGATATCGAGTATCGAAGTTATCAGATCCATCTATAATAATATCATATTGTCT
AATAATGTCGATTGAATTCTCATAGCTTAGTCTTGTTTTGAACACAGTTACTATACACTG
TGGATTTAAATCTAATATTTTTTTTTAGCTATTTCTACTTTTGAATATCCTATATCATGC
ATCGTGTATAAAATTTGCCTCTGTAAATTAGAGAGATCAATTATGTCATCGTCTATAATT
CCAATGCTGCCAATTCCTGCAGCAGCAAGGTAAATGATTCCTGGAGATCCTAGCCCACCA
GCACCAATGAATAAAACTTTTGCTTCTTTTAATCTTTCTTGTCCTTCTAATTGAATTTGT
GGCAATACTAAATGCTTAGAATATCTTGTATATTCTTCTAGTGAGTATTTTGTATTTTCT
GTTTTAAAATTTAGCATAAAAATTTATAGTTTATTTAATAGCACTTAAAAGCTAGCTGAT
AAGTTTATTAAAATGACTTTTGTAAACTTGGTTCTTCTCTATCTAAAATAGCTCCTTGTG
TAGGACAGACCTGAACACAAATACTACAATCAATACAGGCAGAAAAGTCAATCCAATACC
AATTAGTGTTATTATTGTTTTTCCCCTTTCCTTGATGAATACAAGCTACGGGGCAAGCTT
CCACGCATTCAGCAACTCCAATACATTTTTCTGTTACAATTGTATGAGACATATTATTTA
ATTAATAAAAGATAAGCTATTTACATCTAGTAGTTTTGTTGTATACTAAATTTACATATG
CTATCCAGGCCAATATTTAAGGTAGTCTATTTCTACAGTTTACTAATTTGATATGCGCCT
TTAGTTCAGTTGGTAGAACGCAGGTCTCCAAAACCTGATGTCGAGGGTTCAAGTCCTTCA
GGGCGCGTTTAGTTTTATCGTAATTCAATAATATCATTTTATCTTTTTGCAAACATCAAA
ATGATATGATATAAAAGATCATATTTTATACATTGAATATTCTATGTACTAGTACTGTTT
TTTAATATGTTATTATTGATAAGTTAATTATACTAATTCTATTTTTGTAATAAGTAAAAA
CAGTTCTAATTAAGTATGCACGTATTTAATAGTCTTTTGTATATATAACTTGAAAAAATT
TAAACTATGGCTAAAAAAGTTACTGGAATTGTTAAACTAGCATTAAATGCAGGTAAAGCT
ACTCCTGCGCCACCTGTCGGACCTGCTTTAGGACAGCACGGCGTGAATATTGTTATGTTC
TGCAAAGAGTACAATGCACGTACTGCTGACAAGTCAGGACTAGTAATCCCTGTAGAGATC
TCAATATATGAAGATAGAAGTTTTACTTTTATACTTAAAACTCCTCCTGCTTCTGTACTA
ATCGCTAAAGCTGCTGGACTAAATAAAGGTTCGGGTGAACCAAATACCAAAAAAGTAGGC
AGCATAACAAATAAACAGTTAGAATCTATTGCAGAAACAAAACTTCCCGATTTGAACACT
AACAATATACCTCAAGCTATGAAAATAGTTGGAGGTACGGCTAAAAATATGGGAATTCTA
ATTAAAGATTAAAAGTTTCAATTATATTTACTATTTTTATGAAAAAATTTTCACGTCGAC
TTACAACATTAAAATCAAAAGTTGAGCCTAAACTTTACACTATTAATGAAGCAGTATCTA
TATTAAAAGCAACGTCAAATGCTAAATTTAAGGAAACTGCAGAAGCTCATATTGCTCTAG
GTTTAAATCCTAAATATGCAGACCAGCAACTCAGAGCAACAGTTATATTACCGAAAGGTA
CAGGTAAATTGATAAAAGTAGCAGTTATTGCTAAGGGAGAAAAATTAACAGAAGCAATTA
GTGCGGGAGCTGATGTTAGTGGCTCCGAAGAACTAATTGATGAAATACTGAAAGGTAGAT
TAGATTTTGATAAGCTGATAGCAACACCAGATGTTATGCCTTTAATAGCAAAGCTGGGAC
GAGTATTAGGCCCTCGAGGGTTAATGCCTTCTCCTAAAGCAGGCACGGTAACATTGGACG

TAGCAAAAGCCGTGAATGAATTTAAAGGGGGTAAAGTTGAATACAGAGTTGATAGAACAG
GTATAATTCATGTACCTTTTGGAAAATCTAGTTTTTCACAAGAAGATCTAGTTTTGAACC
TCCAAACAATTAAAGAATCGATTGATAGAAACAAGCCTTCTGGAGCAAAAGGGAAGTACT
GGAAAACTTTTTTCTTATCTAGCACCATGGGGCCATCTATTCAAATTGACATCACTAGCC
TTCTATGAAAAATTTTTGTATAATTATAGAACTTGAATAATCTATTATTTCAAATTATAA
TTTGCTAGCATTATTAACGTAACAACTTGCTTACTATATCTTATCTGCATTAAAAACTTT
TTATATATCTTATTAATTATGAGTACAAAGGTTGAAAATATCTTGGAAGAGCTAAAATCT
TTAAACCTTCTAGAGGCTGCTGAACTAGTTAAACAAATAGAAGAAACGTTTGATGTTGAT
GCATCTGCGGCTTCAGGAGGTATGATGATGGCCGCACCAACTTCAGCACCAGCGTCTGCT
GAGGTTGAAGAGAAGACAGAATTTGATGTTGTCCTAGAGGAAGTCCCAGCACCTAAAAAG
ATTTCTGTATTAAAAGCTGTTCGCTCTCTGACTGGTTTGGGTTTAAAAGAAGCAAAAGAT
CTTGTTGAATCTACGCCAAAAGTCTTAAAAGAAGGTGCTTCTAAAGATGATGCAGAAACT
ATGAAAAAACAGCTAGAAGATGCTGGCGCAACAGTTATTGTTAAGTAATGAAGAAGCGCT
TCTGTTTAAGTATAAACAGAAGTGCCTCTTTATTTATTATAAGCAATTATACTAGAAAAG
TCCAAGTGTAATTGCATTGTTGATAGGCATAGTTGCACCAATACCTAGCCAAATACTTAC
AACGGTACCAATTAAGAAAACAGTGGTTGCAATTGGTCTTCTAAATGGATTTTGAAATTT
GTTAACATTTTCAATGAATGGAACAGTAAGTAAACCAGCTGGTACAGCAGCCATACTTAG
AACACCTAATAGTTTATTAGGAATCACTCTTAATAGATTAAATGTTGGAAAGAAGTACCA
TTCTGGTAAAATCTCTAATGGAGTCGCAAATGGATTAGACTTTTCACCTATAGAAGATGG
TTCTAAAATTGCGAGTCCAATACTGCAGGCAAAAGTGCCAATAATTACAACTGGAAAGAC
GTATAATAGATCATTTGGCCAAGCTGGCTCTCCGTAATAATTATGGCCCATTCCTTTTGC
TAATTTAGCTCTCAGCTTCGGATCTGTTAAATCTGGTTTTTTAAGAATTGACATATTGTT
TTTGTGTTTAATATAAAGATTAGTCTAATTACTGTTTTAAAGTGTAATTTTCAACTATTA
TAATGGTCCAGAAATTCCTTGTTTACGTATCATTAAAAAAATGCATAAGCATGAAAACGGC
AGTTAGTAGCGGAAGTACAAAAGTGTGTAAACTATAGAACCTAGTTAAAGTTCCTTGGCC
TACACTGACTCCTCCTCTTAACAATTCTACTATACTCGCTCCGACAACTGGAACAGCATC
AGGGACACCTGTAACAATTTTTACAGCCCAGTATCCGATTTGATCCCATGGCAAAGAATA
ACCTGTTACCCCAAAAGAAACAGTAAGCACTCCTAAAATTACACCTGTTACCCATGTCAA
TTCTCTAGGTTTTTTGAAACCTCCTGTTAAATAAACACGGAATACATGTAGAATCATCAT
CAAGACCATCATGCTCGCTGACCATCTATGAATTGATCTAATAAGCCATCCAAAGTTTAC
ATCAGTCATAATGTACTCTACTGATGTAAAAGCTTCTGCAACTGTAGGTCTATAATAGAA
TGTCATTGCAAATCCAGTTGCAACTTGAATTAGAAAAGATACAAATACAATACCCCCTAA
GCAATAAAAAATATTAACATGAGGTGGTACATACTTACTAGAAATATCGTCAGCAATTGC
TTGAATTTCTAATCTCTCTTCAAACCAGTCATAAATTTTACTCATAAAATAGCTTC-AAA
AGCTCTTCTAATATATTTTGCGTTTAAGCTACTATTAATTCAATAGTTATTGAACTTATA
TTTTTTTATAATCAATACTTATTAACTATTATAACATCTTTTTATTGATTTAAAATTCTC

TGACTTAGTAAAACAGGGCTATGTACTATAATTCTTTTTTTTTGTATAGAGATTAATTTC
GTTTTTAGTAAATTAGCTAAAATTCTTGTTACACTGACTCTATTACTACCTATAATCTGT
GCTAAAATTTTATGTGTAATAGTAAAGTTAAGCATAATACCATTATTAACTTGAGTTCCA
TTTTGCTCAGCTAATAATAATAATAAGCTCGCCAGTCTACTGGTTATACTTTTATGTGAA
ATGATTTCTATAAAATGATAAGCTTTTGCCGAGCAAAATACTAAATGATTTACAAAAAAT
AAATTGAATGGAGCGCAGTTACTACACGCTTTTATAATAGTAGTATAGTTAATACAGGCA
ACTTCTGTTTTATCTATTGCTTCTGCTTCGTAGTAAAAATTATCGTCGACCAATTCTATT
TGTCCAAATGTATCTCCTGTAGTTAATAAGTTAAGAGTTACTTTATGTGTATTTCTCAGG
ACTTTTGTAATAATTAAAGATCCTATCAATATAATGTATAATCTAGAGCTGCTATTAAAC
AAAAGAGTATCATTTTTCTTAAGTGAAAAAATTTGATAATTAATTTTATTACGATTAAAA
AATAGTAGCCATGGATTGCAAGAAAAAAAATCACCTGAACTATTTTCAGAGAATATTGGA
ATACAATTTTCTTGATTTAAACAATTTTTAGTCATAATTACTACTTTATAAAAAAAATATT
CTAATAATTAAGAACTGAAATTAAATGTCATATAAATTGATGTTAGTTGAGAATGACATT
GTATTATCAAAGGCCATTCAAGAATACTTAATAGATCAAGGCTTCAATGTTTATATTGCC
AATAATGGATTAGAAGCACTGAATCTAGCTTATCAATATAATTTCGACTTGATAATTTCT
GATATAATGATGCCGCTAGTTAATGGCTATGAACTATTAGCAAAACTTAAAAAAAACAAA
GCATTATCCAAAATACCTGTTATCTTTTTGACAGCTAAAGGAATGACTAAAGACAGAATA
AAAGGCTACGACATGGGGTGCTATGGTTATTTATCTAAACCATTTGATCCTAATGAGTTG
CTTTCAATTATTAACAACTTAATCGCTAGAGATGTCTTAAAAGAAGCTTCTTTACAAAAC
TTGGCAACATCAAACCAGCAATTAAATCATAAAATACGTTTGACACCTAGAGAAAAAAGT
ATTTTAGATCTTGTTGTTGATGGACTTACTAATAAAGAAATATCTACTATACTGAATACT
AGTGTTAGAAATGTAGAAAAATATGTTAGTCGACTCTTACACAAAACTAATATGAAGAAT
AGGACTTTGCTAGTCAAGTATTCTATAAATAATAATCTATTAAATAATGAGATCAATGAA
AGGGCGAATGACGGGACTCGAACCCGCGAATGATGGAGCCACAACCCATTGCCTTAACCC
CTTGGCCACACTCGCCATATTACACATACCACTATAGCTTGTTTTTTGATATATCGTCTA
GTTTTCGAGTTATATATTTATCTAAAACAATATTTACTTTGAATTTTTATGATACATAGC
AATATTAGTATTCAAATTAATGGGGAACCATTTAATTGCTCAAAGCCCATCTCATTGCAA
TTTTTATTAAATTATCTTGATTTTAATTCCGAGCGTGTAGCAGTCGAGCTAAATGACATT
CTGTTACCAGAGACTCTGTTTCACTCAACTTATTTGAATGATCAAGATAAGCTAGAGGTT
ATTACCATTGTAGGTGGTGGATAATCTATTTGTACATACACAGTTCTCTATCTCAAATGT
TTCATAGATAGAGATAATCTTCCTTGTTTTTTATCTACATGAATAATAACAGCTTTAATA
GTGTCACCTATTTTAAATTGTGATGATATCTGCTCTAATTCTTTGATATTAATCTCAGAA
ATATGGACAAGACCTTTGAGATTTCCTACCTTTATAAATAATCCGTAAGGTGTAATCTGA
TTTATTATGCCTTCAATAATATTACCAACAATTAAATTAGATGATGCTTGAGATATTAGA
GCTCTTCTATGACTCAGTATTAAATTATTAGATTTCTCTTCGACATTGAGTAATTTTAGC
TTAATAAATTTATTATTAAATTGCTCACTTTTTTGAAAATTACCGAGATGAGAATTAGGT

ACAAATCCAGATATACCTTCAAGGTTAATTATCATTCCTCCCTTATTGAATCCTTTTATC
ATTACATTAAGTAAAGAGTCTTCAGCTAATAGTTGCCTAATTCTTTTCCATGCTCTTATA
TATTCAAGGCGACGAATTGATAATATTAATTGTTTCGACTGAATATTATAATCTAATAAA
AAAAATTCTCTTGTGTCATTAATGTTTAAAGAAGTGAAATTATTTAAATCTTGATTACTA
GATACTTCTTGAATAGGCAAGTATGCAGATATTGGTGTTCCTATATCTACTAATACCCCA
TTTAATTCAAAGCTAAATATAGTTCCAGCTACAATATCTCCAAGATTCAAATCATACTTG
TATTTTTGTAAAACAGCTGCAAAATTTCTATGAGTAAATCCTTCATTATTTTTTGTCATA
TTGAATAGTATGAATTAAAGAGTAACTTTGTACAATATATTAAGGTAAATAAATGTTTGT
CGAATTGTATGAAAAAAATTTTAATTTGTTCGGTTGTAATTAAGTATATAAACATATTAAA
ATATCTAGGAGACATGAAAAGTAGACGTAGATAGCTGCAGATTTAATACTATATAATCTG
AGGAAAGTCCGGGCTCCACAAATACAATTTATGCTGGAGAAATCCCAGTGTAGGTAACTG
CGAGGATAGTACCACAGAAAAAAACCGCCAGAATAAGTTATATTAGCTGGTAAGGGTGCA
AAGGCAAGTTAAAAGCTTACCAAAAGTACTGCAAAGTATTTGTTAGGTAAACCCCTAAAA
TGGAGCAAAGCTACTAAACAAATATTTGTGTATATTTTATCTAGTTTAGTTGAAAATACT
GCATGAAGTTATTAGTAACAATAACTCGAGAGGAATAGCTATCCTTTACAATATATCTCG
ATTATATTGAAAGAACAGAACCCGGCTTATGTAGTACTTTTTATGTCTTGTATTATTATG
TCTATTGTCGAATCTTTAAATTTAACTGTTTGACAATTTTTTTTTGTAGCTGATGTTGTT
TATAATCTATTTCTGAATTGGTTAAAGTTTTTTCATTAGATCGATATGTAAATCTTAGAC
CTATACTTTTTTTCTCTTTTCCAATTGATTCATCTATATATTGATCAAATAATGTTGTAC
TTTCTAAATCTTTATCATAAAATTGATCCAATAATTTTAATAAGTATTTAATTTCCATAT
TCTTAGGTATTATTAAAGAGAGATCTCTTATGATCGATGGATACTTAGAGTATGGTTGAA
TCTGATAACTTAAATAATTGAATTCATTATGACAATCTTCTAAAATGTTTAAGTTAACTT
CAAGAAAAAAAAGTTTTGTGTTGAGTCCTAATTCACTATATGTTGCTTGATTAAGTTCAC
TGAATATGCCAATAATAATATTGTTATAGATTAGTGTGGCAAATTTTCCTTTTTGAATGA
ACTTTATTCTACCATCGACTATATCTTTTTTTGTCCACTCAATTTGTCTATTAATCTTTT
GAAAAAAATTTTCTACGATCCCTTTAGCTTCATACCAATTTAAAGAATGCGCAGGGTGTG
ACCATGTAGATCGGATATCTAAATTACCACCCAATATGATAGCTAGATTAGTAGTTTCTA
CAATTTTGTTTTGATCATTATGAAATACTGTTCCTATCTCAAAGCTATCTATAGTTTGGT
TGCTTTGTTTAATATTATAAAGATTTGATTCAATTAATCCTTCCAGCAAACTGCCGCGTA
AATTAGAATAATCTTGAATAAGAGGATTGTTTAAAGCTATATTTCCTTTGGACTTTATTA
AAGAATAATGAACTAATTCAGTTAATCCTAGATTTCTTAAAATACTTCTACTTTTATCTA
TGAATTGTCTCTTTGTGGATAACTTTTTGTTGAATTGAATATTTGGTACTGAGCTCCTGA
ATTTATGATAACCATAAATTCTAGCAATTTCTTCAATAACATCTATTTCTCGAAAAACAT
CTTCTCGTCTATAATTTGGAACAGTAACTTCTATATTTTCCTTATTTTGATAAATAAGAT
CAAAATTTAAAGAGTGAAGAATATTTTGAATTTCTTCAAAGAATAAAAAACGTGTTTGAC
CATTATACATAATTGGTCCTAAAATGTCATGAACTTTTTTAATTGATAAATTAATATTAA

GAGCATGATCATTTATTTTTTCTCGACAGAAAGTCTCTCTAATATTTCCGCCTGTTAGAT
CTGTGATTAAAGCAAGAGCTTCAAAATGTGCATTTTTCCAATTATCTACATTTAGCCCAC
GTTCTTGTCTAATTGAACTTTCTGTTCGAATATTAAGAACTCGAGAAGATTTTCTAACTA
CAGATTGTTTAAATATAGCAGACTCTACAAGTATCGATTTTGTATTATGATCTGTATCAA
AATCAGAGTTGCTTCCAATACCAGCTATACTAGTTACATTTGTATTTACTTGAGTAACTA
AAATATTTTTGTTTAACTCAATATTTTCATTGTTTAACTTAATCTGATCATTACTTCCAA
GAGGAAAATTGCTTCGAATTGTTAGACTATTTTTATGATTCATATTATTAATTTTATTCA
AATCAACAATATTAATAGGCTGTCCCCATTTCAACATAATATAATTGCTTATATCTGTTA
ACAAATTTCTGTGTGTAAACCCAGAAGAGAGCAACCGATTTTTTAGCCAATCTGGAGAAT
CTTTAACTCTTATTTCGTCTATAATGGCAGCAAAATAATTGTCACAATTTAATAAATCGT
GATTACTGATTATAGTCTCCTTTTTAGAGAAATATCTATTATTGGGTTGATGTATAGCTG
ATAACATTGAGGACCCTGTTAAAGCCGAGACTTCTCTTGATAGACCAATCATGCTTAACA
CGTCTGACCTGTTAGCTGTTGATGATATATCTAATATATGATCAATCTCACTTCCAATTA
TAATAGATTCAATAGCTTCAACTTCAAAACCTGCTTGTGTTAGTTGATTAGTTAAATTGT
CTATATCTATTGTTTTGATATTACCAAGTTCTTTCAACCAATTTAAAGAAACTTTCATAA
TTAATTGTCATCTATCTTATAGAAGTATTTATTTTACTTATGATTAAAAATCAATTTAAA
AAATTCATCAAATTAATATAAGTTATTTGATGAATTTGCTTATACAATATATTATTGTGA
TATCTTACTTTTATATGGCAGTTATTAAGAACTAGCTTTTGTAAAAGTTAGCCCATTATC
TTGGGCATATCTTTCCATAAATCTCATGAATCTATCCCAATCATCAGGACTTTTAATAAC
ATGTACAGCTTCTATTGCTTGTGGTTTACCATTAATAAACTTGGCATTAACATCTCTTGT
AATAATTTGTCCCTCTGTGTCCATCAGGTACATACCTGTGATTTCACCTTTGTCTGCCAT
ACTAGCATCTAAAATTTTGGGATTTGTAAACCGAAAAGTTGCTGTTCCTGTACTACCATC
TCGAGACCTTGTTAAACGTACATCTGGTACAACTTCTTCATTGATACCTTGAATAAATTG
AATTGTTGCCATATCAATCTTCTTTATTTGTATATAATTTATATTGGATAAAACTGATTT
CTAGTTCTTGAATGCTTGTTGAGAAACTGTTTTAAAAGCTTATAGTAGCAGTAGATCAAA
TTTTATGTATTATATAAAACTGTAACTTTTCTTTTTTGATCTGTTCAAAGAACTAACTTA
TTATAAGCTAATCATACGAATATTAGCCATATAATGTTCGTAAGTGAAATTAAGGGGTTG
TAGCTCAGAAGGATAGAGCAAGCGCCTCCTAAGCGCTAGGTCAGCGGTTCAAGTCCGCTC
AACCCTGTTAACGAATTAAGTAGTATTATTTAACTTGTTCTGCAAATCAGTATTGATCTA
AGTTAAAGTACTTGACAGCTAAAACAAAAGAATCTGAGAAATATTTTCATTTAAAGAACG
AACAATTATCTTAACTCTAAATATCTGATAATTATTAAAAAATTCTCTTTTTAAAACACA
AGATGATGAAGATATTTAAACTAATAATTGAAATTAAGTTTTTGAAGTTATAGAAGCCAA
TATAATACTTTCTATTAACAGATGCTTGCTTCTATTACTATCTGTTTACAAGAAATTATG
AGTATCAAAATAATTTTAGCTCTACTGACTACTAATAAATATTTATTTTAGTCTAGACAA
GATGAATCTTCATGGTATAACAGAGCTATTCTTTGTGTCATACTTATATAAAATTAGGAT
ACTGTTTTCATTATTATAACTGGGGTGGGAGGATTCGAACCTGCGAATGGCGGAGTCAAA

GTCCGCTGCCTTACCACTTGGCTACACCCCAACATGTGAACATTATAATAGCAGTCGTAT
ACCCTGCCATGTCAATAGACTATTGTCTTTATTTAATAATTTTTGCTGCTCTTGTAGAAT
AAAATCTTTTTTTTCAAATAAGATACGCTATGTTTAAGTTGCACGACTGTTATGTTTTCT
TGCTTTTGACTGAACAGCCACTTGATAGTAATTATTCCTTCTGCAATTTCGTTACTTCCT
ATAATAATACAAGCTACAGCTCTTTTTTTATTTGCTTGCTTAATCTGCTTACTAAAATTA
CTTGAGCTAACATCGATTTCTATTTTAAAAAACTGCTGATGTAAAAAACGCATTATTTGC
ATACCAGTTTCGTTAGCTTTTGTACCTTGTGTAGCAATATAAAAATCGATAGATTGATTA
GGCAAGTCTATATTATCTTTTGCAATCAGTAATAGGCGTTCTAATCCTATTGCACATCCA
ACAGCTGGTGTTGCTTTGCCACCTAATTGGTGAACTAAGCTGTCATATCTTCCACCACCA
CATATAGTATCTTGCCCTTTTGATGTCAATGTCTTAATTTCGAAAGCAGTATCATTATAA
TAATCTAATCCTCGAACTAACTTATTATTTATATTGTAGGGAATATTAAGTAATTTTAGA
TAATTGCAAACGTCACCAAAGTGTTTTTGTGATTCCAAACTTAAAAAATCAGAAATTTTT
GGAGCCTCTGTCAGTATTTTTTGTGTATTAGAATCTTTTGAATCTAAAATTCTAATTGGA
TTACTAGTTAATCTTTTTTGTGAGTCAGTGTCTAAATCATCATGGTACTTTGTAAGATAA
TCTCGTAATTTAACTTGATAAATACTACGATCCTCTACTTTTCCAATTGAATTCAGATCA
AGTTTCAAGTTGTGCAGATTGAGATTGTTAAATATGCTCATAGCTAAATGTATAACTTCA
CTATCTGCTCTTGCATCTAAGCTACCAATAAACTCAATGCCAAGTTGGTGAAATTGTCGT
TGTCGTCCACTTTGTGGTCTTTCGTACCTGAACATTGGACCGCTATACCATAATCTTTGT
AAACTATGGTGGTAACTCATTTTATTTTCAATAAAAGATCTCACAATACCGGCAGTACCT
TCTGGCCTTAAGGTAATATCTCTATTGCTGCGATCATGAAACCGATACATTTCTTTATTT
ACAATATCAGTATCTTCACCAATACCCCTATCATATAATTCACTATTTTCAAAAATAGGT
GTTCTAATTTCTTGGTAATTTGCACATTCTAATAATTTGGAAATTTTATTATGTATAAAC
TGCCAATATTGAATTTCATCGGGAAGAATATCTTTTGTCCCTCTAATAGCCTGAATTTTT
GCCATGATAATTTTTTGATTACCTTCTTAATATGTCTGCATTAAATAGCAGTTGTAGACT
GTTATTCAGTCATGTATTATACATTAGAACATACTGATGTAATTTATAAAGTACGGGCAA
GGAGGGATTCGAACCCCCGACACCATGGTTCGTAGCCATGTGCTCTAATCCACTGAGCTA
CAAGCCCACTTAATAAGTACCTTAACTATATCATTGTTCACTAATGTATACAACTATATT
CTTTTTATCTTACATGTGAAATTTTTGTAAAAGTAAAAAATTATGTTTGTTTTTTACAAT
TGGAGCTATTCATATATAATGCTTAATTTATTATAACTTTGTAAAAATATCTATAAGTTT
AATTGTATGATTCTATTATATTGGTAATCTTGTTATAATTTTGCGCTCTTATTTTTCAAT
TGGCTAGCATTTAATTAACTTATATTTCAAATAAAAAAATCTTATCTAAAATGAGTAAAC
AAATTCTATATCAGGATGATGCCAGAAAAGCATTAGAAAAAGGCATGGATATTTTAACAG
AAGCTGTTTCTGTGACTTTAGGACCAAAAGGAAGAAATGTTGTCTTAGAAAAAAAATTCG
GTGCCCCTCAAATTATTAATGATGGTGTAACGATTGCAAAGGAAATTAGTTTAGAAAATC
ATATTGAAAATACCGGAGTCGCACTGATTAGACAAGCAGCATCTAAAACAAATGATGTAG
CTGGTGATGGTACAACAACAGCTACAGTACTGGCTTCAGCAATTGTTAAGCAAGGAATGA

GAAATGTTGCAGCAGGTTCGAATCCGATGGCTATTAAAAAAGGTATTGAAAAAGCAACAA
ATTTTGTTGTTAGTAAAATAGCTGAGTATGCTAAGCCAGTAGAAGATACAAAAGCTATTA
TACAAGTTGCTTCTTTGTCATCAGGAAATGATATAGAGGTAGGTAAAATGATAGCGAATG
CTATAGAAAAAGTTGGCAGAGAAGGAGTTATTTCTTTAGAAGAAGGGAAATCAACTAATA
CGATTCTTGAGATCACAGAAGGAATGCAGTTTGAAAAAGGCTTTATTTCTCCTTATTTCG
TTACAGATACAGAACGAATGGAAGTTCTTCAAGAAAATCCTTTTATTTTATTTACAGACA
AAAAAATTACTTTGGTGCAGCAAGAACTTGTGCCATTGCTTGAGCAAATTGCAAAAACAT
CTCGGCCTTTATTAATAATAGCTGAAGACATCGAGAAAGAAGCATTAGCCACAATTGTAG
TTAATAAATTAAGAGGGATTTTGAATGTTGTAGCGGTTAGAGCTCCTGGATTCGGTGATA
GAAGAAAATCTTTACTTGAAGATATGAGTATCTTAACGAACGGACAAGTAATTACTGAAG
ATGCAGGTTTATCACTTGATACAGTTCAATTAGATATGTTAGGAAAAGCCCGAAGAGTTA
TTGTTACTAAAGACTCGACAACAATAATTGCAGATGGTCATGAAATTAAAGTTAAATCAA
GATGTGAGCAAATTAAGCGGCAAATAGAGACGAGTGACTCTTTATATGAAAGAGAAAAAT
TGCAAGAACGATTAGCAAAGCTTTCTGGAGGAGTTGCCGTCATTAAAGTTGGTGCAGCTA
CAGAAACAGAGATGAAAGATAAAAAACTAAGACTAGAAGATGCAATTAATGCAACAAAAG
CAGCAATTGAAGAAGGTATTGTACCAGGAGGAGGAGCTACTAATGTCCATATCTCTAGTG
AATTATTTACATGGGCTAAAAACAATTTAGTTGAAGATGAATTAATTGGTGCTTTAATAG
TTGAACGAGCTGTGACCTATCCGTTAAGACGAATAGCTTTTAATGCAGGTGATAATGGAG
CAGTAATAGTGGAAAAAGTTAAAAGTCACGATTTTCATATAGGCTATGATGCAGCAAACG
GTAATATTGTAAATATGTACGATAGAGGTATTATAGATCCAGCGAAAGTAGCTAGATCAG
CTTTGCAAAACGCAGCTTCTATTGCAGCAATGGTTTTAACTACTGAATGTATTGTAGTTG
ATAAAGTCGATGATTGAGCTATAAGATAATAGATAATCTCAAACAAAGAATAATTTCTAA
TTATAGAAATTATTCTTTGTTTCAATTAATCTAATTAATTGATCAAATGTATAATTTTTA
TAATAAAGCCAAAGTTTAAACACGTCTTCTTTTTCAACTATTAAGTAGAGAAGATATAAG
CCCGTAATACCTAAATTATCAATTAAAATATTATTTTCATATATAGATTTTTCTAAAGAT
CTAAAAAATGACATTTTTGAAAAATAATACCGAAATCTTCTCTTATAGTTATTTGCTAAA
TCATTACTGTCTAAAGATTGATTTCCAATTGAATTCAGAACCTTTAACAGAATACTTTTT
AAATCTTTATCTGACTTTATTTGAGAATCAATAATAATTTGATTCAATTGTATGTCAAAA
CTAAGATTAAAAATATTACTTGTTAAAGATATTTGATTTTGAATATCTAGAGATTTTATA
GCCAAGAAGATTAAACTTTTATTGTTCATTGTAAAATATAAGACAATTGATGATGTATTC
TTTAAATACTAAATTTATCTTCTTGTGCTTCTATCGTTTTTTGTAAAATAATTAATGTTT
TAATTTTTTAGTTTTAGTTACTACCCGAAAAAATAAATTCTGGAAATCTAGCTTGAGCTT
TAGCTAGAGAGTGTTTTTTCCCTTCTTCTTGCCAGTAATTGATAACTTCTGTTGTTTTGT
TTAATAACTCAATTCTGTCTCTTTCAGCAATCCACGGCTTGGATTCTAGTTCTGCTTTTA
ATTCTTCCCAAGCATCATTCCTTGGCCAGAAAAAATAAGAAGTAAGTGGACTAGTGCCAT
TTCCAACAATCTGATCAATAGCAATAGCAATATTATTTTCTAACCAAAGTACTTTTAGTG

TAAATTTTGACAAGTTTTTATCTCCATTATTAATATTAAGTATTGGTACAGTAGAATACT
GTACTAGTTATTTTTTTCAGCAACAATTTGAGCTTTGTTTAAAATGTTTTTTACCGTTTT
AGTAGCTTGTGCACCTTGAGATAATCGTTGCAAAATTTTTGAAATGTTAACACGAGTTTC
ATTATTTATAGGATTATAAAATCCTAATTCTTCTATAGCTTTACCATCGCGTTTATTTCT
GCTATCCATTACAACAATTCTATAGCTTGGTTGTTTTTTTCTTCCGTATCGCTTTAACCT
TAACTTGACCATATTAGTCACCTCGCTTATTTTTTATATCTTTATTAAATTATATCTTAC
GACATTATGCAATAATTCAAACAATTAAGCTGATTCAAGTCTAACATTATTAAATATTAC
CATTAAACATTGTAGAAAAATAATACCTAACATAGGTGACATGTCCATTCCAAACATGGG
AGGAATGCTTCCTCTGAATAGTTTTAAGTATGGATCAGTAATTCTATTTAATGAACAGAA
TGGTTCATTGTACCAATTTACAGTTGGAAACCATGCCAATGATAATTTAAGTAAAATTAA
AATTAAATAAATCTCAGAAAAATTAGCTATAGATCCAAGCAATAAATTTAATGTACCAGG
AAGAGTGTTCATACGTATTTTATAATTATTATATACTTCGTTATACTAATAGTTTGATTA
TATATTATAATCGATGTAAATAAATCAGCTAAACAAAAAACTTATCCTTAAAATCATCTT
CTCTTGAGCATTTTTTTTTATCTACATTAGTATTTACTTTTGTCGTATAAATTTCCAGTT
TAGACCAAATATGTCCTAACTGATTTAATTGAGATGTTCCACATTCTAAACAGGCTATTT
TTATATTGCTCAAAACAGCACCTTTTTTTATAAAATCGTTCAATAATGGCGTAATAGTTG
CTTCATCCAAAAATAAATCTAAGACAAGTATTTTTGTAAATGAATTTAATTGATCTGGGA
CATTGTTAATAATATCATTATAATTAACTAAAACTATATTAGCTGTAGGAAGTAAAGATC
GTGCTCCTTCCGCTAAGATAAAGCCATAAGGCATAATAATTACAATAATATATTTATAAT
GACTGCTAATAAGACTTATAGTGGTATTAGCTTTTAATTCGATTGTTTCTGTTACTAGCC
ACTCTCTCATTATTTCGTAAGTAATCCATTTGCCTAGTTCTGAGCAAGCAGTTCTTAAAA
TCGTACCTGGATTATTGTCGTTTTCGAGAATCCCTGACCAATGTTGTATTAATGGATGGG
ATGCTATATTAATTTGGAGTTGCATGTTTTGTTAAATATATTTAACATTTAATTTGTTGA
TTGAATCTTCAATTCCTATTTGTTGAGTATTATTAAAATAGCATATATATTAAAGAAAAT
AATTATAAAAAATGAAAAAAAGTCTTTGGCTGTGGGGTTTTACAGATAGTGCTGAAACTT
GGAATGGTAGATTTGCAATGATTGGTTTTATATCAGTTATTTTTATCGAAGTAGTTACAG
GACAAGGATTATTATATTTAATAGGTATGATGTCATAATAAAAATCCAAAAAAGTTCTTG
TAATTTTTTATAAGAACTTTTTTGGATTTTTATAAATCTAATCTAGCGGTCTCATAGATA
GAACAGGTTCATTCTCTCTATTGATACCTTTTTCAAATCCAGCAGCAGCAGCTCTTGCTC
TGCCTGCATGCCAAAGATGTCCAATAAATAGGAAGAATCCTAGGAAGAAATGAGATGTTG
TTAACCAAGATCTAGGAGATACATAGTTAACTGAATTAATTTCTGTAGCTACACCACCAA
CGGAATTCAAAGAACCTAGTGGTGCGTGAGTCATATATTCTGCGGCACGCCTTTCCTGCC
ACGGTTGAATATCGTTCTTGATTTTATTTAAATCAAGACCATTTGGTCCTCTAAGAGGCT
CAACCCAAGGAGCTCGCAAGTCCCAAAATCTCATGGTTTCACCACCAAAAATAATTTCTC
CACTAGGAGATCTCATTAGGTATTTACCTAATCCAGTAGGTCCTTGAGAAGATGCAACAT
TCGCACCTAGTCTTTGATCTCTAACTAAGAAAGTAAAAGCTTGAGCTTGTGAAGCTTCAG

GACCAGTTGGTCCATAGAATTCACTAGGATAAGCTGTGTTGTTATACCAAACAAAATTAG
AAGCCGTAAGCCCCATGATTGATAAAGCACCTAGACTATAAGATAAGTAAGCCTCACCAG
ACCATACAAAAGCTCTTCTAGCCCAAGCAAAAGGTTTTGTTAGTATATGCCAGATTCCGC
CAGCAATACAAATAATGCCAATCCAGACATGACCACCTACAAGATCTTCCATGTTATTTA
CACTTACGATCCATCCATCACCGCCAAACGGAGACTTTAAGACATAACCAAAGATAACTA
GGGGATTAAGTGTAGGATTGCTAACAAATCTAACATCTCCACCACCTGGAGCCCAAGTGT
CGTATACCCCACCAATAAATAAAGCTTTAATAACTAACAGAAAAGCGCCAATGCCTAATA
ACACTAAGTGTATACCAAGTATTGTTGTCATTTTGTTTTTATCCCGCCAATCGTATCCGA
AGAAAGGAAATGATTCTTCAAGAGTATCAGGGCCAATCAGAGAATGATAAAGACCTCCAA
AGCCAAGAACAGCTGAAGAAATTAAATGCACTACGCCTACTACAAAGTATGGGTATGTAT
TGAAAATCTCGCCGCCTGGACCAACACCCCAGCCTAGTGTAGCTAAGTGCGGAATTAGAA
TAAACCCTTGTTCGTACAAAGGCTTCTCGGGAACAAAGTGTGCTACCTCAAAAAGAGTCA
TGGCACCAGTCCAAAAGACCATTATACCTGCATGAGCAACATGAGCACCAAGCAATTTGC
CAGAAACGTTAATTAAGCGTGCATTGCCAGACCACCAGGCAAACCCGGTAGACTCAATGT
CTCTACCGCCAACACCAACAGTTGTATTAAAGGGCGTTTCCACGTGGTAAAACCTCCTCA
GGGAATATAAAGTTTTCATGAGGTTGATCTTGAGCAGCCATCCAAGAGCGAATACCTTCG
TTCAATAGGATATTTTTAGTATAAAAAGTTTCAAACTCAGGATCTTCCGCAGCTCTCAAC
TCTTGAGAAACAAAATCGTAAGCTCTTAAATTTAGAGCTAGTCCAACAATTCCAAATGCG
CTTGTCCATAATCCAGTTACTGGTACAAATAACATGAAAAAATGTAGCCAACGTTTATTA
GAGAAAGCAACACCAAAAATCTGTGACCAGAATCTATTAGCTGTTACCATCGAATAAGTT
TCTTCAGATTGTGTTGGAGTAAATGCACGAAAAGTATCTGCAGCATCACCATCTTCGAAT
AAGGTATTCTGTACAGTTGCACCGTGAATAGCACATAGTAAAGCACCACCTAAGATTCCT
GCAACGCCCATCATATGGAACGGATTCAGAGTCCAGTTATGAAATCCTTGCAAAAATAGC
AAAAATCTAAAAATTGCAGCAACTCCAAGGCTTGGGGCAAAGAACCAGCTCGCTTGGCCT
AGAGGGTACATTAGAAAGACGGAAACGAATACTGCAATTGGTCCAGAAAATGCAATAGCG
TTGTATGGTCTAAGACCAACTAGCCTAGCAATTTCAAATTGTCTTAAACAGAATCCAATT
AGTCCAAATGATCCATGTAAAGCTATAAATGCCCACAGACCACCAATTTGGCACCAGCGA
GTAAAATCTCCTTGAGCTTCAGGTCCCCAAAGGAAAAGAAGTGAATGTCCCATGCTGTTT
GCTGGGGTAGAAACAGCCGCAGTTAAAAAATTGCATCCTTCTAGATATGAACTGGCTAGC
CCATGAGTATACCAAGAAGTAACAAAAGTAGTCCCAGTTAGCCAGCCACCTACAGCAAGG
TAAGCGCAAGGAAACAGAAGTAGTCCAGACCAACCTACGAATACAAATCGGTCTCTTTTT
AGCCAATCGTCTACAAGATCAAAACCACCACGAGTTTTTTCTTGTCCAATTGCTATGGTC
ATAATTTTAATCTCCAGAGCAAATTTAATAAGTAATTTGTTTAAATAAACACTTTAGATA
TACTCTAAAATTTTACTTTTCTTTACGGTCAATATAATTATAAGTCAAAATTAACCAAAA
TTCACGCTTTTAATAACATTTTTTTAGCTCATTAAGTTAATTTTTTAATATATATAAACAGT
AATAGAGTTATTGTTTAACTTACAATACTCATAATTTATTCATTTCTATAATCCTTTGAA

AAAGATATCCTGTTCCTCGTGCTGTTAAAATCAAGTCGGGATTACTTGGATCATCTTCTA
GTTTAGCTCTCAATCGTGAAATATGAACATCAACAACTCTCGTGTCAACATGCCGTTCGG
GAGTATAGCCCCAAACTTCTTGCAAGATAGAAGCTCTAGAAAAAGGTTCTCCAGCCTTAC
TAACTAAAAGCTCGAGAAGACTGAATTCCATACCTGTTAATCGAACTCTTTCATTATTTT
TATAAACTTGTCTTTTGTTAGTATCTATTTTTAGAAATCCAATACTAATAATCCCAGAGT
TTGGGACTCCAAGACTAGTGGTGATTTTATCAGCTCTTCGAAGAACAGAACGAATGCGAG
CTTCTAGCTCCTTAGGAGAAAATGGTTTAACGACATAGTCATCAGCACCTATTTCTAATC
CGGTAATTCTATCGCAGACTTCACCTAACGCTGTTAGCATTATGATAGGAACATCTGATT
CTTTTCGAAGTTCTTGACAAACACCGTAACCATCTAGTTTAGGCATCATAACATCTAAAA
CAACAAGGCTAGGATACTCTTTTCTAAACACGAGTAAAGCTTCTTCTCCATTTGAAGCAG
TGATAACTTCATAACCTATTATTGTTAGTCTTGTTTCTAAAATTCTTCTTATGCTGGCCT
CATCGTCAACAACGAGAATCTTTTCTTTCTGGTTCTCCAATTTCTTATTTACTCCTATAA
CTCACCGATCATGATTACTATAACCTTAAATTTTTAGCGCTATGAGTATCTTTATTCACT
TTAGAATTTAGTTATTATAAATTTTGCAAGTCTAGTTTACATTAATTAGTACCAGTCAAT
ATTTTTGTTTTAAGTTATGACTATTTTAATCCATAAGTTGACTCTAGTTTGCAGAATTCA
ATATATACATATTTTCTAGCCAAGTAAATTGTATTCTATTTTCTAAATAAAAAAGGGGTT
GACAAACCAGTTGATAGAAAGTATTCTACATTACTATAAGTTAATTCCAACAAAGAAGTT
TTAAATCTTCTTTCGGAGTAATGAATACTTTAAT
>SRR9587922
TATATGATAAATTAAGACGTCTGTTATGTGAGAATTTCTAAAATATACAAAACTTTTTAT
TTAATATAGAAAAAGTAAATTAAAATAAGTTGAACATACTTTGCTCTAATCTAAACCTCT
TAATTGAGATTCTAATTAGAGACAAAAGTATTTACTTATTTTGTATCTATAGGAATAAAA
ATGAAACTTGCAGTTTATGGTAAAGGTGGTATAGGTAAATCTACAACTAGTTGCAATATT
TCTGTAGCACTTTCAAAAAGAGGTAAAAAAGTTCTACAAATTGGCTGCGACCCTAAACAT
GATAGTACATTTACGCTAACAGGTTTTTTAATCCCAACAATTATCGATACTCTTCAATCC
AAGGATTACCACTATGAGGACGTTTGGCCTGAAGATGTAATCTATAAGGGATATGGCGGT
GTAGACTGTGTTGAAGCTGGTGGACCGCCGGCTGGCGCTGGTTGCGGAGGCTACGTTGTA
GGTGAAACAGTTAAACTTTTAAAAGAATTAAATGCTTTTGATGAATACGATATTATCTTA
TTTGATGTTTTAGGTGATGTTGTATGTGGAGGTTTTGCAGCTCCATTAAATTATGCAGAC
TACTGCTTAATCATTACAGACAATGGATTTGATGCTTTATTTGCAGCTAATAGAATAGCA
GCTTCAGTACGAGAAAAAGCTAGAACGCACTCTCTGAGATTAGCTGGACTTGTTGGTAAT
AGAACAGATAAAAGAGATCTAATTGATAAATATATAGATTGTGTTCCAATGCCAGTATTA
GAAGTCTTGCCCTTGATTGAAGATATTAGAGTGTCCAGAGTAAAAGGTAAAACTTTATTT
GAAATGGCAGAAATTGATAAGGATTTAGCATATGTATGCGATTACTATTTGAATATTGCA
GATCAGTTGATTACAAGGCCAGAAGGTGTAGTTCCTAAAGAATCTCCGGATAGAGAATTA
TTTAGTCTTTTATCTGATTTTTACTTAAATCCTAAATCAAAGGTAGGACAAGAAAAAGTA

GATCAAGAAGAATTAGATTTGATGATAGTGTAAATAATATTTTCAGCATAATATAACAAG
GAATAAGATAATGTCTACAGCTCAATCAGATGCTCTTACTTTTGAATGTGAAACAGGTAA
TTATCATACTTTTTGTCCCATCAGTTGCGTTTCTTGGTTATATCAAAAAATTGAAGACAG
TTTTTTCTTAGTTATAGGGACTAAAACTTGTGGTTATTTCTTGCAAAATGCAATGGGAGT
AATGATTTTTGCTGAACCGAGATATGCCATGGCAGAATTAGAGGAAGGAGATATTTCAGC
TAAGCTAAATGATTATGGTGAACTCCGTAGATTATGCTTACAAATAAAAAAAGATAGAAA
TCCTAGTGTTATATTTTGGATTGGAACATGTACAACCGAAATTATAAAAATGGATCTGGA
AGGAATTGCTCCAAAATTGGAAGCAGAAATTCGTGTTCCTATAGTAGTTGCTAGAGCTAA
TGGTTTAGATTATGCCTTTACCCAAGGTGAAGATACAGTTCTAGCAGCTATGGCTCAAAG
GTGTCCATTAAATTTAAAGAATCAATCAGATAACACGAGTCTTAAAACTTCTCCTCATAT
TCCTTTAGTTTTATTTGGATCTCTTCCGGATCCAGTTGTAACCCAGTTAACTATGGAATT
AAAAAAACAAGGTATTTTTGTTTCTGGTTGGTTGCCATCAAAGAGGTATACTGAATTACC
GGTTATAAAAGAAGGATATTATGTTGCGGGAGTTAATCCATTTCTTAGTCGCACAGCTAC
TACACTAATGAGACGCCGAAAAACAAAACTAATTGGTGCACCATTTCCAATAGGTCCAGA
TGGTACTCGAGCCTGGATTGAAAAAATCTGTTCAGTAATGAATGTAAAGCCTATTGGATT
AGAAGATAGAGAAAAAGCAATTTGGGCTTCTTTAGAAGACTATATTTCTTTAATCCGAGG
TAAATCAGTTTTTTTTATGGGTGATAATTTACTAGAAGTATCTCTTGCAAGATTTTTAAC
TAGATGTGGAATGACTGTATATGAAATTGGTATTCCTTATATGGATAAACGCTATCAAGC
AGCAGAGCTAGCTTTATTAAAAGCTACATGTGATAAAATGAACGTTATGATGCCAACAAT
TGTAGAAAAACCAGATAATTATAATCAAGTAGACCGAATCCGTGATCTGAAACCCGATTT
AGTTATCACTGGTATGGCTCATGCAAACCCTTTAGAAGCCAGAGGTATTAATACAAAATG
GTCAGTAGAATTCACATTTGCACAAATTCATGGCTTTACCAATGCAAGAGATATTCTTGA
ACTCGTCACAAGACCATTACGGCGAAATCTTAGTTTGTCAGAATTAGGCTGGGATATTTA
TAGCAAGCAAAGCTAGTATTCCTC----TTTATGACTTACATGGTATAAAAGCCTTGTAA
GTCTTTTATTTTATGTTAAAGAAGCATTTCTTACTCTGCCATCACCAGCCCAAGCTTGTA
AACTTTCTACTTGTTCTTTGTCTGTAAATGCTAAAGGCACAAACTGTTTGATAGCTATTT
TAATATCTTCAGTACTAAATTCTCTCTCTTCACTAAACGCCGTATGCATACTTTCAATAA
TTGCTTGCTCAATTTCAGCGCCAGAAAATTTATTACATAATAAACTCAATTCATGAATAT
CATATTCTTGCCACGATCTTGGTCTAACTTTTGATAAATGTATCTTAAAAATTGACTCTC
TCTCTTGACGATTAGGTAAATCTAAGAAAAAAAATTTCATCGAACCTGCCTTTCCTTAACA
TTTCGGAAGGTAAACTTTGAATTTTATTAGCTGTTGCGACAACAAATACTGGAGCTTTTT
TCTCTGATAGCCAGGTAATAAATGTACCAAATACTCTGGCACTAGTACCACTATCACCTT
GACTATGTAAACCAGAAAAAGCTTTATCTATTTCATCAATCCATAATATGCAAGGCGATA
ATCCTTCAGAGATATTTATCATTTCTCTCATTTTTGATTCCGATTCACCAACTAGTCCAC
CAAACAGTTTTCCCATGTCAAGACGTAGCAGAGGCAGCATCCAATCGCTTGCAATAGCTT
TCGCGGTTAAGGATTTACCCGTCCCCTGTATACCAACTAGCAATAAACCTTTGGGCGAAG

GAATACCGTAATTTAAACTTTGCTTCGAAAATGAGCGAGACCTTTTTTGTAGCCATTGTT
TTAATACATCTAAGCCACCAATATCCCTATTTACTTTACTATAAGGATAAAATTCTAATA
GATGCGTTTGATTAATAATTTGTCTTTTTTCTTCAATAATAATAGGTAAGCTCCGAGAGT
CTATCTGATTATATTGTGCAATAATTTTAGTAATCACTTTGCGGATTCTATCTATCGATA
ATCCTTGGCAGGATTTTGTTATATTATTAACGAGCTCTGAATTCAAATTAAGATTCAAAG
CTTTACTCAATCTTATGATCTCTTTTTTGATTTCTAGAAGACTAGGTAAGGGCAAATCTA
TTACCGTTATGATATCATTCAGAGCAAAAGGTATATTGATTTTGCAAGAAATAATAATAA
TGTGCTTAGATTGCGTTTTGATTATTTTAGCTAAATTGCGAAGTTTCCTAATTAATACTA
TTTCGTTTAGGAAAGAGTCAAAATCTTTTAAAAGAAAAAGATTTAAAGATTCATTATTCA
AGTTTTCAATAAATTCTAATGCTAATAAAGGATTTCTTTTTGCATAACCATTATCGCTAG
GATTGTTAGTGTATCCATCTACAAAGTCCCAGGAATATACTTGTTGACTGTTTGAACAGT
TTAAGCTGTGTTTTATAATATACTCTAGTCTATCTTCTTCTCTAGTATTTATTACAATAA
TAGGATATCGAGATTTTAAAAGTAATCGTAAATCTTGAGTGAAATTCATGTCAATATTTG
TTTCCATCAGACTTTTGTACTTCACCAATTGAAGAAAGACTAAATAAGATTATCTAATAT
TCCTTGGATATATTCAAAAGTCATCTCTCTATTTTCTAAATTATTGCTATTACTAATTTA
ACTTACCATGATTTTTTTTCTTCCTTTTCTTCGTCGACTATTTAATATAGAGCGTCCACA
TGGTGTTTTCATTCTTGCTCTGAAACCAGAAACTCTAATTTTTTTTCTTTTAGATCCTTG
TAGTGTTCTTTTAGTCATGATGTTTCCTCTGTTGTAAAAGATTAAATACTATTTTATCAT
CTTTTATATATCTTTATGATATAGATATGTATTAATATCAAAGTTAACAGCAATAATTTA
TATTAATCAAGAATTATTTATATAATATTAAACATTAAATATTAACTTTATGATATTGGC
TTTGCCCATTTTTTATTTAAGTATTTTGACCATCTTTTTGCTAATCTTAAACTGGTTAAT
TTTTCAGCAGCTAAAAACGATTTTGTTATTAGAATCTCAATTTAAGTATTTTGTTGATAA
AAGCCAAAATCGTAAATTAGAACCCGAAGAAAGTTTTGCTTTTGCAAAAGTATGTGTAGC
TAAAAAATATTTTTCTAAAGCTATTATTGAAGGTCAATTAGCTTTAAAAAACTATCGAGA
CCTGAATATCTTAGATAATAATATAGTAATTGCTAATTTGTATAATATGCTAGGTTTTAT
TTATTTTGAAGCAGGCCAAACAAGTTTTGCAAAGAACTTTTATGAGCAAGCCTTACAAAT
AAACCCTAATTATGTTGTAGCTTTAAATAACCTGGCAAAAATTTACGAAGAGGTAAAAGA
TTTAAAAAAAGCCGAATCTCTATACGATAAAGTGTTAACTCTTAACTTAAATAATAAAAC
TGCTAATAGAAGGAAAGATTTTATAGCAAAAACTAAAAACATCTAATACTATTTGTAATC
GGGATAGCAGGATTTGAACCTGCGACATCCTGCTCCCAAAGCAGGCGCGCTACCAAACTG
CGCTATATCCCGTAAGACATAATTGTTACTATATACCTTTTCTCTAATTATGTCTACTCT
TTCTGTATTTTATCTAGGATACCAAAACATGCCTTTAACTCCATCTGGATCTTTGATAAA
TCCTAATTTTTTATAAAAACTAACAACATCAGGTTCTGCGAATAGTGTAATAGTGCTAAT
TTCTGCCTGTCTCAACTGCTGTATTAATTGATGTATGATTACTTTTCCTAAACCTAAACC
TTGGAAATCTGGATGAATAACTACGTCCCAGATAGTTGCATTAAATCCGTTGTCTGATGT
TGCTCTAGCAAATCCTACAAGTTTACTATTTGCATCTTTTTTTTGTATTAAAGAAATAAT

AATAGAACTATTTTTTAATGCGATCTTTACTTTTTTTAGAGGTCTTTTAACCCATCCGAC
TGAATCACAAAGCTGCTCCAATTCGTATAAATTAATATTCTTATTGCTACTCAGATAGAT
ATCTTTAAATTCGATTTTATCGCAAGTTTTATCTAAAACAATAAGTTTTTTGAAATTTTT
TTCAGAGCTAGCATTGATATCGGAGTTTTGAAAAAAATTTTTCCAGAAGATCATAATGAT
TATGATATAATTAAATAAATATATATATTACAATATTGCTTTAACAAACAATATATTAAT
ATATGCTCAAATTTTTACATGAATCTATTAAGATAGAAGATTGTTAAAATATAGAGAAAT
AAAAATTTTTGTAAAATCTTTCAATAAAATGATATAAAGAGTGCTTTTTAAGACTAACAT
CTATTATTATAAATAAAGTCTCGAAATTGAGATGAAATAACACATTATTCTATGTCTAAT
GCCTTTTAAAGGAGTTATTTGTGAAAAAATCTATGTGTCTAACCTGTTTACTTGCTTTAC
TAATTATGAGCAACCCAATAATAGCTAACGCAGAAGTAGCTGGATTGGTGCCCTGCAAAG
ATTCTGCGGCATTCAATAAGCGTATGGTAAATAGTGTGAAAAAACTTCAAGCCAGATTAG
CTAAATATGATGCAGACACGCCACCAGCGTTAGCTTTAAATAAGCAAATAGAGAAAACTA
AAACTAGATTTGCAACTTATGGTCGAGCAGGTTTGCTGTGTGGTACTGATGGATTGCCAC
ATTTAATTTCTGATGGTCGATGGAGTAGAGCCGGGGACTTTGTTTTTCCAGGACTATTAT
TCTTGTATATTACAGGATGGATTGGCTGGGTAGGTAGAGGATATCTTTTATCTGTTGCTA
AGACTAGTAAGCCAACAGAAAAGGAAATCATTTTAGATGTGCCATTAGCTATCAAATTTA
TGTCATCCGGATTTGCATGGCCGCTAGCGGCTTGGCAAGAATTTAGTAGTGGACAATTAA
TTGCTTCCAATGACGATATTACTGTTTCACCCCGTTAGTAAAAAATTTATATGAATAATA
ATTTTACCAAATACTTATCAACAGCACCTGTAATTGGTGTATTGTGGATGACGTTTACAG
CCGGATTTATTATAGAATTGAACCGCTTTTTCCCAGATGTCTTATACTTTTACTTATAAA
TAAGATCGTATAAAAAACTACATAAATATAGAGAAATACTATTATTTGTGTAGTTTTTAA
TATATAACATAATTATTTATTTTTAAAAATGTTTATCTTTTCGATTATTTTAATATTAGA
TCTTACATAATAAAATAATATCAATATTATGAGCTTAGTAACCCAAATTATAGTTAATGC
CGATGATGAATTAAGATATCCTACAATTGGAGAATTGCAGTCAATTCAAGACTACTTAAC
TACAGGAAGCAATAGAATTAGAATTGCTACTATTATTAGAGATAAAGAAAAGGAGATTAT
ACAGAAAGCTAGTAAGCAAATTTTTCAGTTACATCCAGAATATATAGCTCCAGGTGGTAA
TGCAGCAGGTTCTAGGAAAAGATCGTTATGCTTACGTGATTATGGTTGGTATCTCAGACT
AATTACATACGGAGTATTAGCTGGGGATAAAGATTCTATAGAAACAATTGGTATTATAGG
AGTAAGAGAAATGTACAATTCTTTAGGTGTACCTATAATTGGAATGTTAGATGCAATCCA
GTGTTTAAAGGAAGCATCTTTAGAAATGCTTGGTCAAGATGATATTAGAATCATTTCTCC
TTATTTTGATTATATAATTCGTGGAATGTCATAAATCATAGCTTCAATTAATTAGTTGAA
TAAAAGCTTTTATAATGTTATAATTACAATAAGCCCGAAAGCATATAATTGTAAAAACTA
AATCTTGTCAGAACCGGAAGGTAGCAGCAATAATGTTTCAATACAAAAGTTATGATTTTC
GGGTGTTTATTGTTTTTATCTAATAGTCTCGACTTTTAATTAGTATGCTATGTATAAATC
TGTATATGACATGTAATCCATCAAACCTTCCAGCAAAAATAATTTAATAATTTGATATTA
ATACGTAAAACACCTATACTCTTTTATAAAATTTCTTTATATCTCATCTAAACAATGGAA

ACTTAACTTAATGGGTGTTCATATTTTATCAACAGGTTCGTCTGTCCCAAATTTTTCTGT
AGAGAATCAACAATTTGAAGATATGATCGAAACTTCCGACCATTGGATTTCAACAAGAAC
AGGAATAAAAAAAAGACATCTAGCTCCATCTTCTACTTCTTTAACTAAATTAGCGGCAGA
AGCTGCAAACAAAGCCTTATATGCAGCTAACTTAAAACCTACTGAGATCAGTTTAATTAT
TTTAGCTACGTCTACGCCTGATGATTTATTTGGTAGTGCTAGCCAGTTGCAAGCAGAAAT
AGGTGCAACAACATCGGTAGCTTTTGATATTACGGCTGCCTGCTCCGGTTTTATTGTTGC
TTTAGTAACAGCAGCTCAGTTCATTCAAACTGGTTCCTATGACAATATTTTAGTTGTTGG
AGCAGACACAATGTCTAGATGGATTAATTGGTCAGATAGAACTACCTGTATTTTATTTGG
TGATGGTGCTGGAGCAGTAGTATTGGGGCAAAGCCTCAAAAATAGTATTTTAGGCTTTAA
GTTATGTACAGATGGTCAGCTAAACAGTCATTTACAATTAATGAATAAACCTGTAAATAA
TCAAAAATTTGGTGTTACAGAAATTCCTCATGGAAACTATAATTCCATAACAATGAATGG
CAAGGAAGTGTACAAGTTCGCTGTATTTCAAGTTCCAACAGTAATTAGACAATGTTTGAA
TAATTTAAACATTTCAATAGATGAAGTTGATTGGTTTATATTGCATCAAGCAAACACTAG
AATCATAGAAGCAATTGCGAGCAGATTATCAGTACCTTTTTCTAAAATGATTACGAACTT
AGAGCATTATGGAAATACATCTGCAGCGTCAATCCCTTTAGCGTTAGATGAAGCTATTCA
ATCCAATAAAATTCAACCAGGCCAAATTATTGTTTTATCTGGTTTTGGAGCAGGCTTGAC
TTGGGGAGCAATTGTCTTGAAGTGGTGATTTATATATTGCGGATGACGAGACTCGAACTC
GTAAAGCTTTCGCTACACACCCCTCAAGCGTGCGTGTATACCAATTTCACCACATCCGCA
TTTTTTACCATGAATATTAAAAATTTATAATATTTATCTAAATATTCATATAATGTGTAT
ATATATATACATTCTTGTCAGAAACTCTCTTATATTTTATTTTAAGTGATAAAATTGAGT
TTAGTATAGATAGTAAAATTTTATTTTAAAATCATAAAAATAAATATATAAGGAAAATAA
AATATGACACCATCTTTATCAAGTTTTTTGAATAGTCTTATTCTTGGGGCAGTAATTGTA
GTCGTTCCCATAACTTTGGCTCTTTTATTTGTTAGTCAAAAAGACAGGACAATCCGGTCC
TAAAAATAACTTAGAAAGATATGGAGAAGCAATTTAAAATGAATAATAAAATATTTTTCA
TACTAATAAGCTCTCCATATATTGCTATACTTTAGAAGAATTTTTATTTTTTTATTTTGT
AACATCCTGTAAAGTTTATTCCGTTCTTTCTTTTTCCCATTTACTCTCTTATAATTAATG
ATACAAATGTCAGGTTAATAATAGTTTTTTTATTCTGTATCTTTCATTTTATATACGTAT
TATTATGTTCAATTAATAAAGAAAAGCTCATGTCTTTGTCTAATTGGCCTCTCAAAAAAG
AAAATTCTGAAGCATATAATATTAAGAATTCAAAACAAATCACAATTCCTGATGGTTTAT
GGATAAAATGCTTCGACTGTGGTCTATTAATGTATTCTAAAGTACTGAAGAGAAATTTAA
AAGTTTGCCCTCAATGTAGTTATCATTTTCAAGCTTCTAGTAACGAAAGAATTGATCAAT
TGATAGACCAAGGTAGTTGGCAACCAATGGATGTTCACTTGATCTCTACAGATCCATTAG
GCTTTAAAGACCAAAAGCTTTATAGTCAAAGGTTAAAAGATACTGCTTTCAAGACTGGCC
TGCAAGACGCAGTTCAAACAGGTACTGGGACTATGCAAGGTAAAAAAGTATGCTTAGGTA
TTATGGATTTTAGATTCATGGGGGGAAGCATGGGATCTGTTGTAGGCGAAAAACTAACAA
GACTGCTAGAAAAAGCAACTCAAGAAAAGTTGCCTGCAATTATACTTTGTGCATCAGGCG

GAGCTAGAATGCAAGAAGGTATGTTGAGCTTAATGCAAATGGCAAAAATTTCTTCTGCTC
TAGAAATGCATAAAAAAGAAAATCTACTATATATATCTGTTTTAACCTCTCCCACAACAG
GGGGTGTAACAGCTAGTTTTGCTATGCTTGGAGACTTAATTATTGCAGAGCCAAAAGCTC
TTATTGCATTTGCTGGTAGACGAGTTATAGAACAAACAATCAAAGAAGACTTGCCAGATA
ATTTTCAAAGTTCAGAATATTTATTTGAACATGGTTTCCTAGATTTAATTGTATCCAGAA
CTCAGCTTAGATCAAAGTTAATACAAATTTTGTCCTTACATAATCATAGTAAGTAATGAA
TTGAATATTGACTATAATATCAAAATGCTAGTAAAAATTAAGATATTAAGAAATTTTCAC
TAAAGATATTTGTAGACTAACTAGAATTACAATTACTATTGTAGAAAAATTTGTCTTAAT
GCCATTTTTTCAGTCTTTTAACAAATTGTTTTTATGACTTTAAATAATTGTTAGTTTAAT
ACAAATTTATAAAGAGAATACATACTAAGCTAAATATAGTAATATTATGTTTTCCTATAA
TACTGACCTATCAAATTTCGAGGAAATTTATGCTTAAAAGATCTTCTTGGCTTGCGGCTT
TATTGGGACTATTAACAGTAGTTTCTACAAGTACGCATACATATGCCATAGAGTTAGACG
AGGCAACAAGAACTGTTCCATTAGAATCTTCTGGCAGAACTGTAATTCTTACACCAGAAC
AAGTTAAAAGAGGCAAGCGATTATTTAATAATTCTTGTGCTATTTGCCATAATGGTGGTA
TCACGAAAACAAATCCGAATATTGGACTTGACCCAGAATCTTTAGGATTAGCTACGCCAC
AAAGAGATACCATTGAAGGACTAGTTGACTATATGAAAGATCCGACTAGTTATGATGGTG
CAGAGTCAATCGCAGAATTGCATCCAAGTATTAAAAGTGCTGAAATTTTTCCTAAAATGC
GCAATCTAACCGATGAAGACCTATTTACAATCGCAGGTCATATCTTACTTCAACCTAAAA
TTGTTTCTGAAAAGTGGGGCGGAGGAAAAATTTACTATTAGAACTTCAAAGACCTAAACC
TTGTGAATACTAGTTTATGTATTTGTTGCTTAAGTGTGATTATTTTGTTAGATCATGATA
TATATTAAAGATGGAGTTTGTTGACAGCAAGTTACTTTTTACCCGAACTTGTTTACACTA
TTCGTCTAGTTATTAAAAAGGAGACGTTAAATTGAAGAAGAAGCTTTCAGTTCTTTTCAC
TGTTTTTAGTTTTTTTGTAATAGGTTTCGCACAAATTGCTTTTGCTGCAGATCTAGATAA
TGGAGAAAAAGTTTTTTCTGCTAATTGTGCAGCATGTCATGCTGGCGGTAATAACGCCAT
TATGCCAGATAAAACCTTAAAAAAAGATGTACTTGAAGCTAATAGTATGAATACTATTGA
TGCTATTACTTATCAAGTACAAAATGGTAAAAATGCCATGCCTGCTTTCGGAGGTAGACT
GGTTGATGAAGATATTGAAGATGCAGCAAATTATGTATTATCTCAATCTGAAAAAGGTTG
GTAATTATACTTGATTTTATCCTGTATTAAAGAATAGACAATCTATTTAGTTGTTCATTA
GATTGTCTATTCTTTGTTTATGCTATTATATAAAGATATTTACACAATATTTTATGATGA
AAAGAATACCCGCAATTCTTGTACTAGAAGACGGTGCGTATTATAAAGGATGGTCATTCC
AGCAAGATAAACAAGAGATTACTATTGGTGAAGTAGTTTTTAATACTGGAATGACAGGAT
ATCAAGAAATAATCACAGATCCTAGTTACTTCCATCAAATTGTCGCTTTTACCTACCCGG
AAATTGGGAATACAGGTATTAATAATCAAGATATTGAATCTCACAGTATTAGTATTAAAG
GACTTATTGCAAAAAATATTTGTAAAATTTCAAGCAGCTGGAGAGAGCAGCAATCTTTAG
TTACGTATTTAAGTAGTAATAATATTCCTTTTATTTTCGGAATAGATACAAGGTCTTTAA
CCCAATACTTGCGTCAATTTGGTACAATGAACGGTTGTATCTCTACTGATAATTTAAATC

ATAGTTACTTAAAACAGAAAATTTGTGAGATTCCAAGTATGCAAGGTTTAGATTTAATCC
CGCATGTAACTACAAGAAATGTTTACCCCTGGGATGAAAAAAGTTTTCCAAATTGGTATT
TGACAGATAACATTAGAGTGCACCGAGTTATTCAGTTGAAAGTTATTGTTATAGATTTTG
GAGTTAAACTAAATATACTCAGAAGACTAGCTACACTTGGATGTCAGATAACGGTTGTGC
CTGCCCACACTCCTTTAAAAGATATTTTGGCTTACCAGCCTGATGGTATATTACTCTCTA
ATGGTCCAGGAGATCCATCAGCAGTACATTACGGCATCCAGACAGTTACAAAATTACTAG
ATTACAATGTGCCTATATTTGGGATTTGTATGGGGCATCAAATTTTAAATTTAGCTCTTA
AAGCTAAAACTTTCAAACTTAAATTTGGTCATAGAGGTATTAACCATCCATCCGGATTGA
ACCAGCAAGTTGAAATAACTAGTCAAAATCATGGCTTTGCAGTTGAATTGACTTCGGTTT
TTGAATCTCCTGTAAGAGTGACTCATTTTAATCTAAATGACACTACTATTGCAGGAACTG
GACATAATCAAAGTCCTTATTTTTCTGTGCAATATCATCCAGAATCGAGCCCAGGCCCTC
ATGATGCTGATTATCTATTCGAAAATTTTATAGAAATAATGACAAAGTCCAAGAATAAAG
TTAGTTAGTAATTTTCCCATGCTTTATGAGTGAATAAAGCGGATAGTACTTGTACTCCTC
GTAATTGATTAAAGAGAGGCAAATGTCCTCTGGGGCCATGAGAATTCCATTGAAATTCCT
CAGGATATCTGCAAGGAATTCTATCAACTTCCCAGCCGATTTTTTGCCAGAATTTTCCCC
AATCTTTTCCAACACTAAGCCATATTTGTCTTTGAACAAATAGCCCAAATTTACCTTTTG
AGTGTGTATGCCATAACTTATCAATAGTTTGTAAATCTTGAGCAGGTATTTTTTTTATAT
CTGTAAAATATAGCCAATTACGAGTTTGTGCATTTACACCAGCTAGTTGAATAAGCTTTT
GTTGAGTTAGCTGATCTGCTTTGAGCAAATCCCGATGAGTCAGTAACATTTGTAAATCTT
TGTAATTCATCTGTTGAGCAGAGCGCAATGGAACAATGCCATCGGGACATAAGTTAGAAG
CAAATTTCACTATTTCTTGATTTTTACTGTTAAGTAACTTTTCATAAATTAAACCGTCAA
CACAATTACTTTTATAGTTAGGCCCAGTAATCCTCTCAAAAAATAGATCTGCTAAATCTT
TTAACTCGATAGAGTCTTTGCTATTTATATTCTCAATAATTTCAAGTTGTTGCTTGACAT
TATTAGACTTAGTATTCTTATTCAATTCTAAAAGCTGGGCTCGGATTTGATTTGGCATCT
GATTTGTTTAATTAGTTTTAATAGTCATAAGGTCTTAGAGAGAATTAGATTGTTTTTTGA
AATTAGAAGAGCGCCGCAGTATTTCTAAAGAAGTTCGCATAATTTGATTCAATAAGTTTT
GTCCTGCATAAATTATAATTTGACCAATTAAAAAAATTGTGCTTTTTATTTTAGGAACAA
AAAAATCTTGTACCTCAAGCAGAAAAGTAATTATTATTTGCATTGGTGACAACATCTGTA
ATTCATTCGATCTGCAAGCATATATATACTGACAGTTTAAGCCTTCTTGATAAAATCCCC
ATACTTTGTAACGACTTTCGTAAATTGCTCGTGGTCGCTCAAGATAGTTTTTAATTATAG
TATTCCAAATTAGGTTATTCTTTAATTTTTCTAATTTTCTATCAGATAGAAATTTTACAT
TACAGAGATAACTTGCATTTGTCTCATTTACAGTATTAAAATTTTGAACTAACATATGAG
CAATAATGTTGCTTAGCTGAATTAAATAATTTTCAAGAAAAATTTCGACCTGCTTAACTG
GCATATTGTAAGTAAAGAGATTAAAAGTTTTATTCACAGTTTGAGATGACCCAAAAAGTA
GCTGAATAATTAAAATTTGTAGTAGCATCTTATAGTCGTTCAGTAGATAGTTCAAATCAT
GAATATCTTGGTAAATGTCAATACAGTCCAGTTCATATAACTTACAAAATCTTTTGATAC

ATCTATGGAATAAATCAATTAAAATATTTTCGGTCAAGTTTGTAATATCATTTACATCTA
AATTTGAATTATAAATTTCTAAAAGTATCTTTTCCAGTTCAGCTAATATGATTTTTAATA
AGTTTCTTTTTACTTCAGTGCGAAAAACATCTAAAATTAACACTTCCTGAGAGCAATTAG
TTAATCTCTTATTAATCTTTATAGAAGTTCTAACTAATAGCTCTGCTACCTCTTGATTGA
GTGTTGGTCCCTGAGAGCTTGGCCAATAATTATTCACGTTATACCATAGTAAGAAATATT
GAATTTAATGTTATACGATAACTTTTTAATATACTGAAAAGCAAGTTAATTACTCTATAG
CAACTAGTTTTTACAGATCTTAATAGTTATTTATCAAATAAAGTTATATGATAGTAGCGA
TTGAGCTAATTTTATTAAAATTTATAATAATGACAACTTATTACTTCGCCCTTGCGAGTC
AAAATTTTCTATTAGTACAAGAACCACTAGAAGAAGTTTTTAGAGAGAGAGTTAACTACT
ATCAGTCAAATAATAAAGCAATTGATTTTTGGCTAATACCAAACCCTTCTTTTCTAGAGA
AACCAGAAATGATTTCATTTAAAAACCTTGTACCTAAAGACGCTGTAGCTATAATCTCTA
CTAATCCAATATTTATTAATTGGTTAAAGCTAAGAATCGGCTATATCTGTATCGGGCAAT
TTGAAGATAACCTACAACTTTCTGAAGAATCGTTAAATATTACTGTTTTAACAGACAAAA
TTTAACAATGCTTTTATTATGACTAGTGTTAAATTATTTTGGTTAAGTTATAATTATAGT
CTTTTAGTATTTAGACTCTTATAGTTTATCACTTAGTATTTTATGCTGAGATGGAGTGAC
AAATAGTGTAAGTATTTCTTGGCTAAACGTTTCAGTTGCCTTAGATTTATATCTATTTGG
ATTAACTATAATGGATAGCATTCGTTTTATAGTAACATTTTCAATTTGAGCCCAATGTAC
AATTCCAAGTTCCAATTCCTTAGCAATAGCTGAAACGGAAACAAAGGCAGCTCCTAGCCC
TGATTGCACAGCGTTTTTGATAGCTTCTATAGAATTCAATTCCATCTCTATTTTAAAGCG
ACTGCTGTCAATACCATGTTGACTGAGTACTTTATCTATGACTTTTCTAATTGTTGATTG
AGTATCTAACGCAATAAATCTAAGCCTGTATAAGTCTTCTTTTTGAATGTCTCCTAGTTT
AGAAAAAGGATGTGATTTGGGTAATATAAGTGCTAATTCGTCTTCCGCATAAGAAGTAAC
CTGCAAAACATCTTGCAATTCAGTAGGCACTTCTCCTCCAATAATTGCTAAATCAACTTG
ACCATTAGCTACGCTCCATGAAATAAGTCTAGTTGAATGTACTTGCAATTGAACAGCCAC
TTGTGGATATCTTTGCCTAAATAGTCCGATTAATCTTGGCATCAAATATGTCCCAGTTGT
CTGGCTAGCTCCAATAATTAATGTGCCACCTTGTAAGTTTTGTAAGTCGTCAAGAGCTCG
ACAAGTTTCTTCGCAGAGAGCTAAAATTCTGCCCCCATATCGTAAAAGAAGACTTCCTGC
CTCAGTTAAAGTTGCCTTCTTATTACCTCTTTCAAATAGGGAAACATTCAATTGGCGCTC
TAAATTTTGAATTTGCAAACTAATAGCTGGCTGAGAAACATACAAGCTATTAGCTGCTTT
TTTAAAACTGCCCTCTTTGGCAATTGCTTTTAATATTCTTAACTGATCCAATGTAAATGG
AAGGTCTGTCATTAAAGAATTGTAGTATAATGTATATTATTTAGTATTGATCAATGTATG
CATTTGCCAACTAAAATATTTGTCATTACGCTTCATTATTTTCAAAATAATAAAAAGTAT
AATGTTAAATAAGCTGTATTAAAATTTAGTATCTGTTTGATATTAGATTTATACAAGAGA
TATCGTGAATTTAATTCTAACTATAAAATATAGGAGACAATATGGACTCTAGACTTTTAG
TTGTACTAATACCAGTTTTAGCAGCAGCATCTTGGGCAGTTTACAATATTGGTAGAGTTG
CACTACAGCAATTTAGAAAAATGACATCTTAGTTTTGCTTAGAATATAATTTTTAGACTT

TATGAGATAGAGGACTTTAAAATCCCTATCTCTATTGTATATCTTAATAAAAAAAGAAAG
CCCCCTTGTACTTACTGTTATTACACATTAATATTATTGGTGGTACCTAGATAATAAGTT
TAAGCTAACTTATTTTTTATTTTATAATTAATTAAATTAATAACACAATTATGGCTGTTC
CAAAGAAAAGAACATCTAAAGCTAAAAAAAATGCACGCAAAGCAAATTGGAAAAATCAAG
CAAAAACTGAAGCTCAAAGAGCTTTGTCTTTAGCAAAATCAGTATTAACTAGAAAATCCA
ATGGATTTATTTATAATCTAACTGAAGCATCAGATACTTTTAGCGATTAGAAGTGTTTTT
ACTACAAGTCTGAAAAAGTATTACGCAACAAGTTCTCAGTAATTTTTATCTAGTAATCAT
TTGAAAATCATTCTATTTGATAATAAAATAAATAGCAGATTAAATAAAATCACATTAACA
AATTATGCAGCAAAGCTAGATCAAACTAGTGAAATTTGGTTGTTCAATTGTATTGAAAAT
ATTCAGCATATTTTTTTTAAAAGTCAATTAAAATCAAGTCATATTACTAAAATTTTTATT
TCTGGTACTAGCTTTGAATATACCGCAGGTCTACCAGGATTATTATCCAGCTTAACACTA
AGTGGTAGACTACATCCTATTAGTATATATAGTCCCCAGTCTCTCAAAAAGTATCTTGAA
GCATGTACCAAATATTCCCAAACTAATTTTTCTTTTCCTATTAATTTTCATAATTTACAA
TACGGAGGACAAGTTGTTAACCAATTTTATACAGTAATTTGTTTACCGTTGAGCAAAAAG
AGCCTGCTGTATGGATTTATTATTCTAAAAAAAGAAAAGCAGGGAGTATTTAATTTAGCA
CAAGCTAAAACCTTGAATATTCTTCAAGGACCTATATATGGAAAACTCAAAGAAAAGGAT
AATTTTTTAAGTCCAGATGGTTACTATCTATCCGGCCAAGACTTCTCTTCTAATACAATA
ATGGGACATAAAATATCGCTTCCGTTATTAGTCAGATATTCTAGAATTATTTCTGAGATG
CATTGGTTTTGCTCTTATCCTATTAGATTAAATACTTATTCGCATCAGCAAGGAGCAAAG
TGTTTACCACATAATGTTTTAACTGATATTATGAAATCCCAAATATATCAAGATAACAGT
TTTGTTGAATAATACTTATTCTTGATATATAATGGTCTCGGTAATCTAAATAAAATACTA
GAAATAATTTCTTAAATTTATTTGTCTATATTAAATTTACAATCATTTATGACATACGCA
ATTATTGAAGCAAGTGGCACGCAGCTTTGGATAGAAGAAGGCCGCTACTATGATTTAAAT
CATATACCTGTTGATCCAGGTCAGTCGATTATATTAGGAAAAGTCTTATTATTGAATAAA
AATGGGGAGGTTACTTTAGGCCGCCCTTGTATAGAAGGGGTTACGATAAAGGCTACAGTA
ATGAGGCACTTGCGAGGAAAGAAGATAACTGTTTTCAAAATGAAACCAAAGAAGAAAATG
AGATTAAAAAAAGGTCATCGACAAGAATTGACTCGTTTAATGATCGATTCTATAACATCT
TAGATAAAATCTAGACTCATATTTTTAAAAACTTTTTAATATTTAACTAAAATAGATATA
ATGGCACATAAAAAAGGTAGTGGTAGTACAAGAAATGGCAGAGACTCTAATTCCAAGCGT
TTAGGTGTTAAAAAATATGGTGGAGAGCAAGTAACAGCAGGTAATATTTTAATCAGACAA
CGGGGAACTAAAGTTAAGCCTGGCCAAAATGTTGGAAAAGGAAAAGATGATACATTGTTT
TCTCTAATTGATGGTTTCGTGCTGTTTGAAAAGTCAAATCAAAAGCAAAAAACAATTAGT
GTTTATTCTGCTAAGAAATAGTTAAAACAATTGGTGCAAGATAACTTGAGTGTATTTATT
TCACTTACAGTGGCACCATATATTTGATTTATTAAGAAATGGAGTTCCTTAATGAACCAA
TGAATTTTATTAAAGATTTGCTGTGAAATTTAATGACAAACACTATTGTAATTTCCTGTC
TACACAATATGGCTGCTATTCTATATTGTGGTCAGATACAGAAACTAGTAGTAGCAAATG

CTCATTATCAAGTAAGTGATATCTACCTAGGTTGTGTTGATAAAATCTTCTCAGGAATAA
ATGCGGCATTTATTAACCTAGGAAAGAATGAGTACAGTGGTTTTATACATATCAGTGATA
CCGGTCCGCTTAAAAAGAAATATTATGTCAATAATATTACTAACATTTTAACAATACGGC
AAAAAATTTTAGTACAAATTATTAAAGAGCCAACTTTAAATAAAGGTCCAAGGCTCACTG
CCAATATTACATTATCAGGTCGATATATTGTATTAATGCCTTTTAGTCAATCAATCTGTA
TATCTCGAAAAATATATGATGAAGATGAGCGTCATTATTTGAAGTCTTTAGCTATTTTAA
TTAAACCGGCAACAATGGGCTTGCTATTTAGACCTTCTGCTGTAGGTGTCGATGAGGAAA
TAATATTAAGCGAATTAAAAAATCTAAAAGAACAATGGAACTTTGTTCAAAAATCTGCAA
TTAATAGTTATTCACCTGTTCTTCTATATAAAGATGAAGATATTGTTAAAAAGGTAATCC
GAGATTTTTATAATAATAATACAAACAATATAGTAATTGATTCAAACCTGGGATTAAAAC
AATTAAATTATTATATCCACACTTGGCACTGTAATAACTCTAGCACAGTTCCTAAGATTA
AGCTTTATAGTAATAATCAATGTATACTAGATGCTTTTGGTATCAATCAGGCAATTTCCA
GAGCTCTCATTCCAAAAGTTGATCTTATACTTGGTGGCTATATGTTTATTGAAACTTTAG
AAGCTTTTACTATTATTGACGTTAATTCTGGATCTTTTAATAATTCCACTAGTGCACGAG
AAACAGTTTTAAAAACCAACTGTTCTGCAGCAACAGAAATAGCTTATCAGTTACAAATTA
GAAATATTACTGGTGTAATTATAATCGACTTCATTGATATGGAATCACAAAGAGATCAAT
TGCAATTATTAGAACACTTTAATAAAGAGCTATCACTTGATGATGCTAAACCACAAATTG
TACAGTTATCTGAATTAGGTTTAGTTGAATTGACTAGAAGAAGACAGGGCAAAAGTTTGT
ACGAGTTAATTAGTAGTGATTCTAATTACTTTTATTTTTTCACACAATCAGAGAGATCTC
AGTCTCTTAAGAGATTCGATGATAGACAGCAGAAACAACAGATTTTTAATAAATCTTGGC
TATCTGCAGAGATTAATACTATTAACAAGGTCTTTTTTCAAAAGTCAAATTTGTGCAGAC
CTGCTAACTTTTACCTAATTCGTAATCTCTATATAGTTAAAAGTAGTATTACTTATAAAC
AAAATTATTTATTAACTCACAGATCAAAACTTATTTATTCTAAAGAATACAGCAAAGTAT
TACCAAGCAGTTATTATCTAGCTAGTCTCAATAAGAATAGTAATCAAGAGTTCCTACCTT
AAGATTGTTATATCAACTGTTGAAAAAAAGAAGCTCTCTCTTATATAAGAGAGAGCTTCT
TTTGTAGCTTACTAACTATCTAGTTAGTTAATTAAAAATTAACCGTTAACAGCTGGAGCT
GTTAGAGCTACTGGTAAAGATTCACCAGAAGCTAGATCTAGAGGGAAGTTGTGAGCGTTA
CGTTCGTGCATTACTTCCATACCTAGGTTAGCACGGTTGATGATATCAGCCCATGTGTTA
ATTACACGACCTTGGCTATCAACAACAGATTGGTTAAAGTTAAAACCATTCAAGTTGAAT
GCCATTGTGCTTACAGATAAAGCTGTTAGCCAGATACCAACTACAGGCCATAGACCTAAG
AAGAAATGTAGAGAACGAGAGTTGTTGAAACTAGCATATTGGAAGATTAAACGACCGAAG
TAGCCATGAGCTGCAACGATGTTATAAGTTTCTTCTTCTTGTCCGAATTTGTAACCATAG
TTAGCAGATTCGTTTTCGCTTGTTTCACGAATTAAGCTAGATGTAACTAGGGATCCGTGC
ATAGCACTGAACAGAGAACCACCAAACACACCAGCAACACCTAGTTGGTGGAATGGGTGC
ATTAAAATGTTGTGCTCAGCTTGGAATACAAGCATGAAGTTAAATGTTCCGGAGATACCT
AGAGGCATTCCATCAGAGAAACTACCTTGGCCAATTGGGTATACTAGGAATACTGCTGCT

GCTGCTGCTACTGGAGCAGTAAAAGCAACGGAAATCCATGGACGCATACCTAGGCGGTAG
CTTAGTTCCCACTCACGACCAATGTAGCAAGCTACGCCAGTTAGGAAATGAAGAACAACT
AATTGGTAAGGACCACCGTTGTATAGCCATTCGTCTAAAGAAGCAGCTTCCCAGATTGGG
TAGAAGTGAATACCGATAGCTGCAGAACTTGGAATAACAGCACCAGAAATGATGTTGTTT
CCGTATAGAAGGGAACCAGCAACTGGCTCACGAATTCCATCAATGTCTACTGGAGGTGCA
GCTACGAATGCAATGATGAATACAGATGTGGCAGTTAATAGAGTTGGAATCATTAATACA
CCAAACCAACCAATGTATAGGCGGTTTTCAGTACTAGTAATCCAAGAGCAGAAACGTTCC
CACAAGCTAGCGCTTTCGCGTCTTTGTAAAGTAGCAGTCATAATTTTTTATCAATTTTTA
AGGATTTAACAAAGATGCTTCCCAAACAATAAGTTGTCTGTTATGGAAATTATTACTTAA
GGCCTTTTCAAATGAGAACATTTTTAAAAAAAACATAGCAAAATCTAATCTCAGTAACTA
AAATTTTACTTGTTCATATATTGCTTAATAATTCAATAATAAATTTTTGCTTTTTGCAAA
ATTAACACTATTTTAGGGATATAATAATAATGCATATTAATGCTGCTTATTTTGATTTTA
ATAGTTTATATAATTTTGCCCTACTTAAATACGCATGTCACACTTTACAAAAATTCAGAC
GACCATACAAGATTTAAATCTGTTAAAGCATGCTTTAACAGATTTGGGTTTGATTTGGCA
AATGAATTCTAGTCATATAAAAGTTGGTGAAAATAATCAGCACAAGGTAGATATTTTAAT
TAAACAAGATAATTTGTCACATATTGGCTTTACTTGGAATGATAATAGATATCACTTAGT
TGCCGACTTACAACTTTGGGAACAACCCTGGTCTCTAGAAGTATTTTTGGATAAGTTATC
TCAGAAGTATGCTTACTACTCTATCATCGAAGAAACAAAGAAGCAAGGCTTTGAAAAAAT
GCAGCAGATTTCACAAAAAGATGGATCAATTAAGTTAATTGTGCAACGCTGGAATTATTA
ACTAACATACAAATGCGGGCGGAGAGACTTGAACTCTCACGAGATTATCTCACTAGAACC
TAAATCTAGCGCGTCTACCAATTCCACCACGCCCGCACTGTTGAGACAATATTACTCTAT
CATAATCTATACTAAAACACAAACTAATAGTATAAACTAGCTTCCAAGCTTAAAAGCGTC
TACAAATAGACCGTATGTTATACCAATTTTGAAGTTGTTGAATAAATTAATTTTTATATT
TAATTGCTTGTGGCTAGAGTAGACTATTTTACTAGTCAACTCAGTTGCTGCAACTATTAG
ACTGGCTGCAATAATACCCCAATCTCCTGTTTGTCCAGGAATCGTAGACAAACCTGTTGA
AATAAAAAATCCTAGCAATAAACTAATTAAGCCAGTTGTTAACTCGCTCAAAGAATAATA
AAGCTTATTATTTAAGTTTTTTATTAAGTAAGCAAAAAAAGTTGAAAGTCTAGTTTTAAT
CATAGTATTTAGTAACGGAGTTTTAAAAGGATGACAAGTATTAACAGTCATTAATATTGT
CGTATCCTTTTAAAACTCGTTAATACAAACAGCTGTTCTTGTTTTTAAAGGTCTTGTTCG
CTTAAAGCATTAATAAGATGCTGAAACGGCTCTTGTATAATATCTGTGCGTTTAATTGAT
TGCAAATCTAATTCTTCTTCTATGATTTCTTGTAATAACTGTATGCTTCTTACTGTTGGA
GCAACAGGTACAGATAGTGAATTGTAAGTATCTTTTAGCCCATTTAATACACGCTGATCG
AGAATATTTGTACTACCAGAAACCAAGGCATAACTAGCATACCGTAGGTAATATTCTATA
TCCCTTAGACAAGCTGCATATCTTCTTGTTGTATAAGAATTTCCTCCAGGCCTTAATAAT
TCAGGCTGTTCTTCGTACAACTGAGCTGCGGCCTCTTTTAAAATATTGGTAGCTTGATCA
TTAATAATTTCAGCTATTTTTATTCGATCTAGTCCACTCGAAAAGAAAGATTCTAGTTGT

CCGACAGCTGTCTTATCTAGATAACGACCAGTAAGATCGTAACGATTTAATATTGCTGTT
ATAGCATCTTGCATAAATTAATTCTCCTACTTGCTATCAGAGCGGTATTATAAATGACTA
ATATTGTAATATAATCATTAGTGTTTATGAAATTTATTTAAAACTTATTCTTTATTGCTT
AATGTGCAATTAAAAACTCGCATAGATTTTACACATATAGAGTATACGTAGTTTTTAATT
ATTTTTTTATAAAAACCAAGAAATAAAGCTTTTTTTAAGAGGTTCGTGAATTTGTTAATA
TAACATGAGCTTTCCTCCTAAATAAGTTTTGTAAAGTACAAAGTAAAAAAACGATTAGTC
AATAATAGTAAACAATATTAATATGTATCAAAGTATAAATAAGCTTTTACTCCAGCATCA
AAAAAAATATTTGAGATATGTTGATACAACTGATTATTCAAGTGAAAACTTAGATTTAGA
CCAATTTAGCTATTTTATCATTACAATTAATAAAACTGATAAATGTATTCTTATAGAGCA
ATTTTGTTATAATGCTGAAGGCCATATTTACTCAATATTCTTCAAAGGTCCAACAGCTAA
ACATCTTTCTTCTATAATTTGTCATTTTCAACAATTAAATACAATAATATCTACAGCTCA
TGCCATATATTTAGGACGAGAGCTAATGAAGTCAGAATTAGCTCTCGTGCTAGATCAACA
ATATATTCAAGATTAAATTTTGTAAGTATTAATTATATTTATGCTAGTATAATATTAGTA
AGGGCATGTAACTCAGTGGATAGAGTATCAGATTCCGATTCTGATGGCCGTGGGTTCGAA
TCCCGCCCTGCCCGAGTATTCAGAGTACTATACTAAAACATCTTAATAGCTAGTTTGATA
ATAGTATGGGCATTGGGGCTGCAAGGTTTCTACATTATGAAAAGAAGAAAATATGAAAAA
AAACAAGCTCTCCAAAAGAGCTTTTAGTACAATCAATAAATGCAGAAAACAATATTGTTT
CTTTTTCTCGAAAATTAGCTCTTGCTTAAACACTGTTAATTTTTGGCATTCGACATGTTA
AACTCTTATTCATGGCGAATATTCTGTCAGAGTCGCTCTTAGTTTAAGAAAAGTCAGAAA
AAATATTTATCTTTACGTTTTTACCTATTTTTATTTAAATAAAGGTTAGTATTTTCAACT
TTTATAGTGGACGTGGGTTCGAGTCCCACCAGCTCCATCAATTTGATTTGTATAATTAGC
TAGTTTAACCTTTTTAATAACCAGATATATAATTGGAGGAAATATGGGTTTCATAACAAT
TACTAAGCCCGCTTTAAAGCAAATTGCAATCTTGAAAAATGATCATGAAAATGATGTACA
TCTAAGAATAGGTGTTAGACAAGGCGGATGTTCAGGTATGTCATACTCAATGAATTTTGA
ACATGTTGACAAATTAAAAGATACAGATGAACGGCTTCGTCTAGATAATTTTTCTGTTGT
CTGCGACCCTAAAAGTCTTCTTTATCTTTATGGATTATCATTAGATTTTAGCTCGGAATT
AATAGGAGGAGGATTTCAATTTTCTAACCCGAATGCCAGCCAAACTTGTGGCTGCGGTAA
ATCTTTTTCAGGCTGACCCTCTAACACGTTTTTTATATATTTTGTATTATATATATTATT
TTAGCAAGTTGTTTATTAGAGACAAGCAATGCTCTGTTAAAAACTAATTTCAAGAGTTTT
TATTGTATTATTTTATATATTTATTATCAACTACTTGAAAATAAATTAGCTAAGCAATCT
AATTTGTTATCAGTACCAAACTTTAAATGATTAACTTTTTATTGATATATGCTAATCTTT
ATCAGCCTAAACATATCTGTACATTACAATCTTTTGACATAAAAAGAGTCTTAACATACC
TCTCAAAAATAAATTCAATATTTTATAAATACTCAACATGAATCTGTCACAACTTATCGT
TAATTCTTCTATGATGAAAATTCCTAATAACTTTTATTGCAATATAAAAGCTATTTCTTC
TGAGAATTTAAGTCACTTACAATTATCAAAAACAAATTTATTATCTCCTACAAAAATAAT
TGCTTTTGGACTACCTAAATTTGATTTTATTTCTACACAGATTACAAGTTCTATTAGAGA

AACAGTTGTTGTGTCTACTATTGATGATAATAATCTTTTTGCTGCCCCAACTAATGATTG
GATAAAAAAACACAAGGATAAAAATCATCATTCTTTGTTTTATAAAAAAGTTTTTAAAGC
TTTGCTAAACCATAAAATTAGAGTTGTTGCTGAGCCATTTCATAACTCCGAAATAAATAA
AGAAAAGAGCATAATGGCAAGTCAAAGATACATCTGGGGTAAAAGCTGGAAGCCTTCAAT
TATTTTATCTTGCTTAAAAAACAAAAAAAATAGCAAAATTTCTGATGCATCGAAAAAATT
TTTAACTGAACAGTTATCTGCATCACCAGTATTTGTAGTAAAAAATGGTTTTAATGAAAT
TATTTTAGGTCACCCATTATCTCGTGTTAAAAGAGGAGGAGTTAATAATCTGATGCATGC
GTTTTCTAATTTATTAAATCAGTCTAATGCAACTTATCCTATATCTACTGGTCTATTCTT
TTTTCATCCAGATGATGCATTTGAATTTAAAGACTTTATAATATCGGTCAATCCACTGGC
GGCCAAACATATGGAGATAAGCGTAGAGCCTGTTGGTCTACACTTTGCTTATAAAATGAA
TAGAAATATATCGTCAGATACTCAATTTTCGTTTATTCCAGATTTTAAAGAAGTAGGAGA
TTTATTATTCAAATATAGAAAAGGTAATCATTTAGTTTTTCATAAGAATCAGCATTATGG
CAAAGATTTTTTTCAGGGGCAACCAATATATATGATTCAGCCGATCACTTTTAAAGATCG
AGCCGGAAAGCTAAATATTATCAAATTTACGGGGCTAAATGACACAAGAGAAATTATTTT
TACTAATCTTGAAGCTGCTAATAGATCATGGGCACATTTTATAAAGAGGAATTCACAATT
AAAATCAATTAAAAATCCTACTCTATTAGTCTATAATTTAGAAAGTTTTTTAAAAGATCA
AGAACGATTGGATAATCAAGATTTAAATAAGTTTGTGGTGATCACTAATAAAGAATCTTA
TCTTGCCACAAAAGAGTTAATAGCTTTACCCGATTCTAATAGCTTTTCTAAGCACTTAAA
ATTAAATATAAAGCCTAAACTCTTTTTTGTGAAACTATGGGTAAGACGGTTATTTTCCAC
CTTAACTTATGAATAAATGCTATTGCCTTTATTTTCTCGAATAATATTCTATAACTAGTA
ATTCATTGAGTTGTAAAGCAACCCATTCCCTATCAATGACTCCATTGACTTTCCCTGATA
AATTGGATTTATTCAATTCTAAGTGGCTAGGAATATTAGCTAGTCCAGGGAAAGCTAAGT
AATTTTCTACTAGTTTTCGAGATGCTTCTTGATTTTTTACACTAATTGATTCTCCTGGTT
TACATTGATAACTACAGATAGATACTACTTGTCCATTAATACAAATATGACCATGGTTCA
CTAGTTGTCTAGCGGCAGGAATTGTAGGAGCCATACCAAGTCTAAAAACAGTATTGTCTA
GCCTCATCTCTAGTAGCTGTAACAAAATTTGACCTGTCGACCCTTGTAGTTTTTTAGCTG
CTTTTACGTACTTAAATAACTGTTTCTCACTTAATCCATAATTAAATCGCAACTTCTGTT
TTTCTTCTAATCTTACAGCATACTCAGAAGGTTTTCTGGACTTTTGTCCATGTTCTCCAG
GTGGATAAGATCTTTTAATCGCTTTTCTACTGAGTCCGGGTAAATCACCTAATCTACGGG
AAATGCGTACTCGTGGCCCTCTGTATCTAGACATATTATTTAAATTCTCCTAATGTAAAT
GATTAAGTAATGAGTAGCTATTTTTTAACACAAAAAGAGCATTAAAACAAAGCTTTTGCT
AAAAAATATTTACAAAAAATATAGATTTGGACAAAATACATTACTATGCTTTTTAAGCGG
GTAGCGGGAATCGAACCCGCATCATTAGCTTGGAAGGCTAAGGTTTTACCACTAAACTAT
ACCCGCAATATTCAACAGATTGTCACTAACTCAATATAACATAGTTATGTTAAGTTAAGA
TAATCTTAAATTTAATAGAATTTTAAATTATTTATGCTCCTTCTAAAACCACATCAAGAA
AACGAGCAATTTCGGCAGCTTGTTCTTCAACTTCTGATAATAATAATGGTTGACCGACCC

TAGTCAATGGAATTACTCTTTTATCTTTTGTACATAAATAAATTTCACGTCTTGGATTTA
GCCCCTCTTTAATATCTATTTTTATTGATTTGATTTCTTTTATATTAAACTGAAGACAGA
TTTTTCTATTCTTTCCAGGGAAACCTAGCCGAAAAATTTTTACGATACCTTTATCTTTGT
TAAATTCATTATATCCAGCCCCTATATTCCAAATAATAGTTAGCCACAGAAACAAACTCA
GAAAGACTCCTATACTGCCATAGAATGTCATTACAATACCTTGTGGAATAAATACCAAAT
CGGTTGAATTTGTAAAAGGTAACAAATCAACTTGAAAATAACTTGATAGTCCAGCAAGAA
GAAAGCCCAAAGCCCCAATAAATATTATAGTAGCCCACCAGTAATTACTCAGTCTTCGTG
AACCTAAAATTAAATCTTTTCTAACTGAGTGTATAGATAATGTTTTTTTCATAATTTGTT
CTTTTAAAACAGTATACTAATGCAAAATGGAAAAATTTTGACAATTTTTATGCGCGCTTT
AGATTTTTTAAATAAGCTTGATTGCTTTTAGTCCTAAAAACAGTCCTGTTGCTAACCCAA
AAAAAGCAGCAAGAAATATTATATATCCTAGAAAAACTGACATATTATTATTATTTATTA
TTATTGTTACAATAGTGTTTACAAAAATAGTGAAAATCTATTACTACGGTATTATATTAC
GTTAAATGCCTTGCTGTCTACAGGAAAAAACTTTTTTCAGTTTTTAGCATTGTGTTTATA
TTAGAAATAATATTCTAGTAAATTTATTAAACTTCTGGAGAACAGAATCTTATGTCAGAA
TTTATCAAGCCTTATAACGATGATCCTTTTGTAGGCAATTTGTCCACGCCAGTTAGTACG
TCAAGTTTTAGTAAAGGACTTCTAGGAAATCTACCAGCTTACCGTCGAGGTTTATCTCCG
CTTCTTAGAGGATTAGAAATAGGAATGGCACATGGATACTTTTTAATTGGACCTTTTGAT
AAATTGGGCCCTTTACGAGGTACAGATGTAGCGTTACTAGCGGGATTTCTATCTTCGGTC
GGCCTCATTATTATTCTCACTACATGTTTATCCATGTATGGTAATGTATCTTTTACTAGA
GCAGATTCGAAAGATCCACTACAAACTTCTGAAGGCTGGGGACAATTCACTGCAGGATTT
CTAGTTGGAGCAGTAGGTGGTTCAGGATTTGCTTATTTATTGCTAGCTAATATACCTTTA
TTACAGACTGCAGGTCTTAGTTTATTCTCTTAAGCTAGTAAGGGGACTTGAACCCGTAAC
CTACTGATTACAAATCAGTTGCTCTACCAATTGAGCTATACTAGCATTCAGTTCACAATA
ACACATAAAGATATTTGTTGCAATCTGCTATTTTCAGATGTAACAAATATCTTTTTTATT
TAAACAGAACTGTTATGTTAACACGTATATTAATTAGAATCTGAATTATCTGACTCTGCA
GAAGATTCCTGATCACAGTTTAGATAATAACAAATTGTTTGATCTAGACAAGTTGCAGAC
CAACCAACAGGTGTTTCTCCTGATAATTCTTGCATATCTACAGAATTATAAAAATTATCA
TTTGGCTTATTACTGTTTGCTTGCATAAATATCACTCCCAAAACACTCTGAACTTAATGT
GAAGTAATTTAACTTCTCAATAATATATTAACACAAAATATTAAATATGGTGATTATTCT
TTAATTGTAAAGATGCTGCTAACTTTATAAAGTTTGAGTGAGAGTCTTGATAGCTTTTGT
TGAAATAAGCATTTTAACCCATTTATTTTGCCCTCTCGACCATACTTTTTTGTGTTGTAA
ATTAACTTTTTGTAGTTTTTTTGTTCGTTTATGAGAATGAGATACGGCATATCCATTATT
AGCTACTTTTCCCGTAAGCTGACACTTTTTTGACATAATTTTCTAACTTATATATTTATT
TAGAGTACTTGCTAAAGTTGACTTGGGCACAGCTCCAATCACTGTGTCTACTCTTTCTCC
TGCTTTAAAGATCATTAAAGTAGGTATACTTCTAATGCCATATTCAGCTGCAATAGTAGG
ATTATCGTCTGTGTTTATTTTTACTACCTTAATAGATGATTCATATTCTTCCGCAATTTC

ATCAACTACAGGAGAAACCATTCTACAAGGACCACACCATGGCGCCCAAAAATCTACGAG
TACAGGTAAGTTATTGTTAATAACTTCTTGTTTGAAAGAGGCATCTGTAACTTGAGATAC
TGACATATTCTTTAACCTTTAATATATGTTCCTTGCTAAGCAAATCTTATCACAAATTTC
AAGAAAAATCTTCTATAGTGAATTACTATTAGTGTTGAATTATTTTTTTAAAAATTAGAC
ATTAAAAAAACTTATTACCTATATAAATAAAGTGGTGGCCGAGTGATATCTTGATAGTGA
TAATTTTTATATTAAGGTTGAACACTAGAAATAACTTTTCAAGCAAACTCTTGTTTAAAA
AATTTCTCTGTGACCATAAAAACTTAATTATATTACAATATATTTTATTATTTGTAACTT
AGTTCTGATAATGGTATAAACAACGCAAAAGATACTGCCTTATAATCAAGGAGGAATACA
TGTCTCAATCCGTAGAATCACGGACTAGGATTAAAAGCGAACGTTACGAATCTGGAGTAA
TCCCCTACGCTAAAATGGGCTACTGGGATGCTGACTATGTGATTAAAGAAACAGATATTC
TAGCTCTTTTCAGAATCACTCCTCAACCAGGTGTTGACCCGATTGAAGCATCTGCTGCAA
TTGCAGGTGAATCTTCAACAGCTACTTGGACAGTTGTATGGACTGATTTATTAACAGCTT
GTGACTTATACAGAGCAAAAGCATATCGAGTAGATCCAGTTCCAAACGTGGCAGATCAAT
ATTTTGCTTACATAGCTTATGATATTGATTTGTTTGAAGAAGGTTCCATTGCGAACTTAA
CTGCTTCAATTATTGGTAACGTTTTTGGGTTTAAAGCTGTTAAAGCTCTTCGCTTGGAAG
ATATGCGTATGCCAGTAGCTTATCTAAAAACGTTCCAAGGTCCTGCAACTGGATTGATTG
TAGAACGTGAGCGTATGGATAAGTTCGGTAGACCTTTCTTAGGTGCTACAGTTAAACCTA
AACTAGGTTTATCTGGCAAAAACTACGGAAGAGTTGTATACGAAGGCCTGAAAGGCGGTC
TTGATTTCCTTAAAGATGATGAGAATATTAACTCACAACCATTTATGCGTTGGAGAGAAA
GATTTTTATATTCTATGGAAGGTGTAAATAAAGCATCGGCTTCTGCTGGCGAAATTAAAG
GTCATTACCTTAACGTAACAGCCGCGACAATGGAAGATATGTATGAGAGAGCCGAATTCT
CTAAAGAGGTTGGTAGTATCATTTGTATGATTGACCTTGTGATTGGTTATACTGCGATTC
AAAGTATGGCAATTTGGGCTCGTAAACATGACATGATTTTACATTTACATAGAGCTGGTA
ACTCAACTTACTCTCGTCAAAAAAATCATGGTATGAACTTCCGAGTTATTTGCAAATGGA
TGCGTATGGCTGGTGTTGACCATATTCACGCAGGTACAGTTGTAGGTAAGCTTGAAGGAG
ATCCTTTAATGATTAAAGGCTTCTACAATACTCTACTTGAAAGCGACACAGATATCAACC
TACCTCAAGGTCTGTTCTTTGCTCAAAATTGGGCTTCCCTACGTAAAGTTGTACCAGTAG
CATCTGGTGGTATTCATGCTGGTCAAATGCACCAACTTCTTGATTACTTAGGTGATGATG
TAGTTCTTCAGTTTGGTGGTGGTACAATTGGACATCCTGATGGTATCCAAGCAGGTGCAA
CTGCTAACAGAGTAGCACTAGAGTCCATGGTTATGGCAAGAAATGAAGGCCGTAACTATG
TAGCAGAAGGTCCACAAATCTTGAGGGACGCTGCTAAAACTTGTGGGCCTCTACAAACAG
CTTTAGATTTATGGAAAGATATTAGTTTCAACTACACTTCCACAGATACAGCTGATTTCG
TTGAGACTCCAACAGCAAACATCTAGTTTAATGACTACTTACTGATACTTTAAATAGTCA
ATTGTAAGTGGAATTAACTTATAACAATAAGGAGCATAGAATAGTGAGACTAACACAAGG
GACTTTTTCCTTCCTTCCAGATTTAACTGATGAGCAAATTAATAAACAGCTTGCTTATAT
CGTTTCTAAAGGCTTATCAGCAAACGTTGAGTATACTGACGATCCTCATCCAAGAAACTC

CTATTGGGAACTGTGGGGTTTACCTTTATTTGATGTAAAAGATGCTTCTGCTGTTATGTA
CGAAATTAGCTCATGCAGAAAAGCAAAACCTAATTATTATGTTAAAGTTAACGCTTTTGA
TAATACTAGAGGTATTGAAAGTTGTGTAATGTCTTTCATTGTAAATAGACCTGCTAATGA
ACCAGGATTCTTATTACAACGCCAAGACTTCGAAGGTAGAACTATGAAGTATAGTCTTCA
TAGCTATGCTACTGAAAAGCCTGAAGGAGCTAGGTATTAATATTAATTAAGAATTAATAT
TGGCTAATTATTACCCTCTTAAAAATCATAATTAATTTAGTAATTGTGATTTTTAAGAGG
GATAGCTTCCCAAGTAAATTTTGACTAAATAGATTGAATAATTAAAAATACACAGAAATA
ATGCAATCACAGGATATAATTTCCAACGATACTCTTGTTAATTTACAAGAAGAATATGAT
AGAACACAAATCCAAGAAGTTTTAAATGAGTTAAATCAAGAACTTATAGGATTAGTGCCT
GTAAAGACCAGAATTCGCGAAATTGCTGCGCTATTATTGATTGACAGATTACGCAGAAAA
CTAGAACTAGTTTCTGGTAATCCAGGATTACACATGTCATTTACAGGTAGTCCAGGAACT
GGTAAAACTACAGTTGCTATGAAAATGGCTGATATTTTGCACAGACTTGGATATATAAAA
AAAGGGCATTTGTTGACAGTTACAAGAGATGATCTTGTAGGTCAATATATTGGACATACT
GCCCCTAAAACTAAGGAAGTTCTTAAACAAGCAATGGGAGGAGTTTTATTTATTGACGAA
GCTTACTATCTATATAAAGCAGATAATGAAAGAGACTATGGCTCTGAGGCAATTGAAATT
TTATTACAAGTAATGGAAAACCAAAGAAATGACTTAGTTGTTATCTTTGCTGGATATAAA
GATAGAATGGAAAAATTCTACGAATCCAACCCAGGACTCTCTTCTAGAGTAGCTAATCAT
GTAGACTTCCCAGATTATACTTCAGATGAATTATTACAAATAGCTAAAATGATGATAGAA
GAACAGCAGTACTGTTTTACAGAAGAAGCAGATAAAACTCTTTTAGAGTATACCGAGCGA
AGAATGAAACAGCCTTATTTTGCTAATGCAAGAAGTATTCGCAATGCTATTGACAGGGCT
AGAATGAGACAAGCCAATAGGATTTTTGCCAGTGGAGAAAAAGTATTAACAAAAGCTGAT
TTGGTAACGATTGAAGCAGAAGATATCTTGAAAAGTAGATTATTTTCATTACCTAATGCT
TAATATACACGTGATTTCATGAAACTATATATTATTAAAAAGTTTCATAATCAGTTGCAA
ATTTCTTGAAAAACCTTTATTATTATCTTAATGCAGGTGTGGCGGCATAGCCAAGTGGTA
AGGCAGAGGATTGCAAATCCTTCATCCCCCAGTTCAAATCTGGGTGCCGCCTAGTACTAA
AAAAGGGGGGTGTGGTGGAATGGTAGACACAACAGACTTAAAATCTGTTGATTTTTAGTA
ATCGTGAGGGTTCAAGTCCCTCCACCCCCATATATTGATTTATAAATAAAAAGAGATGTG
TATTTGTATTAATTGCAACCATATTACCAAATGTAATACCTATCACTTAATAGAATCTCA
ACATAAACAGCCTCATCTTACTAGAAGTCCATTGTTTATACCTAAATATCCTGTAGTTCA
TGTTAATATATCTAACAACTGTACCTATAATCAAATTGATTGGGATTTAGTGGAGTGTTT
ATCATTTGTAGAAAAACCGAATAGTTGGAATTTAGACGCTAATTAGTTACAAGAAAAATT
ATGTACCATAAGCAACAACTCTATTTTTTAGATACTCGGTATTAATTTTGGATGTTCTAA
CCAGTAAGATTTTACCCGTTCTTGCTATCTCTATAATGCCAAATTTAGTTAGTAATTGTT
CAATAGCAACAATCTTTCCCGGATCTCCAGTAACCTCTATAATTAAAAGATCTTCTGCAA
TGTCTACTATCTTAGCTCTAAAAATTCTTACAATTTCTAAAGCTTCTGTTCTAGTCTGAG
AATTGATCTGAATCTTAATTAACATTAGTTCTCTTTCAACTGAAGGAATATTTGTTACAT

CTTGAACGTTAAGGATATTTACTAACTTATATAATTGTTTCGTAAGTTGTTCAATAGTTC
TATTGTCTCCTTGAACTACCATTGTGATTCTAGAGACCCCAATTTGCTCTGCTGGTCCAA
CTGCTAAACTTGCGATATTAAACCCTCTCCGGGCAAATAGACCAGATATTCTTGACAGTA
CTCCGGCTTCATCTTGAACTAAAACTGATAAGGTGTGTTTCATGAAAAATTAAATTTAAT
CTATTTGATCTGTTAAGTATATTCTAAGAAAATTACAGAAAAAATACTAGTTAGTCTATA
ACTAAATTATCCAAACTAAACAAAATCCTACTATCAGATAATATATCTACCTTGTAATGC
TATACTATTAACTTAGTATTTAATGATATTCTAATTGCAAATAGAATGCTAATTTTTTAA
AATTTGTTAGGAACTTGTGCTAGAAAAATACAAAAACAGTAAAAAACTCTCTCGAGATTT
ACCTCAAATCAATGATCGCATTAGATTTCCAAAAGTCCGAGTAATTGATGACGAAGGTGA
ACAACTAGGTATTTTTGTGCCTGAAGAAGCTATACAATTAGCTGTCCAACAAGGTTTAGA
CTTAGTTGTTGTTAGTGATAAATCGGACCCGCCAGTATGCCGAATATTAGACTATGGTAA
ATATAAGTTTACACAAGAAAAAAGAGCTAGAGAAGCTAAGAAAAAGCAACATAACAGTAG
TATTAAAGAAGTAAAAATGCGATATAAGATAGAAGAGCATGATTATAAAGTTAGAATAAA
CCAGGCATCCAAATTTATTCAAGCAGGAGATAAAGTAAAAGCAACTATCACATTTCGGGG
GCGTGAAATCCAGCACTCTAATTTAGCTATAGATTTATTGAATAAAATGGCAAGCGATCT
AAATGCAATAGCTGAAATTCAGCAAGCTCCATCAAGAGATGGCAGAAATGTCATAATGCT
CTTATCTCCCAAAAAAGTTAGCTAAACTAATTTTTATTTAAATGCATCTGGCTGGATTCG
AACCAGCGACGTCTCTTTCGAAATGGCGGATTATGAGTCCGCTGCCTTCGGCCCCTCGGC
CACAGATGCATCACTTAAAGTTTATACCTCATAAAGCATATAAAAATCAATGTTTAGCTA
TTTTTAATAAAGTCTATAAGAGTCTATTATTCATTCATATTGTGATATGTCGCAACTGCT
GAAGGGGAAACTTGATTTAAGTATCGAAATATCCAGTATTTAAAAATTGTGTCGAGTATA
ACTGGAAAAGTTGAAATAAATAAAAAAATAAAATCTCGACTTTCCGGAAGTCCTAAATGT
CTTAAAATTACTTCAATAATAACTTCCCATCCATGAGGAGAATGAAAACCCACAAACATA
TCAGTAAAAAGAATGATTAAAAAAGCTTTAGCTGTATCACTAAGACCATAAATAATTTCA
TTTAAAAAAGATTTAACGACTGCAATTTGTCTTTGTCCAGTTATCATTAGTAATATAAAG
ACAAGTATTGATACAAGATCGGATAAAATATTTTTAACAGCATTTGCACTTTCATTTGCA
TAATATTCTCCCAACTCTCTAGCCTTCAGCTGCACTCTTTTTTCAATGATTTCGTAAGAT
ATATCTTCCGAAGGATTCAGAAGAACTTCAAAGTGAATTTTCTCTTCAAATCTTTGTAAT
TCAGCAAAAGCTCTTTCCTCTTGAGAGGAATTTAAAAAAATTTTAGGCTGTTCTTGGTTC
CATAAATAATCAATACAAGGTCCAAATACAAAAAATTTTGAAGCCTGATTAACTAGTACT
GGAGAGATGAGTAATAATAAAATATATTTTACGGAAGTAATAGTTTGGTGTCTAGATATT
CTAAACTCCTCTATAGCTTCAGATTCGCCATTAGGATCTAACTCTTTACGAAACTTTTCA
AAAGTATTAGTAATTGACCTAGGTATTGGCCCAACTTTTTCAAAGGCGAATTGATTATTT
CGTTTTAAATTCCAATATTTCATTTTTTATTGCTGCTAAAATCTCTATTAATCAATAATT
GATCACGTATTCACTATAAATATTAATTCTAGAACTAAACATATAAAACAATATTTATGA
AAAATAAAATACAATTAAAAATTAATTATGCATTAGTTCTATCTGCAGCTACAGCAGTTT

ATGGATATAAATCTTATCAGCCAACTATAAATACACTCTTAATCAAAGATGAAGAAAACT
GGCCTTTTTTCTCTAAAAGGCAGACTGCCTTTATTCCTATAATTGTATTAAAAAAAAATC
ATCAAAATCCCTGAACTCAGAACCTAGAAATAGTAAGTTATATCTTAATGGAATTGGAAG
TAGAAAATTTATCGACAGAATTGTCACTCTACTTGGATATTTAGAAAAACAGGCAAACTA
TACTGAATACTTGAACCAAATAAAAACAAGTGGTTATTTTTCTTTAGTCTACCTAGACAG
TGATATAATGTATAAGAAAAATATTAGTAACATTTTTTTACTACCTAATGATATTCTAAA
AAGAATCTATATTTGCAATAAGAAAAAAAAGCTTATACCGCATTCATTCTTAATAAATTT
ATTTCAGAAACAGATAGGCTATCCAAAAAGTTTTGCTAATTTAAATTGGGCCTTCTCCAA
AATTATAAAATGGTATTATGATAGAGGATACCAATGGTCTCTAGTTGAAGTTAAACAAGC
GTCTGATGCATCTTCTATTGTAATTGATATTCATGAAGGGGTAGTGAAGACAATTATAAC
AGAATACTACACTCTATCTTATAAAAGAGTCTCAGGTATCTTATGCGTAGAGTCAATAGA
GCAGTACCTTGGAGTAAGAGTGGGAGCTCCACTAAATATAATTGATTTACAAAAAAAAAT
TACTTACTTAAAAGATAATCAACTAGTTGGCGACATTATTTATAGTATTGAACGATCAAA
TAATAGCTCAATGAGTTTGGATATCAAATTTCAAATACAAGAATTAAAAGATAAAGAGAT
AATAGTGCTTGCAAAAAGTTCTTCTATTATCTCGCATGCATGTAATTTACTTAATCAATA
TAGAAATAGGCTAGTAGCTTCTAATATAGTTTCACTATCAACTAATAAATTACACGCATG
CTATTTTAATTATAAACTTGACTATCAATATAAGTATATAAACACGTCTAATCTTATAAA
ATTGTTAGCTTATTCTATTTCTTGTAGGAAGACACTACTTATTCATTATTTTAATTTACA
AACTTTAATAAGCTATACAAAAAAAAATACTATTGGATTTCAGCTATATTTACGAAATCT
AAGTTTTGGGAAAGCTTTCTGTGTGCTTAGTATGAAATTTATCAAAAATGGACTTAATAT
AAAAATTTTATATATAAATCCTTCATTGATAGTTGATCAGAATTTTGTATTTCAATTTGC
TATACAAATCATTAAGCAATATCATACTGCTAAACCTCCAGCTTTATTTTTAACGAATCT
AGATCTTGAACAATATGTTGCTGAAAGTTTATTAATGTACCATTTTACATCTTGCTTCTC
AATATCTGAAAAAATATTATTATCTCGAATTATGCATACAGATTCTTTATTTTTCAACTC
TGAAAATTTTCATTTTGATGATAGAACTAACGCGATCAACAGTTATGATACTTTTAAACA
AAATACAAAAATATTTTATCAAGAGTTTCTTTCTTTATTATTAAGTTTACGTTATCAAAA
TTTTAATTATTTAGGTTGGCCTTTAAAAGGCCATTTTTTTGAAATAAAATCTTTATACTT
GGCTCCATTTCAAAAGAGTGATTTTTCTGATAGTCGTAAGACCCTATTCTTCCACAAAAT
GTCGTTAAAGCAAGTATCTAATTTTAATTTACCAATATCTTTTAAAAGCCACCTTAACCA
TATTTTAGTTAGTACTATTAAATGTCAGTCAAATTTGAATATGAGAACTGTATCTTTGCT
ATTAATTGATTCACCTGCTGAATATATGCTATACAAATCTATTCTTAATTTCTCTATTAA
AGTTAGGATGCAGTATTTCATACCTATGAGCAACAATATAAGATTATCTCTATTTTATAA
TTATTTAGATTGTTTTTTAATAAGATCATCTCAGAGTCGCATTCATATATGGCAAGATCT
AAGAACTCTTACACCAATTCAAAATTTTTGGTTAAAAAAATTTTCATATGGAGCTGGAAT
TCAGCTTAAATTACCAATTAAGCAGATGCCACCTCTTTCAATTGAATATACTGTAACTAG
TAGTCGCTACTTTTGTATTTACCTTCGTACTTATTATCAACGATAACTATTACTAAAATG

AAAAATTTAATTTCTTTTTTTGATCGCAGCAAAGGGAAATGGATTTCACAAAGAACAACT
TATGAATTATCTAATAAAAACATGAGTTCGATACAGTCTCAGATGACAATGAAAATAGGC
AACTCATTGTCAGGATCTATAATACTTGCATCGTTAAATTGGGGGGACATTTATAGACAA
GTTGCCCATTACGCTAAAAATCACAGCCGGAGTGAATATGATAATAAGTTTAACTTGCAA
TTCAGTAATCAACTAAATAATCATAAATTATTAACGCTGTGCATAGTGACAGATCCTAGT
CTAATTAGTTTTAAAACTCGCTATGGAAGTACTACCATAGATGAAACATATTGGTTCGCA
ACAAATAATTTACGTCTAAGTACTAGTATTGTTAAACGATTCAACACTTGTGTGGCAGTA
TCTTTTTGTTCAGAAATTAAAGTTTAAACTCTATATAGAAGCAACTAATAGCCTAAAGGC
TATTAGTTGCTTCTATATATCTAAATTTTGTATTAAAAATTATAATACTATATTATTCTA
AGTCTTTACGATTGGGATTCCTGGAAGGATCATTAGAGAGGAACCCAAAGACAAACAAAG
AAATAAAGAAAATAACGGTAGTATATACAAAGATTTTCAAAGTAAACATATTTTCATCCT
TAAAAGCTATTAGTGCGATATTTACATATCTCCACATAATTTTATATCATGATATAAGTT
CCAGCACTATTTTATAAATAAATCAATATCTTTATTTTTTAGTAATGATTTATATTTAAA
TATGCGTGATGTACTAAATACTAAATCTTTTCCGACTTGTTTTGTCTTGTTTGTAAATAT
TTTGTTATTTCTATGGAGTTTACCTTCAATTATCACATAGTCTAACTTCTTTAGCTGTTT
AAAAGTATATGAAGATTTTTTGTTCCAAATAGTTAGATTGATTATAACTAATTGTTTTCT
TTTTAACAGTCTTGCTTTTAACTTAATTATTTGGCTTTTATTTTCACTAATTCTGATACT
CTTACAACTAAGAATTTGGACTAGAAGAGTACAGCTATTCATAATTTATAACAATTTTTT
AAAAAATCTACTAAAATAGATGATTTTACAGAACTAAAATGATATTTCATTATCTTGTTG
ACCTTGCTCATAAGTAATATCTTTCAATACTTTTAATATTTTTCCAAAATACTCTTGCAG
GTATTTCTCTAAAGATATTACTTCCAACCTGTCAATTCCAAGAATACTGTAAACTTCATC
CATCGGAGCTGTAAATTGCTCTCCGCTACTTAAAATTTCGGCAAATGCTAAACGATCAGA
AATATTCCATGTCCATTGAAGAGTTTTAGTAATTCTTCTTAAGGCTTTTAATAATCCAAT
CGGAATTTGTGAGATCTGTGTTTTTTGCCCAGATAATTTTTCACATAATTTAATAATTTC
AGCTGATGTCCAAGCGGTATTACCAACTAACGGCAGGGTCCTATTTTCTGTGGAAGGTAC
TCCAAGACTTTTTATAACTAACTTTGCTGCATCTTGAGTATCAATGTATGCAATGGGTGT
AGATTCTCCTGTAACCCAGACTGATTTTTTGTCTAAAATAGGAATGGCATATTGGTTGAT
TAAACCTTGAAAAAATCCTCCTAAAGAAAACACAGTATATTTCACATTGGACTTCTGAAG
GAAATCTACTACCTGAGACTTTAAGTTCATCAATGGAACATCTGGGTATTTCTCTGAATT
TAATATTGAAAAGAAAATAAATCTTTCAACTTTAGCCGCTTTAGCTGCTTCAATTAATGC
AATTTTCCCATCTAAATCTATTTTTTCTGCATTGTAAGGATCAGTAGGACGAGATGTAGA
AGCATCTATGATTGCTGTTACCCCACAAAAAGATTGCAAAATACTTTCGGGTAATTTTAA
ATCACCATATATAAGTTCTGCTCCCCACTCTTTGAGAAAGGCAGACTTTCTTAAATTTCT
TACCATGCATTTTACATTGTAGCCTTCATCTAAAGCTCGTCTTACAATTTGACGTCCTAA
AGTTCCGGTTGCTCCAATTACTAAAAGAGTCATATTAGTTAAAAATAAGTATGTATAAAG
ACATTGATTTGGTTGACGGCAGAGAGTAAATTAGGTAGAGAGGGAATCGAACCCTCATGA

CCGAAGTCGTCACATTTTGAGTGTGATGCGTATACCAATTTCGCCATCTACCCTATTATA
GACCAACTACTACAAGTAATTTAGTCTTTAGCGTAAAAATAATACTAAGTTTCAATGGAT
ATGTCAACTTTTAAATTTATATTAATAAAAAAACGGAGTCTCTTATGGCCACTTTTGAAT
TAATTCCTTATAGATTGTATTTGAGTCAATCTAACACATGGATCCATAGAATAAAAGCAG
AAATTAAGATATATATATAGTAACGCTTTTATGGATTTCAATTTTTATTTTTTCTTACTTTA
AACTATGTATTATTGCTTTAAGCTTAATTGCCATAAGTTTTACTATAAGAAGTAAACAGA
ATATTATCCAAAAACATTTGTTACAAACGTTATTAATAACGTTCTTAACTACTGTTTTGT
CTTTTAGTGTGGCTATTAGTTATAAACAATATGCAGAACAAGAACAATCGCAGTATTTAT
CTGATTCCAAGAAATATAAAAATTCCAGCAGCTACTATTATATACAAATAGCTAACGATC
AAAGGATCAAAAGACAATACTTAATTACTACTTTAAAGCCTTCCTTGTACTTTTTTATTA
CTATATATTCTATTAAACTAGTTATGATAACAACTTCTCCAGAAGTTTTAGTAATTACTA
TTTATAGATCTAGGATAATAAATAAAATATTTAAAAATGAATTGCTATTTATCTTCCTTC
TTTCGTCACATATTGTTACTAGTATTATTAATAGAATCGATAAAGTGATTCAAGTAACTA
GCTTAAGAGGAAGCTTGAATTTATATAATTCATTGACAAGGCCTTTAATGTTTTCTTTGT
TAATATTTCAAGTCTTCTTTTTGGAGATTATTCGGGAGTCAAAAGAAATAGCTCAAGCTC
TTTATACTAGAAATCTCAATCAAGAAAACAATAATTTTTTGAAAATATATACAGTAAAAT
CTAACTTTAGTGATCGGCTCAATATAATTATTAGCACTTTGTACTTTATTATTTTAGCTC
TAGCGTAATAGAATCCACTTATTCTTCTGACTCTAGTAATACGAAGAGTAATAAAATTAG
TTAGTAGTTTAAATAGATAGAATTAAGATATTATATAAATAATTAAATGTTTCATTATGG
TTAATACTCAAAATCAAATTTCACAAACTTCAGAACTTGATTATATAGTTAATCAACCTT
ATAAATATGGCTTTAAAACTTCTGTTGAATCTGAGCAATTTCCTAGGGGAATAAGTGAAG
ATATTGTCCGCTTGATCTCCAAGAAAAAAGATGAGCCTGAATATCTATTGAATTTTAGGC
TTAAAGCATATAAAAAATGGAAAAAGATGAGTAGTCCGTCATGGGCTCATATTAAGCATC
CGAATATAGACTTTAATACGATTATTTATTACGCTGTTCCTAAATTAAAAAAAGAATTGA
AAAGTCTAGATGAGGTTGATCCAGAAATTCTTGACACTTTTAACAAGTTAGGTATATCTT
TAAATGAGCAAAAGCGAATTTCCAATGTAGCCGTTGATGCTGTTTTTGATAGTGTATCTA
TTGCAACTACTTTTAAGAAAGAACTGTCTGAAGCTGGTGTTATATTCTGTTCAATTTCTG
AAGCTATAAGGGATTATCCAGAATTAATAAAAAAATACTTAGGTACTGTTGTACCAGCTG
GTGATAATTATTTTGCTGCATTGAACTCTGCGGTATTTAGTGATGGCTCTTTTTGCTATA
TCCCTCCTAATACAGTTTGCCCTTTAGAATTATCAACTTACTTTCGTATTAACAACGAAG
AATCTGGGCAATTTGAAAGGACACTAATTATTGCTGATCGTGGCAGTAAAGTAAGTTATC
TTGAAGGTTGTACTGCTCCTCAATTTGACACAAATCAATTACATGCAGCGATTGTAGAAT
TAGTAGCTCTTGAAGGAGCCGAAATTAAATATTCTACAGTACAGAATTGGTATGCTGGTA
ACAAAGAAGGTAAAGGTGGTATATACAATTTTGTTACTAAACGAGGCTTATGCTCGGGTA
ACAATTCAAAAATTTCATGGACTCAAGTAGAAACTGGATCTGCAATTACCTGGAAATATC
CAAGCTGTATTTTAGCTGGTGAAAATTCTCAGGGAGAATTTTACTCTGTAGCTTTAACAA

ATAACTATCAAGAGGCTGATACAGGGACTAAAATGATCCATATTGGTAACAATACAAAAA
GTAGAATTATTTCTAAAGGTATTTCTGCGGGTAGATCGAAAAACAGTTATCGAGGCCTAG
TAAAAGTTGGCCCTCAGTCATTTAATTCTCGTAATTATTCTCAATGCGATTCTTTATTAA
TTGGTCAATCATCTCAAGCTAATACATTTCCCTATATTCAAGTACAGAATCCAACATCAA
AAGTAGAACATGAAGCATCCACTTCAAAAATTAGCGAGGACCAAATTTTTTATTTTTTAC
AAAGAGGGATCAATTTAGAAGAATCTATTGCTCTTATGATCAGTGGTTTTTGTAAAGATG
TCTTTAATGAATTGCCTATGGAATTTGCTACTGAAGCTGATCGTTTACTGAGTTTAAAAT
TAGAGGGAACTGTAGGATGAGCCAGACTATTTTAGAAATCAAAGATTTATATGCTTCTGT
TGGTGAAACAACAATTTTAAAAGGGGTAAACTTATCTATTCGAGCCGGCGAAATACATGC
GATTATGGGGCCTAACGGTTCAGGTAAAAGTACATTATCAAAAGTAATTGCAGGACATCC
AGCGTATTCACTAATAAGCGGAGACATTTTATTTTTTGGACAAAGTATTCTTGAAATGGA
GCCAGACGAGAGGGCAAAAGCAGGTATTTTTTTAGCTTTTCAGTATCCTGTTGAAATTCC
TGGAGTCAGTAATTCTGATTTCTTAAGAATTGCATTAAATGCTCGCAGAAAGTTTCAAGG
ATTATCGGAGTTTAGCCCCTTAGAATTTTTTCAACTAATAACAGAAAAAATAGATCTTGT
TGGCATGCAAGAAAGTTTTTTGACAAGAAATGTTAATGAAGGATTTTCTGGTGGAGAAAA
AAAACGTAACGAAATTCTTCAAATGGCTTTACTAGATAGCAAAATATCTATTTTAGATGA
AACAGATTCTGGACTAGATATTGATGCATTACGAGTAGTTGCAAAAGGAATTAACACTTT
AGCTAAATCAACAAATTCAATTATTTTGATTACTCACTATCAAAGATTATTAGATTATAT
TATTCCAGATTTTGTTCATATTATGAGTAACGGACAAATTGTAAAAACTGGTAGTGTTAC
TCTTGCCCAAGATCTAGAAAAGCACGGATATGACTGGATTACGCAGACATAACTATTTAC
TACAATATTGAGACTAAAGAATATTCTTTAGTCTCAATATTTTTGCAAATGCAAAGTTAA
TTATTTAAGCTTTATCAAAACCTTGCTTAACATCTTCAATTGCTCTTTTTAGCAATTCTT
CACTAACTGGTTCTAATTTTTTAGTACTACGAACATTTTCACCAAATTCTGGTTTAGAAT
TCTTTAAATCTTCTCGAAGTTCTCTAATAAATTCAGCTACTTTTGAAACTTCAATATCAT
CTAGATAGCCATTAATGCCAGTGTAAATAATAGCAGTCTGTTCTTCAACTGGAATAGGAG
AATTTTGAGCTTGTTTCAAAATTTCTCGAAGACGTTGACCTCTTGCTAGTTGATTTTGGG
TTGCTTTATCTAGATCAGATGCAAACTGAGAAAAAGCTTCTAATTCAGCAAATTGTGCTA
ACTCTAGTTTTAACTTGCCTGCCACTTGTTTCATGGCTTTTATTTGCGCAGCAGAGCCTA
CTCTAGAGACTGAGATCCCAACATTAATAGCTGGTCTAATCCCTGAGTTAAATAAGTCAC
CAGATAGAAATATTTGTCCATCTGTAATTGAAATTACATTTGTTGGTATATATGCAGATA
CGTCCCCAGCTTGAGTTTCAATAATAGGGAGAGCTGTCATACTACCTCCACCTAATTCAG
CATTCAATTTAGCTGCTCTCTCTAGCAATCTAGAGTGTAAATAGAATACATCCCCAGGAT
AGGCTTCTCGTCCCGGAGGTCTTCTAAGCAAAAGAGACATCTGACGATAAGCTTGAGCTT
GCTTAGTTAAATCATCATAAATAACTAATGTTGCTTTACCTTTGTACATAAAATATTCAG
CTAAAGCCGCACCTGTATAAGGAGCAATATATTGTAAAGTCGCAGGACTATCTGCATTAG
CTGCAACTATAATAGTATAATCAAGAGCTCCTTTATCCTGTAGTGAAGATACTACTTGGG

CTACTGAAGATGCCTTTTGTCCTATCGCAACATATACACAAACAACATCTTGGCCTTTTT
GATTAATAATTGTGTCCAATGCAACAGCTGTTTTACCTGTTTGACGGTCACCAATAATTA
ATTCTCGCTGACCTCTACCAATTGGGATCATAGAGTCAATAGCAGTTATCCCTGTTTGCA
TAGGTTCACAAACTGATTGCCTTCCAATTATACCAGGGGCCATTGACTCAATTAGCCTTG
TTCCATTACTTGCAGGTTCACCTTTATCATCTATTGGCCGAGCTAATGGATCAACAACTC
TACCTAAAAAAGCATCTCCGACCGGAATTTGAGCAATTCGACCAGTCCCTTTTACGGAAC
TGCCCTCTAAAATATCCCTACCATCTCCCATGAGTACTACACCAACGTTATCACTTTCTA
AATTTAAAGCAACCCCAATAGTTTTATCTTCAAATTCAAGTAGTTCACCAGCCATGACTT
CATCAAGTCCATATACTCGAGCGATACCATCACCAACTTGTAAAACTGTTCCTATATTGG
CTACTTCTACATCCTGATCATACTTTTCAATTTGTTGACGAATAATACTACTTATTTCAT
CAGGTCTAATATTTACCATATTTTTAATATTATTTTTATTAATTGGAAATTCTAAAGCGG
TAATTAGTCAGACTAATGCTATCTGTCTTTTTTACTTTATTTAAGTTACTACAACATCA
AGGTGTGAAGCCATTTGTCTTAGCTGTCCTCTAATACTAGTATCAATGACTTTTGATCCA
ATCTGAATAGTAAAGCCACCAATTAGCTCGGGCTCGACTGAAATAATTAGTTTAACTTCT
TTAGCCTTAGTCATAACCTTGATTTTTTCTGTTAATAGAGTTTCCTGATCAGGGCTTAAA
GCAATAGAAGTATTAATATTTGCAATTGTTAAAGATTCCATTTGGTAAGCCAGTTCCAAA
TACTTACTAGCTATAGCATCTAACATTCCAATTCGCTTGCGATCAACTAAAATCATTAGA
AATGATAATGTATTTTCGTTAATTTGATCGCCAACAGTTGCAATAATTACTTCTTTTTTT
GCCTCTACAGTTTTTAATGGATTAGCTAAAAAAATTTTTAGCTTGTCAGATTGCGCTAAA
ATATTTTGTATTGACTGAATATCTTGGCTCACCTTTTCAGTAACTTTTTTAGTTTTAGCT
AAATCAAGAAGAGCTACTGCATAAGGCTGAGCTATTTTTATAACAACATTGTTACTGCTC
ATAGTTGATCTCCTAACTTAGCAATATTATTATCAATTATACGCAGTTGCATTTCGGAGC
TCATTTGATTTTCCAGCTGCAAAGTAACTCTTTTAAGAGCAAGAAATGTTATTTGTTGCT
GGATTTGCCTTCGGATCTGTTTTTCAGCTGTTTCTATATTTGATTTACCTGTTATGGCAA
GTCTTTCAATATCTAATTGGCCTTGAGCTAATATGGAACTTCTAACTTTTCCAGCTGTTA
GCTGAGCTTCTTTCTTAATTTGATCTATAATGATTTGAGTTTGAGCAAGTTGTTTTTCTG
ATTCTGACAATCTAGCACTTGCTTGCTCTAGTCTTTCTTCGGATTCTTGTATAGCTGCTA
AAACTTTTATCTGTCTTGCATTTAAACTTGATCCGAGAAATTGCTTCAAAACATAAATAA
GACCAAATAAAAGCAACAATATATTAATAACATTTGCTTCAAAAATATCAGAATTAAAGC
CGAATTTGTGTTCACTACTATGTTCTGATAAAATAGTAATTATTTGTGGTATTTTTACTA
TATTATTCATTTAAAATTCTTACTATTTTAATATTTTACTCTGGAATCATGATTTTCAAG
ATTGACTACTTAATAATTTAACTTTAATTTGATCGCTCAAAGTGTCAACCTGATTTTCTA
AAGTTTTAAGAGCTTCTTCTTTTTGAACATTTAACTGTTTCGATGCTTCAGCAATCAGTT
TCTCTGCATTCAATTGAGCTTTTTTTATATCTTCTGAGACTATATTTTGAGCTTCTTTTT
GAGAAGAGGCTATTTTTAATTGTGCATTGCGGCGAGCTTCAGATAAATCTTCTTCATATT
TTGCAGCTAGCTCATCTGCTTTAACAAGCATAGACGATGCAGTAGTCAATGTTGTTCGAA

TATATTCATCTCGTTCGTCTAGTACTTTAGTTACAGGTTTATAAAAAATAGTATTTAATA
AAACCATTAAAGTAAGAAACTGTAATGCCATTAAAGGCAAAGTACCATTGAAGTCAAATA
AGCCACCTTCAATTTCTTCGGCTAATAGAAATGGTAAATCAATCATTTTACTAATTATGT
TTATAGTTAATAATTTTGTTTCAATTAAGAAAAATAACTTTATAGTGCGCTTAGTCTCAT
CAGACTAAGCGCAAATGATTATTTTAACCAACGTATGGATTAGCGAACAATAAAGACAAC
GCAACAACTAGGCCGTAAATTGTTAAAGATTCCATAAATGCTAAACTTAGAAGAAGTGTA
CCCCGAATCTTTCCTTCTACTTCTGGCTGTCTAGCAATACCTTCGACAGCATTAGCTGCT
GCACTACCTTGACCAATGCCTGGGCCTATTGCAGCAAGACCTACAGCAAGACCGGCAGCG
ATAACGGATGCGGCTGAAACGATTGAATCCATAATGAATTTTAATTTTTTGTTATATGAG
TAGAAAATTGACAAATTTCGTATAATACTTAAAATCTATATAATTAAGTATAATAGATTC
TAATATTGTAATTATAGTATATTACTCTTCTCCATGACCTTCCATTGCTTCTCCAATATA
AGCTGCGGACAAAGTAGAAAAGATTAATGCCTGTATTGAACTCGCAAATAATCCGAGTAT
CATAACTGGTAATGGTATTAAAATTGGAATTAGCAAAGTAAATACAGAAACAACTAATTC
ATCTGCTAAGACGTTTCCAAATAATCGAAAACTTAAAGATAGTGGTTTCGTAAAATCTTC
TAGAATATTGATTGGGAGTAATACTGGAGTTGGCTGAATATATCTAGCAAAGTAGCCTAA
CCCTTTTTTACTTAAACCTGCATAAAAATAAGCTAAAGATGTAAGTAAAGACAGGGCCAC
AGTTGTATTAATGTCATTTGTTGGGGCAGCTAGCTCACCTTCAGGCAAGTGAATTAACTT
CCAAGGAATTAATGCACCTGCCCAGTTACAACCTAGAATAAATAGAAATAAAGTTGCAAT
GTAAGGCACCCAAGGGCGATATTCATGCTCACCAATCTGATTTTTTGCAATATCTTGCAG
AAATTCTAATATAAATTCCATAAAATTCTGGAATTTTTCAGGAATTCTTTGTAGATTTCT
AGTACCTAGAAATGATAAAGTTAAAAGTGTAGCAATTACTAACCAAGAGACAATGAAAAC
TTGCCCATGCAATTGTAAGCTACCTATTTTCCAATATAGATGTTTTCCAACTTCTACTGC
TGATAAACTATTATATGGGTTAAAATCTATGAGACTGTTTTGATACATAATAAATATTTT
TTTAAATAGTGCAAGAATCAAAAAATAAGTTTTTGAAGAATTAATATGCAATTAATTTAT
TCTTAAAATTAGACTATTAATAAATTAAAAGCAAAAATAAATTCTAATAGAGATAGCTAT
TTTTAGTAGTGATTATTTTTTGTAAAAAACAATTATTGCTACCTATAATGCTTTTACGTT
TAATTATATACCCATATATTAATTATTTAATATGATTATAGGTATTTTGCTACAAACTTT
ATTGTTTTATCCATTCTAAAGATTGTTATAGTTTATTTTTTATTTAAAATATCTGCGACT
TCATCTGCAAAATTAACTTTCAAATTTTCTTCTCCCCCTCCCAGAAGAAATCTTACAAAT
CTTCTAATTTTAATATTTTCACCTAGAACAGCAATATTTTGGTTTATTAAATCTTCAATA
CTAATATCTTGATTGCGGATAAACATTTGATCTAATAATGAGAGCTCCTTAAGTCTCTTT
TTCATTCGTCCTTCAATGATTGTTTCGATTCTATCAACTGGTTTGTTTTTCAAGTCATCT
TTACCAGCTTCAACTCTTTGTTCCAGATTAATAATTTCACTAGGTATATCCTGTGTTGAT
ACGTATTCCACATTAGGCGATGCCGCTATTTGCATAGCAATATCTTTAGCTAACTTTTGG
AACTCTGGACGTCGTGCTACAAAATCTGTTTCACAGTTGACTTCAACTAATACACCTATT
CTTCCGCCAGTATGAATATAACTCTCTAGTAAACCTTCAATAGCGGTACGATTAGATTTC

TTGTTAGCTGAAGCCAGCCCTTTTTGTCTTAATGATTCTAAAGCTTTTTCTTCATTGCCA
TTATTAGCTTGTAAAGCTTTCTTGCAGTCCATCATACCTGCTCCAGTCTTATCACGTAAG
GCTTTTACGACTTGAGCAGAAATTTGTAGTGTCATATGCTTTCAAAAATTTTTAAGTATT
TAAATTAATTATTATAATTCACTTTGAGTAGGATGAGTTATTTCTTCTAAATCTGTTTGC
CCGTATTTACCATCATAAATTGCATCTGCTATTTTACCTACAATTAGTTTAATTGACCTA
ATGGCATCATCATTAGCTGGGATTGGAATATTAATAATTTCTGGACTACAATTAGTATCA
AGTATACAAATAGTTGGAATACCTAATTTTAAACACTCTTGTATAGCTGTTGTTTCTCTC
TTTTGATCTACAACTACAACAATATCAGGTAAACGAGTCATATTTTTAATACCATTTAGA
TGTTTACGAAGTTTATCTAATTCTCTTCGTAAAACAGCAGCTTCTTTTTTGGGTAACTGA
TCAATCATACCACTTTTATCTTGTTCTTCTAGCTGTCTCAGACGATTAACTCGAGACTTA
ATCGTTACCCAATTAGTTAACATTCCTCCAAGCCATCTCTGGTTAACATAATAAGAATCA
CAGCGTTGTGCTTCCTGTGCCACTATACCAGCGGCTTGTCTTTTTGTTCCTAGAAATAAA
ACTTTTTTACCATCCGACGAAGCCTGTTTTATAAATTCACAAGCTTCTGTTAATAATTGT
GCAGTTTGGACTAGATCAATAATATGTATGCCATTTCTTTCTGTATAAATATATGGAAAC
ATTTTAGGATTCCATCTACGTGCTTGATGTCCAAAATGAACACCTGCTTCTAGTAATTCT
GCTAATGTAACAATAGCCATAAGATTATTAATATTTAAATAATGTAATTATTCGGGAAAT
CCCTTTGTTTAGAAACTGATTCAACTAACTTATTTGAATTTTAGTAAACTAATAATAAAT
TAACACACTTTTAAAGCAGATCAACAAAATAGATCCTGAAGAATTCATTCTACACTTTTA
TTGCTAAAATAATTACGCGCGGTTCTGTCGTCTAAAATGATATCATCTAAATCATCTCGT
ACAGATGAAATAGTACTTTCGTCATTAGTATTTGCAGTTAAATTCTTTTTCTCTAACGAT
GAGCCATTACAGTTATCATACATATTGAAGCCGGTACCAGCCGGTATAAGCCGACCTATA
ATAACATTTTCTTTCAGACCTCTTAACCAATCTAATTTACCAGATATAGCAGCTTCAGTT
AATACTTTTGTAGTTTCTTGAAAACTTGCTGCAGAAATAAAGCTTTCTGTATTTAAGGAA
GCTTGAGTAATACCTAATAGCACAGGCCTATATGATGCATTAATCTTATTTCTCAAGGTT
ATGGCTTTATTAGTCTGTTCAATCTTCTGTAACTCTACAAGCTCTCCAGGTAAATAGCCT
GTTTCTTCACCATTTTCTATTTTCACTTTAGAAGTCATCTGCCGTACTATTACTTCTACA
TGCTTATCAGAAATATTCACTCCTTGAGATTGATATACCAATTGTACTTCTTTAACAAGT
AGTAACTGAATCTCTTGAAAGCTTAATCGAGCAGCTTCATATAAGGCTAATCCACATTCA
ATATACAAATCAAAACTTGCATCGAGGATATCATGAGGATTTAACAATACGTCTGTTTTC
TTTCTTGCTTCAAGAATTTCTTCAATTCTTGGAAGCCCTTGTATAATGTCACCTGTTTTT
GCTCTATCAAAAACCAATATAGCTAATGTTTCTCCCCTTCTAATTAATGCATTGTTATCA
ACATGTAAAATAGCACCATTAGAAACCAAGTAAGGTCTAGCAATTCTTAATGTAACTGAT
CTAGAAGAAATTTCAATAATTTGTCCAGAGTCTAAAGAAGTCATATTTTCAGTAATAAAA
TCACCACAACGAATCCAATCACCCACACTTACTTTAACTATTGATTTTCCTATATTAAAG
GTCTTTTTATCAGAAGATGTCGCAATCAAAATGCGCCTGCTAGTATTCTTTTCAGAATAA
ATCTCCTCAACAGTACCTTCACTCATTGCAATAATCTCAGTACTAGCAACAACTGTAAAT

GGCTTGATATATTCACCATTTTTTACAATAATTCTAGTTTGACTTTGTTGCTTCTCTGAT
TTATTTACATCAATACTTTTAATTGATAATGTTTCAAAAGTTGACAGCCTTAAGTCAAAA
TAATTATTGCCACTTTCTTTAGCAGAAAACTCAATTGAGGATATTAAATTATCATCTCGA
TGCTCAATTTCAGCCACAAGATTAGTTGTTACTAAATGAACTCCTTCTATAGATTTCACT
CTTTCTCCGTCCCGAAAAGGAGTTCTTTTAACTAGCTTTAGTTTAGTTTGACTTGGTTTT
TGTGAAACTAGATTTTCAATTAGACTGGATTTTGTTTCTGGTATTGAATAAACAATAACA
GGTCTAATAAGAATATAGGGCATTTGCTCGTTTTCTATATACTCCCAATAAACTAACTTA
TCTGTAGAAATATTATTATGAAGTCTTTCTCCAGGTCTTAAAAAACCTCTACTTTTATCA
TTATTACTATACACCTCACTAAGTTTATAGATAGATCCAGGCTTAATAATAATTTCTCGA
ACTATTCCATCCTTTTCGATTATTTCAACAATACCTGAGCTTTTAGAAAAAATATTTTTA
ACTAGCTCTGTTCCACTTTCTATAACATCTCCATTATGCACTAGTAGTAGTGATGAATCT
TTATTAATTTCGTGAGTTTCTTCTGAAATCCATAAGATATATCCCGGACTTAAAATTTCG
TAGGCATCTTTATCTAAGCCTGTTTTCTTTTTTGATACATTTAAGTCTAAATATTTAATA
ATACCACCACTTGTCGTTTTATACGTATCAGATATTAGTTCTGCGACTGTGTAACCATCT
TGAATTTTTTGGTTTGGAATAGCTTTGAAAAGAAATTTTTGTTTTTTTTCTGTTTCTAAA
ATATAAGATTCTTTTTTGTGGATTACATCTGTATAAATATAACAACCTGGAATTACAATA
GATTCAGTAATAACTTGTATATTGGTAATACTGCTATTAGTATTCTGGTGTATCCTTACT
TCTCCAGCATAATGATTTATTAATTCTGTCTGTGCAAGTATCGTACCAGCTTTAGTTTTA
TCTTCTTTATGAACAATAACATTAGCAGAATCAGATATACTATAAACTTCACCTGAGAGC
ACCCAGATCAAGCCTCCAGTTTTAGTAATTCTTGTAGTATATTGATTATTATCTGTTTCT
TCAACTGTTAAGTTGGAGAAACAGATTTTGCCAGAGAGATCTGAGACTACATGTTTTTGA
GCTCTTTCTGTCATGAGTCGATTTCTGCGGGGTGATTCAGCAATCACTTGATCCTTCGAT
ACTAATTCACCATCACATATTAAAAGAGTAGTACCTTTACTTAAGTTAATTATTGACTTC
TGATTTTTCTTAGATCTGATTGTAACTTGGGTTGGCTTTTTTGTTATTAATGCTTGCTCA
CCATGTCTGGTACGAACATCTGTATACGATTCAATATCTAGATCTATCAATTGACCATCT
ATAGGTGCGTAGATTTGTTCTGCTAATTCACCGGTAAATACTCCTCCAGTATGAAAGGTC
CTCATTGTCAATTGAGTGCCAGGCTCACCAATAGATTGAGCCGCAATAATACCAACAGCC
TCTCCTAAATCAACTAAGCGGCCATGTGCAAGATTCCATCCGTAACAATACTGACAAACA
GAACTTCTAGAGTTACATGTAACAGGAGATCTTACTAAAACTTTTTTGATACCAGCTTTA
GTTATCTCTTTTGCTAACTTAGGACTGACATCTTGGTTCGTATGAGCAATTAAACAATTT
GTCTCTGGATGGAATACATTTTCTGCTAATACTCGGCCAGATAAAGCTTGTTCTAGATTA
ATTAATATTTTCTGCGTATCTACTAAATCTTCTAGTATGATGCCTTTATTGGTCTTACAG
TCTACTTCTCTAATTATTACATCTTGAGAAACATCGACAAGTCGACGCGTCAAATAGCCG
GAATCTGCTGTTCTTAAAGCAGTATCAACTAATCCTTTTCTTGCTCCGTATGATGATATA
AAATAATCAGTAACAGTTAGTCCTTCTCTAAAATTACTCGATATTGGAAGATCAATAATT
TGTCCCTGTGGATCAGCCATAAGCCCTCTCATGCCCACTAACTGTCTTACTTGAGAAATA

TTACCTCTAGCACCTGAGAACGCCATCATATAAACAGCATTTAGAGGATCTGTCTCTTTA

AAGTATTTAATAACTTCTTGTTTCAAGGATTCACTCGCATTATTCCACGTATCAATAACT

TTTTGAAATCGTTCAACTGCTGTAATCTCACCTCGGCGATATTTGTTTTCAGTGGCCTTA

ATATCTTCAATCGTGCCTAATAAAAGACTTTGCTTACTAGGAGGAATTCTTAAATCTTCT

AAACTTAAAGAAATTCCTGCCTGGGTCGCATAATGAAATCCAAGATCTTTAAGCTTGTCG

GCCATATTGGCTGCTCGGGCTATACCATAATTACGAAAAGCCCAGACAATTAAATTTTTT

AGTTCGTTTTTATCAATAACCTTATTTGAAAAACTTGGCTGTGCAAGACTTCGTCTATTA

TCCACTAAATCTTCTCCATATTAAATGTTCGTTATTAACTATGCTACTAAAGACTCTTGA

ATAATTTTATTAAAGATTATACGGCCAGCTGTTGTTCTAATATATTGCACAATTCTCTGA

TTTTCAGCGTCTTCTTTTATGATATGGTTATTAAAAAATTTTGTTGTACTATTGTCAGAA

TGTTTTTCAATCTTAATCGGATCATGAGGTTGATCACCATCTACTAATCCATCAAAACGA

GCCCATATATAAGAATGTAAATCAATTTTCTTCTGTTCATAAGCCATAACTACATCTTCT

AAGCTTGCAAAATACTGATTAGATCCCTGCTGCTGAGATGGATTATTAGCTGTTAAATAA

TAGCAACCTAAAACCATGTCTTGGCTTGGCATAATAATTGGTTGACCTGTTGCTGGAGAG

AGAAAATTATGAGGGGCTAACATTAGCAATCTAGCTTCGGCTTGAGCCTCTAAAGACAAA

GGAACATGAACAGCCATTTGATCTCCGTCAAAATCAGCGTTAAACGCTGGGCATACTAAT

GGATGAAGCTTAATAGCTCTTCCTTCTACAAGAATAGGTTCAAATGCTTGAATACCTAAT

CTGTGTAGTGTGGGAGCTCTGTTCAATAGTACTGGGTGACCTTGAATAACTTCATTTAAG

ACATTCCAAATAGAAGATTCATTTTTTTGAATCATCTTTTTGGCAGCTTTAATATTGTTA

ACTAATCCCTGCAAAATTAGTCTGTGAATAACAAAAGGCTGAAATAACTCCAAAGCCATT

TCTCGTGGCAATCCACATTGATGTAGTTTTAAATGAGGACCTACAACAATTACAGATCTA

CCGGAATAATCTACTCTTTTACCTAGCAAGTTTTGTCTAAAACGACCTTGTTTTCCTTCT

ATAATATCTGACAAAGATTTTAATGGTCTATTATTTGCACCTACAACAGTTCTACCTCTA

CGACCATTATCCATGAGAGAGTCAACGGCCTCTTGCAGCATTCTTTTTTCATTTCTGATA

ATAATCTCCGGAGCTAAAATTGACTTTAGACGAGATAATCTATTATTTCTATTAATAATT

CGACGATAAAACTCATTCAAATCTGCCGTCGCAAATCTACCTCCATCTAATTGGACCATA

GGCCGTAAGTCTGGAGGTATCACAGGAATGACTGTGAATACCATCCAAGAAGGATCTGCA

CCTGTTGCTATGAAATTTTCAATCAATCTTAAGCGTTTCATTTTTTTATTGAATTTTAAT

GAAGGTGTCTTAAAACTTTTTGGTGGATTTGTAGCTTCAGACCTTAAGGTTTCAGCTATG

TGCTCTAAATCTAAATCTTTCAACAGTTTCTGAATAGCTTCTGCACCTATTCCTACTTCT

ACTTGGTTATTCTCATCTTGATTTTGGTAGATTTCCTCTTCAAGACTTTTCCATTCGTAA

CCTTCGAGAAGTTGCTTGTATTTTAAATTAATATCTGTATTAGATTGCGTAACTACATAA

GAATGAAAATAAACAATTTTTTCTACTTCTTTGACTTTTAAGTCTAATGCTAAAGCAATA

TAGCTTGTACTTCCTTTTAAGTACCAAACATGAGTTACTGGTGAGGCAAGTTCAATATAA

GCCATCCGATGTCTTCTCACGCGTGATTCTGTAACTTCTACGCCACATCGTTCACAAACT

ATACCTTTGTAGCGAAAACGTTTATATTTACCACAGTGACATTCCCAATCCTTAACTGGG

CCAAAAAATTTTCTCACAGAATAAACCATCCATTTCTGGCTTTAAAGTTCTGTAATTAATT
GTTTCAGGCTTTGTAATTTCACCAACAATTTGTCCGTTGGGCAAACTTCTTTCGCCCCAC
TGTCGAATTTTTTCGGGAGAAGCTAAACTAATTTTTACGTAGTCAAAATATTGCTCAAAC
TTTGTCATAAATTTGAATACCTTAAAATCAAATAATTAGCCAATTTATTTACATGCTTCA
TATTGAAAAATGAATATCTTAATAAAGGAATTGTTCAAAATCGTCTACTGGAGGAGTATC
ATAATTAGAACGATCTACTCGGTTATCTTTAGAATCCGACATCAAGTCAACTTCAACTGT
CCGTCTTTGGCCATCTTCAAATAGTTTCAATTTATGTACTGCAATGTCTAAACCTAATGA
CTGCAGCTCTCGCATTAAGACTTTAAAAGATTCTGGAGTTCCAGGCTTCGGTATAGGCTT
CCCTTTAACTATTGCATTTAATGCTTCATTTCTAGCTTGCATATCATCCGACTTTACAGT
TAATAATTCTTGCAAAGTATATGCTGCACCAAAAGCTTCTAAAGCCCACACTTCCATTTC
ACCTAATCTTTGACCTCCATGCTGTGCTCTACCTCCTAATGGCTGCTGCGTAACTAGAGA
GTAAGGACCAGTGGATCTTGCATGGATTTTATCATCAACAAGATGAACAAGCTTAAGCAT
ATAAGCTCTACCAACTGTAACAGGATTATCAAAAGGCTCTCCTGTTCTACCATCAAAAAC
TTGCATTTTACCTGGATGCTGATCATTAAAAAGCCATTTATTGCTAGTAATCAATGATGC
TTCTTTTAATTTTCTATTTACAAGTGCTCTTGATGCTTCTGCTCCATACATTTCATCAAA
AGGTATTATTTTAAATCTCTTTCCTAAATAGCCACCTGCTAAGCCTAGTAGGCACTCAAA
TACTTGACCAACATTCATTCTAGAAGGTACACCTAAAGGATTTAAAACAATGTCCACTGG
TGTTCCATCTGATAAATAAGGCATATCTTGTTTAGGTAAGATCCTAGAAATGATACCTTT
ATTGCCGTGACGACCAGCCATTTTATCACCCACTTGGATTTTTCTCTTCTGCGCAACATA
GACGCGAATCATAGCATTTGTTCCGGGAGGCAGCTCATCTCCTTTCTGCCTAGTAAAAAC
CCTAACCTTAACTACTCTACCTTTTGCAGCATTAGGAAGCCGTAAAGATGTATCTCTCAC
ATCTCGAGCCTTTTCTCCAAAAATAGCTCGCAACAATTTACCTTCTGGTAGTTGATCAGC
TTCACCTTTAGGAGTAATTTTTCCTACTAAAATATCTCCAGCTTCAACCCAAGAACCACC
GACAACAATTCCATTTCTATCTAAATCCTTCAGAGAGTTATCGCTGACGTTAGGAATCTC
TCTAGTAATTTCTTCTGGTCCTAATTTAGTTTGACGGCATTCTACTTCATACTTTTCGAT
ATGGATAGAAGTATACAAGTCATCATAAACTAATCTTTCGCTAATTAAAAATGCATCTTC
GTAGTTATAACCTTCCCAAGGCATATAAGCAACTAGAATATTTCTCCCTAAAGCGATTTC
CCCTCCGTCTGTGGATGCGCCATCAGCTAAAGTTTGGCCAACAACTATTTTTTCTCCTAC
CCAAACAATTGGGCGCTGATTAATACAAGTATCTTGGTTAGACCGATAATATTTTTTTAA
GCGATAATGAACTGTTCTACCACTATTATCCTGAATGCCTATTTTATTAGCAGAAACATA
ATTTACATGACCAGATGTTCTGCTAATAACAACCATACCAGAGTCTCGAGCTATTTTAGT
CTCAAGACCAGTACCAATTATTGGCTTTTCTGGATATAATAATGGAACAGCTTGCCTTTG
CATATTAGATCCCATTAGAGCTCTATTCGCATCATCATGTTCCAAAAAAGGTATTAATGA
TGTTGCAGCTGAAATTACTTGAATAGGTGAAATTGCAATATAATCTACTTGGGTTGGGGT
TGTTGTAATAAACTCTTGACGATAACGTACAGGAATAATATCTCCTTCAATGTAATGTTG
TTTACTAACTTTAACATCTCCTGGAGCTACTCGGAAATCATCTTCCTCATCTGCTGTTAG

ATAGACAGGACTATTGTGATAAATCACTTGACCTTGGTTTACCGGATAAAATGGGGTTTC
TATGAAACCAAAAACATTGACCCGCGCACAGGTTGCTAGTGAACCAATTAAACCTGCGTT
CGGACCTTCCGGAGTTTCAATTGGGCAAATTCTACCATAGTGACTAGGATGTAAATCACG
AACTGCAAAACCTGCCCTGTCCTTATTAAAACCTCCTGGACCTAAGGCACTAATTCTTCT
CTTGTGAGTTAGTTCTGCAACTGGATTAGTTTGGTCCATAAATTGAGAAAGCTGACTAGA
TCCAAAGAATTCTCTCACTGACGCAATTAATGGCTTGGGATTAATTAGATTTGATAAACT
CAATGAATCTATATCACATATCATCATTCTCTCTCTAATAATGCGTTCTAAACGATTGAG
ACCTACTCTGAATTGGTTCTGCAGCAATTCTCCAACTGAACGAACTCTTCTATTACCAAG
ATGATCTATATCATCAAGATTACCTGAGTTTTTATCCTTAATATTAATTAGATAATCAAT
TGAGGATAAGATGTCTTGAGGAGACAATACTCGGAACGTTTTAGGTATATTGAGACCTAG
TTTTTTATTAATTTTATATCTACCAACTTCACCCAAGTCATATCTTTTAGGATCAAAAAA
ACGAGAGTAAAGCATCTGCTTAGCAACAGCAACTGTTGCTGGTTCATTAGGTCTAAGCTT
AGAATAAACTATGAGCAGGGCTTCCTCATCAGTTACTTCTTCAATATTATTTTTTCCAAT
TTCTTTAGCTAATTCTTTAACTGAATATATTTGGGATGCAGAAATAAGAAAAGCATATTT
ACTAAGTCCTTTTTCAATTTCATCTTTATTGAGTCCAATAGCTCGCAGAAAAATATAAGC
ATTTACTTTGTGGGTCTTATCTATTCTTATCCAAATTTCTCCCTTAGGATCAATTTCAAA
TTTTAACCAAGAACCTCTATTAGAAATTAGGCTTGCACTATAAATCTGCTTACCATTTTT
GTCTATCTCTTGTTTATAATAGATTCCTGGACTACGGATTATTTGGTTAATAATAACTCT
TTCCGTCCCAGATACAATAAAAGTTCCTCTATTTGTCATTATTGGCAAGTCTCCAATAAA
AATTAATCTCTTTTTGTATTTTTTATTTTTTATCTGCTTTTCAGCACTGAGCTGTAGATG
GGTTGATGATAAATCTAATGATTTAATATTTTTTTCCTGGTTTTTAGAAGGTAGATCAAT
ATCTTTTCTTGTTAACTTTGCAGGAACATATATTTGAGCACTATAAGTTTTATCCCTATT
TTTTGCCTGTCTGACACTATAACGAGGAAATTTTATCTTGTACTCATTACCAAATAATTG
TAATTCGAGTCGACTAGTAGGGTCTGATATCTTCGGAAAAAATTTCAAGAACTTCCGTCAA
ACCTTCTAATAAAAACCATTTAAAACTGGCCCTCTGAATTTCAACTAAATCTGGAAGAAG
TTTATTTTTTAAGCTTATACGTTGAACCATAGATCTCCTTTATAACAAATTTTGAAATCG
AAGAGTTTAATACTATTAATAGAGTACTGGTAGATAATGCATAAGCATAGTTTGCTACAT
TATTTCTTCTCTAGACATAACTAGAACATCATATTTTATTCAAAAAAAGATTTTTAAAAT
TTTTCTTCACACACACTATCTATAAGTACTTTGCTGCTAAAAGATAGTTCAAGCTATTAA
AAAAAACGAAATTAATACTTGTCTGTCTGTCAATTAATTGACATCAAGATAGACAAGTAT
TATTTATTTAATCAATTCTGTTTTTCTGAGCAAAAGACAATGCTCTCGCAAGTCTAGCTT
TTTTTCTAGCGCCTGTATTAGGGTGAAAGGCCCCTTTTTTTACAGCTTTATCAATCTTAC
TATATACTTGAGAAATACTTGACTGCACATCATTGAAATTATCACTTTCTAAATTATCAA
TATTTAGAAGGCATCTTTTAGTTAAAGTTTTGACAACCGATTTATACTTACGGTTAATGA
GACGATTTCTTTCCGAAGTTTTAATGCGTTTAATCGCAGAAAGGTTCTTAGCCACAGTAA
ATCCAATAAAACTTTTATAAAATATTTTTAGAAACGAAAATACACAAATCTGGGATCTAT

TCAAAAAGGGAATATGCGGCGTATTATAACATTAAAGAAAAACAATAGGCAATAGTTCAC
CAAGAGCTCTTATTATTACACAATAGAGATTGTCATTGCGAAAAACTTTGTAAGATAGTA
TTTAATTAAAATAAACTAACTAGACATATAAATATATATGAAAAAAATTGAAGCTATTAT
TAGACCTTTCAAGCTTAATGAAGTAAAACTTGCTCTAGTCAAGGAAGGTATTGGAGGAAT
GACGGTTATCAAAGTTTCTGGATTTGGGAGACAGAAGGGTCAAACTGAAAGATATAAAGG
ATCCGAATACTCTATTGATATCATTGATAAAATAAAAATTGAAATTATTATTAGCGATGA
TAAAGTAGAGAAAATTGTAGAGACGATTATTAAGGCTTCTAAAACAGGGGAAATTGGAGA
TGGAAAAATATTTATTAGTAGTATTGAAAGAGTAATAAGAATCAGGACTAATGACTTAAA
TTTTGAAGCTTTATAGTATTTTTACTTTTTTAGACTTGATACTTCTTCACTAAACATGAG
ATAATAAGCGTTTATTGTATACTTATAACTAAGGTGTAATACTAATGGCTAAAAGCAAAG
GTGCACGAATCGTAATAACTTTAGAGTGCTCTGATAAAGCTGGAGAGTTTGCTCAAAAAA
GGAAACCTGGCGTTTTTCGATATACAACTACTAAAAATAGACGAAATACACCAAGTAGAA
TTGAATTAAACAAGTTTTGTCCTAATTGTAATCAGCATTGTATCTTCAAAGAAATTAAAT
AGTTATTTATCTTATTAAAATAATCATATTATGGCTATATACAGAAAAAGAATATCTCCA
ATTAAGCCAACAGATGCTGTTGACTACAAAGATATTGACTTGCTAAGAAAATTTATTACA
GAACAAGGCAAAATACTGCCTAAAAGGTCAACTGGATTGACTTCGAAGCAGCAAAAAAAA
CTTACTAAAGCGATTAAACAAGCTAGAATCCTTTCTTTATTACCTTTTTTAAATAAAGAT
TAAATATATAGCCTAACTTCATATTTTTCCATCTAAATATTAAAAATTTCTATAGATTTG
TTTATTAAAATGATCTAACATACTGAATTACTAAGAAGATCTACTCATTACAAGTTTTGT
TCATTTTATAATTAATAATAGCATCATAATTTATTAAGCTAGATTCAGACAAGACATAAA
GGTAACCTCTTTTAATTGATTATATTATATAGTCTATTTAAAAGAGGAAATTTTTTTGCT
TCTAGCAATTCAAGATTCTTTAAGCTTTAACAAGGTATTTAAAACAAAGATTTTTGTTTA
GGTATCAGATCAAATGAATGAATCTTATCTCCCGACTGCCATAATTGAAATTCAGAGATG
AAAATTCCACATTCGTTGCCAGCTTGAATTTCTTCGACATCTTCTCTAACGCGCTTTAAA
GATTCAATTTTTCCTTGATAAATAACTTTTTCTTCTCGAATCACTTTAATCCAAGAGTTT
TTTAATAGCTTATTATTGATCACTCGACATCCTGCTATTTTTCTATTTGCTAACGAGAAT
ACTGTACTTACTTCTGCTTCTCCCACCGGTACTTCTGAATATTCAGGATCAAGTAAGTCT
TCCATCCTTCTTTTAATGTCTTCAATTAAGGCATAAATGATTTGATAGTTTTCTATTAAT
ATATTCGACTTTGCCGATGCTTGCTTTGTTCCTGGAGCAAAGTTGGTATTGAACCCAATG
AGGGTAGAATTTGTGGTTGAAGCTAGTTCAACATCTGTAGCAGTAATTTCTCCAGGCATG
ATTGAGACAACATTGAGTTGAACTTTACTCTGAGGAAATTGAGATAAAGAATCTAGGATT
GCTTCTGTAGAGCCTTGATTATCTGTTTTAATAATTAAAGAAATTTGCTTACTAATATCT
TTAGAATTAGTGTTTTTTAAAGTATCTAATGTAATTCGACTATTAAGTGCTCTTTGTTTC
TGTATAATTGATGAGTCTTTAGAGGTATTTTCAATCGCTTTAAGTTTGGCTTCTTTATCA
CTTTTAACTGCTAAAGCTATTTCTCCTGTAGCTGGTACAGATGACAATCCCCAAATTTCA
ACAACAGATGATGGTATTGCTAAATTAATTTTCTCTTTAGCGTTGTTAATTATAACTCGA

ATCTTTGCGTAAGCTGATCCAATAACCAAATTATCACTAATATTCAATGTACCATTTTGT
ATTAATAGAGTTGCTACTGGACCATGCGATTTATCTAAATGGGCTTCTATTATAATACCT
TGTGCAGGCTGTGTTGGATCAGCTTTCAAATCCTCTAATTCAGCTAACAAGGTAATAGTT
TCTAATAATTTATCAACATTTGACCAGTAAGAGAACTAATAGGAATTATAGGCACTTGG
CCACCAAGTTTCTCCGACATGACATTGTATTTTAATAAATCTTGCTCAATAATATCTGTA
TTAGAACCAGCTTTATCTATCTTAGATATTGCTATGACAAAAGGGACATTCGCTTTTTGA
ATATGATTAATAGCCTCTATGGTTTGAGGTTTAACTCCGTCATCAGCAGCTATAATAATA
ATAGCGACATCTGTTAAATTAGCTCCCCTAGACCTCATACTTGTAAAAGCTTCATGGCCT
GGAGTATCCAAGAAAACAATTTTTTGTTTATTATCTTTCTTAATATACTCAACTTCATAA
GCTGCAATTGCTTGAGTAATTCCTCCAATTTCTTTGTTAGCATTATTAGATTTTCGAATA
TAATCTAATAGCGTTGTTTTGCCATGATCTACATGTCCCATCACTGTGACAATTGGAGGT
CTTTTAATATAATTGCCAGTTTCATAAAGAGCATTAGAGTGATCTAAATTACTAGAAAGT
CCATTATTATTTTCCTTTATATTTGATTCTACTGCTATACCAAAATTATCAGCCACTGAT
GATATTATTGAGGCGTCTATGGTTTGATTCATAGTAACTGATATTCTTTTAAGAAATAAA
TATTTTATAATATCTGTTTCTTGAACACAAATTAGTTTAGATAATTCTTGAATAGTTAGA
GGATTAGTAATACTAATAGACTCTGGCGGAGAATTAGATTGAATGCTTGCTGAATTTTGA
CTAGGAGTTACTGCTACTTTTTTCTGTCTTATAGATTTTTTATTACTAGTCAGTTTTTTT
ACAACTTCCACTTTTGGCTTAGGTGGACGCATTAAAGAGATAGCTAAATCTCCTGCAGTC
TGCGAAACATTAGAATTAGAGTCTCTAAAGTTGTCATCATCATCGTCAATATGTATTTTA
GTTTTTATTTTTTTGTGCTGTCTATTCTTATTTTTTTTACTATCTACTAAGTCGTGAATT
TTATTAAAATTTTTACTTTTTTTATCTAGTTTAGGTGGAGAACTTAATTCTAGATGCTGT
TCACCACCTGTATGTGATTCTGATTTATCAAGGTCTAAATTTAACAGATTATCATTATTG
ACTGACTCTAATCTAATTTTATAGATAATCTGAGGATTTTTCAAATCTACAATTGTTTCA
GAAGAATTGATATTTGAGCTAGATCTAGAGGATCTATTTTCAAAGTTTTGATTATTTAAA
AACATAAATATTGGTTTTATTATCTAATAGTAACTTTCTAATTTTAATACAGAGCTTAAT
ATAAACAAGAATCGAATATCTGTATAAATAATACAAATGTAGAGAATTAATCTATGTTGT
ATTAGATTTAATATAACACTAAAGTCCTTTGTTCGTTATACAAGTTTAAAACTTTTTATG
ATGATAAACACTAGATTCTAGTTTATTGGTATACAATCTATTTATCTAAATAATACTGTT
TTTGTCAGGGCTTTTAAATTATCTAATGTTATGTTCATTATGAAATTCTATTAAAATTTA
AAAACTCAACAATTAAGTAAGAATCGTTAATCACCTCCACGCCACTTATGCCACGATCTC
AAAAAAATGATAATTTTATTGACAAAACTTTTACAGTATTAGCTGATATTGTATTAAAAA
TACTTCCTACAAGTAAAGAGGAAAAAGAAGCTTTCTCTTACTATCGAGATGGAATGTCTG
CACAATCTGAAGGTGAGTATGCGGAAGCTTTAGAAAATTATTATGAGGCTTTAAAACTAG
AGGAAGATCCATATGATAGAAGCTATATTTTATATAATATAGGGCTTATTTATGCTAGTA
ATGGTGAGTATGTAAAAGCTTTAGAATACTACCATCAAGGATTAGAACTAAATTTTAAAT
TACCTCAGGCCCTTAATAATATAGCCGTGATATACCATTACCAAGGAGTTCAAGCAATTG

AGGATAAAGATACAGAGCTATCTAAATTAATGTTTGATAAGGCGGCTCAATATTGGCAGC
AGGCTATTAAGTTAGCTCCTGATAATTATATTGAAGCTCAAAATTGGCTGAAAACGACAG
GACGAATGAGGAATATACAAGGATATTAATATATTTAAGATATAATAAAGATATCAATTA
ATAAATTAAAATTAGTTAAAGAAACTGGTACTATATAGAGTACTTTACTATATATTATAA
TATTATACTAGTTGA------TCTCTAAGACTTAGCTGAATAAACGAAATTAAATCTATT
GACTATATTCATGAATACAAATAAGCATAATGGTTAGTACAACAAAAAGTACCGGATCTG
TTACTCAAATTATTGGACCAGTTTTAGATATTGCATTTCCTAACGGACAGCTTCCGAAAG
TATTCAACGCACTCAAAGTACAAAGCTCAGAAGGAACTATTACTTGTGAAGTACAACAAC
TTTTGGGTGACAACAAGGTACGAGCTGTTTCTATGAGTTCCACTGAAGGACTACAAAGAG
GGGTAGAAGTTATTGATACTGGATCCCCTATATCTGTTCCTGTAGGTACAGATACTCTTG
GACGTATTTTTAATGTTTTAGGTGAACCTGTAGATAATTTGGGTCCCGTTGATTCTGAGA
GTACTTTACCTATCCATCGACCAGCACCTGCTTTTACTAAGTTAGAGACAAAACCAAATA
TTTTTGAAACAGGTATTAAAGTCGTCGATTTACTTGCTCCTTATAGAAGAGGTGGGAAAA
TTGGTTTATTCGGAGGTGCTGGAGTAGGTAAAACTGTATTAATTATGGAACTAATTAATA
ACATCGCTAAAGCTCATGGTGGAGTATCTGTATTTGGAGGCGTTGGTGAAAGAACAAGAG
AAGGAAACGACCTATATATGGAAATGAAAGAGTCTAAAGTAATTGATGCAGATAATCTAA
AAGAATCTAAAGTAGCATTAGTATATGGTCAAATGAATGAACCTCCTGGAGCACGTATGC
GCGTTGGCTTAACTGCATTGACAATGGCAGAATACTTTAGAGATATTAACAAACAAGACG
TTCTATTGTTTATTGATAATATTTTTCGGTTTGTACAAGCTGGATCAGAAGTATCAGCTC
TACTAGGCCGTATGCCATCTGCTGTGGGTTACCAACCAACTCTAGCAACTGAAATGGGAG
CACTTCAAGAAAGAATTACTTCAACAACAGAAGGATCAATTACATCTATTCAAGCTGTAT
ATGTGCCGGCTGATGATTTAACAGACCCAGCTCCGGCCACTACATTTGCACATTTAGATG
CAACAACAGTACTTTCGAGGAACTTGGCGGCAAAAGGAATTTACCCTGCAGTGGATCCAC
TGGATTCAACATCAACAATGCTACAGCCTGGAATTGTTGGAACTGAGCATTATTCTACTG
CTCAAGAGGTAAAATCAACTTTACAAAGATATAAAGAACTGCAAGATATTATTGCTATTC
TTGGTCTTGATGAACTTTCAGAGGAAGACAGACAAACTGTATCAAGAGCAAGAAAAATTG
AAAGATTTTTATCTCAACCTTTTTTCGTGGCAGAAGTGTTTACTGGATCACCTGGGAAAT
ATGTATCTTTGGAAGATGCAATTAAAGGGTTTCAAATGATCTTAAAAGGCAATTTAGACG
ACTTGCCTGAGCAGGCATTTTATCTAGTAGGTGATATAGATGAAGCTATACAAAAAGCTG
ACAGCATGAAAGATTAATATAATTAATGATGACTTTAAATATAAGAATTATTGCTCCTGA
TCGGACTGTTTGGGATGCAGAAGCACAAGAAATTATTTTACCAAGTAGTACAGGGCAACT
TGGTATTTTAACAGGCCATGCACCTTTGCTTACAGCTTTAGATATTGGAGTTATGAGAGT
AAGAGTAGATAAAGAATGGATGCCGATTGTTTTGCTGGGCGGTTTTGCAGAAATAGAGAA
CAATCAATTAACTATTCTGGTTAATGGCGCAGAAGAAGCTAGTCAAATTGATTTATCAGA
AGCAGAAAAAAATTTAGACACTGCAACTCAACTCTTAAGTGATGCCTCGTCTAATAAAGA
AAAAATAGAAGCAACACAAAAAATACGAAAAGCTCGAGCTCGAGTACAAGCTGCAACAGC

AGCAACTTCGTAAAAGATTCCTCTTAAAACATAAAACTTTAGTAATGATTACTAAAGTTT
TATGTTTTATGTTTTATTTGTATGGTTATAGGCAGCTTAAATAAACTTAGCTTAGACCTG
AACAAATATAATCAAAATATAATCCCATCTCTTTACCTGCATCTGGTCCAACTAAACTAA
TAGTAACTTCTTTCATAGCTAAAATAGCTTGAATCGTTGCACCAATAGGAACACCTAGAG
AATTGTATGTTTCTTTTAGACCATTTAATACTCTTTCTTCAAGGATAGATGGATCTCCAG
CTAGCATTCCATAAGTAGCATAGCGTAAGTAGTAGTCTAAATCACGGATGCATGCAGCAT
AACGTCGAGTTGTATACATATTACCACCTGGGCGTGTAATATCCGAATAAAGTAGAGATT
TTGCTACGGATTCTTTAATAATTGTTGCTGCATTAGCTGCAATAGTAGCAGCAGCTCTGA
CTCGTAGCTCACCTGTTTGAAAATAGCCTCTTAATTTTTCAACAGAGCTATCATCTAAAT
ATTTACCTTGGACATCAGCTGCATTAATAACAGAAGTAATTGCGTCTTGCATAACTTTCA
CACATTCCTTAATTTTAAATATCTAATACTAGGGGTATATAACAAAATTACTAGATCTTT
TAGCAATATCAGTATTACTGCATAGCACCCAAAGTATAATCAAAGTAAAAACCAGCTTCT
GCTGAATCTTCACCTGCAAGCAGTGAGCAAGCTACACTTTTCATGCATTTTACACCTTCA
GCAACTCCTGAAATTGGTGTTCCTAAAGAATTATACATTTCTTTAACACCCACTAATCCA
ATTTCTTCAATAGGAGTTATATCGCCAGCAACTATTCCGTAAGTTACTAAGCGAAGATAA
TAGTCCAGATCTCGTAGACAAGTAGCAGTCATCTCTTCGCCATAAGCATTTCCACCAGGA
GAAACTACATCAGGTCTTTTTTGAAACAGTTGTTGGCCGCCTTGCTTTACAATACGCTCA
CGATTATCTGTTAAAATTTGAGCTATTCTTAAACGACGTTGTCCAGATAAAACAAAACTT
TTAATTCTATCTAATTCCCCAGGACTTAAATATCTTGCTTCTGCATCTGCATTTACAATT
GACTTTGTAACAATACTCATGGATAAAACTCCTGTAATGCTTT-AAAAAAAAGCTTAATA
CTAATTATTTATGATATAACTCAGAAAGTAATGTACTTTTTGAGAATTTTATAACCGAAA
CACGTTCATGATATATGAAGCAGGATAGATACTGAGGTAAGTACTAAAACGAGAAAGTTA
TATGTAGGAACTTCTGCTATTGATTTCCTAGTACTTTTTTAAAACTTGGTACCACAATTG
ATATATCCTGCTTAGTCAAACGATTATAGAGTGTTTCTGTATTAGGAAAGTTAGCTGCTG
GTAAAGTCGGAAATCTTCTGTATGGAACAGTATCTTTGCCATATACCGCATTGTATTCAA
CACTTTCAACTAAATTACTTATAAATGCAGATAAGCCTTTTGAAGCTAAAATTTGATTAA
AGAATCTAATTTCTGCCTGATTATTAGGAGCTCTTCCTAAGATATGCTTAGTACCTAATT
CTATAACTTTTGTATTTGGATATGGCTGATAAAATTCTTTACCATATAGTTCAGATAGCG
CCAGTTTTTCTACTAGTTCTTTGACACAGATTTGTCTATTCAAGAAAGCTGACTTAATAT
CCAAGAATTCACCGCCGACACTAAAAGAGTTAAGATCTCTTTCGAAAATTTGACGATATG
CTGCTCTTAAAGCTTGTTCTAACATTTCTTTGTTACTATCACTATTGACTTCAAAGACAA
TAGATTGATCTCTTAAAGAAGTTACTCCCTGTGAAATACGAGACTGAATATCATTACTAC
TCCTCATTTCTTTGACAGTGCCTAATTCAACAAACCTAGCAGATTTACTAGCAATAACTT
TTTTGAATCTCTGGTCAATAATTCCTGGTCTTAAGCTTCTTAAAGCAACACCAGCTGGAG
TACTATATCTTTCATAAGGAACAGTATTATCTCCAAAAGTTTCTGTGTACTCCGAGCTAT
CAATTATTGTGTCTATTACTTGGTAATAGCCTTGCTTATATGCAATATCAAAATATTTAT

TAATTTCTTGCCGACCATATGTAGGGCGACCCAGTAAGCGGTTATGAATATATTCAATAG
CTTTACAAATATATAATGGCTCCCAATAAAGAGATCTAAATATACTTGATTTTGCTAACT
GTCTAACAAACTCGCGAACTGTAATTTGATTATCTTTTAGCTGACTTTCTATTGGCTTGA
GAATTAGTTTTTCTTCTTGATACACTTCTCTGCCAAAAACTCTTAAATAAGCAACTTTTG
TGACAACTTCTACTGAATTTTCAAAATTTTGGGATGAATCTGACTTTATAAGAGCGTTGG
ACAATTTAAAGATTTTTGGTCCCAGGGATCCTGCTGACTTAGGTCTTACCTGTGGATTAC
TAATCTGATTGTAGATTCCTGGCCCACGTCTAACTAATATCCTTCTTGTATCTTTACCAA
AAAGAGCTTGTCTTTTTCTTGGATCTTTATTTTCTTTAGGGAAAATAGCTCCAAATTGAA
TTGATAATGGATCATTACCTATTCCATATGGATGTTGATCAGGTAATGATTGCTTATAAT
CACTAAATAAAGTTATAAATTGAGGAACCTTACGAAAAGGCGCACTATAGGTGAAAAGAT
CTATTTGAGGCCCCCAATTACGGCACTCTTGAGGCTCTTCACCTAAATTTCTAAAGTAAG
GCACAGTCTCTTCACCGAAATAATCTGTATACTCAGAAGAATTTAAAAGTGCATTCACTA
AACCACTTAAACCAGTAGATGATAAAATAGCAAAATATTTTTGAAACTCTTCTAAAGAGC
TAGGACCTCTACCCAAGAAATGTCTAAATGCTAATTCTAATGCTCGACTATTGACAAAAG
GTTCATAAAATTGCTTTCTATAAATACTTGAAGTTCCTAACGAACGAATGAATTCCTTAA
TTGAAATTTGGCCATTTTTTACTTGCGACTCCAAATTCGATAAAGAGAGGTCATATGCTT
TAGCAATATCTCTTTCAAAAATTTGTCTATAACATGCTTTCACAACAATATTTTTCTCAT
CAGCAGATAAACTAGGCTTCATAACAAAACGAGGTGTTGACACTCCTGCCTGTACATAAG
TTTGAGGAAGACGCAAACCTTGTAAATCTCCAGATATTCTTTTTCTTAATTTATCAGTCA
AAGATGGAGCTTCAAATTCAGAAATCACAACATTAAAATACTCTTTAACTAAATCTTGAC
CTTTTATATCTTCCTCAAAGATTAATAATGCTGTCCGTCTCATCTCTCTAAGAGCCACAA
TTGCTGCTGCACTTGAGCAAGCGTTATCAATTAACTCTCTCAATCCTCGTATATTAACAG
ATAAAATATTAGGGTCGCCTGATACAATTGCATATGTCAAATACCGTAAAAACCAGTCTA
AATCTCGAAGAGATTTTCTCATTCTTGTAGTACCGTATCTCAAGACGTTTATAGGTTTAA
ACCCTGGCGGAGTAGCTCCCCCAGCATTAAATAAAGATCGGAAACTTTGGCCAAAATCTC
CTTGGATATTTCCTGATAACTCATTAATTTTATCTTGAGAACTTTGATCTCCTGCAATAA
TAACAGCTGCTTGCGGACGTTCTAAATAAGAAATTGCAGACCCACCTACGAATATTTTAT
CTGCTGCTCGTGCTACCAGAATATTTGCATTTTTAGTTAAAATATCTGCAACTTCCAGTC
TTTTTTGTCCAGAATTTAAAAAAGAAACTAATTGATTTAATTCACCAAGCTGTAAAAATC
GGTCTTGTTGCTCTGCCTGAGTAATAGTTAAAATTGAGGCAGTCCGATAAAGCTGGGGGC
GTGCTAATGGGCTTCCGCCACTTGCTTTGATACTCATTACTTTATTTATCTCCTTAACTG
ATATTGCTTGCTAGCGAATACAAATATAGCTTGATATTTCTAAATATTTAAAGATACTAT
GCAAGCACACTGTTTTCTATATTATTAGGTACTAGTACTTATAGTACAGAGAGTCTTATA
ACTAACTTCTATAAATACAAATTTTGTAATACTTCTATTAACATTTGTCAAAAAAATATT
TAAGTGCTCACTCTTTTATATTAAAATTCAATTAGTGAGAGATTGATGTGAAATAAAGTA
TACTAAGTAAGTTTTATAGATAGGAAAATTAAAAGATTTATTCTACAAACATTAATCACT

TGAATGTTTAGCAATTGTACAATTGTTATTGCTTTTAATTGGTTTAGACTAGTTTTATAT
TTTTTCTAACATAAACAATATTAATGAGCAATTTGTATGAATTTAAAGCCACAAAACAAC
CTATTAACTAACGAAAATATAAATCATGTAGATATACCCGAAAATGATATACCAATGTCA
ATTACGGAACATTTAGAAGAATTAAGACAACGGACTTTATTTGTGTTTTTATTCTTTTTG
TTTGCTACAACTATAAGTTTCACACAAATTAAGATCATTGTTGCAATACTGCAAGCTCCT
GCTGTTGGTATTAAGTTTTTACAACTGGCACCAGGAGAATATTTTTTTTCATCTATTAAG
GTTGCAATATATTGCGGGATTGTTGCAACAACTCCGTTTGCCGTTTATCAAGTTATATTA
TACATACTCCCAGGACTAACTGGAAAAGAAAGAAAAATTATTTTGCCTCTATTAATTAGT
TCTGTATTGCTTTTTATTACAGGTGGTATATTTGCTTACTTTGTTCTCGCACCAGCAGCT
TTAACATTTTTAATTAGCTATGGGTCTGATATTGTAGAACCATTATGGTCTTTCGAACAA
TACTTTGACTTTATTCTATTACTTTTACTTAGTACAGGATTAGCATTTGAGATACCAATC
ATACAATTATTGCTCGGTGTTTCAGGGACATTTTCTTCTAGTCAAATGATACGAGCTTGG
AGATATATTATTATTATAGCAACAATTGCTGGAGCTATTCTGACCCCTTCGACTGATCCC
GTTACACAATTAATAATGTCTTCAGCTGTTTTATTACTTTATTTTGGTGGAATTGTTATA
TTATTAGTCTTAAAAAAGTAGAGTAAATATTTTCTATTAATCCAATATATTTGTTATCTG
GGCAAATAATCTATAAAAACAATTGCTAGATTTAAGTATCAGGCTTCTAAATATTTCAAG
TCTCTTTTTATCATTTAAATACAATCGATACATTGGCTTGTTTTTTCATCAATTATTTGT
GTTGCACACTTTTTAGCACTCTCTTTATTGTTCTTAGAATTATTTCTTTAATCTATTTAT
TAAATAGATATGAAGAGATTTAAGTATTGATTATGGTAATCTATATCAACGTAAAAAGTA
TTTTATTGTGAGTTTTATTTATATAAAGGCTTATTCATTATATAGTTATAACAGTAAGTC
TAATTAATAGTTTTCATGCTTAATTAAAGTAATAACTCTTAATTCTTTTGGCAGAAAAAT
CTGTCTTAATATTTTATCAAAAAAACAGAAAAATGAAGCTAAATAGTCTAATTAACTTAA
TTCAAAAGTCTATATATTCTTGTACACTTTTACTAATTATTTTAAATATTATTTGTGTCG
CACCTAATTCTAGTAATGCATTTCCAATTTATGCGCAACAAGCTTATGAAAGTCCAAGAG
AAGCAACTGGTAGGATAGTATGTGCCAATTGTCATCTTGCTCAAAAGCCTGTGGAGATAG
AAGCGCCTCAAGCAGTACTACCTAATACTGTTTTCGAGACTGTTGTGAAGATTCCATATG
ACAGCAATGCTAAACAGATTTTAGGTAATGGCAGTAAAGGAGGCTTAAATGTTGGAGCCG
TTGTAATATTACCTGAAGGATTTAAGTTAGCTCCTGTTAATAGATTATCTACAGAGTTAA
AAGAAAAGACTAGAAATCTTTACATTCAGCCGTACAGTGCTAAACAAGACAACATTTTAG
TAATTGGACCTATTTCTGGTGATAAAAATAGAGAAATAGTTTTCCCAATACTATCTCCCG
ATCCTGCAAAAGATAAAAAAGCTCATTTTTTCAAGTATCCAATATATGTTGGCGGAAATA
GAGGACGAGGCCAGATTTATCCAACGGGTGACAAAAGCAACAATAATATTGTCTCTGCTT
TAAGCAGTGGTAAAATTAATAAAATTGAATTACTAGACAAAGGTGGATTTATAATACATG
TGACTAACAGTAGCAATGTAGAGTCAACACAGAAGATTTCACCTGGTCTCGAACTTAGAG
TAAAAGAAGGGGATACAATTCAGCTTGATCAAGCTTTGAATAGTGATCCAAATGTAGGTG
GTTTTGGTCAGAACGAAACAGAAATAGTTTTACAGAGTCCAAATAGAATTAAGGGCATGA

TTGTTTTCTTTTTCGCTAGTGTTCTAGCTCAAATTTTCTTCGTATTAAAGAAAAAACAAT
TTGAAAAAGTTCAAGCAGCTGAAATGAATTTTTAAAGCAATATAAAACTGGCACTTACTA
TAAAGAAAGTATAAACACTTTATTCACAGGATGTACCAGTTTTTATGTTATGTAATAATC
GCTTTGACGGAAGTTATGATACTTTTAACAGAATCTACAATCTGACTAGGCTGAATTACT
GTAGCCTGCTCTAAGCTTCCATTATATGGTGTGGGTATATCCTGCGAAGATAGCCTCACG
ACAGGAGCATCTAATTCATCAAAAAGGTACTCATTAATTTGTGCAATTAGCTCTGCTCCA
ATTCCGGCTGTTTTCATACACTCTTCTACAATTAAAACTTTGTGAGTTTTCTTTACTGAG
ATAGATATAGAGTCTATATCCAGAGGTTTTAAAGATATAAGATCTATAACTTCTGGATCA
TAACCTTCTTTTAATAAGGCCGGTAATGCTTGGATAACATGATGCCTCATTCTAGAATAG
GTTAAAATCGTAATATCTTTTCCTTTTCGTACAAATTCAACTTTATTGAGAGGTAAGAAA
TATTCTTCTTGAGGAATCTCTTCTTGTAAATTATAAAGTAGAACGTGCTCAAAGAATACA
ACTGGATTATTGTCTCGAATTGCAGATTTCAGTAATCCTTTTGCATTATAAGGAGTAGAA
CAAGCAACTATTTTTAAGCCGGGGATGGCTTGGAAATAAGCTTCCAGTCTCTGAGAATGC
TCTGCACCTAACTGCCTACCAACTCCTCCCGGTCCTCTAATAACTAAAGGCAATGTAAAA
TTACCTCCAGAAGTATAACGTAACATTCCTGCATTATTAGAAATTTGATTGAATGCTAAT
AATAAAAAACTCATGTTCATACCTTCAACAATTGGTCTTAGCCCTGTTATAGCTGCACCA
ATTGCCATACCAGTAAAGCTATTTTCTGCTATTGGTGTATCAAGAACTCTTAAATCCCCA
TATTTGCTATGCAAATCTTTAGTTACCTTATAAGATCCACCATAGTGACCAACATCTTCT
CCTATTACGCAGACAGTTGGATCTTTTGCCATTTCCTCATCTGTTGCCGCTCGTAAAGCG
TCAAACATAAAGATTTACTCATTTTAATATTTGATTTTTATGATTTGACCACTGTTTTA
AAGTAACAGTTAAGACACCATATTCTATTTAGTTATCTGCAAAAAGATATCGTTTCAATT
CTGACATGTTAGGTTCTGGACTGGAAATAGCAAATTTCACAGCTTGTTCCAATTCGGTTT
TAACAGCATTCTGAATCTCATTAAGTTCACCTATATTAGCAATTTCATTATCTAGAATAT
ATTTTTTGAGTTTTTTGATAGGATCTCTTGCCACCCAAGCCTCTTTTTCTTGTCTTGATC
TTAGTTCATCAGGATCTGCGAGAGAGTGACCACGAAATCTATATGTTAATGCTTCTATTA
AGGTTGGACCATCACCTTGGCGAGCTCTTTGAACTGCTTGTTTTGCAGCTTGTCTTACAG
CTAGCACATCCATTCCATCAACTTCAATCCCAGGAAGCCCAAAAGCTTCTGCTTTTTTAT
GTATTTCAGGTATTGAAGAAGACCGGTGATGTGCCATACCTATAGCCCACTGATTATTTT
CAACAACAAATATAATAGGTAGTTTCCAGAGAACTGCCATATTCAGACATTCAAAAAATT
GCCCATTATTGGTAGTCCCATCACCAAAAAAGCAAGCCGTGACTCTTAAATCCTCTGTTT
CTTTAAGTACTTGCTGGCGGTAGATACTTTGAAAGGCTGCCCCTGTTGCAACCGGTATAC
CTTCCGCAATAAAAGCAAAGCCACCTAAAAAATTGTGAGGCGCAGAAAAAATATGCATCG
AACCTCCTCTGCCTTTACTACAACCAGTCTCTTTTCCAAATAACTCAGCCATCACATTTT
TAGATGGGACGCCTTTACTTAAAGCATGTACATGGTCTCGATAGGTACTGCAAACATAAT
CAGTTGGATTCAGAAGTTTAATTACACCTGTAGAAACAGCTTCTTGACCATTATAAAGAT
GAACAAAACCAAACATCTTTCCTTTATAATACATCTGAGCACACATATCTTCAAAATTTC

TGCCTAACAACATGTCTTCATATAAAACTAATAAATTACTCTTATTAAGATTAAGACCTG
TTGAATTATAGTTAGTCAGCGGCAATTGAACTTTCTTAGGATAACTCATAATTTGTAAAG
AAACCTCTTATTGTAAATAGGAATTTTGAAGAAACAGCTATCAATAGAAAACACACAGAT
AAAAACTTATAAATTATAATATGTTTTTACTAAAGACTTTAATAATTATTTGAAACTAG
TTCAGTATATCAAATTAATTTTGCTTATACTACGGATGTCTAAAACTTATATAAAAAAGT
AGTAAAAAAGTTTATTAAAAGAATAGTTAAAAACAGATATAAATAGAAGTAAACAGAATT
ATTGAAAAAAAATGATTATTATTAATTAATACTTAATATTTCTAAATATTTTACAATTAA
TGGCTATGTCACCATCTTTATTTTATCCTGTTGAACAAGAACTATGTAGTCTTGAAAAAA
ATCTGAAAGCTGTTGCTGGGACTCGTCATCCAATTTTATATGCCGCAGCAAAGCATCTAT
TCGATGCTGGAGGAAAACGAGTTAGACCAGCTCTTGTATTTTTAGTGGCTAAAGCAACCT
CTGAGAAGCAAGATATAAATACTGGACAAAAAAGGCTAGCAGAAATTACTGAAATTATAC
ATACTGCTAGTTTGGTACATGATGATATTATTGACGAGTGCACAACACGTAGAGGAGTCA
AAACTGTACATAATTTATTCAATACTAAGATTGCTGTGCTAGCAGGAGATTTTTTATTTG
CACAGTCTTCTTGGTATTTAGCTAATATTGAAAATTTAGCCGTAGTCAAAGCTATTTCTA
AAGTCATCACCGACTTTGCAGAAGGAGAAATTAGGCAAGGCTTAGTTCATTTCGATCCCA
GTATTTCAATAGATGCTTACATTGAGAAATCATTTTACAAGACTGCTTCACTCATTGCTG
CTAGTTGTCGGGGTGCAGCTATGCTTAATGGTTCCAATCATCAAATAAATAATGATCTTT
ATCTTTACGGTAAACATATGGGATTAGCATTTCAAATTATGGACGATGTTCTAGATATAA
CTGGTTCTACTAAGAGCTTAGGAAAACCTGCTGGCGCTGATCTAATAAATGGAAATTTGA
CCTCTCCTCTCCTTTTTTCACTTACTCAAGAAGCAAGTTTAAATGATCTTATTGATAGGG
AGTTCTGTAATAGTACAGATATAGCCTCAACATTATTTCTTATAAAAAGAAGCGGGGGAA
TTACAAAAGCTAAAGATTTAGCTAAAGAACAGGTGCAGGCGGCACTTTCCTGCCTTCAGT
TTTTACCACAATCTACACCTGTATCTAGTTTAAAAGAATTAACACATTTCATAATCACAA
GATTGTCATAAAGGACTTGCTAAAATTAAAATGTTATTAACTAATTTTTTACTGTTTCTA
AAACAGTCTGAAAAGTTTGAATATCATTACTAGCTAGCTGCGCTAGCATTTTACGATTTA
ACGCAATATTCTCTTTTTTTAATGCACTAATAAAAGTACTATAATTCATGCCTTGATTGT
GAGCAGCAGCATTTATTCTAGTTATCCAAAGACGACGAAAATCTCTCTTTTTCCTTTTTC
GACCAACATAAGAATATCGAAGAGCTTTAAGGACTTGCTGCTTAGCTGTTCGAAATAAAC
ATTTATGTGCACCTTTAAAGCCTTTAGCTAGCTTAAAGATTTTAGCTCGTCTTTTTTTTG
CAACGTTACCTCTTTTAACTCTACTCATAAAATATTACTTAAATTTTAATTATTACCAGC
TTACTAAATTCTTATACTCTTTGATTATAAATAAGGTAATTTCATTGCAATGTTTTTAAT
ATCTTTTAAGTCAACCGAACAAGTAGAAGAAAGATGTCTTCTTTGTTTTGATGACTTTTT
TTGTAATAAATGACTTTTAGAAGCTTTATGTCGAAGAAATTTTCCGGATGAAGAGACTTT
AAATCTTTTTGCTATTGCTTTTGATGTTTTTAACTTAGGCATATTATAAACTTTTAATGA
TTAACAAAAGCATGAAAAGTCTGAGGATATCAATCTTAGACCTTTCAATACTTAGAATAA
TCTTAACACAATTTATTTTACTTACTACATAGGTTAACTGAAACAATTTATATATATAAT

ACTAATCAGTTAATCAACCATAGCTAAAGTGGATTTATATTTTGCTTTGAACTTTTGTAT
AGTATTAGCAATTAGCATTGTTATAATTTTAAGACTGCTTGAATTCAATTCTTCGAACAT
TTTCATATTTAATGTAAATGAAATATTGGCTTCTGAGACAATATTTTGAATTTGAACATC
AGACAAGGGAATCATATCTAATGCAGCTCTATATTGATCTTTAAATAATTTATCGTCTTT
TATTTGGTCAAAATCGTAAAATTTCGTTCCTCCTGAATCTGATAAATTCATAGCCCCTCT
AGCTATTTTTTTTAAAATTTGGCCACCAGAAAGGTCACCCAAATAGCGCGTATAAGCATG
AGCAACTAGTAATTCAGGTTGTTTATGTCCTATAGTGTGAATTCTATCAACATAAATTTT
TGTAGCAGGAGATGGTTCAATAAAATCTAACCAATCTGATCCATAATAATAGTTTAAATC
TTCAGATAGACTCGCTTTCCTATTAAGCTCTGTAAAATATATAGGTTTAATAGCTGGATG
ATTTTTATTAGAAAATAACTCTTCCTCTATTGCACAATAGACAAAGTATAGATTTGCAAC
CAATTTACGATATGACTTCTTATCTACAACTCCTCCCAAAAAAGATTTAACAAAACTAAC
ATTTTCTGCCATACTATGGGACTTAGTAGTACCTTCTCTTAGTTCATTCGCTAAAGTATT
AACCATAATATCAATATTCCATTTTTAAAGATAACAAATTAACAATAAATAGTTATCCGT
ATATTTATAATTGAGTTGCGCTAACTAGGCTGCTGCAAAATAATTTTTTGATTTTATTGG
ATCTGGATTCATTGTTTTGTCACCAGGCTTCCAATTAGCTGGACAAACTTCATCTGGATG
AGATTGTACATATTGAATTGCTTGTAAAACCCTCAAAGTTTCTTCTACACTCCTGCCAAA
CTCTAGATTATTGATTGTAGAGTACTGAATAATTCCTTTCGGGTCTATAATAAATAGTCC
TCTTAGAGCTACACCATCACTATTCAAAACATTATAAGCTGCACTGATCTCTTTTTTTAA
GTCTGATACTAATGGATATGAAAGATCTCCTAATCCACCTGATTCTCTGTCAGTCTGTAA
CCAAGCAAGATGAGAATACTCACTATCAACCGAGACTCCAAGAACTTCTGTATTAAGTTC
CGAAAAAGCATTATACTTATCGCTAAATGCTGTAATTTCTGTAGGGCACACAAATGTAAA
ATCTAAAGGATAAAAGAATAAGACAATATACTTATTCTTGAGATCAGATAATTTTAACGT
TTTAAATTCTTGGTCATAAACAGCTGTAGCTGAAAAATCAGGAGCTAGCTGACCTACTCG
AAGACAATTTGGTCCAGAAATCATTAATTTTTCCCAGAAGAATTGATATAATTTTGTTAT
TTAATAAATAATTATATAACAAAATTTAGTATATATTAAAATACTACCAGTCTTTTTTAA
ATTACTAAATGATATAATCAATACTATCTTTATAGCGGGGAATGGATTTGAACCATTGAC
CTTCGGGTTATGAGCCCGACGAGCTACCAGACTGCTCTACCCCGCGGATAACTAAAATCA
TATTAGTCTACATATATATATAAAAGCAAATGCATAATAGATTTTAGAAGTAAAAAAATA
GAAGAACTGGTCCACTGATCATTAAACTTAGAAACGACAAAGTATTTTTTATCTTATTTA
AAAGAGGAAAGACTTCATATAAACCAATAAATCTATCTTTTAAAAGAATTTCGGACGGCT
TTACCCAAATTTGACCGTCATACCAGCCTGATTCTTCATAAAATACTGTAGCACTCATTA
ATCGTTTTACTACGTATGACCAGCCTAAATATAATCTGATTAAAATAAATCCTGTCATTA
GGCTGGAAGTAATAAATTCTGAAAAGAAAAATTTCAAAGGTAGTTTAGTGATTGGAAAAA
TTGATAATAATATAGGACTAACTAATAAACAGTTAAGTAGTAGTGCAATAGTTATTTTTT
TGTTATAAGATCTATGGCTTAAAGTTGGCCAGCAAAAGAACCAAGAATTTTTCAAAGAAG
TATATTCATGAACAGGTTGCTGCTCCTTTGGCACAGGACATTGAGTATTATATAAATTCA

TTGACATTAAAAAGTATTATAATAAAGACTTTGTTTATATTATCTAACATAGGTACGACA
TTAAATATTAACTAACTGCAATTATAGTAGTGAGTAGAAGAAATAAAACTAAAAATAGCC
AAGTAATTTTATTTAAAGTATTCTCAGTACTACGAGTATTACTAAAGAACTGATTCTGAG
CACCTACACTTCCTAAACCTTCGGATTTAGGATTGTGTATTAGAATAGTGAAGATTAATA
TTATTGTAGATGAGTACCAAAAAAATTTTAAAATTTGTTCCATTATGTAAAAGTTAATAA
AAATAGCACAAAGACAATCGTCTAAAAGATATTGATTGTCTTATCATATTAAAAAGTATT
TATTCACCCTGTACTTTTAAAAGTAAAAATCCTATTGCCAGCCCTACCAAAACTAGGACT
GAACTTAATACAGCACTTTCTACAATTTCTCCGACCATTTTATTTTTCCTTATTATTTTA
TTATTGTCATTCTTTTGACAAGATTATATTGAGCTAACTAAAATCCGTTTCTACCCCAAA
CTACCATTGCAATTGAAAATGTAAACATTGCCATTAGAGCTGCCCACCCCAAACTTAAAA
TATCCATAATTTATAATATTCCTAAAAATAAGATCTCTTTTACTTAAACTATAATATTCT
TAATATTAAAAATATCATTATATAATAAGCTAATTATAACAGAGTACTACTTAATTCGTA
TTTTTAGTGTATTTAAAGAAGATTGAATTTTTTCGAGGTACTGGATAAGTATTTCATCCT
AAGAACCATATTCAACTTTTGATTTATTATAATGCTTAAATCTAAATATATATCACTCAA
GAATCATTAGTAATTATGATGTTTTTAATTATACGATTAACATATTTCAAAATAGAAGAA
TTCATAGCGACTAAGTTAATATGCTAATATAATTTATCAATAAAAAGGAAGTTAGTATTT
CAATTATGCAAACAACTATAAATAATGGGCAAACGTCTAGTAAAGAAACTTTACTAACAC
CTAGATTCTACACGACTGACTTTGAAGAGATGGCTAATATGGATATTTCCGGCAATCAAG
AAGATTTTTTGGCTATCCTCGAAGAATTTCGAGCTGACTATAATAGTGAACATTTTATTA
GAGATGAAGAGTTTAATCAATCTTGGTCTAATTTAGAACATAAAACTAAATCCTTATTTA
TTGAGTTTTTAGAAAGATCTTGTACCGCAGAATTTTCAGGTTTTTTACTATATAAGGAAT
TATCCAGAAAATTAAAAGACAGAAATCCGGTTATAGCTGAGTGCTTTTTATTAATGTCTA
GAGATGAAGCTAGGCATGCCGGTTTTTTAAATAAAGCTATCGGGGACTTCAATTTATCTT
TAGATTTAGGATTTTTAACGAAGAGTCGCAAGTATACTTTTTTCTCACCTAAATTTATTT
TTTATGCAACCTATCTTTCTGAAAAAATTGGATACTGGAGATACATAACTATTTACCGCC
ATCTTGAACAACATCCAGAACACCGTATTTATCCAATCTTTAGATTTTTTGAAAATTGGT
GTCAGGATGAAAATCGTCACGGAGATTTTTTTGCTGCTTTGCTCAAATCCCAACCTCATT
TTTTAAATGACTGGAAAGCAAAAATGTGGTGCAGATTTTTTCTATTAAGTGTATTTGCAA
CAATGTACTTAAACGACTTTCAAAGAATTGATTTTTATAATGCCATAGGCCTAGACTCTA
GACAGTATGATATGCAAGTAATACGAAAAACTAACGAAAGTGCCGCTAGAGTTTTCCCGG
TTGCTTTAGACGTGGACAATCCAAAATTTTTCAAATATTTAGATACTTGTGCATGTGATA
ATAGAGCTCTGATCGATATTGATAATAACAATTCTCCATTATTCATTAAATCTATCGTAA
AGATACCTCTATATTTTTCCTTATTTGCAAATTTACTAAAGATATATTTGATTAAGCCAA
TAGACTCAAAAACAGTATGGAATACAGTTCGATAGTTAAATACAAGGGAATGCTTTTTTC
TTATAAAAAGCTATCTAGTGATTAATATAAAATAAAAGCTTCCTTCTCGATGGAAGGAGC
TTTTATTTTGTCAAGAATATAATGGGATTATATAGATCTAGAACTAAGAACTTACTTTTT

CTTTTGCTTCATACATAACCTCTAAAGTAATTAGATTTTTAGAACAATCACGGGCAAATT
TCTCTGTATTTCTTTTTACTTTACCTCTAACAAACCCAGGAATTTTATTTAATTCTTTCT
GTGCTTCTTCTGACCAATTAATACTATCAACAGCAGAAATTCCTATACTTAATGCTTCTG
TTGTATCATGACCACCAAATATTTCTAGCAAATGATCTTCCATACCAAGTGTAAACGAAT
TATATACTAAATCAGCAATTTGATTTGTTCCCTCATAACCAAGAAATGGACGGTAACTTA
AAGGAAAGTTTTGTATATGCACTGGTGAAGAAATAACGCCACAAGGAATATTTAGACGCT
TACCAATATGTCTTTCCATCTGTGTTCCAAAAATTGCTGCAGGTTCAGTTTTAGCAATTA
AATCTCCAATTAAACCATGATCATCTGAAACGATTACTTCATCGCAGAATTCTTGAACTT
GATCTTTAAACCACTCTTCATCATATTTGCAATAAGTTCCACACCAGGCGACATGTATAC
CCATTTCCTGGTGTAAAATTCGAGTTATTGCTGCAGCATGAGTTGCATCTCCAAATACAA
TAGCCTTTTTACCTGTTAAATTCTGACAATCAATTGATCTTGAAAACCATGCAGATTGTG
AAATAAATCGGGTTTGCTCGTCTATGTATTTTTCGTAGTCTACAGCAGCTCCCAATGCAT
TTACTAATTGCTGTATTGATCGGATGCATGCAGCTGTTTGAACAATACCCATAGGTGTAA
TGTCAACATAAGGCATATTAAATTCTTTCTCTAAATATCGAGCTGTCATTAGTCCAGTTT
CTCTATATGGAATAAAATTAAACCATGCAGAGGGTAATTTTTTTAAATCTTGAACTGATG
CATTTTCTGGAATAATTTGGTTTATTTGGATATCTAGATCTTGAAATAACCTTTTTAATT
CTGCTATATCATGTTGATTATGAAATCCTAAGCTTACTGCTCCAATAATATTAACAGATG
GAGTTTTAGTCTTTTGAGTTAAAACTTGATTATTAGATTTAGCTTTTTCCATATAGAAAG
TGACAATTTGTTCTAACGTTCTATCACCTGCTTGCAGTTCATTTACTCTATAATGATTAA
CATCAGCTAATAAAACATCTGCTTCTGTTTCTATAGATGCTCTGCTAACAAAGTTTTGCA
AATCTTCTTGCAAAATACTCGAAGTACAAGTTGGTGTTAGAATAACTAAATCAGGGCTTT
CTTCTCTGTCTTTTCTAGTAATATTTTCTACAACTTTTTCTTGAGAACCACGGGCTAACA
CATGTCTATCTACAACACTTGCTGTAACAGGAGTAAAATCTCTGTCTCTTTCAAGCATTG
AACGCATTACATTAAAATAATCGTCTCCAAGAGGCGCATGCATAATTGCATGAACTTTTT
TAAAAGAACTCGCAATTCTTAAAGTTCCAATATGAGCAGGGCCTGCATACATCCAATAAG
CTAATTTCATATTAAATTTTCCTATGATTTATTTAGACATAGTAATACTTTTAAAGCTAA
CTAACAAACATTAGCCAAACATCTAATTTAACTCCTTGACAATTTTGTAATATTTTTTAA
ATGAAGCTTATCATTATAAATATTTAAAAAATATAATTCAGAATAGAAATAACTGTTAGA
TCAAGCTTGAAAGTAAAGATTTCTTGCAGTTATAGTTTTTCTTTAAGTAAACATGTAAAA
ATTGTACTATATATAAATGTACTATATATAAACTTTTTTTACTGAATATTGGTAATGCCA
GATACAATTAACTTAAATATGCCTTCCCCAACGTTTGGTGGAAGCACTGGCGGTTGGTTA
AGAGCCGCAGAAGTAGAAGAAAAGTATGCGATAACATGGACAGGCAAAAATGAAAGTAAG
TTTGAAATGCCAACTGGTGGTACCGCAACTATGCGAAATGGAGAAAACTTACTCTATTTA
GCTAAGAAAGAACAGTGTTTAGCTTTAGGTACTCAACTAAAAAGTAAATTTAAAATATCT
GACTATAAGATTTACAGAGTTTTTCCTAACGGAGAAGTGCAATATTTGCATCCTAAAGAT
GGAGTCTTTCCAGAAAAAGTTAATACTGGGAGAGCTAGCGTCAACAGTGTTGATCATTCT

ATTGGACAAAATATTAATCCTGTAGATGTCAAATTCATGAATAAAGCAACTTACGATTAG
TCTCTCAAAAACCTAGACATAAAATAACTTTTATATAGTCTAGGTTTTTGATAATGTATA
CTGATAGTATGGAAGGGTGGCCGAGTGGTTGAAGGCGTCTGATTTGAAATCAGTTGAACT
ATCTGGTTCCGTGGGTTCGAATCCCACCCTTTCCGTTATAAAACTATAGTTGGCTTCTCA
AGGCAAGCTAGCTATACACTGATAGTATTAAGTTTAGACTTAATAAAGTCTACAACTTGT
GACAGGTTAGAAATTTTTTCGGCATCCTCATCTGGTATCTCTATACTAAATTTTTCTTCT
ATGGCCATTACCAGCTCAACTGTATCTAGAGAATCGGCTCCCAGGTCACTTGAAAAATTT
GCTTCCCTAGTCACTATTTTTTTTTCAATTCCTAGCTGTTCTGCTACAATATCTTGAACT
TTTTCAAAGATTTCATTATCTTGCATAATATATTATTTCCTCTAAGGTGTTATCAAAAAT
TTTTCGGCTTATATTATTATCCAACTCTAAAAAATTAGTAATTTTTAGTCGAGATTAAAA
TATTTATACCTATATGATATACTATTATTCTGGATAAATACGAAGTTTACGATATGGCTC
TTCTCCAAAACTACGAGCTCTCAACTGGTAGTTGTCAACTAGATTATGCTGCATTTTTCG
TATATAGGCTGATCGAGGTGTAAGCTGAACTATTGAACTTTCATTCAAGATAATAGTTTC
AATAGCCAATTTAGCTTCTTGAAGAGCCTGGATCTCATCAAAATTTTTATTTTTACAGAG
TTCTACCCAATTAAGACCAGATGAAGTATGAATGTTTAATATTTTTCTGAGTGCACGAGT
AATTTGTGGAACCGTACTATTCTGAATTGTATATATAATAATTTGTTTTGATTTTGCAAT
TTGTCTAAGTTTTGTATTTTGCTTTACTTGATTACGTAGCGCTAGAATAGCATCAGACTT
TTCTATCTCCTTGGTTAAAATAATTGGTAGATCTAGTGAAGAAATAACTGATGTAATATG
CTGCCAACTCAAAGAATAAGCATATAAGTACTGATGAGGTATTTCCAATTGCAAAGATTG
TTTATTAATATTAATAGGGGCATTGAAAGTAGTTGACAATAAAGATGTATCACGATTTTG
TGATTTATTCAAATCTAAGGACTTATTTCTTAATTCTCTATATTGTAAAACCGGAGGCGC
CTGCTGTGCGTTAATTGATTTACGAGCATTAGAGATGTTTACAGGTAAGACTTCTATAGA
TTGCGATGGATAACATTTAATTAAAATTCTACCATTAGCTTGAATTTGTCTTTTTTGCAC
AAAAGGTTGATGGCCTTGCAAAATTTGATCTATTGTCTCTTTAACTTTATTATGAACTAT
CCAAACATTTCGCTCGTGTATTTCGATTGCAATCTGAAAAGCAGGCTCAGCTTTTCTTTC
TAGTATACTCTTTTGCGTACCTCTACGTTTAGCTTCATCGTCACCTAATGTTACATATTG
AATTCCACCAATTAGGTCCGCCAATGTTGGATTTTTAATTAAACTTTCTAAATGATTTCC
ATGCGCTGTACCTACTAATTGAACTCCTCTTTCTGCTATAGTTCGAGCAGCTAACGCTTC
TAATTCCGTGCCAATTTCATCTATAATAATAACCTCGGGCATGTGATTTTCAACAGCTTC
TATCATTACTTGATGTTGCAAATCGGGCCTTGCGACTTGCATTCTTCTAGCTCTACCAAT
AGCTGGATGAGGAATATCTCCGTCACCAGCTATTTCATTTGAAGTATCTATAATAACTAC
TCTTTTTTCCATTTCGTCAGCTAAAACACGGGCCATTTCTCGAACTGCGGTTGTTTTACC
AACTCCTGGTTTTCCTAAGAGTAAAATTGAATCACCTTGCTGTAGTAAATCTCGAATAAT
ACTAATTGTACCAAAGACTGCTCTGCCGACACGACAGGTTAAGCCAATAACACTACCTTC
TCTATTACGCAGTGAACTAATACGATGCAAGGTTTTTTCGATACCAGCTCGATTATCGCC
GCTAAAATTACCAACTTTTTTTACACAATAATCTAAGTCTTGCCAACTAATAGATCTTTG

AGATAAATATTCTGGGTTATCTGGAAATCTAGCTTCTGGACGACGACCTAAATCCATAAC
GACTTCTATTAAATTATTTCTATTAGGATGCTGTTGTAAAGGTTCTTTGACAAAATTTGG
CAAAATTTCTAGCAACTTATCTAAGTCATCTGCAATAAGCATGATTGAGTATATAATAAT
TAATTAATCAAATAGAAGATTCAAGATTCTAAAGATTACAATACATATTAAAGAATATTA
TGATTGGAATCAATTTTATACTTAAATTGAACAGGAAGTTATAGATGTAAAAGAACTGAT
TAAACTTTACGCAATAAATCGATTGGTCAAAATTAATAGTCGTGTTAACATGTTTATTGG
ACAAAAAGTTCGTATTAAATATAGCAAACAAAAAGTTGCTAGTGATATAGCAGATAAAGT
TGGTGAATTAGGAGTTATTAAGGGAATAAAGTTTATTAATAGTCAATGTGTTACTATTAT
TGTTGAGTTTGACAATCACACTAGGCTTTGGATGTTTAGAGAGGAATTAATCTGTCTAAA
TGAATTAAAAACATGAAACATAATTTATTTTATTTATTATACAAAATTTCTTATGAGTCG
TTAAAATAATAAATTTTAAAGGAGTACTTTTAATTGTGCAAATTACTATTAAAAAATTAC
AAGACTTACTTTCGTCTGTACAAAGAAAAAAGATCCAGACATTAAAACTAAAACAAGGTA
AGTTTGAACTTCTATTAAATAAGACCTATAAAAAAGTCAATCAAGAAATTATACCTTCGC
AGAAATCTGCTGTATTACAAAATAGTCCATCTACGATAATTAAATCAATAAATAATACAA
AAAAATCTTCTGTTGTTAATGAAGACCGTACAGAATATGCCACTATTGTTTCTCCAATGG
TTGGAACGTTTTATCATTCACCTGCTCCTGGTGAAAAAATTTTTGTACAAGTTGGCGATG
AGGTCAAATTCAATCAAACAGTCTGTATTATTGAAGCAATGAAGTTAATGAACGAAATCG
AGGCAGAAATTGAAGGCAAGATTATAGAAATTCTTGTTAAAGATGGTGATATAGTAGATT
GCGGGCAAGCCTTAATGAAGGTTGAAACATAATGCTAATTTTTCCATTTTACATATTGTT
AACAGAAATTATTTAGATAGAAAAATTAGCTTATACTATAGTTATGAAAACTTATTTCGC
AAAGGCACTAAAAACTAATATATAAAATTATTTACTTTATCTGCCAAAAATAGAAATAAG
CGTTTTGATTCCTTGTCTCAAGAGAGGAGAATCTCAATGGCAATTAGCTCAAAAGAGCAA
GAGACAAAGAAGGTAAAAATCTCGGTTGATAAAAATCCCGTAGATACTTCTTTCGAAAAG
TGGGCCCAACCAGGCCATTTTTCTCGTACACTAGCAAAAGGACCAAAAACTACTACTTGG
ATTTGGAATCTTCATGCTGATGCTCACGACTTCGATAGTCAAACCAGTTCTTTAGAAGAA
GTTTCACGTAAGATTTTCAGTGCACATTTTGGGCAGCTGTCTGTAATATTTTTATGGCTT
AGTGGAATGTATTTTCACGGAGCCCGCTTCTCTAACTATGTTGCTTGGTTAAGTAATCCA
ACAGGTATTAAGCCAAGTGCGCAGGTTGTTTGGCCTATAGTTGGGCAAGAAATTTTAAAT
GGCGATGTAGGTGGTGGCTTTCAAGGAGTACAAGTTACATCTGGATGGTTCCAACTGTGG
AGAGCATCAGGAATTACTACAGAATTTCAGCTTTACTGTACTGCTATTGGCGGATTAGCT
ATGGCTGCTTTAATGCTGTTTGCAGGATGGTTTCATTATCATAAAGCTGCTCCAAAGTTA
GAATGGTTTCAAAATGTTGAATCAATGATGAATCACCATTTAGCTGGGCTTTTAGGCTTA
GGCTGTTTAGGCTGGGCAGGCCATCAAATCCATTTGTCTTTGCCTATTAATAAGCTACTA
GATTCTGGCGTGTCTCCGCAAGAAATTCCACTACCTCATGAGTTTTTAATTAATAGAGAG
CTTATGGCTCAGCTGTATCCAAGTTTTAGTAAAGGATTAGTTCCATTCTTTACTTTAAAT
TGGGCTGAATATTCCGACTTTTTAACTTTTAAAGGAGGTTTAAACCCTGTTACTGGAGGT

TTATGGCTAAGTGATACTGCTCATCATCATCTAGCATTAGCTGTTCTATTTCTTGCTGCA
GGTCATATGTATAGAACCAATTGGGGTATTGGACATAGCATGAAAGAAATTCTAGAAGCT
CACAAAGGACCTTTTACCGGCAACGGTCACGAAGGTCTATATGAAATTCTTACAACTTCT
TGGCATGCACAGCTTGCAATTAATTTAGCTATGATGGGATCTTTAAGCATCATTGTAGCA
CATCACATGTATGCAATGCCTCCTTATCCATATATTGCTACTGATTACCCGACTCAGTTA
TCGCTCTTCACTCATCATATGTGGATTGGAGGATTTTGTATTGTTGGAGCAGGAGCGCAT
GCTTCTATATTTATGGTAAGGGATTATAATCCTGCAGAAAATTATAACAATCTTTTAGAT
AGAGTCATTAGGCATCGAGATGCTATTGTTTCTCATCTAAATTGGGTATGTATATTTCTT
GGATTCCATTCATTTGGTTTATACATTCACAATGATACTATGCGGGCACTTGGAAGATCT
CAAGATATGTTCTCTGATACAGCTATACAGTTACAACCTATTTTTGCTCAATGGGTACAA
AGTATACACACTTTAGCTCCTGGAAATACAGCTCCAAATGCATTAGCAACAGCTAGTTAT
GCATTTGGAGGAGATATTGTTTCTGTTGGTAACAAAGTTGCAATGATGCCTATTTCTTTA
GGTACTGCAGATTTTTTAGTTCACCATATACATGCATTTACTATTCATGTAACTGTTTTA
ATTTTAGTTAAAGGGTTCCTTTTCTCAAGAAACTCTAGACTAATTCCTGACAAGGCCAAT
CTTGGCTTCAGGTTTCCATGTGATGGACCTGGTAGAGGTGGTACTTGCCAAGTGTCTGGC
TGGGATCATGTTTTTCTTGGCTTATTCTGGATGTACAATTCTCTGTCTGTAGCAATTTTT
CACTTTAGTTGGAAAATGCAATCAGATGTTTGGGGTAGTGTATCTCCGTCTGGAAATGTT
TCTCATATTACTGGCGGTAATTTTGCACAGAGTGCAATTACAATCAATGGATGGCTGAGA
GATTTCCTTTGGGCTCAAGCATCTCAAGTTATTCAATCATACGGTTCTGCGCTATCTGCA
TATGGATTAATTTTCTTAGCAGCACATTTTGTATGGGCATTCAGTTTGATGTTCTTATTT
AGTGGTAGAGGTTATTGGCAAGAGCTTATAGAATCAATCGTATGGGCGCATAATAAGATA
AAAGTTGCTCCTGCAATTCAACCAAGAGCTTTAAGTATTACTCAAGGTAGAGCAGTCGGT
GTTGCACACTACTTATTAGGTGGAATTGGTACAACTTGGGCATTCTTTTTAGCGAGAATT
ATTTCAGTAGGCTAATAGTGAAAATAGGATAAAAAAAACAATTATGGCAACAAAATTTCC
TAAGTTTAGCCAAGCTTTATCACAAGATCCTACAACTAGAAGGATTTGGTATGGTATTGC
TACGGCACATGACTTTGAAAGTCATGATGGAATGACAGAAGAAAATTTATATCAAAAGAT
ATTCGCTTCGCACTTTGGACATCTAGCAATTATCTTCTTATGGACATCTGGTAATTTATT
CCATGTAGCTTGGCAAGGCAACTTTGAACAGTGGGTATTAAATCCTTTGAAAGTTAAACC
AATTGCTCATGCAATTTGGGATCCGCATTTTGGACAACCTGCTTTGAAAGCTTTTAGTAA
AGGTGGCTCAGCTTATCCAGTAAATATAGCATATTCTGGCGTATATCACTGGTGGTATAC
TATTGGTATGAGAAGCAATCAAGACCTGTATTCTGGGGCTTTATTCTTACTAGTTTTATC
AGCTTTACTCTTATTTGGAGGGTGGCTACATCTACAACCAAAATTCAAGCCTGGTTTATC
ATGGTTTAAAAATAACGAATCAAGATTAAATCATCATTTATCTGGATTATTTGGGGTTAG
TTCTTTAGCCTGGACAGGTCATTTAGTACATGTTGCTATACCTGAGGCAAGAGGACAACA
TGTAGGATGGGATAATTTTACAACCGTATTACCTCATCCAGCCGGTTTACAGCCGTTTTT
CAGTGGTAATTGGAGTGTATATGCTCAAAATCCAGATACAGCTCAACATTTATTCGGAAC

TAATGAAGGTGCGGGTACAGCAATTCTGACATTTCTAGGAGGATTTCATCCTCAAAGTCA
GTCTTTGTGGCTAACTGATATGGCTCATCACCATTTGGCTATTGCAGTAGTATTCATTGT
TGCTGGACATATGTATAGGACTAATTGGGGAATTGGGCACAATCTAAAAGATATTTTAGA
TGCTCATAGACCACCTAGTGGTAGATTAGGAGCTGGACATAAAGGGCTATTTGATACTAT
TACTAATTCTTTACACATACAGTTGGGATTAGCATTGGCTTCCCTAGGTGTAATTACTTC
GTTGGTAGCTCAACATATGTATGCTATGCCTCCATATGCTTTCATGGCTAAAGATTTCAC
AACTCAAGCATCTTTGTACACACATCATCAATATATTGCTGGGTTTCTAATGGTTGGAGC
TTTTGCTCATGGGGCAATATTCTTTGTTCGAGACTATGACCCTGAACAGAATAAAGATAA
TGTTTTAGCTCGTATGCTAGAACATAAAGAAGCTATCATTTCTCATTTAAGTTGGGTAAC
TCTATTTTTAGGGTTTCATACATTAGGCCTTTATGTTCACAATGACACAATGATTGCTTT
TGGAACTCCTGAAAAACAAATTCTAATTGAGCCGGTATTTGCTCAATGGATTCAAGCCTC
TTCAGGGAAAGCACTTTATGGGTTTGATGTGTTACTATCATCCTCTACTAATATCGCAAC
ACAAGCTGGTAGCAATATTTGGCTGCCAGGCTGGTTAGAAGCGATTAATAGCGGAAAAAA
TTCATTGTTTTTAACAATTGGTCCTGGTGACTTCTTAGTTCATCATGCAATTGCATTGGG
ATTACATACTACTACGTTAATTTTAGTTAAAGGTGCTTTAGATGCAAGAGGCTCTAAACT
TATGCCGGACAAAAAAGACTTTGGATATAGTTTTCCTTGCGATGGACCTGGCAGAGGCGG
CACCTGTGATATATCTGCATGGGATGCGTTCTATTTAGCTGTATTTTGGATGCTAAATAC
AATAGGTTGGGTAACATTTTATTGGCATTGGAAACATATTACAATATGGCAAGGCAATGC
AACTCAATTCAATGAGTCTTCAACTTATCTAATGGGATGGTTTAGAGATTACTTATGGCT
AAATTCTTCTCCATTAATTAATGGTTATAATCCATATGGCATGAATAATTTATCAGTATG
GTCATGGATGTTCCTATTTGGACATTTAGTATGGGCAACAGGATTTATGTTCTTGATCTC
CTGGCGTGGCTATTGGCAAGAGTTAATTGAAACTCTAGCATGGGCGCATGAACGTACACC
TTTAGCAAACTTGATTCGTTGGAAAGATAAACCTGTTGCATTATCAATTGTACAGGCAAG
ATTAGTAGGTTTAGCACATTTTTCTGTAGGATATGTATTAACTTACGCAGCTTTTGTATT
AGCTTCAACAGCAGGCAAATTTGGTTAGACTAAATTCGAATTAGCT-AAAAAAAGTCAGT
CATAATAGTTTTATGACTGACTTTTTTAGATTTAAAACAAAAACTTACTAAAGTTTCACC
TCAATATCTACACCAGAAGGCAAATTTAACTTCATTAATGCATCTATCGTTTGAGAAGAA
GGTTGATGAATATCAATAATTCTTCTGTGAGATCTTATTTCAAAGTGTTCTCTTGAGTCT
TTATCTACATGTGGAGAACGTAAAACACAATAAATTCTTCTTTTTGTCGGTAAAGGAATT
GGCCCTACTGCAACGGCATTAGTTCTAGATGCGGTATCTAGTATTTTGTCACACGACGTA
TTAAGTATAATAGAATTATATGCTTTCAGTTTAATTCTGATTTTTGTCTGCTGAGTAATT
GTCATGTTAAAAAGATCTTATTATTTAAGAATTTTAGAGACAACACCTGCGCCTACGGTA
CGGCCACCTTCTCTAATAGCGAAACGCATACCTTGCTCAATTGCAATTGCATTAATTAAT
TCAGCAGTCATCTTAATTCTGTCACCAGGCATAACCATTTCTGCATCAGTACCATCATCA
GCAGTAAACTGATTAATAGTACCAGTTACATCAGTTGTTCTAACATAAAACTGAGGTCTA
TATCCTGGAAAAAAATGGAGTATGTCTTCCGCCCTCCTCTTTAGTTAAAATATAAACTTCT

GCTTCAAATTGAGTATGAGGTGTAATTGTACCAGGTTTAGCTAATACCATACCTCTTTCA
ATATCTTTTTTCTGCACACCTCTTAAAAGAATTCCAATATTATCACCCGCTAGACCTTCT
TCTAACGTTTTTTGAAACATTTCTAATCCAGTAATAGTCGTTGTACGAGTTTCTCGTAAA
CCTACAATTTCAATTGTGTCACCAACTTTAATAATGCCTCTTTCAATTCTACCAGTGGCA
ACAGTTCCACGTCCTGTAATAGAAAAAACATCTTCTACAGCCATTAAGAAAGTTTTATCG
ACATCTCTCTCCGGCGTTGGAATATATGTGTCAACTGCTTCCATAAGTGAAAAAATCTTG
TCAACCCATTTATCTTCACCTTGCTTAGTAGCTGGGTTTTTCGTCACAGCTTCTAATGCC
AATAAGGCAGAACCTGCAACAAAAGGAATATCATCTCCAGGAAAGTCGTATTGACTTAAT
AATTCCCTTCCTTCTAATTCTACTAATTCTAGTAGCTCTTCGTCATCTACTTGATCTTCC
TTATTTAGGAATACTACTAATGTAGGAACACCTACTTGTTTTGCTAATAAAATATGTTCA
CGAGTTTGTGGCATTGGACCGTCTGCCGCAGATACAACTAAAATAGCTCCATCCATCTGA
GCAGCACCCGTAATCATATTTTTTACGTAGTCGGCATGGCCTGGACAATCTACGTGAGCA
TAATGACGATTATCTGTTTCGTATTCAACATGAGCAGTATTAATAGTAATACCTCTAGCT
TTTTCTTCTGGAGCGGCATCAATTTCATCAAATTTTTTCGCTGCAGTAGACCCTAAAGTT
GATAAAGTTGCGGAGATCGCTGCTGTTAAAGTTGTCTTACCATGATCAACATGACCAATT
GTGCCAATATTGACATGAGGTTTTTTACGTTCAAATTTAGATCGAGCCATGCTTTTTGTT
TTCCTTATAGTAAAAGTTGCTTTATAGTTACTTTTAACGATAAATCTTGTGAATTGAAAG
ATTTATTATTTAATACAGACAAGATACAATATAACTAATCTAATATCGATAGTGAGCAAA
CGCTTTATTTGCTTCTGCCATTCTATGTGTATCCTCTCTCTTTCTAATAGAATTTCCTGT
TTCATTAGCTGCATCCATAATTTCATTAGCTAATTTCATAGACATACTTTTACCAGATCT
GTCTCTAGAGAATTTAGTAATCCATCTTAGTGCTAAATTCGTACCTCTATAAGCTCGTAC
TTCAATAGGAACTTGGTAAGTAGAACCACCAACTCTTCTTGCTTTTACTTCTACTAGAGG
AGTAATATTTCGGATGGCTTTTTCTAAAATATTTAAAGGATCTGATTCTGTTCTCTCTTT
AACGATATCTAAAGCCTGATATATAATTCTTTGAGATAAAGTTTTTTTACCACTTTTCAA
AATACGAACAGTTAACATACTTACGAGTCTGCTTTTATATAAAGGATCAGGTGATGCAAA
CCTTTTTTTAGCTGTATTACGACGAGACATAGTATTTAAAATTGGTTATTGTAATAATGT
TCTATTAGCAGGTATTTAAGATTTAGGCTTTTTTGTCCCATATTTAGATCGGCTTTTACG
ACGATCTTTTACTCCTGCAGCATCTAAAGTACCACGTACTACATGATATCGGACTCCAGG
CAAATCTTTAATTCGGCCGCCCCTAATCAGTACCACAGAATGCTCTTGAATATTATGACC
TACGCCAGGAATATAAGCTGTAACTTCAAATCCAGATGTTAATCTAACGCGCGCTACTTT
TCTTAAAGCAGAGTTCGGTTTTTTAGGAGTTGTAGTATATACTCTTGTACAAACACCTCT
TCTTTGAGGGCAACTTTGAAGAGCTGGAGATTTTGTTTTTTTATGTATTTTTCGTCTTTC
GGATCTAACAAGTTGTTGAATTGTTGGCATAATAAAATTGATTTAAAGTCTTTAATAGAT
ACTATTTGAACCTGCTTTACATGAATTGGCTAGTTTTTATTTATCTTATACTTACGCATA
AATCTTTCTACTCTACCTTCTGTATCAATAATTCTCTGTGAACCAGTAAAGAATGGATGG
TTTCCTGACCAGATATCTACGTGTAGTTCTGGCTTTGTGGACCCAATCGTCATAATTAAC

TGGCCGTCACAGTAAACTTTTGCTTCTGGATACCAATTTGGATGTATATTATCTTTTGCC
ATTGTTTTTATAATTATATAAATGTATCAATAATAACTATTAACGCTTAGAAAATTGAGG
AGCCTTTCTTGCTTTCTTTAAACCATATTTTTTTCTCTCTTTTACTCTTGGATCTCTTGT
TAAATAGCCTTCAGACTTGAGTGTCGTTCTATTTTCTGGATTAATTGAGCACAATGCTCT
TGCAACACCTAAACGAATTGCATCAGCTTGCCCTGTCAAACCTCCTCCTCTAGCATTTAC
ATGGATATCATACTGGTTTAGTAGTCCTAAAACTTGTAATGGTGCATATGAAACTCTTAA
GTAATTAGGACTAAATTGAAGATAAGACTCTCCTGGTATACCGTTTATAATTAAATTACC
TGACCCTGGGACTAGTCTTACTTGTGCAACAGAACACTTCCGGCGACCTGTTCCAGAATA
GATTGCGCGAGTTTTAATTAATTCTGTCGACATAACATTCCTTGATAACTAAATAAATAT
ACATTCTTTATACTATATACTCTTGTGGCTTTTGCGCGACATGTGGGTGAATTGGGCCAG
AATACACTTTAAGCTTCGTAAATAGTTTTCTGCCTAATGGACCTTTAGGAAGCATACCTT
TAACCGATTTTTCAATAATTCTGTTAGGTAATCTTGTCTGAAGCTGATCAAATGTTTCAA
CTTTTAATCCGCCAGGCTGTCCAGAATGTCTTCTGTATAGTTTTTGATTCGTTTTATTTC
CACTTACAGATACGTGAGCAGAATTAATAACGATTACATAATCTCCAGTATCTAGATAGG
GTGTATAAGAAGGCTTGTTTTTACCTCTTAAGATATTAGAAATATGAGTAGATATTCTAC
CAAGTGTCTGATTTTTAGCGTCTATAACATACCAATGAGAATTAGTATTTAATGAGGGTG
ATTGCGTTTTATTCATGAAAAGATTAATACTTTTGGTTGACAATAAGAGAATATGTAAAT
GGTTTACATTCAATTTCTATACAAGTAGTATTTAAAAATATTATTAACAAAGAGAACTTT
TCTAACAGTTATTTAAATTGTTTGTCAGCAAGAAAATTTCTAGTTATCAATAACTTATAC
TATTTTTAATAATATAAAATATATTAATCACTTTTTTCTTTTGGCAAACTTATACCTAGT
TTTTTTTGCAAGGCATATATAACTTCTTCAGCTGATTTCTGGCCAAAATTTTTAATTTCT
AGCAGTTCTTCTTGAGAGTAATCTAGTAAATCTGCAATAGAATGGATTTGAGCTCGCTTT
AAGCAGTTATAAGCCCTAACAGATAACTGCAGTTCTTCTATTAAAACTTGACTAATTTTT
TTATCTTCTTTGCTACGATAATTATCTGCTGATTTAAAGTCTAAATTTCTTAGTGAACAA
AAAAGATTAGTTAAAACTGTAGCTCCTTGACTTATTGCTTCTTGGGGAGATATGCTCCCA
TTTGTCCAGATTTGTATAATCAATCTATCTTTTATGCTGTTGCTACCAATACGGACTTCT
TCTACTTTATAATTAACTTTATTAACTGGCATAAAAACAGAGTCTACTTGCAAAAAATCC
ACAGATAATTCATCTACAGCTTTTTCAGCTAAGCGATACCCGCAATTTTTTTCAATTTTA
AACTCCATTTCAAATATTGTGTTATTGCAAATAGTTGCAATATACTGTCTAGGATCTACT
ACCTCTATATCAGAAGATAATTCAAATAGGCCGGCTGTAACTATAGCTGGCCCTTGAACT
CTAATTCGACCAATTTGAGATTCTTTGTTATAACTTTTAAATACTACTTCTTTTAGATTA
AGTAATATTTCTAACACATCTTCTCTTACCCCAGGAATTGTGGAAAACTCATGGTTCACT
CCAGCAATCCGTACAGCAACTATAGCAGTACCTTCAAGATCTGACAATATTGATCTTCTC
AAAGCATTACCTAATGTAATACCTTGTCCTTGATTTAATGGTTCTATTACAAAACTACCG
TACTGCCCACGCGCCCCATCTGTTCTTGACTCTATGCATTCAATTTGAAATTGAGCCACC
TAAGAAAGCTCCTTTAAAATAATCAGTGATTAGTAAGTCTCTCTAAAACAAGAAGGAATT

TATACTCGGCGTTTCTTAGGAGGGCGACATCCATTATGAGGTACAGGAGTAATATCTTTT
ATTAGAGTAATCTCTAATCCTGCAGCCTGCAAAGCTCTAATTGCTGTTTCTCGACCCGCT
CCTGGACCATTTACTAGAACTTCAGTTTGGCGCATACCTTGATCCATAGCTTGTCTAGCT
GCTTTTTCAGCTGCTGTTTGAGCTGCAAAAGGTGTTCCTTTTTTAGCTCCCTTAAATCCA
CTTGCACCAGAGGATGACCATGATAATGTTTCTCCTTTTAAATTAGTAATAGTAACAATT
GTATTATTGAATGTAGATTTAATATGTGTAATACCGTTAACTGCATTACGTTTAGTTTTT
CTTGCTCCGGATTTTTTTATTTGTCTAGCCATCGTGTCTCGTCTATTATATAGTTAATTA
AAAGATTATTTTCTTGGAGCTTTTTTCTTACCTGCTACTGTTTTTTTACCTCCTCTGCGT
GTTCTAGCATTAGTTCTAGTTCTTTGTCCTCTCAAAGGAAGACCAAGACGGTGTCTTCTA
CCTCTATAAGTACTAATTTCCATAAGTCTCTTAATGCTCATAGACTCAAAACGTTTGAGA
TCCCCTTCAATCTGATAATTAGACTCAAGAATTTCTCTTATACTGACAACTTGTTGATCA
TGTAAATCTTGACACTTAATATCAGCGTCTATGTTTGTTTTTTCTAATATTTCTTTCGAG
CGAGATAGTCCAATACCATAAATATATGTTAAAGCTATCTCTATTCTTTTGTTTCTTGGA
AGATCTACTCCAGCAATTCTGGCCACTTTGTCTTGTCTCCAATAAATAATATGTATTTTT
ATAACGGTTATTTATAAGCTTAGCCTTGTCTCTGCTTATGTTTAGGATTAGTGCAAATTA
CCATCACCTTTCTGTGACGCCGAATTATTCTACATTTTTCACACATTTTTCGAACAGAAG
GACGAACTTTCATATTAACTCTATATACTAGGAATATTACTATATTAACTCTATTAATTA
TCGACATTAAAAATGTCAACAATATATTGTATTACTTTAACTGTTGTAGATTTTACTTCG
TCATACTATCATATTTTTTTGATATGACGTATGTCTGAATTTGCTTAGCTGTGTCTATTG
CGACTCCAACTAAAATAAGTAAAGACGTAGCCCCGAGACCTCTTAAGTTTTGGATCTGAG
TAACCTTTTCTATTATAAACGGAATCAGCGCTACTGTAAATAAAAACGAGGCTCCTAGGA
ATGTCAGTCTATTTAATATGACTTGTAAATAATCAATAGTCGCTTGACCTGGACGAATAT
TAGGAATGCTTGCACCCATTTTTTTTAAATTTATAGCAATATCTTCTGGATTCATTACTA
TTGATGTATAAAAATAGCTAAAGAAAAGGATCAAAGCACAATAAAGAAGAAGGTATAGTG
AACCATTAGGACAAAACAGGTATAGGATTTGGAGTAATGTTTTATTTTGAATAATTTGGG
TTAGATAAGATGGGAGGGCCATAGATGCAGATGCAAAAACAATAGGCATAACTCCACCTT
GATTTAATTTCAAGGGCAAGTAACTATTTGGATCCAAAATTGAAGATTTTCCTAGCTGTC
TTGCTGAAATAATTTTAATTCTTCTTGTTCCTTCTTGTACACAAATTGTAATTATTATCA
TTAATAAAAAGATCGCTATAAATAATCCGAACTTAAGACTTGCATTACTATAACTAGCAT
CAAAAAATGACTGTGTAAAATTCTTTGGTAGTCCTGACACAATGTTTTGAAAAATAAGTA
AAGAGGCTCCATTACCAATTCCCTTTTCCGTAATTAACTCTGATAACCACATAATAATCA
TAGAGCCTGCTGTTAAAGCTAAAACAGACTCGCAGACAAATGCAAAATTCCAATTAAAAA
CATATGGTTTTACCCATATAGAGATTGCTCCAGATTGTAAAGTCGCCCAACCTAGAGCTA
AGTACCTTGTAATTTGAGTTATTTTTTGGCGGCCCAATTCACCTTCTTCCTTCTGTAATT
TTTCCAGATTGGGGACAATTTTCGTAAGTAGCTGCATTACAATTGAAGAGTTAATATAAG
GAACAATACCTAACGCAAAAATCCCTATTGTTGAAAAACCTCCTCCAGAAAAAATATTCA

GAAAATTTACTAAAGTATTTTTTTCTACACTTGCATAAAAGGCGTCATGATCTATACCTG
GAACAGGTATAAATATTCCTAAACGTGCTAAAACTAATAGAAAGAGAGTAAAGATAATAC
GATTTCTTAGATCACTTTTTTGGCTCATAAATAAATTTTAAATGAAAAGAGATACTTTTT
TATGTAAAAACCCTATTCCAAGACTTGTATAAGTTTAAAATAGGGAAGTAAAATATTATA
GTAATATTAATATAATATTCTATGTAGAATACAGGCTTTCTATTGGAACTCCTCGATCTT
TAGCTGCTTCACTAAAAGTTCTTAATTGAGTTAAGCCATTGAGAACAGCTCGCGCGTTAT
TTAATGTATTATTAGAACCTAATTGCTTAGCTAATATATTCTGTACACCCGACAATTCTA
GGACTGTCCTTACAGAACCTCCGGCAATTACACCTGAGCCAGGTGCAGAGGGCCTTAATA
TAACTTTCGCGGCACCAGAAATTCCGTTAATAGGATGAGGTATAGAATTCGATTTTGTCA
GTGGAACTGTAACTAGATGTTTTTTAGCATCTGTTACTCCTTTTTTTACTGCACCAATTA
CATCGCTTGCTTTTCCCACGCCGACACCAACTTGGCCTTGCTCATTACCAATAACAAGAA
TAACTCGAAAGCTTAGTTTTTTACCTCCTTTTACAACTTTAGTAACTCTTTTAACTTGTA
CGACTCTTTCTTCCCAGCCACTATCTTTATCTTTTCCTTTGCTCTGTTTTTTACGATTGG
CCATTTTAAGAATTATTCCTAATTAGTTAATTACGATTCTAGAAACCCATGCCTGCTTCC
TTAGCAGCTTCAGCCAAGGCCTTAACTCTTCCATGATATAACTTTCCTCCTCTATCGAAG
ACGACATTTTTAATGCCTTCTTTCATAGACTGTTCTGCTAACTGTTTACCAACTACGCGA
GAAGTATCACAGTTTGGTCTTATGTTATCAGATTCTTTATTATTTAAATTAACAGATGAT
GTAGCTACTAATGTAATACCTTGTGTGTCATCAATTATTTGTGCGTATATATGTTTATTA
GATCTAAATACACACAGACGAGGCCTACTTGAGGTTCCTTGAACTTTTTTCCGAACTCTT
TTATGCTTATGAATTCTAGTTTGTTTAGTGTTTAGTTTCATTATTATTTACCTTTTCCAG
CTTTCCCAGCTTTTCTTCTAACAAATTCACCTTGATATCTAATTCCTTTTCCTTTATAAG
GCTCAGGAGGCCTAATAGAACGAATAGTTGAAGCAACCTGACCGACAACTTCTTTATCTA
TGCCAGAGACAGTAATATTTGTGTTGTTTTCAACTTTAATTTCAATATTGGCAGGAGGTT
TAATTTTCACTACATGACTATAGCCAACACTTAAGATTAAATCTTGGTTATCAATTTGAG
AACGATAGCCTACACCTTGTATTTGCAGTTTTTTAAAAAATCCATTAGAAACACCTTCAA
TCATATTACTAATAAGAGTTCTGGATAGCCCATGTAACTGACTAGCCATTTTTGTTTGTC
CACTCGTCTTGACAGCAATTGTGTCGTTAAGTATTTCTAAATTAATACCAGCTGGTAAAG
TTCTGGATAGGGTACCTTTAGGTCCTGTCACGGTGATAGTCTGACCATCGAACTGAGTAC
TAAGATTTGTCGGCAATAAAATTATTTTTTTTCCAATACGAGACATATTCCACCTATAAA
ATAGTTACCAAATATAGCATAATATTTCACCACCAAGACCATCATGACGAGCTTGTCTAT
CTGTCATAACGCCTCTAGAAGTAGAAATAAGAGCGATGCCTAAACCTCCAAGAACTCTGG
GCAACTCTTTATGATTTGCGTAGACTCTTAGTCCGGGTTTACTAATTCTTTTCAGAGCAG
TAATAACTGGTTGACGATTTTTACCATTATACTTAAGAGAAATCATTAAATGAGTCTCTA
TACCTTCACCCATTTGTTCGAAATTTTGAACAAATCCTTCTTCTTTTAGTACTGTTGCCA
TATTGCATGTCATTTTCGTTGCTGGAACTTGCACAATTTGATGTCTTGCTAAGTTTGCGT
TACGAATACGTGTCAGCATATCGGCGATCGTATCGTTGACCACCTTGATCCTCCTTGCGA

TGAATTATTTGAAATATTTTCAAGATTCCCTGAAAGGCATCCCTAGCTTTTTTAACAAAG
CTAGACCTTCTTGATCTGTTTTAGCTGTAGTGACAATTGATATATCTAGACCACGAATTT
GATCAATATTATCATAGTCTATTTCTGGAAAGATCAACTGTTCACGTAAACCTAAATTGT
AATTACCTTTGCCATCAAAACTTCTAGGACTAATTCCTCTAAAGTCTCTAATTCTTGGTA
ATGTTAAATTAATTAATTTCTCTAAAAAAGAATACATCTTGTCTTTTCTTAGATGCACAA
CAATTCCGATAGGAACCTCTTCTCGAATTTTAAATCCTGCAATAGATTTTTTAGCTTTTG
TGACAATTGGTTTTTGTCCAGTTATTAATGTTAATTCTTGAATACTACTTTCAAGAGCCT
TAGCATTCTGAGAAGCTTCACCTAAGCCACGGTTAATAGTAATTTTAGTAAACCTAGGAA
CTTCATGTACATTTTTGTACTGAAATTCATCTTTTAAAGATTGAGTAACAGTTGTTTTAT
ATTTTTCTTTTAATCCTATTGCCATTATATTTTCATCAATTATTTAATAAGTTCGCCAGT
CTTTTTCAGTTTTCGAATTTTTTGGCCTTTATCATTAATTATTACTGAGGATCGACTAGC
AATATTATTTTGTTCACTAAATAACATAACGTTAGAGGTATGTATTGGAGCTTCAAATTT
GATAATTTCTCCCGTTTCTCCTTCTTGCTGAGGTTTTTTATGCTTCACTTTGAGATTAAT
TCCTTTAACAATCACCTTATTTGTTTTATAAATGATTGCAATAATTTCACCTGTTTTTGT
TTTATCACTTCCAGAAATAACTTGAACTAAGTCCCCTTTTTTTAATTTAATTTTTGTATT
ATTTTTGGTTGTTTTAGAAGGACCTTTCATTATACTACCTCCGGTGCAAGAGAAACTATT
TTAGAAAAATTTTTATCTCTTAACTCTCGGGCTATAGGTCCGAAGACTCTTGTGCCACGT
GGATTATTATCTTGATTAATGATAACTGCCGCATTATCTCCAAATCTAATGCTCATACCG
TCAGTTCTCCGTAAAGCTTTACGAGTTCTTACTACAACAGCTCTGACAACATCAGATCGT
TTAACTGGCATATTGGGAGACGCATCTTTGACTACTCCAATAATAACATCTCCAATAGAT
GCATAAGAAGGATTACTAGTGCCTAATACTCTAATACACATTATTTTTCTAGCACCACTA
TTATCTGCAACATTAAGATAGCTTTGAGTCTGTATCATACTTTTATTCTGTATAAGATTA
ATTATCAAAAGACTTAGATAAGATATTAACCATTGTCCAACACTTTGTGCGACTTAAAGG
TCGTGTCTCTTGTATTGTAACAATATCGCCGATTGTGCATTCATTATTTTCATCATGCGC
TTTATATTTCTTTGTCCGGATCATAGTTTTTGCGTACTTTCTATGAGAGATTCTATTTTC
TACAGCTACTACTATAGTTTTATTCATTTTATCGCTTACTACTTTACCTGTTGTTTCTTT
TAAAGGCATAGTCTTTATATTTTTTATATATAATTAAATTTTAAGATTATTACATGGTTG
ACTTAGTACGAGACTTTTCAACAGTTAAAAGTTGGGCTAATCTATGCTTAGAATGTTTAA
ATAGATGAGGCTGGAAATCTTGCCTTGTGGCTCTTTTTAGCCTTAAATCAAAAAGCTCTC
TTTTTATTACAAGGATTTCTTCAGCTAAAGAAGAAGAGTCCAGGTTTGTAACATCTGATA
TTTTAGGGAAAGTCATATTTTAGGATTCTGTTGTATTCCGAACTATAAATTTAGTCTTGA
TTGGTAATTTATAAGAAGCTAATTTCATAGCTTGTTGAGCAGTTTTTTGTGGTACACCTG
TAATTTCAAATAGAATATGTCCAGGCTTAATAACTGCAACCCAATATTCTGGAGCTCCTT
TACCTGACCCCATGCGAGTTTCAGCTGGACGGGCAGTGACTGGTTTATCTGGAAATACTC
TAATCCATAGTTTACCACCTCTTCTCACATATCTAGTAATAGTTCTTCGAGTAGCTTCTA
TTTGTCTAGAAGTTAACCAAACTGGCTCTGTCGCTTGTAATGCATAATCACCGAACGCAA

TTGTGTTACCTTTGCTAGCAGAACCTTTCATTCTACCTCTATGTTGTTTTCTAAATTTTG
TTTTCTTGGGGCTTAGCATAAAAATTAAAAAATAATAATAATTAAGAAGCTGTTGAATCA
GGAGTTTCCTGATTATAAGCAGATTCTAGACTTTTCGATTCTGGCAAAATTTCTCCTTTA
AAAAGCCAAACTTTTACTCCGAGCACACCATACGTAGTGTGAGCTTGGCGATGACAATAA
TCAATATCGGCTCTTAAAGTTTGTAAAGGTACACGACCTTCTCTAACCCATTCACTTCTA
GCTATCTCTGCACCATTAAGCCGACCAGATACTTGAATTTTAACACCCTGTGTATTTGCT
CTTTGAGCTCTTTGAACTGCTTGTCTGACAGCTCTACGAAAGGCCACTCTTTTTTCTAGC
TGTTGAGTAATAAATTCTGCTACTAAGGTTGCTTCAGAATCTGGATCTGCAATCTCTACA
ACATTTACTCTGAGTTGTTTGCTAGGGTCTAGGATTAAGGACAGCGATTTTCTTAAAGAC
TCAATTCCCGCTCCAGATTTTCCAAGTACGATTCCTGGTCGAGCTGTTGCTATGAGAATT
TCTACTTGATCAACTTTACGATTAATTTCAATTTTAGCAATACTAGCATTACTAAGTTTT
GAATGTATAAATGAGCGAATTTTATGATCTTCTTGTAAAAGAACTGGGTAGTCTTTAGAG
TTAGCAAACCATGAAGAACGATGTTTTTGGGTAATGCCTATGCGAAAGCCCAAAGGATGA
ATTTTTTGACCCACAGTATCCTTGATTATAAGTTAAAAGCTATAATGACTAATTGACGTT
AAAAGACTACAGTTCTGAAACACCTAATGTAATATGACAAGTTGGTTTATGTATTGGAAA
GGCTCTTCCTTGTGCTCTAGGCTGAAATCTTTTTAAAGTCGGGCCTTTGTCTGCAAAGGC
TTTACTAACAAACAATTGGTTTTTATTTAATCCATCATTATGCTCAGCATTCGCTGCAGC
AGATTCCAGGATTTGCTTTATATGAGAACAGACTCGGTATGGCATAAATTCCAGAATAAT
TAATGCTTCTTGGTATTTTCTACCTCTAATTTGGTCTAATACACGACGGACTTTATGCGG
AGAAAGACGAATGTATTTCCCTACTGCTTTAGTTTCTTTTACGTTTTTTGTAATACTCAT
AGTTATAGTTTAACGACGGGCTTTTCTGTCACCTTTTACGTGAGTGCGGAAAGTTCTTGT
TGGAACAAACTCTCCTAATTTATGTCCTACCATTTGGTCTGACACAAAGACAGGAAAATG
TTGTTTACCATTATAAACAGCTATTGTATGCCCTACCATATCGGGAATAATAGTAGATGC
TCTCGACCAAGTTTTTAGGACTTCTTTTTTTCCTGAAATATTTAATGCTTCTATACGTTT
AAGAAGACTAACATCAATAAAAGGGCCTTTATGTATAGATCTTGACATAATAATAAATCA
TTAGATTCTTAATAAAATAAATTCTATTTACGGCGACGTAAGACGTACGGGTTACTATAT
TTATTTGGATTTCGTGTTTTAACACCCAATGCAGGTTTACCCCAAGGTGTTACAGGACGA
GCTCGTCCAATTGGAGATTTGCCTTCACCACCACCATGTGGATGGTCTACAGGATTCATG
ACAACACCTCTAACAGTAGGTCTTTTACCTAACCAACGATTTCTACCAGCTTTACCAAGA
GTAATATTACTAGCGTCAATATTACCAACTTGTCCAATAGTAGCGTAGCATTCTTTTCGA
ATCATTCGAACTTCGCTTGAAGGCAATTTTACAGTAACAAAAGTACCTTCTTTTGCTACT
ATTTGAGCATAAGTTCCTGCTGCACGAACAATCTGTCCTCCGCACGAAGGTCTTAATTCT
ATATTATGTACTGCTGTTCCTAAAGGAATACTAGATAAAGGCAAAGCATTTCCAACTTCG
ATAGGGGCAGTAGGACCAGAAAGAACCATAGATCCTACACTGAGAGATCGAGGGTGTAGA
ATATATCTTTTCTCACCATCAAGATAGTGTAATAATGCAATTCTTGCATTTCTATTCGGA
TCATATTCAATAGAAGCAACTTTAGCAACTATATTATGTCTATTTCTTTTAAAGTCAATT

AATCTGTACTGCTGCTTATGTCCACCACCTTTATGACGACAGGTAATGACACCTCTATTA
TTTCGACCTTTGCAGAAATGGTGTTTAACTATTAATGATTTTTCTGGCTTATCAGTAGTA
ATCTCTGAAAAGGTAGAAACCGTTCTATTTCTTGTCCCTGGTGTGTAGGCACGATATAAA
CGAATTGCCATATAAGAAGATTTGAGAAGAGTAAATGTTGAACTATGTGATATATTTAAG
TTTCTGGAAACAAATTAATAGAATCTTCTGAGGCAAGTGTTACAATTGCTTTCTTGTAAT
GTGGTCGCTTCCCTACGAATCTACCGATGCTCCTTTTTTTCTTAGGAGGATGACAAGTGT
TAACACCTGTAACTTGCACGTTGAAAATATACTGTATAGCAGCCTTAATATTAATTTTTG
TTGCTTTGGGATCAACGGCAAAACAATACTGATTTTCTTCTAATAATTTAGTTGTCTTAT
CAGTAATGATGGGATATTTAACCAGATCTAATAAACCTCTTGAATCAATGCTATCCATTA
TATACCTCTTGTATTTTAGATAAAGCATCAACTGTAATAATAATTTTATGTGCAGCTAAC
AAAGCCATAATATTTAGTGTATCAGCTGAAATAAGTTCTACGTTGTGTAGATTGCGAATA
GAAAGATAAACATTCGGATCTTTTTTGTCTACGATGACTAAAACTTTTTTATTTAAATCA
AGATTCCATCGATGTATAGCTTCCATAAATAGTTTAGTCTTGGGCTGATGAAAATAACTG
TTAAAATTTTCTACAATCAGTGTATTAACTGATTTGTTATTTAATGCTGTCCTTAATGCT
AATTGTCTTTCTTTTTTATTCATTTTTTTGGTAAAACTACGAGGTTTTGGCCCAAATATT
ACACCACCACCTCTCCATAGAGGTGAACGGATAGAACCTGCTCTTGCTCGACCAGTCCCT
TTTTGACGCCATGGTTTACGACCTCCACCTCGAACTTCACTTCTTGTTTTAGTATTAGCA
GAACCCTGGCGCTTTTCATTACTTTGCTTTACTAAAGCTCTATGGACTAAGTACATCCCA
GAATCTTGACTGACCTTAAGATTTAAATCAGCGTTGCCACTTACTTGACCTTCCCAATTA
TAGACTTGATAATTTAATTGCGTGTTAACTGTCATGAAATATATCGTATGAAAGAATATT
GCTACTTACTAATTTTTACTAAAGCGCCAGGTTTACCAGGAACAGCTCCCTTAACGATCA
ACAAATCATTCTCTGAGTTAATACTTACAATTTGCAAATTTTTTATTGTAACTTTTTTAT
TACCAAGTTGGCCAGCCATGTTTTTACCTGGATAAACTCTTCCAGGCGTTGTACCGGCTC
CAATAGAGCCAGGTTGCCTATGATTTTTCGAACCATGTGACATTGGGCCTCTACTAAAAT
GATGCCTCTTTTGGTAACCGGAGAAACCTTTACCTACACTTCTAGAAGAGACATTAATCT
TTTGGCCTACTTGAAATAGATCTGTAGATAGTATTTGGCTAACTTCGAAATCATCAGTAG
ATTTCATTTCGTATTCACGTAAATATTTTAGTGGGGGAGCCTGTGATTTTTTTAAGTGTC
CCAACAATGGTTTATTTAATTTTTGTTCAGCAACTTGCTTGTAACCGACTTGAATAGCAT
TGTAGCCATCAGTAGATACAGCTTTAATTTGAGTAATAACACATGGCCCAACTTGAATTA
CAGTAACTGGAATTGATAAACCAGCTTCATCAAAGAATTGGGTCATACCTACTTTAGTAC
CGAGTATACCAACAGACACTAGATTTCTCCTTTTTGAGAGATTTATACTATCATTGTACA
TAAAAATTCTACTTCATAGTTACTGAAGTAAATTAAACTAACAGATGCATGATATTATTC
ATGACAACATAAAGGTTAAAAACTTTACCTATTTAATCTAAAATAGAAACAGCTTTTTGT
CTAGTTACAGCGTATATAAATTAGAAGTATGCTTATTAAATTTTTTATAAACTACTTAGA
TTTAACTGATATTGATATCCGTAACTTACTTGCAGTAATATCAATCAACATGTATAAGTT
TAATTGCCTAAACTAGAACCAGCTATTATTAGAAATAATTCAAGTATAGATAAAAGCGTA

TTTGAACTTTTATATATTGACAAATATAATAGAATATATTGAGGTTCGGTAACTACACCT
TTTTATTTTTACATTTATTGTGCAGTATATATACATCTAATGCAGTAAACATAAGAATAT
AACTGTATAGCAAATGTAATTACACAAATTAGAGAGGTATTAATGGGCAAAGTCGTTGGA
ATTGATCTTGGAACAACGAATTCTGTAATTGCTGTTATGGAAGGAGGTAAACCTACCGTC
ATACCGAATGCAGAAGGTTTTAGAACTACAGCTTCTGTTGTTGCATATACTAAAAGTGGA
GATAAACTTGTAGGACAAATTGCCAGGAGGCAAGCTGTTATTAACCCAGAAAACACTTTC
TACTCTGTCAAAAGATTCATAGGACGTAAACAGAATGAAATTTCGCAAGAGATTCGGCAA
ACATCATATAATGTTAAAACTAGTGGATCAAGCATAAAAATTGCTTGCCCTGCACTGAAT
AAAGATTTTGCTCCAGAAGAAATTTCAGCTCAAGTACTGAGAAAACTTGTTGAAGATGCT
AGTACGTACTTAGGTGAGACTGTTACACAAGCAGTAATAACTGTACCGGCTTACTTTAAC
GATTCACAAAGACAAGCTACTAAAGACGCAGGTAAAATAGCAGGCTTAGATGTATTGAGA
ATTATTAATGAGCCTACCGCAGCTTCTTTGTCTTATGGACTAGACAAACAAAATAATGAA
ACAATACTAGTATTTGACCTTGGTGGGGGCACATTTGATGTATCTGTATTAGAAGTTGGA
GATGGAGTATTTGAAGTACTCTCAACTTCTGGAGATACACATTTAGGCGGAGATGACTTT
GATCAGCAGATTGTAGAATGGCTAATCAAAGATTTTAAACAGAATGAAGGTATTGATCTT
GGTAAAGATAGACAAGCACTTCAGAGATTGACCGAGGCTGCAGAAAAAGCAAAGATAGAA
CTGTCAAATTTAACTCAAACAGAGATCAATCTTCCTTTTATTACTGCCACACAAGATGGT
CCAAAACATTTAGAAAAAACTGTAACTAGAGGGAAGTTTGAAGAACTTTGTTCAAATTTA
ATAGATAAATGTAGTATCCCTGTAAATAATGCTCTGAAAGATGCAAAACTAGAAGCTTCC
AGTATTGATGAAGTTGTTTTAGTTGGTGGATCTACAAGGATTCCAGCCATACAGCAAATG
GTTAAAAGATTAATTGGTAAAGATCCAAACCAAAGTGTCAATCCAGATGAAGTTGTTGCT
ATTGGTGCAGCCGTACAAGCTGGAGTTTTAGCAGGCGAAGTCAAAGATATTCTATTACTA
GATGTGACGCCGTTATCTTTAGGAGTGGAAACTTTGGGTGGCGTGATGACAAAGATTATA
CCAAGAAATACTACTATTCCTACAAAAAAATCAGAAGTATTTTCTACAGCTGTAGATAAT
CAACCAAATGTGGAAATTCAAGTACTTCAAGGCGAAAGAGAACTAACAAAAGACAATAAA
AGCTTAGGGACGTTCCGATTAGATGGCATTATGCCTGCACCTAGAGGAGTTCCTCAAATT
GAAGTTACCTTTGATATTGACGCTAACGGAATTTTATCTGTAAAAGCCAAAGAAAAGGCT
ACTGGTAAAGAGCAATCAATTACTATATCTGGAGCATCAACTTTACCTAAAGATGATGTT
GAAAGAATGGTAAAAGAAGCTGAAGAAAATTTCGATACAGACCAAAAAAGAAGAAAGAAT
ATTGACACAAAGAATCAAGCAGAGTCCCTATGTTACCAAGCTGAAAAGCAAGTTAAAGAG
TTTGAAGACAAAATTAGCCAAGATTTAAAAATAAAAATAGAGGAGCTAATTACAGAGCTT
AGATCTAGTCTAGAGAAAGAAGAATATGACAATATTGAATCCATTTCTCAGCAATTACAA
AATGCTCTGATGGACATTGGAAAAAATGCTGCTCAGACTGAAAGTAAAGATACAAAAGCG
AAGGATGACGACACTGTGATTGACACTGATTTCTCTGAAGCTAAGTAAAAGTAAGCGGGT
AACGCGATTCGAACGCGCGACATCAACCTTGGCAAGGTTGCGCTCTACCACTGAGCTATA
CCCGCATAGATTGTATTATTACAAAAATTAAGATATTTGTCAATCTGTTAATCTATACAT

CTAGACTATTAATTTTGGAATTACATATTAGCTAACTGTTGTAAATATAACAATTGCTAT
AATTTTTTAAAAGTACTTATATCATTATATAAGTACTTTTTATATTCAAAAATACAAATA
AGTATTATAGCTAAACTACTAAGGAGTTAACTAAGCTAGTAACAATTACTAATCCTGTCC
ATAAGCCGGCTCCTGTATATATTAATCCTTTTGATTGCTCCCATTGGCCAGGGGATGCTA
AAACAACAGGTACGCCTACTACTAACACAGTCGATAATGCAATTAATAATAGCACTAATA
ATTGGATCGCAATAATCATTTAATATGTTCTCCTGAAAAATTCAAATGTTAATAATACTG
TATATAATATAATGAAGCTTTTGACCTTCAGCAATTTATCTATGAAGAAATAATTTAAAA
TTATTGTTTTCTAAATAAACCTTAAATTAAGTATTAAAATCTTTATTGTAAACAATAATA
CTATATTTTTTTAGTAGATAAAAAATTTAAAGAAAGTCTTTTTTAAAAAAAAAATTAAGAT
AAGGTGATATGCTGTATAAAAAGCTAAGTATTATTAATTATTAGAGAAATTTAAAATAAG
AGGTAAAACATATGAACTCAGCCCTTTTTTTAGCAAAACTACCAGAAGCTTATGCCATTT
TTAAACCGATCATTGATATCTTACCCGTAATTCCTGTATTTTTTCTTCTTTTAGCTTTTG
TTTGGCAAGCAGCAATTGGTTTTAGATAACATTTAGGTTATAAAAAGAATAACAAATATA
CAGAGTGCCAATTTTTGAGCTCTAATCTTATATCTTACACAATAATAACTATTATGGTTG
AACCCTTACTATCAGGAATTGTTCTTGGACTTATTCCTATTACTTTATTCGGATTATTAG
TAGCAGCTTATTTACAATATCAACGGGGCAACCAATTAGGACTGTAAAGCCTAAATTATA
GATATTTATAAAAACTACAATAATTATAGTTTTTATAAATATCTACAAATAAGCTAATTT
TAAATACAGAAATAATACTATTGAAACTAGAGTGATAACAAAAAAAAATTTATGTAATAAC
AAGCTAATTGTTTACCAGCTCGACTTAGTAACTCCTGGTAAAAGACATTCATGAGACATT
TCTCGAAAAACATGGCGAGATAATCCAAAATCTCGATAATAACCTCTACTTCTACCTGTT
AACCAACATCTATTTCTCCCTCTAACAGCGGCGCTGTTTCTAGGCATTTCTTGGAGTTTT
TGTCTTAAATCCATTTTGTCAGAAAAGCTGCCAGCTTCTTTCAGTTGATCTTTAATAGCC
AGTCTTTTTAAATAATATTTTTTTGCAAGTTTCGATCTCTTAATCTCTCTTTGGATCATA
TTCTTTTTAGCCATGCTGTAAAACTCTCTTTTAAAATTATTTATTCAATATATTAGCTAA
AAAAGCTCCTGCCTGCAACTTTTATATATTTAAAAATAAATAGCATAAAGTAGTGAAATG
CAATTGATATTTAATCAACGATACCACTTTATGCTATTATCAAAAATTTATTTAGTAAAG
TTCTTGTTCAACATGAGTTGAAATAGTGCAATCAGATGAAGGATATGCAATACAGGTTAA
AACATAGCCAGCTAAAAGTTGATCATCATCTAAGAATGATTGATCAGCTTGATCTACAGT
TCCTTCTGTAACTTTACCTGCACAAGTTGAACAAGCTCCAGCTCTACAAGAGTAAGGAAG
TTCAATTCCTTCTTCTTCTGCTGCATCTAGAATATATGTATCCTCTGCACAATTAAATGT
GACATCAATTCCTTCTTCTTCACATAGTAAATGAATTTTATAATCAGCCATTTCTTCTTA
CTCCATAATAAAGATACACTTGAAACTTAATAAAGCAAAATAATTTAAAGAACTTACCAA
AAAATTATATACTAGTTTCATTATGCATGATAGACTGGAAAAAAAGAAAATAGTATACTA
TTCTGAAAAATCAAGCTACATTTCCTACTAAATAGAACTATAGTAGGTCCAAAGTATATA
CATAAATTCATATAGTTAAAATTACTTTTATCATTTAAATAGCTGAACTTGATAGTATAG
ATTGATCATCAAGCTAGTCTTCTGCCAATAAATAATTAATTATGATTTCAATGCTTAATC

AACAAGTAGAACTTAAGCCTATTCTTAAATTTCAGATAACACAAATATATTCGCATTCTC
TTATTCAGGAAATTAAAGCCTTAATAACTAGACTTGTTTTGCAAGTTTGGAGAAGGCCAG
CTACATTGATGGCAGGTATTATCCAACCTTTATTATGGTTAATTCTATTTGGTGGGCTTT
TCTACAATGCTCCTATAAATTTGTTCACTATTAATACAAGCTATAATTGTTTTTTGAGCT
CTGGGATTATAATTTTCACCTCTTTTACTGGAGCTTTGAATTCAGGTCTTCCATTAATGT
TTGATAGAGAATTTGGATTTTTAAATAGATTATTAACGGCTCCCTTAGTATCGAGGACTT
CTATCATTTTATCTTCTGCTACTTTTATGACTTGTATTAGTTTAATACAAGTTGTATTTA
TAGTTACAGCTTCTCTTTTTATGGGAAACCCACCTCTAAATAGCGATAGTACTATGATTT
TTGGACTTATGATTCTATTAGTGACTGTAGGAGTTACAATGCTTAGTTTAGCTTTATCTT
TCACTCTGCCAGGTCATATTGAGCTATTAGCATTTATTTTAGTAGTTAACTTGCCCTTTT
TATTTTCTAGTACGGCTTTAGCTCCTTTATATTTCATGCCGCCATGGCTTCAGTTAATTG
CAAGTCTCAATCCATTGAGTTATGCAATAGAAGGTACAAGATACTTATATTCAAGCGTAA
ACTGGAATTTTACAGAGTGTGTGATTAAGATTAGCTGGGGAGATATTTGTTTAGGGCAAA
TTATTATTTTATTAATCGCTTTAGATATAATGGCAGCTTATCTTGTGTCTAATATATTAA
AAGCTAAACTTAATTAAAATTTTAATAAAAAATTTATTAAATTTATTTTACAAAGATTTT
TACTATATAATACTAATAGTAGTATGGATAATTGAAATAGAAATAGTTTTTCAAAACCAA
AGCTATTATCAACAATGATAAACATTTGTAAGAAAGTCAACAAAGTATGTTCTTATTCAT
AGGAGGCATGTAGTCAATGGGACTACCATGGTACCGTGTACACACGGTTGTTTTAAATGA
TCCTGGACGGTTAATTGCAGTCCACCTAATGCATACTGCACTTGTAGCGGGTTGGGCAGG
ATCTATGGCATTATACGAACTAGCTGTATTTGATCCTTCAGATCCAGTATTAAATCCAAT
GTGGCGACAAGGCATGTTTGTTATGCCATTTATGGCTAGACTGGGCGTAACAGATTCATG
GGGAGGATGGAGTATAACAGGAGAGAGCGTATCCAATCCTGGACTGTGGAGTTTTGAAGG
TGTAGCCTTAACTCATATAGTCCTTTCTGGCATGCTTTTTCTAGCTGCTATTTGGCATTG
GGTTTATTGGGATTTGGAATTATTTAGAGATCCACGAACTGGCGAGCCAGCCTTAGATTT
ACCGAAAATTTTTGGAATTCATTTATTGCTATCAAGTCTACTTTGTTTCGGATTTGGAGC
TTTCCATGTAACTGGACTTTTTGGACCAGGAATGTGGGTATCAGATGGGTACGGAGTAAC
CGGAAAGGTATTACCAGTAGCTCCAGCATGGGGACCAGAAGGATTCAACCCGTTTAATCC
TGGAGGAGTTGCATCTCACCACATTGCCGCAGGTACTGTAGGTATATTAGCTGGTGTTTT
CCATTTAACTGTTAGACCACCACAAAGACTGTATCGTGCTCTAAGAATGGGTAATATTGA
AACTGTATTATCAAGTAGTATCTCTGCTGTTTTTTTCTCAGCTTTTGTGACTTGTGGTAC
GATGTGGTACGGCTCTGCAACTACGCCTATTGAATTATTTGGTCCAACTAGATATCAGTG
GGATAGTGGATATTTTCAGCAAGAAATTGAGAAACGAGTAGAAAATGCTATTGCTGATGG
TGCTGCACCTAGCGAAGCATGGTCAAGAATTCCTGACAAGTTGGCATTCTATGACTATAT
TGGTAATAATCCAGCAAAAGGAGGATTATTCAGAGCGGGCCCTATGAATAAAGGCGACGG
AGTTGCTGAAGCATGGCTTGGACATCCTGTATTCCAAGATAAAGAAGGAAGAGAGCTTAG
TGTTCGCAGAATGCCTGCTTTTTTCGAAACTTTTCCTGTAATTTTAGTTGATAAAGATGG

TATTATACGAGCTGACATTCCATTTAGAAGAGCAGAGTCTAAGTATAGTATTGAACAAGT
AGGTGTAACAGCTAGTTTTTATGGTGGCAAATTAAATGGCCAAGTTTTCAACGATGCTCC
TAGCGTTAAAAAATATGCAAGGAAAGCTCAATTAGGTGAGGTATTTGAATTTGATCGAAC
TACATTAGAATCAGATGGAGTGTTTAGAAGTAGTCCTAGAGGCTGGTTTACATTCGGTCA
TGCTAATTTCGCTTTAATTTTCTTCTTTGGACATCTTTGGCATGGTTCAAGAACTATCTT
CCGGGATGTGTTTGCGGGAATCGGAGCTGAAGTTACTGAACAAGTTGAATTTGGAGCTTT
CCAAAAATTAGGAGATAGAAGTAGTAAAAAACAAGGAGCTGTATAAGACCAAAGTCAAAG
ATAGTCTTTATCAAATATTTCTAATATTAAAATAGTTAAGACAAAGTTTATTATCTAATA
GTTACTAATCAGGAGAATTTATGGAAGCCTTAGTCTATGTATTTTTACTAACAGGAACGC
TAATGGTTATATTCTTTGCGATCTTTTTTAGAGAACCTCCAAGAATAGCAAAGTAACTTG
ATTCCGTTTAAACTTATAATAATAGAACCACTTTCCAACTTATTAGTGGAAAGTGGTTTT
ATTGTAGATTACATTGTATTATTCTTCATGCTCTTCAAAAGGATCTCGGAGATCTTTTGA
AGCAGGACCAAATGCTGTGTATATCGAGTAACCTGTAATACCTAAAAGCAGACTCGAAAT
AAAAATACTAAGAACTGTTGCAGTTTCCATAATTTAAGCAGTATTAAGATGAACTTTTTC
TATAATGATATTATCAATATTAAAACATTTAATTATTAAAATTATCAAAATATACTATGG
CACTTAGAACTAGACTTGGAGAAATTTTAAGACCTTTAAATTCAGAGTATGGAAAAGTTG
CTCCAGGTTGGGGCACAACTCCTATTATGGGAATTTTCATGTTACTATTTTTCTTGTTTT
TATTAATCATTTTACAAATCTATAATTCTTCATTAGTATTAGAAAATGTAGATGTAGATT
GGGCTACTTTAGGTAGCTAACTAGAGAAGTTCTTTGATTACACAATATTAAAGACAACAA
GCGATCGCTATTAGATTATAGTAGCGATCGCTTTAAAAGTATTTTACTATATATCCAGAG
AAAATTATTTACCTAAATTAATTAACTCGGTATCTGCAAAACTATTTGTGTTAACATTAT
TATAGTTAACTTTTTCAAACCTTACTAATACTGGATATTTGATACCACTTTTATCAATTG
CTGCAACAGTACCAATTTCCTGATACCAGTACGATTCTTTTCTCAATATTTTTACTTTTG
AGCCTCTTTCCATATTTTTAGTCCTCAGATAGATTATTAAAGATTATTATATTAAAAAAT
T-TAAAAAACTAAACAAAATATATAAAAATACTACCGACAATTGTAATATATAACTAATA
AAAGGAGAGAGAGGGATTCGAACCCTCGATAACAAAAGTTATGACAATTTTCGAAATTGT
TGCAATAAACCACTCTGCCATCTCTCCTGAATTAAAATACTGTTTTAAAAAATTAGACAA
GAATAACATAGAACTAAGGTAGTTGCCTAGAAAATTAAAACTATGTTAAATATAAATTAT
AATCTTTCTATAGATATATTTAAAAAATATGTACATAGAATCTATATCAGCTTATTTCAA
GATTTTTTAATCTTATACAATTTTTACTAAATAAATAAATTCTCTCATAATATGAAACTA
TCTTGGAAAACACTTCTTCTTTGGTCTTTACCAATATTTGTAATTGGTTTCTTTTTCTGG
CAAGGTTTTTTAGGTCCAACTACTACAGATGTTGGCAGTAATATCGCAAGTTCTAGAATG
ACATATGGACGATTTTTTAGAATATTTAGATATGGGTTGGGTGAAACGGGTTGACCTCTAT
GAAAATAATCATACAGCAATTGTAGAAGCTGTTGGGCCAGAATTAGGAAATAGGGTTCAA
CGAATTCGAGTTGAACTGCCAGCAAGTGCGCCAGAATTAATTACAAAATTACGCAAAGCC
AATGTTGATCTAGATGCTCACCCCCCTAAAAGTACAAGTGCAGTATGGGGACTATTAGGC

AATTTACTATTTCCTTTACTATTAGTTGGCGGGTTAGCTTTCTTATTTAGAAGATCTAAT
AATGCTAGTGGTGGACCTGGTCAAGCAATGTCATTTGGCAAATCGAAAGCTTTATTTCAA
ATGGAGGCTAAAACGGGAGTAGTATTTAATGATGTAGCTGGAGTTGAAGAGGCAAAGGAA
GAATTTCAAGAAGTGGTAACATTTTTAAAACAGCCTGAATCATTTACTGCTGTTGGTGCA
AAAATACCAAAAGGCGTTTTATTAGTTGGACCTCCTGGCACAGGCAAAACATTACTAGCA
AAAGCTATTGCTGGCGAGGCTAGTGTTCCTTTTTTTAGTATCTCAGGCTCAGAATTTGTA
GAAATGTTTGTTGGTGTTGGCGCTTCTCGTGTGAGAGACCTATTCAAGAAAGCAAAAGAC
AATGCGCCTTGTATCGTTTTTATTGATGAAATTGATGCTGTTGGTAGACAACGAGGAACA
GGTGTTGGAGGTGGTAATGATGAAAGGGAACAAACATTAAATCAACTATTGACTGAAATG
GATGGCTTTGAAGGAAATACTGGTGTTATTGTAATTGCCGCTACTAACAGAGCTGATATT
TTAGATTCTGCATTATTAAGACCTGGAAGATTTGATAGACAAGTTTCTGTAGATGTACCA
GATTTTAAAGGCAGGTTAGCAATTCTTGAAGTTCATGCTAAAAATAAGAAAATGGAACCT
AAAGTATCTTTAGAAACCATTGCCAGAAGAACTCCCGGCTTTTCAGGAGCTGATTTAGCT
AACTTACTAAATGAGGCTGCTATCTTAACGGCTCGACGAAGAAAAAATGCAATGACTATG
TCTGAAATTGATACATCAATTGATCGAGTAGTAGCCGGGATGGAAGGCACTCCTTTAATT
GACAGTAAAAGCAAAAGATTAATTGCGTATCACGAAGTGGGTCACGCAATAATAGGCAGT
TTATTAGAGCATCATGATCCTGTGCAAAAAGTTACATTAATACCAAGAGGGCAAGCAAGA
GGCTTAACTTGGTTTACTCCTAGTGATGATCAAAGTCTAATATCAAGATCTCAAATACTA
GCCCGTATCGTAGGTGCTCTTGGTGGCAGAGCTGCAGAAGAAATCATTTTCGGTGACGCA
GAAGTTACTACTGGTGCAAGTAATGATTTACAGCAAGTAACATCAATGGCAAGACAAATG
GTCACTCGATTTGGAATGTCTAAAATTGGACCTTTATCTCTTGAAAGCCAAGGAGGAGAC
CCATTTTTAGGTAGAGGCATGGGAGGAGGCTCAGAATATTCAGATGAAGTTGCAACTAAT
ATTGATAAGCAAGTAAGGGAAATTGTCAGTGAATGCTATGCACAAGCTAAACACATTATT
ATAGATAATCGAGTAGTGATAGATAGATTAGTTGATTTACTAATTGAAAAAGAAACAATT
GAAGGCAATGAATTTAGAGACATCGTTAAGGAATACACTGCAATTCCTGAAAAAAATTAC
TACATATCACAATTTTAAATTAAACGGGACTGACGGGATTCGAACCCGCAACTTCCGCCG
TGACAGGGCGGTGCTCTAACCAGTTGAACTACAGTCCCAAAAAGATGAATACCCAGAGAT
AATCTCATAGTTAGCTTAAACTTGTCAAATTAGAATATTAAATATGAAGAGTATTTGTAA
TAATACTCTTCATATTTAATATAGTAGCTAAAATATTTCTTTTAGAAGGAGAGAGAGGGA
TTCGAACCCTCGGTACGAAGTTAATCGTACAGCAGATTAGCAATCTGCCGCTTTCGACCA
CTCAGCCACCTCTCCATGATATATATTATATATATATCAAATATACTTATATTTTAATAG
GTGTGCATAAATATTTATGGAGCTAAGCGGATTCGAACCGCTGACCCTCTCAATGCCATT
GAGATGCTCTACCAACTGAGCTATAACCCCTTTGGTAAGATATTAAGAAATGGCTCAAGC
GGGATTTGAACCTGCGACCTTGGGCTTATGAATCCCCTGCTCTAACCACTGAGCTACTGA
GCCATAGTATAAAATATTATTAAGTATTATACCATAAATTATGGTCAAATATAAATCAAT
AAAAGAATAATTACTATCATTTACTACTTTTGTGCAAGTTGTAAATATTCGAATAAAATT

TCAAAATTAAAAATGTCTTTAACTTTTTGTTAGAATTATTGATTACGAAAGTATTGATAT
TTAGAATTAACAATAAATTTTGTCTTTATTTGTAGACACAAAAATATTGGAAATTTTCAT
CAATTAATTAGTTAAACTATAACCGACATACAAGAATTATTGAACTACGTTAAATTGTGA
TGCATTGGGCTTGATCAAAACTTCTCTAAAATCAATAGAATATAGTAATTCAAGTAGATC
AAATACTAACAAATGTGACAAACGCTTAAAAAAATAATACAATTAGAATCGTAAAAATTT
GGTTTTTGACTGTGTTAAATAAATTTGATTATCATAGATTTAATATTTAAAAGTGTTCTA
TTACTTGTATTGAGTAATTGTAATATTTAATATCTTCTTTTTTAGATAACAATCATAATA
TAAGCTAAAAAATAGTTTTTATTAAACAACTAGCATAGAGCCAATACCTTGATCAGTTAA
TATTTCTAGCAAGAGAGCATGATCTATTCTACCATCTAAAATATGCGCTGAAGCTACACC
CTGAGCCAAAGAACGAATACAACAATTCACTTTAGGAATCATGCCACCAGAAATTACTGC
TGTTTGAGTTAAATCTCTAGCTTCCTGTATATTCAGATGACTAATTAATGTTGAAGGATC
TGAAGAATTACGTAAAATGCCGGGCGTATCTGTGAGTAGAATTAGTTTTTCGGCATTAAG
AGCGGCTGCTATTTCTCCTGCGACAGTATCAGCATTAATATTATACGATTGGCCTTCCTT
GTCTGCTGCAACACTAGCTATAACAGGTATATAATTATTATTAATTAATATCTTCAGTAA
TTTAGTATCAATATTTTGTACTTCTCCAACAAAACCAAGGTTTGCTTTTCCATTTGGTCT
CGGAGTAATAAGTAATCCATCTTTTCCCGACAAACCTACACTTTTACCACCTTGTTTATT
AATAGTTGCGACAAGATCTTTATTGACTCTTCCAACTAAAACCATTTCCACTATATCCAT
GGTAGGTTGATCTGTTACTCTAACGCCATCATCGAATTTTGGTAATATTTTGAGGCGATC
TAACCAAAAATTAATTTCTGGTCCTCCACCATGAACTAAAATGGGACGTAGTCCTATAAA
AGACAGAAAAACAAGATCACTAATCACTTGATCTTTTAGTTTCTGATTTTTCATAGCCGC
TCCCCCATATTTTATAACAATAATTCTGGAAGAAAATTGCTGAATATAAGGCAGAGCTTC
ACTTAAGACTTTTACTCTTTCTGAATTAGTCAACATAAATAAAGTAGGATTGTGATATAT
AAAATTATATACACAGCTTGTGTTAATTTAAAAGATACCAATGGGTACTTGAAAAAATAG
AAATATTACTAACAGTTAAAACTATAGGGATTATAATTATGAATAAATTTTGGGATAATG
TATTAAGATTTCCTCGATTTTTAGTCAGCGTCATTTTAGGATTAATTTTGATAATTATTA
GTCCTTTTTTCGTGTTATTAAAAAAACCACTGACAAGTTTTTTTTCATCATATCATTGG
CCGGATTAATCACAGTTTTGGCAATAATAATACAAAAAATGATAAATATCGAATGTTGTT
GAGAGAAATAAATAAATTTAATTAAAATTTTTGAACATATATAATATTATATTATCATTA
GATATGATAATTTATTTCCTTTTCTATAAAACCCATAAATATTAAATATTATTTGATAAA
TATTATGCCACTAAAACAAAGAGTTAGCTCTGAAAAGACCGGTGCCTTCGCACTTTTGGA
TAGTATTGTTAGGCACGGAGTTAAACATATATTTGGTTATCCAGGTGGAGCTATTCTTCC
TATTTATGATGAGCTTTATGCTTGGGAAGAAGCCTCTCTAATTAAACATATCCTTGTTCG
TCATGAGCAAGGCGCTGCTCATGCTGCAGATTCTTATTCTAGATCAACAGGAGAGGTTGG
AGTATGCTTCGCTACTTCTGGCCCAGGAGCAACTAATCTTGTCTCAGGTATAGCTACAGC
ACATATTGATTCTGTACCTATATTAGCTATAACAGGTCAAGTTGGGAGAGCTTTTATTGG
TACAGATGCTTTCCAGGAAGTAGATATTTTTGGGATTACACTTCCTATTGTAAAACATTC

ATATGTAGTTCGTGACCCTAGAGACATGTCTAGAATTGTTGCGGAAGCATTTTTTATTTG
TAAACACGGTAGACCAGGTCCAGTATTAATTGATGTTCCTAAAGATGTAGGATTAGAGAA
GTTTAATTATTTTTCTGTTGAGCCCGGAAAAGTTAATATTCCTGGCTGTAGGCCAATTAC
CAGCCTAAAGTCAAGACAAATCCTTATGGCAGCTAAAATGATACAGCAATCTAGCCAGCC
ATTGTTGTATATTGGTGGAGGAGCCATAATCTCTGATTCACATCAAATTATTAAAGAACT
TGTTGATTTTTATAAAATACCTGTTACTACTACTTTGATGGGGAAGGGGATTTTTAATGA
GGATAGCGATTATTGTCTAGGGATGTTAGGTATGCATGGTACTGCGTATGCTAATTTTGC
AGTTAGTGAGTGCGATCTTTTAATTGCTTTAGGAGCTAGATTTGATGATAGAGTTACTGG
AAAATTAGATGAATTTGCTTGTAATGCCCAAGTGATTCACGTAGACATTGATCCTGCTGA
AGTAGGAAAAAATAGGATTCCTCAAGTTGCTATTGTCGGTGACGTAGCAGAAGTTGTTAG
TGAAATATTGAATTTATTAAAGACTTCTTTCCCCCCTTATCCAGAGCAGATTATATCTTG
GCAAGAAAGAATTAATCGTTGGCGTCAACAGTATCCTTTACTGGTTCCTAGAAAATCAAC
AAGCATTTCACCTCAAGAGATTCTTGTTGCAACTAATAAATTAGCCCAAAATGCTTATTT
TACTACAGATGTTGGCCAGCATCAAATGTGGTCAGCTCAATTTCTGAAAGTAAAAGCTAA
GCATTGGCTTTCAAGTGCTGGATTAGGCACGATGGGTTATGGTTTACCTGCAGCAATTGG
CGCTCAAGTAGCACATCCAAATGACGTAGTCATTTGTATTAGTGGTGATTCTAGTTTTCA
AATGAATATGCAAGAGTTAGGAACTATCGCGCAATACCAGTTACCAGTTAAAATTATTAT
TATTAATAATCGATGGCAAGGGATGGTTAGACAGTGGCAACAAGCTTTCTATGGTGAAAG
GTATTCACACTCAAGAATGACAGAAGGAGCACCTGATTTTCAAAAGCTTGCAGAAGCTTT
TGGTATTAAAGCTTTTACTATTAATAATAGGCAAAATATGCAATCTGCTTTACAAGTTGC
TATTGATTATCCTGGTCCAGTTTTATTAGATTGCCAAGTTACAGAAAATGAAAACTGTTA
TCCAATGGTTGCTCCTGGAAAAAGTAATGCACAAATGATAGGTATCGCTAAACCGCAGAG
AGGTACTGCTTCCAACTATATTAATAATAGTGTTTGAAGTAAATAGTTAGTATTTTTTAA
ACAAAGAATAAAGTACATAAATGTATTTTTTTTATACTATTAAAAACTATGTAAGAACAG
CCTTTGACGAGAATTGAACTCGTGACCTTCCCCTTACCAAGGGGATGCTCTACCTCTGAG
CCACAAAGGCTTTTTACTATATTGGGCCGGGTTGGATTTGAACCAACGTAGGCGAAGCCA
GCGGATTTACAGTCCGCCCCCATTAACCACTCGGGCACCGACCCTAATTAAACCTTATTC
AAGATATGACCTATAAGATATCATAACTATGTTGCTAAAAACAAGTACTTTGATAAATAA
AACTGAATAAGTAATGGACATAGCTGGACTTGAACCAGCGACTTTCACGATGTCGACGTG
ACACTCTAACCAACTGAGTTACATGTCCAAAACTTTAATTATCATTATAACACTGTTTAG
TGATTTTATAAGTATCTATTTCTAATTGAAGATTTGTTTTAATCGTCCGGCTTTACCGAG
CAGATCTCTTAGATAATACAGTTTAGCTCTACGTATTTTAGCTCTTCTAATCACTTTGAC
AAAAGTTATTCGCGGTGAATTGAGAAAAAAATACTCTTTCAACTCCAATACCTTGAAATGA
GCATCTTAAAGTTAAACTAGTATTTAAGCTTTTTTTTCTTCTTCTTGCTAATACTACTCC
TTCACATAATTGTTCCCGAGTTTTACTACCTTCCTTAACCATTAGTCCAAGTTGTATTGT
ATCGCCAACTTTAATTTCAGGTACTTCTGTTTTGATAAAAGGTATCTCAACACTTTTCAT

TAGCTGACTTAGCTTTGTACTATTTGCTTTCATATATAGGATTAGTCTTGTGACATGTTT
TGAAATATTATACATTAAAAAAACAAATATGAAAAATTTTAGCGAATAAGCATATTAGTA
TTTATAAAACTCTTTTTATCTCCATTATACAGATCCATTATATTCAACTAGATTCTTTCA
TAGTTTCTTTGATAATCAGTACATCTAGATGATCAAATATTATTTTCAAACATGGCTTAT
GTATGAAAAAGTTCTATAAAAACCTAATGACTAAAATTAGGGCTTAAGTATTTCTTAGTT
TTGTGTTATAATTAGTTACTATCAAAGGTAGTGGCATAACACGAAGCATCAAAATGTTCG
AACGCTTTACTGAAAAAGCTATAAAAGTCATAATGCTAGCACAGGAAGAAGCTAGACGCT
TAGGACATAATTTTGTCGGAACTGAGCAGATACTATTAGGATTAGTTGGTGAAGGTACTG
GAATCGCAGCTCAAGTTTTGAAATCGATGAATGTGAATCTAAAAGATGCAAGAGTTGAAG
TCGAAAAAATTATAGGAAGAGGGTCTGGTTTTGTAGCGGTTGAAATTCCTTTCACTCCTC
GAGCAAAAAGAGTACTAGAATTATCTTTAGAGGAAGCACGTCAACTAGGCCATAACTATA
TTGGCACAGAACACTTGCTAATGGGCTTAGTCCGAGAAGGAGAAGGAGTTGCGGCAAGAG
TTTTAGAAAATTTGGCAGTCGATGTTTCTTCAATTAGAGCTGAAGTTATACAAATGCTCG
GAGAAAATGCGGAAGCCAATGTAAGTGGAAGCAATACTACGCAAGCTAGAAGTAAAACAC
CAACATTAGAAGAGTTCGGATCTAACTTAACTCAAATGGCTATGGAAGGTGGTTTAGATC
CTGTAGTCGGAAGACAAAAGGAAATAGAACGAGTTATTCAAATCTTAGGTAGAAGAACTA
AAAATAATCCTGTCTTAATTGGGGAGCCTGGTGTAGGTAAGACAGCGATTGCGGAAGGAT
TAGCTCAAAGAATTGCTAATAGAGATGTACCTTCTATTTTAGAAGATAAATTAGTTATTA
CTCTTGATGTCGGTCTATTAGTAGCCGGAACTAAATATAGAGGTGAATTTGAAGAAAGAC
TAAAACGTATTATGGATGAGATTAAATCAGCTGATAATGTAATATTAGTGATTGATGAAG
TTCATACATTGATTGGGGCTGGTGCTGCAGAAGGAGCAATAGATGCAGCTAATCTGCTTA
AGCCAGCTTTAGCAAGGGGAGAATTGCAATGTATAGGTGCAACAACTTTAGAAGAATATA
GAAAACATATAGAAAAAGATCCAGCATTAGAAAGAAGATTTCAACCAGTTGTAGTTGGAG
AGCCAAGTGTTGAAGAAACAATTGAAATTTTGTTTGGTCTTAGAGACCGTTATGAAAAGC
ACCATCAATTAACAATGTCAGATGGAGCTTTGGCTGCAGCTGCTAAATACGCTAATCAGT
ATATTTCTGACCGATTTTTGCCAGATAAAGCAATTGATTTAATTGATGAAGCTGGTTCTA
GAGTCCGTTTACTAAATTCTCAATTACCTCCTGCTGCCAGAGAATTAGATAAAGAGTTAA
GAGCTGTATTAAAAACAAAAGATGAAGCTATTAGAGCTCAAAAATATGAAACAGCAGAGC
AGTATAGAGCAAGAGAAATGGAAATTAAAGCTCAAATTGCAGCAATTGCTCAAAGTAAAA
AGAATGAGCCTGATTTAAATTTAGAAGATCCTGTTGTTACAGAAGATGATATTGCTGAAA
TTGTTGCTGCATGGACTGGTATACCAGTAACTAAGCTTACTAAAAGTGAGTCAGAAAAAT
TAATGCACATGGAAGAAACTTTGCATGGACGTATTATTGGTCAAGACGAAGCGGTTGTAG
CTGTCTCTAGAGCGATAAGACGCGCAAGAGTAGGTCTAAAAAATCCTAACAGACCAATTG
CAAGCTTTATTTTTTCCGGACCGACGGGTGTAGGAAAAACAGAATTAACAAAAGCTTTGG
CTTCTTATTTCTTTGGTTCAGAAGCTTCTATGATACGGCTAGATATGTCAGAATACATGG
AAAGACACACTGTATCTAAACTAATTGGTTCTCCTCCAGGATATGTGGGTTATAGTGAAG

GTGGTTATCTAACAGAAGCGGTAAGAAAAAAACCATATACTGTCATCTTATTTGACGAAA
TTGAAAAAGCTCATCCGGATATTTTTAATCTACTTCTTCAAATTTTAGAAGATGGCCGAC
TAACAGATGCAAAAGGCAGAACTATTGATTTTAAGAATACTCTTTTAATTATGACTTCTA
ATATTGGATCTAAAGTTATTGAAAAAGGAGGAGGTAGTTTAGGCTTTGAATTATCAGAAG
ATCAAACAGAATCCCAATATACTAGAGTACGATCTTTAGTAAATGAAGAACTGAAACAAT
ACTTTAGACCAGAGTTTCTAAATCGATTAGATGAAATTATTGTATTTCGTCAGCTTACTA
AAGATGAAGTTAGAGAAATTGCAGAATTAATGCTTAATGAAGTCTTTGCGAGAATTAAGC
AGCAAGATATTCAATTAAATGTAACAGAACGATTTAAGCAACGATTAGTAGAAGAAGGAT
ATAATCCAAGCTATGGAGCTAGACCACTTAGACGAGCAGTAATGAGGCTTTTAGAAGATA
GTCTGGCTGAAGAAGTTTTATCTGGTAAAATTAAAGCTGGTGATAGTGCAGTAGTAGATG
TTACTAATGAAGGAGAAGTTACAGTTTTATTAGGTGAAAAATTAGAACTGTTAACATAAA
AAACAATTTATTAATTAAAGCATTGAAGAGGTTATATTCAATGCTATGAAAAAAATTAAT
AAGATGGGTTGCTATAATTGACAACCTCTGATATTCTTTATTTGTATATAAAGCCGGGAT
AGCTCAGTTGGTAGAGCAGTGGATTGAAAATCCTCGTGTCACCAGTTCAAATCTGGTTCT
TGGCATTTTAAGCATCAGAAAATTTTGCTATCTGAGCATTAAATTTTAATTGAAATGTTC
CTGTGGGACCATTCCTGTGTTTTGCTATTATAACTTCTGTGAAGTCTCTACTTCTAGTTT
CTTGTGTATAGTAACTTTCTCTATATAGCATTATTACTAAATCTGCATCTTGTTCTATAG
AATTATGAACAATAAAGTTATTTGAAATAAAATTGCATAAAGGTTTACATTCTAAATCAA
ACACTATTTGTAAACTTGTAACGTTTATTTTTTGTATACTTTCAAAAGTCAAAGATGAAA
AAGAATTAAAAACATTTTTTTGTTCTTCAAAGTTTTTTATTTGAATAGCAATCATGTCAT
TTTGATTAATTTTATCACATCTTTTCCATCCTTGTGTTGTGAGTAGTTTATGGTTACTTG
TTAATTGTATATATTTCCCAGCTTCGGTAATAATTTTATATACAGTTTTTTTCCCTGTTT
TCGAAATACTACATTTAATTGCTAGAAATAACTGTCGTTTTTGCGCACTAAAGTTAATGA
CTTCAATCTTTTTATAATAGCAATTAAATAATATTTGAGGTTGATTATATAATGATGTCT
GAAGATAATTGAATTTGGATATACACCCACTTTCTCTTAAATCGGATAACAAAGGCCTTT
TGTCACTCCGAGTTTCAAGGTTTCTATTTAGTTGAGATAATGCCAAGATAGGCAAGCTTA
AATCTTTTGCTAATATTTTAAGTGATCGAGTAATTAATGATAGTTCTTGAGATCTATTGT
CAGATTGTCTACTCTCCTGCAGTAATTGAAGATAATCTATAATAATTAATTCTATATTTT
TTCCTTGTAATTTAAAGAATTTTACTTTTGTCTTTATAGTGTCAGTAGAAATCTTAGCGC
TATCATCAATATAAAGATTAAGGTTAGCAAGAGTTTTACTTTTTTGAATAACATATTGCC
ATTCATCATTATTAAGCTGACCAGATTGAATTTTTTGGCCGTTTAAATTACACTCTTGAG
CTAAAATTCTTCTTAGTAGTTGCTCTGTAGACATCTCTAAGCTAAATAAAATAACATAAG
ATCTTTTCTGATTAATTACATATCTAGTTATATTAATAGCAAAAGCAGTTTTCCCCATTG
AAGGTCTTCCTGCAAGAATAATTAAATCTGACTTTTGAAACCCTTGTGTTATAGAGTCGA
GTTCTGTAAAACCAGAAAGTATACTACTATTTATACTAATCTTTTTCCTTGTATCCAAAT
GGACTAATAGTCTTGCAAGAATTGTAGCTAATGTTTGCGTATCTTTTTCGTCAAGTATTT

CATAAGCTTTTGTTAATTGACTAACAATAGAAGTTATGTTTTTTTGTTCAATAAGTTGTT
TTGAGTAGCTAATTAAACATAATGAATCTCCACATGCTAAAAGTAATCTTTTAATGTAGT
TATCTATAACGACTGCTGAATATTCGCTTATAGTATCAGATAGGGGAGCTTGTTCTATTA
GACTAAAAACTTCATTTAGCTCATTCAAGTCTTGAATCATTTTTTCATTTTTTAAGTTAA
TGAAAAAATTTCTTACGCTTTTTGTCTTTGCATGGTTAACATTTTCGAGTGCTGCTCTAT
AAAGTAATGAAGTTGATGTAAAATAAAAGAAATCAGGAGAGATTTTTTCTACTGATTTTA
GTAATGATATCGATTGCTGAGTTAATATTATACTAATTAATATTTTTTCAGCTAAAATAT
TATGAGGAGGTAGATATTTATAAATACTTATTACTTCCTCCTTGTAACATTACTCTTGTG
GTATTAGTTAACTATTGATTTAATTTGATTCTGGAAGAACTTGCAATTTAATATTTGCAG
TTACTTGATGTAATAGCTTAATTTCTACATCGTAAATGCCAATTGTTTTCACGTCTGGTA
AAGAGATACTTTGTTTTTCAATGTCTATGTTGGTAGTGTTTTTAATAATTTGTGAAATTT
CTTTTTCTGTGACACTACCAAAAATATTGTGGCCATCTCCTGTTTTTTTACTAACGCTAA
ACCTTTGTATTTCTTCTAGAAGTTGCTTGACTTTTTTAGCATTTTCTTTAGCTGTTTTTA
ATTTTTCTTCTCTTATAGCTGCATAAAATTTTTGTTGTTTTAAAATACCATTTGTGGCAA
CTGCCGCCATTTTATTCGGGATTAAAAAATTTCTTGCATAACCAGAAGCTACTTTGATAA
CGTCATTACTCTTGCCAAGTTTTTGAATATTCTCTTTTAGCACAACATTTATAACTTTTT
TACTCATCTTAATTCTAAAAAATCTGTATTATCCTATTATACCTGTTGTATACAGATTTT
AATAGGAAGTATTTATTTGTTGATATAATATTCTTGTAAAAAAAGGTAATTTTTTATTTT
TTTCTGATATAATCACTTTCACAATAATTCATTAGGAGAGGTGGCCGAGTGGTTGAAGGC
GCAGCATTGGAAATGCTGTTTAGGAGCAATCTTAACGAGGGTTCGAATCCCTCTCTCTCC
GTACAAAAAATATAAAAAGTTTTATAGAGTCCAATCTAAATCTCCAATAATATTCTTCCA
AGCATTTAATCCACCCCGAACTCTAACAATATTTAGTTTTTGAGCTATTAAAAACTCAGA
AGCAAATATTGATCTAGAATCTAAGCTACAGTAAACAAAACAAATTTTATCTTGTAAATT
GATATGAGAGTAATTCATTCCTTTTATTTTTTGTAAAGGTAGATTTAATGAATATATTAA
ATGACTTTTATGATATTCTTCATGATTTCTTACATCTATTAAAATATATTGTGGATTCCT
ACTAATTAAAAACTTTTGTAATTGAATAACACTAATTTCTCGTACAAATGTATTAGATTT
ACTGCCATAATATTTGTTCCAATGCTTTTTTGTGGATAATACAAATTTAGTATTTATAAT
CTTAAATTTGTTAAATGATGAAGTTAATGAGTTGTACGTTAATATAGTTCCACTTAATAT
AGACTTATAACCCAGAACGATTTTGACAGCTTCAGTTGCTTGAAGTGTACCAACAATACC
CGGTAAGAGACCTAAAACTCCGGAATTACTACAAGTATCTCTTGCGCTCTCTTTATTTTC
AGTTTCACTGTAGAAGTCTCGATAAACAGGTCCACCTTGATAATTAAATACACTAACTTG
TCCTTCAAATTGAAAAATGGCACCATAAATATGTATTTTATTTAACTCTAGGCAAGTATC
ATTCAAGAGATATCGAGTATCGAAGTTATCAGATCCATCTATAATAATATCATATTGTCT
AATAATGTCGATTGAATTCTCATAGCTTAGTCTTGTTTTGAACACAGTTACTATACACTG
TGGATTTAAATCTAATATTTTTTTTTAGCTATTTCTACTTTTGAATATCCTATATCATGC
ATCGTGTATAAAATTTGCCTCTGTAAATTAGAGAGATCAATTATGTCATCGTCTATAATT

CCAATGCTGCCAATTCCTGCAGCAGCAAGGTAAATGATTCCTGGAGATCCTAGCCCACCA
GCACCAATGAATAAAACTTTTGCTTCTTTTAATCTTTCTTGTCCTTCTAATTGAATTTGT
GGCAATACTAAATGCTTAGAATATCTTGTATATTCTTCTAGTGAGTATTTTGTATTTTCT
GTTTTAAAATTTAGCATAAAAATTTATAGTTTATTTAATAGCACTTAAAAGCTAGCTGAT
AAGTTTATTAAAATGACTTTTGTAAACTTGGTTCTTCTCTATCTAAAATAGCTCCTTGTG
TAGGACAGACCTGAACACAAATACTACAATCAATACAGGCAGAAAAGTCAATCCAATACC
AATTAGTGTTATTATTGTTTTTCCCCTTTCCTTGATGAATACAAGCTACGGGGCAAGCTT
CCACGCATTCAGCAACTCCAATACATTTTTCTGTTACAATTGTATGAGACATATTATTTA
ATTAATAAAAGATAAGCTATTTACATCTAGTAGTTTTGTTGTATACTAAATTTACATATG
CTATCCAGGCCAATATTTAAGGTAGTCTATTTCTACAGTTTACTAATTTGATATGCGCCT
TTAGTTCAGTTGGTAGAACGCAGGTCTCCAAAACCTGATGTCGAGGGTTCAAGTCCTTCA
GGGCGCGTTTAGTTTTATCGTAATTCAATAATATCATTTTATCTTTTTGCAAACATCAAA
ATGATATGATATAAAAGATCATATTTTATACATTGAATATTCTATGTACTAGTACTGTTT
TTTAATATGTTATTATTGATAAGTTAATTATACTAATTCTATTTTTGTAATAAGTAAAAA
CAGTTCTAATTAAGTATGCACGTATTTAATAGTCTTTTGTATATATAACTTGAAAAAATT
TAAACTATGGCTAAAAAAGTTACTGGAATTGTTAAACTAGCATTAAATGCAGGTAAAGCT
ACTCCTGCGCCACCTGTCGGACCTGCTTTAGGACAGCACGGCGTGAATATTGTTATGTTC
TGCAAAGAGTACAATGCACGTACTGCTGACAAGTCAGGACTAGTAATCCCTGTAGAGATC
TCAATATATGAAGATAGAAGTTTTACTTTTATACTTAAAACTCCTCCTGCTTCTGTACTA
ATCGCTAAAGCTGCTGGACTAAATAAAGGTTCGGGTGAACCAAATACCAAAAAAGTAGGC
AGCATAACAAATAAACAGTTAGAATCTATTGCAGAAACAAAACTTCCCGATTTGAACACT
AACAATATACCTCAAGCTATGAAAATAGTTGGAGGTACGGCTAAAAATATGGGAATTCTA
ATTAAAGATTAAAAGTTTCAATTATATTTACTATTTTTATGAAAAAATTTTCACGTCGAC
TTACAACATTAAAATCAAAAGTTGAGCCTAAACTTTACACTATTAATGAAGCAGTATCTA
TATTAAAAGCAACGTCAAATGCTAAATTTAAGGAAACTGCAGAAGCTCATATTGCTCTAG
GTTTAAATCCTAAATATGCAGACCAGCAACTCAGAGCAACAGTTATATTACCGAAAGGTA
CAGGTAAATTGATAAAAGTAGCAGTTATTGCTAAGGGAGAAAAATTAACAGAAGCAATTA
GTGCGGGAGCTGATGTTAGTGGCTCCGAAGAACTAATTGATGAAATACTGAAAGGTAGAT
TAGATTTTGATAAGCTGATAGCAACACCAGATGTTATGCCTTTAATAGCAAAGCTGGGAC
GAGTATTAGGCCCTCGAGGGTTAATGCCTTCTCCTAAAGCAGGCACGGTAACATTGGACG
TAGCAAAAGCCGTGAATGAATTTAAAGGGGGTAAAGTTGAATACAGAGTTGATAGAACAG
GTATAATTCATGTACCTTTTGGAAAATCTAGTTTTTCACAAGAAGATCTAGTTTTGAACC
TCCAAACAATTAAAGAATCGATTGATAGAAACAAGCCTTCTGGAGCAAAAGGGAAGTACT
GGAAAACTTTTTTCTTATCTAGCACCATGGGGCCATCTATTCAAATTGATATCACTAGCC
TTCTATGAAAAATTTTTGTATAATTATAGAACTTGAATAATCTATTATTTCAAATTATAA
TTTGCTAGCATTATTAACGTAACAACTTGCTTACTATATCTTATCTGCATTAAAAACTTT

TTATATATCTTATTAATTATGAGTACAAAGGTTGAAAATATCTTGGAAGAGCTAAAATCT
TTAAACCTTCTAGAGGCTGCTGAACTAGTTAAACAAATAGAAGAAACGTTTGATGTTGAT
GCATCTGCGGCTTCAGGAGGTATGATGATGGCCGCACCAACTTCAGCACCAGCGTCTGCT
GAGGTTGAAGAGAAGACAGAATTTGATGTTGTCCTAGAGGAAGTCCCAGCACCTAAAAAG
ATTTCTGTATTAAAAGCTGTTCGCTCTCTGACTGGTTTGGGTTTAAAAGAAGCAAAAGAT
CTTGTTGAATCTACGCCAAAAGTCTTAAAAGAAGGTGCTTCTAAAGATGATGCAGAAACT
ATGAAAAAACAGCTAGAAGATGCTGGCGCAACAGTTATTGTTAAGTAATGAAGAAGCGCT
TCTGTTTAAGTATAAACAGAAGTGCCTCTTTATTTATTATAAGCAATTATACTAGAAAAG
TCCAAGTGTAATTGCATTGTTGATAGGCATAGTTGCACCAATACCTAGCCAAATACTTAC
AACGGTACCAATTAAGAAAACAGTGGTTGCAATTGGTCTTCTAAATGGATTTTGAAATTT
GTTAACATTTTCAATGAATGGAACAGTAAGTAAACCAGCTGGTACAGCAGCCATACTTAG
AACACCTAATAGTTTATTAGGAATCACTCTTAATAGATTAAATGTTGGAAAGAAGTACCA
TTCTGGTAAAATCTCTAATGGAGTCGCAAATGGATTAGACTTTTCACCTATAGAAGATGG
TTCTAAAATTGCGAGTCCAATACTGCAGGCAAAAGTGCCAATAATTACAACTGGAAAGAC
GTATAATAGATCATTTGGCCAAGCTGGCTCTCCGTAATAATTATGGCCCATTCCTTTTGC
TAATTTAGCTCTCAGCTTCGGATCTGTTAAATCTGGTTTTTTAAGAATTGACATATTGTT
TTTGTGTTTAATATAAAGATTAGTCTAATTACTGTTTTAAAGTGTAATTTTCAACTATTA
TAATGGTCCAGAAATTCCTTGTTTACGTATCATTAAAAAAATGCATAAGCATGAAAACGGC
AGTTAGTAGCGGAAGTACAAAAGTGTGTAAACTATAGAACCTAGTTAAAGTTCCTTGGCC
TACACTGACTCCTCCTCTTAACAATTCTACTATACTCGCTCCGACAACTGGAACAGCATC
AGGGACACCTGTAACAATTTTTACAGCCCAGTATCCGATTTGATCCCATGGCAAAGAATA
ACCTGTTACCCCAAAAGAAACAGTAAGCACTCCTAAAATTACACCTGTTACCCATGTCAA
TTCTCTAGGTTTTTTGAAACCTCCTGTTAAATAAACACGGAATACATGTAGAATCATCAT
CAAGACCATCATGCTCGCTGACCATCTATGAATTGATCTAATAAGCCATCCAAAGTTTAC
ATCAGTCATAATGTACTCTACTGATGTAAAAGCTTCTGCAACTGTAGGTCTATAATAGAA
TGTCATTGCAAATCCAGTTGCAACTTGAATTAGAAAAGATACAAATACAATACCCCCTAA
GCAATAAAAAATATTAACATGAGGTGGTACATACTTACTAGAAATATCGTCAGCAATTGC
TTGAATTTCTAATCTCTCTTCAAACCAGTCATAAATTTTACTCATAAAATAGCTTC-AAA
AGCTCTTCTAATATATTTTGCGTTTAAGCTACTATTAATTCAATAGTTATTGAACTTATA
TTTTTTTATAATCAATACTTATTAACTATTATAACATCTTTTTATTGATTTAAAATTCTC
TGACTTAGTAAAACAGGGCTATGTACTATAATTCTTTTTTTTTGTATAGAGATTAATTTC
GTTTTTAGTAAATTAGCTAAAATTCTTGTTACACTGACTCTATTACTACCTATAATCTGT
GCTAAAATTTTATGTGTAATAGTAAAGTTAAGCATAATACCATTATTAACTTGAGTTCCA
TTTTGCTCAGCTAATAATAATAATAAGCTCGCCAGTCTACTGGTTATACTTTTATGTGAA
ATGATTTCTATAAAATGATAAGCTTTTGCCGAGCAAAATACTAAATGATTTACAAAAAAT
AAATTGAATGGAGCGCAGTTACTACACGCTTTTATAATAGTAGTATAGTTAATACAGGCA

ACTTCTGTTTTATCTATTGCTTCTGCTTCGTAGTAAAAATTATCGTCGACCAATTCTATT
TGTCCAAATGTATCTCCTGTAGTTAATAAGTTAAGAGTTACTTTATGTGTATTTCTCAGG
ACTTTTGTAATAATTAAAGATCCTATCAATATAATGTATAATCTAGAGCTGCTATTAAAC
AAAAGAGTATCATTTTTCTTAAGTGAAAAAATTTGATAATTAATTTTATTACGATTAAAA
AATAGTAGCCATGGATTGCAAGAAAAAAAATCACCTGAACTATTTTCAGAGAATATTGGA
ATACAATTTTCTTGATTTAAACAATTTTTAGTCATAATTACTACTTTATAAAAAAATATT
CTAATAATTAAGAACTGAAATTAAATGTCATATAAATTGATGTTAGTTGAGAATGACATT
GTATTATCAAAGGCCATTCAAGAATACTTAATAGATCAAGGCTTCAATGTTTATATTGCC
AATAATGGATTAGAAGCACTGAATCTAGCTTATCAATATAATTTCGACTTGATAATTTCT
GATATAATGATGCCGCTAGTTAATGGCTATGAACTATTAGCAAAACTTAAAAAAAACAAA
GCATTATCCAAAATACCTGTTATCTTTTTGACAGCTAAAGGAATGACTAAAGACAGAATA
AAAGGCTACGACATGGGGTGCTATGGTTATTTATCTAAACCATTTGATCCTAATGAGTTG
CTTTCAATTATTAACAACTTAATCGCTAGAGATGTCTTAAAAGAAGCTTCTTTACAAAAC
TTGGCAACATCAAACCAGCAATTAAATCATAAAATACGTTTGACACCTAGAGAAAAAAGT
ATTTTAGATCTTGTTGTTGATGGACTTACTAATAAAGAAATATCTACTATACTGAATACT
AGTGTTAGAAATGTAGAAAAATATGTTAGTCGACTCTTACACAAAACTAATATGAAGAAT
AGGACTTTGCTAGTCAAGTATTCTATAAATAATAATCTATTAAATAATGAGATCAATGAA
AGGGCGAATGACGGGACTCGAACCCGCGAATGATGGAGCCACAACCCATTGCCTTAACCC
CTTGGCCACACTCGCCATATTACACATACTATTATAGCTTGTTTTTTGATATATCGTCTA
GTTTTCGAGTTATATATTTATCTAAAACAATATTTACTTTGAATTTTTATGATACATAGC
AATATTAGTATTCAAATTAATGGGGAACCATTTAATTGCTCAAAGCCCATCTCATTGCAA
TTTTTATTAAATTATCTTGATTTTAATTCCGAGCGTGTAGCAGTCGAGCTAAATGACATT
CTGTTACCAGAGACTCTGTTTCACTCAACTTATTTGAATGATCAAGATAAGCTAGAGGTT
ATTACCATTGTAGGTGGTGGATAATCTATTTGTACATACACAGTTCTCTATCTCAAATGT
TTCATAGATAGAGATAATCTTCCTTGTTTTTTATCTACATGAATAATAACAGCTTTAATA
GTGTCACCTATTTTAAATTGTGATGATATCTGCTCTAATTCTTTGATATTAATCTCAGAA
ATATGGACAAGACCTTTGAGATTTCCTACCTTTATAAATAATCCGTAAGGTGTAATCTGA
TTTATTATGCCTTCAATAATATTACCAACAATTAAATTAGATGATGCTTGAGATATTAGA
GCTCTTCTATGACTCAGTATTAAATTATTAGATTTCTCTTCGACATTGAGTAATTTTAGC
TTAATAAATTTATTATTAAATTGCTCACTTTTTTGAAAATTACCGAGATGAGAATTAGGT
ACAAATCCAGATATACCTTCAAGGTTAATTATCATTCCTCCCTTATTGAATCCTTTTATC
ATTACATTAAGTAAAGAGTCTTCAGCTAATAGTTGCCTAATTCTTTTCCATGCTCTTATA
TATTCAAGGCGACGAATTGATAATATTAATTGTTTCGACTGAATATTATAATCTAATAAA
AAAAATTCTCTTGTGTCATTAATGTTTAAAGAAGTGAAATTATTTAAATCTTGATTACTA
GATACTTCTTGAATAGGCAAGTATGCAGATATTGGTGTTCCTATATCTACTAATACCCCA
TTTAATTCAAAGCTAAATATAGTTCCAGCTACAATATCTCCAAGATTCAAATCATACTTG

TATTTTTGTAAAACAGCTGCAAAATTTCTATGAGTAAATCCTTCATTATTTTTTGTCATA
TTGAATAGTATGAATTAAAGAGTAACTTTGTACAATATATTAAGGTAAATAAATGTTTGT
CGAATTGTATGAAAAAATTTTAATTTGTTCGGTTGTAATTAAGTATATAAACATATTAAA
ATATCTAGGAGACATGAAAAGTAGACGTAGATAGCTGCAGATTTAATACTATATAATCTG
AGGAAAGTCCGGGCTCCACAAATACAATTTATGCTGGAGAAATCCCAGTGTAGGTAACTG
CGAGGATAGTACCACAGAAAAAAACCGCCAGAATAAGTTATATTAGCTGGTAAGGGTGCA
AAGGCAAGTTAAAAGCTTACCAAAAGTACTGCAAAGTATTTGTTAGGTAAACCCCTAAAA
TGGAGCAAAGCTACTAAACAAATATTTGTGTATATTTTATCTAGTTTAGTTGAAAATACT
GCATGAAGTTATTAGTAACAATAACTCGAGAGGAATAGCTATCCTTTACAATATATCTCG
ATTATATTGAAAGAACAGAACCCGGCTTATGTAGTACTTTTTATGTCTTGTATTATTATG
TCTATTGTCGAATCTTTAAATTTAACTGTTTGACAATTTTTTTTTGTAGCTGATGTTGTT
TATAATCTATTTCTGAATTGGTTAAAGTTTTTTCATTAGATCGATATGTAAATCTTAGAC
CTATACTTTTTTTCTCTTTTCCAATTGATTCATCTATATATTGATCAAATAATGTTGTAC
TTTCTAAATCTTTATCATAAAATTGATCCAATAATTTTAATAAGTATTTAATTTCCATAT
TCTTAGGTATTATTAAAGAGAGATCTCTTATGATCGATGGATACTTAGAGTATGGTTGAA
TCTGATAACTTAAATAATTGAATTCATTATGACAATCTTCTAAAATGTTTAAGTTAACTT
CAAGAAAAAAAAGTTTTGTGTTGAGTCCTAATTCACTATATGTTGCTTGATTAAGTTCAC
TGAATATGCCAATAATAATATTGTTATAGATTAGTGTGGCAAATTTTCCTTTTTGAATGA
ACTTTATTCTACCATCGACTATATCTTTTTTTGTCCACTCAATTTGTCTATTAATCTTTT
GAAAAAAATTTTCTACGATCCCTTTAGCTTCATACCAATTTAAAGAATGCGCAGGGTGTG
ACCATGTAGATCGGATATCTAAATTACCACCCAATATGATAGCTAGATTAGTAGTTTCTA
CAATTTTGTTTTGATCATTATGAAATACTGTTCCTATCTCAAAGCTATCTATAGTTTGGT
TGCTTTGTTTAATATTATAAAGATTTGATTCAATTAATCCTTCCAGCAAACTGCCGCGTA
AATTAGAATAATCTTGAATAAGAGGATTGTTTAAAGCTATATTTCCTTTGGACTTTATTA
AAGAATAATGAACTAATTCAGTTAATCCTAGATTTCTTAAAATACTTCTACTTTTATCTA
TGAATTGTCTCTTTGTGGATAACTTTTTGTTGAATTGAATATTTGGTACTGAGCTCCTGA
ATTTATGATAACCATAAATTCTAGCAATTTCTTCAATAACATCTATTTCTCGAAAAACAT
CTTCTCGTCTATAATTTGGAACAGTAACTTCTATATTTTCCTTATTTTGATAAATAAGAT
CAAAATTTAAAGAGTGAAGAATATTTTGAATTTCTTCAAAGAATAAAAAACGTGTTTGAC
CATTATACATAATTGGTCCTAAAATGTCATGAACTTTTTTAATTGATAAATTAATATTAA
GAGCATGATCATTTATTTTTTCTCGACAGAAAGTCTCTCTAATATTTCCGCCTGTTAGAT
CTGTGATTAAAGCAAGAGCTTCAAAATGTGCATTTTTCCAATTATCTACATTTAGCCCAC
GTTCTTGTCTAATTGAACTTTCTGTTCGAATATTAAGAACTCGAGAAGATTTTCTAACTA
CAGATTGTTTAAATATAGCAGACTCTACAAGTATCGATTTTGTATTATAATCTGTATCAA
AATCAGAGTTGCTTCCAATACCAGCTATACTAGTTACATTTGTATTTACTTGAGTAACTA
AAATATTTTTGTTTAACTCAATATTTTCATTGTTTAACTTAATCTGATCATTACTTCCAA

GAGGAAAATTGCTTCGAATTGTTAGACTATTTTTATGATTCATATTATTAATTTTATTCA
AATCAACAATATTAATAGGCTGTCCCCATTTCAACATAATATAATTGCTTATATCTGTTA
ACAAATTTCTGTGTGTAAACCCAGAAGAGAGCAACCGATTTTTTAGCCAATCTGGAGAAT
CTTTAACTCTTATTTCGTCTATAATGGCAGCAAAATAATTGTCACAATTTAATAAATCGT
GATTACTGATTATAGTCTCCTTTTTAGAGAAATATCTATTATTGGGTTGATGTATAGCTG
ATAACATTGAGGACCCTGTTAAAGCCGAGACTTCTCTTGATAGACCAATCATGCTTAACA
CGTCTGACCTGTTAGCTGTTGATGATATATCTAATATATGATCAATCTCACTTCCAATTA
TAATAGATTCAATAGCTTCAACTTCAAAACCTGCTTGTGTTAGTTGATTAGTTAAATTGT
CTATATCTATTGTTTTGATATTACCAAGTTCTTTCAACCAATTTAAAGAAACTTTCATAA
TTAATTGTCATCTATCTTATAGAAGTATTTATTTTACTTATGATTAAAAATCAATTTAAA
AAATTCATCAAATTAATATAAGTTATTTGATGAATTTGCTTATACAATATATTATTGTGA
TATCTTACTTTTATATGGCAGTTATTAAGAACTAGCTTTTGTAAAAGTTAGCCCATTATC
TTGGGCATATCTTTCCATAAATCTCATGAATCTATCCCAATCATCAGGACTTTTAATAAC
ATGTACAGCTTCTATTGCTTGTGGTTTACCATTAATAAACTTGGCATTAACATCTCTTGT
AATAATTTGTCCCTCTGTGTCCATCAGGTACATACCTGTGATTTCACCTTTGTCTGCCAT
ACTAGCATCTAAAATTTTGGGATTTGTAAACCGAAAAGTTGCTGTTCCTGTACTACCATC
TCGAGACCTTGTTAAACGTACATCTGGTACAACTTCTTCATTGATACCTTGAATAAATTG
AATTGTTGCCATATCAATCTTCTTTATTTGTATATAATTTATATTGGATAAAACTGATTT
CTAGTTCTTGAATGCTTGTTGAGAAACTGTTTTAAAAGCTTATAGTAGCAGTAGATCAAA
TTTTATGTATTATATAAAACTGTAACTTTTCTTTTTTGATCTGTTCAAAGAACTAACTTA
TTATAAGCTAATCATACGAATATTAGCCATATAATGTTCGTAAGTGAAATTAAGGGGTTG
TAGCTCAGAAGGATAGAGCAAGCGCCTCCTAAGCGCTAGGTCAGCGGTTCAAGTCCGCTC
AACCCTGTTAACGAATTAAGTAGTATTATTTAACTTGTTCTGCAAATCAGTATTGATCTA
AGTTAAAGTACTTGACAGCTAAAACAAAAGAATCTGAGAAATATTTTCATTTAAAGAACG
AACAATTATCTTAACTCTAAATATCTGATAATTATTAAAAAATTCTCTTTTTAAAACACA
AGATGATGAAGATATTTAAACTAATAATTGAAATTAAGTTTTTGAAGTTATAGAAGCCAA
TATAATACTTTCTATTAACAGATGCTTGCTTCTATTACTATCTGTTTACAAGAAATTATG
AGTATCAAAATAATTTTAGCTCTACTGACTACTAATAAATATTTATTTTAGTCTAGACAA
GATGAATCTTCATGGTATAACAGAGCTATTCTTTGTGTCATACTTATATAAAATTAGGAT
ACTGTTTTCATTATTATAACTGGGGTGGGAGGATTCGAACCTGCGAATGGCGGAGTCAAA
GTCCGCTGCCTTACCACTTGGCTACACCCCAACATGTGAACATTATAATAGCAGTCGTAT
ACCCTGCCATGTCAATAGACTATTGTCTTTATTTAATAATTTTTGCTGCTCTTGTAGAAT
AAAATCTTTTTTTTCAAATAAGATACGCTATGTTTAAGTTGCACGACTGTTATGTTTTCT
TGCTTTTGACTGAACAGCCACTTGATAGTAATTATTCCTTCTGCAATTTCGTTACTTCCT
ATAATAATACAAGCTACAGCTCTCTTTTTATTTGCTTGCTTAATCTGCTTACTAAAATTA
CTTGAGCTAACATCGATTTCTATTTTAAAAAACTGCTGATGTAAAAAACGCATTATTTGC

ATACCAGTTTCGTTAGCTTTTGTACCTTGTGTAGCAATATAAAAATCGATAGATTGATTA
GGCAAGTCTATATTATCTTTTGCAATCAGTAATAGGCGTTCTAATCCTATTGCACATCCA
ACAGCTGGTGTTGCTTTGCCACCTAATTGGTGAACTAAGCTGTCATATCTTCCACCACCA
CATATAGTATCTTGCCCTTTTGATGTCAATGTCTTAATTTCGAAAGCAGTATCATTATAA
TAATCTAATCCTCGAACTAACTTATTATTTATATTGTAGGGAATATTAAGTAATTTTAGA
TAATTGCAAACGTCACCAAAGTGTTTTTGTGATTCCAAACTTAAAAAATCAGAAATTTTT
GGAGCCTCTGTCAGTATTTTTTGTGTATTAGAATCTTTTGAATCTAAAATTCTAATTGGA
TTACTAGTTAATCTTTTTTGTGAGTCAGTGTCTAAATCATCATGGTACTTTGTAAGATAA
TCTCGTAATTTAACTTGATAAATACTACGATCCTCTACTTTTCCAATTGAATTCAGATCA
AGTTTCAAGTTGTGCAGATTGAGATTGTTAAATATGCTCATAGCTAAATGTATAACTTCA
CTATCTGCTCTTGCATCTAAGCTACCAATAAACTCAATGCCAAGTTGGTGAAATTGTCGT
TGTCGTCCACTTTGTGGTCTTTCGTACCTGAACATTGGACCGCTATACCATAATCTTTGT
AAACTATGGTGGTAACTCATTTTATTTTCAATAAAAGATCTCACAATACCGGCAGTACCT
TCTGGCCTTAAGGTAATATCTCTATTGCTGCGATCATGAAACCGATACATTTCTTTATTT
ACAATATCAGTATCTTCACCAATACCCCTATCATATAATTCACTATTTTCAAAAATAGGT
GTTCTAATTTCTTGGTAATTTGCACATTCTAATAATTTGGAAATTTTATTATGTATAAAC
TGCCAATATTGAATTTCATCGGGAAGAATATCTTTTGTCCCTCTAATAGCCTGAATTTTT
GCCATGATAATTTTTTGATTACCTTCTTAATATGTCTGCATTAAATAGCAGTTGTAGACT
GTTATTCAGTCATGTATTATACATTAGAACATACTGATGTAATTTATAAAGTACGGGCAA
GGAGGGATTCGAACCCCCGACACCATGGTTCGTAGCCATGTGCTCTAATCCACTGAGCTA
CAAGCCCACTTAATAAGTACCTTAACTATATCATTGTTCACTAATGTATACAACTATATT
CTTTTTATCTTACATGTGAAATTTTTGTAAAAGTAAAAAATTATGTTTGTTTTTTACAAT
TGGAGCTATTCATATATAATGCTTAATTTATTATAACTTTGTAAAAATATCTATAAGTTT
AATTGTATGATTCTATTATATTGGTAATCTTGTTATAATTTTGCGCTCTTATTTTTCAAT
TGGCTAGCATTTAATTAACTTATATTTCAAATAAAAAAATCTTATCTAAAATGAGTAAAC
AAATTCTATATCAGGATGATGCCAGAAAAGCATTAGAAAAAGGCATGGATATTTTAACAG
AAGCTGTTTCTGTGACTTTAGGACCAAAAGGAAGAAATGTTGTCTTAGAAAAAAAATTCG
GTGCCCCTCAAATTATTAATGATGGTGTAACGATTGCAAAGGAAATTAGTTTAGAAAATC
ATATTGAAAATACCGGAGTCGCACTGATTAGACAAGCAGCATCTAAAACAAATGATGTAG
CTGGTGATGGTACAACAACAGCTACAGTACTGGCTTCAGCAATTGTTAAGCAAGGAATGA
GAAATGTTGCAGCAGGTTCGAATCCGATGGCTATTAAAAAAGGTATTGAAAAAGCAACAA
ATTTTGTTGTTAGTAAAATAGCTGAGTATGCTAAGCCAGTAGAAGATACAAAAGCTATTA
TACAAGTTGCTTCTTTGTCATCAGGAAATGATATAGAGGTAGGTAAAATGATAGCGAATG
CTATAGAAAAAGTTGGCAGAGAAGGAGTTATTTCTTTAGAAGAAGGGAAATCAACTAATA
CGATTCTTGAGATCACAGAAGGAATGCAGTTTGAAAAAGGCTTTATTTCTCCTTATTTCG
TTACAGATACAGAACGAATGGAAGTTCTTCAAGAAAATCCTTTTATTTTATTTACAGACA

AAAAAATTACTTTGGTGCAGCAAGAACTTGTGCCATTGCTTGAGCAAATTGCAAAAACAT
CTCGGCCTTTATTAATAATAGCTGAAGACATCGAGAAAGAAGCATTAGCCACAATTGTAG
TTAATAAATTAAGAGGGATTTTGAATGTTGTAGCGGTTAGAGCTCCTGGATTCGGTGATA
GAAGAAAATCTTTACTTGAAGATATGAGTATCTTAACGAACGGACAAGTAATTACTGAAG
ATGCAGGTTTATCACTTGATACAGTTCAATTAGATATGTTAGGAAAAGCCCGAAGAGTTA
TTGTTACTAAAGACTCGACAACAATAATTGCAGATGGTCATGAAATTAAAGTTAAATCAA
GATGTGAGCAAATTAAGCGGCAAATAGAGACGAGTGACTCTTTATATGAAAGAGAAAAAT
TGCAAGAACGATTAGCAAAGCTTTCTGGAGGAGTTGCCGTCATTAAAGTTGGTGCAGCTA
CAGAAACAGAGATGAAAGATAAAAAACTAAGACTAGAAGATGCAATTAATGCAACAAAAG
CAGCAATTGAAGAAGGTATTGTACCAGGAGGAGGAGCTACTAATGTCCATATCTCTAGTG
AATTATTTACATGGGCTAAAAACAATTTAGTTGAAGATGAATTAATTGGTGCTTTAATAG
TTGAACGAGCTGTGACCTATCCGTTAAGACGAATAGCTTTTAATGCAGGTGATAATGGAG
CAGTAATAGTGGAAAAAGTTAAAAGTCACGATTTTCATATAGGCTATGATGCAGCAAACG
GTAATATTGTAAATATGTACGATAGAGGTATTATAGATCCAGCGAAAGTAGCTAGATCAG
CTTTGCAAAACGCAGCTTCTATTGCAGCAATGGTTTTAACTACTGAATGTATTGTAGTTG
ATAAAGTCGATGATTGAGCTATAAGATAATAGATAATCTCAAACAAAGAATAATTTCTAA
TTATAGAAATTATTCTTTGTTTCAATTAATCTAATTAATTGATCAAATGTATAATTTTTA
TAATAAAGCCAAAGTTTAAACACGTCTTCTTTTTCAACTATTAAGTAGAGAAGATATAAG
CCCGTAATACCTAAATTATCAATTAAAATATTATTTTCATATATAGATTTTTCTAAAGAT
CTAAAAAATGACATTTTTGAAAAATAATACCGAAATCTTCTCTTATAGTTATTTGCTAAA
TCATTACTGTCTAAAGATTGATTTCCAATTGAATTCAGAACCTTTAACAGAATACTTTTT
AAATCTTTATCTGACTTTATTTGAGAATCAATAATAATTTGATTCAATTGTATGTCAAAA
CTAAGATTAAAAATATTACTTGTTAAAGATATTTGATTTTGAATATCTAGAGATTTTATA
GCCAAGAAGATTAAACTTTTATTGTTCATTGTAAAATATAAGACAATTGATGATGTATTC
TTTAAATACTAAATTTATCTTCTTGTGCTTCTATCGTTTTTTGTAAAATAATTAATGTTT
TAATTTTTTAGTTTTAGTTACTACCCGAAAAAATAAATTCTGGAAATCTAGCTTGAGCTT
TAGCTAGAGAGTGTTTTTTCCCTTCTTCTTGCCAGTAATTGATAACTTCTGTTGTTTTGT
TTAATAACTCAATTCTGTCTCTTTCAGCAATCCACGGCTTGGATTCTAGTTCTGCTTTTA
ATTCTTCCCAAGCATCATTCCTTGGCCAGAAAAAATAAGAAGTAAGTGGACTAGTGCCAT
TTCCAACAATCTGATCAATAGCAATAGCAATATTATTTTCTAACCAAAGTACTTTTAGTG
TAAATTTTGACAAGTTTTTATCTCCATTATTAATATTAAGTATTGGTACAGTAGAATACT
GTACTAGTTATTTTTTTCAGCAACAATTTGAGCTTTGTTTAAAATGTTTTTTACCGTTTT
AGTAGCTTGTGCACCTTGAGATAATCGTTGCAAAATTTTTGAAATGTTAACACGAGTTTC
ATTATTTATAGGATTATAAAATCCTAATTCTTCTATAGCTTTACCATCGCGTTTATTTCT
GCTATCCATTACAACAATTCTATAGCTTGGTTGTTTTTTTCTTCCGTATCGCTTTAACCT
TAACTTGACCATATTAGTCACCTCGCTTATTTTTTATATCTTTATTAAATTATATCTTAC

GACATTATGCAATAATTCAAACAATTAAGCTGATTCAAGTCTAACATTATTAAATATTAC
CATTAAACATTGTAGAAAAATAATACCTAACATAGGTGACATGTCCATTCCAAACATGGG
AGGAATGCTTCCTCTGAATAGTTTTAAGTATGGATCAGTAATTCTATTTAATGAACAGAA
TGGTTCATTGTACCAATTTACAGTTGGAAACCATGCCAATGATAATTTAAGTAAAATTAA
AATTAAATAAATCTCAGAAAAATTAGCTATAGATCCAAGCAATAAATTTAATGTACCAGG
AAGAGTGTTCATACGTATTTTATAATTATTATATACTTCGTTATACTAATAGTTTGATTA
TATATTATAATCGATGTAAATAAATCAGCTAAACAAAAAACTTATCCTTAAAATCATCTT
CTCTTGAGCCTTTTTTTTTATCTACATTAGTATTTACTTTTGTCGTATAAATTTCCAGTT
TAGACCAAATATGTCCTAACTGATTTAATTGAGATGTTCCACATTCTAAACAGGCTATTT
TTATATTGCTCAAAACAGCACCTTTTTTTATAAAATCGTTCAATAATGGCGTAATAGTTG
CTTCATCCAAAAATAAATCTAAGACAAGTATTTTTGTAAATGAATTTAATTGATCTGGGA
CATTGTTAATAATATCATTATAATTAACTAAAACTATATTAGCTGTAGGAAGTAAAGATC
GTGCTCCTTCCGCTAAGATAAAGCCATAAGGCATAATAATTACAATAATATATTTATAAT
GACTGCTAATAAGACTTATAGTGGTATTAGCTTTTAATTCGATTGTTTCTGTTACTAGCC
ACTCTCTCATTATTTCGTAAGTAATCCATTTGCCTAGTTCTGAGCAAGCAGTTCTTAAAA
TCGTACCTGGATTATTGTCGTTTTCGAGAATCCCTGACCAATGTTGTATTAATGGATGGG
ATGCTATATTAATTTGGAGTTGCATGTTTTGTTAAATATATTTAACATTTAATTTGTTGA
TTGAATCTTCAATTCCTATTTGTTGAGTATTATTAAAATAGCATATATATTAAAGAAAAT
AATTATAAAAAATGAAAAAAAGTCTTTGGCTGTGGGGTTTTACAGATAGTGCTGAAACTT
GGAATGGTAGATTTGCAATGATTGGTTTTATATCAGTTATTTTTATCGAAGTAGTTACAG
GACAAGGATTATTATATTTAATAGGTATGATGTCATAATAAAAATCCAAAAAAGTTCTTG
TAATTTTTTATAAGAACTTTTTTGGATTTTTATAAATCTAATCTAGCGGTCTCATAGATA
GAACAGGTTCATTCTCTCTATTGATACCTTTTTCAAATCCAGCAGCAGCAGCTCTTGCTC
TGCCTGCATGCCAAAGATGTCCAATAAATAGGAAGAATCCTAGGAAGAAATGAGATGTTG
TTAACCAAGATCTAGGAGATACATAGTTAACTGAATTAATTTCTGTAGCTACACCACCAA
CGGAATTCAAAGAACCTAGTGGTGCGTGAGTCATATATTCTGCGGCACGCCTTTCCTGCC
ACGGTTGAATATCGTTCTTGATTTTATTTAAATCAAGACCATTTGGTCCTCTAAGAGGCT
CAACCCAAGGAGCTCGCAAGTCCCAAAATCTCATGGTTTCACCACCAAAAATAATTTCTC
CACTAGGAGATCTCATTAGGTATTTACCTAATCCAGTAGGTCCTTGAGAAGATGCAACAT
TCGCACCTAGTCTTTGATCTCTAACTAAGAAAGTAAAAGCTTGAGCTTGTGAAGCTTCAG
GACCAGTTGGTCCATAGAATTCACTAGGATAAGCTGTGTTGTTATACCAAACAAAATTAG
AAGCCGTAAGCCCCATGATTGATAAAGCACCTAGACTATAAGATAAGTAAGCCTCACCAG
ACCATACAAAAGCTCTTCTAGCCCAAGCAAAAGGTTTTGTTAGTATATGCCAGATTCCGC
CAGCAATACAAATAATGCCAATCCAGACATGACCACCTACAAGATCTTCCATGTTATTTA
CACTTACGATCCATCCATCACCGCCAAACGGAGACTTTAAGACATAACCAAAGATAACTA
GGGGATTAAGTGTAGGATTGCTAACAATCTAACATCTCCACCACCTGGAGCCCAAGTGT

CGTATACCCCACCAATAAATAAAGCTTTAATAACTAACAGAAAAGCGCCAATGCCTAATA
ACACTAAGTGTATACCAAGTATTGTTGTCATTTTGTTTTTATCCCGCCAATCGTATCCGA
AGAAAGGAAATGATTCTTCAAGAGTATCAGGGCCAATCAGAGAATGATAAAGACCTCCAA
AGCCAAGAACAGCTGAAGAAATTAAATGCACTACGCCTACTACAAAGTATGGGTATGTAT
TGAAAATCTCGCCGCCTGGACCAACACCCCAGCCTAGTGTAGCTAAGTGCGGAATTAGAA
TAAACCCTTGTTCGTACAAAGGCTTCTCGGGAACAAAGTGTGCTACCTCAAAAAGAGTCA
TGGCACCAGTCCAAAAGACCATTATACCTGCATGAGCAACATGAGCACCAAGCAATTTGC
CAGAAACGTTAATTAAGCGTGCATTGCCAGACCACCAGGCAAACCCGGTAGACTCAATGT
CTCTACCGCCAACACCAACAGTTGTATTAAAGGGCGTTTCCACGTGGTAAAACCTCCTCA
GGGAATATAAAGTTTTCATGAGGTTGATCTTGAGCAGCCATCCAAGAGCGAATACCTTCG
TTCAATAGGATATTTTTAGTATAAAAAGTTTCAAACTCAGGATCTTCCGCAGCTCTCAAC
TCTTGAGAAACAAAATCGTAAGCTCTTAAATTTAGAGCTAGTCCAACAATTCCAAATGCG
CTTGTCCATAATCCAGTTACTGGTACAAATAACATGAAAAAATGTAGCCAACGTTTATTA
GAGAAAGCAACACCAAAAATCTGTGACCAGAATCTATTAGCTGTTACCATCGAATAAGTT
TCTTCAGATTGTGTTGGAGTAAATGCACGAAAAGTATCTGCAGCATCACCATCTTCGAAT
AAGGTATTCTGTACAGTTGCACCGTGAATAGCACATAGTAAAGCACCACCTAAGATTCCT
GCAACGCCCATCATATGGAACGGATTCAGAGTCCAGTTATGAAATCCTTGCAAAAATAGC
AAAAATCTAAAAATTGCAGCAACTCCAAGGCTTGGGGCAAAGAACCAGCTCGCTTGGCCT
AGAGGGTACATTAGAAAGACGGAAACGAATACTGCAATTGGTCCAGAAAATGCAATAGCG
TTGTATGGTCTAAGACCAACTAGCCTAGCAATTTCAAATTGTCTTAAACAGAATCCAATT
AGTCCAAATGATCCATGTAAAGCTATAAATGCCCACAGACCACCAATTTGGCACCAGCGA
GTAAAATCTCCTTGAGCTTCAGGTCCCCAAAGGAAAAGAAGTGAATGTCCCATGCTGTTT
GCTGGGGTAGAAACAGCCGCAGTTAAAAAATTGCATCCTTCTAGATATGAACTGGCTAGC
CCATGAGTATACCAAGAAGTAACAAAAGTAGTCCCAGTTAGCCAGCCACCTACAGCAAGG
TAAGCGCAAGGAAACAGAAGTAGTCCAGACCAACCTACGAATACAAATCGGTCTCTTTTT
AGCCAATCGTCTACAAGATCAAAACCACCACGAGTTTTTTCTTGTCCAATTGCTATGGTC
ATAATTTTAATCTCCAGAGCAAATTTAATAAGTAATTTGTTTAAATAAACACTTTAGATA
TACTCTAAAATTTTACTTTTCTTTACGGTCAATATAATTATAAGTCAAAATTAACCAAAA
TTCACGCTTTTAATAACATTTTTTTAGCTCATTAAGTTAATTTTTTAATATATAAACAGT
AATAGAGTTATTGTTTAACTTACAATACTCATAATTTATTCATTTCTATAATCCTTTGAA
AAAGATATCCTGTTCCTCGTGCTGTTAAAATCAAGTCGGGATTACTTGGATCATCTTCTA
GTTTAGCTCTCAATCGTGAAATATGAACATCAACAACTCTCGTGTCAACATGCCGTTCGG
GAGTATAGCCCCAAACTTCTTGCAAGATAGAAGCTCTAGAAAAAGGTTCTCCAGCCTTAC
TAACTAAAAGCTCGAGAAGACTGAATTCCATACCTGTTAATCGAACTCTTTCATTATTTT
TATAAACTTGTCTTTTGTTAGTATCTATTTTTAGAAATCCAATACTAATAATCCCAGAGT
TTGGGACTCCAAGACTAGTGGTGATTTTATCAGCTCTTCGAAGAACAGAACGAATGCGAG

CTTCTAGCTCCTTAGGAGAAAATGGTTTAACGACATAGTCATCAGCACCTATTTCTAATC
CGGTAATTCTATCGCAGACTTCACCTAACGCTGTTAGCATTATGATAGGAACATCTGATT
CTTTTCGAAGTTCTTGACAAACACCGTAACCATCTAGTTTAGGCATCATAACATCTAAAA
CAACAAGGCTAGGATACTCTTTTCTAAACACGAGTAAAGCTTCTTCTCCATTTGAAGCAG
TGATAACTTCATAACCTATTATTGTTAGTCTTGTTTCTAAAATTCTTCTTATGCTGGCCT
CATCGTCAACAACGAGAATCTTTTCTTTCTGGTTCTCCAATTTCTTATTTACTCCTATAA
CTCACCGATCATGATTACTATAACCTTAAATTTTTAGCGCTATGAGTATCTTTATTCACT
TTAGAATTTAGTTATTATAAATTTTGCAAGTCTAGTTTACATTAATTAGTACCAGTCAAT
ATTTTTGTTTTAAGTTATGACTATTTTAATCCATAAGTTGACTCTAGTTTGCAGAATTCA
ATATATACATATTTTCTAGCCAAGTAAATTGTATTCTATTTTCTAAATAAAAAAGGGGTT
GACAAACCAGTTGATAGAAAGTATTCTACATTACTATAAGTTAATTCCAACAAAGAAGTT
TTAAATCTTCTTTCGGAGTAATGAATACTTTAAT
