# Supplementary figures and images for: Genomic diversity of 39 samples of Pyropia species grown in Japan
Source: PLoS One. 2021 Jun 9;16(6):e0252207. doi: 10.1371/journal.pone.0252207 (PMC8189503; doi:10.1371/journal.pone.0252207)

# Chloroplasts

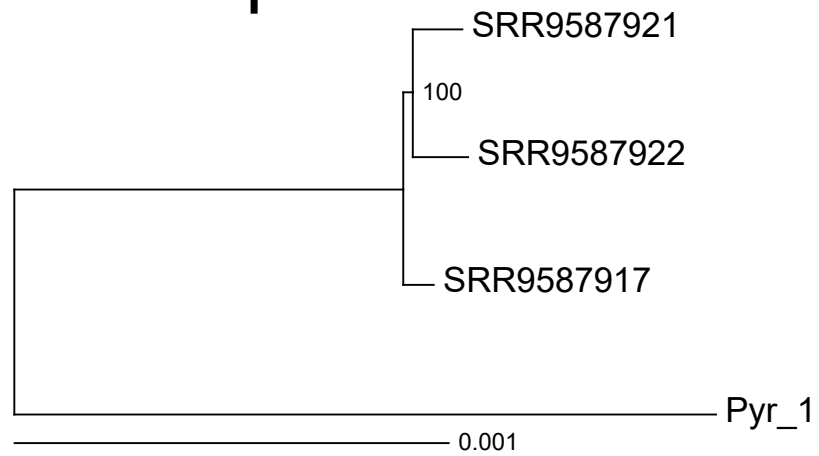

# Mitochondria

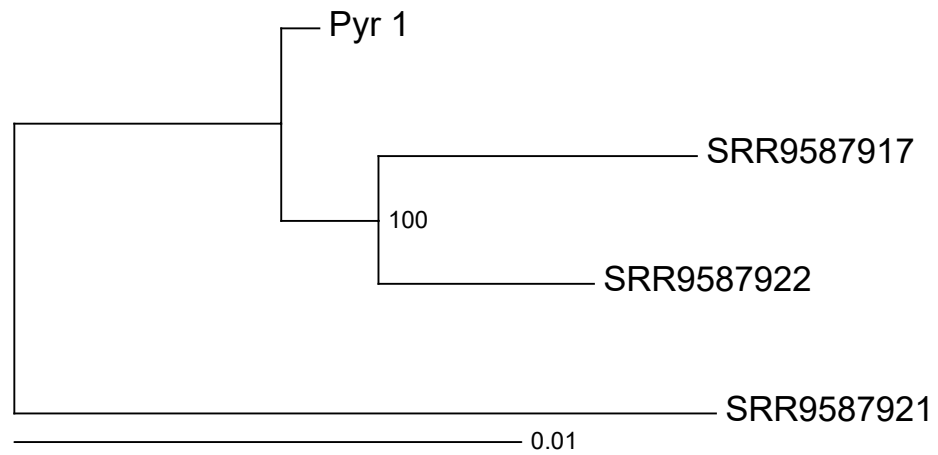

Supplement: S3 Fig — Phylogenetic trees were constructed based on maximum likelihood method. The DNA sequences from the large single copy sections of chloroplast genomes were used to create a chloroplast phylogenetic tree. The DNA sequences from the assembled sequences of mitochondrial genomes were used to create a mitochondrial phylogenetic tree. The numbers at the nodes indicate bootstrap values (% over 1000 replicates). The scale bar shows the number of substitutions per site. In each analysis, the midpoint was used as a root. The parameters for RAxML in the analysis of chloroplast DNA sequences were as follows: -f = a, -x = 12,345, -p = 12,345, -N (bootstrap value) = 1,000, -c = 1 and -m = GTRCATX). The parameters for RAxML in the analysis of mitochondrial DNA sequences were as follows: -f = a, -x = 12,345, -p = 12,345, -N (bootstrap value) = 1,000, and -m = GTRGAMMAX). (PDF) [file pone.0252207.s003.pdf]

## First rRNA repeat

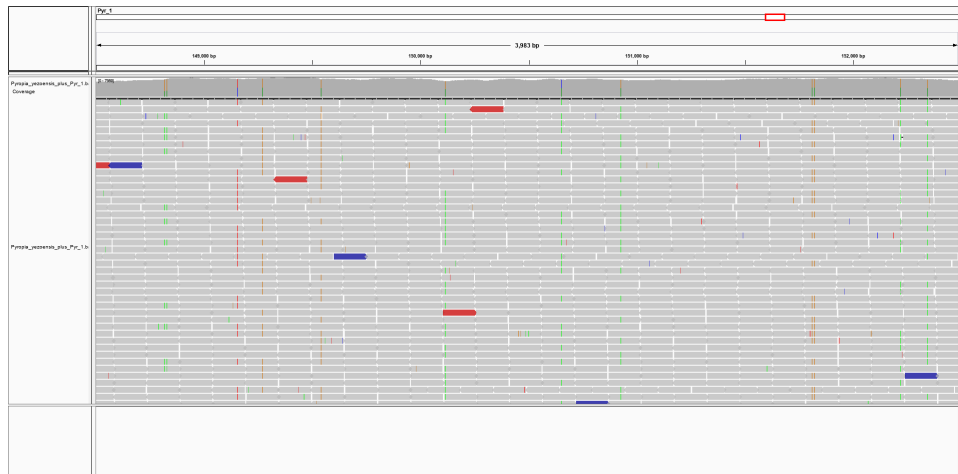

## Second rRNA repeat

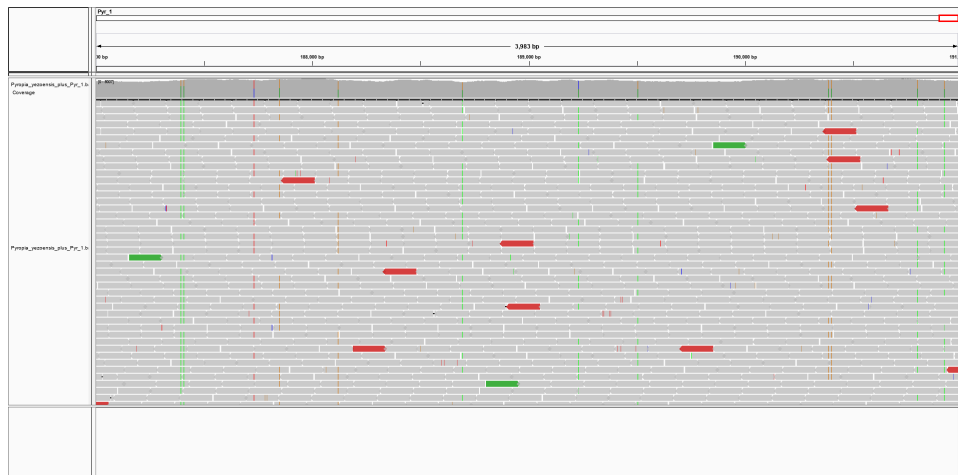

Supplement: S8 Fig — (PDF) [file pone.0252207.s008.pdf]

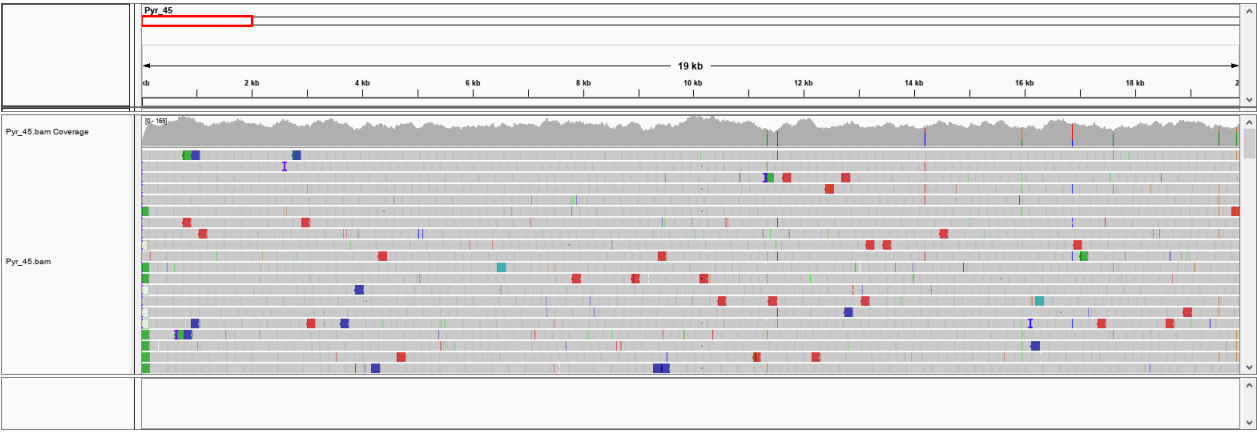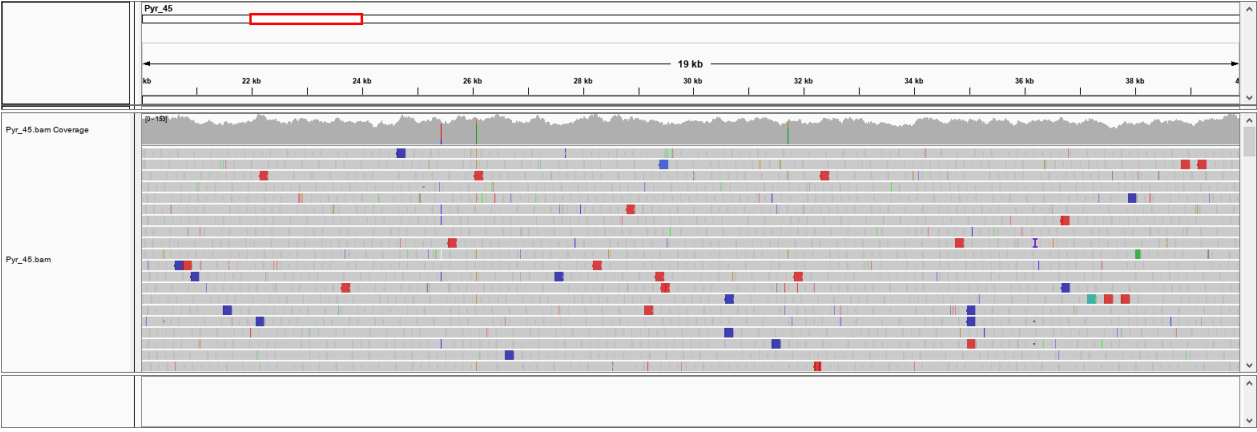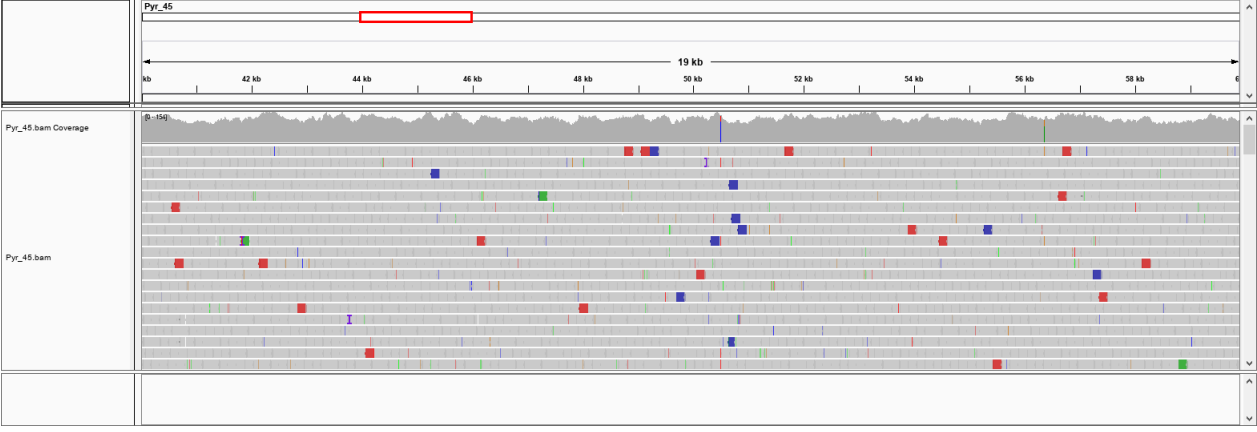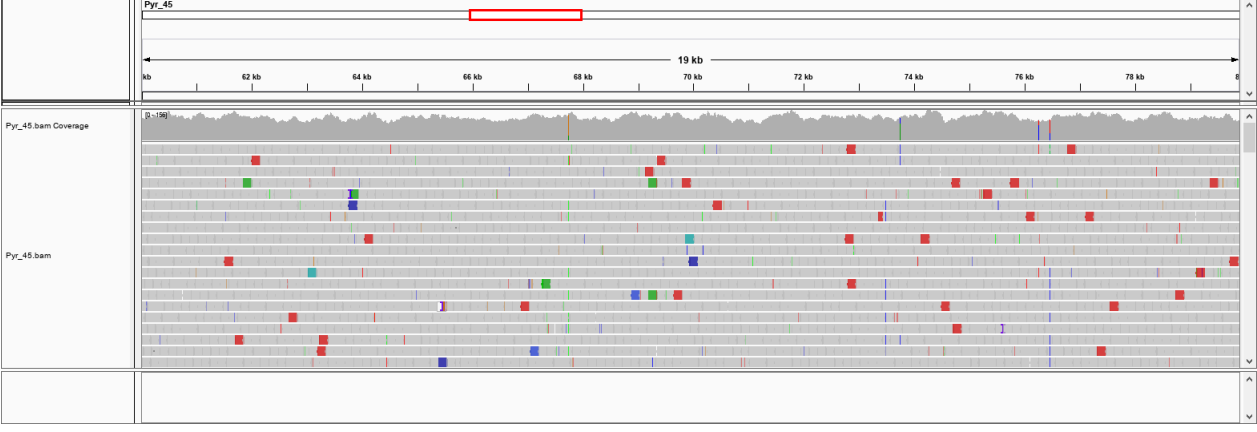

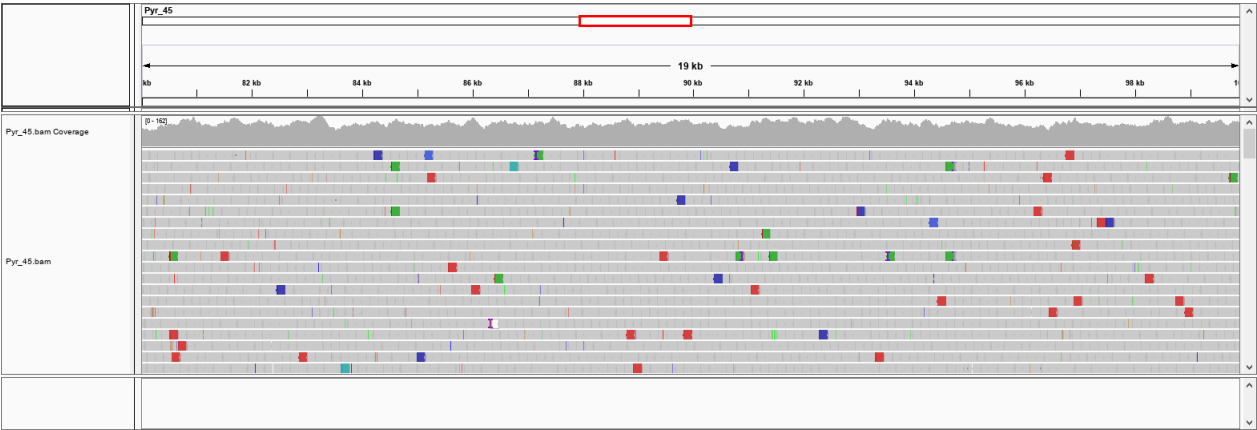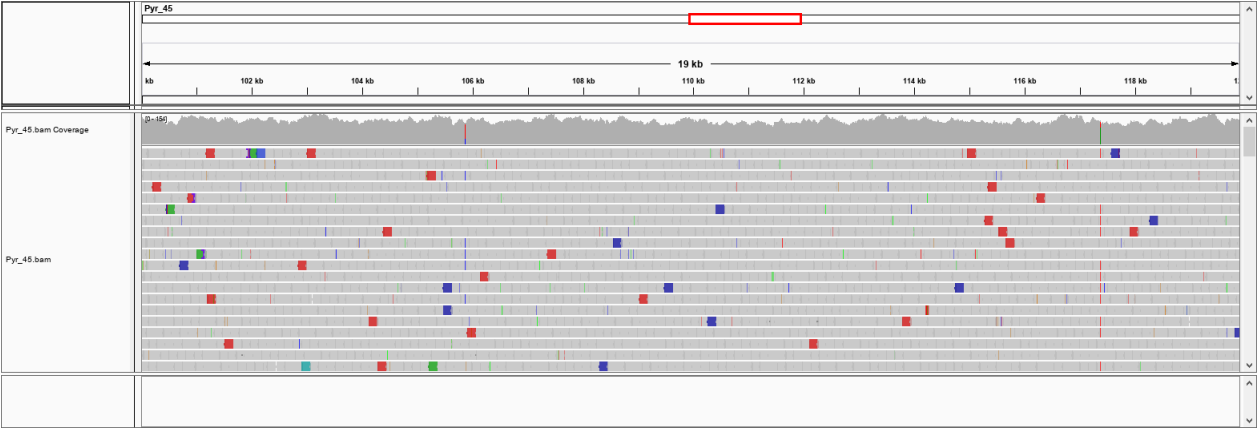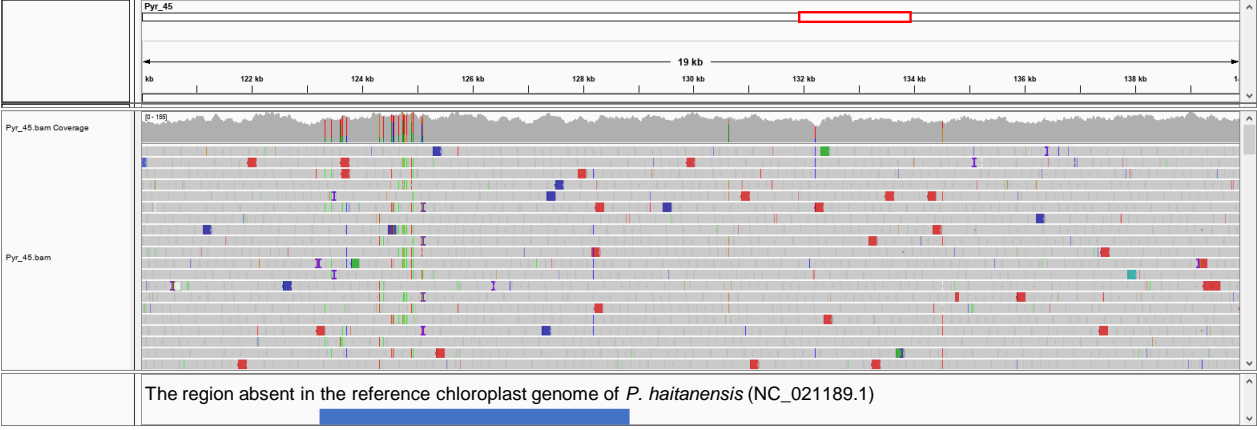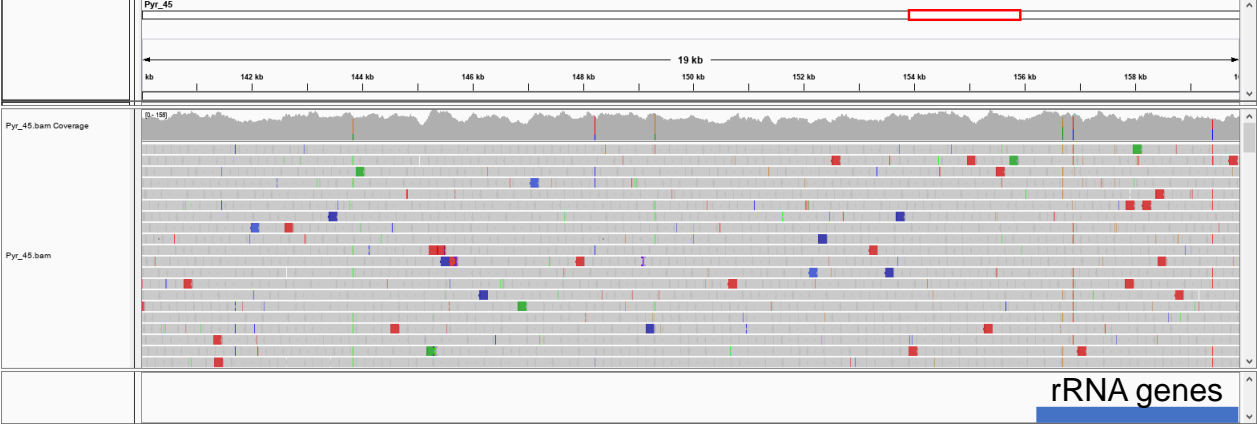

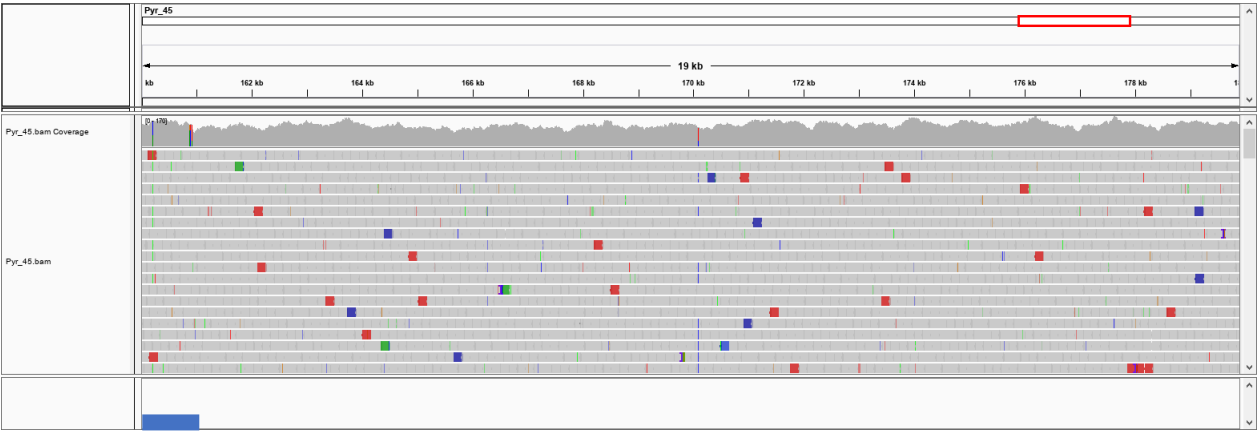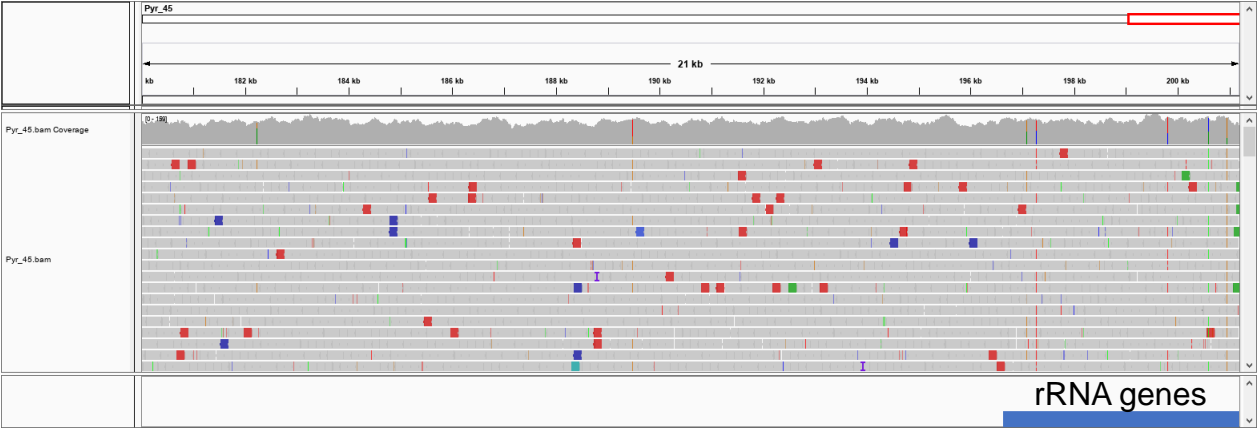

Supplement: S9 Fig — (PDF) [file pone.0252207.s009.pdf]

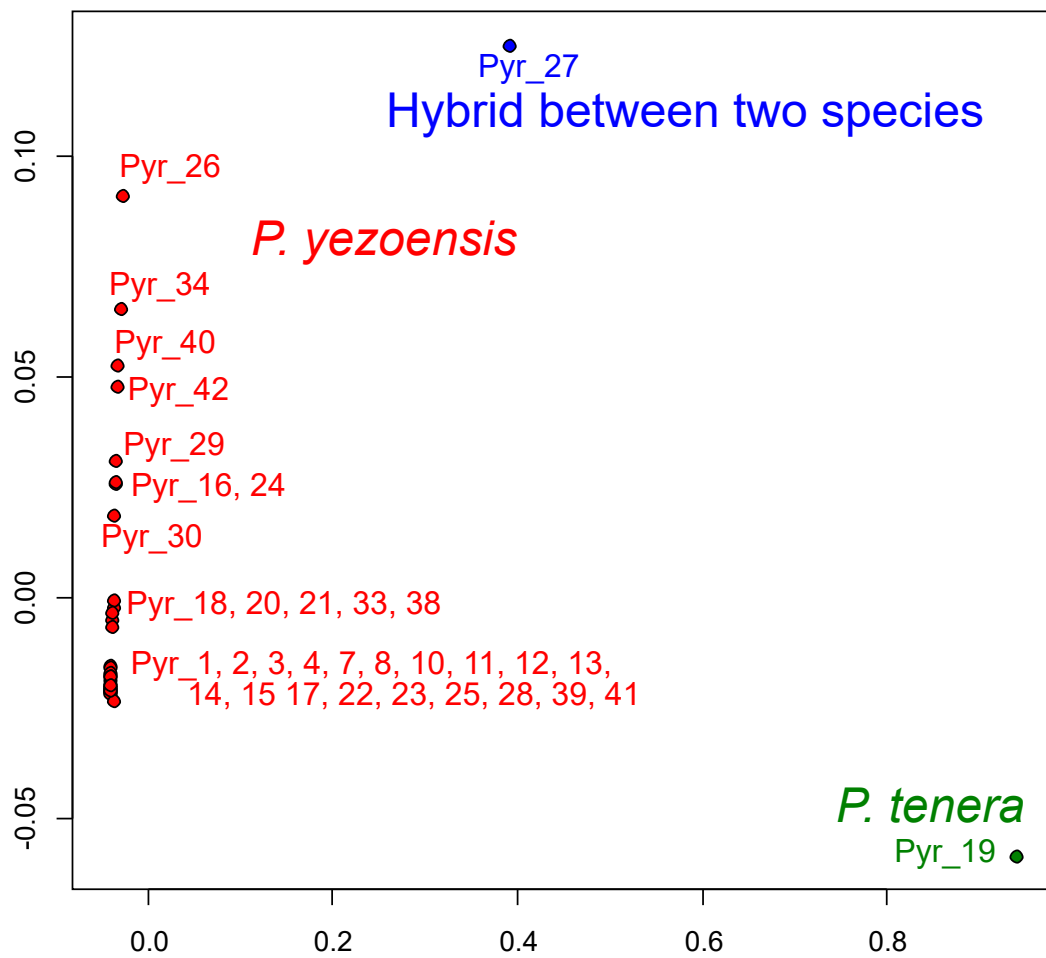

Supplement: S10 Fig — Two-dimensional data were obtained in this analysis. Colors were used to show P. yezoensis samples, P. tenera, and the hybrid between P. yezoensis and P. tenera. (PDF) [file pone.0252207.s010.pdf]

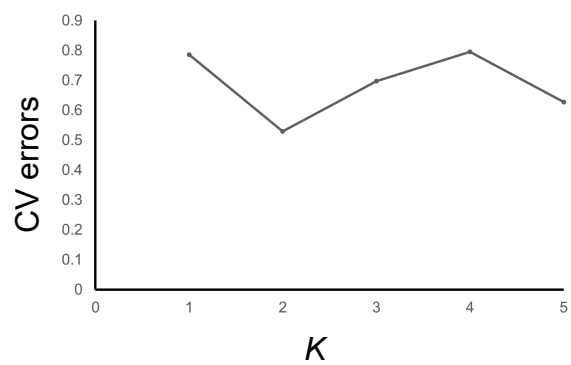

K=3

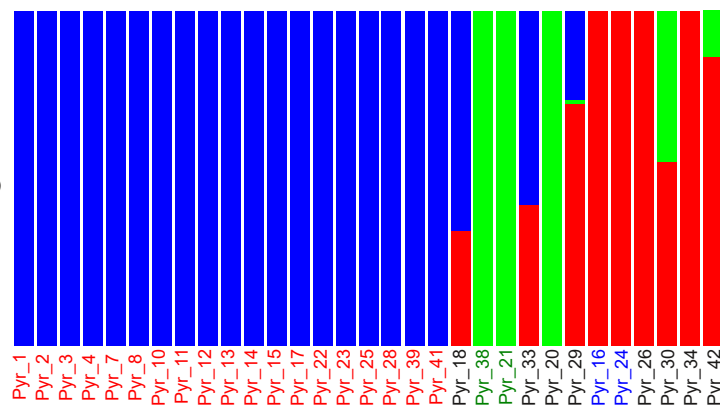

K=4

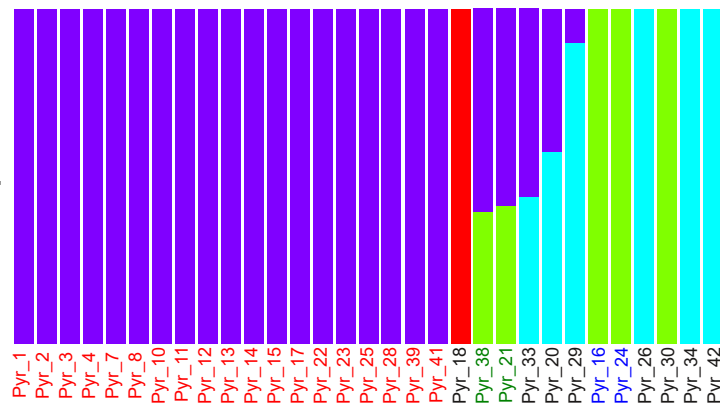

K=5

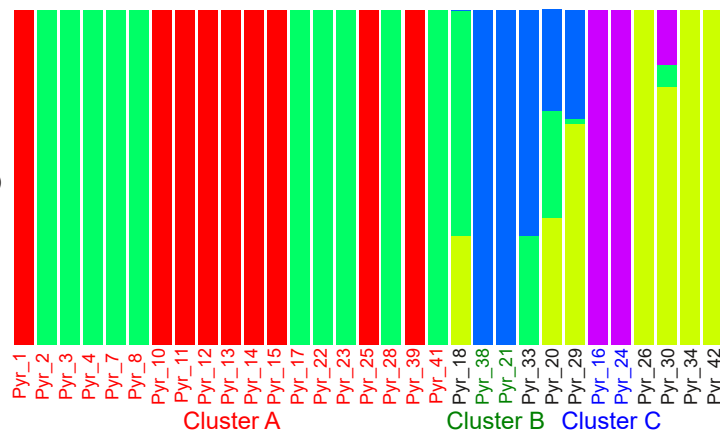

Supplement: S11 Fig — Colors of the sample names were used to show 3 clusters, cluster A, B, and C. (PDF) [file pone.0252207.s011.pdf]
