# Supplementary material for: Genomic diversity of 39 samples of Pyropia species grown in Japan
Source: PLoS One. 2021 Jun 9;16(6):e0252207. doi: 10.1371/journal.pone.0252207 (PMC8189503; doi:10.1371/journal.pone.0252207)
Supplement: S4 Fig — (PDF) [file pone.0252207.s004.pdf]

>Pyr_1
gttttattaattaaaactaccccagtattacacttttaaatatttataagaaataatgca
aatccctttacgctgttatttaatcgaatttaaatttcgattcgtttactttctacttag
tttttttctctcgttgctagtcgttataaactatcatgaggctgtgttttttttttgaaac
gtatactttttttgtttataaacgagggggcattcatcgcgacacatattgctgaactatt
ttcaacttctatgtatattagttttaacctagttttgtgcataaattatccttttgcata
ttatcattgcagccgctttttttaattcgagttgatacaaatcacaggtttggttctttaa
aaatatgcaatcattgacatttattacgtttttagctagtttacttttgtgctactttttt
aattttgccctacacttacgctttttttagacacttgaactgtaacgatagtttatgcatt
taaagtgcagttggaggcaaggatagcaacatatgtttgttgaacactacaaacaatgtg
tttactatctaacattgtctatctcttctttactaggctaatttgtttttacttagtgga
caatatgattaatttgcatttatttttccggaaaaataaaaaatatactttcttttttcgt
atgcttgtcggcttctgtgtgtttaccgcaagaaagttttatacaaattactttcataat
ttctataatagtactatttgagttattctttttggttacatgcttatttttttgcaaaaaa
taatgtctaaaagtggacttgaaccactgacccaaagattttcaatcttttgctctaacc
gctgagctatttagacttaaacttttcttttcccgtatttagagcgaccatttttttcgtt
taacgactgcttgtaaatcgtaaacgcctctcacaatatggtaacgtactccaggtagat
cctttacacgaccacctcttatcagaacaactgaatgttcttgtaaattgtggccttcac
ctcctatatagcctatgatagaacgtccggtactaagacgtattttagctacctttcttt
cggcagaattcggtttttttagggttagtagtatacacttttgtgcaaacaccctttttttt
gaggcgacttattgagagcgggtgtttttgctttggtaattttgtgctttcttggatttt
ttatttttttggtttagtgttgacataatattttttattttaagtaataagggacttgaacc
cctaaccatttcggtgtaaacaaaacgctctacctattgagctaattactttacgtctgg
aaagatttgaactttcaacttttagattcgtaatctaacgctctatccagttaagctaca
gacgtttttctccacttgtatatttagattacttatctatattttggaaatagggacaaa
tattcttgtataatttacttcaggattattcgaaagtataattaaccaattaaacaagta
gaaaacgcatgttttgtattgttactaacttaaaggactattttataataaacttttttat
tgtattatataccaatatttagattgaaaatcgacccaaattcaatgtacttttagcaaa
tgacaccagtgttttagaaaaggtg-ttattttgaaaacatttttggtgtgatttgccct
ttttcagctagtaataatttaattaataacagaaagcgcggtttattgctcgtcgcgttc
aagttattactacggcaacttcctatgcagctgttgggacccccccaactcgtgtaatta
cactttttagttttatttaaattataataaggttatactttatatcttttcttttattta
accccctaagatctttattacgccccaagatggtgtcataaaaaaa-tagcctggagggct
gccaggagtaaaaaaggcccataacggttatttcagcttttatagtgctatttttctttt
ttagagaaggctatttttaattgataactaagtcattttaagtgataattaagctatttt
aattaaaaatgacttattaaaggttttacacagataatacttcttttattaaaaatatac

tacgccgctagaccggaaaaaacccgccatagaacgatgagaggcaaaaagtgtcataaa
ttttaactattttgtaattgtacattttaaaattaaagtttgtcaaacatcaaaactttc
tgttatttcaataatgcgtgtttttaaggcttttaaagcagtttctcgctgatcccgaac
actaaactttttgttaacatgttcttgtaaactgtaattggatgtcataataaccaatgg
attatccggttttaaagtaaaattacctttcattgcaaggcgcatgctatcaccagctaa
aaactggtttaagaattcgatttgtttaccgataaggtgaaattcgtctcatagaataaa
ttgatacaaatgattttcataataatcttatcagttgtcttggggtcacatataacaagc
ggtagtagttttcaagtaatttaacagcgcactttttcccatattaggttctttacttca
tatatggagttggggtttttataatagcgtgaaaaaggactagtattattgtttagtat
gtcgaatatttgttctcataactggatagaacattccgataagttattttattattttg
ccttaggattgttttgcaaaatcgttctatgttgggcttttatttttt-----------

-gtttaatatctggttcatcaaaattaaaaaatattggtgtagtaacaccagattcgatt
tatttcatgatgaagtgctctcttacaagccgctttttagtagattactaaaaattta
ctatgttgatttgatacgcatttttggatgggctcttgcttatttattggatctgaaaag
gagcttggagaattagttaagtagtcaaaatgattcagatttgtgaaattacgtttctta
gaaaattcaatatacgtatataaaatttggtccgagttatttcagctactagaaaagctt
attattattgtttttctctttgtaggagtttgttataaattaaaactaggcaaagaagc
tacatttttgttgtaacttggaaagatttggccgcaacgcgctttttatgtttagttac
ggctggattgagttcaaatactttggacatgagtcgttttaaaaattatttagaaatatg
tgttcgcaaacagattgagtgaaaaaaaa-agtttaaaaaaagcttcataaactttcacc
aaaagtttgatttatatcataaacggagaaaatccaaattgaaaaaactaaaatggtgtg
gcctaaaaaaatacggcgaagtacatatcaacttggttttcagtgaattaaacagaaata
cagtattcccacaagtacgataggaattagaatgatggataaatcaaaattttgatttag
atacgaaaaccatg-------------gtttacaagtaaacttttaattcaatattttg
tgctattagtaaagatttgtgcgactttaaattgaataagctgttaaaataaggtgaatt
taatttaggcaataaagtatcgtacaaatgtttgttctttctatatttgtttagagcttt
cctgtacttttattgtaagcagcaatagatcctatgtgcttgtgttgaaaatctaatct
tttaagtactttgttaaagctcaaaattttattttttaattggttaacag-ttttttacg
ctttctagcatcgtatagtctttcttttagcgtttctagatgagaaggcttttctagtgg
cagttgattgaaatcacttttagtgag-ttttttactatagtacctgtaacaagtgtgct
aacagatactatgacacaaactacttttaagtccaa-ttgctctacaatgcg-tttgatg
aaagtcattttatataaattaatttattaatgtgtaattgtggtatgattttattttgtg
ttaatggtcataattaaatgcatacattatatattatccctccttcggagggata-atat
aataattcttattatataccaatatttagattgaaaatcgtgctattttcaatctaaata
ttggtatataaacaaacctgcacataaataaatatcacaaggacatcaccccaccaaaaa

atcgatattctctagagagcccctataagctttaatttactatctcttgcatgaaattat
accttcttggataggaatcatcgtgaaattgaaaaacattta--gatatgtcttatacaa
ttatgactaaaaaatctacaaaacaaacaactttacacaaatattttcgcaagtcgcata
catgagattgaacttaagaagcctcttttcacaaatcatacagcatttttggcgcttctt
aattctattgcaatattcaatgaaccaaaaacaaagtttttttaaaacgacctgcatgaa
cacaatcttaacaagtgtaaaaaagccctctagc-cccttttttgcaacaccacgagaat
aaaccccggtcggggagaccccgaacccccctcggcgcgggccgtctagcaaagttagacg
gcccgcgcgcgcgttatagattcctatatcgctccattccattccatttcgctattgctg
cgcttcgcttatttttaattctaactgttccttttcgtcataacgattttaggtcgattt
tcaatctaaatatttgtatataaacaattatttatcttgataattatatatctaaaaaag
ggggcatagtttaataggtataatgtatgctttgcaagcataagattaccggttcaaatc
cggttgtctccaaatctaaaaatatcaatgcgttctagaatattacaatacaaaaataac
ttggctgactatgatcttttaactcaattttcgattaaaaatcataatagtatgcctact
ttctcttctcttaatgtcagagtaaaaaatattcaaactagcgacattaagcaggtttgc
ttaaatctaatctctattcaattagtaggaaacaatcatgttgttttcagtagccaaaaa
gacgtttctaatcttatactaaaatgtaacggtattaatacttattttattttagagaat
ttatctttgttaggctacaaaaacaatttaaaaagtgacaaaaacaataaattaactgtg
cacggtaaaaacttcttaaatattattgaatattttgcatctaattcaaatttttcttaag
tccttagaaaaaacatctaataaacatttaagtattgctttttcttatcaacttgctaat
agtaaaaataatgaaatagctttgtatttttttaaaatcattaggatttccgctttaataa
cggtatagttcaattggttagaacaacggaatcataatccgtaagttgtgggttcaagtc
cctctaccgttatggctcttatcgtctaatggttaggacagtgctttttcagggcatcga
cgtgagttcaatcctcactaagagtaacttttttaatataaaaatccttatacaaatacgt
gagcccataagaattcaaaagtaaaaaggatttttctagatctaccaaattattttggga
tccctaggtcgttaaaaatatttaaacaatgtattttattc--atatatatatatactaa
aacttaaaaatataatttagtgcaaaagtctctaaataactgaattttccgttgaatata
ctcgaccaatcataaagatataggtactttatatttaatttttggcgctttctccggtat
cttaggtgcttgcgcgtctatattgatccgaatggaactagcacaaccaggtaatcaact
attattaggcaatcatcaagtgtataacgtactagttacagagcacgcatttttgatgat
tttctttatggttatgcccgtcctaattggaggatttggaaactgattcgtacctattat
gataggtgctccagatatggcctttcctagattaaataatataagtttttgactactacc
tccatcattgtgtcttcttttaggatctgcgatggtagaagtaggcgctggcacaggctg
aactttatatccgcctttgagctctattcagagccattcaggcggtgctgttgatcttgc
tatttttagtttacacttgtcaggtgcttcttctatattaggagctattaatttcattac
gacgatatttaatatgcgcaatccaggacaaagtatgtatcgaataccgctatttgtttg
atctatcctcattactgcgtttcttttactactagcagtacctgtcttggcaggggccat

cacaatgctgttaacagatagaaactttaatacaacatttttttgacccttcaggtggtgg
cgatcctgtattgtatcagcatttattctgattttcggacatccggaagtgtacatttg
tgcgccgtttaattctgttaaataagatggtttattattttaaattacttatctcgaaat
ctttaaactgaatagttaataaagatagacttaattattccttttgtcttaaaacacttt
cccttaataaagttggaaacaattctattaaaagcgggaatgaaagtctcttgcaggaac
ctgaatttcctaaaaatagtaggctagttatgctcatcatgaataagataataaagttga
tacatatcgacggttccacactaaactttatacataacatttcaggtaacagtatttatg
taatatgggtcggtaccagaatgtttaccttcccttgaataattattacacgtcagagcc
atcagctcctaaatgatatgcaacgttctaaaatgagcttgatcgagaaacagaagaact
cgggataccctaccaatcgaaagattcatgagtacggaactctcgtagtaggtggtagaa
gaattcaatcttcttcaaactaccaaaggggagtagaacttagattcttaagtgaaaaac
cctgcattagctcgcaagagtgcgctaggttagtagatttgagaaaagttaattctgaaa
ataaatttcaagttaataagaatactattcatattatatctgatatgaatgtccttattt
tagcatatgaactcataaaaagtaatcctggaaacatgacacctggtgtgaatggttcca
ccttagatgggttagacaagatgtgactgcaaaatattagtaccaaaataaagcaaggta
aatttttattcagccctgggcgtaagaagtacattcctaagcccggttcagcggataaaa
gaccattaggtattgctagcccgaaagaaaaaattgttcaaaaagctattctgctagtac
tagaatcgatttttgaaccaagcttcttgagaaattctcacgggtttcggcctaaccgag
gcaaccataccgctttaaagatggtaaaaagcgagtttcacggagttccctgaattatag
aaggagatatttcgaagtgctttgatgaaattgatcactctattttattggggcttctaa
gcaagaggatatcttgtgataagactttaactttaattaaaagagggttgaaggctgggt
ttatagatttaggaatattcacaagaactaaattgggcacccctcaaggaagcattctga
gtcctatcctatgcaatatctatttgcatgagctagatttatttctacttcaactaaaaa
ttaaattcgatacagggactagtagagcgaagaacccacagttcagaaaactacagtata
aactatccaaccttaaaacgcctctcgagaaaaagcttgtcagaagagacctttgaaaag
tgcatagtctgaacccccctagatcctaacttttgcagaattcactttgttcgatatgcgg
atgattttattgtgggagttacaagctcccatgaagttgctttagaagttaagaatatga
ttaaggaattcctttgtaatcatttgaaattaaatttgaatgagctgaagacacaaatta
ctcatattagagaaaaggatatatttttcctaggtacccttatcaaaggtaactgaaaga
aagagaaacctattcgattgatcaactttccctctagagaaacgtccatcaagacaagag
tcactccgcgtttaagtttgcatgcccctattaaaaaactctttgataaagctactgctg
aaggatttttccgtagggatgggattaattataaacctacctttgtaggtaaattgatta
atatggaccatgcagacattttattttttataattcaatagtaagaggagtactaaatt
actactcatttgtggacaaccacaaaagtttaggatcatgagttcattatatgaaatttt
cctgtgctagaactctagcgttaaagtacaagttacgtttcacatcgaagacttttaaga
aattcggctctaaattggcgtgcccgaatacaaaaaaaagcctgtttttaccaacgagct

ttaagaggacgcaggccttccaaattaatagccctattccttttgagaaaaaattactct
cttgatctaaaaaaattactaaatctaatctgaacaaagtttgtttaatatgtggtagtt
ctctatttgtagaaatgcatcatatacgtagtatagctggcattagaacaagactttgta
gcaataaagcagacttttctctctgcaaatggctgggataaacaggaagcaggttcctc
tttgtcgagaacatcacttgaagctacataataaaacactttcaccagaagaaacgttat
tatttcagaaaaatatgaaggaaatttagatgtatttctttcaattttaactttatttac
aatttaagttcggcgagccgtatgataagaaattatcacgtacggttctgagggcagtta
ttgacgctaaaacaacctcgttgtcaagtttctatttctgacccctactaattttgcctg
gatttggcatcgtcagtcacatcgtttctaccttttctagaaaacctgttttcggttata
taggaatgatttatgctatgctttctataggtattttaggatttatcgtttgagcgcatc
acatgtatactgttggccttgatgtagatacaagagcttacttcacagccgcaaccatga
tcattgctgttcctacaggaataaaaatatttgtgcgccgtttcgtcgattttggaagtg
atcatttagtgattatagacagctggaacatctatttgtcaacttgcttaggtaatatcg
aaaggatattgctgaacacagcatgcaagtaaagcgaagttaacttctaaacatttatga
ataaattttgcaagacaattttctcacggttagggttatgtgacccgaagtgattctact
attgccctcgctttatggggaataccttgaatgtcatggggtatttaaatctacttccga
ttatctgaatattgattagaagaccagctagatttacaaagattaaaataattaatttaa
acgtagacaattcgcaacctaaaagaaataataattccaacgtcggaaatacgggatcac
ctaagtgccgacaggcatatggtgacagagtggacgtagtacgaagaatcaaaaacttcg
aaaggtccacagttaaatcattcttaaacagaaaactttacagtacaggaagtacaatta
atgtagagcgaaagtttgagagttttgttaaaagagcaatggaatttccaaaagaaatta
ttgacagggatctataccgcattttatgtgatgtaaattttctaaatattgcatataaca
atatcaagagcaaacctggtaatatgacttcgggaataactactgaaacgctcgatggta
tatcatacgatatattaaaagatatttccaatagtttaatggaagaatcttttcattta
aaccgggcaggagaattcaaattcccaaaccctccggaggtgaaagatctttaacaatag
catctcctcgagataaaattgtacaggaagctatacgaattattttaaatgcggttttg
aacctacttttctgaagtcttcccatggttttagacccaaaaagagttgccacaccgctt
tggaaaagataaaacttgagtttaagccagtaacttgagttatagaaggagatattacta
agtgctttgacaatattgatcataacttgttgatgaaattaatagaatccaagatttcag
acagacagtttacaaaacttatatcaaaaagtttaaaagcagggtacttcgagaccaaag
ttatttctcataacattgtcggaactcctcaaggatctattataagtcctattttatgca
atatctttatgcatcaattagatgtatttgtagaaaatctcaaaaacgaatttgacaaag
gcgttagagcaaaaaatcttagttcatatgagaactctagatataaaatcaagtgttcta
aacgatcaggagacatgataaaacttaagaaaatttataagatatctcaacgaagtccag
ttatggattttaatgactcctcgtataaaagacttagatatatcaggtatgctgatgact
gaataattgggattaggggtagttttatcgaaacgagacaagttctagaaagggtcagat

ctttttttgaataacgttatgcgcctcgatattaacgactctaagactaaaattactaacc
ttaataaagataaagttgtatttttaggaaccaacatatttcggtctaaacatgtaaaat
atttcgcaaaaagtagtccgtctaaacaaaggcaaaaccttcagcttcaatttcacgtta
gtactgaccgaattagaagtaaacttgcaagtatcagcatgttatcgggcaatgtaccca
agcctagattttttatgattatctttaaatcacgatcagattatacacctttacaactcag
tactaaggggctttataaattactactcttttgtaggtaactatagccaattcgtgtcct
gaataaggtgagtgatatattcatctgtagccaaactattggcaaggaaattcaatctgt
cagttactaaggtttttaaaaagtttggtccaaatctaagttcaggcaagtttgctctat
acgatcctaaatttatagccaacgaacccaggtttaaaaccgatgtatcgcccgtgattc
caaatttatatgctaaatttaaatctacggcaacgctatatgaacttgtatgtgctaaat
gtggatcggactatagagtggaaatgcaccatattagaaaaatgcagaatcttaacccca
aaatttccgaagtggaccgtctaacggttcgggccaatagaaaacaaatttcactttgca
gggaatgtcatatgaagtatcatcgcaacaaaacataaatttaatggagagccgtatgat
gggaaactatcacgtacggttcgggaaagggacctatattgccttcgggggtatgggttc
tagttcatagctggattgctactatgtgagaagggtctatttttttaaaaacccctatgt
tatttgctatagggtttatatttttattcactataggaggacttactggtattatactag
ctaactccggacttgatatatctttacatgatacttattatgtcgtagctcacttccact
atggtgcgccgtctaattgcgtttatcgtcgcttacttcagtgagcatgtattaccattc
ttgaattatataattaaatgttatacaatttatacattaaaacaatcaacttacttattt
ctggccaacagaaagaggactacagcatgttgataaaggagttttgaaatagtaaactcc
gagttatactacacacggttaggctaacgaactccttctacaatcagccaggcggatatg
aagttccgatcattagaaataatgaagtatctcacggtgaaagtttttacatagttagac
ctttacaggaatggtctggtacccaaaatttaagtaaaatttttaaagctgaggagaacc
taaaggtagttattaatggtaagcgtaagaattcgggaaatcctgaaagttgaaaaactg
gaggattcggagggatcgtagtacgaaatataagctgcttagcttatgttaggaagggtc
ctagttcagaagctattctaaattctaagctctcgggttatgaatctatagaagtaggct
taaataatattgacaaacaagtcttagaatatattaaaaccggcaaaagaattgaaggat
taagtagtttacttcggaatccaaattttcttattgcaagttattcaaaaatcaagtcta
ataaaggagctctcactcctggactgagtaatgaaacattggacggcataaaattagaat
gatttgaaaaagctgctgaaagtatagttaatgggtcctatcattttgaacccgttaggc
gaaagtttatacctaaaccgaaaggtggtgaaagacctcttggtatacccaatcccagag
ataaaatcattcaagaaggcatgaggcaactgctggagttagtttacgaaagaatttttg
tagattcctctcatgggtttagacccaacaaaagttgtcacagtgctcttaatcaggtaa
aaatgactatggggtattcctcttgatttattgaggggatatatcaaaatattttgaca
ctgtgaatcatacttatttagtttcaaaaatagcgaaagttattaaagatcaagccttca
ttgatttaatatataaagttttgaaagcaggatacggcttttccaaaagaaatgtcgtaa

gcactagtagaggctttccacaaggtggagttattagtcctatactagccaatatttact
tacatgattttgatttaaaaatattggaaatgtcggaaaactttaatagaggtgtccgtc
gaaaagcaaatcctgaatatactaagatggttagagatggaaaagtagatagaaaaaatt
ttatttatcccgctatgggaaatgatgcttattttaaaagaatgaaatatgtaaggtatg
cggatgatttcttatcggaatcattggttcgaaagcagactgtgaaggaattagaacta
gcatagcgagtattcttaaagaagagttcttacttgaacttaatattgagaaaacaaaaa
tcacgcatgctaaacatgattgcgctttttttcttagggcacaatattcatatatcaatgc
ctcccaaagataaaatacaatatcttccaaaaagaggaaacaagcttgtaagaactacaa
gtcgacctttattggatgctcctattggtaagatagtgttgaaactaggttcggtagggt
attgcaaaactgatggatctcctaggagattcggaaaacttctacatgaaccaatggcgg
aaataatttataggtataaaatactgcaaagtggattattaaattattattctatggcta
ataactatggtcgtctatctgcaagaatacattgaaccttaaaatattcttgtgctctaa
ctatagcttcaaaaatgaaattggggactcttaaaaaagtgtttaaacattatggagcta
atcttgaaataaaaaacgaaaaaggggagattatccaatgctttcctaaaatatcgtatt
ccagacctaagaaccctatcaaaacgaaaatatttgatcctattgatcatatagagaaat
cttccaattatttcaaaagaagtttagcaacttttgagcaagcttgtatgttatgtggaa
agcaagatactactgaaatgcatcacattaataaactgaagaataactcttccacggatt
ggttaacttctcgaatggtcaaaatgaataggaagcaaatacccgtttgtcgaaattgcc
accaacttattcacaagggtaagtatgatggttcaaaaattatttaagtgactcttttga
agtgaaagccatatgcgctgaaaagcgcacgtatggtttggagagaggattttgatactt
aaaaagattagtctcctactctacttctgtctatgggagctgttttcgcaatatttgcag
gcttttattattggtttgaaaagatatccggatttcaatattctgaaatactaggtcaaa
ttcacttttgaggcacttttataggtgtaaatctaacctttttccctatgcactttttag
ggcttgctggtatgcctagacgtattccggattacccagattcttatgccggttggaacg
caatagcttcttacggttcatatgttgcgttatttagcacgctgtttttcttttatcttg
tatttaacacacttgtaacagcaaaaaagacacctgctagaaataacccatggaactttg
aagattcaaaaatgggctcaactacattagaatgagaaatttcttctcctccagcttacc
atacgttcaatgagattccagttataagagaaacagaaacatctttaaaaataaattaac
ttatgataaaaaaaaatactacaatattatttttagggttagctttgctaacccaactac
tacagagtagaatagttattagtgattctgcagaagactggcaattaggcttccaagatc
ctgcaacacctataatggagggaatcattaaccttcatcatgattttatgtttttttatct
gtgctatctcaatttttgtatcttgaatattagcacgcacgttatggcactatcactgaa
caaaaaatgagtacccttctgccacggttcatggaacagccatcgaaataatttggactg
ttactcctagtatcactttgttggcgattgctgtgccttcttttgctctgctatactcta
tggatgaaataattgcgcctgcaataactattaaaacagtaggtcatcaatgatattgaa
gttacgaatactcggactatacaaatgaagacgataacactataatgtttgaaagttata

tgattccagaagaagatttaactttaggtcagttaagattattagaggtagataaccctatggtaatacccgtaaatacacacgtacgtctaatcataacagcagcagatgttttgcaca
gctgagcagtgccttctttaggtataaaatgtgacgctgtaccaggtagattaaaccaaa
gctcgctctttgtaaaacgtgaaggaatattttatggtcaatgtagcgagatttgtggtg
taaatcatggttttatgcctatcgtagttgaagcagtgtctttaccaaattatatttctt
gagtagctaataaacttagcgaataaatagcctaaaaatattctaagcatctgatatgcg
tgtatctctagcccaactatttttctttggttcatctattttttattttattttgatttaa
tcctaaatacttaacttatacaatacgactattaaaaaaaattaaaaagcgttctaaata
ataaagagcgctttaataaaaaacttattatggcaactacgacaaatctaaactttatca
aaacagctaaacaattacaacgccacccttttcatttagttgaccccagcccttggcctg
taacagctgcaatagccgctttttcatgtgctttaggcggagttatgtatatgcatgcat
acagtaatggagggtacctattatttagtggtttttctttactgttatttacaatgttct
catgatggcgcgatgttacaagagaagccactttttcagggcatcatacaggtgctgttc
aaaaaggattgcgttatggtgtaattttatttatagtttcagaaatcctcttctttttttg
ctttttttgagcatttttttcatagtagtctttcaccggctattgacataggttctatgt
gaccaccaaaaggaatagttgtgtttagcccttgagaagttccttttttaaatacaataa
tattattattatctggttgttctgttacatgagcacatcatagcattgtagcaggctata
aaaagcaagcaacgttagctttaataacgacagttatcttagccgctatttttacaggtt
tccaaggttttgaatatagcgtggctaattttacattatccgacggtgtttacggctcca
cattttatatggctacaggctttcatggttttcatgtctttataggtactattttccttg
gtatttgcttacttcgcttattaaaatcacatttgacacaacagcatcattttggttttg
aagcagcagcttgatattgacattttgttgatgttgtatggctttttttatttatttcta
tctactgatgaggtggtacctaatctaaatctctttaatatccaaaaatctatgatgaaa
cttattaatttacctaccataaagctcacttttttgacatgctctagctttcttaattatt
atctgttatcataatatctatatttttacagaagaaagtatactttttattctgttttatt
gcgtgactaaacattacatgaaattatatatctcctcaaattaatgcgtcattatctgaa
agaggagaaaaaatcaattcaaattttcaacatattactaacgataatataataacttga
aaaaaatatagacaaggttattcactaaaaataacacatgttgatattttaaagaactta
atcacatatttaacatgtttaattaagactgtactccttttagaatcaaaaagtagctca
ttacaatctattgcaccttatttaaaaagattacatattgtaaaagacttagaaacaaag
ctgactaaaatttcttatattacgatttgccaacgtatacaggatacagccacaatacgt
agtttttatgctaaccgagtaaaaatcaaatctttccattctgaatctaaattagactta
ttcgaacgtataagaaaattagaagctggttcttagaagtttaagtctatagctcaatgg
ttagagcatacgcttgataagcgtaaggttgattgttcgaatcaatttagacttatacta
tttcaaaaccataatataaaatatgtacaatataaaccaaacatttttttcagcaagccc
tttagaacaatttgaaattatacctttaattcctttagaattatttgggttaaacatgtc

gttaacaaacgcgtccatttttttgatactatctgttgcgttatctatttttttgatccac
tttagtcatatacaaaaataaattagttcctggaaactgacaatctgtaaaagaaatatt
ttatgataccaccttaacgttggtaaaagataatttaggtaaaaaaggttatcgatattt
cccgtttatttttacccttttcacaataatactttattgtaatttaataggtatggtacc
atatagttttactgtaacaagtcatatagctttcacatttggcttagctttagctattta
cataggaattaatattattggcttcagaacccacggtataaagtttttcacaatttttttt
acctaaaggagttcctttatttattgtacctttagtggttgcaatagaattcgtatctta
cgtcgtaaaagttttcacaatatcgataagactttttgcaaatatgacatccgggcatac
tttacttaaaattattgccggatttgtttggacaatgatctcaataggaggcgtgtttgt
atacttacaaataatcccattagttttattactagcgttagtgggtttagaaattggtat
cgctcttttacaagcttacgttttcacattacttacctgcatttacttaaatgatgtttt
agaaatgcactaactaaaaaattatgccacaattagatcgcgttattatttttggtcaaa
tattttgactatttttcaccttttaattgcttatgttgtttatacccatttcatattaa
gtaatttattaaaaatttttttagtccgctgatggaagcttagaaaagatattactcaaa
ttgcattaaagatccgtttaacgagctatttaattgattcaaatattcaaacgttacgta
gaatttattcaacaatcagaaatatactagcttctctaacaaaaagtttattaacaaaaa
gtataagtaagccaaagttagttttaaatgatcttaattctttagttattaaaattagtc
tggaaacgtctttatatggtagcaaaagcatcaccaagtctggaacatattcttattgaa
cttaataaaatatactatgtatttattaataatagctctgcctttagtgggaacattagt
tacagggttaggcggtagatgaatagggcgtaaaggttcaaatttgttttctacaacttg
cgtagtcctgtgtgtcttttttttcttcaatcgctttttttcgaagtaggtctttgtggagt
tccttgttatatatctttgagcccttgaattagttcaggggcactaaatatttcatgagg
ttttttatttgatagtttaacaacaacaatgcttgttgttattacatctatttctagttt
agtccatttgtattctattcaatacatggagcacgaccctcattgccctcggtttatgtc
tttcttggagattttcacatttttttatgatcttattagtaacggctgacaattttgtgca
aatgtttttaggctgagaaggagttggattagcttcttatctattaataaattttgata
cactcgactttgtgcaaatcaagctgcaatcaaagctctggtagtaaatagagtaggtga
ctttggattaagtttaggtattttcacaatttttttatctttttggttctgttgattatga
aatagtattctcttccgcaaacatctacacaaattatagtatttcctttttgtgggttttc
cataaataccttgactttaataggtatttttttattaatagggggctgttggaaagtctgc
acaattaggtctgcatacctggctaccagacgctatggaaggtcctactcctgtttctgc
actcattcatgcggctacaatggtaacagcgggtgtatttttaatagtgcgctgttcacc
tcttattgatttatcctcggatgtcttacttttaattactcttcttggatcaagtacagc
ttttttcgcctctattgttggagtatttcaaaacgatataaagcgggtaattgcttattc
tacttgtagtcaattaggctacatggtctttgtgtgtggtttatcctattataatgtagg
tatgttccatttagtaaatcatgctttttttaaagcattactttttctaagcgctggctc

tgtaatacatgcgctatcaaatgaacaggacatgcgccgaatgggttcgctagcaaatag
cctaccgatcacatatgctgctatgctaattggctctttatccttagcaggattcccttt
tttaacaggttttttattctaaagacttaatcatcgagataacacaaataagttattacag
taatttacagatttcttttggcgtttatgcttgttgacttgctaatatttctgtactctt
cacatcgttttatacatttaggcttatttttctaacttttataaaaaataccaatagcta
tagaaaacacatagaaaatatacacgaatcgccacctttaattctaattcctttaatatt
actcgctatatctagtatttttgtcggtttcttaacaaaagatatattcgtaggaattgg
aactccttttttgaggtaatgctatcaatattctacctacgtcttgtaatctattggaagt
tgaatttatgccttctttaataaaatgacttccgtttgtgttaagttctatgggtgcaat
tctcgcttatacaataaacgtaggtgtactaaaaaataatatacaatttgctcataatca
cttatttagaaaactcgccttttcccttagcaaaaagttatattgagataaattatacaa
ttcattcattgtatctcctttaatgtactttggttataatatttcattcaaaaatcttga
taggggttttatagaattcgtaggtccttatggaatttcgcgtactattaaaaattgatc
cacaaaagtaattaaaatacaaactggtcagctaacccattataccttttttcgtgatttt
tggtttatgttcccttttactactagttcctgtttgagattttctacaatttttagttga
tgtcagattactagtattttgctttatagccctctttgtagcgtagtttacgaaagtttt
aacacttaaatatatgcagataactaatttattattatggacttcacttatccctttgtg
tggcgctatattacttatttttattcctagattttactctcatttaataagaaatattgc
tttcgcaacagcgcagctagcgtttatatactctattttgctatggctttgctttgaatc
aacaacatccttattccaatttatatatacgataaattgatttccctcctataatattta
ttacacaataggtgtagacggtatatctttatttttttatcatacttacaacgtgattaat
tacagtttgtacattaataagttgaaatatgccagacagccaaataaaagaatacttaat
ttgttttcttttgcttgaagctattttaattcaagtttttttgtgttttagatgtcctatt
cttttatatatttttttgaaagtgtccttatccctatgtttttaattataggtgtatgagg
gtcacgggaaagaaaaaattagagctgcgtatcaatttttcatttacacattagctggttc
actgctaatgcttctagcaattttaactatttatttccagcatggtaccacggatatcca
agttttatgaaatataaattttgacgttagaacacaaattttactttggctagcttttttt
cgctagtttagcagtaaaaattcccatgattccttttcatatatgattgcctgaagccca
tgcagaagcacctacagcagggtccgtaattttagcaggtgtgcttttaaaaatgggcgg
gtatggatttttacgtttttctttacctctgtttccggaagcctcactttattttgctcc
attaatttatttactaagtattatagctgctatatatgcttcacttactacaattagaca
agttgacctgaaaaaaataatagcttactcttccgtttcgcatatgggctttgtcacatt
aggtcttttctcttttaactctcaagggatagaaggtagtataatcttgatgcttagcca
cggattagtctctagtgcacttttttttgtgtgtaggtattttatacgataggcataaaac
gcgtcttctcaaatactacggtggtctcgtgcaagttatgcctattttcagcatattact
attattttttactttctctaatatcggttttcctggtacaagcagttttgttggtgaact

attagtgttaatgggagtatttcaatttagtccaatatctacttttctaagtgcattcag
catgattcttggggcagggtattctatttgactattcaatagagtatgttttggtagttt
aaaacttcaatacattacaaattttcaagatatttcaagaagagaattttgtatcctttt
tccgttaagtgtatttgtactctgaatgggtatatatccagaaattttcctatctgaaat
tcactgttcaagttataacctaattgcatattttaactaattttatgttatgaagtttat
taaaacgctaattctagcatttatgaaaaagaagtcctatttttattggtttccacgttt
tttagggctattacttatacctgggtttttatttgataccgagattctagttctctttca
aagccttatcctcttacatgcaagcctaggtttagaagtaatcatagaggactatttaca
cctagaaataataaaacttcagtgtttgtctttaattaaagtacttttaatattattagt
caatcttaatatattatatttattataaaaaatatccttatgttatttatctcctcttat
gatttctacgcattattgacagaaatttactttttaaacgcaatttgtgctttattaatt
tatggtgtaattttaaatacctcatatagaagagggcatccagttattgaacacaatgta
agtggtctctcaactcaaatactaatagtgagtctttggttaacagtttgttcaaatata
ccttgcctaaccagctggaattcacttttagtgcacgatttttttatctttcggtataaaa
agcaccatattagcaatttcgctactttggtctttaattatttttttcttacaatagacta
gaaaaaataaatctctacgagtattgaatcgtgtctatgttggctattgttgccatgctt
tttgtaagttgttcttatgatcttttggcaatgtatttagcaattgaatttcaaagcatt
gcattttatatattagctagttttaaaagaacatctgaattttcaacagaagcgggttta
aaatatttcgtactgggtgcattttcttcagctttgcttcttttaggtatttcactactt
tatggtactactggtttaactaattttggagatctatcaaaatttttttttaggtaccaca
ttggaaaacgcatcatttatcaacataacatttttttggtgtcgttttaatagaagtagct
ctttttttttaagataagtgcagcaccttttcatatgtgatcgccagatgtttatgaaggt
gctcctactaacgttacatcttttttttggtatactgccaaaattagcattagtaagttta
atatttagattcttttatttttgttgtgctgaagttgtgctgttactaaattttacactt
ataatttgtgcgcttttatctatgataatagggacatttggcgctttagcgcaaacaaaa
tgaaaacgtttcattgcgtatagtactataagtcacgtaggatttattgtagctggattt
tcaacgttggaatttaatggtgcatttggtgcgctattttatatcttggtttatacttta
acttctttagccactttttctattgtgctttccttccgatgcttagcatatcctagcaca
taccaattacgctatctaacggatatcgttagtttagtgaagttaaaccctatacttgct
ggtagccttgtagcagttttattttcaatggcaggtatcccgccttttccaggattttt
gctaaagtatttgttttattttcacttttgcaagaacaattaataggattagctataatg
gcaatatttttgagttgtgtttcgtgtttttattatatccgtttgattcaaatgatgtat
tttacacatacaaaaaccatacttatttttttatccaatagaaaagactacatcaactata
ttaagtataactatgttattacttgtacttattttttttgaagatagatctgatttctaat
tttgttcattgtatgttgtttttataaaataaccaattaaaatgttttacaatattgcaa
ttaacattatcaaagtgttgaccattatagtgccacttttaatcgctgtagcttatatga

cactggccgaaagaaaagtgatggcagctatgcaacgacgaaaagggcctaatgtggtag
gtatctttggtcttttacaacccctagcagatgggttaaaacttttctcaaaagaaacta
tactaccttctagtgctaatattttatttttttagctgcacctgtgctaacgtttttgc
tagctttattagcatgatgtgtacttcctctagatgaggggaaagtttttttcggacttaa
atataggtgttttgtatatattagcagtatcatctttaggtgtttatggtattataactg
ctgggtgatctagtaattctaagtatgcttttttaggtgctttgagatcagcagctcaaa
tggtatcttatgaagtttccattggtctaattttaattaatattttattatgcgcaggca
cattaaatttaactcaaattgttctggcgcaacaaaatatgtggtatataatacctctgt
ttcccatatttattatgttttatatttctatattagctgaaactaacagagcccctttcg
atttgccagaagcagaagcagaacttgtagctggttacaatgtagaatactctgcgatgg
ggtttgcgttgttttttttaggcgagtatgcaaatatgatacttatgtgtagtttaacaa
ctattttttttttttggtggttgattacccttagtcaatatgcttcctttttattggattc
cacccgtactttgatttggtttaaaaacaactttacttttatttggttttatttgagtgc
gtgcagcatttccgcgatatagatatgaccaattaatgcgtttaggatgaaaaatatttt
tacctttatcattagggtgagttcttttagtatccgggatactattttctttcgattgat
taccataacaaatgaacgtactttataacgagtattctgctattctcactttttttgcag
tagctttttaatctctctaataatattaatactttcgtatatattaaatcctcaacaaa
gtgatcaagaaaaagtcagcgcctatgagtgtgggtttaatccatttgatgacgcgagag
caacttttgatgttcggttctatttagtcgcaatcctttttttaatatttgatttagaag
taagtttcttatttccttggtcactagtacttgggcagctaccttcttttggattttgat
ctatggttgcctttttagccattttgacattagggtttatttatgaatgaaaaaaaggcg
ctttagaatgagaataatcaaataatttactagagatttttaatttgataatataatata
atatgaacgtaactttacaaagtgcaaaaatgataggagctggactagctactattggtt
taacaggggtaggagctggagtaggaattgttttcggatcgctagtaattgcttattcgc
gtaatccttctctaaaaaatgaattgtttggctacactattttaggattcgctttaacag
aagcgattgcattatttgctcttatgatggctttttttaattttatttacttaatttactt
taattaatgggcgtttataataaacgcccaccttaaaaatacattatgacaagtacaact
ctttttttgaatcttttcaattatttctttaatatccgcttgtatggtggtaagcctgtca
aatgctgtgtattcagttttatttctaattgtagtattttgtaatactgctagtatttta
ttattactaggagcagaattttttatctttttttattttttaatcgtatacgtaggcgcaatt
gcagttttatttttatttgtagttatgatgttaaacgttaaaatagatggagtaaaaatt
aattatagcacaatttttttgattggtattttaataagtctgattttacttattcagatt
tgaactgctctacaattagatattgaagcgtatgataatataggcgtaccactatcccaa
aataactttccaacaataatttcttggatccaagaaaatgaattaccttcaaatacagag
agtattggtttaattttgtatacttcgtatagtttagtatttattatgtgcgcatttata
ctacttttagctatgattggttccattgtactaacaatgaatcaacgtagtggagttaaa

acacaacaaatcacacttcagttatatagaaatcaaaataaagtagttcgatttattgat
ctgagaaaaaattaatttgattgcggatatagatgaattggtacatcagtaattttccac
gttaaaggatatgggttcgagtcccattatccgctcaaattaagagagaatagctcaata
ggtagagcaatagttttcaaaactaaaggttaaaagttcaagtctttttctcttgcaaa
agtgagtctgcttgaacaccttattcagttttaattataattaaaactgaataaggtgtt
caagcagactcacttttgcaagagaaaaaagacttgaactttaaatttatataagaataa
caaccacattttagcatacacaagacgacagccttgtatgcgtactaatatatctctgcg
ggctgtgtagaggcaaaaaaagtttgtgtggggtggctgtgtgacatataaatttaaaat
agttgtcaaaaaaagtttagtttggcgtaaaaggattcgaacctttgaaatcatggtatc
aaaaaccattgccttaccacttggctatacgccaaacaaattgaagataaagtgggattc
gaacccacggtggatagataccacgttagctttcaaaactaaagctttaaaccactcagc
catttatcccggttattaatataaattgtattctaaaatttttagagtctgtttatatga
tagtacgggaataggattcgaacctatatttttagatcatgagcctaatgagttaccttt
ttactctatcccgctattttatctaattaattttgtaacttaataaaaacttctccaaac
tacccagtaaaggtaggatgactaaaaaatataaaaaataataaacgctagctatctgac
ctataattatgtaagggtcttctacaggcataccacctattcaacctaatattaaacaac
aagcagccatgcttcaataaagaccacgatatatgggtctaaaacgcgaactttaaattt
atataagaataacaaccacattttagcacacacaagacgacagccttgtatgcgtactaa
tatatctctgcgggctgtgtagaggtaaaaaaagtttgtgtggggtggctgtgtgacata
taaatttaaaatagttgtcaaaaaaagtttagtttggcgtaaaaggattcgaacctttga
aatcatggtatcaaaaaccattgccttaccacttggctatacgccaaacaaattgaagat
aaagtgggattcgaacccacggtggatagataccacgttagctttcaaaactaaagcttt
aaaccactcagccatttatcccggttattaatataaattgtattctaaaatttttagagt
ctgtttatatgatagtacgggaataggattcgaacctatatttttagatcatgagcctaa
tgagttacctttttactctatcccgctattttatctaattaattttgtaacttaataaaa
acttctccaaactacccagtaaaggtaggatgactaaaaaatataaaaaataataaacgc
tagctatctgacctataattatgtaagggtcttctacaggcataccacctattcaaccta
atattaaacaacaagcagccatgcttcaataaagaccacgatatatgggtctaaaacgcg
aacttctaatttctgtactatgtattcatggtaatagagctaatacaactatagcaaaaa
tcatacatataacgccacctaatttatgtggtatacttcttaagattgcataaaaaggta
agaaatatcactcaggaacaatatgtgctggcgttaccatgggatttgcttcaatgtaat
tatcagggtgacctaaaagattaggcgagaagtatacaaaaaaagaaaagaatataataa
aggccactatccctaacaaatcctttacgatgaaataagggtacataggtaccttatcgc
tgcttgcatcgatacctaaaggatttccagaaccttcttgatgtaaagcggctaaatgca
ctaaagacgctgctgcgataacaaatggtaataaataatgtagactaaagaaacggttta
aagttgcattatcaacagaaaagccacctcaaagccaagcaactatagaatcacctacca

aaggtacagcggatactaaattagtgattacagtagcacctcataagctcatttggcctc
aaggtaatacataacctataaaagcagttattatcattaatagtaaaataattacaccaa
tcactcaaacaaattgtcgaggtgcagcataagaaccataataaagtcccctaaaaatgt
ggatataaactacaataaaaaacattgaagcaccattcgcatgtatatatcgtaaaagtc
aaccaaagttaacatcacgcataatatgctctacactaataaaagctaaatcaacgtgtg
gggtataatgcatagctaggaatattccagtcactatttgtattattaaacacattgcag
aaagaaacccaaaatttcatgcataatgaatattgattggagttggataatctataaggt
gattattaactatattgaaaagaggtttttaattagacgcataaataatgtttttatta
tagatggacgaggaatgggacttgaacccatggcctataaagtcacagtttatcgctcta
ccaaaccgagctctcctcgtcatgttaaaaagttaaatttacggggaaaaagggatttga
accctcactcattgatgtgacaaaccaatattttaacctattaaactacttccccatttt
tattaaatacggatagagggatttgaaccctcatgaataatattcatcaaaacctaaacc
tgacatgtctaccatttccatcatatccgcaaaaaaatgttactattagctttaacggat
aaagagggattcgaacccacggtataatatttcatacgatgatttagcaaaccattgcct
taaaccactcagccatttatcctgtgttttggaagctgcccactaccggacttgaaccgg
taacttaaaaagaacagattttaaatctgtcgtgtttacctatttcaccaaatgggcatt
agctattgctaatgctatgttttattgaagctattgcttttccggggttcaatgatttag
gacacgttctactgcaattcataatggtatggcatttaaaaagttttgatttacctccaa
gtaatgctaaacggtcttgagttttgatatctcgactatcagctaatcatctataggctt
gcaataaaattgcaggacctaaatatttgtcatggtttcaccaataacttgggcaactag
cagaacagcaggcacaaagtatgcactcgtaaataccatttaattctgacctatcctttt
cagactgtagatattctgtttttgaaggtgtattatttataagccacggttttatatatt
tatattgtgcgtaaaaattagataaatcaggaactaaatcttttataatgtacatatggg
gtagcggataaattgtaattgttctagtatttatatttaatggttgcaaacaagctaatg
tattagttccatttatattcattgagcaactaccacaaataccttctctacatgagcgtc
taaaggcgatactcgaatcttgttcgtcttttatttttataagagcatccaataccatag
gtccacaatttttagtatgaataggatgtgtactgaaatgagtaatagttgggtttgatg
gagttcatctatatatacgaaggaattttaagtctaaattattattggatactaattgaa
aagaaatttttttgaataattgcatacttggttttattttgattgctataattatttaag
aacgggttagcccgttcttaaatataagataaaatataatgaacatttcgtatttattta
cttacttttgcaagtattaaataataaaccgatctatctagtaatcttctagaatgttct
ggaaaaagtaagggttatttttgcgcttttaagctgtcagcgcttatataatgttaacgt
ggctactcggctatgcaagaaacaatacaaccgatacactattggttaatatatcttaat
cctctcgtactaaagataaaccctctttttttctttcccacaacagatag----------

--------------------------------ggttatggtaaagaaacctctcacgaat

ttcctcccacttcaaaaccgtacgtgaaggtcacccttcatacggctcctcaaa----at
tatctatag-----------------aaagtgtt--------aaaaaaaaatacacactt
tcctttagtttaaaagtgcttatattagtccacggcttaagtattagctgctttggaatg
tctagtatcgtggcaatgaccatgcataagtcgtaagtttttaagatcagaagatccctt
ttgacttcttggtataatatgatctatttctattctatctgaatctttaaaatataattt
acacatactacactgcggccctttagtttttaatagtcgttttagtagttgcccatactt
attattaacagcgagacgtctggctcaataagtaactcttccgtcatatgggctactata
tccggctacttttgtagatagaataatatttcgttgatcatgacgggttaattttataat
tttgtctccttctttaaggccaaaaactctttctgcactccctataggtattcaatatct
agtgataccaggacctgttcgtttgcgtttcttagctcatttccttagaagataaaacgt
acgtacactacaataactaaaagtttttgtggcactacatacggaaaagtatttagttca
tccgctaataactggtgctagtttactgattaaaactttttgagataatccggtggactt
cgtagtaatacttttttatattagctaaatgagattctatagacttatagctgggttgaca
cctgctttttcatccagtcgcaattccttgattattctttgcggatgtatgtttacctac
cttatagttgacaaaattaaaacctaagaaatctacacctgttattctagacttctct--
------aaactaccagtgtacgttattttttgttttggattccgacaactttagacctaag
ttctgtaaaaagaattcaattttgatttttgctgcaatcaattctttctcttcattacat
aatactaaaaaatcatctgcgtatctaataaggtatactccactttttcctattgcatct
tccattccgtgaagggcaatatttgccaacaagggtgatataatacctccttggggggtt
tcggcttctggtgtgatttcttttatattttctttaaagcctgtaagtattcccgctttt
aatcaagctctcaattgctcttttaaaataggaaacgtgtttacttttagaagtaattta
gagtgatctatattgtcgaagcatccttcaatatctgcatctaatacatgtttaggaagt
tgctgtaaacatttcacgattgcttgtctagcatcgttggaacttcgccctggtctaaat
ccataactgttaggttcgaatatagcttcatattgaggttccaatgcaaactttacaaga
cattgtttagctcgatctcttatagtaggtattcctaaatgcctttctttcccgtttggt
tttaaaattgttactcgacgaattttatccgatttattatcaatttcaatattttgtact
aattccattctttcgtcaggagttaaactactaactccatctactccagctgttcgtttt
cccaaattatcttgagtcacttttcgaacagctaaaaactttgaaaagtcatgctttatg
atttgtttctgtattaagaatacagatctcatattacctttttttgctaaattcaaaaact
ttacactgcaatctatacagtcaaatttcttttatttttcagttcacttgcgttcacttt
ttcataattt------gtttgttggtgtaatcttaacaatgttaaaatttttatttagat
aatt-----ttgtctacacgtctgcatatccataagctttccttacggcattagcttctt
gtagaatcctgatattaatataccttaacactata----------gaaaaacgcccttgt
aaaaaaagaatcaacaaggtttatattaatatttacttcgttccaatattacatacatat
agtcagtaagcacctctattccctctaaaaccggtacccctttctaacgcggccacatta
gattttaccttatgtttagaattcacgctgtttcgtgttaacggggtgggttaacttata

attatcccccgctatgacatatttagggaatgttattccggctaccgtaaaggtaggtta
tacttctataacta-------------cttttcttggcagatttactggtgtatatattc
tcttcaggtttgaatgtttataagtaacattctcccaaatttatgccctgcttatacctt
gtgaataactattttcacactgtatagggtgcactgtttgtaaa-acagctcattgatca
gcattaggagt-aatgcgtaggcccactagttctagagaacttgcttcctacctgtaata
ttagtttaa----------tctctaaagtggagattagctatttacgagaagtacatctc
ttctcttactagcaacgaatcgcacgaccgaactgtctcacgacgttctgaacccagctc
acgtatcttattatttggcgaacaaccatacccttggaacctattgcagctccaggaaaa
gatga---

---------------------------------------gccgacatcgaggtatcaaac
catggcatcgataagaactctcagccatgataaatctgttatccctagagtttctttttt
ccgataagcgacagtatttccatacattactgccggatcattatgaccgactttcgtccc
ggcttgagctgtaactcttaccgtcaagcagattttcatcattacatattatattagcat
atattgccaactaaaatccacttttgtgcacctccgttacagtttaggaggtcctcgtcc
caagtaaactaccacttttacatatgtccttgttagtaagcaatttttatactcagagt
agtttttcaatgacgtacaaatgtacttctacttttactgtactgtgtacaaagttatcg
cgacgtaaaattatagtaaagaatcatagggtctttccgtcttgttgcgggatgtctgca
ttttcacagacaatgtaatttcgctgaggctatactagagacagtggggtagtcgtgacg
ccattcatgcaggacggaatttacccgccaaggaatttcgctacctgaggatccttatag
ttaagaccgccgtttacttgggtttataatatatgcgcaaacttatatttttcactttca
agcactgggcaggcgtcaaaccttatacgtcttcttttgaatttgcaaagttttaagatt
acgataggtagatactcgcatatttcccccgtttagaaccgtacgtgaagattaccctt
catacggctcatccattttaacgtcagaattttctaacgcagctctgtcgcgatgacacg
ttttatgaagaagagtcatatttttaatatatttgtcctccactcactacaggtatga
tatggtgaatttctaattcgtacaatatatctccatattgactatttaaagattccttac
atatggggcacatacccttttgtctttttgccagctttagtcgaatattattcgtttgct
gatttaaacacatattcaaagatcttttattaaaatactcctcaaactcacccaaatagg
gattagcgtcaagttttatcatagtagttcttttaataggagtgtcggatatttgaaaca
aattaattttcttaaatcttttaaattcagtgctagtagatattaatcaatctctattat
ccactttatgataaaatttggatttaagcgctttagcattaagcttcggatgcttaattt
ttatcatgcggtgaatggaatcaaaaacgtatttacttattcgggtataagctaactttg
cagtactagacgagtaataatttgctcagccccttaatattgggtttagttttgagaaga

gcatccacaaaggaagttgcgaatgttctttgacacaaagttttattttagcttttactt
tttgtaagctttcttttgtaggtttatatagaaagatccctttcttttccccgatttctc
gagtagaatccgagaattccctaaagtggaaacctacaaaatcaaatccatcttcaattt
tagtgattttactttttcctatatttaattctaaacctcttatttttagaaatttgttaa
catctagaattaacttttctaaaatatcatagttctgggacactataatgaaatcatcag
catatctcacaagagttgctttgttattagacgcc-tttttatagttttatctaaacca
tcaaggcataaattagctataataggagatattacaccaccctgtggaacaccttcttca
gtatcttgataactgccttcattcataacgccagcttttaacatttttgacaatatcttt
ttatccataggaatattttctaatattcattcatggcatatattatcaaagaaacccttg
atatctccttcgaaaattcatctagggctgcctctctgtctacttaaaagaaatcaaata
tattgacaggcatctttagcacttcgataaggtctatatgcaaaactgcatcgatcagac
ataatttcgcttataggttcaagagccatagcaaacaaagtttgcacaactctatctctt
atagtgggaattcccaaaggccttttctttttgctattcttctttggtataaatactcta
cgtaccgcactgaattcataattttaagatcttgaatttgaattaatatttcatcaata
ctaactttctttcctttagaaccttttaaaacaattttatcaattccgctggtctcacta
cttttacgtttgtgaatttgctttattgcttttattttagcttcttctaaattcactaat
tcattttgtaatttacttcataaacgtttgtttttattcaaataagctattgctattctc
ctttgtaacttataaattttcgtcattatattagtttttattatttt-ccacaatttta
tttaaaaataaactagctggttttgcatgtaatgcaaataaaataaaagttttcttaaag
gacaatacttttaaaacagaacatacataagtcagcaatctttcgattcatgatataatc
actatttcacttattactagcaaacattcgctttttatgagatcttttacctactgaaca
cttgggttgatcgctctttcctgagatatttctatcaattcattaggcttacccttttcc
ttacacataacattaatctaaacaggtaacattctctataccggcagtattatgtctaat
ctataaaataacaaaaagatattttatactactgcttattcctatagctgtataaacagc
taatgatgatcttcggaattctgttttctcgataccttataacaaatgttcacttacgtt
ttacctatttagataagtctagcttttcttagttacatcaagtaagaaactacattgtca
gcaaggcttcatacctccagatcactctagacgcatgcttgcttagacttatattgatta
gataatatagcacttagtgcatttatgtatacggctttaaaggtcgcacttttttgttaaa
cagtcgctacccctaatttttgaaaccttaaaaggtactcctttttgcgaacgtacggag
taaatttgccgagttccttaagtatagttatctcattcgtctttattttctcaataagtt
cacctgtgtcggttttaggtacggtcaaacttcatgtaagttttcctgaaaaattacttt
ttttagcctccagtttagtgggtgtagctattagaagatatctttgtacacaaatacacg
agtattttgcagacacgtggtaatttttcctatcgaatacagttttcactttttttcttaa
gggccgactaactccagttacttaaaattgactggaaacccttgaacaatagacgaccat
gatttattttcaacatggttagcgctactcatgtcagcattagcactcctgattttagat
atgcaatttaacattaacataaaagactacaggacgttccgctaccattaagcttagttt

agcttaattcgaagcttcgatatataaatttaagtctccttacattttaaataggtagaa
caaataaaaatagcgagctaaaacgctttctccaataggtggctgcttctaagcctactc
tgtttattcgaattattccttatttttcactaatttataattttgagatcttagctatc
gattagggttgtttcccttttgacgtaagaccttatcgcccaacgactgtctgctgctat
aaataaaaatatagtttggagtttaataaaatttagcaaaatctaaatttaataagtagc
tctacccacattttaaaaaagcaacgtactactttgatagttttcgcggaaaaccagcta
tcaccaagtttgattggactttcacccctaatcttaagtcatccccgtatttttcaacag
acgtgggttcagtcctccagtactttttaaagcaccttcaacttgcttaagaatagatca
cttggcttcgggtctaatacctgtaactttaagcgccttaatatttttaagcttgctaca
catattaacttactgactcattatgcaaaaggcactttgttgctgtatttgtcagcttca
aataaatataaattaacagattcaaatctttccctcacggtactcgttcactatcgatta
gaaaaggttttagcttagaagatgggactcctattttcatacaaaagtaatcgtactact
ttgtgttactattagtgtcgtaaaggactttataacctactttgggtttagacacttcgc
taagttcgctttcactcaccgttacttacaaattctcgtttgatttttttgctatgttac
taagatgattcaattcacataattatataaatgcatattaaatgcgagttttctaaagag
actcatagttcataggcaggtgcctcgctatgatgtttcgtcgcgcgacgtctttgcttt
tctaccaagatttcctctgataactttttaaatcttttatatttaagaacgggcggaaaa
atgttacccttttgtaagagtcattaactctatagtaattgtgctcctaattcgataata
gacaacaagtattgctagtcctatagaagattccgaagccgctactgttaatatagctaa
agcaaatagttggcctactatattgtctaagtatatagaaaaaaatataaagttaaaact
aactgatagaaacattatttctaaagacattataattattataatactttttttggtttaa
aaaaatgcctaaaactccgactaaaaataaaaaaaaagtataattttcacagtatagttg
ggtaatcatttatttttatatttaacatttgagatagcaaattgtattaaatttttccag
ccgcaggttcccctacggctaccttgttacgacttcactttagtcctttcgctaccatgg
acaaaaaataagatttttgtcttcaagtagagtaaattcccatagtgtgacgggcggtgt
gtacaaaacccgagaacatattcaccgcgacagttctgatccgcgattactagcgattac
gacttcatattctcgagttgcagagaataatccgaattaaagatttttaaagattttgc
tccagctcacgcttttgcttcttattgtaaacattactttgtagcacatgaaagtagccc
aattcataagggtcatgcggacttgacgtcatttttcccttcctcaaggatattccaagc
agtttataatgcattaatgcattatacaaaagtttcgtccgtttgctggaattaaccaaa
cgcctcacggcacggactgacgacagccatgcaacacctgtgacatcttgtgtcatacga
gaattggtaaggttttgcgcgttgtttcgatttaaaccacatgctccaccgcttgtgcgg
gttcccgtcaattcctttgagttttaatcttgcgaccgtaatccccaggcggagtgttta
atgccttagcttcgcctctggaaaattatccaaaaacaaacactcatagttgagggcgta
gactacaggggtatctaatcccttttgatacctacgctttcgtgccttagtgtcagctat
agtccagattattgttttcacttttgaagttcttttgaatatcatcgcattttatcacta

ctttcgaagttccataatcttttcctatgctctagtaaattagtttagtaatctttagta
aacatgaaatttaaaatttttttactacttagtttaccacctacgcaccctttacgccca
gtcaattagaataatacttgctcctcccgttttaccgcggctgctggcacgaaattagcc
ggagctttgattgtaaaatttagtctttgatttttaatctattttacaaagtgatttac
agcctgatagggctttgctctcacatgtggcctggctaggtcaagctttcgctcattgcc
taagattcctcactgctgcctcttaaagagtctggaccttatttctgttccagtgtgact
gatcatcctcaaagaccaattaaggattatgggcttggtaggtcttttaaactaccaact
acctaatcctgcgtagacttttcttaaaccaattattaatttttttaaggcatttaccaca
atttaagataaatttctacgtattactcacccgtacgctatctttttcataagttacgaa
aattattaaacttgcatgtgttaggccgaccactagtattcattcggagccaggatcaaa
ctcttttattattatgttatgtttttatgtttgctatctcagaaattaaatttgattgta
taaagttatccagcgagctaagacagaagctgtaccaatttcatatatatatatgttggt
aatcttccttttggtaatgaggataaaatttgctttacaaaaagttgtatacttttttttg
tttaaatgcggaacatagtttattttctagctttgttccacgtataagaagtctcctata
ttttttttattttattcatacatttgtgtatcaagatgggcagatttgaactaccattccc
ttgcacccaaagcaagtacgttaccattacgctacatcttgatatagtgatattgactct
ttctggataggatttgaacctataaccttgtagttaacagctacctgctctaaccattga
agctaccagaaaaataaaagcgaaaaaagggacttgaacccttataatgaccttggcaag
gtcatgctttgcctattaagctatttccgcacttaaaattgaatataaagttgaataata
ttaacaataagtagaaaaagtaaaagataattgctatctttgtacattttccttgtattt
acatgttttactgaatctcaaaaaaaatggcgtattccattacttatgtggtatagaaat
atagcggacactactcaccggacactagttaaaaaccaaggaaaaaagaggtgatctaca
aaaaaatgtattggcagaatagtaatatataaaaagtatacttgatttagtaaaagaata
gggcctacaattaaaacaaacataataacgccagaaattctatgccaaatagaaaaaata
gaagatctttgcggattgtatatagttaaatgtggagaaattggtctattgatgttctgc
atattatagtttctttttcttgggattttacagccattatacgggcaagttgtaatatc
cattacagagataaatttaagtttagactgttttaatattttaaacacattttttttatt
tttacctcatccttttatccgtacatgtaaagaagtataactgagttctttagtctttat
ctctaagaagtggattatttcggacgaagaaagggacaggcttctttttaccccttttga
ccttgacttgcctgcagttgtccaagatttaattttgcctttacagtctgttaaaagaaa
atgaatattattggaactaaaagataaaaacagtatacccatatgtagtttaattttatg
ttgtagtagtaaagactagggtcagattcgaactgacctaataaagatttgcaatcttac
gcatagccactatgctacctagtcttaaatttagtgaaatctgaagaaagactgtgtttt
aaatgataaagaacttttcacacaagaaagtaattcaagcgattttagctttggtaaatt
ggataactcgaaaagcaactgaccgggccttatataacagcaatgactcactatagctcc
ttttcctttacccatacgggtttctggactaaatttagtaacagcatttttttggtagcat

gtaaaaccacactttgattttacccaaactggactttttttcgatgactttgatctcttt
ctgaattatctttttatattatttatttgtgtgatatctacttgcttgtacgataaaga
gcgtaatccaaattggcctcttttactagatgacttgtttggtttatatatctattttt
ttttttatgctgtcgtgttggattatttttgtattaaatcttttcatattaaattgtgc
ttaaattgttttatatgccattcaaacttgaagattgcaaatcccttgttttgtatataa
tggcgtttcaaaaaattctatataagtttctagtttttgcagtgatactgcgcctaatcg
gaatactatactacttgacatacgacttcggctaccaccgaaacgcccagcgtaccttat
ttttattcctgtaaattcataaaactgtataccacgagcagtttttaatacaattttgtg
ctttttttattgaaaaatagtatttttattgtattaattatcttccggaatgctaggcc
tttttttaggccagattttataaatccacataaaagtgccccgttttgatgtcaaaatgt
tgcattatatttttttcacgtcaagatcgaacattttaaacgtaggtaccaagcaatctga
tttggctaatagaggtacatattgtacattgtattcccatctattgtcatttattttaga
tagcgtatttccttttatgaatgtttttgattcaaaatgcttatttaaaaagtttgtagc
aggtaatactttttttgtatatttgcttttgatttttaaaacctttgccatagcattgact
ttcggtatttcataagtcgttcacacctattcgaaatcctcttggattaactttttgacc
cataatgttttattacttaataacaatatttaaattgaaaatcgaatctaaatagttata
cagaaatacgttttgtaggatttgaacctacactaatcaacttagaaggttgatgtttta
tccaattaaactaaaaacgtttttactttagatgcattgggatttgaacccaaaataagc
agattaaaagtctgttgctgtaccgtttagctatacatctttttaataaagagagtagga
tttgaacctacgatgattttaatcaatagatttacaatctatcgctttcgaccactcagc
catctcttcttcagcggttagatatatgttacaatctgtaataaaataattaattatata
ctattgtttaataacaatatttagattgaaaatcgtacgattttcaatctaaatattgtt
attaaacaat
>SRR9587917
gttttattaattaaaactaccccagtattacacttttaaatatttataagaaataatgca
aatccctttacgctgttatttaatcgaatttaaatttcgattcgtttactttctacttag
tttttttctctcgttgctagtcgttataaactatcatgaggctgtgtttttttttgaaac
gtatactttttttgtttataaacgagggggcattcatcgcgacacatattgctgaactatt
ttcaacttctatgtatattagttttaacctagttttgtgcataaattatccttttgcata
ttatcattgcagccgctttttaaattcgagttgatacaaatcacaggtttggttctttag
aaatatgcaattattgacatttattacgtttttagctagtttacttttgtgctactttttt
aattttgccctacacttacgctttttttagacacttgaactgtaacgatagtttatgcatt
taaagtgcagttggaggcaagaatagcaacatatgtttgttgaacactacaaacaatgtg
tttactatctaacattgtctatctcttctttactaggctaatttgtttttacttagtgga
caatatgattaatttgcatttatttttccggaaaaataaaaaatatactttctttttcgt
atgcttgtcggcttctgtgtgtttaccgcaagaaagttttatacaaattaccttcataat

ttctatagtagtactatttgagttattctttttggttacatgcttatttttttgcaaaaaa
taatgtctaaaagtggacttgaaccactgacccaaagattttcaatcttttgctctaacc
gctgagctatttagacttaaacttttcttttcccgtatttagagcgaccatttttttcgtt
taacgactgcttgtaaatcgtaaacacctctcacaatatggtaacgtactccaggtagat
cctttacacgaccacctcttatcagaacaactgaatgttcttgtaaattgtggccttcac
ctcctatatagcctatgatagaacgtccggtactaagacgtattttagctacctttcttt
cggcagaattcggtttttttagggttagtagtatacacttttgtgcaaacaccctttttt
gaggcgacttattgagagcgggtgttttcgctttggtaattttgtgctttcttggatttt
ttattttttggtttagtgttgacataatatttttattttaagtaataagggacttgaacc
cctaaccatttcggtgtaaacaaaatgctctacctattgagctaattactttacgtctgg
aaagatttgaactttcaacttttagattcgtaatctaacgctctatccaattaagctaca
gacgtttttctccacttgtatatttagattacttatctatattttggaaatagggacaaa
tattcttgtataatttacttcaggattattcgaaagtatagttaaccaattaaacaagta
gaaaacgcatattttgtattgttactaacttaaaggactattttataataaacttttat
tgtattatataccaatatttagattgaaaatcgacctaaattcaatgtacttttagcaaa
tgacaccagtgttttaaaaaaggtgcttattttgaaaacatttttggtgtcatttgctct
ttttcagctaataataatttaattaataacagaaagcgcggtttattgctcgtcgcgttc
cagttattactacggcaacttcctatgcagctgttggga-ccccccaactcgtgtaatta
cactttttagtcttatttaaattataataaggttatactttatatcttttcttttattta
accccctaagatctttattacgccccaaaatggtgtcataaaaaaa-tagcctggagggct
gccaggagtaaaaaagacctataacggttatttcagcttttatagtgctatttttctttt
ttagagaaggctatttttaattggtaactaagtcattttaagtgataattaagctatttt
aattaaaaatgacttattaaaggttttacacagataatacttcttttattaaaaatatac
taagtcgctagaccggaaaaaacccgccatagaacgatgagaggtaaaaagtgtcataaa
ttttaactattttat----------------aattaaagtttgtcaaacatcaaactttc
tgttatttcaataatgcttgtttttaaggcttttaaagcagtttctcgctgatcccgaac
actaaactttttgttaacatgttctcgtaaactgtaattgaatgtcataataaccagtgg
attatcctgttttaaagtaaaattacctttcattgcaaggcgcatgctatcaccagctaa
aaactggtttaagaattcgatttg------------------------------------

--tttcatgataaagtgctctcttacaagccgctttttttagtagattactaaaaatttta
ctatgttgatttgatacgcgtttttagatgggctcttgcttatttattggatctgaaaag
gagcttgaagaattagttaagtagtcaaaataattcggatttgtgaaattacgtttctta
gaaaattcaatatacgtatataaaatttgatccgagttatttcagccactacgaaagctt
attattgttgtttttttctctttgtagaagtttgttataaattaaaattaggcaaagaagc
tacatttt--ttttaacttggaaagatttggccgcaacgcgc-ttttatgtttaattac
ggctggatttagttcaaatactttggacatgagtcgttttaaaaattatttagaaatatg
tgttcgcaaacagattaagtgaaaaaaaagagttttaaaaaagcttcataaactttcacc
aaaagtttgatttatatcataaacggagaaaatccaaattgaaaaaactaaaatggtgtg
gcctaaaaaaatacggcgaagtacatatcaacttgattttcagtgaattaaacagaaata
cagtattcccacaagtacgataggaattaaaatgatggataaatcaaaattttgatttag
atacgaaaaccatggtttactcggttcgtttacaagtaaactttttaattcaatatttg
tgctattagtaaagatttgtgcgactttaaattgaataagctattaaaataaggtgaatt
taatttaggcaataaagtatcgtacaaatgtttgttctttctatatttgtttagagcttt
cctgtactttttattgtaagcagcaatagatcctatgtgcttgtgttgaaaatctaatct
tttaagtactttgttaaagctcaaaattttggtttttaattggttaaccgtttttttacc
ctttctagcatcgtatagtctttcttttagagtttctagatgagaaggcttttctagtgg
cagttgattgaaataacttttagtgagttttttttactatagtacctgtaacaagtgcgct
aacagatactatgacacaaactacttctaagtccaa-ttgctctacaatgtg-tttgatg
aaagtcattttatataaattaatttattaatgtgtaattgtggtatgattttattctgtg
ttaatgggcataattaaatgcatacattatatattatccctccttcggagggaggcatat
aataattcttattatataccaatatttagattgaaaatcgcacgattttcaatctaaata
ttggtatataaacaaacctgcacataaataaatatcactaggacatcaccccaccaaaaa
atcgatattctctagagagcccctataagctttaatttactatctcttgcacgaaattag
accttcttgaataggaatcgtcgtgaaattgaaaaacatttaggggtatatcttatacaa
ttatgaccaaaaaatctacaaaacaaacaactttacacaaatacttttgcaactcgcata
catgagattgaacttaagaaacctcttttcataaatcaggcagcatttttggcgcttctt
aattctattgcaatattcaatgaaccaaaaacaaagttttttt-aaacgacctgcatgaa
cacaatcttaacaagtgtaaaaaagccctctagctcccttttttgcaacaccacgagaat
aaaccccggtcgggcagaccccgaacc---------------------------------
-cgcgcgcgcgcgttatagattcctatatcgc-----tccatttcatttcgctattgctg
cgcttcgcttatttttaattctaactgttctttttcattataacgattttaggtcgattt
tcaatctaaatatttgtatataaacaattatttatcttgataattatatatctaaaaaag
ggggcatagtttaataggtataatgtatgctttgcaagcataaggttaccggttcaaatc
cggttgtctccaaatctaaaaacatcaatgcgttctagaatattacaatacaaaaataac
ttggctgattatgatcttttaactcaattttcgattaaaaatcataatagtatgcctact

ttctcttctcttaatgtcagagtaaaaaatattcaaactagcgacattaagcaggtttgc
ttaaatctaatctctattcaattagtaggaaacaatcatgttgttttcagtagccaaaaa
gacgtttctaatcttatactaaaatgtaacggtattaatacttattttattttagagaat
ttatctttgttaggctacaaaaacaatttaaaaagtgacaaaaacaataaattaactgtg
cacggtaaaaacttcttaaatattattgaatattttgcatccaattcaaattttcttaag
tccttagaaaaaacatctaataaacatttaagtatcgctttttcttatcaacttgctaat
agtaaaaataatgaaatagctttgtatttttttaaaatcattaggatttccgctttaataa
cggtatagttcaattggttagaacaacggaatcataatccgtaagttgtgggttcaagtc
cctctaccgttatggctcttatcgtctaatggttaggacagtgctttttcagggcatcga
cgtgagttcaatcctcactaagagtaacttttttaatataaaaatccttatacaaatacgt
gagcccataagaattcaaaagtaaaaaggatttttctagatctactaaattattttggga
tccctaggtcgttaaaaatatttaaacaatgtattttattcatatatatatatatactaa
aacttaaaaatataatttagtgcaaaagtctctaaataactgaattttccgttgaatata
ctcgaccaatcataaagatataggtactttatatttaatttttggcgctttctccggtat
cttaggtgcttgcgcgtctatattgatccgaatggaactagcacaaccaggtaatcaact
attattaggcaatcatcaagtgtataacgtactagttacagagcacgcatttttgatgat
tttctttatggttatgcccgtcctaattggaggatttggaaactgattcgtacctattat
gataggtgctccagatatggcctttcctagattaaataatataagtttttgactactacc
tccatcattgtgtcttcttttaggatctgcgatggtagaagtaggcgctggcacaggctg
aactttatatccgcctttgagctctattcagagtcattcaggcggtgctgttgatcttgc
tatttttagtttacacttgtcaggtgcttcttctatattaggagctattaatttcattac
gacgatatttaatatgcgcaatccaggacaaagtatgtatcgaataccgctatttgtttg
atctatcctcattactgcgttccttttactactagcagtacctgtcttggcaggggccat
cacaatgctgttaacagatagaaactttaatacaacatttttttgacccttcaggtggtgg
cgatcctgtattgtatcagcatttattctgattcttcggacatccggaagtgtacatttg
tgcgccgtttaattctgttaaataagatggtttattattttaaattacttatctcgaaat
ctttaaactgaatagttaataaagatagacttaattattccttttgtcttaaaacacttt
cccttaataaagttggaaacaattctattaaaagcgggaatgaaagtctcttgcaggaac
ctgaatttcctaaaaatagtaggctagttatgctcatcatgaataagataataaagttga
tacatatcgacggttccacactaaactttatacataacatttcaggtaacagtatttatg
taatatgggtcggtaccagaatgtttaccttcccttgaataattattacacgtcagagcc
atcagctcctaaatgatatgcaacgttctaaaatgagcttgatcgagaaacagaagaact
cgggataccctaccaatcgaaagattcatgagtacggaactctcgtagtaggtggtagaa
gaattcaatcttcttcaaactaccaaaggggagtagaacttagattcttaagtgaaaaac
cctgcattagctcgcaagagtgcgctaggttagtagatttgagaaaagttaattctgaaa
ataaatttcaagttaataagaatactattcatattatatctgatatgaatgtccttattt

tagcatatgaactcataaaaagtaatcctggaaacatgacacctggtgtgaatggttcca

ccttagatgggttagacaagacgtgactgcaaaatattagtaccaaaataaagcaaggta

aatttttattcagccctgggcgtaagaagtacattcctaagcccggttcagcggataaaa

gaccattaggtattgctagcccgaaagaaaaaattgttcaaaaagctattctgctagtac

tagaatcgatttttgaaccaagcttcttgagaaattctcacgggtttcggcctaaccgag

gcaaccataccgctttaaagatggtaaaaagcgagtttcacggagttccctgaattatag

aaggagatatttcgaagtgctttgatgaaattgatcactctattttattggggcttctaa

gcaagaggatatcttgtgataagactttaactttaattaaaagagggttgaaggctgggt

ttatagatttaggaatattcacaagaactaaattgggcacccctcaaggaagcattctga

gtcctatcctatgcaatatctatttgcatgagctagatttatttctacttcaactaaaaa

ttaaattcgatacagggactagtagagcgaagaacccacagttcagaaaactacagtata

aactatctaaccttaaaacgcctctcgagaaaaagcttgtcagaagagacctttgaaaag

tgcatagtctgaacccccctagatcctaacttttgcagaattcactttgttcgatatgcgg

atgattttattgtgggagttacaagctcccatgaagttgctttagaagttaagaatatga

ttaaggaattcctttgtaatcatttgaaattaaatttgaatgagctgaagacacaaatta

ctcatattagagaaaaggatatatttttcctaggtacccttatcaaaggtaactgaaaga

aagagaaacctattcgattgatcaactttccctctagagaaacgtccatcaagacaagag

tcactccgcgtttaagtttgcatgcccctattaaaaaactctttgataaagctactgctg

aaggatttttccgtagggatgggattaattataaacctacctttgtaggtaaattgatta

atatggaccatgcagacattttatttttttataattcaatagtaagaggagtactaaatt

actactcatttgtggacaaccacaaaagtttaggatcatgagtccattatatgaaatttt

cctgtgctagaactctagcgttaaagtacaagttacgtttcacatcgaagacttttaaga

aattcggctctaaattggcgtgcccgaatacaaaaaaaagcctgtttttaccaacgagct

ttaagaggacgcaggccttccaaattaatagccctattccttttgagaaaaaagtactct

cttgatctaaaaaaattactaaatctaatctgaacaaagtttgtttaatatgtggtagtt

ctctatttgtagaaatgcatcatatacgtagtatagctggcattagaacaagactttgta

gcaataaagcagactttttctctctgcaaatggctgggataaacaggaagcaggttcctc

tttgtcgagaacatcacttgaagctacataataaaacactttcaccagaagaaacgttat

tatttcagaaaaatattaaggaaatttagatgtatttctttcaattttaactttatttac

aatttaagttcggcgagccgtatgataagaaattatcacgtacggttctgagggcagtta

ttgacgctaaaacaacctcgttgtcaagtttctatttctgacccctactaatcttgcctg

gatttggcatcgtcagtcacatcgtttccaccttttctagaaaacctgttttcggttata

taggaatgatttatgctatgctttctataggtattttaggatttatcgtttgagcgcatc

acatgtatactgttggccttgatgtagatacaagagcttacttcacagctgcaaccatga

tcattgctgttcctacaggtattaaaatatt-----------------------------

-------tagctggattgctactatgtgagaagggtctatttttttaaaaacccctatgt
tatttgctatagggtttatatttttattcactataggaggacttactggtattatactag
ctaactccggacttgatatatctctacatgatacttattatgtcgtagctcacttccact
atg---

------------------------ttctgtctatgggagctgttttcgcaatatttgcag
gcttttattattggtttgaaaagatatccggatttcaatattctgaaatactaggtcaaa
ttcacttttgaggcacttttataggtgtaaatctaacctttttccctatgcactttttag
ggcttgctggtatgcctagacgtattccggattatccagattcttatgccggttggaacg
caatagcttcttacggttcatatgttgcgttatttagcacgctgttttttttttatcttg
tatttaacacacttgtaacagcaaaaaagacacctgctagaaataacccatggaactttg
aagattcaaagatgggctcaactacattagaatgagaaatttcttctcctccagcttacc
atacgttcaatgagattccagttataagagaaacagaaacatctttaaaaataaattaac
ttatgataaaaaaaaatactacaatattatttttagggttagctttgctaacccaactac
tacagagtagaatagttattagtgattctgcagaagactggcaattaggcttccaagatc
ctgcaacacctataatggagggaatcattaaccttcatcatgattttatgttttttatct
gtgctatctcaatttttgtatcttgaatattagcacgcacgttatggcactaccactgaa
caaaaaatgagtacccttctgccacggttcatggaacagccatcgaaataatttggactg
ttactcctagtatcactttgttggcgattgctgtgccttcttttgctttgctatactcta
tggatgaaataattgcgcctgcaataactattaaaacagtaggtcatcaatgatattgaa
gttacgaatactcggactatacaaatgaagacgataacactataatgtttgaaagttata
tgattccagaagaagatttaactttaggtcagttaagattattagaggtagataacccta
tggtaatacccgtaaatacacacgtacgtctaatcataacagcagcagatgttttgcaca
gctgagcagtgccttctttaggtataaaatgtgacgctgtaccaggtagattaaaccaaa
gctcgctctttgtaaaacgtgaaggaatattttatggtcaatgcagcgagatttgtggtg
taaatcatggttttatgcctatcgtagttgaagcagtgtctttaccaaattatatttctt
gagtagctaataaacttagcgaataaatagcctaaaaatattctaagcatctgatatgcg
tgtatctctagcccaactatttttctttggtttatctatttttattttattttgatttaa
tcctaaatacttaacttatacaatacgactattaaaaaaaattaaaaagcgttctaaata
acaaagagcgctttaataaaaaacttattatggcaactacgacaaatctaaactttatca
aaacagctaaacaattacaacgccacccttttcatttagttgaccccagcccttggcctg
taacagctgcaatagccgctttttcatgtgctttaggcggagttatgtatatgcatgcat
acagtaatggagggtacctattatttagtggtttttctttactgttatttacaatgttct
catgatggcgcgatgttacaagagaagccacttttttcagggcatcatacaggtgctgttc

aaaaaggattgcgttatggtgtaattttatttatagtttcagaaatcctcttctttttg
cttttttttgagcatttttttcatagtagtctttcaccggctattgacataggttctatgt
gaccaccaaaaggaatagttgtgtttagcccttgagaagttcctttttttaaatacaataa
tattattattatctggttgttctgttacatgagcacatcatagcattgtagcaggctata
aaaagcaagcaacgttagctttaataacaacagttatcttagccgctatttttacaggtt
tccaaggttttgaatatagcgtggctaattttacattatccgacggtgtttacggctcca
cattttatatggctacaggctttcatggttttcatgtttttataggtactattttccttg
gtatttgcttacttcgcttattaaaatcacatctgacacaacagcatcattttggttttg
aagcagcagcttgatattgacattttgttgatgttgtatggctttttttatttatttcta
tctactgatgaggtggtacctaatctaaatctctttaacatctaaaaatctatgatgaaa
cttattaatttacctaccataaagctcactttttgacatgctctagctttcttcattatt
atctgttatcataatatctatatttttacagaagaaagtatacttttattctgttttatt
gcgtgactaaacattacatgaaattatatatctcctcaaattaatgcgtcattatctgaa
agaggagaaaaaatcaattcaaattttcaacatattattaacgataatataataacttga
aaaaaatatagacaaggttattcactaaaaataacacatgttgatattttaaagaactta
atcacatatttaacatgtttaattaagactgtactccttttagaatcaaaaagtagctca
ttacaatctattgcaccttatttaaaaagattacatattgtaaaagacttagaaacaaag
ttgactaaaatttcttatattacgatttgccaacgtatacaggatacagccacaatacgt
agtttttatgctaaccgagtaaaaatcaaatctttccattctgaatctaaattagactta
ttcgaacgtataagaaaattagaagctggttcttagaagtttaagtctatagctcaatgg
ttagagcatacgcttgataagcgtaaggttgattgttcgaatcaatttagacttatacta
tttcaaaaccataatataaaatatgtacaatataaaccaaacatttttttcagcaagccc
tttagaacaatttgaaattatacctttaattcctttagaattatttgggttaaacatgtc
gttaacaaacgcgtccatttttttgatactatctgttgcgttatctatttttttgatccac
tttagtcatatacaaaaataaattagttcctggaaactgacaatctgtaaaagaaatatt
ttatgataccaccttaacgttggtaaaagataatttaggtaaaaaaggttatagatattt
cccgtttatttttaccctttttacaataatactttattgtaatttaataggtatggtacc
atatagttttactgtaacaagtcatatagctttcacatttggcttagctttagctattta
cataggaattaatattattggcttcagaacccatggtataaagtttttcacaattttttt
acctaaaggagttcctttatttattgtacctttagtggttgcaatagaattcgtatctta
cgtcgtaaaagttttcacaatatcgataagactttttgcaaatatgacatccgggcatac
tttacttaaaattattgccggatttgtttggacaatgatctcaataggaggcgtgtttgt
atacttacaaataatcccattagttttattactagcgttagtgggtttagaaattggtat
tgctcttttacaagcttacgttttcacattacttacctgcatttacttaaatgatgtttt
agaaatgcactaactaaaaaattatgccacaattagatcgcgttattattttcggtcaaa
tattttgactatttttcaccttttttaattgcttatgttgtttatacccatttcatattaa

gtaatttattaaaaattttcttagtccgctgatggaagcttagaaaagatattactcaaa
ttgcattaaagatccgtttaacgagctatttaattgattcaaatattcaaacgttacgta
gaatttattcaacaatcagaaatatactagcttctctaacaaaaagtttattaacaaaaa
gtataagtaagccaaagttagttttaaatgatcttaattctttagttattaaaattagtc
tggaaacctctttatatggtagcaaaagcatcaccaagtctggaacatattcttattgaa
cttaataaaatatactatgtatttattaataatagctctgcctttagtgggaacattagt
tacagggttaggcggtagatgaatagggcgtaaaggttcaaatttgttttctacaacttg
cgtagtcctgtgtgtctttttttcttcaatcgctttttttcgaagtaggtctttgtggagt
tccttgttatatatctttgagcccttgaattagttcaggggcactaaatatttcatgagg
tttttatttgatagtttaacaacaacaatgcttgttgttattacatctatttctagttt
agtccatttgtattctattcaatacatggagcacgaccctcattgccctcggtttatgtc
tttcttggagattttcacatttttatgatcttattagtaacagctgacaattttgtgca
aatgtttttaggctgagaaggagttggattagcttcttatctattaataaattttgata
cactcgactttgtgcaaatcaagctgcaatcaaagctctggtagtaaatagagtaggtga
ctttggattaagtttaggtattttcacaatttttatctttttggttctgttgattatga
aatagtattctcttccgcaaacatctacacaaattacagtatttcctttttgggttttc
cataaataccttgactttaataggtatttttttattaatagggggctgttggaaagtctgc
acaattaggtctgcatacctggctaccagacgctatggaaggtcctactcctgtttctgc
actcattcatgcggctacaatggtaacagcgggtgtgtttttaatagtgcgctgttcacc
tcttattgatttatcctcggatgtcttacttttaattactcttcttggatcaagtacagc
ttttttcgcctctattgttggagtatttcaaaacgatataaagcgggtaattgcttattc
tacttgtagtcaattaggctacatggtctttgtgtgtggtttatcctattataatgtagg
tatgttccatttagtaaaccatgcttttttttaaagcattactttttctaagcgctggctc
tgtaatacatgcgctatcaaatgaacaggacatgcgccgaatgggttcgctagcaaatag
cctaccgatcacatatgctgctatgctaattggctctttatccttagcaggattcccttt
tttaacaggtttttattctaaagacttaatcatcgagataacacaaataagttattacag
taatttacagatttcttttggcgtttatgcttgttgacttgctaatatttctgtactctt
cacatcgttttatacatttaggcttatttttctaacttttataaaaaataccaatagcta
tagaaaacacatagaaaatatacacgaatcgccacctttaattctaattcctttaatatt
actcgctatatctagtatttttgttggtttcttaacaaaagatatattcgtaggaatcgg
aactcctttttgaggtaatgctatcaatattctacctacgtcttgtaatctattggaagt
tgaatttatgccttctttaataaaatgacttccgtttgtgttaagttctatgggtgcaat
tctcgcttatacaataaatgtaggtgtactaaaaaataatatacaatttgctcataatca
cttatttagaaaacttgccttttcccttagcaaaaagttatattgagataaattatacaa
ttcattcattgtatctcctttaatgtattttggttataatatttcattcaaaaatcttga
taggggttttatagaattcgtaggtccttatggaatttcgcgtactattaaaaactgatc

cacaaaagtaattaaaatacaaactggtcagctaacccattataccttttttcgtgatttt
tggtttatgttcccttttactactagttcctgtttgagattttctacaattttttagttga
tgtcagattactagtattttgctttatagccctctttgtagcgtagtttacgaaagtttt
aacacttaaatatatgcagataactaatttattattatggacttcacttattcctttgtg
tggcgctatattacttattttattcctagattttactctcatttaataagaaatattgc
tttcgcaacagcgcagctagcgtttatatactctattttgctatggctttgctttgaatc
aacaacatccttattccaatttatatatacgataaattgatttccctcctataatattta
ttacacaataggtgtagacggtatatctttatttttttatcatacttacaacgtgattaat
tacagtttgtacattaataagttgaaatatgccagacagccaaataaaagaatacttaat
ttgttttcttttgcttgaagctatttaattcaagtttttttgtgttttagatgtcctatt
cttttatatattttttgaaagtgtccttatccctatgtttttaattataggtgtatgagg
gtcacgggaaagaaaaattagagctgcgtatcaatttttcatttacacattagctggttc
actgctaatgcttctagcaatcttaactatttatttccagcatggtaccacggatatcca
agttttatgaaatataaattttgacgttagaacacaaatttttactttggctagctttttt
cgctagtttagcagtaaaaaattcccatgattccttttcatatatgattgcctgaagccca
tgcagaagcacctacagcagggtccgtaattttagcaggtgtgcttttaaaaatgggcgg
gtatggatttttacgtttttctttacctatgtttccggaagcctcgctttattttgctcc
attaatttatttactaagtattatagctgctatatatgcttcacttactacaattagaca
agttgacttgaaaaaaataatagcttactcttccgtttcgcatatgggctttgtcacatt
aggtcttttctcttttaactctcaagggatagaaggtagtataatcttgatgcttagcca
cggattagtctctagtgcactttttttgtgtgtaggtattttatacgataggcataaaac
gcgtcttctcaaatactacggtggtctcgtgcaagttatgcctattttcagcatattact
attatttttactttctctaatatcggttttcctggtacaagcagttttgttggtgaact
attagtgttaatgggagtatttcaatttagtccaatatctacttttctaagtgcattcag
catgattcttggggcagggtattctatttgactattcaatagagtatgttttggtagttt
aaaacttcaatacattacaaattttcaagatatttcaagaagagaattttgtatcctttt
tccgttaagtgtatttgtactctgaatgggtatatatccagaaattttcctatctgaaat
tcactgttcaagttataacctaattgcatattttaactaattttatgttatgaagtttat
taaaacgctaattctagcatttatgaaaaagaagtcctattttttattggtttccacgttt
tttagggctattacttatacctgggttttttatttgataccgagattctagttctctttca
aagccttatcctcttacatgcaagcctaggtttagaagtaatcatagaggactatttaca
cctagaaataataaaacttcagtgtttgtctttaattaaagtacttttaatattattagt
caatcttaatatattatatttattataaaaaatatccttatgttatttatctcctcttat
gatttctacgcattattgacagaaatttactttttaaacgcaatttgtgctttattaatt
tatggtgtaattttaaatacctcatatagaagagggcatccagttattgaacacaatgta
agtggtctctcaactcaaatactaatagtgagtctttggttaacagtttgttcaaatata

ccttgcctaaccagctggaattcacttttagtgcacgatttttatctttcggtataaaa
agcaccatattagcaatttcgctactttggtctttaattatttttcttacaatagacta
gaaaaaataaatctctacgagtattgaatcgtgtctatgttggctattgttgccatgctt
tttgtaagttgttcttatgatcttttggcaatgtatttagcaattgaattccaaagcatt
gcattttatatattagctagttttaaaagaacatctgaattttcaacagaagcgggttta
aaatatttcgtactgggtgcattttcttcagctttgcttcttttaggtatttcactactt
tatggtactactggtttaactaattttggagatctatcaaaatttttttaggtaccaca
ctggaaaacgcatcatttatcaacataacatttttggtgtcgttttaatagaagtagct
cttttttttaagataagtgcagcaccttttcatatgtgatcgccagatgtttatgaaggt
gctcctactaacgttacatctttttttggtatactgccaaaattagcattagtaagttta
atatttagattcttttatttttgttgtgctgaagttgtgctgctactaaattttacactt
ataatttgtgcgcttttatctatgataatagggacatttggcgctttagcgcaaacaaaa
tgaaaacgtttcattgcgtatagtactataagtcacgtaggatttattgtagctggattt
tcaacgttggaatttaatggtgcatttggtgcgctattttatatcttggtttatacttta
acttctttagccactttttctattgtgctttccttccgatgcttagcatatcctagcaca
taccaattacgctatctaacggatatcgttagtttagtgaagttaaaccctatacttgct
ggtagccttgtagcagttttattttcaatggcaggtataccgccttttccaggattttt
gctaaagtatttgttttattttcacttttgcaagaacaattaataggattagctataatg
gcaatatttttgagttgtgtttcgtgtttttattatatccgtttgattcaaatgatgtat
tttacacatacaaaaaccatacttatttttttatccaatagaaaagactacatcaactata
ttaagtataactatgttattacttgtacttattttttttgaagatagatctgatttctaat
tttgttcattgtatgttgtttttataaaataaccaattaaaatgttttacaatattgcaa
ttaacattatcaaagtgttgaccattatagtgccacttttaatcgctgtagcttatatga
cactggccgaaagaaaagtgatggcagctatgcaacgacgaaaagggcctaatgtggtag
gtatctttggtcttttacaacccctagcagatgggttaaaacttttctcaaaagaaacta
tactaccttctagtgctaatattttatttttttagctgcacctgtgctaacgtttttgc
tagctttattagcatgatgtgtacttcctctagatgaggggaaagtttttttcggacttaa
atataggtgttttgtatatattagcagtatcatctttaggtgtttatggtattataactg
ctgggtgatctagtaattctaagtatgctttttttaggtgctttgagatcagcagctcaaa
tggtatcttatgaagtttccattggtctaattttaattaatatcttattatgcgcaggca
cattaaatttaactcaaattgttctggcgcaacaaaatatgtggtatataatacctctgt
ttcccatatttattatgttttatatttctatattagctgaaactaacagagcccctttcg
atttgccagaagcagaagcagaacttgtagctggttacaatgtagaatactctgcgatgg
ggtttgcgttgtttttttttaggcgagtatgcaaatatgatacttatgtgtagtttaacaa
ctatttttttttttttggtggttgattacccttagtcaatatgcttcctttttattggattc
cacccgtactttgatttggtttaaaaacaactttacttctatttggttttatttgagtac

gcgcagcatttccgcgatatagatatgaccaattaatgcgtttaggatgaaaaatatttt
tacctttatcattagggtgagttcttttagtatccgggatactattttctttcgattgat
taccataacaaatgaacgtactttataacgagtattctgctattctcactttttttgcag
tagctttttttaatctctctaataatattaatactttcgtatatattaaatcctcaacaaa
gtgatcaagaaaaagtcagcgcctatgagtgtgggtttaatccatttgatgacgcgagag
caacttttgatgttcggttctatttagtcgcaatcctttttttaatatttgatctagaag
taagtttcttatttccttggtcactagtacttgggcagctaccttcttttggattttgat
ctatggttgcctttttagccattttgacattagggtttatttatgaatgaaaaaaaggcg
ctttagaatgagaataatcaaataatttactagagatttttaatttgataatataatata
atatgaacgtaactttacaaagtgcaaaaatgataggagctggactagctactattggtt
taacaggggtaggagctggagtaggaattgttttcggatcgctagtaattgcttattcgc
gtaatccttctttaaaaaatgaattgtttggctacactattttaggattcgctttaacag
aagcgattgcattatttgctcttatgatggctttttttaattttatttacttaatttactt
taattaatgggcgtttataataaacgcccaccttaaaaatacattatgacaagtacaact
ctttttttgaatcttttcaattatttctttaatatccgcttgtatggtggtaagcctgtca
aatgctgtgtattcagttttatttctaattgtagtattttgtaatactgctagtatttta
ttattactaggagcagaatttttatctttttttatttttaatcgtatacgtaggcgcaatt
gcagttttatttttgtttgtagttatgatgttaaacgttaaaatagatggagtaaaaatt
aattatagcacaattttttttgattggtattttaataagtctgattttacttattcagatt
tgaactgctctacaattagatattgaagcgtatgataatataggcgtaccactatcccaa
aataactttccaacaatagtttcttggatccaagaaaatgaattaccttcaaatacagag
agtattggtttaattttgtatacttcgtatagtttagtatttattatgtgcgcatttata
ctacttttagctatgattggttccattgtactaacaatgaatcaacgtagtggagttaaa
acacaacaaatcacacttcagttatatagaaatcaaaataaagtagttcgatttattgat
ctgagaaaaaattaatttgattgcggatatagatgaattggtacatcagtaattttccac
gttaaaggatatgggttcgagtcccattatccgctcaaattaagagagaatagctcaata
ggtagagcaatagttttcaaaactaaaggttaaaagttcaagtctttttttctcttgcaaa
agtgagtctgcttgaacaccctattcagttttaattataattaaaactgaatagggtgtt
caagcagactcacttttgcaagagaaaaaagacttgaactttaaattcatataagaataa
caaccacattttagtatacacaagacaacagccttgtatgcgtactaatatatctctgca
ggctgtgtagaggcaaaaaaagtttgtgtgaggtggctgtgtgacatataaattaaaaat
agttgtcaaaaaaagtctagtttggcgtaaaaggattcgaacctttgaaatcatggtatc
aaaaaccattgccttaccacttggctatacgccaaacaaattgaagataaagtgggattc
gaacccacggtggatagataccacgttagctttcaaaactaaagctttaaaccactcagc
catttatcccggttattaatataaattgtattctaaaatttttagagtctgtttatatga
tagtacgggaataggattcgaacctatatttttagatcatgagcctaatgagttaccttt

ttactctatcccgctattttatctaattaattttgtaacttaataaaaacttctccaaac
tacccagtaaaggtaggatgactaaaaaatataaaaaataataaacgctagctatctgac
ctataattatgtaagggtcttctacaggcataccacctattcaacctaatattaaacaac
aagcagccatgcttcaataaagaccacgatatatgggtctaaaacgcgaa----------

--cttctaatttctgtactatgtattcatggtaatagagctaatacaactatagcaaaaa
tcatacatataactccacctaatttatgtggtatacttcttaagattgcataaaaaggta
agaaatatcactcaggaacaatatgtgctggcgttaccatgggatttgcttcaatgtaat
tatcagggtgacctaaaagattaggcgagaagtatacaaaaaaagaaaagaatataataa
aggccactatccctaataaatcctttacgatgaaataagggtacataggtaccttatcgc
tgcttgcatcgatacctaaaggatttccagaaccttcttgatgtaaagcggctaaatgca
ctaaagacgctgctgcgataacaaatggtaataaataatgtagactaaagaaacggttta
aagttgcattatcaacagaaaagccacctcaaagccaagcaactatagaatcacctacca
aaggtacagcggatactaaattagtgattacagtagcacctcataagctcatttgacctc
aaggtaatacataacctataaaagcagttattatcattaatagtaaaatgattacaccaa
tcactcaaacaaattgtcgaggtgcagcataagaaccataataaagtcccctaaaaatgt
ggatataaactacaataaaaaacattgaagcaccattcgcatgtatatatcgtaaaagtc
aaccaaagttaacatcacgcataatatgctctacactaataaaagctaaatcaacgtgtg
gggtataatgcatagctaggaatattccagtcactatttgtattattaaacacattgcag
aaagaaacccaaaatttcatgcataatgaatattaattggagttggataatctataaggt
gattattaactatattgaaaagaggtttttaattagacgcataaataatatttttatta
tagatggacgaggaatgggacttgaacccatggcctataaagtcacagtttatcgctcta
ccaaaccgagctctcctcgtcatgttaaaaagttaaatttacggggaaaaaggatttga
accctcactcattgatgtgacaaaccaatattttaacctattaaactacttccccatttt
tattaaatacggatagagggatttgaaccctcatgaataatattcatcaaaacctaaacc
tgacatgtctaccatttccatcatatccgcaaaaaaatgttactattagctttaacggat

aaagagggattcgaacccacggtataatatttcatacgatgatttagcaaaccattgcct
taaaccactcagccatttatcctgtgttttggaagctgcccactaccggacttgaaccgg
taacttaaaaagaacagattttaaatctgtcgtgtttacctatttcaccaaatgggcatt
agctattgctaatgctatgttttattgaagctattgcttttccggggttcaatgatttag
gacacgttctactgcaattcataatggtatggcatttaaaaagttttgatttacctccaa
gtaatgctaaacggtcttgagttttgatatctcgactatcagctaatcatctataggctt
gcaataaaattgcaggacctaaatatttgtcatggtttcaccaataacttgggcaactag
cagaacagcaggcacaaagtatgcactcgtaaataccatttaattctgacctatcctttt
cagactgtagatattctgtttttgaaggtgtatcatttataagccacggttttatatatt
tatattgtgcgtaaaaattagataaatcaggaactaaatcttttataatgtacatatggg
gtagcggataaattgtaattgtgctagtatttatatttaatggttgcaaacaagctaatg
tattagttccatttatattcattgagcaactaccacaaataccttctctacacgagcgtc
taaaggcgatactcgaatcttgttcgtctttttatttttataagagcatccaataccatag
gtccacaattttttagtatgaataggatgtgtactgaaatgagtgatagttgggtttgatg
gagttcatctatatatacgaaggaattttaagtctaaattattattggatactaattgaa
aagaaattttttttgaataattgcatacttggttttattttgattgctataattatttaag
aacgggttagcccgttcttaaatataagataaaatataatgaacatttcgtatttattta
cttacttttgcaagtattaaataataaaccgatctatctagtaatcttctagaatgttct
ggaaaaagtaaaggttatttttgcgcttttaagctgtcagcgcttatataatgttaacgt
ggctactcggctatgcaagaaacaatacaaccgatacactattggttaatatatcttaat
cctctcgtactaaagataaaccctctttttttctttcccacaacagatag----------

-------------------------------ggttatggtaaagaaacttctcacgaagt
ttcctcccactgcaaaaccgtacgtgaaggtcacccttcatacggctcctcaaa----at
tatctatag-----------------aaagtgt-----------aaaaaaatacacactt
tcctttagtttaaaagtgcttatattagtccgcggcttaagtattagctgctttggaatg
tctagtatcgtggcaatgaccatgcataagtcgtaagtttttaagatcaggagatcccctt
ttgacttcttggtataatatgatctatttctattctatctgaatctttaaaatataattt
acacatactacactgcggccctttagttttaatagtcgttttagtagttgcccatactt
attattaacagcgagacgtctggctcaataagtaactcttccgtcatatgggctactata
tccggctacttttgtagatagaataatatttcgttgatcatgacgggttaattttataat
tttgtctccttctttaaggcctaaaactctttctgcgctccctataggtattcaatatct
agtgataccaggacctgttcgtttgcgtttcttagctcatttccttagaagataaaacgt
acgtacactacaataactaaaagtttttgtggcactacatacggaaaagtatttagttca
tccgctaataactggcgctagtttactgattaaaactttttgagataatccggtggactt
cgtagtaatacttttttatattagctaaatgagattctatagacttatagctgggttcaca

cctgctttttcatccagtcgcaattccttgattattctttgcggatgtatgtttacctac
cttataattgacaaaattaaaacctaagaaatctacacctgttattctagacttctct--
------aaactaccagtgtacgttattttgttttggattccgacaattttagacctaag
ttctgtaaaaagaattcaatttaattttgcttcgatcaattctttctcttcattacat
aatactaaaaatcatctgcgtatctaataaggtatactccactttttcctattgcctct
tccattccgtgaagggcaatatttgccaacaagggtgatatgatatctccttaggggtt
ccggcttctggtgtgatttcttttgtattttcttaaaagcctgtaagtattcccgctttt
aatcaagctctcaattgctcttttaaaacaggaaacgtgtttacttttagaagtagttta
gagtgatcaatattgtcgaagcatccttcaatatctgcatctaatacatgtttagaaagt
tgctttaaacatttcacaattgcttttctagcatcgttagaacttcaccctggtctaaat
ccataactgttaggttcgcatatagcttcatattgaggttccagtgcaaactttacaaga
cattgtttagctcgatctcttatagtaggtattcctaaatgcctttctttcccgtttggt
tttaaaattgttactcgacgaattttatccgatttattatcaattttaatattttgtact
aattccattctttcgtcaggagttaaattactaactctatctactccagctgttcgtttt
cccaaattgtcttgagtcacttttcgaacagctaaaaactttgaaaagtcatgctttatg
atttgtttctgtattaagaatacagatgtcatattacctcttttgctaaattcaaaaact
ttacactgcaatctatacagtcaaatttcttttatttttcagttcacttgcgttcacttt
ttcataattt------gtttgttggtgtaatcttaacaatgttaaaatttttatttagat
aatt-----ttgtctacacgtctgcatatccataagctttccttatggccttggcttctt
gtagaatcctgatattaatataccttaacactata----------gaaaaacgcccttgt
-aaaaaagaatcaacaaggtttatattaatatttacttcgttccaatattacatacatat
agtcagtaagcacctctattccctctaaaaccggtacccctttctaacgcggccacatta
gattttaccttatgtttagaattcacgctgtttcgtgttaacggggtgggttaacttata
attatcccccgctatgacatatttagggaatgttatttcggctaccctaaaggtaggtta
tacttctataacta-------------cttttcttggcagatttactggtgtatatattc
tcttcaggtttaaatgtttataagtaacattctcctgaatttatgccctgcttatacctt
gtgaataactattttcacactgcatagggtgcactgtttgtaaa-acagctcattgatca
gcattaggagt-aatgcgtaggcccactagttctagagaacttgcttcctacctgtaata
ttagtttaa----------tctctaaagtggagattagctatttacgagaagtacatctc
ttctcttactagcaacgaatcgcacgaccgaactgtctcacgacgttctgaacccagctc
acgtatcttattatttggcgaacaaccatacccttggaacctattgcagctccaggaaaa
gatga---

---------------------------------------gccgacatcgaggtatcaaac
catggcatcgataagaactctcagccatgataaatctgttatccctagagtttctttttt
ccgataagcgacagtatttccatacattactgccggatcattatgaccgactttcgtccc
ggcttgagctgtaactcttaccgtcaagcagattttcatcattacatattatattagcat
atattgccaactaaaatccacttttgtgcacctccgttacagtttaggaggtcctcgtcc
caagtaaactaccactttacatatgtccttgttagtaagcaatttttatactcagagt
agtttttcaatggcgtacaaatgtacttctacttttactgtactgtgtacaaagttatcg
cgacgtaaaattatagtaaagaatcatagggtctttccgtcttgttgcaggatgtctgca
ttttcacagacaatgtaatttcgctgaggctatactagagacagtggggtagtcgtgacg
ccattcatgcaggacggaatttacccgccaaggaatttcgctacctgaggatccttatag
ttaagaccgccgtttacttgggtttataatatatgcgcaaacttatatttttcactttca
agcactgggcaggcgtcaaaccttatacgtcttcttttgaatttgcaaagttttaagatt
acgataggtagatactcgcgtattttcccccgtttagaaccgtacgtgaagattaccctt
catacggctcatccattttaacgtcagaatttcctaacgcagctctgtcgcgatgacacg
ttttatgaagaagagtcatatttttaatatatttttacctccactcactacaggtatga
tatggtgaatttctaattcgtacaatatatctccatattgactatttaagaattctttac
atatagggcacatacctttttgtctttttgcccactttaatcgaatattattcgtttgct
gattaaaacacatattcaaagatcttttattaaaatacttctcaaactcacccaaatagg
gattagcgtcaagttttatcatagtagttcttttaataggagtgtcggatatttgaaaca
aattaattttcttaaatcttttaaattcagtgctagtagacattaatcaatctctattat
ccactttgtgataaaatttggatttaagcgcttt-------------ggatgcttaattt
ttatcatgcggtgaatggaatcaaaaacgtatttacttattcgggtataagctaactttg
cagtactagacgagtaataatttgctcagccccttaatattggatttagttttgagaaga
gcattcacagaggaagttgcgaatgttctttgacacaaagttttattttagcttttactt
tttgtaagctttcttctgtaggtttatatagaaaaatccctttcttttccctgatttctc
gagtagaatccgagaattccctaaagtggaaacctacaaaatcaaatccatcttcaattt
tagttattttactttttccatatttaattctaaacctcttatttttagaaatttgttaa
catctagaattaacttatctaaaatatcatagttctgggacactataatgaaatcatcag
catatctcacaagagttgctttgttattagacgcctttttttatagtgttatctaaacca
tcaaggcataaattagctataataggagatattacaccaccctgtggaacaccttcttcg
gtatcttgataactgccttcattcataacgccagcttttaacatttttgacaatatcttt
ttatccataggaatattttctaatattcactcatggcatatattatcaaagaaaccttg
atatctccttcaaaaattcatctagggttgcctctctgtctacttaaaagaaatcaaata
tattgacaggcatctttagcacttcgataaggtctatatgcaaaactgcatctatcagac
acaatttcgcttataggttcaagagccatagcaaacaaagtttgcacaactctatctctt
atagtgggaattcccaaaggccttttcttttcgctattcttctttggtataaatactcta

cgtaccgcactgaattcataatttttaagatcttgaatttgaattaatatttcatcaata
ctaactttctttcctttagaaccttttaaaacaattttgtcaattccgctggtctcacta
cttttacgtttgtgaatttgctttattgcttttattttagcttcttctagattcactaat
tcattttgtaatttacttcataaacgtttgtttttattcaaataagctattgctattctc
ctttgtaacttataaattttcgtcattatattagtttttattattttaccacaattttta
ttt-aaaataaactagctggttttacatgtaatataaataaaat-----ttttcttaaag
gacaatacttttaaaacagaacatacataagtcagcaatctttcgattcatgatataatc
attatttcacttattattagcaaacattcgctttttatgagatcttttacctactgaaca
cttggattgatcgctctttcctgagatatttctatcaattcattaggcttacccttttcc
ttacacataacattaatctaaacaggtaacattctctataccgacagtattatgtctaat
ctataaaataacaaaaagatattttatactactgcttattcctatagctatataaaccgc
taatgatgatcttcggaattctgttttctcgataccttataacaaatgttcacttacgtt
ttacctatttagataagtctagcttttcttagttacatcaagtaagaaactacattgtca
gcaaggcttcatacctccagatcactctagacgcatgcttgcttagacttatattgatta
gataatatagcacttagtgcatttatgtatacggctttaaaggtcgcacttttttgttaaa
cagtcgctacccctaattttttgaaaccttaaaaggtactcctttttgcgaacgtacggag
taaatttgccgagttccttaagtatagttatctcattcgtctttattttctcaataagtt
cacctgtgtcggttttaggtacggtcaaacttcatgtaagttttcctgaaaaatttcttt
ttttagcctccagtttagtgggtgtagctattagaagatatctttgtacacaaatacacg
agtattttgcagacacgtggtaatttttcctatcgaatacagttttcactttttttcttaa
gggccgactaactccagttacttaaaattgactggaaacccttgaacaatagacgaccat
gatttattttcaacatggttagcgctactcatgtcagcattagcactcctgattttagat
atgcaatttaacattaacataaaagactacaggacgttccgctaccattaagcttagttt
agcttaattcgaagcttcgatatataaatttaagtctccttacattttaaataagtagaa
caaataaaaatagcgagctaaaacgctttctccaataggtggctgcttctaagcctactc
tgtttattcgaattattccttatttttttcactaatttataattttgagatcttagctatc
gattagggttgtttcccttttgacgtaagaccttatcgcccaacgactgtctgctgctat
aaataaaaatatagtttggagtttaataaaatttagcaaaatctaaatttaataagtagc
tctacccacattttaaaaaagcaacgtactactttgatagttttcgcggaaaaccagcta
tcaccaagtttgattggactttcacccctaatcttaagtcatccccgtatttttcaacag
acgtgggttcagtcctccagtactttttaaagcaccttcaacttgcttaagaatagatca
cttggcttcgggtctaatacctgtaactttaagcgccttaatatttttaagcttgctaca
catattaacttactgactcattatgcaaaaggcactttgttgctgtatttgtcagcttca
aataaatataaattaacagattcaaatctttccctcacggtactcgttcactatcgatta
gaaaaggttttagcttagaagatgggactcctattttcatacaaaagtaatcgtactact
ttgtgttactattagtgtcgtaaaggactttataacctactttgggtttagacacttcgc

taagttcgctttcactcaccgttacttacaaattctcgtttgatttttttgttatgttac
taagatgattcaattcacataattatataaatgcatattaaatgcgagttttctaaagag
actcatagttcataggcaggtgcctcgctatgatgtttcgtcgcgcgacgtctttgcttt
tctaccaagatttcctctgataacttttaaatcttttatatttaagaacgggcggaaaa
atattacccttttgtaagagtcattaactctatagtaattgtgctcctaattcgataata
gacaacaagtattgctagtcctatagaagattccgaagccgctactgttaatatagctaa
agcaaatagttggcctactatattgtctaagtatatagaaaaaaatataaagttaaaact
aactgatagaaacattatttctaaagacattataattattataatactttttggtttaa
aaaaatgcctaaaactccgactaaaaataaaaaaaaagtataattttcacagtatagttg
agtaatcatttattttatatttaacatttgagatagcaaattgtattaaatttttccag
ccgcaggttcccctacggctaccttgttacgacttcactttagtcctttcgctaccatgg
acaaaagataaaatttttgtcttcaagtagagtaaattcccatagtgtgacgggcggtgt
gtacaaaacccgagaacatattcaccgcgacagttctgatccgcgattactagcgattac
gacttcatattctcgagttgcagagaataatccgaattaaaaatttttaaagattttgc
tccagctcacacttttgcttcttattgtaaacattactttgtagcacatgaaagtagccc
aattcataagggtcatgcggacttgacgtcatttttcccttcctcaaggatattccaagc
agtttataatgcattaatgcattatacaaaagtttcgtccgtttgctggaattaaccaaa
cgcctcacggcacggactgacgacagccatgcaacacctgtgacatcttgtgtcatacga
gaattggtaaggttttgcgcgttgtttcgatttaaaccacatgctccaccgcttgtgcgg
gttcccgtcaattcctttgagttttaatcttgcgaccgtaatccccaggcggagtgttta
atgccttagcttcgcctctggaaaattatccaaaaacaaacactcatagttgagggcgta
gactacaggggtatctaatcccttttgatacctacgctttcgtgccttagtgtcagctat
agtccagattattgttttcacttttgaagttcttttgaatatcatcgcattttatcacta
ctttcgaagttccataatcttttcctatgctctagtaaattagtttagtaatctttagta
aacatgaaatttaaaatttttttactacttagtttaccacctacgcacccttttacgccca
gtcaattagaataatacttgctcctcccgttttaccgcggctgctggcacgaaattagcc
ggagctttgattgtaaaatttagtctccaatttttaatctattttacaaagtgatttac
agcctgatagggctttgctctcacatgtggcctggctaggtcaagctttcgctcattgcc
taagattcctcactgctgcctcttaaagagtctggaccttatttctgttccagtgtgact
gatcatcctcaaagaccaattaaggattatgggcttggtaggtcttttaaactaccaact
acctaatcctgcgtagacttttcttaaaccaattattaatttttaaggcatttaccaca
atttaagataaatttctacgtattactcacccgtacgctatctttttcataagttacgaa
aattattaaacttgcatgtgttaggccgaccactagtattcattcggagccaggatcaaa
ctcttttattattatgttatgtttttatgtttgctatctcagaaattaaatttgattgta
taaagttatccagcgagctaagacagaagctgtaccaatttcatatatatatatgttggt
aatcttccttttggtaatgaggataaaatttgctttacaaaaagttgtatacttttttttg

ttttaatgcggaacatagtttattttctagctttgttctacgtataaaaagtctcctata
tttttttattttattcatacatttgtgtatcaagatgggcagatttgaactaccattccc
ttgcacccaaagcaagtacgttaccattacgctacatcttgatatagtgatattgactct
ttctggataggatttgaacctataaccttgtagttaacagctacctgctctaaccattga
agctaccagaaaaataaaagcgaaaaaaggacttgaacccttataatgaccttggcaag
gtcatgctttgcctattaagctatttccgcacttaaaattgaatataaagttgaataata
ttagcaataagtaaaaaaagtaaaagataattgctatctttgtacattttccttgtattt
acatattttactgaatctcaaaaaaaatggcgtattccattacttatgtggtatagaaat
atagcggacactactcaccggacactagttaaaaaccaaggaaaaaagaggtgatctaca
aaaaaatgtattgacggaatagtaatatataaaaagtatacttgatttagtaaaagaata
gggcctacaattaaaacaaacataataacgccagaaattctatgccaaatagaaaaaata
gaagatctttgcggattgtatatagttaaatgtggagaaattggtctattgatgttctgc
atattatagttttctttttcttgggattttacagccattatgcggacaagttgtaatatc
cattacagagataaatttaagtttagactgttttaatattttaaacacatttttttttatt
tttacctcatccttttatccgtacatgtaaagaagtataactgagtgctttagtctttat
ctctaagaagtgggttatttcggacgaagaaagagacaggcttctttttaccccttttga
ccttgacttgcctgcagttgtccaagatttaattttgcctttacagtctgttaaaagaaa
atgaatattattggaactaaaagataaaaacagtatacccatatgtagtttaattttatg
ttgtagtagtaaagactagggtcagattcgaactgacctaataaagatttgcaatcttac
gcatagccactatgctacctagtcttaaatttagtgaaatctgaagaaagactgtgtttt
aaatgataaagaacctttcacacaagaaagtaattcaagcgattttagctttggtaaatt
ggataactcgaaaagtaactgacccggccttatataacagcaatgactcactatagctcc
ttttcctttacccatacgggtttctggactaaatttggtaacagcatttttttggtagcat
gtaaaaccacactttgattttacccaaactggactttttttcgatgactttgatctcttt
ctgaattatctttttttatattatttatttgtgtgatatctacttgcttgtacgataaaga
gcgtaatccaaattggcctctttttactagatgacttgtttggtttatatatctattttt
ttttttatgctgtcgtgttggattattttttgtattaaatcttttcatattaaattgtgc
ttaaattgttttatatgccattcaaacttgaagattgcaaatcccttgttttgtatataa
tgacgtttcaaaaaattctatataagtttctaatttttgcagtgatactgcgcctaatcg
gaatactatactacttgacatacgacttcggctaccaccgaaacgcccagcgtaccttat
ttttattcctgtaaattcataaaactgtataccacgagcagtttttaatacaattttgtg
ctttttttttattgagaaatagtatttttattgtattaattatcttccggaatgctaggcc
tttttttaggccagattttataaatccacataaaagtgccccgttttgatgtcaaaatgt
tgcattatatttttttcaagtcaagatcgcacattttaaacgtaggtaccgagcaatctga
tttggctaatagaggtacatattgtacattgtattcccatctattgtcatttattttaga
tagcgtatttccttttatgaatgtttttgattcaaaatgcttatttaaaaagtttgtagc

aggtaatactttttttgtatatttgcttttgatttttaaaacctttgccatagcattgact
ttcggtatttcataagtcgttcacgcctattcgaaatcctcttggattaactttttgacc
cataatgttttattacttaataacaatatttaaattgaaaatcgaatctaaatagttgta
caaaaatacgttttgtaggatttgaacctacactaatcaacttagaaggttgatgtttta
tccaattaaactaaaaacgtttttattttagatgcattgggatttgaacccaaaataagc
agattaaaagtctgttgctgtaccgtttagctatacatctttttaataaagagagtagga
tttgaacctacgatgattttaatcaatagatttacaatctatcgctttcgaccactcagc
catctcttcttcagcggttagatatatgttacaatctgtaataaaataattaattatata
ctattgtttaataacaatatttagattgaaaatcgtacgattttcaatctaaatattgtt
attaaacaat
>SRR9587921
gttttattaattaaaactaccccagtattacacttttaaatatttataagaaataatgca
aatccctttacgctgttatttaatcgaatttaaatttcgattcgtttactttctacttag
tttttttctctcgttgctagtcgttataaactatcatgaggctgtgtttttttttgaaac
gtatactttttttgtttataaacgagggggcattcatcgcgacacatattgctgaactatt
ttcaacttctatgtatattagttttaacctagttttgtgcataaattatccttttgcata
ttatcattgcagccgctttttttaattcgagttgatacaaatcacaggtttggttctttaa
aaatatgcaatcattgacatttattacgtttttagctagtttacttttgtgctacttttt
aattttgccctacacttacgctttttttagacacttgaactgtaacgatagtttatgcatt
taaagtgcagttggaggcaaggatagcaacatatgtttgttgaacactacaaacaatgtg
tttactatctaacattgtctatctcttctttactaggctaatttgtttttacttagtgga
caatatgattaatttgcatttatttttccgaaaaaataaaaaatatactttctttttcgt
atgcttgtcggcttctgtgtgtttaccgcaagaaagttttatacaaattactttcataat
ttctataatagtactatttgagttattctttttggttacatgcttatttttttgcaaaaaa
taatgtctaaaagtggacttgaaccactgacccaaagattttcaatcttttgctctaacc
gctgagctatttagacttaaacttttcttttcccgtatttagagcgaccatttttttcgtt
tagcgactgcttgtaaatcgtaaacgcctctcacaatatggtaacgtactccaggtagat
cctttacacgaccacctcttatcagaacaactgaatgttcttgtaaattgtggccttcac
ctcctatatagcctatgatagaacgtccggtactaagacgtattttagctacctttcttt
cggcagaattcggtttttttagggttagtagtatacacttttgtgcaaacacccttttttt
gaggcgacttattgagagcgggtgtttttgctttggtaattttgtgctttcttggatttt
ttattttttggtttagtgttgacataatatttttattttaagtaataagggacttgaacc
cctaaccatttcggtgtaaacaaaacgctctacctattgagctaattactttacgtctgg
aaagatttgaactttcaacttttagattcgtaatctaacgctctatccagttaagctaca
gacgtttttctccacttgtatatttagattacttatctatattttggaaatagggacaaa
tattcttgtataatttacttcaggattattcgaaagtatagttaaccaattaaacaagta

gaaaacgcatgttttgtattgttactaacttaaaggactattttataataaactttttat
tgtattatataccaatatttagattgaaaatcgacccaaattcaatgtacttttagcaaa
tgacaccagtgttttagaaaaggtg-ttattttgaaaacatttttggtgtgatttgtcct
ttttcagctagtaataatttaattaataacagaaagcgcggtttattgctcgtcgcgttc
aagttattactacggcaacttcctatgcagctgttgggacccccccaactcgtgtaatta
cacttttagttttatttaaattataataaggttatactttatatcttttcttttattta
acccctaagatctttattacgccccaaaatggtgtcataaaaaaa-tagcctggagggct
gccaggagt-aaaaaggcccataacggttatttcagcttttatagtgctatttttctttt
ttagagaaggctatttttaattgataactaagtcattttaagtgataattaagctatttt
aattaaaaatgacttattaaaggttttacacagataatacttcttttattaaaaatatac
tacgccgctagaccggaaaaaaccctccatagaacgatgagaggtaaaaagtgtcataaa
ttttaactattttgtaattgtacattttaaaattaaagtttgtcaaacatcaaaactttc
tgttatttcaataatgcgtgtttttaaggcttttaaagcagtttctcgctgatcccgaac
actaaacttttgttaacatgttctcgtaaactgtaattggatgtcataataaccaatgg
attatccggttttaaagtaaaattacctttcattgcaaggcgcatgctatcaccagctaa
aaactggtttaagaattcgatttgtttaccgataaggtgaaattcgtctcatagaataaa
ttgatacaaatgattttcataataatcttatcagttgtcttggggtcacatataacaagc
ggtagtagttttcaagtaatttaacagcgcactttttcccatattaggttctttacttca
tatatagagttggggtttttataatagcgtgaaaaaggactagtattattgtttagtat
gtcgaatatttgttctcataactggatagaacattccgataagttatttttattatttg
ccttaggattgttttgcaaaatcgttctatgttgggcttttatttttttgttttatgtct
aaagaaagttgatgctgtttaacgtaacgatcaaattttatcctgtcattaaacaaagca
cgtttaatatctggttcatcaaaattaaaaaatattggtgtagtaacaccagattcgatt
tatttcatgatgaagtgctctcttacaagccgctttttagtagattactaaaaattta
ctgtgttgatttgatacgcattttggatgggctcttgcttatttattggatctgaaaag
gagcttggagaattagttaagtagtcaaaatgattcagatttgtgaaattacgtttctta
gaaaattcaatatacgtatataaaatttgatccgagttatttcagctactagaaaagctt
attattattgttttttctctttgtaggagtttgttataaattaaaactaggcaaagaagc
tacattttttgttgtaacttggaaagatttggccgcaacgcgctttttatgtttagttac
ggctggatttagttcaaatactttggacatgagtcgttttaaaaattatttagaaatatg
tgttcgcaaacagattgagtgaaaaaaa--agttttaaaaaagcttcataaactttcacc
aaaagtttgatttatatcataaacggagaaaatccaaattgaaaaaactaaaatggtgtg
gcctaaaaaaatacggcgaagtacatatcaacttggttttcagtgaattaaacagaaata
cagtattcccacaagtacgataggaattagaatgatggataaatcaaaattttgatttag
atacgaaaaccatg-------------gtttacaagtaaactttttaattcaatattttg
tgctattagtaaagatttgtgcgactttaaattgaataagctgttaaaataaggtgaatt

taatttaggcaataaagtatcatacaaatgtttgttctttctatatttgtttagagcttt
cctgtacttttttattgtaagcagcaatagatcctatgtgcttgtgttgaaaatctaatct
tttaagtactttgttaaagctcaaaattttagtttttaattggttaacag-ttttttacg
ctttctagcatcgtatagtctttcttttagcgtttctagatgagaaggcttttctagtgg
cagttgattgaaataacttttagtgag-ttttttactatagtacctgtaacaagtgtgct
aacagatactatgacacaaactacttctaagtccaatttgctctacaatgcg-tttgatg
aaagtcattttatataaattaatttattaatgtgtaattgtggtatgattttattttgtg
ttaatggtcataattaaatgcatacattatatattatccctccttcggagggata-atat
aataattcttattatataccaatatttagattgaaaatcgtgctattttcaatctaaata
ttggtatataaacaaacctgcacataaataaatatcacaaggacat----ccaccaaaaa
atcgatattctctagagagcccctataagctttaatttactatctcttgcatgaaattat
accttcttggataggaatcatcgtgaaattgaaaaacattta--gatatgtcttatacaa
ttatgaccaaaaaatctacaaaacaaacaactttacacaaatattttcgcaagtcgcata
catgagattgaacttaagaagcctctttttacaaatcatacagcatttttggcgcttctt
aattctattgcaatattcaatgaaccaaaaacaaagttttttttaaacgacctgcatgaa
cacaatcttaacaagtgtaaaaaagccctctagc-cccttttttgcaacaccacgagaat
aaaccccggtcggggagaccccgaacccccctcggcgcgggccgcctagcaaagttagac-
-ggcccgcgcgcgttatagattcctatatcgctccattccattccatttcgctattgctg
cgcttcgcttatttttaattctaactgttccttttcgttataacgattttaggtcgattt
tcaatctaaatatttgtatataaacaattatttatcttgataattatatatctaaaaagg
ggggcatagtttaataggtataatgtatgctttgcaagcataagattaccggttcaaatc
cggttgtctccaaatctaaaaatatcaatgcgttctagaatattacaatacaaaaataac
ttggctgactatgatcttttaactcaattttcgattaaaaatcataatagtatgcctact
ttctcttctcttaatgtcagagtaaaaaatattcaaactagcgacattaagcaggtttgc
ttaaatctaatctctattcaattagtaggaaacaatcatgttgttttcagtagccaaaaa
gacgtttctaatcttatactaaaatgtaacggtattaatacttattttattttagagaat
ttatctttgttaggctacaaaaacaatttaaaaagtgacaaaaacaataaattaactgtg
cacggtaaaaacttcttaaatattattgaatattttgcatccaattcaaattttcttaag
tccttagaaaaaacatctaataaacatttaagtattgctttttcttatcaacttgctaat
agtaaaaataatgaaatagctttgtatttttttaaaatcattaggatttccgctttaataa
cggtatagttcaattggttagaacaacggaatcataatccgtaagttgtgggttcaagtc
cctctaccgttatggctcttatcgtctaatggttaggacagtgctttttcagggcatcga
cgtgagttcaatcctcactaagagtaacttttttaatataaaaatccttatacaaatacgt
gagcccataagaattcaaaagtaaaaaggatttttctagatctaccaaattattttggga
tccctaggtcgttaaaaatatttaaacaatgtattttattc--atatatatatatactaa
aacttaaaaatataatttagtgcaaaagtctctaaataactgaattttccgttgaatata

ctcgaccaatcataaagatataggtactttatatttaatttttggcgctttctccggtat
cttaggtgcttgcgcgtctatattgatccgaatggaactagcacaaccaggtaatcaact
attattaggcaatcatcaagtgtataacgtactagttacagagcacgcattttttgatgat
tttctttatggttatgcccgtcctaattggaggatttggaaactgattcgtacctattat
gataggtgctccagatatggcctttcctagattaaataatataagtttttgactactacc
tccatcattgtgtcttcttttaggatctgcgatggtagaagtaggcgctggcacaggctg
aactttatatccgcctttgagctctattcagagccattcaggcggtgctgttgatcttgc
catttttagtttacacttgtcaggtgcttcttctatattaggagctattaatttcattac
gacgatatttaatatgcgcaatccaggacaaagtatgtatcgaataccgctatttgtttg
atctatcctcattactgcgtttcttttactactagcagtacctgtcttggcaggggccat
cacaatgctgttaacagatagaaactttaatacaacatttttttgacccttcaggtggtgg
cgatcctgtattgtatcagcatttattctgatttttcggacatccggaagtgtacatttg
tgcgccgtttaattctgttaaataagatggtttattattttaaattacttatctcgaaat
ctttaaactgaatagttaataaagatagacttaattattccttttgtcttaaaacacttt
cccttaataaagttggaaacaattctattaaaagcgggaatgaaagtctcttgcaggaac
ctgaatttcctaaaaatagtaggctagttatgctcatcatgaataagataataaagttga
tacatatcgacggttccacactaaactttatacataacatttcaggtaacagtatttatg
taatatgggtcggtaccagaatgtttaccttcccttgaataattattacacgtcagagcc
atcagctcctaaatgatatgcaacgttctaaaatgagcttgatcgagaaacagaagaact
cgggataccctaccaatcgaaagattcatgagtacggaactctcgtagtaggtggtagaa
gaattcaatcttcttcaaactaccaaaggggagtagaacttagattcttaagtgaaaaac
cctgcattagctcgcaagagtgcgctaggttagtagatttgagaaaagttaattctgaaa
ataaatttcaagttaataagaatactattcatattatatctgatatgaatgtccttattt
tagcatatgaactcataaaaagtaatcctggaaacatgacacctcgtgtgaatggttcca
cattagatgggttagacaagatgtgactgcaaaatattagtaccaaaataaagcaaggta
aatttttattcagccctgggcgtaagaagtacattcctaagcccggttcagcggataaaa
gaccattaggtattgctagcccgaaagaaaaaattgttcaaaaagctattctgctagtac
tagaatcgatttttgaaccaagcttcttgagaaattctcacgggtttcggcctaaccgag
gcaaccataccgctttaaagatggtaaaaagcgagtttcacggagttccctgaattatag
aaggagatatttcgaagtgctttgatgaaattgatcactctattttattggggcttctaa
gcaagaggatatcttgtgataagactttaactttaattaaaagagggttgaaagctgggt
ttatagatttaggaatattcacaagaactaaattgggcacccctcaaggaagcattctga
gtcctatcctatgcaatatctatttgcatgagctagatttatttctacttcaactaaaaa
ttaaattcgatacagggactagtagagcgaagaacccacagttcagaaaactacagtata
aactatctaaccttaaaacgcctctcgagaaaaagcttgtcagaagagacctttgaaaag
tgcatagtctgaaccccctagatcctaacttttgcagaattcactttgttcgatatgcgg

atgattttattgtgggagttacaagctcccatgaagttgctttagaagttaagaatatga
ttaaggaattcctttgtaatcatttgaaattaaatttgaatgagctgaagacacaaatta
ctcatattagagaaaaggatatatttttcctaggtacccttatcaaaggtaactgaaaga
aagagaaacctattcgattgatcaactttccctctagagaaacgtccatcaagacaagag
tcactccgcgtttaagtttgcatgcccctattaaaaaactctttgataaagctactgctg
aaggatttttccgtagggatgggattaattataaacctacctttgtaggtaaattgatta
atatggaccatgcagacattttatttttttataattcaatagtaagaggagtactaaatt
actactcatttgtggacaaccacaaaagtttaggatcatgagtccattatatgaaatttt
cctgtgctagaactctagcgttaaagtacaagttacgtttcacatcgaagacttttaaga
aattcggctctaaattggcgtgcccgaatacaaaaaaaagcctgtttttaccaacgagct
ttaagaggacgcaggccttccaaattaatagccctattccttttgagaaaaaagtactct
cttgatctaaaaaaattactaaatctaatctgaacaaagtttgtttaatatgtggtagtt
ctctatttgtagaaatgcatcatatacgtagtatagctggcattagaacaagactttgta
gcaataaagcagactttttctctctgcaaatggctgggataaacaggaagcaggttcctc
tttgtcgagaacatcacttgaagctacataataaaacactttcaccagaagaaacgttat
tatttcagaaaaatattaaggaaatttagatgtatttctttcaattttaactttatttac
aatttaagttcggcgagccgtatgataagaaattatcacgtacggttctgagggcagtta
ttgacgctaaaacaacctcgttgtcaagtttctatttctgacccctactaatcttgcctg
gatttggcatcgtcagtcacatcgtttctaccttttctagaaaacctgttttcggttata
taggaatgatttatgctatgctttctataggtattttaggatttatcgtttgagcgcatc
acatgtatactgttggccttgatgtagatacaagagcttacttcacagccgcaaccatga
tcattgctgttcctacaggaataaaatatt------------------------------

-------tagctggattgctactatgtgagaagggtctatttttttaaaaacccctatgt
tatttgctatagggtttatatttttattcactataggaggacttactggtattatactag
ctaactccggacttgatatatctttacatgatacttattatgtcgtagctcacttccact
atggtgcgccgtctaattgcgtttatcgtcgcttacttcagtgagcatgtattaccattc
ttgaattatataattaaatgttatacaatttatacattaaaacaatcaacttacttattt
ctggccaacagaaagaggactacagcatgttgataaaggagttttgaaatagtaaactcc
gagttatactacacacggttaggctaacgaactccttctacaatcagccaggcggatatg
aagttccgatcattagaaataatgaagtatctcacggtgaaagcctttacatagttagac
ctttacaggaatggtctggtacccaaaatttaagtaaaatttttaaagctgaggagaacc
taaaggtagttgttaatggtaagcgtaagaattcgggaaatcctgaaagttgaaaaactg
gaggattcggagggatcgtagtacgaaatataagctgcttagcttatgttaggaagggtc
ctagttcagaagctattctaaattctaagctctcgggttatgaatctatagaagtaggct

taaataatattgacaaacaagtcttagaatatattaaaaccggcaaaagaattgaaggat
taagtagtttacttcggaatccaaattttcttattgcaagttattcaaaaatcaagtcta
ataaaggagctctcactcctggactgagtaatgaaacattggacggcataaaattagaat
gatttgaaaaagctgctgaaagtatagttaatgggtcctatcattttgaacccgttaggc
gaaagtttatacctaaaccgaaaggtgatgaaagacctcttggtatacccaatcccagag
ataaaatcattcaagaaggcatgaggcaactgctggagttagtttacgaaagaattttg
tagattcctctcatgggtttagacccaacaaaagttgtcacagtgctcttaatcaggtaa
aaatgactatggggtattcctcttgatttattgaggggatatatcaaaatattttgaca
ctgtgaatcatacttatttagtttcaaaaatagcgaaagttattaaagatcaagccttca
ttgatttaatatataaagttttgaaagcaggatacggcttttccaaaagaaatgtcgtaa
gcactagtagaggctttccacaaggtggagttattagtcctatactagccaatatttact
tacatgattttgatttaaaaatattggaaatgtcggaaaactttaatagaggtgtccgtc
gaaaagcaaatcctgaatatactaagatggttagagatggaaaagtagatagaaaaaatt
ttatttatcccgctatgggaaatgatgcttattttaaaagaatgaaatatgtaaggtatg
cggatgattttcttatcggaatcattggttcgaaagcagactgtgaaggaattagaacta
gcatagcgagtattcttaaagaagagttcttacttgaacttaatattgagaaaacaaaaa
tcacgcatgctaaacatgattgcgctttttttcttagggcacaatattcatatatcaatgc
ctcccaaagataaaatacaatatcttccaaaaagaggaaacaagcttgtaagaactacaa
gtcgacctttattggatgctcctattggtaagatagtgttgaaactaggttcggtagggt
attgcaaaactgatggatctcctaggagattcggaaaacttctacatgaaccaatggcgg
aaataatttataggtataaaatactgcaaagtggattattaaattattattctatggcta
ataactatggtcgtctatctgcaagaatacattgaaccttaaaatattcttgtgctctaa
ctatagcttcaaaaatgaaattggggactcttaaaaaagtgtttaaacattatggagcta
atcttgaaataaaaaacgaaaaaggggagattatccaatgctttcctaaaatatcgtatt
ccagacctaagaaccctatcaaaacgaaaatatttgatcctattgatcatatagagaaat
cttccaatcatttcaaaagaagtttagcaacttttgagcaagcttgtatgttatgtggaa
agcaagatactactgaaatgcatcacattaataaactgaagaataactcttccacggatt
ggttaacttctcgaatggtcaaaatgaataggaagcaaatacccgtttgtcgaaattgcc
accaacttattcacaagggtaagtatgatggttcaaaaattatttaagtgactcttttga
agtgaaagccatatgcgctgaaaagcgcacgtatggtttggagagaggattttgatactt
aaaaagattagtctcctactctacttctgtctatgggagctgttttcgcaatatttgcag
gcttttattattggtttgaaaagatatccggatttcaatattctgaaatactaggtcaaa
ttcacttttgaggcacttttataggtgtaaatctaacctttttccctatgcactttttag
ggcttgctggtatgcctagacgtattccggattacccagattcttatgccggttggaacg
caatagcttcttacggttcatatgttgcgttatttagcacgctgtttttcttttatcttg
tatttaacacacttgtaacagcaaaaaagacacctgctagaaataacccatggaactttg

aagattcaaaaatgggctcaactacattagaatgagaaatttcttctcctccagcttacc
atacgttcaatgagattccagttataagagaaacagaaacatctttaaaaataaattaac
ttatgataaaaaaaaatactacaatattatttttagggttagctttgctaacccaactac
tacagagtagaatagttattagtgattctgcagaagactggcaattaggcttccaagatc
ctgcaacacctataatggagggaatcattaaccttcatcatgattttatgtttttttatct
gtgctatctcaatttttgtatcttgaatattagcacgcacgttatggcactatcactgaa
caaaaaatgagtacccttctgccacggttcatggaacagccatcgaaataatttggactg
ttactcctagtatcactttgttggcgattgctgtgccttcttttgctttgctatactcta
tggatgaaataattgcgcctgcaataactattaaaacagtaggtcatcaatgatattgaa
gttacgaatactcggactatacaaatgaagacgataacactataatgtttgaaagttata
tgattccagaagaagatttaactttaggtcagttaagattattagaggtagataacccta
tggtaatacccgtaaatacacacgtacgtctaatcataacagcagcagatgttttgcaca
gctgagcagtgccttctttaggtataaaatgtgacgctgtaccaggtagattaaaccaaa
gctcgctctttgtaaaacgtgaaggaatattttatggtcaatgtagcgagatttgtggtg
taaatcatggttttatgcctatcgtagttgaagcagtgtctttaccaaattatatttctt
gagtagctaataaacttagcgaataaatagcctaaaaatattctaagcatctgatatgcg
tgtatctctagcccaactatttttctttggtttatctatttttattttattttgatttaa
tcctaaatacttaacttatacaatacgactattaaaaaaaattaaaaagcgttctaaata
ataaagagcgctttaataaaaaacttattatggcaactacgacaaatctaaactttatca
aaacagctaaacaattacaacgccacccttttcatttagttgaccccagcccttggcctg
taacagctgcaatagccgctttttcatgtgctttaggcggagttatgtatatgcatgcat
acagtaatggagggtacctattatttagtggtttttctttactgttatttacaatgttct
catgatggcgcgatgttacaagagaagccacttttttcagggcatcatacaggtgctgttc
aaaaaggattgcgttatggtgtaattttatttatagtttcagaaatcctcttctttttttg
ctttttttgagcatttttcatagtagtctttcaccggctattgacataggttctatgt
gaccaccaaaaggaatagttgtgtttagcccttgagaagttcctttttttaaatacaataa
tattattattatctggttgttctgttacatgagcacatcatagcattgtagcaggctata
aaaagcaagcaacgttagctttaataacgacagttatcttagccgctatttttacaggtt
tccaaggttttgaatatagcgtggctaattttacattatccgacggtgtttacggcgcca
cattttatatggctacaggctttcatggttttcatgtctttataggtactattttccttg
gtatttgcttacttcgcttattaaaatcacatttgacacaacagcatcattttggttttg
aagcagcagcttgatattgacattttgttgatgttgtatggctttttttatttatttcta
tctactgatgaggtggtacctaatctaaatctctttaatatccaaaaatctatgatgaaa
cttattaatttacctaccataaagctcacttttttgacatgctctagctttcttaattatt
atctgttatcataatatctatattttttacagaagaaagtatacttttattctgttttatt
gcgtgactaaacattacatgaaattatatatctcctcaaattaatgcgtcattatctgaa

agaggagaaaaaatcaattcaaattttcaacatattactaacgataatataataacttga
aaaaaatatagacaaggttattcactaaaaataacacatgttgatattttaaagaactta
atcacatatttaacatgtttaattaagactgtactccttttagaatcaaaaagtagctca
ttacaatctattgcaccttatttaaaaagattacatattgtaaaagacttagaaacaaag
ctgactaaaatttcttatattacgatttgccaacgtatacaggatacagccacaatacgt
agtttttatgctaaccgagtaaaaatcaaatctttccattctgaatctaaattagactta
ttcgaacgtataagaaaattagaagctggttcttagaagtttaagtctatagctcaatgg
ttagagcatacgcttgataagcgtaaggttgattgttcgaatcaatttagacttatacta
tttcaaaaccataatataaaatatgtacaatataaaccaaacatttttttcagcaagccc
tttagaacaatttgaaattatacctttaattcctttagaattatttgggttaaacatgtc
gttaacaaacgcgtccatttttttgatactatctgttgcgttatctatttttttgatccac
tttagtcatatacaaaaataaattagttcctggaaactgacaatctgtaaaagaaatatt
ttatgataccaccttaacgttggtaaaagataatttaggtaaaaaaggttatcgatattt
cccgtttattttttaccctttttcacaataatactttattgtaatttaataggtatggtacc
atatagttttactgtaacaagtcatatagctttcacatttggcttagctttagctattta
cataggaattaatattattggcttcagaacccacggtataaagtttttcacaattttttt
acctaaaggagttcctttatttattgtacctttagtggttgcaatagaattcgtatctta
cgtcgtaaaagttttcacaatatcgataagactttttgcaaatatgacatccgggcatac
tttacttaaaattattgccggatttgtttggacaatgatctcaataggaggcgtgtttgt
atacttacaaataatcccattagttttattactagcgttagtgggtttagaaattggtat
cgctcttttacaagcttacgttttcacattacttacctgcatttacttaaatgatgtttt
agaaatgcactaactaaaaaattatgccacaattagatcgcgttattatttttggtcaaa
tattttgactatttttcaccttttttaattgcttatgttgtttatacccatttcatattaa
gtaatttattaaaaattttcttagtccgctgatggaagcttagaaaagatattactcaaa
ttgcattaaagatccgtttaacgagctatttaattgattcaaatattcaaacgttacgta
gaatttattcaacaatcagaaatatactagcttctctaacaaaaagtttattaacaaaaa
gtataagtaagccaaagttagttttaaatgatcttaattctttagttattaaaattagtc
tggaaacgtctttatatggtagcaaaagcatcaccaagtctggaacatattcttattgaa
cttaataaaatatactatgtatttattaataatagctctgcctttagtgggaacattagt
tacagggttaggcggtagatgaatagggcgtaaaggttcaaatttgttttctacaacttg
cgtagtcctgtgtgtctttttttcttcaatagctttttttcgaagtaggtctttgtggagt
tccttgttatatatctttgagcccttgaattagttcaggggcactaaatatttcatgagg
tttttttatttgatagtttaacaacaacaatgcttgttgttattacatctatttctagttt
agtccatttgtattctattcaatacatggagcacgaccctcattgccctcggtttatgtc
tttcttggagattttcacatttttttatgatcttattagtaacggctgacaattttgtgca
aatgtttttaggctgagaaggagttggattagcttcttatctattaataaattttttgata

cactcgactttgtgcaaatcaagctgcaatcaaagctctggtagtaaatagagtaggtga
ctttggattaagtttaggtattttcacaatttttatctttttggttctgttgattatga
aatagtattctcttccgcaaacatctacacaaattatagtatttccttttgtgggttttc
cataaataccttgactttaataggtatttttttattaatagggctgttggaaagtctgc
acaattaggtctgcatacctggctaccagacgctatggaaggtcctactcctgtttctgc
actcattcatgcggctacaatggtaacagcgggtgtatttttaatagtgcgctgttcacc
tcttattgatttatcctcggatgtcttacttttaattactcttcttggatcaagtacagc
ttttttcgcctctattgttggagtatttcaaaacgatataaagcgggtaattgcttattc
tacttgtagtcaattaggctacatggtctttgtgtgtggtttatcctattataatgtagg
tatgttccatttagtaaatcatgctttttttaaagcattactttttctaagcgctggctc
tgtaatacatgcgctatcaaatgaacaggacatgcgccgaatgggttcgctagcaaatag
cctaccgatcacatatgctgctatgctaattggctctttatccttagcaggattcccttt
tttaacaggtttttattctaaagacttaatcatcgaaataacacaaataagttattacag
taatttacagatttcttttggcgtttatgcttgttgacttgctaatatttctgtactctt
cacatcgttttatacatttaggcttatttttctaacttttataaaaaataccaatagcta
tagaaaacacatagaaaatatacacgaatcgccacctttaattctaattcctttaatatt
actcgctatatctagtatttttgtcggtttcttaacaaaagatatattcgtaggaattgg
aactcctttttgaggtaatgctatcaatattctacctacgtcttgtaatctattggaagt
tgaatttatgccttctttaataaaatgacttccgtttgtgttaagttctatgggtgcaat
tctcgcttatacaataaacgtaggtgtactaaaaaataatatacaatttgctcataatca
cttatttagaaaactcgccttttcccttagcaaaaagttatattgagataaattatacaa
ttcattcattgtatctcctttaatgtactttggttataatatttcattcaaaaatcttga
taggggttttatagaattcgtaggtccttatggaatttcgcgtactattaaaaattgatc
cacaaaagtaattaaaatacaaactggtcagctaacccattatacctttttcgtgatttt
tggtttatgttcccttttactactagttcctgtttgagattttctacaatttttagttga
tgtcagattactagtattttgctttatagccctctttgtagcgtagtttacgaaagtttt
aacacttaaatatatgcagataactaatttattattatggacttcacttattcctttgtg
tggcgctatattacttatttttattcctagattttactctcatttaataagaaatattgc
tttcgcaacagcgcagctagcgtttatatactctattttgctatggctttgctttgaatc
aacaacatccttattccaatttatatatacgataaattgatttccctcctataatattta
ttacacaataggtgtagacggtatatctttatttttttatcatacttacaacgtgattaat
tacagtttgtacattaataagttgaaatatgccagacagccaaataaaagaatacttaat
ttgttttcttttgcttgaagctattttaattcaagtttttttgtgttttagatgtcctatt
cttttatatatttttttgaaagtgtccttatccctatgtttttaattataggtgtatgagg
gtcacgggaaagaaaaaattagagctgcgtatcaatttttcatttacacattagctggttc
actgctaatgcttctagcaattttaactatttatttccagcatggtaccacggatatcca

agttttatgaaatataaattttgacgttagaacacaaattttactttggctagctttttt
cgctagtttagcagtaaaaattcccatgattccttttcatatatgattgcctgaagccca
tgcagaagcacctacagcagggtccgtaattttagcaggtgtgcttttaaaaatgggcgg
gtatggattttttacgtttttctttacctctgtttccggaagcctcactttattttgctcc
attaatttatttactaagtattatagctgctatatatgcttcacttactacaattagaca
agttgacttgaaaaaaataatagcttactcttccgtttcgcatatgggctttgtcacatt
aggtcttttctcttttaactctcaagggatagaaggtagtataatcttgatgcttagcca
cggattagtctctagtgcactttttttgtgtgtaggtattttatacgataggcataaaac
gcgtcttctcaaatactacggtggtctcgtgcaagttatgcctattttcagcatattact
attatttttttactttctctaatatcggttttcctggtacaagcagttttgttggtgaact
attagtgttaatgggagtatttcaatttagtccaatatctacttttctaagtgcattcag
catgattcttggggcagggtattctatttgactattcaatagagtatgttttggtagttt
aaaacttcaatacattacaaattttcaagatatttcaagaagagaattttgtatcctttt
tccgttaagtgtatttgtactctgaatgggtatatatccagaaattttcctatctgaaat
tcactgttcaagttataacctaattgcatattttaactaattttatgttatgaagtttat
taaaacgctaattctagcatttatgaaaaagaagtcctatttttattggtttccacgttt
tttagggctattacttatacctgggtttttatttgataccgagattctagttctctttca
aagccttatcctcttacatgcaagcctaggtttagaagtaatcatagaggactatttaca
cctagaaataataaaacttcagtgtttgtctttaattaaagtacttttaatattattagt
caatcttaatatattatatttattataaaaaatatccttatgttatttatctcctcttat
gatttctacgcattattgacagaaatttactttttaaacgcaatttgtgctttattaatt
tatggtgtaattttaaatacctcatatagaagagggcatccagttattgaacacaatgta
agtggtctctcaactcaaatactaatagtgagtctttggttaacagtttgttcaaatata
ccttgcctaaccagctggaattcacttttagtgcacgatttttttatctttcggtataaaa
agcaccatattagcaatttcgctactttggtctttaattatttttttcttacaatagacta
gaaaaaataaatctctacgagtattgaatcgtgtctatgttggctattgttgccatgctt
tttgtaagttgttcttatgatcttttggcaatgtatttagcaattgaatttcaaagcatt
gcattttatatattagctagttttaaaagaacatctgaattttcaacagaagcgggttta
aaatatttcgtactgggtgcattttcttcagctttgcttcttttaggtatttcactactt
tatggtactactggtttaactaattttggagatctatcaaaattttttttaggtaccaca
ttggaaaacgcatcatttatcaacataacatttttttggtgtcgttttaatagaagtagct
ctttttttttaagataagtgcagcaccttttcatatgtgatcgccagatgtttatgaaggt
gctcctactaacgttacatcttttttttggtatactgccaaaattagcattagtaagttta
atatttagattcttttatttttgttgtgctgaagttgtgctgttactaaattttacactt
ataatttgtgcgcttttatctatgataatagggacatttggcgctttagcgcaaacaaaa
tgaaaacgtttcattgcgtatagtactataagtcacgtaggatttattgtagctggattt

tcaacgttggaatttaatggtgcatttggtgcgctattttatatcttggtttatacttta
acttctttagccactttttctattgtgctttccttccgatgcttagcatatcctagcaca
taccaattacgctatctaacggatatcgttagtttagtgaagttaaatcctatacttgct
ggtagccttgtagcagttttattttcaatggcaggtatcccgccttttccaggattttt
gctaaagtatttgttttattttcacttttgcaagaacaattaataggattagctataatg
gcaatatttttgagttgtgtttcgtgtttttattatatccgtttgattcaaatgatgtat
tttacacatacaaaaaccatacttatttttatccaatagaaaagactacatcaactata
ttaagtataactatgttattacttgtacttatttttttgaagatagatctgatttctaat
tttgttcattgtatgttgtttttataaaataaccaattaaaatgttttacaatattgcaa
ttaacattatcaaagtgttgaccattatagtgccacttttaatcgctgtagcttatatga
cactggccgaaagaaaagtgatggcagctatgcaacgacgaaaagggcctaatgtggtag
gtatctttggtcttttacaacccctagcagatgggttaaaacttttctcaaaagaaacta
tactaccttctagtgctaatattttatttttttagctgcacctgtgctaacgtttttgc
tagctttattagcatgatgtgtacttcctctagatgaggggaaagtttttttcggacttaa
atataggtgttttgtatatattagcagtatcatctttaggtgtttatggtattataactg
ctgggtgatctagtaattctaagtatgctttttttaggtgctttgagatcagcagcccaaa
tggtatcttatgaagtttccattggtctaattttaattaatattttattatgcgcaggca
cattaaatttaactcaaattgttctggcgcaacaaaatatgtggtatataatacctctgt
ttcccatatttattatgttttatatttctatattagctgaaactaacagagcccctttcg
atttgccagaagcagaagcagaacttgtagctggttacaatgtagaatactctgcgatgg
ggtttgcgttgttttttttaggcgagtatgcaaatatgatacttatgtgtagtttaacaa
ctatttttttttttggtggttgattacccttagtcaatatgcttccttttttattggattc
cacccgtactttgatttggtttaaaaacaactttactttatttggttttatttgagtgc
gtgcagcatttccgcgatatagatatgaccaattaatgcgtttaggatgaaaaatatttt
tacctttatcattagggtgagttcttttagtatccgggatactattttctttcgattgat
taccataacaaatgaacgtactttataacgagtattctgctattctcactttttttgcag
tagctttttttaatctctctaataatattaatactttcgtatatattaaatcctcaacaaa
gtgatcaagaaaaagtcagcgcctatgagtgtgggtttaatccatttgatgacgcgagag
caactttttgatgttcggttctatttagtcgcaatccttttttttaatatttgatttagaag
taagtttcttatttccttggtcactagtacttgggcagctaccttcttttggattttgat
ctatggttgcctttttagccattttgacattagggtttatttatgaatgaaaaaaaggcg
ctttagaatgagaataatcaaataatttactagagattttttaatttgataatataatata
atatgaacgtaactttacaaagtgcaaaaatgataggagctggactagctactattggtt
taacaggggtaggagctggagtaggaattgttttcggatcgctagtaattgcttattcgc
gtaatccttctctaaaaaatgaattgtttggctacactattttaggattcgctttaacag
aagcgattgcattatttgctcttatgatggcttttttaatttatttacttaatttactt

taattaatgggcgtttataataaacgcccaccttaaaaatacattatgacaagtacaact
ctttttgaatcttttcaattatttctttaatatccgcttgtatggtggtaagcctgtca
aatgctgtgtattcagttttatttctaattgtagtattttgtaatactgctagtatttta
ttattactaggggcagaattttatctttttatttttaatcgtatacgtaggcgcaatt
gcagttttatttttatttgtagttatgatgttaaacgttaaaatagatggagtaaaaatt
aattatagcacaatttttttgattggtattttaataagtctgattttacttattcagatt
tgaactgctctacaattagatattgaagcgtatgataatataggcgtaccactatcccaa
aataactttccaacaataatttcttggatccaagaaaatgaattaccttcaaatacagag
agtattggtttaattttgtatacttcgtatagtttagtatttattatgtgcgcatttata
ctacttttagctatgattggttccattgtactaacaatgaatcaacgtagtggagttaaa
acacaacaaatcacacttcagttatatagaaatcaaaataaagtagttcgatttattgat
ctgagaaaaaattaatttgattgcggatatagatgaattggtacatcagtaattttccac
gttaaaggatatgggttcgagtcccattatccgctcaaattaagagagaatagctcaata
ggtagagcaatagttttcaaaactaaaggttaaaagttcaagtctttttctcttgcaaa
agtgagtctgcttgaacaccttattcagttttaattataattaaaactgaataaggtgtt
caagcagactcacttttgcaagagaaaaaagacttgaacttttaatctatataagaataa
caaccacattttagcatacacaagacgacagccttgtatgcgtactaatatatctctgcg
ggctgtgtagaggcaaaaaaagtttgtgtggggtggctgtgtgacatataaattaaaaat
agttgtcaaaaaaagtttagtttggcgtaaaaggattcgaacctttgaaatcatggtatc
aaaaaccattgccttaccacttggctatacgccaaacaaattgaagataaagtgggattc
gaacccacggtggatagataccacgttagctttcaaaactaaagctttaaaccactcagc
catttatcccggttattaatataaattgtattctaaaatttttagagtctgtttatatga
tagtacgggaataggattcgaacctatatttttagatcatgagcctaatgagttaccttt
ttactctatcccgctattttatctaattaattttgtaacttaataaaaacttctccaaac
tacccagtaaaggtaggatgactaaaaaatataaaaaataataaacgctagctatctgac
ctataattatgtaagggtcttctacaggcataccacctattcaacctaatattaaacaac
aagcagccatgcttcaataaaggccacgatatatgggtctaaaacgcgaa----------

--cttctaatttctgtactatgtattcatggtaatagagctaatacaactatagcaaaaa
tcatacatataacgccacctaatttatgtggtatacttcttaagattgcataaaaaggta
agaaatatcactcaggaacaatatgtgctggcgttaccatgggatttgcttcaatgtaat
tatcagggtgacctaaaagattaggcgagaagtatacaaaaaaagaaaagaatataataa
aggccactatccctaacaaatcctttacgatgaaataagggtacataggtaccttatcgc
tgcttgcatcgatacctaaaggatttccagaaccttcttgatgtaaagcggctaaatgca
ctaaagacgctgctgcgataacaaatggtaataaataatgtagactaaagaaacggttta
aagttgcattatcaacagaaaagccacctcaaagccaagcaactatagaatcacctacca
aaggtacagcggatactaaattagtgattacagtagcacctcataagctcatttggcctc
aaggtaatacataacctataaaagcagttattatcattaatagtaaaataattacaccaa
tcactcaaacaaattgtcgaggtgcagcataagaaccataataaagtcccctaaaaatgt
ggatataaactacaataaaaaacattgaagcaccattcgcatgtatatatcgtaaaagtc
aaccaaagttaacatcacgcataatatgctctacactaataaaagctaaatcaacgtgtg
gggtataatgcatagctaggaatattccagtcactatttgtattattaaacacattgcag
aaagaaacccaaaatttcatgcataatgaatattgattggagttggataatctataaggt
gattattaactatattgaaaagaggtttttaattagacgcataaataatgttttatta
tagatggacgaggaatgggacttgaacccatggcctataaagtcacagtttatcgctcta
ccaaaccgagctctcctcgtcatgttaaaaagttaaatttacggggaaaaagggatttga
accctcactcattgatgtgacaaaccaatattttaacctattaaactacttccccatttt
tattaaatacggatagagggatttgaaccctcatgaataatattcatcaaaacctaaacc
tgacatgtctaccatttccatcatatccgcaaaaaaatgttactattagctttaacggat
aaagagggattcgaacccacggtataatatttcatacgatgatttagcaaaccattgcct
taaaccactcagccatttatcctgtgttttggaagctgcccactaccggacttgaaccgg
taacttaaaaagaacagattttaaatctgtcgtgtttacctatttcaccaaatgggcatt
agctattgctaatgctatgttttattgaagctattgcttttccggggttcaatgatttag
gacacgttctactgcaattcataatggtatggcatttaaaaagttttgatttacctccaa
gtaatgctaaacggtcttgagttttgatatctcgactatcagctaatcatctataggctt
gcaataaaattgcaggacctaaatatttgtcatggtttcaccaataacttgggcaactag
cagaacagcaggcacaaagtatgcactcgtaaataccatttaattctgacctatcctttt
cagactgtagatattctgtttttgaaggtgtattatttataagccacggttttatatatt
tatattgtgcgtaaaaattagataaatcaggaactaaatcttttataatgtacatatggg
gtagcggataaattgtaattgtgctagtatttatatttaatggttgcaaacaagctaatg
tattagttccatttatattcattgagcaactaccacaaataccttctctacatgagcgtc
taaaggcgatactcgaatcttgttcgtcttttatttttataagagcatccaataccatag

gtccacaatttttagtatgaataggatgtgtactgaaatgagtaatagttgggtttgatg
gagttcatctatatatacgaaggaattttaagtctaaattattattggatactaattgaa
aagaaatttttttgaataattgcatacttggttttattttgattgctataattatttaag
aacgggctaacccgttcttaaatataagataaaatataatgaacatttcgtatttattta
cttacttttgcaagtattaaataataaaccgatctatctagtaatcttctagaatgttct
ggaaaaagtaagggttatttttgcgcttttaagctgtcagcgcttatataatgttaacgt
ggctactcggctatgcaagaaacaatacaaccgatacactattggttaatatatcttaat
cctctcgtactaaagataaaccctctttttttctttcccacaacagatagggaccgaact
gtctcacgacgttctgaacccagctcacgtatcttattatttggcgaacaaccataccct
tggaacctattgcagctccaggaaaagatgaggttatggtcagggcattcttcacagaat
ttcctgccactccagaaccgtacatgaaagtcgcccttcatacggctcctcgagccatat
tattaacagttgattatttgttaagcaaaatgtttacataacacaaaaaaatgagctcgt
tact---catttttggaatg
acgcgtgtcatggcaatgaccatgcagaagtctaaggttatctcaatgtgaggttccccc
tttctttcttggtatgatatgatctatttctattctatcggagtcgttaaaatatatttt
gcacatatcacatt-tggccctttcatttttaaaagtgtccttaataattgcccgtactt
gttgtttgatgatagtcttttggatcagtaggtaactctaccatcatacgggctactatc
aagtagtactttagtagatttaatcacatttgtttgatcgtgtctattcaattttataat
tttgtcttcctctatcaatccgaatactcagtttgctgtatcgctcttaattcaatgctt
agaaacaccgatcccggaccgatttttcttctgagatcatctttctagaagataaaatgt
acgcatgctacaaaagctgaaagtttttgttgcattacatactgcaaaatatctggtcca
cccaataataacaggtgccagtttactaattaaaactttttgagataagccacttgacat
tttgataatatttttaatactatctagatgcttattaattgatttaagactaggttgatt
cctagatgttcaaccagttgaatttccatggctatccttggcggctttatgtatacccac
tttgtagttgacaaaattaaaacctaagaaatctataccgct--------atttttcttg
ggagaagaataaacgctatatgttattgtagttttctctttagataattccaatcccata
cctgttaaaaacgcttctattttcagtttggcttctaataactctttctcctcgttacat
aatattaaaaaatcatctgcgtatcttacaagatatacttttcttttactcaccgacttt
tccattccatgtaaagcgatgttggccagcaagggtgatataatacctccttgtggagtt
ccagattctgggataatttctgttttagcaccttgaaaatcaactaaaattccagctact
agtcaagcagaaatctgctctttaagtagagaaaaagttttcagcttttcaagcaactta
gagtggtctatgttatcaaaacatcctttgatatctgcatctaaaatgtatttgggtttt
ctttgtaaacattttacaatagcctttctcgcgtctttagcacttctaccgggtcggaac
ccgtaactgttaggttcaaaaattgcttcatactgcggctcgagtgcaaattttacaagg
cattgcttagctcgatctcttatagtaggtatacccagatttcttacagaaccattcgct
tttggtatagttaccctcaaaatcttatccgaatgtcgatcaatctttatgcttttact

```
aactccaatctttcgtctggggttaataaagaaattttatctactccagctgttcgtttc
tccaaattatcttgggtaactcttctgactgctaaaagttttgcattttcatgttttatg
atctgtttctgtataaaaaaaacacttttcatatcacccatcttacttagattaaatatt
tttatttgtaatttatatagtcaaatttctttatgtttttacacaatgctatttca---t
ttcatgattttattaggtttgtt-------tgttatatatcttcaaaattatatttaaat
taatatgactcgtctacacgtctgcatatccataggctttccctgtggcattggcttctt
gtagaatcctaccataattgtgcattaacgcaataacctacgcccaaataatgcgtttaa
aaaataaacaattatttggttcacaattatagttacttcgttccaatactatatacactt
atttagtaagcccctctgttccctctaaaactggttctccgactcaa-acgtcgtaattg
aatcaaaccttatgtttagatttcacgatgtttcatgttaacggggtgggttaacttata
attgtcccccgctatcctcggtttaggaaactttatttcggata----gacaaaatttta
tttgtccataataatcgacattattggcttttctggtcagatttactaa-ataagtattc
tttctgtagttattattctataagtgaataagctacaaatttatgccctgcttataattt
gtacgt-gttattgatacaaatcatagggttcactgtttgtaaagacagctcattgatca
gcatctggagtcgatgcataggcccactagttcttcagaactagcttcctatctatagta
ttagtttaacataggtctcccttt---------attagctatttacgagaagtacatctc
ttctcttactagcaacgaatcgcac-----------------------------------
------------------------------------------------------------
------------------------------------------------------------
------------------------------------------------------------
------------------------------------------------------------
------------------------------------------------------------
------------------------------------------------------------
------------------------------------------------------------
------------------------------------------------------------
------------------------------------------------------------
------------------------------------------------------------
------------------------------------------------------------
------------------------------------------------------------
------------------------------------------------------------
------------------------------------------------------------
------------------------------------------------------------
------------------------------------------------------------
------------------------------------------------------------
------------------------------------------------------------
------------------------------------------------------------
```


--ccgacatcgaggtatcaaac
catggcatcgataagaactctcagccatgataaatctgttatccctagagtttctttttt
ccgataagcgacagtatttccatacattactgccggatcattatgaccgactttcgtccc
ggcttgagctgtaactcttaccgtcaagcagattttcatcattacatattatattagcat
atattgccaactaaaatccacttttgtgcacctccgttacagtttaggaggtcctcgtcc
caagtaaactaccacttttacatatgtccttgttagtaagcaatttttttatactcagagt
agtttttcaatgacgtacaaatgtacttctacttttactgtactgtgtacaaagttatcg
cgacgtaaaattatagtaaagaatcatagggtctttccgtcttgttgcgggatgtctgca
ttttcacagacaatgtaatttcgctgaggctatactagagacagtggggtagtcgtgacg
ccattcatgcaggacggaatttacccgccaaggaatttcgctacctgaggatccttatag
ttaagaccgccgtttacttgggtttataatatatgcgcaaacttatatttttcactttca
agcactgggcaggcgtcaaaccttatacgtcttcttttgaatttgcaaagttttaagatt
acgatagtagatactcgcatattttcccccgtttagaaccgtacgtgaagattaccctt

catacggctcatccattttaacgtcagaattttctaacgcagctctgtcgcgatgacacg
ttttatgaagaagagtcatattttttaatatatttttgtcctccactcactacaggtatga
tatggtgaatttctaattcgtacaatatatctccatattgactatttaaagattccttac
atatggggcacatacccttttgtctttttgccagctttagtcgaatattattcgtttgct
gatttaaacacatattcaaagatcttttattaaaatactcctcaaactcacccaaatagg
gattagcgtcaagttttatcatagtagttcttttaataggagtgtcggatatttgaaaca
aattaattttcttaaatcttttaaattcagtgctagtagatattaatcaatctctattat
ccactttatgataaaatttggatttaagcgctttagcattaagcttcggatgcttaattt
ttatcatgcggtgaatggaatcaaaaacgtatttacttattcgggtataagctaactttg
cagtactagacgagtaataatttgctcagccccttaatattgggtttagttttgagaaga
gcatccacaaaggaagttgcgaatgttctttgacacaaagttttattttagcttttactt
tttgtaagctttcttttgtaggtttatatagaaagatccctttcttttccccgatttctc
gagtagaatccgagaattccctaaagtggaaacctacaaaatcaaatccatcttcaattt
tagtgattttactttttcccatatttaattctaaacctcttatttttagaaatttgttaa
catctagaattaacttttctaaaatatcatagttctgggacactataatgaaatcagcaa
catatctcacaagagttgctttgttattagacgcc-ttttttatagttttatctaaacca
tcaaggcataaattagctataataggagatattacaccaccctgtggaacaccttcttca
gtatcttgataactgccttcattcataacgccagcttttaacatttttgacaatatcttt
ttatccataggaatattttctaatattcattcatggcatatattatcaaagaaacccttg
atatctccttcgaaaattcatctagggctgcctctctgtctacttaaaagaaatcaaata
tattgacaggcatctttagcacttcgataaggtctatatgcaaaactgcatcgatcagac
ataatttcgcttataggttcaagagccatagcaaacaaagtttgcacaactctatctctt
atagtgggaattcccaaaggccttttctttttgctattcttctttggtataaatactcta
cgtaccgcactgaattcataattttaagatcttgaatttgaattaatatttcatcaata
ctaactttctttcctttagaacctttaaaacaattttatcaattccgctggtctcacta
cttttacgtttgtgaatttgctttattgcttttattttagcttcttctaaattcactaat
tcattttgtaatttacttcataaacgtttgtttttattcaaataagctattgctattctc
ctttgtaacttataaattttcgtcattatattagtttttattatttt-ccacaattttta
tttaaaaataaactagctggttttgcatgtaatgcaaataaaataaaagttttcttaaag
gacaatacttttaaaacagaacatacataagtcagcaatctttcgattcatgatataatc
actatttcacttattactagcaaacattcgctttttatgagatcttttacctactgaaca
cttgggttgatcgctctttcctgagatatttctatcaattcattaggcttacccttttcc
ttacacataacattaatctaaacaggtaacattctctataccggcagtattatgtctaat
ctataaaataacaaaaagatattttatactactgcttattcctatagctgtataaacagc
taatgatgatcttcggaattctgttttctcgataccttataacaaatgttcacttacgtt
ttacctatttagataagtctagcttttcttagttacatcaagtaagaaactacattgtca

gcaaggcttcatacctccagatcactctagacgcatgcttgcttagacttatattgatta
gataatatagcacttagtgcatttatgtatacggctttaaaggtcgcacttttttgttaaa
cagtcgctacccctaatttttgaaaccttaaaaggtactcctttttgcgaacgtacggag
taaatttgccgagttccttaagtatagttatctcattcgtctttatttctcaataagtt
cacctgtgtcggttttaggtacggtcaaacttcatgtaagttttcctgaaaaattacttt
ttttagcctccagtttagtgggtgtagctattagaagatatctttgtacacaaatacacg
agtattttgcagacacgtggtaatttttcctatcgaatacagttttcactttttttcttaa
gggccgactaactccagttacttaaaattgactggaaacccttgaacaatagacgaccat
gatttattttcaacatggttagcgctactcatgtcagcattagcactcctgattttagat
atgcaatttaacattaacataaaagactacaggacgttccgctaccattaagcttagttt
agcttaattcgaagcttcgatatataaatttaagtctccttacattttaaataggtagaa
caaataaaaatagcgagctaaaacgctttctccaataggtggctgcttctaagcctactc
tgtttattcgaattattccttatttttttcactaatttataattttgagatcttagctatc
gattagggttgtttcccttttgacgtaagaccttatcgcccaacgactgtctgctgctat
aaataaaaatatagtttggagtttaataaaatttagcaaaatctaaatttaataagtagc
tctacccacattttaaaaaagcaacgtactactttgatagttttcgcggaaaaccagcta
tcaccaagtttgattggactttcacccctaatcttaagtcatccccgtatttttcaacag
acgtgggttcagtcctccagtacttttttaaagcaccttcaacttgcttaagaatagatca
cttggcttcgggtctaatacctgtaactttaagcgccttaatatttttaagcttgctaca
catattaacttactgactcattatgcaaaaggcactttgttgctgtatttgtcagcttca
aataaatataaattaacagattcaaatctttccctcacggtactcgttcactatcgatta
gaaaaggttttagcttagaagatgggactcctattttcatacaaaagtaatcgtactact
ttgtgttactattagtgtcgtaaaggactttataacctactttgggtttagacacttcgc
taagttcgctttcactcaccgttacttacaaattctcgtttgatttttttgctatgttac
taagatgattcaattcacataattatataaatgcatattaaatgcgagttttctaaagag
actcatagttcataggcaggtgcctcgctatgatgtttcgtcgcgcgacgtctttgcttt
tctaccaagatttcctctgataacttttttaaatcttttatatttaagaacgggcgaaaaa
atgttacccttttgtaagagtcattaactctatagtaattgtgctcctaattcgataata
gacaacaagtattgctagtcctatagaagattccgaagccgctactgttaatatagctaa
agcaaatagttggcctactatattgtctaagtatatagaaaaaaatataaagttaaaact
aactgatagaaacattatttctaaagacattataattattataatactttttttggtttaa
aaaaatgcctaaaactccgactaaaaataaaaaaaaagtataattttcacagtatagttg
ggtaatcatttatttttatatttaacatttgagatagcaaattgtattaaatttttccag
ccgcaggttcccctacggctaccttgttacgacttcactttagtcctttcgctaccatgg
acaaaaaataagatttttgtcttcaagtagagtaaattcccatagtgtgacgggcggtgt
gtacaaaacccgagaacatattcaccgcgacagttctgatccgcgattactagcgattac

gacttcatattctcgagttgcagagaataatccgaattaaagatttttaaagattttgc
tccagctcacgcttttgcttcttattgtaaacattactttgtagcacatgaaagtagccc
aattcataagggtcatgcggacttgacgtcattttcccttcctcaaggatattccaagc
agtttataatgtattaatgcattatacaaaagtttcgtccgtttgctggaattaaccaaa
cgcctcacggcacggactgacgacagccatgcaacacctgtgacatcttgtgtcatacga
gaattggtaaggttttgcgcgttgtttcgatttaaaccacatgctccaccgcttgtgcgg
gttcccgtcaattcctttgagttttaatcttgcgaccgtaatccccaggcggagtgttta
atgccttagcttcgcctctggaaaattatccaaaaacaaacactcatagttgagggcgta
gactacaggggtatctaatccctttgatacctacgctttcgtgccttagtgtcagctat
agtccagattattgttttcacttttgaagttcttttgaatatcatcgcattttatcacta
ctttcgaagttccataatcttttcctatgctctagtaaattagtttagtaatctttagta
aacatgaaatttaaaatttttttactacttagtttaccacctacgcaccctttacgccca
gtcaattagaataatacttgctcctcccgttttaccgcggctgctggcacgaaattagcc
ggagctttgattgtaaaatttagtctttgatttttaatctattttacaaagtgatttac
agcctgatagggctttgctctcacatgtggcctggctaggtcaagctttcgctcattgcc
taagattcctcactgctgcctcttaaagagtctggaccttatttctgttccagtgtgact
gatcatcctcaaagaccaattaaggattatgggcttggtaggtcttttaaactaccaact
acctaatcctgcgtagacttttcttaaaccaattattaatttttaaggcatttaccaca
atttaagataaatttctacgtattactcacccgtacgctatctttttcataagttacgaa
aattattaaacttgcatgtgttaggccgaccactagtattcattcggagccaggatcaaa
ctcttttattattatgttatgtttttatgtttgctatctcagaaattaaatttgattgta
taaagttatccagcgagctaagacagaagctgtaccaatttcatatatatatatgttggt
aatcttccttttggtaatgaggataaaatttgctttacaaaaagttgtatactttttttg
tttaaatgcggaacatagtttattttctagctttgttccacgtataagaagtctcctata
tttttttattttattcatacatttgtgtatcaagatgggcagatttgaactaccattccc
ttgcacccaaagcaagtacgttaccattacgctacatcttgatatagtgatattgactct
ttctggataggatttgaacctataaccttgtagttaacagctacctgctctaaccattga
agctaccagaaaaataaaagcgaaaaaagggacttgaacccttataatgaccttggcaag
gtcatgctttgcctattaagctatttccgcacttaaaattgaatataaagttgaataata
ttaacaataagtagaaaaagtaaaagataattgctatctttgtacattttccttgtattt
acatgttttactgaatctcaaaaaaaatggcgtattccattacttatgtggtatagaaat
atagcggacactactcaccggacactagttaaaaaccaaggaaaaaagaggtgatctaca
aaaaaatgtattggcagaatagtaatatataaaaagtatacttgatttagtaaaagaata
gggcctacaattaaaacaaacataataacgccagaaattctatgccaaatagaaaaaata
gaagatctttgcggattgtatatagttaaatgtggagaaattggtctattgatgttctgc
atattatagttttctttttcttgggattttacagccattatacgggcaagttgtaatatc

cattacagagataaatttaagtttagactgttttaatattttaaacacatttttttatt
tttacctcatccttttatccgtacatgtaaagaagtataactgagttctttagtctttat
ctctaagaagtggattatttcggacgaagaaagggacaggcttctttttaccccttttga
ccttgacttgcctgcagttgtccaagatttaattttgcctttacagtctgttaaaagaaa
atgaatattattggaactaaaagataaaaacagtatacccatatgtagtttaattttatg
ttgtagtagtaaagactagggtcagattcgaactgacctaataaagatttgcaatcttac
gcatagccactatgctacctagtcttaaatttagtgaaatcttaagaaagactgtgtttt
aaatgataaagaacttttcacacaagaaagtaattcaagcgattttagctttggtaaatt
ggataactcgaaaagcaactgaccgggccttatataacagcaatgactcactatagctcc
ttttcctttacccatacgggtttctggactaaatttggtaacagcatttttttggtagcat
gtaaaaccacactttgattttacccaaactggactttttttcgatgactttgatctcttt
ctgaattatctttttttatattatttatttgtgtgatatctacttgcttgtacgataaaga
gcgtaatccaaattggcctctttttactagatgacttgtttggtttatatatctattttt
ttttttatgctgtcgtgttggattattttttgtattaaatcttttcatattaaattgtgc
ttaaattgttttatatgccattcaaacttgaagattgcaaatcccttgttttgtatataa
tggcgtttcaaaaaattctatataagtttctagtttttgcagtgatactgcgcctaatcg
gaatactatactacttgacatacgacttcggctaccaccgaaacgcccagcgtaccttat
ttttattcctgtaaattcataaaactgtataccacgagcagtttttaatacaattttgtg
cttttttttattgaaaaatagtatttttattgtattaattatcttccggaatgctaggcc
ttttttaggccagattttataaatccacataaaagtgccccgttttgatgtcaaaatgt
tgcattatattttttcacgtcaagatcgaacattttaaacgtaggtaccgagcaatctga
tttggctaatagaggtacatattgtacattgtattcccatctattgtaatttattttaga
tagcgtatttccttttatgaatgtttttgattcaaaatgcttatttaaaaagtttgtagc
aggtaatactttttttgtatatttgcttttgatttttaaaacctttgccatagcattgact
ttcggtatttcataagtcgttcacacctattcgaaatcctcttggattaactttttgacc
cataatgttttattacttaataacaatatttaaattgaaaatcgaatctaaatagttata
cagaaatacgttttgtaggatttgaacctacactaatcaacttagaaggttgatgtttta
tccaattaaactaaaaacgtttttactttagatgcattgggatttgaacccaaaataagc
agattaaaagtctgttgctgtaccgtttagctatacatctttttaataaagagagtagga
tttgaacctacgatgattttaatcaatagatttacaatctatcgctttcgaccactcagc
catctcttcttcagcggttagatatatgttacaatctgtaataaaataattaattatata
ctattgtttaataacaatatttagattgaaaatcgtacgattttcaatctaaatattgtt
attaaacaat
>SRR9587922
gttttattaattaaaactaccccagtattacacttttaaatatttataaaaaataatgca
aatccctttacgctgttatttaatcgaatttaaatttcgattcgtttactttctacttag

ttttttctctcgttgctagtcgttataaactatcatgaggctgtgtttttttttgaaac
gtatactttttgtttataaacgagggggcattcatcgcgacacatattgctgaactatt
ttcaacttctatgtatattagttttaacctagttttgtgcataaattatccttttgcata
ttatcattgtagccgctttttaattcgagttgatacaaatcacaggtttggttctttaa
aaatatgcaatcattgacatttattacgtttttagctagtttacttttgtgctactttt
aattttgccctacacttacgctttttagacacttgaactgtaacgatagtttatgcatt
taaagtgcagttggaggcaaggatagcaacatatgtttgttgaacactacaaacaatgtg
tttactgtctaacattgtctatctcttctttactaggctaatttgtttttacttagtgga
caatatgattaatttgcatttatttttccggaaaaataaaaaatatactttctttttcgt
atgcttgtcggcttctgtgtgtttaccgcaagaaagttttatacaaattactttcataat
ttctataatagtactatttgagttattctttttggttacatgcttatttttttgcaaaaaa
taatgtctaaaagtggacttgaaccactgacccaaagattttcaatcttttgctctaacc
gctgagctatttagacttaaacttttcttttcccgtatttagagcgaccatttttttcgtt
taacgactgcttgtaaatcgtaaacgcctctcacaatatggtaacgtactccaggtagat
cctttacacgaccacctcttatcagaacaacggaatgttcttgtaaattgtggccttcac
ctcctatatagcctatgatagaacgtccggtactaagacgtattttagctacctttcttt
cggcagaattcggtttttttagggttagtagtatacacttttgtgcaaacaccctttttttt
gaggcgacttattgagagcaggtgtttttgctttggtaattttgtgctttcttggatttt
ttattttttggtttagtgttgacataatatttttattttaagtaataagggacttgaacc
cctaaccatttcggtgtaaacaaaacgctctacctattgagctaattactttacgtctgg
aaagatttgaactttcaacttttagattcgtaatctaacgctctatccaattaagctaca
gacgtttttctccgcttgtatatttagattacttatctatattttggaaatagggacaaa
tattcttgtataatttacttcaggattattcgaaagtatagttaaccaattaaacaagta
gaaaacgcatattttgtattgttactaacttaaagtactattttataataaacttttat
tgtattatataccaatatttagattgaaaatcgacctaaattcaatgtacttttagcaaa
tgacaccagtgttttaaaaaaggtg-ttattttgaaaacatttttggtgtcatttgccct
ttttcagctaataataatttaattaataacagaaagcgcggtttattgctcgttgcgttc
aagttattactacggcaacttcctatgcagctgttggga-cccccccactcgtgtaatta
cactttttagttttatttaaattataataaggttatactttatatcttttcttttattta
acccctaagatctttattacgccccaaaatagtgttataaaaaaagtagcctggagggct
gccaggagtaaaaaaggcctataacggttatttccacttttatagtgctattt-------
---t
aattaaaaatgacttattaaaggttttacacagataatacttcttttattaaaaatatac
tacgccgctagaccggaaaaaacccgccatagaacgatgagaggtaaaaagtgtcataaa
ttttaactattttataattgtacattttaaaattaaagtttgtcaaacagcaaaactttc
tattatttcaataatgcttgtttttaaggcttttaaagcagtttctcgctgatcccgaac

actaaactttttgttaacatgttctcgtaaactgtaattggatgtcataataaccagtgg

attatccggttttaaagtaaaattacctttcattgcaaggcgcatgctatcaccagctaa

aaactggtttaagaattcgatttgtttaccgataaggtgaaattcgtctcatagaataaa

ttgatacaaatgattttcataataatcttatcagttgtcttgggatcacatataacaagc

ggtagtagttttcaagtaatttaacagcgcactttttcccatattaggttctttactgca

tatatggagttgtggtttttataatagcgtaaaaaggactagtattattatttagtat

gtcgaatatttgttctcataactggatagaacattccgataagttattttttattatttttg

tcgtaggattgttttgcaaaatcgttctatgttgggcttttatttttttgttttatgtct

aaagaaagttgatgctgtttaacgtaacgatcaaattttatgctgtcattaaacaaagca

cgtttaatatctggttcatcaaaattaaaaaacattggtgtagtaacaccagattcgatt

tgtttcatgatgaagtgctctcttacaagccgctttttttagtagattactaaaaattta

ctatgttgatttgatacgcattttttggatggactcttgcttatttattggatctgaaaag

gagcttggagaattagttaagtagtcaaaataattcggatttgtgaaattacgtttctta

gaaaattcaatatacgtatataaaatttgatccgagttatttcagctactagaaaagctt

attattgttgtttttttctctttgtaggagtttgttataaattaaaactaggcaaagaagc

tacatttttttgttgtaacttggaaagatttggccgcaacgcgctttttatgtttagttac

ggctggatttagttcaaatactttggacatgagt--------------------------

------------------------------cgttttaaaaaagcttcataaactttcacc

aaaagtttgatttatatcataaacggagaaaatccaaattgaaaaaactaaaatggtgtg

gcctaaaaaaatacggcgaagtacatatcaacttggttttcagtgaattaaacagaaata

cagtattcccacaagtacgataggaattaaaatgatggataaatcaaaattttgatttag

atacgaaaaccatggtttactcggttcgtttacaagtaaactttttaattcaatatttg

tgctattagtaaagatttgtgcgactttaaattgaataagctgttaaaataaggtgaatt

taatttaggcaataaagtatcgtacaaatgtttgttctttctatatttgtttagagcttt

cctgtactttttattgtaagcagcaatagatcctatgtgcttgtgttgaaaatctaatct

tttaagtactttgttaaagctcaaattttggtttttaattggttaacag-ttttttacg

ctttctagcatcgtatagtctttcttta-------------------gcttttctagtgg

cagttgattgaaataacttttagtgagtttttttactatagtacctgtaacaagtgtgct

aacagatactacgacacaaactactttcaagtccaa-ttgctctgcgatgggtttgatg

aaagtcattttatataaattaatttattaatgtgtaattgtggtataattttattctgtg

ttaatgggcataattaaacgcatacattatatattatccctccttcggagggata-atat

aataattcttattatataccaatatttagattgaaaatcgtgctattttcaatctaaata

ttggtatataaacaaacctgcacataaataaatatcactaggacattatcccaccaaaaa

atcgatattctctagagagcccctataagctttaatttactatctcttgcctgaaattat

accttcttggataggaatcgtcgtgaaattgaaaaacattta--gatatatcttatacaa

ttatgaccaaaaaatctacaaaacaaacaactttacacaaatattttcgcaagtcgcata

catgagattgaacttaagaagcctcttttcataaatcatacagcatttttggcgcttctt
aattctattgcaatattcaatgaaccaaaaacaaagtttttttaaaacgacctgcatgaa
cacaatcttaacaagtgtaaaaaagccctctag--ccccttttggcaacaccacgagaat
aaaccccggtcggggagaccccgaaccccctcggcgcgggctgtctagcaaagttatacg
gcccgcgcgtgcgtt-tgttttcctatatcgc-----tccattccatttcgctattgctg
cgcttcgcttatttttaattctaactgttccttttcgttataacgattttaggtcgattt
tcaatctaaatatttgtatataaacaattatttatcttgataattatatatctaaaaaag
ggggcatagtttaataggtataatgtatgctttgcaagcataaggttaccggttcaaatc
cggttgtctccaaatctaaaaacatcaatgcgttctagaatattacaatacaaaaataac
ttggctgactatgatcttttaactcaattttcgattaaaaatcataatagtatgcctact
ttctcttctcttaatgtcagagtaaaaaatattcaaactagcgacattaagcaggtttgc
ttaaatctaatctctattcaattagtaggaaacaatcatgttgttttcagtagccaaaaa
gacgtttctaatcttatactaaaatgtaacggtattaatacttattttattttagagaat
ttatctttgttaggctacaaaaacaatttaaaaagtgacaaaaacaataaattaactgtg
cacggtaaaaacttcttaaatattattgaatattttgcatccaattcaaattttcttaag
tccttagaaaaaacatctaataaacatttaagtattgctttttcttatcaacttgctaat
agtaaaaataatgaaatagctttgtatttttttaaaatcattaggatttccgctttaataa
cggtatagttcaattggttagaacaacggaatcataatccgtaagttgtgggttcaagtc
cctctaccgttatggctcttatcgtctaatggttaggacagtgctttttcagggcatcga
cgtgagttcaatcctcactaagagtaacttttttaatataaaaatccttatacaaatacgt
gagcccataagaattcaaaagtaaaaaggattattctagatctaccaaattattttggga
tccctaggtcgttaaaaatatttaaacaatgtattttattc--atatatatatatactaa
aacttaaaaatataatttagtgcaaaagtctctaaataactgaattttccgttgaatata
ctcgaccaatcataaagatataggtactttatatttaatttttggcgctttctccggtat
cttaggtgcttgcgcgtctatattgatccgaatggaactagcacaaccaggtaatcaact
attattaggcaatcatcaagtgtataacgtactagttacagagcacgcattttttgatgat
tttctttatggttatgcccgtcctaattggaggatttggaaactgattcgtacctattat
gataggtgctccagatatggcctttcctagattaaataatataagtttttgactactacc
tccatcattgtgtcttcttttaggatctgcgatggtagaagtaggcgctggcacaggctg
aactttatatccgcctttgagctctattcagagccattcaggcggtgctgttgatcttgc
tatttttagtttacacttgtcaggtgcttcttctatattaggagctattaatttcattac
gacgatatttaatatgcgcaatccaggacaaagtatgtatcgaataccgctatttgtttg
atctatccttattactgcgttcctttttactactagcagtacctgtcttggcaggggccat
cacaatgctgttaacagatagaaactttaatacaacatttttttgacccttcaggtggtgg
tgatcctgtattgtatcagcatttattctgatttttcggacatccagaagtgtacatttg
tgcgccgtttaattctgttaaataagatgatttattattttaaattacttatctcgaaat

ctttaaactgaatagttaataaagatagacttaattattctttttgtcttaaaacacttt
cccttaataaagttggaaacaattctattaaaagcgggaatgaaagtctcttgcaggaac
ctgaatttcctaaaaatagtaggctagttatgctcatcatgaataagataataaagttga
tacatatcgacggttccacactaaactttatacataatatttcaggtaacagtatttatg
taatatgggtcggtaccaggatgtttaccttcccttgaataattattacacgtcagagct
atcagctcctaaatgatatgcaacgttctaaaatgagcttgatcgagaaacagaagaact
cgggataccttaccaatcgaaagattcatgggtacggaactctcgtagtaggtggtagaa
gaattcaatcttcttcaaactaccaaaggggagtagaacttagattcttaagtgaaaaac
cctgcattagctcgcaagagtgcgctaggttagtagatttgagaaaagttaattctgaaa
ataaatttcaagttaataagaatactattcatattatatctgatatgaatgtccttattt
tagcatataaactcataaaaagtaatcctggaaacatgacacctggtgtgaatggttcca
ccttagatgggttagacaagaggtgactgcagaatattagtaccaaaataaagcaaggta
aatttttattcagccctgggcgtaagaagtacattcttaagcccggttcagcagataaaa
gactattaggtattgctagcccgaaagaaaaaattgttcaaaaagctattctgctagtac
tagaatcgatttttgaaccaagcttcttgagaaattctcacgggtttcggcctaaccggg
gcaaccataccgctttaaagatggtaaaaagtgagtttcacggagttccttgaattatag
aaggagatatttcgaagtgctttgatgaaattgatcactctattttattggggcttctaa
gcaagaggatatcttgtgataagactttaactttaattaaaagagggttgaaggctgggt
ttatagatttaggaatattcacaagaactaaattgggtacccctcaaggaagccttttga
gtcctatcctatgcaatatctatttacatgagctagatttatttctacttcaacttaaaa
ttaaattcgatacagggactagtagagcgaagaacccacagttcagaaaactacagtata
aactatccaactttaaatcgcctctcgataaaaagcttgtcagaagagacctttgaaaag
tgcatagtctgaacccccctagatcctaattttgcagaattcactttgttcggtatgcgg
atgattttattgtgggagttacaagctcccatgaagttgctttagaagttaagaatatga
ttaaggaatttctttgtaatcatttgaaattaaatttgaatgagctgaagacacacatta
ctcatattagagaaaaggatatatttttcctaggtacccttatcaaaggtaactgaaaga
aagagaaacctattcgattgatcaactttccctctagagaaacgttcatcaagacaagag
tcactccgcgtttaagtttgcatgcccctattaaaaaactctttgataaagctactgctg
aaggattttttccgtagggatgggattaattataaacctacttttgtaggtaaattgatta
atatggaccatgcagacattttagctttttataattcaatattaagaggagtactgaatt
actactcatttgtggacaaccacaaaagtttaggatcatgagtccattatatgaaatttt
cctgtgctagaactctagcgttaaagtacaagttacgtttcacatcgaagacttttaaga
aattcggctctaaattggcgtgcccgaatacaaaaaaaaagcctgtttttaccaacgagct
ttaagagaacgcaggccttccaaattaataaccctattccttttgagaaaaaagtactct
cttgatctaaaaaaattactaaatctaatctaaacaaagtttgtttaatatgtggtagtt
ctctattggtagaaatgcatcatatacgtagtatagctggcattagaacaagacttcgta

gcaataaagcagactttttctctctgcaaattgctgggataaacaggaagcaggttcctc

tttgtcgagaacatcacttgaagctacataataaaacactttcaccagaagaaacgttat

tatttcagaaaaatattaaggaaatttagatgtatttctttcaattttaactttatttac

aatttaagttcggcgagccgtatgataagaaattatcacgtacggttctgagggcagtta

ttgacgctaaaacaacctcgttgtcaagtttccatctctgacccctactaatcttgcctg

gatttggcatcgtcagtcacatcgtttccacctttctagaaaacctgttttcggttata

taggaatgatttatgctatgctttctataggtattttaggatttatcgtttgagcgcatc

acatgtatactgttggccttgatgtagatacaagagcttacttcacagctgcaaccatga

tcattgctgttcctacaggaataaaaatatt-----------------------------

-------tagctggattgctactatgtgagaagggtctatttttttaaaaacccctatgt
tatttgctatagggtttatatttttattcactataggaggacttactggtattatactag
ctaactccggacttgatatatctctacatgatacttattatgtcgtagctcacttccact
atg---

------------------------ttctgtctatgggagctgttttcgcaatatttgcag
gcttttattattggtttgaaaagatatccggatttcaatattctgaaatactaggtcaaa
ttcacttttgaggcacttttataggtgtaaatctaacctttttccctatgcactttttag
ggcttgctggtatgcctagacgtattccggattatccagattcttatgcaggttggaacg
caatagcttcttacggttcatatgttgcgttatttagcacgctgtttttcttttatcttg
tatttaacacacttgtaacagcaaaaaagacacctgctagaaataacccatggaactttg
aagattcaaaaatgggctcaactacattagaatgagaaatttcttctcctccagcttacc
atacgttcaatgagattccagttataagagaaacagaaacatctttaaaaataaattaac
ttatgacaaaaaaaaatactacaatattatttttagggttagctttgctaacccaactac
tacagagcagaatagttattagtgattctgcagaagactggcaattaggcttccaagatc
ctgcaacacctataatggagggaatcattaaccttcatcatgattttatgtttttatct
gtgctatctcaatttttgtatcttgaatattagcacgcacgttatggcactatcactgaa
caaaaaatgagtacccttctgccacggttcatggaacagccatcgaaataatttggactg
ttactcctagtatcactttgttggcgattgctgtgccttcttttgctttgctatactcta
tggatgaaataattgcgcctgcaataactattaaaacagtaggtcatcaatgatattgaa
gttacgaatactcggactatacaaatgaagacgataacactataatgtttgaaagttata
tgattccagaagaagatttaactttaggtcagttaagattattagaggtagataaccta
tggtaatacccgtaaatacacacgtacgtctaattataacagcagcagatgttttgcaca
gctgagcagtgccttctttaggtataaaatgtgacgctgtaccaggtagattaaaccaaa

gctcgctctttgtaaaacgtgaaggaatattttatggtcaatgtagcgagatttgtggtg
taaatcatggttttatgcctatcgtagttgaagcagtgtctttaccaaattatatttctt
gagtagctaataaacttagcgaataaatagtctaaaaatattctaagcatctgatatgcg
tgtatctctagcccaactatttttctttggtttatctattttattttattttgatttaa
tcctaaatatttaacttatacaatacgactattaaaaaaaattaaaaagcgttctaaata
ataaagagcgctttaataaaaaacttattatggcaactacgacaaatctaaactttatca
aaacagctaaacaattacaacgccaccctttttcatttagttgaccccagcccttggcctg
taacagctgcaatagccgctttttcatgtgctttaggcggagttatgtatatgcatgcat
acagtaatggagggtacctattatttagtggtttttctttactgttatttacaatgttct
catgatggcgcgatgttacaagagaagccacttttttcagggcatcatacaggtgctgttc
aaaaaggattgcgttatggtgtaattttatttatagtttcagaaatcctcttctttttttg
ctttttttgagcatttttttcatagtagtctttcaccggctattgacataggttctatgt
gaccaccaaaaggaatagttgtgtttagcccttgagaagttcctttttttaaatacaataa
tattattattatctggttgttctgttacatgagcacatcatagcattgtagcaggctata
aaaagcaagcaacgttagctttaataacgacagttatcttagccgctatttttacaggtt
tccaaggttttgaatatagcgtggctaattttacattatccgacggtgtttacggctcca
cattttatatggctacaggctttcatggttttcatgtctttataggtactattttccttg
gtatttgcttacttcgcttattaaaatcacatttgacacaacagcatcattttggttttg
aagcagcagcttgatattgacattttgttgatgttgtatggcttttttttatttatttcta
tctactgatgaggtggtacctaatctaaatctctttaatatctaaaaatctatgatgaaa
cttattaatttacctaccataaagctcacttttttgacatgctctagctttcttcattatt
atctgttatcataatatctatatttttacagaagaaagtatactttttattctgttttatt
gcgtgactaaacattacatgaaattatatatctcctcaaattaatgcgtcattatctgaa
agaggagaaaaaatcaattcaaattttcaacatattactaacgataatataataacttga
aaaaaatatagacaaggttattcactaaaaataacacatgttgatattttaaagaactta
atcacatatttaacatgcttaattaagactgtactccttttagaatcaaaaagtagctca
ttacaatctattgcaccttatttaaaaagattacatattgtaaaagacttagaaacaaag
ctgactaaaatttcttatattacgatttgccaacgtatacaggatacagctacaatacgt
agtttttatgctaaccgagtaaaaatcaaatctttccattctgaatctaaattagactta
ttcgaacgtataagaaaattagaagctggttcttagaagtttaagtctatagctcaatgg
ttagagcatacgcttgataagcgtaaggttgattgttcgaatcaatttagacttatacta
tttcaaaaccataatataaaatatgtacaatataaaccaaacatttttttcagcaagccc
tttagaacaatttgaaattatacctttaattcctttagaattatttgggttaaacatgtc
gttaacaaacgcgtccatttttttgatactatctgttgcgttatctattttttgatccac
tttagtcatatacaaaaataaattagttcctggaaactgacaatctataaaagaaatatt
ttatgataccaccttaacgttggtaaaagataatttaggtaaaaaaggttatagatattt

cccgtttatttttacccttttacaataatactttattgtaatttaataggtatggtacc
atatagttttactgtaacaagtcatatagctttcacatttggcttagctttagctattta
cataggaattaatattattggcttcagaacccacggtataaagtttttcacaattttttt
acctaaaggagttcctttatttattgtacctttagtggttgcaatagaattcgtatctta
cgtcgtaaaagttttcacaatatcgataagactttttgcaaatatgacatccgggcatac
tttacttaaaattattgccggatttgtttggacaatgatctcaataggaggcgtgtttgt
atacttacaaataatcccattagttttattactagcgttagtgggtttagaaattggtat
tgctcttttacaagcttacgttttcacattacttacctgcatttacttaaatgatgtttt
agaaatgcactaactaaaaaattatgccacaattagatcgcgttattatttttggtcaaa
tattttgactatttttcaccttttaattgcttatgttgtttatacccatttcatattaa
gtaatttattaaaaattttcttagtccgctgatggaagcttagaaaagatattactcaaa
ttgcattaaagatccgtttaacgagctatttaattgattcaaatattcaaacgttacgta
gaatttattcaacaatcagaaatatactagcttctctaacaaaaagtttattaacaaaaa
gtataagtaagccaaagttagttttaaatgatcttaattctttagttattaaaattagtc
tggaaacgtctttatatggtagcaaaagcatcaccaagtctggaacatattcttattgaa
cttaataaaatatactatgtatttattaataatagctctgcctttagtgggaacattagt
tacagggttaggcggtagatgaatagggcgtaaaggttcaaatttgttttctacaacttg
cgtagtcctgtgtgtctttttttcttcaatcgctttttttcgaagtaggtctttgtggagt
tccttgttatatatctttgagcccttgaattagttcaggggcactaaatatttcatgagg
ttttttatttgatagtttaacaacaacaatgcttgttgttattacatctatttctagttt
agtccatttgtattctattcaatacatggagcacgaccctcattgccctcggtttatgtc
tttcttggagattttcacatttttatgatcttattagtaacggctgacaattttgtgca
aatgtttttaggctgagaaggagttggattagcttcttatctattaataaattttgata
cactcgactttgtgcaaatcaagctgcaatcaaagctctggtagtaaatagagtaggtga
ctttggattaagtttaggtattttcacaatttttatctttttggttctgttgattatga
aatagtattctcttccgcaaacatctacacaaattatagtatttcctttttgggttttc
cataaataccttgactttaataggtatttttttattaataggggctgttggaaagtctgc
acaattaggtctgcatacctggctaccagacgctatggaaggtcctactcctgtttctgc
actcattcatgcggctacaatggtaacagcgggtgtatttttaatagtgcgctgttcacc
tcttattgatttatcctcggatgtcttacttttaattactcttcttggatcaagtacagc
ttttttcgcctctattgttggagtatttcaaaacgatataaagcgggtaattgcttattc
tacttgtagtcaattaggctacatggtctttgtgtgtggtttatcctattataatgtagg
tatgttccatttagtaaatcatgctttttttaaagcattactttttctaagcgctggctc
tgtaatacatgcgctatcaaatgaacaggacatgcgccgaatgggttcgctagcaaatag
cctaccgatcacatatgctgctatgctaattggctctttatccttagcaggattcccttt
tttaacaggtttttattctaaagacttaatcatcgagataacacaaataagttattacag

taatttacagatttcttttggcgtttatgcttgttgacttgctaatatttctgtactctt
cacatcgttttatacatttaggcttatttttctaacttttataaaaaataccaatagcta
tagaaaacacatagaaaatatacacgaatcgccacctttaattctaattcctttaatatt
actcgctatatctagtatttttgtcggtttcttaacaaaagatatattcgtaggaatcgg
aactccttttgaggtaatgctatcaatattctacctacgtcttgtaatctattggaagt
tgaatttatgccttctttaataaaatgacttccgtttgtgttaagttctatgggtgcaat
tctcgcttatacaataaacgtaggtgtactaaaaaataatatacaatttgctcataatca
cttatttagaaaactcgccttttcccttagcaaaagttatattgagataaattatacaa
ttcactcattgtatctcctttaatgtactttggttataatatttcattcaaaaatcttga
taggggttttatagaactcgtaggtccttatggaatttcgcgtactattaaaaattgatc
cacaaaagtaattaaaatacaaactggccagctaacccattataccttttttcgtgatttt
tggtttatgttcccttttactactagttcctgtttgagattttctacaatttttagttga
tgtcagattactagtattttgctttatagccctctttgtagcgtagtttacgaaagtttt
aacacttaaatatatgcagataactaatttattattatggacttcacttattcctttgtg
tggcgctatattacttatttttattcctagattttactctcatttaataagaaatattgc
tttcgcaacagcgcagctagcgtttatatactctattttgctatggctttgctttgaatc
aacaacatccttattccaatttatatatacgataaattgatttccctcctataatattta
ttacacaataggtgtagacggtatatctttatttttatcatacttacaacgtgattaat
tacagtttgtacattaataagttgaaatatgccagacagccaaataaaagaatacttaat
ttgttttcttttgcttgaagctattttaattcaagtttttgtgttttagatgtcctatt
cttttatatatttttgaaagtgtccttatccctatgtttttaattataggcgtatgagg
gtcacgggaaagaaaaattagagctgcgtatcaatttttcatttacacattagctggttc
actgctaatgcttctagcaattttaactatttatttccagcatggtaccacggatatcca
agttttatgaaatataaattttgacgttagaacacaaattttactttggctagcttttt
cgctagtttagcagtaaaaattcccatgattccttttcatatatgattacctgaagccca
tgcagaagcacctacagcagggtccgtaattttagcaggtgtgcttttaaaaatgggcgg
gtatggatttttacgtttttctttacctctgtttccggaagcctcactttattttgctcc
attaatttatttactaagtattatagctgctatatatgcttcacttactacaattagaca
agttgacttgaaaaaaataatagcttactcttccgtttcgcatatgggctttgtcacatt
aggtcttttctcttttaactctcaagggatagaaggtagtataatcttgatgcttagcca
cggattagtctctagtgcactttttttgtgtgtaggtattttatacgataggcataaaac
gcgtcttctcaaatactacggtggtctcgtgcaagttatgcctattttcagcatattact
attatttttactttctctaatatcggttttcctggtacaagcagttttgttggtgaact
attagtattaatgggagtatttcaatttagtccaatatctacttttctaagtgcattcag
catgattcttggggcagggtattctatttgactattcaatagagtatgttttggtagttt
aaaacttcaatacattacaaattttcaagatatttcaagaagagaattttgtatcctttt

tccgttaagtgtattcgtactctgaatgggtatatatccagaaattttcctatctgaaat
tcactgttcaagttataacctaattgcatattttaactaattttatgttatgaagtttat
taaaacgctaattctagcatttatgaaaaagaagtcctatttttattggtttccacgttt
tttagggctattacttatacctgggttttatttgataccgagattctagttctctttca
aagccttatcctcttacatgcaagcctaggtttagaagtaatcatagaggactatttaca
cctagaaataataaaacttcagtgtttgtctttaattaaagtacttttaatattattagt
caatcttaatatattatatttattataaaaaatatccttatgttatttatctcctcttat
gatttctacgcattattgacagaaatttactttttaaacgcaatttgtgctttattaatt
tatggtgtaattttaaatacctcatatagaagagggcatccagttattgaacacaatgta
agtggtctctcaactcaaatactaatagtgagtctttggttaacagtttgttcaaatata
ccttgcctaaccagctggaattcacttttagtgcacgatttttttatctttcggtataaaa
agcaccatattagcaatttcgctactttggtctttaattatttttttcttacaatagacta
gaaaaaataaatctctacgagtattgaatcgtgtctatgttggctattgttgccatgctt
tttgtaagttgttcttatgatcttttggcaatgtatttagcaattgaattccaaagcatt
gcattttatatattagctagttttaaaagaacatctgaattttcaacagaagcgggttta
aaatatttcgtactgggtgcattttcttcagctttacttcttttaggtatttcactactt
tatggtactactggtttaactaattttggagatctatcaaaatttttttttaggtaccaca
ttggaaaacgcatcatttatcaacataacatttttttggtgtcgttttaatagaagtagct
cttttttttaagataagtgcagcaccttttcatatgtgatcgccagatgtttatgaaggt
gctcctactaacgttacatcttttttttggtatactgccaaaattagcattagtaagttta
atatttagattcttttatttttgttgtgctgaagttgtgctgttactaaattttacactt
ataatttgtgcgcttttatctatgataatagggacatttggcgctttagcgcaaacaaaa
tgaaaacgtttcattgcgtatagtactataagtcacgtaggatttattgtagctggattt
tcaacgttggaatttaatggtgcatttggtgcgctattttatatcttggtttatacttta
acttctttagccactttttctattgtgctttccttccgatgcttagcatatcctagcaca
taccaattacgctatctaacggatatcgttagtttagtgaagttaaaccctatacttgct
ggtagccttgtagcagttttattttcaatggcaggtataccgccttttccaggattttt
gctaaagtatttgttttattttcacttttgcaagaacaattaataggattagctataatg
gcaatatttttgagttgtgtttcgtgtttttattatatccgtttgattcaaatgatgtat
tttacacatacaaaaaccatacttatttttatccaatagaaaagactacatcaactata
ttaagtataactatgttattacttgtacttatttttttgaagatagatctgatttctaat
tttattcattgtatgttgtttttataaaataaccaattaaaatgttttacaatattgcaa
ttaacattatcaaagtgttgaccattatagtgccacttttaatcgctgtagcttatatga
cactggccgaaagaaaagtgatggcagctatgcaacgacgaaaagggcctaatgtggtag
gtatctttggtcttttacaaccccctagcagatgggttaaaacttttctcaaaagaaacta
tactaccttctagtgctaatatttctatttttttagctgcacctgtgctaacgtttttgc

tagctttattagcatgatgtgtacttcctctagatgaggggaaagtttttttcggacttaa
atataggtgttttgtatatattagcagtatcatctttaggtgtttatggtattataactg
ctgggtgatctagtaattctaagtatgctttttttaggtgctttgagatcagcagctcaaa
tggtatcttatgaagtttccattggtctaattttaattaatattttattatgcgcaggca
cattaaatttaactcaaattgttctggcgcaacaaaatatgtggtatataatacctctat
ttcccatatttattatgttttatatttctatattagctgaaactaacagagcccctttcg
atttgccagaagcagaagcagaacttgtagctggttacaatgtagaatactctgcgatgg
ggtttgcgttgtttttttttaggcgagtatgcaaatatgatacttatgtgtagtttaacaa
ctatttttttttttggtggttgattacccttagtcaatatgcttcctttttattggattc
catccgtactttgatttggtttaaaaacaactttacttttatttggttttatttgagtgc
gtgcagcatttccacgatatagatatgaccaattaatgcgtttaggatgaaaaatatttc
tacctttatcattagggtgagttcttttagtatccgggatactattttctttcgattgat
taccataacaaatgaacgtactttataacgagtattctgctattctcacttttttttgcag
tagctttttttaatctctctaataatattaatactttcgtatatattaaatcctcaacaaa
gtgatcaagaaaaagtcagcgcctatgagtgtgggtttaatccatttgatgacgcgagag
caacttttgatgttcggttctatttagtcgcaatcctttttttaatatttgatttagaaa
taagtttcttatttccttggtcactagtacttgggcagctaccttcttttgggttttgat
ctatggttgccttttttagccattttgacattagggtttatttatgaatgaaaaaaaggcg
ctttagaatgagaataatcaaataatttactagagatttttaatttgataatataatata
atatgaacgtaactttacaaagtgcaaaaatgataggagctggactagctactattggtt
taacaggggtaggagctggagtaggaattgttttcggatcgctagtaattgcttattcgc
gtaatccttctctaaaaaatgaattgtttggctacactattttaggattcgctttaacag
aagcgattgcattatttgctcttatgatggcttttttaattttatttacttaatttactt
taattactgggcgtttataataaacgcccaccttaaaaatacattatgacaagtacaact
ctttttgaatcttttcaattatttctttaatatccgcttgtatggtggtaagcctgtca
aatgctgtgtattcagttttgtttctaattgtagtattttgtaatactgctagtatttta
ttattactaggagcagaatttttatctttttttatttttaatcgtatacgtaggcgcaatt
gcagttttatttttatttgtagttatgatgttaaacgttaaaatagatggagtaaaaatt
aattatagcacaatttttttgattggtattttaataagtctgattttacttattcagatt
tgaactgctctacaattagatattgaagcgtatgataatataggcgtaccactatcccaa
aataactttccaacgataatttcttggatccaagaaaatgaattaccttcaaatacagag
agtattggtttaattttgtatacttcgtatagtttagtatttattatgtgcgcatttata
ctacttttagctatgattggttccattgtactaacaatgaatcaacgtagtggagttaaa
acacaacaaatcacacttcagttatatagaaatcaaaataaagtagttcgatttattgat
ctgagaaaaaattaatttgattgcggatatagatgaattggtacatcagtaattttccac
gttaaaggatatgggttcgagtcccattatccgctcaaattaagagagaatagctcaata

```
ggtagagcaatagttttcaaaactaaaggttaaaagttcaagtctttttctcttgcaaa
agtgagtctgcttgaacaccttattcagttttaattataattaaaactgaataaggtgtt
caagcagactcacttttgcaagagaaaaaaggcttgaactttaaattcatataagaataa
caaccacatttagcatacacaagacgacagccttgtatgtgtactaatatatctctgca
ggctgtgtagagtcgaaaaaagtttgtgtggggtggctgtgtgacatataaattaaaaat
agttgtcaaaaaaagtttagtttggcgtaaaaggattcgaacctttgaaatcatggtatc
aaaaaccattgccttaccacttggctatacgccaaacaaattgaagataaagtgggattc
gaacccacggtggatagataccacgttagctttcaaaactaaagctttaaaccactcagc
catttatcccggttattaatataaattgtattctaaaatttttagagtctgtttatatga
tagtacgggaataggattcgaacctatatttttagatcatgagcctaatgagttaccttt
ttactctatcccgctattttatctaattaattttgtaacttaataaaaacttctccaaac
tacccagtaaaggtaggatgactaaaaaatataaaaaataataaacgctagctatctgac
ctataattatgtaagggtcttctacaggcataccacctattcaacctaatattaaacaac
aagcagccatgcttcaataaagaccacgatatatgggtctaaaacgcgaa----------
------------------------------------------------------------
------------------------------------------------------------
------------------------------------------------------------
------------------------------------------------------------
------------------------------------------------------------
------------------------------------------------------------
------------------------------------------------------------
------------------------------------------------------------
------------------------------------------------------------
------------------------------------------------------------
------------------------------------------------------------
--cttctaatttctgtactatgtattcacggtaatagagctaatacaactatagcaaaaa
tcatacatataacgccacctaatttatgtggtatacttcttaagattgcataaaaaggta
agaaatatcactcaggaacaatatgtgctggcgttaccatgggatttgcttcaatgtaat
tatcagggtgacctaaaagattaggcgagaagtatacaaaaaaagaaaagaatataataa
aggccactatccctaacaaatcctttacgatgaaataagggtacataggtaccttatcgc
tgcttgcatcgatacctaaaggatttccagaaccttcttgatgtaaagcggctaaatgca
ctaaagacgctgctgcgataacaaatggtaataaataatgtagactaaagaaacggttta
aagttgcattatcaacagaaaagccacctcaaagccaagcaactatagaatcacctacca
aaggtacagcggatactaaattagtgattacagtagcacctcataagctcatttggcctc
aaggtaatacataacctataaaagcagttattatcattaatagtaaaataattacaccaa
tcactcaaacaaattgtcgaggtgcagcataagaaccataataaagtcccctaaaaatgt
```

ggatataaactacaataaaaaacattgaagcaccattcgcatgtatatatcgtaaaagtc
aaccaaagttaacatcacgcataatatgctctacactaataaaagctaaatcaacgtgtg
gggtataatgcatagctaggaatattccagtcactatttgtattattaaacacattgcag
aaagaaacccaaaatttcatgcataatgaatattaattggagttggataatctataaggt
gattattaactatattgaagagaggtttttaattagacgcataaataatgtttttatta
tagatggacgaggaatgggacttgaacccatggcctataaagtcacagtttatcgctcta
ccaaaccgagctctcctcgtcatgttaaaaagttaaatttacggggaaaaagggatttga
accctcactcattgatgtgacaaaccaatattttaacctattaaactacttccccatttt
tattaaatacggatagagggatttgaaccctcatgaataatattcatcaaaacctaaacc
tgacatgtctaccatttccatcatatccgcaaaaaaatgttactattagctttaacggat
aaagagggattcgaacccacggtataatatttcatacgatgatttagcaaaccattgcct
taaaccactcagccatttatcctgtgttttggaagctgcccactaccggacttgaaccgg
taacttaaaaagaacagattttaaatctgtcgtgtttacctatttcaccaaatgggcatt
agctattgctaatgctatgttttattgaagctattgcttttccggggttcaatgatttag
gacacgttctactgcaattcataatggtatggcatttaaaaagttttgatttacctccaa
gtaatgctaaacggtcttgagttttgatatctcgactatcagctaatcatctataggctt
gcaataaaattgcaggacctaaatatttgtcatggtttcaccaataacttgggcaactag
cagaacagcaggcacaaagtatgcactcgtaaataccatttaattctgacctatcctttt
cagactgtagatattctgtttttgaaggtgtgttatttataagccacggttttatatatt
tatattgtgcgtaaaaattagataaatcaggaactaaatcttttataatgtacatatggg
gtagcggataaattgtaattgtgctagtatttatatttaatggttgcaaacaagctaatg
tattagttccgtttatattcattgagcaactaccacaaataccttctctacacgagcgtc
taaaggcgatactcgaatcttgttcgtcttttatttttataagagcatccaataccatag
gtccacaattttagtatgaataggatgtgtactgaaatgagtaatagttgggtttgatg
gagttcatctatatatacgaaggaattttaagtctaaattattattggatactaattgaa
aagaaatttttttgaataattgcatacttggttttattttgattgctataattatttaag
aacgggttagcccgttcttaaatataagataaaatataatgaacatttcgtatttattta
cttacttttgcaagtattaaataataaaccgatctatctagtaatcttctagaatgttct
ggaaaaagtaagggttatttttgcgcttttaagctgtcagcgcttatataatgttaacgt
ggctactcggctatgcaagaaacaatacaaccgatacactattggttaatatatcttaat
cctctcgtactaaagataaaccctctttttttctttcccacaacagatag----------

-------------------------------ggttatggtaaagaaacctctcacgaaat
ttcctcccacttcaaaaccgtacgtgaaggtcacccttcatacggctcctcaaa----at
tatctatag-----------------aaagtgtt--------aaaaaaaagtacacactt
tcctttagtttaaaagtgcttatattagtccacggcttaagtattagctgctttggaatg

tctagtatcgtggcaatgaccatgcataagtcgtaagtttttaagatcagaagatcccctt
ttgacttcttggtataatatgatctatttctattctatctgaatctttaaaatataattt
acacatactacactgcggcactttagtttttaatagtcgttttagtagttgcccatactt
attattaacagcgagacgtctggctcaataagtaactcttccgtcatatgggctactatg
tccggctactttttgtagatagaataatatttcgttgatcatgacgggttaattttataat
tttgtctccttctttaaggccaaaaactctttctgcactccctataggtattcaatatct
agtgataccaggacctgttcgtttgcgtttcttagctcatttccttagaagataaaacgt
acgtacactacaataactaaaagtttttgtggcactacatacggaaaagtatttagttca
tccgctaataactggtgctagtttactgattaaaactttttgagataatccggtggactt
cgtagtaatacttttttatattagctaaatgagattctatagacttatagctgggttgaca
cctgctttttcatccagtcgcaattccttgattattctttgcggatgtatgtttacctac
cttatagttgacaaaattaaaacctaagaaatctacacctgttattctagacttctct--
------aaactaccggtgtacgttatttttgttttggattccgacaactttagacctaag
ttctgtaaaaagaattcaatttttgatttttgctgcaatcaattctttctcttcattacat
aatactaaaaaatcatctgcgtatctaataaggtatactccactttttcctattgcatct
tccattccgtgaagggcaatatttgccaacaagggtgatataatacctccttg-ggggtt
ccggcttctggtgtgatttcttttgtattttctttaaagcctgtaagtattcccgctttt
aatcaagctctcaattgctcttttaaaataggaaacgtgtttacctttagaagtaattta
gagtgatcaatattgtcgaagcatccttcaatatctgcatctaatacatgtttaggaagt
tgctgtaaacatttcacgattgcttgtctagcatcgttggaacttcgccctggtctaaat
ccataactgttaggttcgaatatagcttcatattgaggttccaatgcaaactttacaaga
cattgtttagctcgatctcttatagtaggtattcctaaatgcctttctttcccgtttggt
tttaaaattgttactcgacgaattttatccgatttattatcaatttcaatattttgtact
aattccattctttcgtcaggagttaaactactaactccatctactccagctgttcgtttt
cccaaattgtcttgagtcacttttcgaacagctaaaaactttgaaaagtcatgctttatg
atttgtttctgtattaagaatacagatgtcatattaccttttttgctaaattcaaaaact
ttacactgcaatctatacagtcaaatttcttttatttttcagttcacttgcgttcacttt
ttcataattt------gtttgttggtgtaatcttaacaatgttaaaattttttatttagat
aatt-----ttgtctacacgtctgcatatccataagctttccttatggcattggcttctt
gtagaatcctgatattaatataccttaacactata----------gaaaaacgcccttgt
aaaaaaagaatcaacaaggtttatattaatatttacttcgttccaatattacatacatat
agtcagtaagcacctctattccctctaaaaccggtacccctttctaacgcggccacatta
gattttaccttatgtttagaattcacgctgtttcgtgttaacggggtgggttaacttata
attatcccccgctatgacatatttagggaatgttatttcggctaccataagggtaggtta
tacttctataacta-------------cttttcttggcagatttactggtgtatatattc
tcttcaggtttgaatgtttataagtaacattctcccgaatttatgccctgcttataccctt

gtggataactattttcacactgtatagggtgcactgtttgtaaa-acagctcattgatca
gcattaggagt-aatgcgtaggcccactagttctagagaacttgcttcctacctgtaata
ttagtttaa----------tctctaaagtggagattagctatttacgagaagtacatctc
ttctcttactagcaacgaatcgcacgaccgaactgtctcacgacgttctgaacccagctc
acgtatcttattatttggcgaacaaccatacccttggaacctattgcagctccaggaaaa
gatgaggttatggtcagggcattcttcacagaatttcctgccactccagaaccgtacatg
aaaatcgctcttcatacggctcctcgagccatattattaacagttgattatttgttaagc
aaaatgtttacataacacaaaaaataagctcgttacttattttggaatgacgcgtgtca
tgacaatgaccatgcagaagtctaaggttatctcagtgtgaggttcccccttctttctt
ggtatgatatgatctatttatattctatcggagtcattaaaatatattttgcacatatca
catttgggccctttcattttaaaaagtatccttaataattgcccgtacttgttgtttgat
gatagtcttttggatcagtaggtaactctaccatcatacgggctactatcaagtagtact
ttagtagatttaatcacatttgtttgatcgtgtctattcaattttataattttgtcttcc
tctatcaatccgaatactcagtttgcggtatcgctcttaattcaatgcttagaaacaccg
atccaggatcgatttttcttctgagatcatctttctagaagataaaatgtacgcatgcta
caaaagcggaaagtttttgttgcattacatactgcaaaatatctggtccacccaataata
acaggtgccagtttactaattaaaacttttttgagataagccacttgacatttggataata
tttttgatactatctagatgcttattaattgatttaagactaggttgattcctagatgtt
caaccagttgaatttccatggctatccttggcggctttatgtatacccactttgtagttg
acaaaattaaaacctaagaaatctataccgctatttttcttgggagaagaataaacgcta
tatgttattgtagttttctctttgggtaattccaatcccatacctgttaaaaatgcttct
attttcagtttggcttctaataactctttctcctcgttacataatattaaaaaatcatct
gcgtatcttacaagatatactcctcttttactcagcgacttttctattccatgtaaagcg
atgttggccagcaagggttatataatacctccttgtggagttccagattctgggataatt
tctgtttttatcaccttgaaaatcaactaaaattccagctactagtcaagcagaaatctg
ctctttaagtagagggaaagtttccggcttttcaagcaacttagagtggtctatgttatc
aaaacatcttttgatatctgcatttaaaatatatttgggttttctttgtaaacattttac
aatagcctttctcgtgtcgttagcacttctaccgggtcggaacccgtaactgttaggttc
aaaaagtgcttcatactgcggctcgagtgcaaattttacaaggcattgcttagcccgatc
tcttatagtaggtatacccagatttttttacagaaccattcgcttttggtatagttaccct
caaaatcttatccgaatgtcgatcaatctttatgctttttactaactccaatctttcgtc
tggggttaataaagaaattttatctactccagctgttcgtttcccaaaattatcttgggt
aactcttctgactgctaaaaattttgcattttcatgttttatgatctgtttctgtataaa
aaaacacttttcatatcacccatcttacttagattaaatatttttatttgtaatttatat
agtcaaatttctttatgttttcacacaatattatttcatttcatgattttattaggtttg
tttattatatatcttccaaattatatttaaattaatatgactcgtctacacgtctgcata

```
tccataggctttccctgtggcattggcttcttgtagaatcctaccataattgtgcattaa
cgcaataacctgcgcccaaataatgcgttaaaaaaataaacaattatttggttcacaatt
atagttacttcgttccaatactatatacacttatttagtaagcccctctgttccctctaa
aactggttctccgactcaaacgtcgtaattgaatcaaaccttatgtttagatttcacgat
atttcgtgttaacggggtgggttaacttataattgtcccccgctatcctcggtttaggaa
actttatttcggatagacaaaatttttttgtctataataatcgacattattggcttttct
ggtcagatttactaaataagtattctttctatagttattattctataagtgaataagcta
caaatttatgccctgcttataatttgtacgtgttattgatacaaatcatagggttcactg
tttgtaaagacagctcattgatcagcatctggagtcgatgcataggcccactagttctga
agaactagcttcctatctatagtattagtttaatataggtctccctttattagctattta
cgagaagtacatctcttctcttactagcaacgaatcgcacccgacatcgaggtatcaaac
catggcatcgataagaactctcagccatgataaatctgttatccctagagtttcttttt
ccgataagcgacagtatttccatacattactgccggatcattatgaccgactttcgtccc
ggcttgagctgtaactcttaccgtcaagcagattttcatcattacatattatattagcat
atattgccaactaaaatccacttttgtgcacctccgttacagtttaggaggtcctcgtcc
caagtaaactaccacttttacatatgtccttgttagtaagcaatttttatactcagagt
agtttttcaatgacgtacaaatgtacttctacttttactgtactgtgtacaaagttatcg
cgacgtaaaattatagtaaagaatcatagggtctttccgtcttgttgcgggatgtctgca
ttttcacagacaatgtaatttcgctgaggctatactagagacagtggggtagtcgtgacg
ccattcatgcaggacggaatttacccgccaaggaatttcgctacctgaggatccttatag
ttaagaccgccgtttacttgggtttataatatatgcgcaaacttatatttttcactttca
agcactgggcaggcgtcaaaccttatacgtcttcttttgaatttgcaaagttttaag---
------------------------------------------------------------
------------------------------------------------------------
------------------------------------------------------------
------------------------------------------------------------
------------------------------------------------------------
------------------------------------------------------------
------------------------------------------------------------
------------------------------------------------------------
------------------------------------------------------------
------------------------------------------------------------
------------------------------------------------------------
------------------------------------------------------------
------------------------------------------------------------
------------------------------------------------------------
```

```
------------------------------------------------------------
------------------------------------------------------------
------------------------------------------------------------
------------------------------------------------------------
------------------------------------------------------------
------------------------------------------------------------
------------------------------------------------------------
------------------------------------------------------------
------------------------------------------------------------
------------------------------------------------------------
------------------------------------------------------------
------------------------------------------------------------
------------------------------------------------------------
------------------------------------------------------------
------------------------------------------------------------
------------------------------------------------------------
------------------------------------------------------------
------------------------------------------------------------
------------------------------------------------------------
------------------------------------------------------------
------------------------------------------------------------
------------------------------------------------------------
------------------------------------------------------------
------------------------------------------------------------
-------------------------------------------------tttttgttaaa
cagtcgctacccctaatttttgaaaccttaaaaggtactcctttttgcgaacgtacggag
taaatttgccgagttccttaagtatagttatctcattcgtctttattttctcaataagtt
cacctgtgtcggttttaggtacggtcaaacttcatgtaagttttcctgaaaaattacttt
ttttagcctccagtttagtgggtgtagctattagaagatatctttgtacacaaatacacg
agtattttgcagacacgtggtaatttttcctatcgaatacagttttcactttttttcttaa
gggccgactaactccagttacttaaaattgactggaaacccttgaacaatagacgaccat
gatttattttcaacatggttagcgctactcatgtcagcattagcactcctgattttagat
atgcaatttaacattaacataaaagactacaggacgttccgctaccattaagcttagttt
agcttaattcgaagcttcgatatataaatttaagtctccttacattttaaataggtagaa
caaataaaaatagcgagctaaaacgctttctccaataggtggctgcttctaagcctactc
tgtttattcgaattattccttatttttttcactaatttataattttgagatcttagctatc
```

gattagggttgtttcccttttgacgtaagaccttatcgcccaacgactgtctgctgctat
aaataaaaatatagtttggagtttaataaaatttagcaaaatctaaatttaataagtagc
tctacccacattttaaaaaagcaacgtactactttgatagttttcgcggaaaaccagcta
tcaccaagtttgattggactttcaccccctaatcttaagtcatccccgtatttttcaacag
acgtgggttcagtcctccagtacttttttaaagcaccttcaacttgctcaagaatagatca
cttggcttcgggtctaatacctgtaactttaagcgccttagtatttttaagcttgctaca
catattaacttactgactcattatgcaaaaggcactttgttgctgtatttgtcagcttca
aataaatataaattaacagattcaaatctttccctcacggtactcgttcactatcgatta
gaaaaggttttagcttagaagatgggactcctattttcatacaaaagtaatcgtactact
ttgtgttactattagtgtcgtaaaggactttataacctactttgggtttagacacttcgc
taagttcgctttcactcaccgttacttacaaattctcgtttgatttttttgttatgttac
taagatgattcaattcacataattatataaatgcatattaaatgcgagttttctaaagag
actcatagttcataggcaggtgcctcgctatgatgtttcgtcgcgcgacgtctttgcttt
tctaccaagatttcctctgataacttttttaaatcttttatatttaagaacgggcggaaaa
atgttaccctttttgtaagagtcattaactctatagtaattgtactcctaattcgataata
gacaacaagtattgctagtcctatagaagattccgaagccgctactgttaatatagctaa
agcaaatagttggcctactatattgtctaagtatatagaaaaaaatataaagttaaaact
aactgatagaaacattatttctaaagacattataattattataatactttttttggtttaa
aaaaatgcctaaaactccgactaaaaataaaaaaaaaagtataattttcacagtatagttg
ggtaatcatttatttttgtatttaacatttgagatagcaaattgtattaaatttttccag
ccgcaggttcccctacggctaccttgttacgacttcactttagtcctttcgctaccatgg
acaaaaaataagatttttgtcttcaagtagagtaaattcccatagtgtgacgggcggtgt
gtacaaaacccgagaacatattcaccgcgacagttctgatccgcgattactagcgattac
gacttcatattctcgagttgcagagaataatccgaattaaagatttttttaaagattttgc
tccagctcacgcttttgcttcttattgtaaacattactttgtagcacatgaaagtagccc
aattcataagggtcatgcggacttgacgtcatttttcccttcctcaaggatattccaagc
agtttataatgcattaatgcattatacaaaagtttcgtccgtttgctggaattaaccaaa
cgcctcacggcacggactgacgacagccatgcaacacctgtgacatcttgtgtcatacga
gaattggtaaggttttgcgcgttgtttcgatttaaaccacatgctccaccgcttgtgcgg
gttcccgtcaattcctttgagttttaatcttgcgaccgtaatccccaggcggagtgttta
atgccttagcttcgcctctggaaaattatccaaaaacaaacactcatagttgagggcgta
gactacaggggtatctaatcccttttgatacctacgctttcgtgccttagtgtcagctat
agtccagattattgttttcacttttgaagttcttttgaatatcatcgcattttatcacta
ctttcgaagttccataatcttttcctatgctctagtaaattagtttagtaatctttagta
aacatgaaatttaaaattttttttactacttagtttaccacctacgcaccctttacgccca
gtcaattagaataatacttgctcctcccgttttaccgcggctgctggcacgaaattagcc

ggagctttgattgtaaaatttagtctttgatttttaatctattttacaaagtgatttac
agcctgatagggctttgctctcacatgtggcctggctaggtcaagctttcgctcattgcc
taagattcctcactgctgcctcttaaagagtctggaccttatttctgttccagtgtgact
gatcatcctcaaagaccaattaaggattatgggcttggtaggtcttttaaactaccaact
acctaatcctgcgtagacttttcttaaaccaattattaatttttttaaggcatttaccaca
atttaagataaatttctacgtattactcacccgtacgctatctttttcataagttacgaa
aattattaaacttgcatgtgttaggccgaccactagtattcattcggagccaggatcaaa
ctcttttattattatgttatgtttttatgtttgctatctcagaaattaaatttgattgta
taaagttatccagcgagctaagacagaagctgtaccaatttcatatatatatatgttggt
aatcttccttttggtaatgaggataaaatttgctttacaaaaagttgtatactttttttg
ttttaatgcggaacatagtttattttctagctttgttccacgtataagaagtctcctata
tttttttattttattcatacatttgtgtatcaagatgggcagatttgaactaccattccc
ttgcacccaaagcaagtacgttaccattacgctacatcttgatatagtgatattgactct
ttctggataggatttgaacctataaccttgtagttaacagctacctgctctaaccattga
agctaccagaaaaataaaagcgaaaaaagggacttgaacccttataatgaccttggcaag
gtcatgctttgcctattaagctatttccgcacttaaaattgaatataaagttgaataata
ttagcaataagtagaaaaagtaaaagataattgctatctttgtacattttccttgtattt
acatgttttactgaatctcaaaaaaaatggcgtattccattacttatgtggtatagaaat
atagcggacactactcaccggacactagttaaaaaccaaggaaaaaagaggtgatctaca
aaaaaatgtattggcagaatagtaatatataaaaagtatacttgatttagtaaaagaata
gggcctacaattaaaacaaacataataacgccagaaattctatgccaaatagaaaaaata
gaagctctttgcggattgtatatagttaaatgtggagaaattggtctattgatgttctgc
atattatagttttctttttcttgggattttacagccattatgcggacaagttgtaatatc
cattacagagataaatttaagtttagactgttttaatattttaaacacatttttttttatt
tttacctcatccttttatccgtacatgtaaagaagtataactgagttctttagtctttat
ctctaagaagtggattatttcggacgaagaaagggacaggcttctttttaccccttttga
ccttgacttgcctgcagttgtccaagatttaattttgcctttgcagtctgttaaaagaaa
atgaatattattggaactaaaagataaaaacagtatacccatatgtagtttaattttatg
ttgtagtagtaaagactagggtcagattcgaactgacctaataaagatttgcaatcttac
gcatagccactatgctacctagtcttaaatttagtgaaatctgaagaaagactgtgtttt
aaatgataaagaacttttcacacaagaaagtaattcaagcgattttagctttggtaaatt
ggataactcgaaaagcaactgaccgggccttatataacagcaatgactcactatagctcc
ttttcctttacccatacgggtttctggactaaatttggtaacagcatttttttggtagcat
gtaaaaccacactttgattttacccaaactggactttttttcgatgactttgatctcttt
ctgaattatctttttttatattatttatttgtgtgatatctacttgcttgtacgataaaga
gcgtaatccaaattggcctctttttactagatgacttgtttggtttatatatctattttt

ttttttatgctgtcgtgttggattattttttgtattaaatcttttcatattaaattgtgc
ttaaattgttttgtatgccattcaaacttgaagattgcaaatcccttgttttgtgtataa
tggcgtttcaaaaaattctatataagtttctagtttttgcagtgatactgcgcctaatcg
gaatactatactacttgacatacgacttcggctaccaccgaaacgcccagcgtaccttat
ttttattcctgtaaattcataaaactgtataccacgagcagtttttaatacaattttgtg
cttttttttattgaaaaatagtatttttattgtattaattatcttccggaatgctaggcc
tttttttaggccagattttataaatccacataaaagtgccccgttttgatgtcaaaatgt
tgcattatattttttcacgtcaagatcgaacattttaaacgtaggtaccgagcaatctga
tttggctaatagaggtacatattgtacattgtattctcatctattgtcatttattttaga
tagcgtatttccttttatgaatgtttttgattcaaaatgcttatttaaaaagtttgtagc
aggtaatactttttttgtatatttgcttttggtttttaaaacctttgccatagcattgact
ttcggtatttcataagtcgttcacacctattcgaaatcctcttggattaacttttttgacc
cataatgttttattacttaataacaatatttaaattgaaaatcaaatctaaatagttata
caaaaatacgttttgtaggatttgaacctacactaatcaacttagaaggttgatgtttta
tccaattaaactaaaaacgtttttattttagatgcattgggatttgaacccaaaataagc
agattaaaagtctgttgctgtaccgtttagctatacatctttttaataaagagagtagga
tttgaacctacgatgattttaatcaatagatttacaatctatcgctttcgaccactcagc
catctcttcttcagcggttagatatatgttacaatctgtaataaaataattaattatata
ctattgtttaataacaatatttagattgaaaatcgtacgattttcaatctaaatattgtt
attaaacaat
