# Supplementary material for: Genomic diversity of 39 samples of Pyropia species grown in Japan
Source: PLoS One. 2021 Jun 9;16(6):e0252207. doi: 10.1371/journal.pone.0252207 (PMC8189503; doi:10.1371/journal.pone.0252207)
Supplement: S6 Fig — The scale bar shows the number of substitutions per site. The sequences of Pyr_19 (P. tenera) and the hybrid (Pyr_27) between P. yezoensis and P. tenera were used as roots. Colors were used to distinguish between Japanese P. yezoensis and 3 clusters of Chinese P. yezoensis. The parameters for RAxML were as follows: -f = a, -x = 12,345, -p = 12,345, -N (bootstrap value) = 1,000, -c = 1, and -m = GTRCAT). Only variable sites were used in the analysis. (PDF) [file pone.0252207.s006.pdf]

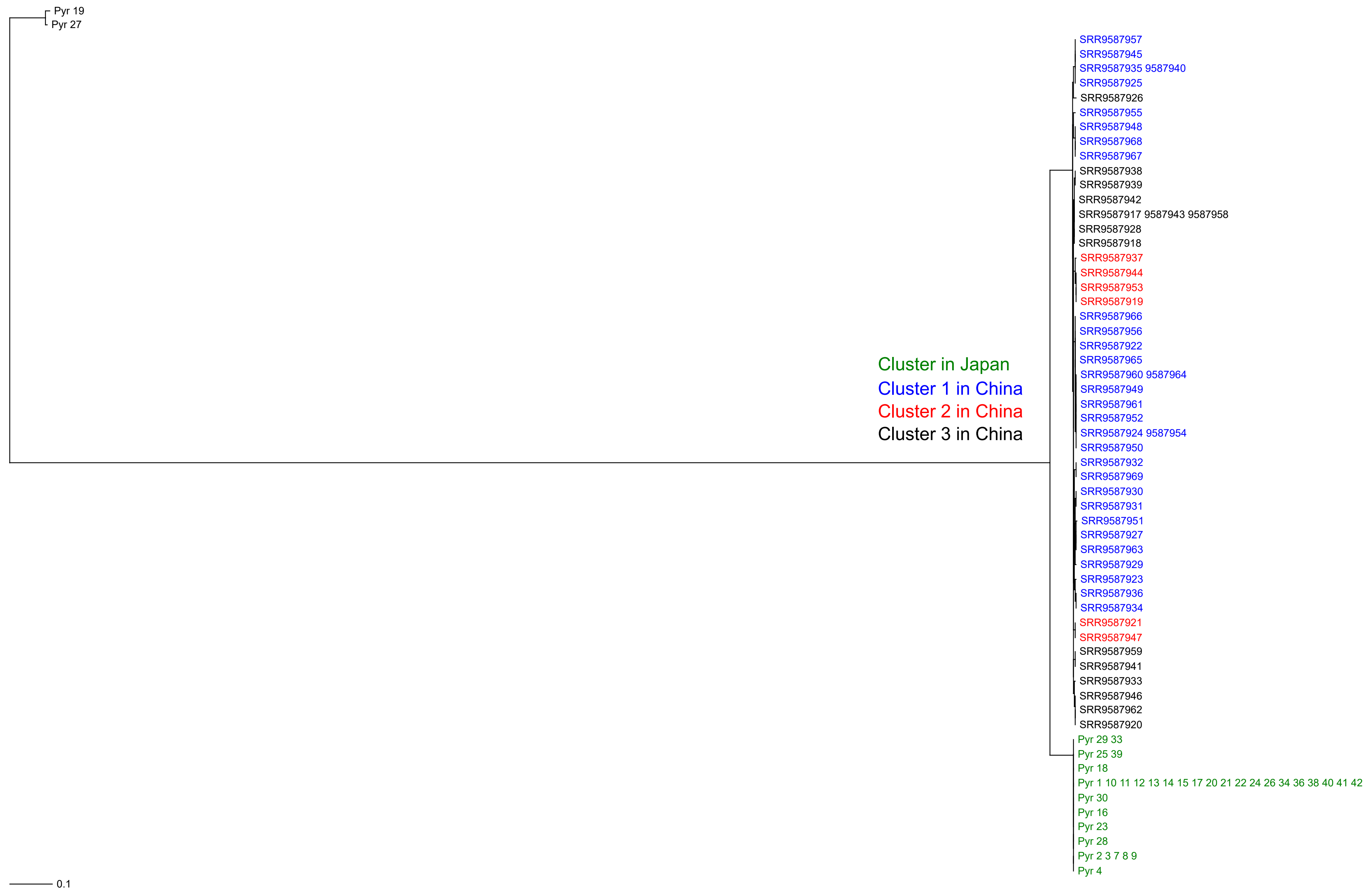

Pyr 19
Pyr 27
SRR9587957
SRR9587945
SRR9587935 9587940
SRR9587925
SRR9587926
SRR9587955
SRR9587948
SRR9587968
SRR9587967
SRR9587938
SRR9587939
SRR9587942
SRR9587917 9587943 9587958
SRR9587928
SRR9587918
SRR9587937
SRR9587944
SRR9587953
SRR9587919
SRR9587966
SRR9587956
SRR9587922
SRR9587965
SRR9587960 9587964
SRR9587949
SRR9587961
SRR9587952
SRR9587924 9587954
SRR9587950
SRR9587932
SRR9587969
SRR9587930
SRR9587931
SRR9587951
SRR9587927
SRR9587963
SRR9587929
SRR9587923
SRR9587936
SRR9587934
SRR9587921
SRR9587947
SRR9587959
SRR9587941
SRR9587933
SRR9587946
SRR9587962
SRR9587920
Pyr 29 33
Pyr 25 39
Pyr 18
Pyr 1 10 11 12 13 14 15 17 20 21 22 24 26 34 36 38 40 41 42
Pyr 30
Pyr 16
Pyr 23
Pyr 28
Pyr 2 3 7 8 9
Pyr 4
Cluster in Japan
Cluster 1 in China
Cluster 2 in China
Cluster 3 in China
0.1
