# Supplementary material for: Genomic diversity of 39 samples of Pyropia species grown in Japan
Source: PLoS One. 2021 Jun 9;16(6):e0252207. doi: 10.1371/journal.pone.0252207 (PMC8189503; doi:10.1371/journal.pone.0252207)
Supplement: S1 Fig — (PDF) [file pone.0252207.s001.pdf]

>P_haitanensis_NC_021189
tatatgatcaataattaaagaatctttttttgtaagaattcctgaaatatacaaaactttt
tatttaatatagaaaaagtaaattagaataagttgaacatatttt-ttctactttaaacc
ttttgattgag------attctaaatagaaataaaaatgttgacatagtttatatctata
ggaataaaaatgaaacttgcagtttatggtaaaggcggtataggtaaatctacaactagt
tgtaatatttctgtagctctttcaaaaagaggcaaaagagttctacaaattggctgtgat
cctaagcatgatagtacatttaccctaacagggtttttaatcccaacaattatcgacacg
ttacaatctaaagattaccattatgaagatgtttggcctgaagatgttatctataaagga
tatggaggtgtagactgtgttgaagctggcggacctccggctggtgctggttgtggaggc
tatgttgtgggtgagacagttaaattattaaaagaacttaatgcttttgatgaatatgat
atcattttatttgatgttttaggtgacgttgtatgtggaggctttgcagctccattaaat
tatgcagattattgcttgattattacagataatgggttcgatgctttgtttgcagccaat
agaattgctgcttcagtacgagaaaaagctagaacacattccttgcgacttgctggactg
gttggtaatagaacagataaaagagatctaatcgataagtatatagattgtgttccaatg
cctgtattagaagtattacccttaattgaagatattcgtgtgtctcgagtaaaaggtaaa
actttatttgaaatgtctgaaactgataaagatttagcatatgtgtgtgattactatcta
aatatcgcagaccaattaattactaggccagaaggcgtagtccctaaagagtctccagac
agagagttgttcagtcttttatctgatttttatttaaatcctaaatctaatgtaggacaa
gctgtcgcagatcaagaagaattagatttaatgatagtgtaaataatattttaagcataa
cataaaaaggaataagataatgtctacagttcaatcagatgctcttacttttgaatgtga
aacagggaattatcatacttttttgtccaattagttgtgtttcttggttatatcaaaaaat
tgaagatagtttttttcttagttattggaactaaaacttgtggttattttttgcaaaacgc
aatgggggtaatgattttcgctgagcctagatatgctatggcagagttagaagaaggaga
tatctcagctaagcttaatgattatggtgaacttcgtagattgtgcttgcaaattaagaa
agatagaaatcctagtgttatttttttggattggaacatgcacaacagaaattataaaaat
ggaccttgaaggaattgctccaaaattagaagcggaaattcgtgttcctattgtagttgc
tagagctaatggcttagattatgcttttacacaaggtgaagacacggttttagcagctat
ggctcagcgttgtccttcaaatttaaataatcaagtaggcagtaatagtcgcgaaatttc
ttcccatatccccttagttttatttggatctctgcccaacccagtggtaacgcagttaac
tatggaattaaaaaaacaaggtattcatgtctctggctggttaccatctaagaggtatac
tgaattaccggttatacaagaaggctattatgttgcaggagttaatccatttcttagtcg
aacagctactacattaatgagacgaagaaaaacaaaattaattggtgcgccatttccaat
aggcccagatggtactagagcctggattgaaaaaatttgctcagtattaaatattcaacc
tattggactagcagatagagagcaagaaatctgggattctttagaagattatattgcttt
aatacgaggcaagtccgtttttttttatgggagataatttattagaggtctccctagcaag
atttcttactcggtgtggaatgactgtgtatgaaatcggtattccatacatggataaacg

ctatcaagcagcagaattagctttattgaaagccacatgtgacgaaatgaatgtcatgat
gccaacgattgtagaaaaacctgataactataatcaagtagatcgaattcgtgatttgaa
accagacttagttattactggtatggctcatgcaaatcctttagaagctagaggcattaa
tacaaaatggtctgtggagtttacctttgcacaaattcatggttttacaaatgcaagaga
tattttagaactagttacaagaccattgcgacgcaatcttagtttgtcagaattaggctg
ggatgtctatagcaagcaaagttagtattagt-------tgcgacttacatggcataaaa
tccttgtaagtct--tctatattatgctaaagaagcatttcttgcccgcccatcttctgc
ccaagcttgtaaattttctatttgttctttatctgtaaatgctaaaggtacaaattgctt
tatagctattttaatatcttctgtattaaattctctctgttcactaaaagctgtatgcat
actttctataatcgcttgctcgatttcagcacctgagaatttattacataataaacttaa
ttgtttaatatcatattcttgccatgattttggtctaacttttgataaatgtatcctaaa
aattgattctctttccttgcgattaggtaagtctaagaaaaaaatctcatcaaaccgtcc
tttccgtagcatttctgaaggtaaactttgaattttattagctgttgcgacgacaaatac
gggagctttttttctgaaagccaagttataaatgttccaaatactcttgcactagtacc
actatctccttggctatacaagccagaaaaagccttatctatttcgtcaatccataatat
acaaggcgataatccttcagcaatatttatcatatccctcattctggattctgactcgcc
aactagtccaccaaataattttccaatatcaagacgtagtaacggcaaagtccaatcctt
ggcaatagctttagctgttaaagattttcctgttccttgtataccaactagcagtaaacc
tttaggggaaggaataccataattcaaactttgtttcgaaaaagagcgagatctttttg
tagccattgctttagtacatctaatccaccaatatctttattcactttattgtaaggata
aaattctagtagacacgtttggttgataatttgtctttttttcttcaataataatcggtaa
gctgcgagaatcgagttgactatattgtgcaataatttttgtaatcactttgcggattct
atctattgatagaccttggcaggatttggtcatattattaactagttctgaatctagatc
aagatttacagctttacttaatcttgtgatttctttttgatttctaaaagacttggtaa
aggtaaatccattattgtcacaatatctcttaaagcaaaaggtatatttattttacaaga
aataataattatatgtttagattgtgctttgattactttagctaaattgcgaagtttcct
agttaggactatttcatttaaaaaagagtcgaagtcttttaaaatgaaaatattcaaaga
ttcattatctaagttttcaataaactctaatgctagtaaaggattccgttttgcataacc
attatcgttagggttgctagtatatccatctacaaaatcccagcaataaacttgttgatt
gtcagagcagtgtaatttatttttataatatattcgagtctttcttcttctctagtatt
tattacaataataggatgtcgagactttaatagtaatcgtaaatcttgtgtgaaattcat
gtcagtatttgtccccatcagacttttgtacttcaccaatta----aagactaaataaga
ttatctaacgttgcatggatatattcaaaagtc------atgtatctattttctaatcta
ttatgatttcagacttaactaaccataattttttttcttcctttgcgtcgtctactattt
aaaatagagcgtccgcatggtgttttcattctagcccggaaaccagaaactttaatttt
tttctttttgatccttgtaatgttctcttagtcattatgtctcctgtattgtaaaagatt

aaatattattttatcatcttttatatatcttgattatatagatctgtattaatatcaaag
tt-aaacgcaataatttatattaatcaagaattatttatataatattaagaattaagtat
----------tagctttatgatattagctctgcctatttttttatttaagtatcctgacag
cgtttttgctaattttgaattggttagttttcagcagctaaaaacaattttgtcattag
aatctcaatttaagtattttattgaaaaaagtcaaaatgatatattggcagtagaagaaa
gctttgcttttgcaaaggtttgtgtagcaaagaagtgcttttctagagcgattatcgaag
gtcaattggcaatgaaaaagagctcagaattaaacgagaaagagagcaatataataattg
ccaatttgcataacatgattggttttatttattctgaagcacagcaactaaatattgcaa
aaaactttatcagcaagccttacaaatagaccctaattatgtcacagctttaaataatt
tagctaaaatttacgaagacaccaaggatttaaaaaagcagaggcactatataataagg
tattaactcttaacttagacaataaaatcgctaatagaaggaaggaatttataacaaaga
tca---------gaaatacttaatattatttataatcgggatagcaggatttgaacctgc
gacatcctgctcccaaagcaggcgcgctaccaaactgcgctatatcccgttagacataat
tagtactatatacctttttctacaattatgtctactatttcagtgttttatctaggatacc
aaaacatgcctttaactccatcaggatctttaatgaatcctaatttttttgtaaaaactaa
ctacatcaggttctgcaaataatgtaatagtgctaatttctgcttgtcttaattgtttta
ttaattggtgtatcacgactttccctaaacctaaaccttgaaaatctggatgaataacaa
catcccagatagtagcattgaatccattatctgatgttgctctcgcaaatcctacgagtt
tagtatttgtatctttcttttgtattaaagaaatgataatcgaactatttttttaatgcga
tttttactttttttagaggtcttttaacccatcctaccgaatcacacagctgttctaatt
cgtataagttaatatttttagtgctactgagatagatatctttgaattcggctctattac
aagttttatctaaaacaacaagtttttttgaaatttttttcagagttactttttgatatgtg
agttttgaaaaaaatttttccagaagatcataatgattatgatagaat----taaatata
tatatattatgatattgccttaatgaacaacatattaatatatgtccagtttttaatatg
gagttattaagaaagaaaattgtttgaaggtatagaaataaaaattttttgtaaaatcttt
caataaaagtatataaagagctcttttaagattaacatctattagtattaatacagtct
cgcaattgagatgaaacaacatattattctatatctaatgccttttaaaggagttatttg
tgaaaaaatctatgtttctaacctgcctacttgctttgttattgcttagtaacccgataa
tagctaatgcagaagtggctgggctagtgccttgtaaagattcggcggcattcaataagc
gtatggttaatagtgttaaaaaacttaaagctagattagctaaatatgatgcagatactc
cgccagcgttagctttaaataagcaaatagaaaaaactaaaactagatttgctacttatg
gtagagcaggcctattgtgtggtacagatggtttacctcatttaatctctgacggtcgat
ggagtagagccggagactttgtttttccaggactgctatttttatacattacaggctgga
ttggttgggtaggtcgaggatatcttctatccgttgctaaaactagcaagcccactgaaa
aagaaattattttagatgtgccattagctattaaattcatgacatctggattcgcatggc
cgttagctgcttggcaagaatttagtagtgggcaactagttgcctccaatgacgatatta

ctgtttcaccccgttagcaaaaaatttatatgaataataattttaccaaatacttatcaa
cagcacctgtagttggcgtgttatggatgacgttcacagctggttttattatagaattaa
accggtttttcccagatgttttatatttttatttgtaaataaataa--aatctcataaa-
---aaccacacaaatatagtaaaacactattatttgtgtggttttt----atatagaata
taactattta-tttttacggaaattctttctttcgactattttaatattagattttacat
aataaaaaa-acagtaatattatgagtttagtaagccaaattatagttaatgctgatgat
gaattaagatatccaacaatcggagaattacagtcaattcaagactacttaagtacagga
agtaatagaattagaatcgcaactattattcgggataaagaaaaagagattatacaaaaa
gcaagtaaacaaatttttcagttacatccagaatatatagctccaggaggtaatgctgca
ggttctagaaaaagatcattatgcctacgtgattatggctggtacttaagattaatcact
tatggagtattagctggtgataaagattctatagaaacgattggtatcatcggtgttaga
gaaatgtataattctttaggtgttcctattattggaatgttagacgcaattcaatgtcta
aaagaagcttctctagaaatgcttggtcaagatgatatcaaaattattgcgccttatttt
gactacataattcgaggcatgtcataaatagtaaattcaattaattagttgaataaaagc
ttgtataatgttatgattagttcaagcccgaaagcatataattgtaaagactaaatcttg
tcagaaccggaaggtagcagcaataatgtttaattacaacagttatga-ttttcgggtgt
ttactatttgtactcaatagtctctcattctaattatgatgctatccataaatt----aa
taatatataatccataaaacctttcagtaaaaataa-ttaataatttgatattaatacgg
aaaacatctatactttttttataaaacttctttatatctcatctaaacaatggaaactta
acttaatgggtgttcatattttatcaacaggttcgtctgtcccaaatttttctgtagaga
ataaacaatttgaagatatgatagaaacttctgactattggattttaacaagaacaggaa
taaaaaaaagacatctagctccttcttctacttctttaactaagttagctgcagaagctg
ctaataatgctttatctgcagctaatttaaaacctaccgatattaatttaattatactag
ccacatctacgcctgatgatttatttggtagtgcaagccagctacaagcagaaatcggtg
caactgcatctgttgcttttgatattacagctgcttgctccgggttcattgttgcattag
taacagcagctcagtttatccagactggttcatatgacaatatcttagtcgttggagcag
atacaatgtcgagatggattaattggtcagatagaactacctgtatcttatttggcgatg
gtgcaggagcagtagtagtgggacaaagccttaaaaatagtattttaggttttaagttat
gtacagatggccagctaaatagtcatttgcaattgatgaatacaccaaaaaataatcaaa
aatttggatttatagatattcctcatggaaattataattctataacaatgaatggtaaag
aagtatacaaatttgctgtatttcaagttccaatggtaattagacaatgtttagataatt
taaatatatcaatagatgaagtagattggtttatattgcatcaggcaaatactagaattt
tagaagcaattgcgagtagattgtcaataccttttttctaaaatgattactaatttagagc
actatggtaatacatctgcagcatcaattcctttagcgttagatgaagccattaaatcca
ataaaattaagccaggtcaaaccattgttttgtcaggttttggagctggtctaacttggg
gtgcgattgtcttgaagtggtaat---tataatattgcggatgacgagaatcgaactcgt

aaagctttcgctacacacccctcaagcgtgcgtgtataccaatttcaccacatccgcact
ttttatcacgaata-ataaaaatttataatatttatttaaatattaatataa--tgtgta
tatatatatacattcttgtcagaaactctcttatattttattttaagtgataaaattgag
t----ttagtatagatgataaaatttttatttacaatcataaaaataaataaatatataa
ggaaaataaaatatgacaccgtccttatcaagctttttaaacagtctaattcttggggcc
gtaattgtagttgtccccataactttagcacttttatttgttagtcaaaaagacagaaca
attcgatcctaacaaagactttgaaacatatggagaagcaatttgagattaataataaac
tatttttcaaacggatatgctctccatatatttctgtatt--ttggaataggttgttt-t
ctttactttgcaatagcctgcacagtgta-----------ttttgttctcatttactctt
tta----taacgatacgtatgtcagg-ttaataatag--tttttttattctgtatctttc
atttaatattaaagtattttatatacatattgttatgttcaagtaataaagaaaagctca
tgtctttgtccaattggcctcttaaaaaagaaaactccgaagcatatactagcaagtata
caaagcaaatcacaatccctgatggattgtggataaaatgcttcgattgcggtctgttaa
tgtattctaaagttctaaaaaggaatttaaaagtttgtcctcaatgtagctatcattttc
aagcttctagcagtgaaagaattgatcagttgatagacaaaggtagctggcgacctatgg
atattcatttaatttctacagatccattaggtttcaaagatcaaaagctttatagtcaaa
ggttaaaagatactgctcttaagactggcttacaagatgcagtccagacgggtattggta
ctatgcaaggtagacaagtctgcttaggtattatggacttccgatttatgggaggaagta
tgggatctgttgtaggcgaaaaattaacaaggctactggaaagagcaactcaagaaaaat
tacctgcaattatactttgtgcatcgggcggcgcaagaatgcaagaaggcatgttaagtt
taatgcagatggcaaaaatttcttctgcgttggaaatgcataaaaaagcaaatctcttgt
atatatctgttttaacatctccaactacaggaggcgtcacagctagctttgctatgctcg
gagatctaattattgcagaaccaaaagctcttattgcatttgctggtagacgagttatag
aacaaactattaaagaagacttgccagataattttcaaagttcagaatatctatttgagc
atggttttctagatttaattgtaccaagaactcagcttagatcaaagctaatacaaattt
tatcattccatactcatactaagtaagtagtttgacattgatcataatattgaaatgcta
gtaaaaattaagatattaagaaattttcactaaagatatttgttgactaactagaattac
aattactattgtagaaaaatttgtcttaattctatttttttcagtcttttaactaattg-
tttttacgactttaaataattcttagtttaatacaaatttataaagagaata-----cat
actaagctaaatatagtaatattatgttttcctataatactgacctatcaaatttcgagg
aattttatgcttaaaagatcttcttggcttgctactttattggggctactaactgtagtt
tctataagtacgcatgcaaatgccatagagctagacgaggcaacaagaactgtcccatta
gaatcttctggaagaactgtagttcttacgccagaacaagttaagcgaggcaagcgatta
ttcaataattcttgtgctatttgtcataatggtggtattacaaaaacaaatccgaatatt
ggacttgacccagagtctttaggcttagcaacgccacaaagagatacaatcgaggggtta
gttgactatatgaaagatccaacaagctatgatggcgcagagtcaattgcagaactgcat

ccaagtattaaaagtgccgaaattttcccaaaaatgcgtaatttaacggatgaagattta
tttacaattgctggtcatatcttgctccaacctaaaattgtttccgaaaaatggggcgga
ggaaaaatttactattaaaggttcaaacacaataatcctacaagtactagtttatgca-t
ttattgcccaagtatgattattttgttagatcatgatatatattaaaggtggagtttgct
aatagtaagtta-tttttgcctaaacttgcttacattattcgtttagttattaaaaagga
gaagttaaattgaaaaagacgctttcagttcttttcactgtatttagtttttttgtgata
ggttttgcacaaattgcttttgctgcagatctagataatggagaaaaagtttttttctgct
aattgtgcggcatgtcatgcgggtggtaacaatgctatcatgccagataaaactttaaaa
aaagatgtgcttgaagctaacagtatgaatagtattgatgctatcacgtatcaagtgaaa
aacggtaaaaatgctatgcctgcttttggaggtagactagttgatgaagatattgaagat
gcagcaaattatgtattatctcaatctgaaaaaggttggtaattatacttaattttgcac
agtattaaaggatagacaatctatttaattattcattagattgtctattctttgtttatg
---ttattatatatagatatttatacaatactga-attatgatgaaaagaatacctgcaa
ttcttgtactagaagatggtacttattacaaaggatggtcatttaagcaaagaaatccag
caattacaattggagaagtggtttttaatactggaatgacaggttatcaagaaatcatta
cagatcctagctacttccagcaaattgtcaccttcacatacccagaaattggtaatacag
gtattaacagccaagatgttgaatctcataatattagtattaaaggcctggtggcaaaaa
atatttgtaaaatttcaagtaactggagagagcaagaatctttagttaaatatttaagcc
gttatgatattcctttcatctttggactagatactcgatccttaactcaatatttacgtc
aatttggcacaatgaatggttgtatttctactaataatttaaatcatagttatttaaagc
aaagaatttctgaagttcctggaatgcaaggcttagatttaattccatatgtaacaacag
acagcatttatccttgggacgaaaaaagtttgcctatctggtatttaaacgataaaagaa
gagtgcaattagatacccagttgaaagttgttgttatagattttggagttaaactaaaca
tactgagacgactagctacacttggatgtcagataactgttgtgcctgcacaaacatctg
tagaagatattttagctaataaacctgatggtatattactttctaatggcccaggtgatc
catctgtagtgtattatggaattaagacagtgaaaaatttattagatcataatgtaccta
ttttcggtatttgtatgggacatcaaattttaaatttggcccttgacgctaaaactttta
aactcaaatttggtcatagaggtattaatcatccatctggattggaacagcaagttgaaa
taactagtcaaaaccatggtttcgcagtcgatttacaatctgtcttcaagtcatctgtac
aagttactcattttaatttaaatgatactactgttgctggaataggccatagtcaaaatc
catatttctctgtacaatatcatccagaatcgagcccgggccctcatgatgctgactatt
tattcgaaaattttgtagaaataatgataaaatctaagcgtaaagttgc-----------
ttaataatcttcccatgctttatgattaaataaagctgataatacttgtactccccgtaa
ttgattgaagagaggtaaatgcccctttggaccataagaattccattgaaattcatcagg
gtatctacaaggaatatgatcaacttcccacccaatcttttgccagaattttccccaatc
ttttccaacactaagccatatttgcctctgaataaaaagcccaaatttacctttttgagtg

tgtatgccacaatttatcaatagtctgaagatcttgggcaggtatttttttatatctgt
aaaatatagccagttgcgattttgtgcattcacaccagctaattgaataagttttgttg
agttaattgatctgcctcttgcaaatcttgttgtgttagcaacacttgtaaatctttata
attcatgtgtttcgcagaacgtaatgggacaataccctcgggacataagtcagtagtaaa
cctaatgatttcttgattttactattcaataacttgttatagatcaaaccatcgacgca
attactttataattgggcctactaatcctttcaagaaatagatccgctaaactttttaa
ttgcatagagtcattattatgtatagtctcaataatatcaagttgttttttga---tatt
atttgaactagtattttcacttaattctaaaaactgaa------------attgcatttg
attt-ttacattaatttaaatagtg----attatttcttaaaaagaatttga-ttttttt
gaaaattagaagaacgacgaattatttctagaagtgttttcataatttgattgaatatgt
ttctccccgcataaatgataatttgaccaattaaaaaaattgcacttttttattttaggta
caaagaagtcttgtacctcaagtagaaatgtaattactatttgcattgatgataatgtat
ataattcatgtgatctacaagcatatatatactgacaatttaaaccttcctgataaaacc
cccatactttgtaacgactttcgtaaattgctcgcggtctctcaatataattttaatta
aagtattccaaactaaattatttttaatttttctagttttctatcagataaaaaatttga
cattacaaagatagcttgcatttgtttcatgtactgtatcaaaattttgaaccaacatat
gtgcaataatattacttaactgaattaaatagttctcaagaaaaaatttcaacttgtttaa
taggtacatttgaagtaaataaattgaaagtcttattcacaccttgagaggatccaaaaa
gtagctgaataattagaatttgtagtaacatcttatagtcatatattaggtagtttaagt
cattaatatcttggcaaatatcattatagtctagttcgtataacctacaaaatttttga
cacatctatgaaataaatcaattaaaatattttcagttagaatcgtaatatcatttaaac
ctaaatttgaattataaatttctaacaggatttttctaattctgttaatattattttta
ataagtttctttttacttcagttcgaaaaacatctaaaattaatacttcctgagaacaat
tagttaattttctattaatttttatagaagttctaactaatagctctgcaacctcttgat
tgagtatgtgtccctgtgagctcggccaataattattcacggtatatcatagtaaggaat
atttaag-----tttatacgataacttttaatacactgaaaagcaagtttatcactgta
tagccattag-------cttttacagatcttaatagttatttttcaaataaagttatatg
atattagcgaccaagctaattttattaaaatttataattaataatgacaatttattactt
tgcacttgccagtcaaaactttctcttagtacaagagcccttggaagaagtttttagaga
gagagttaactattatcagtcaaataataaagcaatcgattttggctagtgccaaatcc
ttctttttagaaaaaccagaaatgatttcgtttaaaaatcttgtacctaatgatgctgt
agccataatctctactaacccagtatttattaattggttaaagttaagaattggttatgt
ctgtgttggacaatttgaagatgatttacaactttctgaagagtctttaaacattactgt
tgcaccagaaaagatttaatcatacttttattgccactaggaat-aattat-----ttaa
acggcaaatataaatt-caaatccttgtttttctatagtttatcgcttagtattttatt
ctgagatggagtgacaaacagtgtaagtatttcttgactaaatgtttcagttgctttgga

tttatacctattagggttgactataatagacagcattcgcttaatagtgacattttcaat
ctgagcccaatgtacaatccctagctctaactcctttgcaatagctgaaacagaaacaaa
agcagctcctaaacctgactgcacggcatttttatagcttctatagaatttaattccat
ctctatttaaagcgactgctatcaataccatgttgactgagcactttatctatgacttt
tctgattgttgattgagtgtctaatgcaataaatttaagtctatacaggtcttcttttg
gatgtcttctaatttagaaaacggatgcgatgttggaagtattagtgctaattcatcttc
ggcataagatgtaacttgcaaaatatcttgtaattcagtaggtacttcccctccaataat
tgctaggtcaacttgaccattagctacgctccaggaaatgagtctagtggaatgtacttg
tagttgaacagctacttgtggatatctttgcctgaatagtcctattaatcttggcatcaa
atatgtcccagttgtctgacttgctccaataattaatgtaccaccttggaggttttgtaa
gtcatcaagagctcggcatgtttcttcacataaagctaaaattcttcctccataccgtaa
aagtaaacttcccgcctcagttaaagttgctttttattacctctctcaaagagggaaac
atttaattggcgctctaaattttgaatttgtaaactaatagctggctgagaaacatataa
gctattagctgccttttaaaactgccttctttggcaattgcttttaatattcttaattg
atccaatgtaaatggaagatctgtcattaaagaattgtagtataatg---------tata
ttatttaatattgatcaatttatgcattcgccaagttaaatatttgctattacgcttcac
tgctttgaaaataataaaaagtataatgttaaatacactgtattagattttaatatctgt
ttaatgttaaatttatataataaatatcg------taattttaattataagatataggag
acaacaatatggactctagacttttaattgtattaataccagttttagcagctgcatctt
gggctatttataatattggtagagtcgcgctacaacaatttaagaaaatgacatcttaat
gatgatgagaatata-atcttaaaattttatgagatagagaactttaaaatctctatctc
tattgtatatcttaataaaaaaagtaagta--acccttgtacttactgttattacctatt
aagatgatgtac-agtacatatatttataataagtttaagctaacttatt-----tttta
taattaactaaattaataacacaattatggctgttccaaagaaaagaacatctaaagcta
aaaaaaatgcacgtaaagcaaattggaaaaatcaagcaagagtcgaagcacaaaaagctt
tgtctttagcaaaatcagtattaactagaaaatcgaatggatttgtttataatccttctg
aaacagcagatacctatactaactaagagtcttt-ttactatcaagctagccaagtatca
aaaaatctattttaagtaatttttatatat----taaacatttggaaatcattctatttg
ataataaaataaatagcagattaaacaaaattacggcaacaaattatgcagcaaagctag
atgaaactaatgaaatttggttatttaattgcatcgaaaatatccagcacactttttga
aaagtcaattaaaatcaagtcatattactaaaattttcgtttctggtactagctttgaat
atgtagcaggtttacctggactattatccagcttaacactaagtggtagaataaatccca
ttagcatatatggcccaaagtctttggaatggtacctgaaagcttctaccaaatattctc
aaactaatttttcttttcctattaattttcataatgtgaaatatggagaacaagttatta
atcaattctatacagtagtttgtttaccgttgagccaggacaatttactgtatggattta
ctatcttaaaaaaagaaaagccaggagcatttaaattagcaaaagctaaaaccttaaata

ttactcaaggtcctatatatggcaaattaaaagaaagaaataattttctaagtcccgatg
gttattatctatgtggcaaagatttctcgttaagtacaacaatgggacacaaaatatctg
ttccactattagtcagatattctagaattatttcagagatgcattggttttgctcttatt
ctattcgatgtaacacttatctacatcagcaaaccataaaacatttgccaaacaatattt
taaccgatactatgaaatctcaaatatctcaagataatagttgcattgaataataattat
tcttgatatataatagtctcgacaatataaataacatacaagagcgaatttcttg-----
----ttatatattaaatttataattatttatgacatacgcaattattgaagcaagtggca
aacaactttgggtagaggaaggacgctattatgatttaaatcgcataccttttgatccag
gccaatcaattatactgggtaaagttttattgttaaataaaaatggacaagtgactttag
gacatccttgcatagcaggagtaacaataaaagctacaataatgaggcatttacgaggaa
aaaaaataactgtttttaaaatgaaacccaagaaaaaaatgagattaaaaaaaggacatc
gacaagaattgactcgtttaatgattaattcaatagtaaactaggtctagatacagttgt
aactgagattgcttcaa-aagattttgaatatttaactacaatacatataatggcacata
aaaaaggtagtggtagcacaagaaatggtagagactctaattctaaacgcttaggagtga
aaaaatatggaagcgaacaagtaagagcaggtaatattttagttagacaacgaggcacga
aagtcaagccaggtcaaaatgttgggagaggaaacgatgacactttattttcgctgatta
atggctttgtatttttcgaaaagtctaaccaaaagcaaaaaacaattagtgtttatgctg
caaaaaaatagccaaaataattggtgcaagatgacctgaat----attttatttcactt
acactggcaccataaacttgatttattaagaaatggagttccttaatgaaccaatgaatt
ttattaaagatttgctttgaaatgtaatgacaaacactattgtaatttcctgtctacaca
atatggctgctattctatattgtggtcagatacagaaactagtagtagcaaatgctcatt
atcaagtaagtgatatttacctaggttgtgtagataaaatcttctcaggaataaatgcag
catttattgacctgggaaaaaatgagtacagtggattcatccatattagtgatactggtc
cgcttaaaaagaaatattatgttaatagtattactaacattttaacaattcgacaaaaaa
ttttagtacaaattactaaagaaccaactttaaataaaggaccaaggcttactgctaata
ttacattatcgggtcgatatattgtactaatgccttttagtcaagcaatttgcatatctc
gaaaaatacatgatgaagatgaacgtcattatttgaaagctttagctattctaattaaac
cagcaacaatgggcttattgttccgaccttctgctgtaggcattgatgaagaaattatac
taagcgaactaaaaaatttaaaagagcaatggaactttgttcaaaaatctgcaatcaaca
gctatgcacctggtctcctatataaggatgaggatattattaaaaaggtaattagagatt
tttatagtaataatacaaataatatcgtaattgattcaaacttgggattaaaacaattga
gttattatatccatacttggcactgcaataattctagtacagctccttcaattaagcttt
atagtaacaatcaatgtatactagatgcttttggtattaatcaagcaatttctagagcgc
taattccaaaagttgatcttatacttggaggctatatgtttattgaaactttagaagctt
ttacaattattgatgtaaattccggatcttttaataactctactagtgcgcgagaaacag
ttttaaaaacaaattgttctgcagcaacagaaatagcttaccagctgcaaattagaaata

ttgcaggtgtaattataattgattttattgatatggaatcacagagagatcaattacaat
tattagagcacttcaataaagagctagcacttgatgatgcaaagcctcaaattgtacagt
tatctgaattaggcctcgttgagttaactagaagaaggcagggcaaaagtttatatgagt
taattagtagtgacttcaattactttcatctccttatgcaatcagagcagactaagtctc
ttgaaagaatagatcaaaaaaagcaaatcttccagactcttaatagatcatggctatctg
tagaaattaatactattaataaagtatttttcaaaaatcaaattttatatacttacca
acttttatttagcacgtgatttatatgtaattaacagtaatctcacttataaacaaaatt
gtttattcactactagattgaaacttatctactctcaagagtataataaaatcttgcctt
gtactggttatagttgtctcatcagttctaatatagactataatcaaaagttccttttgt
gaactgtcggtttaaattgt--
--------------------taaaaaagaagccctctcttgtatgtgagagggcttc-tt
ttttagcttactaactattctagtgaattaattagaaattaaccattgacagctggagct
gctagagctactggcaaggattcaccagaagctagatctagagggaagttatgagcatta
cgttcatgcattacttccatacctaggttagcacggttgatgatatcagcccatgtattg
attacacgaccttggctatcaacaacagactggttaaagttgaaaccattcaagttgaat
gccattgtgcttacagataaagctgttagccagataccaactacaggccatagacctaag
aagaagtgtagagaacgagagttgttgaaactagcgtattggaagattaaacgaccgaag
tagccatgagctgcaacgatgttgtaagtttcttcttcttgtccgaatttgtaaccatag
ttagcagattcattttcgcttgtttcacgaattaagctagatgtaactagggatccgtgc
atagcactgaacagagaaccaccgaatacaccagcaacacctaattggtggaatggatgc
attagaatgttgtgctcagcttggaacacaagcatgaagttaaatgttccggagatacct
agaggcattccatcagagaaactaccttggccaattgggtatactaggaatactgctgct
gctgctgctactggagcagtaaaagcaacggagatccatggacgcatacctaggcggtag
cttagttcccactcacgaccaatgtagcaagctacgccagttaggaaatgaagaacaact
agttggtaaggaccaccgttgtataaccattcgtctaaagaagcagcttcccaaattggg
tagaagtgaataccgatagctgcagaacttggaataacagcaccagaaatgatgttattt
ccgtatagaagggaaccagcaactggttcacgaattccatcaatgtctactggaggtgca
gctacgaatgcaatgatgaatacagatgtggcagttaataaagttggaatcattagaaca
ccaaaccaaccaatgtataggcggttttcagtactagtaatccaagagcagaaacgttcc
cacaagctagcgctttcgcgtctttgtaaagtagcagtcataatttttttatcagtttta
aggatttaactaagatgcttcccaaacaatattttgtctgttaaatatattattacttaa
gatcgttttaaatgagaaca--tttttaaaaaaca-taattaaatttaatctcgataact
aagaaaatataattattgagatattattatataattgaa----------aaaataaattt
tt-act-------------ttttgcagaattagtatgattttaagggtataataatagta
atgcatactaatgctgcttattttgatttcaacagtttacataatcgagctttacttaaa
tacgtatgtcacactttacaaaaattcagacgactatacaagacttaaatctgttaaagc

atgctttaacagatttaggtttaatttggtctatgaattctagtcatatacaagttgatg
aaaatagtcagcacaaagtagatatcttgatcagacaagataatttatcacatattggct
ttatctggaatgataataagtaccacttagttgctgacttgcaactttgggaacaacctt
ggtctttagaagtatttttagataaattatcgcagaagtatgcttaccattctattatcc
aagaaacgaaaaaacaaggctttgaaaaaatggagcaaatctcgcaaaacgatggatcaa
ttaagttagttgtgcaacgttggagttgttaattaatatagaaatgcgggcggagagact
tgaactctcacgagattatctcactagaacctaaatctagcgcgtctaccaattccacca
cgcccgcactgctgtagt--aataatactatatcacaattcatgaataaaaacaaactca
-agaaataaattagctaccaagtttaaatgcatcaacaaatagaccatatgttataccaa
ttttgaagttattaattaaattgatttttatttttaatttttttgattagagtaaacga
tcttactagttaactctgttgctgcaactattagactagctgcaatgattccccagtctc
ctgtttgcccaggaatcgtagataatcctgttgaaataaagaagcctaataacaagctga
ttaaaccagttgttaactcacttaaagaatagtacagtttatcatttaagttttctacta
aatatgcaaaaaaggtagatagtcgagttttaatcataatatttaataacggagttttaa
aaggataataa-----------agattaatgtttttagatccttttgaaacccgtcaata
caaaaattgctttttg-----------tttaaagatcttgctcacttaaagcattaataa
gatgttgaaatggttcttgtataatgtctttacgtttaattgattgtaaatcaagttctt
cttctattatttcttgcaataattgtatgcttcttaccgttggagcaacaggcacagaca
atgaattataagtatctttgaggccgtttaatacacgctgatctagaatattagtgctac
cagagactaatgcataactagcatagcgaaggtagtattctatatctcttagacatgcag
catatcttctagttgtataagaatttcctccaggtcttaataattcgggttgctcttcgt
ataattgagcagcagcctcttttaatatatttgtagcctgattattgataatttcagcta
tttttattctatccaatccacttgaaaagaaagattctatttgtccgacagctgctttat
ctaaataacgaccagtaagatcgtagcgatttaatattgctgttatagcatcttgcataa
attaattctcctac-ttgctatcagagtggtattatgaatgactagtat-----tgtaa-
----------tataatcatcact-------------------------------------
-----------gtttgtataaatagagtatatgtagtttttaattactttttttataaaa
attaagaaataaagatttttt-taataggtttacaattttcttaatataactcaggttct
caaccgagataagttttgtaacacat--aagctaaaataaagatgaatgagtaatcttga

--------------------------ataagaataatatgcatcaa--------------

-----------------agtatagatagtcttttactccagcatcaaaaaagatatttaa
cacatattgaaaaaattgattatgtaagtaaaaatctaaatttagataaaattagctatt
ttataattacaaccaataagaataacaaagatatccttgtagaacaattttgttataatg
cagatggttatacttattcaatattatttaaaggtcaaacagctaaacacctttgttaca
tagttcttcagcttaaacaattaaatataagaatatctacagctcatgctctatatttag
gacgagaactaatgaagtcggaattaactcttatattagatcaacaatatatccaagact
aaa--cttcatagatattaattatatttgtgctaatatagtattaataagggcatgtaac
tcagtggatagagtatcagattccggttctgatggccgtgggttcgaatccctccctgcc
cggcgagtatttagaattct-gtggcaagatatgt-------tagtatctaatttaataa
tattaaaaatattggggctgccaggtttctacattataaaaacaagaaaatatgaaagtt

aaacaagctctttgaaagagcctctagtaaaattaataaatgcagaaaacaatattgttt
ctttttctcgaaaattagctgttgcttaaatactgttaatttttggcattcgacgtgtta
agctcttattcgcggcgaatattctgttagagttgctcttagtataagaaaagtttcaaa
atctatttatatttatgtttttacctatttttatttaaagtaaggttagta-ttttcaa
tttttataatggacgcgggttcgaatcccgccagctccatcaatttaact---------t
tcataattaactaatccaatctttttaagaaccagaaatataattggagcaaatatggat
tttataaaaatcacagaacaagcttttaagcaaattgtagtcttgaaaaatgattacaat
aatgatgtacatttaagaataggtgtcagacaaggtggctgttctggcatgtcatactca
atgaattttgaagatgtagctaacttacaagagaccgatgaaaaactgattttagatgac
ttttctgttgtttgtgatcccaaaagtctttttatatctttatggattgtcattagatttt
agcaacgacttaataggaggagggttccaattctccaacccaaatgccagccagacttgt
ggttgtggtaaatctttctcaggctaaa-------gctactttgttttatattttgtatt
atacgtgttactcttataatcagccattataaatacaaaagtatttttattgaaagctct
ttaaactagtcaaagccttatgaaaactactttaaagaggttttatt-tattatggcagc
ttaaataagtatttatcatcaattgatgaaagt---------acaaaaattcactccctt
gttattgcctaattttaattagttaatttttattgatacatgctaatattt-attagcc
ctgatttattaggaatttataatttctttttataaaagagcttgtcagtttctagcta-
----------------------aaatagattatatatttt-attcatttataaaaactc
aacatgaatcttgctcaagttacttgcaactacttcaatacgaaaactctcaataagttt
tattgcaatacggaatatgtttcctatggtaattttagtcatctcaatttatcaaagata
aatttgccatcttctgccaaaataattgcatttggactccctcaatttgattttattcct
acaaagattaaaaactctatccgagaagcagtggtaatatctaccatcgatgacagtaat
atttttgctgattcaactaataattggataaaaaaacataaagataaaaatcaccactct
cttttctataaaaaaattttaaagctttactgcatcaaaaaataaaagtcgtgacagat
tcagc------taaaaattctaaaatagataaagagaagggcttgattttgagtcaaaaa
tatatattaaagaacagttggaagccatccataatgtcttcttactttgaaaacaaaaaa
aataatgaggttgctaatgtatctaaaaaatttttaatcgatcagctatctgcatcacct
gtgtttgtagtaaaaaaatggttttaatgaaattattttaggccacccagcattccgtata
aagagaggtgtaattagtaattacactcactcgttttcaaatttattaaagcaatctcag
ccaacttatcctatatctactggcttattctttttcatccagatgacgcgtttgaattt
aaaaactttataaagtcggttaatccgttggctagtgagcatatggaaataaatgtagaa
cctgttggcctacactttgcttataaaatgaacagaaatgtatcttctgatactcaattt
ttattcattccagattttaaagaggtaggcgatttattatttaaatataaaaaagataag
cacttaatctttcacaaggatcagtgtcatggtaaaaattttttcaggggcaacctata
tatattattcagcctactattttcaaagatcgaagcggaaacctaaatactattcaattt
actggactaaatgataatagagaagttatttttactagtcttgaagctgctaataaatca

tggacaaattttataaaaaacaattcacaattaaaatcaattagaaaaccgactttatta
gtctataatctagaaagtttttaaaagatcaagaacgattgaataaacaagattttcaa
aaatttgttgtgattactaataaaaaagcttatcttgctacaaaagagttggtagctgta
cccgattctaatagcgttttaaagcatttaaaattaaatatgaagcctaaactcttcttt
ataaaattatgggtaagacggctattttccaccttaacttatgaat-aaatgatgtt-gg
atttattttcttgaatagtattcaataactagtaattcatttagttgtaaagcaacccat
tctctgtcaataactccattgacttttcctgatagattagatttatttaattctaagtgg
ctaggaatgttagctaatccagggaacgctaaatagttttccactagtcttttagatgtt
tcccgagattttacactaattaattctcctggcttacattgataactacatatagatact
acttgtccattaatgcaaatgtgaccatgatttactagctgtcttgcagcaggaattgta
ggcgccattccaagtctaaaaacagtattatcaagtctcatttctagaagctgtaataaa
atttgacctgtagatccttggagtttttttagcggccttaatatacttaaagagttgtttt
tcacttaatccataattaaagcgcaattttttgcttttcttctaatctgacagcatattca
gaaggttttctagatttttgtccgtgctcccctggtggataagattgtttaattgttttt
ctacttagcccaggcaaatcacctagtcgacgggaaatgcgtactcgtggtcctctgtat
ctagacatattttataaattttccgaatattaat-gattaag----------taatgagt
agctattttttaacacaaaaagagtattaaaacaaagc-ttttgctaaaaaattttcatg
aaaaatatag--------------------tgctttac-ttttttaagcgggtagcggaa
tcgaacccgcatcattagcttggaaggctaaggttttaccactaaactatacccgcaata
ttcaataaactgtaacaggcttaatataacataactatcttattcttagatattattaaa
attgacaagaag--caaattatttatgctccttctaaaactacatcaagaaaacgagcaa
tctctgcagcttgctcttcaatttctgaaagcaataatggttgacccactctagttaatg
gaattactttttatcttttgtgcataagtaaatttctcgtcgtggattcaaaccttctt
taatatctatttttattgacttaatatcttttatattgaattgaagacaaatttgtctat
tttttcctgggaaacctaaacgaaaaattttaacagtaccttcatctttattaaactcat
tatatccagcacctatgtcccaaataatagttaaccataaaaataaactaagaaagattc
cgatgctgccatagaatgtcataacaataccttgcggaataaatattaattctgttgaag
tcgtaaaaggcaataaattaatttgaaagtaacttgataatcctgcaagaagaaatcctg
aagctccaacaaagattatggtagcccaccagtaattactaagtcttcttgaacctaaaa
ttagatcttttctaacagagtctatagataatgtttttttcataatttgtt-ttttaaaa
caata-tactaatttgacatataaaaattttgacaatttttttataagcgttttagattt
attaaatgagtttgatagcttttaatcctaaaaataatcctgttgctaaaccaaaaaaag
ctgcaagaaatattatatatcctagaaaaactgaca---tatta----ttattattattg
ttacaatagtgtttacaaaaatagttgaaatctattactaaagtattatattacgttaaa
tgcttcgctgtctacatgaaaaaactttttcagtttttagcgtcgtatttatattagaa
ataatattgtaataattttattaaacttctggagaacagaatcttatggcagaatttatt

aagccttataatgacgatccttttgtaggaaatctatctacaccagttagtacatcgagt
tttagtaaaggattattaggaaatttaccagcttatcgtcgaggtctatctccacttcta
cgaggcctggagataggaatggcacatggatacttttttaattggtccttttgataagtta
ggtcctctacgaggtacagatgtagctttacttgctggattttatcttcagttggcttg
attattattctcactacatgtttatctatgtatggaaacgtatccttttctagaacagat
tcaaaagatccattgcaaacagcggaaggttgggggcagtttactgctggattcttagtt
ggagcagtaggtggctcaggatttgcttacttattgctagctaatattcctgtactccaa
actgcaggtttaagcttgttttcttaagctagtaaggggacttgaacccgtaacctactg
attacaaatcagttgctctaccaattgagctatactagcgctcagctaattgtaacatat
aaagatatttgttgcaatctggtattttcagatgcaacaaatatctttttttatttagaca
gaactgttaaattaatatatgtatgatatgttaattagaatcagagctatctgactcagc
agaatattcttgatcacagtttaaataataacaaattgtttgatctagacatgttgcgga
ccaaccaacaggtgtttctcctgataattcttgcatatctccagaattgtaaaaatcatc
acttggcttattactatttgcttgcataaatatcactcccaaaacactctgaacttaatg
tgaagtaattgaacttctcaataatatattaacacagaatataaaatatggtgcttattc
accaattgtaacaatattatgaaatctacaagttctttgtaagtgttttaatagcttttg
ctgaaacaagcattttaatccatttattcttctctcttgaccatactttttctgttgca
aattaacttttgtaatttttttgttcgtttgtgagaatgagagactgcgtacccattat
tcgctactttccctgtaagctgacatttttttgacataacgtt------attaacttata
tatttatttaaagtacttgccaaagttgactttggtacagcaccaattaccgtgtcaact
ctttctcccgctttaaaaatcattaaagtaggtatacttctaataccatattcagctgca
attgtaggattatcgtctgtgtttatttttactaccttaatagatgactcatattcttct
gcaatctcatccactacaggagagaccattctacaaggaccacaccacggcgcccaaaaa
tctacgagtacaggtaagttattattaataacttcttgtttgaaagaggcatctgtaact
tgagatactgacatatttttttaacctttaatatatgttccttgctaagcaaatcttatca
caaatttcaagaaaaatcttctatagtgaattactattagtgttgaattaa---ttt-tt
tactagacattaaaaaaacttattacctatataaataaagtggtggccgagtgatatctt
gatagtgataatttttctattaaggttgaacactagaaatcatttttcaatcatatattt
ttttgaaaa--acttctttgtgaccatgaaagc-taagtatattacaatatattttatta
tttgtaacttatttcttataatggtataaacaacgcaaaagatactgccttataatcaag
gaggaatacatgtctcaatccgtagaatcacggactaggattaaaagcgaacgttacgaa
tctggagtaatcccttacgctaaaatgggttactgggatgctgactatgtaattaaagaa
acagatgttttagctctgttcagaatcactcctcagccgggtgttgaccctattgaagca
tctgctgcaattgccggtgaatcttcaacagctacttggacagttgtgtggactgacttg
ctaacagcttgcgacttatatagagcaaaagcatatagagtagatccagttccaaacgtt
gcagatcaatactttgcctatattgcttatgatattgatctatttgaagaaggttctatt

gcaaacttaactgcttcaatcattggtaacgtttttggatttaaagctgttaaagctctt
cgtctagaagatatgcgtatgccggtagcttacctaaaaactttccaaggtcctgcaact
ggattaattgtagaacgtgagcgaatggataaatttggtagacctttcttgggtgcaaca
gttaagcctaaactaggtctgtctggtaaaaactatggaagagtagtatacgaaggttta
aaaggtggtcttgacttccttaaagacgatgaaaatattaattctcaaccatttatgcgt
tggaaagaaagattttatattccatggaaggtgtaaataaagcatctgctgctgctggc
gaaattaaaggccattaccttaatgtaacagctgcgaccatggaagatatgtatgaaaga
gcagaattctccaaagaggttggtagtatcatttgtatgattgaccttgtaattggctat
actgctattcaaaccatggcaatatgggctcgtaagcatgatatgattttacatttacat
agagcaggtaactctacttactctcgtcaaaaaaatcatggtatgaacttccgagttatt
tgcaaatggatgcgaatggcaggtgttgaccatattcacgcaggtacagttgtaggtaaa
cttgaaggagatcctttaatgattaaaggcttctacaacactttacttgaaagtgaaaca
gatatcaatctacctcaaggtctattctttgctcaaaactgggcatctctacgtaaagtt
gtaccagtagcatctggtggtattcatgctggtcaaatgcaccaacttcttgattactta
ggcgatgatgtagttcttcagtttggtggtggtacaattggacatcctgatggtatccaa
gcaggtgcgactgcaaatagagtagcattagagtccatggttatggcaagaaatgaaggt
cgtaattatgtagcagaaggtccacaaatcttaagagacgctgctaaaacttgtggacct
ctacaaacagctttagacctatggaaagatattagttttaactatacttccacagacaca
gctgatttcgttgagactccaacagcaaacatctagtttaatgactacttgctaatgctt
aatttggcaaattgtaagtagaattgacttataacaataaggagcatagaatagtgagag
taacacaagggaccttttccttccttccagacctaactgatgaacaaattaataagcaac
ttgcttatatcgtttctaaagggttttcagcaaatgttgagtatactgacgatcctcatc
caagaaactcatattgggaattatggggattacctttatttgatgtaaaagatgcatctg
ctgttatgtatgagattagttcatgcagaaaagcaaaacctaattattatattaaagtta
atgcttttgataatactcgaggtattgaaagttgtgtactatcttttattgtaaatagac
ctattaacgagccagggttcttattacaacgccaagactttgaaggtagaactatgaaat
atagtttacatagttatgctactgaaaagcctgaaggagctagatattaatttcaattaa
tatctaaaacttatgagttagttacccctctttaatatcttaattactaaattaattttg
attttaaagaggggtaaatttcgaaataaattctagttaaattagaattaacaatccaaa
atacacagaaataatgcaatcacaagacataatctccaacgatactcttgttaacttaca
agaagaatatgatagaacacaaatccaggaagttcttaatgaattaaatcaagaacttat
aggattagttcctgtaaaaactagaattcgcgaaattgcagctttattactgattgatag
attgcgcagaaaactagaactagtatctggtaatccaggattacatatgtcatttacagg
tagtcctggaactggcaaaacaacagtagccatgaaaatggcagatattttacatagact
tggctatataaaaaaaggacatttattgacggttacacgagatgatcttgtaggccaata
tattggccatactgctcccaaaactaaagaagttctaaagcaggccatgggaggagtttt

gtttattgatgaagcctattatttatataaagcagataatgaaagagattatggctctga
agcaattgaaattttactacaagtaatggaaaatcagcgaaatgacttggttgtcatctt
tgccgggtacaaagatagaatggaaaaattctatgaatcaaatccgggactttcttctag
agtagctaatcatgtcgactttccagattatacttcggacgagttattacaaatagctaa
aatgatgatagaagaacaacaatattgttttacagaagaggcagataaaactctgctaga
atatactgagcgaaggatgaaacagccatatttgctaatgcgagaagtattcgtaatgc
tattgatagagctagaatgaggcaagccaatagaatttttgcaagtggagaaaaagtttt
gacaaaagctgatttagtaacaattgaagcagaggacatcttgaaaagtagattattttc
attgcccaattcttaataattaagtagcttaatacaacgagatatcataaaaaaactttc
accataagttgcaaatttctttaaaaacctatactattaacatagtaaaggtg-tggcgg
catagccaagtggtaaggcagaggattgcaaatcctttatcccccagttcaaatctgggt
gccgcctagtactaaaaa--gggggtgtggtggaatggtagacacaacagacttaaaatc
tgttga-ttttaataatcgtgagggttcaagtccctccacccccatgtattaacttataa
aaaaaca---atgtgtatttgtattaattgtaaccatattactgaatgtgatacttatca
tttaatagaatctcagcacaaacagctgcatcttactgaaaccccagctttcatacctaa
atgccctgtagtttgtgttaatatatctagtaatagcagccatactcaaatcgattggga
tttagtggaatgcttatcatttgtggaaaaaccaaatagttggaatttaggctgtgatta
gttgtaaaaagaattatgtggtatacgcaataactctatcttttaaatactctgtattaa
ttttagatgttcttaccaacaatatttttcctgttcttgctatctctattataccaaatt
tcgttaataattgttcgatggcaacaatctttcctggatcaccagtaacctctataatta
aaagatcttccgcgatatctactatcttagctctaaaaattttcacaatttctaaagctt
ctgttctggtctgagaattaatcctaatcttaattaacattaattctctttcaactgaag
gaatatttgttacatcttgcacgttcagaatatttactagtttatataattgtttagtga
gttgttcaatagttctattgtctccctgaactaccatcgtaattctagaaactccgattt
gttctgctggtccaactgctaaacttgcaatattgaagcctcgacgtgcaaatagaccag
atattcttgaaagtactcctgcttcatcttgaactaaaactgataaggtatgtttcatga
aattttaatgttatactgcttgatctattacgtatattctatgaaaattacagaaaaaat
gctagttactatataactaaattattccgactaaacaaaatcccaccatcagacggcata
tctattttgtaatgttatac-----------tatttaatgatattctaattctaaataga
atgctaaatttttttaaaatttgttaggaacttgtgctagaaaaatacaaaaacagtaaga
aactctctcgagatctaccccaaatcaacgatcgtattagatttccaaaagttcgagtaa
ttgacgatgaaggtgaacaattaggtatttttgtacctgaagaagctatccaattagctg
cccaacaaggattagatttagttgttgttagtgacaagtctgatccaccagtttgccgaa
tattggactatggtaaatataagtttacacaagaaaaaagagctagagaagccaaaaaaa
aacaacataacagtagtgttaaagaagtcaagatgagatacaagatagaagagcatgatt
ataaagttagaattaatcaagcatctaagttcatccaagcaggagataaagtaaaagtca

ctataacattccgtggtcgcgaaattcagcattctaatttagctatagatttgttaaaca
aaatggcaagcgacttaacttcgatatctgaaattcagcaagctccatcacgagatggaa
gaaatgttataatgcttttatctcccaagaaagcgagtt-aaatgattataatttaaatg
catctggctggattcgaaccagcgacgtctctttcgaaatggcggattatgagtccgctg
ccttcggcccctcggccacagatgcatcacttaaagtttataactcataaagtatataaa
aatcaatgcttgtttctttttcatgacaccta-aaaaaattattattcattcatattatg
ataggtcgcaactgctgaaggggaaacttggtttagatacctaaatatccagtatttaaa
tactgtgtccagtataactggaaaagttgaaataaatagaaaaataaagtcacgactttc
aggtagtcctaaatgccttaaaattacttcaataataacttcccagccatgaggagaatg
aaaaccaacaaacatgtcagtaaaaagaataattagaaaagctttcgcagtatcactaag
gccataaataatttcatttaaaaaagatttaacaattgcaatttgcctttgtccagttat
cattaataagataaaaacaaatattgatacaatatcggacaaaatatttttttacagcgtt
tgcactttcatttgcataatattctcctaattctctagccttcagctgtactctttttttc
tataatttcataagatatatcttctgatgggttcaataaaacttcaaagtgaatttttttc
ttcaaacctttgtagttcagcgaaagctctctcttcttgagatgaatttaaaaaaaatttt
aggttgttcctggttccataaataatcaatacaaggaccaaacacaaaaaaattttgaagc
ttggttcactaatactggagagataaataataataaaatatattttacagaagtaatagt
ttgatgcctagatattctaaattcttctatggcttcagattctccattcggatctaactc
ttttcgaaacttttcaaaagtattagtaatagacctaggtattggcccaactttttcaaa
ggcaaattgattatttcgttttaaattccaatatttcatttttttattgctgctaaaattt
ctattaatcaataattgatcacgtattcactataaatattaattctagaactaaacatat
aaaacaatagttatgaataataaaatacaattaaactttcattatgcattattactatct
tttactacagcaatgtatggatataaatcttatcgtgcaactatgaatacactgttaatc
aaatgtgaagatagccattcattattgtctggtaaacatactgcctttattcctataatt
atattaaaaaaaaatctttaaactccctaaaatcagaacctcacaatactgagttgtgtc
ttaatggaatgggaaatagaaaattcattgacaaaattgcaagtttgctaagccgtttag
aaaaacagatgcactatactgaacatcttaatcaaataaaaactagtgggtacttttctt
tactttatattaacagtaaagcaatctatacaaaaaaaaattagtaatatttttctattac
ctaatgatgttttaaaaagaatttatattcataataagaaaaaaaagcttatacctcata
cattcttaataaaattatttcaaaaacaaataggttacccgaaaaattttgcgaacttaa
acctagccttatcaaaaattatgcaatggtatgctgatagaggatatcaatggtctttag
tggagatcaagcaggctgctgatccatcgtatctcattctgaatatccatgaaggattag
ttaagacaattacaacagagtattatactttgtcttataaaaaattacgcagtatatcaa
gtacggaatcaatagagaagtatcttggagtaacagtaggacttcctttaaatattaatg
ttttgcaaaaaaaaattaactatttaaaagataatcaattagttggcaacattatttata
gtattgagagatcgagcaataactctatgaatttagatatcaaatttcaaatacaagaac

taaaagataaagatttactagtacttgtagaaagatcatctaaaacatctcaaattattt
ctcacgtatatgacttaatagatttagaatgc---agttcctctaattggctagcagctt
ccaatataatgtcactatcgactagcaaaatacaagcatgctattttgatagtaaaatcg
attctgaatacgtctatacagatgctgttaatttaataaagcggttaacttattccattt
ctcatcaaaaaacactaataattgacgatttaatttacaaaatttactaagctggacaa
aaagaaatactattggattccaattgtatttacggaatttggatcatgccaaatcttttt
gtctattcagtctaaaatgcatccagagtggacttaatataaaaatttcgtatctaaatc
cttcattaagcattagtcgtaatttgagttttcaaattgcagtacaaataattaaacaat
atcaaacaagtaaacaatcagctctatctt------------------------------
------tgtttttaaaaagggagatttagcacaattcattttgaaagtatatgttcat
atcattttacatcttatttttcgatatctgaaaagatattactttctcgagttgtgtata
tagattccatattttacaattctgaaaatataggtctttataataataataaaaatcaca
ttataagtagctatgatatctttagacacaataagaaattattatatcaagaatttctga
ccttattgttaagtatacgttatcaaaatttcaattatttagactggcctttaaaaggct
atttatttgaaatgaagtctttatattttaccccatttcagaaaagcaatttttttgaacc
actcttgtcttgacaactacaaaaacctatttttttcataaaatttacttaaaacaaatct
ctcattttagtttaccactgtatttcagagcccatcttaaccatattttagttaatacta
ttaaatgtcagtcgaatttaaatatggaaactatatatctattattaagcgattatccta
ttaaatacactctgcttaagtctattgttactttttctataaaagttaggatggaatatt
taatacctataagccggagcatcagagtatctttgttttataattatctagactgttttt
taatgagacaatctaaacacttagcatatatactggaagatctgtcaggccataaacaat
tccaaagtttttttattggaaaaattttcatggggatttggaattcagcttaaattaccaa
ttaagcagatgccacccctttcaattgaatatactgtaaacagtggtcgccacttttgtc
tttacctgcatatttattatcaaagataattattattaaaatgaaaaatttaatttcttt
ttttgaccgaagcaaaggaaaatggatttcacagcgaacaacctatgaactacgtaataa
gaatatgagttcagtacaatctcaaatgcgaatacaatcaggtaatttgttatcaggatc
tacgcgacttgcatcattgaattggggagaaatttttagacaggttaaccataattctta
ttctcaagatacaagcaaatataatggccagtttaacttagaatttagtaatcatctaaa
taatcgtaggctattgacatcatgcacagtaacagatcctagtctagttaattttaaaac
tcgatatggtagtacaactatagatgaaacatattggtttgcaacaagtaatttacgtct
aagtactagcattattaaaaaatttgatacttgtgttgcagtatctttttgttcagaaat
caaagtttaa-attctgtataaaaacaactaatagctaaaa-agttattagttgctttta
tatatctaatttttgttttagagagtatataaaatattatattattctaaatctttacgg
ttaggatttctagaaggatcattagataagaatccaaaaacaaacagagaaataaagaaa
atgacagtagtataaacaaatattttcaaagtaaacatatttttatccttaaaagctatt
agtgtg----atatttatatatc-acacataattttatatcacgatataagttctggcgc

tattttataaacaaatcaatatctttatttttttagtaatgatttatacttcaatatgcgt
gatgcactaaatactaaatctttttgtacacgcttctcactatttttttaatattttgtga
tttctatgaagtttaccttcaattactacataatctaatttcttcagcaatttaaaagtt
tctaaagacttttttattccaaataattaagttaattaccgctaatctttttctttgtaaa
agcctaactttcaacttaattatttggtgtctactatcactaattttaatactcttacac
ctcaaaatttgaactagtaaggtacagctattcat-attaat-ttaa------tcttgta
actaatctattatgacagataa-atgaaaaaaatcaaaatgaaatttcattatcttgttga
ccttgttcataactaatatcttttaatactttttaatattttcccgaaatactcttgcaaa
tatttttctaacgatattacttctgagctatcaatgcttaaaatactataaacttcattc
atcgaagcagtaaattgctccccactagttaaaatctcggcaaatgctaaacgatcagaa
atattccatgtccattgaagggttttagtgatttttcttaaagctgttaatagtccaatt
ggaatttgtgatatttgtgttttttgcccagataacttttcacataatttaataatttca
gctgatgtccaggccgtgttaccaactaatggcagcactctgttttctgtagaaggcact
ccaagacttttttataactagctttgccgcatcctgagtatcaatatatgcaataggtgta
gactcccctgtaacccatactgatttttttatccaaaataggaattgcatattggttaatt
aatccttgaaaaaaccctcccaaagaaaataccgtgtactttatatcagatttttgaaga
aagtctactacttgtgattttaagttcatcaatggaacagctgggtacttatctgcattc
aatattgaaaagaaaataaatctttcaactttagcagctttagcggcctcaattaaggca
attttaccatctaaatctatttttgctgcattgtaaggatctgtaggacgagatgtagac
gcatctatgactgctgttactccacaaaaagattgcaaaatactttcaggtaattttaaa
tcaccatatacaagttctgcgccccattctttgagaaaagctgactttcgtaagtttctc
accatgcatttcacattgtagccttcatcaagagcacgccttacaatttgacgtcctaga
gttcccgtcgctccaattactagaagagtcatattaattaaaaataagtatgtataaaga
cattggtttggtcgacagcagagattaaattaggtagagagggaatcgaaccctcatgac
cgaagtcgtcacattttgagtgtgatgcgtataccaatttcgccatctaccctattatag
accaactactacaagtaatttcgtctttagcgtaaaaataatactaagtttcaatagata
tgtcaacttttgagtttatattaataaaaaaaccggagtatcttatgtccacttttgaact
aattccttatagattatatttaagtcaatctagtacgtggatgcataggatgaaagcaga
aattaaaatatatatattaacgatattatggatttcaatttttattttctcttactgtaa
actatgcattgttgccctaagtttaattgttcttagccttactgtaaagaataaacaaaa
cattatccagagacatttattgcgaactttagttatgacaatggtaactaccttcttatc
attcagcatagctactaattacaaacaatattctcaaccaaaacaatcacaagatttatc
ttactataatacagctaaaaattcttacatctatcaaaatatattgctacctaatagtcg
taagatcacaacaaaacaactgaggtgcactttaaagcctgcactctatttttttattac
tatatattcaattaaactagtcatgataacgacttccccagaaattttagtgattactgc
ttatagatctaggatctttaatacaatattaaatcgtgaattattatttactttcttatt

gtcatcacatattgttaccaatataattagtaaattagataaacttgttcaagtagctaa
cttaagaggtagcttaaatttatataaatcgtccacaagacttttatattttcttttt
gatgtttcaaatctttttttagagattattaaggaatcaaaagaaatatctcaagctct
ttatactagaaatcttaatcaagaaaacagtaatttttaaaaatatatagagccagatc
gggtattagtgattggttaaatgcaattattgggaccttatattttattattttaggatt
agtataaca-aatctaatcatccttctgactctggtaatacgaatagtaatagacttaag
tcgtggtgtaaatatgaaaaat-tcagatattatataaataattaaatgttttattatgg
tcaatactcaaaatcaaatttcacaaacttcagagcttgattatatagttaatcaacctt
ataaatatggatttaaaacttctgttgaatcagaacaatttcctagaggaatagatgaaa
atatagtaagattaatttctaagaaaaaaaatgagcctgaatatttattaaactttaggc
ttaaagcatacaaaaaaatggaaaaaaatgagtagtccatcatgggccaatctcaagaatc
ctaatatagattttaacagtattatttattacgctgttccgaagttaaagaaggaattaa
acagcttagatgaggttgacccagaaattcttgatacttttaataaattgggcatctctc
tcaatgaacaaaaacgaatttcaaatgtagctgttgacgctgtttttgacagtgtttcta
ttgcaactacttttaagaaagaattatctgcagctggtgttatattctgctcgatttccg
aagctatacgagattatccgaatttaattcataagtatttaggtactgttgtgcccgctg
gggacaattactttgctgcattaaattctgcagtatttagtgatggttctttttgctata
ttcctcctaatactgtttgtcctctagaattatcaacttattttcgtattaataacgaag
aatctggtcaatttgaaagaacactcattgtagctgatcgtggtagtaaagtaagctatt
tagaaggttgtactgcccctcaatttgatacaaaccaattgcatgcagcaattgtagaat
tagtagctcttgatggcgctgaaattaaatactctactgtacaaaattggtatgctggca
atcaagagggcaaaggtggtatatacaatttcgttactaagcgaggcttatgctctggca
ataattctaaaatttcatggacacaagtagaaactgggtcagcaattacctggaaatacc
ccagttgtattttagctggccatagttctcaaggagaattttattctgtagcgttaacaa
ataattatcaagaagctgatactgggactaaaatgattcatattggtaataatactaaaa
gtagaatcatctctaaagggatttctgctggtaaatcaaaaaacagttacagaggactag
taaaaattggcgctcaatcatttaattctcgcaattattctcaatgtgattctttattaa
taggtcaatcgtctcaagctaatacttttccttatattcaagtacagaatccgacatcga
aagtagaacatgaagcatctacttcaaaaattagcgaagatcagatttttttactttttac
aaagaggtatcagtttagaagaatctgtagctcttatgatcagcgggttttgtaaagatg
tattcaatgaacttcctatggagtttgcagctgaagccgatcgtttactgagtttaaaat
tagaaggaactgtaggatgagtcaaactattttagaaattaaagatttgtatgcgtctat
tggtgagacaacaattttaaaggggggtaaacttatctatacaagaaggcgaaatccatgc
gattatgggacctaatggttcgggtaaaagtacactatcaaaagtaattgcagggcatcc
agcttactctttaatcagtggagacatcttatttttttggaaaaagtattcttgaaatgga
gccagatgagagagcaagagctggtatttttcttgcttttcagtatcctgttgaaattcc

aggagtaagcaatgccgatttttttacgcattgcattaaatgctcgtagaaaatttcaagg
attatcagaatttagccccttagaatttttttcaattaataaatgaaaaaataaaccttgt
tggcatgcaagaaagtttcttgacaagaaatgtcaatgaaggattttctggcggagaaaa
aaagcgtaacgaaattcttcaaatggctttgctagatagtaacttatctatttagatga
aacagattctggattagatatagatgcacttagagtagtagcaaaaggaattaatacttt
agcaaaatcaacaaattcaattatttaattactcattatcaaagattattggattatat
tattccagattttgttcatattatgagtaacggaaaaaattgtcaaaactggcaatcttac
tcttgcacaagatctagaaaaaaatgggtatgactggattgtacagagttaaaaatttgt
taaataaggcaatactgagactaaagtatcttctttagtctcaatattattgatcaacag
aaag-----ttatttaggctttactaaaaccttgcttaacatcttcaatggctcttttta
acagttcttcactagatgcgtctaattttttactgctacgaatactttcaccaaattctg
gtttagaattctttaaatcttcccgtaattctttaataaattcagccacttttgatactt
caatatcatctaaataaccgttaattccagtataaataatagctgtttgttcttcaactg
gaataggagaattttgagcctgcttcaaaatttctctaagacgttgacctcttgcaagct
gattttgagttgctttgtctaaatcagatgcgaactgagaaaaagcttctaattcagcaa
attgtgccaactccaattttaatttgcctgccacttgtttcatcgcttttatttgagcag
cagatcctactctagagacagaaataccgacattaatagctggtctaatacctgagttaa
ataaatcacccgataaaaaaaatttgaccatctgtaattgaaattacattcgttggaatat
atgcagacacatctcccgcttgagtttcaataataggaagagcagtcatactaccgcctc
ctagttctgaattcaatttagctgctctttctagcaatctagaatgtaaatagaatacat
cacctggatatgcttcacgtccaggcggtcttcttagtaaaagagacatttgacgatagg
cttgggcttgctttgttaaatcatcataaataaccagggttgctttacctttatacataa
aatattcggctaaagctgcccccgtataaggagcaatatattgtaaagttgcaggactat
ctgcattagctgcgactataatagtataatcaagagctcctttatcttgtagtgaagata
caacttgtgccactgaagatgccttttgtcctatcgcaacatatacacaaacgacatctt
gccctttttgattaataattgtatccagcgcaacagctgttttacctgtttgacggtcac
caataattaattctcgttgacctcgaccaattgggatcatagaatcaatagctgtgattc
ctgtttgcattggttcacaaactgattgacgtccaattataccaggagccatagactcaa
ttaatcttgttgaattacttgcaggttcacctttatcatctattggacgagctaagggat
caactactctacctaaaaaggcgtcaccaactggaatttgagcaattctaccagtccctt
ttacagaactaccctctagaatgtctctaccgtctcccattagtactacacccacattgt
cactttccaaattcaaggcaacaccaatggttttatcttcaaattcaagcaattcaccag
ccataacttcatcgagcccgtacactcgagcaataccatcaccaacttgtaaaactgttc
ctatattggctacttctatatcttgatcatacttttcaatttgttgacgaataatactac
ttatttcatcaggtctaatgtttaccatgtttttaatattctttttattaatgggaaatt
ctaaagtggtaattactcagattaatactatctg--tttttttactgtattttaagttgct

actacatcaaggtgtgaagccatctgccttagttgccctctaatactagtatcaatgact
ttcgatccaatctgaatagtgaaaccaccaattaactctggatcgacagaaacaattagt
ttaacttctttagcctgggtcataaccttaatttttctgttagcagattttcttggtca
gagtttaaagcaatagaagtattaatattggcaattgttagagattccatttgatatgca
agttctaaatacttactagctatagcatccaacattgcaattcttttacgatcaactaaa
atcattagaaatgatagtgtattttcagtaatttgatcaccgaaagtggcaataatgact
tgctttttgcttctgcagtttttaatggattagct---aaaaaatttttaagcttgtca
gattgcaataaaatattttgtattaactttatatctatactcaccttttctgtaactttt
tttgttttagctaattcaagaagagctacagcataaggctgcgcgatttttataacaacg
ttattactgctcatagttggtctcctaacttagcaatattattatcaatgatacgtagtt
gcatttcagagctcatttgattttctagctgcaaagtaactcgtttaagagcaagaaatg
ttatttgctgctggatttgccttcgaatttgtttttcagctgtttcaatatttgacttgc
ctgtcatagcaagtctttcaatatctaattgaccttgagctaatatagagcttctaactt
ttcctgccgttagctgagcttctttttgatttgatctataataatttgagtctgagcaa
gttgtttttctgattctgacaatctagcactagcttgctctagtctttcttctgattctt
gtatagctgctaagacttttatctgtcttgcatttaaactagatccaagaaattgtttca
aaacataaataagaccaaataaaagcaacaatatgttaataacatttgcttcaaaaatat
cagaattaaaaccgaatttatgttcactactatgttctgataaaatagtaattatttgtg
gtatttttactatattattcatttgaaattct----------tactattttaatatttta
cttaggaatcatgattttaaagattgactactcaatagtttagctttaatttggtcgctt
aaagtatccacctgatcttccaaagttttaagagcctcttctttttgaatatttagctgt
tttgatgcttcagcaatcaatttttcagcattcaattgagctttttttaatatcttcagag
actatactttgcgcttctttttgagaagcagctatctttaattgtgcattacgacgagct
tcagataaatcttgctcatattttgcagctaactcatctgctttaacaagcatagaagat
gctgtagtcaaagttgtgcggatatattcatcacgttcatctagtactctagttactggt
ttataaaaaatagtattcagtaaaaccattaacgtaagaaactgtaatgccattaaaggc
aaagtaccattgaagtcaaataagccaccttcaatttcttccgctaatagaaatggtaaa
taaatcatgttactaattatgttgatagttaataatttggtttcaattaagaaaattaac
tttatagtgcgcttagtcttattagactaagcgcaaatgattattttaaccaacgtatgg
attagcaaataatagagacagcgcaacaactagaccataaattgttaaagattccataaa
tgctaaactcagaagcagcgtgcctctaatttttccctctacttctggttgtctagcaat
accttccacagcattagctgctgcactaccttgaccaataccaggacctattgcagcaag
acctaccgcaagaccggcagcaataacagatgcagctgaaacgattgaatccataataaa
ttttgatttttttgttatataagtagaaaattgacaaatttcggataatacttaaaatcta
accaatgaaatattggagattcaaatat-----tgtaattatagtatactactcttctcc
atgaccttccattgcttccccaatataagctgcggataaggtagaaaaaattagtgcttg

gattgaactagcgaataagcctagtatcataactggcaacggtatcaaaattggaattaa
cagagtaaatacagaaacaactaattcatctgctaagacatttccaaatagacgaaaact
taaagataatggtttcgtaaagtcttctaaaatattgataggcaataatactggagtagg
ttgaatgtacctagcaaagtaacctaaaccttttttacttaagcctgcataaaagtaagc
tagagaagttagtaaagataaagctacagttgtattaatatcatttgttggagcagctag
ctcaccttccggaagttgaattaacttccaagggatcaatgcacctgcccaattacaacc
tagaatgaataaaaacagggtagcaatatatggaacccaagggcgatattcatgctctcc
gatctggtttttagcaatatcttgtaagaattctaatataaactccatgaaattttgaaa
tttttcaggaattcgctgaagatttctagtacctaggaaagatatagttaatagtgtagc
aattactaaccaagatacaataaaaacctgaccatgtaattgtagactacctattttcca
atatagatgtttcccgacttctactgctgacaagctagtatatggattaaaatctatgag
actgttttgatacataataaatatttcttttaaatagtacaagaatcaaaaaataagttt
tttgaagaattaatatgcaattagtttatttgcacgattagattttaataaattaaaag
caaaaataaattctaatagagatagctatctttagtagtgattatttttgtaaaaaaca
attattgctacctataatgcttttatgtttaattatatacccatatatgaattatttaat
atgattataggcatttcgctacaaactttattgttttatccatttt----aaaaattctt
ctaatttacttattatttaaaatatctgcaacttcatccgcaaagttagctttcatattt
tcttcgccttctcccaatacaaatctaacaaatcttctaattttaatattttcgcctaaa
acggaaatattttgattaattaaatcttgaatactaatatcttgattgcgaatgaacatt
tgatctaataacgaaagttctttcagcctcttttttcattcgcccttcgataattgctcct
attctatcagctggcttgtttttcaaatcatctttaccagcttcaactcttttttctgaa
ctgataacttcatctggtatatgctgtgttgatacatatgccacatttggcgatgctgct
atttgcatagcgatgtctttagctaatttttgaaattctggacgtcgggccacaaaatca
gtttcacagttcacttcaactaatacaccgattcttcctcctgtgtgaatatagctttct
aatagaccttcaagtgcagtacgattagatttttttgttagctgaagctaatccttttttgt
cttaatgattctaaagcttttcttcattaccattactagcttctaaagctttttttgcag
tccatcatacctgccccagtcttatcacgtaaggcttttacagtttgagctgaaatttgt
aatgtcatatattttcaaaaatttttaagtatttaaattaactaaaacaaattattataa
ttcactttccacagagcgagctacttcctgtacatctgtttgcccatatttaccatcata
aattgcatctgctatcttaccgacaatgagtttaattgatctaatagcatcgtcgttagc
tggaattggaatattaataatttcgggactgcagttcgtgtcaagcatgcaaatagttgg
gatacctaatttcaaacattcttgtatagctgttgtttctctcttttgatctacaactac
aacaatatcaggcaaacgagtcatattttttaataccatttaaatgtttacgaagtttatc
taattctctccgtagaacagcagcttctttttttaggcagttgatcaataataccactttt
gtcttgctcttctaactgtctaaggcggctgactcgggatttgattgtcacccaattagt
taacattcctcctaaccatctttggttaacataataagaatcacatctttgtgcttcctg

agcaactattcctgcggcttgtctttttgttcctaaaaataaaactttcttgccatcgga
cgaagcctttttataaattcacaagcttctgtaagtaattgagcagtctgcactagatc
aataatatgtatgccatttctttctgtataaatatacggaaacattttaggattccatct
gcgagcttgatgtccaaaatgcacgcctgcttctaataactctgctaaggtaacgacagc
cataggattttaata-ttaaataatgtaatttttcgggaaatcccttttc----aaac
taattcaactcatttaacttattgcaattccaataaaccaataataaattaacatatttt
tacagtcgattaag-aa--aaaattgttttactttattcaatctacacttttattgctaa
agtagttacgcgcggttctgtcgtctaaaataatatcatctaaatcatctctcacagacg
acacactactttcgctactgatatctggattccaatcttttttttctaacgacgctctat
tatgattgtcatacgtattaaaaccagtgccagctggtataagtctaccgataataacat
tttctttcaaacctcttaaccaatccagtttaccagatatagcagcttcagttaatactt
tcgtcgtttcttgaaaacttgctgcagaaataaaactttctgtattgagagatgcttgcg
taatacctaacagcacgggtcgataggaagcattcagcttatttcttaaagtcatagctt
tattggtttgctcaatcttttgcaactctacaagttccccaggcaaatagccggtttctt
caccattctctattttcactttagaagtcatctgtcggactatgacttctacatgtttat
cagaaatattaactccttgagattgataaactaattgtacttctttaacaagtaataact
gtatttcttgaaaacttaatctagctgcttcatataaagctagtccgcattcaatatata
ggttgaagcttgcatcaagaatatcatgaggatttaataatacatctgtttttttcgcg
cttcaagaatttcttcaattcttggaaggccttgtataatatcaccagtttttgctctgt
caaatacgagaatagctagtgtttctcctcttctaattaatgcattattatcaacatgta
aaattgccccgctagaaactaggtaaggccgagcaattcttaatgttattgattgagaag
acacgtgagtaatttgtccagagtctaaagaaaccatattttcagcaatcaaatcaccgc
aacgaatccaatcaccaacacatactttagttttagctttttctatattaaaagttttct
tgtcagaagatgttccaattagaattcgtctactagtattcttttcagaataaatttctt
cgaccataccttcactcattgcaataatttctgtactagcaacaaccgtcaatggcttga
tatgttcaccatttttgacaataattctagtttgactttgttgtttttctgatttattga
catcaatacttttaattgataatgtttcaaaagttgaaagcctcaaatcaaaataattag
tatcactttctttattagaaaactcaattgaggatattaaactatcatctgtatgctcaa
tttcagctacaagattggttgtaattaaatgaactccatctatcgatttcaccctttctc
cgtcacgaaaaggtgtccttttaactagctttaatttagtttggctcggtttttgtaatg
ccaaattatcaattaagctagattttatttccggtattgaataaacaataacaggtctaa
tgaggatataaggcatttgctcattttctatatactcccaataaactaacttatctgttg
aaatgttgttatgcaatgtttctccaggtcttaaaaacccccctactttttttcattgttac
tatgtaattcgtttagtttatatatagatccaggcttaataatgatttcacgaactattc
catccttctgaattatctcaacaattccagagcttttagaaaaaatatttttgactagct
cagttccactttctataatatccccattataaactaataatagtgatgcatctttattaa

tttcgtgtgtttcctctgagatccataatatatagccaggactcaaaatttcataagcat
ctttttctgaacccactttctttttgatacattcaagtctaaatatttaataataccac
cacttgtagttttgtaagtatcagatattagctcagcgactgtatagccatcgtaaa---
tttttgattggggacagctttaaaaataaatttctgtttttttctgtttctaaaatgt
aagattctttttattaattaaatctgtataaatataacaaccgggaattacaatagact
cagtaataacttgtatattagtaatgctgctattagcattctgctgtatcctgacttccc
cagcatagtgatttatcaactctgtctgcgcaagtattgtgccagctttagttgtatcct
ctttattaacaatcacattagccgagtcagatatattataaacttctccagagagaaccc
aaatcaacccacctgttttagtgattcgtgtagtatattgattattatctgtttcttcaa
ctgttaagttagagaaacagattttaccagaaaggtctgacactacatgtttttgagccc
tttctgtcatcagtctatttctgcgaggtgattcagcaatcacctgatcttttgatactg
attgcccctcatgtagcagaagagtggttcctttacttaagttaattattgacttttgat
ttcttctagatcgaattgtaacttgggttggattctttgtaattaaggcttgttctccat
gccttgtacgaacatctttatatgattcaatatctagatctgttagctgaccatctatgg
gtgcgtaaatttgctctgccaattcaccagtaaatacaccccccgtatgaaatgttctca
ttgttaactgggtgccaggctccccaatagattgagcagcaataatgccaacagcttctc
ctaaatcaactaagcggccatgagcgagattccatccgtaacaatactgacaaacagaac
ttctcgaattacatgtaacaggtgatctcaccaaaactttttt---gatgccagttttag
ttatctctttggctaatttagggctaatatcttgctttgtgtgagcaatcaaaacatttg
tatctggatggaacacactctctgctaatactcggccagacaaagcttgctctagattga
ttagtattttttgtgtatctactaagtcttctagtattatacctttatttgttccacagt
ctacttctctaattataacgtcttgagaaacgtcaacaagtcgacgcgttaagtagcccg
aatctgctgttctcaaagcagtatcaactaatccttttcttgctccgtatgatgatataa
agtagtccgtaacagttaatccttctctaaaattacttgatattggaaggtcaataattt
gtccttgcggatcagccataagccctctcataccaactaattgtctaacttgagaaatat
tgcctctagctccagagaaagccatcatataaacagcatttaaagggtctgtttctttaa
agtacttaattacttcctgttttaatgattcgcttgcattgttccatgtgtcaataactt
tttgaaatcgttcgaccgctgtaatttcccctcggcggtacttattttcggtagctttaa
tttcttcaattgtacctaataaaagactttgtttgctcggaggaatccttaaatcttcca
aactcaaagagattcctgcctgcgtagcgtaatgaaatccaagatctttaagtttatcag
ccatattagctgctcgtgctataccgtaattacgaaaagcccaaacaattaaatttttaa
gttcgtttttatcaataaccctatttgaaaaacttggctgcgcaagacttcttttactat
ccactcagtgttctccatattaattatttatt----taactacgccactaaagactcttg
aataattttgttgaagattattcggccagctgttgttcgaatatattgcacaattctctg
ctgttcagcatcctcttttataatatggttattataaaattttgttctactattatcact
gtgcttttcgattttaattagctcagtagtttggtcaccatctacaaatccatcaaaacg

agcccatatataagagtgcaaatcaactttcttttgctcataagccatcactacatcttc
taagctagcaaaatattgattcgctcccttctgttcagaaggattattagccgtcaaata
ataacaacccagaaccatatcttgactaggcatgataattggctgacccgtagctggaga
aagaaaattatgaggagccaacattagcaatctagcttcggcttgtgcttctaaagatag
aggaacatgaacagccatttgatcaccatcaaaatcagcgttaaatgcaggacagactaa
gggatgcagcttaatagctcttccttctacaaggattggttcgaatgcttgaatacctaa
tctgtgtagtgttggagctctatttaaaagtacagggtgaccttgaataacttcatttaa
cacattccaaatagaaggttcatttttttgaatcatttttttgcagctttaatattatt
cactaagccctgtaagattaatctatgaattacaaagggttgaaataattcaagagccat
ttctcgcggcaatccacattgatgaagttttaaatgagggcctacgacaattacggatct
tccagaataatccactcttttacctaataagttttgtctaaagcgtccttgtttcccttc
tataatatctgataaggattttagcggtctattattcgcacctacaacagttctgcctct
acgtccattatccatcagagaatcaacagcctcttgtagcatcctttttttcatttctaat
aatgatttctggagctaaaattgacttcagacgcgataatctattgtttctgttaatgat
tctacgataaaactcatttaaatctgccgttgcgaatctccctccatccaattggaccat
aggtcgtaaatctggaggtataacagggattactgtaaataccatccaagagggatctgc
gcctgttgctataaagttctcaatcaatcttaagcgtttcatttttttattaaattttaa
tgaaggtgtcttaaaacttttggtggatttgttgcttcagatcgtaaagtttcagcaat
gtattctaaatctaaatctttcaatagtttttgaatagcttctgcgcctatcccaacttc
tacttgattattttcattttgattttggtagatttcctcttcaaggctcttccattcata
gccttcgagcagttgcttatattttaaattgatatttgtattagattgtgtaactacata
agagtgaaaataaacgatttttttctacctctttaacttttaaatctaatgctaaagcgat
ataacttgtacttccttttaaataccaaacatgagttactggtgatgctaattcaatata
agccatccgatgtcttctcactcgagattctgttacttctactccacatcgctcgcagac
tatacctttatagcgaaagcgtttatatttaccacagtgacattcccaatccttaactgg
gccaaaaatttttttcgcagaataaaccatccatttcaggttttaaagttctataattaat
tgtttctggctttgtaatttcaccaacaatttgtccgttgggtaaacttctttcacccca
atgtcgaattttctcgggagaagctaaactaatctttacatagtcaaaatgttgctcaaa
ctgtgtcataaatttgaataccttgaaattaaataattaaccaacttatttacatatgcc
atattcgaaaatgca----ttaataaagaaattgttcaaaatcatcaactggtggagtat
cataactagtgcgatctgctcgattctctttagtgtcagacatcaaatcgacttcaactg
tacgtctttgaccgtcttcaaatagtttcaatttatgtactgcaatgtctaatcctaatg
actgaagctctcgcattaagaccttaaaagattctggggtcccaggcttaggtataggct
ttcctttcactattgcatttaatgcttcatttctagcttgcatgtcatccgactttacag
ttagtaattcttgtagagtatatgctgctccaaaggcttctaaagcccatacttccattt
cgcctaatctttgacctccatgctgggctctacctcctaatggttgctgtgtaactaaag

agtaaggaccagtagatctcgcatgaatcttatcatctacaaggtgaacaagcttaagca
tataagctctaccaattgtaacaggattatcaaaagattctccagttctgccatcgaaga
cttgcattttaccaggatgcagatcattaaaaagccatttattattagtgagtaatgatg
cttcttttaatttctattaacaagtgctcttgaggcttctgctccatacatctcgtcaa
aaggtattattttaaatcgtttacctaaatagccacctgctagacctagtaagcactcaa
atacttggccaacattcattctggaaggtactcctaaaggatttaaaacaatatctacgg
gggtaccatcagataagtaaggcatgtcttgtttaggtaagattctagaaataatcccct
tattaccatgacgaccagccattttatcgcccacttgaatttttctcttctgggcaacat
aaacgcgaatcatagcattcgtaccaggagggagttcatctcctttttgcctagtaaata
ctctaacattaacgacccttcctttagccgcattaggcaatcttaaagatgtatctctaa
catctctagccttttctccaaaaatagcccttagtaatttaccttcaggtaattgatcag
cttcgcctttaggagtaattttccctactaagatatctcctgcttcaacccaagagccgc
ctaccacaattccatttcgatctaaatcttttaaagagttatcacttacatttgggatct
ctctagtaatttcttctggtcctaatttagtttggcgacattctacttcatacttctcaa
tatgaatagaagtgtataaatcatcataaactagcctttcactaattaagaatgcatctt
cgtagttataaccttcccaaggcatataagcaacgagaatattccttcctagagcaattt
ctcctccatcagtggatgcaccatcagctaaagtttgaccgacaactattttctctccca
cccaaacaattggacgctgattaatacaagtatcttggttagagcgataatatttttca
aacgataatggactgttctaccattattatcttgaatacctattttattagcagaaacat
aattaacataacctgcagttctacttataacaaccatgccagagtctcgcgcaattttag
tctcaagacctgttccgatgattggcttttctggatataataatggcacagcttgtctct
gcatattagaccccattaaggctctatttgcatcatcatgctctaaaaaaggaattaatg
atgttgcagctgaaatgacttgaataggtgaaattgcaatgtaatctacttggctcggtg
ttgttgtaataaattcttgacgataacgtacaggaataatatctccttcaatataatttt
gagaattaactttaacatctcctggagctactcgaaaatcatcttcctcatctgctgtta
agtagatgggactctgttgataaattacttgaccttcatttaccggataaaggggtttt
caataaagccaaaaatattaactctagcacaggttgccagtgagccaattaaacccgcgt
tcggaccttctggtgtttcaattgggcagatccttccatagtgacttggatgtagatctc
gcacagcaaatccagctctatctttattaaaacctcctggacctaaagcactaattcttc
ttttatgggttagttctgcaactggatttgtttgatccataaattgagaaagctgactag
atccaaaaaattctcttactgaggcaattaacggcttaggattaattaggttagataagc
ttaatgagtctatatcacatatcatcattctttctctaataatacgttctaagcgattaa
gacctactctaaattgattttgcagcaactctccaactgaacggacccttcgattaccaa
ggtgatctatatcatcaaggttacctgaatttttatctttaatattaattaaataatcaa
tcgaggataagatatcttgaggagacaacactcgaaaattcttaggtatattaagaccta
atttttgttaattttatatcgaccaacttctcctaagtcatatctctttggatcaaaaa

agcgagaataaagcatctgcttagcaacagcaactgttgctggctcattaggtctaagct
tagagtagactataagcaatgcttcttcatctgttacttcttcgacattgtttttccaa
tttctttagctaattctttaattgaatataattgagatgcagagatgagaaaagcatatt
tactcagccccttttcaatctcgtctttattaaggccaatagcacggagaaaaatataag
catttactttatgggtcttatctattcttatccaaatttctcctttagggtcaatttcaa
atttcaaccaggagcctcgattagaaatcagactagcactgtaaatctgcttgccatttt
tatctatctcttgtttatagtaaattcctgggctacgaattatttgattaataataactc
tttctgtgccagatacaatgaaagtccctctgttagtcattattggcaagtctccaatga
agaccaatctcttcttgtatttttatttttatttccttctcaacactaagctgtaaat
gtgttgatgataaatctagtgacttgatacttttccatgatctttagaaggtagatcaa
tgtcttttcttgtcaacttcgcagggacatatatttgagcactataggttctatctctat
tttttgcctgtctaacactgtaacgagggaattttatttatactcattaccaaacaatt
gcagttcaagccgactagtaggatctaatatatttggaaagaattcaagtacttcagtca
taccttctaatagaaaccatttaaaactggctctttgaatttcaactaaatctggaagaa
gtttattttttaagcttatacgttgaaccatagatctcctttataacaaattctgcaatc
gaagagtttaa-tactatcaacagagtacttataggtaatacctgagtatagttcactac
attatttcttctatagacatgactagaacaaagtattttattcaacaaaaaagattttta
aaattt---ttcacaatcaatatagatataaaaatattacagctattaaaaatag-----
----------------gaagacgaaattaatacttgtctgtctgtcaaatagttgacatc
aagatagacaagtattatttatttaatcaattctg-tttttttgagcgcaagataatgct
ctcgcgagtctagcttttttttctggcaccagtattaggatgaaatgctcctttttttatt
gctttatcaatcttgctatatacttgagaaatacttaattgcacatcgttaagattagca
ctttctagattatcaatatttagcaggcatcttttagttaaagtttttacaaccgattta
tatttacggttaattagacgatttctttccgaagttttaatgcgtttaatcgcagaaagg
ttcttagccacagtaaatccaataaaactttataaaatatttttagaaacgaaaataaa
caaattagagatctattcaaaaagggaatatgcgccgtattataacattaaagaaaaaca
ataggcaatagttcactgagggctcttgttagtacaaactaaatattgtcgttgcataga
a-tttaaaaaatagtattaaat-----taaactaaccagacatatagatatatatgaaaa
aaattgaagctattattagaccttttaaacttaatgaggtaaaacttgccttagtcaggg
aaggtattggagggatgactgttgttaaggtctctggttttggaaggcaaaaaggacaaa
cagaaagatataaaggatctgaatattctatcgatattattgataaaataaaaatcgaaa
tcattattagcgatgataaagtagataaaatcacagagacaattattaaagcctcgaaaa
caggagaaattggagatggaaaaatattcattagtagcgtcgaaaaagtcataagaatta
gaactaatgatttagattccgaagctttataaaactcacactttttttgagacttgatact
tcttcacgaaacatgagataataaacgtttattgtataataataactaaggtataataat
ataatggctaaaagcaaaggtgcccgcatcgtaataactttagagtgctctaataaaact

ggagattttagcaagaagagaaaacctggcgtttttcgatatacaactactaaaaatcga
cgaaatacaccaaacagaattgaattaaaaaaattctgtcctaattgtaatcagcattgt
ctattcaaagagattaagtaattattaattttactaaaat--aatcatattatggccata
tatagaaaaagaatatctccaattaagcccaaagagtctgtagattacaaagatattgac
ttgctaagaaaattcattacagagcaaggtaaaatactacctaagcgatccactggattg
acttcaaaacaacaaaaaaaacttactaaagctattaaacaagccagaatactttcccta
ctaccgtttttaaataaagattaaatatatagaaatatgctttagattgtttcctattaa
catt-------tccatgaatttatttactttaattaccgaatatactgagc---------
----------tactataaagcagctatttactactaattttgttcattttataattaaga
aaaa--atctaacttgttgttaaaatagattt-----agacttataatcgaacctctttt
aatgaattatattacatagtttatttaaaagaggagtcagttttatttgagttaataaat
agtacttttgtattctttagaccagtataaagtatttaagataaagatttttgtttaggt
acaagatcaaacgaatgaatcttatctccgggttgccataattgaaattcagaaatgaaa
attccacattcgttaccagcttggatttcttcaacatcttctctaacccgttttaaagat
tctatctttccctcataaataacttcatcttcccgaatgactttaatccaagagtttttc
aataatttattattcgtgactctacatcctgctatttttctattagccaaagaaaataca
gtgcttacttctgcttctcctactggtacttctgaatattccggatcaagtagatctttc
attcttcctttaatatcttcaatcaatgcataaataatttgataattttcgactagtata
ttaaattttgctgcagcttgcttcgctccaggagcaaaattagtattaaagccaatgaga
ttagcatttgtcgtcgacgctaattcaacatctgtagcagtaatttcgccaggcataatt
gaaacgacatttatttgtactttgctctgaggaaattgtgataaagaatctaggattgct
tctgtagagccttgattatctgttttaataattaaagaaatttgcttgcttagatcttta
gaattagtagttctcaatatatctaacgtaatacgactatttagtgctttctgcttctgt
gcgattaatgaatttttttgaagtattttcaagagctttaagctttgcttctttatcattt
ttcactactaaagctatctctcctatgacaggaacagccgataatccccaaatctcaaca
acagatgatggtattgctgaattaatctttttctgttcattattaatgatagctcgaatc
tttgcataagctgatccaataaccatattgtcgctcgtatgcagcgtaccattttggatc
aaaagtgttgctactggcccatgggatttgtctaagtgagcctcaactataacaccttgt
gcaggctgtgttggatcagcctgtaagttttctaactcagctaataaggtaatggtttct
aataatttatcaatattgtagccggtaacagaactaataggaattacaggaacttgtcca
ccaagtgtctcagacatcacattgtgttttaataagtcctgctcaataatatttgtattt
gtgaagtctttatctatcttagatattgctaccacaaaaggaacatcagctttttgaata
tgatgaatagcctctacggtttgaggtttaataccatcgtctgcagctataataataata
gctacatctgttaatttagctcctctagatctcatacttgtaaaagcttcgtgccctgga
gtatctaaaaagataatttttttgtttattagctttattgatatattcaacttcataagct
gcaatagtttgtgtaattcctccaatttctttattagcattgttagaattatgaatgtaa

tctaataaggttgttttaccatgatctacatgccccataactgtaacaatcggaggtctt
ttaacatagttaccaactttatggagagagctagaagtatctaaattagcaggatacaag
ttattattttcttttgtgtttgagtcaactcctataccaaaattatttgctactgatgct
attattgaagcatctattgtttcattaatagtaactggtattcctttaaggaataaatat
tttatcacatctgtttcttgaacacaaattagtttagataactcttgaatagtaagagga
ttggtaatactaatagattctggtggagaattagataaactgctggtttggtcttggctt
tgagttaatgatatgtttttttgtcttatagacttttttagtattagtaaatttttttata
acttgagcttttggtttaggtggacgcattagagaaatagccaagtctcctgcagtctgc
ggaatcatagaattagaacctctaaagttctct---tcctcatcatcaatatgtattttg
gtctttattttt-tttcgttgtctgttctta--tttttttttattatctgctagatcatga
accttattaaaatttttgtttttttttatctagtttgggtggagaatttaattctaaatgc
ggttcactactagctgtatgcgtatcagattgatcaagatctaagggtaaaacactgtct
ttatgggctgattctaatctaattttatatataatttggggatttttttaaatctataatt
ttttcagaagc---tatatttggactggatgcaggggatctattttcgaaattttgatta
tttaaaaacatgaatattgattttatgatctaatagtaaattttaaatttcaatacatag
ctcaaagtaaagaaaaatcagatatct------ataatactaatat---agaaaattaat
ctttgttgtattagatttaatataacactaaagtcttttgttagttatacaagtttaaaa
ctttt-tgtgatgataaacaacaacttctgctttattgagatacaatcaatttatttaga
tataactgtttttcaa-aggtttttaaa----ttgtctaatattgtgttcgttatgaatt
tttattaaagtttgaaaa-ctataaaatagagtcataatcattaataatctccacgccac
ttatgccacgatctcaaaaaaatgataattttattgacaaaacgtttacagtattagctg
atattgtattaaaaatacttcccacaagcaaagaagaaaaagaagctttttcttactaca
gagacggaatgtctgcacaatctgaaggcgagtatgcagaagctttagaaaactattacg
aagctttaaaactagaagaagatccatatgatagaagttatattttatataatataggtc
ttatttatgctagtaatggtgaatatgtaaaagctttagaatactatcatcaaggattag
aactaaactttaagttacctcaagcactcaacaatatagccgtaatataccattatcaag
gagttcaagcggtcgaagataaagatacagaattatctaaattaatgtttgacaaggccg
ctcagtattggcaacaagcgattaagttagcacctgacaattatattgaagctcagaatt
ggttaaaaacaacaggaaggatgagaaatatacaaggatattaaaatatttaggatataa
t---tatattaattaatgaagcag----aattagagatgcttttaccctataaagcattt
gtagtat--------ataatatactaattac------tgtataaaaatcaacataataaa
cgaaattaaatctattgactatatttatgaatacaaataaacataatggttagtaaaaca
aaaagcatcggatctgtcactcaaatcattggaccagttttagacatagcatttcctaac
ggacaacttccaaaagtattcaatgccctcaaagtagaaagctcagagggaactattact
tgtgaagttcaacaacttttaggtgataacaaagtaagagctgtatctatgagttcaact
gaaggactacaaagaggagttcaagttcttgatacaggatctcctatatccgttccagta

ggtgcagatactcttgggcgcatttttaacgttttaggtgaacctgtagacaatttagga
ccagtaaattctgaaagtactttacctattcatagaccagcacctgcttttactaagtta
gaaacaaagccaaatatttttgaaacaggtattaaggtagtcgatttattagctccttat
agaagaggaggcaaaattggtttatttggggtgccggagtaggtaagacagtattaatt
atggaactgattaataacatcgctaaagctcatggcggtgtatctgtatttggaggtgtt
ggcgaaagaactagagagggtaatgacctctatatggaaatgaaagagtctaaagtaatt
gatgcagacaatttgaaagagtctaaagtcgcactcgtatatggtcaaatgaatgaacct
ccaggggcccgtatgcgtgttggcttgactgcattaacaatggcagaatactttagagat
attaataaacaagacgttctattgtttattgataatatttttcggtttgtccaagcagga
tcagaagtatctgctctactaggacgtatgccatctgctgttggttaccaaccaactttg
gcaaccgagatgggtgcattacaagaaagaattacttcaacaacagaaggttcaattacg
tctattcaagctgtttatgtaccagctgacgatttaacagatccagctcctgcaaccaca
tttgcacatttagatgcaacaacagtgctttcacggaatttagcagcaaaaggaatctat
cccgcagttgatccactagattcaacttcaacaatgctacagcctggaattgttggaact
gagcattattctacagctcaagaggtaaaatccactcttcaaagatataaagagttacaa
gatattatcgccattctaggtcttgacgaactctctgaagaagataggcaaactgtatcc
agagctagaaaaattgaaagatttttatctcagcctttcttcgttgctgaagtatttact
ggctcacctggaaaatatgtatctttagaggatgcaattaaaggttttcagatgattta
aaaggtaaattagatgacttacctgaacaagcattttatctagtaggtgatattgacgaa
gcgatacagaaagcggatagcatgaaagattaatataattaacaatgactttaaatataa
gaattattgcacctgatcggactgtttgggatgcagaagcacaagaaattattttaccaa
gtagcactggacagcttggtattttaacaggacatgcgcctctacttacagctttagata
taggagttatgagagttagagtagataaagaatggatgccgattgttttgatgggtggat
ttgcagaaatagagaacaatcagttaactattcttgtaaatggtgcagaagaagctactc
aaattgacttatcagaagcagaaaaaaatttagaaactgcaactcaattattaaatgatg
cctcttctaataaagagaaaatagaggcaacacaaaaaattagaaaagctagggctagag
tacaagctgcaacagcagctattgtataataacatttt------gctaaaaatataaaac
cttagtaattattactaag-------gttttatgttttatttatgtatttatatacaact
tgagtaagcttagcttaaaccagaacaaatataatcaaaatataaacccatttctttgcc
agcatctggtccaactaaactaatagtaacttctttcatagctaaaatcgcttgaatggt
tgcaccaataggaacacctagagaattataggtttcttttagaccatttaatactctttc
ttccaaaatagatggatctccagctaacattccataagtagcatagcgtaagtaataatc
taaatctcgaatacatgcagcataacgtcgggttgtatacatattacctcctggacgagt
aatatccgagtaaagtaaagattttgctacagattctttaataattgttgctgcattagc
tgcaatagtagcggctgctcttactctaagctcacctgtctgaaaatagcctcttaattt
ttcaacagagctatcatctaaatatttaccttgaacgtccgctgcattaataacagaagt

aattgcgtcttgcataacttgtaaaaattccttaattttaaatatctaatactaaagggg
tatataacaaaataactgaacttttataaataccagtactactgcatggcacccaaagt
ataatcaaagtaaaaaccagcttctgctgaatcttcaccagcaagcagtgagcaagctac
acttttcatacatttcacgccttcagcgactcccgaaattggtgttcctaaagaattata
catttctttaacaccgactagtccaatttcttcaataggcgttacgtctccagcaactat
tccgtaagttactaggcgaagataataatcaaggtctcgtaaacaggtagctgtcatctc
ttctccataagcgtttccaccaggagaaactacatcaggtctttttgaaatagctgttg
accgccttgttttacaatacgctcacgattatctgttaaaatttgagctattcttagacg
acgttgtcctgataagacaaaacttttaattctgtctaattcaccaggacttaaatatct
tgcttctgcatctgcatttacaattgactttgtaacaatactcatggataaaactcctgt
aatgcttt--taaaaaagcttaatactgattatttatgatataactcagaaagtagtacg
ccttttaaaaaaattcatagctatagcacgttggtgatatatgaagtaggacaaatactt
ggataggtactaaaacaagaaagttacttatcgtaccttattttattgattgccgagtac
tttttaaaactaggtactacaattgatatatcttgcttagtaaggcgattatataatgt
ttctgtgttagggaaattagctgctggcaaagttggaaatcttctatacggaacagtata
tttaccatatactgaattatactcagtactttcaactaaagtactaataaatgcagacaa
tccttttgaagctaaaatttggttaaagaatctaatttctgcctgattattaggagctcg
tcctaagatatgcttcgtacctaattctataacttttgtatttggatatggctgataaaa
ctctttaccatataattcagataatgctaatttttctactaattctttaacattaatttc
cctgctcaaaaaggatgcttcaatatctaaaaactctcctccaatactaaaagaattaag
atctctttcaaaaatctggcgatatgcggctcttaaagcttgctctaatacttctttact
actattgtcgtttacttcaaatataatagattgatctcttaaagagctaactccttgaga
aatacgagactggatatcattgccgcttcttatttcttttacagcgcctaattcaacaaa
tctagaagacttgctagtaatgactttcttaaatctttggtcaataatacctggccttaa
gcttcttagagcgataccagctggtgtactatatctttcataaggaacagtactatcacc
aaaagtttctatatactctgagctatcaactattgcatctatcacttgatagtagccctg
cttatatgcaatatcaaaatatttattaatttcttggcggccatatgtaggacgacccaa
taaacggttatggatatactcaatagctttacagatatataatggctcccagtaaagtga
tctaaatatacttgacttcgctaattgtctaacaaattcacgcactgtaatttgaccatc
ttttaactgactttctattggcttaagaagtaatttttcttcttgatatacttctctgcc
aaaaactcttaaataagcaacttttgtaacggcatcgactgaattttcaaaattttcaga
agagtcagcttttacaagaggattggataatttaaaaatttttggacccaaagatcctgc
agacttaggtcttacctgcggattactgatctggttatagattcctgggccacatctaac
cagaattctcctagtatctttaccaaaaagagcttgtcgtttcctaggatccttattttc
tttaggaaagattgctccaaattgaatcgacagtggatcattgcctattccatatggatg
ctgatcaggtaatgcttgcttgtaatcactaaatagggttataaactgaggcaccttacg

aaaaggagcactataattaagaagatctatttgaggtccccaattacgacactcttgagg
ctcttcacctaaatttcggaaataaggtacagtttcttctccaaaataatctgtatattc
agcagaatttaaaattgcatttactagcccacttaaaccagtagctgatagaatagcaaa
atatttttggaattcctctaaagagctgggccctctaccaaggaaatgtctaaatgctaa
ttctaatgctcggctatttacaaaaggctcataaaattgctttctatagatacttgaagt
tcctaaagaacggataaattccttaatggagatttggccatttttacttgcgactctaa
gttagataaagataaatcatatgctttagcaatatctctctcaaagatttgtctgtagca
cgctttaactacagtattttttcatctgcagataaactaggcttcataacaaaacgagg
cgtagaaactcctgcttgtgcataaatctgaggaaggcgcaaaccttgtaaatccccaga
tgttcttttccttaatttatcagtcaaagatggagcttcaaattcagaaattacaacatt
aaaatactctttgaccagatcttgacctttaatatcttcttcaaagattaaaagagctgt
tcgtctcatctctctgagggcaacaattgctgccgcgcttgagcaagcattatcaattaa
ttctcttaatcctctaatattaaccgataaaatattgggatcacctgatacaattgcata
tgttaaataccgtaaaaaccagtctaaatctcgcagagattttctcattcttgtagtccc
atatctcaagacatttatgggtttgaatcctggaggagtagctcctccagcattaaataa
agaacgaaaactctgcccaaaatctccttgaatattgccagatagctcattaattttgtc
ttgagaactttgatctccagcaataataacagctgcttgtggacgttctaaataagaaat
tgcagacccgcctacaaatattttgtctgcagctcgagctactagaatattggcattttt
agttaaaatatctgcaacttctaatctttttttgtccagaatttagaaaagaaactagttg
atttaattctcctagctgtaaaaatcggtcttgctgctctgcctgagtaatagttaaaat
tgaggcagtccgataaagctgggggcgtgctaatgggcttccgccacttgctttgatact
cattactttatttatctccttaacttatattacttgctagctaattcaaatatcacttaa
tatttctaactatttaaagatactacgcaagcacatttgttttctatattattaggtact
agtagttatagtacatagagtcacacaactaacttctataaatacaaattttgtaatact
tctattaacatttatcaaaagtaaatataattgataactcttttatattaaaatgcaatt
attaagagagtgatgctaaataaagtataattagtaagttttatagatagtaaaatt-ca
agatttattctacaaacattaatca------------cttgactgtttgacaattatacg
attgttcttgcttttaattggtttagactagtttcacatttttctaacgtaaacaatat
taataagcaatttgtatgaatttaaaaccacagaataacctattaactaacggaaatata
aaccaacaagatgtacctgtaaatgatatgccaatgtcaattactgagcatttagaggaa
ttaagacaaaggactttatttgtatttttattctttttgcttgctgcaactttgagtttc
acacagatcaaaataattgttgccatattgcaagctccagctattggtattaaattttta
caattagcgccaggagaatatttttttcttctattaaggttgcaatatattcaggaatt
gttgcaacaagtccattcggggtataccaagttatcttgtatatactacctggactaact
ggcagagagcgaaaaattattttgcctatcctaattagttcagtcttactttttataata
ggcggtatatttgcttactttattttagcaccagctgctttaacatttttgatcagctat

ggatctgacattgtcgaaccactatggtcttttgaacaatacttcgactttattttacta
cttttatttagcacaggattagctttcgaaattcctatcatacagttactactaggtgtt
tcaggaacactatcttccaatcagatgattcgagcttggaggtatattattattatatcg
acaattgctggagccattttaactccctcaactgatccagttacacaaatcatcatgtct
tctgctgtcttagtgctttatttagtggaattgtcatattgttacttttaaaaaagtaa
atgtagt--------aaactttcatttcatattatacttttcttgtg-----ctaagcaa
tggagttt--atatagcttttagttcttaacttagatgaatattgaagta--------tt
aagctcttttaaatatttaatttagtcttataatttaaccaatatg------------

---tatatattgg
c---tttttcatcaattatttgcactttacatttctgtacta----ttctatgatgctt
gggactctttatgtaatctgtttactgaat----gacgttgaagaaaattcagcataaat
tttgttaatttataccaatataaaaagtattttattgtgagttttatttatataaaggct
tattcattatatatttataacagtaagtctaattaatagttttttatgcttaattaaagt
aataactcttaatccttttggcagaaaagtctgtcttaatattttatc--aaaaaaacag
aaaaaatgaagctaaatagtctaattaacttaattcaaaagtctatatgttcttgtacat
ttttattaattattttaaatattatttgtgtcgctcctaattctagtaatgcatttccaa
tttatgcacaacaagcttacgaaagtccaagagaagcaactggtcgaatagtatgtgcca
attgtcatcttgctcaaaagccagtagaaatagaagcacctcaatcggtattacctaata
ctgtttttgaaactattgtaaaaattccatatgacagtagtgctaaacagattttaggta
atggaagtaaaggagggttaaatgtcggagctgttgtcatattgcctgaaggatttaagc
tggctcctgctaatagattatctacagagttaaaggaaaaaactaaaaatctatatattc
aaccatatagcgctaaacaagataacatttagtaattggacctattcccggcgacaaga
atagagaaatagtttttccaatactctctcctgatcctgcaaaagataaaaaagctcatt
tttttaagtacccaatatatgttggtggaaatagaggtagggggcaactttatcctacag
gagataaaagcaataacaatgttgttacagctttaagtagtggcaaagttaataagattg
aggttcttgagaaagggggatttgtagttcatgtgactaatagtaacaatatagaatcaa
ctcaaaagatttccccaggtcttgaacttaaggttaaagaaggtgatacagttcaattag
atcaagctttaaatagtgacccgaatgttggtggtttcggccaaaatgaaacagaaatag
ttctacaaagtccaaacagaattaagggcatgattgttttctttttttgctagtgttttag
ctcaaattttctttgtattaaagaaaaaacaattcgaaaaagttcaggcagcagaaatga

atttctaaagcaagataaaactggtacttactataattaaagtg-aaaacactttattca
aagaatgtaccagttttcatgctatataaccgttgcttttgaagacattataatgtcttt
aacagagtctacaatttgactaggctgaattacagtagcctgttctaagctaccattata
tggtgttggtatatcctgcgaagatagtcttactacagggacatctaattcatcaaaaag
gtgttcattgatttgtgcaattaactctgctccaattccagcagttttcatgcattcttc
tacaattaaaactttgtgagttttcttgactgacacagatatagattctatatctagagg
ttttaaagatataaggtctatcacttctggatcgtaaccttcttttagcaaagctggtaa
tgcttgaatcacatgatgcctcattcgagagtaagttaaaatagtaatatcttttcctgt
acgcacaaattcagctttattaagaggtaatagatattcttcttcaggaatttcttcttg
taaattgtaaagcaaaacatgttcaaagaagacaactggattattatctcgaattgcaga
tttcaataatccttttgcattatatggagtagagcaagcaactatttttaaacctggaat
agcttgaaaataagcttctaatctctgagaatgttctgcgcctaattgtctaccaactcc
tcccggtcccctaatgactaaaggtaatgtaaagtttcctccggaggtatagcgcaacat
gcctgcattattagaaatttgattaaatgctaacaataaaaagctcatattcataccttc
aacaattggtcttaaccctgttatagctgcaccaatcgccataccagtaaaactattctc
tgctattggtgtatctaaaactcttaaatccccatacttactatgcaaatctttagttac
cttataagatccgccgtagtgacctacatcttcgcctattacacatacagtcgaatcttt
tgccatttcctcatctgttgccgctcttaatgcgtcaaacataaagattttactcatttt
tgtatttaattttataatttcactactaatctaaattaaaagttaagatattaaatttt
c-tcacgtatctgaaaaaagatatcgtttaagttctgagatattaggctctggactggaa
atagcaaattgcacagattgttctaaatctttttaacagcagtttgaatctcgttgagt
tcacttatgtcggcgatcttattatctaaaatatattttttaagtttttgattggatct
cttgcaatccaagcttctttttcttgtcttgatcttagttcatcaggatctgcaagagag
tgaccacggaatctatatgttaaagcttctattaatgttgggccttcaccatgtctggct
ctctgaactgcttgaatcgctgcttgccttacagctagtacatccattccatcaacttcc
ataccaggaagaccaaaagcttctgcctttttatgtatttccggcatggaagaagatcgg
tgatgtgccatacctatagcccactgattattttcaacaataaatataataggtaacttc
caaagaactgccatatttaaacattcgaaaaattgaccattattagtagtaccatcacca
aaaaagcaagctgtaactctcaaatcttcagtttcttttagtacttgctgacgataaata
ctttggaaagctgcacctgttgcgacaggtataccttctgcaataaaagcaaacccacct
aaaaaattatgaggagcagaaaaaatatgcattgaacctcctctgcctttgctacaacca
gtttcttttccaaatagctcggccattacatttttcgagggaacacctttacttaaagcg
tgtacatgatctcgataagtgctacatacataatctgtagaattgagaagcttaattaca
cctgtagacacagcttcttgaccattataaagatgcacaaaaccaaacatctttcctttg
taatacatctgagcacatatgtcttcaaaatttctgcctaacaacatatcttcatacaag
actaataaagtcttttt------attaagaacagttgaattatagttagttagcggcaat

tgaactttcttaggataactcataatttataaaggaacctct----------tattatac
ataggaattttgaagtaaaagttatcaatagaaaacacacagataaaaatttataaatta
ttatatgc-ttttactaaagacttcaatagttatttgcaactagttcagtatatcaaatt
aattttgcttatactacagatgtctaaagcttagatgaaaagaaa-tagttaaa------
------------------------ataaataaaaataaacagaattattagtgagaaaat
gattattattaatagataattacttaatatttctaaatattttcaaattaatggctatat
catcatctttattttaccctgtcgagaaagagttatacagtctagaaacaaatttgcaag
ctgtggctgggacacgtcatccaattttatacgcagcggcgaaacatctgttcgaagctg
gaggaaaacgagttagaccagctctcgttcttttagtggctaaagcaacatctgagaaac
aagatattaatactggacagagaaggttagcagaaattactgaaattatacatactgcaa
gtttagtacatgacgatattattgacgaatgtacaacgcggagaggagtcaagactgtgc
acaatttgtttaatactaaaattgctgtattagctggagatttttttatttgcacaatctt
cttggtatttagctaatattgaaaatttagcagtagtgaaagctatttctaaagtaatta
cagattttgcagaaggcgaaattaggcaaggattagttcactttgaccctagtatttcaa
tagatgcttacattgaaaagtcattttataagactgcttcacttattgctgctagctgtc
gaggagcagctatgctgaatgattctaatactcaagttaatcatgatctatatctttacg
gtaaacatatgggcttagcatttcaaattatggacgatgttttagatataattggttcta
ctaaaagcttagggaaacctgctggcgcagatctagccaatggtaatttaacatctccta
ttctattttcacttactcaagaaccagatttaaacaatcttattcaaagagaattttgta
ataggacagatgtagcctcagcgttatttcttataaaaaaaagcggaggaattacaaagg
ctcaagacttggccaaagaacaggtacaggcggcactttgctgtctccagtttttaccaa
aatctgcacctgtatctagcttaaaagaattgacacatttcataatcacaagattgtcat
agaacacttgctaaaattaaaatgctattaactaatttttttactatctctacaatagtct
gaaaagtttggatatcattacttgctaactgtgctagcattttacgatttaatgcaatat
tctcttttttttaacgcactaataaaagtactgtacttcatgccttgactatgagctgcag
catttattctagttatccaaagacgacgaaaatctctcttttttccttttttcgaccaacat
aagaatagcgaagagatttgagaacttgctgtttagctgttcggaataaacaattatgtg
cacctttaaagcctttagccagcttaaatatttttgcccgtctttttttttgcaacattac
ctcttttgactctactcataaaatattac-ttacaattaaattattaacagcttcctaga
ttcaaatgcttatcg----attataaataaggtaaatttacagcgatattcttaatatct
cgtgtgtcaactaagcaggtacaagaaagatttcttttttgcttcgatgatttttttttgt
aataaatgacttttagaagctttatgtcgcagaaattttcctgatgaagacactttgaat
ctttttgctatcgcttttgatgtttttaacttaggcatattgtaaacttttaatgattaa
caaaagtatgaaaagtctgaatttatcaaacttagacttttcaatacttaggataatctt
aacacaaattattttacttactatagaggttaacttaaacaatttatatatatataatagca
aataattaatccgtagctactaaagttgatttatatttagctttaaatttttgtatagta

ttagcaattagcattgttattattttaagggtacttgagtttaattcttcgaacattttc
atatttaatgtgaatgatatattcgcttctgacacaatattttgcatttgatcatccgat
aaaggaatcatatccaatgcagctctatacttatctttaaacactttgtcatcttgtatt
tggtcaaagtcataaaacctagtacctctttcatctgataaattcatcgcacctctagcg
attttctttaagatttggcctccagataaatcacctaagtaacgcgtataagcatgagca
actaataattccggctgtttacagcctatactatgaattctatcaacatagattattgtt
gcaggagatggtttaataagatttaaccaatctgctccataataataacttaaatcttct
gacagacttatttttctattgagttctgtaaaatatataggtttaatagctgggtgatct
ttattagaaaacagttcttcttctatagcacaataaacgaaataaagattagcaaccaat
ttacgatatgatttcttgtctacgacacctcccaaaaaagatttcacaaaactaacattt
tctgccatactatgagacttagtagtgccttctcttaattcatttgctaaagtattaacc
ataatatcaatcttccatttta-aaagataacaaattaaac---ataaacagttatctat
atattta------agttgtactaattaagctgctgcaaaataattttttgattttattgg
atctggattcattgttttatcacctggcttccaattagctggacaaacttcatctggatg
agattgtacatattgaattgcttgcagaactctcaaagtttcttctacactcctaccaaa
ctcgagattgttaattgtagaatactgaataattcctttaggatctataataaatagtcc
tcttagcgctacaccatcactgttcaaaacattataagcagcactaatctcttttttaa
gtctgatactaatggataggcaagatctcctaatccaccggattctctgtctgtctgtaa
ccaagcaagatgagaatactcgctatcaaccgaaacacctagaacttctgtgttaagctc
agagaaagcatcatatttatcactaaatgctgtaatttctgtagggcaaacaaatgtaaa
atctaaagggtaaaagaataagataatatacttatttttaaatctgataattttaacgt
tttaaattcttggtcataaacagctgtagctgaaaaatcaggagctagctgacctactcg
aagacaatttggtccagaaatcattaatttttcccagaagaattgatatagttatgttat
ttaataaataattatataacaaaatttagtatatatgaaaatgctatcagtcttttaaaa
attac--------tcaatggcttccaaaatactattttatagcggggaatggatttgaa
ccattgaccttcgggttatgagcccgacgagctaccagactgctctaccccgcggatatt
taatatcatattagtccatctaaatagataaaagcaaatatattatataattcaaaagta
aaagaataagagcacaggagcactaattattaggcttaaaaatgacaaagtatttttat
tttattgagtagaggaaagacttcatataagccgatgaatcgatctttaagaagaacttc
cgatggcttcacccaaatttgaccgtcataccacccagactcttcatagaataccgtagc
actcattaaacgttttactacgtatgaccatcctaaatataatctgattagaataaatcc
agttattaaactcgaggtaataaattctgagaagacaaattttaaaggcagcttggtaat
tggaaaaattgaaagcaatactggactaactaatagaccattaagtagtagtgtaacagt
gattttcctgttatatgattgactacttaaggttggccagcagaagaaccaggaattttt
taaagaagtatactcattaacaggttgctgctcttttggtacaggacactgagtgttata
taaattcattgaaatttaaaattgttatactagaaattgtttttttatctgctttagat-

-ttaggga--------gttaattgattgcaattataatagttagtatcagaaatacgacg
ataaacaaccaagtaactttgtttaatgtgttttcagtactacgagtattgctaaaaaac
tgattctgagctccagcacttcccagaccttcagatttgggattatgtatcagaatagta
aaaaccaatataatagttgatacataccaaaagaattttaaaatttgttccattgtgtaa
aaattaataaaaaatagcacaaagacaatcgtctaaaaaatattgattgtcttattatat
taagaaagatttattctccctgcacttttaaaagtaaaaagcctattgctaaccctacta
aaactaagactgaacttaatacagcactttctacaatttctccgaccatt-gtgtttttc
cttattattttattatta---tcattttgtaaaaaatttgatacctattaaa-ctaaaat
ccatttcttccccaaaccaccatcgcaatagagaaagtaaacattgccattaaagctgcc
catcctaaacttaaaatatccataatttataatattc--ctgaaaataaggtctcttata
cttaaactataatattcttaatattaaaaatatcattatataataagctaattataacag
agtactacttaattcgtattttagtgtatttcaagaagattgac-tttttctaaaatac
ttaacaagtattaaattgtaaaaaatttctgcaat----ttttgcattattatacctatt
aatttggaatgtaaatatcaatttaaaatcgttagtaattttgatttttcaattgtaca
att---aacatatgtaaaaacataaagattcatagcagctaagttga-----tggtctac
tataatttatcaat--aaaaaggaagttagtatttaaattatgcaaacaactataagtaa
tggaaaaacgtctagcaaggaaactctactaacccctaggttctatacgactgattttga
agaaatggctaatatggatatttcagaaaatcaagaagatttctttgctattttagaaga
atttcgagctgattataatagtgagcactttattcgagatgaagaatttaatcaatcttg
gtccaatttagaagataagactaaatctttatttattgaatttctagaaagatcttgcac
tgcagaattttcaggtttttactatacaaagaattatcacgaaaattaaaagatagtaa
tccaattattgctgaatgtttttactaatgtctagagatgaagcccggcatgcaggttt
tttgaataaagctataggagatttcaatttatctttagatttaggctttctaacaaagag
tagaaaatatactttcttctctcctaagtttatttttatgcaacttatctttcagaaaa
aataggatactggagatatataactatttaccgtcatcttgagcagcaccctgagcatcg
catctatccgattttagattctttgaaaattggtgccaagatgaaaatcgtcatggaga
ctttttgccgcattactcaaatcacaaccacatttttaaacgactggaaagctaaaat
gtggtgcagatttttctattaagtgtatttgcaacaatgtatttaaatgattttcagag
aatggattttataatgcaataggtctagattctagacaatatgatatgcaagtaatacg
aaaaactaatgaaagtgctgctagagttttccctgtcgctttagatgtagataacccaaa
attcttcaaatatttagatacttgcgcatgtgacaatagggctctaatagatatcgataa
acaaaatttcccattgttaattaaagctattataaaaatgcctctctattactccctaat
tattagtttgctaaaaatatatttaattaaaccgatcgattcaaagaccgtctggaatac
agttcgataatcaaatcacataaagatatattttcataatacacactatctcattattga
tataaatcaaaatgaaaagctcacttct-----gtcaggaaggagcttttattttattca
caaataaacaatatggttctatagatt-tagaactaagaacttactttttcttttgcttc

atacataacttctaaggtgattagatttttagaacaatcgcgtgcaaatttttctgtgtt
tctttttactttacctctaacaaaacctggaattttacttaattctttctgtgcttcttg
tgaccagttaatactatcaacagcagagatccctacgcttaatgcttccgttgtatcatg
accaccaaatatttctaataagtgatcttccatacccaatgtgaatgagttatagactaa
atcagcaatttgatttgtaccttcatagccaaggaatggacgataactcaaaggaaaatt
ttgtatatgtactggtgaagaaataacaccacaaggtatatttaaacgtttaccaatatg
tctttccatctgtgttccgaaaattgctgcaggctctgttttagcaattaaatctccaat
taaaccatggtcatctgaaataattacttcatcgcagaattcttgaacttgttctttaaa
ccaatcttcatcatatttgcaataagtgccacaccaagtaacatgtatacccatttcctg
gtgtaaaattcgagttattgctgcagcatgagttgcatctccgaatacaatagctttttt
accagttaaattttggcagtcaattgatctagaaaaccaggcagattgcgagataaatct
agtttgttcatctatataattttcgtagtctacaatggctcctaatgtagttaataactg
ttctattgatcggatacatgcagctgtttgtacaatacccataggggtagtatcaacata
aggcatattaaattccttctctaaataacgagctgtcattagtccggtctctctatatgg
gacaaaattaaaccatgcagatggtaaatttttttagatcctgaaccgacgcattttcggg
aataatttgattaattttaatatctaaatcttgaaataatctttttaattctgaaatatc
atgctgattatgaaatcctaaacttacagctccgataatatttacagatggtttttctgt
cttttgagttaaaacctgattattagatttagctttttccatgtaaaaagtaacaatctg
ttccagagttctatcaccagcttgaagttcatttaccctataatgattaacatcagctaa
taaaacatcagcttctgtttctatagaggctctgctaacaaattttgtaaatcttcttg
caatatactagaagtacaagttggtgttaaaataactaaatctgggttttcttctctatc
ttttctagtaatgttttcaacaactttttcttgtgaaccacgtgctagtacatgtctatc
tacaacacttgctgtaacaggcgtaaaatctctgtctctttcaagcattgaacgcataac
attaaaatagtcatccccgagaggcgcatgcataattgcatgaactttttttgaaagaact
agcaattctaagagttccaatatgagcagggcctgcatacatccaataagctaatttcat
attaaattttcctatgatttatttagacatagtaatacttttaaagccaaataataaata
aaattcgaacatctaatttaactccttaatgatttttttcatg-tttttagatagagcgta
acattatatcaagtcaaaatt-------------agagaaaaatgactaatgaataaagc
ttgaaagtaaagatttcttgcagttatagtttttctttaagtaaacatgtaaaaattgta
ctatatataaatgtactatatataaatttttttttactgaatattggtaatgccagataca
attaacttaaatatgccttccccaacgtttggtggaagcactggcggttggttaagagcc
gcagaagtagaagaaaagtatgcaataacatggacaggcaaaaatgaaagtaagtttgaa
atgccgaccggtggtacagcaactatgagaaatggagagaacttactttatttagctaaa
aaagagcagtgtttagctttaggtactcaattaaaaagtaaatttaaaatatctgactat
aaaatttacagagtatttccaaacggagaagtacaatacttacatccgaaagatggtgtt
tttcctgaaaaagttaatgctggaagagctagcgttaacagcgttgatcattctattggg

cgaaatgttaatcctgtagatgtcaaatttacaaataaggcaacttacgattagttgata
aaaaaacctagacataaaataacttttatatagtctaggtttttgataaagtatactaag
agtatggaaaggtggccgagtggttgaaggcgtctgatttgaaatcagttgaactt--ta
tagttccgtgggttcgaatcccaccctttcctttataagact------------ataact
taattattactata----aattatacgctaatagtattcagtttagacttaatgaaatct
acaacttgcgataaattagaaatttttcggcatcttcatctggtatttctataccaaat
ttttcctctattgccattactaactcaactgtatcgagagaatctgctccaagatcactt
gaaaaatttgcttccctggtcactattttttttcaattcctaactgttctgctacaata
tcttgaactttttcaaaaatttcattatcttgcataatatcttgtttcctataaggtgtt
ttcaaaaattttctggctcatattattgtctaactctaattaattagtaattttgagttg
aggtccaaatagttctacctatatgatatactattattctggataaatacgaagtttgcg
gtatggctcttctccaaaactacgagctctcaactggtagttatcaactaaattatgctg
cattttgcgaatataggctgaacgaggtatcaactgaactgtcgatttttcgttcaagat
aatagtttcaatagccagtttagcttcttgaagggcttgaatctcatcaaatttttttatt
ttcacaaagttctacccaattaagatcagaagaagtatgaatatttaatatttttctcag
tgcacgagtaatttgtgggactgtactattctgaattgtatatataataatttgttttga
ttttgcaatttgtcttaattttgtattttgctttacttgattacgcaatgctaaaatagc
atctgactttttctatctctttagttaaaataattggtaaatctaaagaagaaataactga
cgcaatatgctgccaacttaaagaataagcatataaatactgattcggcatttctaaggg
caaagatgggttattaatatttataggagcattaatagtagttgacaataaagatgtatc
acgattttgtaatttattcaaatcggatggcttatttattaattctctatactgcaaaac
agcatgtgacggttgtacactaattggtttacggaaactagaaatatttgacggcaaaac
ttctaaagactgtgacggatagcatttaattaaaatgcggccattagcttgaatttgtct
tttttgtacaaaaggttgatggccctgtaaaatttgatctatagtttctttcactttatt
atggactatccaaacagtgcgttcatggatttcaattgcaatttgaaaagcgggcgcggc
ttttctttctaatatgcttttctgagtacctcttctttttgcttcatcatcacctaatgt
gacatattgaatacctccaattaagtctgacagtgtaggatttttaattaaactttctaa
atgatttccatgtgctgtacctactaattgcactcctctttctgcgatagttcgagcagc
caatgcttctagctctgtaccaatttcatctataataatgacttcaggcatatgattttc
taccgcttctatcattacctgatgctgtaaatcaggccttgcaacttgcattctccgagc
tctacctatagctggatgtggaatgtcaccatcgccagctatttcattagaagtatctat
aataacaactctttttttccatttcatctgctaagacacgagcaatttctcgaacagctgt
tgttttaccaactccaggttttcctaaaagtaaaattgaatcaccttgttctagtaaatc
cctgataatactaatggtgccaaaaactgctcgtccaacacgacaagttaaaccaataat
acttccttctctattacgcatggaactaatacgatgcagggtcttttctataccagctcg
attatctccgctaaaattaccaactttttttacacaataatctaagtcttgccaactaat

agatctttgagataagtattctggcctatctggaaatcttgcttctggacggcgacctaa
atccatcactacttctattaagtgatttctattagggtgttgtttcaggggctctttgac
aaagttcggtaagatttctagtaatttatctaagtcatctgcaataagcatgattgaata
tataataattaactaatcagatagaaaaattaagattctaaagattacaatacatattaa
agaatattgtgctcgtaatcaattttatacttaaattcaataagaaatta-cagatggta
aacaatagattaaactatgtacagtaaataaattggtcaaaattaagagtcatgttaata
tgtttattggacagaaagttcgtattaagtatagtaagcaaaaagttgctaatgacatag
cagataaagtcggtaatcgcggaattattagaggcataaaatttatcaacagtcaatgtg
ttactattattgttgaatttgaaaatcacactagactttggatgtttaaggaggagctaa
tctatttaaatgaat------ttaaacaataagataagattaattt----tattatatat
aaatttttacgagtcgttcaaattatacattttcaaggagtattgttaattgtgcaaatt
actattaaaaaattacaagacttactttcatcagtacaaagaaagaagatacagacattc
aaactgaaacaggataattttgaacttctattaaataaaacttacaaaaaaactaatcca
gaaatattaccttcacagaact---ctgtattacaacacaatcaagctaacataattaag
tcaacacctaataacaatggtccttctgttagtaataataacgttacagaatatgccact
atcgtctctccgatggtggggacattctatcactcacctgctcctggtgaaaaaatttt
gtgcaagttggggatgaagtcaaattcaatcaaacagtttgtattattgaagcaatgaag
ttgatgaatgaaattgaagctgaaattgaaggaaaaattatagaaattctagttaaagat
ggcgatatcgtagattgtggtcaagccttaatgaaagttgaaacataatgccaatatttc
cattttacatattgttaacagaaattatttagatagaaaaattagcttataatataatta
tgaaaacttatttcgtaaaggcac-aaaaagttagtatataaaattatttactttatctg
ccaaacatagaaataagcgttttgattccttgtctcaagagaggagaatctcaatgacaa
ttagctcaaaagagcaagagacaaagaaggtaaaaatctcggttgataaaaatcccgtag
atacttctttcgaaaaatgggcccaaccaggtcatttttctcgtacactagcaaaaggac
caaaaactactacttggatttggaatcttcatgctgatgctcacgacttcgatagtcaaa
ccagttctttagaagaagtttcccgtaaaattttcagcgcccatttggacagctttctg
taatatttttatggcttagtggaatgtattttcacggagcacgtttctctaactatgttg
cttggttaagtaatccaacaggtatcaaaccgagcgcacaagttgtttggcctatagttg
gccaagagattttaaatggagatgtaggtggtggctttcaaggcgtgcaagttacatcag
gatggttccagctttggagagcatcaggaattactacagaatttcaactttactgtactg
ctatcggtggattaggtatggctgctttaatgttgtttgcaggatggttccattaccata
aagcagctccaaagctagagtggttccaaaatgttgaatcaatgatgaatcaccacttag
ctggtcttttaggcttaggctgcttaggatgggcaggccatcagatccatttatctttac
ctattaataaactttagactctggtgtatctcctcaagagattccattgccacatgaat
ttttaattaatagagagcttatggctcagctttacccaagtttcagtaaagggttagttc
catttttcactttaaattgggctgaatattctgactttttgactttcaaggaggtctca

accctgttactggcggtctatggttgagtgatactgctcatcatcatctggcattagctg
ttttatttcttgctgcaggccacatgtatagaaccaattggggtattggacatagtatga
aagaaattttagaagctcacaaaggtccttttactggtaacggtcatgaaggtctctatg
agattcttacaacttcttggcacgcgcagcttgctattaatttagctatgatgggatcat
taagtataatagtggcacatcatatgtatgctatgcctccatatccatatattgctactg
attatccaactcaattatcactattcacccaccatatgtggattggaggattttgtattg
ttggcgcaggagcacatgcttctatctttatggtaagagattataatcctgctgaaaatt
ataataacctcttagatagaattattagacatcgcgatgctattgtctctcatttaaatt
gggtatgcatatttcttggattccattcatttggtttatatatccacaatgatactatga
gagcacttggaagatcccaagacatgttttcggatacagctatacaattgcaacctattt
tcgctcaatgggttcaaagtatacatactctagctcctggtaatacagctcctaatgcat
tagcaacagctagttatgcatttggaggagatgtcgtttctgttggaaataaagtcgcaa
tgatgcctatttctctaggaacagctgattttctggttcaccacatacatgcatttacta
tccacgtaactgtacttatcttagtgaaaggattccttttctctagaaactctcgactaa
ttcctgataaggcaaatcttggctttagatttccatgtgatggacctggcagaggcggta
cttgccaagtgtctggctgggatcatgttttccttggagtattttggatgtacaactctc
tatctgtagcaattttttcactttagttggaaaatgcaatcagatgtttggggtagcgtgt
ctccatccggaaatgtttctcatatcactggtggtaattttgcgcaaagtgcaattacga
tcaatggatggttaagagatttcttatgggctcaagcatctcaggttattcaatcatatg
gttccgcactatctgcatatggactaattttcttggcagcacattttgtgtgggcattta
gcttaatgttttttatttagtggcagaggttattggcaagaacttatagagtcaattgtat
gggcacataataaaataaaagttgctcctgcaatacaaccaagagctttaagtattactc
aaggaagagcagtcggtgttgcacactacttattaggtggaattggtacaacttgggcat
tctttttagcaagaattatttcagtaggctaatagtgaaaataggataaaaa-aacaatt
atggcaacaaaatttcctaagtttagccaagctttatcacaagatcctacaactcgaagg
atttggtatggtattgctacggcgcatgactttgaaagtcatgatggaatgacagaagaa
aatttatatcagaagatatttgcttcacactttgggcatttagcaatcatctttttatgg
acatcgggcaacttattccatgtagcttggcaaggtaactttgaacagtgggtattaaat
cccttaaaagttaaaccaattgctcacgcaatttgggatccacactttgggcaaccagct
ttaaaggcttttagtaaaggtggatcagcatatccagtaaacatagcatactccggagta
tatcactggtggtacacaattggaatgagaagcaatcaagacctgtattctggggcttta
ttcttgctcgttttatcagctttacttttatttggtgggtggctacatctacaaccaaaa
ttcaagcctggtttatcttggtttaaaaataatgaatcaagattaaatcatcatttgtct
ggattattcggagttagctctttagcttggacaggccacctagtacatgttgctatacca
gaagcaagaggtcaacatgtaggatgggataattttacaacagtattgcctcatccagct
ggtttacaaccatttttttagcggtaattggagtgtatatgctcaaagcccagataccgct

caacatatattcggaactaatgaaggggcaggtacagccattttaaccttcttaggtgga
tttcaccctcaaagtcaatctttatggctaactgacatggctcatcaccatttagctatt
gctgtagtcttcattgttgctggacatatgtatagaactaattggggaattggacataat
ttaaaagatatttagatgctcatagaccgcctagtggcagattaggagctggacataaa
ggactatttgataccatcactaattctttacatatacaattaggattagcattagcctca
cttggcgtaattacttctttagtagctcagcatatgtacgctatgcctccatatgctttc
atggctaaagattttaccacacaagcatctttatacactcatcatcaatatattgctgga
tttttaatggtgggagcatttgctcatggtgcaatattctttgttcgagattatgatcca
gaacaaaataagaataatgttttagctcgcatgctagaacataaagaagctatcatttct
catttaagttgggtaactctgtttttagggtttcacaccttaggtctttatgtccataat
gacacaatgattgcttttggtactccagaaaaacaaattttaattgagccagtatttgct
cagtggattcaagcttcttccgggaaagcactttacggttttgatgtgttactatcatct
tctagcaatattgcaacacaagctggtagcaatatttggttaccaggttggctagaggca
attaatagcggaaagaattcattgttcttaacaatcggtcctggtgatttttttagttcat
catgctattgcattaggattacatactactaccttaattttagtgaaaggcgctttagat
gcacgaggatctaagctgatgcctgacaaaaaagatttcggttatagttttccttgcgat
ggccctggtagaggcggtacctgtgatatatctgcatgggatgcattttacttagctgta
ttttggatgttaaataccataggatgggtaacattttattggcattggaaacatattact
atttggcaaggcaatgcaactcaatttaatgagtcttctacatatctaatgggatggttt
agagattacttatggttaaattcttctccattaattaatggttacaatccatatggtatg
aacaatttatcagtatgggcatggatgtttctatttggacatttagtgtgggcaacagga
tttatgttcttaatctcttggcgtggttattggcaagagttaatagagactctagcatgg
gcacatgagcgtacacctttagcgaacttaattcgttggaaagataaacctgttgcgtta
tcaattgttcaagccaggctagtaggtttagcacatt-tttctgtaggatatgtattaac
ttatgcagcttttgttttagcctcaacagcaggcaaatttggttaaagtaga-----att
taattaattgcaagaaaaaaaagtcagtcatgata-gtttcgtgactgactttttttttct
tgacaatagttaattattaaagcttaacttcaatatctacaccagaaggtagatttaact
tcattaatgcatctattgtttgtgaagaaggttgatgaatatcaataatccttctatgag
atctggtttcaaaatgttctcttgagtctttatctacgtgaggagaacgtaagacacagt
aaattcgcctttttgtaggtaaagggattggtcctactgcaatagcattagttctagatg
ccgtatcgactattttatcacaagatgtattaagaatactcgaatcgtatgctttaagct
taatcctaatttttgtctgctgaataagtgtcatattgattaaatcttattatttaagaa
ttttagagacaacacctgcgcctacagtacggccaccttctctaatagcaaaacgcatac
cttgttcaattgcaattgcattaattaattcagcagtcatcttaattctatcaccaggca
taaccatttcagcatcagtaccatcatcagcagtaaactgattaatagttccagttacat
cagttgttctaacataaaactgaggtctatagcctggaaaaaatggagtatgtcttccac

cttcttctttagttaaaatatagacttcagcttcaaactgagtgtgaggagtaattgtac
caggtttagctagcaccattcctctttcaatatctttttctgaacacctctcaaaagaa
ttccaatattatcacctgccagaccttcttctagcgtcttttggaacatttctaatccag
taatagttgtggtccgagtctctctcaagccaacaatttcaattgtatcaccaacttta
taattcctctttcgatcctaccagtagcaacagttccacgtcctgtaatagaaaaaacat
cttctacagccattaagaaagttttgtcgacatctctttctggtgttggaatataagtat
caactgcctccataagtgaaaaaatcttatcaacccatttatcttcacccgctttaatag
tcgggttatttgtgactgcttctaaggctaataaagctgagccggcaacaaaaggaatat
catctccaggaaagtcatactggcttaacaactctctaccttctaattcaactaattcta
acaactcttcatcatccacttgatcttctttattcaagaataccactaatgtaggaacac
caacttgttttgctagtaagatatgttcacgagtttgtggcattggaccatctgctgcag
agactactaaaatagctccatccatttgagctgcacccgtaatcatatttttacatagt
cagcatgacctggacaatccacatgagcatagtgacggttatctgtttcatattctacat
gagcagtattaatagtaatacctctagctttttcttctggcgcagcatcaatttcatcaa
actttttgctgaggtggaacctacagttgataaagtagctgaaattgcagctgttaaag
ttgttttaccatggtcaacatggccaattgttccgatgttaacatgtggtttttacgtt
caaatttagatcgagccatattttctattctccttatagtaaaagttgctttttggttac
ttttaacgataaatcttataaattgaaagacttattatttaatatagataaggtaaaata
agatatagttaatttaatatctatagtgtgcaaacgctttatttgcttccgccatcctat
gtgtttcttctctttttctaatggaactgcctgtttcattagcggcatccatgatttcgt
tggccaattttattgccatacttttaccagatctttctcgagaaaatctagtaatccatc
ttaatgctaaatttgtacctctatatgctcttacctctattggaacttgataagtagatc
cgcctactcttctggctttcacttccactaaaggagtgatatttcgaatagctttttcta
gaatatttaaagggtctgcttctgttctatctttaactatatctaaagcttgatatataa
ttctttgtgctaaagtttttttaccacttttcaaaatacgaacagttagcatactaacca
gtctacttttatataaaggatctggtgatgcgaacctttttttagctgtattacgacggg
acataatattttaaatttgttattgtgataatttgtt-taatagaagatatttaagattt
aggctttttttgtcccatatttagatcgacttttacaacgatcttttactccagcagcatc
taatgtgccacgaactacatgatatcgtactccaggtaaatctttaattctgccccctct
aattagcactactgaatgctcttgaatattatgaccaacaccaggaatataagctgtaac
ttcaaatcctgatgtcaatctcacacgtgcaacttttcttaaagcggaatttggtttctt
gggagtcgtcgtatatactcttgtacagacacctcttctttgagggcaactttgaagagc
tggagattttgtttttttatgtatttttcgtctttcagatctaacaagttgttgaattgt
tggcataataaaattaattttaagtctttaatagatattattcgaacttgctgcgcatga
attaattagcctttatttatcttgtatttacgcataaatctttctactcgcccttctgta
tcaataattctttgtgaaccagtaaaaaatgggtgatttcctgaccaaatatccacatgt

agctcaggctttgttgagccaattgtcataattaattggccgtcacaataaacttttgct
tctgggtaccaactaggatgtatgttatcttttgccattgcttctataattgtataattc
tatcaataataaatattaacgcttagaaaattgaggagcttttcttgctttcttcaaacc
atattttttttctctcttttactcgaggatctcttgttaaatacccttcagatttaagtgc
gtttctatttttctggattaattgagcacaatgctcttgcgacacctaaacgaattgcatc
tgcttgtcctgttaatcctcctcctctagcatttacatgtatatcatactgatttaataa
tcctagaacttgtaacggtgcatacgaaactcttaagtaattaggactaaactgaagata
tgactcaccaggtataccgtttataattaaattgcctgaacctgggaccagtcttacttg
tgcaacagaacacttccggcgacctgttccagaatagattgcacgagttttaattaattc
tgtcgacataatattccttattaactaaataaatatacattctttatactatatactctt
gcggcttttgtgcgatatgtggatgaagtggacctgcatagactttaagcttcgtaaata
attttcttcctaatggacctttaggaagcatacctttaaccgatttctcaataattctat
taggcaatcttctctgtagctgctcaaaggtttctactttagtccaccaggctgcccag
aatgtcttctatataatttctgattagttttatttccgcttacatatatttgagaagaat
taatcacaattacataatctccagtatctagataaggcgtataggaaggcttatttttac
cccttaatatactagaaatatgagtcgatatcctaccaagtgtctgatttttagcatcta
taatataccaatttggattagtatttaatgatggtacttgcgttttgttcatgaaaagat
taatactttttagttaacaataagagagtaggtaaatgctgcacatataatgtcgatacaa
ataatattt-tgtaatatta-------------ttgataaagatagctttcatgacagtt
acttaaattgtttgttagcaagaaaacttatagttatcaataagtgataata-ttgtggc
aatataaaataggttattcactttttttcttttggcaaacttatacctaatttttttttgt-
agagcatatataacttcttcagctgattttttggccaaagttttaatttctagtagttct
tcttgagagtaatctagtaaatctgcaatagaatgaatttgagctcgttttaagcaatta
tatgctcgaacagatagctggagttcttctattaaacttgactaattttttttatcttct
ttgctacgataattatctgctgatttaaagtctaaatttctcagtgagcagaaaaggtta
gttaaaactgtagcaccttggcttatagcttcttggggagagatactcccatttgtccat
atttgtaaaattaacctgtcttttatgctattagtacctatacgtacttcttctacttta
taattgactttattaactggcataaaaactgagtctacttgcagaaaatctacagataat
tcatctactgctttttctgctaaacgatatccacaactttttttcaattttaaactccatt
tcgaatattgtattattgcaaatagttgcaatatactgcctaggatctactacttctata
tcagaagatagttcaaataatccagctgtaactatagctggcccctggactctaattctg
ccaatttgagattctttattatggcttttaaatactacttcttttagattcaatagtatt
tctagcacatcttctcttaccccaggaattgtagagaactcatggtttactcctgcaatg
cgcacagcgactatagcggtaccttcaagatctgataatattgatcttcttaaagcattg
cctaatgtaatgccttgtccttggtttaatgtttctattacaaaactaccgtactgtcca
cgtgctccatctgttcttgattctacacattcaatttgaaattgagccacctaagcaagc

tcc-ttaaaaataatcaatggttagtaactctgcctgcgataagaaagaatctatacacg
gcgtttcttgggggacggcagccattatgaggtacaggagtaatatcttttattaaagt
aatctctaatcctgcagcttgtaatgctcgaattgctgtttcgcgacctgctcctggacc
atttactaaaacttctgtttgacgcagaccttgatccattgcctgtttagcagctttttc
agctgcagtttgagctgcaaaaggtgtaccttttttagctcccttgaatccactagcacc
agaggatgaccaagataacgtttcacctttaaattagtaattgtaacaatcgtgttatt
aaatgtagatttaatatgtgtaacaccattaactgcattacgtctattttttttactcc
agattttttaattgtctagccatcgtgtttcgtctattatgtaattatttaaaatatta
tttccttggagctttttcttacctgctactgtttttttacctcctcttcttgttcttgc
attagttcttgttctttggcctcttaaaggaagaccaagacgatgccttctacctctata
agtactgatctccataagtcttttaatactcatagattcgaaacgtttgaggtcgccttc
aatctgataactagattctaaaatttctcttatactaacaacctgttgatcatttaaatc
ttgacacttaatatcagcatcgacgtttgtttttttcaatatttcttttgagcgagatag
tccaataccataaatatatgttaaagctatttctattctttattccttggaagatctac
tccagcaattctagccacgttgtgctttctccaataaataatatttac-gtttacaacag
aagattaca-gaattagccttgtctttgcttatgtttaggattagtacaaattaccatga
cttttctgtgacgacgaattattctacatttttcacacattttgcgaacagaaggacgaa
ctttcatattaa----ctctatatactaggaatattactg---------tattaattatt
tacatgtgaaatgtcaataatatgtttgattactttaatttctatagctttcacttcgtc
atactatcatatttttttgatatgacatatgtttgaatttgcttagctgtgtctattgcc
actccaactaaaataagtaaagatgtcgctccaagacctctcaagttttgtatctgagta
accttttctattataaacggaatcagcgctactgtaaataaaaacgaagctccgagaaat
gttagtctatttaatataacttgtaaataatcaatggtcgcttgacctggacgaatattc
ggaatacttgcacccattttttttaaatttattgcaatgtcttctggattcattactatc
gatgtataaaaataactaaaaaaaagaattaatgcacaataaagaatgaggtatagcgaa
ccgttaggacaaaatagatatagaatttggagtaacgttttattttgaattatttgagtt
aaataagatggaagagccatagatgcagatgcaaaaacaataggcataactccaccttga
tttaatttcaaaggcaagtaactgttcggatccaaaattgaagattttcctagttgtctt
gctgaaataattttaattcttcttgttccttcttgtacgcaaattgtaattataatcatc
aataagaagatagcaatgaacaatccaaacttaagccccgcattgctatagtttgtatca
aaaaatgactgtgtaaaatttttttggtaatcctgatacaatattttgaaaaataagcaaa
gaggctccattcccaattcctttttctgtaattaactcagataaccacataatgatcatg
gagcctgccgttaaagctaaaacagactcacaaacaaatgtaaaattccagttaaataca
tacggctttacccatatagagattgctccagattgcaaagtggcccatccaagagacaaa
tatcgggtaatttgagttattttttggcgtcctagctcaccttcttctttctgtaatttt
tctaaatcaggaataattttagtgagtagctgcattacaattgaagaattaatataagga

acaatacctaaggcaaagatccctattgttgaaaatcctcctccagaaaaaatattcaaa
aaatttactaaagtattttttctacgcttgcataaaaagcatcatggtctatacctgga
acaggtataaaaattcctaagcgcgctaagactaataaaaaaagagtaaatataatgcga
tttcttaagtcactttttggctcataaataaatt-----ttaaaaaagacaccga--gt
tagttaagaaccctattccaatacttcgacaaatttaaaatagggcgctggcaacaagct
atagaagtaatggca-taatatcttatgttgaataaagactttctatagaaactcctcga
tctttagctgcttcgctaaaagttcttaattgagttaggccatttaaaacagctcttgca
ttatttagtgtattattagatcctaactgtttagctagaatattttgtacacctgataat
tctaatactgtccttacagagccaccagcaattacaccagaaccaggtgcagaaggcctt
agtataactttggctgcaccagaaattccattaataggatgaggtatagaattagatttt
gtaagtggcactgtaactaaatgtttttagcatcagttactcctttttaactgctcca
attacatcactagcttttcctactccaacaccaacttggccctgctcattaccaacaatg
agaataactcgaaagcttagtttcttgcctccttttacaactttagtgactcttttaact
tgtacaactctttcttcccaaccactatctttatctttgcctttactctgtttttacga
ttagccattttaagcgttattcctaa----ttaatta-tgactttaaaaacccatacctg
cttccttagcagcttcagctaaggctttaactcttccatgatataatttccctcctctat
caaagacaacattcgtaataccttctttaatagacttgtgagcgagctgttgaccaacta
cgcgagaagtgtcacaatttgagcctattttatcaaattcttgattatttaaattaacag
atgatgttgagactaatgtgattccttttgtatcatcaattatttgtgcatatatatgtt
tattggatctaaatacgcatagacgcggcctacttgaagttccctgaactttttgcgaa
ctcttttatgcttatgaattctagtctgcttagtatttattttcattattatttaccttt
tccagcttttccagcttttcttctgacaaattcaccttgatatctgattcccttcccctt
gtaaggttcgggaggtctagtagagcggatagttgaagcaacctgacctacaacttcttt
atctatacctgaaacagtaatattggtattattttcaactgtaatttgaatattttcagg
aggttttattttgactacatggctataaccaacgcttaatattaaatcttgattatctat
ctgtgaacgataacctacaccttgtatttgtagtttcttagtaaacccattagaaacacc
ttcaatcatgttactaataagagttctggataatccatgtaattgattagccatttttgt
ctgtttacttgtcctaacagaaattttatcattaagtatctctaagttaataccagccgg
cagagttctagatagagtacccttaggtcctttaaccgtgatggtatgaccatcaaactg
agtactaagattcgccggcaataaaattatttttttccaatacgagacat-ataccacc
tataaaataattaccaaatatagcataatatttcaccaccaagaccatcatgacgagctt
gtctatctgtcataacacctttagaagtagaaataagagcgattcccaaacccccaagaa
ctctaggtagttctttatggtttgcatatactcttaatccaggcttactaattcttttca
gagcagtaataactggttgacgatttttaccgttatatttagagaaatcattaaatgca
tatctatgccttcacccatttgttcaaaattttgaacaaatccttcttcttttagtactg
ttgccatattgcatgtcattttcgttgctgggacttgcacaatttgatgtcttgctaagt

ttgcattacgaatacgtgtcagcatatcggcgatcgtatcgttgaccacc-agatcctcc
ttacgataaattgaattatttaatatgttttaagattccttaaaaggcataccaagctt
tttcagcaaagctagcccttcttgatctgttttggcggtagttacaattgatatatcaga
gccgcgaatttgatcaatattatcgtagtctatttctggaaaaattaactgttcgcgcaa
acctaaattatagttacctttgccatcaaaactttttggactaattcctctgaagtctct
aattcttggcagggttaatttgattagcttctctaaaaaagagaacatcttgtcttttcg
cagatgtactacaatgccaataggaacttcttctcgaattttaaacccagcaatggattt
tttagcttttgtgacaatcggtttttgtccagtgattaatgttaattcttgaatactact
ttcaagagctttagcattctgagaagcttcacctagcccacggttaatagtgattttcgt
aacacgaggaacctcatgtacatttttatattggaattcctcttttaaagcttgaataac
agttgttttatatttttcttttaatcccattactattatgtttt-acaaattatttaata
agttcacccgtcttttttcagttttcgga-tttttttggcctttatcgttaatgattactga
agatctactagcaatattattctgttcactaaatagcatgacattagaactatgtattgg
agcttcaaatttaataatttctccggtctcaccttcttgttgaggtttttttatgttttac
ttttaaattaattcctttaacaaccactttacttgttttgtggatgattgcaataattgc
gcctatttttgttttatcacttccagaaataacttggactaagtcaccctttttcaattt
aatttttgtattaactttagttttttttgaaagaacttctcatcatattacctccggcgca
agagagactatttagaaaaatttttatctctcaactctcgagctataggtccaaatact
cttgtaccacgaggattattatcttgattaataataacggctgcattatctccaaatcta
atactcataccatcagttcttcttaaagctttgcgagttcttactactacagctctgaca
acatcagatcttttaactggcatattaggagatgcatctttaactactccaataatgaca
tcacctatcgatgcataagaaggattactagtacctaatactcgaatacacattattttt
ttagcaccactattatccgcaacattaagatagctttgagtctgtatcatacttttattc
tatataagattaattatcaaaagacttagataagatattaaccatcgtccaacactttgt
acgacttaaaggccgtgtttctcgtattgtaactacatctcctggtgcgcattcattatt
ctcatcatgtactttatatttttttgtgcggatcatagttttttgcatattttctatgagc
aattctattttctacagctactactatagttttattcattttattactgacaactttacc
agttgtttcttttaaaggcatagtttttatc--ttatatatatatacagtgaagttaaat
tataaaataattatgtggctggcttactccgagacttttcaacagttaaaagttgagcta
atctatgcttggaatgcttaaataagtgaggctgaaaatcttgtcttgtagctcttttca
gccttaagtcaaatagcactcttttaattacaagtatttcttcagctaaagaagaattat
ccatttttgtaacatctgatattttagggaaggtcatactttaagattctattgtatttc
gaactataaatttggtctttattggtaatttataagaagctagtttcatagcttgctgag
cagtttttttgtggcacgccagtaatttcaaatagaatgtgaccaggcctaataactgcga
cccaatattccggagctcctttaccagatcccattcgggtttctgcaggacgcgctgtta
caggtttgtcaggaaataccctaatccagagcttacctcctcgcctaacatatctcgtaa

tagttctgcgagtagcctctatttgtctagatgttaaccatactggttctatagcttgca
atgcataatcaccgaatgcgattgtattacctttactagcagaacctttcattctgcctc
tgtgttgttttctaaactttgttttcttgggacttagcataaaaattaaacaatttaaat
aattaagaagctgttagatcagaagtttcctgattatatacagattctgtgcttttcgat
tctggcaaaatttcgcctttaaagagccaaactttaactccaagtacaccatatgtagtg
tgagcttgtcgatgacaataatcaatatcagctcttaaagtttgtaaaggaacacgacct
tctctaacccattcgctcctagcaatctctgcgccattaagacgcccagaaacttgtatt
ttaacgccttgtgtatttgctctttgcgtcctttgaacagcttgtctcacagctcgacgg
aaggcaactctttttctaattgctgagtaataaattctgcgactaaggttgcttcagaa
tcgggatctgcaatctctaaaacattgactctgatttgtttactcgaatctagaattgaa
gataacgattttcgtagagactcaattcctgccccagactttccaagcacaatgccaggt
cgagctgttgctattaaaatttcgacttgatcaactttacgattgatttcgattttagca
atgctagcattactaagtttagagtgtataaacgaacgaattttatgatcttcttgtaaa
agaactggatagtctttagaattggcgaaccatgaagaacgatgtttttgggtaatgcct
attcggaaacccaaaggatgaatttttttgacccacagtatccttttctataaattaaaag
ttataattactaattgactttgaatac----aagactacaattctgagacacataatgta
atgtgacaagttggtttatgtatagggaatgctcttccttgtgctcttggctgaaatctt
tttagcgtagggcctttatctgcaaaagccttactaataaataactcacttttgttcagt
ccatcattatgctcagcattggcagcagcagactctaaaatctgctttatataagaacac
actcgatatggcataaattccagaataattaatgcttcttgatatttcctacctctaatt
tgatctaatacacgacgaactttatgtggagaaagacgaatatatttccctactgcttta
gtttcttttacgtttttttgtaatactcataattatagtttaacgacgcgcttttctatca
ccttttatgtgagtacggaaagttcttgttggaacaaactctcctaatttgtgacctacc
atttgatctgatacaaagacaggaaaatgttgtttaccattataaacagctattgtatgc
cccaccatatctggaataatcgtagatgctcttgaccaagtttttaaaacttctttttttc
ccaacagtatttaatgcttctatacgtttaagaagactcacatcaataaaagggccttta
tgtatagatcttgacataataataaattattagattcttaataaaataaattctatttac
gg-gacgcaaaatgtatggggtgctatacttattgggattccgtgttttaacacctaatg
ctggtttgccccaaggtgttaccggacgagctctcccaattggagatttaccttcaccac
caccatgtgggtggtctactggattcattacgacacctctaacagtaggtcttttaccta
gccaacgatttctaccagctttaccaagagtaatattactagcgtcaatattaccaactt
gccctatagtagcatagcattcttttcgaatcatacgaacttcgcttgagggcagtttca
ccgtgacaaaagagccttctttggctactatttgagcataagttcctgctgcacgaacaa
tttgccctccgcaagaaggtcttaattctatattgtgaacagctgtgcctagaggaatag
ttgataaaggtaatgcattgccaacttcaataggcgcagtgggaccggacagcaccattg
ctccaacacgcaatgatcgaggatgtaaaatgtacctttttttctccgtcaagataatgca

ataatgctattctagcatttctgttcggatcatattcaatagaggcaactttagcaacta
cgttgtgcctgtttcttttaaaatcaattaatctatactgctgtttatgtccaccacctt
tatgacgacacgtaataacacctctattgttgcgacctttgcaaaaatgatgtttaacta
ttaaggattttctggcttatcagtagtaatctctgaaaaagtagaaactgttctatttc
ttgttccaggcgtataggcccggtataaacgaattgccatataggaaaatttaaaaagag
taaatatttta------ctgtgtaatacaattaagtttctgggaataagttaatagaatc
ttctggcgcgagtgttacgattgctctcttatagtgaggccgtttacctacaaatctacc
aatactcctttttttcttaggtggatggcaagtgttaacacctgtaacttgaacattgaa
aatatactgtatcgcagctttaatattaattttagttgctttgggatcaacagcaaaaca
gtactggttttcttctaataattttgtagttttatcagtaatgattgggtatttaactaa
atctaataagcctcttgaatcaatgctatccattatatacctcttgtattttagataaag
catcaactgtaataataattttatgcgcagctaaaagggccatgatattcaaagtgtcag
ctgaaataatttctacattatttaaattgcgaatggaaagatagacattcggatcttttt
tatccactacaactaaaactttttttgtttaaatcaagattccatcggtgaatagcttcca
taaacaatttagtttttggctgctgaaaataactattaaagttttctacaattaacgtat
tgactgatttattatttaacgttgtccttaaagctaattgtctttctttttttattcattt
ttttagtaaaactacgaggcttgggtccaaagattacaccaccacctctccataaaggtg
agcgtatagaaccggctcttgctctaccagttcctttttggcgccatggtttgcggcctc
cacctcggacttcacttcttgttttggtattagcagaaccttgtcgtttttcattccttt
gtcttactaaagctctatgaactaagtacataccagagtcttgactcactttaagattca
aatctgcattaccacttatttcacctttccaattatagacttgataatttaattgtgttt
taactgtcattagatatatcgcgtgaaagaatattgttatttactaattttactaaatt
accaggcttacctgggacagctcctttttacaatcaaaaaatcattttctgagttaatact
tacaatttgcaaatttttttatcgtaactttttttattaccaagttggccagccatattttt
tcctgggtaaaccctcccaggcgtcgtaccagctccaatagaaccaggttgcctatggtt
tttcgagccatgagacattggacctctactaaaatgatgtctcttttgatagcctgaaaa
gcctttacctatgcttttagaagaaacattgattgtctggcctactttaaacatatctgt
agacaagatttgactaacttcaaagtcatcagtagattttactaagtactcacgcaaata
tttcagtggggggagcttgtgacttctttaaatgtcctaataatggtttatttaacttttg
ttcaacaacttgcttgtacccaacttgaatagcattatagccatccttaggtaaagattt
aatttgagtaacaatacacggcccaacctgaattacagtaactggaattgacaagccagc
ttcatcaaagaattgggtcatacctactttagtaccgagtataccaacagacactagatt
tctcctttttgagagatttatactattatcgtacatgaaatttttacttcaaattgcctg
aag--taaactgaactaagagatgtctgagattaatcatgacaacataaagagaaaaaac
tttacttattcaatctaaaatagaaatagtttttttgtccagttacattatgtatagatta
aaggcaaacttattatattatttatacactacg---------------------------

----tgttcaaaataatatgaaccagaagacatgaattcttttag-----atttgtacaa
cattacattattagtactgtctaaaatata----gatagaatattgaatttctacctctc
tagaa-------aaactaaaattgtgttcggtaactacaccttttttatttacatatttat
tgtgcagtatatatgcatatctaatacagcacaggtaaaaatatagc----tgtatagcg
aatatacttacaagaattagagaggtattaatgggtaaagttgttggaattgacttagga
acaaccaattctgtgattgctgttatggaaggaggtaaacctactgtaataccaaacgca
gagggttttagaaccactgcttctgttgttgcatatactaaaagtggagataaacttgtg
ggacaaattgctaggaggcaagctgttattaacccagagaatactttctactcagtcaaa
agatttatagggcgtaaacaaaatgaaatttctcaagaaattagacagacatcatataat
gttaagactagtggatccagcataaaaattgcctgtcctgcacttgataaagattttgca
cccgaagagatttccgctcaagtactcagaaaacttgttgaagatgctagcacatactta
ggcgagactgttacacaagcagttataaccgtacccgcttactttaatgactcacaaaga
caagctacgaaagatgccggtaaaatagccggtttagatgtactaagaattatcaatgaa
cctactgcagcttccttatcttatggactagacaaacaaaataatgaaacaattctagta
tttgaccttggaggcggaacatttgatgtgtccgttttagaagttggagatggagtattt
gaagtactttcaacttctggagatacacatttaggtggagatgactttgaccaacaaatt
gtggaatggttaatcaaagatttcaagcaaaatgaaggtattgatcttgcaaaagataga
caagcacttcaaagattaaccgaagctgcagaaaaagcaaaaatagaactatcgaattta
actcaaacagaaattaatcttccatttattactgctacacaagatggtcccaagcactta
gaaaaaactgtaactaggggtaaatttgaagaactttgctctaatctaatagacaaatgc
agtattcctgtaaataatgctctaaaagatgcaaaattagaagcttccagtattgatgaa
gtagttttagtaggtggatctacaagaattcctgctatacaacagatggttaaaagatta
ataggtaaagatcccaatcaaagtgtaaacccagatgaagttgttgctattggagcagct
gtacaagctggcgttttagctggcgaagtcaaagatattctattattagatgtgacacca
ttgtctttaggagtagaaactttaggtggggtaatgacgaagattataccaagaaacact
actattccgacaaaaaaatcagaagtattttctacagctgtagacaatcaaccaaatgta
gaaatccaagtgcttcaaggtgaaagagagttgacaaaagacaacaaaagcttaggaaca
ttccgattagatggtattatgcctgcacctagaggcgttcctcaaattgaagttacattt
gatattgatgctaatggaattttgtctgttaaagcaaaagagcaagctacaggcaaagaa
caatcaattactatatctggagcttctactttacctaaagatgatgttgaaagaatggta
aaagaagctgaagaaaatttcgatacagaccaaaaaagaagaaagaatattgatactaag
aatcaagcagaatctttatgttaccaggctgaaaaacaaattaaagaatttgaagataaa
atcagccaagatttgaaaacaaagatagaggaactaattacagagcttagatctagttta
gagaaagaagaatatgaaaatattgaatctatctctcaacgactacagaactcattaatg
gatattggaaaaaatggctgctcaggctgaaagtaaaaatacaaacacaaaagatgatggc
actgtaattgacacagatttctctgaagctaaataaaaaatt-agcgggtaacgcgattc

gaacgcgcgacatcaaccttggcaaggttgcgctctaccactgagctatacccgcacaga
atatattattacaaacatggtaacatttgtcaatctgt--------agttatat---agt
tgagctattaatcattaaactatagattacaaacttgttaaaaacaaaaaaactattatc
gtttttaaa-aaaagaagtacttatataag-tatataagtacttcttttattcaaaaa-
-c-aaaatataattagatatcagtt-------ttaaactactaaggaattaaccaagcta
gtaacaattactagtcctgtccataagcctgcccctgtatagattaaccctttttgattgt
tcccattgaccgggtgatgctagaacaacagggacgcctactactaacacagtcgataat
acaattaataatagcactaataattgaattgcaataatcatttattctattctcctaaaa
aattcaaatgtgaataatattgtatataatacaatgaagctttataccttcatcaattta
tctatgaagaaataatttaaaattattgttttctcaataaatattaaattaagtattaaa
atctttattgtaaacaataacactatcttttttttaa-atagataaaaaatttaaagaaag
tctttttaaaaaaaatttagaataaggtgatatgctgtataaaaagctaagtattatta
gttattagagaaattttaaaataagaggtaaaacatatgaattcagccctttttttagcg
aaactaccggaagcttatgcagttttcaaaccgatcattgatattttacctgtaattcct
gtatttttctacttttagcttttgtttggcaagcagcaattggttttagataaccttga
agctataacaaaaatagcaaatataaaaagtaaaatttttttgcgctctaatcttatatct
tacacaataataactattatggttgaacccttactatcaggaattgttcttggccttatt
cctatcactttatttggattattagtagcagcttatctacaatatcaacgtggaaatcaa
ttaggactataaagcctaggttatagatatttataaaagctgtaataattatggtttta
taaatatctacaaaccagctaattataaatacagaaaaaatttattgaaaacagattga
taattggcaatactgattttatagtaagatagcgaatctttcataagataataactattt
taaagttatttgaacccattaaactttctatatgacaagctgcttctttctatacaccaa
cccttcttgaattggatttgctttatttaaaaaagctaatacttgtgaaagatctttaac
actggcttcgctacggaatggattctctaatttactatactcgggtctcttaataagaac
ttttattacagccattagcgaaacatcatttagtgcaatcaatctactggcttcaagaga
tttctcatcaactataggtggatcaaacatctcattaaacttttcggtgtttaaactagc
gaaaatatttatttgactattgttattttttacaatcttcatcatcatcgttagcagct
tttaaaatcacaacgccctctggattagcgctggttaatctataattagtacgcgtatca
tcgctagttattatcgaaaatacttgtggatctctttcgacataaagccaagacttttc
aaaggatatggctccttgtatagcacaagtgtctagaaaacctacagtaagattgtagtt
acgacccattatacaagcacaagcccacacacctagatttataaacgactgttggcttcg
agcattttacaagaaattgctttcagtcgccgatccatagtaattacaccttccgcata
tttagtgctgacagtagcttataaacctcatctttagcaaacccttgtacaattctaat
ataatcggataccaagaagtgatctattatatcaacatgcaaagtagggtaatacggatg
cgggtctataggaaagggatggattttaattctagtaagtagccaaacataataaactt
ggattcaaataaaggtaattttatattaaagtatgatatagatctctttcatatattcta

gacatttgagtgtaagacaatcatttctttactatttataatcttgatttgtgatgaaat
agcaaaattcttatcgccataagcgctcttatatagaaatgtctgagttatgaaatctac
atcgactaataaagaaaaatatatttatttattatttcaatattatcaatgaaaatact
tttagtataaatatactgatttttttctagtaaaattgtttaccagctcgacttagtaac
tccaggtaaaagacattcatgagacatttctcgaaaaacatggcgagataaaccaaaatc
tctataataccctctacttctaccagttaaccagcatctgtttctacctctaacggctgc
gctgtttctaggcatttcttgaagtctttgtcttagttccattttgtcagaaaagcttat
agcttttttagttgatctttaatagccagtcttttaaagagtatttttttttaagctt
ttccctttaagctctctttggatcatattttttttgccatattataa-aacttactta
aaaaattacttaatcaatatactagctaaaaaatctctgaactgcaacatttatatggtc
aaaaacatatagcataaa-------------atagatattttatgaattatact---act
ttatgctattgaaagaaatttatttaataaagttcttgttcaacatgagttgaaatagtg
caatcagatgtaggataagcaatacatgttaaaacataacctgctaaaagctgatcatca
tctaagaatgattgatcacgttggtctacacccccttcagtaactttacctgcgcaagtt
gagcaagctccagctctacaggaatacggaagttcaacaccttcttcttctgctgcatct
aggatgtatgtatcgtccgcacagttaaatgtaacatcaatattttcatcttcacatact
aaatgaattttataatctgcca--tttttttactccagaataaagatatgcttgaaactt
aataa--taaaac-aatttaaaggacttgcccaaa-attgtatattagtttcattatgca
taatagactgcaaaaaaagaaaataatatactattgtaagaaattaagctacatttcata
ctaaatagaactattgcaggttcaaagtatatatataaattcatatagttaaaattattt
ttatcatttaaataactaaacttaatactct-agacctttagattaactgca------ca
gctaatcttttacaataaacaaataagtaattatgacttcaatgataaatcaacaaatag
aactaaagcctattcttcgatttcagataccacatttatattcgagttttcttatacagg
aaatcaaatctttaacaaaaagactcttttttgcaagtttggagaagacctgcgacattga
tggcaggtattattcagcctttattatggctaattctattcggtgggctttttttataatg
ctcccgtgaatttatttactattaacacaagctataactgttttttaagctctggaatta
taattttcacatcgtttactggtgcacttaattcaggtcttcctttaatgtttgatcgag
aattcgggtttttaaatcgcttattaacggctcctttaatatctagaacttctattattt
tatcttctgctacttttatgacttgtattagtttaatacaagttatattcatagttacag
cctcattatttatgggaaattctcccttaaacagtgatagtgcgatagtttttggactta
taattctattagtaactgtgggagttacaatacttagtttagctttatcttttactctgc
ctggtcatattgaactattagcacttattttagtaatcaatttgcctttttttattttcta
gtacggctctggcccctctatatttcatgccaccttggctccagttaattgctagtctaa
atcctttaagctatgcaatagaaggtacaagatatttatattccagcatacattggaatt
ttacagagtctattataaaaaattagttggggagatgtttgtttagggcaaattattgcat
tattagttattctagatataattgcagcctaccttgtctctaagatactaaaagctaaac

ttaatta-----------------aaattttaataaaaaatttattaaatttattttaga
aagattttactatataatactagtagtagtatggataattgaaatagaaatagtttttc
aaaaccaaagctattatcaacaatgataaacatttgtaagaaagtcaacaaagtatgttc
ttattcataggaggcatgtagtcaatgggactaccatggtaccgtgtacacacggttgtt
ttaaatgatcctggacgcttaattgcagtccatctaatgcataccgcacttgtagcaggt
tgggcaggatctatggcattatacgaattagctgtatttgatccttcagatccagtatta
aatccaatgtggagacaaggaatgtttgttatgccattcatggcaaggcttggcgtaaca
gattcttggggaggatggagtattactggagaaagtgtttccaatcctggattatggagc
tttgaaggagtagctttaactcatatagttctttctggaatgctttttctagctgctatt
tggcattgggtttattgggatttagaattatttcgagatccaagaactggagaaccagct
ttagacttgccaaaaattttcggaattcatctattgctatcaagtttactttgttttgga
tttggtgcttttcatgtaactggattattcggaccaggaatgtgggtatctgatggttac
ggagtaacaggtaaagtactgccagtggctccggcgtggggaccagaaggatttaaccca
tttaatcctggaggagttgcgtcacaccacattgctgcaggtactgtaggtatattagct
ggtgtttttcatttaactgtgagaccgccgcaaagactataccgcgctctaagaatgggt
aatatcgaaacagtattatcaagtagtatctctgctgtttttttctcagcttttgttact
tgtggaacaatgtggtacggctctgcaactacacctattgagttatttggtccaactcga
tatcaatgggatagtggatattttcagcaagaaattgaaaaacgagtagagaacgctatt
gctgatggagctgctcctagcgaagcatggtcaagaattccagataagttagccttctat
gattatattggtaataatccagcaaaaggtggtttattcagagctggccctatgaataaa
ggagacggagttgctgaagcatggcttggacatcctgtatttcaagataaagaaggaaga
gaactgagtgtccgcagaatgcctgctttcttcgaaacttttccggtcattttagtcgat
aaagacggtatcatacgagctgacattccatttaggagagcagaatctaagtacagtata
gaacaagtaggtgtaacagctagtttttatggcggaaaattaaatggccaagtatttaat
gatgctcctagcgttaaaaaatatgcaagaaaagcccaattaggggaggtatttgaattt
gaccgaactacactagaatcagatggtgtattcagaagtagtccaagaggctggtttaca
tttggtcatgctaatttcgctttaatatttttctttggacatctttggcatggttcaaga
actatatttagagatgtgtttgcaggaattggagccgaagtaactgaacaagtagagttt
ggagctttccaaaaattaggcgacagaagcagtaagaagcaaggagctgtataatgct-a
agccaaatacagtctttatc---aaatatttccaatattaaaatagttcaggaaaagttt
attgtctaatagttactaattaggagaacctatggaagccttggtttatgtatttttatt
gacaggaacactaatggttatattctttgcgatctttttagagaacctccaagaatagc
taaatagctttattaagtttaaccttacaataatagaaccactttccaacttattagtgg
agagtggttctattgtaggttacagtatattattcttcatgctcttcaaaaggatctctg
agatcttttgaagcaggaccaaatgccgtatatattgaatagcctgtaatacctaaaagc
aaacttgaaataaaaatactaagaactgttgcagtttccataattcaatcagtattaaga

tgaactttctctataatgatattatcaatattaaaacatttaattattaaatttatcaaa
atatattatggcacttagaactagacttggagaaattttaagacccttaaattcagagta
tggaaaagttgctccaggttggggaacaactcctatcatggggattttcatgttactatt
tttcttatttttgttgatcattttgcaaatctacaattcatcactagtactagaaaatgt
agatgtagattgggctactttaggtag----ctaattatacttttttctttaa--------
tttaataacattaaagataat----------------aagcgatcgctattataacataa
tagcgatcgcttaatttctactttactatata--caaaagtaattattttcctaagtcaa
ttaattcgctatttgcaaagctattagtattgacattattataattaactttttcaaatc
ttactagtactggatacttgataccacttttatctattgctgcaacagttccaagttctt
gataccagtatgattcctttctcaatatttttacttttgagccacgttccatatttttag
tcctaagatagattattaaagattagtataatagataaagaaataaaaat-aaaaaaata
cataaaattagtaccgacaat---------taaatagtaaaaggagagagagggattcga
accctcgataactacagttatgacaattttcgaaattgttgcaataaacctctctgccat
ctctcctaaattaaaactctttttccccgaattagataagaataacatagaactaatgta
gttgcctagaaaattaaaactatgttaaatataaattataatctttctatagatctcttt
acaaaatatgtacacagattctatatcagcttatttgaagatttttaatcttatacaat
ttttactaaataaataaactctctcataacatgaaactatcttggaaaacacttcttctt
tggtctttaccaatatttgtcgttgggttctttttctggcaaggtttttaggtccgact
actacagatgttggtagtaatatcgcaagttctagaatgacatatggaagattcttagaa
tatttagatatgggttgggttaaacgagttgacctatatgaaaataatcacacagcaatc
gtcgaagctgttggaccagaattaggcaacagggttcaacgaattcgagttgagctgcca
gcaagtgcaccagaattaattacaaagctacgcaaagctaatgttgatttagatgctcat
cctcctaaaagcacaagtgcagtatggggactattaggtaatttactatttcctctattg
ttagttggaggcttagcttttctatttagaagatctaatagtgctagtggcgggcctggt
caagcaatgtcatttggtaaatcaaaagctttatttcaaatggaagccaaaacaggagta
gtatttaatgatgttgctggagttgaagaagcaaaagaagagtttcaagaagtagtaact
tttttaaaacaacctgaatcatttactgctgttggtgccaaaataccaaaaggcgtttta
ttagttggtcctcctggtacaggaaaaacattattagcaaaggcaattgctggtgaagca
agcgttcctttttttagtatttcaggctcagaatttgtggaaatgtttgttggagttggt
gcttctcgcgtaagagacctattcaaaaaagcaaaagacaatgcaccttgcattgttttt
attgatgaaattgatgctgtcggaagacaaagaggaacaggtgtcggaggtggaaatgat
gaaagggaacaaacattaaatcagctattaactgaaatggatggctttgaagggaatact
ggtgttattgtaattgctgcaactaacagagctgacattctagattctgcattattacgg
ccaggaagatttgacaggcaagtgtctgtggatgttcctgattttaaaggtagattagca
attcttgaggttcatgctaaaaataaaaaaatggagcctaaagtatctttagaaactatt
gcaagacgaactccaggcttttcgggagctgatttagctaacttattaaatgaagctgct

attttaacagctcgaagaagaaagaatgcaatgactatgtcagaaattgatacatcaatt
gatagagtggtagctggaatggaaggcacacctttaattgatagtaaaagtaaaagatta
attgcataccatgaggtaggccatgcaataataggaagcttattagaacatcatgatcct
gttcaaaaagtcaccttaataccaagggggcaagctagaggcttaacatggtttactcct
agcgatgatcaaagtttaatctcaaggtcgcaaatactagcacgtatcgttggtgctctt
ggtggaagagctgcagaagagattatttcggagatgcagaagtaaccactggtgctagt
aatgatttgcagcaagttacatcaatggctagacaaatggtaactcgattcgggatgtct
aaaattggcccattatctcttgaaagtcaaggtggagaccctttcttgggtagaggaatg
ggaggaggctcagaatactcagatgaggttgcaactaatattgataaacaagtacgagaa
attgtgagcgaatgttataagcaggctaaagaaattattgcagataatcgagtagttatg
gatagattagttgatctactaattgaaaaagaaacaattgaaggcaatgaatttagagat
attgttaaagaatacactgcaattccagagaaaaattactataaatctcaattttaa---
---------------atgatacgggactgacgggattcgaacccgcaacttccgccgtga
cagggcggtgctctaaccagttgaactacagtcccaataaaggg---tacagacagataa
tctcataattagcttgtgattgtcaaattatactgtaaaacatgaaaagta---------
--------tcctcaataatatttttcatgttttttattataaagccaattgattaatgca
taaggagagagagggattcgaaccctcggtacgaagttaatcgtacagtagattagcaat
ctaccgctttcgaccactcagccacctctccaaagagtcttacgtactcaatatatcaaa
tattattaaattttaacagctgtgcataaatatttatggagataagcggattcgaaccgc
tgaccctctcaatgccattgagatgctctaccaactgagctataacccc---tttcttaa
ggctattaagaaatggctcaagcgggatttgaacctgcgaccttgggcttatgaatcccc
tgctctaaccactgagctactgagccataataaaaaccattgataaatactataccataa
tttatcttaaaatataaatcaataaaaaagtctttactacaagtacatagatcttatatt
g----------------tccagatt----------------------aacatttcaaaga
tattaaattaccctctacgatatactctcaattcttttcactttat-tggcagtatttca
atattcagcttgtaa---
--------------------aataagt---------------------------------
---aattttttt
g---

-------------------------------caagaatatatagaa--------------
--tcttgctacatatt

------------------tacattattgttaataatt-----------------------

---agcctttgctaaacta---

------taaatctatgaat---

-------------------------------------tttagtagatt------------
-----agactaaataattatgtcttttaaattaggtaaaagtaaagaaagtatgtgagca
aaatatcttaa-----------------ttttt---------------------------

-------------------------------------ttagaaatattttattttcaaca
tttt-tttataatagagcagttaaaaactgaataaatagctaatatc-----ttatacaa
ccaacatcgagccaatgccttgatcagttaatatttctagcaagagagcgtgatcaattc
taccatccaaaatatgagctgaagctactccttgggctaaagagcgaatgcagcaattaa
ctttggggatcatacctccggagatcacctcggtctgagttaagtctctagcttgctgta
tatttaagtgactaatgagtgttgtaggatctgaagagttacgtaaaataccaggagtat
ctgttaacaaaattagtttttccgcattaagggctgctgctacttctcccgctacagtat
cagcattaatattgtatgactgaccttgtttatctgccgcaacactagcaattactggta
tgtagttattatgaattaagatctttaatagcttagtatcaacattctgtacttctccaa
caaagccaagattcgctttaccgctcggtctaggagtaataagtaaaccatctttccctg
acaaacctacactttacctccttgcttattaatcgttgctacaagatccttattgactc
gtccaactaataccatttccacaatatccatagtaggctgatctgtcaccctaacaccgt
tctcaaattgtgataaaatttttagacgatctaaccacaaattaatttcaggacctccac
catgaactaaaataggacgcaaccctataaaagaaagaaaaacgacatcactgattactt
gatcttttaatttttggtttttcatagctgctcccccatatttattacaataattcttg
aggaaaattgttgaatatatggtagggcctcacttaagactttcactctttctgaattag
tcagcatgaataaaatagaattataatatataaatttatttgcaggacttatcttaattt
aatagatactagtaagtactttaaaacttttagaaaataaacatattattaatag-----
----ttaaaagtataaggatgataattatgagtaatttttggaataatatattaaaattt
ccacgatttttagttagtgtcattttcggattagttttaataattattagtccttttttc
gtgttatttaaaaaaccattgacaagtttttttttcatcatatcattaaccggtttaatc

gcaattttagcggcaataa-tacagaaaatgctcaatatcgaatgttgttgagagaatta

atttaatttaattaaagtttttgaacatatataatattatattatcattaactgtgataa

tttaaatttcttttctat--aaaaagtcatacgtattaaatattatttgatagatattat

gttattaaaaccaatagttagctctgaaaaaactggtgcttttgccctttagatagtat

tgttagacatggtgttaagcatatatttggttatccaggcggagctattcttcctatcta

tgatgaactttatgcttgggaagagtcttctttaattaaacatattcttgttcgacatga

gcagggagctgctcacgctgcagattcttattccagatcaacaggagaagttggagtttg

ttttgctacatctggtccaggagcaactaatcttgtttcaggtatagctaccgcgcatat

tgattctgtacctatactagctgttactggtcaagttggaagagcgtttataggtacaga

tgcgttccaggaagtagatatctttggaattactctccctattgtcaagcattcttatgt

agttcgagatcccagagatatgtctaggattgttgcagaatcattctttatttgtaaaca

tggtcgaccaggccctgttttgattgatgtacctaaagatgtaggattagagaagtttaa

ttatttttctattgatccaggcaaagtcaatatcccaggttgtagaccagttactacctt

gaagtctagacaaatacttatggcagctaaaatgatacaacaatctagtcaacctttgtt

gtatataggaggaggagctataatctctgattcacatcaaattattaaagaacttgttga

cttgtataaaatacctgtcactactactttaatggggaaagggattttaatgaagacag

tgaatattgtttgggaatgttgggtatgcatggtaccgcatacgctaattttgcagttag

tgaatgtgatctattaattgcattaggagctagatttgatgatagggtgacagggaagtt

agacgagtttgcttgtaacgcgcaagtaattcatgtagatattgatcctgctgaagtcgg

aaagaatagaattcctcaggttgctatcgttggtgatgttacagaagttgttactgcaat

attaaatctactaaaaacttctttccctccttatccggagcagattgtatcttggcaaga

gcgaattaatcgttggcgcgaacagtatccattactggttcccaaaaaatcaacaagtat

ttcgcctcaagaaatacttgttgcagctaataaactagcccaaaatgcttattttactac

agatgttggtcagcatcaaatgtggtctgctcaattttaaaagttaaagctaaacactg

gctttcaagtgccggtttaggtacaatgggatatggattacctgcagcaattggtgctca

agtagcacatcctaatgatgtagttatttgtattagtggtgattctagctttcaaatgaa

tatgcaagaactaggaactatcgcacaatatcagttacctgtcaaaattattattattaa

taatcgttggcaaggaatggttagacaatggcaacaagctttttatggcgaaagatactc

tcattcccgaatgatagaaggagcaccaaatttccaaaagcttgcagaagcctttggtat

taaggcttttactattaatagtaggcaaagtatgcaatcgtccttagaagctgctatcga

gcatcatggtccagttttgctagactgtcaagttacagaaaatgaaaattgctatccaat

ggttgctccgggtaaaagtaactcacaaatgataggcattgccaaacctcaaagaggtac

tgcctcaaattatattaatgcaaatatctagattag------------------------

```
---------------aaggtctatattccctcaaaaacaaagtg-gattttc----tttt
atgagtacatatttctaatagtatatacaatttttaaaagatagcctttgacgagaatt
gaactcgtgaccttccccttaccaaggggatgctctacctctgagccacaaaggc-tttt
tattatattgggccgggttggatttgaaccaacgtaggcaaagccagcggatttacagtc
cgcccccattaaccactcgggcaccgaccctaattagtttttattcaaaacataacttaa
acagtatcataatttagttgcttaaaacaagtcttttcataaataaaact-attaagtga
tggacatagctggactcgaaccagcgactttcacgatgtcgacgtgacactctaaccagc
tgagttacatgtccaaaaccttaataattattataacactatttattgattttataagta
tctatttttaattaaagatttgctttaaacgacctgctttaccaagaagatctcttagat
aatatagtttagctctccgtactttagctcttctaataactttaataaatgttattcgtg
gagaattgagaaaaaaaacgcgctcaactccaataccttgaaatgagcatcttaaagtta
agctagtatttaagctatttttttcttcttctagccaagattactccttcgcataattgtt
ctctcgttttattaccctccttaaccatcaatccaagttgtattgtatcgccaactctaa
tctcaggtaagtcttgtttaataaaagaagtctccacacttttcatgagcttacttattt
ttgtactatttgctttcatatctagt-ttagccttataatg-ttttttgcaatattatac
attaaaacaataaatatgaaaattattagtagataagcagagtagtatctcagaatcttt
gtattatttattgctagactagatttacttcattttgtgttatcaatgtatttgcttaac
tatcaaaaaattaattcgctaattatagaataatgaccaacttatatttcta----atag
aaatacaaaattcttttatattaaat--taacctttagatatttaattcatt--------
------------------------------------------------------------
------------------------------------------------------------
------------------------------------------------------------
------------------------------------------------------------
------------------------------------------------------------
------------------------------------------------------------
------------------------------------------------------------
------------------------------------------------------------
------------------------------------------------------------
------------------------------------------------------------
------------------------------------------------------------
------------------------------------------------------------
------------------------------------------------------------
------------------------------------------------------------
------------------------------------------------------------
------------------------------------------------------------
------------------------------------------------------------
```



```
------------------------------------------------------------
------------------------------------------------------------
------------------------------------------------------------
------------------------------------------------------------
------------------------------------------------------------
------------------------------------------------------------
------------------------------------------------------------
------------------------------------------------------------
------------------------------------------------------------
------------------------------------------------------------
------------------------------------------------------------
------------------------------------------------------------
------------------------------------------------------------
------------------------------------------------------------
------------------------------------------------------------
------------------------------------------------------------
------------------------------------------------------------
------------------------------------------------------------
------------------------------------------------------------
------------------------------------------------------------
------------------------------------------------------------
---------aattat---------------------------------------------
------------------------------------------------------------
------------------------------------------------------------
------------------------------------------------------------
------------------------------------------------------------
------------------------------------------------------------
------------------------------------------------------------
------------------------------------------------------------
------------------------------------------------------------
------------------------------------------------------------
------------------------------------------------------------
------------------------------------------------------------
------------------------------------------------------------
------------------------------------------------------------
------------------------------------------------------------
```


------tacccgtaatttgttatgatctaagtatctcacccaattattttttattgatg
tta-------------ctatttaaatacttatttttgctaaagaattttacacataa
aacagagtaatcaaaacaacaagattaaggcttaagtatttattagttttgtgttataat
catttactatcaaaggtagtggcataacataaaacataaacaatgtttgaacgctttact
gaaaaagctataaaagtaatcatgttagctcaggaagaagctcgacgcttagggcacaac
tttgtcggaactgagcaaatattattaggattagtaggtgaaggtaccggggttgcagcc
caagtattaaaatctatgaacgtgaatctaaaagatgcaagagtcgaagttgaaaaaatt
ataggaagaggatctggttttgtagcggttgaaattcctttcactcctcgagcaaaaaga
gtactagaattgtctttagaggaagcacgtcaacttggccataattatattggtactgaa
catttgctaatgggcttagtcagagaaggggagggagttgcagcgagagtcttagaaaat
ttagcagttgatgtttcttcaattagagcagaagttatacaaatgctgggagaaaatgca
gaagctaatgtaagtggaagtaatactacacaggctagaagcaaaacacctacattagaa
gagttcggctctaatttaactcaaatggctatggaaggtggtttagatcctgtagttgga
agacaaaaagagatagaacgtgttattcaaattctagggagaaggactaaaaacaatcct
gttctaattggggaaccaggtgtaggtaaaacagcaattgcagaaggcttagctcaaaga
atagctaatagagatgttccttctatattagaagataaattagttattactctagatgtt
ggcttattagttgctggcactaaatatagaggtgaatttgaggaaagactaaaacgtatt
atggacgaaatcaaatcggctgataatgtaatactagttattgatgaagtccatacatta
atcggggctggggctgcagagggcgcaatagatgcagccaatttactaaaacctgctttg
gcacgaggagaattgcaatgtataggtgcaacaactttagaagaatatagaaaacatata
gaaaaagacccagcactagagagaagatttcagcctgttgtagttggagagcctagtgtt
gaagaaacaattgaaatcttatttggtcttcgagaccgttatgaaaaacatcatcaatta
acaatgtctgatggcgctttggcggcagctgcgaaatatgctaatcaatatatttctgat
cgattcttgccagataaagcaattgatttaattgatgaagctggttctagagttcgttta
ctaaattctcaactacctccagctgcaagagaattggataaagagttaagagctgtatta
aaaacaaaagatgaagctattagagctcaaaaatatgaaacggcagaacaatatagagca
agagaaatggaaattaaagctcaaattgcagcaattgctcaaagcaaaaaaaatgagccc
gatttaaacttagaagatcctgttgtaacagaagatgatattgctgaaattgttgctgct
tggactggtataccagtaactaagctgactaaaagtgagtcagaaaaattaatgcacatg
gaagaaactttacatgggcgtatcattggccaagacgaggcagttgtagctgtatctaga

gctataagacgagcaagagtaggtctgaaaaatccgaatagaccaattgcaagtttcatt
ttttctggaccaacaggtgtaggtaaaacagaattgacaaaagctttggcttcttatttt
tttgggtctgaagcttctatgatacgtttagatatgtcagaatatatggaaagacatact
gtatctaaattaattggttctcctcccggctatgtaggttatagtgagggtggttatcta
acagaagcagtaagaaaaaaaccatatactgtaattcttttgatgaaattgaaaaggct
catcctgatattttaatcttcttcttcaaattttagaagatggcagattaacagatgcc
aaaggtcgaactattgattttaagaatactcttctaataatgacttccaatattggatct
aaagtcattgaaaaaggaggaggtagtttaggattcgagttgtcagaagaccaaacagaa
tctcaatacactagagtcagatctttagtaaatgaagaattaaaacaatactttagacca
gagtttctaaaccgattagatgaaattatcgtatttcgtcagcttaccaaagatgaagtt
agagaaattgcagaactgatgttgaatgaagtttttgccagaattaagcaacaggatatt
caactaaatgtaacagaacgttttaaacaacgactagtagaagaaggctataatccaagt
tacggagctcgaccacttagacgagcagtgatgagattattagaagatagtttagcggaa
gaagttttatcaggtaaaattaaagctggcgatagtgctgtagtagatgtcactagcgaa
ggagaagtaacagttttactaggtgaaaaattagaactgctaacgtaaa------aaata
atttattaatctaaaagcattgaatataattt--attcaatgctttt--gaagaaaatta
acaagatgggttgctataattgacaacctctgatattctttatatgtatata---aagcc
gggatagctcagttggtagagcagtggattgaaaatcctcgtgtcaccagttcaaatctg
gttcttggcat-tttaaatatttgaaaagtttgctagatgagcatcaaattttagttgaa
acatacctgttggtccatttctatgttttgctattataatttctgtcaagtctcctccta
cagcttcttgagtataataactctctctatatagcattataactaaatctgcatcttgct
ctatagaattgtgtacgataaagttgttcgcgataaaattatgtaagagttgacattcta
aatcaaatacttgttgcaagctaataaggcgaacatgtttaagattctcaaaaattaaaa
ataaagatggacaagaatttaagagagtttttctttctttaatggcgtt------tattt
gaatagcaagcatattattgtgattaatttcatcgcatcttttccatccttgtgttgtta
atagcttgtgattgcttgttaactttatatacttgccagattgtgtaataatttcataca
cagttttttacctgtcttggaaatattagctttactgtatctaaataagtttgtttttt
gtgcagtaaagctattaacttca-atatgcttatgatgaaatttaaatagaatttg--ag
tctgactatgtaacggtaaatgaagatgactaaatttcgagatacatccactttctctta
gatcagataataaaggtcttttatcgctccgagtttcaagattcctattcagttgagata
atactagtataggtaaatttaagtcttttgctaatatttttaatgatctagtaattagag
atagttcttgagatctattatcagattgtctgctatcttgtaataattgaagatagtcta
taataattagttctatatttttcccttgtaattttaagaattgtactttcgtttttatag
tgtccgtagaaatttttgcgctatcatcaatgtaaagattcagatctgcaagagctttgc
tttttcgaataacatactgccattcattattattaagttggccagattgaatttttttgac
tatttaagtgacattcttgagctaaaatcctccttaggagttgttctatagacatttcta

agctaaataaaataacgaacgatttctctttgctcattatatatctagctacattaatag
caaatgctgttttccccattgaaggtcttcctgcaagaacaatcaaatctgacttttgaa
aaccttgtgtaatagaatctaattctgtaaaaccggaaagtacgctactatttatactaa
ttttttttgttttgtctaaatgaacaagcagattagcaagaatggtagctagtgtttgtg
tatctttctcttcaaggatttcgtaagcttttgttaattggctaacaatagaggttatat
tattctgtctaacaggctggtttgaacagctaattaaaaataatgaatctccagatatta
aaagtaatcttttaatataattatctataataactgacgaatattcatctaaaacatcag
ataaaggtgcttgttctattagattaataacttcgtctagttcattcaagtgtctaatta
tttttctgtttttaagttggctacacaatttcctatagttactcttttatatgattaa
cattttctaggacagctctgtaaagtaatgaattcgatgtaaaataaaaaaaatcaggag
agagtttttctactgattttagtagagagactgattgtttagttaatattgtactaatta
atattttttcagctaaaatattatgaggaggtaaatatttatga------atactgatta
gttcctcc-ttgtaacattattctaata--------agactaattaattactagcttaat
ttgactctggcagaacttgtagttggatgttggcagttacctggtttaatagcttaacct
ctatattataaatgccaactgttttaatctcgggtaaaaggatattttgtttttcaatat
ctatatttgtagcattttttaatagtttgtgaaatttctttttctgtgacgcttccaaaaa
tattttgcccatcccctgtttttttagtcacgctgaacttctgtatttcctctagaagtt
gctgaatttttttgcatcttctttagctatttaaattttcttctttatggctgcgt
ataattttagcttttttaaaataccgtttgttgcgactattgccattttatttgggatta
aaaagttccttgcataaccagcagcaacttttacaagatcattacttttgccaagttttt
gaatattctcgtttagcacaacttttataacttttttacccatatttattctgaattcta
aaaa----atatatataattttattatacttgttgtacccagatttt----aataggaag
tatttacttgttgacatagcatctccgtgaaactgataatgaattttcatctttattctg
gtataataatttatacaacgtttt-----attaggagaggtggccgagtggttgaaggcg
cagcattggaaatgctgtttaggagtaatcttaacgagggttcgaatccctctctctccg
tataa--aaattatgataatctttatatagtccagtcaaaacttccaataatgtttctcc
atgcatttaaaccacctttgactctcacaatattgagcttttgagctattaataacttag
aagcaaatagagatctagaatctaagctacagtatacaaagcaaattttatctttacaat
ttatattagagtaatgtattgttttgagttgttttaaaggtatatttaatgagtgaatta
agtgatttttttatattcttcttgatttctgacatcgattaaaatatattttaggtctt
tgcgatttaaaagtttttgtaattgaacaacattgatttctcttataataagattaggtt
gatcatagctgtatctgttccagtattttttaga-------ggataatacaaatttagta
tttaaaattttaaacttactaaatgataaagttaaagcgttataagttaatatagtaccg
cttaatatagacttgtaaccaagaataattttaatagcctcagttgcttgaagtgtacca
ataatacctggtaataaacctaaaacaccagaattgctgcatgtatctatttcactattc
ctatttacgactttattgtggaagtctcgataacttggacctccttgatagttaaataca

ctaacttgtccttcaaattgaaaaatagccccataaatatgtattttatttaattctagg
caagtatcatttagcagatatcgagtacgaaaattgtctgatccatctataataatatcg
tagtttttaattatatcaaatgagttgttataggttagtcttgcttcaaaagcttttact
gtacattttggattcaaatctaatattttttttttagctatttctactttgagtatcct
atatcgtgaactgtatatataatttgcctttgtaaattagaaagatcaataatatcgtcg
tccataataccgatgcttccaactccagcagctgcaaggtaaattattgcaggagagcca
agcccaccagcaccaataaatagcaccttagctttttttaatctttcttgcccctctaat
tgaatctgtggtaacactaaatgtttagaatatcttgtatattcttctaaagagtatttt
atattatctgctttaaagttcagcat-aaaatttatatgttaattaataacaacta----
--gaaactaattgataagcttattgagatgtttttgtaaactaggttcttctttatcta
aaatagctccttgcgtaggacagacctgaatacaaatactgcagtcaatacaggcagaaa
agtcaatccagtaccaattggtattaatactattttgtccttgtccttggtgaatacaag
ctactggacaagcagatacacattcggcaactccaatgcatttttcggttacaattgtat
gagacatgttatttaattaat-aaaagataaactatttacatctagtagttttattgtat
actaaattgacatacgcta-ccaagccagtaattaaaataatctacttttatagtttact
gatttgatctgcgcctttagttcagctggtagaacgcaggtctccaaaacctgatgtcga
gggttcaagtccttcagggcgcgcttagtttttgttatatttgcatagtagcattttacc
tttttgcaaacatcaaaatgatatgatataaaacatcatattctatatatttgatgtttt
atgtgccagtactg-ttttttatatgttattattggtaagttaattatattaattctat
ttttataatcaataaaaaatagctgaaattaagtgcagattcacttagtga-----tttt
atatatataactaaaaaaattaaaaactatggctaaaaaagttaccggaattgttaagtt
ggcattaaatgcaggtaaagctactcctgcaccacctgtcgggcctgctttaggtcagca
tggtgtaaatattgtcatgttctgtaaagagtataatgcgcgtactgcagataaatcagg
actagtaattcctgtagaaatttcaatatatgaagatagaagtttcacttttatacttaa
aactcctcctgcttctgtactaattgccaaagctgctggcttaaataaaggttcaggcga
accaaacacaaaaaaaaataggtagtataacgtataagcagttagaatctattgcagaaac
taagctaccggatttaaatactaacaatgtacctcaagctatgaaaatagttgaaggtac
agctaaaaatatgggaatcctaattaaagattaaaaaatttcaattataattatta-tt-
-tttatgaaaaaaatttcacgtcgacttaaaatattaaaatctcaagttgagcctaaact
ttataatattgatgaagcaatatcgatgttaaaggcaacatcaaatgctaaatttaagga
aactgcagaagctcatatttgtttagggctaaatcctaagtatgcagatcagcagcttag
agcaacagtcatattaccaaaaggaacaggtaaatcaataaaagtagcagttattgccaa
aggagaaaaattagcagaagcaattagtgcaggagctgatgtaagtggttcagaagaact
catcgatgaaatattgaaaggtagattagattttgacaaattaatagcaacgccagatgt
tatgcctctaatagcgaagcttggccgtgttttaggacctagaggattaatgccttctcc
caaagccggcaccgttaccatagaagtaggcaaggctgtgaatgaatttaaaggcgggaa

agtcgagtatcgagttgatagaactggcataattcatgtaccttttggaaagtccagttt
tcctcaagaagatctagtcttaaatctgcaaacaattaaagaatcaattgatagaaataa
gccttctggtgcaaaagggaagtactggaaaactttttcttatctagtaccatgggacc
atccatccaaatcgatattaatagtcttctatgaaaaatatttatttaattacataactt
gaataatatattatttcaaatta-taattggctagcaatgttaatagaataatttgctta
ttataccttatctgcaccaaaaatttaatatatctcttactaattatgagtacaaaagt
tgaaaatattttagaagaactaaaatctttaaatcttctagaagctgctgaactagtaaa
acagatagaagaaacattcgatgttgatgcgtctgctgctacaggaggcatgatgatgtc
tgcaccagctgtatcaccagccgcatctgaagttgaggagaaaacagaatttgatgttgt
tttagaagaagtacctgcacctaaaaagatttctgttctaaaagttgttcgctctttaac
cggcttaggcttgaaagaagcaaaagatcttgttgaatccacgcctaaagttctaaaaga
aggtgcatctaaagatgattcagaagctatgaaaaaacagttagaagatgctggcgcaac
agttgttattaagtaataaa-t--agtagtgcttttgtttagaagtaaacaaaagcac-t
ttttatttaatagaattaattagactagaaaagtccaagtgtaattgcattattaatagg
catagtcgcaccaatacctaaccaaacacttacaactgtaccaattaagaaaacagttgt
tgcgattggtcgtctaaaaggattttgaaacttattgacattttcaatgaatggaactgt
aagtaaccctgctggtacagctgccatacttagaacacccaatagcttattaggaattac
tcttaacagattaaatgttggaaagaaataccattctggtaaaatttctaatggagtagc
aaatggattagacttttcacctatagatgaaggctctaaaattgctaacccaatactgca
tgcaaaagttccaataattacaactggaaagacatataatagatcatttggccaagctgg
ttctccataataattatggcccattccttttgctaatttagctcttagttttggatctgt
taaatcaggtttttaagaattgacatgttatttttgtgtttaatataaaattgagtct
aattactttatcaaagtgtagttatacattgttataatggtccggaaattccttgtttgc
gtatcattaaaaaatgcataagcatgaaaatagcagttagtaatggaagtacaaaagtat
gtaaactgtagaatcttgttaaagtaccttgtcctacactaactcctcctcttaataatt
ctacaatacttcctccaataactggaacagcatctggaacgcctgtaacaatttttacag
cccaatatccaatttgatcccatggtaaagaataacctgttaccccaaatgaaacagtaa
gtactcctaaaattacacctgtcacccatgtcaattctctaggtttttaaaaccacccg
ttaaatagacacgaaatacatgtaaaatcatcatcaagaccatcatactagctgaccatc
tatgaattgatctaataagccacccgaagttcacatcagtcataatatattctaccgatg
tgaaagcttctgcaactgtaggtctatagtagaatgtcattgcaaatccagttgcaactt
gaattagaaaagatacaaatacaatcccgcctaagcaataaaaaatattaacatgaggtg
gtacatatttgctagaaatatcatcagcaattgcttgaatttctaatctttcttcaaacc
agtcataaattttactcataaaacagcttcaaaaagc-tcttcaatata-tttttgcggt
taagctacttttgattcaatag------ttattgaacttatatt-atttattatctaca
cttattaattattataacatctttctatctattt--------------------aaaatt

ctctgactcaataaaacaggactatgaacgacgatctttttttttctgtatagaaatcaat
ttagtctttagcaattcagataaaatccttgttacacttactctactactccctataatt
tgcgctaacattttatgcgtcatagcaaaattaagcataataccattattactctgagtc
ccattttgctctgctagtaataataataagcttacaagtctacttgtaatacttttgtgt
gacatgatttctacaaaatgataagtttttttctgagcaacatattaaatgatctataaaa
aataaattaaatagtgggtagttactacacgctcttataatagtgttatagttaatacaa
gcaatttttgttttacctattgcttctgcttcatagtagaagttatcattagctaactct
aattgtccgaatgtatctccattagttaataaattgagtgttattttatgtgtgctttta
aagacttttgtaataattaaagatcctattaatataatgtatagtctagagctgctatca
aatagaataatatcattaggctgtagtagaaaaatttgataactaattttattactatta
aaaaatagcaaccatggattataagagggaaaattatttgaactactttcagaaaatgtt
gagatagattttttttgatttaaatgatttttagtcataattcatactttat-a------
---aaaaatactctaataat-taagaactgaaattaaatgtcatacaagttgatgctagt
tgagaatgacattgtattatcaaaagccattcaagaatacttaatagatcaaggatttaa
tgtttacattgccagtaatggattagaagcatttaatttagctaatcaatataatttcaa
tttgataatttctgatataatgatgccattagttaatggctatgaattattagaaaaact
tcaaaatactaaggcgctatcaaagataccagttattttttttgacagccaaaggaatgac
taaagatagaataaaaggttatgacatgggatgttacggttacttatctaaaccatttga
cccagaagagttagtttcacttattaaaaacttaattgctagagacatatcgcaacatac
atctacagaaaaatgtgacatctctaagcagtcaattaaccaaaaaatacatttgactcc
tagagaagcaagtgttttagaccttgtgattgacggactaactaacaaagaaatatctac
aatattagatactagtattagaaatgtagaaaaatatgttagtcgacttttacaaaaaac
taatatgaagaatagaactttactagttaaatattctataaataataaattactagataa
tgtaactaatcaaagggcgaatgacgggactcgaacccgcgaatgatggagccacaaccc
attgccttaaccccttggctacactcgccatattataaatatcactataacttgtttgtt
gatatatcgtctactctgtaagtcatttatttatctaacatattatctaaatttaatctt
ccatgacgtataataatattagtattcaaattaatggagagccgtttaattgctcaaagc
ctatttccttgcaattttttattaaattatcttgattttaattctgagcatatagcagttg
agctaaacgacactttgttacctgagcctctttttcactcaacttacttaaatgatcaag
ataaactagaggttattactattgtaggtggtggataaatttcattaactaa--------
---------ctattttaaatgcttcattgataaggataaacgtccctgcttcttgtccac
atgaataatgacagctttaatagtatcgcctattttaaattgtgacgatatctgatctaa
ttgttttatgttaatttcagagatatgtacaagacctttaagattgccaacttttataaa
taatccgtaaggtgtaatctgatttataattccttcaataatattaccaacaattaaatt
tgatgaggcttgagcgattaaagctcttctgtggcttagtattagattattagatctttc
ttcaacattgagtagctttaacttgataaattcattattagccttatcactttttttggaa

attcccgagatgagaatttggtacaaatccagatataccttcaaggctaatgatcatccc
tcctttattaaatcctttattcgaacattaagtaaagaatcttcagctaataattgtcg
aattcttttccatgctcttatatattcaaggcgccgaattgataaaattaattgttttga
tcgaatattataatctagtaaaaagaattctcttgtatcattaatgtttaaagaagtgaa
attatttaaatcttgattacttgacacctcttgaataggtaagtatgcagatattggtgt
tcctatatcaaccaaaacaccatttaactcaaagctaaatattgtaccagcgacaatatc
gccaagatttaaatcgtatttgtatttttgcaaaacagctgcaaaatttctgtgagtaaa
tccttcattatttttgtcatattgaatagtctgaattaaagattgattttgtacaaaat
attaagttgaataaacttctgtcgtattatatgaaaatttttaaattcgttctgtggtta
ttcctgatctaaacgtattagaatatacggaagacataaaaagtaaacgtagatagctac
agatttaaccaatatacagtctgaggaaagtccgggctccataaatacaatttatgctgg
agagatcccagtgtaggcaattatgaggatagtaccacagaaataaaccgccagaataaa
a----tatactagctggtaagggtgcaaaggcaagttaaaagcttaccaaaaatactgta
aagtatttgttaggtaaacccctaaaatggagcaaagctgctaaacaaatatttgtgtat
attttatatagtttagtttaaaatactgcatgaagttattggtaacaataactctagagg
aatagctatcctttacaatatata-tctattatattgaaagaacagaacccggcttatgt
agtacttttatgtcttacattattctggctattgccgaatctctaagtttaagtgttgt
atgatttttcttgtagctcatgttgtttatcatctatctctaaatttgttaaagttttt
tgactagagcgatatgtaaatctcaaacctatactttttttctgttctccaagggattta
tttgtatattgatcaaataaccttatgctttctaaatctttatcattaaactgatctagt
aattttaataagtaattaaattccatctttttaggtattattaatgaaagatctcttgtg
atagatggatatttagagtaaggtcgaatttgataatttaaataattaaattgattatga
gaatcttctaaaatgtttaaattaatttcaagaacaaaaagttttgcagtaagacctaat
ttactataggttacttgattgagttcactgaatatgccaatattagtattattataaatt
aatgttgcagatctatccttttgaatgaaattcactctactgtcaattatttctggtttt
gtccattcaattttctatttatttttgaaaaaaattttctacaattcctttagcttca
taccaatttaaagagtggactggttgtgaccatgtagatctaatatttaaattaccacct
aatatcatggctagattagttgtttcgacaattttgttgtgaacaggattaaaaactgtt
cctatttcaaaactgtctacagtttgattactttgcttaagattataaagattagattca
attaatccctcgagcaaactatagcgtaaattagagtaatcttggataagaggattattt
agactaacacctcctagagatttcactaaagaataatgaactaattcagttaaccctaaa
tttcttaaaatatttctacttttatctatgaattttctcttttgtgataatttttttacta
aattgaatacttggcactgagcttttaaatttatgatagccataaattcttgcaatctct
tcaataatatctatttctcgaaaaacatcttgtcttcgataatttggaacactaattttt
atactttcttgatttttatgaataatagcaaaatttagagaatgaagaatattttgaatt
tcttgaaagtacaaaaaacgtacctgattattgttactgattggtcctaaaatatcttga

acttttttaattgataaatctatatttagagtatcattcattgttttctctgttgagaat
gtctgcctaatattcccgccagctaagtctattattaaagagatagtttcaagataagca
ttttcccagttgtctgtatttagtcctcgttcctgcctaactgaactttctgtcctaatg
ttaagatctcgggaagactgtctaactatagattgtttaaatatagcagattctacaaat
atagatgttgtattagaatccgtatcaaagttgttattggatcctatgccagccacacta
actacatttgtattcacttgagtcactaaaatcttcttatttaactcaatattttcatta
tttaacttaattttagtactttttccaacattaaagttactttgaattgttatagtatct
tcagg---------agtaatttttattaaaatcaataatattaataggttgtccccatttt
agcataatataattgcttatatctactaacaaatttctatgtgtaaagccagaggacttt
aaacgatttttagccaatttggagaatctataattttaatttcatcgataattgcagaa
aaatagttattacaatgaatcaagctatgattattgagtatatt-ttgttgtctagaaga
gtatttattactaaattgatttatagtttttaacattaaagatccagttaaagcggataa
ctctcttgaaagacctatcatgcttaatacatccgatctattagctgttgatgatatatc
taatacatgatcaatctgattgcctactgtaatagattcaatagcttctacttcaaaacc
tgcctgtgttaattgactagttaaatgaactagatctattgtttctatattagcaagttc
ttttaaccaatttaaagaaactttcataatgaattgccatttatcttatataattattta
ttttacttgtgattaaaaactaatttaaaaaattcatcaaattaatataagttatttgat
gaatttgcttatacgatatattcttgtgctatattactttcaggtcgtagtaattaagaa
ttagcttttgtaaaagtcagtccattatcttgagcatacctttccataaatctcataaat
ctatcccaatcatcaggacttttaatcacatgtacagcttctattgcttgtggtttacca
ttaataaacttagcattgacatctcttgtaataatttgtccctctgtgtccattagatac
atgcctgttatttcacctttatctgccatactagcatctaaaattttgggatttgtaaat
ctgaaagtagctgttccggtactaccatctctagatcttgttaagcgaacatctggcacg
acttcttcattgatgccttgaataaattgaattgttgccatatcaattttt-tttatttgt
atataatgtatattggataaaactgatttctaattctttaatgcttgttgaaaaactgtt
ttaaaagcttatagtagcagcagatcaaattttgtgtactatataaaactgtaacttttc
tttttttg--

----------------------atctgttcaaagaactaacttattataagctaatcata
ctaatattagccatataatgttctgaagt-gaaattaa-gggttgtagctcagatggata
gagcaagcgcctcctaagcgctaggccagcggttcaagtccgctcaaccctgttaataaa
caaaatt---ttgctgaattctacaaataaattagttgaaaaaagaggaaaaaaacaaaa
cagc-----aaatagattctgagtaacacttttattgaa-----aaaaatgatcttaac

tttgataatagaagaattttgtgaaaaaatatttaaaatttgatctctatattcagaaa
aagtttttttaaaatataaactgaaa-----taaaagtcttgagtaacaataggaattaa
attatttaatttttgaaattgatctaaaaaactttatatgaagagaatagttttttct
ctttctctattcagtatttgcctccataattattacttcattcattcacaattaataaaa
tggattttg---

-----------------------------ctttaatgaatgtttatgttatttgattata
ttccatactatataagagtttttctcaacttgtgagagtcaatttgtagttttaatttta
taatatactttatctttggagattgatctacattcaccttcacacatctcttttattgcc
tattaggagaaactatatactgaaatttaacaataattgtgaaaatcgtaggttccttca
acatattattatcctttcatttttgtctccactgctgatcaaagcaacagtgattagact
tattcatttagctggttctagtgagagaagttttatacaagccg--gacacttatttaaa
t---gtgttattaccaaagctgttaaaataatagccattaagattcaatgacttttagt
taatagtttataatcgccatgtttgatttagtactgatatatagttacaagtagttctac
tattgataaaattgacacttttgtgactgtataagctttcgatagaaaaactatgatatt
attcataatgaaaaagcgagtattgaagtttgttgctttcataacaaaaaatatgtttag
tcaatgatgaattttgctagcgatccgagtagtaaatctgtcacatgtaaagatgaaaat
tatatatttgagaatactctctatatctgtatatagcggttggcataacttatcagattt
gatttgcagaatcagtatactaagagatcgcaaaatggttattctaagaaaattttgtct
cagtaatttcacttctaatagatacttgttagtaataaacttttaattcttttatgaaaa
aacttcataatatcaatatttattagaactggggtgggaggattcgaacctgcgaatggc
ggagtcaaagtccgctgccttaccacttggctacaccccaacttatagcaaatataatat
cagtcacataccccgccgtgtcaatagattattcgcattatttaataacttttttagattt
ttgtaaaatagaatctttttttttcaaataagatgtactatgttcaaattgcattatgctc
atgtcttcttgttgttgattggacatccattttatggtcactgttccttgttcaacttct
tgattgcctataataatacaagctacagctcttttcttattagcttgtttgatttgttta
ccaaagttacttgagctaacatctatttctattgtaaaaagtttttgatgtaaaaagcgc
attattttcatgccaacttcattagctttttaccttgtgtagcaatataaaaatcaaca
tatttacttggtaactctatattctctttcgcaagaagtagtaaacgttctaatcctatt
gcacatccgacagctggtgttgctacgcctcctaattgcttcactaagctatcatacctt
cctccaccacaaactgtatcttgaccttttgatgttaatgtcttaatttcaaaagcagta
tcattataatagtctaatcctctaactaacttgtcatttatgttataaggaatatcaagt
aattttaaataattacaaacagcatcaaaatgtctttgtgacgaaatacttaaaaaatta
gaaatcttgggagcctcagctaatatttttttgtgtatttatattttttgaatctagaatc
ctaataggattactaattagtctcttttgtgaatcaggatctaagtcgtcatagtactgt
ttcagatattcttgcaattttgcttgataaatactacgatcttctgcttttccgattgaa

ttaagatcaagctttaagtctttgagattaagattattaaatatacttatagctaaatgt
attacttcactatctgctcttgcatctaggctgccaagaaattcaataccaagttggtga
aattgtctttgtcgtccactttgtggcctttcatacctgaacattggcccgctataccat
aatctttgtaaactatgatgatagttcatttattttcgataaaagctcttacaatgcct
gcagtgccttcaggtcttaatgtaatatctctattgcttcgatcatgaaatctgtacatt
tctttatttacaatatctgtatctcttccaatacctctatcatataaatcgctactttca
aaaataggtgttctaatttcttgatagtttgcacattctaataagttggaaattttgtta
tgtataaattgccagtattgaagctcttcgggcagaatatcttttgttcctctaatagct
tgaatttttgccatgataattttcggatcaccttcttaatgtgtttgcattaaatagcag
ttgtagactgttattcagtcatgtattatacattag--aacatgttgatg-tattttatg
aaatacgggcaaggagggattcgaaccccccgacaccatggttcgtagccatgtgctctaa
tccactgagctacaagcccacttaacaagctatcttaatatatcattctttttcaatgta
tacaactatattttgtct------atttatatttgaaagttgtctgaaagt-aaaaaat
gt----atttatctttaacaattagagttagtcatgtataattttccatttattaaaact
ttgcaca--aatatcgacaaacttaattatgtaattatcttatactgataatttcagaag
aa-cttgtgcttttacttcttgatcga------ctagtatctaattcatctatattccaa
at-aaaaaatcttatctaaaatgagtaagcaaattctatatcaagatgatgcacgtaaag
cattagaaaaaggcatggacattttaacagaagctgtttctgttaccttgggacctaaag
gaagaaacgtcgttttagaaaaaaaatttggggctcctcaaattattaacgatggagtaa
caatcgcaaaagaaattagcctagaaaatcatattgaaaatactggagttgcacttattc
gacaagctgcatctaaaacaaatgatgtcgctggtgatggaacaacaacagctacagtac
tggcatcagcaattgttaaacaaggcatgagaaacgttgctgcagggtcaaatcctatgg
ctataaaaaaaggcatagagaaagcaacaaattttgtagtgagtaaaatagctgaatatg
ctaaaccagtagaagataaaaaagctattatacaagttgcttcgctatcatcgggcaatg
atatagaagtaggtaaaatgatagcgaatgctatagataaagtgggaagggaaggtgtaa
tttctctagaagaaggcaagtcaactaatacgattcttgaaattacagaaggcatgcagt
tcgaaaaaggctttatttctccttattttgttacagataccgagcgaatggaagtccttc
aagaaaatccatttattctctttacagacaagaaaatcactttagtgcagcaagaacttg
ttccactccttgagcaaatagcaaaaacatctcgtcctttattaataatagctgaagata
tagagaaagaagcattagctacgattgtagttaacaaattaagaggcattttaaatgttg
ttgcggttagggctccggggtttggtgatagaagaaaatctttgcttgaagatatgagta
ttttaactaatggacaagtaatcactgaggatgcaggtttatcgcttgatacagttcaat
tagatatgttagggaaagctcgcagagttattgtaactaaagactcaactacaattattg
cagatggtcatgaagttaaagttaaatcaagatgtgaacaaatcaagcggcaaatagaga
cgagcgattctctttatgaaagggaaaaattacaagaacgattagcaaagctttctggtg
gcgtagctgttataaaagttggtgcagctacagaaacagagatgaaagataaaaaattaa

ggctggaagatgccattaatgcaacaaaagctgctattgaagaaggaattgtcccaggag
ggggagctactaatgttcacatttctagcgagttatttacatgggctaaaaacaatttag
ttgaagatgaattaatcggagctctaatagttgaaagagcagttacttatcctttaagac
gtatagcttttaatgcgggagataacggagcagtagtagtagaaaaagttaaaagtaacg
attttcacataggctatgacgcagcaaatggcacaattgtaaatatgtacgatgcaggta
ttatagatcctgcaaaagttgctcgatcagctttacaaaatgcagcttctattgcagcga
tggttttaaccacagaatgtattgtagttgataaagccaatgactaagtta---------
-----tagataatattaaacaaagaataatttccatagctagaaattattctttgtttca
attaagctaattaattgatcaaatgtataatttttataataaagccgaagtttaaatgta
cctctttgctcaactactaagtagagaagatacaagctcgtaattcctaatttatcaatt
aaagtattattatcatatatggatttttcaagagatctaaaaaatgacattttcgtaaaa
taatatcgaaatcttcttttgtagttatttgataaatcattactgtatgtagattgatct
ccaattgcgtttaaaacttttaaaataatgtttttaagtctctatctgactttatctta
gaatcggtaatcaattgatttaattgaatatcaaaactaagattaagaatattacttgtt
acggatgttcgattttgaatatctatagatttgacagccaagaagattaaacttttattg
ttcattgtaaaatgtaagacaattgatattgtattctttaaatacta-atttatcttct
tgtgcgtttatagttttggagaaaaataattaatattatta--------tattaattact
gcccgaaaaaataaattctggaaatctagcttgagcctttgatagtgagtgtttttacc
ttcttcttgccagtaattaataacttctgttgtcttatttagtaattcaattctgtctct
ttcagcaatccatggtttggattctagctcggcttttaattcttcccaagcatcattcct
aggccagaaaaaataagaagtaagtggacttgtaccatttccaacaatttggtcaatagc
aatagcaatattattttccaaccaaagtactttagtgtaaattttgacaagtgattatc
tccattgtaaatagcaaacatatgtatatacagtaaagtactacattagttattttttt
agcaacaagctgagctttatttaaaatattctgtaccgttctagtagtttgtgcaccttg
tgctaatctctgtaaaatttttgaaatatcaatacgagtttcattagttataggattata
gaatcccaattcttctatagccttgccatctcgtttgtttttgctatccattacaacaat
tctgtagcttggctgtttttttcttccatatctctttaatcttaacttgaccatcttagt
cccctcgcttattttttaatatctttattaaattatatcttacgatattatgcaataat
tcgaacaattaagcagactcaagcctgacattattaaatattaccattaaacactggaga
aaaataatacctagcataggtgacatgtccatcccgaacattgggggaatactgcctcta
aagagctttaaatatggatcggtaattcgatttaatgaacagaatggttcattataccag
ttcacagttggaaaccatgccaatgataatttaagtaaaattaaaattaaataaatctca
gaaaaattagctattgatccaagtaataaatttaatgtaccaggaagagtattcatacgt
atttataattattatatagttcattatactaatagtttgagtatatattataagctctg
taaataaat-tagctaaacaaaaaacttattcttaaaaatatcttctcttgaacaatttt
cttcattgatagcactgttaactttcgttgtataaatttctaatttagaccaattatttc

ctagctgatttagttgagatgttctgcattctaaacatgctatttttatattatttaaaa
tagctcctttttttttctaaatcttttaagactggtgtaataattgcttcatctaaaaata
aatccaagatgagaattttttgtaaatgaatttaattgagctggaacattattaacaatgt
tgttgctattaactagagctatattagctgtcggaagtaaagctcttgctccctctgcta
gtataaatccataaggcataataatgacaataatatacttataatgactgttaataagat
ttacattgatattagtttttaattcaatagtttctgttactaaccattctctcattattt
cataagtaatccatttacctagttctgagcaagcagttcttaaaattgtacctggattat
tgctattttcgagaatcccagcccaatgttgtattaaagggtgggaagctatattaattt
tgagttgcatgttttgctaaatatatttaacatttaatttattaattgtcgctatagttt
atgttcactgagtattattaaaatagcat----atatattaaagaaagcaattat-aaaa
aatgaaaaaaactctttggctttgggggttcacagatagtgctgaaacttggaatggtag
atttgcgatgattggttttatagcagttattattattgaagtagttacaggacaaggact
attatatctaataggtgttatatcataataaaaaatcaaaaaagttcttgtaataaatta
caagaac-tttttga-ctgaatgtaaatctaatcgagtggtctcatagatagcactggtt
cattctctctgttaatacctttttcaaatccagcagctgcagctcttgctctaccagcat
gccaaagatgtccaataaataggaagaatcctaagaagaaatgagatgttgttaaccaag
atctaggagatacatagttaactgagttaatttctgtagctacaccgccaacggagttca
atgaacccagtggggcatgagtcatatattctgctgctcgtctttcttgccaaggctgaa
tatcatttttaatttttattcaaatcaagaccatttggtcctctaagaggttcaacccaag
gagctcttaagtcccaaaacctcatagtttcaccaccaaaaataatctctccactaggag
atctcattagatattttcctaagccagtaggcccttgagaagatgcaacattagcaccta
atctttgatctctaactaagaaagtgaaagcttgagcttgtgaagcttcaggaccagtag
gtccataaaactcacttggataagctgtattattataccaaacaaaattagaagctgtaa
gacccatgattgataaagcacctaagctgtaagataagtatgcttcaccagaccatacaa
aagctcttctagcccaagcaaaaggttttgtaagtatgtgccaaattcctccagcgatgc
aaataatgcctatccaaacatgaccaccaacaaggtcttccatgttatttacactaacga
tccagccatcaccgccaaatggagattttaagacgtacccgaaaataactaaaggattaa
gagtaggattgctaacaaatctaacatcaccgccacctggagcccaagtatcatataccc
caccaatgaacagagctttgataactagtaaaaaagctccaatacctagtaaaactaaat
gtataccaagtatagttgtcattttgtttttatctctccaatcatatccgaaaaaaggaa
aagattcttcaagagtatcgggtccgattagtgaatgataaagaccaccaaatccaagaa
cggctgaagaaattaaatgaactacacctactacaaagtatggatatgtattaaaaattt
cgccgcctggacctactccccagcctagtgtagctaagtgtggaattaaaataaatcctt
gttcatacagaggtttttctggcacaaagtgagctacctcaaaaagagtcatagcaccag
tccaaaagaccattataccggcatgagcaacatgagcgccaagcaatttgccagaaacgt
taattaagcgtgcattgccagaccaccaggcaaatccggtagattcaatgtctctaccgc

caacaccaacattcgtattaaagggcgtttccacgtggtaaaacctcctcagggaatata
aagttttcatgaggttgatcctgagcagccatccaagagcgaataccttcgtttaatagg
atgttcttagtataaaaagtttcaaactcaggatcttctgcagctcttagttcttgagaa
acaaaatcgtaagctcttaagttaagagctagtccaacaattccaaatgcacttgtccac
aatccagttactggtacaaatagcatgaaaaaatgtagccaacgtttattggaaaaagct
acaccaaaaatctgtgaccagaatctgttcgctgttaccattgaataagtttcttcagac
tgtgtaggcgtaaatgcccggaaagtatctgcagcatcaccatcttcaaataatgtattt
tgcacggtcgcaccatgaatagcacatagtaaagcaccacctaagatcccggcaacaccc
atcatgtggaatggattcaaagtccaattatgaaatccctgtaagaatagcagaaatcta
aaaattgcagctactccaagacttggagcaaagaaccagcttgcttgacccaaagggtac
attaagaacacagatacaaatactgcaattggtccagaaaaagcaatagcattgtatggt
cttagaccaactaacctagcaatttcaaattgtcttaagcaaaatccaattaatccaaac
gatccatgtaaagcaatgaatgcccataggccgccaatttggcaccagcgagtaaaatct
ccttgagcttcaggtccccaaaggaaaagaagggaatgacccatgctgtttgctggggta
gaaacagcagcagtcaaaaagttacatccttctaggtatgaacttgctagtccatgagta
taccaagaagtcacaaaagtagttccagttagccagccgcctacagcaaggtaagcgcaa
ggaaacagaagtagtccagaccaacccacgaatacgaatcggtctctttttagccaatcg
tctacaagatcaaaaccaccacgagtttttcttgtccaattgctatggtcataatttta
atctccagagcaaatttaataagtaatttgttcaaataaaca---ctttagatctaatct
aaaattttactttctttacggtcaatataattatacgtcaaaatcaacaaaaattcacg
tttttaataaca-tttttttagctcgttaagttaactatctaagaat-------------
taactgtttttaaattaataaaaattataacttgttcatttctataattctctgaaata
gatatcctgttcctcttgctgttaaaatcaagtctggattacttggatcatcttctagtt
tagctcttagccgtgagatatgcacatcaacaactctagtgtctacatgtcgttctggtg
tatatccccagacctcttgtaagatagatgctctagaaaaaggttcgccagccttactca
ctaaaagttcaagaagactaaactccattcctgtcaaacgaactctctcattatttttat
aaacttgtcttttattcgtgtctatctttaaaaatccaatactaattatgccagaatttg
cgacgccaagattagttgttattttatcagctcttctaagaacagatcgaatacgagctt
ccagctctttaggagaaaatggtttaactacataatcatcagcacctatttctagtccag
taatcctgtcgcacacttcgcctaaagctgttaacattattataggaacatcagattctt
ttcgtagttcttgacaaactccgtaaccatcgagtttaggcatcatcacatctaaaacga
caaggcttggatattcttgtctgaacactattaaagcttcctctccgtttgaagctgtta
tgacttcgtaacctattattgttaatcttgtttctaaaattcttcttatgctggcctcat
cgtcaacaacaagaatttttctttctggttctccaa-tttcttatttactcctataagt
caacaagcataactactataatcttaaatttgtacctgtttgagtattctaatttgctgt
aaaa-----cttatttattataaattttataagtt-----tagattaattggtatcagt-

-cattatatcttgaaatatctgtttgtcatttatctc---------tattttaatgcata
agttgattcaagttttcagaattcagtatataaata-ctttttagacaagtctttgacat
tgtattttctgaataaaaaagggggttgacaaaccggctgatagaaagtattctacattga
tataaa----ttactgatcataaacaaacagtaa-----attttttagagtagcataag
tactttaat
>Pyr_45
tatatgatcaataattaaagaatctttttttgtaagaattcctgaaatatacaaaactttt
tatttaatatagaaaaagtaaattagaataagttgaacatatttt-ttctactttaaacc
ttttgattgag------attctaaatagaaataaaaatgttgacatagtttatatctata
ggaataaaaatgaaacttgcagtttatggtaaaggcggtataggtaaatctacaactagt
tgtaatatttctgtagctctttcaaaaagaggcaaaagagttctacaaattggctgtgat
cctaagcatgatagtacatttaccctaacagggtttttaatcccaacaattatcgacacg
ttacaatctaaagattaccattatgaagatgtttggcctgaagatgttatctataaagga
tatggaggtgtagactgtgttgaagctggcggacctccggctggtgctggttgtggaggc
tatgttgtgggtgagacagttaaattattaaaagaacttaatgcttttgatgaatatgat
atcattttatttgatgttttaggtgacgttgtatgtggaggctttgcagctccattaaat
tatgcagattattgcttgattattacagataatgggttcgatgctttgtttgcagccaat
agaattgctgcttcagtacgagaaaaagctagaacacattccctgcgacttgctggactg
gttggtaatagaacagataaaagagatctaatcgataagtatatagattgtgttccaatg
cctgtattagaagtattacccttaattgaagatattcgtgtgtctcgagtaaaaggtaaa
actttatttgaaatgtctgaaactgataaagatttagcatatgtgtgtgattactatcta
aatatcgcagaccaattaattactaggccagaaggcgtagtccctaaagagtctccagac
agagagttgttcagtcttttatctgatttttatttaaatcctaaatctaatgtaggacaa
gctgtcgcagatcaagaagaattagatttaatgatagtgtaaataatattttaagcataa
cataaaaaggaataagataatgtctacagttcaatcagatgctcttacttttgaatgtga
aacagggaattatcatacttttgtccaattagttgtgtttcttggttatatcaaaaaat
tgaagatagtttttcttagttattggaactaaaacttgtggttatttttttgcaaaacgc
aatgggggtaatgattttcgctgagcctagatatgctatggcagagttagaagaaggaga
tatctcagctaagcttaatgattatggtgaacttcgtagattgtgcttgcaaattaagaa
agatagaaatcctagtgttattttttggattggaacatgcacaacagaaattataaaaat
ggaccttgaaggaattgctccaaaattagaagcggaaattcgtgttcctattgtagttgc
tagagctaatggcttagattatgcttttacacaaggtgaagacacggttttagcagctat
ggctcagcgttgtccttcaaatttaaataatcaagtaggcagtaatagtcgcgaaatttc
ttcccatatccccttagttttatttggatctctgcccaacccagtggtaacgcagttaac
tatggaattaaaaaaacaaggtattcatgtctctggctggttaccatctaagaggtatac
tgaattaccggttatacaagaaggctattatgttgcaggagttaatccatttcttagtcg

aacagctactacattaatgagacgaagaaaaacaaaattaattggtgcgccatttccaat
aggcccagatggtactagagcctggattgaaaaaatttgctcagtattaaatattcaacc
tattggactagcagatagagagcaagaaatctgggattctttagaagattatattgcttt
aatacgaggcaagtccgtttttttatgggagataatttattagaggtctccctagcaag
atttcttactcggtgtggaatgactgtgtatgaaatcggtattccatacatggataaacg
ctatcaagcagcagaattagctttattgaaagccacatgtgacgaaatgaatgtcatgat
gccaacgattgtagaaaaacctgataactataatcaagtagatcgaattcgtgatttgaa
accagacttagttattactggtatggctcatgcaaatcctttagaagctagaggcattaa
tacaaaatggtctgtggagtttacctttgcacaaattcatggttttacaaatgcaagaga
tattttagaactagttacaagaccattgcgacgcaatcttagtttgtcagaattaggctg
ggatgtctatagcaagcaaagttagtattagt-------tgcgacttacatggcataaaa
tccttgtaagtct--tctatattatgctaaagaagcatttcttgcccgcccatcttctgc
ccaagcttgtaaattttctatttgttctttatctgtaaatgctaaaggtacaaattgctt
tatagctattttaatatcttctgtattaaattctctctgttcactaaaagctgtatgcat
actttctataatcgcttgctcgatttcagcacctgagaatttattacataataaacttaa
ttgtttaatatcatattcttgccatgattttggtctaacttttgataaatgtatcctaaa
aattgattctctttccttgcgattaggtaagtctaagaaaaaaatctcatcaaaccgtcc
tttccgtagcatttctgaaggtaaactttgaattttattagctgttgcgacgacaaatac
gggagctttttttctgaaagccaagttataaatgttccaaatactcttgcactagtacc
actatctccttggctatacaagccagaaaaagccttatctatttcgtcaatccataatat
acaaggcgataatccttcagcaatatttatcatatccctcattctggattctgactcgcc
aactagtccaccaaataattttccaatatcaagacgtagtaacggcaaagtccaatcctt
ggcaatagctttagctgttaaagattttcctgttccttgtataccaactagcagtaaacc
tttaggggaaggaataccataattcaaactttgtttcgaaaaagagcgagatctttttg
tagccattgctttagtacatctaatccaccaatatctttattcactttattgtaaggata
aaattctagtagacacgtttggttgataatttgtctttttcttcaataataatcggtaa
gctgcgagaatcgagttgactatattgtgcaataatttttgtaatcactttgcggattct
atctattgatagaccttggcaggatttggtcatattattaactagttctgaatctagatc
aagatttacagctttacttaatcttgtgatttctttttgatttctaaaagacttggtaa
aggtaaatccattattgtcacaatatctcttaaagcaaaaggtatatttattttacaaga
aataataattatatgtttagattgtgctttgattactttagctaaattgcgaagtttcct
agttaggactatttcatttaaaaaagagtcgaagtcttttaaaatgaaaatattcaaaga
ttcattatctaagttttcaataaactctaatgctagtaaaggattccgttttgcataacc
attatcgttagggttgctagtatatccatctacaaaatcccagcaataaacttgttgatt
gtcagagcagtgtaatttatttttataatatattcgagtctttcttcttctctagtatt
tattacaataataggatgtcgagactttaatagtaatcgtaaatcttgtgtgaaattcat

gtcagtatttgtccccatcagacttttgtacttcaccaatta----aagactaaataaga
ttatctaacgttgcatggatatattcaaaagtc------atgtatctattttctaatcta
ttatgatttcagacttaactaaccataattttttttcttcctttgcgtcgtctactattt
aaaatagagcgtccgcatggtgttttcattctagcccggaaaccagaaactttaatttt
tttctttttgatccttgtaatgttctcttagtcattatgtctcctgtattgtaaaagatt
aaatattattttatcatcttttatatatcttgattatatagatctgtattaatatcaaag
tt-aaacgcaataatttatattaatcaagaattatttatataatattaagaattaagtat
----------tagctttatgatattagctctgcctatttttatttaagtatcctgacag
cgtttttgctaattttgaattggttagtttttcagcagctaaaaacaattttgtcattag
aatctcaatttaagtattttattgaaaaaagtcaaaatgatatattggcagtagaagaaa
gctttgcttttgcaaaggtttgtgtagcaaagaagtgcttttctagagcgattatcgaag
gtcaattggcaatgaaaaagagctcagaattaaacgagaaagagagcaatataataattg
ccaatttgcataacatgattggttttatttattctgaagcacagcaactaaatattgcaa
aaaacttttatcagcaagccttacaaatagaccctaattatgtcacagctttaaataatt
tagctaaaatttacgaagacaccaaggattttaaaaaagcagaggcactatataataagg
tattaactcttaacttagacaataaaatcgctaatagaaggaaggaatttataacaaaga
tca---------gaaatacttaatattatttataatcgggatagcaggatttgaacctgc
gacatcctgctcccaaagcaggcgcgctaccaaactgcgctatatcccgttagacataat
tagtactatataccttttctacaattatgtctactatttcagtgttttatctaggatacc
aaaacatgcctttaactccatcaggatctttaatgaatcctaatttttttgtaaaaactaa
ctacatcaggttctgcaaataatgtaatagtgctaatttctgcttgtcttaattgtttta
ttaattggtgtatcacgactttccctaaacctaaaccttgaaaatctggatgaataacaa
catcccagatagtagcattgaatccattatctgatgttgctctcgcaaatcctacgagtt
tagtatttgtatctttcttttgtattaaagaaatgataatcgaactatttttaatgcga
tttttactttttttagaggtcttttaacccatcctaccgaatcacacagctgttctaatt
cgtataagttaatatttttagtgctactgagatagatatctttgaattcggctctattac
aagttttatctaaaacaacaagttttttgaaattttttcagagttacttttgatatgtg
agttttgaaaaaaattttccagaagatcataatgattatgatagaat----taaatata
tatatattatgatattgccttaatgaacaacatattaatatatgtccagtttttaatatg
gagttattaagaaagaaaattgtttgaaggtatagaaataaaaattttgtaaaatcttt
caataaaagtatataaagagctcttttttaagattaacatctattagtatcaatacagtct
cgcaattgagatgaaacaacatattattctatatctaatgccttttaaaggagttatttg
tgaaaaaatctatgtttctaacctgcctacttgctttgttattacttagtaacccgataa
tagctaatgcagaagtggctgggctagtgccttgtaaagattcggcggcattcaataagc
gtatggttaatagtgttaaaaaacttaaagctagattagctaaatatgatgcagatactc
cgccagcgttagctttaaataagcaaatagaaaaaactaaaactagatttgctacttatg

gtagagcaggcctattgtgtggtacagatggtttacctcatttaatctctgacggtcgat
ggagtagagccggagactttgtttttccaggactgctatttttatacattacaggctgga
ttggttgggtaggtcgaggatatcttctatccgttgctaaaactagcaagcccactgaaa
aagaaattatttagatgtgccattagctattaaattcatgacatctggattcgcatggc
cgttagctgcttggcaagaatttagtagtgggcaactagttgcctccaatgacgatatta
ctgtttcaccccgttagcaaaaaatttatatgaataataatttaccaaatacttatcaa
cagcacctgtagttggcgtgttatggatgacgttcacagctggttttattatagaattaa
accggtttttcccagatgttttatattttatttgtaaataaataa--aatctcataaa-
---aaccacacaaatatagtaaaacactattatttgtgtggttttt----atatagaata
taactattta-tttttacggaaattctttctttcgactattttaatattagattttacat
aataaaaaa-acagtaatattatgagtttagtaagccaaattatagttaatgctgatgat
gaattaagatatccaacaatcggagaattacagtcaattcaagactacttaagtacagga
agtaatagaattagaatcgcaactattattcgggataaagaaaaagagattatacaaaaa
gcaagtaaacaaatttttcagttacatccagaatatatagctccaggaggtaatgctgca
ggttctagaaaaagatcattatgcctacgtgattatggctggtacttaagattaatcact
tatggagtattagctggtgataaagattctatagaaacgattggtatcatcggtgttaga
gaaatgtataattctttaggtgttcctattattggaatgttagacgcaattcaatgtcta
aaagaagcttctctagaaatgcttggtcaagatgatatcaaaattattgcgccttatttt
gactacataattcgaggcatgtcataaatagtaaattcaattaattagttgaataaaagc
ttgtataatgttatgattagttcaagcccgaaagcatataattgtaaagactaaatcttg
tcagaaccggaaggtagcagcaataatgtttaattacaacagttatga-ttttcgggtgt
ttactatttgtactcaatagtctctcattctaattatgatgctatccataaatt----aa
taatatataatccataaaacctttcagtaaaaataa-ttaataatttgatattaatacgg
aaaacatctatactttttttataaaacttctttatatctcatctaaacaatggaaactta
acttaatgggtgttcatattttatcaacaggttcgtctgtcccaaatttttctgtagaga
ataaacaatttgaagatatgatagaaacttctgactattggattttaacaagaacaggaa
taaaaaaaagacatctagctccttcttctacttctttaactaagttagctgcagaagctg
ctaataatgctttatctgcagctaatttaaaacctaccgatattaatttaattatactag
ccacatctacgcctgatgatttatttggtagtgcaagccagctacaagcagaaatcggtg
caactgcatctgttgcttttgatattacagctgcttgctccgggttcattgttgcattag
taacagcagctcagtttatccagactggttcatatgacaatatcttagtcgttggagcag
atacaatgtcgagatggattaattggtcagatagaactacctgtatcttatttggcgatg
gtgcaggagcagtagtagtgggacaaagccttaaaaatagtattttaggttttaagttat
gtacagatggccagctaaatagtcatttgcaattgatgaatacaccaaaaaataatcaaa
aatttggatttatagatattcctcatggaaattataattctataacaatgaatggtaaag
aagtatacaaatttgctgtatttcaagttccaatggtaattagacaatgtttagataatt

taaatatatcaatagatgaagtagattggtttatattgcatcaggcaaatactagaattt
tagaagcaattgcgagtagattgtcaataccttttctaaaatgattactaatttagagc
actatggtaatacatctgcagcatcaattcctttagcgttagatgaagccattaaatcca
ataaaattaagccaggtcaaaccattgttttgtcaggttttggagctggtctaacttggg
gtgcgattgtcttgaagtggtaat---tataatattgcggatgacgagaatcgaactcgt
aaagctttcgctacacacccctcaagcgtgcgtgtataccaatttcaccacatccgcact
ttttatcacgaata-ataaaaatttataatatttatttaaatattaatataa--tgtgta
tatatatatacattcttgtcagaaactctcttatatttatttaagtgataaaattgag
t----ttagtatagatgataaaattttatttacaatcataaaaataaataaatatataa
ggaaaataaaatatgacaccgtccttatcaagcttttaaacagtctaattcttggggcc
gtaattgtagttgtccccataactttagcacttttatttgttagtcaaaaagacagaaca
attcgatcctaacaaagactttgaaacatatggagaagcaatttgagattaataataaac
tatttttcaaacggatatgctctccatatatttctgtatt--ttggaataggttgttt-t
ctttactttgcaatagcctgcacagtgta-----------ttttgttctcatttactctt
tta----taacgatacgtatgtcagg-ttaataatag--tttttttattctgtatctttc
atttaatattaaagtattttatatacatattgttatgttcaagtaataaagaaaagctca
tgtctttgtccaattggcctcttaaaaaagaaaactccgaagcatatactagcaagtata
caaagcaaatcacaatccctgatggattgtggataaaatgcttcgattgcggtctgttaa
tgtattctaaagttctaaaaaggaatttaaaagtttgtcctcaatgtagctatcattttc
aagcttctagcagtgaaagaattgatcagttgatagacaaaggtagctggcgacctatgg
atattcatttaatttctacagatccattaggtttcaaagatcaaaagctttatagtcaaa
ggttaaaagatactgctcttaagactggcttacaagatgcagtccagacgggtattggta
ctatgcaaggtagacaagtctgcttaggtattatggacttccgatttatgggaggaagta
tgggatctgttgtaggcgaaaaattaacaaggctactggaaagagcaactcaagaaaaat
tacctgcaattatactttgtgcatcgggcggcgcaagaatgcaagaaggcatgttaagtt
taatgcagatggcaaaaatttcttctgcgttggaaatgcataaaaaagcaaatctcttgt
atatatctgttttaacatctccaactacaggaggcgtcacagctagctttgctatgctcg
gagatctaattattgcagaaccaaaagctcttattgcatttgctggtagacgagttatag
aacaaactattaaagaagacttgccagataattttcaaagttcagaatatctatttgagc
atggttttctagatttaattgtaccaagaactcagcttagatcaaagctaatacaaattt
tatcattccatactcatactaagtaagtagtttgacattgatcataatattgaaatgcta
gtaaaaattaagatattaagaaattttcactaaagatatttgttgactaactagaattac
aattactattgtagaaaaatttgtcttaattctattttttcagtcttttaactaattgt
tttttacgactttaaataattcttagtttaatacaaatttataaagagaata-----cat
actaagctaaatatagtaatattatgttttcctataatactgacctatcaaatttcgagg
aattttatgcttaaaagatcttcttggcttgctactttattggggctactaactgtagtt

tctataagtacgcatgcaaatgccatagagctagacgaggcaacaagaactgtcccatta
gaatcttctggaagaactgtagttcttacgccagaacaagttaagcgaggcaagcgatta
ttcaataattcttgtgctatttgtcataatggtggtattacaaaaacaaatccgaatatt
ggacttgacccagagtctttaggcttagcaacgccacaaagagatacaatcgaggggtta
gttgactatatgaaagatccaacaagctatgatggcgcagagtcaattgcagaactgcat
ccaagtattaaaagtgccgaaattttcccaaaaatgcgtaatttaacggatgaagattta
tttacaattgctggtcatatcttgctccaacctaaaattgtttccgaaaaatggggcgga
ggaaaaatttactattaaaggttcaaacacaataatcctacaagtactagtttatgca-t
ttattgcccaagtatgattattttgttagatcatgatatatattaaaggtggagtttgct
aatagtaagtta-tttttgcctaaacttgcttacattattcgtttagttattaaaaagga
gaagttaaattgaaaaagacgctttcagttcttttcactgtatttagtttttttgtgata
ggttttgcacaaattgcttttgctgcagatctagataatggagaaaaagtttttttctgct
aattgtgcggcatgtcatgcgggtggtaacaatgctatcatgccagataaaactttaaaa
aaagatgtgcttgaagctaacagtatgaatagtattgatgctatcacgtatcaagtgaaa
aacggtaaaaaatgctatgcctgcttttggaggtagactagttgatgaagatattgaagat
gcagcaaattatgtattatctcaatctgaaaaaggttggtaattatacttaattttgcac
agtattaaaggatagacaatctatttaattattcattagattgtctattctttatttatg
---ttattatatatagatatttatacaatactga-attatgatgaaaagaatacctgcaa
ttcttgtactagaagatggtacttattacaaaggatggtcatttaagcaaagaaatccag
caattacaattggagaagtggtttttaatactggaatgacaggttatcaagaaattatta
cagatcctagctacttccagcaaattgtcaccttcacatacccagaaattggtaatacag
gtattaacagccaagatgttgaatctcataatattagtattaaaggcctggtggcaaaaa
atatttgtaaaatttcaagtaactggagagagcaagaatctttagttaaatatttaagcc
gttatgatattcctttcatctttggactagatactcgatccttaactcaatatttacgtc
aatttggcacaatgaatggttgtatttctactaataatttaaatcatagttatttaaagc
aaagaatttctgaagttcctggaatgcaaggcttagatttaattccatatgtaacaacag
acagcatttatccttgggacgaaaaaagtttgcctatctggtatttaaacgataaaagaa
gagtgcaattagatacccagttgaaagttgttgttatagattttggagttaaactaaaca
tactgagacgactagctacacttggatgtcagataactgttgtgcctgcacaaacatctg
tagaagatattttagctaataaacctgatggtatattactttctaatggcccaggtgatc
catctgtagtgtattatggaattaagacagtgaaaaatttattagatcataatgtaccta
ttttcggtatttgtatgggacatcaaattttaaatttggcccttgacgctaaaacttttа
aactcaaatttggtcatagaggtattaatcatccatctggattggaacagcaagttgaaa
taactagtcaaaaccatggtttcgcagtcgatttacaatctgtcttcaagtcatctgtac
aagttactcattttaatttaaatgatactactgttgctggaataggccatagtcaaaatc
catatttctctgtacaatatcatccagaatcgagcccgggccctcatgatgctgactatt

tattcgaaaattttgtagaaataatgataaaatctaagcgtaaagttgc-----------
ttaataatcttcccatgctttatgattaaataaagctgataatacttgtactccccgtaa
ttgattgaagagaggtaaatgcccctttggaccataagaattccattgaaattcatcagg
gtatctacaaggaatatgatcaacttcccacccaatcttttgccagaattttccccaatc
ttttccaacactaagccatatttgcctctgaataaaaagcccaaatttacctttgagtg
tgtatgccacaatttatcaatagtctgaagatcttgggcaggtattttttttatatctgt
aaaatatagccagttgcgattttgtgcattcacaccagctaattgaataagtttttgttg
agttaattgatctgcctcttgcaaatcttgttgtgttagcaacacttgtaaatctttata
attcatgtgtttcgcagaacgtaatgggacaataccctcgggacataagtcagtagtaaa
cctaatgatttcttgattttactattcaataacttgttatagatcaaaccatcgacgca
attacttttataattgggcctactaatcctttcaagaaatagatccgctaaactttttaa
ttgcatagagtcattattatgtatagtctcaataatatcaagttgttttttga---tatt
atttgaactagtattttcacttaattctaaaaactgaa------------attgcatttg
attt-ttacattaatttaaatagtg----attatttcttaaaaagaatttga-ttttttt
gaaaattagaagaacgacgaattatttctagaagtgttttcataatttgattgaatatgt
ttctccccgcataaatgataatttgaccaattaaaaaaattgcacttttattttaggta
caaagaagtcttgtacctcaagtagaaatgtaattactatttgcattgatgataatgtat
ataattcatgtgatctacaagcatatatatactgacaatttaaaccttcctgataaaacc
cccatactttgtaacgactttcgtaaattgctcgcggtctctcaatataatttttaatta
aagtattccaaactaaattatttttaatttttctagttttctatcagataaaaaatttga
cattacaaagatagcttgcatttgtttcatgtactgtatcaaaattttgaaccaacatat
gtgcaataatattacttaactgaattaaatagttctcaagaaaaatttcaacttgtttaa
taggtacatttgaagtaaataaattgaaagtcttattcacaccttgagaggatccaaaaa
gtagctgaataattagaatttgtagtaacatcttatagtcatatattaggtagtttaagt
cattaatatcttggcaaatatcattatagtctagttcgtataacctacaaaatttttga
cacatctatgaaataaatcaattaaaatattttcagttagaatcgtaatatcatttaaac
ctaaatttgaattataaatttctaacaggatttttctaattctgttaatattattttta
ataagtttctttttacttcagttcgaaaaacatctaaaattaatacttcctgagaacaat
tagttaatttctattaatttttatagaagttctaactaatagctctgcaacctcttgat
tgagcatgtgtccctgtgagctcggccaataattattcacggtatatcatagtaaggaat
atttaag-----tttatacgataacttttaatacactgaaaagcaagtttatcactgta
tagccattag-------cttttacagatcttaatagttatttttcaaataaagttatatg
atattagcgaccaagctaattttattaaaatttataattaataatgacaatttattactt
tgcacttgccagtcaaaactttctcttagtacaagagcccttggaagaagtttttagaga
gagagttaactattatcagtcaaataataaagcaatcgatttttggctagtgccaaatcc
ttctttttagaaaaaccagaaatgatttcgtttaaaaatcttgtacctaatgatgctgt

agccataatctctactaacccagtatttattaattggttaaagttaagaattggttatgt
ctgtgttggacaatttgaagatgatttacaactttctgaagagtctttaaacattactgt
tgcaccagaaaagatttaatcatactttattgccactaggaat-aattat-----ttaa
acggcaaatataaatt-caaatccttgtttttctatagtttatcgcttagtattttatt
ctgagatggagtgacaaacagtgtaagtatttcttgactaaatgtttcagttgctttgga
tttatacctattagggttgactataatagacagcattcgcttaatagtgacattttcaat
ctgagcccaatgtacaatccctagctctaactcctttgcaatagctgaaacagaaacaaa
agcagctcctaaacctgactgcacggcatttttatagcttctatagaatttaattccat
ctctatttaaagcgactgctatcaataccatgttgactgagcactttatctatgacttt
tctgattgttgattgagtgtctaatgcaataaatttaagtctatacaggtcttcttttg
gatgtcttctaatttagaaaacggatgcgatgttggaagtattagtgctaattcatcttc
ggcataagatgtaacttgcaaaatatcttgtaattcagtaggtacttcccctccaataat
tgctaggtcaacttgaccattagctacgctccaggaaatgagtctagtggaatgtacttg
tagttgaacagctacttgtggatatctttgcctgaatagtcctattaatcttggcatcaa
atatgtcccagttgtctgacttgctccaataattaatgtaccaccttggaggttttgtaa
gtcatcaagagctcggcatgtttcttcacataaagctaaaattcttcctccataccgtaa
aagtaaacttcccgcctcagttaaagttgctttttattacctctctcaaagagggaaac
atttaattggcgctctaaattttgaatttgtaaactaatagctggctgagaaacatataa
gctattagctgcctttttaaaactgccttctttggcaattgcttttaatattcttaattg
atccaatgtaaatggaagatctgtcattaaagaattgtagtataatg---------tata
ttatttaatattgatcaatttatgcattcgccaagttaaatatttgctattacgcttcac
tgctttgaaaataataaaaagtataatgttaaatacactgtattagattttaatatctgt
ttaatgttaaatttatataataaatatcg------taattttaattataagatataggag
acaacaatatggactctagacttttaattgtattaataccagttttagcagctgcatctt
gggctatttataatattggtagagtcgcgctacaacaatttaagaaaatgacatcttaat
gatgatgagaatata-atcttaaaattttatgagatagagaactttaaaatctctatctc
tattgtatatcttaataaaaaaagtaagta--acccttgtacttactgttattacctatt
aagatgatgtac-agtacatatatttataataagtttaagctaacttatt-----tttta
taattaactaaattaataacacaattatggctgttccaaagaaaagaacatctaaagcta
aaaaaaatgcacgtaaagcaaattggaaaaatcaagcaagagtcgaagcacaaaaagctt
tgtctttagcaaaatcagtattaactagaaaatcgaatggatttgtttataatccttctg
aaacagcagatacctatactaactaagagtcttt-ttactatcaagctagccaagtatca
aaaaatctattttaagtaatttttatatat----taaacatttggaaatcattctatttg
ataataaaataaatagcagattaaacaaaattacggcaacaaattatgcagcaaagctag
atgaaactaatgaaatttggttatttaattgcatcgaaaatatccagcacactttttga
aaagtcaattaaaatcaagtcatattactaaaattttcgtttctggtactagctttgaat

atgtagcaggtttacctggactattatccagcttaacactaagtggtagaataaatccca
ttagcatatatggcccaaagtctttggaatggtacctgaaagcttctaccaaatattctc
aaactaatttttcttttcctattaattttataatgtgaaatatggagaacaagttatta
atcaattctatacagtagtttgtttaccgttgagccaggacaatttactgtatggattta
ctatcttaaaaaaagaaaagccaggagcatttaaattagcaaaagctaaaaccttaaata
ttactcaaggtcctatatatggcaaattaaaagaaagaaataatttctaagtcccgatg
gttattatctatgtggcaaagatttctcgttaagtacaacaatgggacacaaaatatctg
ttccactattagtcagatattctagaattatttcagagatgcattggttttgctcttatt
ctattcgatgtaacacttatctacatcagcaaaccataaaacatttgccaaacaatattt
taaccgatactatgaaatctcaaatatctcaagataatagttgcattgaataataattat
tcttgatatataatagtctcgacaatataaataacatacaagagcgaatttcttg-----
----ttatatattaaatttataattatttatgacatacgcaattattgaagcaagtggca
aacaactttgggtagaggaaggacgctattatgatttaaatcgcatacctttttgatccag
gccaatcaattatactgggtaaagttttattgttaaataaaaaatggacaagtgactttag
gacatccttgcatagcaggagtaacaataaaagctacaataatgaggcatttacgaggaa
aaaaaataactgtttttaaaatgaaacccaagaaaaaaatgagattaaaaaaaggacatc
gacaagaattgactcgtttaatgattaattcaatagtaaactaggtctagatacagttgt
aactgagattgcttcaa-aagatttttgaatatttaactacaatacatataatggcacata
aaaaaggtagtggtagcacaagaaatggtagagactctaattctaaacgcttaggagtga
aaaaatatggaagcgaacaagtaagagcaggtaatattttagttagacaacgaggcacga
aagtcaagccaggtcaaaatgttgggagaggaaacgatgacactttattttcgctgatta
atggctttgtatttttcgaaaagtctaaccaaaagcaaaaaacaattagtgtttatgctg
caaaaaaatagccaaaataattggtgcaagatgacctgaat----atttttatttcactt
acactggcaccataaacttgatttattaagaaatggagttccttaatgaaccaatgaatt
ttattaaagatttgctttgaaatgtaatgacaaacactattgtaatttcctgtctacaca
atatggctgctattctatattgtggtcagatacagaaactagtagtagcaaatgctcatt
atcaagtaagtgatatttacctaggttgtgtagataaaatcttctcaggaataaatgcag
catttattgacctgggaaaaaatgagtacagtggattcatccatattagtgatactggtc
cgcttaaaaagaaatattatgttaatagtattactaacattttaacaattcgacaaaaaa
ttttagtacaaattactaaagaaccaactttaaataaaggaccaaggcttactgctaata
ttacattatcgggtcgatatattgtactaatgccttttagtcaagcaatttgcatatctc
gaaaaatacatgatgaagatgaacgtcattatttgaaagctttagctattctaattaaac
cagcaacaatgggcttattgttccgaccttctgctgtaggcattgatgaagaaattatac
taagcgaactaaaaaaatttaaaagagcaatggaactttgttcaaaaatctgcaatcaaca
gctatgcacctggtctcctatataaggatgaggatattattaaaaaggtaattagagatt
tttatagtaataatacaaataatatcgtaattgattcaaacttgggattaaaacaattga

gttattatatccatacttggcactgcaataattctagtacagctccttcaattaagcttt
atagtaacaatcaatgtatactagatgcttttggtattaatcaagcaatttctagagcgc
taattccaaaagttgatcttatacttggaggctatatgtttattgaaactttagaagctt
ttacaattattgatgtaaattccggatcttttaataactctactagtgcgcgagaaacag
ttttaaaaacaaattgttctgcagcaacagaaatagcttaccagctgcaaattagaaata
ttgcaggtgtaattataattgattttattgatatggaatcacagagagatcaattacaat
tattagagcacttcaataaagagctagcacttgatgatgcaaagcctcaaattgtacagt
tatctgaattaggcctcgttgagttaactagaagaaggcagggcaaaagtttatatgagt
taattagtagtgacttcaattactttcatctccttatgcaatcagagcagactaagtctc
ttgaaagaatagatcaaaaaaagcaaatcttccagactcttaatagatcatggctatctg
tagaaattaatactattaataaagtatttttcaaaaatcaaatttttatatacttacca
acttttatttagcacgtgatttatatgtaattaacagtaatctcacttataaacaaaatt
gtttattcactactagattgaaacttatctactctcaagagtataataaaatcttgcctt
gtactggttatagttgtctcatcagttctaatatagactataatcaaaagttccttttgt
gaactgtcggtttaaattgt--
--------------------taaaaaagaagccctctcttgtatgtgagagggcttc-tt
ttttagcttactaactattctagtgaattaattagaaattaaccattgacagctggagct
gctagagctactggcaaggattcaccagaagctagatctagagggaagttatgagcatta
cgttcatgcattacttccatacctaggttagcacggttgatgatatcagcccatgtattg
attacacgaccttggctatcaacaacagactggttaaagttgaaaccattcaagttgaat
gccattgtgcttacagataaagctgttagccagataccaactacaggccatagacctaag
aagaagtgtagagaacgagagttgttgaaactagcgtattggaagattaaacgaccgaag
tagccatgagctgcaacgatgttgtaagtttcttcttcttgtccgaatttgtaaccatag
ttagcagattcattttcgcttgtttcacgaattaagctagatgtaactagggatccgtgc
atagcactgaacagagaaccaccgaatacaccagcaacacctaattggtggaatggatgc
attagaatgttgtgctcagcttggaacacaagcatgaagttaaatgttccggagatacct
agaggcattccatcagagaaactaccttggccaattgggtatactaggaatactgctgct
gctgctgctactggagcagtaaaagcaacggagatccatggacgcatacctaggcggtag
cttagttcccactcacgaccaatgtagcaagctacgccagttaggaaatgaagaacaact
agttggtaaggaccaccgttgtataaccattcgtctaaagaagcagcttcccaaattggg
tagaagtgaataccgatagctgcagaacttggaataacagcaccagaaatgatgttattt
ccgtatagaagggaaccagcaactggttcacgaattccatcaatgtctactggaggtgca
gctacgaatgcaatgatgaatacagatgtggcagttaataaagttggaatcattagaaca
ccaaaccaaccaatgtataggcggttttcagtactagtaatccaagagcagaaacgttcc
cacaagctagcgctttcgcgtctttgtaaagtagcagtcataatttttatcagtttttta
aggatttaactaagatgcttcccaaacaatattttgtctgttaaatatattattacttaa

gatcgttttaaatgagaaca--tttttaaaaaaca-taattaaatttaatctcgataact
aagaaaatataattattgagatattattatataattgaa----------aaaataaattt
tt-act-------------ttttgcagaattagtatgattttaagggtataataatagta
atgcatactaatgctgcttattttgatttcaacagtttacataatcgagctttacttaaa
tacgtatgtcacactttacaaaaattcagacgactatacaagacttaaatctgttaaagc
atgctttaacagatttaggtttaatttggtctatgaattctagtcatatacaagttgatg
aaaatagtcagcacaaagtagatatcttgatcagacaagataatttatcacatattggct
ttatctggaatgataataagtaccacttagttgctgacttgcaactttgggaacaacctt
ggtctttagaagtatttttagataaattatcgcagaagtatgcttaccattctattatcc
aagaaacgaaaaaacaaggctttgaaaaaatggagcaaatctcgcaaaacgatggatcaa
ttaagttagttgtgcaacgttggagttgttaattaatatagaaatgcgggcggagagact
tgaactctcacgagattatctcactagaacctaaatctagcgcgtctaccaattccacca
cgcccgcactgctgtagt--aataatactatatcacaattcatgaataaaaacaaactca
-agaaataaattagctaccaagtttaaatgcatcaacaaatagaccatatgttataccaa
ttttgaagttattaattaaattgatttttatttttaattttttttgattagagtaaacga
tcttactagttaactctgttgctgcaactattagactagctgcaatgattccccagtctc
ctgtttgcccaggaatcgtagataatcctgttgaaataaagaagcctaataacaagctga
ttaaaccagttgttaactcacttaaagaatagtacagtttatcatttaagttttctacta
aatatgcaaaaaaggtagatagtcgagttttaatcataatatttaataacggagttttaa
aaggataataa-----------agattaatgtttttagatccttttgaaacccgtcaata
caaaaattgctttttg-----------tttaaagatcttgctcacttaaagcattaataa
gatgttgaaatggttcttgtataatgtctttacgtttaattgattgtaaatcaagttctt
cttctattatttcttgcaataattgtatgcttcttaccgttggagcaacaggcacagaca
atgaattataagtatctttgaggccgtttaatacacgctgatctagaatattagtgctac
cagagactaatgcataactagcatagcgaaggtagtattctatatctcttagacatgcag
catatcttctagttgtataagaatttcctccaggtcttaataattcgggttgctcttcgt
ataattgagcagcagcctcttttaatatatttgtagcctgattattgataatttcagcta
tttttattctatccaatccacttgaaaagaaagattctatttgtccgacagctgctttat
ctaaataacgaccagtaagatcgtagcgatttaatattgctgttatagcatcttgcataa
attaattctcctac-ttgctatcagagtggtattatgaatgactagtat-----tgtaa-
----------tataatcatcact-------------------------------------
-----------gtttgtataaatagagtatatgtagtttttaattactttttttataaaa
attaagaaataaagatttttt-taataggtttacaatttcttaatataactcaggttct
caaccgagataagttttgtaacacat--aagctaaaataaagatgaatgagtaatcttga


```
------------------------------------------------------------
------------------------------------------------------------
------------------------------------------------------------
------------------------------------------------------------
------------------------------------------------------------
------------------------------------------------------------
------------------------------------------------------------
------------------------------------------------------------
--------------------------ataagaataatatgcatcaa--------------
------------------------------------------------------------
------------------------------------------------------------
------------------------------------------------------------
------------------------------------------------------------
------------------------------------------------------------
------------------------------------------------------------
------------------------------------------------------------
------------------------------------------------------------
------------------------------------------------------------
------------------------------------------------------------
------------------------------------------------------------
------------------------------------------------------------
------------------------------------------------------------
------------------------------------------------------------
------------------------------------------------------------
------------------------------------------------------------
------------------------------------------------------------
------------------------------------------------------------
------------------------------------------------------------
------------------------------------------------------------
------------------------------------------------------------
------------------------------------------------------------
-----------------agtatagatagtcttttactccagcatcaaaaaagatatttaa
cacatattgaaaaaattgattatgtaagtaaaaatctaaatttagataaaattagctatt
ttataattacaaccaataagaataacaaagatatccttgtagaacaattttgttataatg
cagatggttatacttattcaatattatttaaaggtcaaacagctaaacacctttgttaca
tagttcttcagcttaaacaattaaatataagaatatctacagctcatgctctatatttag
```

gacgagaactaatgaagtcggaattaactcttatattagatcaacaatatatccaagact
aaa--cttcatagatattaattatatttgtgctaatatagtattaataagggcatgtaac
tcagtggatagagtatcagattccggttctgatggccgtgggttcgaatccctccctgcc
cggcgagtatttagaattct-gtggcaagatatgt-------tagtatctaatttaataa
tattaaaaatattggggctgccaggtttctacattataaaaacaagaaaatatgaaagtt
aaacaagctctttgaaagagcctctagtaaaattaataaatgcagaaaacaatattgttt
ctttttctcgaaaattagctgttgcttaaatactgttaatttttggcattcgacgtgtta
agctcttattcgcggcgaatattctgttagagttgctcttagtataagaaaagtttcaaa
atctatttatatttatgtttttttacctatttttatttaaagtaaggttagta-ttttcaa
tttttataatggacgcgggttcgaatcccgccagctccatcaatttaact---------t
tcataattaactaatccaatctttttaagaaccagaaatataattggagcaaatatggat
tttataaaaatcacagaacaagcttttaagcaaattgtagtcttgaaaaatgattacaat
aatgatgtacatttaagaataggtgtcagacaaggtggctgttctggcatgtcatactca
atgaattttgaagatgtagctaacttacaagagaccgatgaaaaactgatttagatgac
ttttctgttgtttgtgatcccaaaagtcttttatatctttatggattgtcattagatttt
agcaacgacttaataggaggagggttccaattctccaacccaaatgccagccagacttgt
ggttgtggtaaatctttctcaggctaaa-------gctactttgttttatattttgtatt
atacgtgttactcttataatcagccattataaatacaaaagtatttttattgaaagctct
ttaaactagtcaaagccttatgaaaactactttaaagaggttttatt-tattatggcagc
ttaaataagtatttatcatcaattgatgaaagt---------acaaaaattcactccctt
gttattgcctaattttaattagttaatttttattgatacatgctaatattt-attagcc
ctgatttattaggaatttataatttctttttataaaagagcttgtcagtttctagcta-
----------------------aaatagattatatatttt-attcattttataaaaactc
aacatgaatcttgctcaagttacttgcaactacttcaatacgaaaactctcaataagttt
tattgcaatacggaatatgtttcctatggtaattttagtcatctcaatttatcaaagata
aatttgccatcttctgccaaaataattgcatttggactccctcaatttgattttattcct
acaaagattaaaaactctatccgagaagcagtggtaatatctaccatcgatgacagtaat
atttttgctgattcaactaataattggataaaaaaacataaagataaaaatcaccactct
cttttctataaaaaaattttaaagctttactgcatcaaaaaataaaagtcgtgacagat
tcagc------taaaaattctaaaatagataaagagaagggcttgattttgagtcaaaaa
tatatattaaagaacagttggaagccatccataatgtcttcttactttgaaaacaaaaaa
aataatgaggttgctaatgtatctaaaaaatttttaatcgatcagctatctgcatcacct
gtgtttgtagtaaaaaatggttttaatgaaattattttaggccacccagcattccgtata
aagagaggtgtaattagtaattacactcactcgttttcaaatttattaaagcaatctcag
ccaacttatcctatatctactggcttattctttttttcatccagatgacgcgtttgaattt
aaaaactttataaagtcggttaatccgttggctagtgagcatatggaaataaatgtagaa

cctgttggcctacactttgcttataaaatgaacagaaatgtatcttctgatactcaattt
ttattcattccagattttaaagaggtaggcgatttattatttaaatataaaaaagataag
cacttaatctttcacaaggatcagtgtcatggtaaaaattttttcaggggcaacctata
tatattattcagcctactatttcaaagatcgaagcggaaacctaaatactattcaattt
actggactaaatgataatagagaagttatttttactagtcttgaagctgctaataaatca
tggacaaattttataaaaaacaattcacaattaaaatcaattagaaaaccgactttatta
gtctataatctagaaagtttttttaaaagatcaagaacgattgaataaacaagattttcaa
aaatttgttgtgattactaataaaaaagcttatcttgctacaaaagagttggtagctgta
cccgattctaatagcgttttaaagcatttaaaattaaatatgaagcctaaactcttcttt
ataaaattatgggtaagacggctattttccaccttaacttatgaat-aaatgatgtt-gg
atttattttcttgaatagtattcaataactagtaattcatttagttgtaaagcaacccat
tctctgtcaataactccattgacttttcctgatagattagatttatttaattctaagtgg
ctaggaatgttagctaatccagggaacgctaaatagttttccactagtcttttagatgtt
tcccgagattttacactaattaattctcctggcttacattgataactacatatagatact
acttgtccattaatgcaaatgtgaccatgatttactagctgtcttgcagcaggaattgta
ggcgccattccaagtctaaaaacagtattatcaagtctcatttctagaagctgtaataaa
atttgacctgtagatccttggagtttttttagcggccttaatatacttaaagagttgtttt
tcacttaatccataattaaagcgcaattttttgcttttcttctaatctgacagcatattca
gaaggttttctagatttttgtccgtgctcccctggtggataagattgtttaattgttttt
ctacttagcccaggcaaatcacctagtcgacgggaaatgcgtactcgtggtcctctgtat
ctagacatattttataaattttccgaatattaat-gattaag----------taatgagt
agctatttttaacacaaaaagagtattaaaacaaagc-ttttgctaaaaaattttcatg
aaaaatatag--------------------tgctttac-tttttaagcgggtagcgggaa
tcgaacccgcatcattagcttggaaggctaaggttttaccactaaactatacccgcaata
ttcaataaactgtaacaggcttaatataacataactatcttattcttagatattattaaa
attgacaagaag--caaattatttatgctccttctaaaactacatcaagaaaacgagcaa
tctctgcagcttgctcttcaatttctgaaagcaataatggttgacccactctagttaatg
gaattactttttatcttttgtgcataagtaaatttctcgtcgtggattcaaaccttctt
taatatctatttttattgacttaatatcttttatattgaattgaagacaaatttgtctat
tttttcctgggaaacctaaacgaaaaattttaacagtaccttcatctttattaaactcat
tatatccagcacctatgtcccaaataatagttaaccataaaaataaactaagaaagattc
cgatgctgccatagaatgtcataacaataccttgcggaataaatattaattctgttgaag
tcgtaaaaggcaataaattaatttgaaagtaacttgataatcctgcaagaagaaatcctg
aagctccaacaaagattatggtagcccaccagtaattactaagtcttcttgaacctaaaa
ttagatcttttctaacagagtctatagataatgttttttcataatttgtt-ttttaaaa
caata-tactaatttgacatataaaaattttgacaattttttataagcgttttagattt

attaaatgagtttgatagcttttaatcctaaaaataatcctgttgctaaaccaaaaaaag
ctgcaagaaatattatatatcctagaaaaactgaca---tatta----ttattattattg
ttacaatagtgtttacaaaaatagttgaaatctattactaaagtattatattacgttaaa
tgcttcgctgtctacatgaaaaaactttttcagtttttagcgtcgtatttatattagaa
ataatattgtaataatttttattaaacttctggagaacagaatcttatggcagaatttatt
aagccttataatgacgatccttttgtaggaaatctatctacaccagttagtacatcgagt
tttagtaaaggattattaggaaatttaccagcttatcgtcgaggtctatctccacttcta
cgaggcctggagataggaatggcacatggatacttttaattggtcctttgataagtta
ggtcctctacgaggtacagatgtagctttacttgctggatttttatcttcagttggcttg
attattattctcactacatgtttatctatgtatggaaacgtatcctttttctagaacagat
tcaaaagatccattgcaaacagcggaaggttgggggcagtttactgctggattcttagtt
ggagcagtaggtggctcaggatttgcttacttattgctagctaatattcctgtactccaa
actgcaggtttaagcttgttttcttaagctagtaaggggacttgaacccgtaacctactg
attacaaatcagttgctctaccaattgagctatactagcgctcagctaattgtaacatat
aaagatatttgttgcaatctggtattttcagatgcaacaaatatctttttttatttagaca
gaactgttaaattaatatatgtatgatatgttaattagaatcagagctatctgactcagc
agaatattcttgatcacagtttaaataataacaaattgtttgatctagacatgttgcgga
ccaaccaacaggtgtttctcctgataattcttgcatatctccagaattgtaaaaatcatc
acttggcttattactatttgcttgcataaatatcactcccaaaacactctgaacttaatg
tgaagtaattgaacttctcaataatatattaacacagaatataaaatatggtgcttattc
accaattgtaacaatattatgaaatctacaagttctttgtaagtgttttaatagcttttg
ctgaaacaagcattttaatccatttattcttctctcttgaccatactttttctgttgca
aattaacttttttgtaatttttttgttcgtttgtgagaatgagagactgcgtacccattat
tcgctactttccctgtaagctgacatttttttgacataacgtt------attaacttata
tatttatttaaagtacttgccaaagttgactttggtacagcaccaattaccgtgtcaact
ctttctcccgctttaaaaatcattaaagtaggtatacttctaataccatattcagctgca
attgtaggattatcgtctgtgtttatttttactaccttaatagatgactcatattcttct
gcaatctcatccactacaggagagaccattctacaaggaccacaccacggcgcccaaaaa
tctacgagtacaggtaagttattattaataacttcttgtttgaaagaggcatctgtaact
tgagatactgacatatttttttaacctttaatatatgttccttgctaagcaaatcttatca
caaatttcaagaaaaaatcttctatagtgaattactattagtgttgaattaa---ttt-tt
tactagacattaaaaaaacttattacctatataaataaagtggtggccgagtgatatctt
gatagtgataatttttctattaaggttgaacactagaaatcatttttcaatcatatattt
ttttgaaaa--acttctttgtgaccatgaaagc-taagtatattacaatatattttatta
tttgtaacttatttcttataatggtataaacaacgcaaaagatactgccttataatcaag
gaggaatacatgtctcaatccgtagaatcacggactaggattaaaagcgaacgttacgaa

tctggagtaatcccttacgctaaaatgggttactgggatgctgactatgtaattaaagaa
acagatgttttagctctgttcagaatcactcctcagccgggtgttgaccctattgaagca
tctgctgcaattgccggtgaatcttcaacagctacttggacagttgtgtggactgacttg
ctaacagcttgcgacttatatagagcaaaagcatatagagtagatccagttccaaacgtt
gcagatcaatactttgcctatattgcttatgatattgatctatttgaagaaggttctatt
gcaaacttaactgcttcaatcattggtaacgtttttggatttaaagctgttaaagctctt
cgtctagaagatatgcgtatgccggtagcttacctaaaaactttccaaggtcctgcaact
ggattaattgtagaacgtgagcgaatggataaatttggtagacctttcttgggtgcaaca
gttaagcctaaactaggtctgtctggtaaaaactatggaagagtagtatacgaaggttta
aaaggtggtcttgacttccttaaagacgatgaaaatattaattctcaaccatttatgcgt
tggaaagaaagattttatattccatggaaggtgtaaataaagcatctgctgctgctggc
gaaattaaaggccattaccttaatgtaacagctgcgaccatggaagatatgtatgaaaga
gcagaattctccaaagaggttggtagtatcatttgtatgattgaccttgtaattggctat
actgctattcaaaccatggcaatatgggctcgtaagcatgatatgattttacatttacat
agagcaggtaactctacttactctcgtcaaaaaaatcatggtatgaacttccgagttatt
tgcaaatggatgcgaatggcaggtgttgaccatattcacgcaggtacagttgtaggtaaa
cttgaaggagatcctttaatgattaaaggcttctacaacactttacttgaaagtgaaaca
gatatcaatctacctcaaggtctattctttgctcaaaactgggcatctctacgtaaagtt
gtaccagtagcatctggtggtattcatgctggtcaaatgcaccaacttcttgattactta
ggcgatgatgtagttcttcagtttggtggtggtacaattggacatcctgatggtatccaa
gcaggtgcgactgcaaatagagtagcattagagtccatggttatggcaagaaatgaaggt
cgtaattatgtagcagaaggtccacaaatcttaagagacgctgctaaaacttgtggacct
ctacaaacagctttagacctatggaaagatattagttttaactatacttccacagacaca
gctgatttcgttgagactccaacagcaaacatctagtttaatgactacttgctaatgctt
aatttggcaaattgtaagtagaattgacttataacaataaggagcatagaatagtgagag
taacacaagggaccttttccttccttccagacctaactgatgaacaaattaataagcaac
ttgcttatatcgtttctaaagggttttcagcaaatgttgagtatactgacgatcctcatc
caagaaactcatattgggaattatggggattacctttatttgatgtaaaagatgcatctg
ctgttatgtatgagattagttcatgcagaaaagcaaaacctaattattatattaaagtta
atgcttttgataatactcgaggtattgaaagttgtgtactatcttttattgtaaatagac
ctattaacgagccagggttcttattacaacgccaagactttgaaggtagaactatgaaat
atagtttacatagttatgctactgaaaagcctgaaggagctagatattaatttcaattaa
tatctaaaacttatgagttagttacccctctttaatatcttaattactaaattaattttg
attttaaagaggggtaaatttcgaaataaattctagttaaattagaattaacaatccaaa
atacacagaaataatgcaatcacaagacataatctccaacgatactcttgttaacttaca
agaagaatatgatagaacacaaatccaggaagttcttaatgaattaaatcaagaacttat

aggattagttcctgtaaaaactagaattcgcgaaattgcagctttattactgattgatag
attgcgcagaaaactagaactagtatctggtaatccaggattacatatgtcatttacagg
tagtcctggaactggcaaaacaacagtagccatgaaaatggcagatattttacatagact
tggctatataaaaaaggacatttattgacggttacacgagatgatcttgtaggccaata
tattggccatactgctcccaaaactaaagaagttctaaagcaggccatgggaggagtttt
gtttattgatgaagcctattatttatataaagcagataatgaaagagattatggctctga
agcaattgaaattttactacaagtaatggaaaatcagcgaaatgacttggttgtcatctt
tgccgggtacaaagatagaatggaaaaattctatgaatcaaatccgggactttcttctag
agtagctaatcatgtcgactttccagattatacttcggacgagttattacaaatagctaa
aatgatgatagaagaacaacaatattgttttacagaagaggcagataaaactctgctaga
atatactgagcgaaggatgaaacagccatattttgctaatgcgagaagtattcgtaatgc
tattgatagagctagaatgaggcaagccaatagaattttttgcaagtggagaaaaagtttt
gacaaaagctgatttagtaacaattgaagcagaggacatcttgaaaagtagattattttc
attgcccaattcttaataattaagtagcttaatacaacgagatatcataaaaaaactttc
accataagttgcaaatttctttaaaaacctatactattaacatagtaaaggtg-tggcgg
catagccaagtggtaaggcagaggattgcaaatcctttatcccccagttcaaatctgggt
gccgcctagtactaaaaa--gggggtgtggtggaatggtagacacaacagacttaaaatc
tgttga-ttttaataatcgtgagggttcaagtccctccacccccatgtattaacttataa
aaaaaca---atgtgtatttgtattaattgtaaccatattactgaatgtgatacttatca
tttaatagaatctcagcacaaacagctgcatcttactgaaaccccagctttcatacctaa
atgccctgtagtttgtgttaatatatctagtaatagcagccatactcaaatcgattggga
tttagtggaatgcttatcatttgtggaaaaaccaaatagttggaatttaggctgtgatta
gttgtaaaaagaattatgtggtatacgcaataactctatcttttaaatactctgtattaa
ttttagatgttcttaccaacaatatttttcctgttcttgctatctctattataccaaatt
tcgttaataattgttcgatggcaacaatctttcctggatcaccagtaacctctataatta
aaagatcttccgcgatatctactatcttagctctaaaaattttcacaatttctaaagctt
ctgttctggtctgagaattaatcctaatcttaattaacattaattctctttcaactgaag
gaatatttgttacatcttgcacgttcagaatatttactagtttatataattgtttagtga
gttgttcaatagttctattgtctccctgaactaccatcgtaattctagaaactccgattt
gttctgctggtccaactgctaaacttgcaatattgaagcctcgacgtgcaaatagaccag
atattcttgaaagtactcctgcttcatcttgaactaaaactgataaggtatgtttcatga
aattttaatgttatactgcttgatctattacgtatattctatgaaaattacagaaaaaat
gctagttactatataactaaattattccgactaaacaaaatcccaccatcagacggcata
tctattttgtaatgttatac-----------tatttaatgatattctaattctaaataga
atgctaaatttttttaaaatttgttaggaacttgtgctagaaaaatacaaaaacagtaaga
aactctctcgagatctaccccaaatcaacgatcgtattagatttccaaaagttcgagtaa

ttgacgatgaaggtgaacaattaggtatttttgtacctgaagaagctatccaattagctg
cccaacaaggattagatttagttgttgttagtgacaagtctgatccaccagtttgccgaa
tattggactatggtaaatataagtttacacaagaaaaaagagctagagaagccaaaaaaa
aacaacataacagtagtgttaaagaagtcaagatgagatacaagatagaagagcatgatt
ataaagttagaattaatcaagcatctaagttcatccaagcaggagataaagtaaaagtca
ctataacattccgtggtcgcgaaattcagcattctaatttagctatagattgttaaaca
aaatggcaagcgacttaacttcgatatctgaaattcagcaagctccatcacgagatggaa
gaaatgttataatgcttttatctcccaagaaagcgagtt-aaatgattataatttaaatg
catctggctggattcgaaccagcgacgtctctttcgaaatggcggattatgagtccgctg
ccttcggcccctcggccacagatgcatcacttaaagtttataactcataaagtatataaa
aatcaatgcttgtttctttttcatgacaccta-aaaaaattattattcattcatattatg
ataggtcgcaactgctgaaggggaaacttggtttagatacctaaatatccagtatttaaa
tactgtgtccagtataactggaaaagttgaaataaatagaaaaataaagtcacgactttc
aggtagtcctaaatgccttaaaattacttcaataataacttcccagccatgaggagaatg
aaaaccaacaaacatgtcagtaaaaagaataattagaaaagctttcgcagtatcactaag
gccataaataatttcatttaaaaaagatttaacaattgcaatttgcctttgtccagttat
cattaataagataaaaacaaatattgatacaatatcggacaaaatattttttacagcgtt
tgcactttcatttgcataatattctcctaattctctagccttcagctgtactctttttc
tataatttcataagatatatcttctgatgggttcaataagacttcaaagtgaatttttc
ttcaaacctttgtagttcagcgaaagctctctcttcttgagatgaatttaaaaaaatttt
aggttgttcctggttccataaataatcaatacaaggaccaaacacaaaaaaatttgaagc
ttggttcactaatactggagagataaataataataaaatatattttacagaagtaatagt
ttgatgcctagatattctaaattcttctatggcttcagattctccattcggatctaactc
ttttcgaaacttttcaaaagtattagtaatagacctaggtattggcccaactttttcaaa
ggcaaattgattatttcgttttaaattccaatatttcatttttattgctgctaaaattt
ctattaatcaataattgatcacgtattcactataaatattaattctagaactaaacatat
aaaacaatagttatgaataataaaatacaattaaactttcattatgcattattactatct
tttactacagcaatgtatggatataaatcttatcgtgcaactatgaatacactgttaatc
aaatgtgaagatagccattcattattgtctggtaaacatactgcctttattcctataatt
atattaaaaaaaaatctttaaactccctaaaatcagaacctcacaatactgagttgtgtc
ttaatggaatgggaaatagaaaattcattgacaaaattgcaagtttgctaagccgtttag
aaaaacagatgcactatactgaacatcttaatcaaataaaaactagtgggtacttttctt
tactttatattaacagtaaagcaatctatacaaaaaaaattagtaatatttttctattac
ctaatgatgttttaaaaagaatttatattcataataagaaaaaaaagcttatacctcata
cattcttaataaaattatttcaaaaacaaataggttacccgaaaaattttgcgaacttaa
acctagccttatcaaaaattatgcaatggtatgctgatagaggatatcaatggtctttag

tggagatcaagcaggctgctgatccatcgtatctcattctgaatatccatgaaggattag
ttaagacaattacaacagagtattatactttgtcttataaaaaattacgcagtatatcaa
gtacggaatcaatagagaagtatcttggagtaacagtaggacttcctttaaatattaatg
ttttgcaaaaaaaaattaactatttaaaagataatcaattagttggcaacattatttata
gtattgagagatcgagcaataactctatgaatttagatatcaaatttcaaatacaagaac
taaaagataaagatttactagtacttgtagaaagatcatctaaaacatctcaaattattt
ctcacgtatatgacttaatagatttagaatgc---agttcctctaattggctagcagctt
ccaatataatgtcactatcgactagcaaaatacaagcatgctattttgatagtaaaatcg
attctgaatacgtctatacagatgctgttaatttaataaagcggttaacttattccattt
ctcatcaaaaaacactaataattgacgatttaatttacaaaatttactaagctggacaa
aaagaaatactattggattccaattgtatttacggaatttggatcatgccaaatcttttt
gtctattcagtctaaaatgcatccagagtggacttaatataaaaatttcgtatctaaatc
cttcattaagcattagtcgtaatttgagttttcaaattgcagtacaaataattaaacaat
atcaaacaagtaaacaatcagctctatctt------------------------------
------tgtttttaaaaagggagatttagcacaattcatttttgaaagtatatgttcat
atcattttacatcttatttttcgatatctgaaaagatattactttctcgagttgtgtata
tagattccatattttacaattctgaaaatataggtctttataataataataaaaatcaca
ttataagtagctatgatatctttagacacaataagaaattattatatcaagaatttctga
ccttattgttaagtatacgttatcaaaatttcaattatttagactggcctttaaaaggct
atttatttgaaatgaagtctttatatttaccccatttcagaaaagcaatttttttgaacc
actcttgtcttgacaactacaaaaacctatttttttcataaaatttacttaaaacaaatct
ctcattttagtttaccactgtatttcagagcccatcttaaccatatttagttaatacta
ttaaatgtcagtcgaatttaaatatggaaactatatatctattattaagcgattatccta
ttaaatacactctgcttaagtctattgttactttttctataaaagttaggatggaatatt
taatacctataagccggagcatcagagtatctttgttttataattatctagactgttttt
taatgagacaatctaaacacttagcatatatactggaagatctgtcaggccataaacaat
tccaaagtttttttattggaaaaattttcatggggatttggaattcagcttaaattaccaa
ttaagcagatgccacccctttcaattgaatatactgtaaacagtggtcgccacttttgtc
tttacctgcatatttattatcaaagataattattattaaaatgaaaaatttaatttcttt
ttttgaccgaagcaaaggaaaatggatttcacagcgaacaacctatgaactacgtaataa
gaatatgagttcagtacaatctcaaatgcgaatacaatcaggtaatttgttatcaggatc
tacgcgacttgcatcattgaattggggagaaatttttagacaggttaaccataattctta
ttctcaagatacaagcaaatataatggccagtttaacttagaatttagtaatcatctaaa
taatcgtaggctattgacatcatgcacagtaacagatcctagtctagttaattttaaaac
tcgatatggtagtacaactatagatgaaacatattggtttgcaacaagtaatttacgtct
aagtactagcattattaaaaaatttgatacttgtgttgcagtatctttttgttcagaaat

caaagtttaa-attctgtataaaaacaactaatagctaaaa-agttattagttgcttta
tatatctaatttttgttttagagagtatataaaatattatattattctaaatctttacgg
ttaggatttctagaaggatcattagataagaatccaaaaacaaacagagaaataaagaaa
atgacagtagtataaacaaatatttcaaagtaaacatatttttatccttaaaagctatt
agtgtg----atatttatatatc-acacataattttatatcacgatataagttctggcgc
tattttataaacaaatcaatatctttatttttagtaatgatttatacttcaatatgcgt
gatgcactaaatactaaatctttttgtacacgcttctcactatttttaatattttgtga
tttctatgaagtttaccttcaattactacataatctaatttcttcagcaatttaaaagtt
tctaaagactttttattccaaataattaagttaattaccgctaatctttttctttgtaaa
agcctaactttcaacttaattatttggtgtctactatcactaatttaatactcttacac
ctcaaaatttgaactagtaaggtacagctattcat-attaat-ttaa------tcttgta
actaatctattatgacagataa-atgaaaaaatcaaaatgaaatttcattatcttgttga
ccttgttcataactaatatctttaatacttttaatattttcccgaaatactcttgcaaa
tatttttctaacgatattacttctgagctatcaatgcttaaaatactataaacttcattc
atcgaagcagtaaattgctccccactagttaaaatctcggcaaatgctaaacgatcagaa
atattccatgtccattgaagggttttagtgatttttcttaaagctgttaatagtccaatt
ggaatttgtgatatttgtgtttttttgcccagataacttttcacataatttaataatttca
gctgatgtccaggccgtgttaccaactaatggcagcactctgttttctgtagaaggcact
ccaagactttttataactagctttgccgcatcctgagtatcaatatatgcaataggtgta
gactcccctgtaacccatactgatttttttatccaaaataggaattgcatattggttaatt
aatccttgaaaaaaccctcccaaagaaaataccgtgtactttatatcagatttttgaaga
aagtctactacttgtgattttaagttcatcaatggaacagctgggtacttatctgcattc
aatattgaaaagaaaataaatctttcaactttagcagctttagcggcctcaattaaggca
attttaccatctaaatctatttttgctgcattgtaaggatctgtaggacgagatgtagac
gcatctatgactgctgttactccacaaaaagattgcaaaatactttcaggtaattttaaa
tcaccatatacaagttctgcgccccattctttgagaaaagctgactttcgtaagtttctc
accatgcatttcacattgtagccttcatcaagagcacgccttacaatttgacgtcctaga
gttcccgtcgctccaattactagaagagtcatattaattaaaaataagtatgtataaaga
cattggtttggtcgacagcagagattaaattaggtagagagggaatcgaaccctcatgac
cgaagtcgtcacattttgagtgtgatgcgtataccaatttcgccatctaccctattatag
accaactactacaagtaatttcgtctttagcgtaaaaataatactaagtttcaatagata
tgtcaacttttgagtttatattaataaaaaaccggagtatcttatgtccacttttgaact
aattccttatagattatatttaagtcaatctagtacgtggatgcataggatgaaagcaga
aattaaaatatatatattaacgatattatggatttcaattttattttctcttactgtaa
actatgcattgttgccctaagtttaattgttcttagccttactgtaaagaataaacaaaa
cattatccagagacatttattgcgaactttagttatgacaatggtaactaccttcttatc

attcagcatagctactaattacaaacaatattctcaaccaaaacaatcacaagatttatc
ttactataatacagctaaaaattcttacatctatcaaaatatattgctacctaatagtcg
taagatcacaacaaaacaactgaggtgcactttaaagcctgcactctattttttattac
tatatattcaattaaactagtcatgataacgacttccccagaaattttagtgattactgc
ttatagatctaggatctttaatacaatattaaatcgtgaattattatttactttcttatt
gtcatcacatattgttaccaatataattagtaaattagataaacttgttcaagtagctaa
cttaagaggtagcttaaatttatataaatcgtccacaagacttttttatattttcttttt
gatgtttcaaatctttttttagagattattaaggaatcaaaagaaatatctcaagctct
ttatactagaaatcttaatcaagaaaacagtaatttttaaaaatatatagagccagatc
gggtattagtgattggttaaatgcaattattgggaccttatattttattattttaggatt
agtataaca-aatctaatcatccttctgactctggtaatacgaatagtaatagacttaag
tcgtggtgtaaatatgaaaaat-tcagatattatataaataattaaatgttttattatgg
tcaatactcaaaatcaaatttcacaaacttcagagcttgattatatagttaatcaacctt
ataaatatggatttaaaacttctgttgaatcagaacaatttcctagaggaatagatgaaa
atatagtaagattaatttctaagaaaaaaaatgagcctgaatatttattaaactttaggc
ttaaagcatacaaaaaatggaaaaaaatgagtagtccatcatgggccaatctcaagaatc
ctaatatagattttaacagtattatttattacgctgttccgaagttaaagaaggaattaa
acagcttagatgaggttgacccagaaattcttgatacttttaataaattgggcatctctc
tcaatgaacaaaaacgaatttcaaatgtagctgttgacgctgtttttgacagtgtttcta
ttgcaactacttttaagaaagaattatctgcagctggtgttatattctgctcgatttccg
aagctatacgagattatccgaatttaattcataagtatttaggtactgttgtgcccgctg
gggacaattactttgctgcattaaattctgcagtatttagtgatggttctttttgctata
ttcctcctaatactgtttgtcctctagaattatcaacttattttcgtattaataacgaag
aatctggtcaatttgaaagaacactcattgtagctgatcgtggtagtaaagtaagctatt
tagaaggttgtactgcccctcaatttgatacaaaccaattgcatgcagcaattgtagaat
tagtagctcttgatggcgctgaaattaaatactctactgtacaaaattggtatgctggca
atcaagagggcaaaggtggtatatacaatttcgttactaagcgaggcttatgctctggca
ataattctaaaatttcatggacacaagtagaaactgggtcagcaattacctggaaatacc
ccagttgtattttagctggccatagttctcaaggagaattttattctgtagcgttaacaa
ataattatcaagaagctgatactgggactaaaatgattcatattggtaataatactaaaa
gtagaatcatctctaaagggatttctgctggtaaatcaaaaaaacagttacagaggactag
taaaaattggcgctcaatcatttaattctcgcaattattctcaatgtgattctttattaa
taggtcaatcgtctcaagctaatactttttccttatattcaagtacagaatccgacatcga
aagtagaacatgaagcatctacttcaaaaattagcgaagatcagatttttttactttttac
aaagaggtatcagtttagaagaatctgtagctcttatgatcagcgggttttgtaaagatg
tattcaatgaacttcctatggagtttgcagctgaagccgatcgtttactgagtttaaaat

tagaaggaactgtaggatgagtcaaactattttagaaattaaagatttgtatgcgtctat
tggtgagacaacaattttaaaggggtaaacttatctatacaagaaggcgaaatccatgc
gattatgggacctaatggttcgggtaaaagtacactatcaaaagtaattgcagggcatcc
agcttactctttaatcagtggagacatcttatttttggaaaaagtattcttgaaatgga
gccagatgagagagcaagagctggtatttttcttgcttttcagtatcctgttgaaattcc
aggagtaagcaatgccgatttttttacgcattgcattaaatgctcgtagaaaatttcaagg
attatcagaatttagccccttagaatttttttcaattaataaatgaaaaaataaaccttgt
tggcatgcaagaaagtttcttgacaagaaatgtcaatgaaggattttctggcggagaaaa
aaagcgtaacgaaattcttcaaatggctttgctagatagtaacttatctattttagatga
aacagattctggattagatatagatgcacttagagtagtagcaaaaggaattaatacttt
agcaaaatcaacaaattcaattattttaattactcattatcaaagattattggattatat
tattccagattttgttcatattatgagtaacggaaaaattgtcaaaactggcaatcttac
tcttgcacaagatctagaaaaaaaatgggtatgactggattgtacagagttaaaaatttgt
taaataaggcaatactgagactaaagtatcttctttagtctcaatattattgatcaacag
aaag-----ttatttaggctttactaaaaccttgcttaacatcttcaatggctcttttta
acagttcttcactagatgcgtctaatttttactgctacgaatactttcaccaaattctg
gtttagaattctttaaatcttcccgtaattctttaataaattcagccacttttgatactt
caatatcatctaaataaccgttaattccagtataaataatagctgtttgttcttcaactg
gaataggagaattttgagcctgcttcaaaatttctctaagacgttgacctcttgcaagct
gattttgagttgctttgtctaaatcagatgcgaactgagaaaaagcttctaattcagcaa
attgtgccaactccaattttaatttgcctgccacttgtttcatcgcttttatttgagcag
cagatcctactctagagacagaaataccgacattaatagctggtctaatacctgagttaa
ataaatcacccgataaaaaaaatttgaccatctgtaattgaaattacattcgttggaatat
atgcagacacatctcccgcttgagtttcaataataggaagagcagtcatactaccgcctc
ctagttctgaattcaatttagctgctctttctagcaatctagaatgtaaatagaatacat
cacctggatatgcttcacgtccaggcggtcttcttagtaaaagagacatttgacgatagg
cttgggcttgctttgttaaatcatcataaataaccagggttgctttacctttatacataa
aatattcggctaaagctgcccccgtataaggagcaatatattgtaaagttgcaggactat
ctgcattagctgcgactataatagtataatcaagagctcctttatcttgtagtgaagata
caacttgtgccactgaagatgccttttgtcctatcgcaacatatacacaaacgacatctt
gcccttttttgattaataattgtatccagcgcaacagctgttttacctgtttgacggtcac
caataattaattctcgttgacctcgaccaattgggatcatagaatcaatagctgtgattc
ctgtttgcattggttcacaaactgattgacgtccaattataccaggagccatagactcaa
ttaatcttgttgaattacttgcaggttcacctttatcatctattggacgagctaagggat
caactactctacctaaaaaggcatcaccaactggaatttgagcaattctaccagtccctt
ttacagaactaccctctagaatgtctctaccgtctcccattagtactacacccacattgt

cactttccaaattcaaggcaacaccaatggttttatcttcaaattcaagcaattcaccag
ccataacttcatcgagcccgtacactcgagcaataccatcaccaacttgtaaaactgttc
ctatattggctacttctatatcttgatcatacttttcaatttgttgacgaataatactac
ttatttcatcaggtctaatgtttaccatgttttaatattctttttattaatgggaaatt
ctaaagtggtaattactcagattaatactatctg--tttttactgtattttaagttgct
actacatcaaggtgtgaagccatctgccttagttgccctctaatactagtatcaatgact
ttcgatccaatctgaatagtgaaaccaccaattaactctggatcgacagaaacaattagt
ttaacttctttagcctgggtcataaccttaatttttctgttagcagattttcttggtca
gagtttaaagcaatagaagtattaatattggcaattgttagagattccatttgatatgca
agttctaaatacttactagctatagcatccaacattgcaattcttttacgatcaactaaa
atcattagaaatgatagtgtattttcagtaatttgatcaccgaaagtggcaataatgact
tgctttttgcttctgcagtttttaatggattagct---aaaaaattttaagcttgtca
gattgcaataaaatattttgtattaactttatatctatactcacctttctgtaactttt
tttgttttagctaattcaagaagagctacagcataaggctgcgcgattttataacaacg
ttattactgctcatagttggtctcctaacttagcaatattattatcaatgatacgtagtt
gcatttcagagctcatttgattttctagctgcaaagtaactcgtttaagagcaagaaatg
ttatttgctgctggatttgccttcgaatttgtttttcagctgtttcaatatttgacttgc
ctgtcatagcaagtctttcaatatctaattgaccttgagctaatatagagcttctaactt
ttcctgccgttagctgagcttctttttgatttgatctataataatttgagtctgagcaa
gttgtttttctgattctgacaatctagcactagcttgctctagtctttcttctgattctt
gtatagctgctaagacttttatctgtcttgcatttaaactagatccaagaaattgtttca
aaacataaataagaccaaataaaagcaacaatatgttaataacatttgcttcaaaaatat
cagaattaaaaccgaatttatgttcactactatgttctgataaaatagtaattatttgtg
gtatttttactatattattcatttgaaattct----------tactattttaatatttta
cttaggaatcatgattttaaagattgactactcaatagtttagctttaatttggtcgctt
aaagtatccacctgatcttccaaagttttaagagcctcttcttttgaatatttagctgt
tttgatgcttcagcaatcaatttttcagcattcaattgagctttttaatatcttcagag
actatactttgcgcttctttttgagaagcagctatctttaattgtgcattacgacgagct
tcagataaatcttgctcatattttgcagctaactcatctgctttaacaagcatagaagat
gctgtagtcaaagttgtgcggatatattcatcacgttcatctagtactctagttactggt
ttataaaaaatagtattcagtaaaaccattaacgtaagaaactgtaatgccattaaaggc
aaagtaccattgaagtcaaataagccaccttcaatttcttccgctaatagaaatggtaaa
taaatcatgttactaattatgttgatagttaataatttggtttcaattaagaaaattaac
tttatagtgcgcttagtcttattagactaagcgcaaatgattattttaaccaacgtatgg
attagcaaataatagagacagcgcaacaactagaccataaattgttaaagattccataaa
tgctaaactcagaagcagcgtgcctctaatttttccctctacttctggttgtctagcaat

accttccacagcattagctgctgcactaccttgaccaataccaggacctattgcagcaag
acctaccgcaagaccggcagcaataacagatgcagctgaaacgattgaatccataataaa
ttttgatttttttgttatataagtagaaaattgacaaatttcggataatacttaaaatcta
accaatgaaatattggagattcaaatat-----tgtaattatagtatactactcttctcc
atgaccttccattgcttccccaatataagctgcggataaggtagaaaaaattagtgcttg
gattgaactagcgaataagcctagtatcataactggcaacggtatcaaaattggaattaa
cagagtaaatacagaaacaactaattcatctgctaagacatttccaaatagacgaaaact
taaagataatggtttcgtaaagtcttctaaaatattgataggcaataatactggagtagg
ttgaatgtacctagcaaagtaacctaaaccttttttacttaagcctgcataaaagtaagc
tagagaagttagtaaagataaagctacagttgtattaatatcatttgttggagcagctag
ctcaccttccggaagttgaattaacttccaagggatcaatgcacctgcccaattacaacc
tagaatgaataaaaacagggtagcaatatatggaacccaagggcgatattcatgctctcc
gatctggtttttagcaatatcttgtaagaattctaatataaactccatgaaattttgaaa
tttttcaggaattcgctgaagatttctagtacctaggaaagatatagttaatagtgtagc
aattactaaccaagatacaataaaaacctgaccatgtaattgtagactacctattttcca
atatagatgtttcccgacttctactgctgacaagctagtatatggattaaaatctatgag
actgttttgatacataataaatatttcttttaaatagtacaagaatcaaaaaataagttt
tttgaagaattaatatgcaattagtttatttgcacgattagattttaataaattaaaag
caaaaataaattctaatagagatagctatctttagtagtgattattttttgtaaaaaaca
attattgctacctataatgcttttatgtttaattatatacccatatatgaattatttaat
atgattataggcatttcgctacaaactttattgttttatccatttt----aaaaaattctt
ctaatttacttattatttaaaatatctgcaacttcatccgcaaagttagctttcatattt
tcttcgccttctcccaatacaaatctaacaaatcttctaattttaatattttcgcctaaa
acggaaatattttgattaattaaatcttgaatactaatatcttgattgcgaatgaacatt
tgatctaataacgaaagttctttcagcctctttttcattcgcccttcgataattgctcct
attctatcagctggcttgtttttcaaatcatctttaccagcttcaactctttttttctgaa
ctgataacttcatctggtatatgctgtgttgatacatatgccacatttggcgatgctgct
atttgcatagcgatgtctttagctaattttttgaaattctggacgtcgggccacaaaatca
gtttcacagttcacttcaactaatacaccgattcttcctcctgtgtgaatatagctttct
aatagaccttcaagtgcagtacgattagatttttttgttagctgaagctaatccttttttgt
cttaatgattctaaagctttttcttcattaccattactagcttctaaagcttttttttgcag
tccatcatacctgccccagtcttatcacgtaaggcttttacagtttgagctgaaatttgt
aatgtcatatattttcaaaaatttttaagtatttaaattaactaaaacaaattatataa
ttcactttccacagagcgagctacttcctgtacatctgtttgcccatatttaccatcata
aattgcatctgctatcttaccgacaattagtttaattgatctaatagcatcgtcgttagc
tggaattggaatattaataatttcgggactgcagttcgtgtcaagcatgcaaatagttgg

gatacctaatttcaaacattcttgtatagctgttgtttctctcttttgatctacaactac
aacaatatcaggcaaacgagtcatattttaataccatttaaatgtttacgaagtttatc
taattctctccgtagaacagcagcttctttttaggcagttgatcaataataccactttt
gtcttgctcttctaactgtctaaggcggctgactcgggatttgattgtcacccaattagt
taacattcctcctaaccatctttggttaacataataagaatcacatctttgtgcttcctg
agcaactattcctgcggcttgtctttttgttcctaaaaataaaactttcttgccatcgga
cgaagcctttttataaattcacaagcttctgtaagtaattgagcagtctgcactagatc
aataatatgtatgccatttctttctgtataaatatacggaaacattttaggattccatct
gcgagcttgatgtccaaaatgcacgcctgcttctaataactctgctaaggtaacgacagc
cataggattttaata-ttaaataatgtaatttttcgggaaatcccttttc----aaac
taattcaactcatttaacttattgcaattccaataaaccaataataaattaacatatttt
tacagtcgattaag-aa--aaaattgttttactttattcaatctacacttttattgctaa
agtagttacgcgcggttctgtcgtctaaaataatatcatctaaatcatctctcacagacg
acacactactttcgctactgatatctggattccaatctttttttctaacgacgctctat
tatgattgtcatacgtattaaaaccagtgccagctggtataagtctaccgataataacat
tttctttcaaacctcttaaccaatccagtttaccagatatagcagcttcagttaatactt
tcgtcgtttcttgaaaacttgctgcagaaataaaactttctgtattgagagatgcttgcg
taatacctaacagcacgggtcgataggaagcattcagcttatttcttaaagtcatagctt
tattggtttgctcaatcttttgcaactctacaagttccccaggcaaatagccggtttctt
caccattctctattttcactttagaagtcatctgtcggactatgacttctacatgtttat
cagaaatattaactccttgagattgataaactaattgtacttctttaacaagtaataact
gtatttcttgaaaacttaatctagctgcttcatataaagctagtccgcattcaatatata
ggttgaagcttgcatcaagaatatcatgaggatttaataatacatctgtttttttcgcg
cttcaagaatttcttcaattcttggaaggccttgtataatatcaccagtttttgctctgt
caaatacgagaatagctagtgtttctcctcttctaattaatgcattattatcaacatgta
aaattgccccgctagaaactaggtaaggccgagcaattcttaatgttattgattgagaag
acacgtgagtaatttgtccagagtctaaagaaaccatattttcagcaatcaaatcaccgc
aacgaatccaatcaccaacacatactttagttttagctttttctatattaaaagttttct
tgtcagaagatgttccaattagaattcgtctactagtattcttttcagaataaatttctt
cgaccataccttcactcattgcaataatttctgtactagcaacaaccgtcaatggcttga
tatgttcaccatttttgacaataattctagtttgactttgttgtttttctgatttattga
catcaatacttttaattgataatgtttcaaaagttgaaagcctcaaatcaaaataattag
tatcactttctttattagaaaactcaattgaggatattaaactatcatctgtatgctcaa
tttcagctacaagattggttgtaattaaatgaactccatctatcgatttcaccctttctc
cgtcacgaaaaggtgtccttttaactagctttaatttagtttggctcggtttttgtaatg
ccaaattatcaattaagctagatttatttccggtattgaataaacaataacaggtctaa

tgaggatataaggcatttgctcattttctatatactcccaataaactaacttatctgttg
aaatgttgttatgcaatgtttctccaggtcttaaaaacccccctactttttttcattgttac
tatgtaattcgtttagtttatatatagatccaggcttaataatgatttcacgaactattc
catccttctgaattatctcaacaattccagagcttttagaaaaaatattttttgactagct
cagttccactttctataatatccccattataaactaataatagtgatgcatctttattaa
tttcgtgtgtttcctctgagatccataatatatagccaggactcaaaatttcataagcat
ctttttctgaacccactttctttttttgatacattcaagtctaaatatttaataataccac
cacttgtagttttgtaagtatcagatattagctcagcgactgtatagccatcgtaaa---
ttttttgattggggacagctttaaaaataaatttctgtttttttctgtttctaaaatgt
aagattctttttattaattaaatctgtataaatataacaaccgggaattacaatagact
cagtaataacttgtatattagtaatgctgctattagcattctgctgtatcctgacttccc
cagcatagtgatttatcaactctgtctgcgcaagtattgtgccagctttagttgtatcct
ctttattaacaatcacattagccgagtcagatatattataaacttctccagagagaaccc
aaatcaacccacctgtttagtgattcgtgtagtatattgattattatctgtttcttcaa
ctgttaagttagagaaacagattttaccagaaaggtctgacactacatgtttttgagccc
tttctgtcatcagtctatttctgcgaggtgattcagcaatcacctgatcttttgatactg
attgaccctcatgtagcagaagagtggttcctttacttaagttaattattgacttttgat
ttcttctagatcgaattgtaacttgggttggattctttgtaattaaggcttgttctccat
gccttgtacgaacatctttatatgattcaatatctagatctgttagctgaccatctatgg
gtgcgtaaatttgctctgccaattcaccagtaaatacacccccccgtatgaaatgttctca
ttgttaactgggtgccaggctccccaatagattgagcagcaataatgccaacagcttctc
ctaaatcaactaagcggccatgagcgagattccatccgtaacaatactgacaaacagaac
ttctcgaattacatgtaacaggtgatctcaccaaaactttttt---gatgccagttttag
ttatctctttggctaatttagggctaatatcttgctttgtgtgagcaatcaaaacatttg
tatctggatggaacacactctctgctaatactcggccagacaaagcttgctctagattga
ttagtatttttttgtgtatctactaagtcttctagtattatacctttatttgttccacagt
ctacttctctaattataacgtcttgagaaacgtcaacaagtcgacgcgttaagtagcccg
aatctgctgttctcaaagcagtatcaactaatccttttcttgctccgtatgatgatataa
agtagtccgtaacagttaatccttctctaaaattacttgatattggaaggtcaataattt
gtccttgcggatcagccataagccctctcataccaactaattgtctaacttgagaaatat
tgcctctagctccagagaaagccatcatataaacagcatttaaagggtctgtttctttaa
agtacttaattacttcctgttttaatgattcgcttgcattgttccatgtgtcaataactt
tttgaaatcgttcgaccgctgtaatttcccctcggcggtacttattttcggtagctttaa
tttcttcaattgtacctaataaaagactttgtttgctcggaggaatccttaaatcttcca
aactcaaagagattcctgcctgcgtagcgtaatgaaatccaagatctttaagtttatcag
ccatattagctgctcgtgctataccgtaattacgaaaagcccaaacaattaaatttttaa

gttcgtttttatcaataaccctatttgaaaaacttggctgcgcaagacttcttttactat
ccactcagtgttctccatattaattatttatt----taactacgccactaaagactcttg
aataatttttgttgaagattattcggccagctgttgttcgaatatattgcacaattctctg
ctgttcagcatcctcttttataatatggttattataaaatttgttctactattatcact
gtgcttttcgattttaattagctcagtagtttggtcaccatctacaaatccatcaaaacg
agcccatatataagagtgcaaatcaactttcttttgctcataagccatcactacatcttc
taagctagcaaaatattgattcgctcccttctgttcagaaggattattagccgtcaaata
ataacaacccagaaccatatcttgactaggcatgataattggctgacccgtagctggaga
aagaaaattatgaggagccaacattagcaatctagcttcggcttgtgcttctaaagatag
aggaacatgaacagccatttgatcaccatcaaaatcagcgttaaatgcaggacagactaa
gggatgcagcttaatagctcttccttctacaaggattggttcgaatgcttgaatacctaa
tctgtgtagtgttggagctctatttaaaagtacagggtgaccttgaataacttcatttaa
cacattccaaatagaaggttcatttttttgaatcatttttttttgcagctttaatattatt
cactaagccctgtaagattaatctatgaattacaaagggttgaaataattcaagagccat
ttctcgcggcaatccacattgatgaagttttaaatgagggcctacgacaattacggatct
tccagaataatccactcttttacctaataagttttgtctaaagcgtccttgtttcccttc
tataatatctgataaggattttagcggtctattattcgcacctacaacagttctgcctct
acgtccattatccatcagagaatcaacagcctcttgtagcatcctttttttcatttctaat
aatgatttctggagctaaaattgacttcagacgcgataatctattgtttctgttaatgat
tctacgataaaactcatttaaatctgccgttgcgaatctccctccatccaattggaccat
aggtcgtaaatctggaggtataacagggattactgtaaataccatccaagagggatctgc
gcctgttgctataaagttctcaatcaatcttaagcgtttcatttttttattaaattttaa
tgaaggtgtcttaaaactttttggtggatttgttgcttcagatcgtaaagtttcagcaat
gtattctaaatctaaatctttcaatagtttttgaatagcttctgcgcctatcccaacttc
tacttgattattttcattttgattttggtagatttcctcttcaaggctcttccattcata
gccttcgagcagttgcttatatttaaattgatatttgtattagattgtgtaactacata
agagtgaaaataaacgatttttttctacctctttaacttttaaatctaatgctaaagcgat
ataacttgtacttcctttaaataccaaacatgagttactggtgatgctaattcaatata
agccatccgatgtcttctcactcgagattctgttacttctactccacatcgctcgcagac
tatacctttatagcgaaagcgtttatatttaccacagtgacattcccaatccttaactgg
gccaaaaatttttttcgcagaataaaccatccatttcaggttttaaagttctataattaat
tgtttctggctttgtaatttcaccaacaatttgtccgttgggtaaacttctttcacccca
atgtcgaattttctcgggagaagctaaactaatctttacatagtcaaaatgttgctcaaa
ctgtgtcataaatttgaataccttgaaattaaataattaaccaacttatttacatatgcc
atattcgaaaatgca----ttaataaagaaattgttcaaaatcatcaactggtggagtat
cataactagtgcgatctgctcgattctctttagtgtcagacatcaaatcgacttcaactg

tacgtctttgaccgtcttcaaatagtttcaatttatgtactgcaatgtctaatcctaatg
actgaagctctcgcattaagaccttaaaagattctggggtcccaggcttaggtataggct
ttcctttcactattgcatttaatgcttcatttctagcttgcatgtcatccgactttacag
ttagtaattcttgtagagtatatgctgctccaaaggcttctaaagcccatacttccattt
cgcctaatctttgacctccatgctgggctctacctcctaatggttgctgtgtaactaaag
agtaaggaccagtagatctcgcatgaatcttatcatctacaaggtgaacaagcttaagca
tataagctctaccaattgtaacaggattatcaaaagattctccagttctgccatcgaaga
cttgcatttaccaggatgcagatcattaaaaagccatttattattagtgagtaatgatg
cttcttttaattttctattaacaagtgctcttgaggcttctgctccatacatctcgtcaa
aaggtattattttaaatcgtttacctaaatagccacctgctagacctagtaagcactcaa
atacttggccaacattcattctggaaggtactcctaaaggatttaaaacaatatctacgg
gggtaccatcagataagtaaggcatgtcttgtttaggtaagattctagaaataatcccct
tattaccatgacgaccagccattttatcgcccacttgaattttctcttctgggcaacat
aaacgcgaatcatagcattcgtaccaggagggagttcatctccttttgcctagtaaata
ctctaacattaacgacccttcctttagccgcattaggcaatcttaaagatgtatctctaa
catctctagccttttctccaaaaatagcccttagtaatttaccttcaggtaattgatcag
cttcgcctttaggagtaattttccctactaagatatctcctgcttcaacccaagagccac
ctaccacaattccatttcgatctaaatcttttaaagagttatcacttacatttgggatct
ctctagtaatttcttctggtcctaatttagtttggcgacattctacttcatacttctcaa
tatgaatagaagtgtataaatcatcataaactagcctttcactaattaagaatgcatctt
cgtagttataaccttcccaaggcatataagcaacgagaatattccttcctagagcaattt
ctcctccatcagtggatgcaccatcagctaaagtttgaccgacaactattttctctccca
cccaaacaattggacgctgattaatacaagtatcttggttagagcgataatattttttca
aacgataatggactgttctaccattattatcttgaatacctattttattagcagaaacat
aattaacataacctgcagttctacttataacaaccatgccagagtctcgcgcaattttag
tctcaagacctgttccgatgattggcttttctggatataataatggcacagcttgtctct
gcatattagaccccattaaggctctatttgcatcatcatgctctaaaaaaggaattaatg
atgttgcagctgaaatgacttgaataggtgaaattgcaatgtaatctacttggctcggtg
ttgttgtaataaattcttgacgataacgtacaggaataatatctccttcaatataatttt
gagaattaactttaacatctcctggagctactcgaaaatcatcttcctcatctgctgtta
agtagatgggactctgttgataaattacttgaccttcatttaccggataaaaggggggttt
caataaagccaaaaatattaactctagcacaggttgccagtgagccaattaaacccgcgt
tcggaccttctggtgtttcaattgggcagatccttccatagtgacttggatgtagatctc
gcacagcaaatccagctctatctttattaaaacctcctggacctaaagcactaattcttc
ttttatgggttagttctgcaactggatttgtttgatccataaattgagaaagctgactag
atccaaaaaattctcttactgaggcaattaacggcttaggattaattaggttagataagc

ttaatgagtctatatcacatatcatcattctttctctaataatacgttctaagcgattaa
gacctactctaaattgattttgcagcaactctccaactgaacggacccttcgattaccaa
ggtgatctatatcatcaaggttacctgaatttttatctttaatattaattaaataatcaa
tcgaggataagatatcttgaggagacaacactcgaaaattcttaggtatattaagaccta
attttttgttaattttatatcgaccaacttctcctaagtcatatctctttggatcaaaaa
agcgagaataaagcatctgcttagcaacagcaactgttgctggctcattaggtctaagct
tagagtagactataagcaatgcttcttcatctgttacttcttcgacattgtttttttccaa
tttctttagctaattctttaattgaatataattgagatgcagagatgagaaaagcatatt
tactcagccccttttcaatctcgtctttattaaggccaatagcacggagaaaaatataag
catttactttatgggtcttatctattcttatccaaatttctcctttagggtcaatttcaa
atttcaaccaggagcctcgattagaaatcagactagcactgtaaatctgcttgccatttt
tatctatctcttgtttatagtaaattcctgggctacgaattatttgattaataataactc
tttctgtgccagatacaatgaaagtccctctgttagtcattattggcaagtctccaatga
agaccaatctcttcttgtatttttatttttatttccttctcaacactaagctgtaaat
gtgttgatgataaatctagtgacttgatactttttccatgatctttagaaggtagatcaa
tgtcttttcttgtcaacttcgcagggacatatatttgagcactataggttctatctctat
tttttgcctgtctaacactgtaacgagggaattttattttatactcattaccaaacaatt
gcagttcaagccgactagtaggatctaatatatttggaaagaattcaagtacttcagtca
taccttctaatagaaaccatttaaaactggctctttgaatttcaactaaatctggaagaa
gtttatttttttaagcttatacgttgaaccatagatctcctttataacaaattctgcaatc
gaagagtttaa-tactatcaacagagtacttataggtaatacctgagtatagttcactac
attatttcttctatagacatgactagaacaaagtattttattcaacaaaaaagattttta
aaattt---ttcacaatcaatatagatataaaaatattacagctattaaaaatag-----
----------------gaagacgaaattaatacttgtctgtctgtcaaatagttgacatc
aagatagacaagtattatttatttaatcaattctgttttttttgagcgcaagataatgct
ctcgcgagtctagcttttttttctggcaccagtattaggatgaaatgctcctttttttatt
gctttatcaatcttgctatatacttgagaaatacttaattgcacatcgttaagattagca
ctttctagattatcaatatttagcaggcatcttttagttaaagtttttacaaccgattta
tatttacggttaattagacgatttctttccgaagttttaatgcgtttaatcgcagaaagg
ttcttagccacagtaaatccaataaaactttttataaaatatttttagaaacgaaaataca
caaattagagatctattcaaaaagggaatatgcgccgtattataacattaaagaaaaaca
ataggcaatagttcactgagggctcttgttagtacaaactaaatattgtcgttgcataga
a-tttaaaaaaatagtattaaat-----taaactaaccagacatatagatatatatgaaaa
aaattgaagctattattagacctttttaaacttaatgaggtaaaacttgccttagtcaggg
aaggtattggagggatgactgttgttaaggtctctggttttggaaggcaaaaaggacaaa
cagaaagatataaaggatctgaatattctatcgatattattgataaaataaaaatcgaaa

tcattattagcgatgataaagtagataaaatcacagagacaattattaaagcctcgaaaa
caggagaaattggagatggaaaaatattcattagtagcgtcgaaaaagtcataagaatta
gaactaatgatttagattccgaagctttataaaactcacactttttgagacttgatact
tcttcacgaaacatgagataataaacgtttattgtataataataactaaggtataataat
ataatggctaaaagcaaaggtgcccgcatcgtaataactttagagtgctctaataaaact
ggagatttagcaagaagagaaaacctggcgtttttcgatatacaactactaaaaatcga
cgaaatacaccaaacagaattgaattaaaaaaattctgtcctaattgtaatcagcattgt
ctattcaaagagattaagtaattattaattttactaaaat--aatcatattatggccata
tatagaaaaagaatatctccaattaagcccaaagagtctgtagattacaaagatattgac
ttgctaagaaaattcattacagagcaaggtaaaatactacctaagcgatccactggattg
acttcaaaacaacaaaaaaaacttactaaagctattaaacaagccagaatactttcccta
ctaccgtttttaaataaagattaaatatatacaaatatgctttagattgtttcctattaa
catt-------tccatgaatttatttactttaattaccgaatatactgagc---------
----------tactataaagcagctatttactactaattttgttcattttataattaaga
aaaa--atctaacttgttgttaaaatagattt-----agacttataatcgaacctctttt
aatgaattatattacatagtttatttaaaagaggagtcagttttatttgagttaataaat
agtacttttgtattctttagaccagtataaagtatttaagataaagatttttgtttaggt
acaagatcaaacgaatgaatcttatctccgggttgccataattgaaattcagaaatgaaa
attccacattcgttaccagcttggatttcttcaacatcttctctaacccgttttaaagat
tctatctttccctcataaataacttcatcttcccgaatgactttaatccaagagtttttc
aataatttattattcgtgactctacatcctgctatttttctattagccaaagaaaataca
gtgcttacttctgcttctcctactggtacttctgaatattccggatcaagtagatctttc
attcttcctttaatatcttcaatcaatgcataaataatttgataattttcgactagtata
ttaaattttgctgcagcttgcttcgctccaggagcaaaattagtattaaagccaatgaga
ttagcatttgtcgtcgacgctaattcaacatctgtagcagtaatttcgccaggcataatt
gaaacgacatttaattgtactttgctctgaggaaattgtgataaagaatctaggattgct
tctgtagagccttgattatctgttttaataattaaagaaatttgcttgcttagatcttta
gaattagtagttctcaatatatctaacgtaatacgactatttagtgctttctgcttctgt
gcgattaatgaatttttttgaagtattttcaagagctttaagctttgcttctttatcattt
ttcactactaaagctatctctcctatgacaggaacagccgataatccccaaatctcaaca
acagatgatggtattgctgaattaatctttttctgttcattattaatgatagctcgaatc
tttgcataagctgatccaataaccatattgtcgctcgtatgcagcgtaccattttggatc
aaaagtgttgctactggcccatgggatttgtctaagtgagcctcaactataacaccttgt
gcaggctgtgttggatcagcctgtaagttttctaactcagctaataaggtaatggtttct
aataatttatcaatattgtagccggtaacagaactaataggaattacaggaacttgtcca
ccaagtgtctcagacatcacattgtgttttaataagtcctgctcaataatatttgtattt

gtgaagtctttatctatcttagatattgctaccacaaaaggaacatcagctttttgaata
tgatgaatagcctctacggtttgaggtttaataccatcgtctgcagctataataataata
gctacatctgttaatttagctcctctagatctcatacttgtaaaagcttcgtgccctgga
gtatctaaaaagataatttttgtttattagctttattgatatattcaacttcataagct
gcaatagtttgtgtaattcctccaatttctttattagcattgttagaattatgaatgtaa
tctaataaggttgttttaccatgatctacatgccccataactgtaacaatcggaggtctt
ttaacatagttaccaactttatggagagagctagaagtatctaaattagcaggatacaag
ttattatttctttgtgtttgagtcaactcctataccaaaattatttgctactgatgct
attattgaagcatctattgtttcattaatagtaactggtattcctttaaggaataaatat
tttatcacatctgtttcttgaacacaaattagtttagataactcttgaatagtaagagga
ttggtaatactaatagattctggtggagaattagataaactgctggtttggtcttggctt
tgagttaatgatatgtttttttgtcttatagacttttagtattagtaaatttttttata
acttgagcttttggtttaggtggacgcattagagaaatagccaagtctcctgcagtctgc
ggaatcatagaattagaacctctaaagttctct---tcctcatcatcaatatgtattttg
gtctttattttt-tttcgttgtctgttctta--ttttttttattatctgctagatcatga
accttattaaaattttttgttttttttatctagtttgggtggagaatttaattctaaatgc
ggttcactactagctgtatgcgtatcagattgatcaagatctaagggtaaaacactgtct
ttatgggctgattctaatctaatttatatataatttggggatttttaaatctataatt
ttttcagaagc---tatatttggactggatgcaggggatctattttcgaaattttgatta
tttaaaaacatgaatattgattttatgatctaatagtaaattttaaatttcaatacatag
ctcaaagtaaagaaaaatcagatatct------ataatactaatat---agaaaattaat
ctttgttgtattagatttaatataacactaaagtcttttgttagttatacaagtttaaaa
ctttt-tgtgatgataaacaacaacttctgctttattgagatacaatcaatttatttaga
tataactgtttttcaa-aggtttttaaa----ttgtctaatattgtgttcgttatgaatt
tttattaaagtttgaaaa-ctataaaatagagtcataatcattaataatctccacgccac
ttatgccacgatctcaaaaaaatgataatttattgacaaaacgtttacagtattagctg
atattgtattaaaaatacttcccacaagcaaagaagaaaaagaagctttttcttactaca
gagacggaatgtctgcacaatctgaaggcgagtatgcagaagctttagaaaactattacg
aagctttaaaactagaagaagatccatatgatagaagttatattttatataatataggtc
ttatttatgctagtaatggtgaatatgtaaaagctttagaatactatcatcaaggattag
aactaaactttaagttacctcaagcactcaacaatatagccgtaatataccattatcaag
gagttcaagcggtcgaagataaagatacagaattatctaaattaatgtttgacaaggccg
ctcagtattggcaacaagcgattaagttagcacctgacaattatattgaagctcagaatt
ggttaaaaacaacaggaaggatgagaaatatacaaggatattaaaatatttaggatataa
t---tatattaattaatgaagcag----aattagagatgcttttaccctataaagcattt
gtagtat--------ataatatactaattac------tgtataaaaatcaacataataaa

cgaaattaaatctattgactatatttatgaatacaaataaacataatggttagtaaaaca
aaaagcatcggatctgtcactcaaatcattggaccagttttagacatagcatttcctaac
ggacaacttccaaaagtattcaatgccctcaaagtagaaagctcagagggaactattact
tgtgaagttcaacaacttttaggtgataacaaagtaagagctgtatctatgagttcaact
gaaggactacaaagaggagttcaagttcttgatacaggatctcctatatccgttccagta
ggtgcagatactcttgggcgcatttttaacgttttaggtgaacctgtagacaatttagga
ccagtaaattctgaaagtactttacctattcatagaccagcacctgcttttactaagtta
gaaacaaagccaaatattttgaaacaggtattaaggtagtcgatttattagctccttat
agaagaggaggcaaaattggtttatttggggtgccggagtaggtaagacagtattaatt
atggaactgattaataacatcgctaaagctcatggcggtgtatctgtatttggaggtgtt
ggcgaaagaactagagagggtaatgacctctatatggaaatgaaagagtctaaagtaatt
gatgcagacaatttgaaagagtctaaagtcgcactcgtatatggtcaaatgaatgaacct
ccaggggcccgtatgcgtgttggcttgactgcattaacaatggcagaatactttagagat
attaataaacaagacgttctattgtttattgataatatttttcggtttgtccaagcagga
tcagaagtatctgctctactaggacgtatgccatctgctgttggttaccaaccaactttg
gcaaccgagatgggtgcattacaagaaagaattacttcaacaacagaaggttcaattacg
tctattcaagctgtttatgtaccagctgacgatttaacagatccagctcctgcaaccaca
tttgcacatttagatgcaacaacagtgctttcacggaatttagcagcaaaaggaatctat
cccgcagttgatccactagattcaacttcaacaatgctacagcctggaattgttggaact
gagcattattctacagctcaagaggtaaaatccactcttcaaagatataaagagttacaa
gatattatcgccattctaggtcttgacgaactctctgaagaagataggcaaactgtatcc
agagctagaaaaattgaaagatttttatctcagcctttcttcgttgctgaagtatttact
ggctcacctggaaaatatgtatctttagaggatgcaattaaaggttttcagatgatttta
aaaggtaaattagatgacttacctgaacaagcattttatctagtaggtgatattgacgaa
gcgatacagaaagcggatagcatgaaagattaatataattaacaatgactttaaatataa
gaattattgcacctgatcggactgtttgggatgcagaagcacaagaaattatttaccaa
gtagcactggacagcttggtattttaacaggacatgcgcctctacttacagctttagata
taggagttatgagagttagagtagataaagaatggatgccgattgttttgatgggtggat
ttgcagaaatagagaacaatcagttaactattcttgtaaatggtgcagaagaagctactc
aaattgacttatcagaagcagaaaaaaatttagaaactgcaactcaattattaaatgatg
cctcttctaataaagagaaaatagaggcaacacaaaaaattagaaaagctagggctagag
tacaagctgcaacagcagctattgtataataacatttt------gctaaaaatataaaac
cttagtaattattactaag-------gttttatgttttatttatgtatttatatacaact
tgagtaagcttagcttaaaccagaacaaatataatcaaaatataaacccatttctttgcc
agcatctggtccaactaaactaatagtaacttctttcatagctaaaatcgcttgaatggt
tgcaccaataggaacacctagagaattataggtttcttttagaccatttaatactctttc

ttccaaaatagatggatctccagctaacattccataagtagcatagcgtaagtaataatc
taaatctcgaatacatgcagcataacgtcgggttgtatacatattacctcctggacgagt
aatatccgagtaaagtaaagattttgctacagattctttaataattgttgctgcattagc
tgcaatagtagcggctgctcttactctaagctcacctgtctgaaaatagcctcttaattt
ttcaacagagctatcatctaaatatttaccttgaacgtccgctgcattaataacagaagt
aattgcgtcttgcataacttgtaaaaattccttaattttaaatatctaatactaaagggg
tatataacaaaataactgaactttttataaataccagtactactgcatggcacccaaagt
ataatcaaagtaaaaaccagcttctgctgaatcttcaccagcaagcagtgagcaagctac
acttttcatacatttcacgccttcagcgactcccgaaattggtgttcctaaagaattata
catttctttaacaccgactagtccaatttcttcaataggcgttacgtctccagcaactat
tccgtaagttactaggcgaagataataatcaaggtctcgtaaacaggtagctgtcatctc
ttctccataagcgtttccaccaggagaaactacatcaggtctttttttgaaatagctgttg
accgccttgttttacaatacgctcacgattatctgttaaaatttgagctattcttagacg
acgttgtcctgataagacaaaactttttaattctgtctaattcaccaggacttaaatatct
tgcttctgcatctgcatttacaattgactttgtaacaatactcatggataaaactcctgt
aatgcttt--taaaaaagcttaatactgattatttatgatataactcagaaagtagtacg
ccttttaaaaaaattcatagctatagcacgttggtgatatatgaagtaggacaaatactt
ggataggtactaaaacaagaaagttacttatcgtaccttattttattgattgccgagtac
tttttaaaactaggtactacaattgatatatcttgcttagtaaggcgattatataatgt
ttctgtgttagggaaattagctgctggcaaagttggaaatcttctatacggaacagtata
tttaccatatactgaattatactcagtactttcaactaaagtactaataaatgcagacaa
tccttttgaagctaaaatttggttaaagaatctaatttctgcctgattattaggagctcg
tcctaagatatgcttcgtacctaattctataacttttgtatttggatatggctgataaaa
ctctttaccatataattcagataatgctaatttttctactaattctttaacattaatttc
cctgctcaaaaaggatgcttcaatatctaaaaactctcctccaatactaaaagaattaag
atctctttcaaaaatctggcgatatgcggctcttaaagcttgctctaatacttctttact
actattgtcgtttacttcaaatataatagattgatctcttaaagagctaactccttgaga
aatacgagactggatatcattgccgcttcttatttcttttacagcgcctaattcaacaaa
tctagaagacttgctagtaatgactttcttaaatctttggtcaatgatacctggccttaa
gcttcttagagcgataccagctggtgtactatatctttcataaggaacagtactatcacc
aaaagtttctatatactctgagctatcaactattgcatctatcacttgatagtagccctg
cttatatgcaatatcaaaatatttattaatttcttggcggccatatgtaggacgacccaa
taaacggttatggatatactcaatagctttacagatatataatggctcccagtaaagtga
tctaaatatacttgacttcgctaattgtctaacaaattcacgcactgtaatttgaccatc
ttttaactgactttctattggcttaagaagtaatttttcttcttgatatacttctctgcc
aaaaactcttaaataagcaacttttgtaacggcatcgactgaattttcaaaattttcaga

agagtcagcttttacaagaggattggataatttaaaaatttttggacccaaagatcctgc
agacttaggtcttacctgcggattactgatctggttatagattcctgggccacatctaac
cagaattctcctagtatctttaccaaaaagagcttgtcgtttcctaggatccttattttc
tttaggaaagattgctccaaattgaatcgacagtggatcattgcctattccatatggatg
ctgatcaggtaatgcttgcttgtaatcactaaatagggttataaactgaggcaccttacg
aaaaggagcactataattaagaagatctatttgaggtccccaattacgacactcttgagg
ctcttcacctaaatttcggaaataaggtacagtttcttctccaaaataatctgtatattc
agcagaatttaaaattgcatttactagcccacttaaaccagtagctgatagaatagcaaa
atatttttggaattcctctaaagagctgggccctctaccaaggaaatgtctaaatgctaa
ttctaatgctcggctatttacaaaaggctcataaaattgctttctatagatacttgaagt
tcctaaagaacggataaattccttaatggagatttggccatttttttacttgcgactctaa
gttagataaagataaatcatatgctttagcaatatctctctcaaagatttgtctgtagca
cgctttaactacagtattttttttcatctgcagataaactaggcttcataacaaaacgagg
cgtagaaactcctgcttgtgcataaatctgaggaaggcgcaaaccttgtaaatccccaga
tgttcttttccttaatttatcagtcaaagatggagcttcaaattcagaaattacaacatt
aaaatactctttgaccagatcttgacctttaatatcttcttcaaagattaaaagagctgt
tcgtctcatctctctgagggcaacaattgctgccgcgcttgagcaagcattatcaattaa
ttctcttaatcctctaatattaaccgataaaatattgggatcacctgatacaattgcata
tgttaaataccgtaaaaaccagtctaaatctcgcagagattttctcattcttgtagtccc
atatctcaagacatttatgggtttgaatcctggaggagtagctcctccagcattaaataa
agaacgaaaactctgcccaaaatctccttgaatattgccagatagctcattaattttgtc
ttgagaactttgatctccagcaataataacagctgcttgtggacgttctaaataagaaat
tgcagacccgcctacaaatattttgtctgcagctcgagctactagaatattggcattttt
agttaaaatatctgcaacttctaatctttttttgtccagaatttagaaaagaaactagttg
atttaattctcctagctgtaaaaatcggtcttgctgctctgcctgagtaatagttaaaat
tgaggcagtccgataaagctgggggcgtgctaatgggcttccgccacttgctttgatact
cattactttatttatctccttaacttatattacttgctagctaattcaaatatcacttaa
tatttctaactatttaaagatactacgcaagcacatttgttttctatattattaggtact
agtagttatagtacatagagtcacacaactaacttctataaatacaaattttgtaatact
tctattaacatttatcaaaagtaaatataattgataactcttttatattaaaatgcaatt
attaagagagtgatgctaaataaagtataattagtaagttttatagatagtaaaatt-ca
agatttattctacaaacattaatca------------cttgactgtttgacaattatacg
attgttcttgcttttaattggtttagactagtttcacatttttttctaacgtaaacaatat
taataagcaatttgtatgaatttaaaaccacagaataacctattaactaacggaaatata
aaccaacaagatgtacctgtaaatgatatgccaatgtcaattactgagcatttagaggaa
ttaagacaaaggactttatttgtatttttattctttttgcttgctgcaactttgagtttc

acacagatcaaaataattgttgccatattgcaagctccagctattggtattaaattttta
caattagcgccaggagaatattttttttcttctattaaggttgcaatatattcaggaatt
gttgcaacaagtccattcggggtataccaagttatcttgtatatactacctggactaact
ggcagagagcgaaaaattattttgcctatcctaattagttcagtcttacttttataata
ggcggtatatttgcttactttattttagcaccagctgctttaacattttgatcagctat
ggatctgacattgtcgaaccactgtggtcttttgaacaatacttcgactttattttacta
cttttatttagcacaggattagctttcgaaattcctatcatacagttactactaggtgtt
tcaggaacactatcttccaatcagatgattcgagcttggaggtatattattattatatcg
acaattgctggagccattttaactccctcaactgatccagttacacaaatcatcatgtct
tctgctgtcttagtgctttattttagtggaattgtcatattgttacttttaaaaaagtaa
atgtagt--------aaactttcatttcatattatacttttcttgtg-----ctaagcaa
tggagttt--atatagcttttagttcttaacttagatgaatattgaagta--------tt
aagctcttttaaatatttaatttagtcttataatttaaccaatatg--------------

--tatatattgg
c---ttttttcatcaattatttgcactttacatttctgtacta----ttctatgatgctt
gggactctttatgtaatctgtttactgaat----gacgttgaagaaaattcagcataaat
tttgttaatttataccaatataaaaagtattttattgtgagttttatttatataaaggct
tattcattatatatttataacagtaagtctaattaatagtttttttatgcttaattaaagt
aataactcttaatccttttggcagaaaagtctgtcttaatattttatc--aaaaaaacag
aaaaaatgaagctaaatagtctaattaacttaattcaaaagtctatatgttcttgtacat
ttttattaattattttaaatattatttgtgtcgctcctaattctagtaatgcatttccaa
tttatgcacaacaagcttacgaaagtccaagagaagcaactggtcgaatagtatgtgcca
attgtcatcttgctcaaaagccagtagaaatagaagcacctcaatcggtattacctaata
ctgtttttgaaactattgtaaaaattccatatgacagtagtgctaaacagattttaggta
atggaagtaaaggagggttaaatgtcggagctgttgtcatattgcctgaaggatttaagc
tggctcctgctaatagattatctacagagttaaaggaaaaactaaaaatctatatattc
aaccatatagcgctaaacaagataacattttagtaattggacctattcccggcgacaaga
atagagaaatagtttttccaatactctctcctgatcctgcaaaagataaaaaagctcatt
tttttaagtacccaatatatgttggtggaaatagaggtagggggcaactttatcctacag
gagataaaagcaataacaatgttgttacagctttaagtagtggcaaagttaataagattg

aggttcttgagaaaggggggatttgtagttcatgtgactaatagtaacaatatagaatcaa
ctcaaaagatttccccaggtcttgaacttaaggttaaagaaggtgatacagttcaattag
atcaagctttaaatagtgacccgaatgttggtggtttcggccaaaatgaaacagaaatag
ttctacaaagtccaaacagaattaagggcatgattgttttctttttttgctagtgttttag
ctcaaattttctttgtattaaagaaaaaacaattcgaaaaagttcaggcagcagaaatga
atttctaaagcaagataaaactggtacttactataattaaagtg-aaaacactttattca
aagaatgtaccagttttcatgctatataaccgttgcttttgaagacattataatgtcttt
aacagagtctacaatttgactaggctgaattacagtagcctgttctaagctaccattata
tggtgttggtatatcctgcgaagatagtcttactacagggacatctaattcatcaaaaag
gtgttcattgatttgtgcaattaactctgctccaattccagcagttttcatgcattcttc
tacaattaaaactttgtgagttttcttgactgacacagatatagattctatatctagagg
ttttaaagatataaggtctatcacttctggatcgtaaccttcttttagcaaagctggtaa
tgcttgaatcacatgatgcctcattcgagagtaagttaaaatagtaatatcttttcctgt
acgcacaaattcagctttattaagaggtaatagatattcttcttcaggaatttcttcttg
taaattgtaaagcaaaacatgttcaaagaagacaactggattattatctcgaattgcaga
tttcaataatccttttgcattatatggagtagagcaagcaactatttttaaacctggaat
agcttgaaaataagcttctaatctctgagaatgttctgcgcctaattgtctaccaactcc
tcccggtcccctaatgactaaaggtaatgtaaagtttcctccggaggtatagcgcaacat
gcctgcattattagaaatttgattaaatgctaacaataaaaagctcatattcataccttc
aacaattggtcttaaccctgttatagctgcaccaatcgccataccagtaaaactattctc
tgctattggtgtatctaaaactcttaaatccccatacttactatgcaaatctttagttac
cttataagatccgccgtagtgacctacatcttcgcctattacacatacagtcgaatcttt
tgccatttcctcatctgttgccgctcttaatgcgtcaaacataaagatttactcatttt
tgtatttaatttttataatttcactactaatctaaattaaaagttaagatattaaatttt
c-tcacgtatctgaaaaaagatattgtttaagttctgagatattaggctctggactggaa
atagcaaattgcacagattgttctaaatctttttaacagcagtttgaatctcgttgagt
tcacttatgtcggcgatcttattatctaaaatatattttttaagtttttttgattggatct
cttgcaatccaagcttctttttcttgtcttgatcttagttcatcaggatctgcaagagag
tgaccacggaatctatatgttaaagcttctattaatgttgggccttcaccatgtctggct
ctctgaactgcttgaatcgctgcttgccttacagctagtacatccattccatcaacttcc
ataccaggaagaccaaaagcttctgccttttttatgtatttccggcatggaagaagatcgg
tgatgtgccatacctatagcccactgattattttcaacaataaatataataggtaacttc
caaagaactgccatatttaaacattcgaaaaattgaccattattagtagtaccatcacca
aaaaagcaagctgtaactctcaaatcttcagtttcttttagtacttgctgacgataaata
ctttggaaagctgcacctgttgcgacaggtataccttctgcaataaaagcaaacccacct
aaaaaattatgaggagcagaaaaaatatgcattgaacctcctctgcctttgctacaacca

gtttcttttccaaatagctcggccattacatttttcgagggaacacctttacttaaagcg
tgtacatgatctcgataagtgctacatacataatctgtagaattgagaagcttaattaca
cctgtagacacagcttcttgaccattataaagatgcacaaaaccaaacatctttcctttg
taatacatctgagcacatatgtcttcaaaatttctgcctaacaacatatcttcatacaag
actaataaagtcttttt------attaagaacagttgaattatagttagttagcggcaat
tgaactttcttaggataactcataatttataaaggaacctct----------tattatac
ataggaattttgaagtaaaagttatcaatagaaaacacacagataaaaatttataaatta
ttatatgc-ttttactaaagacttcaatagttatttgcaactagttcagtatatcaaatt
aattttgcttatactacagatgtctaaagcttagatgaaaagaaa-tagttaaa------
------------------------ataaataaaaataaacagaattattagtgagaaaat
gattattattaatagataattacttaatatttctaaatattttcaaattaatggctatat
catcatctttattttaccctgtcgagaaagagttatacagtctagaaacaaatttgcaag
ctgtggctgggacacgtcatccaattttatacgcagcggcgaaacatctgttcgaagctg
gaggaaaacgagttagaccagctctcgttcttttagtggctaaagcaacatctgagaaac
aagatattaatactggacagagaaggttagcagaaattactgaaattatacatactgcaa
gtttagtacatgacgatattattgacgaatgtacaacgcggagaggagtcaagactgtgc
acaatttgtttaatactaaaattgctgtattagctggagatttttttatttgcacaatctt
cttggtatttagctaatattgaaaatttagcagtagtgaaagctatttctaaagtaatta
cagattttgcagaaggcgaaattaggcaaggattagttcactttgaccctagtatttcaa
tagatgcttacattgaaaagtcattttataagactgcttcacttattgctgctagctgtc
gaggagcagctatgctgaatgattctaatactcaagttaatcatgatctatatctttacg
gtaaacatatgggcttagcatttcaaattatggacgatgttttagatataattggttcta
ctaaaagcttagggaaacctgctggcgcagatctagccaatggtaatttaacatctccta
ttctattttcacttactcaagaaccagatttaaacaatcttattcaaagagaattttgta
ataggacagatgtagcctcagcgttatttcttataaaaaaaagcggaggaattacaaagg
ctcaagacttggccaaagaacaggtacaggcggcactttgctgtctccagtttttaccaa
aatctgcacctgtatctagcttaaaagaattgacacatttcataatcacaagattgtcat
agaacacttgctaaaattaaaatgctattaactaatttttttactatctctacaatagtct
gaaaagtttggatatcattacttgctaactgtgctagcattttacgatttaatgcaatat
tctctttttttaacgcactaataaaagtactgtacttcatgccttgactatgagctgcag
catttattctagttatccaaagacgacgaaaatctctcttttttcctttttcgaccaacat
aagaatagcgaagagatttgagaacttgctgtttagctgttcggaataaacaattatgtg
cacctttaaagcctttagccagcttaaatatttttgcccgtcttttttttgcaacattac
ctcttttgactctactcataaaatattac-ttacaattaaattattaacagcttcctaga
ttcaaatgcttatcg----attataaataaggtaaatttacagcgatattcttaatatct
cgtgtgtcaactaagcaggtacaagaaagattcctttttttgcttcgatgatttttttttgt

aataaatgacttttagaagctttatgtcgcagaaattttcctgatgaagacactttgaat
ctttttgctatcgcttttgatgtttttaacttaggcatattgtaaacttttaatgattaa
caaaagtatgaaaagtctgaatttatcaaacttagacttttcaatacttaggataatctt
aacacaaattattttacttactatagaggttaacttaaacaatttatatatataatagca
aataattaatccgtagctactaaagttgatttatatttagctttaaatttttgtatagta
ttagcaattagcattgttattattttaagggtacttgagtttaattcttcgaacattttc
atatttaatgtgaatgatatattcgcttctgacacaatattttgcatttgatcatccgat
aaaggaatcatatccaatgcagctctatacttatctttaaacactttgtcatcttgtatt
tggtcaaagtcataaaacctagtacctctttcatctgataaattcatcgcacctctagcg
atttctttaagatttggcctccagataaatcacctaagtaacgcgtataagcatgagca
actaataattccggctgtttacagcctatactatgaattctatcaacatagattattgtt
gcaggagatggtttaataagatttaaccaatctgctccataataataacttaaatcttct
gacagacttattttttctattgagttctgtaaaatatataggtttaatagctgggtgatct
ttattagaaaacagttcttcttctatagcacaataaacgaaataaagattagcaaccaat
ttacgatatgatttcttgtctacgacacctcccaaaaaagatttcacaaaactaacattt
tctgccatactatgagacttagtagtgccttctcttaattcatttgctaaagtattaacc
ataatatcaatcttccatttta-aaagataacaaattaaac---ataaacagttatctat
atattta------agttgtactaattaagctgctgcaaaataatttttttgattttattgg
atctggattcattgttttatcacctggcttccaattagctggacaaacttcatctggatg
agattgtacatattgaattgcttgcagaactctcaaagtttcttctacactcctaccaaa
ctcgagattgttaattgtagaatactgaataattcctttaggatctataataaatagtcc
tcttagcgctacaccatcactgttcaaaacattataagcagcactaatctctttttttaa
gtctgatactaatggataggcaagatctcctaatccaccggattctctgtctgtctgtaa
ccaagcaagatgagaatactcgctatcaaccgaaacacctagaacttctgtgttaagctc
agagaaagcatcatatttatcactaaatgctgtaatttctgtagggcaaacaaatgtaaa
atctaaagggtaaaagaataagataatatacttatttttaaatctgataattttaacgt
tttaaattcttggtcataaacagctgtagctgaaaaatcaggagctagctgacctactcg
aagacaatttggtccagaaatcattaatttttcccagaagaattgatatagttatgttat
ttaataaataattatataacaaaatttagtatatatgaaaatgctatcagtcttttaaaa
attac--------tcaatggcttccaaaatactatttttatagcggggaatggatttgaa
ccattgaccttcgggttatgagcccgacgagctaccagactgctctaccccgcggatatt
taatatcatattagtccatctaaatagataaaagcaaatatattatataattcaaaagta
aaagaataagagcacaggagcactaattattaggcttaaaaatgacaaagtatttttttat
tttattgagtagaggaaagacttcatataagccgatgaatcgatctttaagaagaacttc
cgatggcttcacccaaatttgaccgtcataccacccagactcttcatagaataccgtagc
actcattaaacgttttactacgtatgaccatcctaaatataatctgattagaataaatcc

agttattaaactcgaggtaataaattctgagaagacaaattttaaaggcagcttggtaat
tggaaaaattgaaagcaatactggactaactaatagaccattaagtagtagtgtaacagt
gattttcctgttatatgattgactacttaaggttggccagcagaagaaccaggaattttt
taaagaagtatactcattaacaggttgctgctcttttggtacaggacactgagtgttata
taaattcattgaaatttaaaattgttatactagaaattgtttttttatctgctttagat-
-ttaggga--------gttaattgattgcaattataatagttagtatcagaaatacgacg
ataaacaaccaagtaactttgtttaatgtgttttcagtactacgagtattgctaaaaaac
tgattctgagctccagcacttcccagaccttcagatttgggattatgtatcagaatagta
aaaaccaatataatagttgatacataccaaaagaattttaaaatttgttccattgtgtaa
aaattaataaaaaatagcacaaagacaatcgtctaaaaaatattgattgtcttattatat
taagaaagatttattctccctgcacttttaaaagtaaaaagcctattgctaaccctacta
aaactaagactgaacttaatacagcactttctacaatttctccgaccatt-gtgtttttc
cttattattttattatta---tcattttgtaaaaaatttgatacctattaaa-ctaaaat
ccatttcttccccaaaccaccatcgcaatagagaaagtaaacattgccattaaagctgcc
catcctaaacttaaaatatccataatttataatattc--ctgaaaataaggtctcttata
cttaaactataatattcttaatattaaaaatatcattatataataagctaattataacag
agtactacttaattcgtatttttagtgtatttcaagaagattgac-tttttctaaaatac
ttaacaagtattaaattgtaaaaaatttctgcaat----ttttgcattattatacctatt
aatttggaatgtaaatatcaatttaaaatcgttagtaattttgatttttttcaattgtaca
att---aacatatgtaaaaacataaagattcatagcagctaagttga-----tggtctac
tataatttatcaat--aaaaaggaagttagtatttaaattatgcaaacaactataagtaa
tggaaaaacgtctagcaaggaaactctactaacccctaggttctatacgactgattttga
agaaatggctaatatggatatttcagaaaatcaagaagatttctttgctattttagaaga
atttcgagctgattataatagtgagcactttattcgagatgaagaatttaatcaatcttg
gtccaatttagaagataagactaaatctttatttattgaatttctagaaagatcttgcac
tgcagaattttcaggtttttttactatacaaagaattatcacgaaaattaaaagatagtaa
tccaattattgctgaatgtttttttactaatgtctagagatgaagcccggcatgcaggttt
tttgaataaagctataggagatttcaatttatctttagatttaggctttctaacaaagag
tagaaaatatactttcttctctcctaagtttatttttttatgcaacttatctttcagaaaa
aataggatactggagatatataactatttaccgtcatcttgagcagcaccctgagcatcg
catctatccgatttttagattctttgaaaattggtgccaagatgaaaatcgtcatggaga
ctttttttgccgcattactcaaatcacaaccacatttttttaaacgactggaaagctaaaat
gtggtgcagatttttttctattaagtgtatttgcaacaatgtatttaaatgattttcagag
aatggatttttataatgcaataggtctagattctagacaatatgatatgcaagtaatacg
aaaaactaatgaaagtgctgctagagttttccctgtcgctttagatgtagataacccaaa
attcttcaaatatttagatacttgcgcatgtgacaatagggctctaatagatatcgataa

acaaaatttcccattgttaattaaagctattataaaaatgcctctctattactccctaat
tattagtttgctaaaaatatatttaattaaaccgatcgattcaaagaccgtctggaatac
agttcgataatcaaatcacataaagatatatttcataatacacactatctcattattga
tataaatcaaaatgaaaagctcacttct-----gtcaggaaggagcttttattttattca
caaataaacaatatggttctatagatt-tagaactaagaacttactttttcttttgcttc
atacataacttctaaggtgattagattttagaacaatcgcgtgcaaatttttctgtgtt
tctttttactttacctctaacaaaacctggaattttacttaattctttctgtgcttcttg
tgaccagttaatactatcaacagcagagatccctacgcttaatgcttccgttgtatcatg
accaccaaatatttctaataagtgatcttccatacccaatgtgaatgagttatagactaa
atcagcaatttgatttgtaccttcatagccaaggaatggacgataactcaaaggaaaatt
ttgtatatgtactggtgaagaaataacaccacaaggtatatttaaacgtttaccaatatg
tctttccatctgtgttccgaaaattgctgcaggctctgttttagcaattaaatctccaat
taaaccatggtcatctgaaataattacttcatcgcagaattcttgaacttgttctttaaa
ccaatcttcatcatatttgcaataagtgccacaccaagtaacatgtatacccatttcctg
gtgtaaaattcgagttattgctgcagcatgagttgcatctccgaatacaatagcttttt
accagttaaattttggcagtcaattgatctagaaaaccaggcagattgcgagataaatct
agtttgttcatctatataattttcgtagtctacaatggctcctaatgtagttaataactg
ttctattgatcggatacatgcagctgtttgtacaatacccatagggtagtatcaacata
aggcatattaaattccttctctaaataacgagctgtcattagtccggtctctctatatgg
gacaaaattaaaccatgcagatggtaaatttttagatcctgaaccgacgcattttcggg
aataatttgattaattttaatatctaaatcttgaaataatctttttaattctgaaatatc
atgctgattatgaaatcctaaacttacagctccgataatatttacagatggtttttctgt
cttttgagttaaaacctgattattagatttagctttttccatgtaaaaagtaacaatctg
ttccagagttctatcaccagcttgaagttcatttaccctataatgattaacatcagctaa
taaaacatcagcttctgtttctatagaggctctgctaacaaaatttgtaaatcttcttg
caatatactagaagtacaagttggtgttaaaataactaaatctgggtttcttctctatc
ttttctagtaatgttttcaacaactttttcttgtgaaccacgtgctagtacatgtctatc
tacaacacttgctgtaacaggcgtaaaatctctgtctctttcaagcattgaacgcataac
attaaaatagtcatccccgagaggcgcatgcataattgcatgaactttttgaaagaact
agcaattctaagagttccaatatgagcagggcctgcatacatccaataagctaatttcat
attaaattttcctatgatttatttagacatagtaatacttttaaagccaaataataaata
aaattcgaacatctaatttaactccttaatgatttttcatg-tttttagatagagcgta
acattatatcaagtcaaaatt-------------agagaaaaatgactaatgaataaagc
ttgaaagtaaagatttcttgcagttatagtttttctttaagtaaacatgtaaaaattgta
ctatatataaatgtactatatataaattttttttactgaatattggtaatgccagataca
attaacttaaatatgccttccccaacgtttggtggaagcactggcggttggttaagagcc

gcagaagtagaagaaaagtatgcaataacatggacaggcaaaaatgaaagtaagtttgaa
atgccgaccggtggtacagcaactatgagaaatggagagaacttactttatttagctaaa
aaagagcagtgtttagctttaggtactcaattaaaaagtaaatttaaaatatctgactat
aaaatttacagagtatttccaaacggagaagtacaatacttacatccgaaagatggtgtt
tttcctgaaaaagttaatgctggaagagctagcgttaacagcgttgatcattctattggg
cgaaatgttaatcctgtagatgtcaaatttacaaataaggcaacttacgattagttgata
aaaaaacctagacataaaataacttttatatagtctaggtttttgataaagtatactaag
agtatggaaaggtggccgagtggttgaaggcgtctgatttgaaatcagttgaactt--ta
tagttccgtgggttcgaatcccaccctttcctttataagact------------ataact
taattattactata----aattatacgctaatagtattcagtttagacttaatgaaatct
acaacttgcgataaattagaaatttttcggcatcttcatctggtatttctataccaaat
ttttcctctattgccattactaactcaactgtatcgagagaatctgctccaagatcactt
gaaaaatttgcttccctggtcactatttttttttcaattcctaactgttctgctacaata
tcttgaacttttttcaaaaatttcattatcttgcataatatcttgtttcctataaggtgtt
ttcaaaaattttctggctcatattattgtctaactctaattaattagtaattttgagttg
aggtccaaatagttctacctatatgatatactattattctggataaatacgaagtttgcg
gtatggctcttctccaaaactacgagctctcaactggtagttatcaactaaattatgctg
cattttgcgaatataggctgaacgaggtatcaactgaactgtcgatttttcgttcaagat
aatagtttcaatagccagtttagcttcttgaagggcttgaatctcatcaaatttttttatt
ttcacaaagttctacccaattaagatcagaagaagtatgaatatttaatatttttctcag
tgcacgagtaatttgtgggactgtactattctgaattgtatatataataatttgttttga
ttttgcaatttgtcttaattttgtattttgctttacttgattacgcaatgctaaaatagc
atctgacttttctatctctttagttaaaataattggtaaatctaaagaagaaataactga
cgcaatatgctgccaacttaaagaataagcatataaatactgattcggcatttctaaggg
caaagatgggttattaatatttataggagcattaatagtagttgacaataaagatgtatc
acgattttgtaatttattcaaatcggatggcttatttattaattctctatactgcaaaac
agcatgtgacggttgtacactaattggtttacggaaactagaaatatttgacggcaaaac
ttctaaagactgtgacggatagcatttaattaaaatgcggccattagcttgaatttgtct
tttttgtacaaaaggttgatggccctgtaaaatttgatctatagtttctttcactttatt
atggactatccaaacagtgcgttcatggatttcaattgcaatttgaaaagcgggcgcggc
ttttctttctaatatgcttttctgagtacctcttctttttgcttcatcatcacctaatgt
gacatattgaatacctccaattaagtctgacagtgtaggattttttaattaaactttctaa
atgatttccatgtgctgtacctactaattgcactcctctttctgcgatagttcgagcagc
caatgcttctagctctgtaccaatttcatctataataatgacttcaggcatatgattttc
taccgcttctatcattacctgatgctgtaaatcaggccttgcaacttgcattctccgagc
tctacctatagctggatgtggaatgtcaccatcgccagctatttcattagaagtatctat

aataacaactctttttttccatttcatctgctaagacacgagcaatttctcgaacagctgt
tgttttaccaactccaggttttcctaaaagtaaaattgaatcaccttgttctagtaaatc
cctgataatactaatggtgccaaaaactgctcgtccaacacgacaagttaaaccaataat
acttccttctctattacgcatggaactaatacgatgcagggtcttttctataccagctcg
attatctccgctaaaattaccaactttttttacacaataatctaagtcttgccaactaat
agatctttgagataagtattctggcctatctggaaatcttgcttctggacggcgacctaa
atccatcactacttctattaagtgatttctattagggtgttgtttcaggggctctttgac
aaagttcggtaagatttctagtaatttatctaagtcatctgcaataagcatgattgaata
tataataattaactaatcagatagaaaaattaagattctaaagattacaatacatattaa
agaatattgtgctcgtaatcaattttatacttaaattcaataagaaatta-cagatggta
aacaatagattaaactatgtacagtaaataaattggtcaaaattaagagtcatgttaata
tgtttattggacagaaagttcgtattaagtatagtaagcaaaaagttgctaatgacatag
cagataaagtcggtaatcgcggaattattagaggcataaaatttatcaacagtcaatgtg
ttactattattgttgaatttgaaaatcacactagactttggatgtttaaggaggagctaa
tctatttaaatgaat------ttaaacaataagataagattaattt----tattatatat
aaatttttacgagtcgttcaaattatacattttcaaggagtattgttaattgtgcaaatt
actattaaaaaattacaagacttactttcatcagtacaaagaaagaagatacagacattc
aaactgaaacaggataattttgaacttctattaaataaaacttacaaaaaaactaatcca
gaaatattaccttcacagaact---ctgtattacaacacaatcaagctaacataattaag
tcaacacctaataacaatggtccttctgttagtaataataacgttacagaatatgccact
atcgtctctccgatggtggggacattctatcactcacctgctcctggtgaaaaaatttt
gtgcaagttggggatgaagtcaaattcaatcaaacagtttgtattattgaagcaatgaag
ttgatgaatgaaattgaagctgaaattgaaggaaaaaattatagaaattctagttaaagat
ggcgatatcgtagattgtggtcaagccttaatgaaagttgaaacataatgccaatatttc
catttacatattgttaacagaaattatttagatagaaaaattagcttataatataatta
tgaaaacttatttcgtaaaggcac-aaaaagttagtatataaaattatttactttatctg
ccaaacatagaaataagcgttttgattccttgtctcaagagaggagaatctcaatgacaa
ttagctcaaaagagcaagagacaaagaaggtaaaaatctcggttgataaaaatcccgtag
atacttctttcgaaaaatgggcccaaccaggtcatttttctcgtacactagcaaaaggac
caaaaactactacttggatttggaatcttcatgctgatgctcacgacttcgatagtcaaa
ccagttctttagaagaagtttcccgtaaaattttcagcgcccattttggacagctttctg
taatatttttatggcttagtggaatgtattttcacggagcacgtttctctaactatgttg
cttggttaagtaatccaacaggtatcaaaccgagcgcacaagttgtttggcctatagttg
gccaagagatttaaatggagatgtaggtggtggctttcaaggcgtgcaagttacatcag
gatggttccagctttggagagcatcaggaattactacagaatttcaactttactgtactg
ctatcggtggattaggtatggctgctttaatgttgtttgcaggatggttccattaccata

aagcagctccaaagctagagtggttccaaaatgttgaatcaatgatgaatcaccacttag
ctggtcttttaggcttaggctgcttaggatgggcaggccatcagatccatttatctttac
ctattaataaacttttagactctggtgtatctcctcaagagattccattgccacatgaat
ttttaattaatagagagcttatggctcagctttacccaagtttcagtaaagggttagttc
catttttcactttaaattgggctgaatattctgactttttgactttcaagggaggtctca
accctgttactggcggtctatggttgagtgatactgctcatcatcatctggcattagctg
ttttatttcttgctgcaggccacatgtatagaaccaattggggtattggacatagtatga
aagaaattttagaagctcacaaaggtccttttactggtaacggtcatgaaggtctctatg
agattcttacaacttcttggcacgcgcagcttgctattaatttagctatgatgggatcat
taagtataatagtggcacatcatatgtatgctatgcctccatatccatatattgctactg
attatccaactcaattatcactattcacccaccatatgtggattggaggattttgtattg
ttggcgcaggagcacatgcttctatctttatggtaagagattataatcctgctgaaaatt
ataataacctcttagatagaattattagacatcgcgatgctattgtctctcatttaaatt
gggtatgcatatttcttggattccattcatttggtttatatatccacaatgatactatga
gagcacttggaagatcccaagacatgttttcggatacagctatacaattgcaacctattt
tcgctcaatgggttcaaagtatacatactctagctcctggtaatacagctcctaatgcat
tagcaacagctagttatgcatttggaggagatgtcgtttctgttggaaataaagtcgcaa
tgatgcctatttctctaggaacagctgattttctggttcaccacatacatgcatttacta
tccacgtaactgtacttatcttagtgaaaggattccttttctctagaaactctcgactaa
ttcctgataaggcaaatcttggctttagatttccatgtgatggacctggcagaggcggta
cttgccaagtgtctggctgggatcatgttttccttggagtattttggatgtacaactctc
tatctgtagcaatttttcactttagttggaaaatgcaatcagatgtttggggtagcgtgt
ctccatccggaaatgtttctcatatcactggtggtaattttgcgcaaagtgcaattacga
tcaatggatggttaagagatttcttatgggctcaagcatctcaggttattcaatcatatg
gttccgcactatctgcatatggactaattttcttggcagcacattttgtgtgggcattta
gcttaatgttttttatttagtggcagaggttattggcaagaacttatagagtcaattgtat
gggcacataataaaataaaagttgctcctgcaatacaaccaagagctttaagtattactc
aaggaagagcagtcggtgttgcacactacttattaggtggaattggtacaacttgggcat
tctttttagcaagaattatttcagtaggctaatagtgaaaataggataaaaa-aacaatt
atggcaacaaaatttcctaagtttagccaagctttatcacaagatcctacaactcgaagg
atttggtatggtattgctacggcgcatgactttgaaagtcatgatggaatgacagaagaa
aatttatatcagaagatatttgcttcacactttgggcatttagcaatcatctttttatgg
acatcgggcaacttattccatgtagcttggcaaggtaactttgaacagtgggtattaaat
cccttaaaagttaaaccaattgctcacgcaatttgggatccacactttgggcaaccagct
ttaaaggcttttagtaaaggtggatcagcatatccagtaaacatagcatactccggagta
tatcactggtggtacacaattggaatgagaagcaatcaagacctgtattctggggcttta

ttcttgctcgttttatcagctttactttatttggtgggtggctacatctacaaccaaaa
ttcaagcctggtttatcttggtttaaaaataatgaatcaagattaaatcatcatttgtct
ggattattcggagttagctctttagcttggacaggccacctagtacatgttgctatacca
gaagcaagaggtcaacatgtaggatgggataatttacaacagtattgcctcatccagct
ggtttacaaccatttttagcggtaattggagtgtatatgctcaaagcccagataccgct
caacatatattcggaactaatgaaggggcaggtacagccattttaaccttcttaggtgga
tttcaccctcaaagtcaatctttatggctaactgacatggctcatcaccatttagctatt
gctgtagtcttcattgttgctggacatatgtatagaactaattggggaattggacataat
ttaaaagatattttagatgctcatagaccgcctagtggcagattaggagctggacataaa
ggactatttgataccatcactaattctttacatatacaattaggattagcattagcctca
cttggcgtaattacttctttagtagctcagcatatgtacgctatgcctccatatgctttc
atggctaaagatttaccacacaagcatctttatacactcatcatcaatatattgctgga
tttttaatggtgggagcatttgctcatggtgcaatattctttgttcgagattatgatcca
gaacaaaataagaataatgttttagctcgcatgctagaacataaagaagctatcatttct
catttaagttgggtaactctgtttttagggtttcacaccttaggtctttatgtccataat
gacacaatgattgcttttggtactccagaaaaacaaattttaattgagccagtatttgct
cagtggattcaagcttcttccgggaaagcactttacggttttgatgtgttactatcatct
tctagcaatattgcaacacaagctggtagcaatatttggttaccaggttggctagaggca
attaatagcggaaagaattcattgttcttaacaatcggtcctggtgatttttagttcat
catgctattgcattaggattacatactactaccttaattttagtgaaaggcgctttagat
gcacgaggatctaagctgatgcctgacaaaaaagatttcggttatagttttccttgcgat
ggccctggtagaggcggtacctgtgatatatctgcatgggatgcattttacttagctgta
ttttggatgttaaataccataggatgggtaacattttattggcattggaaacatattact
atttggcaaggcaatgcaactcaatttaatgagtcttctacatatctaatgggatggttt
agagattacttatggttaaattcttctccattaattaatggttacaatccatatggtatg
aacaatttatcagtatgggcatggatgtttctatttggacatttagtgtgggcaacagga
tttatgttcttaatctcttggcgtggttattggcaagagttaatagagactctagcatgg
gcacatgagcgtacacctttagcgaacttaattcgttggaaagataaacctgttgcgtta
tcaattgttcaagccaggctagtaggtttagcacatt-tttctgtaggatatgtattaac
ttatgcagcttttgttttagcctcaacagcaggcaaatttggttaaagtaga-----att
taattaattgcaagaaaaaaaagtcagtcatgata-gtttcgtgactgacttttttttct
tgacaatagttaattattaaagcttaacttcaatatctacaccagaaggtagatttaact
tcattaatgcatctattgtttgtgaagaaggttgatgaatatcaataatccttctatgag
atctggtttcaaaatgttctcttgagtctttatctacgtgaggagaacgtaagacacagt
aaattcgcctttttgtaggtaaagggattggtcctactgcaatagcattagttctagatg
ccgtatcgactattttatcacaagatgtattaagaatactcgaatcgtatgctttaagct

taatcctaatttttgtctgctgaataagtgtcatattgattaaatcttattatttaagaa
ttttagagacaacacctgcgcctacagtacggccaccttctctaatagcaaaacgcatac
cttgttcaattgcaattgcattaattaattcagcagtcatcttaattctatcaccaggca
taaccatttcagcatcagtaccatcatcagcagtaaactgattaatagttccagttacat
cagttgttctaacataaaactgaggtctatagcctggaaaaaatggagtatgtcttccac
cttcttctttagttaaaatatagacttcagcttcaaactgagtgtgaggagtaattgtac
caggtttagctagcaccattcctctttcaatatctttttctgaacacctctcaaaagaa
ttccaatattatcacctgccagaccttcttctagcgtcttttggaacatttctaatccag
taatagttgtggtccgagtctctctcaagccaacaatttcaattgtatcaccaactttta
taattcctctttcgatcctaccagtagcaacagttccacgtcctgtaatagaaaaaacat
cttctacagccattaagaaagttttgtcgacatctctttctggtgttggaatataagtat
caactgcctccataagtgaaaaaatcttatcaacccatttatcttcacccgctttaatag
tcgggttatttgtgactgcttctaaggctaataaagctgagccggcaacaaaaggaatat
catctccaggaaagtcatactggcttaacaactctctaccttctaattcaactaattcta
acaactcttcatcatccacttgatcttctttattcaagaataccactaatgtaggaacac
caacttgttttgctagtaagatatgttcacgagtttgtggcattggaccatctgctgcag
agactactaaaatagctccatccatttgagctgcacccgtaatcatatttttttacatagt
cagcatgacctggacaatccacatgagcatagtgacggttatctgtttcatattctacat
gagcagtattaatagtaatacctctagctttttcttctggcgcagcatcaatttcatcaa
actttttgctgaggtggaacctacagttgataaagtagctgaaattgcagctgttaaag
ttgttttaccatggtcaacatggccaattgttccgatgttaacatgtggtttttttacgtt
caaatttagatcgagccatatttctattctccttatagtaaaagttgctttttggttac
ttttaacgataaatcttataaattgaaagacttattatttaatatagataaggtaaaata
agatatagttaatttaatatctatagtgtgcaaacgctttatttgcttccgccatcctat
gtgtttcttctctttttctaatggaactgcctgtttcattagcggcatccatgatttcgt
tggccaattttattgccatacttttaccagatctttctcgagaaaatctagtaatccatc
ttaatgctaaatttgtacctctatatgctcttacctctattggaacttgataagtagatc
cgcctactcttctggctttcacttccactaaaggagtgatatttcgaatagctttttcta
gaatatttaaagggtctgcttctgttctatctttaactatatctaaagcttgatatataa
ttctttgtgctaaagtttttttaccacttttcaaaatacgaacagttagcatactaacca
gtctacttttatataaaggatctggtgatgcgaacctttttttagctgtattacgacggg
acataatattttaaatttgttattgtgataatttgtt-taatagaagatatttaagattt
aggctttttttgtcccatatttagatcgacttttacaacgatcttttactccagcagcatc
taatgtgccacgaactacatgatatcgtactccaggtaaatctttaattctgccccctct
aattagcactactgaatgctcttgaatattatgaccaacaccaggaatataagctgtaac
ttcaaatcctgatgtcaatctcacacgtgcaacttttcttaaagcggaatttggtttctt

gggagtcgtcgtatatactcttgtacagacacctcttctttgagggcaactttgaagagc
tggagatttttgtttttttatgtatttttcgtctttcagatctaacaagttgttgaattgt
tggcataataaaattaattttaagtctttaatagatattattcgaacttgctgcgcatga
attaattagcctttatttatcttgtatttacgcataaatctttctactcgcccttctgta
tcaataattctttgtgaaccagtaaaaaatgggtgatttcctgaccaaatatccacatgt
agctcaggctttgttgagccaattgtcataattaattggccgtcacaataaacttttgct
tctgggtaccaactaggatgtatgttatcttttgccattgcttctataattgtataattc
tatcaataataaatattaacgcttagaaaattgaggagcttttcttgctttcttcaaacc
atattttttctctctttactcgaggatctcttgttaaatacccttcagatttaagtgc
gtttctattttctggattaattgagcacaatgctcttgcgacacctaaacgaattgcatc
tgcttgtcctgttaatcctcctcctctagcatttacatgtatatcatactgatttaataa
tcctagaacttgtaacggtgcatacgaaactcttaagtaattaggactaaactgaagata
tgactcaccaggtataccgtttataattaaattgcctgaacctgggaccagtcttacttg
tgcaacagaacacttccggcgacctgttccagaatagattgcacgagttttaattaattc
tgtcgacataatattccttattaactaaataaatatacattctttatactatatactctt
gcggcttttgtgcgatatgtggatgaagtggacctgcatagactttaagcttcgtaaata
attttcttcctaatggacctttaggaagcatacctttaaccgatttctcaataattctat
taggcaatcttctctgtagctgctcaaaggtttctacttttagtccaccaggctgcccag
aatgtcttctatataatttctgattagttttatttccgcttacatatatttgagaagaat
taatcacaattacataatctccagtatctagataaggcgtataggaaggcttatttttac
cccttaatatactagaaatatgagtcgatatcctaccaagtgtctgattttagcatcta
taatataccaatttggattagtatttaatgatggtacttgcgttttgttcatgaaaagat
taatacttttagttaacaataagagagtaggtaaatgctgcacatataatgtcgatacaa
ataatattt-tgtaatatta-------------ttgataaagatagctttcatgacagtt
acttaaattgtttgttagcaagaaaacttatagttatcaataagtgataata-ttgtggc
aatataaaataggttactcactttttctttggcaaacttatacctaatttttttgt-
agagcatatataacttcttcagctgatttttggccaaagtttttaatttctagtagttct
tcttgagagtaatctagtaaatctgcaatagaatgaatttgagctcgttttaagcaatta
tatgctcgaacagatagctggagttcttctattaaaacttgactaatttttttatcttct
ttgctacgataattatctgctgatttaaagtctaaatttctcagtgagcagaaaaggtta
gttaaaactgtagcaccttggcttatagcttcttggggagagatactcccatttgtccat
atttgtaaaattaacctgtcttttatgctattagtacctatacgtacttcttctacttta
taattgactttattaactggcataaaaactgagtctacttgcagaaaatctacagataat
tcatctactgctttttctgctaaacgatatccacaactttttttcaattttaaactccatt
tcgaatattgtattattgcaaatagttgcaatatactgcctaggatctactacttctata
tcagaagatagttcaaataatccagctgtaactatagctggcccctggactctaattctg

ccaatttgagattctttattatggcttttaaatactacttcttttagattcaatagtatt
tctagcacatcttctcttaccccaggaattgtagagaactcatggtttactcctgcaatg
cgcacagcgactatagcggtaccttcaagatctgataatattgatcttcttaaagcattg
cctaatgtaatgccttgtccttggtttaatgtttctattacaaaactaccgtactgtcca
cgtgctccatctgttcttgattctacacattcaatttgaaattgagccacctaagcaagc
tcc-ttaaaaataatcaatggttagtaactctgcctgcgataagaaagaatctatacacg
gcgtttcttgggggggacggcagccattatgaggtacaggagtaatatcttttattaaagt
aatctctaatcctgcagcttgtaatgctcgaattgctgtttcgcgacctgctcctggacc
atttactaaaacttctgtttgacgcagaccttgatccattgcctgtttagcagctttttc
agctgcagtttgagctgcaaaaggtgtacctttttagctcccttgaatccactagcacc
agaggatgaccaagataacgtttcaccttttaaattagtaattgtaacaatcgtgttatt
aaatgtagatttaatatgtgtaacaccattaactgcattacgtctatttttttttactcc
agattttttaattgtctagccatcgtgtttcgtctattatgtaattatttaaaatatta
tttccttggagctttttcttacctgctactgtttttttacctcctcttcttgttcttgc
attagttcttgttctttggcctcttaaaggaagaccaagacgatgccttctacctctata
agtactgatctccataagtcttttaatactcatagattcgaaacgtttgaggtcgccttc
aatctgataactagattctaaaatttctcttatactaacaacctgttgatcatttaaatc
ttgacacttaatatcagcatcgacgtttgtttttttcaatatttcttttgagcgagatag
tccaataccataaatatatgttaaagctatttctattcttttattccttggaagatctac
tccagcaattctagccacgttgtgctttctccaataaataatatttac-gtttacaacag
aagattaca-gaattagccttgtctttgcttatgtttaggattagtacaaattaccatga
cttttctgtgacgacgaattattctacatttttcacacattttgcgaacagaaggacgaa
ctttcatattaa----ctctatatactaggaatattactg---------tattaattatt
tacatgtgaaatgtcaataatatgtttgattactttaatttctatagctttcacttcgtc
atactatcatatttttttgatatgacatatgtttgaatttgcttagctgtgtctattgcc
actccaactaaaataagtaaagatgtcgctccaagacctctcaagttttgtatctgagta
accttttctattataaacggaatcagcgctactgtaaataaaaaacgaagctccgagaaat
gttagtctatttaatataacttgtaaataatcaatggtcgcttgacctggacgaatattc
ggaatacttgcacccattttttttaaatttattgcaatgtcttctggattcattactatc
gatgtataaaaataactaaaaaaaagaattaatgcacaataaagaatgaggtatagcgaa
ccgttaggacaaaatagatatagaatttggagtaacgttttattttgaattatttgagtt
aaataagatggaagagccatagatgcagatgcaaaaacaataggcataactccaccttga
tttaatttcaaaggcaagtaactgttcggatccaaaattgaagattttcctagttgtctt
gctgaaataattttaattcttcttgttccttcttgtacgcaaattgtaattataatcatc
aataagaagatagcaatgaacaatccaaacttaagccccgcattgctatagtttgtatca
aaaaatgactgtgtaaaatttttttggtaatcctgatacaatattttgaaaaataagcaaa

gaggctccattcccaattcctttttctgtaattaactcagataaccacataatgatcatg
gagcctgccgttaaagctaaaacagactcacaaacaaatgtaaaattccagttaaataca
tacggctttacccatatagagattgctccagattgcaaagtggcccatccaagagacaaa
tatcgggtaatttgagttatttttggcgtcctagctcaccttcttctttctgtaatttt
tctaaatcaggaataattttagtgagtagctgcattacaattgaagaattaatataagga
acaatacctaaggcaaagatccctattgttgaaaatcctcctccagaaaaaatattcaaa
aaatttactaaagtattttttctacgcttgcataaaaagcatcatggtctatacctgga
acaggtataaaaattcctaagcgcgctaagactaataaaaaaagagtaaatataatgcga
tttcttaagtcactttttggctcataaataaatt-----ttaaaaaagacaccga--gt
tagttaagaaccctattccaatacttcgacaaatttaaaatagggcgctggcaacaagct
atagaagtaatggca-taatatcttatgttgaataaagactttctatagaaactcctcga
tctttagctgcttcgctaaaagttcttaattgagttaggccatttaaaacagctcttgca
ttatttagtgtattattagatcctaactgtttagctagaatattttgtacacctgataat
tctaatactgtccttacagagccaccagcaattacaccagaaccaggtgcagaaggcctt
agtataactttggctgcaccagaaattccattaataggatgaggtatagaattagatttt
gtaagtggcactgtaactaaatgtttttagcatcagttactcctttttaactgctcca
attacatcactagcttttcctactccaacaccaacttggccctgctcattaccaacaatg
agaataactcgaaagcttagtttcttgcctccttttacaactttagtgactcttttaact
tgtacaactctttcttcccaaccactatctttatctttgcctttactctgtttttacga
ttagccattttaagcgttattcctaa----ttaatta-tgactttaaaaacccatacctg
cttccttagcagcttcagctaaggctttaactcttccatgatataatttccctcctctat
caaagacaacattcgtaataccttctttaatagacttgtgagcgagctgttgaccaacta
cgcgagaagtgtcacaatttgagcctattttatcaaattcttgattatttaaattaacag
atgatgttgagactaatgtgattccttttgtatcatcaattatttgtgcatatatatgtt
tattggatctaaatacgcatagacgcggcctacttgaagttccctgaactttttgcgaa
ctcttttatgcttatgaattctagtctgcttagtatttattttcattattatttacttt
tccagcttttccagcttttcttctgacaaattcaccttgatatctgattcccttcccctt
gtaaggttcgggaggtctagtagagcggatagttgaagcaacctgacctacaacttcttt
atctatacctgaaacagtaatattggtattattttcaactgtaatttgaatattttcagg
aggttttattttgactacatggctataaccaacgcttaatattaaatcttgattatctat
ctgtgaacgataacctacaccttgtatttgtagtttcttagtaaacccattagaaacacc
ttcaatcatgttactaataagagttctggataatccatgtaattgattagccatttttgt
ctgtttacttgtcctaacagaaattttatcattaagtatctctaagttaataccagccgg
cagagttctagatagagtacccttaggtcctttaaccgtgatggtatgaccatcaaactg
agtactaagattcgccggcaataaaattattttttttccaatacgagacat-ataccacc
tataaaataattaccaaatatagcataatatttcaccaccaagaccatcatgacgagctt

gtctatctgtcataacacctttagaagtagaaataagagcgattcccaaacccccaagaa
ctctaggtagttctttatggtttgcatatactcttaatccaggcttactaattcttttca
gagcagtaataactggttgacgattttaccgttatatttagagaaatcattaaatgca
tatctatgccttcacccatttgttcaaaatttgaacaaatccttcttctttagtactg
ttgccatattgcatgtcattttcgttgctgggacttgcacaatttgatgtcttgctaagt
ttgcattacgaatacgtgtcagcatatcggcgatcgtatcgttgaccacc-agatcctcc
ttacgataaattgaattatttaatatgtttttaagattccttaaaaggcataccaagctt
tttcagcaaagctagcccttcttgatctgttttggcggtagttacaattgatatatcaga
gccgcgaatttgatcaatattatcgtagtctatttctggaaaaattaactgttcgcgcaa
acctaaattatagttacctttgccatcaaaactttttggactaattcctctgaagtctct
aattcttggcagggttaatttgattagcttctctaaaaaagagaacatcttgtcttttcg
cagatgtactacaatgccaataggaacttcttctcgaattttaaacccagcaatggattt
tttagcttttgtgacaatcggtttttgtccagtgattaatgttaattcttgaatactact
ttcaagagctttagcattctgagaagcttcacctagcccacggttaatagtgattttcgt
aacacgaggaacctcatgtacattttatatattggaattcctcttttaaagcttgaataac
agttgttttatatttttcttttaatcccattactattatgtttt-acaaattatttaata
agttcacccgtctttttcagttttcgga-ttttttggcctttatcgttaatgattactga
agatctactagcaatattattctgttcactaaatagcatgacattagaactatgtattgg
agcttcaaatttaataatttctccggtctcaccttcttgttgaggtttttttatgttttac
ttttaaattaattcctttaacaaccactttacttgttttgtggatgattgcaataattgc
gcctattttttgttttatcacttccagaaataacttggactaagtcacccttttttcaattt
aatttttgtattaactttagtttttttgaaagaacttctcatcatattacctccggcgca
agagagactattttagaaaaatttttatctctcaactctcgagctataggtccaaatact
cttgtaccacgaggattattatcttgattaataataacggctgcattatctccaaatcta
atactcataccatcagttcttcttaaagctttgcgagttcttactactacagctctgaca
acatcagatcttttaactggcatattaggagatgcatctttaactactccaataatgaca
tcacctatcgatgcataagaaggattactagtacctaatactcgaatacacattattttt
ttagcgccactattatccgcaacattaagatagctttgagtctgtatcatacttttattc
tatataagattaattatcaaaagacttagataagatattaaccatcgtccaacactttgt
acgacttaaaggccgtgtttctcgtattgtaactacatctcctggtgcgcattcattatt
ctcatcatgtactttatattttttttgtgcggatcatagtttttgcatattttctatgagc
aattctattttctacagctactactatagttttattcattttattactgacaactttacc
agttgtttcttttaaaggcatagtttttatc--ttatatatatatacagtgaagttaaat
tataaaataattatgtggctggcttactccgagacttttcaacagttaaaagttgagcta
atctatgcttggaatgcttaaataagtgaggctgaaaatcttgtcttgtagctcttttca
gccttaagtcaaatagcactcttttaattacaagtatttcttcagctaaagaagaattat

ccatttttgtaacatctgatattttagggaaggtcatactttaagattctattgtatttc
gaactataaatttggtctttattggtaatttataagaagctagtttcatagcttgctgag
cagtttttttgtggcacgccagtaatttcaaatagaatgtgaccaggcctaataactgcga
cccaatattccggagctcctttaccagatcccattcgggtttctgcaggacgcgctgtta
caggtttgtcaggaaataccctaatccagagcttacctcctcgcctaacatatctcgtaa
tagttctgcgagtagcctctatttgtctagatgttaaccatactggttctatagcttgca
atgcataatcaccgaatgcgattgtattacctttactagcagaacctttcattctgcctc
tgtgttgttttctaaactttgttttcttgggacttagcataaaaattaaacaatttaaat
aattaagaagctgttagatcagaagtttcctgattatatacagattctgtgcttttcgat
tctggcaaaatttcgcctttaaagagccaaactttaactccaagtacaccatatgtagtg
tgagcttgtcgatgacaataatcaatatcagctcttaaagtttgtaaaggaacacgacct
tctctaacccattcgctcctagcaatctctgcgccattaagacgcccagaaacttgtatt
ttaacgccttgtgtatttgctctttgcgtcctttgaacagcttgtctcacagctcgacgg
aaggcaactctttttctaattgctgagtaataaattctgcgactaaggttgcttcagaa
tcgggatctgcaatctctaaaacattgactctgatttgtttactcgaatctagaattgaa
gataacgattttcgtagagactcaattcctgccccagactttccaagcacaatgccaggt
cgagctgttgctattaaaatttcgacttgatcaactttacgattgatttcgattttagca
atgctagcattactaagtttagagtgtataaacgaacgaattttatgatcttcttgtaaa
agaactggatagtctttagaattggcgaaccatgaagaacgatgtttttgggtaatgcct
attcggaaacccaaaggatgaatttttgacccacagtatccttttctataaattaaaag
ttataattactaattgactttgaatac----aagactacaattctgagacacataatgta
atgtgacaagttggtttatgtatagggaatgctcttccttgtgctcttggctgaaatctt
tttagcgtagggcctttatctgcaaaagccttactaataaataactcacttttgttcagt
ccatcattatgctcagcattggcagcagcagactctaaaatctgctttatataagaacac
actcgatatggcataaattccagaataattaatgcttcttgatatttcctacctctaatt
tgatctaatacacgacgaactttatgtggagaaagacgaatatatttccctactgcttta
gtttcttttacgtttttttgtaatactcataattatagtttaacgacgcgcttttctatca
ccttttatgtgagtacggaaagttcttgttggaacaaactctcctaatttgtgacctacc
atttgatctgatacaaagacaggaaaatgttgtttaccattataaacagctattgtatgc
cccaccatatctggaataatcgtagatgctcttgaccaagtttttaaaacttctttttttc
ccaacagtatttaatgcttctatacgtttaagaagactcacatcaataaaagggccttta
tgtatagatcttgacataataataaattattagattcttaataaaataaattctatttac
ggcgacgcaaaatgtatggggtgctatacttattgggattccgtgttttaacacctaatg
ctggtttgccccaaggtgttaccggacgagctctcccaattggagatttaccttcaccac
caccatgtgggtggtctactggattcattacgacacctctaacagtaggtcttttaccta
gccaacgatttctaccagctttaccaagagtaatattactagcgtcaatattaccaactt

gccctatagtagcatagcattcttttcgaatcatacgaacttcgcttgagggcagtttca
ccgtgacaaaagagccttctttggctactatttgagcataagttcctgctgcacgaacaa
tttgccctccgcaagaaggtcttaattctatattgtgaacagctgtgcctagaggaatag
ttgataaaggtaatgcattgccaacttcaataggcgcagtgggaccggacagcaccattg
ctccaacacgcaatgatcgaggatgtaaaatgtacctttttctccgtcaagataatgca
ataatgctattctagcatttctgttcggatcatattcaatagaggcaactttagcaacta
cgttgtgcctgtttcttttaaaatcaattaatctatactgctgtttatgtccaccacctt
tatgacgacacgtaataacacctctattgttgcgacctttgcaaaaatgatgtttaacta
ttaaggatttttctggcttatcagtagtaatctctgaaaaagtagaaactgttctatttc
ttgttccaggcgtataggcccggtataaacgaattgccatataggaaaatttaaaaagag
taaatattta------ctgtgtaatacaattaagtttctgggaataagttaatagaatc
ttctggcgcgagtgttacgattgctctcttatagtgaggccgtttacctacaaatctacc
aatactcctttttttcttaggtggatggcaagtgttaacacctgtaacttgaacattgaa
aatatactgtatcgcagctttaatattaattttagttgctttgggatcaacagcaaaaca
gtactggttttcttctaataattttgtagttttatcagtaatgattgggtatttaactaa
atctaataagcctcttgaatcaatgctatccattatatacctcttgtattttagataaag
catcaactgtaataataattttatgcgcagctaaaagggccatgatattcaaagtgtcag
ctgaaataatttctacattatttaaattgcgaatggaaagatagacattcggatcttttt
tatccactacaactaaaactttttttgtttaaatcaagattccatcggtgaatagcttcca
taaacaatttagtttttggctgctgaaaataactattaaagttttctacaattaacgtat
tgactgatttattatttaacgttgtccttaaagctaattgtctttctttttattcattt
ttttagtaaaactacgaggcttgggtccaaagattacaccaccacctctccataaaggtg
agcgtatagaaccggctcttgctctaccagttcctttttggcgccatggtttgcggcctc
cacctcggacttcacttcttgttttggtattagcagaaccttgtcgtttttcattccttt
gtcttactaaagctctatgaactaagtacataccagagtcttgactcactttaagattca
aatctgcattaccacttatttcacctttccaattatagacttgataatttaattgtgttt
taactgtcattagatatatcgcgtgaaagaatattgttatttactaatttttactaaatt
accaggcttacctgggacagctccttttacaatcaacaaatcattttctgagttaatact
tataatttgcaaatttttttatcgtaactttttttattaccaagttggccagccatattttt
tcctgggtaaaccctcccaggcgtcgtaccagctccaatagaaccaggttgcctatggtt
tttcgagccatgagacattggacctctactaaaatgatgtctcttttgatagcctgaaaa
gcctttacctatgcttttagaagaaacattgattgtctggcctactttaaacatatctgt
agacaagatttgactaacttcaaagtcatcagtagattttactaagtactcacgcaaata
tttcagtgggggagcttgtgacttctttaaatgtcctaataatggtttatttaactttg
ttcaacaacttgcttgtacccaacttgaatagcattatagccatccttaggtaaagattt
aatttgagtaacaatacacggcccaacctgaattacagtaactggaattgacaagccagc

ttcatcaaagaattgggtcatacctactttagtaccgagtataccaacagacactagatt
tctccttttttgagagatttatactattatcgtacatgaaatttttacttcaaattgcctg
aag--taaactgaactaagagatgtctgagattaatcatgacaacataaagagaaaaaac
tttacttattcaatctaaaatagaaatagtttttttgtccagttacattatgtatagatta
aaggcaaacttattatattatttatacactacg---------------------------
----tgttcaaaataatatgaaccagaagacatgaattcttttag-----atttgtacaa
cattacattattagtactgtctaaaatata----gatagaatattgaatttctacctctc
tagaa-------aaactaaaattgtgttcggtaactacaccttttttatttacatatttat
tgtgcagtatatatgcatatctaatacagcacaggtaaaaatatagc----tgtatagcg
aatatacttacaagaattagagaggtattaatgggtaaagttgttggaattgacttagga
acaaccaattctgtgattgctgttatggaaggaggtaaacctactgtaataccaaacgca
gagggttttagaaccactgcttctgttgttgcatatactaaaagtggagataaacttgtg
ggacaaattgctaggaggcaagctgttattaacccagagaatactttctactcagtcaaa
agatttatagggcgtaaacaaaatgaaatttctcaagaaattagacagacatcatataat
gttaagactagtggatccagcataaaaattgcctgtcctgcacttgataaagattttgca
cccgaagagatttccgctcaagtactcagaaaacttgttgaagatgctagcacatactta
ggcgagactgttacacaagcagttataaccgtacccgcttactttaatgactcacaaaga
caagctacgaaagatgccggtaaaatagccggtttagatgtactaagaattattaatgaa
cctactgcagcttccttatcttatggactagacaaacaaaataatgaaacaattctagta
tttgaccttggaggcggaacatttgatgtgtccgttttagaagttggagatggagtattt
gaagtactttcaacttctggagatacacatttaggtggagatgactttgaccaacaaatt
gtggaatggttaatcaaagatttcaagcaaaatgaaggtattgatcttgcaaaagataga
caagcacttcaaagattaaccgaagctgcagaaaaagcaaaaatagaactatcgaattta
actcaaacagaaattaatcttccatttattactgctacacaagatggtcccaagcactta
gaaaaaactgtaactaggggtaaatttgaagaactttgctctaatctaatagacaaatgc
agtattcctgtaaataatgctctaaaagatgcaaaattagaagcttccagtattgatgaa
gtagttttagtaggtggatctacaagaattcctgctatacaacagatggttaaaagatta
ataggtaaagatcccaatcaaagtgtaaacccagatgaagttgttgctattggagcagct
gtacaagctggcgttttagctggcgaagtcaaagatattctattattagatgtgacacca
ttgtctttaggagtagaaactttaggtggggtaatgacgaagattataccaagaaacact
actattccgacaaaaaaatcagaagtattttctacagctgtagacaatcaaccaaatgta
gaaatccaagtgcttcaaggtgaaagagagttgacaaaagacaacaaaagcttaggaaca
ttccgattagatggtattatgcctgcacctagaggcgttcctcaaattgaagttacattt
gatattgatgctaatggaattttgtctgttaaagcaaaagagaaagctacaggcaaagaa
caatcaattactatatctggagcttctactttacctaaagatgatgttgaaagaatggta
aaagaagctgaagaaaatttcgatacagaccaaaaaagaagaaagaatattgatactaag

aatcaagcagaatctttatgttaccaggctgaaaaacaaattaaagaatttgaagataaa
atcagccaagatttgaaaacaaagatagaggaactaattacagagcttagatctagttta
gagaaagaagaatatgaaaatattgaatctatctctcaacgactacagaactcattaatg
gatattggaaaaatggctgctcaggctgaaagtaaaaatacaaacacaaaagatgatggc
actgtaattgacacagatttctctgaagctaaataaaaaatt-agcgggtaacgcgattc
gaacgcgcgacatcaaccttggcaaggttgcgctctaccactgagctatacccgcacaga
atatattattacaaacatggtaacatttgtcaatctgt--------agttatat---agt
tgagctattaatcattaaactatagattacaaacttgttaaaaacaaaaaaactattatc
gttttttaaa-aaaagaagtacttatataag-tatataagtacttcttttattcaaaaa-
-c-aaaatataattagatatcagtt-------ttaaactactaaggaattaaccaagcta
gtaacaattactagtcctgtccataagcctgcccctgtatagattaacccttttgattgt
tcccattgaccgggtgatgctagaacaacagggacgcctactactaacacagtcgataat
acaattaataatagcactaataattgaattgcaataatcatttattctattctcctaaaa
aattcaaatgtgaataatattgtatataatacaatgaagctttataccttcatcaattta
tctatgaagaaataatttaaaattattgttttctcaataaatattaaattaagtattaaa
atctttattgtaaacaataacactatcttttttaa-atagataaaaaatttaaagaaag
tctttttaaaaaaaatttagaataaggtgatatgctgtataaaaagctaagtattatta
gttattagagaaattttaaaataagaggtaaaacatatgaattcagccctttttttagcg
aaactaccggaagcttatgcagttttcaaaccgatcattgatattttacctgtaattcct
gtatttttctacttttagcttttgtttggcaagcagcaattggttttagataaccttga
agctataacaaaaatagcaaatataaaaagtaaaatttttttgcgctctaatcttatatct
tacacaataataactattatggttgaacccttactatcaggaattgttcttggccttatt
cctatcactttatttggattattagtagcagcttatctacaatatcaacgtggaaatcaa
ttaggactataaagcctaggttatagatatttataaaagctgtaataattatggtttta
taaatatctacaaaccagctaattataaatacagaaaaaatttattgaaaacagattga
taattggcaatactgattttatagtaagatagcgaatctttcataagataataactattt
taaagttatttgaacccattaaactttctatatgacaagctgcttctttctatacaccaa
cccttcttgaattggatttgctttatttaaaaaagctaatacttgtgaaagatctttaac
actggcttcgctacggaatggattctctaatttactatactcgggtctcttaataagaac
ttttattacagccattagcgaaacatcatttagtgcaatcaatctactggcttcaagaga
tttctcatcaactataggtggatcaaacatctcattaaacttttcggtgtttaaactagc
gaaaatatttatttgactattgttatttttttacaatcttcatcatcatcgttagcagct
tttaaaatcacaacgccctctggattagcgctggttaatctataattagtacgcgtatca
tcgctagttattatcgaaaatacttgtggatctctttcgacataaagccaagactttttc
aaaggatatggctccttgtatagcacaagtgtctagaaaacctacagtaagattgtagtt
acgacccattatacaagcacaagcccccacacctagatttataaacgactgttggcttcg

agcatttttacaagaaattgctttcagtcgccgatccatagtaattacaccttccgcata
tttagtgctgacagtagcttataaaacctcatctttagcaaacccttgtacaattctaat
ataatcggataccaagaagtgatctattatatcaacatgcaaagtagggtaatacggatg
cggttctataggaaagggatggattttaattctagtaagtagccaaacataataaactt
ggattcaaataaaggtaattttatattaaagtatgatatagatctctttcatatattcta
gacatttgagtgtaagacaatcatttctttactatttataatcttgatttgtgatgaaat
agcaaaattcttatcgccataagcgctcttatatagaaatgtctgagttatgaaatctac
atcgactaataaagaaaaatatatttatttattattttcaatattatcaatgaaaatact
tttagtataaatatactgatttttttctagtaaaattgtttaccagctcgacttagtaac
tccaggtaaaagacattcatgagacatttctcgaaaaacatggcgagataaaccaaaatc
tctataataccctctacttctaccagttaaccagcatctgtttctacctctaacggctgc
gctgtttctaggcatttcttgaagtctttgtcttagttccattttgtcagaaaagcttat
agcttttttagttgatctttaatagccagtctttttaaagagtattttttttaagctt
ttccctttaagctctctttggatcatatttttttttgccatattataa-aacttactta
aaaaattacttaatcaatatactagctaaaaaatctctgaactgcaacatttatatggtc
aaaaacatatagcataaa-------------atagatattttatgaattatact---act
ttatgctattgaaagaaatttatttaataaagttcttgttcaacatgagttgaaatagtg
caatcagatgtaggataagcaatacatgttaaaacataacctgctaaaagctgatcatca
tctaagaatgattgatcacgttggtctacacccccttcagtaactttacctgcgcaagtt
gagcaagctccagctctacaggaatacggaagttcaacaccttcttcttctgctgcatct
aggatgtatgtatcgtccgcacagttaaatgtaacatcaatattttcatcttcacatact
aaatgaattttataatctgcca--tttttttactccagaataaagatatgcttgaaactt
aataa--taaaac-aatttaaaggacttgcccaaa-attgtatattagtttcattatgca
taatagactgcaaaaaaagaaaataatatactattgtaagaaattaagctacatttcata
ctaaatagaactattgcaggttcaaagtatatatataaattcatatagttaaaattattt
ttatcatttaaataactaaacttaatactct-agacctttagattaactgca------ca
gctaatcttttacaataaacaaataagtaattatgacttcaatgataaatcaacaaatag
aactaaagcctattcttcgatttcagataccacatttatattcgagttttcttatacagg
aaatcaaatctttaacaaaaagactctttttgcaagtttggagaagacctgcgacattga
tggcaggtattattcagcctttattatggctaattctattcggtgggctttttttataatg
ctcccgtgaatttatttactattaacacaagctataactgtttttttaagctctggaatta
taattttcacatcgtttactggtgcacttaattcaggtcttcctttaatgtttgatcgag
aattcgggtttttaaatcgcttattaacggctcctttaatatctagaacttctattattt
tatcttctgctacttttatgacttgtattagtttaatacaagttatattcatagttacag
cctcattatttatgggaaattctcccttaaacagtgatagtgcgatagtttttggactta
taattctattagtaactgtgggagttacaatacttagtttagctttatcttttactctgc

ctggtcatattgaactattagcacttattttagtaatcaatttgcctttttttattttcta
gtacggctctggcccctctatatttcatgccaccttggctccagttaattgctagtctaa
atcctttaagctatgcaatagaaggtacaagatatttatattccagcatacattggaatt
ttacagagtctattataaaaattagttggggagatgtttgtttagggcaaattattgcat
tattagttattctagatataattgcagcctaccttgtctctaagatactaaaagctaaac
ttaatta-----------------aaatttaataaaaaatttattaaatttattttaga
aagattttactatataatactagtagtagtatggataattgaaatagaaatagtttttc
aaaaccaaagctattatcaacaatgataaacatttgtaagaaagtcaacaaagtatgttc
ttattcataggaggcatgtagtcaatgggactaccatggtaccgtgtacacacggttgtt
ttaaatgatcctggacgcttaattgcagtccatctaatgcataccgcacttgtagcaggt
tgggcaggatctatggcattatacgaattagctgtatttgatccttcagatccagtatta
aatccaatgtggagacaaggaatgtttgttatgccattcatggcaaggcttggcgtaaca
gattcttggggaggatggagtattactggagaaagtgtttccaatcctggattatggagc
tttgaaggagtagctttaactcatatagttctttctggaatgctttttctagctgctatt
tggcattgggtttattgggatttagaattatttcgagatccaagaactggagaaccagct
ttagacttgccaaaaattttcggaattcatctattgctatcaagtttactttgttttgga
tttggtgcttttcatgtaactggattattcggaccaggaatgtgggtatctgatggttac
ggagtaacaggtaaagtactgccagtggctccggcgtggggaccagaaggatttaaccca
tttaatcctggaggagttgcgtcacaccacattgctgcaggtactgtaggtatattagct
ggtgtttttcatttaactgtgagaccgccgcaaagactataccgcgctctaagaatgggt
aatatcgaaacagtattatcaagtagtatctctgctgtttttttctcagcttttgttact
tgtggaacaatgtggtacggctctgcaactacacctattgagttatttggtccaactcga
tatcaatgggatagtggatattttcagcaagaaattgaaaaacgagtagagaacgctatt
gctgatggagctgctcctagcgaagcatggtcaagaattccagataagttagccttctat
gattatattggtaataatccagcaaaaggtggtttattcagagctggccctatgaataaa
ggagacggagttgctgaagcatggcttggacatcctgtatttcaagataaagaaggaaga
gaactgagtgtccgcagaatgcctgctttcttcgaaacttttccggtcattttagtcgat
aaagacggtatcatacgagctgacattccatttaggagagcagaatctaagtacagtata
gaacaagtaggtgtaacagctagtttttatggcggaaaattaaatggccaagtatttaat
gatgctcctagcgttaaaaaatatgcaagaaaagcccaattaggggaggtatttgaattt
gaccgaactacactagaatcagatggtgtattcagaagtagtccaagaggctggtttaca
tttggtcatgctaatttcgctttaatatttttctttggacatctttggcatggttcaaga
actatatttagagatgtgtttgcaggaattggagccgaagtaactgaacaagtagagttt
ggagctttccaaaaattaggcgacagaagcagtaagaagcaaggagctgtataatgct-a
agccaaatacagtctttatc---aaatatttccaatattaaaatagttcaggaaaagttt
attgtctaatagttactaattaggagaacctatggaagccttggtttatgtattttttatt

gacaggaacactaatggttatattctttgcgatctttttagagaacctccaagaatagc
taaatagctttattaagtttaaccttacaataatagaaccactttccaacttattagtgg
agagtggttctattgtaggttacagtatattattcttcatgctcttcaaaaggatctctg
agatcttttgaagcaggaccaaatgccgtatatattgaatagcctgtaatacctaaaagc
aaacttgaaataaaaatactaagaactgttgcagtttccataattcaatcagtattaaga
tgaactttctctataatgatattatcaatattaaaacatttaattattaaatttatcaaa
atatattatggcacttagaactagacttggagaaattttaagacccttaaattcagagta
tggaaaagttgctccaggttggggaacaactcctatcatggggattttcatgttactatt
tttcttatttttgttgatcattttgcaaatctacaattcatcactagtactagaaaatgt
agatgtagattgggctactttaggtag----ctaattatacttttttctttaa--------
tttaataacattaaagataat----------------aagcgatcgctattataacataa
tagcgatcgcttaatttctactttactatata--caaaagtaattattttcctaagtcaa
ttaattcgctatttgcaaagctattagtattgacattattataattaactttttcaaatc
ttactagtactggatacttgataccacttttatctattgctgcaacagttccaagttctt
gataccagtatgattcctttctcaatatttttacttttgagccacgttccatatttttag
tcctaagatagattattaaagattagtataatagataaagaaataaaaat-aaaaaaata
cataaaattagtaccgacaat---------taaatagtaaaaggagagagagaggattcga
accctcgataactacagttatgacaattttcgaaattgttgcaataaacctctctgccat
ctctcctaaattaaaactcttttttccccgaattagataagaataacatagaactaatgta
gttgcctagaaaattaaaactatgttaaatataaattataatctttctatagatctcttt
acaaaatatgtacacagattctatatcagcttatttgaagatttttaatcttatacaat
ttttactaaataaataaactctctcataacatgaaactatcttggaaaacacttcttctt
tggtctttaccaatatttgtcgttgggttctttttctggcaaggttttttaggtccgact
actacagatgttggtagtaatatcgcaagttctagaatgacatatggaagattcttagaa
tatttagatatgggttgggttaaacgagttgacctatatgaaaataatcacacagcaatc
gtcgaagctgttggaccagaattaggcaacagggttcaacgaattcgagttgagctgcca
gcaagtgcaccagaattaattacaaagctacgcaaagctaatgttgatttagatgctcat
cctcctaaaagcacaagtgcagtatggggactattaggtaatttactatttcctctattg
ttagttggaggcttagcttttctatttagaagatctaatagtgctagtggcgggcctggt
caagcaatgtcatttggtaaatcaaaagctttatttcaaatggaagccaaaacaggagta
gtatttaatgatgttgctggagttgaagaagcaaaagaagagtttcaagaagtagtaact
tttttaaaacaacctgaatcatttactgctgttggtgccaaaataccaaaaggcgtttta
ttagttggtcctcctggtacaggaaaaacattattagcaaaggcaattgctggtgaagca
agcgttccttttttttagtatttcaggctcagaatttgtggaaatgtttgttggagttggt
gcttctcgcgtaagagacctattcaaaaaagcaaaagacaatgcaccttgcattgttttt
attgatgaaattgatgctgtcggaagacaaagaggaacaggtgtcggaggtggaaatgat

gaaagggaacaaacattaaatcagctattaactgaaatggatggctttgaagggaatact
ggtgttattgtaattgctgcaactaacagagctgacattctagattctgcattattacgg
ccaggaagatttgacaggcaagtgtctgtggatgttcctgattttaaaggtagattagca
attcttgaggttcatgctaaaaataaaaaaatggagcctaaagtatctttagaaactatt
gcaagacgaactccaggcttttcgggagctgatttagctaacttattaaatgaagctgct
atttaacagctcgaagaagaaagaatgcaatgactatgtcagaaattgatacatcaatt
gatagagtggtagctggaatggaaggcacacctttaattgatagtaaaagtaaaagatta
attgcataccatgaggtaggccatgcaataataggaagcttattagaacatcatgatcct
gttcaaaaagtcaccttaataccaagggggcaagctagaggcttaacatggtttactcct
agcgatgatcaaagtttaatctcaaggtcgcaaatactagcacgtatcgttggtgctctt
ggtggaagagctgcagaagagattattttcggagatgcagaagtaaccactggtgctagt
aatgatttgcagcaagttacatcaatggctagacaaatggtaactcgattcgggatgtct
aaaattggcccattatctcttgaaagtcaaggtggagaccctttcttgggtagaggaatg
ggaggaggctcagaatactcagatgaggttgcaactaatattgataaacaagtacgagaa
attgtgagcgaatgttataagcaggctaaagaaattattgcagataatcgagtagttatg
gatagattagttgatctactaattgaaaaagaaacaattgaaggcaatgaatttagagat
attgttaaagaatacactgcaattccagagaaaaattactataaatctcaattttaa---
---------------atgatacgggactgacgggattcgaacccgcaacttccgccgtga
cagggcggtgctctaaccagttgaactacagtcccaataaaggg---tacagacagataa
tctcataattagcttgtgattgtcaaattatactgtaaaacatgaaaagta---------
--------tcctcaataatatttttcatgtttttattataaagccaattgattaatgca
taaggagagagagggattcgaaccctcggtacgaagttaatcgtacagtagattagcaat
ctaccgctttcgaccactcagccacctctccaaagagtcttacgtactcaatatatcaaa
tattattaaattttaacagctgtgcataaatatttatggagataagcggattcgaaccgc
tgaccctctcaatgccattgagatgctctaccaactgagctataacccc---tttcttaa
ggctattaagaaatggctcaagcgggatttgaacctgcgaccttgggcttatgaatcccc
tgctctaaccactgagctactgagccataataaaaaccattgataaatactataccataa
tttatcttaaaatataaatcaataaaaaagtctttactacaagtacatagatcttatatt
g----------------tccagatt---------------------aacatttcaaaga
tattaaattaccctctacgatatactctcaattcttttcactttat-tggcagtatttca
atattcagcttgtaa---
--------------------aataagt---------------------------------
---aattttttt
g---

-------------------------------caagaatatatagaa--------------
--tcttgctacatatt

------------------tacattattgttaataatt-----------------------

---agcctttgctaaacta---

------taaatctatgaat---

-------------------------------------tttagtagatt------------
-----agactaaataattatgtcttttaaattaggtaaaagtaaagaaagtatgtgagca
aaatatcttaa-----------------ttttt---------------------------

-------------------------------------ttagaaatattttattttcaaca
tttt-tttataatagagcagttaaaaactgaataaatagctaatatc-----ttatacaa
ccaacatcgagccaatgccttgatcagttaatatttctagcaagagagcgtgatcaattc
taccatccaaaatatgagctgaagctactccttgggctaaagagcgaatgcagcaattaa
ctttggggatcatacctccggagatcacctcggtctgagttaagtctctagcttgctgta
tatttaagtgactaatgagtgttgtaggatctgaagagttacgtaaaataccaggagtat
ctgttaacaaaattagtttttccgcattaagggctgctgctacttctcccgctacagtat
cagcattaatattgtatgactgaccttgtttatctgccgcaacactagcaattactggta
tgtagttattatgaattaagatctttaatagcttagtatcaacattctgtacttctccaa
caaagccaagattcgctttaccgctcggtctaggagtaataagtaaaccatctttccctg
acaaacctacacttttacctccttgcttattaatcgttgctacaagatccttattgactc
gtccaactaataccatttccacaatatccatagtaggctgatctgtcaccctaacaccgt
tctcaaattgtgataaaatttttagacgatctaaccacaaattaatttcaggacctccac
catgaactaaaataggacgcaaccctataaaagaaagaaaaacgacatcactgattactt
gatcttttaatttttggtttttcatagctgctcccccatattttattacaataattcttg
aggaaaattgttgaatatatggtagggcctcacttaagactttcactctttctgaattag

tcagcatgaataaaatagaattataatatataaatttatttgcaggacttatcttaattt
aatagatactagtaagtactttaaaacttttagaaaataaacatattattaatag-----
----ttaaaagtataaggatgataattatgagtaatttttggaataatatattaaaattt
ccacgattttagttagtgtcattttcggattagttttaataattattagtcctttttc
gtgttatttaaaaaaccattgacaagtttttttttcatcatatcattaaccggtttaatc
gcaattttagcggcaataa-tacagaaaatgctcaatatcgaatgttgttgagagaatta
atttaatttaattaaagtttttgaacatatataatattatattatcattaactgtgataa
tttaaatttcttttctat--aaaaagtcatacgtattaaatattatttgatagatattat
gttattaaaaccaatagttagctctgaaaaaactggtgcttttgccctttagatagtat
tgttagacatggtgttaagcatatatttggttatccaggcggagctattcttcctatcta
tgatgaactttatgcttgggaagagtcttctttaattaaacatattcttgttcgacatga
gcagggagctgctcacgctgcagattcttattccagatcaacaggagaagttggagtttg
ttttgctacatctggtccaggagcaactaatcttgtttcaggtatagctaccgcgcatat
tgattctgtacctatactagctgttactggtcaagttggaagagcgtttataggtacaga
tgcgttccaggaagtagatatctttggaattactctccctattgtcaagcattcttatgt
agttcgagatcccagagatatgtctaggattgttgcagaatcattctttatttgtaaaca
tggtcgaccaggccctgttttgattgatgtacctaaagatgtaggattagagaagtttaa
ttatttttctattgatccaggcaaagtcaatatcccaggttgtagaccagttactacctt
gaagtctagacaaatacttatggcagctaaaatgatacaacaatctagtcaacctttgtt
gtatataggaggaggagctataatctctgattcacatcaaattattaaagaacttgttga
cttgtataaaatacctgtcactactactttaatggggaaagggattttaatgaagacag
tgaatattgtttgggaatgttgggtatgcatggtaccgcatacgctaattttgcagttag
tgaatgtgatctattaattgcattaggagctagatttgatgatagggtgacagggaagtt
agacgagtttgcttgtaacgcgcaagtaattcatgtagatattgatcctgctgaagtcgg
aaagaatagaattcctcaggttgctatcgttggtgatgttacagaagttgttactgcaat
attaaatctactaaaaacttctttccctccttatccggagcagattgtatcttggcaaga
gcgaattaatcgttggcgcgaacagtatccattactggttcccaaaaaatcaacaagtat
ttcgcctcaagaaatacttgttgcagctaataaactagcccaaaatgcttattttactac
agatgttggtcagcatcaaatgtggtctgctcaattttttaaaagttaaagctaaacactg
gctttcaagtgccggtttaggtacaatgggatatggattacctgcagcaattggtgctca
agtagcacatcctaatgatgtagttatttgtattagtggtgattctagctttcaaatgaa
tatgcaagaactaggaactatcgcacaatatcagttacctgtcaaaattattattattaa
taatcgttggcaaggaatggttagacaatggcaacaagctttttatggcgaaagatactc
tcattcccgaatgatagaaggagcaccaaatttccaaaagcttgcagaagcctttggtat
taaggcttttactattaatagtaggcaaagtatgcaatcgtccttagaagctgctatcga
gcatcatggtccagttttgctagactgtcaagttacagaaaatgaaaattgctatccaat

ggttgctccgggtaaaagtaactcacaaatgataggcattgccaaacctcaaagaggtac
tgcctcaaattatattaatgcaaatatctagattag-----------------------

---------------aaggtctatattccctcaaaaacaaagtg-gattttc----tttt
atgagtacatatttctaatagtatatacaatttttaaaagatagcctttgacgagaatt
gaactcgtgaccttccccttaccaaggggatgctctacctctgagccacaaaggc-tttt
tattatattgggccgggttggatttgaaccaacgtaggcaaagccagcggatttacagtc
cgcccccattaaccactcgggcaccgaccctaattagtttttattcaaaacataacttaa
acagtatcataatttagttgcttaaaacaagtcttttcataaataaaact-attaagtga
tggacatagctggactcgaaccagcgactttcacgatgtcgacgtgacactctaaccagc
tgagttacatgtccaaaaccttaataattattataacactatttattgattttataagta
tctatttttaattaaagatttgctttaaacgacctgctttaccaagaagatctcttagat
aatatagtttagctctccgtactttagctcttctaataactttaataaatgttattcgtg
gagaattgagaaaaaaaacgcgctcaactccaataccttgaaatgagcatcttaaagtta
agctagtatttaagctatttttttcttcttctagccaagattactccttcgcataattgtt
ctctcgttttattaccctccttaaccatcaatccaagttgtattgtatcgccaactctaa
tctcaggtaagtcttgtttaataaaagaagtctccacacttttcatgagcttacttattt
ttgtactatttgctttcatatctagt-ttagctttataatg-ttttttgcaatattatac
attaaaacaataaatatgaaaattattagtagataagcagagtagtatctcagaatcttt
gtattatttattgctagactagatttacttcattttgtgttatcaatgtatttgcttaac
tatcaaaaaattaattcgctaattatagaataatgaccaacttatatttcta----atag
aaatacaaaattcttttatattaaat--taacctttagatatttaattcattgaaaagac
attaggatcaccgtccgaatacatacctgaaagttttaaatttggtatggcaaacgcaca
gatgaacatagaacagctcacaaaacgtttatataatttagtcatatatgtattaataaa
gatgcatcgctattaccggtgctcgatagactaatttgctacgaggctttgaggtcttta
acttagttttattagacttaccacttttcatcagtttagttcccttgccatgatcaacaa
cgcgcgaagtgtgtacatatgaaacactaactggtggcattgctgactgaaatttgtgag
cttttaaaaagacttgttcacaatcagaaaatgcaagtaaaacttcatcatcaacatcga
ttgagctagaagtatttatagatgtatcgccggacactagctcgttagagaacacgttat
cgggtattccgtaaatattaattgtggctggactactaagtcctataaaacgcaatgata
cttttagattatgtataaatcctgtagtagagtcatttagtccacgtgtatttggtattg
gcaacattaacaaattattatgactattcttaggataaagtatactaataaatttccatt
ttataataacagactgcaacctacgtataggcatcaaaaatttaaacaatagatcgttta
agaaagtttggacggatagacctgtaaaaactgattgcttgctattattcttaaaaacta

aaaccagcgagtaatctgtaaccgaatcaacaaaaccataaacaagtttaattttcacat
atttagtaggcgacatatatgtataagcaagctgatcaaacatcgtgtaatttaagaatg
atttatagacgaatgaattgtatttattaatctggcatatttcgaggtggtaagcgcaaa
cgacgatctgaagatgggacatcaatcgtaacaagattttgattgagcgcagcagcctca
cgtgccaacctatatctataattacaatagcgaataaatctgtagactagataaataata
aaaacaatgagtagtgaaaatccaaagtgtacaactaaacgaatgtatcggcataactga
taataggtcacttcattaagcgctatactaaaaactttaccggtcattccttcgcaatta
tcgtaaagtgttttcccatcaatcaaaccaaaagtcagatcaaaaggtttcttcatttca
ggcgtaatacgatccacagctggattcctaggaggaacaccttttaagagatcaataacc
ccattagtagtattgctaacaaattcatacgtatttgcagcaggattcgcagaagaggta
acagcaccatcgttcatcatagtttcatacaattgtgaaatcgtataagcacagaccggg
cgttctacctccttcgataagagtgtacctgctttggtaccaccttcgagaacaatttt
cgggcatcggtacgagttatagtcggattggcttttaggaaagctgtaactgcttttcgc
ttggtataagtatctgttgcgagtccagcaactatttgggctagaggtaacaccgtagct
ttaatagggaccggtataagaaatgatgctacagattttagacgtttatgttgatctggc
ataagagtacccttgattttaggtataaaaattgcggggacaaattatttacaaaatcac
ctattttcatattttgtcccagtagccttactaagcaacgcccgtcaaatgacgtaaaac
aggtgtattttaggggattaagccccaacccggcaaaatttgctaaattttcaattaaaa
tctcctttcaaaacatttggtttttatattctattactataacttcatttttgggttttc
ttaaggcctaaccgcgacttatttatattcactattcaaacttacttcaattcatttgtt
agtgttacactcgagccgtcattagcgatgtttcgctagtgtgtgtgaattattgcatca
gtgtttatacctttagattgctattgtgttttactagttcactagttgacatgcgattgt
aaacgtgttatcatgtcttatttcctttattgttagaacataatatgagcataaatttag
cactgcaaaatcaaccagttcgtggtaggcgccttttgcatgatcattcatatcgccaac
gcaaagctgcggcaggacaatgtcaattaagtgtctgggtacctaaaaatcactacgatg
gtttaagagtcttattgacagatcatgatgtattatctaaagttctatcttattactata
gtattcaatcgaatccaccacacattaagcgaaatattcgtacaacatcacctaaagtta
ttgaaagaacactcggtagttaccagtctagcaagattagtcgccatactaatgattatc
aaataagtgatatgtctattaaaccagaagaaaaatttgataatgagacgaatttcataa
cggaagaattagctgataacaagttaaataaattatcagaagaatttgatgcaaagctta
taaatctatctacaaatattgatagtagatttaatcttttatctgaacaatttaccgcgt
taaacactagcgttttaacgctaacttcaaaagttgcggagatttcctcacaacaagttg
tgctaagacttgattgtgagaagaattttaacaagctaaatgataaaataaatgaacaat
cttttacgggtgaaactggtatcgatatgtttgataatacgaaaactcattcaaataaca
atgattcaacagaattagattctgatttagaaggatatgatgtgtgtgaattccctattg
tggataaatacgatctaatagatctttcaaaaatctcaggcaatacgttgcgagcgtata

atataagtcgatttacagatgcaagtaaatctactcagacaaagtttttagcagattata
agttggcatgtttttgtaaacatattatggagaaaatggttgcaaacgaagagccaacac
cctcattaagagcgttggctcgtgttgcaacatctaaaggctatggatatataaatccca
agactggaaagcccagtcttatttataaaagtagagttaaaagagttatgcaatctttca
atattccgtttaatacttaatagccaacagtttgcattccaaatttattatgcgttgttt
ataatttatataatgtgttctttatatctttttctaaatctacaactaatttattcctag
gatcttcacagaattgtctatgaatatgagctctttcaacttttgttagtgttgatctat
aggaacgtttaccagctatgatagccatgccaatcactaaaatagctctttgcaaatcaa
gattatttcctgtttgaaagaacatttcatagccttgcttatatttactgtataattgag
cttcctcccttttccaagaggattcctgagtttcgctttcaaactgaaactgtgataatg
ataattctcgaagctttttaacagattgtatcgattgtccaggatgtatacagttacgat
tgtatttgccaaggttttgtagatactgtttagtttttaatattcgactttcgacaatcg
gtttgattcgaggtatgtcagtaggattttcgttaatggctttaaataaattttgtaccg
gattgtaatgaactaatttattatcactagttattacataatcttgattttttgtatcctc
tcatttttattgaagggactatatttttatgtcgagtttgataatttgccttaccatgtg
ttgccaattcggtgaatatagcttcgacttcgacatcaaatagttttataatcgttagat
ctggtaacacgattcttaaatgctcggtaatcatttttgagattgttgaattatccagat
caataaatcttaatttattagtatcgccatcataaattaaacgggttttataatcaatgt
ctcccaaaggttttagagatatttgtctggtttttctactgtgttcaagatcatataaac
ttacggcagaaagtttgcgatctttttcgaatgtacaatttttcaaggtctaatgaca
cactatcagttatagtttgtgtaccattcagggtttctcaagataccaagccatagctc
tggctctcgccgtaacattgttcccgacaacggctttactaaccggaaattttggtgaaa
ccaatataccgtaaatcgtgttgattaatagtttaattaacttattcattgaactagtgt
ccggattatctttaggatacttttctcttaaatcaatcagacgtcctagtatcaatttat
caatatttacactaaaccaattatgatcttgtttattagatgttataacttcaaaattct
ctttgtcttgtttcaatttccaagtgttaacacctgtatgtagtttcaccttttcttcat
agtccgaaaaattattaactcttgcagattttggataaaatacacccgatataacttta
agcggtctaaatattcttttcgctgagctggtttacaaaccttatccaaccactcaagat
cggcgtatgtaaaaggagctaaatgaacttctcgactataatacttagtagattcatctc
taaatacattgtctgaatcttgataatcagtatcactcaattctaaatctgttaggtttt
taggcggataccaagacataaaccagtcatttttatgttctaataaatatcctgggtctg
ttgaaacacgaaaataccaaagaccatctactaactcatgtccaatatctgctaaagttt
ctctaagagttttatactgattgttctgagcagatttcttatattctataagtatcggtc
ggccaataggaaattcttgtacttttaaacaattaccataacagcttttgatatcaaccc
tggcaaaaatacctcgggacgctgtgtgcaatggtttattattaaagcagcgtccgccat
tagtttttgaaagataaatcccagtttgatttttatttgcaaacaggcttgtagtagaac

ctttcttgatatgtgacatcattgtacgcgaactaattttaagttcattttgcaatgcaa
ctgtaaataattcagctacggtcgtaccaatggtcaaccctgtacgcttatataacttgc
caaaacccaactccttataaatctttttaataagatgttcttgatttaatagcgcttcat
agacaagtagatcgcttgtagcgtattttataaaacgatttggatctgttattagtacat
ctaacatgttactcttttcagatactgttataagttctttatctataatagatgtacccg
tgttggctaagaagcttcgtaaagatatagctccttggatagcgcaggtatctgtgaatc
ctagctttatattgtactccttgccaaatatacgtattgcaataggtatatctatatatg
gaatacaccttctagaactaactctagtatatgctttaaacgcctttccattaatatac
aaccatgattctcgattttgttcgcagttgcttctaataatctttccttaacacttccgc
taacaatacgataacaatcagccaataagaaatgtccgtatatatctatatatatagttt
gattatctgactgtggtttaacagtttctgctgtataatttaatttgtttaaatagtcta
atactatacccgtaatttgttatgatctaagtatctcacccaattatttttttattgatg
tta-------------ctatatttaaatacttatttttcgctaaagaattttacacataa
aacagagtaatcaaaacaacaagattaaggcttaagtatttattagttttgtgttataat
catttactatcaaaggtagtggcataacataaaacataaacaatgtttgaacgctttact
gaaaaagctataaaagtaatcatgttagctcaggaagaagctcgacgcttagggcacaac
tttgtcggaactgagcaaatattattaggattagtaggtgaaggtaccggggttgcagcc
caagtattaaaatctatgaacgtgaatctaaaagatgcaagagtcgaagttgaaaaaatt
ataggaagaggatctggttttgtagcggttgaaattcctttcactcctcgagcaaaaaga
gtactagaattgtctttagaggaagcacgtcaacttggccataattatattggtactgaa
catttgctaatgggcttagtcagagaaggggagggagttgcagcgagagtcttagaaaat
ttagcagttgatgtttcttcaattagagcagaagttatacaaatgctgggagaaaatgca
gaagctaatgtaagtggaagtaatactacacaggctagaagcaaaacacctacattagaa
gagttcggctctaatttaactcaaatggctatggaaggtggtttagatcctgtagttgga
agacaaaaagagatagaacgtgttattcaaattctagggagaaggactaaaaacaatcct
gttctaattggggaaccaggtgtaggtaaaacagcaattgcagaaggcttagctcaaaga
atagctaatagagatgttccttctatattagaagataaattagttattactctagatgtt
ggcttattagttgctggcactaaatatagaggtgaatttgaggaaagactaaaacgtatt
atggacgaaatcaaatcggctgataatgtaatactagttattgatgaagtccatacatta
atcggggctggggctgcagagggcgcaatagatgcagccaatttactaaaacctgctttg
gcacgaggagaattgcaatgtataggtgcaacaactttagaagaatatagaaaacatata
gaaaaagacccagcactagagagaagatttcagcctgttgtagttggagagcctagtgtt
gaagaaacaattgaaatcttatttggtcttcgagaccgttatgaaaaacatcatcaatta
acaatgtctgatggcgctttggcggcagctgcgaaatatgctaatcaatatatttctgat
cgattcttgccagataaagcaattgatttaattgatgaagctggttctagagttcgttta
ctaaattctcaactacctccagctgcaagagaattggataaagagttaagagctgtatta

aaaacaaaagatgaagctattagagctcaaaaatatgaaacggcagaacaatatagagca
agagaaatggaaattaaagctcaaattgcagcaattgctcaaagcaaaaaaaatgagccc
gatttaaacttagaagatcctgttgtaacagaagatgatattgctgaaattgttgctgct
tggactggtataccagtaactaagctgactaaaagtgagtcagaaaaattaatgcacatg
gaagaaactttacatgggcgtatcattggccaagacgaggcagttgtagctgtatctaga
gctataagacgagcaagagtaggtctgaaaaatccgaatagaccaattgcaagtttcatt
ttttctggaccaacaggtgtaggtaaaacagaattgacaaaagctttggcttcttatttt
tttgggtctgaagcttctatgatacgtttagatatgtcagaatatatggaaagacatact
gtatctaaattaattggttctcctcccggctatgtaggttatagtgagggtggttatcta
acagaagcagtaagaaaaaaaccatatactgtaattctttttgatgaaattgaaaaggct
catcctgatattttttaatcttcttcttcaaattttagaagatggcagattaacagatgcc
aaaggtcgaactattgattttaagaatactcttctaataatgacttccaatattggatct
aaagtcattgaaaaaggaggaggtagtttaggattcgagttgtcagaagaccaaacagaa
tctcaatacactagagtcagatctttagtaaatgaagaattaaaacaatactttagacca
gagtttctaaaccgattagatgaaattatcgtatttcgtcagcttaccaaagatgaagtt
agagaaattgcagaactgatgttgaatgaagtttttgccagaattaagcaacaggatatt
caactaaatgtaacagaacgttttaaacaacgactagtagaagaaggctataatccaagt
tacggagctcgaccacttagacgagcagtgatgagattattagaagatagtttagcggaa
gaagttttatcaggtaaaattaaagctggcgatagtgctgtagtagatgtcactagcgaa
ggagaagtaacagttttactaggtgaaaaattagaactgctaacgtaaa------aaata
atttattaatctaaaagcattgaatataattt--attcaatgctttt--gaagaaaatta
acaagatgggttgctataattgacaacctctgatattctttatatgtatata---aagcc
gggatagctcagttggtagagcagtggattgaaaatcctcgtgtcaccagttcaaatctg
gttcttggcat-tttaaatatttgaaaagtttgctagatgagcatcaaattttagttgaa
acatacctgttggtccatttctatgttttgctattataatttctgtcaagtctcctccta
cagcttcttgagtataataactctctctatatagcattataactaaatctgcatcttgct
ctatagaattgtgtacgataaagttgttcgcgataaaattatgtaagagttgacattcta
aatcaaatacttgttgcaagctaataaggcgaacatgtttaagattctcaaaaattaaaa
ataaagatggacaagaatttaagagagtttttctttctttaatggcgtt------tattt
gaatagcaagcatattattgtgattaatttcatcgcatcttttccatccttgtgttgtta
atagcttgtgattgcttgttaactttatatacttgccagattgtgtaataatttcataca
cagtttttttacctgtcttggaaatattagctttactgtatctaaataagtttgtttttt
gtgcagtaaagctattaacttca-atatgcttatgatgaaatttaaatagaatttg--ag
tctgactatgtaacggtaaatgaagatgactaaatttcgagatacatccactttctctta
gatcagataataaaggtcttttatcgctccgagtttcaagattcctattcagttgagata
atactagtataggtaaatttaagtcttttgctaatatttttaatgatctagtaattagag

atagttcttgagatctattatcagattgtctgctatcttgtaataattgaagatagtcta
taataattagttctatatttttcccttgtaattttaagaattgtactttcgtttttatag
tgtccgtagaaatttttgcgctatcatcaatgtaaagattcagatctgcaagagctttgc
tttttcgaataacatactgccattcattattattaagttggccagattgaatttttttgac
tatttaagtgacattcttgagctaaaatcctccttaggagttgttctatagacatttcta
agctaaataaaataacgaacgatttctctttgctcattatatatctagctacattaatag
caaatgctgttttccccattgaaggtcttcctgcaagaacaatcaaatctgacttttgaa
aaccttgtgtaatagaatctaattctgtaaaaccggaaagtacgctactatttatactaa
ttttttttgttttgtctaaatgaacaagcagattagcaagaatggtagctagtgtttgtg
tatctttctcttcaaggatttcgtaagcttttgttaattggctaacaatagaggttatat
tattctgtctaacaggctggtttgaacagctaattaaaaataatgaatctccagatatta
aaagtaatcttttaatataattatctataataactgacgaatattcatctaaaacatcag
ataaaggtgcttgttctattagattaataacttcgtctagttcattcaagtgtctaatta
ttttttctgtttttaagttggctacacaatttcctatagttactctttttatatgattaa
cattttctaggacagctctgtaaagtaatgaattcgatgtaaaataaaaaaaaatcaggag
agagtttttctactgattttagtagagagactgattgtttagttaatattgtactaatta
atattttttcagctaaaatattatgaggaggtaaatatttatga------atactgatta
gttcctcc-ttgtaacattattctaata--------agactaattaattactagcttaat
ttgactctggcagaacttgtagttggatgttggcagttacctggtttaatagcttaacct
ctatattataaatgccaactgttttaatctcgggtaaaaggatattttgtttttcaatat
ctatatttgtagcatttttaatagtttgtgaaatttctttttctgtgacgcttccaaaaa
tattttgcccatcccctgtttttttagtcacgctgaacttctgtatttcctctagaagtt
gctgaatttttttttgcatcttctttagctatttaaatttttcttcttttatggctgcgt
ataattttagctttttttaaaataccgtttgttgcgactattgccattttatttgggatta
aaaagttccttgcataaccagcagcaacttttacaagatcattacttttgccaagtttttt
gaatattctcgtttagcacaacttttataactttttttacccatatttattctgaattcta
aaaa----atatatataattttattatacttgttgtacccagatttt----aataggaag
tatttacttgttgacatagcatctccgtgaaactgataatgaattttcatctttattctg
gtataataatttatacaacgtttt-----attaggagaggtggccgagtggttgaaggcg
cagcattggaaatgctgtttaggagtaatcttaacgagggttcgaatccctctctctccg
tataa--aaattatgataatctttatatagtccagtcaaaacttccaataatgtttctcc
atgcatttaaaccacctttgactctcacaatattgagcttttgagctattaataacttag
aagcaaatagagatctagaatctaagctacagtatacaaagcaaattttatctttacaat
ttatattagagtaatgtattgttttgagttgttttaaaggtatatttaatgagtgaatta
agtgatttttttgatattcttcttgatttctgacatcgattaaaatatattttaggtctt
tgcgatttaaaagtttttgtaattgaacaacattgatttctcttataataagattaggtt

gatcatagctgtatctgttccagtatttttaga-------ggataatacaaatttagta
tttaaaattttaaacttactaaatgataaagttaaagcgttataagttaatatagtaccg
cttaatatagacttgtaaccaagaataattttaatagcctcagttgcttgaagtgtacca
ataatacctggtaataaacctaaaacaccagaattgctgcatgtatctatttcactattc
ctatttacgactttattgtggaagtctcgataacttggacctccttgatagttaaataca
ctaacttgtccttcaaattgaaaaatagccccataaatatgtattttatttaattctagg
caagtatcatttagcagatatcgagtacgaaaattgtctgatccatctataataatatcg
tagtttttaattatatcaaatgagttgttataggttagtcttgcttcaaaagcttttact
gtacattttggattcaaatctaatatttttttttagctatttctacttttgagtatcct
atatcgtgaactgtatatataatttgcctttgtaaattagaaagatcaataatatcgtcg
tccataataccgatgcttccaactccagcagctgcaaggtaaattattgcaggagagcca
agcccaccagcaccaataaatagcaccttagctttttttaatctttcttgcccctctaat
tgaatctgtggtaacactaaatgtttagaatatcttgtatattcttctaaagagtatttt
atattatctgctttaaagttcagcat-aaaatttatatgttaattaataacaacta----
--gaaactaattgataagcttattgagatgtttttgtaaactaggttcttctttatcta
aaatagctccttgcgtaggacagacctgaatacaaatactgcagtcaatacaggcagaaa
agtcaatccagtaccaattggtattaatactattttgtccttgtccttggtgaatacaag
ctactggacaagcagatacacattcggcaactccaatgcatttttcggttacaattgtat
gagacatgttatttaattaat-aaaagataaactatttacatctagtagttttattgtat
actaaattgacatacgcta-ccaagccagtaattaaaataatctacttttatagtttact
gatttgatctgcgcctttagttcagctggtagaacgcaggtctccaaaacctgatgtcga
gggttcaagtccttcagggcgcgcttagtttttgttatatttgcatagtagcattttacc
tttttgcaaacatcaaaatgatatgatataaaacatcatattctatatatttgatgtttt
atgtgccagtactg-tttttttatatgttattattggtaagttaattatattaattctat
ttttataatcaataaaaaatagctgaaattaagtgcagattcacttagtga-----tttt
atatatataactaaaaaaattaaaaactatggctaaaaaagttaccggaattgttaagtt
ggcattaaatgcaggtaaagctactcctgcaccacctgtcgggcctgctttaggtcagca
tggtgtaaatattgtcatgttctgtaaagagtataatgcgcgtactgcagataaatcagg
actagtaattcctgtagaaatttcaatatatgaagatagaagtttcacttttatacttaa
aactcctcctgcttctgtactaattgccaaagctgctggcttaaataaaggttcaggcga
accaaacacaaaaaaaaataggtagtataacgtataagcagttagaatctattgcagaaac
taagctaccggatttaaatactaacaatgtacctcaagctatgaaaatagttgaaggtac
agctaaaaatatgggaatcctaattaaagattaaaaaatttcaattataattatta-tt-
-tttatgaaaaaaatttcacgtcgacttaaaatattaaaatctcaagttgagcctaaact
ttataatattgatgaagcaatatcgatgttaaaggcaacatcaaatgctaaatttaagga
aactgcagaagctcatatttgtttagggctaaatcctaagtatgcagatcagcagcttag

agcaacagtcatattaccaaaaggaacaggtaaatcaataaaagtagcagttattgccaa
aggagaaaaattagcagaagcaattagtgcaggagctgatgtaagtggttcagaagaact
catcgatgaaatattgaaaggtagattagattttgacaaattaatagcaacgccagatgt
tatgcctctaatagcgaagcttggccgtgttttaggacctagaggattaatgccttctcc
caaagccggcaccgttaccatagaagtaggcaaggctgtgaatgaatttaaaggcgggaa
agtcgagtatcgagttgatagaactggcataattcatgtaccttttggaaagtccagttt
tcctcaagaagatctagtcttaaatctgcaaacaattaaagaatcaattgatagaaataa
gccttctggtgcaaaagggaagtactggaaaactttttcttatctagtaccatgggacc
atccatccaaatcgatattaatagtcttctatgaaaaatatttatttaattacataactt
gaataatatattatttcaaatta-taattggctagcaatgttaatagaataatttgctta
ttataccttatctgcaccaaaaattttaatatatctcttactaattatgagtacaaaagt
tgaaaatattttagaagaactaaaatctttaaatcttctagaagctgctgaactagtaaa
acagatagaagaaacattcgatgttgatgcgtctgctgctacaggaggcatgatgatgtc
tgcaccagctgtatcaccagccgcatctgaagttgaggagaaaacagaatttgatgttgt
tttagaagaagtacctgcacctaaaaagatttctgttctaaaagttgttcgctctttaac
cggcttaggcttgaaagaagcaaaagatcttgttgaatccacgcctaaagttctaaaaga
aggtgcatctaaagatgattcagaagctatgaaaaaacagttagaagatgctggcgcaac
agttgttattaagtaataaa-t--agtagtgcttttgtttagaagtaaacaaaagcac-t
ttttatttaatagaattaattagactagaaaagtccaagtgtaattgcattattaatagg
catagtcgcaccaatacctaaccaaacacttacaactgtaccaattaagaaaacagttgt
tgcgattggtcgtctaaaaggattttgaaacttattgacattttcaatgaatggaactgt
aagtaaccctgctggtacagctgccatacttagaacacccaatagcttattaggaattac
tcttaacagattaaatgttggaaagaaataccattctggtaaaatttctaatggagtagc
aaatggattagacttttcacctatagatgaaggctctaaaattgctaacccaatactgca
tgcaaaagttccaataattacaactggaaagacatataatagatcatttggccaagctgg
ttctccataataattatggcccattcctttgctaatttagctcttagttttggatctgt
taaatcaggtttttttaagaattgacatgttatttttttgtgtttaatataaaattgagtct
aattactttatcaaagtgtagttatacattgttataatggtccggaaattccttgtttgc
gtatcattaaaaaatgcataagcatgaaaatagcagttagtaatggaagtacaaaagtat
gtaaactgtagaatcttgttaaagtaccttgtcctacactaactcctcctcttaataatt
ctacaatacttcctccaataactggaacagcatctggaacgcctgtaacaatttttacag
cccaatatccaatttgatcccatggtaaagaataacctgttaccccaaatgaaacagtaa
gtactcctaaaattacacctgtcacccatgtcaattctctaggtttttttaaaaccacccg
ttaaatagacacgaaatacatgtaaaatcatcatcaagaccatcatactagctgaccatc
tatgaattgatctaataagccacccgaagttcacatcagtcataatatattctaccgatg
tgaaagcttctgcaactgtaggtctatagtagaatgtcattgcaaatccagttgcaactt

gaattagaaaagatacaaatacaatcccgcctaagcaataaaaaatattaacatgaggtg
gtacatatttgctagaaatatcatcagcaattgcttgaatttctaatctttcttcaaacc
agtcataaattttactcataaaacagcttcaaaaagc-tcttcaatata-tttttgcggt
taagctacttttgattcaatag------ttattgaacttatatt-attttattatctaca
cttattaattattataacatctttctatctattt--------------------aaaatt
ctctgactcaataaaacaggactatgaacgacgatctttttttctgtatagaaatcaat
ttagtctttagcaattcagataaaatccttgttacacttactctactactccctataatt
tgcgctaacattttatgcgtcatagcaaaattaagcataataccattattactctgagtc
ccattttgctctgctagtaataataataagcttacaagtctacttgtaatacttttgtgt
gacatgatttctacaaaatgataagtttttctgagcaacatattaaatgatctataaaa
aataaattaaatagtgggtagttactacacgctcttataatagtgttatagttaatacaa
gcaattttgttttacctattgcttctgcttcatagtagaagttatcattagctaactct
aattgtccgaatgtatctccattagttaataaattgagtgttattttatgtgtgctttta
aagacttttgtaataattaaagatcctattaatataatgtatagtctagagctgctatca
aatagaataatatcattaggctgtagtagaaaaatttgataactaattttattactatta
aaaaatagcaaccatggattataagagggaaaattatttgaactactttcagaaaatgtt
gagatagattttttttgatttaaatgatttttagtcataattcatactttat-a------
---aaaaatactctaataat-taagaactgaaattaaatgtcatacaagttgatgctagt
tgagaatgacattgtattatcaaaagccattcaagaatacttaatagatcaaggatttaa
tgtttacattgccagtaatggattagaagcatttaatttagctaatcaatataatttcaa
tttgataatttctgatataatgatgccattagttaatggctatgaattattagaaaaact
tcaaaatactaaggcgctatcaaagataccagttattttttgacagccaaaggaatgac
taaagatagaataaaaggttatgacatgggatgttacggttacttatctaaaccatttga
cccagaagagttagtttcacttattaaaaacttaattgctagagacatatcgcaacatac
atctacagaaaaatgtgacatctctaagcagtcaattaaccaaaaaatacatttgactcc
tagagaagcaagtgttttagaccttgtgattgacggactaactaacaaagaaatatctac
aatattagatactagtattagaaatgtagaaaaatatgttagtcgacttttacaaaaaac
taatatgaagaatagaactttactagttaaatattctataaataataaattactagataa
tgtaactaatcaaagggcgaatgacgggactcgaacccgcgaatgatggagccacaaccc
attgccttaaccccttggctacactcgccatattataaatatcactataacttgtttgtt
gatatatcgtctactctgtaagtcatttatttatctaacatattatctaaatttaatctt
ccatgacgtataataatattagtattcaaattaatggagagccgtttaattgctcaaagc
ctatttccttgcaatttttattaaattatcttgattttaattctgagcatatagcagttg
agctaaacgacactttgttacctgagcctctttttcactcaacttacttaaatgatcaag
ataaactagaggttattactattgtaggtggtggataaatttcattaactaa--------
---------ctattttaaatgcttcattgataaggataaacgtccctgcttcttgtccac

atgaataatgacagctttaatagtatcgcctattttaaattgtgacgatatctgatctaa
ttgttttatgttaatttcagagatatgtacaagacctttaagattgccaacttttataaa
taatccgtaaggtgtaatctgatttataattccttcaataatattaccaacaattaaatt
tgatgaggcttgagcgattaaagctcttctgtggcttagtattagattattagatctttc
ttcaacattgagtagctttaacttgataaattcattattagccttatcactttttttggaa
attcccgagatgagaatttggtacaaatccagatataccttcaaggctaatgatcatccc
tcctttattaaatccttttattcgaacattaagtaaagaatcttcagctaataattgtcg
aattcttttccatgctcttatatattcaaggcgccgaattgataaaattaattgttttga
tcgaatattataatctagtaaaaagaattctcttgtatcattaatgtttaaagaagtgaa
attatttaaatcttgattacttgacacctcttgaataggtaagtatgcagatattggtgt
tcctatatcaaccaaaacaccatttaactcaaagctaaatattgtaccagcgacaatatc
gccaagatttaaatcgtatttgtatttttgtaaaacagctgcaaaatttctgtgagtaaa
tccttcattattttttgtcatattgaatagtctgaattaaagattgattttgtacaaaat
attaagttgaataaacttctgtcgtattatatgaaaatttttaaattcgttctgtggtta
ttcctgatctaaacgtattagaatatacggaagacataaaaagtaaacgtagatagctac
agatttaaccaatatacagtctgaggaaagtccgggctccataaatacaatttatgctgg
agagatcccagtgtaggcaattatgaggatagtaccacagaaataaaccgccagaataaa
a----tatactagctggtaagggtgcaaaggcaagttaaaagcttaccaaaaatactgta
aagtatttgttaggtaaacccctaaaatggagcaaagctgctaaacaaatatttgtgtat
attttatatagtttagtttaaaatactgcatgaagttattggtaacaataactctagagg
aatagctatcctttacaatatata-tctattatattgaaagaacagaacccggcttatgt
agtacttttatgtcttacattattctggctattgccgaatctctaagtttaagtgttgt
atgatttttcttgtagctcatgttgtttatcatctatctctaaatttgttaaagttttt
tgactagagcgatatgtaaatctcaaacctatactttttttctgttctccaagggattta
tttgtatattgatcaaataaccttatgctttctaaatctttatcattaaactgatctagt
aattttaataagtaattaaattccatcttttaggtattattaatgaaagatctcttgtg
atagatggatatttagagtaaggtcgaatttgataatttaaataattaaattgattatga
gaatcttctaaaatgtttaaattaatttcaagaacaaaaagttttgcagtaagacctaat
ttactataggttacttgattgagttcactgaatatgccaatattagtattattataaatt
aatgttgcagatctatccttttgaatgaaattcactctactgtcaattatttctggtttt
gtccattcaattttctatttattttttgaaaaaaattttctacaattcctttagcttca
taccaatttaaagagtggactggttgtgaccatgtagatctaatatttaaattaccacct
aatatcatggctagattagttgtttcgacaattttgttgtgaacaggattaaaaactgtt
cctatttcaaaactgtctacagtttgattactttgcttaagattataaagattagattca
attaatccctcgagcaaactatagcgtaaattagagtaatcttggataagaggattattt
agactaacacctcctagagatttcactaaagaataatgaactaattcagttaaccctaaa

tttcttaaaatatttctacttttatctatgaattttctcttttgtgataatttttacta
aattgaatacttggcactgagcttttaaatttatgatagccataaattcttgcaatctct
tcaataatatctatttctcgaaaaacatcttgtcttcgataatttggaacactaatttt
atactttcttgattttatgaataatagtaaaatttagagaatgaagaatattttgaatt
tcttgaaagtacaaaaaacgtacctgattattgttactgattggtcctaaaatatcttga
actttttaattgataaatctatatttagagtatcattcattgtttctctgttgagaat
gtctgcctaatattcccgccagctaagtctattattaaagagatagtttcaagataagca
ttttcccagttgtctgtatttagtcctcgttcctgcctaactgaactttctgtcctaatg
ttaagatctcgggaagactgtctaactatagattgtttaaatatagcagattctacaaat
atagatgttgtattagaatccgtatcaaagttgttattggatcctatgccagccacacta
actacatttgtattcacttgagtcactaaaatcttcttatttaactcaatattttcatta
tttaacttaatttagtactttttccaacattaaagttactttgaattgttatagtatct
tcagg---------agtaattttattaaaatcaataatattaataggttgtccccatttt
agcataatataattgcttatatctactaacaaatttctatgtgtaaagccagaggacttt
aaacgatttttagccaatttggagaatctataattttaatttcatcgataattgcagaa
aaatagttattacaatgaatcaagctatgattattgagtatatt-ttgttgtctagaaga
gtatttattactaaattgatttatagtttttaacattaaagatccagttaaagcggataa
ctctcttgaaagacctatcatgcttaatacatccgatctattagctgttgatgatatatc
taatacatgatcaatctgattgcctactgtaatagattcaatagcttctacttcaaaacc
tgcctgtgttaattgactagttaaatgaactagatctattgtttctatattagcaagttc
ttttaaccaatttaaagaaactttcataatgaattgccatttatcttatataattattta
ttttacttgtgattaaaaactaatttaaaaaattcatcaaattaatataagttatttgat
gaatttgcttatacgatatattcttgtgctatattactttcaggtcgtagtaattaagaa
ttagcttttgtaaaagtcagtccattatcttgagcatacctttccataaatctcataaat
ctatcccaatcatcaggacttttaatcacatgtacagcttctattgcttgtggtttacca
ttaataaacttagcattgacatctcttgtaataatttgtccctctgtgtccattagatac
atgcctgttatttcacctttatctgccatactagcatctaaaattttgggatttgtaaat
ctgaaagtagctgttccggtactaccatctctagatcttgttaagcgaacatctggcacg
acttcttcattgatgccttgaataaattgaattgttgccatatcaatttt-tttatttgt
atataatgtatattggataaaactgatttctaattctttaatgcttgttgaaaaactgtt
ttaaaagcttatagtagcagcagatcaaattttgtgtactatataaaactgtaactttc
tttttttg--

----------------------atctgttcaaagaactaacttattataagctaatcata
ctaatattagccatataatgttctgaagt-gaaattaa-gggttgtagctcagatggata
gagcaagcgcctcctaagcgctaggccagcggttcaagtccgctcaaccctgttaataaa
caaaatt---ttgctgaattctacaaataaattagttgaaaaagaggaaaaaaacaaaa
cagc-----aaatagattctgagtaacacttttttattgaa-----aaaaatgatcttaac
tttgataatagaagaattttgtgaaaaaatatttaaaattttgatctctatattcagaaa
aagtttttttaaaatataaactgaaa-----taaaagtcttgagtaacaataggaattaa
attatttaatttttttgaaattgatctaaaaaactttatatgaagagaatagtttttttct
ctttctctattcagtatttgcctccataattattacttcattcattcacaattaataaaa
tggatttg--

-----------------------------ctttaatgaatgtttatgttatttgattata
ttccatactatataagagtttttctcaacttgtgagagtcaatttgtagttttaatttta
taatatactttatctttggagattgatctacattcaccttcacacatctcttttattgcc
tattaggagaaactatatactgaaatttaacaataattgtgaaaatcgtaggttccttca
acatattattatcctttcatttttgtctccactgctgatcaaagcaacagtgattagact
tattcatttagctggttctagtgagagaagttttatacaagccg--gacacttatttaaa
t---gtgttattaccaaagctgttaaaataatagccattaagattcaataacttttttagt
taatagtttataatcgccatgtttgatttagtactgatatatagttacaagtagttctac
tattgataaaattgacacttttgtgactgtataagctttcgatagaaaaactatgatatt
attcataatgaaaaagcgagtattgaagtttgttgctttcataacaaaaaatatgtttag
tcaatgatgaattttgctagcgatccgagtagtaaatctgtcacatgtaaagatgaaaat
tatatatttgagaatactctctatatctgtatatagcggttggcataacttatcagattt
gatttgcagaatcagtatactaagagatcgcaaaatggttattctaagaaaattttgtct
cagtaatttcacttctaatagatacttgttagtaataaacttttaattcttttatgaaaa
aacttcataatatcaatatttattagaactggggtgggaggattcgaacctgcgaatggc
ggagtcaaagtccgctgccttaccacttggctacaccccaacttatagcaaatataatat
cagtcacataccccgccgtgtcaatagattattcgcattatttaataacttttttagattt
ttgtaaaatagaatcttttttttcaaataagatgtactatgttcaaattgcattatgctc
atgtcttcttgttgttgattggacatccattttatggtcactgttccttgttcaacttct
tgattgcctataataatacaagctacagctcttttcttattagcttgtttgatttgttta
ccaaagttacttgagctaacatctatttctattgtaaaaagtttttgatgtaaaaagcgc
attattttcatgccaacttcattagctttttttaccttgtgtagcaatataaaaatcaaca
tatttacttggtaactctatattctctttcgcaagaagtagtaaacgttctaatcctatt
gcacatccgacagctggtgttgctacgcctcctaattgcttcactaagctatcatacctt
cctccaccacaaactgtatcttgaccttttgatgttaatgtcttaatttcaaaagcagta

tcattataatagtctaatcctctaactaacttgtcatttatgttataaggaatatcaagt
aattttaaataattacaaacagcatcaaaatgtctttgtgacgaaatacttaaaaaatta
gaaatcttgggagcctcagctaatatttttgtgtatttatatttttgaatctagaatc
ctaataggattactaattagtctcttttgtgaatcaggatctaagtcgtcatagtactgt
ttcagatattcttgcaattttgcttgataaatactacgatcttctgcttttccgattgaa
ttaagatcaagctttaagtctttgagattaagattattaaatatacttatagctaaatgt
attacttcactatctgctcttgcatctaggctgccaagaaattcaataccaagttggtga
aattgtctttgtcgtccactttgtggcctttcatacctgaacattggcccgctataccat
aatctttgtaaactatgatgatagttcattttattttcgataaaagctcttacaatgcct
gcagtgccttcaggtcttaatgtaatatctctattgcttcgatcatgaaatctgtacatt
tctttatttacaatatctgtatctcttccaatacctctatcatataaatcgctactttca
aaaataggtgttctaatttcttgatagtttgcacattctaataagttggaaattttgtta
tgtataaattgccagtattgaagctcttcgggcagaatatcttttgttcctctaatagct
tgaatttttgccatgataattttcggatcaccttcttaatgtgtttgcattaaatagcag
ttgtagactgttattcagtcatgtattatacattag--aacatgttgatg-tattttatg
aaatacgggcaaggagggattcgaacccccgacaccatggttcgtagccatgtgctctaa
tccactgagctacaagcccacttaacaagctatcttaatatatcattctttttcaatgta
tacaactatattttgtct------atttatatttgaaagttgtctgaaagt-aaaaaat
gt----atttatctttaacaattagagttagtcatgtataattttccatttattaaaact
ttgcaca--aatatcgacaaacttaattatgtaattatcttatactgataatttcagaag
aa-cttgtgcttttacttcttgatcga------ctagtatctaattcatctatattccaa
at-aaaaaatcttatctaaaatgagtaagcaaattctatatcaagatgatgcacgtaaag
cattagaaaaaggcatggacattttaacagaagctgtttctgttaccttgggacctaaag
gaagaaacgtcgttttagaaaaaaaatttggggctcctcaaattattaacgatggagtaa
caatcgcaaaagaaattagcctagaaaatcatattgaaaatactggagttgcacttattc
gacaagctgcatctaaaacaaatgatgtcgctggtgatggaacaacaacagctacagtac
tggcatcagcaattgttaaacaaggcatgagaaacgttgctgcagggtcaaatcctatgg
ctataaaaaaaggcatagagaaagcaacaaattttgtagtgagtaaaatagctgaatatg
ctaaaccagtagaagataaaaaagctattatacaagttgcttcgctatcatcgggcaatg
atatagaagtaggtaaaatgatagcgaatgctatagataaagtgggaagggaaggtgtaa
tttctctagaagaaggcaagtcaactaatacgattcttgaaattacagaaggcatgcagt
tcgaaaaaggctttatttctccttattttgttacagataccgagcgaatggaagtccttc
aagaaaatccatttattctctttacagacaagaaaatcactttagtgcagcaagaacttg
ttccactccttgagcaaatagcaaaaacatctcgtcctttattaataatagctgaagata
tagagaaagaagcattagctacgattgtagttaacaaattaagaggcattttaaatgttg
ttgcggttagggctccggggtttggtgatagaagaaaatctttgcttgaagatatgagta

ttttaactaatggacaagtaatcactgaggatgcaggtttatcgcttgatacagttcaat
tagatatgttagggaaagctcgcagagttattgtaactaaagactcaactacaattattg
cagatggtcatgaagttaaagttaaatcaagatgtgaacaaatcaagcggcaaatagaga
cgagcgattctctttatgaaagggaaaaattacaagaacgattagcaaagctttctggtg
gcgtagctgttataaaagttggtgcagctacagaaacagagatgaaagataaaaaattaa
ggctggaagatgccattaatgcaacaaaagctgctattgaagaaggaattgtcccaggag
ggggagctactaatgttcacatttctagcgagttatttacatgggctaaaaacaatttag
ttgaagatgaattaatcggagctctaatagttgaaagagcagttacttatcctttaagac
gtatagcttttaatgcgggagataacggagcagtagtagtagaaaaagttaaaagtaacg
attttcacataggctatgacgcagcaaatggcacaattgtaaatatgtacgatgcaggta
ttatagatcctgcaaaagttgctcgatcagctttacaaaatgcagcttctattgcagcga
tggttttaaccacagaatgtattgtagttgataaagccaatgactaagtta---------
-----tagataatattaaacaaagaataatttccatagctagaaattattctttgtttca
attaagctaattaattgatcaaatgtataatttttataataaagccgaagtttaaatgta
cctctttgctcaactactaagtagagaagatacaagctcgtaattcctaatttatcaatt
aaagtattattatcatatatggatttttcaagagatctaaaaaatgacattttcgtaaaa
taatatcgaaatcttcttttgtagttatttgataaatcattactgtatgtagattgatct
ccaattgcgtttaaaacttttaaaataatgtttttaagtctctatctgactttatctta
gaatcggtaatcaattgatttaattgaatatcaaaactaagattaagaatattacttgtt
acggatgttcgattttgaatatctatagatttgacagccaagaagattaaacttttattg
ttcattgtaaaatgtaagacaattgatattgtattctttaaatacta-atttatcttct
tgtgcgtttatagttttggagaaaaataattaatattatta--------tattaattact
gcccgaaaaaataaattctggaaatctagcttgagcctttgatagtgagtgtttttttacc
ttcttcttgccagtaattaataacttctgttgtcttatttagtaattcaattctgtctct
ttcagcaatccatggtttggattctagctcggcttttaattcttcccaagcatcattcct
aggccagaaaaaataagaagtaagtggacttgtaccatttccaacaatttggtcaatagc
aatagcaatattattttccaaccaaagtacttttagtgtaaattttgacaagtgattatc
tccattgtaaatagcaaacatatgtatatacagtaaagtactacattagttattttttt
agcaacaagctgagctttatttaaaatattctgtaccgttctagtagtttgtgcaccttg
tgctaatctctgtaaaatttttgaaatatcaatacgagtttcattagttataggattata
gaatcccaattcttctatagccttgccatctcgtttgtttttgctatccattacaacaat
tctgtagcttggctgtttttttcttccatatctctttaatcttaacttgaccatcttagt
cccctcgcttattttttaatatctttattaaattatatcttacgatattatgcaataat
tcgaacaattaagcagactcaagcctgacattattaaatattaccattaaacactggaga
aaaataatacctagcataggtgacatgtccatcccgaacattgggggaatactgcctcta
aagagctttaaatatggatcggtaattcgatttaatgaacagaatggttcattataccag

ttcacagttggaaaccatgccaatgataatttaagtaaaattaaaattaaataaatctca
gaaaaattagctattgatccaagtaataaatttaatgtaccaggaagagtattcatacgt
attttataattattatatagttcattatactaatagtttgagtatatattataagctctg
taaataaat-tagctaaacaaaaaacttattcttaaaaatatcttctcttgaacaatttt
cttcattgatagcactgttaactttcgttgtataaatttctaatttagaccaattatttc
ctagctgatttagttgagatgttctgcattctaaacatgctatttttatattatttaaaa
tagctccttttttttctaaatcttttaagactggtgtaataattgcttcatctaaaaata
aatccaagatgagaatttttgtaaatgaatttaattgagctggaacattattaacaatgt
tgttgctattaactagagctatattagctgtcggaagtaaagctcttgctccctctgcta
gtataaatccataaggcataataatgacaataatatacttataatgactgttaataagat
ttacattgatattagtttttaattcaatagtttctgttactaaccattctctcattattt
cataagtaatccatttacctagttctgagcaagcagttcttaaaattgtacctggattat
tgctattttcgagaatcccagcccaatgttgtattaaagggtgggaagctatattaattt
tgagttgcatgttttgctaaatatatttaacatttaatttattaattgtcgctatagttt
atgttcactgagtattattaaaatagcat----atatattaaagaaagcaattat-aaaa
aatgaaaaaaactctttggctttgggggttcacagatagtgctgaaacttggaatggtag
atttgcgatgattggttttatagcagttattattattgaagtagttacaggacaaggact
attatatctaataggtgttatatcataataaaaaatcaaaaaagttcttgtaataaatta
caagaactttttga-ctgaatgtaaatctaatcgagtggtctcatagatagcactggtt
cattctctctgttaataccttttcaaatccagcagctgcagctcttgctctaccagcat
gccaaagatgtccaataaataggaagaatcctaagaagaaatgagatgttgttaaccaag
atctaggagatacatagttaactgagttaatttctgtagctacaccgccaacggagttca
atgaacccagtggggcatgagtcatatattctgctgctcgtctttcttgccaaggctgaa
tatcattttaatttattcaaatcaagaccatttggtcctctaagaggttcaacccaag
gagctcttaagtcccaaaacctcatagtttcaccaccaaaaataatctctccactaggag
atctcattagatattttcctaagccagtaggcccttgagaagatgcaacattagcaccta
atctttgatctctaactaagaaagtgaaagcttgagcttgtgaagcttcaggaccagtag
gtccataaaactcacttggataagctgtattattataccaaacaaaattagaagctgtaa
gacccatgattgataaagcacctaagctgtaagataagtatgcttcaccagaccatacaa
aagctcttctagcccaagcaaaaggttttgtaagtatgtgccaaattcctccagcgatgc
aaataatgcctatccaaacatgaccaccaacaaggtcttccatgttatttacactaacga
tccagccatcaccgccaaatggagattttaagacgtacccgaaaataactaaaggattaa
gagtaggattgctaacaaatctaacatcaccgccacctggagcccaagtatcatataccc
caccaatgaacagagctttgataactagtaaaaaagctccaatacctagtaaaactaaat
gtataccaagtatagttgtcattttgtttttatctctccaatcatatccgaaaaaaggaa
aagattcttcaagagtatcgggtccgattagtgaatgataaagaccaccaaatccaagaa

cggctgaagaaattaaatgaactacacctactacaaagtatggatatgtattaaaaattt
cgccgcctggacctactccccagcctagtgtagctaagtgtggaattaaaataaatcctt
gttcatacagaggtttttctggcacaaagtgagctacctcaaaaagagtcatagcaccag
tccaaaagaccattataccggcatgagcaacatgagcgccaagcaatttgccagaaacgt
taattaagcgtgcattgccagaccaccaggcaaatccggtagattcaatgtctctaccgc
caacaccaacattcgtattaaagggcgtttccacgtggtaaaacctcctcagggaatata
aagttttcatgaggttgatcctgagcagccatccaagagcgaataccttcgtttaatagg
atgttcttagtataaaaagtttcaaactcaggatcttctgcagctcttagttcttgagaa
acaaaatcgtaagctcttaagttaagagctagtccaacaattccaaatgcacttgtccac
aatccagttactggtacaaatagcatgaaaaaatgtagccaacgtttattggaaaaagct
acaccaaaaatctgtgaccagaatctgttcgctgttaccattgaataagtttcttcagac
tgtgtaggcgtaaatgcccggaaagtatctgcagcatcaccatcttcaaataatgtattt
tgcacggtcgcaccatgaatagcacatagtaaagcaccacctaagatcccggcaacaccc
atcatgtggaatggattcaaagtccaattatgaaatccctgtaagaatagcagaaatcta
aaaattgcagctactccaagacttggagcaaagaaccagcttgcttgacccaaagggtac
attaagaacacagatacaaatactgcaattggtccagaaaaagcaatagcattgtatggt
cttagaccaactaacctagcaatttcaaattgtcttaagcaaaatccaattaatccaaac
gatccatgtaaagcaatgaatgcccataggccgccaatttggcaccagcgagtaaaatct
ccttgagcttcaggtccccaaaggaaaagaagggaatgacccatgctgtttgctggggta
gaaacagcagcagtcaaaaagttacatccttctaggtatgaacttgctagtccatgagta
taccaagaagtcacaaaagtagttccagttagccagccgcctacagcaaggtaagcgcaa
ggaaacagaagtagtccagaccaacccacgaatacgaatcggtctcttttagccaatcg
tctacaagatcaaaaccaccacgagtttttttcttgtccaattgctatggtcataatttta
atctccagagcaaatttaataagtaatttgttcaaataaaca---ctttagatctaatct
aaaattttacttttctttacggtcaatataattatacgtcaaaatcaacaaaaattcacg
tttttaataaca-ttttttagctcgttaagttaactatctaagaat------------
taactgtttttaaattaataaaaattataacttgttcatttctataattctctgaaata
gatatcctgttcctcttgctgttaaaatcaagtctggattacttggatcatcttctagtt
tagctcttagccgtgagatatgcacatcaacaactctagtgtctacatgtcgttctggtg
tatatccccagacctcttgtaagatagatgctctagaaaaaggttcgccagccttactca
ctaaaagttcaagaagactaaactccattcctgtcaaacgaactctctcattatttttat
aaacttgtcttttattcgtgtctatctttaaaaatccaatactaattatgccagaatttg
cgacgccaagattagttgttattttatcagctcttctaagaacagatcgaatacgagctt
ccagctctttaggagaaaatggtttaactacataatcatcagcacctatttctagtccag
taatcctgtcgcacacttcgcctaaagctgttaacattattataggaacatcagattctt
ttcgtagttcttgacaaactccgtaaccatcgagtttaggcatcatcacatctaaaacga

caaggcttggatattcttgtctgaacactattaaagcttcctctccgtttgaagctgtta
tgacttcgtaacctattattgttaatcttgtttctaaaattcttcttatgctggcctcat
cgtcaacaacaagaatttttctttctggttctccaa-tttcttatttactcctataagt
caacaagcataactactataatcttaaatttgtacctgtttgagtattctaatttgctgt
aaaa-----cttatttattataaattttataagtt-----tagattaattggtatcagt-
-cattatatcttgaaatatctgtttgtcatttatctc---------tattttaatgcata
agttgattcaagttttcagaattcagtatataaata-ctttttagacaagtctttgacat
tgtattttctgaataaaaaaggggttgacaaaccggctgatagaaagtattctacattga
tataaa----ttactgatcataaacaaacagtaa-----atttttttagagtagcataag
tactttaat
>Pyr_35
tatatgattaataattgcaacatcttttatgtaagaattcctaaaatatacaaaactttt
tatttaatatataaaaagtaaattagaataagttgagcatattttgttctactttagagc
ttttgattgag------attctaaatagaaacaaagatgtttacatagtttatatctata
ggaataaaaatgaaacttgcagtttatggtaaaggtggtataggcaaatctacaactagt
tgtaatatttctgtagctctttcaaaaagaggcaaaaaagttctacaaattggctgtgat
cctaagcatgatagtacatttactctaacagggtttttaatcccaacaattatcgacacg
ttacaatctaaagattaccactacgaggatgtttggcctgaagatgtgatttataaagga
tatggaggtgtagactgtgttgaagctggcgggcctcccgctggtgctggttgtggaggc
tatgttgtaggtgaaacagttaaactcttaaaagaacttaatgcttttgatgaatatgat
attattttatttgatgttttaggtgatgttgtatgtggaggctttgcagctccattaaac
tatgcagattattgtctgattattacagataatgggttcgatgctttatttgcagccaat
agaattgctgcttcagtacgagaaaaagctagaacacattctttgcgacttgctggatta
gttggtaatagaacagataaaagagatctaattgataagtatatagattgtgttccaatg
cctgtattagaagttttaccattgattgaagatattcgtgtgtcccgagtaaaaggtaaa
actttatttgaaatgtctgaaactgaccaagatttagcgtatgtatgtgattactatcta
aatattgcagaccaattaattactaggccagaaggcgtagtccctaaagagtctcctgac
agagagctgtttagtcttttatctgatttttacttaaatcctaaatctaatgtaggacaa
actgtagtagatcaagaagaattagatttaatgatagtgtaaataatattttaagcataa
gataaaaaggaataagacaatgtctaaaattcaatcagacgctcttactttgaatgtga
aacaggaaactatcatactttttgtccaattagttgtgtttcttggttatatcaaaaaat
tgaagatagtttttcttagttataggaacgaaaacttgtggttattttttgcaaaacgc
aatgggggtaatgattttcgctgagcctaggtatgctatggcagaattagaagaaggaga
catttcagctaagctcaatgactatggtgaacttcgtagattgtgcttgcaaattaagaa
agatagaaatcccagcgttatttttggattggaacatgtacaacagaaattataaaaat
ggaccttgaaggaattgctccaaaattagaagcagaaattcgcgttcctattgtagttgc

tagagctaatggtttagattatgcttttacacaaggtgaagacactgttttagcagctat
ggctcagcgttgtccttcaaatttaaatatgcaagcaggtagtaatagtcatgaaaattc
ttctcatattcctttagttttatttggctctcttcctgacccagtggtaactcaactaac
tatggaattaaaaaaacaaggtattcatgtttctggctggttgccatctaagaggtatac
tgaattaccggttatacaagaaggctattatgttgctggagtcaatccatttcttagtcg
aacagctactacattaatgagacgaagaaaaacaaaattaattggtgcaccatttccaat
aggcccagatggtactagagcctggattgaaaaaatttgctcagtattaagtattcaacc
tgttgggctagcagatagagagcaagaaatctgggactctttagaagattatattacttt
aatacgaggcaagtctgtctttttttatgggagataatttattagaagtctccttagcaag
atttcttactcggtgtggaatgactgtatatgaaattggtattccatacatggataaacg
ttatcaagcagcagaattagctttattaaaaaccacatgtgataaaatgaaggtcatgat
gccaacaattgtagaaaagccagataactataatcaagtagatcgaattcgtgatttaaa
gcctgatttagttattactggtatggctcatgcaaatcctttagaagctagaggaattaa
tacaaaatggtctgtggagtttacctttgcgcaaattcatggttttacgaatgcaagaga
tattttagaactagttacaagaccattgcgacgcaatcttagtttatcagaattaggctg
ggacgtttatagtaagcaaaggtagtattaatt---aattgcgacttacatggtataaaa
tccttgtaagtct--tatatattatgctaaagaagcatttcttgctcggccatcttctgc
ccaagcttgtaaattctctacttgttctttatctgtaaatgctaaaggtataaactgctt
tatagctattttaatatcttctgtattaaattctctttgctcactaaaagctgtatgcat
actttctataattgcttgctcaatttcagccccagagaatttattacataataaacttaa
ttgcttaatatcatattcttgccatgattttggtcgaacttttgataaatggatcctaaa
aattgactctctttccttgcgattaggtaaatctaagaaaaaaatttcatcaaaccttcc
ctttcttagcatttcagaaggtaaactttgaattttattagctgttgcgacaacaaatac
gggagtcttttttttctgagagccaagttatgaatgttccgaatactcttgcactagtacc
actatcaccttggctatacaagccagaaaaagctttatctatttcgtcaatccataatat
acaaggcgataatccttctgcaatgtttatcatttctctcattttttgattccgactcgcc
aactaatcctccgaataattttccaatgtcaagacgtagtaaaggtaaagtccaatcgtt
tgcaatggctttagcggttaaagatttgcctgtgccttgtataccaactagtagtaaccc
tttaggtgaaggaataccataatttaaactttgttttgaaaaagagcgagaccttttttg
tagccattgctttagtacatctaatccaccaatatctctatttactttattatagggata
aaattctagtagacgtgtttggttgataatttgtctttttttcttcaataataatcggtaa
gctgcgagaatctatttgattatattgtgcaataattttggtaattactttgcggatttt
atctattgatagaccttggcaagattttgtcatattattaactagttctgaatcaagatc
aagatttaccgctttacttaatcttgtgatttctttttttgatttctagaagacttggtaa
aggcaaatctattattgttacaatatctcttaacgcaaaaggtatatttattttacaaga
aataataattatgtgtttagcttgtgtttttattacttttgccaaattgcgaactttcct

agttagaactatttcatttaagaaagaatcgaaatcttttaaaataaaaatattcaaaga
ttcactatctaaattttcaataaactctaatgctagcaaaggattccgttttgcataacc
attatcgttagggttactagtgtatccatctacaaaatcccagcaataaacttgctgatt
gtcagaacaatgtaatttatttttataatatattctagtctttcttcttctctagtatt
tattacaataacaggatatcgagactttaatagtaatcgtaaatcttgcgtgaaattcat
gtcaatatttttccccattagactttatacttcaccaatta----aagactaaataaga
ttatctaatattgcatgtatatattcaaaagcc------atgtatctattttctaatcta
ttgtgatttccaacctaactagtcatgatttttttcttcctttgcgccgtctactattt
aaaatagagcgtccacatggtgttttcattctggcgcggaaaccggaaactctgattttt
tttcttttcgatccctgtagtgttctcttagtcattatgtctcctgtgttgtaaaagatt
aaatattattttatcatcttttatatgtcttgataatatatctctgtattaatatcaaag
tt-aaatgcaataatttatattaatcaagaattatttatataatattaagaattaattat
----------tagctttatgatattagctttgcctatttttttatttaagtatcctgacaa
tgtttttgctaattttgaattggttaatctttcagcagctaaaaataatttgtcattag
aatcacaatttaaatattttattgaaaaaagccaaaatgatatattatcagtagaagaaa
gctttgcctttgcaaaagcttgtgtagcaaagaagtgcttttctagagctattatcgaag
gccaattagcgataaaaaagagcttacaattaaatgataaagagggcagtataataattg
ctaatttgcataatatgcttggttttatttattctgaagcaaaacaactaaatatcgcaa
aaagcttttaccagcaagctttacaaataaaccctaattatgttgtagctttaaataatt
tagctaaagtttacgaagataccaaagatttgacaaaagcagaggccatatatgataagg
tattaactctcaacttagataataaaatagctaatagaaggaaaaactatataacaaaaa
aca---------gaaatgcttaatgttatttacaatcgggatagcaggatttgaacctgc
gacatcctgctcccaaagcaggcgcgctaccaaactgcgctatatcccgttagacataac
tagtactatataccttttatgtaattatgtctaccgtttcagtgttttatctaggatacc
aaaacatgcctttaactccatctggatctttaatgaatcctaatttttttataaaaactaa
ctacatcaggttctgcaaataatgtaatagtgctaatttctgcttgtctcaattgtttta
ttaattgatgtataactacttttcctaaacctacgccctgaaaatcaggatggataacaa
catcccagatagtagcattgaatccattatctgatgttgctctagcaaatcccactagtc
gagtattcgcatcttttttttgtattaaagaaatgataatcgaactatttttaatgcga
tttttaccttttttagaggtcttttaacccatcctaccgaatcacaaagctgctccaatt
cgtataagttaatatttttagtgctactcagatagatatctttaaattcggctctattac
aagttttgtctaaaacgacaagtttttttgaaatttttttcagaattacttttgatatgtg
agttttgaaaaaaatttttccagaagatcataatgattatgatagaattaaataaatata
tatatattatgatattgccttaataaacaacatattaatatatgtccagtttttaatacg
aagctattaagaaagaagattgttttaagatatagaaataaaaatttttgtaaaatcttt
taagaaaagtatataaagagctctttttaagattaacatctattagtataaataaagtct

tgcaattgagatgagacaacatattattctatatctaatgccttttaaaggagttatttg
tgaaaaaatccatgtttctaacctgcctacttgctttattactgcttagcaacccgataa
tagctaatgcagaagtagctgggctagtgccttgtaaagattcggcggcattcaataagc
gtatggttaatagtgttaaaaaacttaaagctagattagctaaatacgatgcagatactc
ccccagcgttagctttaaataagcaaatagaaaaaactaaaacaagatttgctacttatg
gtagagcaggtctattatgtggcacagatggcttacctcatttaatttctgacggtcgat
ggagtagagccggagacttcgtttttcctggattactattcttatatattacaggctgga
ttggttgggtaggtagaggatatcttttatctgttgctaaaactagcaagcctactgaga
aagaaattattttagatgtgccattagctattaaatttatgacatctggattcgcatggc
cattagctgcttggcaagaatttagtagtgggcaactgattgcctctaatgatgatatta
ctgtttcaccccgttagtaaaaaatttatatgaataataattttaccaaatatttatcaa
cagcacctgtagttggtgtgttatggatgacatttacagcaggttttattatagaattaa
accgtttttcccagatgttttatatttttatttataaataaataa--aatcttataaa-
--gaaccacacaaatatagtaaaacactattatttgtgtggttctt----atataaaaca
tcattatttatttttactgattttttttctttcgactattttaatattagattttatat
aataaaaaa-acatcaatattatgagtttagtaagccaaattatagttaatgctgatgat
gaattaagatatccaacaatcggagaattgcagtcaattcaagactatttaagtacaggc
agtaataggattagaattgcaactattattagggataaagaacaagaaattgtacaaaga
gcgagtaaacaaatttttcagctacatccagaatatatagctccaggaggtaatgctgca
ggttccagaaaaagatcattatgcttgcgtgattacggttggtacttaagactaattacg
tatggagtattagctggtgataaagattctatagaaacgattggtatcataggtgttaga
gaaatgtataattctttaggtgttcctataattggaatgttagatgcaattcaatgttta
aaagaagcttctctagagatgcttggtcaagatgacattagaattatttctccttatttt
gattacataattcgaggtatgtcataaatagtaaattgaattatttggttgaataaaagc
ctatacaatgctataatcagtttaagcccgaaagcatataattgtaaaaactaaatcttg
tcagaaccggaaggtagcagcaataatgtttaattacaaaagttatga-ttttcgggtgt
ttactatttgtacttaatagtctctaattctaattatgatattatccataaatc----aa
taatagataactcatgaaacctttcagtaaaaataa-ttaataatttgatattaatacgg
aaaccacctatac-ttttttataaaatttctttatatctcatctaaacaatggaaactta
acttaatgggtgttcatatttatcaacaggttcgtctgtcccaaattttctgtagaga
atcaacaatttgaagatatgatagagacttctgaccattggatttcaacaagaacaggaa
taaaaaaaagacatctagctccttcttctacttctttaactaagttagctgcagaagccg
ctaataacgccttatctgcagctaatttaaaacctactgatattaatttaattatactag
ctacatctacgcctgatgatttatttggtagtgcaagccagctacaagcagaaataggtg
caaccgcatctgttgcttttgatattacagccgcctgctcaggattcattgtggcattag
taacagcagctcagtttattgagactggttcatatgacaatattttagttgttggagcag

atataatgtcgagatggattaattggtcagataggactacctgtattttatttggtgatg
gtgcaggagcagtagtggtgggacaaagcttaaaaaacagtattttaggttttaagttat
gtacagatggccagctaaatagtcatttgcaattgatgaatactccagaaaataatcaaa
aatttggattcatagatattcctcatggaaattataattctataacaatgaatggaaaag
aagtatacaaatttgctgtatttcaagttccaatagtaattagacaatgtttagataatt
taaatatttcaatagatgaagtggattggtttatattacatcaggcaaatactagaattt
tagaagcaattgcgagtagattgtcaataccttttctaagatgattactaatttagaga
actatggtaatacatctgcagcatcaattcctttagcgttagatgaagctattaaatcta
ataaaattaaaccgggtcaaactattgttttgtcaggtttcggagctggtttaacttggg
gcgcgattgtcttgaagtggtaat---tagaatattgcggatgacgagactcgaactcgt
acagctttcgctacacacccctcaagcgtgcgtgtataccaatttcaccacatccgcatt
ttttattatgaata-ataaaaatttatcacatttatttaaatattaatataa--tgtgta
tatatatatacattcttgtcagaaactctcttatattttcttttaagtgataaaattgag
t----ttagtatagataataaaatttatttacaatcataa----aaataaatatataa
ggaaaataaaatatgacaccgtccttatcaagctttttaaatagtctaattcttggggca
gtaattgtagttgttcccataacgttagcacttttatttgttagtcaaaaagacagaaca
attcgatcctaacaaggacttggaaacatatggagaagcaatttaagattaataataaac
tattttttaaatgtatatgctctccatatatttctacatt--ttgaaagaatttttgc-t
ttttactttgcaatagccttcacagtgtattctgtt-tttttttgtcctcacttactctt
tta----taaagatacaaatgtcagg-ttgataatagt-ttttttattctgtatctttc
atttaatatcaaagtattttatatacatattattatgttcaattaataaagaaaagctca
tgtctttgtctaattggcctctcaaaaaagaaaactccgaagcatatactactaagaatt
caaagcaaatcacaattcctgatggattgtggataaaatgctttgattgcggtctgttaa
tgtattctaaagttttaaaacgaaatttaaaagtttgtcctcaatgtagctatcattttc
aagcttctagcaatgaaagaattgatcaattaatagacagaggtagctggcgacctatgg
atgttcatttaatttctacagatccattaggttttaaagatcaaaagctttatagtcaaa
ggttaaaagatacagctctcaagactgggttacaagatgcagtccagacgggcattggta
atatgcaaggtagacaagtctgtttaggcattatggacttccggtttatgggaggaagta
tgggatctgtcgtaggcgaaaaattaacaaggctactggaaaaagcaactcaagaaaaat
tacctgcaattatactctgtgcatctggcggagcaagaatgcaggaaggcatgctaagtt
taatgcagatggcgaaaatttcttctgcactagaaatgcataaaaaaagcaaatctcttgt
atatctctgttttaacatctccaacgacagggggtgttacggctagctttgctatgcttg
gagatctaattattgcagaaccaaaagctcttattgcatttgctggcagacgagttatag
agcaaactattaaagaagacttgccagacaattttcaaagttcagaatatctatttgagc
atggtttcctagatttaattgtaccaagaactcagcttagatcaaagctaatacaaattt
tatcattacacaaccaaagtaagtaattagtgtcacattgatcgtaacattaaagtgcga

atataaattaagatattaagaaattttcactaaagatatttgttgactaactagaattac
aattactattgtagaaaaatttgtcttaattcta-ttttttcagtcttttaactaattg-
tttctatgactttaaataattgttagtttaatacaaatttataaagagaata-----cat
actaagctaaatatagtaatattatgtttccctataatactgacctatcaaatttcgagg
aattttatgcttaaaagatcttcttggcttgctactatattgggactactaactgtagtt
tctctaagtacgcatgcaaatgccatagagctagacgaggcaacaagaactgtcccatta
gaatcttctggaagaactgtagttcttacgccagaacaagttaagcgaggcaagcgatta
ttcaataattcttgtgctatttgtcataatggtggaatcacaaaaacaaatccaaatatt
ggacttgacccagaatctttaggcttagcaacgccacaaagagatacaattgaaggttta
gttgattatatgaaagatccaacaagttatgatggcgcagagtcaattgcagaattacac
ccaagtattaaaagtgccgaaatttttcccaaaatgcgtaatttaacggatgaagattta
tttacaattgctggtcatatcttgcttcaacctaaaattgtttccgaaaaatggggagga
ggaaaaatttactattaagggttcaaacgcaatagtcttataagtactagtttatgta-t
ttatttcctaagtgtgattattttgttagatcgtgatatatattaaagatggagtttgtt
gatagtaagttatttttttgcctaaacttacttgcattattcgtctagttattaaaaagga
gaagttaaattgaaaaagacgctttcagttcttttcactgtgtttagttttttttatgata
ggttttgcacaaattgtttttgctgcagatctagataatggagaaaaagtttttttctgct
aattgcgcggcatgtcatgcgggtggtaataatgctatcatgccagataaaactttaaaa
aaagatgtacttgaagctaatagtatgaatagtattgatgctatcacttaccaagtgaaa
aatggcaaaaatgctatgcctgcttttggaggaagactagttgatgaagatattgaagat
gcagcaaattatgtattatctcaatctgaaaaaggttggtaattatacttgattttgctc
aatattaaaggatagacaatctatttagttgttcattagattgtctattctttgtttctg
---ttattatatatagatatttacacaatactga-attatgatgaaaagaataccagcaa
ttctcgtactagaagatggtacttattataaggggtggtcatttaagcaaagaaagccag
taatgacaattggagaagtagtttttaatactggaatgacaggatatcaagaaataatta
cagatcctagctacttccagcaaattgtcactttcacatacccagaaattggtaatacag
gtattaataatcaagatgttgaatctcataatattagcattaaaggtcttgttgcgaaaa
atatttgtaaaatttcaagtagctggagggagcaagaatctttagtcaaatatttaagcc
gcaatgatattcctttcattttttggaatagatactcgatctgtaactcaatatttgcgtc
aatttggcacaatgaatggctgtatttctactaataatttaaatcatagttatttaaagc
aaagactttctcaagttcctggaatgcagggttcagatttaattccatatgtaacaacac
aaagcatatatccttgggatgaaaaaagtttgcccagctggtatttaactgataaaagta
gagtcgcactcgatacccaattgaaagtaattgttatagattttggagttaaactcaaca
tactaagacgattagctacactgggatgtcagataactgttgtgcctgcgcaaacatctg
tagaagatattttagctaataaacctgacggtatcctactttctaatggtccaggagatc
catctgtagtgtattatggaattaaaacagtaaaaaatttgttaaatcacaatgtaccta

tttttggtatttgtatgggacatcaaattttaaatttggcccttaacgctaaaactttta
aacttaaatttggtcatagaggtattaaccatccatctggattggaacagcaagttgaaa
taactagtcaaaaccacggtttcgcagttgacttgcaatctgtcttcaaatcatctgtac
aagttactcatttaatttaaatgataccactgttgctggaataggtcatagtcaaagtc
catatttctctgtacaatatcatccagaatcgagtccaggccctcatgatgccgattatt
tatttgaaaattttgtagaaataatgataaaatctaagcgtaaagttaa-----------
ttaataatcttcccatgcttcatgattaaatagcgctgataatacttgtactcctcgtaa
ttgattgaagagaggtagatgccctcttggaccataagaattccattgaaattcatcagg
atatctacaaggaatattatcaacttcccacccaatcttttgccagaacttttcccaatc
tttgccgacactaagccatatttgcctttgaataaaaagcccaaatttacctttgagta
tgtatgccataatttatcaatagtctgaagatcttgagcaggtatttttttatatctgt
aaaatataaccaattgcgattttgtgcattcacgccagctaattgaataagcttttgttg
agttaattgatctgctttgagcaaatcttgttgtgttagcaactcttgtaaatctttgta
attcatatgtttagcagaatttaatggaacaataccctcgggacacaagctagtagtcaa
tctgacgatttcttgatttttactatttaataacttgttgtagattaagccatcgacgca
attacttttataatcgggtctattaatcctctcaaaaaatagatcagctaaacctttttaa
ttgtataaagtcattattatgtatagtctcaataatatcaagttgtcttttga---tatt
atttgacctagtactcttacttagttctaaaagctgaactcgaatttgattttgcatttg
attt-ttaaattaacttaaatagta----attagttcttaaagagaatttgattttttt
gaaaattagaagaacgacgaattatttctaaaagtgttttcataatttgattgaatatgt
tttgtcccgtataaatgataatttgaccaattagaaagactgtgcttttttatttaggaa
caaagaagtcttgtaactcaagtagaaatgtaatcaatatttgtattgatgataatgtat
ataattcattcgatctgcaagcatatatatactgacaatttaaaccttcctggtaaaacc
cccatactttgtaacgactttcataaattgctcgaggtctttcaatataattcttaatta
aagtattccaaattaaattatttttaatttttctagttttctatcagataaaaatttga
cattgcaaagatagctcgcatttgtttcatgtactgtatcaaaattttgaactaacatat
gtgcaataatattgcttaactgaattaaatagttttcaagaaaaacctcaacttgtttaa
ttggtacatttgaagtaaatagattaaaagtcttatttacacccttagaggatccaaaaa
gtagctgaataattagaatttgtagtaacatcctatagtcgtatattaggtagtttaaat
cattaatatcttggcaaatattattatagttaagttcatacaaactacaaaatcttttga
cacatctatgaaacaaatcaattaaaatattctcagttagagtcgtaatctcatttaaac
ctagatttgaattataaatttctaaaaggattttctctaactctgacaatattattttta
ataagtttctttttacttcagttcgaaaaacatctaaaattaacacttcctgagaacaat
tagttaatttctattaatttttatagaagttctaactaatagttctgcgacttcttgat
tgagtgtctgtccctgcgagctcggccaataattattcacgttatgtcatagtaagaaat
atttaaattaattttatacgataactttttaatacaccgaaaagcaagtttattactgca

tagctattag-------gttttacagatcttaatagttatttttcaaataaaggtatatg
atattagcgactaagctaattttattgaaattta----taataatgacaatttactactt
tgcacttgccagtcaaaactttcttttagtacaagagcccttggaagaagtttttagaga
gagagttaattattaccagtcaaacaataaagtaatcgattttggctaataccaaatcc
ttctttcttagaaaagccagaaatgatttcgtttaaagatcttgtacctaatgatgctgt
agctatagtctctactaacccagtatttattaattggttaaagcttagaattggttatgt
ctgtattggacaattcgaagatgatctacagctgtccgaagaatctttgaacataactgt
tacacaggaaaaaatttaatcatgcttctattaccactagtaac-aaata------ttaa
acggcaaatataatttacaaacacttatttttcttatagtttatcgcttattattttatg
ttgagatggagtgacaaacagtgtcagtatttcttgactaaatgtttcagttgctttgga
tttatatctgttggggttgactataatagacagcattcgttttatagtgacattttcaat
ctgagcccaatgtacaatcccaagttctaactccttggcaatagctgaaacagaaacaaa
agccgctcccaaacctgattgcactgcatttttatagcttctatagaatttaattccat
ttctattttgaaacgactgctatcaataccatgttgacttagcactttatctatgacttt
tctgattgttgactgagtatctaatgcaataaatttaagtctgtatagatcttcttttg
tatgtcttctagtttagaaaatggatgtgatgttggaagtattagtgctaattcatcttc
agcataagatgtaacttgaagaacatcttgcaattcagtaggtacttcccctccaataat
tgctagatcaacttggccattagctacgctccaagaaatgagcctagtggaatgtacttg
taattgaacagctacttgcggatatctttgcctaaatagtcctattaatcttggcatcaa
atatgtcccagttgtttgactagctccaataattaatgtgccaccttgtaagttctgtag
atcatcaagagctcgacatgtttcttcacataaagctaaaattctacctccatatcgtaa
aagtagatttcctgcctcagttaaagttgctttttattgcctctttcaaagagggaaac
atttaattggcgctctaaattttgaatttgtaaactaatagctggctgagaaacatataa
gctattagctgccttttaaaactgccttctttggcaattgcttttaatattcttaattg
atccaatgtaaatggaagatctgtcattaaagaattgtagtataatg---------tata
ttatttagtattgatcaatttatgcattcgccaagttaaatatttgctattacgcttcac
tattttgaaaataataaaaagtataatgttaaatacactgtattagattttaacatctgt
ttgatgttaaatttatacaatagatatcgtaaatttaattttaactataaaatataggag
aaaac---atggactctagacttttaattgtattaataccagttttagcagctgcatctt
gggcagtttataatattggtagagttgcactgcaacaatttaggaaaatgacatcttagt
gatgctcagactata-attttaaaattttatgagatagagaacttgaagatctctatctc
tattgtataccttaataaaaaaagtcagta--actcttgtacttactgttattacctatt
aagatgatgtac-agtaaatacattcctaataagtttaagctaacttatt-----tttta
taattaattaaattaataacacaattatggctgttccaaagaaaagaacatctaaagcta
aaaaaaatgcacgtaaagcaaattggaaaaaccaagcaaaaggcgaagcacaaaaagcct
tgtctttagcaaaatcagtattaactagaaaatctaatggatttgtttataatctttctg

agacagcagataattctactgactaggagtattt-ctactattaagctaactaagtatcc
aaaaaaacatttaagtaatttttatatat----taaacacttggaaatcattctatttg
ataataaaataaatagcagattaaacaaaattacagcaacaaattatgcagcaaaactag
atcaaactagtgagatctggttatttaattgtatcgaaaatattcaacatactttttga
aaagtcaattaaaatcgaatcatattactaaaattttcatttctggtactagttttgaat
atgcggcaggtttacctggactattatctagcttaacactaagtggtagaataaatccaa
ttagtatatacggcccaaagtctctgaaacggtatcttcaagcttgtaccaaatattctc
aaactaatttttcttttcctattaattttcataatgtaaaatatggagaacaagttgtcg
atgaactgtatacagtagtttgtttacctctaagtaagaaaagtttactgtatggattca
ctatcttaaaaaaagaaaagcagggagcatttaaagtagcaaaagctaaaatcttaaaca
ttactcaaggtcctatatatggcaaattaaaagaaagagataattttctaagtccagatg
gttattatttatgtggtaatgatttctcgtcaagcacaacaatgggacataaaatatctc
ttccactattagtcaggtattctagaattatttcagagatgcattggttttgctcttatc
ctattagatttaacacgtattcacatcagcaagccataaaatatttgccgcacaatattt
taactgatattatgaaatcccaaatatctcaagataatagtttcgttgaataatacttat
tcttgatatataatagtctcggcaatctaaataacatacaagaaagaatctcttg-----
----ttctatattaaatttataatcacttatgacgtacgcaattattgaagcaagtggca
aacaactttgggtagaggaaggacgctattatgatttaaatcgtatacctgttgatccag
gccaatcgattatactgggtaaagttttattgttaaacaagaatggagaagtgactttag
gacgtccgtgtatagcgggagttaccataaaagccacagtaatgaggcatttaagaggaa
aaaaattaactgtttttaaaatgaaaccaaagaaaaaaatgagattgaaaaaaggccatc
gacaagaattaactcgtttaatgattgattcaataataaactagg------tacagttga
gactaagatttttttcaacaagagtttgaacatttaactaaaatatatataatggcacata
aaaaaggtagtggtagcacaagaaatggtagagactctaattctaagcgcttgggagtga
aaaaatatggcggcgagcaagtaatagcaggtaatattttaattagacaacgtggaacta
aagttaagccgggccaaaatgttggtaaaggaaaagatgacactttgttttcactgatta
acggcttcgtgtttttttgaaaagtctaaccaaaagcaaaaaaacaattagtgtttattctg
caaaaaaatagccaaaataattggtgcaagctgacttgaat----atttttatttcactt
acactagcaccataaacttgatttattaagaaatggagttccttaatgaaccaatgaatt
ttattaaagatttgctttaaaatgtaatgacaaacactattgtaatttcctgtctacaca
atatggctgctattctatattgtggtcagatacagaaactagtagtagcaaatgctcatt
atcaagtaagtgatatttacctaggttgtgtagataaaatcttctcaggaataaatgcag
catttattgacctgggaaagaatgagtacagtggcttcatccatattagtgacactggtc
cgcttaaaaagaaatattatgttaatagtattactaacattttaacaattcgacaaaaaa
ttttagtacaaattactaaagagccaactttaaataaaggaccaaggctcactgctaata
ttacattgtcaggccgatatattgtactaatgccttttagccaagcaatttgcatatctc

gaaaaatacatgatgaagatgaacgtcattatttaaagtctttagctatcctaatcaagc
cagcaacaatgggcttactatttagaccttctgctgtaggtattgatgaagaaattatat
taagtgaactaaaaaatttaaaagagcaatggaactttgttcaaaaatctgcaatcaata
gctatgcacctgtccttctatataaagatgaagatattattaaaaaggtaattagagatt
tttataataataatacaaataatattgtaattgattcaaatttaggattaaaacaattga
attattatatccatacctggcattgtaataattcaagtacagctccttcaattaagcttt
atagtaataatcaatgtatattagatgcttttggtattaatcaagcaatttctcgagctc
taattccaaaagttgatcttatacttggaggctatatgtttattgaaacattagaagctt
ttacaattattgatgtcaattctggatcttttaataactctactagtgcgcgagaaacag
tcttaaaaacaaattgttctgcagcaacagaaatagcttaccagttacaaattagaaata
ttgcgggtgtaattataattgattttattgatatggaatcacagagagatcaattgcagt
tattagagcactttaataaagagttagcacttgatgatgcaaaacctcaaattgtacagt
tatctgaattgggtttagttgaattaactagaagaagacaaggcaaaagcttatatgagt
taattagtagtgactttaattactttcatttctttatccaagcagagcgaattaagtctt
ttaaaagaatagatcacaaaaagcaaacacttcagactcttaatagatcatggttatcta
cagaaattaatactattaataaagtatttttcaaaaatccaattttatatactggcta
acttttacttggcgcgcgatttacatatagttgatagtaatcttacttataagcaaaatc
atttattaactactagattgaaactaatttactctcaagattataacaaaatcttgcctt
ttactatccatagccatctaatcagctcgagtatagactataatcagaagtttttgttgt
aaaatatttgtttaaattgt--
--------------------taaaaaagaagccctctcttttataagagagggcttc-tt
ttttagcttactaactattctagctaattaattagaaattaaccgttaacagctggagct
gttagagctactggtaaagattcgccagaagctagatctagagggaagttatgagcatta
cgttcatgcattacttccatacctaggttagcacggttgatgatatcagcccatgtattg
attacacgaccttggctatcaacaacggattggttaaagttaaaaccatttaagttgaat
gccattgtgcttacagataaagctgttagccagataccaactacaggccatagacctaag
aagaaatgtagagaacgagagttgttgaaactagcgtattggaagattaaacgaccgaag
tagccatgagctgcaacgatgttataagtttcttcttcttgtccgaatttgtaaccgtag
ttagcagattcgttttcgcttgtttcacgaattaagctagatgtaactagggatccgtgc
atagcactgaacagagaaccaccgaatacaccagcaacacctaattggtggaatgggtgc
attaaaatgttgtgctcagcttggaacacaagcatgaagttaaatgttccggagatacct
agaggcattccatcagagaaactaccttggccaattgggtatactaggaatactgctgct
gctgctgctactggagcagtaaaagcaacggaaatccatggacgcatacctaggcggtag
cttagttcccactcacgaccaatatagcaagctacgccagttaggaaatgaagaacaact
agttggtaaggaccaccgttgtataaccattcgtctaaagaagcagcttcccaaattggg
tagaagtgaataccgatagctgcagaacttggaataacagcaccagaaatgatgttgttt

ccgtatagaagggaaccagcaactggttcacgaattccatcaatgtctactggaggtgca
gctacgaatgcaatgatgaatacagatgtggcagttaataaagttggaatcattagcaca
ccgaaccaaccaatgtataggcgattttcagtactagtaatccaagagcagaaacgttcc
cacaagctagcgctttcgcgtctttgtaaagtagcagtcataatttttatcaattttta
aggatttaactaagatgcttcccaaacaatattttgtctgttagatatattattacttag
aatcttgtaaaatgagaacgt-tttttcaaaaata-taattaaatttagtctcaataact
a--aaaatgtacttattaagctactattatatacttgaa----------gaaataaattt
ttcact-------------ttttctaaaattagtataattttaaggatataataatagta
atgcatactaatgctgcttattttgatttcaacagtttacataattgagccttacttaaa
tacgcatgtcacactttacaaaaattcagacgacaatacaagacttaaatctgttaaagc
atgctttaacagatttaggtttgacttggtgtatgaattctagtcatatacaagttggtg
aaaatagtcaacacaaagtagatatattgattaaacaggataatctatcgcatattggct
ttatctggaatgataataaatataacctagttgctgatttacaactttgggaacaacctt
ggtctttagaagtctttttagataaattgtcccaaaaatatgcttaccattctattatta
aagaaacaaagaagcaaggttttgaaaaaatggagcaaacctcgcaaaacgatggatcaa
ttaagttagttgtgcaacgttggagctgttaattactatataaatgcgggcggagagact
tgaactctcacgagattacctcactagaacctaaatctagcgcgtctaccaattccacca
cgcccgcattgacatgac--aatagcattgtatcatagttaatctgaaaaaacaaactaa
-aaagataaactagctgccaagtttaaatgcatcaacaaataggccgtatgttataccaa
tcttgaagttattaattaaattaatctttatatttaatttttttcgattagagtaaacga
ttttactagttaactctgttgctgcaactattaaactggctgcaatgattccccaatctc
ctgtttgcccaggaattgtagataatcctgttgaaataaagaagcctaataacagactga
ttaagccagttgttaactcatttagagaatagtaaagtttatcacttaagtttttttactg
aatacgcaagaaaggtagataatctagttttaatcataatatctaataacggagttttaa
aaggataacaa-----------agatgaatgtttttaaatccttttgaaacccgtcaata
caaaaattactttttg-----------tttaaaggtcttgttcactcaaagcattaatca
gatgttgaaatggttcttgtataatgtctttacgtttaattgactgtaagtctagttctt
cttctattatttcttgcaataattgtatgcttcttactgttggagcaataggtacagata
atgaattgtaagtatctttgagcccgtttaatacacgctgatctagaatgttggtgctac
cagagactagtgcataactagcataacgaagatagtattctatgtctcttaaacacgcgg
catatcttcttgtggtataagaatttcctccaggtcttaataattcgggttgctcttcgt
acaattgagcggcagcctctttcaagatgttcgtagcttgattattaataatttcagcta
tttttattctatctagcccacttgagaagaaagactccatttgtccgacagctgctttat
ctagataacgaccagtaagatcgtaacgatttaatattgctgttatcgcatcttgcataa
attaattctcctac-ttgctatcagagcggtattatgaatgacttatat-----tataat
catgaatgtttataatatttatttaaaatctattctggattgatcagtttgcaattaaaa

acagatatagattttatataaatagagtatacgtagtttttaattaattttttgataaaa
attaagaaatacagattttttt-taataggtttgtaattttcttaatataactcaggttct
cgaccgaaataagttttgtaacacat--aagctagaacaaagatgagtaggtagtatt--

---------------------------taataataatatgcatcaa--------------

-----------------agtataaataatctcttaatacagcatcaaaaaaggtatttaa
cacatattgaaaaatttgattatttaaataaaaacttaaatttagataagattagctatt
ttataattacaactaataaaaatgataaatacatccttgtagaacaattctgttataatg
cagacggttctgtttattcaatattatttaaaggtcagacagctaaacacctttgctata
taattcttcagcttaaacaattaaatataagaatatcttcagctcatgctttatatttag
gaagagaactaatgaagtctgaattaactcttgtattagatcaacaatatatccaagact
aaa--ctgcatagatattaattgtatttgtgctagtataatattactaagggcatgtaac
tcagtggatagagtatcagattccggttctgatagccgtgggttcgaatccctccttgcc
cgaagagtatttagaatact-gtaacaagatacat-------tgggatcttgtttagtaa
tagtaaacatgttggggctgcaaggtttctacattatgaaaataagaaaatatgaaagta
agacaagctcttt-agagagtttctagtaaaattaataaatgcagaaaacaatattgttt
ctttttctcgaaaattagctattgcttaaatactgttaatttttgacattcgaagcgtta
aactcttatgcgtgacgaatattctgttagagttgctcttagtataagaaaagttataaa
atttatttatatttatgtttttacctatttttatttaaagtaaggttagta-ttttcaa
tttttataatggacgtgggttcgagtcccaccagctccatcaatttaact---------t
tcataattaactaatccaatcttttaataaccagaaatataattggagcaaatatggat
ttcataaaaattacagaacaagcttttaagcaaattacagtcctgaaaagcaattatgat
caggatgtacatttaagaataggtgtaagacaaggaggctgctctggtatgtcatactca
atgaattttgaagatgtagttaatctaaaagagactgatgaaaaacttattttagatgac
ttttctgttgtttgtgatcccaaaagccttttgtatctttacggattatcattggatttt
agcaacgatttaataggaggagggtttcaattttccaacccaaatgctagccagacttgt
ggttgcggtaaatctttctctgggtaaac-attgtgctattt--ttttatatttgtact
gtacgtgttactcttgtaattag-catcataaatacacaaatatctttattgaaagctct
ttaaactagtcaaagacttataaaaactactttaaggagactttatt-tattcttgtagc
ttaagtaaatatttatgattaaatgatgaaagt---------acgaaaattcactttatc
attattgtctaattttaattagttaa-tttttattgatatatgctaatcttt-attagac
tcgatttattgaaaatttatag-ttctttttctaagaaagtctgtaaatttctagcta-
----------------------aaataaattatatatttt-attcattttataaaaaatc
aacatgaatcttgctcaagttacttgcgagtactctagtacaaaaattcttaataagttt
tattgcaatacggaatgtgtttcccctggtgatttagccacctcaaattat--------
-------catcctctgcaaaaataattgcttttggaattcctcaatttgattttatccct
acaaaaattaaaaactctattagagaagcagtagtcatatctactgtcgatgatagtaat
atttttgctgattcaactaataattggataaaaaaacataaagataaaaatcaccattct
cttttctataaaaaaatttttaaagctttactaaatcaaaaaatcaaagtagttatagat
cgagc------taataatactacaatagataaagagaagaattcaattatgagtcaaaaa
tatgtttggggtaatagttggaagccatcaataatatcatcttacttcgaaaacaaaaaa

aatcatgaagtttctaatgcatctaaaaagtttctaattgatcagttgtcggcatcacct
gtctttgtagtaaaaaatggttttaatgaaattatttaggccacccagcattccgtatt
aagagaggaagcattagcaatcaaactcactcgttttaaatttattaaagcaatctcag
caaaattatcctatatctactggtttattctttttacatccagatgacgcatttgaattt
aaaaactttataaaatctgttaatccgttggctagtcagcagatggagataaatgtagaa
cctgtcggccttcactttgcttataaaatgagcagaaatctatcttctgatactcaattt
ttgttcattccagattttaaagaggtaggcgatttattatttaaacatcaaggcaataag
aacttaatctttcacaaggatcagcgtcacggcaaacatttttttcagggacaacctata
tatattattcaacctactactttaaagatcgaagtggcaagctaaatactattcaattt
actggactaagtgataacagagaagttgttttcactagtcttgaagccgctaataaatca
tggacaaattttataaaaaataattcacaattaaaatcaatcagtaagccgactttatta
gtctataatctagaaagtttttaaaagatcaagaacgattgaataaacaaaattttcaa
aaattcgttgtagttactaataaaaaagcttatcttactacaagagagttaataactgca
ccagattctaatagcgttttaaagcatttaaaattaaatatgaagcctaaactcttcttt
gtgaaattatgggtaagacggctattttccaccttgacttatgaat-aaatgacgtt-gt
atttattttcttgaatagtattcgataactaatagttcatttagttgtaaagcaacccat
tctctgtcaataactccattgactttccctgataaattagatttattcaattctaagtgg
ctaggaatattagctagtccaggaaacgctaaatagttttctactagtcttttagatgtt
tgctgatcttttacactaattaattctcctggtttacattgataactgcatatagatact
acttgttcgttaatacaaatgtgaccatgattcactagctgtcttgcagcaggaatggtg
ggtgccattccaagtctaaaaacagtattgtcaagtctcatttctaagagctgtaataaa
atttgacctgtagatccttgaagtttttagcagccttaacatacttaaatagttgtttt
tcacttagtccataattaaagcgtaacttctgtttttcttctaatcttacggcatactca
gaaggttttctagattttgtccatgttcccctggtggataagaccttttaattgctttt
ctgctaagtccaggtaaatcgcctagtcgacgggaaatgcgcactcgtggtcctctatat
ctagacatattttataaattctccgaatattaat-gattaag----------taatgagt
agctatttttaacacaaaaagagtattaaaaccaagc-ttttactaaaaaatctttata
caaaatagag--------------------tgctttactttttaagcgggtagcgggaa
tcgaacccgcatcattagcttggaaggctaaggttttaccactaaactatacccgcaatg
ttcaataaattgtgacgcgcttaatataacataattatcttatgtttagatattatctaa
tctagaaaagaggtgaaattatttatgctccttctaaaactacatcaagaaagcgagcaa
tctctgcagcctgctcttcaacttctgaaagtaataatggctgacccactctagttaacg
gaattactcttttatcttttgtgcataaataaatttctcgtcgtggatttaaaccttctt
taatatctattcttattgatttaatttcttttatattcaactgaagacaaatttgtctat
ttttgcctggaaaacctaaccgaaaaattttaacagtaccttcatctttattaaattcat
tgtacccagcacctatgtcccaaataatagttagccacaaaaataaactaagaaagattc

cgatgctgccatagaatgtcataacaataccttgcggaataaatactaattctgttgaag
ttgtaaaaggcaataaatcaatttgaaagtaacttgataatcccgcaaggagaaaccctg
aagctccaataaagattattgtagcccaccaataattactaaatcttcgggaacccaaaa
ttaaatcttttctaacagagtttatagataatgctttttcataatttgttactttaaaa
caata-tact---------------aattttgacaatttttt--tcagtgttttagattt
gttaaatgagtttgatagcttttaatcctaaaaataatcctgttgctaaaccaaaaaaag
cagcaagaaatattgtgtaccctagaaaaactgacatattatta----ttattattattg
ttacaatagtgtttacaaaaatagttgaaatctattactagagtattatattacgttaaa
tgcttcgctgtctacatgaaaaaactttttttcagtttttagcattgtatttacattagaa
ataatattgtaataatttattaaacttctggagaacagaatcttatggcagaatttatc
aagccttataatgatgacccctttgtaggaaatttatctacaccaattagtacatcaagc
tttagtaaaggactattagggaatctaccagcttatcgtcgaggcctatctccacttcta
cgaggactagaaatcggaatggcacacggatacttttttaattggtccttttgataagtta
ggtcctttgcgaggtacagatgtagccttactagcaggatttttatcttcagttggctta
attattattttgactacatgcttatctatgtacggaaacgtatcttttactagaacagat
tcaaaagatccactgcaaacagctgaaggttggggtcagtttactgctggattcttagtt
ggagcagtaggcggctcaggatttgcttacttgttgctagctaatattcctgtacttcaa
acggcaggtttgagcttattttcttaagctagtaaggggacttgaacccgtaacctactg
attacaaatcagttgctctaccaattgagctatactagcacctaattaattataacatat
aaagatatttgttgcaatctggtattttcagatgcaacaaatatctttttttatttagaca
gagctgttaaattagtatatctataatatgttaattagaatctgaactatctgactcagc
agaatattcttgatcacagtttaaataataacaaattgtttgatctagacatgttgcgga
ccaaccaacaggtgtttctcctgataattcttgcatatctccagaactataaaaatcatc
actcggcttattactatttgcttgcataaatatcactcccaaaacactcttaacttaatt
tgaagtaatttaacttctcaataatatattaacatataatacaaaacatggtgcttattc
attaattgtaacaataatatgaaatttacaaggtctgagtaagtgttttaatggcttttg
ttgaaacaagcattttaatccatttattcttctctcttgaccatactttttttatgttgta
aattaacttttttgtaatttttttttgttcgcttgtgagaatgagaaactgcgtacccattat
tagctactttccctgtaagctgacatttttttcgacataatttt------attaacttata
tatttatttaaagtacttgctagagttgactttggcacggcaccaattaccgtatcaact
ctttctccagctttaaaaatcattaaagtaggtatgcttctaataccatattctgctgca
attgttggattatcgtccgtgtttattttttactaccttaatagatgactcgtattcttct
gcaatttcatcaactacaggagaaaccattctacaaggaccacaccatggcgcccaaaaa
tctacgagtacaggtaagttattattaataacttcttgtttgaaagaggcatctgtaact
tgagatactgacatattttttaacctttaatatatgttccttgctaagcaaatcttatca
caaattttaggaaaaaatcttctatagttaattactattagtgttgaattaattgttt-tt

aattagacattaaaaaaacttattacctatataaataaagtggtggccgagtgatatctt
gatagtgatagtttttctattaaggttgaacaatagaaataatttttcaagcatata-tt
ttttgaaaa--acttctttgtgaccatgaaagcttaattatattacaatatattttatta
tttgtaacttagttctgataatggtataaacaacgcaaaagatactgccttataatcaag
gaggaatacatgtctcaatccgtagaatcacggactaggattaaaagcgaacgttacgaa
tctggagtaatcccttacgctaaaatgggttactgggatgctgactatgtaattaaagaa
acagatattttagctctgtttagaattactcctcagccgggtgttgaccctattgaagca
tctgctgcaattgcaggtgaatcttcaacagctacttggacagttgtatggactgatttg
ctaacagcttgcgatttatatagagcaaaagcatatagagtagatccagttccaaacgtt
gcagatcaatactttgcttatattgcttatgatattgatttatttgaagaaggttctatt
gcaaacttaactgcttcaattattggtaacgtttttggatttaaagctgttaaagctctt
cgtctagaagatatgcgtatgccagtagcttacttaaaaactttccaaggtcctgcaact
ggactaattgtagagcgtgagcgtatggataaatttggcaggcctttcttgggtgcgaca
gttaagcctaaactaggtttgtctggtaaaaattatggaagagtagtatacgaaggtcta
aaaggtggtcttgatttccttaaagatgatgaaaatattaactcacaaccatttatgcgt
tggagagaaagattttatattccatggaaggtgtaaataaagcatctgcttcggctggt
gaaattaaaggtcattaccttaacgtaacagctgcgactatggaagatatgtatgaaaga
gcagaattctccaaagaagtcggcagtatcatttgcatgattgaccttgtaattggttat
actgctattcaaagcatggcgatatgggctcgtaagcatgacatgattttacatttacat
agagcaggtaactctacttactctcgtcaaaaaaatcatggtatgaacttccgagttatt
tgcaaatggatgcgtatggcaggtgttgaccatatccatgcaggtacggttgtaggtaag
cttgaaggagatcctttaatgattaaaggtttctacaatactttacttgaaagtgaaaca
gatatcaatttacctcaaggtctattctttgctcaaaactgggcatctctacgtaaagtt
gtgccagtagcatctggtggtattcatgctggtcaaatgcaccagcttcttgattactta
ggtgacgatgtagttcttcagtttggtggtggtacaattggacatcctgatggtatccaa
gcaggcgcgactgcaaatagagtagccttagagtccatggttatggcaagaaatgaaggt
cgtaactatgtagcagaaggtcctcaaatcttaagagacgctgctaaaacttgtggacct
ctacaaacagctttagatttatggaaagatattagttttaactatacttccacagataca
gctgatttcgttgagactccaacagcaaacatctagtttaatgactacttgctaatgctt
gatttagcaaattgtaagtagaattaacttataataataaggagcatagaatagtgagac
taacacaagggactttttccttccttccagatctaactgatgagcaaattaataaacagc
ttacttacatcgtttctaaaggtttatcagcaaacgttgagtacactgatgatcctcatc
caagaaattcctattgggaattgtggggtttacctttatttgatgtaaaagatgcatctg
ctgttatgtatgagattagctcatgcagaaaagcaaaacctaattattatgttaaagtta
atgcttttgataatactcgaggtattgaaagttgtgtaatgtcttttatcgtaaatagac
ctgctaatgagccaggattcttattacaacgccaagactttgaaggtagaactatgaaat

atagtcttcatagctatgctactgaaaagcctgaaggagctagatattaattttaattaa
tagctaaaacctattaactggttacccctctttgatatcttaattactaaattaattttg
attttaaggaggggcaaatttcgaaataaattctagttaaattagaatttacaattaaaa
atacacagaaataatgcaatcacaagatataatctccaacgatactcttgttaacttaca
agaagaatatgatagaacacaaatccaagaagttcttaacgaattaaatcaagaacttat
aggattagttcctgtaaaaactagaattcgcgaaattgcagctctattactaattgatag
actacgtagacaactagaactggtatctggtaatccagggttacatatgtcatttacagg
tagcccagggactggcaaaacaacagtggccatgaaaatggcggatattttgcatagact
tggctatataaaaaaagggcatttattaacagttacacgagacgatcttgtggggcaata
tattggtcatactgctcccaaaactaaagaagttctaaagcaggcaatgggaggagttct
gtttattgatgaagcttattatttatataaggcagacaatgaaagagattatggctctga
agcaatcgaaattttactacaagtaatggaaaatcaacgaaatgacttagttgtcatttt
tgctggttacaaagacagaatggaaaaattctatgaatcaaatccaggactttcttctag
agtagctaatcatgtcgatttcccagattacacttctgatgagctattgcaaatagctaa
aatgatgatagaggaacagcaatattgttttacggaagaggctgataaaactcttttaga
atatactgagcgaagaatgaaacagccatattttgctaatgcgaggagtattcgcaatgc
tattgatagagctagaatgaggcaagccaatagaatttttgcaagtggagaaaaagtttt
gacaaaagctgatttagtaacaattgaagcagaggatatcttgaaaagtagattattttc
attacctaattcttagtgattaagtagtttgataaaactgtatattatgaaaaaactttt
ataataagttgcaaatttctttgaaaacctatactattaatgtattataggtg-tggcgg
catagccaagtggtaaggcagaggattgcaaatccttcatcccccagttcaaatctgggt
gccgcctagtactaaaaa--gggggtgtggtggaatggtagacacaacagacttaaaatc
tgttga-ttttaataatcgtgagggttcaagtccctccacccccacatattaacttataa
caaaaaacagatgtgtatttgtattaattgtaaccatattactgaatgtgatacttatca
tttaatagaatcccagcacaaacaacttcatctaactgagactccatcattcatacctaa
atgccctgttgtttgtgttaatatatctaacaatagtagcaatatccaaattgattggga
tttagtcgaatgcttatcatttgtggagaaaccgaatagttggaatttaggctgtgatta
gttgtgcaaaaaattatgtggtgtacgcaataactctatcttttagatactctgtattaa
ttttagatgttcttactaacagaattttccctgttcttgctatctctataataccgaatt
tagttaataattgttcaatggcaacaatctttcctggatcgccagtaacctctataatta
aaaagttttccgcgacatctactatcttagctctaaaaattttcacaatttctaaagctt
ctgttctggtctgagaattaattctaatcttaattaacattaattctctttcaactgaag
gaatattggttacgtcttgtacgttaagaatatttactaatttatataattgtttggtga
gttgttcaatagttctgttgtctccctgaactaccatcgtaattctagaaactccgattt
gttctgctggtccaactgctaaacttgcaatattaaagcctctgcgggcaaatagaccag
atattcttgaaagtactcccgcttcatcttgaactaaaactgataaggtgtgtttcatga

aattttaatattatactatttgatctattaaatatattctatgaaaattatagaaaaaat
actagttaatatatagcgaaattattcaaactaaacaaaatcctattatcagatagcata
ttttttttgtaatgttatactattaatctagtatttaatgatattctaattctaaataga
atgctaaatttttaaaatttgttaggaacttgtgttagaaaaatacaaaaacagtaaaa
aactctctcgagatctaccccaaatcaacgatcgtattagatttccaaaagtccgagtaa
ttgacgatgaaggtgaacagttaggtatttttgtacctgaagaagctatgcaattagctg
cccaacaagggttagacttagttgttgttagtgataagtctgatccaccagtttgcagaa
tattggattatggtaaatataagtttacacaggaaaaaagagccagagaagctaagaaaa
aacaacataatagtagtgtaaaagaagtaaagatgcgatataagatagaagagcatgatt
ataaagttagaattaatcaagcatctaagtttatccaagcaggagataaagtaaaagtaa
ctataacatttcgtggtcgcgaaattcagcattctaatttagctatagatttgttaaaca
aaatggcaagcgacttgacttcgatatctgaaattcagcaagctccatcacgagatggaa
gaaacgttataatgcttttatctcccaagaaagtgagct-aaatgatttaatttaaatg
catctggctggattcgaaccagcgacgtctctttcgaaatggcggattatgagtccgctg
ccttcggcccctcggccacagatgcatcacttaaagtttataactcataaagtatct-aa
aatcaatatctatttcttgttcatgacatcta-aaaaaattattactcattcatattgtg
atatgtcgcaactgctgacggagaaacttggttcaagtagcgaaatatccaatatttaaa
tattgtgtctagtatgactggaaaagttgaaataaatagaaaaataaagtcacgactttc
aggtaatcctagatgccttaaaattacttcaataataacttcccagccatgaggagaatg
aaaaccaacaaacatatcggtgaaaagaatgattagaaaagcttttgcagtgtcactaag
gccataaataatttcatttaaaaaagatttgacaattgcaatttgtctttgtccagttat
cattaataatataaaaacaaatattgatacaatatcggacaaaatgtttttcacagcatt
cgcactttcatttgcatagtattctcctagttctctggccttcagctgcactctttttc
tataatttcataagatatatcttccgatgggttcaataagacttcaaagtgaattttctc
ttcaaacctttgtagttcagcaaaagctctctcttcttgagatgaatttaaaaaaatttt
aggttgttcttggttccataaataatcaatacaaggaccaaacacaaaaaactttgaagc
ttgattaactaggactggagagataaataataataaaatatattttacggaagtaatagt
ttgatgtctagatattctaaactcttctatggcttcggattctccatttggatctaactc
tttgcgaaacttttcaaaagtattagtaattgacctaggtattggtccaactttttcaaa
ggcaaattgattatttcgttttaaattccaatatttcatttttattgctgctaaaattt
ctattaatcaataattggtcacgtattcactataaatattaattctagaactaaacatat
aaaacaatagttatgaataataaaatacaattaagctttaattacgcattgttactatct
cttgctacaacaatatatggatgtagatcttatcgtgaaactatgagtacactattaata
aaatatgaagatagctatccattattgtctgatagacatactgcctttattcctataatt
gtattaaaaaaaaatctttaaactccctaaaatcagaacctcacaatactaagttgtgtc
ttaatggaatgggaaatagaaaatttattgataaaattgcaaatttgctaagccgtttag

aaaaacagatacactatactgaacatattaatcaaataaaaactagtgggtacttttctt
tactttatgttaacagtaaagcaatgtatacaaaagaaattagcaatatctttatattgc
caaatgatgttctaaaaagaatttatattcagaataaacaaaaaaagcttatacctgata
cattcttaataaaattatttcaaaaacaagtaggttacccgagaaattttgcaaacttaa
acctagccttgtcaaaaattatgcaatggtattctgatagaggatatcaatggtctttag
tggaaattaaacaagctactgacccatctcatctgatccttaatatccatgaaggattag
ttaaaacaattacaacagagtattatactttgtctcgtaaaaaatcacctagcatctcta
gtacagaattaatagagaagtatcttggagtaaaagtcggacttcctttaaatattaatt
ttttacaaaaaaaaattaactatttaaaagataatcaattagttggaaacattatttata
gtattgagcgatcagccaataactctatgaatctagatattaaatttcaaatacaagaac
taaaagataaagatttactagtacttgtagaaagatcatccaaaacttctcaaattatct
ctcacgcatatgacttactaggtgtagaacgcaggagttctcctaattgcctggcagatt
ccacaataatgtcattatcgactattaaattacaagcatgctattttaattataaaattg
atttgaacagaaatatatagatgctattaatctaatcaaactgttaacttattccattt
ttcgtcaaaaaacactaataagtgatagttttaatttacagactttagtcagctggataa
aaagaaatactattggatttcaactgtatttacgaaatttggatcgtgcaaaatcttttt
gtctattcagtctgaaatgtattcagagtggactgaatataaagatttcatatctaaatc
cttcattaatagttaatcgaaatttgagttttcagcttgcaatacaaataattaagcaat
atcacacgaatcaacaatcagttccatctt------------------------------
------tgtttttagcacaaggagatctggtacaattaatttttgaaagtatatgtacat
atcattttacatcttgtttttcgatatctgaaaaaatattactttctcgagttgtttata
tagattccatattttacaattctgaaaatttaagtctttgtaacaagaataaaaatcaat
ttgtaaatagctgtgatatcttgagacaaaataaaaaattattgcagcaaaaatttctta
ctttattgttaagcatccattatcaaaattttaattatttggattggcctttaaaaggcc
atttatttgaaatgaggtttttatactttaccccatttcagaacagcaatttttttaaact
actcttttcttgataactacaaaaacctgtttttcataaaatttatttaaaacaaatct
ctcactttaatttaccattgtgttttagagaccatcttaaccatattttagttagtacta
ttcaatgtcagtcaaatttagatatggagactgtttatctattgttaagcaattatcctc
ttgaatatactctatttaagtctattgttactttttctcttaaagttaggatggaatatt
taatacctataagcaggagtattagagtatctttgttttataattatctagattgttttt
tcataaaacaatctaaaaaagtagcatatgcgctccaagatctatcgagcagtaaacaaa
ttcgaagtttttattggaaaaattttcatggggatttggaattcagctaagattaccaa
ttaaacagatgccacccctttcaattgaatacactttaaacagtggtaggcactttgta
tttatctacatatttactattaaagatggctagtattaaaatgaaaaatttaatttcttt
ttttaaccgaagcaaaggaaaatggatttcacaaagaacaacttatgaactatataataa
gaatatgagttcagtacaatctcaaatgcaaataaagtcaaatgaattattgttagaatc

tgcaggacttgcgtcactaaattggggagagatttctagacaagttaaccataattctta
ttctcaaaatacaatcagaaataataataagtttaacttagaatttagtaatcagctaaa
taatcgtaagctattgacattatgcacggtaacagatcctagtctaattaattttaaaac
tcgatatggtagtactactgtggatgaaacatattggtttgcaacaagtaaattgcgcct
aagtaccagtattattaaaaaatttgatacttgtattgcagtatctttttgttcagaaat
caaagtttaa-attctgtataaaagcaactaatagctgaaa-agttattagttgcttta
tggatctaagttttgttttatagagtatataaaataatatattattctaaatctttacgg
ttaggatttctagaaggatcattagataaaaatccaaaaacaaacagagaaataaagaaa
atcacagtagtataaacaaatattttcaaagtaaacatatttacatccttaaaagctatt
agtgtg----atatttatatatc-acacataattttatatcacgatatgagtctcggtgc
tattttataaacaaatcaatatctttatttttttagcaatgatttatactttaatatgcgt
gatacactaaatattaaatctttttgtatacgcttcttactattttaaatactttatta
tttctatggagtttaccttcaattattacgtagtctagtttcttcagtaatttgaaaatc
tctaaagatttttattccaaataattaagttgattgtagctaatcttttcctttgtaaa
agcctgacttccaacttaatgattttgtttctattatcactaattttaatacttttacac
cttaaaatttgaactagcaatgtacagctattcat-atttgtattaa------tctcata
actaatctattatgccagataa-atgaaaaaattaaaatgagatctcgttatcttgttga
acttgttcataactaatatcttttaaaacctttaatattttcccgaaatactcttgcaaa
tatttttctagcgatattacttctgagctatcaatgcctaaaatactgtaaacttcattc
atggaagcagtaaattgctctccactagttaaaatctctgcaaatgctagacgatcagaa
atattccatgtccattgaagggttttagtaattttccttaaagctgttaataatccaatt
ggaatttgtgatatttgtgtttttttgtccagataacttttcacataatttaataatttcc
gctgatgtccaagctgtgttaccaactagtggcagtattctattttctgtggaaggcact
ccaagacttttttataactaattttgctgcatcctgagtatcaatatatgcaatcggcgta
gactctcctgtaacccatactgatttcttatccaaaataggaattgcatattggttaatt
aatccctgaaaaaatcctcccagagaaaataccgtgtactttatattagattttcgaaga
aaatctactacttgcgacttcaagttcattaatggaacagctgggtatttatctgcattt
aatattgaaaagaaaataaatctttcaacttcagcagctttggctgcctcaattaaggca
attttaccatctaaatctatttttgctgcattataaggatctgtaggacgagatgtagaa
gcatctatgactgctgttactccacaaaaagattgcaaaatactttcaggtaattttaaa
tcaccataaacaagttctgccccccattctttgagaaaagctgactttcgtaaatttctt
accatgcattttacattatagccttcatctagagcacgccttacaatttgacgtcctaga
gtccctgttgctccaattactagaagagtcatattaattaaaaataagtatgtataaaga
cattgatttcatcgtcgacggagagtacattaggtagagagggaatcgaaccctcatgac
cgaagtcgtcacattttgagtgtgatgcgtataccaatttcgccatctaccctattatag
accaactactacaagtaatttcgtctttagcgtaaaaataatactaagttttaatagata

tgtcaactttttagtctatattaacaaaaaacaggagtatcttatgtctacttttgaatt
aattccttatagattatatttaagtcaatctagtacatggatgcatatgatgaaagcaga
aattaaaatctatatattaacactattatggatttcagttttcatttttttcttacttaa
actatttattattgcgctaagtttaattatgattagcctgactataagaaataaacaaaa
tattatacaaaaacatttcttgcaaaccttaatcatgacagtggtaactaccttcttgtc
atttagcttgactactagttacaaacaatattcgcaactaaaacaatcacaaaacttatc
ttacaataataaagctaaagatccttacaattatcaaaatatacaaatatctagtggtct
taagattcgaaaaaaacaattaagatgcagtttaaagcctgcactctatttttttattac
tatatattcaatgaaactagtcatgataacgacttccccagaaattttagtgattactgc
ttatagatctaggatcttcagtgtgctatttaataatgaattattatttaccttcttatt
gtcgtcacatattgttactaatgtaattagtaaattaaataaaatcattcaagtagctag
tttaagaggtagcttaaatttatataaatcatccacaaaactttaatatttctttttt
aatatttcaagtctttttttagagatcattaaggaatcaaaagaaatatctcaagctct
ttatactagaaatcttaatcaagaaaacagtaatttttttaaaaatatatagggccaaatc
taatattaatgattgtttaattgcgattattggaaccttatatttcattattttaggagt
agcataaca-aaccgaattatctttccgactctagtaatacgaatagtaatagacttaag
tagtagttagaatatgtaaaat-tcaaatattatataaataattaaatgttttattatgg
tcaatactcaaaatccaattgcacaaacttcagagcttgattatattgtcaatcaacctt
acaaatacgggtttaaaacttctgttgaatcagaacaatttcctagaggcattaatgaaa
atatcgtaagattaatttctaagaagaaaaatgagcccgagtatttattaaacttcaggc
ttaaagcatacaaaaaaatggaaaaaaatgagcagtccatcatgggctcatcttaagcatc
cgaatatagacttcaacaatattatttattatgctgttccgaaattaaagaaagaactaa
atagtttagatgaagttgatccagaaattcttgacacttttaacaaattgggtatctctc
tcaatgaacaaaaacgaatttccaatgtagccgttgacgctgtttttgatagtgtttcta
ttgcaactactttaagaaagaattatctgaagctggtgttatattctgttcaatttctg
aagctatacgggattatcccaatttaattaataagtatttgggtactgttgtaccagctg
gggacaattattttgctgcattaaattctgcagtatttagtgatggctctttttgctata
ttcctcctgatacagtttgtcctctagaattatcaacttattttcgcattaataacgaag
aatctggtcaatttgaaagaacattaattgtagctgatcgtggtagtaaagtgagttatt
tagaaggttgtaccgcgcctcaatttgatacaaaccaattgcatgcagcaattgtagaat
tagtagctcttgatggtgccgaaattaaatactctaccgtacaaaattggtatgctggta
atcaagaaggcgaaggtggcatatataattttgttactaaaagaggcttatgctctggca
ataactctaaaatttcatggacacaagtagaaactgggtcagcaattacctggaaatacc
ctagctgtattttagctggccatagttctcaaggagaattttattctgtagctttaacaa
ataattatcaagaagctgatacaggtactaagatgattcacattggtaataatactaaaa
gtagaatcatctctaaggggatttctgctggtaaatcgaaaaacagctatagaggactag

ttaaaattggccctcagtcatttaattctcgtaattactctcaatgtgattctttattaa
taggtcaatcgtctcaggctaatactttccttatattcaagtacagaatccaacatcaa
aagtagaacatgaagcatctacttcaaaaattagcgaagatcagatttttatttttac
aaagaggtattagtttagaagaatctgtagctcttatgatcagcgggtttgcaaagatg
tatttaatgaacttcctatggagtttgcagctgaagccgatcgtttacttagtttaaaat
tagaaggaactgtaggatgagtcaaaccattttagaaattaaagatttatatgcgtctat
tggagaaacagtaattttaaagggtgtaaacttatctatacaagaaggcgaaatccatgc
aattatgggacctaatggttccggtaaaagtaccctatccaaagtaattgcagggcatcc
agcatactctttaataagtggagacatcttatttttgggaaaagtattcttgcaatgga
gccagatgagagagcaagagctggtatctttcttgcttttcagtatcccgtcgaaattcc
aggagtcagtaatgcagatttttacgaattgcattaaacgctcgtagaaaattcaaagg
attatcagaatttagcccctagaatttttcaattaataaatgaaaagataaagcttgt
tggcatgcaagaaagtttcttgacaagaaatgttaatgaaggattttctggcggagagaa
aaagcgtaacgaaattcttcaaatggctttgctagatagcaacttatctattctagatga
aacagattctgggttagatatagatgcacttagagtagtcgcaaaaggcattaatacttt
agcaaactcaacaaattcaattatttaattactcattatcaaagattattggattatat
cattccagattttgttcatattatgagtaacggcaaaattgtaaaaaccggcaatctcac
tcttgcaaaagatctggaaaagcatgggtatgactggattgtacagagctaaatatttgt
taaatacaaaaatactgagactaaagtattttctttagtctcaatatttttgaccaacaa
aaag-----ttatttaggctttatcgaaaccttgtttaacatcttcaattgcttttttta
gcagttcttcactagatgcgtctaatttttagtgctacgaatactttccccaaattctg
gtttagaattctttaaatcttctctcaattctttgatgaactcagccacttttgacacct
caatgtcatctaaataaccgttaattccagtataaataatagctgtttgttcttcaactg
gaataggagagttctgagcttgctttaaaatttctctaagacgttgacctcgtgccagtt
gattttgagttgctttgtctaaatcagatgcgaattgagaaaaagcttctaattcagcaa
attgtgctaactccaattttaatttgcctgccacttgtttcatcgcttttatttgagcag
cagatcctactctagagacagaaataccgacgttaatagcgggcctaatacctgagttaa
acaaatcgcccgatagaaaaatttgaccatccgtaattgaaattacatttgttggtatat
atgcggatacatccccggcttgagtttcaataattggaagggcagtcatactaccgcctc
ctaattctgagttcaatttagctgctctttctagcaatctagagtgtaaataaaatacat
cacctggatatgcttcacgcccaggaggtcttctcagtagaagagacatttgacgatagg
cttgcgcttgctttgttaaatcatcataaataactagtgtcgctttacctttatacataa
aatactctgctaaagctgcacctgtataaggagcaatatattgcaacgttgctggactat
ctgcattagctgcaactataatagtataatcaagagctcctttatcttgtagtgaagata
caacttgagccactgacgatgctttttgtcctattgcaacatatacacaaacgacatctt
gccctttctggttaatgattgtatccagcgcaacagctgttttacctgtttgacggtcac

caataattaattctctttgacctcgaccaattgggatcatagaatcaatagctgtgattc
ctgtttgcataggttcacaaactgattgacgtccaattatgccaggggccatagactcaa
ttaatcttgttgcgttacttgcaggttcacctttgtcatctattggacgagctaagggat
caaccactctacctaaaaaagcatcgccaactggaatttgagcaattctaccagttcctt
ttacagagctgccctctaaaatatctctaccatctcccatcagtacaacacccacgttat
cactttctaaatttaaggcgacaccaatggttttgtcttcaaattcaagcaattcaccag
ccataacttcatcaagcccatatactcgagcaataccatcaccaacttgtaaaactgttc
ctatattggctacttctacatcttgatcatacttttcaatttgttgacgaataatactac
ttatttcatcaggtctaatgtttaccatgtttttaatattctttttattaatgggaaatt
ctaaagtggtaattactcagattaatactatctg--tcttttactgcattttaagttact
actacattaaggtgtgaagccatctgccttagttgtcctctgatactagtatcaataact
ttcgatccaatctgaatagtgaaaccaccaattagctcgggatcgacagagacaattagt
ttgacttctttagactgggtcatcaccttgatttttctgtcagcagattttcttgatca
gagcttaaagcaatagaagtattaatattagcaattgttagagattccatttgatatgca
agttcgagatacttactagctatagcatctaacattgcaattcttttgcgatcaactaaa
atcagtaaaaatgataatgtattttcgctaatttgatcactgaaagtggcaataatgact
tgctttttgcttctgcagtttttaatggattagct---aaaaagtttttcagcttgtca
gattgcaataaaatatttgtattgactgtatatcttgactcacctttctgtaactttt
tttattttagctaactcaagaagagctacggcataaggctgagctattttcataacaatg
ttattactgctcatagttgatctcctaacttagcaatattattatcagtgatacgtagct
gcatttcagagcttatttgattttctagctgcacagtaactcgtttaagagcaagaaatg
ttatttgctgctggatttgcctgcgaatttgtttttcagctgtttcgatatttgacttgc
ctgtcatagccagtctttcaatatctaattgaccttgagctaatatagaacttctaactt
ttcctgctgtcagctgagcctctttttgatttgatctataataatttgagtctgagcaa
gttgcttttctgattctgataatctagcacttgcttgctctagtctttcttctgattctt
gtatagctgctaagacttttatctgtcttgcatttaaactagatccaagaaattgtttca
gcacgtaaataagaccaaataaaagcaacaagatattgataacattcgcttcaaaaatat
cagaattaaagccgaatttatgttcactactatgttctgataaaatagtaattatttgtg
gtatgtttactatattattcatttagaattct----------tactattttaatatttta
cttaggagtcatgattttaaagattgactacttaatagtttggctttaatttggtcgctt
aaagtatccacttgatcttccaaagttttaagagcttcttctttttgagtgtttagctgt
tttgatgcttcagcaattaatttttcagcattcaattgagctttttttaatatcttcagag
actatattttgcgcttctttttgagaagaagctatctttagttgtgcattacgacgagct
tcggataaatcttgttcatatttgcagctaactcgtctgctttaacaagcatagaagat
gcagtagttaaagttgtgcgaatatattcatcacgttcatctagtactctagttactggt
ttataaaaaatagtatttaataaaaccattaaagtaagaaactgtaatgccattaaaggc

aaagtaccattgaagtcaaataagccccttcaatttcttccgctaatagaaatggtaaa
taaatcattgtactaattatgtttacagttaataatttggtctcaattaagaaaattaac
tttatagtgcgcttagtctgattagactaagcgcaaatgattattttaaccaacgtatgg
attagcaaataatagagacagtgcaacaactagaccataaattgttaaagattccataaa
tgctaaacttagaagtaatgtgcctctaattttccttctacttctggttgtctagcaat
accttccacagcattagcggctgcactaccttgaccaataccaggacctattgcagcaag
acctacagcaagaccggcagcgataacggatgcagctgaaacgattgaatccataatgaa
ttttgatttttgttatataagtagaaaattgacaaatttcggataatatttaaaatcta
aacaatagaatatcgtagattcaaatat-----tgtaattatagtatactactcttctcc
atgaccttccattgcttccccaatataagctgcagacaaagtggaaaaaattagtgcttg
gattgaactagcaaataatcctaataccataactggcaacggtatcaaaattggaattag
caaagtaaatacagcgacaactaattcatctgctaagacatttccgaatagacgaaaact
taaagataatggcttagtaaaatcttctaaaatattgataggtaataatactggagtagg
ttgaatatacctggcaaagtaacctaacccttttttacttaagcctgcataaaagtaagc
taaagaagttagtaaagataatgctacagttgtattaatatcatttgttggagcagctag
ctcaccttctggaagttgaattaacttccaaggaattaatgcgcctgcccaattacaacc
tagaatgaataaaaatagagtagcaatatatggaacccaaggacgatattcatgctctcc
aatctgattttagcaatgtcttgtaagaattctaatataaactccataaaattttgaaa
tttttcaggaattctttgaagattcctggtacccaggaaagatattgttaatagtgtagc
aattactaaccaagacacaataaaaacttgaccatgtaattgtaaactacctattttcca
atatagatgttttccgacttctactgctgacaagctagtatatggattaaaatctatgag
actgttttgatacataataaatatttcttttaaatagtgcaagaatcaaaaaataagttt
tttgaagaattaatatgcaattagtttattggcaagattaggtttttaataaattaagag
caaaaataaattctaatagagatgggtatctttagtagtgattattttttgtaaaaaaca
attattgctacctataatgcttttatgtttaattatatacccatatattaattatttaat
atgattataagtatttcgctacaaactttattgttttatctattct----aggtgttatt
ctaactcatttcttatttaaaatatctgcaacttcatcggcaaaattagctctggtattt
tcttctcctccacctaatacgaatctaacaaatcttctgattttatattctcacctaaa
acggaaatattttgattgattaaatcttcaatactaatatcttgattgcgaatgaacatt
tggtctaataatgaaagttctttcagcctctttttcattcgcccttcaataattccgtct
attctatcggcgggcttgtttttcaagtcatctttaccagcttcaactctttttctaaa
ctgataacttcagctggtatgtgctgtgttgatacgtattccacatttggcgatgctgct
atttgcatagcaatatctttagctaattttgaaattctggacggcgtgccacaaaatcc
gtttcacagtttacttcaactaatacacctattcttcctccggtgtgaatatagctttct
aatagaccttcaattgcagttcgactagatttcttattagctgaagctaacccttttgt
cttaatgattctaaagctttttcttcattaccattattagcttctaaagctttttacag

tccatcatgcctgcaccagttttatcacgtaaggctttcacagtttgagcagaaatttgt
agtgtcatatattttcaaaaatttttaagtatttaaattaactaaaacaaattattataa
tttactttcaacagagctagctatttcttctacatccgtctgcccatatttgccatcata
aattgcgtctgctatttaccaacaattagtttaattgatctaatagcatcatcattagc
tggaattggaatattaataatttcgggactgcagttagtgtcaagcatgcaaatagttgg
gatacccaatttcaaacactcttgtatagctgttgtttctcgcttttgatctataactac
aacgacatcaggcagacgagtcatattttaataccgtttaaatgtttacgaagtttatc
taactctctccgtagaacagcagcttctttttaggtagttgatcaataataccacttgt
atcttgctcttctaactgtctaagacgactgactcgagatttgattgttacccaattagt
taacattcctcctaaccatctttggttaatgtaatacgaatcacatctttgtgcttcttg
ggcaactattcctgccgcttgtctttttgttcctaaaaataaaactttcttaccatcaga
cgatgcctttttataaactcacaagcttctgtcagtaattgagcagtctgcactagatc
aataatatgtataccatttctttctgtatagatatacggaaacattttagggttccatct
acgagcttgatgtccaaaatgcacgcctgcttctaataattctgctaaggtaacaacagc
cataagattattaata-ttaaataatgtgattattcgggaaatcccttttttt----aaac
tgat-----tcacttaacttattgcaattctaatcaaccgataataaattaacacacttt
tacagtagattaagaaa--aaaatctttttactttatttaatctacacttttattactaa
aatagttacgcgcggttctgtcgtctaaaataatatcgtctaaatcatctctaacggatg
acacactactcccgctactggtatctgaattccaggctttttatctaacgacactttat
tatgattgtcgtacgtattaaagccagtaccagctggtataagtctaccgataataacat
tttctttcaaacctctcaaccaatccaatttaccagatatagcagcttcagttaatactt
ttgtggtttcttggaaacttgctgcagaaataaaactttctgtattgagagatgcttgcg
taatacctaataacacggggcgatatgaggcattcaacttgtttcttagtgtcatagctt
tatttgtttgttcaatcttttgcaactctacaagttctccaggtaaatagccggtttctt
caccattttctattttcactttagaggtcatctgtcggactataacttctacatgtttat
cagaaatattaactccttgggattgataaactaattgtacttctttaacaagtagtaact
gtatctcttgaaaacttaatctagctgcttcatatagagccagtccgcattcaatatata
gattgaaactcgcatcaagaatatcatgaggatttaataacacatccgtctttttttcgtg
cttcaagaatttcttcaattcttggaaggccttgtataatatcaccagtttttgctctat
caaatacgagaatagctaatgtttctcctcttctaattaatgcattattgtcaacatgta
gaattgcgccgttagaaactaaatagggcctagcaattcttaatgtaattgattgagaag
atacttgaataatttgtccagagtctaaagaaaccatattttcagtaatagaatcgccgc
aacgaatccaatcgccaacatttattttagttttggcttcttctatattaaaaactttt
tatcagaagatgttgcaactagaatccgtctactagtattcttttcagaataaatttctt
caacgacaccttcactaattgcaataatttctgtgcttgcaacaaccgttaatggtttaa
tatattgaccatttttaacaataattctagtttgactttgttgtttttctgatttattga

catcaatacttttaattgatagtgtctcaaaagttgaaagcctcaagtcaaaataattat
taccgctttctttagcagagaattcaattgaagatattaaactatcatctgcatgctcaa
tttcagctacaagattggttgtaactaaatgaactccatctatcgatttcaccctttctc
cgtcacgaaaagctgttcttttaactagctttaatttagtttgactaggtttttgtgatg
ccaaattatcaattaagctagattttgtttccggtattgaataaacaataacaggtctaa
taaggatataaggcatttgctcatttctatatattcccaataaactaacttatctgtgg
aaatattattatgcaatgtttctccaggtcttaaaaacccccctactttttcattattac
tatacacttcatttagtttatagatagatcctggcttaataatgatttcacgaactattc
catccttctggattatctcaacaattcctgagcttttagaaaaaacattttgaccaact
cagttccactttctataatatccccattataaactagcaaaagtgatgcatctttattaa
tctcatgtgtttcttctgagatccataatatgtagccagggctcaaaatttcataagcat
ctttctctgaacccactttctttttgaaacatttaaatctaaatatttaatgatgccac
cacttgtagttttataagtatcagatattagctcagcaactgtatcaccgtcgtgaa---
ttttttgatttggaacagctttgaaaataaattttgtttttttctgtttctaaaatat
aagattctttttattaattaaatctgtgtaaatataacaacctggaattataatagact
ctgtaataacttgtatattagtaatgctgctattagcatttttttgtatcctgacttccc
ccgcatagtgatttattaactctgtctgcgcgagtattgtgccagctgtaattttatctt
ctttatcaacaatcacgttggccgagtcagatatactataaacttctcctgagagaaccc
aaatcaatccacccgttttagtaattcgtgtagtatattgattattatctgtttcttcaa
ctgttaagttagagaaacagattttaccagacaggtcggatactacatgtttttgagccc
tttctgtcatcagtctatttctacgaggtgattcagcaatcacctgatcttttgatacta
attggccatcacgcaataagagagtagttcctttacttaagttaattattgacttctgat
ttcttctagatcgaattgtaacttgcgttggatttttttgtaattaaggcttgctctccat
gccttgtgcgaacatttatatatgattcaatatctagatccgttagctggccatctatgg
gagcataaatttgttctgctaattcaccagtaaatacacccccagtgtggaacgttctca
ttgttaactgagtgccgggttccccaatagattgagcagcaataatgcccacagcttctc
ccaaatcaactaagcgaccatgagcgagattccatccgtaacaatactgacaaacagaac
ttctcgaattacatgtcacaggtgatctaaccagaacttttttt---aataccagctctag
ttatctccctggctaatttagggctaatatcttgctttgtatgagcaatcaaaacattcg
tatctggatgaaacacatttctgctaatactcgaccagacaaagcttgttctaaattga
ttaatgttttttgtgtgtctactaaatcttctagtattataccttttatttgttctacagt
ctacttctctaatgataacgtcttgagaaacgtcaacaagtcgacgcgttaagtagccgg
aatctgctgttctcaaagcagtatcaactaatccttttcttgctccgtacgatgatataa
aatagtctgtaacagttaatccttctctaaaattacttgatattggtaagtcaataattt
gtccttgcggatcagccataagccctctcataccaaccagttgtctgacttgagaaatat
tgcctctagctccagagaaagccatcatataaacagcatttaagggatctgtctctttaa

agtacttaattacttcttgttttaatgattcgcttgcattgttccatgtatcaataactt
tttgaaatcgttcgactgctgtaatttcccctcggcggtatttattttcggtagctttaa
tttcttcaattgtgcccaataaaagactttgtttgctaggaggaatccttaaatcttcca
gacttaacgagattcctgcctgcgtagcgtaatggaatccaagatctttaagcttatcag
ccatattagctgctcgtgctataccatagttacgaaaagcccaaacaattaaattcttaa
gttcattttatcaataaccttatttgaaaaacttggctgtgcaagacttcttctattat
ccactcaatcttctccatattaattatgtatt----taactacgccactaaagattcttg
aataatttgttaaagattatccgaccagctgttgttcgaatatattgcacaattctatg
ttggtcagcatcttcttttatgatgtgattattaaaaaattttgtgctactattatcact
gtgtttttcaattttaattggctcagtagtttgatcaccatctacaaatccatcaaaacg
agcccatatataagaatgcaagtcaactttcttctgctcataagccatcactacatcttc
taagctagcaaaatattgatttgccccctgctgttgacacggattattagcagtcaaata
gtaacaacccagaaccatatcttgactaggcatgataattggctgacctgtagctggaga
aagaaaattatgaggagctaacattagcaatctagcttcggcttgtgcttctagagatag
aggaacatgcacggccatttgatctccatcaaaatcagcattaaatgcaggacaaactaa
gggatgaagcttaatagctcttccttctacaagaataggttcaaatgcttgaatacctaa
tctgtgtaatgtcggagctctattcaacagtacagggtgaccttgaataacttcatttaa
aacattccaaatagaaggttcattttctgaatcatttttttagctgctttaatattatt
aactaagccctgtaagattaatctgtggatcacaaagggttgaaataactctagagccat
ttctcgtggcaatccacattgatgaagttttaaatgaggacctacaacaattacagatct
tcccgagtaatctactcttttgcctaataagttttgtctaaagcgtccttgtttaccttc
tataatatctgataaggattttaaaggcctattattcgcacccacaacagtcctacctct
acgtccattatccatcagagagtcaacagcctcttgtagcattcttttctcgtttctaat
aataatttccggagccaaaattgatttcagacgtgataatctattatttctattaatgat
tcgacgataaaactcatttaaatctgccgttgcaaatctgcctccatccaattggaccat
aggtcgtaaatctggaggtataacagggattactgtaaataccatccaagaaggatctgc
acctgttgctataaaattctcaatcaatcttaagcgtttcatttttttattaaattttaa
agaaggtgtcttgaaacttttggtggatttgttgcttcagatcgtaaattttctgcaat
gtattctaaatctaaatctttcaacaatttctgaatagcttctgcacctattccaacttc
tacttgattactttcattttgattttggtaaatttcctcttcaagacttttccattcata
accttctagcagctgcttgtattttaaattgatatttgtattagattgtgtaactacata
ggagtgaaaatagacaatttttttctacttcttttacttttaaatctaatgctaaagcgat
atagcttgtacttccctttaaataccaaacatgagttactggtgatgctaattcaatata
agccatccgatgtcttcgcactcgagattctgttacttctactccacaccgttcacagac
tataccttttatagcgaaagcgtttatatttaccacaatgacattcccaatctttaactgg
gccaaaaatttttttcgcagaataaaccatccatttctggttttaaagttctataattaat

tgtttctggttttgtaatctcaccaacaatttgtccattgggcaaacttctttcacccca
ttgacgaatttttttcgggagaagctagactaatctttacatagtcaaaatattgttcaaa
ctgtgtcataaatttgaataccttaaaattaagtaattaaccaaattatttacatatgcc
atattcaaaaatgaa----ttaataaagaaattgttcaaaatcatctactgggggagtat
cataattgctccgatctgctcgattgtctttagagtcagacatcaaatcgacttcgactg
tccgtctttgaccatcttcaaatagtttcaatttatgtactgcaatatctaatcctagtg
actgaagctctcgcattaaaaccttaaaagattcgggggtccctggcttagggataggct
ttcctttaactattgcattcaatgcttcatttctagcttgcatatcatctgactttacag
ttaataattcttgaagagtatatgctgctccaaaagcttctaaagcccatacttccattt
ctcccaatctttgacctccatgctgagctctacctcctaatggttgctgcgtaactaaag
agtaaggaccagtggatctcgcatgaatcttatcatctacaaggtgaacaagtttaagca
tataagctctaccaattgtaacaggattatcaaaacactctcctgttctgccgtcgaaga
cttgcattttaccaggatgctgatcattaaaaagccatttattattagtcagtaatgaag
cttcttttaactttctattcacaagtgctcttgaggcttccgctccatacatctcgtcaa
aaggtattattttaaatcgtttacctaaatatccacctgctagtcctaataagcactcaa
atacttggccgacattcattctagaaggaacacctaaaggatttaaaacaatatctacag
gggtaccatcagataggtaaggcatatcttgtttaggtagaattctagaaataattccct
tattgccatgacgaccagccattttatcgcctacttgaattttcctcttttgcgcaacgt
aaacacgaatcatcgcatttgtaccaggaggaagttcatctccttttttgcctagtaaata
ccctaacattaaccacccttcctttagccgcgttaggtaatcttagcgatgtatctctga
catctctagccttttctccaaaaatagcccttaataatttaccttcaggtaattgatctg
cttcgcctttaggagtaatttttcctactaaaacatctcctgcttcaacccaagaaccac
ctaccacgattccatttctatctaaatcttttaaagagttatcacttacattagggattt
ccctagtgatttcttctggtcctaatttagtttggcgacattctacttcatacttttcga
tatgaatagaagtgtataaatcatcataaactagcctttcactaattaaaaatgcatctt
cgtagttgtaaccttcccaaggcatgtaagcaacgaggacatttcttcctagagcaattt
ccccgccatcagtagatgcaccatcagctaaagtttggccaacaactatttttttctccca
cccacacgattggacgctgattaatacaagtatcttggttagagcgataatattttttca
aacgataatggactgttctaccattattatcttggatgcctattttattagcagaaacat
aattaacataacctgcagttctactgataacaaccatcccagagtctcgagcaattttag
tctcaaggcctgttccaatgattggcttttctggatataacaatggtacagcctgtcttt
gcatattagaccccattaaggccctatttgcatcatcatgctccaaaaaaaggaattaatg
atgttgcagctgaaatgacttgaataggtgaaattgcaatataatctacttgacttggtg
ttgttgtaataaattcttgacggtaacgtacaggaataatatcgccttcaatataatttt
gagaattaactttaacatcgcctggagcaactctaaaatcatcttcctcatctgctgtta
aatacataggactatcttgataaattacttgaccttcgtgtaccagataaaaaggagttt

cgataaagccaaaaatattaactctggcacaggttgccagcgagccaattaaacctgcgt
ttggaccttcaggtgtttcgattgggcagatccttccatagtgacttgggtgcagatcgc
gaacagcaaatcctgctctatctttattaaagcctcctggacctagagcactaattcttc
gtttatgggttaattctgcaactggatttgtttgatccataaattgagaaagttgactag
atccaaaaaattctcttactgatgcaattaacggcttgggattaattagattagataaac
tcagcgagtctatatcgcatatcatcattctctctctaataatacgttctaaacgattga
gacctactctaaactgattttgcaataactctccaactgaacgaacccgtcgattgccaa
gatgatctatatcatcaaggttacctgagtttttatctttaatattaattaaataatcaa
tcgacgataaaatatcttgaggggagagcacacgaaaagttttaggtatattaagaccta
attttttgttaatcttatgtcgacccacttctcctaagtcatatctctttgggtcgaaaa
agcgagaataaagcatttgcttagcaacagcaactgttgctggctcattcggtctcagtt
tagagtagactataagcagtgcttcttcatctgttacttcttcgatattatttttttccaa
tttctttagctaattctttaactgaatatggttgggatgcagagataagaaaggcgtatt
tgctcagtcctttttcaatctcgtctttattaaggccaatagcgcgaagaaaaatataag
catttactttatgagtcttatctattcttatccaaatttctcctttaggatcaatttcaa
attttaaccaagagcctcgattagaaatcaggctagcactgtaaatttgcttgccatttt
tatctatctcttgtttatagtaaattcctgggctgcggattatttgattaataataactc
tttctgtaccagatacaataaaagttcctctgttagtcattattggcaagtctccaatga
agactaatcttttcttgtatttttttgtttttatttgcttttcagtactaagctgtaaat
gtgttgatgataaatctagtgatttgatatttttttccatgatctttagaaggtaaatcaa
tatcttttctcgtcaactttgcaggaacgtatatttgggcactataagttttatctctat
tttttgcctgtctaacactgtaacgaggaaattttatttttatactcattaccaaatagtt
gcagttcgagtcgattagtaggatctgatatattcggaaagaattcaagtacttcagtca
taccttctaatagaaaccatttaaaactggctctctgaatttcgactaaatctggaagaa
gtttattttttaagcttatacgttgaaccatagatctcctttataacaaattttgaaatt
gaagagtttaa-tactatcaatagagtacttgcacgtcatacctgagcatagttcactac
attctttcttctctagacacaactagaacactatattttattcaacaaaaaagattttta
aaattt---tt-----acaatatagatataaaagtccttcggctatcaaaaaccgttgaa
gttattaaaa-acacaaaagacgaaattaatacttgtccgtctgtcaagtatttgacatc
aagatagacaagtattatttatttaatcaattctg-tttttttgagcgtaagataatgct
ctcgcaagtctagctttttttctagcgccagtattagggtggaatgctcctttttttatt
gctttatcaatcttgctatatacttgagaaacacttgattgtacatcattaagattagaa
ctttctagattatcaatatttagcaggcatcttttagttaaagtttttacaaccgattta
tacttacggttaattagacgatttctttccgaagttttaatgcgtttaatcgcagaaagg
ttcttagccacagtaaatccaataaaacttttataaaatatttttagaaacgaaaataca
caaatttaagatctattcaaaaaaggaatatgcgccgtattataacattaaagaaaaaca

ataggcaatagttcactaagagctcttgttagtacaaaataaatattgtcattgcacaaa
a-tttacaaaatagtattaaat-----taaactaaccagacatataaatatatatgaaaa
aaattgaagctattattagaccttttaaacttaatgaagtaaaacttgccttagttaagg
aaggtattgggggatgactgttgttaatgtctctggttttggaagacaaaaaggccaaa
cagaaaggtataaaggatctgaatattctattgatattattgataaaataaaaatcgaaa
tcattattagcgatgacaaagtagataaaatcacagagacaattattaaagcttcgaaaa
caggagaaattggagatggaaaaatatttattagtagcatcgaaaaagtcataagaatta
gaactaatgatttggattccgaagctttataaaatttacactttttgagacttgatact
ttttcatgaaacatgagataataagcgtttattgtataaaaacaactaaggtataata--
ataatggctaaaagcaaaggtgcccgcatcgtaataactttagagtgctctgataaagct
ggcgattttagtcataaaaggaaacctggcgttttccgatatacaactactaaaaatcga
cgaaacacaccaagtagaattgaattaagaaaattctgccctaattgtaatcagcattgt
ctattcaaagagattaaatagttattaatttattaaaat--aatcatattatggccgtg
tatagaaaaagaatatctccaattaagcccacagagtctgtagattacaaagatattgac
ttgctaaggaaattcattacagagcaaggcaaaatactaccaaaaagatccaccggatta
acttcgaaacaacaaaaaaaacttactaaagcgattaaacaagccaggatactttctcta
ttgccttttttaaataaagattaaatatataccattatgcttcatattgtttcctattaa
tatt-------tttatggatttatttactgtaagtatcgaatatactgggt---------
----------tgcgacaaaaaagctgtttattaccaattttgttcattttataattaata
aaaa--atccaacttactattaaactagattc-----agacttatagcagtacctctttt
aatgaattatattgtacaattcatttaaaagaggagttcattttattctagttaataaat
aatcctcctgtctttttttatgcctatacacagttttaaaataaagatttttgtttaggt
ataagatcaaatgaatgaatcctatcaccggattgccataattgaaattcagagataaaa
attccacattcattaccagcttggatttcttcaacatcttctctaacccgctttaaagac
tctatttttccttcataaataacttcatgttccctaataactttaatccaagaattttc
agtaatttattattagtgactctacatcctgctatttttctattagccaaagaaaagaca
gtgcttacttcggcttctcctactggcacttctgaatattcagggtcaagtagctcttcc
attcttcctgtaacatcttcaatcaatgcataaataatttgataattttcaactagtata
ttcaattttgctgcagcttgctttgctccaggagcaaaattagtattgaaaccaagaaga
ttagcatttgttgtcgatgctaattcaacatctgtagcagtaatttcgccaggcataatt
aaaacaacgtttaattgcaccttactttggggaaattgtgataaagaatctaagattgct
tctgtagagccttgattatctgttttaataattaaagaaatttgcttacttaaatcttta
gaattagtggctcccaaggtgtctaacgtaatacgactattaagtgctttctgtttctgg
acgattagtgaattttttgaagtattttcaagagctttaagctttgcttctttatcattc
ttaactactagagctatctctcctgtgacaggaacagccgacagtccccaaatttcaaca
acagatgatggtgttgctgaattaatctttttttgttgattattaataatagctcgaatc

tttgcgtaagctgatccaataaccatattatcgctcgtatgcagtgtaccattttggatt
aaaagggttgctactggcccatgagatttgtctaagtgagcctcgactataacacctttc
gcaggctgtgttggatcagcctgcaagttttctaactcagctaataaggtaatggtttct
aataatttatcaatattgtagccagtaatagaactgataggaattacaggaacttgtcca
ccaagtgtctcagacatcacattgtgttttaataagtcctgttcgataatatctgtgttt
gcagagtctttatctatcttagatattgctaccacaaaaggaacattagctttttgaata
tgctgaatagcctctacagtctgaggtttaatgccatcgtctgcagctataataataata
gctacatctgttaaattagctcctctagatctcatacttgtaaaagcttcatgacctgga
gtgtctaaaaaaataatttttgcttatgctcattattgatatactcaacttcataagct
gcaatagtttgtgtaattcctccgatttctttattggcgttgttagagttatgaatataa
tctagtaaggttgttttgccatggtctacatgccccattactgtaacaatcggaggtctc
ctaatatagttgcaagcttcattaagagagctagaagtatctaaattggcagaatataag
ttattattttcttttatttctgagtcaactgatataccaaaattattcgctactgatgat
attattgaagtatctattgtttgattaatagtaactgatattccttgaaggaataaatac
tttataaatatctgtttcttgaacacaaattagtttagataactcttgaatagtaagagga
ttagtaataataatagattctggtggagaatcagatacactgatggcttgattttggctt
tgagttaatgatatttttttttgttttgtagactttttagtattagtaattttttttata
acttgagcttgtggtttaggtggacgcattagagagatagctaaatctcctgcagtctgc
ggaatactagaattagcacctctaaaattctct---tcctcatcatcaatatgtattttg
gtctttattttc-tttcgttgtctgtttttg--tttttttattatctgctaaatcatga
accttattgaaatttttattttttttatctagtttaggtggagaattcaactctaaatga
ggttcactactcgctgtatgggtatctgatttatcaagatctaagtgcaacaaattatct
gtatgggctgattctaatctaattttatatataatttggggactttttaaatctataatt
ttttcagaaga---tatatttggactaggtgcaagagatctattttcgaaattttgatta
tttaaaaacatgactgttgagtttatgatctaatagtaaattttcaatttcaatacatag
cttaaagtaaagaagaatcacatatct------ataatactaatat---agaaaattaat
ttttgttgtattagatttaatataacactaaagtcttttgttagttatacaagtttaaaa
ctttt-tataatgataaacactacattctagtttattgatatacaatctatttatttaaa
tagtactgtttttcaa-aagtttttaaa----ttatctaatattgtgttcgttatgaatt
tttattaaagtttgaaag-ctatacaattaagtaataatcattaataatctccacgccac
ttatgccacgatctcaaaaaaatgataattttattgacaaaacgtttacagtattagctg
atattgtattaaaaatacttcctacaagcaaagaagaaaaagaagccttttcttactaca
gagacggcatgtctgcacaatctgaaggagagtatgctgaagctctagaaaactattatg
aagctttaaaactagaagaagatccatatgatagaagttatattttatataatataggtc
tcatttatgctagtaatggtgaatatgtaaaagctttagaatattatcatcaaggattag
aactaaactttaagttgcctcaagctctcaacaatatagccgtaatataccattatcaag

gagttcaagcagttgaagataaagatacagagctatctaaattaatgtttgacaaggctg
ctcagtattggcagcaagccattaagttagcaccggataattacattgaagctcagaatt
ggttaaaaacaacaggaaggatgagaaatatacaaggatattaaaatatttaggatataa
t---tatattaattaatgactcag----aattaaggactcttttactctatagcacattt
gtattat--------ataatatactaattgc------tgtataagaatcaacgtaataaa
cgaaattaaatctattgactatatttatgaatacaaataagcataatggttagtaaaaca
aaaagtactggatctgttactcaaatcattggaccagttttagatattgcatttcctaat
ggacaacttccaaaagtattcaatgcactcaaagtagaaagctcagaaggaactattact
tgtgaagttcaacaacttttaggtgacaacaaagtacgagctgtgtctatgagctcaact
gaaggactacaaagaggagtgaaagttcttgataccggatctcctatagccgttccagta
ggcgccgatactcttgggcgtatttttaacgttttaggggaacctgtagacaatttagga
ccagtcaattctgaaagtactttacctattcatagacccgcaccagcttttactaagtta
gaaacaaagccaaatattttcgaaacaggcattaaggtcgtcgacttattggctccttat
agaagaggaggaaaaattggcttatttggaggtgctggagtaggcaagactgtattaatt
atggaactgattaataacattgctaaagctcatggtggtgtatctgtatttggtggtgtt
ggcgagagaactagagaaggaaatgatctttatatggagatgaaagagtcgaaagtaatt
aatgcagataatttgaaagagtcaaaagtggcactcgtatatggtcaaatgaatgaacct
ccaggagcacgtatgcgtgttggcttaactgcattaacaatggcagaatactttagagat
attaataaacaagacgttctattgtttattgataatatttttcgatttgtccaagcagga
tcagaagtatctgctctactaggacgtatgccatctgctgttggttaccaaccaactttg
gcaaccgaaatgggtgcattacaagaaagaattacttcaacaacagaaggatcgattaca
tctattcaagctgtttatgtaccagctgatgatttaacagatccagctcctgcaactaca
tttgcacatttagatgcaacaacagtgctttcacgaaatttagcagcaaagggcatttat
cccgcagttgatccgttagattcaacttcaacaatgctacagcctggaattgttggcact
gagcattattctacagctcaagaggtaaaatcaactcttcaacgatataaagaattacaa
gatattattgccattctaggtcttgacgaactttctgaagaagataggcaaactgtatcc
agagctagaaaaattgaaagatttttatctcaacctttcttcgttgctgaagtatttact
ggatcacctggaaaatatgtatctttagaagatgcaattaaaggttttcaaatgatttta
aaaggcaaattggatgacttacctgaacaagcattttatctagtaggtgatattgatgaa
gcgatacagaaagcggatagcatgaaagattaacataattaacaatgactttaaatataa
gaattattgcacctgaccggactgtttgggatgcagaggcacaagaaattattttaccca
gtagcactggacaacttggtattttaacaggccatgcccctctacttactgctttagata
taggagttatgagagttagagtagataaggaatggatgccgattgttttgatgggtggat
tcgcagaaatagaaaacaatcagttaactattcttgtaaatggcgcagaagaagctaatc
aaattgatttatcagaagcagaaaaaaatttagaaaatgcaactcaattattaagtgatg
cctcttctaataaagaaaaaatagaggcaacacaaaaaattcgaaaagctagagctagag

tacaggctgcaacagcagctattgtataa-aacatttt------gctgaaaatataaaac
cttagtaataactactaag--------------gttttatttgtgtatttataggcaact
taagtaaggttagcttaaaccagaacaaatataatcaaaatataaacccatctctttacc
cgcatctggtccaactaaactaatagtaacttctttcatagctaaaattgcttggattgt
tgcaccaatagggacacctaaagaattatatgtctcttttagaccatttaatactctttc
ttccaagatagatggatctccagctaacatcccataagtagcatagcgtaaataataatc
taaatcgcgaatgcatgcagcataacgtcgagttgtgtacatattacctcctgggcgggt
aatatccgagtaaagtaaagattttgctacagattctttaataattgttgctgcattagc
tgcgatagtagcagcagctcttactcttagttcacctgtctgaaaatagcctcttaattt
ttcaacagagctatcatctaagtatttcccttgaacatcggctgcattaataacagaagt
aattgcgtcttgcataaattgtaaaaattccttaattttaaatatttaatactaaagggg
tatataacaaaataactgaacttttatatataccagtattactgcatagcacccaaagt
ataatcaaagtaaaaaccagcttctgctgaatcttcaccagcaagtaatgagcaagcttc
acttttcatacatttgacaccttcagcgactcccgaaattggtgttcctaaagaattata
catttctttaacgccgactagtccaatttcttcaataggcgtcacatctccagcaactat
tccataagttactagacgaagataataatcaaggtctcttaaacaggtagctgtcatctc
ttctccataagcatttccaccgggagagacgacatcaggtctttttttgaaacagctgttg
accgccctgctttacaatacgctcacgattatctgttaaaatttgagctattcttaaacg
acgttgtccagataagacaaaacttttaattctatctaattcaccaggacttaaatatct
tgcttctgcatctgcatttacaattgacttggtaacaatactcatggataaaactcctgt
aatgcttt--ttaaaaagcttaatactaattatttatgatataactcagaaagtagtgcg
cattttagaaaaatttatagctacagcacgttggtgatgtgtaaagcagcacaaatactt
atgtaagtactaaaacaagaaagttgcatgtcgtaccttattttattggttaccaagtac
tttttttaaaactaggtaccacaattgatacatcttgtttagtaaggcgattatataatgt
ttccgtattcgggaaattagctgccggcaaagttggaaatcttctataaggaacagtata
tttgccatatactgaattatactcagtactttcaactaaattactaatgaatgcagataa
tccttttgaagctaaaatttggttaaagaatctaatttctgcctgattattaggagctcg
tcctaagatatgcttcgtacctagttctataacttttgtatttggatatggctgataaaa
ttctttaccatataattcagataatgctaatttttctactaattctttaacattgatttt
cttattcaagaaggatgcttcaatatctaaaaactctcctccgatactaaaagaatttag
atctctttcaaaaatctggcgatatgtcgctcttaaagcttgctctaatacttcttgatt
actattattgtttacttcaaatataatagattgatctcttaaagagctaactccttgcga
gatacgagactggatgtcattgctacttcttatttcttttacagtacctagttcaacaaa
tctagcagacttgctagtaatgactttttaaatctctggtcaataatgcctggtcttaa
gcttcttagagcaataccggctggtgtactatatctttcataaggcacagtactatcacc
aaaagtttctatatactcagaactatcaattattgcatctatcacttgatagtaaccctg

tttatatgcaatatcaaaatacttattaatttcttgacggccatatgtaggacgacccaa
taagcggttgtgaatatactcaatagctttgcagatatataatggctcccagtaaagtga
tctaaatatactcgatttggctaattgtctaacaaattcacgaactgtaatttgaccatc
ttttagttgactttctattggcttaagaagtagttttcttcttgatacacgtctctacc
aaagactcttaaataagcaactttcgtaacaacattgactgaattttcaaaattttcaga
agagtcagcttttacgagagtgttggataatttaaaaattttggggcccaaagatcctgc
agacttaggtcttacctgtggattactaatctggttgtagattccggggccacgcctaac
taaaattcttctagtatctttgccaaaaagagcttgtctttttctaggatccttattttc
tttaggaaaaattgctccaaattgaatcgacaatggatcattgcctattccatatggatg
ctgatcaggtaatgcttgtttgtaatcactaaataatgttataaactgaggtaccttacg
aaaaggagcactatagttaagaagatctatttgcggcccccaattacgacactcttgagg
ctcttcgcctaaattccggaagtaaggtacagtttcttctccaaaataatctgtatattc
agaagaatttaaaattgcatttactagtccacttaaaccagtagctgacagaatagcaaa
atatttttgaaattcctccaaagagctaggccctctaccaagaaaatgtctaaatgctaa
ttctagtgctcggctatttacaaaaggctcatagaattgctttctataaatacttgaggt
tcctaacgaacggacaaattctttaatggaaatttgaccatttttacttgcgactctaa
gttagataaagacaagtcatatgctttggcaatatctctttcaaagatttgtctataaca
tgctttcactacggtattttttcgtctgcagacaaactaggcttcataacaaaacgagg
cgtagaaactcctgcctgtgcgtaaatttgaggaaggcgcaaaccttgtaaatctccaga
tgttctttttcttaacttatcggtcaaagatggagcttcaaattcagaaattacaacatt
aaaatactctttgactaaatcttgacctttaatatcttcttcaaatatcaatagagctgt
tcgtctcatctctctaagggccacaattgctgctgcgcttgagcaagcattatcaattaa
ttctcttagtcctctaatattaacagataaaatattgggatcacctgatacaattgcata
tgttaagtaccgtaaaaaccagtctaaatctcttaaagattttctcattcttgtagtacc
gtatctcaagacatttataggtttaaatccaggaggggtagctcctccagcattaaataa
agaacgaaaactctgcccaaaatctccttgaatatttccagatagctcattaatttgtc
ttgagaactttgatctcctgcaatcataacagctgcctgtggacgctctaaataagaaat
tgcagacccacctacaaatattttatctgcagctcgagctactagaatattggcattttt
agttaaaatgtctgcaacttctaatctttttttgcccagaatttagaaaagaaactagttg
atttaattctcccagctgtaaaaatcggtcttgctgctctgcctgagtaatagttaaaat
tgaggcagtccgataaagctgggggcgtgctaatgggcttccgccacttgctttaatact
cattactttatttatctccttaactgatattacttgctagcgaattcaaatatcacctga
tatttctaactatttaaagatactatgcaagtacatttgttttctatattattaggtact
agtagttatagtacatagagtcgcgcaactaacttctataaatacaaatttttgtaatact
tctattaacatttatcaaaagtgaatataattgatgactcttttatattaaaatgcaatt
agtgagagattaattctaaataaagtatacttagtaagttttatagatagtaaaatt-ca

agacttattctacaaacattaatca------------cttgactgtttgacaattatatg
attgttcttgcttttaattggtttagactagtttcacatttttctaacgtaaacaatat
caataagcaatttgtatgaatttagaaccacagaataacctattaactaacaacaatata
aaccaacaagatatacccgaaaatgatataccaatgtcaattacggaacatttagaggaa
ttgagacaaagaactttatttgtattttattctttttgcttgctgcaactttaagtttc
acacagattaaaataattgtcgctatactacaagctccagctattggtattaaattttta
caattagcgccaggggaatatttttttcgtctattaaagttgcaatatattctggaatt
gttgcaacagctccgttcggtttataccaagttatcttatatatactacctggactaact
ggcagagagcgaaaaataattttacctatactaattagttcagtcttccttttgtaaca
ggcggtatatttgcttactttatttggcaccagctgctttaacctttttgatcagctat
ggatctgacattgtagaaccattgtggtcttttgaacaatatttggactttattttacta
cttttatttagcacaggattagctttcgagatccctatcatacaattgttactaggtgtt
tcaggtgcattatcttccaatcagatgatccgagcttggaggtatattattattatatcg
acaatagctggagccattttaactccttcaactgatccagttactcaaattatcatgtct
tctgctgttctagtgctttattttagtggaatcgttatattactacttttaaaaaagtaa
atatagt--------aagcttttatttcatactatatttttcttatc-----ctaaaaaa
tggagtct--atataacttgtggttcttaacttagattaatattgaagcattcggctttt
agactctttaaaaatatttagtcttattatggaactaatataatatg------------

---tatatatcga
t---tttttcatcaattatttgtgattttcatttttgtacta----ctttatgattctt
gcaactatttatgtaatctatttactgaat----aataataaagaaaattcagcgtaaat
tatgataatctataccaatataaaaagtattttattgtgagttttatttatataaaggct
tattcattatatatttataacagtaagtctaattaatagtttttcatgcttaattaaagt
aataactcttaatccttttggcaaaacagttcgtcttaatattttatc---aaaaaacag
aaaaaatgaagctaaatagtctaattaacttaattcaaaagtctatatgttcttgtacac
ttttattaattattttaaacattatttgtgtcgcacctaattctagtagtgcatttccaa
tctatgcacaacaagcttacgaaagtccaagagaagcaactggtagaatagtatgtgcca
attgtcatcttgctcaaaagccagtagaaatagaagcgcctcaagcggtactacctaata
ctgtttttgaaactgttgtaaaaattccatatgacagtagtgctaagcaaattttaggta
atggaagtaaaggagggttaaatgtcggagctgttgtaatattacctgaaggatttaagt

tggctcctgctaatagattatctacagagttaaaggaaaaaaccaaaaatctatacattc
aaccatatagctctaaacaaaataatatttttagtaatcggacctattcctggtgacaaga
atagagaaatagtttttccaatactctctcctgatcctgcaaaagataaaaaagctcatt
tttttaagtacccaatatatgttggtggaaatagaggtcggggtcaactttatcctacag
gagataagagcaataacaatgttgttgcagctttaagtagtggtaaagttaataagattg
aggtccttgagaaaggtggatttatagttcatgtgactaacagtaacaatgtagaatcaa
ctcaaaagatttcccaaggtcttgaacttaaggttaaagaaggtgatacagttcaattag
atcaggctttaaatagtgatcctaatgtcggtggtttcggtcaaaatgaaacagaaatag
ttctacaaagtccgaacagaattaaggggatgattgttttctttttttgctagtgttttag
ctcaaattttctttgtattaaagaaaaaacaattcgaaaaagttcaggcagcagaaatga
atttttaacgcaagataaaactggtacttgctataattaaagtg-taaacactttattca
aagaatgtaccagtttttatattatataaccattgtttttgaagacagtataatattttt
aacagattctataatttgactaggttgaattacagtagcttgttctaagctaccattata
tggcgttggtatatcctgcgaagatagtcttactacaggaacatctaaatcgtcaaaaag
atgttcattgatttgtgcaattagctccgctccaattccagctgttttcatacattcttc
tacaatcaaaactttgtgagttttcttgactgagacagatatagactctatgtctagagg
ttttaaagatataagatctataacttctggatcgtaaccttcttctagcaaagctggtag
tgcttgaataacatgatgacgcattctagagtaagttagaatagtaatatcttttcctat
acgcacaaattcagctttgttaagaggcaataggtattcttctttaggaatttcttcttg
taaattgtaaagtaaaacatgctcaaagaagacaactggattattatctcgaattgcaga
tttcagtaatccttttgcattatatggagtagagcaagcaactattttttaaacctggaat
agcttgaaaataagcttctaatctctgagaatgttctgcgcctaattgtcgaccaactcc
tcccggtcccctaatgactaaaggcaacgtaaagtttcctccggaagtatagcgcaacat
gcctgcattattagaaatttggttaaatgctaataacagaaagctcatattcatgccttc
aacaatcggtcttaaccctgttatggctgcaccaatcgccatacccgtaaaactattctc
tgctattggtgtatctagaactcttaaatctccatacttgctgtgcaaatctttagtcac
cttataagagccaccatagtgacctacatcttctcctatcacacacacagtcgaatcgtt
ttccatttcctcatctgttgccgctcttagtgcgtcaaacataaagattttactcatttt
tatatttagtttttatggtttaactactactcagaagtaaaagttaagatatccgatttt
cttcaggtatctgaaaaaagatatcgtttaagttctgagatattaggctctggactggaa
atagcaaatttcacagattgctctaaatctttttttaacagaagtttgaatttcattgagt
tcatttatgtcggcaatcttattatctaaaatatatttttttaagtttttttgattggatct
cttgcaatccaagcttctttttcttgtcttgatcttaattcatcaggatctgcaagagaa
tgaccgcggaatctatatgttaaagcttctattaatgtcgggccttcaccctgtctagct
ctctgaactgcttgaatcgctgcttgccttacagctagtacatccattccatcaacttca
ataccaggaagaccaaaagcttctgcctttttatgtatttctggtattgaggaagatcgg

tggtgtgccatacctatagcccactgattattttcaacaataaatataataggtaacttc
caaagaactgccatatttaaacattcaaaaaattgaccgttattagtagtcccatcaccg
aaaaagcaagctgtaactctcaaatcttcagtttcttttaacacttgctgacgataaata
ctttggaaagctgcacctgtcgcaacaggtatgccttctgcaataaaagcaaatccacct
aaaaaattatgaggtgcagaaaaaatatgcattgaacctcctctgcctttgctacaacca
gtttcttttccaaatagctcagccattacattttagatggaacgcctttacttaaagca
tgtacatgatctctataagtactacagacataatctgtagaattaagaaccttaattaca
cctgtagaaacagcttcttgaccattgtaaagatgcacaaagccaaacatctttcctttg
taatacatttgagcacacatatcttcaaaatttctacctaacaacatatcttcatataag
actaataaagtattctt------attaagaccagttgaattatagttagttagcggcaat
tgaactttcttaggataactcataatttataaagaaacctct----------tattatac
ataggaattttgaagtaaaagttatcaatagaaaacacacagataaaaattcataaatta
ttatatgcttttattaaagactttaataattatttgcaactagttcagtatatcaaatt
aattttgcttatactacaggtgtctaaaacttagattacaaggga-tagtggaaaagtgc
ttaaaagaatag-taaaaacaaatagaaatagaaataaacagaattatgcgtaagaaaat
gactattattaatagataattacttaatatttctaaatattttcaaattaatggctatat
catcatctttattttaccctgttgagaaagaattgtacagtttggaaacaaatctaaaag
ctgtagccggaacacgtcatccaattctatacgcagcagcaaaacatctgttcgaagctg
gaggaaaacgagttagaccagctctcgtttttttagtggctaaagcaacatcagagaaac
aagatattaatactggacagagaaggttagcagaaattaccgaaattatacataccgcaa
gtttagtacatgacgatattattgacgaatgtacaacgcgtagaggagtcaagactgtgc
ataatttatttaatacgaagattgctgtattagctggagatttttatttgcacaatctt
cttggtatttagctaatattgaaaatttagcagtcgtaaaagctatttctaaagtaatta
cagattttgcagaaggcgagatcaggcagggcttagttcattttgaccctagtatttcaa
tagatgcttacattgaaaaatcattttataagactgcttcactcattgctgctagctgtc
gaggagcagctatgctgaatggttctagtactcaggttaataatgatttatatctttacg
gtaaacatatgggcctagcatttcaaattatggatgacgttttagatataactggttcta
ctaaaagcttggggaaacctgccggtgcagatctaactaatggtaatttaacatctccta
ttctgttttcacttactaaagaattagatttaaaccgccttattcaaagagaattttgta
acagtacagatgtagcctcagcattatttcttattaaaaaaagcggagggattacaaaag
ctcaagacttggctaaagaacaggtacaggcagcactctactgcctaaagtttttaccaa
aatctgcacctgtatctagcttaaaagaattgacacatttcataatcacaagattgtcat
agaatacttgctaaaattaaaatgctattaactaatttttcactgtttctaaaatagtct
gaaaagtttgaatatcattacttgctaactgtgctagcattttacgatttaatgcaatat
tctctttttcaatgcactaataaaagtactatacttcataccttgactatgagctgcgg
catttattctggttatccaaaggcgacgaaaatctctctttttcctttttcgaccaacat

aagaatatctcagcgctttaagaacttgctgtttagctgttcgaaataaacacttatgtg
cccctttaaagcctttagctagcttaaatatttcgctcgtctttttttgcaacattac
ctcttttaactctactcataaaatattac-ttaaaattaatttattaccggcttcctcag
tttgaattgttatcg----attataaataaggcaaatttatagcgatatttttaatatct
cttgaatcagctgaacatgtagaagaaagatgtcttctttgctttgacgacttcttttgt
aataaatgactttagaagctttatgtcgcaaaaatttcccgatgaagacactttaaat
ctttttgctatcgcttttgatgtttttaacttaggcatattataaacttttaatgattaa
caaaagtatgaaaagtctaaagatattaaacttagacttttcaatacttaagataatctt
aacacaaattattttacttactatagagatttactgaaataatttatgtatataacagta
agtaattaatcagtagctgctaaagttgatttatatttagctttaaatttttgtatagta
ttagcaattagcattgttattattttaagagtacttgaatttaattcttcgaacattctc
atatttaatgtgaatgaaatatttgcttctgatacaatattttgtatttgctcctccgat
aaaggaatcatattcagtgaagctctatacttagctttaaagactttgtcatcttgtatt
tggtcaaagtcataaaacctagtacctctttcatcggataaattcatcgcacttctagcg
atttttttaagatttggcctccagataaatcacctaaatagcgggtataagcatgagca
actaataattctggctgtttacagcctatactatgaattctatcaacatatattcttgtt
gcaggagatggtttaataagattcaaccaatctgctccataataataacttaaatcttct
gacagacttattttctattaagttctgtgaaatatataggttggatagctgggtgatct
ttattagaaaatagttcttcttctatagcgcaataaacgaaataaagattagcaactaat
ttacgatatgatttcttatctacgacacctcccaaaaaagatttcacaaaactaacattt
tctgccatactatgagacttagtagtgccttctcttaattcattcgctaaagtattaacc
ataatatcaatcttccatttta-aaagataaccgattaaat---ataaaaagttatctat
atattta------cgttttactaattaagctgctgcaaaataatttttgattttattgg
gtctggattcatcgttttatcaccaggcttccaattagctggacacacttcatctggatg
agattgtacatattgaattgcttgtaggactctcaaagtttcttctacactcctaccaaa
ctcgagattgttaattgtagaatactgaataattcctttgggatctataataaatagtcc
tcttaacgctataccatcactgtttaaaacattataagcagcactaatctcttttttaa
gtctgatactagtggatatgcaagatctcctaagccaccagattctctatctgtttgtaa
ccaggcaagatgagaatactcgctatccaccgaaacacctagaacttctgtgttgagctc
ggaaaaagcatcatacttatcactaaatgctgtaatttctgtaggacaaacaaatgtaaa
atctaaaggataaaaaaataagataatgtacttatttttaaatctgataattttaaagt
tttaaattcttggtcataaacagctgtagctgaaaaatcaggagctagctgacctactcg
aagacaatttggtccagaaatcattaatttttcccagaagaattgatataattgtgttat
ttaataaataattatataacaaaatttagtatatattaaaatgctatcagtctttttaa
attac--------tcaatgactctacgaatactatttttatagcggggaatggatttgaa
ccattgaccttcgggttatgagcccgacgagctaccagactgctctaccccgcggataat

taaaatcatattaatccatctaaatagataaaagcaaatatattataagattcaaaaata
aaagaataaaagcacaggagcactaattaataagctcaaaaatgataaagtatttttat
tttatttaacagagggaaaacttcatataagccgataaagcgatctttaagaagaatttc
cgatggctttacccaaatttgaccgtcataccaaccagattcttcatagaataccgtagc
actcattaaacgttttactacgtatgaccatcctaaatatagtctgattaaaataaatcc
agttattaaacttgaggtaataaattctgaaaaaacaaatttcagaggcagcttggtaat
tggaaaaattgaaagcaatactggactaactaataaaccattaagtagcagtgtaaccgt
aatttttctgttgtataattgactgcttagagttggccaacaaaaaaaccaagaatttct
tagagaagtgtattcgttaacaggttgctgctctttgggtacaggacactgagtattata
taaattcattgaaatttaaaattattatactagaaattgtttttttatctgatctaaatg
attagcga--------gttaattaattgcaattatagtagttagtatcagaaataaaact
agaaataaccaagtaactttatttaatgcattttcagtactacgagtattgctaaaaaat
tgattctgagctccgacacttcctagaccttcagatttgggattatgtatcaaaatagta
aaaactaatataattgttactgcataccacaagaatttcaaaatttgttccattatatac
aagttaataaaaaatagcacaaagacaatcgtctaaaagatattgattgtcttatcatat
tgaaaaatatttattcaccctgtacttttaacagtaaaaatcctattgccaaccctacta
aaactaaaactgaacttaagacagcactttctacaatttctccgaccatt-atatttttc
cttattattttattatta---taattttataaaaaagttgatacctattaaa-ctagaag
ccgtttcttccccaaactaccattgcaatagaaaaggtaaacattgccattagagctgcc
catcctaaacttaaaatatccataatttataatattc--ctgaaaataaggtctcttgta
cttaaactataatattcttaatattaaaaatatcattatataataagctaattataacag
agtactactgaattagtacttttagtgcatttaaagaagattgaa-tttttgcgaagtac
ttagcaagtattaatttgtaaaaaatttctgccat----ttttgcattattatacctatt
aaattgaaatgtaagtattactcaaaaatcgttagtaattttgatttttttcaattgtacc
att---aacatatgtaaaaacataaagattcatataagctaagttaa-----tgttctac
tataatttattaat---aaaaggaagctggtattaaaatcatgcaaacaactataagtaa
tgggaaaacgtctagcaaagaaactctactaacacctcgattttatacgactgattttga
agaaatggctaatatggatatttcagaaaatcaacaagatttctttgctattttagaaga
atttcgagctgattataatagtgaacattttattcgagacgaagaatttaatcagtcttg
gtccagcttagaagataagactaaatctttatttattgaatttctagaaagatcttgcac
cgcagaattttcaggtttttttgctatacaaagaattatcacgaaaattaaaagatagtaa
tccgattattgccgaatgctttttgctaatgtctagagatgaagcacgccatgcaggttt
tttgaataaagctataggagatttcaatttatctttagatttaggctttctaacgaagag
tcgaaaatataccttcttttctcctaagtttatttttttatgcaacttatctttcagaaaa
aataggatactggagatacataactatttatcgtcatcttgagcagcatcctgaacatcg
catctatccaatctttagattctttgaaaattggtgccaagatgaaaatcgtcatggaga

cttttttgccgcattacttaaatcgcaaccacatttttttgaacgactggaaagctaaaat
gtggtgcagatttttttctattaagtgtatttgcaacaatgtatctaaatgattttcaaag
aattgatttttataatgcaataggcctagattctagacagtatgatatgcaagtaatacg
aaaaactaatgaaagtgctgctagagtttttcctgttgctttagatgtagataatccaaa
gttctttaaatatttagatacttgcgcatgtgacaatagagctctaatagatatcgataa
gcaaaatttcccattattaattaaagctattataaaaactcctctatattgctccctaac
tattagtttactaaaaatatatttgattaaaccgattgattcgaaaacagtgtggaatac
agttcgataatcaaataacataagaataaatcttaatgacgcaggctatctagttattaa
tataacgcagactgaaaagctcacttct-----gccaagaaggagctt---ttttattaa
caaatagacaatatggttctataaatt-tagaactaggaacttacttttttcttttgcttc
atacataacttctagggtgattagattttttggaacaatcacgtgcaaatttttctgtatt
tctttttactttacctctaacaaaacccggaatttactcaattctttctgtgcttcttg
tgaccagttaatactatcaacagcagagattcctacacttaatgcttctgttgtatcatg
accaccaaatatttctaataagtgatcttccatgcccaacgtgaatgagttgtacactaa
atctgcaatttgatttgtaccttcatagccaaggaatggacgataacttaagggaaaatt
ttgtatatgtactggtgaagaaataaccccacaaggtatatttaatcgtttaccaatatg
tctttccatctgtgttccaaaaattgctgcaggttctgttttagcaattaaatctccaat
taaaccatgatcatcggaaataattacttcatcacagaattcttggacttgttctttaaa
ccactcttcgtcatatttgcaataagtgccacaccaagtaacatgtatgcccatttcttg
atgtaaaattcgagttattgctgcagcatgagttgcatctccgaatacaatagccttttt
gccagttagattttggcagtcaattgatctagaaaaccaagcagattgagagataaatct
agtttgttcatctatatacttttcatagtctacaatagctcctaatgtagttaataactt
ttgtattgagcggatacatgctgctgtttgcacaattcccataggtgtagtatcaacata
aggcatgttaaattctttttctaaataacgagctgtcattagcccggtctctctataggg
aacaaaattaaaccatgcagatggtaaatttttttaaatcttgaacggatgcattttcggg
aataatttgattaatcttaatatctagatcttgaaatagtctttttaattctgaaatatc
atgctgattatgaaatcctaaacttacagctccaataatattaacagatggggtttctgt
ctttcgaattaaaactttattgttagattgcgctttttccatataaaaagtaacaatttg
ttccaaagttctatcaccagcttgaagttcgtttactctgtaatgattaacgtcagctaa
taaaacatcagcttctgtttctatagaggctctgctaacaaaatttttgtaaatcttcttg
taagatactagaagtacatgttggcgtcaaaataactaagtctgggttttcttctctgtc
ttttctagtaatattctcaacaactttttcttgcgaaccacgtgctagtacatgtctatc
tacaacactcgctgtaacaggcgtaaagtctctgtctcgttcaagcattgaacgcatgac
attaaaatagtcgtctccgagaggtgcatgcataattgcatgaactttttttgaaagagct
agcaattctaagagttccaatatgggcagggcctgcatacatccaataagctaatttcat
attaaattttcctatgatttatttagacatagtaatacttttaaagccaaataataaata

aaagtcaaacatctaatttaactctttaataatttttttcatg-tttttagataaagatta
acattacattgtagtcaaatc-------------agaaataaatgagtaatgaataaagc
ttgaaagtaaagatttcttgcagttatagtttttctttaagtaaacatgtcaaaattgta
ctatatataaatgtactatatataaactttttttactgaatattggtaatgccagataca
attaacttaaatatgccttccccaacgtttggcggaagcactggcggttggttaagagcc
gcagaagtagaagaaaagtatgcaataacatggaccggcaaaaatgacagtaagtttgaa
atgccaaccggtggcacagcaactatgagaaatggggagaacttgctttatttagctaaa
aaagagcagtgtttagctttaggtactcaattaaaaagtaaatttaaaatatctgactat
aaaatttacagagtgttcccgaacggagaagtgcaatatttacatcctaaagatggtgtt
tttcctgaaaaagttaatgctggaagagctagtgttaacagcgttgatcattctattggg
aaaaatgttaatcctgtagatgtcaaatttacaaataaggcaacttacgattagtcaatc
aaaaaacctagacataaaattactttatatagtctaggtttttgatagagtatactagt
attatggaaaggtggccgagtggttgaaggcgtctgatttgaaatcagttgaactg--ta
tcgttccgtgggttcgaatcccaccctttcctttataaagct------------ataat-
--------------------ttatacactaatagtattaagtttagacttaataaaatct
acaacttgcgataaattagaaatttttcggcgtcttcatctggtatttctataccgaat
ttttcctctattgccattactaactctactgtatctaaagaatctgcgccaaggtcgctt
gaaaaatttgcttctctagttacgattttctttcgattcctaactgctctgctacaata
tcttgaactttttcaaaaatttcattatcttgcataatatttgttttcctctaaggtgtt
atcaaaaatttttcggctcatattattatctaactctaatcaattagtaattttgagtta
agatctaaatatttctacctatatgatatactattattctggataaatacgaagtttgcg
atatggctcttctccaaaactacgagctctcaactggtagttatcaactaaattatgttg
catttttcgaatataagctgaacgaggtatcaactgaactgttgaattttcgttcaagat
aatagtttcaatagccaatttagcttcttgaagggcttgaatttcatcaaatttttttatt
tttacaaagttctacccaattaagatcagaagaagtatgaatatttaatatttttctcag
tgcacgagtaatttgtgggactgtactattctgaattgtatatataataatttgttttga
ttttgcaatttgtcttaattttgtattttgctttacttgattacgcaatgctagaatagc
atctgacttttctatctctttagttaaaataattggtaaatctaaagaagaaataactga
tgcaatatgatgccaactcaaagaataagcatataaatactgattaggcacttctaatgg
------tggtgtattaatatttataggagcgttaatagtagttgacaataaagatgtatc
acgattttgtaatttattcaaatcggatgacttattttttaattctctgtactgcagaac
aggatgtgcctgttgtatattaattggttggcggaagctagaaatatttgatggcaaaac
ttctaaagattgtgatggatagcatttaattaaaatgcggccattagcttgaatttgtct
tttttgtacaaaaggttgatggccctgtaaaatttgatctatagtttctcttacttgatt
atggactatccaaacagttcgttcatgaatttcaattgcaatttgaaaagcaggcgcggc
ttttctttctagtatgcttttctgagtacctcttctttttgcttcatcatcacctaatgt

aacatattgaattcctccaattaagtctgctaatgtaggatttttaattaaactttctaa
atgatttccatgtgctgtacctactaattggactcctctttctgctatagttcgagcggc
taatgcttccagctctgtaccaatttcatctataataatcacttcgggcatatgattttc
taccgcttctatcattacctgatgctgtaaatccggccttgcaacttgcattctcctggc
tctacctatagctggatgtggaatatcaccatcaccagctatttcattagaagtatctat
aatgacaactctttttccatttcatctgctaagacacgagcaatttctcgaacagctgt
tgttttaccaactccaggttttcctaaaagtaaaattgaatcaccttgttctagtaaatc
tctaataatactaattgtaccgaaaactgctcgtccaacacgacaggttaaaccaataat
acttccttctctattacgcatggaactaatacgatgcaaggtttttctatgccagctcg
attatctccgctaaaattaccaactttttttacacagtagtctaagtcttgccaactaat
agatctttgagataagtattctggcttatctggaaatcttgcttccggacggcgacctaa
atccatcactacttctattaaatgatttctactagggtgctgtttaaggggctctttgac
aaagttcggtaagatttctagcaacttatctaagtcatctgcaataagcatgattgaata
tataataattaattaatcagatagaaaaattaagattctaaagattacaatacatattaa
agaatattatgctcgtaatcaattttatacttaaattaaataagaaattg-cagatggaa
aagactagattaaaccgtatacagtaaatagattggtcaaaattaatagtcatgttaata
tgtttattggacagaaagttcgtattaagtatagcaagcaaaaagttgctaatgacatag
cagataaggttggtgatcgaggaattattagaggcgtaaagtttatcaatagtcaatgtg
ttactgttattgttgaatttgacaatcatactagactgtggatgtttagagaggagctaa
tttgtttaaatgaat------ttaaaaaatgatataagattaattt----tattatatag
aaattttcatgagtcgttcaaattatacattttaaaggagtattgttaattgtgcaaatt
actattaaaaaattacaagacttactttcatcagtacaaagaaaaaagatacagacattt
aaactaaaacaggatgattttgaacttctattaaataaaacttataagaaaactaattca
gaaatattacctttacaaaact---ctgtattacagcatagtcaagctgcaataattaaa
tcaacatctaataacactagtcgatctattagtaacaatgatgttacagaatatgccact
atcgtctctccaatggtgggaacattttatcactcacctgctcctggtgagaaaattttt
gtgcaagttggagatgaagtcaaattcaatcaaacagtttgtattattgaagcaatgaag
ttgatgaatgaaatagaagctgaagttgaagggaaaattatcgaaattcttgttaaagat
ggcgatatcgtcgattgtggtcaagccttaatgaaggttgaaacatgatgacaatctttc
cattttacatattgttaacagaaattatttagatagaaaaattagtttatactatagtta
tgaaaacttatttcgtaaagacac-ataaagttattatgtaaaataatttactttatctg
ccaaacatagaaataagcgttttgattccttgtctcaagagaggagaatctcaatgacaa
ttagctcaaaagagcaagagacaaagaaggtaaaaatctcggttgataaaaatcccgtag
atacttctttcgaaaaatgggcccaaccaggtcatttttctcgtacactagcaaaaggac
caaaaactactacttggatttggaatcttcatgctgatgctcacgacttcgatagtcaaa
ccagttctttagaagaagtttctcgtaaaatttttagcgcccattttggacaactttctg

taatatttttatggcttagtggaatgtattttcacggagcacgcttctctaactatgttg
cttggttaagtaatccaacaggtattaaaccaagcgctcaagttgtttggcctatagttg
gccaagagattttaaatggtgatgtcggtggtggctttcaaggcgtgcaagttacatcag
gctggttccagctatggagagcatccggaattactacggaattccaactttactgtactg
ctattggcggattagctatggctgctttaatgttgtttgcaggatggtttcattaccaca
aagcagctccaaagttagagtggtttcaaaatgttgaatcaatgatgaatcaccacttag
ctggtcttttagggttaggctgcttaggctgggcaggccatcaaatccatttatctttac
caattaataaactcttagactccggtgtatctccgcaagagattccactgccacatgaat
ttttaattaatagagagctgatggctcagctttacccaagttttagtaaaggattagttc
cgtttttcactttaaactgggctgaatattctgactttctaactttcaaaggagggctta
accctgtcactggcggtttatggctaagtgatactgctcatcatcatctggcattagctg
ttctatttcttgctgcaggccacatgtatagaaccaattggggtattgggcacagtatga
aagaaattttagaagctcacaaaggtccttttactggtaacggtcatgaggggctctatg
aaattcttacaacttcttggcacgcgcaacttgctattaatttagctatgatgggatcat
taagcatcatagtggcacatcatatgtatgctatgcctccatatccatatattgctactg
attacccaactcaattatcactatttactcaccatatgtggattggaggattttgtgtcg
ttggcgcaggagcacatgcttctatatttatggtaagagattataatcctgctgaaaatt
ataataatttgttggatagagttattaggcatcgcgatgctattgtctctcatttaaatt
gggtatgcatatttcttgggttccattcatttggtttatatatccataacgatactatga
gagcactaggaagatcccaagacatgttctcagatacagctatacaattgcaacctattt
tcgctcaatgggtacaaagtatacatactttagctcctggtaatacagctcccaatgcgt
tagcaacagctagttatgcattcggaggagatgtcgtttctgttggaaataaagttgcaa
tgatgccgatttctttaggaacagctgattttctagttcaccatatacatgcatttacta
ttcacgtaactgtacttatcttagttaaaggtttccttttctcaagaaactctcgactaa
ttcctgataaggcaaatcttggctttagatttccatgtgatggccctggtagaggcggta
cttgccaagtatctggctgggatcatgttttccttggactattctggatgtataattctc
tatctgtagcaattttttcactttagttggaaaatgcaatcagatgtttggggtagcgtgt
ctccatctggaaatgtttcccacatcactggtggtaattttgcgcaaagcgcaattacaa
tcaatggatggttaagagatttcttatgggctcaagcatctcaggttattcagtcatatg
gttcggcactatctgcatatggactaattttcttggcagcacattttgtatgggcattta
gtttgatgttcttatttagtggtaggggttattggcaagaacttatagagtcaatcgtat
gggcacataacaaaataaaagtcgctcctgcaattcaaccaagagccttaagcattactc
aaggaagagcagtcggtgttgcacactacttactaggtggaattggtacaacttgggcat
tctttttagcaagaattatttcagtaggctaatagtgaaaataggataaaaaaaacaatt
atggcaacaaaatttcctaagtttagccaagctttatcacaagatcctacaactcgcagg
atttggtatggtattgctacggcgcatgactttgaaagtcatgatggaatgacagaagaa

aatttatatcaaaagatatttgcttcgcactttggacatctagcaatcatctttttatgg
acatccggcaatttatttcacgtcgcttggcagggcaactttgaacagtgggtgttaaat
cctttaaaagttaagccaattgctcacgcaatttgggatccacactttggacaaccagct
ttaaaagcttttagtaaaggtgggtcagcatatccagtaaatatcgcatactccggcgta
tatcactggtggtatacaattggaatgagaagcaatcaagacctatattctggggcttta
ttcttacttgttttatcagctttacttctatttggtgggtggctacatctacaacccaaa
tttaagcctggtttgtcttggtttaagaacaatgaatcaagattaaatcatcatttatct
ggattgtttggcgttagctctttagcttggacaggtcacttagtacacgttgctatacca
gaagcaagaggacaacatgtaggatgggataattttacaacagtattacctcatccagct
ggtctacaaccatttttttagcggcaactggagtgtgtatgctcaaagcccagatacagct
caacacgtattcggcactaatgagggtgcaggtacagctattttaactttttttaggtgga
tttcaccctcaaagtcagtctttatggctaactgacatggcccatcaccatttagctatt
gctgtagtcttcattgttgctggacatatgtatagaactaattggggggattggacataat
ttaaaagatatttttagatgctcatagaccgcctagtggcagattaggagctggacataaa
ggactatttgataccattactaattctttacatatacaactgggattagcattagcttca
cttggcgtaattacttctttagtagctcaacatatgtatgctatgcctccatatgctttc
atggctaaagattttactacgcaagcatctttgtacactcatcatcaatatattgctgga
tttttaatggtaggagcatttgctcatggtgcaatatttttttgttcgagattatgatcca
gaacaaaataagaataatgttttagctcgcatgctagaacataaagaagctatcatttct
catttaagttgggtaactctatttttagggtttcacacattaggtctttatgtacacaat
gacactatgattgcttttggtacaccagaaaaacaaattttaattgagccagtatttgct
caatggattcaagcttcttcaggaaaagcactttacggttttgatgtattactatcatct
tctagcaatattgcaacacaagctggtagtaacatttggctaccaggttggctagaggca
attaatagcggaaagaattcattgttcttaacaatcggtcctggtgatttttttagttcat
catgctattgcattaggcttacatacaactaccttaattttggtgaaaggcgctttagat
gcgagaggatctaaacttatgcctgacaaaaaagatttcggctatagtttcccttgtgat
ggtcctggtagaggcggtacctgtgatatatctgcatgggatgcattttacttggctgtc
ttttggatgctaaataccataggctgggtaacattttattggcattggaaacatattact
atttggcaaggtaatgcaactcaatttaatgagtcttctacctatctaatgggatggttt
agagattacttatggttaaattcttctccattaattaatggttacaatccatatggtatg
aacaatttatcagtatggtcatggatgttcctatttggacatttagtatgggcaacagga
tttatgttcttaatctcttggcgtggttattggcaagagttaatagaaactctggcatgg
gcacatgagcgtacacctttagcaaacttaattcgttggaaagataaacctgtggcatta
tcaattgttcaggccagattagtaggtttagcacatt-tttcagtaggatatgtattaac
ttatgcagcttttgtattagcttcaacagcaggcaaatttggttaaggtaaa-----att
taattaattgca--ataaaaaagtcagtcatgaat-ctatcatgactgactttttttat

tgacaatagttacttattaaagtttaacttcaatatctataccagaaggtagatttaact
tcattaatgcatctattgtttgagaagaaggttgatgaatatcgataatccttctgtgag
atcttatttcaaaatgttctcttgagtctttatctacatgaggagaacgtaagacgcagt
agattcttctttttgtaggtaaagggatcggccctactgcaatagcgttagttctagatg
ctgtgtcgactattttatcacaagatgcattaaggatactcgaattatatgctttaagct
taatcctaattttttgtttgctgagtaagtgtcatattgagtaaatcttattatttaagaa
ttttggagacaacacctgcacctacagtgcggccaccttctctaatagcaaaacgcatac
cttgctcaattgcaattgcattaattaattcagcagtcattttaattctatcaccaggca
tcaccatttcagcatcggtaccatcatcagcagtaaactgattaatagtacccgttacat
cagttgttctaacataaaactgaggtctgtagcctggaaaaaatggagtatgtcttccac
cttcttctttagttaaaatataaacttcagcttcaaactgagtgtgaggtgtaattgtcc
caggtttagctaataccattcctctttcaatgtctttttctgaacgcctcgcaaaagaa
ttccaatattatcacctgctagaccttcttctagcgtcttttggaacatttctaatccag
taatagttgttgtccgagtctctctcaagccaacaatttcaattgtatcaccgactttta
taattcctctttcgatcctaccagtagcaacagttccacgtcctgtaatagaaaagacat
cttctacagccattaagaaagttttgtcgacatctctttcaggtgttggaatgtaagtat
caactgcgtccataagtgaaaaaatcttatcaacccatttatcttcccccctgcttaatag
tcgggttctttgtaactgcttctagggctaacaaagcagagccagcaacaaaaggaatgt
catctccaggaaagtcatattggcttaacagttctctaccttctaattcaactaattcta
acaactcttcatcatctacttgatcttctttatttaagaatactactaatgtaggaacac
caacttgttttgctaacaagatatgttcacgagtttgtggcattggaccatctgctgcag
aaacaactaaaatagctccatccatttgagcagcacccgtaatcatatttttttacatagt
cagcatgacctggacaatccacatgagcatagtgacggttatctgtttcatattccacat
gagcagtattaatagtaatacctctagctttttcttctggcgcagcatcaatttcatcaa
actttttgctgaggtggaacctaaagttgataaagtcgctgagattgcagctgttagag
ttgttttaccatggtcaacgtgaccaattgttccgatattaacatgtggtttttttacgtt
caaatttagatcgagccatattttttgctttccttatagtacaagttgcttaatggttac
ttttaacgatagatcttacagattgaaagatttattatttgatatagacagtg-----ca
agggatggttaatctaatatcgatagtgtgcaaacgctttatttgcttccgccatcctat
gtgtttcttctctttttctaatagagctacctgtttcattggctgcatccataatttcgt
tagccaattttattgccatacttttaccagatctttctcgagaaaatctagtaatccatc
ttaatgctaaatttgtacctctatatgctcttacttctattggtacttgataagtagagc
ctcctacccttctggctttcacttccactaaaggagtgatatttcgaatagctttttcta
aaatatttaaaggatctaattcagtcctatctttaactatatctaaagcttgatatataa
ttctttgtgccaaagtttttttaccactttttaaaatacgaacagttagcatactaacaa
gtctgcttttatatagagggtctggtgacgcgaaccttttttttagctgtattacgacgag

acataatatttgaaatttgttattgtgataatttgtt-taatagaagatatttaagattt
aggctttttgtaccatatttagatcgacttttacaacgatccttaactccagcagcatc
taatgtaccacgcactacatgatatcgtactccaggtaaatctttaattctccctcccct
aattagcactactgaatgctcttgaatattgtgaccaacaccaggaatataagctgtaac
ttcaaatcctgatgtcaatctcacacgtgcaactttcttaaagcagaattgggtttctt
aggagtcgtagtatatactcttgtgcagacacctcttctttgaggacaactttgaagagc
tggagattttgtttttttatgtattttacgtctttcagatctaacaagttgttgaattgt
tggcataataaaattgatttaagtctttaatagatattatttgaacttgctacacgtga
attaattagtctttatttatcttgtatttacgcataaatctttctactcgaccttctgtg
tcaataattctttgtgaaccagtaaagaacggatgatttcctgaccagatatccacatgt
aactcaggctttgttgaaccaattgtcataattaattggccatcacaatagacttttgct
tctgggtaccaatttggatgtatgttatttttttgccattgcttatataattg--------
tatcaataataaatattaacgcttagaaaattgaggagcttttcttgctttctttaaacc
atattttttttctctcctttactcgtggatctcttgttaaatacccttcagatttaagtgc
gtttctattctctggattaattgagcacaatgctcttgcgacacctaaacgaattgcatc
tgcttgccccgttaatcctcctcctctagcatttacatggatatcatactgatttaataa
tcctagaacctgcaatggtgcatgcgatactcttaagtaattaggactaaattgaagata
tgactcaccaggtataccgtttataattagattacctgaacctgggactaatcttacctg
tgcaacagaacacttgcggcgacctgttccagaatagattgcacgagttttaattaattc
tgtcgacataatattcctttataactaaataaatatacattctttatactatatactctt
gtggcttttgcgcgatatgtgggtgaattggaccagcataaactttaagcttcgtaaata
atttcttcctaatggacctttaggaagcatgcctttaaccgatttttcaataattctat
taggcaatcttgtctggagctgctcaaatgtttctacttttagtccaccaggctgtccag
aatgtcttctatatagtttttgattagttttattgccgcttatatgtacttgagaagagt
taatcacaattacataatctccagtatctagataaggcgtataagaaggcttatttttac
cccttaagatattagaaatatgagtagatatcctacctaatgtctgattttagcgtcta
taatataccaatttggattagtatttaatgatggtgcttgcgttttgttcatgaaaagat
taatacttttagttggcaataagagaataggtaaatgctgtacatgtaatttcgatacaa
ataatacttgtaaaatatta-------------ttgacaaagagaactgtcgtgacagtt
atttaaattatttgttagcaagaaaacttctaattatcagtaatttataata-ttgtggt
aatataaaacaggttaatcactttttttcttttggcaaacttatacctaattttttttgt-
agggcatatattacttcttcagctgatttttggccaaagtttttaatttctagcagttct
tcttgagagtaatctagtaaatctgcaatagaatgaatttgagctcgctttaagcaattg
tatgctctaacagacaactgcagttcttcgattaaaacttgactaatttttttatcttct
ttactacgatagttatctgctgatttaaagtccaaatttcttagtgagcagaaaaggttc
gttaaaactgtggcaccttggcttatagcttcttggggagaaatactcccatttgtccat

atttgtaaaattaaccgatcttttatactattagtacctatacgtacttcttctacttta
taattgactttattaactggcataaagactgaatctacttgcaagaaatctaaagataat
tcatctactgctttttctgctaaacgatatccacagtttttttcaattttaaactccatt
tcgaatattgtattattgcaaatagttgcaatatactgcctaggatctactacttctata
tcagaagataattcaaacaatccagctgtaacgatagctggtccctggactctaattcta
ccaatttgagattcttgattatgacttttaaatactacttcttttaggttcaataatatt
tccaaaacatcttctcttactccaggaattgtagaaaactcatggtttactcctgcaatg
cgcacagcaactatagcagtaccttcaagatctgataatattgatcttcttaaagcatta
cctaatgtaatgccttgtccttggtttaatgcttctattacaaaactaccgtactgtcca
cgtgctccatctgttcttgattctacacattcaatttgaaattgagccacctaagcaagc
tcc-ttaaaaataatcaatgattagtaattttgtcgacaataagaaagaatctatacacg
gcgtttcttaggaggacggcatccattgtgaggcacaggagtaatatcctttattaaagt
aatctctaaacctgcggcttgtaatgctcgaattgctgtttctcgacctgcacccggacc
atttactaaaacttcagtttgacgcataccttggtccattgcctgtttagcagctttttc
agctgcagtttgagctgcaaaaggtgtacctttttttagctcctttgaatccactagaacc
agaggatgaccaagataatgtttcaccttttaaattagtaatagtaacaatcgtgttatt
aaatgtagatttaatatgtgtaacaccattaactgcattgcgtttgttctttcttactcc
agatttttttaattgtctagccatcttgtttcgtctattatataattagttacaatatta
tttccttggagctttttttcttgcctgctactgtttttttacctcctcttcgtgttctcgc
attagttcttgttctttggcctcttaaaggaagaccaagacgatgccttctacctctata
agtactaatttccataagtcttttaatactcatcgattcaaaacgtttgagatctccttc
aatctgataactagattctaaaatttctcttatgctaacaacctgttgatcatttagatc
ttgacacttagtatcagcatcaatgttggtttttttttaatatttcttttgaacgagatag
cccaatgccataaatatatgttaaagctatctctattcttttgtttttttggaagatctac
tccagcaattcgagccacgttgtgctttctccaataaataatgtttac-gtttatgacag
aagattaca-ggattagccttgtctttgcttgtgtttaggattagtgcaaattaccatga
cttttctgtgacgacgaattattctacatttttcacacattttgcggacagaaggacgaa
ctttcatattaa----ctctatatactaggaatattactg---------tattaattatt
tacatcaaaaatgttaataatatatttgattactttaattgttatagctttcacttcgtc
atactatcatatttttttgatatgacatatgtttgaatttgcttagccgtgtctattgcg
actccaactaaaataagtaaagatgtcgctccaagacctcttaagttttggatctgagta
acctttctattataaacggaatcagcgctactgtaaataagaacgaggctccgagaaat
gtcagtctatttaatataacttgtaaataatcaatagtcgcttgacctggacgaatattc
ggaatactggcacccatttttttttaaatttattgcgatgtcttctggattcattactatt
gatgtataaaaatagctaaaaaaaagaattaacgcacagtaaagaataaggtacagcgaa
ccgttgggacaaaatagatatagaatttggagtattgttttattttgaattatttgagtt

aaataagatggtagagccatagatgcagatgcaaaaacaataggcataactccaccttga
tttaatttcaaaggcaagtaactgttcggatccagaattgaggattttcctaactgcctt
gctgaaataattttaattcttcttgttccttcttgtacacaaattgtaattacaatcatc
aataaaaatataacaatgaacaatccaaacttaagacccggattactatagtttgcatca
aaaaatgactgtgtaaagtttttggtagtcctgatacaatattttggaaaataagcaaa
gaggctccatttccgattcctttttctgtaattaactcagataaccacataatgatcatg
gaacctgccgttaaagccaaaacagactcacaaacaaatgtaaagttccaattaaataca
tacggctttacccatatagatattgctccagattgtaaagtagcccatccaagagacaaa
tatcgggtaatttgagtgatttttttggcgtcccagctcaccttcttctttctgtaatttt
tctaaatcaggaataattttcgtgagtagttgcattacaattgaagaattaatataagga
acaatacccaaagcaaaaatccctattgttgagaatcctcctccagaaaagatattcagg
aaatttactaaagtgttcttttctacgcttgcataaaaagcatcatggtctatacctgga
acaggtataaaaatgcctaagcgcgctaagactaatagaaaaagagtaaatataatgcga
tttcttaaatcactttttttgtctcataaataaatta---aaaaaaaaagacactga--gt
tagataaaaaccctattccaatacttgtataagtttaaaatagggaaatgg---caagct
atagaagtagcgcta-taatatcttatgtcgaataaagactttctataggaactcctcga
tctttagctgcttcgctaaaagttcttaattgagttaggccgtttaaaacagctctggca
ttgttgagtgtattattagatcccaactgtttagctagaatgttctgtacgcctgataat
tctaatactgtccttacagaacctccagcaattacaccagaaccaggcgcggaaggcctt
agaataacttttgctgcaccagaaattccgttaataggatgaggtatagaattaaatttt
gtaagtggcactgtaactaaatgtttttttggcatcagttactcctttttttaactgctcca
attacatcactagcttttcctactccgacaccaacttgcccctgctcattaccaacaata
agaataactcgaaagcttagcttcttgcctcctttttacaactttagtaactcttttaact
tgtacaactctttcttcccaaccactatctttatctttgcctttactctgtttttttacga
ttagccattttaagcactattcctaa----ttaatta-tgactttaaaaacccatacctg
cttccttagcagcttcagctaaggctttaactcttccatgatataattttcctcctctgt
caaagacaacatttgtaatgccttctttcatagacttttcagctagctgtttaccaacta
ctcgagaagtgtcacaatttgagcctattttatcaagttcttgattatttaaattaacag
atgacgttgcgactaatgtaattccttgtgtatcatcaattatttgtgcatatatgtgtt
tattagatctaaatatacatagacgcggcctacttgaagttccttgaactttttttcgaa
ctcttttatgcttatgaattctagtctgcttagtatttattttcattattatttaccttt
tccggcttttccggcttttcttctgacaaattcgccttggtatctaattcctttaccctt
ataaggttccggaggtctagtggatcgaatagttgaagcaacttgcccaacaacttcttt
gtctatacctgaaacagtaatatttgtgttattttcaactgtaattttaatattttcggg
aggttgtattttgactacatggctatagccaacgcttaatattaaatcttgattatctat
ttgtgagcgataacctacaccttgtatttgtagcttcttagtaaacccattagaaacacc

ttcaatcatattactaataagagttctggataatccatgtaattgactagccatttttgt
ctgcccacttgtcttgacagaaatttatcattaaatatctctaagttaataccatccgg
caaagttcgcgatagagtacccttcggtcctttcaccgtgatggtacggccatcaaactc
agtactaagatttgtcggcaataaaattatttttttccaatacgagacat-attccacc
tataaaatagttaccaaatatagcataatatttcaccaccaagaccatcatgacgggctt
gtctatctgtcataacacctttagaagtagaaataagagcgattcctaaacccccaagaa
ctctaggtagttccttgtgatttgcatagactcttaatccaggcttactaattcttttca
gagcagtaataactggttgacgatttttaccgttatactttagagaaatcattaaatgtg
tctctatgccttcacccatttgttcaaaattttgaacaaatccttcttcttttagtactg
ttgccatattgcatgtcattttcgttgctggaacttgcacaatttgatgtcttgctaagt
ttgcgttacgaatacgtgttagcatatcggcgatcgtatcgttgaccacc-agatcctcc
ttacgataaactgaattatttaatatttttcaagattccttaaaaggcataccaagctt
ttttaacaaagctaacccttcttgatctgttttggctgtagttacaattgatatatcaag
acctcgaatttgatcaatattgtcgtagtctatttctggaaaaattaattgttcgcgcaa
acctaaattataattacctttgccatcaaagctttttggactaattcctctgaaatctct
aattcttggcaaggttaaattgattagcttctctaaaaaagaatacatcttatcttttcg
tagatgtactacaattccaataggcacttcttctcgaattttaaaaccagcaatagattt
tttagcttttgtaacaattggtttttgtccagttattagtgttaattctttaatactact
ttccaaagctttagcattttgagaagcttcacctaaaccacgattaatagtgattttcgt
aaaacgaggaacttcatgtacattttatattggaattcctcttttaaagcttgagtaac
agttgttttatatttttcttttaatcctattaccattatctttt-acaaattatttaata
agttcacctgtctttttcaatttccgga-tttttgacctttatcgttaattatgactga
aaatctactggcaatattattttgttcactaaacaacatgacattagaactatgtattgg
agcttcaaatttgataacttctcctgtctcaccttcttgctgaggtttttatgttttac
ttttagattaattcctttaacaattactttacttgtcttatggatgattgcaataatttc
gcctgtcttggttttatcgcttccagaaataacttgaactaaatcacccttttttcaattt
aatttttgcattaactttagtttttttcaaagaacttttcattatattacctccggtgca
agagagactattttagaaaaatttttatctctcaactctcgagctataggtccaaatact
cttgtgccgcggggattattatcctgattaataataacagctgcattatctccgaatcta
atactcataccgtcagttcttctcaaggctttgcgagttcttactactacagctctaaca
acatcagatcttttaactggcatattaggtgaagcatctttaactactccgatgatgaca
tcacctatagatgcataagcaggattactagtacctaatactctaatacacattattttt
ttagcgccactattatccgcaacattaagatagctttgagtctgtatcatacttttattc
tatataagattaattatcaaaagacttagataagatattaaccatcgtccaacacttcgt
gcgacttaaaggccgtgtttcttgtattgtaactatatctcctgctgcgcattcgttatt
ttcatcatgtactttatattttttttgtgcggatcatggttttcgcatattttctatgagc

aattctattttctacagctactactatagttttattcatttattacttacaactttacc
agttgtttcttttaaaggcatagtttttatc--ttttatatat----aatgaaattaagt
tataaagtaattatatagctggtttatttcgagacttttcaatagttaaaagttgagcta
atctatgcttagaatgtttgaataagtgaggctgaaaatcttgtcttgtagctcttttca
gtcttaactcaaaaagcgttcttttaattacaagtatttcttcagctaaggaagaatcat
ccatttgtgtaacatctgatatttagggaaagtcatgctttaagattctattatatttc
gaactataaatttggtcttcattggtaatttataagaagctagtttcatagcttgctgag
cagttctttgtggcacaccagtaatttcaaataaaatgtgaccaggcctaataactgcga
cccaatattctggagctcctttaccagatcccattctagtttctgcaggacgcgccgtta
caggtttgtctggaaacaccctaatccatagcttacctcctcgtcttacatatctagtga
tagttcttctagtagcctctatttgtctcgaggttaaccatactggttctatagcttgca
atgcataatcaccgaatgcaattgtattgcctttactagcagaacctttcattctgcctc
tgtgttgttttctgaactttgttttcttggggcttagcataagaattaaacaatttaaat
aattaagaagctgttggatcagaagtttcctgattatatacagattctgtgctctttgat
tctggcaaaatttctcctttaaagagccacactttaactccaagtactccatatgtagtg
tgagcttgtcgatgacaataatcaatgtcagctcttaaagtttgtaaagggacgcggcct
tctctaacccattcacttctagcaatctctgcgccattaagacgtccagacacttgtatt
ttaacaccttgtgtatttgctctttgtgccctttgaacagcctgtctcacagctcgacga
aaggcaactctttttttctaactgctgagtaataaattctgcgactaaggttgcttcagaa
tctggatctgcaatttctacaacattgactctaatttgtttgctcggatctaaaattgaa
gataataattttcttaaagattcaattcctgctccagattttccaagtacaataccaggt
cgagctgttgctattaaaatttcgacttgatcaactttacgattaatttcgattttagca
atgctagcattactaagtttagaatatataaacgaacgaattttatgatcttcttctaaa
agaactggatagtctttagagttggcgaaccatgaagaacgatgtttttgggtaatgcct
attcggaaacccaaaggatgaatttttttgacccacagtatccttttctataaattaaaag
ttgtgattactaattgacttgaaatac----aagactacaactctgagacacataacgta
atgtgacaagttggtttatgtataggaaatgctcttccttgtgctcttggttgaaatctt
tttagcgtagggcctttatctgcaaaagccttgctaataaataactgacttttattcagg
ccatcattatgctcagcatttgcagcagcagactctaaaatttgctttatataagagcac
actcgatatggcataaattctaaaataattaatgcttcttgatatttcctgcctctaatt
tgatctaatacacgacggactttatgtggagaaagacgaatgtatttccctactgcttta
gtttcttttacgtttttttgtaatgctcataattatagtttaacgacgggcttttctatca
ccttttacgtgggtacggaaagttcttgttggaacaaactcccctaatttatggcctacc
atttggtcagatacaaagacaggaaaatgttgtttaccattgtaaacagctattgtatgc
ccaaccatatctggaataatagtagatgctctcgaccaggtttttaaaacttctttttttc
cccaaagtatttaatgcttctatgcggttaagaagactcacatcaataaaagggccttta

tgtatagatcttgacataataataaattattagattctaaatgaaataaattctatttac
ggcgacgcaaaacgtatgggttgctatatttattgggatttctcgttttaacacccaatg
ctggtttgccccaaggtgttactggacgagctctcccaattggagatttaccttcaccac
caccatgtgggtgatctactggattcattacaacacctctaactgtaggtcttttaccta
accaacgatttctaccagctttaccaagagtgatattactagcatcaatattgccaactt
gcccaatagtagcatagcattcttttctaatcatacgaacttcgcttgagggtagtttca
cagtgacaaaagagccttccttggctactatttgagcataagttcctgctgcacgaacaa
tttgtcctccgcaagaaggtcgtaattctatattgtgaacggcggttcctagaggaatac
tggataaaggtaatgcatttccaacttcaataggcgcagtgggaccagaaagcactattg
ctccaacacgcaatgatcgagggtgcaaaatataccttttttctccatcaagataatgca
ataatgctattctagcatttctattagggtcatattcaatagaggccactttagcaacta
tattatgcctgtttcttttaaaatcaattaatctatactgctgcttatgtccgccacctt
tatgacgacatgtaataacacctctattgttgcgacctttgcaaaaatgatgtttaacta
ttaaagatttctctggcttatcagtagtaatctctgaaaaagtagaaactgttctatttc
ttgtcccaggcgtataggcccggtataaacgaattgccatataaggaaatttaaaaagag
taaatatgtga------ctgtgtaatacgattaagtttctggaaataaattaatagaatc
ttctggcacaagtgttacgattgctctcttgtaatgaggtcttctacctacaaatctacc
aatacttcttttttcttaggtggatggcaagtgttaacacctttgacttgaacattaaa
aatatactgtatggcagctttaatattaattttggttgctttgggatcaacagcaaaaca
gtactgattttcttctaataattttgttgttttatcagtaatgattggatatttaactaa
atccaataaacctcttgaatcaatgctatccattatatacctcttgtattttagataaag
catcaactgtaataatgattttatgcgcagctaaaagggccatgatattcaaagtatcag
ctgaaataatttctacattatgtaaattgcgaatggaaagataaacattcggatcttttt
tatccaccacaactagaactttttatttaaatcaagattccatcggtgaatagcttcca
taaacagtttagtctttggctgctgaaagtagctattaaagttttctacaatcaaagtat
taactgatttattatttaatgccgtccttaaagctaattgtctttctttttttgttcattt
ttttagtaaaactgcgaggctttggtccaaatattactccaccgcctctccataagggtg
agcggatagaaccagctcttgctctacctgttcctttttggcgccatggtttgcggcctc
caccctgaacttcacttcttgttttagtattagcagaaccttgccgtttttcgctacttt
gttttactaaagctctatgaactaaatacataccagagtcttggcttaccttaagattta
aatctgcgctaccacttatttgaccttcccaattatagacttgataatttaattgtgttt
taactgtcatgagatatatcgagtgaaagaatattgttatttactaattttactaaagt
tccaggtttacctgggacagctccttttacaatcaacaaatcattctttgagttaatact
tacaatttgcaaatttttttaccgtaactttttttattaccaagttgaccagccatattttt
tcctgggtagaccctcccaggcgttgtcccagcaccaatagaaccgggctgcctatgatt
tttcgaaccatgagacattggacctctactaaaatgatgcctcttttgatagcctgaaaa

gcctttacctatgcttttagaagaaacatcgatttttgacccactttaaacatatctgt
ggacaaaatttgactgacttcaaagtcatcagtagatttcactaagtactcactcaaata
ttttagtgggggagcttgtgatttttttaaatgtcctaatagtggtttatttaacttttg
ttcaacaacttgcttataaccaatttgaatagcgttgtagccatcgcgaggtaaggcttt
aatttgagtaacaacacatggcccaacttgaattacagtaactggaattgacaagccatc
ttcatcaaagaattgagtcatacctactttagtaccgagtataccaacagacactagatt
tctcctttttgagagatttatactatcatcatacataaaa-ttttactccaaattgcttg
aag--taaaccaaactaagagatgcatgacattaatcatggcaacataaagag-aaaaac
tttacttattcaatctaaaatagaaacagtttttttgtccagttacattatatataaatta
aaagtaagcttattatattatttacacactac----------------------------
-------------taatatgaagaacaaggaatgtcttcttttag-----ttttacacaa
cattgcattattagtactgtctcaaatataaatgaataaaatattaaaatttcacctctc
tagagaaactacaaactacaattgtgttcggtaactacaccttttttatttacatatttat
tgtgcagtatatatgcatatatagtacagcgcacgtaaaaatatagc----tgtataaca
aatatacttataagaattaaagaggtattaatgggtaaagttgttggaatcgacttagga
acaaccaattcagtgattgctgttatggagggaggtaaaccgactgtaataccaaacgca
gaaggttttagaactactgcttctgttgttgcatatactaaaagtggagataaacttgtg
ggacaaattgctagaaggcaggctgttattaacccagagaatactttctactcagtcaaa
aggtttataggacgtaaacaaaatgagatttcccaagaaattagacagacatcatataat
gttaagactagtggatcaagcataaaaaattgcctgtcctgcacttaataaagattttgca
cccgaagaaatttctgctcaagtactgagaaaacttgttgaagatgctagtacatactta
ggcgagactgttacacaagcagttattaccgtacctgcatactttaatgactcacaaaga
caagctaccaaagatgcaggtaaaatagccgggttagatgtactaaggattattaacgaa
cctactgcagcctccttgtcttatggactagacaaacaaaataatgaaacaattttagta
tttgatcttggaggaggaacattcgacgtgtccgttttagaagttggagatggagtattt
gaagtgctttcaacttctggagatacgcatttaggtggagatgactttgaccaacaaatt
gtggaatggctaataaaagattttaaacaaaatgaaggtattgatcttggtaaagataga
caagcacttcaaagattaaccgaggctgcagaaaaagcaaaaatagaactatcaaattta
actcaaacagaaattaatcttccatttattactgctacacaagatggtccaaaacatttg
gaaaaaactgtaactaggggggaaatttgaagaactttgctcgaatctaatagacaaatgc
agtattcctgtaaataatgctctaaaagatgcaaaactagaagcttccagtattgatgaa
gttgttttagtaggtggatctacaagaattccagctatacaacaaatggtcaaaagatta
ataggtaaagatcccaatcaaagtgtaaacccagatgaagtggttgctattggagcagct
gtacaagctggtgttttagctggtgaagttaaagatattctactattagatgtgacaccg
ttatctttaggagtagaaactttaggtggggtgatgacgaagattataccaagaaatact
actattccgacaaaaaaatcagaagtgttttctacagctgtagacaatcaaccaaatgta

gaaatccaagtacttcaaggcgaaagagagttaaccaaagacaacaaaagcttaggaacg
tttcgactagatggtattatgcctgcacctagaggcgttcctcagattgaagttacattt
gatattgatgctaatggaattttatctgttaaagcaaaagaaaaagctacaggcaaagaa
cagtcaattactatatctggagcttctactttacctaaagatgatgttgatagaatggta
aaagaagctgaagagaattttgatacggaccaaaaaagaagaaaaaatattgatattaag
aatcaagcagaatctttatcttaccaggctgaaaaacaaattaaggaatttgaagataaa
attagccaagatttaaaaacaaagatagaagggctaattatagaccttagagctagttta
gagaaagaagaatatgagaaaattgaatctatctcccaacaactacaaaactcgttaatg
gatattggcaaaatggctgctcagtctgaaagtaaaaatacaaacacaaaagatgatgac
actgtaattgacacagatttctctgaagctaaataagaaatt-agcgggtaacgcgattc
gaacgcgcgacatcaaccttggcaaggttgcgctctaccactgagctatacccgcataga
gtgtattattacaaatatggtaacatttgtcaatctgt--------aattatat---agt
tgagttactaattcttaaactataaattaaacacctggtaaaaatacaaa----attttt
gtttttgaat-aaaagaagtacttatataac-gatataagtacttctttattcaaaag-
-caaaaatataattaaatatcagtg-------ttaaactactaaggaatttactaagcta
gtaacaattactaatccagtccataggcctgctcctgtgtagattaacccttttgattgt
tcccattgaccaggtgatgctagaacaacaggtacgcctactactaacacggtcgacaga
acaattaataatagcactaataattgaatcgcaataatcatttattatattctcctgaaa
aattcaaatgtgaataatattgtatataatacaatgaagctttataccttcattaattta
tctatgaagaaataatttaaaattattgttttctcaataaatattaaattaagtattaaa
atctttattgtaaacaataacactatcttttttttaa-atagataaaaaatttaaagaaag
tctttttaaaaaaaatttaggataaggtgatatgctgtataaaaagttaagtattatta
gttattagagaaatttcaaaataagaggtaaaccatatgaactcagcccttttttttagca
aaactacccgaagcttatgcagttttcaaaccaatcattgatattttgcctgtaattcct
gtgttctttctacttctagcttttgtttggcaagcagcaatcggttttagataacttta
agttatgacaaaaatagcaaatataaagagtgaaatttttaagctctaatcgtatatct
tacacaataataactattatggttgaaccattactatcaggaattgttcttggccttatt
cctatcactttattcggattattagtagcagcttatctacaatatcaacgtggaaatcaa
ttaggactttaaagcctaaattatagatatttataaaaactataattattgtagtcttta
taaatatctacaaaccagctaattataaatacagaaaaaatttattgaaaccagattaa
taattgacaaaattgatatgatagtataa------------------------------


```
------------------------------------------------------------
------------------------------------------------------------
------------------------------------------------------------
------------------------------------------------------------
------------------------------------------------------------
------------------------------------------------------------
------------------------------------------------------------
------------------------------------------------------------
------------------------------------------------------------
------------------------------------------------------------
------------------------------------------------------------
------------------------------------------------------------
------------------------------------------------------------
----------------------------taataaaaattttaccaactcgacttagtaac
tccaggtaaaagacattcatgagacatttctcgaaacacatggcgagataacccaaaatc
tctgtaatacccccctacttctaccagttaaccagcacctatttctacctctaacggctgc
gctgtttctaggcatttcttgaagtctttgtcttagttccattttgtcagaaaagcttat
agcttctttcagctgatctttaatagcgagtcttttcaagtagtattttttttcaagctt
ttctcttttaagctctctttggatcatattttttttttgccatattgtaa-aatttacttt
aaaaattatttaatcaatatactagctaaaaaatctctgaactgcaacatttatataatc
aaaaacatatagcataaagcaatgccataatataaatatttaattaatggtact---act
ttatgctattgacccaaatttatttaataaagttcttgctcaacatgagttgaaatagtg
caatcagattgaggataagcaatacatgttaaaacataacctgctaacatctgatcatca
tctaagaatgattgatcagattggtctacaagaccttccgtaactttgcctgcgcaagtt
gaacaagctccagctctacaggaatatggaagttcaataccttcttcttctgctgcatct
aggatatatgtatcatctgcacatttaaatgtaacatcaatgttttcgtcttcgcatact
aaatgaattttatactctgcca---tttttactccataataaagatacacttgaaactt
aataaagcaaaat-aatctaaaggatttacccaaagattgtatattagtttcattatgca
taatagactgcaaaaaaaggaaataatagactattttgagaaatcaggctacatttcata
ctaaatagaactatagtaggttcaaagtatatatataaattcatatagttaaaattattt
ttatcatttaaataactaaacttaacaatct-aaacttttagattaatttac------ta
tttaatcttttacaataagcaaataagtaattatgacttcaatgatgaatcaacaaatag
agctgaagcctattcttcgatttcagataccatatttatattcaggcttccttatacagg
aaattaaggctttaacgaccagactttttctgcaagtttggagaagacctgctacattga
tggcaggtattattcagcctttattatggttaattttatttgggggtctttttttataatg
ctccagtgaatttatttactattaacacgagctataactgtttcttaagctctggaatta
```

taattttcacatcgtttactggtgcactcaattcaggccttcctttaatgtttgacagag
aattcggattttaaatcgattattaacagctcctttagtgtctagaacttctattattt
tatcttctgctacttttatgacttgtattagcttaatacaagttatatttatagttacag
cttcattatttatgggaaattctcctttaaacagtgatagcgctataattttggattta
taattctattagtaactgtgggagttacaatgcttagtttagctttatctttactctgc
ctggtcatattgaactattagcacttatttagtaattaatttgccttttctattttcta
gtacggctttggctcctctgtatttcatgccaccttggctgcagttgattgctagtctga
atcctttaagttacgcaatagaaggtacaagatatttatattccagcatacattggaact
ttacagagtgcattatcaaaattagttggggagatgtttgtttaggacagattatttcac
tattagttattttagatgtaattgcagcctatgttgtttctaagatattaaaagctaaac
ttaatta-----------------aaattttaataaaaaatttattaaatttattttaga
aagattttactatataatactattagtagtatggataattgaaataggaatagtttttc
aaaaccaaagctattatcaacaatgataaacatttgtaagaaagtcaacaaagtatgttc
ttattcataggaggcatgtagtcaatgggactaccatggtatcgtgtacacacggttgtt
ttaaatgatcctggacgcttaattgcagtacatctaatgcatactgcacttgtagcaggt
tgggcaggatctatggcattatacgaattggctgtatttgatccttcagatccagtatta
aatccaatgtggagacaaggaatgtttgttatgccattcatggcaaggcttggcgtaaca
gattcttggggaggatggagtattactggagaaagcgtctccaatcctggattgtggagt
tttgaaggagtagctttaacccatatagttctttctggaatgctttttctggctgctatt
tggcattgggtttattgggatttagaattatttcgagatccaagaactggcgaaccagct
ttagacttgccaaaaatttttgggattcatctattgctatcaagtttactgtgctttgga
ttcggtgcttttcatgtaactggattatttggaccaggaatgtgggtatctgatggttac
ggagtaacaggtaaagtgctgccagtggctccggcgtggggaccagaaggatttaacccg
tttaatcccggaggagttgcatcacaccacattgctgcaggtactgtaggtatattagct
ggtgtttttcatttaactgttagaccaccgcaaagattataccgcgctctaagaatgggt
aatatcgaaactgtattatcgagtagtatctcggctgtttttttctcagcttttgttact
tgcggaacaatgtggtatggctctgcaactacacctgtcgagttatttggtccaactaga
tatcaatgggatagtggatattttcaacaagaaattgaaaaacgagtcgaaaacgctatt
gctgatggggctgctcctagcgaagcatggtcaagaattccagataagttagctttctat
gactatattggtaacaatccagcaaaaggtggtttattcagagctggccctatgaataaa
ggagacggagttgctgaagcatggcttggacatcctgtatttcaggataaagaagggaga
gaactaagtgttcgcagaatgcctgctttcttcgaaacgtttcccgtcattttagttgat
aaagatggcatcatacgagctgacattccatttaggagagcagagtctaagtacagtatc
gaacaagtgggtgtaaccgctagtttttatggcggcaaattaaatggccaagtatttaac
gatgctcctagcgttaagaagtatgcaaggaaagctcaattaggtgaagttttcgaattt
gaccgaactacactagaatcagacggtgtattcagaagtagtccaagaggctggtttaca

tttggtcatgctaatttcgctttaatctttttctttggacatctttggcatggttcacga
actatctttagagatgtattcgcaggaatcggagctgaagtaactgaacaagtagaattt
ggagctttccaaaaattaggcgacagaagcagtaaaaagcaaggcgctgtataagact-a
catcaaacacagtccttatt---caatatttccagtattaaaataggtcaaaaaaagttt
attatctaatagttactaattaggagaacatatggaagccttggtttatgtatttttatt
gacaggaacactaatggttatattcttcgcgatctttttagagaacctccaagaatagc
taaataactttattaggtttccccttacaataatagaaccactttccagcttattagtgg
aaagtggttctattgtaagttacagtatattattcttcatgctcttcaaaaggatctcta
agatcttttgaagcaggaccaaatgccgtatatattgaataacctgtaatacctaaaagc
aaactcgaaatgaaaatactaagaactgttgcagtttccataattcaatcagtattaagg
tgaactttttctataatgatattatcaatattaaaacatttaattattaaatttatcaaa
atatattatggcacttagaactagacttggagagatcttaagacctttaaattcagagta
tggaaaagttgctccaggttggggaacgactcctatcatggggattttatgttactatt
tttcttatttttgttgatcatttacaaatctacaattcatcactagtactagagaatgt
agatgtagattgggctactttaggtagctaactaattacactgttgctttaa--------
tttaataacattaaaaataat----------------aagcgatcactattatgtcataa
tagcgatcgctcaaattgtgttttactatata--caaaagtaattattttcctaagtcaa
ttaattcgctatctgcaaaactattcgtattaacattattataattaacttttcaaatc
ttactagtactggatacttgataccgcttttatccattgctgcaactgtcccaacttctt
gataccagtatgattcctttctcaatattttactttgagcctcgttccatatttttaa
tcctaagataggttattaaagatcagtataatagataaagaaataaaattaaaaaaaata
catgaaattagtaccgacaat---------ttaatcataaaaggagagagagggattcga
accctcgataactacagttatgacaattttcgaaattgttgcaataaaccactctgccat
ctctcctaaactaaaactcttttttcggaattagataagaataacatagaacgaaggta
gttgcctagaaaattaaaactatgttaaatataaattataatctttctatagatctcttt
acaaaatatgtacacagattctatatcagcttatttcaagatttttaatcttacacaat
ttttactaaataaataaactctctcataatatgaaactatcttggaaaacacttcttctt
tggtctttaccaatatttgtcgttggtttctttttctggcaaggtttttaggtccgact
actacagatgttggtagtaatattgcaagttctagaatgacatacggaagattcttagaa
tatttggatatgggttgggttaaacgagttgacctatacgagaataaccacacagcaatc
gtagaagctgttggtccggaattaggcaatagagttcaacgaattcgagttgagctgcca
gcgagtgcaccggagttaattaccaaactacggaaggctaatgttgatttagatgctcat
cctcctaaaagtacaagtgctgtatggggactattaggtaatttattatttccgctcttg
ttggttggaggcttagcttttctatttagaagatctaatagtgctagtggtggcctggt
caagcaatgtcatttggtaaatcgaaagctctgtttcagatggaagccaaaacaggagtc
gtatttaatgatgttgctggagttgaagaggccaaagaagagtttcaagaagtagtaact

tttttaaaacaacctgaatcatttactgctgttggtgcaaaaataccaaaaggtgttttg
ttagttggtcctcctggtacaggaaaaacgttattagcaaaagcaattgctggtgaagca
agtgttcctttttttagtatctcaggctcagaatttgtagaaatgtttgttggggttggt
gcttctcgcgtaagagacctgttcaaaaaagcaaaagacaatgcaccttgcattgttttt
attgatgaaattgatgctgtgggcagacaaagaggaacaggtgtcggaggtggaaatgat
gaaagagagcaaacattaaatcagctattaactgaaatggatggctttgaagggaatact
ggtgttattgtaattgctgctactaatagagctgatattctagattctgcattattaagg
ccaggaagatttgacaggcaagtatctgtggatgttcctgactttaaaggtagattagca
attcttgaggtgcatgctaaaaataaaaaaatggaacctaaagtatctttagaaactatt
gcaagacgaactccaggcttttcgggagctgatttagctaatttattaaatgaagctgct
attttaactgctcgaagaagaaaggatgccatgaccatgtcagaaattgatacatcaatt
gatagagtggtagctggaatggaaggcacgcctttaattgatagtaaaagtaaaagatta
attgcatatcatgaggtaggccacgcaataataggaagcttattagagcatcatgatcct
gttcaaaaagtcaccttaataccaagggggcaagctagaggcttaacatggtttactcct
agcgatgatcaaagtttaatctcaaggtcacaaatactagcacgtatcgtcggtgctctt
ggtggaagagctgcagaagaaattattttcggagatgcagaagttactactggtgcgagt
aatgatttacagcaagttacatcaatggctagacaaatggtaactcgattcggatgtct
aaaattggcccgttatctcttgaaagtcaaggtggagaccctttcttaggtagaggaatg
ggaggaggttcagaatactcagatgaagtcgcaactaatattgataaacaagtacgagaa
attgtcagtgaatgctataaacaggctaaagaaattgttgcagataatcgagtagttatg
gatagattagttgatctactaattgaaaaagaaacaatcgaaggcaatgaatttagggat
attgttaaagaatacactgctattccagaaaaaaattattataaatctcaattttaa---
---------------atagtacgggactgacgggattcgaacccgcaacttccgccgtga
cagggcggtgctctaaccagttgaactacagtccc-accaagac---tataaataaataa
tctcacaattagcttgtaattgtcaaattatactattaaacatgagaagta---------
-----------ttattaatacttttcatgttctttattacagagtcaattaatccattta
caaggagagagagagggattcgaaccctcggtacgaaattaatcgtacagcagattagcaat
ctgccgctttcgaccactcagccacctctccaaaaatttttatatactgaatatagcaaa
tattattaagttttaacagatatgcataaacatttatggagctaagcggattcgaaccgc
tgaccctctcaatgccattgagatgctctaccaactgagctataacccc---tttttga
ggctattaagaaatggctcaagcgggatttgaacctgcgaccttgggcttatgaatcccc
tgctctaaccactgagctactgagccataatcaaaactattactaaatactataccatag
attactttaaaatataaatcaatgaaaaaatatttactacaagtgcctcgattttatatt
g----------------accaaatt---------------------agcatttaaaaga
tatccaaat-ttttttacgatatgctatcagttcttttcatttaat-taggagtatttca
atgttcaacttccaaagactttgtatcttgttatatatttaatagaattacactcttttt

ttctgacataaactattaataataagtttcttattttgataaaaaatcttgttttctccc
agaaaaacaagattttgggtgttttaacgctaactgacgccagagaaaaaaattttttt
gccgctgcgtggcggggctttggtaacattaagccgcccccgcgcctgtaacaaatttat
tttgcaacatttttaacgctacaaacgccacactataaaaagttttcaaaaattacaag
tataagctcttattttagacttaattattccaaaaagaagggtcaaaaaattcgattaaa
tctgttaatagaatttttgtgaaaacctttaatatcatatgtttcaactatattatcc
gccaactttctctctctaatttgattcaacgctattagcaaagagctcaaataagcagaa
ttaattctaggcatagttctttgccttgtcagtttaacaattgcagatgaagttttattc
aacacctcactcaactctctactactgatatcaagattgtacataacatcttgaagcttc
actcctaaagctctaaatacggcattcttatctagttcaactttggttttggtacatttt
ttaggatacgttagaaataatttttagcatttattctaaatttttcttttgtgacgga
tttcttttttttctgacataaactattaataataagttttctattttgataaaaaattct
gtttttctgggaaaaaacagaattttggcatttttagcgccagctgacgccggaaaaaa
aatttttttcgccgctgcgcggcggggctttggtagcattaagccccgcccacgctcgta
acaagtttttctgaaatatttttaacgccacgaacactacactatgaaaagttttcaa
aaattacaagtataggctcttattttagacttaattatctgaaaagaaaggctcaaaaat
ttctattaaatctattgatagaatttttgtgaaaactttttaatatcatacgtttcatc
catattactcttcaacttcttttatataactggattcaacgctattagccaagagctcaa
ataagcagaattaattctaggcatagtttttagccttttcagcttaacaattgcagataa
agttttatttaaagcctcacctagctctttactactaatatcaagattgtacataatatc
ttgaagcttccaaagcacctgaacagctgcttcaaccttcaatgaatccttattcctgtc
tattaagattgaataattatgtctctttaattagccaaaagcaaaggaaatatctaagta
aaatatcttac-----------------tttcttataaagac------------aattta
tttaacagcatatt----------gtacaagcaaaattacatagtcaatatatatttctg
aaatcaagatgttgcaagtcttatttttatatatattaaatagacttaagggttatgaa
ttctgtaattaaaagacatttattgacttcg-----cttagtaatatttgattcttagca
ttgt-ttaaatacagattagttagaaactgaataaatagataatatc-----ttatacaa
ccagcatcgagccaatgccttgatcagttaatatttctagcaagagagcatgatcaattc
taccatctaaaatatgcgctgaagctactccttgagctaaagaacgaatacagcagttaa
cttttgggatcattcccccgtaaatcacttctgtctgggttaagtctctagcttgctgta
tattcaagtgactaattagtgttgtaggatctgaagaattacgtaaaatgccaggagtat
ctgttagtaaaattaatttttcagcattaagggctgctgctatttctcccgccacagtat
cagcattaatattgtatgactgaccttgtttatccgctgcaacactagcaattacgggta
tatagttattattaattaagatcttcaatagcttagtatcaacattttgcacttctccaa
caaagccaagatttgctttaccacttggtctcggagtaatgagtaacccatctttaccag
acaagcctacacttttacctccttgcttattaatcgttgctacaagatctttattgactc

gtccaactaataccatttctacaatatccatagtaggctgatctgtcacccttacaccat
tatcaaattgtggtaaaattttagacgatctagccagaaattaatttctggacctccac
catgaactaaaataggacgcaacccgataaaagaaagaaaaaccagatcactgattactt
gatcttttaattttgattttcatagctgctcccccgtatttatcacaataattcttg
aagaaaattgttgaatatacggtaaggcctcacttaatactttcactctttctgaattag
tcagcatgaataaaatagaattataatatgtaaatttatttgcagagcttatattaattt
aacagatactaacaagctattcaaaactttcagagaataaacctattattaataattaaa
atatttaaaaatataaggataataattatgagtaattttggaataatatattaaaattt
ccacgattttagttagtgtcatttttggattagttttaataattattagtcctttttc
gtgttatttaaaaaaccaatgacaagtttttttttcatcatatcattagccggtttaatc
gcagttttagcggtaataa-taaaaaaaaatgctcaatatcgaatgttgttgagaggatta
atttaatttaattaaaatttttgaacatatataatattatattatcattaactatgataa
tttaagtttcttttctata-aaaaaatcctacatattaaatattacttgatagatattat
gtcattaaaacaaatagttggttctgaaaaaactggtgcatttgctcttcttgatagtat
tgttagacatgggattaaacatatatttggttacccaggcggagctatccttcctatata
tgatgaactttatgtttgggaagaatcttccctaattaaacatattcttgttcgacatga
gcaaggagctgctcatgctgcagattcttattctagatcaacaggacaagttggtgtttg
ttttgctacatcaggtccgggagcaaccaatcttgtttctggtatagctacggcacatat
tgactctgtacccatactagctgtcactggtcaagttgggagagcatttataggtacaga
tgcctttcaagaagtagatatttttggtattactcttcctattgtgaagcattcttatgt
agttcgtgatcctagagatatggctagaattgttgcggaagcatttttatttgcaaaca
tggtcgaccaggacctgttttgattgatgtccctaaagatgtaggcctagaaaagtttaa
ttatttttctattgatccagggaaagtgaatattccaggttgtagaccgattactacttt
gaagtctagacaaatacttatggcagctaagatgatacaacaatctagtcaacctttgct
gtacataggaggaggagcgataatctctgactctcaccaaattattaaagaacttgttga
tttatataaaatacctgttactactactctaatgggtaaaggtatttttaatgaagatag
tgaatattgcttgggaatgttgggtatgcatggtactgcatatgctaattttgcagttag
cgaatgtgacctcttaattgcattaggggctagatttgatgatagagtaacaggaaaact
agacgagtttgcttgcaatgctcaagtaattcatgtagatattgatcctgccgaagtcgg
aaagaatagaattcctcaagttgctattgttggtgatgttacagaagttgttactgcaat
cttaaatttactaaaaacttcttttcctccttatccggagcagattgtatcttggcaaga
gcgaattaaccgttggcgccaacagtatccattgctggttccaaaaaaatcaacaagtat
ttcacctcaagaaatacttgttgcagctaataaattagctcaaaatgcttattttactac
agatgttggtcagcatcaaatgtggtctgctcaattttttaaaagtgaaggctaagcactg
gctttcaagtgctgggttaggtacgatgggatatggattgcctgcagcaattggtgctca
agtagcacatcctaatgatgtagttatttgtattagtggtgattctagttttcaaatgaa

tatgcaagaactaggaactattgcgcaatatcagttacctgtaaaaattattattattaa
taatcgttggcaaggaatggttagacaatggcagcaggctttttatggtgaaagatactc
tcattcacgcatgacagaaggagccccaaattttcaaaagcttgcagaagctttcgggat
taaagcttttactattaatagtaggcaaaatatgcaatcttcattagaagctgctattga
gcatcatggtccagttttgctagactgtcaagttacagaaaatgaaaattgctacccaat
ggttgctccaggtaaaagtaatgcacaaatgataggtattgctaaacctcaaagaggtac
tgcctcaaactatgttaatacaaatatctaaataga------------------------

---------------aagattcgtattttctcaaaaatataatgaaaatctt----tttt
ctgagtatatatttctaacattatat-taaatttttagacgatagcctttgacgagaatt
gaactcgtgaccttccccttaccaaggggatgctctacctctgagccacaaaggcttttt
tgttatattgggccgggttggatttgaaccaacgtaggcgaagccagcggatttacagtc
cgcccccattaaccactcgggcaccgaccctaattaatttttatgtaaaatataacttaa
acaatatcataagttagttgcttaaaacaagtcttttcataaataaaact-agtaagtaa
tggacatagctggactcgaaccagcgactttcacgatgtcgacgtgacactctaaccagc
tgagttacatgtccaaaaccttaataattattataacactatttattgattgtataagta
tctatttctaattaaagatttgttttaagcgacctgctttaccgagaagatctcttagat
aatatagcttagctctgcgtactttagctcttctaataactttaacaaatgttatgcgtg
gtgaattaagaaaaaaaaacgcgctcaactccaataccttgaaatgagcatcttaaagtta
aactagtatttaagctatttttttcttcttctggccaagattactccttcgcataattgtt
cccgggtttttttaccctctttaaccatcaatccaagttgtactgtgtcgccaactctaa
tttcaggcacgtcttgtttaataaaaggattctctacacttttcatgagcttacttatct
tggtattatttgctttcatatatagcattagcctcataatg-ttttttgcaatattatac
attaaaacaatcaatatgaaaattattagtaaataagcaaaataacatcgagatagtctt
---t
catctaaaaacc------------------ttataagcaactgttcattaca----gtgc
aaatctgggaaatttttaaaacaaatagtataaagtggcagtctaactcact--------

-------------------------------agtatcacgtccttt------------tt
ctttta--
---actcatagtattttatt
ttatcgcgac--

--------------taaaacttcagttgta------------------------------

--------------tttgagagttatattcaacactttgtt-------------------

---------------------------taaaatttagcattatataa-------------
---tttaatttttttagtaaaagaatgagcattttg------------------------

--------------------caattcggcaact---------------------------

--------------------ttgctatctttcacactcctgacttcatttt---------

------------------------------cttcaataaagta-----------------
-----------------------------tttactatataactaaatga-----------

---ggtcat-------------
---catagaaatcacta-----
--gatt
ctataagattgtattcatcatatatt-------------gtatcgtgttacctg------
-------------tgatagtcatgtatct-------------------------------
--atttgtagagcgatcataacactcaaggaataacttcgttgcaggataaattt-----

-----actagctctctagcggtg-----------------------ttcacagtaagcaa
tgata------------------------------cttgccaataagat------gtaag
cctttactactaatgctcctttcgacatatttgtc-------------------------

-------atgaaatttacttattatttccgaaattttagg--------------------

---agaatcatataat------
--------tttattaattaatag-------------------------------------
--------------gtgctctgtatttc--------------------------------

----------------------------gacaatcactaaaac-----------------

--ta
ataatttctca---
--tg
agttaactccatctatatcaataaaa--------------------------aagtacgg
tgttctagtgaa--

-gacaaatcctaatctaataataaca----------------------------------

----------------------------------tcttctagctgcttatttaaatgaa-

---cat
agtccgacaagttaa------------atttagtacaaaa--------------------

---tttcgttatataaaattat--------------------------------------

--------------ttttcacata------------------------------------

---ttc
gatggacgaattat--
------gaactgtaatttactataatttaattcttttgtcctatta-ttgattggcactg
tta-------------ctatctttaaatatttattctccactcaagaattgtatacatac
aactgaatgatcaaaacaataaaattaaggcttaagtattgattagttttgtgttataat
tagttactatcaaaggtagtggcataacacaaagcataaacaatgtttgaacgctttact
gaaaaagctataaaagtaataatgctagctcaggaagaagctagacgcttaggacataac
tttgtcggaactgagcaaatactattaggattagtaggtgaaggtactgggattgcagcc
caagtcttaaaatctatgaacgtaaatttaaaagatgcaagagttgaagtagaaaaaatt
ataggaagaggatctggttttgtagcggttgaaattcctttcactcctcgagcaaaaaga
gtactagaattatctttagaagaagcacgtcaacttggccataattatattggtaccgaa
catttgttaatgggtttagtcagagaaggagaaggagttgcagcgagagtcttagaaaat
ttagcagttgatgtttcttcgattagggcagaagttatacaaatgctaggagaaaatgca
gaagctaacgtaagtggaagtaatactacacaggctagaagcaagacacctacattagaa
gagtttggctctaatctaactcagatggctatggaaggtggcttagatcctgtagttgga
agacaaaaagagatagaacgtgttattcaaattttagggcgaagaactaaaaacaatcct
gttctaattggcgaaccaggtgtaggtaaaacagcaattgcagaaggtttagctcaaaga
atagctaatagagatgttccttctatattagaagacaaattagttattactctcgatgtt
ggtttattagttgctggcactaaatatagaggtgaatttgaggaaagactaaaacgtatt
atggatgaaatcaaatcagctgataatgtaatactagttattgatgaagtccatacatta
atcggagctggggctgcagagggagcaatagatgcagccaatctactgaagcctgcttta
gcacgaggagaattgcagtgtataggcgcaacaaccttggaagaatataggaagcatata

gaaaaagatccagcattagagcgaagatttcagccggttgtagttggagagccaagtgtt
gaagaaacaatcgaaatcttatttggtcttcgagaccgttatgaaaaacatcatcaatta
acaatgtctgacggcgctttggctgctgctgctaaatatgctaaccaatatatttctgat
cgatttttgccagataaagcaattgatttaattgatgaagctggttctagggttcgttta
ctaaattctcaattacctccagctgcaagagaattagataaagagttaagagctgtattg
aaaacaaaagatgaagctattagggctcaaaagtatgaaacagcagagcaatatagagca
agagaaatggaaattaaagctcaaattgcagcaattgctcaaagcaaaaaaaatgagcct
gatttaaacttagaagatcctgttgtgaccgaagatgatattgctgaaattgttgctgct
tggactggtataccagtaactaagttaactaaaagtgagtcagaaaaattaatgcacatg
gaagaaactttacatgggcggatcattggccaagatgaagcagttgtagctgtatctaga
gctataagacgagcaagagtaggtctaaaaaatcctaatagaccaattgcaagcttcatt
ttctctggaccaacaggtgtaggtaaaacagaattaacaaaagctttggcttcttatttt
tttgggtctgaagcttctatgatacgtttagatatgtcagaatatatggaaagacatact
gtatctaaactaattggttctcctcccggctatgtaggttatagtgaaggtgggtatctg
acagaagcagtaagaaaaaaaccatatactgtaattctttttgatgaaattgaaaaagct
catcctgatattttaatcttcttcttcaaattttagaagatggcagattaacagatgca
aaaggtcgaactattgattttaagaatacccttctaataatgacttccaatattggatct
aaagtcattgaaaaaggaggaggtagtctaggattcgagttatcagaagatcaaacagaa
tctcaatatactagagtcaggtctttagtaaatgaagaattaaaacaatactttagacca
gagtttttaaaccgcttagatgaaattatcgtatttcgtcagcttactaaagatgaagtg
agagaaattgcagaactgatgttgaatgaagtttttgccagaattaagcaacaagatatt
cagctaaatgtaacagaacgttttaagcagcgactagtagaagaaggttacaatccaagt
tatggagctcgaccacttagacgagcagtgatgaggttattagaagatagtttagccgaa
gaagttttgtcaggtaaaattaaagctggtgatagtgctgtagtagatgttactaatgaa
ggagaggtgaccgttttattaggtgaaaaactagaactactaacttaaa------aaata
atttgttaatcggaaagcattgaataaactat--attcaatgctttt-agaagaaatta
ataagatgggttgctataattgacaacctctgatattctttatatgtatata---aagcc
gggatagctcagttggtagagcagtggattgaaaatcctcgtgtcaccagttcaaatctg
gttcttggcat-tttaaacgtttgaaaagtttgctagatcagcatcaaattttagttgga
atatacctgtagggccatttctatgttttgctattataatttctgtcagatctcctccta
cagtttcttgagtgtagtaactttctctatacagcattataaccaaatctgcatcttgct
ctatagaattatgtactataaagttactcgcgataaaattacgtaagtgctgacagtcta
aatcgaatactgtttgtaagttaataacacttatgtgtttaagattttcaaaaactaaaa
ataaagaaggaaaagaattgcagatagactttttttctttaatagtatt------cattt
gaatagcgagcatattatcctgatcaatttcatcacatcttttccatccttgcgttgtta
atagcttgtgattgcttgttaaatgtatatactttccagattgagtaataatttcatata

tagtctttttttcctgtttttgaaatactagctttactagttttaaataagtttgattttt
gtacagtaaagctagtaacttca-atacgcttatgatgaaaattcaatagaatttg--ag
tttgaccatgtaacggtacatgaagatgactaaattttgatatacacccactttctctta
aatcagacaataaaggtcttttatcacttcgagtttcaagattcctattcagttgagata
atactagtataggtaaatttaagtcttttgctaatatttttagtgatcgagtaattaatg
atagttcttgagatctattatcagattgcctgctatcttgtaataattgaagatagtcta
taataattagttctatatttttttccttgtaattttaagaattgtacttttgtttttatag
tgtccgtagaaattttttgcactatcatcaatgtaaagattgagatctgcaagagctttac
ttctttcaataacatattgccattcatcattattaagttgacctgattgaatttttttgac
cgtttaagtgacattcttgagctaaaattcttcttagtagttgttctatcgacatttcta
agctaaataaaataacgaacgattcctctttgttaattatatatctagctacattaatag
caaatgccgttttgcccattgaaggtcttcctgcaagaataattaaatcggacttttgaa
aaccttgtgtaatagaatccagttctgtaaaaccggaaagtacgctactatttatactaa
ttttttttgttgtgtctaaatgaacaagtagattagcaagaattgtagctagtgtctgag
tatctttctcttcaaggatttcgtaagcttttgttaattgactaacaatagaggttatat
tattttgcttaacaggctggtttgaacagctaattaaaaataatgaatctccagatatta
gaagtaatcttttaatataattgtcgataatgattgatgaatattcatctacgatatcag
ataaaggtgcttgttctattagactaagaacttcgtttagttcactcaagtgcctgagta
ttttctctgtttttaagttaactaaaaaaatttcctatacttatctttttttgtatgattga
tattttctagggcagctttgtaaagtaatgaatttgatgtaaaataaaaaaaaatcaggag
agagcttatctactgattttagtaaagagattgattgcttagttaatattgtagtaatta
atattttttcagctaaaatattatgaggaggcaaatatttgtaa------atactgataa
gttcctcc-ttgtaacattattctaaca--------atactaattaattactaacttaat
ttgattctggaagaacttgtagttgaacgttggcagttacctgacttaaaagtttaatct
ctatattataaattccaactgtgttaatctcaggtaaaaagatattttgtttttcaatgt
ctatatttgtagcatttttaataatttgtgcaatctctttttctgtgacactaccaaaaa
tatgttgcccatctcctgttttttttacttacgctgaacttttgtatttcttctagaagtt
gttgaatttttttttgcatcttccttagctattttttaattttcttcctttatggctgcgt
aaaattttttgctgttttaaaataccatttgttgcgattatcgccattttatttggaatta
aaaaatttcttgcataaccagcagcaacttttacaagatcattactttttaccaagttttt
gaatattctcttttaatacaacttttataactttttttacccatctttactctgaattcta
aaaa----atctatataatttattatacctgttgtgcccatattttgacagacagaaag
tattcacttgttgccataatatctgtgtaaagcggataatgaattttcatctttattctg
gtataataagtcgtacaacattat-----gttaggagaggtggccgagtggttgaaggcg
cagcattggaaatgctgtttaggagcaatcttaacgagggttcgaatccctctctctccg
tacaa--aaatgattataatacttatatagtccagtcaaaatttccaataatgtttctcc

atgcatttaaaccaccttgtactctcacaatatttagtttttgagctattagcaactgag
aagcaaataaagatctagaatctaagctgcagtacacaaagcaaactttattttctaaat
ttatattagagtaatgtatttttttgagttgttctaaaggtatatttaatgcgtgagtta
agtgatttttttatattcgtcttgatttctgacatcgattaaaatatatttaggtctt
tgcgatttaaaagtttttgcaattgcacaacattaatctctcttataataagattaagtt
tatcaaagctgtatttgttccagtgttgtttttt-------agataatacaaatttagta
tttaaaattttaaacttgctaaatgataaagttaaggcgttgtaggttaatatcatacca
cttaatatacacttatagccaagaataattttaatagcttcagttgcttgaagtgtacca
ataatacctggcaatagtcctaaaacaccagaattgctacatgtatctatttcactatct
tcatttttttactttattgtaaaagtctcgataacttggacctccttgatagttaaataca
ctaatttgtccttcaaattgaaaaatagctccataaatatgtattttattcaactcaagg
caagtgtcattcagcaggtatcgagtgttgaaattgtctgatccatctataataatatcg
tatttttttaacgatgtcaattgagttttgagaggttagtcttgttgcaaaaaacttttact
ttacatttcggatttaaatctaata-tttttttttagctatttccacttttgaatatcct
atatcgtgaattgtatatataatctgtctctgtaaattagaaagatcaataatgtcgtcg
tctataataccgatgctgccaactccagcagcagcaaggtaaattatgccaggagaaccc
aggcctccggcaccaacgaataacaccttcgcttttttttaatctttcttgtccctctaat
tgaacttgtggtaacaataagtgtttagaatatcttgtatattcgtctaaagagtatttt
atattctctactttaaagttcagcataaaaaatttataagttaattaataccgacta----
--gaaactaattgataagcttattaagatgacttttgtaaactaggttcttctgtatcca
aaatagcgccttgtgtaggacagacttgaatacaaatactacagtcaatacaggcagaaa
agtcaatccagtaccaattagtgttaatactatttttttccttgtccctggtgaatacaag
ctactggacaagcggctacacattcagcaactccaatgcatttttcggttacaattgtat
gagacatgttatttaattaat-aaaagataaactgtttacatctagtagttttattgtat
actagattggcatatgctatccaagtcaataattaaagtaatctactttttatagcttact
aatttgatctgcgcctttagttcagttggtagaacgcaggtctccaaaacctgatgtcga
gggttcaagtccttcagggcgcgcttagttgtttttacagtctcatagtagcattttatc
tttttgcaagcatcaaaataatatgatataaaacatcatattcaatatattggatatttt
atgtgctaggactg-ttttttttatatgttattattggtaagttgattatattaattccat
ttgtataatcaat-aaaaataactgaaattaaatgcagatgtactcaatagtc-tttttt
atatataaaacttgaaaaattaaaaactatggctaaaaaagttaccggaattgttaaatt
ggcattaaacgcagggaaagctactcctgcaccacctgtagggcctgctttaggtcagca
tggcgtaaatattgttatgttctgtaaagagtataatgcgcgtactgcagataaatcagg
actggtaattcctgtcgaaatttcaatatatgaagacagaagctttactttatacttaa
aactccgccggcctctgtactaattgctaaagctgctggcttaaataaaggttcgggagc
accaaacacaaaaaaagtaggtagtataacatataagcagttagaagctattgcagaaac

gaaactacctgatttaaatactaacaatataccccaagctatgaaaatagttggaggtac
agctaaaaatatgggaattctaattaaagattaaaaaatttcaattatatttatta-tt-
-tttatgaaaaaaatttcacgccgacttaaaatattaaaatctcaagttgaacctaaact
tcatactattgatgaagcaatatctatagtaaaagcaacatcaaatgctaaatttaaaga
aactgcagaagcacatatttgtttaggtttaaaccctaaatacgcagatcagcagcttag
agcaacagtcatattaccaaaaggaacaggaaaattaataaaagtagcagttattgctaa
aggagaaaaattaacagaagcaattagtgcaggagctgatgtaagtggttcagaagacct
aatcgatgaaatactaaaaggtagattagattttgataaattaatagcaactccagacgt
tatgcctctgatagcaaagcttggccgtgttttaggacccagaggattaatgccttcccc
taaagccggcaccgttaccacagaagtgggcaaggctgtaaatgaatttaaaggcggaaa
agtcgaatatcgagttgatagaactggcataattcatgtaccttttggaaagtcaagttt
tcccaaagaagatttagtcttaaatcttcaaacaattaaagaatcaattgataggaataa
gccttctggtgcaaaaggaaagtactggaaaactttttttcttatctagtactatgggacc
gtctatccaaatcgatattaatagtcttttatgaaaact-tttgtgtaattacataactt
gaataatatattatttcaatta-taatttattagcaatgttaataaaatagtctgctta
ttatatcttatctgcgctaaaaatttaatatatatcttactaattatgagtacaaaggt
tgaaaatatcttagaagaactaaaatctttaaatcttctagaagctgctgaactagtaaa
acaaatagaaaaaacattcgatgttgatgcatctgcttcttcaggaggtatgatgatgtc
tgcgccagctgtatcaccagctgcatctgaagtcgaagagaaaacggaatttgatgttgt
tctagaagaagtgcctgcacctaaaaagatttctgtcctaaaagttgttcgttctctgac
cggcttaggcttgaaagaagcaaaagatcttgttgaatccacgcctaaagttctgaaaga
aggtgcgtctaaagatgactcagaagctatgaaaaaacagttagaagatgctggtgcaac
ggttgttgttaagtaataca-t--aaaagtgcttttgtttgtaaataaacaaaagcac-t
ttttatttaatagaacaaattagactagaaaagcccaagtgtaattgcattattaatagg
catagttgcaccaatacctaaccaaatactgacaactgtgccaattaagaaaacagttgt
tgcgattggtcttctaaaaggattttgaaacttgttgacgttttcaataaatggaacagt
aagcaatcctgctggtacagctgccatacttagaacccctaatagcttattcggaatcac
tcttaacagattaaatgttggaaagaaataccattctggtaaaatttccaatggagtagc
aaatggattagacttttcacctatagacgaaggctctaaaattgctagtccaatactaca
tgcaaaagttccaataattacaactggaaagacgtataataggtcatttggccaagctgg
ttcaccataataattatgacccattccttttgctaatttagctcttaattttggatctgt
taaatcaggtttttttaagaattgacatgttatttttttgtgtttaatataaaattgagttt
aattacttgatcaaagtgtaactatacattgttacagtggtccggaaattccttgcttgc
gtatcattaaaaaatgcataagcatgaaaactgcagttagtaatggaagtacaaaagtat
gtaaactgtagaatcttgttaaagttccttgtcctacactgactcctcctcttaataatt
ctacgatgctccctccgacaactggaacagcatctggaacacctgtaacaatttttacag

cccaatatccaatttgatcccatggtaaagaataacctgttacaccaaatgaaacagtaa
gtactcctaaaattacacctgttacccatgtcaattctctaggctttttaaagccacctg
ttaaatagacacggaatacatgtaaaatcatcatcaagaccatcatactagctgaccatc
tatgaattgatctaataagccatccaaagtttacatcagtcataatatactctactgacg
tgaaagcttctgcaactgtaggtctatagtagaatgtcattgcaaatccagttgcaactt
gaattagaaaagatacaaatacaatcccgcctaagcaataaaaaatattaacatgaggtg
gtacatatttgctagaaatatcatcggcaattgcttgaatttctagtctttcttcaaacc
agtcataaattttactcataaaatagcttcaaaaagctttttaatata-tttttgcggt
taagctac-tttgctttagtag------ttattgaacttatatt-attttattgtccata
cttactaattattataacaccttttttatttattt-------------------aaaatt
ctttgactcaataaaacaggactatgaactacgatcttttttttctgcatagagattaat
ttggttttcagtaatccagataaaattcttgtcacacttactctactacttcctataatt
tgtgccaacattttgtgtgtcatagcaaaattaaggataataccattattactttgcgtt
ccattttgctctgctaataataataataagcttacaagtctgcttgtaatacttttatgt
gaaatgatttctgtaaaatgataagctttttctgagcaatatattaaatgatttacaaaa
aacaaattaaatggcgggtagttactgcatgctcttataatagtattatagttaatacaa
gcaattttagttttacctattgcttctgcttcatagtaaaagttgtcattagctaattct
aattgtccgaatgtatctccagtagttaataaattaagtgttattttatgggtactttta
aagacttttgtaataattaaagaacctattaatataatatatagtctagaactgctatca
aacaaaataatatcattgggcttaagtaaaaaaacttgataactaattttattactatta
aaaaatagtagccatggattataagaaaaaaatttccccgaactactttctgaaaatatt
gaaatagattttttttggatttaaatgatttttagtcataattactactttataa------
---aaaaatactctaataat-taataacggaaattaaatgtcatacaagttgatgctcgt
tgagaatgacattgtattatcaaaagccattcaagaatacttaacagatcaaggatttaa
cgtttacactgccaaaaatggattagaagcatttgagttagctaatcaatataatttcaa
tttgataatttctgatataatgatgccactagttaatggttatgaattattagaaaaact
tcaaaatactaagtctttatcaaaaataccagttatttttttgacagctaaaggaatgac
taaagatagaataaaaggttatgacatgggatgttatggttatttgtctaaaccatttga
cccagaagagctagtttcacttataaaaaatttaattgctagagatgtattgcaaaatag
atcgatagaaaaaggtaacatcccaaagcattcaattaacaataaaatacatttaacgcc
tagagaagcaagtattttagaccttgtgattgatggactaactaataaagaaatatctat
catattagatactagtatcagaaatgtagaaaaatatgttagtcgacttttacaaaaaac
taatatgaagaatagaaccttactagtgaaatattctataaataataatctaatagataa
ttcagttaatcaaagggcgaatgacgggactcgaacccgcgaatgatggagccacaaccc
attgccttaaccccttggccacactcgccatatcataaatatcactataacttgtttgct
gacatatcgtctactctctaagtggtttatttatctaacatagtatctaaatttaatcct

ttatgatgcataataatattagtattcaaattaatggagagccatttaattgctcagagc
ctatctcattgcaatttttattaaattatcttgatttaattctgggcgtgtagcagttg
agctaaacgatactttattacctgagtctcttttcactcaacttacttgaatgatcaag
ataaactagaagttattactattgtcggcggtggataaattctatcaactaa--------
---------ttatttagatgcttcattgatagagataaacgcccttgtttcttatccac
atgaataataacagctttaatagtatcgcctatttaaattgtgatgatatctggtctaa
ttgttttatattaatttcagagatatgcacaagccctttaagattgccaactcttataaa
taatccgtaaggtgtaatctgatttataattccttcgataatattgccaacaattaaatt
tgatgaggcttgggctattaaagctcttctgtggcttagtattaaattattagatttctc
ttcaacattgagcagctttagcttgataaattcattattagcttgatcatttttttgaaa
attaccaagatgagaattgggcacaaatcctgatataccttcaaggctaatgatcatccc
tcctttattaaatcctttgattctaacattaagtaaagaatcttcagctaataattgtct
gattcttttccaagctcttatatattcgagacgtcgaattgataaaattaattgctttga
ttgaatattataatctagtaaaaaaaattctcttgtatcattaatgtttaaagaagtaaa
attatttaaatcttgattacttgagacctcttgaatcggtaagtacgccgatattggtgt
tcctatatcaaccaacacgccatttagctcaaagctaaatatagtgccagctacaatatc
gccaagatttaaatcatacttatatttttgcaaaacagcagcaaaatttctgtgtgtaaa
tccttcattatttttttgtcatattgaatagtctgaattaaagattaattttgtacagcat
attaagttaaataaacttttattgcacgatatgaaaatttttttaatttgctctgttgtca
ttccttatttaaacgtattagaatatacggaagacatgaaaagtaaacgtagatagctac
agatttaa-taatacacagtctgaggaaagtccgggctccataaatacaatttatgctgg
agaaagcccagtgtaagaaattatgaggatagtaccacagaaataaaccaccagcgtaag
g----tatattagctggtaagggtgcaaaggcaagttaaaagcttaccaaaaatactgta
aagtatttgttaggtaaacccctaaaatggagcaaagctactaaacaaatatttgtgtat
attttgtatagtttagtttaaaatactgcatgaagttattggtaacaataactctagagg
aatagctatcctttacaatatatc-tatattatattgaaagaacagaacccggcttatgt
agtactttttatgtcttatatttctctggttattgccgaatctctaaatttaagtgttgt
ataattttttttttgtagctcatgttgtttattatctatatctaaattagttaaagttttt
tgattagagcgatatgtaaatctcaaacctatactttttttctgttctccaagggattta
tttatatattgatcaaataaccttgtgctttctaaatctttatcattaaacttatctaat
aattttaataagtaattaacttccatatttttaggtattattaatgaaagatcccttata
atagatggatatttggagtaaggtcgaatttgataatttaaataattaaattgattatca
gaatcttctaaaatgcttaaattaatttcaagaacaaaaatttttgtagtaacacccaaa
ttactataagtcaccttgttgagttcactgaatatgccaatattagtattattataaagt
aatgttgcagatctattcctatgaattaaatttactctactatcaaccacttcttgtttt
ttccattcaatttgtctattcatttttttgaaaaaaattctccacgattcctttagcctca

taccaattcaacgagtgggctggttgtgaccatgtagatctaatatctaaattaccacct
aatatcatagctagattagttgtttcgacaatttcgtcttcaacagtattaaaaactgtt
cctatttcaaaactatctatagtttgattactttgtttaagattataaagattagattca
attaatccttcgagtaaactatagcgtaaattagaatggtcttggataagaggattattg
agactaatctctccgagggatttaactaaagaataatgaactaactcagttaaccctaaa
tttcttaaaatatttctactttatctataaatttctttttgcgatactttttacta
aattgaacggttggcactacgcttttaaatttatgatagccataaattcgtgcaatttct
tcaattatatctatttctcgaaaaacatcttgtcttctataatttggaacactaattctt
atattttcttgatctttatgaataatagtaaaatttagagagtaaagaatatcttcaatt
tcttgaaagtataagaaacgcacctgaccattattattgattggccctaaaatatcttga
actttttaagtgatacatctatatttagaatctttttattgttttctctgtggagaat
gtttctcgaatgttcccaccagctaagtctattatcaaagacatagtctcaagataagca
tttttccaattatctgtatttagtcctcgttcttgcctaactgaactttctgtcctgata
ttaagatctcgggaagattgtctcactagagactgtttaaatatagcagattctataaat
atagacgttgtagtagaacttgtatcaaagatgttgttggaccctacaccagctacgcta
attacatctgcatttatttgagtcactaaaatatttttatttagcttaatattttcatta
tttaactgaattttagtattttttccatcataaaagttgctttgaattgttatgttatgt
tcagg---------agtaagtttattaaaatcaataatattaataggttgtccccatttc
agcataatataattgcttatatccactaataaatttttattcgtaaagccgcaggattgt
aggcgatttttttagccaatttggagaatctgtaatttcaattttatcaataattgcagaa
aaatagttattacaatgaatcaagctatgattggtaagtatatt-ttgtttttttagagaa
gtgcttaccactaggttgatttatagtttgcaacattgaagatccagttatagccaagac
ttctcttgaaagacctatcatgcttaatacgtctgatctattagctgttgatgagatatc
taatatatgatcaatttgattatctactataatagattcaacagcctctacttcaaaacc
tgcctgcgtcaattgattagttaaattatctagatctattgtttctagattagcaagttc
tttcaaccaatttaaagaaactttcataattaattgccatctatcttatatatatattta
-tttacttgtgactaaaaactaatttaaaaaattcatcaaattaatataagttatttgat
gaatttgcttatacaatatattattgtgctatcttacttttggatggtagtaattaagaa
ctagcttttgtaaaagttagcccgttatcttgagcatacctttccataaatctcataaat
ctatcccaatcatcaggacttttaattacatgtacagcttctattgcttgcggtttacca
ttaataaacttagcattaacatctctggtaataatttgtccctctgtgtccattagatac
atacctgttatttcacctttgtctgccatactagcatctaaaattttgggatttgtaaat
ctgaaagtagcggttcccgtactaccatctctagatcttgttaaacgaacatctggcaca
acttcttcgttgatgccttgaataaattgaattgttgccatatcaatttt-tttatttgt
atataatgtatattggataaaactgatttctaattctttaatgcttgttgaaaaactgtt
ttaaaagcttatagtagcagtagatcaaattttatgtattatataaaactgtaactttc

-----ttg--

----------------------atctgttcaaagaactaacttattataagctaatcata
ctaatattagccatataatgttctgaagtagaaattag-gggttgtagctcagatggata
gagcaagcgcctcctaagcgctaggccagtggttcaagtccgctcaaccctgttaataaa
caaagtt---ttgctaaattttaccgataaatcagttgaaaaaagattaagagcacaaaa
cagg-----aaacagattctaaatatcacttttattgaaa---gaaaagcgatcttaac
tctgacaatagaagaatttttttg-aaaaatatactaagtttcaatgtttatatttagaag
aaa-tttttaaaaacgtaaacagaaa-----ta----ttttgagtaatactgaaaattaa
attatttaatttcttgaaattaatttaaaaaactttatatgaaatgaactatcttttt---
--ctctttattcagtatttgtctacacaattatta----attaattcaaaattaatcaaa
tggcttttgggttgaagatgaatttttaaactatagcaatagatcagtaactcgattatt
attctatatagattctgtttagaatttagtttgctctttaaaaaagaagcacttattatt
tgctagatagaaattcctaaagataatatttacaatgaatgtccgtgttatttaactaca
ttccatactttatagtaaattttgccaatttacgagag-------------ttaattcta
caacat-cttggttcctggaagttgaattttat---ctttttttacatttctcttttcgtc
tattacgagaaatcatatatctaaatttaatgataatagtaagaatcatggatttcttaa
ctatattactatccttttacctttattcctattgctaatcacagcagcag----------
---ttatttagtcaattctaatgctagaaatttaatacaagccagcgataattctttaaa
tttgttgttgttatcgaagttgttaacataataaccattaagattcaatgatttgttagt
tgatattctgcatctgcaattcttgacataatactgttagatagttgtaagtagttttat
tattgatataattgatattatcttgattgtatattaatttgata----------------
---------gaaaaaacgagtatttaagtttcctgctttcatgtcaaaaaatatgtttag
ttaacgatgaattttactagcaagccgattagtaactttatcatatgtaaaaataaagtt
tgtatattctagaatattctcta------tatattgcggcttgcaaaacttctcagagtt
gatttacagagccagtgggctaaaag-taacaaaatggtaattccacgaaaattttgtac
cagtgatatgacttctaataaacacttaagagtaaacaattcttaatttttgtattaaga
agcttcatagtattattaattgttagaactggggtgggaggattcgaacctgcgaatggc
ggagtcaaagtccgctgccttaccacttggctacaccccaacttatagaaactataatag
cagtcacataccctaccatgtcaagagactattctccttatttaataactttttagactt
ttgtaaaatagaattttctttttcaaataagatgtactatttttaaactgtatcattctc
atgtcttcttgcttctgattcgacatccatttgatggtaactgtttcttgttcaacttct
ttattgcctataataatacaagctacagctttttttcttattggcttgtttgatttgttta

ctaaagttacttgagctaatatctatctctatcgtaaaaaattgttgacgtaaaaatcgc
attattctcataccaacctcattagctttttaccctgtgtagcaatgtaaaaatcaata
tacttatttggtaactctattttttctttagcaaggagtagtaatcgctctaatcctatt
gcacatccaacagctggtgttgctgcgcctcctaactgctgcactaggctatcatatctt
cctccaccacacactgtatcttgaccttttgatgttaatgtcttaatttcaaaggcagta
tcattataatagtctaatcctctaactaacttatcatttatgttataaggaatatcaagt
aaattcaaataatgacaaacatcatcaaaatatttttgtgacgaaagacttaaaaaatca
gaaatcttgggagcctcagttagtatttttgtgtattagtatttttgaatctagaatc
ctaataggattactaattagtctcttctgtgaatcaggatctaattcatcatagtactga
ttaagatattcttgtaattttgcttgataaatactacgatcttctaccttgccaattgaa
ttaagatcaagcttcaagtctgtgagattaagactcttaaatatacttgtagccaaatgt
attacttcactatctgctcttgcatctaggctaccaagaaattcaataccaagttggtga
aattgtcgttgtcgtccgctttgtggtctttcatatctaaacattggcccgctataccat
aatctctgtaacctgtgatgatagctcatttattttcaatgaaagctcttacaatgctt
gcagtaccttcaggtctcaatgtaatatctctattgctgcgatcgttaaatctgtacatt
tctttatttacaatatccgtatctctaccaatacttctatcatataaatcgctgttttca
aaaataggtgttctgatttcttgatagtttgcacattctaataagttggaaattttgttg
tgtatgaactgccaatattgaagctcttcgggcagaatgtcttttgttcctctaatagct
tgaatttttgccatgataattttcggatcaccttcttaatgtgtctgcactaaatagtag
ttgtagactattattcagtcatgtattatacattag--aacatgttgatg-tattttata
aaatacgggcaaggagggattcgaaccccccgacaccatggttcgtagccatgtgctctaa
tccactgagctacaagcccacttaacaagttacctaactatatcactgttctttcatgta
tacaactatattttgtct------atttatatttgaaagttttgtcaaagtaaaaaaat
tt----atttatatttaatagttaaagttagtcatttataattcctcatttattacaact
ttgtaca--aatatcggcaaacttaattatgaaattatcttatactgataatttcctcat
aa-ctcgtgcttttactttttgatcga------ctagtatctaattaatgtatatttcaa
at-aaaaaatcttacctaaaatgagcaagcaaattctatatcaagatgatgcacgtaaag
cattagaaaaaggcatggacattttaaccgaagctgtttctgttacattaggacctaagg
gaagaaatgttgtcttagaaaaaaaatttggagctcctcaaattattaacgatggtgtaa
ccatcgcaaaagaaattagtctagaaaatcatattgaaaatactggagttgcacttattc
gacaagctgcatctaaaacaaatgacgttgctggtgatggaacaacaacagctacagtac
tggcatcagcaattgtcaaacaaggcatgagaaacgttgctgcaggttcaaatcctatgg
ctattaaaaaaggcatagaaaaagcaacaaattttgtagtgagcaaaatagctgaatatg
ctaaaccagtagaagataaaaaagcaattatacaagttgcttctctatcatcaggcaatg
atatggaagtagggaaaatgatagcgaatgctatagataaagtcggccgagaaggcgtaa
tttctttagaagaaggcaagtcaactaatacggttcttgaaattacagaaggaatgcagt

ttgaaaaaggctttatttctccgtactttgtgaccgacaccgagcgaatggaagtccttc
aagaaaatccatttattctatttacagacaagaaaattactttagtgcagcaagaacttg
ttccacttcttgaacaaatagcaaaaacatctcgccctttattaataatagctgaagata
tagagaaagaagcattagccacgattgtagttaacaaattaagaggtattttaaatgttg
tcgcggttagggctcctggatttggtgacagaagaaaatctttacttgaagatatgagta
ttttaactaatgggcaagtcgtcactgaagatgcaggtttatcactggatacagttcaac
tagatatgctaggaaaagctcgcagagttattataactaaagattcaaccacaattattg
cagatggccatgaaattaaagtaaaatcaagatgtgaacaaattaagcgacaaatagaga
caagcgattctctttatgaaagggaaaaattgcaagaacgattagcaaagctttctggtg
gtgtagctgttataaaagttggtgcagctactgaaacagagatgaaagataaaaaattaa
ggttagaagatgcaattaatgcaacaaaagctgctattgaagaaggaattgttccaggag
ggggagctactaatgtccacatctctagcgagttatttacatgggcgaaaaacaatttag
ttgaagacgaattaatcggagctctaatagttgaaagagcaattacttatcctttaagac
gtatagcttttaatgcaggagataatggagcagtagcggtggagaaagttaaaagtaatg
attttcatataggctataatgccgcaaatggcgcaattgtaaacatgtatgatgcaggta
ttatagatcctgcaaaagttgctagatcagctctgcaaaatgcagcctctattgcagcaa
tggttttaactacagaatgtattgttgttgataaagctaatgactaagttt---------
-----taaataatattaaacaaagaataatttctatctataggcattattctttgttcta
attaagttaattaattgagcaaatgtataattttataataaagccaaagcttaaatgtg
cctctttgctcaactattaagtagaggagatacaagcctgtaattcctaaattatcaatt
aaagcagtattattatctatcgatttctcaaaagatctaaaaaatgacattttgtaaaa
tagtatcggaatcttcttttgtagttatttgataaatcattactacatttagattgatct
gcaactgcatttaaaacttttaaaataatgctttttaagtctttatctgactgtatctta
gaatcgctaataaattgagttaattgaatatcaaaactacaattaagaaaattactggcg
acagatgtctgattttgaatatctatagatttgatagccaagaagattaaatttttattg
ttcattgtaaaatgtaagacaattaatgttgtattctttaaatacta-atttatcttct
tgtgtatttatagttttggataaaaataattaatattatta--------tattaattact
tcccgaaaaaataaattcaggaaatctagcctgagcctttgatagtgagtgtttttacc
ttcttcttgccagtaattaataacttctgttgttttatttaatagctcaatcctatctct
ttcagcaatccatggcttagattctagttcggcttttaattcttcccaagcatcatttct
tggccagaaaaaataagaagtaagtggacttgtaccatttccaacaatttgatcaattgc
aatagcaatattgttttctaaccaaagtacctttagtgtaaattttgacaagtgattatc
cccattataaatagtaaatatatgtatatacagtaaaatgctgtattagttgttctcttt
agcaacaatctgagctttatttaaaatattttgtaccgttttagtagcttgtgcaccttg
tgctaatctctgtaaaatttttgaaatgtcaatacgagtttcattagtgattggattata
aaatcctaattcttctatagctttgccatctcgtttatttctgctatccattacgacaat

tctgtagcttggttgtttttttcttccatatctttttaatctcaacttaaccatcttagt
caccccgcttatttttt--atatctttattaaattatatcttacgatattatgcaataat
tcgaacaattaagcagactcaagcctgacattattaaatattaccattaaacactggaga
aaaataatacctagcataggtgacatgtccattccaaacattggaggaatactgcctcta
aagagctttaaatatggatcagtaattcggtttaaggaacagaatggttcattataccag
ttcacagttggaaaccatgccaatgacaatttaagtaaaattaaaattaaataaatctca
gaaaaattagctattgatccaagtaataaatttaatgtaccaggaagagtattcatacgt
attttataattattatatagttcattatactaatagtttgagtatatatcataagctcta
taaataaat-tagctaaacaaaaaacttattcttaaaaatatcttctcttgaacaatctt
cttcattgatagtactgttaacttttgttgtataaacttctaatttagaccaattatgtc
ctaactgatttagctgagatgttccgcattctaaacatgctatttttacattatttaaaa
taacaccttttttactaaatcttttaaaactggtgtaataattgtttcatctaaaaata
aatctaagattagaacttttgtaaatgaatttaactgatctgggacattattagtaatat
tattgccattaactaaagctatactagctgtaggaagcaaagctcgcgctccttctgcta
gtataaatccataaggcataataatgacaataatatacttataatgactgttaataagat
ttatattgatattagtttttaattcaatagtttctgttactaaccattctctcattattt
cataagtaatccacttacctaattctgagcaagcagtccttaaaatcgtacttggattat
tgttattttcgagaataccagcccaatgttgtattagagggtgcgaagctatattaattt
tgagttgcatgttttgttaaatatatttaacatttaatttgttaattgtgtctttggttt
ttgttcactgagtattattaaaatagcat----atatattaaagaaaacaattat-ataa
aatgaaaaaaactctttggctttggggtttcacagatagtgctgaaacttggaatggtag
atttgcaatgattggttttatagcagttatctttatcgaaatattgacgggacaaggact
attatatttaataggtgttatctaataataaaaaatcaaaaaagttcttgtaatacatta
caagaactttttga-atgaatataaatctaatcgagcggtctcatagatagtactggtt
cattttctctattaataccttttcaaatccagcagcagcagctcttgctctaccagcat
gccaaagatgtccaataaataggaaaaatcctaagaagaaatgagatgttgttaaccaag
atctaggagatacatagttaactgagttaatttctgtagctacaccgccaacagagttca
aagaacctagtggcgcatgagtcatatattctgctgcacgtctttcttgccaaggttgaa
tatcattttaattttattcaaatcaagaccatttggtcctctaagaggttcaacccaag
gtgcacgcaagtcccaaaatctcatagtttcaccaccaaaaataatctctccactaggag
atctcattaagtatttgcccaagccagtaggtccttgagaagacgcaacattagcaccta
atctttgatctctaactaagaaagtaaaagcttgagcttgtgaagcttcaggaccagtag
gtccataaaattcacttggataagctgtattattgtaccaaacaaaattagaagctgtaa
ggcccatgattgataaagcacccaaactgtaagataagtatgcttcaccagaccatacaa
aagctcttctagcccaagcaaaaggttttgtaagtatgtgccaaattcctccagcaatac
aaataatacctatccaaacatgaccaccaacaaggtcttccatgttatttacactaacga

tccatccatcgccgccaaatggagatttcaagacgtatccgaaaataactaaaggattaa
gagtaggattgctgacaaatctaacatctccaccacctggagcccaagtatcatacactc
caccaatgaacagagctttaatcactaataagaaagctccaatgcctagtaacactaagt
gtataccaagtatggttgtcattttattttatctctccaatcatatccaaagaaaggga
aagattcttcaagagtatcaggtccgattagtgaatgataaagaccaccaaatccaagaa
cggctgaagaaattaaatgcactacacctactacaaagtatgggtatgtattaaaaattt
cgccgcctggacccacaccccagcctagtgtagctaagtgcggaattagaataaatcctt
gctcatacagaggcttttctggtacaaagtgagctacctcaaaaagagtcatagcaccag
tccaaaataccattatacctgcatgagcaacatgagcgccaagtaatttgccagaaacgt
taattaaacgtgcattgccagaccaccaggcaaatccggtagattcaatgtctctaccgc
caacaccaacatttgtattaaagggcgtttccacgtggtaaaacctcctcagggaatata
aagttttcatgaggttgatcctgagcagccatccaagagcgaataccttcatttagcagg
atattcttagtatagaaagtttcaaactcagggtcttctgcagctctcagttcttgagaa
acaaaatcgtaagctcttaagttaagagctagtccaacaattccaaacgcacttgtccac
aatccagttacaggtacaaatagcatgaaaaaatgtagccagcgtttattagaaaaagct
acaccaaaaatttgtgaccagaatctgttcgctgttaccattgaataagtttcttcagac
tgtgtaggtgtaaatgcacgaaaagtatctgcagcatcaccatcttcaaacagtgtattt
tgtacggtcgcaccatgaatagcgcataataaagcaccacctaagattccggcaacaccc
atcatgtggaatggatttagcgtccaattatgaaatccctgtaagaatagcaaaaatcta
aaaattgcagctacgccaagacttggagcaaaaaaccaacttgcctgacccaaagggtac
attaagaaaacagatacaaatactgcaatcggtccagaaaaagcaatagcattgtatggt
ctaagaccaactaatctagcaatttcaaattgtcttaagcaaaatccaattaatccaaaa
gatccatgtaaagcaataaatgcccataagccgccaatttggcaccagcgagtaaaatct
ccttgagcttcaggtccccaaaggaaaagaagggaatgccccatgctgtttgctggcgta
gaaacagcagcagtcaagaagttgcatccttctaggtatgaactagctagtccatgagta
taccaagaagttacaaaagtagtcccagttagccagccgcctacagcaaggtaagcgcaa
ggaaacagaagtagtccagaccaacccacgaatacgaatcggtctctttttagccaatcg
tctacaagatcaaaaccgccacgagtttttcttgtccaattgctatggtcataattta
atctccagagcaaatttaataagtaatttgtttaaataaaca---ctttagatctagtct
aaaatttacttttctttacggtcaatataattataagtcaaaatcaacaaaaattcacg
tttttaacaata-ttttttagctcgttaagttaattattttaaagt-------------
taactattttttaattaatcaaaatcatagtttgttcatttctataattctttgaaata
aatatcctgttcctcttgctgttaaaattaagtctggattacttggatcatcttctagtt
tagctcttaggcgtgaaatgtgcacgtctacaactcttgtgtctacgtgtcgttctggcg
tatatccccaaacctcttgtaaaatagatgctctagaaaaaggttcgccagccttactca
ctaaaagttcaagaagactgaactccattcctgttaagcgaactcgttcattgtttttgt

aaacttgtcttttattggtatctatctttaaaaatccaatactaattatgccagaatttg
ggactccaagattagctgttattttatcagcccttctaagaacagatcgaatgcgagctt
ctagctctttaggagaaaatggtttaactacataatcatcagcacctatttctagtccag
taattctgtcacacacttcgcctaaagctgttaacattattataggaacatcagattctt
tccgtagttcctggcaaactccgtaaccatcgagtttaggcatcattacatctaaaacga
caaggctaggatattcttttcgaaatactattaaagcttcctctccgtttgaagctgtta
tgacttcgtaacctattattgttaatcttgtttctaaaattcttcttatgctggcttcat
cgtcaacaacaagaatttttctttctggttctccaa-tttcttatttactcctataagt
caataagcacaactactataatcttaaatttgtaactgtttgagtatcttaatttgctgc
aaaa-----cttatttactataaattgtataattc-----tatattaattggtatcagt-
-cattatacctgcaaatagccgtttgtcagttatgtc---------tattttaatgcata
agttaattcaagttttcagaattcagtatataaata-ctttttagacaagccttttacat
tctattttctaaataaaaaagggttgacaaactagctgatagaaagtattctacattaa
tataaataatttactgattataaa-gagcagtaa-----atttctttagagtattacaat
tgctttaat
>Pyr_19
tatatgata---aattaagacgtctcttatgtgagaatttctaaaatatacaaaactttt
tatttaatatagaaaaactaaattaaaataagttgaacataccttgctctaatctaaacc
tcttaattgag------attctaattagagataaaagtatttactta--ttgtatctata
ggaataaaaatgaaacttgcagtttatggtaaaggtggtatcggtaaatctacaactagt
tgcaatatttctgtagcactttcaaaaagaggtaaaaaagttctacaaattggctgcgac
cctaaacatgacagtacgtttacactaacgggattttaatcccaacaattatcgatact
cttcaatccaaggattaccactatgaggacgtttggcctgaagatgtaatctataaagga
tatggcggtgtagactgtgttgaagctggtggaccgccggctggcgctggttgcggaggc
tacgttgtaggtgaaacagttaaacttttaaaagaattaaatgcttttgatgaatatgat
attatcttatttgatgttttaggtgatgttgtatgtggaggttttgcagctccattaaat
tatgcagactactgcttaatcattacagacaacggatttgatgctttatttgcagctaat
agaatagcagcttcagtacgagaaaaagctagaacgcactctctgagattagctggactc
gttggtaatagaacagataaaagagatctaattgataaatatatagattgtgttccaatg
ccagttttagaagtcttgcccttgattgaagatattagagtgtccagagtaaaaggtaaa
actttatttgaaatggcagaaattgataaggatttagcatatgtatgcgattactatttg
aatattgcagatcagttgattacaaggccagaaggtgtagttcctaaagaatctcccgat
agagaattatttagtcttttatctgatttttacttaaatcctaaatcaaatgtaggacaa
gacaaagtagatcaagaagaattagatttgatgatagtgtaaataatattttcagaataa
tataacaaggaataagataatgtctacagctcaatcagatgctcttacttttgaatgtga
aacaggtaattatcatactttttgtcccatcagttgtgtttcttggttatatcaaaaaat

tgaagacagtttttttcttagttataggaactaaaacttgtggctatttcttgcaaaatgc
aatgggagtaatgatttttgctgaaccgagatatgccatggcagaattagaagaaggaga
tatttcagctaagctaaatgattatggtgaactacgtagattatgcttacaaataaaaaa
agatagaaatcctagtgttatatttggattggaacatgtacaacagaaattataaaaat
ggatctggaaggaattgctccaaaattggaagcagaaattcgtgtccctatagtagttgc
tagagctaatggtttagattatgcctttactcaaggtgaagatacagttctagcagctat
ggctcaaaggtgtccattaaatttaaataatcaatcagataatacgagtcttaaaatttc
tcctcatattcctttggttttatttggatctcttccggattcagttgtaacccagttaac
tatggaattaaaaaaacaaggtattttttgtttctggttggttgccatcaaagaggtatac
tgaattaccggttataaaagaaggatattatgttgcgggagttaatccatttcttagtcg
cacagctactacactaatgagacgccgaaaaacgaaactaattggtgcaccatttccaat
aggtccagatggtactcgggcctggattgaaaaaatctgttcagtaatgaatgtaaagcc
tattggcttagaagacagagaaaaagaaatttgggcttctttagaagactatatttcttt
aatacgaggtaaatcagtttttttttatgggtgataatttactagaagtttctcttgcaag
atttttaactagatgtggaatgaccgtatatgaaattggtatcccttatatggataaacg
ctatcaagcagcagagctagctttattaaaagctacatgtgataaaatgaacgttatgat
gccaacaattgtagaaaaaccagataattataaccaagtagaccgaatccgtgatctgaa
acccgacctagttatcactggtatggctcatgcaaatcctttagaagccagaggtattaa
tacaaaatggtcagtagaatttacatttgcacaaattcatggctttaccaatgcaagaga
tattcttgaactcgtcacaagaccattacgacgaaatcttagtttatcagaattaggctg
ggatgtgtatagcaagcaaagctagtatgcttc----tttatgacttacatggtataaaa
gccttgtaagtct--tttattttatgtcaaagaagcatttcttgctctgccatcaccagc
ccaagcttgtaaactttctacttgttctttatctgtaaatgctaaaggcacaaattgttt
gatagctattttaatatcttcagtactaaattctctctcttcactaaacgctgtatgcat
actttcaataattgcttgctcaatttcagcgccagaaaatttattacataataaactcaa
ttcatgaatatcatattcttgccacgatcttggtctaacttttgataaatgtatcttaaa
aattgactctctctcttgacgattaggtaaatctaagaaaaaaatttcatcgaacctgcc
tttccttaacatttcggaaggtaaactctgaattttattagcagttgcgacaacaaatac
tggagctttttttctctgatagccaggtaataaatgtgccaaacactcttgcactagtacc
actatcaccttggctatataaaccagaaaaagctttatctatttcatcaatccataatat
gcaaggcgataatccttcagagatatttatcatttctctcattttggattccgattcacc
aactagtccaccaaacagttttcccatgtcaagacgtagcagaggcaacatccaatcgct
tgcaatagctttcgcggttaaagatttacccgtcccctgtataccaactagcaataaacc
tttgggcgaaggaatgccgtaatttaaactttgcttcgaaaatgagcgagaccttttttg
tagccattgttttaatatatctaagccaccaatatctctatttactgtattataaggata
aaattctaatagacacgtttgattaataatttgcctttttttcttcaataataataggtaa

gctccgagagtctatctgattatattgtgcaataattttagtaatcactttgcggattct
atctatcgataatccttggcaggattttgttatattattaacgagctctgaattcaaatt
aagattcaaagttttactcaatctcctgatctctttttgatttctagaagactaggtaa
gggtaaatctattactgttatgatatcgcttagagcaaaaggtatattgattttgcagga
aataataacaatgtgcttagattgcgttttgattattttagctaaattgcgaagtttcct
aattaatactatttcatttaggaaagagtcaaaatcttttaaaagaaaaagatttaaaga
ttcattatttaagttctcaataaattctaatgctaataaaggatttctttttgcataacc
attatcgctaggattgttagtgtatccatctacaaagtcccagcaatacacttgttgatt
atttgaacagtttaagctgtgttttataatgtactctagtctatcttcttctctagtatt
tactacaataataggatatcgagattttaaaagtaatcgtaaatcttgagtgaaattcat
gtcaatatttttttccatcagacttttgtacttcaccaattg----aagactaaataaga
ttatctaatattgcttggatatattcaaaagtc------atctctctatttctaaatta
ttgctattattaacttaacttaccatgatttttttcttccttttcttcgtcgactattt
aatatagagcgtccacatggtgttttcattcttgctctgaaaccagaaactctaatttt
tttcttttagatccttgtagtgttcttttagtcatgatgtttcctctgttgtaaaagatt
aaatactattttatcatcttttatatatctttatgatatagatatatattaatatcaaag
tt-aacagcaataatttatattaatcaagaactatttatataatattaaccattaaatat
----------taactttatgatattggctttgcccatttttatttaagtattttgacca
tctttttgctaatcttaaactggttaattttccagcagctaaaaacgattttgtcattag
aatctcaatttaagtattttgttgataaaagccaaaatggtaaattagaacccgacgaaa
gttttgcttttgcaaaagtatgtgtagctaaaaaatttttttccaaagctattattgaag
gtcaattagctttgaaaaaatatcgagaccttaataccttagataataatatagtaattg
ccaatttatataatatgctaggttttatttattttgaagcaggccaaataagttttgcca
agaacttttatgagcaagccttacaaataaaccctaattatgttgtagctttaaataacc
tggcaaaaatttatgaagaagtaaaagatttaaaaaagctgaatctctatatgataaag
tgttaattcttaacttaaataataaaactgctaatagaaggaaaaatttatagcaaaga
cta---------aaaatgtctaatgcggtttgtaatcgggatagcaggatttgaacctgc
gacatcctgctcccaaagcaggcgcgctaccaaactgcgctatatcccgtaagacataat
tagtactatatacctttatctaattatgtctactctttctgtattttatctaggatacc
aaaacatgcctttaactccatctggatccttaataaatcctaatttttttataaaaactaa
caacatcaggctctgcaaatagtgtaatagtgctaatttctgcttgtctcaattgctgta
ttaattgatgtatgactactttccctaaacctaaaccttggaaatctggatgaataacta
cgtcccagatagttgcattaaatccgttgtctgatgttgctctagcaaatcctacaagtt
tactatttgcatcttttttttgtattaaagaaataataatagaactatttttaatgcga
tctttactttttttagaggtcttttaacccatccgactgaatcacagagctgctccaatt
cgtataaattaatatttttattgctactcagatagatatctttaaattcgattctatcgc

aagttttatctaaaacaataagtttttttgaaatttttttcagagctagcattgatatcgg
agttttgaaaaaaatttttccagaagatcataatgattatgatataatta--aataaata
tatatattacaatattgctttaacaaacaatatattaatatatgctcaaatttttacatg
aatctattaagatagaagattgttaaaatatagagaaataaaaattttgtaaaatcttt
caataaaatcatataaagagtgctttttaagactaacatctattattataaataaagtct
cgcaattgagatgaaataacacattattctatgtctaatgccttttagaggagttatttg
tgaaaaaatctatgtttctaaccggtttacttgctttactaattatgagcaacccaataa
tagctaacgcagaagtagctggattggtaccatgcaaagattctgcggcattcaacaagc
gtatggtaaatagtgttaaaaaacttcaagccagattatctaaatatgatgcagataccc
caccagcgttagctttaaataagcaaatagagaaaactaaaactagatttgcaacttatg
gtcgagcaggtttgttgtgtggtactgatggattgccacatttaatttctgatggtagat
ggagtagagccggggactttgtttttccaggactattattcttgtatattacaggatgga
ttggctgggtaggtagaggatatcttttatctgttgctaagactagtaagccaacagaaa
aggaaatcatttttagatgtgccattagctatcaaatttatgtcatccggatttgcatggc
cgctagcggcttggcaagaatttagtagtggacaattaattgcttccaatgacgatatta
ctgtttcaccccgttagtaaaaaatttatatgaataataattttaccaaatacttatcaa
cagcacctgtaattggtgtattgtggatgacgtttacagccggatttattatagaattga
accgctttttcccagatgtcttatacttttactt----ataaataa--gatcttataaa-
--aaactacaccaatatagagaaatactattatttgtgtagtttttaa-tatatataaca
taattattta-tttttaaaaatatttatcttttcgattattttaagattagatcttacat
aataaaata-atatcaatattatgagcttagtaacccaaattatagttaatgctgatgat
gaattaagatatcctacaattggagaattgcagtcaattcaagactacttaactacaggg
agtaatagaattagaattgctactattattagagataaagaaaaggagattatacagaaa
gctagtaaacaaatttttcagttacatccagaatatatagctccaggtggtaatgcagca
ggttctaggaaaagatcgttatgcttacgtgattatggttggtatctcagactaattaca
tatggcgtattagctggggataaagattctatagaaacaattggtattataggtgtaaga
gaaatgtacaattctttaggcgtacctataattggaatgttagatgcgatccaatgttta
aaggaagcatctttagaactacttggtcaagatgatattagaatcattgctccttatttt
gattatataattcgtggaatgtcataaatcgtagcttcaattaattagttgaataaaagc
ttttataatgttataattacaataagcccgaaagcatataattgtaaaaactaaatcttg
tcagaaccggaaggtagcagcaataatgtttaaatacaaaagttatga-ttttcgggtgt
ttactgttttcatctaatagtctcgacttttaattagtatgctatgtataaatctgtata
tgatatgtaattcatcaaacctttcagcaaaaacaatttaataatttgatattaatacgt
aaaacacctatac-tttttttataaaatttctttatatctcatctaaacaatggaaactta
acttaatgggtgttcatattttatcaacaggttcgtctgtcccaaattttctgtagaga
atcaacaatttgaagatatgatcgaaacttccgaccattggatttcaacaagaacaggaa

taaaaaaaaagacatctagctccatcttctatttctttaactaaattagcagcagaagctg
ccaacaaagccttatatgcagctaacttaaaacctactgagatcagtttaattattttag
ctacgtctacgcctgatgatttatttggtagtgctagtcagttgcaagcagaaataggtg
caacaacatcggtagcttttgatattacggctgcctgctccggtttcattgttgctttag
taacagcagctcagttcattcaaactggttcctatgacaatattttagttgttggagcag
atacaatgtctagatggatcaattggtcagatagaactacctgtattttatttggtgatg
gtgctggagcagtagtattggggcaaagccgcaaaaatagtattttaggctttaagttat
gtacagacggtcagctaaacagtcatttacaattaatgaataaacctgtaaataatcaaa
agtttggtgttacagaaattcctcatggaaactataattccataacaatgaatggcaagg
aagtttacaagttcgctgtatttcaagttccaacagtaattagacaatgtttgaataatt
taaatatttcaatagatgaagttgattggtttatattgcatcaagcaaacactagaatta
tagaggcaattgcgagcagattatcagtaccttttctaaaatgattacgaacctagagc
attatggaaatacatctgcagcgtcaattcctttagcgttagatgaagctattcaatcca
ataaaattcaaccaggccaaattattgttttatctggttttggagccggcttgacttggg
gagcaattgtcttgaagtggtgat---ttatatattgcggatgacgagactcgaactcgt
aaagctttcgctacacacccctcaagcgtgcgtgtataccaatttcaccacatccgcatt
ttttatcatgaata-ttaaaaatttataatatttatctaaatattcatataatgtgtgta
tatatatatacattcttgtcagaaactctcttatatttttatttaagtgataaaattgag
t----ttagtatagatagtaaaattttattttaaaatcataa----aaataaatatataa
ggaaactaaaatatgacaccgtccttatcaagtttttttgaatagtcttattcttggggca
gtaattgtagtcgttcccataactttggctcttttatttgttagtcaaaaagacaggaca
attcggtcctaaaaataacttagaaagatatggagaagcaatttaagatgaataatgaaa
tatttttcataataatacgctctccatacattgctatact--ttagaagaa--------t
ttttattttgtaacatcctgtaaagtttattccgctctttctttttcct-atttactctc
tta----taatgatacaaatgtcagg-ttaataatag--ttttttttattttttatctttc
atttatatcaaagtattttatatacgtattattatgttcaattaataaagaaaagctca
tgtctttgtctaattggcctctcaaaaaaagaaaattctgaagcatataatattaagaatt
caaaacaaatcacaattcctgatggtttatgggtaaaatgcttcgactgtggtctattaa
tgtattctaaagtactaaagcgaaatttaaaagtttgccctcaatgtagttaccattttc
aagcttctagtaacgaaagaattgatcaattgatagaccaaggtagttggcaaccaatgg
atgttcacttgatctctacagatccattaggttttaaagatcaaaagctttatagtcaaa
ggttaaaagatactgctctgaagactggcctgcaagatgcagttcaaacaggtactggta
ctatgcaaggtaaaaaagtatgcttaggtatcatggattttagattcatgggggggaagca
tgggatctgttgtaggcgaaaaactaacaagactactagaaaaagcaactcaagataagt
tgcctgcaattatactttgtgcatcaggcggagctagaatgcaagaaggtatgttgagct
taatgcaaatggcaaaaatttcttctgctctagaaatgcataaaaaagaaaatttactat

atatatctgttttaacctctcctacaacaggggggtgtaacagccagttttgctatgcttg
gagacttaattattgcagagccgaaagctcttattgcatttgctggtagacgagttatag
aacaaacaatcaaagaagacttgccagataattttcaaagttcagaatatttatttgaac
atggtttcctagatttaattgtacccagaactcagcttagatcaaagttaatccaaattt
tgtccttacataatcatagtaagtaattaat----tattgactataatataaaaatgcta
gtaaaaattaagatattaagaaattttcactaaagatatttgtagactaactagaattac
aattactattgtagaaaaatttgtcttaatgtca-t-ttttcagtctttgaacaaattg-
tttttaggactttaaataattgttagtttaatacaaatttataaagaaaata-----cat
actaagctaaatatagtaatattatgttttcctataatactgacctatcaaatttcgagg
aaatttatgcttaaaagatcttcttggcttgcggctttattgggactattaacagtggtt
tctacaagtacgcatacatatgccatagagttagacgaggcaacaagaactgttccatta
gaatcttctggcagaactgtaattcttacaccagaacaagttaaaagaggtaagcgatta
tttaataattcttgtgctatttgccataatggtggtatcacaaaaacaaatccgaatatt
ggacttgacccagaatctttaggattagctacgccacaaagagataccattgagggacta
gttgactatatgaaagatccgactagttatgatggtgcagagtcaatcgcagaattgcat
ccaagtattaaaagtgctgaaattttcctaaaatgcgtaatctaaccgatgaagacta
ttcacaattgctggtcatatcttgctccaacctaaaattgtttctgaaaagtggggcgga
ggaaaaatttattattagaacttcaaagaactaaatcttgtgaatactagtttatgta-t
ttgttgcttaagtgtgattattttgttagatcatgatatatattaaagatggagtttgtt
gaaagcaagttatttcttagccgaacttgcttacactattcgtctagttattaaaaagga
gacgttaaattgaaaaagaagttttcagttcttttcactgtttttagtttttttgtaata
ggtttcgcacaaattgcttttgctgcagatctagataatggagaaaaagtttttttctgct
aattgtgcagcatgtcatgctggcggtaataacgctattatgccagataaaaccttaaaa
aaagatgtacttgaagctaatagtatgaatactattgatgctattacttatcaagtacaa
aatggtaaaaatgccatgcctgctttcggaggtagactggttgatgaagatattgaagat
gcagcaaattatgtattatctcaatctgaaaaaggttggtaattatacttgattttatcc
tgtattaaagaatagacaatctatttagttattcattagattgtctattctttgtttatg
---ctattatatagagatatttacacaatatt----ttatgatgaaaagaatacctgcaa
ttcttgtactagaagacggtacttattataaaggatggtcattccagcaagataaacaag
agattactattggtgaagtagtttttaatactggaatgacaggatatcaagaaataatca
cagatcctagttacttccatcaaattgtcgcttttacttaccccgaaattgggaatacag
gtattaataatcaagatattgaatctcacagtattagtattaaaggacttattgcaaaaa
acatttgtaaaatttcaagcagctggagagagcagcaatctttatttaagtatttaagta
gtaataatattccttttatttttggaatagatacaaggtctttaacccaatacttgcgtc
aatttggtacaatgaacggttgtatctctactgataatctaaaccatagttacttaaaac
agaaaatttgcgagattccaagtatgcaaggtttagatttaatcccgcatgtaactacaa

gaaatgtttacccctgggatgaaaaaagtttgccgaattggtatttgacagataacatga
gagtgcaccgagttattcagttgaaagttattgttatagattttggagttaaactaaata
tactcagaagactagctacacttggatgtcagataacggttgtgcctgcccacactcctt
taaaagatattttggcttaccaacctgatggcatattactctctaatggtccaggagatc
catctgcagtacattacgggatccagacagttacaaacttactagattacaatgtgccta
tatttgggatttgtatggggcatcaaattttaaatttagctcttaaagctaaaactttca
aacttaaatttggtcatagaggtattaaccatccatccggattgaaccagcaagttgaaa
taactagtcaaaaccatggctttgcagttgacttgacttcggtttttgaatctcctgtaa
gagtgactcattttaatctaaatgacactactattgcaggaactggacataatcaaagtc
cttatttttctgtgcaatatcatccagaatcgagcccaggccctcatgatgctgattatc
tattcgaaagttttatagaaataatgacaaagtccaagaagaaagttag-----------
ttagtaattttcccatgctttatgagtgaataaagcggatagtacttgtactcctcgtaa
ttgattgaagagaggtaaatgccctttggggccatgagaattccattgaaattcttcagg
atatctgcaaggaattctatcaacttcccagccgatttttttgccagaatttttcccaatc
ttttccaacactaagccatacttgtctttgaacaaataacccaaatttaccctttgagtg
tgtatgccataaattatcaatagtttgtaaatcttgagcaggtatttttttatatctgt
aaaatatagccaattacgagtttgtgcatttacaccagctagttgaataagtttttgttg
agttagctgatctgctttgagcaaatcccgatgagtcagtaacacttgtaaatctttgta
attcatctgttgagcagagcgcaatggaacaatgccatcgggaaataagttagaggcaaa
tttcactatttcttgattcttactgttgagtaacttttcataaattaaaccgtcaacaca
attacttttatagttaggcccagtaatcctctcaaacaatagatctgctaaatcttttaa
ctcgatagagtcgttgctatttatattctcaataatttcaagttgttgcttga---catt
attagacttagtatccttattcaattctaaaagctgggctcggatttgatttggcatctg
ttttgtttaattagttttaatagtcataaataaggtcttatagagaattagattgttttt
tgaaattagaagagcgccgcagtatttctaaagaagttcgcataatttgattcaataagt
tttgtcctgcataaattataatttgaccaattaaaaaaattgtgtttttttatttaggaa
caaaaaaatcttgtacctcaagcagaaaagtaattagtatttgcattgatgacaacatct
gtaattcattcgatctgcaagcatatatatactgacagtttaagccttcttgataaaatc
cccatactttgtaacgactttcgtaaattgctcgtggtcgctcaagatagtttttaatta
tagtattccaaattaggttattctttaattttctaattttctatcagatagaaatttta
cattacagagataacttgcatttgtctcatttacagtattgaaattttgaactaacatat
gagcaataatgttgcttagctgaattaaataattttcaagaaaaatttcgacctgcttaa
ctggcatattgtaagtaaagagattaaaagttttattcacagtttgggatgacccaaaaa
gtagttgaataattagaatttgtagtagcatcttataatcgttcagtaggtagttcaaat
cattaatatcttggtaaatatcaatatagtccagttcatataacttacaaaatcttttga
cgcatctatggaataaatcaattaaaatattttcggtcaagtttgtaatatcatttacat

ctaaatttgaattataaatttctaaaagtatcttttccagttcagctaatataattttta
ataagttcctttttacttcggtgcgaaaaacatctaaaattaacacttcctgagaacaat
tagttaatctcttattaatctttatagaagttctaactaacagctccgctacctcctgat
tgagtgttggtccctgagagcttggccaataattattcacgttataccatagtaagaaat
attgaatttaatgttatacgataactttttaatatactgaaaagcaagttagttactcta
tagcaattag-------gttttacagatcttaatacttatttatcaaataaagttatatt
atagtagcgactgagctaattttattaaaattta----taataatgacaacttattactt
tgcccttgcgagtcaaaatttctattagtacaagaaccactagaagaagtctttagaga
gagagttaactactatcagtcaaataataaagcaattgattttggctaataccaaaccc
ttctttttagagaaaccagaaatgatttcatttaagaatcttgtacctaaagaggctgt
agctataatctctactaatccaatatttattaattggttaaagctaagaattggctatat
ttgtattggacaatttgaagataacctacaactttctgaagaatcgttaaatattactgt
tttaaaagacaaaatttaacaatgcttttattataactagtgtt-aattattttggttaa
gttatagttatagtcttttagtattta-gatgcttatagtttatcacttagtattttatg
ctgagatggagtgacaaatagtgtaagtatttcttggctaaatgtttcagttgccttaga
tttatatctatttggattaactataatagatagcattcgttttatagtaacattttcaat
ttgagcccaatgtacaattccaagttccaattccttagcaatagctgaaacggaaacaaa
ggcagctcctagccctgattgcacagcgtttttgatagcttctatagaattcaattccat
ctctattttaaagcgactgctgtcaataccatgttgactgagtactttatctatgacttt
tctaattgttgattgagtatctaacgcaataaatctaagcctgtataagtcttctttttg
aatgtctcctagtttagaaaaaggatgtgattttggtaatatcagtgctaattcgtcttc
tgcataagaagtaacttgcaaaacatcttgtaattcagtaggcacttctcctccaataat
tgctaaatcaacttggccattagctacgctccatgaaataagtctagttgaatgtacttg
caattgaacagccacttgtggatatctttgcctaaatagtccgattaatcttggcatcaa
atatgtcccagttgtctggctagccccaataattaatgtgccaccttgtaagttttgtaa
gtcgtcaagagctcgacaagtttcttcgcagagggctaaaattctgcccccgtatcgtaa
aagaagacttcctgcctcagttaaagttgccttcttattacctctttcaaatagggaaac
atttaattggcgctctaaattttgaatttgcaaactaatagccggctgagaaacatacaa
gctattagctgctttttaaaactgccctctttggcaattgcttttaatattcttaactg
atccaatgtaaatggaaggtctgtcattaaagaattgtagtataatg---------tata
ttatttagtattgatcaatgtatgcatttgccaaccaaaatatttgttattacgcttcat
tatttttaaaataataaaaagtataatgttaaataagctgtattaaaatttaatatctgt
ttgatattaaatttatactaaagatatcgtaaatttaattctaactataaaatataggag
acaat---atggactctagacttttagttgtactaataccagttttagcagcagcatctt
gggcagtttacaatattggtagagttgcattacagcaatttagaaaaatgacatcttagt
tttgcttagaatata-atttttagactttatgagatagaggactttaaaatccctatctc

tattgtatatcttaataaaaaaat-caggc--ccccttgtacttactgttattacacatt
aagattattgat-agtaccta----gataataagtttaagctaacttatttttttatttta
taattaattaaattaataacacaattatggctgttccaaagaaaagaacatctaaagcta
aaaaaaatgcacgcaaagcaaattggaaaaatcaagcaaaaactgaagcacaaaaagctt
tatctttagcaaaatcagtattaactagaaaatccaatggatttatttataatctaactg
aagcgtcagatacttttagtgattagaagtgttt-ttactacaagtctgaaaaagtatta
cgcaacaagttctaagtaatttttatctat----taatcatttgaaaatcattctatttg
ataataaaataaatagcagattaaataaaattacagtaacaaattatgcagcaaagctag
atcaaactagtgaaatttggttgttcaattgtattgaaaatattcagcatatttttttaa
aaagtcaattaaaatcaagtcatattactaaaattttatttctggtactagctttgaat
ataccgcaggtctaccaggactattatccagcttaacactaagtggtagactacatccta
ttagtatatatagtcctcagtctctcaaaaagtatcttgaagtatgtaccaaatattccc
aaactaatttttcttttcctattaatttcataatttacaatacggagaacaagttgtta
gccaattttatacagtaatttgtttaccgttgagtaaaaagagcttgctgtatggattta
ttattttaaaaaaagaaaagcatggagtatttaaattagcacaagctaaaaccttgaata
ttcttcaaggacctatatatggaaaactcaaagaaaaagataatttttaagtccagatg
gttactatctatccggccaagacttctcttctaatacaataatgggacataaaatatcgc
ttcctttattagtcagatattctagaattatttctgagatgcattggttttgctcttatc
ctattagattaaatacttattcgcatcagcaagtagtcaagtgtttaccacataatgttt
taactgatattatgaagtcgcaaatatatcaagataacagttttgttgaataatacttat
tcttgatatataatggtctcggtaatccaaataacatactagaaataatttcttgaattt
atttgttcatattaaatttacaatcatttatgacatacgcaattattgaagcaagtggca
cccagctttggatagaagaaggccgctactatgatttaaatcatatacctgttgatccag
gtcagtcgattatattaggaaaagtcttattattaaataaaaatgggcaggttactttag
gacgtccttgtatagaaggtgttactataaaagctacagtaatgaggcacttgcgaggaa
agaaaataactgttttcaagatgaaaccaaagaagaaaatgagattaaaaaaaggtcatc
gacaagaattgactcgtttaatgatcgattctataacctcttaga------taaaatcta
gactcatattttt--aa-aaacttttttaatacttaactaaaatagatataatggcacata
aaaaaggtagtggtagtacgagaaatggcagagactctaatgccaagcgtttaggtgtta
aaaaatatggtggagagcaagtaacagcaggtaatattttaatcagacaacggggaacta
aagttaagcctggccaaaatgttggaaaaggaaaagatgatacattattttcgctaatcg
atggtttcgtgctgtttgaaaagtcaaatcaaaagcaaaaaacaattagtgtttattctg
ctaagaaatagttaaaacaattggtgcaagataacttgagt----gtatttattgcactt
acagtggcaccatatatttgatttattaagaaatggagttccttaatgaaccaatgaatt
ttattaaagatttgctgtgaaatttaatgacaaacactattgtaatttcctgtctacaca
atatggctgctattctatattgtggtcagatacagaaactagtagtagcaaatgctcatt

atcaagtaagtgatatctacctaggttgtgttgataaaatcttctcaggaataaatgcgg
catttattaacctaggaaagaatgagtacagtggttttatacatatcagtgatacgggtc
cgcttaaaaagaaatattatgtcaataatattactaacattttaacaatacgacaaaaaa
ttttagtacaaatcattaaagagccaactttaaataaaggtccaaggctcactgccaata
ttacattatcaggtcgatatattgtactaatgccttttagtcaatcaatctgcatatctc
gaaaaatacatgatgaagatgagcgtcattatttgaaatctttagctattttaattaaac
cagcaacaatgggcttgctatttagaccttctgctgtaggcgtcgatgaggaaattatat
taagcgaattaaaaaatttaaaagaacaatggaactttgttcaaaaatctgcaattaata
gtcattcacctgttcttctatataaagatgaagatattgttaaaaaggtaatccgagatt
tttataataataatacaaacaatatagtaattgattcaaacctgggattaaaacaattaa
attattatatccacacttggcactgtaataactctagtacagttcccaagatgaagcttt
atagtaataatcaatgtatactagatgcttttggtatcaatcaggcaatttccagagctc
ttattccaaaagttgatcttatacttggtggctatatgtttattgaaactttagaagctt
ttactattattgacgtcaattctggatctttcaataattctactagtgcacgagaaacag
ttttaaaaaccaattgttctgcagcaacagaaatagcttatcagttacaaattagaaata
ttactggtgtaattataatcgacttcattgatatggaatcacaaagagatcaattgcagt
tattagaacactttaataaagagctggcacttgatgatgctaaaccacaaattgtacagt
tatctgaattaggtctagttgaattgactagaagaagacagggcaaaagtttatacgagc
taattagtagtgattctaattacttttatttttcacacaatcagagagatcccagtctc
ttaagagatttaatgatagacaacagaaacagcagacttttaatagatcttggctatcta
tagagattaatactattaacaaggtctttttcaaaagtcaaatttgtgcagacctgcta
acttttacctaattcgtaatctctatatagttaaaagtagtattacttataaacaaaatt
atttattaactcacagatcaaaacttatttattctcaagaatacaacaaagtattaccaa
gca------gttattatctagctagtctcaacaggaatagtaatcaggagttcctatctt
aagatttttatatcaactgtt---------------------------------------
-------------------gaaaaaaagaagctctctcttatataagagagagcttcttt
ttttagcttactaacta-tctagttagttaattaaaaattaaccgtaaacagctggagct
gttagagctactggtaaagattcaccagaagctagatctagagggaagttgtgagcgtta
cgttcgtgcattacttccatacctaggttagcacggttgatgatatcagcccatgtgtta
attacacgaccttggctatcaacaacagattggttaaagttaaaaccattcaagttaaat
gccattgtgcttacagataaagctgttagccagataccaactacaggccatagacctaag
aagaaatgtagagaacgagagttgttgaaactagcatattggaagattaaacgaccgaag
tagccatgagctgcaacgatgttataagtttcttcttcttgtccgaatttgtaaccatag
ttagcagattcgttttcgcttgtttcacgaattaagctagatgtaactagggatccgtgc
atagcactgaacaaagaaccaccaaacacaccagcaacacctagttggtggaatgggtgc
attaaaatgttgtgctcagcttggaatacaagcatgaagttaaatgttccggagatacct

agaggcattccatcagagaaactaccttggccaattgggtatactaggaatactgctgct
gctgctgctactggagcagtaaaagcaacggaaatccatggacgcatacctaggcggtag
cttagttcccactcacgaccaatgtagcaagctacgccagttagaaaatgaagaacaact
aattggtaaggaccaccgttgtatagccattcgtctaaagaagcagcttcccagattggg
tagaagtgaataccgatagctgcagaacttggaataacagcaccagaaataatgttgttt
ccgtatagaagggaaccagcaactggctcacgaattccatcaatgtctactggaggtgca
gctacgaatgcaatgatgaatacagatgtggcagttaatagagttggaatcattaataca
ccaaaccaaccaatgtataggcggttttcagtactagtaatccaagagcagaaacgttcc
cacaagctagcgctttcgcgtctttgtaaagtagcagtcataatttttttatcaattttta
aggagttaacaaagatgcttcccaaacaataatttgtctgttatggaaattattacttaa
ggccttttcaaatgagaacattttaaaaaaaaaca-tagcaaaatctaatctcagtaact
aa-aaatt-taattgttgatatattgcttaatacttcaa-----------taataaattt
tt-gct-------------ttttacaaaattaacaccattttagggatataataataata
atgcatattaatgctgcttattttgattttaatagtttatataattttgccttacttaca
tacgcatgtcacactttacaaaaattcagacgaccatacaagatttaaatctgttaaagc
atgctttaacagatttgggtttgacttggcaaatgaattctagtcatataaaagttggtg
aaaataatcagcataaggtagatatcttaattaaacaagataatttgtcacatattggct
ttatttggaatgataataaataccacttagttgccgacttgcaactttgggaacaaccct
ggtctctagaagtatttttggataaattatctcagaagtatgcttaccactctattatcg
aagaaacaaagaagcaaggctttgaaaaaaatgcagcagatttcacaaaaagatggatcaa
ttaagttaattgtgcaacgctggaattattaactaaaatataaatgcgggcggagagact
tgaactctcacgagattatctcactagaacctaaatctagcgcgtctaccaattccacca
cgcccgcactgttaaaac--aatattactttatcataatctatactaaaacacaaactaa
-tagtataaactagcttccaagcttaaaagcgtctacaaataaaccatatgttataccaa
ttttgaagttgttgaataaattaatttttatattgaattgcttttggctagagtagacta
ttttactagtcaattcagttgctgcaactattagactggctgcaataataccccaatctc
ctgtttgtccaggaattgtagataaacctgttgaaataaaaaaatcctaacaataaactaa
ttaagccagttgttaactcgctcaaagaataataaagcttattatttaagtttttttatta
agtaagcaaaaaaagttgaaagtctagttttaatcatagtatttagtaacggagttttaa
aaggatgacaagtattaacag-tcattaatattgtcgtatccttttaaaactcgttaaga
caaaaagctgctcttgt---------ttttaaaggtcttgttcgcttaaagcattaataa
gatgctgaaacggctcttgtataatatctttgcgtttaattgattgcaaatctaattctt
cttctatgatttcttgtaataactgtatgcttcttactgttggagcaacaggtacagata
gtgaattgtaagtatcttttagcccatttaatacacgctgatcgagaatatttgtactac
cagaaaccaaggcataactagtataccgtaggtaatattctatatctcttagacaagctg
catatcttcttgttgtataagaatttcctccaggccttaataattcaggctgttcttcgt

acaactgagctgcggcctcttttaaaatattggtagcttgatcattaataatttcagcta
tttttattcgatctagtccacttgaaaagaaagattctagttgtccgacagctgctttat
ctagataacgaccagtaagatcgtaacgatttaatattgctgttatagcatcttgcataa
attaattctcctac-ttgctatcagagcggtattataaatgactaatattgtaatataat
cattagtgtttatgaaatttatttaaaacttattctttattgcttaatgtgcaattaaaa
actcgcatagattttacatatatagagtatacgtagtttttaatta-tttttttataaaa
attaagaaataaagctttttt-taagaggttcgtgaatttcttaatacaatctaagcttt
ccgcccaaataagttttgtaaagtac--------aaaaaaagattattcaataatagtaa

--------------------------acaatattaatatgtatcaa--------------

-----------------agtataaataatctttactccagcatcaaaaaaaatatttaa
catatgttgatacaattgattattcaagtgaaaacttagatttagaccaatttagctatt
ttatcattacaattaataaagctgataaatgtattcttatagagcaattttgttataatg
ctgaaggtcatatttactcaatattcttcaaaggtccaacagctaaacacctttcttcca
taatttgtcattttcaacaattaaatacaataatatccacagctcatgcaatatatttag
gaagagagctaatgaagtcagaattagctctcgtgctagatcaacaatatattcaagatt
aaatttttttaagtattaattatatttatgctagtataatattggtaagggcatgtaac
tcagtggatagagtatcagattccgattctgatggccgtgggttcgaatcccgccctgcc
c---gagtattcagattact-acactaaaacatct-------taatagctagtttgataa
tagtatgggcattggggctgcaaggtttctacattatgaaaagaagaaaatata--aaaa
aaacaagctctccaaaagagcttttagtacaattaataaatgcagaaaacaatattgttt
ctttttctcgaaaattagctcttgcttaaatactgttaattttggtattcgacatgtta
aactcttattcatggcgaatattctgtcagagtcgctcttagtttaagaaaagtcag-aa
aaatatttatctttacgtttt-tacctatttttatttaaataaaggttagta-ttttcaa
cttttatagtggacgtgggttcgagtcccaccagctccatcaatttgatt---------t
gtataattagctagttcaacctttttaataaccagatatataattggaggaaatatggat
tttataacaattactaagcccgctttaaagcaaattgcaatcttgaaaaatgatcatgaa
aatgatgtacatctaagaataggtgttagacaaggcggatgttcaggtatgtcatactca
atgaattttgaacatgttgacaatttaaaagatacagatgaacggcttcttctagataat
ttttctgttgtctgcgaccctaaaagtcttctttatctttatggcttatcattagatttt
agctcggaattaataggaggaggatttcaatttctaacccgaatgctagccaaacttgt
ggctgtggtaaatctttttcaggctaaca-ctctaacaggtttttatatattttgtatt
acacatagtattttagcaagtcg-tttaatagagacaagcaatgctctgttaaaaac---
----------------------------taatttcaagagttttattgtattatt-----
----ttatatatttattatcaactacttgaaaatggattagttaagcaatctaatttgtt
atcagtatcaaactttaagtgattaactttttattgatatatgctaatcttt-ataagcc
taaacgtatctgtacattacaatttttgaaataaaaagagtcttaacataactgtcaa-
----------------------gaagaaattata----------tattttataaatactc
aacatgaatctgtcacaacttattgttaattcctctatgatgaaaattcctaataacttt
tattgcaatacgaaatttatttcttctgagaatttaagtcacttacaattatcaaaaaca
aatttattatctcctacaaaaataattgcttttggactacctaattttgatttcatctct

acacagattacaagttctattagggaatcagttgttgtatctagtattgatgataataat
ctttttgctgccccaactaatgattggataaaaaaacacaaggataaaaatcatcattct
ttgttttataagaaaatctttaaagctttgctaaaccgtaaaatcagagttgttgatgag
ccatt------ttataactctgaaataaataaagaaaagagcataatgacaagtcaaaga
tacgtctggggtaaaagctggaagccttcaattattttatcttgcttaaaaaacaaaaaa
aatagcaaaatttctgacgcatcgaaaaaattttaactgaacagttatctgcatcacca
gtatttgtggtaaaaaatggttttaatgaaattattttaggtcacccattatctcgtgtt
aaaacaggaagagttaataatctgacgcatgcattttctaatttattaaatcagtctaat
gaaacttatcctatatctaccggtttattctttttcatccagatgatgcatttgaattc
aaagactttataatatcggttaatccactggcggccaaacatatggagataagcgtagaa
cccgttggtttacactttgcttataaaatgaatagagatagatcgtcagatactcaattt
tcgtttattccagatttaaagaagtaggagatttattattcaaatatagaaaagatcat
catttagtttttcataagaatcagcattatggcaaagatttttttcaggggcaaccaata
tatatgattcagccgatcactttaaagatcgatccggaaagctgaatattatcaaattt
actgggctaaatgacacaagagaaattatttttactaatcttgaagctgctaatagatca
tggacacattttataaagaggaatccacaattaaaatcagttaaaaatcctactttatta
gtttataatctagaaagtttttaaaagatcaagaacgattggataatcaagatttacac
aaatttgttgtgatcactaataaatcatcttatcttaccacaaaagagttaatagcttta
accgattctaatagcttttttaagcacttaaaattaaatataaagcctaaactcttttt
gtgaaactatggggaagacggttattttccaccttaacttatgaat-aaatgctatt-gc
ctttatttcctcgaataatattctataactagtaattcattgagttgtaaagcgacccat
tccctatcaatgactccattgactttccctgataaattagatttatttaattctaagtgg
ctaggaatattagctaatccagggaaagctaagtaattttcgactagttttcgagatgct
tcttgatttttcacactaattgattctcctggtttacattgataactacagatagatact
acttgtccattaatacaaatatgaccatggttcactagttgtctagcggcaggaattgta
ggagccataccaagtctaaaaacagtattgtctagtctcatctctagtagttgtaacaaa
atttgacctgtcgacccttgtaatttttagctgcttttacgtacttaaataattgtttc
tcacttaatccataattaaatcgcaacttctgtttttcctctaatcttacagcatactca
gaaggttttctagacttttgtccatgttctccaggtggataagatcgcttaattgctttt
ctactgagtccgggtaaatcacctaatctacgggaaatgcgtactcgtggccctctgtat
ctagacatattatttaaattctcctaatgtaaatggattaag----------taatgagt
agctatttttaacacaaaaagagcattaaaacaaagc-ttttgctaaaaaatctttata
agaaatataggcttggacgaaa---cacactatgatgc-tttttaagcgggtagcgggaa
tcgaacccgcatcattagcttggaaggctaaggttttaccactaaactatacccgcaata
ttaaacagattgtcactaactcaatataacatagttatgttaagttaagataatcttaaa
tttaatagaaat-tcaaattatttacgctccttctaaaactacatcaagaaaacgagcaa

tttcggcagcttgttcttcaacttctgataataataatggttgaccaaccctagttaatg
gaattactcttttatcttttgtacataaataaatttcacgtcttggattgagcccctctt
taatatctatttttattgatttgatttcttttatactaaactgaagacagatttttctat
tctttccagggaaaccaagccgaaaaatttttacaatacctttatctttgttgaattcat
tatatccagcccctatattccaaataatagttagccatagaaacaaactcaggaagattc
ctatactgccatagaatgtcattacaataccttgtggaataaataccaaatcggttgaag
ttgtaaagggtaacaaatcaacttgaaaataacttgatagtccagcaagaagaaagccca
aagccccaataaatattatagtagcccaccagtaattactcagtcttcgtgaacctaaaa
ttaaatcttttctaactgagtgtatagataatgtttttttcataatttgttcttttaaaa
cagta-tactaatgcaaaatggaaaaattttgacaattttt--attagcgctttagattt
tttaaataagcttgattgcttttagccctaaaaatagtcctgttgctaacccaaaaaaag
cagcaagaaatattgtatatcctaaaaaaactgaca---tatta----ttattattattg
ttacaatagtgtttacaaaaatagtggaaatctattactagggtattatattacgttaaa
tgccttgctgtctacatgaaaaaactttttcagtttttagcattgtgttcatattagaa
ataatattctagtcaatttattaaacttctggagaacagaatcttatgtcagaatttatc
aagccttataacgatgatccttttgtaggcaatttgtccacgccagttagtacatcaagt
tttagtaaaggacttctaggaaatctaccagcttaccgtcgaggtttatctccgcttctt
agaggattagagataggaatggcacatggatacttttaattggaccttttgataaattg
ggccctttacgaggtacagatgtagcattactagcaggatttctatcttcggtcggtctc
attattattctcaccacatgtttatccatgtatggtaatgtatcttttactagagcagac
tcgaaagatccattacaaacttctgaaggctggggacaattcactgcaggatttctagtt
ggagcagtaggtggttcaggatttgcttatttattgctagctaatatacctgtattacag
actgcaggtcttagtttattttcttaagctagtaaggggacttgaacccgtaacctactg
attacaaatcagttgctctaccaattgagctatactagcaccttttttcatcataacatat
aaagatatttgttgcaatctgttattttcagatgtaacaaatatctttttttatttaaaca
gaactgttatattaacac-------gtatattaattagaatctgaattatctgactctgc
agaatattcttgatcacagtttagataataacaaattgtttgatctagacaagttgcaga
ccaaccaacaggtgtttcacctgataattcttgcatatctccagaattgtaaaaatcatc
atttggcttattactgtttgcttgcataaatatcactcccaaaacactctgaacttaatg
tgaagtaatttaacttctctataatatattaacacaaaatattaaatatggtgattattc
tttaattgtaaagatgcggctaactttataaagtttgagtgagagtcttgatagcttttg
ttgaaataaacattttaatccatttattttgctctctcgaccatactttttttatgttgta
aattaactttttgtagtttttttgttcgtttatgagaatgagatactgcatatccattat
tagctactttccccgtaagctgacactttttttgacatgatttt-------ttaacttata
tatttatttagagtacttgctaaagttgactttggcacagctccaatcactgtgtcaact
ctttctcctgctttaaagatcattaaagtaggtatacttctaatgccatattcagctgca

atagtaggattttcgtctgtgtttatttttactaccttaatagatgattcatattcttcc
gcaatttcatcaactacaggagagaccattctacaaggaccacaccatgacgcccaaaaa
tctacgagtacaggtaagttattgttaataacttcttgtttgaaagaggcatctgtaact
tgagatactgacatattctttaacctttaatatatgttccttgctaagcaaatcttatca
caaatttcaagaaaaatcttctatagtgaattactattagtgttgaattaattttttaaa
aattagacattaaaaaaacttattacctatataaataaagtggtggccgagtgatatctt
gatagtgatagtttttatattaaggttgaacactagaaataacttttcaagcaaattttt
atttgaaaa--acttctctgtgaccataaaaacttaattatattacaatatattttatta
tttgtaacttagttctgataatggtataaacaacgcaaaagatactgccttataatcaag
gaggaatacatgtctcaatccgtagaatcacggactaggattaaaagcgaacgttacgaa
tctggagtaatcccttacgctaaaatgggctactgggatgctgactatgtaattaaagaa
acagatattttagctcttttcagaatcactcctcaaccaggtgttgacccgattgaagca
tctgctgcaattgcaggtgaatcttcaacagctacttggacagttgtatggactgattta
ttaacagcttgtgacttatacagagcaaaagcatatcgagtagatccagttccaaacgtg
gcagatcaatattttgcttacatagcttatgatattgatttatttgaagaaggttccatt
gcgaacttaactgcttcaattattggtaatgtttttggttttaaagctgttaaagctctt
cgtttggaagacatgcgcatgccagtagcttatctaaaaacgttccaaggtcctgcaact
ggattgattgtagaacgtgagcgtatggataagttcggtagacctttcttaggtgctaca
gttaagcctaaactaggtttatctggcaaaaactacggaagagttgtatacgaaggctta
aaaggaggtcttgatttcctgaaagatgatgagaatattaactcacaaccatttatgcgt
tggagagaaagattttttatattctatggaaggtgtaaataaagcatctgcttctgctggc
gaaattaaaggtcattaccttaacgtaacagctgcgacaatggaagatatgtatgagaga
gccgaattctctaaagaggttggtagtatcatttgtatgattgaccttgtgattggttat
actgcgattcaaagtatggcaatttgggctcgtaaacatgacatgattttacatttacat
agagctggtaactcaacttactctcgtcaaaaaaatcatggtatgaacttccgagttatt
tgcaaatggatgcgtatggctggtgttgaccatattcacgcaggtacagttgtaggtaag
ctggaaggagatcctttaatgattaaaggcttctacaatactctacttgaaagcgaaaca
gatataaacctacctcaaggtctgttctttgctcaaaattgggcttccctacgtaaagtt
gtaccagtagcatctggtggtattcatgctggtcaaatgcaccaacttcttgattactta
ggtgatgatgtggttcttcagtttggtggtggtacaattggacatcctgatggtatccaa
gcaggtgcaactgctaacagagtagcactagagtccatggttatggcaagaaatgaaggc
cgtaactatgtagcagaaggtccacaaatcttgagggacgctgctaaaacttgtgggcct
ctacaaacagctttagatttatggaaagatattagttttaactatacttccacagataca
gctgatttcgttgagactccaacagcaaacatctagtttaatgacgacttactaatactt
taaacagtcaattgtaagtagaattaacttataacaataaggagcacagaatagtgagac
taacacaagggacttttttccttccttccagatttaactgatgagcaaattaataaacagc

tttcttatatcgtttctaaaggcttatcagcaaacgttgagtatactgacgatcctcatc
caagaaactcctattgggaactgtggggtttacctttatttgatgtaaaagatgcttctg
ccgttatgtatgaaattagctcatgcagaaaagcaaaacctaattattatgttaaagtta
acgcttttgataatactagaggtattgaaagttgtgtaatgtctttcattgtaaatagac
ctgctaatgaaccaggattcttattacaacgccaagacttcgaaggtagaactatgaagt
acagtcttcatagctatgctactgaaaagcctgaaggagctag------atattaattaa
gaattaatattggctaatta--ctcccctctttaaaatcacaattgctaagttaattatg
atttaaagaggggtaatttcccaagtaaattttgactaaa-tagattggataattaaaa
atacacagaaataatgcaatcacaggatataatttccaacgatactcttgttaatttaca
agaagaatatgatagaacacaaatccaagaagttttaaatgagttaaatcaagaacttat
aggattagtacctgtaaagaccagaattcgcgaaattgctgcgctattattgattgacag
attacgtagaaaactagaactagtttctggtaatccaggattacacatgtcatttacagg
tagtccaggaactggtaaaactacagttgctatgaaaatggcggatattttgcatagact
tggatatataaaaaaaggacatttgttaacagttacaagagatgatcttgtaggtcaata
tattggacatactgcacctaaaaccaaggaagttcttaagcaagcaatgggcggagtttt
atttattgacgaagcttactatctatataaagcagataatgaaagagactatggctctga
agcaattgagattttattacaagtaatggaaaaccaaagaaatgacttagttgttatctt
tgctggatataaagatagaatggaaaaattctacgaatccaatccaggactctcttctag
agtagctaatcatgtagactttccagattatacttcagatgaattattacaaatagctaa
aatgatgatagaagaacagcagtactgttttacagaagaagcagacaaaactcttttaga
gtatactgagcgaagaatgaaacagccttattttgctaatgcaagaagcattcgcaatgc
tattgacagggctagaatgagacaagccaataggattttttgccagtggagaaaaagtatt
aacaaaagctgatttagtaacgattgaagcagaagatatcttgaaaagtagattattttc
attacctgatgcttaatattcacgtgatttaatgaaactatatattattaaaaag-cttc
ataatcagttgcaatttttttgaaaaacccttattatcatcttaatgaaggtg-tggcgg
catagccaagtggtaaggcagaggattgcaaatccttcatcccccagttcaaatctgggt
gccgcctagtactaaaaa--gggggtgtggtggaatggtagacacaacagacttaaaatc
tgttgatttttagtaatcgtgagggttcaagtccctccacccccatatattaacttataa
ataaaaagagatgtgtatttgtattaattgcaaccatattacaaaatgtaagacctatca
cttaatagaatctcaacacaagcagcctcatattactagaagtccattgtttatacctaa
atatcctgtagttcatgttaatatatctaacaatggtacctataatcaaattgattggga
tttagtggagtgtttatcatttgtagaaaaaccgaatagttggaatttagacgctaatta
gttacaagaaaaattatgtactataagcaacaactctatttttttagatactcggtattga
ttttggatgttctaaccaataagattttacctgttcttgctatctctataatgccaaatt
tagttagtaattgttcaatagcaacaatctttcctggatctccagtaacttctataatta
aaagatcttctgcaatatctactatcttagctctaaaaatttttacaatttctaaagctt

ctgttctagtctgagaattgatcttaatcttaattaacattagttctctttcaactgaag
gaatatttgttacatcttgaacattaaggatatttactaacttatataattgtttagtaa
gttgttcaatagttctattgtctccttgaactaccatcgtgattctagagaccccaattt
gctctgctggtccgactgctaaacttgcgatattaaagcctctccgggcaaatagaccag
atattcttgacagtactccggcttcatcttgaactaaaactgataaggtgtgtttcatga
aaaattaattttaatctatttgatctattaagtatattctaagaaaattacagaaaaaat
actagttagtctacaactaaattatctaaactaaacaaaatcctactatcagataatata
tctaccttgtaatgctatactattaacttagtatttaatgatattctaattgcaaataga
atgct-aatttttaaaatttgttaggaacttgtgctagaaaaatacaaaaacagtaaaa
aactctctcgagatttacctcaaatcaatgatcgcatcagatttccgaaagtccgagtaa
ttgatgacgaaggtgaacaactaggtatttttgtgcctgaagaagctatacaattagctg
tccaacaaggtttagacttagttgttgttagtgataaatcggacccgccagtatgccgaa
tattagactatggtaaatataagtttacacaagaaaaaagagctagagaagctaagaaaa
agcaacataacagtagtattaaagaagtgaaaatgcgatacaagatagaagagcatgatt
ataaagttagaataaatcaggcatctaaatttattcaagcaggagacaaagtaaaagcaa
ctatcacatttcgggggcgtgaaatccagcactctaatttagctatagatttattgaata
aaatggcaagcgatctaaatacaatagctgaaattcagcaagctccatcaagagatggca
gaaatgttataatgctcttatctcccaaaaaagttggctaaactaatttttatttaaatg
catctggctggattcgaaccagcgacgtctctttcgaaatggcggattatgagtccgctg
ccttcggcccctcggccacagatgcatcacttaaagtttataactcataaagcatataaa
aatcaatgtttagctatttttaataaagtctataagagtctattattcattcatattgtg
atatgtagcaactgctgaaggggaaacttgatttaagtatcgaaatatccagtatttaaa
aattgtgtcgagtatgactggaaaagttgaaataaataaaaaaataaaatctcgactttc
tggaagtcctaaatgtcttaaaattacttcaataataacttcccatccatgaggagaatg
aaaacccacaaacatatcagtaaaaagaatgattaaaaaagctttagctgtatcactaag
accataaataatttcatttaaaaaagatttgacgactgcaatttgtctttgtccagttat
cattagtaatataaagacaagtattgatacaagatcggataaaatatttttaacagcatt
tgcactttcgtttgcataatattcgcccaattctctagccttcagctgtactctttttc
aatgatttcgtaagatatatcttccgaaggattcagaagaacttcaaagtgaattttctc
ttcaaatctttgtaattcagcaaaagctctttcctcttgagaggaatttaaaaaaatttt
aggctgttcttggttccataaataatcaatacaaggtccaaatacaaaaaactttgaagc
ctgattaacgagtactggagagatgaataatagtaaaatatattttacggaagtaatagt
ttggtgcctagatattctaaactcctctatagcttcagattcgccattaggatctaactc
tttacgaaacttttcaaaagtattagtaattgacctaggtattggcccaacctttcaaa
ggcgaattgattatttcgttttaaattccaatatttcatttttattgctgctaaaattt
ctattaatcaataattgatcacgtattcactataaatattaattctagaactaaacatat

aaaacaatatttatgaaaaataaaatacaattaaaaattaattatgcattagttctatct
gtggctacagcaatgtatggatataaatcttatcagccaactataaatacactcttaatt
aaagatgaaaaaaactggccttgtttctctaaaaggcatactgcctttattcctataatt
gtattaaaaaaaaatcatcaaaatccctgaactcagaacctagaaatagtaagttatatt
ttaatggaattggaagtagaaaatttatcgagagaattgttaatctacttgaatatttag
aaaaacaggcaaactatactgaatatctgaaccaaattaaaactagtggttatttttctt
tactctaccaggacagcaatacaatgtataagaaaaatattagtaatatttttttattac
ctaatgatattctaaaaagaatctatatttgcaataagaaaaaaaagcttataccacatg
cattcttaataaatttatttcggaaacagataggctatccaaaaagttttgctaattta
attgggccttctccaaaattatgaaatggtattatgatagaggataccaatggtctctag
ttgaagttaaacaagcgtctgatgcatcttctattgtaattgatattcatgaaggggtag
tgaagacaattataacagaatactacactttatcttataaaagactctcaggtatctcat
acatagagtcaatagagcagtatcttggagtaagagtcggagctccattaaatataattg
atttacaaaaaaagattagttacttaaaagataaccaactagttggcgacattatttata
gtattgaacgatcaaataataactcaatgagtttggatgtcaaatttcagatacaagaat
taaaagataaagagataatagtgcttgcagaaagttcttcta------------ttatct
cgcatgcatgtgatttactgaatc---aatataga---------aataggctagtagctt
ctaatataatttcactatcaactaataaattacacgcatgctattttaattataaactcg
actatcaatataagtatataaacacctctaatctcgtacaattgttaacttactctattt
cttgtaggaagacactacttagccattattttaatttacaaactttaataagctggacaa
aaaaaaatactattggatttcagctatatttacgaaatctaagctttggaaaagcttttt
gtgtgttcagtatgaaatttatcaaaaatggacttaatataagaattttatatataaatc
cttcattgatagttgatcagaattttatctttcaatttgctatacaaataattaagcaat
atcatactgcta------aaccaccagcct------------------------------
------tatttttaagcaatctagatcttgaacaatatgttgctgaaagtctattaatgt
accatttacatcttgcttctcaatatctgaaaaaatattattatctcggattatgcata
cagattctttatttttcaactctgagaatctccattttcattttgatgatagaaatcacg
cgatcaacagttatgataattttaaacaaaatacaaaaatatttatcaagagtttctct
ctttattatttagtttacgttatcaaaatttttaattatttaggttggccttcaaaaggcc
attttttgaaataaaatctttatacttggctccatttcaaaagagtg------------
---atttttctgatagtcgtaagaccctattcttccacaaaatgtcgttaaagcaagtat
ctaattttaatttaccaatatcttttagaagccactttaaccatattttagttagtacta
ttaaatgccagtcaaatttgaatatgagaactgtatctttgctattaattgattcccctg
ctgaatatatgctatacaaatctattcttaatttctctattaaagttaggatgcagtatt
tcatacctatgagcaacagtataagagtatctctattctataattatttagattgttttt
taataagatcatctcagagtttcattcacatatggcaagatttaagaactcttaaaccaa

ttccaaatttttggttaaaaaaattttcatatggagttggaattcagcttaaattaccaa
ttaaacagatgccacctctttcaattgaatatactataactagtagtcgctacttttgta
tttaccttcgtacttattatcaacgataactattactaaaatgaaaaatttaatttcttt
ttttgatcgcagcaaagggaagtggatttcacaaagaacaacttatgaattatctaataa
aaacatgagttcagcacagtctcagatgataatgaaaataggcaaatcattgtcaggatc
tataatacttgcatcgttaaattggggagacatttatagacaagttgcccattacgctaa
aagtcatagccggagtgaatataataataagtttaacttacaatttaataatcaactaaa
taaccataaattattgacgctgtgcatagtgacagatcctagtctaatcagttttaaaac
tcgctatggaagtactacaatagatgaaacatattggtttgcaacaaataatttacgtct
aagtactagtattgttaaacgattcaatacttgtgtggcagtatctttttgttcagaaat
taaagtttaa-actctatatagaagcaactaataacctttta-ggctattagttgcttcta
tatatctaaattttctattaaaaattat----aatactatattattctaagtctttacga
ttaggattcctagaaggatcattagagaggaagccaaagacaaacaaagaaataaagaaa
ataacggtagtatatacaaagattttcaaagtaaacatattttcatccttaaaagctatt
agtgcg----atatttacatatctccacataattttatatcatgatatgagttccagtac
tattttataaataaatcaatatctttatttttagtaatgatttatatttgaatatgcgt
gatgtactaaatactaaatcttttccgatttgttttgtcttgtttgtaaatattttgtta
tttctatggagtttaccttcaattatcacatagtctaacttctttagctgtctaaaagta
tatgaagatttttgttccaaatagttagattgattataactaatcgttttctttttaac
agtcttgcttttaacttaattatttggcttttattttcactaattctgatactcttacaa
ctaagaatttggactagcagagtacagctattcataatttataacaa------tttttta
aagaatctactaaaatagatgattttacaaaactaaaatgatatttcattatcttgttga
ccttgctcataagtaatgtctttcaatacttttaatatttttccaaagtactcttgcagg
tatttttctaaagatattacttccaacctgtcaattccaagaatactgtaaacttcatcc
atcggagctgtaaattgatctccgctagttaaaatttcagcaaatgctaaacgatcagaa
atattccatgtccattgaagagttttagtaatttttcttaaggcttttaataatccaatc
ggaatttgtgagatctgtgtttttttgcccagataatttttcacataatttaataatttca
gctgatgtccaagcggtattaccaactagcggcaggattctattttctgtggaaggtact
ccaagacttttataactaacttggctgcatcctgagtatcaatgtatgcaatgggcgta
gattctcctgtaacccagactgacttcttgtctaaaataggaatggcatactggttgatt
aaaccttgaaaaaatcctcctaaagaaaacacagtatatttcacattggacttctgaagg
aaatctactacctgagattttaagctcattaatggaacatctgggtatttctctgaattt
agtattgaaaagaaaataaatctttcaactttagccgctttagctgcttcaattaatgca
atttttccatctaaatctatttttttctgcattgtaaggatcagtaggacgagatgtggaa
gcatctatgactgctgttactccacaaaaagattgcaaaatactttcgggtaattttaaa
tcaccatatataagttctgctccccactctttgagaaaagcagactttcttaaatttctt

accatgcattttacattgtagccttcatctaaagctcgtcttacaatttgacgtcctaaa
gttccggttgctccaattactaaaagagtcatattagttaaaaataagtatgtataaaga
cattgatttggttgacggcagagagtaaattaggtagagagggaatcgaaccctcatgac
cgaagtcgtcacattttgagtgtgatgcgtataccaatttcgccatctaccctattatag
accaactactacaagtaatttagtctttagcgtaaagataatactaagtttcaatagata
tgtcaacttttaaatttatattaataaaaaaacggagtctcttatgaccacttttgaatt
aattccttatagattgtatttaagtcaatctaacacatggatgcatagaataaaagcaga
aattaagatatatatagtaatgcttttatggatttcaattttactttttcttactttaa
actatgtattattgctttaagcttaattgcaataagttttactataagaagtaaacagaa
tattattcaacaacatttgttacagacgttattaatgacgttcttaactactgttttgtc
ttttagtgtggctattaattataaacaatatgcagaacaagaacaatctcagtatttatc
tgattccaagaaatataaaaattctaggagctactattatatacaaatagctaacgatca
aaggatcaaaagacaatacttaattactactttaaagccttccttgtactttttttattac
tatatattctattaaactagttatgataacaacctctccagaagttttagtaattactat
ttatagatctagtataataaataaaatatttaaaaatgaattgctgtttatcttccttct
ttcgtcacatattgttactagcattattaatagagttgataaagttattcaagtaactag
cttaagaggaagcttgagtttatataattcatttacaaggcttttaatgttttctttgtt
aatatttcaagtcttctttttagagattatccgggaatcaaaagagatagctcaagctct
ttatactagaaatctcaatcaagaaaacaataatttttttgaaaatatatacagtaaaatc
tcactttagtgatcggctaaatataattattagcactttgtactttattattttagctat
agcgtaataaaatccacttattcttctgactctagtaatacgaagagtaataaaattagt
tagtagtttaaatagatagaat-taacatattatataaataattaaatgtttcattatgg
tcaatactcaaaatcaaatttcacaaacttcagaacttgattatatagttaatcaacctt
ataaatatggctttaaaacttctgttgaatctgagcaatttcctaggggaataagtgaag
atattgtccgcttgatctcaaagaaaaaagatgagcctgaatatctgttgaattttaggc
ttagagcatataaaaaatggaaaaagatgagtaatccgtcatgggctcatattaagcatc
cgaatatagactttaataatattatttattatgctgttcctaaattaaaaaaagaattga
aaagtctagatgaggttgacccagaaattcttgacacttttaataagttaggtatatctt
taaatgagcaaaagcgaatttccaatgtagccgttgatgctgtttttgatagtgtatcta
ttgcaactacttttaagaaagaactgtctgaagctggtgttatattctgttcaatttctg
aagctataagggattatccagaattaattaaaaaatacttaggtactgttgtcccagctg
gtgataattattttgctgcattgaactctgcggtatttagtgatggctctttttgctata
tccccccctaatacagtttgccctttagaattatcaacttactttcgtattaacaacgaag
aatctgggcaatttgaaaggacactaattattgctgatcgtggcagtaaagtaagttatc
ttgaaggttgtaccgctcctcaatttgacacaaatcaattacatgcagcgattgtagaat
taatagctcttgaaggagccgaaattaaatattctacagtacagaattggtatgctggta

acaaagaaggtaaaggcggtatatacaattttgttactaaacgaggcttctgctcgggta
ataattcaaaaatttcatggactcaagtagaaactggatctgcaattacctggaaatatc
caagctgtattttagctggtgataattctcagggagaattttactctgtagctttaacaa
ataattatcaagaggctgatacagggactaaaatgatccatattggcaacaatacaaaaa
gtagaattatttctaaaggtatttctgcgggtagatcgaaaaacagttatcgaggcctag
taaaaattggccctcagtcatttaattcacgtaattattctcaatgcgattctttattaa
ttggtcaatcatctcaagctaatacatttccttatattcaagtacagaatccaacatcaa
aagtagaacatgaagcatccacttcaaaaattagcgaggaccaaatttttatttttttac
aaagagggatcaatttagaagaatctattgctcttatgatcagtggtttttgtaaagatg
tctttaatgaattgcctatggaatttgctactgaagctgatcgtttactgagtttaaaat
tagagggaactgtaggatgagccagattattttagagatcaaagatctatatgcttctgt
tggtaaaacaacaattttaaaaggggtaaacttatctattcgagccggcgagatacatgc
gattatgggtcctaacggttcaggtaaaagtacattatcaaaagtaattgcaggacatcc
agcatattcattaataagcggagacattttatttttttggacacagtattcttgaaatgga
gccagacgagagggcaagagctggtatttttttagcttttcagtatcctgttgaaattcc
gggagtcagtaattctgatttcctaagaattgcattaaatgctcgcagaaagtttcaagg
attatcggagtttagccccttagaatttttttcaactaataatagaaaaagtagatcttgt
tggcatgcaagaaagtttttttgacaagaaatgttaatgaaggattttctggtggagaaaa
aaaacgtaacgaaattcttcaaatggctttactagatagcaacttatctattttagacga
aacagattctggactagatattgatgcattacgagtagttgcaaaaggaattaacacttt
agctaaatcaacaaattcaattattttgattactcactatcaaagattactagattatat
tattccagattttgttcatattatgagtaacggaaaaattgtaaaaactggtaatgttac
tcttgcccaagatctagaaaagcacggatatgactggattacgcaaacatagctatttag
tag-------aatattgagactaaagaatattctttagtctcaatatt-tttgcaaatac
-aagtt-aattatttaagctttgtcaaaaccttgcttaacatcttcaattgctcttttta
gcaattcttcgctagctggttctaatttttttagtactacgaacattttcaccaaattctg
gtttagaatttttttaaatcttctcgaagttctctaataaattcagccacttttgaaactt
caatatcatctagatagccattaattccagtataaataatagcagtctgttcttcaactg
gaataggagaattttgagcttgcttcaaaatttctcgaagacgttgacctcttgctagtt
gattttgggttgctttatctagatcagatgcaaactgagaaaaagcttctaattcagcaa
attgtgctaactctagttttaatttgcctgctacttgtttcatggcttttatttgcgcag
cagagcctactctggagactgagataccaacattaatagctggtctaatccctgagttaa
ataagtctccagataaaaatatttgtccatctgtaattgaaattacatttgttggtatat
atgcagatacgtccccagcttgagtttcaatgataggaagagctgtcatactacctccac
ctaattcagcattcaatttagctgctctctctagcaatctagagtgtaaatagaatacat
caccaggataggcttcccgtcccggaggtcttctaagcaaaagagacatctgacgataag

cttgagcttgcttagttaaatcatcataaataactaatgttgctttacctttgtacataa
aatattcagctaaagccgcacctgtataaggagcaatatattgtaaagtcgcaggactgt
ctgcattagctgcaactataatagtataatcgagagctcctttatcctgtagtgaagata
ctacttgagctactgaagatgccttttgtcctatcgcaacatatacacaaacaacatctt
gaccttttttgattaataatcgtgtccaatgcaacagctgttttacctgtttgacggtcac
caataattaattctcgctgacctctaccaattgggatcatagagtcaatagcagttatcc
ctgtttgcataggctcacaaactgattgccttccaattataccaggggccattgactcaa
ttagccttgttccattactcgcaggttcacctttatcatctattggccgagctaatggat
caacaactctacctaaaaaagcatctccgaccggaatttgagcaatgcgaccagtacctt
ttacagaactgccctctaaaatatccctaccatctcccatgagtactacaccaacgttat
cactttctaaatttaaagcaactccaatagttttatcttcaaattcaagtaattcaccag
ccataacttcatcaagtccatatactcgagcaataccatcaccaacttgcaaaactgttc
ctatattggctacttctacatcctgatcatacttttcaatttgttgacgaataatactac
ttatttcatcaggtctaatatttaccatattttaatattatttttattaattggaaatt
ctaaagcggtaattagtcagactaatgctatctgtctttttttactttattttaagttgct
acaacatcaaggtgtgaagccatctgtcttagctggcctctaatactagtatcaataact
ttcgatccaatctgaatagtaaagccaccaattagctcgggatcgactgaaataattagt
ttaacttctttagctttagtcataaccttgatttttttctgttaataaagtttcctgatca
gggcttaaagcaatagaagtattaatattcgcaattgttaaagattccatttggtacgcc
agttccaaatatttgctagctatagcatctaacattccaattcgcttgcgatcaactaaa
atcattagaaatgataatgtattttcgctaatttgatcaccaacagttgcaataattact
tctttttttgactctacagtttttagtggattagct---aaaaaatttttagcttgtca
gattgcgctaaaatattttgtattgactgaatatcttggctcaccttttcagtaactttt
ttagttttagctaaatcaagaagagctactgcataaggctgagctatttttataacaaca
ttgttactgctcatagttgatctcctaacttagcaatattattatcaattatacgtagtt
gcatttcggagctcatttgattttccagctgcaaagtaactcttttaagagcaagaaatg
ttatttgctgctggatttgccttcggatctgttgttcagctgtttctatatttgatttac
ctgttatagcaagtctttcaatgtccaattggccttgagctaatatggaacttctaactt
ttccagctgttagctgagcttctttcttaatttgatctataataatttgagtttgagcaa
gttgtttttctgattctgacaatctagcacttgcttgctctagtctttcttcggattctt
ggatagctgctaaaacttttatctgtcttgcacttaaacttgatccgagaaattgcttca
aaacataaataagaccaaataaaagcaacaatatattaataacatttgcttcaaaaatat
cagaattaaagccgaatttgtgttcactactatgttctgataaaatagtaattatttgtg
gtatttttactatattattcatttaaaattca------------tactattttaatatttta
ctctggaatcatgattttaaagattgaccacttaatagtttagctttaatttgatcgctc
aaagtatcaacctgattttctaaagttttaagagcttcttctttttgaatatttaactgt

ttcgatgcttcagcaatcagtttctctgcgttcaattgagctttttttatatcttctgag
actatatttgagcttctttttgagaagaggctatttttaattgtgcattgcggcgtgct
tcagatagatcttcttcatattttgcagctagctcatctgctttaacaagcatagacgat
gcagtagtcaatgttgttcgaatatattcgtctcgttcatctagtactttagttacaggt
ttgtaaaaaatattatttagtaaaaccattaaagtaagaaactgtaatgccattaaaggc
aaagtaccattgaagtcaaataagccaccttcaatttcttcggctaatagaaatggtaaa
tcaatcattttactaattatgtttatagttaataatttggtttcaattaagaaaaataac
tttatagtgcgcttagtctcatcagactaagcgcaaatgattattttaaccaacgtatgg
attagcgaacaataaagacagcgcaacaaccaggccgtaaattgttaaagattccataaa
tgctaaacttagaagaagtgtacctctaatctttccttctacttctggctgtctagcaat
accttcgacagcattagctgctgcactaccttgaccaatgcctgggcctattgccgcaag
acctacagcaagaccagcagcgataacggatgcggctgaaacgattgaatccataatgaa
ttttaatttttttgttatataagtagaaaattgacaaatttcgtataatacttaaaatcta
tataattaagtataatagattctaatat-----tgtaattatagtatattactcttctcc
atgaccttccattgcttctccaatataagctgcagacagagtagaaaaaaattagtgcttg
tattgaactcgcaaataatccgagtatcataactggtaatggtattaaaattggaattag
cagagtaaatacagaaacaactaattcatctgctaagacgtttccaaacaatcgaaaact
taaagatagtggtttcgtaaaatcttctagaatattaattggtagtaatactggagttgg
ctgaatatatctagcaaagtaacctaacccttttttacttaaacctgcgtaaaaataagc
tacagatgtgagtagagacaaggccacagttgtattaatgtcatttgttggggcagctag
ctcaccttcaggaaggtgaattaacttccaaggaattaatgcacctgcccagttacagcc
tagaataaatagaaataaagttgcaatgtaaggcacccacgggcgatattcatgctcacc
aatctgatttttttgcaatatcttgcagaaattctaatataaattccataaaattctgaaa
tttttcaggaattctttgtagatttctagtacctagaaatgataaagttaaaagtgtagc
aattactagccaagagacaatgaaaacttgcccatgcaattgtaggctacctattttcca
atatagatgttttccaacttctactgctgataagctagtatatgggttaagatctatgag
actgttttgatacataataaatatttcttttaaatagtgcaagaatcaaaaaaataag-tt
tttgaagaattaacatgccattaatttattcttaaaattagattattaataaattaagag
caaaaataaattctaatagagatagctatctttagtagtgattattttttgtaaaaaaca
attattgctacctataatgcttttacgtttaattatatacccatatattaattatttaat
atgattataggtattttgctacaaactttattgttttatccattct----agagattgtt
atagtttatttttttatttaacatacctgcgacttcatctgcaaaattaactttcaaattt
tcttctccccctcccagaagaaatcttacaaatcttctaattttaatattttcacctaga
acagcaatattttggtttattaaatcttcaatactaatatcttgattgcgaataaacatt
tgatctaataatgaaagctccttaagtctctttttcattcgtccttcaatgattgtttcg
attctatcaactggtttgtttttcaagtcatctttaccagcttcaattctttgttccaaa

tcaataatttcactaggtatatcctctgttgatacgtattccacattaggcgatgccgct
atttgcatagcaatatctttagctaactttggaactctggacgtcgtgctacaaaatct
gtttcacaattgacttcaactaatacacctattcttccgcctgtatgaatataactctct
agtaaaccttcaatagcggtacgattagatttttgttagctgaagctagccctttttgt
cttaatgattctaaagctttttcttcattgccattattagcttgtaaagctttcttgcag
tccatcatacctgctccagtcttatcacgtaaggcttttacgatttgagcagaaatttgt
agtgtcatatgctttcaaaaatttttaagtatttaaatt----------aattattataa
ttcactttgagtaggatgagttatttcttctaaatctgtttgcccgtatttgccatcata
aattgcatctgctattttacctacaattagtttaatggacctaatggcatcatcattagc
tgggattggaatattaataatttctggactacagttagtatcaagtatacaaatagttgg
aatacctaattttaaacactcttgtatagctgttgtttctctcttttgatctacaactac
aacaatatcaggtaaacgagtcatattttttaataccatttagatgtttgcgaagtttatc
taattctcttcgtaaaacagcagcttcttttttcggtaactgatcaatcataccactttt
atcttgttcttctagctgtctcagacgactaactcgagatttaatcgttacccaattagt
taacatccccccaagccatctctggttaacataataagaatcacagcgttgtgcttcctg
tgccactataccagctgcttgtctttttgttcctagaaataaaactttttaccatccga
cgaagcctgttttataaattcacaagcttctgttaataattgtgcagtttggactagatc
aataatatgtatgccatttctttctgtataaatatagggaaacattttaggattccatct
acgtgcttgatgtccaaaatgaacacctgcttctagtaattctgctaatgtaacaatagc
cataagattattaatatttaaataatgtaattattcgggaaatcccttttttttagaaac
tgat-----tcaactaacttatttgaattttagtaaactaataataaattaacacacttt
taaagcagatcaataaaatagatcctgaagaattcat----tctacacttttattgctaa
aataattacgcgcggttctgtcgtctaaaatgatatcatctaaatcatctcgtacagatg
aaatagtactttcgtcatcagtatttggagttaaattctttttctctaacgatgagctat
tatagttatcatacatattgaaagcagtaccagctggtataagtctacctataataacat
tttctttcagacctcttaaccaatctaatttaccagatatagcagcttcagttaatactt
ttgtagtttcttgaaaacttgctgcagaaataaagctttctgtatttaaggaagcttgag
taatacctaatagtacaggcctatatgatgcattaatcttatgtctcaaagtcatggctt
tattagtctgttcaatcttttgtaactctacaagctctccaggtaaatagcctgtttctt
caccattttctattttcactttagaagtcatctgccgtactattacttctacatgcttat
cagaaatattgactccttgagattgatatactaattgtacttctttaacaagtagtaact
gaatctcttgaaagcttaatcgagcagcttcatataaggctaatccacattcaatataca
aattaaaacttgcgtctaggatatcatgaggattcaacaatacgtctgttttctttcttg
cttcaagaatttcttcaattcttggaagaccttgtataatgtcacctgtttttgctctat
caaaaaccaacacagctaatgtttctccccttctaattaatgcattgttattaacatgta
aaatggcaccattagaaaccaagtaaggtctagcaattcttaatgtaactgatctagaag

atatttcaataatttgtccagagtctaaagaagtcatattttcagtaataaaatcaccgc
agcgaatccaatcacccacacttactttaactattgattttcctatattaaaggtctttt
tatcagaagatgtcgcaatcaaaatgcgcctactagtattcttttcagaataaatttcct
caacagtaccttcactcattgcaataatctcagtactagcaacaactgtaaatggcttga
tatattcaccatttttacaataattctagtttgactttgttgcttctctgatttattta
catcgatacttttaattgataatgtttcaaaagttgacagccttaagtcaaaataattat
tgccactttctttagcagaaaactcaattgaggatattaaagtatcatctcgatgctcaa
tttcagccacaagattagttgttactaaatgaactccttctatagatttcactctttctc
cgtcccgaaaaggagttcttttaactagctttagtttagtttgacttggtttctgtgaaa
ctagattctcaattagactggatttgtttctggtattgaataaacaataacaggtctaa
taagaatataaggcatttgctcgttttctatatactcccaataaactaacttatctgtag
aaatattattatgaagtgtttctccaggtcttaaaaaacctctactttatcattattac
tatacacctcactaagtttatagatagatccaggcttaataataatttctcgaactattc
catccttttggattatttcaacaatacctgagcttttagaaaaaatattttaactagct
ctgttccactttctataacatctccattatgcactagtagtagtgatgaatctttattaa
tttcgtgagtttcttctgaaatccataagatatatcccggacttaaaatttcgtaagcat
ctttatctgaacctgttttctttttgatacatttaagtctaaatacttaataatacccc
cacttgccgttttgtacgtatcagatattagttctgcgactgtgtaaccatcttgaa---
ttttttggtttgggatagctttgaaaagaaatttttgtttttttctgtttctaaaatat
acgattctttttgtggattacatctgtataaatataacaacctggaattacaatagatt
cagtaataacttgtatattagtaatactgctattagtattctggtgtatccttacttctc
cagcataatgatttattaattctgtctgcgcaagtatcgtaccagctttagttttatctt
ctttatgaacaataacattagcggaatcagatatactataaacttcacctgagagcaccc
agatcaagcctccagttttagtaattcttgtagtatattgattattatctgtttcttcaa
ctgttaagttggagaaacatattttgccagagagatctgaaactacatgtttttgagctc
tttctgtcatgagtcgatttctacggggtgattcagcaatcacttgatccttcgatacta
attcaccatcacatattaaaagagtagtatctttacttaagttaattattgacttttgat
ttttcttagatctgattgtaacttgggttggctttttttgttattaatgcttgctcaccat
gtctagtacgaacatctgtatacgattcaatatctagatctatcagttgaccatctatag
gtgagtaaatttgttctgctaattcaccggtaaatactcctccagtatgaaaggtcctca
ttgtcaattgagtgccaggctcaccaatagattgagccgcaataataccaacagcctctc
ctaaatcaactaagcgaccatgtgcaagattccatccgtaacaatactgacaaacagaac
ttctagagttacatgtaacaggagatcttactaaaactttttt---gataccagctttag
ttatctcttttgctaacttaggactgacatcttggttcgtatgagcaattaaacaatttg
tctctggatggaatacattttctgctaatactcggccagataaagcttgctctagattaa
ttaatattttctgcgtatctactaaatcttctagtatgatgcctttattggtcttacagt

ctacttctctaattattacatcttgagaaacatcaacaagtcgacgcgtcaaatagccgg
aatctgctgttcttaaagcagtatcaactaatcctttccttgctccgtatgatgatataa
aataatcagtaacagttagtccttctctaaaattactcgatattggaagatcaataattt
gtccctgtggatcagccataagccccctcatccccactaactgtcttacttgagaaatat
tacctctagcacctgagaacgccatcatataaacagcatttagaggatctgtctctttaa
agtatttaataacttcttgtttcaaggattcactcgcattattccacgtatcaataactt
tttgaaatcgttcaactgctgtaatctcgcctcggcgatatttgttttcagtggccttaa
tatcttcaatcgtgcctaataagagactttgcttgctgggaggaattcttaaatcttcta
aacttaaagaaattcctgcttgggtcgcataatgaaatccaagatctttaagcttgtcgg
ccatattggctgctcgggctataccgtaattacgaaaagcccagacaattaaatttttta
gttcgtttttatcaataaccttatttgaaaaacttggctgtgcaagacttcgtctattat
ccactaaatcttctccatattaaatatttgtt--attaactatgctactaaagactcttg
aataattttattaaagattatacggccagctgttgttctaatatattgcacaattctctg
attttcagcatcttctttatgatatggttattaaaaaattttgttgtactcttgtcaga
atgtttttcaatcttaattggatcatgaggttgatcaccatctactaatccatcaaaacg
agcccatatataagaatgtaaatcaagtttcttttgctcataagccataactacatcttc
taagcttgcaaaatactgattagatccctgctgctgagatggattattagctgtcaaata
atagcaacctaaaaccatgtcttggcttggcataataattggttgacctgttgctggaga
gagaaaattatgaggggctaacattagcaatctagcttcagcttgagcttctaaagacaa
aggaacatgaacagccatttgatctccgtcaaaatcagcgttaaacgctgggcatactaa
tggatgcagcttaatagctcttccttctacaagaataggttcaaatgcttgaatacctaa
tctgtgtagcgtgggagctctattcaatagtactgggtgcccttgaataacttcatttaa
aacattccaaatagaagattcatttttttgaatcatctttttggcagctttaatattgtt
aactaatccctgcaaaattagtctgtggataacaaaaggctgaaataactccaaagccat
ttctcgtggcaatccacattgatgtagttttaaatgaggacctacaactattacagatct
accagaataatctactcttttacccaacaagttttgtctaaaacgaccttgttttccctc
tataatatctgataaagattttaatggtctgttatttgcacctacaacagttctacctct
acgaccattatccatgagagagtcaacggcctcttgcagcattctttttttcatttctgat
aataatctccggagctaaaattgactttagacgagataatctattatttctattaataat
tcgacgataaaactcatttaaatctgccgtcgcaaatctacctccatctaattggaccat
aggccgtaagtcgggaggtatcacaggaataactgtgaataccatccaagaaggatctgc
accggttgctatgaaattttcaatcaatcttaagcgtttcatttttttattaaattttaa
tgaaggtgtcttaaaactttttggtggatttgtagcttcaaaccttaaggtttcagctat
gtcctctaaatctaaatctttcaacagtttttgaatagcttctgcacctattcctacttc
tacttggttattctcatcttgattttggtagatttcctcttcaaggctcttccattcgta
accttcgagaagctgcttgtattttaaattaatatctgtattagattgcgtaactacata

agaatgaaaataaacaatttttctacttctttgacttttaagtctaatgctaaagcaat
atagcttgtacttccttttaagtaccaaacatgagttactggtgaggaaagttcaatgta
agccatccgatgtcttctcacgcgtgattctgtaacttctacgccacatcgttcacaaac
tatgcctttgtagcgaaaacgtttatatttaccacagtgacattcccaatccttaactgg
gccaaaaattttctcacagaataaaccatccatttctggctttaaagttctgtaattaat
tgtttcaggctttgtgatttcaccaacaatttgtccgttaggcaaacttctttcgcccca
ctgtcgaatttttcgggagaagctaaactaatttttacgtagtcaaaatattgctcaaa
ctttgtcataaatttgaataccttaaaatcaaataattagccaatttatttacatgcttc
atattcaaaaatgaatatcttaataaaggaattgttcaaaatcgtctactggaggagtat
cataattagaacgatctgctcggttatctttagaatccgacatcaagtcaacttcaactg
ttcgcctttggccatcttcaaatagtttcaatttatgtactgcaatgtctaaacctaatg
attgcagctctcgcattaagactttaaaagattccggtgttccaggcttcggtataggct
tccctttaactattgcgtttaatgcttcatttctagcttgcatatcatccgactttacag
ttaataattcttgcaaagtatatgctgcaccaaaagcttctaaagcccacacttccattt
cacctaatctttgacctccatgctgtgctctacctcccaatggctgctgcgtaaccagag
agtagggaccagtggatcttgcgtggatttatcatcaacaagatgaacaagcttaagca
tataagctctaccaactgtaacaggattatcgaaaggctctcctgttctaccatcaaaaa
cttgcattttacctggatgctgatcattaaaaagccatttattgctagtaatcaatgagg
cttcttttaattttctatttacaagtgctcttgatgcttctgctccatacatctcatcaa
aaggtattattttaaatctttttcctaaatagccacctgctaaacccagtaagcactcaa
atacttgaccaacattcattctagaagggacacctaaaggatttaaaacaatgtctactg
gtgttccatctgataaataaggcatatcttgtttaggtaagatcctagaaatgatacctt
tattgccgtgacgaccagccattttatcacccacttggatttttctcttctgcgcaacat
agacgcgaatcatagcatttgttccgggaggcagctcatctcctttctgcctagtaaaga
ccctaaccttaactactctaccttttgcagcattaggaagccgtaaagatgtatctctca
catcccgagccttttctccgaaaatagctcgcaacaatttaccttctggtaattgatcag
cttcacctttaggagtaatttttcctactaaaatatctccagcttcaacccaagaaccac
cgacaacaattccatttctatctaaatccttcagagagttatcgctgacgttaggaatct
ctctagtgatttcttctggtcctaatttagtttgccggcattctacttcatacttttcga
tatgaatagaagtatacaagtcatcataaactaatctttcgctaattaaaaacgcatctt
cgtagttataaccttcccaaggcatataagcaacaagaatatttctccctaaagcgattt
ctcctccgtctgtagatgcgccatcagctaaagtttggccaacaactatttttttctccta
cccaaacaattggacgctgattaatacaagtatcttggttagaccgataatatttttttta
agcgataatgaactgttctaccattagtatcctgaatgcctattttattagcagaaacat
aatttacataaccagatgttctgctaataacaaccataccagagtctcgagctattttag
tttcaagaccagtaccaattattggcttttctggatataataatggaacagcttgccttt

gcatattagatcccattagagctctattcgcatcgtcatgttccaaaaaaggtattaatg
atgttgcagctgaaattacttgaataggtgaaattgcaatataatctacttgggttggcg
ttgttgtaataaactcttgacgataacgtacaggaataatatctccttcaatgtaatatt
gtttactaactttaacatctcctggagctactcgaaaatcatcttcctcatctgctgtta
gatagacaggactattgtgataaatcacttgaccttggtgtaccggataaaatggggttt
ctatgaaaccaaaaacattgacccgcgcacaagttgctagtgaaccaattaaacctgcgt
tcggaccttccggagtttcaattgggcaaattctaccatagtgactaggatgtaaatcac
gaactgcaaaacctgccctgtctttattaaaacctcctggacctaaggcactaattcttc
tcttgtgagttagttctgcaactggattagtttggtccataaattgagaaagctgactag
atccaaagaattctctcactgacgcaattaatggcttgggattaattagatttgataaac
tcaatgaatctatatcacatatcatcattctttctctaataatgcgttctaaacgattga
gacctactctgaattggttctgcagcaattctccaactgaacgaactcttctattaccaa
gatgatctatatcatcaagattacctgagtttttatccttaatattaattagataatcaa
ttgaggataagatatcttgaggagacaatactcggaacgttttaggtatattgagaccta
gtttttttattaattttatatctaccgacttcacctaagtcatatcttttaggatcaaaaa
aacgagagtaaagcatctgcttagcaacagcaactgttgctggttcattaggtctaagct
tagaataaactatgagaagggcttcctcatcagttacttcttcaatattattttttccaa
tttctttagctaattctttaactgaatatatttgggatgcagaaataagaaaagcatatt
tactaagtcctttttcaatttcatctttattgagcccaatagctcgtagaaaaatataag
catttactttgtgggttttatctattcttatccaaatttctcccttaggatcaatttcga
attttaaccaagaacctctattagaaattaggcttgcactataaatctgcttaccatttt
tgtctatctcttgtttataatagattcctggactacggattatttggttaataataactc
tttctgtaccagatacaataaaagttcctctatttgtcattattggcaagtctccaataa
aaaccaatctctttttgtacttttttatttttttatctgcttttcagcactgagctgtagat
gggttgatgataaatctaatgatttaatattttttttcctgatctttagaaggtagatcaa
tatcttttcttgttaactttacaggaacatatatttgagcactataagttttatccctat
tttttgcctgtctgacactataacgaggaaattttatcttatattcattaccaaataatt
gtaattcgagtcgactagtagggtctgatatcttcggaaaaaaattcaagaacttccgtca
aaccttctaataaaaaccatttaaaactggccctctgaatttcaactaaatctggaagaa
gtttatttttttaagcttatacgttggaccatagatctcctttataacaaattttgaaatc
gaagagtttaa-tactattaatagagtactggtagataatgtataagcatattttgctac
attatttcttctctagacataactagaacataatattttattcaa--aaaaagattttaa
aaatt----ttcac--acactatatatataagcactttgctgcta--aaagaaagttcaa
gctatataa-------aaaaacgaaattaatacttgtctgtctgtcaagtaattgacatc
aagatagacaagtattatttatttaatcaattctg-tttttttgagcaaaagacaatgct
ctcgcaagtctagctttttttctagcgcctgtattagggtgaaatgccccttttttttaca

gctttgtcaatcttgctatatacttgagaaatacttgactgcacatcattgaaattatca
ctttctaaattatcaatatttagaaggcatcttttagttaaagttttgacaaccgattta
tacttacggttaatgagacgatttctttccgaagttttaatgcgtttaatcgcagaaagg
ttcttagccacagtaaatccaataaaactttataaaatatttttagaaacgaaaataca
caaatctgagatctattcaaaaagggaatatgcggcgtattataacattaaagaaaaaca
ataggcaatagttcaccaagagctcttgttattacacaatagagattgtcattgcgaaaa
actttgtaagatagtatttaattagaataaactaactagacatataaatatatatgaaaa
aaattgaagctattattagaccttttaagcttaatgaagtaaaatttgcgctagtcaagg
aaggtattggaggaatgactgttatcaaagtttctggatttgggagacagaagggtcaaa
ctgaaagatataaaggatctgaatactctattgatatcattgataaaataaaaattgaaa
ttattattaacgatgataaagtagagaaaattgtagagacgattattaaggcttctaaaa
caggggaaattggagatgggaaaatatttattagtagtattgaaagagtaataagaatca
ggactaatgacttagattttgaagctttatagtatttttacttt-tttagacttgatact
tcttcactaaacatgagataataagcgcttattgtatacttataactaaggtgtaata--
ctaatggctaaaagtaaaggtgcacggatcgtaataactttagagtgctctgataaagct
ggagattttgctcaaaaaaggaaacctggcgtttttcgatatacaactactaaaaataga
cgaaatacaccaaatagaattgaattaaacaagttttgtcctaattgcaatcaacattgt
atcttcaaagaaattaaatagttatttatcttattaaaat--aatcatattatggctgta
tatagaaaaagaatatctccaattaaaccaacagaggctgttgactacaaagatattgac
ttgctaagaaaatttattacagaacagggcaaaatactgcctaaacggtcaaccggattg
acttcgaagcagcaaaaaaaacttactaaagcgattaaacaagctagaatcctttcttta
ttacctttttaaataaagattaa--atatatggcctaacttcacatttttcatctaaa
tattagaaacttctatagatttgtttagcaaaatgatctaacatactgaat---------
----------tagtactaaaa-tctaatcattataagttttgttcattttataattaata
atagcactataattta---ttaagctagattc-----agacataaagtgcaacctctttt
aattgattatattatatagtctatttaaaagaggaaatttttttgcttc----------t
agcaattcaagattctttcagctttaacgatatatttaaaacaaagatttttgtttaggt
atcagatcaaatgagtgaattttatctcccgactgccataattgaaattcagagatgaaa
attccacattcgttaccagcttgaatttcttcgacatcttctctaacgcgttttaaagat
tcaatttttccttgataaataactttttcttcccggatcactttaatccaagagtttttt
aatagcttattattgatcactcgacatcctgctatttttctatttgctaacgagaatact
gtgcttacttctgcttctcccaccggtacttctgagtattcaggatcaagtaagtcttcc
atccttcttttaatgtcttcaattaaggcataaatgatttgatagttttctattaatata
ttcaactttgccgatgcttgctttgttcctggagcaaagttagtattgaacccaatgaga
atagaatttgtggttgaagcaagttcaacatctgtagcagtaatctctccaggcatgatt
gacacgacattgagttgaactttactctgaggaaattgagataaagaatctaggattgct

tctgtagagccttgattatctgttttaataactaaagaaatttgcttactaatatcttta
gaattagtgtttttaaagtatctaatgtaattcgactattaagtgctctttgtttctgt
ataattgatgagtctttagaggtattttcaatcgctttaagtttggcttctttatcactt
ttaactgctaaagctatttctcctgtagctggtacagatgacaatccccaaatttcaacg
acggatgagggtattgctaaattaatttctctttagcgttgctaattatagctcgaatc
tttgcgtaagctgatccaataaccaaattatcactaatattcaatgtaccattttgtatt
aatagagttgctactggaccatgcgatttatctaagtgggcttctattataataccttgt
gcaggctgtgttgggtcagctttcaaattctctaattcagctaacaaggtaatagtttct
aataatttatcaacattttgaccagtaagagaactaataggaattataggcacttgtcca
ccgagtttctcagacatgacattgtgttttaataaatcttgctcaataatatctatattc
gaaccagctttatctatcttagatattgctacgacaaaagggacattcgctttttgaata
tgatggatagcctctatggtttgaggtttaactccgtcatcagcagctataataataata
gcgacatctgttaaattagctcctctggacctcatacttgtgaaagcttcatggcctgga
gtatccaagaaaacaatttttttgtttattagctttcttaatatactcaacttcataagct
gcaatggcttgagtgattcctccaatttctttattagcattattagattttcgaatataa
tctaatagcgttgttttgccatgatctacatgtcccatcactgtgacaattggaggtctt
ttaatataattgccagtttcataaagaacattagagttatctaaattactaggaagtcca
ttattatttcctctacatttgattctactgctatcccaaaattatcagctactgatgat
attattgaggcgtctatggtttgattcatagtaactgatattcttttaagaaataaatat
tttataatatctgtttcttgaacacaaattagtttagataattcttgaatagttagagga
ttagtaatactaatagactctggcggagaatttgattgactgcttgttgaattttgacca
ggagttaatgctactttttttctgtcttatagatttttattattagttagtttttttgcg
acttccacttttggcttaggtggacgcattaaagagatagctaaatctcctgcagtctgc
gcaacattagaattagagtctctaaagttgtca---tcatcatcgtcaatatgtattttc
gtttttagtttt-ttgcgctgtctattctta--tttttttactatctactaagtcgtga
atttattaaaatttttactttttttatctagtttgggtggagaacttaattctagatgc
tgttcaccacc---tgtatgtgattctgatttatcaaggtctaaatgtaacagatcatca
ttattaactgactctaatctaattttatagataatctgagggtttttcaaatctataatt
gtttcagaagagttgatatttgagctagatcgagaggatctattttcaaagttttgatta
tttaaaaacataaatgttggttttattatctaatagtaactttctaattttaatacagag
cttaatataaacaaaaatcgaatatctgtataaataatacaaatgt---agagaattaat
ctctgttgtattagatttaatataacactaaagtcgtttgttcgttatacaagtttaaaa
ctttt-tatgatgataaacactagattctagtttattggtatacaatctatttatctaaa
taatactgtttttgtc-gggcgtttaaa----ttatctaatgttatattcattatgaaat
tctattgaaatttaaaaa-ctcaacaattaagtaagaatcgttaatcacctccacgccac
ttatgccacgatctcaaaaaaaatgataattttattgacaaaacttttacagtattagctg

atattgtattaaaaatacttcctacaagtaaagaggaaaaggaagcttttcttactatc
gagatggaatgtctgcacaatctgaaggtgagtatgcagaagctttagaaaattattatg
aggctttaaaactagaagaagatccatatgatagaagctatatttatacaatatagggc
ttatttatgctagtaatggagagtatgtaaaagctttagaatactaccatcaaggattag
aactaaattttaaattacctcaggcccttaatatatagccgtgatatatcattaccaag
gagttcaagcaattgaggataaagatacagagctatctaaattaatgtttgacaaggcag
ctcaatattggcaacaggctattaagttagctcctgataattatattgaagctcaaaatt
ggctgaaaacaacaggacgaatgaggaatatacaaggatattaatatatttgagatataa
taaagatatcaattaataaattaaaattagttaaagaaactgatactatatagagtactt
-tgatat--------atgttatactagttga------tctctaagacttagctcaataac
cgaaattaaatctattgactatattaatgaatacaaataagcataatggttagtacaaca
aaaagtaccggatctgttactcaaattatcggaccagttttagatattgcatttcctaac
ggacagcttccgaaagtattcaatgcactcaaagtacaaagctcagaagcaactattact
tgtgaagtacaacaacttttgggtgacaacaaggtacgagctgtttctatgagttccact
gaaggactacaaagaggggtcgaagttattgatactggatcccctatatctgttcctgta
ggtacagatactcttggacgtatttttaatgttttaggtgaacctgtagataatttgggt
cccgttaattctgagagtactttacctatccatcgaccagcacctgcttttactaagtta
gagacaaaaccaaatatttttgaaacaggtattaaagttgtcgatttacttgctccctat
agaagaggtgggaaaattggtttatttggaggtgctggagtaggtaaaactgtattaatt
atggaactaattaataacatcgctaaagctcatggtggagtatctgtatttggaggcgtt
ggtgaaagaacaagagaaggaaacgacctatatatggaaatgaaggagtctaaagtaatt
aatgcagataacctgaaagaatctaaagtagcattagtatatggtcaaatgaatgaacct
cctggagcgcgtatgcgcgttggcttaactgcattgacaatggcagagtactttagagat
attaacaaacaagacgttctattgtttattgataatatttttcggtttgtacaagctgga
tcagaagtgtcagctctactaggccgtatgccatctgctgtgggttaccagccaactcta
gcaactgaaatgggagcactgcaagaaagaattacttcaacaacagaaggatcaattaca
tctattcaagctgtatatgtaccggctgatgatttaacagacccagctccagccactaca
tttgcacatttagatgcaacaacagtactttcgaggaacttggcagcaaaaggaatttat
cctgcagttgatccactggattcaacatcaacaatgctacagcctggaattgttggaact
gagcattattctactgctcaagaggtaaaatcaactttacaaagatataaagaactgcaa
gatattattgctattcttggtcttgatgaactttcagaggaagatagacagactgtctca
agagcgagaaaaattgaaagatttttatctcaacctttttttcgtggcggaagtgtttact
ggatcacctgggaaatatgtatctttggaagatgcaattaaagggtttcaaatgatctta
aaaggcaatttagacgacttgcctgagcaggcattttatctagtaggtgatatagatgaa
gctatacaaaaagctgacagcatgaaagattaatataattaacgatgactttaaatataa
gaattattgctcctgatcggactgtttgggatgcagaagcacaagaaattattttaccaa

gtagtacaggacaacttggtattttaacaggccatgcacctttgcttacagctttagata
ttggagttatgagagtaagagtagataaagaatggatgccgattgttttgctgggcgggt
ttgcagaaatagagaacaatcaattaactattctagtcaatggggcagaagaagctagtc
aaattgatttatcagaagcagaaaaaaatttagacactgcaactcaacttttaagtgatg
cctcatctaataaagaaaaaaatagaagcaacacaaaaaaatacgaaaagctagagctcgag
tacaagctgcaacagctgcaactccataa-aagattcc------tcttaaaacataaaac
tttagtaattattactaaa-------gttttatgttttatttgtatggttataggcaact
taaataaacttagcttagacctgaacaaatataatcaaaatataatcccatctctttacc
tgcatctggtccaactaaactaatagtaacttctttcatagctaaaatagcttgaatcgt
tgcaccaataggaacacctagggaattgtatgtttcttttagaccatttaatactctttc
ttcaagaatagatggatctccagctagcattccataagtagcatagcgtaagtagtagtc
taaatcacgaatgcatgcagcataacgtcgagttgtatacatgttaccacctgggcgtgt
aatatccgaataaagtagagattttgctacggattctttaataattgttgctgcattagc
tgcaatagtagcagcagctcttactcgtagctcacctgtttgaaaatagcctcttaattt
ttcaacagagctatcatctaaatatttaccttggacatcagctgcattaataacagaagt
aattgcgtcttgcataactttcacaaattccttaattttaaatatctaatacta-ggggg
tatataacaaaattactagatcctttagcgatatcagtattactgcatagcacccaaagt
ataatcaaagtaaaaaccagcttctgctgaatcttcacctgcaagcagtgagcaagctac
acttttcatgcattttacgccttcagcaactcctgaaattggtgttcctaaagaattata
catttctttaacacccactaatccaatttcttcaataggagttacatcgccagcaactat
tccgtaagttactaagcgaagataatagtcaagatctcgtagacaagtagcagtcatctc
ttcgccgtaagcatttccaccaggagaaactacatcaggtctttttgaaacagctgttg
gccgccttgctttacaatacgctcacgattatctgttaaaatttgagctattcttaaacg
acgttgtccagataaaacaaaacttttaattctatctaattccccaggacttaaatatct
tgcttctgcatctgcatttacaattgactttgtaacaatactcatggataaaactcctgt
aatgcttt-aaaaaaaagcttaatactaattatttatgatataactcagaaagtaatgta
ctttttgag-aattttataaccggaacacgttcatgatatacgaggcaggataggtactg
aggtaagtactaaaacgagaaagttatatgtaggaacttctgttattgatttcctagtac
tttttaaaacttggtaccacaattgatatatcctgcttagtcaaacgattataaagtgt
ttgtgtattaggaaagttagctgctggtaaagttggaaatcttctgtatggaacagtatc
tttaccatataccgcattgtattcatcactttcaactaaattacttataaatgcagataa
gccttttgaagctaaaatctgattcaagaatctaatttctgcctgattattaggagctcg
tcctaagatatgcttagtacctaattctataacttttgtatttggatatggctgataaaa
ttctttaccatatagttcagatagcgccaatttttctacgagttctttgacacagatttg
tctattcaagaaagctgactcaatatccaagaattcaccgccgatactaaaagagttaag
atctctttcgaaaatttgacgatatgctgctcttaaagcttgttctaacatttctttgtt

actatcactattcacttcaaagacaatagattgatctcttaaagaagttactccttgtga
aatacgagacttaatatcattactactcctcatttctttgacagcacctaattcaacaaa
cctagcagatttactagcaataactttttgaatctctggtcaataattcctggtcttaa
gcttcttaaagcaacaccagctggcgtgctatatctttcatacggaacagtattatctcc
aaaagtttctgtgtactcagagctatcaagtattgcgtctattacttggtaatagccttg
cttatatgcaatatcaaaatatttattaatttcttgccgaccatatgtagggcgacccag
taagcggttatgaatatattcaatagctttacaaatatataatggctcccaataaagaga
tctaaatatacttgattttgctaactgtctaacaaattcgcgaactgtaatttgattgtc
ttttagctgactttctattggcttgagaattagtttttcttcttgatacacttctctacc
aaaaactcttaaataagcaacttttgtgacaacttctactgaattttcaaaattttggga
tgaatctgactttataagagcactggataatttaaagatttttggtcccagggatcctgc
tgacttaggtcttacctgtggattactaatctgattgtagattcctggcccgcgtctaac
tagtatccttcttgtatctttaccaaaaagggcttgtctttttcttggatctttattttc
tttagggaaaatagctccaaattgaattgataatggatcattacctatgccatatggatg
ttgatcaggtaatgattgcttataatcactaaataaagttataaattgaggaaccttacg
aaaaggcgcactataactgagaagatctatttgaggcccccaattacggcactcttgggg
ctcttcacctaaatttctaaagtaaggcacagtctcttcgccgaaataatctgtatactc
agaagaatttaaaattgcatttactaaaccacttaaaccagtagatgataaaatagcaaa
atatttttgaaactcttctagagagctaggacctctacccaagaaatgtctaaatgctaa
ttctaatgctcgactattgacaaaaggttcataaaattgctttctataaatacttgaagt
tcctaacgaacgaataaattccttaattgaaatttggccatttttttacttgtgactccaa
attcgataaagagaggtcatatgctttcgcaatatctctttcaaaaatttgtctataaca
tgctttcacaacagtatttttctcatcagcagataaactaggcttcataacaaagcgagg
tgttgacactcctgcctgtacataagtctgaggaagacgtaaaccttgtaaatctccaga
tattctttttcttaatttatcagtcaaagatggagcttcaaattcagaaatcacaacatt
aaaatactctttaactaaatcttgaccttttatatcttcctcaaagattagtaaggctgt
ccgtctcatctctctaagagccacaattgctgctgcacttgagcaagcattatcaattag
ctctctcaatcctcgtatattaacagataaaatattagggtcgcctgatacaattgcata
tgtcaaataccgtaaaaaccagtctaaatctcgaagagattttctcattcttgtggtgcc
gtatctcaagacgtttataggtttaaatcctggcggagtagctcccccagcattaaataa
agatcggaaactttggccaaaatctccttggatatttcctgataactcattaattttatc
ttgagaactttgatcacctgcgataataacagctgcttgaggacgttctaaataagaaat
tgcagacccacctacgaatattttatctgctgctcgtgctacgagaatatttgcattttt
agttaaaatatctgcaacttccagtctttttttgtccagaatttaaaaaagcaactaattg
attcaattcaccaagctgtaaaaatcggtcttgttgctctgcctgagtaatagttaaaat
tgaggcagtccgataaagctgggggcgtgctaatgggcttccgccacttgctttgatact

cattactttatttatctccttaactgatattgcttgctagcgaatacaaatatgacttga
tatttctaaatatttaaagatactatgcaagcaca-ctgttttctatattattaggcact
agtacttatagtacaaagagtcttacaactaacttctataaatacaaattttgtaatact
tctattaacatttatcaaaaatatatttaagtgctcactcttttatattaaaattcaatt
agtgagagattgacgtgaaataaagtatactaagtaagttttatagataggaaaattaaa
agatttattctacaaacattaatca------------cttgaatgtttagcaattataca
attgttattgcttttaattggtttagactagtttt-tattttttctaacataaacaatat
taatgagcaatttgtatgaatttaaagccacaaaacaacctattaactaacgaaaatata
aatcacatagatatacccgaaaatgatataccaatgtcaattacggaacatttagaagaa
ttaagacaacgaactttatttgtgtttttattctttttatttgctacaactataagtttc
acacaaattaagataattgttgcaatactgcaagctcctgcttttggtattaaattttta
caattggcaccaggagaatattttttttcatctattaaagttgcaatatattgcgggatt
gttgcaacaactccgtttgccgtttatcaagttatattatacatactcccaggactaact
cgaaaagaaagaaaaattatttacctctattaattagttctgtattgctttttatcaca
ggtgggatatttgcttactttgttctcgcaccagcagctttaacatttttaattagctat
gggtctgatattgtagaaccactatggtctttcgaacaatactttgactttattctatta
cttttacttagtacaggattagcatttgagataccagtcatacaattattgctcggtgtt
tctgggacatttcttctaatcaaatgatacgagcttggagatatattattattatagca
acaattgctggagctattttgaccccttcaactgatcccgttacacaattaataatgtct
tcagctgttttattactttattttggtggaattgttatattattagtcttaaaaaagtag
agtaaat---------atttcta-ttaatccaatatatttgttatc-----tgggcaac
taatctataaaaacaatttct------------------agatttaagtatcaggcttct
aaatatttcaa---------gtctcttttatcatttaaataaaatc-------------

--gatacattgg
cttgtttttccatcaattatttgtattgtgcac-tttttagcactatctttattattctt
agaattatttccttaatctatttattaaat----agatatgaagagatttcagtattgat
tatggtaatctatatcaacgtaaaaagtattttcttatgagttttatttatataaaggct
tattcattatatagttataacagtaagtctaattaatag-ttttcatgcttaattaaagt
aataactcttaattcttttggcagaaaaatctgtcttaatattttatc--aaaaaaatag
-aaaaatgaagctaaatagtctaattaacttaattcaaaagtctatatattcttgtacac

ttttattaattatttttaaatattatttgtgttgcacctaattctagtaatgcatttccaa
tttatgcgcagcaagcttatgaaagtccaagagaagcaactggtaggatagtatgtgcca
attgtcatcttgctcaaaagcctgtggagatagaagcgcctcaagcggtactacctaata
ctgttttcgagactgttgtgaagattccatatgacagcaatgctaaacagattttaggta
atggcagtaaaggaggcttaaatgttggagcggttgtaatattacctgaaggatttaagt
tagctcctgctaatagattatctacagagttaaaagaaaagactaaaaatctttacattc
agccgtacagtgctaaacaagataatattttagtaattggacctatttctggtgataaaa
atagagaaatagttttcccaatactatctcccgatcctgcaaaagataaaaaagctcatt
ttttcaagtatccaatatatgttggcggaaatagaggacgaggccagatttatccaacgg
gtgataaaagcaacaataatattgtctctgctttaagcagtggtaaaattaataaaattg
aatcactagacaagggtggatttataatacatgtgactaacagtagcaatgtagagtcaa
cacaaaagatttcacctggtctcgaacttaaagtgaaagaaggggatacaattcagcttg
atcaagctttgaatagtgatccaaatgtaggtggttttggccagaacgaaacagaaatag
ttttacagagtccaaatagaattaagggcatgattgttttctttttcgctagtgttctag
ctcaaattttcttcgtattaaagaaaaaacaatttgaaaaagttcaagcagctgaaatga
atttttaaaggaatataaaactggcacttactataaataaagta-taaacactttattca
caggatgtaccagtctttatgttatgtaatactcgctttgacggaggttatgatactttt
aacagaatctacaatttggctaggctgaattactgtagcctgctctaagcttccattata
tggtgtgggtagatcctgcgaagatagcctcacgacaggagcatctaattcatcaaaaag
atattcattaatttgtgcaattagctctgctccaattccggctgttttcatacactcttc
tacaattaaaactttgtgagttttctttactgagatagatatagagtctatatccagagg
ttttaaagatataagatctataacttctggatcataaccttcttttaataaggccggtaa
tgcttggataacatgatgcctcattctggaataggttaaaatcgtaatatcttttccttt
tcgtacaaattcagctttattaagaggtaagaaatattcttcttgaggaatctcttcttg
taaattgtaaagtagaacgtgctcaaagaatacaactggattattgtctcgaattgcaga
tttcagtaatccttttgcgttataaggagtagaacaagcaactattttttaagccggggat
ggcttggaaataagcttccagtctctgagaatgctctgcacctaactgtctacccacacc
tcccggtcctctaataactaaaggcaacgtaaaattacctccagaagtataacgtaacat
tcctgcattattagaaatttgattgaatgctaataataaaaaactcatattcataccttc
aacaattggtcttagccctgttatagctgcaccaattgccataccagtaaagctattttc
tgctattggtgtatcaaggactcttaaatccccatatttactatgcaaatctttagttac
cttataagatccaccatagtgaccaacatcttcgcctattacgcagacagttggatcttt
ttccatttcttcatctgttgccgctcgtaaagcgtcaaacataaagattttactcatttt
aatatttgatttttatgatttgaccactgttttaaagtaacagttaagactccatattct
atttagttatctgcaaaaagatatcgtttcaattctgaaatgttaggttctggactggaa
atagcaaatttcacggcttgttccaattctgttttaacagcagcctgaatcccattaagt

tcacctatatcagcaatttcattatctagaatatatttttttgagtttttttgataggatct
cttgccacccaagcctctttttcttgtcttgatcttagttcatcaggatctgccagagag
tgaccacgaaatctatatgttaatgcttctattaaggttggaccatcaccttggcgagct
ctttgaactgcttgttttgcagcttgtcttacagctagcacatccattccatcaacttca
atcccaggaagcccaaaagcttccgctttttttatgtatttcaggtattgaagaagaccgg
tgatgtgccatacctatagcccactgattattttcaacaacaaatataataggtagtttc
cagagaactgccatattcaaacattcaaaaaattgcccattgttggtagtcccgtcacca
aaaaagcaagccgtgactcttaaatcgtctgtttctttaagtacttgctgacggtagata
ctttgaaaagctgcccctgttgcaactggtataccttctgcaataaaagcaaagccaccc
aaaaaattgtgaggcgcagaaaaaatatgcatcgaacctcctctgcctttactacaacca
gtctcttttccaaacaactcagccattacatttttagatgggacgcctttacttaaagcg
tgtacatggtctcgataggtactgcaaacataatcagtcggattcagaagtttaattaca
cctgtagaaacagcttcttgaccattataaagatgaacaaaaccaaacatctttccttta
taatacatctgagcacacatatcttcaaaatttctgcctaacaacatatcttcatataaa
actaataaattactcttattaagattaagacctgttgaattataattagtcagcggcaat
tgaactttcttaggataactcataatttgtaaagaaacctct----------tattgtaa
ataggaattttgaagaaaaagctatcaatagaaaacatacagataaaaacttataaatta
ttgtatgtttttttactaaagactttaataattatttgacactagttcagtatatcaaatt
aattttgcttatactacagatgtctgaaacttagacaaaaaagtagtaa-aaaaaattta
ttaaaagaatagttaaaaacagatataaatagaagtaaacagaattatt-gaaaaaaaat
gattattattaattaa----tacttaatatttctaaatattttacaattaatggctatgt
caccatctttattttatcctgttgaacaagaactatgtagtcttgaaaaaaatctgaaag
ctgttgccgggactcgtcatccaattttatatgccgcagcaaagcatctattcgatgctg
gaggaaaacgagttagaccagctcttgtgtttttagtggctaaagcaacctctgagaagc
aagatataaatactggacaaaaaaggctagcagaaattactgaaattatacatactgcta
gtttggtacatgatgatattatcgacgagtgcacaacacgtagaggagtcaaaactgtac
ataatttattcaatactaagattgctgtgctagcaggagatttttttatttgcacagtctt
cttggtatttagctaatattgaaaatttagcagtagtcaaagctatttctaaagtcatta
ccgactttgccgaaggagaaattaggcaaggcttagttcatttcgatcctaatatttcaa
tagatgcttacattgagaaatcattttacaagactgcttcactcattgctgctagttgtc
gcggtgcagctatgcttaatggttgcaatcatcaaataaataatgatctttatctttacg
gtaaacatatgggattagcatttcaaattatggacgatgttttagatataactggttcta
ctcaaagcttagggaaacctgctggcgctgatctaataaatgggaatttaacctctccta
tcctttttttcacttactcaagaaacaagtttaaatgatcttattgatagagagttctgta
atagtacagacgtagcctcagcattatttcttataaaaagaagcggaggaattacaaaag
ctaaagatttagctaaagaacaggtgcaggcggcactttcctgccttcagtttttaccac

aatctacacctgtatctagtttaaaagaattaacacatttcataatcacaagattgtcgt
agaggacttgctaaaattaaaatgttattaactaatttttactgtttctaaaatagtct
gaaaagtttgaatatcattactagctagctgcgctagcattttacgatttaacgcaatat
tctctttttttaatgcactaataaaagtactataattcatgccttgattgtgagcagcag
catttattctagttatccaaagacgacgaaaatctctcttttcctttttgcgaccaacat
aagaatatcgaagagctttaaggacttgctgcttagctgttcgaaataaacatttatgtg
cacctttaaagcctttagctagcttaaagattttagctcgtctttttttttgcaacgttac
ctcttttaactctactcataaaatattac-ttaaattttaattattaccagcttactaaa
ttcgtatactctttg----attataaataaggtaattttattgcaatatttttaatatcc
tttaagtcaaccaaacaagtagaagagagatgtcttctttgttttgatgactttttttgt
aataaatgacttttagaagctttatgtcgaagaaattttccggatgaagagactttaaat
ctttttgctattgcttttgatgtttttaacttaggcatattataaactttttaatgattaa
caaaagcatgaaaagtctgaggatattaatcttagacctttcaatacttagaataatctt
aacacaatttattttacttactacataggttaactgaaacaatttatatatataatacta
atcagttaatcaaccgcagctaaagtggatttgtattttgctttgaacttttgtatagta
ttagcaattagcattgttataattttaagactacttgaatttaattcttcgaacattttc
atatttaatgtaaatgaaatattagcttctgagacaatattttgaatttgaacatcagac
aaaggaatcatatctaatgcagctctatattgatctttaaataatttatcgtcttttatt
tggtcaaaatcgtaaaattttgttcctcctgaatctgataaattcatagcacctctagct
atttttttaaaatttggccgccagaaagatcacccaaatagcgcgtataagcatgagcg
actaataattcaggttgtttatgtcctataatgtgaattctatcaacatagattttttgta
gcaggagatggttcaataaaatctgaccaatctgatccgtaataataatttaaatcttca
gatagactctctttcctattgagctctgtaaaatatataggtttaatagctggatgatct
ttattagaaaataactcttcctctattgcacaatagacaaagtatagatttgcaaccaat
ttacgatatgacttcttatctacaactcctcccaaaaaagatttaacaaaactaacattt
tctgccatactatgggacttagtagtaccttctcttagttcattcgctaaagtattaatc
ataatatcaatattccatttttt-aaagacaacaaattaaca---ataaatagttatccgt
atatttatacttgagttgtgctaactaggctgctgcaaaataattttttgattttattgg
atctggattcattgttttgtcaccaggcttccaattagctggacaaacttcatctggatg
agattgtacatattgaattgcttgtaacacccttaaagtttcttctacactcctgccaaa
ctctaaattattgattgtagagtactgaataattcctttcgggtctataataaatagtcc
tcttagagctacaccatcactattcaaaacattataagccgcactgatctcttttttttaa
gtctgatactaatggatatgaaagatctcctaatccacccgattctctgtcagtctgtaa
ccaggcaagatgagaatactcactatcaaccgagactccaagaacttctgtattaagttc
ggaaaaagcattatacttatcgctaaatgctgtaatttctgtagggcatacaaatgtaaa
atctaaaggataaaagaataagacaatatacttgttcttgaaatcagataattttaacgt

tttaaattcttggtcataaacagctgtagctgaaaaatcaggagctagctgacctactcg
aagacaatttggtccagaaatcattaatttttcccagaagaattgatataatttttgttat
ttaataaataattatataacaaaatttagtatatattaaaatactaccagtcttttttaa
attac--------taaatgatataatcaatactatctttatagcggggaatggatttgaa
ccattgaccttcgggttatgagcccgacgagctaccagactgctctaccccgcggataac
tagaattatattagtctagatatatatataaaagcaaatgcataatagatttagaagta
aaaaaatagaagaacaggtccactgatcattaaacttagaaacgataaagtatttttat
cttatttaaaagaggaaagacttcatataaaccaataaatctatcttttaaaagaatttc
ggacggctttacccaaatttgaccatcataccagcctgattcttcataaaatactgtagc
actcattaatcgttttactacgtatgaccagcctaaatataatctgattaaaataaatcc
tgtcattaggctggaagtaataaattctgaaaagaaaaatttcagaggtagtttattgat
tggaaaaattgataataatataggactaattaataaacaattaattagtagtgcaatagt
tattttttattataagatctgcgacttaaagttggccagcaaaagaaccaagaattttt
caaagaagtatattcatgaacaggttgctgctcctttgggacaggacattgagtattata
taaattcattgacattaaaaagtattatactaaagac--tttctttatagtatctaatat
aggtacgacattaaatattaactaactgcaattatagtagtgagtaaaagaaataaaact
aaaaatagccaagtaattttatttaaagtactctcagtactacgagtattactaaagaac
tgattctgagcaccgacacttcctaagccttcagatttaggattatgtattaaaatagtg
aagattaatattattgttgatgaataccaaaaaaatttaaaatttgttccattatgtaa
gagttaat-aaaaatagcacaaagacaatcgtctaaaaggtattgattgtcttatcatat
taaaaagtatttattctccctgtacttttaaaagtaaaaatcctattgccagccctacca
aaactaggactgaacttaatacggcactttctacaatttctccgaccatt-ttatttttc
cttattactttattattg---tcattcttttgacaagattgtattgagctaa-ctaaaat
ccgtttctaccccaaactaccattgcaattgaaaatgtaaacattgccattagagctgcc
catcccaaacttaaaatatccataatttataatactc--ctaaaaataagatctcttta
cttaaactataatattcttaatattaaaaatatcattatataataagctaattataacag
agtactacttaattcgtatttttggtgtatttaaagaagattgaatttttttcgaggtac
tggataagtatttcatcctaagaaccatctttaac----ttttgatttattatactgctt
aaatctaaatata--tagcactcaagaatcattagtaattatgatgtttttaattatacg
attaacaacatatgtcaaaatagaagaattcatagcgactcagttaa-----tatgctaa
tataatttatcaat--aaaaaggaagttagtatttcaattatgcaaacaactataaataa
tgggcaaccgtctagtaaagaaactttactaacacctagattctacacgactgactttga
agagatggctaatatggatatttccggcaatcaagaagatttttttggctatcctcgaaga
atttcgagctgactataatagtgaacactttattagagatgaagagtttaatcaatcttg
gtctaatttagaacataaaactaaatccttatttattgagtttttagaaagatcttgtac
cgcagaattttcaggtttttactatataaagaattatccagaaaattaaaagacagaaa

tccggttatagctgagtgctttttattgatgtctagagatgaagctaggcatgctggttt
tttaaataaagctatcggagacttcaatttatctttagatttgggattttaacgaagag
tcgcaagtatactttttctctcctaaatttatttttatgcaacctatctttctgaaaa
aattggatactggagatacataactatttaccgccatcttgaacaatatccagaacaccg
tatttatccaatctttagatttttgaaaattggtgtcaggatgaaaatcgtcatggtga
tttttttgctgctttgcttaaatctcaacctcatttttaaatgactggaaagcaaaaat
gtggtgcaggtttttctattaagtgtatttgcaactatgtacttgaacgactttcaaag
aattgattttataatgccataggcctagactctagacagtatgatatgcaagtaatacg
caaaaccaacgaaagtgccgctagagttttccctgttgctttagacgtggacaatccaaa
atttttcaaatatttagatacttgtgcatgtgataatagagctctgatagatattgacaa
taacaattctccattattcattaaatctattgtaaagatacctctatatttttccttatt
tacaaatttactgaagatatatttgattaagccaatagactcaaaaacagtatggaatac
agttcgatagttaaatacaagggaatactttttattataaaaagctatctaattattaa
tacaagat------aaaagcttccttct-----cgatggaaggagcttttatttttgtcaa
aaa-tatataatgggattatatagatc-tagaactaagaacttactttttcttttgcttc
atacataacctctaaagtaattatattttagaacaatcacgggcaaatttctctgtatt
tctttttactttacctctaacaaacccaggaattttatttaattctttctgtgcttcttc
tgaccaattaatactatcaacagcagaaattcctacacttaatgcttctgttgtgtcatg
accaccaaatatttccagcaaatgatcttccataccaagtgtaaatgaattatatactaa
gtcagcaatttgatttgttccctcataaccaagaaatggacggtaacttaaaggaaagtt
ctggatatgcactggtgaagaaataacgccacaagggatatttaaacgcttaccaatatg
tctttccatctgtgttccaaaaattgctgcaggttcagttttagcaattaaatctccaat
taaaccatgatcatctgaaacaattacttcatcgcaaaattcttgaacttgatctttaaa
ccactcttcatcatatttgcaataagttccacaccaagcgacatttatacccatttcctg
gtgtaaaattcgagttattgctgcagcatgagttgcatctccaaatacaatagccttttt
acctgttaaattctgacaatcaattgatctggaaaaccatgctgattgtgaaataaatcg
ggtttgttcatctatgtatttttcgtaatctacagcagctcccgatgcatttaataattg
ctgtattgatcggatgcatgcagctgtttgaacaatacccataggtgtaatgtcaacata
aggcatattaaactctttctctaaatatcgagctgtcattagtccagtttctctatatgg
aataaaattaaaccatgcagatggtaatttttttaaatcttgaactgatgcattttctgg
aataatctggtttatttggatatctagatcttgaaataacctttttaattctgctatatc
atgctgattgtgaaatcctaagcttactgctccaataatattaacagatggagttttcgt
cttttgagttaaaacttgattattagatttagctttttccatatagaaagcgacaatttg
ttctaacgttctatcacctgcttgcagttcatttactctataatgattaacatcagctaa
taaaacatctgcttctgtttctatagatgctctgctaacaaagttttgcaaatcctcttg
caaaatactcgaagtgcaagttggtgttaaaataactaaatcagggctttcttctctgtc

ttttctagtgatattttctacaactttttcttgcgaaccacgggctaacacatgtctatc
cacaacacttgctgtaacaggagtaaaatctctgtctctttcaagcattgaacgcattac
attaaaataatcgtctccgagaggcgcatgcataattgcatgaactttttttaaaagaact
ggcaattcttaaagttccaatatgagcagggcctgcatacatccaataagctaatttcat
attaaattttcctatgatttatttagacatagtaatactttttaaagctaactaacaaaca
ttagaaaaacatctaatttaactccttgataattttgtaatattttttagatgaagctta
tcattataaatatttaaaaaatataattcagaatagaaattaataactgttagatcaagc
ttgaaagtaaagatttcttgcagttatagtttttctttaagtaaacatgtaaaaattgta
ctatatataaatgtactatatataaacttttttttactgaatattggtaatgccagataca
attaacttaaatatgccttccccaacgtttggtggaagcactggcggttggttaagagcc
gcagaagtagaagaaaagtatgcgataacatggacaggtaaaaatgaaagtaagtttgaa
atgccaaccggtggtaccgcaactatgcgaaatggagaaaacttactttatttagctaag
aaagaacaatgtttagctttaggtactcaactaaaaagtaaatttaaaatatctgactat
aagatttacagagtttttcctaacggagaagtgcaatatttgcatcctaaagatggagtc
ttcccagaaaaagttaatgctggaagagctagcgtcaacagtgttgatcattccattgga
caaaatattaatcctgtagatgtcaaattcatgaataaagcaacttacgattagtctctc
-aaaaacctagacataaaataacttttatatagtctaggtttttgataaggtatactgat
agtatggaagggtggccgagtggttgaaggcgtctgatttgaaatcagttgaacta--tc
tggttccgtgggttcgaatcccaccctttccgttataaaact------------atagtt
tgcttctcaaggcaagctagctatacactgatagtattaagtttagacttaataaagtct
acaacttgtgacaggttagaaatttttttcggcatcctcatctggtatctctatactaaat
ttttcttctatggccattactagctcaactgtgtctaaagaatcggctcccaggtcactt
gaaaaatttgcttccctggtcactattttttttttcaattcctagctgttctgctacaata
tcttgaacttttttcaaagatttcattatcttgcataatatattatttcctctaaggtgtt
atcaaaaatttttttggcttatattattacccaactctaacaaattagtaatttttagtcg
agattaaaatatttatacctatatgatatactattattctggataaatacgaagtttacg
atatggctcttctccgaaactacgagctctcaactggtagttgtcaactagattatgctg
catttttcggatataggctgaccgaggtgtaagctgaactattgaactttcattcaagat
aatagtttcaacagccaatttagcttcttgaagagcctggatctcatcaaacttttttatt
tttacagagttctacccaattaagaccagatgaagtatgaatgtttaatatttttctgag
tgcacaagtaatttgtggaaccgtactgttctgaattgtatatataataatttgttttga
ttttgcaatttgtctaagttttgtattttgctttacttgattacgtagcgctagaatagc
atcagacttttctatctccttggttaaaataattggtaaatctagtgaagaaataactga
tgtaatatgctgccaactcaaagaataagcatataagtactgatgaggtatttctaattg
caaagattgtttattaatattaatagggggcattgaaagtagttgacaataaagatgtatc
acgattttgtgatttattcaaatctaaggacttatttcttaattctctatattgtaaaac

aggagacgcctgctgtgtgttaattgatttacgactattagagatgtttacaggtaagac
ttctacagattgcgatggataacatttaattaaaattctaccattagcttgaatttgtct
tttttgcacaaaaggttgatggccttgcaaaatttgatctattgtctctttaactttatt
atgaactatccaaacatttcgctcatgtatttcgatcgcaatctgaaaagctggcgatgc
ttttctttctagtatactcttctgcgtacctctacgtttagcttcatcgtcacccaatgt
tacatattgaattccgccgattaggtccgctaatgttggattttttaattaaactttctaa
atgatttccatgcgctgtacctactaattggactcctctttctgctatagttcgagcagc
taacgcttctagctccgttccaatttcatctataataataacctcgggcatgtgattctc
aacagcttctatcattacttgatgttgcaaatcgggccttgcgacttgcattcttctagc
tctaccaatagccgggtgaggaatatctccgtcaccagctatttcattcgaagtatctat
aataactactctttttttccatttcatcagctaaaacacgagccatttctcgaactgcggt
tgttttaccaactcctggttttcctaagagtaaaattgaatcacctcgctctagtaaatc
tcgaataatactaattgtaccaaaaactgctctgccaacacgacaggttaagccgataac
actaccctctctattacgcagtgaactaatacgatgcaatgtttttttcgataccagctcg
attatcgccgctaaaattaccaatttttttttacacagtaatctaagtcttgccaactaat
agatctttgagataaatattctggattatctggaaatctggcttctggacgtcgacctaa
atccataacaacttctattaaattatttctattgggatgctgttttaaaggttctttgac
aaaatttggcaaaatttctagcaacttatctaagtcatctgcaataagcatgattgagta
tataataattaattaatcaaatagaagattcaagattctaaagattacaataaatattaa
agaatattataattggaatcaattttatatttaaattgaacaggaattta-tagatgtaa
aagaactgattaaactttacgcaataaatcgattggtcaaaattaatagtcgtattaaca
tgtttattggacaaaaagttcgtattaaatatagcaaacaaaaagttgctagtgatatag
cagataaagttggtgaattaggagttattaagggaataaagtttattaatagtcaatgtg
ttactattattgttgagtttgacaatcatactaggctttggatgtttagagaggaattaa
tctgtctaaatgaat------taa-------aaacataatttattttatttattatacag
aatttcttatgagtcgttaacataataaattttaaaggagtacttttaattgtgcaaatt
actattaaaaaattacaagacttactttcgtctgtacaaagaaaaaagatccagacatta
aaactaaaacaagataagtttgaacttctattaaataagacctataaaaaggtcaatcaa
gaaataataccttcgcagaaatctgctgtattacaaaatagtccatctacgacaattaaa
tcaataaatagtacaaaaaaatcttctgttgtcaatgaagaccgtacagaatatgccact
attgtttctccaatggttggaacgttttatcattcacctgctcctggtgaaaaaatttt
gtacgagttggcgatgaggtcaaattcaatcaaacagtttgtattattgaggcaatgaag
ttaatgaacgaaatcgaagcggaaattgaaggaaaaattatagaaattcttgttaaagat
ggtgatatagtagattgcgggcaagctttaatgaaggttgaaatataatgctaattttcc
cattttacatattgttaacagaaattatttagatagaaaaattagcttatactatagtta
tgaaaacttatttcgcaaaggcacaaaaaaactaatatataaaattattt-ttttatctg

ccaaaaatagaagtaagcgttttgattccttgtctcaagagaggagaatctcaatggcaa
ttagctcaaaagagcaagagacaaagaaggtaaaaatctcggttgataaaaatcccgtag
atacttctttcgaaaaatgggcccaaccaggccatttttctcgtacactagcaaaaggac
caaaaactactacttggatttggaatcttcatgctgatgctcacgacttcgatagtcaaa
ccagttctttagaagaagtttcacgtaagattttcagtgcacattttgggcaactgtctg
taatatttttatggcttagtggaatgtattttcacggagcccgcttctctaactatgttg
cttggttaagtaatccaacaggtattaagccaagtgcacaggttgtttggcctatagttg
gacaagaaattttaaatggcgatgtaggtggtggctttcaaggagtacaagttacatctg
gatggttccaactgtggagagcatcaggaattactacagaatttcagctttactgtactg
ctattggcggattagctatggctgctttaatgctgtttgcaggatggtttcattatcata
aagcagctccaaagttagaatggtttcaaaatgttgagtcaatgatgaatcaccatttag
ctgggcttttaggcttaggctgtttaggctgggcaggtcatcaaatccatttgtctttgc
ctattaataagctactagattctggtgtgtctccgcaagaaatcccactacctcatgaat
ttttaattaatagagagcttatggctcagctgtatccaagttttagtaaaggattagttc
cattctttactttaaattgggctgaatattccgactttttaacttttaaaggaggtttaa
atcctgttactggaggtttatggctaagtgatactgctcatcatcatctagcattagctg
ttctatttcttgctgcaggtcatatgtatagaaccaattggggtattggacatagcatga
aagaaattctagaagctcataaaggacctttaccggcaacggtcacgaaggtctatatg
aaattcttacaacttcttggcatgcacagcttgcaattaatttagctatgatgggatctt
taagcataattgtagcacatcacatgtacgctatgcctccttatccatatattgctactg
attacccgactcaattatcgcttttcactcatcatatgtggattggaggattttgtattg
ttggagcaggagcgcatgcttctatatttatggtaagggattataatcctgcagaaaatt
ataacaatcttttagatagagttattaggcatcgagatgctattgtttctcatctaaatt
gggtatgtatatttcttggattccattcatttggtttatacattcacaatgatactatgc
gggcacttggaagatctcaagatatgttctctgatacagctatacagttacaacctattt
ttgctcaatgggtacaaagtatacacactttagctcctggaaatacagctccaaatgcgt
tagcaacagctagttatgcatttggaggagatgttgtttctgttggtaacaaagttgcaa
tgatgcctatttctttaggtactgcagatttttagttcaccatatacatgcatttacta
ttcatgtaactgttttaattttagttaaaggattccttttctcgagaaactctagactaa
ttcctgataaggccaatcttggcttcagatttccatgtgatggacctggtagaggtggta
cttgccaagtatctggctgggatcatgtttttcttggcttattctggatgtacaattctc
tgtctgtagcaatttttcactttagttggaaaatgcaatccgatgtttggggtagtgtat
ctccgtccggaaatgtttctcatattactggtggtaattttgcacaaagtgcaattacaa
tcaatggatggttaagagatttcctttgggctcaagcatctcaggttattcaatcatacg
gttctgcgctatctgcatatggattaatttcttagcagcacattttgtatgggcattca
gtttgatgttcttatttagtggtagaggttattggcaagagcttatagaatcaatcgtat

gggcgcataataaaataaaagttgctcctgcaattcaaccaagagctttaagtattactc
aaggtagagcagtcggtgttgcacactacttattaggtggaattggtacaacttgggcat
tctttttagcaagaattatttcagtaggctaatagtgaaaataggat-aaaaaaacaatt
atggcaacaaaatttcctaagtttagccaagctttatcacaagatcctacaactagaagg
atttggtatggtattgctacggcacatgactttgaaagtcatgatggaatgacagaagaa
aatttatatcagaagatatttgcttcgcactttggacatctagcaattatcttcttatgg
acatctggtaatttattccatgtagcttggcaaggcaactttgaacagtgggtattaaat
cctttgaaagttaaaccaattgctcatgcaatttgggatccacattttggacaaccagct
ttaaaagcttttagtaaaggggctcagcttatccagtaaatatagcatattctggcgta
tatcactggtggtacactattggtatgagaagcaatcaagacctatattctggggcttta
ttcttactagttttatcagctttactcttatttggagggtggctacatctacaaccaaaa
ttcaagcctggtttatcctggtttaaaaataacgaatcaagattaaatcatcatttatct
ggattatttggggttagttctttagcctggacaggtcatttagtacatgttgctatacct
gaggcaagaggacaacatgtaggatgggataattttacaacagtattacctcatccagcc
ggtttacaaccatttttcagtggtaattggagtgtatatgctcaaaatccagatacagct
caacatttattcggaactaatgaaggtgcgggtacagcaattttaacatttctgggggga
ttccatcctcaaagtcagtctttatggctaactgacatggctcatcaccatttggctatt
gcagtagtattcattgttgctggacatatgtataggactaattggggggattgggcacaat
ctaaaagatattttagatgctcatagaccacctagtggtagattaggagctggacataaa
gggttatttgatactattactaattctttacacatgcagttaggattagcattagcttcc
ctaggtgtaattacttcgttggtagctcaacatatgtatgctatgcctccatatgctttc
atggctaaagatttcacaactcaagcgtctttgtacacacatcatcaatatattgctggg
tttctaatggttggagcttttgctcatggggcaatattctttgttcgagactatgaccct
gaacagaataaagataatgttttagctcgcatgctagaacataaagaagctatcatttct
catctaagttgggtaactctattttagggtttcatacattaggcctttatgttcacaac
gacacaatgattgcttttggaactcctgaaaaacaaattttaattgagccggtatttgct
caatggattcaagcttcttcaggaaaagcactttacggttttgatgtgttactatcatcc
tctactaatgtcgcaacacaagctggtagcaatatttggctgccaggctggttagaagca
attaatagcggaaaaaattcattgtttttaacaattggtcctggtgacttcttagttcat
catgcaattgcattgggattacatactactacgttaattttagttaaaggtgctttagat
gcgagaggctctaaacttatgccggacaaaaaagactttggttatagttttccttgcgat
ggacctggcagaggtggcacctgtgatatatctgcatgggatgcgttctatttagctgta
ttttggatgctgaatacgataggttgggtaacattttattggcattggaaacatattaca
atatggcaaggcaatgcaactcaattcaatgagtcttcaacttatctaatgggatggttt
agagattacttatggctaaattcttctccattaattaatggttataatccatatggcatg
aataatttatcagtatggtcatggatgttcttatttgggcatttagtatgggcaacagga

tttatgttcttgatctcctggcgtggctattggcaagagttaattgaaactctagcctgg
gcgcatgaacgtacacctttagcaaacttgattcgttggaaagataaacctgttgcgcta
tcaattgtacaggcaagattagtaggtttagcacatt-tttctgtaggatatgtattaac
ttacgcagcttttgtattagcttcaacagcaggcaaatttggttagactaaa-----ttc
aaattagctaa-----aaaaaagtcagtcataata-gttttatgactgacttttttggat
ttaaaacaaaaacttactaaagtttcacctcaatatctacaccggaaggcaaatttaact
tcattaatgcatctatcgtttgagaagaaggttgatgaatatcaataattcttctatgag
atcttatttcaaaatgttctcttgagtctttatctacatgtggagaacgtaagacacaat
aaattcttcttcttgtaggtaaaggaattggccctactgcaacagcattagttctagatg
cggtatctagtattttgtcacacgacgtattaagtatactagaattatatgctttcagtt
taattctgatttttgtctgctgagtaattgtcatgttaaaaagatcttattatttaagaa
ttttagagacaacacctgcacctacggtacggccaccttctctaatagcgaaacgcatac
cttgctcaattgcaattgcattaattaattcagcagtcatcttaattctgtcaccaggca
tgaccatttccgcatcagtaccatcatcagcagtaaactgattaatagtaccagttacat
cagttgttctaacataaaactgaggtctatatcctgggaaaaatggagtatgtcttccgc
cctcctctttagttagaatataaacttctgcttcaaattgagtatgaggtgtaattgtac
caggtttagctaataccatacctctttcaatatctttttctgcacgcctcttaaaagaa
ttccaatattgtcacccgctagaccttcttctagcgtttttgaaacatttctaatccag
taatagtcgttgtacgagtttctcgtaaacctacaatttcaattgtatcaccaactttaa
taatgcctctttcaattctaccagtggcaacagttccacgtcctgtgatagaaaaaacat
cttctacagccattaagaaagttttatcgacatctctctccggtgttggaatatacgtgt
caactgcttccataagtgaaaaaatcttgtcaacccatttatcttcaccttgcttaatag
ctgggtttttcgtcacagcttctaatgctaataaggcagaacctgcaacaaaaggaatat
catctccaggaaagtcgtattgacttaataattcccttccttctaattctactaattcta
gtagctcttcgtcatctacttgatcttccttatttaggaatactactaatgtgggaacac
ctacttgttttgctaataaaatatgttcacgagtttgtggcattggaccatctgccgcag
atacaactaaaatagctccatccatctgagcagcacctgtaatcatatttttttacgtaat
cggcatggcctggacaatctacgtgagcataatgacgattatctgtttcgtattcaacat
gagcagtattaatagtaatacctctagctttttcttctggagcggcatcaatttcatcaa
atttttttgctgcagtagaacctaaagttgataaagtcgcagagatcgcagctgttaaag
ttgtcttaccatgatcaacatgaccaattgttccgatattgacatgaggtttttttacgtt
caaatttagatcgagccatgctttttgttttccttatagtaaaagttgctttatagttac
ttttaacgataaatcttgtgaattgaaagatttattatttaatacagacaaga-----ta
aaatataactaatctaatatcgatagtgagcaaacgctttatttgcttctgccattctat
gtgtctcctctctctttctaatagaatttcctgtttcattagctgcatccataatttcgt
tagctaattttatagacatactttaccagatctttctctagaaaatttagtgatccatc

ttaacgctaaattcgtacctctataagctcgtacttcaataggaacttggtaagtagaac
caccaactcttctagcttttacttctactagaggagtaatatttcggatagctttttcta
aaatatttaaaggatctgattctgttctctctttaacgatatctaaagcctgatatataa
ttctttgagctaaagtttttttaccacttttcaaaatacgaacagttaacatacttacga
gtctgcttttatataaaggatcaggtgatgcgaaccttttttagctgtattacgacgag
acatagtatttaaaattggttattgtaataa-tgttc-tattagaagttatttaagattt
aggctttttttgtcccatatttagatcggcttttacgacgatcctttactcctgcagcatc
taaagtaccacgtactacatgatatcggactccaggcaaatctttaattcggccacccct
aatcagtaccacagaatgctcttgaatattatgacctacgccaggaatataagctgtaac
ttcaaatccagatgttaatctaacgcgcgctacttttcttaaagcagagttcggtttttt
aggagtcgtagtatatactcttgtacaaacacctcttctttgagggcaactttgaagagc
tggagattttgttttttatgtatttttcgtctttcggatctaacaagttgttgaatggt
tggcataataaaattgatttaaagtctttaatagatactatttgaacctgctttagatga
cttggctagtttttatttatcttatacttacgcataaatctttctactctaccttctgta
tcaataattctctgtgaaccagtaaagaatggatggtttcctgaccagatatctacgtgt
agttctggctttgtggacccaatcgtcataattaactggccgtcacaataaacttttgct
tctggataccaatttggatgtatattatcttttgccatggtttctataattatataaatg
tatcaataataactattaacgcttagaaaattgaggagcctttcttgctttctttaaacc
atattttttctctcttttactcttggatctcttgttaaataaccctcagacttgagtgt
cgttctattttctggattaattgagcacaatgctcttgcaacacctaaacgaattgcatc
ggcttgccctgtcaaacctcctcctctagcattcacatgtatatcatactgatttaatag
tcctaaaacttgtaatggtgcatatgaaactcttaagtaattaggactaaattgaagata
agactctcctggtataccgtttataattaaattacctgatcctgggactagtcttacttg
tgcaacagaacacttccggcgacctgttccagaatagattgcacgagttttaattaattc
tgtcgacataacattccttgataactaaataaatatacattctttatactatatactctt
gtggcttttgcgcgacatgtgggtgaattgggccagaatatactttaagcttcgtaaata
gttttctgcctaatggacctttaggaagcatacctttaaccgatttttcaataattctgt
taggtaatcttgtctgaagctgataaaatgtttcaacttttaatccgccaggttgtccag
aatgtcttctgtatagtttttgattcgttttattgccacttacagatacgtgagcagagt
taataacaattacataatctccagtgtctagatagggtgtataagaaggcttgtttttgc
ctcttaagatattagaaatatgagtagatattctaccaagtgtctgatttttagcgtcta
taacataccaatgagaattagtatttaatgagggtgattgcgttttattcatgaaaagat
taatacttttggttgacaataagagaatatgtaaatggtttacattcaatttctatacaa
gtaatattt-aaaaatatta-------------ttaacaaagagaacttttctaacagtt
atttcaagtgtttgtcagcaagaaaatttctagttctcaataacttatactatttttaat
aatataaaatatattaatcactttttttcttttggcaaacttatacctagtttttttttgc-

aaggcatatataacttcttcagctgatttttggccaaaatttttaatttctagcagttct
tcttgagagtaatctagtaaatctgcaatagaatggatttgagctcgctttaagcagtta
taagccctaacagataactgcagttcttctattaaaacttgactaatttttttatcttct
ttactacgataattatctgctgatttaaagtctaaatttcttagtgaacaaaaaagatta
gttaaaactgtagctccttgacttattgcttcttgaggagagatgctcccatttgtccag
atttgtataatcaatctatcttttatgctgttgctaccaatacggacttcttctacttta
taattaactttattaactggcataaaaacagagtctacttgcaaaaaatccacagataat
tcatctactgctttttcagctaagcgatacccgcaatttttttcaattttaaactccatt
tcaaatattgtattattgcaaatagttgcaatatactgtctaggatctactacctctata
tcagaagataattcaaataggccggctgtaactatagctggcccttgaactctaattcga
ccaatttgagattctttgttataacttttaaatactacttcttttagattaagtaatatt
tctaacacatcttctcttaccccagggattgtagaaaactcatggttcactccagcaatg
cgtacagcaactatagcagtaccttcaagatctgacaatattgatcttcttaaagcatta
cctaatgtaataccttgtccttgatttaatggttctattacaaaactaccgtactgccca
cgcgccccatgtgttcttgactctacgcattcaatttgaaattgagccacctaagaaagc
tcc-tttaaaataatcagtgattagtaagcctctctaaaataagaaggaatttatactcg
gcgtttcttaggaggacgacatccattatgaggtacaggagtaatatcttttattagagt
aatctctaatcctgcagcctgcaaagctctaattgctgtttctcgacccgctcctggacc
atttactaaaacttcggtttggcgcataccttgatccatagcttgtttagctgctttttc
agctgcggtttgagctgcaaaaggtgttcctttttagctcccttaaatccacttgcacc
agaggatgaccatgataatgtctctcctttcaaattagtaatagtaacaattgtattatt
gaatgtagatttaatatgtgtaacaccgttaactgcattacgtttagtttttcttgctcc
ggattttttatttgtctagccatcgtgtctcgtctattatatagttaattagaagatta
ttttcttggagctttttcttacctgctactgtttttttacctcctctgcgtgttctagc
attagttctagttctttgtcctctcaaaggaagaccaagacggtgtcttctacctctata
agtactaatttccataagtctcttaatgctcatagactcaaaacgtttgagatccccttc
aatctgatagttagactcaagaatttctcttatactgacaacttgttgatcatttaaatc
ttgacacttaatatcaacatctatctttgtttttctaatatttctttcgaacgcgatag
tccaataccataaatatatgttaaagctatctctattcttttgtttcttggaagatctac
tccagcaattctggccactttgtattgtctccaataaataatatgtat-ttttataacgg
tagtttata-agcttagccttgtctctgcttatgtttaggattagtgcaaattaccatca
cctttctgtgacgccgaattattctacatttttcacacatttttcgaacagaaggacgaa
ctttcatattaa----ttctatatactaggatattactatattaactctattaattatc
gacattaaaaacgtcaataatatattgtattactttaactattttagattttacttggtc
atactatcatatttttttgatatgacgtatgtctgaatttgcttagctgtgtctattgcg
actccaactaaaataagtaaagaagtagccccaagacctcttaagttttggatctgagta

acctttttctattataaacggaatcagcgctactgtaaataaaaatgaggctcctaggaat
gtcagtctatttaatataacttgtaaataatcaatagtcgcttgacctggacgaatatta
ggaatgcttgcacccatttttttttagatttgtagcaatatcttctggattcattactatt
gatgtataaaaatagctaaagaaaaggatcaaagcacaataaagaagaaggtatagtgaa
ccattaggacaaaacaagtataggatttggagtaatgttttattttgaataatttgggtt
agataagatgggagggccatagatgcagatgcaaaaacaataggcataactccaccttga
tttaatttcaagggcaagtaactatttggatccaaaattgaagattttcctagctgtctt
gctgaaataattttaattcttcttgttccttcttgtacacaaattgtaattattatcatt
aataaaaagatcgctataaataatccaaacttgagacttgcattactataattagcatca
aaaaatgactgtgtaaaattctttggtagtcctgacacaatgttttgaaaaataagcaaa
gaggctccattaccaattcctttttctgtaattaactctgataaccacataataatcata
gagccagctgttaaagctaaaacagactcgcagacaaattcgacattccaattaaaaaca
tatggttttacccatatagagattgctccagattgtaaagtcgcccaacctagagctaag
taccttgtaatttgagttatttttttggcggcccaattcaccctcttccttctgtaatttt
tccagattggggacaattttcgtaagtagctgcattacaattgaagagttaatataagga
acaatacctaacgcaaaaatccctattgttgaaaaacctcctccagaaaaaatatttaga
aaatttactaaagtatttttttctacacttgcataaaaggcgtcatgatctatacctgga
acaggtataaatattcctaaacgtgctaaaactaatagaaaaagggtaaagataatacga
tttctcaggtcactttttttggctcataaataaattttaaatgaaaagagatactttt--tt
tatgtaaaaaccctattccaagacttatataagtttaaaatagggaagtaa---aatatt
atag------taata-taatattctatgtagaatacaagctttctataggcactcctcga
tctttagctgcttcactaaaagttcttaattgagttaagccatttagaacagctcgcgcg
ttatttaatgtattattagaacctaattgcttagctaatatattctgtacacccgacaat
tctaggactgtccttacagaacctccggcaattacacctgaaccaggtgcagagggcctt
aatataactttcgcggcaccagaaattccattaataggatgaggtatagagttcgatttt
gtcagtggaactgtaactagatgtttttttagcatctgttactcctttttttactgcacca
attacatcgcttgcttttcccacgccgacaccgacttggccttgctcgttaccaacaaca
agaataactcgaaagcttagtttttttacctcctttttacaactttagtaactcttttaact
tgtacgactctttcttcccagccactatctttatcttttcctttgctctgtttttttacga
ttggccattttaagaattattcctaattagttaatta-cgcttctagaaacccatgcctg
cttccttagcagcttcagccaaggccttaactcttccatgatataactttccacctctat
cgaagacaacatttttaatgccttctttcatagactgttctgctaactgtttaccaacta
cgcgagaagtatcacagtttggtcttatattatcggattctttactatttaaattaacag
atgatgtagctactaatgtaataccttgtgtatcatcaattatttgtgcgtatatatgtt
tgttagatctaaatacacacaggcgtggcctacttgaggttccttgaactttttttcgaa
ctcttttatgcttatgaattctagtttgtttagtgtttagtttcattattatttaccttt

tccagctttcccagcttttcttctaacaaattcaccttgatatctaattccttttccttt
ataaggctcaggaggcctaatagaacgaatagttgaagcaacctgaccgacaacttcttt
atctatgccagagacagtaatatttgtattattttcaactttaatttcaatattagcagg
aggtttaattttcactacatgactatagccaacatttaagattaaatcttggttatcaat
ttgagaacgatagcctacaccttgtatttgcagtttttttaaaaaatccattagaaacacc
ttcaatcatattactaataagagttctggatagcccatgtaactgactagccatttttgt
ttgtccactcgtcttgacagcaatcgtgtcattaagtatttctaaattaataccagctgg
taaagttctggatagggtacctttaggtcctgtcacagtgatagtctgaccatcaaactg
agtactaagatttgtcggcaataaaattattttttttccaatacgagacat-attccacc
tataaaatagttaccaaatatagcataatatttcaccaccaagaccatcatgacgagctt
gtctatctgtcataacacctctagaagtagaaataagagcgatgcctaaacctccaagaa
ctctgggtagctctttgtgatttgcgtagactcttagtccgggtttactaattcttttca
gagcagtaataactggttgacgattttaccattatacttaagagaaatcattaaattag
tctctataccttcacctatttgttcgaaattttgaacaaatccttcttcttttagtactg
ttgccatattgcatgtcattttcgttgctggaacttgcacaatttgatgtcttgctaagt
ttgcgttacgaatacgtgtcagcatatcggcgatcgtatcgttgaccaccttgatcctcc
ttgcga-----tgaattatttaaaatattttcaagattccctgaaaggcatccctagctt
ttttaacaaagctagaccttcttgatctgttttagctgtagtgacaattgatatatctag
accacgaatttgatcaatattatcatagtctatttctggaaagatcaactgttcacgtaa
acctaaattgtaattacctttgccgtcaaaactttaggactaattcctctgaagtctct
aattcttggtaatgttaaattaattagtttctctaaaaaagaatacatcttgtcttttct
tagatgcacaacaattccaataggaacctcttctcgaattttaaatcctgcaatagattt
tttagcttttgtgacaatcggtttttgtccagttattaatgttaattcttgaatactact
ttcaagagccttagcattctgagaagcttcacctaagccacggttaatagtaattttagt
aaacctaggaacttcatgtacatttttgtactgaaattcatcttttaaagattgagtaac
agttgttttatattttctttaatcctattgccatggtatttttatcaattatttaata
agttcgccagtctttttcagttttcgaa-tttttggcctttatcattaattattactga
ggatcgactagcaatattattttgctcactgaataacataacattagaggtatgtattgg
agcttcaaatttgataatttctcccgtttctccttcttgttgaggtttttttatgcttcac
tttgagattaattcctttaacaatcaccttacttgttttatgaatgattgcaataatttc
acctgtttttgttttatcacttccagaaataacttgaactaagtcccctttttttaattt
aatttttgtattattttttgattgttttagaagaacttttcattatactacctccggtgca
agagacactatttttagaaaaattttatctcttaactctcgggctataggtccgaagact
cttgtgccacgtggattattatcttgattaatgataactgccgcattatctccaaatcta
atgctcataccgtcagttctccttaaagctttacgagttcttactacaacagctctgaca
acatcagatcgtttaactggcatattgggagacgcatctttgaccactccaataataaca

tccccaatagatgcataagaaggattactagtgcctaatactctaatacacattattttt
ttagcaccactattatctgcaacattaagatagctttgagtctgtatcatacttttattc
tgtataaaattaattatcaaaagacttagataagatattaaccattgtccagcactttgt
gcgacttaaaggtcttgtctcttgtattgtaacaatatcaccgattgtgcattcattatt
ttcatcatgcgctttatatttctttgtccggatcatagtttttgcgtactttctatgaga
gattctatttctacagctactactatagttttattcatttatcgcttactactttacc
tgttgtttcttttaaaggcatagtctttatattttttatatat---------aattaaat
tttaagattattgcacggttgacttattacgagacttttcaacagttaaaagttgagcta
atctatgcttagaatgtttaaatagatgaggctgaaaatcttgccttgtggctcttttta
gccttaaatcaaaaagctctcttttattacaaggatttcttcagctaaagaagaagagt
ccatgtttgtaacatctgatattttagggaaagtcatgttttaggattctgttgtattcc
gaactataaatttggtcttgattggtaatttataagaagctaatttcatagcttgttgag
cagtttttgtggtacacctgtaatttcaaatagaatatgtccaggcttaataactgcaa
cccaatattctggagctcctttacctgaccccatgcgagtttcagctggacgggcagtga
ctggtttatctggaaatactctaatccataatttgccacctcttctaacatatctagtaa
tagttcttcgagtagcttctatttgtctagaagttaaccaaactggctctgtcgcttgta
acgcataatcaccgaacgcaattgtgttgcctttactagcagaacctttcattctacctc
tgtgttgttttctaaattttgttttcttggggcttagcataaaaattaaataataataat
aattaagaagctgttgaatcaggagtttcctgattataagcagattctagacttttcgat
tctggcaaaatttctcctttaaaaagccaaactttactccgagcacaccatacgtagtg
tgagcttggcgatgacaataatcaatatcagctcttaaagtttgtaaaggtacacgacct
tctctaacccattcacttctagctatctctgcaccattaagccgaccagatacttgaatt
ttaacaccctgtgtatttgctctttgagctctttgaactgcttgtctaacagctctacga
aaggcaactctttttttctagctgttgagtaataaattctgctactaaggttgcttcagaa
tctggatctgcaatctctataacattgactctgagttgtttgctagggcctagaattaag
gacaacgattttcttaaagactcaattccagctccagattttccaagtacaattccaggt
cgagctgttgctatgagaatttctacttgatcaactttacgattgatttcaattttagca
atactagcattactaagttttgaatgtataaatgagcgaattttatgatcttcttgtaaa
agaactgggtagtctttagagttagcaaaccatgaagaacgatgtttttgggtaatgcct
atgcgaaaacccaaaggatgaatttttttgacccacagtatccttgattataaattaaaag
ctataatgactaattgacgttaag--------agactacagttctgagacacctaatgta
atatgacaagttggcttatgtattggaaaggctcttccttgggctctaggctgaaatctt
tttaaagtcgggcctttgtctgcaaaggctttactaacaaataattggtttttatttaat
ccatcattatgctcagcattcgctgcagcagattccaggatttgctttatatgagaacag
actcggtatggcataaattccagaataattaatgcttcttggtattttctacctctaatt
tggtctaatacacggcggactttatgcggagaaagacgaatgtatttccctactgcttta

gtttcttttacgtttttttgtaatactcatatttatagtttaacgacgggcttttctgtca
ccttttatgtgagtgcggaaagttcttgttgggacaaactctcctaatttatgtcctacc
atttggtctgacacaaagacaggaaaatgttgtttgccattataaacagctattgtatgc
cctaccatatcgggaataatagtagatgctctagaccaagtttttaggacttcttttttt
cctgaaatatttaatgcttctatacgtttaagaagactaacatcaataaaagggccttta
tgtatagatcttgacataataataaatcattagattttgataaaataaattctatttac
ggcgacgtaagacgtacgggttactatatttatttggatttcgtgttttaacacccaatg
caggtttaccccaaggtgttacaggacgagctcttccaattggagatttgccttcaccac
caccatgtggatggtctacaggattcataacaacacctctaacagtaggtcttttacta
accaacgatttctaccagctttaccaagagtaatattactagcatcaatattaccaactt
gtccaatagtagcatagcattcttttcgaatcattcgaacttcacttgaaggcaattta
cagtaacaaaagcaccttcttttgctactatttgagcataagttcctgctgcacgaacaa
tctgtcctccgcacgaaggtcttagttctatattatgtactgctgttcctaaaggaatac
tagataaaggcaaagcatttccaacttcgataggggcagtagaaccagaaagaaccatag
atcctacacttagagatcgagggtgcagaatgtatcttttctccccatcaagatagtgta
ataatgcaattcttgcatttctattcggatcatattcaatagaagcaactttagcagcta
tattatgcctatttcttttaaagtcaattaatctgtactgctgcttatgtccaccacctt
tatgacgacaggtaatgacacctctattatttcgacctttgcagaaatggtgtttaacta
ttaatgatttttctggcttatcagtagtaatctctgaaaaggtagaaactgttctatttc
ttgttcctggtgtgtaggcacgatataaacgaattgccatataagaagatttgagaagag
taaatgttgaa------ctatgtaatatatttaagtttctggaaacaaattgatagaatc
ttttgaggcaagtgtcacaattgctttcttgtaatgtggtcgcttgcctacgaatctgcc
gatgctcctttttttcttaggaggatggcaagtgttaacacctgtaacttgcacgttgaa
aatatactgtatagcagccttaatattaattttgttgctttgggatcaacagcaaaaca
atactgatttcttctaataatttagttgtcttatcagtaatgatgggatatttaactag
atctaataaacctcttgaatcaatgctatccattatatacctcttgtattttagataaag
catcaactgtaataataattttatgtgcagctaacaaagccataatatttagtgtatcag
ctgaaataagttctacgttgtgtagattgcgaatagaaagataaacgttcggatcttttt
tgtctactatgactaagactttttgtttaaatcaagattccatcgatgtatagcttcca
taaatagtttagtcctgggctgatgaaaataactgttaaaattttctacaatcaatgtat
taactgatttgttatttaatgctgtccttaatgctaattgtctttctttttattcattt
ttttggtaaaactacgaggttttggcccaaatattacaccaccgcctctccatagaggtg
aacggatagaacctgctcttgctcgaccagtcccttttttgacgccatggtttacgacctc
cacctcgaacttcacttcttgttttagtattcgcagaaccctgacgcttttcattacttt
gctttactaacgctctatggactaagtacatccctgaatcttgactaaccttaagattta
aatcagcgttgccacttacttgaccttcccaattatagacttgataatttaattgtgtgt

taactgtcatgaaatatatcgtatgaaagaatattgctacttactaattttactaaagc
gccaggtttaccaggaacagctcccttaacgatcaacaaatcattctctgagttaatact
tacaatttgcaaatttttattgtaactttttattaccaagttggccagccatgttttt
acctggataaactcttccaggcgttgtaccagctccaatagaaccaggttgtctatgatt
tttcgaaccatgtgacattgggcctctactaaaatgatgcctcttttggtaaccggagaa
acctttacctacacttctagaagagacattaatcttttggcctacttgaaatagatctgt
agatagtatttggctaacttcgaaatcatcagtagatttcacttcgtattcacgtaagta
ttttagtgggggagcctgtgatttttttaagtgtcccaacaatggtttatttaactttg
ttcagcaacttgcttataaccgacttgaatagcattatagccatcagtagatatagcttt
aatttgagtaataatacatggcccaacttgaattacagtaactggaattgataaaccagc
ttcatcaaagaattgggtcatacctactttagtaccgagtataccaacagacactagatt
tctcctttttgagagatttatactatcattgtacataaaaattctacttcatattgactg
aag--taaattaaactgactgatgcatgatattattcatgacaacataaaggtttaaaac
tttacctatttaatctaaaatagaaacagctttttgtccagttacagcgtatataaatta
gaagcatgcttattaaatttttatagactact-gaaatttcactgatattgatattcgt
aacttacttgcagtaat--------atcaacatgtataagtttaattgcctagactagaa
ccagctattattagaaatgattcaagtatagataaaagcgcatttgaacttttatctatt
gacaaatatagtagaatatattgaggttcggtaactacaccttttttattttcacatttat
tgtgca--gtatatatacatctaatgcagtaaacataagaatataac----tgtatagca
aatgtaattacaaaaattagagaggtattaatgggcaaagtcgttggaattgatcttgga
acaacgaattctgtaattgctgttatggaaggaggtaaacctaccgtcataccgaatgca
gaaggttttagaactacagcttctgttgttgcatatactaaaagtggagataaacttgtc
ggacaaattgccaggaggcaagctgttattaacccagaaaacactttctactctgtcaaa
agattcataggacgtaaacagaatgaaatttcgcaagagattcggcaaacatcatataat
gttaaaactagtggatcaagcataaaaattgcttgccctgcactgaataaagattttgct
ccagaagaaatttcagctcaagtactgagaaaacttgttgaagatgctagtacctactta
ggtgagactgttacacaagccgtaataactgtaccggcttactttaacgattcacaaaga
caagctactaaagacgcaggtaaaatagcaggcttagatgtattgagaattattaatgag
cctactgcagcttctttgtcttatggactagacaaacaaaataatgaaacaatactagta
tttgaccttggcgggggcacatttgatgtatctgtattagaagttggagatggagtattt
gaagtactctcaacttctggagatacacatttaggtggagatgactttgatcagcagatt
gtagaatggctaatcaaagattttaaacaaaatgaaggtattgatcttggtaaagataga
caagcacttcaaagattaaccgaagctgcagaaaaagcaaagatagaactatcaaattta
actcaaacagagattaatcttcctttttattactgctacacaagatggtccaaagcattta
gaaaagactgtaactagaggtaagtttgaagaactttgttcaaatttaatagacaaatgt
agtattcctgtaaacaatgctctgaaagatgcaaaactagaagcttccagtattgatgaa

gttgttttagttggtggatctacaaggattccagccatacagcaaatggttaaaagatta
attggtaaagatccaaaccagagcgtcaatccagatgaagttgttgctattggtgcagcc
gtacaagctggagttttagcaggcgaagtcaaagatattctattactagatgtgacgccg
ttatctttaggagtggaaactttaggtggcgtgatgacaaagattataccaagaaacact
actattcctacaaaaaaatcagaagtattttctacagctgtagataatcaaccaaatgtg
gaaattcaagtacttcaaggcgaaagagaactaacaaaagacaataagagcttagggacg
ttccgattagatggcattatgcctgcacctaggggggttcctcaaattgaagttacgttt
gacattgacgctaacggaattttatctgtaaaagccaaagaaaaggctactggtaaagag
caatcaattactatatctggagcatcaactctacctaaagatgatgttgaaagaatggta
aaagaagccgaagaaaatttcgatacagaccaaaaaagaagaaagaacattgacacaaag
aatcaagcagagtccctatgttaccaagctgaaaagcaagttaaagagtttgaagacaaa
attagccaagatttaaaaataaaaatagaggagctaattacagagcttagatctagtcta
gaaaaagaagaatatgacaatatcgaatccatttctcagcaattacaaaattctctgatg
gacattggaaaaaatgctgctcagactgaaagtaaagatgcaaaagcaaaggatgacaac
accgtgattgacactgatttctctgaagctaagt-aaaagta-agcgggtaacgcgattc
gaacgcgcgacatcaaccttggcaaggttgcgctctaccactgagctatacccgcatatg
ttgtattattacaaaaattaagatatttgtcaatctgtta------atctatac---atc
tagactattaatttccgaatcacatattagctaaatgttgtaaatataacaattgctata
actttttaa-------aagtacttatatcat-tatataagtactttttatgtt-------
-caaaaatacaaataagtattatag-------ctaaactactaaggaattaactaagcta
gtaacaattactaatcctgtccataagccagctcccgtatatattaatccttttgattgc
tcccattgacctggggatgctaaaacaacaggtacacctactactaacacggtcgacaat
gcaattaataatagcactaataattggatcgcaataatcatttaatatgttctcctgaaa
aatttaaatgttaataatactgtatataatacaatgaagcttttgaccttcagcaattta
tctatgaagaaataatttaaaattattgttttctaaataaaccttaaattaagtattaaa
atctttattgtaaataataatactatattttttta---tagataaaaaatttaaagaaag
tctttttaaaaaaaaattaagataagatgatatgctgtataaaaagctaagtattatta
attattagagaaatttaaa-ataagaggtaaaacatatgaactcagcccttttttggca
aaactaccagaagcttatgccgttttaaaccgatcattgatatcttacccgtaattcct
gtatttttcttcttttagcttttgtttggcaagcagcaattggttttagataaaattta
ggttataaaaagaataacaaatatacaaagtgacatttttgagctctaatcttatatct
tacacaataataactattatggttgaacccttactatcaggaattgttcttggacttatt
cctattactttattcggattattagtagcagcttatttacaatatcaacggggcaaccaa
ttaggactgtaaacgctaagtcatagatatttataaaaactacaataattatagtttttta
taaatatctacaaataagctaattttaaatacaaagataatattattgaaactagagtga
taacgaaaaagatttatataa---taa---------------------------------

---------------------------caaactaattgtttaccagctcgacttagtaac
tcctggtaaaagacattcatgagacatttccctaaaaacatggcgagataatccaaaatc
tctataataccctctacttctcccggttaaccaacatctatttctccctctaacaggggc
gctgtttctaggcatttcttgaagtttttgtcttaaatccattttgtcagaaaagctgcc
agcttctttcagctgatctttaatagccaatctctttaagtaatattttttttgcaagttt
cgatctcttaatctctctttggatcatattctttttagccatgctgtag-aactctcttt
taaaattatttattcagtatattagctaaaaaagctcctatctgcaacttttatctattt
aaaaataaatagcataaagtagtaaaatgcaattgatatttaatcaacgatacc---act
ttatgctattatcaaaaatttatttaataaagttcttgttcaacatgagttgaaatagtg
caatcagatgaagggtatgcaatacaggttaacacataaccagctaaaagttgatcatca
tctaagaatgattgatcagcttgatctacagttccttctgtaactttacctgcacaagtt
gaacaagctccagctctacaagagtaaggcagttcaattccttcttcttctgctgcatct
aaaatatatgtatcctctgcacaattaaatgtaacatcaattccttcttcttcacatagt
aaatgaattttataatcagccatttctttttactccataataaagatacacttgaaactt
aataaagcaaaat-aatttaaagaacttaccaaaaaattatatactagtttcattatgca
tgatagactggaaaaaaagaaaataatatactattctgaaaaatcaggctacatttccta
ctaaatagaactatagtaggtccaaagtatatacataaattcatatagttaaaattattt
ttatcatttaaatagctgaacttaatagta---------taggttgatcatc------aa

gctagtcttctgc---caataaataattaattatgatttcaatgcttaatcaacaagtag
aacttaagcctattcttaaattccagataacacaaatatattcacattctcttattcagg
aaattaaagcattaataaccagacttgttttgcaagtttggaggaggccggctacattga
tggcaggtattatgcaacctttgttatggttaattctatttggtgggcttttctacaatg
ctcctataaatttgtttactatcaatacaaactataattgtttttttgagctctgggatta
taattttcacatctttactggagctttaaattcaggtcttccattaatgtttgatagag
aatttggatttttaaatagattattaacggctccgttagtatcgagggcttctatcattt
tatcttctgctacttttatgacttgtattagtttaatacaagttatatttatagttacag
cttctctttttatgggaaactcacctctaaatagcgatagtgctatgatttttggactta
taatcctattagtgactgtaggagttacaatgcttagtttagctttatctttcactctgc
caggtcatattgagctattagcatttattttagtagttaacttacccttttttattttcta
gtacggctttagctcctttatatttcatgccaccatggcttcagttaattgcaagtctca
atccattgagttatgccatagaaggtacaagatatttatattcaagcgtaaactggaatt
ttacagagtgtgtgattaagattagctggggagatatttgtttagggcagattattgttt
tattaatcgctttagatataatggcagcttatgttgtgtctaagatattaaaagctaaac
ttaatta-----------------aaatttaataaaaaatttattaaatttatttaaca
aagattttactatataatactaatagtagtatggataattgaaatagaaatagtttttc
aaaaccaaagctattatcaacaatgataaacatttgtaagaaagtcaacaaagtatgttc
ttattcataggaggcatgtagtcaatgggactaccatggtaccgtgtacacacggttgtt
ttaaatgatcctggacgcttaattgcagtccacctaatgcatactgcacttgtagcaggt
tgggcagggtctatggcattatacgaactagctgtatttgatccttcagatccagtacta
aatccaatgtggcgacaaggcatgtttgttatgccatttatggctagactgggcgtaaca
gattcatggggaggatggagtataacaggagaaagcgtatccaatcctggactgtggagt
tttgaaggtgtcgcgttaactcatatagtcctttctggcatgcttttcctagctgctatt
tggcattgggtttattgggatttagaattatttagagatccacgaactggcgaaccagct
ttagatttaccgaaaatttttggaattcatttattgctatcaagtctactttgtttcgga
tttggagctttccatgtgactggacttttggaccaggaatgtgggtatcagatgggtat
ggagtaaccggaaaggtgctaccagtagctccagcatggggaccagaaggattcaatccg
tttaatcctggaggagttgcatctcaccacattgccgcaggtactgtaggtatattagct
ggtgttttccatttaactgttagaccaccacaaagactgtatcgtgctctaagaatgggt
aatattgaaactgtattatcaagtagtatctctgctgtttttttctcagcttttgtgact
tgtggtacgatgtggtacggctctgcaactacgcctattgaattatttggtccaactaga
tatcagtgggatagtggatattttcagcaagaaattgagaaacgagtagaaaatgctatt
gctgatggtgctgcacctagcgaagcatggtcaaggattcctgacaagttggcattctat
gactatattggtaataatccagcaaaaggaggattattcagagcaggccctatgaataaa
ggcgacggagttgcagaagcatggcttggacatcctgtattccaggataaagaaggaaga

gagcttagtgttcgcagaatgcctgctttttttcgaaactttcctgtaattttagttgat
aaagatggtattatacgagctgacattccgtttagaagagcagagtccaagtatagtatt
gaacaagtaggtgtaacagctagtttttatggtggcaaattaaatggccaagttttcaac
gatgctcctagcgttaaaaaatatgcaaggaaagctcaattaggtgaggtatttgaattt
gatcgaactacattagaatcagatggagtatttaggagtagtcctagaggctggtttaca
ttcggtcatgctaatttcgctttaatttcttctttggacatctttggcatggttcaaga
actatcttccgggatgtatttgcgggaatcggagctgaagttactgaacaagttgaattt
ggagctttccaaaaattaggagatagaagtagtaaaaaacaaggagctgtataagaccaa
agtcaaagacagtatttgtc---aaatatttccaatattaaaatagttaagacaaagttt
attatctaatagttactaatcaggagaatttatggaagccttagtctatgtatttttact
aacaggaacgctgatggttatattctttgcgatctttttagagaacctccaagaatagc
aaagtaacttgattccgtttaaacttataataatagaaccactttccaacttattagtgg
aaagtggttctattgtagattacattgtattattcttcatgctcttcaaaaggatctcga
aggtcttttgaagcaggaccaaatgctgtgtatatcgagtaacctgtaatacctaaaagc
agactcgaaataaaaatactaagaactgttgcagtttccataattcaagcagtattaaga
tgaacttttttctataatgatattatcaatattaaaacatttaattattaaaattatcaaa
atatactatggcacttagaactagacttggagaaattttaagacccttaaattcagagta
tggaaaagttgctccaggttggggcacaactcctattatgggaattttcatgttactatt
tttcttgtttttattaattattttacaaatctataattcttcattagtattagaaaatgt
agatgtagattgggctactttaggtag----ctaattagataagttctttga--------
ttaaacaatattaaagataac----------------aagcgatcgctattagattatag
tagcgatcgcttaaaaaatgttttactgtatatccagagaaaattatttacctaaattaa
ttaactcgctatctgcaaaactatttgtattaacattattatagttaactttttcaaacc
ttactaatactggatatttgataccacttttatcaattgctgcaacagtaccaatttcct
gataccagtatgattcctttctcaatatttttacttttgagcctcgttccatatttttag
tccttagatagattattaaagattattatattgaagaattttaaaaaactagacaaaata
tataaaagtactaccgacaattgtaatacctaactaataaaaggagagagagagggattcga
accctcgataacaaaagttatgacaattttcgaaattgttgcaataaaccgctctgccat
ctctcctaaattaaaatactcttttagaaaattagacaagaataacatagaactaaggta
gttgcctagaaaattaaaaatatgttaaatataaattataatctttctatagatatattt
acaaaatatgtacatagaatctatatcagcttatttcaagatttttttaatcttatacaat
ttttactaaataaataaattctctcataatatgaaactatcttggaaaacacttcttctt
tggtctttaccaatatttgtcgttggtttcttttttctggcaaggtttttttaggtccaact
actacagatgttggcagtaatatcgcaagttctagaatgacatatggacgatttttagaa
tatttagatatgggttgggtgaaacgggttgacctctatgaaaataatcatacagcaatt
gtagaagctgttggtccagaattaggaaatagagttcaacgaattcgagttgaactgcca

gcaagtgcgccagaattaattacaaaattacgcaaagccaatgttgatctagatgctcat
ccccctaaaagtacaagtgcagtatggggactattaggcaatttactatttcctttacta
ttagttggcggattagctttcttatttagaagatctaataatgctagtggtgggcctggt
caagcaatgtcatttggcaaatcgaaagctttgtttcaaatggaggctaaaacaggagtg
gtatttaatgatgtagctggagttgaagaggcaaaggaggaatttcaagaagtagtaaca
tttttaaaacagcctgaatcatttactgctgttggtgcaaaaataccaaaaggcgtttta
ttagttggtcctcctggcacaggcaaaacattactagcaaaagctattgctggcgaggca
agtgttcctttttttagtatctcaggctcagaatttgtagaaatgtttgttggtgttggc
gcttctcgtgtgagagacctattcaagaaagcaaaagacaatgcgccttgtatcgttttt
attgatgaaattgatgctgttggtagacaaagaggaacaggcgttggaggtggtaatgat
gaaagggaacaaacattaaatcagctattaactgaaatggatggctttgaaggaaatact
ggtgttattgtaattgccgctactaacagagctgatattttggattctgcattattaaga
cctggaagatttgatagacaagtttctgtagatgtaccagattttaaaggcaggttagca
attcttgaagttcatgctaaaaataagaaaatggaacctaaagtatctttagaaactatt
gccagaagaactcccggcttttcaggagctgatttagctaacttactaaatgaggctgct
atcttaacggctcgacgaagaaaaaatgcaatgactatgtctgaaattgatacatcaatt
gatcgagtagtagctgggatggaaggcactcctttaattgatagtaaaagcaagagatta
attgcatatcacgaagtgggtcacgcaataataggcagtttattagagcatcatgatcct
gtgcaaaaagttacattaataccaagagggcaagcaagaggcttaacttggtttactcct
agtgatgatcaaagtctaatatcaagatcccaaatactagcccgtatcgtaggtgctctt
ggtggtagagctgcagaagaaatcattttcggcgacgcagaagttaccacgggtgcaagt
aatgatttgcagcaagtaacatcaatggccagacaaatggtcactcgatttggcatgtct
aaaattggacctttatctcttgaaagtcaaggaggagacccatttttaggtagaggcatg
ggaggaggctcagaatattcagatgaagttgcaactaatattgataaacaagtaagggaa
attgtaagtgaatgctatgcgcaagctaaaaacattattatagataatcgagtagtgatg
gatagattagttgatttactaattgagaaagaaacaattgaaggcaatgaatttagagac
atcgttaaggaatatacagcaattcctgaaaaaaattactacatatcacaattttaa---
---------------attagacgggactgacgggattcgaacccgcaacttccgccgtga
cagggcggtgctctaaccagttgaactacagtccc-aaaaagataaatacaaatagataa
tctcatagttagcttaaacttgtcaaattagaatattaagtatgaagagta---------
--------tttgtaatagtactcttcatatttaata-tagtagcgaaaatatttctttta
gaaggagagagagggattcgaaccctcggtacgaaattaatcgtacagcagattagcaat
ctgccgctttcgaccactcagccacctctccatga---tctatatataaaatatatcaaa
tatacttatatttaataggtgtgcataaatatttatggagctaagcggattcgaaccgc
tgaccctctcaatgccattgagatgctctaccaactgagctataaccc----ctttatga
ggatattaagaaatggctcaagcgggatttgaacctgcgaccttgggcttatgaatcccc

tgctctaaccactgagctactgagccatagtataaaatattattaattattataccataa
attatgctcaaatataaatcaataaaagaataattactatca-ttcactactttta----
-----------------tacaagttgtaaatattcggacaagatttcaaaattaaaaatg
tctttaacttttgtttga--------attatttgtttacgaaa-----------atattg
gtatttaaaattaac---
--------------------aataaatttg------------------------------
---tctttattt
g---

-------tacaca---
-------------------------------caaaaatattggaaa--------------
------------------------ttttcatcaatt--------aattatactaattagt

------------------tagactataaccgacgtac-----------------------

---aagaattatttaacta---

---cgttaaat---

-------------------------------------tgtgatgcatt------------
-----gggcttgatcaaaacttctctaaaatcaacaaaatctagt---------------
---aattcaa
gtaaatcaaatact----------acagacgcttaaaaaaataata--------------
caattagaatcgtaaacatttagtttttga-ttatgttaaataaatttgattatcataga
tttaatatttaaaagtgttctattacttgta-----ttgagtaactgt-aatatttaatg
tcttcttttttagataataatcataatataagctaaaaaatattttt---tattaaacaa
ctagcatagagccaataccttgatcagttaatatttctagcaagagagcatgatctattc
taccatctagaatatgcgctgaagctacaccctgagctaaagaacgaatacaacaattca
ctttaggaatcatgccaccagaaattacttctgtttgagttaaatctctagcttcctgta
tattcagatgactaattaacgttgaaggatctgaagaattacgtaaaataccaggcgtat

ctgtgagtagaattaattttttcggcattaagagcggctgctatttctcctgcgacagtat
cagcattaatattatacgattggccttgcttgtctgctgcaacactagctataacaggta
tataattattattaattaatatcttcagtaatttagtatcaatattttgtacttctccaa
caaagccaaggtttgcttttccatttggtcttggagtaataagtaatccatctttccccg
acaaacctacactttaccaccttgtttattaatagttgcgacaagatctttgttgactc
ttccaactaaaaccatttccactatatccatagtaggttgatctgttactctaacgccgt
catcgaattttggtaatattttaaggcgatctaaccaaaaattaatttctggtcctccac
catgaactaaaatgggacgtagtcctataaaggacagaaacacaagatcactaatcactt
gatctttttgtttttgatttttcatagccgatcccccatattttataacaataattctgg
aagaaaattgctgaatataaggcagagcttcacttaagactcttactctttctgaattag
tcaacataaataaagtaggattatgatatataaaattatttgcacaacttgtgttaattt
aaaaagtaccagtaggtacttc-aaaacttgaaaaaatagacatgttactaaaaa-----
----ttaaaactataaggattataattatgaataaattttgggataatgttttaagattt
ccacgatttttagtcagcgtcattttaggattagttttgataattattagtcctttttttc
gtgttattaaaaaaaccattgacaagttttttttttcatcatatcattgacgggattaatc
acagttttagcgataataa-tacaaaaaaatgctaaatatcgaatgttgttgagagaaata
aataaatttaattaaaatttttgaacatatataatattatattatcatttaatatgataa
tct-attttcttttccat---aaaacttataagtattaaatattatttgataaatattat
gccactaaaacaaatagttagttctgaaaaaaactggtgcctttgcacttctggatagtat
tgttaggcacggagttaaacatatatttggttatccaggtggagccattcttcctatcta
tgatgagctttacgcttgggaggaagcctatctaattaaacacattcttgttcgccatga
gcaaggcgctgctcatgctgcagattcttattctaggtcaacgggagaggttggagtatg
ctttgccacttctggtccaggggcaactaatcttgtctcgggtatagctacagcacacat
tgattctgtacctatattagctataacaggtcaagttggaagagcttttattggcacaga
tgcttttcaagaagtagatatttttgggattacacttcctattgtgaaacattcatatgt
agtccgtgatcctagagacctgtctagaattgttgcggaagcatttttttatttgtaaaca
tggtagaccaggtccagtattaattgatgttcctaaagatgtaggattagagaagtttaa
ttatttttctgttgaacccggaaaggttaatattcctggctgtaggccaattaccagcct
aaagtcaagacaaatccttatggcagctaaaatgatacagcaatccagccaaccattgtt
gtatattggtggaggagctataatctctgattcacatcaaattattaaagaacttgttga
tttttataaaatacctgttactactactttgatggggaaggggattttttaatgaggacag
tgactattgtctaggaatgttaggtatgcatggtactgcgtatgctaattttgcagttag
tgagtgtgatcttttaattgctttaggagctagatttgatgatagagttactggaaaatt
agatgaatttgcttgtaatgcccaagtgattcacgtagatattgatcctgctgaagtagg
aaaaaaataggattcctcaagtcgctattgtcggtgacgtagcagaagttgttagtgcaat
attaaatttattaaagacttcttttccccccttatccagatcagattacatcttggcaaga

aagaattaatcgttggcgtcaacagtatcctttactggttcctagaaaatcaacaagcat
ttcacctcaagagattcttgttgcaactaataaattagcccaaaatgcttattttactac
agatgttggccagcatcaaatgtggtcagctcaatttctgaaagtaaaagctaagcattg
gctttcaagtgctggattaggcacgatgggttatggtttacctgcagcaattggtgctca
agtagcacatcctaatgacgtagttatttgtattagtggtgattctagttttcaaatgaa
tatgcaagagttaggaactatcgcgcaataccagctaccggttaaaattattattattaa
taatcgatggcaagggatggttagacaatggcaacaggctttctatggtgaaagatattc
acactcaagaatgacagaaggagcacctgattttcaaaagcttgcagaagcttttggtat
taaagcttttactattaataataggcaaaatatgcaatctgctttacaagctgctattga
tcatcctggtccagttttattagattgccaagttacagaaaatgaaaactgttatccaat
ggttgctcctggaaaaagtaatgcacaaatgataggtatagctaaaccgcagagaggaac
tgcttccaactatattaataacagcgttggaagtaa------------------------

---------------atagttagtattttctt-aaaacagaata-aagtaaatgaagtat
ctaaatgtaattttcttatactatta-aaaactatgtaagaaaagcctttgacgagaatt
gaactcgtgaccttccccttaccaaggggatgctctacctctgagccacaaaggc-tttt
tactatattgggccgggttggatttgaaccaacgtaggcgaagccagcggatttacagtc
cgcccccattaaccactcgggcaccgaccctagttaaaccttatttaagctatcatatac
aagatatcataattatgttgctaaaaacaagtacttttataaataaaactgaataagtaa
tggacatagctggacttgaaccagcgactttcacgatgtcgacgtgacactctaaccaac
tgagttacatgtccaaaactttaatcattattataacactgtttagtgattttataagta
tttatttctaattgaagatttgttttaatcggccggctttaccgagcagatctcttagat
aatacagtttagctctacgtattttagctcttctaataactctgacaaaagttattcgtg
gtgaattaagaaaaaatactctttcaactccaataccttgaaatgagcatcttaaagtta
aactagtatttaagctttttttcttcttctggctaatactactccttcacataattgtt
cacgagttttactaccttccttaaccattagtcccagttgtattgtatcgccaactttaa
tttcagatacttctgttttgacaagaggtatctcaacacttttcattagctgacttagct
ttgtattatttgctttcatatataggattagtcttgtgaca-tgttttgaaatattatac
attaaaaaaacaaatatgaaaaattttagcgaataagcatattagtatttataaaacttt
tttttattt---
---cgattata--------
----------------catatccatt------------atattcaact------------

----------acattcttccatagtttc--------------------------------

--------tttgataatcagtaa-------------------------------------

---------------------------atcta----------------------------

----gatgatcacatctcattttcaaacatggcttatgtatgaaaaagttctata---aa
aatctaatgactaaaataataaaattaaggcttaagtatttcttagttttgtgttataat
tagttactatcaaaggtagtggcataacacgaagcataaa-aatgttcgaacgctttact
gaaaaagctataaaagtcataatgctagcacaggaagaagctagacgcttaggacataat
tttgtcggaactgagcagatattattaggattagttggcgaaggtactggaatcgcagct
caagtcttgaaatcgatgaatgtgaatctaaaagatgcaagagttgaagttgaaaaaatt
ataggaagagggtctggctttgtagcggttgaaattcctttcactcctcgagcaaaaaga
gtactagaattatccttagaggaagcacgccaactaggccataattatattggtacagaa
cacttgctaatgggcttagtccgagaaggagaaggagttgcggcaagagttttagaaaat
ttggcagttgatgtttcttcaattagagctgaagttatacaaatgcttggagaaaatgcg
gaagccaatgtaagtggaagcaatactacgcaggctagaagtaaaacaccaacactagaa
gagtttggatctaacttaactcaaatggctattgaaggtggtttagatcctgtagtcgga
agacaaaaggaaatagaacgagttatccaaatcttaggtagaagaactaaaaataatccc
gtcttaattggggagcctggtgtaggtaagacagcgattgcggaaggattagctcaaaga

attgctaatagagacgtaccttctattttagaagataaattagttattactcttgatgtt
ggtttattagtagctggaactaaatatagaggtgaatttgaagaaagactaaaacgtatt
atggatgagattaaatcagctgataatgtaatattagtgattgatgaagttcatacattg
attggagctggtgctgcagaaggagcaatagatgcagctaatctgcttaagccagcttta
gcaaggggagaattgcaatgtataggtgcaacaactttagaagaatatagaaaacatata
gaaaaagatccagcattagaaagaagatttcaaccagttgtagttggagagccaagtgtt
gaagaaacaattgaaattttatttggtcttagagaccgttatgaaaagcaccatcaatta
acaatgtcagatggagctttggctgcagctgctaaatatgctaatcagtatatttctgac
cgcttttttgccagataaagcaattgacttaattgatgaggctggttctagagttcgttta
ctaaattctcaattacctccggctgctagagaattagataaagagttaagagctgtatta
aaaacaaaagatgaagctattagagctcaaaaatatgaaacagcagagcagtatagagca
agagaaatggaaattaaagctcaaattgcagcaattgctcaaagtaaaaagaatgagcct
gatttaaatttagaagatcctgttgttacagaagatgatattgctgaaattgttgctgca
tggactggtataccagtaactaagcttactaaaagtgagtcagaaaaattaatgcacatg
gaagaaactttgcatggacgcattattggtcaagatgaagcggttgtagctgtctctaga
gcaataagacgcgcaagagtaggtctaaaaaatcctaacagaccaattgcaagctttatt
ttttccggaccgacgggtgttggaaaaacagaattaacaaaagctttagcttcttatttc
ttcggttcagaagcttctatgatacggctagatatgtcagaatacatggaaagacacact
gtatctaaactaattggttctcccccaggatatgtaggttatagtgaaggtggttatcta
acagaagcggtaagaaaaaaaccatatactgttattttatttgacgaaattgaaaaagct
catccggatatttttaatctacttcttcaaattttagaagatggccgactaacagatgca
aaaggtagaactattgatttcaagaatactcttttgattatgacttctaacattggatct
aaagttattgaaaaaggaggaggaggtttaggctttgaattatcagaagatcaaacagaa
tcccaatatactagagtacgatctttagtaaatgaagaattgaaacaatactttagacca
gagtttctaaatcgattagatgaaattattgtatttcgtcagcttactaaagatgaagtt
agagaaattgcagaattaatgctcaatgaagtctttgcgagaattaagcagcaagatatt
caattaaatgtaacagaacgatttaagcaacgattagtagaagaaggatataacccaagc
tatggagctagaccacttagacgagcagtaatgaggcttttagaagatagtttggctgaa
gaagttttatctggtaaaattaaagctggtgacagtgcagtagtagatgttactaatgaa
ggtgaagttacagttttattaggtgaaaaattagaactgttaacataaa------aaaca
atttattaat--taaagcattgaagaggttat--attcaatgctat---gaaaaaaaata
ataagatgggttgctataattgacaacctctgatactctgtatttgtatata---aagcc
gggatagctcagttggtagagcagtggattgaaaatcctcgtgtcaccagttcaaatctg
gttcttggcat-tttaagcattagaaaattttgctatctgagcattaaattttaattgaa
atgtccctgtgggaccattcctatgttttgctattataacttctgtgaagtctctatttg
tagtttcttgtgtatagtaactttctctatatagcattattactaaatctgcatcttgtt

ctatagaattatgaacaataaaattatttgaaataaaattgcacaaaggtttacattcta
aatcaaacactatttgtaaacttataatgtttattttttgtatattttcaaaagtcacag
atgaaa------aagaatgaaaaatattttttttgttcttcaaagttttt------tattt
gaatagcaatcatatcattttgattaatttcatcacatcttttccacccttgtgttgtga
atagtttatggttacttgttaattgtatatatttgccagctccagtaataatttttatata
cagtttttttttcctgtttttgaaatattagatttaattgctagaaataactgtagtttgt
gcgcagtaaagttaacgacttca-atctttttataatagcaatcaaataatatttg--ag
gttgattatataatgatgtctgaagataattgaatttagatatacacccactttctctta
aatcggataacaaaggccttttgtcacttcgagtttcaaggtttctatttagttgagata
atactaagataggtaagcttaaatctttgctaatattttaagtgatcgagtaattaatg
atagttcttgagatctattgtcagattttctactctcctgtagtaattgaagataatcta
taataattaattctatatttttttccttgcaatttaaagaatttcactttttgtctttatag
tttcagtagaaatcttagcgctatcatcaatataaaggttaaggttagcaagatttttac
ttttttgaataacatattgccagtcatcattattaagctgaccagattgaattttttggc
cgtttaaattacactcttgagctaaaattcttcttagtagttgctctgtcgacatctcca
agctaaataaaataacataagatttttttctgattgatgacatatctagttatattaatag
caaaagcagttttccccattgaaggtcttcctgcaaggataattaaatctgacttttgaa
acccttgtgttatagagtcgagttctgtaaaaccagaaagtatactactatttatactaa
tctttttcctggtatccaaatggactaatagacttgcaagaattgtagctaatgtttgcg
tatctttttcgtcaagtatttcataagcttctgttaattgactaacaatagaagctatat
ttttttgttcaataagttgttttgagcagctaatcaaacataatgaatctccacatgcta
aaagtaatcttttaatgtagttatctataacgaccgctgaatattcgcttatagcatcag
ataggggagcttgttctattagactaaaaacttcatttagctcatccaagtcttgaatca
ttttttcatttttttaagttaatgaaaaaaatttcttacatttttttgtctttgcatggttaa
tattttcgagtgctgctctataaagtagtgaagttgatgtaaaataaaaaaaaatcaggag
agatttttttctactgattttagtaatgatatcgattgctgagttaatattatactaatta
atattttttcagctaaaatattatgaggaggtagatatttataa------atacttatta
attcctcc-ttgtaacatcactcttatggtattagtgtattagttaactattgatttaat
tggattctggaagaacttgcaatttaatatttgcagttacttgatgcaatagcttaattt
ctacatcgtaaatgccaattgttttcacctctggtaaagagatactttgtttttcaatgt
ctatgttggtagtgtttttaataatttgtgaaatttctttttctgtgacactaccaaaaa
tattttggccatctcctgtttttttactaacgctaaacttttgtatttcttctagaagtt
gcttaacctttttagcatcttctttagctatttttaatttttcttctcttatagctgcat
gaaatctttgttgttttaaaataccatttgtggcaactaccgccattttattcgggatta
aaaaatttcttgcataaccagaagctactttgataaggtcattactcttgccaagctttt
gaatattctcttttagcacaacatttacaactttttttactcatctt-------aattcta

aaaa----atctatattatcctattatacctgttgtatacagatttt----aataggaat
tatttatttgttgagataatattcttgtaaaaacaaaggtgatttttattttt-ttctg
atataattattttcacaataattc-----attaggagaggtggccgagtggttgaaggcg
cagcattggaaatgctgtttaggagcaatcttaacgagggttcgaatccctctctctccg
tgcaa--aaaatataaaaagttttatagagtccaatctaaatctccaataacattcttcc
aagcatttaatccaccccgaactctaacaatatttagtttttgagctattaaaaattcag
aagcaaatattgatcgagaatctaagctacagtaaacaaaacaaattttatcttgtaaat
tgatattagagtaattcatttcttttatttttgtaaaggtagatttaatgaatgtatta
aatgacttttatgatactcttcatgatttcttacatctattaaaatatattgtggattcc
tactaattaaaaacctttgtaattgaataacactaatttctcgtacaaatgtactaaatt
tactgccataatattcgttccaatgcttttgtgt-------ggataatacaaatttagta
tttataatcttaaatttcttaaatgatgaagttaatgcgttgtacgttaatatagtccca
cttaatatagacttataacccagaacgattttgacagcttcagtggcttgaagtgtacca
ataatacctggtaatagacctaaaactccggaattactacaagtatctcttgcactctct
ttattttcaatttcactgtagaagtctcgataaacaggtccaccttgataattaaataca
ctaacttgtccttcaaattgaaaaatggcaccataaatatgtattttatttaactcaagg
caagtattattcaagacatatcgagtatcgaagttatcagatccatctataataatatca
tattctctaataatgtcgattgaattctcgtagcttagtcttgtgttgaacacagttact
atacactgtggatttaaatctaata-tttttttttagctatttctacttttgaatatcct
atatcatgcatcgtgtataaaatttgcctctgtaaattagaaagatcaactatgtcatca
tccataatgccaatgctcccaattcccacagcagcaaggtaaataattcctggagatcct
agcccaccagcaccaatgaataaaacttttgctttttttaatctttcttgtccttctaat
tgaatttgtggcaatactaaatgcttagaatatcttgtatattcctctaaggagtatttt
gtattgtctgttttaaaatttagcataaaaatttatagtttatttaatagtactta----
--aaagctagctgataagtttattaaaatgacttttgtaaacttggttcttctctatcta
aaatagctccttgtgtaggacagacctgaacacaaatactacaatcaatacaggcagaaa
agtcaatccaataccaattagtattactattgtttttcccctttccttgatgaatacaag
ctactgggcaagcttccacgcattcagcaactccaatacatttttctgttacaattgtat
gagacatattatttaattaat-aaaagataagctatttacatctagtagttttgttgtat
actaaatttacatatgctatccagtccaatatttaaggtagtctatttttacagtttact
aatttgatatgcgcctttagttcagttggtagaacgcaggtctccaaaacctgatgtcga
gggttcaagtccttcagggcgcgtttagtttttatcgtaattcaataatatcattttatc
tttttgcaaacatcaaaatgatatgatataaaagatcatattttatacattgaatattct
atgtactagtactg-ttttttaatatgttattattgataagttaattatactaattctat
ttttgtaataagt-aaaaacagttctaattaagtatgaacgtatttaatagtc-ttttgt
atatac--aacttaaaaaattttaaactatggctaaaaaagttactggaattgttaaact

agcattaaatgcaggtaaagctactcctgcgccacctgtcggacctgctttaggacagca
tggcgtgaatattgttatgttctgcaaagagtacaatgcacgtaccgctgagaagtcagg
actagtgattcctgtagagatctcaatatatgaagatagaagttttacttttatacttaa
aactcctcctgcttctgtactaattgctaaagctgctggactaaataaaggttcaggtga
accaaataccaaaaaagtaggcagcataacaaataaacagttagaatctattgcagaaac
aaaactgcccgatttgaacactaacaatatacctcaagctatgaaaatagttggaggtac
ggctaaaaatatgggaattctgattaaagatt-aaaagtttcaattatatttatta-ta-
-tttatgaaaaaaatttcacgtcgacttaaaacattacaatcaaaagttgagcctaaact
ttacactattaatgaagcagtatctatattaaaagaaacatcaaatgctaaatttaagga
aactgcagaagctcatattgctctaggtttaaatcctaaatatgcagaccagcaactcag
agcaacagttatattacctaaaggcacaggcaaattgataaaagtagcagttattactaa
gggagaaaaattaacagaagcaattagtgcgggagctgatgttagtggttccgaagaact
aattgatgaaatactgaaaggtagattagattttgataagctaatagcaacaccagatgt
tatgcctttaatagcaaagttgggaagagtattaggacctcgagggttaatgccttctcc
taaagcaggcacggtaacattgaacgtagcaaaagccgtaaatgaatttaaaggggtaa
agttgaatacagagttgatagaacaggtataattcatgtgccttttggaaaatctagttt
ttcacaagaagatctagttttgaacctccaaacaattaaagaatcgattgatagaaataa
gccttctggagcaaaagggaagtactggaaaactttttcttatctagcaccatggggcc
atctgttcaaatcgatatcactagccttctatgaaaaattttgtatagttatagaactt
gaataatctattatttcaaatta-taatttgctagcattattaacgtaataatttgctta
ctatatcttatctgcattaaaaacttt-ttatatatcttattaattatgagtacaaaggt
tgaaaatatcttggaagagttaaaatctttaaaccttctagaggctgctgaactagttaa
acaaatagaagaaacgtttgatgttgatgcatctgcggcttcaggaggtatgatgatggc
cgcaccaacttcagcaccagcatctgctgaggttgaagagaagacagaatttgatgttgt
cctagaggaagtcccagcccctaaaaagatttctgtattaaaagttgttcgctctctaac
tggtttgggtttaaaagaagcaaaagatcttgttgaatctacgccaaaagtcttaaaaga
agctgcatctaaagacgatgcagaaactatgaaaaaacagctagaagatgctggtgcgac
agttattgttaagtaataa-----agaggcgcttctgtttatacttaaacagaagcgcct
ctttatttattataggcaattatactagaaaagtccaagtgtaattgcattgttgatagg
catagttgcaccaatacctagccaaatacttacaactgtaccaattaaaaaaacagtggt
tgcaattggtcttctaaatggattttgaaatttgttaacattttcaatgaatggaacagt
aagtaaaccagctggtacagcagccatacttagaacacctaatagtttattaggaattac
tcttaatagattaaatgttggaaagaagtaccattctggtaaaatctctaatggagttgc
aaatggattcgacttctcacctatagaagatggttctaaaattgcgagtccaatactaca
ggcaaaagtgccaataattacaactggaaagacatataatagatcatttggccaagctgg
ctctccgtaataattatggcccattccttttgttaatttagctctcagctttggatctgt

taaatctggtttttttaagaattgacatatta-ttttttttgtttaatataaagattagtct
aatcactgttttaaagcgtaattttcaactattataatggtccagaaattccttgtttac
gtatcattaaaaaatgcataagcatgaaaacggcagttagtagcggaagtacaaaagtgt
gtaaactatagaacctagttaaagttccttggcctacactaactcctcctcttaacagtt
ctactatactcgctccaacaactggaacagcatcaggaacaccagtaacaatttttacag
cccagtatccgatttgatcccacggcaaagaataacctgttaccccaaaagaaacagtaa
gcactcctaaaattacacctgttacccatgtcaattctctaggttttttgaaacctcctg
ttaaataaacccggaatacatgtagaatcatcatcaagaccatcatgctcgctgaccatc
tatgaattgatctaataagccatccaaagtttacatcagtcatgatgtactctactgatg
taaaagcttctgcaactgtaggtctataatagaatgtcattgcaaatccagttgcaactt
gaattagaaaagatacaaatacaataccccctaagcaataaaaaatattaacatgaggtg
gtacatacttactagaaatatcgtcagcaattgcttgaatttctaatctctcttcaaacc
agtcataaattttactcataaaatagcttcaaaaagctcttctaatata--ttttgcgtt
taagctactattaattcaatag------ttattgaacttatattgtttttataatcaata
cctattaaatattataacatctttttattgattt--------------------aaaatt
ctctgactcagtaaaacagggctatgcactataattcttttttttttgtatagagattaat
ttcgtttttagtaatttagctaaaattcttgttacactgactctattactacctataatc
tgtgctaaaattttatgtgtaatagtaaagttaagcataataccattattaacttgagtt
ccattttgctcagataataataataataagctcacaagtctactggttatacttttatgt
gaaataacttctataaaatgataagcttttgccgagcaaaatattaaatgatttacaaaa
aatagattgaatggagcgcagttactacatgcttttataatagtagtatagttaatacag
gcaacttctgttttatctattgcttctgcttcgtagtaaaaattatcgtcgaccaattct
atttgtccaaatgtatctcctgtagttaataagttaagagttactttatgtgtatttctc
aggacttttgtaataattaaagatcctatcagtataatgtataatctagagctgctatta
aacaaaagagtatcattttgtctaagtgaaaaaatttgataattaatttttattacgatta
aaaaatagtagccatggattgcaagaaaaaacatcttctgaactactttcagagaatatt
ggaacaaaattttcttgatttaaacaatttttagtcataattactactttataa------
---aaaaatattctaataat-taagaactgaaattaaatgttatataaattgatgttagt
tgagaatgacattgcattatcaaaggccattcaagaatacttaatagatcaaggcttcaa
tgtttatattgccaataatggattagaagcactgaatctagcttatcaatataatttcaa
tttgataatttctgatataatgatgcccctagttaatggctatgaactattagcggaact
tcaaaaaaacaaagcgttatccaaaatacctgttattttttttgacagctaaaggaatgac
gaaagacagaataaaaggttacgacatagggtgctatggttatttatctaaaccatttga
ccctaatgagttactctcaattattaacaacttaatcgctagagatgccttaaaagaagc
ttctttacaaaactcggaaatatcaaaccaggaattaaatcataaaatacgtttgacgcc
tagagaaaaaaagtattttagatcttgttgttgatggacttactaataaagaaatatctac

tatattgaatactagtgttagaaatgtagaaaaatatgttagtcgactcttacacaaaac
taatatgaagaataggactttgctagttaagtattctataaataataatttattaaataa
taagatcaatgaaagggcgaatgacgggactcgaacccgcgaatgatggagccacaaccc
attgccttaaccccttggccacactcgccatattacatatatcactatagctcgtttttt
gatatatcgtctagttttcgagttatatatttatataaaacaatatttactttgaatttt
-tatgatacatagcaatattagtattcaaattaatggagaaccatttaattgctcaaagc
ccatctcattgcaatttttattaaattatcttgactttaattccgagcgtgtagcagtcg
agctaaatgacattctgttaccagagactctgtttcactcaacttatttgaatgatcaag
ataagctagaggttattaccattgtaggtggtggataatctatttttacat---------
---agttctctatttcaaatgtttcatagatagagataatcttccttgtttttatctac
atgaataataacagctttaatagtatcacctattttaaattgtgatgatatctgctctag
ttctttgatattaatctcagaaatatggacaagacctttgagatttcctacctttataaa
taacccgtaaggtgtaatctgatttattatgccttcaataatattaccaacaattaaatt
agatgatgcttgagatattagagctcttctatgactgagtattaaattattagatttctc
ttcaacattgagtaattttagcttaataaatttattattaaattgctcactttttttgaaa
attaccaagatgagaattaggtacaaatccagatataccttcaaggttaattatcattcc
tcctttattgaatccttttatcattacattaagtaaagagtcttcagctaatagttgcct
aattcttttccatgctcttatatattcaaggcgacgaattgataagattaattgtttcga
ctgaatattataatctaataaaaaaaattctcttgtatcattaatatttaaagaagtgaa
attatttaaatcttgattactagatacttcttgaataggcaagtatgcagatattggtgt
tcctatatctactaataccccatttaattcaaagctaaatatagttccagctacaatatc
tccaagattcaaatcatacttgtatttttgtaaaacagctgcaaaatttctatgagtaaa
tccgtcattatttttttgtcatattgaatagtatgaattaaagagtaactttgtacaatat
attaaggtaaacaaatgtttgtcgaattgtatg-aagaattttaatttattcggttgtaa
ttaaacatataaacatattaaaatatttaggagacataaaaagtagacgtagatagctgc
agatttaa-taatatataatctgaggaaagtccgggctccacaaatacaatttatgctgg
agaaatcccagtgtaggtaactgcgaggatagtaccacagaaaaaaaccgccagaataag
t----tatattagctggtaagggtgcaaaggtaagttaaaagcttaccaaaagtactgca
aagtatttgttaggtaaaccccctaaaatggagcaaagctactaaacaaatatttgtgtat
attttatctagtttagttgaaaatactgcatgaagttattagtaacaataactcgagagg
aatagctatcctttacaatatatc-tctattatattgaaagaacagaacccggcttatgt
aatacttattatgtcttgtattattatgtctattgtcgaatctctaaatttaactgtttg
acaatttttttctgtagctgatgttgtttataatctatttctgaattggttaaagttttc
tcattagatcgatatgtaaatcttagacctatacttttttttcccttttccaattgattta
tctatatattgatcaaataatgttgtactttctaaatctttatcataaaattgatccaat
gattttaataagtatttaatttccatattcttaggtattattaaagagagatctcttatg

atcgatggatacttagagtatggtttaatctgataatttaaataattgaattcattatga
caatcttctaaaatgtttaagttaacttcaagaacaaaaagttttgtgttaagtcctaat
tcactatattttgcttgattaagttcactgaatatgccaataacgttattattatagatt
agtgtggcaaatttcccttttgaatgaactttattctaccatcgactatattttttttt
gtccactcaatttgtctattaatcttttgaaaaaaattttctacgatccctttagcttca
taccaatttaaagaatgcgcaggatgtgaccatgtagatcggatatctagattaccaccc
aatatgatagctagattagtagtttccataattttgttttgatcattatgaaatactgtt
cctatctcaaagctatctatagtttggttgctttttttaatattataaagatttgattca
attaatccttccagcaaactgctgcgtaaattagaataatcttgaataagaggattgttt
aaagttatatttcctttggactttattaaagaataatgaactaattcagttagtcctaga
tttcttaaaatacttctacttttatctatgaattgtctctttgtcgataactttttgttg
aattgaatatttggtactgagctcctgaatttatgataaccataaattctggcaatttct
tcaataatatctatttctcgaaaaacatcttgttgtctataatttggaacagtaacttct
atattttccttatttgataaataagatcaaaatttaaagagtggagaatattttgaatt
tcttcaaagaataaaaaacgtgtttgaccattatatataattggtcctaaaatgtcatga
actttttaattgataaattaatattaagagcattatcagttgtttttctcgatagaaa
gtctctctaacatttccgcatgttaaatctgtgattaaagaaagagcttcaaaatgtgca
tttttccaattatctacatttagcccacgttcttgtctaattgaactttctgttcgaata
ttaagaactcgagaagactttctaaccacagattgtttaaatatagcagactctacaagt
atcgatgttgtattataatctgtatcaagattagagttgcttccaataccagctatacta
attatatttgtatttacttgagtaaccaaaatatttttgtttaacccaatattttcattg
tttaacttaatctgatcattattttcaataggaaaatcgcttcgaattgttagactattt
tcatgattcatattattaattttattcaaatcaacaacattaatgggctgtccccatttc
aacataatataattgcttatatctgttaacaaatttctgtgtgtaaacccagaagagagc
aatcgatttttttagccaatctggagaatctttaattcttatttcgtctataatggcagca
aaataattgtcgcaatttaataaattgtgatcacttattatagt-ctccttttttagagaa
atatctattattgggttgatatatagctgataacattgaggaccctgttaaagccgagac
ttctcttgatagaccaatcatgcttaacacgtctgacctgttagctgttgatgatatatc
taatacatgatcaatctcacctccaattgtaatagattcaatagcttcaacttcaaaacc
tgcttgtgttaattgattagttaaattatctatatctattgttttgatattaacaagttc
tttcaaccaatttaaagaaactttcataattaattgtcatctatcttatagaagtattta
ttttacttatgattaaaaatcaatttaaaaaattcatcaaattaatataagttatttgat
gaatttgcttatacaatatattattgtgatatcttacttttatatggcacttattaagaa
ttagcttttgtaaaagttagcccattatcttgggcatatctctccataaatctcatgaat
ctatcccaatcatcaggacttttaataacatggacagcttctattgcttgtggcttacca
ttaataaacttggcattaacatctcttgtaataatttgtccctctgtgtccatcagatac

atacctgtgatttcacctttatctgccatactagcatctaaaattttgggatttgtaaac
cgaaaagttgctgttcctgtactaccatctcgagaccttgttaaacgtacatctggtaca
acttcttcattgataccttgaataaattgaattgttgccatatcaatcttctttatttat
atataatttatattggataaaactgatttctagttcttgaatgcttgttgagaaactgtt
ttaaaagcttatagtagcagtagatcaaattttatgtattatataaaactgtaacttttc
-ttttttg--

----------------------atctgttcaaagaattaacttattataagctaatcata
cgaatattagccatataatgtttgtaagt-gaaattaaggggttgtagctcagaaggata
gagcaagcgcctcctaagcgctaggtcagcggttcaagtccgctcaaccctgttaacaag
ttaagtagtattatttaacttgttctgcaaatcagtattgatttaagttaaagttcttga
tagctaaaacaaaagaatctgagaaatatttttattta----------aattatcttaac
tctgaatatctgataattattaa-aaagttct----------------------------
----ctttttaagacacaaggtgatgaagataagatatttaaactaataatggaaattaa
gtttttgaagttatagaagccaat--ataatactttctattaacaga-----------cg
cttgcttctattcctatttgtttacaaga--aatt----atgaatatcaaaata------
--attttagctcca-------------------------------------ctgat----

-----------------------------tactaataaata-------------------
------------------tttatttcagtctagacaag------atgaatcttcatggta
taagag--

--------------agctattctctgtgtcatacttatataaaatcaggat-------ac
tgtt--

---------------gtcattattataactggggtgggaggattcgaacctgcgaatggc

ggagtcaaagtccgctgccttaccacttggctacaccccaacatgtgaatattataatag
cagtcgtataccctgccatgtcaatagactgttgtctttatttaataattttgctgctc
ttgtagaataaaatcttttttttcaaataagatacgctatgtttaagttgaacgactgtt
atgttttcttgcttttgactaattagccacttgatagtaattattccttctgcaatttcg
ttacttcctataataatacaagctacagctctttttttatttgcttgcttaatctgttta
ctaaaattacttgagctaacatctatttctattgtgaaaaactgctgatgtaaaaaacgc
attacttgcataccagtttcgttagctttttcaccttgtgtagcaatataaaaatcgata
gatttattaggcaagtttatattatcctttgcaagcagtaataggcgttctaatcctatt
gcacatccaacagctggtgttgctttgccacctaattggtgaactaagctgtcatatctt
ccaccaccgcatatagtatcttgcccttttgatgtcaatgtcttaatttcgaaagcagta
tcattataataatctaatccgcgaactaacttattatttatattgtagggaatatcaagt
aattttagataattgcaaacgtcatcaaagtgtttttgtgattccaaacttaaaaaatca
gaaatttttggagcctctgttagtatttttttgtgtattagcatcttttgaatctaaaatt
ctaattggattactagttaatctttttttgtgagtcagtgtctaaatcatcatggtacttt
gtaagataatctagtaatttaacttgataaatactacgatcctctacttttccaattgaa
ttcagatcaagttttaagttatgcagattgagattgttaaagatgctcatagctaaatgt
ataacttcactatctgctcttgcgtctaggctaccaataaactcaatgccaagttggtga
aattgtcgttgtcgtccactttgcggtctttcgtacctgaacattggaccgctataccat
aatctttgtaaactatgttggtaactcattttattttcaataaaagatctcacaatcccg
gcagtaccttctggccttaaggtaatatctctattgctgcgatcatgaaaccggtacatt
tctttatttacaatatcagtattttcaccaatactcctatcatataattcactattttca
aaaataggtgttctaatttcttggtaatttgcacattctaataatttggaaattttatta
tgtataaactgccaatattgaatttcatccggaagaatatcttttgtccctctaatagcc
tgaatttttgccatgataatttttgattaccttcttaatatttctgcattaaatagcag
ttgtagactggtattcagtcatgtattatacattag--aacatactggtg-tattttata
aaatacgggcaaggagggattcgaaccccccgacaccatggttcgtagccatgtgctctaa
tccactgagctacaagcccacttaataagtacgttaactatatcattattctctaatgta
tacaactatattctttttt------atcttacatttgaaatttttgtaaaagtgaaaaatt
at----gtttgtttttacaattggaactattcatatataatgcttaatttattataact
ttgtaaa--aatatctataagtttaattgtatgattctactatatt-gtaatcttgtcat
aattttgcgctcttatttttcaattag------ctagcatctaaataacttatatttcaa
ataaaaaaaatcttatctaaaatgagtaaacaaattctatatcaggatgatgcaagaaaag
cactagaaaaaggcatggatattttaacagaagctgtttctgtgactttaggaccaaaag
gaagaaatgttgtcttggaaaaaaaaatttggtgcccctcaaattattaatgatggtgtaa
cgattgcaaaagaaattagtttagaaaatcatattgaaaatactggagtcgcactgatta
gacaagcagcatctaaaacaaatgatgtagccggtgatggtacaacaacagctacagtac

ttgcttcagcaattgttaagcaaggcatgagaaatgttgcagcaggttcaaatcctatgg
ctattaaaaaaggtattgaaaaagcaacaaattttgttgtcagtaaaatagctgaatatg
ctaagccagtagaagatacaaaagctattatacaagttgcttctttgtcatcaggaaatg
atatagaggtaggtaaaatgatagcgaatgctatagaaaaagttggaagagaaggagtta
tttctttagaagaaggaaaatcaactaatacgattcttgagatcacagaaggaatgcagt
ttgaaaaaggctttatttctccttatttcgttacagatacagaacgaatggaagttcttc
aagaaaacccttttattttatttacagacaaaaaaattactttggtacagcaagaacttg
ttccattgcttgagcaaattgcaaaaacgtctcggcctttattaataatagctgaagaca
tcgagaaagaagcattagccacaattgtagttaataaattaagagggattttgaatgttg
tagcggttagagctcctggatttggtgatagaagaaaatctttacttgaagatatgagta
tcttaacgaatggacaagtaattactgaagatgcaggtttatcacttgatacagttcaat
tagatatgttaggaaaagcccgaagagttattgttactaaagattcaacaacaataattg
cagatggtcatgaaattaaagttaaatcaagatgtgagcaaattaagcggcaaatagaga
cgagtgactctttatatgaaagagaaaaattgcaagaacgattagcaaagctttctggag
gagttgccgtcattaaagttggtgcagctacagaaacagagatgaaagataaaaaactaa
gactagaagatgcaattaatgcaacaaaagcagcaattgaagaaggtattgtaccaggag
gaggcgctactaatgtccatatctctagtgaattatttacatgggctaaaaacaatttaa
ctgaagatgaattaattggtgctttaatagttgaacgagctgtgacctatcctttaagac
gaatagcttttaatgcaggtgacaatggagcagtaatagtagaaaaagttaaaagtcatg
atttccatataggctacgatgcagcaaacagtaatattgtaaatatgtatgatagaggta
ttatagatccagcgaaagtagctagatcagctttgcaaaacgcagcttctattgcagcaa
tggttttaactactgaatgtattgtagttgataaagttgatgattgatttata------a
gataatagataatctcaaacaaagaataatttctaattatagaaattattctttgtttca
attaagctaattaattgatcaaatgtataattttataataaaaccaaagtttaaacaca
tcttctttttcaactattaagtagagaagatataagcccgtaatacctaaattatcaatt
aaaatattattttcatatatagattttctaaagatctaaaaaatgacattttttgaaaaa
taataccgaaatcttcttttgtagttatttgctaactcattactgcctaaagattgattt
ccaattgaattcagaacctttaacagaacacttttttaaatctttatctgactttatttta
gaatcaataataatttgattcaattgcatgtcaaaactaagattaaaaatactacttgtt
aaagatatttgattttgaatatctagagattttatagccaagaagattaaactttattg
ttcattgtaaaatataagacaattaatgatgtattctttaaatacta-aatttatcttct
tgtgcttctatcgtttttttgt-aaaataattaatgctttaatttttttagttttagttact
acccgaaaaaataaattctggaaatctagcttgagctttagctagagagtgtttttttcc
ttcttcttgccagtaattgataacttctgttgttttgtttaataactcaattctatctct
ttcagcaatccacggcttagattctagttctgcttttaattcttcccaagcatcatttct
tggccagaaaaaaataagaagtaagtggactagtgccattcccaacaatttgatcaatagc

aatagcaatattattttctaaccaaagtacttttagtgtaaattttgacaagtttttatc
tccattattaatattaagtgttgg----tacagtagaatactgtactagttattttttc
agcaagaatttgagctttgtttaaaatattttgtactgttttagtagcttgtgcaccttg
agacaatcgttgcaaaattttgaaatgtcaacgcgagtttcattagttataggattata
aaatcctaattcctctatagctttaccatcgcgtttatttctgctatccattacaacaat
tctatagcttggttgtttttttcttccgtatcgttttaaccttaacttgaccatattatt
cacctcgcttattttttt--atatctttattaaattatatcttacgacattatgcaataat
tcaaacaattaagcagattcaagtctaacgttattaaatattaccattaaacattgtaga
aaaataatacctaacataggtgacatgtccatgccaaacataggaggaatgcttcctctg
aatagttttaaatatggatcagtaattctatttaatgaacagaatggctcattgtaccaa
tttacagttggaaaccatgccaatgataatttaagtaaaattaaaattaaataaatctca
gaaaaattagctattgatccaagtaataaatttaatgtaccaggaagagtattcatacgt
attttataattattatatacttcattatactaatagtttgattatatattataatctctg
taaataaat-cagctaaacaaaaaacttatccttaaagctatcttctcgcgagcattttt
ctttatctacattagtatttacttttgtcgtataaatttctagtttagaccaattttgtc
ctaacttatttaattgagatgttccacattctaaacatgctatttttatattgctcaaaa
cagcacctttttgtataaaatcattcaataatggagtaatagttgcttcatccaaaaata
aatctaagacaaggattttttgtaaatgaatttaattgagctgggacattgttaataatat
tattataattaactaaaactatattagctgtaggaagtaaagctcgtgctccttccgcta
atataaagccataaggcataataattacaataatatatttataatgactgttaataagac
ttatagtggtattagcttttaattcgattgtttctgttactaaccactctctcattattt
cgtaagtgatccacttacctagttctgagcaagcagttcttaaaattgtacctggattat
tgttgttttcgagaacccctgaccaatgttgtattaatggatgggatgctatattaattt
ggagttgcatgttttgttaaatatatttaacatttaatgtgttgattgaaacttcaattt
ctatttgttgagtattattaaaatatcat----atatattaaagaaaataattat-aaaa
aatgaaaaaaagtctttggttgtggggttttacagatagtgctgaaacttggaatggtag
attcgcaatgattggttttatatcagttatttttatcgaagtagttacagggcaaggatt
attatatttaataggtatgatgtcataataaaaatccaaaaaagttcttgtaatgtttta
caagaactttttttgg-atttttataaatctaatctagcggtctcatagatagaacaggtt
cattctctctattgatacctttttcaaatccagcagcagcagctcttgctctgcctgcat
gccaaagatgtccaataaataggaagaatcctaggaagaaatgagatgttgttaaccaag
atctaggagatacatagttaactgaattaatttctgtagctacaccaccaacagaattca
aagaacctagtggtgcgtgagtcatgtactctgcagcacgcctttcctgccacggttgaa
tatcgttcttgattttattcaaatcaagaccatttggtcctctaagaggttcaacccaag
gagctcgcaagtcccaaaatctcatggtttcaccaccaaaaataatttctccactaggag
atctcattaggtatttacctaatccagtaggtccttgagaagacgcaacattggcaccta

gtctttgatctctaactaagaaagtaaaagcttgagcttgtgaagcttcaggaccagtag
gtccatagaattcactaggataagctgtgttgttataccaaacaaaattagaagctgtaa
gacccatgattgataaagcacctagactataagacaagtaagcctcaccagaccatacaa
aagctcttctagcccaagcaaaaggttttgttagtatatgccaaattccgccagcaatac
aaataatgccaatccagacatgaccacctacaagatcttccatgttatttacacttacga
tccatccatcaccgccaaacggagactttaaaacataaccaaagataactaggggattaa
gtgtaggattgctaacaaatctaacatctccaccacctggagcccaagtgtcgtataccc
caccaacaaataaagctttaataactaatagaaaagcgccaatgcctagtaatactaaat
gtataccaagtattgttgtcattttgtttttatctcgccaatcgtatccgaagaaaggaa
atgattcttcaagagtatcagggccaatcagagaatgataaagaccaccaaagccaagaa
cagctgaagagattaaatgcactacgcctactacaaagtatgggtatgtattgaaaattt
cgccgcctggaccaacaccccagcctagtgtagctaagtgcggaattagaataaaccctt
gttcgtacaaaggcttctcgggaacaaagtgcgctacctcaaaaagagtcatggcaccag
tccaaaagaccattatacctgcatgagcaacgtgagcaccaagtaatttgccagaaacgt
taattaagcgtgcattgccagaccaccaggcaaacccggtagactcaatgtctctaccgc
caacaccaacagttgtattaaagggcgtttccacgtggtaaaacctcctcagggaatata
aagttttcatgaggttgatcctgagcagccatccaagagcgaataccttcgttcaataga
atgtttttagtataaaaagtttcaaactcaggatcttccgcagctctcaactcttgagaa
acaaaatcgtaagctcttagatttagagctagcccaacaattccaaatgcgcttgtccat
aatccagttactggcacaaataacatgaaaaaatgtagccaacgtttattagaaaaagca
acaccaaaaatctgtgaccagaatctgttagctgtcaccatcgaataagtttcttcagat
tgtgttggagtaaatgcgcgaaaagtatctgcagcatccccatcttcgaataaggtattc
tgtacagttgcaccgtgaatagcacatagtagagcaccacctaagattcctgcaacgccc
atcatatggaacggattcagagtccagttatgaaatccttgcaaaaatagcaaaaatcta
aaaattgcagcaactccaaggcttggggcaaagaaccagcttgcttggcctagagggtac
attagaaagacggagacgaatactgcaattggcccagaaaacgcaatagcgttgtatggt
ctaagaccaactagcctagcaatttcaaattgtcttaaacagaatccaattagtccaaat
gatccatgtaaagctataaatgcccacagaccaccaatttggcaccagcgagtaaaatct
ccttgagcttcaggtccccaaaggaaaagaagtgaatgtcccatgctgtttgctggcgtg
gaaacagccgcagttaaaaaattgcatccttctagatatgaactggctagcccatgagta
taccaagaagtaacaaaagtagttccagttagccagccacctacagcaaggtaagcgcaa
ggaaacagaagtagtccagaccaacctacgaatacaaatcggtctctttttagccaatcg
tctacaagatcaaaaccaccacgagtttttttcttgtccaattgctatggtcataattta
atctccagagcaaatttaataagtaatttgtttaaataaaca---ctttagatatattct
aaaattttacttttctttacggtcaatataattataagtcaaaactaaccaaaattcacg
tttttaataaca-ttttttagctcattaagttaattttttttaatatatgaacataaata

gagttattgttt-aactcaaagtactcataatttattcatttctataatcctttgaaaga
gatatcctgttcctcgtgctgttaaaatcaagtcgggactacttggatcatcttctagtt
tagctctcaatcgtgaaatatgaacatcaacaactctcgtgtcaacatgtcgttcgggag
tatagccccagacttcttgcaaaatagaagctctagaaaaaggttctccagccttactaa
ctaaaagctcgagaagactgaattccatacctgttaagcgaactctttcattatttttat
aaacttgtcttttattggtatctattttagaaatccaatactaataatcccagagtttg
ggactccaagactagtggtgattttatcagctcttctaagaacagaacgaatgcgagcct
ctagttccttaggagaaaatggtttaacgacatagtcatcagcacctatttctaatccgg
taattctatcgcatacttcacctaacgctgttagcattattataggaacatctgattctt
ttcgaagttcttgacaaacaccgtaaccatctaatttaggcatcataacatctaaaacaa
caaggctaggatactcttttctaaacactagtaaagcttcttctccatttgaagcagtta
taacttcgtaacctattattgttagtcttgtttctaaaatccttcttatgctggcctcat
catcaacaacgagaatcttttctttctggttctccaatttttttatttactcctataaat
cactgataatgattactataaccttaaatttttagctctatgagtatcttcatttctttt
agaacttagtttagttattataaattctgcaagtctagtttgcattaattagtaccagt-
-aaatatttttgt-----------tttaagttatgac---------tattttaatccata
agttgactctagtttgcagaattcaatatatacata-ttttctagccaagtaatttgtat
tctattttctaaataaaaaagggggttgacaaactagttgatagaaagtattctacattac
tataag----ttaattctaaaaaagaaattctaa-----atcttctttcggagtaataaa
tactttaat
>Pyr_27
tatatgata---aattaagacgtctcttatgtgagaatttctaaaatatacaaaactttt
tatttaatatagaaaaactaaattaaaataagttgaacataccttgctctaatctaaacc
tcttaattgag------attctaattagagataaaagtatttactta--ttgtatctata
ggaataaaaatgaaacttgcagtttatggtaaaggtggtatcggtaaatctacaactagt
tgcaatatttctgtagcactttcaaaaagaggtaaaaaagttctacaaattggctgcgac
cctaaacatgacagtacgtttacactaacgggattttttaatcccaacaattatcgatact
cttcaatccaaggattaccactatgaggacgtttggcctgaagatgtaatctataaagga
tatggcggtgtagactgtgttgaagctggtggaccgccggctggcgctggttgcggaggc
tacgttgtaggtgaaacagttaaactttttaaaagaattaaatgcttttgatgaatatgat
attatcttatttgatgttttaggtgatgttgtatgtggaggttttgcagctccattaaat
tatgcagactactgcttaatcattacagacaacggatttgatgctttatttgcagctaat
agaatagcagcttcagtacgagaaaaagctagaacgcactctctgagattagctggactc
gttggtaatagaacagataaaagagatctaattgataaatatatagattgtgttccaatg
ccagttttagaagtcttgcccttgattgaagatattagagtgtccagagtaaaaggtaaa
actttatttgaaatggcagaaattgataaggatttagcatatgtatgcgattactatttg

aatattgcagatcagttgattacaaggccagaaggtgtagttcctaaagaatctcccgat
agagaattatttagtctgttatctgattttacttaaatcctaaatcaaatgtaggacaa
gacaaagtagatcaagaagaattagatttgatgatagtgtaaataatattttcagaataa
tataacaaggaataagataatgtctacagctcaatcagatgctcttacttttgaatgtga
aacaggtaattatcatacttttttgtcccatcagttgtgtttcttggttatatcaaaaaat
tgaagacagtttttttcttagttataggaactaaaacttgtggctatttcttgcaaaatgc
aatgggagtaatgatttttgctgaaccgagatatgccatggcagaattagaagaaggaga
tatttcagctaagctaaatgattatggtgaactacgtagattatgcttacaaataaaaaa
agatagaaatcctagtgttatattttggattggaacatgtacaacagaaattataaaaat
ggatctggaaggaattgctccaaaattggaagcagaaattcgtgtccctatagtagttgc
tagagctaatggtttagattatgcctttactcaaggtgaagatacagttctagcagctat
ggctcaaaggtgtccattaaatttaaataatcaatcagataatacgagtcttaaaatttc
tcctcatattcctttggttttatttggatctcttccggattcagttgtaacccagttaac
tatggaattaaaaaaacaaggtatttttgtttctggttggttgccatcaaagaggtatac
tgaattaccggttataaaagaaggatattatgttgcgggagttaatccatttcttagtcg
cacagctactacactaatgagacgccgaaaaacgaaactaattggtgcaccatttccaat
aggtccagatggtactcgggcctggattgaaaaaatctgttcagtaatgaatgtaaagcc
tattggcttagaagacagagaaaaagaaatttgggcttctttagaagactatatttcttt
aatacgaggtaaatcagtttttttttatgggtgataatttactagaagtttctcttgcaag
atttttaactagatgtggaatgaccgtatatgaaattggtatcccttatatggataaacg
ctatcaagcagcagagctagctttattaaaagctacatgtgataaaatgaacgttatgat
gccaacaattgtagaaaaaccagataattataaccaagtagaccgaatccgtgatctgaa
acccgacctagttatcactggtatggctcatgcaaatcctttagaagccagaggtattaa
tacaaaatggtcagtagaatttacatttgcacaaattcatggctttaccaatgcaagaga
tattcttgaactcgtcacaagaccattacgacgaaatcttagtttatcagaattaggctg
ggatgtgtatagcaagcaaagctagtatgcttc----tttatgacttacatggtataaaa
gccttgtaagtct--tttattttatgtcaaagaagcatttcttgctctgccatcaccagc
ccaagcttgtaaactttctacttgttctttatctgtaaatgctaaaggcacaaattgttt
gatagctattttaatatcttcagtactaaattctctctcttcactaaacgctgtatgcat
actttcaataattgcttgctcaatttcagcgccagaaaatttattacataataaactcaa
ttcatgaatatcatattcttgccacgatcttggtctaacttttgataaatgtatcttaaa
aattgactctctctcttgacgattaggtaaatctaagaaaaaaatttcatcgaacctgcc
tttccttaacatttcggaaggtaaactctgaattttattagcagttgcgacaacaaatac
tggagctttttctctgatagccaggtaataaatgtgccaaacactcttgcactagtacc
actatcaccttggctatataaaccagaaaaagctttatctatttcatcaatccataatat
gcaaggcgataatccttcagagatatttatcatttctctcattttggattccgattcacc

aactagtccaccaaacagttttcccatgtcaagacgtagcagaggcaacatccaatcgct
tgcaatagctttcgcggttaaagatttacccgtcccctgtataccaactagcaataaacc
tttgggcgaaggaatgccgtaatttaaactttgcttcgaaaatgagcgagaccttttttg
tagccattgttttaatatatctaagccaccaatatctctatttactgtattataaggata
aaattctaatagacacgtttgattaataatttgcctttttttcttcaataataataggtaa
gctccgagagtctatctgattatattgtgcaataatttagtaatcactttgcggattct
atctatcgataatccttggcaggattttgttatattattaacgagctctgaattcaaatt
aagattcaaagttttactcaatctcctgatctctttttttgatttctagaagactaggtaa
gggtaaatctattactgttatgatatcgcttagagcaaaaggtatattgattttgcagga
aataataacaatgtgcttagattgcgttttgattatttagctaaattgcgaagtttcct
aattaatactatttcatttaggaaagagtcaaaatcttttaaaagaaaaagatttaaaga
ttcattatttaagttctcaataaattctaatgctaataaaggatttctttttgcataacc
attatcgctaggattgttagtgtatccatctacaaagtcccagcaatacacttgttgatt
atttgaacagtttaagctgtgttttataatgtactctagtctatcttcttctctagtatt
tactacaataataggatatcgagattttaaaagtaatcgtaaatcttgagtgaaattcat
gtcaatattttttttccatcagacttttgtacttcaccaattg----aagactaaataaga
ttatctaatattgcttggatatattcaaaagtc------atctctctattttctaaatta
ttgctattattaacttaacttaccatgatttttttttcttccttttcttcgtcgactattt
aatatagagcgtccacatggtgttttcattcttgctctgaaaccagaaactctaattttt
tttcttttagatccttgtagtgttcttttagtcatgatgtttcctctgttgtaaaagatt
aaatactattttatcatcttttatatatctttatgatatagatatatattaatatcaaag
tt-aacagcaataatttatattaatcaagaactatttatataatattaaccattaaatat
----------taactttatgatattggctttgcccatttttttatttaagtattttgacca
tctttttgctaatcttaaactggttaattttccagcagctaaaaacgattttgtcattag
aatctcaatttaagtattttgttgataaaagccaaaatggtaaattagaacccgacgaaa
gttttgcttttgcaaaagtatgtgtagctaaaaaattttttttccaaagctattattgaag
gtcaattagctttgaaaaaaatatcgagaccttaataccttagataataatatagtaattg
ccaatttatataatatgctaggttttatttattttgaagcaggccaaataagttttgcaa
agaacttttatgagcaagccttacaaataaaccctaattatgttgtagctttaaataacc
tggcaaaaaatttatgaagaagtaaaagatttaaaaaagctgaatctctatatgataaag
tgttaattcttaacttaaataataaaactgctaatagaaggaaaaaattttatagcaaaga
cta---------aaaatgtctaatgcggtttgtaatcgggatagcaggatttgaacctgc
gacatcctgctcccaaagcaggcgcgctaccaaactgcgctatatcccgtaagacataat
tagtactatatacctttttatctaattatgtctactctttctgtattttatctaggatacc
aaaacatgcctttaactccatctggatccttaataaatcctaatttttttataaaaactaa
caacatcaggctctgcaaatagtgtaatagtgctaatttctgcttgtctcaattgctgta

ttaattgctgtatgactactttccctaaacctaaaccttggaaatctggatgaataacta
cgtcccagatagttgcattaaatccgttgtctgatgttgctctagcaaatcctacaagtt
tactatttgcatctttttttgtattaaagaaataataatagaactatttttaatgcga
tctttactttttttagaggtcttttaacccatccgactgaatcacagagctgctccaatt
cgtataaattaatatttttattgctactcagatagatatctttaaattcgattctatcgc
aagttttatctaaaacaataagtttttgaaatttttttcagagctagcattgatatcgg
agttttgaaaaaaatttttccagaagatcataatgattatgatataatta--aataaata
tatatattacaatattgctttaacaaacaatatattaatatatgctcaaatttttacatg
aatctattaagatagaagattgttaaaatatagagaaataaaaattttgtaaaatcttt
caataaaatcatataaagagtgctttttaagactaacatctattattataaataaagtct
cgcaattgagatgaaataacacattattctatgtctaatgccttttagaggagttatttg
tgaaaaaatctatgtttctaaccggtttacttgctttactaattatgagcaacccaataa
tagctaacgcagaagtagctggattggtaccatgcaaagattctgcggcattcaacaagc
gtatggtaaatagtgttaaaaaacttcaagccagattatctaaatatgatgcagataccc
caccagcgttagctttaaataagcaaatagagaaaactaaaactagatttgcaacttatg
gtcgagcaggtttgttgtgtggtactgatggattgccacatttaatttctgatggtagat
ggagtagagccggggactttgtttttccaggactattattcttgtatattacaggatgga
ttggctgggtaggtagaggatatcttttatctgttgctaagactagtaagccaacagaaa
aggaaatcattttagatgtgccattagctatcaaatttatgtcatccggatttgcatggc
cgctagcggcttggcaagaatttagtagtggacaattaattgcttccaatgacgatatta
ctgtttcaccccgttagtaaaaaatttatatgaataataattttaccaaatacttatcaa
cagcacctgtaattggtgtattgtggatgacgtttacagccggatttattatagaattga
accgctttttcccagatgtcttatacttttactt----ataaataa--gatcttataaa-
--aaactacaccaatatagagaaatactattatttgtgtagtttttaa-tatatataaca
taattattta-ttttaaaaatatttatcttttcgattattttaagattagatcttacat
aataaaata-atatcaatattatgagcttagtaacccaaattatagttaatgctgatgat
gaattaagatatcctacaattggagaattgcagtcaattcaagactacttaactacaggg
agtaatagaattagaattgctactattattagagataaagaaaaggagattatacagaaa
gctagtaaacaaatttttcagttacatccagaatatatagctccaggtggtaatgcagca
ggttctaggaaaagatcgttatgcttacgtgattatggttggtatctcagactaattaca
tatggcgtattagctggggataaagattctatagaaacaattggtattataggtgtaaga
gaaatgtacaattctttaggcgtacctataattggaatgttagatgcgatccaatgttta
aaggaagcatctttagaactacttggtcaagatgatattagaatcattgctccttatttt
gattatataattcgtggaatgtcataaatcgtagcttcaattaattagttgaataaaagc
ttttataatgttataattacaataagcccgaaagcatataattgtaaaaactaaatcttg
tcagaaccggaaggtagcagcaataatgtttaaatacaaaagttatga-ttttcgggtgt

ttactgttttcatctaatagtctcgacttttaattagtatgctatgtataaatctgtata
tgatatgtaattcatcaaacctttcagcaaaaacaatttaataatttgatattaatacgt
aaaacacctatac-tttttataaaatttctttatatctcatctaaacaatggaaactta
acttaatgggtgttcatatttatcaacaggttcgtctgtcccaaatttttctgtagaga
atcaacaatttgaagatatgatcgaaacttccgaccattggatttcaacaagaacaggaa
taaaaaaaagacatctagctccatcttctatttctttaactaaattagcagcagaagctg
ccaacaaagccttatatgcagctaacttaaaacctactgagatcagtttaattattttag
ctacgtctacgcctgatgatttatttggtagtgctagtcagttgcaagcagaaataggtg
caacaacatcggtagcttttgatattacggctgcctgctccggtttcattgttgctttag
taacagcagctcagttcattcaaactggttcctatgacaatatttagttgttggagcag
atacaatgtctagatggatcaattggtcagatagaactacctgtattttatttggtgatg
gtgctggagcagtagtattggggcaaagccgcaaaaatagtattttaggctttaagttat
gtacagacggtcagctaaacagtcatttacaattaatgaataaacctgtaaataatcaaa
agtttggtgttacagaaattcctcatggaaactataattccataacaatgaatggcaagg
aagtttacaagttcgctgtatttcaagttccaacagtaattagacaatgtttgaataatt
taaatatttcaatagatgaagttgattggtttatattgcatcaagcaaacactagaatta
tagaggcaattgcgagcagattatcagtacctttttctaaaatgattacgaacctagagc
attatggaaatacatctgcagcgtcaattcctttagcgttagatgaagctattcaatcca
ataaaattcaaccaggccaaattattgttttatctggttttggagccggcttgacttggg
gagcaattgtcttgaagtggtgat---ttatatattgcggatgacgagactcgaactcgt
aaagctttcgctacacacccctcaagcgtgcgtgtataccaatttcaccacatccgcatt
ttttatcatgaata-ttaaaaatttataatatttatctaaatattcatataatgtgtgta
tatatatatacattcttgtcagaaactctcttatattttattttaagtgataaaattgag
t----ttagtatagatagtaaaattttattttaaaatcataa----aaataaatatataa
ggaaactaaaatatgacaccgtccttatcaagtttttgaatagtcttattcttggggca
gtaattgtagtcgttcccataactttggctcttttatttgttagtcaaaaagacaggaca
attcggtcctaaaaataacttagaaagatatggagaagcaatttaagatgaataatgaaa
tatttttcataataatacgctctccatacattgctatact--ttagaagaa--------t
ttttattttgtaacatcctgtaaagtttattccgctctttctttttcct-atttactctc
tta----taatgatacaaatgtcagg-ttaataatag--ttttttatttttttatctttc
attttatatcaaagtattttatatacgtattattatgttcaattaataaagaaaagctca
tgtctttgtctaattggcctctcaaaaaagaaaattctgaagcatataacattaagaatt
caaaacaaatcacaattcctgatggtttatgggtaaaatgcttcgactgtggtctattaa
tgtattctaaagtactaaagcgaaatttaaaagtttgccctcaatgtagttaccattttc
aagcttctagtaacgaaagaattgatcaattgatagaccaaggtagttggcaaccaatgg
atgttcacttgatctctacagatccattaggttttaaagatcaaaagctttatagtcaaa

ggttaaaagatactgctctgaagactggcctgcaagatgcagttcaaacaggtactggta
ctatgcaaggtaaaaaagtatgcttaggtatcatggattttagattcatggggggaagca
tgggatctgttgtaggcgaaaaactaacaagactactagaaaaagcaactcaagataagt
tgcctgcaattatactttgtgcatcaggcggagctagaatgcaagaaggtatgttgagct
taatgcaaatggcaaaaatttcttctgctctagaaatgcataaaaaagaaaatttactat
atatatctgttttaacctctcctacaacaggggggtgtaacagccagttttgctatgcttg
gagacttaattattgcagagccgaaagctcttattgcatttgctggtagacgagttatag
aacaaacaatcaaagaagacttgccagataattttcaaagttcagaatatttatttgaac
atggtttcctagatttaattgtacccagaactcagcttagatcaaagttaatccaaattt
tgtccttacataatcatagtaagtaattaat----tattgactataatataaaaatgcta
gtaaaaattaagatattaagaaattttcactaaagatatttgtagactaactagaattac
aattactattgtagaaaaatttgtcttaatgtca-t-ttttcagtctttgaacaaattg-
tttttaggactttaaataattgttagtttaatacaaatttataaagaaaata-----cat
actaagctaaatatagtaatattatgttttcctataatactgacctatcaaatttcgagg
aaatttatgcttaaaagatcttcttggcttgcggctttattgggactattaacagtggtt
tctacaagtacgcatacatatgccatagagttagacgaggcaacaagaactgttccatta
gaatcttctggcagaactgtaattcttacaccagaacaagttaaaagaggtaagcgatta
tttaataattcttgtgctatttgccataatggtggtatcacaaaaacaaatccgaatatt
ggacttgacccagaatctttaggattagctacgccacaaagagataccattgagggacta
gttgactatatgaaagatccgactagttatgatggtgcagagtcaatcgcagaattgcat
ccaagtattaaaagtgctgaaatttttcctaaaatgcgtaatctaaccgatgaagaccta
ttcacaattgctggtcatatcttgctccaacctaaaattgtttctgaaaagtggggcgga
ggaaaaatttattattagaacttcaaagaactaaatcttgtgaatactagtttatgta-t
ttgttgcttaagtgtgattattttgttagatcatgatatatattaaagatggagtttgtt
gaaagcaagttatttcttagccgaacttgcttacactattcgtctagttattaaaaagga
gacgttaaattgaaaaagaagttttcagttcttttcactgtttttagttttttttgtaata
ggtttcgcacaaattgcttttgctgcagatctagataatggagaaaaagtttttttctgct
aattgtgcagcatgtcatgctggcggtaataacgctattatgccagataaaaccttaaaa
aaagatgtacttgaagctaatagtatgaatactattgatgctattacttatcaagtacaa
aatggtaaaaatgccatgcctgctttcggaggtagactggttgatgaagatattgaagat
gcagcaaattatgtattatctcaatctgaaaaaggttggtaattatacttgattttatcc
tgtattaaagaatagacaatctatttagttattcattagattgtctattctttgtttatg
---ctattatatagagatatttacacaatatt----ttatgatgaaaagaatacctgcaa
ttcttgtactagaagacggtacttattataaaggatggtcattccagcaagataaacaag
agattactattggtgaagtggtttttaatactggaatgacaggatatcaagaaataatca
cagatcctagttacttccatcaaattgtcgcttttacttaccccgaaattgggaatacag

gtattaataatcaagatattgaatctcacagtattagtattaaaggacttattgcaaaaa
acatttgtaaaatttcaagcagctggagagagcagcaatctttatttaagtatttaagta
gtaataatattccttttatttttggaatagatacaaggtctttaacccaatacttgcgtc
aatttggtacaatgaacggttgtatctctactgataatctaaaccatagttacttaaaac
agaaaatttgcgagattccaagtatgcaaggtttagatttaatcccgcatgtaactacaa
gaaatgtttacccctgggatgaaaaaagtttgccgaattggtatttgacagataacatga
gagtgcaccgagttattcagttgaaagttattgttatagattttggagttaaactaaata
tactcagaagactagctacacttggatgtcagataacggttgtgcctgcccacactcctt
taaaagatattttggcttaccaacctgatggcatattactctctaatggtccaggagatc
catctgcagtacattacgggatccagacagttacaaacttactagattacaatgtgccta
tatttgggatttgtatggggcatcaaattttaaatttagctcttaaagctaaaactttca
aacttaaatttggtcatagaggtattaaccatccatccggattgaaccagcaagttgaaa
taactagtcaaaaccatggctttgcagttgacttgacttcggtttttgaatctcctgtaa
gagtgactcatttaatctaaatgacactactattgcaggaactggacataatcaaagtc
cttatttttctgtgcaatatcatccagaatcgagcccaggccctcatgatgctgattatc
tattcgaaagttttatagaaataatgacaaagtccaagaagaaagttag-----------
ttagtaattttcccatgctttatgagtgaataaagcggatagtacttgtactcctcgtaa
ttgattgaagagaggtaaatgccctttggggccatgagaattccattgaaattcttcagg
atatctgcaaggaattctatcaacttcccagccgatttttttgccagaatttttcccaatc
ttttccaacactaagccatacttgtctttgaacaaataacccaaatttaccctttgagtg
tgtatgccataaattatcaatagtttgtaaatcttgagcaggtattttttttatatctgt
aaaatatagccaattacgagtttgtgcatttacaccagctagttgaataagtttttgttg
agttagctgatctgctttgagcaaatcccgatgagtcagtaacacttgtaaatctttgta
attcatctgttgagcagagcgcaatggaacaatgccatcgggaaataagttagaggcaaa
tttcactatttcttgattcttactgttgagtaacttttcataaattaaaccgtcaacaca
attacttttatagttaggcccagtaatcctctcaaacaatagatctgctaaatcttttaa
ctcgatagagtcgttgctatttatattctcaataatttcaagttgttgcttga---catt
attagacttagtatccttattcaattctaaaagctgggctcggatttgatttggcatctg
ttttgtttaattagttttaatagtcataaataaggtcttatagagaattagattgttttt
tgaaattagaagagcgccgcagtatttctaaagaagttcgcataatttgattcaataagt
tttgtcctgcataaattataatttgaccaattaaaaaaattgtgtttttttattttaggaa
caaaaaaatcttgtacctcaagcagaaaagtaattagtatttgcattgatgacaacatct
gtaattcattcgatctgcaagcatatatatactgacagtttaagccttcttgataaaatc
cccatactttgtaacgactttcgtaaattgctcgtggtcgctcaagatagtttttaatta
tagtattccaaattaggttattctttaatttttctaattttctatcagatagaaatttta
cattacagagataacttgcatttgtctcatttacagtattgaaattttgaactaacatat

gagcaataatgttgcttagctgaattaaataattttcaagaaaaatttcgacctgcttaa
ctggcatattgtaagtaaagagattaaaagttttattcacagtttgggatgacccaaaaa
gtagttgaataattagaatttgtagtagcatcttataatcgttcagtaggtagttcaaat
cattaatatcttggtaaatatcaatatagtccagttcatataacttacaaaatcttttga
cgcatctatggaataaatcaattaaaatattttcggtcaagtttgtaatatcatttacat
ctaaatttgaattataaatttctaaaagtatcttttctagttcagctaatataattttta
ataagttcctttttacttcggtgcgaaaaacatctaaaattaacacttcctgagaacaat
tagttaatctcttattaatctttatagaagttctaactaacagctccgctacctcctgat
tgagtgttggtccctgagagcttggccaataattattcacgttataccatagtaagaaat
attgaatttaatgttatacgataacttttaatatactgaaaagcaagttagttactcta
tagcaattag-------gttttacagatcttaatacttatttatcaaataaagttatatt
atagtagcgactgagctaattttattaaaattta----taataatgacaacttattactt
tgcccttgcgagtcaaaattttctattagtacaagaaccactagaagaagtctttagaga
gagagttaactactatcagtcaaataataaagcaattgatttttggctaataccaaaccc
ttctttttagagaaaccagaaatgatttcatttaagaatcttgtacctaaagaggctgt
agctataatctctactaatccaatatttattaattggttaaagctaagaattggctatat
ttgtattggacaatttgaagataacctacaactttctgaagaatcgttaaatattactgt
tttaaaagacaaaatttaacaatgcttttattataactagtgtt-aattattttggttaa
gttatagttatagtcttttagtattta-gatgcttatagtttatcacttagtattttatg
ctgagatggagtgacaaatagtgtaagtatttcttggctaaatgtttcagttgccttaga
tttatatctatttggattaactataatagatagcattcgttttatagtaacattttcaat
ttgagcccaatgtacaattccaagttccaattccttagcaatagctgaaacggaaacaaa
ggcagctcctagccctgattgcacagcgtttttgatagcttctatagaattcaattccat
ctctattttaaagcgactgctgtcaataccatgttgactgagtactttatctatgacttt
tctaattgttgattgagtatctaacgcaataaatctaagcctgtataagtcttctttttg
aatgtctcctagtttagaaaaaggatgtgattttggtaatatcagtgctaattcgtcttc
tgcataagaagtaacttgcaaaacatcttgtaattcagtaggcacttctcctccaataat
tgctaaatcaacttggccattagctacgctccatgaaataagtctagttgaatgtacttg
caattgaacagccacttgtggatatctttgcctaaatagtccgattaatcttggcatcaa
atatgtcccagttgtctggctagccccaataattaatgtgccaccttgtaagttttgtaa
gtcgtcaagagctcgacaagtttcttcgcagagggctaaaattctgcccccgtatcgtaa
aagaagacttcctgcctcagttaaagttgccttcttattacctctttcaaatagggaaac
atttaattggcgctctaaattttgaatttgcaaactaatagccggctgagaaacatacaa
gctattagctgctttttaaaactgccctctttggcaattgcttttaatattcttaactg
atccaatgtaaatggaaggtctgtcattaaagaattgtagtataatg---------tata
ttatttagtattgatcaatgtatgcatttgccaaccaaaatatttgttattacgcttcat

tatttttaaaataataaaaagtataatgttaaataagctgtattaaaatttaatatctgt
ttgatattaaatttatactaaagatatcgtaaatttaattctaactataaaatataggag
acaat---atggactctagacttttagttgtactaataccagttttagcagcagcatctt
gggcagtttacaatattggtagagttgcattacagcaatttagaaaaatgacatcttagt
tttgcttagaatata-atttttagactttatgagatagaggactttaaaatccctatctc
tattgtatatcttaataaaaaaat-caggc--ccccttgtacttactgttattacacatt
aagattattgat-agtaccta----gataataagtttaagctaacttatttttttatttta
taattaattaaattaataacacaattatggctgttccaaagaaaagaacatctaaagcta
aaaaaaatgcacgcaaagcaaattggaaaaatcaagcaaaaactgaagcacaaaaagctt
tatctttagcaaaatcagtattaactagaaaatccaatggatttatttataatctaactg
aagcgtcagatacttttagtgattagaagtgttt-ttactacaagtctgaaaaagtatta
cgcaacaagttctaagtaatttttatctat----taatcatttgaaaatcattctatttg
ataataaaataaatagcagattaaataaaattacagtaacaaattatgcagcaaagctag
atcaaactagtgaaatttggttgttcaattgtattgaaaatattcagcatattttttaa
aaagtcaattaaaatcaagtcatattactaaaatttttatttctggtactagctttgaat
ataccgcaggtctaccaggactattatccagcttaacactaagtggtagactacatccta
ttagtatatatagtcctcagtctctcaaaaagtatcttgaagtatgtaccaaatattccc
aaactaatttttcttttcctattaatttcataatttacaatacggagaacaagttgtta
gccaattttatacagtaatttgtttaccgttgagtaaaaagagcttgctgtatggattta
ttattttaaaaaaagaaaagcatggagtatttaaattagcacaagctaaaaccttgaata
ttcttcaaggacctatatatggaaaactcaaagaaaaagataatttttaagtccagatg
gttactatctatccggccaagacttctcttctaatacaataatgggacataaaatatcgc
ttcctttattagtcagatattctagaattatttctgagatgcattggttttgctcttatc
ctattagattaaatacttattcgcatcagcaagtagtcaagtgtttaccacataatgttt
taactgatattatgaagtcgcaaatatatcaagataacagttttgttgaataatacttat
tcttgatatataatggtctcggtaatccaaataacatactagaaataatttcttgaattt
atttgttcatattaaatttacaatcatttatgacatacgcaattattgaagcaagtggca
cccagctttggatagaagaaggccgctactatgatttaaatcatatacctgttgatccag
gtcagtcgattatattaggaaaagtcttattattaaataaaaaatgggcaggttactttag
gacgtccttgtatagaaggtgttactataaaagctacagtaatgaggcacttgcgaggaa
agaaaataactgttttcaagatgaaaccaaagaagaaaatgagattaaaaaaaggtcatc
gacaagaattgactcgtttaatgatcgattctataacctcttaga------taaaatcta
gactcatattttt--aa-aaactttttaatacttaactaaaatagatataatggcacata
aaaaaggtagtggtagtacgagaaatggcagagactctaatgccaagcgtttaggtgtta
aaaaatatggtggagagcaagtaacagcaggtaatattttaatcagacaacggggaacta
aagttaagcctggccaaaatgttggaaaaggaaaagatgatacattattttcgctaatcg

atggtttcgtgctgtttgaaaagtcaaatcaaaagcaaaaaacaattagtgtttattctg
ctaagaaatagttaaaacaattggtgcaagataacttgagt----gtatttattgcactt
acagtggcaccatatatttgatttattaagaaatggagttccttaatgaaccaatgaatt
ttattaaagatttgctgtgaaatttaatgacaaacactattgtaatttcctgtctacaca
atatggctgctattctatattgtggtcagatacagaaactagtagtagcaaatgctcatt
atcaagtaagtgatatctacctaggttgtgttgataaaatcttctcaggaataaatgcgg
catttattaacctaggaaagaatgagtacagtggttttatacatatcagtgatacgggtc
cgcttaaaaagaaatattatgtcaataatattactaacattttaacaatacgacaaaaaa
ttttagtacaaatcattaaagagccaactttaaataaaggtccaaggctcactgccaata
ttacattatcaggtcgatatattgtactaatgccttttagtcaatcaatctgcatatctc
gaaaaatacatgatgaagatgagcgtcattatttgaaatctttagctattttaattaaac
cagcaacaatgggcttgctatttagaccttctgctgtaggcgtcgatgaggaaattatat
taagcgaattaaaaaatttaaaagaacaatggaactttgttcaaaaatctgcaattaata
gtcattcacctgttcttctatataaagatgaagatattgttaaaaaggtaatccgagatt
tttataataataatacaaacaatatagtaattgattcaaacctgggattaaaacaattaa
attattatatccacacttggcactgtaataactctagtacagttcccaagatgaagcttt
atagtaataatcaatgtatactagatgcttttggtatcaatcaggcaatttccagagctc
ttattccaaaagttgatcttatacttggtggctatatgtttattgaaactttagaagctt
ttactattattgacgtcaattctggatctttcaataattctactagtgcacgagaaacag
ttttaaaaaccaattgttctgcagcaacagaaatagcttatcagttacaaattagaaata
ttactggtgtaattataatcgacttcattgatatggaatcacaaagagatcaattgcagt
tattagaacactttaataaagagctggcacttgatgatgctaaaccacaaattgtacagt
tatctgaattaggtctagttgaattgactagaagaagacagggcaaaagtttatacgagc
taattagtagtgattctaattacttttatttttcacacaatcagagagatcccagtctc
ttaagagatttaatgatagacaacagaaacagcagacttttaatagatcttggctatcta
tagagattaatactattaacaaggtctttttcaaaagtcaaatttgtgcagacctgcta
acttttacctaattcgtaatctctatatagttaaaagtagtattacttataaacaaaatt
atttattaactcacagatcaaaacttatttattctcaagaatacaacaaagtattaccaa
gca------gttattatctagctagtctcaacaggaatagtaatcaggagttcctatctt
aagattttatatcaactgtt--
-------------------gaaaaaaagaagctctctcttatataagagagagcttcttt
ttttagcttactaacta-tctagttagttaattaaaaattaaccgtaaacagctggagct
gttagagctactggtaaagattcaccagaagctagatctagagggaagttgtgagcgtta
cgttcgtgcattacttccatacctaggttagcacggttgatgatatcagcccatgtgtta
attacacgaccttggctatcaacaacagattggttaaagttaaaaccattcaagttaaat
gccattgtgcttacagataaagctgttagccagataccaactacaggccatagacctaag

aagaaatgtagagaacgagagttgttgaaactagcatattggaagattaaacgaccgaag
tagccatgagctgcaacgatgttataagtttcttcttcttgtccgaatttgtaaccatag
ttagcagattcgttttcgcttgtttcacgaattaagctagatgtaactagggatccgtgc
atagcactgaacaaagaaccaccaaacacaccagcaacacctagttggtggaatgggtgc
attaaaatgttgtgctcagcttggaatacaagcatgaagttaaatgtcccggagatacct
agaggcattccatcagagaaactaccttggccaattgggtatactaggaatactgctgct
gctgctgctactggagcagtaaaagcaacggaaatccatggacgcatacctaggcggtag
cttagttcccactcacgaccaatgtagcaagctacgccagttagaaaatgaagaacaact
aattggtaaggaccaccgttgtatagccattcgtctaaagaagcagcttcccagattggg
tagaagtgaataccgatagctgcagaacttggaataacagcaccagaaataatgttgttt
ccgtatagaagggaaccagcaactggctcacgaattccatcaatgtctactggaggtgca
gctacgaatgcaatgatgaatacagatgtggcagttaatagagttggaatcattaataca
ccaaaccaaccaatgtataggcggttttcagtactagtaatccaagagcagaaacgttcc
cacaagctagcgctttcgcgtctttgtaaagtagcagtcataatttttatcaattttta
aggagttaacaaagatgcttcccaaacaataatttgtctgttatggaaattattacttaa
ggccttttcaaatgagaacattttaaaaaaaaaca-tagcaaaatctaatctcagtaact
aa-aaatt-taattgttgatatattgcttaatacttcaa-----------taataaattt
tt-gct-------------ttttacaaaattaacaccattttagggat---ataataata
atgcatattaatgctgcttattttgattttaatagtttatataattttgccttacttaca
tacgcatgtcacactttacaaaaattcagacgaccatacaagatttaaatctgttaaagc
atgctttaacagatttgggtttgacttggcaaatgaattctagtcatataaaagttggtg
aaaataatcagcataaggtagatatcttaattaaacaagataatttgtcacatattggct
ttatttggaatgataataaataccacttagttgccgacttgcaactttgggaacaaccct
ggtctctagaagtatttttggataaattatctcagaagtatgcttaccactctattatcg
aagaaacaaagaagcaaggctttgaaaaaatgcagcagatttcacaaaaagatggatcaa
ttaagttaattgtgcaacgctggaattattaactaaaatataaatgcgggcggagagact
tgaactctcacgagattatctcactagaacctaaatctagcgcgtctaccaattccacca
cgcccgcactgttaaaac--aatattactttatcataatctatactaaaacacaaactaa
-tagtataaactagcttccaagcttaaaagcgtctacaaataaaccatatgttataccaa
ttttgaagttgttgaataaattaatttttatattgaattgcttttggctagagtagacta
ttttactagtcaattcagttgctgcaactattagactggctgcaataataccccaatctc
ctgtttgtccaggaattgtagataaacctgttgaaataaaaaatcctaacaataaactaa
ttaagccagttgttaactcgctcaaagaataataaagcttattatttaagtttttttatta
agtaagcaaaaaaagttgaaagtctagttttaatcatagtatttagtaacggagttttaa
aaggatgacaagtattaacag-tcattaatattgtcgtatccttttaaaactcgttaaga
caaaaagctgctcttgt---------ttttaaaggtcttgttcgcttaaagcattaataa

gatgctgaaacggctcttgtataatatctttgcgtttaattgattgcaaatctaattctt
cttctatgatttcttgtaataactgtatgcttcttactgttggagcaacaggtacagata
gtgaattgtaagtatcttttagcccatttaatacacgctgatcgagaatatttgtactac
cagaaaccaaggcataactagtataccgtaggtaatattctatatctcttagacaagctg
catatcttcttgttgtataagaatttcctccaggccttaataattcaggctgttcttcgt
acaactgagctgcggcctcttttaaaatattggtagcttgatcattaataatttcagcta
tttttattcgatctagtccacttgaaaagaaagattctagttgtccgacagctgctttat
ctagataacgaccagtaagatcgtaacgatttaatattgctgttatagcatcttgcataa
attaattctcctac-ttgctatcagagcggtattataaatgactaatattgtaatataat
cattagtgtttatgaaatttatttaaaacttattctttattgcttaatgtgcaattaaaa
actcgcatagattttacatatatagagtatacgtagtttttaatta-tttttttataaaa
attaagaaataaagctttttt-taagaggttcgtgaatttcttaatacaatctaagcttt
ccgcccaaataagttttgtaaagtac--------aaaaaaagattattcaataatagtaa

--------------------------acaatattaatatgtatcaa--------------

-----------------agtataaataatcttttactccagcatcaaaaaaaatatttaa
catatgttgatacaattgattattcaagtgaaaacttagatttagaccaatttagctatt
ttatcattacaattaataaagctgataaatgtattcttatagagcaattttgttataatg
ctgaaggtcatatttactcaatattcttcaaaggtccaacagctaaacacctttcttcca
taatttgtcattttcaacaattaaatacaataatatccacagctcatgcaatatatttag
gaagagagctaatgaagtcagaattagctctcgtgctagatcaacaatatattcaagatt
aaatttttttaagtattaattatatttatgctagtataatattggtaagggcatgtaac
tcagtggatagagtatcagattccgattctgatggccgtgggttcgaatcccgccctgcc
c---gagtattcagattact-acactaaaacatct-------taatagctagtttgataa
tagtatgggcattggggctgcaaggtttctacattatgaaaagaagaaaatata--aaaa
aaacaagctctccaaaagagcttttagtacaattaataaatgcagaaaacaatattgttt
ctttttctcgaaaattagctcttgcttaaatactgttaatttttggtattcgacatgtta
aactcttattcatggcgaatattctgtcagagtcgctcttagtttaagaaaagtcag-aa
aaatatttatctttacgtttt-tacctattttatttaaataaaggttagta-ttttcaa
cttttatagtggacgtgggttcgagtcccaccagctccatcaatttgatt---------t
gtataattagctagttcaacctttttaataaccagatatataattggaggaaatatggat
tttataacaattactaagcccgctttaaagcaaattgcaatcttgaaaaatgatcatgaa
aatgatgtacatctaagaataggtgttagacaaggcggatgttcaggtatgtcatactca
atgaattttgaacatgttgacaatttaaaagatacagatgaacggcttcttctagataat
ttttctgttgtctgcgaccctaaaagtcttctttatctttatggcttatcattagatttt
agctcggaattaataggaggaggatttcaattttctaacccgaatgctagccaaacttgt
ggctgtggtaaatctttttcaggctaaca-ctctaacaggtttttttatatattttgtatt
acacatagtattttagcaagtcg-tttaatagagacaagcaatgctctgttaaaaac---
----------------------------taatttcaagagtttttattgtattatt-----
----ttatatatttattatcaactacttgaaaatggattagttaagcaatctaatttgtt
atcagtatcaaactctaagtgattaacttttttattgatatatgctaatcttt-ataagcc

taaacgtatctgtacattacaatttttgacataaaaagagtcttaacataactgtcaa-
----------------------gaagaaattata----------tattttataaatactc
aacatgaatctgtcacaacttattgttaattcctctatgatgaaaattcctaataacttt
tattgcaatacgaaatttatttcttctgagaatttaagtcacttacaattatcaaaaaca
aatttattatctcctacaaaaataattgcttttggactacctaattttgatttcatctct
acacagattacaagttctattagggaatcagttgttgtatctagtattgatgataataat
ctttttgctgccccaactaatgattggataaaaaaacacaaggataaaaatcatcattct
ttgttttataagaaaatctttaaagctttgctaaaccgtaaaatcagagttgttgatgag
ccatt------ttataactctgaaataaataaagaaaagagcataatgacaagtcaaaga
tacgtctggggtaaaagctggaagccttcaattattttatcttgcttaaaaaacaaaaaa
aatagcaaaatttctgacgcatcgaaaaaatttttaactgaacagttatctgcatcacca
gtatttgtggtaaaaaatggttttaatgaaattattttaggtcacccattatctcgtgtt
aaaacaggaagagttaataatctgacgcatgcattttctaatttattaaatcagtctaat
gaaacttatcctatatctaccggtttattctttttcatccagatgatgcatttgaattc
aaagactttataatatcggttaatccactggcggccaaacatatggagataagcgtagaa
cccgttggtttacactttgcttataaaatgaatagagatagatcgtcagatactcaattt
tcgtttattccagattttaaagaagtaggagatttattattcaaatatagaaaagatcat
catttagtttttcataagaatcagcattatggcaaagatttttttcaggggcaaccaata
tatatgattcagccgatcacttttaaagatcgatccggaaagctgaatattatcaaattt
actgggctaaatgacacaagagaaattatttttactaatcttgaagctgctaatagatca
tggacacattttataaagaggaatccacaattaaaatcagttaaaaatcctactttatta
gtttataatctagaaagtttttttaaaagatcaagaacgattggataatcaagatttacac
aaatttgttgtgatcactaataaatcatcttatcttaccacaaaagagttaatagcttta
accgattctaatagctttttttaagcacttaaaattaaatataaagcctaaactcttttt
gtgaaactatggggaagacggttattttccaccttaacttatgaat-aaatgctatt-gc
ctttatttcctcgaataatattctataactagtaattcattgagttgtaaagcgacccat
tccctatcaatgactccattgactttccctgataaattagatttatttaattctaagtgg
ctaggaatattagctaatccagggaaagctaagtaattttcgactagttttcgagatgct
tcttgatttttcacactaattgattctcctggtttacattgataactacagatagatact
acttgtccattaatacaaatatgaccatggttcactagttgtctagcggcaggaattgta
ggagccataccaagtctaaaaacagtattgtctagtctcatctctagtagttgtaacaaa
atttgacctgtcgacccttgtaatttttttagctgcttttacgtacttaaataattgtttc
tcacttaatccataattaaatcgcaacttctgtttttcctctaatcttacagcatactca
gaaggttttctagacttttgtccatgttctccaggtggataagatcgcttaattgctttt
ctactgagtccgggtaaatcacctaatctacgggaaatgcgtactcgtggccctctgtat
ctagacatattatttaaattctcctaatgtaaatggattaag----------taatgagt

agctatttttaacacaaaaagagcattaaaacaaagc-ttttgctaaaaaatctttata
agaaatataggcttggacgaaa---cacactatgatgc-tttttaagcgggtagcgggaa
tcgaacccgcatcattagcttggaaggctaaggttttaccactaaactatacccgcaata
ttaaacagattgtcactaactcaatataacatagttatgttaagttaagataatcttaaa
tttaatagaaat-tcaaattatttacgctccttctaaaactacatcaagaaaacgagcaa
tttcggcagcttgttcttcaacttctgataataataatggttgaccaaccctagttaatg
gaattactcttttatcttttgtacataaataaatttcacgtcttggattgagcccctctt
taatatctatttttattgatttgatttcttttatactaaactgaagacagatttttctat
tctttccagggaaacctagccgaaaaatttttacaatacctttatctttgttgaattcat
tatatccagcccctatattccaaataatagttagccatagaaacaaactcaggaagattc
ctatactgccatagaatgtcattacaataccttgtggaataaataccaaatcggttgaag
ttgtaaagggtaacaaatcaacttgaaaataacttgatagtccagcaagaagaaagccca
aagccccaataaatattatagtagcccaccagtaattactcagtcttcgtgaacctaaaa
ttaaatcttttctaactgagtgtatagataatgtttttttcataatttgttcttttaaaa
cagta-tactaatgcaaaatggaaaaattttgacaattttt--attagcgctttagattt
tttaaataagcttgattgcttttagccctaaaaatagtcctgttgctaacccaaaaaaag
cagcaagaaatattgtatatcctaaaaaaactgaca---tatta----ttattattattg
ttacaatagtgtttacaaaaatagtggaaatctattactagggtattatattacgttaaa
tgccttgctgtctacatgaaaaaactttttttcagtttttagcattgtgttcatattagaa
ataatattctagtcaatttattaaacttctggagaacagaatcttatgtcagaatttatc
aagccttataacgatgatccttttgtaggcaatttgtccacgccagttagtacatcaagt
tttagtaaaggacttctaggaaatctaccagcttaccgtcgaggtttatctccgcttctt
agaggattagagataggaatggcacatggatactttttaattggaccttttgataaattg
ggccctttacgaggtacagatgtagcattactagcaggatttctatcttcggtcggtctc
attattattctcaccacatgtttatccatgtatggtaatgtatcttttactagagcagac
tcgaaagatccattacaaacttctgaaggctggggacaattcactgcaggatttctagtt
ggagcagtaggtggttcaggatttgcttatttattgctagctaatatacctgtattacag
actgcaggtcttagtttattttcttaagctagtaaggggacttgaacccgtaacctactg
attacaaatcagttgctctaccaattgagctatactagcacctttttcatcataacatat
aaagatatttgttgcaatctgttattttcagatgtaacaaatatctttttttatttaaaca
gaactgttatattaacac-------gtatattaattagaatctgaattatctgactctgc
agaatattcttgatcacagtttagataataacaaattgtttgatctagacaagttgcaga
ccaaccaacaggtgtttcacctgataattcttgcatatctccagaattgtaaaaatcatc
atttggcttattactgtttgcttgcataaatatcactcccaaaacactctgaacttaatg
tgaagtaatttaacttctctataatatattaacacaaaatattaaatatggtgattattc
tttaattgtaaagatgcggctaactttataaagtttgagtgagagtcttgatagcttttg

ttgaaataaacattttaatccatttattttgctctctcgaccatactttttttatgttgta
aattaacttttttgtagtttttttgttcgtttatgagaatgagatactgcatatccattat
tagctactttccccgtaagctgacactttttttgacatgatttt-------ttaacttata
tatttatttagagtacttgctaaagttgactttggcacagctccaatcactgtgtcaact
ctttctcctgctttaaagatcattaaagtaggtatacttctaatgccatattcagctgca
atagtaggattttcgtctgtgtttatttttactaccttaatagatgattcatattcttcc
gcaatttcatcaactacaggagagaccattctacaaggaccacaccatgacgcccaaaaa
tctacgagtacaggtaagttattgttaataacttcttgtttgaaagaggcatctgtaact
tgagatactgacatattctttaacctttaatatatgttccttgctaagcaaatcttatca
caaatttcaagaaaaatcttctatagtgaattactattagtgttgaattaatttttttaaa
aattagacattaaaaaaacttattacctatataaataaagtggtggccgagtgatatctt
gatagtgatagtttttatattaaggttgaacactagaaataacttttcaagcaaattttt
atttgaaaa--acttctctgtgaccataaaaacttaattatattacaatatattttatta
tttgtaacttagttctgataatggtataaacaacgcaaaagatactgccttataatcaag
gaggaatacatgtctcaatccgtagaatcacggactaggattaaaagcgaacgttacgaa
tctggagtaatcccttacgctaaaatgggctactgggatgctgactatgtaattaaagaa
acagatattttagctcttttcagaatcactcctcaaccaggtgttgacccgattgaagca
tctgctgcaattgcaggtgaatcttcaacagctacttggacagttgtatggactgattta
ttaacagcttgtgacttatacagagcaaaagcatatcgagtagatccagttccaaacgtg
gcagatcaatattttgcttacatagcttatgatattgatttatttgaagaaggttccatt
gcgaacttaactgcttcaattattggtaatgtttttggttttaaagctgttaaagctctt
cgtttggaagacatgcgcatgccagtagcttatctaaaaacgttccaaggtcctgcaact
ggattgattgtagaacgtgagcgtatggataagttcggtagacctttcttaggtgctaca
gttaagcctaaactaggtttatctggcaaaaactacggaagagttgtatacgaaggctta
aaaggaggtcttgatttcctgaaagatgatgagaatattaactcacaaccatttatgcgt
tggagagaaagatttttatattctatggaaggtgtaaataaagcatctgcttctgctggc
gaaattaaaggtcattaccttaacgtaacagctgcgacaatggaagatatgtatgagaga
gccgaattctctaaagaggttggtagtatcatttgtatgattgaccttgtgattggttat
actgcgattcaaagtatggcaatttgggctcgtaaacatgacatgattttacatttacat
agagctggtaactcaacttactctcgtcaaaaaaatcatggtatgaacttccgagttatt
tgcaaatggatgcgtatggctggtgttgaccatattcacgcaggtacagttgtaggtaag
ctggaaggagatcctttaatgattaaaggcttctacaatactctacttgaaagcgaaaca
gatataaacctacctcaaggtctgttctttgctcaaaattgggcttccctacgtaaagtt
gtaccagtagcatctggtggtattcatgctggtcaaatgcaccaacttcttgattactta
ggtgatgatgtggttcttcagtttggtggtggtacaattggacatcctgatggtatccaa
gcaggtgcaactgctaacagagtagcactagagtccatggttatggcaagaaatgaaggc

cgtaactatgtagcagaaggtccacaaatcttgagggacgctgctaaaacttgtgggcct
ctacaaacagctttagatttatggaaagatattagttttaactatacttccacagataca
gctgatttcgttgagactccaacagcaaacatctagtttaatgacgacttactaatactt
taaacagtcaattgtaagtagaattaacttataacaataaggagcacagaatagtgagac
taacacaagggactttttccttccttccagatttaactgatgagcaaattaataaacagc
tttcttatatcgtttctaaaggcttatcagcaaacgttgagtatactgacgatcctcatc
caagaaactcctattgggaactgtggggtttacctttatttgatgtaaaagatgcttctg
ccgttatgtacgaaattagctcatgcagaaaagcaaaacctaattattatgttaaagtta
acgcttttgataatactagaggtattgaaagttgtgtaatgtctttcattgtaaatagac
ctgctaatgaaccaggattcttattacaacgccaagacttcgaaggtagaactatgaagt
acagtcttcatagctatgctactgaaaagcctgaaggagctag------atattaattaa
gaattaatattggctaatta--ctcccctctttaaaatcacaattactaagttaattatg
attttaaagaggggtaatttcccaagtaaattttgactaaa-tagattggataattaaaa
atacacagaaataatgcaatcacaggatataatttccaacgatactcttgttaatttaca
agaagaatatgatagaacacaaatccaagaagttttaaatgagttaaatcaagaacttat
aggattagtacctgtaaagaccagaattcgcgaaattgctgcgctattattgattgacag
attacgtagaaaactagaactagtttctggtaatccaggattacacatgtcatttacagg
tagtccaggaactggtaaaactacagttgctatgaaaatggcggatattttgcatagact
tggatatataaaaaaaggacatttgttaacagttacaagagatgatcttgtaggtcaata
tattggacatactgcacctaaaaccaaggaagttcttaagcaagcaatgggcggagtttt
atttattgacgaagcttactatctatataaagcagataatgaaagagactatggctctga
agcaattgagattttattacaagtaatggaaaaccaaagaaatgacttagttgttatctt
tgctggatataaagatagaatggaaaaattctacgaatccaatccaggactctcttctag
agtagctaatcatgtagactttccagattatacttcagatgaattattacaaatagctaa
aatgatgatagaagaacagcagtactgttttacagaagaagcagacaaaactcttttaga
gtatactgagcgaagaatgaaacagccttattttgctaatgcaagaagcattcgcaatgc
tattgacagggctagaatgagacaagccaataggattttttgccagtggagaaaaagtatt
aacaaaagctgatttagtaacgattgaagcagaagatatcttgaaaagtagattattttc
attacctgatgcttaatattcacgtgatttaatgaaactatatattattaaaaag-cttc
ataatcagttgcaaatttttttgaaaaacccttattatcatcttaatgaaggtg-tggcgg
catagccaagtggtaaggcagaggattgcaaatccttcatcccccagttcaaatctgggt
gccgcctagtactaaaaa--gggggtgtggtggaatggtagacacaacagacttaaaatc
tgttgatttttagtaatcgtgagggttcaagtccctccacccccatatattaacttataa
ataaaaagagatgtgtatttgtattaattgcaaccatattacaaaatgtaagacctatca
cttaatagaatctcaacacaagcagcctcatattactagaagtccattgtttatacctaa
atatcctgtagttcatgttaatatatctaacaatggtacctataatcaaattgattggga

tttagtggagtgtttatcatttgtagaaaaaccgaatagttggaatttagacgctaatta
gttacaagaaaaattatgtactataagcaacaactctattttttagatactcggtattga
ttttggatgttctaaccaataagattttacctgttcttgctatctctataatgccaaatt
tagttagtaattgttcaatagcaacaatctttcctggatctccagtaacttctataatta
aaagatcttctgcaatatctactatcttagctctaaaaattttacaatttctaaagctt
ctgttctagtctgagaattgatcttaatcttaattaacattagttctctttcaactgaag
gaatatttgttacatcttgaacattaaggatatttactaacttatataattgtttagtaa
gttgttcaatagttctattgtctccttgaactaccatcgtgattctagagaccccaattt
gctctgctggtccgactgctaaacttgcgatattaaagcctctccgggcaaatagaccag
atattcttgacagtactccggcttcatcttgaactaaaactgataaggtgtgtttcatga
aaaattaattttaatctatttgatctattaagtatattctaagaaaattacagaaaaaat
actagttagtctacaactaaattatctaaactaaacaaaatcctactatcagataatata
tctaccttgtaatgctatactattaacttagtatttaatgatattctaattgcaaataga
atgct-aattttttaaaatttgttaggaacttgtgctagaaaaatacaaaaacagtaaaa
aactctctcgagatttacctcaaatcaatgatcgcatcagatttccgaaagtccgagtaa
ttgatgacgaaggtgaacaactaggtatttttgtgcctgaagaagctatacaattagctg
tccaacaaggtttagacttagttgttgttagtgataaatcggacccgccagtatgccgaa
tattagactatggtaaatataagtttacacaagaaaaaagagctagagaagctaagaaaa
agcaacataacagtagtattaaagaagtgaaaatgcgatacaagatagaagagcatgatt
ataaagttagaataaatcaggcatctaaatttattcaagcaggagacaaagtaaaagcaa
ctatcacatttcgggggcgtgaaatccagcactctaatttagctatagatttattgaata
aaatggcaagcgatctaaatacaatagctgaaattcagcaagctccatcaagagatggca
gaaatgttataatgctcttatctcccaaaaaagttggctaaactaattttttatttaaatg
catctggctggattcgaaccagcgacgtctctttcgaaatggcggattatgagtccgctg
ccttcggcccctcggccacagatgcatcacttaaagtttataactcataaagcatataaa
aatcaatgtttagctatttttaataaagtctataagagtctattattcattcatattgtg
atatgtagcaactgctgaaggggaaacttgatttaagtatcgaaatatccagtatttaaa
aattgtgtcgagtatgactggaaaagttgaaataaataaaaaaataaaatctcgactttc
tggaagtcctaaatgtcttaaaattacttcaataataacttcccatccatgaggagaatg
aaaacccacaaacatatcagtaaaaagaatgattaaaaaagctttagctgtatcactaag
accataaataatttcatttaaaaaagatttgacgactgcaatttgtctttgtccagttat
cattagtaatataaagacaagtattgatacaagatcggataaaatatttttaacagcatt
tgcactttcgtttgcataatattcgcccaattctctagccttcagctgtactctttttc
aatgatttcgtaagatatatcttccgaaggattcagaagaacttcaaagtgaattttctc
ttcaaatctttgtaattcagcaaaagctctttcctcttgagaggaatttaaaaaaatttt
aggctgttcttggttccataaataatcaatacaaggtccaaatacaaaaaactttgaagc

ctgattaacgagtactggagagatgaataatagtaaaatatattttacggaagtaatagt
ttggtgcctagatattctaaactcctctatagcttcagattcgccattaggatctaactc
tttacgaaacttttcaaaagtattagtaattgacctaggtattggcccaaccttttcaaa
ggcgaattgattatttcgttttaaattccaatatttcatttttattgctgctaaaattt
ctattaatcaataattgatcacgtattcactataaatattaattctagaactaaacatat
aaaacaatatttatgaaaaataaaatacaattaaaaattaattatgcattagttctatct
gtggctacagcaatgtatggatataaatcttatcagccaactataaatacactcttaatt
aaagatgaaaaaaactggccttgtttctctaaaaggcatactgcctttattcctataatt
gtattaaaaaaaaatcatcaaaatccctgaactcagaacctagaaatagtaagttatatt
ttaatggaattggaagtagaaaatttatcgagagaattgttaatctacttgaatatttag
aaaaacaggcaaactatactgaatatctgaaccaaattaaaactagtggttatttttctt
tactctaccaggacagcaatacaatgtataagaaaaatattagtaatatttttttattac
ctaatgatattctaaaaagaatctatatttgcaataagaaaaaaaaagcttataccacatg
cattcttaataaatttatttcggaaacagataggctatccaaaaagttttgctaatttta
attgggccttctccaaaattatgaaatggtattatgatagaggataccaatggtctctag
ttgaagttaaacaagcgtctgatgcatcttctattgtaattgatattcatgaaggggtag
tgaagacaattataacagaatactacactttatcttataaaagactctcaggtatctcat
acatagagtcaatagagcagtatcttggagtaagagtcggagctccattaaatataattg
atttacaaaaaaagattagttacttaaaagataaccaactagttggcgacattatttata
gtattgaacgatcaaataataactcaatgagtttggatgtcaaatttcagatacaagaat
taaaagataaagagataatagtgcttgcagaaagttcttcta------------ttatct
cgcatgcatgtgatttactgaatc---aatataga---------aataggctagtagctt
ctaatataatttcactatcaactaataaattacacgcatgctattttaattataaactcg
actatcaatataagtatataaacacctctaatctcgtacaattgttaacttactctattt
cttgtaggaagacactacttagccattattttaatttacaaactttaataagctggacaa
aaaaaaatactattggatttcagctatatttacgaaatctaagctttggaaaagcttttt
gtgtgttcagtatgaaatttatcaaaaatggacttaatataagaattttatatataaatc
cttcattgatagttgatcagaattttatctttcaatttgctatacaaataattaagcaat
atcatactgcta------aaccaccagcct------------------------------
------tatttttaagcaatctagatcttgaacaatatgttgctgaaagtctattaatgt
accattttacatcttgcttctcaatatctgaaaaaatattattatctcggattatgcata
cagattctttatttttcaactctgagaatctccattttcattttgatgatagaaatcacg
cgatcaacagttatgataattttaaacaaaatacaaaaatattttatcaagagtttctct
ctttattatttagtttacgttatcaaaattttaattatttaggttggccttcaaaaggcc
attttttgaaataaaatctttatacttggctccatttcaaaagagtg------------
---atttttctgatagtcgtaagaccctattcttccacaaaatgtcgttaaagcaagtat

ctaattttaatttaccaatatcttttagaagccactttaaccatattttagttagtacta
ttaaatgccagtcaaatttgaatatgagaactgtatctttgctattaattgattcccctg
ctgaatatatgctatacaaatctattcttaatttctctattaaagttaggatgcagtatt
tcatacctatgagcaacagtataagagtatctctattctataattatttagattgttttt
taataagatcatctcagagtttcattcacatatggcaagatttaagaactcttaaaccaa
ttccacatttttggttaaaaaaattttcatatggagttggaattcagcttaaattaccaa
ttaaacagatgccacctctttcaattgaatatactataactagtagtcgctacttttgta
tttaccttcgtacttattatcaacgataactattactaaaatgaaaaatttaatttcttt
ttttgatcgcagcaaagggaagtggatttcacaaagaacaacttatgaattatctaataa
aaacatgagttcagcacagtctcagatgataatgaaaataggcaaatcattgtcaggatc
tataatacttgcatcgttaaattggggagacatttatagacaagttgcccattacgctaa
aagtcatagccggagtgaatataataataagtttaacttacaatttaataatcaactaaa
taaccataaattattgacgctgtgcatagtgacagatcctagtctaatcagttttaaaac
tcgctatggaagtactacaatagatgaaacatattggtttgcaacaaataatttacgtct
aagtactagtattgttaaacgattcaatacttgtgtggcagtatctttttgttcagaaat
taaagtttaa-actctatatagaagcaactaataaccttta-ggctattagttgcttcta
tatatctaaattttctattaaaaattat----aatactatattattctaagtctttacga
ttaggattcctagaaggatcattagagaggaagccaaagacaaacaaagaaataaagaaa
ataacggtagtatatacaaagattttcaaagtaaacatattttcatccttaaaagctatt
agtgcg----atatttacatatctccacataattttatatcatgatatgagttccagtac
tattttataaataaatcaatatctttatttttagtaatgatttatatttgaatatgcgt
gatgtactaaatactaaatcttttccgatttgttttgtcttgtttgtaaatattttgtta
tttctatggagtttaccttcaattatcacatagtctaacttctttagctgtctaaaagta
tatgaagatttttttgttccaaatagttagattgattataactaatcgttttctttttaac
agtcttgcttttaacttaattatttggcttttattttcactaattctgatactcttacaa
ctaagaatttggactagcagagtacagctattcataatttataacaa------tttttta
aagaatctactaaaatagatgattttacaaaactaaaatgatatttcattatcttgttga
ccttgctcataagtaatgtctttcaatacttttaatatttttccaaagtactcttgcagg
tatttttctaaagatattacttccaacctgtcaattccaagaatactgtaaacttcatcc
atcggagctgtaaattgatctccgctagttaaaatttcagcaaatgctaaacgatcagaa
atattccatgtccattgaagagttttagtaatttttcttaaggcttttaataatccaatc
ggaatttgtgagatctgtgtttttttgcccagataatttttcacataatttaataatttca
gctgatgtccaagcggtattaccaactagcggcaggattctattttctgtggaaggtact
ccaagacttttataactaacttggctgcatcctgagtatcaatgtatgcaatgggcgta
gattctcctgtaacccagactgacttcttgtctaaaataggaatggcatactggttgatt
aaaccttgaaaaaatcctcctaaagaaaacacagtatatttcacattggacttctgaagg

aaatctactacctgagattttaagctcattaatggaacatctgggtatttctctgaattt
agtattgaaaagaaaataaatctttcaactttagccgctttagctgcttcaattaatgca
atttttccatctaaatctatttttttctgcattgtaaggatcagtaggacgagatgtggaa
gcatctatgactgctgttactccacaaaaagattgcaaaatactttcgggtaattttaaa
tcaccatatataagttctgctccccactctttgagaaaagcagactttcttaaatttctt
accatgcattttacattgtagccttcatctaaagctcgtcttacaatttgacgtcctaaa
gttccggttgctccaattactaaaagagtcatattagttaaaaataagtatgtataaaga
cattgatttggttgacagcagagagtaaattaggtagagagggaatcgaaccctcatgac
cgaagtcgtcacattttgagtgtgatgcgtataccaatttcgccatctaccctattatag
accaactactacaagtaatttagtctttagcgtaaagataatactaagtttcaatagata
tgtcaacttttaaatttatattaataaaaaaacggagtctcttatgaccacttttgaatt
aattccttatagattgtatttaagtcaatctaacacatggatgcatagaataaaagcaga
aattaagatatatatagtaatgcttttatggatttcaatttttactttttcttactttaa
actatgtattattgctttaagcttaattgcaataagttttactataagaagtaaacagaa
tattattcaacaacatttgttacagacgttattaatgacgttcttaactactgttttgtc
ttttagtgtggctattaattataaacaatatgcagaacaagaacaatctcagtatttatc
tgattccaagaaatataaaaattctaggagctactattatatacaaatagctaacgatca
aaggatcaaaagacaatacttaattactactttaaagccttccttgtactttttattac
tatatattctattaaactagttatgataacaacctctccagaagttttagtaattactat
ttatagatctagtataataaataaaatatttaaaaatgaattgctgtttatcttccttct
ttcgtcacatattgttactagcattattaatagagttgataaagttattcaagtaactag
cttaagaggaagcttgagtttatataattcatttacaaggcttttaatgttttctttgtt
aatatttcaagtcttctttttagagattatccgggaatcaaaagagatagctcaagctct
ttatactagaaatctcaatcaagaaaacaataatttttttgaaaatatatacagtagaatc
tcactttagtgatcggctaaatataattattagcactttgtactttattattttagctat
agcgtaataaaatccacttattcttctgactctagtaatacgaagagtaataaaattagt
tagtagtttaaatagatagaat-taacatattatataaataattaaatgtttcattatgg
tcaatactcaaaatcaaatttcacaaacttcagaacttgattatatagttaatcaacctt
ataaatatggctttaaaacttctgttgaatctgagcaatttcctaggggaataagtgaag
atattgtccgcttgatctcaaagaaaaaagatgagcctgaatatctgttgaattttaggc
ttagagcatataaaaaatggaaaaagatgagtaatccgtcatgggctcatattaagcatc
cgaatatagactttaataatattatttattatgctgttcctaaattaaaaaaagaattga
aaagtctagatgaggttgacccagaaattcttgacacttttaataagttaggtatatctt
taaatgagcaaaagcgaatttccaatgtagccgttgatgctgtttttgatagtgtatcta
ttgcaactacttttaagaaagaactgtctgaagctggtgttatattctgttcaatttctg
aagctataagggattatccagaattaattaaaaaatacttaggtactgttgtcccagctg

gtgataattattttgctgcattgaactctgcggtatttagtgatggctctttttgctata
tccccccctaatacagtttgccctttagaattatcaacttactttcgtattaacaacgaag
aatctgggcaatttgaaaggacactaattattgctgatcgtggcagtaaagtaagttatc
ttgaaggttgtaccgctcctcaatttgacacaaatcaattacatgcagcgattgtagaat
taatagctcttgaaggagccgaaattaaatattctacagtacagaattggtatgctggta
acaaagaaggtaaaggcggtatatacaattttgttactaaacgaggcttctgctcgggta
ataattcaaaaatttcatggactcaagtagaaactggatctgcaattacctggaaatatc
caagctgtattttagctggtgataattctcagggagaattttactctgtagctttaacaa
ataattatcaagaggctgatacagggactaaaatgatccatattggcaacaatacaaaaa
gtagaattatttctaaaggtatttctgcgggtagatcgaaaaacagttatcgaggcctag
taaaaattggccctcagtcatttaattcacgtaattattctcaatgcgattctttattaa
ttggtcaatcatctcaagctaatacatttccttatattcaagtacagaatccaacatcaa
aagtagaacatgaagcatccacttcaaaaattagcgaggaccaaatttttttatttttttac
aaagagggatcaatttagaagaatctattgctcttatgatcagtggtttttgtaaagatg
tctttaatgaattgcctatggaatttgctactgaagctgatcgtttactgagtttaaaat
tagagggaactgtaggatgagccagattattttagagatcaaagatctatatgcttctgt
tggtaaaacaacaattttaaaaggggtaaacttatctattcgagccggcgagatacatgc
gattatgggtcctaacggttcaggtaaaagtacattatcaaaagtaattgcaggacatcc
agcatattcattaataagcggagacattttatttttttggacacagtattcttgaaatgga
gccagacgagagggcaagagctggtatttttttagcttttcagtatcctgttgaaattcc
gggagtcagtaattctgatttcctaagaattgcattaaatgctcgcagaaagtttcaagg
attatcggagtttagccccttagaatttttcaactaataatagaaaaagtagatcttgt
tggcatgcaagaaagtttttttgacaagaaatgttaatgaaggattttctggtggagaaaa
aaaacgtaacgaaattcttcaaatggctttactagatagcaacttatctattttagacga
aacagattctggactagatattgatgcattacgagtagttgcaaaaggaattaacacttt
agctaaatcaacaaattcaattattttgattactcactatcaaagattactagattatat
tattccagattttgttcatattatgagtaacggaaaaattgtaaaaactggtaatgttac
tcttgcccaagatctagaaaagcacggatatgactggattacgcaaacatagctatttag
tag-------aatattgagactaaagaatattctttagtctcaatatt-tttgcaaatac
-aagtt-aattatttaagctttgtcaaaaccttgcttaacatcttcaattgctcttttta
gcaattcttcgctagctggttctaatttttttagtactacgaacattttcaccaaattctg
gtttagaatttttttaaatcttctcgaagttctctaataaattcagccacttttgaaactt
caatatcatctagatagccattaattccagtataaataatagcagtctgttcttcaactg
gaataggagaattttgagcttgcttcaaaatttctcgaagacgttgacctcttgctagtt
gattttgggttgctttatctagatcagatgcaaactgagaaaaagcttctaattcagcaa
attgtgctaactctagttttaatttgcctgctacttgtttcatggcttttatttgcgcag

cagagcctactctggagactgagataccaacattaatagctggtctaatccctgagttaa
ataagtctccagataaaaatatttgtccatctgtaattgaaattacatttgttggtatat
atgcagatacgtccccagcttgagtttcaatgataggaagagctgtcatactacctccac
ctaattcagcattcaatttagctgctctctctagcaatctagagtgtaaatagaatacat
caccaggataggcttcccgtcccggaggtcttctaagcaaaagagacatctgacgataag
cttgagcttgcttagttaaatcatcataaataactaatgttgctttacctttgtacataa
aatattcagctaaagccgcacctgtataaggagcaatatattgtaaagtcgcaggactgt
ctgcattagctgcaactataatagtataatcgagagctcctttatcctgtagtgaagata
ctacttgagctactgaagatgccttttgtcctatcgcaacatatacacaaacaacatctt
gaccttttgattaataatcgtgtccaatgcaacagctgttttacctgtttgacggtcac
caataattaattctcgctgacctctaccaattgggatcatagagtcaatagcagttatcc
ctgtttgcataggctcacaaactgattgccttccaattataccaggggccattgactcaa
ttagccttgttccattactcgcaggttcacctttatcatctattggccgagctaatggat
caacaactctacctaaaaaagcatctccgaccggaatttgagcaatgcgaccagtacctt
ttacagaactgccctctaaaatatccctaccatctcccatgagtactacaccaacgttat
cactttctaaatttaaagcaactccaatagttttatcttcaaattcaagtaattcaccag
ccataacttcatcaagtccatatactcgagcaataccatcaccaacttgcaaaactgttc
ctatattggctacttctacatcctgatcatacttttcaatttgttgacgaataatactac
ttatttcatcaggtctaatatttaccatatttttaatattatttttattaattggaaatt
ctaaagcggtaattagtcagactaatgctatctgtctttttttactttattttaagttgct
acaacatcaaggtgtgaagccatctgtcttagctggcctctaatactagtatcaataact
ttcgatccaatctgaatagtaaagccaccaattagctcgggatcgactgaaataattagt
ttaacttctttagctttagtcataaccttgatttttttctgttaataaagtttcctgatca
gggcttaaagcaatagaagtattaatattcgcaattgttaaagattccatttggtacgcc
agttccaaatatttgctagctatagcatctaacattccaattcgcttgcgatcaactaaa
atcattagaaatgataatgtattttcgctaatttgatcaccaacagttgcaataattact
tcttttttgactctacagtttttagtggattagct---aaaaaatttttttagcttgtca
gattgcgctaaaatattttgtattgactgaatatcttggctcaccttttcagtaactttt
ttagttttagctaaatcaagaagagctactgcataaggctgagctatttttataacaaca
ttgttactgctcatagttgatctcctaacttagcaatattattatcaattatacgtagtt
gcatttcggagctcatttgattttccagctgcaaagtaactcttttaagagcaagaaatg
ttatttgctgctggatttgccttcggatctgttgttcagctgtttctatatttgatttac
ctgttatagcaagtctttcaatgtccaattggccttgagctaatatggaacttctaactt
ttccagctgttagctgagcttctttcttaatttgatctataataatttgagtttgagcaa
gttgtttttctgattctgacaatctagcacttgcttgctctagtctttcttcggattctt
ggatagctgctaaaacttttatctgtcttgcacttaaacttgatccgagaaattgcttca

aaacataaataagaccaaataaaagcaacaatatattaataacatttgcttcaaaaatat
cagaattaaagccgaatttgtgttcactactatgttctgataaaatagtaattatttgtg
gtatttttactatattattcatttaaaattca----------tactattttaatatttta
ctctggaatcatgatttaaagattgaccacttaatagtttagctttaatttgatcgctc
aaagtatcaacctgattttctaaagttttaagagcttcttcttttgaatatttaactgt
ttggatgcttcagcaatcagtttctctgcgttcaattgagctttttttatatcttctgag
actatattttgagcttcttttgagaagaggctatttttaattgtgcattgcggcgtgct
tcagatagatcttcttcatattttgcagctagctcatctgctttaacaagcatagacgat
gcagtagtcaatgttgttcgaatatattcgtctcgttcatctagtactttagttacaggt
ttgtaaaaaatattatttagtaaaaccattaaagtaagaaactgtaatgccattaaaggc
aaagtaccattgaagtcaaataagccaccttcaatttcttcggctaatagaaatggtaaa
tcaatcattttactaattatgtttatagttaataatttggtttcaattaagaaaaataac
tttatagtgcgcttagtctcatcagactaagcgcaaatgattattttaaccaacgtatgg
attagcgaacaataaagacagcgcaacaaccaggccgtaaattgttaaagattccataaa
tgctaaacttagaagaagtgtacctctaatctttccttctacttctggctgtctagcaat
accttcgacagcattagctgctgcactaccttgaccaatgcctgggcctattgccgcaag
acctacagcaagaccagcagcgataacggatgcagctgaaacgattgaatccataatgaa
ttttaatttttttgttatataagtagaaaattgacaaatttcgtataatacttaaaatcta
tataattaagtataatagattctaatat-----tgtaattatagtatattactcttctcc
atgaccttccattgcttctccaatataagctgcagacagagtagaaaaaattagtgcttg
tattgaactcgcaaataatccgagtatcataactggtaatggtattaaaattggaattag
cagagtaaatacagaaacaactaattcatctgctaagacgtttccaaacaatcgaaaact
taaagatagtggtttcgtaaaatcttctagaatattaattggtagtaatactggagttgg
ctgaatatatctagcaaagtaacctaaccctttttacttaaacctgcgtaaaaataagc
tacagatgtgagtagagacaaggccacagttgtattaatgtcatttgttggggcagctag
ctcaccttcaggaaggtgaattaacttccaaggaattaatgcacctgcccagttacaacc
tagaataaatagaaataaagttgcaatgtaaggcacccacgggcgatattcatgctcacc
aatctgatttttttgcaatatcttgcagaaattctaatataaattccataaaattctgaaa
tttttcaggaattctttgtagatttctagtacctagaaatgataaagttaaaagtgtagc
aattactagccaagagacaatgaaaacttgcccatgcaattgtaggctacctattttcca
atatagatgttttccaacttctactgctgataagctagtatatgggttaagatctatgag
actgttttgatacataataaatatttcttttaaatagtgcaagaatcaaaaaaataag-tt
tttgaagaattaacatgccattaatttattcttaaaattagattattaataaattaagag
caaaaataaattctaatagagatagctatctttagtagtgattatttttttgtaaaaaaca
attattgctacctataatgcttttacgtttaattatatacccatatattaattatttaat
atgattataggtattttgctacaaactttattgttttatccattct----agagattgtt

atagtttatttttatttaacatacctgcgacttcatctgcaaaattaactttcaaattt
tcttctccccctcccagaagaaatcttacaaatcttctaattttaatattttcacctaga
acagcaatattttggtttattaaatcttcaatactaatatcttgattgcgaataaacatt
tgatctaataatgaaagctccttaagtctcttttcattcgtccttcaatgattgtttcg
attctatcaactggtttgtttttcaagtcatctttaccagcttcaattctttgttccaaa
tcaataatttcactaggtatatcctctgttgatacgtattccacattaggcgatgccgct
atttgcatagcaatatctttagctaacttttggaactctggacgtcgtgctacaaaatct
gtttcacaattgacttcaactaatacacctattcttccgcctgtatgaatataactctct
agtaaaccttcaatagcggtacgattagatttttgttagctgaagctagccctttttgt
cttaatgattctaaagctttttcttcattgccattattagcttgtaaagctttcttgcag
tccatcatacctgctccagtcttatcacgtaaggcttttacgatttgagcagaaatttgt
agtgtcatatgctttcaaaaattttaagtatttaaatt----------aattattataa
ttcactttgagtaggatgagttatttcttctaaatctgtttgcccgtatttgccatcata
aattgcatctgctattttacctacaattagtttaatggacctaatggcatcatcattagc
tgggattggaatattaataatttctggactacagttagtatcaagtatacaaatagttgg
aatacctaattttaaacactcttgtatagctgttgtttctctcttttgatctacaactac
aacaatatcaggtaaacgagtcatattttaataccatttagatgtttgcgaagtttatc
taattctcttcgtaaaacagcagcttctttttcggtaactgatcaatcataccactttt
atcttgttcttctagctgtctcagacgactaactcgagatttaatcgttacccaattagt
taacatcccccaagccatctctggttaacataataagaatcacagcgttgtgcttcctg
tgccactataccagctgcttgtctttttgttcctagaaataaaactttttaccatccga
cgaagcctgttttataaattcacaagcttctgttaataattgtgcagtttggactagatc
aataatatgtatgccatttctttctgtataaatatagggaaacattttaggattccatct
acgtgcttgatgtccaaaatgaacacctgcttctagtaattctgctaatgtaacaatagc
cataagattattaatatttaaataatgtaattattcgggaaatcccttttttttagaaac
tgat-----tcaactaacttatttgaattttagtaaactaataataaattaacacacttt
taaagcagatcaataaaatagatcctgaagaattcat----tctacacttttattgctaa
aataattacgcgcggttctgtcgtctaaaatgatatcatctaaatcatctcgtacagatg
aaatagtactttcgtcatcagtatttggagttaaattctttttctctaacgatgagctat
tatagttatcatacatattgaaaccagtaccagctggtataagtctacctataataacat
tttctttcagacctcttaaccaatctaatttaccagatatagcagcttcagttaatactt
ttgtagtttcttgaaaacttgctgcagaaataaagctttctgtatttaaggaagcttgag
taatacctaatagtacaggcctatatgatgcattaatcttatgtctcaaagtcatggctt
tattagtctgttcaatcttttgtaactctacaagctctccaggtaaatagcctgtttctt
caccattttctattttcactttagaagtcatctgccgtactattacttctacatgcttat
cagaaatattgactccttgagattgatatactaattgtacttctttaacaagtagtaact

gaatctcttgaaagcttaatcgagcagcttcatataaggctaatccacattcaatataca
aattaaaacttgcgtctaggatatcatgaggattcaacaatacgtctgttttctttcttg
cttcaagaatttcttcaattcttggaagaccttgtataatgtcacctgtttttgctctat
caaaaaccaacacagctaatgtttctccccttctaattaatgcattgttattaacatgta
aaatggcaccattagaaaccaagtaaggtctagcaattcttaatgtaactgatctagaag
atatttcaataatttgtccagagtctaaagaagtcatattttcagtaataaaatcaccgc
agcgaatccaatcacccacacttactttaactattgattttcctatattaaaggtctttt
tatcagaagatgtcgcaatcaaaatgcgcctactagtattcttttcagaataaatttcct
caacagtaccttcactcattgcaataatctcagtactagcaacaactgtaaatggcttga
tatattcaccatttttacaataattctagtttgactttgttgcttctctgatttattta
catcgatacttttaattgataatgtttcaaaagttgacagccttaagtcaaaataattat
tgccactttctttagcagaaaactcaattgaggatattaaagtatcatctcgatgctcaa
tttcagccacaagattagttgttactaaatgaactccttctatagatttcactctttctc
cgtcccgaaaaggagttcttttaactagctttagtttagtttgacttggtttctgtgaaa
ctagattctcaattagactggattttgtttctggtattgaataaacaataacaggtctaa
taagaatataaggcatttgctcgttttctatatactcccaataaactaacttatctgtag
aaatattattatgaagtgtttctccaggtcttaaaaaacctctacttttatcattattac
tatacacctcactaagtttatagatagatccaggcttaataataatttctcgaactattc
catccttttggattatttcaacaatacctgagcttttagaaaaaatatttttaactagct
ctgttccactttctataacatctccattatgcactagtagtagtgatgaatctttattaa
tttcgtgagtttcttctgaaatccataagatatatcccggacttaaaatttcgtaagcat
ctttatctgaacctgttttctttttgatacatttaagtctaaatacttaataatacccc
cacttgccgttttgtacgtatcagatattagttctgcgactgtgtaaccatcttgaa---
ttttttggtttgggatagctttgaaaagaaattttgtttttttctgtttctaaaatat
acgattctttttgtggattacatctgtataaatataacaacctggaattacaatagatt
cagtaataacttgtatattagtaatactgctattagtattctggtgtatccttacttctc
cagcataatgatttattaattctgtctgcgcaagtatcgtaccagctttagttttatctt
ctttatgaacaataacattagcggaatcagatatactataaacttcacctgagagcaccc
agatcaagcctccagttttagtaattcttgtagtatattgattattatctgtttcttcaa
ctgttaagttggagaaacatattttgccagagagatctgaaactacatgtttttgagctc
tttctgtcatgagtcgatttctacggggtgattcagcaatcacttgatccttcgatacta
attcaccatcacatattaaaagagtagtatctttacttaagttaattattgacttttgat
ttttcttagatctgattgtaacttgggttggctttttgttattaatgcttgctcaccat
gtctagtacgaacatctgtatacgattcaatatctagatctatcagttgaccatctatag
gtgagtaaatttgttctgctaattcaccggtaaatactcctccagtatgaaaggtcctca
ttgtcaattgagtgccaggctcaccaatagattgagccgcaataataccaacagcctctc

ctaaatcaactaagcgaccatgtgcaagattccatccgtaacaatactgacaaacagaac
ttctagagttacatgtaacaggagatcttactaaaactttttt---gataccagctttag
ttatctcttttgctaacttaggactgacatcttggttcgtatgagcaattaaacaatttg
tctctggatggaatacattttctgctaatactcggccagataaagcttgctctagattaa
ttaatattttctgcgtatctactaaatcttctagtatgatgcctttattggtcttacagt
ctacttctctaattattacatcttgagaaacatcaacaagtcgacgcgtcaaatagccgg
aatctgctgttcttaaagcagtatcaactaatcctttccttgctccgtatgatgatataa
aataatcagtaacagttagtccttctctaaaattactcgatattggaagatcaataattt
gtccctgtggatcagccataagccccctcatccccactaactgtcttacttgagaaatat
tacctctagcacctgagaacgccatcatataaacagcatttagaggatctgtctctttaa
agtatttaataacttcttgtttcaaggattcactcgcattattccacgtatcaataactt
tttgaaatcgttcaactgctgtaatctcgcctcggcgatatttgttttcagtggccttaa
tatcttcaatcgtgcctaataagagactttgcttgctgggaggaattcttaaatcttcta
aacttaaagaaattcctgcttgggtcgcataatgaaatccaagatctttaagcttgtcgg
ccatattggctgctcgggctataccgtaattacgaaaagcccagacaattaaattttta
gttcgtttttatcaataaccttatttgaaaaacttggctgtgcaagacttcgtctattat
ccactaaatcttctccatattaaatatttgtt--attaactatgctactaaagactcttg
aataatttattaaagattatacggccagctgttgttctaatatattgcacaattctctg
attttcagcatcttcttttatgatatggttattaaaaaattttgttgtactcttgtcaga
atgtttttcaatcttaattggatcatgaggttgatcaccatctactaatccatcaaaacg
agcccatatataagaatgtaaatcaagtttcttttgctcataagccataactacatcttc
taagcttgcaaaatactgattagatccctgctgctgagatggattattagctgtcaaata
atagcaacctaaaaccatgtcttggcttggcataataattggttgacctgttgctggaga
gagaaaattatgaggggctaacattagcaatctagcttcagcttgagcttctaaagacaa
aggaacatgaacagccatttgatctccgtcaaaatcagcgttaaacgctgggcatactaa
tggatgcagcttaatagctcttccttctacaagaataggttcaaatgcttgaatacctaa
tctgtgtagcgtgggagctctattcaatagtactgggtgcccttgaataacttcatttaa
aacattccaaatagaagattcattttttgaatcatctttttggcagctttaatattgtt
aactaatccctgcaaaattagtctgtggataacaaaaggctgaaataactccaaagccat
ttctcgtggcaatccacattgatgtagttttaaatgaggacctacaactattacagatct
accggaataatctactcttttacccaacaagttttgtctaaaacgaccttgttttccctc
tataatatctgataaagatttaatggtctgttatttgcacctacaacagttctacctct
acgaccattatccatgagagagtcaacggcctcttgcagcattctttttttcatttctgat
aataatctccggagctaaaattgactttagacgagataatctattatttctattaataat
tcgacgataaaactcatttaaatctgccgtcgcaaatctacctccatctaattggaccat
aggccgtaagtcgggaggtatcacaggaataactgtgaataccatccaagaaggatctgc

accggttgctatgaaattttcaatcaatcttaagcgtttcatttttttattaaattttaa
tgaaggtgtcttaaaactttttggtggatttgtagcttcaaaccttaaggtttcagctat
gtcctctaaatctaaatctttcaacagtttttgaatagcttctgcacctattcctacttc
tacttggttattctcatcttgattttggtagatttcctcttcaaggctcttccattcgta
accttcgagaagctgcttgtattttaaattaatatctgtattagattgcgtaactacata
agaatgaaaataaacaatttttttctacttctttgacttttaagtctaatgctaaagcaat
atagcttgtacttccttttaagtaccaaacatgagttactggtgaggaaagttcaatgta
agccatccgatgtcttctcacgcgtgattctgtaacttctacgccacatcgttcacaaac
tatgcctttgtagcgaaaacgtttatatttaccacagtgacattcccaatccttaactgg
gccaaaaattttctcacagaataaaccatccatttctggctttaaagttctgtaattaat
tgtttcaggctttgtgatttcaccaacaatttgtccgttaggcaaacttctttcgcccca
ctgtcgaatttttttcgggagaagctaaactaattttttacgtagtcaaaatattgctcaaa
ctttgtcataaatttgaataccttaaaatcaaataattagccaatttatttacatgcttc
atattcaaaaatgaatatcttaataaaggaattgttcaaaatcgtctactggaggagtat
cataattagaacgatctgctcggttatctttagaatccgacatcaagtcaacttcaactg
ttcgcctttggccatcttcaaatagtttcaatttatgtactgcaatgtctaaacctaatg
attgcagctctcgcattaagactttaaaagattccggtgttccaggcttcggtataggct
tccctttaactattgcgtttaatgcttcatttctagcttgcatatcatccgactttacag
ttaataattcttgcaaagtatatgctgcaccaaaagcttctaaagcccacacttccattt
cacctaatctttgacctccatgctgtgctctacctcccaatggctgctgcgtaaccagag
agtagggaccagtggatcttgcgtggattttatcatcaacaagatgaacaagcttaagca
tataagctctaccaactgtaacaggattatcgaaaggctctcctgttctaccatcaaaaa
cttgcattttacctggatgctgatcattaaaaagccatttattgctagtaatcaatgatg
cttcttttaattttctatttacaagtgctcttgatgcttctgctccatacatctcatcaa
aaggtattattttaaatctttttcctaaatagccacctgctaaacccagtaagcactcaa
atacttgaccaacattcattctagaagggacacctaaaggatttaaaacaatgtctactg
gtgttccatctgataaataaggcatatcttgtttaggtaagatcctagaaatgatacctt
tattgccgtgacgaccagccattttatcacccacttggatttttctcttctgcgcaacat
agacgcgaatcatagcatttgttccgggaggcagctcatctcctttctgcctagtaaaga
ccctaaccttaactactctaccttttgcagcattaggaagccgtaaagatgtatctctca
catcccgagccttttctccgaaaatagctcgcaacaatttaccttctggtaattgatcag
cttcacctttaggagtaatttttcctactaaaatatctccagcttcaacccaagaaccac
cgacaacaattccatttctatctaaatccttcagagagttatcgctgacgttaggaatct
ctctagtgatttcttctggtcctaatttagtttgccggcattctacttcatacttttcga
tatgaatagaagtatacaagtcatcataaactaatctttcgctaattaaaaacgcatctt
cgtagttataaccttcccaaggcatataagcaacaagaatatttctccctaaagcgattt

ctcctccgtctgtagatgcgccatcagctaaagtttggccaacaactattttttctccta
cccaaacaattggacgctgattaatacaagtatcttggttagaccgataatattttttta
agcgataatgaactgttctaccattagtatcctgaatgcctattttattagcagaaacat
aatttacataaccagatgttctgctaataacaaccataccagagtctcgagctattttag
tttcaagaccagtaccaattattggcttttctggatataataatggaacagcttgccttt
gcatattagatcccattagagctctattcgcatcgtcatgttccaaaaaaggtattaatg
atgttgcagctgaaattacttgaataggtgaaattgcaatataatctacttgggttggcg
ttgttgtaataaactcttgacgataacgtacaggaataatatctccttcaatgtaatatt
gtttactaactttaacatctcctggagctactcgaaaatcatcttcctcatctgctgtta
gatagacaggactattgtgataaatcacttgaccttggtgtaccggataaaatggggttt
ctatgaaaccaaaaacattgacccgcgcacaagttgctagtgaaccaattaaacctgcgt
tcggaccttccggagtttcaattgggcaaattctaccatagtgactaggatgtaaatcac
gaactgcaaaacctgccctgtctttattaaaacctcctggacctaaggcactaattcttc
tcttgtgagttagttctgcaactggattagtttggtccataaattgagaaagctgactag
atccaaagaattctctcactgacgcaattaatggcttgggattaattagatttgataaac
tcaatgaatctatatcacatatcatcattctttctctaataatgcgttctaaacgattga
gacctactctgaattggttctgcagcaattctccaactgaacgaactcttctattaccaa
gatgatctatatcatcaagattacctgagtttttatccttaatattgattagataatcaa
ttgaggataagatatcttgaggagacaatactcggaacgttttaggtatattgagaccta
gtttttattaattttatatctaccgacttcacctaagtcatatcttttaggatcaaaaa
aacgagagtaaagcatctgcttagcaacagcaactgttgctggttcattaggtctaagct
tagaataaactatgagaagggcttcctcatcagttacttcttcaatattatttttccaa
tttctttagctaattctttaactgaatatatttgggatgcagaaataagaaaagcatatt
tactaagtccttttttcaatttcatctttattgagcccaatagctcgtagaaaaatataag
catttactttgtgggttttatctattcttatccaaatttctcccttaggatcaatttcga
attttaaccaagaacctctattagaaattaggcttgcactataaatctgcttaccatttt
tgtctatctcttgtttataatagattcctggactacggattatttggttaataataactc
tttctgtaccagatacaataaaagttcctctatttgtcattattggcaagtctccaataa
aaaccaatctctttttgtacttttttatttttatctgcttttcagcactgagctgtagat
gggttgatgataaatctaatgatttaatatttttttcctggtctttagaaggtagatcaa
tatcttttcttgttaactttacaggaacatatatttgagcactataagttttatccctat
tttttgcctgtctgacactataacgaggaaattttatcttatattcattaccaaataatt
gtaattcgagtcgactagtagggtctgatatcttcggaaaaaaattcaagaacttccgtca
aaccttctaataaaaaccatttaaaactggccctctgaatttcaactaaatctggaagaa
gtttatttttttaagcttatacgttggaccatagatctcctttataacaaattttgaaatc
gaagagtttaa-tactattaatagagtactggtagataatgtataagcatattttgctac

attatttcttctctagacataactagaacataatattttattcaa--aaaaagattttaa
aaatt----ttcac--acactatatatataagcactttgctgcta--aaagaaagttcaa
gctatataa-------aaaaacgaaattaatacttgtctgtctgtcaagtaattgacatc
aagatagacaagtattatttatttaatcaattctg-tttttttgagcaaaagacaatgct
ctcgcaagtctagcttttttttctagcgcctgtattagggtgaaatgcccctttttttaca
gctttgtcaatcttgctatatacttgagaaatacttgactgcacatcattgaaattatca
ctttctaaattatcaatatttagaaggcatcttttagttaaagttttgacaaccgattta
tacttacggttaatgagacgatttctttccgaagttttaatgcgtttaatcgcagaaagg
ttcttagccacagtaaatccaataaaactttttataaaatatttttagaaacgaaaataca
caaatctgagatctattcaaaaagggaatatgcggcgtattataacattaaagaaaaaca
ataggcaatagttcaccaagagctcttgttattacacaatagagattgtcattgcgaaaa
actttgtaagatagtatttaattagaataaactaactagacatataaatatatatgaaaa
aaattgaagctattattagaccttttaagcttaatgaagtaaaatttgcgctagtcaagg
aaggtattggaggaatgactgttatcaaagtttctggatttgggagacagaagggtcaaa
ctgaaagatataaaggatctgaatactctattgatatcattgataaaataaaaaattgaaa
ttattattaacgatgataaagtagagaaaattgtagagacgattattaaggcttctaaaa
caggggaaattggagatgggaaaatatttattagtagtattgaaagagtaataagaatca
ggactaatgacttagattttgaagctttatagtatttttacttt-tttagacttgatact
tcttcactaaacatgagataataagcgcttattgtatacttataactaaggtgtaata--
ctaatggctaaaagtaaaggtgcacggatcgtaataactttagagtgctctgataaagct
ggagattttgctcaaaaaaggaaacctggcgtttttcgatatacaactactaaaaataga
cgaaatacaccaaatagaattgaattaaacaagttttgtcctaattgcaatcaacattgt
atcttcaaagaaattaaatagttatttatcttattaaaat--aatcatattatggctgta
tatagaaaaagaatatctccaattaaaccaacagaggctgttgactacaaagatattgac
ttgctaagaaaatttattacagaacagggcaaaatactgcctaaacggtcaaccggattg
acttcgaagcagcaaaaaaaacttactaaagcgattaaacaagctagaatcctttcttta
ttaccttttttaaataaagattaa--atatatggcctaacttcacatttttttcatctaaa
tattagaaacttctatagatttgtttagcaaaatgatctaacatactgaat---------
----------tagtactaaaa-tctaatcattataagttttgttcatttataattaata
atagcactataattta---ttaagctagattc-----agacataaagtgcaacctctttt
aattgattatattatatagtctatttaaaagaggaaattttttttgcttc----------t
agcaattcaagattctttcagctttaacgatatatttaaaacaaagatttttgtttaggt
atcagatcaaatgagtgaattttatctcccgactgccataattgaaattcagagatgaaa
attccacattcgttaccagcttgaatttcttcgacatcttctctaacgcgttttaaagat
tcaatttttccttgataaataactttttcttcccggatcactttaatccaagagtttttt
aatagcttattattgatcactcgacatcctgctatttttctatttgctaacgagaatact

gtgcttacttctgcttctcccaccggtacttctgagtattcaggatcaagtaagtcttcc
atccttcttttaatgtcttcaattaaggcataaatgatttgatagttttctattaatata
ttcaactttgccgatgcttgctttgttcctggagcaaagttggtattgaacccaatgaga
atagaatttgtggttgaagcaagttcaacatctgtagcagtaatctctccaggcatgatt
gacacgacattgagttgaactttactctgaggaaattgagataaagaatctaggattgct
tctgtagagccttgattatctgttttaataactaaagaaatttgcttactaatatcttta
gaattagtgtttttttaaagtatctaatgtaattcgactattaagtgctctttgtttctgt
ataattgatgagtctttagaggtattttcaatcgctttaagtttggcttctttatcactt
ttaactgctaaagctatttctcctgtagctggtacagatgacaatccccaaatttcaacg
acggatgagggtattgctaaattaatttctctttagcgttgctaattatagctcgaatc
tttgcgtaagctgatccaataaccaaattatcactaatattcaatgtaccattttgtatt
aatagagttgctactggaccatgcgatttatctaagtgggcttctattataataccttgt
gcaggctgtgttgggtcagctttcaaattctctaattcagctaacaaggtaatagtttct
aataatttatcaacatttgaccagtaagagaactaataggaattataggcacttgtcca
ccgagtttctcagacatgacattgtgttttaataaatcttgctcaataatatctatattc
gaaccagctttatctatcttagatattgctacgacaaaagggacattcgctttttgaata
tgatggatagcctctatggtttgaggtttaactccgtcatcagcagctataataataata
gcgacatctgttaaattagctcctctggacctcatacttgtgaaagcttcatggcctgga
gtatccaagaaaacaatttttttgtttattagctttcttaatatactcaacttcataagct
gcaatggcttgagtgattcctccaatttctttattagcattattagattttcgaatataa
tctaatagcgttgttttgccatgatctacatgtcccatcactgtgacaattggaggtctt
ttaatataattgccagtttcataaagaacattagagttatctaaattactaggaagtcca
ttattattttcctctacatttgattctactgctatcccaaaattatcagctactgatgat
attattgaggcgtctatggtttgattcatagtaactgatattcttttaagaaataaatat
tttataatatctgtttcttgaacacaaattagtttagataattcttgaatagttagagga
ttagtaatactaatagactctggcggagaatttgattgactgcttgttgaattttgacca
ggagttaatgctactttttttctgtcttatagatttttttattattagttagtttttttgcg
acttccacttttggcttaggtggacgcattaaagagatagctaaatctcctgcagtctgc
gcaacattagaattagagtctctaaagttgtca---tcatcatcgtcaatatgtattttc
gtttttagtttt-ttgcgctgtctattctta--tttttttttactatctactaagtcgtga
attttattaaaatttttactttttttatctagtttgggtggagaacttaattctagatgc
tgttcaccacc---tgtatgtgattctgatttatcaaggtctaaatgtaacagatcatca
ttattaactgactctaatctaattttatagataatctgagggtttttcaaatctataatt
gtttcagaagagttgatatttgagctagatcgagaggatctattttcaaagttttgatta
tttaaaaacataaatgttggttttattatctaatagtaactttctaattttaatacagag
cttaatataaacaaaaatcgaatatctgtataaataatacaaatgt---agagaattaat

ctctgttgtattagatttaatataacactaaagtcgtttgttcgttatacaagtttaaaa
ctttt-tatgatgataaacactagattctagtttattggtatacaatctatttatctaaa
taatactgtttttgtc-gggcgtttaaa----ttatctaatgttatattcattatgaaat
tctattgaaatttaaaaa-ctcaacaattaagtaagaatcgttaatcacctccacgccac
ttatgccacgatctcaaaaaaatgataattttattgacaaaacttttacagtattagctg
atattgtattaaaaatacttcctacaagtaaagaggaaaaggaagctttttcttactatc
gagatggaatgtctgcacaatctgaaggtgagtatgcagaagctttagaaaattattatg
aggctttaaaactagaagaagatccatatgatagaagctatattttatacaatatagggc
ttatttatgctagtaatggagagtatgtaaaagctttagaatactaccatcaaggattag
aactaaattttaaattacctcaggcccttaataatatagccgtgatatatcattaccaag
gagttcaagcaattgaggataaagatacagagctatctaaattaatgtttgacaaggcag
ctcaatattggcaacaggctattaagttagctcctgataattatattgaagctcaaaatt
ggctgaaaacaacaggacgaatgaggaatatacaaggatattaatatatttgagatataa
taaagatatcaattaataaattaaaattagttaaagaaactgatactatatagagtactt
-tgatat--------atgttatactagttga------tctctaagacttagctcaataac
cgaaattaaatctattgactatattaatgaatacaaataagcataatggttagtacaaca
aaaagtaccggatctgttactcaaattatcggaccagttttagatattgcatttcctaac
ggacagcttccgaaagtattcaatgcactcaaagtacaaagctcagaagcaactattact
tgtgaagtacaacaactgttgggtgacaacaaggtacgagctgtttctatgagttccact
gaaggactacaaagaggggtcgaagttattgatactggatcccctatatctgttcctgta
ggtacagatactcttggacgtatttttaatgttttaggtgaacctgtagataatttgggt
cccgttaattctgagagtactttacctatccatcgaccagcacctgcttttactaagtta
gagacaaaaccaaatatttttgaaacaggtattaaagttgtcgatttacttgctccctat
agaagaggtgggaaaattggtttatttggaggtgctggagtaggtaaaactgtattaatt
atggaactaattaataacatcgctaaagctcatggtggagtatctgtatttggaggcgtt
ggtgaaagaacaagagaaggaaacgacctatatatggaaatgaaggagtctaaagtaatt
aatgcagataacctgaaagaatctaaagtagcattagtatatggtcaaatgaatgaacct
cctggagcgcgtatgcgcgttggcttaactgcattgacaatggcagagtactttagagat
attaacaaacaagacgttctattgtttattgataatatttttcggtttgtacaagctgga
tcagaagtgtcagctctactaggccgtatgccatctgctgtgggttaccagccaactcta
gcaactgaaatgggagcactgcaagaaagaattacttcaacaacagaaggatcaattaca
tctattcaagctgtatatgtaccggctgatgatttaacagacccagctccagccactaca
tttgcacatttagatgcaacaacagtactttcgaggaacttggcagcaaaaggaatttat
cctgcagttgatccactggattcaacatcaacaatgctacagcctggaattgttggaact
gagcattattctactgctcaagaggtaaaatcaactttacaaagatataaagaactgcaa
gatattattgctattcttggtcttgatgaactttcagaggaagatagacagactgtctca

agagcgagaaaaattgaaagatttttatctcaaccttttttcgtggcggaagtgtttact
ggatcacctgggaaatatgtatctttggaagatgcaattaaagggtttcaaatgatctta
aaaggcaatttagacgacttgcctgagcaggcattttatctagtaggtgatatagatgaa
gctatacaaaaagctgacagcatgaaagattaatataattaacgatgactttaaatataa
gaattattgctcctgatcggactgtttgggatgcagaagcacaagaaattatttaccaa
gtagtacaggacaacttggtattttaacaggccatgcacctttgcttacagctttagata
ttggagttatgagagtaagagtagataaagaatggatgccgattgttttgctgggcgggt
ttgcagaaatagagaacaatcaattaactattctagtcaatggggcagaagaagctagtc
aaattgatttatcagaagcagaaaaaaatttagacactgcaactcaacttttaagtgatg
cctcatctaataaagaaaaaatagaagcaacacaaaaaatacgaaaagctagagctcgag
tacaagctgcaacagctgcaactccataa-aagattcc------tcttaaaacataaaac
tttagtaattattactaaa-------gttttatgttttatttgtatggttataggcaact
taaataaacttagcttagacctgaacaaatataatcaaaatataatcccatctctttacc
tgcatctggtccaactaaactaatagtaacttctttcatagctaaaatagcttgaatcgt
tgcaccaataggaacacctagggaattgtatgtttcttttagaccatttaatactctttc
ttcaagaatagatggatctccagctagcattccataagtagcatagcgtaagtagtagtc
taaatcacgaatgcatgcagcataacgtcgagttgtatacatgttaccacctgggcgtgt
aatatccgaataaagtagagattttgctacggattctttaataattgttgctgcattagc
tgcaatagtagcagcagctcttactcgtagctcacctgtttgaaaatagcctcttaattt
ttcaacagagctatcatctaaatatttaccttggacatcagctgcattaataacagaagt
aattgcgtcttgcataactttcacaaattccttaattttaaatatctaatacta-ggggg
tatataacaaaattactagatcctttagcgatatcagtattactgcatagcacccaaagt
ataatcaaagtaaaaaccagcttctgctgaatcttcacctgcaagcagtgagcaagctac
acttttcatgcattttacgccttcagcaactcctgaaattggtgttcctaaagaattata
catttctttaacacccactaatccaatttcttcaataggagttacatcgccagcaactat
tccgtaagttactaagcgaagataatagtcaagatctcgtagacaagtagcagtcatctc
ttcgccgtaagcatttccaccaggagaaactacatcaggtctttttttgaaacagttgttg
gccgccttgctttacaatacgctcacgattatctgttaaaatttgagctattcttaaacg
acgttgtccagataaaacaaaactttttaattctatctaattccccaggacttaaatatct
tgcttctgcatctgcatttacaattgactttgtaacaatactcatggataaaactcctgt
aatgcttt-aaaaaaaagcttaatactaattatttatgatataactcagaaagtaatgta
ctttttgag-aattttataaccgaaacacgttcatgatatacgaggcaggataggtactg
aggtaagtactaaaacgagaaagttatatgtaggaacttctgttattgatttcctagtac
tttttttaaaacttggtaccacaattgatatatcctgcttagtcaaacgattataaagtgt
ttctgtattaggaaagttagctgctggtaaagttggaaatcttctgtatggaacagtatc
tttaccatataccgcattgtattcatcactttcaactaaattacttataaatgcagataa

gccttttgaagctaaaatctgattcaagaatctaatttctgcctgattattaggagctcg
tcctaagatatgcttagtacctaattctataacttttgtatttggatatggctgataaaa
ttctttaccatatagttcagatagcgccaatttttctacgagttctttgacacagatttg
tctattcaagaaagctgactcaatatccaagaattcaccgccgatactaaaagagttaag
atctctttcgaaaatttgacgatatgctgctcttaaagcttgttctaacatttctttgtt
actatcactattcacttcaaagacaatagattgatctcttaaagaagttactccttgtga
aatacgagacttaatatcattactactcctcatttctttgacagcacctaattcaacaaa
cctagcagatttactagcaataactttttgaatctctggtcaataattcctggtcttaa
gcttcttaaagcaacaccagctggcgtactatatctttcatacggaacagtattatctcc
aaaagtttctgtgtactcagagctatcaagtattgcgtctattacttggtaatagccttg
cttatatgcaatatcaaaatatttattaatttcttgccgaccatatgtagggcgacccag
taagcggttatgaatatattcaatagctttacaaatatataatggctcccaataaagaga
tctaaatatacttgattttgctaactgtctaacaaattcgcgaactgtaatttgattgtc
ttttagctgactttctattggcttgagaattagtttttcttcttgatacacttctctacc
aaaaactcttaaataagcaacttttgtgacaacttctactgaattttcaaaattttggga
tgaatctgactttataagagcactggataatttaaagattttggtcccagggatcctgc
tgacttaggtcttacctgtggattactaatctgattgtagattcctggcccgcgtctaac
tagtatccttcttgtatctttaccaaaaagggcttgtcttttcttggatctttattttc
tttagggaaaatagctccaaattgaattgataatggatcattacctatgccatatggatg
ttgatcaggtaatgattgcttataatcactaaataaagttataaattgaggaaccttacg
aaaaggcgcactataactgagaagatctatttgaggcccccaattacggcactcttgggg
ctcttcacctaaatttctaaagtaaggcacagtctcttcgccgaaataatctgtatactc
agaagaatttaaaattgcatttactaaaccacttaaaccagtagatgataaaatagcaaa
atatttttgaaactcttctagagagctaggacctctacccaagaaatgtctaaatgctaa
ttctaatgctcgactattgacaaaaggttcataaaattgctttctataaatacttgaagt
tcctaacgaacgaataaattccttaattgaaatttggccatttttacttgtgactccaa
attcgataaagagaggtcatatgctttcgcaatatctctttcaaaaatttgtctataaca
tgctttcacaacagtatttttctcatcagcagataaactaggcttcataacaaagcgagg
tgttgacactcctgcctgtacataagtctgaggaagacgtaaaccttgtaaatctccaga
tattctttttcttaatttatcagtcaaagatggagcttcaaattcagaaatcacaacatt
aaaatactctttaactaaatcttgaccttttatatcttcctcaaagattagtaaggctgt
ccgtctcatctctctaagagccacaattgctgctgcacttgagcaagcattatcaattag
ctctctcaatcctcgtatattaacagataaaatattagggtcgcctgatacaattgcata
tgtcaaataccgtaaaaaccagtctaaatctcgaagagattttctcattcttgtggtgcc
gtatctcaagacgtttataggtttaaatcctggcggagtagctcccccagcattaaataa
agatcggaaactttggccaaaatctccttggatatttcctgataactcattaattttatc

ttgagaactttgatcacctgcgataataacagctgcttgaggacgttctaaataagaaat
tgcagacccacctacgaatattttatctgctgctcgtgctacgagaatatttgcattttt
agttaaaatatctgcaacttccagtctttttttgtccagaatttaaaaaagcaactaattg
attcaattcaccaagctgtaaaaatcggtcttgttgctctgcctgagtaatagttaaaat
tgaggcagtccgataaagctgggggcgtgctaatgggcttccgccacttgctttgatact
cattactttatttatctccttaactgatattgcttgctagcgaatacaaatatgacttga
tatttctaaatatttaaagatactatgcaagcaca-ctgttttctatattattaggtact
agtacttatagtacaaagagtcttacaactaacttctataaatacaaattttgtaatact
tctattaacatttatcaaaaatatatttaagtgctcactcttttatattaaaattcaatt
agtgagagattgacgtgaaataaagtatactaagtaagttttatagataggaaaattaaa
agatttattctacaaacattaatca------------cttgaatgtttagcaattataca
attgttattgcttttaattggtttagactagtttt-tatttttctaacataaacaatat
taatgagcaatttgtatgaatttaaagccacaaaacaacctattaactaacgaaaatata
aatcacatagatatacccgaaaatgatataccaatgtcaattacggaacatttagaagaa
ttaagacaacgaactttatttgtgtttttattctttttatttgctacaactataagtttc
acacaaattaagataattgttgcaatactgcaagctcctgcttttggtattaaattttta
caattggcaccaggagaatattttttttcatctattaaagttgcaatatattgcgggatt
gttgcaacaactccgtttgccgtttatcaagttatattatacatactcccaggactaact
cgaaaagaaagaaaaattattttacctctattaattagttctgtattgctttttatcaca
ggtgggatatttgcttactttgttctcgcaccagcagctttaacattttttaattagctat
gggtctgatattgtagaaccactatggtctttcgaacaatactttgactttattctatta
cttttacttagtacaggattagcatttgagataccagtcatacaattattgctcggtgtt
tctgggacattttcttctaatcaaatgatacgagcttggagatatattattattatagca
acaattgctggagctattttgaccccttcaactgatcccgttacacaattaataatgtct
tcagctgttttattactttattttggtggaattgttatattattagtcttaaaaaagtag
agtaaat---------attttcta-ttaatccaatatatttgttatc-----tgggcaac
taatctataaaaacaatttct------------------agatttaagtatcaggcttct
aaatatttcaa---------gtctcttttatcatttaaataaaatc------------

--gatacattgg
cttgtttttccatcaattatttgtattgtgcac-tttttagcactatctttattattctt

agaattatttccttaatctatttattaaat----agatatgaagagatttcagtattgat
tatggtaatctatatcaacgtaaaaagtattttcttatgagttttatttatataaaggct
tattcattatatagttataacagtaagtctaattaatag-ttttcatgcttaattaaagt
aataactcttaattcttttggcagaaaaatctgtcttaatattttatc--aaaaaaatag
-aaaaatgaagctaaatagtctaattaacttaattcaaaagtctatatattcttgtacac
ttttattaattattttaaatattatttgtgttgcacctaattctagtaatgcatttccaa
tttatgcgcagcaagcttatgaaagtccaagagaagcaactggtaggatagtatgtgcca
attgtcatcttgctcaaaagcctgtggagatagaagcgcctcaagcggtactacctaata
ctgttttcgagactgttgtgaagattccatatgacagcaatgctaaacagattttaggta
atggcagtaaaggaggcttaaatgttggagcggttgtaatattacctgaaggatttaagt
tagctcctgctaatagattatctacagagttaaaagaaaagactaaaaatctttacattc
agccgtacagtgctaaacaagataatatttagtaattggacctatttctggtgataaaa
atagagaaatagttttcccaatactatctcccgatcctgcaaaagataaaaaagctcatt
ttttcaagtatccaatatatgttggcggaaatagaggacgaggccagatttatccaacgg
gtgataaaagcaacaataatattgtctctgctttaagcagtggtaaaattaataaaattg
aatcactagacaagggtggatttataatacatgtgactaacagtagcaatgtagagtcaa
cacaaaagatttcacctggtctcgaacttaaagtgaaagaaggggatacaattcagcttg
atcaagctttgaatagtgatccaaatgtaggtggttttggccagaacgaaacagaaatag
ttttacagagtccaaatagaattaagggcatgattgttttctttttcgctagtgttctag
ctcaaatttcttcgtattaaagaaaaaacaatttgaaaaagttcaagcagctgaaatga
atttttaaaggaatataaaactggcacttactataaataaagta-taaacactttattca
caggatgtaccagtctttatgttatgtaatactcgctttgacggaggttatgatactttt
aacagaatctacaatttggctaggctgaattactgtagcctgctctaagcttccattata
tggtgtgggtagatcctgcgaagatagcctcacgacaggagcatctaattcatcaaaaag
atattcattaatttgtgcaattagctctgctccaattccggctgttttcatacactcttc
tacaattaaaactttgtgagttttctttactgagatagatatagagtctatatccagagg
ttttaaagatataagatctataacttctggatcataaccttcttttaataaggccggtaa
tgcttggataacatgatgcctcattctggaataggttaaaatcgtaatatcttttccttt
tcgtacaaattcagctttattaagaggtaagaaatattcttcttgaggaatctcttcttg
taaattgtaaagtagaacgtgctcaaagaatacaactggattattgtctcgaattgcaga
tttcagtaatccttttgcgttataaggagtagaacaagcaactatttttaagccggggat
ggcttggaaataagcttccagtctctgagaatgctctgcacctaactgtctacccacacc
tcccggtcctctaataactaaaggcaacgtaaaattacctccagaagtataacgtaacat
tcctgcattattagaaatttgattgaatgctaataataaaaaactcatattcataccttc
aacaattggtcttagccctgttatagctgcaccaattgccataccagtaaagctattttc
tgctattggtgtatcaaggactcttaaatccccatatttactatgcaaatctttagttac

cttataagatccaccatagtgaccaacatcttcgcctattacgcagacagttggatcttt
ttccatttcttcatctgttgccgctcgtaaagcgtcaaacataaagatttactcatttt
aatatttgatttttatgatttgaccactgttttaaagtaacagttaagactccatattct
atttagttatctgcaaaaagatatcgtttcaattctgaaatgttaggttctggactggaa
atagcaaatttcacggcttgttccaattctgttttaacagcagcctgaatcccattaagt
tcacctatatcagcaatttcattatctagaatatatttttttgagtttttgataggatct
cttgccacccaagcctctttttcttgtcttgatcttagttcatcaggatctgccagagag
tgaccacgaaatctatatgttaatgcttctattaaggttggaccatcaccttggcgagct
ctttgaactgcttgttttgcagcttgtcttacagctagcacatccattccatcaacttca
atcccaggaagcccaaaagcttccgctttttttatgtatttcaggtattgaagaagaccgg
tgatgtgccatacctatagcccactgattattttcaacaacaaatataataggtagtttc
cagagaactgccatattcaaacattcaaaaaattgcccattgttggtagtcccgtcacca
aaaaagcaagccgtgactcttaaatcgtctgtttctttaagtacttgctgacggtagata
ctttgaaaagctgcccctgttgcaactggtataccttctgcaataaaagcaaagccaccc
aaaaaattgtgaggcgcagaaaaaatatgcatcgaacctcctctgcctttactacaacca
gtctcttttccaaacaactcagccattacattttagatgggacgcctttacttaaagcg
tgtacatggtctcgataggtactgcaaacataatcagtcggattcagaagtttaattaca
cctgtagaaacagcttcttgaccattataaagatgaacaaaaccaaacatctttccttta
taatacatctgagcacacatatcttcaaaatttctgcctaacaacatatcttcatataaa
actaataaattactcttattaagattaagacctgttgaattataattagtcagcggcaat
tgaactttcttaggataactcataatttgtaaagaaacctct----------tattgtaa
ataggaattttgaagaaaaagctatcaatagaaaacatacagataaaaacttataaatta
ttgtatgtttttttactaaagactttaataattatttgacactagttcagtatatcaaatt
aattttgcttatactacagatgtctgaaacttagacaaaaaagtagtaa-aaaaaattta
ttaaaagaatagttaaaaacagatataaatagaagtaaacagaattatt-gaaaaaaaat
gattattattaattaa----tacttaatatttctaaatattttacaattaatggctatgt
caccatctttattttatcctgttgaacaagaactatgtagtcttgaaaaaaatctgaaag
ctgttgccgggactcgtcatccaattttatatgccgcagcaaagcatctattcgatgctg
gaggaaaacgagttagaccagctcttgtgtttttagtggctaaagcaacctctgagaagc
aagatataaatactggacaaaaaaggctagcagaaattactgaaattatacatactgcta
gtttggtacatgatgatattatcgacgagtgcacaacacgtagaggagtcaaaactgtac
ataatttattcaatactaagattgctgtgctagcaggagatttttttatttgcacagtctt
cttggtatttagctaatattgaaaatttagcagtagtcaaagctatttctaaagtcatta
ccgactttgccgaaggagaaattaggcaaggcttagttcatttcgatcctaatatttcaa
tagatgcttacattgagaaatcattttacaagactgcttcactcattgctgctagttgtc
gcggtgcagctatgcttaatggttgcaatcatcaaataaataatgatctttatctttacg

gtaaacatatgggattagcatttcaaattatggacgatgttttagatataactggttcta
ctcaaagcttagggaaacctgctggcgctgatctaataaatgggaatttaacctctccta
tcctttttcacttactcaagaaacaagtttaaatgatcttattgatagagagttctgta
atagtacagacgtagcctcagcattatttcttataaaaagaagcggaggaattacaaaag
ctaaagatttagctaaagaacaggtgcaggcggcactttcctgccttcagtttttaccac
aatctacacctgtatctagtttaaaagaattaacacatttcataatcacaagattgtcgt
agaggacttgctaaaattaaaatgttattaactaatttttttactgtttctaaaatagtct
gaaaagtttgaatatcattactagctagctgcgctagcattttacgatttaacgcaatat
tctctttttttaatgcactaataaaagtactataattcatgccttgattgtgagcagcag
catttattctagttatccaaagacgacgaaaatctctctttttcctttttgcgaccaacat
aagaatatcgaagagctttaaggacttgctgcttagctgttcgaaataaacatttatgtg
cacctttaaagcctttagctagcttaaagatttagctcgtcttttttttgcaacgttac
ctcttttaactctactcataaaatattac-ttaaattttaattattaccagcttactaaa
ttcgtatactctttg----attataaataaggtaattttattgcaatatttttaatatcc
tttaagtcaaccaaacaagtagaagagagatgtcttctttgttttgatgactttttttgt
aataaatgactttagaagctttatgtcgaagaaattttccggatgaagagactttaaat
ctttttgctattgcttttgatgtttttaacttaggcatattataaacttttaatgattaa
caaaagcatgaaaagtctgaggatattaatcttagacctttcaatacttagaataatctt
aacacaatttattttacttactacataggttaactgaaacaatttatatatataatacta
atcagttaatcaaccgcagctaaagtggatttgtattttgctttgaacttttgtatagta
ttagcaattagcattgttataattttaagactacttgaatttaattcttcgaacattttc
atatttaatgtaaatgaaatattagcttctgagacaatattttgaatttgaacatcagac
aaaggaatcatatctaatgcagctctatattgatctttaaataatttatcgtcttttatt
tggtcaaaatcgtaaaattttgttcctcctgaatctgataaattcatagcacctctagct
atttttttaaaatttggccgccagaaagatcacccaaatagcgcgtataagcatgagcg
actaataattcaggttgtttatgtcctataatgtgaattctatcaacatagatttttgta
gcaggagatggttcaataaaatctgaccaatctgatccgtaataataatttaaatcttca
gatagactctctttcctattgagctctgtaaaatatataggtttaatagctggatgatct
ttattagaaaataactcttcctctattgcacaatagacaaagtatagatttgcaaccaat
ttacgatatgacttcttatctacaactcctcccaaaaaagatttaacaaaactaacattt
tctgccatactatgggacttagtagtaccttctcttagttcattcgctaaagtattaatc
ataatatcaatattccattttt-aaagacaacaaattaaca---ataaatagttatccgt
atatttatacttgagttgtgctaactaggctgctgcaaaataattttttgattttattgg
atctggattcattgttttgtcaccaggcttccaattagctggacaaacttcatctggatg
agattgtacatattgaattgcttgtaacacccttaaagtttcttctacactcctgccaaa
ctctaaattattgattgtagagtactgaataattcctttcgggtctataataaatagtcc

tcttagagctacaccatcactattcaaaacattataagccgcactgatctctttttttaa
gtctgatactaatggatatgaaagatctcctaatccacccgattctctgtcagtctgtaa
ccaggcaagatgagaatactcgctatcaaccgagactccaagaacttctgtattaagttc
ggaaaaagcattatacttatcgctaaatgctgtaatttctgtagggcatacaaatgtaaa
atctaaaggataaaagaataagacaatatacttgttcttgaaatcagataattttaacgt
tttaaattcttggtcataaacagctgtagctgaaaaatcaggagctagctgacctactcg
aagacaatttggtccagaaatcattaatttttcccagaagaattgatataattttgttat
ttaataaataattatataacaaaatttagtatatattaaaatactaccagtctttttaa
attac--------taaatgatataatcaatactatctttatagcggggaatggatttgaa
ccattgaccttcgggttatgagcccgacgagctaccagactgctctaccccgcggataac
tagaattatattagtctagatatatatataaaagcaaatgcataatagattttagaagta
aaaaaatagaagaacaggtccactgatcattaaacttagaaacgataaagtatttttat
cttatttaaaagaggaaagacttcatataaaccaataaatctatcttttaaaagaatttc
ggacggctttacccaaatttgaccatcataccagcctgattcttcataaaatactgtagc
actcattaatcgttttactacgtatgaccagcctaaatataatctgattaaaataaatcc
tgtcattaggctggaagtaataaattctgaaaagaaaaatttcagaggtagtttattgat
tggaaaaattgataataatataggactaattaataaacaattaattagtagtgcaatagt
tatttttttattataagatctgcgacttaaagttggccaacaaaagaaccaagaattttt
caaagaagtatattcatgaacaggttgctgctcctttgggacaggacattgagtattata
taaattcattgacattaaaaagtattatactaaagac--tttctttatagtatctaatat
aggtacgacattaaatattaactaactgcaattatagtagtgagtaaaagaaataaaact
aaaaatagccaagtaattttatttaaagtactctcagtactacgagtattactaaagaac
tgattctgagcaccgacacttcctaagccttcagatttaggattatgtattaaaatagtg
aagattaatattattgttgatgaataccaaaaaaattttaaaatttgttccattatgtaa
gagttaat-aaaaatagcacaaagacaatcgtctaaaaggtattgattgtcttatcatat
taaaaagtatttattctccctgtacttttaaaagtaaaaatcctattgccagccctacca
aaactaggactgaacttaatacggcactttctacaatttctccgaccatt-ttatttttc
cttattactttattattg---tcattcttttgacaagattgtattgagctaa-ctaaaat
ccgtttctaccccaaactaccattgcaattgaaaatgtaaacattgccattagagctgcc
catcccaaacttaaaatatccataatttataatactc--ctaaaaataagatctctttta
cttaaactataatattcttaatattaaaaatatcattatataataagctaattataacag
agtactacttaattcgtatttttggtgtatttaaagaagattgaatttttttcgaggtac
tggataagtatttcatcctaagaaccatctttaac----ttttgatttattatactgctt
aaatctaaatata--tagcactcaagaatcattagtaattatgatgtttttaattatacg
attaacaacatatgtcaaaatagaagaattcatagcgactcagttaa-----tatgctaa
tataatttatcaat--aaaaaggaagttagtatttcaattatgcaaacaactataaataa

tgggcaaccgtctagtaaagaaactttactaacacctagattctacacgactgactttga
agagatggctaatatggatatttccggcaatcaagaagatttttggctatcctcgaaga
atttcgagctgactataatagtgaacactttattagagatgaagagtttaatcaatcttg
gtctaatttagaacataaaactaaatccttatttattgagtttttagaaagatcttgtac
cgcagaattttcaggtttttactatataaagaattatccagaaaattaaaagacagaaa
tccggttatagctgagtgctttttattgatgtctagagatgaagctaggcatgctggttt
tttaaataaagctatcggagacttcaatttatctttagatttgggatttttaacgaagag
tcgcaagtatactttttctctcctaaatttatttttatgcaacctatctttctgaaaa
aattggatactggagatacataactatttaccgccatcttgaacaatatccagaacaccg
tatttatccaatctttagatttttgaaaattggtgtcaggatgaaaatcgtcatggtga
ttttttgctgctttgcttaaatctcaacctcatttttaaatgactggaaagcaaaaat
gtggtgcaggtttttctattaagtgtatttgcaactatgtacttgaacgactttcaaag
aattgatttttataatgccataggcctagactctagacagtatgatatgcaagtaatacg
caaaaccaacgaaagtgccgctagagttttccctgttgctttagacgtggacaatccaaa
atttttcaaatatttagatacttgtgcatgtgataatagagctctgatagatattgacaa
taacaattctccattattcattaaatctattgtaaagatacctctatattttccttatt
tacaaatttactgaagatatatttgattaagccaatagactcaaaaacagtatggaatac
agttcgatagttaaatacaagggaatactttttattataaaaagctatctaattattaa
tacaagat------aaaagcttccttct-----cgatggaaggagcttttattttgtcaa
aaa-tatataatgggattatatagatc-tagaactaagaacttacttttcttttgcttc
atacataacctctaaagtaattatatttttagaacaatcacgggcaaatttctctgtatt
tctttttactttacctctaacaaacccaggaatttatttaattctttctgtgcttcttc
tgaccaattaatactatcaacagcagaaattcctacacttaatgcttctgttgtgtcatg
accaccaaatatttccagcaaatgatcttccataccaagtgtaaatgaattatatactaa
gtcagcaatttgatttgttccctcataaccaagaaatggacggtaacttaaaggaaagtt
ctggatatgcactggtgaagaaataacgccacaagggatatttaaacgcttaccaatatg
tctttccatctgtgttccaaaaattgctgcaggttcagttttagcaattaaatctccaat
taaaccatgatcatctgaaacaattacttcatcgcaaaattcttgaacttgatctttaaa
ccactcttcatcatatttgcaataagttccacaccaagcgacatttatacccatttcctg
gtgtaaaattcgagttattgctgcagcatgagttgcatctccaaatacaatagccttttt
acctgttaaattctgacaatcaattgatctggaaaaccatgctgattgtgaaataaatcg
ggtttgttcatctatgtatttttcgtaatctacagcagctcccgatgcatttaataattg
ctgtattgatcggatgcatgcagctgtttgaacaatacccataggtgtaatgtcaacata
aggcatattaaactctttctctaaatatcgagctgtcattagtccagtttctctatatgg
aataaaattaaaccatgcagatggtaatttttttaaatcttgaactgatgcattttctgg
aataatctggtttatttggatatctagatcttgaaataacctttttaattctgctatatc

atgctgattgtgaaatcctaagcttactgctccaataatattaacagatggagttttcgt
cttttgagttaaaacttgattattagatttagctttttccatatagaaagcgacaatttg
ttctaacgttctatcacctgcttgcagttcatttactctataatgattaacatcagctaa
taaaacatctgcttctgtttctatagatgctctgctaacaaagttttgcaaatcctcttg
caaaatactcgaagtgcaagttggtgttaaaataactaaatcagggctttcttctctgtc
ttttctagtgatattttctacaactttttcttgcgaaccacgggctaacacatgtctatc
cacaacacttgctgtaacaggagtaaaatctctgtctctttcaagcattgaacgcattac
attaaaataatcgtctccgagaggcgcatgcataattgcatgaacttttttaaaagaact
ggcaattcttaaagttccaatatgagcagggcctgcatacatccaataagctaatttcat
attaaattttcctatgatttatttagacatagtaatactttttaaagctaactaacaaaca
ttagaaaaacatctaatttaactccttgataattttgtaatattttttagatgaagctta
tcattataaatatttaaaaaatataattcagaatagaaattaataactgttagatcaagc
ttgaaagtaaagatttcttgcagttatagtttttctttaagtaaacatgtaaaaattgta
ctatatataaatgtactatatataaactttttttactgaatattggtaatgccagataca
attaacttaaatatgccttccccaacgtttggtggaagcactggcggttggttaagagcc
gcagaagtagaagaaaagtatgcgataacatggacaggtaaaaatgaaagtaagtttgaa
atgccaaccggtggtaccgcaactatgcgaaatggagaaaacttactttatttagctaag
aaagaacaatgtttagctttaggtactcaactaaaaagtaaatttaaaatatctgactat
aagatttacagagtttttcctaacggagaagtgcaatatttgcatcctaaagatggagtc
ttcccagaaaaagttaatgctggaagagctagcgtcaacagtgttgatcattccattgga
caaaatattaatcctgtagatgtcaaattcatgaataaagcaacttacgattagtctctc
-aaaaacctagacataaaataactttatatagtctaggtttttgataaggtatactgat
agtatggaagggtggccgagtggttgaaggcgtctgatttgaaatcagttgaacta--tc
tggttccgtgggttcgaatcccacccttccgttataaaact------------atagtt
tgcttctcaaggcaagctagctatacactgatagtattaagtttagacttaataaagtct
acaacttgtgacaggttagaaatttttttcggcatcctcatctggtatctctatactaaat
ttttcttctatggccattactagctcaactgtgtctaaagaatcggctcccaggtcactt
gaaaaatttgcttccctggtcactatttttttttcaattcctagttgttctgctacaata
tcttgaacttttttcaaagatttcattatcttgcataatatattatttcctctaaggtgtt
atcaaaaatttttttggcttatattattacccaactctaacaaattagtaatttttagtcg
agattaaaatatttatacctatatgatatactattattctggataaatacgaagtttacg
atatggctcttctccgaaactacgagctctcaactggtagttgtcaactagattatgctg
catttttcggatataggctgaccgaggtgtaagctgaactattgaactttcattcaagat
aatagtttcaacagccaatttagcttcttgaagagcctggatctcatcaaacttttttatt
tttacagagttctacccaattaagaccagatgaagtatgaatgtttaatatttttctgag
tgcacgagtaatttgtggaaccgtactgttctgaattgtatatatataataatttgttttga

ttttgcaatttgtctaagttttgtattttgctttacttgattacgtagcgctagaatagc
atcagacttttctatctccttggttaaaataattggtaaatctagtgaagaaataactga
tgtaatatgctgccaactcaaagaataagcatataagtactgatgaggtatttctaattg
caaagattgtttattaatattaataggggcattgaaagtagttgacaataaagatgtatc
acgattttgtgatttattcaaatctaaggacttatttcttaattctctatattgtaaaac
aggagacgcctgctgtgtgttaattgatttacgactattagagatgtttacaggtaagac
ttctacagattgcgatggataacatttaattaaaattctaccattagcttgaatttgtct
tttttgcacaaaaggttgatggccttgcaaaatttgatctattgtctctttaactttatt
atgaactatccaaacatttcgctcatgtatttcgatcgcaatctgaaaagctggcgatgc
ttttctttctagtatactcttctgcgtacctctacgtttagcttcatcgtcacccaatgt
tacatattgaattccgccgattaggtccgctaatgttggatttttaattaaactttctaa
atgatttccatgcgctgtacctactaattggactcctctttctgctatagttcgagctgc
taacgcttctagctccgttccaatttcatctataataataacctcgggcatgtgattctc
aacagcttctatcattacttgatgttgcaaatcgggccttgcgacttgcattcttctagc
tctaccaatagccgggtgaggaatatctccgtcaccagctatttcattcgaagtatctat
aataactactctttttttccatttcatcagctaaaacacgagccatttctcgaactgcggt
tgttttaccaactcctggttttcctaagagtaaaattgaatcacctcgctctagtaaatc
tcgaataatactaattgtaccaaaaactgctctgccaacacgacaggttaagccgataac
actaccctctctattacgcagtgaactaatacgatgcaatgtttttttcgataccagctcg
attatcgccgctaaaattaccaatttttttacacagtaatctaagtcttgccaactaat
agatctttgagataaatattctggattatctggaaatctggcttctggacgtcgacctaa
atccataacaacttctattaaattatttctattgggatgctgttttaaaggttctttgac
aaaatttggcaaaatttctagcaacttatctaagtcatctgcaataagcatgattgagta
tataataattaattaatcaaatagaagattcaagattctaaagattacaatacatattaa
agaatattataattggaatcaattttatatttaaattgaacaggaattta-tagatgtaa
aagaactgattaaactttacgcaataaatcgattggtcaaaattaatagtcgtattaaca
tgtttattggacaaaaagttcgtattaaatatagcaaacaaaaagttgctagtgatatag
cagataaagttggtgaattaggagttattaagggaataaagtttattaatagtcaatgtg
ttactattattgttgagtttgacaatcatactaggctttggatgtttagagaggaattaa
tctgtctaaatgaat------taa-------aaacataatttattttatttattatacag
aatttcttatgagtcgttaacataataaattttaaaggagtacttttaattgtgcaaatt
actattaaaaaattacaagacttactttcgtctgtacaaagaaaaaagatccagacatta
aaactaaaacaagataagtttgaacttctattaaataagacctataaaaaggtcaatcaa
gaaataataccttcgcagaaatctgctgtattacaaaatagtccatctacgacaattaaa
tcaataaatagtacaaaaaaatcttctgttgtcaatgaagaccgtacagaatatgccact
attgtttctccaatggttggaacgttttatcattcacctgctcctggtgaaaaaatttt

gtacgagttggcgatgaggtcaaattcaatcaaacagtttgtattattgaggcaatgaag
ttaatgaacgaaatcgaagcggaaattgaaggaaaaattatagaaattcttgttaaagat
ggtgatatagtagattgcgggcaagctttaatgaaggttgaaatataatgctaattttcc
cattttacatattgttaacagaaattatttagatagaaaaattagcttatactatagtta
tgaaaacttatttcgcaaaggcacaaaaaaactaatatataaaattattt-ttttatctg
ccaaaaatagaagtaagcgttttgattccttgtctcaagagaggagaatctcaatggcaa
ttagctcaaaagagcaagagacaaagaaggtaaaaatctcggttgataaaaatcccgtag
atacttctttcgaaaaatgggcccaaccaggccatttttctcgtacactagcaaaaggac
caaaaactactacttggatttggaatcttcatgctgatgctcacgacttcgatagtcaaa
ccagttctttagaagaagtttcacgtaagattttcagtgcacattttgggcaactgtctg
taatatttttatggcttagtggaatgtattttcacggagcccgcttctctaactatgttg
cttggttaagtaatccaacaggtattaagccaagtgcacaggttgtttggcctatagttg
gacaagaaattttaaatggcgatgtaggtggtggctttcaaggagtacaagttacatctg
gatggttccaactgtggagagcatcaggaattactacagaatttcagctttactgtactg
ctattggcggattagctatggctgctttaatgctgtttgcaggatggtttcattatcata
aagcagctccaaagttagaatggtttcaaaatgttgagtcaatgatgaatcaccatttag
ctgggcttttaggcttaggctgtttaggctgggcaggtcatcaaatccatttgtctttgc
ctattaataagctactagattctggtgtgtctccgcaagaaatcccactacctcatgaat
ttttaattaatagagagcttatggctcagctgtatccaagttttagtaaaggattagttc
cattctttactttaaattgggctgaatattccgacttttttaactttaaaggaggtttaa
atcctgttactggaggtttatggctaagtgatactgctcatcatcatctagcattagctg
ttctatttcttgctgcaggtcatatgtatagaaccaattggggtattggacatagcatga
aagaaattctagaagctcataaaggacctttaccggcaacggtcacgaaggtctatatg
aaattcttacaacttcttggcatgcacagcttgcaattaatttagctatgatgggatctt
taagcataattgtagcacatcacatgtacgctatgcctccttatccatatattgctactg
attacccgactcaattatcgcttttcactcatcatatgtggattggaggatttgtattg
ttggagcaggagcgcatgcttctatatttatggtaagggattataatcctgcagaaaatt
ataacaatcttttagatagagttattaggcatcgagatgctattgtttctcatctaaatt
gggtatgtatatttcttggattccattcatttggtttatacattcacaatgatactatgc
gggcacttggaagatctcaagatatgttctctgatacagctatacagttacaacctattt
ttgctcaatgggtacaaagtatacacactttagctcctggaaatacagctccaaatgcgt
tagcaacagctagttatgcatttggaggagatgttgtttctgttggtaacaaagttgcaa
tgatgcctatttctttaggtactgcagatttttagttcaccatatacatgcatttacta
ttcatgtaactgttttaattttagttaaaggattccttttctcgagaaactctagactaa
ttcctgataaggccaatcttggcttcagatttccatgtgatggacctggtagaggtggta
cttgccaagtatctggctgggatcatgtttttcttggcttattctggatgtacaattctc

tgtctgtagcaatttttcactttagttggaaaatgcaatccgatgtttggggtagtgtat
ctccgtccggaaatgtttctcatattactggtggtaattttgcacaaagtgcaattacaa
tcaatggatggttaagagatttcctttgggctcaagcatctcaggttattcaatcatacg
gttctgcgctatctgcatatggattaattttcttagcagcacattttgtatgggcattca
gtttgatgttcttatttagtggtagaggttattggcaagagcttatagaatcaatcgtat
gggcgcataataaaataaaagttgctcctgcaattcaaccaagagctttaagtattactc
aaggtagagcagtcggtgttgcacactacttattaggtggaattggtacaacttgggcat
tctttttagcaagaattatttcagtaggctaatagtgaaaataggat-aaaaaaacaatt
atggcaacaaaatttcctaagtttagccaagctttatcacaagatcctacaactagaagg
atttggtatggtattgctacggcacatgactttgaaagtcatgatggaatgacagaagaa
aatttatatcagaagatatttgcttcgcactttggacatctagcaattatcttcttatgg
acatctggtaatttattccatgtagcttggcaaggcaactttgaacagtgggtattaaat
cctttgaaagttaaaccaattgctcatgcaatttgggatccacattttggacaaccagct
ttaaaagcttttagtaaaggggctcagcttatccagtaaatatagcatattctggcgta
tatcactggtggtacactattggtatgagaagcaatcaagacctatattctggggcttta
ttcttactagttttatcagctttactcttatttggagggtggctacatctacaaccaaaa
ttcaagcctggtttatcctggtttaaaaataacgaatcaagattaaatcatcatttatct
ggattatttggggttagttctttagcctggacaggtcatttagtacatgttgctatacct
gaggcaagaggacaacatgtaggatgggataattttacaacagtattacctcatccagcc
ggtttacaaccatttttcagtggtaattggagtgtatatgctcaaaatccagatacagct
caacatttattcggaactaatgaaggtgcgggtacagcaattttaacatttctgggggga
ttccatcctcaaagtcagtctttatggctaactgacatggctcatcaccatttggctatt
gcagtagtattcattgttgctggacatatgtataggactaattggggggattgggcacaat
ctaaaagatattttagatgctcatagaccacctagtggtagattaggagctggacataaa
gggttatttgatactattactaattctttacacatgcagttaggattagcattagcttcc
ctaggtgtaattacttcgttggtagctcaacatatgtatgctatgcctccatatgctttc
atggctaaagatttcacaactcaagcgtctttgtacacacatcatcaatatattgctggg
tttctaatggttggagcttttgctcatggggcaatattctttgttcgagactatgaccct
gaacagaataaagataatgttttagctcgcatgctagaacataaagaagctatcatttct
catctaagttgggtaactctatttttagggtttcatacattaggcctttatgttcacaac
gacacaatgattgcttttggaactcctgaaaaacaaattttaattgagccggtatttgct
caatggattcaagcttcttcaggaaaagcactttacggttttgatgtgttactatcatcc
tctactaatgtcgcaacacaagctggtagcaatatttggctgccaggctggttagaagca
attaatagcggaaaaaattcattgtttttaacaattggtcctggtgacttcttagttcat
catgcaattgcattgggattacatactactacgttaattttagttaaaggtgctttagat
gcgagaggctctaaacttatgccggacaaaaaagactttggttatagttttccttgcgat

ggacctggcagaggtggcacctgtgatatatctgcatgggatgcgttctatttagctgta
ttttggatgctgaatacgataggttgggtaacattttattggcattggaaacatattaca
atatggcaaggcaatgcaactcaattcaatgagtcttcaacttatctaatgggatggttt
agagattacttatggctaaattcttctccattaattaatggttataatccatatggcatg
aataatttatcagtatggtcatggatgttcttatttgggcatttagtatgggcaacagga
tttatgttcttgatctcctggcgtggctattggcaagagttaattgaaactctagcctgg
gcgcatgaacgtacacctttagcaaacttgattcgttggaaagataaacctgttgcgcta
tcaattgtacaggcaagattagtaggtttagcacatt-tttctgtaggatatgtattaac
ttacgcagcttttgtattagcttcaacagcaggcaaatttggttagactaaa-----ttc
aaattagctaa-----aaaaaagtcagtcataata-gttttatgactgactttttggat
ttaaaacaaaaacttactaaagtttcacctcaatatctacaccggaaggcaaatttaact
tcattaatgcatctatcgtttgagaagaaggttgatgaatatcaataattcttctatgag
atcttatttcaaaatgttctcttgagtctttatctacatgtggagaacgtaagacacaat
aaattcttcttcttgtaggtaaaggaattggccctactgcaacagcattagttctagatg
cggtatctagtattttgtcacacgacgtattaagtatactagaattatatgctttcagtt
taattctgatttttgtctgctgagtaattgtcatgttaaaaagatcttattatttaagaa
ttttagagacaacacctgcacctacggtacggccaccttctctaatagcgaaacgcatac
cttgctcaattgcaattgcattaattaattcagcagtcatcttaattctgtcaccaggca
tgaccatttccgcatcagtaccatcatcagcagtaaactgattaatagtaccagttacat
cagttgttctaacataaaactgaggtctatatcctgggaaaaatggagtatgtcttccgc
cctcctctttagttagaatataaacttctgcttcaaattgagtatgaggtgtaattgtac
caggtttagctaataccatacctctttcaatatctttttttctgcacgcctcttaaaagaa
ttccaatattgtcacccgctagaccttcttctagcgtttttttgaaacatttctaatccag
taatagtcgttgtacgagtttctcgtaaacctacaatttcaattgtatcaccaactttaa
taatgcctctttcaattctaccagtggcaacagttccacgtcctgtgatagaaaaaacat
cttctacagccattaagaaagttttatcgacatctctctccggtgttggaatatacgtgt
caactgcttccataagtgaaaaaatcttgtcaacccatttatcttcaccttgcttaatag
ctgggtttttcgtcacagcttctaatgctaataaggcagaacctgcaacaaaaggaatat
catctccaggaaagtcgtattgacttaataattcccttccttctaattctactaattcta
gtagctcttcgtcatctacttgatcttccttatttaggaatactactaatgtgggaacac
ctacttgttttgctaataaaatatgttcacgagtttgtggcattggaccatctgccgcag
atacaactaaaatagctccatccatctgagcagcacctgtaatcatattttttacgtaat
cggcatggcctggacaatctacgtgagcataatgacgattatctgtttcgtattcaacat
gagcagtattaatagtaatacctctagctttttcttctggagcggcatcaatttcatcaa
atttttttgctgcagtagaacctaaagttgataaagtcgcagagatcgcagctgttaaag
ttgtcttaccatgatcaacatgaccaattgttccgatattgacatgaggtttttttacgtt

caaatttagatcgagccatgctttttgttttccttatagtaaaagttgctttatagttac
ttttaacgataaatcttgtgaattgaaagatttattatttaatacagacaaga-----ta
aaatataactaatctaatatcgatagtgagcaaacgctttatttgcttctgccattctat
gtgtatcctctctctttctaatagaatttcctgtttcattagctgcatccataatttcgt
tagctaattttatagacatacttttaccagatctttctctagaaaatttagtgatccatc
ttaacgctaaattcgtacctctataagctcgtacttcaataggaacttggtaagtagaac
caccaactcttctagcttttacttctactagaggagtaatatttcggatagctttttcta
aaatatttaaaggatctgattctgttctctctttaacgatatctaaagcctgatatataa
ttctttgagctaaagtttttttaccacttttcaaaatacgaacagttaacatacttacga
gtctgcttttatataaaggatcaggtgatgcgaaccttttttttagctgtattacgacgag
acatagtatttaaaattggttattgtaataa-tgttc-tattagaagttatttaagattt
aggctttttttgtcccatatttagatcggcttttacgacgatcctttactcctgcagcatc
taaagtaccacgtactacatgatatcggactccaggcaaatctttaattcggccacccct
aatcagtaccacagaatgctcttgaatattatgacctacgccaggaatataagctgtaac
ttcaaatccagatgttaatctaacgcgcgctacttttcttaaagcagagttcggtttttt
aggagtcgtagtatatactcttgtacaaacacctcttctttgagggcaactttgaagagc
tggagattttgttttttttatgtatttttcgtctttcggatctaacaagttgttgaatggt
tggcataataaaattgatttaaagtctttaatagatactatttgaacctgctttagatga
cttggctagtttttatttatcttatacttacgcataaatctttctactctaccttctgta
tcaataattctctgtgaaccagtaaagaatggatggtttcctgaccagatatctacgtgt
agttctggctttgtggacccaatcgtcataattaactggccgtcacaataaacttttgct
tctggataccaatttggatgtatattatcttttgccatggtttctataattatataaatg
tatcaataataactattaacgcttagaaaattgaggagcctttcttgctttctttaaacc
atatttttttctctcttttactcttggatctcttgttaaataaccctcagacttgagtgt
cgttctattttctggattaattgagcacaatgctcttgcaacacctaaacgaattgcatc
ggcttgccctgtcaaacctcctcctctagcattcacatgtatatcatactgatttaatag
tcctaaaacttgtaatggtgcatatgaaactcttaagtaattaggactaaattgaagata
agactctcctggtataccgtttataattaaattacctgatcctgggactagtcttacttg
tgcaacagaacacttccggcgacctgttccagaatagattgcacgagttttaattaattc
tgtcgacataacattccttgataactaaataaatatacattctttatactatatactctt
gtggcttttgcgcgacatgtgggtgaattgggccagaatatactttaagcttcgtaaata
gttttctgcctaatggacctttaggaagcatacctttaaccgatttttcaataattctgt
taggtaatcttgtctgaagctgataaaatgtttcaacttttaatccgccaggttgtccag
aatgtcttctgtatagtttttgattcgttttattgccacttacagatacgtgagcagagt
taataacaattacataatctccagtgtctagatagggtgtataagaaggcttgtttttgc
ctcttaagatattagaaatatgagtagatattctaccaagtgtctgatttttagcgtcta

taacataccaatgagaattagtatttaatgagggtgattgcgttttattcatgaaaagat
taatacttttggttgacaataagagaatatgtaaatggtttacattcaatttctatacaa
gtaatattt-aaaaatatta-------------ttaacaaagagaacttttctaacagtt
atttcaagtgtttgtcagcaagaaaatttctagttctcaataacttatactatttttaat
aatataaaatatattaatcacttttttcttttggcaaacttatacctagttttttttgc-
aaggcatatataacttcttcagctgattttggccaaaattttaatttctagcagttct
tcttgagagtaatctagtaaatctgcaatagaatggatttgagctcgctttaagcagtta
taagccctaacagataactgcagttcttctattaaaacttgactaatttttttatcttct
ttactacgataattatctgctgatttaaagtctaaatttcttagtgaacaaaaaagatta
gttaaaactgtagctccttgacttattgcttcttgaggagagatgctcccatttgtccag
atttgtataatcaatctatcttttatgctgttgctaccaatacggacttcttctactta
taattaactttattaactggcataaaaacagagtctacttgcaaaaaatccacagataat
tcatctactgctttttcagctaagcgatacccgcaatttttttcaattttaaactccatt
tcaaatattgtattattgcaaatagttgcaatatactgtctaggatctactacctctata
tcagaagataattcaaataggccggctgtaactatagctggcccttgaactctaattcga
ccaatttgagattctttgttataacttttaaatactacttcttttagattaagtaatatt
tctaacacatcttctcttaccccagggattgtagaaaactcatggttcactccagcaatg
cgtacagcaactatagcagtaccttcaagatctgacaatattgatcttcttaaagcatta
cctaatgtaataccttgtccttgatttaatggttctattacaaaactaccgtactgccca
cgcgccccatgtgttcttgactctacgcattcaatttgaaattgagccacctaagaaagc
tcc-tttaaaataatcagtgattagtaagcctctctaaaataagaaggaatttatactcg
gcgtttcttaggaggacgacatccattatgaggtacaggagtaatatcttttattagagt
aatctctaatcctgcagcctgcaaagctctaattgctgtttctcgacccgctcctggacc
atttactaaaacttcggtttggcgcataccttgatccatagcttgtttagctgcttttc
agctgcggtttgagctgcaaaaggtgttcctttttagctcccttaaatccacttgcacc
agaggatgaccatgataatgtctctcctttcaaattagtaatagtaacaattgtattatt
gaatgtagatttaatatgtgtaacaccgttaactgcattacgtttagtttttcttgctcc
ggatttttttatttgtctagccatcgtgtctcgtctattatatagttaattagaagatta
ttttcttggagctttttcttacctgctactgtttttttacctcctctgcgtgttctagc
attagttctagttctttgtcctctcaaaggaagaccaagacggtgtcttctacctctata
agtactaatttccataagtctcttaatgctcatagactcaaaacgtttgagatcccttc
aatctgatagttagactcaagaatttctcttatactgacaacttgttgatcatttaaatc
ttgacacttaatatcaacatctatctttgtttttctaatatttctttcgaacgagatag
tccaataccataaatatatgttaaagctatctctattcttttgtttcttggaagatctac
tccagcaattctggccactttgtattgtctccaataaataatatgtat-ttttataacgg
tagtttata-agcttagccttgtctctgcttatgtttaggattagtgcaaattaccatca

cctttctgtgacgccgaattattctacatttttcacacatttttcgaacagaaggacgaa
ctttcatattaa----ttctatatactaggatattactatattaactctattaattatc
gacattaaaaacgtcaataatatattgtattactttaactattttagattttacttggtc
atactatcatatttttttgatatgacgtatgtctgaatttgcttagctgtgtctattgcg
actccaactaaaataagtaaagaagtagccccaagacctcttaagttttggatctgagta
acctttttctattataaacggaatcagcgctactgtaaataaaaatgaggctcctaggaat
gtcagtctatttaatataacttgtaaataatcaatagtcgcttgacctggacgaatatta
ggaatgcttgcacccattttttttagatttgtagcaatatcttctggattcattactatt
gatgtataaaaatagctaaagaaaaggatcaaagcacaataaagaagaaggtatagtgaa
ccattaggacaaaacaagtataggatttggagtaatgttttattttgaataatttgggtt
agataagatgggagggccatagatgcagatgcaaaaacaataggcataactccaccttga
tttaatttcaagggcaagtaactatttggatccaaaattgaagattttcctagctgtctt
gctgaaataattttaattcttcttgttccttcttgtacacaaattgtaattattatcatt
aataaaaagatcgctataaataatccaaacttgagacttgcattactataattagcatca
aaaaatgactgtgtaaaattctttggtagtcctgacacaatgttttgaaaaataagcaaa
gaggctccattaccaattcctttttctgtaattaactctgataaccacataataatcata
gagccagctgttaaagctaaaacagactcgcagacaaattcgacattccaattaaaaaca
tatggttttacccatatagagattgctccagattgtaaagtcgcccaacctagagctaag
taccttgtaatttgagttatttttttggcggcccaattcaccctcttccttctgtaatttt
tccagattggggacaattttcgtaagtagctgcattacaattgaagagttaatataagga
acaatacctaacgcaaaaatccctattgttgaaaaacctcctccagaaaaaatatttaga
aaatttactaaagtatttttttctacacttgcataaaaggcgtcatgatctatacctgga
acaggtataaatattcctaaacgtgctaaaactaatagaaaaagggtaaagataatacga
tttctcaggtcactttttttggctcataaataaattttaaatgaaaagagatacttt--tt
tatgtaaaaaccctattccaagacttatataagtttaaaatagggaagtaa---aatatt
atag------taata-taatattctatgtagaatacaagctttctataggcactcctcga
tctttagctgcttcactaaaagttcttaattgagttaagccatttagaacagctcgcgcg
ttatttaatgtattattagaacctaattgcttagctaatatattctgtacacccgacaat
tctaggactgtccttacagaacctccggcaattacacctgaaccaggtgcagagggcctt
aatataactttcgcggcaccagaaattccattaataggatgaggtatagagttcgatttt
gtcagtggaactgtaactagatgtttttttagcatctgttactcctttttttactgcacca
attacatcgcttgcttttcccacgccgacaccgacttggccttgctcgttaccaacaaca
agaataactcgaaagcttagtttttttacctccttttacaactttagtaactcttttaact
tgtacgactctttcttcccagccactatctttatcttttcctttgctctgtttttttacga
ttggccattttaagaattattcctaattagttaatta-cgcttctagaaacccatgcctg
cttccttagcagcttcagccaaggccttaactcttccatgatataactttccacctctat

cgaagacaacatttttaatgccttctttcatagactgttctgctaactgtttaccaacta
cgcgagaagtatcacagtttggtcttatattatcggattctttactatttaaattaacag
atgatgtagctactaatgtaataccttgtgtatcatcaattatttgtgcgtatatatgtt
tgttagatctaaatacacacaggcgtggcctacttgaggttccttgaactttttttcgaa
ctcttttatgcttatgaattctagtttgtttagtgtttagtttcattattatttaccttt
tccagctttcccagcttttcttctaacaaattcaccttgatatctaattccttttccttt
ataaggctcaggaggcctaatagaacgaatagttgaagcaacctgaccgacaacttcttt
atctatgccagagacagtaatatttgtattattttcaactttaatttcaatattagcagg
aggtttaattttcactacatgactatagccaacatttaagattaaatcttggttatcaat
ttgagaacgatagcctacaccttgtatttgcagtttttttaaaaaatccattagaaacacc
ttcaatcatattactaataagagttctggatagcccatgtaactgactagccatttttgt
ttgtccactcgtcttgacagcaatcgtgtcattaagtatttctaaattaataccagctgg
taaagttctggatagggtacctttaggtcctgtcacagtgatagtctgaccatcaaactg
agtactaagatttgtcggcaataaaattatttttttttccaatacgagacat-attccacc
tataaaatagttaccaaatatagcataatatttcaccaccaagaccatcatgacgagctt
gtctatctgtcataacacctctagaagtagaaataagagcgatgcctaaacctccaagaa
ctctgggtagctctttgtgatttgcgtagactcttagtccgggtttactaattcttttca
gagcagtaataactggttgacgattttttaccattatacttaagagaaatcattaaattag
tctctataccttcacctatttgttcgaaattttgaacaaatccttcttcttttagtactg
ttgccatattgcatgtcattttcgttgctggaacttgcacaatttgatgtcttgctaagt
ttgcgttacgaatacgtgtcagcatatcggcgatcgtatcgttgaccaccttgatcctcc
ttgcga-----tgaattatttaaaatattttcaagattccctgaaaggcatccctagctt
ttttaacaaagctagaccttcttgatctgttttagctgtagtgacaattgatatatctag
accacgaatttgatcaatattatcatagtctatttctggaaagatcaactgttcacgtaa
acctaaattgtaattacctttgccgtcaaaacttttaggactaattcctctgaagtctct
aattcttggtaatgttaaattaattagtttctctaaaaaagaatacatcttgtcttttct
tagatgcacaacaattccaataggaacctcttctcgaattttaaatcctgcaatagattt
tttagcttttgtgacaatcggtttttgtccagttattaatgttaattcttgaatactact
ttcaagagccttagcattctgagaagcttcacctaagccacggttaatagtaattttagt
aaacctaggaacttcatgtacatttttgtactgaaattcatcttttaaagattgagtaac
agttgttttatatttttcttttaatcctattgccatggtatttttatcaattatttaata
agttcgccagtctttttcagttttcgaa-ttttttggcctttatcattaattattactga
ggatcgactagcaatattattttgctcactgaataacataacattagaggtatgtattgg
agcttcaaatttgataatttctcccgtttctccttcttgttgaggtttttatgcttcac
tttgagattaattcctttaacaatcaccttacttgttttatgcatgattgcaataatttc
acctgtttttgttttatcacttccagaaataacttgaactaagtcccctttttttaattt

aatttttgtattattttgattgttttagaagaacttttcattatactacctccggtgca
agagacactatttagaaaaatttttatctcttaactctcgggctataggtccgaagact
cttgtgccacgtggattattatcttgattaatgataactgccgcattatctccaaatcta
atgctcataccgtcagttctccttaaagctttacgagttcttactacaacagctctgaca
acatcagatcgtttaactggcatattgggagacgcatctttgaccactccaataataaca
tccccaatagatgcataagaaggattactagtgcctaatactctaatacacattattttt
ttagcaccactattatctgcaacattaagatagctttgagtctgtatcatacttttattc
tgtataaaattaattatcaaaagacttagataagatattaaccattgtccagcactttgt
gcgacttaaaggtcttgtctcttgtattgtaacaatatcaccgattgtgcattcattatt
ttcatcatgcgctttatatttctttgtccggatcatagtttttgcgtactttctatgaga
gattctattttctacagctactactatagttttattcattttatcgcttactactttacc
tgttgtttcttttaaaggcatagtctttatattttttatatat---------aattaaat
tttaagattattgcacggttgacttattacgagacttttcaacagttaaaagttgagcta
atctatgcttagaatgtttaaatagatgaggctgaaaatcttgccttgtggctcttttta
gccttaaatcaaaaagctctctttttattacaaggatttcttcagctaaagaagaagagt
ccatgtttgtaacatctgatattttagggaaagtcatgttttaggattctgttgtattcc
gaactataaatttggtcttgattggtaatttataagaagctaatttcatagcttgttgag
cagtttttgtggtacacctgtaatttcaaatagaatatgtccaggcttaataactgcaa
cccaatattctggagctcctttacctgaccccatgcgagtttcagctggacgggcagtga
ctggtttatctggaaatactctaatccataatttgccacctcttctaacatatctagtaa
tagttcttcgagtagcttctatttgtctagaagttaaccaaactggctctgtcgcttgta
acgcataatcaccgaacgcaattgtgttgcctttactagcagaacctttcattctacctc
tgtgttgttttctaaattttgttttcttggggcttagcataaaaattaaataataataat
aattaagaagctgttgaatcaggagtttcctgattataagcagattctagacttttcgat
tctggcaaaatttctcctttaaaaagccaaacttttactccgagcacaccatacgtagtg
tgagcttggcgatgacaataatcaatatcagctcttaaagtttgtaaaggtacacgacct
tctctaacccattcacttctagctatctctgcaccattaagccgaccagatacttgaatt
ttaacaccctgtgtatttgctctttgagctctttgaactgcttgtctaacagctctacga
aaggcaactctttttttctagctgttgagtaataaattctgctactaaggttgcttcagaa
tctggatctgcaatctctataacattgactctgagttgtttgctagggcctagaattaag
gacaacgattttcttaaagactcaattccagctccagattttccaagtacaattccaggt
cgagctgttgctatgagaatttctacttgatcaactttacgattgatttcaattttagca
atactagcattactaagttttgaatgtataaatgagcgaattttatgatcttcttgtaaa
agaactgggtagtctttagagttagcaaaccatgaagaacgatgtttttgggtaatgcct
atgcgaaaacccaaaggatgaatttttttgacccacagtatccttgattataaattaaaag
ctataatgactaattgacgttaag--------agactacagttctgagacacctaatgta

atatgacaagttggcttatgtattggaaaggctcttccttgggctctaggctgaaatctt
tttaaagtcgggcctttgtctgcaaaggctttactaacaaataattggttttatttaat
ccatcattatgctcagcattcgctgcagcagattccaggatttgctttatatgagaacag
actcggtatggcataaattccagaataattaatgcttcttggtattttctacctctaatt
tggtctaatacacggcggactttatgcggagaaagacgaatgtatttccctactgcttta
gtttcttttacgtttttgtaatactcatatttatagtttaacgacgggcttttctgtca
ccttttatgtgagtgcggaaagttcttgttgggacaaactctcctaatttatgtcctacc
atttggtctgacacaaagacaggaaaatgttgtttgccattataaacagctattgtatgc
cctaccatatcgggaataatagtagatgctctagaccaagtttttaggacttcttttttt
cctgaaatatttaatgcttctatacgtttaagaagactaacatcaataaaagggccttta
tgtatagatcttgacataataataaatcattagatttttgataaaataaattctatttac
ggcgacgtaagacgtacgggttactatatttatttggatttcgtgttttaacacccaatg
caggtttaccccaaggtgttacaggacgagctcttccaattggagatttgccttcaccac
caccatgtggatggtctacaggattcataacaacacctctaacagtaggtcttttaccta
accaacgatttctaccagctttaccaagagtaatattactagcatcaatattaccaactt
gtccaatagtagcatagcattcttttcgaatcattcgaacttcacttgaaggcaatttta
cagtaacaaaagcaccttcttttgctactatttgagcataagttcctgctgcacgaacaa
tctgtcctccgcacgaaggtcttagttctatattatgtactgctgttcctaaaggaatac
tagataaaggcaaagcatttccaacttcgataggggcagtagaaccagaaagaaccatag
atcctacacttagagatcgagggtgcagaatgtatcttttctccccatcaagatagtgta
ataatgcaattcttgcatttctattcggatcatattcaatagaagcaactttagcagcta
tattatgcctatttcttttaaagtcaattaatctgtactgctgcttatgtccaccacctt
tatgacgacaggtaatgacacctctattatttcgacctttgcagaaatggtgtttaacta
ttaatgatttttctggcttatcagtagtaatctctgaaaaggtagaaactgttctatttc
ttgttcctggtgtgtaggcacgatataaacgaattgccatataagaagatttgagaagag
taaatgttgaa------ctatgtaatatatttaagtttctggaaacaaattgatagaatc
ttttgaggcaagtgtcacaattgctttcttgtaatgtggtcgcttgcctacgaatctgcc
gatgctcctttttttcttaggaggatggcaagtgttaacacctgtaacttgcacgttgaa
aatatactgtatagcagccttaatattaatttttgttgctttgggatcaacagcaaaaca
atactgattttcttctaataatttagttgtcttatcagtaatgatgggatatttaactaa
atctaataaacctcttgaatcaatgctatccattatatacctcttgtattttagataaag
catcaactgtaataataattttatgtgcagctaacaaagccataatatttagtgtatcag
ctgaaataagttctacgttgtgtagattgcgaatagaaagataaacgttcggatcttttt
tgtctactatgactaagactttttttgtttaaatcaagattccatcgatgtatagcttcca
taaatagtttagtcctgggctgatgaaaataactgttaaaattttctacaatcaatgtat
taactgatttgttatttaatgctgtccttaatgctaattgtctttctttttttattcattt

ttttggtaaaactacgaggttttggcccaaatattacaccaccgcctctccatagaggtg
aacggatagaacctgctcttgctcgaccagtcccttttttgacgccatggtttacgacctc
cacctcgaacttcacttcttgttttagtattcgcagaaccctgacgcttttcattacttt
gctttactaacgctctatggactaagtacatccctgaatcttgactaaccttaagattta
aatcagcgttgccacttacttgaccttcccaattatagacttgataatttaattgtgtgt
taactgtcatgaaatatatcgtatgaaagaatattgctacttactaatttttactaaagc
gccaggtttaccaggaacagctcccttaacgatcaacaaatcattctctgagttaatact
tacaatttgcaaatttttattgtaactttttattaccaagttggccagccatgttttt
acctggataaactcttccaggcgttgtaccagctccaatagaaccaggttgtctatgatt
tttcgaaccatgtgacattgggcctctactaaaatgatgcctcttttggtaaccggagaa
acctttacctacacttctagaagagacattaatcttttggcctacttgaaatagatctgt
agatagtatttggctaacttcgaaatcatcagtagatttcacttcgtattcacgtaagta
ttttagtgggggagcctgtgatttttttaagtgtcccaacaatggtttatttaacttttg
ttcagcaacttgcttataaccgacttgaatagcattatagccatcagtagatatagcttt
aatttgagtaataatacatggcccaacttgaattacagtaactggaattgataaaccagc
ttcatcaaagaattgggtcatacctactttagtaccgagtataccaacagacactagatt
tctccttttttgagagatttatactatcattgtacataaaaattctacttcatattgactg
aag--taaattaaactgactgatgcatgatattattcatgacaacataaaggtttaaaac
tttacctatttaatctaaaatagaaacagctttttgtccagttacagcgtatataaatta
gaagcatgcttattaaatttttatagactact-gaaatttcactgatattgatattcgt
aacttacttgcagtaat--------atcaacatgtataagtttaattgcctagactagaa
ccagctattattagaaatgattcaagtatagataaaagcgcatttgaacttttatctatt
gacaaatatagtagaatatattgaggttcggtaactacaccttttttattttcacatttat
tgtgca--gtatatatacatctaatgcagtaaacataggaatataac----tgtatagca
aatgtaattacaaaaattagagaggtattaatgggcaaagtcgttggaattgatcttgga
acaacgaattctgtaattgctgttatggaaggaggtaaacctaccgtcataccgaatgca
gaaggttttagaactacagcttctgttgttgcatatactaaaagtggagataaacttgtc
ggacaaattgccaggaggcaagctgttattaacccagaaaacactttctactctgtcaaa
agattcataggacgtaaacagaatgaaatttcgcaagagattcggcaaacatcatataat
gttaaaactagtggatcaagcataaaaattgcttgccctgcactgaataaagattttgct
ccagaagaaatttcagctcaagtactgagaaaacttgttgaagatgctagtacctactta
ggtgagactgttacacaagccgtaataactgtaccggcttactttaacgattcacaaaga
caagctactaaagacgcaggtaaaatagcaggcttagatgtattgagaattattaatgag
cctactgcagcttctttgtcttatggactagacaaacaaaataatgaaacaatactagta
tttgaccttggcgggggcacatttgatgtatctgtattagaagttggagatggagtattt
gaagtactctcaacttctggagatacacatttaggtggagatgactttgatcagcagatt

gtagaatggctaatcaaagattttaaacaaaatgaaggtattgatcttggtaaagataga
caagcacttcaaagattaaccgaagctgcagaaaaagcaaagatagaactatcaaattta
actcaaacagagattaatcttccttttattactgctacacaagatggtccaaagcattta
gaaaagactgtaactagaggtaagtttgaagaactttgttcaaatttaatagacaaatgt
agtattcctgtaaacaatgctctgaaagatgcaaaactagaagcttccagtattgatgaa
gttgttttagttggtggatctacaaggattccagccatacagcaaatggttaaaagatta
attggtaaagatccaaaccagagcgtcaatccagatgaagttgttgctattggtgcagcc
gtacaagctggagttttagcaggcgaagtcaaagatattctattactagatgtgacgccg
ttatctttaggagtggaaactttaggtggcgtgatgacaaagattataccaagaaacact
actattcctacaaaaaaatcagaagtattttctacagctgtagataatcaaccaaatgtg
gaaattcaagtacttcaaggcgaaagagaactaacaaaagacaataagagcttagggacg
ttccgattagatggcattatgcctgcacctaggggggttcctcaaattgaagttacgttt
gacattgacgctaacggaattttatctgtaaaagccaaagaaaaggctactggtaaagag
caatcaattactatatctggagcatcaactctacctaaagatgatgttgaaagaatggta
aaagaagccgaagaaaatttcgatacagaccaaaaaagaagaaagaacattgacacaaag
aatcaagcagagtccctatgttaccaagctgaaaagcaagttaaagagtttgaagacaaa
attagccaagatttaaaaataaaaatagaggagctaattacagagcttagatctagtcta
gaaaaagaagaatatgacaatatcgaatccatttctcagcaattacaaaattctctgatg
gacattggaaaaaatgctgctcagactgaaagtaaagatgcaaaagcaaaggatgacaac
accgtgattgacactgatttctctgaagctaagt-aaaagta-agcgggtaacgcgattc
gaacgcgcgacatcaaccttggcaaggttgcgctctaccactgagctatacccgcatatg
ttgtattattacaaaaattaagatatttgtcaatctgtta------atctatac---atc
tagactattaatttccgaatcacatattagctaaatgttgtaaatataacaattgctata
actttttaa-------aagtacttatatcat-tatataagtactttttatgtt-------
-caaaaatacaaataagtattatag-------ctaaactactaaggaattaactaagcta
gtaacaattactaatcctgtccataagccagctcccgtatatattaatccttttgattgc
tcccattgacctggggatgctaaaacaacaggtacacctactactaacacggtcgacaat
gcaattaataatagcactaataattggatcgcaataatcatttaatatgttctcctgaaa
aatttaaatgttaataatactgtatataatacaatgaagcttttgaccttcagcaattta
tctatgaagaaataatttaaaattattgttttataaataaaccttaaattaagtattaaa
atctttattgtaaataataatactatattttttta---tagataaaaaatttaaagaaag
tctttttttaaaaaaaaattaagataagatgatatgctgtataaaaagctaagtattatta
attattagagaaatttaaa-ataagaggtaaaacatatgaactcagccctttttttggca
aaactaccagaagcttatgccgtttttaaaccgatcattgatatcttacccgtaattcct
gtatttttttcttcttttagcttttgtttggcaagcagcaattggttttagataaaattta
ggttataaaaagaataacaaatatacagagtgacatttttttgagctctaatcttatatct

tacacaataataactattatggttgaacccttactatcaggaattgttcttggacttatt
cctattactttattcggattattagtagcagcttatttacaatatcaacggggcaaccaa
ttaggactgtaaacgctaaatcatagatatttataaaaactacaataattatagtttttta
taaatatctacaaataagctaattttaaatacaaagataatattattgaaactagagtga
taacgaaaaagatttatataa---taa---------------------------------

---------------------------caaactaattgtttaccagctcgacttagtaac
tcctggtaaaagacattcatgagacatttccctaaaaacatggcgagataatccaaaatc
tctataataccctctacttctcccggttaaccaacatctatttctccctctaacaggggc
gctgtttctaggcatttcttgaagtttttgtcttaaatccattttgtcagaaaagctgcc
agcttctttcagctgatctttaatagccaatctctttaagtaatatttttttgcaagttt
cgatctcttaatctctctttggatcatattctttttagccatgctgtag-aactctcttt
taaaattatttattcagtatattagctaaaaaagctcctatctgcaacttttatctattt
aaaaataaatagcataaagtagtaaaatgcaattgatatttaatcaacgatacc---act
ttatgctattatcaaaaatttatttaataaagttcttgttcaacatgagttgaaatagtg
caatcagatgaagggtatgcaatacaggttaacacataaccagctaaaagttgatcatca
tctaagaatgattgatcagcttgatctacagttccttctgtaactttacctgcacaagtt
gaacaagctccagctctacaagagtaaggcagttcaattccttcttcttctgctgcatct
aaaatatatgtatcctctgcacaattaaatgtaacatcaattccttcttcttcacatagt

aaatgaattttataatcagccatttctttttactccataataaagatacacttgaaactt
aataaagcaaaat-aatttaaagaacttaccaaaaaattatatactagtttcattatgca
tgatagactggaaaaaaagaaaataatatactattctgaaaaatcaggctacatttccta
ctaaatagaactatagtaggtccaaagtatatacataaattcatatagttaaaattattt
ttatcatttaaatagctgaacttaatagta---------taggttgatcatc------aa
gctagtcttctgc---caataaataattaattatgatttcaatgcttaatcaacaagtag
aacttaagcctattcttaaattccagataacacaaatatattcacattctcttattcagg
aaattaaagcattaataaccagacttgttttgcaagtttggaggaggccggctacattga
tggcaggtattatgcaacctttgttatggttaattctatttggtgggcttttctacaatg
ctcctataaatttgtttactatcaatacaaactataattgtttttttgagctctgggatta
taattttcacatcttttactggagctttaaattcaggtcttccattaatgtttgatagag
aatttggattttttaaatagattattaacggctccgttagtatcgagggcttctatcattt
tatcttctgctacttttatgacttgtattagtttaatacaagttatatttatagttacag
cttctctttttatgggaaactcacctctaaatagcgatagtgctatgatttttggactta
taatcctattagtaactgtaggagttacaatgcttagtttagctttatctttcactctgc
caggtcatattgagctattagcatttatttagtagttaacttaccctttttattttcta
gtacggctttagctcctttatatttcatgccaccatggcttcagttaattgcaagtctca
atccattgagttatgccatagaaggtacaagatatttatattcaagcgtaaactggaatt
ttacagagtgtgtgattaagattagctggggagatatttgtttagggcagattattgttt
tattaatcgctttagatataatggcagcttatgttgtgtctaagatattaaaagctaaac
ttaatta----------------aaattttaataaaaaatttattaaatttatttaaca
aagattttttactatataatactaatagtagtatggataattgaaatagaaatagtttttc
aaaaccaaagctattatcaacaatgataaacatttgtaagaaagtcaacaaagtatgttc
ttattcataggaggcatgtagtcaatgggactaccatggtaccgtgtacacacggttgtt
ttaaatgatcctggacgcttaattgcagtccacctaatgcatactgcacttgtagcaggt
tgggcagggtctatggcattatacgaactagctgtatttgatccttcagatccagtacta
aatccaatgtggcgacaaggcatgtttgttatgccatttatggctagactgggcgtaaca
gattcatggggaggatggagtataacaggagaaagcgtatccaatcctggactgtggagt
tttgaaggtgtcgcgttaactcatatagtcctttctggcatgcttttcctagctgctatt
tggcattgggtttattgggatttagaattatttagagatccacgaactggcgaaccagct
ttagatttaccgaaaatttttggaattcatttattgctatcaagtctactttgtttcgga
tttggagctttccatgtgactggacttttggaccaggaatgtgggtatcagatgggtat
ggagtaaccggaaaggtgctaccagtagctccagcatggggaccagaaggattcaatccg
tttaatcctggaggagttgcatctcaccacattgccgcaggtactgtaggtatattagct
ggtgttttccatttaactgttagaccaccacaaagactgtatcgtgctctaagaatgggt
aatattgaaactgtattatcaagtagtatctctgctgtttttttctcagcttttgtgact

tgtggtacgatgtggtacggctctgcaactacgcctattgaattatttggtccaactaga
tatcagtgggatagtggatattttcagcaagaaattgagaaacgagtagaaaatgctatt
gctgatggtgctgcacctagcgaagcatggtcaaggattcctgacaagttggcattctat
gactatattggtaataatccagcaaaaggaggattattcagagcaggccctatgaataaa
ggcgacggagttgcagaagcatggcttggacatcctgtattccaggataaagaaggaaga
gagcttagtgttcgcagaatgcctgctttttcgaaactttcctgtaattttagttgat
aaagatggtattatacgagctgacattccgtttagaagagcagagtccaagtatagtatt
gaacaagtaggtgtaacagctagtttttatggtggcaaattaaatggccaagttttcaac
gatgctcctagcgttaaaaaatatgcaaggaaagctcaattaggtgaggtatttgaattt
gatcgaactacattagaatcagatggagtatttaggagtagtcctagaggctggtttaca
ttcggtcatgctaatttcgctttaattttcttctttggacatctttggcatggttcaaga
actatcttccgggatgtatttgcgggaatcggagctgaagttactgaacaagttgaattt
ggagctttccaaaaattaggagatagaagtagtaaaaaacaaggagctgtataagaccaa
agtcaaagacagtatttgtc---aaatatttccaatattaaaatagttaagacaaagttt
attatctaatagttactaatcaggagaatttatggaagccttagtctatgtatttttact
aacaggaacgctgatggttatattctttgcgatctttttagagaacctccaagaatagc
aaagtaacttgattccgtttaaacttataataatagaaccactttccaacttattagtgg
aaagtggttctattgtagattacattgtattattcttcatgctcttcaaaaggatctcga
aggtcttttgaagcaggaccaaatgctgtgtatatcgagtaacctgtaatacctaaaagc
agactcgaaataaaaatactaagaactgttgcagtttccataattcaagcagtattaaga
tgaacttttctataatgatattatcaatattaaaacatttaattattaaaattatcaaa
atatactatggcacttagaactagacttggagaaattttaagacccttaaattcagagta
tggaaaagttgctccaggttggggcacaactcctattatgggaattttcatgttactatt
tttcttgttttattaattattttacaaatctataattcttcattagtattagaaaatgt
agatgtagattgggctactttaggtag----ctaattagataagttctttga--------
ttaaacaatattaaagataac----------------aagcgatcgctattagattatag
tagcgatcgcttaaaaaatgttttactgtatatccagagaaaattatttacctaaattaa
ttaactcgctatctgcaaaactatttgtattaacattattatagttaactttttcaaacc
ttactaatactggatatttgataccacttttatcaattgctgcaacagtaccaatttcct
gataccagtatgattcctttctcaatatttttacttttgagcctcgttccatatttttag
tccttagatagattattaaagattattatattgaagaattttaaaaaactagacaaaata
tataaaagtactaccgacaattgtaatacctaactaataaaaggagagagagagggattcga
accctcgataacaaaagttatgacaattttcgaaattgttgcaataaaccgctctgccat
ctctcctaaattaaaatactcttttagaaaattagacaagaataacatagaactaaggta
gttgcctagaaaattaaaaatatgttaaatataaattataatctttctatagatatattt
acaaaatatgtacatagaatctatatcagcttatttcaagatttttaatcttatacaat

ttttactaaataaataaattctctcataatatgaaactatcttggaaaacacttcttctt
tggtctttaccaatatttgtcgttggtttctttttctggcaaggtttttaggtccaact
actacagatgttggcagtaatatcgcaagttctagaatgacatatggacgatttttagaa
tatttagatatgggttgggtgaaacgggttgacctctatgaaaataatcatacagcaatt
gtagaagctgttggtccagaattaggaaatagagttcaacgaattcgagttgaactgcca
gcaagtgcgccagaattaattacaaaattacgcaaagccaatgttgatctagatgctcat
ccccctaaaagtacaagtgcagtatggggactattaggcaatttactatttcctttacta
ttagttggcggattagctttcttatttagaagatctaataatgctagtggtgggcctggt
caagcaatgtcatttggcaaatcgaaagctttgtttcaaatggaggctaaaacaggagtg
gtatttaatgatgtagctggagttgaagaggcaaaggaggaatttcaagaagtagtaaca
tttttaaaacagcctgaatcatttactgctgttggtgcaaaaataccaaaaggcgtttta
ttagttggtcctcctggcacaggcaaaacattactagcaaaagctattgctggcgaggca
agtgttccttttttagtatctcaggctcagaatttgtagaaatgtttgttggtgttggc
gcttctcgtgtgagagacctattcaagaaagcaaaagacaatgcgccttgtatcgttttt
attgatgaaattgatgctgttggtagacaaagaggaacaggcgttggaggtggtaatgat
gaaagggaacaaacattaaatcagctattaactgaaatggatggctttgaaggaaatact
ggtgttattgtaattgccgctactaacagagctgatattttggattctgcattattaaga
cctggaagatttgatagacaagtttctgtagatgtaccagattttaaaggcaggttagca
attcttgaagttcatgctaaaaataagaaaatggaacctaaagtatctttagaaactatt
gccagaagaactcccggcttttcaggagctgatttagctaacttactaaatgaggctgct
atcttaacggctcgacgaagaaaaaatgcaatgactatgtctgaaattgatacatcaatt
gatcgagtagtagctgggatggaaggcactcctttaattgatagtaaaagcaagagatta
attgcatatcacgaagtgggtcacgcaataataggcagtttattagagcatcatgatcct
gtgcaaaaagttacattaataccaagagggcaagcaagaggcttaacttggtttactcct
agtgatgatcaaagtctaatatcaagatcccaaatactagcccgtatcgtaggtgctctt
ggtggtagagctgcagaagaaatcattttcggcgacgcagaagttaccacgggtgcaagt
aatgatttgcagcaagtaacatcaatggccagacaaatggtcactcgatttggcatgtct
aaaattggacctttatctcttgaaagtcaaggaggagacccattttaggtagaggcatg
ggaggaggctcagaatattcagatgaagttgcaactaatattgataaacaagtaagggaa
attgtaagtgaatgctatgcgcaagctaaaaacattattatagataatcgagtagtgatg
gatagattagttgatttactaattgagaaagaaacaattgaaggcaatgaatttagagac
atcgttaaggaatatacagcaattcctgaaaaaaattactacatatcacaattttaa---
---------------atgagacgggactgacgggattcgaacccgcaacttccgccgtga
cagggcggtgctctaaccagttgaactacagtccc-aaaaagataaatacaaatagataa
tctcatagttagcttaaacttgtcaaattagaatattaagtatgaagagta---------
--------tttgtaatagtactcttcatatttaata-tagtagcgaaaatatttcttta

gaaggagagagagggattcgaaccctcggtacgaaattaatcgtacagcagattagcaat
ctgccgctttcgaccactcagccacctctccatga---tctatatataaaatatatcaaa
tatacttatatttaataggtgtgcataaatatttatggagctaagcggattcgaaccgc
tgaccctctcaatgccattgagatgctctaccaactgagctataaccc----ctttatga
ggatattaagaaatggctcaagcgggatttgaacctgcgaccttgggcttatgaatcccc
tgctctaaccactgagctactgagccatagtataaaatattattaattattataccataa
attatgctcaaatataaatcaataaaagaataattactatca-ttcactactttta----
-----------------tacaagttgtaaatattcggacaagatttcaaaattaaaaatg
tctttaactttttgtttga-------attatttgtttacgaaa-----------atattg
gtatttaaaattaac---
--------------------aataaattttg-----------------------------
---tctttattt
a---

-------tacaca---
-------------------------------caaaaatattggaaa--------------
------------------------ttttcatcaatt--------aattatactaattagt

------------------tagactataaccgacgtac-----------------------

---aagaattatttaacta---

---cgttaaat---

-------------------------------------tgtgatgcatt------------
-----gggcttgatcaaaacttctctaaaatcaacaaaatctagt---------------
---aattcaa
gtaaatcaaatact----------acagacgcttaaaaaaataata--------------
caattagaatcgtaaacatttagtttttga-ttatgttaaataaatttgattatcataga
tttaatatttaaaagtgttctattacttgta-----ttgagtaactgt-aatatttaatg

tcttctttttagataataatcataatataagctaaaaaatattttt---tattaaacaa
ctagcatagagccaataccttgatcagttaatatttctagcaagagagcatgatctattc
taccatctagaatatgcgctgaagctacaccctgagctaaagaacgaatacaacaattca
ctttaggaatcatgccaccagaaattacttctgtttgagttaaatctctagcttcctgta
tattcagatgactaattaacgttgaaggatctgaagaattacgtaaaataccaggcgtat
ctgtgagtagaattaatttttcggcattaagagcggctgctatttctcctgcgacagtat
cagcattaatattatacgattggccttgcttgtctgctgcaacactagctataacaggta
tataattattattaattaatatcttcagtaatttagtatcaatattttgtacttctccaa
caaagccaaggtttgcttttccatttggtcttggagtaataagtaatccatctttccccg
acaaacctacacttttaccaccttgtttattaatagttgcgacaagatctttgttgactc
ttccaactaaaaccatttccactatatccatagtaggttgatctgttactctaacgccgt
catcgaattttggtaatattttaaggcgatctaaccaaaaattaatttctggtcctccac
catgaactaaaatgggacgtagtcctataaaggacagaaacacaagatcactaatcactt
gatcttttgtttttgatttttcatagccgatcccccatattttataacaataattctgg
aagaaaattgctgaatataaggcagagcttcacttaagactcttactctttctgaattag
tcaacataaataaagtaggattatgatatataaaattatttgcacaacttgtgttaattt
aaaaagtaccagtaggtacttc-aaaacttgaaaaaatagacatgttactaaaaa-----
----ttaaaactataaggattataattatgaataaattttgggataatgttttaagattt
ccacgatttttagtcagcgtcattttaggattagttttgataattattagtcctttttc
gtgttattaaaaaaaccattgacaagttttttttcatcatatcattgacgggattaatc
acagttttagcgataataa-tacaaaaaatgctaaatatcgaatgttgttgagagaaata
aataaatttaattaaatttttgaacatatataatattatattatcatttaatatgataa
tct-attttcttttccat---aaaacttataagtattaaatattatttgataaatattat
gccactaaaacaaatagttagttctgaaaaaactggtgcctttgcacttctggatagtat
tgttaggcacggagttaaacatatatttggttatccaggtggagccattcttcctatcta
tgatgagctttacgcttgggaggaagcctatctaattaaacacattcttgttcgccatga
gcaaggcgctgctcatgctgcagattcttattctaggtcaacgggagaggttggagtatg
ctttgccacttctggtccaggggcaactaatcttgtctcgggtatagctacagcacacat
tgattctgtacctatattagctataacaggtcaagttggaagagcttttattggcacaga
tgcttttcaagaagtagatatttttgggattacacttcctattgtgaaacattcatatgt
agtccgtgatcctagagacctgtctagaattgttgcggaagcatttttatttgtaaaca
tggtagaccaggtccagtattaattgatgttcctaaagatgtaggattagagaagtttaa
ttatttttctgttgaacccggaaaggttaatattcctggctgtaggccaattaccagcct
aaagtcaagacaaatccttatggcagctaaaatgatacagcaatccagccaaccattgtt
gtatattggtggaggagctataatctctgattcacatcaaattattaaagaacttgttga
tttctataaaatacctgttactactactttgatggggaaggggattttaatgaggacag

tgactattgtctaggaatgttaggtatgcatggtactgcgtatgctaattttgcagttag
tgagtgtgatcttttaattgctttaggagctagatttgatgatagagttactggaaaatt
agatgaatttgcttgtaatgcccaagtgattcacgtagatattgatcctgctgaagtagg
aaaaaataggattcctcaagtcgctattgtcggtgacgtagcagaagttgttagtgcaat
attaaatttattaaagacttcttttccccttatccagatcagattacatcttggcaaga
aagaattaatcgttggcgtcaacagtatcctttactggttcctagaaaatcaacaagcat
ttcacctcaagagattcttgttgcaactaataaattagcccaaaatgcttattttactac
agatgttggccagcatcaaatgtggtcagctcaatttctgaaagtaaaagctaagcattg
gctttcaagtgctggattaggcacgatgggttatggtttacctgcagcaattggtgctca
agtagcacatcctaatgacgtagttatttgtattagtggtgattctagttttcaaatgaa
tatgcaagagttaggaactatcgcgcaataccagctaccggttaaaattattattattaa
taatcgatggcaagggatggttagacaatggcaacaggctttctatggtgaaagatattc
acactcaagaatgacagaaggagcacctgattttcaaaagcttgcagaagcttttggtat
taaagcttttactattaataataggcaaaatatgcaatctgctttacaagctgctattga
tcatcctggtccagttttattagattgccaagttacagaaaatgaaaactgttatccaat
ggttgctcctggaaaaagtaatgcacaaatgataggtatagctaaaccgcagagaggaac
tgcttccaactatattaataacagcgttggaagtaa------------------------

---------------atagttagtattttctt-aaaacagaata-aagtaaatgaagtat
ctaaatgtaattttcttatactatta-aaaactatgtaagaaaagcctttgacgagaatt
gaactcgtgaccttccccttaccaaggggatgctctacctctgagccacaaaggc-tttt
tactatattgggccgggttggatttgaaccaacgtaggcgaagccagcggatttacagtc
cgcccccattaaccactcgggcaccgaccctagttaaaccttatttaagctatcatatac
aagatatcataattatgtttctaaaaacaagtacttttataaataaaactgaataagtaa
tggacatagctggacttgaaccagcgactttcacgatgtcgacgtgacactctaaccaac
tgagttacatgtccaaaactttaatcattattataacactgtttagtgattttataagta
tttatttctaattgaagatttgttttaatcggccggctttaccgagcagatctcttagat
aatacagtttagctctacgtattttagctcttctaataactctgacaaaagttattcgtg
gtgaattaagaaaaaatactctttcaactccaataccttgaaatgagcatcttaaagtta
aactagtatttaagcttttttttcttcttctggctaatactactccttcacataattgtt
cacgagttttactaccttccttaaccattagtcccagttgtattgtatcgccaactttaa
tttcagatacttctgttttgacaagaggtatctcaacacttttcattagctgacttagct
ttgtattatttgctttcatatataggattagtcttgtgaca-tgttttgaaatattatac
attaaaaaaacaaatatgaaaaattttagcgaataagcatattagtatttataaacttt

tttttattt---
--cgattata--------
----------------catatccatt------------atattcaact------------

----------acattcttccatagtttc--------------------------------

--------tttgataatcagtaa-------------------------------------

---------------------------atcta----------------------------

----gatgatcacatctcattttcaaacatggcttatgtatgaaaaagttctata---aa
aatctaatgactaaaataataaaattaaggcttaagtatttcttagttttgtgttataat
tagttactatcaaaggtagtggcataacacgaagcataaa-aatgttcgaacgctttact
gaaaaagctataaaagtcataatgctagcacaggaagaagctagacgcttaggacataat
tttgtcggaactgagcagatattattaggattagttggcgaaggtactggaatcgcagct
caagtcttgaaatcgatgaatgtgaatctaaaagatgcaagagttgaagttgaaaaaatt
ataggaagagggtctggctttgtagcggttgaaattcctttcactcctcgagcaaaaaga
gtactagaattatccttagaggaagcacgccaactaggccataattatattggtacagaa
cacttgctaatgggcttagtccgagaaggagaaggagttgcggcaagagttttagaaaat

ttggcagttgatgtttcttcaattagagctgaagttatacaaatgcttggagaaaatgcg
gaagccaatgtaagtggaagcaatactacgcaggctagaagtaaaacaccaacactagaa
gagtttggatctaacttaactcaaatggctattgaaggtggtttagatcctgtagtcgga
agacaaaaggaaatagaacgagttatccaaatcttaggtagaagaactaaaaataatccc
gtcttaattggggagcctggtgtaggtaagacagcgattgcggaaggattagctcaaaga
attgctaatagagacgtaccttctatttagaagataaattagttattactcttgatgtt
ggtttattagtagctggaactaaatatagaggtgaatttgaagaaagactaaaacgtatt
atggatgagattaaatcagctgataatgtaatattagtgattgatgaagttcatacattg
attggagctggtgctgcagaaggagcaatagatgccgctaatctgcttaagccagcttta
gcaaggggagaattgcaatgtataggtgcaacaactttagaagaatatagaaaacatata
gaaaaagatccagcattagaaagaagatttcaaccagttgtagttggagagccaagtgtt
gaagaaacaattgaaattttatttggtcttagagaccgttatgaaaagcaccatcaatta
acaatgtcagatggagctttggctgcagctgctaaatatgctaatcagtatatttctgac
cgctttttgccagataaagcaattgacttaattgatgaggctggttctagagtccgttta
ctaaattctcaattacctccggctgctagagaattagataaagagttaagagctgtatta
aaaacaaaagatgaagctattagagctcaaaaatatgaaacagcagagcagtatagagca
agagaaatggaaattaaagctcaaattgcagcaattgctcaaagtaaaaagaatgagcct
gatttaaatttagaagatcctgttgttacagaagatgatattgctgaaattgttgctgca
tggactggtataccagtaactaagcttactaaaagtgagtcagaaaaattaatgcacatg
gaagaaactttgcatggacgcattattggtcaagatgaagcggttgtagctgtctctaga
gcaataagacgcgcaagagtaggtctaaaaaatcctaacagaccaattgcaagctttatt
ttttccggaccgacgggtgttggaaaaacagaattaacaaaagctttagcttcttatttc
ttcggttcagaagcttctatgatacggctagatatgtcagaatacatggaaagacacact
gtatctaaactaattggttctcccccaggatatgtaggttatagtgaaggtggttatcta
acagaagcggtaagaaaaaaaccatatactgttattttatttgacgaaattgaaaaagct
catccggatattttaatctacttcttcaaattttagaagatggccgactaacagatgca
aaaggtagaactattgatttcaagaatactcttttgattatgacttctaacattggatct
aaagttattgaaaaaggaggaggaggtttaggctttgaattatcagaagatcaaacagaa
tcccaatatactagagtacgatctttagtaaatgaagaattgaaacaatactttagacca
gagtttctaaatcgattagatgaaattattgtatttcgtcagcttactaaagatgaagtt
agagaaattgcagaattaatgctcaatgaagtctttgcgagaattaagcagcaagatatt
caattaaatgtaacagaacgatttaagcaacgattagtagaagaaggatataacccaagc
tatggagctagaccacttagacgagcagtaatgaggcttttagaagatagtttggctgaa
gaagttttatctggtaaaattaaagctggtgacagtgcagtagtagatgttactaatgaa
ggtgaagttacagttttattaggtgaaaaattagaactgttaacataaa------aaaca
atttattaat--taaagcattgaagaggttat--attcaatgctat---gaaaaaaaata

ataagatgggttgctataattgacaacctctgatactctgtatttgtatata---aagcc
gggatagctcagttggtagagcagtggattgaaaatcctcgtgtcaccagttcaaatctg
gttcttggcat-tttaagcattagaaaattttgctatctgagcattaaattttaattgaa
atgtccctgtgggaccattcctatgttttgctattataacttctgtgaagtctctacttg
tagtttcttgtgtatagtaactttctctatatagcattattactaaatctgcatcttgtt
ctatagaattatgaacaataaaattatttgaaataaaattgcacaaaggtttacattcta
aatcaaacactatttgtaaacttataatgtttatttttttgtatattttcaaaagtcacag
atgaaa------aagaatgaaaaatatttttttgttcttcaaagttttt------tattt
gaatagcaatcatatcattttgattaatttcatcacatcttttccacccttgtgttgtga
atagtttatggttacttgttaattgtatatatttgccagctccagtaataatttttatata
cagtttttttttcctgtttttgaaatattagatttaattgctagaaataactgtagtttgt
gcgcagtaaagttaacgacttca-atcttttataatagcaatcaaataatatttg--ag
gttgattatataatgatgtctgaagataattgaatttagatatacacccactttctctta
aatcggataacaaaggccttttgtcacttcgagtttcaaggtttctatttagttgagata
atactaagataggtaagcttaaatcttttgctaatattttaagtgatcgagtaattaatg
atagttcttgagatctattgtcagattttctactctcctgtagtaattgaagataatcta
taataattaattctatatttttttccttgcaatttaaagaatttcacttttgtctttatag
tttcagtagaaatcttagcgctatcatcaatataaaggttaaggttagcaagatttttac
ttttttgaataacatattgccagtcatcattattaagctgaccagattgaatttttttggc
cgtttaaattacactcttgagctaaaattcttcttagtagttgctctgtcgacatctcca
agctaaataaaataacataagatttttttctgattgatgacatatctagttatattaatag
caaaagcagttttccccattgaaggtcttcctgcaaggataattaaatctgacttttgaa
acccttgtgttatagagtcgagttctgtaaaaccagaaagtatactattatttatactaa
tctttttcctggtatccaaatggactaatagacttgcaagaattgtagctaatgtttgcg
tatctttttcgtcaagtatttcataagcttctgttaattgactaacaatagaagctatat
ttttttgttcaataagttgttttgagcagctaatcaaacataatgaatctccacatgcta
aaagtaatcttttaatgtagttatctataacgaccgctgaatattcgcttatagcatcag
ataggggagcttgttctattagactaaaaacttcatttagctcatccaagtcttgaatca
ttttttcatttttttaagttaatgaaaaaaatttcttacatttttttgtctttgcatggttaa
tattttcgagtgctgctctataaagtagtgaagttgatgtaaaataaaaaaaaatcaggag
agatttttttctactgattttagtaatgatatcgattgctgagttaatattatactaatta
atattttttcagctaaaatattatgaggaggtagatatttataa------atacttatta
attcctcc-ttgtaacatcactcttatggtattagtgtattagttaactattgatttaat
tggattctggaagaacttgcaatttaatatttgcagttacttgatgcaatagcttaattt
ctacatcgtaaatgccaattgttttcacctctggtaaagagatactttgtttttcaatgt
ctatgttggtagtgtttttaataatttgtgaaatttctttttctgtgacactaccaaaaa

tattttggccatctcctgtttttttactaacgctaaactttgtatttcttctagaagtt
gcttaaccttttagcatcttctttagctatttttaatttttcttctcttatagctgcat
gaaatctttgttgttttaaaataccatttgtggcaactaccgccattttattcgggatta
aaaaatttcttgcataaccagaagctactttgataaggtcattactcttgccaagctttt
gaatattctcttttagcacaacatttacaactttttactcatctt-------aattcta
aaaa----atctatattatcctattatacctgttgtatacagatttt----aataggaat
tatttatttgttgagataatattcttgtaaaaacaaaggtgatttttttattttt-ttctg
atataattattttcacaataattc-----attaggagaggtggccgagtggttgaaggcg
cagcattggaaatgctgtttaggagcaatcttaacgagggttcgaatccctctctctccg
tgcaa--aaaatataaaaagttttatagagtccaatctaaatctccaataacattcttcc
aagcatttaatccaccccgaactctaacaatatttagtttttgagctattaaaaattcag
aagcaaatattgatcgagaatctaagctacagtaaacaaaacaaatttatcttgtaaat
tgatattagagtaattcatttcttttatttttttgtaaaggtagatttaatgaatgtatta
aatgacttttatgatactcttcatgatttcttacatctattaaaatatattgtggattcc
tactaattaaaaacctttgtaattgaataacactaatttctcgtacaaatgtactaaatt
tactgccataatattcgttccaatgctttttttgt-------ggataatacaaatttagta
tttataatcttaaatttcttaaatgatgaagttaatgcgttgtacgttaatatagtccca
cttaatatagacttataacccagaacgattttgacagcttcagtggcttgaagtgtacca
ataatacctggtaatagacctaaaactccggaattactacaagtatctcttgcactctct
ttattttcaatttcactgtagaagtctcgataaacaggtccaccttgataattaaataca
ctaacttgtccttcaaattgaaaaatggcaccataaatatgtattttatttaactcaagg
caagtattattcaagacatatcgagtatcgaagttatcagatccatctataataatatca
tattctctaataatgtcgattgaattctcgtagcttagtcttgtgttgaacacagttact
atacactgtggatttaaatctaata-tttttttttagctatttctactttgaatatcct
atatcatgcatcgtgtataaaatttgcctctgtaaattagaaagatcaactatgtcatca
tccataatgccaatgctcccaattcccgcagcagcaaggtaaataattcctggagatcct
agcccaccagcaccaatgaataaaacttttgctttttttaatctttcttgtccttctaat
tgaatttgtggcaatactaaatgcttagaatatcttgtatattcctctaaggagtatttt
gtattgtctgttttaaaatttagcataaaaatttatagtttatttaatagtactta----
--aaagctagctgataagtttattaaaatgacttttgtaaacttggttcttctctatcta
aaatagctccttgtgtaggacagacctgaacacaaatactacaatcaatacaggcagaaa
agtcaatccaataccaattagtattactattgtttttcccctttccttgatgaatacaag
ctactgggcaagcttccacgcattcagcaactccaatacatttttctgttacaattgtat
gagacatattatttaattaat-aaaagataagctatttacatctagtagttttgttgtat
actaaatttacatatgctatccagtccaatatttaaggtagtctatttttacagtttact
aatttgatatgcgcctttagttcagttggtagaacgcaggtctccaaaacctgatgtcga

gggttcaagtccttcagggcgcgtttagtttttatcgtaattcaataatatcattttatc
tttttgcaaacatcaaaatgatatgatataaaagatcatattttatacattgaatattct
atgtactagtactg-ttttttaatatgttattattgataagttaattatactaattctat
ttttgtaataagt-aaaaacagttctaattaagtatgaacgtatttaatagtc-ttttgt
atatac--aacttaaaaaattttaaactatggctaaaaaagttactggaattgttaaact
agcattaaatgcaggtaaagctactcctgcgccacctgtcggacctgctttaggacagca
tggcgtgaatattgttatgttctgcaaagagtacaatgcacgtaccgctgagaagtcagg
actagtgattcctgtagagatctcaatatatgaagatagaagttttacttttatacttaa
aactcctcctgcttctgtactaattgctaaagctgctggactaaataaaggttcaggtga
accaaataccaaaaaagtaggcagcataacaaataaacagttagaatctattgcagaaac
aaaactgcccgatttgaacactaacaatatacctcaagctatgaaaatagttggaggtac
ggctaaaaatatgggaattctgattaaagatt-aaaagtttcaattatatttatta-ta-
-tttatgaaaaaaatttcacgtcgacttaaaacattaaaatcaaaagttgagcctaaact
ttacactattaatgaagcagtatctatattaaaagaaacatcaaatgctaaatttaagga
aactgcagaagctcatattgctctaggtttaaatcctaaatatgcagaccagcaactcag
agcaacagttatattacctaaaggcacaggcaaattgataaaagtagcagttattactaa
gggagaaaaattaacagaagcaattagtgcgggagctgatgttagtggttccgaagaact
aattgatgaaatactgaaaggtagattagattttgataagctaatagcaacaccagatgt
tatgcctttaatagcaaagttgggaagagtattaggacctcgagggttaatgccttctcc
taaagcaggcacggtaacattgaacgtagcaaaagccgtaaatgaatttaaaggggggtaa
agttgaatacagagttgatagaacaggtataattcatgtgccttttggaaaatctagttt
ttcacaagaagatctagttttgaacctccaaacaattaaagaatcgattgatagaaataa
gccttctggagcaaaagggaagtactggaaaactttttttcttatctagcaccatggggcc
atctgttcaaatcgatatcactagccttctatgaaaaatttttgtatagttatagaactt
gaataatctattatttcaaatta-taatttgctagcattattaacgtaataatttgctta
ctatatcttatctgcattaaaaacttt-ttatatatcttattaattatgagtacaaaggt
tgaaaatatcttggaagagttaaaatctttaaaccttctagaggctgctgaactagttaa
acaaatagaagaaacgtttgatgttgatgcatctgcggcttcaggaggtatgatgatggc
cgcaccaacttcagcaccagcatctgctgaggttgaagagaagacagaatttgatgttgt
cctagaggaagtcccagcccctaaaaagatttctgtattaaaagttgttcgctctctaac
tggtttgggtttaaaagaagcaaaagatcttgttgaatctacgccaaaagtcttaaaaga
agctgcatctaaagacgatgcagaaactatgaaaaaacagctagaagatgctggtgcgac
agttattgttaagtaataa-----agaggcgcttctgtttatacttaaacagaagcgcct
ctttatttattataggcaattatactagaaaagtccaagtgtaattgcattgttgatagg
catagttgcaccaatacctagccaaatacttacaactgtaccaattaaaaaaacagtggt
tgcaattggtcttctaaatggattttgaaatttgttaacattttcaatgaatggaacagt

aagtaaaccagctggtacagcagccatacttagaacacctaatagtttattaggaattac
tcttaatagattaaatgttggaaagaagtaccattctggtaaaatctctaatggagttgc
aaatggattcgacttctcacctatagaagatggttctaaaattgcgagtccaatactaca
ggcaaaagtgccaataattacaactggaaagacatataatagatcatttggccaagctgg
ctctccgtaataattatggcccattccttttgctaatttagctctcagctttggatctgt
taaatctggtttttaagaattgacatatta-tttttttgtttaatataaagattagtct
aatcactgttttaaagcgtaattttcaactattataatggtccagaaattccttgtttac
gtatcattaaaaaatgcataagcatgaaaacggcagttagtagcggaagtacaaaagtgt
gtaaactatagaacctagttaaagttccttggcctacactaactcctcctcttaacagtt
ctactatactcgctccaacaactggaacagcatcaggaacaccagtaacaatttttacag
cccagtatccgatttgatcccacggcaaagaataacctgttaccccaaaagaaacagtaa
gcactcctaaaattacacctgttacccatgtcaattctctaggtttttttgaaacctcctg
ttaaataaacccggaatacatgtagaatcatcatcaagaccatcatgctcgctgaccatc
tatgaattgatctaataagccatccaaagtttacatcagtcatgatgtactctactgatg
taaaagcttctgcaactgtaggtctataatagaatgtcattgcaaatccagttgcaactt
gaattagaaaagatacaaatacaatacccccctaagcaataaaaaatattaacatgaggtg
gtacatacttactagaaatatcgtcagcaattgcttgaatttctaatctctcttcaaacc
agtcataaattttactcataaaatagcttcaaaaagctcttctaatata--ttttgcgtt
taagctactattaattcaatag------ttattgaacttatattatttttataatcaata
cctattaaatattataacatctttttattgattt--------------------aaaatt
ctctgactcagtaaaacagggctatgcactataattcttttttttttgtatagagattaat
ttcgtttttagtaatttagctaaaattcttgttacactgactctattactacctataatc
tgtgctaaaattttatgtgtaatagtaaagttaagcataataccattattaacttgagtt
ccattttgctcagataataataataataagctcacaagtctactggttatacttttatgt
gaaataacttctataaaatgataagcttttgccgagcaaaatattaaatgatttacaaaa
aatagattgaatggagcgcagttactacatgcttttataatagtagtatagttaatacag
gcaacttctgttttatctattgcttctgcttcgtagtaaaaattatcgtcgaccaattct
atttgtccaaatgtatttcctgtagttaataagttaagagttactttatgtgtatttctc
aggacttttgtaataattaaagatcctatcagtataatgtataatctagagctgctatta
aacaaaagagtatcattttgtctaagtgaaaaaatttgataattaatttttattacgatta
aaaaatagtagccatggattgcaagaaaaaacatcttctgaactactttcagagaatatt
ggaacaaaattttcttgatttaaacaatttttagtcataattactactttataa------
---aaaaatattctaataat-taagaactgaaattaaatgttatataaattgatgttagt
tgagaatgacattgcattatcaaaggccattcaagaatacttaatagatcaaggcttcaa
tgtttatattgccaataatggattagaagcactgaatctagcttatcaatataatttcaa
tttgataatttctgatataatgatgcccctagttaatggctatgaactattagcggaact

tcaaaaaaacaaagcgttatccaaaatacctgttattttttgacagctaaaggaatgac
gaaagacagaataaaaggttacgacatagggtgctatggttatttatctaaaccatttga
ccctaatgagttactctcaattattaacaacttaatcgctagagatgccttaaaagaagc
ttctttacaaaactcggaaatatcaaaccaggaattaaatcataaaatacgtttgacgcc
tagagaaaaaagtattttagatcttgttgttgatggacttactaataaagaaatatctac
tatattgaatactagtgttagaaatgtagaaaaatatgttagtcgactcttacacaaaac
taatatgaagaataggactttgctagttaagtattctataaataataatttattaaataa
taagatcaatgaaagggcgaatgacgggactcgaacccgcgaatgatggagccacaaccc
attgccttaaccccttggccacactcgccatattacatatatcactatagctcgtttttt
gatatatcgtctagttttcgagttatatatttatataaaacaatatttactttgaatttt
-tatgatacatagcaatattagtattcaaattaatggagaaccatttaattgctcaaagc
ccatctcattgcaattttttattaaattatcttgactttaattccgagcgtgtagcagtcg
agctaaatgacattctgttaccagagactctgtttcactcaacttatttgaatgatcaag
ataagctagaggttattaccattgtaggtggtggataatctatttttacat---------
---agttctctatttcaaatgtttcatagatagagataatcttccttgtttttatctac
atgaataataacagctttaatagtatcacctattttaaattgtgatgatatctgctctag
ttctttgatattaatctcagaaatatggacaagacctttgagatttcctacctttataaa
taacccgtaaggtgtaatctgatttattatgccttcaataatattaccaacaattaaatt
agatgatgcttgagatattagagctcttctatgactgagtattaaattattagatttctc
ttcaacattgagtaattttagcttaataaatttattattaaattgctcactttttttgaaa
attaccaagatgagaattaggtacaaatccagatataccttcaaggttaattatcattcc
tcctttattgaatccttttatcattacattaagtaaagagtcttcagctaatagttgcct
aattcttttccatgctcttatatattcaaggcgacgaattgataagattaattgtttcga
ctgaatattataatctaataaaaaaaattctcttgtatcattaatatttaaagaagtgaa
attatttaaatcttgattactagatacttcttgaataggcaagtatgcagatattggtgt
tcctatatctactaataccccatttaattcaaagctaaatatagttccagctacaatatc
tccaagattcaaatcatacttgtatttttgtaaaacagctgcaaaatttctatgagtaaa
tccgtcattatttttttgtcatattgaatagtatgaattaaagagtaactttgtacaatat
attaaggtaaacaaatgtttgtcgaattgtatg-aagaattttaatttattcggttgtaa
ttaaacatataaacatattaaaatatttaggagacataaaaagtagacgtagatagctgc
agatttaa-taatatataatctgaggaaagtccgggctccacaaatacaatttatgctgg
agaaatcccagtgtaggtaactgcgaggatagtaccacagaaaaaaaccgccagaataag
t----tatattagctggtaagggtgcaaaggtaagttaaaagcttaccaaaagtactgca
aagtatttgttaggtaaaccccctaaaatggagcaaagctactaaacaaatatttgtgtat
attttatctagtttagttgaaaatactgcatgaagttattagtaacaataactcgagagg
aatagctatcctttacaatatatc-tctattatattgaaagaacagaacccggcttatgt

aatacttattatgtcttgtattattatgtctattgtcgaatctctaaatttaactgtttg
acaatttttttctgtagctgatgttgtttataatctatttctgaattggttaaagttttc
tcattagatcgatatgtaaatcttagacctatactttttttcccttttccaattgattta
tctatatattgatcaaataatgttgtactttctaaatctttatcataaaattgatccaat
gattttaataagtatttaatttccatattcttaggtattattaaagagagatctcttatg
atcgatggatacttagagtatggtttaatctgataatttaaataattgaattcattatga
caatcttctaaaatgtttaagttaacttcaagaacaaaaagttttgtgttaagtcctaat
tcactatattttgcttgattaagttcactgaatatgccaataacgttattattatagatt
agtgtggcaaatttccctttttgaatgaactttattctaccatcgactatattttttttt
gtccactcaatttgtctattaatcttttgaaaaaaattttctacgatccctttagcttca
taccaatttaaagaatgcgcaggatgtgaccatgtagatcggatatctagattaccaccc
aatatgatagctagattagtagtttccataattttgttttgatcattatgaaatactgtt
cctatctcaaagctatctatagtttggttgctttttttaatattataaagatttgattca
attaatccttccagcaaactgctgcgtaaattagaataatcttgaataagaggattgttt
aaagttatatttcctttggactttattaaagaataatgaactaattcagttagtcctaga
tttcttaaaatacttctactttatctatgaattgtctctttgtcgataactttttgttg
aattgaatatttggtactgagctcctgaatttatgataaccataaattctggcaatttct
tcaataatatctatttctcgaaaaacatcttgtcgtctataatttggaacagtaacttct
atattttccttattttgataaataagatcaaaatttaaagagtggagaatattttgaatt
tcttcaaagaataaaaaacgtgtttgaccattatatataattggtcctaaaatgtcatga
actttttaattgataaattaatattaagagcattatcagttgtttttctcgatagaaa
gtctctctaacatttccgcatgttaaatctgtgattaaagaaagagcttcaaaatgtgca
tttttccaattatctacatttagcccacgttcttgtctaattgaactttctgttcgaata
ttaagaactcgagaagactttctaaccacagattgtttaaatatagcagactctacaagt
atcgatgttgtattataatctgtatcaagattagagttgcttccaataccagctatacta
attatatttgtatttacttgagtaaccaaaatatttttgtttaacccaatattttcattg
tttaacttaatctgatcattattttcaataggaaaatcgcttcgaattgttagactattt
tcatgattcatattattaattttattcaaatcaacaacattaatgggctgtccccatttc
aacataatataattgcttatatctgttaacaaatttctgtgtgtaaacccagaagagagc
aatcgatttttttagccaatctggagaatctttaattcttatttcgtctataatggcagca
aaataattgtcgcaatttaataaattgtgatcacttattatagt-ctccttttttagagaa
atatctattattgggttgatatatagctgataacattgaggaccctgttaaagccgagac
ttctcttgatagaccaatcatgcttaacacgtctgacctgttagctgttgatgatatatc
taatacatgatcaatctcacctccaattgtaatagattcaatagcttcaacttcaaaacc
tgcttgtgttaattgattagttaaattatctatatctattgttttgatattaacaagttc
tttcaaccaatttaaagaactttcataattaattgtcatctatcttatagaagtattta

```
ttttacttatgattaaaaatcaatttaaaaaattcatcaaattaatataagttatttgat
gaatttgcttatacaatatattattgtgatatcttacttttatatggcacttattaagaa
ttagcttttgtaaaagttagcccattatcttgggcatatctctccataaatctcatgaat
ctatcccaatcatcaggacttttaataacatggacagcttctattgcttgtggcttacca
ttaataaacttggcattaacatctcttgtaataatttgtccctctgtgtccatcagatac
atacctgtgatttcacctttatctgccatactagcatctaaaattttgggatttgtaaac
cgaaaagttgctgttcctgtactaccatctcgagaccttgttaaacgtacatctggtaca
acttcttcattgataccttgaataaattgaattgttgccatatcaatcttctttatttat
atataatttatattggataaaactgatttctagttcttgaatgcttgttgagaaactgtt
ttaaaagcttatagtagcagtagatcaaattttatgtattatataaaactgtaacttttc
-ttttttg----------------------------------------------------
------------------------------------------------------------
------------------------------------------------------------
------------------------------------------------------------
------------------------------------------------------------
----------------------atctgttcaaagaattaacttattataagctaatcata
cgaatattagccatataatgtttgtaagt-gaaattaaggggttgtagctcagaaggata
gagcaagcgcctcctaagcgctaggtcagcggttcaagtccgctcaaccctgttaacaag
ttaagtagtattatttaacttgttctgcaaatcagtattgatttaagttaaagttcttga
tagctaaaacaaaagaatctgagaaatatttttattta----------aattatcttaac
tctgaatatctgataattattaa-aaagttct----------------------------
----ctttttaagacacaaggtgatg-----aagatatttaaactaataatggaaattaa
gtttttgaagttatagaagccaat--ataatactttctattaacaga-----------cg
cttgcttctattcctatttgtttacaaga--aatt----atgaatatcaaaata------
--attttagctcca-------------------------------------ctgat----
------------------------------------------------------------
-----------------------------tactaataaata-------------------
------------------tttatttcagtctagacaag------atgaatcttcatggta
taagag------------------------------------------------------
------------------------------------------------------------
------------------------------------------------------------
------------------------------------------------------------
------------------------------------------------------------
--------------agctattctctgtgtcatacttatataaaatcaggat-------ac
tgtt--------------------------------------------------------
------------------------------------------------------------
```


---------------gtcattattataactggggtgggaggattcgaacctgcgaatggc
ggagtcaaagtccgctgccttaccacttggctacaccccaacatgtgaatattataatag
cagtcgtataccctgccatgtcaatagactgttgtctttatttaataatttttgctgctc
ttgtagaataaaatcttttttttcaaataagatacgctatgtttaagttgaacgactgtt
atgttttcttgcttttgactaattagccacttgatagtaattattccttctgcaatttcg
ttacttcctataataatacaagctacagctctttttttatttgcttgcttaatctgttta
ctaaaattacttgagctaacatctatttctattgtgaaaaactgctgatgtaaaaaacgc
attacttgcataccagtttcgttagctttttcaccttgtgtagcaatataaaaatcgata
gatttattaggcaagtttatattatcctttgcaagcagtaataggcgttctaatcctatt
gcacatccaacagctggtgttgctttgccacctaattggtgaactaagctgtcatatctt
ccaccaccgcatatagtatcttgcccttttgatgtcaatgtcttaatttcgaaagcagta
tcattataataatctaatccgcgaactaacttattatttatattgtagggaatatcaagt
aatttagataattgcaaacgtcatcaaagtgtttttgtgattccaaacttaaaaaatca
gaaattttggagcctctgttagtattttttgtgtattagcatcttttgaatctaaaatt
ctaattggattactagttaatctttttttgtgagtcagtgtctaaatcatcatggtacttt
gtaagataatctagtaatttaacttgataaatactacgatcctctacttttccaattgaa
ttcagatcaagttttaagttatgcagattgagattgttaaagatgctcatagctaaatgt
ataacttcactatctgctcttgcgtctaggctaccaataaactcaatgccaagttggtga
aattgtcgttgtcgtccactttgcggtctttcgtacctgaacattggaccgctataccat
aatctttgtaaactatgttggtaactcatttatttcaataaaagatctcacaatcccg
gcagtaccttctggccttaaggtaatatctctattgctgcgatcatgaaaccggtacatt
tctttatttacaatatcagtattttcaccaatactcctatcatataattcactattttca
aaaataggtgttctaatttcttggtaatttgcacattctaataatttggaaattttatta
tgtataaactgccaatattgaatttcatccggaagaatatcttttgtccctctaatagcc
tgaatttttgccatgataatttttttgattaccttcttaatatttctgcattaaatagcag
ttgtagactggtattcagtcatgtattatacattag--aacatactggtg-tattttata
aaatacgggcaaggagggattcgaacccccgacaccatggttcgtagccatgtgctctaa
tccactgagctacaagcccacttaataagtacgttaactatatcattattctctaatgta
tacaactatattctttt------atcttacatttgaaattttttgtaaaagtgaaaaatt
at----gtttgtttttttacaattggaactattcatatataatgcttaatttattataact
ttgtaaa--aatatctataagtttaattgtatgattctactatatt-gtaatcttgtcat
aattttgcgctcttatttttcaattag------ctagcatctaaataacttatatttcaa

ataaaaaaatcttatctaaaatgagtaaacaaattctatatcaggatgatgcaagaaaag
cactagaaaaaggcatggatattttaacagaagctgtttctgtgactttaggaccaaaag
gaagaaatgttgtcttggaaaaaaaatttggtgcccctcaaattattaatgatggtgtaa
cgattgcaaaagaaattagtttagaaaatcatattgaaaatactggagtcgcactgatta
gacaagcagcatctaaaacaaatgatgtagccggtgatggtacaacaacagctacagtac
ttgcttcagcaattgttaagcaaggcatgagaaatgttgcagcaggttcaaatcctatgg
ctattaaaaaaggtattgaaaaagcaacaaattttgttgtcagtaaaatagctgaatatg
ctaagccagtagaagatacaaaagctattatacaagttgcttctttgtcatcaggaaatg
atatagaggtaggtaaaatgatagcgaatgctatagaaaaagttggaagagaaggagtta
tttctttagaagaaggaaaatcaactaatacgattcttgagatcacagaaggaatgcagt
ttgaaaaaggctttatttctccttatttcgttacagatacagaacgaatggaagttcttc
aagaaaaccctttttatttatttacagacaaaaaaattactttggtacagcaagaacttg
ttccattgcttgagcaaattgcaaaaacgtctcggcctttattaataatagctgaagaca
tcgagaaagaagcattagccacaattgtagttaataaattaagagggatttgaatgttg
tagcggttagagctcctggatttggtgatagaagaaaatctttacttgaagatatgagta
tcttaacgaatggacaagtaattactgaagatgcaggtttatcacttgatacagttcaat
tagatatgttaggaaaagcccgaagagttattgttactaaagactcaacaacaataattg
cagatggtcatgaaattaaagttaaatcaagatgtgagcaaattaagcggcaaatagaga
cgagtgactctttatatgaaagagaaaaattgcaagaacgattagcaaagctttctggag
gagttgccgtcattaaagttggtgcagctacagaaacagagatgaaagataaaaaactaa
gactagaagatgcaattaatgcaacaaaagcagcaattgaagaaggtattgtaccaggag
gaggagctactaatgtccatatctctagtgaattatttacatgggctaaaaacaatttaa
ctgaagatgaattaattggtgctttaatagttgaacgagctgtgacctatcctttaagac
gaatagcttttaatgcaggtgacaatggagcagtaatagtagaaaaagttaaaagtcatg
atttccatataggctacgatgcagcaaacagtaatattgtaaatatgtatgatagaggta
ttatagatccagcgaaagtagctagatcagctttgcaaaacgcagcttctattgcagcaa
tggttttaactactgaatgtattgtagttgataaagttgatgattgatttata------a
gataatagataatctcaaacaaagaataatttctaattatagaaattattctttgtttca
attaagctaattaattgatcaaatgtataatttttataataaaaccaaagtttaaacaca
tcttctttttcaactattaagtagagaagatataagcccgtaatacctaaattatcaatt
aaaatattattttcatatatagatttttctaaagatctaaaaaaatgacatttttgaaaaa
taataccgaaatcttcttttgtagttatttgctaactcattactgcctaaagattgattt
ccaattgaattcagaacctttaacagaacacttttttaaatctttatctgactttatttta
gaatcaataataatttgattcaattgcatgtcaaaactaagattaaaaatactacttgtt
aaagatatttgattttgaatatctagagattttatagccaagaagattaaacttttattg
ttcattgtaaaatataagacaattaatgatgtattctttaaatacta-aatttatcttct

tgtgcttctatcgtttttttgt-aaaataattaatgctttaatttttttagttttagttact
acccgaaaaaaataaattctggaaatctagcttgagctttagctagagagtgtttttttcc
ttcttcttgccagtaattgataacttctgttgttttgtttaataactcaattctatctct
ttcagcaatccacggcttagattctagttctgcttttaattcttcccaagcatcatttct
tggccagaaaaaaataagaagtaagtggactagtgccattcccaacaatttgatcaatagc
aatagcaatattattttctaaccaaagtactttagtgtaaattttgacaagtttttatc
tccattattaatattaagtgttgg----tacagtagaatactgtactagttatttttttc
agcaagaatttgagctttgtttaaaatatttgtactgttttagtagcttgtgcaccttg
agacaatcgttgcaaaatttttgaaatgtcaacgcgagtttcattagttataggattata
aaatcctaattcctctatagctttaccatcgcgtttatttctgctatccattacaacaat
tctatagcttggttgtttttttcttccgtatcgttttaaccttaacttgaccatattatt
cacctcgcttatttttt--atatctttattaaattatatcttacgacattatgcaataat
tcaaacaattaagcagattcaagtctaacgttattaaatattaccattaaacattgtaga
aaaataatacctaacataggtgacatgtccatgccaaacataggaggaatgcttcctctg
aatagttttaaatatggatcagtaattctatttaatgaacagaatggctcattgtaccaa
tttacagttggaaaccatgccaatgataatttaagtaaaattaaaattaaataaatctca
gaaaaattagctattgatccaagtaataaatttaatgtaccaggaagagtattcatacgt
atttataattattatatacttcattatactaatagtttgattatatattataatctctg
taaataaat-cagctaaacaaaaaacttatccttaaagctatcttctcgcgagcattttt
ctttatctacattagtatttacttttgtcgtataaatttctagtttagaccaattttgtc
ctaacttatttaattgagatgttccacattctaaacatgctatttttatattgctcaaaa
cagcacctttttgtataaaatcattcaataatggcgtaatagttgcttcatccaaaaata
aatctaagacaaggatttttgtaaatgaatttaattgagctgggacattgttaataatat
tattataattaactaaaactatattagctgtaggaagtaaagctcgtgctccttccgcta
atataaagccataaggcataataattacaataatatatttataatgactgttaataagac
ttatagtggtattagcttttaattcgattgtttctgttactaaccactctctcattattt
cgtaagtgatccacttacctagttctgagcaagcagttcttaaaattgtacctggattat
tgttgttttcgagaacccctgaccaatgttgtattaatggatgggatgctatattaattt
ggagttgcatgttttgttaaatatatttaacatttaatgtgttgattgaaacttcaattt
ctatttgttgagtattattaaaatatcat----atatattaaagaaaataattat-aaaa
aatgaaaaaaagtctttggttgtggggttttacagatagtgctgaaacttggaatggtag
attcgcaatgattggttttatatcagttatttttatcgaagtagttacagggcaaggatt
attatatttaataggtatgatgtcataataaaaatccaaaaaagttcttgtaatgtttta
caagaactttttttgg-atttttataaatctaatctagcggtctcatagatagaacaggtt
cattctctctattgataccttttcaaatccagcagcagcagctcttgctctgcctgcat
gccaaagatgtccaataaataggaagaatcctaggaagaaatgagatgttgttaaccaag

atctaggagatacatagttaactgaattaatttctgtagctacaccaccaacagaattca
aagaacctagtggtgcgtgagtcatgtactctgcagcacgcctttcctgccacggttgaa
tatcgttcttgattttattcaaatcaagaccatttggtcctctaagaggttcaacccaag
gagctcgcaagtcccaaaatctcatggtttcaccaccaaaaataatttctccactaggag
atctcattaggtatttacctaatccagtaggtccttgagaagacgcaacattggcaccta
gtctttgatctctaactaagaaagtaaaagcttgagcttgtgaagcttcaggaccagtag
gtccatagaattcactaggataagctgtgttgttataccaaacaaaattagaagctgtaa
gacccatgattgataaagcacctagactataagacaagtaagcctcaccagaccatacaa
aagctcttctagcccaagcaaaaggttttgttagtatatgccaaattccgccagcaatac
aaataatgccaatccagacatgaccacctacaagatcttccatgttatttacacttacga
tccatccatcaccgccaaacggagactttaaaacataaccaaagataactaggggattaa
gtgtaggattgctaacaaatctaacatctccaccacctggagcccaagtgtcgtataccc
caccaacaaataaagctttaataactaatagaaaagcgccaatgcctagtaatactaaat
gtataccaagtattgttgtcattttgttttatctcgccaatcgtatccgaagaaaggaa
atgattcttcaagagtatcagggccaatcagagaatgataaagaccaccaaagccaagaa
cagctgaagagattaaatgcactacgcctactacaaagtatgggtatgtattgaaaattt
cgccgcctggaccaacaccccagcctagtgtagctaagtgcggaattagaataaaccctt
gttcgtacaaaggcttctcgggaacaaagtgcgctacctcaaaaagagtcatggcaccag
tccaaaagaccattatacctgcatgagcaacgtgagcaccaagtaatttgccagaaacgt
taattaagcgtgcattgccagaccaccaggcaaacccggtagactcaatgtctctaccgc
caacaccaacagttgtattaaagggcgtttccacgtggtaaaacctcctcagggaatata
aagttttcatgaggttgatcctgagcagccatccaagagcgaataccttcgttcaataga
atgtttttagtataaaaagtttcaaactcaggatcttccgcagctctcaactcttgagaa
acaaaatcgtaagctcttagatttagagctagcccaacaattccaaatgcgcttgtccat
aatccagttactggcacaaataacatgaaaaaatgtagccaacgtttattagaaaaagca
acaccaaaaatctgtgaccagaatctgttagctgtcaccatcgaataagtttcttcagat
tgtgttggagtaaatgcgcgaaaagtatctgcagcatccccatcttcgaataaggtattc
tgtacagttgcaccgtgaatagcacatagtagagcaccacctaagattcctgcaacgccc
atcatatggaacggattcagagtccagttatgaaatccttgcaaaaatagcaaaaatcta
aaaattgcagcaactccaaggcttggggcaaagaaccagcttgcttggcctagagggtac
attagaaagacggagacgaatactgcaattggcccagaaaacgcaatagcgttgtatggt
ctaagaccaactagcctagcaatttcaaattgtcttaaacagaatccaattagtccaaat
gatccatgtaaagctataaatgcccacagaccaccaatttggcaccagcgagtaaaatct
ccttgagcttcaggtccccaaaggaaaagaagtgaatgtcccatgctgtttgctggcgtg
gaaacagccgcagttaaaaaaattgcatccttctagatatgaactggctagcccatgagta
taccaagaagtaacaaaagtagttccagttagccagccacctacagcaaggtaagcgcaa

ggaaacagaagtagtccagaccaacctacgaatacaaatcggtctctttttagccaatcg
tctacaagatcaaaaccaccacgagtttttcttgtccaattgctatggtcataatttta
atctccagagcaaatttaataagtaatttgtttaaataaaca---ctttagatatattct
aaaatttactttctttacggtcaatataattataagtcaaaactaaccaaaattcacg
tttttaataaca-tttttttagctcattaagttaatttttttaatatatgaacataaata
gagttattgttt-aactcaaagtactcataatttattcatttctataatcctttgaaaga
gatatcctgttcctcgtgctgttaaaatcaagtcgggactacttggatcatcttctagtt
tagctctcaatcgtgaaatatgaacatcaacaactctcgtgtcaacatgtcgttcgggag
tatagccccagacttcttgcaaaatagaagctctagaaaaaggttctccagccttactaa
ctaaaagctcgagaagactgaattccatacctgttaagcgaactctttcattatttttat
aaacttgtcttttattggtatctatttttagaaatccaatactaataatcccagagtttg
ggactccaagactagtggtgattttatcagctcttctaagaacagaacgaatgcgagcct
ctagttccttaggagaaaatggtttaacgacatagtcatcagcacctatttctaatccgg
taattctatcgcatacttcacctaacgctgttagcattattataggaacatctgattctt
ttcgaagttcttgacaaacaccgtaaccatctaatttaggcatcataacatctaaaacaa
caaggctaggatactcttttctaaacactagtaaagcttcttctccatttgaagcagtta
taacttcgtaacctattattgttagtcttgtttctaaaatccttcttatgctggcctcat
catcaacaacgagaatcttttctttctggttctccaatttttttatttactcctataaat
cactgataatgattactataaccttaaatttttagctctatgagtatcttcatttctttt
agaacttagtttagttattataaattctgcaagtctagtttgcattaattagtaccagt-
-aaatatttttgt-----------tttaagttatgac---------tattttaatccata
agttgactctagtttgcagaattcaatatatacata-ttttctagccaagtaatttgtat
tctattttctaaataaaaaagggggttgacaaactagttgatagaaagtattctacattac
tataag----ttaattctaaaaaagaaattctaa-----atcttctttcggagtaataaa
tactttaat
>Pyr_1_10_11_12_13_14_15_16_17_18_20_21_22_24_26_28_30_34_36_38_40_
41_42_P_yezoensis_MK695880
tatatgata---aattaagacatctgttatgtgagaatttctaaaatatacaaaactttt
tatttaatatagaaaaagtaaattaaaataagttgaacatactttgctctaatctaaacc
tcttaattgag------attctaattagagacaaaagtatttacttattttgtatctata
ggaataaaaatgaaacttgcagtttatggtaaaggtggtataggtaaatctacaactagt
tgcaatatttctgtagcactttcaaaaagaggtaaaaaagttctacaaattggctgcgac
cctaaacatgatagtacatttacgctaacaggtttttttaatcccaacaattatcgatact
cttcaatccaaggattaccactatgaggacgtttggcctgaagatgtaatctataaggga
tatggcggtgtagactgtgttgaagctggtggaccgccggctggcgctggttgcggaggc
tacgttgtaggtgaaacagttaaacttttaaaagaattaaatgcttttgatgaatacgat

attatcttatttgatgttttaggtgatgttgtatgtggaggttttgcagctccattaaat
tatgcagactactgcttaatcattacagacaatggatttgatgctttatttgcagctaat
agaatagcagcttcagtacgagaaaaagctagaacgcactctctgagattagctggactt
gttggtaatagaacagataaaagagatctaattgataaatatatagattgtgttccaatg
ccagtattagaagtcttgcccttgattgaagatattagagtgtccagagtaaaaggtaaa
actttatttgaaatggcagaaattgataaggatttagcatatgtatgcgattactatttg
aatattgcagatcagttgattacaaggccagaaggtgtagttcctaaagaatctccggat
agagaattatttagtcttttatctgattttacttaaatcctaaatcaaaggtaggacaa
gaaaaagtagatcaagaagaattagatttgatgatagtgtaaataatattttcagcataa
tataacaaggaataagataatgtctacagctcaatcagatgctcttacttttgaatgtga
aacaggtaattatcatactttttgtcccatcagttgcgtttcttggttatatcaaaaaat
tgaagacagtttttttcttagttatagggactaaaacttgtggttatttcttgcaaaatgc
aatgggagtaatgatttttgctgaaccgagatatgctatggcagaattagaggaaggaga
tatttcagctaagctaaatgattatggtgaactccgtagattatgcttacaaataaaaaa
agatagaaatcctagtgttatattttggattggaacatgtacaaccgaaattataaaaat
ggatctggaaggaattgctccaaaattggaagcagaaattcgtgttcctatagtagttgc
tagagctaatggtttagattatgcctttacccaaggtgaagatacagttctagcagctat
ggctcaaaggtgtccattaaatttaaagaatcaatcagataacacgagtcttaaaacttc
tcctcatattcctttagttttatttggatctcttccggatccagttgtaacccagttaac
tatggaattaaaaaaacaaggtatttttgtttctggttggttgccatcaaagaggtatac
tgaattaccggttataaaagaaggatattatgttgcgggagttaatccatttcttagtcg
cacagctactacactaatgagacgccgaaaaacaaaactaattggtgcaccatttccaat
aggtccagatggtactcgagcctggattgaaaaaatctgttcagtaatgaatgtaaagcc
tattggattagaagatagagaaaaagcaatttgggcttctttagaagactatatttcttt
aatccgaggtaaatcagtttttttttatgggtgataatttactagaagtatctcttgcaag
atttttaactagatgtggaatgactgtatatgaaattggtattccttatatggataaacg
ctatcaagcagcagagctagctttattaaaagctacatgtgataaaatgaacgttatgat
gccaacaattgtagaaaaaccagataattataatcaagtagaccgaatccgtgatctgaa
acccgatttagttatcactggtatggctcatgcaaatcctttagaagccagaggtattaa
tacaaaatggtcagtagaattcacatttgcacaaattcatggctttaccaatgcaagaga
tattcttgaactcgtcacaagaccattacggcgaaatcttagtttgtcagaattaggctg
ggatgtttatagcaagcaaagctagtattcctc----tttatgacttacatggtataaaa
gccttgtaagtct--tttattttatgttaaagaagcatttcttgctctgccatcaccagc
ccaagcttgtaaactttctacttgttctttgtctgtaaatgctaaaggcacaaattgttt
gatagctattttaatatcttcagtactaaattctctctcttcactaaacgccgtatgcat
actttcaataattgcttgctcaatttcagcgccagaaaatttattacataataaactcaa

ttcatgaatatcatatttttgccacgatcttggtctaacttttgataaatgtatcttaaa
aattgattctctctcttgacgattaggtaaatctaagaaaaaaatttcatcgaacctgcc
tttccttaacatttcggaaggtaaactttgaattttattagctgttgcgacaacaaatac
tggagctttttctctgatagccaggtaataaatgtaccaaatactctggcactagtacc
actatcaccttgactatgtaaaccagaaaaagctttatctatttcatcaatccataatat
gcaaggtgataatccttcagagatatttatcatttctctcattttgattccgattcacc
aactagtccaccaaacagttttcccatgtcaagacgtagcagaggcagcatccaatcgct
tgcaatagctttcgcggttaaggatttacccgtcccctgtataccaactagcaataaacc
tttgggcgaaggaataccgtaatttaaactttgcttcgaaaatgagcgagaccttttttg
tagccattgttttaatacatctaagccaccaatatccctatttactttactataaggata
aaattctaatagatgcgtttgattaataatttgcctttttttcttcaataataataggtaa
gctccgagagtctatctgattatattgtgcaataattttagtaatcactttgcggattct
atctatcgataatccttggcaggattttgttatattattaacgagctctgaattcaaatt
aagattcaaagctttactcaatcttatgatctctttttttgatttctagaagactaggtaa
gggcaaatctattactgttatgatatcattcagagcaaaaggtatattgattttgcaaga
aataataataatgtgcttagattgcgttttgattattttagctaaattgcgaagtttcct
aattaatactatttcgtttaggaaagagtcaaaatcttttaaaagaaaaagatttaaaga
ttcattattcaagttttcaataaattctaatgctaataaaggatttctttttgcataacc
attatcgctaggattgttagtgtatccatctacaaagtcccaggaatatacttgctgact
gtttgaacagtttaagctgtgttttataatatactctagtctatcttcttctctagtatt
tattacaataataggatatcgagattttaaaagtaatcgtaaatcttgagtgaaattcat
gtcaatatttgtttccatcagacttttgtacttcaccaattgaagaaagactaaataaga
ttatctaatattccttggatatattcaaaagtc------atctctctattttctaaatta
ttgctattactaatttaacttaccatgatttttttcttccttttcttcgtcgactattt
aatatagagcgtccacatggtgttttcattcttgctctgaaaccagaaactctaatttttt
tttcttttagatccttgtagtgttctttagtcatgatgtctcctctgttgtaaaagatt
aaatactattttatcatcttttatatatctttatgatatagatatgtattaatatcaaag
tt-aacagcaataatttatattaatcaagaactatttatataatattaaacattaaatat
----------taactttatgatattggctttgcccatttttttatttaagtattttgacca
tctttttgctaatcttaaactggttaatttttcagcagctaaaaacgattttgttattag
aatctcaatttaagtattttgttgataaaagccaaaatcgtaaattagaacccgacgaaa
gttttgcttttgcaaaagtatgtgtagctaaaaaatatttttctaaagctattattgaag
gtcaattagctttaaaaaactatcgagacctgaatatcttagataataatatagtaattg
ctaatttgtataatatgctaggttttatttattttgaagcaggccaaacaagttttgcaa
agaacttttatgagcaagccttacaaataaaccctaattatgttgtagctttaaataacc
tggcaaaaatttatgaagaggtaaaagatttaaaaaaagccgaatctctatacgataaag

tgttaactcttaacttaaataataaaactgctaatagaaggaaagattttatagcaaaaa
cta---------aaaacatctaatactatttgtaatcgggatagcaggatttgaacctgc
gacatcctgctcccaaagcaggcgcgctaccaaactgcgctatatcccgtaagacataat
tattactatatacctttctctaattatgtctactctttctgtattttatctaggatacc
aaaacatgcctttaactccatctggatctttgataaatcctaatttttataaaaactaa
caacatcaggttctgcgaatagtgtaatagtgctaatttctgcctgtctcaattgctgta
ttaattggtgtatgactacttttcctaaacctaaaccttggaaatctggatgaataacta
cgtcccagatagttgcattaaatccgttgtctgatgttgctctagcaaatcctacaagtt
tactatttgcatcttttttttgtattaaagaaataataatagaactatttttaatgcga
tctttactttttttagaggtcttttaacccatccgactgaatcacaaagctgctccaatt
catataaattaatattcttattgctactcagatagatatctttaaattcgattttatcgc
aagttttatctaaaacaataagtttttgaaatttttttcagagctagcattgatattgg
agttttgaaaaaaatttttccagaagatcataatgattatgatataatta--aataaata
tatatattacaatattgctttaacaaacaatatattaatatatgctcaaattttacatg
aatctattaagatagaagattgttaaaatatagagaaataaaaatttttgtaaaatcttt
caataaaatgatataaagagtgcttttaagactaacatctattattataaataaagtct
cgaaattgagatgaaataacacattattctatgtctaatgccttttaaaggagttatttg
tgaaaaaatctatgtgtctaacctgtttacttgctttactaattatgagcaacccaataa
tagctaacgcagaagtagctggattggtgccctgcaaagattctgcggcattcaataagc
gtatggtaaatagtgtgaaaaaacttcaagccagattagctaaatatgatgcagacacgc
caccagcgttagctttaaataagcaaatagagaaaactaaaactagatttgcaacttatg
gtcgagcaggtttgctgtgtggtactgatggattgccacatttaatttctgatggtcgat
ggagtagagccggggactttgtttttccaggactattattcttgtatattacaggatgga
ttggctgggtaggtagaggatatcttttatctgttgctaagactagtaagccaacagaaa
aggaaatcattttagatgtgccattagctatcaaatttatgtcatccggatttgcatggc
cgctagcggcttggcaagaatttagtagtggacaattaattgcttccaatgacgatatta
ctgtttcaccccgttagtaaaaaatttatatgaataataattttaccaaatacttatcaa
cagcacctgtaattggtgtattgtggatgacgtttacagccggatttattatagaattga
accgcttttcccagatgtcttatacttttactt----ataaataa--gatcgtataaa-
--aaactacataaatatagagagatactattatttgtgtagtttta---atatataaca
taattattta-tttttaaaaatgtttatcttttcgattattttaatattagatcttacat
aataaaata-atatcaatattatgagcttagtaacccaaattatagttaatgccgatgat
gaattaagatatcctacaattggagaattgcagtcaattcaagactacttaactacagga
agcaatagaattagaattgctactattattagagataaagaaaaggagattatacagaaa
gctagtaagcaaatttttcagttacatccagaatatatagctccaggtggtaatgcagca
ggttctaggaaaagatcgttatgcttacgtgattatggttggtatctcagactaattaca

tacggagtattagctggggataaagattctatagaaacaattggtattataggagtaaga
gaaatgtacaattctttaggtgtacctataattggaatgttagatgcaatccagtgttta
aaggaagcatctttagaaatgcttggtcaagatgatattagaatcatttctccttatttt
gattatataattcgtggaatgtcataaatcatagcttcaattaattagttgaataaaagc
ttttataatgttataattacaataagcccgagagcatataattgtaaaaactaaatcttg
tcagaaccggaaggtagcagcaataatgtttcaatacaaaagttatga-ttttcgggtgt
ttattgtttttatctaatagtctcgacttttaattagtatgctatgtataaatctgtata
tgacatgtaatccatcaaaccttccagcaaaaataatttaataatttgatattaatacgt
aaaacacctatac-tcttttataaaatttctttatatctcatctaaacaatggaaactta
acttaatgggtgttcatattttatcaacaggttcgtctgtcccaaattttctgtagaga
atcaacaatttgaagacatgatcgaaacttccgaccattggatttcaacaagaacaggaa
taaaaaaaagacatctagctccatcttctacttctttaactaaattagcggcagaagctg
caaacaaagccttatatgcagctaacttaaaacctactgagatcagtttaattattttag
ctacgtctacgcctgatgatttatttggtagtgctagccagttgcaagcggaaataggtg
caacaacatcagtagcttttgatattacggctgcctgctccggttttattgttgctttag
taacagcagctcagttcattcaaactggttcctatgacaatattttagttgttggagcag
acacaatgtctagatggattaattggtcagatagaactacctgtattttatttggtgatg
gtgctggagcagtagtattggggcaaagcctcaaaaatagtattttaggctttaagttat
gtacagacggtcagctaaacagtcatttacaattaatgaataaacctgtaaataatcaaa
aatttggtggtacagaaattcctcatggaaactataattccataacaatgaatggcaagg
aagtgtacaagttcgctgtatttcaagttccaacagtaattagacaatgtttgaataatt
taaacatttcaatagatgaagttgattggtttatattgcatcaagcaaacactagaatca
tagaagcaattgcgagcagattatcagtaccttttctaaaatgattacgaacttagagc
attatggaaatacatctgcagcgtcaatccctttagcgttagatgaagctattcaatcca
ataaaattcaaccaggtcaaattattgttttatctggttttggagcaggcttgacttggg
gagcaattgtcttgaagtggtgat---ttatatattgcggatgacgagactcgaactcgt
aaagctttcgctacacacccctcaagcgtgcgtgtataccaatttcaccacatccgcatt
ttttaccatgaata-ttaaaaatttataatatttatctaaatattcatataa--tgtgta
tatatatatacattcttgtcagaaactctcttatattttattttaagtgataaaattgag
t----ttagtatagatagtaaaattttattttaaaatcataa----aaataaatatataa
ggaaaataaaatatgacaccatctttatcaagtttttttgaatagtcttattcttggggca
gtaattgtagtcgttcccataactttggctcttttatttgttagtcaaaaagacaggaca
atccggtcctaaaaataacttagaaagatatggagaagcaatttaagatgaataatgaaa
tatttttcatactaataagctctccatatattgctatact--ttagaagaattttattt
ttttattttgtaacatcctgtaaagtttattccgttctttctttttccc-atttactctc
ttataattaatgatacaaatgtcagg-ttaataatag---tttttattctgtatctttc

---------------atttatatacgtattattatgttcaattaataaagaaaagctca
tgtctttgtctaattggcctctcaaaaaagaaaattctgaagcatataatattaagaatt
caaaacaaatcacaattcctgatggtttatggataaaatgcttcgactgtggtctattaa
tgtattctaaagtactgaagagaaatttaaaagtttgccctcaatgtagttatcattttc
aagcttctagtaacgaaagaattgatcaattgatagaccaaggtagttggcaaccaatgg
atgttcacttgatctctacagatccattaggctttaaagaccaaaagctttatagtcaaa
ggttaaaagatactgctttcaagactggcctgcaagacgcagttcaaacaggtactggga
ctatgcaaggtaaaaaagtatgcttaggtattatggattttagattcatgggggaagca
tgggatctgttgtaggcgaaaaactaacaagactgctagaaaaagcaactcaagaaaagt
tgcctgcaattatactttgtgcatcaggcggagctagaatgcaagaaggtatgttgagct
taatgcaaatggcaaaaatttcttctgctctagaaatgcataaaaaagaaaatctactat
atatatctgttttaacctctcccacaacagggggtgtaacagctagttttgctatgcttg
gagacttaattattgcagagccaaaagctcttattgcatttgctggtagacgagttatag
aacaaacaatcaaagaagacttgccagataattttcaaagttcagaatatttatttgaac
atggtttcctagatttaattgtatccagaactcagcttagatcaaagttaatacaaattt
tgtccttacataatcatagtaagtaatgaattgaatattgactataatatcaaaatgcta
gtaaaaattaagatattaagaaattttcactaaagatatttgtagactaactagaattac
aattactattgtagaaaaatttgtcttaatgcca-ttttttcagtcttttaacaaattg-
tttttatgactttaaataattgttagtttaatacaaatttataaagagaata-----cat
actaagctaaatatagtaatattatgttttcctataatactgacctatcaaatttcgagg
aaatttatgcttaaaagatcttcttggcttgcggctttattgggactattaacagtagtt
tctacaagtacgcatacatatgccatagagttagacgaggcaacaagaactgttccatta
gaatcttctggcagaactgtaattcttacaccagaacaagttaaaagaggcaagcgatta
tttaataattcttgtgctatttgccataatggtggtatcacgaaaacaaatccgaatatt
ggacttgacccagaatctttaggattagctacgccacaaagagataccattgaaggacta
gttgactatatgaaagatccgactagttatgatggtgcagagtcaatcgcagaattgcat
ccaagtattaaaagtgctgaaattttcctaaaatgcgcaatctaaccgatgaagacccta
tttacaatcgcaggtcatatcttacttcaacctaaaattgtttctgaaaagtggggcgga
ggaaaaatttactattagaacttcaaagacctaaaccttgtgaatactagtttatgta-t
ttgttgcttaagtgtgattattttgttagatcatgatatatattaaagatggagtttgtt
gacagcaagttacttttttacccgaacttgtttacactattcgtctagttattaaaaagga
gacgttaaattgaagaagaagctttcagttcttttcactgtttttagtttttttgtaata
ggtttcgcacaaattgcttttgctgcagatctagataatggagaaaaagtttttttctgct
aattgtgcagcatgtcatgctggcggtaataacgccattatgccagataaaaccttaaaa
aaagatgtacttgaagctaatagtatgaatactattgatgctattacttatcaagtacaa
aatggtaaaaaatgccatgcctgctttcggaggtagactggttgatgaagatattgaagat

gcagcaaattatgtattatctcaatctgaaaaaggttggtaattatacttgattttatcc
tgtattaaagaatagacaatctatttagttgttcattagattgtctattctttgtttatg
---ctattatataaagatatttacacaatatt----ttatgatgaaaagaatacccgcaa
ttcttgtactagaagacggtgcatattataaaggatggtcattccagcaagataaacaag
agattactattggtgaagtagtttttaatactggaatgacaggatatcaagaaataatca
cagatcctagttacttccatcaaattgtcgcttttacctacccggaaattgggaatacag
gtattaataatcaagatattgaatctcacagtattagtattaaaggacttattgcaaaaa
atatttgtaaaatttcaagcagctggagagagcagcaatctttagttaagtatttaagta
gtaataatattccttttattttcggaatagatacaaggtctttaacccaatacttgcgtc
aatttggtacaatgaacggttgtatctctactgataatttaaatcatagttacttaaaac
agaaaatttgtgagattccaagtatgcaaggtttagatttaatcccgcatgtaactacaa
gaaatgtttacccctgggatgaaaaaagttttccgaattggtatttgacagataacatta
gagtacaccgagttattcagttgaaagttattgttatagattttggagttaaactaaata
tactcagaagactagctacacttggatgtcagataacggttgtgcctgcccacactcctt
taaaagatattttggcttaccagcctgatggtatattactctctaatggtccaggagatc
catcagcagtacattacggcatccagacagttacaaatttactagattacaatgtgccta
tatttgggatttgtatggggcatcaaattttaaatttagctcttaaagctaaaactttca
aacttaaatttggccatagaggtattaaccatccatccggattgaaccagcaagttgaaa
taactagtcaaaatcatggctttgcagttgaattgacttcggtttttgaatctcctgtaa
gagtgactcattttaatctaaatgacactactattgcaggaactggacataatcaaagtc
cttatttttctgtgcaatatcatccagaatcgagcccaggccctcatgatgctgattatc
tattcgaaagttttatagaaataatgacaaagtccaagaataaagttag-----------
ttagtaattttcccatgctttatgagtgaataaagcggatagtacttgtactcctcgtaa
ttgattaaagagaggcaaatgtcctctggggccatgagaattccattgaaattcctcagg
atatctgcaaggaattctatcaacttcccagccgatttttgccagaattttccccaatc
ttttccaacactaagccatatttgtctttgaacaaatagcccaaatttacctttgagtg
tgtatgccataacttatcaatagtttgtaaatcttgagcaggtattttttttatatctgt
aaaatatagccaattacgagtttgtgcatttacaccagctagttgaataagcttttgttg
agttagctgatctgctttgagcaaatcccgatgagtcagtaacatttgtaaatctttgta
attcatctgttgagcagagcgcaatggaacaatgccatcgggacataagttagaagcaaa
tttcactatttcttgatttttactgttaagtaacttttcataaattaaaccgtcaacaca
attacttttatagttaggcccagtaatcctctcaaaaaatagatctgctaaatcttttaa
ctcgatagagtcgttgctatttatattctcaataatttcaagttgttgcttga---catt
attagacttagtattcttattcaattctaaaagctgggctcggatttgatttggcatctg
atttgtttaattagttttaatagtc----ataaggtcttagagagaattagattgttttt
tgaaattagaagagcgccgcagtatttctaaagaagttcgcataatttgattcaataagt

tttgtcctgcataaattataatttgaccaattaaaaaaattgtgctttttatttttaggaa
caaaaaaatcttgtacctcaagcagaaaagtaattattatttgcattggtgacaacatct
gtaattcattcgatctgcaagcatatatatactgacagtttaagccttcttgataaaatc
cccatactttgtaacgactttcgtaaattgctcgtggtcgctcaagatagtttttaatta
tagtattccaaattaggttattctttaatttttctaattttctatcagatagaaatttta
cattacagagataacttgcatttgtctcatttacagtattaaaattttgaactaacatat
gagcaataatgttgcttagctgaattaaataattttcaagaaaaatttcgacctgtttaa
ctggcatattgtaagtaaagagattaaaagttttattcacagtttgagatgacccaaaaa
gtagctgaataattaaaatttgtagtagcattttatagtcgttcagtagatagttcaaat
catgaatatcttggtaaatgtcaatacagtccagttcatataacttacaaaatcttttga
tacacctatggaataaatcaattaaaatattttcggtcaagtttgtaatatcatttacat
ctaaatttgaattataaatttctaaaagtatcttttccagttcagctaatatgatttttta
ataagtttctttttacttcagtgcgaaaaacatctaaaattaacacttcctgagagcaat
tagttaatctcttattaatctttatagaagttctaactaatagctctgctacctcttgat
tgagtgttggtccctgagagcttggccaataattattcacgttataccatagtaagaaat
attgaatttaatgttatacgataacttttttaatatactgaaaagcaagttaattactcta
tagcaactag-------tttttacagatcttaatagttatttatcaaataaagttatatg
atagtagcgattgagctaattttattaaaattta----taataatgacaacttattactt
cgcccttgcgagtcaaaattttctattagtacaagaaccactagaagaagtttttagaga
gagagttaactactatcagtcaaataataaagcaattgatttttggctaataccaaaccc
ttcttttctagagaaaccagaaatgatttcatttaaaaaccttgtacctaaagacgctgt
agctataatctctactaatccaatatttattaattggttaaagctaagaattggctatat
ctgtattggacaatttgaagataacctacaactttctgaagaatcgttaaatattactgt
tttaacagacaaaatttaacaatgcttttattatgactagtgttaaattattttggttaa
gttataattatagtcttttagtattta-gactcttatagtttatcacttagtattttatg
ctgagatggagtgacaaatagtgtaagtatttcttggctaaacgtttcagttgccttaga
tttatatctatttggattaactataatggatagcattcgttttatagtaacattttcaat
ttgagcccaatgtacaattccaagttccaattccttagcaatagctgaaacggaaacaaa
ggcagctcctagccctgattgcacagcgtttttgatagcttctatagaattcaattccat
ctctattttaaagcgactgctgtcaataccatgttgactgagtactttatctatcacttt
tctaattgttgattgagtatctaatgcaataaatctaagcctgtataagtcttctttttg
aatgtctcctagtttagaaaaaggatgtgatttgggtaatataagtgctaattcgtcttc
cgcataagaagtaacctgcaaaacatcttgcaattcagtaggcacttctcctccaataat
tgctaaatcaacttgaccattagctacgctccatgaaataagtctagttgaatgtacttg
caattgaacagccacttgtggatatctttgcctaaatagtccgattaatcttggcatcaa
atatgtcccagttgtctggctagctccaataattaatgtgccaccttgtaagttttgtaa

gtcgtcaagagctcgacaagtttcttcgcagagagctaaaattctgcccccgtatcgtaa
aagaagacttcctgcctcagttaaagttgccttcttattacctctttcaaatagggaaac
atttaattggcgctctaaattttgaatttgcaaactaatagctggctgagaaacatacaa
gctattagctgctttttaaaactgccctctttggcaattgcttttaatattcttaactg
atccaatgtaaatggaaggtctgtcattaaagaattgtagtataatg---------tata
ttatttagtattgatcaatgtatgcatttgccaactaaaatatttgtcattacgcttcat
tattttcaaaataataaaaagtataatgttaaataagctgtattaaaatttagtatctgt
ttgatattaaatttatacaagagatatcgtgaatttaattctaactataaaatataggag
acaat---atggactctagacttttagttgtactaataccagttttagcagcagcatctt
gggcagtttacaatattggtagagttgcactacagcaatttagaaaaatgacatcttagt
tttgcttagaatata-atttttagactttatgagatagaggactttaaaatccctatctc
tattgtatatcttaataaaaaaag-aaagc--ccccttgtacttactgttattacacatt
aatattattggt-ggtaccta----gataataagtttaagctaacttatttttttattta
taattaattaaattaataacacaattatggctgttccaaagaaaagaacatctaaagcta
aaaaaaatgcacgcaaagcaaattggaaaaatcaagcaaaaactgaagctcaaagagctt
tgtctttagcaaaatcagtattaactagaaaatccaatggatttatttataatctaactg
aagcatcagatacttttagcgattagaagtgttt-ttactacaagtctgaaaaagtatta
cgcaacaagttctcagtaatttttatctat----taatcatttgaaaatcattctatttg
ataataaaataaatagcagattaaataaaatcacattaacaaattatgcagcaaagctag
atcaaactagtgaaatttggttgttcaattgtattgaaaatattcagcatattttttta
aaagtcaattaaaatcaagtcatattactaaaattttttatttctggtactagctttgaat
ataccgcaggtctaccaggattattatccagcttaacactaagtggtagactacatccta
ttagtatatatagtccccagtctctcaaaaagtatcttgaagcatgtaccaaatattccc
aaactaatttttcttttcctattaattttcataatttacaatacggaggacaagttgtta
accaattttatacagtaatttgtttaccgttgagcaaaaagagcctgctgtatggattta
ttattctaaaaaaagaaaagcagggagtatttaatttagcacaagctaaaaccttgaata
ttcttcaaggacctatatatggaaaactcaaagaaaaggataatttttttaagtccagatg
gttactatctatccggccaagacttctcttctaatacaataatgggacataaaatatcgc
ttccgttattagtcagatattctagaattatttctgagatgcattggttttgctcttatc
ctattagattaaatacttattcgcatcagcaaggagtaaagtgtttaccacataatgttt
taactgatattatgaaatcccaaatatatcaagataacagttttgttgaataatacttat
tcttgatatataatggtctcggtaatctaaataaaatactagaaataatttcttaaattt
atttgtctatattaaatttacaatcatttatgacatacgcaattattgaagcaagtggca
cgcagctttggatagaagaaggccgctactatgatttaaatcatatacctgttgatccag
gtcagtcgattatattaggaaaagtcttattattgaataaaaaatggggaggttactttag
gacgcccttgtatagaaggggttacgataaaggctacagtaatgaggcacttgcgaggaa

agaagataactgttttcaaaatgaaaccaaagaagaaaatgagattaaaaaaaggtcatc
gacaagaattgactcgtttaatgatcgattctataacatcttaga------taaaatcta
gactcatattttt--aa-aaactttttaatatttaactaaaatagatataatggcacata
aaaaaggtagtggtagtacaagaaatggcagagactctaattccaagcgtttaggtgtta
aaaaatatggtggagagcaagtaacagcaggtaatattttaatcagacaacggggaacta
aagttaagcctggccaaaatgttggaaaaggaaaagatgatacattgttttctctaattg
atggtttcgtgctgtttgaaaagtcaaatcaaaagcaaaaaacaattagtgtttattctg
ctaagaaatagttaaaacaattggtgcaagataacttgagt----gtatttatttcactt
acagtggcaccatatatttgatttattaagaaatggagttccttaatgaaccaatgaatt
ttattaaagatttgctgtgaaatttaatgacaaacactattgtaatttcctgtctacaca
atatggctgctattctatattgtggtcagatacagaaactagtagtagcaaatgctcatt
atcaagtaagtgatatctacctaggttgtgttgataaaatcttctcaggaataaatgcgg
catttattaacctaggaaagaatgagtacagtggttttatacatatcagtgataccggtc
cgcttaaaaagaaatattatgtcaataatattactaacattttaacaatacggcaaaaaa
ttttagtacaaatcattaaagagccaactttaaataaaggtccaaggctcactgccaata
ttacattatcaggtcgatatattgtattaatgccttttagtcaatcaatctgtatatctc
gaaaaatatatgatgaagatgagcgtcattatttgaaatctttagctattttaattaaac
cggcaacaatgggcttgctatttagaccttctgctgtaggtgtcgatgaggaaataatat
taagcgaattaaaaaatctaaaagaacaatggaactttgttcaaaaatctgcaattaata
gttattcacctgttcttctatataaagatgaagatattgttaaaaaagtaatccgagatt
tttataataataatacaaacaatatagtaattgattcaaacctgggattaaaacaattaa
attattatatccacacttggcactgtaataactctagcacagttcctaagattaagcttt
atagtaataatcaatgtatactagatgcttttggtatcaatcaggcaatttccagagctc
tcattccaaaagttgatcttatacttggtggctatatgtttattgaaactttagaagctt
ttactattattgacgttaattctggatcttttaataattccactagtgcacgagaaacag
ttttaaaaaccaactgttctgcagcaacagaaatagcttatcagttacaaattagaaata
ttactggtgtaattataatcgacttcattgatatggaatcacaaagagatcaattgcaat
tattagaacactttaataaagagctatcacttgatgatgctaaaccacaaattgtacagt
tatctgaattaggtttagttgaattgactagaagaagacagggcaaaagtttgtacgagt
taattagtagtgattctaattactttttatttttcacacaatcagagagatctcagtctc
ttaagagattcgatgatagacagcagaaacaacagatttttaataaatcttggctatctg
cagagattaatactattaacaaggtctttttttcaaaagtcaaatttgtgcagacctgcta
acttttacctaattcgtaatctctatatagttaaaagtagtattacttataaacaaaatt
atttattaactcacagatcaaaacttatttattctaaagaatacagcaaagtattaccaa
gca------gttattatctagctagtctcaataagaatagtaatcaagagttcctatctt
aagattgttatatcaactgtt---------------------------------------

--------------------gaaaaaaagaagctctctcttatataagagagagcttc-tt
ttatagcttactaacta-tctagttagttaattaaaaattaaccgttaacagctggagct
gttagagctactggtaaagattcaccagaagctagatctagagggaagttgtgagcgtta
cgttcgtgcattacttccatacctaggttagcacggttgatgatatcagcccatgtgtta
attacacgaccttggctatcaacaacagattggttaaagttaaaaccattcaagttgaat
gccattgtgcttacagataaagctgttagccagataccaactacaggccatagacctaag
aagaaatgtagagaacgagagttgttgaaactagcatattggaagattaaacgaccgaag
tagccatgagctgcaacgatgttataagtttcttcttcttgtccgaatttgtaaccatag
ttagcagattcgttttcgcttgtttcacgaattaagctagatgtaactagggatccgtgc
atagcactgaacagagaaccaccaaacacaccagcaacacctagttggtggaatgggtgc
attaaaatgttgtgctcagcttggaatacaagcatgaagttaaatgttccggagatacct
agaggcattccatcagagaaactaccttggccaattgggtatactaggaatactgctgct
gctgctgctactggagcagtaaaagcaacggaaatccatggacgcatacctaggcggtag
cttagttcccactcacgaccaatgtagcaagctacgccagttaggaaatgaagaacaact
aattggtaaggaccaccgttgtatagccattcgtctaaagaagcagcttcccagattggg
tagaagtgaataccgatagctgcagaacttggaataacagcaccagaaatgatgttgttt
ccgtatagaagggaaccagcaactggctcacgaattccatcaatgtctactggaggtgca
gctacgaatgcaatgatgaatacagatgtggcagttaatagagttggaatcattaataca
ccaaaccaaccaatgtataggcggttttcagtactagtaatccaagagcagaaacgttcc
cacaagctagcgctttcgcgtctttgtaaagtagcagtcataatttttatcaattttta
aggatttaacaaagatgcttcccaaacaataagttgtctgttatgaaaattattacttaa
ggccttttcaaatgagaacattttaaaaaaaaca-tagcaaaatctaatctcagtaact
aa-aattt-tacttgttcatatattgcttaataattcaa-----------taataaattt
tt-gct-------------ttttgcaaaattaacactattttagggat---ataataata
atgcatattaatgctgcttattttgattttaatagtttatataattttgccctacttaaa
tacgcatgtcacactttacaaaaattcagacgaccatacaagatttaaatctgttaaagc
atgctttaacagatttgggtttgatttggcaaatgaattctagtcatataaaagttggtg
aaaataatcagcataaggtagatattttaattaaacaagataacttgtcacatattggct
ttacttggaatgataatagatatcacttagttgccgacttacaactttgggaacaaccct
ggtctctagaagtatttttggataagttatctcagaagtatgcttactactctatcatcg
aagaaacaaagaagcaaggctttgaaaaaaatgcagcagatttcacaaaaagatggatcaa
ttaagttaattgtgcaacgctggaattattaactaacatacaaatgcgggcggagagact
tgaactctcacgagattatctcactagaacctaaatctagcgcgtctaccaattccacca
cgcccgcactgttgagac--aatattattctatcataatctatactaaaacacaaactaa
-tagtataaactagcttccaagcttaaaagcgtctacaaatagaccgtatgttataccaa
ttttgaagttgttgaataaattaatttttatatttaattgcttgtggctagagtagacta

ttttactagtcaactcagttgctgcaactattagactggctgcaataataccccaatctc
ctgtttgtccaggaatcgtagacaaacctgttgaaataaaaaatcctagcaataaactaa
ttaagccagttgttaactcgctcaaagaataataaagcttattatttaagtttttatta
agtaagcaaaaaaagttgaaagtctagttttaatcatagtatttagtaacggagttttaa
aaggatgacaagtattaacag-tcattaatattgtcgtatccttttaaaactcgttaata
caaacagctgttcttgt---------ttttaaaggtcttgttcgcttaaagcattaataa
gatgctgaaacggctcttgtataatatctgtgcgtttaattgattgcaaatctaattctt
cttctatgatttcttgtaataactgtatgcttcttactgttggagcaacaggtacagata
gtgaattgtaagtatcttttagcccatttaatacacgctgatcgagaatatttgtactac
cagaaaccaaggcataactagcataccgtaggtaatattctatatcccttagacaagctg
catatcttcttgttgtataagaatttcctccaggccttaataattcaggctgttcttcgt
acaactgagctgcggcctcttttaaaatattggtagcttgatcattaataatttcagcta
tttttattcgatctagtccactcgaaaagaaagattctagttgtccgacagctgtcttat
ctagataacgaccagtaagatcgtaacgatttaatattgctgttatagcatcttgcataa
attaattctcctac-ttgctatcagagcggtattataaatgactaatattgtaatataat
cattagtgtttatgaaatttatttaaaacttattctttattgcttaatgtgcaattaaaa
actcgcatagattttacacatatagagtatacgtagtttttaatta-tttttttataaaa
accaagaaataaagctttttt-taagaggttcgtgaatttgttaatataacatgagcttt
cctcctaaataagttttgtaaagtac--aaagtaaaaaaacgattagtcaataatagtaa

--------------------------acaatattaatatgtatcaa--------------

-----------------agtataaataagcttttactccagcatcaaaaaaaatatttga
gatatgttgatacaactgattattcaagtgaaaacttagatttagaccaatttagctatt
ttatcattacaattaataaaactgataaatgtattcttatagagcaattttgttataatg
ctgaaggccatatttactcaatattcttcaaaggtccaacagctaaacatctttcttcta
taatttgtcattttcaacaattaaatacaataatatctacagctcatgccatatatttag
gacgagagctaatgaagtcagaattagctctcgtgctagatcaacaatatattcaagatt
aaa--ttttgtaagtattaattatatttatgctagtataatattagtaagggcatgtaac
tcagtggatagagtatcagattccgattctgatggccgtgggttcgaatcccgccctgcc
c---gagtactcagagtact-atactaaaacatct-------taatagctagtttgataa
tagtatgggcattggggctgcaaggtttctacattatgaaaagaagaaaatatg--aaaa
aaacaagctctccaaaagagcttttagtacaatcaataaatgcagaaaacaatattgttt
ctttttctcgaaaattagcttttgcttaaacactgttaattttggcattcgacatgtta
aactcttattcatggcgaatattctgtcagagtcgctcttagtttaagaaaagtcagaaa
aaatatttatctttacgtttt-tacctatttttatttaaataaaggttagta-ttttcaa
cttttatagtggacgtgggttcgagtcccaccagctccatcaatttgatt---------t
gtataattagctagtttaacctttttaataaccagatatataattggaggaaatatgggt
ttcataacaattactaagcccgctttaaagcaaattgcaatcttgaaaaatgatcatgaa
aatgatgtacatctaagaataggtgttagacaaggcggatgttcaggtatgtcatactca
atgaattttgaacatgttgacaaattaaaagatacagatgaacggcttcgtctagataat
ttttctgttgtctgcgaccctaaaagtcttctttatctttatggattatcattagatttt

agctcggaattaataggaggaggatttcaattttctaacccgaatgccagccaaacttgt
ggctgcggtaaatctttttcaggctgacc-ctcgaacacgtttttttatatattttgtatt
atatatattattttagcaagtcg-tttattagagacaagcaatgctctgttaaaaac---
---------------------------taatttcaagagtttttattgtattatt-----
----ttatatatttattatcaactacttgaaaataaattagctaagcaatctaatttgtt
atcagtaccaaactttaaatgattaacttttttattgatatatgctaatcttt-atcagcc
taaacatatctgtacattacaatcttttgacataaaaagagtcttagcatacctctcaa-
----------------------aaataaattcaa----------tattttataaatactc
aacatgaatctgtcacaacttatcgttaattcttctatgatgaaaattcctaataacttt
tattgcaatataaaagctatttcttctgagaatttaagtcacttacaattatcaaaaaca
aatttattatctcctacaaaaataattgcttttggactacctaaatttgattttatttct
acacagattacaagttctattagagaaacagttgttgtgtctactattgatgataataat
ctttttgctgccccaactaatgattggataaaaaaacacaaggataaaaatcatcattct
ttgttttataaaaaagtttttaaagctttgctaaaccataaaattagagttgttgctgag
ccact------tcataactccgaaataaataaagaaaagagcataatggcaagtcaaaga
tacatctggggtaaaagctggaagccttcaattattttatcttgcttaaaaaacaaaaaa
aatagcaaaatttctgatgcatcgaaaaaatttttaactgaacagttatctgcatcacca
gtatttgtggtaaaaaatggttttaatgaaattattttaggtcacccattatctcgtgtt
aaaagaggaagagttaataatctgatgcatgcgttttctaatttattaaatcagtctaat
gcaacttatcctatatctactggtctattctttttcatccagatgatgcatttgaattt
aaagactttataatatcggtcaatccactggcggccaaacatatggagataagcgtagag
cctgttggtctacactttgcttataaaatgaatagaaatatatcgtcagatactcaattt
tcgtttattccagattttaaagaagtaggagatttattattcaaatatagaaaaggtaat
catttagtttttcataagaatcagcattatggcaaagatttttttcaggggcaaccaata
tatatgattcagccgatcacttttaaagatcgagccggaaagctaaatattatcaaattt
acggggctaaatgacacaagagaaattattttttactaatcttgaagctgctaatagatca
tgggcacattttataaagaggaattcacaattaaaatcaattaaaaatcctactctatta
gtctataatttagaaagtttttttaaaagatcaagaacgattggataatcaagatttaaat
aagtttgtggtgatcactaataaagaatcttatcttgccacaaaagagttaatagcttta
cccgattctaatagcttttctaagcacttaaaattaaatataaagcctaaactctttttt
gtgaaactatgggtaagacggttattttccaccttaacttatgaat-aaatgctatt-gc
ctttattttctcgaataatattctataactagtaattcattgagttgtaaagcaacccat
tccctatcaatgactccattgactttccctgataaattggatttattcaattctaagtgg
ctaggaatattagctagtccagggaaagctaagtaattttctactagttttcgagatgct
tcttgatttttttacactaattgattctcctggtttacattgataactacagatagatact
acttgtccattaatacaaatatgaccatggttcactagttgtctagcggcaggaattgta

ggagccataccaagtctaaaaacagtattgtctagcctcatctctagtagctgtaacaaa
atttgacctgtcgacccttgtagtttttttagctgcttttacgtacttaaataactgtttc
tcacttaatccataattaaatcgcaacttctgtttttcttctaatcttacagcatactca
gaaggttttctggacttttgtccatgttctccaggtggataagatcttttaatcgctttt
ctactgagtccgggtaaatcacctaatctacgggaaatgcgtactcgtggtcctctgtat
ctagacatattatttaaattctcctaatgtaaat-gattaag----------taatgagt
agctatttttaacacaaaaagagcattaaaacaaagc-ttttgctaaaaaatatttaca
aaaaatataggtttggacaaaa---tacattactatgc-tttttaagcgggtagcgggaa
tcgaacccgcatcattagcttggaaggctaaggttttaccactaaactatacccgcaata
ttcaacagattgtcactaactcaatataacatagttatgttaagttaagataatcttaaa
tttaatagaatt-ttaaattatttatgctccttctaaaaccacatcaagaaaacgagcaa
tttcggcagcttgttcttcaacttctgataataataatggttgaccgaccctagttaatg
gaattactcttttatcttttgtacataaataaatttcacgtcttggatttagcccctctt
taatatctatttttattgatttgatttctttatattaaactgaagacagatttttctat
tctttccagggaaacctagccgaaaaattttacaatacctttatctttgttaaattcat
tatatccagcccctatattccaaataatagttagccacagaaacaaactcagaaagactc
ctatactgccatagaatgtcattacaataccttgtggaataaataccaaatcggttgaat
ttgtaaaaggtaacaaatcaacttgaaaataacttgatagtccagcaagaagaaagccca
aagccccaataaatattatagtagcccaccagtaattactcagtcttcgtgaacctaaaa
ttaaatcttttctaactgagtgtatagataatgtttttttcataatttgttcttttaaaa
cagta-tactaatgcaaaatggaaaaattttgacaatttt--atgcgcgctttagattt
tttaaataagcttgattgcttttagtcctaaaaacagtcctgttgctaacccaaaaaaag
cagcaagaaatattatatatcctagaaaaactgaca---tattattatttattattattg
ttacaatagtgtttacaaaaatagtgaaaatctattactacggtattatattacgttaaa
tgccttgctgtctacaggaaaaaactttttcagtttttagcattgtgtttatattagaa
ataatattctagtaaatttattaaacttctggagaacagaatcttatgtcagaatttatc
aagccttataacgatgatccttttgtaggcaatttgtccacgccagttagtacgtcaagt
tttagtaaaggacttctaggaaatctaccagcttaccgtcgaggtttatctccgcttctt
agaggattagaaataggaatggcacatggatacttttaattggaccttttgataaattg
ggccctttacgaggtacagatgtagcgttactagcgggatttctatcttcggtcggcctc
attattattctcactacatgtttatccatgtatggtaatgtatcttttactagagcagat
tcgaaagatccactacaaacttctgaaggctggggacaattcactgcaggatttctagtt
ggagcagtaggtggttcaggatttgcttatttattgctagctaatatacctgtattacag
actgcaggtcttagtttattctcttaagctagtaaggggacttgaacccgtaacctactg
attacaaatcagttgctctaccaattgagctatactagcattcagttcataataacatat
aaagatatttgttgcaatctgctattttcagatgtaacaaatatctttttttatttaaaca

gaactgttatgttaacac-------gtatattaattagaatctgaattatctgactctgc
agaagattcctgatcacagtttagataataacaaattgtttgatctagacaagttgcaga
ccaaccaacaggtgtttctcctgataattcttgcatatctacagaattataaaaattatc
atttggcttattactgtttgcttgcataaatatcactcccaaaacactctgaacttaatg
tgaagtaatttaacttctcaataatatattaacacaaaatattaaatatggtgattattc
tttaattgtaaagatgctgctaactttataaagtttgagtgagagtcttgatagcttttg
ttgaaataagcattttaacccatttattttgcccgctcgaccatactttttttgtgttgta
aattaactttttgtagtttttttgttcgtttatgagaatgagatacggcatatccattat
tagctacttttcccgtaagctgacactttttgacataatttt-------ctaacttata
tatttatttagagtacttgctaaagttgacttgggcacagctccaatcactgtgtctact
ctttctcctgctttaaagatcattaaagtaggtatacttctaatgccatattcagccgca
atagtaggattatcgtctgtgtttatttttactaccttaatagatgattcatattcttcc
gcaatttcatcaactacaggagaaaccattctacaaggaccacaccatggcgcccaaaaa
tctacgagtacaggtaagttattgttaataacttcttgtttgaaagaggcatctgtaact
tgagatactgacatattctttaacctttaatatatgttccttgctaagcaaatcttatca
caaatttcaagaaaaaatcttctatagtgaattactattagtgttgaattattttttttaaa
aattagacattaaaaaaacttattacctatataaataaagtggtggccgagtgatatctt
gatagtgataatttttatattaaggttgaacactagaaataacttttcaagcaaactctt
atttgaaaa--atttctctgtgaccataaaaacttaattatattacaatatattttatta
tttgtaacttagttctgataatggtataaacaacgcaaaagatactgccttataatcaag
gaggaatacatgtctcaatccgtagaatcacggactaggattaaaagcgaacgttacgaa
tctggagtaatcccttacgctaaaatgggctactgggatgctgactatgtgattaaagaa
acagatattctagctcttttcagaatcactcctcaaccaggtgttgacccgattgaagca
tctgctgcaattgcaggtgaatcttcaacagctacttggacagttgtatggactgattta
ttaacagcttgtgacttatacagagcaaaagcatatcgagtagatccagttccaaacgtg
gcagatcaatattttgcttacatagcttatgatattgatttgtttgaagaaggttccatt
gcgaacttaactgcttcaattattggtaacgtttttgggtttaaagctgttaaagctctt
cgcttggaagatatgcgtatgccagtagcttatctaaaaacgttccaaggtcctgcaact
ggattgattgtagaacgtgagcgtatggataagttcggtagacctttcttaggtgctaca
gttaaacctaaactaggtttatctggcaaaaactacggaagagttgtatacgaaggcctg
aaaggcggtcttgatttccttaaagatgatgagaatattaactcacaaccatttatgcgt
tggagagaaagattttatattctatggaaggtgtaaataaagcatcggcttctgctggc
gaaattaaaggtcattaccttaacgtaacagccgcgacaatggaagatatgtatgagaga
gccgaattctctaaagaggttggtagtatcatttgtatgattgaccttgtgattggttat
actgcgattcaaagtatggcaatttgggctcgtaaacatgacatgattttacatttacat
agagctggtaactcaacttactctcgtcaaaaaaatcatggtatgaacttccgagttatt

tgcaaatggatgcgtatggctggtgttgaccatattcacgcaggtacagttgtaggtaag
cttgaaggagatcctttaatgattaaaggcttctacaatactctacttgaaagcgacaca
gatatcaacctacctcaaggtctgttctttgctcaaaattgggcttccctacgtaaagtt
gtaccagtagcatctggtggtattcatgctggtcaaatgcaccaacttcttgattactta
ggtgatgatgtagttcttcagtttggtggtggtacaattggacatcctgatggtatccaa
gcaggtgcaactgctaacagagtagcactagagtccatggttatggcaagaaatgaaggc
cgtaactatgtagcagaaggtccacaaatcttgagggacgctgctaaaacttgtgggcct
ctacaaacagctttagatttatggaaagatattagttttaactacacttccacagataca
gctgatttcgttgagactccaacagcaaacatctagtttaatgactacttactgatactt
taaatagttaattgtaagtgaaattaacttataacaataaggagcatagaatagtgagac
taacacaagggactttttccttccttccagatttaactgatgagcaaattaataaacagc
ttgcttatatcgtttctaaaggcttatcagcaaacgttgagtatactgacgatcctcatc
caagaaactcctattgggaactgtggggtttacctttatttgatgtaaaagatgcttctg
ctgttatgtacgaaattagctcatgcagaaaagcaaaacctaattattatgttaaagtta
acgcttttgataatactagaggtattgaaagttgtgtaatgtctttcattgtaaatagac
ctgctaatgaaccaggattcttattacaacgccaagacttcgaaggtagaactatgaagt
atagtcttcatagctatgctactgaaaagcctgaaggagctaggtattaatattaattct
taattaatattggctaatta--ttcccctcttaaaaatcataattaatttagtaattgtg
atttttaagagggatagcttcccaagtaaattttgactaaa-tagattgaataattaaaa
atacacagaaataatgcaatcacaggatataatttccaacgatactcttgttaatttaca
agaagaatatgatagaacacaaatccaagaagttttaaatgagttaaatcaagaacttat
aggattagtgcctgtaaagaccagaattcgcgaaattgctgcgctattattgattgacag
attacgcagaaaactagaactagtttctggtaatccaggattacacatgtcatttacagg
tagtccaggaactggtaaaactacagttgctatgaaaatggctgatattttgcacagact
tggatatataaaaaaagggcatttgttgacagttacaagagatgatcttgtaggtcaata
tattggacatactgcccctaaaactaaggaagttcttaaacaagcaatgggaggagtttt
atttattgacgaagcttactatctatataaagcagataatgaaagagactatggctctga
ggcaattgaaattttattacaagtaatggaaaaccaaagaaatgacttagttgttatctt
tgctggatataaagatagaatggaaaaattctacgaatccaacccaggactctcttctag
agtagctaatcatgtagacttcccagattatacttcagatgaattattacaaatagctaa
aatgatgatagaagaacagcagtactgttttacagaagaagcagataaaactcttttaga
gtataccgagcgaagaatgaaacagccttattttgctaatgcaagaagtattcgcaatgc
tattgacagggctagaatgagacaagccaataggatttttgccagtggagaaaaagtatt
aacaaaagctgatttggtaacgattgaagcagaagatatcttgaaaagtagattattttc
attacctaatgcttaatatacacgtgatttcatgaaactatatattattaaaaag-tttc
ataatcagttgcaaatttcttgaaaaacctttattattatcttaatgtaggtg-tggcgg

catagccaagtggtaaggcagaggattgcaaatccttcatcccccagttcaaatctgggt
gccgcctagtactaaaaaaggggggtgtggtggaatggtagacacaacagacttaaaatc
tgttgatttttagtaatcgtgagggttcaagtccctccacccccatatattgatttataa
ataaaaagagatgtgtatttgtattaattgcaaccatattaccaaatgtaatacctatca
cttaatagaatctcaacataaacagcctcatcttactagaagtccattgtttatacctaa
atatcctgtagttcatgttaatatatctaacaactgtacctataatcaaattgattggga
tttagtggagtgtttatcatttgtagaaaaaccgaatagttggaatttagacgctaatta
gttacaagaaaaattatgtaccataagcaacaactctatttttagatactcggtattaa
ttttggatgttctaaccagtaagattttacccgttcttgctatctctataatgccaaatt
tagttagtaattgttcaatagcaacaatctttcccggatctccagtaacctctataatta
aaagatcttctgcaatgtctactatcttagctctaaaaattcttacaatttctaaagctt
ctgttctagtctgagaattgatctgaatcttaattaacattagttctctttcaactgaag
gaatatttgttacatcttgaacgttaaggatatttactaacttatataattgtttcgtaa
gttgttcaatagttctattgtctccttgaactaccattgtgattctagagaccccaattt
gctctgctggtccaactgctaaacttgcgatattaaagcctctccgggcaaatagaccag
atattcttgacagtactccggcttcatcttgaactaaaactgataaggtgtgtttcatga
aaaattaaatttaatctatttgatctattaagtatattctaagaaaattacagaaaaaat
actagttagtctataactaaattatccaaactaaacaaaatcctactatcagataatata
tctaccttgtaatgctatactattaacttagtatttaatgatattctaattgcaaataga
atgct-aatttttttaaatttgttaggaacttgtgctagaaaaatacaaaaacagtaaaa
aactctctcgagatttacctcaaatcaatgatcgcattagatttccaaaagtccgagtaa
ttgatgacgaaggtgaacaactaggtatttttgtgcctgaagaagctatacaattagctg
tccaacaaggtttagacttagttgttgttagtgataaatcggacccgccagtatgccgaa
tattagactatggtaaatataagtttacacaagaaaaaagagctagagaagctaagaaaa
agcaacataacagtagtattaaagaagtaaaaatgcgatataagatagaagagcatgatt
ataaagttagaataaaccaggcatccaaatttattcaagcaggagataaagtaaaagcaa
ctatcacatttcgggggcgtgaaatccagcactctaatttagctatagatttattgaata
agatggcaagcgatctaaatgcaatagctgaaattcagcaagctccatcaagagatggca
gaaatgtcataatgctcttatctcccaaaaaagttagctaaactaatttttatttaaatg
catctggctggattcgaaccagcgacgtctctttcgaaatggcggattatgagtccgctg
ccttcggcccctcggccacagatgcatcacttaaagtttataactcataaagcatataaa
aatcaatgtttagctatttttaataaagtctataaaagtctattattcattcatattgtg
atatgtcgcaactgctgaaggggaaacttgatttaagtatcgaaatatccagtatttaaa
aattgtgtcgagtataactggaaaagttgaaataaataaaaaaataaaatctcgactttc
cggaagtcctaaatgtcttaaaattacttcaataataacttcccatccatgaggagaatg
aaaacccacaaacatatcagtaaaaagaatgattaaaaaagctttagctgtatcactaag

accataaataatttcatttaaaaaagatttaacgactgcaatttgtctttgtccagttat
cattagtaatataaagacaagtattgatacaagatcggataaaatattttaacagcatt
tgcactttcgtttgcataatattctcccaactctctagccttcagctgcactctttttc
aatgatttcgtaagatatatcttccgaaggattcagaagaacttcaaagtgaattttctc
ttcaaatctttgtaattcagcaaaagctctttcctcttgagaggaatttaaaaaaatttt
aggctgttcttggttccataaataatcaatacaaggtccaaatacaaaaaatttgaagc
ctgattaactagtactggagagatgaataataataaaatatattttacggaagtaatagt
ttggtgtctagatattctaaactcctctatagcttcagattcgccattaggatctaactc
tttacgaaacttttcaaaagtattagtaattgacctaggtattggcccaactttttcaaa
ggcgaattgattatttcgttttaaattccaatatttcatttttattgctgctaaaattt
ctattaatcaataattgatcacgtattcactataaatattaattctagaactaaacatat
aaaacaatatttatgaaaaataaaatacaattaaaaattaattatgcattagttctatct
gcagctacagcagtttatggatataaatcttatcagccaactataaatacactcttaatc
aaagatgaagaaaactggcctttttctctaaaaggcagactgcctttattcctataatt
gtattaaaaaaaaatcatcaaaatccctgaactcagaacctagaaatagtaagttatatc
ttaatggaattggaagtagaaaatttatcgacagaattgtcactctacttggatatttag
aaaaacaggcaaactatactgaatacttgaaccaaataaaaacaagtggttatttttctt
tagtctacctagacagtgatataatgtataagaaaaatattagtaacatttttttactac
ctaatgatattctaaaaagaatctatatttgcaataagaaaaaaaaagcttataccgcatt
cattcttaataaatttatttcagaaacagataggctatccaaaaagttttgctaatttaa
attgggccttctctaaaattataaaatggtattatgatagaggataccaatggtctctag
ttgaagttaaacaagcgtctgatgcatcttctattgtaattgatattcatgaaggggtag
tgaagacaattataacagaatactacactctatcttataaaagagtctcaggtatcttat
gcgtagagtcaatagagcagtaccttggagtaagagtgggagctccactaaatataattg
atttacaaaaaaaaattacttacttaaaagataatcaactagttggcgacattatttata
gtattgaacgatcaaataatagctcaatgagtttggatatcaaatttcaaatacaagaat
taaaagataaagagataatagtgcttgcagaaagttcttcta------------ttatct
cgcatgcatgtaatttactcaatc---aatataga---------aataggctagtagctt
ctaatatagtttcactatcaactaataaattacacgcatgctattttaattataaacttg
actatcaatataagtatataaacacctctaatcttataaaattgttagcttattctattt
cttgtaggaagacactacttattcattattttaatttacaaactttaataagctgtacaa
aaaaaaatactattggatttcagctatatttacgaaatctaagttttgggaaagctttct
gtgtgcttagtatgaaatttatcaaaaatggacttaatataaaaattttatatataaatc
cttcattgatagttgatcagaattttgtatttcaatttgctatacaaatcattaagcaat
atcatactgcta------aacctccagctt------------------------------
------tatttttaacgaatctagatcttgaacaatatgttgctgaaagtttattaatgt

accattttacatcttgcttctcaatatctgaaaaaatattattatctcgaattatgcata
cagattctttatttttcaactctgaaa------attttcattttgatgatagaactaacg
cgatcaacagttatgatacttttaaacaaaatacaaaaatatttatcaagagtttcttt
ctttattattaagtttacgttatcaaaatttaattatttaggttggcctttaaaaggcc
atttttttgaaataaaatctttatacttggctccatttcaaaagagtg------------
---atttttctgatagtcgtaagacgctattcttccacaaaatgtcgttaaagcaagtat
ctaattttaatttaccagtatcttttaaaagccaccttaaccatattttagttagtacta
ttaaatgtcagtcaaatttgaatatgagaactgtatctttgctattaattgattcacctg
ctgaatatctgctatacaaatctattcttaatttctctattaaagttaggatgcagtatt
tcatacctatgagcaacaatataagattatctctatttttataattatttagattgttttt
taataagatcatctcagagttgcattcacatatggcaagatctaagaactcttacaccaa
ttcaaaatttttggttaaaaaaatttcatatggagctggaattcagcttaaattaccaa
ttaagcagatgccacctctttcaattgaatatactgtaactagtagtcgctacttttgta
tttaccttcgtacttattatcaacgataactattactaaaatgaaaaatttaatttcttt
ttttgatcgcagcaaagggaaatggatttcacaaagaacaacttatgaattatctaataa
aaacatgagttcggtacagtctcagatgacaatgaaaataggcaactcattgtcaggatc
tataatacttgcatcgttaaattgggggacatttatagacaagttgcccattacgctaa
aaatcacagccggagtgaatatgataataagtttaacttgcaattcggtaatcaactaaa
taatcataaattattaacgctgtgcatagtgacagatcctagtctaattagttttaaaac
tcgctatggaagtactaccatagatgaaacatattggttcgcaacaaataatttacgtct
aagtactagtattgttaaacgattcaacacttgtgtggcagtatctttttgttcagaaat
taaagtttaa-actctatatagaagcaactaatagcctttta-ggctattagttgcttcta
tgtatctaaattttgtattaaaaattat----aatactatattattctaaatctttacga
ttgggattcctggaaggatcattagagaggaacccaaagacaaacaaagaaataaagaaa
ataacggtagtatatacaaagattttcaaagtaaacatattttcatccttaaaagctatt
agtgcg----atatttacatatctccacataattttatatcatgatataagttccagcac
tattttataaataaatcaatatctttatttttttagtaatgatttatatttaaatatgcgt
gatgtactaaatactaaatcttttccgacttgttttgtcttgtttgtaaatattttgtta
tttctatggagtttaccttcaattatcacatagtctaacttctttagctgtttaaaagta
tatgaagatttttttgttccaaatagttagattgattataactaatcgttttctttttaac
agtcttgcttttaacttaattatttggcttttattttcactaattctgatactcttacaa
ctaagaatttggactagaagagtacagctattcataatttataacaa------tttttta
aaaaatctactaaaatagatgattttacagaactaaaatgatatttcattatcttgttga
ccttgctcataagtaatatctttcaatacttttaatatttttccaaaatactcttgcagg
tatttttctaaagatattacttccaacctgtcaattccaagaatactgtaaacttcatcc
atcggagctgtaaattgctctccgctacttaaaatttcggcaaatgctaaacgatcagaa

atattccatgtccattgaagagttttagtaattcttcttaaggcttttaataatccaatc
ggaatttgtgagatctgtgttttttgcccagataatttttcacataatttaataatttca
gctgatgtccaagcggtattaccaactaacggcagggtcctattttctgtggaaggtact
ccaagacttttataactaacttggctgcatcttgagtatcaatgtatgcaatgggtgta
gattctcctgtaacccagactgatttcttgtctaaaataggaatggcatattggttgatt
aaaccttgaaaaaatcctcctaaagaaaacacagtatatttcacattggacttctgaagg
aaatctactacctgagactttaagttcatcaatggaacatctgggtatttctctgaattt
aatattgaaaagaaaataaatctttcaactttagccgctttagctgcttcaattaatgca
attttcccatctaaatctatttttttctgcattataaggatcagtaggacgagatgtagaa
gcatctatgattgctgttaccccacaaaaagattgcaaaatactttcgggtaattttaaa
tcaccatatataagttctgctccccactctttgagaaaggcagactttcttaaatttctt
accatgcattttacattgtagccttcatctaaagctcgtcttacaatttgacgtcctaaa
gttccggttgctccaattactaaaagagtcatattagttaaaaataagtatgtataaaga
cattgatttggttgacggcagagagtaaattaggtagagagggaatcgaaccctcatgac
cgaagtcgtcacattttgagtgtgatgcgtataccaatttcgccatctaccctattatag
accaactactacaagtaatttagtctttagcgtaaaaataatactaagtttcaatagata
tgtcaacttttaaatttatattaataaaaaaacggagtctcttatggccacttttgaatt
aattccttatagattgtatttgagtcaatctaacacatggatccatagaataaaagcaga
aattaagatatatatagtagcgcttttatggatttcaattttatttttttcttactttaa
actatgtattattgctttaagcttaattgccataagttttactataagaagtaaacagaa
tattattcaaaaacatttgttacaaacgttattaatgacgttcttaactactgttttgtc
ttttagtgtggctattagttataaacaatatgcagaacaagaacaatcgcagtatttatc
tgattccaagaaatataaaaattccagcagctactattatatacaaatagcgaacgatca
aaggatcaaaagacaatacttaattactactttaaagccttccttgtactttttttattac
tatatattctattaaactagttatgataacaacctctccagaagttttagtaattactat
ttatagatctaggataataaataaaatatttaaaaatgaattgctatttatcttccttct
ttcgtcacatattgttactagtattattaatagaatcgataaagtgattcaagtaactag
cttaagaggaagcttgaatttatataattcattgacaaggcctttaatgttttctttgtt
aatatttcaagtcttctttttggagattattcgggagtcaaaagaaatagctcaagctct
ttatactagaaatctcaatcaagaaaacaataatttttttgaaaatatatacagtaaaatc
taactttagtgatcggctcaatataattattagcactttgtactttattattttagctct
agcgtaatagaatccacttattcttctgactctagtaatacgaagagtaataaaattagt
tagtagtttaaatagatagaat-taagatattatataaataattaaatgtttcattatgg
tcaatactcaaaatcaaatttcacaaacttcagaacttgattatatagttaatcaacctt
ataaatatggctttaaaacttctgttgaatctgagcaatttcctaggggaataagtgaag
atattgtccgcttgatctccaagaaaaaagatgagcctgaatatctattgaattttaggc

ttaaagcatataaaaaatggaaaaagatgagtagtccgtcatgggctcatattaagcatc
cgaatatagactttaatacgattatttattacgctgttcctaaattaaaaaaagaattga
aaagtctagatgaggttgatccagaaattcttgacacttttaacaagttaggtatatctt
taaatgagcaaaagcgaatttccaatgtagctgttgatgctgtttttgatagtgtatcta
ttgcaactacttttaagaaagaactgtctgaagctggtgttatattctgttcaatttctg
aagctataagggattatccagaattaataaaaaaatacttaggtactgttgtaccagctg
gtgataattattttgctgcattgaactctgcggtatttagtgatggctctttttgctata
tccctcctaatacagtttgccctttagaattatcaacttactttcgtattaacaacgaag
aatctgggcaatttgaaaggacactaattattgctgatcgtggcagtaaagtaagttatc
ttgaaggttgtactgctcctcaatttgacacaaatcaattacatgcagcgattgtagaat
tagtagctcttgaaggagccgaaattaaatattctacagtacagaattggtatgctggta
acaaagaaggtaaaggtggtatatacaatttgttactaaacgaggcttatgctcgggta
acaattcaaaaatttcatggactcaagtagaaactggatctgcaattacctggaaatatc
caagctgtattttagctggtgaaaattctcagggagaatttactctgtagctttaacaa
ataactatcaagaggctgatacagggactaaaatgatccatattggtaacaatacaaaaa
gtagaattatttctaaaggtatttctgcgggtagatcgaaaaacagttatcgaggcctag
taaaagttggccctcagtcatttaattctcgtaattattctcaatgcgattctttattaa
ttggtcaatcatctcaagctaatacatttccttatattcaagtacagaatccaacatcaa
aagtagaacatgaagcatccacttcaaaaattagcgaggaccaaatttttatttttac
aaagagggatcaatttagaagaatctattgctcttatgatcagtggtttttgtaaagatg
tctttaatgaattgcctatggaatttgctactgaagctgatcgtttactgagtttaaaat
tagagggaactgtaggatgagccagactattttagaaatcaaagatttatatgcttctgt
tggtgaaacaacaattttaaaaggggtaaacttatctattcgagccggcgaaatacatgc
gattatggggcctaacggttcaggtaaaagtacattatcaaaagtaattgcaggacatcc
agcgtattcactaataagcggagatattttatttttggacaaagtattcttgaaatgga
gccagacgagagggcaaaagcaggtatttttttagcttttcagtatcctgttgaaattcc
tggagtcagtaattctgatttcttaagaattgcattaaatgctcgcagaaagtttcaagg
attatcggagtttagccccttagaatttttcaactaataacagaaaaaatagatcttgt
tggcatgcaagaaagtttttttgacaagaaatgttaatgaaggattttctggtggagaaaa
aaaacgtaacgaaattcttcaaatggctttactagatagcaaaatatctattttagatga
aacagattctggactagatattgatgcattacgagtagttgcaaaaggaattaacacttt
agctaaatcaacaaattcaattattttgattactcactatcaaagattattagattatat
tattccagattttgttcatattatgagtaacgggcaaattgtaaaaactggtagtgttac
tcttgcccaagatctagaaaagcacggatatgactggattacgcagacataactatttac
tac-------aatattgagactaaagaatattctttagtctcaatatt-tttgcaaatgc
aaagtt-aattatttaagctttatcaaaaccttgcttaacatcttcaattgctcttttta

gcaattcttcactaactggttctaatttttagtactacgaacattttcaccaaattctg
gtttagaattctttaaatcttctcgaagttctctaataaattcagctacttttgaaactt
caatatcatctagatagccattaattccagtgtaaataatagcagtctgttcttcaactg
gaataggagaattttgggcttgcttcaaaatttctcgaagacgttgacctcttgctagtt
gattttgggttgctttatctagatcagatgcaaactgagaaaaagcttctaattcagcaa
attgtgctaactctagttttaacttgcctgccacttgtttcatggcttttatttgcgcag
cagagcctactctagagactgagatcccaacattaatagctggtctaatccctgagttaa
ataagtcaccagatagaaatatttgtccatctgtaattgaaattacatttgttggtatat
atgcagatacgtccccagcttgagtttcaataatagggagagctgtcatactacctccac
ctaattcagcattcaatttagctgctctctctagcaatctagagtgtaaatagaatacat
ccccaggataggcttctcgtcccggaggtcttctaagcaaaagagacatctgacgataag
cttgagcttgcttagttaaatcatcataaataactaatgttgctttacctttgtacataa
aatattcagctaaagccgcacctgtataaggagcaatatattgtaaagtcgcaggactat
ctgcattagctgcaactataatagtataatcaagagctcctttatcctgtagtgaagata
ctacttgggctactgaagatgccttttgtcctatcgcaacatatacacaaacaacatctt
ggccttttttgattaataattgtgtccaatgcaacagctgttttacctgtttgacggtcac
caataattaattctcgctgacctctaccaattgggatcatagagtcaatagcagttatcc
ctgtttgcataggttcacacactgattgccttccaattataccaggggccattgactcaa
ttagccttgttccattacttgcaggttcacctttatcatctattggccgagctaatggat
caacaactctacctaaaaaagcatctccgaccggaatttgagcaattcgaccagtccctt
ttacggaactgccctctaaaatatccctaccatctcccatgagtactacaccaacgttat
cactttctaaatttaaagcaaccccaatagttttatcttcaaattcaagtagttcaccag
ccatgacttcatcaagtccatatactcgagcgataccatcaccaacttgtaaaactgttc
ctatattggctacttctacatcctgatcatacttttcaatttgttgacgaataatactac
ttatttcatcaggtctaatatttaccatattttaatattatttttattaattggaaatt
ctaaagcggtaattagtcagactaatgctatctgtctttttttactttattttaagttact
acaacatcaaggtgtgaagccatttgtcttagctgtcctctaatactagtatcaatgact
tttgatccaatctgaatagtaaagccaccaattagctcgggctcgactgaaataattagt
ttaacttctttagccttagtcataaccttgatttttttctgttaatagagtttcctgatca
gggcttaaagcaatagaagtattaatatttgcaattgttaaagattccatttggtaagcc
agttccaaatacttactagctatagcatctaacattccaattcgcttgcgatcaactaaa
atcattagaaatgataatgtattttcgttaatttgatcgccaacagttgcaataattact
tcttttttttgcctctacagtttttaatggattagct---aaaaaaattttttagcttgtca
gattgcgctaaaatattttgtattgactgaatatcttggctcacctttttcagtaactttt
ttagttttagctaaatcaagaagagctactgcataaggctgagctatttttataacaaca
ttgttactgctcatagttgatctcctaacttagcaatattattatcaattatacgcagtt

gcatttcggagctcatttgattttccagctgcaaagtaactcttttaagagcaagaaatg
ttatttgttgctggatttgccttcggatctgtttttcagctgtttctatatttgatttac
ctgttatggcaagtctttcaatatctaattggccttgagctaatatggaacttctaactt
ttccagctgttagctgagcttctttcttaatttgatctataatgatttgagtttgagcaa
gttgtttttctgattctgacaatctagcacttgcttgctctagtctttcttcggattctt
gtatagctgctaaaacttttatctgtcttgcatttaaacttgatccgagaaattgcttca
aaacataaataagaccaaataaaagcaacaatatattaataacatttgcttcaaaaatat
cagaattaaagccgaatttgtgttcactactatgttctgataaaatagtaattatttgtg
gtatttttactatattattcatttaaaattct----------tactattttaatatttta
ctctggaatcatgattttcaagattgactacttaataatttaactttaatttgatcgctc
aaagtgtcaacctgattttctaaagttttaagagcttcttcttttttgaacatttaactgt
ttcgatgcttcagcaatcagtttctctgcattcaattgagctttttttatatcttctgag
actatattttgagcttctttttgagaagaggctattttttaattgtgcattgcggcgagct
tcagatagatcttcttcatatttgcagctagctcatctgctttaacaagcatagacgat
gcagtagtcaatgttgttcgaatatattcatctcgttcgtctagtactttagttacaggt
ttataaaaaatagtatttaataaaaccattaaagtaagaaactgtaatgccattaaaggc
aaagtaccattgaagtcaaataagccaccttcaatttcttcggctaatagaaatggtaaa
tcaatcattttactaattatgtttatagttaataattttgtttcaattaagaaaaataac
tttatagtgcgcttagtctcatcagactaagcgcaaatgattattttaaccaacgtatgg
attagcgaacaataaagacaacgcaacaactaggccgtaaattgttaaagattccataaa
tgctaaacttagaagaagtgtaccccgaatctttccttctacttctggctgtctagcaat
accttcgacagcattagctgctgcactaccttgaccaatgcctgggcctattgcagcaag
acctacagcaagaccggcagcgataacggatgcggctgaaacgattgaatccataatgaa
ttttaatttttttgttatatgagtagaaaattgacaaatttcgtataatacttaaaatcta
tataattaagtataatagattctaatat-----tgtaattatagtatattactcttctcc
atgaccttccattgcttctccaatataagctgcggacaaagtagaaaagattaatgcctg
tattgaactcgcaaataatccgagtatcataactggtaatggtattaaaattggaattag
caaagtaaatacagaaacaactaattcatctgctaagacgtttccaaataatcgaaaact
taaagatagtggtttcgtaaaatcttctagaatattgattggtagtaatactggagttgg
ctgaatatatctagcaaagtagcctaaccctttttttacttaaacctgcataaaaaataagc
taaagatgtaagtaaagacagggccacagttgtattaatgtcatttgttggggcagctag
ctcaccttcaggcaagtgaattaacttccaaggaattaatgcacctgcccagttacaacc
tagaataaatagaaataaagttgcaatgtaaggcacccaagggcgatattcatgctcacc
aatctgatttttttgcaatatcttgcagaaattctaatataaattccataaaattctgaaa
tttttcaggaattctttgtagatttctagtacctagaaatgataaagttaaaagtgtagc
aattactaaccaagagacaatgaaaacttgcccatgcaattgtaagctacctattttcca

atatagatgttttccaacttctactgctgataaactattatatgggttaaaatctatgag
actgttttgatacataataaatattt-ttttaaatagtgcaagaatcaaaaaataag-tt
tttgaagaattaatatgcaattaatttattcttaaaattagactattaataaattaaaag
caaaaataaattctaatagagatagctatttttagtagtgattattttttgtaaaaaaca
attattgctacctataatgctttttacgtttaattatatacccatatattaattatttaat
atgattataggtattttgctacaaactttattgttttatccattct----aaagattgtt
atagtttatttttttatttaaaatatctgcgacttcatctgcaaaattaactttcaaattt
tcttctcccccctcccagaagaaatcttacaaatcttctaatttaatattttcacctaga
acagcaatattttggtttattaaatcttcaatactaatatcttgattgcgaataaacatt
tgatctaataatgatagctccttaagtctctttttcattcgtccttcaatgattgtttcg
attctatcaactggtttgtttttcaagtcatctttaccagcttcaactctttgttccaga
ttaataatttcactaggtatatcctgtgttgatacgtattccacattaggcgatgccgct
atttgcatagcaatatctttagctaacttttggaactctggacgtcgtgctacaaaatct
gtttcacagttgacttcaactaatacacctattcttccgccagtatgaatataactctct
agtaaaccttcaatagcggtacgattagatttcttgttagctgaagccagccctttttgt
cttaatgattctaaagctttttcttcattgccattattagcttgtaaagctttcttgcag
tccatcatacctgctccagtcttatcacgtaaggcttttacgacttgagcagaaatttgt
agtgtcatatgctttcaaaaatttttaagtatttaaatt----------aattattataa
ttcactttgagtaggatgagttatttcttctaaatctgtttgcccgtatttaccatcata
aattgcatctgctattttacctacaattagtttaattgacctaatggcatcatcattagc
tgggattggaatattaataatttctggactacaattagtatcaagtatacaaatagttgg
aatacctaattttaaacactcttgtatagctgttgtttctctcttttgatctacaactac
aacaatatcaggtaaacgagtcatatttttaataccatttagatgtttacgaagtttatc
taattctcttcgtaaaacagcggcttctttttttgggtaactgatcaatcataccactttt
atcttgttcttctagctgtctcagacgactaactcgagacttaatcgttacccaattagt
taacattcctccaagccatctctggttaacataataagaatcacagcgttgtgcttcctg
tgccactataccagcggcttgtctttttgttcctagaaataaaactttttttaccatccga
cgaagcctgttttataagttcacaagcttctgttaataattgtgcagtttggactagatc
aataatatgtatgccatttctttctgtataaatatatggaaacattttaggattccatct
acgtgcttgatgtccaaaatgaacacctgcttctagtaattctgctaatgtaacaatagc
cataagattattaatatttaaataatgtaattattcgggaaatccc-tttgtttagaaac
tgat-----tcaactaacttatttgaatttagtaaactaataataaattaacacacttt
taaagcagatcaacaaaatagatcctgaagaattcat----tctacacttttattgctaa
aataattacgcgcggttctgtcgtctaaaatgatatcatctaaatcatctcgtacagatg
aaatagtactttcgtcattagtatttgcagttaaattctttttctctaacgatgagccat
tacagttatcatacatattgaagccggtaccagctggtataagccgacctataataacat

tttctttcagacctcttaaccaatctaatttaccagatatagcagcttcagttaatactt
ttgtagtttcttgaaaacttgctgcagaaataaagctttctgtatttaaggaagcttgag
taatacctaatagcacaggcctatatgatgcattaatcttatttctcaaggttatggctt
tattagtctgttcaatcttctgtaactctacaagctctccaggtaaatagcctgtttctt
caccattttctattttcactttagaagtcatctgccgtactattacttctacatgcttat
cagaaatattcactccttgagattgatataccaattgtacttctttaacaagtagtaact
gaatctcttgaaagcttaatcgagcagcttcatataaggctaatccacattcaatataca
aatcaaaacttgcatcgaggatatcatgaggatttaacaatacgtctgttttctttcttg
cttcaagaatttcttcaattcttggaagcccttgtataatgtcacctgtttttgctctat
caaaaaccaatatagctaatgtttctccccttctaattaatgcattgttatcaacatgta
aaatagcaccattagaaaccaagtaaggtctagcaattcttaatgtaactgatctagaag
aaatttcaataatttgtccagagtctaaagaagtcatattttcagtaataaaatcaccac
aacgaatccaatcacccacacttactttaactgttgattttcctatattaaaggtctttt
tatcagaagatgtcgcaatcaaaatgcgcctgctagtattcttttcagaataaatctcct
caacagtaccttcactcattgcaataatctcagtactagcaacaactgtaaatggcttga
tatattcaccatttttacaataattctagtttgactttgttgcttctctgatttattta
catcaatacttttaattgataatgtttcaaaagttgacagccttaagtcaaaataattat
tgccactttctttagcagaaaactcaattgaggatattaaattatcatctcgatgctcaa
tttcagccacaagattagttgttactaaatgaactccttctatagatttcactctttctc
cgtcccgaaaaggagttcttttaactagctttagtttagtttgacttggtttttgtgaaa
ctagattttcaattagactggattttgtttctggtattgaataaacaataacaggtctaa
taagaatatagggcatttgctcgttttctatatactcccaataaactaacttatctgtag
aaatattattatgaagtctttctccaggtcttaaaaaacctctactttatcattattac
tatacacctcactaagtttatagatagatccaggcttaataataatttctcgaactattc
catccttttcgattatttcaacaatacctgagcttttagaaaaaatattttaactagct
ctgttccactttctataacatctccattatgcactagtattagtgatgaatctttattaa
tttcgtgagtttcttctgaaatccataagatatatcccggacttaaaatttcgtaggcat
ctttatctaagcctgttttctttttgatacatttaagtctaaatatttaataataccac
cacttgtcgttttatacgtatcagatattagttctgcgactgtgtaaccatcttgaa---
ttttttggtttggaatagctttgaaaagaaattttgttttttttctgtttctaaaatat
aagattctttttgtggattacatctgtataaatataacaacctggaattacaatagatt
cagtaataacttgtatattggtaatactgctattagtattctggtgtatccttacttctc
cagcataatgatttattaattctgtctgtgcaagtatagtaccagctttagttttatctt
ctttatgaacaataacattagcagaatcagatatactataaacttcacctgagagcaccc
agatcaagcctccagttttagtaattcttgtagtatattgattattatctgtttcttcaa
ctgttaaattggagaaacagattttgccagagagatctgaaactacatgtttttgagctc

tttctgtcatgagtcgatttctgcggggtgattcagcaatcacttgatccttcgatacta
attcaccatcacatattaaaagagtagtacctttacttaagttaattattgacttctgat
ttttcttagatctgattgtaacttgggttggctttttttgttattaatgcttgctcaccat
gtctggtacgaacatctgtatacgattcaatatctagatctatcaattgaccatctatag
gtgcgtagatttgttctgctaattcaccggtaaatactcctccagtatgaaaggtcctca
ttgtcaattgagtgccaggctcaccaatagattgagccgcaataataccaacagcctctc
ctaaatcaactaagcggccatgtgcaagattccatccgtaacaatactgacaaacagaac
ttctagagttacatgtaacaggagatcttactaaaactttttt---gataccagctttag
ttatctcttttgctaacttaggactgacatcttggttcgtatgagcaattaaacaatttg
tctctggatggaatacatttctgctaatactcggccagataaagcttgttctagattaa
ttaatattttctgcgtatctactaaatcttctagtatgatgcctttattggtcttacagt
ctacttctctaattattacatcttgagaaacatcgacaagtcgacgcgtcaaatagccgg
aatctgctgttcttaaagcagtatcaactaatccttttcttgctccgtatgatgatataa
aataatcagtaacagttagtccttctctaaaattactcgatattggaagatcaataattt
gtccctgtggatcagccataagccctctcatgcccactaactgtcttacttgagaaatat
tacctctagcacctgagaacgccatcatataaacagcatttagaggatctgtctctttaa
agtatttaataacttcttgtttcaaggattcactcgcattattccacgtatcaataactt
tttgaaatcgttcaactgctgtaatctcacctcggcgatatttgttttcagtggccttaa
tatcttcaatcgtgcctaataaaagactttgcttactaggaggaattcttaaatcttcta
aacttaaagaaattcctgcctgggtcgcataatgaaatccaagatctttaagcttgtcgg
ccatattggctgctcgggctataccataattacgaaaagcccagacaattaaatttttta
gttcgttttatcaataaccttatttgaaaaacttggctgtgcaagacttcgtctattat
ccactaaatcttctccatattaaatgttcgtt--attaactatgctactaaagactcttg
aataattttattaaagattatacggccagctgttgttctaatatattgcacaattctctg
attttcagcgtcttcttttatgatatggttattaaaaaattttgttgtactattgtcaga
atgtttttcaatcttaatcggatcatgaggttgatcaccatctactaatccatcaaaacg
agcccatatataagaatgtaaatcaattttcttctgttcataagccataactacatcttc
taagcttgcaaaatactgattagatccctgctgctgagatggattattagctgttaaata
atagcaacctaaaaccatgtcttggcttggcataataattggttgacctgttgctggaga
gagaaaattatgaggggctaacattagcaatctagcttcggcttgagcctctaaagacaa
aggaacatgaacagccatttgatctccgtcaaaatcagcgttaaacgctgggcatactaa
tggatgaagcttaatagctcttccttctacaagaataggttcaaatgcttgaatacctaa
tctgtgtagtgtgggagctctgttcaatagtactgggtgaccttgaataacttcatttaa
gacattccaaatagaagattcatttttttgaatcatctttttggcagctttaatattgtt
aactaatccctgcaaaattagtctgtgaataacaaaaggctgaaataactccaaagccat
ttctcgtggcaatccacattgatgtagttttaaatgaggacctacaacaattacagatct

accggaataatctactcttttacctagcaagttttgtctaaaacgaccttgttttccttc
tataatatctgacaaagatttaatggtctattatttgcacctacaacagttctacctct
acgaccattatccatgagagagtcaacggcctcttgcagcattctttttcatttctgat
aataatctccggagctaaaattgactttagacgagataatctattatttctattaataat
tcgacgataaaactcattcaaatctgccgtcgcaaatctacctccatctaattggaccat
aggccgtaagtctggaggtatcacaggaatgactgtgaataccatccaagaaggatctgc
acctgttgctatgaaattttcaatcaatcttaagcgtttcattttttattgaattttaa
tgaaggtgtcttaaaactttttggtggatttgtagcttcagaccttaaggtttcagctat
gtgctctaaatctaaatctttcaacagtttctgaatagcttctgcacctattcctacttc
tacttggttattctcatcttgattttggtagatttcctcttcaagacttttccattcgta
accttcgagcagttgcttgtattttaaattaatatctgtattagattgcgtaactacata
agaatgaaaataaacaatttttctacttctttgacttttaagtctaatgctaaagcaat
atagcttgtacttccttttaagtaccaaacatgagttactggtgaggcaagttcaatata
agccatccgatgtcttctcacgcgtgattctgtaacttctacgccacatcgttcacaaac
tatacctttgtagcgaaaacgtttatatttaccacagtgacattcccaatccttaactgg
gccaaaaattttctcacagaataaaccatccatttctggctttaaagttctgtaattaat
tgtttcaggctttgtaatttcaccaacaatttgtccgttgggcaaacttctttcgcccca
ctgtcgaatttttcgggagaagctaaactaatttttacgtagtcaaaatattgctcaaa
ctttgtcataaatttgaataccttaaaatcaaataattagccaatttatttacatgcttc
atattgaaaaatgaatatcttaataaaggaattgttcaaaatcgtctactggaggagtat
cataattagaacgatctactcggttatctttagaatccgacatcaagtcaacttcaactg
tccgtctttggccatcttcaaatagtttcaatttatgtactgcaatgtctaaacctaatg
actgcagctctcgcattaagactttaaaagattctggagttccaggcttcggtataggct
tccctttaactattgcatttaatgcttcatttctagcttgcatatcatccgactttacag
ttaataattcttgcaaagtatatgctgcaccaaaagcttctaaagcccacacttccattt
cacctaatctttgacctccatgctgtgctctacctcctaatggctgctgcgtaactagag
agtaaggaccagtggatcttgcatggattttatcatcaacaagatgaacaagcttaagca
tataagctctaccaactgtaacaggattatcaaaaggctctcctgttctaccatcaaaaa
cttgcattttacctggatgctgatcattaaaaagccatttattgctagtaatcaatgatg
cttcttttaattttctatttacaagtgctcttgatgcttctgctccatacatttcatcaa
aaggtattattttaaatctctttcctaaatagccacctgctaagcctagtaggcactcaa
atacttgaccaacattcattctagaaggtacacctaaaggatttaaaacaatgtccactg
gtgttccatctgataaataaggcatatcttgtttaggtaagatcctagaaatgatacctt
tattgccgtgacgaccagccattttatcacccacttggatttttctcttctgcgcaacat
agacgcgaatcatagcatttgttccgggaggcagctcatctcctttctgcctagtaaaaa
ccctaaccttaactactctacctttttgcagcattaggaagccgtaaagatgtatctctca

catctcgagccttttctccaaaaatagctcgcaacaatttaccttctggtagttgatcag
cttcacctttaggagtaatttttcctactaaaatatctccagcttcaacccaagaaccac
cgacaacaattccatttctatctaaatccttcagagagttatcgctgacgttaggaatct
ctctagtaatttcttctggtcctaatttagtttgacggcattctacttcatacttttcga
tatggatagaagtatacaagtcatcataaactaatctttcgctaattaaaaatgcatctt
cgtagttataaccttcccaaggcatataagcaactagaatatttctccctaaagcgattt
cccctccgtctgtggatgcgccatcagctaaagtttggccaacaactatttttttctccca
cccaaacaattgggcgctgattaatacaagtatcttggttagaccgataatatttttta
agcgataatgaactgttctaccactattatcctgaatgcctattttattagcagaaacat
aatttacatgaccagatgttctgctaataacaaccataccagagtctcgagctatttag
tctcaagaccagtaccaattattggcttttctggatataataatggaacagcttgccttt
gcatattagatcccattagagctctattcgcatcatcatgttctaaaaaaggtattaatg
atgttgcagctgaaattacttgaataggtgaaattgcaatataatctacttgggttgggg
ttgttgtaataaactcttgacgataacgtacaggaataatatctccttcaatgtaatgtt
gcttactaactttaacatctcctggagctactcggaaatcatcttcctcatctgctgtta
gatagacaggactattgtgataaatcacttgaccttggtttaccggataaaatggggttt
ctatgaaaccaaaaacattgacccgcgcacaggttgctagtgaaccaattaaacctgcgt
tcggaccttccggagtttcaattgggcaaattctaccatagtgactaggatgtaaatcac
gaactgcaaaacctgccctgtctttattaaaacctcctggacctaaggcactaattcttc
tcttgtgagttagttctgcaactggattagtttggtccataaattgagaaagctgactag
atccaaagaattctctcactgacgcaattaatggcttgggattaattagatttgataaac
tcaatgaatctatatcacatatcatcattctctctctaataatgcgttctaaacgattga
gacctactctgaattggttctgcagcaattctccaactgaacgaactcttctattaccaa
gatgatctatatcatcaagattacctgagtttttatccttaatattaattagataatcaa
ttgaggataagatgtcttgaggagacaatactcggaacgttttaggtatattgagaccta
gtttttattaatttatatctaccaacttcacctaagtcatatcttttaggatcaaaaa
aacgagagtaaagcatctgcttagcaacagcaactgttgctggttcattaggtctaagct
tagaataaactatgagcagggcttcctcatcagttacttcttcaatattatttttttccaa
tttctttagctaattctttaactgaatatatttgggatgcagaaataagaaaagcatatt
tactaagtcctttttcaatttcatctttattgagtccaatagctcgcagaaaaatataag
catttactttgtgggtcttatctattcttatccaaatttctcccttaggatcaatttcaa
attttaaccaagaacctctattagaaattaggcttgcactataaatctgcttaccatttt
tgtctatctcttgtttataatagattcctggactacggattatttggttaataataactc
tttctgtcccagatacaataaaagttcctctatttgtcattattggcaagtctccaataa
aaactaatctctttttgtactttttatttttttatctgcttttcagcactgagctgtagat
gggttgatgataaatctaatgatttaatattttttttcttggtttttagaaggtagatcaa

tatcttttcttgttaactttgcaggaacatatatttgagcactataagttttatccctat
tttttgcctgtctgacactataacgaggaaattttatcttgtactcattaccaaataatt
gtaattcgagtcgactagtagggtctgatatcttcggaaaaatttcaagaacttccgtca
aaccttctaataaaaaccatttaaaactggccctctgaatttcaactaaatctggaagaa
gtttatttttttaagcttatacgttgaaccatagatctcctttataacaaattttgaaatc
gaagagtttaa-tactattaatagagtactggtagataatgcataagcatagtttgctac
attatttcttctctagacataactagaacatcatattttattcaa--aaaaagattttta
aaatttttcttcac--acacactatctataagtactttgctgcta--aaagatagttcaa
gctattaaa-------aaaaacgaaattaatacttgtctgtctgtcaattaattgacatc
aagatagacaagtattatttatttaatcaattctg-tttttctgagcaaaagacaatgct
ctcgcaagtctagcttttttttctagcgcctgtattagggtgaaatgccccttttttttaca
gctttatcaatcttactatatacttgagaaatacttgactgcacatcattgaaattatca
ctttctaaattatcaatatttagaaggcatcttttagttaaagttttgacaaccgattta
tacttacggttaatgagacgatttctttccgaagttttaatgcgtttaatcgcagaaagg
ttcttagccacagtaaatccaataaaacttttataaaatatttttagaaacgaaaataca
caaatctgggatctattcaaaaagggaatatgcggcgtattataacattaaagaaaaaca
ataggcaatagttcaacaagagctcttgttattacacaatagagattgtcattgcgaaaa
actttgtaagatagtatttaattaaaataaactaactagacatataaatatatatgaaaa
aaattgaagctattattagacctttcaagcttaatgaagtaaaacttgctctagtcaagg
aaggtattggaggaatgacggttatcaaagtttctggatttgggagacagaagggtcaaa
ctgaaagatataaaggatccgaatactctattgatatcattgataaaataaaaattgaaa
ttattattagcgatgataaagtagagaaaattgtagagacgattattaaggcttctaaaa
caggggaaattggagatggaaaaatatttattagtagtattgaaagagtaataagaatca
ggactaatgacttaaattttgaagctttatagtatttttacttt-tttagacttgatact
tcttcactaaacatgagataataagcgtttattgtatacttataactaaggtgtaata--
ctaatggctaaaagcaaaggtgcacgaatcgtaataactttagagtgctctgataaggct
ggagagtttgctcaaaaaaggaaacctggcgtttttcgatatacaactactaaaaataga
cgaaatacaccaagtagaattgaattaaacaagttttgtcctaattgtaatcagcattgt
atcttcaaagaaattaaatagttatttatcttattaaaat--aatcatattatggctata
tatagaaaaagaatatctccaattaagccaacagatgctgttgactacaaagatattgac
ctgctaagaaaatttattacagaacaaggcaaaatactgcctaaaaggtcaactggattg
acttcgaagcagcaaaaaaaacttactaaagcgattaaacaagctagaatcctttcttta
ttaccttttttaaataaagattaa--atatatagcctaacttcatatttttccatctaaa
tattaaaaatttctatagatttgtttattaaaatgatctaacatactgaat---------
----------tactaagaaga-tctactcattacaagttttgttcattttataattaata
atagcattataattta---ttaagctagattc-----agacataaag-gtaacctctttt

aattgattatattatatagtctatttaaaagaggaaatttttttgcttc----------t
agcaattcaagattctttaagctttaacaaggtatttaaaacaaagatttttgtttaggt
atcagatcaaatgaatgaatcttatctcccgactgccataattgaaattcagagatgaaa
attccacattcgttgccagcttgaatttcttcgacatcttctctaacgcgctttaaagat
tcaatttttccttgataaataactttttcttctcgaatcactttaatccaagagtttttt
aatagcttattattgatcactcgacatcctgctatttttctatttgctaacgagaatact
gtacttacttctgcttctcccaccggtacttctgaatattcaggatcaagtaagtcttcc
atccttcttttaatgtcttcaattaaggcataaatgatttgatagttttctattaatata
ttcgactttgccgatgcttgctttgttcctggagcaaagttggtattgaacccaatgaga
gtagaatttgtggttgaagctagttcaacatctgtagcagtaatttctccaggcatgatt
gagacaacattgagttgaactttactctgaggaaattgagataaagaatctaggattgct
tctgtagagccttgattatctgttttaataattaaagaaatttgcttactaatatcttta
gaattagtgtttttttaaagtatctaatgtaattcgactattaagtgctctttgtttctgt
ataattgatgagtctttagaggtattttcaatcgctttaagtttggcttctttatcactt
ttaactgctaaagctatttctcctgtagctggtacagatgacaatccccaaatttcaaca
acagatgatggtattgctaaattaattttctctttagcgttgttaattataactcgaatc
tttgcgtaagctgatccaataaccaaattatcactaatattcaatgtaccattttgtatt
aatagagttgctactggaccatgcgatttatctaaatgggcttctattataataccttgt
gcaggctgtgttggatcagctttcaaatcctctaattcagctaacaaggtaatagtttct
aataatttatcaacattttgaccagtaagagaactaataggaattataggcacttggcca
ccaagtttctccgacatgacattgtattttaataaatcttgctcaataatatctgtatta
gaaccagctttatctatcttagatattgctatgacaaaagggacattcgctttttgaata
tgattaatagcctctatggtttgaggtttaactccgtcatcagcagctataataataata
gcgacatctgttaaattagctcccctagacctcatacttgtaaaagcttcatggcctgga
gtatccaagaaaacaatttttttgtttattatctttcttaatatactcaacttcataagct
gcaatggcttgagtaattcctccaatttctttgttagcattattagattttcgaatataa
tctaatagcgttgttttgccatgatctacatgtcccataactgtgacaattggaggtctt
ttaatataattgccagtttcataaaaagcattagagttatctaaattactagaaagtcca
ttattattttcctttacatttgattctactgctataccaaaattatcagccactgatgat
attattgaggcgtctatggtttgattcatagtaactgatattcttttaagaaataaatat
tttataaatatctgtttcttgaacacaaattagtttagataattcttgaatagttagagga
ttagtaatactaatagactctggcggagaattagattgaatgcttgctgaattttgacta
ggagttacggctactttttttctgtcttatagatttttttattactagtcagtttttttaca
acttccacttttggcttaggtggacgcattaaagagatagctaaatctcctgcagtctgc
gaaacattagaattagagtctctaaagttgtca---tcatcatcgtcaatatgtatttta
gtttttattttt-ttgcgctgtctattctta--tttttttttactatctactaagtcgtga

attttattaaaatttttactttttttatctagtttaggtggagaacttaattctagatgc
tgttcaccacc---tgtatgtgattctgatttatcaaggtctaaatttaacagattatca
ttattgactgactctaatctaattttatagataatctgaggatttttcaaatctacaatt
gtttcagaagaattgatatttgagctagatctagaggatctattttcaaagttttgatta
tttaaaaacataaatattggttttattatctaatagtaactttctaattttaatacagag
cttaatataaacaagaatcgaatatctgtataaataatacaaatgt---agagaattaat
ctatgttgtattagatttaatataacactaaagtcctttgttcgttatacaagtttaaaa
ctttt-tatgatgataaacactagattctagtttattggtatacaatctatttatctaaa
taatactgtttttgtcagggcttttaaa----ttatctaatgttatgttcattatgaaat
tctattaaaatttaaaaa-ctcaacaattaagtaagaatcgttaatcacctccacgccac
ttatgccacgatctcaaaaaaaatgataattttattgacaaaacttttacagtattagctg
atattgtattaaaaatacttcctacaagtaaagaggaaaaagaagctttctcttactatc
gagatggaatgtctgcacaatctgaaggtgagtatgcggaagctttagaaaattattatg
aggctttaaaactagaagaagatccatatgatagaagctatattttatataatatagggc
ttatttatgctagtaatggagagtatgtgaaagctttagaatactaccatcaaggattag
aactaaattttaaattacctcaggcccttaataatatagccgtgatataccattaccaag
gagttcaagcaattgaggataaagatacagagctatctaaattaatgtttgataaggcgg
ctcaatattggcagcaggctattaagttagctcctgataattatattgaagctcaaaatt
ggctgaaaacgacaggacgaatgaggaatatacaaggatattaatatatttaagatataa
taaagatatcaattaataaattaaaattagttaaagaaactggtactatatagagtactt
-tactatatattataatattatactagttgatctctatctctaagacttagctgaataac
cgaaattaaatctattgactatattcatgaatacaaataagcataatggttagtacaaca
aaaagtaccggatctgttactcaaattatcggaccagttttagatattgcatttcctaac
ggacagcttccgaaagtattcaacgcactcaaagtacaaagctcagaaggaactattact
tgtgaagtacaacaacttttgggtgacaacaaggtacgagctgtttctatgagttccact
gaaggactacaaagaggggtagaagttattgatactggatcccctatatctgttcctgta
ggtacagatactcttggacgtatttttaatgttttaggtgaacctgtagataatttgggt
cccgttgattctgagagtactttacctatccatcgaccagcacctgcttttactaagcta
gagacaaaaccaaatatttttgaaacaggtattaaagtcgtcgatttacttgctccttat
agaagaggtgggaaaattggtttattcggaggtgctggagtaggtaaaactgtattaatt
atggaactaattaataacatcgctaaagctcatggtggagtatctgtatttggaggcgtt
ggtgaaagaacaagagaaggaaacgacctatatatggaaatgaaagagtctaaagtaatt
gatgcagataatctgaaagaatctaaagtagcattagtatatggtcaaatgaatgaacct
cctggagcacgtatgcgcgttggcttaactgcattgacaatggcagaatactttagagat
attaacaaacaagacgttctattgtttattgataatatttttcggtttgtacaagctgga
tcagaagtatcagctctactaggccgtatgccatctgctgtgggttaccaaccaactcta

gcaactgaaatgggagcactgcaagaaagaattacttcaacaacagaaggatcaattaca
tctattcaagctgtatatgtgccggctgatgatttaacagacccagctccggccactaca
tttgcacatttagatgcaacaacagtactttcgaggaacttggcggcaaaaggaatttac
cctgcagtggatccactggattcaacatcaacaatgctacagcctggaattgttggaact
gagcattattctactgctcaagaggtaaaatcaactttacaaagatataaagaactgcaa
gatattattgctattcttggtcttgatgaactttcagaggaagacagacaaactgtatca
agagcaagaaaaattgaaagattttatctcaaccttttttcgtggcagaagtgtttact
ggatcacctgggaaatatgtatctttggaagatgcaattaaagggtttcaaatgatctta
aaaggcaatttagacgacttgcctgagcaggcattttatctagtaggtgatatagatgaa
gctatacaaaaagctgacagcatgaaagattaatataattaacgatgactttaaatataa
gaattattgctcctgatcggactgtttgggatgcagaagcacaagaaattattttaccaa
gtagtacagggcaacttggtattttaacaggccatgcacctttgcttacagctttagata
ttggagttatgagagtaagagtagataaagaatggatgccgattgttttgctgggcggtt
ttgcagaaatagagaacaatcaattaactattctggttaatggcgcagaagaagctagtc
aaattgatttatcagaagcagaaaaaaatttagacactgcaactcaactcttaagtgatg
cctcgtctaataaagaaaaaatagaagcaacacaaaaaatacgaaaagctcgagctcgag
tacaagctgcaacagcagcaacttcgtaa-aagattcc------tcttaaaacataaaac
tttagtaattattactaaa-------gttttatgttttatttgtatggttataggcagct
taaataaacttagcttagacctgaacaaatataatcaaaatataatcccatctctttacc
tgcatctggtccaactaaactaatagtaacttctttcatagctaaaatagcttgaatcgt
tgcaccaataggaacacctagagaattgtatgtttcttttagaccatttaatactctttc
ttcaaggatagatggatctccagctagcattccataagtagcatagcgtaagtagtagtc
taaatcacggatgcatgcagcataacgtcgagttgtatacatattaccacctgggcgtgt
aatatccgaataaagtagagattttgctacggattctttaataattgttgctgcattagc
tgcaatagtagcagcagctctgactcgtagctcacctgtttgaaaatagcctcttaattt
ttcaacagagctatcatctaaatatttaccttggacatcagctgcattaataacagaagt
aattgcgtcttgcataactttcacacattccttaattttaaatatctaatacta--gggg
tatataacaaaattactagatcttttagcgatatcagtattactgcatagcacccaaagt
ataatcaaagtaaaaaccagcttctgctgaatcttcacctgcaagcagtgagcaagctac
acttttcatgcattttacaccttcagcaactcctgaaattggtgttcctaaagaattata
catttctttaacacccactaatccaatttcttcaataggagttacatcgccagcaactat
tccgtaagttactaagcgaagataatagtccagatctcgtagacaagtagcagtcatctc
ttcgccataagcatttccaccaggagaaactacatcaggtctttttttgaaacagttgttg
gccgccttgctttacaatacgctcacgattatctgttaaaatttgagctattcttaaacg
acgttgtccagataaaacaaaacttttaattctatctaattccccaggacttaaatatct
tgcttctgcatctgcatttacaattgactttgtaacaatactcatggataaaactcctgt

aatgcttt--aaaaaaagcttaatactaattatttatgatataactcagaaagtaatgta
ctttttgag-aattttctaaccgaaacacgttcatgatatatgaagcaggatagatactg
aggtaagtactaaaacgagaaagttatatgtaggaacttctgttattgatttcctagtac
tttttaaaacttggtaccacaattgatatatcctgcttagtcaaacgattatagagtgt
ttctgtattaggaaagttagctgctggtaaagtcggaaatcttctgtatggaacagtatc
tttgccgtataccgcattgtattcaacactttcaactaaattacttataaatgcagataa
gccttttgaagctaaaatttgattaaagaatctaatttctgcctgattattaggagctct
tcctaagatatgcttagtacctaattctataacttttgtatttggatatggctgataaaa
ttctttaccatatagttcagatagcgccagtttttctactagttctttgacacagatttg
tctattcaagaaagctgacttaatatccaagaattcaccgccgacactaaaagagttaag
atctctttcgaaaatttgacgatatgctgctcttaaagcttgttctaacatttctttgtt
actatcactattcacttcaaagacaatagattgatctcttaaagaagttactccttgtga
aatacgagactgaatatcattactactcctcatttctttgacagtgcctaattcaacaaa
cctagcagatttactagcaataactttttgaatctctggtcaataattcctggtcttaa
gcttcttaaagcaacaccagctggagtactatatctttcataaggaacagtattatctcc
aaaagtttctgtgtactctgagctatcaattattgtgtctattacttggtaatagccttg
cttatatgcaatatcaaaatatttattaatttcttgccgaccatatgtagggcgacccag
taagcggttatgaatatattcaatagctttacaaatatataatggctcccaataaagaga
tctaaatatacttgattttgctaactgtctaacaaactcgcgaactgtaatttgattatc
ttttagctgactttctattggcttgagaattagtttttcttcttgatacacttctctgcc
aaaaactcttaaataagcaacttttgtgacaacttctactgaattttcaaaattttggga
tgaatctgactttataagagcgttggacaatttaaagatttttggtcccagggatcctgc
tgacttaggtcttacctgtggattactaatctgattgtagattcctggcccacgtctaac
taatatccttcttgtatctttaccaaaaagagcttgtctttttcttggatctttattttc
tttagggaaaatagctccaaattgaattgataatggatcattacctattccatatggatg
ttgatcaggtaatgattgcttataatcactaaataaagttataaattgaggaaccttacg
aaaaggcgcactataggtgagaagatctatttgaggcccccaattacggcactcttgagg
ctcttcacctaaatttctaaagtaaggcacagtctcttcaccgaaataatctgtatactc
agaagaatttaaaagtgcattcactaaaccacttaaaccagtagatgataaaatagcaaa
atatttttgaaactcttctaaagagctaggacctctacccaagaaatgtctaaatgctaa
ttctaatgctcgactattgacaaaaggttcataaaattgctttctataaatacttgaagt
tcctaacgaacgaataaattccttaattgaaatttggccatttttttacttgcgactccaa
attcgataaagagaggtcatatgctttagcaatatctctttcaaaaatttgtctataaca
tgctttcacaacaatatttttctcatcagcagataaactaggcttcataacaaaacgagg
tgttgacactcctgcctgtacataagtctgaggaagacgcaaaccttgtaaatctccaga
tattctttttcttaatttatcagtcaaagatggagcttcaaattcagaaatcacaacatt

aaaatactctttaactaaatcttgaccttttatatcttcctcaaagattaataatgctgt
ccgtctcatctctctaagagccacaattgctgctgcacttgagcaagcgttatcaattaa
ctctctcaatcctcgtatattaacagataaaatattagggtcgcctgatacaattgcata
tgtcaaataccgtaaaaaccagtctaaatctcgaagagattttctcattcttgtagtacc
gtatctcaagacgtttataggtttaaaccctggcggagtagctcccccagcattaaataa
agatcggaaactttggccaaaatctccttggatatttcctgataactcattaattttatc
ttgagaactttgatctcctgcaataataacagctgcttgcggacgttctaaataagaaat
tgcagacccacctacgaatattttatctgctgctcgtgctaccagaatatttgcattttt
agttaaaatatctgcaacttccagtctttttgtccagaatttaaaaaagaaactaattg
atttaattcaccaagctgtaaaaatcggtcttgttgctctgcctgagtaatagttaaaat
tgaggcagtccgataaagctgggggcgtgctaatgggcttccgccacttgctttgatact
cattactttatttatctccttaactgatattgcttgctagcgaatacaaatatagcttga
tatttctaaatatttaaagatactatgcaagcaca-ctgttttctatattattaggtact
agtacttatagtacagagagtcttataactaacttctataaatacaaatttgtaatact
tctattaacatttgtcaaaaaaatatttaagtgctcactcttttatattaaaattcaatt
agtgagaggttgatgtgaaataaagtatactaagtaagttttatagataggaaaattaaa
agatttattctacaaacattaatca------------cttgaatgtttagcaattgtaca
attgttattgcttttaattggtttagactagttttatattttttctaacataaacaatat
taatgagcaatttgtatgaatttaaagccacaaaacaacctattaactaacgaaaatata
gatcatgtagatatacccgaaaatgatataccaatgtcaattacggaacatttagaagaa
ttaagacaacggactttatttgtgtttttattctttttgtttgctacaactataagtttc
acacaaattaagatcattgttgcaatactgcaagctcctgctgttggtattaagtttta
caactggcaccaggagaatattttttttcatctattaaggttgcaatatattgcgggatt
gttgcaacaactccgtttgccgtttatcaagttatattatacatactcccaggactaact
ggaaaagaaagaaaaattattttgcctctattaattagttctgtattgctttttattaca
ggtggtatatttgcttactttgttctcgcaccagcagctttaacattttttaattagctat
gggtctgatattgtagaaccattatggtctttcgaacaatactttgactttattctatta
cttttacttagtacaggattagcatttgagataccaatcatacaattattgctcggtgtt
tcagggacattttcttctagtcaaatgatacgagcttggagatatattattattatagca
acaattgctggagctattctgaccccttcgactgatcccgttacacaattaataatgtct
tcagctgttttattactttattttggtggaattgttatattattagtcttaaaaaagtag
agtaaat---------attttcta-ttaatccaatatatttgttatc-----tgggcaaa
taatctat-aaaacaattgct------------------agatttaagtatcaggcttct
aaatatttcaa---------gtctcttttatcattgaaatacaatc-------------

--gatacattgg
cttgtttttcatcaattatttgtgttgcacac-ttttagtactctctttattgttctt
agaattatttctttaatctatttattaaat----agatatgaagagatttaagtattgat
tatggtaatctatatcaacgtaaaaagtattttattgtgagttttatttatataaaggct
tattcattatatagttataacagtaagtctaattaatag-ttttcatgcttaattaaagt
aataactcttaattcttttggcagaaaaatctgtcttaatattttatc--aaaaaaacag
-aaaaatgaagctaaatagtctaattaacttaattcaaaagtctatatattcttgtacac
ttttactaattatttaaatattatttgtgtcgcacctaattctagtaatgcatttccaa
tttatgcgcaacaagcttatgaaagtccaagagaagcaactggtaggatagtatgtgcca
attgtcatcttgctcaaaagcctgtggagatagaagcgcctcaagcagtactacctaata
ctgttttcgagactgttgtgaagattccatatgacagcaatgctaaacagattttaggta
atggcagtaaaggaggcttaaatgttggagccgttgtaatattacctgaaggatttaagt
tagctcctgttaatagattatctacagagttaaaagaaaagactagaaatctttacattc
agccgtacagtgctaaacaagacaacatttagtaattggacctatttctggtgataaaa
atagagaaatagttttcccaatactatctcccgatcctgcaaaagataaaaaagctcatt
ttttcaagtatccaatatatgttggcggaaatagaggacgaggccagatttatccaacgg
gtgacaaaagcaacaataatattgtctctgctttaagcagtggtaaaattaataaaattg
aattactagacaaaggtggatttataatacatgtgactaacagtagcaatgtggagtcaa
cacagaagatttcacctggtctcgaacttagagtaaaagaaggggatacaattcagcttg
atcaagctttgaatagtgatccaaatgtaggtggttttggtcagaacgaaacagaaatag
ttttacagagtccaaatagaattaagggcatgattgttttctttttcgctagtgttctag
ctcaaatttttcttcgtattaaagaaaaaacaatttgaaaaagttcaagcagctgaaatga
atttttaaagcaatataaaactggcacttactataaagaaagta-taaacactttattca
caggatgtaccagtttttatgttatgtaataatcgctttgacggaagttatgatactttt
aacagaatctacaatctgactaggctgaattactgtagcctgctctaagcttccattata
tggtgtgggtatatcctgcgaagatagcctcacgacaggagcatctaattcatcaaaaag
atactcattaatttgtgcaattagctctgctccaattccggctgttttcatacactcttc
tacaattaaaactttgtgagttttctttactgagatagatatagagtctatatccagagg
ttttaaagatataagatctataacttctggatcataaccttcttttaataaggccggtaa
tgcttggataacatgatgcctcattctagaataggttaaaatcgtaatatcttttccttt
tcgtacaaattcaactttattgagaggtaagaaatattcttcttgaggaatctcttcttg
taaattataaagtagaacgtgctcaaagaatacaactggattattgtctcgaattgcaga

tttcagtaatccttttgcattataaggagtagaacaagcaactatttttaagccggggat
ggcttggaaataagcttccagtctctgagaatgctctgcacctaactgcctaccaactcc
tcccggtcctctaataactaaaggcaatgtaaaattacctccagaagtataacgtaacat
tcctgcattattagaaatttgattgaatgctaataataaaaaactcatgttcataccttc
aacaattggtcttagccctgttatagctgcaccaattgccataccagtaaagctattttc
tgctattggtgtatcaagaactctcaaatccccatatttgctatgcaaatctttagttac
cttataagatccaccatagtgaccaacatcttctcctattacgcagacagttggatcttt
tgccatttcctcatctgttgccgctcgtaaagcgtcaaacataaagatttactcatttt
aatatttgatttttatgatttgaccactgttttaaagtaacagttaagacaccatattct
atttagttatctgcaaaaagatatcgtttcaattctgacatgttaggttctggactggaa
atagcaaatttcacagcttgttccaattcggttttaacagcattctgaatctcattaagt
tcacctatattagcaatttcattatctagaatatatttttttgagtttttttgataggatct
cttgccacccaagcctctttttcttgtcttgatcttagttcatcaggatctgcgagagag
tgaccacgaaatctatatgttaatgcttctattaaggttggaccatcaccttggcgagct
ctttgaactgcttgttttgcagcttgtcttacagctagcacatccattccatcaacttca
atcccaggaagcccaaaagcttctgctttttatgtatttcaggtattgaagaagaccgg
tgatgtgccatacctatagcccattgattattttcaacaacaaatataataggtagtttc
cagagaactgccatattcagacattcaaaaaattgcccattattggtagtcccatcacca
aaaaagcaagccgtgactcttaaatcctctgtttctttaagtacttgctggcggtagata
ctttgaaaggctgcccctgttgcaaccggtataccttccgcaataaaagcaaagccacct
aaaaaattgtgaggcgcagaaaaaatatgcatcgaacctcctctgcctttactacaacca
gtctcttttccaaataactcagccatcacattttagatgggacgcctttacttaaagca
tgtacatggtctcgataggtactgcaaacataatcagttggattcagaagtttaattaca
cctgtagaaacagcttcttgaccattataaagatgaacaaaaccgaacatctttccttta
taatacatctgagcacacatatcttcaaaatttctgcctaacaacatgtcttcatataaa
actaataaattactcttattaagattaagacctgttgaattatagttagtcagcggcaat
tgaactttcttaggataactcataatttgtaaagaaacctct----------tattgtaa
ataggaattttgaagaaacagctatcaatagaaaacacacagataaaaacttataaatta
taatatgtttttttactaaagactttaataattatttgaaactagttcagtatatcaaatt
aattttgcttatactacggatgtctgaaacttatataaaaaagtagt-a-aaaaagttta
ttaaaagaatagttaaaaacagatataaatagaagtaaacagaattatt-gaaaaaaaat
gattattattaattaa----tacttaatatttctaaatattttacaattaatggctatgt
caccatctttattttatcctgttgaacaagaactatgtagtcttgaaaaaaatctgaaag
ctgttgctgggactcgtcatccaattttatatgccgcagcaaagcatctattcgatgctg
gaggaaaacgagttagaccagctcttgtatttttagtggctaaagcaacctctgagaagc
aagatataaatactggacaaaaaaggctagcagaaattactgaaattatacatactgcta

gtttggtacatgatgatattattgacgagtgcacaacacgtagaggagtcaaaactgtac
ataatttattcaatactaagattgctgtgctagcaggagatttttatttgcacagtctt
cttggtatttagctaatattgaaaatttagccgtagtcaaagctatttctaaagtcatca
ccgactttgcagaaggagaaattaggcaaggcttagttcatttcgatcccagtatttcaa
tagatgcttacattgagaaatcattttacaagactgcttcactcattgctgctagttgtc
ggggtgcagctatgcttaatggttccaatcatcaaataaataatgatctttatctttacg
gtaaacatatgggattagcatttcaaattatggacgatgttctagatataactggttcta
ctaagagcttagggaaacctgctggcgctgatctaataaatggaaatttgacctctcctc
tcctttttcacttactcaagaagcaagtttaaatgatcttattgatagggagttctgta
atagtacagatatagcctcaacattatttcttataaaaagaagcgggggaattacaaaag
ctaaagatttagctaaagaacaggtgcaggcggcactttcctgccttcagtttttaccac
aatctacacctgtatctagtttaaaagaattaacacatttcataatcacaagattgtcat
aaaggacttgctaaaattaaaatgttattaactaatttttttactgtttctaaaacagtct
gaaaagtttgaatatcattactagctagctgcgctagcattttacgatttaacgcaatat
tctcttttttaatgcactaataaaagtactataattcatgccttgattgtgagcagcag
catttattctagttatccaaagacgacgaaaatctctcttttccttttcgaccaacat
aagaatatcgaagagctttaaggacttgctgcttagctgttcgaaataaacatttatgtg
cacctttaaagcctttagctagcttaaagatttagctcgtctttttttgcaacgttac
ctcttttaactctactcataaaatattac-ttaaattttaattattaccagcttactaaa
ttcttatactctttg----attataaataaggtaatttcattgcaatgtttttaatatct
tttaagtcaaccgaacaagtagaagaaagatgtcttctttgttttgatgactttttttgt
aataaatgacttttagaagctttatgtcgaagaaattttccggatgaagagactttaaat
ctttttgctattgcttttgatgtttttaacttaggcatattataaacttttaatgattaa
caaaagcatgaaaagtctgaggatatcaatcttagacctttcaatacttagaataatctt
aacacaatttattttacttactacataggttaactgaaacaatttatatatataatacta
atcagttaatcaaccatagctaaagtggatttatattttgctttgaacttttgtatagta
ttagcaattagcattgttataattttaagactgcttgaattcaattcttcgaacattttc
atatttaatgtaaatgaaatattggcttctgagacaatattttgaatttgaacatcagac
aagggaatcatatctaatgcagctctatattgatctttaaataatttatcgtcttttatt
tggtcaaaatcgtaaaatttcgttcctcctgaatctgataaattcatagcccctctagct
atttttttaaaatttggccgccagaaaggtcacccaaatagcgcgtataagcatgagca
actaataattcaggttgtttatgtcctatagtgtgaattctatcaacataaatttttgta
gcaggagatggttcaataaaatctaaccaatctgatccataataatagtttaaatcttca
gatagactcgctttcctattaagctctgtaaaatatataggtttaatagctggatgattt
ttattagaaaataactcttcctctattgcacaatagacaaagtatagatttgcaaccaat
ttacgatatgacttcttatctacaactcctcccaaaaaaagatttaacaaaactaacattt

tctgccatactatgggacttagtagtaccttctcttagttcattcgctaaagtattaacc
ataatatcaatattccattttt-aaagataacaaattaaca---ataaatagttatccgt
atatttataattgagttgcgctaactaggctgctgcaaaataatttttttgattttattgg
atctggattcattgttttgtcaccaggcttccaattagctggacaaacttcatctggatg
agattgtacatattgaattgcttgtaaaaccctcaaagtttcttctacactcctgccaaa
ctctagattattgattgtagagtactgaataattcctttcgggtctataataaatagtcc
tcttagagctacaccatcactattcaaaacattataagctgcactgatctcttttttttaa
gtctgatactaatggatatgaaagatctcctaatccacctgattctctgtcagtctgtaa
ccaagcaagatgagaatactcactatcaaccgagactccaagaacttctgtattaagttc
cgaaaaagcattatacttatcgctaaatgctgtaatttctgtagggcacacaaatgtaaa
atctaaaggataaaagaataagacaatatacttattcttgagatcagataattttaacgt
tttaaattcttggtcataaacagctgtagctgaaaaatcaggagctagctgacctactcg
aagacaatttggtccagaaatcattaatttttcccagaagaattgatataattttgttat
ttaataaataattatataacaaaatttagtatatattaaaatactacaagtctttttttaa
attac--------taaatgatataatcaatactatctttatagcggggaatggatttgaa
ccattgaccttcgggttatgagcccgacgagctaccagactgctctaccccgcggataac
taaaatcatattagtctacatatatatataaaagcaaatgcataatagattttagaagta
aaaaaatagaagaactggtccactgatcattaaacttagaaacgacaaagtatttttttat
cttatttaaaagagggaagacttcatataaaccaataaatctatcttttaaaagaatttc
ggacggctttacccaaatttgaccgtcataccagcctgattcttcataaaatactgtagc
actcattaatcgttttactacgtatgaccagcctaaatataatctgattaaaataaatcc
tgtcattaggctggaagtaataaattctgaaaagaaaaatttcaaaggtagtttagtgat
tggaaaaattgataataatataggactaactaataaacagttaagtagtagtgcaatagt
tattttttattataagatctacggcttaaagttggccagcaaaagaaccaagaattttt
caaagaagtatattcatgaacaggttgctgctcctttggcacaggacattgagtattata
taaattcattgacattaaaaagtattataataaagac--tttgtttatattatctaacat
aggtacgacattaaatattaactaactgcaattatagtagtgagtagaagaaataaaact
aaaaatagccaagtaattttatttaaagtattctcagtactacgagtattactaaagaac
tgattctgagcacctacacttcctaaaccttcggatttaggattgtgtattagaatagtg
aagattaatattattgtagatgagtaccaaaaaaatttttaaaatttgttccattatgtaa
aagttaat-aaaaatagcacaaagacaatcgtctaaaagatattgattgtcttatcatat
taaaaagtatttattcaccctgtacttttaaaagtaaaaatcctattgccagccctacca
aaactaggactgaacttaatacagcactttctacaatttctccgaccatt-ttatttttc
cttattattttattattg---tcattcttttgacaagattatattgagctaa-ctaaaat
ccgtttctaccccaaactaccattgcgattgaaaatgtaaacattgccattagagctgcc
catcccaaacttaaaatatccataatttataatattc--ctaaaaataagatctctttta

cttaaactataatattcttaatattaaaaatatcattatataataagctaattataacag
agtactacttaattcgtattttagtgtatttaaagaagattgaa-ttttttcgaggtac
tggataagtatttcatcctaagaaccatagtcaac----ttttgatttattataatgctt
aaatctaaatata--tatcactcaagaatcattagtaattatgatgtttttaattatacg
att---aacatatttcaaaatagaagaattcatagcgactaagttaa-----tatgctaa
tataatttatcaat--aaaaaggaagttagtatttcaattatgcaaacaactataaataa
tgggcaaacgtctagtaaagaaactttactaacacctagattctatacgactgactttga
agagatggctaatatggatatttccggcaatcaagaagatttttggctatcctcgaaga
atttcgagctgactataatagtgaacattttattagagacgaagagtttaatcaatcttg
gtctaatttagaacataaaactaaatccttatttattgagtttttagaaagatcttgtac
cgcagaattttcaggtttttactatataaggaattatccagaaaattaaaagacagaaa
tccggttatagctgagtgctttttattaatgtctagagatgaagctaggcatgccggttt
tttaaataaagctatcggggacttcaatttatctttagatttaggatttttaacgaagag
tcgcaagtatactttttctcacctaaatttatttttatgcaacctatctttctgaaaa
aattggatactggagatacataactatttaccgccatcttgaacaacatccagaacaccg
tatttatccaatctttagatttttgaaaattggtgtcaggatgaaaatcgtcacggaga
ttttttgctgctttgctcaaatcccaacctcatttttaaatgactggaaagcaaaaat
gtggtgcagatttttctattaagtgtatttgcaacaatgtacttaaacgactttcaaag
aattgatttttataatgccataggcctagactctagacagtatgatatgcaagtaatacg
aaaaactaacgaaagtgccgctagagttttcccggttgctttagacgtggacaatccaaa
atttttcaaatatttagatacttgtgcatgtgataatagagctctgatcgatattgataa
taacaattctccattattcattaaatctatcgtaaagatacctctatatttttccttatt
tgcaaatttactaaagatatatttgattaagccaatagactcaaaaacagtatggaatac
agttcgatagttaaatacaagggaatgctttttcttataaaaagctatctagtgattaa
tataaaat------aaaagcttccttct-----cgatggaaggagcttttatttgtcaa
ga---atataatgggattatatagatc-tagaactaagaacttacttttcttttgcttc
atacataacctctaaagtaattagattttagaacaatcacgggcaaatttctctgtatt
tctttttactttacctctaacaaacccaggaatttatttaattctttctgtgcttcttc
tgaccaattaatactatcaacagcagaaattcctatacttaatgcttctgttgtatcatg
accaccaaatatttctagcaaatgatcttccataccaagtgtaaacgaattatatactaa
atcagcaatttgatttgttccctcataaccaagaaatggacggtaacttaaaggaaagtt
ttgtatatgcactggtgaagaaataacgccacaaggaatatttagacgcttaccaatatg
tctttccatctgtgttccaaaaattgctgcaggttcagttttagcaattaaatctccaat
taaaccatgatcatctgaaacgattacttcatcgcagaattcttgaacttgatctttaaa
ccactcttcatcatatttgcaataagttccacaccaggcgacatgtatacccatttcctg
gtgtaaaattcgagttattgctgcagcatgagttgcatctccaaatacaatagccttttt

acctgttaaattctgacaatcaattgatcttgaaaaccatgcagattgtgaaataaatcg
ggtttgctcgtctatgtatttttcgtagtctacagcagctcccaatgcatttactaattg
ctgtattgatcggatgcatgcagctgtttgaacaatacccataggtgtaatgtcaacata
aggcatattaaattctttctctaaatatcgagctgtcattagtccagtttctctatatgg
aataaaattaaaccatgcagagggtaattttttaaatcttgaactgatgcattttctgg
aataatttggtttatttggatatctagatcttgaaataaccttttaattctgctatatc
atgttgattatgaaatcctaagcttactgctccaataatattaacagatggagttttagt
cttttgagttaaaacttgattattagatttagcttttccatatagaaagtgacaatttg
ttctaatgttctatcacctgcttgcagttcatttactctataatgattaacatcagctaa
taaaacatctgcttctgtttctatagatgctctgctaacaaagttttgcaaatcttcttg
caaaatactcgaagtacaagttggtgttagaataactaaatcagggctttcttctctgtc
ttttctagtaatattttctacaacttttttcttgagaaccacgggctaacacatgtctatc
tacaacacttgctgtaacaggagtaaaatctctgtctctttcaagcattgaacgcattac
attaaaataatcgtctccgagaggcgcatgcataattgcatgaactttttttaaaagaact
ggcaattcttaaagttccaatatgagcagggcctgcatacatccaataagctaatttcat
attaaattttcctatgatttatttagacatagtaatactttttaaagctaactaacaaaca
ttagccaaacatctaatttaactccttgacaattttgtaatattttttaaatgaagctta
tcattataaatatttaaaaaatataattcagaataga----aataactgttagatcaagc
ttgaaagtaaagatttcttgcagttatagtttttctttaagtaaacatgtaaaaattgta
ctatatataaatgtactatatataaacttttttttactgaatattggtaatgccagataca
attaacttaaatatgccttccccaacgtttggtggaagcactggcggttggttaagagcc
gcagaagtagaagaaaagtatgcgataacatggacaggcaaaaatgaaagtaagtttgaa
atgccaactggtggtaccgcaactatgcgaaatggagaaaacttactctatttagctaag
aaagaacaatgtttagctttaggtactcaactaaaaagtaaatttaaaatatctgactat
aagatttacagagtttttcctaacggagaagtgcaatatttgcatcctaaagatggagtc
tttccagaaaaagttaatactgggagagctagcgtcaacagtgttgatcattctattgga
caaaatattaatcctgtagatgtcaaattcatgaataaagcaacttacgattagtctctc
-aaaaacctagacataaaataactttatatagtctaggtttttgataatgtatactgat
agtatggaagggtggccgagtggttgaaggcgtctgatttgaaatcagttgaacta--tc
tggttccgtgggttcgaatcccaccctttccgttataaaact------------atagtt
ggcttctcaaggcaagctagctatacactgatagtattaagtttagacttaataaagtct
acaacttgtgacaggttagaaatttttttcggcatcctcatctggtatctctatactaaat
ttttcttctatggccattaccagctcaactgtatctagagaatcggctcccaggtcactt
gaaaaatttgcttccctagtcactatttttttttcaattcctagctgttctgctacaata
tcttgaactttttcaaagatttcattatcttgcataatatattatttcctctaaggtgtt
atcaaaaatttttcggcttatattattatccaactctaaaaaattagtaatttttagtcg

agattaaaatatttatacctatatgatatactattattctggataaatacgaagtttacg
atatggctcttctccaaaactacgagctctcaactggtagttgtcaactagattatgctg
catttttcgtatataggctgatcgaggtgtaagctgaactattgaactttcattcaagat
aatagtttcaatagccaatttagcttcttgaagagcctggatctcatcaaaatttttatt
tttacagagttctacccaattaagaccagatgaagtatgaatgtttaatatttttctgag
tgcacgagtaatttgtggaaccgtactattctgaattgtatatataataatttgttttga
ttttgcaatttgtctaagttttgtattttgctttacttgattacgtagcgctagaatagc
atcagacttttctatctccttggttaaaataattggtagatctagtgaagaaataactga
tgtaatatgctgccaactcaaagaataagcatataagtactgatgaggtatttccaattg
caaagattgtttattaatattaataggggcattgaaagtagttgacaataaagatgtatc
acgattttgtgatttattcaaatctaaggacttatttcttaattctctatattgtaaaac
cggaggcgcctgctgtgcgttaattgattttcgagcattagagatgtttacaggtaagac
ttctatagattgcgatggataacatttaattaaaattctaccattagcttgaatttgtct
tttttgcacaaaaggttgatggccttgcaaaatttgatctattgtctctttaactttatt
atgaactatccaaacatttcgctcgtgtatttcgatcgcaatctgaaaagcaggctcagc
ttttctttctagtatactcttttgcgtacctctacgtttagcttcatcgtcacctaatgt
tacatattgaattccaccaattaggtccgccaatgttggatttttaattaaactttctaa
atgatttccatgcgctgtacctactaattgaactcctctttctgctatagttcgagcagc
taacgcttctaattccgtgccaatttcatctataataataacctcgggcatgtgattttc
aacagcttctatcattacttgatgttgcaaatcgggccttgcgacttgcattcttctagc
tctaccaatagctggatgaggaatatctccgtcaccagctatttcatttgaagtatctat
aataaccactctttttttccatttcgtcagctaaaacacgggccatttctcgaactgcggt
tgttttaccaactcctggttttcctaagagtaaaattgaatcaccttgctgtagtaaatc
tcgaataatactaattgtaccaaagactgctctgccgacacgacaggttaagccaataac
actaccttctctattacgcagtgaactaatacgatgcaaggtttttttcgataccagctcg
attatcgccgctaaaattaccaactttttttacacaataatctaagtcttgccaactaat
agatctttgagataaatattctgggttatctggaaatctagcttctggacgacgacctaa
atccataacgacttctattaaattatttctattaggatgctgttgtaaaggttctttgac
aaaatttggcaaaatttctagcaacttatctaagtcatctgcaataagcatgattgagta
tataataattaattaatcaaatagaagattcaagattctaaagattacaatacatattaa
agaatattatgattggaatcaattttatacttaaattgaacaggaagtta-tagatgtaa
aagaactgattaaactttacgcaataaatcgattggtcaaaattaatagtcgtgttaaca
tgtttattggacaaaaagttcgtattaaatatagcaaacaaaaagttgctagtgatatag
cagataaagttggtgaattaggagttattaagggaataaagtttattaatagtcaatgtg
ttactattattgttgagtttgacaatcacactaggctttggatgtttagagaggaattaa
tctgtctaaatgaat------taaaaacatgaaacataatttattttatttattatacaa

aatttcttatgagtcgttaaaataataaattttaaaggagtacttttaattgtgcaaatt
actattaaaaaattacaagacttactttcgtctgtacaaagaaaaaagatccagacatta
aaactaaaacaaggtaagtttgaacttctattaaataagacctataaaaaagtcaatcaa
gaaattataccttcgcagaaatctgctgtattacaaaatagtccatctacgataattaaa
tcaataaataatacaaaaaaatcttctgttgtcaatgaagaccgtacagaatatgccact
attgtttctccaatggttggaacgttttatcattcacctgctcctggtgaaaaaattttt
gtacaagttggcgatgaggtcaaattcaatcaaacagtctgtattattgaagcaatgaag
ttaatgaacgaaatcgaagcagaaattgaaggcaagattatagaaattcttgttaaagat
ggtgatatagtagattgcgggcaagccttaatgaaggttgaaacataatgctaatttttc
catttacatattgttaacagaaattatttagatagaaaaattagcttatactatagtta
tgaaaacttatttcgcaaaggcac-taaaaactaatatataaaattatttactttatctg
ccaaaaatagaaataagcgttttgattccttgtctcaagagaggagaatctcaatggcaa
ttagctcaaaagagcaagagacaaagaaggtaaaaatctcggttgataaaaatcccgtag
atacttctttcgaaaagtgggcccaaccaggccatttttctcgtacactagcaaaaggac
caaaaactactacttggatttggaatcttcatgctgatgctcacgacttcgatagtcaaa
ccagttctttagaagaagtttcacgtaagattttcagtgcacattttgggcagctgtctg
taatatttttatggcttagtggaatgtattttcacggagcccgcttctctaattatgttg
cttggttaagtaatccaacaggtattaagccaagtgcgcaggttgtttggcctatagttg
ggcaagaaattttaaatggcgatgtaggtggtggctttcaaggagtacaagttacatctg
gatggttccaactgtggagagcatcaggaattactacagaatttcagctttactgtactg
ctattggcggattagctatggctgctttaatgctgtttgcaggatggtttcattatcata
aagctgctccaaagttagaatggtttcaaaatgttgaatcaatgatgaatcaccatttag
ctgggcttttaggcttaggctgtttaggctggacaggccatcaaatccatttgtctttgc
ctattaataagctactagattctggcgtgtctccgcaagaaattccactacctcatgagt
ttttaattaatagagagcttatggctcagctgtatccaagttttagtaaaggattagttc
cattctttactttaaattgggctgaatattccgactttttaacttttaaaggaggtttaa
accctgttactggaggtttatggctaagtgatactgctcatcatcatctagcattagctg
ttctatttcttgctgcaggtcatatgtatagaaccaattggggtattggacatagcatga
aagaaattctagaagctcacaaaggaccttttaccggcaacggtcacgaaggtctatatg
aaattcttacaacttcttggcatgcacagcttgcaattaatttagctatgatgggatctt
taagcatcattgtagcacatcacatgtatgcaatgcctccttatccatatattgctactg
attacccaactcagttatcgctcttcactcatcatatgtggattggaggattttgtattg
ttggagcaggagcgcatgcttctatatttatggtaagagattataaccctgcagaaaatt
ataacaatcttttagatagagtcattaggcatcgagatgctattgtttctcatctaaatt
gggtatgtatatttcttggattccattcatttggtttatacattcacaatgatactatgc
gggcacttggaagatctcaagatatgttctctgatacagctatacagttacaacctattt

ttgctcaatgggtacaaagtatacacactttagctcctggaaatacagctccaaatgcat
tagcaacagctagttatgcatttggaggagatattgtttctgttggtaacaaagttgcaa
tgatgcctatttctttaggtactgcagatttttttagttcaccatatacatgcatttacta
ttcatgtaactgttttaattttagttaaagggttcctttctcaagaaactctagactaa
ttcctgacaaggccaatcttggcttcaggtttccatgtgatggacctggtagaggtggta
cttgccaagtgtctggctgggatcatgtttttcttggcttattctggatgtacaattctc
tgtctgtagcaatttttcactttagttggaaaatgcaatcagatgtttggggtagtgtat
ctccgtctggaaatgtttctcatattactggcggtaattttgcacagagtgcaattacaa
tcaatggatggctgagagatttcctttgggctcaagcatctcaagttattcaatcatacg
gttctgcgctatctgcatatggattaatttcttagcagcacattttgtatgggcattca
gtttgatgttcttatttagtggtagaggttattggcaagagcttatagaatcaatcgtat
gggcgcacaataagataaaagttgctcctgcaattcaaccaagagctttaagtattactc
aaggtagagcagtcggtgttgcacactacttattaggtggaattggtacaacttgggcat
tctttttagcgagaattatttcagtaggctaatagtgaaaataggataaaaaaaacaatt
atggcaacaaaatttcctaagtttagccaagctttatcacaagatcctacaactagaagg
atttggtatggtattgctacggcacatgactttgaaagtcatgatggaatgacagaagaa
aatttatatcaaaagatattcgcttcgcactttggacatctagcaattatcttcttatgg
acatctggtaatttattccatgtagcttggcaaggcaactttgaacagtgggtattaaat
cctttgaaagttaaaccaattgctcatgcaatttgggatccgcattttggacaacctgct
ttgaaagcttttagtaaaggtggctcagcttatccagtaaatatagcatattctggcgta
taccactggtggtatactattggtatgagaagcaatcaagacctgtattctggggcttta
ttcttactagttttatcagctttactcttatttggagggtggctacatctacaaccaaaa
ttcaagcctggtttatcatggtttaaaaataacgaatcaagattaaatcatcatttatct
ggattatttggggttagttctttagcctggacaggtcatttagtacatgttgctatacct
gaggcaagaggacaacatgtaggatgggataattttacaaccgtattacctcatccagcc
ggtttacagccgtttttcagtggtaattggagtgtatatgctcaaaatccagatacagct
caacatttattcggaactaatgaaggtgcgggtacagcaattctgacatttctaggagga
tttcatcctcaaagtcagtctttgtggctaactgatatggctcatcaccatttggctatt
gcagtagtattcattgttgctggacatatgtataggactaattggggaattgggcacaat
ctaaaagatattttagatgctcatagaccacctagtggtagattaggagctggacataaa
gggctatttgatactattactaattctttacacatacagttgggattagcattggcttcc
ctaggtgtaattacttcgttggtagctcaacatatgtatgctatgcctccatatgctttc
atggctaaagatttcacaactcaagcatctttgtacacacatcatcaatatattgctggg
tttctaatggttggagcttttgctcatggggcaatattctttgttcgagactatgaccct
gaacagaataaagataatgttttggctcgtatgctagaacataaagaagctatcatttct
catttaagttgggtaactctatttttagggtttcatacattaggcctttatgttcacaat

gatacaatgattgcttttggaactcctgaaaaacaaattctaattgagccggtatttgct
caatggattcaagcctcttcagggaaagcactttatgggtttgatgtgttactatcatcc
tctactaatatcgcaacacaagctggtagcaatatttggctgccaggctggttagaagcg
attaatagcggaaaaaattcattgtttttaacaattggtcctggtgacttcttagttcat
catgcaattgcattgggattacatactactacgttaattttagttaaaggtgctttagat
gcaagaggctctaaacttatgccggacaaaaaagactttggatatagttttccttgcgat
ggacctggcagaggcggcacctgtgatatatctgcatgggatgcgttctatttagctgta
ttttggatgctaaatacaataggttgggtaacattttattggcattggaaacatattaca
atatggcaaggcaatgcaactcaattcaatgagtcttcaacttatctaatgggatggttt
agagattacttatggctaaattcttctccattaattaatggttataatccatatggcatg
aataatttatcagtatggtcatggatgttcctatttggacatttagtatgggcaacagga
tttatgttcttgatctcctggcgtggctattggcaagagttaattgaaactctagcatgg
gcgcatgaacgtacacctttagcaaacttgattcgttggaaagataaacctgttgcatta
tcaattgtacaggcaagattagtaggtttagcacatt-tttctgtaggatatgtattaac
ttacgcagcttttgtattagcttcaacagcaggcaaatttggttagactaaa-----ttc
gaattagctaa-----aaaaaagtcagtcataata-gttttatgactgactttttagat
ttaaaacaaaaacttactaaagtttcacctcaatatctacaccagaaggcaaatttaact
tcattaatgcatctatcgtttgagaagaaggttgatgaatatcaataattcttctgtgag
atcttatttcaaagtgttctcttgagtctttatctacatgtggagaacgtaaaacacaat
aaattcttctttttgtcggtaaaggaattggccctactgcaacggcattagttctagatg
cggtatctagtattttgtcacacgacgtattaagtataatagaattatatgctttcagtt
taattctgatttttgtctgctgagtaattgtcatgttaaaaagatcttattatttaagaa
ttttagagacaacacctgcgcctacggtacggccaccttctctaatagcgaaacgcatac
cttgctcaattgcaattgcattaattaattcagcagtcatcttaattctgtcaccaggca
taaccatttctgcatcagtaccatcatcagcagtaaactgattaatagtaccagttacat
cagttgttctaacataaaactgaggtctatatcctggaaaaaatggagtatgtcttccgc
cctcctctttagttagaatataaacttctgcttcaaattgagtatgaggtgtaattgtac
caggtttagctaataccatacctctttcaatatctttttctgcacacctcttaaaagaa
ttccaatattatcacccgctagaccttcttctaacgtttttttgaaacatttctaatccag
taatagtcgttgtacgagtttctcgtaaacctacaatttcaattgtgtcaccaactttaa
taatgcctctttcaattctaccagtggcaacagttccacgtcctgtaatagaaaaaacat
cttctacagccattaagaaagttttatcgacatctctctccggcgttggaatatatgtgt
caactgcttccataagtgaaaaaatcttgtcaacccatttatcttcaccttgcttaatag
ctgggtttttcgtcacagcttctaatgccaataaggcagaacctgcaacaaaaggaatat
catctccaggaaagtcgtattgacttaataattcccttccttctaattctactaattcta
gtagctcttcgtcatctacttgatcttccttatttaggaatactactaatgtaggaacac

ctacttgttttgctaataaaatatgttcacgagtttgtggcattggaccgtctgccgcag
atacaactaaaatagctccatccatctgagcagcacccgtaatcatattttttacgtagt
cggcatggcctggacaatctacgtgagcataatgacgattatctgtttcgtattcaacat
gagcagtattaatagtaatacctctagctttttcttctggagcggcatcaatttcatcaa
attttttcgctgcagtagaccctaaagttgataaagtcgcagagatcgctgctgttaaag
ttgtcttaccatgatcaacatgaccaattgtgccaatattgacatgaggtttttttacgtt
caaatttagatcgagccatgctttttgttttccttatagtaaaagttgctttatagttac
ttttaacgataaatcttgtggattgaaagatttattatttaatacagacaaga-----ta
caatataactaatctaatatcgatagtgagcaaacgctttatttgcttctgccattctat
gtgtatcctctctctttctaatagaatttcctgtttcattagctgcatccataatttcat
tagctaatttcatagacatacttttaccagatctgtctctagagaatttagtaatccatc
ttagtgctaaattcgtacctctataagctcgtacttcaataggaacttggtaagtagaac
caccaactcttcttgcttttacttctactagaggagtaatatttcggatggctttttcta
aaatatttaaaggatctgattctgttctctctttaacgatatctaaagcctgatatataa
ttctttgagataaagtttttttaccacttttcaaaatacgaacagttaacatacttacga
gtctgcttttatataaaggatcaggtgatgcaaacctttttttagctgtattacgacgag
acatagtatttaaaattggttattgtaataa-tgttc-tattagcaggtatttaagattt
aggctttttttgtcccatatttagatcggcttttacgacgatcttttactcctgcagcatc
taaagtaccacgtactacatgatatcggactccaggcaaatctttaattcggccgcccct
aatcagtaccacagaatgctcttgaatattatgacctacgccaggaatataagctgtaac
ttcaaatccagatgttaatctaacacgcgctacttttcttaaagcagagttcggtttttt
aggagttgtagtatatactcttgtacaaacacctcttctttgagggcaactttgaagagc
tggagattttgtttttttatgtatttttcgtctttcggatctaacaagttgttgaattgt
tggcataataaaattgatttaaagtctttaatagatactatttgaacttgctttacatga
attggctagtttttatttatcttatacttacgcataaatctttctactctaccttctgta
tcaataattctctgtgaaccagtaaagaatggatggtttcctgaccagatatctacgtgt
agttctggctttgtggacccaatcgtcataattaactggccgtcacagtaaacttttgct
tctggataccaatttggatgtatattatcttttgccattgtttttataattatataaatg
tatcaataataactattaacgcttagaaaattgaggagcctttctggctttctttaaacc
atatttttttctctcttttactcttggatctcttgttaaatagccttcagacttaagtgt
cgttctattttctggattaattgagcacaatgctcttgcaacacctaaacgaattgcatc
agcttgccctgtcaaacctcctcctctagcatttacatggatatcatactggtttagtag
tcctaaaacttgtaatggtgcatatgaaactcttaagtaattaggactaaattgaagata
agactctcctggtataccgtttataattaaattacctgaccctgggactagtcttacttg
tgcaacagaacacttccggcgacctgttccagaatagattgcgcgagttttaattaattc
tgtcgacataacattccttgataactaaataaatatacattctttatactatatactctt

gtggcttttgcgcgacatgtgggtgaattgggccagaatacactttaagcttcgtaaata
gttttctgcctaatggacctttaggaagcatacctttaaccgatttttcaataattctgt
taggtaatcttgtctgaagctgatcaaatgtttcaacttttaatccgccaggctgtccag
aatgtcttctgtatagttttgattcgttttatttccacttacagatacgtgagcagaat
taataacgattacataatctccagtatctagatagggtgtataagaaggcttgttttac
ctcttaagatattagaaatatgagtagatattctaccaagtgtctgattttagcgtcta
taacataccaatgagaattagtatttaatgagggtgattgcgttttattcatgaaaagat
taatacttttggttgacaataagagaatatgtaaatggtttacattcaatttctatacaa
gtagtattt-aaaaatatta-------------ttaacaaagagaacttttctaacagtt
atttaaattgtttgtcagcaagaaaatttctagttatcaataacttatactatttttaat
aatataaaatatattaatcactttttcttttggcaaacttatacctagtttttttgc-
aaggcatatataacttcttcagctgatttctggccaaaattttaatttctagcagttct
tcttgagagtaatctagtaaatctgcaatagaatggatttgagctcgctttaagcagtta
taagccctaacagataactgcagttcttctattaaaacttgactaatttttttatcttct
ttgctacgataattatctgctgatttaaagtctaaatttcttagtgaacaaaaaagatta
gttaaaactgtagctccttgacttattgcttcttgaggagagatgctcccatttgtccag
atttgtataatcaatctatcttttatgctgttgctaccaatacggacttcttctacttta
taattaactttattaactggcataaaaacagagtctacttgcaaaaaatccacagataat
tcatctacagctttttcagctaagcgatacccgcaatttttttcaattttaaactccatt
tcaaatattgtattattgcaaatagttgcaatatactgtctaggatctactacctctata
tcagaagataattcaaataggccggctgtaactatagctggcccttgaactctaattcga
ccaatttgagattctttgttataactttaaatactacttcttttagattaagtaatatt
tctaacacatcttctcttaccccaggaattgtagaaaactcatggttcactccagcaatc
cgtacagcaactatagcagtaccttcaagatctgacaatattgatcttctcaaagcatta
cctaatgtaataccttgtccttgatttaatggttctattacaaaactaccgtactgccca
cgcgccccatctgttcttgactctatgcattcaatttgaaattgagccacctaagaaagc
tcc-tttaaaataatcagtgattagtaagtctctctaaaacaagaaggaatttatactcg
gcgtttcttaggagggcgacatccattatgaggtacaggagtaatatcttttattagagt
aatctctaatcctgcagcctgcaaagctctaattgctgtttctcgacccgctcctggacc
atttactagaacttcagtttggcgcataccttgatccatagcttgtctagctgcttttc
agctgctgtttgagctgcaaaaggtgttccttttttagctcccttaaatccacttgcacc
agaggatgaccatgataatgtttctccttttaaattagtaatagtaacaattgtattatt
gaatgtagatttaatatgtgtaataccgttaactgcattacgtttagtttttcttgctcc
ggatttttttatttgtctagccatcgtgtctcgtctattatatagttaattaaaagatta
ttttcttggagctttttcttacctgctactgtttttttacctcctctgcgtgttctagc
attagttctagttctttgtcctctcaaaggaagaccaagacggtgtcttctacctctata

agtactaatttccataagtctcttaatgctcatagactcaaaacgtttgagatccccttc
aatctgataattagactcaagaatttctcttatactgacaacttgttgatcatttaaatc
ttgacacttaatatcagcgtctatgtttgtttttctaatatttctttcgagcgagatag
tccaataccataaatatatgttaaagctatctctattcttttgtttcttggaagatctac
tccagcaattctggccactttgtcttgtctccaataaataatatgtat-ttttataacgg
ttgtttata-agcttagccttgtctctgcttatgtttaggattagtgcaaattaccatca
cctttctgtgacgccgaattattctacatttttcacacatttttcgaacagaaggacgaa
ctttcatattaa----ctctatatactaggaatattactatattaactctattaattatc
gacattaaaaatgtcaacaatatattgtactactttaactgttgtagattttacttcgtc
atactatcatattttttgatatgacgtatgtctgaatttgcttagctgtgtctattgcg
actccaactaaaataagtaaagacgtagccccgagacctcttaagttttggatctgagta
accttttctattataaacggaatcagcgctactgtaaataaaaacgaggctcctaggaat
gtcagtctatttaatatgacttgtaaataatcaatagtcgcttgacctggacgaatatta
ggaatgcttgcacccatttttttaaatttatagcaatatcttctggattcattactatt
gatgtataaaaatagctaaagaaaaggatcaaagcacaataaagaagaaggtatagtgaa
ccattaggacaaaacaggtataggatttggagtaatgttttattttgaataatttgggtt
agataagatgggagggccatagatgcagatgcaaaaacaataggcataactccaccttga
tttaatttcaagggcaagtaactatttggatccaaaattgaagattttcctagctgtctt
gctgaaataattttaattcttcttgttccttcttgtacacaaattgtaattattatcatt
aataaaaagatcgctataaataatccgaacttaagacttgcattactataactagcatca
aaaaatgactgtgtaaaattctttggtagtcctgacacaatgttttgaaaaataagtaaa
gaggctccattaccaattcccttttccgtaattaactctgataaccacataataatcata
gaacctgctgttaaagctaaaacagactcgcagacaaatgcaaaattccaattaaaaaca
tatggttttacccatatagagattgctccagattgtaaagtcgcccaacctagagctaag
taccttgtaatttgagttatttttggcggcccaattcaccttcttccttctgtaatttt
tccagattggggacaattttcgtaagtagctgcattacaattgaagagttaatataagga
acaatacctaacgcaaaaatccctattgttgaaaaacctcctccagaaaaaatattcaga
aaatttactaaagtattttttctacacttgcataaaaggcgtcatgatctatacctgga
acaggtataaatattcctaaacgtgctaaaactaatagaaagagagtaaagataatacga
tttcttagatcactttttggctcataaataaattttaaatgaaaagagatactttt--tt
tatgtaaaaaccctattccaagacttgtataagtttaaaatagggaagtaa---aatatt
atagtaatattaata-taatattctatgtagaatacaggctttctattggaactcctcga
tctttagctgcttcactaaaagttcttaattgagttaagccattgagaacagctcgcgcg
ttatttaatgtattattagaacctaattgcttagctaatatattctgtacacccgacaat
tcgaggactgtccttacagaacctccggcaattacacctgagccaggtgcagagggcctt
aatataactttcgcggcaccagaaattccgttaataggatgaggtatagaattcgatttt

gtcagtggaactgtaactagatgtttttttagcatctgttactcctttttttactgcacca
attacatcgcttgcttttcccacgccgacaccaacttggccttgctcattaccaataaca
agaataactcgaaagcttagtttttttacctccttttacaactttagtaactcttttaact
tgtacgactctttcttcccagccactatctttatcttttcctttgctctgtttttttacga
ttggccattttaagaattattcctaattagttaatta-cgattctagaaacccatgcctg
cttccttagcagcttcagccaaggccttaactcttccatgatataactttcctcctctat
cgaagacgacatttttaatgccttctttcatagactgttctgctaactgtttaccaacta
cgcgagaagtatcacagtttggtcttatgttatcagattctttattatttaaattaacag
atgatgtagctactaatgtaataccttgtgtgtcatcaattatttgtgcgtatatatgtt
tattagatctaaatacacacagacgaggcctacttgaggttccttgaactttttttccgaa
ctcttttatgcttatgaattctagtttgtttagtgtttagtttcattattatttaccttt
tccagctttcccagcttttcttctaacaaattcaccttgatatctaattccttttccttt
ataaggctcaggaggcctaatagaacgaatagttgaagcaacctgaccgacaacttcttt
atctatgccagagacagtaatatttgtgttgttttcaactttaatttcaatattggcagg
aggtttaattttcactacatgactatagccaacacttaagattaaatcttggttatcaat
ttgagaacgatagcctacaccttgtatttgcagtttttttaaaaaatccattagaaacacc
ttcaatcatattactaataagagttctggatagcccatgtaactgactagccatttttgt
ttgtccactcgtcttgacagcaattgtgtcgttaagtatttctaaattaataccagctgg
taaagttctggatagggtacctttaggtcctgtcacggtgatagtctgaccatcgaactg
agtactaagatttgtcggcaataaaattatttttttttccaatacgagacat-attccacc
tataaaatagttaccaaatatagcataatatttcaccaccaagaccatcatgacgagctt
gtctatctgtcataacgcctctagaagtagaaataagagcgatgcctaaacctccaagaa
ctctgggcaactctttatgatttgcgtagactcttagtccgggtttactaattcttttca
gagcagtaataactggttgacgatttttaccattatacttaagagaaatcattaaatgag
tctctataccttcacccatttgttcgaaattttgaacaaatccttcttctttcagtactg
ttgccatattgcatgtcattttcgttgctggaacttgcacaatttgatgtcttgctaagt
ttgcgttacgaatacgtgtcagcatatcggcgatcgtatcgttgaccaccttgatcctcc
ttgcga-----tgaattatttgaaatattttcaagattccctgaaaggcatccctagctt
ttttaacaaagctagaccttcttgatctgttttagctgtagtgacaattgatatatctaa
accacgaatttgatcaatattatcatagtctatttctggaaagatcaactgttcacgtaa
acctaaattgtaattacctttgccgtcaaaacttctaggactaattcctctgaagtctct
aattcttggtaatgttaaattaattaatttctctaaaaaagaatacatcttgtcttttct
tagatgcacaacaattccgataggaacctcttctcgaattttaaatcctgcaatagattt
tttagcttttgtgacaattggttttttgtccagttattaatgtcaattcttgaatactact
ttcaagagccttagcattctgagaagcttcacctaagccacggttaatagtaattttagt
aaacctaggaacttcatgtacatttttgtactgaaattcatcttttaaagattgagtaac

agttgttttatatttttcttttaatcctattgccattatattttcatcaattatttaata
agttcgccagtctttttcagttttcgaa-ttttttggcctttatcattaattattactga
ggatcgactagcaatattattttgttcactaaataacataacgttagaggtatgtattgg
agcttcaaatttgataatttctcccgtttctccttcttgttgaggtttttatgcttcac
tttgagattaattcctttaacaatcaccttatttgttttataaataattgcaataatttc
acctgtttttgttttatcacttccagaaataacttgaactaagtcccctttttttaattt
aatttttgtattatttttggttgttttagaaggacctttcattatactacctccggtgca
agagaaactatttagaaaaatttttatctcttaactctcgggctataggtccgaagact
cttgtgccacgtggattattatcttgattaatgataactgccgcattatctccaaatcta
atgctcataccgtcagttctccgtaaagctttacgagttcttactacaacggctctgaca
acatcagatcgtttaactggcatattgggagacgcatctttgactactccaataataaca
tctccaatagatgcataagaaggattactagtgcctagtactctaatacacattattttt
ctagcaccactattatctgcaacattaagatagctttgagtctgtatcatacttttattc
tgtataagattaattatcaaaagacttagataagatattaaccattgtccaacactttgt
gcgacttaaaggtcgtgtctcttgtattgtaacaatatcgccgattgtgcattcattatt
ttcatcatgcgctttatatttctttgtccggatcatagtttttgcgtactttctatgaga
gattctattttctacagctactactatagttttattcattttatcgcttactactttacc
tgttgtttcttttaaaggcatagtctttatatttttttatatat---------aattaaat
tttaagattattacatggttgacttagtacgagacttttcaacagttaaaagttgggcta
atctatgcttagaatgtttaaatagatgaggctggaaatcttgccttgtggctcttttta
gccttaaatcaaaaagctctcttttttattacaaggatttcttcagctaaagaagaagagt
ccaggtttgtaacatctgatattttagggaaagtcatatttttaggattctgttgtattcc
gaactataaatttagtcttgattggtaatttataagaagctaatttcatagcttgttgag
cagtttttttgtggtacacctgtaatttcaaatagaatatgtccaggcttaataactgcaa
cccaatattctggagctcctttacctgaccccatgcgagtttcagctggacggcagtga
ctggtttatctggaaatactctaatccatagtttaccacctcttctcacatatctagtaa
tagttcttcgagtagcttctatttgtctagaagttaaccaaactggctctgtcgcttgta
atgcataatcaccgaacgcaattgtgttacctttgctagcagaacctttcattctacctc
tatgttgttttctaaattttgttttcttggggcttagcataaaaattaaaaaataataat
aattaagaagctgttgaatcaggagtttcctgattataagcagattctagacttttcgat
tctggcaaaatttctcctttaaaaagccaaacttttactccgagcacaccatacgtagtg
tgagcttggcgatgacaataatcaatatcggctcttaaagtttgtaaaggtacacgacct
tctctaacccattcacttctagctatctctgcaccattaagccgaccagatacttgaatt
ttaacaccctgtgtatttgctctttgagctctttgaactgcttgtctgacagctctacga
aaggccactctttttttctagctgttgagtaataaattctgctactaaggttgcttcagaa
tctggatctgcaatctctacaacatttactctgagttgtttgctagggtctaggattaag

gacagcgattttcttaaagactcaattcccgctccagattttccaagtacgattcctggt
cgagctgttgctatgagaatttctacttgatcaactttacgattaatttcaattttagca
atactagcattactaagttttgaatgtataaatgagcgaattttatgatcttcttgtaaa
agaactgggtagtctttagagttagcaaaccatgaagaacgatgtttttgggtaatgcct
atgcgaaagcccaaaggatgaatttttttgacccacagtatccttgattataagttaaaag
ctataatgactaattgacgttaaa--------agactacagttctgaaacacctaatgta
atatgacaagttggtttatgtattggaaaggctcttccttgtgctctaggctgaaatctt
tttaaagtcgggcctttgtctgcaaaggctttactaacaaacaattggtttttatttaat
ccatcattatgctcagcattcgctgcagcagattccaggatttgctttatatgagaacag
actcggtatggcataaattccagaataattaatgcttcttggtattttctacctctaatt
tggtctaatacacgacggactttatgcggagaaagacgaatgtatttccctactgcttta
gtttcttttacgtttttgtaatactcatagttatagtttaacgacgggcttttctgtca
ccttttacgtgagtgcggaaagttcttgttggaacaaactctcctaatttatgtcctacc
atttggtctgacacaaagacaggaaaatgttgtttaccattataaacagctattgtatgc
cctaccatatcgggaataatagtagatgctctcgaccaagtttttaggacttcttttttt
cctgaaatatttaatgcttctatacgtttaagaagactaacatcaataaaagggccttta
tgtatagatcttgacataataataaatcattagattcttaataaaataaattctatttac
ggcgacgtaagacgtacgggttactatatttatttggatttcgtgttttaacacccaatg
caggtttaccccaaggtgttacaggacgagctcgtccaattggagatttgccttcaccac
caccatgtggatggtctacaggattcatgacaacacctctaacagtaggtcttttaccta
accaacgatttctaccagctttaccaagagtaatattactagcatcaatattaccaactt
gtccaatagtagcgtagcattcttttcgaatcattcgaacttcgcttgaaggcaatttta
cagtaacaaaagtaccttcttttgctactatttgagcataagttcctgctgcacgaacaa
tctgtcctccgcacgaaggtcttaattctatattatgtactgctgttcctaaaggaatac
tagataaaggcaaagcatttccaacttcgataggggcagtaggaccagaaagaaccatag
atcctacactgagagatcgagggtgtagaatatatcttttctcaccatcaagatagtgta
ataatgcaattcttgcatttctattcggatcatattcaatagaagcaactttagcaacta
tattatgtctatttcttttaaagtcaattaatctgtactgctgcttatgtccaccacctt
tatgacgacaggtaatgacacctctattatttcgacctttgcagaaatggtgtttaacta
ttaatgatttttctggcttatcagtagtaatctctgaaaaggtagaaaccgttctatttc
ttgtccctggtgtgtaggcacgatataaacgaattgccatataagaagatttgagaagag
taaatgttgaa------ctatgtgatatatttaagtttctggaaacaaattaatagaatc
ttctgaggcaagtgttacaattgctttcttgtaatgtggtcgcttccctacgaatctacc
gatgctcctttttttcttaggaggatgacaagtgttaacacctgtaacttgcacgttgaa
aatatactgtatagcagccttaatattaatttttgttgctttgggatcaacggcaaaaca
atactgattctcttctaataatttagttgtcttatcagtaatgatgggatatttaaccag

atctaataaacctcttgaatcaatgctatccattatatacctcttgtattttagataaag
catcaactgtaataataattttatgtgcagctaacaaagccataatatttagtgtatcag
ctgaaataagttctacgttgtgtagattgcgaatagaaagataaacattcggatcttttt
tgtctactatgactaaaactttttatttaaatcaagattccatcgatgtatagcttcca
taaatagtttagtcttgggctgatgaaaataactgttaaaattttctacaatcagtgtat
taactgatttgttatttaatgctgtccttaatgctaattgtctttctttttattcattt
ttttggtaaaactacgaggttttggcccaaatattacaccaccacctctccatagaggtg
aacggatagaacctgctcttgctcgaccagtccctttttgacgccatggtttacgacctc
cacctcgaacttcacttcttgttttagtattagcagaaccctggcgcttttcattacttt
gctttactaaagctctatggactaagtacatcccagaatcttgactgaccttaagattta
aatcagcgttgccacttacttgaccttcccaattatagacttgataatttaattgcgtgt
taactgtcatgaaatatatcgtatgaaagaatattgctacttactaattttaccaaagc
gccaggtttaccaggaacagctcccttaacgatcaacaaatcattctctgagttaatact
tacaatttgcaaatttttattgtaacttttttattaccaagttggccagccatgtttttt
acctggataaactcttccaggcgttgtaccggctccaatagagccaggttgcctatgatt
tttcgaaccatgtgacattgggcctctactaaaatgatgcctcttttggtaaccggagaa
acctttacctacacttctagaagagacattaatcttttggcctacttgaaatagatctgt
agatagtatttggctaacttcgaaatcatcagtagatttcatttcgtattcacgtaaata
ttttagtggggagcctgtgatttttttaagtgtcccaacaatggtttatttaatttttg
ttcagcaacttgcttgtaaccgacttgaatagcattgtagccatcagtagatacagcttt
aatttgagtaataacacatggcccaacttgaattacagtaactggaattgataaaccagc
ttcatcaaagaattgggtcatacctactttagtaccgagtataccaacagacactagatt
tctcctttttgagagatttatactatcattgtacataaaaattctacttcatagttactg
aag--taaattaaactaacagatgcatgatattattcatgacaacataaaggttaaaaac
tttacctatttaatctaaaatagaaacagctttttgtctagttacagcgtatataaatta
gaagtatgcttattaaatttttttataaactact-tagatttaactgatattgatatccgt
aacttacttgcagtaatatc----aatcaacatgtataagtttaattgcctaaactagaa
ccagctattattagaaataattcaagtatagataaaagcgtatttgaacttttatatatt
gacaaatataatggaatatattgaggttcggtaactacaccttttttatttttacatttat
tgtgca--gtatatatacatctaatgcagtaaacataagaatataac----tgtatagca
aatgtaattacacaaattagagaggtattaatgggcaaagtcgttggaattgatcttgga
acaacgaattctgtaattgctgttatggaaggaggtaaacctaccgtcataccgaatgca
gaaggttttagaactacagcttctgttgttgcatatactaaaagtggagataaacttgta
ggacaaattgccaggaggcaagctgttattaacccagaaaacactttctactctgtcaaa
agattcataggacgtaaacagaatgaaatttcgcaagagattcggcaaacatcatataat
gttaaaactagtggatcaagcataaaaattgcttgccctgcactgaataaagattttgct

ccagaagaaatttcagctcaagtactgagaaaacttgttgaagatgctagtacgtactta
ggtgagactgttacacaagcagtaataactgtacctgcttactttaacgattcacaaaga
caagctactaaagacgcaggtaaaatagcaggcttagatgtattgagaattattaatgag
cctaccgcagcttctttgtcttatggactagacaagcaaaataatgaaacaatactagta
tttgaccttggtgggggcacatttgatgtatctgtattagaagttggagatggagtattt
gaagtactctcaacttctggagatacacatttaggcggagatgactttgatcagcagatt
gtagaatggctaatcaaagattttaaacagaatgaaggtattgatcttggtaaagataga
caagcacttcagagattgaccgaggctgcagaaaaagcaaagatagagctgtcaaattta
actcaaacagagatcaatcttccttttattactgccacacaagatggtccaaaacattta
gaaaaaactgtaactagagggaagtttgaagaactttgttcaaatttaatagataaatgt
agtatccctgtaaataatgctctgaaagatgcaaaactagaagcttccagtattgatgaa
gttgttttagttggtggatctacaaggattccagccatacagcaaatggttaaaagatta
attggtaaagatccaaaccaaagtgtcaatccagatgaagttgttgctattggtgcagcc
gtacaagctggagttttagcaggcgaagtcaaagatattctattactagatgtgacgccg
ttatctttaggagtggaaactctgggtggcgtgatgacaaagattataccaagaaatact
actattcctacaaaaaaatcagaagtattttctacagctgtagataatcaaccaaatgtg
gaaattcaagtacttcaaggcgaaagagaactaacaaaagacaataaaagcttaggacg
ttccgattagatggcattatgcctgcacctagaggagttcctcaaattgaagttaccttt
gatattgacgctaacggaattttatctgtaaaagccaaagaaaaggctactggtaaagag
caatcaattactatatctggagcatcaactttacctaaagatgatgttgaaagaatggta
aaagaagctgaagaaaatttcgatacagaccaaaaaagaagaaagaatattgacacaaag
aatcaagcagagtccctatgttaccaagctgaaaagcaagttaaagagtttgaagacaaa
attagccaagatttaaaaataaaaatagaagagctaattacagagcttagatctagtcta
gagaaagaagaatatgacaatattgaatctatttctcagcaattacaaaatgctctgatg
gacattggaaaaaatgctgctcagactgaaagtaaagatacaaaagcgaaggatgacgac
actgtgattgacactgatttctctgaagctaagt-aaaagta-agcgggtaacgcgattc
gaacgcgcgacatcaaccttggcaaggttgcgctctaccactgagctatacccgcataga
ttgtattattacaaaaattaagatatttgtcaatctgtta------atctatac---atc
tagactattaattttggaattacatattagctaactgttgtaaatataacaattgctata
attttttaa-------aagtacttatatcat-tatataagtacttttatatt-------
-caaaaatacaaataagtattatag-------ctaaactactaaggaattaactaagcta
gtaacaattactaatcctgtccataagccggctcctgtatatattaatccttttgattgc
tcccattggccaggggatgctaaaacaacaggtacgcctactactaacacagtcgataat
gcaattaataatagcactaataattggatcgcaataatcatttaatatgttctcctgaaa
aattcaaatgttaataatactgtatataatacaatgaagcttttgaccttcagcaattta
tctatgaagaaataatttaaaattattgttttctaaataaaccttaaattaagtattaaa

atctttattgtaaacaataatactatattttttta--gtagataaaaaatttaaagaaag
tctttttaaaaaaaaattaagataaggtgatatgctgtataaaaagctaagtattatta
attattagagaaatttaaa-ataagaggtaaaacatatgaactcagccctttttttagca
aaactaccagaagcttatgccattttaaaccgatcattgatatcttacccgtaattcct
gtatttttcttcttttagcttttgtttggcaagcagcaattggttttagataacattta
ggttataaaaagaataacaaatatacagagtgccaattttgagctctaatcttatatct
tacacaataataactattatggttgaacccttactatcaggaattgttcttggacttatt
cctattactttattcggattattagtagcagcttatttacaatatcaacggggcaaccaa
ttaggactgtaaagcctaaattatagatatttataaaaactacaataattatagtttta
taaatatctacaaataagctaattttaaatacagaaataatactattgaaactagagtga
taacaaaaaaaatttatgtaa---taa---------------------------------

---------------------------caagctaattgtttaccagcttgacttagtaac
tcctggtaaaagacattcatgagacatttctcgaaaaacatggcgagataatccaaaatc
tcgataataacctctacttctacctgttaaccaacatctatttctccctctaacagcggc
gctgtttctaggcatttcttggagtttttgtcttaaatccattttgtcagaaaagctgcc
agcttctttcagttgatctttaatagccagtctttttaaataatattttttgcaagttt
cgatctcttaatctctctttggatcatattctttttagccatgctgtaa-aactctcttt
taaaattatttattcaatatattagctaaaaagctcctgcctgcaacttttatatattt

aaaaataaatagcataaagtagtgaaatgcaattgatatttaatcaacgatacc---act
ttatgctattatcaaaaatttatttagtaaagttcttgttcaacatgagttgaaatagtg
caatcagatgaaggatatgcaatacaggttaaaacatagccagctaaaagttgatcatca
tctaagaatgattgatcagcttgatctacagttccttctgtaactttacctgcacaagtt
gaacaagctccagctctacaagagtaaggaagttcaattccttcttcttctgctgcatct
agaatatatgtatcctccgcacaattaaatgtgacatcaattccttcttcttcacatagt
aaatgaattttataatcagccatttcttcttactccttaataaagatacacttgaaactt
aataaagcaaaat-aatttaaagaacttaccaaaaaattatatactagtttcattatgca
tgatagactggaaaaaaagaaaatagtatactattctgaaaaatcaagctacatttccta
ctaaatagaactatagtaggtccaaagtatatacataaattcatatagttaaaattactt
ttatcatttaaatagctgaacttgatagta---------tagattgatcatc------aa
gctagtcttctgc---caataaataattaattatgatttcaatgcttaatcaacaagtag
aacttaagcctattcttaaatttcagataacacaaatatattcgcattctcttattcagg
aaattaaagccttaataactagacttgttttgcaagtttggagaaggccggctacattga
tggcaggtattatccaacctttattatggttaattctatttggtgggcttttctacaatg
ctcctataaatttgttcactattaatacaagctataattgtttttttgagctctgggatta
taatttttacctcttttactggagctttgaattcaggtcttccattaatgtttgatagag
aatttggattttttaaatagattattaacggctcctttagtatcgaggacttctatcattt
tatcttctgctacttttatgacttgtattagtttaatacaagttgtatttatagttacag
cttctcttttttatgggaaactcacctctaaatagcgatagtactatgatttttggactta
tgattctattagtgactgtaggagttacaatgcttagtttagctttatctttcactctgc
caggtcatattgagctattagcatttatttagtagttaacttgcccttttttattttcta
gtacggctttagctcctttatatttcatgccgccatggcttcagttaattgcaagtctca
atccattgagttatgcaatagaaggtacaagatacttatattcaagcgtaaactggaatt
ttacagagtgtgtgattaagattagctggggagatatttgtttagggcaaattattattt
tattaatcgctttagatataatggcagcttatcttgtgtctaatatattaaaagctaaac
ttaatta-----------------aaattttaataaaaaatttattaaatttattttaca
aagattttactatataatactaatagtagtatggataattgaaatagaaatagtttttc
aaaaccaaagctattatcaacaatgataaacatttgtaagaaagtcaacaaagtatgttc
ttattcataggaggcatgtagtcaatgggactaccatggtaccgtgtacacacggttgtt
ttaaatgatcctggacggttaattgcagtccacctaatgcatactgcacttgtagcgggt
tgggcaggatctatggcattatacgaactagctgtatttgatccttcagatccagtatta
aatccaatgtggcgacaaggcatgtttgttatgccatttatggctagactgggcgtaaca
gattcatggggaggatggagtataacaggagagagcgtatccaatcctggactgtggagt
tttgaaggtgtagccttaactcatatagtcctttctggcatgctttttctagctgctatt
tggcattgggtttattgggatttggaattatttagagatccacgaactggcgagccagcc

ttagatttaccgaaaatttttggaattcatttattgctatcaagtctactttgtttcgga
tttggagctttccatgtaactggacttttggaccaggaatgtgggtatcagatgggtac
ggagtaaccggaaaggtattaccagtagctccagcatggggaccagaaggattcaacccg
tttaatcctggaggggttgcatctcaccacattgccgcaggtactgtaggtatattagct
ggtgttttccatttaactgttagaccaccacaaagactgtatcgtgctctaagaatgggt
aatattgaaactgtattatcaagtagtatctctgctgtttttttctcagcttttgtgact
tgtggtacgatgtggtacggttctgcaactacgcctattgaattatttggtccaactaga
tatcagtgggatagtggatattttcagcaagaaattgagaaacgagtagaaaatgctatt
gctgatggtgctgcacctagcgaagcatggtcaagaattcctgacaagttggcattctat
gactatattggtaataatccagcaaaaggaggattattcagagcgggccctatgaataaa
ggcgacggagttgctgaagcatggcttggacatcctgtattccaagataaagaaggaaga
gagcttagtgttcgcagaatgcctgctttttcgaaacttttcctgtaatttagttgat
aaagatggtattatacgagctgacattccatttagaagagcagagtctaagtatagtatt
gaacaagtaggtgtaacagctagtttttatggtggcaaattaaatggccaagttttcaac
gatgctcctagcgttaaaaaatatgcaaggaaagctcaattaggtgaggtatttgaattt
gatcgaactacattagaatcagatggagtgtttagaagtagtcctagaggctggtttaca
ttcggtcatgctaatttcgctttaatttcttctttggacatctttggcatggttcaaga
actatcttccgggatgtgtttgcgggaatcggagctgaagttactgaacaagttgaattt
ggagctttccaaaaaattaggagatagaagtagtaaaaaacaaggagctgtataagaccaa
agtcaaagatagtctttatc---aaatatttctaatattaaaatagttaagacaaagttt
attatctaatagttactaatcaggagaatttatggaagccttagtctatgtatttttact
aacaggaacgctaatggttatattctttgcgatctttttagagaacctccaagaatagc
aaagtaacttgattccgtttaaacttataataatagaaccactttccaacttattagtgg
aaagtggttttattgtagattacattgtattattcttcatgctcttcaaaaggatctcgg
agatcttttgaagcaggaccaaatgctgtgtatatcgagtaacctgtaatacctaaaagc
agactcgaaataaaaatactaagaactgttgcagtttccataatttaagcagtattaaga
tgaactttttctataatgatattatcaatattaaaacatttaattattaaaattatcaaa
atatactatggcacttagaactagacttggagaaattttaagacccttaaattcagagta
tggaaaagttgctccaggttggggcacaactcctattatgggaattttcatgttactatt
tttcttgttttattaatcattttacaaatctataattcttcattagtattagaaaatgt
agatgtagattgggctactttaggtag----ctaactagataagttctttga--------
ttacacaatattaaagacaac----------------aagcgatcgctattagattatag
tagcgatcgctttaaaagtattttactatatatccagagaaaattatttacctaaattaa
ttaactcggtatctgcaaaactatttgtgttaacattattatagttaactttttcaaacc
ttactaatactggatatttgataccacttttatcaattgctgcaacagtaccaatttcct
gataccagtacgattcttttctcaatatttttacttttgagcctctttccatatttttag

tcctcagatagattattaaagattattatattaaaaaattaaaaaaaactaaacaaaata
tataaaaatactaccgacaattgtaatatataactaataaaaggagagagagggattcga
accctcgataacaaaagttatgacaattttcgaaattgttgcaataaaccactctgccat
ctctcctgaattaaaatactgttttaaaaaattagacaagaataacatagaactaaggta
gttgcctagaaaattaaaactatgttaaatataaattataatctttctatagatatattt
aaaaaatatgtacatagaatctatatcagcttacttcaagatttttaatcttatacaat
ttttactaaataaataaattctctcataatatgaaactatcttggaaaacacttcttctt
tggtctttaccaatatttgtaattggtttctttttctggcaaggtttttaggtccaact
actacagatgttggcagtaatatcgcaagttctagaatgacatatggacgatttttagaa
tatttagatatgggttgggtgaaacgggttgacctctatgaaaataatcatacagcaatt
gtagaagctgttgggccagaattaggaaacagggttcaacgaattcgagttgaactgcca
gcaagtgcgccagaattaattacaaaattacgcaaagccaatgttgatctagatgctcac
ccccctaaaagtacaagtgcagtatggggactattaggcaatttactatttcctttacta
ttagttggcgggttagctttcttatttagaagatctaataatgctagtggtggacctggt
caagcaatgtcatttggcaaatcgaaagctttatttcaaatggaggctaaaacgggagta
gtatttaatgatgtagctggagttgaagaggcaaaggaagaatttcaagaagtggtaaca
tttttaaaacagcctgaatcatttactgctgttggtgcaaaaataccaaaaggcgtttta
ttagttggacctcctggcacaggcaaaacattactagcaaaagctattgctggcgaggct
agtgttcctttttttagtatctcaggctcagaatttgtagaaatgtttgttggtgttggc
gcttctcgtgtgagagacctattcaagaaagcaaaagacaatgcgccttgtatcgttttt
attgatgaaattgatgctgttggtagacaacgaggaacaggtgttggaggtggtaatgat
gaaagggaacaaacattaaatcaactattgactgaaatggatggctttgaaggaaatact
ggtgttattgtaattgccgctactaacagagctgatattttagattctgcattattaaga
cctggaagatttgatagacaagtttctgtagatgtaccagattttaaaggcaggttagca
attcttgaagttcatgctaaaaataagaaaatggaacctaaagtatctttagaaaccatt
gccagaagaactcccggcttttcaggagctgatttagctaacttactaaatgaggctgct
atcttaacggctcgacgaagaaaaaatgcaatgactatgtctgaaattgatacatcaatt
gatcgagtagtagctgggatggaaggcactcctttaattgacagtaaaagcaaaagatta
attgcgtatcacgaagtgggtcacgcaataataggcagtttattagagcatcatgatcct
gtgcaaaaagttacattaataccaagagggcaagcaagaggcttaacttggtttactcct
agtgatgatcaaagtctaatatcaagatctcaaatactagcccgtatcgtaggtgctctt
ggtggcagagctgcagaagaaatcattttcggtgacgcagaagttactactggtgcaagt
aatgatttacagcaagtaacatcaatggcaagacaaatggtcactcgatttggaatgtct
aaaattggacctttatctcttgaaagccaaggaggagacccatttttaggtagaggcatg
ggaggaggctcagaatattcagatgaagttgcaactaatattgataagcaagtaagggaa
attgtcagtgaatgctatgcacaagctaaacacattattatagataatcgagtagtgata

gatagattagttgatttactaattgaaaaagaaacaattgaaggcaatgaatttagagac
atcgttaaggaatacactgcaattcctgaaaaaaattactacatatcacaattttaa---
---------------attaaacgggactgacgggattcgaacccgcaacttccgccgtga
cagggcggtgctctaaccagttgaactacagtccc-aaaaagatgaatacccagagataa
tctcatagttagcttaaacttgtcaaattagaatattaaatatgaagagta---------
--------tttgtaataatactcttcatatttgata-tagtagctaaaatatttctttta
gaaggagagagagggattcgaaccctcggtacgaagttaatcgtacagcagattagcaat
ctgccgctttcgaccactcagccacctctccatga---tatatattatatatatatcaaa
tatacttatattttaataggtgtgcataaatatttatggagctaagcggattcgaaccgc
tgaccctctcaatgccattgagatgctctaccaactgagctataaccc----ctttggta
agatattaagaaatggctcaagcgggatttgaacctgcgaccttgggcttatgaatcccc
tgctctaaccactgagctactgagccatagtataaaatactattaaatattataccataa
attatggtcaaatataaatcaataaaagaataattactatca-tttactacttttg----
-----------------tgcaagttgtaaatattcgaataaaatttcaaaattaaaaatg
tctttaactttttgttaga-------atta-ttgattacgaaa-----------atattg
atatttagaattaac---
--------------------aataaatttg------------------------------
---tctttattt
g---

-------tagaca---
-------------------------------caaaaatattggaaa--------------
------------------------ttttcatcaat-----------------taattagt

------------------taaactataaccgacgtac-----------------------

---aagaattattgaacta---

---cgttaaat---

-----------------------------------tgtgatgcatt------------
-----gggcttgatcaaaacttctctaaaatcaatagaatatagt---------------
---aattcaa
gtaaatcaaatactaacaaatgtgacaaacgcttaaaaaaataata--------------
caattagaatcgtaaaaatttggtttttga-ctgtgttaaataaatttgattatcataga
tttaatatttaaaagtgttctattacttgta-----ttgagtaattgt-aatatttaata
tcttctcttttagataataatcataatataagctaaaaaatagtttt---tattaaacaa
ctagcatagagccaataccttgatcagttaatatttctagcaagagagcatgatctattc
taccatctaaaatatgcgctgaagctacaccctgagctaaagaacgaatacaacaattca
ctttaggaatcatgccaccagaaattactgctgtttgagttaaatctctagcttcctgta
tattcagatgactaattaatgttgaaggatctgaagaattacgtaaaatgccgggcgtat
ctgtgagtagaattagtttttcggcattaagagcggctgctatttctcctgcgacagtat
cagcattaatattatacgattggccttccttgtctgctgcaacactagctataacaggta
tataattattattaattaatatcttcagtaatttagtatcaatattttgtacttctccaa
caaagccaaggtttgcttttccatttggtctcggagtaataagtaatccatcttttcccg
acaaacctacacttttaccaccttgtttattaatagttgcgacaagatctttattgactc
ttccaactaaaaccatttccactatatccatggtaggttgatctgttactctaacgccat
catcgaattttggtaatattttgaggcgatctaaccaaaaattaatttctggtcctccac
catgaactaaaatgggacgtagtcctataaaagacagaaaaacaagatcactaatcactt
gatcttttagtttctgatttttcatagccgctcccccatatttataacaataattctgg
aagaaaattgctgaatataaggcagagcttcacttaagacttttactctttctgaattag
tcaacataaataaagtaggattgtgatatataaaattatatacacagcttgtgttaattt
aaaagataccaatgggt---------acttgaaaaaatagaaatattactaacag-----
----ttaaaactatagggattataattatgaataaattttgggataatgtattaagattt
ccacgattttagtcagcgtcattttaggattaattttgataattattagtcctttttc
gtgttattaaaaaaaccactgacaagttttttttcatcatatcattggccggattaatc
acagttttggcaataataa-tacaaaaaatgataaatatcgaatgttgttgagagaaata
aataaatttaattaaaattttgaacatatataatattatattatcattaaatatgataa
ttt-atttcctttctat---aaaacccataaatattaaatattatttgataaatattat
gccactaaaacaaagagttagctctgaaaagaccggtgccttcgcacttttggatagtat
tgttaggcacggagttaaacatatatttggttatccaggtggagctattcttcctattta
tgatgagctttatgcttgggaagaagcctctctaattaaacatatccttgttcgtcatga
gcaaggcgctgctcatgctgcagattcttattctagatcaacaggagaggttggagtatg
cttcgctacttctggcccaggagcaactaatcttgtctcaggtatagctacagcacatat
tgattctgtacctatattagctataacaggtcaagttgggagagcttttattggtacaga
tgctttccaagaagtagatatttttgggattacacttcctattgtaaaacattcatatgt

agttcgtgaccctagagacatgtctagaattgttgcggaagcatttttatttgtaaaca
cggtagaccaggtccagtattaattgatgttcctaaagatgtaggattagagaagtttaa
ttatttttctgttgagcccggaaaagttaatattcctggctgtaggccaattaccagcct
aaagtcaagacaaatccttatggcagctaaaatgatacagcaatctagccagccattgtt
gtatattggtggaggagccataatctctgattcacatcaaattattaaagaacttgttga
tttttataaaatacctgttactactactttgatggggaaggggattttaatgaggatag
cgactattgtctagggatgttaggtatgcatggtactgcgtatgctaattttgcagttag
tgagtgcgatcttttaattgctttaggagctagatttgatgatagagttactggaaaatt
agatgaatttgcttgtaatgcccaagtgattcacgtagatattgatcctgctgaagtagg
aaaaaataggattcctcaagttgctattgtcggtgacgtagcagaagttgttagtgaaat
attgaatttattaaagacttctttccccccttatccagagcagattatatcttggcaaga
aagaattaatcgttggcgtcaacagtatcctttactggttcctagaaaatcaacaagcat
ttcacctcaagagattcttgttgcaactaataaattagcccaaaatgcttattttactac
agatgttggccagcatcaaatgtggtcagctcaatttctgaaagtaaaagctaagcattg
gctttcaagtgctggattaggcacgatgggttatggtttacctgcagcaattggcgctca
agtagcacatccaaatgacgtagtcatttgtattagtggtgattctagttttcaaatgaa
tatgcaagagttaggaactatcgcgcaataccagttaccagttaaaattattattattaa
taatcgatggcaagggatggttagacaatggcaacaagctttctatggtgaaaggtattc
acactcaagaatgacagaaggagcacctgattttcaaaagcttgcagaagcttttggtat
taaagcttttactattaataataggcaaaatatgcaatctgctttacaagttgctattga
ttatcctggtccagttttattagattgccaagttacagaaaatgaaaactgttatccaat
ggttgctcctggaaaaagtaatgcacaaatgataggtatcgctaaaccgcagagaggtac
tgcttccaactatattaataatagtgtttaaagtaa------------------------

---------------atagttagtatttttta-aacaaagaata-aagtaca--------
-taaatgtaatttttttatactatta-aaaactatgtaagaacagcctttgacgagaatt
gaactcgtgaccttccccttaccaaggggatgctctacctctgagccacaaaggc-tttt
tactatattgggccgggttggatttgaaccaacgtaggcgaagccagcggatttacagtc
cgcccccattaaccactcgggcaccgaccctaattaaaccttattcaagatatgacctat
aagatatcataactatgttgctaaaaacaagtactttgataaataaaactgaataagtaa
tggacatagctggacttgaaccagcgactttcacgatgtcgacgtgacactctaaccaac
tgagttacatgtccaaaactttaattattattataacactgtttagtgattttataagta
tctatttctaattgaagatttgttttaatcgtccggctttaccgagcagatctcttagat
aatacagtttagctctacgtattttagctcttctaatcactttgacaaaagttattcgcg

gtgaattgagaaaaaatactctttcaactccaataccttgaaatgagcatcttaaagtta
aactagtatttaagcttttttttcttcttctcgctaatactactccttcacataattgtt
cccgagttttactaccttccttaaccattagtccaagttgtattgtatcgccaactttaa
tttcaggtacttctgttttgataaaaggtatctcaacacttttcattagctgacttagct
ttgtactatttgctttcatatataggattagtcttgtgaca-tgttttgaaatattatac
attaaaaaaacaaatatgaaaaattttagcgaataagcatactagtatttataaaac-tc
tttttatct---
--ccattata--------
----------------cagatccatt------------atattcaact------------

----------agattcttttatagtttc--------------------------------

--------tttgataatcagtac-------------------------------------

---------------------------atcta----------------------------

----gatgatcaaatattattttcaaacatggcttatgtatgaaaaagttctata---aa
aacctaatgac--------taaaattagggcttaagtatttcttagttttgtgttataat
tagttactatcaaaggtagtggcataacacgaagcatcaa-aatgttcgaacgctttact

gaaaaagctataaaagtcataatgctagcacaggaagaagctagacgcttaggacataat
tttgtcggaactgagcagatactattaggattagttggtgaaggtactggaatcgcagct
caagttttgaaatcgatgaatgtgaatctaaaagatgcaagagttgaagtcgaaaaaatt
ataggaagagggtctggttttgtagcggttgaaattcctttcactcctcgagcaaaaaga
gtactagaattatctttagaggaagcacgtcaactaggccataactatattggcacagaa
cacttgctaatgggcttagtccgagaaggagaaggagttgcggcaagagttttagaaaat
ctggcagtcgatgtttcttcaattagagctgaagttatacaaatgctcggagaaaatgcg
gaagccaatgtaagtggaagcaatactacgcaagctagaagtaaaacaccaacattagaa
gagttcggatctaacttaactcaaatggctatggaaggtggtttagatcctgtagtcgga
agacaaaaggaaatagaacgagttattcaaatcttaggtagaagaactaaaaataatcct
gtcttaattggggagcctggtgtaggtaagacagcgattgcggaaggattagctcaaaga
attgctaatagagatgtaccttctatttagaagataaattagttattactcttgatgta
ggtctattagtagccggaactaaatatagaggtgaatttgaagaaaggctaaaacgtatt
atggatgagattaaatcagctgataatgtaatattagtgattgatgaagttcatacattg
attggggctggtgctgcagaaggagcaatagatgcagctaatctgcttaagccagcttta
gcaaggggagaattgcaatgtataggtgcaacaactttagaagaatatagaaaacatata
gaaaaagatccagcattagaaagaagatttcaaccagttgtagttggagagccaagtgtt
gaagaaacaattgaaattttgtttggtcttagagaccgttatgaaaagcaccatcaatta
acaatgtcagatggagctttggctgcagctgctaaatacgctaatcagtatatttctgac
cgatttttgccagataaagcaattgatttaattgatgaagctggttctagagttcgttta
ctaaattctcaattacctcctgctgccagagaattagataaagagttaagagctgtatta
aaaacaaaagatgaagctattagagctcaaaaatatgaaacagcagagcagtatagagca
agagaaatggaaattaaagctcaaattgcagcaattgctcaaagtaaaaagaatgagcct
gatttaaatttagaagatcctgttgttacagaagatgatattgctgaaattgttgctgca
tggactggtataccagtaactaagcttactaaaagtgagtcagaaaaattaatgcacatg
gaagaaactttgcatggacgtattattggtcaagacgaagcggttgtagctgtctctaga
gcgataagacgcgcaagagtaggtctaaaaaatcctaacagaccaattgcaagctttatt
ttttccggaccgacgggtgtaggaaaaacagaattaacaaaagctttggcttcttatttc
tttggttcagaagcttctatgatacggctagatatgtcagaatacatggaaagacacact
gtatctaaactaattggttctcctccaggatatgtgggttatagtgaaggtggttatcta
acagaagcggtaagaaaaaaaccatatactgtcatcttatttgacgaaattgaaaaagct
catccggatatttttaatctacttcttcaaattttagaagatggccgactaacagatgca
aaaggtagaactattgattttaagaatactcttttaattatgacttctaatattggatct
aaagttattgaaaaaggaggaggtagtttaggctttgaattatcagaagatcaaacagaa
tcccaatatactagagtacgatctttagtaaatgaagaactgaaacaatactttagacca
gagtttctaaatcgattagatgaaattattgtatttcgtcagcttactaaagatgaagtt

agagaaattgcagaattaatgcttaatgaagtctttgcgagaattaagcagcaagatatt
caattaaatgtaacagaacgatttaagcaacgattagtagaagaaggatataatccaagc
tatggagctagaccacttagacgagcagtaatgaggcttttagaagatagtctggctgaa
gaagttttatctggtaaaattaaagctggtgatagtgcagtagtagatgttactaatgaa
ggagaagttacagttttattaggtgaaaaattagaactgttaacataaa------aaaca
atttattaat--taaagcattgaagaggttat--attcaatgctat---gaaaaaaatta
ataagatgggttgctataattgacaacctctgatactctttatttgtatata---aagcc
gggatagctcagttggtagagcagtggattgaaaatcctcgtgtcaccagttcaaatctg
gttcttggcat-tttaagcatcagaaaattttgctatctgagcattaaattttaattgaa
atgttcctgtcggaccattcctgtgttttgctattataacttctgtgaagtctctacttc
tagtttcttgtgtatagtaactttctctatatagcattattactaaatctgcatcttgtt
ctatagaattatgaacaataaagttgtttgaaataaaattgcataaaggtttacattcta
aatcaaacactatttgtaaacttgtaacgtttatttttttgtatactttcaaaagtcaaag
atgaaa------aagaattaaaaacatttttttgttcttcaaagttttt------tattt
gaatagcaatcatgtcattttgattaattttatcacatcttttccatccttgtgttgtga
gtagtttatggttacttgttaattgtatatatttcccagcttcggtaataattttatata
cagtttttttccctgttttcgaaatactacatttaattgctagaaataactgttgtttttt
gcgcactaaagttaatgacttca-atctttttataatagcaattaaataatatttg--aa
gttgattatataatgatgtctgaagataattgaatttggatatacacccactttctctta
aatcggataacaaaggccttttgtcactccgagtttcaaggtttctatttagttgagata
atgccaagataggcaagcttaaatcttttgctaatattttaagtgatcgagtaattaatg
atagttcttgagatctattgtcagattgtctactctcctgcagcaattgaagataatcta
taataattaattctatatttttttccttgtaatttaaagaattttacttttgtctttatag
tgtcagtagaaatcttagcgctatcatcaatataaagattaaggttagcaagagttttac
ttttttgaataacatattgccattcatcattattaagctgaccagattgaattttttggc
cgtttaaattacactcttgagctaaaattcttcttagtagttgctctgtagacatctcta
agctaaataaaataacataagatcttttctgattaattacatatctagttatattaatag
caaaagcagttttccccattgaaggtcttcctgcaagaataattaaatctgacttttgaa
acccttgtgttatagagtcgagttctgtaaaaccagaaagtatactactatttatactaa
tctttttccttgtatccaaatggactaataggtttgcaagaattgtagctaatgtttgcg
tatctttttcgtcaagtatttcataagcttttgttaattgactaacaatagaagttatgt
ttttttgttcaataagttgttttgagcagctaattaaacataatgaatctccacatgcta
aaagtaatcttttaatgtagttatctataacgactgctgaatattcgcttatagtatcag
ataggggagcttgttctattagactaaaaacttcatttagctcattcaaatcttgaatca
ttttttcatttttaagttaatgaaaaaatttcttacgctttttgtctttgcatggttaa
cattttcgagtgctgctctataaagtaatgaagttgatgtaaaataaaaaaaatcaggag

agatttttctactgattttagtaatgatatcgattgctgagttaatattatactaatta
atattttttcagctaaaatattatgaggaggtagatatttataa------atacttatta
cttcctcc-ttgtaacattactcttgtg--------gtattagttaactattgatttaat
ttgattctggaagaacttgcaatttaatatttgcagttacttgatgtaatagcttaattt
ctacatcgtaaatgccaattgttttcacgtctggtaaagagatactttgtttttcaatgt
ctatgttagtagtgtttttaataatttgtgaaatttctttttctgtgacactaccaaaaa
tattgtggccatctcctgtttttttactaacgctaaacctttgtatttcttctagaagtt
gcttgactttttagcattttctttagctgtttttaattttcttctcttatagctgcat
aaaatttttgttgttttaaaataccatttgtggcaactgccgccattttattcgggatta
aaaaatttcttgcataaccagaagctactttgataacgtcattactcttgccaagttttt
gaatattctcttttagcacaacatttataactttttttactcatctt-------aattcta
aaaa----atctgtattatcctattatacctgttgtatacagatttt----aatagtaag
tatttatttgttgatgtaatattcttgtaaaa--aaaggtaatttttttattttt-ttctg
atataatcactttcacaataattc-----attaggagaggtggccgagtggttgaaggcg
cagcattggaaatgctgtttaggagcaatcttaacgagggttcgaatccctctctctccg
tacaa--aaaatataaaaagttttatagagtccaatctaaatctccaataatattcttcc
aagcatttaatccaccccgaactctaacaatatttagtttttgagctattaaaaacttag
aagcaaatattgatctagaatctaagctacagtaaacaaaacaaattttatcttgtaaat
tgatatgagagtaattcattccttttatttttttgtaaaggtagatttaatgaatgtatta
aatgacttttatgatattcttcatgatttcttacatctattaaaatatattgtggattcc
tactaattaaaaacttttgtaattgaataacactaatttctcgtacaaatgtactagatt
tactgccataatatttgttccaatgctttttttgt-------ggataatacaaatttagta
tttataatcttaaatttgttaaatgatgaagttaatgagttgtacgttaatatagttcca
cttaatatagacttataacccagaacgattttgacagcttcagttgcttgaagtgtacca
acaatacccggtaatagacctaaaactccggaattactacaagtatctcttgcgctctct
ttattttcagtttcactgtagaagtctcgataaacaggtccaccttgataattaaataca
ctaacttgtccttcaaattgaaaaaatggcaccataaatatgtattttatttaactctagg
caagtatcattcaagagatatcgagtatcgaagttatcagatccatctataataatatca
tattgtctaataatgtcgattgaattctcatagcttagtcttgttttgaacacagttact
atacactgtggatttaaatctaata-tttttttttagctatttctacttttgaatatcct
atatcatgcatcgtgtataaaatttgcctctgtaaattagagagatcaattatgtcatcg
tctataattccaatgctgccaattcctgcagcagcaaggtaaatgattcctggagatcct
agcccaccagccccaatgaataaaacttttgcttcttttaatctttcttgtccttctaat
tgaatttgtggcaatactaaatgcttagaatatcttgtatattcttctagtgagtatttt
gtattttctgttttaaaatttagcataaaaatttatagtttatttaatagcactta----
--aaagctagctgataagtttattaaaatgacttttgtaaacttggttcttctctatcta

aaatagctccttgtgtaggacagacctgaacacaaatactacaatcaatacaggcagaaa
agtcaatccaataccaattagtgttattattgtttttccctttccttgatgaatacaag
ctacggggcaagcttccacgcattcagaaactccaatacatttttctgttacaattgtat
gagacatattatttaattaat-aaaagataagctatttacatctagtagttttgttgtat
actaaatttacatatgctatccaggccaatatttaaggtagtctatttctacagtttact
aatttgatatgcgcctttagttcagttggtagaacgcaggtctccaaaacctgatgtcga
gggttcaagtccttcagggcgcgtttag-ttttatcgtaattcaataatatcattttatc
tttttgcaaacatcaaaatgatatgatataaaagatcatatttatacattgaatattct
atgtactagtactg-ttttttaatatgttattattgataagttaattatactaattctat
ttttgtaataagt-aaaaacagttctaattaagtatgcacgtatttaatagtc-ttttgt
atatat--aacttgaaaaaatttaaactatggctaaaaaagttactggaattgttaaact
agcattaaatgcaggtaaagctactcctgcgccacctgtcggacctgctttaggacagca
cggcgtgaatattgttatgttctgcaaagagtacaatgcacgtactgctgacaagtcagg
actagtaatccctgtagagatctcaatatatgaagatagaagttttactttatacttaa
aactcctcctgcttctgtactaatcgctaaagctgctggactaaataaaggttcgggtga
accaaataccaaaaaagtaggcagcataacaaataaacagttagaatctattgcagaaac
aaaacttcccgatttgaacactaacaatatacctcaagctatgaaaatagttggaggtac
ggctaaaaatatgggaattctaattaaagatt-aaaagtttcaattatatttacta-tt-
-tttatgaaaaaattttcacgtcgacttacaacattaaaatcaaaagttgagcctaaact
ttacactattaatgaagcagtatctatattaaaagcaacgtcaaatgctaaatttaagga
aactgcagaagctcatattgctctaggtttaaatcctaaatatgcagaccagcaactcag
agcaacagttatattaccgaaaggtacaggtaaattgataaaagtagcagttattgctaa
gggagaaaaattaacagaagcaattagtgcgggagctgatgtcagtggctccgaagaact
aattgatgaaatactgaaaggtagattagattttgataagctgatagcaacaccagatgt
tatgcctttaatagcaaagctgggacgagtattaggccctcgagggttaatgccttctcc
taaagcaggcacggtaacattggacgtagcaaaagccgtgaatgaatttaaaggggtaa
agttgaatacagagttgatagaacaggtataattcatgtaccttttggaaaatctagttt
ttcacaagaagatctagttttgaacctccaaacaattaaagaatcgattgatagaaacaa
gccttctggagcaaaagggaagtactggaaaactttttcttatctagcaccatggggcc
atctattcaaattgacatcactagccttctatgaaaaattttgtataattatagaactt
gaataatctattatttcaaatta-taatttgctagcattattaacgtaacaacttgctta
ctatatcttatctgcattaaaaacttt-ttatatatcttattaattatgagtacaaaggt
tgaaaatatcttggaagagctaaaatctttaaaccttctagaggctgctgaactagttaa
acaaatagaagaaacgtttgatgttgatgcatctgcggcttcaggaggtatgatgatggc
cacaccaacttcagcaccagcgtctgctgaggttgaagagaagacagaatttgatgttgt
cctagaggaagtcccagcacctaaaaagatttctgtattaaaagttgttcgctctctgac

tggtttgggtttaaaagaagcaaaagatcttgttgaatctactccaaaagtcttaaaaga
aggtgcttctaaagatgatgcagaaactatgaaaaaacagctagaagatgctggcgcaac
agttattgttaagtaatga-----agaagcgcttctgtttaagtataaacagaagtgcct
ctttatttattataagcaattatactagaaaagtccaagtgtaattgcattgttgatagg
catagttgcaccaatacctagccaaatacttacaacggtaccaattaagaaaacagtggt
tgcaattggtcttctaaatggattttgaaatttgttaacattttcaatgaatggaacagt
aagtaaaccagctggtacagcagccatacttagaacacctaatagtttattaggaatcac
tcttaatagattaaatgttggaaagaagtaccattctggtaaaatctctaatggagtcgc
aaatggattagacttttcacctatagaagatggttctaaaattgcgagtccaatactgca
ggcaaaagtgccaataattacaactggaaagacgtataatagatcatttggccaagctgg
ctctccgtaataattatggcccattccttttgctaatttagctctcagcttcggatctgt
taaatctggtttttaagaattgacatattg-tttttgtgtttaatataaagattagtct
aattactgttttaaagtgtaattttcaactattataatggtccagaaattccttgtttac
gtatcattaaaaaatgcataagcatgaaaacggcagttagtagcggaagtacaaaagtgt
gtaaactatagaacctagttaaagttccttggcctacactaactcctcctcttaacaatt
ctactatactcgctccgacaactggaacagcatcagggacacctgtaacaatttttacag
cccagtatccgatttgatcccatggcaaagaataacctgttaccccaaaagaaacagtaa
gcactcctaaaattacacctgttacccatgtcaattctctaggtttttgaaacctcctg
ttaaataaacacggaatacatgtagaatcatcatcaagaccatcatgctcgctgaccatc
tatgaattgatctaataagccatccaaagtttacatcagtcataatgtactctactgatg
taaaagcttctgcaactgtaggtctataatagaatgtcattgcaaatccagttgcaactt
gaattagaaaagatacaaatacaatacccccctaagcaataaaaaatattaacatgaggtg
gtacatacttactagaaatatcgtcagcaattgcttgaatttctaatctctcttcaaacc
agtcataaattttactcataaaatagcttcaaaaagctcttctaatata--ttttgcgtt
taagctactattaattcaatag------ttattgaacttatatt-tttttataatcaata
cttattaactattataacatctttttattgattt--------------------aaaatt
ctctgacttagtaaaacagggctatgtactataattctttttttttgtatagagattaat
ttcgtttttagtaaattagctaaaattcttgttacactgactctattactacctataatc
tgtgctaaaattttatgtgtaatagtaaagttaagcataataccattattaacttgagtt
ccattttgctcagctaataataataataagctcgccagtctactggttatacttttatgt
gaaatgatttctataaaatgataagcttttgccgagcaaaatactaaatgatttacaaaa
aataaattgaatggagcgcagttactacatgcttttataatagtagtatagttaatacag
gcaacttctgttttatctattgcttctgcttcgtagtaaaaattatcgtcgaccaattct
atttgtccaaatgtatctcctgtagttaataagttaagagttactttatgtgtatttctc
aggacttttgtaataattaaagatcctatcaatataatgtataatctagagctgctatta
aacaaaagagtatcatttttcttaagtgaaaaaatttgataattaattttattacgatta

aaaaatagtagccatggattgcaagaaaaaaaatcacctgaactattttcagagaatatt
ggaatacaattttcttgatttaaacaatttttagtcataattactactttataa------
---aaaaatattctaataat-taagaactgaaattaaatgtcatataaattgatgttagt
tgagaatgacattgtattatcaaaggccattcaagaatacttaatagatcaaggcttcaa
tgtttatattgccaataatggattagaagcactgaatctagcttatcaatataatttcga
cttgataatttctgatataatgatgccgctagttaatggctatgaactattagcaaaact
taaaaaaaacaaagcattatccaaaatacctgttatctttttgacagctaaaggaatgac
taaagacagaataaaaggctacgacatggggtgctatggttatttatctaaaccatttga
tcctaatgagttgctttcaattattaacaacttaatcgctagagatgtcttaaaagaagc
ttctttacaaaactcggcaacatcaaaccagcaattaaatcataaaatacgtttgacacc
tagagaaaaaagtattttagatcttgttgttgatggacttactaataaagaaatatctac
tatactgaatactagtgttagaaatgtagaaaaatatgttagtcgactcttacacaaaac
taatatgaagaataggactttgctagtcaagtattctataaataataatctattaaataa
tgagatcaatgaaagggcgaatgacgggactcgaacccgcgaatgatggagccacaaccc
attgccttaacccccttggccacactcgccatattacatacaccactatagcttgtttttt
gatatatcgtctagttttcgagttatatatttatctaaaacaatatttactttgaatttt
-tatgatacatagcaatattagtattcaaattaatggggaaccatttaattgctcaaagc
ccatctcattgcaatttttattaaattatcttgattttaattccgagcgtgtagcagtcg
agctaaatgacattcttttaccagagactctgtttcactcaacttatttgaatgatcaag
ataagctagaggttattaccattgtaggtggtggataatctatttgtacatac-------
-acagttttctatctcaaatgtttcatagatagagataatcttccttgtttttttatctac
atgaataataacagctttaatagtgtcacctattttaaattgtgatgatatctgctctaa
ttctttgatattaatctcagaaatatggacaagacctttgagatttcctacctttataaa
taatccgtaaggtgtaatctgatttattatgccttcaataatattaccaacaattaaatt
agatgatgcttgagatattagagctcttctatgactcagtattaaattattagatttctc
ttcgacattgagtaattttagcttaataaatttattattaaattgctcactttttttgaaa
attaccgagatgagaattaggtacaaatccagatataccttcaaggttaattatcattcc
tcccttattgaatccttttatcattacattaagtaaagagtcttcagctaatagttgcct
aattcttttccatgctcttatatattcaaggcgacgaattgataatattaattgtttcga
ctgaatattataatctaataaaaaaaattctcttgtgtcattaatgtttaaagaagtgaa
attatttaaatcttgattactagatacttcttgaataggcaagtatgcagatattggtgt
tcctatatctactaataccccatttaattcaaagctaaatatagttccagctacaatatc
tccaagattcaaatcatacttgtatttttgtaaaacagctgcaaaatttctatgagtaaa
tccttcattatttttttgtcatattgaatagtatgaattaaagagtaactttgtacaatat
attaaggtaaataaatgtttgtcgaattgtatgaaaaaattttaatttgttcggttgtaa
ttaagtatataaacatattaaaatatctaggagacatgaaaagtagacgtagatagctgc

agatttaa-tactatataatctgaggaaagtccgggctccacaaatacaatttatgctgg
agaaatcccagtgtaggtaactgcgaggatagtaccacagaaaaaaaccgccagaataag
t----tatattagctggtaagggtgcaaaggcaagttaaaagcttaccaaaagtactgca
aagtatttgttaggtaaacccctaaaatggagcaaagctactaaacaaatatttgtgtat
attttatctagtttagttgaaaatactgcatgaagttattagtaacaataactcgagagg
aatagctatcctttacaatatatc-tcgattatattgaaagaacagaacccggcttatgt
agtactttttatgtcttgtattattatgtctattgtcgaatctttaaatttaactgtttg
acaatttttttttgtagctgatgttgtttataatctatttctgaattggttaaagttttt
tcattagatcgatatgtaaatcttagacctatactttttttctcttttccaattgattca
tctatatattgatcaaataatgttgtactttctaaatctttatcataaaattgatctaat
aattttaataagtatttaatttccatattcttaggtattattaaagagagatctcttatg
atcgatggatacttagagtatggttgaatctgataacttaaataattgaattcattatga
caatcttctaaaatgtttaagttaacttcaagaaaaaaaagttttgtattgagtcctaat
tcactatatgttgcttgattaagttcactgaatatgccaataataatattgttatagatt
agtgtggcaaattttcctttttgaatgaactttattctaccatcgactatatcttttttt
gtccactcaatttgtctattaatcttttgaaaaaaatttctacgatccctttagcttca
taccaatttaaagaatgcgcagggtgtgaccatgtagatcggatatctagattaccaccc
aatatgatagctagattagtagtttctacaattttgttttgatcattatgaaatactgtt
cctatctcaaagctatctatagtttggttgctttgtttaatattataaagatttgattca
attaatccttccagcaaactgccgcgtaaattagaataatcttgaataagaggattgttt
aaagctatatttcctttggactttattaaagaataatgaactaattcagttaatcctaga
tttcttaaaatacttctacttttgtctatgaatttctctttgtggataaccttttgttg
aattgaatatttggtactgagctcctgaatttatgataaccataaattctagcaatttct
tcaataacatctatttctcgaaaaacatcttctcgtctataatttggaacagtaacttct
atattttccttattttgataaataagatcaaaatttaaagagtgaagaatattttgaatt
tcttcaaagaataaaaaacgtgtttgaccattatacataattggtcctaaaatgtcatga
actttttaattgataaattaatattaagagcatgatcagttattttttctcgacagaaa
gtatctctaatatttccgcatgttaaatctgtgattaaagcaagagcttcaaaatgtgca
tttttccaattatctacatttagcccacgttcttgtctaattgaactttctgttcgaata
ttaagaactcgagaagactttctaactacagattgtttaaatatagcagactctacaagt
atcgattttgtattataatctgtatcaaaatcagagttgcttccaataccagctatacta
gttacatttgtatttacttgagtaactaaaatatttttgtttaactcaatattttcattg
tttaacttaatctgatcattacttccaagaggaaaattgcttcgaattgttagactattt
ttatgattcatattattaattttattcaaatcaataatattaataggctgtccccatttc
aacataatataattgcttatatctgttaacaaatttctgtgtgtaaacccagaagagagc
aaccgatttttttagccaatctggagaatctttaactcttatttcgtctataatggcagca

aaataattgtcacaatttaataaatcgtgattactgattatagt-ctcctttttagagaa
atatctattattgggttgatgtatagctgataacattgaggaccctgttaaagccgagac
ttctcttgatagaccaatcatgcttaacacgtctgacctgttagctgttgatgatatatc
taatatatgatcaatctcacctccaattataatagattcaatagcttcaacttcaaaacc
tgcttgtgttagttgattagttaaattgtctatatctattgttttgatattaccaagttc
tttcaaccaatttaaagaaactttcataattaattgtcatctatcttatagaagtattta
ttttacttatgattaaaaatcaatttaaaaaattcatcaaattaatataagttatttgat
gaatttgcttatacaatatattattgtgatatcttacttttatatggcagttattaagaa
ctagcttttgtaaaagttagcccattatcttgggcatatctttccataaatctcatgaat
ctatcccaatcatcaggacttttaataacatgtacagcttctattgcttgtggtttacca
ttaataaacttggcattaacatctcttgtaataatttgtccctctgtgtccatcaggtac
atacctgtgatttcacctttgtctgccatactagcatctaaaattttgggatttgtaaac
cgaaaagttgctgttcctgtactaccatctcgagaccttgttaaacgtacatctggtaca
acttcttcattgataccttgaataaattgaattgttgccatatcaatcttctttatttgt
atataatttatattggataaaactgatttctagttcttgaatgcttgttgagaaactgtt
ttaaaagcttatagtatcagtagatcaaattttatgtattatataaaactgtaacttttc
-ttttttg---

----------------------atctgttcaaagaactaacttattataagctaatcata
cgaatattagccatataatgttcgtaagt-gaaattaaggggttgtagctcagaaggata
gagcaagcgcctcctaagcgctaggtcagcggttcaagtccgctcaaccctgttaacgaa
ttaagtagtattatttaacttgttctgcaaatcagtattgatctaagttaaagtacttga
cagctaaaacaaaagaatctgagaaatatttcatttaaagaacgaacaattatcttaac
tctaaatatctgataattagtaa-aaaattct----------------------------
----ctttttaaaacacaagatgatg-----aagatatttaaactaataattgaaattaa
gtttttgaagttatagaagccaat--ataatactttctattaacaga-----------tg
cttgcttctattactatctgtttacaaga--aatt----atgagtatcaaaata------
--attttagctcca-------------------------------------ctgac----

-----------------------------tactaataaata-------------------
------------------tttattttagtctagacaag------atgaatcttcatggta
taacag--

--------------agctattctttgtgtcatacttatataaaattaggat-------ac
tgtt--

---------------ttcattaccataactggggtgggaggattcgaacctgcgaatggc
ggagtcaaagtccgctgccttaccacttggctacaccccaacatgtgaacattataatag
cagtcgtataccctgccatgtcaatagactattgtctttatttaataatttttgctgctc
ttgtagaataaaatctttttttcaaataagatacgctatgtttaagttgcacgactgtt
atgttttcttgcttttgactgaacagccacttgatagtaattattccttctgcaatttcg
ttacttcctataataatacaagctacagctctttttttatttgcttgcttaatctgctta
ctaaaattacttgagctaacatcgatttctattttaaaaaactgctgatgtaaaaaacgc
attatttgcataccagtttcgttagcttttgtaccttgtgtagcaatataaaaatcgata
gatttattaggcaagtctatattatcttttgcaatcagtaataggcgttctaatcctatt
gcacatccaacagctggtgttgctttgccacctaattggtgaactaagctgtcatatctt
ccaccaccacatatagtatcttgcccttttgatgtcaatgtcttaatttcgaaagcagta
tcattataataatctaatcctcgaactaacttattatttatattgtagggaatattaagt
aattttagataattgcaaacgtcaccaaagtgttgttgtgattccaaacttaaaaaatca
gaaatttttggagcctctgtcagtatttttgtgtattagaatcttttgaatctaaaatt
ctaattggattactagttaatctttttgtgagtcagtgtctaaatcatcatggtacttt
gtaagataatctcgtaatttaacttgataaatactacgatcctctacttttccaattgaa
ttcagatcaagtttcaagttgtgcagattgagattgttaaatatgctcatagctaaatgt
ataacttcactatctgctcttgcatctaagctaccaataaactcaatgccaagttggtga
aattgtcgttgtcgtccactttgtggtctttcgtacctgaacattggaccgctataccat
aatctttgtaaactatggtggtaactcatttatttcaataaaagatctcacaataccg
gcagtaccttctggccttaaggtaatatctctattgctgcgatcatgaaaccgatacatt
tctttatttacaatatcagtatcttcaccaatacccctatcatataattcactattttca
aaaataggtgttctaatttcttggtaatttgcacattctaataatttggaaattttatta
tgtataaactgccaatattgaatttcatcgggaagaatatcttttgtccctctaatagcc
tgaatttttgccatgataatttttttgattaccttcttaatatgtctgcattaaatagcag
ttgtagactgttattcagtcatgtattatacattag--aacatactgatg-taatttata

aagtacgggcaaggagggattcgaacccccgacaccatggttcgtagccatgtgctctaa
tccactgagctacaagcccacttaataagtaccttaactatatcattgttcactaatgta
tacaactatattcttttt------atcttacatgtgaaatttttgtaaaagtaaaaaatt
at----gtttgtttttacaattggaactattcatatataatgcttaatttattataact
ttgtaaa--aatatctataagtttaattgtatgattctattatattggtaatcttgttat
aattttgcgctcttatctttcaattgg------ctagcatctaattaacttatatttcaa
ataaaaaaatcttatctaaaatgagtaaacaaattctatatcaggatgatgccagaaaag
cattagaaaaaggcatggatattttaacagaagctgtttctgtgactttaggaccaaaag
gaagaaatgttgtcttagaaaaaaaattcggtgcccctcaaattattaatgatggtgtaa
cgattgcaaaggaaattagtttagaaaatcatattgaaaataccggagtcgcactgatta
gacaagcagcatctaaaacaaatgatgtagctggtgatggtacaacaacagctacagtac
tggcttcagcaattgttaagcaaggaatgagaaatgttgcagcaggttcgaatccgatgg
ctattaaaaaaggtattgaaaaagcaacaaattttgttgttagtaaaatagctgagtatg
ctaagccagtagaagatacaaaagctattatacaagttgcttctttgtcatcaggaaatg
atatagaggtaggtaaaatgatagcgaatgctatagaaaaagttggcagagaaggagtta
tttctttagaagaagggaaatcaactaatacgattcttgagatcacagaaggaatgcagt
ttgaaaaaggctttatttctccttatttcgttacagatacagaacgaatggaagttcttc
aagaaaatccttttatttatttacagacaaaaaaattactttggtgcagcaagaacttg
tgccattgcttgagcaaattgcaaaaacatctcggcctttattaataatagctgaagaca
tcgagaaagaagcattagccacaattgtagttaataaattaagagggattttgaatgttg
tagcggttagagctcctggattcggtgatagaagaaaatctttacttgaagatatgagta
tcttaacgaacggacaagtaattactgaagatgcaggtttatcacttgatacagttcaat
tagatatgttaggaaaagcccgaagagttattgttactaaagactcgacaacaataattg
cagatggtcatgaaattaaagttaaatcaagatgtgagcaaattaagcggcaaatagaga
cgagtgactctttatatgaaagagaaaaattgcaagaacgattagcaaagctttctggag
gagttgccgtcattaaagttggtgcagctacagaaacagagatgaaagataaaaaactaa
gactagaagatgcaattaatgcaacaaaagcagcaattgaagaaggtattgtaccaggag
gaggagctactaatgtccatatctctagtgaattatttacatgggctaaaaacaatttag
ttgaagatgaattaattggtgctttaatagttgaacgagctgtgacctatccgttaagac
gaatagcttttaatgcaggtgataatggagcagtaatagtggaaaaagttaaaagtcacg
attttcatataggctatgatgcagcaaacggtaatattgtaaatatgtacgatagaggta
ttatagatccagcgaaagtagctagatcagctttgcaaaacgcagcttctattgcagcaa
tggttttaactactgaatgtattgtagttgataaagtcgatgattgagctata------a
gataatagataatctcaaacaaagaataatttctaattatagaaattattctttgtttca
attaatctaattaattgatcaaatgtataatttttataataaagccaaagtttaaacacg
tcttctttttcaactattaagtagagaagatataagcccgtaatacctaaattatcaatt

aaaatattattctcatatatagatttttctaaagatctaaaaaatgacatttttgaaaaa
taataccgaaatcttctcttatagttatttgctaaatcattactgtctaaagattgattt
ccaattgaattcagaacctttaacagaatactttttaaatctttatctgactttatttga
gaatcaataataatttgattcaattgtatgtcaaaactaagattaaaaatattacttgtt
aaagatatttgattttgaatatctagagattttatagccaagaagattaaacttttattg
ttcattgtaaaatataagacaattgatgatgtattctttaaatacta-aatttatcttct
tgtgcttctatcgttttttgt-aaaataattaatgttttaatttttttagttttagttact
acccgaaaaaataaattctggaaatctagcttgagctttagctagagagtgtttttttccc
ttcttcttgccagtaattgataacttctgttgttttgtttaataactcaattctgtctct
ttcagcaatccacggcttggattctagttctgcttttaattcttcccaagcatcattcct
tggccagaaaaaataagaagtaagtggactagtgccatttccaacaatctgatcaatagc
aatagcaatattattttctaaccaaagtacttttagtgtaaattttgacaagtttttatc
tccattattaatattaagtattgg----tacagtagaatactgtactagttatttttttc
agcaacaatttgagctttgtttaaaatgtttttttaccgtttagtagcttgtgcaccttg
agataatcgttgcaaaatttttgaaatgttaacacgagtttcattatttataggattata
aaatcctaattcttctatagctttaccatcgcgtttatttctgctatccattacaacaat
tctatagcttggttgtttttttcttccgtatcgctttaaccttaacttgaccatattagt
cacctcgcttatttttt--atatctttattaaattatatcttacgacattatgcaataat
tcaaacaattaagctgattcaagtctaacattattaaatattaccattaaacattgtaga
aaaataatacctaacataggtgacatgtccattccaaacatgggaggaatgcttcctctg
aatagttttaagtatggatcagtaattctatttaatgaacagaatggttcattgtaccaa
tttacagttggaaaccatgccaatgataatttaagtaaaattaaaattaaataaatctca
gaaaaattagctatcgatccaagcaataaatttaatgtaccaggaagagtgttcatacgt
attttataattattatatacttcgttatactaatagtttgattatatattataatcgatg
taaataaat-cagctaaacaaaaaacttatccttaaaatcatcttctcttgagcactttt
ctttatctacattagtatttacttttgtcgtataaatttccagtttagaccaaatctgtc
ctaactgatttaattgagatgttccacattctaaacaggctatttttatattgctcaaaa
cagcacctttttttataaaatcgttcaataatggcgtaatagttgcttcatccaaaaata
aatctaagacaagtatttttgtaaatgaatttaattgatctgggacattgttaataatat
cattataattaactaaaactatattagctgtaggaagtaaagatcgtgctccttccgcta
agataaagccataaggcataataattacaataatatatttataatgactgctaataagac
ttatagtggtattagcttttaattcgattgtttctgttactagccactctctcattattt
cgtaagtaatccatttgcctagttctgagcaagcagttcttaaaatcgtacctggattat
tgtcgtttttgagaatccctgaccaatgttgtattaatggatgggatgctatattaattt
ggagttgcatgttttgttaaatatatttaacatttaatttgttgattgaatcttcaattc
ctatttgttgagtattattaaaatagcat----atatattaaagaaaataattat-aaaa

aatgaaaaaaagtctttggctgtggggttttacagatagtgctgaaacttggaatggtag
atttgcaatgattggttttatatcagttatttttatcgaagtagttacaggacaaggatt
attatatttaataggtatgatgtcataataaaaatccaaaaaagttcttgtaatttttta
taagaactttttgg-attttttataaatctaatctagcggtctcatagatagaacaggtt
cattctctctattgatacctttttcaaatccagcagcagcagctcttgctctgcctgcat
gccaaagatgtccaataaataggaagaatcctaggaagaaatgagatgttgttaaccaag
atctaggagatacatagttaactgaattaatttctgtagctacaccaccaacggaattca
aagaacctagtggtgcgtgagtcatatattctgcggcacgcctttcctgccacggttgaa
tatcgttcttgattttatttaaatcaagaccatttggtcctctaagaggctcaacccaag
gagctcgcaagtcccaaaatctcatggtttcaccaccaaaaataatttctccactaggag
atctcattaggtatttacctaatccagtaggtccttgagaagatgcaacattcgcaccta
gtctttgatctctaactaagaaagtaaaagcttgagcttgtgaagcttcaggaccagttg
gtccatagaattcactaggataagctgtgttgttataccaaacaaaattagaagccgtaa
gccccatgattgataaagcacctagactataagataagtaagcctcaccagaccatacaa
aagctcttctagcccaagcaaaaggttttgttagtatatgccagattccgccagcaatac
aaataatgccaatccagacatgaccacctacaagatcttccatgttatttacacttacga
tccatccatcaccgccaaacggagactttaagacataaccaaagataactaggggattaa
gtgtaggattgctaacaaatctaacatctccaccacctggagcccaagtgtcgtataccc
caccaataaataaagctttaataactaacagaaaagcgccaatgcctaataacactaagt
gtataccaagtattgttgtcattttgtttttatcccgccaatcgtatccgaagaaaggaa
atgattcttcaagagtatcagggccaatcagagaatgataaagacctccaaagccaagaa
cagctgaagaaattaaatgcactacgcctactacaaagtatgggtatgtattgaaaatct
cgccgcctggaccaacaccccagcctagtgtagctaagtgcggaattagaataaaccctt
gttcgtacaaaggcttctcgggaacaaaatgtgctacctcaaaaagagtcatggcaccag
tccaaaagaccattatacctgcatgagcaacatgagcaccaagcaatttgccagaaacgt
taattaagcgtgcattgccagaccaccaggcaaacccggtagactcaatgtctctaccgc
caacaccaacagttgtattaaagggcgtttccacgtggtaaaacctcctcagggaatata
aagttttcatgaggttgatcttgagcagccatccaagagcgaataccttcgttcaatagg
atatttttagtataaaaagtttcaaactcaggatcttccgcagctctcaactcttgagaa
acaaaatcgtaagctcttaaatttagagctagtccaacaattccaaatgcgcttgtccat
aatccagttactggtacaaataacatgaaaaaatgtagccaacgtttattagaaaaagca
acaccaaaaatctgtgaccagaatctattagctgttaccatcgaataagtttcttcagat
tgtgttggagtaaatgcacgaaaagtatctgcagcatcaccatcttcgaataaggtattc
tgtacagttgcaccgtgaatagcacatagtaaagcaccacctaagattcctgcaacgccc
atcatatggaacggattcagggtccagttatgaaatccttgcaaaaatagcaaaaatcta
aaaattgcagcaactccaaggcttggggcaaagaaccagctcgcttggcctagagggtac

attagaaagacggaaacgaatactgcaattggtccagaaaatgcaatagcgttgtatggt
ctaagaccaactagcctagcaatttcaaattgtcttaaacagaatccaattagtccaaat
gatccatgtaaagctataaatgcccacagaccaccaatttggcaccagcgagtaaaatct
ccttgagcttcaggtccccaaaggaaaagaagtgaatgtcccatgctgtttgctggggta
gaaacagccgcagttaaaaaattgcatccttctagatatgaactggctagcccatgagta
taccaagaagtaacaaaagtagtcccagttagccagccacctacagcaaggtaagcgcaa
ggaaacagaagtagtccagaccaacctacgaatacaaatcggtctctttttagccaatcg
tctacaagatcaaaaccaccacgagtttttttcttgtccaattgctatggtcataattta
atctccagagcaaatttaataagtaatttgtttaaataaaca---ctttagatatactct
aaaattttacttttctttacggtcaatataattataagtcaaaagtaaccaaaattcacg
cttttaataaca-tttttttagctcattaagttaa-ttttttaatatataaacagtaata
gagttattgttt-aacttacaatactcacaatttattcatttctataatcctttgaaaaa
gatatcctgttcctcgtgctgttaaaatcaagtcgggattacttggatcatcttctagtt
tagctctcaatcgtgaaatatgaacatcaacaactctcgtgtcaacatgccgttcgggag
tatagccccaaacttcttgcaagatagaagctctagaaaaaggttctccagccttactaa
ctaaaagctcgagaagactgaattccatacctgttaatcgaactctttcattatttttat
aaacttgtcttttgttagtatctatttttaaaaatccaatactaataatcccagagtttg
ggactccaagactagtggtgattttatcagctcttcgaagaacagaacgaatgcgagctt
ctagctccttaggagaaaatggtttaacgacatagtcatcagcacctatttctaatccgg
taattctatcgcagacttcacctaacgctgttagcattataataggaacatctgattctt
ttcgaagttcttgacaaacaccgtaaccatctagtttaggcatcataacatctaaaacaa
caaggctaggatactcttttctaaacacgagtaaagcttcttctccatttgaagcagtga
taacttcataacctattattgttagtcttgtttctaaaattcttcttatgctggcctcat
cgtcaacaacgagaatcttttctttctggttctccaa-tttcttatttactcctgtaact
caccgatcatgattaccataaccttaaattttagcgctatgagtatctttattcacttt
agaa-----tttagttattataaattttgcaagtctagtttacattaattagtaccagt-
-caatatttttgt-----------tttaagttatgac---------tattttaatccata
agttgactctagtttgcagaattcaatatatacata-ttttctacccaagtaaattgtat
tctattttctaaataaaaaagggggttgacaaaccagttgatagaaagtattctacattac
tataag----ttaattccaacaaagaagttttaa-----atcttctttcggagtaatgaa
tactttaat
>Pyr_23
tatatgata---aattaagacatctgttatgtgagaatttctaaaatatacaaaactttt
tatttaatatagaaaaagtaaattaaaataagttgaacatactttgctctaatctaaacc
tcttaattgag------attctaattagagacaaaagtatttacttattttgtatctata
ggaataaaaatgaaacttgcagtttatggtaaaggtggtataggtaaatctacaactagt

tgcaatatttctgtagcactttcaaaaagaggtaaaaaagttctacaaattggctgcgac
cctaaacatgatagtacatttacgctaacaggtttttaatcccaacaattatcgatact
cttcaatccaaggattaccactatgaggacgtttggcctgaagatgtaatctataaggga
tatggcggtgtagactgtgttgaagctggtggaccgccggctggcgctggttgcggaggc
tacgttgtaggtgaaacagttaaactttaaaagaattaaatgcttttgatgaatacgat
attatcttatttgatgttttaggtgatgttgtatgtggaggttttgcagctccattaaat
tatgcagactactgcttaatcattacagacaatggatttgatgctttatttgcagctaat
agaatagcagcttcagtacgagaaaaagctagaacgcactctctgagattagctggactt
gttggtaatagaacagataaaagagatctaattgataaatatatagattgtgttccaatg
ccagtattagaagtcttgcccttgattgaagatattagagtgtccagagtaaaaggtaaa
actttatttgaaatggcagaaattgataaggatttagcatatgtatgcgattactatttg
aatattgcagatcagttgattacaaggccagaaggtgtagttcctaaagaatctccggat
agagaattatttagtcttttatctgattttacttaaatcctaaatcaaaggtaggacaa
gaaaaagtagatcaagaagaattagatttgatgatagtgtaaataatattttcagcataa
tataacaaggaataagataatgtctacagctcaatcagatgctcttacttttgaatgtga
aacaggtaattatcatactttttgtcccatcagttgcgtttcttggttatatcaaaaaat
tgaagacagtttttttcttagttatagggactaaaacttgtggttatttcttgcaaaatgc
aatgggagtaatgatttttgctgaaccgagatatgctatggcagaattagaggaaggaga
tatttcagctaagctaaatgattatggtgaactccgtagattatgcttacaaataaaaaa
agatagaaatcctagtgttatattttggattggaacatgtacaaccgaaattataaaaat
ggatctggaaggaattgctccaaaattggaagcagaaattcgtgttcctatagtagttgc
tagagctaatggtttagattatgcctttacccaaggtgaagatacagttctagcagctat
ggctcaaaggtgtccattaaatttaaagaatcaatcagataacacgagtcttaaaacttc
tcctcatattcctttagttttatttggatctcttccggatccagttgtaacccagttaac
tatggaattaaaaaaacaaggtatttttgtttctggttggttgccatcaaagaggtatac
tgaattaccggttataaaagaaggatattatgttgcgggagttaatccatttcttagtcg
cacagctactacactaatgagacgccgaaaaacaaaactaattggtgcaccatttccaat
aggtccagatggtactcgagcctggattgaaaaaatctgttcagtaatgaatgtaaagcc
tattggattagaagatagagaaaaagcaatttgggcttctttagaagactatatttcttt
aatccgaggtaaatcagttttttttatgggtgataatttactagaagtatctcttgcaag
atttttaactagatgtggaatgactgtatatgaaattggtattccttatatggataaacg
ctatcaagcagcagagctagctttattaaaagctacatgtgataaaatgaacgttatgat
gccaacaattgtagaaaaaccagataattataatcaagtagaccgaatccgtgatctgaa
acccgatttagttatcactggtatggctcatgcaaatcctttagaagccagaggtattaa
tacaaaatggtcagtagaattcacatttgcacaaattcatggctttaccaatgcaagaga
tattcttgaactcgtcacaagaccattacggcgaaatcttagtttgtcagaattaggctg

ggatgtttatagcaagcaaagctagtattcctc----tttatgacttacatggtataaaa
gccttgtaagtct--tttattttatgttaaagaagcatttcttgctctgccatcaccagc
ccaagcttgtaaactttctacttgttctttgtctgtaaatgctaaaggcacaaattgttt
gatagctattttaatatcttcagtactaaattctctctcttcactaaacgccgtatgcat
actttcaataattgcttgctcaatttcagcgccagaaaatttattacataataaactcaa
ttcatgaatatcatatttttgccacgatcttggtctaacttttgataaatgtatcttaaa
aattgattctctctcttgacgattaggtaaatctaagaaaaaaatttcatcgaacctgcc
tttccttaacatttcggaaggtaaactttgaattttattagctgttgcgacaacaaatac
tggagctttttctctgatagccaggtaataaatgtaccaaatactctggcactagtacc
actatcaccttgactatgtaaaccagaaaaagctttatctatttcatcaatccataatat
gcaaggtgataatccttcagagatatttatcatttctctcatttttgattccgattcacc
aactagtccaccaaacagttttcccatgtcaagacgtagcagaggcagcatccaatcgct
tgcaatagctttcgcggttaaggatttacccgtcccctgtataccaactagcaataaacc
tttgggcgaaggaataccgtaatttaaactttgcttcgaaaatgagcgagaccttttttg
tagccattgttttaatacatctaagccaccaatatccctatttactttactataaggata
aaattctaatagatgcgtttgattaataatttgcctttttttcttcaataataataggtaa
gctccgagagtctatctgattatattgtgcaataattttagtaatcactttgcggattct
atctatcgataatccttggcaggattttgttatattattaacgagctctgaattcaaatt
aagattcaaagctttactcaatcttatgatctctttttttgatttctagaagactaggtaa
gggcaaatctattactgttatgatatcattcagagcaaaaggtatattgattttgcaaga
aataataataatgtgcttagattgcgttttgattattttagctaaattgcgaagtttcct
aattaatactatttcgtttaggaaagagtcaaaatcttttaaaagaaaaagatttaaaga
ttcattattcaagttttcaataaattctaatgctaataaaggatttctttttgcataacc
attatcgctaggattgttagtgtatccatctacaaagtcccaggaatatacttgctgact
gtttgaacagtttaagctgtgttttataatatactctagtctatcttcttctctagtatt
tattacaataataggatatcgagattttaaaagtaatcgtaaatcttgagtgaaattcat
gtcaatatttgtttccatcagacttttgtacttcaccaattgaagaaagactaaataaga
ttatctaatattccttggatatattcaaaagtc------atctctctattttctaaatta
ttgctattactaatttaacttaccatgatttttttcttccttttcttcgtcgactattt
aatatagagcgtccacatggtgttttcattcttgctctgaaaccagaaactctaatttt
tttcttttagatccttgtagtgttcttttagtcatgatgtctcctctgttgtaaaagatt
aaatactattttatcatcttttatatatctttatgatatagatatgtattaatatcaaag
tt-aacagcaataatttatattaatcaagaactatttatataatattaaacattaaatat
----------taactttatgatattggctttgcccatttttttatttaagtattttgacca
tctttttgctaatcttaaactggttaatttttcagcagctaaaaacgattttgttattag
aatctcaatttaagtattttgttgataaaagccaaaatcgtaaattagaacccgacgaaa

gttttgcttttgcaaaagtatgtgtagctaaaaaatatttttctaaagctattattgaag
gtcaattagctttaaaaaactatcgagacctgaatatcttagataataatatagtaattg
ctaatttgtataatatgctaggttttatttattttgaagcaggccaaacaagttttgcaa
agaacttttatgagcaagccttacaaataaaccctaattatgttgtagctttaaataacc
tggcaaaaatttatgaagaggtaaaagatttaaaaaaagccgaatctctatacgataaag
tgttaactcttaacttaaataataaaactgctaatagaaggaaagatttatagcaaaaa
cta---------aaaacatctaatactatttgtaatcgggatagcaggatttgaacctgc
gacatcctgctcccaaagcaggcgcgctaccaaactgcgctatatcccgtaagacataat
tattactatatacctttctctaattatgtctactctttctgtattttatctaggatacc
aaaacatgcctttaactccatctggatctttgataaatcctaatttttataaaaactaa
caacatcaggttctgcgaatagtgtaatagtgctaatttctgcctgtctcaattgctgta
ttaattggtgtatgactacttttcctaaacctaaaccttggaaatctggatgaataacta
cgtcccagatagttgcattaaatccgttgtctgatgttgctctagcaaatcctacaagtt
tactatttgcatcttttttttgtattaaagaaataataatagaactatttttaatgcga
tctttactttttttagaggtcttttaacccatccgactgaatcacaaagctgctccaatt
catataaattaatattcttattgctactcagatagatatctttaaattcgattttatcgc
aagttttatctaaaacaataagtttttgaaatttttttcagagctagcattgatattgg
agttttgaaaaaaatttttccagaagatcataatgattatgatataatta--aataaata
tatatattacaatattgctttaacaaacaatatattaatatatgctcaaatttttacatg
aatctattaagatagaagattgttaaaatatagagaaataaaaatttttgtaaaatcttt
caataaaatgatataaagagtgcttttaagactaacatctattattataaataaagtct
cgaaattgagatgaaataacacattattctatgtctaatgccttttaaaggagttatttg
tgaaaaaatctatgtgtctaacctgtttacttgctttactaattatgagcaacccaataa
tagctaacgcagaagtagctggattggtgccctgcaaagattctgcggcattcaataagc
gtatggtaaatagtgtgaaaaaacttcaagccagattagctaaatatgatgcagacacgc
caccagcgttagctttaaataagcaaatagagaaaactaaaactagatttgcaacttatg
gtcgagcaggtttgctgtgtggtactgatggattgccacatttaatttctgatggtcgat
ggagtagagccggggactttgtttttccaggactattattcttgtatattacaggatgga
ttggctgggtaggtagaggatatcttttatctgttgctaagactagtaagccaacagaaa
aggaaatcatttagatgtgccattagctatcaaatttatgtcatccggatttgcatggc
cgctagcggcttggcaagaatttagtagtggacaattaattgcttccaatgacgatatta
ctgtttcaccccgttagtaaaaaatttatatgaataataattttaccaaatacttatcaa
cagcacctgtaattggtgtattgtggatgacgtttacagccggatttattatagaattga
accgctttttcccagatgtcttatacttttactt----ataaataa--gatcgtataaa-
--aaactacataaatatagagagatactattatttgtgtagtttta---atatataaca
taattattta-tttttaaaaatgtttatcttttcgattattttaatattagatcttacat

aataaaata-atatcaatattatgagcttagtaacccaaattatagttaatgccgatgat
gaattaagatatcctacaattggagaattgcagtcaattcaagactacttaactacagga
agcaatagaattagaattgctactattattagagataaagaaaaggagattatacagaaa
gctagtaagcaaatttttcagttacatccagaatatatagctccaggtggtaatgcagca
ggttctaggaaaagatcgttatgcttacgtgattatggttggtatctcagactaattaca
tacggagtattagctggggataaagattctatagaaacaattggtattataggagtaaga
gaaatgtacaattctttaggtgtacctataattggaatgttagatgcaatccagtgttta
aaggaagcatctttagaaatgcttggtcaagatgatattagaatcatttctccttatttt
gattatataattcgtggaatgtcataaatcatagcttcaattaattagttgaataaaagc
ttttataatgttataattacaataagcccgagagcatataattgtaaaaactaaatcttg
tcagaaccggaaggtagcagcaataatgtttcaatacaaaagttatga-ttttcgggtgt
ttattgtttttatctaatagtctcgacttttaattagtatgctatgtataaatctgtata
tgacatgtaatccatcaaaccttccagcaaaaataatttaataatttgatattaatacgt
aaaacacctatac-tcttttataaaatttctttatatctcatctaaacaatggaaactta
acttaatgggtgttcatattttatcaacaggttcgtctgtcccaaatttttctgtagaga
atcaacaatttgaagacatgatcgaaacttccgaccattggatttcaacaagaacaggaa
taaaaaaaagacatctagctccatcttctacttctttaactaaattagcggcagaagctg
caaacaaagccttatatgcagctaacttaaaacctactgagatcagtttaattattttag
ctacgtctacgcctgatgatttatttggtagtgctagccagttgcaagcggaaataggtg
caacaacatcagtagcttttgatattacggctgcctgctccggttttattgttgctttag
taacagcagctcagttcattcaaactggttcctatgacaatattttagttgttggagcag
acacaatgtctagatggattaattggtcagatagaactacctgtattttatttggtgatg
gtgctggagcagtagtattggggcaaagcctcaaaaatagtattttaggctttaagttat
gtacagacggtcagctaaacagtcatttacaattaatgaataaacctgtaaataatcaaa
aatttggtggtacagaaattcctcatggaaactataattccataacaatgaatggcaagg
aagtgtacaagttcgctgtatttcaagttccaacagtaattagacaatgtttgaataatt
taaacatttcaatagatgaagttgattggtttatattgcatcaagcaaacactagaatca
tagaagcaattgcgagcagattatcagtaccttttctaaaatgattacgaacttagagc
attatggaaatacatctgcagcgtcaatccctttagcgttagatgaagctattcaatcca
ataaaattcaaccaggtcaaattattgttttatctggttttggagcaggcttgacttggg
gagcaattgtcttgaagtggtgat---ttatatattgcggatgacgagactcgaactcgt
aaagctttcgctacacacccctcaagcgtgcgtgtataccaatttcaccacatccgcatt
ttttaccatgaata-ttaaaaatttataatatttatctaaatattcatataa--tgtgta
tatatatatacattcttgtcagaaactctcttatattttattttaagtgataaaattgag
t----ttagtatagatagtaaaattttattttaaaatcataa----aaataaatatataa
ggaaaataaaatatgacaccatctttatcaagtttttttgaatagtcttattcttggggca

gtaattgtagtcgttcccataactttggctcttttatttgttagtcaaaaagacaggaca
atccggtcctaaaaataacttagaaagatatggagaagcaatttaagatgaataatgaaa
tatttttcatactaataagctctccatatattgctatact--ttagaagaatttttattt
ttttatttgtaacatcctgtaaagtttattccgttctttctttttccc-atttactctc
ttataattaatgatacaaatgtcagg-ttaataatag---ttttttattctgtatctttc
---------------atttatatacgtattattatgttcaattaataaagaaaagctca
tgtctttgtctaattggcctctcaaaaaagaaaattctgaagcatataatattaagaatt
caaaacaaatcacaattcctgatggtttatggataaaatgcttcgactgtggtctattaa
tgtattctaaagtactgaagagaaatttaaaagtttgccctcaatgtagttatcattttc
aagcttctagtaacgaaagaattgatcaattgatagaccaaggtagttggcaaccaatgg
atgttcacttgatctctacagatccattaggctttaaagaccaaaagctttatagtcaaa
ggttaaaagatactgctttcaagactggcctgcaagacgcagttcaaacaggtactggga
ctatgcaaggtaaaaaagtatgcttaggtattatggattttagattcatgggggggaagca
tgggatctgttgtaggcgaaaaactaacaagactgctagaaaaagcaactcaagaaaagt
tgcctgcaattatactttgtgcatcaggcggagctagaatgcaagaaggtatgttgagct
taatgcaaatggcaaaaatttcttctgctctagaaatgcataaaaaagaaaatctactat
atatatctgttttaacctctcccacaacaggggggtgtaacagctagttttgctatgcttg
gagacttaattattgcagagccaaaagctcttattgcatttgctggtagacgagttatag
aacaaacaatcaaagaagacttgccagataattttcaaagttcagaatatttatttgaac
atggtttcctagatttaattgtatccagaactcagcttagatcaaagttaatacaaattt
tgtccttacataatcatagtaagtaatgaattgaatattgactataatatcaaaatgcta
gtaaaaattaagatattaagaaattttcactaaagatatttgtagactaactagaattac
aattactattgtagaaaaatttgtcttaatgcta-ttttttcagtcttttaacaaattg-
tttttatgactttaaataattgttagtttaatacaaatttataaagagaata-----cat
actaagctaaatatagtaatattatgttttcctataatactgacctatcaaatttcgagg
aaatttatgcttaaaagatcttcttggcttgcggctttattgggactattaacagtagtt
tctacaagtacgcatacatatgccatagagttagacgaggcaacaagaactgttccatta
gaatcttctggcagaactgtaattcttacaccagaacaagttaaaagaggcaagcgatta
tttaataattcttgtgctatttgccataatggtggtatcacgaaaacaaatccgaatatt
ggacttgacccagaatctttaggattagctacgccacaaagagataccattgaaggacta
gttgactatatgaaagatccgactagttatgatggtgcagagtcaatcgcagaattgcat
ccaagtattaaaagtgctgaaatttttcctaaaatgcgcaatctaaccgatgaagaccta
tttacaatcgcaggtcatatcttacttcaacctaaaattgtttctgaaaagtggggcgga
ggaaaaatttactattagaacttcaaagacctaaaccttgtgaatactagtttatgta-t
ttgttgcttaagtgtgattattttgttagatcatgatatatattaaagatggagtttgtt
gacagcaagttacttttacccgaacttgtttacactattcgtctagttattaaaaagga

gacgttaaattgaagaagaagctttcagttcttttcactgtttttagtttttttgtaata
ggtttcgcacaaattgcttttgctgcagatctagataatggagaaaaagtttttttctgct
aattgtgcagcatgtcatgctggcggtaataacgccattatgccagataaaaccttaaaa
aaagatgtacttgaagctaatagtatgaatactattgatgctattacttatcaagtacaa
aatggtaaaaatgccatgcctgctttcggaggtagactggttgatgaagatattgaagat
gcagcaaattatgtattatctcaatctgaaaaaggttggtaattatacttgattttatcc
tgtattaaagaatagacaatctatttagttgttcattagattgtctattctttgtttatg
---ctattatataaagatatttacacaatatt----ttatgatgaaaagaatacccgcaa
ttcttgtactagaagacggtgcatattataaaggatggtcattccagcaagataaacaag
agattactattggtgaagtagtttttaatactggaatgacaggatatcaagaaataatca
cagatcctagttacttccatcaaattgtcgcttttacctacccggaaattgggaatacag
gtattaataatcaagatattgaatctcacagtattagtattaaaggacttattgcaaaaa
atatttgtaaaatttcaagcagctggagagagcagcaatctttagttaagtatttaagta
gtaataatattccttttattttcggaatagatacaaggtctttaacccaatacttgcgtc
aatttggtacaatgaacggttgtatctctactgataatttaaatcatagttacttaaaac
agaaaatttgtgagattccaagtatgcaaggtttagatttaatcccgcatgtaactacaa
gaaatgtttacccctgggatgaaaaaagttttccgaattggtatttgacagataacatta
gagtacaccgagttattcagttgaaagttattgttatagattttggagttaaactaaata
tactcagaagactagctacacttggatgtcagataacggttgtgcctgcccacactcctt
taaaagatattttggcttaccagcctgatggtatattactctctaatggtccaggagatc
catcagcagtacattacggcatccagacagttacaaatttactagattacaatgtgccta
tatttgggatttgtatggggcatcaaattttaaatttagctcttaaagctaaaactttca
aacttaaatttggccatagaggtattaaccatccatccggattgaaccagcaagttgaaa
taactagtcaaaatcatggctttgcagttgaattgacttcggtttttgaatctcctgtaa
gagtgactcattttaatctaaatgacactactattgcaggaactggacataatcaaagtc
cttatttttctgtgcaatatcatccagaatcgagcccaggccctcatgatgctgattatc
tattcgaaagttttatagaaataatgacaaagtccaagaataaagttag-----------
ttagtaattttcccatgctttatgagtgaataaagcggatagtacttgtactcctcgtaa
ttgattaaagagaggcaaatgtcctctggggccatgagaattccattgaaattcctcagg
atatctgcaaggaattctatcaacttcccagccgatttttttgccagaattttccccaatc
ttttccaacactaagccatatttgtctttgaacaaatagcccaaatttaccttttgagtg
tgtatgccataacttatcaatagtttgtaaatcttgagcaggtatttttttttatatctgt
aaaatatagccaattacgagtttgtgcatttacaccagctagttgaataagcttttgttg
agttagctgatctgctttgagcaaatcccgatgagtcagtaacatttgtaaatctttgta
attcatctgttgagcagagcgcaatggaacaatgccatcgggacataagttagaagcaaa
tttcactatttcttgatttttactgttaagtaacttttcataaattaaaccgtcaacaca

attacttttatagttaggcccagtaatcctctcaaaaaatagatctgctaaatcttttaa
ctcgatagagtcgttgctatttatattctcaataatttcaagttgttgcttga---catt
attagacttagtattcttattcaattctaaaagctgggctcggatttgatttggcatctg
atttgtttaattagttttaatagtc----ataaggtcttagagagaattagattgttttt
tgaaattagaagagcgccgcagtatttctaaagaagttcgcataatttgattcaataagt
tttgtcctgcataaattataatttgaccaattaaaaaaattgtgcttttttatttaggaa
caaaaaaatcttgtacctcaagcagaaaagtaattattatttgcattggtgacaacatct
gtaattcattcgatctgcaagcatatatatactgacagtttaagccttcttgataaaatc
cccatactttgtaacgactttcgtaaattgctcgtggtcgctcaagatagtttttaatta
tagtattccaaattaggttattctttaatttttctaattttctatcagatagaaatttta
cattacagagataacttgcatttgtctcatttacagtattaaaattttgaactaacatat
gagcaataatgttgcttagctgaattaaataattttcaagaaaaatttcgacctgtttaa
ctggcatattgtaagtaaagagattaaaagttttattcacagtttgagatgacccaaaaa
gtagctgaataattaaaatttgtagtagcattttatagtcgttcagtagatagttcaaat
catgaatatcttggtaaatgtcaatacagtccagttcatataacttacaaaatcttttga
tacacctatggaataaatcaattaaaatattttcggtcaagtttgtaatatcatttacat
ctaaatttgaattataaatttctaaaagtatcttttccagttcagctaatatgattttta
ataagtttctttttacttcagtgcgaaaaacatctaaaattaacacttcctgagagcaat
tagttaatctcttattaatctttatagaagttctaactaatagctctgctacctcttgat
tgagtgttggtccctgagagcttggccaataattattcacgttataccatagtaagaaat
attgaatttaatgttatacgataacttttttaatatactgaaaagcaagttaattactcta
tagcaactag-------tttttacagatcttaatagttatttatcaaataaagttatatg
atagtagcgattgagctaattttattaaaattta----taataatgacaacttattactt
cgcccttgcgagtcaaaatttctattagtacaagaaccactagaagaagtttttagaga
gagagttaactactatcagtcaaataataaagcaattgattttggctaataccaaaccc
ttcttttctagagaaaccagaaatgatttcatttaaaaaccttgtacctaaagacgctgt
agctataatctctactaatccaatatttattaattggttaaagctaagaattggctatat
ctgtattggacaatttgaagataacctacaactttctgaagaatcgttaaatattactgt
tttaacagacaaaatttaacaatgcttttattatgactagtgttaaattattttggttaa
gttataattatagtcttttagtattta-gactcttatagtttatcacttagtattttatg
ctgagatggagtgacaaatagtgtaagtatttcttggctaaacgtttcagttgccttaga
tttatatctatttggattaactataatggatagcattcgttttatagtaacattttcaat
ttgagcccaatgtacaattccaagttccaattccttagcaatagctgaaacggaaacaaa
ggcagctcctagccctgattgcacagcgtttttgatagcttctatagaattcaattccat
ctctattttaaagcgactgctgtcaataccatgttgactgagtactttatctatcacttt
tctaattgttgattgagtatctaatgcaataaatctaagcctgtataagtcttctttttg

aatgtctcctagtttagaaaaaggatgtgatttgggtaatataagtgctaattcgtcttc
cgcataagaagtaacctgcaaaacatcttgcaattcagtaggcacttctcctccaataat
tgctaaatcaacttgaccattagctacgctccatgaaataagtctagttgaatgtacttg
caattgaacagccacttgtggatatctttgcctaaatagtccgattaatcttggcatcaa
atatgtcccagttgtctggctagctccaataattaatgtgccaccttgtaagttttgtaa
gtcgtcaagagctcgacaagtttcttcgcagagagctaaaattctgcccccgtatcgtaa
aagaagacttcctgcctcagttaaagttgccttcttattacctctttcaaatagggaaac
atttaattggcgctctaaattttgaatttgcaaactaatagctggctgagaaacatacaa
gctattagctgctttttttaaaactgccctctttggcaattgcttttaatattcttaactg
atccaatgtaaatggaaggtctgtcattaaagaattgtagtataatg---------tata
ttatttagtattgatcaatgtatgcatttgccaactaaaatatttgtcattacgcttcat
tattttcaaaataataaaaagtataatgttaaataagctgtattaaaatttagtatctgt
ttgatattaaatttatacaagagatatcgtgaatttaattctaactataaaatataggag
acaat---atggactctagacttttagttgtactaataccagttttagcagcagcatctt
gggcagtttacaatattggtagagttgcactacagcaatttagaaaaatgacatcttagt
tttgcttagaatata-atttttagactttatgagatagaggactttaaaatccctatctc
tattgtatatcttaataaaaaaag-aaagc--ccccttgtacttactgttattacacatt
aatattattggt-ggtaccta----gataataagtttaagctaacttatttttatttta
taattaattaaattaataacacaattatggctgttccaaagaaaagaacatctaaagcta
aaaaaaatgcacgcaaagcaaattggaaaaatcaagcaaaaactgaagctcaaagagctt
tgtctttagcaaaatcagtattaactagaaaatccaatggatttatttataatctaactg
aagcatcagatacttttagcgattagaagtgttt-ttactacaagtctgaaaaagtatta
cgcaacaagttctcagtaatttttatctat----taatcatttgaaaatcattctatttg
ataataaaataaatagcagattaaataaaatcacattaacaaattatgcagcaaagctag
atcaaactagtgaaatttggttgttcaattgtattgaaaatattcagcatattttttta
aaagtcaattaaaatcaagtcatattactaaaattttatttctggtactagctttgaat
ataccgcaggtctaccaggattattatccagcttaacactaagtggtagactacatccta
ttagtatatatagtccccagtctctcaaaaagtatcttgaagcatgtaccaaatattccc
aaactaatttttcttttcctattaattttcataatttacaatacggaggacaagttgtta
accaattttatacagtaatttgtttaccgttgagcaaaaagagcctgctgtatggattta
ttattctaaaaaaagaaaagcagggagtatttaatttagcacaagctaaaaccttgaata
ttcttcaaggacctatatatggaaaactcaaagaaaaggataatttttttaagtccagatg
gttactatctatccggccaagacttctcttctaatacaataatgggacataaaatatcgc
ttccgttattagtcagatattctagaattatttctgagatgcattggttttgctcttatc
ctattagattaaatacttattcgcatcagcaaggagtaaagtgtttaccacataatgttt
taactgatattatgaaatcccaaatatatcaagataacagttttgttgaataatacttat

tcttgatatataatggtctcggtaatctaaataaaatactagaaataatttcttaaattt
atttgtctatattaaatttacaatcatttatgacatacgcaattattgaagcaagtggca
cgcagctttggatagaagaaggccgctactatgatttaaatcatatacctgttgatccag
gtcagtcgattatattaggaaaagtcttattattgaataaaaatggggaggttactttag
gacgcccttgtatagaaggggttacgataaaggctacagtaatgaggcacttgcgaggaa
agaagataactgttttcaaaatgaaaccaaagaagaaaatgagattaaaaaaaggtcatc
gacaagaattgactcgtttaatgatcgattctataacatcttaga------taaaatcta
gactcatatttt--aa-aaacttttaatatttaactaaaatagatataatggcacata
aaaaaggtagtggtagtacaagaaatggcagagactctaattccaagcgtttaggtgtta
aaaaatatggtggagagcaagtaacagcaggtaatattttaatcagacaacggggaacta
aagttaagcctggccaaaatgttggaaaaggaaaagatgatacattgttttctctaattg
atggtttcgtgctgtttgaaaagtcaaatcaaaagcaaaaaacaattagtgtttattctg
ctaagaaatagttaaaacaattggtgcaagataacttgagt----gtatttatttcactt
acagtggcaccatatatttgatttattaagaaatggagttccttaatgaaccaatgaatt
ttattaaagatttgctgtgaaatttaatgacaaacactattgtaatttcctgtctacaca
atatggctgctattctatattgtggtcagatacagaaactagtagtagcaaatgctcatt
atcaagtaagtgatatctacctaggttgtgttgataaaatcttctcaggaataaatgcgg
catttattaacctaggaaagaatgagtacagtggttttatacatatcagtgataccggtc
cgcttaaaaagaaatattatgtcaataatattactaacattttaacaatacggcaaaaaa
ttttagtacaaatcattaaagagccaactttaaataaaggtccaaggctcactgccaata
ttacattatcaggtcgatatattgtattaatgccttttagtcaatcaatctgtatatctc
gaaaaatatatgatgaagatgagcgtcattatttgaaatctttagctattttaattaaac
cggcaacaatgggcttgctatttagaccttctgctgtaggtgtcgatgaggaaataatat
taagcgaattaaaaaatctaaaagaacaatggaactttgttcaaaaatctgcaattaata
gttattcacctgttcttctatataaagatgaagatattgttaaaaaagtaatccgagatt
tttataataataatacaaacaatatagtaattgattcaaacctgggattaaaacaattaa
attattatatccacacttggcactgtaataactctagcacagttcctaagattaagcttt
atagtaataatcaatgtatactagatgcttttggtatcaatcaggcaatttccagagctc
tcattccaaaagttgatcttatacttggtggctatatgtttattgaaactttagaagctt
ttactattattgacgttaattctggatcttttaataattccactagtgcacgagaaacag
ttttaaaaaccaactgttctgcagcaacagaaatagcttatcagttacaaattagaaata
ttactggtgtaattataatcgacttcattgatatggaatcacaaagagatcaattgcaat
tattagaacactttaataaagagctatcacttgatgatgctaaaccacaaattgtacagt
tatctgaattaggtttagttgaattgactagaagaagacagggcaaaagtttgtacgagt
taattagtagtgattctaattacttttattttttcacacaatcagagagatctcagtctc
ttaagagattcgatgatagacagcagaaacaacagatttttaataaatcttggctatctg

cagagattaatactattaacaaggtctttttttcaaaagtcaaatttgtgcagacctgcta
acttttacctaattcgtaatctctatatagttaaaagtagtattacttataaacaaaatt
atttattaactcacagatcaaaacttatttattctaaagaatacagcaaagtattaccaa
gca------gttattatctagctagtctcaataagaatagtaatcaagagttcctatctt
aagattgttatatcaactgtt---------------------------------------
-------------------gaaaaaaagaagctctctcttatataagagagagcttc-tt
ttatagcttactaacta-tctagttagttaattaaaaattaaccgttaacagctggagct
gttagagctactggtaaagattcaccagaagctagatctagagggaagttgtgagcgtta
cgttcgtgcattacttccatacctaggttagcacggttgatgatatcagcccatgtgtta
attacacgaccttggctatcaacaacagattggttaaagttaaaaccattcaagttgaat
gccattgtgcttacagataaagctgttagccagataccaactacaggccatagacctaag
aagaaatgtagagaacgagagttgttgaaactagcatattggaagattaaacgaccgaag
tagccatgagctgcaacgatgttataagtttcttcttcttgtccgaatttgtaaccatag
ttagcagattcgttttcgcttgtttcacgaattaagctagatgtaactagggatccgtgc
atagcactgaacagagaaccaccaaacacaccagcaacacctagttggtggaatgggtgc
attaaaatgttgtgctcagcttggaatacaagcatgaagttaaatgttccggagatacct
agaggcattccatcagagaaactaccttggccaattgggtatactaggaatactgctgct
gctgctgctactggagcagtaaaagcaacggaaatccatggacgcatacctaggcggtag
cttagttcccactcacgaccaatgtagcaagctacgccagttaggaaatgaagaacaact
aattggtaaggaccaccgttgtatagccattcgtctaaagaagcagcttcccagattggg
tagaagtgaataccgatagctgcagaacttggaataacagcaccagaaatgatgttgttt
ccgtatagaagggaaccagcaactggctcacgaattccatcaatgtctactggaggtgca
gctacgaatgcaatgatgaatacagatgtggcagttaatagagttggaatcattaataca
ccaaaccaaccaatgtataggcggttttcagtactagtaatccaagagcagaaacgttcc
cacaagctagcgctttcgcgtctttgtaaagtagcagtcataatttttttatcaattttta
aggatttaacaaagatgcttcccaaacaataagttgtctgttatgaaaattattacttaa
ggccttttcaaatgagaacatttttaaaaaaaaca-tagcaaaatctaatctcagtaact
aa-aattt-tacttgttcatatattgcttaataattcaa-----------taataaattt
tt-gct-------------ttttgcaaaattaacactattttagggat---ataataata
atgcatattaatgctgcttattttgattttaatagtttatataattttgccctacttaaa
tacgcatgtcacactttacaaaaattcagacgaccatacaagatttaaatctgttaaagc
atgctttaacagatttgggtttgatttggcaaatgaattctagtcatataaaagttggtg
aaaataatcagcataaggtagatattttaattaaacaagataacttgtcacatattggct
ttacttggaatgataatagatatcacttagttgccgacttacaactttgggaacaaccct
ggtctctagaagtatttttggataagttatctcagaagtatgcttactactctatcatcg
aagaaacaaagaagcaaggctttgaaaaaatgcagcagatttcacaaaaagatggatcaa

ttaagttaattgtgcaacgctggaattattaactaacatacaaatgcgggcggagagact
tgaactctcacgagattatctcactagaacctaaatctagcgcgtctaccaattccacca
cgcccgcactgttgagac--aatattattctatcataatctatactaaaacacaaactaa
-tagtataaactagcttccaagcttaaaagcgtctacaaatagaccgtatgttataccaa
ttttgaagttgttgaataaattaattttatatttaattgcttgtggctagagtagacta
ttttactagtcaactcagttgctgcaactattagactggctgcaataataccccaatctc
ctgtttgtccaggaatcgtagacaaacctgttgaaataaaaaatcctagcaataaactaa
ttaagccagttgttaactcgctcaaagaataataaagcttattatttaagtttttatta
agtaagcaaaaaaagttgaaagtctagttttaatcatagtatttagtaacggagttttaa
aaggatgacaagtattaacag-tcattaatattgtcgtatccttttaaaactcgttaata
caaacagctgttcttgt---------ttttaaaggtcttgttcgcttaaagcattaataa
gatgctgaaacggctcttgtataatatctgtgcgtttaattgattgcaaatctaattctt
cttctatgatttcttgtaataactgtatgcttcttactgttggagcaacaggtacagata
gtgaattgtaagtatcttttagcccatttaatacacgctgatcgagaatatttgtactac
cagaaaccaaggcataactagcataccgtaggtaatattctatatcccttagacaagctg
catatcttcttgttgtataagaatttcctccaggccttaataattcaggctgttcttcgt
acaactgagctgcggcctcttttaaaatattggtagcttgatcattaataatttcagcta
tttttattcgatctagtccactcgaaaagaaagattctagttgtccgacagctgtcttat
ctagataacgaccagtaagatcgtaacgatttaatattgctgttatagcatcttgcataa
attaattctcctac-ttgctatcagagcggtattataaatgactaatattgtaatataat
cattagtgtttatgaaatttatttaaaacttattctttattgcttaatgtgcaattaaaa
actcgcatagattttacacatatagagtatacgtagtttttaatta-tttttttataaaa
accaagaaataaagctttttt-taagaggttcgtgaatttgttaatataacatgagcttt
cctcctaaataagttttgtaaagtac--aaagtaaaaaaacgattagtcaataatagtaa

--------------------------acaatattaatatgtatcaa--------------

-----------------agtataaataagcttttactccagcatcaaaaaaaatatttga
gatatgttgatacaactgattattcaagtgaaaacttagatttagaccaatttagctatt
ttatcattacaattaataaaactgataaatgtattcttatagagcaattttgttataatg
ctgaaggccatatttactcaatattcttcaaaggtccaacagctaaacatctttcttcta
taatttgtcattttcaacaattaaatacaataatatctacagctcatgccatatatttag
gacgagagctaatgaagtcagaattagctctcgtgctagatcaacaatatattcaagatt
aaa--ttttgtaagtattaattatatttatgctagtataatattagtaagggcatgtaac
tcagtggatagagtatcagattccgattctgatggccgtgggttcgaatcccgccctgcc
c---gagtactcagagtact-atactaaaacatct-------taatagctagtttgataa
tagtatgggcattggggctgcaaggtttctacattatgaaaagaagaaaatatg--aaaa
aaacaagctctccaaaagagcttttagtacaatcaataaatgcagaaaacaatattgttt
ctttttctcgaaaattagcttttgcttaaacactgttaatttttggcattcgacatgtta
aactcttattcatggcgaatattctgtcagagtcgctcttagtttaagaaaagtcagaaa
aaatatttatctttacgtttt-tacctatttttatttaaataaaggttagta-ttttcaa
cttttatagtggacgtgggttcgagtcccaccagctccatcaatttgatt---------t

gtataattagctagtttaacctttttaataaccagatatataattggaggaaatatgggt
ttcataacaattactaagcccgctttaaagcaaattgcaatcttgaaaaatgatcatgaa
aatgatgtacatctaagaataggtgttagacaaggcggatgttcaggtatgtcatactca
atgaattttgaacatgttgacaaattaaaagatacagatgaacggcttcgtctagataat
ttttctgttgtctgcgaccctaaaagtcttctttatctttatggattatcattagatttt
agctcggaattaataggaggaggatttcaattttctaacccgaatgccagccaaacttgt
ggctgcggtaaatctttttcaggctgacc-ctcgaacacgtttttttatatattttgtatt
atatatattattttagcaagtcg-tttattagagacaagcaatgctctgttaaaaac---
----------------------------taatttcaagagttttattgtattatt-----
----ttatatatttattatcaactacttgaaaataaattagctaagcaatctaatttgtt
atcagtaccaaactttaaatgattaactttttattgatatatgctaatcttt-atcagcc
taaacatatctgtacattacaatcttttgacataaaaagagtcttagcatacctctcaa-
----------------------aaataaattcaa----------tattttataaatactc
aacatgaatctgtcacaacttatcgttaattcttctatgatgaaaattcctaataacttt
tattgcaatataaaagctatttcttctgagaatttaagtcacttacaattatcaaaaaca
aatttattatctcctacaaaaataattgcttttggactacctaaatttgattttatttct
acacagattacaagttctattagagaaacagttgttgtgtctactattgatgataataat
ctttttgctgccccaactaatgattggataaaaaaacacaaggataaaaatcatcattct
ttgttttataaaaaagtttttaaagctttgctaaaccataaaattagagttgttgctgag
ccact------tcataactccgaaataaataaagaaaagagcataatggcaagtcaaaga
tacatctggggtaaaagctggaagccttcaattattttatcttgcttaaaaaacaaaaaa
aatagcaaaatttctgatgcatcgaaaaaatttttaactgaacagttatctgcatcacca
gtatttgtggtaaaaaatggttttaatgaaattattttaggtcacccattatctcgtgtt
aaaagaggaagagttaataatctgatgcatgcgttttctaatttattaaatcagtctaat
gcaacttatcctatatctactggtctattctttttttcatccagatgatgcatttgaattt
aaagactttataatatcggtcaatccactggcggccaaacatatggagataagcgtagag
cctgttggtctacactttgcttataaaatgaatagaaatatatcgtcagatactcaattt
tcgtttattccagattttaaagaagtaggagatttattattcaaatatagaaaaggtaat
catttagtttttcataagaatcagcattatggcaaagatttttttcaggggcaaccaata
tatatgattcagccgatcacttttaaagatcgagccggaaagctaaatattatcaaattt
acggggctaaatgacacaagagaaattatttttactaatcttgaagctgctaatagatca
tgggcacattttataaagaggaattcacaattaaaatcaattaaaaatcctactctatta
gtctataatttagaaagtttttttaaaagatcaagaacgattggataatcaagatttaaat
aagtttgtggtgatcactaataaagaatcttatcttgccacaaaagagttaatagcttta
cccgattctaatagcttttctaagcacttaaaattaaatataaagcctaaactcttttttt
gtgaaactatgggtaagacggttattttccaccttaacttatgaat-aaatgctatt-gc

ctttattttctcgaataatattctataactagtaattcattgagttgtaaagcaacccat
tccctatcaatgactccattgactttccctgataaattggatttattcaattctaagtgg
ctaggaatattagctagtccagggaaagctaagtaattttctactagttttcgagatgct
tcttgatttttacactaattgattctcctggtttacattgataactacagatagatact
acttgtccattaatacaaatatgaccatggttcactagttgtctagcggcaggaattgta
ggagccataccaagtctaaaaacagtattgtctagcctcatctctagtagctgtaacaaa
atttgacctgtcgacccttgtagtttttagctgcttttacgtacttaaataactgtttc
tcacttaatccataattaaatcgcaacttctgtttttcttctaatcttacagcatactca
gaaggttttctggacttttgtccatgttctccaggtggataagatcttttaatcgctttt
ctactgagtccgggtaaatcacctaatctacgggaaatgcgtactcgtggtcctctgtat
ctagacatattatttaaattctcctaatgtaaat-gattaag----------taatgagt
agctatttttaacacaaaaagagcattaaaacaaagc-ttttgctaaaaaatatttaca
aaaaatataggtttggacaaaa---tacattactatgc-tttttaagcgggtagcgggaa
tcgaacccgcatcattagcttggaaggctaaggttttaccactaaactatacccgcaata
ttcaacagattgtcactaactcaatataacatagttatgttaagttaagataatcttaaa
tttaatagaatt-ttaaattatttatgctccttctaaaaccacatcaagaaaacgagcaa
tttcggcagcttgttcttcaacttctgataataataatggttgaccgaccctagttaatg
gaattactcttttatcttttgtacataaataaatttcacgtcttggatttagcccctctt
taatatctatttttattgatttgatttcttttatattaaactgaagacagatttttctat
tctttccagggaaacctagccgaaaaattttacaatacctttatctttgttaaattcat
tatatccagcccctatattccaaataatagttagccacagaaacaaactcagaaagactc
ctatactgccatagaatgtcattacaataccttgtggaataaataccaaatcggttgaat
ttgtaaaaggtaacaaatcaacttgaaaataacttgatagtccagcaagaagaaagccca
aagccccaataaatattatagtagcccaccagtaattactcagtcttcgtgaacctaaaa
ttaaatcttttctaactgagtgtatagataatgtttttttcataatttgttcttttaaaa
cagta-tactaatgcaaaatggaaaaatttgacaattttt--atgcgcgctttagattt
tttaaataagcttgattgcttttagtcctaaaaacagtcctgttgctaacccaaaaaaag
cagcaagaaatattatatatcctagaaaaactgaca---tattattatttattattattg
ttacaatagtgtttacaaaaatagtgaaaatctattactacggtattatattacgttaaa
tgccttgctgtctacaggaaaaaactttttcagtttttagcattgtgtttatattagaa
ataatattctagtaaatttattaaacttctggagaacagaatcttatgtcagaatttatc
aagccttataacgatgatccttttgtaggcaatttgtccacgccagttagtacgtcaagt
tttagtaaaggacttctaggaaatctaccagcttaccgtcgaggtttatctccgcttctt
agaggattagaaataggaatggcacatggatacttttaattggaccttttgataaattg
ggccctttacgaggtacagatgtagcgttactagcgggatttctatcttcggtcggcctc
attattattctcactacatgtttatccatgtatggtaatgtatcttttactagagcagat

tcgaaagatccactacaaacttctgaaggctggggacaattcactgcaggatttctagtt
ggagcagtaggtggttcaggatttgcttatttattgctagctaatatacctgtattacag
actgcaggtcttagtttattctcttaagctagtaaggggacttgaacccgtaacctactg
attacaaatcagttgctctaccaattgagctatactagcattcagttcataataacatat
aaagatatttgttgcaatctgctattttcagatgtaacaaatatctttttttatttaaaca
gaactgttatgttaacac-------gtatattaattagaatctgaattatctgactctgc
agaagattcctgatcacagtttagataataacaaattgtttgatctagacaagttgcaga
ccaaccaacaggtgtttctcctgataattcttgcatatctacagaattataaaaattatc
atttggcttattactgtttgcttgcataaatatcactcccaaaacactctgaacttaatg
tgaagtaatttaacttctcaataatatattaacacaaaatattaaatatggtgattattc
tttaattgtaaagatgctgctaactttataaagtttgagtgagagtcttgatagcttttg
ttgaaataagcattttaacccatttatttgcccgctcgaccatactttttttgtgttgta
aattaactttttgtagttttttttgttcgtttatgagaatgagatacggcatatccattat
tagctacttttcccgtaagctgacactttttttgacataatttt-------ctaacttata
tatttatttagagtacttgctaaagttgacttgggcacagctccaatcactgtgtctact
ctttctcctgctttaaagatcattaaagtaggtatacttctaatgccatattcagccgca
atagtaggattatcgtctgtgtttatttttactaccttaatagatgattcatattcttcc
gcaatttcatcaactacaggagaaaccattctacaaggaccacaccatggcgcccaaaaa
tctacgagtacaggtaagttattgttaataacttcttgtttgaaagaggcatctgtaact
tgagatactgacatattctttaacctttaatatatgttccttgctaagcaaatcttatca
caaatttcaagaaaaatcttctatagtgaattactattagtgttgaattattttttttaaa
aattagacattaaaaaaacttattacctatataaataaagtggtggccgagtgatatctt
gatagtgataatttttatattaaggttgaacactagaaataacttttcaagcaaactctt
atttgaaaa--atttctctgtgaccataaaaacttaattatattacaatatattttatta
tttgtaacttagttctgataatggtataaacaacgcaaaagatactgccttataatcaag
gaggaatacatgtctcaatccgtagaatcacggactaggattaaaagcgaacgttacgaa
tctggagtaatcccttacgctaaaatgggctactgggatgctgactatgtgattaaagaa
acagatattctagctcttttcagaatcactcctcaaccaggtgttgacccgattgaagca
tctgctgcaattgcaggtgaatcttcaacagctacttggacagttgtatggactgattta
ttaacagcttgtgacttatacagagcaaaagcatatcgagtagatccagttccaaacgtg
gcagatcaatattttgcttacatagcttatgatattgatttgtttgaagaaggttccatt
gcgaacttaactgcttcaattattggtaacgtttttgggtttaaagctgttaaagctctt
cgcttggaagatatgcgtatgccagtagcttatctaaaaacgttccaaggtcctgcaact
ggattgattgtagaacgtgagcgtatggataagttcggtagacctttcttaggtgctaca
gttaaacctaaactaggtttatctggcaaaaactacggaagagttgtatacgaaggcctg
aaaggcggtcttgatttccttaaagatgatgagaatattaactcacaaccatttatgcgt

tggagagaaagatttttatattctatggaaggtgtaaataaagcatcggcttctgctggc
gaaattaaaggtcattaccttaacgtaacagccgcgacaatggaagatatgtatgagaga
gccgaattctctaaagaggttggtagtatcatttgtatgattgaccttgtgattggttat
actgcgattcaaagtatggcaatttgggctcgtaaacatgacatgattttacatttacat
agagctggtaactcaacttactctcgtcaaaaaaatcatggtatgaacttccgagttatt
tgcaaatggatgcgtatggctggtgttgaccatattcacgcaggtacagttgtaggtaag
cttgaaggagatcctttaatgattaaaggcttctacaatactctacttgaaagcgacaca
gatatcaacctacctcaaggtctgttctttgctcaaaattgggcttccctacgtaaagtt
gtaccagtagcatctggtggtattcatgctggtcaaatgcaccaacttcttgattactta
ggtgatgatgtagttcttcagtttggtggtggtacaattggacatcctgatggtatccaa
gcaggtgcaactgctaacagagtagcactagagtccatggttatggcaagaaatgaaggc
cgtaactatgtagcagaaggtccacaaatcttgagggacgctgctaaacttgtgggcct
ctacaaacagctttagatttatggaaagatattagttttaactacacttccacagataca
gctgatttcgttgagactccaacagcaaacatctagtttaatgactacttactgatactt
taaatagttaattgtaagtgaaattaacttataacaataaggagcatagaatagtgagac
taacacaagggactttttccttccttccagatttaactgatgagcaaattaataaacagc
ttgcttatatcgtttctaaaggcttatcagcaaacgttgagtatactgacgatcctcatc
caagaaactcctattgggaactgtggggtttacctttatttgatgtaaaagatgcttctg
ctgttatgtacgaaattagctcatgcagaaaagcaaaacctaattattatgttaaagtta
acgcttttgataatactagaggtattgaaagttgtgtaatgtctttcattgtaaatagac
ctgctaatgaaccaggattcttattacaacgccaagacttcgaaggtagaactatgaagt
atagtcttcatagctatgctactgaaaagcctgaaggagctaggtattaatattaattct
taattaatattggctaatta--ttcccctcttaaaaatcataattaatttagtaattgtg
atttttaagagggatagcttcccaagtaaattttgactaaa-tagattgaataattaaaa
atacacagaaataatgcaatcacaggatataatttccaacgatactcttgttaatttaca
agaagaatatgatagaacacaaatccaagaagttttaaatgagttaaatcaagaacttat
aggattagtgcctgtaaagaccagaattcgcgaaattgctgcgctattattgattgacag
attacgcagaaaactagaactagtttctggtaatccaggattacacatgtcatttacagg
tagtccaggaactggtaaaactacagttgctatgaaaatggctgatattttgcacagact
tggatatataaaaaaagggcatttgttgacagttacaagagatgatcttgtaggtcaata
tattggacatactgcccctaaaactaaggaagttcttaaacaagcaatgggaggagtttt
atttattgacgaagcttactatctatataaagcagataatgaaagagactatggctctga
ggcaattgaaattttattacaagtaatggaaaaccaaagaaatgacttagttgttatctt
tgctggatataaagatagaatggaaaaattctacgaatccaacccaggactctcttctag
agtagctaatcatgtagacttcccagattatacttcagatgaattattacaaatagctaa
aatgatgatagaagaacagcagtactgttttacagaagaagcagataaaactcttttaga

gtataccgagcgaagaatgaaacagccttattttgctaatgcaagaagtattcgcaatgc
tattgacagggctagaatgagacaagccaataggattttgccagtggagaaaaagtatt
aacaaaagctgatttggtaacgattgaagcagaagatatcttgaaaagtagattattttc
attacctaatgcttaatatacacgtgatttcatgaaactatatattattaaaaag-tttc
ataatcagttgcaaatttcttgaaaaacctttattattatcttaatgtaggtg-tggcgg
catagccaagtggtaaggcagaggattgcaaatccttcatcccccagttcaaatctgggt
gccgcctagtactaaaaaagggggtgtggtggaatggtagacacaacagacttaaaatc
tgttgattttagtaatcgtgagggttcaagtccctccacccccatatattgatttataa
ataaaaagagatgtgtatttgtattaattgcaaccatattaccaaatgtaatacctatca
cttaatagaatctcaacataaacagcctcatcttactagaagtccattgtttatacctaa
atatcctgtagttcatgttaatatatctaacaactgtacctataatcaaattgattggga
tttagtggagtgtttatcatttgtagaaaaaccgaatagttggaatttagacgctaatta
gttacaagaaaaattatgtaccataagcaacaactctatttttagatactcggtattaa
ttttggatgttctaaccagtaagattttacccgttcttgctatctctataatgccaaatt
tagttagtaattgttcaatagcaacaatctttcccggatctccagtaacctctataatta
aaagatcttctgcaatgtctactatcttagctctaaaaattcttacaatttctaaagctt
ctgttctagtctgagaattgatctgaatcttaattaacattagttctctttcaactgaag
gaatatttgttacatcttgaacgttaaggatatttactaacttatataattgtttcgtaa
gttgttcaatagttctattgtctccttgaactaccattgtgattctagagaccccaattt
gctctgctggtccaactgctaaacttgcgatattaaagcctctccgggcaaatagaccag
atattcttgacagtactccggcttcatcttgaactaaaactgataaggtgtgtttcatga
aaaattaaatttaatctatttgatctattaagtatattctaagaaaattacagaaaaaat
actagttagtctataactaaattatccaaactaaacaaaatcctactatcagataatata
tctaccttgtaatgctatactattaacttagtatttaatgatattctaattgcaaataga
atgct-aattttttaaatttgttaggaacttgtgctagaaaaatacaaaaacagtaaaa
aactctctcgagatttacctcaaatcaatgatcgcattagatttccaaaagtccgagtaa
ttgatgacgaaggtgaacaactaggtatttttgtgcctgaagaagctatacaattagctg
tccaacaaggtttagacttagttgttgttagtgataaatcggacccgccagtatgccgaa
tattagactatggtaaatataagtttacacaagaaaaaagagctagagaagctaagaaaa
agcaacataacagtagtattaaagaagtaaaaatgcgatataagatagaagagcatgatt
ataaagttagaataaaccaggcatccaaatttattcaagcaggagataaagtaaaagcaa
ctatcacatttcgggggcgtgaaatccagcactctaatttagctatagatttattgaata
agatggcaagcgatctaaatgcaatagctgaaattcagcaagctccatcaagagatggca
gaaatgtcataatgctcttatctcccaaaaaagttagctaaactaattttatttaaatg
catctggctggattcgaaccagcgacgtctctttcgaaatggcggattatgagtccgctg
ccttcggcccctcggccacagatgcatcacttaaagtttataactcataaagcatataaa

aatcaatgtttagctatttttaataaagtctataaaagtctattattcattcatattgtg
atatgtcgcaactgctgaaggggaaacttgatttaagtatcgaaatatccagtatttaaa
aattgtgtcgagtataactggaaaagttgaaataaataaaaaaataaaatctcgactttc
cggaagtcctaaatgtcttaaaattacttcaataataacttcccatccatgaggagaatg
aaaacccacaaacatatcagtaaaaagaatgattaaaaaagctttagctgtatcactaag
accataaataatttcatttaaaaaagatttaacgactgcaatttgtctttgtccagttat
cattagtaatataaagacaagtattgatacaagatcggataaaatatttttaacagcatt
tgcactttcgtttgcataatattctcccaactctctagccttcagctgcactctttttc
aatgatttcgtaagatatatcttccgaaggattcagaagaacttcaaagtgaattttctc
ttcaaatctttgtaattcagcaaaagctctttcctcttgagaggaatttaaaaaaatttt
aggctgttcttggttccataaataatcaatacaaggtccaaatacaaaaaaattttgaagc
ctgattaactagtactggagagatgaataataataaaatatattttacggaagtaatagt
ttggtgtctagatattctaaactcctctatagcttcagattcgccattaggatctaactc
tttacgaaacttttcaaaagtattagtaattgacctaggtattggcccaactttttcaaa
ggcgaattgattatttcgttttaaattccaatatttcatttttattgctgctaaaattt
ctattaatcaataattgatcacgtattcactataaatattaattctagaactaaacatat
aaaacaatatttatgaaaaataaaatacaattaaaaattaattatgcattagttctatct
gcagctacagcagtttatggatataaatcttatcagccaactataaatacactcttaatc
aaagatgaagaaaactggcctttttctctaaaaggcagactgcctttattcctataatt
gtattaaaaaaaaatcatcaaaatccctgaactcagaacctagaaatagtaagttatatc
ttaatggaattggaagtagaaaatttatcgacagaattgtcactctacttggatatttag
aaaaacaggcaaactatactgaatacttgaaccaaataaaaacaagtggttatttttctt
tagtctacctagacagtgatataatgtataagaaaaatattagtaacatttttttactac
ctaatgatattctaaaaagaatctatatttgcaataagaaaaaaaagcttataccgcatt
cattcttaataaatttatttcagaaacagataggctatccaaaaagttttgctaatttaa
attgggccttctctaaaattataaaatggtattatgatagaggataccaatggtctctag
ttgaagttaaacaagcgtctgatgcatcttctattgtaattgatattcatgaaggggtag
tgaagacaattataacagaatactacactctatcttataaaagagtctcaggtatcttat
gcgtagagtcaatagagcagtaccttggagtaagagtgggagctccactaaatataattg
atttacaaaaaaaaattacttacttaaaagataatcaactagttggcgacattatttata
gtattgaacgatcaaataatagctcaatgagtttggatatcaaatttcaaatacaagaat
taaaagataaagagataatagtgcttgcagaaagttcttcta------------ttatct
cgcatgcatgtaatttactcaatc---aatataga---------aataggctagtagctt
ctaatatagtttcactatcaactaataaattacacgcatgctattttaattataaacttg
actatcaatataagtatataaacacctctaatcttataaaattgttagcttattctattt
cttgtaggaagacactacttattcattattttaatttacaaactttaataagctgtacaa

aaaaaaatactattggatttcagctatatttacgaaatctaagttttgggaaagctttct
gtgtgcttagtatgaaatttatcaaaaatggacttaatataaaaatttatatataaatc
cttcattgatagttgatcagaattttgtatttcaatttgctatacaaatcattaagcaat
atcatactgcta------aacctccagctt------------------------------
------tatttttaacgaatctagatcttgaacaatatgttgctgaaagtttattaatgt
accattttacatcttgcttctcaatatctgaaaaaatattattatctcgaattatgcata
cagattctttatttttcaactctgaaa------attttcattttgatgatagaactaacg
cgatcaacagttatgatactttttaaacaaaatacaaaaatattttatcaagagtttcttt
ctttattattaagtttacgttatcaaaattttaattatttaggttggcctttaaaaggcc
attttttgaaataaaatctttatacttggctccatttcaaaagagtg-------------
---atttttctgatagtcgtaagacgctattcttccacaaaatgtcgttaaagcaagtat
ctaattttaatttaccagtatcttttaaaagccaccttaaccatattttagttagtacta
ttaaatgtcagtcaaatttgaatatgagaactgtatctttgctattaattgattcacctg
ctgaatatctgctatacaaatctattcttaatttctctattaaagttaggatgcagtatt
tcatacctatgagcaacaatataagattatctctattttataattatttagattgttttt
taataagatcatctcagagttgcattcacatatggcaagatctaagaactcttacaccaa
ttcaaaattttggttaaaaaaattttcatatggagctggaattcagcttaaattaccaa
ttaagcagatgccacctctttcaattgaatatactgtaactagtagtcgctacttttgta
tttaccttcgtacttattatcaacgataactattactaaaatgaaaaatttaatttcttt
ttttgatcgcagcaaagggaaatggatttcacaaagaacaacttatgaattatctaataa
aaacatgagttcggtacagtctcagatgacaatgaaaataggcaactcattgtcaggatc
tataatacttgcatcgttaaattgggggacatttatagacaagttgcccattacgctaa
aaatcacagccggagtgaatatgataataagtttaacttgcaattcggtaatcaactaaa
taatcataaattattaacgctgtgcatagtgacagatcctagtctaattagttttaaaac
tcgctatggaagtactaccatagatgaaacatattggttcgcaacaaataatttacgtct
aagtactagtattgttaaacgattcaacacttgtgtggcagtatctttttgttcagaaat
taaagtttaa-actctatatagaagcaactaatagcctttta-ggctattagttgcttcta
tgtatctaaattttgtattaaaaattat----aatactatattattctaaatctttacga
ttgggattcctggaaggatcattagagaggaacccaaagacaaacaaagaaataaagaaa
ataacggtagtatatacaaagattttcaaagtaaacatattttcatccttaaaagctatt
agtgcg----atatttacatatctccacataattttatatcatgatataagttccagcac
tattttataaataaatcaatatctttatttttttagtaatgatttatatttaaatatgcgt
gatgtactaaatactaaatcttttccgacttgttttgtcttgtttgtaaatattttgtta
tttctatggagtttaccttcaattatcacatagtctaacttctttagctgtttaaaagta
tatgaagatttttttgttccaaatagttagattgattataactaatcgttttctttttaac
agtcttgcttttaacttaattatttggcttttattttcactaattctgatactcttacaa

ctaagaatttggactagaagagtacagctattcataatttataacaa------tttttta
aaaaatctactaaaatagatgattttacagaactaaaatgatatttcattatcttgttga
ccttgctcataagtaatatctttcaatacttttaatatttttccaaaatactcttgcagg
tatttttctaaagatattacttccaacctgtcaattccaagaatactgtaaacttcatcc
atcggagctgtaaattgctctccgctacttaaaatttcggcaaatgctaaacgatcagaa
atattccatgtccattgaagagttttagtaattcttcttaaggcttttaataatccaatc
ggaatttgtgagatctgtgtttttttgcccagataatttttcacataatttaataatttca
gctgatgtccaagcggtattaccaactaacggcagggtcctattttctgtggaaggtact
ccaagacttttataactaacttggctgcatcttgagtatcaatgtatgcaatgggtgta
gattctcctgtaacccagactgatttcttgtctaaaataggaatggcatattggttgatt
aaaccttgaaaaaatcctcctaaagaaaacacagtatatttcacattggacttctgaagg
aaatctactacctgagactttaagttcatcaatggaacatctgggtatttctctgaattt
aatattgaaaagaaaataaatctttcaactttagccgctttagctgcttcaattaatgca
atttcccatctaaatctatttttctgcattataaggatcagtaggacgagatgtagaa
gcatctatgattgctgttaccccacaaaaagattgcaaaatactttcgggtaattttaaa
tcaccatatataagttctgctccccactctttgagaaaggcagactttcttaaatttctt
accatgcattttacattgtagccttcatctaaagctcgtcttacaatttgacgtcctaaa
gttccggttgctccaattactaaaagagtcatattagttaaaaataagtatgtataaaga
cattgatttggttgacggcagagagtaaattaggtagagagggaatcgaaccctcatgac
cgaagtcgtcacattttgagtgtgatgcgtataccaatttcgccatctaccctattatag
accaactactacaagtaatttagtctttagcgtaaaaataatactaagtttcaatagata
tgtcaacttttaaatttatattaataaaaaaacggagtctcttatggccactttgaatt
aattccttatagattgtatttgagtcaatctaacacatggatccatagaataaaagcaga
aattaagatatatatagtagcgcttttatggatttcaattttattttttcttactttaa
actatgtattattgctttaagcttaattgccataagttttactataagaagtaaacagaa
tattattcaaaaacatttgttacaaacgttattaatgacgttcttaactactgttttgtc
ttttagtgtggctattagttataaacaatatgcagaacaagaacaatcgcagtatttatc
tgattccaagaaatataaaaattccagcagctactattatatacaaatagcgaacgatca
aaggatcaaaagacaatacttaattactactttaaagccttccttgtactttttttattac
tatatattctattaaactagttatgataacaacctctccagaagttttagtaattactat
ttatagatctaggataataaataaaatatttaaaaatgaattgctatttatcttccttct
ttcgtcacatattgttactagtattattaatagaatcgataaagtgattcaagtaactag
cttaagaggaagcttgaatttatataattcattgacaaggcctttaatgttttctttgtt
aatatttcaagtcttctttttggagattattcgggagtcaaaagaaatagctcaagctct
ttatactagaaatctcaatcaagaaaacaataatttttttgaaaatatatacagtaaaatc
taactttagtgatcggctcaatataattattagcactttgtactttattattttagctct

agcgtaatagaatccacttattcttctgactctagtaatacgaagagtaataaaattagt
tagtagtttaaatagatagaat-taagatattatataaataattaaatgtttcattatgg
tcaatactcaaaatcaaatttcacaaacttcagaacttgattatatagttaatcaacctt
ataaatatggctttaaaacttctgttgaatctgagcaatttcctaggggaataagtgaag
atattgtccgcttgatctccaagaaaaaagatgagcctgaatatctattgaattttaggc
ttaaagcatataaaaaatggaaaaagatgagtagtccgtcatgggctcatattaagcatc
cgaatatagactttaatacgattatttattacgctgttcctaaattaaaaaaagaattga
aaagtctagatgaggttgatccagaaattcttgacacttttaacaagttaggtatatctt
taaatgagcaaaagcgaatttccaatgtagctgttgatgctgtttttgatagtgtatcta
ttgcaactacttttaagaaagaactgtctgaagctggtgttatattctgttcaatttctg
aagctataagggattatccagaattaataaaaaaatacttaggtactgttgtaccagctg
gtgataattattttgctgcattgaactctgcggtatttagtgatggctctttttgctata
tccctcctaatacagtttgccctttagaattatcaacttactttcgtattaacaacgaag
aatctgggcaatttgaaaggacactaattattgctgatcgtggcagtaaagtaagttatc
ttgaaggttgtactgctcctcaatttgacacaaatcaattacatgcagcgattgtagaat
tagtagctcttgaaggagccgaaattaaatattctacagtacagaattggtatgctggta
acaaagaaggtaaaggtggtatatacaattttgttactaaacgaggcttatgctcgggta
acaattcaaaaatttcatggactcaagtagaaactggatctgcaattacctggaaatatc
caagctgtattttagctggtgaaaattctcagggagaattttactctgtagctttaacaa
ataactatcaagaggctgatacagggactaaaatgatccatattggtaacaatacaaaaa
gtagaattatttctaaaggtatttctgcgggtagatcgaaaaacagttatcgaggcctag
taaaagttggccctcagtcatttaattctcgtaattattctcaatgcgattctttattaa
ttggtcaatcatctcaagctaatacatttccttatattcaagtacagaatccaacatcaa
aagtagaacatgaagcatccacttcaaaaattagcgaggaccaaatttttatttttttac
aaagagggatcaatttagaagaatctattgctcttatgatcagtggtttttgtaaagatg
tctttaatgaattgcctatggaatttgctactgaagctgatcgtttactgagtttaaaat
tagagggaactgtaggatgagccagactattttagaaatcaaagatttatatgcttctgt
tggtgaaacaacaattttaaaagggtaaacttatctattcgagccggcgaaatacatgc
gattatggggcctaacggttcaggtaaaagtacattatcaaaagtaattgcaggacatcc
agcgtattcactaataagcggagatattttatttttggacaaagtattcttgaaatgga
gccagacgagagggcaaaagcaggtattttttagcttttcagtatcctgttgaaattcc
tggagtcagtaattctgatttcttaagaattgcattaaatgctcgcagaaagtttcaagg
attatcggagtttagccccttagaatttttcaactaataacagaaaaaatagatcttgt
tggcatgcaagaaagtttttgacaagaaatgttaatgaaggattttctggtggagaaaa
aaaacgtaacgaaattcttcaaatggctttactagatagcaaaatatctattttagatga
aacagattctggactagatattgatgcattacgagtagttgcaaaaggaattaacacttt

agctaaatcaacaaattcaattattttgattactcactatcaaagattattagattatat
tattccagattttgttcatattatgagtaacgggcaaattgtaaaaactggtagtgttac
tcttgcccaagatctagaaaagcacggatatgactggattacgcagacataactatttac
tac-------aatattgagactaaagaatattctttagtctcaatatt-tttgcaaatgc
aaagtt-aattatttaagctttatcaaaaccttgcttaacatcttcaattgctcttttta
gcaattcttcactaactggttctaatttttagtactacgaacattttcaccaaattctg
gtttagaattctttaaatcttctcgaagttctctaataaattcagctacttttgaaactt
caatatcatctagatagccattaattccagtgtaaataatagcagtctgttcttcaactg
gaataggagaattttgggcttgcttcaaaatttctcgaagacgttgacctcttgctagtt
gattttgggttgctttatctagatcagatgcaaactgagaaaaagcttctaattcagcaa
attgtgctaactctagttttaacttgcctgccacttgtttcatggcttttatttgcgcag
cagagcctactctagagactgagatcccaacattaatagctggtctaatccctgagttaa
ataagtcaccagatagaaatatttgtccatctgtaattgaaattacatttgttggtatat
atgcagatacgtccccagcttgagtttcaataatagggagagctgtcatactacctccac
ctaattcagcattcaatttagctgctctctctagcaatctagagtgtaaatagaatacat
ccccaggataggcttctcgtcccggaggtcttctaagcaaaagagacatctgacgataag
cttgagcttgcttagttaaatcatcataaataactaatgttgctttacctttgtacataa
aatattcagctaaagccgcacctgtataaggagcaatatattgtaaagtcgcaggactat
ctgcattagctgcaactataatagtataatcaagagctcctttatcctgtagtgaagata
ctacttgggctactgaagatgccttttgtcctatcgcaacatatacacaaacaacatctt
ggcctttttgattaataattgtgtccaatgcaacagctgttttacctgtttgacggtcac
caataattaattctcgctgacctctaccaattgggatcatagagtcaatagcagttatcc
ctgtttgcataggttcacacactgattgccttccaattataccaggggccattgactcaa
ttagccttgttccattacttgcaggttcacctttatcatctattggccgagctaatggat
caacaactctacctaaaaaagcatctccgaccggaatttgagcaattcgaccagtccctt
ttacggaactgccctctaaaatatccctaccatctcccatgagtactacaccaacgttat
cactttctaaatttaaagcaaccccaatagttttatcttcaaattcaagtagttcaccag
ccatgacttcatcaagtccatatactcgagcgataccatcaccaacttgtaaaactgttc
ctatattggctacttctacatcctgatcatacttttcaatttgttgacgaataatactac
ttatttcatcaggtctaatatttaccatattttaatattatttttattaattggaaatt
ctaaagcggtaattagtcagactaatgctatctgtctttttttactttattttaagttact
acaacatcaaggtgtgaagccatttgtcttagctgtcctctaatactagtatcaatgact
tttgatccaatctgaatagtaaagccaccaattagctcgggctcgactgaaataattagt
ttaacttctttagccttagtcataaccttgatttttctgttaatagagtttcctgatca
gggcttaaagcaatagaagtattaatatttgcaattgttaaagattccatttggtaagcc
agttccaaatacttactagctatagcatctaacattccaattcgcttgcgatcaactaaa

atcattagaaatgataatgtattttcgttaatttgatcgccaacagttgcaataattact
tctttttttgcctctacagtttttaatggattagct---aaaaaaattttttagcttgtca
gattgcgctaaaatattttgtattgactgaatatcttggctcaccttttcagtaactttt
ttagttttagctaaatcaagaagagctactgcataaggctgagctatttttataacaaca
ttgttactgctcatagttgatctcctaacttagcaatattattatcaattatacgcagtt
gcatttcggagctcatttgattttccagctgcaaagtaactcttttaagagcaagaaatg
ttatttgttgctggatttgccttcggatctgtttttcagctgtttctatatttgatttac
ctgttatggcaagtctttcaatatctaattggccttgagctaatatggaacttctaactt
ttccagctgttagctgagcttctttcttaatttgatctataatgatttgagtttgagcaa
gttgtttttctgattctgacaatctagcacttgcttgctctagtctttcttcggattctt
gtatagctgctaaaacttttatctgtcttgcatttaaacttgatccgagaaattgcttca
aaacataaataagaccaaataaaagcaacaatatattaataacatttgcttcaaaaatat
cagaattaaagccgaatttgtgttcactactatgttctgataaaatagtaattatttgtg
gtatttttactatattattcatttaaaattct----------tactattttaatatttta
ctctggaatcatgattttcaagattgactacttaataatttaactttaatttgatcgctc
aaagtgtcaacctgattttctaaagttttaagagcttcttctttttgaacatttaactgt
ttcgatgcttcagcaatcagtttctctgcattcaattgagctttttttatatcttctgag
actatattttgagcttctttttgagaagaggctatttttaattgtgcattgcggcgagct
tcagatagatcttcttcatattttgcagctagctcatctgctttaacaagcatagacgat
gcagtagtcaatgttgttcgaatatattcatctcgttcgtctagtactttagttacaggt
ttataaaaaatagtatttaataaaaccattaaagtaagaaactgtaatgccattaaaggc
aaagtaccattgaagtcaaataagccaccttcaatttcttcggctaatagaaatggtaaa
tcaatcattttactaattatgtttatagttaataattttgtttcaattaagaaaaataac
tttatagtgcgcttagtctcatcagactaagcgcaaatgattattttaaccaacgtatgg
attagcgaacaataaagacaacgcaacaactaggccgtaaattgttaaagattccataaa
tgctaaacttagaagaagtgtaccccgaatctttccttctacttctggctgtctagcaat
accttcgacagcattagctgctgcactaccttgaccaatgcctgggcctattgcagcaag
acctacagcaagaccggcagcgataacggatgcggctgaaacgattgaatccataatgaa
ttttaatttttttgttatatgagtagaaaattgacaaatttcgtataatacttaaaatcta
tataattaagtataatagattctaatat-----tgtaattatagtatattactcttctcc
atgaccttccattgcttctccaatataagctgcggacaaagtagaaaagattaatgcctg
tattgaactcgcaaataatccgagtatcataactggtaatggtattaaaattggaattag
caaagtaaatacagaaacaactaattcatctgctaagacgtttccaaataatcgaaaact
taaagatagtggtttcgtaaaatcttctagaatattgattggtagtaatactggagttgg
ctgaatatatctagcaaagtagcctaaccctttttttacttaaacctgcataaaaataagc
taaagatgtaagtaaagacagggccacagttgtattaatgtcatttgttggggcagctag

ctcaccttcaggcaagtgaattaacttccaaggaattaatgcacctgcccagttacaacc
tagaataaatagaaataaagttgcaatgtaaggcacccaagggcgatattcatgctcacc
aatctgatttttgcaatatcttgcagaaattctaatataaattccataaaattctgaaa
tttttcaggaattctttgtagatttctagtacctagaaatgataaagttaaaagtgtagc
aattactaaccaagagacaatgaaaacttgcccatgcaattgtaagctacctattttcca
atatagatgttttccaacttctactgctgataaactattatatgggttaaaatctatgag
actgttttgatacataataaatattt-ttttaaatagtgcaagaatcaaaaaataag-tt
tttgaagaattaatatgcaattaatttattcttaaaattagactattaataaattaaaag
caaaaataaattctaatagagatagctatttttagtagtgattatttttttgtaaaaaaca
attattgctacctataatgcttttacgtttaattatatacccatatattaattatttaat
atgattataggtattttgctacaaactttattgttttatccattct----aaagattgtt
atagtttatttttatttaaaatatctgcgacttcatctgcaaaattaactttcaaattt
tcttctcccccctcccagaagaaatcttacaaatcttctaattttaatattttcacctaga
acagcaatattttggtttattaaatcttcaatactaatatcttgattgcgaataaacatt
tgatctaataatgatagctccttaagtctctttttcattcgtccttcaatgattgtttcg
attctatcaactggtttgtttttcaagtcatctttaccagcttcaactctttgttccaga
ttaataatttcactaggtatatcctgtgttgatacgtattccacattaggcgatgccgct
atttgcatagcaatatctttagctaacttttggaactctggacgtcgtgctacaaaatct
gtttcacagttgacttcaactaatacacctattcttccgccagtatgaatataactctct
agtaaaccttcaatagcggtacgattagatttcttgttagctgaagccagcccttttttgt
cttaatgattctaaagctttttcttcattgccattattagcttgtaaagctttcttgcag
tccatcatacctgctccagtcttatcacgtaaggcttttacgacttgagcagaaatttgt
agtgtcatatgctttcaaaaatttttaagtatttaaatt----------aattattataa
ttcactttgagtaggatgagttatttcttctaaatctgtttgcccgtatttaccatcata
aattgcatctgctattttacctacaattagtttaattgacctaatggcatcatcattagc
tgggattggaatattaataatttctggactacaattagtatcaagtatacaaatagttgg
aatacctaattttaaacactcttgtatagctgttgtttctctcttttgatctacaactac
aacaatatcaggtaaacgagtcatatttttaataccatttagatgtttacgaagtttatc
taattctcttcgtaaaacagcggcttctttttttgggtaactgatcaatcataccactttt
atcttgttcttctagctgtctcagacgactaactcgagacttaatcgttacccaattagt
taacattcctccaagccatctctggttaacataataagaatcacagcgttgtgcttcctg
tgccactataccagcggcttgtctttttgttcctagaaataaaactttttttaccatccga
cgaagcctgttttataagttcacaagcttctgttaataattgtgcagtttggactagatc
aataatatgtatgccatttctttctgtataaatatatggaaacattttaggattccatct
acgtgcttgatgtccaaaatgaacacctgcttctagtaattctgctaatgtaacaatagc
cataagattattaatatttaaataatgtaattattcgggaaatccc-tttgtttagaaac

tgat-----tcaactaacttatttgaattttagtaaactaataataaattaacacacttt
taaagcagatcaacaaaatagatcctgaagaattcat----tctacacttttattgctaa
aataattacgcgcggttctgtcgtctaaaatgatatcatctaaatcatctcgtacagatg
aaatagtactttcgtcattagtatttgcagttaaattctttttctctaacgatgagccat
tacagttatcatacatattgaagccggtaccagctggtataagccgacctataataacat
tttctttcagacctcttaaccaatctaatttaccagatatagcagcttcagttaatactt
ttgtagtttcttgaaaacttgctgcagaaataaagctttctgtatttaaggaagcttgag
taatacctaatagcacaggcctatatgatgcattaatcttatttctcaaggttatggctt
tattagtctgttcaatcttctgtaactctacaagctctccaggtaaatagcctgtttctt
caccattttctattttcactttagaagtcatctgccgtactattacttctacatgcttat
cagaaatattcactccttgagattgatataccaattgtacttctttaacaagtagtaact
gaatctcttgaaagcttaatcgagcagcttcatataaggctaatccacattcaatataca
aatcaaaacttgcatcgaggatatcatgaggatttaacaatacgtctgttttctttcttg
cttcaagaatttcttcaattcttggaagcccttgtataatgtcacctgtttttgctctat
caaaaaccaatatagctaatgtttctccccttctaattaatgcattgttatcaacatgta
aaatagcaccattagaaaccaagtaaggtctagcaattcttaatgtaactgatctagaag
aaatttcaataatttgtccagagtctaaagaagtcatattttcagtaataaaatcaccac
aacgaatccaatcacccacacttactttaactgttgattttcctatattaaaggtctttt
tatcagaagatgtcgcaatcaaaatgcgcctgctagtattcttttcagaataaatctcct
caacagtaccttcactcattgcaataatctcagtactagcaacaactgtaaatggcttga
tatattcaccatttttacaataattctagtttgactttgttgcttctctgatttattta
catcaatacttttaattgataatgtttcaaaagttgacagccttaagtcaaaataattat
tgccactttctttagcagaaaactcaattgaggatattaaattatcatctcgatgctcaa
tttcagccacaagattagttgttactaaatgaactccttctatagatttcactctttctc
cgtcccgaaaaggagttcttttaactagctttagtttagtttgacttggtttttgtgaaa
ctagattttcaattagactggattttgtttctggtattgaataaacaataacaggtctaa
taagaatatagggcatttgctcgttttctatatactcccaataaactaacttatctgtag
aaatattattatgaagtctttctccaggtcttaaaaaacctctactttatcattattac
tatacacctcactaagtttatagatagatccaggcttaataataatttctcgaactattc
catccttttcgattatttcaacaatacctgagctttagaaaaaatatttttaactagct
ctgttccactttctataacatctccattatgcactagtattagtgatgaatctttattaa
tttcgtgagtttcttctgaaatccataagatatatcccggacttaaaatttcgtaggcat
ctttatctaagcctgtttttcttttttgatacatttaagtctaaatatttaataataccac
cacttgtcgttttatacgtatcagatattagttctgcgactgtgtaaccatcttgaa---
ttttttggtttggaatagctttgaaaagaaatttttgtttttttttctgtttctaaaatat
aagattctttttttgtggattacatctgtataaatataacaacctggaattacaatagatt

cagtaataacttgtatattggtaatactgctattagtattctggtgtatccttacttctc
cagcataatgatttattaattctgtctgtgcaagtatagtaccagctttagttttatctt
ctttatgaacaataacattagcagaatcagatatactataaacttcacctgagagcaccc
agatcaagcctccagttttagtaattcttgtagtatattgattattatctgtttcttcaa
ctgttaaattggagaaacagattttgccagagagatctgaaactacatgtttttgagctc
tttctgtcatgagtcgatttctgcggggtgattcagcaatcacttgatccttcgatacta
attcaccatcacatattaaaagagtagtacctttacttaagttaattattgacttctgat
ttttcttagatctgattgtaacttgggttggctttttttgttattaatgcttgctcaccat
gtctggtacgaacatctgtatacgattcaatatctagatctatcaattgaccatctatag
gtgcgtagatttgttctgctaattcaccggtaaatactcctccagtatgaaaggtcctca
ttgtcaattgagtgccaggctcaccaatagattgagccgcaataataccaacagcctctc
ctaaatcaactaagcggccatgtgcaagattccatccgtaacaatactgacaaacagaac
ttctagagttacatgtaacaggagatcttactaaaactttttt---gataccagctttag
ttatctcttttgctaacttaggactgacatcttggttcgtatgagcaattaaacaatttg
tctctggatggaatacattttctgctaatactcggccagataaagcttgttctagattaa
ttaatattttctgcgtatctactaaatcttctagtatgatgcctttattggtcttacagt
ctacttctctaattattacatcttgagaaacatcgacaagtcgacgcgtcaaatagccgg
aatctgctgttcttaaagcagtatcaactaatccttttcttgctccgtatgatgatataa
aataatcagtaacagttagtccttctctaaaattactcgatattggaagatcaataattt
gtccctgtggatcagccataagccctctcatgcccactaactgtcttacttgagaaatat
tacctctagcacctgagaacgccatcatataaacagcatttagaggatctgtctctttaa
agtatttaataacttcttgtttcaaggattcactcgcattattccacgtatcaataactt
tttgaaatcgttcaactgctgtaatctcacctcggcgatatttgttttcagtggccttaa
tatcttcaatcgtgcctaataaaagactttgcttactaggaggaattcttaaatcttcta
aacttaaagaaattcctgcctgggtcgcataatgaaatccaagatctttaagcttgtcgg
ccatattggctgctcgggctataccataattacgaaaagcccagacaattaaatttttta
gttcgtttttatcaataaccttatttgaaaaacttggctgtgcaagacttcgtctattat
ccactaaatcttctccatattaaatgttcgtt--attaactatgctactaaagactcttg
aataattttattaaagattatacggccagctgttgttctaatatattgcacaattctctg
attttcagcgtcttcttttatgatatggttattaaaaaattttgttgtactattgtcaga
atgtttttcaatcttaatcggatcatgaggttgatcaccatctactaatccatcaaaacg
agcccatatataagaatgtaaatcaattttcttctgttcataagccataactacatcttc
taagcttgcaaaatactgattagatccctgctgctgagatggattattagctgttaaata
atagcaacctaaaaccatgtcttggcttggcataataattggttgacctgttgctggaga
gagaaaattatgaggggctaacattagcaatctagcttcggcttgagcctctaaagacaa
aggaacatgaacagccatttgatctccgtcaaaatcagcgttaaacgctgggcatactaa

tggatgaagcttaatagctcttccttctacaagaataggttcaaatgcttgaatacctaa
tctgtgtagtgtgggagctctgttcaatagtactgggtgaccttgaataacttcatttaa
gacattccaaatagaagattcatttttttgaatcatctttttggcagctttaatattgtt
aactaatccctgcaaaattagtctgtgaataacaaaaggctgaaataactccaaagccat
ttctcgtggcaatccacattgatgtagttttaaatgaggacctacaacaattacagatct
accggaataatctactcttttacctagcaagttttgtctaaaacgaccttgttttccttc
tataatatctgacaaagattttaatggtctattatttgcacctacaacagttctacctct
acgaccattatccatgagagagtcaacggcctcttgcagcattctttttttcatttctgat
aataatctccggagctaaaattgactttagacgagataatctattatttctattaataat
tcgacgataaaactcattcaaatctgccgtcgcaaatctacctccatctaattggaccat
aggccgtaagtctggaggtatcacaggaatgactgtgaataccatccaagaaggatctgc
acctgttgctatgaaattttcaatcaatcttaagcgtttcatttttttattgaattttaa
tgaaggtgtcttaaaactttttggtggatttgtagcttcagaccttaaggtttcagctat
gtgctctaaatctaaatctttcaacagtttctgaatagcttctgcacctattcctacttc
tacttggttattctcatcttgattttggtagatttcctcttcaagacttttccattcgta
accttcgagcagttgcttgtattttaaattaatatctgtattagattgcgtaactacata
agaatgaaaataaacaatttttttctacttctttgacttttaagtctaatgctaaagcaat
atagcttgtacttccttttaagtaccaaacatgagttactggtgaggcaagttcaatata
agccatccgatgtcttctcacgcgtgattctgtaacttctacgccacatcgttcacaaac
tatacctttgtagcgaaaacgtttatatttaccacagtgacattcccaatccttaactgg
gccaaaaattttctcacagaataaaccatccatttctggctttaaagttctgtaattaat
tgtttcaggctttgtaatttcaccaacaatttgtccgttgggcaaacttctttcgcccca
ctgtcgaatttttttcgggagaagctaaactaatttttacgtagtcaaaatattgctcaaa
ctttgtcataaatttgaataccttaaaatcaaataattagccaatttatttacatgcttc
atattgaaaaatgaatatcttaataaaggaattgttcaaaatcgtctactggaggagtat
cataattagaacgatctactcggttatctttagaatccgacatcaagtcaacttcaactg
tccgtctttggccatcttcaaatagtttcaatttatgtactgcaatgtctaaacctaatg
actgcagctctcgcattaagactttaaaagattctggagttccaggcttcggtataggct
tccctttaactattgcatttaatgcttcatttctagcttgcatatcatccgactttacag
ttaataattcttgcaaagtatatgctgcaccaaaagcttctaaagcccacacttccattt
cacctaatctttgacctccatgctgtgctctacctcctaatggctgctgcgtaactagag
agtaaggaccagtggatcttgcatggattttatcatcaacaagatgaacaagcttaagca
tataagctctaccaactgtaacaggattatcaaaaggctctcctgttctaccatcaaaaa
cttgcattttacctggatgctgatcattaaaaagccatttattgctagtaatcaatgatg
cttcttttaattttctatttacaagtgctcttgatgcttctgctccatacatttcatcaa
aaggtattattttaaatctctttcctaaatagccacctgctaagcctagtaggcactcaa

atacttgaccaacattcattctagaaggtacacctaaaggatttaaaacaatgtccactg
gtgttccatctgataaataaggcatatcttgtttaggtaagatcctagaaatgatacctt
tattgccgtgacgaccagccattttatcacccacttggatttttctcttctgcgcaacat
agacgcgaatcatagcatttgttccgggaggcagctcatctcctttctgcctagtaaaaa
ccctaaccttaactactctaccttttgcagcattaggaagccgtaaagatgtatctctca
catctcgagccttttctccaaaaatagctcgcaacaatttaccttctggtagttgatcag
cttcacctttaggagtaatttttcctactaaaatatctccagcttcaacccaagaaccac
cgacaacaattccatttctatctaaatccttcagagagttatcgctgacgttaggaatct
ctctagtaatttcttctggtcctaatttagtttgacggcattctacttcatacttttcga
tatggatagaagtatacaagtcatcataaactaatctttcgctaattaaaaatgcatctt
cgtagttataaccttcccaaggcatataagcaactagaatatttctccctaaagcgattt
cccctccgtctgtggatgcgccatcagctaaagtttggccaacaactatttttttctccca
cccaaacaattgggcgctgattaatacaagtatcttggttagaccgataatatttttttta
agcgataatgaactgttctaccactattatcctgaatgcctattttattagcagaaacat
aatttacatgaccagatgttctgctaataacaaccataccagagtctcgagctattttag
tctcaagaccagtaccaattattggcttttctggatataataatggaacagcttgccttt
gcatattagatcccattagagctctattcgcatcatcatgttctaaaaaaggtattaatg
atgttgcagctgaaattacttgaataggtgaaattgcaatataatctacttgggttgggg
ttgttgtaataaactcttgacgataacgtacaggaataatatctccttcaatgtaatgtt
gcttactaactttaacatctcctggagctactcggaaatcatcttcctcatctgctgtta
gatagacaggactattgtgataaatcacttgaccttggtttaccggataaaatggggttt
ctatgaaaccaaaaacattgacccgcgcacaggttgctagtgaaccaattaaacctgcgt
tcggaccttccggagtttcaattgggcaaattctaccatagtgactaggatgtaaatcac
gaactgcaaaacctgccctgtctttattaaaacctcctggacctaaggcactaattcttc
tcttgtgagttagttctgcaactggattagtttggtccataaattgagaaagctgactag
atccaaagaattctctcactgacgcaattaatggcttgggattaattagatttgataaac
tcaatgaatctatatcacatatcatcattctctctctaataatgcgttctaaacgattga
gacctactctgaattggttctgcagcaattctccaactgaacgaactcttctattaccaa
gatgatctatatcatcaagattacctgagtttttatccttaatattaattagataatcaa
ttgaggataagatgtcttgaggagacaatactcggaacgttttaggtatattgagaccta
gtttttttattaatttatatctaccaacttcacctaagtcatatcttttaggatcaaaaa
aacgagagtaaagcatctgcttagcaacagcaactgttgctggttcattaggtctaagct
tagaataaactatgagcagggcttcctcatcagttacttcttcaatattatttttttccaa
tttctttagctaattctttaactgaatatatttgggatgcagaaataagaaaagcatatt
tactaagtcctttttcaatttcatctttattgagtccaatagctcgcagaaaaatataag
catttactttgtgggtcttatctattcttatccaaatttctcccttaggatcaatttcaa

attttaaccaagaacctctattagaaattaggcttgcactataaatctgcttaccatttt
tgtctatctcttgtttataatagattcctggactacggattatttggttaataataactc
tttctgtcccagatacaataaaagttcctctatttgtcattattggcaagtctccaataa
aaactaatctctttttgtacttttttatttttttatctgcttttcagcactgagctgtagat
gggttgatgataaatctaatgatttaatatttttttcttggtttttagaaggtagatcaa
tatcttttcttgttaactttgcaggaacatatatttgagcactataagttttatccctat
tttttgcctgtctgacactataacgaggaaattttatcttgtactcattaccaaataatt
gtaattcgagtcgactagtagggtctgatatcttcggaaaaatttcaagaacttccgtca
aaccttctaataaaaaccatttaaaactggccctctgaatttcaactaaatctggaagaa
gtttatttttaagcttatacgttgaaccatagatctcctttataacaaattttgaaatc
gaagagtttaa-tactattaatagagtactggtagataatgcataagcatagtttgctac
attatttcttctctagacataactagaacatcatattttattcaa--aaaaagattttta
aaatttttcttcac--acacactatctataagtactttgctgcta--aaagatagttcaa
gctattaaa-------aaaaacgaaattaatacttgtctgtctgtcaattaattgacatc
aagatagacaagtattatttatttaatcaattctg-tttttctgagcaaaagacaatgct
ctcgcaagtctagcttttttctagcgcctgtattagggtgaaatgcccctttttttaca
gctttatcaatcttactatatacttgagaaatacttgactgcacatcattgaaattatca
ctttctaaattatcaatatttagaaggcatcttttagttaaagttttgacaaccgattta
tacttacggttaatgagacgatttctttccgaagttttaatgcgtttaatcgcagaaagg
ttcttagccacagtaaatccaataaaactttataaaatatttttagaaacgaaaataca
caaatctgggatctattcaaaaagggaatatgcggcgtattataacattaaagaaaaaca
ataggcaatagttcaacaagagctcttgttattacacaatagagattgtcattgcgaaaa
actttgtaagatagtatttaattaaaataaactaactagacatataaatatatatgaaaa
aaattgaagctattattagacctttcaagcttaatgaagtaaaacttgctctagtcaagg
aaggtattggaggaatgacggttatcaaagtttctggatttgggagacagaagggtcaaa
ctgaaagatataaaggatccgaatactctattgatatcattgataaaataaaaattgaaa
ttattattagcgatgataaagtagagaaaattgtagagacgattattaaggcttctaaaa
caggggaaattggagatggaaaaatatttattagtagtattgaaagagtaataagaatca
ggactaatgacttaaattttgaagctttatagtatttttactttt-tttagacttgatact
tcttcactaaacatgagataataagcgtttattgtatacttataactaaggtgtaata--
ctaatggctaaaagcaaaggtgcacgaatcgtaataactttagagtgctctgataaggct
ggagagtttgctcaaaaaaggaaacctggcgtttttcgatatacaactactaaaaataga
cgaaatacaccaagtagaattgaattaaacaagttttgtcctaattgtaatcagcattgt
atcttcaaagaaattaaatagttatttatcttattaaaat--aatcatattatggctata
tatagaaaaagaatatctccaattaagccaacagatgctgttgactacaaagatattgac
ctgctaagaaaatttattacagaacaaggcaaaatactgcctaaaaggtcaactggattg

acttcgaagcagcaaaaaaaacttactaaagcgattaaacaagctagaatcctttcttta
ttacctttttaaataaagattaa--atatatagcctaacttcatattttccatctaaa
tattaaaaatttctatagatttgtttattaaaatgatctaacatactgaat---------
----------tactaagaaga-tctactcattacaagttttgttcattttataattaata
atagcattataattta---ttaagctagattc-----agacataaag-gtaacctctttt
aattgattatattatatagtctatttaaaagaggaaattttttgcttc----------t
agcaattcaagattctttaagctttaacaaggtatttaaaacaaagatttttgtttaggt
atcagatcaaatgaatgaatcttatctcccgactgccataattgaaattcagagatgaaa
attccacattcgttgccagcttgaatttcttcgacatcttctctaacgcgctttaaagat
tcaatttttccttgataaataactttttcttctcgaatcactttaatccaagagtttttt
aatagcttattattgatcactcgacatcctgctatttttctatttgctaacgagaatact
gtacttacttctgcttctcccaccggtacttctgaatattcaggatcaagtaagtcttcc
atccttcttttaatgtcttcaattaaggcataaatgatttgatagttttctattaatata
ttcgactttgccgatgcttgctttgttcctggagcaaagttggtattgaacccaatgaga
gtagaatttgtggttgaagctagttcaacatctgtagcagtaatttctccaggcatgatt
gagacaacattgagttgaactttactctgaggaaattgagataaagaatctaggattgct
tctgtagagccttgattatctgttttaataattaaagaaatttgcttactaatatcttta
gaattagtgtttttaaagtatctaatgtaattcgactattaagtgctctttgtttctgt
ataattgatgagtctttagaggtattttcaatcgctttaagtttggcttctttatcactt
ttaactgctaaagctatttctcctgtagctggtacagatgacaatccccaaatttcaaca
acagatgatggtattgctaaattaatttctctttagcgttgttaattataactcgaatc
tttgcgtaagctgatccaataaccaaattatcactaatattcaatgtaccattttgtatt
aatagagttgctactggaccatgcgatttatctaaatgggcttctattataataccttgt
gcaggctgtgttggatcagctttcaaatcctctaattcagctaacaaggtaatagtttct
aataatttatcaacattttgaccagtaagagaactaataggaattataggcacttggcca
ccaagtttctccgacatgacattgtattttaataaatcttgctcaataatatctgtatta
gaaccagctttatctatcttagatattgctatgacaaaagggacattcgctttttgaata
tgattaatagcctctatggtttgaggtttaactccgtcatcagcagctataataataata
gcgacatctgttaaattagctcccctagacctcatacttgtaaaagcttcatggcctgga
gtatccaagaaaacaatttttttgtttattatctttcttaatatactcaacttcataagct
gcaatggcttgagtaattcctccaatttctttgttagcattattagattttcgaatataa
tctaatagcgttgttttgccatgatctacatgtcccataactgtgacaattggaggtctt
ttaatataattgccagtttcataaaaagcattagagttatctaaattactagaaagtcca
ttattattttcctttacatttgattctactgctataccaaaattatcagccactgatgat
attattgaggcgtctatggtttgattcatagtaactgatattcttttaagaaataaatat
tttataatatctgtttcttgaacacaaattagtttagataattcttgaatagttagagga

ttagtaatactaatagactctggcggagaattagattgaatgcttgctgaattttgacta
ggagttacggctactttttctgtcttatagatttttattactagtcagtttttttaca
acttccacttttggcttaggtggacgcattaaagagatagctaaatctcctgcagtctgc
gaaacattagaattagagtctctaaagttgtca---tcatcatcgtcaatatgtatttta
gtttttattttt-ttgcgctgtctattctta--ttttttttactatctactaagtcgtga
atttattaaaatttttactttttttatctagtttaggtggagaacttaattctagatgc
tgttcaccacc---tgtatgtgattctgatttatcaaggtctaaatttaacagattatca
ttattgactgactctaatctaatttatagataatctgaggatttttcaaatctacaatt
gtttcagaagaattgatatttgagctagatctagaggatctattttcaaagttttgatta
tttaaaaacataaatattggtttattatctaatagtaactttctaattttaatacagag
cttaatataaacaagaatcgaatatctgtataaataatacaaatgt---agagaattaat
ctatgttgtattagatttaatataacactaaagtcctttgttcgttatacaagtttaaaa
ctttt-tatgatgataaacactagattctagtttattggtatacaatctatttatctaaa
taatactgtttttgtcagggcttttaaa----ttatctaatgttatgttcattatgaaat
tctattaaaatttaaaaa-ctcaacaattaagtaagaatcgttaatcacctccacgccac
ttatgccacgatctcaaaaaaatgataattttattgacaaaacttttacagtattagctg
atattgtattaaaaatacttcctacaagtaaagaggaaaaagaagctttctcttactatc
gagatggaatgtctgcacaatctgaaggtgagtatgcggaagctttagaaaattattatg
aggctttaaaactagaagaagatccatatgatagaagctatattttatataatatagggc
ttatttatgctagtaatggagagtatgtgaaagctttagaatactaccatcaaggattag
aactaaattttaaattacctcaggcccttaataatatagccgtgatataccattaccaag
gagttcaagcaattgaggataaagatacagagctatctaaattaatgtttgataaggcgg
ctcaatattggcagcaggctattaagttagctcctgataattatattgaagctcaaaatt
ggctgaaaacgacaggacgaatgaggaatatacaaggatattaatatatttaagatataa
taaagatatcaattaataaattaaaattagttaaagaaactggtactatatagagtactt
-tactatatattataatattatactagttgatctctatctctaagacttagctgaataac
cgaaattaaatctattgactatattcatgaatacaaataagcataatggttagtacaaca
aaaagtaccggatctgttactcaaattatcggaccagttttagatattgcatttcctaac
ggacagcttccgaaagtattcaacgcactcaaagtacaaagctcagaaggaactattact
tgtgaagtacaacaacttttgggtgacaacaaggtacgagctgtttctatgagttccact
gaaggactacaaagaggggtagaagttattgatactggatcccctatatctgttcctgta
ggtacagatactcttggacgtatttttaatgttttaggtgaacctgtagataatttgggt
cccgttgattctgagagtactttacctatccatcgaccagcacctgcttttactaagcta
gagacaaaaccaaatatttttgaaacaggtattaaagtcgtcgatttacttgctccttat
agaagaggtgggaaaattggtttattcggaggtgctggagtaggtaaaactgtattaatt
atggaactaattaataacatcgctaaagctcatggtggagtatctgtatttggaggcgtt

ggtgaaagaacaagagaaggaaacgacctatatatggaaatgaaagagtctaaagtaatt
gatgcagataatctgaaagaatctaaagtagcattagtatatggtcaaatgaatgaacct
cctggagcacgtatgcgcgttggcttaactgcattgacaatggcagaatactttagagat
attaacaaacaagacgttctattgtttattgataatatttttcggtttgtacaagctgga
tcagaagtatcagctctactaggccgtatgccatctgctgtgggttaccaaccaactcta
gcaactgaaatgggagcactgcaagaaagaattacttcaacaacagaaggatcaattaca
tctattcaagctgtatatgtgccggctgatgatttaacagacccagctccggccactaca
tttgcacatttagatgcaacaacagtactttcgaggaacttggcggcaaaaggaatttac
cctgcagtggatccactggattcaacatcaacaatgctacagcctggaattgttggaact
gagcattattctactgctcaagaggtaaaatcaactttacaaagatataaagaactgcaa
gatattattgctattcttggtcttgatgaactttcagaggaagacagacaaactgtatca
agagcaagaaaaattgaaagattttatctcaacctttttcgtggcagaagtgtttact
ggatcacctgggaaatatgtatctttggaagatgcaattaaagggtttcaaatgatctta
aaaggcaatttagacgacttgcctgagcaggcattttatctagtaggtgatatagatgaa
gctatacaaaaagctgacagcatgaaagattaatataattaacgatgactttaaatataa
gaattattgctcctgatcggactgtttgggatgcagaagcacaagaaattattttaccaa
gtagtacagggcaacttggtattttaacaggccatgcacctttgcttacagctttagata
ttggagttatgagagtaagagtagataaagaatggatgccgattgttttgctgggcggtt
ttgcagaaatagagaacaatcaattaactattctggttaatggcgcagaagaagctagtc
aaattgatttatcagaagcagaaaaaaatttagacactgcaactcaactcttaagtgatg
cctcgtctaataaagaaaaaatagaagcaacacaaaaaatacgaaaagctcgagctcgag
tacaagctgcaacagcagcaacttcgtaa-aagattcc------tcttaaaacataaaac
tttagtaattattactaaa-------gttttatgttttatttgtatggttataggcagct
taaataaacttagcttagacctgaacaaatataatcaaaatataatcccatctctttacc
tgcatctggtccaactaaactaatagtaacttctttcatagctaaaatagcttgaatcgt
tgcaccaataggaacacctagagaattgtatgtttcttttagaccatttaatactctttc
ttcaaggatagatggatctccagctagcattccataagtagcatagcgtaagtagtagtc
taaatcacggatgcatgcagcataacgtcgagttgtatacatattaccacctgggcgtgt
aatatccgaataaagtagagattttgctacggattctttaataattgttgctgcattagc
tgcaatagtagcagcagctctgactcgtagctcacctgtttgaaaatagcctcttaattt
ttcaacagagctatcatctaaatatttaccttggacatcagctgcattaataacagaagt
aattgcgtcttgcataactttcacacattccttaattttaaatatctaatacta--gggg
tatataacaaaattactagatcttttagcgatatcagtattactgcatagcacccaaagt
ataatcaaagtaaaaaccagcttctgctgaatcttcacctgcaagcagtgagcaagctac
acttttcatgcattttacaccttcagcaactcctgaaattggtgttcctaaagaattata
catttctttaacacccactaatccaatttcttcaataggagttacatcgccagcaactat

tccgtaagttactaagcgaagataatagtccagatctcgtagacaagtagcagtcatctc
ttcgccataagcatttccaccaggagaaactacatcaggtctttttttgaaacagttgttg
gccgccttgctttacaatacgctcacgattatctgttaaaatttgagctattcttaaacg
acgttgtccagataaaacaaaacttttaattctatctaattccccaggacttaaatatct
tgcttctgcatctgcatttacaattgactttgtaacaatactcatggataaaactcctgt
aatgcttt--aaaaaaagcttaatactaattatttatgatataactcagaaagtaatgta
ctttttgag-aattttctaaccgaaacacgttcatgatatatgaagcaggatagatactg
aggtaagtactaaaacgagaaagttatatgtaggaacttctgttattgatttcctagtac
tttttaaaacttggtaccacaattgatatatcctgcttagtcaaacgattatagagtgt
ttctgtattaggaaagttagctgctggtaaagtcggaaatcttctgtatggaacagtatc
tttgccgtataccgcattgtattcaacactttcaactaaattacttataaatgcagataa
gccttttgaagctaaaatttgattaaagaatctaatttctgcctgattattaggagctct
tcctaagatatgcttagtacctaattctataacttttgtatttggatatggctgataaaa
ttctttaccatatagttcagatagcgccagtttttctactagttctttgacacagatttg
tctattcaagaaagctgacttaatatccaagaattcaccgccgacactaaaagagttaag
atctctttcgaaaatttgacgatatgctgctcttaaagcttgttctaacatttctttgtt
actatcactattcacttcaaagacaatagattgatctcttaaagaagttactccttgtga
aatacgagactgaatatcattactactcctcatttctttgacagtgcctaattcaacaaa
cctagcagatttactagcaataactttttttgaatctctggtcaataattcctggtcttaa
gcttcttaaagcaacaccagctggagtactatatctttcataaggaacagtattatctcc
aaaagtttctgtgtactctgagctatcaattattgtgtctattacttggtaatagccttg
cttatatgcaatatcaaaatatttattaatttcttgccgaccatatgtagggcgacccag
taagcggttatgaatatattcaatagctttacaaatatataatggctcccaataaagaga
tctaaatatacttgattttgctaactgtctaacaaactcgcgaactgtaatttgattatc
ttttagctgactttctattggcttgagaattagtttttcttcttgatacacttctctgcc
aaaaactcttaaataagcaacttttgtgacaacttctactgaattttcaaaattttggga
tgaatctgactttataagagcgttggacaatttaaagatttttggtcccagggatcctgc
tgacttaggtcttacctgtggattactaatctgattgtagattcctggcccacgtctaac
taatatccttcttgtatctttaccaaaaagagcttgtctttttcttggatctttattttc
tttagggaaaatagctccaaattgaattgataatggatcattacctattccatatggatg
ttgatcaggtaatgattgcttataatcactaaataaagttataaattgaggaaccttacg
aaaaggcgcactataggtgagaagatctatttgaggcccccaattacggcactcttgagg
ctcttcacctaaatttctaaagtaaggcacagtctcttcaccgaaataatctgtatactc
agaagaatttaaaagtgcattcactaaaccacttaaaccagtagatgataaaatagcaaa
atatttttgaaactcttctaaagagctaggacctctacccaagaaatgtctaaatgctaa
ttctaatgctcgactattgacaaaaggttcataaaattgctttctataaatacttgaagt

tcctaacgaacgaataaattccttaattgaaatttggccatttttttacttgcgactccaa
attcgataaagagaggtcatatgctttagcaatatctctttcaaaaatttgtctataaca
tgctttcacaacaatatttttctcatcagcagataaactaggcttcataacaaaacgagg
tgttgacactcctgcctgtacataagtctgaggaagacgcaaaccttgtaaatctccaga
tattctttttcttaatttatcagtcaaagatggagcttcaaattcagaaatcacaacatt
aaaatactctttaactaaatcttgacctttttatatcttcctcaaagattaataatgctgt
ccgtctcatctctctaagagccacaattgctgctgcacttgagcaagcgttatcaattaa
ctctctcaatcctcgtatattaacagataaaatattagggtcgcctgatacaattgcata
tgtcaaataccgtaaaaaccagtctaaatctcgaagagattttctcattcttgtagtacc
gtatctcaagacgtttataggtttaaaccctggcggagtagctcccccagcattaaataa
agatcggaaactttggccaaaatctccttggatatttcctgataactcattaattttatc
ttgagaactttgatctcctgcaataataacagctgcttgcggacgttctaaataagaaat
tgcagacccacctacgaatattttatctgctgctcgtgctaccagaatatttgcattttt
agttaaaatatctgcaacttccagtctttttgtccagaatttaaaaaagaaactaattg
atttaattcaccaagctgtaaaaatcggtcttgttgctctgcctgagtaatagttaaaat
tgaggcagtccgataaagctgggggcgtgctaatgggcttccgccacttgctttgatact
cattactttatttatctccttaactgatattgcttgctagcgaatacaaatatagcttga
tatttctaaatatttaaagatactatgcaagcaca-ctgttttctatattattaggtact
agtacttatagtacagagagtcttataactaacttctataaatacaaattttgtaatact
tctattaacatttgtcaaaaaaatatttaagtgctcactcttttatattaaaattcaatt
agtgagaggttgatgtgaaataaagtatactaagtaagttttatagataggaaaattaaa
agatttattctacaaacattaatca------------cttgaatgtttagcaattgtaca
attgttattgcttttaattggtttagactagttttatatttttctaacataaacaatat
taatgagcaatttgtatgaatttaaagccacaaaacaacctattaactaacgaaaatata
gatcatgtagatatacccgaaaatgatataccaatgtcaattacggaacatttagaagaa
ttaagacaacggactttatttgtgtttttattctttttgtttgctacaactataagtttc
acacaaattaagatcattgttgcaatactgcaagctcctgctgttggtattaagtttttta
caactggcaccaggagaatattttttttcatctattaaggttgcaatatattgcgggatt
gttgcaacaactccgtttgccgtttatcaagttatattatacatactcccaggactaact
ggaaaagaaagaaaaattattttgcctctattaattagttctgtattgctttttattaca
ggtggtatatttgcttactttgttctcgcaccagcagctttaacatttttaattagctat
gggtctgatattgtagaaccattatggtctttcgaacaatactttgactttattctatta
cttttacttagtacaggattagcattgagataccaatcatacaattattgctcggtgtt
tcagggacatttcttctagtcaaatgatacgagcttggagatatattattattatagca
acaattgctggagctattctgaccccttcgactgatcccgttacacaattaataatgtct
tcagctgttttattactttattttggtggaattgttatattattagtcttaaaaaagtag

agtaaat---------attttcta-ttaatccaatatatttgttatc-----tgggcaaa
taatctat-aaaacaattgct-----------------agatttaagtatcaggcttct
aaatatttcaa---------gtctcttttatcattgaaatacaatc------------

--gatacattgg
cttgtttttcatcaattatttgtgttgcacac-tttttagtactctctttattgttctt
agaattatttctttaatctatttattaaat----agatatgaagagatttaagtattgat
tatggtaatctatatcaacgtaaaaagtattttattgtgagttttatttatataaaggct
tattcattatatagttataacagtaagtctaattaatag-ttttcatgcttaattaaagt
aataactcttaattcttttggcagaaaaatctgtcttaatattttatc--aaaaaaacag
-aaaaatgaagctaaatagtctaattaacttaattcaaaagtctatatattcttgtacac
ttttactaattattttaaatattatttgtgtcgcacctaattctagtaatgcatttccaa
tttatgcgcaacaagcttatgaaagtccaagagaagcaactggtaggatagtatgtgcca
attgtcatcttgctcaaaagcctgtggagatagaagcgcctcaagcagtactacctaata
ctgttttcgagactgttgtgaagattccatatgacagcaatgctaaacagattttaggta
atggcagtaaaggaggcttaaatgttggagccgttgtaatattacctgaaggatttaagt
tagctcctgttaatagattatctacagagttaaaagaaaagactagaaatctttacattc
agccgtacagtgctaaacaagacaacattttagtaattggacctatttctggtgataaaa
atagagaaatagttttcccaatactatctcccgatcctgcaaaagataaaaaagctcatt
ttttcaagtatccaatatatgttggcggaaatagaggacgaggccagatttatccaacgg
gtgacaaaagcaacaataatattgtctctgctttaagcagtggtaaaattaataaaattg
aattactagacaaaggtggatttataatacatgtgactaacagtagcaatgtggagtcaa
cacagaagatttcacctggtctcgaacttagagtaaaagaaggggatacaattcagcttg
atcaagctttgaatagtgatccaaatgtaggtggttttggtcagaacgaaacagaaatag
ttttacagagtccaaatagaattaagggcatgattgttttctttttcgctagtgttctag
ctcaaattttcttcgtattaaagaaaaaacaatttgaaaaagttcaagcagctgaaatga
atttttaaagcaatataaaactggcacttactataaagaaagta-taaacactttattca
caggatgtaccagtttttatgttatgtaataatcgctttgacggaagttatgatactttt
aacagaatctacaatctgactaggctgaattactgtagcctgctctaagcttccattata
tggtgtgggtatatcctgcgaagatagcctcacgacaggagcatctaattcatcaaaaag
atactcattaatttgtgcaattagctctgctccaattccggctgttttcatacactcttc

tacaattaaaactttgtgagttttctttactgagatagatatagagtctatatccagagg
ttttaaagatataagatctataacttctggatcataaccttcttttaataaggccggtaa
tgcttggataacatgatgcctcattctagaataggttaaaatcgtaatatcttttccttt
tcgtacaaattcaactttattgagaggtaagaaatattcttcttgaggaatctcttcttg
taaattataaagtagaacgtgctcaaagaatacaactggattattgtctcgaattgcaga
tttcagtaatccttttgcattataaggagtagaacaagcaactattttaagccggggat
ggcttggaaataagcttccagtctctgagaatgctctgcacctaactgcctaccaactcc
tcccggtcctctaataactaaaggcaatgtaaaattacctccagaagtataacgtaacat
tcctgcattattagaaatttgattgaatgctaataataaaaaactcatgttcataccttc
aacaattggtcttagccctgttatagctgcaccaattgccataccagtaaagctattttc
tgctattggtgtatcaagaactctcaaatccccatatttgctatgcaaatctttagttac
cttataagatccaccatagtgaccaacatcttctcctattacgcagacagttggatcttt
tgccatttcctcatctgttgccgctcgtaaagcgtcaaacataaagattttactcatttt
aatatttgatttttatgatttgaccactgttttaaagtaacagttaagacaccatattct
atttagttatctgcaaaaagatatcgtttcaattctgacatgttaggttctggactggaa
atagcaaatttcacagcttgttccaattcggttttaacagcattctgaatctcattaagt
tcacctatattagcaatttcattatctagaatatatttttgagtttttgataggatct
cttgccacccaagcctctttttcttgtcttgatcttagttcatcaggatctgcgagagag
tgaccacgaaatctatatgttaatgcttctattaaggttggaccatcaccttggcgagct
ctttgaactgcttgttttgcagcttgtcttacagctagcacatccattccatcaacttca
atcccaggaagcccaaaagcttctgctttttatgtatttcaggtattgaagaagaccgg
tgatgtgccatacctatagcccattgattattttcaacaacaaatataataggtagtttc
cagagaactgccatattcagacattcaaaaaattgcccattattggtagtcccatcacca
aaaaagcaagccgtgactcttaaatcctctgtttctttaagtacttgctggcggtagata
ctttgaaaggctgcccctgttgcaaccggtataccttccgcaataaaagcaaagccacct
aaaaaattgtgaggcgcagaaaaaatatgcatcgaacctcctctgcctttactacaacca
gtctcttttccaaataactcagccatcacatttttagatgggacgcctttacttaaagca
tgtacatggtctcgataggtactgcaaacataatcagttggattcagaagtttaattaca
cctgtagaaacagcttcttgaccattataaagatgaacaaaaccgaacatctttccttta
taatacatctgagcacacatatcttcaaaatttctgcctaacaacatgtcttcatataaa
actaataaattactcttattaagattaagacctgttgaattatagttagtcagcggcaat
tgaactttcttaggataactcataatttgtaaagaaacctct----------tattgtaa
ataggaattttgaagaaacagctatcaatagaaaacacacagataaaaacttataaatta
taatatgtttttactaaagactttaataattatttgaaactagttcagtatatcaaatt
aattttgcttatactacggatgtctgaaacttatataaaaaagtagt-a-aaaaagttta
ttaaaagaatagttaaaaacagatataaatagaagtaaacagaattatt-gaaaaaaat

gattattattaattaa----tacttaatatttctaaatattttacaattaatggctatgt
caccatctttattttatcctgttgaacaagaactatgtagtcttgaaaaaaatctgaaag
ctgttgctgggactcgtcatccaattttatatgccgcagcaaagcatctattcgatgctg
gaggaaaacgagttagaccagctcttgtattttagtggctaaagcaacctctgagaagc
aagatataaatactggacaaaaaaggctagcagaaattactgaaattatacatactgcta
gtttggtacatgatgatattattgacgagtgcacaacacgtagaggagtcaaaactgtac
ataatttattcaatactaagattgctgtgctagcaggagatttttttatttgcacagtctt
cttggtatttagctaatattgaaaatttagccgtagtcaaagctatttctaaagtcatca
ccgactttgcagaaggagaaattaggcaaggcttagttcatttcgatcccagtatttcaa
tagatgcttacattgagaaatcatttacaagactgcttcactcattgctgctagttgtc
ggggtgcagctatgcttaatggttccaatcatcaaataaataatgatctttatctttacg
gtaaacatatgggattagcatttcaaattatggacgatgttctagatataactggttcta
ctaagagcttagggaaacctgctggcgctgatctaataaatggaaatttgacctctcctc
tcctttttcacttactcaagaagcaagtttaaatgatcttattgatagggagttctgta
atagtacagatatagcctcaacattatttcttataaaaagaagcgggggaattacaaaag
ctaaagatttagctaaagaacaggtgcaggcggcactttcctgccttcagtttttaccac
aatctacacctgtatctagtttaaaagaattaacacatttcataatcacaagattgtcat
aaaggacttgctaaaattaaaatgttattaactaatttttttactgtttctaaaacagtct
gaaaagtttgaatatcattactagctagctgcgctagcattttacgatttaacgcaatat
tctctttttttaatgcactaataaaagtactataattcatgccttgattgtgagcagcag
catttattctagttatccaaagacgacgaaaatctctctttttcctttttcgaccaacat
aagaatatcgaagagctttaaggacttgctgcttagctgttcgaaataaacatttatgtg
cacctttaaagcctttagctagcttaaagatttagctcgtctttttttttgcaacgttac
ctcttttaactctactcataaaatattac-ttaaattttaattattaccagcttactaaa
ttcttatactctttg----attataaataaggtaatttcattgcaatgtttttaatatct
tttaagtcaaccgaacaagtagaagaaagatgtcttctttgttttgatgactttttttgt
aataaatgacttttagaagctttatgtcgaagaaattttccggatgaagagactttaaat
ctttttgctattgcttttgatgtttttaacttaggcatattataaactttttaatgattaa
caaaagcatgaaaagtctgaggatatcaatcttagacctttcaatacttagaataatctt
aacacaatttattttacttactacataggttaactgaaacaatttatatatataatacta
atcagttaatcaaccatagctaaagtggatttatattttgctttgaacttttgtatagta
ttagcaattagcattgttataattttaagactgcttgaattcaattcttcgaacattttc
atatttaatgtaaatgaaatattggcttctgagacaatattttgaatttgaacatcagac
aagggaatcatatctaatgcagctctatattgatctttaaataatttatcgtcttttatt
tggtcaaaatcgtaaaatttcgttcctcctgaatctgataaattcatagcccctctagct
atttttttaaaatttggccgccagaaaggtcacccaaatagcgcgtataagcatgagca

actaataattcaggttgtttatgtcctatagtgtgaattctatcaacataaattttttgta
gcaggagatggttcaataaaatctaaccaatctgatccataataatagtttaaatcttca
gatagactcgctttcctattaagctctgtaaaatatataggtttaatagctggatgattt
ttattagaaaataactcttcctctattgcacaatagacaaagtatagatttgcaaccaat
ttacgatatgacttcttatctacaactcctcccaaaaaagatttaacaaaactaacattt
tctgccatactatgggacttagtagtaccttctcttagttcattcgctaaagtattaacc
ataatatcaatattccattttt-aaagataacaaattaaca---ataaatagttatccgt
atatttataattgagttgcgctaactaggctgctgcaaaataatttttttgattttattgg
atctggattcattgttttgtcaccaggcttccaattagctggacaaacttcatctggatg
agattgtacatattgaattgcttgtaaaaccctcaaagtttcttctacactcctgccaaa
ctctagattattgattgtagagtactgaataattcctttcgggtctataataaatagtcc
tcttagagctacaccatcactattcaaaacattataagctgcactgatctcttttttttaa
gtctgatactaatggatatgaaagatctcctaatccacctgattctctgtcagtctgtaa
ccaagcaagatgagaatactcactatcaaccgagactccaagaacttctgtattaagttc
cgaaaaagcattatacttatcgctaaatgctgtaatttctgtagggcacacaaatgtaaa
atctaaaggataaaagaataagacaatatacttattcttgagatcagataattttaacgt
tttaaattcttggtcataaacagctgtagctgaaaaatcaggagctagctgacctactcg
aagacaatttggtccagaaatcattaatttttcccagaagaattgatataattttgttat
ttaataaataattatataacaaaatttagtatatattaaaatactacaagtcttttttaa
attac--------taaatgatataatcaatactatctttatagcggggaatggatttgaa
ccattgaccttcgggttatgagcccgacgagctaccagactgctctaccccgcggataac
taaaatcatattagtctacatatatatataaaagcaaatgcataatagattttagaagta
aaaaaatagaagaactggtccactgatcattaaacttagaaacgacaaagtatttttttat
cttatttaaaagagggaagacttcatataaaccaataaatctatcttttaaaagaatttc
ggacggctttacccaaatttgaccgtcataccagcctgattcttcataaaatactgtagc
actcattaatcgttttactacgtatgaccagcctaaatataatctgattaaaataaatcc
tgtcattaggctggaagtaataaattctgaaaagaaaaatttcaaaggtagtttagtgat
tggaaaaattgataataatataggactaactaataaacagttaagtagtagtgcaatagt
tattttttttattataagatctacggcttaaagttggccagcaaaagaaccaagaattttt
caaagaagtatattcatgaacaggttgctgctcctttggcacaggacattgagtattata
taaattcattgacattaaaaagtattataataaagac--tttgtttatattatctaacat
aggtacgacattaaatattaactaactgcaattatagtagtgagtagaagaaataaaact
aaaaatagccaagtaattttatttaaagtattctcagtactacgagtattactaaagaac
tgattctgagcacctacacttcctaaaccttcggatttaggattgtgtattagaatagtg
aagattaatattattgtagatgagtaccaaaaaaaattttaaaatttgttccattatgtaa
aagttaat-aaaaatagcacaaagacaatcgtctaaaagatattgattgtcttatcatat

taaaaagtatttattcaccctgtacttttaaaagtaaaaatcctattgccagccctacca
aaactaggactgaacttaatacagcactttctacaatttctccgaccatt-ttattttc
cttattattttattattg---tcattcttttgacaagattatattgagctaa-ctaaaat
ccgtttctaccccaaactaccattgcgattgaaaatgtaaacattgccattagagctgcc
catcccaaacttaaaatatccataatttataatattc--ctaaaaataagatctctttta
cttaaactataatattcttaatattaaaaatatcattatataataagctaattataacag
agtactacttaattcgtatttttagtgtatttaaagaagattgaa-ttttttcgaggtac
tggataagtatttcatcctaagaaccatagtcaac----ttttgatttattataatgctt
aaatctaaatata--tatcactcaagaatcattagtaattatgatgtttttaattatacg
att---aacatatttcaaaatagaagaattcatagcgactaagttaa-----tatgctaa
tataatttatcaat--aaaaaggaagttagtatttcaattatgcaaacaactataaataa
tgggcaaacgtctagtaaagaaactttactaacacctagattctatacgactgactttga
agagatggctaatatggatatttccggcaatcaagaagatttttttggctatcctcgaaga
atttcgagctgactataatagtgaacatttttattagagacgaagagtttaatcaatcttg
gtctaatttagaacataaaactaaatccttatttattgagtttttagaaagatcttgtac
cgcagaattttcaggtttttactatataaggaattatccagaaaattaaaagacagaaa
tccggttatagctgagtgctttttattaatgtctagagatgaagctaggcatgccggttt
tttaaataaagctatcggggacttcaatttatctttagatttaggattttaacgaagag
tcgcaagtatactttttctcacctaaatttatttttatgcaacctatctttctgaaaa
aattggatactggagatacataactatttaccgccatcttgaacaacatccagaacaccg
tatttatccaatctttagatttttgaaaattggtgtcaggatgaaaatcgtcacggaga
ttttttgctgctttgctcaaatcccaacctcatttttaaatgactggaaagcaaaaat
gtggtgcagatttttctattaagtgtatttgcaacaatgtacttaaacgactttcaaag
aattgattttataatgccataggcctagactctagacagtatgatatgcaagtaatacg
aaaaactaacgaaagtgccgctagagttttcccggttgctttagacgtggacaatccaaa
atttttcaaatatttagatacttgtgcatgtgataatagagctctgatcgatattgataa
taacaattctccattattcattaaatctatcgtaaagatacctctatatttttccttatt
tgcaaatttactaaagatatatttgattaagccaatagactcaaaaacagtatggaatac
agttcgatagttaaatacaagggaatgctttttcttataaaaagctatctagtgattaa
tataaaat------aaaagcttccttct-----cgatggaaggagcttttatttgtcaa
ga---atataatgggattatatagatc-tagaactaagaacttactttttcttttgcttc
atacataacctctaaagtaattagattttagaacaatcacgggcaaatttctctgtatt
tctttttactttacctctaacaaacccaggaattttatttaattctttctgtgcttcttc
tgaccaattaatactatcaacagcagaaattcctatacttaatgcttctgttgtatcatg
accaccaaatatttctagcaaatgatcttccataccaagtgtaaacgaattatatactaa
atcagcaatttgatttgttccctcataaccaagaaatggacggtaacttaaaggaaagtt

ttgtatatgcactggtgaagaaataacgccacaaggaatatttagacgcttaccaatatg
tctttccatctgtgttccaaaaattgctgcaggttcagttttagcaattaaatctccaat
taaaccatgatcatctgaaacgattacttcatcgcagaattcttgaacttgatctttaaa
ccactcttcatcatatttgcaataagttccacaccaggcgacatgtatacccatttcctg
gtgtaaaattcgagttattgctgcagcatgagttgcatctccaaatacaatagccttttt
acctgttaaattctgacaatcaattgatcttgaaaaccatgcagattgtgaaataaatcg
ggtttgctcgtctatgtatttttcgtagtctacagcagctcccaatgcatttactaattg
ctgtattgatcggatgcatgcagctgtttgaacaatacccataggtgtaatgtcaacata
aggcatattaaattctttctctaaatatcgagctgtcattagtccagtttctctatatgg
aataaaattaaaccatgcagagggtaatttttttaaatcttgaactgatgcattttctgg
aataatttggtttatttggatatctagatcttgaaataacctttttaattctgctatatc
atgttgattatgaaatcctaagcttactgctccaataatattaacagatggagttttagt
cttttgagttaaaacttgattattagatttagctttttccatatagaaagtgacaatttg
ttctaatgttctatcacctgcttgcagttcatttactctataatgattaacatcagctaa
taaaacatctgcttctgtttctatagatgctctgctaacaaagttttgcaaatcttcttg
caaaatactcgaagtacaagttggtgttagaataactaaatcagggctttcttctctgtc
ttttctagtaatattttctacaactttttcttgagaaccacgggctaacacatgtctatc
tacaacacttgctgtaacaggagtaaaatctctgtctctttcaagcattgaacgcattac
attaaaataatcgtctccgagaggcgcatgcataattgcatgaactttttttaaaagaact
ggcaattcttaaagttccaatatgagcagggcctgcatacatccaataagctaatttcat
attaaattttcctatgatttatttagacatagtaatactttttaaagctaactaacaaaca
ttagccaaacatctaatttaactccttgacaattttgtaatattttttaaatgaagctta
tcattataaatatttaaaaaatataattcagaataga----aataactgttagatcaagc
ttgaaagtaaagatttcttgcagttatagtttttctttaagtaaacatgtaaaaattgta
ctatatataaatgtactatatataaactttttttactgaatattggtaatgccagataca
attaacttaaatatgccttccccaacgtttggtggaagcactggcggttggttaagagcc
gcagaagtagaagaaaagtatgcgataacatggacaggcaaaaaatgaaagtaagtttgaa
atgccaactggtggtaccgcaactatgcgaaatggagaaaacttactctatttagctaag
aaagaacaatgtttagctttaggtactcaactaaaaagtaaatttaaaatatctgactat
aagatttacagagtttttcctaacggagaagtgcaatatttgcatcctaaagatggagtc
tttccagaaaaagttaatactgggagagctagcgtcaacagtgttgatcattctattgga
caaaatattaatcctgtagatgtcaaattcatgaataaagcaacttacgattagtctctc
-aaaaacctagacataaaataacttttatatagtctaggtttttgataatgtatactgat
agtatggaagggtggccgagtggttgaaggcgtctgatttgaaatcagttgaacta--tc
tggttccgtgggttcgaatcccaccctttccgttataaaact------------atagtt
ggcttctcaaggcaagctagctatacactgatagtattaagtttagacttaataaagtct

acaacttgtgacaggttagaaatttttttcggcatcctcatctggtatctctatactaaat
ttttcttctatggccattaccagctcaactgtatctagagaatcggctcccaggtcactt
gaaaaatttgcttccctagtcactattttttttcaattcctagctgttctgctacaata
tcttgaacttttcaaagatttcattatcttgcataatatattatttcctctaaggtgtt
atcaaaaattttcggcttatattattatccaactctaaaaaattagtaatttttagtcg
agattaaaatatttatacctatatgatatactattattctggataaatacgaagtttacg
atatggctcttctccaaaactacgagctctcaactggtagttgtcaactagattatgctg
catttttcgtatataggctgatcgaggtgtaagctgaactattgaactttcattcaagat
aatagtttcaatagccaatttagcttcttgaagagcctggatctcatcaaaatttttatt
tttacagagttctacccaattaagaccagatgaagtatgaatgtttaatatttttctgag
tgcacgagtaatttgtggaaccgtactattctgaattgtatatataataatttgttttga
ttttgcaatttgtctaagttttgtattttgctttacttgattacgtagcgctagaatagc
atcagacttttctatctccttggttaaaataattggtagatctagtgaagaaataactga
tgtaatatgctgccaactcaaagaataagcatataagtactgatgaggtatttccaattg
caaagattgtttattaatattaatagggggcattgaaagtagttgacaataaagatgtatc
acgattttgtgatttattcaaatctaaggacttatttcttaattctctatattgtaaaac
cggaggcgcctgctgtgcgttaattgattttcgagcattagagatgtttacaggtaagac
ttctatagattgcgatggataacatttaattaaaattctaccattagcttgaatttgtct
tttttgcacaaaaggttgatggccttgcaaaatttgatctattgtctctttaactttatt
atgaactatccaaacatttcgctcgtgtatttcgatcgcaatctgaaaagcaggctcagc
ttttctttctagtatactcttttgcgtacctctacgtttagcttcatcgtcacctaatgt
tacatattgaattccaccaattaggtccgccaatgttggatttttaattaaactttctaa
atgatttccatgcgctgtacctactaattgaactcctctttctgctatagttcgagcagc
taacgcttctaattccgtgccaatttcatctataataataacctcgggcatgtgattttc
aacagcttctatcattacttgatgttgcaaatcgggccttgcgacttgcattcttctagc
tctaccaatagctggatgaggaatatctccgtcaccagctatttcatttgaagtatctat
aataaccactctttttttccatttcgtcagctaaaacacgggccatttctcgaactgcggt
tgttttaccaactcctggttttcctaagagtaaaattgaatcaccttgctgtagtaaatc
tcgaataatactaattgtaccaaagactgctctgccgacacgacaggttaagccaataac
actaccttctctattacgcagtgaactaatacgatgcaaggtttttttcgataccagctcg
attatcgccgctaaaattaccaactttttttacacaataatctaagtcttgccaactaat
agatctttgagataaatattctgggttatctggaaatctagcttctggacgacgacctaa
atccataacgacttctattaaattatttctattaggatgctgttgtaaaggttctttgac
aaaatttggcaaaatttctagcaacttatctaagtcatctgcaataagcatgattgagta
tataataattaattaatcaaatagaagattcaagattctaaagattacaatacatattaa
agaatattatgattggaatcaattttatacttaaattgaacaggaagtta-tagatgtaa

aagaactgattaaactttacgcaataaatcgattggtcaaaattaatagtcgtgttaaca
tgtttattggacaaaaagttcgtattaaatatagcaaacaaaaagttgctagtgatatag
cagataaagttggtgaattaggagttattaagggaataaagtttattaatagtcaatgtg
ttactattattgttgagtttgacaatcacactaggctttggatgtttagagaggaattaa
tctgtctaaatgaat------taaaaacatgaaacataatttattttatttattatacaa
aatttcttatgagtcgttaaaataataaattttaaaggagtacttttaattgtgcaaatt
actattaaaaaattacaagacttactttcgtctgtacaaagaaaaaagatccagacatta
aaactaaaacaaggtaagtttgaacttctattaaataagacctataaaaaagtcaatcaa
gaaattataccttcgcagaaatctgctgtattacaaaatagtccatctacgataattaaa
tcaataaataatacaaaaaaatcttctgttgtcaatgaagaccgtacagaatatgccact
attgtttctccaatggttggaacgttttatcattcacctgctcctggtgaaaaaattttt
gtacaagttggcgatgaggtcaaattcaatcaaacagtctgtattattgaagcaatgaag
ttaatgaacgaaatcgaagcagaaattgaaggcaagattatagaaattcttgttaaagat
ggtgatatagtagattgcgggcaagccttaatgaaggttgaaacataatgctaattttc
cattttacatattgttaacagaaattatttagatagaaaaattagcttatactatagtta
tgaaaacttatttcgcaaaggcac-taaaaactaatatataaaattatttactttatctg
ccaaaaatagaaataagcgttttgattccttgtctcaagagaggagaatctcaatggcaa
ttagctcaaaagagcaagagacaaagaaggtaaaaatctcggttgataaaaatcccgtag
atacttctttcgaaaagtgggcccaaccaggccatttttctcgtacactagcaaaaggac
caaaaactactacttggatttggaatcttcatgctgatgctcacgacttcgatagtcaaa
ccagttctttagaagaagtttcacgtaagattttcagtgcacattttgggcagctgtctg
taatatttttatggcttagtggaatgtattttcacggagcccgcttctctaattatgttg
cttggttaagtaatccaacaggtattaagccaagtgcgcaggttgtttggcctatagttg
ggcaagaaattttaaatggcgatgtaggtggtggctttcaaggagtacaagttacatctg
gatggttccaactgtggagagcatcaggaattactacagaatttcagctttactgtactg
ctattggcggattagctatggctgctttaatgctgtttgcaggatggtttcattatcata
aagctgctccaaagttagaatggtttcaaaatgttgaatcaatgatgaatcaccatttag
ctgggcttttaggcttaggctgtttaggctggacaggccatcaaatccatttgtctttgc
ctattaataagctactagattctggcgtgtctccgcaagaaattccactacctcatgagt
ttttaattaatagagagcttatggctcagctgtatccaagttttagtaaaggattagttc
cattctttactttaaattgggctgaatattccgactttttaacttttaaaggaggtttaa
accctgttactggaggtttatggctaagtgatactgctcatcatcatctagcattagctg
ttctatttcttgctgcaggtcatatgtatagaaccaattggggtattggacatagcatga
aagaaattctagaagctcacaaaggaccttttaccggcaacggtcacgaaggtctatatg
aaattcttacaacttcttggcatgcacagcttgcaattaatttagctatgatgggatctt
taagcatcattgtagcacatcacatgtatgcaatgcctccttatccatatattgctactg

attacccaactcagttatcgctcttcactcatcatatgtggattggaggattttgtattg
ttggagcaggagcgcatgcttctatatttatggtaagagattataaccctgcagaaaatt
ataacaatcttttagatagagtcattaggcatcgagatgctattgtttctcatctaaatt
gggtatgtatatttcttggattccattcatttggtttatacattcacaatgatactatgc
gggcacttggaagatctcaagatatgttctctgatacagctatacagttacaacctattt
ttgctcaatgggtacaaagtatacacactttagctcctggaaatacagctccaaatgcat
tagcaacagctagttatgcatttggaggagatattgtttctgttggtaacaaagttgcaa
tgatgcctatttctttaggtactgcagatttttagttcaccatatacatgcatttacta
ttcatgtaactgttttaattttagttaaagggttccttttctcaagaaactctagactaa
ttcctgacaaggccaatcttggcttcaggtttccatgtgatggacctggtagaggtggta
cttgccaagtgtctggctgggatcatgtttttcttggcttattctggatgtacaattctc
tgtctgtagcaatttttcactttagttggaaaatgcaatcagatgtttggggtagtgtat
ctccgtctggaaatgtttctcatattactggcggtaattttgcacagagtgcaattacaa
tcaatggatggctgagagatttcctttgggctcaagcatctcaagttattcaatcatacg
gttctgcgctatctgcatatggattaattttcttagcagcacattttgtatgggcattca
gtttgatgttcttatttagtggtagaggttattggcaagagcttatagaatcaatcgtat
gggcgcacaataagataaaagttgctcctgcaattcaaccaagagctttaagtattactc
aaggtagagcagtcggtgttgcacactacttattaggtggaattggtacaacttgggcat
tctttttagcgagaattatttcagtaggctaatagtgaaaataggataaaaaaaacaatt
atggcaacaaaatttcctaagtttagccaagctttatcacaagatcctacaactagaagg
atttggtatggtattgctacggcacatgactttgaaagtcatgatggaatgacagaagaa
aatttatatcaaaagatattcgcttcgcactttggacatctagcaattatcttcttatgg
acatctggtaatttattccatgtagcttggcaaggcaactttgaacagtgggtattaaat
cctttgaaagttaaaccaattgctcatgcaatttgggatccgcattttggacaacctgct
ttgaaagcttttagtaaaggtggctcagcttatccagtaaatatagcatattctggcgta
taccactggtggtatactattggtatgagaagcaatcaagacctgtattctggggcttta
ttcttactagttttatcagctttactcttatttggagggtggctacatctacaaccaaaa
ttcaagcctggtttatcatggtttaaaaataacgaatcaagattaaatcatcatttatct
ggattatttggggttagttctttagcctggacaggtcatttagtacatgttgctatacct
gaggcaagaggacaacatgtaggatgggataattttacaaccgtattacctcatccagcc
ggtttacagccgtttttcagtggtaattggagtgtatatgctcaaaatccagatacagct
caacatttattcggaactaatgaaggtgcgggtacagcaattctgacatttctaggagga
tttcatcctcaaagtcagtctttgtggctaactgatatggctcatcaccatttggctatt
gcagtagtattcattgttgctggacatatgtataggactaattggggaattgggcacaat
ctaaaagatattttagatgctcatagaccacctagtggtagattaggagctggacataaa
gggctatttgatactattactaattctttacacatacagttgggattagcattggcttcc

ctaggtgtaattacttcgttggtagctcaacatatgtatgctatgcctccatatgctttc
atggctaaagatttcacaactcaagcatctttgtacacacatcatcaatatattgctggg
tttctaatggttggagcttttgctcatggggcaatattctttgttcgagactatgaccct
gaacagaataaagataatgttttggctcgtatgctagaacataaagaagctatcatttct
catttaagttgggtaactctattttagggtttcatacattaggcctttatgttcacaat
gatacaatgattgcttttggaactcctgaaaaacaaattctaattgagccggtatttgct
caatggattcaagcctcttcagggaaagcactttatgggtttgatgtgttactatcatcc
tctactaatatcgcaacacaagctggtagcaatatttggctgccaggctggttagaagcg
attaatagcggaaaaaattcattgtttttaacaattggtcctggtgacttcttagttcat
catgcaattgcattgggattacatactactacgttaattttagttaaaggtgctttagat
gcaagaggctctaaacttatgccggacaaaaaagactttggatatagttttccttgcgat
ggacctggcagaggcggcacctgtgatatatctgcatgggatgcgttctatttagctgta
ttttggatgctaaatacaataggttgggtaacattttattggcattggaaacatattaca
atatggcaaggcaatgcaactcaattcaatgagtcttcaacttatctaatgggatggttt
agagattacttatggctaaattcttctccattaattaatggttataatccatatggcatg
aataatttatcagtatggtcatggatgttcctatttggacatttagtatgggcaacagga
tttatgttcttgatctcctggcgtggctattggcaagagttaattgaaactctagcatgg
gcgcatgaacgtacacctttagcaaacttgattcgttggaaagataaacctgttgcatta
tcaattgtacaggcaagattagtaggtttagcacatt-tttctgtaggatatgtattaac
ttacgcagcttttgtattagcttcaacagcaggcaaatttggttagactaaa-----ttc
gaattagctaa-----aaaaaagtcagtcataata-gttttatgactgactttttagat
ttaaaacaaaaacttactaaagtttcacctcaatatctacaccagaaggcaaatttaact
tcattaatgcatctatcgtttgagaagaaggttgatgaatatcaataattcttctgtgag
atcttatttcaaagtgttctcttgagtctttatctacatgtggagaacgtaaaacacaat
aaattcttctttttgtcggtaaaggaattggccctactgcaacggcattagttctagatg
cggtatctagtattttgtcacacgacgtattaagtataatagaattatatgctttcagtt
taattctgatttttgtctgctgagtaattgtcatgttaaaaagatcttattatttaagaa
ttttagagacaacacctgcgcctacggtacggccaccttctctaatagcgaaacgcatac
cttgctcaattgcaattgcattaattaattcagcagtcatcttaattctgtcaccaggca
taaccatttctgcatcagtaccatcatcagcagtaaactgattaatagtaccagttacat
cagttgttctaacataaaactgaggtctatatcctggaaaaaatggagtatgtcttccgc
cctcctctttagttagaatataaacttctgcttcaaattgagtatgaggtgtaattgtac
caggtttagctaataccatacctctttcaatatctttttctgcacacctcttaaaagaa
ttccaatattatcacccgctagaccttcttctaacgtttttgaaacatttctaatccag
taatagtcgttgtacgagtttctcgtaaacctacaatttcaattgtgtcaccaactttaa
taatgcctctttcaattctaccagtggcaacagttccacgtcctgtaatagaaaaaacat

cttctacagccattaagaaagttttatcgacatctctctccggcgttggaatatatgtgt
caactgcttccataagtgaaaaaatcttgtcaacccatttatcttcaccttgcttaatag
ctgggtttttcgtcacagcttctaatgccaataaggcagaacctgcaacaaaaggaatat
catctccaggaaagtcgtattgacttaataattcccttccttctaattctactaattcta
gtagctcttcgtcatctacttgatcttccttatttaggaatactactaatgtaggaacac
ctacttgttttgctaataaaatatgttcacgagtttgtggcattggaccgtctgccgcag
atacaactaaaatagctccatccatctgagcagcacccgtaatcatatttttttacgtagt
cggcatggcctggacaatctacgtgagcataatgacgattatctgtttcgtattcaacat
gagcagtattaatagtaatacctctagctttttcttctggagcggcatcaatttcatcaa
attttttcgctgcagtagaccctaaagttgataaagtcgcagagatcgctgctgttaaag
ttgtcttaccatgatcaacatgaccaattgtgccaatattgacatgaggtttttttacgtt
caaatttagatcgagccatgctttttgttttccttatagtaaaagttgctttatagttac
ttttaacgataaatcttgtggattgaaagatttattatttaatacagacaaga-----ta
caatataactaatctaatatcgatagtgagcaaacgctttatttgcttctgccattctat
gtgtatcctctctctttctaatagaatttcctgtttcattagctgcatccataatttcat
tagctaatttcatagacatacttttaccagatctgtctctagagaatttagtaatccatc
ttagtgctaaattcgtacctctataagctcgtacttcaataggaacttggtaagtagaac
caccaactcttcttgcttttacttctactagaggagtaatatttcggatggctttttcta
aaatatttaaaggatctgattctgttctctctttaacgatatctaaagcctgatatataa
ttctttgagataaagtttttttaccacttttcaaaatacgaacagttaacatacttacga
gtctgcttttatataaaggatcaggtgatgcaaacctttttttagctgtattacgacgag
acatagtatttaaaattggttattgtaataa-tgttc-tattagcaggtatttaagattt
aggctttttttgtcccatatttagatcggcttttacgacgatcttttactcctgcagcatc
taaagtaccacgtactacatgatatcggactccaggcaaatctttaattcggccgcccct
aatcagtaccacagaatgctcttgaatattatgacctacgccaggaatataagctgtaac
ttcaaatccagatgttaatctaacacgcgctactttcttaaagcagagttcggtttttt
aggagttgtagtatatactcttgtacaaacacctcttctttgagggcaactttgaagagc
tggagattttgtttttttatgtatttttcgtctttcggatctaacaagttgttgaattgt
tggcataataaaattgatttaaagtctttaatagatactatttgaacttgctttacatga
attggctagttttatttatcttatacttacgcataaatctttctactctaccttctgta
tcaataattctctgtgaaccagtaaagaatggatggtttcctgaccagatatctacgtgt
agttctggctttgtggacccaatcgtcataattaactggccgtcacagtaaacttttgct
tctggataccaatttggatgtatattatcttttgccattgtttttataattatataaatg
tatcaataataactattaacgcttagaaaattgaggagcctttctggctttctttaaacc
atatttttttctctcttttactcttggatctcttgttaaatagccttcagacttaagtgt
cgttctattttctggattaattgagcacaatgctcttgcaacacctaaacgaattgcatc

agcttgccctgtcaaacctcctcctctagcatttacatggatatcatactggtttagtag
tcctaaaacttgtaatggtgcatatgaaactcttaagtaattaggactaaattgaagata
agactctcctggtataccgtttataattaaattacctgaccctgggactagtcttacttg
tgcaacagaacacttccggcgacctgttccagaatagattgcgcgagttttaattaattc
tgtcgacataacattccttgataactaaataaatatacattctttatactatatactctt
gtggcttttgcgcgacatgtgggtgaattgggccagaatacactttaagcttcgtaaata
gttttctgcctaatggacctttaggaagcatacctttaaccgatttttcaataattctgt
taggtaatcttgtctgaagctgatcaaatgtttcaacttttaatccgccaggctgtccag
aatgtcttctgtatagtttttgattcgttttatttccacttacagatacgtgagcagaat
taataacgattacataatctccagtatctagatagggtgtataagaaggcttgttttac
ctcttaagatattagaaatatgagtagatattctaccaagtgtctgatttttagcgtcta
taacataccaatgagaattagtatttaatgagggtgattgcgttttattcatgaaaagat
taatacttttggttgacaataagagaatatgtaaatggtttacattcaatttctatacaa
gtagtattt-aaaaatatta-------------ttaacaaagagaacttttctaacagtt
atttaaattgtttgtcagcaagaaaatttctagttatcaataacttatactatttttaat
aatataaaatatattaatcactttttctttggcaaacttatacctagtttttttgc-
aaggcatatataacttcttcagctgatttctggccaaaattttaatttctagcagttct
tcttgagagtaatctagtaaatctgcaatagaatggatttgagctcgctttaagcagtta
taagccctaacagataactgcagttcttctattaaaacttgactaatttttttatcttct
ttgctacgataattatctgctgatttaaagtctaaatttcttagtgaacaaaaaagatta
gttaaaactgtagctccttgacttattgcttcttgaggagagatgctcccatttgtccag
atttgtataatcaatctatcttttatgctgttgctaccaatacggacttcttctacttta
taattaactttattaactggcataaaaacagagtctacttgcaaaaaatccacagataat
tcatctacagctttttcagctaagcgatacccgcaatttttttcaattttaaactccatt
tcaaatattgtattattgcaaatagttgcaatatactgtctaggatctactacctctata
tcagaagataattcaaataggccggctgtaactatagctggcccttgaactctaattcga
ccaatttgagattctttgttataacttttaaatactacttcttttagattaagtaatatt
tctaacacatcttctcttaccccaggaattgtagaaaactcatggttcactccagcaatc
cgtacagcaactatagcagtaccttcaagatctgacaatattgatcttctcaaagcatta
cctaatgtaataccttgtccttgatttaatggttctattacaaaactaccgtactgccca
cgcgccccatctgttcttgactctatgcattcaatttgaaattgagccacctaagaaagc
tcc-tttaaaataatcagtgattagtaagtctctctaaaacaagaaggaatttatactcg
gcgtttcttaggagggcgacatccattatgaggtacaggagtaatatcttttattagagt
aatctctaatcctgcagcctgcaaagctctaattgctgtttctcgacccgctcctggacc
atttactagaacttcagtttggcgcataccttgatccatagcttgtctagctgctttttc
agctgctgtttgagctgcaaaaggtgttcctttttttagctcccttaaatccacttgcacc

agaggatgaccatgataatgtttctccttttaaattagtaatagtaacaattgtattatt
gaatgtagatttaatatgtgtaataccgttaactgcattacgtttagtttttcttgctcc
ggatttttttatttgtctagccatcgtgtctcgtctattatatagttaattaaaagatta
ttttcttggagctttttcttacctgctactgtttttttacctcctctgcgtgttctagc
attagttctagttctttgtcctctcaaaggaagaccaagacggtgtcttctacctctata
agtactaatttccataagtctcttaatgctcatagactcaaaacgtttgagatccccttc
aatctgataattagactcaagaatttctcttatactgacaacttgttgatcatttaaatc
ttgacacttaatatcagcgtctatgtttgtttttctaatatttctttcgagcgagatag
tccaataccataaatatatgttaaagctatctctattcttttgtttcttggaagatctac
tccagcaattctggccactttgtcttgtctccaataaataatatgtat-ttttataacgg
ttgtttata-agcttagccttgtctctgcttatgtttaggattagtgcaaattaccatca
cctttctgtgacgccgaattattctacatttttcacacatttttcgaacagaaggacgaa
ctttcatattaa----ctctatatactaggaatattactatattaactctattaattatc
gacattaaaaatgtcaacaatatattgtactactttaactgttgtagatttacttcgtc
atactatcatatttttttgatatgacgtatgtctgaatttgcttagctgtgtctattgcg
actccaactaaaataagtaaagacgtagccccgagacctcttaagttttggatctgagta
accttttctattataaacggaatcagcgctactgtaaataaaaaacgaggctcctaggaat
gtcagtctatttaatatgacttgtaaataatcaatagtcgcttgacctggacgaatatta
ggaatgcttgcacccattttttttaaatttatagcaatatcttctggattcattactatt
gatgtataaaaatagctaaagaaaaggatcaaagcacaataaagaagaaggtatagtgaa
ccattaggacaaaacaggtataggatttggagtaatgttttattttgaataatttgggtt
agataagatgggagggccatagatgcagatgcaaaaacaataggcataactccaccttga
tttaatttcaagggcaagtaactatttggatccaaaattgaagattttcctagctgtctt
gctgaaataattttaattcttcttgttccttcttgtacacaaattgtaattattatcatt
aataaaaagatcgctataaataatccgaacttaagacttgcattactataactagcatca
aaaaatgactgtgtaaaattctttggtagtcctgacacaatgttttgaaaaataagtaaa
gaggctccattaccaattcccttttccgtaattaactctgataaccacataataatcata
gaacctgctgttaaagctaaaacagactcgcagacaaatgcaaaattccaattaaaaaca
tatggttttacccatatagagattgctccagattgtaaagtcgcccaacctagagctaag
taccttgtaatttgagttatttttggcggcccaattcaccttcttccttctgtaatttt
tccagattggggacaattttcgtaagtagctgcattacaattgaagagttaatataagga
acaatacctaacgcaaaaatccctattgttgaaaaacctcctccagaaaaaatattcaga
aaatttactaaagtatttttttctacacttgcataaaaggcgtcatgatctatacctgga
acaggtataaatattcctaaacgtgctaaaactaatagaaagagagtaaagataatacga
tttcttagatcactttttttggctcataaataaattttaaatgaaaagagatactttt--tt
tatgtaaaaaccctattccaagacttgtataagtttaaaatagggaagtaa---aatatt

atagtaatattaata-taatattctatgtagaatacaggctttctattggaactcctcga
tctttagctgcttcactaaaagttcttaattgagttaagccattgagaacagctcgcgcg
ttatttaatgtattattagaacctaattgcttagctaatatattctgtacacccgacaat
tcgaggactgtccttacagaacctccggcaattacacctgagccaggtgcagagggcctt
aatataactttcgcggcaccagaaattccgttaataggatgaggtatagaattcgatttt
gtcagtggaactgtaactagatgtttttagcatctgttactcctttttttactgcacca
attacatcgcttgcttttcccacgccgacaccaacttggccttgctcattaccaataaca
agaataactcgaaagcttagtttttacctcctttacaactttagtaactcttttaact
tgtacgactctttcttcccagccactatctttatcttttcctttgctctgtttttttacga
ttggccattttaagaattattcctaattagttaatta-cgattctagaaacccatgcctg
cttccttagcagcttcagccaaggccttaactcttccatgatataactttcctcctctat
cgaagacgacattttaatgccttctttcatagactgttctgctaactgtttaccaacta
cgcgagaagtatcacagtttggtcttatgttatcagattctttattatttaaattaacag
atgatgtagctactaatgtaataccttgtgtgtcatcaattatttgtgcgtatatatgtt
tattagatctaaatacacacagacgaggcctacttgaggttccttgaactttttttccgaa
ctcttttatgcttatgaattctagtttgtttagtgtttagtttcattattatttaccttt
tccagctttcccagcttttcttctaacaaattcaccttgatatctaattccttttccttt
ataaggctcaggaggcctaatagaacgaatagttgaagcaacctgaccgacaacttcttt
atctatgccagagacagtaatatttgtgttgttttcaactttaatttcaatattggcagg
aggtttaattttcactacatgactatagccaacacttaagattaaatcttggttatcaat
ttgagaacgatagcctacaccttgtatttgcagtttttttaaaaaatccattagaaacacc
ttcaatcatattactaataagagttctggatagcccatgtaactgactagccatttttgt
ttgtccactcgtcttgacagcaattgtgtcgttaagtatttctaaattaataccagctgg
taaagttctggatagggtacctttaggtcctgtcacggtgatagtctgaccatcgaactg
agtactaagatttgtcggcaataaaattattttttttccaatacgagacat-attccacc
tataaaatagttaccaaatatagcataatatttcaccaccaagaccatcatgacgagctt
gtctatctgtcataacgcctctagaagtagaaataagagcgatgcctaaacctccaagaa
ctctgggcaactctttatgatttgcgtagactcttagtccgggtttactaattcttttca
gagcagtaataactggttgacgatttttaccattatacttaagagaaatcattaaatgag
tctctataccttcacccatttgttcgaaattttgaacaaatccttcttctttcagtactg
ttgccatattgcatgtcattttcgttgctggaacttgcacaatttgatgtcttgctaagt
ttgcgttacgaatacgtgtcagcatatcggcgatcgtatcgttgaccaccttgatcctcc
ttgcga-----tgaattatttgaaatattttcaagattccctgaaaggcatccctagctt
ttttaacaaagctagaccttcttgatctgttttagctgtagtgacaattgatatatctaa
accacgaatttgatcaatattatcatagtctatttctggaaagatcaactgttcacgtaa
acctaaattgtaattacctttgccgtcaaaacttctaggactaattcctctgaagtctct

aattcttggtaatgttaaattaattaatttctctaaaaaagaatacatcttgtcttttct
tagatgcacaacaattccgataggaacctcttctcgaattttaaatcctgcaatagattt
tttagcttttgtgacaattggtttttgtccagttattaatgtcaattcttgaatactact
ttcaagagccttagcattctgagaagcttcacctaagccacggttaatagtaattttagt
aaacctaggaacttcatgtacatttttgtactgaaattcatcttttaaagattgagtaac
agttgttttatatttttcttttaatcctattgccattatatttcatcaattatttaata
agttcgccagtctttttcagttttcgaa-ttttttggcctttatcattaattattactga
ggatcgactagcaatattatttgttcactaaataacataacgttagaggtatgtattgg
agcttcaaatttgataatttctcccgtttctccttcttgttgaggtttttttatgcttcac
tttgagattaattcctttaacaatcaccttatttgttttataaataattgcaataatttc
acctgtttttgttttatcacttccagaaataacttgaactaagtccccttttttttaattt
aatttttgtattattttttggttgttttagaaggacctttcattatactacctccggtgca
agagaaactatttagaaaaattttttatctcttaactctcgggctataggtccgaagact
cttgtgccacgtggattattatcttgattaatgataactgccgcattatctccaaatcta
atgctcataccgtcagttctccgtaaagctttacgagttcttactacaacggctctgaca
acatcagatcgtttaactggcatattgggagacgcatctttgactactccaataataaca
tctccaatagatgcataagaaggattactagtgcctagtactctaatacacattattttt
ctagcaccactattatctgcaacattaagatagctttgagtctgtatcatacttttattc
tgtataagattaattatcaaaagacttagataagatattaaccattgtccaacactttgt
gcgacttaaaggtcgtgtctcttgtattgtaacaatatcgccgattgtgcattcattatt
ttcatcatgcgctttatatttctttgtccggatcatagtttttgcgtactttctatgaga
gattctattttctacagctactactatagttttattcattttatcgcttactactttacc
tgttgtttcttttaaaggcatagtctttatattttttatatat---------aattaaat
tttaagattattacatggttgacttagtacgagacttttcaacagttaaaagttgggcta
atctatgcttagaatgtttaaatagatgaggctggaaatcttgccttgtggctcttttta
gccttaaatcaaaaagctctcttttttattacaaggatttcttcagctaaagaagaagagt
ccaggtttgtaacatctgatattttagggaaagtcatattttaggattctgttgtattcc
gaactataaatttagtcttgattggtaatttataagaagctaatttcatagcttgttgag
cagtttttttgtggtacacctgtaatttcaaatagaatatgtccaggcttaataactgcaa
cccaatattctggagctcctttacctgaccccatgcgagtttcagctggacgggcagtga
ctggtttatctggaaatactctaatccatagtttaccacctcttctcacatatctagtaa
tagttcttcgagtagcttctatttgtctagaagttaaccaaactggctctgtcgcttgta
atgcataatcaccgaacgcaattgtgttacctttgctagcagaacctttcattctacctc
tatgttgttttctaaattttgttttcttggggcttagcataaaaaattaaaaaaataataat
aattaagaagctgttgaatcaggagtttcctgattataagcagattctagacttttcgat
tctggcaaaatttctcctttaaaaagccaaacttttactccgagcacaccatacgtagtg

tgagcttggcgatgacaataatcaatatcggctcttaaagtttgtaaaggtacacgacct
tctctaacccattcacttctagctatctctgcaccattaagccgaccagatacttgaatt
ttaacaccctgtgtatttgctctttgagctctttgaactgcttgtctgacagctctacga
aaggccactctttttctagctgttgagtaataaattctgctactaaggttgcttcagaa
tctggatctgcaatctctacaacatttactctgagttgtttgctagggtctaggattaag
gacagcgattttcttaaagactcaattcccgctccagattttccaagtacgattcctggt
cgagctgttgctatgagaatttctacttgatcaactttacgattaatttcaattttagca
atactagcattactaagttttgaatgtataaatgagcgaattttatgatcttcttgtaaa
agaactgggtagtctttagagttagcaaaccatgaagaacgatgtttttgggtaatgcct
atgcgaaagcccaaaggatgaatttttttgacccacagtatccttgattataagttaaaag
ctataatgactaattgacgttaaa--------agactacagttctgaaacacctaatgta
atatgacaagttggtttatgtattggaaaggctcttccttgtgctctaggctgaaatctt
tttaaagtcgggcctttgtctgcaaaggctttactaacaaacaattggtttttatttaat
ccatcattatgctcagcattcgctgcagcagattccaggatttgctttatatgagaacag
actcggtatggcataaattccagaataattaatgcttcttggtattttctacctctaatt
tggtctaatacacgacggactttatgcggagaaagacgaatgtatttccctactgcttta
gtttcttttacgtttttttgtaatactcatagttatagtttaacgacgggcttttctgtca
ccttttacgtgagtgcggaaagttcttgttggaacaaactctcctaatttatgtcctacc
atttggtctgacacaaagacaggaaaatgttgtttaccattataaacagctattgtatgc
cctaccatatcgggaataatagtagatgctctcgaccaagtttttaggacttcttttttt
cctgaaatatttaatgcttctatacgtttaagaagactaacatcaataaaagggccttta
tgtatagatcttgacataataataaatcattagattcttaataaaataaattctatttac
ggcgacgtaagacgtacgggttactatatttatttggatttcgtgttttaacacccaatg
caggtttaccccaaggtgttacaggacgagctcgtccaattggagatttgccttcaccac
caccatgtggatggtctacaggattcatgacaacacctctaacagtaggtcttttaccta
accaacgatttctaccagctttaccaagagtaatattactagcatcaatattaccaactt
gtccaatagtagcgtagcattcttttcgaatcattcgaacttcgcttgaaggcaatttta
cagtaacaaaagtaccttcttttgctactatttgagcataagttcctgctgcacgaacaa
tctgtcctccgcacgaaggtcttaattctatattatgtactgctgttcctaaaggaatac
tagataaaggcaaagcatttccaacttcgataggggcagtaggaccagaaagaaccatag
atcctacactgagagatcgagggtgtagaatatatcttttctcaccatcaagatagtgta
ataatgcaattcttgcatttctattcggatcatattcaatagaagcaactttagcaacta
tattatgtctatttcttttaaagtcaattaatctgtactgctgcttatgtccaccacctt
tatgacgacaggtaatgacacctctattatttcgacctttgcagaaatggtgtttaacta
ttaatgatttttctggcttatcagtagtaatctctgaaaaggtagaaaccgttctatttc
ttgtccctggtgtgtaggcacgatataaacgaattgccatataagaagatttgagaagag

taaatgttgaa------ctatgtgatatatttaagtttctggaaacaaattaatagaatc
ttctgaggcaagtgttacaattgctttcttgtaatgtggtcgcttccctacgaatctacc
gatgctcctttttttcttaggaggatgacaagtgttaacacctgtaacttgcacgttgaa
aatatactgtatagcagccttaatattaattttgttgctttgggatcaacggcaaaaca
atactgattctcttctaataatttagttgtcttatcagtaatgatgggatatttaaccag
atctaataaacctcttgaatcaatgctatccattatatacctcttgtattttagataaag
catcaactgtaataataattttatgtgcagctaacaaagccataatatttagtgtatcag
ctgaaataagttctacgttgtgtagattgcgaatagaaagataaacattcggatcttttt
tgtctactatgactaaaactttttatttaaatcaagattccatcgatgtatagcttcca
taaatagtttagtcttgggctgatgaaaataactgttaaaattttctacaatcagtgtat
taactgatttgttatttaatgctgtccttaatgctaattgtctttctttttattcattt
ttttggtaaaactacgaggttttggcccaaatattacaccaccacctctccatagaggtg
aacggatagaacctgctcttgctcgaccagtcccttttttgacgccatggtttacgacctc
cacctcgaacttcacttcttgttttagtattagcagaaccctggcgcttttcattacttt
gctttactaaagctctatggactaagtacatcccagaatcttgactgaccttaagattta
aatcagcgttgccacttacttgaccttcccaattatagacttgataatttaattgcgtgt
taactgtcatgaaatatatcgtatgaaagaatattgctacttactaattttaccaaagc
gccaggtttaccaggaacagctcccttaacgatcaacaaatcattctctgagttaatact
tacaatttgcaaatttttttattgtaactttttattaccaagttggccagccatgttttt
acctggataaactcttccaggcgttgtaccggctccaatagagccaggttgcctatgatt
tttcgaaccatgtgacattgggcctctactaaaatgatgcctcttttggtaaccggagaa
acctttacctacacttctagaagagacattaatcttttggcctacttgaaatagatctgt
agatagtatttggctaacttcgaaatcatcagtagatttcatttcgtattcacgtaaata
ttttagtgggggagcctgtgatttttttaagtgtcccaacaatggtttatttaatttttg
ttcagcaacttgcttgtaaccgacttgaatagcattgtagccatcagtagatacagcttt
aatttgagtaataacacatggcccaacttgaattacagtaactggaattgataaaccagc
ttcatcaaagaattgggtcatacctactttagtaccgagtataccaacagacactagatt
tctccttttttgagagatttatactatcattgtacataaaaaattctacttcatagttactg
aag--taaattaaactaacagatgcatgatattattcatgacaacataaaggttaaaaac
tttacctatttaatctaaaatagaaacagctttttgtctagttacagcgtatataaatta
gaagtatgcttattaaatttttttataaactact-tagatttaactgatattgatatccgt
aacttacttgcagtaatatc----aatcaacatgtataagtttaattgcctaaactagaa
ccagctattattagaaataattcaagtatagataaaagcgtatttgaacttttatatatt
gacaaatataatggaatatattgaggttcggtaactacacctttttattttttacatttat
tgtgca--gtatatatacatctaatgcagtaaacataagaatataac----tgtatagca
aatgtaattacacaaattagagaggtattaatgggcaaagtcgttggaattgatcttgga

acaacgaattctgtaattgctgttatggaaggaggtaaacctaccgtcataccgaatgca
gaaggttttagaactacagcttctgttgttgcatatactaaaagtggagataaacttgta
ggacaaattgccaggaggcaagctgttattaacccagaaaacactttctactctgtcaaa
agattcataggacgtaaacagaatgaaatttcgcaagagattcggcaaacatcatataat
gttaaaactagtggatcaagcataaaaattgcttgccctgcactgaataaagattttgct
ccagaagaaatttcagctcaagtactgagaaaacttgttgaagatgctagtacgtactta
ggtgagactgttacacaagcagtaataactgtacctgcttactttaacgattcacaaaga
caagctactaaagacgcaggtaaaatagcaggcttagatgtattgagaattattaatgag
cctaccgcagcttctttgtcttatggactagacaagcaaaataatgaaacaatactagta
tttgaccttggtgggggcacatttgatgtatctgtattagaagttggagatggagtattt
gaagtactctcaacttctggagatacacatttaggcggagatgactttgatcagcagatt
gtagaatggctaatcaaagattttaaacagaatgaaggtattgatcttggtaaagataga
caagcacttcagagattgaccgaggctgcagaaaaagcaaagatagagctgtcaaattta
actcaaacagagatcaatcttcctttattactgccacacaagatggtccaaaacattta
gaaaaaactgtaactagagggaagtttgaagaactttgttcaaatttaatagataaatgt
agtatccctgtaaataatgctctgaaagatgcaaaactagaagcttccagtattgatgaa
gttgttttagttggtggatctacaaggattccagccatacagcaaatggttaaaagatta
attggtaaagatccaaaccaaagtgtcaatccagatgaagttgttgctattggtgcagcc
gtacaagctggagttttagcaggcgaagtcaaagatattctattactagatgtgacgccg
ttatctttaggagtggaaactctgggtggcgtgatgacaaagattataccaagaaatact
actattcctacaaaaaaatcagaagtattttctacagctgtagataatcaaccaaatgtg
gaaattcaagtacttcaaggcgaaagagaactaacaaaagacaataaaagcttagggacg
ttccgattagatggcattatgcctgcacctagaggagttcctcaaattgaagttaccttt
gatattgacgctaacggaattttatctgtaaaagccaaagaaaaggctactggtaaagag
caatcaattactatatctggagcatcaactttacctaaagatgatgttgaaagaatggta
aaagaagctgaagaaaatttcgatacagaccaaaaaagaagaaagaatattgacacaaag
aatcaagcagagtccctatgttaccaagctgaaaagcaagttaaagagtttgaagacaaa
attagccaagatttaaaaataaaaatagaagagctaattacagagcttagatctagtcta
gagaaagaagaatatgacaatattgaatctatttctcagcaattacaaaatgctctgatg
gacattggaaaaaatgctgctcagactgaaagtaaagatacaaaagcgaaggatgacgac
actgtgattgacactgatttctctgaagctaagt-aaaagta-agcgggtaacgcgattc
gaacgcgcgacatcaaccttggcaaggttgcgctctaccactgagctatacccgcataga
ttgtattattacaaaaattaagatatttgtcaatctgtta------atctatac---atc
tagactattaattttggaattacatattagctaactgttgtaaatataacaattgctata
attttttaa-------aagtacttatatcat-tatataagtacttttatatt-------
-caaaaatacaaataagtattatag-------ctaaactactaaggaattaactaagcta

gtaacaattactaatcctgtccataagccggctcctgtatatattaatccttttgattgc
tcccattggccaggggatgctaaaacaacaggtacgcctactactaacacagtcgataat
gcaattaataatagcactaataattggatcgcaataatcatttaatatgttctcctgaaa
aattcaaatgttaataatactgtatataatacaatgaagcttttgaccttcagcaattta
tctatgaagaaataatttaaaattattgttttctaaataaaccttaaattaagtattaaa
atctttattgtaaacaataatactatatttttttta--gtagataaaaaatttaaagaaag
tcttttttaaaaaaaaattaagataaggtgatatgctgtataaaaagctaagtattatta
attattagagaaatttaaa-ataagaggtaaaacatatgaactcagcccttttttttagca
aaactaccagaagcttatgccatttttaaaccgatcattgatatcttacccgtaattcct
gtattttttcttcttttagcttttgtttggcaagcagcaattggttttagataacattta
ggttataaaaagaataacaaatatacagagtgccaatttttgagctctaatcttatatct
tacacaataataactattatggttgaacccttactatcaggaattgttcttggacttatt
cctattactttattcggattattagtagcagcttatttacaatatcaacggggcaaccaa
ttaggactgtaaagcctaaattatagatatttataaaaactacaataattatagtttttta
taaatatctacaaataagctaattttaaatacagaaataatactattgaaactagagtga
taacaaaaaaaatttatgtaa---taa---------------------------------

---------------------------caagctaattgtttaccagcttgacttagtaac
tcctggtaaaagacattcatgagacatttctcgaaaaacatggcgagataatccaaaatc

tcgataataacctctacttctacctgttaaccaacatctatttctccctctaacagcggc
gctgtttctaggcatttcttggagtttttgtcttaaatccattttgtcagaaaagctgcc
agcttctttcagttgatctttaatagccagtctttttaaataatatttttttgcaagttt
cgatctcttaatctctctttggatcatattctttttagccatgctgtaa-aactctcttt
taaaattatttattcaatatattagctaaaaaagctcctgcctgcaacttttatatattt
aaaaataaatagcataaagtagtgaaatgcaattgatatttaatcaacgatacc---act
ttatgctattatcaaaaatttatttagtaaagttcttgttcaacatgagttgaaatagtg
caatcagatgaaggatatgcaatacaggttaaaacatagccagctaaaagttgatcatca
tctaagaatgattgatcagcttgatctacagttccttctgtaactttacctgcacaagtt
gaacaagctccagctctacaagagtaaggaagttcaattccttcttcttctgctgcatct
agaatatatgtatcctccgcacaattaaatgtgacatcaattccttcttcttcacatagt
aaatgaattttataatcagccatttcttcttactccttaataaagatacacttgaaactt
aataaagcaaaat-aatttaaagaacttaccaaaaaattatatactagtttcattatgca
tgatagactggaaaaaaagaaaatagtatactattctgaaaaatcaagctacatttccta
ctaaatagaactatagtaggtccaaagtatatacataaattcatatagttaaaattactt
ttatcatttaaatagctgaacttgatagta---------tagattgatcatc------aa
gctagtcttctgc---caataaataattaattatgatttcaatgcttaatcaacaagtag
aacttaagcctattcttaaatttcagataacacaaatatattcgcattctcttattcagg
aaattaaagccttaataactagacttgttttgcaagtttggagaaggccggctacattga
tggcaggtattatccaacctttattatggttaattctatttggtgggcttttctacaatg
ctcctataaatttgttcactattaatacaagctataattgtttttttgagctctgggatta
taatttttacctcttttactggagctttgaattcaggtcttccattaatgtttgatagag
aatttggatttttaaatagattattaacggctcctttagtatcgaggacttctatcattt
tatcttctgctacttttatgacttgtattagtttaatacaagttgtatttatagttacag
cttctcttttttatgggaaactcacctctaaatagcgatagtactatgatttttggactta
tgattctattagtgactgtaggagttacaatgcttagtttagctttatctttcactctgc
caggtcatattgagctattagcatttattttagtagttaacttgcccttttttattttcta
gtacggctttagctcctttatatttcatgccgccatggcttcagttaattgcaagtctca
atccattgagttatgcaatagaaggtacaagatacttatattcaagcgtaaactggaatt
ttacagagtgtgtgattaagattagctggggagatatttgtttagggcaaattattattt
tattaatcgctttagatataatggcagcttatcttgtgtctaatatattaaaagctaaac
ttaatta-----------------aaatttaataaaaaatttattaaatttattttaca
aagatttttactatataatactaatagtagtatggataattgaaatagaaatagtttttc
aaaaccaaagctattatcaacaatgataaacatttgtaagaaagtcaacaaagtatgttc
ttattcataggaggcatgtagtcaatgggactaccatggtaccgtgtacacacggttgtt
ttaaatgatcctggacggttaattgcagtccacctaatgcatactgcacttgtagcgggt

tgggcaggatctatggcattatacgaactagctgtatttgatccttcagatccagtatta
aatccaatgtggcgacaaggcatgtttgttatgccatttatggctagactgggcgtaaca
gattcatggggaggatggagtataacaggagagagcgtatccaatcctggactgtggagt
tttgaaggtgtagccttaactcatatagtcctttctggcatgctttttctagctgctatt
tggcattgggtttattgggatttggaattatttagagatccacgaactggcgagccagcc
ttagatttaccgaaaatttttggaattcatttattgctatcaagtctactttgtttcgga
tttggagctttccatgtaactggactttttggaccaggaatgtgggtatcagatgggtac
ggagtaaccggaaaggtattaccagtagctccagcatggggaccagaaggattcaacccg
tttaatcctggaggggttgcatctcaccacattgccgcaggtactgtaggtatattagct
ggtgttttccatttaactgttagaccaccacaaagactgtatcgtgctctaagaatgggt
aatattgaaactgtattatcaagtagtatctctgctgtttttttctcagcttttgtgact
tgtggtacgatgtggtacggttctgcaactacgcctattgaattatttggtccaactaga
tatcagtgggatagtggatattttcagcaagaaattgagaaacgagtagaaaatgctatt
gctgatggtgctgcacctagcgaagcatggtcaagaattcctgacaagttggcattctat
gactatattggtaataatccagcaaaaggaggattattcagagcgggccctatgaataaa
ggcgacggagttgctgaagcatggcttggacatcctgtattccaagataaagaaggaaga
gagcttagtgttcgcagaatgcctgctttttcgaaacttttcctgtaattttagttgat
aaagatggtattatacgagctgacattccatttagaagagcagagtctaagtatagtatt
gaacaagtaggtgtaacagctagtttttatggtggcaaattaaatggccaagttttcaac
gatgctcctagcgttaaaaaatatgcaaggaaagctcaattaggtgaggtatttgaattt
gatcgaactacattagaatcagatggagtgtttagaagtagtcctagaggctggtttaca
ttcggtcatgctaatttcgctttaatttcttctttggacatctttggcatggttcaaga
actatcttccgggatgtgtttgcgggaatcggagctgaagttactgaacaagttgaattt
ggagctttccaaaaattaggagatagaagtagtaaaaaacaaggagctgtataagaccaa
agtcaaagatagtctttatc---aaatatttctaatattaaaatagttaagacaaagttt
attatctaatagttactaatcaggagaatttatggaagccttagtctatgtatttttact
aacaggaacgctaatggttatattctttgcgatctttttagagaacctccaagaatagc
aaagtaacttgattccgtttaaacttataataatagaaccactttccaacttattagtgg
aaagtggttttattgtagattacattgtattattcttcatgctcttcaaaaggatctcgg
agatcttttgaagcaggaccaaatgctgtgtatatcgagtaacctgtaatacctaaaagc
agactcgaaataaaaatactaagaactgttgcagtttccataatttaagcagtattaaga
tgaactttttctataatgatattatcaatattaaaacatttaattattaaaattatcaaa
atatactatggcacttagaactagacttggagaaattttaagacccttaaattcagagta
tggaaaagttgctccaggttggggcacaactcctattatgggaattttcatgttactatt
tttcttgtttttattaatcattttacaaatctataattcttcattagtattagaaaatgt
agatgtagattgggctactttaggtag----ctaactagataagttctttga--------

ttacacaatattaaagacaac----------------aagcgatcgctattagattatag
tagcgatcgctttaaaagtattttactatatatccagagaaaattatttacctaaattaa
ttaactcggtatctgcaaaactatttgtgttaacattattatagttaactttttcaaacc
ttactaatactggatatttgataccactttatcaattgctgcaacagtaccaatttcct
gataccagtacgattcttttctcaatatttttacttttgagcctctttccatatttttag
tcctcagatagattattaaagattattatattaaaaaattaaaaaaaactaaacaaaata
tataaaaatactaccgacaattgtaatatataactaataaaaggagagagagggattcga
accctcgataacaaaagttatgacaattttcgaaattgttgcaataaaccactctgccat
ctctcctgaattaaaatactgttttaaaaaattagacaagaataacatagaactaaggta
gttgcctagaaaattaaaactatgttaaatataaattataatctttctatagatatattt
aaaaaatatgtacatagaatctatatcagcttacttcaagatttttaatcttatacaat
ttttactaaataaataaattctctcataatatgaaactatcttggaaaacacttcttctt
tggtctttaccaatatttgtaattggtttctttttctggcaaggtttttaggtccaact
actacagatgttggcagtaatatcgcaagttctagaatgacatatggacgatttttagaa
tatttagatatgggttgggtgaaacgggttgacctctatgaaaataatcatacagcaatt
gtagaagctgttgggccagaattaggaaacagggttcaacgaattcgagttgaactgcca
gcaagtgcgccagaattaattacaaaattacgcaaagccaatgttgatctagatgctcac
ccccctaaaagtacaagtgcagtatggggactattaggcaatttactatttcctttacta
ttagttggcgggttagctttcttatttagaagatctaataatgctagtggtggacctggt
caagcaatgtcatttggcaaatcgaaagctttatttcaaatggaggctaaaacgggagta
gtatttaatgatgtagctggagttgaagaggcaaaggaagaatttcaagaagtggtaaca
tttttaaaacagcctgaatcatttactgctgttggtgcaaaaataccaaaaggcgtttta
ttagttggacctcctggcacaggcaaaacattactagcaaaagctattgctggcgaggct
agtgttcctttttttagtatctcaggctcagaatttgtagaaatgtttgttggtgttggc
gcttctcgtgtgagagacctattcaagaaagcaaaagacaatgcgccttgtatcgttttt
attgatgaaattgatgctgttggtagacaacgaggaacaggtgttggaggtggtaatgat
gaaagggaacaaacattaaatcaactattgactgaaatggatggctttgaaggaaatact
ggtgttattgtaattgccgctactaacagagctgatattttagattctgcattattaaga
cctggaagatttgatagacaagtttctgtagatgtaccagattttaaaggcaggttagca
attcttgaagttcatgctaaaaataagaaaatggaacctaaagtatctttagaaaccatt
gccagaagaactcccggcttttcaggagctgatttagctaacttactaaatgaggctgct
atcttaacggctcgacgaagaaaaaatgcaatgactatgtctgaaattgatacatcaatt
gatcgagtagtagctgggatggaaggcactcctttaattgacagtaaaagcaaaagatta
attgcgtatcacgaagtgggtcacgcaataataggcagtttattagagcatcatgatcct
gtgcaaaaagttacattaataccaagagggcaagcaagaggcttaacttggtttactcct
agtgatgatcaaagtctaatatcaagatctcaaatactagcccgtatcgtaggtgctctt

ggtggcagagctgcagaagaaatcattttcggtgacgcagaagttactactggtgcaagt
aatgatttacagcaagtaacatcaatggcaagacaaatggtcactcgatttggaatgtct
aaaattggacctttatctcttgaaagccaaggaggagacccatttttaggtagaggcatg
ggaggaggctcagaatattcagatgaagttgcaactaatattgataagcaagtaagggaa
attgtcagtgaatgctatgcacaagctaaacacattattatagataatcgagtagtgata
gatagattagttgatttactaattgaaaaagaaacaattgaaggcaatgaatttagagac
atcgttaaggaatacactgcaattcctgaaaaaaattactacatatcacaattttaa---
---------------attaaacgggactgacgggattcgaacccgcaacttccgccgtga
cagggcggtgctctaaccagttgaactacagtccc-aaaaagatgaatacccagagataa
tctcatagttagcttaaacttgtcaaattagaatattaaatatgaagagta---------
--------tttgtaataatactcttcatatttgata-tagtagctaaaatatttctttta
gaaggagagagagggattcgaaccctcggtacgaagttaatcgtacagcagattagcaat
ctgccgctttcgaccactcagccacctctccatga---tatatattatatatatatcaaa
tatacttatatttaataggtgtgcataaatatttatggagctaagcggattcgaaccgc
tgaccctctcaatgccattgagatgctctaccaactgagctataaccc----ctttggta
agatattaagaaatggctcaagcgggatttgaacctgcgaccttgggcttatgaatcccc
tgctctaaccactgagctactgagccatagtataaaatactattaaatattataccataa
attatggtcaaatataaatcaataaaagaataattactatca-tttactacttttg----
-----------------tgcaagttgtaaatattcgaataaaatttcaaaattaaaaatg
tctttaactttttgttaga-------atta-ttgattacgaaa------------atattg
atatttagaattaac---
--------------------aataaatttg------------------------------
---tctttattt
g---

-------tagaca---
-------------------------------caaaaatattggaaa--------------
------------------------ttttcatcaat-----------------taattagt

------------------taaactataaccgacgtac-----------------------

---aagaattattgaacta---

---cgttaaat---

-------------------------------------tgtgatgcatt------------
-----gggcttgatcaaaacttctctaaaatcaatagaatatagt---------------
--aattcaa
gtaaatcaaatactaacaaatgtgacaaacgcttaaaaaaataata--------------
caattagaatcgtaaaaatttggtttttga-ctgtgttaaataaatttgattatcataga
tttaatatttaaaagtgttctattacttgta-----ttgagtaattgt-aatatttaata
tcttctcttttagataataatcataatataagctaaaaaatagtttt---tattaaacaa
ctagcatagagccaataccttgatcagttaatatttctagcaagagagcatgatctattc
taccatctaaaatatgcgctgaagctacaccctgagctaaagaacgaatacaacaattca
ctttaggaatcatgccaccagaaattactgctgtttgagttaaatctctagcttcctgta
tattcagatgactaattaatgttgaaggatctgaagaattacgtaaaatgccgggcgtat
ctgtgagtagaattagtttttcggcattaagagcggctgctatttctcctgcgacagtat
cagcattaatattatacgattggccttccttgtctgctgcaacactagctataacaggta
tataattattattaattaatatcttcagtaatttagtatcaatattttgtacttctccaa
caaagccaaggtttgcttttccatttggtctcggagtaataagtaatccatcttttcccg
acaaacctacacttttaccaccttgtttattaatagttgcgacaagatctttattgactc
ttccaactaaaaccatttccactatatccatggtaggttgatctgttactctaacgccat
catcgaattttggtaatattttgaggcgatctaaccaaaaattaatttctggtcctccac
catgaactaaaatgggacgtagtcctataaaagacagaaaaacaagatcactaatcactt
gatcttttagtttctgatttttcatagccgctcccccatattttataacaataattctgg
aagaaaattgctgaatataaggcagagcttcacttaagacttttactctttctgaattag
tcaacataaataaagtaggattgtgatatataaaattatatacacagcttgtgttaattt
aaaagataccaatgggt---------acttgaaaaaatagaaatattactaacag-----
----ttaaaactatagggattataattatgaataaattttgggataatgtattaagattt
ccacgatttttagtcagcgtcattttaggattaattttgataattattagtcctttttttc
gtgttattaaaaaaaccactgacaagtttttttttcatcatatcattggccggattaatc
acagttttggcaataataa-tacaaaaaatgataaatatcgaatgttgttgagagaaata
aataaatttaattaaaatttttgaacatatataatattatattatcattaaatatgataa
ttt-atttccttttctat---aaaacccataaatattaaatattatttgataaatattat
gccactaaaacaaagagttagctctgaaaagaccggtgccttcgcacttttggatagtat
tgttaggcacggagttaaacatatatttggttatccaggtggagctattcttcctattta

tgatgagctttatgcttgggaagaagcctctctaattaaacatatccttgttcgtcatga
gcaaggcgctgctcatgctgcagattcttattctagatcaacaggagaggttggagtatg
cttcgctacttctggcccaggagcaactaatcttgtctcaggtatagctacagcacatat
tgattctgtacctatattagctataacaggtcaagttgggagagctttattggtacaga
tgctttccaagaagtagatattttgggattacacttcctattgtaaaacattcatatgt
agttcgtgaccctagagacatgtctagaattgttgcggaagcatttttatttgtaaaca
cggtagaccaggtccagtattaattgatgttcctaaagatgtaggattagagaagtttaa
ttatttttctgttgagcccggaaaagttaatattcctggctgtaggccaattaccagcct
aaagtcaagacaaatccttatggcagctaaaatgatacagcaatctagccagccattgtt
gtatattggtggaggagccataatctctgattcacatcaaattattaaagaacttgttga
tttttataaaatacctgttactactactttgatggggaaggggatttttaatgaggatag
cgactattgtctagggatgttaggtatgcatggtactgcgtatgctaattttgcagttag
tgagtgcgatcttttaattgctttaggagctagatttgatgatagagttactggaaaatt
agatgaatttgcttgtaatgcccaagtgattcacgtagatattgatcctgctgaagtagg
aaaaaataggattcctcaagttgctattgtcggtgacgtagcagaagttgttagtgaaat
attgaatttattaaagacttctttccccccttatccagagcagattatatcttggcaaga
aagaattaatcgttggcgtcaacagtatcctttactggttcctagaaaatcaacaagcat
ttcacctcaagagattcttgttgcaactaataaattagcccaaaatgcttatttactac
agatgttggccagcatcaaatgtggtcagctcaatttctgaaagtaaaagctaagcattg
gctttcaagtgctggattaggcacgatgggttatggtttacctgcagcaattggcgctca
agtagcacatccaaatgacgtagtcatttgtattagtggtgattctagttttcaaatgaa
tatgcaagagttaggaactatcgcgcaataccagttaccagttaaaattattattattaa
taatcgatggcaagggatggttagacaatggcaacaagctttctatggtgaaaggtattc
acactcaagaatgacagaaggagcacctgattttcaaaagcttgcagaagcttttggtat
taaagcttttactattaataataggcaaaatatgcaatctgctttacaagttgctattga
ttatcctggtccagttttattagattgccaagttacagaaaatgaaaactgttatccaat
ggttgctcctggaaaaagtaatgcacaaatgataggtatcgctaaaccgcagagaggtac
tgcttccaactatattaataatagtgtttaaagtaa------------------------

---------------atagttagtatttttta-aacaaagaata-aagtaca--------
-taaatgtaatttttttatactatta-aaaactatgtaagaacagcctttgacgagaatt
gaactcgtgaccttccccttaccaaggggatgctctacctctgagccacaaaggc-tttt
tactatattgggccgggttggatttgaaccaacgtaggcgaagccagcggatttacagtc
cgcccccattaaccactcgggcaccgaccctaattaaaccttattcaagatatgacctat

aagatatcataactatgttgctaaaaacaagtactttgataaataaaactgaataagtaa
tggacatagctggacttgaaccagcgactttcacgatgtcgacgtgacactctaaccaac
tgagttacatgtccaaaactttaattattattataacactgtttagtgattttataagta
tctatttctaattgaagatttgttttaatcgtccggctttaccgagcagatctcttagat
aatacagtttagctctacgtattttagctcttctaatcactttgacaaaagttattcgcg
gtgaattgagaaaaaatactctttcaactccaataccttgaaatgagcatcttaaagtta
aactagtatttaagcttttttttcttcttctcgctaatactactccttcacataattgtt
cccgagttttactaccttccttaaccattagtccaagttgtattgtatcgccaactttaa
tttcaggtacttctgttttgataaaaggtatctcaacacttttcattagctgacttagct
ttgtactatttgctttcatatataggattagtcttgtgaca-tgttttgaaatattatac
attaaaaaaacaaatatgaaaaattttagcgaataagcatactagtatttataaaac-tc
tttttatct---
--ccattata--------
----------------cagatccatt------------atattcaact------------

----------agattcttttatagtttc--------------------------------

--------tttgataatcagtac-------------------------------------

---------------------------atcta----------------------------

----gatgatcaaatattattttcaaacatggcttatgtatgaaaaagttctata---aa
aacctaatgac--------taaaattagggcttaagtatttcttagttttgtgttataat
tagttactatcaaaggtagtggcataacacgaagcatcaa-aatgttcgaacgctttact
gaaaaagctataaaagtcataatgctagcacaggaagaagctagacgcttaggacataat
tttgtcggaactgagcagatactattaggattagttggtgaaggtactggaatcgcagct
caagttttgaaatcgatgaatgtgaatctaaaagatgcaagagttgaagtcgaaaaaatt
ataggaagagggtctggttttgtagcggttgaaattcctttcactcctcgagcaaaaaga
gtactagaattatctttagaggaagcacgtcaactaggccataactatattggcacagaa
cacttgctaatgggcttagtccgagaaggagaaggagttgcggcaagagttttagaaaat
ctggcagtcgatgtttcttcaattagagctgaagttatacaaatgctcggagaaaatgcg
gaagccaatgtaagtggaagcaatactacgcaagctagaagtaaaacaccaacattagaa
gagttcggatctaacttaactcaaatggctatggaaggtggtttagatcctgtagtcgga
agacaaaaggaaatagaacgagttattcaaatcttaggtagaagaactaaaaataatcct
gtcttaattggggagcctggtgtaggtaagacagcgattgcggaaggattagctcaaaga
attgctaatagagatgtaccttctattttagaagataaattagttattactcttgatgta
ggtctattagtagccggaactaaatatagaggtgaatttgaagaaaggctaaaacgtatt
atggatgagattaaatcagctgataatgtaatattagtgattgatgaagttcatacattg
attggggctggtgctgcagaaggagcaatagatgcagctaatctgcttaagccagcttta
gcaaggggagaattgcaatgtataggtgcaacaactttagaagaatatagaaaacatata
gaaaaagatccagcattagaaagaagatttcaaccagttgtagttggagagccaagtgtt
gaagaaacaattgaaattttgtttggtcttagagaccgttatgaaaagcaccatcaatta
acaatgtcagatggagctttggctgcagctgctaaatacgctaatcagtatatttctgac
cgatttttgccagataaagcaattgatttaattgatgaagctggttctagagttcgttta
ctaaattctcaattacctcctgctgccagagaattagataaagagttaagagctgtatta
aaaacaaaagatgaagctattagagctcaaaaatatgaaacagcagagcagtatagagca
agagaaatggaaattaaagctcaaattgcagcaattgctcaaagtaaaaagaatgagcct
gatttaaatttagaagatcctgttgttacagaagatgatattgctgaaattgttgctgca
tggactggtataccagtaactaagcttactaaaagtgagtcagaaaaattaatgcacatg
gaagaaactttgcatggacgtattattggtcaagacgaagcggttgtagctgtctctaga
gcgataagacgcgcaagagtaggtctaaaaaatcctaacagaccaattgcaagctttatt
ttttccggaccgacgggtgtaggaaaaacagaattaacaaaagctttggcttcttatttc
tttggttcagaagcttctatgatacggctagatatgtcagaatacatggaaagacacact
gtatctaaactaattggttctcctccaggatatgtgggttatagtgaaggtggttatcta
acagaagcggtaagaaaaaaaccatatactgtcatcttatttgacgaaattgaaaaagct

catccggatatttttaatctacttcttcaaattttagaagatggccgactaacagatgca
aaaggtagaactattgattttaagaatactcttttaattatgacttctaatattggatct
aaagttattgaaaaaggaggaggtagtttaggctttgaattatcagaagatcaaacagaa
tcccaatatactagagtacgatctttagtaaatgaagaactgaaacaatactttagacca
gagtttctaaatcgattagatgaaattattgtatttcgtcagcttactaaagatgaagtt
agagaaattgcagaattaatgcttaatgaagtctttgcgagaattaagcagcaagatatt
caattaaatgtaacagaacgatttaagcaacgattagtagaagaaggatataatccaagc
tatggagctagaccacttagacgagcagtaatgaggcttttagaagatagtctggctgaa
gaagttttatctggtaaaattaaagctggtgatagtgcagtagtagatgttactaatgaa
ggagaagttacagttttattaggtgaaaaattagaactgttaacataaa------aaaca
atttattaat--taaagcattgaagaggttat--attcaatgctat---gaaaaaaatta
ataagatgggttgctataattgacaacctctgatactctttatttgtatata---aagcc
gggatagctcagttggtagagcagtggattgaaaatcctcgtgtcaccagttcaaatctg
gttcttggcat-tttaagcatcagaaaatttgctatctgagcattaaattttaattgaa
atgttcctgtcggaccattcctgtgttttgctattataacttctgtgaagtctctacttc
tagtttcttgtgtatagtaactttctctatatagcattattactaaatctgcatcttgtt
ctatagaattatgaacaataaagttgtttgaaataaaattgcataaaggtttacattcta
aatcaaacactatttgtaaacttgtaacgtttatttttttgtatactttcaaaagtcaaag
atgaaa------aagaattaaaaacatttttttgttcttcaaagttttt------tattt
gaatagcaatcatgtcattttgattaattttatcacatcttttccatccttgtgttgtga
gtagtttatggttacttgttaattgtatatatttcccagcttcggtaataatttttatata
cagtttttttccctgttttcgaaatactacatttaattgctagaaataactgttgttttt
gcgcactaaagttaatgacttca-atctttttataatagcaattaaataatatttg--aa
gttgattatataatgatgtctgaagataattgaatttggatatacacccactttctctta
aatcggataacaaaggccttttgtcactccgagtttcaaggtttctatttagttgagata
atgccaagataggcaagcttaaatcttttgctaatattttaagtgatcgagtaattaatg
atagttcttgagatctattgtcagattgtctactctcctgcagcaattgaagataatcta
taataattaattctatatttttttccttgtaatttaaagaattttacttttgtctttatag
tgtcagtagaaatcttagcgctatcatcaatataaagattaaggttagcaagagttttac
ttttttgaataacatattgccattcatcattattaagctgaccagattgaatttttttggc
cgtttaaattacactcttgagctaaaattcttcttagtagttgctctgtagacatctcta
agctaaataaaataacataagatcttttctgattaattacatatctagttatattaatag
caaaagcagttttccccattgaaggtcttcctgcaagaataattaaatctgacttttgaa
acccttgtgttatagagtcgagttctgtaaaaccagaaagtatactactatttatactaa
tctttttccttgtatccaaatggactaataggtttgcaagaattgtagctaatgtttgcg
tatctttttcgtcaagtatttcataagcttttgttaattgactaacaatagaagttatgt

ttttttgttcaataagttgttttgagcagctaattaaacataatgaatctccacatgcta
aaagtaatcttttaatgtagttatctataacgactgctgaatattcgcttatagtatcag
ataggggagcttgttctattagactaaaaacttcatttagctcattcaaatcttgaatca
tttttcatttttaagttaatgaaaaaatttcttacgcttttgtctttgcatggttaa
cattttcgagtgctgctctataaagtaatgaagttgatgtaaaataaaaaaaatcaggag
agatttttctactgattttagtaatgatatcgattgctgagttaatattatactaatta
atattttttcagctaaaatattatgaggaggtagatatttataa------atacttatta
cttcctcc-ttgtaacattactcttgtg--------gtattagttaactattgatttaat
ttgattctggaagaacttgcaatttaatatttgcagttacttgatgtaatagcttaattt
ctacatcgtaaatgccaattgttttcacgtctggtaaagagatactttgtttttcaatgt
ctatgttagtagtgtttttaataatttgtgaaatttctttttctgtgacactaccaaaaa
tattgtggccatctcctgtttttttactaacgctaaacctttgtatttcttctagaagtt
gcttgactttttagcatttctttagctgtttttaatttttcttctcttatagctgcat
aaaattttgttgttttaaaataccatttgtggcaactgccgccattttattcgggatta
aaaaatttcttgcataaccagaagctactttgataacgtcattactcttgccaagttttt
gaatattctcttttagcacaacatttataactttttttactcatctt-------aattcta
aaaa----atctgtattatcctattatacctgttgtatacagatttt----aatagtaag
tatttatttgttgatgtaatattcttgtaaaa--aaaggtaatttttatttttt-ttctg
atataatcactttcacaataattc-----attaggagaggtggccgagtggttgaaggcg
cagcattggaaatgctgtttaggagcaatcttaacgagggttcgaatccctctctctccg
tacaa--aaaatataaaaagttttatagagtccaatctaaatctccaataatattcttcc
aagcatttaatccaccccgaactctaacaatatttagtttttgagctattaaaaacttag
aagcaaatattgatctagaatctaagctacagtaaacaaaacaaattttatcttgtaaat
tgatatgagagtaattcattcctttatttttttgtaaaggtagatttaatgaatgtatta
aatgacttttatgatattcttcatgatttcttacatctattaaaatatattgtggattcc
tactaattaaaaactttgtaattgaataacactaatttctcgtacaaatgtactagatt
tactgccataatatttgttccaatgctttttttgt-------ggataatacaaatttagta
tttataatcttaaatttgttaaatgatgaagttaatgagttgtacgttaatatagttcca
cttaatatagacttataacccagaacgattttgacagcttcagttgcttgaagtgtacca
acaatacccggtaatagacctaaaactccggaattactacaagtatctcttgcgctctct
ttattttcagtttcactgtagaagtctcgataaacaggtccaccttgataattaaataca
ctaacttgtccttcaaattgaaaaatggcaccataaatatgtattttatttaactctagg
caagtatcattcaagagatatcgagtatcgaagttatcagatccatctataataatatca
tattgtctaataatgtcgattgaattctcatagcttagtcttgttttgaacacagttact
atacactgtggatttaaatctaata-tttttttttagctatttctacttttgaatatcct
atatcatgcatcgtgtataaaatttgcctctgtaaattagagagatcaattatgtcatcg

tctataattccaatgctgccaattcctgcagcagcaaggtaaatgattcctggagatcct
agcccaccagccccaatgaataaaacttttgcttcttttaatctttcttgtccttctaat
tgaatttgtggcaatactaaatgcttagaatatcttgtatattcttctagtgagtatttt
gtattttctgttttaaaatttagcataaaaatttatagtttatttaatagcactta----
--aaagctagctgataagtttattaaaatgacttttgtaaacttggttcttctctatcta
aaatagctccttgtgtaggacagacctgaacacaaatactacaatcaatacaggcagaaa
agtcaatccaataccaattagtgttattattgtttttccctttccttgatgaatacaag
ctacggggcaagcttccacgcattcagaaactccaatacattttctgttacaattgtat
gagacatattatttaattaat-aaaagataagctatttacatctagtagttttgttgtat
actaaatttacatatgctatccaggccaatatttaaggtagtctatttctacagtttact
aatttgatatgcgcctttagttcagttggtagaacgcaggtctccaaaacctgatgtcga
gggttcaagtccttcagggcgcgtttag-ttttatcgtaattcaataatatcattttatc
tttttgcaaacatcaaaatgatatgatataaaagatcatattttatacattgaatattct
atgtactagtactg-tttttaatatgttattattgataagttaattatactaattctat
ttttgtaataagt-aaaaacagttctaattaagtatgcacgtatttaatagtc-ttttgt
atatat--aacttgaaaaaatttaaactatggctaaaaaagttactggaattgttaaact
agcattaaatgcaggtaaagctactcctgcgccacctgtcggacctgctttaggacagca
cggcgtgaatattgttatgttctgcaaagagtacaatgcacgtactgctgacaagtcagg
actagtaatccctgtagagatctcaatatatgaagatagaagttttacttttatacttaa
aactcctcctgcttctgtactaatcgctaaagctgctggactaaataaaggttcgggtga
accaaataccaaaaaagtaggcagcataacaaataaacagttagaatctattgcagaaac
aaaacttcccgatttgaacactaacaatatacctcaagctatgaaaatagttggaggtac
ggctaaaaatatgggaattctaattaaagatt-aaaagtttcaattatatttacta-tt-
-tttatgaaaaaattttcacgtcgacttacaacattaaaatcaaaagttgagcctaaact
ttacactattaatgaagcagtatctatattaaaagcaacgtcaaatgctaaatttaagga
aactgcagaagctcatattgctctaggtttaaatcctaaatatgcagaccagcaactcag
agcaacagttatattaccgaaaggtacaggtaaattgataaaagtagcagttattgctaa
gggagaaaaattaacagaagcaattagtgcgggagctgatgtcagtggctccgaagaact
aattgatgaaatactgaaaggtagattagattttgataagctgatagcaacaccagatgt
tatgcctttaatagcaaagctgggacgagtattaggccctcgagggttaatgccttctcc
taaagcaggcacggtaacattggacgtagcaaaagccgtgaatgaatttaaaggggggtaa
agttgaatacagagttgatagaacaggtataattcatgtaccttttggaaaatctagttt
ttcacaagaagatctagttttgaacctccaaacaattaaagaatcgattgatagaaacaa
gccttctggagcaaaagggaagtactggaaaactttttcttatctagcaccatggggcc
atctattcaaattgacatcactagccttctatgaaaaattttgtataattatagaactt
gaataatctattatttcaaatta-taatttgctagcattattaacgtaacaacttgctta

ctatatcttatctgcattaaaaacttt-ttatatatcttattaattatgagtacaaaggt
tgaaaatatcttggaagagctaaaatctttaaaccttctagaggctgctgaactagttaa
acaaatagaagaaacgtttgatgttgatgcatctgcggcttcaggaggtatgatgatggc
cacaccaacttcagcaccagcgtctgctgaggttgaagagaagacagaatttgatgttgt
cctagaggaagtcccagcacctaaaaagatttctgtattaaaagttgttcgctctctgac
tggtttgggtttaaaagaagcaaaagatcttgttgaatctactccaaaagtcttaaaaga
aggtgcttctaaagatgatgcagaaactatgaaaaaacagctagaagatgctggcgcaac
agttattgttaagtaatga-----agaagcgcttctgtttaagtataaacagaagtgcct
ctttatttattataagcaattatactagaaaagtccaagtgtaattgcattgttgatagg
catagttgcaccaatacctagccaaatacttacaacggtaccaattaagaaaacagtggt
tgcaattggtcttctaaatggattttgaaatttgttaacattttcaatgaatggaacagt
aagtaaaccagctggtacagcagccatacttagaacacctaatagtttattaggaatcac
tcttaatagattaaatgttggaaagaagtaccattctggtaaaatctctaatggagtcgc
aaatggattagacttttcacctatagaagatggttctaaaattgcgagtccaatactgca
ggcaaaagtgccaataattacaactggaaagacgtataatagatcatttggccaagctgg
ctctccgtaataattatggcccattccttttgctaatttagctctcagcttcggatctgt
taaatctggtttttaagaattgacatattg-tttttgtgtttaatataaagattagtct
aattactgttttaaagtgtaattttcaactattataatggtccagaaattccttgtttac
gtatcattaaaaaatgcataagcatgaaaacggcagttagtagcggaagtacaaaagtgt
gtaaactatagaacctagttaaagttccttggcctacactaactcctcctcttaacaatt
ctactatactcgctccgacaactggaacagcatcagggacacctgtaacaatttttacag
cccagtatccgatttgatcccatggcaaagaataacctgttaccccaaaagaaacagtaa
gcactcctaaaattacacctgttacccatgtcaattctctaggtttttttgaaacctcctg
ttaaataaacacggaatacatgtagaatcatcatcaagaccatcatgctcgctgaccatc
tatgaattgatctaataagccatccaaagtttacatcagtcataatgtactctactgatg
taaaagcttctgcaactgtaggtctataatagaatgtcattgcaaatccagttgcaactt
gaattagaaaagatacaaatacaatacccccctaagcaataaaaaatattaacatgaggtg
gtacatacttactagaaatatcgtcagcaattgcttgaatttctaatctctcttcaaacc
agtcataaattttactcataaaatagcttcaaaaagctcttctaatata--ttttgcgtt
taagctactattaattcaatag------ttattgaacttatatt-tttttataatcaata
cttattaactattataacatctttttattgattt--------------------aaaatt
ctctgacttagtaaaacagggctatgtactataattcttttttttttgtatagagattaat
ttcgtttttagtaaattagctaaaattcttgttacactgactctattactacctataatc
tgtgctaaaattttatgtgtaatagtaaagttaagcataataccattattaacttgagtt
ccattttgctcagctaataataataataagctcgccagtctactggttatacttttatgt
gaaatgatttctataaaatgataagcttttgccgagcaaaatactaaatgatttacaaaa

aataaattgaatggagcgcagttactacatgcttttataatagtagtatagttaatacag
gcaacttctgttttatctattgcttctgcttcgtagtaaaaattatcgtcgaccaattct
atttgtccaaatgtatctcctgtagttaataagttaagagttactttatgtgtatttctc
aggacttttgtaataattaaagatcctatcaatataatgtataatctagagctgctatta
aacaaaagagtatcatttttcttaagtgaaaaaatttgataattaatttttattacgatta
aaaaatagtagccatggattgcaagaaaaaaaatcacctgaactattttcagagaatatt
ggaatacaattttcttgatttaaacaatttttagtcataattactactttataa------
---aaaaatattctaataat-taagaactgaaattaaatgtcatataaattgatgttagt
tgagaatgacattgtattatcaaaggccattcaagaatacttaatagatcaaggcttcaa
tgtttatattgccaataatggattagaagcactgaatctagcttatcaatataatttcga
cttgataatttctgatataatgatgccgctagttaatggctatgaactattagcaaaact
taaaaaaaacaaagcattatccaaaatacctgttatcttttttgacagctaaaggaatgac
taaagacagaataaaaggctacgacatggggtgctatggttatttatctaaaccatttga
tcctaatgagttgctttcaattattaacaacttaatcgctagagatgtcttaaaagaagc
ttctttacaaaactcggcaacatcaaaccagcaattaaatcataaaatacgtttgacacc
tagagaaaaaagtatttagatcttgttgttgatggacttactaataaagaaatatctac
tatactgaatactagtgttagaaatgtagaaaaatatgttagtcgactcttacacaaaac
taatatgaagaataggactttgctagtcaagtattctataaataataatctattaaataa
tgagatcaatgaaagggcgaatgacgggactcgaacccgcgaatgatggagccacaaccc
attgccttaaccccttggccacactcgccatattacatacaccactatagcttgtttttt
gatatatcgtctagttttcgagttatatatttatctaaaacaatatttactttgaatttt
-tatgatacatagcaatattagtattcaaattaatggggaaccatttaattgctcaaagc
ccatctcattgcaatttttattaaattatcttgattttaattccgagcgtgtagcagtcg
agctaaatgacattcttttaccagagactctgtttcactcaacttatttgaatgatcaag
ataagctagaggttattaccattgtaggtggtggataatctatttgtacatac-------
-acagttttctatctcaaatgtttcatagatagagataatcttccttgtttttttatctac
atgaataataacagctttaatagtgtcacctattttaaattgtgatgatatctgctctaa
ttctttgatattaatctcagaaatatggacaagacctttgagatttcctacctttataaa
taatccgtaaggtgtaatctgatttattatgccttcaataatattaccaacaattaaatt
agatgatgcttgagatattagagctcttctatgactcagtattaaattattagatttctc
ttcgacattgagtaattttagcttaataaatttattattaaattgctcactttttttgaaa
attaccgagatgagaattaggtacaaatccagatataccttcaaggttaattatcattcc
tcccttattgaatccttttatcattacattaagtaaagagtcttcagctaatagttgcct
aattcttttccatgctcttatatattcaaggcgacgaattgataatattaattgtttcga
ctgaatattataatctaataaaaaaaaattctcttgtgtcattaatgtttaaagaagtgaa
attatttaaatcttgattactagatacttcttgaataggcaagtatgcagatattggtgt

tcctatatctactaataccccatttaattcaaagctaaatatagttccagctacaatatc
tccaagattcaaatcatacttgtatttttgtaaaacagctgcaaaatttctatgagtaaa
tccttcattatttttgtcatattgaatagtatgaattaaagagtaactttgtacaatat
attaaggtaaataaatgtttgtcgaattgtatgaaaaaatttaatttgttcggttgtaa
ttaagtatataaacatattaaaatatctaggagacatgaaaagtagacgtagatagctgc
agatttaa-tactatataatctgaggaaagtccgggctccacaaatacaatttatgctgg
agaaatcccagtgtaggtaactgcgaggatagtaccacagaaaaaaaccgccagaataag
t----tatattagctggtaagggtgcaaaggcaagttaaaagcttaccaaaagtactgca
aagtatttgttaggtaaacccctaaaatggagcaaagctactaaacaaatatttgtgtat
attttatctagtttagttgaaaatactgcatgaagttattagtaacaataactcgagagg
aatagctatcctttacaatatatc-tcgattatattgaaagaacagaacccggcttatgt
agtacttttatgtcttgtattattatgtctattgtcgaatctttaaatttaactgtttg
acaatttttttttgtagctgatgttgtttataatctatttctgaattggttaaagttttt
tcattagatcgatatgtaaatcttagacctatactttttttctcttttccaattgattca
tctatatattgatcaaataatgttgtactttctaaatctttatcataaaattgatctaat
aattttaataagtatttaatttccatattcttaggtattattaaagagagatctcttatg
atcgatggatacttagagtatggttgaatctgataacttaaataattgaattcattatga
caatcttctaaaatgtttaagttaacttcaagaaaaaaaagttttgtattgagtcctaat
tcactatatgttgcttgattaagttcactgaatatgccaataataatattgttatagatt
agtgtggcaaattttcctttttgaatgaactttattctaccatcgactatatcttttttt
gtccactcaatttgtctattaatcttttgaaaaaaattttctacgatccctttagcttca
taccaatttaaagaatgcgcagggtgtgaccatgtagatcggatatctagattaccaccc
aatatgatagctagattagtagtttctacaattttgttttgatcattatgaaatactgtt
cctatctcaaagctatctatagtttggttgctttgtttaatattataaagatttgattca
attaatccttccagcaaactgccgcgtaaattagaataatcttgaataagaggattgttt
aaagctatatttcctttggactttattaaagaataatgaactaattcagttaatcctaga
tttcttaaaatacttctacttttgtctatgaattttctctttgtggataaccttttgttg
aattgaatatttggtactgagctcctgaatttatgataaccataaattctagcaatttct
tcaataacatctatttctcgaaaaacatcttctcgtctataatttggaacagtaacttct
atattttccttattttgataaataagatcaaaatttaaagagtgaagaatattttgaatt
tcttcaaagaataaaaaacgtgtttgaccattatacataattggtcctaaaatgtcatga
actttttaattgataaattaatattaagagcatgatcagttatttttctcgacagaaa
gtatctctaatatttccgcatgttaaatctgtgattaaagcaagagcttcaaaatgtgca
tttttccaattatctacatttagcccacgttcttgtctaattgaactttctgttcgaata
ttaagaactcgagaagactttctaactacagattgtttaaatatagcagactctacaagt
atcgattttgtattataatctgtatcaaaatcagagttgcttccaataccagctatacta

gttacatttgtatttacttgagtaactaaaatatttttgtttaactcaatattttcattg
tttaacttaatctgatcattacttccaagaggaaaattgcttcgaattgttagactattt
ttatgattcatattattaattttattcaaatcaataatattaataggctgtccccatttc
aacataatataattgcttatatctgttaacaaatttctgtgtgtaaacccagaagagagc
aaccgatttttagccaatctggagaatctttaactcttatttcgtctataatggcagca
aaataattgtcacaatttaataaatcgtgattactgattatagt-ctcctttttagagaa
atatctattattgggttgatgtatagctgataacattgaggaccctgttaaagccgagac
ttctcttgatagaccaatcatgcttaacacgtctgacctgttagctgttgatgatatatc
taatatatgatcaatctcacctccaattataatagattcaatagcttcaacttcaaaacc
tgcttgtgttagttgattagttaaattgtctatatctattgttttgatattaccaagttc
tttcaaccaatttaaagaaactttcataattaattgtcatctatcttatagaagtattta
ttttacttatgattaaaaatcaatttaaaaaattcatcaaattaatataagttatttgat
gaatttgcttatacaatatattattgtgatatcttacttttatatggcagttattaagaa
ctagcttttgtaaaagttagcccattatcttgggcatatctttccataaatctcatgaat
ctatcccaatcatcaggacttttaataacatgtacagcttctattgcttgtggtttacca
ttaataaacttggcattaacatctcttgtaataatttgtccctctgtgtccatcaggtac
atacctgtgatttcacctttgtctgccatactagcatctaaaattttgggatttgtaaac
cgaaaagttgctgttcctgtactaccatctcgagaccttgttaaacgtacatctggtaca
acttcttcattgataccttgaataaattgaattgttgccatatcaatcttctttatttgt
atataatttatattggataaaactgatttctagttcttgaatgcttgttgagaaactgtt
ttaaaagcttatagtatcagtagatcaaattttatgtattatataaaactgtaacttttc
-ttttttg---

----------------------atctgttcaaagaactaacttattataagctaatcata
cgaatattagccatataatgttcgtaagt-gaaattaaggggttgtagctcagaaggata
gagcaagcgcctcctaagcgctaggtcagcggttcaagtccgctcaaccctgttaacgaa
ttaagtagtattatttaacttgttctgcaaatcagtattgatctaagttaaagtacttga
cagctaaaacaaaagaatctgagaaatattttcatttaaagaacgaacaattatcttaac
tctaaatatctgataattagtaa-aaaattct----------------------------
----ctttttaaaacacaagatgatg-----aagatatttaaactaataattgaaattaa
gtttttgaagttatagaagccaat--ataatactttctattaacaga-----------tg
cttgcttctattactatctgtttacaaga--aatt----atgagtatcaaaata------
--attttagctcca-------------------------------------ctgac----

-----------------------------tactaataaata-------------------
------------------tttattttagtctagacaag------atgaatcttcatggta
taacag--

--------------agctattctttgtgtcatacttatataaaattaggat-------ac
tgtt--

---------------ttcattaccataactggggtgggaggattcgaacctgcgaatggc
ggagtcaaagtccgctgccttaccacttggctacaccccaacatgtgaacattataatag
cagtcgtataccctgccatgtcaatagactattgtctttatttaataattttttgctgctc
ttgtagaataaaatcttttttttcaaataagatacgctatgtttaagttgcacgactgtt
atgttttcttgcttttgactgaacagccacttgatagtaattattccttctgcaatttcg
ttacttcctataataatacaagctacagctctttttttatttgcttgcttaatctgctta
ctaaaattacttgagctaacatcgatttctatttaaaaaactgctgatgtaaaaaacgc
attatttgcataccagtttcgttagcttttgtaccttgtgtagcaatataaaaatcgata
gatttattaggcaagtctatattatcttttgcaatcagtaataggcgttctaatcctatt
gcacatccaacagctggtgttgctttgccacctaattggtgaactaagctgtcatatctt
ccaccaccacatatagtatcttgcccttttgatgtcaatgtcttaatttcgaaagcagta
tcattataataatctaatcctcgaactaacttattatttatattgtagggaatattaagt
aattttagataattgcaaacgtcaccaaagtgttgttgtgattccaaacttaaaaaatca
gaaatttttggagcctctgtcagtatttttgtgtattagaatcttttgaatctaaaatt
ctaattggattactagttaatctttttgtgagtcagtgtctaaatcatcatggtacttt
gtaagataatctcgtaatttaacttgataaatactacgatcctctacttttccaattgaa
ttcagatcaagtttcaagttgtgcagattgagattgttaaatatgctcatagctaaatgt
ataacttcactatctgctcttgcatctaagctaccaataaactcaatgccaagttggtga
aattgtcgttgtcgtccactttgtggtctttcgtacctgaacattggaccgctataccat
aatctttgtaaactatggtggtaactcattttattttcaataaaagatctcacaataccg
gcagtaccttctggccttaaggtaatatctctattgctgcgatcatgaaaccgatacatt

tctttatttacaatatcagtatcttcaccaataccсctatcatataattcactattttca
aaaataggtgttctaatttcttggtaatttgcacattctaataatttggaaattttatta
tgtataaactgccaatattgaatttcatcgggaagaatatcttttgtccctctaatagcc
tgaatttttgccatgataatttttgattaccttcttaatatgtctgcattaaatagcag
ttgtagactgttattcagtcatgtattatacattag--aacatactgatg-taatttata
aagtacgggcaaggagggattcgaacccccgacaccatggttcgtagccatgtgctctaa
tccactgagctacaagcccacttaataagtaccttaactatatcattgttcactaatgta
tacaactatattcttttt------atcttacatgtgaaattttttgtaaaagtaaaaaatt
at----gtttgtttttacaattggaactattcatatataatgcttaatttattataact
ttgtaaa--aatatctataagtttaattgtatgattctattatattggtaatcttgttat
aattttgcgctcttatctttcaattgg------ctagcatctaattaacttatatttcaa
ataaaaaaatcttatctaaaatgagtaaacaaattctatatcaggatgatgccagaaaag
cattagaaaaaggcatggatattttaacagaagctgtttctgtgactttaggaccaaaag
gaagaaatgttgtcttagaaaaaaaattcggtgcccctcaaattattaatgatggtgtaa
cgattgcaaaggaaattagtttagaaaatcatattgaaaataccggagtcgcactgatta
gacaagcagcatctaaaacaaatgatgtagctggtgatggtacaacaacagctacagtac
tggcttcagcaattgttaagcaaggaatgagaaatgttgcagcaggttcgaatccgatgg
ctattaaaaaaggtattgaaaaagcaacaaattttgttgttagtaaaatagctgagtatg
ctaagccagtagaagatacaaaagctattatacaagttgcttctttgtcatcaggaaatg
atatagaggtaggtaaaatgatagcgaatgctatagaaaaagttggcagagaaggagtta
tttctttagaagaagggaaatcaactaatacgattcttgagatcacagaaggaatgcagt
ttgaaaaaggctttatttctccttatttcgttacagatacagaacgaatggaagttcttc
aagaaaatcctttttatttatttacagacaaaaaaattactttggtgcagcaagaacttg
tgccattgcttgagcaaattgcaaaaacatctcggcctttattaataatagctgaagaca
tcgagaaagaagcattagccacaattgtagttaataaattaagagggattttgaatgttg
tagcggttagagctcctggattcggtgatagaagaaaatctttacttgaagatatgagta
tcttaacgaacggacaagtaattactgaagatgcaggtttatcacttgatacagttcaat
tagatatgttaggaaaagcccgaagagttattgttactaaagactcgacaacaataattg
cagatggtcatgaaattaaagttaaatcaagatgtgagcaaattaagcggcaaatagaga
cgagtgactctttatatgaaagagaaaaattgcaagaacgattagcaaagctttctggag
gagttgccgtcattaaagttggtgcagctacagaaacagagatgaaagataaaaaactaa
gactagaagatgcaattaatgcaacaaaagcagcaattgaagaaggtattgtaccaggag
gaggagctactaatgtccatatctctagtgaattatttacatgggctaaaaacaatttag
ttgaagatgaattaattggtgctttaatagttgaacgagctgtgacctatccgttaagac
gaatagcttttaatgcaggtgataatggagcagtaatagtggaaaaagttaaaagtcacg
attttcatataggctatgatgcagcaaacggtaatattgtaaatatgtacgatagaggta

ttatagatccagcgaaagtagctagatcagctttgcaaaacgcagcttctattgcagcaa
tggttttaactactgaatgtattgtagttgataaagtcgatgattgagctata------a
gataatagataatctcaaacaaagaataatttctaattatagaaattattctttgtttca
attaatctaattaattgatcaaatgtataattttataataaagccaaagtttaaacacg
tcttctttttcaactattaagtagagaagatataagcccgtaatacctaaattatcaatt
aaaatattattctcatatatagattttctaaagatctaaaaaatgacattttgaaaaa
taataccgaaatcttctcttatagttatttgctaaatcattactgtctaaagattgattt
ccaattgaattcagaacctttaacagaatacttttaaatctttatctgactttatttga
gaatcaataataatttgattcaattgtatgtcaaaactaagattaaaaatattacttgtt
aaagatatttgattttgaatatctagagattttatagccaagaagattaaacttttattg
ttcattgtaaaatataagacaattgatgatgtattctttaaatacta-aatttatcttct
tgtgcttctatcgtttttgt-aaaataattaatgttttaatttttagttttagttact
acccgaaaaaataaattctggaaatctagcttgagctttagctagagagtgtttttccc
ttcttcttgccagtaattgataacttctgttgttttgtttaataactcaattctgtctct
ttcagcaatccacggcttggattctagttctgcttttaattcttcccaagcatcattcct
tggccagaaaaaataagaagtaagtggactagtgccatttccaacaatctgatcaatagc
aatagcaatattattttctaaccaaagtacttttagtgtaaattttgacaagtttttatc
tccattattaatattaagtattgg----tacagtagaatactgtactagttattttttc
agcaacaatttgagctttgtttaaaatgtttttaccgttttagtagcttgtgcaccttg
agataatcgttgcaaaattttgaaatgttaacacgagtttcattatttataggattata
aaatcctaattcttctatagctttaccatcgcgtttatttctgctatccattacaacaat
tctatagcttggttgtttttttcttccgtatcgctttaaccttaacttgaccatattagt
cacctcgcttattttt--atatctttattaaattatatcttacgacattatgcaataat
tcaaacaattaagctgattcaagtctaacattattaaatattaccattaaacattgtaga
aaaataatacctaacataggtgacatgtccattccaaacatgggaggaatgcttcctctg
aatagttttaagtatggatcagtaattctatttaatgaacagaatggttcattgtaccaa
tttacagttggaaaccatgccaatgataatttaagtaaaattaaaattaaataaatctca
gaaaaattagctatcgatccaagcaataaatttaatgtaccaggaagagtgttcatacgt
attttataattattatatacttcgttatactaatagtttgattatatattataatcgatg
taaataaat-cagctaaacaaaaaacttatccttaaaatcatcttctcttgagcactttt
ctttatctacattagtatttacttttgtcgtataaatttccagtttagaccaaatctgtc
ctaactgatttaattgagatgttccacattctaaacaggctatttttatattgctcaaaa
cagcacctttttttataaaatcgttcaataatggcgtaatagttgcttcatccaaaaata
aatctaagacaagtatttttgtaaatgaatttaattgatctgggacattgttaataatat
cattataattaactaaaactatattagctgtaggaagtaaagatcgtgctccttccgcta
agataaagccataaggcataataattacaataatatatttataatgactgctaataagac

ttatagtggtattagcttttaattcgattgtttctgttactagccactctctcattattt
cgtaagtaatccatttgcctagttctgagcaagcagttcttaaaatcgtacctggattat
tgtcgtttttgagaatccctgaccaatgttgtattaatggatgggatgctatattaattt
ggagttgcatgttttgttaaatatatttaacatttaatttgttgattgaatcttcaattc
ctatttgttgagtattattaaaatagcat----atatattaaagaaaataattat-aaaa
aatgaaaaaaagtctttggctgtggggttttacagatagtgctgaaacttggaatggtag
atttgcaatgattggttttatatcagttatttttatcgaagtagttacaggacaaggatt
attatatttaataggtatgatgtcataataaaaatccaaaaaagttcttgtaattttta
taagaactttttgg-atttttataaatctaatctagcggtctcatagatagaacaggtt
cattctctctattgatacctttttcaaatccagcagcagcagctcttgctctgcctgcat
gccaaagatgtccaataaataggaagaatcctaggaagaaatgagatgttgttaaccaag
atctaggagatacatagttaactgaattaatttctgtagctacaccaccaacggaattca
aagaacctagtggtgcgtgagtcatatattctgcggcacgcctttcctgccacggttgaa
tatcgttcttgatttatttaaatcaagaccatttggtcctctaagaggctcaacccaag
gagctcgcaagtcccaaaatctcatggtttcaccaccaaaaaataatttctccactaggag
atctcattaggtatttacctaatccagtaggtccttgagaagatgcaacattcgcaccta
gtctttgatctctaactaagaaagtaaaagcttgagcttgtgaagcttcaggaccagttg
gtccatagaattcactaggataagctgtgttgttataccaaacaaaattagaagccgtaa
gccccatgattgataaagcacctagactataagataagtaagcctcaccagaccatacaa
aagctcttctagcccaagcaaaaggttttgttagtatatgccagattccgccagcaatac
aaataatgccaatccagacatgaccacctacaagatcttccatgttatttacacttacga
tccatccatcaccgccaaacggagactttaagacataaccaaagataactaggggattaa
gtgtaggattgctaacaaatctaacatctccaccacctggagcccaagtgtcgtataccc
caccaataaataaagctttaataactaacagaaaagcgccaatgcctaataacactaagt
gtataccaagtattgttgtcattttgtttttatcccgccaatcgtatccgaagaaaggaa
atgattcttcaagagtatcagggccaatcagagaatgataaagacctccaaagccaagaa
cagctgaagaaattaaatgcactacgcctactacaaagtatgggtatgtattgaaaatct
cgccgcctggaccaacaccccagcctagtgtagctaagtgcggaattagaataaaccctt
gttcgtacaaaggcttctcgggaacaaaatgtgctacctcaaaaagagtcatggcaccag
tccaaaagaccattatacctgcatgagcaacatgagcaccaagcaatttgccagaaacgt
taattaagcgtgcattgccagaccaccaggcaaacccggtagactcaatgtctctaccgc
caacaccaacagttgtattaaagggcgtttccacgtggtaaaacctcctcagggaatata
aagttttcatgaggttgatcttgagcagccatccaagagcgaataccttcgttcaatagg
atatttttagtataaaaagtttcaaactcaggatcttccgcagctctcaactcttgagaa
acaaaatcgtaagctcttaaatttagagctagtccaacaattccaaatgcgcttgtccat
aatccagttactggtacaaataacatgaaaaaatgtagccaacgtttattagaaaaagca

acaccaaaaatctgtgaccagaatctattagctgttaccatcgaataagtttcttcagat
tgtgttggagtaaatgcacgaaaagtatctgcagcatcaccatcttcgaataaggtattc
tgtacagttgcaccgtgaatagcacatagtaaagcaccacctaagattcctgcaacgccc
atcatatggaacggattcagggtccagttatgaaatccttgcaaaaatagcaaaaatcta
aaaattgcagcaactccaaggcttggggcaaagaaccagctcgcttggcctagagggtac
attagaaagacggaaacgaatactgcaattggtccagaaaatgcaatagcgttgtatggt
ctaagaccaactagcctagcaatttcaaattgtcttaaacagaatccaattagtccaaat
gatccatgtaaagctataaatgcccacagaccaccaatttggcaccagcgagtaaaatct
ccttgagcttcaggtccccaaaggaaaagaagtgaatgtcccatgctgtttgctggggta
gaaacagccgcagttaaaaaattgcatccttctagatatgaactggctagcccatgagta
taccaagaagtaacaaaagtagtcccagttagccagccacctacagcaaggtaagcgcaa
ggaaacagaagtagtccagaccaacctacgaatacaaatcggtctcttttagccaatcg
tctacaagatcaaaaccaccacgagtttttcttgtccaattgctatggtcataattta
atctccagagcaaatttaataagtaatttgtttaaataaaca---ctttagatatactct
aaaattttacttttctttacggtcaatataattataagtcaaaagtaaccaaaattcacg
cttttaataaca-ttttttagctcattaagttaa-ttttttaatatataaacagtaata
gagttattgttt-aacttacaatactcacaatttattcatttctataatcctttgaaaaa
gatatcctgttcctcgtgctgttaaaatcaagtcgggattacttggatcatcttctagtt
tagctctcaatcgtgaaatatgaacatcaacaactctcgtgtcaacatgccgttcgggag
tatagccccaaacttcttgcaagatagaagctctagaaaaaggttctccagccttactaa
ctaaaagctcgagaagactgaattccatacctgttaatcgaactctttcattatttttat
aaacttgtcttttgttagtatctatttttaaaaatccaatactaataatcccagagtttg
ggactccaagactagtggtgattttatcagctcttcgaagaacagaacgaatgcgagctt
ctagctccttaggagaaaatggtttaacgacatagtcatcagcacctatttctaatccgg
taattctatcgcagacttcacctaacgctgttagcattataataggaacatctgattctt
ttcgaagttcttgacaaacaccgtaaccatctagtttaggcatcataacatctaaaacaa
caaggctaggatactcttttctaaacacgagtaaagcttcttctccatttgaagcagtga
taacttcataacctattattgttagtcttgtttctaaaattcttcttatgctggcctcat
cgtcaacaacgagaatcttttctttctggttctccaa-tttcttatttactcctgtaact
caccgatcatgattaccataaccttaaattttttagcgctatgagtatctttattcacttt
agaa-----tttagttattataaattttgcaagtctagtttacattaattagtaccagt-
-caatatttttgt-----------tttaagttatgac---------tattttaatccata
agttgactctagtttgcagaattcaatatatacata-ttttctacccaagtaaattgtat
tctattttctaaataaaaaaggggttgacaaaccagttgatagaaagtattctacattac
tataag----ttaattccaacaaagaagttttaa-----atcttctttcggagtaatgaa
tactttaat

>Pyr_25_39
tatatgata---aattaagacatctgttatgtgagaatttctaaaatatacaaaactttt
tatttaatatagaaaaagtaaattaaaataagttgaacatactttgctctaatctaaacc
tcttaattgag------attctaattagagacaaaagtatttacttattttgtatctata
ggaataaaaatgaaacttgcagtttatggtaaaggtggtataggtaaatctacaactagt
tgcaatatttctgtagcactttcaaaaagaggtaaaaaagttctacaaattggctgcgac
cctaaacatgatagtacatttacgctaacaggtttttttaatcccaacaattatcgatact
cttcaatccaaggattaccactatgaggacgtttggcctgaagatgtaatctataaggga
tatggcggtgtagactgtgttgaagctggtggaccgccggctggcgctggttgcggaggc
tacgttgtaggtgaaacagttaaacttttaaaagaattaaatgcttttgatgaatacgat
attatcttatttgatgttttaggtgatgttgtatgtggaggttttgcagctccattaaat
tatgcagactactgcttaatcattacagacaatggatttgatgctttatttgcagctaat
agaatagcagcttcagtacgagaaaaagctagaacgcactctctgagattagctggactt
gttggtaatagaacagataaaagagatctaattgataaatatatagattgtgttccaatg
ccagtattagaagtcttgcccttgattgaagatattagagtgtccagagtaaaaggtaaa
actttatttgaaatggcagaaattgataaggatttagcatatgtatgcgattactatttg
aatattgcagatcagttgattacaaggccagaaggtgtagttcctaaagaatctccggat
agagaattatttagtcttttatctgatttttacttaaatcctaaatcaaaggtaggacaa
gaaaaagtagatcaagaagaattagatttgatgatagtgtaaataatattttcagcataa
tataacaaggaataagataatgtctacagctcaatcagatgctcttacttttgaatgtga
aacaggtaattatcatactttttgtcccatcagttgcgtttcttggttatatcaaaaaat
tgaagacagtttttttcttagttatagggactaaaacttgtggttatttcttgcaaaatgc
aatgggagtaatgatttttgctgaaccgagatatgctatggcagaattagaggaaggaga
tatttcagctaagctaaatgattatggtgaactccgtagattatgcttacaaataaaaaa
agatagaaatcctagtgttatattttggattggaacatgtacaaccgaaattataaaaat
ggatctggaaggaattgctccaaaattggaagcagaaattcgtgttcctatagtagttgc
tagagctaatggtttagattatgcctttacccaaggtgaagatacagttctagcagctat
ggctcaaaggtgtccattaaatttaaagaatcaatcagataacacgagtcttaaaacttc
tcctcatattcctttagttttatttggatctcttccggatccagttgtaacccagttaac
tatggaattaaaaaaacaaggtatttttgtttctggttggttgccatcaaagaggtatac
tgaattaccggttataaaagaaggatattatgttgcgggagttaatccatttcttagtcg
cacagctactacactaatgagacgccgaaaaacaaaactaattggtgcaccatttccaat
aggtccagatggtactcgagcctggattgaaaaaatctgttcagtaatgaatgtaaagcc
tattggattagaagatagagaaaaagcaatttgggcttctttagaagactatatttcttt
aatccgaggtaaatcagtttttttttatgggtgataatttactagaagtatctcttgcaag
atttttaactagatgtggaatgactgtatatgaaattggtattccttatatggataaacg

ctatcaagcagcagagctagctttattaaaagctacatgtgataaaatgaacgttatgat
gccaacaattgtagaaaaaccagataattataatcaagtagaccgaatccgtgatctgaa
acccgatttagttatcactggtatggctcatgcaaatcctttagaagccagaggtattaa
tacaaaatggtcagtagaattcacatttgcacaaattcatggctttaccaatgcaagaga
tattcttgaactcgtcacaagaccattacggcgaaatcttagtttgtcagaattaggctg
ggatgtttatagcaagcaaagctagtattcctc----tttatgacttacatggtataaaa
gccttgtaagtct--tttattttatgttaaagaagcatttcttgctctgccatcaccagc
ccaagcttgtaaactttctacttgttctttgtctgtaaatgctaaaggcacaaattgttt
gatagctattttaatatcttcagtactaaattctctctcttcactaaacgccgtatgcat
actttcaataattgcttgctcaatttcagcgccagaaaatttattacataataaactcaa
ttcatgaatatcatatttttgccacgatcttggtctaacttttgataaatgtatcttaaa
aattgattctctctcttgacgattaggtaaatctaagaaaaaaatttcatcgaacctgcc
tttccttaacatttcggaaggtaaactttgaattttattagctgttgcgacaacaaatac
tggagctttttctctgatagccaggtaataaatgtaccaaatactctggcactagtacc
actatcaccttgactatgtaaaccagaaaaagctttatctatttcatcaatccataatat
gcaaggtgataatccttcagagatatttatcatttctctcattttgattccgattcacc
aactagtccaccaaacagttttcccatgtcaagacgtagcagaggcagcatccaatcgct
tgcaatagctttcgcggttaaggatttacccgtcccctgtataccaactagcaataaacc
tttgggcgaaggaataccgtaatttaaactttgcttcgaaaatgagcgagaccttttttg
tagccattgttttaatacatctaagccaccaatatccctatttactttactataaggata
aaattctaatagatgcgtttgattaataatttgcctttttttcttcaataataataggtaa
gctccgagagtctatctgattatattgtgcaataattttagtaatcactttgcggattct
atctatcgataatccttggcaggattttgttatattattaacgagctctgaattcaaatt
aagattcaaagctttactcaatcttatgatctctttttttgatttctagaagactaggtaa
gggcaaatctattactgttatgatatcattcagagcaaaaggtatattgattttgcaaga
aataataataatgtgcttagattgcgttttgattattttagctaaattgcgaagtttcct
aattaatactatttcgtttaggaaagagtcaaaatcttttaaaagaaaaagatttaaaga
ttcattattcaagttttcaataaattctaatgctaataaaggatttctttttgcataacc
attatcgctaggattgttagtgtatccatctacaaagtcccaggaatatacttgctgact
gtttgaacagtttaagctgtgttttataatatactctagtctatcttcttctctagtatt
tattacaataataggatatcgagattttaaaagtaatcgtaaatcttgagtgaaattcat
gtcaatatttgtttccatcagacttttgtacttcaccaattgaagaaagactaaataaga
ttatctaatattccttggatatattcaaaagtc------atctctctattttctaaatta
ttgctattactaatttaacttaccatgatttttttttcttccttttcttcgtcgactattt
aatatagagcgtccacatggtgttttcattcttgctctgaaaccagaaactctaattttt
tttcttttagatccttgtagtgttcttttagtcatgatgtctcctctgttgtaaaagatt

aaatactattttatcatctttatatatctttatgatatagatatgtattaatatcaaag
tt-aacagcaataatttatattaatcaagaactatttatataatattaaacattaaatat
----------taactttatgatattggctttgcccatttttatttaagtattttgacca
tctttttgctaatcttaaactggttaattttcagcagctaaaaacgattttgttattag
aatctcaatttaagtattttgttgataaaagccaaaatcgtaaattagaacccgacgaaa
gttttgcttttgcaaaagtatgtgtagctaaaaaatatttttctaaagctattattgaag
gtcaattagctttaaaaaactatcgagacctgaatatcttagataataatatagtaattg
ctaatttgtataatatgctaggttttatttattttgaagcaggccaaacaagttttgcaa
agaacttttatgagcaagccttacaaataaaccctaattatgttgtagctttaaataacc
tggcaaaaatttatgaagaggtaaaagatttaaaaaaagccgaatctctatacgataaag
tgttaactcttaacttaaataataaaactgctaatagaaggaaagattttatagcaaaaa
cta---------aaaacatctaatactatttgtaatcgggatagcaggatttgaacctgc
gacatcctgctcccaaagcaggcgcgctaccaaactgcgctatatcccgtaagacataat
tattactatataccttttctctaattatgtctactctttctgtattttatctaggatacc
aaaacatgcctttaactccatctggatctttgataaatcctaatttttttataaaaaactaa
caacatcaggttctgcgaatagtgtaatagtgctaatttctgcctgtctcaattgctgta
ttaattggtgtatgactacttttcctaaacctaaaccttggaaatctggatgaataacta
cgtcccagatagttgcattaaatccgttgtctgatgttgctctagcaaatcctacaagtt
tactatttgcatcttttttttgtattaaagaaataataatagaactatttttttaatgcga
tctttactttttttagaggtcttttaacccatccgactgaatcacaaagctgctccaatt
catataaattaatattcttattgctactcagatagatatctttaaattcgattttatcgc
aagttttatctaaaacaataagtttttttgaaattttttcagagctagcattgatattgg
agttttgaaaaaaatttttccagaagatcataatgattatgatataatta--aataaata
tatatattacaatattgctttaacaaacaatatattaatatatgctcaaatttttacatg
aatctattaagatagaagattgttaaaatatagagaaataaaaattttgtaaaatcttt
caataaaatgatataaagagtgcttttaagactaacatctattattataaataaagtct
cgaaattgagatgaaataacacattattctatgtctaatgccttttaaaggagttatttg
tgaaaaaatctatgtgtctaacctgtttacttgctttactaattatgagcaacccaataa
tagctaacgcagaagtagctggattggtgccctgcaaagattctgcggcattcaataagc
gtatggtaaatagtgtgaaaaaacttcaagccagattagctaaatatgatgcagacacgc
caccagcgttagctttaaataagcaaatagagaaaactaaaactagatttgcaacttatg
gtcgagcaggtttgctgtgtggtactgatggattgccacatttaatttctgatggtcgat
ggagtagagccggggactttgtttttccaggactattattcttgtatattacaggatgga
ttggctgggtaggtagaggatatcttttatctgttgctaagactagtaagccaacagaaa
aggaaatcattttagatgtgccattagctatcaaatttatgtcatccggatttgcatggc
cgctagcggcttggcaagaatttagtagtggacaattaattgcttccaatgacgatatta

ctgtttcaccccgttagtaaaaaatttatatgaataataattttaccaaatacttatcaa
cagcacctgtaattggtgtattgtggatgacgtttacagccggatttattatagaattga
accgctttttcccagatgtcttatacttttactt----ataaataa--gatcgtataaa-
--aaactacataaatatagagagatactattatttgtgtagttttta---atatataaca
taattattta-tttttaaaaatgtttatcttttcgattattttaatattagatcttacat
aataaaata-atatcaatattatgagcttagtaacccaaattatagttaatgccgatgat
gaattaagatatcctacaattggagaattgcagtcaattcaagactacttaactacagga
agcaatagaattagaattgctactattattagagataaagaaaaggagattatacagaaa
gctagtaagcaaatttttcagttacatccagaatatatagctccaggtggtaatgcagca
ggttctaggaaaagatcgttatgcttacgtgattatggttggtatctcagactaattaca
tacggagtattagctggggataaagattctatagaaacaattggtattataggagtaaga
gaaatgtacaattctttaggtgtacctataattggaatgttagatgcaatccagtgttta
aaggaagcatctttagaaatgcttggtcaagatgatattagaatcatttctccttatttt
gattatataattcgtggaatgtcataaatcatagcttcaattaattagttgaataaaagc
ttttataatgttataattacaataagcccgagagcatataattgtaaaaactaaatcttg
tcagaaccggaaggtagcagcaataatgtttcaatacaaaagttatga-ttttcgggtgt
ttattgtttttatctaatagtctcgacttttaattagtatgctatgtataaatctgtata
tgacatgtaatccatcaaaccttccagcaaaaataatttaataatttgatattaatacgt
aaaacacctatac-tcttttataaaatttctttatatctcatctaaacaatggaaactta
acttaatgggtgttcatattttatcaacaggttcgtctgtcccaaatttttctgtagaga
atcaacaatttgaagacatgatcgaaacttccgaccattggatttcaacaagaacaggaa
taaaaaaaagacatctagctccatcttctacttctttaactaaattagcggcagaagctg
caaacaaagccttatatgcagctaacttaaaacctactgagatcagtttaattattttag
ctacgtctacgcctgatgatttatttggtagtgctagccagttgcaagcggaaataggtg
caacaacatcagtagcttttgatattacggctgcctgctccggttttattgttgctttag
taacagcagctcagttcattcaaactggttcctatgacaatattttagttgttggagcag
acacaatgtctagatggattaattggtcagatagaactacctgtattttatttggtgatg
gtgctggagcagtagtattggggcaaagcctcaaaaatagtattttaggctttaagttat
gtacagacggtcagctaaacagtcatttacaattaatgaataaacctgtaaataatcaaa
aatttggtggtacagaaattcctcatggaaactataattccataacaatgaatggcaagg
aagtgtacaagttcgctgtatttcaagttccaacagtaattagacaatgtttgaataatt
taaacatttcaatagatgaagttgattggtttatattgcatcaagcaaacactagaatca
tagaagcaattgcgagcagattatcagtaccttttttctaaaatgattacgaacttagagc
attatggaaatacatctgcagcgtcaatccctttagcgttagatgaagctattcaatcca
ataaaattcaaccaggtcaaattattgttttatctggttttggagcaggcttgacttggg
gagcaattgtcttgaagtggtgat---ttatatattgcggatgacgagactcgaactcgt

aaagctttcgctacacacccctcaagcgtgcgtgtataccaatttcaccacatccgcatt
ttttaccatgaata-ttaaaaatttataatatttatctaaatattcatataa--tgtgta
tatatatatacattcttgtcagaaactctcttatattttattttaagtgataaaattgag
t----ttagtatagatagtaaaatttttatttaaaatcataa----aaataaatatataa
ggaaaataaaatatgacaccatctttatcaagtttttttgaatagtcttattcttggggca
gtaattgtagtcgttcccataactttggctcttttatttgttagtcaaaaagacaggaca
atccggtcctaaaaataacttagaaagatatggagaagcaatttaagatgaataatgaaa
tatttttcatactaataagctctccatatattgctatact--ttagaagaatttttattt
ttttattttgtaacatcctgtaaagtttattccgttctttctttttccc-atttactctc
ttataattaatgatacaaatgtcagg-ttaataatag---tttttttattctgtatctttc
---------------atttatatacgtattattatgttcaattaataaagaaaagctca
tgtctttgtctaattggcctctcaaaaaagaaaattctgaagcatataatattaagaatt
caaaacaaatcacaattcctgatggtttatggataaaatgcttcgactgtggtctattaa
tgtattctaaagtactgaagagaaatttaaaagtttgccctcaatgtagttatcattttc
aagcttctagtaacgaaagaattgatcaattgatagaccaaggtagttggcaaccaatgg
atgttcacttgatctctacagatccattaggctttaaagaccaaaagctttatagtcaaa
ggttaaaagatactgctttcaagactggcctgcaagacgcagttcaaacaggtactggga
ctatgcaaggtaaaaaagtatgcttaggtattatggattttagattcatggggggaagca
tgggatctgttgtaggcgaaaaactaacaagactgctagaaaaagcaactcaagaaaagt
tgcctgcaattatactttgtgcatcaggcggagctagaatgcaagaaggtatgttgagct
taatgcaaatggcaaaaatttcttctgctctagaaatgcataaaaaagaaaatctactat
atatatctgttttaacctctcccacaacaggggggtgtaacagctagttttgctatgcttg
gagacttaattattgcagagccaaaagctcttattgcatttgctggtagacgagttatag
aacaaacaatcaaagaagacttgccagataattttcaaagttcagaatatttatttgaac
atggtttcctagatttaattgtatccagaactcagcttagatcaaagttaatacaaattt
tgtccttacataatcatagtaagtaatgaattgaatattgactataatatcaaaatgcta
gtaaaaattaagatattaagaaattttcactaaagatatttgtagactaactagaattac
aattactattgtagaaaaatttgtcttaatgcca-tttttttcagtcttttaacaaattg-
tttttatgactttaaataattgttagtttaatacaaatttataaagagaata-----cat
actaagctaaatatagtaatattatgttttcctataatactgacctatcaaatttcgagg
aaatttatgcttaaaagatcttcttggcttgcggctttattgggactattaacagtagtt
tctacaagtacgcatacatatgccatagagttagacgaggcaacaagaactgttccatta
gaatcttctggcagaactgtaattcttacaccagaacaagttaaaagaggcaagcgatta
tttaataattcttgtgctatttgccataatggtggtatcacgaaaacaaatccgaatatt
ggacttgacccagaatctttaggattagctacgccacaaagagataccattgaaggacta
gttgactatatgaaagatccgactagttatgatggtgcagagtcaatcgcagaattgcat

ccaagtattaaaagtgctgaaatttttcctaaaatgcgcaatctaaccgatgaagaccta
tttacaatcgcaggtcatatcttacttcaacctaaaattgtttctgaaaagtggggcgga
ggaaaaatttactattagaacttcaaagacctaaaccttgtgaatactagtttatgta-t
ttgttgcttaagtgtgattattttgttagatcatgatatatattaaagatggagtttgtt
gacagcaagttacttttacccgaacttgtttacactattcgtctagttattaaaaagga
gacgttaaattgaagaagaagctttcagttcttttcactgtttttagtttttttgtaata
ggtttcgcacaaattgcttttgctgcagatctagataatggagaaaaagtttttttctgct
aattgtgcagcatgtcatgctggcggtaataacgccattatgccagataaaaccttaaaa
aaagatgtacttgaagctaatagtatgaatactattgatgctattacttatcaagtacaa
aatggtaaaaatgccatgcctgctttcggaggtagactggttgatgaagatattgaagat
gcagcaaattatgtattatctcaatctgaaaaaggttggtaattatacttgattttatcc
tgtattaaagaatagacaatctatttagttgttcattagattgtctattctttgtttatg
---ctattatataaagatatttacacaatatt----ttatgatgaaaagaatacccgcaa
ttcttgtactagaagacggtgcatattataaaggatggtcattccagcaagataaacaag
agattactattggtgaagtagtttttaatactggaatgacaggatatcaagaaataatca
cagatcctagttacttccatcaaattgtcgcttttacctacccggaaattgggaatacag
gtattaataatcaagatattgaatctcacagtattagtattaaaggacttattgcaaaaa
atatttgtaaaatttcaagcagctggagagagcagcaatctttagttaagtatttaagta
gtaataatattccttttattttcggaatagatacaaggtctttaacccaatacttgcgtc
aatttggtacaatgaacggttgtatctctactgataatttaaatcatagttacttaaaac
agaaaatttgtgagattccaagtatgcaaggtttagatttaatcccgcatgtaactacaa
gaaatgtttacccctgggatgaaaaaagttttccgaattggtatttgacagataacatta
gagtacaccgagttattcagttgaaagttattgttatagattttggagttaaactaaata
tactcagaagactagctacacttggatgtcagataacggttgtgcctgcccacactcctt
taaaagatattttggcttaccagcctgatggtatattactctctaatggtccaggagatc
catcagcagtacattacggcatccagacagttacaaatttactagattacaatgtgccta
tatttgggatttgtatggggcatcaaattttaaatttagctcttaaagctaaaactttca
aacttaaatttggccatagaggtattaaccatccatccggattgaaccagcaagttgaaa
taactagtcaaaatcatggctttgcagttgaattgacttcggtttttgaatctcctgtaa
gagtgactcatttaatctaaatgacactactattgcaggaactggacataatcaaagtc
cttatttttctgtgcaatatcatccagaatcgagcccaggccctcatgatgctgattatc
tattcgaaagttttatagaaataatgacaaagtccaagaataaagttag-----------
ttagtaattttcccatgctttatgagtgaataaagcggatagtacttgtactcctcgtaa
ttgattaaagagaggcaaatgtcctctggggccatgagaattccattgaaattcctcagg
atatctgcaaggaattctatcaacttcccagccgatttttttgccagaattttccccaatc
ttttccaacactaagccatatttgtctttgaacaaatagcccaaatttaccttttgagtg

tgtatgccataacttatcaatagtttgtaaatcttgagcaggtatttttttatatctgt
aaaatatagccaattacgagtttgtgcatttacaccagctagttgaataagcttttgttg
agttagctgatctgctttgagcaaatcccgatgagtcagtaacatttgtaaatctttgta
attcatctgttgagcagagcgcaatggaacaatgccatcgggacataagttagaagcaaa
tttcactatttcttgattttactgttaagtaacttttcataaattaaaccgtcaacaca
attacttttatagttaggcccagtaatcctctcaaaaaatagatctgctaaatcttttaa
ctcgatagagtcgttgctatttatattctcaataatttcaagttgttgcttga---catt
attagacttagtattcttattcaattctaaaagctgggctcggatttgatttggcatctg
atttgtttaattagttttaatagtc----ataaggtcttagagagaattagattgttttt
tgaaattagaagagcgccgcagtatttctaaagaagttcgcataatttgattcaataagt
tttgtcctgcataaattataatttgaccaattaaaaaaattgtgctttttattttaggaa
caaaaaaatcttgtacctcaagcagaaaagtaattattatttgcattggtgacaacatct
gtaattcattcgatctgcaagcatatatatactgacagtttaagccttcttgataaaatc
cccatactttgtaacgactttcgtaaattgctcgtggtcgctcaagatagtttttaatta
tagtattccaaattaggttattctttaatttttctaattttctatcagatagaaatttta
cattacagagataacttgcatttgtctcatttacagtattaaaattttgaactaacatat
gagcaataatgttgcttagctgaattaaataattttcaagaaaaatttcgacctgtttaa
ctggcatattgtaagtaaagagattaaaagttttattcacagtttgagatgacccaaaaa
gtagctgaataattaaaatttgtagtagcattttatagtcgttcagtagatagttcaaat
catgaatatcttggtaaatgtcaatacagtccagttcatataacttacaaaatcttttga
tacacctatggaataaatcaattaaaatattttcggtcaagtttgtaatatcatttacat
ctaaatttgaattataaatttctaaaagtatcttttccagttcagctaatatgattttta
ataagtttctttttacttcagtgcgaaaaacatctaaaattaacacttcctgagagcaat
tagttaatctcttattaatctttatagaagttctaactaatagctctgctacctcttgat
tgagtgttggtccctgagagcttggccaataattattcacgttataccatagtaagaaat
attgaatttaatgttatacgataacttttaatatactgaaaagcaagttaattactcta
tagcaactag-------tttttacagatcttaatagttatttatcaaataaagttatatg
atagtagcgattgagctaattttattaaaattta----taataatgacaacttattactt
cgcccttgcgagtcaaaattttctattagtacaagaaccactagaagaagtttttagaga
gagagttaactactatcagtcaaataataaagcaattgatttttggctaataccaaaccc
ttcttttctagagaaaccagaaatgatttcatttaaaaaccttgtacctaaagacgctgt
agctataatctctactaatccaatatttattaattggttaaagctaagaattggctatat
ctgtattggacaatttgaagataacctacaactttctgaagaatcgttaaatattactgt
tttaacagacaaaatttaacaatgcttttattatgactagtgttaaattattttggttaa
gttataattatagtcttttagtattta-gactcttatagtttatcacttagtattttatg
ctgagatggagtgacaaatagtgtaagtatttcttggctaaacgtttcagttgccttaga

tttatatctatttggattaactataatggatagcattcgttttatagtaacattttcaat
ttgagcccaatgtacaattccaagttccaattccttagcaatagctgaaacggaaacaaa
ggcagctcctagccctgattgcacagcgtttttgatagcttctatagaattcaattccat
ctctatttaaagcgactgctgtcaataccatgttgactgagtactttatctatcacttt
tctaattgttgattgagtatctaatgcaataaatctaagcctgtataagtcttcttttg
aatgtctcctagtttagaaaaaggatgtgatttgggtaatataagtgctaattcgtcttc
cgcataagaagtaacctgcaaaacatcttgcaattcagtaggcacttctcctccaataat
tgctaaatcaacttgaccattagctacgctccatgaaataagtctagttgaatgtacttg
caattgaacagccacttgtggatatctttgcctaaatagtccgattaatcttggcatcaa
atatgtcccagttgtctggctagctccaataattaatgtgccaccttgtaagttttgtaa
gtcgtcaagagctcgacaagtttcttcgcagagagctaaaattctgcccccgtatcgtaa
aagaagacttcctgcctcagttaaagttgccttcttattacctctttcaaatagggaaac
atttaattggcgctctaaattttgaatttgcaaactaatagctggctgagaaacatacaa
gctattagctgctttttaaaactgccctctttggcaattgcttttaatattcttaactg
atccaatgtaaatggaaggtctgtcattaaagaattgtagtataatg---------tata
ttatttagtattgatcaatgtatgcatttgccaactaaaatatttgtcattacgcttcat
tattttcaaaataataaaaagtataatgttaaataagctgtattaaaatttagtatctgt
ttgatattaaatttatacaagagatatcgtgaatttaattctaactataaaatataggag
acaat---atggactctagacttttagttgtactaataccagttttagcagcagcatctt
gggcagtttacaatattggtagagttgcactacagcaatttagaaaaatgacatcttagt
tttgcttagaatata-atttttagactttatgagatagaggactttaaaatccctatctc
tattgtatatcttaataaaaaaag-aaagc--ccccttgtacttactgttattacacatt
aatattattggt-ggtaccta----gataataagtttaagctaacttatttttatttta
taattaattaaattaataacacaattatggctgttccaaagaaaagaacatctaaagcta
aaaaaaatgcacgcaaagcaaattggaaaaatcaagcaaaaactgaagctcaaagagctt
tgtctttagcaaaatcagtattaactagaaaatccaatggatttatttataatctaactg
aagcatcagatacttttagcgattagaagtgttt-ttactacaagtctgaaaaagtatta
cgcaacaagttctcagtaatttttatctat----taatcatttgaaaatcattctatttg
ataataaaataaatagcagattaaataaaatcacattaacaaattatgcagcaaagctag
atcaaactagtgaaatttggttgttcaattgtattgaaaatattcagcatattttttta
aaagtcaattaaaatcaagtcatattactaaaattttttatttctggtactagctttgaat
ataccgcaggtctaccaggattattatccagcttaacactaagtggtagactacatccta
ttagtatatatagtccccagtctctcaaaaagtatcttgaagcatgtaccaaatattccc
aaactaatttttcttttcctattaattttcataatttacaatacggaggacaagttgtta
accaattttatacagtaatttgtttaccgttgagcaaaaagagcctgctgtatggattta
ttattctaaaaaaagaaaagcagggagtatttaatttagcacaagctaaaaccttgaata

ttcttcaaggacctatatatggaaaactcaaagaaaaggataatttttttaagtccagatg
gttactatctatccggccaagacttctcttctaatacaataatgggacataaaatatcgc
ttccgttattagtcagatattctagaattatttctgagatgcattggttttgctcttatc
ctattagattaaatacttattcgcatcagcaaggagtaaagtgtttaccacataatgttt
taactgatattatgaaatcccaaatatatcaagataacagttttgttgaataatacttat
tcttgatatataatggtctcggtaatctaaataaaatactagaaataatttcttaaattt
atttgtctatattaaatttacaatcatttatgacatacgcaattattgaagcaagtggca
cgcagctttggatagaagaaggccgctactatgatttaaatcatatacctgttgatccag
gtcagtcgattatattaggaaaagtcttattattgaataaaaaatggggaggttactttag
gacgcccttgtatagaaggggttacgataaaggctacagtaatgaggcacttgcgaggaa
agaagataactgttttcaaaatgaaaccaaagaagaaaatgagattaaaaaaaaggtcatc
gacaagaattgactcgtttaatgatcgattctataacatcttaga------taaaatcta
gactcatattttt--aa-aaactttttaatatttaactaaaatagatataatggcacata
aaaaaggtagtggtagtacaagaaatggcagagactctaattccaagcgtttaggtgtta
aaaaatatggtggagagcaagtaacagcaggtaatattttaatcagacaacggggaacta
aagttaagcctggccaaaatgttggaaaaggaaaagatgatacattgttttctctaattg
atggtttcgtgctgtttgaaaagtcaaatcaaaagcaaaaaacaattagtgtttattctg
ctaagaaatagttaaaacaattggtgcaagataacttgagt----gtatttatttcactt
acagtggcaccatatatttgatttattaagaaatggagttccttaatgaaccaatgaatt
ttattaaagatttgctgtgaaatttaatgacaaacactattgtaatttcctgtctacaca
atatggctgctattctatattgtggtcagatacagaaactagtagtagcaaatgctcatt
atcaagtaagtgatatctacctaggttgtgttgataaaatcttctcaggaataaatgcgg
catttattaacctaggaaagaatgagtacagtggttttatacatatcagtgataccggtc
cgcttaaaaagaaatattatgtcaataatattactaacattttaacaatacggcaaaaaa
ttttagtacaaatcattaaagagccaactttaaataaaggtccaaggctcactgccaata
ttacattatcaggtcgatatattgtattaatgccttttagtcaatcaatctgtatatctc
gaaaaatatatgatgaagatgagcgtcattatttgaaatctttagctattttaattaaac
cggcaacaatgggcttgctatttagaccttctgctgtaggtgtcgatgaggaaataatat
taagcgaattaaaaaaatctaaaagaacaatggaactttgttcaaaaatctgcaattaata
gttattcacctgttcttctatataaagatgaagatattgttaaaaaagtaatccgagatt
tttataataataatacaaacaatatagtaattgattcaaacctgggattaaaacaattaa
attattatatccacacttggcactgtaataactctagcacagttcctaagattaagcttt
atagtaataatcaatgtatactagatgcttttggtatcaatcaggcaatttccagagctc
tcattccaaaagttgatcttatacttggtggctatatgtttattgaaactttagaagctt
ttactattattgacgttaattctggatcttttaataattccactagtgcacgagaaacag
ttttaaaaaccaactgttctgcagcaacagaaatagcttatcagttacaaattagaaata

ttactggtgtaattataatcgacttcattgatatggaatcacaaagagatcaattgcaat
tattagaacactttaataaagagctatcacttgatgatgctaaaccacaaattgtacagt
tatctgaattaggtttagttgaattgactagaagaagacagggcaaaagtttgtacgagt
taattagtagtgattctaattactttatttttcacacaatcagagagatctcagtctc
ttaagagattcgatgatagacagcagaaacaacagattttaataaatcttggctatctg
cagagattaatactattaacaaggtctttttcaaaagtcaaatttgtgcagacctgcta
acttttacctaattcgtaatctctatatagttaaaagtagtattacttataaacaaaatt
atttattaactcacagatcaaacttatttattctaaagaatacagcaaagtattaccaa
gca------gttattatctagctagtctcaataagaatagtaatcaagagttcctatctt
aagattgttatatcaactgtt---------------------------------------
-------------------gaaaaaaagaagctctctcttatataagagagagcttc-tt
ttatagcttactaacta-tctagttagttaattaaaaattaaccgttaacagctggagct
gttagagctactggtaaagattcaccagaagctagatctagagggaagttgtgagcgtta
cgttcgtgcattacttccatacctaggttagcacggttgatgatatcagcccatgtgtta
attacacgaccttggctatcaacaacagattggttaaagttaaaaccattcaagttgaat
gccattgtgcttacagataaagctgttagccagataccaactacaggccatagacctaag
aagaaatgtagagaacgagagttgttgaaactagcatattggaagattaaacgaccgaag
tagccatgagctgcaacgatgttataagtttcttcttcttgtccgaatttgtaaccatag
ttagcagattcgttttcgcttgtttcacgaattaagctagatgtaactagggatccgtgc
atagcactgaacagagaaccaccaaacacaccagcaacacctagttggtggaatgggtgc
attaaaatgttgtgctcagcttggaatacaagcatgaagttaaatgttccggagatacct
agaggcattccatcagagaaactaccttggccaattgggtatactaggaatactgctgct
gctgctgctactggagcagtaaaagcaacggaaatccatggacgcatacctaggcggtag
cttagttcccactcacgaccaatgtagcaagctacgccagttaggaaatgaagaacaact
aattggtaaggaccaccgttgtatagccattcgtctaaagaagcagcttcccagattggg
tagaagtgaataccgatagctgcagaacttggaataacagcaccagaaatgatgttgttt
ccgtatagaagggaaccagcaactggctcacgaattccatcaatgtctactggaggtgca
gctacgaatgcaatgatgaatacagatgtggcagttaatagagttggaatcattaataca
ccaaaccaaccaatgtataggcggttttcagtactagtaatccaagagcagaaacgttcc
cacaagctagcgctttcgcgtctttgtaaagtagcagtcataatttttttatcaattttta
aggatttaacaaagatgcttcccaaacaataagttgtctgttatgaaaattattacttaa
ggccttttcaaatgagaacattttaaaaaaaaca-tagcaaaatctaatctcagtaact
aa-aattt-tacttgttcatatattgcttaataattcaa-----------taataaattt
tt-gct-------------ttttgcaaaattaacactattttagggat---ataataata
atgcatattaatgctgcttattttgattttaatagtttatataattttgccctacttaaa
tacgcatgtcacactttacaaaaattcagacgaccatacaagatttaaatctgttaaagc

atgctttaacagatttgggtttgatttggcaaatgaattctagtcatataaaagttggtg
aaaataatcagcataaggtagatattttaattaaacaagataacttgtcacatattggct
ttacttggaatgataatagatatcacttagttgccgacttacaactttgggaacaaccct
ggtctctagaagtattttttggataagttatctcagaagtatgcttactactctatcatcg
aagaaacaaagaagcaaggctttgaaaaaaatgcagcagatttcacaaaaagatggatcaa
ttaagttaattgtgcaacgctggaattattaactaacatacaaatgcgggcggagagact
tgaactctcacgagattatctcactagaacctaaatctagcgcgtctaccaattccacca
cgcccgcactgttgagac--aatattattctatcataatctatactaaaacacaaactaa
-tagtataaactagcttccaagcttaaaagcgtctacaaatagaccgtatgttataccaa
ttttgaagttgttgaataaattaattttatatttaattgcttgtggctagagtagacta
ttttactagtcaactcagttgctgcaactattagactggctgcaataataccccaatctc
ctgtttgtccaggaatcgtagacaaacctgttgaaataaaaaatcctagcaataaactaa
ttaagccagttgttaactcgctcaaagaataataaagcttattatttaagtttttatta
agtaagcaaaaaaagttgaaagtctagttttaatcatagtatttagtaacggagttttaa
aaggatgacaagtattaacag-tcattaatattgtcgtatccttttaaaactcgttaata
caaacagctgttcttgt---------ttttaaaggtcttgttcgcttaaagcattaataa
gatgctgaaacggctcttgtataatatctgtgcgtttaattgattgcaaatctaattctt
cttctatgatttcttgtaataactgtatgcttcttactgttggagcaacaggtacagata
gtgaattgtaagtatcttttagcccatttaatacacgctgatcgagaatatttgtactac
cagaaaccaaggcataactagcataccgtaggtaatattctatatcccttagacaagctg
catatcttcttgttgtataagaatttcctccaggccttaataattcaggctgttcttcgt
acaactgagctgcggcctcttttaaaatattggtagcttgatcattaataatttcagcta
tttttattcgatctagtccactcgaaaagaaagattctagttgtccgacagctgtcttat
ctagataacgaccagtaagatcgtaacgatttaatattgctgttatagcatcttgcataa
attaattctcctac-ttgctatcagagcggtattataaatgactaatattgtaatataat
cattagtgtttatgaaatttatttaaaacttattctttattgcttaatgtgcaattaaaa
actcgcatagattttacacatatagagtatacgtagtttttaatta-tttttttataaaa
accaagaaataaagctttttt-taagaggttcgtgaatttgttaatataacatgagcttt
cctcctaaataagttttgtaaagtac--aaagtaaaaaaaacgattagtcaataatagtaa
----

--------------------------acaatattaatatgtatcaa--------------

-----------------agtataaataagcttttactccagcatcaaaaaaaatatttga
gatatgttgatacaactgattattcaagtgaaaacttagatttagaccaatttagctatt
ttatcattacaattaataaaactgataaatgtattcttatagagcaattttgttataatg
ctgaaggccatatttactcaatattcttcaaaggtccaacagctaaacatctttcttcta
taatttgtcattttcaacaattaaatacaataatatctacagctcatgccatatatttag
gacgagagctaatgaagtcagaattagctctcgtgctagatcaacaatatattcaagatt
aaa--ttttgtaagtattaattatatttatgctagtataatattagtaagggcatgtaac
tcagtggatagagtatcagattccgattctgatggccgtgggttcgaatcccgccctgcc
c---gagtactcagagtact-atactaaaacatct-------taatagctagtttgataa
tagtatgggcattggggctgcaaggtttctacattatgaaaagaagaaaatatg--aaaa

aaacaagctctccaaaagagcttttagtacaatcaataaatgcagaaaacaatattgttt
ctttttctcgaaaattagcttttgcttaaacactgttaatttttggcattcgacatgtta
aactcttattcatggcgaatattctgtcagagtcgctcttagtttaagaaaagtcagaaa
aaatatttatctttacgtttt-tacctattttatttaaataaaggttagta-ttttcaa
cttttatagtggacgtgggttcgagtcccaccagctccatcaatttgatt---------t
gtataattagctagtttaaccttttaataaccagatatataattggaggaaatatgggt
ttcataacaattactaagcccgctttaaagcaaattgcaatcttgaaaaatgatcatgaa
aatgatgtacatctaagaataggtgttagacaaggcggatgttcaggtatgtcatactca
atgaattttgaacatgttgacaaattaaaagatacagatgaacggcttcgtctagataat
ttttctgttgtctgcgaccctaaaagtcttctttatctttatggattatcattagatttt
agctcggaattaataggaggaggatttcaattttctaacccgaatgccagccaaacttgt
ggctgcggtaaatctttttcaggctgacc-ctcgaacacgtttttttatatattttgtatt
atatatattattttagcaagtcg-tttattagagacaagcaatgctctgttaaaaac---
---------------------------taatttcaagagttttattgtattatt-----
----ttatatatttattatcaactacttgaaaataaattagctaagcaatctaatttgtt
atcagtaccaaactttaaatgattaacttttattgatatatgctaatcttt-atcagcc
taaacatatctgtacattacaatcttttgacataaaaagagtcttagcatacctctcaa-
----------------------aaataaattcaa----------tattttataaatactc
aacatgaatctgtcacaacttatcgttaattcttctatgatgaaaattcctaataacttt
tattgcaatataaaagctatttcttctgagaatttaagtcacttacaattatcaaaaaca
aatttattatctcctacaaaaataattgcttttggactacctaaatttgattttatttct
acacagattacaagttctattagagaaacagttgttgtgtctactattgatgataataat
ctttttgctgccccaactaatgattggataaaaaaacacaaggataaaaatcatcattct
ttgttttataaaaaagtttttaaagctttgctaaaccataaaattagagttgttgctgag
ccact------tcataactccgaaataaataaagaaaagagcataatggcaagtcaaaga
tacatctggggtaaaagctggaagccttcaattattttatcttgcttaaaaaacaaaaaa
aatagcaaaatttctgatgcatcgaaaaaatttttaactgaacagttatctgcatcacca
gtatttgtggtaaaaaatggttttaatgaaattattttaggtcacccattatctcgtgtt
aaaagaggaagagttaataatctgatgcatgcgttttctaatttattaaatcagtctaat
gcaacttatcctatatctactggtctattctttttttcatccagatgatgcatttgaattt
aaagactttataatatcggtcaatccactggcggccaaacatatggagataagcgtagag
cctgttggtctacactttgcttataaaatgaatagaaatatatcgtcagatactcaattt
tcgtttattccagattttaaagaagtaggagatttattattcaaatatagaaaaggtaat
catttagtttttcataagaatcagcattatggcaaagatttttttcaggggcaaccaata
tatatgattcagccgatcacttttaaagatcgagccggaaagctaaatattatcaaattt
acggggctaaatgacacaagagaaattatttttactaatcttgaagctgctaatagatca

tgggcacattttataaagaggaattcacaattaaaatcaattaaaaatcctactctatta
gtctataatttagaaagtttttaaaagatcaagaacgattggataatcaagatttaaat
aagtttgtggtgatcactaataaagaatcttatcttgccacaaaagagttaatagcttta
cccgattctaatagcttttctaagcacttaaaattaaatataaagcctaaactcttttt
gtgaaactatgggtaagacggttattttccaccttaacttatgaat-aaatgctatt-gc
ctttattttctcgaataatattctataactagtaattcattgagttgtaaagcaacccat
tccctatcaatgactccattgactttccctgataaattggatttattcaattctaagtgg
ctaggaatattagctagtccagggaaagctaagtaattttctactagttttcgagatgct
tcttgatttttacactaattgattctcctggtttacattgataactacagatagatact
acttgtccattaatacaaatatgaccatggttcactagttgtctagcggcaggaattgta
ggagccataccaagtctaaaaacagtattgtctagcctcatctctagtagctgtaacaaa
atttgacctgtcgacccttgtagtttttagctgcttttacgtacttaaataactgtttc
tcacttaatccataattaaatcgcaacttctgtttttcttctaatcttacagcatactca
gaaggttttctggacttttgtccatgttctccaggtggataagatcttttaatcgctttt
ctactgagtccgggtaaatcacctaatctacgggaaatgcgtactcgtggtcctctgtat
ctagacatattatttaaattctcctaatgtaaat-gattaag----------taatgagt
agctatttttaacacaaaaagagcattaaaacaaagc-ttttgctaaaaaatatttaca
aaaaatataggtttggacaaaa---tacattactatgc-tttttaagcgggtagcgggaa
tcgaacccgcatcattagcttggaaggctaaggttttaccactaaactatacccgcaata
ttcaacagattgtcactaactcaatataacatagttatgttaagttaagataatcttaaa
tttaatagaatt-ttaaattatttatgctccttctaaaaccacatcaagaaaacgagcaa
tttcggcagcttgttcttcaacttctgataataataatggttgaccgaccctagttaatg
gaattactcttttatcttttgtacataaataaatttcacgtcttggatttagcccctctt
taatatctatttttattgatttgatttcttttatattaaactgaagacagatttttctat
tctttccagggaaacctagccgaaaaatttttacaatacctttatctttgttaaattcat
tatatccagcccctatattccaaataatagttagccacagaaacaaactcagaaagactc
ctatactgccatagaatgtcattacaataccttgtggaataaataccaaatcggttgaat
ttgtaaaaggtaacaaatcaacttgaaaataacttgatagtccagcaagaagaaagccca
aagccccaataaatattatagtagcccaccagtaattactcagtcttcgtgaacctaaaa
ttaaatcttttctaactgagtgtatagataatgtttttttcataatttgttcttttaaaa
cagta-tactaatgcaaaatggaaaaattttgacaattttt--atgcgcgctttagattt
tttaaataagcttgattgcttttagtcctaaaaacagtcctgttgctaacccaaaaaaag
cagcaagaaatattatatatcctagaaaaactgaca---tattattatttattattattg
ttacaatagtgtttacaaaaatagtgaaaatctattactacggtattatattacgttaaa
tgccttgctgtctacaggaaaaaactttttcagtttttagcattgtgtttatattagaa
ataatattctagtaaatttattaaacttctggagaacagaatcttatgtcagaatttatc

aagccttataacgatgatccttttgtaggcaatttgtccacgccagttagtacgtcaagt
tttagtaaaggacttctaggaaatctaccagcttaccgtcgaggtttatctccgcttctt
agaggattagaaataggaatggcacatggatacttttttaattggaccttttgataaattg
ggccctttacgaggtacagatgtagcgttactagcgggatttctatcttcggtcggcctc
attattattctcactacatgtttatccatgtatggtaatgtatcttttactagagcagat
tcgaaagatccactacaaacttctgaaggctggggacaattcactgcaggatttctagtt
ggagcagtaggtggttcaggatttgcttatttattgctagctaatatacctgtattacag
actgcaggtcttagtttattctcttaagctagtaaggggacttgaacccgtaacctactg
attacaaatcagttgctctaccaattgagctatactagcattcagttcataataacatat
aaagatatttgttgcaatctgctattttcagatgtaacaaatatcttttttatttaaaca
gaactgttatgttaacac-------gtatattaattagaatctgaattatctgactctgc
agaagattcctgatcacagtttagataataacaaattgtttgatctagacaagttgcaga
ccaaccaacaggtgtttctcctgataattcttgcatatctacagaattataaaaattatc
atttggcttattactgtttgcttgcataaatatcactcccaaaacactctgaacttaatg
tgaagtaatttaacttctcaataatatattaacacaaaatattaaatatggtgattattc
tttaattgtaaagatgctgctaactttataaagtttgagtgagagtcttgatagcttttg
ttgaaataagcattttaacccatttattttgcccgctcgaccatacttttttgtgttgta
aattaactttttgtagtttttttgttcgtttatgagaatgagatacggcatatccattat
tagctacttttcccgtaagctgacactttttttgacataatttt-------ctaacttata
tatttatttagagtacttgctaaagttgacttgggcacagctccaatcactgtgtctact
ctttctcctgctttaaagatcattaaagtaggtatacttctaatgccatattcagccgca
atagtaggattatcgtctgtgtttatttttactaccttaatagatgattcatattcttcc
gcaatttcatcaactacaggagaaaccattctacaaggaccacaccatggcgcccaaaaa
tctacgagtacaggtaagttattgttaataacttcttgtttgaaagaggcatctgtaact
tgagatactgacatattctttaacctttaatatatgttccttgctaagcaaatcttatca
caaatttcaagaaaaatcttctatagtgaattactattagtgttgaattattttttttaaa
aattagacattaaaaaaacttattacctatataaataaagtggtggccgagtgatatctt
gatagtgataattttttatattaaggttgaacactagaaataacttttcaagcaaactctt
atttgaaaa--atttctctgtgaccataaaaacttaattatattacaatatattttatta
tttgtaacttagttctgataatggtataaacaacgcaaaagatactgccttataatcaag
gaggaatacatgtctcaatccgtagaatcacggactaggattaaaagcgaacgttacgaa
tctggagtaatcccttacgctaaaatgggctactgggatgctgactatgtgattaaagaa
acagatattctagctcttttcagaatcactcctcaaccaggtgttgacccgattgaagca
tctgctgcaattgcaggtgaatcttcaacagctacttggacagttgtatggactgattta
ttaacagcttgtgacttatacagagcaaaagcatatcgagtagatccagttccaaacgtg
gcagatcaatattttgcttacatagcttatgatattgatttgtttgaagaaggttccatt

gcgaacttaactgcttcaattattggtaacgtttttgggtttaaagctgttaaagctctt
cgcttggaagatatgcgtatgccagtagcttatctaaaaacgttccaaggtcctgcaact
ggattgattgtagaacgtgagcgtatggataagttcggtagacctttcttaggtgctaca
gttaaacctaaactaggtttatctggcaaaaactacggaagagttgtatacgaaggcctg
aaaggcggtcttgatttccttaaagatgatgagaatattaactcacaaccatttatgcgt
tggagagaaagattttatattctatggaaggtgtaaataaagcatcggcttctgctggc
gaaattaaaggtcattaccttaacgtaacagccgcgacaatggaagatatgtatgagaga
gccgaattctctaaagaggttggtagtatcatttgtatgattgaccttgtgattggttat
actgcgattcaaagtatggcaatttgggctcgtaaacatgacatgattttacatttacat
agagctggtaactcaacttactctcgtcaaaaaaatcatggtatgaacttccgagttatt
tgcaaatggatgcgtatggctggtgttgaccatattcacgcaggtacagttgtaggtaag
cttgaaggagatcctttaatgattaaaggcttctacaatactctacttgaaagcgacaca
gatatcaacctacctcaaggtctgttctttgctcaaaattgggcttccctacgtaaagtt
gtaccagtagcatctggtggtattcatgctggtcaaatgcaccaacttcttgattactta
ggtgatgatgtagttcttcagtttggtggtggtacaattggacatcctgatggtatccaa
gcaggtgcaactgctaacagagtagcactagagtccatggttatggcaagaaatgaaggc
cgtaactatgtagcagaaggtccacaaatcttgagggacgctgctaaaacttgtgggcct
ctacaaacagctttagatttatggaaagatattagttttaactacacttccacagataca
gctgatttcgttgagactccaacagcaaacatctagtttaatgactacttactgatactt
taaatagttaattgtaagtgaaattaacttataacaataaggagcatagaatagtgagac
taacacaagggacttttccttccttccagatttaactgatgagcaaattaataaacagc
ttgcttatatcgtttctaaaggcttatcagcaaacgttgagtatactgacgatcctcatc
caagaaactcctattgggaactgtggggtttacctttatttgatgtaaaagatgcttctg
ctgttatgtacgaaattagctcatgcagaaaagcaaaacctaattattatgttaaagtta
acgcttttgataatactagaggtattgaaagttgtgtaatgtctttcattgtaaatagac
ctgctaatgaaccaggattcttattacaacgccaagacttcgaaggtagaactatgaagt
atagtcttcatagctatgctactgaaaagcctgaaggagctaggtattaatattaattct
taattaatattggctaatta--ttcccctcttaaaaatcataattaatttagtaattgtg
atttttaagagggatagcttcccaagtaaattttgactaaa-tagattgaataattaaaa
atacacagaaataatgcaatcacaggatataatttccaacgatactcttgttaatttaca
agaagaatatgatagaacacaaatccaagaagttttaaatgagttaaatcaagaacttat
aggattagtgcctgtaaagaccagaattcgcgaaattgctgcgctattattgattgacag
attacgcagaaaactagaactagtttctggtaatccaggattacacatgtcatttacagg
tagtccaggaactggtaaaactacagttgctatgaaaatggctgatattttgcacagact
tggatatataaaaaaagggcatttgttgacagttacaagagatgatcttgtaggtcaata
tattggacatactgcccctaaaactaaggaagttcttaaacaagcaatgggaggagtttt

atttattgacgaagcttactatctatataaagcagataatgaaagagactatggctctga
ggcaattgaaattttattacaagtaatggaaaaccaaagaaatgacttagttgttatctt
tgctggatataaagatagaatggaaaaattctacgaatccaacccaggactctcttctag
agtagctaatcatgtagacttcccagattatacttcagatgaattattacaaatagctaa
aatgatgatagaagaacagcagtactgttttacagaagaagcagataaaactcttttaga
gtataccgagcgaagaatgaaacagccttatttgctaatgcaagaagtattcgcaatgc
tattgacagggctagaatgagacaagccaataggatttttgccagtggagaaaaagtatt
aacaaaagctgatttggtaacgattgaagcagaagatatcttgaaaagtagattattttc
attacctaatgcttaatatacacgtgatttcatgaaactatatattattaaaaag-tttc
ataatcagttgcaaatttcttgaaaaacctttattattatcttaatgtaggtg-tggcgg
catagccaagtggtaaggcagaggattgcaaatccttcatcccccagttcaaatctgggt
gccgcctagtactaaaaaagggggtgtggtggaatggtagacacaacagacttaaaatc
tgttgattttagtaatcgtgagggttcaagtccctccaccccatatattgatttataa
ataaaaagagatgtgtatttgtattaattgcaaccatattaccaaatgtaatacctatca
cttaatagaatctcaacataaacagcctcatcttactagaagtccattgtttatacctaa
atatcctgtagttcatgttaatatatctaacaactgtacctataatcaaattgattggga
tttagtggagtgtttatcatttgtagaaaaaccgaatagttggaatttagacgctaatta
gttacaagaaaaattatgtaccataagcaacaactctatttttagatactcggtattaa
ttttggatgttctaaccagtaagattttacccgttcttgctatctctataatgccaaatt
tagttagtaattgttcaatagcaacaatctttcccggatctccagtaacctctataatta
aaagatcttctgcaatgtctactatcttagctctaaaaattcttacaatttctaaagctt
ctgttctagtctgagaattgatctgaatcttaattaacattagttctctttcaactgaag
gaatatttgttacatcttgaacgttaaggatatttactaacttatataattgtttcgtaa
gttgttcaatagttctattgtctccttgaactaccattgtgattctagagaccccaattt
gctctgctggtccaactgctaaacttgcgatattaaagcctctccgggcaaatagaccag
atattcttgacagtactccggcttcatcttgaactaaaactgataaggtgtgtttcatga
aaaattaaatttaatctatttgatctattaagtatattctaagaaaattacagaaaaaat
actagttagtctataactaaattatccaaactaaacaaaatcctactatcagataatata
tctaccttgtaatgctatactattaacttagtatttaatgatattctaattgcaaataga
atgct-aatttttttaaatttgttaggaacttgtgctagaaaaatacaaaaacagtaaaa
aactctctcgagatttacctcaaatcaatgatcgcattagatttccaaaagtccgagtaa
ttgatgacgaaggtgaacaactaggtatttttgtgcctgaagaagctatacaattagctg
tccaacaaggtttagacttagttgttgttagtgataaatcggacccgccagtatgccgaa
tattagactatggtaaatataagtttacacaagaaaaaagagctagagaagctaagaaaa
agcaacataacagtagtattaaagaagtaaaaatgcgatataagatagaagagcatgatt
ataaagttagaataaaccaggcatccaaatttattcaagcaggagataaagtaaaagcaa

ctatcacatttcgggggcgtgaaatccagcactctaatttagctatagatttattgaata
agatggcaagcgatctaaatgcaatagctgaaattcagcaagctccatcaagagatggca
gaaatgtcataatgctcttatctcccaaaaaagttagctaaactaattttatttaaatg
catctggctggattcgaaccagcgacgtctctttcgaaatggcggattatgagtccgctg
ccttcggcccctcggccacagatgcatcacttaaagtttataactcataaagcatataaa
aatcaatgtttagctattttaataaagtctataaaagtctattattcattcatattgtg
atatgtcgcaactgctgaaggggaaacttgatttaagtatcgaaatatccagtatttaaa
aattgtgtcgagtataactggaaaagttgaaataaataaaaaaataaaatctcgactttc
cggaagtcctaaatgtcttaaaattacttcaataataacttcccatccatgaggagaatg
aaaacccacaaacatatcagtaaaaagaatgattaaaaaagctttagctgtatcactaag
accataaataatttcatttaaaaaagatttaacgactgcaatttgtctttgtccagttat
cattagtaatataaagacaagtattgatacaagatcggataaaatatttttaacagcatt
tgcactttcgtttgcataatattctcccaactctctagccttcagctgcactctttttc
aatgatttcgtaagatatatcttccgaaggattcagaagaacttcaaagtgaattttctc
ttcaaatctttgtaattcagcaaaagctctttcctcttgagaggaatttaaaaaaatttt
aggctgttcttggttccataaataatcaatacaaggtccaaatacaaaaaattttgaagc
ctgattaactagtactggagagatgaataataataaaatatattttacggaagtaatagt
ttggtgtctagatattctaaactcctctatagcttcagattcgccattaggatctaactc
tttacgaaacttttcaaaagtattagtaattgacctaggtattggcccaactttttcaaa
ggcgaattgattatttcgttttaaattccaatatttcatttttattgctgctaaaattt
ctattaatcaataattgatcacgtattcactataaatattaattctagaactaaacatat
aaaacaatatttatgaaaaataaaatacaattaaaaattaattatgcattagttctatct
gcagctacagcagtttatggatataaatcttatcagccaactataaatacactcttaatc
aaagatgaagaaaactggcctttttctctaaaaggcagactgcctttattcctataatt
gtattaaaaaaaaatcatcaaaatccctgaactcagaacctagaaatagtaagttatatc
ttaatggaattggaagtagaaaatttatcgacagaattgtcactctacttggatatttag
aaaaacaggcaaactatactgaatacttgaaccaaataaaaacaagtggttatttttctt
tagtctacctagacagtgatataatgtataagaaaaatattagtaacattttttactac
ctaatgatattctaaaaagaatctatatttgcaataagaaaaaaaagcttataccgcatt
cattcttaataaatttatttcagaaacagataggctatccaaaaagttttgctaatttaa
attgggccttctctaaaattataaaatggtattatgatagaggataccaatggtctctag
ttgaagttaaacaagcgtctgatgcatcttctattgtaattgatattcatgaaggggtag
tgaagacaattataacagaatactacactctatcttataaaagagtctcaggtatcttat
gcgtagagtcaatagagcagtaccttggagtaagagtgggagctccactaaatataattg
atttacaaaaaaaaattacttacttaaaagataatcaactagttggcgacattatttata
gtattgaacgatcaaataatagctcaatgagtttggatatcaaatttcaaatacaagaat

taaaagataaagagataatagtgcttgcagaaagttcttcta------------ttatct
cgcatgcatgtaatttactcaatc---aatataga---------aataggctagtagctt
ctaatatagtttcactatcaactaataaattacacgcatgctattttaattataaacttg
actatcaatataagtatataaacacctctaatcttataaaattgttagcttattctattt
cttgtaggaagacactacttattcattattttaatttacaaactttaataagctgtacaa
aaaaaaatactattggatttcagctatatttacgaaatctaagttttgggaaagctttct
gtgtgcttagtatgaaatttatcaaaaatggacttaatataaaaatttatatataaatc
cttcattgatagttgatcagaattttgtatttcaatttgctatacaaatcattaagcaat
atcatactgcta------aacctccagctt------------------------------
------tatttttaacgaatctagatcttgaacaatatgttgctgaaagtttattaatgt
accattttacatcttgcttctcaatatctgaaaaaatattattatctcgaattatgcata
cagattctttattttcaactctgaaa------attttcattttgatgatagaactaacg
cgatcaacagttatgatacttttaaacaaaatacaaaaatatttatcaagagtttcttt
ctttattattaagtttacgttatcaaaatttaattatttaggttggcctttaaaaggcc
attttttgaaataaaatctttatacttggctccatttcaaaagagtg------------
---attttctgatagtcgtaagacgctattcttccacaaaatgtcgttaaagcaagtat
ctaattttaatttaccagtatcttttaaaagccaccttaaccatattttagttagtacta
ttaaatgtcagtcaaatttgaatatgagaactgtatctttgctattaattgattcacctg
ctgaatatctgctatacaaatctattcttaatttctctattaaagttaggatgcagtatt
tcatacctatgagcaacaatataagattatctctattttataattatttagattgttttt
taataagatcatctcagagttgcattcacatatggcaagatctaagaactcttacaccaa
ttcaaaatttttggttaaaaaaattttcatatggagctggaattcagcttaaattaccaa
ttaagcagatgccacctctttcaattgaatatactgtaactagtagtcgctacttttgta
tttaccttcgtacttattatcaacgataactattactaaaatgaaaaatttaatttcttt
ttttgatcgcagcaaagggaaatggatttcacaaagaacaacttatgaattatctaataa
aaacatgagttcggtacagtctcagatgacaatgaaaataggcaactcattgtcaggatc
tataatacttgcatcgttaaattgggggacatttatagacaagttgcccattacgctaa
aaatcacagccggagtgaatatgataataagtttaacttgcaattcggtaatcaactaaa
taatcataaattattaacgctgtgcatagtgacagatcctagtctaattagttttaaaac
tcgctatggaagtactaccatagatgaaacatattggttcgcaacaaataatttacgtct
aagtactagtattgttaaacgattcaacacttgtgtggcagtatctttttgttcagaaat
taaagtttaa-actctatatagaagcaactaatagcctttta-ggctattagttgcttcta
tgtatctaaattttgtattaaaaattat----aatactatattattctaaatctttacga
ttgggattcctggaaggatcattagagaggaacccaaagacaaacaaagaaataaagaaa
ataacggtagtatatacaaagattttcaaagtaaacatattttcatccttaaaagctatt
agtgcg----atatttacatatctccacataattttatatcatgatataagttccagcac

tattttataaataaatcaatatctttatttttagtaatgatttatatttaaatatgcgt
gatgtactaaatactaaatcttttccgacttgttttgtcttgtttgtaaatattttgtta
tttctatggagtttaccttcaattatcacatagtctaacttctttagctgtttaaaagta
tatgaagatttttgttccaaatagttagattgattataactaatcgttttctttttaac
agtcttgcttttaacttaattatttggctttttatttcactaattctgatactcttacaa
ctaagaatttggactagaagagtacagctattcataatttataacaa------tttttta
aaaaatctactaaaatagatgattttacagaactaaaatgatatttcattatcttgttga
ccttgctcataagtaatatctttcaatacttttaatatttttccaaaatactcttgcagg
tatttttctaaagatattacttccaacctgtcaattccaagaatactgtaaacttcatcc
atcggagctgtaaattgctctccgctacttaaaatttcggcaaatgctaaacgatcagaa
atattccatgtccattgaagagttttagtaattcttcttaaggcttttaataatccaatc
ggaatttgtgagatctgtgtttttttgcccagataatttttcacataatttaataatttca
gctgatgtccaagcggtattaccaactaacggcagggtcctattttctgtggaaggtact
ccaagacttttttataactaacttggctgcatcttgagtatcaatgtatgcaatgggtgta
gattctcctgtaacccagactgatttcttgtctaaaataggaatggcatattggttgatt
aaaccttgaaaaaatcctcctaaagaaaacacagtatatttcacattggacttctgaagg
aaatctactacctgagactttaagttcatcaatggaacatctgggtatttctctgaattt
aatattgaaaagaaaataaatctttcaactttagccgctttagctgcttcaattaatgca
attttcccatctaaatctatttttttctgcattataaggatcagtaggacgagatgtagaa
gcatctatgattgctgttaccccacaaaaagattgcaaaatactttcgggtaattttaaa
tcaccatatataagttctgctccccactctttgagaaaggcagactttcttaaatttctt
accatgcattttacattgtagccttcatctaaagctcgtcttacaatttgacgtcctaaa
gttccggttgctccaattactaaaagagtcatattagttaaaaataagtatgtataaaga
cattgatttggttgacggcagagagtaaattaggtagagagggaatcgaaccctcatgac
cgaagtcgtcacattttgagtgtgatgcgtataccaatttcgccatctaccctattatag
accaactactacaagtaatttagtctttagcgtaaaaataatactaagtttcaatagata
tgtcaacttttaaatttatattaataaaaaaacggagtctcttatggccacttttgaatt
aattccttatagattgtatttgagtcaatctaacacatggatccatagaataaaagcaga
aattaagatatatatagtagcgcttttatggatttcaattttattttttcttactttaa
actatgtattattgctttaagcttaattgccataagttttactataagaagtaaacagaa
tattattcaaaaacatttgttacaaacgttattaatgacgttcttaactactgttttgtc
ttttagtgtggctattagttataaacaatatgcagaacaagaacaatcgcagtatttatc
tgattccaagaaatataaaaattccagcagctactattatatacaaatagcgaacgatca
aaggatcaaaagacaatacttaattactactttaaagccttccttgtactttttttattac
tatatattctattaaactagttatgataacaacctctccagaagttttagtaattactat
ttatagatctaggataataaataaaatatttaaaaatgaattgctatttatcttccttct

ttcgtcacatattgttactagtattattaatagaatcgataaagtgattcaagtaactag
cttaagaggaagcttgaatttatataattcattgacaaggcctttaatgttttctttgtt
aatatttcaagtcttctttttggagattattcgggagtcaaaagaaatagctcaagctct
ttatactagaaatctcaatcaagaaaacaataatttttgaaaatatatacagtaaaatc
taactttagtgatcggctcaatataattattagcactttgtactttattattttagctct
agcgtaatagaatccacttattcttctgactctagtaatacgaagagtaataaaattagt
tagtagtttaaatagatagaat-taagatattatataaataattaaatgtttcattatgg
tcaatactcaaaatcaaatttcacaaacttcagaacttgattatatagttaatcaacctt
ataaatatggctttaaaacttctgttgaatctgagcaatttcctaggggaataagtgaag
atattgtccgcttgatctccaagaaaaaagatgagcctgaatatctattgaattttaggc
ttaaagcatataaaaaatggaaaaagatgagtagtccgtcatgggctcatattaagcatc
cgaatatagactttaatacgattatttattacgctgttcctaaattaaaaaaagaattga
aaagtctagatgaggttgatccagaaattcttgacacttttaacaagttaggtatatctt
taaatgagcaaaagcgaatttccaatgtagctgttgatgctgtttttgatagtgtatcta
ttgcaactacttttaagaaagaactgtctgaagctggtgttatattctgttcaatttctg
aagctataagggattatccagaattaataaaaaaatacttaggtactgttgtaccagctg
gtgataattattttgctgcattgaactctgcggtatttagtgatggctctttttgctata
tccctcctaatacagtttgccctttagaattatcaacttactttcgtattaacaacgaag
aatctgggcaatttgaaaggacactaattattgctgatcgtggcagtaaagtaagttatc
ttgaaggttgtactgctcctcaatttgacacaaatcaattacatgcagcgattgtagaat
tagtagctcttgaaggagccgaaattaaatattctacagtacagaattggtatgctggta
acaaagaaggtaaaggtggtatatacaattttgttactaaacgaggcttatgctcgggta
acaattcaaaaatttcatggactcaagtagaaactggatctgcaattacctggaaatatc
caagctgtattttagctggtgaaaattctcagggagaattttactctgtagctttaacaa
ataactatcaagaggctgatacagggactaaaatgatccatattggtaacaatacaaaaa
gtagaattatttctaaaggtatttctgcgggtagatcgaaaaacagttatcgaggcctag
taaaagttggccctcagtcatttaattctcgtaattattctcaatgcgattctttattaa
ttggtcaatcatctcaagctaatacatttccttatattcaagtacagaatccaacatcaa
aagtagaacatgaagcatccacttcaaaaattagcgaggaccaaatttttttattttttac
aaagagggatcaatttagaagaatctattgctcttatgatcagtggtttttgtaaagatg
tctttaatgaattgcctatggaatttgctactgaagctgatcgtttactgagtttaaaat
tagagggaactgtaggatgagccagactattttagaaatcaaagatttatatgcttctgt
tggtgaaacaacaattttaaaaggggtaaacttatctattcgagccggcgaaatacatgc
gattatggggcctaacggttcaggtaaaagtacattatcaaaagtaattgcaggacatcc
agcgtattcactaataagcggagatattttattttttggacaaagtattcttgaaatgga
gccagacgagagggcaaaagcaggtatttttttagcttttcagtatcctgttgaaattcc

tggagtcagtaattctgatttcttaagaattgcattaaatgctcgcagaaagtttcaagg
attatcggagtttagccccttagaatttttcaactaataacagaaaaaatagatcttgt
tggcatgcaagaaagtttttgacaagaaatgttaatgaaggattttctggtggagaaaa
aaaacgtaacgaaattcttcaaatggctttactagatagcaaaatatctatttagatga
aacagattctggactagatattgatgcattacgagtagttgcaaaaggaattaacacttt
agctaaatcaacaaattcaattattttgattactcactatcaaagattattagattatat
tattccagattttgttcatattatgagtaacgggcaaattgtaaaaactggtagtgttac
tcttgcccaagatctagaaaagcacggatatgactggattacgcagacataactatttac
tac-------aatattgagactaaagaatattctttagtctcaatatt-tttgcaaatgc
aaagtt-aattatttaagctttatcaaaaccttgcttaacatcttcaattgctcttttta
gcaattcttcactaactggttctaatttttagtactacgaacattttcaccaaattctg
gtttagaattctttaaatcttctcgaagttctctaataaattcagctacttttgaaactt
caatatcatctagatagccattaattccagtgtaaataatagcagtctgttcttcaactg
gaataggagaattttgggcttgcttcaaaatttctcgaagacgttgacctcttgctagtt
gattttgggttgctttatctagatcagatgcaaactgagaaaaagcttctaattcagcaa
attgtgctaactctagttttaacttgcctgccacttgtttcatggcttttatttgcgcag
cagagcctactctagagactgagatcccaacattaatagctggtctaatccctgagttaa
ataagtcaccagatagaaatatttgtccatctgtaattgaaattacatttgttggtatat
atgcagatacgtccccagcttgagtttcaataatagggagagctgtcatactacctccac
ctaattcagcattcaatttagctgctctctctagcaatctagagtgtaaatagaatacat
ccccaggataggcttctcgtcccggaggtcttctaagcaaaagagacatctgacgataag
cttgagcttgcttagttaaatcatcataaataactaatgttgctttacctttgtacataa
aatattcagctaaagccgcacctgtataaggagcaatatattgtaaagtcgcaggactat
ctgcattagctgcaactataatagtataatcaagagctcctttatcctgtagtgaagata
ctacttgggctactgaagatgccttttgtcctatcgcaacatatacacaaacaacatctt
ggcctttttgattaataattgtgtccaatgcaacagctgttttacctgtttgacggtcac
caataattaattctcgctgacctctaccaattgggatcatagagtcaatagcagttatcc
ctgtttgcataggttcacacactgattgccttccaattataccaggggccattgactcaa
ttagccttgttccattacttgcaggttcacctttatcatctattggccgagctaatggat
caacaactctacctaaaaaagcatctccgaccggaatttgagcaattcgaccagtccctt
ttacggaactgccctctaaaatatccctaccatctcccatgagtactacaccaacgttat
cactttctaaatttaaagcaaccccaatagttttatcttcaaattcaagtagttcaccag
ccatgacttcatcaagtccatatactcgagcgataccatcaccaacttgtaaaactgttc
ctatattggctacttctacatcctgatcatacttttcaatttgttgacgaataatactac
ttatttcatcaggtctaatatttaccatatttttaatattatttttattaattggaaatt
ctaaagcggtaattagtcagactaatgctatctgtctttttttactttattttaagttact

acaacatcaaggtgtgaagccatttgtcttagctgtcctctaatactagtatcaatgact
tttgatccaatctgaatagtaaagccaccaattagctcgggctcgactgaaataattagt
ttaacttctttagccttagtcataaccttgatttttctgttaatagagtttcctgatca
gggcttaaagcaatagaagtattaatatttgcaattgttaaagattccatttggtaagcc
agttccaaatacttactagctatagcatctaacattccaattcgcttgcgatcaactaaa
atcattagaaatgataatgtattttcgttaatttgatcgccaacagttgcaataattact
tctttttttgcctctacagtttttaatggattagct---aaaaaaatttttagcttgtca
gattgcgctaaaatattttgtattgactgaatatcttggctcaccttttcagtaactttt
ttagttttagctaaatcaagaagagctactgcataaggctgagctatttttataacaaca
ttgttactgctcatagttgatctcctaacttagcaatattattatcaattatacgcagtt
gcatttcggagctcatttgattttccagctgcaaagtaactcttttaagagcaagaaatg
ttatttgttgctggatttgccttcggatctgtttttcagctgtttctatatttgatttac
ctgttatggcaagtctttcaatatctaattggccttgagctaatatggaacttctaactt
ttccagctgttagctgagcttctttcttaatttgatctataatgatttgagtttgagcaa
gttgtttttctgattctgacaatctagcacttgcttgctctagtctttcttcggattctt
gtatagctgctaaaacttttatctgtcttgcatttaaacttgatccgagaaattgcttca
aaacataaataagaccaaataaaagcaacaatatattaataacatttgcttcaaaaatat
cagaattaaagccgaatttgtgttcactactatgttctgataaaatagtaattatttgtg
gtatttttactatattattcatttaaaattct----------tactattttaatatttta
ctctggaatcatgattttcaagattgactacttaataatttaactttaatttgatcgctc
aaagtgtcaacctgattttctaaagttttaagagcttcttctttttgaacatttaactgt
ttcgatgcttcagcaatcagtttctctgcattcaattgagctttttttatatcttctgag
actatattttgagcttctttttgagaagaggctatttttaattgtgcattgcggcgagct
tcagatagatcttcttcatattttgcagctagctcatctgctttaacaagcatagacgat
gcagtagtcaatgttgttcgaatatattcatctcgttcgtctagtactttagttacaggt
ttataaaaaatagtatttaataaaaccattaaagtaagaaactgtaatgccattaaaggc
aaagtaccattgaagtcaaataagccaccttcaatttcttcggctaatagaaatggtaaa
tcaatcattttactaattatgtttatagttaataattttgtttcaattaagaaaaataac
tttatagtgcgcttagtctcatcagactaagcgcaaatgattattttaaccaacgtatgg
attagcgaacaataaagacaacgcaacaactaggccgtaaattgttaaagattccataaa
tgctaaacttagaagaagtgtaccccgaatctttccttctacttctggctgtctagcaat
accttcgacagcattagctgctgcactaccttgaccaatgcctgggcctattgcagcaag
acctacagcaagaccggcagcgataacggatgcggctgaaacgattgaatccataatgaa
ttttaatttttttgttatatgagtagaaaattgacaaatttcgtataatacttaaaatcta
tataattaagtataatagattctaatat-----tgtaattatagtatattactcttctcc
atgaccttccattgcttctccaatataagctgcggacaaagtagaaaagattaatgcctg

tattgaactcgcaaataatccgagtatcataactggtaatggtattaaaattggaattag
caaagtaaatacagaaacaactaattcatctgctaagacgtttccaaataatcgaaaact
taaagatagtggtttcgtaaaatcttctagaatattgattggtagtaatactggagttgg
ctgaatatatctagcaaagtagcctaacccttttttacttaaacctgcataaaaataagc
taaagatgtaagtaaagacagggccacagttgtattaatgtcatttgttggggcagctag
ctcaccttcaggcaagtgaattaacttccaaggaattaatgcacctgcccagttacaacc
tagaataaatagaaataaagttgcaatgtaaggcacccaagggcgatattcatgctcacc
aatctgatttttgcaatatcttgcagaaattctaatataaattccataaaattctgaaa
tttttcaggaattctttgtagatttctagtacctagaaatgataaagttaaaagtgtagc
aattactaaccaagagacaatgaaaacttgcccatgcaattgtaagctacctattttcca
atatagatgttttccaacttctactgctgataaactattatatgggttaaaatctatgag
actgttttgatacataataaatattt-ttttaaatagtgcaagaatcaaaaaataag-tt
tttgaagaattaatatgcaattaatttattcttaaaattagactattaataaattaaaag
caaaaataaattctaatagagatagctatttttagtagtgattatttttttgtaaaaaaca
attattgctacctataatgcttttacgtttaattatatacccatatattaattatttaat
atgattataggtattttgctacaaactttattgttttatccattct----aaagattgtt
atagtttatttttatttaaaatatctgcgacttcatctgcaaaattaactttcaaattt
tcttctcccctcccagaagaaatcttacaaatcttctaatttaatattttcacctaga
acagcaatattttggtttattaaatcttcaatactaatatcttgattgcgaataaacatt
tgatctaataatgatagctccttaagtctcttttcattcgtccttcaatgattgtttcg
attctatcaactggtttgtttttcaagtcatctttaccagcttcaactctttgttccaga
ttaataatttcactaggtatatcctgtgttgatacgtattccacattaggcgatgccgct
atttgcatagcaatatctttagctaacttttggaactctggacgtcgtgctacaaaatct
gtttcacagttgacttcaactaatacacctattcttccgccagtatgaatataactctct
agtaaaccttcaatagcggtacgattagatttcttgttagctgaagccagccctttttgt
cttaatgattctaaagctttttcttcattgccattattagcttgtaaagctttcttgcag
tccatcatacctgctccagtcttatcacgtaaggcttttacgacttgagcagaaatttgt
agtgtcatatgctttcaaaaatttttaagtatttaaatt----------aattattataa
ttcactttgagtaggatgagttatttcttctaaatctgtttgcccgtatttaccatcata
aattgcatctgctattttacctacaattagtttaattgacctaatggcatcatcattagc
tgggattggaatattaataatttctggactacaattagtatcaagtatacaaatagttgg
aatacctaattttaaacactcttgtatagctgttgtttctctcttttgatctacaactac
aacaatatcaggtaaacgagtcatattttaataccatttagatgtttacgaagtttatc
taattctcttcgtaaaacagcggcttctttttgggtaactgatcaatcataccactttt
atcttgttcttctagctgtctcagacgactaactcgagacttaatcgttacccaattagt
taacattcctccaagccatctctggttaacataataagaatcacagcgttgtgcttcctg

tgccactataccagcggcttgtctttttgttcctagaaataaaactttttaccatccga
cgaagcctgttttataagttcacaagcttctgttaataattgtgcagtttggactagatc
aataatatgtatgccatttctttctgtataaatatatggaaacattttaggattccatct
acgtgcttgatgtccaaaatgaacacctgcttctagtaattctgctaatgtaacaatagc
cataagattattaatatttaaataatgtaattattcgggaaatccc-tttgtttagaaac
tgat-----tcaactaacttatttgaattttagtaaactaataataaattaacacacttt
taaagcagatcaacaaaatagatcctgaagaattcat----tctacacttttattgctaa
aataattacgcgcggttctgtcgtctaaaatgatatcatctaaatcatctcgtacagatg
aaatagtactttcgtcattagtatttgcagttaaattctttttctctaacgatgagccat
tacagttatcatacatattgaagccggtaccagctggtataagccgacctataataacat
tttctttcagacctcttaaccaatctaatttaccagatatagcagcttcagttaatactt
ttgtagtttcttgaaaacttgctgcagaaataaagctttctgtatttaaggaagcttgag
taatacctaatagcacaggcctatatgatgcattaatcttatttctcaaggttatggctt
tattagtctgttcaatcttctgtaactctacaagctctccaggtaaatagcctgtttctt
caccattttctattttcactttagaagtcatctgccgtactattacttctacatgcttat
cagaaatattcactccttgagattgatataccaattgtacttctttaacaagtagtaact
gaatctcttgaaagcttaatcgagcagcttcatataaggctaatccacattcaatataca
aatcaaaacttgcatcgaggatatcatgaggatttaacaatacgtctgttttctttcttg
cttcaagaatttcttcaattcttggaagcccttgtataatgtcacctgtttttgctctat
caaaaaccaatatagctaatgtttctccccttctaattaatgcattgttatcaacatgta
aaatagcaccattagaaaccaagtaaggtctagcaattcttaatgtaactgatctagaag
aaatttcaataatttgtccagagtctaaagaagtcatattttcagtaataaaatcaccac
aacgaatccaatcacccacacttactttaactgttgattttcctatattaaaggtctttt
tatcagaagatgtcgcaatcaaaatgcgcctgctagtattcttttcagaataaatctcct
caacagtaccttcactcattgcaataatctcagtactagcaacaactgtaaatggcttga
tatattcaccatttttacaataattctagtttgactttgttgcttctctgatttattta
catcaatacttttaattgataatgtttcaaaagttgacagccttaagtcaaaataattat
tgccactttctttagcagaaaactcaattgaggatattaaattatcatctcgatgctcaa
tttcagccacaagattagttgttactaaatgaactccttctatagatttcactctttctc
cgtcccgaaaaggagttcttttaactagctttagtttagtttgacttggtttttgtgaaa
ctagattttcaattagactggattttgtttctggtattgaataaacaataacaggtctaa
taagaatatagggcatttgctcgttttctatatactcccaataaactaacttatctgtag
aaatattattatgaagtctttctccaggtcttaaaaaacctctacttttatcattattac
tatacacctcactaagtttatagatagatccaggcttaataataatttctcgaactattc
catccttttcgattatttcaacaatacctgagcttttagaaaaaatatttttaactagct
ctgttccactttctataacatctccattatgcactagtattagtgatgaatctttattaa

tttcgtgagtttcttctgaaatccataagatatatcccggacttaaaatttcgtaggcat
ctttatctaagcctgttttctttttgatacatttaagtctaaatatttaataataccac
cacttgtcgttttatacgtatcagatattagttctgcgactgtgtaaccatcttgaa---
tttttggtttggaatagctttgaaaagaaattttgtttttttctgtttctaaaatat
aagattctttttgtggattacatctgtataaatataacaacctggaattacaatagatt
cagtaataacttgtatattggtaatactgctattagtattctggtgtatccttacttctc
cagcataatgatttattaattctgtctgtgcaagtatagtaccagctttagttttatctt
ctttatgaacaataacattagcagaatcagatatactataaacttcacctgagagcaccc
agatcaagcctccagttttagtaattcttgtagtatattgattattatctgtttcttcaa
ctgttaaattggagaaacagattttgccagagagatctgaaactacatgtttttgagctc
tttctgtcatgagtcgatttctgcggggtgattcagcaatcacttgatccttcgatacta
attcaccatcacatattaaaagagtagtacctttacttaagttaattattgacttctgat
ttttcttagatctgattgtaacttgggttggctttttttgttattaatgcttgctcaccat
gtctggtacgaacatctgtatacgattcaatatctagatctatcaattgaccatctatag
gtgcgtagatttgttctgctaattcaccggtaaatactcctccagtatgaaaggtcctca
ttgtcaattgagtgccaggctcaccaatagattgagccgcaataataccaacagcctctc
ctaaatcaactaagcggccatgtgcaagattccatccgtaacaatactgacaaacagaac
ttctagagttacatgtaacaggagatcttactaaaactttttt---gataccagctttag
ttatctcttttgctaacttaggactgacatcttggttcgtatgagcaattaaacaatttg
tctctggatggaatacattttctgctaatactcggccagataaagcttgttctagattaa
ttaatattttctgcgtatctactaaatcttctagtatgatgcctttattggtcttacagt
ctacttctctaattattacatcttgagaaacatcgacaagtcgacgcgtcaaatagccgg
aatctgctgttcttaaagcagtatcaactaatccttttcttgctccgtatgatgatataa
aataatcagtaacagttagtccttctctaaaattactcgatattggaagatcaataattt
gtccctgtggatcagccataagccctctcatgcccactaactgtcttacttgagaaatat
tacctctagcacctgagaacgccatcatataaacagcatttagaggatctgtctctttaa
agtatttaataacttcttgtttcaaggattcactcgcattattccacgtatcaataactt
tttgaaatcgttcaactgctgtaatctcacctcggcgatatttgttttcagtggccttaa
tatcttcaatcgtgcctaataaaagactttgcttactaggaggaattcttaaatcttcta
aacttaaagaaattcctgcctgggtcgcataatgaaatccaagatctttaagcttgtcgg
ccatattggctgctcgggctataccataattacgaaaagcccagacaattaaattttta
gttcgtttttatcaataaccttatttgaaaaacttggctgtgcaagacttcgtctattat
ccactaaatcttctccatattaaatgttcgtt--attaactatgctactaaagactcttg
aataattttattaaagattatacggccagctgttgttctaatatattgcacaattctctg
attttcagcgtcttcttttatgatatggttattaaaaaattttgttgtactattgtcaga
atgtttttcaatcttaatcggatcatgaggttgatcaccatctactaatccatcaaaacg

agcccatatataagaatgtaaatcaattttcttctgttcataagccataactacatcttc
taagcttgcaaaatactgattagatccctgctgctgagatggattattagctgttaaata
atagcaacctaaaaccatgtcttggcttggcataataattggttgacctgttgctggaga
gagaaaattatgaggggctaacattagcaatctagcttcggcttgagcctctaaagacaa
aggaacatgaacagccatttgatctccgtcaaaatcagcgttaaacgctgggcatactaa
tggatgaagcttaatagctcttccttctacaagaataggttcaaatgcttgaatacctaa
tctgtgtagtgtgggagctctgttcaatagtactgggtgaccttgaataacttcatttaa
gacattccaaatagaagattcatttttttgaatcatctttttggcagctttaatattgtt
aactaatccctgcaaaattagtctgtgaataacaaaaggctgaaataactccaaagccat
ttctcgtggcaatccacattgatgtagttttaaatgaggacctacaacaattacagatct
accggaataatctactcttttacctagcaagttttgtctaaaacgaccttgttttccttc
tataatatctgacaaagatttaatggtctattatttgcacctacaacagttctacctct
acgaccattatccatgagagagtcaacggcctcttgcagcattctttttttcatttctgat
aataatctccggagctaaaattgactttagacgagataatctattatttctattaataat
tcgacgataaaactcattcaaatctgccgtcgcaaatctacctccatctaattggaccat
aggccgtaagtctggaggtatcacaggaatgactgtgaataccatccaagaaggatctgc
acctgttgctatgaaattttcaatcaatcttaagcgtttcattttttttattgaattttaa
tgaaggtgtcttaaaacttttggtggatttgtagcttcagaccttaaggtttcagctat
gtgctctaaatctaaatctttcaacagtttctgaatagcttctgcacctattcctacttc
tacttggttattctcatcttgattttggtagatttcctcttcaagacttttccattcgta
accttcgagcagttgcttgtattttaaattaatatctgtattagattgcgtaactacata
agaatgaaaataaacaatttttttctacttctttgacttttaagtctaatgctaaagcaat
atagcttgtacttccttttaagtaccaaacatgagttactggtgaggcaagttcaatata
agccatccgatgtcttctcacgcgtgattctgtaacttctacgccacatcgttcacaaac
tatacctttgtagcgaaaacgtttatatttaccacagtgacattcccaatccttaactgg
gccaaaaattttctcacagaataaaccatccatttctggctttaaagttctgtaattaat
tgtttcaggctttgtaatttcaccaacaatttgtccgttgggcaaacttctttcgcccca
ctgtcgaatttttttcgggagaagctaaactaatttttacgtagtcaaaatattgctcaaa
ctttgtcataaatttgaataccttaaaatcaaataattagccaatttatttacatgcttc
atattgaaaaatgaatatcttaataaaggaattgttcaaaatcgtctactggaggagtat
cataattagaacgatctactcggttatctttagaatccgacatcaagtcaacttcaactg
tccgtctttggccatcttcaaatagtttcaatttatgtactgcaatgtctaaacctaatg
actgcagctctcgcattaagactttaaaagattctggagttccaggcttcggtataggct
tccctttaactattgcatttaatgcttcatttctagcttgcatatcatccgactttacag
ttaataattcttgcaaagtatatgctgcaccaaaagcttctaaagcccacacttccattt
cacctaatctttgacctccatgctgtgctctacctcctaatggctgctgcgtaactagag

agtaaggaccagtggatcttgcatggattttatcatcaacaagatgaacaagcttaagca
tataagctctaccaactgtaacaggattatcaaaaggctctcctgttctaccatcaaaaa
cttgcattttacctggatgctgatcattaaaaagccatttattgctagtaatcaatgatg
cttcttttaatttctatttacaagtgctcttgatgcttctgctccatacatttcatcaa
aaggtattattttaaatctctttcctaaatagccacctgctaagcctagtaggcactcaa
atacttgaccaacattcattctagaaggtacacctaaaggatttaaaacaatgtccactg
gtgttccatctgataaataaggcatatcttgtttaggtaagatcctagaaatgatacctt
tattgccgtgacgaccagccattttatcacccacttggatttttctcttctgcgcaacat
agacgcgaatcatagcatttgttccgggaggcagctcatctcctttctgcctagtaaaaa
ccctaaccttaactactctaccttttgcagcattaggaagccgtaaagatgtatctctca
catctcgagccttttctccaaaaatagctcgcaacaatttaccttctggtagttgatcag
cttcacctttaggagtaatttttcctactaaaatatctccagcttcaacccaagaaccac
cgacaacaattccatttctatctaaatccttcagagagttatcgctgacgttaggaatct
ctctagtaatttcttctggtcctaatttagtttgacggcattctacttcatacttttcga
tatggatagaagtatacaagtcatcataaactaatctttcgctaattaaaaatgcatctt
cgtagttataaccttcccaaggcatataagcaactagaatatttctccctaaagcgattt
cccctccgtctgtggatgcgccatcagctaaagtttggccaacaactatttttttctccca
cccaaacaattgggcgctgattaatacaagtatcttggttagaccgataatatttttttta
agcgataatgaactgttctaccactattatcctgaatgcctattttattagcagaaacat
aatttacatgaccagatgttctgctaataacaaccataccagagtctcgagctattttag
tctcaagaccagtaccaattattggcttttctggatataataatggaacagcttgccttt
gcatattagatcccattagagctctattcgcatcatcatgttctaaaaaaggtattaatg
atgttgcagctgaaattacttgaataggtgaaattgcaatataatctacttgggttgggg
ttgttgtaataaactcttgacgataacgtacaggaataatatctccttcaatgtaatgtt
gcttactaactttaacatctcctggagctactcggaaatcatcttcctcatctgctgtta
gatagacaggactattgtgataaatcacttgaccttggtttaccggataaaatggggttt
ctatgaaaccaaaaacattgacccgcgcacaggttgctagtgaaccaattaaacctgcgt
tcggaccttccggagtttcaattgggcaaattctaccatagtgactaggatgtaaatcac
gaactgcaaaacctgccctgtctttattaaaacctcctggacctaaggcactaattcttc
tcttgtgagttagttctgcaactggattagtttggtccataaattgagaaagctgactag
atccaaagaattctctcactgacgcaattaatggcttgggattaattagatttgataaac
tcaatgaatctatatcacatatcatcattctctctctaataatgcgttctaaacgattga
gacctactctgaattggttctgcagcaattctccaactgaacgaactcttctattaccaa
gatgatctatatcatcaagattacctgagtttttatccttaatattaattagataatcaa
ttgaggataagatgtcttgaggagacaatactcggaacgttttaggtatattgagaccta
gtttttattaattttatatctaccaacttcacctaagtcatatcttttaggatcaaaaa

aacgagagtaaagcatctgcttagcaacagcaactgttgctggttcattaggtctaagct
tagaataaactatgagcagggcttcctcatcagttacttcttcaatattatttttccaa
tttctttagctaattctttaactgaatatatttgggatgcagaaataagaaaagcatatt
tactaagtcctttttcaatttcatctttattgagtccaatagctcgcagaaaaatataag
catttactttgtgggtcttatctattcttatccaaatttctcccttaggatcaatttcaa
attttaaccaagaacctctattagaaattaggcttgcactataaatctgcttaccatttt
tgtctatctcttgtttataatagattcctggactacggattatttggttaataataactc
tttctgtcccagatacaataaaagttcctctatttgtcattattggcaagtctccaataa
aaactaatctctttttgtacttttatttttatctgcttttcagcactgagctgtagat
gggttgatgataaatctaatgatttaatatttttttcttggtttttagaaggtagatcaa
tatcttttcttgttaactttgcaggaacatatatttgagcactataagttttatccctat
tttttgcctgtctgacactataacgaggaaattttatcttgtactcattaccaaataatt
gtaattcgagtcgactagtagggtctgatatcttcggaaaaatttcaagaacttccgtca
aaccttctaataaaaaccatttaaaactggccctctgaatttcaactaaatctggaagaa
gtttattttttaagcttatacgttgaaccatagatctcctttataacaaattttgaaatc
gaagagtttaa-tactattaatagagtactggtagataatgcataagcatagtttgctac
attatttcttctctagacataactagaacatcatattttattcaa--aaaaagattttta
aaatttttcttcac--acacactatctataagtactttgctgcta--aaagatagttcaa
gctattaaa-------aaaaacgaaattaatacttgtctgtctgtcaattaattgacatc
aagatagacaagtattatttatttaatcaattctg-tttttctgagcaaaagacaatgct
ctcgcaagtctagctttttttctagcgcctgtattagggtgaaatgccccttttttaca
gctttatcaatcttactatatacttgagaaatacttgactgcacatcattgaaattatca
ctttctaaattatcaatatttagaaggcatcttttagttaaagttttgacaaccgattta
tacttacggttaatgagacgatttctttccgaagttttaatgcgtttaatcgcagaaagg
ttcttagccacagtaaatccaataaaactttataaaatatttttagaaacgaaaataca
caaatctgggatctattcaaaaagggaatatgcggcgtattataacattaaagaaaaaca
ataggcaatagttcaacaagagctcttgttattacacaatagagattgtcattgcgaaaa
actttgtaagatagtatttaattaaaataaactaactagacatataaatatatatgaaaa
aaattgaagctattattagacctttcaagcttaatgaagtaaaacttgctctagtcaagg
aaggtattggaggaatgacggttatcaaagtttctggatttgggagacagaagggtcaaa
ctgaaagatataaaggatccgaatactctattgatatcattgataaaataaaaattgaaa
ttattattagcgatgataaagtagagaaaattgtagagacgattattaaggcttctaaaa
caggggaaattggagatggaaaaatatttattagtagtattgaaagagtaataagaatca
ggactaatgacttaaattttgaagctttatagtatttttacttt-tttagacttgatact
tcttcactaaacatgagataataagcgtttattgtatacttataactaaggtgtaata--
ctaatggctaaaagcaaaggtgcacgaatcgtaataactttagagtgctctgataaggct

ggagagtttgctcaaaaaaggaaacctggcgtttttcgatatacaactactaaaaataga
cgaaatacaccaagtagaattgaattaaacaagttttgtcctaattgtaatcagcattgt
atcttcaaagaaattaaatagttatttatcttattaaaat--aatcatattatggctata
tatagaaaaagaatatctccaattaagccaacagatgctgttgactacaaagatattgac
ctgctaagaaaatttattacagaacaaggcaaaatactgcctaaaaggtcaactggattg
acttcgaagcagcaaaaaaaacttactaaagcgattaaacaagctagaatcctttcttta
ttaccttttttaaataaagattaa--atatatagcctaacttcatatttttccatctaaa
tattaaaaatttctatagatttgtttattaaaatgatctaacatactgaat---------
----------tactaagaaga-tctactcattacaagttttgttcattttataattaata
atagcattataattta---ttaagctagattc-----agacataaag-gtaacctctttt
aattgattatattatatagtctatttaaaagaggaaattttttttgcttc----------t
agcaattcaagattctttaagctttaacaaggtatttaaaacaaagattttttgtttaggt
atcagatcaaatgaatgaatcttatctcccgactgccataattgaaattcagagatgaaa
attccacattcgttgccagcttgaatttcttcgacatcttctctaacgcgctttaaagat
tcaatttttccttgataaataactttttcttctcgaatcactttaatccaagagtttttt
aatagcttattattgatcactcgacatcctgctatttttctatttgctaacgagaatact
gtacttacttctgcttctcccaccggtacttctgaatattcaggatcaagtaagtcttcc
atccttcttttaatgtcttcaattaaggcataaatgatttgatagtttctattaatata
ttcgactttgccgatgcttgctttgttcctggagcaaagttggtattgaacccaatgaga
gtagaatttgtggttgaagctagttcaacatctgtagcagtaatttctccaggcatgatt
gagacaacattgagttgaactttactctgaggaaattgagataaagaatctaggattgct
tctgtagagccttgattatctgttttaataattaaagaaatttgcttactaatatcttta
gaattagtgtttttttaaagtatctaatgtaattcgactattaagtgctctttgtttctgt
ataattgatgagtctttagaggtattttcaatcgctttaagtttggcttctttatcactt
ttaactgctaaagctatttctcctgtagctggtacagatgacaatccccaaatttcaaca
acagatgatggtattgctaaattaattttctctttagcgttgttaattataactcgaatc
tttgcgtaagctgatccaataaccaaattatcactaatattcaatgtaccattttgtatt
aatagagttgctactggaccatgcgatttatctaaatgggcttctattataataccttgt
gcaggctgtgttggatcagctttcaaatcctctaattcagctaacaaggtaatagtttct
aataatttatcaacattttgaccagtaagagaactaataggaattataggcacttggcca
ccaagtttctccgacatgacattgtattttaataaatcttgctcaataatatctgtatta
gaaccagctttatctatcttagatattgctatgacaaaagggacattcgctttttgaata
tgattaatagcctctatggtttgaggtttaactccgtcatcagcagctataataataata
gcgacatctgttaaattagctccccctagacctcatacttgtaaaagcttcatggcctgga
gtatccaagaaaacaatttttttgtttattatctttcttaatatactcaacttcataagct
gcaatggcttgagtaattcctccaatttctttgttagcattattagattttcgaatataa

tctaatagcgttgttttgccatgatctacatgtcccataactgtgacaattggaggtctt
ttaatataattgccagtttcataaaaagcattagagttatctaaattactagaaagtcca
ttattattttcctttacatttgattctactgctataccaaaattatcagccactgatgat
attattgaggcgtctatggtttgattcatagtaactgatattcttttaagaaataaatat
tttataatatctgtttcttgaacacaaattagtttagataattcttgaatagttagagga
ttagtaatactaatagactctggcggagaattagattgaatgcttgctgaattttgacta
ggagttacggctactttttttctgtcttatagatttttttattactagtcagtttttttaca
acttccacttttggcttaggtggacgcattaaagagatagctaaatctcctgcagtctgc
gaaacattagaattagagtctctaaagttgtca---tcatcatcgtcaatatgtatttta
gtttttattttt-ttgcgctgtctattctta--ttttttttactatctactaagtcgtga
attttattaaaatttttactttttttatctagtttaggtggagaacttaattctagatgc
tgttcaccacc---tgtatgtgattctgatttatcaaggtctaaatttaacagattatca
ttattgactgactctaatctaattttatagataatctgaggatttttcaaatctacaatt
gtttcagaagaattgatatttgagctagatctagaggatctattttcaaagttttgatta
tttaaaaacataaatattggttttattatctaatagtaactttctaattttaatacagag
cttaatataaacaagaatcgaatatctgtataaataatacaaatgt---agagaattaat
ctatgttgtattagatttaatataacactaaagtcctttgttcgttatacaagtttaaaa
ctttt-tatgatgataaacactagattctagtttattggtatacaatctatttatctaaa
taatactgtttttgtcagggcttttaaa----ttatctaatgttatgttcattatgaaat
tctattaaaatttaaaaa-ctcaacaattaagtaagaatcgttaatcacctccacgccac
ttatgccacgatctcaaaaaaatgataattttattgacaaaacttttacagtattagctg
atattgtattaaaaatacttcctacaagtaaagaggaaaaagaagctttctcttactatc
gagatggaatgtctgcacaatctgaaggtgagtatgcggaagctttagaaaattattatg
aggctttaaaactagaagaagatccatatgatagaagctatattttatataatatagggc
ttatttatgctagtaatggagagtatgtgaaagctttagaatactaccatcaaggattag
aactaaattttaaattacctcaggcccttaataatatagccgtgatataccattaccaag
gagttcaagcaattgaggataaagatacagagctatctaaattaatgtttgataaggcgg
ctcaatattggcagcaggctattaagttagctcctgataattatattgaagctcaaaatt
ggctgaaaacgacaggacgaatgaggaatatacaaggatattaatatatttaagatataa
taaagatatcaattaataaattaaaattagttaaagaaactggtactatatagagtactt
-tactatatattataatattatactagttgatctctatctctaagacttagctgaataac
cgaaattaaatctattgactatattcatgaatacaaataagcataatggttagtacaaca
aaaagtaccggatctgttactcaaattatcggaccagttttagatattgcatttcctaac
ggacagcttccgaaagtattcaacgcactcaaagtacaaagctcagaaggaactattact
tgtgaagtacaacaacttttgggtgacaacaaggtacgagctgtttctatgagttccact
gaaggactacaaagaggggtagaagttattgatactggatcccctatatctgttcctgta

ggtacagatactcttggacgtatttttaatgttttaggtgaacctgtagataatttgggt
cccgttgattctgagagtactttacctatccatcgaccagcacctgcttttactaagcta
gagacaaaaccaaatatttttgaaacaggtattaaagtcgtcgatttacttgctccttat
agaagaggtgggaaaattggtttattcggaggtgctggagtaggtaaaactgtattaatt
atggaactaattaataacatcgctaaagctcatggtggagtatctgtatttggaggcgtt
ggtgaaagaacaagagaaggaaacgacctatatatggaaatgaaagagtctaaagtaatt
gatgcagataatctgaaagaatctaaagtagcattagtatatggtcaaatgaatgaacct
cctggagcacgtatgcgcgttggcttaactgcattgacaatggcagaatactttagagat
attaacaaacaagacgttctattgtttattgataatatttttcggtttgtacaagctgga
tcagaagtatcagctctactaggccgtatgccatctgctgtgggttaccaaccaactcta
gcaactgaaatgggagcactgcaagaaagaattacttcaacaacagaaggatcaattaca
tctattcaagctgtatatgtgccggctgatgatttaacagacccagctccggccactaca
tttgcacatttagatgcaacaacagtactttcgaggaacttggcggcaaaaggaatttac
cctgcagtggatccactggattcaacatcaacaatgctacagcctggaattgttggaact
gagcattattctactgctcaagaggtaaaatcaactttacaaagatataaagaactgcaa
gatattattgctattcttggtcttgatgaactttcagaggaagacagacaaactgtatca
agagcaagaaaaattgaaagatttttatctcaacctttttttcgtggcagaagtgtttact
ggatcacctgggaaatatgtatctttggaagatgcaattaaagggtttcaaatgatctta
aaaggcaatttagacgacttgcctgagcaggcattttatctagtaggtgatatagatgaa
gctatacaaaaagctgacagcatgaaagattaatataattaacgatgactttaaatataa
gaattattgctcctgatcggactgtttgggatgcagaagcacaagaaattattttaccaa
gtagtacagggcaacttggtattttaacaggccatgcacctttgcttacagctttagata
ttggagttatgagagtaagagtagataaagaatggatgccgattgttttgctgggcggtt
ttgcagaaatagagaacaatcaattaactattctggttaatggcgcagaagaagctagtc
aaattgatttatcagaagcagaaaaaaatttagacactgcaactcaactcttaagtgatg
cctcgtctaataaagaaaaaatagaagcaacacaaaaaatacgaaaagctcgagctcgag
tacaagctgcaacagcagcaacttcgtaa-aagattcc------tcttaaaacataaaac
tttagtaattattactaaa-------gttttatgttttatttgtatggttataggcagct
taaataaacttagcttagacctgaacaaatataatcaaaatataatcccatctctttacc
tgcatctggtccaactaaactaatagtaacttctttcatagctaaaatagcttgaatcgt
tgcaccaataggaacacctagagaattgtatgtttcttttagaccatttaatactctttc
ttcaaggatagatggatctccagctagcattccataagtagcatagcgtaagtagtagtc
taaatcacggatgcatgcagcataacgtcgagttgtatacatattaccacctgggcgtgt
aatatccgaataaagtagagattttgctacggattctttaataattgttgctgcattagc
tgcaatagtagcagcagctctgactcgtagctcacctgtttgaaaatagcctcttaattt
ttcaacagagctatcatctaaatatttaccttggacatcagctgcattaataacagaagt

aattgcgtcttgcataactttcacacattccttaattttaaatatctaatacta--gggg
tatataacaaaattactagatcttttagcgatatcagtattactgcatagcacccaaagt
ataatcaaagtaaaaaccagcttctgctgaatcttcacctgcaagcagtgagcaagctac
acttttcatgcattttacaccttcagcaactcctgaaattggtgttcctaaagaattata
catttctttaacacccactaatccaatttcttcaataggagttacatcgccagcaactat
tccgtaagttactaagcgaagataatagtccagatctcgtagacaagtagcagtcatctc
ttcgccataagcatttccaccaggagaaactacatcaggtctttttgaaacagttgttg
gccgccttgctttacaatacgctcacgattatctgttaaaatttgagctattcttaaacg
acgttgtccagataaaacaaaacttttaattctatctaattccccaggacttaaatatct
tgcttctgcatctgcatttacaattgactttgtaacaatactcatggataaaactcctgt
aatgcttt--aaaaaaagcttaatactaattatttatgatataactcagaaagtaatgta
ctttttgag-aattttctaaccgaaacacgttcatgatatatgaagcaggatagatactg
aggtaagtactaaaacgagaaagttatatgtaggaacttctgttattgatttcctagtac
ttttttaaaacttggtaccacaattgatatatcctgcttagtcaaacgattatagagtgt
ttctgtattaggaaagttagctgctggtaaagtcggaaatcttctgtatggaacagtatc
tttgccgtataccgcattgtattcaacactttcaactaaattacttataaatgcagataa
gccttttgaagctaaaatttgattaaagaatctaatttctgcctgattattaggagctct
tcctaagatatgcttagtacctaattctataacttttgtatttggatatggctgataaaa
ttctttaccatatagttcagatagcgccagtttttctactagttctttgacacagatttg
tctattcaagaaagctgacttaatatccaagaattcaccgccgacactaaaagagttaag
atctctttcgaaaatttgacgatatgctgctcttaaagcttgttctaacatttctttgtt
actatcactattcacttcaaagacaatagattgatctcttaaagaagttactccttgtga
aatacgagactgaatatcattactactcctcatttctttgacagtgcctaattcaacaaa
cctagcagatttactagcaataactttttgaatctctggtcaataattcctggtcttaa
gcttcttaaagcaacaccagctggagtactatatctttcataaggaacagtattatctcc
aaaagtttctgtgtactctgagctatcaattattgtgtctattacttggtaatagccttg
cttatatgcaatatcaaaatatttattaatttcttgccgaccatatgtagggcgacccag
taagcggttatgaatatattcaatagctttacaaatatataatggctcccaataaagaga
tctaaatatacttgattttgctaactgtctaacaaactcgcgaactgtaatttgattatc
ttttagctgactttctattggcttgagaattagtttttcttcttgatacacttctctgcc
aaaaactcttaaataagcaacttttgtgacaacttctactgaattttcaaaattttggga
tgaatctgactttataagagcgttggacaatttaaagatttttggtcccagggatcctgc
tgacttaggtcttacctgtggattactaatctgattgtagattcctggcccacgtctaac
taatatccttcttgtatctttaccaaaaagagcttgtctttttcttggatctttattttc
tttagggaaaatagctccaaattgaattgataatggatcattacctattccatatggatg
ttgatcaggtaatgattgcttataatcactaaataaagttataaattgaggaaccttacg

aaaaggcgcactataggtgagaagatctatttgaggcccccaattacggcactcttgagg
ctcttcacctaaatttctaaagtaaggcacagtctcttcaccgaaataatctgtatactc
agaagaatttaaaagtgcattcactaaaccacttaaaccagtagatgataaaatagcaaa
atattttgaaactcttctaaagagctaggacctctacccaagaaatgtctaaatgctaa
ttctaatgctcgactattgacaaaaggttcataaaattgctttctataaatacttgaagt
tcctaacgaacgaataaattccttaattgaaatttggccatttttacttgcgactccaa
attcgataaagagaggtcatatgctttagcaatatctctttcaaaaatttgtctataaca
tgctttcacaacaatatttttctcatcagcagataaactaggcttcataacaaaacgagg
tgttgacactcctgcctgtacataagtctgaggaagacgcaaaccttgtaaatctccaga
tattctttttcttaatttatcagtcaaagatggagcttcaaattcagaaatcacaacatt
aaaatactctttaactaaatcttgaccttttatatcttcctcaaagattaataatgctgt
ccgtctcatctctctaagagccacaattgctgctgcacttgagcaagcgttatcaattaa
ctctctcaatcctcgtatattaacagataaaatattagggtcgcctgatacaattgcata
tgtcaaataccgtaaaaaccagtctaaatctcgaagagattttctcattcttgtagtacc
gtatctcaagacgtttataggtttaaaccctggcggagtagctcccccagcattaaataa
agatcggaaactttggccaaaatctccttggatatttcctgataactcattaattttatc
ttgagaactttgatctcctgcaataataacagctgcttgcggacgttctaaataagaaat
tgcagacccacctacgaatatttatctgctgctcgtgctaccagaatatttgcattttt
agttaaaatatctgcaacttccagtctttttttgtccagaatttaaaaaagaaactaattg
atttaattcaccaagctgtaaaaatcggtcttgttgctctgcctgagtaatagttaaaat
tgaggcagtccgataaagctgggggcgtgctaatgggcttccgccacttgctttgatact
cattactttatttatctccttaactgatattgcttgctagcgaatacaaatatagcttga
tatttctaaatatttaaagatactatgcaagcaca-ctgttttctatattattaggtact
agtacttatagtacagagagtcttataactaacttctataaatacaaattttgtaatact
tctattaacatttgtcaaaaaaatatttaagtgctcactcttttatattaaaattcaatt
agtgagaggttgatgtgaaataaagtatactaagtaagtttttatagataggaaaattaaa
agatttattctacaaacattaatca------------cttgaatgtttagcaattgtaca
attgttattgcttttaattggtttagactagttttatatttttctaacataaacaatat
taatgagcaatttgtatgaatttaaagccacaaaacaacctattaactaacgaaaatata
gatcatgtagatatacccgaaaatgatataccaatgtcaattacggaacatttagaagaa
ttaagacaacggactttatttgtgtttttattctttttgtttgctacaactataagtttc
acacaaattaagatcattgttgcaatactgcaagctcctgctgttggtattaagtttta
caactggcaccaggagaatattttttttcatctattaaggttgcaatatattgcgggatt
gttgcaacaactccgtttgccgtttatcaagttatattatacatactcccaggactaact
ggaaaagaaagaaaaattattttgcctctattaattagttctgtattgctttttattaca
ggtggtatatttgcttactttgttctcgcaccagcagctttaacatttttaattagctat

gggtctgatattgtagaaccattatggtctttcgaacaatactttgactttattctatta
cttttacttagtacaggattagcatttgagataccaatcatacaattattgctcggtgtt
tcagggacattttcttctagtcaaatgatacgagcttggagatatattattattatagca
acaattgctggagctattctgaccccttcgactgatcccgttacacaattaataatgtct
tcagctgttttattactttattttggtggaattgttatattattagtcttaaaaaagtag
agtaaat---------attttcta-ttaatccaatatatttgttatc-----tgggcaaa
taatctat-aaaacaattgct------------------agatttaagtatcaggcttct
aaatatttcaa---------gtctctttttatcattgaaatacaatc-------------

--gatacattgg
cttgttttttcatcaattatttgtgttgcacac-tttttagtactctctttattgttctt
agaattatttctttaatctatttattaaat----agatatgaagagatttaagtattgat
tatggtaatctatatcaacgtaaaaagtattttattgtgagttttatttatataaaggct
tattcattatatagttataacagtaagtctaattaatag-ttttcatgcttaattaaagt
aataactcttaattcttttggcagaaaaatctgtcttaatattttatc--aaaaaaacag
-aaaaatgaagctaaatagtctaattaacttaattcaaaagtctatatattcttgtacac
ttttactaattattttaaatattatttgtgtcgcacctaattctagtaatgcatttccaa
tttatgcgcaacaagcttatgaaagtccaagagaagcaactggtaggatagtatgtgcca
attgtcatcttgctcaaaagcctgtggagatagaagcgcctcaagcagtactacctaata
ctgttttcgagactgttgtgaagattccatatgacagcaatgctaaacagattttaggta
atggcagtaaaggaggcttaaatgttggagccgttgtaatattacctgaaggatttaagt
tagctcctgttaatagattatctacagagttaaaagaaaagactagaaatctttacattc
agccgtacagtgctaaacaagacaacattttagtaattggacctatttctggtgataaaa
atagagaaatagttttcccaatactatctcccgatcctgcaaaagataaaaaagctcatt
ttttcaagtatccaatatatgttggcggaaatagaggacgaggccagatttatccaacgg
gtgacaaaagcaacaataatattgtctctgctttaagcagtggtaaaattaataaaattg
aattactagacaaaggtggatttataatacatgtgactaacagtagcaatgtggagtcaa
cacagaagatttcacctggtctcgaacttagagtaaaagaaggggatacaattcagcttg
atcaagctttgaatagtgatccaaatgtaggtggttttggtcagaacgaaacagaaatag
ttttacagagtccaaatagaattaagggcatgattgttttctttttcgctagtgttctag
ctcaaatttcttcgtattaaagaaaaaacaatttgaaaaagttcaagcagctgaaatga

atttttaaagcaatataaaactggcacttactataaagaaagta-taaacactttattca
caggatgtaccagtttttatgttatgtaataatcgctttgacggaagttatgatactttt
aacagaatctacaatctgactaggctgaattactgtagcctgctctaagcttccattata
tggtgtgggtatatcctgcgaagatagcctcacgacaggagcatctaattcatcaaaaag
atactcattaatttgtgcaattagctctgctccaattccggctgttttcatacactcttc
tacaattaaaactttgtgagttttctttactgagatagatatagagtctatatccagagg
ttttaaagatataagatctataacttctggatcataaccttcttttaataaggccggtaa
tgcttggataacatgatgcctcattctagaataggttaaaatcgtaatatcttttccttt
tcgtacaaattcaactttattgagaggtaagaaatattcttcttgaggaatctcttcttg
taaattataaagtagaacgtgctcaaagaatacaactggattattgtctcgaattgcaga
tttcagtaatccttttgcattataaggagtagaacaagcaactatttttaagccggggat
ggcttggaaataagcttccagtctctgagaatgctctgcacctaactgcctaccaactcc
tcccggtcctctaataactaaaggcaatgtaaaattacctccagaagtataacgtaacat
tcctgcattattagaaatttgattgaatgctaataataaaaaactcatgttcataccttc
aacaattggtcttagccctgttatagctgcaccaattgccataccagtaaagctattttc
tgctattggtgtatcaagaactctcaaatccccatatttgctatgcaaatctttagttac
cttataagatccaccatagtgaccaacatcttctcctattacgcagacagttggatcttt
tgccatttcctcatctgttgccgctcgtaaagcgtcaaacataaagatttactcatttt
aatatttgatttttatgatttgaccactgttttaaagtaacagttaagacaccatattct
atttagttatctgcaaaaagatatcgtttcaattctgacatgttaggttctggactggaa
atagcaaatttcacagcttgttccaattcggttttaacagcattctgaatctcattaagt
tcacctatattagcaatttcattatctagaatatatttttgagtttttgataggatct
cttgccacccaagcctcttttcttgtcttgatcttagttcatcaggatctgcgagagag
tgaccacgaaatctatatgttaatgcttctattaaggttggaccatcaccttggcgagct
ctttgaactgcttgttttgcagcttgtcttacagctagcacatccattccatcaacttca
atcccaggaagcccaaaagcttctgctttttatgtatttcaggtattgaagaagaccgg
tgatgtgccatacctatagcccattgattattttcaacaacaaatataataggtagtttc
cagagaactgccatattcagacattcaaaaaattgcccattattggtagtcccatcacca
aaaaagcaagccgtgactcttaaatcctctgtttctttaagtacttgctggcggtagata
ctttgaaaggctgcccctgttgcaaccggtataccttccgcaataaaagcaaagccacct
aaaaaattgtgaggcgcagaaaaaatatgcatcgaacctcctctgcctttactacaacca
gtctcttttccaaataactcagccatcacatttttagatgggacgcctttacttaaagca
tgtacatggtctcgataggtactgcaaacataatcagttggattcagaagtttaattaca
cctgtagaaacagcttcttgaccattataaagatgaacaaaaccgaacatctttccttta
taatacatctgagcacacatatcttcaaaatttctgcctaacaacatgtcttcatataaa
actaataaattactcttattaagattaagacctgttgaattatagttagtcagcggcaat

tgaactttcttaggataactcataatttgtaaagaaacctct----------tattgtaa
ataggaattttgaagaaacagctatcaatagaaaacacacagataaaaacttataaatta
taatatgtttttactaaagactttaataattatttgaaactagttcagtatatcaaatt
aattttgcttatactacggatgtctgaaacttatataaaaaagtagt-a-aaaaagttta
ttaaaagaatagttaaaaacagatataaatagaagtaaacagaattatt-gaaaaaaaat
gattattattaattaa----tacttaatatttctaaatattttacaattaatggctatgt
caccatctttattttatcctgttgaacaagaactatgtagtcttgaaaaaaatctgaaag
ctgttgctgggactcgtcatccaattttatatgccgcagcaaagcatctattcgatgctg
gaggaaaacgagttagaccagctcttgtatttttagtggctaaagcaacctctgagaagc
aagatataaatactggacaaaaaaggctagcagaaattactgaaattatacatactgcta
gtttggtacatgatgatattattgacgagtgcacaacacgtagaggagtcaaaactgtac
ataatttattcaatactaagattgctgtgctagcaggagatttttttatttgcacagtctt
cttggtatttagctaatattgaaaatttagccgtagtcaaagctatttctaaagtcatca
ccgactttgcagaaggagaaattaggcaaggcttagttcatttcgatcccagtatttcaa
tagatgcttacattgagaaatcattttacaagactgcttcactcattgctgctagttgtc
ggggtgcagctatgcttaatggttccaatcatcaaataaataatgatctttatctttacg
gtaaacatatgggattagcatttcaaattatggacgatgttctagatataactggttcta
ctaagagcttagggaaacctgctggcgctgatctaataaatggaaatttgacctctcctc
tcctttttcacttactcaagaagcaagtttaaatgatcttattgatagggagttctgta
atagtacagatatagcctcaacattatttcttataaaaagaagcgggggaattacaaaag
ctaaagatttagctaaagaacaggtgcaggcggcactttcctgccttcagtttttaccac
aatctacacctgtatctagtttaaaagaattaacacatttcataatcacaagattgtcat
aaaggacttgctaaaattaaaatgttattaactaatttttttactgtttctaaaacagtct
gaaaagtttgaatatcattactagctagctgcgctagcattttacgatttaacgcaatat
tctcttttttaatgcactaataaaagtactataattcatgccttgattgtgagcagcag
catttattctagttatccaaagacgacgaaaatctctcttttcctttttcgaccaacat
aagaatatcgaagagctttaaggacttgctgcttagctgttcgaaataaacatttatgtg
cacctttaaagcctttagctagcttaaagatttagctcgtcttttttttgcaacgttac
ctcttttaactctactcataaaatattac-ttaaattttaattattaccagcttactaaa
ttcttatactctttg----attataaataaggtaatttcattgcaatgtttttaatatct
tttaagtcaaccgaacaagtagaagaaagatgtcttctttgttttgatgactttttttgt
aataaatgacttttagaagctttatgtcgaagaaattttccggatgaagagactttaaat
ctttttgctattgcttttgatgtttttaacttaggcatattataaacttttaatgattaa
caaaagcatgaaaagtctgaggatatcaatcttagacctttcaatacttagaataatctt
aacacaatttattttacttactacataggttaactgaaacaatttatatatataatacta
atcagttaatcaaccatagctaaagtggatttatattttgctttgaacttttgtatagta

ttagcaattagcattgttataattttaagactgcttgaattcaattcttcgaacattttc
atatttaatgtaaatgaaatattggcttctgagacaatattttgaatttgaacatcagac
aagggaatcatatctaatgcagctctatattgatctttaaataatttatcgtcttttatt
tggtcaaaatcgtaaaatttcgttcctcctgaatctgataaattcatagcccctctagct
atttttttaaaatttggccgccagaaaggtcacccaaatagcgcgtataagcatgagca
actaataattcaggttgtttatgtcctatagtgtgaattctatcaacataaattttgta
gcaggagatggttcaataaaatctaaccaatctgatccataataatagtttaaatcttca
gatagactcgctttcctattaagctctgtaaaatatataggtttaatagctggatgattt
ttattagaaaataactcttcctctattgcacaatagacaaagtatagatttgcaaccaat
ttacgatatgacttcttatctacaactcctcccaaaaaagatttaacaaaactaacattt
tctgccatactatgggacttagtagtaccttctcttagttcattcgctaaagtattaacc
ataatatcaatattccattttt-aaagataacaaattaaca---ataaatagttatccgt
atatttataattgagttgcgctaactaggctgctgcaaaataatttttttgattttattgg
atctggattcattgttttgtcaccaggcttccaattagctggacaaacttcatctggatg
agattgtacatattgaattgcttgtaaaaccctcaaagtttcttctacactcctgccaaa
ctctagattattgattgtagagtactgaataattcctttcgggtctataataaatagtcc
tcttagagctacaccatcactattcaaaacattataagctgcactgatctcttttttttaa
gtctgatactaatggatatgaaagatctcctaatccacctgattctctgtcagtctgtaa
ccaagcaagatgagaatactcactatcaaccgagactccaagaacttctgtattaagttc
cgaaaaagcattatacttatcgctaaatgctgtaatttctgtagggcacacaaatgtaaa
atctaaaggataaaagaataagacaatatacttattcttgagatcagataattttaacgt
tttaaattcttggtcataaacagctgtagctgaaaaatcaggagctagctgacctactcg
aagacaatttggtccagaaatcattaatttttcccagaagaattgatataattttgttat
ttaataaataattatataacaaaatttagtatatattaaaatactacaagtctttttttaa
attac--------taaatgatataatcaatactatctttatagcggggaatggatttgaa
ccattgaccttcgggttatgagcccgacgagctaccagactgctctaccccgcggataac
taaaatcatattagtctacatatatatataaaagcaaatgcataatagattttagaagta
aaaaaatagaagaactggtccactgatcattaaacttagaaacgacaaagtatttttttat
cttatttaaaagagggaagacttcatataaaccaataaatctatcttttaaaagaatttc
ggacggctttacccaaatttgaccgtcataccagcctgattcttcataaaatactgtagc
actcattaatcgttttactacgtatgaccagcctaaatataatctgattaaaataaatcc
tgtcattaggctggaagtaataaattctgaaaagaaaaatttcaaaggtagtttagtgat
tggaaaaattgataataatataggactaactaataaacagttaagtagtagtgcaatagt
tattttttttattataagatctacggcttaaagttggccagcaaaagaaccaagaattttt
caaagaagtatattcatgaacaggttgctgctcctttggcacaggacattgagtattata
taaattcattgacattaaaaagtattataataaagac--tttgtttatattatctaacat

aggtacgacattaaatattaactaactgcaattatagtagtgagtagaagaaataaaact
aaaaatagccaagtaattttatttaaagtattctcagtactacgagtattactaaagaac
tgattctgagcacctacacttcctaaaccttcggatttaggattgtgtattagaatagtg
aagattaatattattgtagatgagtaccaaaaaaattttaaaatttgttccattatgtaa
aagttaat-aaaaatagcacaaagacaatcgtctaaaagatattgattgtcttatcatat
taaaaagtatttattcaccctgtacttttaaaagtaaaaatcctattgccagccctacca
aaactaggactgaacttaatacagcactttctacaatttctccgaccatt-ttatttttc
cttattattttattattg---tcattcttttgacaagattatattgagctaa-ctaaaat
ccgtttctaccccaaactaccattgcgattgaaaatgtaaacattgccattagagctgcc
catcccaaacttaaaatatccataatttataatattc--ctaaaaataagatctctttta
cttaaactataatattcttaatattaaaaatatcattatataataagctaattataacag
agtactacttaattcgtattttagtgtatttaaagaagattgaa-tttttcgaggtac
tggataagtatttcatcctaagaaccatagtcaac----ttttgatttattataatgctt
aaatctaaatata--tatcactcaagaatcattagtaattatgatgttttaattatacg
att---aacatatttcaaaatagaagaattcatagcgactaagttaa-----tatgctaa
tataatttatcaat--aaaaaggaagttagtatttcaattatgcaaacaactataaataa
tgggcaaacgtctagtaaagaaactttactaacacctagattctatacgactgactttga
agagatggctaatatggatatttccggcaatcaagaagatttttggctatcctcgaaga
atttcgagctgactataatagtgaacattttattagagacgaagagtttaatcaatcttg
gtctaatttagaacataaaactaaatccttatttattgagtttttagaaagatcttgtac
cgcagaattttcaggtttttactatataaggaattatccagaaaattaaaagacagaaa
tccggttatagctgagtgctttttattaatgtctagagatgaagctaggcatgccggttt
tttaaataaagctatcggggacttcaatttatctttagatttaggattttaacgaagag
tcgcaagtatactttttctcacctaaatttatttttatgcaacctatctttctgaaaa
aattggatactggagatacataactatttaccgccatcttgaacaacatccagaacaccg
tatttatccaatctttagatttttgaaaattggtgtcaggatgaaaatcgtcacggaga
tttttttgctgctttgctcaaatcccaacctcatttttaaatgactggaaagcaaaaat
gtggtgcagatttttctattaagtgtatttgcaacaatgtacttaaacgactttcaaag
aattgattttataatgccataggcctagactctagacagtatgatatgcaagtaatacg
aaaaactaacgaaagtgccgctagagttttcccggttgctttagacgtggacaatccaaa
atttttcaaatatttagatacttgtgcatgtgataatagagctctgatcgatattgataa
taacaattctccattattcattaaatctatcgtaaagatacctctatatttttccttatt
tgcaaatttactaaagatatatttgattaagccaatagactcaaaaacagtatggaatac
agttcgatagttaaatacaagggaatgctttttcttataaaagctatctagtgattaa
tataaaat------aaaagcttccttct-----cgatggaaggagcttttattttgtcaa
ga---atataatgggattatatagatc-tagaactaagaacttactttttcttttgcttc

atacataacctctaaagtaattagatttttagaacaatcacgggcaaatttctctgtatt
tctttttactttacctctaacaaacccaggaatttatttaattctttctgtgcttcttc
tgaccaattaatactatcaacagcagaaattcctatacttaatgcttctgttgtatcatg
accaccaaatatttctagcaaatgatcttccataccaagtgtaaacgaattatatactaa
atcagcaatttgatttgttccctcataaccaagaaatggacggtaacttaaaggaaagtt
ttgtatatgcactggtgaagaaataacgccacaaggaatatttagacgcttaccaatatg
tctttccatctgtgttccaaaaaattgctgcaggttcagttttagcaattaaatctccaat
taaaccatgatcatctgaaacgattacttcatcgcagaattcttgaacttgatctttaaa
ccactcttcatcatatttgcaataagttccacaccaggcgacatgtatacccatttcctg
gtgtaaaattcgagttattgctgcagcatgagttgcatctccaaatacaatagccttttt
acctgttaaattctgacaatcaattgatcttgaaaaccatgcagattgtgaaataaatcg
ggtttgctcgtctatgtatttttcgtagtctacagcagctcccaatgcatttactaattg
ctgtattgatcggatgcatgcagctgtttgaacaatacccataggtgtaatgtcaacata
aggcatattaaattctttctctaaatatcgagctgtcattagtccagtttctctatatgg
aataaaattaaaccatgcagagggtaattttttaaatcttgaactgatgcattttctgg
aataatttggtttatttggatatctagatcttgaaataacctttttaattctgctatatc
atgttgattatgaaatcctaagcttactgctccaataatattaacagatggagttttagt
cttttgagttaaaacttgattattagatttagctttttccatatagaaagtgacaatttg
ttctaatgttctatcacctgcttgcagttcatttactctataatgattaacatcagctaa
taaaacatctgcttctgtttctatagatgctctgctaacaaagttttgcaaatcttcttg
caaaatactcgaagtacaagttggtgttagaataactaaatcagggctttcttctctgtc
ttttctagtaatattttctacaactttttcttgagaaccacgggctaacacatgtctatc
tacaacacttgctgtaacaggagtaaaatctctgtctctttcaagcattgaacgcattac
attaaaataatcgtctccgagaggcgcatgcataattgcatgaactttttttaaaagaact
ggcaattcttaaagttccaatatgagcagggcctgcatacatccaataagctaatttcat
attaaattttcctatgatttatttagacatagtaatacttttaaagctaactaacaaaca
ttagccaaacatctaatttaactccttgacaattttgtaatattttttaaatgaagctta
tcattataaatatttaaaaaatataattcagaataga----aataactgttagatcaagc
ttgaaagtaaagatttcttgcagttatagtttttctttaagtaaacatgtaaaaattgta
ctatatataaatgtactatatataaactttttttactgaatattggtaatgccagataca
attaacttaaatatgccttccccaacgtttggtggaagcactggcggttggttaagagcc
gcagaagtagaagaaaagtatgcgataacatggacaggcaaaaatgaaagtaagtttgaa
atgccaactggtggtaccgcaactatgcgaaatggagaaaacttactctatttagctaag
aaagaacaatgtttagctttaggtactcaactaaaaagtaaatttaaaatatctgactat
aagatttacagagtttttcctaacggagaagtgcaatatttgcatcctaaagatggagtc
tttccagaaaaagttaatactgggagagctagcgtcaacagtgttgatcattctattgga

caaaatattaatcctgtagatgtcaaattcatgaataaagcaacttacgattagtctctc
-aaaaacctagacataaaataactttttatatagtctaggtttttgataatgtatactgat
agtatggaagggtggccgagtggttgaaggcgtctgatttgaaatcagttgaacta--tc
tggttccgtgggttcgaatcccaccctttccgttataaaact------------atagtt
ggcttctcaaggcaagctagctatacactgatagtattaagtttagacttaataaagtct
acaacttgtgacaggttagaaatttttcggcatcctcatctggtatctctatactaaat
ttttcttctatggccattaccagctcaactgtatctagagaatcggctcccaggtcactt
gaaaaatttgcttccctagtcactatttttttttcaattcctagctgttctgctacaata
tcttgaacttttcaaagatttcattatcttgcataatatattatttcctctaaggtgtt
atcaaaaatttttcggcttatattattatccaactctaaaaaattagtaatttttagtcg
agattaaaatatttatacctatatgatatactattattctggataaatacgaagtttacg
atatggctcttctccaaaactacgagctctcaactggtagttgtcaactagattatgctg
catttttcgtatataggctgatcgaggtgtaagctgaactattgaactttcattcaagat
aatagtttcaatagccaatttagcttcttgaagagcctggatctcatcaaaatttttatt
tttacagagttctacccaattaagaccagatgaagtatgaatgtttaatatttttctgag
tgcacgagtaatttgtggaaccgtactattctgaattgtatatataataatttgttttga
ttttgcaatttgtctaagttttgtattttgctttacttgattacgtagcgctagaatagc
atcagacttttctatctccttggttaaaataattggtagatctagtgaagaaataactga
tgtaatatgctgccaactcaaagaataagcatataagtactgatgaggtatttccaattg
caaagattgtttattaatattaataggggcattgaaagtagttgacaataaagatgtatc
acgattttgtgatttattcaaatctaaggacttatttcttaattctctatattgtaaaac
cggaggcgcctgctgtgcgttaattgatttcgagcattagagatgtttacaggtaagac
ttctatagattgcgatggataacatttaattaaaattctaccattagcttgaatttgtct
tttttgcacaaaaggttgatggccttgcaaaatttgatctattgtctctttaactttatt
atgaactatccaaacatttcgctcgtgtatttcgatcgcaatctgaaaagcaggctcagc
ttttctttctagtatactcttttgcgtacctctacgtttagcttcatcgtcacctaatgt
tacatattgaattccaccaattaggtccgccaatgttggatttttaattaaactttctaa
atgatttccatgcgctgtacctactaattgaactcctctttctgctatagttcgagcagc
taacgcttctaattccgtgccaatttcatctataataataacctcgggcatgtgattttc
aacagcttctatcattacttgatgttgcaaatcgggccttgcgacttgcattcttctagc
tctaccaatagctggatgaggaatatctccgtcaccagctatttcatttgaagtatctat
aataaccactctttttttccatttcgtcagctaaaacacgggccatttctcgaactgcggt
tgttttaccaactcctggttttcctaagagtaaaattgaatcaccttgctgtagtaaatc
tcgaataatactaattgtaccaaagactgctctgccgacacgacaggttaagccaataac
actaccttctctattacgcagtgaactaatacgatgcaaggttttttcgataccagctcg
attatcgccgctaaaattaccaactttttttacacaataatctaagtcttgccaactaat

agatctttgagataaatattctgggttatctggaaatctagcttctggacgacgacctaa
atccataacgacttctattaaattatttctattaggatgctgttgtaaaggttctttgac
aaaatttggcaaaatttctagcaacttatctaagtcatctgcaataagcatgattgagta
tataataattaattaatcaaatagaagattcaagattctaaagattacaatacatattaa
agaatattatgattggaatcaattttatacttaaattgaacaggaagtta-tagatgtaa
aagaactgattaaactttacgcaataaatcgattggtcaaaattaatagtcatgttaaca
tgtttattggacaaaaagttcgtattaaatatagcaaacaaaaagttgctagtgatatag
cagataaagttggtgaattaggagttattaagggaataaagtttattaatagtcaatgtg
ttactattattgttgagtttgacaatcacactaggctttggatgtttagagaggaattaa
tctgtctaaatgaat------taaaaacatgaaacataatttatttatttattatacaa
aatttcttatgagtcgttaaaataataaattttaaaggagtacttttaattgtgcaaatt
actattaaaaaattacaagacttactttcgtctgtacaaagaaaaaagatccagacatta
aaactaaaacaaggtaagtttgaacttctattaaataagacctataaaaaagtcaatcaa
gaaattataccttcgcagaaatctgctgtattacaaaatagtccatctacgataattaaa
tcaataaataatacaaaaaaatcttctgttgtcaatgaagaccgtacagaatatgccact
attgtttctccaatggttggaacgttttatcattcacctgctcctggtgaaaaaatttt
gtacaagttggcgatgaggtcaaattcaatcaaacagtctgtattattgaagcaatgaag
ttaatgaacgaaatcgaagcagaaattgaaggcaagattatagaaattcttgttaaagat
ggtgatatagtagattgcgggcaagccttaatgaaggttgaaacataatgctaatttttc
cattttacatattgttaacagaaattatttagatagaaaaattagcttatactatagtta
tgaaaacttatttcgcaaaggcac-taaaaactaatatataaaattatttactttatctg
ccaaaaatagaaataagcgttttgattccttgtctcaagagaggagaatctcaatggcaa
ttagctcaaaagagcaagagacaaagaaggtaaaaatctcggttgataaaaatcccgtag
atacttctttcgaaaagtgggcccaaccaggccatttttctcgtacactagcaaaaggac
caaaaactactacttggatttggaatcttcatgctgatgctcacgacttcgatagtcaaa
ccagttctttagaagaagtttcacgtaagattttcagtgcacattttgggcagctgtctg
taatatttttatggcttagtggaatgtattttcacggagcccgcttctctaattatgttg
cttggttaagtaatccaacaggtattaagccaagtgcgcaggttgtttggcctatagttg
ggcaagaaattttaaatggcgatgtaggtggtggctttcaaggagtacaagttacatctg
gatggttccaactgtggagagcatcaggaattactacagaatttcagctttactgtactg
ctattggcggattagctatggctgctttaatgctgtttgcaggatggtttcattatcata
aagctgctccaaagttagaatggtttcaaaatgttgaatcaatgatgaatcaccatttag
ctgggcttttaggcttaggctgtttaggctggacaggccatcaaatccatttgtctttgc
ctattaataagctactagattctggcgtgtctccgcaagaaattccactacctcatgagt
ttttaattaatagagagcttatggctcagctgtatccaagttttagtaaaggattagttc
cattctttactttaaattgggctgaatattccgactttttaacttttaaaggaggtttaa

accctgttactggaggtttatggctaagtgatactgctcatcatcatctagcattagctg
ttctatttcttgctgcaggtcatatgtatagaaccaattggggtattggacatagcatga
aagaaattctagaagctcacaaaggaccttttaccggcaacggtcacgaaggtctatatg
aaattcttacaacttcttggcatgcacagcttgcaattaatttagctatgatgggatctt
taagcatcattgtagcacatcacatgtatgcaatgcctccttatccatatattgctactg
attacccaactcagttatcgctcttcactcatcatatgtggattggaggattttgtattg
ttggagcaggagcgcatgcttctatatttatggtaagagattataaccctgcagaaaatt
ataacaatcttttagatagagtcattaggcatcgagatgctattgtttctcatctaaatt
gggtatgtatatttcttggattccattcatttggtttatacattcacaatgatactatgc
gggcacttggaagatctcaagatatgttctctgatacagctatacagttacaacctattt
ttgctcaatgggtacaaagtatacacactttagctcctggaaatacagctccaaatgcat
tagcaacagctagttatgcatttggaggagatattgtttctgttggtaacaaagttgcaa
tgatgcctatttctttaggtactgcagatttttttagttcaccatatacatgcatttacta
ttcatgtaactgttttaattttagttaaagggttccttttctcaagaaactctagactaa
ttcctgacaaggccaatcttggcttcaggtttccatgtgatggacctggtagaggtggta
cttgccaagtgtctggctgggatcatgtttttcttggcttattctggatgtacaattctc
tgtctgtagcaattttttcactttagttggaaaatgcaatcagatgtttggggtagtgtat
ctccgtctggaaatgtttctcatattactggcggtaattttgcacagagtgcaattacaa
tcaatggatggctgagagatttcctttgggctcaagcatctcaagttattcaatcatacg
gttctgcgctatctgcatatggattaatttcttagcagcacattttgtatgggcattca
gtttgatgttcttatttagtggtagaggttattggcaagagcttatagaatcaatcgtat
gggcgcacaataagataaaagttgctcctgcaattcaaccaagagctttaagtattactc
aaggtagagcagtcggtgttgcacactacttattaggtggaattggtacaacttgggcat
tctttttagcgagaattatttcagtaggctaatagtgaaaataggataaaaaaaacaatt
atggcaacaaaatttcctaagtttagccaagctttatcacaagatcctacaactagaagg
atttggtatggtattgctacggcacatgactttgaaagtcatgatggaatgacagaagaa
aatttatatcaaaagatattcgcttcgcactttggacatctagcaattatcttcttatgg
acatctggtaatttattccatgtagcttggcaaggcaactttgaacagtgggtattaaat
cctttgaaagttaaaccaattgctcatgcaatttgggatccgcattttggacaacctgct
ttgaaagcttttagtaaaggtggctcagcttatccagtaaatatagcatattctggcgta
taccactggtggtatactattggtatgagaagcaatcaagacctgtattctggggcttta
ttcttactagttttatcagctttactcttatttggagggtggctacatctacaaccaaaa
ttcaagcctggtttatcatggtttaaaaataacgaatcaagattaaatcatcatttatct
ggattatttggggttagttctttagcctggacaggtcatttagtacatgttgctatacct
gaggcaagaggacaacatgtaggatgggataattttacaaccgtattacctcatccagcc
ggtttacagccgtttttcagtggtaattggagtgtatatgctcaaaatccagatacagct

caacatttattcggaactaatgaaggtgcgggtacagcaattctgacatttctaggagga
tttcatcctcaaagtcagtctttgtggctaactgatatggctcatcaccatttggctatt
gcagtagtattcattgttgctggacatatgtataggactaattggggaattgggcacaat
ctaaaagatatttagatgctcatagaccacctagtggtagattaggagctggacataaa
gggctatttgatactattactaattctttacacatacagttgggattagcattggcttcc
ctaggtgtaattacttcgttggtagctcaacatatgtatgctatgcctccatatgctttc
atggctaaagatttcacaactcaagcatctttgtacacacatcatcaatatattgctggg
tttctaatggttggagcttttgctcatggggcaatattctttgttcgagactatgaccct
gaacagaataaagataatgttttggctcgtatgctagaacataaagaagctatcatttct
catttaagttgggtaactctattttagggtttcatacattaggcctttatgttcacaat
gatacaatgattgcttttggaactcctgaaaaacaaattctaattgagccggtatttgct
caatggattcaagcctcttcagggaaagcactttatgggtttgatgtgttactatcatcc
tctactaatatcgcaacacaagctggtagcaatatttggctgccaggctggttagaagcg
attaatagcggaaaaaattcattgttttaacaattggtcctggtgacttcttagttcat
catgcaattgcattgggattacatactactacgttaattttagttaaaggtgctttagat
gcaagaggctctaaacttatgccggacaaaaaagactttggatatagttttccttgcgat
ggacctggcagaggcggcacctgtgatatatctgcatgggatgcgttctatttagctgta
ttttggatgctaaatacaataggttgggtaacattttattggcattggaaacatattaca
atatggcaaggcaatgcaactcaattcaatgagtcttcaacttatctaatgggatggttt
agagattacttatggctaaattcttctccattaattaatggttataatccatatggcatg
aataatttatcagtatggtcatggatgttcctatttggacatttagtatgggcaacagga
tttatgttcttgatctcctggcgtggctattggcaagagttaattgaaactctagcatgg
gcgcatgaacgtacacctttagcaaacttgattcgttggaaagataaacctgttgcatta
tcaattgtacaggcaagattagtaggtttagcacatt-tttctgtaggatatgtattaac
ttacgcagcttttgtattagcttcaacagcaggcaaatttggttagactaaa-----ttc
gaattagctaa-----aaaaaagtcagtcataata-gttttatgactgactttttttagat
ttaaaacaaaaacttactaaagtttcacctcaatatctacaccagaaggcaaatttaact
tcattaatgcatctatcgtttgagaagaaggttgatgaatatcaataattcttctgtgag
atcttatttcaaagtgttctcttgagtctttatctacatgtggagaacgtaaaacacaat
aaattcttcttttgtcggtaaaggaattggccctactgcaacggcattagttctagatg
cggtatctagtattttgtcacacgacgtattaagtataatagaattatatgctttcagtt
taattctgatttttgtctgctgagtaattgtcatgttaaaaagatcttattatttaagaa
ttttagagacaacacctgcgcctacggtacggccaccttctctaatagcgaaacgcatac
cttgctcaattgcaattgcattaattaattcagcagtcatcttaattctgtcaccaggca
taaccatttctgcatcagtaccatcatcagcagtaaactgattaatagtaccagttacat
cagttgttctaacataaaactgaggtctatatcctggaaaaaatggagtatgtcttccgc

cctcctctttagttagaatataaacttctgcttcaaattgagtatgaggtgtaattgtac
caggtttagctaataccatacctctttcaatatctttttctgcacacctcttaaaagaa
ttccaatattatcacccgctagaccttcttctaacgtttttgaaacatttctaatccag
taatagtcgttgtacgagtttctcgtaaacctacaatttcaattgtgtcaccaacttaa
taatgcctctttcaattctaccagtggcaacagttccacgtcctgtaatagaaaaaacat
cttctacagccattaagaaagttttatcgacatctctctccggcgttggaatatatgtgt
caactgcttccataagtgaaaaaatcttgtcaacccatttatcttcaccttgcttaatag
ctgggtttttcgtcacagcttctaatgccaataaggcagaacctgcaacaaaaggaatat
catctccaggaaagtcgtattgacttaataattcccttccttctaattctactaattcta
gtagctcttcgtcatctacttgatcttccttatttaggaatactactaatgtaggaacac
ctacttgttttgctaataaaatatgttcacgagtttgtggcattggaccgtctgccgcag
atacaactaaaatagctccatccatctgagcagcacccgtaatcatatttttacgtagt
cggcatggcctggacaatctacgtgagcataatgacgattatctgtttcgtattcaacat
gagcagtattaatagtaatacctctagctttttcttctggagcggcatcaatttcatcaa
attttttcgctgcagtagaccctaaagttgataaagtcgcagagatcgctgctgttaaag
ttgtcttaccatgatcaacatgaccaattgtgccaatattgacatgaggtttttacgtt
caaatttagatcgagccatgctttttgttttccttatagtaaaagttgctttatagttac
ttttaacgataaatcttgtggattgaaagatttattatttaatacagacaaga-----ta
caatataactaatctaatatcgatagtgagcaaacgctttatttgcttctgccattctat
gtgtatcctctctctttctaatagaatttcctgtttcattagctgcatccataatttcat
tagctaatttcatagacatacttttaccagatctgtctctagagaatttagtaatccatc
ttagtgctaaattcgtacctctataagctcgtacttcaataggaacttggtaagtagaac
caccaactcttcttgcttttacttctactagaggagtaatatttcggatggctttttcta
aaatatttaaaggatctgattctgttctctctttaacgatatctaaagcctgatatataa
ttctttgagataaagtttttttaccacttttcaaaatacgaacagttaacatacttacga
gtctgcttttatataaaggatcaggtgatgcaaaccttttttttagctgtattacgacgag
acatagtatttaaaattggttattgtaataa-tgttc-tattagcaggtatttaagattt
aggctttttttgtcccatatttagatcggcttttacgacgatcttttactcctgcagcatc
taaagtaccacgtactacatgatatcggactccaggcaaatctttaattcggccgcccct
aatcagtaccacagaatgctcttgaatattatgacctacgccaggaatataagctgtaac
ttcaaatccagatgttaatctaacacgcgctacttttcttaaagcagagttcggtttttt
aggagttgtagtatatactcttgtacaaacacctcttctttgagggcaactttgaagagc
tggagattttgtttttttatgtatttttcgtctttcggatctaacaagttgttgaattgt
tggcataataaaattgatttaaagtctttaatagatactatttgaacttgctttacatga
attggctagtttttatttatcttatacttacgcataaatctttctactctaccttctgta
tcaataattctctgtgaaccagtaaagaatggatggtttcctgaccagatatctacgtgt

agttctggctttgtggacccaatcgtcataattaactggccgtcacagtaaacttttgct
tctggataccaatttggatgtatattatcttttgccattgtttttataattatataaatg
tatcaataataactattaacgcttagaaaattgaggagcctttctggctttctttaaacc
atatttttttctctcttttactcttggatctcttgttaaatagccttcagacttaagtgt
cgttctattttctggattaattgagcacaatgctcttgcaacacctaaacgaattgcatc
agcttgccctgtcaaacctcctcctctagcatttacatggatatcatactggtttagtag
tcctaaaacttgtaatggtgcatatgaaactcttaagtaattaggactaaattgaagata
agactctcctggtataccgtttataattaaattacctgaccctgggactagtcttacttg
tgcaacagaacacttccggcgacctgttccagaatagattgcgcgagttttaattaattc
tgtcgacataacattccttgataactaaataaatatacattctttatactatatactctt
gtggcttttgcgcgacatgtgggtgaattgggccagaatacactttaagcttcgtaaata
gttttctgcctaatggacctttaggaagcatacctttaaccgatttttcaataattctgt
taggtaatcttgtctgaagctgatcaaatgtttcaacttttaatccgccaggctgtccag
aatgtcttctgtatagtttttgattcgttttatttccacttacagatacgtgagcagaat
taataacgattacataatctccagtatctagatagggtgtataagaaggcttgtttttac
ctcttaagatattagaaatatgagtagatattctaccaagtgtctgattttagcgtcta
taacataccaatgagaattagtatttaatgagggtgattgcgttttattcatgaaaagat
taatacttttggttgacaataagagaatatgtaaatggtttacattcaatttctatacaa
gtagtattt-aaaaatatta-------------ttaacaaagagaacttttctaacagtt
atttaaattgtttgtcagcaagaaaatttctagttatcaataacttatactatttttaat
aatataaaatatattaatcactttttttcttttggcaaacttatacctagtttttttttgc-
aaggcatatataacttcttcagctgatttctggccaaaatttttaatttctagcagttct
tcttgagagtaatctagtaaatctgcaatagaatggatttgagctcgctttaagcagtta
taagccctaacagataactgcagttcttctattaaaacttgactaattttttatcttct
ttgctacgataattatctgctgatttaaagtctaaatttcttagtgaacaaaaaagatta
gttaaaactgtagctccttgacttattgcttcttgaggagagatgctcccatttgtccag
atttgtataatcaatctatcttttatgctgttgctaccaatacggacttcttctacttta
taattaactttattaactggcataaaaacagagtctacttgcaaaaaatccacagataat
tcatctacagcttttcagctaagcgatacccgcaatttttttcaattttaaactccatt
tcaaatattgtattattgcaaatagttgcaatatactgtctaggatctactacctctata
tcagaagataattcaaataggccggctgtaactatagctggcccttgaactctaattcga
ccaatttgagattctttgttataacttttaaatactacttcttttagattaagtaatatt
tctaacacatcttctcttaccccaggaattgtagaaaactcatggttcactccagcaatc
cgtacagcaactatagcagtaccttcaagatctgacaatattgatcttctcaaagcatta
cctaatgtaataccttgtccttgatttaatggttctattacaaaactaccgtactgccca
cgcgccccatctgttcttgactctatgcattcaatttgaaattgagccacctaagaaagc

tcc-tttaaaataatcagtgattagtaagtctctctaaaacaagaaggaatttatactcg
gcgtttcttaggagggcgacatccattatgaggtacaggagtaatatcttttattagagt
aatctctaatcctgcagcctgcaaagctctaattgctgtttctcgacccgctcctggacc
atttactagaacttcagtttggcgcataccttgatccatagcttgtctagctgctttttc
agctgctgtttgagctgcaaaaggtgttcctttttagctcccttaaatccacttgcacc
agaggatgaccatgataatgtttctcctttaaattagtaatagtaacaattgtattatt
gaatgtagatttaatatgtgtaataccgttaactgcattacgtttagtttttcttgctcc
ggatttttttatttgtctagccatcgtgtctcgtctattatatagttaattaaaagatta
ttttcttggagctttttcttacctgctactgtttttttacctcctctgcgtgttctagc
attagttctagttctttgtcctctcaaaggaagaccaagacggtgtcttctacctctata
agtactaatttccataagtctcttaatgctcatagactcaaaacgtttgagatccccttc
aatctgataattagactcaagaatttctcttatactgacaacttgttgatcatttaaatc
ttgacacttaatatcagcgtctatgtttgtttttctaatatttctttcgagcgagatag
tccaataccataaatatatgttaaagctatctctattcttttgtttcttggaagatctac
tccagcaattctggccactttgtcttgtctccaataaataatatgtat-ttttataacgg
ttgtttata-agcttagccttgtctctgcttatgtttaggattagtgcaaattaccatca
cctttctgtgacgccgaattattctacatttttcacacatttttcgaacagaaggacgaa
ctttcatattaa----ctctatatactaggaatattactatattaactctattaattatc
gacattaaaaatgtcaacaatatattgtactactttaactgttgtagattttacttcgtc
atactatcatattttttgatatgacgtatgtctgaatttgcttagctgtgtctattgcg
actccaactaaaataagtaaagacgtagccccgagacctcttaagttttggatctgagta
accttttctattataaacggaatcagcgctactgtaaataaaaacgaggctcctaggaat
gtcagtctatttaatatgacttgtaaataatcaatagtcgcttgacctggacgaatatta
ggaatgcttgcacccatttttttaaatttatagcaatatcttctggattcattactatt
gatgtataaaaatagctaaagaaaaggatcaaagcacaataaagaagaaggtatagtgaa
ccattaggacaaaacaggtataggatttggagtaatgttttattttgaataatttgggtt
agataagatgggagggccatagatgcagatgcaaaaacaataggcataactccaccttga
tttaatttcaagggcaagtaactatttggatccaaaattgaagattttcctagctgtctt
gctgaaataattttaattcttcttgttccttcttgtacacaaattgtaattattatcatt
aataaaaagatcgctataaataatccgaacttaagacttgcattactataactagcatca
aaaaatgactgtgtaaaattctttggtagtcctgacacaatgttttgaaaaataagtaaa
gaggctccattaccaattcccttttccgtaattaactctgataaccacataataatcata
gaacctgctgttaaagctaaaacagactcgcagacaaatgcaaaattccaattaaaaaca
tatggttttacccatatagagattgctccagattgtaaagtcgcccaacctagagctaag
taccttgtaatttgagttatttttttggcggcccaattcaccttcttccttctgtaatttt
tccagattggggacaattttcgtaagtagctgcattacaattgaagagttaatataagga

acaatacctaacgcaaaaatccctattgttgaaaaacctcctccagaaaaaatattcaga
aaatttactaaagtatttttttctacacttgcataaaaggcgtcatgatctatacctgga
acaggtataaatattcctaaacgtgctaaaactaatagaaagagagtaaagataatacga
tttcttagatcactttttggctcataaataaattttaaatgaaaagagatacttt--tt
tatgtaaaaaccctattccaagacttgtataagtttaaaatagggaagtaa---aatatt
atagtaatattaata-taatattctatgtagaatacaggctttctattggaactcctcga
tctttagctgcttcactaaaagttcttaattgagttaagccattgagaacagctcgcgcg
ttatttaatgtattattagaacctaattgcttagctaatatattctgtacacccgacaat
tcgaggactgtccttacagaacctccggcaattacacctgagccaggtgcagagggcctt
aatataactttcgcggcaccagaaattccgttaataggatgaggtatagaattcgatttt
gtcagtggaactgtaactagatgtttttagcatctgttactcctttttttactgcacca
attacatcgcttgcttttcccacgccgacaccaacttggccttgctcattaccaataaca
agaataactcgaaagcttagtttttacctccttttacaactttagtaactcttttaact
tgtacgactctttcttcccagccactatctttatcttttcctttgctctgtttttacga
ttggccattttaagaattattcctaattagttaatta-cgattctagaaacccatgcctg
cttccttagcagcttcagccaaggccttaactcttccatgatataactttcctcctctat
cgaagacgacattttaatgccttctttcatagactgttctgctaactgtttaccaacta
cgcgagaagtatcacagtttggtcttatgttatcagattctttattatttaaattaacag
atgatgtagctactaatgtaataccttgtgtgtcatcaattatttgtgcgtatatatgtt
tattagatctaaatacacacagacgaggcctacttgaggttccttgaactttttccgaa
ctcttttatgcttatgaattctagtttgtttagtgtttagtttcattattatttaccttt
tccagctttcccagcttttcttctaacaaattcaccttgatatctaattccttttccttt
ataaggctcaggaggcctaatagaacgaatagttgaagcaacctgaccgacaacttcttt
atctatgccagagacagtaatatttgtgttgttttcaactttaatttcaatattggcagg
aggtttaattttcactacatgactatagccaacacttaagattaaatcttggttatcaat
ttgagaacgatagcctacaccttgtatttgcagtttttaaaaaatccattagaaacacc
ttcaatcatattactaataagagttctggatagcccatgtaactgactagccatttttgt
ttgtccactcgtcttgacagcaattgtgtcgttaagtatttctaaattaataccagctgg
taaagttctggatagggtacctttaggtcctgtcacggtgatagtctgaccatcgaactg
agtactaagatttgtcggcaataaaattattttttttccaatacgagacat-attccacc
tataaaatagttaccaaatatagcataatatttcaccaccaagaccatcatgacgagctt
gtctatctgtcataacgcctctagaagtagaaataagagcgatgcctaaacctccaagaa
ctctgggcaactctttatgatttgcgtagactcttagtccgggtttactaattcttttca
gagcagtaataactggttgacgattttaccattatacttaagagaaatcattaaatgag
tctctataccttcacccatttgttcgaaattttgaacaaatccttcttctttcagtactg
ttgccatattgcatgtcattttcgttgctggaacttgcacaatttgatgtcttgctaagt

ttgcgttacgaatacgtgtcagcatatcggcgatcgtatcgttgaccaccttgatcctcc
ttgcga-----tgaattatttgaaatattttcaagattccctgaaaggcatccctagctt
ttttaacaaagctagaccttcttgatctgttttagctgtagtgacaattgatatatctaa
accacgaatttgatcaatattatcatagtctatttctggaaagatcaactgttcacgtaa
acctaaattgtaattacctttgccgtcaaaacttctaggactaattcctctgaagtctct
aattcttggtaatgttaaattaattaatttctctaaaaaagaatacatcttgtcttttct
tagatgcacaacaattccgataggaacctcttctcgaattttaaatcctgcaatagattt
tttagcttttgtgacaattggtttttgtccagttattaatgtcaattcttgaatactact
ttcaagagccttagcattctgagaagcttcacctaagccacggttaatagtaattttagt
aaacctaggaacttcatgtacattttttgtactgaaattcatcttttaaagattgagtaac
agttgttttatatttttcttttaatcctattgccattatattttcatcaattatttaata
agttcgccagtctttttcagttttcgaa-tttttggcctttatcattaattattactga
ggatcgactagcaatattattttgttcactaaataacataacgttagaggtatgtattgg
agcttcaaatttgataatttctcccgtttctccttcttgttgaggtttttatgcttcac
tttgagattaattcctttaacaatcaccttatttgttttataaataattgcaataatttc
acctgtttttgttttatcacttccagaaataacttgaactaagtccccttttttttaattt
aatttttgtattattttttggttgttttagaaggacctttcattatactacctccggtgca
agagaaactatttagaaaaattttatctcttaactctcgggctataggtccgaagact
cttgtgccacgtggattattatcttgattaatgataactgccgcattatctccaaatcta
atgctcataccgtcagttctccgtaaagctttacgagttcttactacaacggctctgaca
acatcagatcgtttaactggcatattgggagacgcatctttgactactccaataataaca
tctccaatagatgcataagaaggattactagtgcctagtactctaatacacattattttt
ctagcaccactattatctgcaacattaagatagctttgagtctgtatcatacttttattc
tgtataagattaattatcaaaagacttagataagatattaaccattgtccaacactttgt
gcgacttaaaggtcgtgtctcttgtattgtaacaatatcgccgattgtgcattcattatt
ttcatcatgcgctttatatttctttgtccggatcatagttttttgcgtactttctatgaga
gattctattttctacagctactactatagttttattcattttatcgcttactactttacc
tgttgtttcttttaaaggcatagtctttatattttttatatat---------aattaaat
tttaagattattacatggttgacttagtacgagacttttcaacagttaaaagttgggcta
atctatgcttagaatgtttaaatagatgaggctggaaatcttgccttgtggctcttttta
gccttaaatcaaaaagctctctttttattacaaggatttcttcagctaaagaagaagagt
ccaggtttgtaacatctgatattttagggaaagtcatattttaggattctgttgtattcc
gaactataaatttagtcttgattggtaatttataagaagctaatttcatagcttgttgag
cagtttttttgtggtacacctgtaatttcaaatagaatatgtccaggcttaataactgcaa
cccaatattctggagctcctttacctgaccccatgcgagtttcagctggacgggcagtga
ctggtttatctggaaatactctaatccatagtttaccacctcttctcacatatctagtaa

tagttcttcgagtagcttctatttgtctagaagttaaccaaactggctctgtcgcttgta
atgcataatcaccgaacgcaattgtgttacctttgctagcagaacctttcattctacctc
tatgttgttttctaaattttgttttcttggggcttagcataaaaattaaaaaataataat
aattaagaagctgttgaatcaggagtttcctgattataagcagattctagacttttcgat
tctggcaaaatttctcctttaaaaagccaaacttttactccgagcacaccatacgtagtg
tgagcttggcgatgacaataatcaatatcggctcttaaagtttgtaaaggtacacgacct
tctctaacccattcacttctagctatctctgcaccattaagccgaccagatacttgaatt
ttaacaccctgtgtatttgctctttgagctctttgaactgcttgtctgacagctctacga
aaggccactctttttctagctgttgagtaataaattctgctactaaggttgcttcagaa
tctggatctgcaatctctacaacatttactctgagttgtttgctagggtctaggattaag
gacagcgattttcttaaagactcaattcccgctccagattttccaagtacgattcctggt
cgagctgttgctatgagaatttctacttgatcaactttacgattaatttcaattttagca
atactagcattactaagttttgaatgtataaatgagcgaattttatgatcttcttgtaaa
agaactgggtagtctttagagttagcaaaccatgaagaacgatgtttttgggtaatgcct
atgcgaaagcccaaaggatgaatttttttgacccacagtatccttgattataagttaaaag
ctataatgactaattgacgttaaa--------agactacagttctgaaacacctaatgta
atatgacaagttggtttatgtattggaaaggctcttccttgtgctctaggctgaaatctt
tttaaagtcgggcctttgtctgcaaaggctttactaacaaacaattggttttatttaat
ccatcattatgctcagcattcgctgcagcagattccaggatttgctttatatgagaacag
actcggtatggcataaattccagaataattaatgcttcttggtattttctacctctaatt
tggtctaatacacgacggactttatgcggagaaagacgaatgtatttccctactgcttta
gtttcttttacgtttttttgtaatactcatagttatagtttaacgacgggcttttctgtca
ccttttacgtgagtgcggaaagttcttgttggaacaaactctcctaatttatgtcctacc
atttggtctgacacaaagacaggaaaatgttgtttaccattataaacagctattgtatgc
cctaccatatcgggaataatagtagatgctctcgaccaagtttttaggacttcttttttt
cctgaaatatttaatgcttctatacgtttaagaagactaacatcaataaaagggccttta
tgtatagatcttgacataataataaatcattagattcttaataaaataaattctatttac
ggcgacgtaagacgtacgggttactatatttatttggatttcgtgttttaacacccaatg
caggtttaccccaaggtgttacaggacgagctcgtccaattggagatttgccttcaccac
caccatgtggatggtctacaggattcatgacaacacctctaacagtaggtcttttaccta
accaacgatttctaccagctttaccaagagtaatattactagcatcaatattaccaactt
gtccaatagtagcgtagcattcttttcgaatcattcgaacttcgcttgaaggcaatttta
cagtaacaaaagtaccttcttttgctactatttgagcataagttcctgctgcacgaacaa
tctgtcctccgcacgaaggtcttaattctatattatgtactgctgttcctaaaggaatac
tagataaaggcaaagcatttccaacttcgataggggcagtaggaccagaaagaaccatag
atcctacactgagagatcgagggtgtagaatatatcttttctcaccatcaagatagtgta

ataatgcaattcttgcatttctattcggatcatattcaatagaagcaactttagcaacta
tattatgtctatttcttttaaagtcaattaatctgtactgctgcttatgtccaccacctt
tatgacgacaggtaatgacacctctattatttcgacctttgcagaaatggtgtttaacta
ttaatgatttttctggcttatcagtagtaatctctgaaaaggtagaaaccgttctatttc
ttgtccctggtgtgtaggcacgatataaacgaattgccatataagaagatttgagaagag
taaatgttgaa------ctatgtgatatatttaagtttctggaaacaaattaatagaatc
ttctgaggcaagtgttacaattgctttcttgtaatgtggtcgcttccctacgaatctacc
gatgctcctttttttcttaggaggatgacaagtgttaacacctgtaacttgcacgttgaa
aatatactgtatagcagccttaatattaatttttgttgctttgggatcaacggcaaaaca
atactgattctcttctaataatttagttgtcttatcagtaatgatgggatatttaaccag
atctaataaacctcttgaatcaatgctatccattatatacctcttgtattttagataaag
catcaactgtaataataattttatgtgcagctaacaaagccataatatttagtgtatcag
ctgaaataagttctacgttgtgtagattgcgaatagaaagataaacattcggatcttttt
tgtctactatgactaaaactttttatttaaatcaagattccatcgatgtatagcttcca
taaatagtttagtcttgggctgatgaaaataactgttaaaattttctacaatcagtgtat
taactgatttgttatttaatgctgtccttaatgctaattgtctttctttttattcattt
ttttggtaaaactacgaggttttggcccaaatattacaccaccacctctccatagaggtg
aacggatagaacctgctcttgctcgaccagtccctttttgacgccatggtttacgacctc
cacctcgaacttcacttcttgttttagtattagcagaaccctggcgcttttcattacttt
gctttactaaagctctatggactaagtacatcccagaatcttgactgaccttaagattta
aatcagcgttgccacttacttgaccttcccaattatagacttgataatttaattgcgtgt
taactgtcatgaaatatatcgtatgaaagaatattgctacttactaattttaccaaagc
gccaggtttaccaggaacagctcccttaacgatcaacaaatcattctctgagttaatact
tacaatttgcaaatttttattgtaactttttattaccaagttggccagccatgttttt
acctggataaactcttccaggcgttgtaccggctccaatagagccaggttgcctatgatt
tttcgaaccatgtgacattgggcctctactaaaatgatgcctcttttggtaaccggagaa
acctttacctacacttctagaagagacattaatcttttggcctacttgaaatagatctgt
agatagtatttggctaacttcgaaatcatcagtagatttcatttcgtattcacgtaaata
ttttagtgggggagcctgtgatttttttaagtgtcccaacaatggtttatttaattttg
ttcagcaacttgcttgtaaccgacttgaatagcattgtagccatcagtagatacagcttt
aatttgagtaataacacatggcccaacttgaattacagtaactggaattgataaaccagc
ttcatcaaagaattgggtcatacctactttagtaccgagtataccaacagacactagatt
tctcctttttgagagatttatactatcattgtacataaaaattctacttcatagttactg
aag--taaattaaactaacagatgcatgatattattcatgacaacataaaggttaaaaac
tttacctatttaatctaaaatagaaacagctttttgtctagttacagcgtatataaatta
gaagtatgcttattaaatttttataaactact-tagatttaactgatattgatatccgt

aacttacttgcagtaatatc----aatcaacatgtataagtttaattgcctaaactagaa
ccagctattattagaaataattcaagtatagataaaagcgtatttgaacttttatatatt
gacaaatataatggaatatattgaggttcggtaactacaccttttttattttttacatttat
tgtgca--gtatatatacatctaatgcagtaaacataagaatataac----tgtatagca
aatgtaattacacaaattagagaggtattaatgggcaaagtcgttggaattgatcttgga
acaacgaattctgtaattgctgttatggaaggaggtaaacctaccgtcataccgaatgca
gaaggttttagaactacagcttctgttgttgcatatactaaaagtggagataaacttgta
ggacaaattgccaggaggcaagctgttattaacccagaaaacactttctactctgtcaaa
agattcataggacgtaaacagaatgaaatttcgcaagagattcggcaaacatcatataat
gttaaaactagtggatcaagcataaaaattgcttgccctgcactgaataaagattttgct
ccagaagaaatttcagctcaagtactgagaaaacttgttgaagatgctagtacgtactta
ggtgagactgttacacaagcagtaataactgtacctgcttactttaacgattcacaaaga
caagctactaaagacgcaggtaaaatagcaggcttagatgtattgagaattattaatgag
cctaccgcagcttctttgtcttatggactagacaagcaaaataatgaaacaatactagta
tttgaccttggtgggggcacatttgatgtatctgtattagaagttggagatggagtattt
gaagtactctcaacttctggagatacacatttaggcggagatgactttgatcagcagatt
gtagaatggctaatcaaagattttaaacagaatgaaggtattgatcttggtaaagataga
caagcacttcagagattgaccgaggctgcagaaaaagcaaagatagagctgtcaaattta
actcaaacagagatcaatcttccttttattactgccacacaagatggtccaaaacattta
gaaaaaactgtaactagagggaagtttgaagaactttgttcaaatttaatagataaatgt
agtatccctgtaaataatgctctgaaagatgcaaaactagaagcttccagtattgatgaa
gttgttttagttggtggatctacaaggattccagccatacagcaaatggttaaaagatta
attggtaaagatccaaaccaaagtgtcaatccagatgaagttgttgctattggtgcagcc
gtacaagctggagttttagcaggcgaagtcaaagatattctattactagatgtgacgccg
ttatctttaggagtggaaactctgggtggcgtgatgacaaagattataccaagaaatact
actattcctacaaaaaaatcagaagtattttctacagctgtagataatcaaccaaatgtg
gaaattcaagtacttcaaggcgaaagagaactaacaaaagacaataaaagcttagggacg
ttccgattagatggcattatgcctgcacctagaggagttcctcaaattgaagttaccttt
gatattgacgctaacggaattttatctgtaaaagccaaagaaaaggctactggtaaagag
caatcaattactatatctggagcatcaactttacctaaagatgatgttgaaagaatggta
aaagaagctgaagaaaatttcgatacagaccaaaaaagaagaaagaatattgacacaaag
aatcaagcagagtccctatgttaccaagctgaaaagcaagttaaagagtttgaagacaaa
attagccaagatttaaaaataaaaatagaagagctaattacagagcttagatctagtcta
gagaaagaagaatatgacaatattgaatctatttctcagcaattacaaaatgctctgatg
gacattggaaaaaaatgctgctcagactgaaagtaaagatacaaaagcgaaggatgacgac
actgtgattgacactgatttctctgaagctaagt-aaaagta-agcgggtaacgcgattc

gaacgcgcgacatcaaccttggcaaggttgcgctctaccactgagctatacccgcataga
ttgtattattacaaaaattaagatatttgtcaatctgtta------atctatac---atc
tagactattaattttggaattacatattagctaactgttgtaaatataacaattgctata
attttttaa-------aagtacttatatcat-tatataagtacttttatatt-------
-caaaaatacaaataagtattatag-------ctaaactactaaggaattaactaagcta
gtaacaattactaatcctgtccataagccggctcctgtatatattaatccttttgattgc
tcccattggccaggggatgctaaaacaacaggtacgcctactactaacacagtcgataat
gcaattaataatagcactaataattggatcgcaataatcatttaatatgttctcctgaaa
aattcaaatgttaataatactgtatataatacaatgaagcttttgaccttcagcaattta
tctatgaagaaataatttaaaattattgttttctaaataaaccttaaattaagtattaaa
atctttattgtaaacaataatactatatttttta--gtagataaaaaatttaaagaaag
tctttttaaaaaaaaattaagataaggtgatatgctgtataaaaagctaagtattatta
attattagagaaatttaaa-ataagaggtaaaacatatgaactcagcccttttttagca
aaactaccagaagcttatgccattttaaaccgatcattgatatcttacccgtaattcct
gtatttttcttcttttagcttttgtttggcaagcagcaattggttttagataacattta
ggttataaaaagaataacaaatatacagagtgccaattttgagctctaatcttatatct
tacacaataataactattatggttgaacccttactatcaggaattgttcttggacttatt
cctattactttattcggattattagtagcagcttatttacaatatcaacggggcaaccaa
ttaggactgtaaagcctaaattatagatatttataaaaactacaataattatagtttta
taaatatctacaaataagctaattttaaatacagaaataatactattgaaactagagtga
taacaaaaaaaatttatgtaa---taa---------------------------------

---------------------------caagctaattgtttaccagcttgacttagtaac
tcctggtaaaagacattcatgagacatttctcgaaaaacatggcgagataatccaaaatc
tcgataataacctctacttctacctgttaaccaacatctatttctccctctaacagcggc
gctgtttctaggcatttcttggagtttttgtcttaaatccattttgtcagaaaagctgcc
agcttctttcagttgatctttaatagccagtctttttaaataatatttttttgcaagttt
cgatctcttaatctctctttggatcatattctttttagccatgctgtaa-aactctcttt
taaaattatttattcaatatattagctaaaaaagctcctgcctgcaacttttatatattt
aaaaataaatagcataaagtagtgaaatgcaattgatatttaatcaacgatacc---act
ttatgctattatcaaaaatttatttagtaaagttcttgttcaacatgagttgaaatagtg
caatcagatgaaggatatgcaatacaggttaaaacatagccagctaaaagttgatcatca
tctaagaatgattgatcagcttgatctacagttccttctgtaactttacctgcacaagtt
gaacaagctccagctctacaagagtaaggaagttcaattccttcttcttctgctgcatct
agaatatatgtatcctccgcacaattaaatgtgacatcaattccttcttcttcacatagt
aaatgaattttataatcagccatttcttcttactccttaataaagatacacttgaaactt
aataaagcaaaat-aatttaaagaacttaccaaaaaattatatactagtttcattatgca
tgatagactggaaaaaaagaaaatagtatactattctgaaaaatcaagctacatttccta
ctaaatagaactatagtaggtccaaagtatatacataaattcatatagttaaaattactt
ttatcatttaaatagctgaacttgatagta---------tagattgatcatc------aa
gctagtcttctgc---caataaataattaattatgatttcaatgcttaatcaacaagtag
aacttaagcctattcttaaatttcagataacacaaatatattcgcattctcttattcagg
aaattaaagccttaataactagacttgttttgcaagtttggagaaggccggctacattga
tggcaggtattatccaacctttattatggttaattctatttggtgggcttttctacaatg
ctcctataaatttgttcactattaatacaagctataattgtttttttgagctctgggatta
taatttttacctcttttactggagctttgaattcaggtcttccattaatgtttgatagag
aatttggatttttaaatagattattaacggctcctttagtatcgaggacttctatcattt
tatcttctgctacttttatgacttgtattagtttaatacaagttgtatttatagttacag
cttctctttttatgggaaactcacctctaaatagcgatagtactatgatttttggactta
tgattctattagtgactgtaggagttacaatgcttagtttagctttatctttcactctgc
caggtcatattgagctattagcatttattttagtagttaacttgcccttttttattttcta
gtacggctttagctcctttatatttcatgccgccatggcttcagttaattgcaagtctca
atccattgagttatgcaatagaaggtacaagatacttatattcaagcgtaaactggaatt
ttacagagtgtgtgattaagattagctggggagatatttgtttagggcaaattattattt
tattaatcgctttagatataatggcagcttatcttgtgtctaatatattaaaagctaaac

ttaatta-----------------aaattttaataaaaaatttattaaatttattttaca
aagattttactatataatactaatagtagtatggataattgaaatagaaatagtttttc
aaaaccaaagctattatcaacaatgataaacatttgtaagaaagtcaacaaagtatgttc
ttattcataggaggcatgtagtcaatgggactaccatggtaccgtgtacacacggttgtt
ttaaatgatcctggacggttaattgcagtccacctaatgcatactgcacttgtagcgggt
tgggcaggatctatggcattatacgaactagctgtatttgatccttcagatccagtatta
aatccaatgtggcgacaaggcatgtttgttatgccatttatggctagactgggcgtaaca
gattcatggggaggatggagtataacaggagagagcgtatccaatcctggactgtggagt
tttgaaggtgtagccttaactcatatagtcctttctggcatgctttttctagctgctatt
tggcattgggtttattgggatttggaattatttagagatccacgaactggcgagccagcc
ttagatttaccgaaaatttttggaattcatttattgctatcaagtctactttgtttcgga
tttggagctttccatgtaactggacttttggaccaggaatgtgggtatcagatgggtac
ggagtaaccggaaaggtattaccagtagctccagcatggggaccagaaggattcaacccg
tttaatcctggaggggttgcatctcaccacattgccgcaggtactgtaggtatattagct
ggtgttttccatttaactgttagaccaccacaaagactgtatcgtgctctaagaatgggt
aatattgaaactgtattatcaagtagtatctctgctgtttttttctcagcttttgtgact
tgtggtacgatgtggtacggttctgcaactacgcctattgaattatttggtccaactaga
tatcagtgggatagtggatattttcagcaagaaattgagaaacgagtagaaaatgctatt
gctgatggtgctgcacctagcgaagcatggtcaagaattcctgacaagttggcattctat
gactatattggtaataatccagcaaaaggaggattattcagagcgggccctatgaataaa
ggcgacggagttgctgaagcatggcttggacatcctgtattccaagataaagaaggaaga
gagcttagtgttcgcagaatgcctgctttttcgaaacttttcctgtaattttagttgat
aaagatggtattatacgagctgacattccatttagaagagcagagtctaagtatagtatt
gaacaagtaggtgtaacagctagtttttatggtggcaaattaaatggccaagttttcaac
gatgctcctagcgttaaaaaatatgcaaggaaagctcaattaggtgaggtatttgaattt
gatcgaactacattagaatcagatggagtgtttagaagtagtcctagaggctggtttaca
ttcggtcatgctaatttcgctttaattttcttctttggacatctttggcatggttcaaga
actatcttccgggatgtgtttgcgggaatcggagctgaagttactgaacaagttgaattt
ggagctttccaaaaattaggagatagaagtagtaaaaaacaaggagctgtataagaccaa
agtcaaagatagtctttatc---aaatatttctaatattaaaatagttaagacaaagttt
attatctaatagttactaatcaggagaatttatggaagccttagtctatgtatttttact
aacaggaacgctaatggttatattctttgcgatctttttagagaacctccaagaatagc
aaagtaacttgattccgtttaaacttataataatagaaccactttccaacttattagtgg
aaagtggttttattgtagattacattgtattattcttcatgctcttcaaaaggatctcgg
agatcttttgaagcaggaccaaatgctgtgtatatcgagtaacctgtaatacctaaaagc
agactcgaaataaaaatactaagaactgttgcagtttccataatttaagcagtattaaga

tgaactttttctataatgatattatcaatattaaaacatttaattattaaaattatcaaa
atatactatggcacttagaactagacttggagaaattttaagacccttaaattcagagta
tggaaaagttgctccaggttggggcacaactcctattatgggaattttcatgttactatt
tttcttgttttattaatcattttacaaatctataattcttcattagtattagaaaatgt
agatgtagattgggctactttaggtag----ctaactagataagttctttga--------
ttacacaatattaaagacaac----------------aagcgatcgctattagattatag
tagcgatcgctttaaaagtattttactatatatccagagaaaattatttacctaaattaa
ttaactcggtatctgcaaaactatttgtgttaacattattatagttaactttttcaaacc
ttactaatactggatatttgataccacttttatcaattgctgcaacagtaccaatttcct
gataccagtacgattcttttctcaatatttttacttttgagcctctttccatatttttag
tcctcagatagattattaaagattattatattaaaaaattaaaaaaaactaaacaaaata
tataaaaatactaccgacaattgtaatatataactaataaaggagagagagagggattcga
accctcgataacaaaagttatgacaattttcgaaattgttgcaataaaccactctgccat
ctctcctgaattaaaatactgttttaaaaaattagacaagaataacatagaactaaggta
gttgcctagaaaattaaaactatgttaaatataaattataatctttctatagatatattt
aaaaaatatgtacatagaatctatatcagcttacttcaagatttttaatcttatacaat
ttttactaaataaataaattctctcataatatgaaactatcttggaaaacacttcttctt
tggtctttaccaatatttgtaattggtttctttttctggcaaggtttttaggtccaact
actacagatgttggcagtaatatcgcaagttctagaatgacatatggacgatttttagaa
tatttagatatgggttgggtgaaacgggttgacctctatgaaaataatcatacagcaatt
gtagaagctgttgggccagaattaggaaacagggttcaacgaattcgagttgaactgcca
gcaagtgcgccagaattaattacaaaattacgcaaagccaatgttgatctagatgctcac
ccccctaaaagtacaagtgcagtatggggactattaggcaatttactatttcctttacta
ttagttggcgggttagctttcttatttagaagatctaataatgctagtggtggacctggt
caagcaatgtcatttggcaaatcgaaagctttatttcaaatggaggctaaaacgggagta
gtatttaatgatgtagctggagttgaagaggcaaaggaagaatttcaagaagtggtaaca
tttttaaaacagcctgaatcatttactgctgttggtgcaaaaataccaaaaggcgtttta
ttagttggacctcctggcacaggcaaaacattactagcaaaagctattgctggcgaggct
agtgttcctttttttagtatctcaggctcagaatttgtagaaatgtttgttggtgttggc
gcttctcgtgtgagagacctattcaagaaagcaaaagacaatgcgccttgtatcgttttt
attgatgaaattgatgctgttggtagacaacgaggaacaggtgttggaggtggtaatgat
gaaagggaacaaacattaaatcaactattgactgaaatggatggctttgaaggaaatact
ggtgttattgtaattgccgctactaacagagctgatattttagattctgcattattaaga
cctggaagatttgatagacaagtttctgtagatgtaccagattttaaaggcaggttagca
attcttgaagttcatgctaaaaataagaaaatggaacctaaagtatctttagaaaccatt
gccagaagaactcccggcttttcaggagctgatttagctaacttactaaatgaggctgct

atcttaacggctcgacgaagaaaaaatgcaatgactatgtctgaaattgatacatcaatt
gatcgagtagtagctgggatggaaggcactcctttaattgacagtaaaagcaaaagatta
attgcgtatcacgaagtgggtcacgcaataataggcagtttattagagcatcatgatcct
gtgcaaaaagttacattaataccaagagggcaagcaagaggcttaacttggtttactcct
agtgatgatcaaagtctaatatcaagatctcaaatactagcccgtatcgtaggtgctctt
ggtggcagagctgcagaagaaatcattttcggtgacgcagaagttactactggtgcaagt
aatgatttacagcaagtaacatcaatggcaagacaaatggtcactcgatttggaatgtct
aaaattggacctttatctcttgaaagccaaggaggagacccattttaggtagaggcatg
ggaggaggctcagaatattcagatgaagttgcaactaatattgataagcaagtaagggaa
attgtcagtgaatgctatgcacaagctaaacacattattatagataatcgagtagtgata
gatagattagttgatttactaattgaaaaagaaacaattgaaggcaatgaatttagagac
atcgttaaggaatacactgcaattcctgaaaaaaattactacatatcacaattttaa---
---------------attaaacgggactgacgggattcgaacccgcaacttccgccgtga
cagggcggtgctctaaccagttgaactacagtccc-aaaaagatgaatacccagagataa
tctcatagttagcttaaacttgtcaaattagaatattaaatatgaagagta---------
--------tttgtaataatactcttcatatttgata-tagtagctaaaatatttctttta
gaaggagagagagagggattcgaaccctcggtacgaagttaatcgtacagcagattagcaat
ctgccgctttcgaccactcagccacctctccatga---tatatattatatatatatcaaa
tatacttatattttaataggtgtgcataaatatttatggagctaagcggattcgaaccgc
tgaccctctcaatgccattgagatgctctaccaactgagctataaccc----ctttggta
agatattaagaaatggctcaagcgggatttgaacctgcgaccttgggcttatgaatcccc
tgctctaaccactgagctactgagccatagtataaaatactattaaatattataccataa
attatggtcaaatataaatcaataaaagaataattactatca-tttactacttttg----
------------------tgcaagttgtaaatattcgaataaaatttcaaaattaaaaatg
tctttaactttttgttaga-------atta-ttgattacgaaa-----------atattg
atatttagaattaac---
--------------------aataaattttg-----------------------------
---tctttattt
g---

-------tagaca---
-------------------------------caaaaatattggaaa--------------
------------------------tttcatcaat-----------------taattagt

------------------taaactataaccgacgtac-----------------------

---aagaattattgaacta---

---cgttaaat---

-------------------------------------tgtgatgcatt------------
-----gggcttgatcaaaacttctctaaaatcaatagaatatagt---------------
---aattcaa
gtaaatcaaatactaacaaatgtgacaaacgcttaaaaaaataata--------------
caattagaatcgtaaaaatttggtttttga-ctgtgttaaataaatttgattatcataga
tttaatatttaaaagtgttctattacttgta-----ttgagtaattgt-aatatttaata
tcttctcttttagataataatcataatataagctaaaaaatagtttt---tattaaacaa
ctagcatagagccaataccttgatcagttaatatttctagcaagagagcatgatctattc
taccatctaaaatatgcgctgaagctacaccctgagctaaagaacgaatacaacaattca
ctttaggaatcatgccaccagaaattactgctgtttgagttaaatctctagcttcctgta
tattcagatgactaattaatgttgaaggatctgaagaattacgtaaaatgccgggcgtat
ctgtgagtagaattagtttttcggcattaagagcggctgctatttctcctgcgacagtat
cagcattaatattatacgattggccttccttgtctgctgcaacactagctataacaggta
tataattattattaattaatatcttcagtaatttagtatcaatattttgtacttctccaa
caaagccaaggtttgcttttccatttggtctcggagtaataagtaatccatcttttcccg
acaaacctacactttaccaccttgtttattaatagttgcgacaagatctttattgactc
ttccaactaaaaccatttccactatatccatggtaggttgatctgttactctaacgccat
catcgaattttggtaatattttgaggcgatctaaccaaaaattaatttctggtcctccac
catgaactaaaatgggacgtagtcctataaaagacagaaaaacaagatcactaatcactt
gatcttttagtttctgatttttcatagccgctcccccatatttataacaataattctgg
aagaaaattgctgaatataaggcagagcttcacttaagacttttactctttctgaattag
tcaacataaataaagtaggattgtgatatataaaattatatacacagcttgtgttaattt
aaaagataccaatgggt---------acttgaaaaaatagaaatattactaacag-----
----ttaaaactatagggattataattatgaataaattttgggataatgtattaagattt
ccacgatttttagtcagcgtcattttaggattaattttgataattattagtcctttttc
gtgttattaaaaaaaccactgacaagtttttttttcatcatatcattggccggattaatc

acagttttggcaataataa-tacaaaaaatgataaatatcgaatgttgttgagagaaata
aataaatttaattaaaattttgaacatatataatattatattatcattaaatatgataa
ttt-atttccttttctat---aaaacccataaatattaaatattatttgataaatattat
gccactaaaacaaagagttagctctgaaaagaccggtgccttcgcacttttggatagtat
tgttaggcacggagttaaacatatatttggttatccaggtggagctattcttcctattta
tgatgagctttatgcttgggaagaagcctctctaattaaacatatccttgttcgtcatga
gcaaggcgctgctcatgctgcagattcttattctagatcaacaggagaggttggagtatg
cttcgctacttctggcccaggagcaactaatcttgtctcaggtatagctacagcacatat
tgattctgtacctatattagctataacaggtcaagttgggagagcttttattggtacaga
tgctttccaagaagtagatatttttgggattacacttcctattgtaaaacattcatatgt
agttcgtgaccctagagacatgtctagaattgttgcggaagcatttttatttgtaaaca
cggtagaccaggtccagtattaattgatgttcctaaagatgtaggattagagaagtttaa
ttatttttctgttgagcccggaaaagttaatattcctggctgtaggccaattaccagcct
aaagtcaagacaaatccttatggcagctaaaatgatacagcaatctagccagccattgtt
gtatattggtggaggagccataatctctgattcacatcaaattattaaagaacttgttga
tttttataaaatacctgttactactactttgatggggaaggggattttaatgaggatag
cgactattgtctagggatgttaggtatgcatggtactgcgtatgctaattttgcagttag
tgagtgcgatcttttaattgctttaggagctagatttgatgatagagttactggaaaatt
agatgaatttgcttgtaatgcccaagtgattcacgtagatattgatcctgctgaagtagg
aaaaaataggattcctcaagttgctattgtcggtgacgtagcagaagttgttagtgaaat
attgaatttattaaagacttctttccccccttatccagagcagattatatcttggcaaga
aagaattaatcgttggcgtcaacagtatcctttactggttcctagaaaatcaacaagcat
ttcacctcaagagattcttgttgcaactaataaattagcccaaaatgcttattttactac
agatgttggccagcatcaaatgtggtcagctcaatttctgaaagtaaaagctaagcattg
gctttcaagtgctggattaggcacgatgggttatggtttacctgcagcaattggcgctca
agtagcacatccaaatgacgtagtcatttgtattagtggtgattctagttttcaaatgaa
tatgcaagagttaggaactatcgcgcaataccagttaccagttaaaattattattattaa
taatcgatggcaagggatggttagacaatggcaacaagctttctatggtgaaaggtattc
acactcaagaatgacagaaggagcacctgattttcaaaagcttgcagaagcttttggtat
taaagcttttactattaataataggcaaaatatgcaatctgctttacaagttgctattga
ttatcctggtccagttttattagattgccaagttacagaaaatgaaaactgttatccaat
ggttgctcctggaaaaagtaatgcacaaatgataggtatcgctaaaccgcagagaggtac
tgcttccaactatattaataatagtgtttaaagtaa------------------------

---------------atagttagtatttttta-aacaaagaata-aagtaca--------
-taaatgtaatttttttatactatta-aaaactatgtaagaacagcctttgacgagaatt
gaactcgtgaccttccccttaccaaggggatgctctacctctgagccacaaaggc-tttt
tactatattgggccgggttggatttgaaccaacgtaggcgaagccagcggatttacagtc
cgcccccattaaccactcgggcaccgaccctaattaaaccttattcaagatatgacctat
aagatatcataactatgttgctaaaaacaagtactttgataaataaaactgaataagtaa
tggacatagctggacttgaaccagcgactttcacgatgtcgacgtgacactctaaccaac
tgagttacatgtccaaactttaattattattataacactgtttagtgattttataagta
tctatttctaattgaagatttgttttaatcgtccggctttaccgagcagatctcttagat
aatacagtttagctctacgtattttagctcttctaatcactttgacaaaagttattcgcg
gtgaattgagaaaaaatactctttcaactccaataccttgaaatgagcatcttaaagtta
aactagtatttaagcttttttttcttcttctcgctaatactactccttcacataattgtt
cccgagttttactaccttccttaaccattagtccaagttgtattgtatcgccaactttaa
tttcaggtacttctgttttgataaaaggtatctcaacacttttcattagctgacttagct
ttgtactatttgctttcatatataggattagtcttgtgaca-tgttttgaaatattatac
attaaaaaaacaaatatgaaaaattttagcgaataagcatactagtatttataaaac-tc
tttttatct---
--ccattata--------
----------------cagatccatt------------atattcaact------------


```
------------------------------------------------------------
------------------------------------------------------------
------------------------------------------------------------
------------------------------------------------------------
------------------------------------------------------------
------------------------------------------------------------
------------------------------------------------------------
------------------------------------------------------------
------------------------------------------------------------
------------------------------------------------------------
------------------------------------------------------------
------------------------------------------------------------
------------------------------------------------------------
------------------------------------------------------------
------------------------------------------------------------
------------------------------------------------------------
------------------------------------------------------------
------------------------------------------------------------
------------------------------------------------------------
------------------------------------------------------------
------------------------------------------------------------
------------------------------------------------------------
------------------------------------------------------------
------------------------------------------------------------
------------------------------------------------------------
------------------------------------------------------------
------------------------------------------------------------
------------------------------------------------------------
------------------------------------------------------------
------------------------------------------------------------
----------agattcttttatagtttc--------------------------------
------------------------------------------------------------
------------------------------------------------------------
------------------------------------------------------------
------------------------------------------------------------
--------tttgataatcagtac-------------------------------------
```

```
------------------------------------------------------------
------------------------------------------------------------
------------------------------------------------------------
------------------------------------------------------------
------------------------------------------------------------
------------------------------------------------------------
------------------------------------------------------------
------------------------------------------------------------
------------------------------------------------------------
------------------------------------------------------------
------------------------------------------------------------
------------------------------------------------------------
------------------------------------------------------------
------------------------------------------------------------
------------------------------------------------------------
------------------------------------------------------------
------------------------------------------------------------
------------------------------------------------------------
------------------------------------------------------------
------------------------------------------------------------
------------------------------------------------------------
---------------------------atcta----------------------------
------------------------------------------------------------
------------------------------------------------------------
------------------------------------------------------------
------------------------------------------------------------
------------------------------------------------------------
------------------------------------------------------------
------------------------------------------------------------
------------------------------------------------------------
------------------------------------------------------------
------------------------------------------------------------
------------------------------------------------------------
------------------------------------------------------------
------------------------------------------------------------
------------------------------------------------------------
```


----gatgatcaaatattatttcaaacatggcttatgtatgaaaaagttctata---aa
aacctaatgac--------taaaattagggcttaagtatttcttagttttgtgttataat
tagttactatcaaaggtagtggcataacacgaagcatcaa-aatgttcgaacgctttact
gaaaaagctataaaagtcataatgctagcacaggaagaagctagacgcttaggacataat
tttgtcggaactgagcagatactattaggattagttggtgaaggtactggaatcgcagct
caagttttgaaatcgatgaatgtgaatctaaaagatgcaagagttgaagtcgaaaaaatt
ataggaagagggtctggttttgtagcggttgaaattcctttcactcctcgagcaaaaaga
gtactagaattatctttagaggaagcacgtcaactaggccataactatattggcacagaa
cacttgctaatgggcttagtccgagaaggagaaggagttgcggcaagagttttagaaaat
ctggcagtcgatgtttcttcaattagagctgaagttatacaaatgctcggagaaaatgcg
gaagccaatgtaagtggaagcaatactacgcaagctagaagtaaaacaccaacattagaa
gagttcggatctaacttaactcaaatggctatggaaggtggtttagatcctgtagtcgga
agacaaaaggaaatagaacgagttattcaaatcttaggtagaagaactaaaaataatcct
gtcttaattggggagcctggtgtaggtaagacagcgattgcggaaggattagctcaaaga
attgctaatagagatgtaccttctatttagaagataaattagttattactcttgatgta
ggtctattagtagccggaactaaatatagaggtgaatttgaagaaaggctaaaacgtatt
atggatgagattaaatcagctgataatgtaatattagtgattgatgaagttcatacattg
attggggctggtgctgcagaaggagcaatagatgcagctaatctgcttaagccagcttta
gcaaggggagaattgcaatgtataggtgcaacaactttagaagaatatagaaaacatata
gaaaaagatccagcattagaaagaagatttcaaccagttgtagttggagagccaagtgtt
gaagaaacaattgaaattttgtttggtcttagagaccgttatgaaaagcaccatcaatta
acaatgtcagatggagctttggctgcagctgctaaatacgctaatcagtatatttctgac
cgatttttgccagataaagcaattgatttaattgatgaagctggttctagagttcgttta
ctaaattctcaattacctcctgctgccagagaattagataaagagttaagagctgtatta
aaaacaaaagatgaagctattagagctcaaaaatatgaaacagcagagcagtatagagca
agagaaatggaaattaaagctcaaattgcagcaattgctcaaagtaaaaagaatgagcct
gatttaaatttagaagatcctgttgttacagaagatgatattgctgaaattgttgctgca
tggactggtataccagtaactaagcttactaaaagtgagtcagaaaaattaatgcacatg
gaagaaactttgcatggacgtattattggtcaagacgaagcggttgtagctgtctctaga

gcgataagacgcgcaagagtaggtctaaaaaatcctaacagaccaattgcaagctttatt
ttttccggaccgacgggtgtaggaaaaacagaattaacaaaagctttggcttcttatttc
tttggttcagaagcttctatgatacggctagatatgtcagaatacatggaaagacacact
gtatctaaactaattggttctcctccaggatatgtgggttatagtgaaggtggttatcta
acagaagcggtaagaaaaaaaccatatactgtcatcttatttgacgaaattgaaaaagct
catccggatatttttaatctacttcttcaaattttagaagatggccgactaacagatgca
aaaggtagaactattgattttaagaatactcttttaattatgacttctaatattggatct
aaagttattgaaaaaggaggaggtagtttaggctttgaattatcagaagatcaaacagaa
tcccaatatactagagtacgatctttagtaaatgaagaactgaaacaatactttagacca
gagtttctaaatcgattagatgaaattattgtatttcgtcagcttactaaagatgaagtt
agagaaattgcagaattaatgcttaatgaagtctttgcgagaattaagcagcaagatatt
caattaaatgtaacagaacgatttaagcaacgattagtagaagaaggatataatccaagc
tatggagctagaccacttagacgagcagtaatgaggcttttagaagatagtctggctgaa
gaagttttatctggtaaaattaaagctggtgatagtgcagtagtagatgttactaatgaa
ggagaagttacagttttattaggtgaaaaattagaactgttaacataaa------aaaca
atttattaat--taaagcattgaagaggttat--attcaatgctat---gaaaaaaatta
ataagatgggttgctataattgacaacctctgatactctttatttgtatata---aagcc
gggatagctcagttggtagagcagtggattgaaaatcctcgtgtcaccagttcaaatctg
gttcttggcat-tttaagcatcagaaaattttgctatctgagcattaaattttaattgaa
atgttcctgtcggaccattcctgtgttttgctattataacttctgtgaagtctctacttc
tagtttcttgtgtatagtaactttctctatatagcattattactaaatctgcatcttgtt
ctatagaattatgaacaataaagttgtttgaaataaaattgcataaaggtttacattcta
aatcaaacactatttgtaaacttgtaacgtttatttttttgtatactttcaaaagtcaaag
atgaaa------aagaattaaaaacatttttttgttcttcaaagttttt------tattt
gaatagcaatcatgtcattttgattaattttatcacatcttttccatccttgtgttgtga
gtagtttatggttacttgttaattgtatatatttcccagcttcggtaataattttatata
cagtttttttccctgttttcgaaatactacatttaattgctagaaataactgttgttttt
gcgcactaaagttaatgacttca-atcttttttataatagcaattaaataatatttg--aa
gttgattatataatgatgtctgaagataattgaatttggatatacacccactttctctta
aatcggataacaaaggccttttgtcactccgagtttcaaggtttctatttagttgagata
atgccaagataggcaagcttaaatcttttgctaatattttaagtgatcgagtaattaatg
atagttcttgagatctattgtcagattgtctactctcctgcagcaattgaagataatcta
taataattaattctatatttttttccttgtaatttaaagaattttacttttgtctttatag
tgtcagtagaaatcttagcgctatcatcaatataaagattaaggttagcaagagttttac
ttttttgaataacatattgccattcatcattattaagctgaccagattgaattttttggc
cgtttaaattacactcttgagctaaaattcttcttagtagttgctctgtagacatctcta

agctaaataaaataacataagatcttttctgattaattacatatctagttatattaatag
caaaagcagttttccccattgaaggtcttcctgcaagaataattaaatctgacttttgaa
acccttgtgttatagagtcgagttctgtaaaaccagaaagtatactactatttatactaa
tctttttccttgtatccaaatggactaataggtttgcaagaattgtagctaatgtttgcg
tatctttttcgtcaagtatttcataagcttttgttaattgactaacaatagaagttatgt
tttttgttcaataagttgttttgagcagctaattaaacataatgaatctccacatgcta
aaagtaatcttttaatgtagttatctataacgactgctgaatattcgcttatagtatcag
ataggggagcttgttctattagactaaaaacttcatttagctcattcaaatcttgaatca
ttttttcatttttaagttaatgaaaaaatttcttacgctttttgtctttgcatggttaa
cattttcgagtgctgctctataaagtaatgaagttgatgtaaaataaaaaaaatcaggag
agatttttctactgattttagtaatgatatcgattgctgagttaatattatactaatta
atatttttcagctaaaatattatgaggaggtagatatttataa------atacttatta
cttcctcc-ttgtaacattactcttgtg--------gtattagttaactattgatttaat
ttgattctggaagaacttgcaatttaatatttgcagttacttgatgtaatagcttaattt
ctacatcgtaaatgccaattgttttcacgtctggtaaagagatactttgtttttcaatgt
ctatgttagtagtgtttttaataatttgtgaaatttctttttctgtgacactaccaaaaa
tattgtggccatctcctgtttttttactaacgctaaacctttgtatttcttctagaagtt
gcttgactttttttagcattttctttagctgtttttaatttttcttctcttatagctgcat
aaaattttttgttgttttaaaataccatttgtggcaactgccgccattttattcgggatta
aaaaatttcttgcataaccagaagctactttgataacgtcattactcttgccaagttttt
gaatattctcttttagcacaacatttataactttttttactcatctt-------aattcta
aaaa----atctgtattatcctattatacctgttgtatacagatttt----aatagtaag
tatttatttgttgatgtaatattcttgtaaaa--aaaggtaattttttattttt-ttctg
atataatcactttcacaataattc-----attaggagaggtggccgagtggttgaaggcg
cagcattggaaatgctgtttaggagcaatcttaacgagggttcgaatccctctctctccg
tacaa--aaaatataaaaagttttatagagtccaatctaaatctccaataatattcttcc
aagcatttaatccaccccgaactctaacaatatttagtttttgagctattaaaaacttag
aagcaaatattgatctagaatctaagctacagtaaacaaaacaaattttatcttgtaaat
tgatatgagagtaattcattcctttttatttttttgtaaaggtagatttaatgaatgtatta
aatgacttttatgatattcttcatgatttcttacatctattaaaatatattgtggattcc
tactaattaaaaacttttgtaattgaataacactaatttctcgtacaaatgtactagatt
tactgccataatatttgttccaatgctttttttgt-------ggataatacaaatttagta
tttataatcttaaatttgttaaatgatgaagttaatgagttgtacgttaatatagttcca
cttaatatagacttataacccagaacgattttgacagcttcagttgcttgaagtgtacca
acaatacccggtaatagacctaaaactccggaattactacaagtatctcttgcgctctct
ttattttcagtttcactgtagaagtctcgataaacaggtccaccttgataattaaataca

ctaacttgtccttcaaattgaaaaatggcaccataaatatgtattttatttaactctagg
caagtatcattcaagagatatcgagtatcgaagttatcagatccatctataataatatca
tattgtctaataatgtcgattgaattctcatagcttagtcttgttttgaacacagttact
atacactgtggatttaaatctaata-tttttttttagctatttctactttgaatatcct
atatcatgcatcgtgtataaaatttgcctctgtaaattagagagatcaattatgtcatcg
tctataattccaatgctgccaattcctgcagcagcaaggtaaatgattcctggagatcct
agcccaccagccccaatgaataaaacttttgcttcttttaatctttcttgtccttctaat
tgaatttgtggcaatactaaatgcttagaatatcttgtatattcttctagtgagtatttt
gtattttctgttttaaaatttagcataaaaatttatagtttatttaatagcactta----
--aaagctagctgataagtttattaaaatgacttttgtaaacttggttcttctctatcta
aaatagctccttgtgtaggacagacctgaacacaaatactacaatcaatacaggcagaaa
agtcaatccaataccaattagtgttattattgtttttccctttccttgatgaatacaag
ctacggggcaagcttccacgcattcagaaactccaatacatttttctgttacaattgtat
gagacatattatttaattaat-aaaagataagctatttacatctagtagttttgttgtat
actaaatttacatatgctatccaggccaatatttaaggtagtctatttctacagtttact
aatttgatatgcgcctttagttcagttggtagaacgcaggtctccaaaacctgatgtcga
gggttcaagtccttcagggcgcgtttag-ttttatcgtaattcaataatatcattttatc
tttttgcaaacatcaaaatgatatgatataaaagatcatatttatacattgaatattct
atgtactagtactg-ttttttaatatgttattattgataagttaattatactaattctat
ttttgtaataagt-aaaaacagttctaattaagtatgcacgtatttaatagtc-ttttgt
atatat--aacttgaaaaaatttaaactatggctaaaaaagttactggaattgttaaact
agcattaaatgcaggtaaagctactcctgcgccacctgtcggacctgctttaggacagca
cggcgtgaatattgttatgttctgcaaagagtacaatgcacgtactgctgacaagtcagg
actagtaatccctgtagagatctcaatatatgaagatagaagttttacttttatacttaa
aactcctcctgcttctgtactaatcgctaaagctgctggactaaataaaggttcgggtga
accaaataccaaaaaagtaggcagcataacaaataaacagttagaatctattgcagaaac
aaaacttcccgatttgaacactaacaatatacctcaagctatgaaaatagttggaggtac
ggctaaaaatatgggaattctaattaaagatt-aaaagtttcaattatatttacta-tt-
-tttatgaaaaaattttcacgtcgacttacaacattaaaatcaaaagttgagcctaaact
ttacactattaatgaagcagtatctatattaaaagcaacgtcaaatgctaaatttaagga
aactgcagaagctcatattgctctaggtttaaatcctaaatatgcagaccagcaactcag
agcaacagttatattaccgaaaggtacaggtaaattgataaaagtagcagttattgctaa
gggagaaaaattaacagaagcaattagtgcgggagctgatgtcagtggctccgaagaact
aattgatgaaatactgaaaggtagattagattttgataagctgatagcaacaccagatgt
tatgcctttaatagcaaagctgggacgagtattaggccctcgagggttaatgccttctcc
taaagcaggcacggtaacattggacgtagcaaaagccgtgaatgaatttaaaggggggtaa

agttgaatacagagttgatagaacaggtataattcatgtaccttttggaaaatctagttt
ttcacaagaagatctagttttgaacctccaaacaattaaagaatcgattgatagaaacaa
gccttctggagcaaaagggaagtactggaaaactttttcttatctagcaccatggggcc
atctattcaaattgacatcactagccttctatgaaaaattttgtataattatagaactt
gaataatctattatttcaaatta-taatttgctagcattattaacgtaacaacttgctta
ctatatcttatctgcattaaaaacttt-ttatatatcttattaattatgagtacaaaggt
tgaaaatatcttggaagagctaaaatctttaaaccttctagaggctgctgaactagttaa
acaaatagaagaaacgtttgatgttgatgcatctgcggcttcaggaggtatgatgatggc
cacaccaacttcagcaccagcgtctgctgaggttgaagagaagacagaatttgatgttgt
cctagaggaagtcccagcacctaaaaagatttctgtattaaaagttgttcgctctctgac
tggtttgggtttaaaagaagcaaaagatcttgttgaatctactccaaaagtcttaaaaga
aggtgcttctaaagatgatgcagaaactatgaaaaaacagctagaagatgctggcgcaac
agttattgttaagtaatga-----agaagcgcttctgtttaagtataaacagaagtgcct
ctttatttattataagcaattatactagaaaagtccaagtgtaattgcattgttgatagg
catagttgcaccaatacctagccaaatacttacaacggtaccaattaagaaaacagtggt
tgcaattggtcttctaaatggattttgaaatttgttaacattttcaatgaatggaacagt
aagtaaaccagctggtacagcagccatacttagaacacctaatagtttattaggaatcac
tcttaatagattaaatgttggaaagaagtaccattctggtaaaatctctaatggagtcgc
aaatggattagacttttcacctatagaagatggttctaaaattgcgagtccaatactgca
ggcaaaagtgccaataattacaactggaaagacgtataatagatcatttggccaagctgg
ctctccgtaataattatggcccattccttttgctaatttagctctcagcttcggatctgt
taaatctggtttttaagaattgacatattg-tttttgtgtttaatataaagattagtct
aattactgttttaaagtgtaattttcaactattataatggtccagaaattccttgtttac
gtatcattaaaaaatgcataagcatgaaaacggcagttagtagcggaagtacaaaagtgt
gtaaactatagaacctagttaaagttccttggcctacactaactcctcctcttaacaatt
ctactatactcgctccgacaactggaacagcatcagggacacctgtaacaattttacag
cccagtatccgatttgatcccatggcaaagaataacctgttaccccaaaagaaacagtaa
gcactcctaaaattacacctgttacccatgtcaattctctaggtttttgaaacctcctg
ttaaataaacacggaatacatgtagaatcatcatcaagaccatcatgctcgctgaccatc
tatgaattgatctaataagccatccaaagtttacatcagtcataatgtactctactgatg
taaaagcttctgcaactgtaggtctataatagaatgtcattgcaaatccagttgcaactt
gaattagaaaagatacaaatacaatacccccctaagcaataaaaaatattaacatgaggtg
gtacatacttactagaaatatcgtcagcaattgcttgaatttctaatctctcttcaaacc
agtcataaattttactcataaaatagcttcaaaaagctcttctaatata--ttttgcgtt
taagctactattaattcaatag------ttattgaacttatatt-tttttataatcaata
cttattaactattataacatctttttattgattt--------------------aaaatt

ctctgacttagtaaaacagggctatgtactataattcttttttttgtatagagattaat
ttcgttttagtaaattagctaaaattcttgttacactgactctattactacctataatc
tgtgctaaaattttatgtgtaatagtaaagttaagcataataccattattaacttgagtt
ccattttgctcagctaataataataataagctcgccagtctactggttatactttatgt
gaaatgatttctataaaatgataagcttttgccgagcaaaatactaaatgatttacaaaa
aataaattgaatggagcgcagttactacatgcttttataatagtagtatagttaatacag
gcaacttctgttttatctattgcttctgcttcgtagtaaaaattatcgtcgaccaattct
atttgtccaaatgtatctcctgtagttaataagttaagagttactttatgtgtatttctc
aggacttttgtaataattaaagatcctatcaatataatgtataatctagagctgctatta
aacaaaagagtatcattttcttaagtgaaaaaatttgataattaatttattacgatta
aaaaatagtagccatggattgcaagaaaaaaaatcacctgaactattttcagagaatatt
ggaatacaattttcttgatttaaacaattttagtcataattactactttataa------
---aaaaatattctaataat-taagaactgaaattaaatgtcatataaattgatgttagt
tgagaatgacattgtattatcaaaggccattcaagaatacttaatagatcaaggcttcaa
tgtttatattgccaataatggattagaagcactgaatctagcttatcaatataatttcga
cttgataatttctgatataatgatgccgctagttaatggctatgaactattagcaaaact
taaaaaaaacaaagcattatccaaaatacctgttatctttttgacagctaaaggaatgac
taaagacagaataaaaggctacgacatggggtgctatggttatttatctaaaccatttga
tcctaatgagttgctttcaattattaacaacttaatcgctagagatgtcttaaaagaagc
ttctttacaaaactcggcaacatcaaaccagcaattaaatcataaaatacgtttgacacc
tagagaaaaaagtattttagatcttgttgttgatggacttactaataaagaaatatctac
tatactgaatactagtgttagaaatgtagaaaaatatgttagtcgactcttacacaaaac
taatatgaagaataggactttgctagtcaagtattctataaataataatctattaaataa
tgagatcaatgaaagggcgaatgacgggactcgaacccgcgaatgatggagccacaaccc
attgccttaaccccttggccacactcgccatattacatacaccactatagcttgtttttt
gatatatcgtctagttttcgagttatatatttatctaaaacaatatttactttgaatttt
-tatgatacatagcaatattagtattcaaattaatggggaaccatttaattgctcaaagc
ccatctcattgcaattttttattaaattatcttgattttaattccgagcgtgtagcagtcg
agctaaatgacattcttttaccagagactctgtttcactcaacttatttgaatgatcaag
ataagctagaggttattaccattgtaggtggtggataatctatttgtacatac-------
-acagttttctatctcaaatgtttcatagatagagataatcttccttgtttttatctac
atgaataataacagctttaatagtgtcacctattttaaattgtgatgatatctgctctaa
ttctttgatattaatctcagaaatatggacaagacctttgagatttcctacctttataaa
taatccgtaaggtgtaatctgatttattatgccttcaataatattaccaacaattaaatt
agatgatgcttgagatattagagctcttctatgactcagtattaaattattagatttctc
ttcgacattgagtaattttagcttaataaatttattattaaattgctcactttttttgaaa

attaccgagatgagaattaggtacaaatccagatataccttcaaggttaattatcattcc
tcccttattgaatcctttatcattacattaagtaaagagtcttcagctaatagttgcct
aattcttttccatgctcttatatattcaaggcgacgaattgataatattaattgtttcga
ctgaatattataatctaataaaaaaattctcttgtgtcattaatgtttaaagaagtgaa
attatttaaatcttgattactagatacttcttgaataggcaagtatgcagatattggtgt
tcctatatctactaataccccatttaattcaaagctaaatatagttccagctacaatatc
tccaagattcaaatcatacttgtatttttgtaaaacagctgcaaaatttctatgagtaaa
tccttcattattttttgtcatattgaatagtatgaattaaagagtaactttgtacaatat
attaaggtaaataaatgtttgtcgaattgtatgaaaaaattttaatttgttcggttgtaa
ttaagtatataaacatattaaaatatctaggagacatgaaaagtagacgtagatagctgc
agatttaa-tactatataatctgaggaaagtccgggctccacaaatacaatttatgctgg
agaaatcccagtgtaggtaactgcgaggatagtaccacagaaaaaaaccgccagaataag
t----tatattagctggtaagggtgcaaaggcaagttaaaagcttaccaaaagtactgca
aagtatttgttaggtaaaccccctaaaatggagcaaagctactaaacaaatatttgtgtat
attttatctagtttagttgaaaatactgcatgaagttattagtaacaataactcgagagg
aatagctatcctttacaatatatc-tcgattatattgaaagaacagaacccggcttatgt
agtacttttatgtcttgtattattatgtctattgtcgaatctttaaatttaactgtttg
acaattttttttgtagctgatgttgtttataatctatttctgaattggttaaagttttt
tcattagatcgatatgtaaatcttagacctatactttttttctcttttccaattgattca
tctatatattgatcaaataatgttgtactttctaaatctttatcataaaattgatctaat
aattttaataagtatttaatttccatattcttaggtattattaaagagagatctcttatg
atcgatggatacttagagtatggttgaatctgataacttaaataattgaattcattatga
caatcttctaaaatgtttaagttaacttcaagaaaaaaaagttttgtattgagtcctaat
tcactatatgttgcttgattaagttcactgaatatgccaataataatattgttatagatt
agtgtggcaaattttcctttttgaatgaactttattctaccatcgactatatcttttttt
gtccactcaatttgtctattaatcttttgaaaaaaattttctacgatccctttagcttca
taccaatttaaagaatgcgcagggtgtgaccatgtagatcggatatctagattaccaccc
aatatgatagctagattagtagtttctacaattttgttttgatcattatgaaatactgtt
cctatctcaaagctatctatagtttggttgctttgtttaatattataaagatttgattca
attaatccttccagcaaactgccgcgtaaattagaataatcttgaataagaggattgttt
aaagctatatttcctttggactttattaaagaataatgaactaattcagttaatcctaga
tttcttaaaatacttctacttttgtctatgaattttctctttgtggataaccttttgttg
aattgaatatttggtactgagctcctgaatttatgataaccataaattctagcaatttct
tcaataacatctatttctcgaaaaacatcttctcgtctataatttggaacagtaacttct
atattttccttattttgataaataagatcaaaatttaaagagtgaagaatattttgaatt
tcttcaaagaataaaaaacgtgtttgaccattatacataattggtcctaaaatgtcatga

actttttaattgataaattaatattaagagcatgatcagttattttttctcgacagaaa
gtatctctaatatttccgcatgttaaatctgtgattaaagcaagagcttcaaaatgtgca
tttttccaattatctacatttagcccacgttcttgtctaattgaactttctgttcgaata
ttaagaactcgagaagactttctaactacagattgtttaaatatagcagactctacaagt
atcgattttgtattataatctgtatcaaaatcagagttgcttccaataccagctatacta
gttacatttgtatttacttgagtaactaaaatatttttgtttaactcaatattttcattg
tttaacttaatctgatcattacttccaagaggaaaattgcttcgaattgttagactattt
ttatgattcatattattaatttattcaaatcaataatattaataggctgtccccatttc
aacataatataattgcttatatctgttaacaaatttctgtgtgtaaacccagaagagagc
aaccgatttttagccaatctggagaatctttaactcttatttcgtctataatggcagca
aaataattgtcacaatttaataaatcgtgattactgattatagt-ctcctttttagagaa
atatctattattgggttgatgtatagctgataacattgaggaccctgttaaagccgagac
ttctcttgatagaccaatcatgcttaacacgtctgacctgttagctgttgatgatatatc
taatatatgatcaatctcacctccaattataatagattcaatagcttcaacttcaaaacc
tgcttgtgttagttgattagttaaattgtctatatctattgttttgatattaccaagttc
tttcaaccaatttaaagaaactttcataattaattgtcatctatcttatagaagtattta
ttttacttatgattaaaaatcaatttaaaaaattcatcaaattaatataagttatttgat
gaatttgcttatacaatatattattgtgatatcttacttttatatggcagttattaagaa
ctagcttttgtaaaagttagcccattatcttgggcatatctttccataaatctcatgaat
ctatcccaatcatcaggacttttaataacatgtacagcttctattgcttgtggtttacca
ttaataaacttggcattaacatctcttgtaataatttgtccctctgtgtccatcaggtac
atacctgtgatttcacctttgtctgccatactagcatctaaaattttgggatttgtaaac
cgaaaagttgctgttcctgtactaccatctcgagaccttgttaaacgtacatctggtaca
acttcttcattgataccttgaataaattgaattgttgccatatcaatcttctttatttgt
atataatttatattggataaaactgatttctagttcttgaatgcttgttgagaaactgtt
ttaaaagcttatagtatcagtagatcaaattttatgtattatataaaactgtaactttc
-tttttg---

----------------------atctgttcaaagaactaacttattataagctaatcata
cgaatattagccatataatgttcgtaagt-gaaattaaggggttgtagctcagaaggata
gagcaagcgcctcctaagcgctaggtcagcggttcaagtccgctcaaccctgttaacgaa
ttaagtagtattatttaacttgttctgcaaatcagtattgatctaagttaaagtacttga
cagctaaaacaaaagaatctgagaaatattttcatttaaagaacgaacaattatcttaac

tctaaatatctgataattagtaa-aaaattct----------------------------
----ctttttaaaacacaagatgatg-----aagatatttaaactaataattgaaattaa
gtttttgaagttatagaagccaat--ataatactttctattaacaga-----------tg
cttgcttctattactatctgtttacaaga--aatt----atgagtatcaaaata------
--atttagctcca--------------------------------------ctgac----

-----------------------------tactaataaata-------------------
------------------tttattttagtctagacaag------atgaatcttcatggta
taacag--

--------------agctattctttgtgtcatacttatataaaattaggat-------ac
tgtt--

---------------ttcattaccataactggggtgggaggattcgaacctgcgaatggc
ggagtcaaagtccgctgccttaccacttggctacaccccaacatgtgaacattataatag
cagtcgtataccctgccatgtcaatagactattgtctttatttaataatttttgctgctc
ttgtagaataaaatctttttttttcaaataagatacgctatgtttaagttgcacgactgtt
atgttttcttgcttttgactgaacagccacttgatagtaattattccttctgcaatttcg
ttacttcctataataatacaagctacagctctttttttatttgcttgcttaatctgctta
ctaaaattacttgagctaacatcgatttctattttaaaaaactgctgatgtaaaaaacgc
attatttgcataccagtttcgttagcttttgtaccttgtgtagcaatataaaaatcgata
gatttattaggcaagtctatattatcttttgcaatcagtaataggcgttctaatcctatt
gcacatccaacagctggtgttgctttgccacctaattggtgaactaagctgtcatatctt
ccaccaccacatatagtatcttgcccttttgatgtcaatgtcttaatttcgaaagcagta
tcattataataatctaatcctcgaactaacttattatttatattgtagggaatattaagt
aattttagataattgcaaacgtcaccaaagtgttgttgtgattccaaacttaaaaaatca
gaaatttttggagcctctgtcagtatttttttgtgtattagaatcttttgaatctaaaatt
ctaattggattactagttaatctttttttgtgagtcagtgtctaaatcatcatggtacttt
gtaagataatctcgtaatttaacttgataaatactacgatcctctacttttccaattgaa

ttcagatcaagtttcaagttgtgcagattgagattgttaaatatgctcatagctaaatgt
ataacttcactatctgctcttgcatctaagctaccaataaactcaatgccaagttggtga
aattgtcgttgtcgtccactttgtggtctttcgtacctgaacattggaccgctataccat
aatctttgtaaactatggtggtaactcatttattttcaataaaagatctcacaataccg
gcagtaccttctggccttaaggtaatatctctattgctgcgatcatgaaaccgatacatt
tctttatttacaatatcagtatcttcaccaatacccctatcatataattcactattttca
aaaataggtgttctaatttcttggtaatttgcacattctaataatttggaaattttatta
tgtataaactgccaatattgaatttcatcgggaagaatatcttttgtccctctaatagcc
tgaatttttgccatgataatttttgattaccttcttaatatgtctgcattaaatagcag
ttgtagactgttattcagtcatgtattatacattag--aacatactgatg-taatttata
aagtacgggcaaggagggattcgaacccccgacaccatggttcgtagccatgtgctctaa
tccactgagctacaagcccacttaataagtaccttaactatatcattgttcactaatgta
tacaactatattctttt------atcttacatgtgaaattttgtaaaagtaaaaaatt
at----gtttgtttttacaattggaactattcatatataatgcttaatttattataact
ttgtaaa--aatatctataagtttaattgtatgattctattatattggtaatcttgttat
aattttgcgctcttatctttcaattgg------ctagcatctaattaacttatatttcaa
ataaaaaaatcttatctaaaatgagtaaacaaattctatatcaggatgatgccagaaaag
cattagaaaaaggcatggatattttaacagaagctgtttctgtgactttaggaccaaaag
gaagaaatgttgtcttagaaaaaaaattcggtgcccctcaaattattaatgatggtgtaa
cgattgcaaaggaaattagtttagaaaatcatattgaaaataccggagtcgcactgatta
gacaagcagcatctaaaacaaatgatgtagctggtgatggtacaacaacagctacagtac
tggcttcagcaattgttaagcaaggaatgagaaatgttgcagcaggttcgaatccgatgg
ctattaaaaaaggtattgaaaaagcaacaaattttgttgttagtaaaatagctgagtatg
ctaagccagtagaagatacaaaagctattatacaagttgcttctttgtcatcaggaaatg
atatagaggtaggtaaaatgatagcgaatgctatagaaaaagttggcagagaaggagtta
tttctttagaagaagggaaatcaactaatacgattcttgagatcacagaaggaatgcagt
ttgaaaaaggctttatttctccttatttcgttacagatacagaacgaatggaagttcttc
aagaaaatcctttttattttatttacagacaaaaaaattactttggtgcagcaagaacttg
tgccattgcttgagcaaattgcaaaaacatctcggcctttattaataatagctgaagaca
tcgagaaagaagcattagccacaattgtagttaataaattaagagggattttgaatgttg
tagcggttagagctcctggattcggtgatagaagaaaatctttacttgaagatatgagta
tcttaacgaacggacaagtaattactgaagatgcaggtttatcacttgatacagttcaat
tagatatgttaggaaaagcccgaagagttattgttactaaagactcgacaacaataattg
cagatggtcatgaaattaaagttaaatcaagatgtgagcaaattaagcggcaaatagaga
cgagtgactctttatatgaaagagaaaaattgcaagaacgattagcaaagctttctggag
gagttgccgtcattaaagttggtgcagctacagaaacagagatgaaagataaaaaactaa

gactagaagatgcaattaatgcaacaaaagcagcaattgaagaaggtattgtaccaggag
gaggagctactaatgtccatatctctagtgaattatttacatgggctaaaaacaatttag
ttgaagatgaattaattggtgctttaatagttgaacgagctgtgacctatccgttaagac
gaatagcttttaatgcaggtgataatggagcagtaatagtggaaaaagttaaaagtcacg
attttcatataggctatgatgcagcaaacggtaatattgtaaatatgtacgatagaggta
ttatagatccagcgaaagtagctagatcagctttgcaaaacgcagcttctattgcagcaa
tggttttaactactgaatgtattgtagttgataaagtcgatgattgagctata------a
gataatagataatctcaaacaaagaataatttctaattatagaaattattctttgtttca
attaatctaattaattgatcaaatgtataatttttataataaagccaaagtttaaacacg
tcttctttttcaactattaagtagagaagatataagcccgtaatacctaaattatcaatt
aaaatattattctcatatatagatttttctaaagatctaaaaaatgacatttttgaaaaa
taataccgaaatcttctcttatagttatttgctaaatcattactgtctaaagattgattt
ccaattgaattcagaacctttaacagaatacttttaaatctttatctgactttatttga
gaatcaataataatttgattcaattgtatgtcaaaactaagattaaaaatattacttgtt
aaagatatttgattttgaatatctagagattttatagccaagaagattaaacttttattg
ttcattgtaaaatataagacaattgatgatgtattctttaaatacta-aatttatcttct
tgtgcttctatcgtttttttgt-aaaataattaatgttttaattttttagttttagttact
acccgaaaaaataaattctggaaatctagcttgagctttagctagagagtgtttttttccc
ttcttcttgccagtaattgataacttctgttgttttgtttaataactcaattctgtctct
ttcagcaatccacggcttggattctagttctgcttttaattcttcccaagcatcattcct
tggccagaaaaaaataagaagtaagtggactagtgccatttccaacaatctgatcaatagc
aatagcaatattattttctaaccaaagtactttttagtgtaaattttgacaagtttttatc
tccattattaatattaagtattgg----tacagtagaatactgtactagttattttttttc
agcaacaatttgagctttgtttaaaatgtttttttaccgttttagtagcttgtgcaccttg
agataatcgttgcaaaatttttgaaatgttaacacgagtttcattatttataggattata
aaatcctaattcttctatagctttaccatcgcgtttatttctgctatccattacaacaat
tctatagcttggttgtttttttttcttccgtatcgctttaaccttaacttgaccatattagt
cacctcgcttattttttt--atatctttattaaattatatcttacgacattatgcaataat
tcaaacaattaagctgattcaagtctaacattattaaatattaccattaaacattgtaga
aaaataatacctaacataggtgacatgtccattccaaacatgggaggaatgcttcctctg
aatagttttaagtatggatcagtaattctatttaatgaacagaatggttcattgtaccaa
tttacagttggaaaccatgccaatgataatttaagtaaaattaaaattaaataaatctca
gaaaaattagctatcgatccaagcaataaatttaatgtaccaggaagagtgttcatacgt
attttataattattatatacttcgttatactaatagtttgattatatattataatcgatg
taaataaat-cagctaaacaaaaaacttatccttaaaatcatcttctcttgagcactttt
ctttatctacattagtatttacttttgtcgtataaatttccagtttagaccaaatctgtc

ctaactgatttaattgagatgttccacattctaaacaggctatttttatattgctcaaaa
cagcaccttttttttataaaatcgttcaataatggcgtaatagttgcttcatccaaaaata
aatctaagacaagtatttttgtaaatgaatttaattgatctgggacattgttaataatat
cattataattaactaaaactatattagctgtaggaagtaaagatcgtgctccttccgcta
agataaagccataaggcataataattacaataatatatttataatgactgctaataagac
ttatagtggtattagcttttaattcgattgtttctgttactagccactctctcattattt
cgtaagtaatccatttgcctagttctgagcaagcagttcttaaaatcgtacctggattat
tgtcgtttttgagaatccctgaccaatgttgtattaatggatgggatgctatattaattt
ggagttgcatgttttgttaaatatatttaacatttaatttgttgattgaatcttcaattc
ctatttgttgagtattattaaaatagcat----atatattaaagaaaataattat-aaaa
aatgaaaaaaagtctttggctgtggggttttacagatagtgctgaaacttggaatggtag
atttgcaatgattggttttatatcagttatttttatcgaagtagttacaggacaaggatt
attatatttaataggtatgatgtcataataaaaatccaaaaaagttcttgtaattttta
taagaactttttttgg-atttttataaatctaatctagcggtctcatagatagaacaggtt
cattctctctattgataccttttttcaaatccagcagcagcagctcttgctctgcctgcat
gccaaagatgtccaataaataggaagaatcctaggaagaaatgagatgttgttaaccaag
atctaggagatacatagttaactgaattaatttctgtagctacaccaccaacggaattca
aagaacctagtggtgcgtgagtcatatattctgcggcacgcctttcctgccacggttgaa
tatcgttcttgatttatttaaatcaagaccatttggtcctctaagaggctcaacccaag
gagctcgcaagtcccaaaatctcatggtttcaccaccaaaaataatttctccactaggag
atctcattaggtatttacctaatccagtaggtccttgagaagatgcaacattcgcaccta
gtctttgatctctaactaagaaagtaaaagcttgagcttgtgaagcttcaggaccagttg
gtccatagaattcactaggataagctgtgttgttataccaaacaaaattagaagccgtaa
gccccatgattgataaagcacctagactataagataagtaagcctcaccagaccatacaa
aagctcttctagcccaagcaaaaggttttgttagtatatgccagattccgccagcaatac
aaataatgccaatccagacatgaccacctacaagatcttccatgttatttacacttacga
tccatccatcaccgccaaacggagactttaagacataaccaaagataactaggggattaa
gtgtaggattgctaacaaatctaacatctccaccacctggagcccaagtgtcgtataccc
caccaataaataaagctttaataactaacagaaaagcgccaatgcctaataacactaagt
gtataccaagtattgttgtcattttgttttatcccgccaatcgtatccgaagaaaggaa
atgattcttcaagagtatcagggccaatcagagaatgataaagacctccaaagccaagaa
cagctgaagaaattaaatgcactacgcctactacaaagtatgggtatgtattgaaaatct
cgccgcctggaccaacaccccagcctagtgtagctaagtgcggaattagaataaaccctt
gttcgtacaaaggcttctcgggaacaaaatgtgctacctcaaaaagagtcatggcaccag
tccaaaagaccattatacctgcatgagcaacatgagcaccaagcaatttgccagaaacgt
taattaagcgtgcattgccagaccaccaggcaaacccggtagactcaatgtctctaccgc

caacaccaacagttgtattaaagggcgtttccacgtggtaaaacctcctcagggaatata
aagttttcatgaggttgatcttgagcagccatccaagagcgaataccttcgttcaatagg
atatttttagtataaaaagtttcaaactcaggatcttccgcagctctcaactcttgagaa
acaaaatcgtaagctcttaaatttagagctagtccaacaattccaaatgcgcttgtccat
aatccagttactggtacaaataacatgaaaaaatgtagccaacgtttattagaaaaagca
acaccaaaaatctgtgaccagaatctattagctgttaccatcgaataagtttcttcagat
tgtgttggagtaaatgcacgaaaagtatctgcagcatcaccatcttcgaataaggtattc
tgtacagttgcaccgtgaatagcacatagtaaagcaccacctaagattcctgcaacgccc
atcatatggaacggattcagggtccagttatgaaatccttgcaaaaatagcaaaaatcta
aaaattgcagcaactccaaggcttggggcaaagaaccagctcgcttggcctagagggtac
attagaaagacggaaacgaatactgcaattggtccagaaaatgcaatagcgttgtatggt
ctaagaccaactagcctagcaatttcaaattgtcttaaacagaatccaattagtccaaat
gatccatgtaaagctataaatgcccacagaccaccaatttggcaccagcgagtaaaatct
ccttgagcttcaggtccccaaaggaaaagaagtgaatgtcccatgctgtttgctggggta
gaaacagccgcagttaaaaaattgcatccttctagatatgaactggctagcccatgagta
taccaagaagtaacaaaagtagtcccagttagccagccacctacagcaaggtaagcgcaa
ggaaacagaagtagtccagaccaacctacgaatacaaatcggtctctttttagccaatcg
tctacaagatcaaaaccaccacgagtttttcttgtccaattgctatggtcataattta
atctccagagcaaatttaataagtaatttgtttaaataaaca---ctttagatatactct
aaaattttactttctttacggtcaatataattataagtcaaaagtaaccaaaattcacg
cttttaataaca-ttttttagctcattaagttaa-ttttttaatatataaacagtaata
gagttattgttt-aacttacaatactcacaatttattcatttctataatcctttgaaaaa
gatatcctgttcctcgtgctgttaaaatcaagtcgggattacttggatcatcttctagtt
tagctctcaatcgtgaaatatgaacatcaacaactctcgtgtcaacatgccgttcgggag
tatagccccaaacttcttgcaagatagaagctctagaaaaaggttctccagccttactaa
ctaaaagctcgagaagactgaattccatacctgttaatcgaactctttcattatttttat
aaacttgtcttttgttagtatctatttttaaaaatccaatactaataatcccagagtttg
ggactccaagactagtggtgattttatcagctcttcgaagaacagaacgaatgcgagctt
ctagctccttaggagaaaatggtttaacgacatagtcatcagcacctatttctaatccgg
taattctatcgcagacttcacctaacgctgttagcattataataggaacatctgattctt
ttcgaagttcttgacaaacaccgtaaccatctagtttaggcatcataacatctaaaacaa
caaggctaggatactcttttctaaacacgagtaaagcttcttctccatttgaagcagtga
taacttcataacctattattgttagtcttgtttctaaaattcttcttatgctggcctcat
cgtcaacaacgagaatcttttctttctggttctccaa-tttcttatttactcctgtaact
caccgatcatgattaccataaccttaaatttttagcgctatgagtatctttattcacttt
agaa-----tttagttattataaattttgcaagtctagtttacattaattagtaccagt-

-caatatttttgt-----------tttaagttatgac---------tattttaatccata
agttgactctagtttgcagaattcaatatatacata-ttttctacccaagtaaattgtat
tctattttctaaataaaaaagggggttgacaaaccagttgatagaaagtattctacattac
tataag----ttaattccaacaaagaagttttaa-----atcttctttcggagtaatgaa
tactttaat
>Pyr_29_33
tatatgata---aattaagacatctgttatgtgagaatttctaaaatatacaaaactttt
tatttaatatagaaaaagtaaattaaaataagttgaacatactttgctctaatctaaacc
tcttaattgag------attctaattagagacaaaagtatttacttattttgtatctata
ggaataaaaatgaaacttgcagtttatggtaaaggtggtataggtaaatctacaactagt
tgcaatatttctgtagcactttcaaaaagaggtaaaaaagttctacaaattggctgcgac
cctaaacatgatagtacatttacgctaacaggtttttaatcccaacaattatcgatact
cttcaatccaaggattaccactatgaggacgtttggcctgaagatgtaatctataaggga
tatggcggtgtagactgtgttgaagctggtggaccgccggctggcgctggttgcggaggc
tacgttgtaggtgaaacagttaaacttttaaaagaattaaatgcttttgatgaatacgat
attatcttatttgatgttttaggtgatgttgtatgtggaggttttgcagctccattaaat
tatgcagactactgcttaatcattacagacaatggatttgatgctttatttgcagctaat
agaatagcagcttcagtacgagaaaaagctagaacgcactctctgagattagctggactt
gttggtaatagaacagataaaagagatctaattgataaatatatagattgtgttccaatg
ccagtattagaagtcttgcccttgattgaagatattagagtgtccagagtaaaaggtaaa
actttatttgaaatggcagaaattgataaggatttagcatatgtatgcgattactatttg
aatattgcagatcagttgattacaaggccagaaggtgtagttcctaaagaatctccggat
agagaattatttagtcttttatctgatttttacttaaatcctaaatcaaaggtaggacaa
gaaaaagtagatcaagaagaattagatttgatgatagtgtaaataatattttcagcataa
tataacaaggaataagataatgtctacagctcaatcagatgctcttacttttgaatgtga
aacaggtaattatcatactttttgtcccatcagttgcgtttcttggttatatcaaaaaat
tgaagacagtttttttcttagttatagggactaaaacttgtggttatttcttgcaaaatgc
aatgggagtaatgatttttgctgaaccgagatatgctatggcagaattagaggaaggaga
tatttcagctaagctaaatgattatggtgaactccgtagattatgcttacaaataaaaaa
agatagaaatcctagtgttatatttggattggaacatgtacaaccgaaattataaaaat
ggatctggaaggaattgctccaaaattggaagcagaaattcgtgttcctatagtagttgc
tagagctaatggtttagattatgcctttacccaaggtgaagatacagttctagcagctat
ggctcaaaggtgtccattaaatttaaagaatcaatcagataacacgagtcttaaaacttc
tcctcatattcctttagttttatttggatctcttccggatccagttgtaacccagttaac
tatggaattaaaaaaacaaggtatttttgtttctggttggttgccatcaaagaggtatac
tgaattaccggttataaaagaaggatattatgttgcgggagttaatccatttcttagtcg

cacagctactacactaatgagacgccgaaaaacaaaactaattggtgcaccatttccaat
aggtccagatggtactcgagcctggattgaaaaaatctgttcagtaatgaatgtaaagcc
tattggattagaagatagagaaaaagcaatttgggcttctttagaagactatatttcttt
aatccgaggtaaatcagttttttttatgggtgataatttactagaagtatctcttgcaag
atttttaactagatgtggaatgactgtatatgaaattggtattccttatatggataaacg
ctatcaagcagcagagctagctttattaaaagctacatgtgataaaatgaacgttatgat
gccaacaattgtagaaaaaccagataattataatcaagtagaccgaatccgtgatctgaa
acccgatttagttatcactggtatggctcatgcaaatcctttagaagccagaggtattaa
tacaaaatggtcagtagaattcacatttgcacaaattcatggctttaccaatgcaagaga
tattcttgaactcgtcacaagaccattacggcgaaatcttagtttgtcagaattaggctg
ggatgtttatagcaagcaaagctagtattcctc----tttatgacttacatggtataaaa
gccttgtaagtct--tttattttatgttaaagaagcatttcttgctctgccatcaccagc
ccaagcttgtaaactttctacttgttctttgtctgtaaatgctaaaggcacaaattgttt
gatagctattttaatatcttcagtactaaattctctctcttcactaaacgccgtatgcat
actttcaataattgcttgctcaatttcagcgccagaaaatttattacataataaactcaa
ttcatgaatatcatatttttgccacgatcttggtctaacttttgataaatgtatcttaaa
aattgattctctctcttgacgattaggtaaatctaagaaaaaaatttcatcgaacctgcc
tttccttaacatttcggaaggtaaactttgaattttattagctgttgcgacaacaaatac
tggagctttttctctgatagccaggtaataaatgtaccaaatactctggcactagtacc
actatcaccttgactatgtaaaccagaaaaagctttatctatttcatcaatccataatat
gcaaggtgataatccttcagagatatttatcatttctctcatttttgattccgattcacc
aactagtccaccaaacagttttcccatgtcaagacgtagcagaggcagcatccaatcgct
tgcaatagctttcgcggttaaggatttacccgtcccctgtataccaactagcaataaacc
tttgggcgaaggaataccgtaatttaaactttgcttcgaaaatgagcgagaccttttttg
tagccattgttttaatacatctaagccaccaatatccctatttactttactataaggata
aaattctaatagatgcgtttgattaataatttgcctttttttcttcaataataataggtaa
gctccgagagtctatctgattatattgtgcaataattttagtaatcactttgcggattct
atctatcgataatccttggcaggattttgttatattattaacgagctctgaattcaaatt
aagattcaaagctttactcaatcttatgatctctttttttgatttctagaagactaggtaa
gggcaaatctattactgttatgatatcattcagagcaaaaggtatattgattttgcaaga
aataataataatgtgcttagattgcgttttgattattttagctaaattgcgaagtttcct
aattaatactatttcgtttaggaaagagtcaaaatcttttaaaagaaaaagatttaaaga
ttcattattcaagttttcaataaattctaatgctaataaaggatttctttttgcataacc
attatcgctaggattgttagtgtatccatctacaaagtcccaggaatatacttgctgact
gtttgaacagtttaagctgtgttttataatatactctagtctatcttcttctctagtatt
tattacaataataggatatcgagattttaaaagtaatcgtaaatcttgagtgaaattcat

gtcaatatttgtttccatcagacttttgtacttcaccaattgaagaaagactaaataaga
ttatctaatattccttggatatattcaaaagtc------atctctctatttctaaatta
ttgctattactaatttaacttaccatgatttttttcttccttttcttcgtcgactattt
aatatagagcgtccacatggtgttttcattcttgctctgaaaccagaaactctaatttt
tttcttttagatccttgtagtgttctttagtcatgatgtctcctctgttgtaaaagatt
aaatactatttatcatcttttatatatctttatgatatagatatgtattaatatcaaag
tt-aacagcaataatttatattaatcaagaactatttatataatattaaacattaaatat
----------taactttatgatattggctttgcccatttttatttaagtattttgacca
tctttttgctaatcttaaactggttaatttttcagcagctaaaaacgatttgttattag
aatctcaatttaagtattttgttgataaaagccaaaatcgtaaattagaacccgacgaaa
gttttgcttttgcaaaagtatgtgtagctaaaaaatatttttctaaagctattattgaag
gtcaattagctttaaaaaactatcgagacctgaatatcttagataataatatagtaattg
ctaatttgtataatatgctaggttttatttattttgaagcaggccaaacaagttttgcaa
agaacttttatgagcaagccttacaaataaaccctaattatgttgtagctttaaataacc
tggcaaaaatttatgaagaggtaaaagatttaaaaaaagccgaatctctatacgataaag
tgttaactcttaacttaaataataaaactgctaatagaaggaaagatttatagcaaaaa
cta---------aaaacatctaatactatttgtaatcgggatagcaggatttgaacctgc
gacatcctgctcccaaagcaggcgcgctaccaaactgcgctatatcccgtaagacataat
tattactatataccttttctctaattatgtctactctttctgtattttatctaggatacc
aaaacatgcctttaactccatctggatctttgataaatcctaatttttataaaaactaa
caacatcaggttctgcgaatagtgtaatagtgctaatttctgcctgtctcaattgctgta
ttaattggtgtatgactacttttcctaaacctaaaccttggaaatctggatgaataacta
cgtcccagatagttgcattaaatccgttgtctgatgttgctctagcaaatcctacaagtt
tactatttgcatctttttttgtattaaagaaataataatagaactatttttaatgcga
tctttactttttttagaggtcttttaacccatccgactgaatcacaaagctgctccaatt
catataaattaatattcttattgctactcagatagatatctttaaattcgattttatcgc
aagttttatctaaaacaataagtttttgaaatttttttcagagctagcattgatattgg
agttttgaaaaaaatttttccagaagatcataatgattatgatataatta--aataaata
tatatattacaatattgctttaacaaacaatatattaatatatgctcaaatttttacatg
aatctattaagatagaagattgttaaaatatagagaaataaaaatttttgtaaaatcttt
caataaaatgatataaagagtgctttttaagactaacatctattattataaataaagtct
cgaaattgagatgaaataacacattattctatgtctaatgccttttaaaggagttatttg
tgaaaaaatctatgtgtctaacctgtttacttgctttactaattatgagcaacccaataa
tagctaacgcagaagtagctggattggtgccctgcaaagattctgcggcattcaataagc
gtatggtaaatagtgtgaaaaaacttcaagccagattagctaaatatgatgcagacacgc
caccagcgttagctttaaataagcaaatagagaaaactaaaactagatttgcaacttatg

gtcgagcaggtttgctgtgtggtactgatggattgccacatttaatttctgatggtcgat
ggagtagagccggggactttgtttttccaggactattattcttgtatattacaggatgga
ttggctgggtaggtagaggatatcttttatctgttgctaagactagtaagccaacagaaa
aggaaatcatttagatgtgccattagctatcaaatttatgtcatccggatttgcatggc
cgctagcggcttggcaagaatttagtagtggacaattaattgcttccaatgacgatatta
ctgtttcaccccgttagtaaaaaatttatatgaataataatttaccaaatacttatcaa
cagcacctgtaattggtgtattgtggatgacgtttacagccggatttattatagaattga
accgctttttcccagatgtcttatacttttactt----ataaataa--gatcgtataaa-
--aaactacataaatatagagagatactattatttgtgtagtttttta---atatataaca
taattattta-tttttaaaaatgtttatcttttcgattattttaatattagatcttacat
aataaaata-atatcaatattatgagcttagtaacccaaattatagttaatgccgatgat
gaattaagatatcctacaattggagaattgcagtcaattcaagactacttaactacagga
agcaatagaattagaattgctactattattagagataaagaaaaggagattatacagaaa
gctagtaagcaaattttttcagttacatccagaatatatagctccaggtggtaatgcagca
ggttctaggaaaagatcgttatgcttacgtgattatggttggtatctcagactaattaca
tacggagtattagctggggataaagattctatagaaacaattggtattataggagtaaga
gaaatgtacaattctttaggtgtacctataattggaatgttagatgcaatccagtgttta
aaggaagcatctttagaaatgcttggtcaagatgatattagaatcatttctccttatttt
gattatataattcgtggaatgtcataaatcatagcttcaattaattagttgaataaaagc
ttttataatgttataattacaataagcccgagagcatataattgtaaaaactaaatcttg
tcagaaccggaaggtagcagcaataatgtttcaatacaaaagttatga-ttttcgggtgt
ttattgtttttatctaatagtctcgacttttaattagtatgctatgtataaatctgtata
tgacatgtaatccatcaaaccttccagcaaaaataatttaataatttgatattaatacgt
aaaacacctatac-tcttttataaaatttctttatatctcatctaaacaatggaaactta
acttaatgggtgttcatattttatcaacaggttcgtctgtcccaaattttttctgtagaga
atcaacaatttgaagacatgatcgaaacttccgaccattggatttcaacaagaacaggaa
taaaaaaaagacatctagctccatcttctacttctttaactaaattagcggcagaagctg
caaacaaagccttatatgcagctaacttaaaacctactgagatcagtttaattattttag
ctacgtctacgcctgatgatttatttggtagtgctagccagttgcaagcggaaataggtg
caacaacatcagtagcttttgatattacggctgcctgctccggttttattgttgctttag
taacagcagctcagttcattcaaactggttcctatgacaatattttagttgttggagcag
acacaatgtctagatggattaattggtcagatagaactacctgtattttatttggtgatg
gtgctggagcagtagtattggggcaaagcctcaaaaatagtattttaggctttaagttat
gtacagacggtcagctaaacagtcatttacaattaatgaataaacctgtaaataatcaaa
aatttggtggtacagaaattcctcatggaaactataattccataacaatgaatggcaagg
aagtgtacaagttcgctgtatttcaagttccaacagtaattagacaatgtttgaataatt

taaacatttcaatagatgaagttgattggtttatattgcatcaagcaaacactagaatca
tagaagcaattgcgagcagattatcagtaccttttctaaaatgattacgaacttagagc
attatggaaatacatctgcagcgtcaatccctttagcgttagatgaagctattcaatcca
ataaaattcaaccaggtcaaattattgttttatctggttttggagcaggcttgacttggg
gagcaattgtcttgaagtggtgat---ttatatattgcggatgacgagactcgaactcgt
aaagctttcgctacacacccctcaagcgtgcgtgtataccaatttcaccacatccgcatt
ttttaccatgaata-ttaaaaatttataatatttatctaaatattcatataa--tgtgta
tatatatatacattcttgtcagaaactctcttatatttatttaagtgataaaattgag
t----ttagtatagatagtaaaattttatttaaaatcataa----aaataaatatataa
ggaaaataaaatatgacaccatctttatcaagtttttgaatagtcttattcttggggca
gtaattgtagtcgttcccataactttggctcttttatttgttagtcaaaaagacaggaca
atccggtcctaaaaataacttagaaagatatggagaagcaatttaagatgaataatgaaa
tatttttcatactaataagctctccatatattgctatact--ttagaagaatttttattt
ttttatttgtaacatcctgtaaagtttattccgttctttctttttccc-atttactctc
ttataattaatgatacaaatgtcagg-ttaataatag---ttttttattctgtatctttc
---------------atttatatacgtattattatgttcaattaataaagaaaagctca
tgtctttgtctaattggcctctcaaaaaagaaaattctgaagcatataatattaagaatt
caaaacaaatcacaattcctgatggtttatggataaaatgcttcgactgtggtctattaa
tgtattctaaagtactgaagagaaatttaaaagtttgccctcaatgtagttatcattttc
aagcttctagtaacgaaagaattgatcaattgatagaccaaggtagttggcaaccaatgg
atgttcacttgatctctacagatccattaggctttaaagaccaaaagctttatagtcaaa
ggttaaaagatactgctttcaagactggcctgcaagacgcagttcaaacaggtactggga
ctatgcaaggtaaaaaagtatgcttaggtattatggattttagattcatgggggggaagca
tgggatctgttgtaggcgaaaaactaacaagactgctagaaaaagcaactcaagaaaagt
tgcctgcaattatactttgtgcatcaggcggagctagaatgcaagaaggtatgttgagct
taatgcaaatggcaaaaatttcttctgctctagaaatgcataaaaaagaaaatctactat
atatatctgttttaacctctcccacaacaggggggtgtaacagctagttttgctatgcttg
gagacttaattattgcagagccaaaagctcttattgcatttgctggtagacgagttatag
aacaaacaatcaaagaagacttgccagataattttcaaagttcagaatatttatttgaac
atggtttcctagatttaattgtatccagaactcagcttagatcaaagttaatacaaattt
tgtccttacataatcatagtaagtaatgaattgaatattgactataatatcaaaatgcta
gtaaaaattaagatattaagaaattttcactaaagatatttgtagactaactagaattac
aattactattgtagaaaaatttgtcttaatgcca-tttttttcagtcttttaacaaattg-
tttttatgactttaaataattgttagtttaatacaaatttataaagagaata-----cat
actaagctaaatatagtaatattatgttttcctataatactgacctatcaaatttcgagg
aaatttatgcttaaaagatcttcttggcttgcggctttattgggactattaacagtagtt

tctacaagtacgcatacatatgccatagagttagacgaggcaacaagaactgttccatta
gaatcttctggcagaactgtaattcttacaccagaacaagttaaaagaggcaagcgatta
tttaataattcttgtgctatttgccataatggtggtatcacgaaaacaaatccgaatatt
ggacttgacccagaatctttaggattagctacgccacaaagagataccattgaaggacta
gttgactatatgaaagatccgactagttatgatggtgcagagtcaatcgcagaattgcat
ccaagtattaaaagtgctgaaattttcctaaaatgcgcaatctaaccgatgaagacctta
tttacaatcgcaggtcatatcttacttcaacctaaaattgtttctgaaaagtggggcgga
ggaaaaatttactattagaacttcaaagacctaaaccttgtgaatactagtttatgta-t
ttgttgcttaagtgtgattattttgttagatcatgatatatattaaagatggagtttgtt
gacagcaagttacttttttacccgaacttgtttacactattcgtctagttattaaaaagga
gacgttaaattgaagaagaagctttcagttcttttcactgtttttagtttttttgtaata
ggtttcgcacaaattgcttttgctgcagatctagataatggagaaaaagtttttttctgct
aattgtgcagcatgtcatgctggcggtaataacgccattatgccagataaaaccttaaaa
aaagatgtacttgaagctaatagtatgaatactattgatgctattacttatcaagtacaa
aatggtaaaaatgccatgcctgctttcggaggtagactggttgatgaagatattgaagat
gcagcaaattatgtattatctcaatctgaaaaaggttggtaattatacttgattttatcc
tgtattaaagaatagacaatctatttagttgttcattagattgtctattctttgtttatg
---ctattatataaagatatttacacaatatt----ttatgatgaaaagaatacccgcaa
ttcttgtactagaagacggtgcatattataaaggatggtcattccagcaagataaacaag
agattactattggtgaagtagtttttaatactggaatgacaggatatcaagaaataatca
cagatcctagttacttccatcaaattgtcgcttttacctacccggaaattgggaatacag
gtattaataatcaagatattgaatctcacagtattagtattaaaggacttattgcaaaaa
atatttgtaaaatttcaagcagctggagagagcagcaatctttagttaagtatttaagta
gtaataatattccttttattttcggaatagatacaaggtctttaacccaatacttgcgtc
aatttggtacaatgaacggttgtatctctactgataatttaaatcatagttacttaaaac
agaaaatttgtgagattccaagtatgcaaggtttagatttaatcccgcatgtaactacaa
gaaatgtttaccccctgggatgaaaaaagttttccgaattggtatttgacagataacatta
gagtacaccgagttattcagttgaaagttattgttatagattttggagttaaactaaata
tactcagaagactagctacacttggatgtcagataacggttgtgcctgcccacactcctt
taaaagatattttggcttaccagcctgatggtatattactctctaatggtccaggagatc
catcagcagtacattacggcatccagacagttacaaatttactagattacaatgtgccta
tatttgggatttgtatggggcatcaaattttaaatttagctcttaaagctaaaactttca
aacttaaatttggccatagaggtattaaccatccatccggattgaaccagcaagttgaaa
taactagtcaaaatcatggctttgcagttgaattgacttcggtttttgaatctcctgtaa
gagtgactcattttaatctaaatgacactactattgcaggaactggacataatcaaagtc
cttatttttctgtgcaatatcatccagaatcgagcccaggccctcatgatgctgattatc

tattcgaaaattttatagaaataatgacaaagtccaagaataaagttag-----------
ttagtaattttcccatgctttatgagtgaataaagcggatagtacttgtactcctcgtaa
ttgattaaagagaggcaaatgtcctctggggccatgagaattccattgaaattcctcagg
atatctgcaaggaattctatcaacttcccagccgatttttgccagaattttccccaatc
ttttccaacactaagccatatttgtctttgaacaaatagcccaaatttacctttgagtg
tgtatgccataacttatcaatagtttgtaaatcttgagcaggtattttttttatatctgt
aaaatatagccaattacgagtttgtgcatttacaccagctagttgaataagcttttgttg
agttagctgatctgctttgagcaaatcccgatgagtcagtaacatttgtaaatctttgta
attcatctgttgagcagagcgcaatggaacaatgccatcgggacataagttagaagcaaa
tttcactatttcttgattttactgttaagtaacttttcataaattaaaccgtcaacaca
attacttttatagttaggcccagtaatcctctcaaaaaatagatctgctaaatcttttaa
ctcgatagagtcgttgctatttatattctcaataatttcaagttgttgcttga---catt
attagacttagtattcttattcaattctaaaagctgggctcggatttgatttggcatctg
atttgtttaattagttttaatagtc----ataaggtcttagagagaattagattgttttt
tgaaattagaagagcgccgcagtatttctaaagaagttcgcataatttgattcaataagt
tttgtcctgcataaattataatttgaccaattaaaaaaattgtgcttttatttttaggaa
caaaaaaatcttgtacctcaagcagaaaagtaattattatttgcattggtgacaacatct
gtaattcattcgatctgcaagcatatatatactgacagtttaagccttcttgataaaatc
cccatactttgtaacgactttcgtaaattgctcgtggtcgctcaagatagtttttaatta
tagtattccaaattaggttattctttaatttttctaatttctatcagatagaaatttta
cattacagagataacttgcatttgtctcatttacagtattaaaattttgaactaacatat
gagcaataatgttgcttagctgaattaaataattttcaagaaaaatttcgacctgtttaa
ctggcatattgtaagtaaagagattaaaagttttattcacagtttgagatgacccaaaaa
gtagctgaataattaaaatttgtagtagcattttatagtcgttcagtagatagttcaaat
catgaatatcttggtaaatgtcaatacagtccagttcatataacttacaaaatcttttga
tacacctatggaataaatcaattaaaatattttcggtcaagtttgtaatatcatttacat
ctaaatttgaattataaatttctaaaagtatcttttccagttcagctaatatgattttta
ataagtttctttttacttcagtgcgaaaaacatctaaaattaacacttcctgagagcaat
tagttaatctcttattaatctttatagaagttctaactaatagctctgctacctcttgat
tgagtgttggtccctgagagcttggccaataattattcacgttataccatagtaagaaat
attgaatttaatgttatacgataacttttttaatatactgaaaagcaagttaattactcta
tagcaactag-------tttttacagatcttaatagttatttatcaaataaagttatatg
atagtagcgattgagctaattttattaaaattta----taataatgacaacttattactt
cgcccttgcgagtcaaaattttctattagtacaagaaccactagaagaagtttttagaga
gagagttaactactatcagtcaaataataaagcaattgatttttggctaataccaaaccc
ttcttttctagagaaaccagaaatgatttcatttaaaaaccttgtacctaaagacgctgt

agctataatctctactaatccaatatttattaattggttaaagctaagaattggctatat
ctgtattggacaatttgaagataacctacaactttctgaagaatcgttaaatattactgt
tttaacagacaaaatttaacaatgcttttattatgactagtgttaaattattttggttaa
gttataattatagtcttttagtattta-gactcttatagtttatcacttagtattttatg
ctgagatggagtgacaaatagtgtaagtatttcttggctaaacgtttcagttgccttaga
tttatatctatttggattaactataatggatagcattcgttttatagtaacattttcaat
ttgagcccaatgtacaattccaagttccaattccttagcaatagctgaaacggaaacaaa
ggcagctcctagccctgattgcacagcgtttttgatagcttctatagaattcaattccat
ctctatttaaagcgactgctgtcaataccatgttgactgagtactttatctatcacttt
tctaattgttgattgagtatctaatgcaataaatctaagcctgtataagtcttcttttg
aatgtctcctagtttagaaaaaggatgtgatttgggtaatataagtgctaattcgtcttc
cgcataagaagtaacctgcaaaacatcttgcaattcagtaggcacttctcctccaataat
tgctaaatcaacttgaccattagctacgctccatgaaataagtctagttgaatgtacttg
caattgaacagccacttgtggatatctttgcctaaatagtccgattaatcttggcatcaa
atatgtcccagttgtctggctagctccaataattaatgtgccaccttgtaagttttgtaa
gtcgtcaagagctcgacaagtttcttcgcagagagctaaaattctgcccccgtatcgtaa
aagaagacttcctgcctcagttaaagttgccttcttattacctctttcaaatagggaaac
atttaattggcgctctaaattttgaatttgcaaactaatagctggctgagaaacatacaa
gctattagctgctttttaaaactgccctctttggcaattgcttttaatattcttaactg
atccaatgtaaatggaaggtctgtcattaaagaattgtagtataatg---------tata
ttatttagtattgatcaatgtatgcatttgccaactaaaatatttgtcattacgcttcat
tattttcaaaataataaaaagtataatgttaaataagctgtattaaaatttagtatctgt
ttgatattaaatttatacaagagatatcgtgaatttaattctaactataaaatataggag
acaat---atggactctagacttttagttgtactaataccagttttagcagcagcatctt
gggcagtttacaatattggtagagttgcactacagcaatttagaaaaatgacatcttagt
tttgcttagaatata-atttttagactttatgagatagaggactttaaaatccctatctc
tattgtatatcttaataaaaaaag-aaagc--ccccttgtacttactgttattacacatt
aatattattggt-ggtaccta----gataataagtttaagctaacttatttttttattta
taattaattaaattaataacacaattatggctgttccaaagaaaagaacatctaaagcta
aaaaaaatgcacgcaaagcaaattggaaaaatcaagcaaaaactgaagctcaaagagctt
tgtctttagcaaaatcagtattaactagaaaatccaatggatttatttataatctaactg
aagcatcagatacttttagcgattagaagtgttt-ttactacaagtctgaaaaagtatta
cgcaacaagttctcagtaatttttatctat----taatcatttgaaaatcattctatttg
ataataaaataaatagcagattaaataaaatcacattaacaaattatgcagcaaagctag
atcaaactagtgaaatttggttgttcaattgtattgaaaatattcagcatattttttta
aaagtcaattaaaatcaagtcatattactaaaatttttatttctggtactagctttgaat

ataccgcaggtctaccaggattattatccagcttaacactaagtggtagactacatccta
ttagtatatatagtccccagtctctcaaaaagtatcttgaagcatgtaccaaatattccc
aaactaatttttcttttcctattaattttcataatttacaatacggaggacaagttgtta
accaatttatacagtaatttgtttaccgttgagcaaaaagagcctgctgtatggattta
ttattctaaaaaaagaaaagcagggagtatttaatttagcacaagctaaaaccttgaata
ttcttcaaggacctatatatggaaaactcaaagaaaaggataatttttaagtccagatg
gttactatctatccggccaagacttctcttctaatacaataatgggacataaaatatcgc
ttccgttattagtcagatattctagaattatttctgagatgcattggttttgctcttatc
ctattagattaaatacttattcgcatcagcaaggagtaaagtgtttaccacataatgttt
taactgatattatgaaatcccaaatatatcaagataacagttttgttgaataatacttat
tcttgatatataatggtctcggtaatctaaataaaatactagaaataatttcttaaattt
atttgtctatattaaatttacaatcatttatgacatacgcaattattgaagcaagtggca
cgcagctttggatagaagaaggccgctactatgatttaaatcatatacctgttgatccag
gtcagtcgattatattaggaaaagtcttattattgaataaaaatggggaggttactttag
gacgcccttgtatagaaggggttacgataaaggctacagtaatgaggcacttgcgaggaa
agaagataactgttttcaaaatgaaaccaaagaagaaaatgagattaaaaaaaggtcatc
gacaagaattgactcgtttaatgatcgattctataacatcttaga------taaaatcta
gactcatatttt--aa-aaacttttaatatttaactaaaatagatataatggcacata
aaaaaggtagtggtagtacaagaaatggcagagactctaattccaagcgtttaggtgtta
aaaaatatggtggagagcaagtaacagcaggtaatattttaatcagacaacggggaacta
aagttaagcctggccaaaatgttggaaaaggaaaagatgatacattgttttctctaattg
atggtttcgtgctgtttgaaaagtcaaatcaaaagcaaaaaacaattagtgtttattctg
ctaagaaatagttaaaacaattggtgcaagataacttgagt----gtatttatttcactt
acagtggcaccatatatttgatttattaagaaatggagttccttaatgaaccaatgaatt
ttattaaagatttgctgtgaaatttaatgacaaacactattgtaatttcctgtctacaca
atatggctgctattctatattgtggtcagatacagaaactagtagtagcaaatgctcatt
atcaagtaagtgatatctacctaggttgtgttgataaaatcttctcaggaataaatgcgg
catttattaacctaggaaagaatgagtacagtggttttatacatatcagtgataccggtc
cgcttaaaaagaaatattatgtcaataatattactaacattttaacaatacggcaaaaaa
ttttagtacaaatcattaaagagccaactttaaataaaggtccaaggctcactgccaata
ttacattatcaggtcgatatattgtattaatgccttttagtcaatcaatctgtatatctc
gaaaaatatatgatgaagatgagcgtcattatttgaaatctttagctattttaattaaac
cggcaacaatgggcttgctatttagaccttctgctgtaggtgtcgatgaggaaataatat
taagcgaattaaaaaatctaaaagaacaatggaactttgttcaaaaatctgcaattaata
gttattcacctgttcttctatataaagatgaagatattgttaaaaaagtaatccgagatt
tttataataataatacaaacaatatagtaattgattcaaacctgggattaaaacaattaa

attattatatccacacttggcactgtaataactctagcacagttcctaagattaagcttt
atagtaataatcaatgtatactagatgcttttggtatcaatcaggcaatttccagagctc
tcattccaaaagttgatcttatacttggtggctatatgtttattgaaactttagaagctt
ttactattattgacgttaattctggatcttttaataattccactagtgcacgagaaacag
ttttaaaaaccaactgttctgcagcaacagaaatagcttatcagttacaaattagaaata
ttactggtgtaattataatcgacttcattgatatggaatcacaaagagatcaattgcaat
tattagaacactttaataaagagctatcacttgatgatgctaaaccacaaattgtacagt
tatctgaattaggtttagttgaattgactagaagaagacagggcaaaagtttgtacgagt
taattagtagtgattctaattactttatttttcacacaatcagagagatctcagtctc
ttaagagattcgatgatagacagcagaaacaacagattttaataaatcttggctatctg
cagagattaatactattaacaaggtctttttcaaaagtcaaatttgtgcagacctgcta
acttttacctaattcgtaatctctatatagttaaaagtagtattacttataaacaaaatt
atttattaactcacagatcaaaacttatttattctaaagaatacagcaaagtattaccaa
gca------gttattatctagctagtctcaataagaatagtaatcaagagttcctatctt
aagattgttatatcaactgtt---------------------------------------
-------------------gaaaaaaagaagctctctcttatataagagagagcttc-tt
ttatagcttactaacta-tctagttagttaattaaaaattaaccgttaacagctggagct
gttagagctactggtaaagattcaccagaagctagatctagagggaagttgtgagcgtta
cgttcgtgcattacttccatacctaggttagcacggttgatgatatcagcccatgtgtta
attacacgaccttggctatcaacaacagattggttaaagttaaaaccattcaagttgaat
gccattgtgcttacagataaagctgttagccagataccaactacaggccatagacctaag
aagaaatgtagagaacgagagttgttgaaactagcatattggaagattaaacgaccgaag
tagccatgagctgcaacgatgttataagtttcttcttcttgtccgaatttgtaaccatag
ttagcagattcgttttcgcttgtttcacgaattaagctagatgtaactagggatccgtgc
atagcactgaacagagaaccaccaaacacaccagcaacacctagttggtggaatgggtgc
attaaaatgttgtgctcagcttggaatacaagcatgaagttaaatgttccggagatacct
agaggcattccatcagagaaactaccttggccaattgggtatactaggaatactgctgct
gctgctgctactggagcagtaaaagcaacggaaatccatggacgcatacctaggcggtag
cttagttcccactcacgaccaatgtagcaagctacgccagttaggaaatgaagaacaact
aattggtaaggaccaccgttgtatagccattcgtctaaagaagcagcttcccagattggg
tagaagtgaataccgatagctgcagaacttggaataacagcaccagaaatgatgttgttt
ccgtatagaagggaaccagcaactggctcacgaattccatcaatgtctactggaggtgca
gctacgaatgcaatgatgaatacagatgtggcagttaatagagttggaatcattaataca
ccaaaccaaccaatgtataggcggttttcagtactagtaatccaagagcagaaacgttcc
cacaagctagcgctttcgcgtctttgtaaagtagcagtcataatttttatcaatttta
aggatttaacaaagatgcttcccaaacaataagttgtctgttatgaaaattattacttaa

ggccttttcaaatgagaacatttttaaaaaaaaca-tagcaaaatctaatctcagtaact
aa-aattt-tacttgttcatatattgcttaataattcaa-----------taataaattt
tt-gct-------------ttttgcaaaattaacactattttagggat---ataataata
atgcatattaatgctgcttattttgattttaatagtttatataattttgccctacttaaa
tacgcatgtcacactttacaaaaattcagacgaccatacaagatttaaatctgttaaagc
atgctttaacagatttgggtttgatttggcaaatgaattctagtcatataaaagttggtg
aaaataatcagcataaggtagatattttaattaaacaagataacttgtcacatattggct
ttacttggaatgataatagatatcacttagttgccgacttacaactttgggaacaaccct
ggtctctagaagtatttttggataagttatctcagaagtatgcttactactctatcatcg
aagaaacaaagaagcaaggctttgaaaaaatgcagcagatttcacaaaaagatggatcaa
ttaagttaattgtgcaacgctggaattattaactaacatacaaatgcgggcggagagact
tgaactctcacgagattatctcactagaacctaaatctagcgcgtctaccaattccacca
cgcccgcactgttgagac--aatattattctatcataatctatactaaaacacaaactaa
-tagtataaactagcttccaagcttaaaagcgtctacaaatagaccgtatgttataccaa
ttttgaagttgttgaataaattaatttttatatttaattgcttgtggctagagtagacta
ttttactagtcaactcagttgctgcaactattagactggctgcaataataccccaatctc
ctgtttgtccaggaatcgtagacaaacctgttgaaataaaaaatcctagcaataaactaa
ttaagccagttgttaactcgctcaaagaataataaagcttattatttaagtttttatta
agtaagcaaaaaaagttgaaagtctagttttaatcatagtatttagtaacggagttttaa
aaggatgacaagtattaacag-tcattaatattgtcgtatccttttaaaactcgttaata
caaacagctgttcttgt---------ttttaaaggtcttgttcgcttaaagcattaataa
gatgctgaaacggctcttgtataatatctgtgcgtttaattgattgcaaatctaattctt
cttctatgatttcttgtaataactgtatgcttcttactgttggagcaacaggtacagata
gtgaattgtaagtatcttttagcccatttaatacacgctgatcgagaatatttgtactac
cagaaaccaaggcataactagcataccgtaggtaatattctatatcccttagacaagctg
catatcttcttgttgtataagaatttcctccaggccttaataattcaggctgttcttcgt
acaactgagctgcggcctcttttaaaatattggtagcttgatcattaataatttcagcta
tttttattcgatctagtccactcgaaaagaaagattctagttgtccgacagctgtcttat
ctagataacgaccagtaagatcgtaacgatttaatattgctgttatagcatcttgcataa
attaattctcctac-ttgctatcagagcggtattataaatgactaatattgtaatataat
cattagtgtttatgaaatttatttaaaacttattctttattgcttaatgtgcaattaaaa
actcgcatagattttacacatatagagtatacgtagtttttaatta-tttttttataaaa
accaagaaataaagctttttt-taagaggttcgtgaatttgttaatataacatgagcttt
cctcctaaataagttttgtaaagtac--aaagtaaaaaaacgattagtcaataatagtaa

--------------------------acaatattaatatgtatcaa--------------

-----------------agtataaataagcttttactccagcatcaaaaaaaatatttga
gatatgttgatacaactgattattcaagtgaaaacttagatttagaccaatttagctatt
ttatcattacaattaataaaactgataaatgtattcttatagagcaattttgttataatg
ctgaaggccatatttactcaatattcttcaaaggtccaacagctaaacatctttcttcta
taatttgtcattttcaacaattaaatacaataatatctacagctcatgccatatatttag

gacgagagctaatgaagtcagaattagctctcgtgctagatcaacaatatattcaagatt
aaa--ttttgtaagtattaattatatttatgctagtataatattagtaagggcatgtaac
tcagtggatagagtatcagattccgattctgatggccgtgggttcgaatcccgccctgcc
c---gagtactcagagtact-atactaaaacatct-------taatagctagtttgataa
tagtatgggcattggggctgcaaggtttctacattatgaaaagaagaaaatatg--aaaa
aaacaagctctccaaaagagcttttagtacaatcaataaatgcagaaaacaatattgttt
ctttttctcgaaaattagcttttgcttaaacactgttaatttttggcattcgacatgtta
aactcttattcatggcgaatattctgtcagagtcgctcttagtttaagaaaagtcagaaa
aaatatttatctttacgtttt-tacctatttttatttaaataaaggttagta-ttttcaa
cttttatagtggacgtgggttcgagtcccaccagctccatcaatttgatt---------t
gtataattagctagtttaacctttttaataaccagatatataattggaggaaatatgggt
ttcataacaattactaagcccgctttaaagcaaattgcaatcttgaaaaatgatcatgaa
aatgatgtacatctaagaataggtgttagacaaggcggatgttcaggtatgtcatactca
atgaattttgaacatgttgacaaattaaaagatacagatgaacggcttcgtctagataat
ttttctgttgtctgcgaccctaaaagtcttctttatctttatggattatcattagatttt
agctcggaattaataggaggaggatttcaattttctaacccgaatgccagccaaacttgt
ggctgcggtaaatctttttcaggctgacc-ctcgaacacgtttttttatatatttgtatt
atatatattattttagcaagtcg-tttattagagacaagcaatgctctgttaaaaac---
--------------------------taatttcaagagtttttattgtattatt-----
----ttatatatttattatcaactacttgaaaataaattagctaagcaatctaatttgtt
atcagtaccaaactttaaatgattaactttttattgatatatgctaatcttt-atcagcc
taaacatatctgtacattacaatcttttgacataaaaagagtcttagcatacctctcaa-
----------------------aaataaattcaa----------tattttataaatactc
aacatgaatctgtcacaacttatcgttaattcttctatgatgaaaattcctaataacttt
tattgcaatataaaagctatttcttctgagaatttaagtcacttacaattatcaaaaaca
aatttattatctcctacaaaaataattgcttttggactacctaaatttgattttatttct
acacagattacaagttctattagagaaacagttgttgtgtctactattgatgataataat
ctttttgctgccccaactaatgattggataaaaaaacacaaggataaaaatcatcattct
ttgttttataaaaaaagtttttaaagctttgctaaaccataaaattagagttgttgctgag
ccact------tcataactccgaaataaataaagaaaagagcataatggcaagtcaaaga
tacatctggggtaaaagctggaagccttcaattattttatcttgcttaaaaaacaaaaaa
aatagcaaaatttctgatgcatcgaaaaaatttttaactgaacagttatctgcatcacca
gtatttgtggtaaaaaatggttttaatgaaattattttaggtcacccattatctcgtgtt
aaaagaggaagagttaataatctgatgcatgcgttttctaatttattaaatcagtctaat
gcaacttatcctatatctactggtctattctttttttcatccagatgatgcatttgaattt
aaagactttataatatcggtcaatccactggcggccaaacatatggagataagcgtagag

cctgttggtctacactttgcttataaaatgaatagaaatatatcgtcagatactcaattt
tcgtttattccagattttaaagaagtaggagatttattattcaaatatagaaaaggtaat
catttagtttttcataagaatcagcattatggcaaagatttttttcaggggcaaccaata
tatatgattcagccgatcacttttaaagatcgagccggaaagctaaatattatcaaattt
acggggctaaatgacacaagagaaattatttttactaatcttgaagctgctaatagatca
tgggcacattttataaagaggaattcacaattaaaatcaattaaaaatcctactctatta
gtctataatttagaaagtttttttaaaagatcaagaacgattggataatcaagatttaaat
aagtttgtggtgatcactaataaagaatcttatcttgccacaaaagagttaatagcttta
cccgattctaatagcttttctaagcacttaaaattaaatataaagcctaaactctttttt
gtgaaactatgggtaagacggttattttccaccttaacttatgaat-aaatgctatt-gc
ctttattttctcgaataatattctataactagtaattcattgagttgtaaagcaacccat
tccctatcaatgactccattgactttccctgataaattggatttattcaattctaagtgg
ctaggaatattagctagtccagggaaagctaagtaattttctactagttttcgagatgct
tcttgatttttttacactaattgattctcctggtttacattgataactacagatagatact
acttgtccattaatacaaatatgaccatggttcactagttgtctagcggcaggaattgta
ggagccataccaagtctaaaaacagtattgtctagcctcatctctagtagctgtaacaaa
atttgacctgtcgacccttgtagtttttttagctgcttttacgtacttaaataactgtttc
tcacttaatccataattaaatcgcaacttctgtttttcttctaatcttacagcatactca
gaaggttttctggacttttgtccatgttctccaggtggataagatcttttaatcgctttt
ctactgagtccgggtaaatcacctaatctacgggaaatgcgtactcgtggtcctctgtat
ctagacatattatttaaattctcctaatgtaaat-gattaag----------taatgagt
agctatttttttaacacaaaaagagcattaaaacaaagc-ttttgctaaaaaatatttaca
aaaaatataggtttggacaaaa---tacattactatgc-tttttaagcgggtagcgggaa
tcgaacccgcatcattagcttggaaggctaaggttttaccactaaactatacccgcaata
ttcaacagattgtcactaactcaatataacatagttatgttaagttaagataatcttaaa
tttaatagaatt-ttaaattatttatgctccttctaaaaccacatcaagaaaacgagcaa
tttcggcagcttgttcttcaacttctgataataataatggttgaccgaccctagttaatg
gaattactcttttatcttttgtacataaataaatttcacgtcttggatttagcccctctt
taatatctatttttattgatttgatttcttttatattaaactgaagacagatttttctat
tctttccagggaaacctagccgaaaaatttttacaatacctttatctttgttaaattcat
tatatccagcccctatattccaaataatagttagccacagaaacaaactcagaaagactc
ctatactgccatagaatgtcattacaataccttgtggaataaataccaaatcggttgaat
ttgtaaaaggtaacaaatcaacttgaaaataacttgatagtccagcaagaagaaagccca
aagccccaataaatattatagtagcccaccagtaattactcagtcttcgtgaacctaaaa
ttaaatcttttctaactgagtgtatagataatgttttttttcataatttgttcttttaaaa
cagta-tactaatgcaaaatggaaaaattttgacaattttt--atgcgcgctttagattt

tttaaataagcttgattgcttttagtcctaaaaacagtcctgttgctaacccaaaaaaag
cagcaagaaatattatatatcctagaaaaactgaca---tattattatttattattattg
ttacaatagtgtttacaaaaatagtgaaaatctattactacggtattatattacgttaaa
tgccttgctgtctacaggaaaaaactttttcagttttagcattgtgtttatattagaa
ataatattctagtaaatttattaaacttctggagaacagaatcttatgtcagaatttatc
aagccttataacgatgatccttttgtaggcaatttgtccacgccagttagtacgtcaagt
tttagtaaaggacttctaggaaatctaccagcttaccgtcgaggtttatctccgcttctt
agaggattagaaataggaatggcacatggatacttttaattggaccttttgataaattg
ggccctttacgaggtacagatgtagcgttactagcgggatttctatcttcggtcggcctc
attattattctcactacatgtttatccatgtatggtaatgtatcttttactagagcagat
tcgaaagatccactacaaacttctgaaggctggggacaattcactgcaggatttctagtt
ggagcagtaggtggttcaggatttgcttatttattgctagctaatatacctgtattacag
actgcaggtcttagtttattctcttaagctagtaaggggacttgaacccgtaacctactg
attacaaatcagttgctctaccaattgagctatactagcattcagttcataataacatat
aaagatatttgttgcaatctgctattttcagatgtaacaaatatctttttatttaaaca
gaactgttatgttaacac-------gtatattaattagaatctgaattatctgactctgc
agaagattcctgatcacagtttagataataacaaattgtttgatctagacaagttgcaga
ccaaccaacaggtgtttctcctgataattcttgcatatctacagaattataaaaattatc
atttggcttattactgtttgcttgcataaatatcactcccaaaacactctgaacttaatg
tgaagtaatttaacttctcaataatatattaacacaaaatattaaatatggtgattattc
tttaattgtaaagatgctgctaactttataaagtttgagtgagagtcttgatagcttttg
ttgaaataagcattttaacccatttattttgcccgctcgaccatactttttgtgttgta
aattaactttttgtagtttttttgttcgtttatgagaatgagatacggcatatccattat
tagctacttttcccgtaagctgacactttttgacataatttt-------ctaacttata
tatttatttagagtacttgctaaagttgacttgggcacagctccaatcactgtgtctact
ctttctcctgctttaaagatcattaaagtaggtatacttctaatgccatattcagccgca
atagtaggattatcgtctgtgtttatttttactaccttaatagatgattcatattcttcc
gcaatttcatcaactacaggagaaaccattctacaaggaccacaccatggcgcccaaaaa
tctacgagtacaggtaagttattgttaataacttcttgtttgaaagaggcatctgtaact
tgagatactgacatattctttaacctttaatatatgttccttgctaagcaaatcttatca
caaatttcaagaaaaatcttctatagtgaattactattagtgttgaattattttttaaa
aattagacattaaaaaaacttattacctatataaataaagtggtggccgagtgatatctt
gatagtgataattttatattaaggttgaacactagaaataacttttcaagcaaactctt
atttgaaaa--atttctctgtgaccataaaaacttaattatattacaatatattttatta
tttgtaacttagttctgataatggtataaacaacgcaaaagatactgccttataatcaag
gaggaatacatgtctcaatccgtagaatcacggactaggattaaaagcgaacgttacgaa

tctggagtaatcccttacgctaaaatgggctactgggatgctgactatgtgattaaagaa
acagatattctagctcttttcagaatcactcctcaaccaggtgttgacccgattgaagca
tctgctgcaattgcaggtgaatcttcaacagctacttggacagttgtatggactgattta
ttaacagcttgtgacttatacagagcaaaagcatatcgagtagatccagttccaaacgtg
gcagatcaatattttgcttacatagcttatgatattgatttgtttgaagaaggttccatt
gcgaacttaactgcttcaattattggtaacgtttttgggtttaaagctgttaaagctctt
cgcttggaagatatgcgtatgccagtagcttatctaaaaacgttccaaggtcctgcaact
ggattgattgtagaacgtgagcgtatggataagttcggtagacctttcttaggtgctaca
gttaaacctaaactaggtttatctggcaaaaactacggaagagttgtatacgaaggcctg
aaaggcggtcttgatttccttaaagatgatgagaatattaactcacaaccatttatgcgt
tggagagaaagattttttatattctatggaaggtgtaaataaagcatcggcttctgctggc
gaaattaaaggtcattaccttaacgtaacagccgcgacaatggaagatatgtatgagaga
gccgaattctctaaagaggttggtagtatcatttgtatgattgaccttgtgattggttat
actgcgattcaaagtatggcaatttgggctcgtaaacatgacatgatttacatttacat
agagctggtaactcaacttactctcgtcaaaaaaaatcatggtatgaacttccgagttatt
tgcaaatggatgcgtatggctggtgttgaccatattcacgcaggtacagttgtaggtaag
cttgaaggagatcctttaatgattaaaggcttctacaatactctacttgaaagcgacaca
gatatcaacctacctcaaggtctgttctttgctcaaaattgggcttccctacgtaaagtt
gtaccagtagcatctggtggtattcatgctggtcaaatgcaccaacttcttgattactta
ggtgatgatgtagttcttcagtttggtggtggtacaattggacatcctgatggtatccaa
gcaggtgcaactgctaacagagtagcactagagtccatggttatggcaagaaatgaaggc
cgtaactatgtagcagaaggtccacaaatcttgagggacgctgctaaaacttgtgggcct
ctacaaacagctttagatttatggaaagatattagttttaactacacttccacagataca
gctgatttcgttgagactccaacagcaaacatctagtttaatgactacttactgatactt
taaatagttaattgtaagtgaaattaacttataacaataaggagcatagaatagtgagac
taacacaagggacttttccttccttccagatttaactgatgagcaaattaataaacagc
ttgcttatatcgtttctaaaggcttatcagcaaacgttgagtatactgacgatcctcatc
caagaaactcctattgggaactgtggggtttacctttatttgatgtaaaagatgcttctg
ctgttatgtacgaaattagctcatgcagaaaagcaaaacctaattattatgttaaagtta
acgcttttgataatactagaggtattgaaagttgtgtaatgtctttcattgtaaatagac
ctgctaatgaaccaggattcttattacaacgccaagacttcgaaggtagaactatgaagt
atagtcttcatagctatgctactgaaaagcctgaaggagctaggtattaatattaattct
taattaatattggctaatta--ttcccctcttaaaaatcataattaatttagtaattgtg
atttttaagagggatagcttcccaagtaaattttgactaaa-tagattgaataattaaaa
atacacagaaataatgcaatcacaggatataatttccaacgatactcttgttaatttaca
agaagaatatgatagaacacaaatccaagaagttttaaatgagttaaatcaagaacttat

aggattagtgcctgtaaagaccagaattcgcgaaattgctgcgctattattgattgacag
attacgcagaaaactagaactagtttctggtaatccaggattacacatgtcatttacagg
tagtccaggaactggtaaaactacagttgctatgaaaatggctgatattttgcacagact
tggatatataaaaaaagggcatttgttgacagttacaagagatgatcttgtaggtcaata
tattggacatactgccccctaaaactaaggaagttcttaaacaagcaatgggaggagtttt
atttattgacgaagcttactatctatataaagcagataatgaaagagactatggctctga
ggcaattgaaattttattacaagtaatggaaaaccaaagaaatgacttagttgttatctt
tgctggatataaagatagaatggaaaaattctacgaatccaacccaggactctcttctag
agtagctaatcatgtagacttcccagattatacttcagatgaattattacaaatagctaa
aatgatgatagaagaacagcagtactgttttacagaagaagcagataaaactcttttaga
gtataccgagcgaagaatgaaacagccttattttgctaatgcaagaagtattcgcaatgc
tattgacagggctagaatgagacaagccaataggattttgccagtggagaaaaagtatt
aacaaaagctgatttggtaacgattgaagcagaagatatcttgaaaagtagattattttc
attacctaatgcttaatatacacgtgatttcatgaaactatatattattaaaaag-tttc
ataatcagttgcaaatttcttgaaaaacctttattattatcttaatgtaggtg-tggcgg
catagccaagtggtaaggcagaggattgcaaatccttcatcccccagttcaaatctgggt
gccgcctagtactaaaaaagggggtgtggtggaatggtagacacaacagacttaaaatc
tgttgatttttagtaatcgtgagggttcaagtccctccacccccatatattgatttataa
ataaaaagagatgtgtatttgtattaattgcaaccatattaccaaatgtaatacctatca
cttaatagaatctcaacataaacagcctcatcttactagaagtccattgtttatacctaa
atatcctgtagttcatgttaatatatctaacaactgtacctataatcaaattgattggga
tttagtggagtgtttatcatttgtagaaaaaccgaatagttggaatttagacgctaatta
gttacaagaaaaattatgtaccataagcaacaactctatttttttagatactcggtattaa
ttttggatgttctaaccagtaagattttacccgttcttgctatctctataatgccaaatt
tagttagtaattgttcaatagcaacaatctttcccggatctccagtaacctctataatta
aaagatcttctgcaatgtctactatcttagctctaaaaattcttacaatttctaaagctt
ctgttctagtctgagaattgatctgaatcttaattaacattagttctctttcaactgaag
gaatatttgttacatcttgaacgttaaggatatttactaacttatataattgtttcgtaa
gttgttcaatagttctattgtctccttgaactaccattgtgattctagagaccccaattt
gctctgctggtccaactgctaaacttgcgatattaaagcctctccgggcaaatagaccag
atattcttgacagtactccggcttcatcttgaactaaaactgataaggtgtgtttcatga
aaaattaaatttaatctatttgatctattaagtatattctaagaaaattacagaaaaaat
actagttagtctataactaaattatccaaactaaacaaaatcctactatcagataatata
tctaccttgtaatgctatactattaacttagtatttaatgatattctaattgcaaataga
atgct-aattttttttaaatttgttaggaacttgtgctagaaaaatacaaaaacagtaaaa
aactctctcgagatttacctcaaatcaatgatcgcattagatttccaaaagtccgagtaa

ttgatgacgaaggtgaacaactaggtatttttgtgcctgaagaagctatacaattagctg
tccaacaaggtttagacttagttgttgttagtgataaatcggacccgccagtatgccgaa
tattagactatggtaaatataagtttacacaagaaaaaagagctagagaagctaagaaaa
agcaacataacagtagtattaaagaagtaaaaatgcgatataagatagaagagcatgatt
ataaagttagaataaaccaggcatccaaatttattcaagcaggagataaagtaaaagcaa
ctatcacatttcgggggcgtgaaatccagcactctaatttagctatagatttattgaata
agatggcaagcgatctaaatgcaatagctgaaattcagcaagctccatcaagagatggca
gaaatgtcataatgctcttatctcccaaaaaagttagctaaactaattttttatttaaatg
catctggctggattcgaaccagcgacgtctctttcgaaatggcggattatgagtccgctg
ccttcggcccctcggccacagatgcatcacttaaagtttataactcataaagcatataaa
aatcaatgtttagctatttttaataaagtctataaaagtctattattcattcatattgtg
atatgtcgcaactgctgaaggggaaacttgatttaagtatcgaaatatccagtatttaaa
aattgtgtcgagtataactggaaaagttgaaataaataaaaaaataaaatctcgactttc
cggaagtcctaaatgtcttaaaattacttcaataataacttcccatccatgaggagaatg
aaaacccacaaacatatcagtaaaaagaatgattaaaaaagctttagctgtatcactaag
accataaataatttcatttaaaaaagatttaacgactgcaatttgtctttgtccagttat
cattagtaatataaagacaagtattgatacaagatcggataaaatatttttaacagcatt
tgcactttcgtttgcataatattctcccaactctctagccttcagctgcactctttttc
aatgatttcgtaagatatatcttccgaaggattcagaagaacttcaaagtgaattttctc
ttcaaatctttgtaattcagcaaaagctctttcctcttgagaggaatttaaaaaaatttt
aggctgttcttggttccataaataatcaatacaaggtccaaatacaaaaaaatttgaagc
ctgattaactagtactggagagatgaataataataaaatatattttacggaagtaatagt
ttggtgtctagatattctaaactcctctatagcttcagattcgccattaggatctaactc
tttacgaaacttttcaaaagtattagtaattgacctaggtattggcccaactttttcaaa
ggcgaattgattatttcgttttaaattccaatatttcatttttattgctgctaaaattt
ctattaatcaataattgatcacgtattcactataaatattaattctagaactaaacatat
aaaacaatatttatgaaaaataaaatacaattaaaaattaattatgcattagttctatct
gcagctacagcagtttatggatataaatcttatcagccaactataaatacactcttaatc
aaagatgaagaaaactggcctttttttctctaaaaggcagactgcctttattcctataatt
gtattaaaaaaaaatcatcaaaatccctgaactcagaacctagaaatagtaagttatatc
ttaatggaattggaagtagaaaatttatcgacagaattgtcactctacttggatatttag
aaaaacaggcaaactatactgaatacttgaaccaaataaaaacaagtggttatttttctt
tagtctacctagacagtgatataatgtataagaaaaatattagtaacatttttttactac
ctaatgatattctaaaaagaatctatatttgcaataagaaaaaaaagcttataccgcatt
cattcttaataaatttatttcagaaacagataggctatccaaaaaagttttgctaatttaa
attgggccttctctaaaattataaaatggtattatgatagaggataccaatggtctctag

ttgaagttaaacaagcgtctgatgcatcttctattgtaattgatattcatgaaggggtag
tgaagacaattataacagaatactacactctatcttataaaagagtctcaggtatcttat
gcgtagagtcaatagagcagtaccttggagtaagagtgggagctccactaaatataattg
atttacaaaaaaaaattacttacttaaaagataatcaactagttggcgacattatttata
gtattgaacgatcaaataatagctcaatgagtttggatatcaaatttcaaatacaagaat
taaaagataaagagataatagtgcttgcagaaagttcttcta------------ttatct
cgcatgcatgtaatttactcaatc---aatataga---------aataggctagtagctt
ctaatatagtttcactatcaactaataaattacacgcatgctattttaattataaacttg
actatcaatataagtatataaacacctctaatcttataaaattgttagcttattctattt
cttgtaggaagacactacttattcattatttaatttacaaactttaataagctgtacaa
aaaaaaatactattggatttcagctatatttacgaaatctaagttttgggaaagctttct
gtgtgcttagtatgaaatttatcaaaaatggacttaatataaaaatttatatataaatc
cttcattgatagttgatcagaattttgtatttcaatttgctatacaaatcattaagcaat
atcatactgcta------aacctccagctt------------------------------
------tatttttaacgaatctagatcttgaacaatatgttgctgaaagtttattaatgt
accattttacatcttgcttctcaatatctgaaaaaatattattatctcgaattatgcata
cagattctttatttttcaactctgaaa------attttcattttgatgatagaactaacg
cgatcaacagttatgatactttaaacaaaatacaaaaatatttatcaagagtttcttt
ctttattattaagtttacgttatcaaaattttaattatttaggttggcctttaaaaggcc
attttttgaaataaaatctttatacttggctccatttcaaaagagtg------------
---atttttctgatagtcgtaagacgctattcttccacaaaatgtcgttaaagcaagtat
ctaattttaatttaccagtatcttttaaaagccaccttaaccatattttagttagtacta
ttaaatgtcagtcaaatttgaatatgagaactgtatctttgctattaattgattcacctg
ctgaatatctgctatacaaatctattcttaatttctctattaaagttaggatgcagtatt
tcatacctatgagcaacaatataagattatctctattttataattatttagattgttttt
taataagatcatctcagagttgcattcacatatggcaagatctaagaactcttacaccaa
ttcaaaatttttggttaaaaaaattttcatatggagctggaattcagcttaaattaccaa
ttaagcagatgccacctctttcaattgaatatactgtaactagtagtcgctacttttgta
tttaccttcgtacttattatcaacgataactattactaaaatgaaaaatttaatttcttt
ttttgatcgcagcaaagggaaatggatttcacaaagaacaacttatgaattatctaataa
aaacatgagttcggtacagtctcagatgacaatgaaaataggcaactcattgtcaggatc
tataatacttgcatcgttaaattgggggacatttatagacaagttgcccattacgctaa
aaatcacagccggagtgaatatgataataagtttaacttgcaattcggtaatcaactaaa
taatcataaattattaacgctgtgcatagtgacagatcctagtctaattagttttaaaac
tcgctatggaagtactaccatagatgaaacatattggttcgcaacaaataatttacgtct
aagtactagtattgttaaacgattcaacacttgtgtggcagtatctttttgttcagaaat

taaagtttaa-actctatatagaagcaactaatagcctttta-ggctattagttgcttcta
tgtatctaaattttgtattaaaaattat----aatactatattattctaaatctttacga
ttgggattcctggaaggatcattagagaggaacccaaagacaaacaaagaaataaagaaa
ataacggtagtatatacaaagattttcaaagtaaacatattttcatccttaaaagctatt
agtgcg----atatttacatatctccacataattttatatcatgatataagttccagcac
tattttataaataaatcaatatctttatttttagtaatgatttatatttaaatatgcgt
gatgtactaaatactaaatcttttccgacttgttttgtcttgtttgtaaatattttgtta
tttctatggagtttaccttcaattatcacatagtctaacttctttagctgtttaaaagta
tatgaagatttttgttccaaatagttagattgattataactaatcgttttctttttaac
agtcttgcttttaacttaattatttggcttttattttcactaattctgatactcttacaa
ctaagaatttggactagaagagtacagctattcataatttataacaa------tttttta
aaaaatctactaaaatagatgatttacagaactaaaatgatatttcattatcttgttga
ccttgctcataagtaatatctttcaatacttttaatatttttccaaaatactcttgcagg
tatttttctaaagatattacttccaacctgtcaattccaagaatactgtaaacttcatcc
atcggagctgtaaattgctctccgctacttaaaatttcggcaaatgctaaacgatcagaa
atattccatgtccattgaagagttttagtaattcttcttaaggcttttaataatccaatc
ggaatttgtgagatctgtgtttttttgcccagataatttttcacataatttaataatttca
gctgatgtccaagcggtattaccaactaacggcagggtcctattttctgtggaaggtact
ccaagactttttataactaacttggctgcatcttgagtatcaatgtatgcaatgggtgta
gattctcctgtaacccagactgatttcttgtctaaaataggaatggcatattggttgatt
aaaccttgaaaaaatcctcctaaagaaaacacagtatatttcacattggacttctgaagg
aaatctactacctgagactttaagttcatcaatggaacatctgggtatttctctgaattt
aatattgaaaagaaaataaatctttcaactttagccgctttagctgcttcaattaatgca
atttttcccatctaaatctatttttttctgcattataaggatcagtaggacgagatgtagaa
gcatctatgattgctgttaccccacaaaaagattgcaaaatactttcgggtaattttaaa
tcaccatatataagttctgctccccactctttgagaaaggcagactttcttaaatttctt
accatgcattttacattgtagccttcatctaaagctcgtcttacaatttgacgtcctaaa
gttccggttgctccaattactaaaagagtcatattagttaaaaataagtatgtataaaga
cattgatttggttgacggcagagagtaaattaggtagagagggaatcgaaccctcatgac
cgaagtcgtcacattttgagtgtgatgcgtataccaatttcgccatctaccctattatag
accaactactacaagtaatttagtctttagcgtaaaaataatactaagtttcaatagata
tgtcaacttttaaatttatattaataaaaaaacggagtctcttatggccacttttgaatt
aattccttatagattgtatttgagtcaatctaacacatggatccatagaataaaagcaga
aattaagatatatatagtagcgcttttatggatttcaattttattttttcttactttaa
actatgtattattgctttaagcttaattgccataagttttactataagaagtaaacagaa
tattattcaaaaacatttgttacaaacgttattaatgacgttcttaactactgttttgtc

ttttagtgtggctattagttataaacaatatgcagaacaagaacaatcgcagtatttatc
tgattccaagaaatataaaaattccagcagctactattatatacaaatagcgaacgatca
aaggatcaaaagacaatacttaattactactttaaagccttccttgtactttttttattac
tatatattctattaaactagttatgataacaacctctccagaagttttagtaattactat
ttatagatctaggataataaataaaatatttaaaaatgaattgctatttatcttccttct
ttcgtcacatattgttactagtattattaatagaatcgataaagtgattcaagtaactag
cttaagaggaagcttgaatttatataattcattgacaaggcctttaatgttttctttgtt
aatatttcaagtcttctttttggagattattcgggagtcaaaagaaatagctcaagctct
ttatactagaaatctcaatcaagaaaacaataatttttttgaaaatatatacagtaaaatc
taactttagtgatcggctcaatataattattagcactttgtactttattattttagctct
agcgtaatagaatccacttattcttctgactctagtaatacgaagagtaataaaattagt
tagtagtttaaatagatagaat-taagatattatataaataattaaatgtttcattatgg
tcaatactcaaaatcaaatttcacaaacttcagaacttgattatatagttaatcaacctt
ataaatatggctttaaaacttctgttgaatctgagcaatttcctaggggaataagtgaag
atattgtccgcttgatctccaagaaaaaagatgagcctgaatatctattgaattttaggc
ttaaagcatataaaaaatggaaaaagatgagtagtccgtcatgggctcatattaagcatc
cgaatatagactttaatacgattatttattacgctgttcctaaattaaaaaaagaattga
aaagtctagatgaggttgatccagaaattcttgacacttttaacaagttaggtatatctt
taaatgagcaaaagcgaatttccaatgtagctgttgatgctgtttttgatagtgtatcta
ttgcaactacttttaagaaagaactgtctgaagctggtgttatattctgttcaatttctg
aagctataagggattatccagaattaataaaaaaatacttaggtactgttgtaccagctg
gtgataattattttgctgcattgaactctgcggtatttagtgatggctctttttgctata
tccctcctaatacagtttgccctttagaattatcaacttactttcgtattaacaacgaag
aatctgggcaatttgaaaggacactaattattgctgatcgtggcagtaaagtaagttatc
ttgaaggttgtactgctcctcaatttgacacaaatcaattacatgcagcgattgtagaat
tagtagctcttgaaggagccgaaattaaatattctacagtacagaattggtatgctggta
acaaagaaggtaaaggtggtatatacaattttgttactaaacgaggcttatgctcgggta
acaattcaaaaatttcatggactcaagtagaaactggatctgcaattacctggaaatatc
caagctgtattttagctggtgaaaattctcagggagaattttactctgtagctttaacaa
ataactatcaagaggctgatacagggactaaaatgatccatattggtaacaatacaaaaa
gtagaattatttctaaaggtatttctgcgggtagatcgaaaaacagttatcgaggcctag
taaaagttggccctcagtcatttaattctcgtaattattctcaatgcgattctttattaa
ttggtcaatcatctcaagctaatacatttccttatattcaagtacagaatccaacatcaa
aagtagaacatgaagcatccacttcaaaaattagcgaggaccaaatttttttatttttac
aaagagggatcaatttagaagaatctattgctcttatgatcagtggtttttgtaaagatg
tctttaatgaattgcctatggaatttgctactgaagctgatcgtttactgagtttaaaat

tagagggaactgtaggatgagccagactattttagaaatcaaagatttatatgcttctgt
tggtgaaacaacaattttaaaaggggtaaacttatctattcgagccggcgaaatacatgc
gattatggggcctaacggttcaggtaaaagtacattatcaaaagtaattgcaggacatcc
agcgtattcactaataagcggagatattttatttttggacaaagtattcttgaaatgga
gccagacgagagggcaaaagcaggtatttttttagcttttcagtatcctgttgaaattcc
tggagtcagtaattctgatttcttaagaattgcattaaatgctcgcagaaagtttcaagg
attatcggagtttagccccttagaatttttcaactaataacagaaaaaatagatcttgt
tggcatgcaagaaagtttttgacaagaaatgttaatgaaggattttctggtggagaaaa
aaaacgtaacgaaattcttcaaatggctttactagatagcaaaatatctattttagatga
aacagattctggactagatattgatgcattacgagtagttgcaaaaggaattaacacttt
agctaaatcaacaaattcaattattttgattactcactatcaaagattattagattatat
tattccagattttgttcatattatgagtaacgggcaaattgtaaaaactggtagtgttac
tcttgcccaagatctagaaaagcacggatatgactggattacgcagacataactatttac
tac-------aatattgagactaaagaatattctttagtctcaatatt-tttgcaaatgc
aaagtt-aattatttaagctttatcaaaaccttgcttaacatcttcaattgctcttttta
gcaattcttcactaactggttctaatttttagtactacgaacattttcaccaaattctg
gtttagaattctttaaatcttctcgaagttctctaataaattcagctacttttgaaactt
caatatcatctagatagccattaattccagtgtaaataatagcagtctgttcttcaactg
gaataggagaattttgggcttgcttcaaaatttctcgaagacgttgacctcttgctagtt
gattttgggttgctttatctagatcagatgcaaactgagaaaaagcttctaattcagcaa
attgtgctaactctagttttaacttgcctgccacttgtttcatggcttttatttgcgcag
cagagcctactctagagactgagatcccaacattaatagctggtctaatccctgagttaa
ataagtcaccagatagaaatatttgtccatctgtaattgaaattacatttgttggtatat
atgcagatacgtccccagcttgagtttcaataatagggagagctgtcatactacctccac
ctaattcagcattcaatttagctgctctctctagcaatctagagtgtaaatagaatacat
ccccaggataggcttctcgtcccggaggtcttctaagcaaaagagacatctgacgataag
cttgagcttgcttagttaaatcatcataaataactaatgttgctttacctttgtacataa
aatattcagctaaagccgcacctgtataaggagcaatatattgtaaagtcgcaggactat
ctgcattagctgcaactataatagtataatcaagagctcctttatcctgtagtgaagata
ctacttgggctactgaagatgccttttgtcctatcgcaacatatacacaaacaacatctt
ggcctttttgattaataattgtgtccaatgcaacagctgttttacctgtttgacggtcac
caataattaattctcgctgacctctaccaattgggatcatagagtcaatagcagttatcc
ctgtttgcataggttcacacactgattgccttccaattataccaggggccattgactcaa
ttagccttgttccattacttgcaggttcacctttatcatctattggccgagctaatggat
caacaactctacctaaaaaagcatctccgaccggaatttgagcaattcgaccagtccctt
ttacggaactgccctctaaaatatccctaccatctcccatgagtactacaccaacgttat

cactttctaaatttaaagcaaccccaatagttttatcttcaaattcaagtagttcaccag
ccatgacttcatcaagtccatatactcgagcgataccatcaccaacttgtaaaactgttc
ctatattggctacttctacatcctgatcatacttttcaatttgttgacgaataatactac
ttatttcatcaggtctaatatttaccatattttaatattatttttattaattggaaatt
ctaaagcggtaattagtcagactaatgctatctgtctttttttactttattttaagttact
acaacatcaaggtgtgaagccatttgtcttagctgtcctctaatactagtatcaatgact
tttgatccaatctgaatagtaaagccaccaattagctcgggctcgactgaaataattagt
ttaacttctttagccttagtcataaccttgatttttctgttaatagagtttcctgatca
gggcttaaagcaatagaagtattaatatttgcaattgttaaagattccatttggtaagcc
agttccaaatacttactagctatagcatctaacattccaattcgcttgcgatcaactaaa
atcattagaaatgataatgtattttcgttaatttgatcgccaacagttgcaataattact
tcttttttttgcctctacagttttaatggattagct---aaaaaaattttagcttgtca
gattgcgctaaaatattttgtattgactgaatatcttggctcacctttttcagtaactttt
ttagttttagctaaatcaagaagagctactgcataaggctgagctatttttataacaaca
ttgttactgctcatagttgatctcctaacttagcaatattattatcaattatacgcagtt
gcatttcggagctcatttgattttccagctgcaaagtaactcttttaagagcaagaaatg
ttatttgttgctggatttgccttcggatctgtttttcagctgtttctatatttgatttac
ctgttatggcaagtctttcaatatctaattggccttgagctaatatggaacttctaactt
ttccagctgttagctgagcttctttcttaatttgatctataatgatttgagtttgagcaa
gttgtttttctgattctgacaatctagcacttgcttgctctagtctttcttcggattctt
gtatagctgctaaaacttttatctgtcttgcatttaaacttgatccgagaaattgcttca
aaacataaataagaccaaataaaagcaacaatatattaataacatttgcttcaaaaatat
cagaattaaagccgaatttgtgttcactactatgttctgataaaatagtaattatttgtg
gtatttttactatattattcatttaaaattct----------tactattttaatatttta
ctctggaatcatgattttcaagattgactacttaataatttaactttaatttgatcgctc
aaagtgtcaacctgattttctaaagttttaagagcttcttctttttgaacatttaactgt
ttcgatgcttcagcaatcagtttctctgcattcaattgagctttttttatatcttctgag
actatattttgagcttctttttgagaagaggctatttttaattgtgcattgcggcgagct
tcagatagatcttcttcatattttgcagctagctcatctgctttaacaagcatagacgat
gcagtagtcaatgttgttcgaatatattcatctcgttcgtctagtactttagttacaggt
ttataaaaaaatagtatttaataaaaccattaaagtaagaaactgtaatgccattaaaggc
aaagtaccattgaagtcaaataagccaccttcaatttcttcggctaatagaaatggtaaa
tcaatcattttactaattatgtttatagttaataattttgtttcaattaagaaaaataac
tttatagtgcgcttagtctcatcagactaagcgcaaatgattattttaaccaacgtatgg
attagcgaacaataaagacaacgcaacaactaggccgtaaattgttaaagattccataaa
tgctaaacttagaagaagtgtaccccgaatctttccttctacttctggctgtctagcaat

accttcgacagcattagctgctgcactaccttgaccaatgcctgggcctattgcagcaag
acctacagcaagaccggcagcgataacggatgcggctgaaacgattgaatccataatgaa
ttttaatttttgttatatgagtagaaaattgacaaatttcgtataatacttaaaatcta
tataattaagtataatagattctaatat-----tgtaattatagtatattactcttctcc
atgaccttccattgcttctccaatataagctgcggacaaagtagaaaagattaatgcctg
tattgaactcgcaaataatccgagtatcataactggtaatggtattaaaattggaattag
caaagtaaatacagaaacaactaattcatctgctaagacgtttccaaataatcgaaaact
taaagatagtggtttcgtaaaatcttctagaatattgattggtagtaatactggagttgg
ctgaatatatctagcaaagtagcctaaccctttttacttaaacctgcataaaaataagc
taaagatgtaagtaaagacagggccacagttgtattaatgtcatttgttggggcagctag
ctcaccttcaggcaagtgaattaacttccaaggaattaatgcacctgcccagttacaacc
tagaataaatagaaataaagttgcaatgtaaggcacccaagggcgatattcatgctcacc
aatctgatttttgcaatatcttgcagaaattctaatataaattccataaaattctgaaa
tttttcaggaattctttgtagatttctagtacctagaaatgataaagttaaaagtgtagc
aattactaaccaagagacaatgaaaacttgcccatgcaattgtaagctacctattttcca
atatagatgttttccaacttctactgctgataaactattatatgggttaaaatctatgag
actgttttgatacataataaatattt-ttttaaatagtgcaagaatcaaaaaataag-tt
tttgaagaattaatatgcaattaatttattcttaaaattagactattaataaattaaaag
caaaaataaattctaatagagatagctatttttagtagtgattattttttgtaaaaaaca
attattgctacctataatgcttttacgtttaattatatacccatatattaattatttaat
atgattataggtattttgctacaaactttattgttttatccattct----aaagattgtt
atagtttatttttatttaaaatatctgcgacttcatctgcaaaattaactttcaaattt
tcttctccccctcccagaagaaatcttacaaatcttctaattttaatattttcacctaga
acagcaatattttggtttattaaatcttcaatactaatatcttgattgcgaataaacatt
tgatctaataatgatagctccttaagtctctttttcattcgtccttcaatgattgtttcg
attctatcaactggtttgtttttcaagtcatctttaccagcttcaactctttgttccaga
ttaataatttcactaggtatatcctgtgttgatacgtattccacattaggcgatgccgct
atttgcatagcaatatctttagctaacttttggaactctggacgtcgtgctacaaaatct
gtttcacagttgacttcaactaatacacctattcttccgccagtatgaatataactctct
agtaaaccttcaatagcggtacgattagatttcttgttagctgaagccagcccttttttgt
cttaatgattctaaagctttttcttcattgccattattagcttgtaaagctttcttgcag
tccatcatacctgctccagtcttatcacgtaaggcttttacgacttgagcagaaatttgt
agtgtcatatgctttcaaaaatttttaagtatttaaatt----------aattattataa
ttcactttgagtaggatgagttatttcttctaaatctgtttgcccgtatttaccatcata
aattgcatctgctattttacctacaattagtttaattgacctaatggcatcatcattagc
tgggattggaatattaataatttctggactacaattagtatcaagtatacaaatagttgg

aatacctaattttaaacactcttgtatagctgttgtttctctcttttgatctacaactac
aacaatatcaggtaaacgagtcatattttaataccatttagatgtttacgaagtttatc
taattctcttcgtaaaacagcggcttctttttgggtaactgatcaatcataccactttt
atcttgttcttctagctgtctcagacgactaactcgagacttaatcgttacccaattagt
taacattcctccaagccatctctggttaacataataagaatcacagcgttgtgcttcctg
tgccactataccagcggcttgtctttttgttcctagaaataaaactttttaccatccga
cgaagcctgttttataagttcacaagcttctgttaataattgtgcagtttggactagatc
aataatatgtatgccatttctttctgtataaatatatggaaacattttaggattccatct
acgtgcttgatgtccaaaatgaacacctgcttctagtaattctgctaatgtaacaatagc
cataagattattaatatttaaataatgtaattattcgggaaatccc-tttgtttagaaac
tgat-----tcaactaacttatttgaattttagtaaactaataataaattaacacacttt
taaagcagatcaacaaaatagatcctgaagaattcat----tctacacttttattgctaa
aataattacgcgcggttctgtcgtctaaaatgatatcatctaaatcatctcgtacagatg
aaatagtactttcgtcattagtatttgcagttaaattctttttctctaacgatgagccat
tacagttatcatacatattgaagccggtaccagctggtataagccgacctataataacat
tttctttcagacctcttaaccaatctaatttaccagatatagcagcttcagttaatactt
ttgtagtttcttgaaaacttgctgcagaaataaagctttctgtatttaaggaagcttgag
taatacctaatagcacaggcctatatgatgcattaatcttatttctcaaggttatggctt
tattagtctgttcaatcttctgtaactctacaagctctccaggtaaatagcctgtttctt
caccattttctattttcactttagaagtcatctgccgtactattacttctacatgcttat
cagaaatattcactccttgagattgatataccaattgtacttctttaacaagtagtaact
gaatctcttgaaagcttaatcgagcagcttcatataaggctaatccacattcaatataca
aatcaaaacttgcatcgaggatatcatgaggatttaacaatacgtctgttttctttcttg
cttcaagaatttcttcaattcttggaagcccttgtataatgtcacctgtttttgctctat
caaaaaccaatatagctaatgtttctccccttctaattaatgcattgttatcaacatgta
aaatagcaccattagaaaccaagtaaggtctagcaattcttaatgtaactgatctagaag
aaatttcaataatttgtccagagtctaaagaagtcatattttcagtaataaaatcaccac
aacgaatccaatcacccacacttactttaactgttgattttcctatattaaaggtctttt
tatcagaagatgtcgcaatcaaaatgcgcctgctagtattcttttcagaataaatctcct
caacagtaccttcactcattgcaataatctcagtactagcaacaactgtaaatggcttga
tatattcaccatttttttacaataattctagtttgactttgttgcttctctgatttattta
catcaatacttttaattgataatgtttcaaaagttgacagccttaagtcaaaataattat
tgccactttctttagcagaaaactcaattgaggatattaaattatcatctcgatgctcaa
tttcagccacaagattagttgttactaaatgaactccttctatagatttcactctttctc
cgtcccgaaaaggagttcttttaactagctttagtttagtttgacttggtttttgtgaaa
ctagattttcaattagactggattttgtttctggtattgaataaacaataacaggtctaa

taagaatatagggcatttgctcgttttctatatactcccaataaactaacttatctgtag
aaatattattatgaagtctttctccaggtcttaaaaaacctctactttatcattattac
tatacacctcactaagtttatagatagatccaggcttaataataatttctcgaactattc
catccttttcgattatttcaacaatacctgagcttttagaaaaaatattttaactagct
ctgttccactttctataacatctccattatgcactagtattagtgatgaatctttattaa
tttcgtgagtttcttctgaaatccataagatatatcccggacttaaaatttcgtaggcat
ctttatctaagcctgttttctttttgatacatttaagtctaaatatttaataataccac
cacttgtcgttttatacgtatcagatattagttctgcgactgtgtaaccatcttgaa---
ttttttggtttggaatagctttgaaaagaaattttgtttttttctgtttctaaaatat
aagattctttttgtggattacatctgtataaatataacaacctggaattacaatagatt
cagtaataacttgtatattggtaatactgctattagtattctggtgtatccttacttctc
cagcataatgatttattaattctgtctgtgcaagtatagtaccagctttagttttatctt
ctttatgaacaataacattagcagaatcagatatactataaacttcacctgagagcaccc
agatcaagcctccagttttagtaattcttgtagtatattgattattatctgtttcttcaa
ctgttaaattggagaaacagattttgccagagagatctgaaactacatgtttttgagctc
tttctgtcatgagtcgatttctgcggggtgattcagcaatcacttgatccttcgatacta
attcaccatcacatattaaaagagtagtacctttacttaagttaattattgacttctgat
ttttcttagatctgattgtaacttgggttggctttttgttattaatgcttgctcaccat
gtctggtacgaacatctgtatacgattcaatatctagatctatcaattgaccatctatag
gtgcgtagatttgttctgctaattcaccggtaaatactcctccagtatgaaaggtcctca
ttgtcaattgagtgccaggctcaccaatagattgagccgcaataataccaacagcctctc
ctaaatcaactaagcggccatgtgcaagattccatccgtaacaatactgacaaacagaac
ttctagagttacatgtaacaggagatcttactaaaactttttt---gataccagctttag
ttatctcttttgctaacttaggactgacatcttggttcgtatgagcaattaaacaatttg
tctctggatggaatacatttctgctaatactcggccagataaagcttgttctagattaa
ttaatattttctgcgtatctactaaatcttctagtatgatgcctttattggtcttacagt
ctacttctctaattattacatcttgagaaacatcgacaagtcgacgcgtcaaatagccgg
aatctgctgttcttaaagcagtatcaactaatccttttcttgctccgtatgatgatataa
aataatcagtaacagttagtccttctctaaaattactcgatattggaagatcaataattt
gtccctgtggatcagccataagccctctcatgcccactaactgtcttacttgagaaatat
tacctctagcacctgagaacgccatcatataaacagcatttagaggatctgtctctttaa
agtatttaataacttcttgtttcaaggattcactcgcattattccacgtatcaataactt
tttgaaatcgttcaactgctgtaatctcacctcggcgatatttgttttcagtggccttaa
tatcttcaatcgtgcctaataaaagactttgcttactaggaggaattcttaaatcttcta
aacttaaagaaattcctgcctgggtcgcataatgaaatccaagatctttaagcttgtcgg
ccatattggctgctcgggctataccataattacgaaaagcccagacaattaaattttta

gttcgtttttatcaataaccttatttgaaaaacttggctgtgcaagacttcgtctattat
ccactaaatcttctccatattaaatgttcgtt--attaactatgctactaaagactcttg
aataattttattaaagattatacggccagctgttgttctaatatattgcacaattctctg
attttcagcgtcttcttttatgatatggttattaaaaaatttttgttgtactattgtcaga
atgtttttcaatcttaatcggatcatgaggttgatcaccatctactaatccatcaaaacg
agcccatatataagaatgtaaatcaattttcttctgttcataagccataactacatcttc
taagcttgcaaaatactgattagatccctgctgctgagatggattattagctgttaaata
atagcaacctaaaaccatgtcttggcttggcataataattggttgacctgttgctggaga
gagaaaattatgaggggctaacattagcaatctagcttcggcttgagcctctaaagacaa
aggaacatgaacagccatttgatctccgtcaaaatcagcgttaaacgctgggcatactaa
tggatgaagcttaatagctcttccttctacaagaataggttcaaatgcttgaatacctaa
tctgtgtagtgtgggagctctgttcaatagtactgggtgaccttgaataacttcatttaa
gacattccaaatagaagattcatttttttgaatcatctttttggcagctttaatattgtt
aactaatccctgcaaaattagtctgtgaataacaaaaggctgaaataactccaaagccat
ttctcgtggcaatccacattgatgtagttttaaatgaggacctacaacaattacagatct
accggaataatctactcttttacctagcaagttttgtctaaaacgaccttgttttccttc
tataatatctgacaaagattttaatggtctattatttgcacctacaacagttctacctct
acgaccattatccatgagagagtcaacggcctcttgcagcattctttttttcatttctgat
aataatctccggagctaaaattgactttagacgagataatctattatttctattaataat
tcgacgataaaactcattcaaatctgccgtcgcaaatctacctccatctaattggaccat
aggccgtaagtctggaggtatcacaggaatgactgtgaataccatccaagaaggatctgc
acctgttgctatgaaattttcaatcaatcttaagcgtttcattttttttattgaattttaa
tgaaggtgtcttaaaactttttggtggatttgtagcttcagaccttaaggtttcagctat
gtgctctaaatctaaatctttcaacagtttctgaatagcttctgcacctattcctacttc
tacttggttattctcatcttgattttggtagatttcctcttcaagacttttccattcgta
accttcgagcagttgcttgtattttaaattaatatctgtattagattgcgtaactacata
agaatgaaaataaacaatttttttctacttctttgacttttaagtctaatgctaaagcaat
atagcttgtacttccttttaagtaccaaacatgagttactggtgaggcaagttcaatata
agccatccgatgtcttctcacgcgtgattctgtaacttctacgccacatcgttcacaaac
tatacctttgtagcgaaaacgtttatatttaccacagtgacattcccaatccttaactgg
gccaaaaattttctcacagaataaaccatccatttctggctttaaagttctgtaattaat
tgtttcaggctttgtaatttcaccaacaatttgtccgttgggcaaacttctttcgcccca
ctgtcgaatttttttcgggagaagctaaactaatttttacgtagtcaaaatattgctcaaa
ctttgtcataaatttgaataccttaaaatcaaataattagccaatttatttacatgcttc
atattgaaaaatgaatatcttaataaaggaattgttcaaaatcgtctactggaggagtat
cataattagaacgatctactcggttatctttagaatccgacatcaagtcaacttcaactg

tccgtctttggccatcttcaaatagtttcaatttatgtactgcaatgtctaaacctaatg
actgcagctctcgcattaagactttaaaagattctggagttccaggcttcggtataggct
tccctttaactattgcatttaatgcttcatttctagcttgcatatcatccgactttacag
ttaataattcttgcaaagtatatgctgcaccaaaagcttctaaagcccacacttccattt
cacctaatctttgacctccatgctgtgctctacctcctaatggctgctgcgtaactagag
agtaaggaccagtggatcttgcatggatttatcatcaacaagatgaacaagcttaagca
tataagctctaccaactgtaacaggattatcaaaaggctctcctgttctaccatcaaaaa
cttgcattttacctggatgctgatcattaaaaagccatttattgctagtaatcaatgatg
cttcttttaatttctatttacaagtgctcttgatgcttctgctccatacatttcatcaa
aaggtattattttaaatctctttcctaaatagccacctgctaagcctagtaggcactcaa
atacttgaccaacattcattctagaaggtacacctaaaggatttaaaacaatgtccactg
gtgttccatctgataaataaggcatatcttgtttaggtaagatcctagaaatgatacctt
tattgccgtgacgaccagccattttatcacccacttggattttctcttctgcgcaacat
agacgcgaatcatagcatttgttccgggaggcagctcatctcctttctgcctagtaaaaa
ccctaaccttaactactctaccttttgcagcattaggaagccgtaaagatgtatctctca
catctcgagccttttctccaaaaatagctcgcaacaatttaccttctggtagttgatcag
cttcacctttaggagtaatttttcctactaaaatatctccagcttcaacccaagaaccac
cgacaacaattccatttctatctaaatccttcagagagttatcgctgacgttaggaatct
ctctagtaatttcttctggtcctaatttagtttgacggcattctacttcatacttttcga
tatggatagaagtatacaagtcatcataaactaatctttcgctaattaaaaatgcatctt
cgtagttataaccttcccaaggcatataagcaactagaatatttctccctaaagcgattt
cccctccgtctgtggatgcgccatcagctaaagtttggccaacaactatttttttctccca
cccaaacaattgggcgctgattaatacaagtatcttggttagaccgataatatttttttta
agcgataatgaactgttctaccactattatcctgaatgcctattttattagcagaaacat
aatttacatgaccagatgttctgctaataacaaccataccagagtctcgagctattttag
tctcaagaccagtaccaattattggcttttctggatataataatggaacagcttgccttt
gcatattagatcccattagagctctattcgcatcatcatgttctaaaaaaggtattaatg
atgttgcagctgaaattacttgaataggtgaaattgcaatataatctacttgggttgggg
ttgttgtaataaactcttgacgataacgtacaggaataatatctccttcaatgtaatgtt
gcttactaactttaacatctcctggagctactcggaaatcatcttcctcatctgctgtta
gatagacaggactattgtgataaatcacttgaccttggtttaccggataaaatggggttt
ctatgaaaccaaaaacattgacccgcgcacaggttgctagtgaaccaattaaacctgcgt
tcggaccttccggagtttcaattgggcaaattctaccatagtgactaggatgtaaatcac
gaactgcaaaacctgccctgtctttattaaaacctcctggacctaaggcactaattcttc
tcttgtgagttagttctgcaactggattagtttggtccataaattgagaaagctgactag
atccaaagaattctctcactgacgcaattaatggcttgggattaattagatttgataaac

tcaatgaatctatatcacatatcatcattctctctctaataatgcgttctaaacgattga
gacctactctgaattggttctgcagcaattctccaactgaacgaactcttctattaccaa
gatgatctatatcatcaagattacctgagtttttatccttaatattaattagataatcaa
ttgaggataagatgtcttgaggagacaatactcggaacgttttaggtatattgagaccta
gtttttattaatttatatctaccaacttcacctaagtcatatcttttaggatcaaaaa
aacgagagtaaagcatctgcttagcaacagcaactgttgctggttcattaggtctaagct
tagaataaactatgagcagggcttcctcatcagttacttcttcaatattattttttccaa
tttctttagctaattctttaactgaatatatttgggatgcagaaataagaaaagcatatt
tactaagtccttttttcaatttcatctttattgagtccaatagctcgcagaaaaatataag
catttactttgtgggtcttatctattcttatccaaatttctcccttaggatcaatttcaa
attttaaccaagaacctctattagaaattaggcttgcactataaatctgcttaccatttt
tgtctatctcttgtttataatagattcctggactacggattatttggttaataataactc
tttctgtcccagatacaataaaagttcctctatttgtcattattggcaagtctccaataa
aaactaatctctttttgtacttttttatttttatctgcttttcagcactgagctgtagat
gggttgatgataaatctaatgatttaatatttttttcttggtttttagaaggtagatcaa
tatcttttcttgttaactttgcaggaacatatatttgagcactataagttttatccctat
tttttgcctgtctgacactataacgaggaaattttatcttgtactcattaccaaataatt
gtaattcgagtcgactagtagggtctgatatcttcggaaaaatttcaagaacttccgtca
aaccttctaataaaaaccatttaaaactggccctctgaatttcaactaaatctggaagaa
gtttattttttaagcttatacgttgaaccatagatctcctttataacaaattttgaaatc
gaagagtttaa-tactattaatagagtactggtagataatgcataagcatagtttgctac
attatttcttctctagacataactagaacatcatattttattcaa--aaaaagattttta
aaatttttcttcac--acacactatctataagtactttgctgcta--aaagatagttcaa
gctattaaa-------aaaaacgaaattaatacttgtctgtctgtcaattaattgacatc
aagatagacaagtattatttatttaatcaattctg-tttttctgagcaaaagacaatgct
ctcgcaagtctagctttttttctagcgcctgtattagggtgaaatgcccctttttttaca
gctttatcaatcttactatatacttgagaaatacttgactgcacatcattgaaattatca
ctttctaaattatcaatatttagaaggcatcttttagttaaagttttgacaaccgattta
tacttacggttaatgagacgatttctttccgaagttttaatgcgtttaatcgcagaaagg
ttcttagccacagtaaatccaataaaactttttataaaatatttttagaaacgaaaataca
caaatctgggatctattcaaaaagggaatatgcggcgtattataacattaaagaaaaaca
ataggcaatagttcaacaagagctcttgttattacacaatagagattgtcattgcgaaaa
actttgtaagatagtatttaattaaaataaactaactagacatataaatatatatgaaaa
aaattgaagctattattagacctttcaagcttaatgaagtaaaacttgctctagtcaagg
aaggtattggaggaatgacggttatcaaagtttctggatttgggagacagaagggtcaaa
ctgaaagatataaaggatccgaatactctattgatatcattgataaaataaaaattgaaa

ttattattagcgatgataaagtagagaaaattgtagagacgattattaaggcttctaaaa
caggggaaattggagatggaaaaatatttattagtagtattgaaagagtaataagaatca
ggactaatgacttaaattttgaagctttatagtatttttacttt-tttagacttgatact
tcttcactaaacatgagataataagcgtttattgtatacttataactaaggtgtaata--
ctaatggctaaaagcaaaggtgcacgaatcgtaataactttagagtgctctgataaggct
ggagagtttgctcaaaaaaggaaacctggcgtttttcgatatacaactactaaaaataga
cgaaatacaccaagtagaattgaattaaacaagttttgtcctaattgtaatcagcattgt
atcttcaaagaaattaaatagttatttatcttattaaaat--aatcatattatggctata
tatagaaaaagaatatctccaattaagccaacagatgctgttgactacaaagatattgac
ctgctaagaaaatttattacagaacaaggcaaaatactgcctaaaaggtcaactggattg
acttcgaagcagcaaaaaaaacttactaaagcgattaaacaagctagaatcctttcttta
ttacctttttaaataaagattaa--atatatagcctaacttcatattttccatctaaa
tattaaaaatttctatagatttgtttattaaaatgatctaacatactgaat---------
----------tactaagaaga-tctactcattacaagttttgttcatttataattaata
atagcattataattta---ttaagctagattc-----agacataaag-gtaacctctttt
aattgattatattatatagtctatttaaaagaggaaattttttgcttc----------t
agcaattcaagattctttaagctttaacaaggtatttaaaacaaagattttgtttaggt
atcagatcaaatgaatgaatcttatctcccgactgccataattgaaattcagagatgaaa
attccacattcgttgccagcttgaatttcttcgacatcttctctaacgcgctttaaagat
tcaatttttccttgataaataactttttcttctcgaatcactttaatccaagagtttttt
aatagcttattattgatcactcgacatcctgctattttttctatttgctaacgagaatact
gtacttacttctgcttctcccaccggtacttctgaatattcaggatcaagtaagtcttcc
atccttcttttaatgtcttcaattaaggcataaatgatttgatagttttctattaatata
ttcgactttgccgatgcttgctttgttcctggagcaaagttggtattgaacccaatgaga
gtagaatttgtggttgaagctagttcaacatctgtagcagtaatttctccaggcatgatt
gagacaacattgagttgaactttactctgaggaaattgagataaagaatctaggattgct
tctgtagagccttgattatctgttttaataattaaagaaatttgcttactaatatcttta
gaattagtgtttttttaaagtatctaatgtaattcgactattaagtgctctttgtttctgt
ataattgatgagtctttagaggtattttcaatcgctttaagtttggcttctttatcactt
ttaactgctaaagctatttctcctgtagctggtacagatgacaatccccaaatttcaaca
acagatgatggtattgctaaattaattttctctttagcgttgttaattataactcgaatc
tttgcgtaagctgatccaataaccaaattatcactaatattcaatgtaccattttgtatt
aatagagttgctactggaccatgcgatttatctaaatgggcttctattataataccttgt
gcaggctgtgttggatcagctttcaaatcctctaattcagctaacaaggtaatagtttct
aataatttatcaacattttgaccagtaagagaactaataggaattataggcacttggcca
ccaagtttctccgacatgacattgtattttaataaatcttgctcaataatatctgtatta

gaaccagctttatctatcttagatattgctatgacaaaagggacattcgctttttgaata
tgattaatagcctctatggtttgaggtttaactccgtcatcagcagctataataataata
gcgacatctgttaaattagctcccctagacctcatacttgtaaaagcttcatggcctgga
gtatccaagaaaacaatttttttgtttattatctttcttaatatactcaacttcataagct
gcaatggcttgagtaattcctccaatttctttgttagcattattagattttcgaatataa
tctaatagcgttgttttgccatgatctacatgtcccataactgtgacaattggaggtctt
ttaatataattgccagtttcataaaaagcattagagttatctaaattactagaaagtcca
ttattattttcctttacatttgattctactgctataccaaaattatcagccactgatgat
attattgaggcgtctatggtttgattcatagtaactgatattcttttaagaaataaatat
tttataatatctgtttcttgaacacaaattagtttagataattcttgaatagttagagga
ttagtaatactaatagactctggcggagaattagattgaatgcttgctgaattttgacta
ggagttacggctactttttctgtcttatagatttttattactagtcagttttttaca
acttccacttttggcttaggtggacgcattaaagagatagctaaatctcctgcagtctgc
gaaacattagaattagagtctctaaagttgtca---tcatcatcgtcaatatgtatttta
gtttttattttt-ttgcgctgtctattctta--tttttttactatctactaagtcgtga
atttattaaaatttttactttttttatctagtttaggtggagaacttaattctagatgc
tgttcaccacc---tgtatgtgattctgatttatcaaggtctaaatttaacagattatca
ttattgactgactctaatctaattttatagataatctgaggatttttcaaatctacaatt
gtttcagaagaattgatatttgagctagatctagaggatctattttcaaagttttgatta
tttaaaaacataaatattggttttattatctaatagtaactttctaattttaatacagag
cttaatataaacaagaatcgaatatctgtataaataatacaaatgt---agagaattaat
ctatgttgtattagatttaatataacactaaagtcctttgttcgttatacaagtttaaaa
ctttt-tatgatgataaacactagattctagtttattggtatacaatctatttatctaaa
taatactgtttttgtcagggcttttaaa----ttatctaatgttatgttcattatgaaat
tctattaaaatttaaaaa-ctcaacaattaagtaagaatcgttaatcacctccacgccac
ttatgccacgatctcaaaaaaatgataatttttattgacaaaacttttacagtattagctg
atattgtattaaaaatacttcctacaagtaaagaggaaaaagaagctttctcttactatc
gagatggaatgtctgcacaatctgaaggtgagtatgcggaagctttagaaaattattatg
aggctttaaaactagaagaagatccatatgatagaagctatattttatataatatagggc
ttatttatgctagtaatggagagtatgtgaaagctttagaatactaccatcaaggattag
aactaaattttaaattacctcaggcccttaataatatagccgtgatataccattaccaag
gagttcaagcaattgaggataaagatacagagctatctaaattaatgtttgataaggcgg
ctcaatattggcagcaggctattaagttagctcctgataattatattgaagctcaaaatt
ggctgaaaacgacaggacgaatgaggaatatacaaggatattaatatatttaagatataa
taaagatatcaattaataaattaaaattagttaaagaaactggtactatatagagtactt
-tactatatattataatattatactagttgatctctatctctaagacttagctgaataac

cgaaattaaatctattgactatattcatgaatacaaataagcataatggttagtacaaca
aaaagtaccggatctgttactcaaattatcggaccagttttagatattgcatttcctaac
ggacagcttccgaaagtattcaacgcactcaaagtacaaagctcagaaggaactattact
tgtgaagtacaacaacttttgggtgacaacaaggtacgagctgtttctatgagttccact
gaaggactacaaagaggggtagaagttattgatactggatcccctatatctgttcctgta
ggtacagatactcttggacgtatttttaatgttttaggtgaacctgtagataatttgggt
cccgttgattctgagagtactttacctatccatcgaccagcacctgcttttactaagcta
gagacaaaaccaaatattttgaaacaggtattaaagtcgtcgatttacttgctccttat
agaagaggtgggaaaattggtttattcggaggtgctggagtaggtaaaactgtattaatt
atggaactaattaataacatcgctaaagctcatggtggagtatctgtatttggaggcgtt
ggtgaaagaacaagagaaggaaacgacctatatatggaaatgaaagagtctaaagtaatt
gatgcagataatctgaaagaatctaaagtagcattagtatatggtcaaatgaatgaacct
cctggagcacgtatgcgcgttggcttaactgcattgacaatggcagaatactttagagat
attaacaaacaagacgttctattgtttattgataatatttttcggtttgtacaagctgga
tcagaagtatcagctctactaggccgtatgccatctgctgtgggttaccaaccaactcta
gcaactgaaatgggagcactgcaagaaagaattacttcaacaacagaaggatcaattaca
tctattcaagctgtatatgtgccggctgatgatttaacagacccagctccggccactaca
tttgcacatttagatgcaacaacagtactttcgaggaacttggcggcaaaaggaatttac
cctgcagtggatccactggattcaacatcaacaatgctacagcctggaattgttggaact
gagcattattctactgctcaagaggtaaaatcaactttacaaagatataaagaactgcaa
gatattattgctattcttggtcttgatgaactttcagaggaagacagacaaactgtatca
agagcaagaaaaattgaaagattttatctcaacctttttcgtggcagaagtgtttact
ggatcacctgggaaatatgtatctttggaagatgcaattaaagggtttcaaatgatctta
aaaggcaatttagacgacttgcctgagcaggcattttatctagtaggtgatatagatgaa
gctatacaaaaagctgacagcatgaaagattaatataattaacgatgactttaaatataa
gaattattgctcctgatcggactgtttgggatgcagaagcacaagaaattatttaccaa
gtagtacagggcaacttggtattttaacaggccatgcacctttgcttacagctttagata
ttggagttatgagagtaagagtagataaagaatggatgccgattgttttgctgggcggtt
ttgcagaaatagagaacaatcaattaactattctggttaatggcgcagaagaagctagtc
aaattgatttatcagaagcagaaaaaaatttagacactgcaactcaactcttaagtgatg
cctcgtctaataaagaaaaaatagaagcaacacaaaaaaatacgaaaagctcgagctcgag
tacaagctgcaacagcagcaacttcgtaa-aagattcc------tcttaaaacataaaac
tttagtaattattactaaa-------gttttatgttttatttgtatggttataggcagct
taaataaacttagcttagacctgaacaaatataatcaaaatataatcccatctctttacc
tgcatctggtccaactaaactaatagtaacttctttcatagctaaaatagcttgaatcgt
tgcaccaataggaacacctagagaattgtatgtttcttttagaccatttaatactctttc

ttcaaggatagatggatctccagctagcattccataagtagcatagcgtaagtagtagtc
taaatcacggatgcatgcagcataacgtcgagttgtatacatattaccacctgggcgtgt
aatatccgaataaagtagagattttgctacggattctttaataattgttgctgcattagc
tgcaatagtagcagcagctctgactcgtagctcacctgtttgaaaatagcctcttaattt
ttcaacagagctatcatctaaatatttaccttggacatcagctgcattaataacagaagt
aattgcgtcttgcataactttcacacattccttaattttaaatatctaatacta--gggg
tatataacaaaattactagatcttttagcgatatcagtattactgcatagcacccaaagt
ataatcaaagtaaaaaccagcttctgctgaatcttcacctgcaagcagtgagcaagctac
acttttcatgcattttacaccttcagcaactcctgaaattggtgttcctaaagaattata
catttctttaacacccactaatccaatttcttcaataggagttacatcgccagcaactat
tccgtaagttactaagcgaagataatagtccagatctcgtagacaagtagcagtcatctc
ttcgccataagcatttccaccaggagaaactacatcaggtctttttgaaacagttgttg
gccgccttgctttacaatacgctcacgattatctgttaaaatttgagctattcttaaacg
acgttgtccagataaaacaaaactttaattctatctaattccccaggacttaaatatct
tgcttctgcatctgcatttacaattgactttgtaacaatactcatggataaaactcctgt
aatgcttt--aaaaaaagcttaatactaattatttatgatataactcagaaagtaatgta
ctttttgag-aattttctaaccgaaacacgttcatgatatatgaagcaggatagatactg
aggtaagtactaaaacgagaaagttatatgtaggaacttctgttattgatttcctagtac
tttttaaaacttggtaccacaattgatatatcctgcttagtcaaacgattatagagtgt
ttctgtattaggaaagttagctgctggtaaagtcggaaatcttctgtatggaacagtatc
tttgccgtataccgcattgtattcaacactttcaactaaattacttataaatgcagataa
gccttttgaagctaaaatttgattaaagaatctaatttctgcctgattattaggagctct
tcctaagatatgcttagtacctaattctataacttttgtatttggatatggctgataaaa
ttctttaccatatagttcagatagcgccagtttttctactagttctttgacacagatttg
tctattcaagaaagctgacttaatatccaagaattcaccgccgacactaaaagagttaag
atctctttcgaaaatttgacgatatgctgctcttaaagcttgttctaacatttctttgtt
actatcactattcacttcaaagacaatagattgatctcttaaagaagttactccttgtga
aatacgagactgaatatcattactactcctcatttctttgacagtgcctaattcaacaaa
cctagcagatttactagcaataactttttgaatctctggtcaataattcctggtcttaa
gcttcttaaagcaacaccagctggagtactatatctttcataaggaacagtattatctcc
aaaagtttctgtgtactctgagctatcaattattgtgtctattacttggtaatagccttg
cttatatgcaatatcaaaatatttattaatttcttgccgaccatatgtagggcgacccag
taagcggttatgaatatattcaatagctttacaaatatataatggctcccaataaagaga
tctaaatatacttgattttgctaactgtctaacaaactcgcgaactgtaatttgattatc
ttttagctgactttctattggcttgagaattagtttttcttcttgatacacttctctgcc
aaaaactcttaaataagcaacttttgtgacaacttctactgaattttcaaaattttggga

tgaatctgactttataagagcgttggacaatttaaagatttttggtcccagggatcctgc
tgacttaggtcttacctgtggattactaatctgattgtagattcctggcccacgtctaac
taatatccttcttgtatctttaccaaaaagagcttgtctttttcttggatctttattttc
tttagggaaaatagctccaaattgaattgataatggatcattacctattccatatggatg
ttgatcaggtaatgattgcttataatcactaaataaagttataaattgaggaaccttacg
aaaaggcgcactataggtgagaagatctatttgaggcccccaattacggcactcttgagg
ctcttcacctaaatttctaaagtaaggcacagtctcttcaccgaaataatctgtatactc
agaagaatttaaaagtgcattcactaaaccacttaaaccagtagatgataaaatagcaaa
atatttttgaaactcttctaaagagctaggacctctacccaagaaatgtctaaatgctaa
ttctaatgctcgactattgacaaaaggttcataaaattgctttctataaatacttgaagt
tcctaacgaacgaataaattccttaattgaaatttggccatttttttacttgcgactccaa
attcgataaagagaggtcatatgctttagcaatatctctttcaaaaatttgtctataaca
tgctttcacaacaatatttttctcatcagcagataaactaggcttcataacaaaacgagg
tgttgacactcctgcctgtacataagtctgaggaagacgcaaaccttgtaaatctccaga
tattctttttcttaatttatcagtcaaagatggagcttcaaattcagaaatcacaacatt
aaaatactctttaactaaatcttgaccttttatatcttcctcaaagattaataatgctgt
ccgtctcatctctctaagagccacaattgctgctgcacttgagcaagcgttatcaattaa
ctctctcaatcctcgtatattaacagataaaatattagggtcgcctgatacaattgcata
tgtcaaataccgtaaaaaccagtctaaatctcgaagagattttctcattcttgtagtacc
gtatctcaagacgtttataggtttaaaccctggcggagtagctcccccagcattaaataa
agatcggaaactttggccaaaatctccttggatatttcctgataactcattaattttatc
ttgagaactttgatctcctgcaataataacagctgcttgcggacgttctaaataagaaat
tgcagacccacctacgaatattttatctgctgctcgtgctaccagaatatttgcattttt
agttaaaatatctgcaacttccagtctttttttgtccagaatttaaaaaagaaactaattg
atttaattcaccaagctgtaaaaatcggtcttgttgctctgcctgagtaatagttaaaat
tgaggcagtccgataaagctgggggcgtgctaatgggcttccgccacttgctttgatact
cattactttatttatctccttaactgatattgcttgctagcgaatacaaatatagcttga
tatttctaaatatttaaagatactatgcaagcaca-ctgttttctatattattaggtact
agtacttatagtacagagagtcttataactaacttctataaatacaaattttgtaatact
tctattaacatttgtcaaaaaaatatttaagtgctcactcttttatattaaaattcaatt
agtgagaggttgatgtgaaataaagtatactaagtaagttttatagataggaaaattaaa
agatttattctacaaacattaatca------------cttgaatgtttagcaattgtaca
attgttattgcttttaattggtttagactagttttatatttttttctaacataaacaatat
taatgagcaatttgtatgaatttaaagccacaaaacaacctattaactaacgaaaatata
gatcatgtagatatacccgaaaatgatataccaatgtcaattacggaacatttagaagaa
ttaagacaacggactttatttgtgtttttattctttttgtttgctacaactataagtttc

acacaaattaagatcattgttgcaatactgcaagctcctgctgttggtattaagtttta
caactggcaccaggagaatattttttttcatctattaaggttgcaatatattgcgggatt
gttgcaacaactccgtttgccgtttatcaagttatattatacatactcccaggactaact
ggaaaagaaagaaaaattattttgcctctattaattagttctgtattgctttttattaca
ggtggtatatttgcttactttgttctcgcaccagcagctttaacattttaattagctat
gggtctgatattgtagaaccattatggtctttcgaacaatactttgactttattctatta
cttttacttagtacaggattagcatttgagataccaatcatacaattattgctcggtgtt
tcagggacattttcttctagtcaaatgatacgagcttggagatatattattattatagca
acaattgctggagctattctgacccttcgactgatcccgttacacaattaataatgtct
tcagctgttttattactttattttggtggaattgttatattattagtcttaaaaaagtag
agtaaat---------attttcta-ttaatccaatatatttgttatc-----tgggcaaa
taatctat-aaaacaattgct------------------agatttaagtatcaggcttct
aaatatttcaa---------gtctcttttatcattgaaatacaatc--------------

--gatacattgg
cttgttttttcatcaattatttgtgttgcacac-tttttagtactctctttattgttctt
agaattatttctttaatctatttattaaat----agatatgaagagatttaagtattgat
tatggtaatctatatcaacgtaaaaagtattttattgtgagttttatttatataaaggct
tattcattatatagttataacagtaagtctaattaatag-ttttcatgcttaattaaagt
aataactcttaattcttttggcagaaaaatctgtcttaatattttatc--aaaaaaacag
-aaaaatgaagctaaatagtctaattaacttaattcaaaagtctatatattcttgtacac
ttttactaattattttaaatattatttgtgtcgcacctaattctagtaatgcatttccaa
tttatgcgcaacaagcttatgaaagtccaagagaagcaactggtaggatagtatgtgcca
attgtcatcttgctcaaaagcctgtggagatagaagcgcctcaagcagtactacctaata
ctgttttcgagactgttgtgaagattccatatgacagcaatgctaaacagattttaggta
atggcagtaaaggaggcttaaatgttggagccgttgtaatattacctgaaggatttaagt
tagctcctgttaatagattatctacagagttaaaagaaaagactagaaatctttacattc
agccgtacagtgctaaacaagacaacattttagtaattggacctatttctggtgataaaa
atagagaaatagttttcccaatactatctcccgatcctgcaaaagataaaaaagctcatt
ttttcaagtatccaatatatgttggcggaaatagaggacgaggccagatttatccaacgg
gtgacaaaagcaacaataatattgtctctgctttaagcagtggtaaaattaataaaattg

aattactagacaaaggtggatttataatacatgtgactaacagtagcaatgtggagtcaa
cacagaagatttcacctggtctcgaacttagagtaaaagaaggggatacaattcagcttg
atcaagctttgaatagtgatccaaatgtaggtggttttggtcagaacgaaacagaaatag
ttttacagagtccaaatagaattaagggcatgattgttttctttttcgctagtgttctag
ctcaaattttcttcgtattaaagaaaaaacaatttgaaaaagttcaagcagctgaaatga
atttttaaagcaatataaaactggcacttactataaagaaagta-taaacactttattca
caggatgtaccagtttttatgttatgtaataatcgctttgacggaagttatgatactttt
aacagaatctacaatctgactaggctgaattactgtagcctgctctaagcttccattata
tggtgtgggtatatcctgcgaagatagcctcacgacaggagcatctaattcatcaaaaag
atactcattaatttgtgcaattagctctgctccaattccggctgttttcatacactcttc
tacaattaaaactttgtgagttttctttactgagatagatatagagtctatatccagagg
ttttaaagatataagatctataacttctggatcataaccttcttttaataaggccggtaa
tgcttggataacatgatgcctcattctagaataggttaaaatcgtaatatcttttccttt
tcgtacaaattcaactttattgagaggtaagaaatattcttcttgaggaatctcttcttg
taaattataaagtagaacgtgctcaaagaatacaactggattattgtctcgaattgcaga
tttcagtaatccttttgcattataaggagtagaacaagcaactatttttaagccggggat
ggcttggaaataagcttccagtctctgagaatgctctgcacctaactgcctaccaactcc
tcccggtcctctaataactaaaggcaatgtaaaattacctccagaagtataacgtaacat
tcctgcattattagaaatttgattgaatgctaataataaaaaactcatgttcataccttc
aacaattggtcttagccctgttatagctgcaccaattgccataccagtaaagctattttc
tgctattggtgtatcaagaactctcaaatccccatatttgctatgcaaatctttagttac
cttataagatccaccatagtgaccaacatcttctcctattacgcagacagttggatcttt
tgccatttcctcatctgttgccgctcgtaaagcgtcaaacataaagatttactcatttt
aatatttgattttatgatttgaccactgttttaaagtaacagttaagacaccatattct
atttagttatctgcaaaaagatatcgtttcaattctgacatgttaggttctggactggaa
atagcaaatttcacagcttgttccaattcggttttaacagcattctgaatctcattaagt
tcacctatattagcaatttcattatctagaatatatttttgagtttttgataggatct
cttgccacccaagcctctttttcttgtcttgatcttagttcatcaggatctgcgagagag
tgaccacgaaatctatatgttaatgcttctattaaggttggaccatcaccttggcgagct
ctttgaactgcttgttttgcagcttgtcttacagctagcacatccattccatcaacttca
atcccaggaagcccaaaagcttctgctttttatgtatttcaggtattgaagaagaccgg
tgatgtgccatacctatagcccattgattattttcaacaacaaatataataggtagtttc
cagagaactgccatattcagacattcaaaaaattgcccattattggtagtcccatcacca
aaaaagcaagccgtgactcttaaatcctctgtttctttaagtacttgctggcggtagata
ctttgaaaggctgcccctgttgcaaccggtataccttccgcaataaaagcaaagccacct
aaaaaattgtgaggcgcagaaaaaatatgcatcgaacctcctctgcctttactacaacca

gtctcttttccaaataactcagccatcacatttttagatgggacgcctttacttaaagca
tgtacatggtctcgataggtactgcaaacataatcagttggattcagaagtttaattaca
cctgtagaaacagcttcttgaccattataaagatgaacaaaaccgaacatctttccttta
taatacatctgagcacacatatcttcaaaatttctgcctaacaacatgtcttcatataaa
actaataaattactcttattaagattaagacctgttgaattatagttagtcagcggcaat
tgaactttcttaggataactcataatttgtaaagaaacctct----------tattgtaa
ataggaattttgaagaaacagctatcaatagaaaacacacagataaaaacttataaatta
taatatgtttttactaaagactttaataattatttgaaactagttcagtatatcaaatt
aattttgcttatactacggatgtctgaaacttatataaaaaagtagt-a-aaaaagttta
ttaaaagaatagttaaaaacagatataaatagaagtaaacagaattatt-gaaaaaaaat
gattattattaattaa----tacttaatatttctaaatattttacaattaatggctatgt
caccatctttattttatcctgttgaacaagaactatgtagtcttgaaaaaaatctgaaag
ctgttgctgggactcgtcatccaattttatatgccgcagcaaagcatctattcgatgctg
gaggaaaacgagttagaccagctcttgtattttagtggctaaagcaacctctgagaagc
aagatataaatactggacaaaaaaggctagcagaaattactgaaattatacatactgcta
gtttggtacatgatgatattattgacgagtgcacaacacgtagaggagtcaaaactgtac
ataatttattcaatactaagattgctgtgctagcaggagatttttttatttgcacagtctt
cttggtatttagctaatattgaaaatttagccgtagtcaaagctatttctaaagtcatca
ccgactttgcagaaggagaaattaggcaaggcttagttcatttcgatcccagtatttcaa
tagatgcttacattgagaaatcattttacaagactgcttcactcattgctgctagttgtc
ggggtgcagctatgcttaatggttccaatcatcaaataaataatgatctttatctttacg
gtaaacatatgggattagcatttcaaattatggacgatgttctagatataactggttcta
ctaagagcttagggaaacctgctggcgctgatctaataaatggaaatttgacctctcctc
tcctttttcacttactcaagaagcaagtttaaatgatcttattgatagggagttctgta
atagtacagatatagcctcaacattatttcttataaaaagaagcggggaattacaaaag
ctaaagatttagctaaagaacaggtgcaggcggcactttcctgccttcagtttttaccac
aatctacacctgtatctagtttaaaagaattaacacatttcataatcacaagattgtcat
aaaggacttgctaaaattaaaatgttattaactaatttttactgtttctaaaacagtct
gaaaagtttgaatatcattactagctagctgcgctagcattttacgatttaacgcaatat
tctcttttttaatgcactaataaaagtactataattcatgccttgattgtgagcagcag
catttattctagttatccaaagacgacgaaaatctctcttttcctttttcgaccaacat
aagaatatcgaagagctttaaggacttgctgcttagctgttcgaaataaacatttatgtg
cacctttaaagcctttagctagcttaaagattttagctcgtcttttttttgcaacgttac
ctcttttaactctactcataaaatattac-ttaaattttaattattaccagcttactaaa
ttcttatactctttg----attataaataaggtaatttcattgcaatgtttttaatatct
tttaagtcaaccgaacaagtagaagaaagatgtcttctttgttttgatgacttttttgt

aataaatgacttttagaagctttatgtcgaagaaattttccggatgaagagactttaaat
ctttttgctattgcttttgatgtttttaacttaggcatattataaacttttaatgattaa
caaaagcatgaaaagtctgaggatatcaatcttagacctttcaatacttagaataatctt
aacacaatttatttacttactacataggttaactgaaacaatttatatatataatacta
atcagttaatcaaccatagctaaagtggatttatattttgctttgaacttttgtatagta
ttagcaattagcattgttataattttaagactgcttgaattcaattcttcgaacattttc
atatttaatgtaaatgaaatattggcttctgagacaatattttgaatttgaacatcagac
aagggaatcatatctaatgcagctctatattgatctttaaataatttatcgtcttttatt
tggtcaaaatcgtaaaatttcgttcctcctgaatctgataaattcatagcccctctagct
atttttttaaaatttggccgccagaaaggtcacccaaatagcgcgtataagcatgagca
actaataattcaggttgtttatgtcctatagtgtgaattctatcaacataaatttttgta
gcaggagatggttcaataaaatctaaccaatctgatccataataatagtttaaatcttca
gatagactcgctttcctattaagctctgtaaaatatataggtttaatagctggatgattt
ttattagaaaataactcttcctctattgcacaatagacaaagtatagatttgcaaccaat
ttacgatatgacttcttatctacaactcctcccaaaaaagatttaacaaaactaacattt
tctgccatactatgggacttagtagtaccttctcttagttcattcgctaaagtattaacc
ataatatcaatattccattttt-aaagataacaaattaaca---ataaatagttatccgt
atatttataattgagttgcgctaactaggctgctgcaaaataatttttgatttattgg
atctggattcattgttttgtcaccaggcttccaattagctggacaaacttcatctggatg
agattgtacatattgaattgcttgtaaaaccctcaaagtttcttctacactcctgccaaa
ctctagattattgattgtagagtactgaataattcctttcgggtctataataaatagtcc
tcttagagctacaccatcactattcaaaacattataagctgcactgatctcttttttaa
gtctgatactaatggatatgaaagatctcctaatccacctgattctctgtcagtctgtaa
ccaagcaagatgagaatactcactatcaaccgagactccaagaacttctgtattaagttc
cgaaaaagcattatacttatcgctaaatgctgtaatttctgtagggcacacaaatgtaaa
atctaaaggataaaagaataagacaatatacttattcttgagatcagataattttaacgt
tttaaattcttggtcataaacagctgtagctgaaaaatcaggagctagctgacctactcg
aagacaatttggtccagaaatcattaatttttcccagaagaattgatataattttgttat
ttaataaataattatataacaaaatttagtatatattaaaatactacaagtctttttaa
attac--------taaatgatataatcaatactatctttatagcggggaatggatttgaa
ccattgaccttcgggttatgagcccgacgagctaccagactgctctaccccgcggataac
taaaatcatattagtctacatatatatataaaagcaaatgcataatagatttagaagta
aaaaaatagaagaactggtccactgatcattaaacttagaaacgacaaagtatttttat
cttatttaaaagagggaagacttcatataaaccaataaatctatcttttaaaagaatttc
ggacggctttacccaaatttgaccgtcataccagcctgattcttcataaaatactgtagc
actcattaatcgttttactacgtatgaccagcctaaatataatctgattaaaataaatcc

tgtcattaggctggaagtaataaattctgaaaagaaaaatttcaaaggtagtttagtgat
tggaaaaattgataataatataggactaactaataaacagttaagtagtagtgcaatagt
tattttttattataagatctacggcttaaagttggccagcaaaagaaccaagaattttt
caaagaagtatattcatgaacaggttgctgctcctttggcacaggacattgagtattata
taaattcattgacattaaaaagtattataataaagac--tttgtttatattatctaacat
aggtacgacattaaatattaactaactgcaattatagtagtgagtagaagaaataaaact
aaaaatagccaagtaattttatttaaagtattctcagtactacgagtattactaaagaac
tgattctgagcacctacacttcctaaaccttcggatttaggattgtgtattagaatagtg
aagattaatattattgtagatgagtaccaaaaaaattttaaaatttgttccattatgtaa
aagttaat-aaaaatagcacaaagacaatcgtctaaaagatattgattgtcttatcatat
taaaaagtatttattcaccctgtacttttaaaagtaaaaatcctattgccagccctacca
aaactaggactgaacttaatacagcactttctacaatttctccgaccatt-ttatttttc
cttattattttattattg---tcattcttttgacaagattatattgagctaa-ctaaaat
ccgtttctaccccaaactaccattgcgattgaaaatgtaaacattgccattagagctgcc
catcccaaacttaaaatatccataatttataatattc--ctaaaaataagatctctttta
cttaaactataatattcttaatattaaaaatatcattatataataagctaattataacag
agtactacttaattcgtattttagtgtatttaaagaagattgaa-ttttttcgaggtac
tggataagtatttcatcctaagaaccatagtcaac----ttttgatttattataatgctt
aaatctaaatata--tatcactcaagaatcattagtaattatgatgtttttaattatacg
att---aacatatttcaaaatagaagaattcatagcgactaagttaa-----tatgctaa
tataatttatcaat--aaaaaggaagttagtatttcaattatgcaaacaactataaataa
tgggcaaacgtctagtaaagaaactttactaacacctagattctatacgactgactttga
agagatggctaatatggatatttccggcaatcaagaagatttttttggctatcctcgaaga
atttcgagctgactataatagtgaacattttattagagacgaagagtttaatcaatcttg
gtctaatttagaacataaaactaaatccttatttattgagtttttagaaagatcttgtac
cgcagaattttcaggtttttactatataaggaattatccagaaaattaaaagacagaaa
tccggttatagctgagtgctttttattaatgtctagagatgaagctaggcatgccggttt
tttaaataaagctatcggggacttcaatttatctttagatttaggatttttaacgaagag
tcgcaagtatactttttctcacctaaatttattttttatgcaacctatctttctgaaaa
aattggatactggagatacataactatttaccgccatcttgaacaacatccagaacaccg
tatttatccaatctttagatttttttgaaaattggtgtcaggatgaaaatcgtcacggaga
tttttttgctgctttgctcaaatcccaacctcatttttttaaatgactggaaagcaaaaat
gtggtgcagatttttttctattaagtgtatttgcaacaatgtacttaaacgactttcaaag
aattgatttttataatgccataggcctagactctagacagtatgatatgcaagtaatacg
aaaaactaacgaaagtgccgctagagttttcccggttgctttagacgtggacaatccaaa
atttttcaaatatttagatacttgtgcatgtgataatagagctctgatcgatattgataa

taacaattctccattattcattaaatctatcgtaaagatacctctatatttttccttatt
tgcaaatttactaaagatatatttgattaagccaatagactcaaaaacagtatggaatac
agttcgatagttaaatacaagggaatgctttttcttataaaaagctatctagtgattaa
tataaaat------aaaagcttccttct-----cgatggaaggagcttttattttgtcaa
ga---atataatgggattatatagatc-tagaactaagaacttactttttcttttgcttc
atacataacctctaaagtaattagattttagaacaatcacgggcaaatttctctgtatt
tctttttactttacctctaacaaacccaggaattttatttaattctttctgtgcttcttc
tgaccaattaatactatcaacagcagaaattcctatacttaatgcttctgttgtatcatg
accaccaaatatttctagcaaatgatcttccataccaagtgtaaacgaattatatactaa
atcagcaatttgatttgttccctcataaccaagaaatggacggtaacttaaaggaaagtt
ttgtatatgcactggtgaagaaataacgccacaaggaatatttagacgcttaccaatatg
tctttccatctgtgttccaaaaattgctgcaggttcagttttagcaattaaatctccaat
taaaccatgatcatctgaaacgattacttcatcgcagaattcttgaacttgatctttaaa
ccactcttcatcatatttgcaataagttccacaccaggcgacatgtatacccatttcctg
gtgtaaaattcgagttattgctgcagcatgagttgcatctccaaatacaatagccttttt
acctgttaaattctgacaatcaattgatcttgaaaaccatgcagattgtgaaataaatcg
ggtttgctcgtctatgtatttttcgtagtctacagcagctcccaatgcatttactaattg
ctgtattgatcggatgcatgcagctgtttgaacaatacccataggtgtaatgtcaacata
aggcatattaaattctttctctaaatatcgagctgtcattagtccagtttctctatatgg
aataaaattaaaccatgcagagggtaatttttttaaatcttgaactgatgcattttctgg
aataatttggtttatttggatatctagatcttgaaataacctttttaattctgctatatc
atgttgattatgaaatcctaagcttactgctccaataatattaacagatggagttttagt
cttttgagttaaaacttgattattagatttagctttttccatatagaaagtgacaatttg
ttctaatgttctatcacctgcttgcagttcatttactctataatgattaacatcagctaa
taaaacatctgcttctgtttctatagatgctctgctaacaaagttttgcaaatcttcttg
caaaatactcgaagtacaagttggtgttagaataactaaatcagggctttcttctctgtc
ttttctagtaatattttctacaactttttcttgagaaccacgggctaacacatgtctatc
tacaacacttgctgtaacaggagtaaaatctctgtctctttcaagcattgaacgcattac
attaaaataatcgtctccgagaggcgcatgcataattgcatgaactttttttaaaagaact
ggcaattcttaaagttccaatatgagcagggcctgcatacatccaataagctaatttcat
attaaattttcctatgatttatttagacatagtaatacttttaaagctaactaacaaaca
ttagccaaacatctaatttaactccttgacaattttgtaatatttttttaaatgaagctta
tcattataaatatttaaaaaatataattcagaataga----aataactgttagatcaagc
ttgaaagtaaagatttcttgcagttatagtttttctttaagtaaacatgtaaaaattgta
ctatatataaatgtactatatataaactttttttactgaatattggtaatgccagataca
attaacttaaatatgccttccccaacgtttggtggaagcactggcggttggttaagagcc

gcagaagtagaagaaaagtatgcgataacatggacaggcaaaaatgaaagtaagtttgaa
atgccaactggtggtaccgcaactatgcgaaatggagaaaacttactctatttagctaag
aaagaacaatgtttagctttaggtactcaactaaaaagtaaatttaaaatatctgactat
aagatttacagagtttttcctaacggagaagtgcaatatttgcatcctaaagatggagtc
tttccagaaaaagttaatactgggagagctagcgtcaacagtgttgatcattctattgga
caaaatattaatcctgtagatgtcaaattcatgaataaagcaacttacgattagtctctc
-aaaaacctagacataaaataacttttatatagtctaggtttttgataatgtatactgat
agtatggaagggtggccgagtggttgaaggcgtctgatttgaaatcagttgaacta--tc
tggttccgtgggttcgaatcccaccctttccgttataaaact------------atagtt
ggcttctcaaggcaagctagctatacactgatagtattaagtttagacttaataaagtct
acaacttgtgacaggttagaaatttttcggcatcctcatctggtatctctatactaaat
ttttcttctatggccattaccagctcaactgtatctagagaatcggctcccaggtcactt
gaaaaatttgcttccctagtcactattttttttcaattcctagctgttctgctacaata
tcttgaacttttcaaagatttcattatcttgcataatatattatttcctctaaggtgtt
atcaaaaatttttcggcttatattattatccaactctaaaaaattagtaatttttagtcg
agattaaaatatttatacctatatgatatactattattctggataaatacgaagtttacg
atatggctcttctccaaaactacgagctctcaactggtagttgtcaactagattatgctg
catttttcgtatataggctgatcgaggtgtaagctgaactattgaactttcattcaagat
aatagtttcaatagccaatttagcttcttgaagagcctggatctcatcaaaatttttatt
tttacagagttctacccaattaagaccagatgaagtatgaatgtttaatatttttctgag
tgcacgagtaatttgtggaaccgtactattctgaattgtatatataataatttgttttga
ttttgcaatttgtctaagttttgtattttgctttacttgattacgtagcgctagaatagc
atcagacttttctatctccttggttaaaataattggtagatctagtgaagaaataactga
tgtaatatgctgccaactcaaagaataagcatataagtactgatgaggtatttccaattg
caaagattgtttattaatattaatagggcattgaaagtagttgacaataaagatgtatc
acgattttgtgatttattcaaatctaaggacttatttcttaattctctatattgtaaaac
cggaggcgcctgctgtgcgttaattgattttcgagcattagagatgtttacaggtaagac
ttctatagattgcgatggataacatttaattaaaattctaccattagcttgaatttgtct
tttttgcacaaaaggttgatggccttgcaaaatttgatctattgtctctttaactttatt
atgaactatccaaacatttcgctcgtgtatttcgatcgcaatctgaaaagcaggctcagc
ttttctttctagtatactcttttgcgtacctctacgtttagcttcatcgtcacctaatgt
tacatattgaattccaccaattaggtccgccaatgttggattttttaattaaactttctaa
atgatttccatgcgctgtacctactaattgaactcctctttctgctatagttcgagcagc
taacgcttctaattccgtgccaatttcatctataataataacctcgggcatgtgattttc
aacagcttctatcattacttgatgttgcaaatcgggccttgcgacttgcattcttctagc
tctaccaatagctggatgaggaatatctccgtcaccagctatttcatttgaagtatctat

aataaccactctttttttccatttcgtcagctaaaacacgggccatttctcgaactgcggt
tgttttaccaactcctggttttcctaagagtaaaattgaatcaccttgctgtagtaaatc
tcgaataatactaattgtaccaaagactgctctgccgacacgacaggttaagccaataac
actaccttctctattacgcagtgaactaatacgatgcaaggtttttttcgataccagctcg
attatcgccgctaaaattaccaactttttttacacaataatctaagtcttgccaactaat
agatctttgagataaatattctgggttatctggaaatctagcttctggacgacgacctaa
atccataacgacttctattaaattatttctattaggatgctgttgtaaaggttctttgac
aaaatttggcaaaatttctagcaacttatctaagtcatctgcaataagcatgattgagta
tataataattaattaatcaaatagaagattcaagattctaaagattacaatacatattaa
agaatattatgattggaatcaattttatacttaaattgaacaggaagtta-tagatgtaa
aagaactgattaaactttacgcaataaatcgattggtcaaaattaatagtcgtgttaaca
tgtttattggacaaaaagttcgtattaaatatagcaaacaaaaagttgctagtgatatag
cagataaagttggtgaattaggagttattaagggaataaagtttattaatagtcaatgtg
ttactattattgttgagtttgacaatcacactaggctttggatgtttagagaggaattaa
tctgtctaaatgaat------taaaaacatgaaacataatttattttatttattatacaa
aatttcttatgagtcgttaaaataataaattttaaaggagtacttttaattgtgcaaatt
actattaaaaaattacaagacttactttcgtctgtacaaagaaaaaagatccagacatta
aaactaaaacaaggtaagtttgaacttctattaaataagacctataaaaaagtcaatcaa
gaaattataccttcgcagaaatctgctgtattacaaaatagtccatctacgataattaaa
tcaataaataatacaaaaaaatcttctgttgtcaatgaagaccgtacagaatatgccact
attgtttctccaatggttggaacgttttatcattcacctgctcctggtgaaaaaattttt
gtacaagttggcgatgaggtcaaattcaatcaaacagtctgtattattgaagcaatgaag
ttaatgaacgaaatcgaagcagaaattgaaggcaagattatagaaattcttgttaaagat
ggtgatatagtagattgcgggcaagccttaatgaaggttgaaacataatgctaatttttc
catttacatattgttaacagaaattatttagatagaaaaattagcttatactatagtta
tgaaaacttatttcgcaaaggcac-taaaaactaatatataaaattatttactttatctg
ccaaaaatagaaataagcgttttgattccttgtctcaagagaggagaatctcaatggcaa
ttagctcaaaagagcaagagacaaagaaggtaaaaatctcggttgataaaaatcccgtag
atacttctttcgaaaagtgggcccaaccaggccatttttctcgtacactagcaaaaggac
caaaaactactacttggatttggaatcttcatgctgatgctcacgacttcgatagtcaaa
ccagttctttagaagaagtttcacgtaagattttcagtgcacattttgggcagctgtctg
taatatttttatggcttagtggaatgtattttcacggagcccgcttctctaattatgttg
cttggttaagtaatccaacaggtattaagccaagtgcgcaggttgtttggcctatagttg
ggcaagaaattttaaatggcgatgtaggtggtggctttcaaggagtacaagttacatctg
gatggttccaactgtggagagcatcaggaattactacagaatttcagctttactgtactg
ctattggcggattagctatggctgctttaatgctgtttgcaggatggtttcattatcata

aagctgctccaaagttagaatggtttcaaaatgttgaatcaatgatgaatcaccatttag
ctgggcttttaggcttaggctgtttaggctggacaggccatcaaatccatttgtctttgc
ctattaataagctactagattctggcgtgtctccgcaagaaattccactacctcatgagt
ttttaattaatagagagcttatggctcagctgtatccaagttttagtaaaggattagttc
cattctttactttaaattgggctgaatattccgacttttttaacttttaaaggaggtttaa
accctgttactggaggtttatggctaagtgatactgctcatcatcatctagcattagctg
ttctatttcttgctgcaggtcatatgtatagaaccaattggggtattggacatagcatga
aagaaattctagaagctcacaaaggacctttaccggcaacggtcacgaaggtctatatg
aaattcttacaacttcttggcatgcacagcttgcaattaatttagctatgatgggatctt
taagcatcattgtagcacatcacatgtatgcaatgcctccttatccatatattgctactg
attacccaactcagttatcgctcttcactcatcatatgtggattggaggattttgtattg
ttggagcaggagcgcatgcttctatatttatggtaagagattataaccctgcagaaaatt
ataacaatcttttagatagagtcattaggcatcgagatgctattgtttctcatctaaatt
gggtatgtatatttcttggattccattcatttggtttatacattcacaatgatactatgc
gggcacttggaagatctcaagatatgttctctgatacagctatacagttacaacctattt
ttgctcaatgggtacaaagtatacacactttagctcctggaaatacagctccaaatgcat
tagcaacagctagttatgcatttggaggagatattgtttctgttggtaacaaagttgcaa
tgatgcctatttctttaggtactgcagatttttagttcaccatatacatgcatttacta
ttcatgtaactgttttaattttagttaaagggttccttttctcaagaaactctagactaa
ttcctgacaaggccaatcttggcttcaggtttccatgtgatggacctggtagaggtggta
cttgccaagtgtctggctgggatcatgtttttcttggcttattctggatgtacaattctc
tgtctgtagcaatttttcactttagttggaaaatgcaatcagatgtttggggtagtgtat
ctccgtctggaaatgtttctcatattactggcggtaattttgcacagagtgcaattacaa
tcaatggatggctgagagatttcctttgggctcaagcatctcaagttattcaatcatacg
gttctgcgctatctgcatatggattaattttcttagcagcacattttgtatgggcattca
gtttgatgttcttatttagtggtagaggttattggcaagagcttatagaatcaatcgtat
gggcgcacaataagataaaagttgctcctgcaattcaaccaagagctttaagtattactc
aaggtagagcagtcggtgttgcacactacttattaggtggaattggtacaacttgggcat
tcttttttagcgagaattatttcagtaggctaatagtgaaaataggataaaaaaaacaatt
atggcaacaaaatttcctaagtttagccaagctttatcacaagatcctacaactagaagg
atttggtatggtattgctacggcacatgactttgaaagtcatgatggaatgacagaagaa
aatttatatcaaaagatattcgcttcgcactttggacatctagcaattatcttcttatgg
acatctggtaatttattccatgtagcttggcaaggcaactttgaacagtgggtattaaat
cctttgaaagttaaaccaattgctcatgcaatttgggatccgcattttggacaacctgct
ttgaaagcttttagtaaaggtggctcagcttatccagtaaatatagcatattctggcgta
taccactggtggtatactattggtatgagaagcaatcaagacctgtattctggggcttta

ttcttactagttttatcagctttactcttatttggagggtggctacatctacaaccaaaa
ttcaagcctggtttatcatggtttaaaaataacgaatcaagattaaatcatcatttatct
ggattatttggggttagttctttagcctggacaggtcatttagtacatgttgctatacct
gaggcaagaggacaacatgtaggatgggataatttacaaccgtattacctcatccagcc
ggtttacagccgtttttcagtggtaattggagtgtatatgctcaaaatccagatacagct
caacatttattcggaactaatgaaggtgcgggtacagcaattctgacatttctaggagga
tttcatcctcaaagtcagtctttgtggctaactgatatggctcatcaccatttggctatt
gcagtagtattcattgttgctggacatatgtataggactaattggggaattgggcacaat
ctaaaagatatttagatgctcatagaccacctagtggtagattaggagctggacataaa
gggctatttgatactattactaattctttacacatacagttgggattagcattggcttcc
ctaggtgtaattacttcgttggtagctcaacatatgtatgctatgcctccatatgctttc
atggctaaagatttcacaactcaagcatctttgtacacacatcatcaatatattgctggg
tttctaatggttggagcttttgctcatggggcaatattctttgttcgagactatgaccct
gaacagaataaagataatgttttggctcgtatgctagaacataaagaagctatcatttct
catttaagttgggtaactctatttttagggtttcatacattaggcctttatgttcacaat
gatacaatgattgcttttggaactcctgaaaaacaaattctaattgagccggtatttgct
caatggattcaagcctcttcagggaaagcactttatgggtttgatgtgttactatcatcc
tctactaatatcgcaacacaagctggtagcaatatttggctgccaggctggttagaagcg
attaatagcggaaaaaattcattgtttttaacaattggtcctggtgacttcttagttcat
catgcaattgcattgggattacatactactacgttaattttagttaaaggtgctttagat
gcaagaggctctaaacttatgccggacaaaaaagactttggatatagttttccttgcgat
ggacctggcagaggcggcacctgtgatatatctgcatgggatgcgttctatttagctgta
ttttggatgctaaatacaataggttgggtaacattttattggcattggaaacatattaca
atatggcaaggcaatgcaactcaattcaatgagtcttcaacttatctaatgggatggttt
agagattacttatggctaaattcttctccattaattaatggttataatccatatggcatg
aataatttatcagtatggtcatggatgttcctatttggacatttagtatgggcaacagga
tttatgttcttgatctcctggcgtggctattggcaagagttaattgaaactctagcatgg
gcgcatgaacgtacacctttagcaaacttgattcgttggaaagataaacctgttgcatta
tcaattgtacaggcaagattagtaggtttagcacatt-tttctgtaggatatgtattaac
ttacgcagcttttgtattagcttcaacagcaggcaaatttggttagactaaa-----ttc
gaattagctaa-----aaaaaagtcagtcataata-gttttatgactgactttttagat
ttaaaacaaaaacttactaaagtttcacctcaatatctacaccagaaggcaaatttaact
tcattaatgcatctatcgtttgagaagaaggttgatgaatatcaataattcttctgtgag
atcttatttcaaagtgttctcttgagtctttatctacatgtggagaacgtaaaacacaat
aaattcttctttttgtcggtaaaggaattggccctactgcaacggcattagttctagatg
cggtatctagtattttgtcacacgacgtattaagtataatagaattatatgctttcagtt

taattctgatttttgtctgctgagtaattgtcatgttaaaaagatcttattatttaagaa
ttttagagacaacacctgcgcctacggtacggccaccttctctaatagcgaaacgcatac
cttgctcaattgcaattgcattaattaattcagcagtcatcttaattctgtcaccaggca
taaccatttctgcatcagtaccatcatcagcagtaaactgattaatagtaccagttacat
cagttgttctaacataaaactgaggtctatatcctggaaaaaatggagtatgtcttccgc
cctcctctttagttagaatataaacttctgcttcaaattgagtatgaggtgtaattgtac
caggtttagctaataccatacctctttcaatatctttttctgcacacctcttaaaagaa
ttccaatattatcacccgctagaccttcttctaacgtttttgaaacatttctaatccag
taatagtcgttgtacgagtttctcgtaaacctacaatttcaattgtgtcaccaactttaa
taatgcctctttcaattctaccagtggcaacagttccacgtcctgtaatagaaaaaacat
cttctacagccattaagaaagttttatcgacatctctctccggcgttggaatatatgtgt
caactgcttccataagtgaaaaaatcttgtcaacccatttatcttcaccttgcttaatag
ctgggtttttcgtcacagcttctaatgccaataaggcagaacctgcaacaaaaggaatat
catctccaggaaagtcgtattgacttaataattcccttccttctaattctactaattcta
gtagctcttcgtcatctacttgatcttccttatttaggaatactactaatgtaggaacac
ctacttgttttgctaataaaatatgttcacgagtttgtggcattggaccgtctgccgcag
atacaactaaaatagctccatccatctgagcagcacccgtaatcatattttttacgtagt
cggcatggcctggacaatctacgtgagcataatgacgattatctgtttcgtattcaacat
gagcagtattaatagtaatacctctagctttttcttctggagcggcatcaatttcatcaa
atttttcgctgcagtagaccctaaagttgataaagtcgcagagatcgctgctgttaaag
ttgtcttaccatgatcaacatgaccaattgtgccaatattgacatgaggtttttttacgtt
caaatttagatcgagccatgctttttgttttccttatagtaaaagttgctttatagttac
ttttaacgataaatcttgtggattgaaagatttattatttaatacagacaaga-----ta
caatataactaatctaatatcgatagtgagcaaacgctttatttgcttctgccattctat
gtgtatcctctctctttctaatagaatttcctgtttcattagctgcatccataatttcat
tagctaatttcatagacatactttaccagatctgtctctagagaatttagtaatccatc
ttagtgctaaattcgtacctctataagctcgtacttcaataggaacttggtaagtagaac
caccaactcttcttgcttttacttctactagaggagtaatatttcggatggctttttcta
aaatatttaaaggatctgattctgttctctctttaacgatatctaaagcctgatatataa
ttctttgagataaagtttttttaccacttttcaaaatacgaacagttaacatacttacga
gtctgcttttatataaaggatcaggtgatgcaaacctttttttagctgtattacgacgag
acatagtatttaaaattggttattgtaataa-tgttc-tattagcaggtatttaagattt
aggctttttttgtcccatatttagatcggcttttacgacgatcttttactcctgcagcatc
taaagtaccacgtactacatgatatcggactccaggcaaatctttaattcggccgcccct
aatcagtaccacagaatgctcttgaatattatgacctacgccaggaatataagctgtaac
ttcaaatccagatgttaatctaacacgcgctacttttcttaaagcagagttcggtttttt

aggagttgtagtatatactcttgtacaaacacctcttctttgagggcaactttgaagagc
tggagattttgtttttttatgtatttttcgtctttcggatctaacaagttgttgaattgt
tggcataataaaattgatttaaagtctttaatagatactatttgaacttgctttacatga
attggctagttttatttatcttatacttacgcataaatctttctactctaccttctgta
tcaataattctctgtgaaccagtaaagaatggatggtttcctgaccagatatctacgtgt
agttctggctttgtggacccaatcgtcataattaactggccgtcacagtaaacttttgct
tctggataccaatttggatgtatattatcttttgccattgtttttataattatataaatg
tatcaataataactattaacgcttagaaaattgaggagcctttctggctttctttaaacc
atatttttttctctctttttactcttggatctcttgttaaatagccttcagacttaagtgt
cgttctattttctggattaattgagcacaatgctcttgcaacacctaaacgaattgcatc
agcttgccctgtcaaacctcctcctctagcatttacatggatatcatactggtttagtag
tcctaaaacttgtaatggtgcatatgaaactcttaagtaattaggactaaattgaagata
agactctcctggtataccgtttataattaaattacctgaccctgggactagtcttacttg
tgcaacagaacacttccggcgacctgttccagaatagattgcgcgagttttaattaattc
tgtcgacataacattccttgataactaaataaatatacattctttatactatatactctt
gtggcttttgcgcgacatgtgggtgaattgggccagaatacactttaagcttcgtaaata
gttttctgcctaatggacctttaggaagcatacctttaaccgatttttcaataattctgt
taggtaatcttgtctgaagctgatcaaatgtttcaacttttaatccgccaggctgtccag
aatgtcttctgtatagtttttgattcgttttatttccacttacagatacgtgagcagaat
taataacgattacataatctccagtatctagatagggtgtataagaaggcttgtttttac
ctcttaagatattagaaatatgagtagatattctaccaagtgtctgattttagcgtcta
taacataccaatgagaattagtatttaatgagggtgattgcgttttattcatgaaaagat
taatacttttggttgacaataagagaatatgtaaatggtttacattcaatttctatacaa
gtagtattt-aaaaatatta-------------ttaacaaagagaactttctaacagtt
atttaaattgtttgtcagcaagaaaatttctagttatcaataacttatactatttttaat
aatataaaatatattaatcactttttctttggcaaacttatacctagtttttttgc-
aaggcatatataacttcttcagctgatttctggccaaaatttttaatttctagcagttct
tcttgagagtaatctagtaaatctgcaatagaatggatttgagctcgctttaagcagtta
taagccctaacagataactgcagttcttctattaaaacttgactaatttttttatcttct
ttgctacgataattatctgctgatttaaagtctaaatttcttagtgaacaaaaaagatta
gttaaaactgtagctccttgacttattgcttcttgaggagagatgctcccatttgtccag
atttgtataatcaatctatcttttatgctgttgctaccaatacggacttcttctacttta
taattaactttattaactggcataaaaacagagtctacttgcaaaaaatccacagataat
tcatctacagctttttcagctaagcgatacccgcaatttttttcaattttaaactccatt
tcaaatattgtattattgcaaatagttgcaatatactgtctaggatctactacctctata
tcagaagataattcaaataggccggctgtaactatagctggcccttgaactctaattcga

ccaatttgagattctttgttataacttttaaatactacttcttttagattaagtaatatt
tctaacacatcttctcttaccccaggaattgtagaaaactcatggttcactccagcaatc
cgtacagcaactatagcagtaccttcaagatctgacaatattgatcttctcaaagcatta
cctaatgtaataccttgtccttgatttaatggttctattacaaaactaccgtactgccca
cgcgccccatctgttcttgactctatgcattcaatttgaaattgagccacctaagaaagc
tcc-tttaaaataatcagtgattagtaagtctctctaaaacaagaaggaatttatactcg
gcgtttcttaggagggcgacatccattatgaggtacaggagtaatatcttttattagagt
aatctctaatcctgcagcctgcaaagctctaattgctgtttctcgacccgctcctggacc
atttactagaacttcagtttggcgcataccttgatccatagcttgtctagctgctttttc
agctgctgtttgagctgcaaaaggtgttcctttttttagctcccttaaatccacttgcacc
agaggatgaccatgataatgtttctccttttaaattagtaatagtaacaattgtattatt
gaatgtagatttaatatgtgtaataccgttaactgcattacgtttagtttttcttgctcc
ggatttttttatttgtctagccatcgtgtctcgtctattatatagttaattaaaagatta
ttttcttggagctttttttcttacctgctactgtttttttacctcctctgcgtgttctagc
attagttctagttctttgtcctctcaaaggaagaccaagacggtgtcttctacctctata
agtactaatttccataagtctcttaatgctcatagactcaaaacgtttgagatccccttc
aatctgataattagactcaagaatttctcttatactgacaacttgttgatcatttaaatc
ttgacacttaatatcagcgtctatgtttgtttttttctaatatttctttcgagcgagatag
tccaataccataaatatatgttaaagctatctctattcttttgtttcttggaagatctac
tccagcaattctggccactttgtcttgtctccaataaataatatgtat-ttttataacgg
ttgtttata-agcttagccttgtctctgcttatgtttaggattagtgcaaattaccatca
cctttctgtgacgccgaattattctacatttttcacacatttttcgaacagaaggacgaa
ctttcatattaa----ctctatatactaggaatattactatattaactctattaattatc
gacattaaaaatgtcaacaatatattgtactactttaactgttgtagattttacttcgtc
atactatcatattttttttgatatgacgtatgtctgaatttgcttagctgtgtctattgcg
actccaactaaaataagtaaagacgtagccccgagacctcttaagttttggatctgagta
accttttctattataaacggaatcagcgctactgtaaataaaaacgaggctcctaggaat
gtcagtctatttaatatgacttgtaaataatcaatagtcgcttgacctggacgaatatta
ggaatgcttgcacccattttttttaaatttatagcaatatcttctggattcattactatt
gatgtataaaaatagctaaagaaaaggatcaaagcacaataaagaagaaggtatagtgaa
ccattaggacaaaacaggtataggatttggagtaatgttttattttgaataatttgggtt
agataagatgggagggccatagatgcagatgcaaaaacaataggcataactccaccttga
tttaatttcaagggcaagtaactatttggatccaaaattgaagattttcctagctgtctt
gctgaaataattttaattcttcttgttccttcttgtacacaaattgtaattattatcatt
aataaaaagatcgctataaataatccgaacttaagacttgcattactataactagcatca
aaaaatgactgtgtaaaattctttggtagtcctgacacaatgttttgaaaaataagtaaa

gaggctccattaccaattcccttttccgtaattaactctgataaccacataataatcata
gaacctgctgttaaagctaaaacagactcgcagacaaatgcaaaattccaattaaaaaca
tatggttttacccatatagagattgctccagattgtaaagtcgcccaacctagagctaag
taccttgtaatttgagttattttttggcggcccaattcaccttcttccttctgtaatttt
tccagattggggacaattttcgtaagtagctgcattacaattgaagagttaatataagga
acaatacctaacgcaaaaatccctattgttgaaaaacctcctccagaaaaaatattcaga
aaatttactaaagtatttttttctacacttgcataaaaggcgtcatgatctatacctgga
acaggtataaatattcctaaacgtgctaaaactaatagaaagagagtaaagataatacga
tttcttagatcactttttttggctcataaataaatttaaatgaaaagagatactttt--tt
tatgtaaaaaccctattccaagacttgtataagtttaaaatagggaagtaa---aatatt
atagtaatattaata-taatattctatgtagaatacaggctttctattggaactcctcga
tctttagctgcttcactaaaagttcttaattgagttaagccattgagaacagctcgcgcg
ttatttaatgtattattagaacctaattgcttagctaatatattctgtacacccgacaat
tcgaggactgtccttacagaacctccggcaattacacctgagccaggtgcagagggcctt
aatataactttcgcggcaccagaaattccgttaataggatgaggtatagaattcgatttt
gtcagtggaactgtaactagatgtttttttagcatctgttactcctttttttactgcacca
attacatcgcttgcttttcccacgccgacaccaacttggccttgctcattaccaataaca
agaataactcgaaagcttagtttttttacctccttttacaactttagtaactcttttaact
tgtacgactctttcttcccagccactatctttatcttttcctttgctctgtttttttacga
ttggccattttaagaattattcctaattagttaatta-cgattctagaaacccatgcctg
cttccttagcagcttcagccaaggccttaactcttccatgatataactttcctcctctat
cgaagacgacatttttaatgccttctttcatagactgttctgctaactgtttaccaacta
cgcgagaagtatcacagtttggtcttatgttatcagattctttattatttaaattaacag
atgatgtagctactaatgtaataccttgtgtgtcatcaattatttgtgcgtatatatgtt
tattagatctaaatacacacagacgaggcctacttgaggttccttgaactttttttccgaa
ctcttttatgcttatgaattctagtttgtttagtgtttagtttcattattatttaccttt
tccagctttcccagcttttcttctaacaaattcaccttgatatctaattccttttccttt
ataaggctcaggaggcctaatagaacgaatagttgaagcaacctgaccgacaacttcttt
atctatgccagagacagtaatatttgtgttgttttcaactttaatttcaatattggcagg
aggtttaattttcactacatgactatagccaacacttaagattaaatcttggttatcaat
ttgagaacgatagcctacaccttgtatttgcagtttttttaaaaaaatccattagaaacacc
ttcaatcatattactaataagagttctggatagcccatgtaactgactagccatttttgt
ttgtccactcgtcttgacagcaattgtgtcgttaagtatttctaaattaataccagctgg
taaagttctggatagggtacctttaggtcctgtcacggtgatagtctgaccatcgaactg
agtactaagatttgtcggcaataaaattattttttttccaatacgagacat-attccacc
tataaaatagttaccaaatatagcataatatttcaccaccaagaccatcatgacgagctt

gtctatctgtcataacgcctctagaagtagaaataagagcgatgcctaaacctccaagaa
ctctgggcaactctttatgatttgcgtagactcttagtccgggtttactaattcttttca
gagcagtaataactggttgacgattttaccattatacttaagagaaatcattaaatgag
tctctataccttcacccatttgttcgaaattttgaacaaatccttcttctttcagtactg
ttgccatattgcatgtcattttcgttgctggaacttgcacaatttgatgtcttgctaagt
ttgcgttacgaatacgtgtcagcatatcggcgatcgtatcgttgaccaccttgatcctcc
ttgcga-----tgaattatttgaaatattttcaagattccctgaaaggcatccctagctt
ttttaacaaagctagaccttcttgatctgttttagctgtagtgacaattgatatatctaa
accacgaatttgatcaatattatcatagtctatttctggaaagatcaactgttcacgtaa
acctaaattgtaattacctttgccgtcaaaacttctaggactaattcctctgaagtctct
aattcttggtaatgttaaattaattaatttctctaaaaaagaatacatcttgtcttttct
tagatgcacaacaattccgataggaacctcttctcgaattttaaatcctgcaatagattt
tttagcttttgtgacaattggtttttgtccagttattaatgtcaattcttgaatactact
ttcaagagccttagcattctgagaagcttcacctaagccacggttaatagtaattttagt
aaacctaggaacttcatgtacatttttgtactgaaattcatcttttaaagattgagtaac
agttgttttatattttctttttaatcctattgccattatattttcatcaattatttaata
agttcgccagtctttttcagttttcgaa-ttttttggcctttatcattaattattactga
ggatcgactagcaatattatttgttcactaaataacataacgttagaggtatgtattgg
agcttcaaatttgataatttctcccgtttctccttcttgttgaggtttttttatgcttcac
tttgagattaattcctttaacaatcaccttatttgttttataaataattgcaataatttc
acctgtttttgttttatcacttccagaaataacttgaactaagtccccttttttttaattt
aatttttgtattattttttggttgttttagaaggacctttcattatactacctccggtgca
agagaaactattttagaaaaatttttatctcttaactctcgggctataggtccgaagact
cttgtgccacgtggattattatcttgattaatgataactgccgcattatctccaaatcta
atgctcataccgtcagttctccgtaaagctttacgagttcttactacaacggctctgaca
acatcagatcgtttaactggcatattgggagacgcatctttgactactccaataataaca
tctccaatagatgcataagaaggattactagtgcctagtactctaatacacattattttt
ctagcaccactattatctgcaacattaagatagctttgagtctgtatcatacttttattc
tgtataagattaattatcaaaagacttagataagatattaaccattgtccaacactttgt
gcgacttaaaggtcgtgtctcttgtattgtaacaatatcgccgattgtgcattcattatt
ttcatcatgcgctttatatttctttgtccggatcatagtttttgcgtactttctatgaga
gattctattttctacagctactactatagttttattcattttatcgcttactactttacc
tgttgtttcttttaaaggcatagtctttatattttttatatat---------aattaaat
tttaagattattacatggttgacttagtacgagacttttcaacagttaaaagttgggcta
atctatgcttagaatgtttaaatagatgaggctggaaatcttgccttgtggctcttttta
gccttaaatcaaaaagctctctttttattacaaggatttcttcagctaaagaagaagagt

ccaggtttgtaacatctgatattttagggaaagtcatattttaggattctgttgtattcc
gaactataaatttagtcttgattggtaatttataagaagctaatttcatagcttgttgag
cagtttttgtggtacacctgtaatttcaaatagaatatgtccaggcttaataactgcaa
cccaatattctggagctcctttacctgaccccatgcgagtttcagctggacgggcagtga
ctggtttatctggaaatactctaatccatagtttaccacctcttctcacatatctagtaa
tagttcttcgagtagcttctatttgtctagaagttaaccaaactggctctgtcgcttgta
atgcataatcaccgaacgcaattgtgttacctttgctagcagaacctttcattctacctc
tatgttgttttctaaattttgttttcttggggcttagcataaaaattaaaaaataataat
aattaagaagctgttgaatcaggagtttcctgattataagcagattctagacttttcgat
tctggcaaaatttctcctttaaaaagccaaacttttactccgagcacaccatacgtagtg
tgagcttggcgatgacaataatcaatatcggctcttaaagtttgtaaaggtacacgacct
tctctaacccattcacttctagctatctctgcaccattaagccgaccagatacttgaatt
ttaacaccctgtgtatttgctctttgagctctttgaactgcttgtctgacagctctacga
aaggccactctttttctagctgttgagtaataaattctgctactaaggttgcttcagaa
tctggatctgcaatctctacaacatttactctgagttgtttgctagggtctaggattaag
gacagcgattttcttaaagactcaattcccgctccagattttccaagtacgattcctggt
cgagctgttgctatgagaatttctacttgatcaactttacgattaatttcaattttagca
atactagcattactaagttttgaatgtataaatgagcgaattttatgatcttcttgtaaa
agaactgggtagtctttagagttagcaaaccatgaagaacgatgtttttgggtaatgcct
atgcgaaagcccaaaggatgaatttttttgacccacagtatccttgattataagttaaaag
ctataatgactaattgacgttaaa--------agactacagttctgaaacacctaatgta
atatgacaagttggtttatgtattggaaaggctcttccttgtgctctaggctgaaatctt
tttaaagtcgggcctttgtctgcaaaggctttactaacaaacaattggtttttatttaat
ccatcattatgctcagcattcgctgcagcagattccaggatttgctttatatgagaacag
actcggtatggcataaattccagaataattaatgcttcttggtattttctacctctaatt
tggtctaatacacgacggactttatgcggagaaagacgaatgtatttccctactgcttta
gtttcttttacgtttttttgtaatactcatagttatagtttaacgacgggcttttctgtca
ccttttacgtgagtgcggaaagttcttgttggaacaaactctcctaatttatgtcctacc
atttggtctgacacaaagacaggaaaatgttgtttaccattataaacagctattgtatgc
cctaccatatcgggaataatagtagatgctctcgaccaagtttttaggacttcttttttt
cctgaaatatttaatgcttctatacgtttaagaagactaacatcaataaaagggccttta
tgtatagatcttgacataataataaatcattagattcttaataaaataaattctatttac
ggcgacgtaagacgtacgggttactatatttatttggatttcgtgttttaacacccaatg
caggtttaccccaaggtgttacaggacgagctcgtccaattggagatttgccttcaccac
caccatgtggatggtctacaggattcatgacaacacctctaacagtaggtcttttaccta
accaacgatttctaccagctttaccaagagtaatattactagcatcaatattaccaactt

gtccaatagtagcgtagcattcttttcgaatcattcgaacttcgcttgaaggcaatttta
cagtaacaaaagtaccttcttttgctactatttgagcataagttcctgctgcacgaacaa
tctgtcctccgcacgaaggtcttaattctatattatgtactgctgttcctaaaggaatac
tagataaaggcaaagcatttccaacttcgataggggcagtaggaccagaaagaaccatag
atcctacactgagagatcgagggtgtagaatatatcttttctcaccatcaagatagtgta
ataatgcaattcttgcatttctattcggatcatattcaatagaagcaactttagcaacta
tattatgtctatttcttttaaagtcaattaatctgtactgctgcttatgtccaccacctt
tatgacgacaggtaatgacacctctattatttcgacctttgcagaaatggtgtttaacta
ttaatgatttttctggcttatcagtagtaatctctgaaaaggtagaaaccgttctatttc
ttgtccctggtgtgtaggcacgatataaacgaattgccatataagaagatttgagaagag
taaatgttgaa------ctatgtgatatatttaagtttctggaaacaaattaatagaatc
ttctgaggcaagtgttacaattgctttcttgtaatgtggtcgcttccctacgaatctacc
gatgctcctttttttcttaggaggatgacaagtgttaacacctgtaacttgcacgttgaa
aatatactgtatagcagccttaatattaatttttgttgctttgggatcaacggcaaaaca
atactgattctcttctaataatttagttgtcttatcagtaatgatgggatatttaaccag
atctaataaacctcttgaatcaatgctatccattatatacctcttgtattttagataaag
catcaactgtaataataattttatgtgcagctaacaaagccataatatttagtgtatcag
ctgaaataagttctacgttgtgtagattgcgaatagaaagataaacattcggatcttttt
tgtctactatgactaaaactttttatttaaatcaagattccatcgatgtatagcttcca
taaatagtttagtcttgggctgatgaaaataactgttaaaatttctacaatcagtgtat
taactgatttgttatttaatgctgtccttaatgctaattgtctttctttttattcattt
ttttggtaaaactacgaggttttggcccaaatattacaccaccacctctccatagaggtg
aacggatagaacctgctcttgctcgaccagtccctttttgacgccatggtttacgacctc
cacctcgaacttcacttcttgttttagtattagcagaaccctggcgcttttcattacttt
gctttactaaagctctatggactaagtacatcccagaatcttgactgaccttaagattta
aatcagcgttgccacttacttgaccttcccaattatagacttgataatttaattgcgtgt
taactgtcatgaaatatatcgtatgaaagaatattgctacttactaattttaccaaagc
gccaggtttaccaggaacagctcccttaacgatcaacaaatcattctctgagttaatact
tacaatttgcaaatttttttattgtaactttttttattaccaagttggccagccatgttttt
acctggataaactcttccaggcgttgtaccggctccaatagagccaggttgcctatgatt
tttcgaaccatgtgacattgggcctctactaaaatgatgcctcttttggtaaccggagaa
acctttacctacacttctagaagagacattaatcttttggcctacttgaaatagatctgt
agatagtatttggctaacttcgaaatcatcagtagatttcatttcgtattcacgtaaata
ttttagtgggggagcctgtgatttttttaagtgtcccaacaatggtttatttaattttg
ttcagcaacttgcttgtaaccgacttgaatagcattgtagccatcagtagatacagcttt
aatttgagtaataacacatggcccaacttgaattacagtaactggaattgataaaccagc

ttcatcaaagaattgggtcatacctactttagtaccgagtataccaacagacactagatt
tctccttttgagagatttatactatcattgtacataaaaattctacttcatagttactg
aag--taaattaaactaacagatgcatgatattattcatgacaacataaaggttaaaaac
tttacctatttaatctaaaatagaaacagcttttgtctagttacagcgtatataaatta
gaagtatgcttattaaatttttataaactact-tagatttaactgatattgatatccgt
aacttacttgcagtaatatc----aatcaacatgtataagtttaattgcctaaactagaa
ccagctattattagaaataattcaagtatagataaaagcgtatttgaacttttatatatt
gacaaatataatggaatatattgaggttcggtaactacaccttttattttacatttat
tgtgca--gtatatatacatctaatgcagtaaacataagaatataac----tgtatagca
aatgtaattacacaaattagagaggtattaatgggcaaagtcgttggaattgatcttgga
acaacgaattctgtaattgctgttatggaaggaggtaaacctaccgtcataccgaatgca
gaaggttttagaactacagcttctgttgttgcatatactaaaagtggagataaacttgta
ggacaaattgccaggaggcaagctgttattaacccagaaaacactttctactctgtcaaa
agattcataggacgtaaacagaatgaaatttcgcaagagattcggcaaacatcatataat
gttaaaactagtggatcaagcataaaaattgcttgccctgcactgaataaagattttgct
ccagaagaaatttcagctcaagtactgagaaaacttgttgaagatgctagtacgtactta
ggtgagactgttacacaagcagtaataactgtacctgcttactttaacgattcacaaaga
caagctactaaagacgcaggtaaaatagcaggcttagatgtattgagaattattaatgag
cctaccgcagcttctttgtcttatggactagacaagcaaaataatgaaacaatactagta
tttgaccttggtgggggcacatttgatgtatctgtattagaagttggagatggagtattt
gaagtactctcaacttctggagatacacatttaggcggagatgactttgatcagcagatt
gtagaatggctaatcaaagatttaaacagaatgaaggtattgatcttggtaaagataga
caagcacttcagagattgaccgaggctgcagaaaaagcaaagatagagctgtcaaattta
actcaaacagagatcaatcttccttttattactgccacacaagatggtccaaaacattta
gaaaaaactgtaactagagggaagtttgaagaactttgttcaaatttaatagataaatgt
agtatccctgtaaataatgctctgaaagatgcaaaactagaagcttccagtattgatgaa
gttgttttagttggtggatctacaaggattccagccatacagcaaatggttaaaagatta
attggtaaagatccaaaccaaagtgtcaatccagatgaagttgttgctattggtgcagcc
gtacaagctggagttttagcaggcgaagtcaaagatattctattactagatgtgacgccg
ttatctttaggagtggaaactctgggtggcgtgatgacaaagattataccaagaaatact
actattcctacaaaaaaatcagaagtattttctacagctgtagataatcaaccaaatgtg
gaaattcaagtacttcaaggcgaaagagaactaacaaaagacaataaaagcttagggacg
ttccgattagatggcattatgcctgcacctagaggagttcctcaaattgaagttaccttt
gatattgacgctaacggaattttatctgtaaaagccaaagaaaaggctactggtaaagag
caatcaattactatatctggagcatcaactttacctaaagatgatgttgaaagaatggta
aaagaagctgaagaaaatttcgatacagaccaaaaaagaagaaagaatattgacacaaag

aatcaagcagagtccctatgttaccaagctgaaaagcaagttaaagagtttgaagacaaa
attagccaagatttaaaaataaaaatagaagagctaattacagagcttagatctagtcta
gagaaagaagaatatgacaatattgaatctatttctcagcaattacaaaatgctctgatg
gacattggaaaaaatgctgctcagactgaaagtaaagatacaaaagcgaaggatgacgac
actgtgattgacactgatttctctgaagctaagt-aaaagta-agcgggtaacgcgattc
gaacgcgcgacatcaaccttggcaaggttgcgctctaccactgagctatacccgcataga
ttgtattattacaaaaattaagatatttgtcaatctgtta------atctatac---atc
tagactattaattttggaattacatattagctaactgttgtaaatataacaattgctata
attttttaa-------aagtacttatatcat-tatataagtactttttatatt-------
-caaaaatacaaataagtattatag-------ctaaactactaaggaattaactaagcta
gtaacaattactaatcctgtccataagccggctcctgtatatattaatccttttgattgc
tcccattggccaggggatgctaaaacaacaggtacgcctactactaacacagtcgataat
gcaattaataatagcactaataattggatcgcaataatcatttaatatgttctcctgaaa
aattcaaatgttaataatactgtatataatacaatgaagcttttgaccttcagcaattta
tctatgaagaaataatttaaaattattgttttctaaataaaccttaaattaagtattaaa
atctttattgtaaacaataatactatattttttta--gtagataaaaaatttaaagaaag
tctttttaaaaaaaaattaagataaggtgatatgctgtataaaaagctaagtattatta
attattagagaaatttaaa-ataagaggtaaaacatatgaactcagcccttttttagca
aaactaccagaagcttatgccattttaaaccgatcattgatatcttacccgtaattcct
gtatttttcttcttttagcttttgtttggcaagcagcaattggttttagataacattta
ggttataaaaagaataacaaatatacagagtgccaattttgagctctaatcttatatct
tacacaataataactattatggttgaacccttactatcaggaattgttcttggacttatt
cctattactttattcggattattagtagcagcttatttacaatatcaacggggcaaccaa
ttaggactgtaaagcctaaattatagatatttataaaaactacaataattatagtttta
taaatatctacaaataagctaattttaaatacagaaataatactattgaaactagagtga
taacaaaaaaaatttatgtaa---taa--------------------------------

---------------------------caagctaattgtttaccagcttgacttagtaac
tcctggtaaaagacattcatgagacatttctcgaaaaacatggcgagataatccaaaatc
tcgataataacctctacttctacctgttaaccaacatctatttctccctctaacagcggc
gctgtttctaggcatttcttggagtttttgtcttaaatccatttgtcagaaaagctgcc
agcttctttcagttgatctttaatagccagtctttttaaataatattttttgcaagttt
cgatctcttaatctctctttggatcatattctttttagccatgctgtaa-aactctcttt
taaaattatttattcaatatattagctaaaaaagctcctgcctgcaacttttatatattt
aaaaataaatagcataaagtagtgaaatgcaattgatatttaatcaacgatacc---act
ttatgctattatcaaaaatttatttagtaaagttcttgttcaacatgagttgaaatagtg
caatcagatgaaggatatgcaatacaggttaaaacatagccagctaaaagttgatcatca
tctaagaatgattgatcagcttgatctacagttccttctgtaactttacctgcacaagtt
gaacaagctccagctctacaagagtaaggaagttcaattccttcttcttctgctgcatct
agaatatatgtatcctccgcacaattaaatgtgacatcaattccttcttcttcacatagt
aaatgaattttataatcagccatttcttcttactccttaataaagatacacttgaaactt
aataaagcaaaat-aatttaaagaacttaccaaaaaattatatactagtttcattatgca
tgatagactggaaaaaaagaaaatagtatactattctgaaaaatcaagctacatttccta
ctaaatagaactatagtaggtccaaagtatatacataaattcatatagttaaaattactt
ttatcatttaaatagctgaacttgatagta---------tagattgatcatc------aa
gctagtcttctgc---caataaataattaattatgatttcaatgcttaatcaacaagtag
aacttaagcctattcttaaatttcagataacacaaatatattcgcattctcttattcagg
aaattaaagccttaataactagacttgttttgcaagtttggagaaggccggctacattga
tggcaggtattatccaacctttattatggttaattctatttggtgggcttttctacaatg
ctcctataaatttgttcactattaatacaagctataattgtttttttgagctctgggatta
taatttttacctcttttactggagctttgaattcaggtcttccattaatgtttgatagag
aatttggatttttaaatagattattaacggctcctttagtatcgaggacttctatcattt
tatcttctgctacttttatgacttgtattagtttaatacaagttgtatttatagttacag
cttctctttttatgggaaactcacctctaaatagcgatagtactatgatttttggactta
tgattctattagtgactgtaggagttacaatgcttagtttagctttatctttcactctgc

caggtcatattgagctattagcatttattttagtagttaacttgcccttttattttcta
gtacggctttagctcctttatatttcatgccgccatggcttcagttaattgcaagtctca
atccattgagttatgcaatagaaggtacaagatacttatattcaagcgtaaactggaatt
ttacagagtgtgtgattaagattagctggggagatatttgtttagggcaaattattattt
tattaatcgctttagatataatggcagcttatcttgtgtctaatatattaaaagctaaac
ttaatta-----------------aaattttaataaaaaatttattaaatttattttaca
aagattttactatataatactaatagtagtatggataattgaaatagaaatagtttttc
aaaaccaaagctattatcaacaatgataaacatttgtaagaaagtcaacaaagtatgttc
ttattcataggaggcatgtagtcaatgggactaccatggtaccgtgtacacacggttgtt
ttaaatgatcctggacggttaattgcagtccacctaatgcatactgcacttgtagcgggt
tgggcaggatctatggcattatacgaactagctgtatttgatccttcagatccagtatta
aatccaatgtggcgacaaggcatgtttgttatgccatttatggctagactgggcgtaaca
gattcatggggaggatggagtataacaggagagagcgtatccaatcctggactgtggagt
tttgaaggtgtagccttaactcatatagtcctttctggcatgctttttctagctgctatt
tggcattgggtttattgggatttggaattatttagagatccacgaactggcgagccagcc
ttagatttaccgaaaatttttggaattcatttattgctatcaagtctactttgtttcgga
tttggagctttccatgtaactggactttttggaccaggaatgtgggtatcagatgggtac
ggagtaaccggaaaggtattaccagtagctccagcatggggaccagaaggattcaacccg
tttaatcctggagggttgcatctcaccacattgccgcaggtactgtaggtatattagct
ggtgttttccatttaactgttagaccaccacaaagactgtatcgtgctctaagaatgggt
aatattgaaactgtattatcaagtagtatctctgctgtttttttctcagcttttgtgact
tgtggtacgatgtggtacggttctgcaactacgcctattgaattatttggtccaactaga
tatcagtgggatagtggatattttcagcaagaaattgagaaacgagtagaaaatgctatt
gctgatggtgctgcacctagcgaagcatggtcaagaattcctgacaagttggcattctat
gactatattggtaataatccagcaaaaggaggattattcagagcgggccctatgaataaa
ggcgacggagttgctgaagcatggcttggacatcctgtattccaagataaagaaggaaga
gagcttagtgttcgcagaatgcctgctttttttcgaaacttttcctgtaattttagttgat
aaagatggtattatacgagctgacattccatttagaagagcagagtctaagtatagtatt
gaacaagtaggtgtaacagctagtttttatggtggcaaattaaatggccaagttttcaac
gatgctcctagcgttaaaaaatatgcaaggaaagctcaattaggtgaggtatttgaattt
gatcgaactacattagaatcagatggagtgtttagaagtagtcctagaggctggtttaca
ttcggtcatgctaatttcgctttaattttcttctttggacatctttggcatggttcaaga
actatcttccgggatgtgtttgcgggaatcggagctgaagttactgaacaagttgaattt
ggagctttccaaaaattaggagatagaagtagtaaaaaacaaggagctgtataagaccaa
agtcaaagatagtctttatc---aaatatttctaatattaaaatagttaagacaaagttt
attatctaatagttactaatcaggagaatttatggaagccttagtctatgtattttact

aacaggaacgctaatggttatattctttgcgatctttttagagaacctccaagaatagc
aaagtaacttgattccgtttaaacttataataatagaaccactttccaacttattagtgg
aaagtggttttattgtagattacattgtattattcttcatgctcttcaaaaggatctcgg
agatcttttgaagcaggaccaaatgctgtgtatatcgagtaacctgtaatacctaaaagc
agactcgaaataaaaatactaagaactgttgcagtttccataatttaagcagtattaaga
tgaactttttctataatgatattatcaatattaaaacatttaattattaaaattatcaaa
atatactatggcacttagaactagacttggagaaattttaagacccttaaattcagagta
tggaaaagttgctccaggttggggcacaactcctattatgggaattttcatgttactatt
tttcttgtttttattaatcatttacaaatctataattcttcattagtattagaaaatgt
agatgtagattgggctactttaggtag----ctaactagataagttctttga--------
ttacacaatattaaagacaac----------------aagcgatcgctattagattatag
tagcgatcgctttaaaagtattttactatatatccagagaaaattatttacctaaattaa
ttaactcggtatctgcaaaactatttgtgttaacattattatagttaactttttcaaacc
ttactaatactggatatttgataccactttatcaattgctgcaacagtaccaatttcct
gataccagtacgattcttttctcaatatttttacttttgagcctctttccatatttttag
tcctcagatagattattaaagattattatattaaaaaattaaaaaaaactaaacaaaata
tataaaaatactaccgacaattgtaatatataactaataaaaggagagagagaggattcga
accctcgataacaaaagttatgacaattttcgaaattgttgcaataaaccactctgccat
ctctcctgaattaaaatactgttttaaaaaattagacaagaataacatagaactaaggta
gttgcctagaaaattaaaactatgttaaatataaattataatctttctatagatatattt
aaaaaatatgtacatagaatctatatcagcttacttcaagatttttttaatcttatacaat
ttttactaaataaataaattctctcataatatgaaactatcttggaaaacacttcttctt
tggtctttaccaatatttgtaattggtttctttttctggcaaggtttttttaggtccaact
actacagatgttggcagtaatatcgcaagttctagaatgacatatggacgatttttagaa
tatttagatatgggttgggtgaaacgggttgacctctatgaaaataatcatacagcaatt
gtagaagctgttgggccagaattaggaaacagggttcaacgaattcgagttgaactgcca
gcaagtgcgccagaattaattacaaaattacgcaaagccaatgttgatctagatgctcac
ccccctaaaagtacaagtgcagtatggggactattaggcaatttactatttcctttacta
ttagttggcgggttagctttcttatttagaagatctaataatgctagtggtggacctggt
caagcaatgtcatttggcaaatcgaaagctttatttcaaatggaggctaaaacgggagta
gtatttaatgatgtagctggagttgaagaggcaaaggaagaatttcaagaagtggtaaca
tttttaaaacagcctgaatcatttactgctgttggtgcaaaaataccaaaaggcgtttta
ttagttggacctcctggcacaggcaaaacattactagcaaaagctattgctggcgaggct
agtgttccttttttttagtatctcaggctcagaatttgtagaaatgtttgttggtgttggc
gcttctcgtgtgagagacctattcaagaaagcaaaagacaatgcgccttgtatcgttttt
attgatgaaattgatgctgttggtagacaacgaggaacaggtgttggaggtggtaatgat

gaaagggaacaaacattaaatcaactattgactgaaatggatggctttgaaggaaatact
ggtgttattgtaattgccgctactaacagagctgatatttagattctgcattattaaga
cctggaagatttgatagacaagtttctgtagatgtaccagattttaaaggcaggttagca
attcttgaagttcatgctaaaaataagaaaatggaacctaaagtatctttagaaaccatt
gccagaagaactcccggcttttcaggagctgatttagctaacttactaaatgaggctgct
atcttaacggctcgacgaagaaaaaatgcaatgactatgtctgaaattgatacatcaatt
gatcgagtagtagctgggatggaaggcactcctttaattgacagtaaaagcaaaagatta
attgcgtatcacgaagtgggtcacgcaataataggcagtttattagagcatcatgatcct
gtgcaaaaagttacattaataccaagagggcaagcaagaggcttaacttggtttactcct
agtgatgatcaaagtctaatatcaagatctcaaatactagcccgtatcgtaggtgctctt
ggtggcagagctgcagaagaaatcattttcggtgacgcagaagttactactggtgcaagt
aatgatttacagcaagtaacatcaatggcaagacaaatggtcactcgatttggaatgtct
aaaattggacctttatctcttgaaagccaaggaggagacccatttttaggtagaggcatg
ggaggaggctcagaatattcagatgaagttgcaactaatattgataagcaagtaagggaa
attgtcagtgaatgctatgcacaagctaaacacattattatagataatcgagtagtgata
gatagattagttgatttactaattgaaaaagaaacaattgaaggcaatgaatttagagac
atcgttaaggaatacactgcaattcctgaaaaaaattactacatatcacaattttaa---
---------------attaaacgggactgacgggattcgaacccgcaacttccgccgtga
cagggcggtgctctaaccagttgaactacagtccc-aaaaagatgaatacccagagataa
tctcatagttagcttaaacttgtcaaattagaatattaaatatgaagagta---------
--------tttgtaataatactcttcatatttgata-tagtagctaaaatatttctttta
gaaggagagagagggattcgaaccctcggtacgaagttaatcgtacagcagattagcaat
ctgccgctttcgaccactcagccacctctccatga---tatatattatatatatatcaaa
tatacttatatttaataggtgtgcataaatatttatggagctaagcggattcgaaccgc
tgaccctctcaatgccattgagatgctctaccaactgagctataaccc----ctttggta
agatattaagaaatggctcaagcgggatttgaacctgcgaccttgggcttatgaatcccc
tgctctaaccactgagctactgagccatagtataaaatactattaaatattataccataa
attatggtcaaatataaatcaataaaagaataattactatca-tttactacttttg----
------------------tgcaagttgtaaatattcgaataaaatttcaaaattaaaaatg
tctttaactttttgttaga-------atta-ttgattacgaaa-----------atattg
atatttagaattaac---
--------------------aataaatttg------------------------------
--tctttattt
g---

-------tagaca---
-------------------------------caaaaatattggaaa--------------
------------------------ttttcatcaat-----------------taattagt

------------------taaactataaccgacgtac-----------------------

---aagaattattgaacta---

---cgttaaat---

-------------------------------------tgtgatgcatt------------
-----gggcttgatcaaaacttctctaaaatcaatagaatatagt---------------
---aattcaa
gtaaatcaaatactaacaaatgtgacaaacgcttaaaaaaataata--------------
caattagaatcgtaaaaatttggtttttga-ctgtgttaaataaatttgattatcataga
tttaatatttaaaagtgttctattacttgta-----ttgagtaattgt-aatatttaata
tcttctcttttagataataatcataatataagctaaaaaatagtttt---tattaaacaa
ctagcatagagccaataccttgatcagttaatatttctagcaagagagcatgatctattc
taccatctaaaatatgcgctgaagctacaccctgagctaaagaacgaatacaacaattca
ctttaggaatcatgccaccagaaattactgctgtttgagttaaatctctagcttcctgta
tattcagatgactaattaatgttgaaggatctgaagaattacgtaaaatgccgggcgtat
ctgtgagtagaattagtttttcggcattaagagcggctgctatttctcctgcgacagtat
cagcattaatattatacgattggccttccttgtctgctgcaacactagctataacaggta
tataattattattaattaatatcttcagtaatttagtatcaatattttgtacttctccaa
caaagccaaggtttgcttttccatttggtctcggagtaataagtaatccatcttttcccg
acaaacctacactttaccaccttgtttattaatagttgcgacaagatctttattgactc
ttccaactaaaaccatttccactatatccatggtaggttgatctgttactctaacgccat
catcgaattttggtaatattttgaggcgatctaaccaaaaattaatttctggtcctccac
catgaactaaaatgggacgtagtcctataaaagacagaaaaacaagatcactaatcactt
gatcttttagtttctgatttttcatagccgctcccccatattttataacaataattctgg
aagaaaattgctgaatataaggcagagcttcacttaagacttttactctttctgaattag

tcaacataaataaagtaggattgtgatatataaaattatatacacagcttgtgttaattt
aaaagataccaatgggt---------acttgaaaaaatagaaatattactaacag-----
----ttaaaactatagggattataattatgaataaattttgggataatgtattaagattt
ccacgattttagtcagcgtcattttaggattaattttgataattattagtcctttttc
gtgttattaaaaaaaccactgacaagtttttttttcatcatatcattggccggattaatc
acagttttggcaataataa-tacaaaaaatgataaatatcgaatgttgttgagagaaata
aataaatttaattaaaatttttgaacatatataatattatattatcattaaatatgataa
ttt-atttcctttctat---aaaacccataaatattaaatattatttgataaatattat
gccactaaaacaaagagttagctctgaaaagaccggtgccttcgcacttttggatagtat
tgttaggcacggagttaaacatatatttggttatccaggtggagctattcttcctattta
tgatgagctttatgcttgggaagaagcctctctaattaaacatatccttgttcgtcatga
gcaaggcgctgctcatgctgcagattcttattctagatcaacaggagaggttggagtatg
cttcgctacttctggcccaggagcaactaatcttgtctcaggtatagctacagcacatat
tgattctgtacctatattagctataacaggtcaagttgggagagcttttattggtacaga
tgctttccaagaagtagatatttttgggattacacttcctattgtaaaacattcatatgt
agttcgtgaccctagagacatgtctagaattgttgcggaagcatttttttatttgtaaaca
cggtagaccaggtccagtattaattgatgttcctaaagatgtaggattagagaagtttaa
ttatttttctgttgagcccggaaaagttaatattcctggctgtaggccaattaccagcct
aaagtcaagacaaatccttatggcagctaaaatgatacagcaatctagccagccattgtt
gtatattggtggaggagccataatctctgattcacatcaaattattaaagaacttgttga
tttttataaaatacctgttactactactttgatggggaaggggattttaatgaggatag
cgactattgtctagggatgttaggtatgcatggtactgcgtatgctaattttgcagttag
tgagtgcgatcttttaattgctttaggagctagatttgatgatagagttactggaaaatt
agatgaatttgcttgtaatgcccaagtgattcacgtagatattgatcctgctgaagtagg
aaaaaataggattcctcaagttgctattgtcggtgacgtagcagaagttgttagtgaaat
attgaatttattaaagacttctttcccccttatccagagcagattatatcttggcaaga
aagaattaatcgttggcgtcaacagtatcctttactggttcctagaaaatcaacaagcat
ttcacctcaagagattcttgttgcaactaataaattagcccaaaatgcttattttactac
agatgttggccagcatcaaatgtggtcagctcaatttctgaaagtaaaagctaagcattg
gctttcaagtgctggattaggcacgatgggttatggtttacctgcagcaattggcgctca
agtagcacatccaaatgacgtagtcatttgtattagtggtgattctagttttcaaatgaa
tatgcaagagttaggaactatcgcgcaataccagttaccagttaaaattattattattaa
taatcgatggcaagggatggttagacaatggcaacaagctttctatggtgaaaggtattc
acactcaagaatgacagaaggagcacctgattttcaaaagcttgcagaagcttttggtat
taaagcttttactattaataataggcaaaatatgcaatctgctttacaagttgctattga
ttatcctggtccagttttattagattgccaagttacagaaaatgaaaactgttatccaat

ggttgctcctggaaaaagtaatgcacaaatgataggtatcgctaaaccgcagagaggtac
tgcttccaactatattaataatagtgtttaaagtaa------------------------

---------------atagttagtatttttta-aacaaagaata-aagtaca--------
-taaatgtaatttttttatactatta-aaaactatgtaagaacagcctttgacgagaatt
gaactcgtgaccttccccttaccaaggggatgctctacctctgagccacaaaggc-tttt
tactatattgggccgggttggatttgaaccaacgtaggcgaagccagcggatttacagtc
cgcccccattaaccactcgggcaccgaccctaattaaaccttattcaagatatgacctat
aagatatcataactatgttgctaaaaacaagtactttgataaataaaactgaataagtaa
tggacatagctggacttgaaccagcgactttcacgatgtcgacgtgacactctaaccaac
tgagttacatgtccaaaactttaattattattataacactgtttagtgattttataagta
tctatttctaattgaagatttgttttaatcgtccggctttaccgagcagatctcttagat
aatacagtttagctctacgtattttagctcttctaatcactttgacaaaagttattcgcg
gtgaattgagaaaaaatactctttcaactccaataccttgaaatgagcatcttaaagtta
aactagtatttaagcttttttttcttcttctcgctaatactactccttcacataattgtt
cccgagttttactaccttccttaaccattagtccaagttgtattgtatcgccaactttaa
tttcaggtacttctgttttgataaaaggtatctcaacacttttcattagctgacttagct
ttgtactatttgctttcatatataggattagtcttgtgaca-tgttttgaaatattatac
attaaaaaaacaaatatgaaaaattttagcgaataagcatactagtatttataaaac-tc
tttttatct---
--ccattata--------
----------------cagatccatt------------atattcaact------------

----------agattcttttatagtttc--------------------------------

--------tttgataatcagtac-------------------------------------

---------------------------atcta----------------------------

----gatgatcaaatattattttcaaacatggcttatgtatgaaaaagttctata---aa
aacctaatgac--------taaaattagggcttaagtatttcttagttttgtgttataat
tagttactatcaaaggtagtggcataacacgaagcatcaa-aatgttcgaacgctttact
gaaaaagctataaaagtcataatgctagcacaggaagaagctagacgcttaggacataat
tttgtcggaactgagcagatactattaggattagttggtgaaggtactggaatcgcagct
caagttttgaaatcgatgaatgtgaatctaaaagatgcaagagttgaagtcgaaaaaatt
ataggaagagggtctggttttgtagcggttgaaattcctttcactcctcgagcaaaaaga
gtactagaattatctttagaggaagcacgtcaactaggccataactatattggcacagaa
cacttgctaatgggcttagtccgagaaggagaaggagttgcggcaagagttttagaaaat
ctggcagtcgatgtttcttcaattagagctgaagttatacaaatgctcggagaaaatgcg
gaagccaatgtaagtggaagcaatactacgcaagctagaagtaaaacaccaacattagaa
gagttcggatctaacttaactcaaatggctatggaaggtggtttagatcctgtagtcgga
agacaaaaggaaatagaacgagttattcaaatcttaggtagaagaactaaaaataatcct
gtcttaattggggagcctggtgtaggtaagacagcgattgcggaaggattagctcaaaga
attgctaatagagatgtaccttctatttagaagataaattagttattactcttgatgta
ggtctattagtagccggaactaaatatagaggtgaatttgaagaaaggctaaaacgtatt
atggatgagattaaatcagctgataatgtaatattagtgattgatgaagttcatacattg
attggggctggtgctgcagaaggagcaatagatgcagctaatctgcttaagccagcttta
gcaaggggagaattgcaatgtataggtgcaacaactttagaagaatatagaaaacatata
gaaaaagatccagcattagaaagaagatttcaaccagttgtagttggagagccaagtgtt
gaagaaacaattgaaattttgtttggtcttagagaccgttatgaaaagcaccatcaatta
acaatgtcagatggagctttggctgcagctgctaaatacgctaatcagtatatttctgac
cgatttttgccagataaagcaattgatttaattgatgaagctggttctagagttcgttta
ctaaattctcaattacctcctgctgccagagaattagataaagagttaagagctgtatta

aaaacaaaagatgaagctattagagctcaaaaatatgaaacagcagagcagtatagagca
agagaaatggaaattaaagctcaaattgcagcaattgctcaaagtaaaaagaatgagcct
gatttaaatttagaagatcctgttgttacagaagatgatattgctgaaattgttgctgca
tggactggtataccagtaactaagcttactaaaagtgagtcagaaaaattaatgcacatg
gaagaaactttgcatggacgtattattggtcaagacgaagcggttgtagctgtctctaga
gcgataagacgcgcaagagtaggtctaaaaaatcctaacagaccaattgcaagctttatt
ttttccggaccgacgggtgtaggaaaaacagaattaacaaaagctttggcttcttatttc
tttggttcagaagcttctatgatacggctagatatgtcagaatacatggaaagacacact
gtatctaaactaattggttctcctccaggatatgtgggttatagtgaaggtggttatcta
acagaagcggtaagaaaaaaaccatatactgtcatcttatttgacgaaattgaaaaagct
catccggatatttttaatctacttcttcaaattttagaagatggccgactaacagatgca
aaaggtagaactattgattttaagaatactcttttaattatgacttctaatattggatct
aaagttattgaaaaaggaggaggtagtttaggctttgaattatcagaagatcaaacagaa
tcccaatatactagagtacgatctttagtaaatgaagaactgaaacaatactttagacca
gagtttctaaatcgattagatgaaattattgtatttcgtcagcttactaaagatgaagtt
agagaaattgcagaattaatgcttaatgaagtctttgcgagaattaagcagcaagatatt
caattaaatgtaacagaacgatttaagcaacgattagtagaagaaggatataatccaagc
tatggagctagaccacttagacgagcagtaatgaggcttttagaagatagtctggctgaa
gaagttttatctggtaaaattaaagctggtgatagtgcagtagtagatgttactaatgaa
ggagaagttacagttttattaggtgaaaaattagaactgttaacataaa------aaaca
atttattaat--taaagcattgaagaggttat--attcaatgctat---gaaaaaaatta
ataagatgggttgctataattgacaacctctgatactctttatttgtatata---aagcc
gggatagctcagttggtagagcagtggattgaaaatcctcgtgtcaccagttcaaatctg
gttcttggcat-tttaagcatcagaaaattttgctatctgagcattaaattttaattgaa
atgttcctgtcggaccattcctgtgttttgctattataacttctgtgaagtctctacttc
tagtttcttgtgtatagtaactttctctatatagcattattactaaatctgcatcttgtt
ctatagaattatgaacaataaagttgtttgaaataaaattgcataaaggtttacattcta
aatcaaacactatttgtaaacttgtaacgtttatttttttgtatactttcaaaagtcaaag
atgaaa------aagaattaaaaacatttttttgttcttcaaagttttt------tattt
gaatagcaatcatgtcattttgattaattttatcacatcttttccatccttgtgttgtga
gtagtttatggttacttgttaattgtatatatttcccagcttcggtaataattttatata
cagtttttttccctgttttcgaaatactacatttaattgctagaaataactgttgttttt
gcgcactaaagttaatgacttca-atctttttataatagcaattaaataatatttg--aa
gttgattatataatgatgtctgaagataattgaatttggatatacacccactttctctta
aatcggataacaaaggccttttgtcactccgagtttcaaggtttctatttagttgagata
atgccaagataggcaagcttaaatcttttgctaatattttaagtgatcgagtaattaatg

atagttcttgagatctattgtcagattgtctactctcctgcagcaattgaagataatcta
taataattaattctatatttttccttgtaatttaaagaatttactttgtctttatag
tgtcagtagaaatcttagcgctatcatcaatataaagattaaggttagcaagagttttac
tttttgaataacatattgccattcatcattattaagctgaccagattgaatttttggc
cgtttaaattacactcttgagctaaaattcttcttagtagttgctctgtagacatctcta
agctaaataaaataacataagatcttttctgattaattacatatctagttatattaatag
caaaagcagttttccccattgaaggtcttcctgcaagaataattaaatctgacttttgaa
acccttgtgttatagagtcgagttctgtaaaaccagaaagtatactactatttatactaa
tctttttccttgtatccaaatggactaataggtttgcaagaattgtagctaatgtttgcg
tatctttttcgtcaagtatttcataagcttttgttaattgactaacaatagaagttatgt
ttttttgttcaataagttgttttgagcagctaattaaacataatgaatctccacatgcta
aaagtaatcttttaatgtagttatctataacgactgctgaatattcgcttatagtatcag
ataggggagcttgttctattagactaaaaacttcatttagctcattcaaatcttgaatca
tttttcatttttaagttaatgaaaaaatttcttacgctttttgtctttgcatggttaa
cattttcgagtgctgctctataaagtaatgaagttgatgtaaaataaaaaaaaatcaggag
agatttttctactgattttagtaatgatatcgattgctgagttaatattatactaatta
atattttttcagctaaaatattatgaggaggtagatatttataa------atacttatta
cttcctcc-ttgtaacattactcttgtg--------gtattagttaactattgatttaat
ttgattctggaagaacttgcaatttaatatttgcagttacttgatgtaatagcttaattt
ctacatcgtaaatgccaattgttttcacgtctggtaaagagatactttgtttttcaatgt
ctatgttagtagtgtttttaataatttgtgaaatttctttttctgtgacactaccaaaaa
tattgtggccatctcctgtttttttactaacgctaaacctttgtatttcttctagaagtt
gcttgactttttttagcattttctttagctgttttttaatttttcttctcttatagctgcat
aaaattttttgttgttttaaaataccatttgtggcaactgccgccattttattcgggatta
aaaaatttcttgcataaccagaagctactttgataacgtcattactcttgccaagttttt
gaatattctcttttagcacaacatttataactttttttactcatctt-------aattcta
aaaa----atctgtattatcctattatacctgttgtatacagatttt----aatagtaag
tatttatttgttgatgtaatattcttgtaaaa--aaaggtaatttttttatttttt-ttctg
atataatcactttcacaataattc-----attaggagaggtggccgagtggttgaaggcg
cagcattggaaatgctgtttaggagcaatcttaacgagggttcgaatccctctctctccg
tacaa--aaaatataaaaagttttatagagtccaatctaaatctccaataatattcttcc
aagcatttaatccaccccgaactctaacaatatttagtttttgagctattaaaaacttag
aagcaaatattgatctagaatctaagctacagtaaacaaaacaaattttatcttgtaaat
tgatatgagagtaattcattcctttttattttttgtaaaggtagatttaatgaatgtatta
aatgacttttatgatattcttcatgatttcttacatctattaaaatatattgtggattcc
tactaattaaaaacttttgtaattgaataacactaatttctcgtacaaatgtactagatt

tactgccataatatttgttccaatgctttttttgt-------ggataatacaaatttagta
tttataatcttaaatttgttaaatgatgaagttaatgagttgtacgttaatatagttcca
cttaatatagacttataacccagaacgattttgacagcttcagttgcttgaagtgtacca
acaatacccggtaatagacctaaaactccggaattactacaagtatctcttgcgctctct
ttattttcagtttcactgtagaagtctcgataaacaggtccaccttgataattaaataca
ctaacttgtccttcaaattgaaaaatggcaccataaatatgtattttatttaactctagg
caagtatcattcaagagatatcgagtatcgaagttatcagatccatctataataatatca
tattgtctaataatgtcgattgaattctcatagcttagtcttgttttgaacacagttact
atacactgtggatttaaatctaata-tttttttttagctatttctacttttgaatatcct
atatcatgcatcgtgtataaaatttgcctctgtaaattagagagatcaattatgtcatcg
tctataattccaatgctgccaattcctgcagcagcaaggtaaatgattcctggagatcct
agcccaccagccccaatgaataaaacttttgcttcttttaatctttcttgtccttctaat
tgaatttgtggcaatactaaatgcttagaatatcttgtatattcttctagtgagtatttt
gtattttctgttttaaaatttagcataaaaatttatagtttatttaatagcactta----
--aaagctagctgataagtttattaaaatgacttttgtaaacttggttcttctctatcta
aaatagctccttgtgtaggacagacctgaacacaaatactacaatcaatacaggcagaaa
agtcaatccaataccaattagtgttattattgtttttttccctttccttgatgaatacaag
ctacggggcaagcttccacgcattcagaaactccaatacatttttctgttacaattgtat
gagacatattatttaattaat-aaaagataagctatttacatctagtagttttgttgtat
actaaatttacatatgctatccaggccaatatttaaggtagtctatttctacagtttact
aatttgatatgcgcctttagttcagttggtagaacgcaggtctccaaaacctgatgtcga
gggttcaagtccttcagggcgcgtttag-ttttatcgtaattcaataatatcattttatc
tttttgcaaacatcaaaatgatatgatataaaagatcatattttatacattgaatattct
atgtactagtactg-ttttttaatatgttattattgataagttaattatactaattctat
ttttgtaataagt-aaaaacagttctaattaagtatgcacgtatttaatagtc-ttttgt
atatat--aacttgaaaaaatttaaactatggctaaaaaagttactggaattgttaaact
agcattaaatgcaggtaaagctactcctgcgccacctgtcggacctgctttaggacagca
cggcgtgaatattgttatgttctgcaaagagtacaatgcacgtactgctgacaagtcagg
actagtaatccctgtagagatctcaatatatgaagatagaagttttactttttatacttaa
aactcctcctgcttctgtactaatcgctaaagctgctggactaaataaaggttcgggtga
accaaataccaaaaaaagtaggcagcataacaaataaacagttagaatctattgcagaaac
aaaacttcccgatttgaacactaacaatatacctcaagctatgaaaatagttggaggtac
ggctaaaaatatgggaattctaattaaagatt-aaaagtttcaattatatttacta-tt-
-tttatgaaaaaattttcacgtcgacttacaacattaaaatcaaaagttgagcctaaact
ttacactattaatgaagcagtatctatattaaaagcaacgtcaaatgctaaatttaagga
aactgcagaagctcatattgctctaggtttaaatcctaaatatgcagaccagcaactcag

agcaacagttatattaccgaaaggtacaggtaaattgataaaagtagcagttattgctaa
gggagaaaaattaacagaagcaattagtgcgggagctgatgtcagtggctccgaagaact
aattgatgaaatactgaaaggtagattagattttgataagctgatagcaacaccagatgt
tatgcctttaatagcaaagctgggacgagtattaggccctcgagggttaatgccttctcc
taaagcaggcacggtaacattggacgtagcaaaagccgtgaatgaatttaaaggggggtaa
agttgaatacagagttgatagaacaggtataattcatgtaccttttggaaaatctagttt
ttcacaagaagatctagttttgaacctccaaacaattaaagaatcgattgatagaaacaa
gccttctggagcaaaagggaagtactggaaaactttttcttatctagcaccatggggcc
atctattcaaattgacatcactagccttctatgaaaaatttttgtataattatagaactt
gaataatctattatttcaaatta-taatttgctagcattattaacgtaacaacttgctta
ctatatcttatctgcattaaaaacttt-ttatatatcttattaattatgagtacaaaggt
tgaaaatatcttggaagagctaaaatctttaaaccttctagaggctgctgaactagttaa
acaaatagaagaaacgtttgatgttgatgcatctgcggcttcaggaggtatgatgatggc
cacaccaacttcagcaccagcgtctgctgaggttgaagagaagacagaatttgatgttgt
cctagaggaagtcccagcacctaaaaagatttctgtattaaaagttgttcgctctctgac
tggtttgggtttaaaagaagcaaaagatcttgttgaatctactccaaaagtcttaaaaga
aggtgcttctaaagatgatgcagaaactatgaaaaaacagctagaagatgctggcgcaac
agttattgttaagtaatga-----agaagcgcttctgtttaagtataaacagaagtgcct
ctttatttattataagcaattatactagaaaagtccaagtgtaattgcattgttgatagg
catagttgcaccaatacctagccaaatacttacaacggtaccaattaagaaaacagtggt
tgcaattggtcttctaaatggattttgaaatttgttaacattttcaatgaatggaacagt
aagtaaaccagctggtacagcagccatacttagaacacctaatagtttattaggaatcac
tcttaatagattaaatgttggaaagaagtaccattctggtaaaatctctaatggagtcgc
aaatggattagacttttcacctatagaagatggttctaaaattgcgagtccaatactgca
ggcaaaagtgccaataattacaactggaaagacgtataatagatcatttggccaagctgg
ctctccgtaataattatggcccattccttttgctaatttagctctcagcttcggatctgt
taaatctggtttttttaagaattgacatattg-tttttgtgtttaatataaagattagtct
aattactgttttaaagtgtaattttcaactattataatggtccagaaattccttgtttac
gtatcattaaaaaaatgcataagcatgaaaacggcagttagtagcggaagtacaaaagtgt
gtaaactatagaacctagttaaagttccttggcctacactaactcctcctcttaacaatt
ctactatactcgctccgacaactggaacagcatcagggacacctgtaacaatttttacag
cccagtatccgatttgatcccatggcaaagaataacctgttaccccaaaagaaacagtaa
gcactcctaaaattacacctgttacccatgtcaattctctaggtttttgaaacctcctg
ttaaataaacacggaatacatgtagaatcatcatcaagaccatcatgctcgctgaccatc
tatgaattgatctaataagccatccaaagtttacatcagtcataatgtactctactgatg
taaaagcttctgcaactgtaggtctataatagaatgtcattgcaaatccagttgcaactt

gaattagaaaagatacaaatacaataccccctaagcaataaaaaatattaacatgaggtg
gtacatacttactagaaatatcgtcagcaattgcttgaatttctaatctctcttcaaacc
agtcataaattttactcataaaatagcttcaaaaagctcttctaatata--ttttgcgtt
taagctactattaattcaatag------ttattgaacttatatt-tttttataatcaata
cttattaactattataacatctttttattgattt-------------------aaaatt
ctctgacttagtaaaacagggctatgtactataattcttttttttgtatagagattaat
ttcgtttttagtaaattagctaaaattcttgttacactgactctattactacctataatc
tgtgctaaaattttatgtgtaatagtaaagttaagcataataccattattaacttgagtt
ccattttgctcagctaataataataataagctcgccagtctactggttatacttttatgt
gaaatgatttctataaaatgataagcttttgccgagcaaaatactaaatgatttacaaaa
aataaattgaatggagcgcagttactacatgcttttataatagtagtatagttaatacag
gcaacttctgttttatctattgcttctgcttcgtagtaaaaattatcgtcgaccaattct
atttgtccaaatgtatctcctgtagttaataagttaagagttactttatgtgtatttctc
aggacttttgtaataattaaagatcctatcaatataatgtataatctagagctgctatta
aacaaaagagtatcatttttcttaagtgaaaaaatttgataattaattttattacgatta
aaaaatagtagccatggattgcaagaaaaaaaatcacctgaactattttcagagaatatt
ggaatacaattttcttgatttaaacaatttttagtcataattactactttataa------
---aaaaatattctaataat-taagaactgaaattaaatgtcatataaattgatgttagt
tgagaatgacattgtattatcaaaggccattcaagaatacttaatagatcaaggcttcaa
tgtttatattgccaataatggattagaagcactgaatctagcttatcaatataatttcga
cttgataatttctgatataatgatgccgctagttaatggctatgaactattagcaaaact
taaaaaaaacaaagcattatccaaaatacctgttatctttttgacagctaaaggaatgac
taaagacagaataaaaggctacgacatggggtgctatggttatttatctaaaccatttga
tcctaatgagttgctttcaattattaacaacttaatcgctagagatgtcttaaaagaagc
ttctttacaaaactcggcaacatcaaaccagcaattaaatcataaaatacgtttgacacc
tagagaaaaaagtattttagatcttgttgttgatggacttactaataaagaaatatctac
tatactgaatactagtgttagaaatgtagaaaaatatgttagtcgactcttacacaaaac
taatatgaagaataggactttgctagtcaagtattctataaataataatctattaaataa
tgagatcaatgaaagggcgaatgacgggactcgaacccgcgaatgatggagccacaaccc
attgccttaaccccttggccacactcgccatattacatacaccactatagcttgtttttt
gatatatcgtctagttttcgagttatatatttatctaaaacaatatttactttgaatttt
-tatgatacatagcaatattagtattcaaattaatggggaaccatttaattgctcaaagc
ccatctcattgcaatttttattaaattatcttgattttaattccgagcgtgtagcagtcg
agctaaatgacattcttttaccagagactctgtttcactcaacttatttgaatgatcaag
ataagctagaggttattaccattgtaggtggtggataatctatttgtacatac-------
-acagttttctatctcaaatgtttcatagatagagataatcttccttgtttttttatctac

atgaataataacagctttaatagtgtcacctattttaaattgtgatgatatctgctctaa
ttctttgatattaatctcagaaatatggacaagacctttgagatttcctacctttataaa
taatccgtaaggtgtaatctgatttattatgccttcaataatattaccaacaattaaatt
agatgatgcttgagatattagagctcttctatgactcagtattaaattattagatttctc
ttcgacattgagtaatttagcttaataaatttattattaaattgctcactttttttgaaa
attaccgagatgagaattaggtacaaatccagatataccttcaaggttaattatcattcc
tcccttattgaatccttttatcattacattaagtaaagagtcttcagctaatagttgcct
aattcttttccatgctcttatatattcaaggcgacgaattgataatattaattgtttcga
ctgaatattataatctaataaaaaaaattctcttgtgtcattaatgtttaaagaagtgaa
attatttaaatcttgattactagatacttcttgaataggcaagtatgcagatattggtgt
tcctatatctactaataccccatttaattcaaagctaaatatagttccagctacaatatc
tccaagattcaaatcatacttgtattttgtaaaacagctgcaaaatttctatgagtaaa
tccttcattatttttgtcatattgaatagtatgaattaaagagtaactttgtacaatat
attaaggtaaataaatgtttgtcgaattgtatgaaaaaattttaatttgttcggttgtaa
ttaagtatataaacatattaaaatatctaggagacatgaaaagtagacgtagatagctgc
agatttaa-tactatataatctgaggaaagtccgggctccacaaatacaatttatgctgg
agaaatcccagtgtaggtaactgcgaggatagtaccacagaaaaaaaccgccagaataag
t----tatattagctggtaagggtgcaaaggcaagttaaaagcttaccaaaagtactgca
aagtatttgttaggtaaacccctaaaatggagcaaagctactaaacaaatatttgtgtat
attttatctagtttagttgaaaatactgcatgaagttattagtaacaataactcgagagg
aatagctatcctttacaatatatc-tcgattatattgaaagaacagaacccggcttatgt
agtacttttatgtcttgtattattatgtctattgtcgaatctttaaatttaactgtttg
acaatttttttttgtagctgatgttgtttataatctatttctgaattggttaaagttttt
tcattagatcgatatgtaaatcttagacctatactttttttctcttttccaattgattca
tctatatattgatcaaataatgttgtactttctaaatctttatcataaaattgatctaat
aattttaataagtatttaatttccatattcttaggtattattaaagagagatctcttatg
atcgatggatacttagagtatggttgaatctgataacttaaataattgaattcattatga
caatcttctaaaatgtttaagttaacttcaagaaaaaaaagttttgtattgagtcctaat
tcactatatgttgcttgattaagttcactgaatatgccaataataatattgttatagatt
agtgtggcaaattttccttttgaatgaactttattctaccatcgactatatcttttttt
gtccactcaatttgtctattaatcttttgaaaaaaattttctacgatccctttagcttca
taccaatttaaagaatgcgcagggtgtgaccatgtagatcggatatctagattaccaccc
aatatgatagctagattagtagtttctacaattttgttttgatcattatgaaatactgtt
cctatctcaaagctatctatagtttggttgctttgtttaatattataaagatttgattca
attaatccttccagcaaactgccgcgtaaattagaataatcttgaataagaggattgttt
aaagctatatttcctttggactttattaaagaataatgaactaattcagttaatcctaga

tttcttaaaatacttctacttttgtctatgaattttctctttgtggataaccttttgttg
aattgaatatttggtactgagctcctgaatttatgataaccataaattctagcaatttct
tcaataacatctatttctcgaaaaacatcttctcgtctataatttggaacagtaacttct
atattttccttattttgataaataagatcaaaatttaaagagtgaagaatattttgaatt
tcttcaaagaataaaaaacgtgtttgaccattatacataattggtcctaaaatgtcatga
actttttaattgataaattaatattaagagcatgatcagttattttttctcgacagaaa
gtatctctaatatttccgcatgttaaatctgtgattaaagcaagagcttcaaaatgtgca
tttttccaattatctacatttagcccacgttcttgtctaattgaactttctgttcgaata
ttaagaactcgagaagactttctaactacagattgtttaaatatagcagactctacaagt
atcgattttgtattataatctgtatcaaaatcagagttgcttccaataccagctatacta
gttacatttgtatttacttgagtaactaaaatatttttgtttaactcaatattttcattg
tttaacttaatctgatcattacttccaagaggaaaattgcttcgaattgttagactattt
ttatgattcatattattaattttattcaaatcaataatattaataggctgtccccatttc
aacataatataattgcttatatctgttaacaaatttctgtgtgtaaacccagaagagagc
aaccgatttttttagccaatctggagaatctttaactcttatttcgtctataatggcagca
aaataattgtcacaatttaataaatcgtgattactgattatagt-ctccttttagagaa
atatctattattgggttgatgtatagctgataacattgaggaccctgttaaagccgagac
ttctcttgatagaccaatcatgcttaacacgtctgacctgttagctgttgatgatatatc
taatatatgatcaatctcacctccaattataatagattcaatagcttcaacttcaaaacc
tgcttgtgttagttgattagttaaattgtctatatctattgttttgatattaccaagttc
tttcaaccaatttaaagaaactttcataattaattgtcatctatcttatagaagtattta
ttttacttatgattaaaaatcaatttaaaaaattcatcaaattaatataagttatttgat
gaatttgcttatacaatatattattgtgatatcttacttttatatggcagttattaagaa
ctagcttttgtaaaagttagcccattatcttgggcatatctttccataaatctcatgaat
ctatcccaatcatcaggacttttaataacatgtacagcttctattgcttgtggtttacca
ttaataaacttggcattaacatctcttgtaataatttgtccctctgtgtccatcaggtac
atacctgtgatttcacctttgtctgccatactagcatctaaaattttgggatttgtaaac
cgaaaagttgctgttcctgtactaccatctcgagaccttgttaaacgtacatctggtaca
acttcttcattgataccttgaataaattgaattgttgccatatcaatcttctttatttgt
atataatttatattggataaaactgatttctagttcttgaatgcttgttgagaaactgtt
ttaaaagcttatagtatcagtagatcaaattttatgtattatataaaactgtaacttttc
-ttttttg---

----------------------atctgttcaaagaactaacttattataagctaatcata
cgaatattagccatataatgttcgtaagt-gaaattaaggggttgtagctcagaaggata
gagcaagcgcctcctaagcgctaggtcagcggttcaagtccgctcaaccctgttaacgaa
ttaagtagtattatttaacttgttctgcaaatcagtattgatctaagttaaagtacttga
cagctaaaacaaaagaatctgagaaatattttcatttaaagaacgaacaattatcttaac
tctaaatatctgataattagtaa-aaaattct----------------------------
----ctttttaaaacacaagatgatg-----aagatatttaaactaataattgaaattaa
gtttttgaagttatagaagccaat--ataatactttctattaacaga-----------tg
cttgcttctattactatctgtttacaaga--aatt----atgagtatcaaaata------
--attttagctcca-------------------------------------ctgac----

-----------------------------tactaataaata-------------------
------------------tttattttagtctagacaag------atgaatcttcatggta
taacag--

--------------agctattctttgtgtcatacttatataaaattaggat-------ac
tgtt--

---------------ttcattaccataactggggtgggaggattcgaacctgcgaatggc
ggagtcaaagtccgctgccttaccacttggctacaccccaacatgtgaacattataatag
cagtcgtataccctgccatgtcaatagactattgtctttatttaataatttttgctgctc
ttgtagaataaaatcttttttttcaaataagatacgctatgtttaagttgcacgactgtt
atgttttcttgcttttgactgaacagccacttgatagtaattattccttctgcaatttcg
ttacttcctataataatacaagctacagctctttttttatttgcttgcttaatctgctta
ctaaaattacttgagctaacatcgatttctattttaaaaaactgctgatgtaaaaaacgc
attatttgcataccagtttcgttagcttttgtaccttgtgtagcaatataaaaatcgata
gatttattaggcaagtctatattatcttttgcaatcagtaataggcgttctaatcctatt
gcacatccaacagctggtgttgctttgccacctaattggtgaactaagctgtcatatctt
ccaccaccacatatagtatcttgcccttttgatgtcaatgtcttaatttcgaaagcagta

tcattataataatctaatcctcgaactaacttattatttatattgtagggaatattaagt
aattttagataattgcaaacgtcaccaaagtgttgttgtgattccaaacttaaaaaatca
gaaatttttggagcctctgtcagtatttttgtgtattagaatcttttgaatctaaaatt
ctaattggattactagttaatctttttgtgagtcagtgtctaaatcatcatggtacttt
gtaagataatctcgtaatttaacttgataaatactacgatcctctacttttccaattgaa
ttcagatcaagtttcaagttgtgcagattgagattgttaaatatgctcatagctaaatgt
ataacttcactatctgctcttgcatctaagctaccaataaactcaatgccaagttggtga
aattgtcgttgtcgtccactttgtggtctttcgtacctgaacattggaccgctataccat
aatctttgtaaactatggtggtaactcattttattttcaataaaagatctcacaataccg
gcagtaccttctggccttaaggtaatatctctattgctgcgatcatgaaaccgatacatt
tctttatttacaatatcagtatcttcaccaatacccctatcatataattcactattttca
aaaataggtgttctaatttcttggtaatttgcacattctaataatttggaaattttatta
tgtataaactgccaatattgaatttcatcgggaagaatatcttttgtccctctaatagcc
tgaatttttgccatgataatttttgattaccttcttaatatgtctgcattaaatagcag
ttgtagactgttattcagtcatgtattatacattag--aacatactgatg-taatttata
aagtacgggcaaggagggattcgaacccccgacaccatggttcgtagccatgtgctctaa
tccactgagctacaagcccacttaataagtaccttaactatatcattgttcactaatgta
tacaactatattctttt------atcttacatgtgaaatttttgtaaaagtaaaaaatt
at----gtttgtttttacaattggaactattcatatataatgcttaatttattataact
ttgtaaa--aatatctataagtttaattgtatgattctattatattggtaatcttgttat
aattttgcgctcttatctttcaattgg------ctagcatctaattaacttatatttcaa
ataaaaaaatcttatctaaaatgagtaaacaaattctatatcaggatgatgccagaaaag
cattagaaaaaggcatggatattttaacagaagctgtttctgtgactttaggaccaaaag
gaagaaatgttgtcttagaaaaaaaattcggtgcccctcaaattattaatgatggtgtaa
cgattgcaaaggaaattagtttagaaaatcatattgaaaataccggagtcgcactgatta
gacaagcagcatctaaaacaaatgatgtagctggtgatggtacaacaacagctacagtac
tggcttcagcaattgttaagcaaggaatgagaaatgttgcagcaggttcgaatccgatgg
ctattaaaaaaggtattgaaaaagcaacaaattttgttgttagtaaaatagctgagtatg
ctaagccagtagaagatacaaaagctattatacaagttgcttctttgtcatcaggaaatg
atatagaggtaggtaaaatgatagcgaatgctatagaaaaagttggcagagaaggagtta
tttctttagaagaagggaaatcaactaatacgattcttgagatcacagaaggaatgcagt
ttgaaaaaggctttatttctccttatttcgttacagatacagaacgaatggaagttcttc
aagaaaatcctttttatttatttacagacaaaaaaattactttggtgcagcaagaacttg
tgccattgcttgagcaaattgcaaaaacatctcggcctttattaataatagctgaagaca
tcgagaaagaagcattagccacaattgtagttaataaattaagagggattttgaatgttg
tagcggttagagctcctggattcggtgatagaagaaaatctttacttgaagatatgagta

tcttaacgaacggacaagtaattactgaagatgcaggtttatcacttgatacagttcaat
tagatatgttaggaaaagcccgaagagttattgttactaaagactcgacaacaataattg
cagatggtcatgaaattaaagttaaatcaagatgtgagcaaattaagcggcaaatagaga
cgagtgactctttatatgaaagagaaaaattgcaagaacgattagcaaagctttctggag
gagttgccgtcattaaagttggtgcagctacagaaacagagatgaaagataaaaaactaa
gactagaagatgcaattaatgcaacaaaagcagcaattgaagaaggtattgtaccaggag
gaggagctactaatgtccatatctctagtgaattatttacatgggctaaaaacaatttag
ttgaagatgaattaattggtgctttaatagttgaacgagctgtgacctatccgttaagac
gaatagcttttaatgcaggtgataatggagcagtaatagtggaaaaagttaaaagtcacg
attttcatataggctatgatgcagcaaacggtaatattgtaaatatgtacgatagaggta
ttatagatccagcgaaagtagctagatcagctttgcaaaacgcagcttctattgcagcaa
tggttttaactactgaatgtattgtagttgataaagtcgatgattgagctata------a
gataatagataatctcaaacaaagaataatttctaattatagaaattattctttgtttca
attaatctaattaattgatcaaatgtataatttttataataaagccaaagtttaaacacg
tcttctttttcaactattaagtagagaagatataagcccgtaatacctaaattatcaatt
aaaatattattctcatatatagattttctaaagatctaaaaaatgacattttgaaaaa
taataccgaaatcttctcttatagttatttgctaaatcattactgtctaaagattgattt
ccaattgaattcagaacctttaacagaatactttttaaatctttatctgactttatttga
gaatcaataataatttgattcaattgtatgtcaaaactaagattaaaaatattacttgtt
aaagatatttgattttgaatatctagagattttatagccaagaagattaaacttttattg
ttcattgtaaaatataagacaattgatgatgtattctttaaatacta-aatttatcttct
tgtgcttctatcgtttttgt-aaaataattaatgttttaatttttagttttagttact
acccgaaaaaataaattctggaaatctagcttgagctttagctagagagtgtttttccc
ttcttcttgccagtaattgataacttctgttgttttgtttaataactcaattctgtctct
ttcagcaatccacggcttggattctagttctgcttttaattcttcccaagcatcattcct
tggccagaaaaaataagaagtaagtggactagtgccatttccaacaatctgatcaatagc
aatagcaatattattttctaaccaaagtacttttagtgtaaattttgacaagtttttatc
tccattattaatattaagtattgg----tacagtagaatactgtactagttattttttc
agcaacaatttgagctttgtttaaaatgtttttaccgttttagtagcttgtgcaccttg
agataatcgttgcaaaatttttgaaatgttaacacgagtttcattatttataggattata
aaatcctaattcttctatagctttaccatcgcgtttatttctgctatccattacaacaat
tctatagcttggttgtttttttcttccgtatcgctttaaccttaacttgaccatattagt
cacctcgcttatttttt--atatctttattaaattatatcttacgacattatgcaataat
tcaaacaattaagctgattcaagtctaacattattaaatattaccattaaacattgtaga
aaaataatacctaacataggtgacatgtccattccaaacatgggaggaatgcttcctctg
aatagttttaagtatggatcagtaattctatttaatgaacagaatggttcattgtaccaa

tttacagttggaaaccatgccaatgataatttaagtaaaattaaaattaaataaatctca
gaaaaattagctatcgatccaagcaataaatttaatgtaccaggaagagtgttcatacgt
attttataattattatatacttcgttatactaatagtttgattatatattataatcgatg
taaataaat-cagctaaacaaaaaacttatccttaaaatcatcttctcttgagcactttt
ctttatctacattagtatttacttttgtcgtataaatttccagtttagaccaaatctgtc
ctaactgatttaattgagatgttccacattctaaacaggctatttttatattgctcaaaa
cagcacctttttttataaaatcgttcaataatggcgtaatagttgcttcatccaaaaata
aatctaagacaagtatttttgtaaatgaatttaattgatctgggacattgttaataatat
cattataattaactaaaactatattagctgtaggaagtaaagatcgtgctccttccgcta
agataaagccataaggcataataattacaataatatatttataatgactgctaataagac
ttatagtggtattagcttttaattcgattgtttctgttactagccactctctcattattt
cgtaagtaatccatttgcctagttctgagcaagcagttcttaaaatcgtacctggattat
tgtcgtttttgagaatccctgaccaatgttgtattaatggatgggatgctatattaattt
ggagttgcatgttttgttaaatatatttaacatttaatttgttgattgaatcttcaattc
ctatttgttgagtattattaaaatagcat----atatattaaagaaaataattat-aaaa
aatgaaaaaaagtctttggctgtggggttttacagatagtgctgaaacttggaatggtag
atttgcaatgattggttttatatcagttatttttatcgaagtagttacaggacaaggatt
attatatttaataggtatgatgtcataataaaaatccaaaaaagttcttgtaattttta
taagaactttttgg-atttttataaatctaatctagcggtctcatagatagaacaggtt
cattctctctattgataccttttcaaatccagcagcagcagctcttgctctgcctgcat
gccaaagatgtccaataaataggaagaatcctaggaagaaatgagatgttgttaaccaag
atctaggagatacatagttaactgaattaatttctgtagctacaccaccaacggaattca
aagaacctagtggtgcgtgagtcatatattctgcggcacgcctttcctgccacggttgaa
tatcgttcttgatttatttaaatcaagaccatttggtcctctaagaggctcaacccaag
gagctcgcaagtcccaaaatctcatggtttcaccaccaaaaataatttctccactaggag
atctcattaggtatttacctaatccagtaggtccttgagaagatgcaacattcgcaccta
gtctttgatctctaactaagaaagtaaaagcttgagcttgtgaagcttcaggaccagttg
gtccatagaattcactaggataagctgtgttgttataccaaacaaaattagaagccgtaa
gccccatgattgataaagcacctagactataagataagtaagcctcaccagaccatacaa
aagctcttctagcccaagcaaaaggttttgttagtatatgccagattccgccagcaatac
aaataatgccaatccagacatgaccacctacaagatcttccatgttatttacacttacga
tccatccatcaccgccaaacggagactttaagacataaccaaagataactaggggattaa
gtgtaggattgctaacaaatctaacatctccaccacctggagcccaagtgtcgtataccc
caccaataaataaagctttaataactaacagaaaagcgccaatgcctaataacactaagt
gtataccaagtattgttgtcattttgtttttatcccgccaatcgtatccgaagaaaggaa
atgattcttcaagagtatcagggccaatcagagaatgataaagacctccaaagccaagaa

cagctgaagaaattaaatgcactacgcctactacaaagtatgggtatgtattgaaaatct
cgccgcctggaccaacaccccagcctagtgtagctaagtgcggaattagaataaaccctt
gttcgtacaaaggcttctcgggaacaaaatgtgctacctcaaaaagagtcatggcaccag
tccaaaagaccattatacctgcatgagcaacatgagcaccaagcaatttgccagaaacgt
taattaagcgtgcattgccagaccaccaggcaaacccggtagactcaatgtctctaccgc
caacaccaacagttgtattaaagggcgtttccacgtggtaaaacctcctcagggaatata
aagttttcatgaggttgatcttgagcagccatccaagagcgaataccttcgttcaatagg
atatttttagtataaaaagtttcaaactcaggatcttccgcagctctcaactcttgagaa
acaaaatcgtaagctcttaaatttagagctagtccaacaattccaaatgcgcttgtccat
aatccagttactggtacaaataacatgaaaaaatgtagccaacgtttattagaaaaagca
acaccaaaaatctgtgaccagaatctattagctgttaccatcgaataagtttcttcagat
tgtgttggagtaaatgcacgaaaagtatctgcagcatcaccatcttcgaataaggtattc
tgtacagttgcaccgtgaatagcacatagtaaagcaccacctaagattcctgcaacgccc
atcatatggaacggattcagggtccagttatgaaatccttgcaaaaatagcaaaaatcta
aaaattgcagcaactccaaggcttggggcaaagaaccagctcgcttggcctagagggtac
attagaaagacggaaacgaatactgcaattggtccagaaaatgcaatagcgttgtatggt
ctaagaccaactagcctagcaatttcaaattgtcttaaacagaatccaattagtccaaat
gatccatgtaaagctataaatgcccacagaccaccaatttggcaccagcgagtaaaatct
ccttgagcttcaggtccccaaaggaaaagaagtgaatgtcccatgctgtttgctggggta
gaaacagccgcagttaaaaaattgcatccttctagatatgaactggctagcccatgagta
taccaagaagtaacaaaagtagtcccagttagccagccacctacagcaaggtaagcgcaa
ggaaacagaagtagtccagaccaacctacgaatacaaatcggtctctttttagccaatcg
tctacaagatcaaaaccaccacgagtttttttcttgtccaattgctatggtcataattta
atctccagagcaaatttaataagtaatttgtttaaataaaca---ctttagatatactct
aaaattttactttctttacggtcaatataattataagtcaaaagtaaccaaaattcacg
cttttaataaca-ttttttagctcattaagttaa-ttttttaatatataaacagtaata
gagttattgttt-aacttacaatactcacaatttattcatttctataatcctttgaaaaa
gatatcctgttcctcgtgctgttaaaatcaagtcgggattacttggatcatcttctagtt
tagctctcaatcgtgaaatatgaacatcaacaactctcgtgtcaacatgccgttcgggag
tatagccccaaacttcttgcaagatagaagctctagaaaaaggttctccagccttactaa
ctaaaagctcgagaagactgaattccatacctgttaatcgaactctttcattatttttat
aaacttgtcttttgttagtatctatttttaaaaatccaatactaataatcccagagtttg
ggactccaagactagtggtgattttatcagctcttcgaagaacagaacgaatgcgagctt
ctagctccttaggagaaaatggtttaacgacatagtcatcagcacctatttctaatccgg
taattctatcgcagacttcacctaacgctgttagcattataataggaacatctgattctt
ttcgaagttcttgacaaacaccgtaaccatctagtttaggcatcataacatctaaaacaa

caaggctaggatactcttttctaaacacgagtaaagcttcttctccatttgaagcagtga
taacttcataacctattattgttagtcttgtttctaaaattcttcttatgctggcctcat
cgtcaacaacgagaatcttttctttctggttctccaa-tttcttatttactcctgtaact
caccgatcatgattaccataaccttaaattttagcgctatgagtatctttattcacttt
agaa-----tttagttattataaattttgcaagtctagtttacattaattagtaccagt-
-caatatttttgt-----------tttaagttatgac---------tattttaatccata
agttgactctagtttgcagaattcaatatatacata-ttttctacccaagtaaattgtat
tctattttctaaataaaaaaggggttgacaaaccagttgatagaaagtattctacattac
tataag----ttaattccaacaaagaagttttaa-----atcttctttcggagtaatgaa
tactttaat
>Pyr_2_3_4_7_8_9
tatatgata---aattaagacatctgttatgtgagaattctaaaatatacaaaactttt
tatttaatatagaaaaagtaaattaaaataagttgaacatactttgctctaatctaaacc
tcttaattgag------attctaattagagacaaaagtatttacttattttgtatctata
ggaataaaaatgaaacttgcagtttatggtaaaggtggtataggtaaatctacaactagt
tgcaatatttctgtagcactttcaaaaagaggtaaaaaagttctacaaattggctgcgac
cctaaacatgatagtacatttacgctaacaggtttttaatcccaacaattatcgatact
cttcaatccaaggattaccactatgaggacgtttggcctgaagatgtaatctataaggga
tatggcggtgtagactgtgttgaagctggtggaccgccggctggcgctggttgcggaggc
tacgttgtaggtgaaacagttaaacttttaaaagaattaaatgcttttgatgaatacgat
attatcttatttgatgttttaggtgatgttgtatgtggaggttttgcagctccattaaat
tatgcagactactgcttaatcattacagacaatggatttgatgctttatttgcagctaat
agaatagcagcttcagtacgagaaaaagctagaacgcactctctgagattagctggactt
gttggtaatagaacagataaaagagatctaattgataaatatatagattgtgttccaatg
ccagtattagaagtcttgcccttgattgaagatattagagtgtccagagtaaaaggtaaa
actttatttgaaatggcagaaattgataaggatttagcatatgtatgcgattactatttg
aatattgcagatcagttgattacaaggccagaaggtgtagttcctaaagaatctccggat
agagaattatttagtcttttatctgatttttacttaaatcctaaatcaaaggtaggacaa
gaaaaagtagatcaagaagaattagatttgatgatagtgtaaataatattttcagcataa
tataacaaggaataagataatgtctacagctcaatcagatgctcttactttgaatgtga
aacaggtaattatcatactttttgtcccatcagttgcgtttcttggttatatcaaaaaat
tgaagacagtttttttcttagttatagggactaaaacttgtggttatttcttgcaaaatgc
aatgggagtaatgatttttgctgaaccgagatatgctatggcagaattagaggaaggaga
tatttcagctaagctaaatgattatggtgaactccgtagattatgcttacaaataaaaaa
agatagaaatcctagtgttatattttggattggaacatgtacaaccgaaattataaaaat
ggatctggaaggaattgctccaaaattggaagcagaaattcgtgttcctatagtagttgc

tagagctaatggtttagattatgcctttacccaaggtgaagatacagttctagcagctat
ggctcaaaggtgtccattaaatttaaagaatcaatcagataacacgagtcttaaaacttc
tcctcatattcctttagttttatttggatctcttccggatccagttgtaacccagttaac
tatggaattaaaaaaacaaggtattttgtttctggttggttgccatcaaagaggtatac
tgaattaccggttataaaagaaggatattatgttgcgggagttaatccatttcttagtcg
cacagctactacactaatgagacgccgaaaaacaaaactaattggtgcaccatttccaat
aggtccagatggtactcgagcctggattgaaaaaatctgttcagtaatgaatgtaaagcc
tattggattagaagatagagaaaaagcaatttgggcttctttagaagactatatttcttt
aatccgaggtaaatcagtttttttatgggtgataatttactagaagtatctcttgcaag
atttttaactagatgtggaatgactgtatatgaaattggtattccttatatggataaacg
ctatcaagcagcagagctagctttattaaaagctacatgtgataaaatgaacgttatgat
gccaacaattgtagaaaaaccagataattataatcaagtagaccgaatccgtgatctgaa
acccgatttagttatcactggtatggctcatgcaaatcctttagaagccagaggtattaa
tacaaaatggtcagtagaattcacatttgcacaaattcatggctttaccaatgcaagaga
tattcttgaactcgtcacaagaccattacggcgaaatcttagtttgtcagaattaggctg
ggatgtttatagcaagcaaagctagtattcctc----tttatgacttacatggtataaaa
gccttgtaagtct--tttattttatgttaaagaagcatttcttgctctgccatcaccagc
ccaagcttgtaaactttctacttgttctttgtctgtaaatgctaaaggcacaaattgttt
gatagctattttaatatcttcagtactaaattctctctcttcactaaacgccgtatgcat
actttcaataattgcttgctcaatttcagcgccagaaaatttattacataataaactcaa
ttcatgaatatcatatttttgccacgatcttggtctaacttttgataaatgtatcttaaa
aattgattctctctcttgacgattaggtaaatctaagaaaaaaatttcatcgaacctgcc
tttccttaacatttcggaaggtaaactttgaattttattagctgttgcgacaacaaatac
tggagctttttctctgatagccaggtaataaatgtaccaaatactctggcactagtacc
actatcaccttgactatgtaaaccagaaaaagctttatctatttcatcaatccataatat
gcaaggtgataatccttcagagatatttatcatttctctcattttttgattccgattcacc
aactagtccaccaaacagttttcccatgtcaagacgtagcagaggcagcatccaatcgct
tgcaatagctttcgcggttaaggatttacccgtcccctgtataccaactagcaataaacc
tttgggcgaaggaataccgtaatttaaactttgcttcgaaaatgagcgagaccttttttg
tagccattgttttaatacatctaagccaccaatatccctatttactttactataaggata
aaattctaatagatgcgtttgattaataatttgcctttttttcttcaataataataggtaa
gctccgagagtctatctgattatattgtgcaataattttagtaatcactttgcggattct
atctatcgataatccttggcaggattttgttatattattaacgagctctgaattcaaatt
aagattcaaagctttactcaatcttatgatctctttttttgatttctagaagactaggtaa
gggcaaatctattactgttatgatatcattcagagcaaaaggtatattgattttgcaaga
aataataataatgtgcttagattgcgttttgattattttagctaaattgcgaagtttcct

aattaatactatttcgtttaggaaagagtcaaaatcttttaaaagaaaaagatttaaaga
ttcattattcaagttttcaataaattctaatgctaataaaggatttctttttgcataacc
attatcgctaggattgttagtgtatccatctacaaagtcccaggaatatacttgctgact
gtttgaacagtttaagctgtgttttataatatactctagtctatcttcttctctagtatt
tattacaataataggatatcgagattttaaaagtaatcgtaaatcttgagtgaaattcat
gtcaatatttgtttccatcagacttttgtacttcaccaattgaagaaagactaaataaga
ttatctaatattccttggatatattcaaaagtc------atctctctattttctaaatta
ttgctattactaatttaacttaccatgatttttttcttcctttcttcgtcgactattt
aatatagagcgtccacatggtgttttcattcttgctctgaaaccagaaactctaatttttt
tttcttttagatccttgtagtgttctttagtcatgatgtctcctctgttgtaaaagatt
aaatactattttatcatcttttatatatctttatgatatagatatgtattaatatcaaag
tt-aacagcaataatttatattaatcaagaactatttatataatattaaacattaaatat
----------taactttatgatattggctttgcccatttttatttaagtattttgacca
tctttttgctaatcttaaactggttaatttttcagcagctaaaaacgatttgttattag
aatctcaatttaagtattttgttgataaaagccaaaatcgtaaattagaacccgacgaaa
gttttgcttttgcaaaagtatgtgtagctaaaaaatatttttctaaagctattattgaag
gtcaattagctttaaaaaactatcgagacctgaatatcttagataataatatagtaattg
ctaatttgtataatatgctaggttttatttattttgaagcaggccaaacaagttttgcaa
agaacttttatgagcaagccttacaaataaaccctaattatgttgtagctttaaataacc
tggcaaaaatttatgaagaggtaaaagatttaaaaaaagccgaatctctatacgataaag
tgttaactcttaacttaaataataaaactgctaatagaaggaaagatttatagcaaaaa
cta---------aaaacatctaatactatttgtaatcgggatagcaggatttgaacctgc
gacatcctgctcccaaagcaggcgcgctaccaaactgcgctatatcccgtaagacataat
tattactatataccttttctctaattatgtctactctttctgtattttatctaggatacc
aaaacatgcctttaactccatctggatctttgataaatcctaatttttttataaaaactaa
caacatcaggttctgcgaatagtgtaatagtgctaatttctgcctgtctcaattgctgta
ttaattggtgtatgactacttttcctaaacctaaaccttggaaatctggatgaataacta
cgtcccagatagttgcattaaatccgttgtctgatgttgctctagcaaatcctacaagtt
tactatttgcatcttttttttgtattaaagaaataataatagaactatttttttaatgcga
tctttacttttttagaggtcttttaacccatccgactgaatcacaaagctgctccaatt
catataaattaatattcttattgctactcagatagatatctttaaattcgattttatcgc
aagttttatctaaaacaataagtttttttgaaatttttttcagagctagcattgatattgg
agttttgaaaaaaatttttccagaagatcataatgattatgatataatta--aataaata
tatatattacaatattgctttaacaaacaatatattaatatatgctcaaatttttacatg
aatctattaagatagaagattgttaaaatatagagaaataaaaatttttgtaaaatcttt
caataaaatgatataaagagtgctttttaagactaacatctattattataaataaagtct

cgaaattgagatgaaataacacattattctatgtctaatgccttttaaaggagttatttg
tgaaaaaatctatgtgtctaacctgtttacttgctttactaattatgagcaacccaataa
tagctaacgcagaagtagctggattggtgccctgcaaagattctgcggcattcaataagc
gtatggtaaatagtgtgaaaaaacttcaagccagattagctaaatatgatgcagacacgc
caccagcgttagctttaaataagcaaatagagaaaactaaaactagatttgcaacttatg
gtcgagcaggtttgctgtgtggtactgatggattgccacatttaatttctgatggtcgat
ggagtagagccggggactttgtttttccaggactattattcttgtatattacaggatgga
ttggctgggtaggtagaggatatcttttatctgttgctaagactagtaagccaacagaaa
aggaaatcattttagatgtgccattagctatcaaatttatgtcatccggatttgcatggc
cgctagcggcttggcaagaatttagtagtggacaattaattgcttccaatgacgatatta
ctgtttcaccccgttagtaaaaaatttatatgaataataattttaccaaatacttatcaa
cagcacctgtaattggtgtattgtggatgacgtttacagccggatttattatagaattga
accgctttttcccagatgtcttatacttttactt----ataaataa--gatcgtataaa-
--aaactacataaatatagagagatactattatttgtgtagtttttta---atatataaca
taattattta-tttttaaaaatgtttatcttttcgattattttaatattagatcttacat
aataaaata-atatcaatattatgagcttagtaacccaaattatagttaatgccgatgat
gaattaagatatcctacaattggagaattgcagtcaattcaagactacttaactacagga
agcaatagaattagaattgctactattattagagataaagaaaaggagattatacagaaa
gctagtaagcaaatttttcagttacatccagaatatatagctccaggtggtaatgcagca
ggttctaggaaaagatcgttatgcttacgtgattatggttggtatctcagactaattaca
tacggagtattagctggggataaagattctatagaaacaattggtattataggagtaaga
gaaatgtacaattctttaggtgtacctataattggaatgttagatgcaatccagtgttta
aaggaagcatctttagaaatgcttggtcaagatgatattagaatcatttctccttatttt
gattatataattcgtggaatgtcataaatcatagcttcaattaattagttgaataaaagc
ttttataatgttataattacaataagcccgagagcatataattgtaaaaactaaatcttg
tcagaaccggaaggtagcagcaataatgtttcaatacaaaagttatga-ttttcgggtgt
ttattgtttttatctaatagtctcgacttttaattagtatgctatgtataaatctgtata
tgacatgtaatccatcaaaccttccagcaaaaataatttaataatttgatattaatacgt
aaaacacctatac-tcttttataaaatttctttatatctcatctaaacaatggaaactta
acttaatgggtgttcatattttatcaacaggttcgtctgtcccaaattttttctgtagaga
atcaacaatttgaagacatgatcgaaacttccgaccattggatttcaacaagaacaggaa
taaaaaaaaagacatctagctccatcttctacttctttaactaaattagcggcagaagctg
caaacaaagccttatatgcagctaacttaaaacctactgagatcagtttaattattttag
ctacgtctacgcctgatgatttatttggtagtgctagccagttgcaagcggaaataggtg
caacaacatcagtagcttttgatattacggctgcctgctccggttttattgttgctttag
taacagcagctcagttcattcaaactggttcctatgacaatattttagttgttggagcag

acacaatgtctagatggattaattggtcagatagaactacctgtattttatttggtgatg
gtgctggagcagtagtattggggcaaagcctcaaaaatagtattttaggctttaagttat
gtacagacggtcagctaaacagtcatttacaattaatgaataaacctgtaaataatcaaa
aatttggtggtacagaaattcctcatggaaactataattccataacaatgaatggcaagg
aagtgtacaagttcgctgtatttcaagttccaacagtaattagacaatgtttgaataatt
taaacatttcaatagatgaagttgattggtttatattgcatcaagcaaacactagaatca
tagaagcaattgcgagcagattatcagtaccttttctaaaatgattacgaacttagagc
attatggaaatacatctgcagcgtcaatccctttagcgttagatgaagctattcaatcca
ataaaattcaaccaggtcaaattattgttttatctggttttggagcaggcttgacttggg
gagcaattgtcttgaagtggtgat---ttatatattgcggatgacgagactcgaactcgt
aaagctttcgctacacacccctcaagcgtgcgtgtataccaatttcaccacatccgcatt
ttttaccatgaata-ttaaaaatttataatatttatctaaatattcatataa--tgtgta
tatatatatacattcttgtcagaaactctcttatattttattttaagtgataaaattgag
t----ttagtatagatagtaaaatttatttaaaatcataa----aaataaatatataa
ggaaaataaaatatgacaccatctttatcaagtttttgaatagtcttattcttggggca
gtaattgtagtcgttcccataactttggctcttttatttgttagtcaaaaagacaggaca
atccggtcctaaaaataacttagaaagatatggagaagcaatttaagatgaataatgaaa
tatttttcatactaataagctctccatatattgctatact--ttagaagaattttattt
ttttattttgtaacatcctgtaaagtttattccgttctttctttttccc-atttactctc
ttataattaatgatacaaatgtcagg-ttaataatag---ttttttattctgtatctttc
---------------attttatatacgtattattatgttcaattaataaagaaaagctca
tgtctttgtctaattggcctctcaaaaaagaaaattctgaagcatataatattaagaatt
caaaacaaatcacaattcctgatggtttatggataaaatgcttcgactgtggtctattaa
tgtattctaaagtactgaagagaaatttaaaagtttgccctcaatgtagttatcattttc
aagcttctagtaacgaaagaattgatcaattgatagaccaaggtagttggcaaccaatgg
atgttcacttgatctctacagatccattaggctttaaagaccaaaagctttatagtcaaa
ggttaaaagatactgctttcaagactggcctgcaagacgcagttcaaacaggtactggga
ctatgcaaggtaaaaaagtatgcttaggtattatggattttagattcatgggggggaagca
tgggatctgttgtaggcgaaaaactaacaagactgctagaaaaagcaactcaagaaaagt
tgcctgcaattatactttgtgcatcaggcggagctagaatgcaagaaggtatgttgagct
taatgcaaatggcaaaaatttcttctgctctagaaatgcataaaaaagaaaatctactat
atatatctgttttaacctctcccacaacagggggtgtaacagctagttttgctatgcttg
gagacttaattattgcagagccaaaagctcttattgcatttgctggtagacgagttatag
aacaaacaatcaaagaagacttgccagataattttcaaagttcagaatatttatttgaac
atggtttcctagatttaattgtatccagaactcagcttagatcaaagttaatacaaattt
tgtccttacataatcatagtaagtaatgaattgaatattgactataatatcaaaatgcta

gtaaaaattaagatattaagaaattttcactaaagatatttgtagactaactagaattac
aattactattgtagaaaaatttgtcttaatgcca-ttttttcagtcttttaacaaattg-
tttttatgactttaaataattgttagtttaatacaaatttataaagagaata-----cat
actaagctaaatatagtaatattatgttttcctataatactgacctatcaaatttcgagg
aaatttatgcttaaaagatcttcttggcttgcggctttattgggactattaacagtagtt
tctacaagtacgcatacatatgccatagagttagacgaggcaacaagaactgttccatta
gaatcttctggcagaactgtaattcttacaccagaacaagttaaaagaggcaagcgatta
tttaataattcttgtgctatttgccataatggtggtatcacgaaaacaaatccgaatatt
ggacttgacccagaatctttaggattagctacgccacaaagagataccattgaaggacta
gttgactatatgaaagatccgactagttatgatggtgcagagtcaatcgcagaattgcat
ccaagtattaaaagtgctgaaatttttcctaaaatgcgcaatctaaccgatgaagaccta
tttacaatcgcaggtcatatcttacttcaacctaaaattgtttctgaaaagtggggcgga
ggaaaaatttactattagaacttcaaagacctaaaccttgtgaatactagtttatgta-t
ttgttgcttaagtgtgattattttgttagatcatgatatatattaaagatggagtttgtt
gacagcaagttacttttttacccgaacttgtttacactattcgtctagttattaaaaagga
gacgttaaattgaagaagaagctttcagttcttttcactgtttttagttttttttgtaata
ggtttcgcacaaattgcttttgctgcagatctagataatggagaaaaagtttttttctgct
aattgtgcagcatgtcatgctggcggtaataacgccattatgccagataaaaccttaaaa
aaagatgtacttgaagctaatagtatgaatactattgatgctattacttatcaagtacaa
aatggtaaaaatgccatgcctgctttcggaggtagactggttgatgaagatattgaagat
gcagcaaattatgtattatctcaatctgaaaaaggttggtaattatacttgattttatcc
tgtattaaagaatagacaatctatttagttgttcattagattgtctattctttgtttatg
---ctattatataaagatatttacacaatatt----ttatgatgaaaagaatacccgcaa
ttcttgtactagaagacggtgcatattataaaggatggtcattccagcaagataaacaag
agattactattggtgaagtagtttttaatactggaatgacaggatatcaagaaataatca
cagatcctagttacttccatcaaattgtcgcttttacctacccggaaattgggaatacag
gtattaataatcaagatattgaatctcacagtattagtattaaaggacttattgcaaaaa
atatttgtaaaatttcaagcagctggagagagcagcaatctttagttaagtatttaagta
gtaataatattccttttattttcggaatagatacaaggtctttaacccaatacttgcgtc
aatttggtacaatgaacggttgtatctctactgataatttaaatcatagttacttaaaac
agaaaatttgtgagattccaagtatgcaaggtttagatttaatcccgcatgtaactacaa
gaaatgtttacccctgggatgaaaaaagttttccgaattggtatttgacagataacatta
gagtacaccgagttattcagttgaaagttattgttatagattttggagttaaactaaata
tactcagaagactagctacacttggatgtcagataacggttgtgcctgcccacactcctt
taaaagatattttggcttaccagcctgatggtatattactctctaatggtccaggagatc
catcagcagtacattacggcatccagacagttacaaatttactagattacaatgtgccta

tatttgggatttgtatggggcatcaaattttaaatttagctcttaaagctaaaactttca
aacttaaatttggccatagaggtattaaccatccatccggattgaaccagcaagttgaaa
taactagtcaaaatcatggctttgcagttgaattgacttcggtttttgaatctcctgtaa
gagtgactcatttaatctaaatgacactactattgcaggaactggacataatcaaagtc
cttatttttctgtgcaatatcatccagaatcgagcccaggccctcatgatgctgattatc
tattcgaaagttttatagaaataatgacaaagtccaagaataaagttag-----------
ttagtaattttcccatgctttatgagtgaataaagcggatagtacttgtactcctcgtaa
ttgattaaagagaggcaaatgtcctctggggccatgagaattccattgaaattcctcagg
atatctgcaaggaattctatcaacttcccagccgatttttgccagaattttccccaatc
ttttccaacactaagccatatttgtctttgaacaaatagcccaaatttacctttgagtg
tgtatgccataacttatcaatagtttgtaaatcttgagcaggtatttttttatatctgt
aaaatatagccaattacgagtttgtgcatttacaccagctagttgaataagcttttgttg
agttagctgatctgctttgagcaaatcccgatgagtcagtaacatttgtaaatctttgta
attcatctgttgagcagagcgcaatggaacaatgccatcgggacataagttagaagcaaa
tttcactatttcttgatttttactgttaagtaacttttcataaattaaaccgtcaacaca
attacttttatagttaggcccagtaatcctctcaaaaaatagatctgctaaatcttttaa
ctcgatagagtcgttgctatttatattctcaataatttcaagttgttgcttga---catt
attagacttagtattcttattcaattctaaaagctgggctcggatttgatttggcatctg
atttgtttaattagttttaatagtc----ataaggtcttagagagaattagattgttttt
tgaaattagaagagcgccgcagtatttctaaagaagttcgcataatttgattcaataagt
tttgtcctgcataaattataatttgaccaattaaaaaaattgtgctttttattttaggaa
caaaaaaatcttgtacctcaagcagaaaagtaattattatttgcattggtgacaacatct
gtaattcattcgatctgcaagcatatatatactgacagtttaagccttcttgataaaatc
cccatactttgtaacgactttcgtaaattgctcgtggtcgctcaagatagtttttaatta
tagtattccaaattaggttattctttaatttttctaattttctatcagatagaaatttta
cattacagagataacttgcatttgtctcatttacagtattaaaattttgaactaacatat
gagcaataatgttgcttagctgaattaaataattttcaagaaaaatttcgacctgtttaa
ctggcatattgtaagtaaagagattaaaagttttattcacagtttgagatgacccaaaaa
gtagctgaataattaaaatttgtagtagcattttatagtcgttcagtagatagttcaaat
catgaatatcttggtaaatgtcaatacagtccagttcatataacttacaaaatcttttga
tacacctatggaataaatcaattaaaatattttcggtcaagtttgtaatatcatttacat
ctaaatttgaattataaatttctaaaagtatcttttccagttcagctaatatgattttta
ataagtttctttttacttcagtgcgaaaaacatctaaaattaacacttcctgagagcaat
tagttaatctcttattaatctttatagaagttctaactaatagctctgctacctcttgat
tgagtgttggtccctgagagcttggccaataattattcacgttataccatagtaagaaat
attgaatttaatgttatacgataacttttaatatactgaaaagcaagttaattactcta

tagcaactag-------tttttacagatcttaatagttatttatcaaataaagttatatg
atagtagcgattgagctaatttttattaaaattta----taataatgacaacttattactt
cgcccttgcgagtcaaaattttctattagtacaagaaccactagaagaagtttttagaga
gagagttaactactatcagtcaaataataaagcaattgatttttggctaataccaaaccc
ttcttttctagagaaaccagaaatgatttcatttaaaaaccttgtacctaaagacgctgt
agctataatctctactaatccaatatttattaattggttaaagctaagaattggctatat
ctgtattggacaatttgaagataacctacaactttctgaagaatcgttaaatattactgt
tttaacagacaaaatttaacaatgcttttattatgactagtgttaaattattttggttaa
gttataattatagtcttttagtattta-gactcttatagtttatcacttagtattttatg
ctgagatggagtgacaaatagtgtaagtatttcttggctaaacgtttcagttgccttaga
tttatatctatttggattaactataatggatagcattcgttttatagtaacattttcaat
ttgagcccaatgtacaattccaagttccaattccttagcaatagctgaaacggaaacaaa
ggcagctcctagccctgattgcacagcgtttttgatagcttctatagaattcaattccat
ctctatttaaagcgactgctgtcaataccatgttgactgagtactttatctatcacttt
tctaattgttgattgagtatctaatgcaataaatctaagcctgtataagtcttctttttg
aatgtctcctagtttagaaaaaggatgtgatttgggtaatataagtgctaattcgtcttc
cgcataagaagtaacctgcaaaacatcttgcaattcagtaggcacttctcctccaataat
tgctaaatcaacttgaccattagctacgctccatgaaataagtctagttgaatgtacttg
caattgaacagccacttgtggatatctttgcctaaatagtccgattaatcttggcatcaa
atatgtcccagttgtctggctagctccaataattaatgtgccaccttgtaagttttgtaa
gtcgtcaagagctcgacaagtttcttcgcagagagctaaaattctgcccccgtatcgtaa
aagaagacttcctgcctcagttaaagttgccttcttattacctctttcaaatagggaaac
atttaattggcgctctaaattttgaatttgcaaactaatagctggctgagaaacatacaa
gctattagctgctttttttaaaactgccctctttggcaattgcttttaatattcttaactg
atccaatgtaaatggaaggtctgtcattaaagaattgtagtataatg---------tata
ttatttagtattgatcaatgtatgcatttgccaactaaaatatttgtcattacgcttcat
tattttcaaaataataaaaagtataatgttaaataagctgtattaaaatttagtatctgt
ttgatattaaatttatacaagagatatcgtgaatttaattctaactataaaatataggag
acaat---atggactctagacttttagttgtactaataccagttttagcagcagcatctt
gggcagtttacaatattggtagagttgcactacagcaatttagaaaaatgacatcttagt
tttgcttagaatata-atttttagactttatgagatagaggactttaaaatccctatctc
tattgtatatcttaataaaaaaag-aaagc--ccccttgtacttactgttattacacatt
aatattattggt-ggtaccta----gataataagtttaagctaacttattttttatttta
taattaattaaattaataacacaattatggctgttccaaagaaaagaacatctaaagcta
aaaaaaatgcacgcaaagcaaattggaaaaatcaagcaaaaactgaagctcaaagagctt
tgtctttagcaaaatcagtattaactagaaaatccaatggatttatttataatctaactg

aagcatcagatacttttagcgattagaagtgttt-ttactacaagtctgaaaaagtatta
cgcaacaagttctcagtaatttttatctat----taatcatttgaaaatcattctatttg
ataataaaataaatagcagattaaataaaatcacattaacaaattatgcagcaaagctag
atcaaactagtgaaatttggttgttcaattgtattgaaaatattcagcatattttttta
aaagtcaattaaaatcaagtcatattactaaaattttatttctggtactagctttgaat
ataccgcaggtctaccaggattattatccagcttaacactaagtggtagactacatccta
ttagtatatatagtccccagtctctcaaaaagtatcttgaagcatgtaccaaatattccc
aaactaatttttcttttcctattaattttcataatttacaatacggaggacaagttgtta
accaatttatacagtaatttgtttaccgttgagcaaaaagagcctgctgtatggattta
ttattctaaaaaaagaaaagcagggagtatttaatttagcacaagctaaaaccttgaata
ttcttcaaggacctatatatggaaaactcaaagaaaaggataatttttaagtccagatg
gttactatctatccggccaagacttctcttctaatacaataatgggacataaaatatcgc
ttccgttattagtcagatattctagaattatttctgagatgcattggttttgctcttatc
ctattagattaaatacttattcgcatcagcaaggagtaaagtgtttaccacataatgttt
taactgatattatgaaatcccaaatatatcaagataacagttttgttgaataatacttat
tcttgatatataatggtctcggtaatctaaataaaatactagaaataatttcttaaattt
atttgtctatattaaatttacaatcatttatgacatacgcaattattgaagcaagtggca
cgcagctttggatagaagaaggccgctactatgatttaaatcatatacctgttgatccag
gtcagtcgattatattaggaaaagtcttattattgaataaaaaatggggaggttactttag
gacgcccttgtatagaaggggttacgataaaggctacagtaatgaggcacttgcgaggaa
agaagataactgttttcaaaatgaaaccaaagaagaaaatgagattaaaaaaaggtcatc
gacaagaattgactcgtttaatgatcgattctataacatcttaga------taaaatcta
gactcatatttt--aa-aaacttttaatatttaactaaaatagatataatggcacata
aaaaaggtagtggtagtacaagaaatggcagagactctaattccaagcgtttaggtgtta
aaaaatatggtggagagcaagtaacagcaggtaatattttaatcagacaacggggaacta
aagttaagcctggccaaaatgttggaaaaggaaaagatgatacattgttttctctaattg
atggtttcgtgctgtttgaaaagtcaaatcaaaagcaaaaaacaattagtgtttattctg
ctaagaaatagttaaaacaattggtgcaagataacttgagt----gtatttatttcactt
acagtggcaccatatatttgatttattaagaaatggagttccttaatgaaccaatgaatt
ttattaaagatttgctgtgaaatttaatgacaaacactattgtaatttcctgtctacaca
atatggctgctattctatattgtggtcagatacagaaactagtagtagcaaatgctcatt
atcaagtaagtgatatctacctaggttgtgttgataaaatcttctcaggaataaatgcgg
catttattaacctaggaaagaatgagtacagtggttttatacatatcagtgataccggtc
cgcttaaaaagaaatattatgtcaataatattactaacattttaacaatacggcaaaaaa
ttttagtacaaatcattaaagagccaactttaaataaaggtccaaggctcactgccaata
ttacattatcaggtcgatatattgtattaatgccttttagtcaatcaatctgtatatctc

gaaaaatatatgatgaagatgagcgtcattatttgaaatctttagctattttaattaaac
cggcaacaatgggcttgctatttagaccttctgctgtaggtgtcgatgaggaaataatat
taagcgaattaaaaaatctaaaagaacaatggaactttgttcaaaaatctgcaattaata
gttattcacctgttcttctatataaagatgaagatattgttaaaaaagtaatccgagatt
tttataataataatacaaacaatatagtaattgattcaaacctgggattaaaacaattaa
attattatatccacacttggcactgtaataactctagcacagttcctaagattaagcttt
atagtaataatcaatgtatactagatgcttttggtatcaatcaggcaatttccagagctc
tcattccaaaagttgatcttatacttggtggctatatgtttattgaaactttagaagctt
ttactattattgacgttaattctggatcttttaataattccactagtgcacgagaaacag
ttttaaaaaccaactgttctgcagcaacagaaatagcttatcagttacaaattagaaata
ttactggtgtaattataatcgacttcattgatatggaatcacaaagagatcaattgcaat
tattagaacactttaataaagagctatcacttgatgatgctaaaccacaaattgtacagt
tatctgaattaggtttagttgaattgactagaagaagacagggcaaaagtttgtacgagt
taattagtagtgattctaattactttatttttcacacaatcagagagatctcagtctc
ttaagagattcgatgatagacagcagaaacaacagatttttaataaatcttggctatctg
cagagattaatactattaacaaggtctttttcaaaagtcaaatttgtgcagacctgcta
acttttacctaattcgtaatctctatatagttaaaagtagtattacttataaacaaaatt
atttattaactcacagatcaaaacttatttattctaaagaatacagcaaagtattaccaa
gca------gttattatctagctagtctcaataagaatagtaatcaagagttcctatctt
aagattgttatatcaactgtt---------------------------------------
-------------------gaaaaaaagaagctctctcttatataagagagagcttc-tt
ttatagcttactaacta-tctagttagttaattaaaaattaaccgttaacagctggagct
gttagagctactggtaaagattcaccagaagctagatctagagggaagttgtgagcgtta
cgttcgtgcattacttccatacctaggttagcacggttgatgatatcagcccatgtgtta
attacacgaccttggctatcaacaacagattggttaaagttaaaaccattcaagttgaat
gccattgtgcttacagataaagctgttagccagataccaactacaggccatagacctaag
aagaaatgtagagaacgagagttgttgaaactagcatattggaagattaaacgaccgaag
tagccatgagctgcaacgatgttataagtttcttcttcttgtccgaatttgtaaccatag
ttagcagattcgttttcgcttgtttcacgaattaagctagatgtaactagggatccgtgc
atagcactgaacagagaaccaccaaacacaccagcaacacctagttggtggaatgggtgc
attaaaatgttgtgctcagcttggaatacaagcatgaagttaaatgttccggagatacct
agaggcattccatcagagaaactaccttggccaattgggtatactaggaatactgctgct
gctgctgctactggagcagtaaaagcaacggaaatccatggacgcatacctaggcggtag
cttagttcccactcacgaccaatgtagcaagctacgccagttaggaaatgaagaacaact
aattggtaaggaccaccgttgtatagccattcgtctaaagaagcagcttcccagattggg
tagaagtgaataccgatagctgcagaacttggaataacagcaccagaaatgatgttgttt

ccgtatagaagggaaccagcaactggctcacgaattccatcaatgtctactggaggtgca
gctacgaatgcaatgatgaatacagatgtggcagttaatagagttggaatcattaataca
ccaaaccaaccaatgtataggcggttttcagtactagtaatccaagagcagaaacgttcc
cacaagctagcgctttcgcgtctttgtaaagtagcagtcataatttttatcaattttta
aggatttaacaaagatgcttcccaaacaataagttgtctgttatgaaaattattacttaa
ggccttttcaaatgagaacattttaaaaaaaaca-tagcaaaatctaatctcagtaact
aa-aattt-tacttgttcatatattgcttaataattcaa-----------taataaattt
tt-gct-------------ttttgcaaaattaacactattttagggat---ataataata
atgcatattaatgctgcttattttgattttaatagtttatataattttgccctacttaaa
tacgcatgtcacactttacaaaaattcagacgaccatacaagatttaaatctgttaaagc
atgctttaacagatttgggtttgatttggcaaatgaattctagtcatataaaagttggtg
aaaataatcagcataaggtagatattttaattaaacaagataacttgtcacatattggct
ttacttggaatgataatagatatcacttagttgccgacttacaactttgggaacaaccct
ggtctctagaagtatttttggataagttatctcagaagtatgcttactactctatcatcg
aagaaacaaagaagcaaggctttgaaaaaaatgcagcagatttcacaaaaagatggatcaa
ttaagttaattgtgcaacgctggaattattaactaacatacaaatgcgggcggagagact
tgaactctcacgagattatctcactagaacctaaatctagcgcgtctaccaattccacca
cgcccgcactgttgagac--aatattattctatcataatctatactaaaacacaaactaa
-tagtataaactagcttccaagcttaaaagcgtctacaaatagaccgtatgttataccaa
ttttgaagttgttgaataaattaattttatatttaattgcttgtggctagagtagacta
ttttactagtcaactcagttgctgcaactattagactggctgcaataataccccaatctc
ctgtttgtccaggaatcgtagacaaacctgttgaaataaaaaatcctagcaataaactaa
ttaagccagttgttaactcgctcaaagaataataaagcttattatttaagtttttttatta
agtaagcaaaaaaagttgaaagtctagttttaatcatagtatttagtaacggagttttaa
aaggatgacaagtattaacag-tcattaatattgtcgtatccttttaaaactcgttaata
caaacagctgttcttgt---------ttttaaaggtcttgttcgcttaaagcattaataa
gatgctgaaacggctcttgtataatatctgtgcgtttaattgattgcaaatctaattctt
cttctatgatttcttgtaataactgtatgcttcttactgttggagcaacaggtacagata
gtgaattgtaagtatcttttagcccatttaatacacgctgatcgagaatatttgtactac
cagaaaccaaggcataactagcataccgtaggtaatattctatatcccttagacaagctg
catatcttcttgttgtataagaatttcctccaggccttaataattcaggctgttcttcgt
acaactgagctgcggcctcttttaaaatattggtagcttgatcattaataatttcagcta
tttttattcgatctagtccactcgaaaagaaagattctagttgtccgacagctgtcttat
ctagataacgaccagtaagatcgtaacgatttaatattgctgttatagcatcttgcataa
attaattctcctac-ttgctatcagagcggtattataaatgactaatattgtaatataat
cattagtgtttatgaaatttatttaaaacttattctttattgcttaatgtgcaattaaaa

```
actcgcatagattttacacatatagagtatacgtagtttttaatta-tttttttataaaa
accaagaaataaagctttttt-taagaggttcgtgaatttgttaatataacatgagcttt
cctcctaaataagttttgtaaagtac--aaagtaaaaaaacgattagtcaataatagtaa
------------------------------------------------------------
------------------------------------------------------------
------------------------------------------------------------
------------------------------------------------------------
------------------------------------------------------------
------------------------------------------------------------
------------------------------------------------------------
------------------------------------------------------------
------------------------------------------------------------
------------------------------------------------------------
--------------------------acaatattaatatgtatcaa--------------
------------------------------------------------------------
------------------------------------------------------------
------------------------------------------------------------
------------------------------------------------------------
------------------------------------------------------------
------------------------------------------------------------
------------------------------------------------------------
------------------------------------------------------------
------------------------------------------------------------
------------------------------------------------------------
------------------------------------------------------------
------------------------------------------------------------
------------------------------------------------------------
------------------------------------------------------------
------------------------------------------------------------
------------------------------------------------------------
------------------------------------------------------------
------------------------------------------------------------
------------------------------------------------------------
------------------------------------------------------------
------------------------------------------------------------
------------------------------------------------------------
```

-----------------agtataaataagcttttactccagcatcaaaaaaaatatttga
gatatgttgatacaactgattattcaagtgaaaacttagatttagaccaatttagctatt
ttatcattacaattaataaaactgataaatgtattcttatagagcaattttgttataatg
ctgaaggccatatttactcaatattcttcaaaggtccaacagctaaacatctttcttcta
taatttgtcattttcaacaattaaatacaataatatctacagctcatgccatatatttag
gacgagagctaatgaagtcagaattagctctcgtgctagatcaacaatatattcaagatt
aaa--ttttgtaagtattaattatatttatgctagtataatattagtaagggcatgtaac
tcagtggatagagtatcagattccgattctgatggccgtgggttcgaatcccgccctgcc
c---gagtactcagagtact-atactaaaacatct-------taatagctagtttgataa
tagtatgggcattggggctgcaaggtttctacattatgaaaagaagaaaatatg--aaaa
aaacaagctctccaaaagagcttttagtacaatcaataaatgcagaaaacaatattgttt
ctttttctcgaaaattagcttttgcttaaacactgttaatttttggcattcgacatgtta
aactcttattcatggcgaatattctgtcagagtcgctcttagtttaagaaaagtcagaaa
aaatatttatctttacgtttt-tacctatttttatttaaataaaggttagta-ttttcaa
cttttatagtggacgtgggttcgagtcccaccagctccatcaatttgatt---------t
gtataattagctagtttaaccttttaataaccagatatataattggaggaaatatgggt
ttcataacaattactaagcccgctttaaagcaaattgcaatcttgaaaaatgatcatgaa
aatgatgtacatctaagaataggtgttagacaaggcggatgttcaggtatgtcatactca
atgaattttgaacatgttgacaaattaaaagatacagatgaacggcttcgtctagataat
ttttctgttgtctgcgaccctaaaagtcttctttatctttatggattatcattagatttt
agctcggaattaataggaggaggatttcaatttctaacccgaatgccagccaaacttgt
ggctgcggtaaatctttttcaggctgacc-ctcgaacacgtttttatatatttttgtatt
atatatattattttagcaagtcg-tttattagagacaagcaatgctctgttaaaaac---
---------------------------taatttcaagagttttattgtattatt-----
----ttatatatttattatcaactacttgaaaataaattagctaagcaatctaatttgtt
atcagtaccaaactttaaatgattaacttttattgatatatgctaatcttt-atcagcc
taaacatatctgtacattacaatcttttgacataaaaagagtcttagcatacctctcaa-
----------------------aaataaattcaa----------tattttataaatactc
aacatgaatctgtcacaacttatcgttaattcttctatgatgaaaattcctaataacttt
tattgcaatataaaagctatttcttctgagaatttaagtcacttacaattatcaaaaaca
aatttattatctcctacaaaaataattgcttttggactacctaaatttgattttatttct
acacagattacaagttctattagagaaacagttgttgtgtctactattgatgataataat
ctttttgctgccccaactaatgattggataaaaaaacacaaggataaaaatcatcattct
ttgttttataaaaaagtttttaaagctttgctaaaccataaaattagagttgttgctgag
ccact------tcataactccgaaataaataaagaaaagagcataatggcaagtcaaaga
tacatctggggtaaaagctggaagccttcaattattttatcttgcttaaaaaacaaaaaa

aatagcaaaatttctgatgcatcgaaaaaatttttaactgaacagttatctgcatcacca
gtatttgtggtaaaaaatggttttaatgaaattatttaggtcacccattatctcgtgtt
aaaagaggaagagttaataatctgatgcatgcgttttctaatttattaaatcagtctaat
gcaacttatcctatatctactggtctattctttttcatccagatgatgcatttgaattt
aaagactttataatatcggtcaatccactggcggccaaacatatggagataagcgtagag
cctgttggtctacactttgcttataaaatgaatagaaatatatcgtcagatactcaattt
tcgtttattccagattttaaagaagtaggagatttattattcaaatatagaaaaggtaat
catttagtttttcataagaatcagcattatggcaaagatttttttcaggggcaaccaata
tatatgattcagccgatcacttttaaagatcgagccggaaagctaaatattatcaaattt
acggggctaaatgacacaagagaaattattttttactaatcttgaagctgctaatagatca
tgggcacattttataaagaggaattcacaattaaaatcaattaaaaatcctactctatta
gtctataatttagaaagtttttttaaaagatcaagaacgattggataatcaagatttaaat
aagtttgtggtgatcactaataaagaatcttatcttgccacaaaagagttaatagcttta
cccgattctaatagcttttctaagcacttaaaattaaatataaagcctaaactctttttt
gtgaaactatgggtaagacggttattttccaccttaacttatgaat-aaatgctatt-gc
ctttattttctcgaataatattctataactagtaattcattgagttgtaaagcaacccat
tccctatcaatgactccattgactttccctgataaattggatttattcaattctaagtgg
ctaggaatattagctagtccagggaaagctaagtaattttctactagttttcgagatgct
tcttgattttttacactaattgattctcctggtttacattgataactacagatagatact
acttgtccattaatacaaatatgaccatggttcactagttgtctagcggcaggaattgta
ggagccataccaagtctaaaaacagtattgtctagcctcatctctagtagctgtaacaaa
atttgacctgtcgacccttgtagtttttttagctgcttttacgtacttaaataactgtttc
tcacttaatccataattaaatcgcaacttctgtttttcttctaatcttacagcatactca
gaaggttttctggacttttgtccatgttctccaggtggataagatcttttaatcgctttt
ctactgagtccgggtaaatcacctaatctacgggaaatgcgtactcgtggtcctctgtat
ctagacatattatttaaattctcctaatgtaaat-gattaag----------taatgagt
agctatttttttaacacaaaaagagcattaaaacaaagc-ttttgctaaaaaatatttaca
aaaaatataggtttggacaaaa---tacattactatgc-tttttaagcgggtagcgggaa
tcgaacccgcatcattagcttggaaggctaaggttttaccactaaactatacccgcaata
ttcaacagattgtcactaactcaatataacatagttatgttaagttaagataatcttaaa
tttaatagaatt-ttaaattatttatgctccttctaaaaccacatcaagaaaacgagcaa
tttcggcagcttgttcttcaacttctgataataataatggttgaccgaccctagttaatg
gaattactcttttatcttttgtacataaataaatttcacgtcttggatttagcccctctt
taatatctatttttattgatttgatttcttttatattaaactgaagacagattttctat
tctttccagggaaacctagccgaaaaatttttacaatacctttatctttgttaaattcat
tatatccagcccctatattccaaataatagttagccacagaaacaaactcagaaagactc

ctatactgccatagaatgtcattacaataccttgtggaataaataccaaatcggttgaat
ttgtaaaaggtaacaaatcaacttgaaaataacttgatagtccagcaagaagaaagccca
aagccccaataaatattatagtagcccaccagtaattactcagtcttcgtgaacctaaaa
ttaaatcttttctaactgagtgtatagataatgtttttttcataatttgttcttttaaaa
cagta-tactaatgcaaaatggaaaaattttgacaattttt--atgcgcgctttagattt
tttaaataagcttgattgcttttagtcctaaaaacagtcctgttgctaacccaaaaaaag
cagcaagaaatattatatatcctagaaaaactgaca---tattattatttattattattg
ttacaatagtgtttacaaaaatagtgaaaatctattactacggtattatattacgttaaa
tgccttgctgtctacaggaaaaaactttttttcagtttttagcattgtgtttatattagaa
ataatattctagtaaatttattaaacttctggagaacagaatcttatgtcagaatttatc
aagccttataacgatgatccttttgtaggcaatttgtccacgccagttagtacgtcaagt
tttagtaaaggacttctaggaaatctaccagcttaccgtcgaggtttatctccgcttctt
agaggattagaaataggaatggcacatggatactttttaattggaccttttgataaattg
ggccctttacgaggtacagatgtagcgttactagcgggatttctatcttcggtcggcctc
attattattctcactacatgtttatccatgtatggtaatgtatcttttactagagcagat
tcgaaagatccactacaaacttctgaaggctggggacaattcactgcaggatttctagtt
ggagcagtaggtggttcaggatttgcttatttattgctagctaatatacctgtattacag
actgcaggtcttagtttattctcttaagctagtaaggggacttgaacccgtaacctactg
attacaaatcagttgctctaccaattgagctatactagcattcagttcataataacatat
aaagatatttgttgcaatctgctattttcagatgtaacaaatatctttttttatttaaaca
gaactgttatgttaacac-------gtatattaattagaatctgaattatctgactctgc
agaagattcctgatcacagtttagataataacaaattgtttgatctagacaagttgcaga
ccaaccaacaggtgtttctcctgataattcttgcatatctacagaattataaaaattatc
atttggcttattactgtttgcttgcataaatatcactcccaaaacactctgaacttaatg
tgaagtaatttaacttctcaataatatattaacacaaaatattaaatatggtgattattc
tttaattgtaaagatgctgctaactttataaagtttgagtgagagtcttgatagcttttg
ttgaaataagcattttaacccatttattttgcccgctcgaccatactttttttgtgttgta
aattaacttttttgtagttttttttgttcgtttatgagaatgagatacggcatatccattat
tagctacttttcccgtaagctgacactttttttgacataatttt-------ctaacttata
tatttatttagagtacttgctaaagttgacttgggcacagctccaatcactgtgtctact
ctttctcctgctttaaagatcattaaagtaggtatacttctaatgccatattcagccgca
atagtaggattatcgtctgtgtttatttttactaccttaatagatgattcatattcttcc
gcaatttcatcaactacaggagaaaccattctacaaggaccacaccatggcgcccaaaaa
tctacgagtacaggtaagttattgttaataacttcttgtttgaaagaggcatctgtaact
tgagatactgacatattctttaacctttaatatatgttccttgctaagcaaatcttatca
caaatttcaagaaaaaatcttctatagtgaattactattagtgttgaattattttttttaaa

aattagacattaaaaaaacttattacctatataaataaagtggtggccgagtgatatctt
gatagtgataatttttatattaaggttgaacactagaaataacttttcaagcaaactctt
atttgaaaa--atttctctgtgaccataaaaacttaattatattacaatatattttatta
tttgtaacttagttctgataatggtataaacaacgcaaaagatactgccttataatcaag
gaggaatacatgtctcaatccgtagaatcacggactaggattaaaagcgaacgttacgaa
tctggagtaatcccttacgctaaaatgggctactgggatgctgactatgtgattaaagaa
acagatattctagctcttttcagaatcactcctcaaccaggtgttgacccgattgaagca
tctgctgcaattgcaggtgaatcttcaacagctacttggacagttgtatggactgattta
ttaacagcttgtgacttatacagagcaaaagcatatcgagtagatccagttccaaacgtg
gcagatcaatattttgcttacatagcttatgatattgatttgtttgaagaaggttccatt
gcgaacttaactgcttcaattattggtaacgtttttgggtttaaagctgttaaagctctt
cgcttggaagatatgcgtatgccagtagcttatctaaaaacgttccaaggtcctgcaact
ggattgattgtagaacgtgagcgtatggataagttcggtagacctttcttaggtgctaca
gttaaacctaaactaggtttatctggcaaaaactacggaagagttgtatacgaaggcctg
aaaggcggtcttgatttccttaaagatgatgagaatattaactcacaaccatttatgcgt
tggagagaaagattttatattctatggaaggtgtaaataaagcatcggcttctgctggc
gaaattaaaggtcattaccttaacgtaacagccgcgacaatggaagatatgtatgagaga
gccgaattctctaaagaggttggtagtatcatttgtatgattgaccttgtgattggttat
actgcgattcaaagtatggcaatttgggctcgtaaacatgacatgattttacatttacat
agagctggtaactcaacttactctcgtcaaaaaaatcatggtatgaacttccgagttatt
tgcaaatggatgcgtatggctggtgttgaccatattcacgcaggtacagttgtaggtaag
cttgaaggagatcctttaatgattaaaggcttctacaatactctacttgaaagcgacaca
gatatcaacctacctcaaggtctgttctttgctcaaaattgggcttccctacgtaaagtt
gtaccagtagcatctggtggtattcatgctggtcaaatgcaccaacttcttgattactta
ggtgatgatgtagttcttcagtttggtggtggtacaattggacatcctgatggtatccaa
gcaggtgcaactgctaacagagtagcactagagtccatggttatggcaagaaatgaaggc
cgtaactatgtagcagaaggtccacaaatcttgagggacgctgctaaaacttgtgggcct
ctacaaacagctttagatttatggaaagatattagttttaactacacttccacagataca
gctgatttcgttgagactccaacagcaaacatctagtttaatgactacttactgatactt
taaatagttaattgtaagtgaaattaacttataacaataaggagcatagaatagtgagac
taacacaagggactttttccttccttccagatttaactgatgagcaaattaataaacagc
ttgcttatatcgtttctaaaggcttatcagcaaacgttgagtatactgacgatcctcatc
caagaaactcctattgggaactgtggggtttacctttatttgatgtaaaagatgcttctg
ctgttatgtacgaaattagctcatgcagaaaagcaaaacctaattattatgttaaagtta
acgcttttgataatactagaggtattgaaagttgtgtaatgtctttcattgtaaatagac
ctgctaatgaaccaggattcttattacaacgccaagacttcgaaggtagaactatgaagt

atagtcttcatagctatgctactgaaaagcctgaaggagctaggtattaatattaattct
taattaatattggctaatta--ttcccctcttaaaaatcataattaatttagtaattgtg
atttttaagagggatagcttcccaagtaaattttgactaaa-tagattgaataattaaaa
atacacagaaataatgcaatcacaggatataatttccaacgatactcttgttaatttaca
agaagaatatgatagaacacaaatccaagaagttttaaatgagttaaatcaagaacttat
aggattagtgcctgtaaagaccagaattcgcgaaattgctgcgctattattgattgacag
attacgcagaaaactagaactagtttctggtaatccaggattacacatgtcatttacagg
tagtccaggaactggtaaaactacagttgctatgaaaatggctgatattttgcacagact
tggatatataaaaaaagggcatttgttgacagttacaagagatgatcttgtaggtcaata
tattggacatactgcccctaaaactaaggaagttcttaaacaagcaatgggaggagtttt
atttattgacgaagcttactatctatataaagcagataatgaaagagactatggctctga
ggcaattgaaattttattacaagtaatggaaaaccaaagaaatgacttagttgttatctt
tgctggatataaagatagaatggaaaaattctacgaatccaacccaggactctcttctag
agtagctaatcatgtagacttcccagattatacttcagatgaattattacaaatagctaa
aatgatgatagaagaacagcagtactgttttacagaagaagcagataaaactcttttaga
gtataccgagcgaagaatgaaacagccttattttgctaatgcaagaagtattcgcaatgc
tattgacagggctagaatgagacaagccaataggatttttgccagtggagaaaaagtatt
aacaaaagctgatttggtaacgattgaagcagaagatatcttgaaaagtagattattttc
attacctaatgcttaatatacacgtgatttcatgaaactatatattattaaaaag-tttc
ataatcagttgcaaatttcttgaaaaacctttattattatcttaatgtaggtg-tggcgg
catagccaagtggtaaggcagaggattgcaaatccttcatcccccagttcaaatctgggt
gccgcctagtactaaaaaaggggggtgtggtggaatggtagacacaacagacttaaaatc
tgttgatttttagtaatcgtgagggttcaagtccctccacccccatatattgatttataa
ataaaaagagatgtgtatttgtattaattgcaaccatattaccaaatgtaatacctatca
cttaatagaatctcaacataaacagcctcatcttactagaagtccattgtttatacctaa
atatcctgtagttcatgttaatatatctaacaactgtacctataatcaaattgattggga
tttagtggagtgtttatcatttgtagaaaaaccgaatagttggaatttagacgctaatta
gttacaagaaaaattatgtaccataagcaacaactctatttttagatactcggtattaa
ttttggatgttctaaccagtaagattttacccgttcttgctatctctataatgccaaatt
tagttagtaattgttcaatagcaacaatctttcccggatctccagtaacctctataatta
aaagatcttctgcaatgtctactatcttagctctaaaaattcttacaatttctaaagctt
ctgttctagtctgagaattgatctgaatcttaattaacattagttctctttcaactgaag
gaatatttgttacatcttgaacgttaaggatatttactaacttatataattgtttcgtaa
gttgttcaatagttctattgtctccttgaactaccattgtgattctagagaccccaattt
gctctgctggtccaactgctaaacttgcgatattaaagcctctccgggcaaatagaccag
atattcttgacagtactccggcttcatcttgaactaaaactgataaggtgtgtttcatga

aaaattaaatttaatctatttgatctattaagtatattctaagaaaattacagaaaaaat
actagttagtctataactaaattatccaaactaaacaaaatcctactatcagataatata
tctaccttgtaatgctatactattaacttagtatttaatgatattctaattgcaaataga
atgct-aattttttttaaatttgttaggaacttgtgctagaaaaatacaaaaacagtaaaa
aactctctcgagatttacctcaaatcaatgatcgcattagatttccaaaagtccgagtaa
ttgatgacgaaggtgaacaactaggtatttttgtgcctgaagaagctatacaattagctg
tccaacaaggtttagacttagttgttgttagtgataaatcggacccgccagtatgccgaa
tattagactatggtaaatataagtttacacaagaaaaaagagctagagaagctaagaaaa
agcaacataacagtagtattaaagaagtaaaaatgcgatataagatagaagagcatgatt
ataaagttagaataaaccaggcatccaaatttattcaagcaggagataaagtaaaagcaa
ctatcacatttcgggggcgtgaaatccagcactctaatttagctatagatttattgaata
agatggcaagcgatctaaatgcaatagctgaaattcagcaagctccatcaagagatggca
gaaatgtcataatgctcttatctcccaaaaaagttagctaaactaatttttatttaaatg
catctggctggattcgaaccagcgacgtctctttcgaaatggcggattatgagtccgctg
ccttcggcccctcggccacagatgcatcacttaaagtttataactcataaagcatataaa
aatcaatgtttagctatttttaataaagtctataaaagtctattattcattcatattgtg
atatgtcgcaactgctgaaggggaaacttgatttaagtatcgaaatatccagtatttaaa
aattgtgtcgagtataactggaaaagttgaaataaataaaaaaataaaatctcgactttc
cggaagtcctaaatgtcttaaaattacttcaataataacttcccatccatgaggagaatg
aaaacccacaaacatatcagtaaaaagaatgattaaaaaagctttagctgtatcactaag
accataaataatttcatttaaaaaagatttaacgactgcaatttgtctttgtccagttat
cattagtaatataaagacaagtattgatacaagatcggataaaatattttttaacagcatt
tgcactttcgtttgcataatattctcccaactctctagccttcagctgcactcttttttc
aatgatttcgtaagatatatcttccgaaggattcagaagaacttcaaagtgaattttctc
ttcaaatctttgtaattcagcaaaagctctttcctcttgagaggaatttaaaaaaatttt
aggctgttcttggttccataaataatcaatacaaggtccaaatacaaaaaaatttgaagc
ctgattaactagtactggagagatgaataataataaaatatattttacggaagtaatagt
ttggtgtctagatattctaaactcctctatagcttcagattcgccattaggatctaactc
tttacgaaacttttcaaaagtattagtaattgacctaggtattggcccaactttttcaaa
ggcgaattgattatttcgttttaaattccaatatttcatttttttattgctgctaaaattt
ctattaatcaataattgatcacgtattcactataaatattaattctagaactaaacatat
aaaacaatatttatgaaaaataaaatacaattaaaaattaattatgcattagttctatct
gcagctacagcagtttatggatataaatcttatcagccaactataaatacactcttaatc
aaagatgaagaaaactggcctttttttctctaaaaggcagactgcctttattcctataatt
gtattaaaaaaaaatcatcaaaatccctgaactcagaacctagaaatagtaagttatatc
ttaatggaattggaagtagaaaatttatcgacagaattgtcactctacttggatatttag

aaaaacaggcaaactatactgaatacttgaaccaaataaaaacaagtggttatttttctt
tagtctacctagacagtgatataatgtataagaaaaatattagtaacatttttttactac
ctaatgatattctaaaaagaatctatatttgcaataagaaaaaaaagcttataccgcatt
cattcttaataaatttatttcagaaacagataggctatccaaaaagttttgctaatttaa
attgggccttctctaaaattataaaatggtattatgatagaggataccaatggtctctag
ttgaagttaaacaagcgtctgatgcatcttctattgtaattgatattcatgaaggggtag
tgaagacaattataacagaatactacactctatcttataaaagagtctcaggtatcttat
gcgtagagtcaatagagcagtaccttggagtaagagtgggagctccactaaatataattg
atttacaaaaaaaaattacttacttaaaagataatcaactagttggcgacattatttata
gtattgaacgatcaaataatagctcaatgagtttggatatcaaatttcaaatacaagaat
taaaagataaagagataatagtgcttgcagaaagttcttcta------------ttatct
cgcatgcatgtaatttactcaatc---aatataga---------aataggctagtagctt
ctaatatagtttcactatcaactaataaattacacgcatgctattttaattataaacttg
actatcaatataagtatataaacacctctaatcttataaaattgttagcttattctattt
cttgtaggaagacactacttattcattattttaatttacaaactttaataagctgtacaa
aaaaaaatactattggatttcagctatatttacgaaatctaagttttgggaaagctttct
gtgtgcttagtatgaaatttatcaaaaatggacttaatataaaaatttatatataaatc
cttcattgatagttgatcagaatttgtatttcaatttgctatacaaatcattaagcaat
atcatactgcta------aacctccagctt------------------------------
------tatttttaacgaatctagatcttgaacaatatgttgctgaaagtttattaatgt
accattttacatcttgcttctcaatatctgaaaaaatattattatctcgaattatgcata
cagattctttatttttcaactctgaaa------attttcattttgatgatagaactaacg
cgatcaacagttatgatacttttaaacaaaatacaaaaatattttatcaagagtttcttt
ctttattattaagtttacgttatcaaaattttaattatttaggttggcctttaaaaggcc
attttttgaaataaaatctttatacttggctccatttcaaaagagtg------------
---attttctgatagtcgtaagacgctattcttccacaaaatgtcgttaaagcaagtat
ctaattttaatttaccagtatcttttaaaagccaccttaaccatattttagttagtacta
ttaaatgtcagtcaaatttgaatatgagaactgtatctttgctattaattgattcacctg
ctgaatatctgctatacaaatctattcttaatttctctattaaagttaggatgcagtatt
tcatacctatgagcaacaatataagattatctctattttataattatttagattgttttt
taataagatcatctcagagttgcattcacatatggcaagatctaagaactcttacaccaa
ttcaaaatttttggttaaaaaaattttcatatggagctggaattcagcttaaattaccaa
ttaagcagatgccacctctttcaattgaatatactgtaactagtagtcgctacttttgta
tttaccttcgtacttattatcaacgataactattactaaaatgaaaaatttaatttcttt
ttttgatcgcagcaaagggaaatggatttcacaaagaacaacttatgaattatctaataa
aaacatgagttcggtacagtctcagatgacaatgaaaataggcaactcattgtcaggatc

tataatacttgcatcgttaaattgggggacatttatagacaagttgcccattacgctaa
aaatcacagccggagtgaatatgataataagtttaacttgcaattcggtaatcaactaaa
taatcataaattattaacgctgtgcatagtgacagatcctagtctaattagttttaaaac
tcgctatggaagtactaccatagatgaaacatattggttcgcaacaaataatttacgtct
aagtactagtattgttaaacgattcaacacttgtgtggcagtatctttttgttcagaaat
taaagtttaa-actctatatagaagcaactaatagccttta-ggctattagttgcttcta
tgtatctaaattttgtattaaaaattat----aatactatattattctaaatctttacga
ttgggattcctggaaggatcattagagaggaacccaaagacaaacaaagaaataaagaaa
ataacggtagtatatacaaagattttcaaagtaaacatattttcatccttaaaagctatt
agtgcg----atatttacatatctccacataattttatatcatgatataagttccagcac
tattttataaataaatcaatatctttattttttagtaatgatttatatttaaatatgcgt
gatgtactaaatactaaatcttttccgacttgttttgtcttgtttgtaaatattttgtta
tttctatggagtttaccttcaattatcacatagtctaacttctttagctgtttaaaagta
tatgaagatttttgttccaaatagttagattgattataactaatcgttttctttttaac
agtcttgcttttaacttaattatttggcttttattttcactaattctgatactcttacaa
ctaagaatttggactagaagagtacagctattcataatttataacaa------tttttta
aaaaatctactaaaatagatgattttacagaactaaaatgatatttcattatcttgttga
ccttgctcataagtaatatctttcaatacttttaatatttttccaaaatactcttgcagg
tatttttctaaagatattacttccaacctgtcaattccaagaatactgtaaacttcatcc
atcggagctgtaaattgctctccgctacttaaaatttcggcaaatgctaaacgatcagaa
atattccatgtccattgaagagttttagtaattcttcttaaggcttttaataatccaatc
ggaatttgtgagatctgtgtttttttgcccagataatttttcacataatttaataatttca
gctgatgtccaagcggtattaccaactaacggcagggtcctattttctgtggaaggtact
ccaagacttttataactaacttggctgcatcttgagtatcaatgtatgcaatgggtgta
gattctcctgtaacccagactgatttcttgtctaaaataggaatggcatattggttgatt
aaaccttgaaaaaatcctcctaaagaaaacacagtatatttcacattggacttctgaagg
aaatctactacctgagactttaagttcatcaatggaacatctgggtatttctctgaattt
aatattgaaaagaaaataaatctttcaactttagccgctttagctgcttcaattaatgca
attttcccatctaaatctatttttttctgcattataaggatcagtaggacgagatgtagaa
gcatctatgattgctgttaccccacaaaaagattgcaaaatactttcgggtaattttaaa
tcaccatatataagttctgctccccactctttgagaaaggcagactttcttaaatttctt
accatgcattttacattgtagccttcatctaaagctcgtcttacaatttgacgtcctaaa
gttccggttgctccaattactaaaagagtcatattagttaaaaataagtatgtataaaga
cattgatttggttgacggcagagagtaaattaggtagagagggaatcgaaccctcatgac
cgaagtcgtcacattttgagtgtgatgcgtataccaatttcgccatctaccctattatag
accaactactacaagtaatttagtctttagcgtaaaaataatactaagtttcaatagata

tgtcaacttttaaatttatattaataaaaaaacggagtctcttatggccacttttgaatt
aattccttatagattgtatttgagtcaatctaacacatggatccatagaataaaagcaga
aattaagatatatatagtagcgcttttatggatttcaatttttattttttcttacttttaa
actatgtattattgctttaagcttaattgccataagttttactataagaagtaaacagaa
tattattcaaaaacatttgttacaaacgttattaatgacgttcttaactactgttttgtc
ttttagtgtggctattagttataaacaatatgcagaacaagaacaatcgcagtatttatc
tgattccaagaaatataaaaattccagcagctactattatatacaaatagcgaacgatca
aaggatcaaaagacaatacttaattactactttaaagccttccttgtactttttttattac
tatatattctattaaactagttatgataacaacctctccagaagttttagtaattactat
ttatagatctaggataataaataaaatatttaaaaatgaattgctatttatcttccttct
ttcgtcacatattgttactagtattattaatagaatcgataaagtgattcaagtaactag
cttaagaggaagcttgaatttatataattcattgacaaggcctttaatgttttctttgtt
aatatttcaagtcttctttttggagattattcgggagtcaaaagaaatagctcaagctct
ttatactagaaatctcaatcaagaaaacaataatttttttgaaaatatatacagtaaaatc
taactttagtgatcggctcaatataattattagcactttgtactttattattttagctct
agcgtaatagaatccacttattcttctgactctagtaatacgaagagtaataaaattagt
tagtagtttaaatagatagaat-taagatattatataaataattaaatgtttcattatgg
tcaatactcaaaatcaaatttcacaaacttcagaacttgattatatagttaatcaacctt
ataaatatggctttaaaacttctgttgaatctgagcaatttcctaggggaataagtgaag
atattgtccgcttgatctccaagaaaaaagatgagcctgaatatctattgaattttaggc
ttaaagcatataaaaaatggaaaaagatgagtagtccgtcatgggctcatattaagcatc
cgaatatagactttaatacgattatttattacgctgttcctaaattaaaaaaagaattga
aaagtctagatgaggttgatccagaaattcttgacacttttaacaagttaggtatatctt
taaatgagcaaaagcgaatttccaatgtagctgttgatgctgtttttgatagtgtatcta
ttgcaactacttttaagaaagaactgtctgaagctggtgttatattctgttcaatttctg
aagctataagggattatccagaattaataaaaaaatacttaggtactgttgtaccagctg
gtgataattattttgctgcattgaactctgcggtatttagtgatggctctttttgctata
tccctcctaatacagtttgccctttagaattatcaacttactttcgtattaacaacgaag
aatctgggcaatttgaaaggacactaattattgctgatcgtggcagtaaagtaagttatc
ttgaaggttgtactgctcctcaatttgacacaaatcaattacatgcagcgattgtagaat
tagtagctcttgaaggagccgaaattaaatattctacagtacagaattggtatgctggta
acaaagaaggtaaaggtggtatatacaattttgttactaaacgaggcttatgctcgggta
acaattcaaaaatttcatggactcaagtagaaactggatctgcaattacctggaaatatc
caagctgtattttagctggtgaaaattctcagggagaattttactctgtagctttaacaa
ataactatcaagaggctgatacagggactaaaatgatccatattggtaacaatacaaaaa
gtagaattatttctaaaggtatttctgcgggtagatcgaaaaacagttatcgaggcctag

taaaagttggccctcagtcatttaattctcgtaattattctcaatgcgattctttattaa
ttggtcaatcatctcaagctaatacatttccttatattcaagtacagaatccaacatcaa
aagtagaacatgaagcatccacttcaaaaattagcgaggaccaaatttttttattttttac
aaagagggatcaatttagaagaatctattgctcttatgatcagtggtttttgtaaagatg
tctttaatgaattgcctatggaatttgctactgaagctgatcgtttactgagtttaaaat
tagagggaactgtaggatgagccagactattttagaaatcaaagatttatatgcttctgt
tggtgaaacaacaattttaaaaggggtaaacttatctattcgagccggcgaaatacatgc
gattatggggcctaacggttcaggtaaaagtacattatcaaaagtaattgcaggacatcc
agcgtattcactaataagcggagatattttattttttggacaaagtattcttgaaatgga
gccagacgagagggcaaaagcaggtatttttttagcttttcagtatcctgttgaaattcc
tggagtcagtaattctgatttcttaagaattgcattaaatgctcgcagaaagtttcaagg
attatcggagtttagccccttagaatttttcaactaataacagaaaaaatagatcttgt
tggcatgcaagaaagtttttttgacaagaaatgttaatgaaggattttctggtggagaaaa
aaaacgtaacgaaattcttcaaatggctttactagatagcaaaatatctattttagatga
aacagattctggactagatattgatgcattacgagtagttgcaaaaggaattaacacttt
agctaaatcaacaaattcaattattttgattactcactatcaaagattattagattatat
tattccagattttgttcatattatgagtaacgggcaaattgtaaaaactggtagtgttac
tcttgcccaagatctagaaaagcacggatatgactggattacgcagacataactatttac
tac-------aatattgagactaaagaatattctttagtctcaatatt-tttgcaaatgc
aaagtt-aattatttaagctttatcaaaaccttgcttaacatcttcaattgctcttttta
gcaattcttcactaactggttctaatttttttagtactacgaacattttcaccaaattctg
gtttagaattctttaaatcttctcgaagttctctaataaattcagctacttttgaaactt
caatatcatctagatagccattaattccagtgtaaataatagcagtctgttcttcaactg
gaataggagaattttgggcttgcttcaaaatttctcgaagacgttgacctcttgctagtt
gattttgggttgctttatctagatcagatgcaaactgagaaaaagcttctaattcagcaa
attgtgctaactctagttttaacttgcctgccacttgtttcatggcttttatttgcgcag
cagagcctactctagagactgagatcccaacattaatagctggtctaatccctgagttaa
ataagtcaccagatagaaatatttgtccatctgtaattgaaattacatttgttggtatat
atgcagatacgtccccagcttgagtttcaataatagggagagctgtcatactacctccac
ctaattcagcattcaatttagctgctctctctagcaatctagagtgtaaatagaatacat
ccccaggataggcttctcgtcccggaggtcttctaagcaaaagagacatctgacgataag
cttgagcttgcttagttaaatcatcataaataactaatgttgctttacctttgtacataa
aatattcagctaaagccgcacctgtataaggagcaatatattgtaaagtcgcaggactat
ctgcattagctgcaactataatagtataatcaagagctcctttatcctgtagtgaagata
ctacttgggctactgaagatgccttttgtcctatcgcaacatatacacaaacaacatctt
ggcctttttgattaataattgtgtccaatgcaacagctgttttacctgtttgacggtcac

caataattaattctcgctgacctctaccaattgggatcatagagtcaatagcagttatcc
ctgtttgcataggttcacacactgattgccttccaattataccaggggccattgactcaa
ttagccttgttccattacttgcaggttcacctttatcatctattggccgagctaatggat
caacaactctacctaaaaaagcatctccgaccggaatttgagcaattcgaccagtccctt
ttacggaactgccctctaaaatatccctaccatctcccatgagtactacaccaacgttat
cactttctaaatttaaagcaaccccaatagttttatcttcaaattcaagtagttcaccag
ccatgacttcatcaagtccatatactcgagcgataccatcaccaacttgtaaaactgttc
ctatattggctacttctacatcctgatcatacttttcaatttgttgacgaataatactac
ttatttcatcaggtctaatatttaccatattttaatattatttttattaattggaaatt
ctaaagcggtaattagtcagactaatgctatctgtctttttttactttattttaagttact
acaacatcaaggtgtgaagccatttgtcttagctgtcctctaatactagtatcaatgact
tttgatccaatctgaatagtaaagccaccaattagctcgggctcgactgaaataattagt
ttaacttctttagccttagtcataaccttgatttttctgttaatagagtttcctgatca
gggcttaaagcaatagaagtattaatatttgcaattgttaaagattccattggtaagcc
agttccaaatacttactagctatagcatctaacattccaattcgcttgcgatcaactaaa
atcattagaaatgataatgtattttcgttaatttgatcgccaacagttgcaataattact
tctttttttgcctctacagtttttaatggattagct---aaaaaaatttttagcttgtca
gattgcgctaaaatattttgtattgactgaatatcttggctcaccttttcagtaactttt
ttagttttagctaaatcaagaagagctactgcataaggctgagctatttttataacaaca
ttgttactgctcatagttgatctcctaacttagcaatattattatcaattatacgcagtt
gcatttcggagctcatttgattttccagctgcaaagtaactcttttaagagcaagaaatg
ttatttgttgctggatttgccttcggatctgtttttcagctgtttctatatttgatttac
ctgttatggcaagtctttcaatatctaattggccttgagctaatatggaacttctaactt
ttccagctgttagctgagcttctttcttaatttgatctataatgatttgagtttgagcaa
gttgtttttctgattctgacaatctagcacttgcttgctctagtctttcttcggattctt
gtatagctgctaaaacttttatctgtcttgcatttaaacttgatccgagaaattgcttca
aaacataaataagaccaaataaaagcaacaatatattaataacatttgcttcaaaaatat
cagaattaaagccgaatttgtgttcactactatgttctgataaaatagtaattatttgtg
gtatttttactatattattcatttaaaattct----------tactattttaatatttta
ctctggaatcatgattttcaagattgactacttaataatttaactttaatttgatcgctc
aaagtgtcaacctgattttctaaagttttaagagcttcttctttttgaacatttaactgt
ttcgatgcttcagcaatcagtttctctgcattcaattgagctttttttatatcttctgag
actatattttgagcttctttttgagaagaggctatttttaattgtgcattgcggcgagct
tcagatagatcttcttcatattttgcagctagctcatctgctttaacaagcatagacgat
gcagtagtcaatgttgttcgaatatattcatctcgttcgtctagtactttagttacaggt
ttataaaaaatagtatttaataaaaccattaaagtaagaaactgtaatgccattaaaggc

aaagtaccattgaagtcaaataagccaccttcaatttcttcggctaatagaaatggtaaa
tcaatcattttactaattatgtttatagttaataattttgtttcaattaagaaaaataac
tttatagtgcgcttagtctcatcagactaagcgcaaatgattattttaaccaacgtatgg
attagcgaacaataaagacaacgcaacaactaggccgtaaattgttaaagattccataaa
tgctaaacttagaagaagtgtaccccgaatctttccttctacttctggctgtctagcaat
accttcgacagcattagctgctgcactaccttgaccaatgcctgggcctattgcagcaag
acctacagcaagaccggcagcgataacggatgcggctgaaacgattgaatccataatgaa
ttttaatttttgttatatgagtagaaaattgacaaatttcgtataatacttaaaatcta
tataattaagtataatagattctaatat-----tgtaattatagtatattactcttctcc
atgaccttccattgcttctccaatataagctgcggacaaagtagaaaagattaatgcctg
tattgaactcgcaaataatccgagtatcataactggtaatggtattaaaattggaattag
caaagtaaatacagaaacaactaattcatctgctaagacgtttccaaataatcgaaaact
taaagatagtggtttcgtaaaatcttctagaatattgattggtagtaatactggagttgg
ctgaatatatctagcaaagtagcctaacccttttttacttaaacctgcataaaaataagc
taaagatgtaagtaaagacagggccacagttgtattaatgtcatttgttggggcagctag
ctcaccttcaggcaagtgaattaacttccaaggaattaatgcacctgcccagttacaacc
tagaataaatagaaataaagttgcaatgtaaggcacccaagggcgatattcatgctcacc
aatctgatttttttgcaatatcttgcagaaattctaatataaattccataaaattctgaaa
tttttcaggaattctttgtagatttctagtacctagaaatgataaagttaaaagtgtagc
aattactaaccaagagacaatgaaaacttgcccatgcaattgtaagctacctattttcca
atatagatgttttccaacttctactgctgataaactattatatgggttaaaatctatgag
actgttttgatacataataaatattt-ttttaaatagtgcaagaatcaaaaaataag-tt
tttgaagaattaatatgcaattaatttattcttaaaattagactattaataaattaaaag
caaaaataaattctaatagagatagctatttttagtagtgattatttttttgtaaaaaaca
attattgctacctataatgcttttacgtttaattatatacccatatattaattatttaat
atgattataggtattttgctacaaactttattgttttatccattct----aaagattgtt
atagtttatttttttatttaaaatatctgcgacttcatctgcaaaattaactttcaaattt
tcttctccccctcccagaagaaatcttacaaatcttctaatttttaatattttcacctaga
acagcaatattttggtttattaaatcttcaatactaatatcttgattgcgaataaacatt
tgatctaataatgatagctccttaagtctcttttttcattcgtccttcaatgattgtttcg
attctatcaactggtttgtttttcaagtcatctttaccagcttcaactctttgttccaga
ttaataatttcactaggtatatcctgtgttgatacgtattccacattaggcgatgccgct
atttgcatagcaatatctttagctaacttttggaactctggacgtcgtgctacaaaatct
gtttcacagttgacttcaactaatacacctattcttccgccagtatgaatataactctct
agtaaaccttcaatagcggtacgattagatttcttgttagctgaagccagcccttttttgt
cttaatgattctaaagctttttcttcattgccattattagcttgtaaagctttcttgcag

tccatcatacctgctccagtcttatcacgtaaggcttttacgacttgagcagaaatttgt
agtgtcatatgctttcaaaaatttttaagtatttaaatt----------aattattataa
ttcactttgagtaggatgagttatttcttctaaatctgtttgcccgtatttaccatcata
aattgcatctgctatttacctacaattagtttaattgacctaatggcatcatcattagc
tgggattggaatattaataatttctggactacaattagtatcaagtatacaaatagttgg
aatacctaattttaaacactcttgtatagctgttgtttctctcttttgatctacaactac
aacaatatcaggtaaacgagtcatatttttaataccatttagatgtttacgaagtttatc
taattctcttcgtaaaacagcggcttctttttgggtaactgatcaatcataccactttt
atcttgttcttctagctgtctcagacgactaactcgagacttaatcgttacccaattagt
taacattcctccaagccatctctggttaacataataagaatcacagcgttgtgcttcctg
tgccactataccagcggcttgtctttttgttcctagaaataaaactttttaccatccga
cgaagcctgttttataagttcacaagcttctgttaataattgtgcagtttggactagatc
aataatatgtatgccatttctttctgtataaatatatggaaacattttaggattccatct
acgtgcttgatgtccaaaatgaacacctgcttctagtaattctgctaatgtaacaatagc
cataagattattaatatttaaataatgtaattattcgggaaatccc-tttgtttagaaac
tgat-----tcaactaacttatttgaatttagtaaactaataataaattaacacacttt
taaagcagatcaacaaaatagatcctgaagaattcat----tctacacttttattgctaa
aataattacgcgcggttctgtcgtctaaaatgatatcatctaaatcatctcgtacagatg
aaatagtactttcgtcattagtatttgcagttaaattctttttctctaacgatgagccat
tacagttatcatacatattgaagccggtaccagctggtataagccgacctataataacat
tttctttcagacctcttaaccaatctaatttaccagatatagcagcttcagttaatactt
ttgtagtttcttgaaaacttgctgcagaaataaagctttctgtatttaaggaagcttgag
taatacctaatagcacaggcctatatgatgcattaatcttatttctcaaggttatggctt
tattagtctgttcaatcttctgtaactctacaagctctccaggtaaatagcctgtttctt
caccattttctattttcactttagaagtcatctgccgtactattacttctacatgcttat
cagaaatattcactccttgagattgatataccaattgtacttctttaacaagtagtaact
gaatctcttgaaagcttaatcgagcagcttcatataaggctaatccacattcaatataca
aatcaaaacttgcatcgaggatatcatgaggatttaacaatacgtctgttttctttcttg
cttcaagaatttcttcaattcttggaagcccttgtataatgtcacctgtttttgctctat
caaaaaccaatatagctaatgtttctccccttctaattaatgcattgttatcaacatgta
aaatagcaccattagaaaccaagtaaggtctagcaattcttaatgtaactgatctagaag
aaatttcaataatttgtccagagtctaaagaagtcatattttcagtaataaaatcaccac
aacgaatccaatcacccacacttactttaactgttgattttcctatattaaaggtctttt
tatcagaagatgtcgcaatcaaaatgcgcctgctagtattcttttcagaataaatctcct
caacagtaccttcactcattgcaataatctcagtactagcaacaactgtaaatggcttga
tatattcaccatttttacaataattctagtttgactttgttgcttctctgatttatta

catcaatacttttaattgataatgtttcaaaagttgacagccttaagtcaaaataattat
tgccactttctttagcagaaaactcaattgaggatattaaattatcatctcgatgctcaa
tttcagccacaagattagttgttactaaatgaactccttctatagatttcactctttctc
cgtcccgaaaaggagttcttttaactagctttagtttagtttgacttggtttttgtgaaa
ctagattttcaattagactggattttgtttctggtattgaataaacaataacaggtctaa
taagaatatagggcatttgctcgttttctatatactcccaataaactaacttatctgtag
aaatattattatgaagtctttctccaggtcttaaaaaacctctacttttatcattattac
tatacacctcactaagtttatagatagatccaggcttaataataatttctcgaactattc
catccttttcgattatttcaacaatacctgagcttttagaaaaaatatttttaactagct
ctgttccactttctataacatctccattatgcactagtattagtgatgaatctttattaa
tttcgtgagtttcttctgaaatccataagatatatcccggacttaaaatttcgtaggcat
ctttatctaagcctgttttctttttgatacatttaagtctaaatatttaataataccac
cacttgtcgttttatacgtatcagatattagttctgcgactgtgtaaccatcttgaa---
ttttttggtttggaatagctttgaaaagaaattttttgtttttttctgtttctaaaatat
aagattctttttgtggattacatctgtataaatataacaacctggaattacaatagatt
cagtaataacttgtatattggtaatactgctattagtattctggtgtatccttacttctc
cagcataatgatttattaattctgtctgtgcaagtatagtaccagctttagttttatctt
ctttatgaacaataacattagcagaatcagatatactataaacttcacctgagagcaccc
agatcaagcctccagttttagtaattcttgtagtatattgattattatctgtttcttcaa
ctgttaaattggagaaacagattttgccagagagatctgaaactacatgtttttgagctc
tttctgtcatgagtcgatttctgcggggtgattcagcaatcacttgatccttcgatacta
attcaccatcacatattaaaagagtagtacctttacttaagttaattattgacttctgat
ttttcttagatctgattgtaacttgggttggctttttttgttattaatgcttgctcaccat
gtctggtacgaacatctgtatacgattcaatatctagatctatcaattgaccatctatag
gtgcgtagatttgttctgctaattcaccggtaaatactcctccagtatgaaaggtcctca
ttgtcaattgagtgccaggctcaccaatagattgagccgcaataataccaacagcctctc
ctaaatcaactaagcggccatgtgcaagattccatccgtaacaatactgacaaacagaac
ttctagagttacatgtaacaggagatcttactaaaactttttt---gataccagctttag
ttatctcttttgctaacttaggactgacatcttggttcgtatgagcaattaaacaatttg
tctctggatggaatacattttctgctaatactcggccagataaagcttgttctagattaa
ttaatattttctgcgtatctactaaatcttctagtatgatgcctttattggtcttacagt
ctacttctctaattattacatcttgagaaacatcgacaagtcgacgcgtcaaatagccgg
aatctgctgttcttaaagcagtatcaactaatccttttcttgctccgtatgatgatataa
aataatcagtaacagttagtccttctctaaaattactcgatattggaagatcaataattt
gtccctgtggatcagccataagccctctcatgcccactaactgtcttacttgagaaatat
tacctctagcacctgagaacgccatcatataaacagcatttagaggatctgtctctttaa

agtatttaataacttcttgtttcaaggattcactcgcattattccacgtatcaataactt
tttgaaatcgttcaactgctgtaatctcacctcggcgatatttgttttcagtggccttaa
tatcttcaatcgtgcctaataaaagactttgcttactaggaggaattcttaaatcttcta
aacttaaagaaattcctgcctgggtcgcataatgaaatccaagatctttaagcttgtcgg
ccatattggctgctcgggctataccataattacgaaaagcccagacaattaaatttttta
gttcgttttatcaataaccttatttgaaaaacttggctgtgcaagacttcgtctattat
ccactaaatcttctccatattaaatgttcgtt--attaactatgctactaaagactcttg
aataatttattaaagattatacggccagctgttgttctaatatattgcacaattctctg
attttcagcgtcttcttttatgatatggttattaaaaaattttgttgtactattgtcaga
atgtttttcaatcttaatcggatcatgaggttgatcaccatctactaatccatcaaaacg
agcccatatataagaatgtaaatcaattttcttctgttcataagccataactacatcttc
taagcttgcaaaatactgattagatccctgctgctgagatggattattagctgttaaata
atagcaacctaaaaccatgtcttggcttggcataataattggttgacctgttgctggaga
gagaaaattatgaggggctaacattagcaatctagcttcggcttgagcctctaaagacaa
aggaacatgaacagccatttgatctccgtcaaaatcagcgttaaacgctgggcatactaa
tggatgaagcttaatagctcttccttctacaagaataggttcaaatgcttgaatacctaa
tctgtgtagtgtgggagctctgttcaatagtactgggtgaccttgaataacttcatttaa
gacattccaaatagaagattcatttttttgaatcatctttttggcagctttaatattgtt
aactaatccctgcaaaattagtctgtgaataacaaaaggctgaaataactccaaagccat
ttctcgtggcaatccacattgatgtagttttaaatgaggacctacaacaattacagatct
accggaataatctactcttttacctagcaagttttgtctaaaacgaccttgttttccttc
tataatatctgacaaagattttaatggtctattatttgcacctacaacagttctacctct
acgaccattatccatgagagagtcaacggcctcttgcagcattctttttttcatttctgat
aataatctccggagctaaaattgactttagacgagataatctattatttctattaataat
tcgacgataaaactcattcaaatctgccgtcgcaaatctacctccatctaattggaccat
aggccgtaagtctggaggtatcacaggaatgactgtgaataccatccaagaaggatctgc
acctgttgctatgaaattttcaatcaatcttaagcgtttcatttttttattgaattttaa
tgaaggtgtcttaaaactttttggtggatttgtagcttcagaccttaaggtttcagctat
gtgctctaaatctaaatctttcaacagtttctgaatagcttctgcacctattcctacttc
tacttggttattctcatcttgattttggtagatttcctcttcaagacttttccattcgta
accttcgagcagttgcttgtattttaaattaatatctgtattagattgcgtaactacata
agaatgaaaataaacaatttttttctacttctttgacttttaagtctaatgctaaagcaat
atagcttgtacttccttttaagtaccaaacatgagttactggtgaggcaagttcaatata
agccatccgatgtcttctcacgcgtgattctgtaacttctacgccacatcgttcacaaac
tatacctttgtagcgaaaacgtttatatttaccacagtgacattcccaatccttaactgg
gccaaaaattttctcacagaataaaccatccatttctggctttaaagttctgtaattaat

tgtttcaggctttgtaatttcaccaacaatttgtccgttgggcaaacttctttcgcccca
ctgtcgaatttttttcgggagaagctaaactaatttttacgtagtcaaaatattgctcaaa
ctttgtcataaatttgaataccttaaaatcaaataattagccaatttatttacatgcttc
atattgaaaaatgaatatcttaataaaggaattgttcaaaatcgtctactggaggagtat
cataattagaacgatctactcggttatctttagaatccgacatcaagtcaacttcaactg
tccgtctttggccatcttcaaatagtttcaatttatgtactgcaatgtctaaacctaatg
actgcagctctcgcattaagactttaaaagattctggagttccaggcttcggtataggct
tccctttaactattgcatttaatgcttcatttctagcttgcatatcatccgactttacag
ttaataattcttgcaaagtatatgctgcaccaaaagcttctaaagcccacacttccattt
cacctaatctttgacctccatgctgtgctctacctcctaatggctgctgcgtaactagag
agtaaggaccagtggatcttgcatggattttatcatcaacaagatgaacaagcttaagca
tataagctctaccaactgtaacaggattatcaaaaggctctcctgttctaccatcaaaaa
cttgcattttacctggatgctgatcattaaaaagccatttattgctagtaatcaatgatg
cttcttttaatttctatttacaagtgctcttgatgcttctgctccatacatttcatcaa
aaggtattattttaaatctctttcctaaatagccacctgctaagcctagtaggcactcaa
atacttgaccaacattcattctagaaggtacacctaaaggatttaaaacaatgtccactg
gtgttccatctgataaataaggcatatcttgtttaggtaagatcctagaaatgatacctt
tattgccgtgacgaccagccattttatcacccacttggatttttctcttctgcgcaacat
agacgcgaatcatagcatttgttccgggaggcagctcatctcctttctgcctagtaaaaa
ccctaaccttaactactctaccttttgcagcattaggaagccgtaaagatgtatctctca
catctcgagccttttctccaaaaatagctcgcaacaatttaccttctggtagttgatcag
cttcacctttaggagtaatttttcctactaaaatatctccagcttcaacccaagaaccac
cgacaacaattccatttctatctaaatccttcagagagttatcgctgacgttaggaatct
ctctagtaatttcttctggtcctaatttagtttgacggcattctactgcatacttttcga
tatggatagaagtatacaagtcatcataaactaatctttcgctaattaaaaatgcatctt
cgtagttataaccttcccaaggcatataagcaactagaatatttctccctaaagcgattt
cccctccgtctgtggatgcgccatcagctaaagtttggccaacaactatttttttctccca
cccaaacaattgggcgctgattaatacaagtatcttggttagaccgataatattttttta
agcgataatgaactgttctaccactattatcctgaatgcctattttattagcagaaacat
aatttacatgaccagatgttctgctaataacaaccataccagagtctcgagctattttag
tctcaagaccagtaccaattattggcttttctggatataataatggaacagcttgccttt
gcatattagatcccattagagctctattcgcatcatcatgttctaaaaaaggtattaatg
atgttgcagctgaaattacttgaataggtgaaattgcaatataatctacttgggttgggg
ttgttgtaataaactcttgacgataacgtacaggaataatatctccttcaatgtaatgtt
gcttactaactttaacatctcctggagctactcggaaatcatcttcctcatctgctgtta
gatagacaggactattgtgataaatcacttgaccttggtttaccggataaaatggggttt

ctatgaaaccaaaaacattgacccgcgcacaggttgctagtgaaccaattaaacctgcgt
tcggaccttccggagtttcaattgggcaaattctaccatagtgactaggatgtaaatcac
gaactgcaaaacctgccctgtctttattaaaacctcctggacctaaggcactaattcttc
tcttgtgagttagttctgcaactggattagtttggtccataaattgagaaagctgactag
atccaaagaattctctcactgacgcaattaatggcttgggattaattagatttgataaac
tcaatgaatctatatcacatatcatcattctctctctaataatgcgttctaaacgattga
gacctactctgaattggttctgcagcaattctccaactgaacgaactcttctattaccaa
gatgatctatatcatcaagattacctgagttttatccttaatattaattagataatcaa
ttgaggataagatgtcttgaggagacaatactcggaacgttttaggtatattgagaccta
gtttttattaatttatatctaccaacttcacctaagtcatatcttttaggatcaaaaa
aacgagagtaaagcatctgcttagcaacagcaactgttgctggttcattaggtctaagct
tagaataaactatgagcagggcttcctcatcagttacttcttcaatattattttttccaa
tttctttagctaattctttaactgaatatatttgggatgcagaaataagaaaagcatatt
tactaagtccttttttcaatttcatctttattgagtccaatagctcgcagaaaaatataag
catttactttgtgggtcttatctattcttatccaaatttctcccttaggatcaatttcaa
attttaaccaagaacctctattagaaattaggcttgcactataaatctgcttaccatttt
tgtctatctcttgtttataatagattcctggactacggattatttggttaataataactc
tttctgtcccagatacaataaaagttcctctatttgtcattattggcaagtctccaataa
aaactaatctctttttgtactttttatttttatctgcttttcagcactgagctgtagat
gggttgatgataaatctaatgatttaatatttttttcttggtttttagaaggtagatcaa
tatcttttcttgttaactttgcaggaacatatatttgagcactataagttttatccctat
tttttgcctgtctgacactataacgaggaaattttatcttgtactcattaccaaataatt
gtaattcgagtcgactagtagggtctgatatcttcggaaaaaatttcaagaacttccgtca
aaccttctaataaaaaccatttaaaactggccctctgaatttcaactaaatctggaagaa
gtttattttttaagcttatacgttgaaccatagatctcctttataacaaattttgaaatc
gaagagtttaa-tactattaatagagtactggtagataatgcataagcatagtttgctac
attatttcttctctagacataactagaacatcatattttattcaa--aaaaagattttta
aaatttttcttcac--acacactatctataagtactttgctgcta--aaagatagttcaa
gctattaaa-------aaaaacgaaattaatacttgtctgtctgtcaattaattgacatc
aagatagacaagtattatttatttaatcaattctg-tttttctgagcaaaagacaatgct
ctcgcaagtctagcttttttttctagcgcctgtattagggtgaaatgcccctttttttaca
gctttatcaatcttactatatacttgagaaatacttgactgcacatcattgaaattatca
ctttctaaattatcaatatttagaaggcatcttttagttaaagttttgacaaccgattta
tacttacggttaatgagacgatttctttccgaagttttaatgcgtttaatcgcagaaagg
ttcttagccacagtaaatccaataaaacttttataaaatatttttagaaacgaaaataca
caaatctgggatctattcaaaaagggaatatgcggcgtattataacattaaagaaaaaca

ataggcaatagttcaacaagagctcttgttattacacaatagagattgtcattgcgaaaa
actttgtaagatagtatttaattaaaataaactaactagacatataaatatatatgaaaa
aaattgaagctattattagacctttcaagcttaatgaagtaaaacttgctctagtcaagg
aaggtattggaggaatgacggttatcaaagtttctggatttgggagacagaagggtcaaa
ctgaaagatataaaggatccgaatactctattgatatcattgataaaataaaaattgaaa
ttattattagcgatgataaagtagagaaaattgtagagacgattattaaggcttctaaaa
caggggaaattggagatggaaaaatatttattagtagtattgaaagagtaataagaatca
ggactaatgacttaaattttgaagctttatagtatttttacttt-tttagacttgatact
tcttcactaaacatgagataataagcgtttattgtatacttataactaaggtgtaata--
ctaatggctaaaagcaaaggtgcacgaatcgtaataactttagagtgctctgataaggct
ggagagtttgctcaaaaaaggaaacctggcgtttttcgatatacaactactaaaaataga
cgaaatacaccaagtagaattgaattaaacaagttttgtcctaattgtaatcagcattgt
atcttcaaagaaattaaatagttatttatcttattaaaat--aatcatattatggctata
tatagaaaaagaatatctccaattaagccaacagatgctgttgactacaaagatattgac
ctgctaagaaaatttattacagaacaaggcaaaatactgcctaaaaggtcaactggattg
acttcgaagcagcaaaaaaaacttactaaagcgattaaacaagctagaatcctttcttta
ttaccttttttaaataaagattaa--atatatagcctaacttcatatttttccatctaaa
tattaaaaatttctatagatttgtttattaaaatgatctaacatactgaat---------
----------tactaagaaga-tctactcattacaagttttgttcattttataattaata
atagcattataattta---ttaagctagattc-----agacataaag-gtaacctctttt
aattgattatattatatagtctatttaaaagaggaaattttttttgcttc----------t
agcaattcaagattctttaagctttaacaaggtatttaaaacaaagatttttgtttaggt
atcagatcaaatgaatgaatcttatctcccgactgccataattgaaattcagagatgaaa
attccacattcgttgccagcttgaatttcttcgacatcttctctaacgcgctttaaagat
tcaatttttccttgataaataacttttttcttctcgaatcactttaatccaagagtttttt
aatagcttattattgatcactcgacatcctgctatttttctatttgctaacgagaatact
gtacttacttctgcttctcccaccggtacttctgaatattcaggatcaagtaagtcttcc
atccttcttttaatgtcttcaattaaggcataaatgatttgatagttttctattaatata
ttcgactttgccgatgcttgctttgttcctggagcaaagttggtattgaacccaatgaga
gtagaatttgtggttgaagctagttcaacatctgtagcagtaatttctccaggcatgatt
gagacaacattgagttgaactttactctgaggaaattgagataaagaatctaggattgct
tctgtagagccttgattatctgttttaataattaaagaaatttgcttactaatatcttta
gaattagtgtttttttaaagtatctaatgtaattcgactattaagtgctctttgtttctgt
ataattgatgagtctttagaggtattttcaatcgctttaagtttggcttctttatcactt
ttaactgctaaagctatttctcctgtagctggtacagatgacaatccccaaatttcaaca
acagatgatggtattgctaaattaattttctctttagcgttgttaattataactcgaatc

tttgcgtaagctgatccaataaccaaattatcactaatattcaatgtaccattttgtatt
aatagagttgctactggaccatgcgatttatctaaatgggcttctattataataccttgt
gcaggctgtgttggatcagctttcaaatcctctaattcagctaacaaggtaatagtttct
aataatttatcaacattttgaccagtaagagaactaataggaattataggcacttggcca
ccaagtttctccgacatgacattgtattttaataaatcttgctcaataatatctgtatta
gaaccagctttatctatcttagatattgctatgacaaaagggacattcgctttttgaata
tgattaatagcctctatggtttgaggtttaactccgtcatcagcagctataataataata
gcgacatctgttaaattagctcccctagacctcatacttgtaaaagcttcatggcctgga
gtatccaagaaaacaatttttttgtttattatctttcttaatatactcaacttcataagct
gcaatggcttgagtaattcctccaatttctttgttagcattattagattttcgaatataa
tctaatagcgttgttttgccatgatctacatgtcccataactgtgacaattggaggtctt
ttaatataattgccagtttcataaaaagcattagagttatctaaattactagaaagtcca
ttattattttcctttacatttgattctactgctataccaaaattatcagccactgatgat
attattgaggcgtctatggtttgattcatagtaactgatattcttttaagaaataaatat
tttataatatctgtttcttgaacacaaattagtttagataattcttgaatagttagagga
ttagtaatactaatagactctggcggagaattagattgaatgcttgctgaattttgacta
ggagttacggctactttttctgtcttatagatttttattactagtcagtttttttaca
acttccacttttggcttaggtggacgcattaaagagatagctaaatctcctgcagtctgc
gaaacattagaattagagtctctaaagttgtca---tcatcatcgtcaatatgtatttta
gttttattttt-ttgcgctgtctattctta--tttttttactatctactaagtcgtga
attttattaaaattttactttttttatctagtttaggtggagaacttaattctagatgc
tgttcaccacc---tgtatgtgattctgatttatcaaggtctaaatttaacagattatca
ttattgactgactctaatctaattttatagataatctgaggatttttcaaatctacaatt
gtttcagaagaattgatatttgagctagatctagaggatctattttcaaagttttgatta
tttaaaaacataaatattggtttattatctaatagtaactttctaattttaatacagag
cttaatataaacaagaatcgaatatctgtataaataatacaaatgt---agagaattaat
ctatgttgtattagatttaatataacactaaagtcctttgttcgttatacaagtttaaaa
ctttt-tatgatgataaacactagattctagtttattggtatacaatctatttatctaaa
taatactgtttttgtcagggcttttaaa----ttatctaatgttatgttcattatgaaat
tctattaaaatttaaaaa-ctcaacaattaagtaagaatcgttaatcacctccacgccac
ttatgccacgatctcaaaaaaatgataattttattgacaaaacttttacagtattagctg
atattgtattaaaaatacttcctacaagtaaagaggaaaaagaagctttctcttactatc
gagatggaatgtctgcacaatctgaaggtgagtatgcggaagctttagaaaattattatg
aggctttaaaactagaagaagatccatatgatagaagctatattttatataatatagggc
ttatttatgctagtaatggagagtatgtgaaagctttagaatactaccatcaaggattag
aactaaattttaaattacctcaggcccttaataatatagccgtgatataccattaccaag

gagttcaagcaattgaggataaagatacagagctatctaaattaatgtttgataaggcgg
ctcaatattggcagcaggctattaagttagctcctgataattatattgaagctcaaaatt
ggctgaaaacgacaggacgaatgaggaatatacaaggatattaatatatttaagatataa
taaagatatcaattaataaattaaaattagttaaagaaactggtactatatagagtactt
-tactatatattataatattatactagttgatctctatctctaagacttagctgaataac
cgaaattaaatctattgactatattcatgaatacaaataagcataatggttagtacaaca
aaaagtaccggatctgttactcaaattatcggaccagttttagatattgcatttcctaac
ggacagcttccgaaagtattcaacgcactcaaagtacaaagctcagaaggaactattact
tgtgaagtacaacaacttttgggtgacaacaaggtacgagctgtttctatgagttccact
gaaggactacaaagaggggtagaagttattgatactggatcccctatatctgttcctgta
ggtacagatactcttggacgtatttttaatgttttaggtgaacctgtagataatttgggt
cccgttgattctgagagtactttacctatccatcgaccagcacctgcttttactaagcta
gagacaaaaccaaatatttttgaaacaggtattaaagtcgtcgatttacttgctccttat
agaagaggtgggaaaattggtttattcggaggtgctggagtaggtaaaactgtattaatt
atggaactaattaataacatcgctaaagctcatggtggagtatctgtatttggaggcgtt
ggtgaaagaacaagagaaggaaacgacctatatatggaaatgaaagagtctaaagtaatt
gatgcagataatctgaaagaatctaaagtagcattagtatatggtcaaatgaatgaacct
cctggagcacgtatgcgcgttggcttaactgcattgacaatggcagaatactttagagat
attaacaaacaagacgttctattgtttattgataatatttttcggtttgtacaagctgga
tcagaagtatcagctctactaggccgtatgccatctgctgtgggttaccaaccaactcta
gcaactgaaatgggagcactgcaagaaagaattacttcaacaacagaaggatcaattaca
tctattcaagctgtatatgtgccggctgatgatttaacagacccagctccggccactaca
tttgcacatttagatgcaacaacagtactttcgaggaacttggcggcaaaaggaatttac
cctgcagtggatccactggattcaacatcaacaatgctacagcctggaattgttggaact
gagcattattctactgctcaagaggtaaaatcaactttacaaagatataaagaactgcaa
gatattattgctattcttggtcttgatgaactttcagaggaagacagacaaactgtatca
agagcaagaaaaattgaaagatttttatctcaacctttttttcgtggcagaagtgtttact
ggatcacctgggaaatatgtatctttggaagatgcaattaaagggtttcaaatgatctta
aaaggcaatttagacgacttgcctgagcaggcattttatctagtaggtgatatagatgaa
gctatacaaaaagctgacagcatgaaagattaatataattaacgatgactttaaatataa
gaattattgctcctgatcggactgtttgggatgcagaagcacaagaaattattttaccaa
gtagtacagggcaacttggtattttaacaggccatgcacctttgcttacagctttagata
ttggagttatgagagtaagagtagataaagaatggatgccgattgttttgctgggcggtt
ttgcagaaatagagaacaatcaattaactattctggttaatggcgcagaagaagctagtc
aaattgatttatcagaagcagaaaaaaatttagacactgcaactcaactcttaagtgatg
cctcgtctaataaagaaaaaatagaagcaacacaaaaaaatacgaaaagctcgagctcgag

tacaagctgcaacagcagcaacttcgtaa-aagattcc------tcttaaaacataaaac
tttagtaattattactaaa-------gttttatgttttatttgtatggttataggcagct
taaataaacttagcttagacctgaacaaatataatcaaaatataatcccatctctttacc
tgcatctggtccaactaaactaatagtaacttctttcatagctaaaatagcttgaatcgt
tgcaccaataggaacacctagagaattgtatgtttcttttagaccatttaatactctttc
ttcaaggatagatggatctccagctagcattccataagtagcatagcgtaagtagtagtc
taaatcacggatgcatgcagcataacgtcgagttgtatacatattaccacctgggcgtgt
aatatccgaataaagtagagattttgctacggattctttaataattgttgctgcattagc
tgcaatagtagcagcagctctgactcgtagctcacctgtttgaaaatagcctcttaattt
ttcaacagagctatcatctaaatatttaccttggacatcagctgcattaataacagaagt
aattgcgtcttgcataactttcacacattccttaattttaaatatctaatacta--gggg
tatataacaaaattactagatcttttagcgatatcagtattactgcatagcacccaaagt
ataatcaaagtaaaaaccagcttctgctgaatcttcacctgcaagcagtgagcaagctac
acttttcatgcattttacaccttcagcaactcctgaaattggtgttcctaaagaattata
catttctttaacacccactaatccaatttcttcaataggagttacatcgccagcaactat
tccgtaagttactaagcgaagataatagtccagatctcgtagacaagtagcagtcatctc
ttcgccataagcatttccaccaggagaaactacatcaggtctttttttgaaacagttgttg
gccgccttgctttacaatacgctcacgattatctgttaaaatttgagctattcttaaacg
acgttgtccagataaaacaaaacttttaattctatctaattccccaggacttaaatatct
tgcttctgcatctgcatttacaattgactttgtaacaatactcatggataaaactcctgt
aatgcttt--aaaaaaagcttaatactaattatttatgatataactcagaaagtaatgta
ctttttgag-aattttctaaccgaaacacgttcatgatatatgaagcaggatagatactg
aggtaagtactaaaacgagaaagttatatgtaggaacttctgttattgatttcctagtac
ttttttaaaacttggtaccacaattgatatatcctgcttagtcaaacgattatagagtgt
ttctgtattaggaaagttagctgctggtaaagtcggaaatcttctgtatggaacagtatc
tttgccgtataccgcattgtattcaacactttcaactaaattacttataaatgcagataa
gccttttgaagctaaaatttgattaaagaatctaatttctgcctgattattaggagctct
tcctaagatatgcttagtacctaattctataacttttgtatttggatatggctgataaaa
ttctttaccatatagttcagatagcgccagtttttctactagttctttgacacagatttg
tctattcaagaaagctgacttaatatccaagaattcaccgccgacactaaaagagttaag
atctctttcgaaaatttgacgatatgctgctcttaaagcttgttctaacatttctttgtt
actatcactattcacttcaaagacaatagattgatctcttaaagaagttactccttgtga
aatacgagactgaatatcattactactcctcatttctttgacagtgcctaattcaacaaa
cctagcagatttactagcaataactttttttgaatctctggtcaataattcctggtcttaa
gcttcttaaagcaacaccagctggagtactatatctttcataaggaacagtattatctcc
aaaagtttctgtgtactctgagctatcaattattgtgtctattacttggtaatagccttg

cttatatgcaatatcaaaatatttattaatttcttgccgaccatatgtagggcgacccag
taagcggttatgaatatattcaatagctttacaaatatataatggctcccaataaagaga
tctaaatatacttgattttgctaactgtctaacaaactcgcgaactgtaatttgattatc
ttttagctgactttctattggcttgagaattagtttttcttcttgatacacttctctgcc
aaaaactcttaaataagcaacttttgtgacaacttctactgaattttcaaaattttggga
tgaatctgactttataagagcgttggacaatttaaagattttggtcccagggatcctgc
tgacttaggtcttacctgtggattactaatctgattgtagattcctggcccacgtctaac
taatatccttcttgtatctttaccaaaaagagcttgtctttttcttggatctttattttc
tttagggaaaatagctccaaattgaattgataatggatcattacctattccatatggatg
ttgatcaggtaatgattgcttataatcactaaataaagttataaattgaggaaccttacg
aaaaggcgcactataggtgagaagatctatttgaggcccccaattacggcactcttgagg
ctcttcacctaaatttctaaagtaaggcacagtctcttcaccgaaataatctgtatactc
agaagaatttaaaagtgcattcactaaaccacttaaaccagtagatgataaaatagcaaa
atatttttgaaactcttctaaagagctaggacctctacccaagaaatgtctaaatgctaa
ttctaatgctcgactattgacaaaaggttcataaaattgctttctataaatacttgaagt
tcctaacgaacgaataaattccttaattgaaatttggccatttttacttgcgactccaa
attcgataaagagaggtcatatgctttagcaatatctctttcaaaaatttgtctataaca
tgctttcacaacaatatttttctcatcagcagataaactaggcttcataacaaaacgagg
tgttgacactcctgcctgtacataagtctgaggaagacgcaaaccttgtaaatctccaga
tattctttttcttaatttatcagtcaaagatggagcttcaaattcagaaatcacaacatt
aaaatactctttaactaaatcttgaccttttatatcttcctcaaagattaataatgctgt
ccgtctcatctctctaagagccacaattgctgctgcacttgagcaagcgttatcaattaa
ctctctcaatcctcgtatattaacagataaaatattagggtcgcctgatacaattgcata
tgtcaaataccgtaaaaaccagtctaaatctcgaagagattttctcattcttgtagtacc
gtatctcaagacgtttataggtttaaaccctggcggagtagctcccccagcattaaataa
agatcggaaactttggccaaaatctccttggatatttcctgataactcattaattttatc
ttgagaactttgatctcctgcaataataacagctgcttgcggacgttctaaataagaaat
tgcagacccacctacgaatattttatctgctgctcgtgctaccagaatatttgcattttt
agttaaaatatctgcaacttccagtctttttgtccagaatttaaaaaagaaactaattg
atttaattcaccaagctgtaaaaatcggtcttgttgctctgcctgagtaatagttaaaat
tgaggcagtccgataaagctgggggcgtgctaatgggcttccgccacttgctttgatact
cattactttatttatctccttaactgatattgcttgctagcgaatacaaatatagcttga
tatttctaaatatttaaagatactatgcaagcaca-ctgttttctatattattaggtact
agtacttatagtacagagagtcttataactaacttctataaatacaaatttgtaatact
tctattaacatttgtcaaaaaaatatttaagtgctcactcttttatattaaaattcaatt
agtgagaggttgatgtgaaataaagtatactaagtaagttttatagataggaaaattaaa

agatttattctacaaacattaatca------------cttgaatgtttagcaattgtaca
attgttattgcttttaattggtttagactagttttatatttttttctaacataaacaatat
taatgagcaatttgtatgaatttaaagccacaaaacaacctattaactaacgaaaatata
gatcatgtagatatacccgaaaatgatataccaatgtcaattacggaacatttagaagaa
ttaagacaacggactttatttgtgtttttattctttttgtttgctacaactataagtttc
acacaaattaagatcattgttgcaatactgcaagctcctgctgttggtattaagtttta
caactggcaccaggagaatatttttttttcatctattaaggttgcaatatattgcgggatt
gttgcaacaactccgtttgccgtttatcaagttatattatacatactcccaggactaact
ggaaaagaaagaaaaattattttgcctctattaattagttctgtattgcttttattaca
ggtggtatatttgcttactttgttctcgcaccagcagctttaacatttttaattagctat
gggtctgatattgtagaaccattatggtctttcgaacaatactttgactttattctatta
cttttacttagtacaggattagcatttgagataccaatcatacaattattgctcggtgtt
tcagggacatttcttctagtcaaatgatacgagcttggagatatattattattatagca
acaattgctggagctattctgaccccttcgactgatcccgttacacaattaataatgtct
tcagctgttttattactttattttggtggaattgttatattattagtcttaaaaaagtag
agtaaat---------atttcta-ttaatccaatatatttgttatc-----tggcaaa
taatctat-aaaacaattgct------------------agatttaagtatcaggcttct
aaatatttcaa---------gtctcttttatcattgaaatacaatc--------------

--gatacattgg
cttgttttttcatcaattatttgtgttgcacac-tttttagtactctctttattgttctt
agaattatttctttaatctatttattaaat----agatatgaagagatttaagtattgat
tatggtaatctatatcaacgtaaaaagtattttattgtgagttttatttatataaaggct
tattcattatatagttataacagtaagtctaattaatag-ttttcatgcttaattaaagt
aataactcttaattcttttggcagaaaaatctgtcttaatattttatc--aaaaaaacag
-aaaaatgaagctaaatagtctaattaacttaattcaaaagtctatatattcttgtacac
ttttactaattattttaaatattatttgtgtcgcacctaattctagtaatgcatttccaa
tttatgcgcaacaagcttatgaaagtccaagagaagcaactggtaggatagtatgtgcca
attgtcatcttgctcaaaagcctgtggagatagaagcgcctcaagcagtactacctaata
ctgttttcgagactgttgtgaagattccatatgacagcaatgctaaacagattttaggta
atggcagtaaaggaggcttaaatgttggagccgttgtaatattacctgaaggatttaagt

tagctcctgttaatagattatctacagagttaaaagaaaagactagaaatctttacattc
agccgtacagtgctaaacaagacaacatttagtaattggacctatttctggtgataaaa
atagagaaatagttttcccaatactatctcccgatcctgcaaaagataaaaaagctcatt
ttttcaagtatccaatatatgttggcggaaatagaggacgaggccagatttatccaacgg
gtgacaaaagcaacaataatattgtctctgctttaagcagtggtaaaattaataaaattg
aattactagacaaaggtggatttataatacatgtgactaacagtagcaatgtggagtcaa
cacagaagatttcacctggtctcgaacttagagtaaaagaaggggatacaattcagcttg
atcaagctttgaatagtgatccaaatgtaggtggttttggtcagaacgaaacagaaatag
ttttacagagtccaaatagaattaagggcatgattgttttctttttcgctagtgttctag
ctcaaattttcttcgtattaaagaaaaaacaatttgaaaaagttcaagcagctgaaatga
atttttaaagcaatataaaactggcacttactataaagaaagta-taaacactttattca
caggatgtaccagtttttatgttatgtaataatcgctttgacggaagttatgatactttt
aacagaatctacaatctgactaggctgaattactgtagcctgctctaagcttccattata
tggtgtgggtatatcctgcgaagatagcctcacgacaggagcatctaattcatcaaaaag
atactcattaatttgtgcaattagctctgctccaattccggctgttttcatacactcttc
tacaattaaaactttgtgagttttctttactgagatagatatagagtctatatccagagg
ttttaaagatataagatctataacttctggatcataaccttcttttaataaggccggtaa
tgcttggataacatgatgcctcattctagaataggttaaaatcgtaatatcttttccttt
tcgtacaaattcaactttattgagaggtaagaaatattcttcttgaggaatctcttcttg
taaattataaagtagaacgtgctcaaagaatacaactggattattgtctcgaattgcaga
tttcagtaatccttttgcattataaggagtagaacaagcaactattttaagccggggat
ggcttggaaataagcttccagtctctgagaatgctctgcacctaactgcctaccaactcc
tcccggtcctctaataactaaaggcaatgtaaaattacctccagaagtataacgtaacat
tcctgcattattagaaatttgattgaatgctaataataaaaaactcatgttcataccttc
aacaattggtcttagccctgttatagctgcaccaattgccataccagtaaagctattttc
tgctattggtgtatcaagaactctcaaatccccatatttgctatgcaaatctttagttac
cttataagatccaccatagtgaccaacatcttctcctattacgcagacagttggatcttt
tgccatttcctcatctgttgccgctcgtaaagcgtcaaacataaagatttactcatttt
aatatttgatttttatgatttgaccactgttttaaagtaacagttaagacaccatattct
atttagttatctgcaaaaagatatcgtttcaattctgacatgttaggttctggactggaa
atagcaaatttcacagcttgttccaattcggttttaacagcattctgaatctcattaagt
tcacctatattagcaatttcattatctagaatatatttttgagtttttgataggatct
cttgccacccaagcctctttttcttgtcttgatcttagttcatcaggatctgcgagagag
tgaccacgaaatctatatgttaatgcttctattaaggttggaccatcaccttggcgagct
ctttgaactgcttgttttgcagcttgtcttacagctagcacatccattccatcaacttca
atcccaggaagcccaaaagcttctgctttttttatgtatttcaggtattgaagaagaccgg

tgatgtgccatacctatagcccattgattattttcaacaacaaatataataggtagtttc
cagagaactgccatattcagacattcaaaaaattgcccattattggtagtcccatcacca
aaaaagcaagccgtgactcttaaatcctctgtttctttaagtacttgctggcggtagata
ctttgaaaggctgcccctgttgcaaccggtataccttccgcaataaaagcaaagccacct
aaaaaattgtgaggcgcagaaaaaatatgcatcgaacctcctctgcctttactacaacca
gtctcttttccaaataactcagccatcacattttagatgggacgcctttacttaaagca
tgtacatggtctcgataggtactgcaaacataatcagttggattcagaagtttaattaca
cctgtagaaacagcttcttgaccattataaagatgaacaaaaccgaacatctttccttta
taatacatctgagcacacatatcttcaaaatttctgcctaacaacatgtcttcatataaa
actaataaattactcttattaagattaagacctgttgaattatagttagtcagcggcaat
tgaactttcttaggataactcataatttgtaaagaaacctct----------tattgtaa
ataggaattttgaagaaacagctatcaatagaaaacacacagataaaaacttataaatta
taatatgtttttactaaagactttaataattatttgaaactagttcagtatatcaaatt
aattttgcttatactacggatgtctgaaacttatataaaaaagtagt-a-aaaaagttta
ttaaaagaatagttaaaaacagatataaatagaagtaaacagaattatt-gaaaaaaaat
gattattattaattaa----tacttaatatttctaaatattttacaattaatggctatgt
caccatctttattttatcctgttgaacaagaactatgtagtcttgaaaaaaatctgaaag
ctgttgctgggactcgtcatccaattttatatgccgcagcaaagcatctattcgatgctg
gaggaaaacgagttagaccagctcttgtatttttagtggctaaagcaacctctgagaagc
aagatataaatactggacaaaaaaggctagcagaaattactgaaattatacatactgcta
gtttggtacatgatgatattattgacgagtgcacaacacgtagaggagtcaaaactgtac
ataatttattcaatactaagattgctgtgctagcaggagatttttttatttgcacagtctt
cttggtatttagctaatattgaaaatttagccgtagtcaaagctatttctaaagtcatca
ccgactttgcagaaggagaaattaggcaaggcttagttcatttcgatcccagtatttcaa
tagatgcttacattgagaaatcattttacaagactgcttcactcattgctgctagttgtc
ggggtgcagctatgcttaatggttccaatcatcaaataaataatgatctttatctttacg
gtaaacatatgggattagcatttcaaattatggacgatgttctagatataactggttcta
ctaagagcttagggaaacctgctggcgctgatctaataaatggaaatttgacctctcctc
tcctttttcacttactcaagaagcaagtttaaatgatcttattgatagggagttctgta
atagtacagatatagcctcaacattatttcttataaaaagaagcgggggaattacaaaag
ctaaagatttagctaaagaacaggtgcaggcggcactttcctgccttcagtttttaccac
aatctacacctgtatctagtttaaaagaattaacacatttcataatcacaagattgtcat
aaaggacttgctaaaattaaaatgttattaactaatttttttactgtttctaaaacagtct
gaaaagtttgaatatcattactagctagctgcgctagcattttacgatttaacgcaatat
tctctttttttaatgcactaataaaagtactataattcatgccttgattgtgagcagcag
catttattctagttatccaaagacgacgaaaatctctctttttcctttttcgaccaacat

aagaatatcgaagagctttaaggacttgctgcttagctgttcgaaataaacatttatgtg
cacctttaaagcctttagctagcttaaagatttagctcgtctttttttgcaacgttac
ctcttttaactctactcataaaatattac-ttaaattttaattattaccagcttactaaa
ttcttatactctttg----attataaataaggtaatttcattgcaatgtttttaatatct
tttaagtcaaccgaacaagtagaagaaagatgtcttctttgttttgatgactttttttgt
aataaatgactttagaagctttatgtcgaagaaatttccggatgaagagactttaaat
ctttttgctattgcttttgatgtttttaacttaggcatattataaacttttaatgattaa
caaaagcatgaaaagtctgaggatatcaatcttagacctttcaatacttagaataatctt
aacacaatttattttacttactacataggttaactgaaacaatttatatatataatacta
atcagttaatcaaccatagctaaagtggatttatatttgctttgaactttgtatagta
ttagcaattagcattgttataattttaagactgcttgaattcaattcttcgaacattttc
atatttaatgtaaatgaaatattggcttctgagacaatattttgaatttgaacatcagac
aagggaatcatatctaatgcagctctatattgatctttaaataatttatcgtcttttatt
tggtcaaaatcgtaaaatttcgttcctcctgaatctgataaattcatagcccctctagct
atttttttaaaatttggccgccagaaaggtcacccaaatagcgcgtataagcatgagca
actaataattcaggttgtttatgtcctatagtgtgaattctatcaacataaatttttgta
gcaggagatggttcaataaaatctaaccaatctgatccataataatagtttaaatcttca
gatagactcgctttcctattaagctctgtaaaatatataggtttaatagctggatgattt
ttattagaaaataactcttcctctattgcacaatagacaaagtatagatttgcaaccaat
ttacgatatgacttcttatctacaactcctcccaaaaaagatttaacaaaactaacattt
tctgccatactatgggacttagtagtaccttctcttagttcattcgctaaagtattaacc
ataatatcaatattccattttt-aaagataacaaattaaca---ataaatagttatccgt
atatttataattgagttgcgctaactaggctgctgcaaaataatttttttgattttattgg
atctggattcattgttttgtcaccaggcttccaattagctggacaaacttcatctggatg
agattgtacatattgaattgcttgtaaaaccctcaaagtttcttctacactcctgccaaa
ctctagattattgattgtagagtactgaataattcctttcgggtctataataaatagtcc
tcttagagctacaccatcactattcaaaacattataagctgcactgatctcttttttaa
gtctgatactaatggatatgaaagatctcctaatccacctgattctctgtcagtctgtaa
ccaagcaagatgagaatactcactatcaaccgagactccaagaacttctgtattaagttc
cgaaaaagcattatacttatcgctaaatgctgtaatttctgtagggcacacaaatgtaaa
atctaaaggataaaagaataagacaatatacttattcttgagatcagataattttaacgt
tttaaattcttggtcataaacagctgtagctgaaaaatcaggagctagctgacctactcg
aagacaatttggtccagaaatcattaatttttcccagaagaattgatataattttgttat
ttaataaataattatataacaaaatttagtatatattaaaatactacaagtcttttttaa
attac--------taaatgatataatcaatactatctttatagcggggaatggatttgaa
ccattgaccttcgggttatgagcccgacgagctaccagactgctctaccccgcggataac

taaaatcatattagtctacatatatatataaaagcaaatgcataatagattttagaagta
aaaaaatagaagaactggtccactgatcattaaacttagaaacgacaaagtatttttat
cttatttaaaagagggaagacttcatataaaccaataaatctatcttttaaaagaatttc
ggacggctttacccaaatttgaccgtcataccagcctgattcttcataaaatactgtagc
actcattaatcgttttactacgtatgaccagcctaaatataatctgattaaaataaatcc
tgtcattaggctggaagtaataaattctgaaaagaaaaatttcaaaggtagtttagtgat
tggaaaaattgataataatataggactaactaataaacagttaagtagtagtgcaatagt
tattttttattataagatctacggcttaaagttggccagcaaaagaaccaagaattttt
caaagaagtatattcatgaacaggttgctgctcctttggcacaggacattgagtattata
taaattcattgacattaaaaagtattataataaagac--tttgtttatattatctaacat
aggtacgacattaaatattaactaactgcaattatagtagtgagtagaagaaataaaact
aaaaatagccaagtaattttatttaaagtattctcagtactacgagtattactaaagaac
tgattctgagcacctacacttcctaaaccttcggatttaggattgtgtattagaatagtg
aagattaatattattgtagatgagtaccaaaaaaatttaaaatttgttccattatgtaa
aagttaat-aaaaatagcacaaagacaatcgtctaaaagatattgattgtcttatcatat
taaaaagtatttattcaccctgtacttttaaaagtaaaaatcctattgccagccctacca
aaactaggactgaacttaatacagcactttctacaatttctccgaccatt-ttattttc
cttattatttattattg---tcattcttttgacaagattatattgagctaa-ctaaaat
ccgtttctaccccaaactaccattgcgattgaaaatgtaaacattgccattagagctgcc
catcccaaacttaaaatatccataatttataatattc--ctaaaaataagatctcttta
cttaaactataatattcttaatattaaaaatatcattatataataagctaattataacag
agtactacttaattcgtattttagtgtatttaaagaagattgaa-ttttttcgaggtac
tggataagtatttcatcctaagaaccatagtcaac----ttttgatttattataatgctt
aaatctaaatata--tatcactcaagaatcattagtaattatgatgtttttaattatacg
att---aacatatttcaaaatagaagaattcatagcgactaagttaa-----tatgctaa
tataatttatcaat--aaaaaggaagttagtatttcaattatgcaaacaactataaataa
tgggcaaacgtctagtaaagaaactttactaacacctagattctatacgactgactttga
agagatggctaatatggatatttccggcaatcaagaagatttttttggctatcctcgaaga
atttcgagctgactataatagtgaacattttattagagacgaagagtttaatcaatcttg
gtctaatttagaacataaaactaaatccttatttattgagtttttagaaagatcttgtac
cgcagaattttcaggtttttttactatataaggaattatccagaaaattaaaagacagaaa
tccggttatagctgagtgcttttttattaatgtctagagatgaagctaggcatgccggttt
tttaaataaagctatcggggacttcaatttatctttagatttaggattttttaacgaagag
tcgcaagtatactttttttctcacctaaatttattttttatgcaacctatctttctgaaaa
aattggatactggagatacataactatttaccgccatcttgaacaacatccagaacaccg
tatttatccaatctttagatttttttgaaaattggtgtcaggatgaaaatcgtcacggaga

ttttttgctgctttgctcaaatcccaacctcatttttaaatgactggaaagcaaaaat
gtggtgcagatttttctattaagtgtatttgcaacaatgtacttaaacgactttcaaag
aattgatttttataatgccataggcctagactctagacagtatgatatgcaagtaatacg
aaaaactaacgaaagtgccgctagagttttcccggttgctttagacgtggacaatccaaa
atttttcaaatatttagatacttgtgcatgtgataatagagctctgatcgatattgataa
taacaattctccattattcattaaatctatcgtaaagatacctctatatttttccttatt
tgcaaatttactaaagatatatttgattaagccaatagactcaaaaacagtatggaatac
agttcgatagttaaatacaagggaatgctttttcttataaaaagctatctagtgattaa
tataaaat------aaaagcttccttct-----cgatggaaggagcttttatttgtcaa
ga---atataatgggattatatagatc-tagaactaagaacttacttttcttttgcttc
atacataacctctaaagtaattagatttttagaacaatcacgggcaaatttctctgtatt
tctttttactttacctctaacaaacccaggaatttatttaattctttctgtgcttcttc
tgaccaattaatactatcaacagcagaaattcctatacttaatgcttctgttgtatcatg
accaccaaatatttctagcaaatgatcttccataccaagtgtaaacgaattatatactaa
atcagcaatttgatttgttccctcataaccaagaaatggacggtaacttaaaggaaagtt
ttgtatatgcactggtgaagaaataacgccacaaggaatatttagacgcttaccaatatg
tctttccatctgtgttccaaaaattgctgcaggttcagttttagcaattaaatctccaat
taaaccatgatcatctgaaacgattacttcatcgcagaattcttgaacttgatctttaaa
ccactcttcatcatatttgcaataagttccacaccaggcgacatgtatacccatttcctg
gtgtaaaattcgagttattgctgcagcatgagttgcatctccaaatacaatagccttttt
acctgttaaattctgacaatcaattgatcttgaaaaccatgcagattgtgaaataaatcg
ggtttgctcgtctatgtatttttcgtagtctacagcagctcccaatgcatttactaattg
ctgtattgatcggatgcatgcagctgtttgaacaatacccataggtgtaatgtcaacata
aggcatattaaattctttctctaaatatcgagctgtcattagtccagtttctctatatgg
aataaaattaaaccatgcagagggtaatttttttaaatcttgaactgatgcattttctgg
aataatttggtttatttggatatctagatcttgaaataacctttttaattctgctatatc
atgttgattatgaaatcctaagcttactgctccaataatattaacagatggagttttagt
cttttgagttaaaacttgattattagatttagctttttccatatagaaagtgacaatttg
ttctaatgttctatcacctgcttgcagttcatttactctataatgattaacatcagctaa
taaaacatctgcttctgtttctatagatgctctgctaacaaagttttgcaaatcttcttg
caaaatactcgaagtacaagttggtgttagaataactaaatcagggctttcttctctgtc
ttttctagtaatattttctacaactttttcttgagaaccacgggctaacacatgtctatc
tacaacacttgctgtaacaggagtaaaatctctgtctctttcaagcattgaacgcattac
attaaaataatcgtctccgagaggcgcatgcataattgcatgaactttttttaaaagaact
ggcaattcttaaagttccaatatgagcagggcctgcatacatccaataagctaatttcat
attaaattttcctatgatttatttagacatagtaatacttttaaagctaactaacaaaca

ttagccaaacatctaatttaactccttgacaattttgtaatatttttaaatgaagctta
tcattataaatatttaaaaaatataattcagaataga----aataactgttagatcaagc
ttgaaagtaaagatttcttgcagttatagtttttctttaagtaaacatgtaaaaattgta
ctatatataaatgtactatatataaactttttttactgaatattggtaatgccagataca
attaacttaaatatgccttccccaacgtttggtggaagcactggcggttggttaagagcc
gcagaagtagaagaaaagtatgcgataacatggacaggcaaaaatgaaagtaagtttgaa
atgccaactggtggtaccgcaactatgcgaaatggagaaaacttactctatttagctaag
aaagaacaatgtttagctttaggtactcaactaaaaagtaaatttaaaatatctgactat
aagatttacagagtttttcctaacggagaagtgcaatatttgcatcctaaagatggagtc
tttccagaaaaagttaatactgggagagctagcgtcaacagtgttgatcattctattgga
caaaatattaatcctgtagatgtcaaattcatgaataaagcaacttacgattagtctctc
-aaaaacctagacataaaataactttatatagtctaggtttttgataatgtatactgat
agtatggaagggtggccgagtggttgaaggcgtctgatttgaaatcagttgaacta--tc
tggttccgtgggttcgaatcccaccctttccgttataaaact------------atagtt
ggcttctcaaggcaagctagctatacactgatagtattaagtttagacttaataaagtct
acaacttgtgacaggttagaaatttttcggcatcctcatctggtatctctatactaaat
ttttcttctatggccattaccagctcaactgtatctagagaatcggctcccaggtcactt
gaaaaatttgcttccctagtcactatttttttttcaattcctagctgttctgctacaata
tcttgaactttttcaaagatttcattatcttgcataatatattatttcctctaaggtgtt
atcaaaaatttttcggcttatattattatccaactctaaaaaattagtaatttttagtcg
agattaaaatatttatacctatatgatatactattattctggataaatacgaagtttacg
atatggctcttctccaaaactacgagctctcaactggtagttgtcaactagattatgctg
catttttcgtatataggctgatcgaggtgtaagctgaactattgaactttcattcaagat
aatagtttcaatagccaatttagcttcttgaagagcctggatctcatcaaaatttttatt
tttacagagttctacccaattaagaccagatgaagtatgaatgtttaatatttttctgag
tgcacgagtaatttgtggaaccgtactattctgaattgtatatataataatttgttttga
ttttgcaatttgtctaagttttgtattttgctttacttgattacgtagcgctagaatagc
atcagacttttctatctccttggttaaaataattggtagatctagtgaagaaataactga
tgtaatatgctgccaactcaaagaataagcatataagtactgatgaggtatttccaattg
caaagattgtttattaatattaataggggcattgaaagtagttgacaataaagatgtatc
acgattttgtgatttattcaaatctaaggacttatttcttaattctctatattgtaaaac
cggaggcgcctgctgtgcgttaattgattttcgagcattagagatgtttacaggtaagac
ttctatagattgcgatggataacatttaattaaaattctaccattagcttgaatttgtct
tttttgcacaaaaggttgatggccttgcaaaatttgatctattgtctctttaactttatt
atgaactatccaaacatttcgctcgtgtatttcgatcgcaatctgaaaagcaggctcagc
ttttctttctagtatactcttttgcgtacctctacgtttagcttcatcgtcacctaatgt

tacatattgaattccaccaattaggtccgccaatgttggattttaattaaactttctaa
atgatttccatgcgctgtacctactaattgaactcctctttctgctatagttcgagcagc
taacgcttctaattccgtgccaatttcatctataataataacctcgggcatgtgattttc
aacagcttctatcattacttgatgttgcaaatcgggccttgcgacttgcattcttctagc
tctaccaatagctggatgaggaatatctccgtcaccagctatttcatttgaagtatctat
aataaccactctttttccatttcgtcagctaaaacacgggccatttctcgaactgcggt
tgttttaccaactcctggttttcctaagagtaaaattgaatcaccttgctgtagtaaatc
tcgaataatactaattgtaccaaagactgctctgccgacacgacaggttaagccaataac
actaccttctctattacgcagtgaactaatacgatgcaaggtttttcgataccagctcg
attatcgccgctaaaattaccaactttttttacacaataatctaagtcttgccaactaat
agatctttgagataaatattctgggttatctggaaatctagcttctggacgacgacctaa
atccataacgacttctattaaattatttctattaggatgctgttgtaaaggttctttgac
aaaatttggcaaaatttctagcaacttatctaagtcatctgcaataagcatgattgagta
tataataattaattaatcaaatagaagattcaagattctaaagattacaatacatattaa
agaatattatgattggaatcaattttatacttaaattgaacaggaagtta-tagatgtaa
aagaactgattaaactttacgcaataaatcgattggtcaaaattaatagtcgtgttaaca
tgtttattggacaaaaagttcgtattaaatatagcaaacaaaaagttgctagtgatatag
cagataaagttggtgaattaggagttattaagggaataaagtttattaatagtcaatgtg
ttactattattgttgagtttgacaatcacactaggctttggatgtttagagaggaattaa
tctgtctaaatgaat------taaaaacatgaaacataatttattttatttattatacaa
aatttcttatgagtcgttaaaataataaattttaaaggagtacttttaattgtgcaaatt
actattaaaaaattacaagacttactttcgtctgtacaaagaaaaaagatccagacatta
aaactaaaacaaggtaagtttgaacttctattaaataagacctataaaaaagtcaatcaa
gaaattataccttcgcagaaatctgctgtattacaaaatagtccatctacgataattaaa
tcaataaataatacaaaaaaatcttctgttgtcaatgaagaccgtacagaatatgccact
attgtttctccaatggttggaacgttttatcattcacctgctcctggtgaaaaaatttt
gtacaagttggcgatgaggtcaaattcaatcaaacagtctgtattattgaagcaatgaag
ttaatgaacgaaatcgaagcagaaattgaaggcaagattatagaaattcttgttaaagat
ggtgatatagtagattgcgggcaagccttaatgaaggttgaaacataatgctaattttc
cattttacatattgttaacagaaattatttagatagaaaaattagcttatactatagtta
tgaaaacttatttcgcaaaggcac-taaaaactaatatataaaattatttactttatctg
ccaaaaatagaaataagcgttttgattccttgtctcaagagaggagaatctcaatggcaa
ttagctcaaaagagcaagagacaaagaaggtaaaaatctcggttgataaaaatcccgtag
atacttctttcgaaaagtgggcccaaccaggccatttttctcgtacactagcaaaaggac
caaaaactactacttggatttggaatcttcatgctgatgctcacgacttcgatagtcaaa
ccagttctttagaagaagtttcacgtaagattttcagtgcacattttgggcagctgtctg

taatatttttatggcttagtggaatgtattttcacggagcccgcttctctaattatgttg
cttggttaagtaatccaacaggtattaagccaagtgcgcaggttgtttggcctatagttg
ggcaagaaattttaaatggcgatgtaggtggtggctttcaaggagtacaagttacatctg
gatggttccaactgtggagagcatcaggaattactacagaatttcagctttactgtactg
ctattggcggattagctatggctgctttaatgctgtttgcaggatggtttcattatcata
aagctgctccaaagttagaatggtttcaaaatgttgaatcaatgatgaatcaccatttag
ctgggcttttaggcttaggctgtttaggctggacaggccatcaaatccatttgtctttgc
ctattaataagctactagattctggcgtgtctccgcaagaaattccactacctcatgagt
ttttaattaatagagagcttatggctcagctgtatccaagttttagtaaaggattagttc
cattctttactttaaattgggctgaatattccgactttttaacttttaaaggaggtttaa
accctgttactggaggtttatggctaagtgatactgctcatcatcatctagcattagctg
ttctatttcttgctgcaggtcatatgtatagaaccaattggggtattggacatagcatga
aagaaattctagaagctcacaaaggaccttttaccggcaacggtcacgaaggtctatatg
aaattcttacaacttcttggcatgcacagcttgcaattaatttagctatgatgggatctt
taagcatcattgtagcacatcacatgtatgcaatgcctccttatccatatattgctactg
attacccaactcagttatcgctcttcactcatcatatgtggattggaggattttgtattg
ttggagcaggagcgcatgcttctatatttatggtaagagattataaccctgcagaaaatt
ataacaatcttttagatagagtcattaggcatcgagatgctattgtttctcatctaaatt
gggtatgtatatttcttggattccattcatttggtttatacattcacaatgatactatgc
gggcacttggaagatctcaagatatgttctctgatacagctatacagttacaacctattt
ttgctcaatgggtacaaagtatacacactttagctcctggaaatacagctccaaatgcat
tagcaacagctagttatgcatttggaggagatattgtttctgttggtaacaaagttgcaa
tgatgcctatttctttaggtactgcagatttttttagttcaccatatacatgcatttacta
ttcatgtaactgttttaattttagttaaagggttccttttctcaagaaactctagactaa
ttcctgacaaggccaatcttggcttcaggtttccatgtgatggacctggtagaggtggta
cttgccaagtgtctggctgggatcatgtttttcttggcttattctggatgtacaattctc
tgtctgtagcaattttttcactttagttggaaaatgcaatcagatgtttggggtagtgtat
ctccgtctggaaatgtttctcatattactggcggtaattttgcacagagtgcaattacaa
tcaatggatggctgagagatttcctttgggctcaagcatctcaagttattcaatcatacg
gttctgcgctatctgcatatggattaatttcttagcagcacatttgtatgggcattca
gtttgatgttcttatttagtggtagaggttattggcaagagcttatagaatcaatcgtat
gggcgcacaataagataaaagttgctcctgcaattcaaccaagagctttaagtattactc
aaggtagagcagtcggtgttgcacactacttattaggtggaattggtacaacttgggcat
tctttttagcgagaattatttcagtaggctaatagtgaaaataggataaaaaaaacaatt
atggcaacaaaatttcctaagtttagccaagctttatcacaagatcctacaactagaagg
atttggtatggtattgctacggcacatgactttgaaagtcatgatggaatgacagaagaa

aatttatatcaaaagatattcgcttcgcactttggacatctagcaattatcttcttatgg
acatctggtaatttattccatgtagcttggcaaggcaactttgaacagtgggtattaaat
cctttgaaagttaaaccaattgctcatgcaatttgggatccgcattttggacaacctgct
ttgaaagcttttagtaaaggtggctcagcttatccagtaaatatagcatattctggcgta
taccactggtggtatactattggtatgagaagcaatcaagacctgtattctggggcttta
ttcttactagttttatcagctttactcttatttggagggtggctacatctacaaccaaaa
ttcaagcctggtttatcatggtttaaaaataacgaatcaagattaaatcatcatttatct
ggattatttggggttagttctttagcctggacaggtcatttagtacatgttgctatacct
gaggcaagaggacaacatgtaggatgggataattttacaaccgtattacctcatccagcc
ggtttacagccgtttttcagtggtaattggagtgtatatgctcaaaatccagatacagct
caacatttattcggaactaatgaaggtgcgggtacagcaattctgacatttctaggagga
tttcatcctcaaagtcagtctttgtggctaactgatatggctcatcaccatttggctatt
gcagtagtattcattgttgctggacatatgtataggactaattggggaattgggcacaat
ctaaaagatatttagatgctcatagaccacctagtggtagattaggagctggacataaa
gggctatttgatactattactaattctttacacatacagttgggattagcattggcttcc
ctaggtgtaattacttcgttggtagctcaacatatgtatgctatgcctccatatgctttc
atggctaaagatttcacaactcaagcatctttgtacacacatcatcaatatattgctggg
tttctaatggttggagcttttgctcatggggcaatattctttgttcgagactatgaccct
gaacagaataaagataatgttttggctcgtatgctagaacataaagaagctatcatttct
catttaagttgggtaactctattttagggtttcatacattaggcctttatgttcacaat
gatacaatgattgcttttggaactcctgaaaaacaaattctaattgagccggtatttgct
caatggattcaagcctcttcagggaaagcactttatgggtttgatgtgttactatcatcc
tctactaatatcgcaacacaagctggtagcaatatttggctgccaggctggttagaagcg
attaatagcggaaaaaattcattgtttttaacaattggtcctggtgacttcttagttcat
catgcaattgcattgggattacatactactacgttaattttagttaaaggtgctttagat
gcaagaggctctaaacttatgccggacaaaaaagactttggatatagttttccttgcgat
ggacctggcagaggcggcacctgtgatatatctgcatgggatgcgttctatttagctgta
ttttggatgctaaatacaataggttgggtaacattttattggcattggaaacatattaca
atatggcaaggcaatgcaactcaattcaatgagtcttcaacttatctaatgggatggttt
agagattacttatggctaaattcttctccattaattaatggttataatccatatggcatg
aataatttatcagtatggtcatggatgttcctatttggacatttagtatgggcaacagga
tttatgttcttgatctcctggcgtggctattggcaagagttaattgaaactctagcatgg
gcgcatgaacgtacacctttagcaaacttgattcgttggaaagataaacctgttgcatta
tcaattgtacaggcaagattagtaggtttagcacatt-tttctgtaggatatgtattaac
ttacgcagcttttgtattagcttcaacagcaggcaaatttggttagactaaa-----ttc
gaattagctaa-----aaaaaagtcagtcataata-gttttatgactgactttttagat

ttaaaacaaaaacttactaaagtttcacctcaatatctacaccagaaggcaaatttaact
tcattaatgcatctatcgtttgagaagaaggttgatgaatatcaataattcttctgtgag
atcttatttcaaagtgttctcttgagtctttatctacatgtggagaacgtaaaacacaat
aaattcttctttttgtcggtaaaggaattggccctactgcaacggcattagttctagatg
cggtatctagtattttgtcacacgacgtattaagtataatagaattatatgctttcagtt
taattctgatttttgtctgctgagtaattgtcatgttaaaaagatcttattatttaagaa
ttttagagacaacacctgcgcctacggtacggccaccttctctaatagcgaaacgcatac
cttgctcaattgcaattgcattaattaattcagcagtcatcttaattctgtcaccaggca
taaccatttctgcatcagtaccatcatcagcagtaaactgattaatagtaccagttacat
cagttgttctaacataaaactgaggtctatatcctggaaaaaatggagtatgtcttccgc
cctcctctttagttagaatataaacttctgcttcaaattgagtatgaggtgtaattgtac
caggtttagctaataccatacctctttcaatatctttttctgcacacctcttaaaagaa
ttccaatattatcacccgctagaccttcttctaacgtttttttgaaacatttctaatccag
taatagtcgttgtacgagtttctcgtaaacctacaatttcaattgtgtcaccaactttaa
taatgcctctttcaattctaccagtggcaacagttccacgtcctgtaatagaaaaaacat
cttctacagccattaagaaagttttatcgacatctctctccggcgttggaatatatgtgt
caactgcttccataagtgaaaaaatcttgtcaacccatttatcttcaccttgcttaatag
ctgggtttttcgtcacagcttctaatgccaataaggcagaacctgcaacaaaaggaatat
catctccaggaaagtcgtattgacttaataattcccttccttctaattctactaattcta
gtagctcttcgtcatctacttgatcttccttatttaggaatactactaatgtaggaacac
ctacttgttttgctaataaaatatgttcacgagtttgtggcattggaccgtctgccgcag
atacaactaaaatagctccatccatctgagcagcacccgtaatcatatttttttacgtagt
cggcatggcctggacaatctacgtgagcataatgacgattatctgtttcgtattcaacat
gagcagtattaatagtaatacctctagctttttcttctggagcggcatcaatttcatcaa
attttttcgctgcagtagaccctaaagttgataaagtcgcagagatcgctgctgttaaag
ttgtcttaccatgatcaacatgaccaattgtgccaatattgacatgaggtttttttacgtt
caaatttagatcgagccatgctttttgttttccttatagtaaaagttgctttatagttac
ttttaacgataaatcttgtggattgaaagatttattatttaatacagacaaga-----ta
caatataactaatctaatatcgatagtgagcaaacgctttatttgcttctgccattctat
gtgtatcctctctctttctaatagaatttcctgtttcattagctgcatccataatttcat
tagctaatttcatagacatacttttaccagatctgtctctagagaatttagtaatccatc
ttagtgctaaattcgtacctctataagctcgtacttcaataggaacttggtaagtagaac
caccaactcttcttgcttttacttctactagaggagtaatatttcggatggcttttttcta
aaatatttaaaggatctgattctgttctctctttaacgatatctaaagcctgatatataa
ttctttgagataaagtttttttaccacttttcaaaatacgaacagttaacatacttacga
gtctgcttttatataaaggatcaggtgatgcaaaccttttttttagctgtattacgacgag

acatagtatttaaaattggttattgtaataa-tgttc-tattagcaggtatttaagattt
aggctttttgtcccatatttagatcggcttttacgacgatcttttactcctgcagcatc
taaagtaccacgtactacatgatatcggactccaggcaaatctttaattcggccgcccct
aatcagtaccacagaatgctcttgaatattatgacctacgccaggaatataagctgtaac
ttcaaatccagatgttaatctaacacgcgctactttcttaaagcagagttcggtttttt
aggagttgtagtatatactcttgtacaaacacctcttctttgagggcaactttgaagagc
tggagattttgtttttttatgtatttttcgtctttcggatctaacaagttgttgaattgt
tggcataataaaattgatttaaagtctttaatagatactatttgaacttgctttacatga
attggctagtttttatttatcttatacttacgcataaatctttctactctaccttctgta
tcaataattctctgtgaaccagtaaagaatggatggtttcctgaccagatatctacgtgt
agttctggctttgtggacccaatcgtcataattaactggccgtcacagtaaacttttgct
tctggataccaatttggatgtatattatcttttgccattgtttttataattatataaatg
tatcaataataactattaacgcttagaaaattgaggagcctttctggctttctttaaacc
atatttttttctctctttactcttggatctcttgttaaatagccttcagacttaagtgt
cgttctattttctggattaattgagcacaatgctcttgcaacacctaaacgaattgcatc
agcttgccctgtcaaacctcctcctctagcatttacatggatatcatactggtttagtag
tcctaaaacttgtaatggtgcatatgaaactcttaagtaattaggactaaattgaagata
agactctcctggtataccgtttataattaaattacctgaccctgggactagtcttacttg
tgcaacagaacacttccggcgacctgttccagaatagattgcgcgagttttaattaattc
tgtcgacataacattccttgataactaaataaatatacattctttatactatatactctt
gtggcttttgcgcgacatgtgggtgaattgggccagaatacactttaagcttcgtaaata
gttttctgcctaatggacctttaggaagcatacctttaaccgatttttcaataattctgt
taggtaatcttgtctgaagctgatcaaatgtttcaacttttaatccgccaggctgtccag
aatgtcttctgtatagtttttgattcgttttatttccacttacagatacgtgagcagaat
taataacgattacataatctccagtatctagatagggtgtataagaaggcttgttttac
ctcttaagatattagaaatatgagtagatattctaccaagtgtctgattttagcgtcta
taacataccaatgagaattagtatttaatgagggtgattgcgttttattcatgaaaagat
taatacttttggttgacaataagagaatatgtaaatggtttacattcaatttctatacaa
gtagtattt-aaaaatatta-------------ttaacaaagagaacttttctaacagtt
atttaaattgtttgtcagcaagaaaatttctagttatcaataacttatactatttttaat
aatataaaatatattaatcactttttcttttggcaaacttatacctagtttttttgc-
aaggcatatataacttcttcagctgatttctggccaaaatttttaatttctagcagttct
tcttgagagtaatctagtaaatctgcaatagaatggatttgagctcgctttaagcagtta
taagccctaacagataactgcagttcttctattaaaacttgactaatttttttatcttct
ttgctacgataattatctgctgatttaaagtctaaatttcttagtgaacaaaaaagatta
gttaaaactgtagctccttgacttattgcttcttgaggagagatgctcccatttgtccag

atttgtataatcaatctatcttttatgctgttgctaccaatacggacttcttctacttta
taattaactttattaactggcataaaaacagagtctacttgcaaaaaatccacagataat
tcatctacagctttttcagctaagcgatacccgcaatttttttcaattttaaactccatt
tcaaatattgtattattgcaaatagttgcaatatactgtctaggatctactacctctata
tcagaagataattcaaataggccggctgtaactatagctggcccttgaactctaattcga
ccaatttgagattctttgttataactttaaatactacttctttagattaagtaatatt
tctaacacatcttctcttaccccaggaattgtagaaaactcatggttcactccagcaatc
cgtacagcaactatagcagtaccttcaagatctgacaatattgatcttctcaaagcatta
cctaatgtaataccttgtccttgatttaatggttctattacaaaactaccgtactgccca
cgcgccccatctgttcttgactctatgcattcaatttgaaattgagccacctaagaaagc
tcc-tttaaaataatcagtgattagtaagtctctctaaaacaagaaggaatttatactcg
gcgtttcttaggagggcgacatccattatgaggtacaggagtaatatcttttattagagt
aatctctaatcctgcagcctgcaaagctctaattgctgtttctcgacccgctcctggacc
atttactagaacttcagtttggcgcataccttgatccatagcttgtctagctgctttttc
agctgctgtttgagctgcaaaaggtgttccttttttagctcccttaaatccacttgcacc
agaggatgaccatgataatgtttctccttttaaattagtaatagtaacaattgtattatt
gaatgtagatttaatatgtgtaataccgttaactgcattacgtttagtttttcttgctcc
ggatttttttatttgtctagccatcgtgtctcgtctattatatagttaattaaaagatta
ttttcttggagctttttcttacctgctactgtttttttacctcctctgcgtgttctagc
attagttctagttctttgtcctctcaaaggaagaccaagacggtgtcttctacctctata
agtactaatttccataagtctcttaatgctcatagactcaaaacgtttgagatccccttc
aatctgataattagactcaagaatttctcttatactgacaacttgttgatcatttaaatc
ttgacacttaatatcagcgtctatgtttgtttttctaatatttctttcgagcgagatag
tccaataccataaatatatgttaaagctatctctattcttttgtttcttggaagatctac
tccagcaattctggccactttgtcttgtctccaataaataatatgtat-ttttataacgg
ttgtttata-agcttagccttgtctctgcttatgtttaggattagtgcaaattaccatca
cctttctgtgacgccgaattattctacatttttcacacatttttcgaacagaaggacgaa
ctttcatattaa----ctctatatactaggaatattactatattaactctattaattatc
gacattaaaaatgtcaacaatatattgtactactttaactgttgtagattttacttcgtc
atactatcatatttttttgatatgacgtatgtctgaatttgcttagctgtgtctattgcg
actccaactaaaataagtaaagacgtagccccgagacctcttaagttttggatctgagta
accttttctattataaacggaatcagcgctactgtaaataaaaacgaggctcctaggaat
gtcagtctatttaatatgacttgtaaataatcaatagtcgcttgacctggacgaatatta
ggaatgcttgcacccatttttttaaatttatagcaatatcttctggattcattactatt
gatgtataaaaatagctaaagaaaaggatcaaagcacaataaagaagaaggtatagtgaa
ccattaggacaaaacaggtataggatttggagtaatgttttattttgaataatttgggtt

agataagatgggagggccatagatgcagatgcaaaaacaataggcataactccaccttga
tttaatttcaagggcaagtaactatttggatccaaaattgaagattttcctagctgtctt
gctgaaataattttaattcttcttgttccttcttgtacacaaattgtaattattatcatt
aataaaaagatcgctataaataatccgaacttaagacttgcattactataactagcatca
aaaaatgactgtgtaaaattctttggtagtcctgacacaatgttttgaaaaataagtaaa
gaggctccattaccaattcccttttccgtaattaactctgataaccacataataatcata
gaacctgctgttaaagctaaaacagactcgcagacaaatgcaaaattccaattaaaaaca
tatggttttacccatatagagattgctccagattgtaaagtcgcccaacctagagctaag
taccttgtaatttgagttatttttggcggcccaattcaccttcttccttctgtaatttt
tccagattggggacaattttcgtaagtagctgcattacaattgaagagttaatataagga
acaatacctaacgcaaaaatccctattgttgaaaaacctcctccagaaaaaatattcaga
aaatttactaaagtatttttttctacacttgcataaaaggcgtcatgatctatacctgga
acaggtataaatattcctaaacgtgctaaaactaatagaaagagagtaaagataatacga
tttcttagatcactttttttggctcataaataaattttaaatgaaaagagatactttt--tt
tatgtaaaaaccctattccaagacttgtataagtttaaaatagggaagtaa---aatatt
atagtaatattaata-taatattctatgtagaatacaggctttctattggaactcctcga
tctttagctgcttcactaaaagttcttaattgagttaagccattgagaacagctcgcgcg
ttatttaatgtattattagaacctaattgcttagctaatatattctgtacacccgacaat
tcgaggactgtccttacagaacctccggcaattacacctgagccaggtgcagagggcctt
aatataactttcgcggcaccagaaattccgttaataggatgaggtatagaattcgatttt
gtcagtggaactgtaactagatgtttttttagcatctgttactcctttttttactgcacca
attacatcgcttgcttttcccacgccgacaccaacttggccttgctcattaccaataaca
agaataactcgaaagcttagtttttttacctccttttacaactttagtaactcttttaact
tgtacgactctttcttcccagccactatctttatcttttcctttgctctgtttttttacga
ttggccattttaagaattattcctaattagttaatta-cgattctagaaacccatgcctg
cttccttagcagcttcagccaaggccttaactcttccatgatataactttcctcctctat
cgaagacgacattttttaatgccttctttcatagactgttctgctaactgtttaccaacta
cgcgagaagtatcacagtttggtcttatgttatcagattctttattatttaaattaacag
atgatgtagctactaatgtaataccttgtgtgtcatcaattatttgtgcgtatatatgtt
tattagatctaaatacacacagacgaggcctacttgaggttccttgaactttttttccgaa
ctcttttatgcttatgaattctagtttgtttagtgtttagtttcattattatttaccttt
tccagctttcccagcttttcttctaacaaattcaccttgatatctaattccttttccttt
ataaggctcaggaggcctaatagaacgaatagttgaagcaacctgaccgacaacttcttt
atctatgccagagacagtaatatttgtgttgttttcaactttaatttcaatattggcagg
aggtttaattttcactacatgactatagccaacacttaagattaaatcttggttatcaat
ttgagaacgatagcctacaccttgtatttgcagtttttttaaaaaatccattagaaacacc

ttcaatcatattactaataagagttctggatagcccatgtaactgactagccatttttgt
ttgtccactcgtcttgacagcaattgtgtcgttaagtatttctaaattaataccagctgg
taaagttctggatagggtacctttaggtcctgtcacggtgatagtctgaccatcgaactg
agtactaagatttgtcggcaataaaattatttttttccaatacgagacat-attccacc
tataaaatagttaccaaatatagcataatatttcaccaccaagaccatcatgacgagctt
gtctatctgtcataacgcctctagaagtagaaataagagcgatgcctaaacctccaagaa
ctctgggcaactctttatgatttgcgtagactcttagtccgggtttactaattcttttca
gagcagtaataactggttgacgatttttaccattatacttaagagaaatcattaaatgag
tctctataccttcacccatttgttcgaaattttgaacaaatccttcttctttcagtactg
ttgccatattgcatgtcattttcgttgctggaacttgcacaatttgatgtcttgctaagt
ttgcgttacgaatacgtgtcagcatatcggcgatcgtatcgttgaccaccttgatcctcc
ttgcga-----tgaattatttgaaatattttcaagattccctgaaaggcatccctagctt
ttttaacaaagctagaccttcttgatctgttttagctgtagtgacaattgatatatctaa
accacgaatttgatcaatattatcatagtctatttctggaaagatcaactgttcacgtaa
acctaaattgtaattacctttgccgtcaaaacttctaggactaattcctctgaagtctct
aattcttggtaatgttaaattaattaatttctctaaaaaagaatacatcttgtcttttct
tagatgcacaacaattccgataggaacctcttctcgaattttaaatcctgcaatagattt
tttagcttttgtgacaattggtttttgtccagttattaatgtcaattcttgaatactact
ttcaagagccttagcattctgagaagcttcacctaagccacggttaatagtaattttagt
aaacctaggaacttcatgtacattttttgtactgaaattcatcttttaaagattgagtaac
agttgttttatatttttcttttaatcctattgccattatattttcatcaattatttaata
agttcgccagtctttttcagttttcgaa-ttttttggcctttatcattaattattactga
ggatcgactagcaatattattttgttcactaaataacataacgttagaggtatgtattgg
agcttcaaatttgataatttctcccgtttctccttcttgttgaggtttttttatgcttcac
tttgagattaattcctttaacaatcaccttatttgttttataaataattgcaataatttc
acctgtttttgttttatcacttccagaaataacttgaactaagtcccctttttttaattt
aatttttgtattatttttggttgttttagaaggacctttcattatactacctccggtgca
agagaaactattttagaaaaatttttatctcttaactctcgggctataggtccgaagact
cttgtgccacgtggattattatcttgattaatgataactgccgcattatctccaaatcta
atgctcataccgtcagttctccgtaaagctttacgagttcttactacaacggctctgaca
acatcagatcgtttaactggcatattgggagacgcatctttgactactccaataataaca
tctccaatagatgcataagaaggattactagtgcctagtactctaatacacattattttt
ctagcaccactattatctgcaacattaagatagctttgagtctgtatcatacttttattc
tgtataagattaattatcaaaagacttagataagatattaaccattgtccaacactttgt
gcgacttaaaggtcgtgtctcttgtattgtaacaatatcgccgattgtgcattcattatt
ttcatcatgcgctttatatttctttgtccggatcatagtttttgcgtactttctatgaga

gattctattttctacagctactactatagttttattcattttatcgcttactactttacc
tgttgtttcttttaaaggcatagtctttatatttttatatat---------aattaaat
tttaagattattacatggttgacttagtacgagacttttcaacagttaaaagttgggcta
atctatgcttagaatgtttaaatagatgaggctggaaatcttgccttgtggctcttttta
gccttaaatcaaaaagctctcttttattacaaggatttcttcagctaaagaagaagagt
ccaggtttgtaacatctgatatttagggaaagtcatatttaggattctgttgtattcc
gaactataaatttagtcttgattggtaatttataagaagctaatttcatagcttgttgag
cagtttttgtggtacacctgtaatttcaaatagaatatgtccaggcttaataactgcaa
cccaatattctggagctcctttacctgaccccatgcgagtttcagctggacggcagtga
ctggtttatctggaaatactctaatccatagtttaccacctcttctcacatatctagtaa
tagttcttcgagtagcttctatttgtctagaagttaaccaaactggctctgtcgcttgta
atgcataatcaccgaacgcaattgtgttacctttgctagcagaacctttcattctacctc
tatgttgttttctaaattttgttttcttggggcttagcataaaaattaaaaaataataat
aattaagaagctgttgaatcaggagtttcctgattataagcagattctagacttttcgat
tctggcaaaatttctcctttaaaaagccaaacttttactccgagcacaccatacgtagtg
tgagcttggcgatgacaataatcaatatcggctcttaaagtttgtaaaggtacacgacct
tctctaacccattcacttctagctatctctgcaccattaagccgaccagatacttgaatt
ttaacaccctgtgtatttgctctttgagctctttgaactgcttgtctgacagctctacga
aaggccactcttttttctagctgttgagtaataaattctgctactaaggttgcttcagaa
tctggatctgcaatctctacaacatttactctgagttgtttgctagggtctaggattaag
gacagcgattttcttaaagactcaattcccgctccagattttccaagtacgattcctggt
cgagctgttgctatgagaatttctacttgatcaactttacgattaatttcaattttagca
atactagcattactaagttttgaatgtataaatgagcgaattttatgatcttcttgtaaa
agaactgggtagtctttagagttagcaaaccatgaagaacgatgtttttgggtaatgcct
atgcgaaagcccaaaggatgaatttttttgacccacagtatccttgattataagttaaaag
ctataatgactaattgacgttaaa--------agactacagttctgaaacacctaatgta
atatgacaagttggtttatgtattggaaaggctcttccttgtgctctaggctgaaatctt
tttaaagtcgggcctttgtctgcaaaggctttactaacaaacaattggtttttatttaat
ccatcattatgctcagcattcgctgcagcagattccaggatttgctttatatgagaacag
actcggtatggcataaattccagaataattaatgcttcttggtattttctacctctaatt
tggtctaatacacgacggactttatgcggagaaagacgaatgtatttccctactgcttta
gtttcttttacgtttttttgtaatactcatagttatagtttaacgacgggcttttctgtca
ccttttacgtgagtgcggaaagttcttgttggaacaaactctcctaatttatgtcctacc
atttggtctgacacaaagacaggaaaatgttgtttaccattataaacagctattgtatgc
cctaccatatcgggaataatagtagatgctctcgaccaagtttttaggacttcttttttt
cctgaaatatttaatgcttctatacgtttaagaagactaacatcaataaaagggccttta

tgtatagatcttgacataataataaatcattagattcttaataaaataaattctatttac
ggcgacgtaagacgtacgggttactatatttatttggatttcgtgttttaacacccaatg
caggtttaccccaaggtgttacaggacgagctcgtccaattggagatttgccttcaccac
caccatgtggatggtctacaggattcatgacaacacctctaacagtaggtcttttaccta
accaacgatttctaccagctttaccaagagtaatattactagcatcaatattaccaactt
gtccaatagtagcgtagcattcttttcgaatcattcgaacttcgcttgaaggcaatttta
cagtaacaaaagtaccttcttttgctactatttgagcataagttcctgctgcacgaacaa
tctgtcctccgcacgaaggtcttaattctatattatgtactgctgttcctaaaggaatac
tagataaaggcaaagcatttccaacttcgataggggcagtaggaccagaaagaaccatag
atcctacactgagagatcgagggtgtagaatatatcttttctcaccatcaagatagtgta
ataatgcaattcttgcatttctattcggatcatattcaatagaagcaactttagcaacta
tattatgtctatttcttttaaagtcaattaatctgtactgctgcttatgtccaccacctt
tatgacgacaggtaatgacacctctattatttcgacctttgcagaaatggtgtttaacta
ttaatgatttttctggcttatcagtagtaatctctgaaaaggtagaaaccgttctatttc
ttgtccctggtgtgtaggcacgatataaacgaattgccatataagaagatttgagaagag
taaatgttgaa------ctatgtgatatatttaagtttctggaaacaaattaatagaatc
ttctgaggcaagtgttacaattgctttcttgtaatgtggtcgcttccctacgaatctacc
gatgctcctttttttcttaggaggatgacaagtgttaacacctgtaacttgcacgttgaa
aatatactgtatagcagccttaatattaatttttgttgctttgggatcaacggcaaaaca
atactgattctcttctaataatttagttgtcttatcagtaatgatgggatatttaaccag
atctaataaacctcttgaatcaatgctatccattatatacctcttgtattttagataaag
catcaactgtaataataattttatgtgcagctaacaaagccataatatttagtgtatcag
ctgaaataagttctacgttgtgtagattgcgaatagaaagataaacattcggatcttttt
tgtctactatgactaaaactttttatttaaatcaagattccatcgatgtatagcttcca
taaatagtttagtcttgggctgatgaaaataactgttaaaattttctacaatcagtgtat
taactgatttgttatttaatgctgtccttaatgctaattgtctttctttttattcattt
ttttggtaaaactacgaggttttggcccaaatattacaccaccacctctccatagaggtg
aacggatagaacctgctcttgctcgaccagtccctttttgacgccatggtttacgacctc
cacctcgaacttcacttcttgttttagtattagcagaaccctggcgcttttcattacttt
gctttactaaagctctatggactaagtacatcccagaatcttgactgaccttaagattta
aatcagcgttgccacttacttgaccttcccaattatagacttgataatttaattgcgtgt
taactgtcatgaaatatatcgtatgaaagaatattgctacttactaatttttaccaaagc
gccaggtttaccaggaacagctcccttaacgatcaacaaatcattctctgagttaatact
tacaatttgcaaatttttttattgtaactttttttattaccaagttggccagccatgttttt
acctggataaactcttccaggcgttgtaccggctccaatagagccaggttgcctatgatt
tttcgaaccatgtgacattgggcctctactaaaatgatgcctcttttggtaaccggagaa

acctttacctacacttctagaagagacattaatcttttggcctacttgaaatagatctgt
agatagtatttggctaacttcgaaatcatcagtagatttcatttcgtattcacgtaaata
ttttagtgggggagcctgtgatttttttaagtgtcccaacaatggtttatttaattttttg
ttcagcaacttgcttgtaaccgacttgaatagcattgtagccatcagtagatacagcttt
aatttgagtaataacacatggcccaacttgaattacagtaactggaattgataaaccagc
ttcatcaaagaattgggtcatacctactttagtaccgagtataccaacagacactagatt
tctcctttttgagagatttatactatcattgtacataaaaattctacttcatagttactg
aag--taaattaaactaacagatgcatgatattattcatgacaacataaaggttaaaaac
tttacctatttaatctaaaatagaaacagctttttgtctagttacagcgtatataaatta
gaagtatgcttattaaatttttttataaactact-tagatttaactgatattgatatccgt
aacttacttgcagtaatatc----aatcaacatgtataagtttaattgcctaaactagaa
ccagctattattagaaataattcaagtatagataaaagcgtatttgaacttttatatatt
gacaaatataatggaatatattgaggttcggtaactacaccttttttattttttacatttat
tgtgca--gtatatatacatctaatgcagtaaacataagaatataac----tgtatagca
aatgtaattacacaaattagagaggtattaatgggcaaagtcgttggaattgatcttgga
acaacgaattctgtaattgctgttatggaaggaggtaaacctaccgtcataccgaatgca
gaaggttttagaactacagcttctgttgttgcatatactaaaagtggagataaacttgta
ggacaaattgccaggaggcaagctgttattaacccagaaaacactttctactctgtcaaa
agattcataggacgtaaacagaatgaaatttcgcaagagattcggcaaacatcatataat
gttaaaactagtggatcaagcataaaaattgcttgccctgcactgaataaagattttgct
ccagaagaaatttcagctcaagtactgagaaaacttgttgaagatgctagtacgtactta
ggtgagactgttacacaagcagtaataactgtacctgcttactttaacgattcacaaaga
caagctactaaagacgcaggtaaaatagcaggcttagatgtattgagaattattaatgag
cctaccgcagcttctttgtcttatggactagacaagcaaaataatgaaacaatactagta
tttgaccttggtgggggcacatttgatgtatctgtattagaagttggagatggagtattt
gaagtactctcaacttctggagatacacatttaggcggagatgactttgatcagcagatt
gtagaatggctaatcaaagattttaaacagaatgaaggtattgatcttggtaaagataga
caagcacttcagagattgaccgaggctgcagaaaaagcaaagatagagctgtcaaattta
actcaaacagagatcaatcttcctttttattactgccacacaagatggtccaaaacattta
gaaaaaactgtaactagagggaagtttgaagaactttgttcaaatttaatagataaatgt
agtatccctgtaaataatgctctgaaagatgcaaaactagaagcttccagtattgatgaa
gttgttttagttggtggatctacaaggattccagccatacagcaaatggttaaaagatta
attggtaaagatccaaaccaaagtgtcaatccagatgaagttgttgctattggtgcagcc
gtacaagctggagttttagcaggcgaagtcaaagatattctattactagatgtgacgccg
ttatctttaggagtggaaactctgggtggcgtgatgacaaagattataccaagaaatact
actattcctacaaaaaaaatcagaagtattttctacagctgtagataatcaaccaaatgtg

gaaattcaagtacttcaaggcgaaagagaactaacaaaagacaataaaagcttagggacg
ttccgattagatggcattatgcctgcacctagaggagttcctcaaattgaagttaccttt
gatattgacgctaacggaattttatctgtaaaagccaaagaaaaggctactggtaaagag
caatcaattactatatctggagcatcaactttacctaaagatgatgttgaaagaatggta
aaagaagctgaagaaaatttcgatacagaccaaaaaagaagaaagaatattgacacaaag
aatcaagcagagtccctatgttaccaagctgaaaagcaagttaaagagtttgaagacaaa
attagccaagatttaaaaataaaaatagaagagctaattacagagcttagatctagtcta
gagaaagaagaatatgacaatattgaatctatttctcagcaattacaaaatgctctgatg
gacattggaaaaaatgctgctcagactgaaagtaaagatacaaaagcgaaggatgacgac
actgtgattgacactgatttctctgaagctaagt-aaaagta-agcgggtaacgcgattc
gaacgcgcgacatcaaccttggcaaggttgcgctctaccactgagctatacccgcataga
ttgtattattacaaaaattaagatatttgtcaatctgtta------atctatac---atc
tagactattaattttggaattacatattagctaactgttgtaaatataacaattgctata
attttttaa-------aagtacttatatcat-tatataagtacttttatatt-------
-caaaaatacaaataagtattatag-------ctaaactactaaggaattaactaagcta
gtaacaattactaatcctgtccataagccggctcctgtatatattaatccttttgattgc
tcccattggccaggggatgctaaaacaacaggtacgcctactactaacacagtcgataat
gcaattaataatagcactaataattggatcgcaataatcatttaatatgttctcctgaaa
aattcaaatgttaataatactgtatataatacaatgaagcttttgaccttcagcaattta
tctatgaagaaataatttaaaattattgttttctaaataaaccttaaattaagtattaaa
atctttattgtaaacaataatactatattttttta--gtagataaaaaatttaaagaaag
tctttttaaaaaaaaattaagataaggtgatatgctgtataaaaagctaagtattatta
attattagagaaatttaaa-ataagaggtaaaacatatgaactcagcccttttttagca
aaactaccagaagcttatgccattttaaaccgatcattgatatcttacccgtaattcct
gtatttttcttcttttagcttttgtttggcaagcagcaattggttttagataacattta
ggttataaaaagaataacaaatatacagagtgccaattttgagctctaatcttatatct
tacacaataataactattatggttgaacccttactatcaggaattgttcttggacttatt
cctattactttattcggattattagtagcagcttatttacaatatcaacggggcaaccaa
ttaggactgtaaagcctaaattatagatatttataaaaactacaataattatagtttta
taaatatctacaaataagctaattttaaatacagaaataatactattgaaactagagtga
taacaaaaaaaatttatgtaa---taa---------------------------------

---------------------------caagctaattgtttaccagcttgacttagtaac
tcctggtaaaagacattcatgagacatttctcgaaaaacatggcgagataatccaaaatc
tcgataataacctctacttctacctgttaaccaacatctatttctccctctaacagcggc
gctgtttctaggcatttcttggagtttttgtcttaaatccattttgtcagaaaagctgcc
agcttctttcagttgatctttaatagccagtctttttaaataatatttttttgcaagttt
cgatctcttaatctctctttggatcatattctttttagccatgctgtaa-aactctcttt
taaaattatttattcaatatattagctaaaaaagctcctgcctgcaacttttatatattt
aaaaataaatagcataaagtagtgaaatgcaattgatatttaatcaacgatacc---act
ttatgctattatcaaaaatttatttagtaaagttcttgttcaacatgagttgaaatagtg
caatcagatgaaggatatgcaatacaggttaaaacatagccagctaaaagttgatcatca
tctaagaatgattgatcagcttgatctacagttccttctgtaactttacctgcacaagtt
gaacaagctccagctctacaagagtaaggaagttcaattccttcttcttctgctgcatct
agaatatatgtatcctccgcacaattaaatgtgacatcaattccttcttcttcacatagt
aaatgaattttataatcagccatttcttcttactccttaataaagatacacttgaaactt
aataaagcaaaat-aatttaaagaacttaccaaaaaattatatactagtttcattatgca
tgatagactggaaaaaaagaaaatagtatactattctgaaaaatcaagctacatttccta
ctaaatagaactatagtaggtccaaagtatatacataaattcatatagttaaaattactt
ttatcatttaaatagctgaacttgatagta---------tagattgatcatc------aa
gctagtcttctgc---caataaataattaattatgatttcaatgcttaatcaacaagtag
aacttaagcctattcttaaatttcagataacacaaatatattcgcattctcttattcagg
aaattaaagccttaataactagacttgttttgcaagtttggagaaggccggctacattga
tggcaggtattatccaacctttattatggttaattctatttggtgggcttttctacaatg
ctcctataaatttgttcactattaatacaagctataattgtttttttgagctctgggatta

taatttttacctcttttactggagctttgaattcaggtcttccattaatgtttgatagag
aatttggattttttaaatagattattaacggctcctttagtatcgaggacttctatcattt
tatcttctgctacttttatgacttgtattagtttaatacaagttgtatttatagttacag
cttctcttttttatgggaaactcacctctaaatagcgatagtactatgattttggactta
tgattctattagtgactgtaggagttacaatgcttagtttagctttatctttcactctgc
caggtcatattgagctattagcatttattttagtagttaacttgccctttttattttcta
gtacggctttagctcctttatatttcatgccgccatggcttcagttaattgcaagtctca
atccattgagttatgcaatagaaggtacaagatacttatattcaagcgtaaactggaatt
ttacagagtgtgtgattaagattagctggggagatatttgtttagggcaaattattattt
tattaatcgctttagatataatggcagcttatcttgtgtctaatatattaaaagctaaac
ttaatta-----------------aaattttaataaaaaatttattaaatttattttaca
aagattttactatataatactaatagtagtatggataattgaaatagaaatagtttttc
aaaaccaaagctattatcaacaatgataaacatttgtaagaaagtcaacaaagtatgttc
ttattcataggaggcatgtagtcaatgggactaccatggtaccgtgtacacacggttgtt
ttaaatgatcctggacggttaattgcagtccacctaatgcatactgcacttgtagcgggt
tgggcaggatctatggcattatacgaactagctgtatttgatccttcagatccagtatta
aatccaatgtggcgacaaggcatgtttgttatgccatttatggctagactgggcgtaaca
gattcatggggaggatggagtataacaggagagagcgtatccaatcctggactgtggagt
tttgaaggtgtagccttaactcatatagtcctttctggcatgctttttctagctgctatt
tggcattgggtttattgggatttggaattatttagagatccacgaactggcgagccagcc
ttagatttaccgaaaatttttggaattcatttattgctatcaagtctactttgtttcgga
tttggagctttccatgtaactggacttttggaccaggaatgtgggtatcagatgggtac
ggagtaaccggaaaggtattaccagtagctccagcatggggaccagaaggattcaacccg
tttaatcctggaggggttgcatctcaccacattgccgcaggtactgtaggtatattagct
ggtgttttccatttaactgttagaccaccacaaagactgtatcgtgctctaagaatgggt
aatattgaaactgtattatcaagtagtatctctgctgtttttttctcagcttttgtgact
tgtggtacgatgtggtacggttctgcaactacgcctattgaattatttggtccaactaga
tatcagtgggatagtggatattttcagcaagaaattgagaaacgagtagaaaatgctatt
gctgatggtgctgcacctagcgaagcatggtcaagaattcctgacaagttggcattctat
gactatattggtaataatccagcaaaaggaggattattcagagcgggccctatgaataaa
ggcgacggagttgctgaagcatggcttggacatcctgtattccaagataaagaaggaaga
gagcttagtgttcgcagaatgcctgctttttttcgaaacttttcctgtaattttagttgat
aaagatggtattatacgagctgacattccatttagaagagcagagtctaagtatagtatt
gaacaagtaggtgtaacagctagtttttatggtggcaaattaaatggccaagttttcaac
gatgctcctagcgttaaaaaatatgcaaggaaagctcaattaggtgaggtatttgaattt
gatcgaactacattagaatcagatggagtgtttagaagtagtcctagaggctggtttaca

ttcggtcatgctaatttcgctttaatttcttctttggacatctttggcatggttcaaga
actatcttccgggatgtgtttgcgggaatcggagctgaagttactgaacaagttgaattt
ggagctttccaaaaattaggagatagaagtagtaaaaaacaaggagctgtataagaccaa
agtcaaagatagtctttatc---aaatatttctaatattaaaatagttaagacaaagttt
attatctaatagttactaatcaggagaatttatggaagccttagtctatgtatttttact
aacaggaacgctaatggttatattctttgcgatctttttagagaacctccaagaatagc
aaagtaacttgattccgtttaaacttataataatagaaccactttccaacttattagtgg
aaagtggttttattgtagattacattgtattattcttcatgctcttcaaaaggatctcgg
agatcttttgaagcaggaccaaatgctgtgtatatcgagtaacctgtaatacctaaaagc
agactcgaaataaaaatactaagaactgttgcagtttccataatttaagcagtattaaga
tgaactttttctataatgatattatcaatattaaaacatttaattattaaaattatcaaa
atatactatggcacttagaactagacttggagaaattttaagacccttaaattcagagta
tggaaaagttgctccaggttggggcacaactcctattatgggaattttcatgttactatt
tttcttgttttattaatcatttacaaatctataattcttcattagtattagaaaatgt
agatgtagattgggctactttaggtag----ctaactagataagttctttga--------
ttacacaatattaaagacaac----------------aagcgatcgctattagattatag
tagcgatcgctttaaaagtatttactatatatccagagaaaattatttacctaaattaa
ttaactcggtatctgcaaaactatttgtgttaacattattatagttaactttttcaaacc
ttactaatactggatatttgataccacttttatcaattgctgcaacagtaccaatttcct
gataccagtacgattcttttctcaatatttttacttttgagcctctttccatatttttag
tcctcagatagattattaaagattattatattaaaaaattaaaaaaaaactaaacaaaata
tataaaaatactaccgacaattgtaatatataactaataaaaggagagagagggattcga
accctcgataacaaaagttatgacaattttcgaaattgttgcaataaaccactctgccat
ctctcctgaattaaaatactgttttaaaaaattagacaagaataacatagaactaaggta
gttgcctagaaaattaaaactatgttaaatataaattataatctttctatagatatattt
aaaaaatatgtacatagaatctatatcagcttacttcaagatttttaatcttatacaat
ttttactaaataaataaattctctcataatatgaaactatcttggaaaacacttcttctt
tggtctttaccaatatttgtaattggtttctttttctggcaaggtttttaggtccaact
actacagatgttggcagtaatatcgcaagttctagaatgacatatggacgatttttagaa
tatttagatatgggttgggtgaaacgggttgacctctatgaaaataatcatacagcaatt
gtagaagctgttgggccagaattaggaaacagggttcaacgaattcgagttgaactgcca
gcaagtgcgccagaattaattacaaaattacgcaaagccaatgttgatctagatgctcac
ccccctaaaagtacaagtgcagtatggggactattaggcaatttactatttcctttacta
ttagttggcgggttagctttcttatttagaagatctaataatgctagtggtggacctggt
caagcaatgtcatttggcaaatcgaaagctttatttcaaatggaggctaaaacgggagta
gtatttaatgatgtagctggagttgaagaggcaaaggaagaatttcaagaagtggtaaca

tttttaaaacagcctgaatcatttactgctgttggtgcaaaaataccaaaaggcgtttta
ttagttggacctcctggcacaggcaaaacattactagcaaaagctattgctggcgaggct
agtgttcctttttttagtatctcaggctcagaatttgtagaaatgtttgttggtgttggc
gcttctcgtgtgagagacctattcaagaaagcaaaagacaatgcgccttgtatcgttttt
attgatgaaattgatgctgttggtagacaacgaggaacaggtgttggaggtggtaatgat
gaaagggaacaaacattaaatcaactattgactgaaatggatggctttgaaggaaatact
ggtgttattgtaattgccgctactaacagagctgatattttagattctgcattattaaga
cctggaagatttgatagacaagtttctgtagatgtaccagattttaaaggcaggttagca
attcttgaagttcatgctaaaaataagaaaatggaacctaaagtatctttagaaaccatt
gccagaagaactcccggcttttcaggagctgatttagctaacttactaaatgaggctgct
atcttaacggctcgacgaagaaaaaatgcaatgactatgtctgaaattgatacatcaatt
gatcgagtagtagctgggatggaaggcactcctttaattgacagtaaaagcaaaagatta
attgcgtatcacgaagtgggtcacgcaataataggcagtttattagagcatcatgatcct
gtgcaaaaagttacattaataccaagagggcaagcaagaggcttaacttggtttactcct
agtgatgatcaaagtctaatatcaagatctcaaatactagcccgtatcgtaggtgctctt
ggtggcagagctgcagaagaaatcattttcggtgacgcagaagttactactggtgcaagt
aatgatttacagcaagtaacatcaatggcaagacaaatggtcactcgatttggaatgtct
aaaattggacctttatctcttgaaagccaaggaggagacccatttttaggtagaggcatg
ggaggaggctcagaatattcagatgaagttgcaactaatattgataagcaagtaagggaa
attgtcagtgaatgctatgcacaagctaaacacattattatagataatcgagtagtgata
gatagattagttgatttactaattgaaaaagaaacaattgaaggcaatgaatttagagac
atcgttaaggaatacactgcaattcctgaaaaaaattactacatatcacaattttaa---
---------------attaaacgggactgacgggattcgaacccgcaacttccgccgtga
cagggcggtgctctaaccagttgaactacagtccc-aaaaagatgaatacccagagataa
tctcatagttagcttaaacttgtcaaattagaatattaaatatgaagagta---------
--------tttgtaataatactcttcatatttgata-tagtagctaaaatatttctttta
gaaggagagagagggattcgaaccctcggtacgaagttaatcgtacagcagattagcaat
ctgccgctttcgaccactcagccacctctccatga---tatatattatatatatatcaaa
tatacttatatttaataggtgtgcataaatatttatggagctaagcggattcgaaccgc
tgaccctctcaatgccattgagatgctctaccaactgagctataaccc----ctttggta
agatattaagaaatggctcaagcgggatttgaacctgcgaccttgggcttatgaatcccc
tgctctaaccactgagctactgagccatagtataaaatactattaaatattataccataa
attatggtcaaatataaatcaataaaagaataattactatca-tttactactttg----
------------------tgcaagttgtaaatattcgaataaaatttcaaaattaaaaatg
tctttaactttttgttaga-------atta-ttgattacgaaa-----------atattg
atatttagaattaac---

--------------------aataaatttg------------------------------
---tctttattt
g---

-------tagaca---
-------------------------------caaaaatattggaaa--------------
------------------------ttttcatcaat-----------------taattagt

------------------taaactataaccgacgtac-----------------------

---aagaattattgaacta---

---cgttaaat---

-------------------------------------tgtgatgcatt------------
-----gggcttgatcaaaacttctctaaaatcaatagaatatagt---------------
---aattcaa
gtaaatcaaatactaacaaatgtgacaaacgcttaaaaaaataata--------------
caattagaatcgtaaaaatttggtttttga-ctgtgttaaataaatttgattatcataga
tttaatatttaaaagtgttctattacttgta-----ttgagtaattgt-aatatttaata
tcttctcttttagataataatcataatataagctaaaaaatagtttt---tattaaacaa
ctagcatagagccaataccttgatcagttaatatttctagcaagagagcatgatctattc
taccatctaaaatatgcgctgaagctacaccctgagctaaagaacgaatacaacaattca
ctttaggaatcatgccaccagaaattactgctgtttgagttaaatctctagcttcctgta
tattcagatgactaattaatgttgaaggatctgaagaattacgtaaaatgccgggcgtat
ctgtgagtagaattagtttttcggcattaagagcggctgctatttctcctgcgacagtat
cagcattaatattatacgattggccttccttgtctgctgcaacactagctataacaggta
tataattattattaattaatatcttcagtaatttagtatcaatattttgtacttctccaa
caaagccaaggtttgcttttccatttggtctcggagtaataagtaatccatcttttcccg
acaaacctacacttttaccaccttgtttattaatagttgcgacaagatctttattgactc

ttccaactaaaaccatttccactatatccatggtaggttgatctgttactctaacgccat
catcgaattttggtaatatttgaggcgatctaaccaaaaattaatttctggtcctccac
catgaactaaaatgggacgtagtcctataaaagacagaaaaacaagatcactaatcactt
gatcttttagtttctgattttcatagccgctcccccatatttataacaataattctgg
aagaaaattgctgaatataaggcagagcttcacttaagacttttactctttctgaattag
tcaacataaataaagtaggattgtgatatataaaattatatacacagcttgtgttaattt
aaaagataccaatgggt---------acttgaaaaaatagaaatattactaacag-----
----ttaaaactatagggattataattatgaataaattttgggataatgtattaagattt
ccacgattttagtcagcgtcattttaggattaattttgataattattagtcctttttc
gtgttattaaaaaaaccactgacaagtttttttcatcatatcattggccggattaatc
acagttttggcaataataa-tacaaaaaatgataaatatcgaatgttgttgagagaaata
aataaatttaattaaaattttgaacatatataatattatattatcattaaatatgataa
ttt-atttcctttctat---aaaacccataaatattaaatattatttgataaatattat
gccactaaaacaaagagttagctctgaaaagaccggtgccttcgcacttttggatagtat
tgttaggcacggagttaaacatatatttggttatccaggtggagctattcttcctattta
tgatgagctttatgcttgggaagaagcctctctaattaaacatatccttgttcgtcatga
gcaaggcgctgctcatgctgcagattcttattctagatcaacaggagaggttggagtatg
cttcgctacttctggcccaggagcaactaatcttgtctcaggtatagctacagcacatat
tgattctgtacctatattagctataacaggtcaagttgggagagcttttattggtacaga
tgctttccaagaagtagatatttttgggattacacttcctattgtaaaacattcatatgt
agttcgtgaccctagagacatgtctagaattgttgcggaagcatttttatttgtaaaca
cggtagaccaggtccagtattaattgatgttcctaaagatgtaggattagagaagtttaa
ttatttttctgttgagcccggaaaagttaatattcctggctgtaggccaattaccagcct
aaagtcaagacaaatccttatggcagctaaaatgatacagcaatctagccagccattgtt
gtatattggtggaggagccataatctctgattcacatcaaattattaaagaacttgttga
tttttataaaatacctgttactactactttgatggggaaggggattttaatgaggatag
cgactattgtctagggatgttaggtatgcatggtactgcgtatgctaattttgcagttag
tgagtgcgatcttttaattgctttaggagctagatttgatgatagagttactggaaaatt
agatgaatttgcttgtaatgcccaagtgattcacgtagatattgatcctgctgaagtagg
aaaaaataggattcctcaagttgctattgtcggtgacgtagcagaagttgttagtgaaat
attgaatttattaaagacttctttccccccttatccagagcagattatatcttggcaaga
aagaattaatcgttggcgtcaacagtatcctttactggttcctagaaaatcaacaagcat
ttcacctcaagagattcttgttgcaactaataaattagcccaaaatgcttattttactac
agatgttggccagcatcaaatgtggtcagctcaatttctgaaagtaaaagctaagcattg
gctttcaagtgctggattaggcacgatgggttatggtttacctgcagcaattggcgctca
agtagcacatccaaatgacgtagtcatttgtattagtggtgattctagttttcaaatgaa

tatgcaagagttaggaactatcgcgcaataccagttaccagttaaaattattattattaa
taatcgatggcaagggatggttagacaatggcaacaagctttctatggtgaaaggtattc
acactcaagaatgacagaaggagcacctgattttcaaaagcttgcagaagcttttggtat
taaagcttttactattaataataggcaaaatatgcaatctgctttacaagttgctattga
ttatcctggtccagttttattagattgccaagttacagaaaatgaaaactgttatccaat
ggttgctcctggaaaaagtaatgcacaaatgataggtatcgctaaaccgcagagaggtac
tgcttccaactatattaataatagtgtttaaagtaa------------------------

---------------atagttagtatttttta-aacaaagaata-aagtaca--------
-taaatgtaatttttttatactatta-aaaactatgtaagaacagcctttgacgagaatt
gaactcgtgaccttccccttaccaaggggatgctctacctctgagccacaaaggc-tttt
tactatattgggccgggttggatttgaaccaacgtaggcgaagccagcggatttacagtc
cgcccccattaaccactcgggcaccgaccctaattaaaccttattcaagatatgacctat
aagatatcataactatgttgctaaaaacaagtactttgataaataaaactgaataagtaa
tggacatagctggacttgaaccagcgactttcacgatgtcgacgtgacactctaaccaac
tgagttacatgtccaaaactttaattattattataacactgtttagtgattttataagta
tctatttctaattgaagatttgttttaatcgtccggctttaccgagcagatctcttagat
aatacagtttagctctacgtattttagctcttctaatcactttgacaaaagttattcgcg
gtgaattgagaaaaaatactctttcaactccaataccttgaaatgagcatcttaaagtta
aactagtatttaagctttttttttcttcttctcgctaatactactccttcacataattgtt
cccgagttttactaccttccttaaccattagtccaagttgtattgtatcgccaactttaa
tttcaggtacttctgttttgataaaaggtatctcaacacttttcattagctgacttagct
ttgtactatttgctttcatatataggattagtcttgtgaca-tgttttgaaatattatac
attaaaaaaacaaatatgaaaaattttagcgaataagcatactagtatttataaaac-tc
tttttatct---
--ccattata--------
----------------cagatccatt------------atattcaact------------

----------agattcttttatagtttc--------------------------------

--------tttgataatcagtac-------------------------------------

---------------------------atcta----------------------------

----gatgatcaaatattattttcaaacatggcttatgtatgaaaaagttctata---aa
aacctaatgac--------taaaattagggcttaagtatttcttagttttgtgttataat
tagttactatcaaaggtagtggcataacacgaagcatcaa-aatgttcgaacgctttact
gaaaaagctataaaagtcataatgctagcacaggaagaagctagacgcttaggacataat
tttgtcggaactgagcagatactattaggattagttggtgaaggtactggaatcgcagct
caagttttgaaatcgatgaatgtgaatctaaaagatgcaagagttgaagtcgaaaaaatt
ataggaagagggtctggttttgtagcggttgaaattcctttcactcctcgagcaaaaaga
gtactagaattatctttagaggaagcacgtcaactaggccataactatattggcacagaa
cacttgctaatgggcttagtccgagaaggagaaggagttgcggcaagagttttagaaaat
ctggcagtcgatgtttcttcaattagagctgaagttatacaaatgctcggagaaaatgcg
gaagccaatgtaagtggaagcaatactacgcaagctagaagtaaaacaccaacattagaa
gagttcggatctaacttaactcaaatggctatggaaggtggtttagatcctgtagtcgga
agacaaaaggaaatagaacgagttattcaaatcttaggtagaagaactaaaaataatcct
gtcttaattggggagcctggtgtaggtaagacagcgattgcggaaggattagctcaaaga
attgctaatagagatgtaccttctattttagaagataaattagttattactcttgatgta
ggtctattagtagccggaactaaatatagaggtgaatttgaagaaaggctaaaacgtatt
atggatgagattaaatcagctgataatgtaatattagtgattgatgaagttcatacattg
attggggctggtgctgcagaaggagcaatagatgcagctaatctgcttaagccagcttta
gcaaggggagaattgcaatgtataggtgcaacaactttagaagaatatagaaaacatata

gaaaaagatccagcattagaaagaagatttcaaccagttgtagttggagagccaagtgtt
gaagaaacaattgaaattttgtttggtcttagagaccgttatgaaaagcaccatcaatta
acaatgtcagatggagctttggctgcagctgctaaatacgctaatcagtatatttctgac
cgattttgccagataaagcaattgatttaattgatgaagctggttctagagttcgttta
ctaaattctcaattacctcctgctgccagagaattagataaagagttaagagctgtatta
aaaacaaaagatgaagctattagagctcaaaaatatgaaacagcagagcagtatagagca
agagaaatggaaattaaagctcaaattgcagcaattgctcaaagtaaaaagaatgagcct
gatttaaatttagaagatcctgttgttacagaagatgatattgctgaaattgttgctgca
tggactggtataccagtaactaagcttactaaaagtgagtcagaaaaaattaatgcacatg
gaagaaactttgcatggacgtattattggtcaagacgaagcggttgtagctgtctctaga
gcgataagacgcgcaagagtaggtctaaaaaatcctaacagaccaattgcaagctttatt
ttttccggaccgacgggtgtaggaaaaacagaattaacaaaagctttggcttcttatttc
tttggttcagaagcttctatgatacggctagatatgtcagaatacatggaaagacacact
gtatctaaactaattggttctcctccaggatatgtgggttatagtgaaggtggttatcta
acagaagcggtaagaaaaaaaccatatactgtcatcttatttgacgaaattgaaaaagct
catccggatatttttaatctacttcttcaaattttagaagatggccgactaacagatgca
aaaggtagaactattgattttaagaatactcttttaattatgacttctaatattggatct
aaagttattgaaaaaggaggaggtagtttaggctttgaattatcagaagatcaaacagaa
tcccaatatactagagtacgatctttagtaaatgaagaactgaaacaatactttagacca
gagtttctaaatcgattagatgaaattattgtatttcgtcagcttactaaagatgaagtt
agagaaattgcagaattaatgcttaatgaagtctttgcgagaattaagcagcaagatatt
caattaaatgtaacagaacgatttaagcaacgattagtagaagaaggatataatccaagc
tatggagctagaccacttagacgagcagtaatgaggcttttagaagatagtctggctgaa
gaagttttatctggtaaaattaaagctggtgatagtgcagtagtagatgttactaatgaa
ggagaagttacagttttattaggtgaaaaattagaactgttaacataaa------aaaca
atttattaat--taaagcattgaagaggttat--attcaatgctat---gaaaaaaatta
ataagatgggttgctataattgacaacctctgatactctttatttgtatata---aagcc
gggatagctcagttggtagagcagtggattgaaaatcctcgtgtcaccagttcaaatctg
gttcttggcat-tttaagcatcagaaaattttgctatctgagcattaaattttaattgaa
atgttcctgtcggaccattcctgtgttttgctattataacttctgtgaagtctctacttc
tagtttcttgtgtatagtaactttctctatatagcattattactaaatctgcatcttgtt
ctatagaattatgaacaataaagttgtttgaaataaaattgcataaaggtttacattcta
aatcaaacactatttgtaaacttgtaacgtttattttttgtatactttcaaaagtcaaag
atgaaa------aagaattaaaaacatttttttgttcttcaaagttttt------tattt
gaatagcaatcatgtcattttgattaattttatcacatcttttccatccttgtgttgtga
gtagtttatggttacttgttaattgtatatatttcccagcttcggtaataattttatata

cagttttttttccctgttttcgaaatactacatttaattgctagaaataactgttgttttt
gcgcactaaagttaatgacttca-atctttttataatagcaattaaataatatttg--aa
gttgattatataatgatgtctgaagataattgaatttggatatacacccactttctctta
aatcggataacaaaggccttttgtcactccgagtttcaaggtttctatttagttgagata
atgccaagataggcaagcttaaatcttttgctaatattttaagtgatcgagtaattaatg
atagttcttgagatctattgtcagattgtctactctcctgcagcaattgaagataatcta
taataattaattctatattttttccttgtaatttaaagaattttacttttgtctttatag
tgtcagtagaaatcttagcgctatcatcaatataaagattaaggttagcaagagttttac
ttttttgaataacatattgccattcatcattattaagctgaccagattgaatttttttggc
cgtttaaattacactcttgagctaaaattcttcttagtagttgctctgtagacatctcta
agctaaataaaataacataagatcttttctgattaattacatatctagttatattaatag
caaaagcagttttccccattgaaggtcttcctgcaagaataattaaatctgacttttgaa
acccttgtgttatagagtcgagttctgtaaaaccagaaagtatactactatttatactaa
tctttttccttgtatccaaatggactaataggtttgcaagaattgtagctaatgtttgcg
tatctttttcgtcaagtatttcataagcttttgttaattgactaacaatagaagttatgt
ttttttgttcaataagttgttttgagcagctaattaaacataatgaatctccacatgcta
aaagtaatcttttaatgtagttatctataacgactgctgaatattcgcttatagtatcag
ataggggagcttgttctattagactaaaaacttcatttagctcattcaaatcttgaatca
ttttttcatttttttaagttaatgaaaaaatttcttacgctttttgtctttgcatggttaa
cattttcgagtgctgctctataaagtaatgaagttgatgtaaaataaaaaaaatcaggag
agatttttttctactgattttagtaatgatatcgattgctgagttaatattatactaatta
atattttttcagctaaaatattatgaggaggtagatatttataa------atacttatta
cttcctcc-ttgtaacattactcttgtg--------gtattagttaactattgatttaat
ttgattctggaagaacttgcaatttaatatttgcagttacttgatgtaatagcttaattt
ctacatcgtaaatgccaattgttttcacgtctggtaaagagatactttgtttttcaatgt
ctatgttagtagtgtttttaataatttgtgaaatttctttttctgtgacactaccaaaaa
tattgtggccatctcctgtttttttactaacgctaaacctttgtatttcttctagaagtt
gcttgactttttttagcattttctttagctgtttttaatttttcttctcttatagctgcat
aaaatttttgttgttttaaaataccatttgtggcaactgccgccattttattcgggatta
aaaaatttcttgcataaccagaagctactttgataacgtcattactcttgccaagttttt
gaatattctcttttagcacaacatttataactttttttactcatctt-------aattcta
aaaa----atctgtattatcctattatacctgttgtatacagatttt----aatagtaag
tatttatttgttgatgtaatattcttgtaaaa--aaaggtaatttttttatttttt-ttctg
atataatcactttcacaataattc-----attaggagaggtggccgagtggttgaaggcg
cagcattggaaatgctgtttaggagcaatcttaacgagggttcgaatccctctctctccg
tacaa--aaaatataaaaagttttatagagtccaatctaaatctccaataatattcttcc

aagcatttaatccaccccgaactctaacaatatttagtttttgagctattaaaaacttag
aagcaaatattgatctagaatctaagctacagtaaacaaaacaaatttatcttgtaaat
tgatatgagagtaattcattccttttatttttttgtaaaggtagatttaatgaatgtatta
aatgacttttatgatattcttcatgatttcttacatctattaaaatatattgtggattcc
tactaattaaaaacttttgtaattgaataacactaatttctcgtacaaatgtactagatt
tactgccataatatttgttccaatgctttttgt-------ggataatacaaatttagta
tttataatcttaaatttgttaaatgatgaagttaatgagttgtacgttaatatagttcca
cttaatatagacttataacccagaacgattttgacagcttcagttgcttgaagtgtacca
acaatacccggtaatagacctaaaactccggaattactacaagtatctcttgcgctctct
ttattttcagtttcactgtagaagtctcgataaacaggtccaccttgataattaaataca
ctaacttgtccttcaaattgaaaaatggcaccataaatatgtattttatttaactctagg
caagtatcattcaagagatatcgagtatcgaagttatcagatccatctataataatatca
tattgtctaataatgtcgattgaattctcatagcttagtcttgttttgaacacagttact
atacactgtggatttaaatctaata-tttttttttagctatttctacttttgaatatcct
atatcatgcatcgtgtataaaatttgcctctgtaaattagagagatcaattatgtcatcg
tctataattccaatgctgccaattcctgcagcagcaaggtaaatgattcctggagatcct
agcccaccagccccaatgaataaaacttttgcttcttttaatctttcttgtccttctaat
tgaatttgtggcaatactaaatgcttagaatatcttgtatattcttctagtgagtatttt
gtattttctgttttaaaatttagcataaaaatttatagtttatttaatagcactta----
--aaagctagctgataagtttattaaaatgacttttgtaaacttggttcttctctatcta
aaatagctccttgtgtaggacagacctgaacacaaatactacaatcaatacaggcagaaa
agtcaatccaataccaattagtgttattattgtttttttccctttccttgatgaatacaag
ctacggggcaagcttccacgcattcagaaactccaatacatttttctgttacaattgtat
gagacatattatttaattaat-aaaagataagctatttacatctagtagttttgttgtat
actaaatttacatatgctatccaggccaatatttaaggtagtctatttctacagtttact
aatttgatatgcgcctttagttcagttggtagaacgcaggtctccaaaacctgatgtcga
gggttcaagtccttcagggcgcgtttag-ttttatcgtaattcaataatatcattttatc
tttttgcaaacatcaaaatgatatgatataaaagatcatattttatacattgaatattct
atgtactagtactg-tttttaatatgttattattgataagttaattatactaattctat
ttttgtaataagt-aaaaacagttctaattaagtatgcacgtatttaatagtc-ttttgt
atatat--aacttgaaaaaatttaaactatggctaaaaaagttactggaattgttaaact
agcattaaatgcaggtaaagctactcctgcgccacctgtcggacctgctttaggacagca
cggcgtgaatattgttatgttctgcaaagagtacaatgcacgtactgctgacaagtcagg
actagtaatccctgtagagatctcaatatatgaagatagaagttttacttttatacttaa
aactcctcctgcttctgtactaatcgctaaagctgctggactaaataaaggttcgggtga
accaaataccaaaaaagtaggcagcataacaaataaacagttagaatctattgcagaaac

aaaacttcccgatttgaacactaacaatatacctcaagctatgaaaatagttggaggtac
ggctaaaaatatgggaattctaattaaagatt-aaaagtttcaattatatttacta-tt-
-tttatgaaaaaattttcacgtcgacttacaacattaaaatcaaaagttgagcctaaact
ttacactattaatgaagcagtatctatattaaaagcaacgtcaaatgctaaatttaagga
aactgcagaagctcatattgctctaggtttaaatcctaaatatgcagaccagcaactcag
agcaacagttatattaccgaaaggtacaggtaaattgataaaagtagcagttattgctaa
gggagaaaaattaacagaagcaattagtgcgggagctgatgtcagtggctccgaagaact
aattgatgaaatactgaaaggtagattagattttgataagctgatagcaacaccagatgt
tatgcctttaatagcaaagctgggacgagtattaggccctcgagggttaatgccttctcc
taaagcaggcacggtaacattggacgtagcaaaagccgtgaatgaatttaaaggggtaa
agttgaatacagagttgatagaacaggtataattcatgtaccttttggaaaatctagttt
ttcacaagaagatctagttttgaacctccaaacaattaaagaatcgattgatagaaacaa
gccttctggagcaaaagggaagtactggaaaactttttttcttatctagcaccatggggcc
atctattcaaattgacatcactagccttctatgaaaaattttttgtataattatagaactt
gaataatctattatttcaaatta-taatttgctagcattattaacgtaacaacttgctta
ctatatcttatctgcattaaaaacttt-ttatatatcttattaattatgagtacaaaggt
tgaaaatatcttggaagagctaaaatctttaaaccttctagaggctgctgaactagttaa
acaaatagaagaaacgtttgatgttgatgcatctgcggcttcaggaggtatgatgatggc
cacaccaacttcagcaccagcgtctgctgaggttgaagagaagacagaatttgatgttgt
cctagaggaagtcccagcacctaaaaagatttctgtattaaaagttgttcgctctctgac
tggtttgggtttaaaagaagcaaaagatcttgttgaatctactccaaaagtcttaaaaga
aggtgcttctaaagatgatgcagaaactatgaaaaaacagctagaagatgctggcgcaac
agttattgttaagtaatga-----agaagcgcttctgtttaagtataaacagaagtgcct
ctttatttattataagcaattatactagaaaagtccaagtgtaattgcattgttgatagg
catagttgcaccaatacctagccaaatacttacaacggtaccaattaagaaaacagtggt
tgcaattggtcttctaaatggattttgaaatttgttaacattttcaatgaatggaacagt
aagtaaaccagctggtacagcagccatacttagaacacctaatagtttattaggaatcac
tcttaatagattaaatgttggaaagaagtaccattctggtaaaatctctaatggagtcgc
aaatggattagacttttcacctatagaagatggttctaaaattgcgagtccaatactgca
ggcaaaagtgccaataattacaactggaaagacgtataatagatcatttggccaagctgg
ctctccgtaataattatggcccattccttttgctaatttagctctcagcttcggatctgt
taaatctggtttttttaagaattgacatattg-tttttgtgtttaatataaagattagtct
aattactgttttaaagtgtaattttcaactattataatggtccagaaattccttgtttac
gtatcattaaaaaatgcataagcatgaaaacggcagttagtagcggaagtacaaaagtgt
gtaaactatagaacctagttaaagttccttggcctacactaactcctcctcttaacaatt
ctactatactcgctccgacaactggaacagcatcagggacacctgtaacaatttttacag

cccagtatccgatttgatcccatggcaaagaataacctgttaccccaaaagaaacagtaa
gcactcctaaaattacacctgttacccatgtcaattctctaggtttttgaaacctcctg
ttaaataaacacggaatacatgtagaatcatcatcaagaccatcatgctcgctgaccatc
tatgaattgatctaataagccatccaaagtttacatcagtcataatgtactctactgatg
taaaagcttctgcaactgtaggtctataatagaatgtcattgcaaatccagttgcaactt
gaattagaaaagatacaaatacaataccccctaagcaataaaaaatattaacatgaggtg
gtacatacttactagaaatatcgtcagcaattgcttgaatttctaatctctcttcaaacc
agtcataaattttactcataaaatagcttcaaaaagctcttctaatata--ttttgcgtt
taagctactattaattcaatag------ttattgaacttatatt-tttttataatcaata
cttattaactattataacatctttttattgattt--------------------aaaatt
ctctgacttagtaaaacagggctatgtactataattctttttttttgtatagagattaat
ttcgtttttagtaaattagctaaaattcttgttacactgactctattactacctataatc
tgtgctaaaattttatgtgtaatagtaaagttaagcataataccattattaacttgagtt
ccattttgctcagctaataataataataagctcgccagtctactggttatacttttatgt
gaaatgatttctataaaatgataagcttttgccgagcaaaatactaaatgatttacaaaa
aataaattgaatggagcgcagttactacatgcttttataatagtagtatagttaatacag
gcaacttctgttttatctattgcttctgcttcgtagtaaaaattatcgtcgaccaattct
atttgtccaaatgtatctcctgtagttaataagttaagagttactttatgtgtatttctc
aggacttttgtaataattaaagatcctatcaatataatgtataatctagagctgctatta
aacaaaagagtatcatttttcttaagtgaaaaaatttgataattaattttattacgatta
aaaaatagtagccatggattgcaagaaaaaaaatcacctgaactattttcagagaatatt
ggaatacaattttcttgatttaaacaatttttagtcataattactactttataa------
---aaaaatattctaataat-taagaactgaaattaaatgtcatataaattgatgttagt
tgagaatgacattgtattatcaaaggccattcaagaatacttaatagatcaaggcttcaa
tgtttatattgccaataatggattagaagcactgaatctagcttatcaatataatttcga
cttgataatttctgatataatgatgccgctagttaatggctatgaactattagcaaaact
taaaaaaaacaaagcattatccaaaatacctgttatctttttgacagctaaaggaatgac
taaagacagaataaaaggctacgacatggggtgctatggttatttatctaaaccatttga
tcctaatgagttgctttcaattattaacaacttaatcgctagagatgtcttaaaagaagc
ttctttacaaaactcggcaacatcaaaccagcaattaaatcataaaatacgtttgacacc
tagagaaaaaaagtattttagatcttgttgttgatggacttactaataaagaaatatctac
tatactgaatactagtgttagaaatgtagaaaaatatgttagtcgactcttacacaaaac
taatatgaagaataggactttgctagtcaagtattctataaataataatctattaaataa
tgagatcaatgaaagggcgaatgacgggactcgaacccgcgaatgatggagccacaaccc
attgccttaaccccttggccacactcgccatattacatacaccactatagcttgtttttt
gatatatcgtctagttttcgagttatatatttatctaaaacaatatttactttgaatttt

-tatgatacatagcaatattagtattcaaattaatggggaaccatttaattgctcaaagc
ccatctcattgcaatttttattaaattatcttgattttaattccgagcgtgtagcagtcg
agctaaatgacattcttttaccagagactctgtttcactcaacttatttgaatgatcaag
ataagctagaggttattaccattgtaggtggtggataatctatttgtacatac-------
-acagttttctatctcaaatgtttcatagatagagataatcttccttgtttttatctac
atgaataataacagctttaatagtgtcacctattttaaattgtgatgatatctgctctaa
ttctttgatattaatctcagaaatatggacaagacctttgagatttcctacctttataaa
taatccgtaaggtgtaatctgatttattatgccttcaataatattaccaacaattaaatt
agatgatgcttgagatattagagctcttctatgactcagtattaaattattagatttctc
ttcgacattgagtaattttagcttaataaatttattattaaattgctcactttttttgaaa
attaccgagatgagaattaggtacaaatccagatataccttcaaggttaattatcattcc
tcccttattgaatccttttatcattacattaagtaaagagtcttcagctaatagttgcct
aattcttttccatgctcttatatattcaaggcgacgaattgataatattaattgtttcga
ctgaatattataatctaataaaaaaaattctcttgtgtcattaatgtttaaagaagtgaa
attatttaaatcttgattactagatacttcttgaataggcaagtatgcagatattggtgt
tcctatatctactaataccccatttaattcaaagctaaatatagttccagctacaatatc
tccaagattcaaatcatacttgtatttttgtaaaacagctgcaaaatttctatgagtaaa
tccttcattatttttttgtcatattgaatagtatgaattaaagagtaactttgtacaatat
attaaggtaaataaatgtttgtcgaattgtatgaaaaaattttaatttgttcggttgtaa
ttaagtatataaacatattaaaatatctaggagacatgaaaagtagacgtagatagctgc
agatttaa-tactatataatctgaggaaagtccgggctccacaaatacaatttatgctgg
agaaatcccagtgtaggtaactgcgaggatagtaccacagaaaaaaaccgccagaataag
t----tatattagctggtaagggtgcaaaggcaagttaaaagcttaccaaaagtactgca
aagtatttgttaggtaaaccccctaaaatggagcaaagctactaaacaaatatttgtgtat
attttatctagtttagttgaaaatactgcatgaagttattagtaacaataactcgagagg
aatagctatcctttacaatatatc-tcgattatattgaaagaacagaacccggcttatgt
agtactttttatgtcttgtattattatgtctattgtcgaatctttaaatttaactgtttg
acaatttttttttgtagctgatgttgtttataatctatttctgaattggttaaagttttt
tcattagatcgatatgtaaatcttagacctatactttttttctcttttccaattgattca
tctatatattgatcaaataatgttgtactttctaaatctttatcataaaattgatctaat
aattttaataagtatttaatttccatattcttaggtattattaaagagagatctcttatg
atcgatggatacttagagtatggttgaatctgataacttaaataattgaattcattatga
caatcttctaaaatgtttaagttaacttcaagaaaaaaaagttttgtattgagtcctaat
tcactatatgttgcttgattaagttcactgaatatgccaataataatattgttatagatt
agtgtggcaaattttcctttttgaatgaactttattctaccatcgactatatcttttttt
gtccactcaatttgtctattaatcttttgaaaaaaatttctacgatccctttagcttca

taccaatttaaagaatgcgcagggtgtgaccatgtagatcggatatctagattaccaccc
aatatgatagctagattagtagtttctacaattttgttttgatcattatgaaatactgtt
cctatctcaaagctatctatagtttggttgctttgtttaatattataaagatttgattca
attaatccttccagcaaactgccgcgtaaattagaataatcttgaataagaggattgttt
aaagctatatttcctttggactttattaaagaataatgaactaattcagttaatcctaga
tttcttaaaatacttctacttttgtctatgaatttctctttgtggataaccttttgttg
aattgaatatttggtactgagctcctgaatttatgataaccataaattctagcaatttct
tcaataacatctatttctcgaaaaacatcttctcgtctataatttggaacagtaacttct
atattttccttattttgataaataagatcaaaatttaaagagtgaagaatattttgaatt
tcttcaaagaataaaaaacgtgtttgaccattatacataattggtcctaaaatgtcatga
actttttaattgataaattaatattaagagcatgatcagttatttttctcgacagaaa
gtatctctaatatttccgcatgttaaatctgtgattaaagcaagagcttcaaaatgtgca
tttttccaattatctacatttagcccacgttcttgtctaattgaactttctgttcgaata
ttaagaactcgagaagactttctaactacagattgtttaaatatagcagactctacaagt
atcgattttgtattataatctgtatcaaaatcagagttgcttccaataccagctatacta
gttacatttgtatttacttgagtaactaaaatatttttgtttaactcaatattttcattg
tttaacttaatctgatcattacttccaagaggaaaattgcttcgaattgttagactattt
ttatgattcatattattaattttattcaaatcaataatattaataggctgtccccatttc
aacataatataattgcttatatctgttaacaaatttctgtgtgtaaacccagaagagagc
aaccgatttttttagccaatctggagaatctttaactcttatttcgtctataatggcagca
aaataattgtcacaatttaataaatcgtgattactgattatagt-ctcctttttagagaa
atatctattattgggttgatgtatagctgataacattgaggaccctgttaaagccgagac
ttctcttgatagaccaatcatgcttaacacgtctgacctgttagctgttgatgatatatc
taatatatgatcaatctcacctccaattataatagattcaatagcttcaacttcaaaacc
tgcttgtgttagttgattagttaaattgtctatatctattgttttgatattaccaagttc
tttcaaccaatttaaagaaactttcataattaattgtcatctatcttatagaagtattta
ttttacttatgattaaaaatcaatttaaaaaattcatcaaattaatataagttatttgat
gaatttgcttatacaatatattattgtgatatcttacttttatatggcagttattaagaa
ctagcttttgtaaaagttagcccattatcttgggcatatctttccataaatctcatgaat
ctatcccaatcatcaggacttttaataacatgtacagcttctattgcttgtggtttacca
ttaataaacttggcattaacatctcttgtaataatttgtccctctgtgtccatcaggtac
atacctgtgatttcacctttgtctgccatactagcatctaaaattttgggatttgtaaac
cgaaaagttgctgttcctgtactaccatctcgagaccttgttaaacgtacatctggtaca
acttcttcattgataccttgaataaattgaattgttgccatatcaatcttctttatttgt
atataatttatattggataaaactgatttctagttcttgaatgcttgttgagaaactgtt
ttaaaagcttatagtatcagtagatcaaattttatgtattatataaaactgtaacttttc

-ttttttg--

----------------------atctgttcaaagaactaacttattataagctaatcata
cgaatattagccatataatgttcgtaagt-gaaattaaggggttgtagctcagaaggata
gagcaagcgcctcctaagcgctaggtcagcggttcaagtccgctcaaccctgttaacgaa
ttaagtagtattatttaacttgttctgcaaatcagtattgatctaagttaaagtacttga
cagctaaaacaaaagaatctgagaaatattttcatttaaagaacgaacaattatcttaac
tctaaatatctgataattagtaa-aaaattct----------------------------
----ctttttaaaacacaagatgatg-----aagatatttaaactaataattgaaattaa
gtttttgaagttatagaagccaat--ataatactttctattaacaga-----------tg
cttgcttctattactatctgtttacaaga--aatt----atgagtatcaaaata------
--attttagctcca-------------------------------------ctgac----

-----------------------------tactaataaata-------------------
------------------tttattttagtctagacaag------atgaatcttcatggta
taacag--

--------------agctattctttgtgtcatacttatataaaattaggat-------ac
tgtt--

---------------ttcattaccataactggggtgggaggattcgaacctgcgaatggc
ggagtcaaagtccgctgccttaccacttggctacaccccaacatgtgaacattataatag
cagtcgtataccctgccatgtcaatagactattgtctttatttaataatttttgctgctc
ttgtagaataaaatcttttttttcaaataagatacgctatgtttaagttgcacgactgtt
atgttttcttgcttttgactgaacagccacttgatagtaattattccttctgcaatttcg
ttacttcctataataatacaagctacagctctttttttatttgcttgcttaatctgctta

ctaaaattacttgagctaacatcgatttctattttaaaaaactgctgatgtaaaaaacgc
attatttgcataccagtttcgttagcttttgtaccttgtgtagcaatataaaaatcgata
gatttattaggcaagtctatattatcttttgcaatcagtaataggcgttctaatcctatt
gcacatccaacagctggtgttgctttgccacctaattggtgaactaagctgtcatatctt
ccaccaccacatatagtatcttgcccttttgatgtcaatgtcttaatttcgaaagcagta
tcattataataatctaatcctcgaactaacttattatttatattgtagggaatattaagt
aattttagataattgcaaacgtcaccaaagtgttgttgtgattccaaacttaaaaaatca
gaaattttggagcctctgtcagtatttttgtgtattagaatcttttgaatctaaaatt
ctaattggattactagttaatctttttgtgagtcagtgtctaaatcatcatggtacttt
gtaagataatctcgtaatttaacttgataaatactacgatcctctacttttccaattgaa
ttcagatcaagtttcaagttgtgcagattgagattgttaaatatgctcatagctaaatgt
ataacttcactatctgctcttgcatctaagctaccaataaactcaatgccaagttggtga
aattgtcgttgtcgtccactttgtggtctttcgtacctgaacattggaccgctataccat
aatctttgtaaactatggtggtaactcatttatttcaataaaagatctcacaataccg
gcagtaccttctggccttaaggtaatatctctattgctgcgatcatgaaaccgatacatt
tctttatttacaatatcagtatcttcaccaataccccctatcatataattcactattttca
aaaataggtgttctaatttcttggtaatttgcacattctaataatttggaaattttatta
tgtataaactgccaatattgaatttcatcgggaagaatatcttttgtccctctaatagcc
tgaatttttgccatgataattttttgattaccttcttaatatgtctgcattaaatagcag
ttgtagactgttattcagtcatgtattatacattag--aacatactgatg-taatttata
aagtacgggcaaggagggattcgaacccccgacaccatggttcgtagccatgtgctctaa
tccactgagctacaagcccacttaataagtaccttaactatatcattgttcactaatgta
tacaactatattcttttt------atcttacatgtgaaatttttgtaaaagtaaaaaatt
at----gtttgtttttacaattggaactattcatatataatgcttaatttattataact
ttgtaaa--aatatctataagtttaattgtatgattctattatattggtaatcttgttat
aattttgcgctcttatctttcaattgg------ctagcatctaattaacttatatttcaa
ataaaaaaatcttatctaaaatgagtaaacaaattctatatcaggatgatgccagaaaag
cattagaaaaaggcatggatattttaacagaagctgtttctgtgactttaggaccaaaag
gaagaaatgttgtcttagaaaaaaaaattcggtgcccctcaaattattaatgatggtgtaa
cgattgcaaaggaaattagtttagaaaatcatattgaaaataccggagtcgcactgatta
gacaagcagcatctaaaacaaatgatgtagctggtgatggtacaacaacagctacagtac
tggcttcagcaattgttaagcaaggaatgagaaatgttgcagcaggttcgaatccgatgg
ctattaaaaaaggtattgaaaaagcaacaaattttgttgttagtaaaatagctgagtatg
ctaagccagtagaagatacaaaagctattatacaagttgcttctttgtcatcaggaaatg
atatagaggtaggtaaaatgatagcgaatgctatagaaaaagttggcagagaaggagtta
tttctttagaagaagggaaatcaactaatacgattcttgagatcacagaaggaatgcagt

ttgaaaaaggctttatttctccttatttcgttacagatacagaacgaatggaagttcttc
aagaaaatccttttatttatttacagacaaaaaaattactttggtgcagcaagaacttg
tgccattgcttgagcaaattgcaaaaacatctcggcctttattaataatagctgaagaca
tcgagaaagaagcattagccacaattgtagttaataaattaagagggattttgaatgttg
tagcggttagagctcctggattcggtgatagaagaaaatctttacttgaagatatgagta
tcttaacgaacggacaagtaattactgaagatgcaggtttatcacttgatacagttcaat
tagatatgttaggaaaagcccgaagagttattgttactaaagactcgacaacaataattg
cagatggtcatgaaattaaagttaaatcaagatgtgagcaaattaagcggcaaatagaga
cgagtgactctttatatgaaagagaaaaattgcaagaacgattagcaaagctttctggag
gagttgccgtcattaaagttggtgcagctacagaaacagagatgaaagataaaaaactaa
gactagaagatgcaattaatgcaacaaaagcagcaattgaagaaggtattgtaccaggag
gaggagctactaatgtccatatctctagtgaattatttacatgggctaaaaacaatttag
ttgaagatgaattaattggtgctttaatagttgaacgagctgtgacctatccgttaagac
gaatagcttttaatgcaggtgataatggagcagtaatagtggaaaaagttaaaagtcacg
attttcatataggctatgatgcagcaaacggtaatattgtaaatatgtacgatagaggta
ttatagatccagcgaaagtagctagatcagctttgcaaaacgcagcttctattgcagcaa
tggttttaactactgaatgtattgtagttgataaagtcgatgattgagctata------a
gataatagataatctcaaacaaagaataatttctaattatagaaattattctttgtttca
attaatctaattaattgatcaaatgtataatttttataataaagccaaagtttaaacacg
tcttctttttcaactattaagtagagaagatataagcccgtaatacctaaattatcaatt
aaaatattattctcatatatagatttttctaaagatctaaaaaatgacattttgaaaaa
taataccgaaatcttctcttatagttatttgctaaatcattactgtctaaagattgattt
ccaattgaattcagaacctttaacagaatactttttaaatctttatctgactttatttga
gaatcaataataatttgattcaattgtatgtcaaaactaagattaaaaatattacttgtt
aaagatatttgattttgaatatctagagattttatagccaagaagattaaacttttattg
ttcattgtaaaatataagacaattgatgatgtattctttaaatacta-aatttatcttct
tgtgcttctatcgtttttttgt-aaaataattaatgttttaatttttttagttttagttact
acccgaaaaaataaattctggaaatctagcttgagctttagctagagagtgtttttttccc
ttcttcttgccagtaattgataacttctgttgttttgtttaataactcaattctgtctct
ttcagcaatccacggcttggattctagttctgcttttaattcttcccaagcatcattcct
tggccagaaaaaataagaagtaagtggactagtgccatttccaacaatctgatcaatagc
aatagcaatattattttctaaccaaagtacttttagtgtaaattttgacaagtttttatc
tccattattaatattaagtattgg----tacagtagaatactgtactagttattttttttc
agcaacaatttgagctttgtttaaaatgtttttttaccgttttagtagcttgtgcaccttg
agataatcgttgcaaaatttttgaaatgttaacacgagtttcattatttataggattata
aaatcctaattcttctatagctttaccatcgcgtttatttctgctatccattacaacaat

tctatagcttggttgtttttttcttccgtatcgctttaaccttaacttgaccatattagt
cacctcgcttatttttt--atatctttattaaattatatcttacgacattatgcaataat
tcaaacaattaagctgattcaagtctaacattattaaatattaccattaaacattgtaga
aaaataatacctaacataggtgacatgtccattccaaacatgggaggaatgcttcctctg
aatagttttaagtatggatcagtaattctatttaatgaacagaatggttcattgtaccaa
tttacagttggaaaccatgccaatgataatttaagtaaaattaaaattaaataaatctca
gaaaaattagctatcgatccaagcaataaatttaatgtaccaggaagagtgttcatacgt
atttataattattatatacttcgttatactaatagtttgattatatattataatcgatg
taaataaat-cagctaaacaaaaaacttatccttaaaatcatcttctcttgagcactttt
ctttatctacattagtatttacttttgtcgtataaatttccagtttagaccaaatctgtc
ctaactgatttaattgagatgttccacattctaaacaggctatttttatattgctcaaaa
cagcacctttttttataaaatcgttcaataatggcgtaatagttgcttcatccaaaaata
aatctaagacaagtatttttgtaaatgaatttaattgatctgggacattgttaataatat
cattataattaactaaaactatattagctgtaggaagtaaagatcgtgctccttccgcta
agataaagccataaggcataataattacaataatatatttataatgactgctaataagac
ttatagtggtattagcttttaattcgattgtttctgttactagccactctctcattattt
cgtaagtaatccatttgcctagttctgagcaagcagttcttaaaatcgtacctggattat
tgtcgtttttgagaatccctgaccaatgttgtattaatggatgggatgctatattaattt
ggagttgcatgttttgttaaatatatttaacatttaatttgttgattgaatcttcaattc
ctatttgttgagtattattaaaatagcat----atatattaaagaaaataattat-aaaa
aatgaaaaaaagtctttggctgtggggttttacagatagtgctgaaacttggaatggtag
atttgcaatgattggttttatatcagttatttttatcgaagtagttacaggacaaggatt
attatatttaataggtatgatgtcataataaaaatccaaaaaagttcttgtaattttta
taagaactttttgg-atttttataaatctaatctagcggtctcatagatagaacaggtt
cattctctctattgataccttttcaaatccagcagcagcagctcttgctctgcctgcat
gccaaagatgtccaataaataggaagaatcctaggaagaaatgagatgttgttaaccaag
atctaggagatacatagttaactgaattaatttctgtagctacaccaccaacggaattca
aagaacctagtggtgcgtgagtcatatattctgcggcacgcctttcctgccacggttgaa
tatcgttcttgatttatttaaatcaagaccatttggtcctctaagaggctcaacccaag
gagctcgcaagtcccaaaatctcatggtttcaccaccaaaaataatttctccactaggag
atctcattaggtatttacctaatccagtaggtccttgagaagatgcaacattcgcaccta
gtctttgatctctaactaagaaagtaaaagcttgagcttgtgaagcttcaggaccagttg
gtccatagaattcactaggataagctgtgttgttataccaaacaaaattagaagccgtaa
gccccatgattgataaagcacctagactataagataagtaagcctcaccagaccatacaa
aagctcttctagcccaagcaaaaggttttgttagtatatgccagattccgccagcaatac
aaataatgccaatccagacatgaccacctacaagatcttccatgttatttacacttacga

tccatccatcaccgccaaacggagactttaagacataaccaaagataactaggggattaa
gtgtaggattgctaacaaatctaacatctccaccacctggagcccaagtgtcgtataccc
caccaataaataaagctttaataactaacagaaaagcgccaatgcctaataacactaagt
gtataccaagtattgttgtcattttgtttttatcccgccaatcgtatccgaagaaaggaa
atgattcttcaagagtatcagggccaatcagagaatgataaagacctccaaagccaagaa
cagctgaagaaattaaatgcactacgcctactacaaagtatgggtatgtattgaaaatct
cgccgcctggaccaacaccccagcctagtgtagctaagtgcggaattagaataaaccctt
gttcgtacaaaggcttctcgggaacaaaatgtgctacctcaaaaagagtcatggcaccag
tccaaaagaccattatacctgcatgagcaacatgagcaccaagcaatttgccagaaacgt
taattaagcgtgcattgccagaccaccaggcaaacccggtagactcaatgtctctaccgc
caacaccaacagttgtattaaagggcgtttccacgtggtaaaacctcctcagggaatata
aagttttcatgaggttgatcttgagcagccatccaagagcgaataccttcgttcaatagg
atatttttagtataaaaagtttcaaactcaggatcttccgcagctctcaactcttgagaa
acaaaatcgtaagctcttaaatttagagctagtccaacaattccaaatgcgcttgtccat
aatccagttactggtacaaataacatgaaaaaatgtagccaacgtttattagaaaaagca
acaccaaaaatctgtgaccagaatctattagctgttaccatcgaataagtttcttcagat
tgtgttggagtaaatgcacgaaaagtatctgcagcatcaccatcttcgaataaggtattc
tgtacagttgcaccgtgaatagcacatagtaaagcaccacctaagattcctgcaacgccc
atcatatggaacggattcagggtccagttatgaaatccttgcaaaaatagcaaaaatcta
aaaattgcagcaactccaaggcttggggcaaagaaccagctcgcttggcctagagggtac
attagaaagacggaaacgaatactgcaattggtccagaaaatgcaatagcgttgtatggt
ctaagaccaactagcctagcaatttcaaattgtcttaaacagaatccaattagtccaaat
gatccatgtaaagctataaatgcccacagaccaccaatttggcaccagcgagtaaaatct
ccttgagcttcaggtccccaaaggaaaagaagtgaatgtcccatgctgtttgctggggta
gaaacagccgcagttaaaaaattgcatccttctagatatgaactggctagcccatgagta
taccaagaagtaacaaaagtagtcccagttagccagccacctacagcaaggtaagcgcaa
ggaaacagaagtagtccagaccaacctacgaatacaaatcggtctctttttagccaatcg
tctacaagatcaaaaccaccacgagtttttttcttgtccaattgctatggtcataattta
atctccagagcaaatttaataagtaatttgtttaaataaaca---ctttagatatactct
aaaatttacttttctttacggtcaatataattataagtcaaaagtaaccaaaattcacg
cttttaataaca-ttttttagctcattaagttaa-ttttttaatatataaacagtaata
gagttattgttt-aacttacaatactcacaatttattcatttctataatcctttgaaaaa
gatatcctgttcctcgtgctgttaaaatcaagtcgggattacttggatcatcttctagtt
tagctctcaatcgtgaaatatgaacatcaacaactctcgtgtcaacatgccgttcgggag
tatagccccaaacttcttgcaagatagaagctctagaaaaaggttctccagccttactaa
ctaaaagctcgagaagactgaattccatacctgttaatcgaactctttcattatttttat

aaacttgtcttttgttagtatctatttttaaaaatccaatactaataatcccagagtttg
ggactccaagactagtggtgattttatcagctcttcgaagaacagaacgaatgcgagctt
ctagctccttaggagaaaatggtttaacgacatagtcatcagcacctatttctaatccgg
taattctatcgcagacttcacctaacgctgttagcattataataggaacatctgattctt
ttcgaagttcttgacaaacaccgtaaccatctagtttaggcatcataacatctaaaacaa
caaggctaggatactcttttctaaacacgagtaaagcttcttctccatttgaagcagtga
taacttcataacctattattgttagtcttgtttctaaaattcttcttatgctggcctcat
cgtcaacaacgagaatcttttctttctggttctccaa-tttcttatttactcctgtaact
caccgatcatgattaccataaccttaaatttttagcgctatgagtatctttattcacttt
agaa-----tttagttattataaattttgcaagtctagtttacattaattagtaccagt-
-caatatttttgt-----------tttaagttatgac---------tattttaatccata
agttgactctagtttgcagaattcaatatatacata-ttttctacccaagtaaattgtat
tctattttctaaataaaaaaggggttgacaaaccagttgatagaaagtattctacattac
tataag----ttaattccaacaaagaagttttaa-----atcttctttcggagtaatgaa
tactttaat
>SRR9587917
tatatgata---aattaagacgtctgttatgtgagaatttctaaaatatacaaaactttt
tatttaatatagaaaaagtaaattaaaataagttgaacatactttgctctaatctaaacc
tcttaattgag------attctaattagagacaaaagtatttacttattttgtatctata
ggaataaaaatgaaacttgcagtttatggtaaaggtggtataggtaaatctacaactagt
tgcaatatttctgtagcactttcaaaaagaggtaaaaaagttctacaaattggctgcgac
cctaaacatgatagtacatttacgctaacaggtttttaatcccaacaattatcgatact
cttcaatccaaggattaccactatgaggacgtttggcctgaagatgtaatctataaggga
tatggcggtgtagactgtgttgaagctggtggaccgccggctggcgctggttgcggaggc
tacgttgtaggtgaaacagttaaacttttaaaagaattaaatgcttttgatgaatacgat
attatcttatttgatgttttaggtgatgttgtatgtggaggttttgcagctccattaaat
tatgcagactactgcttaatcattacagacaatggatttgatgctttatttgcagctaat
agaatagcagcttcagtacgagaaaaagctagaacgcactctctgagattagctggactt
gttggtaatagaacagataaaagagatctaattgataaatatatagattgtgttccaatg
ccagtattagaagtcttgcccttgattgaagatattagagtgtccagagtaaaaggtaaa
actttatttgaaatggcagaaattgataaggatttagcatatgtatgcgattactatttg
aatattgcagatcagttgattacaaggccagaaggtgtagttcctaaagaatctccggat
agagaattatttagtcttttatctgatttttacttaaatcctaaatcaaaggtaggacaa
gaaaaagtagatcaagaagaattagatttgatgatagtgtaaataatattttcagcataa
tataacaaggaataagataatgtctacagctcaatcagatgctcttacttttgaatgtga
aacaggtaattatcatactttttgtcccatcagttgcgtttcttggttatatcaaaaaat

tgaagacagtttttttcttagttatagggactaaaacttgtggttatttcttgcaaaatgc
aatgggagtaatgatttttgctgaaccgagatatgccatggcagaattagaggaaggaga
tatttcagctaagctaaatgattatggtgaactccgtagattatgcttacaaataaaaaa
agatagaaatcctagtgttatatttggattggaacatgtacaaccgaaattataaaaat
ggatctggaaggaattgctccaaaattggaagcagaaattcgtgttcctatagtagttgc
tagagctaatggtttagattatgcctttacccaaggtgaagatacagttctagcagctat
ggctcaaaggtgtccattaaatttaaagaatcaatcagataacacgagtcttaaaacttc
tcctcatattcctttagttttatttggatctcttccggatccagttgtaacccagttaac
tatggaattaaaaaaacaaggtatttttgtttctggttggttgccatcaaagaggtatac
tgaattaccggttataaaagaaggatattatgttgcgggagttaatccatttcttagtcg
cacagctactacactaatgagacgccgaaaaacaaaactaattggtgcaccatttccaat
aggtccagatggtactcgagcctggattgaaaaaatctgttcagtaatgaatgtaaagcc
tattggattagaagatagagaaaaagcaatttgggcttctttagaagactatatttcttt
aatccgaggtaaatcagttttttttatgggtgataatttactagaagtatctcttgcaag
atttttaactagatgtggaatgactgtatatgaaattggtattccttatatggataaacg
ctatcaagcagcagagctagctttattaaaagctacatgtgataaaatgaacgttatgat
gccaacaattgtagaaaaaccagataattataatcaagtagaccgaatccgtgatctgaa
acccgatttagttatcactggtatggctcatgcaaaccctttagaagccagaggtattaa
tacaaaatggtcagtagaattcacatttgcacaaattcatggctttaccaatgcaagaga
tattcttgaactcgtcacaagaccattacggcgaaatcttagtttgtcagaattaggctg
ggatgtttatagcaagcaaagctagtattcctctttatttatgacttacatggtataaaa
gccttgtaagtct--tttattttatgttaaagaagcatttcttactctgccatcaccagc
ccaagcttgtaaactttctacttgttctttgtctgtaaatgctaaaggcacaaactgttt
gatagctattttaatatcttcagtactaaattctctctcttcactaaacgccgtatgcat
actttcaataattgcttgctcaatttcagcgccagaaaatttattacataataaactcaa
ttcatgaatatcatattcttgccacgatcttggtctaacttttgataaatgtatcttaaa
aattgactctctctcttgacgattaggtaaatctaagaaaaaaatttcatcgaacctgcc
tttccttaacatttcggaaggtaaactttgaattttattagctgttgcgacaacaaatac
tggagctttttttctctgatagccaggtaataaatgtaccaaatactctggcactagtacc
actatcaccttgactatgtaaaccagaaaaagctttatctatttcatcaatccataatat
gcaaggcgataatccttcagagatatttatcatttctctcatttttgattccgattcacc
aactagtccaccaaacagttttcccatgtcaagacgtagcagaggcagcatccaatcgct
tgcaatagctttcgcggttaaggatttacccgtcccctgtataccaactagcaataaacc
tttgggcgaaggaataccgtaatttaaactttgcttcgaaaatgagcgagacctttttttg
tagccattgttttaatacatctaagccaccaatatccctatttactttactataaggata
aaattctaatagatgcgtttgattaataatttgtctttttttcttcaataataataggtaa

gctccgagagtctatctgattatattgtgcaataattttagtaatcactttgcggattct
atctatcgataatccttggcaggattttgttatattattaacgagctctgaattcaaatt
aagattcaaagctttactcaatcttatgatctctttttgatttctagaagactaggtaa
gggcaaatctattaccgttatgatatcattcagagcaaaaggtatattgattttgcaaga
aataataataatgtgcttagattgcgttttgattattttagctaaattgcgaagtttcct
aattaatactatttcgtttaggaaagagtcaaaatcttttaaaagaaaaagatttaaaga
ttcattattcaagttttcaataaattctaatgctaataaaggatttctttttgcataacc
attatcgctaggattgttagtgtatccatctacaaagtcccaggaatatacttgttgact
gtttgaacagtttaagctgtgttttataatatactctagtctatcttcttctctagtatt
tattacaataataggatatcgagattttaaaagtaatcgtaaatcttgagtgaaattcat
gtcaatatttgtttccatcagacttttgtacttcaccaattgaagaaagactaaataaga
ttatctaatattccttggatatattcaaaagtc------atctctctatttctaaatta
ttgctattactaatttaacttaccatgatttttttcttccttttcttcgtcgactattt
aatatagagcgtccacatggtgttttcattcttgctctgaaaccagaaactctaatttt
tttcttttagatccttgtagtgttcttttagtcatgatgtttcctctgttgtaaaagatt
aaatactattttatcatcttttatatatctttatgatatagatatgtattaatatcaaag
tt-aacagcaataatttatattaatcaagaattatttatataatattaaacattaaatat
----------taactttatgatattggctttgcccatttttatttaagtattttgacca
tctttttgctaatcttaaactggttaatttttcagcagctaaaaacgattttgttattag
aatctcaatttaagtattttgttgataaaagccaaaatcgtaaattagaacccgaagaaa
gttttgcttttgcaaaagtatgtgtagctaaaaaatatttttctaaagctattattgaag
gtcaattagctttaaaaaactatcgagacctgaatatcttagataataatatagtaattg
ctaatttgtataatatgctaggttttatttattttgaagcaggccaaacaagttttgcaa
agaacttttatgagcaagccttacaaataaaccctaattatgttgtagctttaaataacc
tggcaaaaatttacgaagaggtaaaagatttaaaaaaagccgaatctctatacgataaag
tgttaactcttaacttaaataataaaactgctaatagaaggaaagatttatagcaaaaa
cta---------aaaacatctaatactatttgtaatcgggatagcaggatttgaacctgc
gacatcctgctcccaaagcaggcgcgctaccaaactgcgctatatcccgtaagacataat
tattactatatacctttctctaattatgtctactctttctgtattttatctaggatacc
aaaacatgcctttaactccatctggatctttgataaatcctaatttttataaaaactaa
caacatcaggttctgcgaatagtgtaatagtgctaatttctgcctgtctcaactgctgta
ttaattgatgtatgattacttttcctaaacctaaaccttggaaatctggatgaataacta
cgtcccagatagttgcattaaatccgttgtctgatgttgctctagcaaatcctacaagtt
tactatttgcatctttttttgtattaaagaaataataatagaactatttttaatgcga
tctttactttttttagaggtcttttaacccatccgactgaatcacaaagctgctccaatt
cgtataaattaatattcttattgctactcagatagatatctttaaattcgattttatcgc

aagttttatctaaaacaataagtttttgaaatttttttcagagctagcattgatatcgg
agttttgaaaaaaatttttccagaagatcataatgattatgatataatta--aataaata
tatatattacaatattgctttaacaaacaatatattaatatatgctcaatttttacatg
aatctattaagatagaagattgttaaaatatagagaaataaaaattttgtaaaatcttt
caataaaatgatataaagagtgctttttaagactaacatctattattataaataaagtct
cgaaattgagatgaaataacacattattctatgtctaatgccttttaaaggagttatttg
tgaaaaaatctatgtgtctaacctgtttacttgctttactaattatgagcaacccaataa
tagctaacgcagaagtagctggattggtgccctgcaaagattctgcggcattcaataagc
gtatggtaaatagtgtgaaaaaacttcaagccagattagctaaatatgatgcagacacgc
caccagcgttagctttaaataagcaaatagagaaaactaaaactagatttgcaacttatg
gtcgagcaggtttgctgtgtggtactgatggattgccacatttaatttctgatggtcgat
ggagtagagccggggactttgtttttccaggactattattcttgtatattacaggatgga
ttggctgggtaggtagaggatatcttttatctgttgctaagactagtaagccaacagaaa
aggaaatcattttagatgtgccattagctatcaaatttatgtcatccggatttgcatggc
cgctagcggcttggcaagaatttagtagtggacaattaattgcttccaatgacgatatta
ctgtttcaccccgttagtaaaaaatttatatgaataataattttaccaaatacttatcaa
cagcacctgtaattggtgtattgtggatgacgtttacagccggatttattatagaattga
accgctttttcccagatgtcttatacttttactt----ataaataa--gatcgtataaa-
--aaactacataaatatagagaaatactattatttgtgtagtttta---atatataaca
taattattta-tttttaaaaatgtttatcttttcgattattttaatattagatcttacat
aataaaata-atatcaatattatgagcttagtaacccaaattatagttaatgccgatgat
gaattaagatatcctacaattggagaattgcagtcaattcaagactacttaactacagga
agcaatagaattagaattgctactattattagagataaagaaaaggagattatacagaaa
gctagtaagcaaatttttcagttacatccagaatatatagctccaggtggtaatgcagca
ggttctaggaaaagatcgttatgcttacgtgattatggttggtatctcagactaattaca
tacggagtattagctggggataaagattctatagaaacaattggtattataggagtaaga
gaaatgtacaattctttaggtgtacctataattggaatgttagatgcaatccagtgttta
aaggaagcatctttagaaatgcttggtcaagatgatattagaatcatttctccttatttt
gattatataattcgtggaatgtcataaatcatagcttcaattaattagttgaataaaagc
ttttataatgttataattacaataagcccgaaagcatataattgtaaaaactaaatcttg
tcagaaccggaaggtagcagcaataatgtttcaatacaaaagttatga-ttttcgggtgt
ttattgtttttatctaatagtctcgacttttaattagtatgctatgtataaatctgtata
tgacatgtaatccatcaaaccttccagcaaaaataatttaataatttgatattaatacgt
aaaacacctatac-tcttttataaaatttctttatatctcatctaaacaatggaaactta
acttaatgggtgttcatattttatcaacaggttcgtctgtcccaaattttctgtagaga
atcaacaatttgaagatatgatcgaaacttccgaccattggatttcaacaagaacaggaa

taaaaaaaaagacatctagctccatcttctacttctttaactaaattagcggcagaagctg
caaacaaagccttatatgcagctaacttaaaacctactgagatcagtttaattattttag
ctacgtctacgcctgatgatttatttggtagtgctagccagttgcaagcagaaataggtg
caacaacatcggtagcttttgatattacggctgcctgctccggttttattgttgctttag
taacagcagctcagttcattcaaactggttcctatgacaatattttagttgttggagcag
acacaatgtctagatggattaattggtcagatagaactacctgtattttatttggtgatg
gtgctggagcagtagtattggggcaaagcctcaaaaatagtattttaggctttaagttat
gtacagatggtcagctaaacagtcatttacaattaatgaataaacctgtaaataatcaaa
aatttggtgttacagaaattcctcatggaaactataattccataacaatgaatggcaagg
aagtgtacaagttcgctgtatttcaagttccaacagtaattagacaatgtttgaataatt
taaacatttcaatagatgaagttgattggtttatattgcatcaagcaaacactagaatca
tagaagcaattgcgagcagattatcagtaccttttctaaaatgattacgaacttagagc
attatggaaatacatctgcagcgtcaatccctttagcgttagatgaagctattcaatcca
ataaaattcaaccaggccaaattattgttttatctggttttggagcaggcttgacttggg
gagcaattgtcttgaagtggtgat---ttatatattgcggatgacgagactcgaactcgt
aaagctttcgctacacacccctcaagcgtgcgtgtataccaatttcaccacatccgcatt
ttttaccatgaata-ttaaaaatttataatatttatctaaatattcatataa--tgtgta
tatatatatacattcttgtcagaaactctcttatatttatttaagtgataaaattgag
t----ttagtatagatagtaaaattttattttaaaatcataa----aaataaatatataa
ggaaaataaaatatgacaccatctttatcaagtttttgaatagtcttattcttggggca
gtaattgtagtcgttcccataactttggctcttttatttgttagtcaaaaagacaggaca
atccggtcctaaaaataacttagaaagatatggagaagcaatttaaaatgaataataaaa
tatttttcatactaataagctctccatatattgctatact--ttagaagaatttttattt
ttttatttgtaacatcctgtaaagtttattccgttctttctttttccc-atttactctc
ttataattaatgatacaaatgtcagg-ttaataatag--tttttttattctgtatctttc
---------------attttatatacgtattattatgttcaattaataaagaaaagctca
tgtctttgtctaattggcctctcaaaaaagaaaattctgaagcatataatattaagaatt
caaaacaaatcacaattcctgatggtttatggataaaatgcttcgactgtggtctattaa
tgtattctaaagtactgaagagaaatttaaaagtttgccctcaatgtagttatcattttc
aagcttctagtaacgaaagaattgatcaattgatagaccaaggtagttggcaaccaatgg
atgttcacttgatctctacagatccattaggctttaaagaccaaaagctttatagtcaaa
ggttaaaagatactgctttcaagactggcctgcaagacgcagttcaaacaggtactggga
ctatgcaaggtaaaaaagtatgcttaggtattatggattttagattcatgggggaagca
tgggatctgttgtaggcgaaaaactaacaagactgctagaaaaagcaactcaagaaaagt
tgcctgcaattatactttgtgcatcaggcggagctagaatgcaagaaggtatgttgagct
taatgcaaatggcaaaaatttcttctgctctagaaatgcataaaaaagaaaatctactat

atatatctgttttaacctctcccacaacaggggggtgtaacagctagttttgctatgcttg
gagacttaattattgcagagccaaaagctcttattgcatttgctggtagacgagttatag
aacaaacaatcaaagaagacttgccagataattttcaaagttcagaatatttatttgaac
atggtttcctagatttaattgtatccagaactcagcttagatcaaagttaatacaaattt
tgtccttacataatcatagtaagtaatgaattgaatattgactataatatcaaaatgcta
gtaaaaattaagatattaagaaattttcactaaagatatttgtagactaactagaattac
aattactattgtagaaaaatttgtcttaatgcca-ttttttcagtcttttaacaaattg-
tttttatgactttaaataattgttagtttaatacaaatttataaagagaata-----cat
actaagctaaatatagtaatattatgttttcctataatactgacctatcaaatttcgagg
aaatttatgcttaaaagatcttcttggcttgcggctttattgggactattaacagtagtt
tctacaagtacgcatacatatgccatagagttagacgaggcaacaagaactgttccatta
gaatcttctggcagaactgtaattcttacaccagaacaagttaaaagaggcaagcgatta
tttaataattcttgtgctatttgccataatggtggtatcacgaaaacaaatccgaatatt
ggacttgacccagaatctttaggattagctacgccacaaagagataccattgaaggacta
gttgactatatgaaagatccgactagttatgatggtgcagagtcaatcgcagaattgcat
ccaagtattaaaagtgctgaaattttcctaaaatgcgcaatctaaccgatgaagacctta
tttacaatcgcaggtcatatcttacttcaacctaaaattgtttctgaaaagtggggcgga
ggaaaaatttactattagaacttcaaagacctaaaccttgtgaatactagtttatgta-t
ttgttgcttaagtgtgattattttgttagatcatgatatatattaaagatggagtttgtt
gacagcaagttacttttttacccgaacttgtttacactattcgtctagttattaaaaagga
gacgttaaattgaagaagaagctttcagttcttttcactgtttttagtttttttgtaata
ggtttcgcacaaattgcttttgctgcagatctagataatggagaaaaagtttttttctgct
aattgtgcagcatgtcatgctggcggtaataacgccattatgccagataaaaccttaaaa
aaagatgtacttgaagctaatagtatgaatactattgatgctattacttatcaagtacaa
aatggtaaaaatgccatgcctgctttcggaggtagactggttgatgaagatattgaagat
gcagcaaattatgtattatctcaatctgaaaaaggttggtaattatacttgattttatcc
tgtattaaagaatagacaatctatttagttgttcattagattgtctattctttgtttatg
---ctattatataaagatatttacacaatatt----ttatgatgaaaagaatacccgcaa
ttcttgtactagaagacggtgcgtattataaaggatggtcattccagcaagataaacaag
agattactattggtgaagtagtttttaatactggaatgacaggatatcaagaaataatca
cagatcctagttacttccatcaaattgtcgcttttacctacccggaaattgggaatacag
gtattaatatcaagatattgaatctcacagtattagtattaaaggacttattgcaaaaa
atatttgtaaaatttcaagcagctggagagagcagcaatctttagttaagtatttaagta
gtaataatattccttttattttcggaatagatacaaggtctttaacccaatacttgcgtc
aatttggtacaatgaacggttgtatctctactgataatttaaatcatagttacttaaaac
agaaaatttgtgagattccaagtatgcaaggtttagatttaatcccgcatgtaactacaa

gaaatgtttacccctgggatgaaaaaagttttccaaattggtatttgacagataacatta
gagtgcaccgagttattcagttgaaagttattgttatagattttggagttaaactaaata
tactcagaagactagctacacttggatgtcagataacggttgtgcctgcccacactcctt
taaaagatattttggcttaccagcctgatggtatattactctctaatggtccaggagatc
catcagcagtacattacggcatccagacagttacaaaattactagattacaatgtgccta
tatttgggatttgtatggggcatcaaattttaaatttagctcttaaagctaaaactttca
aacttaaatttggtcatagaggtattaaccatccatccggattgaaccagcaagttgaaa
taactagtcaaaatcatggctttgcagttgaattgacttcggtttttgaatctcctgtaa
gagtgactcattttaatctaaatgacactactattgcaggaactggacataatcaaagtc
cttatttttctgtgcaatatcatccagaatcgagcccaggccctcatgatgctgattatc
tattcgaaaattttatagaaataatgacaaagtccaagaataaagttag-----------
ttagtaattttcccatgctttatgagtgaataaagcggatagtacttgtactcctcgtaa
ttgattaaagagaggcaaatgtcctctggggccatgagaattccattgaaattcctcagg
atatctgcaaggaattctatcaacttcccagccgatttttttgccagaattttccccaatc
ttttccaacactaagccatatttgtctttgaacaaatagcccaaatttaccttttgagtg
tgtatgccataacttatcaatagtttgtaaatcttgagcaggtatttttttttatatctgt
aaaatatagccaattacgagtttgtgcatttacaccagctagttgaataagcttttgttg
agttagctgatctgctttgagcaaatcccgatgagtcagtaacatttgtaaatctttgta
attcatctgttgagcagagcgcaatggaacaatgccatcgggacataagttagaagcaaa
tttcactatttcttgatttttactgttaagtaacttttcataaattaaaccgtcaacaca
attacttttatagttaggcccagtaatcctctcaaaaaatagatctgctaaatcttttaa
ctcgatagagtctttgctatttatattctcaataatttcaagttgttgcttga---catt
attagacttagtattcttattcaattctaaaagctgggctcggatttgatttggcatctg
atttgtttaattagttttaatagtc----ataaggtcttagagagaattagattgttttt
tgaaattagaagagcgccgcagtatttctaaagaagttcgcataatttgattcaataagt
tttgtcctgcataaattataatttgaccaattaaaaaaattgtgcttttttattttaggaa
caaaaaaatcttgtacctcaagcagaaaagtaattattatttgcattggtgacaacatct
gtaattcattcgatctgcaagcatatatatactgacagtttaagccttcttgataaaatc
cccatactttgtaacgactttcgtaaattgctcgtggtcgctcaagatagtttttaatta
tagtattccaaattaggttattctttaatttttctaattttctatcagatagaaattta
cattacagagataacttgcatttgtctcatttacagtattaaaattttgaactaacatat
gagcaataatgttgcttagctgaattaaataattttcaagaaaaatttcgacctgcttaa
ctggcatattgtaagtaaagagattaaaagttttattcacagtttgagatgacccaaaaa
gtagctgaataattaaaatttgtagtagcatcttatagtcgttcagtagatagttcaaat
catgaatatcttggtaaatgtcaatacagtccagttcatataacttacaaaatcttttga
tacatctatggaataaatcaattaaaatattttcggtcaagtttgtaatatcatttacat

ctaaatttgaattataaatttctaaaagtatcttttccagttcagctaatatgattttta
ataagtttctttttacttcagtgcgaaaaacatctaaaattaacacttcctgagagcaat
tagttaatctcttattaatctttatagaagttctaactaatagctctgctacctcttgat
tgagtgttggtccctgagagcttggccaataattattcacgttataccatagtaagaaat
attgaatttaatgttatacgataactttttaatatactgaaaagcaagttaattactcta
tagcaactag-------tttttacagatcttaatagttatttatcaaataaagttatatg
atagtagcgattgagctaattttattaaaattta----taataatgacaacttattactt
cgcccttgcgagtcaaaatttctattagtacaagaaccactagaagaagtttttagaga
gagagttaactactatcagtcaaataataaagcaattgatttttggctaataccaaaccc
ttcttttctagagaaaccagaaatgatttcatttaaaaaccttgtacctaaagacgctgt
agctataatctctactaatccaatatttattaattggttaaagctaagaatcggctatat
ctgtattgggcaatttgaagataacctacaactttctgaagaatcgttaaatattactgt
tttaacagacaaaatttaacaatgcttttattatgactagtgttaaattattttggttaa
gttataattatagtcttttagtattta-gactcttatagtttatcacttagtattttatg
ctgagatggagtgacaaatagtgtaagtatttcttggctaaacgtttcagttgccttaga
tttatatctatttggattaactataatggatagcattcgttttatagtaacattttcaat
ttgagcccaatgtacaattccaagttccaattccttagcaatagctgaaacggaaacaaa
ggcagctcctagccctgattgcacagcgtttttgatagcttctatagaattcaattccat
ctctattttaaagcgactgctgtcaataccatgttgactgagtactttatctatgacttt
tctaattgttgattgagtatctaacgcaataaatctaagcctgtataagtcttcttttg
aatgtctcctagtttagaaaaaggatgtgatttgggtaatataagtgctaattcgtcttc
cgcataagaagtaacctgcaaaacatcttgcaattcagtaggcacttctcctccaataat
tgctaaatcaacttgaccattagctacgctccatgaaataagtctagttgaatgtacttg
caattgaacagccacttgtggatatctttgcctaaatagtccgattaatcttggcatcaa
atatgtcccagttgtctggctagctccaataattaatgtgccaccttgtaagttttgtaa
gtcgtcaagagctcgacaagtttcttcgcagagagctaaaattctgccccccatatcgtaa
aagaagacttcctgcctcagttaaagttgccttcttattacctctttcaaatagggaaac
attcaattggcgctctaaattttgaatttgcaaactaatagctggctgagaaacatacaa
gctattagctgctttttaaaactgccctctttggcaattgcttttaatattcttaactg
atccaatgtaaatggaaggtctgtcattaaagaattgtagtataatg---------tata
ttatttagtattgatcaatgtatgcatttgccaactaaaatatttgtcattacgcttcat
tattttcaaaataataaaaagtataatgttaaataagctgtattaaaatttagtatctgt
ttgatattagatttatacaagagatatcgtgaatttaattctaactataaaatataggag
acaat---atggactctagacttttagttgtactaataccagttttagcagcagcatctt
gggcagtttacaatattggtagagttgcactacagcaatttagaaaaatgacatcttagt
tttgcttagaatata-atttttagactttatgagatagaggactttaaaatccctatctc

tattgtatatcttaataaaaaaag-aaagc--ccccttgtacttactgttattacacatt
aatattattggt-ggtaccta----gataataagtttaagctaacttatttttattta
taattaattaaattaataacacaattatggctgttccaaagaaaagaacatctaaagcta
aaaaaaatgcacgcaaagcaaattggaaaaatcaagcaaaaactgaagctcaaagagctt
tgtctttagcaaaatcagtattaactagaaaatccaatggatttatttataatctaactg
aagcatcagatacttttagcgattagaagtgttt-ttactacaagtctgaaaaagtatta
cgcaacaagttctcagtaatttttatctat----taatcatttgaaaatcattctatttg
ataataaaataaatagcagattaaataaaatcacattaacaaattatgcagcaaagctag
atcaaactagtgaaatttggttgttcaattgtattgaaaatattcagcatattttttta
aaagtcaattaaaatcaagtcatattactaaaattttatttctggtactagctttgaat
ataccgcaggtctaccaggattattatccagcttaacactaagtggtagactacatccta
ttagtatatatagtccccagtctctcaaaaagtatcttgaagcatgtaccaaatattccc
aaactaatttttcttttcctattaattttcataatttacaatacggaggacaagttgtta
accaattttatacagtaatttgtttaccgttgagcaaaaagagcctgctgtatggattta
ttattctaaaaaaagaaaagcagggagtatttaatttagcacaagctaaaaccttgaata
ttcttcaaggacctatatatggaaaactcaaagaaaaggataatttttaagtccagatg
gttactatctatccggccaagacttctcttctaatacaataatgggacataaaatatcgc
ttccgttattagtcagatattctagaattatttctgagatgcattggttttgctcttatc
ctattagattaaatacttattcgcatcagcaaggagcaaagtgtttaccacataatgttt
taactgatattatgaaatcccaaatatatcaagataacagttttgttgaataatacttat
tcttgatatataatggtctcggtaatctaaataaaatactagaaataatttcttaaattt
atttgtctatattaaatttacaatcatttatgacatacgcaattattgaagcaagtggca
cgcagctttggatagaagaaggccgctactatgatttaaatcatatacctgttgatccag
gtcagtcgattatattaggaaaagtcttattattgaataaaaatggggaggttactttag
gccgccccttgtatagaaggggttacgataaaggctacagtaatgaggcacttgcgaggaa
agaagataactgttttcaaaatgaaaccaaagaagaaaatgagattaaaaaaaggtcatc
gacaagaattgactcgtttaatgatcgattctataacatcttaga------taaaatcta
gactcatatttt--aa-aaacttttaatatttaactaaaatagatataatggcacata
aaaaaggtagtggtagtacaagaaatggcagagactctaattccaagcgtttaggtgtta
aaaaatatggtggagagcaagtaacagcaggtaatattttaatcagacaacggggaacta
aagttaagcctggccaaaatgttggaaaaggaaaagatgatacattgttttctctaattg
atggtttcgtgctgtttgaaaagtcaaatcaaaagcaaaaaacaattagtgtttattctg
ctaagaaatagttaaaacaattggtgcaagataacttgagt----gtatttatttcactt
acagtggcaccatatatttgatttattaagaaatggagttccttaatgaaccaatgaatt
ttattaaagatttgctgtgaaatttaatgacaaacactattgtaatttcctgtctacaca
atatggctgctattctatattgtggtcagatacagaaactagtagtagcaaatgctcatt

atcaagtaagtgatatctacctaggttgtgttgataaaatcttctcaggaataaatgcgg
catttattaacctaggaaagaatgagtacagtggttttatacatatcagtgataccggtc
cgcttaaaaagaaatattatgtcaataatattactaacattttaacaatacggcaaaaaa
ttttagtacaaattattaaagagccaactttaaataaaggtccaaggctcactgccaata
ttacattatcaggtcgatatattgtattaatgccttttagtcaatcaatctgtatatctc
gaaaaatatatgatgaagatgagcgtcattatttgaagtctttagctattttaattaaac
cggcaacaatgggcttgctatttagaccttctgctgtaggtgtcgatgaggaaataatat
taagcgaattaaaaaatctaaaagaacaatggaactttgttcaaaaatctgcaattaata
gttattcacctgttcttctatataaagatgaagatattgttaaaaaggtaatccgagatt
tttataataataatacaaacaatatagtaattgattcaaacctgggattaaaacaattaa
attattatatccacacttggcactgtaataactctagcacagttcctaagattaagcttt
atagtaataatcaatgtatactagatgcttttggtatcaatcaggcaatttccagagctc
tcattccaaaagttgatcttatacttggtggctatatgtttattgaaactttagaagctt
ttactattattgacgttaattctggatcttttaataattccactagtgcacgagaaacag
ttttaaaaaccaactgttctgcagcaacagaaatagcttatcagttacaaattagaaata
ttactggtgtaattataatcgacttcattgatatggaatcacaaagagatcaattgcaat
tattagaacactttaataaagagctatcacttgatgatgctaaaccacaaattgtacagt
tatctgaattaggtttagttgaattgactagaagaagacagggcaaaagtttgtacgagt
taattagtagtgattctaattactttttatttttttcacacaatcagagagatctcagtctc
ttaagagattcgatgatagacagcagaaacaacagatttttaataaatcttggctatctg
cagagattaatactattaacaaggtcttttttcaaaagtccaatttgtgcagacctgcta
acttttacctaattcgtaatctctatatagttaaaagtagtattacttataaacaaaatt
atttattaactcacagatcaaaacttatttattctaaagaatacagcaaagtattaccaa
gca------gttattatctagctagtctcaataagaatagtaatcaagagttcctacctt
aagattgttatatcaactgtt---------------------------------------
-------------------gaaaaaaagaagctctctcttatataagagagagcttc-tt
ttgtagcttactaacta-tctagttagttaattaaaaattaaccgttaacagctggagct
gttagagctactggtaaagattcaccagaagctagatctagagggaagttgtgagcgtta
cgttcgtgcattacttccatacctaggttagcacggttgatgatatcagcccatgtgtta
attacacgaccttggctatcaacaacagattggttaaagttaaaaccattcaagttgaat
gccattgtgcttacagataaagctgttagccagataccaactacaggccatagacctaag
aagaaatgtagagaacgagagttgttgaaactagcatattggaagattaaacgaccgaag
tagccatgagctgcaacgatgttataagtttcttcttcttgtccgaatttgtaaccatag
ttagcagattcgttttcgcttgtttcacgaattaagctagatgtaactagggatccgtgc
atagcactgaacagagaaccaccaaacacaccagcaacacctagttggtggaatgggtgc
attaaaatgttgtgctcagcttggaatacaagcatgaagttaaatgttccggagatacct

agaggcattccatcagagaaactaccttggccaattgggtatactaggaatactgctgct
gctgctgctactggagcagtaaaagcaacggaaatccatggacgcatacctaggcggtag
cttagttcccactcacgaccaatgtagcaagctacgccagttaggaaatgaagaacaact
aattggtaaggaccaccgttgtatagccattcgtctaaagaagcagcttcccagattggg
tagaagtgaataccgatagctgcagaacttggaataacagcaccagaaatgatgttgttt
ccgtatagaagggaaccagcaactggctcacgaattccatcaatgtctactggaggtgca
gctacgaatgcaatgatgaatacagatgtggcagttaatagagttggaatcattaataca
ccaaaccaaccaatgtataggcggttttcagtactagtaatccaagagcagaaacgttcc
cacaagctagcgctttcgcgtctttgtaaagtagcagtcataatttttatcaatttta
aggatttaacaaagatgcttcccaaacaataagttgtctgttatggaaattattacttaa
ggccttttcaaatgagaacattttaaaaaaaaca-tagtaaaatctaatctcagtaact
aa-aattt-tacttgttcatatattgcttaataattcaa-----------taataaattt
tt-gct-------------ttttgcaaaattaacactattttagggat---ataataata
atgcatattaatgctgcttattttgattttaatagtttatataattttgccctacttaaa
tacgcatgtcacactttacaaaaattcagacgaccatacaagatttaaatctgttaaagc
atgctttaacagatttgggtttgatttggcaaatgaattctagtcatataaaagttggtg
aaaataatcagcacaaggtagatattttaattaaacaagataatttgtcacatattggct
ttacttggaatgataatagatatcacttagttgccgacttacaactttgggaacaaccct
ggtctctagaagtatttttggataagttatctcagaagtatgcttactactctatcatcg
aagaaacaaagaagcaaggctttgaaaaaatgcagcagatttcacaaaaagatggatcaa
ttaagttaattgtgcaacgctggaattattaactaacatacaaatgcgggcggagagact
tgaactctcacgagattatctcactagaacctaaatctagcgcgtctaccaattccacca
cgcccgcactgttgagac--aatattactctatcataatctatactaaaacacaaactaa
-tagtataaactagcttccaagcttaaaagcgtctacaaatagaccgtatgttataccaa
ttttgaagttgttgaataaattaatttttatatttaattgcttgtggctagagtagacta
ttttactagtcaactcagttgctgcaactattagactggctgcaataataccccaatctc
ctgtttgtccaggaatcgtagacaaacctgttgaaataaaaaatcctagcaataaactaa
ttaagccagttgttaactcgctcaaagaataataaagcttattatttaagtttttatta
agtaagcaaaaaaagttgaaagtctagttttaatcatagtatttagtaacggagttttaa
aaggatgacaagtattaacag-tcattaatattgtcgtatccttttaaaactcgttaata
caaacagctgttcttgt---------ttttaaaggtcttgttcgcttaaagcattaataa
gatgctgaaacggctcttgtataatatctgtgcgtttaattgattgcaaatctaattctt
cttctatgatttcttgtaataactgtatgcttcttactgttggagcaacaggtacagata
gtgaattgtaagtatcttttagcccatttaatacacgctgatcgagaatatttgtactac
cagaaaccaaggcataactagcataccgtaggtaatattctatatccctcagacaagctg
catatcttcttgttgtataagaatttcctccaggccttaataattcaggctgttcttcgt

acaactgagctgcggcctcttttaaaatattggtagcttgatcattaataatttcagcta

tttttattcgatctagtccactcgaaaagaaagattctagttgtccgacagctgtcttat

ctagataacgaccagtaagatcgtaacgatttaatattgctgttatagcatcttgcataa

attaattctcctac-ttgctatcagagcggtattataaatgactaatattgtaatataat

cattagtgtttatgaaatttatttaaaacttattctttattgcttaatgtgcaattaaaa

actcgcatagattttacacatatagagtatacgtagtttttaatta-tttttttataaaa

accaagaaataaagctttttt-taagaggttcgtgaatttgttaatataacatgagcttt

cctcctaaataagttttgtaaagtac--aaagtaaaaaaacgattagtcaataatagtaa

--------------------------acaatattaatatgtatcaa--------------

-----------------agtataaataagcttttactccagcatcaaaaaaaatatttga
gatatgttgatacaactgattattcaagtgaaaacttagatttagaccaatttagctatt
ttatcattacaattaataaaactgataaatgtattcttatagagcaattttgttataatg
ctgaaggccatatttactcaatattcttcaaaggtccaacagctaaacatctttcttcta
taatttgtcattttcaacaattaaatacaataatatctacagctcatgccatatatttag
gacgagagctaatgaagtcagaattagctctcgtgctagatcaacaatatattcaagatt
aaa--ttttgtaagtattaattatatttatgctagtataatattagtaagggcatgtaac
tcagtggatagagtatcagattccgattctgatggccgtgggttcgaatcccgccctgcc
c---gagtattcagagtact-atactaaaacatct-------taatagctagtttgataa
tagtatgggcattggggctgcaaggtttctacattatgaaaagaagaaaatatg-aaaaa
aaacaagctctccaaaagagcttttagtacaatcaataaatgcagaaaacaatattgttt
ctttttctcgaaaattagctcttgcttaaacactgttaatttttggcattcgacatgtta
aactcttattcatggcgaatattctgtcagagtcgctcttagtttaagaaaagtcagaaa
aaatatttatctttacgtttt-tacctattttatttaaataaaggttagta-ttttcaa
cttttatagtggacgtgggttcgagtcccaccagctccatcaatttgatt---------t
gtataattagctagtttaaccttttaataaccagatatataattggaggaaatatgggt
ttcataacaattactaagcccgctttaaagcaaattgcaatcttgaaaaatgatcatgaa
aatgatgtacatctaagaataggtgttagacaaggcggatgttcaggtatgtcatactca
atgaattttgaacatgttgacaaattaaaagatacagatgaacggcttcgtctagataat
ttttctgttgtctgcgaccctaaaagtcttctttatctttatggattatcattagatttt
agctcggaattaataggaggaggatttcaatttctaacccgaatgccagccaaacttgt
ggctgcggtaaatctttttcaggctgacc-ctctaacacgtttttttatatattttgtatt
atatatattattttagcaagttg-tttattagagacaagcaatgctctgttaaaaac---
----------------------------taatttcaagagttttattgtattatt-----
----ttatatatttattatcaactacttgaaaataaattagctaagcaatctaatttgtt
atcagtaccaaactttaaatgattaacttttattgatatatgctaatcttt-atcagcc
taaacatatctgtacattacaatcttttgacataaaaagagtcttaacatacctctcaa-
----------------------aaataaattcaa----------tattttataaatactc
aacatgaatctgtcacaacttatcgttaattcttctatgatgaaaattcctaataacttt
tattgcaatataaaagctatttcttctgagaatttaagtcacttacaattatcaaaaaca
aatttattatctcctacaaaaataattgcttttggactacctaaatttgattttatttct

acacagattacaagttctattagagaaacagttgttgtgtctactattgatgataataat
ctttttgctgccccaactaatgattggataaaaaaacacaaggataaaaatcatcattct
ttgttttataaaaaagtttttaaagctttgctaaaccataaaattagagttgttgctgag
ccatt------tcataactccgaaataaataaagaaaagagcataatggcaagtcaaaga
tacatctggggtaaaagctggaagccttcaattattttatcttgcttaaaaaacaaaaaa
aatagcaaaatttctgatgcatcgaaaaaatttttaactgaacagttatctgcatcacca
gtatttgtagtaaaaaatggttttaatgaaattattttaggtcacccattatctcgtgtt
aaaagaggaggagttaataatctgatgcatgcgttttctaatttattaaatcagtctaat
gcaacttatcctatatctactggtctattctttttcatccagatgatgcatttgaattt
aaagactttataatatcggtcaatccactggcggccaaacatatggagataagcgtagag
cctgttggtctacactttgcttataaaatgaatagaaatatatcgtcagatactcaattt
tcgtttattccagatttaaagaagtaggagatttattattcaaatatagaaaaggtaat
catttagtttttcataagaatcagcattatggcaaagatttttttcaggggcaaccaata
tatatgattcagccgatcacttttaaagatcgagccggaaagctaaatattatcaaattt
acggggctaaatgacacaagagaaattatttttactaatcttgaagctgctaatagatca
tgggcacattttataaagaggaattcacaattaaaatcaattaaaaatcctactctatta
gtctataatttagaaagtttttaaaagatcaagaacgattggataatcaagatttaaat
aagtttgtggtgatcactaataaagaatcttatcttgccacaaaagagttaatagcttta
cccgattctaatagcttttctaagcacttaaaattaaatataaagcctaaactcttttt
gtgaaactatgggtaagacggttattttccaccttaacttatgaat-aaatgctatt-gc
ctttattttctcgaataatattctataactagtaattcattgagttgtaaagcaacccat
tccctatcaatgactccattgactttccctgataaattggatttattcaattctaagtgg
ctaggaatattagctagtccagggaaagctaagtaattttctactagttttcgagatgct
tcttgatttttacactaattgattctcctggtttacattgataactacagatagatact
acttgtccattaatacaaatatgaccatggttcactagttgtctagcggcaggaattgta
ggagccataccaagtctaaaaacagtattgtctagcctcatctctagtagctgtaacaaa
atttgacctgtcgacccttgtagtttttagctgcttttacgtacttaaataactgtttc
tcacttaatccataattaaatcgcaacttctgtttttcttctaatcttacagcatactca
gaaggttttctggacttttgtccatgttctccaggtggataagatcttttaatcgctttt
ctactgagtccgggtaaatcacctaatctacgggaaatgcgtactcgtggccctctgtat
ctagacatattatttaaattctcctaatgtaaat-gattaag----------taatgagt
agctatttttaacacaaaaagagcattaaaacaaagc-ttttgctaaaaaatatttaca
aaaaatatagatttggacaaaa---tacattactatgc-tttttaagcgggtagcgggaa
tcgaacccgcatcattagcttggaaggctaaggttttaccactaaactatacccgcaata
ttcaacagattgtcactaactcaatataacatagttatgttaagttaagataatcttaaa
tttaatagaatt-ttaaattatttatgctccttctaaaaccacatcaagaaaacgagcaa

tttcggcagcttgttcttcaacttctgataataataatggttgaccgaccctagtcaatg
gaattactcttttatcttttgtacataaataaatttcacgtcttggatttagcccctctt
taatatctatttttattgatttgatttcttttatattaaactgaagacagatttttctat
tctttccagggaaacctagccgaaaaattttacgatacctttatctttgttaaattcat
tatatccagcccctatattccaaataatagttagccacagaaacaaactcagaaagactc
ctatactgccatagaatgtcattacaataccttgtggaataaataccaaatcggttgaat
ttgtaaaaggtaacaaatcaacttgaaaataacttgatagtccagcaagaagaaagccca
aagccccaataaatattatagtagcccaccagtaattactcagtcttcgtgaacctaaaa
ttaaatcttttctaactgagtgtatagataatgtttttttcataatttgttcttttaaaa
cagta-tactaatgcaaaatggaaaaatttttgacaattttt--atgcgcgctttagattt
tttaaataagcttgattgcttttagtcctaaaaacagtcctgttgctaacccaaaaaaag
cagcaagaaatattatatatcctagaaaaactgacatattattattatttattattattg
ttacaatagtgtttacaaaaatagtgaaaatctattactacggtattatattacgttaaa
tgccttgctgtctacaggaaaaaactttttcagtttttagcattgtgtttatattagaa
ataatattctagtaaatttattaaacttctggagaacagaatcttatgtcagaatttatc
aagccttataacgatgatccttttgtaggcaatttgtccacgccagttagtacgtcaagt
tttagtaaaggacttctaggaaatctaccagcttaccgtcgaggtttatctccgcttctt
agaggattagaaataggaatggcacatggatacttttaattggaccttttgataaattg
ggccctttacgaggtacagatgtagcgttactagcgggatttctatcttcggtcggcctc
attattattctcactacatgtttatccatgtatggtaatgtatcttttactagagcagat
tcgaaagatccactacaaacttctgaaggctggggacaattcactgcaggatttctagtt
ggagcagtaggtggttcaggatttgcttatttattgctagctaatatacctgtattacag
actgcaggtcttagtttattctcttaagctagtaaggggacttgaacccgtaacctactg
attacaaatcagttgctctaccaattgagctatactagcattcagttcacaataacacat
aaagatatttgttgcaatctgctattttcagatgtaacaaatatctttttttatttaaaca
gaactgttatgttaacac-------gtatattaattagaatctgaattatctgactctgc
agaagattcctgatcacagtttagataataacaaattgtttgatctagacaagttgcaga
ccaaccaacaggtgtttctcctgataattcttgcatatctacagaattataaaaattatc
atttggcttattactgtttgcttgcataaatatcactcccaaaacactctgaacttaatg
tgaagtaatttaacttctcaataatatattaacacaaaatattaaatatggtgattattc
tttaattgtaaagatgctgctaactttataaagtttgagtgagagtcttgatagcttttg
ttgaaataagcattttaacccatttatttgccctctcgaccatactttttttgtgttgta
aattaactttttgtagtttttttgttcgtttatgagaatgagatacggcatatccattat
tagctacttttcccgtaagctgacactttttttgacataatttt-------ctaacttata
tatttatttagagtacttgctaaagttgacttgggcacagctccaatcactgtgtctact
ctttctcctgctttaaagatcattaaagtaggtatacttctaatgccatattcagctgca

atagtaggattatcgtctgtgtttatttttactaccttaatagatgattcatattcttcc
gcaatttcatcaactacaggagaaaccattctacaaggaccacaccatggcgcccaaaaa
tctacgagtacaggtaagttattgttaataacttcttgtttgaaagaggcatctgtaact
tgagatactgacatattctttaacctttaatatatgttccttgctaagcaaatcttatca
caaatttcaagaaaaatcttctatagtgaattactattagtgttgaattattttttttaaa
aattagacattaaaaaaacttattacctatataaataaagtggtggccgagtgatatctt
gatagtgataatttttatattaaggttgaacactagaaataacttttcaagcaaactctt
gtttaaaaa--atttctctgtgaccataaaaacttaattatattacaatatattttatta
tttgtaacttagttctgataatggtataaacaacgcaaaagatactgccttataatcaag
gaggaatacatgtctcaatccgtagaatcacggactaggattaaaagcgaacgttacgaa
tctggagtaatcccctacgctaaaatgggctactgggatgctgactatgtgattaaagaa
acagatattctagctcttttcagaatcactcctcaaccaggtgttgacccgattgaagca
tctgctgcaattgcaggtgaatcttcaacagctacttggacagttgtatggactgattta
ttaacagcttgtgacttatacagagcaaaagcatatcgagtagatccagttccaaacgtg
gcagatcaatattttgcttacatagcttatgatattgatttgtttgaagaaggttccatt
gcgaacttaactgcttcaattattggtaacgtttttgggtttaaagctgttaaagctctt
cgcttggaagatatgcgtatgccagtagcttatctaaaaacgttccaaggtcctgcaact
ggattgattgtagaacgtgagcgtatggataagttcggtagacctttcttaggtgctaca
gttaaacctaaactaggtttatctggcaaaaactacggaagagttgtatacgaaggcctg
aaaggcggtcttgatttccttaaagatgatgagaatattaactcacaaccatttatgcgt
tggagagaaagatttttatattctatggaaggtgtaaataaagcatcggcttctgctggc
gaaattaaaggtcattaccttaacgtaacagccgcgacaatggaagatatgtatgagaga
gccgaattctctaaagaggttggtagtatcatttgtatgattgaccttgtgattggttat
actgcgattcaaagtatggcaatttgggctcgtaaacatgacatgattttacatttacat
agagctggtaactcaacttactctcgtcaaaaaaatcatggtatgaacttccgagttatt
tgcaaatggatgcgtatggctggtgttgaccatattcacgcaggtacagttgtaggtaag
cttgaaggagatcctttaatgattaaaggcttctacaatactctacttgaaagcgacaca
gatatcaacctacctcaaggtctgttctttgctcaaaattgggcttccctacgtaaagtt
gtaccagtagcatctggtggtattcatgctggtcaaatgcaccaacttcttgattactta
ggtgatgatgtagttcttcagtttggtggtggtacaattggacatcctgatggtatccaa
gcaggtgcaactgctaacagagtagcactagagtccatggttatggcaagaaatgaaggc
cgtaactatgtagcagaaggtccacaaatcttgagggacgctgctaaaacttgtgggcct
ctacaaacagctttagatttatggaaagatattagtttcaactacacttccacagataca
gctgatttcgttgagactccaacagcaaacatctagtttaatgactacttactgatactt
taaatagtcaattgtaagtggaattaacttataacaataaggagcatagaatagtgagac
taacacaagggactttttccttccttccagatttaactgatgagcaaattaataaacagc

ttgcttatatcgtttctaaaggcttatcagcaaacgttgagtatactgacgatcctcatc
caagaaactcctattgggaactgtggggtttacctttatttgatgtaaaagatgcttctg
ctgttatgtacgaaattagctcatgcagaaaagcaaaacctaattattatgttaaagtta
acgcttttgataatactagaggtattgaaagttgtgtaatgtctttcattgtaaatagac
ctgctaatgaaccaggattcttattacaacgccaagacttcgaaggtagaactatgaagt
atagtcttcatagctatgctactgaaaagcctgaaggagctaggtattaatattaattaa
gaattaatattggctaatta--ttaccctcttaaaaatcataattaatttagtaattgtg
atttttaagagggatagcttcccaagtaaattttgactaaa-tagattgaataattaaaa
atacacagaaataatgcaatcacaggatataatttccaacgatactcttgttaatttaca
agaagaatatgatagaacacaaatccaagaagttttaaatgagttaaatcaagaacttat
aggattagtgcctgtaaagaccagaattcgcgaaattgctgcgctattattgattgacag
attacgcagaaaactagaactagtttctggtaatccaggattacacatgtcatttacagg
tagtccaggaactggtaaaactacagttgctatgaaaatggctgatattttgcacagact
tggatatataaaaaaagggcatttgttgacagttacaagagatgatcttgtaggtcaata
tattggacatactgcccctaaaactaaggaagttcttaaacaagcaatgggaggagtttt
atttattgacgaagcttactatctatataaagcagataatgaaagagactatggctctga
ggcaattgaaattttattacaagtaatggaaaaccaaagaaatgacttagttgttatctt
tgctggatataaagatagaatggaaaaattctacgaatccaacccaggactctcttctag
agtagctaatcatgtagacttcccagattatacttcagatgaattattacaaatagctaa
aatgatgatagaagaacagcagtactgttttacagaagaagcagataaaactcttttaga
gtataccgagcgaagaatgaaacagccttattttgctaatgcaagaagtattcgcaatgc
tattgacagggctagaatgagacaagccaataggatttttgccagtggagaaaaagtatt
aacaaaagctgatttggtaacgattgaagcagaagatatcttgaaaagtagattattttc
attacctaatgcttaatatacacgtgatttcatgaaactatatattattaaaaag-tttc
ataatcagttgcaaatttcttgaaaaacctttattattatcttaatgcaggtg-tggcgg
catagccaagtggtaaggcagaggattgcaaatccttcatcccccagttcaaatctgggt
gccgcctagtactaaaaaaggggggtgtggtggaatggtagacacaacagacttaaaatc
tgttgatttttagtaatcgtgagggttcaagtccctccacccccatatattgatttataa
ataaaaagagatgtgtatttgtattaattgcaaccatattaccaaatgtaatacctatca
cttaatagaatctcaacataaacagcctcatcttactagaagtccattgtttatacctaa
atatcctgtagttcatgttaatatatctaacaactgtacctataatcaaattgattggga
tttagtggagtgtttatcatttgtagaaaaaccgaatagttggaatttagacgctaatta
gttacaagaaaaattatgtaccataagcaacaactctatttttagatactcggtattaa
ttttggatgttctaaccagtaagattttacccgttcttgctatctctataatgccaaatt
tagttagtaattgttcaatagcaacaatctttcccggatctccagtaacctctataatta
aaagatcttctgcaatgtctactatcttagctctaaaaattcttacaatttctaaagctt

ctgttctagtctgagaattgatctgaatcttaattaacattagttctctttcaactgaag
gaatatttgttacatcttgaacgttaaggatatttactaacttatataattgtttcgtaa
gttgttcaatagttctattgtctccttgaactaccattgtgattctagagaccccaattt
gctctgctggtccaactgctaaacttgcgatattaaaccctctccgggcaaatagaccag
atattcttgacagtactccggcttcatcttgaactaaaactgataaggtgtgtttcatga
aaaattaaatttaatctatttgatctgttaagtatattctaagaaaattacagaaaaaat
actagttagtctataactaaattatccaaactaaacaaaatcctactatcagataatata
tctaccttgtaatgctatactattaacttagtatttaatgatattctaattgcaaataga
atgct-aatttttaaaatttgttaggaacttgtgctagaaaaatacaaaaacagtaaaa
aactctctcgagatttacctcaaatcaatgatcgcattagatttccaaaagtccgagtaa
ttgatgacgaaggtgaacaactaggtatttttgtgcctgaagaagctatacaattagctg
tccaacaaggtttagacttagttgttgttagtgataaatcggacccgccagtatgccgaa
tattagactatggtaaatataagtttacacaagaaaaaagagctagagaagctaagaaaa
agcaacataacagtagtattaaagaagtaaaaatgcgatataagatagaagagcatgatt
ataaagttagaataaaccaggcatccaaatttattcaagcaggagataaagtaaaagcaa
ctatcacatttcgggggcgtgaaatccagcactctaatttagctatagatttattgaata
aaatggcaagcgatctaaatgcaatagctgaaattcagcaagctccatcaagagatggca
gaaatgtcataatgctcttatctcccaaaaaagttagctaaactaatttttatttaaatg
catctggctggattcgaaccagcgacgtctctttcgaaatggcggattatgagtccgctg
ccttcggcccctcggccacagatgcatcacttaaagtttatacctcataaagcatataaa
aatcaatgtttagctatttttaataaagtctataagagtctattattcattcatattgtg
atatgtcgcaactgctgaaggggaaacttgatttaagtatcgaaatatccagtatttaaa
aattgtgtcgagtataactggaaaagttgaaataaataaaaaaataaaatctcgactttc
cggaagtcctaaatgtcttaaaattacttcaataataacttcccatccatgaggagaatg
aaaacccacaaacatatcagtaaaaagaatgattaaaaaagctttagctgtatcactaag
accataaataatttcatttaaaaaagatttaacgactgcaatttgtctttgtccagttat
cattagtaatataaagacaagtattgatacaagatcggataaaatatttttaacagcatt
tgcactttcatttgcataatattctcccaactctctagccttcagctgcactctttttc
aatgatttcgtaagatatatcttccgaaggattcagaagaacttcaaagtgaattttctc
ttcaaatctttgtaattcagcaaaagctctttcctcttgagaggaatttaaaaaaatttt
aggctgttcttggttccataaataatcaatacaaggtccaaatacaaaaaaattttgaagc
ctgattaactagtactggagagatgaataataataaaatatattttacggaagtaatagt
ttggtgtctagatattctaaactcctctatagcttcagattcgccattaggatctaactc
tttacgaaacttttcaaaagtattagtaattgacctaggtattggcccaactttttcaaa
ggcgaattgattatttcgttttaaattccaatatttcatttttattgctgctaaaatct
ctattaatcaataattgatcacgtattcactataaatattaattctagaactaaacatat

aaaacaatatttatgaaaaataaaatacaattaaaaattaattatgcattagttctatct
gcagctacagcagtttatggatataaatcttatcagccaactataaatacactcttaatc
aaagatgaagaaaactggcctttttctctaaaaggcagactgcctttattcctataatt
gtattaaaaaaaaatcatcaaaatccctgaactcagaacctagaaatagtaagttatatc
ttaatggaattggaagtagaaaatttatcgacagaattgtcactctacttggatatttag
aaaaacaggcaaactatactgaatacttgaaccaaataaaaacaagtggttatttttctt
tagtctacctagacagtgatataatgtataagaaaaatattagtaacatttttttactac
ctaatgatattctaaaaagaatctatatttgcaataagaaaaaaaagcttataccgcatt
cattcttaataaatttatttcagaaacagataggctatccaaaaagttttgctaatttaa
attgggccttctccaaaattataaaatggtattatgatagaggataccaatggtctctag
ttgaagttaaacaagcgtctgatgcatcttctattgtaattgatattcatgaaggggtag
tgaagacaattataacagaatactacactctatcttataaaagagtctcaggtatcttat
gcgtagagtcaatagagcagtaccttggagtaagagtgggagctccactaaatataattg
atttacaaaaaaaaattacttacttaaaagataatcaactagttggcgacattatttata
gtattgaacgatcaaataatagctcaatgagtttggatatcaaatttcaaatacaagaat
taaaagataaagagataatagtgcttgcaaaaagttcttcta------------ttatct
cgcatgcatgtaatttacttaatc---aatataga---------aataggctagtagctt
ctaatatagtttcactatcaactaataaattacacgcatgctattttaattataaacttg
actatcaatataagtatataaacacgtctaatcttataaaattgttagcttattctattt
cttgtaggaagacactacttattcattattttaatttacaaactttaataagctatacaa
aaaaaaatactattggatttcagctatatttacgaaatctaagttttgggaaagctttct
gtgtgcttagtatgaaatttatcaaaaatggacttaatataaaaatttatatataaatc
cttcattgatagttgatcagaattttgtatttcaatttgctatacaaatcattaagcaat
atcatactgcta------aacctccagctt------------------------------
------tatttttaacgaatctagatcttgaacaatatgttgctgaaagtttattaatgt
accatttacatcttgcttctcaatatctgaaaaaatattattatctcgaattatgcata
cagattctttatttttcaactctgaaa------attttcattttgatgatagaactaacg
cgatcaacagttatgatactttttaaacaaaatacaaaaatatttatcaagagtttcttt
ctttattattaagtttacgttatcaaaatttaattatttaggttggcctttaaaaggcc
attttttgaaataaaatctttatacttggctccatttcaaaagagtg------------
---atttttctgatagtcgtaagaccctattcttccacaaaatgtcgttaaagcaagtat
ctaattttaatttaccaatatcttttaaaagccaccttaaccatattttagttagtacta
ttaaatgtcagtcaaatttgaatatgagaactgtatctttgctattaattgattcacctg
ctgaatatatgctatacaaatctattcttaatttctctattaaagttaggatgcagtatt
tcatacctatgagcaacaatataagattatctctattttataattatttagattgttttt
taataagatcatctcagagtcgcattcatatatggcaagatctaagaactcttacaccaa

ttcaaaatttttggttaaaaaaattttcatatggagctggaattcagcttaaattaccaa
ttaagcagatgccacctctttcaattgaatatactgtaactagtagtcgctacttttgta
tttaccttcgtacttattatcaacgataactattactaaaatgaaaaatttaatttcttt
ttttgatcgcagcaaagggaaatggatttcacaaagaacaacttatgaattatctaataa
aaacatgagttcgatacagtctcagatgacaatgaaaataggcaactcattgtcaggatc
tataatacttgcatcgttaaattgggggacatttatagacaagttgcccattacgctaa
aaatcacagccggagtgaatatgataataagtttaacttgcaattcagtaatcaactaaa
taatcataaattattaacgctgtgcatagtgacagatcctagtctaattagttttaaaac
tcgctatggaagtactaccatagatgaaacatattggttcgcaacaaataatttacgtct
aagtactagtattgttaaacgattcaacacttgtgtggcagtatctttttgttcagaaat
taaagtttaa-actctatatagaagcaactaatagcctaaa-ggctattagttgcttcta
tatatctaaattttgtattaaaaattat----aatactatattattctaagtctttacga
ttgggattcctggaaggatcattagagaggaacccaaagacaaacaaagaaataaagaaa
ataacggtagtatatacaaagattttcaaagtaaacatattttcatccttaaaagctatt
agtgcg----atatttacatatctccacataattttatatcatgatataagttccagcac
tattttataaataaatcaatatctttatttttagtaatgatttatatttaaatatgcgt
gatgtactaaatactaaatcttttccgacttgttttgtcttgtttgtaaatattttgtta
tttctatggagtttaccttcaattatcacatagtctaacttctttagctgtttaaaagta
tatgaagatttttttgttccaaatagttagattgattataactaattgttttctttttaac
agtcttgcttttaacttaattatttggcttttattttcactaattctgatactcttacaa
ctaagaatttggactagaagagtacagctattcataatttataacaa------tttttta
aaaaatctactaaaatagatgattttacagaactaaaatgatatttcattatcttgttga
ccttgctcataagtaatatctttcaatacttttaatatttttccaaaatactcttgcagg
tatttctctaaagatattacttccaacctgtcaattccaagaatactgtaaacttcatcc
atcggagctgtaaattgctctccgctacttaaaatttcggcaaatgctaaacgatcagaa
atattccatgtccattgaagagttttagtaattcttcttaaggcttttaataatccaatc
ggaatttgtgagatctgtgtttttttgcccagataatttttcacataatttaataatttca
gctgatgtccaagcggtattaccaactaacggcagggtcctattttctgtggaaggtact
ccaagacttttttataactaactttgctgcatcttgagtatcaatgtatgcaatgggtgta
gattctcctgtaacccagactgatttttttgtctaaaataggaatggcatattggttgatt
aaaccttgaaaaaaatcctcctaaagaaaacacagtatatttcacattggacttctgaagg
aaatctactacctgagactttaagttcatcaatggaacatctgggtatttctctgaattt
aatattgaaaagaaaataaatctttcaactttagccgctttagctgcttcaattaatgca
attttcccatctaaatctatttttttctgcattgtaaggatcagtaggacgagatgtagaa
gcatctatgattgctgttaccccacaaaaagattgcaaaatactttcgggtaattttaaa
tcaccatatataagttctgctccccactctttgagaaaggcagactttcttaaatttctt

accatgcattttacattgtagccttcatctaaagctcgtcttacaatttgacgtcctaaa
gttccggttgctccaattactaaaagagtcatattagttaaaaataagtatgtataaaga
cattgatttggttgacggcagagagtaaattaggtagagagggaatcgaaccctcatgac
cgaagtcgtcacattttgagtgtgatgcgtataccaatttcgccatctaccctattatag
accaactactacaagtaatttagtctttagcgtaaaaataatactaagtttcaatggata
tgtcaacttttaaatttatattaataaaaaaacggagtctcttatggccacttttgaatt
aattccttatagattgtatttgagtcaatctaacacatggatccatagaataaaagcaga
aattaagatatatatagtaacgcttttatggatttcaattttatttttttcttacttaa
actatgtattattgctttaagcttaattgccataagttttactataagaagtaaacagaa
tattatccaaaaacatttgttacaaacgttattaataacgttcttaactactgttttgtc
ttttagtgtggctattagttataaacaatatgcagaacaagaacaatcgcagtatttatc
tgattccaagaaatataaaaattccagcagctactattatatacaaatagctaacgatca
aaggatcaaaagacaatacttaattactactttaaagccttccttgtactttttttattac
tatatattctattaaactagttatgataacaacttctccagaagttttagtaattactat
ttatagatctaggataataaataaaatatttaaaaatgaattgctatttatcttccttct
ttcgtcacatattgttactagtattattaatagaatcgataaagtaattcaagtaactag
cttaagaggaagcttgaatttatataattcattgacaaggcctttaatgttttctttgtt
aatatttcaagtcttctttttggagattattcgggagtcaaaagaaatagctcaagctct
ttatactagaaatctcaatcaagaaaacaataatttttttgaaaatatatacagtaaaatc
taactttagtgatcggctcaatataattattagcactttgtactttattattttagctct
agcgtaatagaatccacttattcttctgactctagtaatacgaagagtaataaaattagt
tagtagtttaaatagatagaat-taagatattatataaataattaaatgtttcattatgg
ttaatactcaaaatcaaatttcacaaacttcagaacttgattatatagttaatcaacctt
ataaatatggctttaaaacttctgttgaatctgagcaatttcctaggggaataagtgaag
atattgtccgcttgatctccaagaaaaaagatgagcctgaatatctattgaattttaggc
ttaaagcatataaaaaatggaaaaagatgagtagtccgtcatgggctcatattaagcatc
cgaatatagactttaatacgattatttattacgctgttcctaaattaaaaaaagaattga
aaagtctagatgaggttgatccagaaattcttgacacttttaacaagttaggtatatctt
taaatgagcaaaagcgaatttccaatgtagccgttgatgctgtttttgatagtgtatcta
ttgcaactacttttaagaaagaactgtctgaagctggtgttatattctgttcaatttctg
aagctataagggattatccagaattaataaaaaaatacttaggtactgttgtaccagctg
gtgataattattttgctgcattgaactctgcggtatttagtgatggctctttttgctata
tccctcctaatacagtttgccctttagaattatcaacttactttcgtattaacaacgaag
aatctgggcaatttgaaaggacactaattattgctgatcgtggcagtaaagtaagttatc
ttgaaggttgtactgctcctcaatttgacacaaatcaattacatgcagcgattgtagaat
tagtagctcttgaaggagccgaaattaaatattctacagtacagaattggtatgctggta

acaaagaaggtaaaggtggtatatacaattttgttactaaacgaggcttatgctcgggta
acaattcaaaaatttcatggactcaagtagaaactggatctgcaattacctggaaatatc
caagctgtattttagctggtgaaaattctcagggagaattttactctgtagctttaacaa
ataactatcaagaggctgatacagggactaaaatgatccatattggtaacaatacaaaaa
gtagaattatttctaaaggtatttctgcgggtagatcgaaaaacagttatcgaggcctag
taaaagttggccctcagtcatttaattctcgtaattattctcaatgcgattctttattaa
ttggtcaatcatctcaagctaatacatttccctatattcaagtacagaatccaacatcaa
aagtagaacatgaagcatccacttcaaaaattagcgaggaccaaatttttatttttttac
aaagagggatcaatttagaagaatctattgctcttatgatcagtggtttttgtaaagatg
tctttaatgaattgcctatggaatttgctactgaagctgatcgtttactgagtttaaaat
tagagggaactgtaggatgagccagactattttagaaatcaaagatttatatgcttctgt
tggtgaaacaacaattttaaaaggggtaaacttatctattcgagccggcgaaatacatgc
gattatggggcctaacggttcaggtaaaagtacattatcaaaagtaattgcaggacatcc
agcgtattcactaataagcggagacattttatttttggacaaagtattcttgaaatgga
gccagacgagagggcaaaagcaggtatttttttagcttttcagtatcctgttgaaattcc
tggagtcagtaattctgatttcttaagaattgcattaaatgctcgcagaaagtttcaagg
attatcggagtttagccccttagaatttttcaactaataacagaaaaaatagatcttgt
tggcatgcaagaaagtttttgacaagaaatgttaatgaaggatttctggtggagaaaa
aaaacgtaacgaaattcttcaaatggctttactagatagcaaaatatctattttagatga
aacagattctggactagatattgatgcattacgagtagttgcaaaaggaattaacacttt
agctaaatcaacaaattcaattattttgattactcactatcaaagattattagattatat
tattccagattttgttcatattatgagtaacggacaaattgtaaaaactggtagtgttac
tcttgcccaagatctagaaaagcacggatatgactggattacgcagacataactatttac
tac-------aatattgagactaaagaatattctttagtctcaatatt-tttgcaaatgc
aaagtt-aattatttaagctttatcaaaaccttgcttaacatcttcaattgctcttttta
gcaattcttcactaactggttctaatttttagtactacgaacattttcaccaaattctg
gtttagaattctttaaatcttctcgaagttctctaataaattcagctacttttgaaactt
caatatcatctagatagccattaatgccagtgtaaataatagcagtctgttcttcaactg
gaataggagaattttgagcttgtttcaaaatttctcgaagacgttgacctcttgctagtt
gattttgggttgctttatctagatcagatgcaaactgagaaaaagcttctaattcagcaa
attgtgctaactctagttttaacttgcctgccacttgtttcatggcttttatttgcgcag
cagagcctactctagagactgagatcccaacattaatagctggtctaatccctgagttaa
ataagtcaccagatagaaatatttgtccatctgtaattgaaattacatttgttggtatat
atgcagatacgtccccagcttgagtttcaataatagggagagctgtcatactacctccac
ctaattcagcattcaatttagctgctctctctagcaatctagagtgtaaatagaatacat
ccccaggataggcttctcgtcccggaggtcttctaagcaaaagagacatctgacgataag

cttgagcttgcttagttaaatcatcataaataactaatgttgctttacctttgtacataa
aatattcagctaaagccgcacctgtataaggagcaatatattgtaaagtcgcaggactat
ctgcattagctgcaactataatagtataatcaagagctcctttatcctgtagtgaagata
ctacttgggctactgaagatgccttttgtcctatcgcaacatatacacaaacaacatctt
ggcctttttgattaataattgtgtccaatgcaacagctgttttacctgtttgacggtcac
caataattaattctcgctgacctctaccaattgggatcatagagtcaatagcagttatcc
ctgtttgcataggttcacaaactgattgccttccaattataccaggggccattgactcaa
ttagccttgttccattacttgcaggttcacctttatcatctattggccgagctaatggat
caacaactctacctaaaaaagcatctccgaccggaatttgagcaattcgaccagtccctt
ttacggaactgccctctaaaatatccctaccatctcccatgagtactacaccaacgttat
cactttctaaatttaaagcaaccccaatagttttatcttcaaattcaagtagttcaccag
ccatgacttcatcaagtccatatactcgagcgataccatcaccaacttgtaaaactgttc
ctatattggctacttctacatcctgatcatacttttcaatttgttgacgaataatactac
ttatttcatcaggtctaatatttaccatattttaatattattttttattaattggaaatt
ctaaagcggtaattagtcagactaatgctatctgtctttttttactttattttaagttact
acaacatcaaggtgtgaagccatttgtcttagctgtcctctaatactagtatcaatgact
tttgatccaatctgaatagtaaagccaccaattagctcgggctcgactgaaataattagt
ttaacttctttagccttagtcataaccttgatttttctgttaatagagtttcctgatca
gggcttaaagcaatagaagtattaatatttgcaattgttaaagattccatttggtaagcc
agttccaaatacttactagctatagcatctaacattccaattcgcttgcgatcaactaaa
atcattagaaatgataatgtattttcgttaatttgatcgccaacagttgcaataattact
tctttttttgcctctacagtttttaatggattagct---aaaaaaattttagcttgtca
gattgcgctaaaatattttgtattgactgaatatcttggctcaccttttcagtaactttt
ttagttttagctaaatcaagaagagctactgcataaggctgagctatttttataacaaca
ttgttactgctcatagttgatctcctaacttagcaatattattatcaattatacgcagtt
gcatttcggagctcatttgattttccagctgcaaagtaactcttttaagagcaagaaatg
ttatttgttgctggatttgccttcggatctgtttttcagctgtttctatatttgatttac
ctgttatggcaagtctttcaatatctaattggccttgagctaatatggaacttctaactt
ttccagctgttagctgagcttctttcttaatttgatctataatgatttgagtttgagcaa
gttgtttttctgattctgacaatctagcacttgcttgctctagtctttcttcggattctt
gtatagctgctaaaacttttatctgtcttgcatttaaacttgatccgagaaattgcttca
aaacataaataagaccaaataaaagcaacaatatattaataacatttgcttcaaaaatat
cagaattaaagccgaatttgtgttcactactatgttctgataaaatagtaattatttgtg
gtatttttactatattattcatttaaaattct----------tactattttaatatttta
ctctggaatcatgattttcaagattgactacttaataatttaactttaatttgatcgctc
aaagtgtcaacctgattttctaaagttttaagagcttcttctttttgaacatttaactgt

ttcgatgcttcagcaatcagtttctctgcattcaattgagcttttttatatcttctgag
actatatttgagcttctttttgagaagaggctatttttaattgtgcattgcggcgagct
tcagataaatcttcttcatattttgcagctagctcatctgctttaacaagcatagacgat
gcagtagtcaatgttgttcgaatatattcatctcgttcgtctagtactttagttacaggt
ttataaaaaatagtatttaataaaaccattaaagtaagaaactgtaatgccattaaaggc
aaagtaccattgaagtcaaataagccaccttcaatttcttcggctaatagaaatggtaaa
tcaatcattttactaattatgtttatagttaataattttgtttcaattaagaaaaataac
tttatagtgcgcttagtctcatcagactaagcgcaaatgattattttaaccaacgtatgg
attagcgaacaataaagacaacgcaacaactaggccgtaaattgttaaagattccataaa
tgctaaacttagaagaagtgtaccccgaatctttccttctacttctggctgtctagcaat
accttcgacagcattagctgctgcactaccttgaccaatgcctgggcctattgcagcaag
acctacagcaagaccggcagcgataacggatgcggctgaaacgattgaatccataatgaa
ttttaatttttttgttatatgagtagaaaattgacaaatttcgtataatacttaaaatcta
tataattaagtataatagattctaatat-----tgtaattatagtatattactcttctcc
atgaccttccattgcttctccaatataagctgcggacaaagtagaaaagattaatgcctg
tattgaactcgcaaataatccgagtatcataactggtaatggtattaaaattggaattag
caaagtaaatacagaaacaactaattcatctgctaagacgtttccaaataatcgaaaact
taaagatagtggtttcgtaaaatcttctagaatattgattgggagtaatactggagttgg
ctgaatatatctagcaaagtagcctaacccttttttacttaaacctgcataaaaataagc
taaagatgtaagtaaagacagggccacagttgtattaatgtcatttgttggggcagctag
ctcaccttcaggcaagtgaattaacttccaaggaattaatgcacctgcccagttacaacc
tagaataaatagaaataaagttgcaatgtaaggcacccaagggcgatattcatgctcacc
aatctgatttttttgcaatatcttgcagaaattctaatataaattccataaaattctggaa
tttttcaggaattctttgtagatttctagtacctagaaatgataaagttaaaagtgtagc
aattactaaccaagagacaatgaaaacttgcccatgcaattgtaagctacctattttcca
atatagatgttttccaacttctactgctgataaactattatatgggttaaaatctatgag
actgttttgatacataataaatattt-ttttaaatagtgcaagaatcaaaaaataag-tt
tttgaagaattaatatgcaattaatttattcttaaaattagactattaataaattaaaag
caaaaataaattctaatagagatagctatttttagtagtgattatttttttgtaaaaaaca
attattgctacctataatgcttttacgtttaattatatacccatatattaattatttaat
atgattataggtattttgctacaaactttattgttttatccattct----aaagattgtt
atagtttatttttttatttaaaatatctgcgacttcatctgcaaaattaactttcaaattt
tcttctccccctcccagaagaaatcttacaaatcttctaattttaatattttcacctaga
acagcaatattttggtttattaaatcttcaatactaatatcttgattgcggataaacatt
tgatctaataatgagagctccttaagtctctttttcattcgtccttcaatgattgtttcg
attctatcaactggtttgtttttcaagtcatctttaccagcttcaactctttgttccaga

ttaataatttcactaggtatatcctgtgttgatacgtattccacattaggcgatgccgct
atttgcatagcaatatctttagctaacttttggaactctggacgtcgtgctacaaaatct
gtttcacagttgacttcaactaatacacctattcttccgccagtatgaatataactctct
agtaaaccttcaatagcggtacgattagatttcttgttagctgaagccagccctttttgt
cttaatgattctaaagctttttcttcattgccattattagcttgtaaagctttcttgcag
tccatcatacctgctccagtcttatcacgtaaggcttttacgacttgagcagaaatttgt
agtgtcatatgctttcaaaaatttttaagtatttaaatt----------aattattataa
ttcactttgagtaggatgagttatttcttctaaatctgtttgcccgtatttaccatcata
aattgcatctgctattttacctacaattagtttaattgacctaatggcatcatcattagc
tgggattggaatattaataatttctggactacaattagtatcaagtatacaaatagttgg
aatacctaattttaaacactcttgtatagctgttgtttctctcttttgatctacaactac
aacaatatcaggtaaacgagtcatattttaataccatttagatgtttacgaagtttatc
taattctcttcgtaaaacagcagcttctttttgggtaactgatcaatcataccactttt
atcttgttcttctagctgtctcagacgattaactcgagacttaatcgttacccaattagt
taacattcctccaagccatctctggttaacataataagaatcacagcgttgtgcttcctg
tgccactataccagcggcttgtctttttgttcctagaaataaaactttttaccatccga
cgaagcctgttttataaattcacaagcttctgttaataattgtgcagtttggactagatc
aataatatgtatgccatttctttctgtataaatatatggaaacattttaggattccatct
acgtgcttgatgtccaaaatgaacacctgcttctagtaattctgctaatgtaacaatagc
cataagattattaatatttaaataatgtaattattcgggaaatccc-tttgtttagaaac
tgat-----tcaactaacttatttgaattttagtaaactaataataaattaacacacttt
taaagcagatcaacaaaatagatcctgaagaattcat----tctacacttttattgctaa
aataattacgcgcggttctgtcgtctaaaatgatatcatctaaatcatctcgtacagatg
aaatagtactttcgtcattagtatttgcagttaaattctttttctctaacgatgagccat
tacagttatcatacatattgaagccggtaccagccggtataagccgacctataataacat
tttctttcagacctcttaaccaatctaatttaccagatatagcagcttcagttaatactt
ttgtagtttcttgaaaacttgctgcagaaataaagctttctgtatttaaggaagcttgag
taatacctaatagcacaggcctatatgatgcattaatcttatttctcaaggttatggctt
tattagtctgttcaatcttctgtaactctacaagctctccaggtaaatagcctgtttctt
caccattttctattttcactttagaagtcatctgccgtactattacttctacatgcttat
cagaaatattcactccttgagattgatataccaattgtacttctttaacaagtagtaact
gaatctcttgaaagcttaatcgagcagcttcatataaggctaatccacattcaatataca
aatcaaaacttgcatcgaggatatcatgaggatttaacaatacgtctgttttctttcttg
cttcaagaatttcttcaattcttggaagcccttgtataatgtcacctgtttttgctctat
caaaaaccaatatagctaatgtttctccccttctaattaatgcattgttatcaacatgta
aaatagcaccattagaaaccaagtaaggtctagcaattcttaatgtaactgatctagaag

aaatttcaataatttgtccagagtctaaagaagtcatattttcagtaataaaatcaccac
aacgaatccaatcacccacacttactttaactattgattttcctatattaaaggtctttt
tatcagaagatgtcgcaatcaaaatgcgcctgctagtattcttttcagaataaatctcct
caacagtaccttcactcattgcaataatctcagtactagcaacaactgtaaatggcttga
tatattcaccatttttacaataattctagtttgactttgttgcttctctgatttattta
catcaatacttttaattgataatgtttcaaaagttgacagccttaagtcaaaataattat
tgccactttctttagcagaaaactcaattgaggatattaaattatcatctcgatgctcaa
tttcagccacaagattagttgttactaaatgaactccttctatagatttcactctttctc
cgtcccgaaaaggagttcttttaactagctttagtttagtttgacttggtttttgtgaaa
ctagattttcaattagactggatttgtttctggtattgaataaacaataacaggtctaa
taagaatatagggcatttgctcgttttctatatactcccaataaactaacttatctgtag
aaatattattatgaagtctttctccaggtcttaaaaaacctctactttatcattattac
tatacacctcactaagtttatagatagatccaggcttaataataatttctcgaactattc
catccttttcgattatttcaacaatacctgagctttagaaaaaatattttaactagct
ctgttccactttctataacatctccattatgcactagtagtagtgatgaatctttattaa
tttcgtgagtttcttctgaaatccataagatatatcccggacttaaaatttcgtaggcat
ctttatctaagcctgttttctttttgatacatttaagtctaaatatttaataataccac
cacttgtcgttttatacgtatcagatattagttctgcgactgtgtaaccatcttgaa---
ttttttggtttggaatagctttgaaaagaaatttttgtttttttctgtttctaaaatat
aagattctttttgtggattacatctgtataaatataacaacctggaattacaatagatt
cagtaataacttgtatattggtaatactgctattagtattctggtgtatccttacttctc
cagcataatgatttattaattctgtctgtgcaagtatcgtaccagctttagttttatctt
ctttatgaacaataacattagcagaatcagatatactataaacttcacctgagagcaccc
agatcaagcctccagttttagtaattcttgtagtatattgattattatctgtttcttcaa
ctgttaagttggagaaacagattttgccagagagatctgagactacatgtttttgagctc
tttctgtcatgagtcgatttctgcggggtgattcagcaatcacttgatccttcgatacta
attcaccatcacatattaaaagagtagtacctttacttaagttaattattgacttctgat
ttttcttagatctgattgtaacttgggttggctttttttgttattaatgcttgctcaccat
gtctggtacgaacatctgtatacgattcaatatctagatctatcaattgaccatctatag
gtgcgtagatttgttctgctaattcaccggtaaatactcctccagtatgaaaggtcctca
ttgtcaattgagtgccaggctcaccaatagattgagccgcaataataccaacagcctctc
ctaaatcaactaagcggccatgtgcaagattccatccgtaacaatactgacaaacagaac
ttctagagttacatgtaacaggagatcttactaaaactttttt---gataccagctttag
ttatctcttttgctaacttaggactgacatcttggttcgtatgagcaattaaacaatttg
tctctggatggaatacattttctgctaatactcggccagataaagcttgttctagattaa
ttaatattttctgcgtatctactaaatcttctagtatgatgcctttattggtcttacagt

ctacttctctaattattacatcttgagaaacatcgacaagtcgacgcgtcaaatagccgg
aatctgctgttcttaaagcagtatcaactaatccttttcttgctccgtatgatgatataa
aataatcagtaacagttagtccttctctaaaattactcgatattggaagatcaataattt
gtccctgtggatcagccataagccctctcatgcccactaactgtcttacttgagaaatat
tacctctagcacctgagaacgccatcatataaacagcatttagaggatctgtctctttaa
agtatttaataacttcttgtttcaaggattcactcgcattattccacgtatcaataactt
tttgaaatcgttcaactgctgtaatctcacctcggcgatatttgttttcagtggccttaa
tatcttcaatcgtgcctaataaaagactttgcttactaggaggaattcttaaatcttcta
aacttaaagaaattcctgcctgggtcgcataatgaaatccaagatctttaagcttgtcgg
ccatattggctgctcgggctataccataattacgaaaagcccagacaattaaatttttta
gttcgtttttatcaataaccttatttgaaaaacttggctgtgcaagacttcgtctattat
ccactaaatcttctccatattaaatgttcgtt--attaactatgctactaaagactcttg
aataattttattaaagattatacggccagctgttgttctaatatattgcacaattctctg
attttcagcgtcttcttttatgatatggttattaaaaaattttgttgtactattgtcaga
atgtttttcaatcttaatcggatcatgaggttgatcaccatctactaatccatcaaaacg
agcccatatataagaatgtaaatcaattttcttctgttcataagccataactacatcttc
taagcttgcaaaatactgattagatccctgctgctgagatggattattagctgttaaata
atagcaacctaaaaccatgtcttggcttggcataataattggttgacctgttgctggaga
gagaaaattatgaggggctaacattagcaatctagcttcggcttgagcctctaaagacaa
aggaacatgaacagccatttgatctccgtcaaaatcagcgttaaacgctgggcatactaa
tggatgaagcttaatagctcttccttctacaagaataggttcaaatgcttgaatacctaa
tctgtgtagtgtgggagctctgttcaatagtactgggtgaccttgaataacttcatttaa
gacattccaaatagaagattcatttttttgaatcatctttttggcagctttaatattgtt
aactaatccctgcaaaattagtctgtgaataacaaaaggctgaaataactccaaagccat
ttctcgtggcaatccacattgatgtagttttaaatgaggacctacaacaattacagatct
accggaataatctactcttttacctagcaagttttgtctaaaacgaccttgttttccttc
tataatatctgacaaagattttaatggtctattatttgcacctacaacagttctacctct
acgaccattatccatgagagagtcaacggcctcttgcagcattctttttttcatttctgat
aataatctccggagctaaaattgactttagacgagataatctattatttctattaataat
tcgacgataaaactcattcaaatctgccgtcgcaaatctacctccatctaattggaccat
aggccgtaagtctggaggtatcacaggaatgactgtgaataccatccaagaaggatctgc
acctgttgctatgaaattttcaatcaatcttaagcgtttcatttttttattgaattttaa
tgaaggtgtcttaaaactttttggtggatttgtagcttcagaccttaaggtttcagctat
gtgctctaaatctaaatctttcaacagtttctgaatagcttctgcacctattcctacttc
tacttggttattctcatcttgattttggtagatttcctcttcaagacttttccattcgta
accttcgagaagttgcttgtattttaaattaatatctgtattagattgcgtaactacata

agaatgaaaataaacaatttttttctacttctttgacttttaagtctaatgctaaagcaat
atagcttgtacttccttttaagtaccaaacatgagttactggtgaggcaagttcaatata
agccatccgatgtcttctcacgcgtgattctgtaacttctacgccacatcgttcacaaac
tatacctttgtagcgaaaacgtttatatttaccacagtgacattcccaatccttaactgg
gccaaaaattttctcacagaataaaccatccatttctggctttaaagttctgtaattaat
tgtttcaggctttgtaatttcaccaacaatttgtccgttgggcaaacttctttcgcccca
ctgtcgaatttttttcgggagaagctaaactaatttttacgtagtcaaaatattgctcaaa
ctttgtcataaatttgaataccttaaaatcaaataattagccaatttatttacatgcttc
atattgaaaaatgaatatcttaataaaggaattgttcaaaatcgtctactggaggagtat
cataattagaacgatctactcggttatctttagaatccgacatcaagtcaacttcaactg
tccgtctttggccatcttcaaatagtttcaatttatgtactgcaatgtctaaacctaatg
actgcagctctcgcattaagactttaaaagattctggagttccaggcttcggtataggct
tccctttaactattgcatttaatgcttcatttctagcttgcatatcatccgactttacag
ttaataattcttgcaaagtatatgctgcaccaaaagcttctaaagcccacacttccattt
cacctaatctttgacctccatgctgtgctctacctcctaatggctgctgcgtaactagag
agtaaggaccagtggatcttgcatggatttatcatcaacaagatgaacaagcttaagca
tataagctctaccaactgtaacaggattatcaaaaggctctcctgttctaccatcaaaaa
cttgcattttacctggatgctgatcattaaaaagccatttattgctagtaatcaatgatg
cttcttttaattttctatttacaagtgctcttgatgcttctgctccatacatttcatcaa
aaggtattattttaaatctctttcctaaatagccacctgctaagcctagtaggcactcaa
atacttgaccaacattcattctagaaggtacacctaaaggatttaaaacaatgtccactg
gtgttccatctgataaataaggcatatcttgtttaggtaagatcctagaaatgatacctt
tattgccgtgacgaccagccattttatcacccacttggatttttctcttctgcgcaacat
agacgcgaatcatagcatttgttccgggaggcagctcatctcctttctgcctagtaaaaa
ccctaaccttaactactctaccttttgcagcattaggaagccgtaaagatgtatctctca
catctcgagccttttctccaaaaatagctcgcaacaatttaccttctggtagttgatcag
cttcacctttaggagtaatttttcctactaaaatatctccagcttcaacccaagaaccac
cgacaacaattccatttctatctaaatccttcagagagttatcgctgacgttaggaatct
ctctagtaatttcttctggtcctaatttagtttgacggcattctacttcatacttttcga
tatggatagaagtatacaagtcatcataaactaatctttcgctaattaaaaatgcatctt
cgtagttataaccttcccaaggcatataagcaactagaatatttctccctaaagcgattt
cccctccgtctgtggatgcgccatcagctaaagtttggccaacaactatttttttctccta
cccaaacaattgggcgctgattaatacaagtatcttggttagaccgataatattttttta
agcgataatgaactgttctaccactattatcctgaatgcctattttattagcagaaacat
aatttacatgaccagatgttctgctaataacaaccataccagagtctcgagctattttag
tctcaagaccagtaccaattattggcttttctggatataataatggaacagcttgccttt

gcatattagatcccattagagctctattcgcatcatcatgttccaaaaaaggtattaatg
atgttgcagctgaaattacttgaataggtgaaattgcaatataatctacttgggttgggg
ttgttgtaataaactcttgacgataacgtacaggaataatatctccttcaatgtaatgtt
gtttactaactttaacatctcctggagctactcggaaatcatcttcctcatctgctgtta
gatagacaggactattgtgataaatcacttgaccttggtttaccggataaaatggggttt
ctatgaaaccaaaaacattgacccgcgcacaggttgctagtgaaccaattaaacctgcgt
tcggaccttccggagtttcaattgggcaaattctaccatagtgactaggatgtaaatcac
gaactgcaaaacctgccctgtctttattaaaacctcctggacctaaggcactaattcttc
tcttgtgagttagttctgcaactggattagtttggtccataaattgagaaagctgactag
atccaaagaattctctcactgacgcaattaatggcttgggattaattagatttgataaac
tcaatgaatctatatcacatatcatcattctctctctaataatgcgttctaaacgattga
gacctactctgaattggttctgcagcaattctccaactgaacgaactcttctattaccaa
gatgatctatatcatcaagattacctgagtttttatccttaatattaattagataatcaa
ttgaggataagatgtcttgaggagacaatactcggaacgttttaggtatattgagaccta
gtttttattaattttatatctaccaacttcacccaagtcatatcttttaggatcaaaaa
aacgagagtaaagcatctgcttagcaacagcaactgttgctggttcattaggtctaagct
tagaataaactatgagcagggcttcctcatcagttacttcttcaatattattttttccaa
tttctttagctaattctttaactgaatatatttgggatgcagaaataagaaaagcatatt
tactaagtcctttttcaatttcatctttattgagtccaatagctcgcagaaaaatataag
catttactttgtgggtcttatctattcttatccaaatttctcccttaggatcaatttcaa
attttaaccaagaacctctattagaaattaggcttgcactataaatctgcttaccatttt
tgtctatctcttgtttataatagattcctggactacggattatttggttaataataactc
tttccgtcccagatacaataaaagttcctctatttgtcattattggcaagtctccaataa
aaattaatctctttttgtatttttttatttttatctgcttttcagcactgagctgtagat
gggttgatgataaatctaatgatttaatatttttttcctggtttttagaaggtagatcaa
tatcttttcttgttaactttgcaggaacatatatttgagcactataagttttatccctat
tttttgcctgtctgacactataacgaggaaattttatcttgtactcattaccaaataatt
gtaattcgagtcgactagtagggtctgatatcttcggaaaaatttcaagaacttccgtca
aaccttctaataaaaaccatttaaaactggccctctgaatttcaactaaatctggaagaa
gtttatttttttaagcttatacgttgaaccatagatctcctttataacaaattttgaaatc
gaagagtttaa-tactattaatagagtactggtagataatgcataagcatagtttgctac
attatttcttctctagacataactagaacatcatattttattcaa--aaaaagattttta
aaatttttcttcac--acacactatctataagtactttgctgcta--aaagatagttcaa
gctattaaa-------aaaaacgaaattaatacttgtctgtctgtcaattaattgacatc
aagatagacaagtattatttatttaatcaattctg-tttttctgagcaaaagacaatgct
ctcgcaagtctagctttttttctagcgcctgtattagggtgaaaggcccctttttttaca

gctttatcaatcttactatatacttgagaaatacttgactgcacatcattgaaattatca
ctttctaaattatcaatatttagaaggcatcttttagttaaagttttgacaaccgattta
tacttacggttaatgagacgatttctttccgaagttttaatgcgtttaatcgcagaaagg
ttcttagccacagtaaatccaataaaactttataaaatattttagaaacgaaaataca
caaatctgggatctattcaaaaagggaatatgcggcgtattataacattaaagaaaaaca
ataggcaatagttcaccaagagctcttattattacacaatagagattgtcattgcgaaaa
actttgtaagatagtatttaattaaaataaactaactagacatataaatatatatgaaaa
aaattgaagctattattagacctttcaagcttaatgaagtaaaacttgctctagtcaagg
aaggtattggaggaatgacggttatcaaagtttctggatttgggagacagaagggtcaaa
ctgaaagatataaaggatccgaatactctattgatatcattgataaaataaaaattgaaa
ttattattagcgatgataaagtagagaaaattgtagagacgattattaaggcttctaaaa
caggggaaattggagatggaaaaatatttattagtagtattgaaagagtaataagaatca
ggactaatgacttaaattttgaagctttatagtatttttacttt-tttagacttgatact
tcttcactaaacatgagataataagcgtttattgtatacttataactaaggtgtaata--
ctaatggctaaaagcaaaggtgcacgaatcgtaataactttagagtgctctgataaagct
ggagagtttgctcaaaaaaggaaacctggcgtttttcgatatacaactactaaaaataga
cgaaatacaccaagtagaattgaattaaacaagttttgtcctaattgtaatcagcattgt
atcttcaaagaaattaaatagttatttatcttattaaaat--aatcatattatggctata
tacagaaaaagaatatctccaattaagccaacagatgctgttgactacaaagatattgac
ttgctaagaaaatttattacagaacaaggcaaaatactgcctaaaaggtcaactggattg
acttcgaagcagcaaaaaaaacttactaaagcgattaaacaagctagaatcctttcttta
ttacctttttaaataaagattaa--atatatagcctaacttcatattttccatctaaa
tattaaaaatttctatagatttgtttattaaaatgatctaacatactgaat---------
----------tactaagaaga-tctactcattacaagttttgttcattttataattaata
atagcatcataattta---ttaagctagattcagacaagacataaag-gtaacctctttt
aattgattatattatatagtctatttaaaagaggaaattttttgcttc----------t
agcaattcaagattctttaagctttaacaaggtatttaaaacaaagatttttgtttaggt
atcagatcaaatgaatgaatcttatctcccgactgccataattgaaattcagagatgaaa
attccacattcgttgccagcttgaatttcttcgacatcttctctaacgcgctttaaagat
tcaatttttccttgataaataactttttcttctcgaatcactttaatccaagagtttttt
aatagcttattattgatcactcgacatcctgctatttttctatttgctaacgagaatact
gtacttacttctgcttctcccaccggtacttctgaatattcaggatcaagtaagtcttcc
atccttcttttaatgtcttcaattaaggcataaatgatttgatagttttctattaatata
ttcgactttgccgatgcttgctttgttcctggagcaaagttggtattgaacccaatgagg
gtagaatttgtggttgaagctagttcaacatctgtagcagtaatttctccaggcatgatt
gagacaacattgagttgaactttactctgaggaaattgagataaagaatctaggattgct

tctgtagagccttgattatctgttttaataattaaagaaatttgcttactaatatcttta
gaattagtgtttttaaagtatctaatgtaattcgactattaagtgctctttgtttctgt
ataattgatgagtctttagaggtattttcaatcgctttaagtttggcttctttatcactt
ttaactgctaaagctatttctcctgtagctggtacagatgacaatccccaaatttcaaca
acagatgatggtattgctaaattaatttctctttagcgttgttaattataactcgaatc
tttgcgtaagctgatccaataaccaaattatcactaatattcaatgtaccattttgtatt
aatagagttgctactggaccatgcgatttatctaaatgggcttctattataataccttgt
gcaggctgtgttggatcagctttcaaatcctctaattcagctaacaaggtaatagtttct
aataatttatcaacattttgaccagtaagagaactaataggaattataggcacttggcca
ccaagtttctccgacatgacattgtattttaataaatcttgctcaataatatctgtatta
aaaccagctttatctatcttagatattgctatgacaaaagggacattcgctttttgaata
tgattaatagcctctatggtttgaggtttaactccgtcatcagcagctataataataata
gcgacatctgttaaattagctcccctagacctcatacttgtaaaagcttcatggcctgga
gtatccaagaaaacaatttttttgtttattatctttcttaatatactcaacttcataagct
gcaattgcttgagtaattcctccaatttctttgttagcattattagattttcgaatataa
tctaatagcgttgttttgccatgatctacatgtcccatcactgtgacaattggaggtctt
ttaatataattgccagtttcataaagagcattagagtgatctaaattactagaaagtcca
ttattattttcctttacatttgattctactgctataccaaaattatcagccactgatgat
attattgaggcgtctatggtttgattcatagtaactgatattcttttaagaaataaatat
tttataatatctgtttcttgaacacaaattagtttagataattcttgaatagttagagga
ttagtaatactaatagactctggcggagaattagattgaatgcttgctgaattttgacta
ggagttactgctactttttctgtcttatagatttttattactagtcagtttttttaca
acttccacttttggcttaggtggacgcattaaagagatagctaaatctcctgcagtctgc
gaaacattagaattagagtctctaaagttgtca---tcatcatcgtcaatatgtatttta
gtttttattttt-ttgcgctgtctattctta--tttttttactatctactaagtcgtga
atttattaaaattttactttttttatctagtttaggtggagaacttaattctagatgc
tgttcaccacc---tgtatgtgattctgatttatcaaggtctaaatttaacagattatca
ttattgactgactctaatctaattttatagataatctgaggattttcaaatctacaatt
gtttcagaagaattgatatttgagctagatctagaggatctattttcaaagttttgatta
tttaaaaacataaatattggtttattatctaatagtaactttctaattttaatacagag
cttaatataaacaagaatcgaatatctgtataaataatacaaatgt---agagaattaat
ctatgttgtattagatttaatataacactaaagtcctttgttcgttatacaagtttaaaa
ctttt-tatgatgataaacactagattctagtttattggtatacaatctatttatctaaa
taatactgtttttgtcagggcttttaaa----ttatctaatgttatgttcattatgaaat
tctattaaaatttaaaaa-ctcaacaattaagtaagaatcgttaatcacctccacgccac
ttatgccacgatctcaaaaaaatgataattttattgacaaaacttttacagtattagctg

atattgtattaaaaatacttcctacaagtaaagaggaaaaagaagctttctcttactatc
gagatggaatgtctgcacaatctgaaggtgagtatgcggaagctttagaaaattattatg
aggctttaaaactagaggaagatccatatgatagaagctatattttatataatatagggc
ttatttatgctagtaatggtgagtatgtaaaagctttagaatactaccatcaaggattag
aactaaattttaaattacctcaggcccttaataatatagccgtgatataccattaccaag
gagttcaagcaattgaggataaagatacagagctatctaaattaatgtttgataaggcgg
ctcaatattggcagcaggctattaagttagctcctgataattatattgaagctcaaaatt
ggctgaaaacgacaggacgaatgaggaatatacaaggatattaatatatttaagatataa
taaagatatcaattaataaattaaaattagttaaagaaactggtactatatagagtactt
-tactatatattataatattatactagttga------tctctaagacttagctgaataaa
cgaaattaaatctattgactatattcatgaatacaaataagcataatggttagtacaaca
aaaagtaccggatctgttactcaaattattggaccagttttagatattgcatttcctaac
ggacagcttccgaaagtattcaacgcactcaaagtacaaagctcagaaggaactattact
tgtgaagtacaacaacttttgggtgacaacaaggtacgagctgtttctatgagttccact
gaaggactacaaagaggggtagaagttattgatactggatcccctatatctgttcctgta
ggtacagatactcttggacgtatttttaatgttttaggtgaacctgtagataatttgggt
cccgttgattctgagagtactttacctatccatcgaccagcacctgcttttactaagtta
gagacaaaaccaaatatttttgaaacaggtattaaagtcgtcgatttacttgctccttat
agaagaggtgggaaaattggtttattcggaggtgctggagtaggtaaaactgtattaatt
atggaactaattaataacatcgctaaagctcatggtggagtatctgtatttggaggcgtt
ggtgaaagaacaagagaaggaaacgacctctatatggaaatgaaagagtctaaagtaatt
gatgcagataatctgaaagaatctaaagtagcattagtatatggtcaaatgaatgaacct
cctggagcacgtatgcgcgttggcttaactgcattgacaatggcagaatactttagagat
attaacaaacaagacgttctattgtttattgataatatttttcggtttgtacaagctgga
tcagaagtatcagctctactaggccgtatgccatctgctgtgggttaccaaccaactcta
gcaactgaaatgggagcacttcaagaaagaattacttcaacaacagaaggatcaattaca
tctattcaagctgtatatgtgccggctgatgatttaacagacccagctccggccactaca
tttgcacatttagatgcaacaacagtactttcgaggaacttggcggcaaaaggaatttac
cctgcagtggatccactggattcaacatcaacaatgctacagcctggaattgttggaact
gagcattattctactgctcaagaggtaaaatcaactttacaaagatataaagaactgcaa
gatattattgctattcttggtcttgatgaactttcagaggaagacagacaaactgtatca
agagcaagaaaaattgaaagatttttatctcaaccttttttcgtggcagaagtgtttact
ggatcacctgggaaatatgtatctttggaagatgcaattaaagggtttcaaatgatctta
aaaggcaatttagacgacttgcctgagcaggcattttatctagtaggtgatatagatgaa
gctatacaaaaagctgacagcatgaaagattaatataattaatgatgactttaaatataa
gaattattgctcctgatcggactgtttgggatgcagaagcacaagaaattattttaccaa

gtagtacagggcaacttggtattttaacaggccatgcacctttgcttacagctttagata
ttggagttatgagagtaagagtagataaagaatggatgccgattgttttgctgggcggtt
ttgcagaaatagagaacaatcaattaactattctggttaatggcgcagaagaagctagtc
aaattgatttatcagaagcagaaaaaaatttagacactgcaactcaactcttaagtgatg
cctcgtctaataaagaaaaaaatagaagcaacacaaaaaatacgaaaagctcgagctcgag
tacaagctgcaacagcagcaacttcgtaa-aagattcc------tcttaaaacataaaac
tttagtaatgattactaaa-------gttttatgttttatttgtatggttataggcagct
taaataaacttagcttagacctgaacaaatataatcaaaatataatcccatctctttacc
tgcatctggtccaactaaactaatagtaacttctttcatagctaaaatagcttgaatcgt
tgcaccaataggaacacctagagaattgtatgtttcttttagaccatttaatactctttc
ttcaaggatagatggatctccagctagcattccataagtagcatagcgtaagtagtagtc
taaatcacggatgcatgcagcataacgtcgagttgtatacatattaccacctgggcgtgt
aatatccgaataaagtagagattttgctacggattctttaataattgttgctgcattagc
tgcaatagtagcagcagctctgactcgtagctcacctgtttgaaaatagcctcttaattt
ttcaacagagctatcatctaaatatttaccttggacatcagctgcattaataacagaagt
aattgcgtcttgcataactttcacacattccttaattttaaatatctaatacta--gggg
tatataacaaaattactagatcttttagcaatatcagtattactgcatagcacccaaagt
ataatcaaagtaaaaaccagcttctgctgaatcttcacctgcaagcagtgagcaagctac
acttttcatgcattttacaccttcagcaactcctgaaattggtgttcctaaagaattata
catttctttaacacccactaatccaatttcttcaataggagttacatcgccagcaactat
tccgtaagttactaagcgaagataatagtccagatctcgtagacaagtagcagtcatctc
ttcgccataagcatttccaccaggagaaactacatcaggtctttttgaaacagttgttg
gccgccttgctttacaatacgctcacgattatctgttaaaatttgagctattcttaaacg
acgttgtccagataaaacaaaacttttaattctatctaattccccaggacttaaatatct
tgcttctgcatctgcatttacaattgactttgtaacaatactcatggataaaactcctgt
aatgctttaaaaaaaaagcttaatactaattatttatgatataactcagaaagtaatgta
ctttttgag-aattttataaccgaaacacgttcatgatatatgaagcaggatagatactg
aggtaagtactaaaacgagaaagttatatgtaggaacttctgctattgatttcctagtac
tttttaaaacttggtaccacaattgatatatcctgcttagtcaaacgattatagagtgt
ttctgtattaggaaagttagctgctggtaaagtcggaaatcttctgtatggaacagtatc
tttgccatataccgcattgtattcaacactttcaactaaattacttataaatgcagataa
gccttttgaagctaaaatttgattaaagaatctaatttctgcctgattattaggagctct
tcctaagatatgcttagtacctaattctataactttgtatttggatatggctgataaaa
ttctttaccatatagttcagatagcgccagtttttctactagttctttgacacagatttg
tctattcaagaaagctgacttaatatccaagaattcaccgccgacactaaaagagttaag
atctctttcgaaaatttgacgatatgctgctcttaaagcttgttctaacatttctttgtt

actatcactattgacttcaaagacaatagattgatctcttaaagaagttactccctgtga
aatacgagactgaatatcattactactcctcatttctttgacagtgcctaattcaacaaa
cctagcagatttactagcaataactttttttgaatctctggtcaataattcctggtcttaa
gcttcttaaagcaacaccagctggagtactatatctttcataaggaacagtattatctcc
aaaagtttctgtgtactccgagctatcaattattgtgtctattacttggtaatagccttg
cttatatgcaatatcaaaatatttattaatttcttgccgaccatatgtagggcgacccag
taagcggttatgaatatattcaatagctttacaaatatataatggctcccaataaagaga
tctaaatatacttgattttgctaactgtctaacaaactcgcgaactgtaatttgattatc
ttttagctgactttctattggcttgagaattagtttttcttcttgatacacttctctgcc
aaaaactcttaaataagcaacttttgtgacaacttctactgaattttcaaaattttggga
tgaatctgactttataagagcgttggacaatttaaagatttttggtcccagggatcctgc
tgacttaggtcttacctgtggattactaatctgattgtagattcctggcccacgtctaac
taatatccttcttgtatctttaccaaaaagagcttgtctttttcttggatctttattttc
tttagggaaaatagctccaaattgaattgataatggatcattacctattccatatggatg
ttgatcaggtaatgattgcttataatcactaaataaagttataaattgaggaaccttacg
aaaaggcgcactataggtgaaaagatctatttgaggcccccaattacggcactcttgagg
ctcttcacctaaatttctaaagtaaggcacagtctcttcaccgaaataatctgtatactc
agaagaatttaaaagtgcattcactaaaccacttaaaccagtagatgataaaatagcaaa
atatttttgaaactcttctaaagagctaggacctctacccaagaaatgtctaaatgctaa
ttctaatgctcgactattgacaaaaggttcataaaattgctttctataaatacttgaagt
tcctaacgaacgaatgaattccttaattgaaatttggccatttttttacttgcgactccaa
attcgataaagagaggtcatatgctttagcaatatctctttcaaaaatttgtctataaca
tgctttcacaacaatatttttctcatcagcagataaactaggcttcataacaaaacgagg
tgttgacactcctgcctgtacataagtttgaggaagacgcaaaccttgtaaatctccaga
tattctttttcttaatttatcagtcaaagatggagcttcaaattcagaaatcacaacatt
aaaatactctttaactaaatcttgaccttttatatcttcctcaaagattaataatgctgt
ccgtctcatctctctaagagccacaattgctgctgcacttgagcaagcgttatcaattaa
ctctctcaatcctcgtatattaacagataaaatattagggtcgcctgatacaattgcata
tgtcaaataccgtaaaaaccagtctaaatctcgaagagattttctcattcttgtagtacc
gtatctcaagacgtttataggtttaaaccctggcggagtagctcccccagcattaaataa
agatcggaaactttggccaaaatctccttggatatttcctgataactcattaattttatc
ttgagaactttgatctcctgcaataataacagctgcttgcggacgttctaaataagaaat
tgcagacccacctacgaatattttatctgctgctcgtgctaccagaatatttgcattttt
agttaaaaatatctgcaacttccagtctttttttgtccagaatttaaaaaagaaactaattg
atttaattcaccaagctgtaaaaatcggtcttgttgctctgcctgagtaatagttaaaat
tgaggcagtccgataaagctgggggcgtgctaatgggcttccgccacttgctttgatact

cattactttatttatctccttaactgatattgcttgctagcgaatacaaatatagcttga
tatttctaaatatttaaagatactatgcaagcaca-ctgttttctatattattaggtact
agtacttatagtacagagagtcttataactaacttctataaatacaaattttgtaatact
tctattaacatttgtcaaaaaaatatttaagtgctcactcttttatattaaaattcaatt
agtgagagattgatgtgaaataaagtatactaagtaagttttatagataggaaaattaaa
agatttattctacaaacattaatca------------cttgaatgtttagcaattgtaca
attgttattgcttttaattggtttagactagttttatatttttttctaacataaacaatat
taatgagcaatttgtatgaatttaaagccacaaaacaacctattaactaacgaaaatata
aatcatgtagatatacccgaaaatgatataccaatgtcaattacggaacatttagaagaa
ttaagacaacggactttatttgtgtttttattctttttgtttgctacaactataagtttc
acacaaattaagatcattgttgcaatactgcaagctcctgctgttggtattaagttttta
caactggcaccaggagaatatttttttttcatctattaaggttgcaatatattgcggatt
gttgcaacaactccgtttgccgtttatcaagttatattatacatactcccaggactaact
ggaaaagaaagaaaaattattttgcctctattaattagttctgtattgcttttttattaca
ggtggtatatttgcttactttgttctcgcaccagcagctttaacatttttaattagctat
gggtctgatattgtagaaccattatggtctttcgaacaatactttgactttattctatta
cttttacttagtacaggattagcatttgagataccaatcatacaattattgctcggtgtt
tcagggacatttcttctagtcaaatgatacgagcttggagatatattattattatagca
acaattgctggagctattctgaccccttcgactgatcccgttacacaattaataatgtct
tcagctgttttattactttattttggtggaattgttatattattagtcttaaaaaagtag
agtaaat----------atttcta-ttaatccaatatatttgttatc-----tgggcaaa
taatctataaaaacaattgct------------------agatttaagtatcaggcttct
aaatatttcaa---------gtctcttttatcatttaaatacaatc--------------

--gatacattgg
cttgttttttcatcaattatttgtgttgcacac-tttttagcactctctttattgttctt
agaattatttctttaatctatttattaaat----agatatgaagagatttaagtattgat
tatggtaatctatatcaacgtaaaaagtattttattgtgagttttatttatataaaggct
tattcattatatagttataacagtaagtctaattaatag-ttttcatgcttaattaaagt
aataactcttaattcttttggcagaaaaatctgtcttaatattttatc--aaaaaaacag
-aaaaatgaagctaaatagtctaattaacttaattcaaaagtctatatattcttgtacac

ttttactaattattttaaatattatttgtgtcgcacctaattctagtaatgcatttccaa
tttatgcgcaacaagcttatgaaagtccaagagaagcaactggtaggatagtatgtgcca
attgtcatcttgctcaaaagcctgtggagatagaagcgcctcaagcagtactacctaata
ctgttttcgagactgttgtgaagattccatatgacagcaatgctaaacagattttaggta
atggcagtaaaggaggcttaaatgttggagccgttgtaatattacctgaaggatttaagt
tagctcctgttaatagattatctacagagttaaaagaaaagactagaaatctttacattc
agccgtacagtgctaaacaagacaacattttagtaattggacctatttctggtgataaaa
atagagaaatagttttcccaatactatctcccgatcctgcaaaagataaaaaagctcatt
ttttcaagtatccaatatatgttggcggaaatagaggacgaggccagatttatccaacgg
gtgacaaaagcaacaataatattgtctctgctttaagcagtggtaaaattaataaaattg
aattactagacaaaggtggatttataatacatgtgactaacagtagcaatgtagagtcaa
cacagaagatttcacctggtctcgaacttagagtaaaagaaggggatacaattcagcttg
atcaagctttgaatagtgatccaaatgtaggtggttttggtcagaacgaaacagaaatag
ttttacagagtccaaatagaattaagggcatgattgttttctttttcgctagtgttctag
ctcaaattttcttcgtattaaagaaaaaacaatttgaaaaagttcaagcagctgaaatga
atttttaaagcaatataaaactggcacttactataaagaaagta-taaacactttattca
caggatgtaccagtttttatgttatgtaataatcgctttgacggaagttatgatactttt
aacagaatctacaatctgactaggctgaattactgtagcctgctctaagcttccattata
tggtgtgggtatatcctgcgaagatagcctcacgacaggagcatctaattcatcaaaaag
gtactcattaatttgtgcaattagctctgctccaattccggctgttttcatacactcttc
tacaattaaaactttgtgagttttctttactgagatagatatagagtctatatccagagg
ttttaaagatataagatctataacttctggatcataaccttcttttaataaggccggtaa
tgcttggataacatgatgcctcattctagaataggttaaaatcgtaatatcttttccttt
tcgtacaaattcaactttattgagaggtaagaaatattcttcttgaggaatctcttcttg
taaattataaagtagaacgtgctcaaagaatacaactggattattgtctcgaattgcaga
tttcagtaatccttttgcattataaggagtagaacaagcaactattttttaagccggggat
ggcttggaaataagcttccagtctctgagaatgctctgcacctaactgcctaccaactcc
tcccggtcctctaataactaaaggcaatgtaaaattacctccagaagtataacgtaacat
tcctgcattattagaaatttgattgaatgctaataataaaaaactcatgttcataccttc
aacaattggtcttagccctgttatagctgcaccaattgccataccagtaaagctattttc
tgctattggtgtatcaagaactcttaaatccccatatttgctatgcaaatctttagttac
cttataagatccaccatagtgaccaacatcttctcctattacgcagacagttggatcttt
tgccatttcctcatctgttgccgctcgtaaagcgtcaaacataaagattttactcatttt
aatatttgatttttatgatttgaccactgttttaaagtaacagttaagacaccatattct
atttagttatctgcaaaaagatatcgtttcaattctgacatgttaggttctggactggaa
atagcaaatttcacagcttgttccaattcggttttaacagcattctgaatctcattaagt

tcacctatattagcaatttcattatctagaatatattttttgagtttttttgataggatct
cttgccacccaagcctctttttcttgtcttgatcttagttcatcaggatctgcgagagag
tgaccacgaaatctatatgttaatgcttctattaaggttggaccatcaccttggcgagct
ctttgaactgcttgttttgcagcttgtcttacagctagcacatccattccatcaacttca
atcccaggaagcccaaaagcttctgctttttttatgtatttcaggtattgaagaagaccgg
tgatgtgccatacctatagcccactgattattttcaacaacaaatataataggtagtttc
cagagaactgccatattcagacattcaaaaaattgcccattattggtagtcccatcacca
aaaaagcaagccgtgactcttaaatcctctgtttctttaagtacttgctggcggtagata
ctttgaaaggctgcccctgttgcaaccggtataccttccgcaataaaagcaaagccacct
aaaaaattgtgaggcgcagaaaaaatatgcatcgaacctcctctgcctttactacaacca
gtctcttttccaaataactcagccatcacatttttagatgggacgcctttacttaaagca
tgtacatggtctcgataggtactgcaaacataatcagttggattcagaagtttaattaca
cctgtagaaacagcttcttgaccattataaagatgaacaaaaccaaacatctttccttta
taatacatctgagcacacatatcttcaaaatttctgcctaacaacatgtcttcatataaa
actaataaattactcttattaagattaagacctgttgaattatagttagtcagcggcaat
tgaactttcttaggataactcataatttgtaaagaaacctct----------tattgtaa
ataggaattttgaagaaacagctatcaatagaaaacacacagataaaaacttataaatta
taatatgtttttactaaagactttaataattatttgaaactagttcagtatatcaaatt
aattttgcttatactacggatgtctaaaacttatataaaaaagtagt-a-aaaaagttta
ttaaaagaatagttaaaaacagatataaatagaagtaaacagaattatt-gaaaaaaaat
gattattattaattaa----tacttaatatttctaaatattttacaattaatggctatgt
caccatctttattttatcctgttgaacaagaactatgtagtcttgaaaaaaatctgaaag
ctgttgctgggactcgtcatccaattttatatgccgcagcaaagcatctattcgatgctg
gaggaaaacgagttagaccagctcttgtatttttagtggctaaagcaacctctgagaagc
aagatataaatactggacaaaaaaggctagcagaaattactgaaattatacatactgcta
gtttggtacatgatgatattattgacgagtgcacaacacgtagaggagtcaaaactgtac
ataatttattcaatactaagattgctgtgctagcaggagatttttttatttgcacagtctt
cttggtatttagctaatattgaaaatttagccgtagtcaaagctatttctaaagtcatca
ccgactttgcagaaggagaaattaggcaaggcttagttcatttcgatcccagtatttcaa
tagatgcttacattgagaaatcattttacaagactgcttcactcattgctgctagttgtc
ggggtgcagctatgcttaatggttccaatcatcaaataaataatgatctttatctttacg
gtaaacatatgggattagcatttcaaattatggacgatgttctagatataactggttcta
ctaagagcttaggaaaacctgctggcgctgatctaataaatggaaatttgacctctcctc
tcctttttcacttactcaagaagcaagtttaaatgatcttattgatagggagttctgta
atagtacagatatagcctcaacattatttcttataaaaagaagcgggggaattacaaaag
ctaaagatttagctaaagaacaggtgcaggcggcactttcctgccttcagtttttaccac

aatctacacctgtatctagtttaaaagaattaacacatttcataatcacaagattgtcat
aaaggacttgctaaaattaaaatgttattaactaatttttttactgtttctaaaacagtct
gaaaagtttgaatatcattactagctagctgcgctagcattttacgatttaacgcaatat
tctcttttttaatgcactaataaaagtactataattcatgccttgattgtgagcagcag
catttattctagttatccaaagacgacgaaaatctctcttttcctttttcgaccaacat
aagaatatcgaagagctttaaggacttgctgcttagctgttcgaaataaacatttatgtg
cacctttaaagcctttagctagcttaaagatttagctcgtctttttttgcaacgttac
ctcttttaactctactcataaaatattac-ttaaattttaattattaccagcttactaaa
ttcttatactctttg----attataaataaggtaatttcattgcaatgtttttaatatct
tttaagtcaaccgaacaagtagaagaaagatgtcttctttgttttgatgactttttttgt
aataaatgacttttagaagctttatgtcgaagaaattttccggatgaagagactttaaat
ctttttgctattgcttttgatgtttttaacttaggcatattataaactttaatgattaa
caaaagcatgaaaagtctgaggatatcaatcttagacctttcaatacttagaataatctt
aacacaatttattttacttactacataggttaactgaaacaatttatatatataatacta
atcagttaatcaaccatagctaaagtggatttatattttgctttgaacttttgtatagta
ttagcaattagcattgttataattttaagactgcttgaattcaattcttcgaacattttc
atatttaatgtaaatgaaatattggcttctgagacaatattttgaatttgaacatcagac
aagggaatcatatctaatgcagctctatattgatctttaaataatttatcgtcttttatt
tggtcaaaatcgtaaaatttcgttcctcctgaatctgataaattcatagcccctctagct
atttttttaaaatttggccaccagaaaggtcacccaaatagcgcgtataagcatgagca
actagtaattcaggttgtttatgtcctatagtgtgaattctatcaacataaattttgta
gcaggagatggttcaataaaatctaaccaatctgatccataataatagtttaaatcttca
gatagactcgctttcctattaagctctgtaaaatatataggtttaatagctggatgattt
ttattagaaaataactcttcctctattgcacaatagacaaagtatagatttgcaaccaat
ttacgatatgacttcttatctacaactcctcccaaaaaagatttaacaaaactaacattt
tctgccatactatgggacttagtagtaccttctcttagttcattcgctaaagtattaacc
ataatatcaatattccattttt-aaagataacaaattaaca---ataaatagttatccgt
atatttataattgagttgcgctaactaggctgctgcaaaataatttttttgattttattgg
atctggattcattgttttgtcaccaggcttccaattagctggacaaacttcatctggatg
agattgtacatattgaattgcttgtaaaaccctcaaagtttcttctacactcctgccaaa
ctctagattattgattgtagagtactgaataattcctttcgggtctataataaatagtcc
tcttagagctacaccatcactattcaaaacattataagctgcactgatctctttttttaa
gtctgatactaatggatatgaaagatctcctaatccacctgattctctgtcagtctgtaa
ccaagcaagatgagaatactcactatcaaccgagactccaagaacttctgtattaagttc
cgaaaaagcattatacttatcgctaaatgctgtaatttctgtagggcacacaaatgtaaa
atctaaaggataaaagaataagacaatatacttattcttgagatcagataattttaacgt

tttaaattcttggtcataaacagctgtagctgaaaaatcaggagctagctgacctactcg
aagacaatttggtccagaaatcattaatttttcccagaagaattgatataattttgttat
ttaataaataattatataacaaaatttagtatatattaaaatactaccagtctttttaa
attac--------taaatgatataatcaatactatctttatagcggggaatggatttgaa
ccattgaccttcgggttatgagcccgacgagctaccagactgctctaccccgcggataac
taaaatcatattagtctacatatatatataaaagcaaatgcataatagatttagaagta
aaaaaatagaagaactggtccactgatcattaaacttagaaacgacaaagtatttttat
cttatttaaaagaggaaagacttcatataaaccaataaatctatcttttaaaagaatttc
ggacggctttacccaaatttgaccgtcataccagcctgattcttcataaaatactgtagc
actcattaatcgttttactacgtatgaccagcctaaatataatctgattaaaataaatcc
tgtcattaggctggaagtaataaattctgaaaagaaaaatttcaaaggtagtttagtgat
tggaaaaattgataataatataggactaactaataaacagttaagtagtagtgcaatagt
tatttttttgttataagatctatggcttaaagttggccagcaaaagaaccaagaattttt
caaagaagtatattcatgaacaggttgctgctcctttggcacaggacattgagtattata
taaattcattgacattaaaaagtattataataaagac--tttgtttatattatctaacat
aggtacgacattaaatattaactaactgcaattatagtagtgagtagaagaaataaaact
aaaaatagccaagtaattttatttaaagtattctcagtactacgagtattactaaagaac
tgattctgagcacctacacttcctaaaccttcggatttaggattgtgtattagaatagtg
aagattaatattattgtagatgagtaccaaaaaaaattttaaaatttgttccattatgtaa
aagttaat-aaaaatagcacaaagacaatcgtctaaaagatattgattgtcttatcatat
taaaaagtatttattcaccctgtacttttaaaagtaaaaatcctattgccagccctacca
aaactaggactgaacttaatacagcactttctacaatttctccgaccatt-ttattttc
cttattattttattattg---tcattcttttgacaagattatattgagctaa-ctaaaat
ccgtttctaccccaaactaccattgcaattgaaaatgtaaacattgccattagagctgcc
caccccaaacttaaaatatccataatttataatattc--ctaaaaataagatctcttta
cttaaactataatattcttaatattaaaaatatcattatataataagctaattataacag
agtactacttaattcgtatttttagtgtatttaaagaagattgaa-ttttttcgaggtac
tggataagtatttcatcctaagaaccatattcaac----ttttgatttattataatgctt
aaatctaaatata--tatcactcaagaatcattagtaattatgatgtttttaattatacg
att---aacatatttcaaaatagaagaattcatagcgactaagttaa-----tatgctaa
tataatttatcaat--aaaaaggaagttagtatttcaattatgcaaacaactataaataa
tgggcaaacgtctagtaaagaaactttactaacacctagattctacacgactgactttga
agagatggctaatatggatatttccggcaatcaagaagatttttttggctatcctcgaaga
atttcgagctgactataatagtgaacattttattagagatgaagagtttaatcaatcttg
gtctaatttagaacataaaactaaatccttatttattgagtttttagaaagatcttgtac
cgcagaattttcaggtttttactatataaggaattatccagaaaattaaaagacagaaa

tccggttatagctgagtgctttttattaatgtctagagatgaagctaggcatgccggttt
tttaaataaagctatcggggacttcaatttatctttagatttaggattttaacgaagag
tcgcaagtatactttttctcacctaaatttatttttatgcaacctatctttctgaaaa
aattggatactggagatacataactatttaccgccatcttgaacaacatccagaacaccg
tatttatccaatctttagatttttgaaaattggtgtcaggatgaaaatcgtcacggaga
tttttttgctgctttgctcaaatcccaacctcatttttaaatgactggaaagcaaaaat
gtggtgcagatttttctattaagtgtatttgcaacaatgtacttaaacgactttcaaag
aattgattttataatgccataggcctagactctagacagtatgatatgcaagtaatacg
aaaaactaacgaaagtgccgctagagttttcccggttgctttagacgtggacaatccaaa
atttttcaaatatttagatacttgtgcatgtgataatagagctctgatcgatattgataa
taacaattctccattattcattaaatctatcgtaaagatacctctatatttttccttatt
tgcaaatttactaaagatatatttgattaagccaatagactcaaaaacagtatggaatac
agttcgatagttaaatacaagggaatgctttttttcttataaaaagctatctagtgattaa
tataaaat------aaaagcttccttct-----cgatggaaggagcttttattttgtcaa
ga---atataatgggattatatagatc-tagaactaagaacttactttttcttttgcttc
atacataacctctaaagtaattagattttagaacaatcacgggcaaatttctctgtatt
tctttttactttacctctaacaaacccaggaattttatttaattctttctgtgcttcttc
tgaccaattaatactatcaacagcagaaattcctatacttaatgcttctgttgtatcatg
accaccaaatatttctagcaaatgatcttccataccaagtgtaaacgaattatatactaa
atcagcaatttgatttgttccctcataaccaagaaatggacggtaacttaaaggaaagtt
ttgtatatgcactggtgaagaaataacgccacaaggaatatttagacgcttaccaatatg
tctttccatctgtgttccaaaaattgctgcaggttcagttttagcaattaaatctccaat
taaaccatgatcatctgaaacgattacttcatcgcagaattcttgaacttgatctttaaa
ccactcttcatcatatttgcaataagttccacaccaggcgacatgtatacccatttcctg
gtgtaaaattcgagttattgctgcagcatgagttgcatctccaaatacaatagccttttt
acctgttaaattctgacaatcaattgatcttgaaaaccatgcagattgtgaaataaatcg
ggtttgctcgtctatgtatttttcgtagtctacagcagctcccaatgcatttactaattg
ctgtattgatcggatgcatgcagctgtttgaacaatacccataggtgtaatgtcaacata
aggcatattaaattctttctctaaatatcgagctgtcattagtccagtttctctatatgg
aataaaattaaaccatgcagagggtaattttttttaaatcttgaactgatgcattttctgg
aataatttggtttatttggatatctagatcttgaaataacctttttaattctgctatatc
atgttgattatgaaatcctaagcttactgctccaataatattaacagatggagttttagt
cttttgagttaaaacttgattattagatttagctttttccatatagaaagtgacaatttg
ttctaacgttctatcacctgcttgcagttcatttactctataatgattaacatcagctaa
taaaacatctgcttctgtttctatagatgctctgctaacaaagttttgcaaatcttcttg
caaaatactcgaagtacaagttggtgttagaataactaaatcagggctttcttctctgtc

ttttctagtaatattttctacaactttttcttgagaaccacgggctaacacatgtctatc
tacaacacttgctgtaacaggagtaaaatctctgtctctttcaagcattgaacgcattac
attaaaataatcgtctccaagaggcgcatgcataattgcatgaacttttttaaaagaact
cgcaattcttaaagttccaatatgagcagggcctgcatacatccaataagctaatttcat
attaaattttcctatgatttatttagacatagtaatacttttaaagctaactaacaaaca
ttagccaaacatctaatttaactccttgacaattttgtaatatttttttaaatgaagctta
tcattataaatatttaaaaaatataattcagaataga----aataactgttagatcaagc
ttgaaagtaaagatttcttgcagttatagttttctttaagtaaacatgtaaaaattgta
ctatatataaatgtactatatataaactttttttactgaatattggtaatgccagataca
attaacttaaatatgccttccccaacgtttggtggaagcactggcggttggttaagagcc
gcagaagtagaagaaaagtatgcgataacatggacaggcaaaaatgaaagtaagtttgaa
atgccaactggtggtaccgcaactatgcgaaatggagaaaacttactctatttagctaag
aaagaacagtgtttagctttaggtactcaactaaaaagtaaatttaaaatatctgactat
aagatttacagagtttttcctaacggagaagtgcaatatttgcatcctaaagatggagtc
tttccagaaaaagttaatactgggagagctagcgtcaacagtgttgatcattctattgga
caaaatattaatcctgtagatgtcaaattcatgaataaagcaacttacgattagtctctc
-aaaaacctagacataaaataacttttatatagtctaggtttttgataatgtatactgat
agtatggaagggtggccgagtggttgaaggcgtctgatttgaaatcagttgaacta--tc
tggttccgtgggttcgaatcccaccctttccgttataaaact------------atagtt
ggcttctcaaggcaagctagctatacactgatagtattaagtttagacttaataaagtct
acaacttgtgacaggttagaaatttttttcggcatcctcatctggtatctctatactaaat
ttttcttctatggccattaccagctcaactgtatctagagaatcggctcccaggtcactt
gaaaaatttgcttccctagtcactatttttttttcaattcctagctgttctgctacaata
tcttgaacttttttcaaagatttcattatcttgcataatatattatttcctctaaggtgtt
atcaaaaatttttcggcttatattattatccaactctaaaaaattagtaatttttagtcg
agattaaaatatttatacctatatgatatactattattctggataaatacgaagtttacg
atatggctcttctccaaaactacgagctctcaactggtagttgtcaactagattatgctg
catttttcgtatataggctgatcgaggtgtaagctgaactattgaactttcattcaagat
aatagtttcaatagccaatttagcttcttgaagagcctggatctcatcaaaatttttatt
tttacagagttctacccaattaagaccagatgaagtatgaatgtttaatatttttctgag
tgcacgagtaatttgtggaaccgtactattctgaattgtatatataataatttgttttga
ttttgcaatttgtctaagttttgtattttgctttacttgattacgtagcgctagaatagc
atcagacttttctatctccttggttaaaataattggtagatctagtgaagaaataactga
tgtaatatgctgccaactcaaagaataagcatataagtactgatgaggtatttccaattg
caaagattgtttattaatattaatagggggcattgaaagtagttgacaataaagatgtatc
acgattttgtgatttattcaaatctaaggacttatttcttaattctctatattgtaaaac

cggaggcgcctgctgtgcgttaattgatttacgagcattagagatgtttacaggtaagac
ttctatagattgcgatggataacatttaattaaaattctaccattagcttgaatttgtct
tttttgcacaaaaggttgatggccttgcaaaatttgatctattgtctctttaactttatt
atgaactatccaaacatttcgctcgtgtatttcgatcgcaatctgaaaagcaggctcagc
ttttctttctagtatactcttttgcgtacctctacgtttagcttcatcgtcacctaatgt
tacatattgaattccaccaattaggtccgccaatgttggatttttaattaaactttctaa
atgatttccatgcgctgtacctactaattgaactcctctttctgctatagttcgagcagc
taacgcttctaattccgtgccaatttcatctataataataacctcgggcatgtgattttc
aacagcttctatcattacttgatgttgcaaatcgggccttgcgacttgcattcttctagc
tctaccaatagctggatgaggaatatctccgtcaccagctatttcatttgaagtatctat
aataactactctttttttccatttcgtcagctaaaacacgggccatttctcgaactgcggt
tgttttaccaactcctggttttcctaagagtaaaattgaatcaccttgctgtagtaaatc
tcgaataatactaattgtaccaaagactgctctgccgacacgacaggttaagccaataac
actaccttctctattacgcagtgaactaatacgatgcaaggtttttttcgataccagctcg
attatcgccgctaaaattaccaacttttttttacacaataatctaagtcttgccaactaat
agatctttgagataaatattctgggttatctggaaatctagcttctggacgacgacctaa
atccataacgacttctattaaattatttctattaggatgctgttgtaaaggttctttgac
aaaatttggcaaaatttctagcaacttatctaagtcatctgcaataagcatgattgagta
tataataattaattaatcaaatagaagattcaagattctaaagattacaatacatattaa
agaatattatgattggaatcaattttatacttaaattgaacaggaagtta-tagatgtaa
aagaactgattaaactttacgcaataaatcgattggtcaaaattaatagtcgtgttaaca
tgtttattggacaaaaagttcgtattaaatatagcaaacaaaaagttgctagtgatatag
cagataaagttggtgaattaggagttattaagggaataaagtttattaatagtcaatgtg
ttactattattgttgagtttgacaatcacactaggctttggatgtttagagaggaattaa
tctgtctaaatgaat------taaaaacatgaaacataatttattttatttattatacaa
aatttcttatgagtcgttaaaataataaattttaaaggagtacttttaattgtgcaaatt
actattaaaaaattacaagacttactttcgtctgtacaaagaaaaaagatccagacatta
aaactaaaacaaggtaagtttgaacttctattaaataagacctataaaaaagtcaatcaa
gaaattataccttcgcagaaatctgctgtattacaaaatagtccatctacgataattaaa
tcaataaataatacaaaaaaatcttctgttgttaatgaagaccgtacagaatatgccact
attgtttctccaatggttggaacgttttatcattcacctgctcctggtgaaaaaaatttt
gtacaagttggcgatgaggtcaaattcaatcaaacagtctgtattattgaagcaatgaag
ttaatgaacgaaatcgaggcagaaattgaaggcaagattatagaaattcttgttaaagat
ggtgatatagtagattgcgggcaagccttaatgaaggttgaaacataatgctaatttttc
cattttacatattgttaacagaaattatttagatagaaaaattagcttatactatagtta
tgaaaacttatttcgcaaaggcac-taaaaactaatatataaaattatttactttatctg

ccaaaaaatagaaataagcgttttgattccttgtctcaagagaggagaatctcaatggcaa
ttagctcaaaagagcaagagacaaagaaggtaaaaatctcggttgataaaaatcccgtag
atacttctttcgaaaagtgggcccaaccaggccatttttctcgtacactagcaaaaggac
caaaaactactacttggatttggaatcttcatgctgatgctcacgacttcgatagtcaaa
ccagttctttagaagaagtttcacgtaagattttcagtgcacattttgggcagctgtctg
taatatttttatggcttagtggaatgtattttcacggagcccgcttctctaactatgttg
cttggttaagtaatccaacaggtattaagccaagtgcgcaggttgtttggcctatagttg
ggcaagaaattttaaatggcgatgtaggtggtggctttcaaggagtacaagttacatctg
gatggttccaactgtggagagcatcaggaattactacagaatttcagctttactgtactg
ctattggcggattagctatggctgctttaatgctgtttgcaggatggtttcattatcata
aagctgctccaaagttagaatggtttcaaaatgttgaatcaatgatgaatcaccatttag
ctgggcttttaggcttaggctgtttaggctgggcaggccatcaaatccatttgtctttgc
ctattaataagctactagattctggcgtgtctccgcaagaaattccactacctcatgagt
ttttaattaatagagagcttatggctcagctgtatccaagttttagtaaaggattagttc
cattctttactttaaattgggctgaatattccgactttttaacttttaaaggaggtttaa
accctgttactggaggtttatggctaagtgatactgctcatcatcatctagcattagctg
ttctatttcttgctgcaggtcatatgtatagaaccaattggggtattggacatagcatga
aagaaattctagaagctcacaaaggacctttaccggcaacggtcacgaaggtctatatg
aaattcttacaacttcttggcatgcacagcttgcaattaatttagctatgatgggatctt
taagcatcattgtagcacatcacatgtatgcaatgcctccttatccatatattgctactg
attacccgactcagttatcgctcttcactcatcatatgtggattggaggattttgtattg
ttggagcaggagcgcatgcttctatatttatggtaagggattataatcctgcagaaaatt
ataacaatcttttagatagagtcattaggcatcgagatgctattgtttctcatctaaatt
gggtatgtatatttcttggattccattcatttggtttatacattcacaatgatactatgc
gggcacttggaagatctcaagatatgttctctgatacagctatacagttacaacctattt
ttgctcaatgggtacaaagtatacacactttagctcctggaaatacagctccaaatgcat
tagcaacagctagttatgcatttggaggagatattgtttctgttggtaacaaagttgcaa
tgatgcctatttctttaggtactgcagatttttagttcaccatatacatgcatttacta
ttcatgtaactgttttaattttagttaaagggttccttttctcaagaaactctagactaa
ttcctgacaaggccaatcttggcttcaggtttccatgtgatggacctggtagaggtggta
cttgccaagtgtctggctgggatcatgtttttcttggcttattctggatgtacaattctc
tgtctgtagcaatttttcactttagttggaaaatgcaatcagatgtttggggtagtgtat
ctccgtctggaaatgtttctcatattactggcggtaattttgcacagagtgcaattacaa
tcaatggatggctgagagatttcctttgggctcaagcatctcaagttattcaatcatacg
gttctgcgctatctgcatatggattaattttcttagcagcacattttgtatgggcattca
gtttgatgttcttatttagtggtagaggttattggcaagagcttatagaatcaatcgtat

gggcgcataataagataaaagttgctcctgcaattcaaccaagagctttaagtattactc
aaggtagagcagtcggtgttgcacactacttattaggtggaattggtacaacttgggcat
tctttttagcgagaattatttcagtaggctaatagtgaaaataggataaaaaaaacaatt
atggcaacaaaatttcctaagtttagccaagctttatcacaagatcctacaactagaagg
atttggtatggtattgctacggcacatgactttgaaagtcatgatggaatgacagaagaa
aatttatatcaaaagatattcgcttcgcactttggacatctagcaattatcttcttatgg
acatctggtaatttattccatgtagcttggcaaggcaactttgaacagtgggtattaaat
cctttgaaagttaaaccaattgctcatgcaatttgggatccgcattttggacaacctgct
ttgaaagcttttagtaaaggtggctcagcttatccagtaaatatagcatattctggcgta
tatcactggtggtatactattggtatgagaagcaatcaagacctgtattctggggcttta
ttcttactagttttatcagctttactcttatttggagggtggctacatctacaaccaaaa
ttcaagcctggtttatcatggtttaaaaataacgaatcaagattaaatcatcatttatct
ggattatttggggttagttctttagcctggacaggtcatttagtacatgttgctatacct
gaggcaagaggacaacatgtaggatgggataattttacaaccgtattacctcatccagcc
ggtttacagccgtttttcagtggtaattggagtgtatatgctcaaaatccagatacagct
caacatttattcggaactaatgaaggtgcgggtacagcaattctgacatttctaggagga
tttcatcctcaaagtcagtctttgtggctaactgatatggctcatcaccatttggctatt
gcagtagtattcattgttgctggacatatgtataggactaattggggaattgggcacaat
ctaaaagatattttagatgctcatagaccacctagtggtagattaggagctggacataaa
gggctatttgatactattactaattctttacacatacagttgggattagcattggcttcc
ctaggtgtaattacttcgttggtagctcaacatatgtatgctatgcctccatatgctttc
atggctaaagatttcacaactcaagcatctttgtacacacatcatcaatatattgctggg
tttctaatggttggagcttttgctcatggggcaatattctttgttcgagactatgaccct
gaacagaataaagataatgttttagctcgtatgctagaacataaagaagctatcatttct
catttaagttgggtaactctattttttagggtttcatacattaggcctttatgttcacaat
gacacaatgattgcttttggaactcctgaaaaacaaattctaattgagccggtatttgct
caatggattcaagcctcttcagggaaagcactttatgggtttgatgtgttactatcatcc
tctactaatatcgcaacacaagctggtagcaatatttggctgccaggctggttagaagcg
attaatagcggaaaaaattcattgtttttaacaattggtcctggtgacttcttagttcat
catgcaattgcattgggattacatactactacgttaattttagttaaaggtgctttagat
gcaagaggctctaaacttatgccggacaaaaaagactttggatatagttttccttgcgat
ggacctggcagaggcggcacctgtgatatatctgcatgggatgcgttctatttagctgta
ttttggatgctaaatacaataggttgggtaacattttattggcattggaaacatattaca
atatggcaaggcaatgcaactcaattcaatgagtcttcaacttatctaatgggatggttt
agagattacttatggctaaattcttctccattaattaatggttataatccatatggcatg
aataatttatcagtatggtcatggatgttcctatttggacatttagtatgggcaacagga

tttatgttcttgatctcctggcgtggctattggcaagagttaattgaaactctagcatgg
gcgcatgaacgtacacctttagcaaacttgattcgttggaaagataaacctgttgcatta
tcaattgtacaggcaagattagtaggtttagcacatt-tttctgtaggatatgtattaac
ttacgcagcttttgtattagcttcaacagcaggcaaatttggttagactaaa-----ttc
gaattagct-a-----aaaaaagtcagtcataata-gttttatgactgactttttagat
ttaaaacaaaaacttactaaagtttcacctcaatatctacaccagaaggcaaatttaact
tcattaatgcatctatcgtttgagaagaaggttgatgaatatcaataattcttctgtgag
atcttatttcaaagtgttctcttgagtctttatctacatgtggagaacgtaaaacacaat
aaattcttctttttgtcggtaaaggaattggccctactgcaacggcattagttctagatg
cggtatctagtattttgtcacacgacgtattaagtataatagaattatatgctttcagtt
taattctgatttttgtctgctgagtaattgtcatgttaaaaagatcttattatttaagaa
ttttagagacaacacctgcgcctacggtacggccaccttctctaatagcgaaacgcatac
cttgctcaattgcaattgcattaattaattcagcagtcatcttaattctgtcaccaggca
taaccatttctgcatcagtaccatcatcagcagtaaactgattaatagtaccagttacat
cagttgttctaacataaaactgaggtctatatcctggaaaaaatggagtatgtcttccgc
cctcctctttagttaaaatataaacttctgcttcaaattgagtatgaggtgtaattgtac
caggtttagctaataccatacctctttcaatatctttttttctgcacacctcttaaaagaa
ttccaatattatcacccgctagaccttcttctaacgtttttttgaaacatttctaatccag
taatagtcgttgtacgagtttctcgtaaacctacaatttcaattgtgtcaccaactttaa
taatgcctctttcaattctaccagtggcaacagttccacgtcctgtaatagaaaaaacat
cttctacagccattaagaaagttttatcgacatctctctccggcgttggaatatatgtgt
caactgcttccataagtgaaaaaatcttgtcaacccatttatcttcaccttgcttagtag
ctgggtttttcgtcacagcttctaatgccaataaggcagaacctgcaacaaaaggaatat
catctccaggaaagtcgtattgacttaataattcccttccttctaattctactaattcta
gtagctcttcgtcatctacttgatcttccttatttaggaatactactaatgtaggaacac
ctacttgttttgctaataaaatatgttcacgagtttgtggcattggaccgtctgccgcag
atacaactaaaatagctccatccatctgagcagcacccgtaatcatatttttttacgtagt
cggcatggcctggacaatctacgtgagcataatgacgattatctgtttcgtattcaacat
gagcagtattaatagtaatacctctagctttttcttctggagcggcatcaatttcatcaa
attttttcgctgcagtagaccctaaagttgataaagtcgcggagatcgctgctgttaaag
ttgtcttaccatgatcaacatgaccaattgtgccaatattgacatgaggtttttttacgtt
caaatttagatcgagccatgctttttgttttccttatagtaaaagttgctttatagttac
ttttaacgataaatcttgtgaattgaaagatttattatttaatacagacaaga-----ta
caatataactaatctaatatcgatagtgagcaaacgctttatttgcttctgccattctat
gtgtatcctctctctttctaatagaatttcctgtttcattagctgcatccataatttcat
tagctaatttcatagacatacttttaccagatctgtctctagagaatttagtaatccatc

ttagtgctaaattcgtacctctataagctcgtacttcaataggaacttggtaagtagaac
caccaactcttcttgcttttacttctactagaggagtaatatttcggatggctttttcta
aaatatttaaaggatctgattctgttctctctttaacgatatctaaagcctgatatataa
ttctttgagataaagtttttttaccacttttcaaaatacgaacagttaacatacttacga
gtctgcttttatataaaggatcaggtgatgcaaacctttttttagctgtattacgacgag
acatagtatttaaaattggttattgtaataa-tgttc-tattagcaggtatttaagattt
aggctttttttgtcccatatttagatcggcttttacgacgatcttttactcctgcagcatc
taaagtaccacgtactacatgatatcggactccaggcaaatctttaattcggccgcccct
aatcagtaccacagaatgctcttgaatattatgacctacgccaggaatataagctgtaac
ttcaaatccagatgttaatctaacgcgcgctacttttcttaaagcagagttcggtttttt
aggagttgtagtatatactcttgtacaaacacctcttctttgagggcaactttgaagagc
tggagattttgtttttttatgtatttttcgtctttcggatctaacaagttgttgaattgt
tggcataataaaattgatttaaagtctttaatagatactatttgaacctgctttacatga
attggctagtttttatttatcttatacttacgcataaatctttctactctaccttctgta
tcaataattctctgtgaaccagtaaagaatggatggtttcctgaccagatatctacgtgt
agttctggctttgtggacccaatcgtcataattaactggccgtcacagtaaacttttgct
tctggataccaatttggatgtatattatcttttgccattgtttttataattatataaatg
tatcaataataactattaacgcttagaaaattgaggagcctttcttgctttctttaaacc
atattttttttctctcttttactcttggatctcttgttaaatagccttcagacttgagtgt
cgttctattttctggattaattgagcacaatgctcttgcaacacctaaacgaattgcatc
agcttgccctgtcaaacctcctcctctagcatttacatggatatcatactggtttagtag
tcctaaaacttgtaatggtgcatatgaaactcttaagtaattaggactaaattgaagata
agactctcctggtataccgtttataattaaattacctgaccctgggactagtcttacttg
tgcaacagaacacttccggcgacctgttccagaatagattgcgcgagttttaattaattc
tgtcgacataacattccttgataactaaataaatatacattctttatactatatactctt
gtggcttttgcgcgacatgtgggtgaattgggccagaatacactttaagcttcgtaaata
gttttctgcctaatggacctttaggaagcatacctttaaccgatttttcaataattctgt
taggtaatcttgtctgaagctgatcaaatgtttcaacttttaatccgccaggctgtccag
aatgtcttctgtatagtttttgattcgttttatttccacttacagatacgtgagcagaat
taataacgattacataatctccagtatctagatagggtgtataagaaggcttgtttttac
ctcttaagatattagaaatatgagtagatattctaccaagtgtctgatttttagcgtcta
taacataccaatgagaattagtatttaatgagggtgattgcgttttattcatgaaaagat
taatacttttggttgacaataagagaatatgtaaatggtttacattcaatttctatacaa
gtagtattt-aaaaatatta-------------ttaacaaagagaacttttctaacagtt
atttaaattgtttgtcagcaagaaaatttctagttatcaataacttatactatttttaat
aatataaaatatattaatcactttttttcttttggcaaacttatacctagtttttttttgc-

aaggcatatataacttcttcagctgatttctggccaaaattttaatttctagcagttct
tcttgagagtaatctagtaaatctgcaatagaatggatttgagctcgctttaagcagtta
taagccctaacagataactgcagttcttctattaaaacttgactaatttttttatcttct
ttgctacgataattatctgctgatttaaagtctaaatttcttagtgaacaaaaaagatta
gttaaaactgtagctccttgacttattgcttcttggggagatatgctcccatttgtccag
atttgtataatcaatctatcttttatgctgttgctaccaatacggacttcttctacttta
taattaactttattaactggcataaaaacagagtctacttgcaaaaaatccacagataat
tcatctacagctttttcagctaagcgatacccgcaatttttttcaattttaaactccatt
tcaaatattgtgttattgcaaatagttgcaatatactgtctaggatctactacctctata
tcagaagataattcaaataggccggctgtaactatagctggcccttgaactctaattcga
ccaatttgagattctttgttataacttttaaatactacttcttttagattaagtaatatt
tctaacacatcttctcttaccccaggaattgtggaaaactcatggttcactccagcaatc
cgtacagcaactatagcagtaccttcaagatctgacaatattgatcttctcaaagcatta
cctaatgtaataccttgtccttgatttaatggttctattacaaaactaccgtactgccca
cgcgccccatctgttcttgactctatgcattcaatttgaaattgagccacctaagaaagc
tcc-tttaaaataatcagtgattagtaagtctctctaaaacaagaaggaatttatactcg
gcgtttcttaggagggcgacatccattatgaggtacaggagtaatatcttttattagagt
aatctctaatcctgcagcctgcaaagctctaattgctgtttctcgacccgctcctggacc
atttactagaacttcagtttggcgcataccttgatccatagcttgtctagctgcttttc
agctgctgtttgagctgcaaaaggtgttcctttttagctcccttaaatccacttgcacc
agaggatgaccatgataatgtttctccttttaaattagtaatagtaacaattgtattatt
gaatgtagatttaatatgtgtaataccgttaactgcattacgtttagtttttcttgctcc
ggatttttttatttgtctagccatcgtgtctcgtctattatatagttaattaaaagatta
ttttcttggagctttttcttacctgctactgtttttttacctcctctgcgtgttctagc
attagttctagttctttgtcctctcaaaggaagaccaagacggtgtcttctacctctata
agtactaatttccataagtctcttaatgctcatagactcaaaacgtttgagatccccttc
aatctgataattagactcaagaatttctcttatactgacaacttgttgatcatttaaatc
ttgacacttaatatcagcgtctatgtttgtttttctaatatttctttcgagcgagatag
tccaataccataaatatatgttaaagctatctctattcttttgtttcttggaagatctac
tccagcaattctggccactttgtcttgtctccaataaataatatgtat-ttttataacgg
ttatttata-agcttagccttgtctctgcttatgtttaggattagtgcaaattaccatca
cctttctgtgacgccgaattattctacatttttcacacatttttcgaacagaaggacgaa
ctttcatattaa----ctctatatactaggaatattactatattaactctattaattatc
gacattaaaaatgtcaacaatatattgtattactttaactgttgtagattttacttcgtc
atactatcatatttttttgatatgacgtatgtctgaatttgcttagctgtgtctattgcg
actccaactaaaataagtaaagacgtagccccgagacctcttaagttttggatctgagta

acctttctattataaacggaatcagcgctactgtaaataaaaacgaggctcctaggaat
gtcagtctatttaatatgacttgtaaataatcaatagtcgcttgacctggacgaatatta
ggaatgcttgcacccatttttttaaatttatagcaatatcttctggattcattactatt
gatgtataaaaatagctaaagaaaaggatcaaagcacaataaagaagaaggtatagtgaa
ccattaggacaaaacaggtataggatttggagtaatgttttattttgaataatttgggtt
agataagatgggagggccatagatgcagatgcaaaaacaataggcataactccaccttga
tttaatttcaagggcaagtaactatttggatccaaaattgaagattttcctagctgtctt
gctgaaataatttaattcttcttgttccttcttgtacacaaattgtaattattatcatt
aataaaaagatcgctataaataatccgaacttaagacttgcattactataactagcatca
aaaaatgactgtgtaaaattctttggtagtcctgacacaatgttttgaaaaataagtaaa
gaggctccattaccaattccctttccgtaattaactctgataaccacataataatcata
gagcctgctgttaaagctaaaacagactcgcagacaaatgcaaaattccaattaaaaaca
tatggttttacccatatagagattgctccagattgtaaagtcgcccaacctagagctaag
taccttgtaatttgagttatttttggcggcccaattcaccttcttccttctgtaatttt
tccagattggggacaattttcgtaagtagctgcattacaattgaagagttaatataagga
acaatacctaacgcaaaaatccctattgttgaaaaacctcctccagaaaaaatattcaga
aaatttactaaagtatttttttctacacttgcataaaaggcgtcatgatctatacctgga
acaggtataaatattcctaaacgtgctaaaactaatagaaagagagtaaagataatacga
tttcttagatcactttttggctcataaataaattttaaatgaaaagagatacttt--tt
tatgtaaaaaccctattccaagacttgtataagtttaaaatagggaagtaa---aatatt
atagtaatattaata-taatattctatgtagaatacaggctttctattggaactcctcga
tctttagctgcttcactaaaagttcttaattgagttaagccattgagaacagctcgcgcg
ttatttaatgtattattagaacctaattgcttagctaatatattctgtacacccgacaat
tctaggactgtccttacagaacctccggcaattacacctgagccaggtgcagagggcctt
aatataactttcgcggcaccagaaattccgttaataggatgaggtatagaattcgatttt
gtcagtggaactgtaactagatgtttttagcatctgttactcctttttttactgcacca
attacatcgcttgcttttcccacgccgacaccaacttggccttgctcattaccaataaca
agaataactcgaaagcttagtttttacctccttttacaactttagtaactcttttaact
tgtacgactctttcttcccagccactatctttatcttttcctttgctctgtttttacga
ttggccattttaagaattattcctaattagttaatta-cgattctagaaacccatgcctg
cttccttagcagcttcagccaaggccttaactcttccatgatataactttcctcctctat
cgaagacgacatttttaatgccttctttcatagactgttctgctaactgtttaccaacta
cgcgagaagtatcacagtttggtcttatgttatcagattctttattatttaaattaacag
atgatgtagctactaatgtaataccttgtgtgtcatcaattatttgtgcgtatatatgtt
tattagatctaaatacacacagacgaggcctacttgaggttccttgaactttttccgaa
ctcttttatgcttatgaattctagtttgtttagtgtttagtttcattattatttaccttt

tccagctttcccagcttttcttctaacaaattcaccttgatatctaattccttttccttt
ataaggctcaggaggcctaatagaacgaatagttgaagcaacctgaccgacaacttcttt
atctatgccagagacagtaatatttgtgttgttttcaactttaatttcaatattggcagg
aggtttaattttcactacatgactatagccaacacttaagattaaatcttggttatcaat
ttgagaacgatagcctacaccttgtatttgcagtttttttaaaaaatccattagaaacacc
ttcaatcatattactaataagagttctggatagcccatgtaactgactagccatttttgt
ttgtccactcgtcttgacagcaattgtgtcgttaagtatttctaaattaataccagctgg
taaagttctggatagggtacctttaggtcctgtcacggtgatagtctgaccatcgaactg
agtactaagatttgtcggcaataaaattatttttttccaatacgagacat-attccacc
tataaaatagttaccaaatatagcataatatttcaccaccaagaccatcatgacgagctt
gtctatctgtcataacgcctctagaagtagaaataagagcgatgcctaaacctccaagaa
ctctgggcaactctttatgatttgcgtagactcttagtccgggtttactaattcttttca
gagcagtaataactggttgacgattttaccattatacttaagagaaatcattaaatgag
tctctataccttcacccatttgttcgaaattttgaacaaatccttcttcttttagtactg
ttgccatattgcatgtcattttcgttgctggaacttgcacaatttgatgtcttgctaagt
ttgcgttacgaatacgtgtcagcatatcggcgatcgtatcgttgaccaccttgatcctcc
ttgcga-----tgaattatttgaaatattttcaagattccctgaaaggcatccctagctt
ttttaacaaagctagaccttcttgatctgttttagctgtagtgacaattgatatatctag
accacgaatttgatcaatattatcatagtctatttctggaaagatcaactgttcacgtaa
acctaaattgtaattacctttgccatcaaaacttctaggactaattcctctaaagtctct
aattcttggtaatgttaaattaattaatttctctaaaaaagaatacatcttgtcttttct
tagatgcacaacaattccgataggaacctcttctcgaattttaaatcctgcaatagattt
tttagcttttgtgacaattggtttttgtccagttattaatgttaattcttgaatactact
ttcaagagccttagcattctgagaagcttcacctaagccacggttaatagtaattttagt
aaacctaggaacttcatgtacatttttgtactgaaattcatcttttaaagattgagtaac
agttgttttatattttttcttttaatcctattgccattatatttcatcaattatttaata
agttcgccagtctttttcagttttcgaa-ttttttggcctttatcattaattattactga
ggatcgactagcaatattattttgttcactaaataacataacgttagaggtatgtattgg
agcttcaaatttgataatttctcccgtttctccttcttgctgaggtttttttatgcttcac
tttgagattaattcctttaacaatcaccttatttgttttataaatgattgcaataatttc
acctgtttttgttttatcacttccagaaataacttgaactaagtcccctttttttaattt
aatttttgtattatttttggttgttttagaaggacctttcattatactacctccggtgca
agagaaactatttttagaaaaatttttatctcttaactctcgggctataggtccgaagact
cttgtgccacgtggattattatcttgattaatgataactgccgcattatctccaaatcta
atgctcataccgtcagttctccgtaaagctttacgagttcttactacaacagctctgaca
acatcagatcgtttaactggcatattgggagacgcatctttgactactccaataataaca

tctccaatagatgcataagaaggattactagtgcctaatactctaatacacattattttt
ctagcaccactattatctgcaacattaagatagctttgagtctgtatcatacttttattc
tgtataagattaattatcaaaagacttagataagatattaaccattgtccaacactttgt
gcgacttaaaggtcgtgtctcttgtattgtaacaatatcgccgattgtgcattcattatt
ttcatcatgcgctttatatttctttgtccggatcatagtttttgcgtactttctatgaga
gattctatttctacagctactactatagttttattcatttatcgcttactactttacc
tgttgtttcttttaaaggcatagtctttatatttttatatat---------aattaaat
tttaagattattacatggttgacttagtacgagacttttcaacagttaaaagttgggcta
atctatgcttagaatgtttaaatagatgaggctggaaatcttgccttgtggctcttttta
gccttaaatcaaaaagctctcttttattacaaggatttcttcagctaaagaagaagagt
ccaggtttgtaacatctgatattttagggaaagtcatattttaggattctgttgtattcc
gaactataaatttagtcttgattggtaatttataagaagctaatttcatagcttgttgag
cagttttttgtggtacacctgtaatttcaaatagaatatgtccaggcttaataactgcaa
cccaatattctggagctcctttacctgaccccatgcgagtttcagctggacgggcagtga
ctggtttatctggaaatactctaatccatagtttaccacctcttctcacatatctagtaa
tagttcttcgagtagcttctatttgtctagaagttaaccaaactggctctgtcgcttgta
atgcataatcaccgaacgcaattgtgttacctttgctagcagaacctttcattctacctc
tatgttgttttctaaattttgttttcttggggcttagcataaaaattaaaaaataataat
aattaagaagctgttgaatcaggagtttcctgattataagcagattctagacttttcgat
tctggcaaaatttctcctttaaaaagccaaactttactccgagcacaccatacgtagtg
tgagcttggcgatgacaataatcaatatcggctcttaaagtttgtaaaggtacacgacct
tctctaacccattcacttctagctatctctgcaccattaagccgaccagatacttgaatt
ttaacaccctgtgtatttgctctttgagctctttgaactgcttgtctgacagctctacga
aaggccactctttttttctagctgttgagtaataaattctgctactaaggttgcttcagaa
tctggatctgcaatctctacaacatttactctgagttgtttgctagggtctaggattaag
gacagcgattttcttaaagactcaattcccgctccagattttccaagtacgattcctggt
cgagctgttgctatgagaatttctacttgatcaactttacgattaatttcaattttagca
atactagcattactaagttttgaatgtataaatgagcgaattttatgatcttcttgtaaa
agaactgggtagtctttagagttagcaaaccatgaagaacgatgtttttgggtaatgcct
atgcgaaagcccaaaggatgaatttttttgacccacagtatccttgattataagttaaaag
ctataatgactaattgacgttaaa--------agactacagttctgaaacacctaatgta
atatgacaagttggtttatgtattggaaaggctcttccttgtgctctaggctgaaatctt
tttaaagtcgggcctttgtctgcaaaggctttactaacaaacaattggtttttatttaat
ccatcattatgctcagcattcgctgcagcagattccaggatttgctttatatgagaacag
actcggtatggcataaattccagaataattaatgcttcttggtattttctacctctaatt
tggtctaatacacgacggactttatgcggagaaagacgaatgtatttccctactgcttta

gtttcttttacgtttttttgtaatactcatagttatagtttaacgacgggcttttctgtca
ccttttacgtgagtgcggaaagttcttgttggaacaaactctcctaatttatgtcctacc
atttggtctgacacaaagacaggaaaatgttgtttaccattataaacagctattgtatgc
cctaccatatcgggaataatagtagatgctctcgaccaagtttttaggacttcttttttt
cctgaaatatttaatgcttctatacgtttaagaagactaacatcaataaaagggccttta
tgtatagatcttgacataataataaatcattagattcttaataaaataaattctatttac
ggcgacgtaagacgtacgggttactatatttatttggatttcgtgttttaacacccaatg
caggtttaccccaaggtgttacaggacgagctcgtccaattggagatttgccttcaccac
caccatgtggatggtctacaggattcatgacaacacctctaacagtaggtcttttaccta
accaacgatttctaccagctttaccaagagtaatattactagcgtcaatattaccaactt
gtccaatagtagcgtagcattcttttcgaatcattcgaacttcgcttgaaggcaatttta
cagtaacaaaagtaccttcttttgctactatttgagcataagttcctgctgcacgaacaa
tctgtcctccgcacgaaggtcttaattctatattatgtactgctgttcctaaaggaatac
tagataaaggcaaagcatttccaacttcgataggggcagtaggaccagaaagaaccatag
atcctacactgagagatcgagggtgtagaatatatcttttctcaccatcaagatagtgta
ataatgcaattcttgcatttctattcggatcatattcaatagaagcaactttagcaacta
tattatgtctatttcttttaaagtcaattaatctgtactgctgcttatgtccaccacctt
tatgacgacaggtaatgacacctctattatttcgacctttgcagaaatggtgtttaacta
ttaatgatttttctggcttatcagtagtaatctctgaaaaggtagaaaccgttctatttc
ttgtccctggtgtgtaggcacgatataaacgaattgccatataagaagatttgagaagag
taaatgttgaa------ctatgtgatatatttaagtttctggaaacaaattaatagaatc
ttctgaggcaagtgttacaattgctttcttgtaatgtggtcgcttccctacgaatctacc
gatgctcctttttttcttaggaggatgacaagtgttaacacctgtaacttgcacgttgaa
aatatactgtatagcagccttaatattaattttgttgctttgggatcaacggcaaaaca
atactgatttcttctaataatttagttgtcttatcagtaatgatgggatatttaaccag
atctaataaacctcttgaatcaatgctatccattatatacctcttgtattttagataaag
catcaactgtaataataattttatgtgcagctaacaaagccataatatttagtgtatcag
ctgaaataagttctacgttgtgtagattgcgaatagaaagataaacattcggatcttttt
tgtctacgatgactaaaactttttatttaaatcaagattccatcgatgtatagcttcca
taaatagtttagtcttgggctgatgaaaataactgttaaaattttctacaatcagtgtat
taactgatttgttatttaatgctgtccttaatgctaattgtctttctttttttattcattt
ttttggtaaaactacgaggttttggcccaaatattacaccaccacctctccatagaggtg
aacggatagaacctgctcttgctcgaccagtcccttttttgacgccatggtttacgacctc
cacctcgaacttcacttcttgttttagtattagcagaaccctggcgcttttcattacttt
gctttactaaagctctatggactaagtacatcccagaatcttgactgaccttaagattta
aatcagcgttgccacttacttgaccttcccaattatagacttgataatttaattgcgtgt

taactgtcatgaaatatatcgtatgaaagaatattgctacttactaattttactaaagc
gccaggtttaccaggaacagctcccttaacgatcaacaaatcattctctgagttaatact
tacaatttgcaaatttttattgtaactttttattaccaagttggccagccatgttttt
acctggataaactcttccaggcgttgtaccggctccaatagagccaggttgcctatgatt
tttcgaaccatgtgacattgggcctctactaaaatgatgcctcttttggtaaccggagaa
acctttacctacacttctagaagagacattaatcttttggcctacttgaaatagatctgt
agatagtatttggctaacttcgaaatcatcagtagatttcatttcgtattcacgtaaata
ttttagtgggggagcctgtgatttttttaagtgtcccaacaatggtttatttaattttttg
ttcagcaacttgcttgtaaccgacttgaatagcattgtagccatcagtagatacagcttt
aatttgagtaataacacatggcccaacttgaattacagtaactggaattgataaaccagc
ttcatcaaagaattgggtcatacctactttagtaccgagtataccaacagacactagatt
tctccttttttgagagatttatactatcattgtacataaaaaattctacttcatagttactg
aag--taaattaaactaacagatgcatgatattattcatgacaacataaaggttaaaaac
tttacctatttaatctaaaatagaaacagctttttgtctagttacagcgtatataaatta
gaagtatgcttattaaatttttataaactact-tagatttaactgatattgatatccgt
aacttacttgcagtaatatc----aatcaacatgtataagtttaattgcctaaactagaa
ccagctattattagaaataattcaagtatagataaaagcgtatttgaacttttatatatt
gacaaatataatagaatatattgaggttcggtaactacacctttttattttttacatttat
tgtgca--gtatatatacatctaatgcagtaaacataagaatataac----tgtatagca
aatgtaattacacaaattagagaggtattaatgggcaaagtcgttggaattgatcttgga
acaacgaattctgtaattgctgttatggaaggaggtaaacctaccgtcataccgaatgca
gaaggttttagaactacagcttctgttgttgcatatactaaaagtggagataaacttgta
ggacaaattgccaggaggcaagctgttattaacccagaaaacactttctactctgtcaaa
agattcataggacgtaaacagaatgaaatttcgcaagagattcggcaaacatcatataat
gttaaaactagtggatcaagcataaaaattgcttgccctgcactgaataaagattttgct
ccagaagaaatttcagctcaagtactgagaaaacttgttgaagatgctagtacgtactta
ggtgagactgttacacaagcagtaataactgtaccggcttactttaacgattcacaaaga
caagctactaaagacgcaggtaaaatagcaggcttagatgtattgagaattattaatgag
cctaccgcagcttctttgtcttatggactagacaaacaaaataatgaaacaatactagta
tttgaccttggtgggggcacatttgatgtatctgtattagaagttggagatggagtattt
gaagtactctcaacttctggagatacacatttaggcggagatgactttgatcagcagatt
gtagaatggctaatcaaagattttaaacagaatgaaggtattgatcttggtaaagataga
caagcacttcagagattgaccgaggctgcagaaaaagcaaagatagaactgtcaaattta
actcaaacagagatcaatcttcctttttattactgccacacaagatggtccaaaacattta
gaaaaaaactgtaactagagggaagtttgaagaactttgttcaaatttaatagataaatgt
agtatccctgtaaataatgctctgaaagatgcaaaactagaagcttccagtattgatgaa

gttgttttagttggtggatctacaaggattccagccatacagcaaatggttaaaagatta
attggtaaagatccaaaccaaagtgtcaatccagatgaagttgttgctattggtgcagcc
gtacaagctggagttttagcaggcgaagtcaaagatattctattactagatgtgacgccg
ttatctttaggagtggaaactttgggtggcgtgatgacaaagattataccaagaaatact
actattcctacaaaaaaatcagaagtattttctacagctgtagataatcaaccaaatgtg
gaaattcaagtacttcaaggcgaaagagaactaacaaaagacaataaaagcttagggacg
ttccgattagatggcattatgcctgcacctagaggagttcctcaaattgaagttaccttt
gatattgacgctaacggaattttatctgtaaaagccaaagaaaaggctactggtaaagag
caatcaattactatatctggagcatcaactttacctaaagatgatgttgaaagaatggta
aaagaagctgaagaaaatttcgatacagaccaaaaaagaagaaagaatattgacacaaag
aatcaagcagagtccctatgttaccaagctgaaaagcaagttaaagagtttgaagacaaa
attagccaagatttaaaaataaaaatagaggagctaattacagagcttagatctagtcta
gagaaagaagaatatgacaatattgaatccatttctcagcaattacaaaatgctctgatg
gacattggaaaaaatgctgctcagactgaaagtaaagatacaaaagcgaaggatgacgac
actgtgattgacactgatttctctgaagctaagt-aaaagta-agcgggtaacgcgattc
gaacgcgcgacatcaaccttggcaaggttgcgctctaccactgagctatacccgcataga
ttgtattattacaaaaattaagatatttgtcaatctgtta------atctatac---atc
tagactattaattttggaattacatattagctaactgttgtaaatataacaattgctata
attttttaa-------aagtacttatatcat-tatataagtactttttatatt-------
-caaaaatacaaataagtattatag-------ctaaactactaaggagttaactaagcta
gtaacaattactaatcctgtccataagccggctcctgtatatattaatccttttgattgc
tcccattggccaggggatgctaaaacaacaggtacgcctactactaacacagtcgataat
gcaattaataatagcactaataattggatcgcaataatcatttaatatgttctcctgaaa
aattcaaatgttaataatactgtatataatataatgaagcttttgaccttcagcaattta
tctatgaagaaataatttaaaattattgttttctaaataaaccttaaattaagtattaaa
atctttattgtaaacaataatactatattttttta--gtagataaaaaatttaaagaaag
tctttttttaaaaaaaaattaagataaggtgatatgctgtataaaaagctaagtattatta
attattagagaaatttaaa-ataagaggtaaaacatatgaactcagcccttttttttagca
aaactaccagaagcttatgccattttaaaccgatcattgatatcttacccgtaattcct
gtatttttcttcttttagcttttgtttggcaagcagcaattggttttagataacattta
ggttataaaaagaataacaaatatacagagtgccaatttttgagctctaatcttatatct
tacacaataataactattatggttgaacccttactatcaggaattgttcttggacttatt
cctattactttattcggattattagtagcagcttatttacaatatcaacggggcaaccaa
ttaggactgtaaagcctaaattatagatatttataaaaactacaataattatagtttttta
taaatatctacaaataagctaattttaaatacagaaataatactattgaaactagagtga
taacaaaaaaaatttatgtaa---taa---------------------------------

---------------------------caagctaattgtttaccagctcgacttagtaac
tcctggtaaaagacattcatgagacatttctcgaaaaacatggcgagataatccaaaatc
tcgataataacctctacttctacctgttaaccaacatctatttctccctctaacagcggc
gctgtttctaggcatttcttggagtttttgtcttaaatccattttgtcagaaaagctgcc
agcttctttcagttgatctttaatagccagtctttttaaataatattttttttgcaagttt
cgatctcttaatctctctttggatcatattctttttagccatgctgtaa-aactctcttt
taaaattatttattcaatatattagctaaaaaagctcctgcctgcaacttttatatattt
aaaaataaatagcataaagtagtgaaatgcaattgatatttaatcaacgatacc---act
ttatgctattatcaaaaatttatttagtaaagttcttgttcaacatgagttgaaatagtg
caatcagatgaaggatatgcaatacaggttaaaacatagccagctaaaagttgatcatca
tctaagaatgattgatcagcttgatctacagttccttctgtaactttacctgcacaagtt
gaacaagctccagctctacaagagtaaggaagttcaattccttcttcttctgctgcatct
agaatatatgtatcctctgcacaattaaatgtgacatcaattccttcttcttcacatagt
aaatgaattttataatcagccatttcttcttactccataataaagatacacttgaaactt
aataaagcaaaat-aatttaaagaacttaccaaaaaattatatactagtttcattatgca
tgatagactggaaaaaaagaaaatagtatactattctgaaaaatcaagctacatttccta
ctaaatagaactatagtaggtccaaagtatatacataaattcatatagttaaaattactt
ttatcatttaaatagctgaacttgatagta---------tagattgatcatc------aa

gctagtcttctgc---caataaataattaattatgatttcaatgcttaatcaacaagtag
aacttaagcctattcttaaatttcagataacacaaatatattcgcattctcttattcagg
aaattaaagccttaataactagacttgttttgcaagtttggagaaggccagctacattga
tggcaggtattatccaacctttattatggttaattctatttggtgggcttttctacaatg
ctcctataaatttgttcactattaatacaagctataattgtttttgagctctgggatta
taattttcacctcttttactggagctttgaattcaggtcttccattaatgtttgatagag
aatttggatttttaaatagattattaacggctcccttagtatcgaggacttctatcattt
tatcttctgctacttttatgacttgtattagtttaatacaagttgtatttatagttacag
cttctctttttatgggaaacccacctctaaatagcgatagtactatgatttttggactta
tgattctattagtgactgtaggagttacaatgcttagtttagctttatctttcactctgc
caggtcatattgagctattagcatttattttagtagttaacttgcccttttttattttcta
gtacggctttagctcctttatatttcatgccgccatggcttcagttaattgcaagtctca
atccattgagttatgcaatagaaggtacaagatacttatattcaagcgtaaactggaatt
ttacagagtgtgtgattaagattagctggggagatatttgtttagggcaaattattattt
tattaatcgctttagatataatggcagcttatcttgtgtctaatatattaaaagctaaac
ttaatta-----------------aaattttaataaaaaatttattaaatttattttaca
aagattttactatataatactaatagtagtatggataattgaaatagaaatagtttttc
aaaaccaaagctattatcaacaatgataaacatttgtaagaaagtcaacaaagtatgttc
ttattcataggaggcatgtagtcaatgggactaccatggtaccgtgtacacacggttgtt
ttaaatgatcctggacggttaattgcagtccacctaatgcatactgcacttgtagcgggt
tgggcaggatctatggcattatacgaactagctgtatttgatccttcagatccagtatta
aatccaatgtggcgacaaggcatgtttgttatgccatttatggctagactgggcgtaaca
gattcatggggaggatggagtataacaggagagagcgtatccaatcctggactgtggagt
tttgaaggtgtagccttaactcatatagtcctttctggcatgctttttctagctgctatt
tggcattgggtttattgggatttggaattatttagagatccacgaactggcgagccagcc
ttagatttaccgaaaatttttggaattcatttattgctatcaagtctactttgtttcgga
tttggagctttccatgtaactggacttttggaccaggaatgtgggtatcagatgggtac
ggagtaaccggaaaggtattaccagtagctccagcatggggaccagaaggattcaacccg
tttaatcctggaggagttgcatctcaccacattgccgcaggtactgtaggtatattagct
ggtgttttccatttaactgttagaccaccacaaagactgtatcgtgctctaagaatgggt
aatattgaaactgtattatcaagtagtatctctgctgtttttttctcagcttttgtgact
tgtggtacgatgtggtacggctctgcaactacgcctattgaattatttggtccaactaga
tatcagtgggatagtggatattttcagcaagaaattgagaaacgagtagaaaatgctatt
gctgatggtgctgcacctagcgaagcatggtcaagaattcctgacaagttggcattctat
gactatattggtaataatccagcaaaaggaggattattcagagcgggccctatgaataaa
ggcgacggagttgctgaagcatggcttggacatcctgtattccaagataaagaaggaaga

gagcttagtgttcgcagaatgcctgctttttcgaaacttttcctgtaattttagttgat
aaagatggtattatacgagctgacattccatttagaagagcagagtctaagtatagtatt
gaacaagtaggtgtaacagctagtttttatggtggcaaattaaatggccaagttttcaac
gatgctcctagcgttaaaaaatatgcaaggaaagctcaattaggtgaggtatttgaattt
gatcgaactacattagaatcagatggagtgtttagaagtagtcctagaggctggtttaca
ttcggtcatgctaatttcgctttaatttcttctttggacatctttggcatggttcaaga
actatcttccgggatgtgtttgcgggaatcggagctgaagttactgaacaagttgaattt
ggagctttccaaaaattaggagatagaagtagtaaaaaacaaggagctgtataagaccaa
agtcaaagatagtctttatc---aaatatttctaatattaaaatagttaagacaaagttt
attatctaatagttactaatcaggagaatttatggaagccttagtctatgtatttttact
aacaggaacgctaatggttatattctttgcgatctttttagagaacctccaagaatagc
aaagtaacttgattccgtttaaacttataataatagaaccactttccaacttattagtgg
aaagtggttttattgtagattacattgtattattcttcatgctcttcaaaaggatctcgg
agatcttttgaagcaggaccaaatgctgtgtatatcgagtaacctgtaatacctaaaagc
agactcgaaataaaaaatactaagaactgttgcagtttccataatttaagcagtattaaga
tgaactttttctataatgatattatcaatattaaaacatttaattattaaaattatcaaa
atatactatggcacttagaactagacttggagaaattttaagacctttaaattcagagta
tggaaaagttgctccaggttggggcacaactcctattatgggaattttcatgttactatt
tttcttgtttttattaatcattttacaaatctataattcttcattagtattagaaaatgt
agatgtagattgggctactttaggtag----ctaactagagaagttctttga--------
ttacacaatattaaagacaac----------------aagcgatcgctattagattatag
tagcgatcgctttaaaagtattttactatatatccagagaaaattatttacctaaattaa
ttaactcggtatctgcaaaactatttgtgttaacattattatagttaactttttcaaacc
ttactaatactggatatttgataccacttttatcaattgctgcaacagtaccaatttcct
gataccagtacgattcttttctcaatatttttacttttgagcctctttccatatttttag
tcctcagatagattattaaagattattatattaaaaaatt-taaaaaactaaacaaaata
tataaaaatactaccgacaattgtaatatataactaataaaaggagagagagagggattcga
accctcgataacaaaagttatgacaattttcgaaattgttgcaataaaccactctgccat
ctctcctgaattaaaatactgttttaaaaaattagacaagaataacatagaactaaggta
gttgcctagaaaattaaaactatgttaaatataaattataatctttctatagatatattt
aaaaaatatgtacatagaatctatatcagcttatttcaagatttttaatcttatacaat
ttttactaaataaataaattctctcataatatgaaactatcttggaaaacacttcttctt
tggtctttaccaatatttgtaattggtttctttttctggcaaggtttttaggtccaact
actacagatgttggcagtaatatcgcaagttctagaatgacatatggacgatttttagaa
tatttagatatgggttgggtgaaacgggttgacctctatgaaaataatcatacagcaatt
gtagaagctgttgggccagaattaggaaatagggttcaacgaattcgagttgaactgcca

gcaagtgcgccagaattaattacaaaattacgcaaagccaatgttgatctagatgctcac
ccccctaaaagtacaagtgcagtatggggactattaggcaatttactatttcctttacta
ttagttggcgggttagctttcttatttagaagatctaataatgctagtggtggacctggt
caagcaatgtcatttggcaaatcgaaagctttatttcaaatggaggctaaaacgggagta
gtatttaatgatgtagctggagttgaagaggcaaaggaagaatttcaagaagtggtaaca
tttttaaaacagcctgaatcatttactgctgttggtgcaaaaataccaaaaggcgtttta
ttagttggacctcctggcacaggcaaaacattactagcaaaagctattgctggcgaggct
agtgttcctttttttagtatctcaggctcagaatttgtagaaatgtttgttggtgttggc
gcttctcgtgtgagagacctattcaagaaagcaaaagacaatgcgccttgtatcgttttt
attgatgaaattgatgctgttggtagacaacgaggaacaggtgttggaggtggtaatgat
gaaagggaacaaacattaaatcaactattgactgaaatggatggctttgaaggaaatact
ggtgttattgtaattgccgctactaacagagctgatattttagattctgcattattaaga
cctggaagatttgatagacaagtttctgtagatgtaccagattttaaaggcaggttagca
attcttgaagttcatgctaaaaataagaaaatggaacctaaagtatctttagaaaccatt
gccagaagaactcccggcttttcaggagctgatttagctaacttactaaatgaggctgct
atcttaacggctcgacgaagaaaaaatgcaatgactatgtctgaaattgatacatcaatt
gatcgagtagtagccgggatggaaggcactcctttaattgacagtaaaagcaaaagatta
attgcgtatcacgaagtgggtcacgcaataataggcagtttattagagcatcatgatcct
gtgcaaaaagttacattaataccaagagggcaagcaagaggcttaacttggtttactcct
agtgatgatcaaagtctaatatcaagatctcaaatactagcccgtatcgtaggtgctctt
ggtggcagagctgcagaagaaatcattttcggtgacgcagaagttactactggtgcaagt
aatgatttacagcaagtaacatcaatggcaagacaaatggtcactcgatttggaatgtct
aaaattggacctttatctcttgaaagccaaggaggagacccatttttaggtagaggcatg
ggaggaggctcagaatattcagatgaagttgcaactaatattgataagcaagtaagggaa
attgtcagtgaatgctatgcacaagctaaacacattattatagataatcgagtagtgata
gatagattagttgatttactaattgaaaaagaaacaattgaaggcaatgaatttagagac
atcgttaaggaatacactgcaattcctgaaaaaaattactacatatcacaattttaa---
---------------attaaacgggactgacgggattcgaacccgcaacttccgccgtga
cagggcggtgctctaaccagttgaactacagtccc-aaaaagatgaatacccagagataa
tctcatagttagcttaaacttgtcaaattagaatattaaatatgaagagta---------
--------tttgtaataatactcttcatatttaata-tagtagctaaaatatttctttta
gaaggagagagagagggattcgaaccctcggtacgaagttaatcgtacagcagattagcaat
ctgccgctttcgaccactcagccacctctccatga---tatatattatatatatatcaaa
tatacttatatttaataggtgtgcataaatatttatggagctaagcggattcgaaccgc
tgaccctctcaatgccattgagatgctctaccaactgagctataaccc----ctttggta
agatattaagaaatggctcaagcgggatttgaacctgcgaccttgggcttatgaatcccc

tgctctaaccactgagctactgagccatagtataaaatattattaagtattataccataa
attatggtcaaatataaatcaataaaagaataattactatca-tttactacttttg----
-----------------tgcaagttgtaaatattcgaataaaatttcaaaattaaaaatg
tctttaactttttgttaga-------atta-ttgattacgaaa-----------gtattg
atatttagaattaac---
--------------------aataaatttg------------------------------
---tctttattt
g---

-------tagaca---
-------------------------------caaaaatattggaaa--------------
------------------------ttttcatcaat-----------------taattagt

------------------taaactataaccgacatac-----------------------

---aagaattattgaacta---

---cgttaaat---

-------------------------------------tgtgatgcatt------------
-----gggcttgatcaaaacttctctaaaatcaatagaatatagt---------------
---aattcaa
gtagatcaaatactaacaaatgtgacaaacgcttaaaaaaataata--------------
caattagaatcgtaaaaatttggtttttga-ctgtgttaaataaatttgattatcataga
tttaatatttaaaagtgttctattacttgta-----ttgagtaattgt-aatatttaata
tcttcttttttagataacaatcataatataagctaaaaaatagtttt---tattaaacaa
ctagcatagagccaataccttgatcagttaatatttctagcaagagagcatgatctattc
taccatctaaaatatgcgctgaagctacaccctgagccaaagaacgaatacaacaattca
ctttaggaatcatgccaccagaaattactgctgtttgagttaaatctctagcttcctgta
tattcagatgactaattaatgttgaaggatctgaagaattacgtaaaatgccgggcgtat

ctgtgagtagaattagtttttcggcattaagagcggctgctatttctcctgcgacagtat
cagcattaatattatacgattggccttccttgtctgctgcaacactagctataacaggta
tataattattattaattaatatcttcagtaatttagtatcaatattttgtacttctccaa
caaaaccaaggtttgcttttccatttggtctcggagtaataagtaatccatcttttcccg
acaaacctacacttttaccaccttgtttattaatagttgcgacaagatctttattgactc
ttccaactaaaaccatttccactatatccatggtaggttgatctgttactctaacgccat
catcgaattttggtaatattttgaggcgatctaaccaaaaattaatttctggtcctccac
catgaactaaaatgggacgtagtcctataaaagacagaaaaacaagatcactaatcactt
gatcttttagtttctgatttttcatagccgctcccccatattttataacaataattctgg
aagaaaattgctgaatataaggcagagcttcacttaagacttttactctttctgaattag
tcaacataaataaagtaggattgtgatatataaaattatatacacagcttgtgttaattt
aaaagataccaatgggt---------acttgaaaaaatagaaatattactaacag-----
----ttaaaactatagggattataattatgaataaattttgggataatgtattaagattt
cctcgattttagtcagcgtcattttaggattaattttgataattattagtcctttttc
gtgttattaaaaaaaccactgacaagtttttttttcatcatatcattggccggattaatc
acagttttggcaataataa-tacaaaaaatgataaatatcgaatgttgttgagagaaata
aataaatttaattaaaatttttgaacatatataatattatattatcattagatatgataa
ttt-atttccttttctat---aaaacccataaatattaaatattatttgataaatattat
gccactaaaacaaagagttagctctgaaaagaccggtgccttcgcacttttggatagtat
tgttaggcacggagttaaacatatatttggttatccaggtggagctattcttcctattta
tgatgagctttatgcttgggaagaagcctctctaattaaacatatccttgttcgtcatga
gcaaggcgctgctcatgctgcagattcttattctagatcaacaggagaggttggagtatg
cttcgctacttctggcccaggagcaactaatcttgtctcaggtatagctacagcacatat
tgattctgtacctatattagctataacaggtcaagttgggagagcttttattggtacaga
tgctttccaagaagtagatatttttgggattacacttcctattgtaaaacattcatatgt
agttcgtgaccctagagacatgtctagaattgttgcggaagcatttttttatttgtaaaca
cggtagaccaggtccagtattaattgatgttcctaaagatgtaggattagagaagtttaa
ttatttttctgttgagcccggaaaagttaatattcctggctgtaggccaattaccagcct
aaagtcaagacaaatccttatggcagctaaaatgatacagcaatctagccagccattgtt
gtatattggtggaggagccataatctctgattcacatcaaattattaaagaacttgttga
tttttataaaatacctgttactactactttgatggggaaggggatttttaatgaggatag
cgattattgtctagggatgttaggtatgcatggtactgcgtatgctaattttgcagttag
tgagtgcgatcttttaattgctttaggagctagatttgatgatagagttactggaaaatt
agatgaatttgcttgtaatgcacaagtgattcacgtagacattgatcctgctgaagtagg
aaaaaataggattcctcaagttgctattgtcggtgacgtagcagaagttgttagtgaaat
attgaatttattaaagacttctttccccccttatccagagcagattatatcttggcaaga

aagaattaatcgttggcgtcaacagtatcctttactggttcctagaaaatcaacaagcat
ttcacctcaagagattcttgttgcaactaataaattagcccaaaatgcttattttactac
agatgttggccagcatcaaatgtggtcagctcaatttctgaaagtaaaagctaagcattg
gctttcaagtgctggattaggcacgatgggttatggtttacctgcagcaattggcgctca
agtagcacatccaaatgacgtagtcatttgtattagtggtgattctagttttcaaatgaa
tatgcaagagttaggaactatcgcgcaataccagttaccagttaaaattattattattaa
taatcgatggcaagggatggttagacagtggcaacaagctttctatggtgaaaggtattc
acactcaagaatgacagaaggagcacctgattttcaaaagcttgcagaagcttttggtat
taaagcttttactattaataataggcaaaatatgcaatctgctttacaagttgctattga
ttatcctggtccagttttattagattgccaagttacagaaaatgaaaactgttatccaat
ggttgctcctggaaaaagtaatgcacaaatgataggtatcgctaaaccgcagagaggtac
tgcttccaactatattaataatagtgtttgaagtaa------------------------

---------------atagttagtatttttta-aacaaagaata-aagtaca--------
-taaatgtattttttttatactatta-aaaactatgtaagaacagcctttgacgagaatt
gaactcgtgaccttccccttaccaaggggatgctctacctctgagccacaaaggc-tttt
tactatattgggccgggttggatttgaaccaacgtaggcgaagccagcggatttacagtc
cgcccccattaaccactcgggcaccgaccctaattaaaccttattcaagatatgacctat
aagatatcataactatgttgctaaaaacaagtactttgataaataaaactgaataagtaa
tggacatagctggacttgaaccagcgactttcacgatgtcgacgtgacactctaaccaac
tgagttacatgtccaaaactttaattatcattataacactgtttagtgattttataagta
tctatttctaattgaagatttgttttaatcgtccggctttaccgagcagatctcttagat
aatacagtttagctctacgtattttagctcttctaatcactttgacaaaagttattcgcg
gtgaattgagaaaaaatactctttcaactccaataccttgaaatgagcatcttaaagtta
aactagtatttaagctttttttttcttcttcttgctaatactactccttcacataattgtt
cccgagttttactaccttccttaaccattagtccaagttgtattgtatcgccaactttaa
tttcaggtacttctgttttgataaaaggtatctcaacacttttcattagctgacttagct
ttgtactatttgctttcatatataggattagtcttgtgaca-tgttttgaaatattatac
attaaaaaaacaaatatgaaaaattttagcgaataagcatattagtatttataaaac-tc
tttttatct---
---ccattata--------
----------------cagatccatt------------atattcaact------------

----------agattctttcatagtttc--------------------------------

--------tttgataatcagtac-------------------------------------

---------------------------atcta----------------------------

----gatgatcaaatattattttcaaacatggcttatgtatgaaaaagttctata---aa
aacctaatgac--------taaaattagggcttaagtatttcttagttttgtgttataat
tagttactatcaaaggtagtggcataacacgaagcatcaa-aatgttcgaacgctttact
gaaaaagctataaaagtcataatgctagcacaggaagaagctagacgcttaggacataat
tttgtcggaactgagcagatactattaggattagttggtgaaggtactggaatcgcagct
caagttttgaaatcgatgaatgtgaatctaaaagatgcaagagttgaagtcgaaaaaatt
ataggaagagggtctggttttgtagcggttgaaattcctttcactcctcgagcaaaaaga
gtactagaattatctttagaggaagcacgtcaactaggccataactatattggcacagaa
cacttgctaatgggcttagtccgagaaggagaaggagttgcggcaagagttttagaaaat
ttggcagtcgatgtttcttcaattagagctgaagttatacaaatgctcggagaaaatgcg
gaagccaatgtaagtggaagcaatactacgcaagctagaagtaaaacaccaacattagaa
gagttcggatctaacttaactcaaatggctatggaaggtggtttagatcctgtagtcgga
agacaaaaggaaatagaacgagttattcaaatcttaggtagaagaactaaaaataatcct
gtcttaattggggagcctggtgtaggtaagacagcgattgcggaaggattagctcaaaga

attgctaatagagatgtaccttctattttagaagataaattagttattactcttgatgtc
ggtctattagtagccggaactaaatatagaggtgaatttgaagaaagactaaaacgtatt
atggatgagattaaatcagctgataatgtaatattagtgattgatgaagttcatacattg
attggggctggtgctgcagaaggagcaatagatgcagctaatctgcttaagccagcttta
gcaaggggagaattgcaatgtataggtgcaacaactttagaagaatatagaaaacatata
gaaaaagatccagcattagaaagaagatttcaaccagttgtagttggagagccaagtgtt
gaagaaacaattgaaattttgtttggtcttagagaccgttatgaaaagcaccatcaatta
acaatgtcagatggagctttggctgcagctgctaaatacgctaatcagtatatttctgac
cgatttttgccagataaagcaattgatttaattgatgaagctggttctagagtccgttta
ctaaattctcaattacctcctgctgccagagaattagataaagagttaagagctgtatta
aaaacaaaagatgaagctattagagctcaaaaatatgaaacagcagagcagtatagagca
agagaaatggaaattaaagctcaaattgcagcaattgctcaaagtaaaaagaatgagcct
gatttaaatttagaagatcctgttgttacagaagatgatattgctgaaattgttgctgca
tggactggtataccagtaactaagcttactaaaagtgagtcagaaaaattaatgcacatg
gaagaaactttgcatggacgtattattggtcaagacgaagcggttgtagctgtctctaga
gcgataagacgcgcaagagtaggtctaaaaaatcctaacagaccaattgcaagctttatt
ttttccggaccgacgggtgtaggaaaaacagaattaacaaaagctttggcttcttatttc
tttggttcagaagcttctatgatacggctagatatgtcagaatacatggaaagacacact
gtatctaaactaattggttctcctccaggatatgtgggttatagtgaaggtggttatcta
acagaagcggtaagaaaaaaaccatatactgtcatcttatttgacgaaattgaaaaagct
catccggatatttttaatctacttcttcaaattttagaagatggccgactaacagatgca
aaaggcagaactattgattttaagaatactcttttaattatgacttctaatattggatct
aaagttattgaaaaaggaggaggtagtttaggctttgaattatcagaagatcaaacagaa
tcccaatatactagagtacgatctttagtaaatgaagaactgaaacaatactttagacca
gagtttctaaatcgattagatgaaattattgtatttcgtcagcttactaaagatgaagtt
agagaaattgcagaattaatgcttaatgaagtctttgcgagaattaagcagcaagatatt
caattaaatgtaacagaacgatttaagcaacgattagtagaagaaggatataatccaagc
tatggagctagaccacttagacgagcagtaatgaggcttttagaagatagtctggctgaa
gaagttttatctggtaaaattaaagctggtgatagtgcagtagtagatgttactaatgaa
ggagaagttacagttttattaggtgaaaaattagaactgttaacataaa------aaaca
atttattaat--taaagcattgaagaggttat--attcaatgctat---gaaaaaaatta
ataagatgggttgctataattgacaacctctgatattctttatttgtatata---aagcc
gggatagctcagttggtagagcagtggattgaaaatcctcgtgtcaccagttcaaatctg
gttcttggcat-tttaagcatcagaaaattttgctatctgagcattaaattttaattgaa
atgttcctgtgggaccattcctgtgttttgctattataacttctgtgaagtctctacttc
tagtttcttgtgtatagtaactttctctatatagcattattactaaatctgcatcttgtt

ctatagaattatgaacaataaagttatttgaaataaaattgcataaaggtttacattcta
aatcaaacactatttgtaaacttgtaacgtttatttttttgtatactttcaaaagtcaaag
atgaaa------aagaattaaaaacatttttttgttcttcaaagttttt------tattt
gaatagcaatcatgtcattttgattaattttatcacatcttttccatccttgtgttgtga
gtagtttatggttacttgttaattgtatatatttcccagcttcggtaataattttatata
cagtttttttccctgttttcgaaatactacatttaattgctagaaataactgtcgttttt
gcgcactaaagttaatgacttca-atctttttataatagcaattaaataatatttg--ag
gttgattatataatgatgtctgaagataattgaatttggatatacacccactttctctta
aatcggataacaaaggccttttgtcactccgagtttcaaggtttctatttagttgagata
atgccaagataggcaagcttaaatcttttgctaatattttaagtgatcgagtaattaatg
atagttcttgagatctattgtcagattgtctactctcctgcagtaattgaagataatcta
taataattaattctatattttttccttgtaatttaaagaattttacttttgtctttatag
tgtcagtagaaatcttagcgctatcatcaatataaagattaaggttagcaagagttttac
ttttttgaataacatattgccattcatcattattaagctgaccagattgaatttttttggc
cgtttaaattacactcttgagctaaaattcttcttagtagttgctctgtagacatctcta
agctaaataaaataacataagatcttttctgattaattacatatctagttatattaatag
caaaagcagttttccccattgaaggtcttcctgcaagaataattaaatctgacttttgaa
acccttgtgttatagagtcgagttctgtaaaaccagaaagtatactactatttatactaa
tctttttccttgtatccaaatggactaatagtcttgcaagaattgtagctaatgtttgcg
tatctttttcgtcaagtatttcataagcttttgttaattgactaacaatagaagttatgt
ttttttgttcaataagttgttttgagtagctaattaaacataatgaatctccacatgcta
aaagtaatcttttaatgtagttatctataacgactgctgaatattcgcttatagtatcag
ataggggagcttgttctattagactaaaaacttcatttagctcattcaagtcttgaatca
ttttttcatttttttaagttaatgaaaaaatttcttacgctttttgtctttgcatggttaa
cattttcgagtgctgctctataaagtaatgaagttgatgtaaaataaaaaaaatcaggag
agatttttttctactgattttagtaatgatatcgattgctgagttaatattatactaatta
atattttttcagctaaaatattatgaggaggtagatatttataa------atacttatta
cttcctcc-ttgtaacattactcttgtg--------gtattagttaactattgatttaat
ttgattctggaagaacttgcaatttaatatttgcagttacttgatgtaatagcttaattt
ctacatcgtaaatgccaattgttttcacgtctggtaaagagatactttgtttttcaatgt
ctatgttggtagtgtttttaataatttgtgaaatttctttttctgtgacactaccaaaaa
tattgtggccatctcctgtttttttactaacgctaaacctttgtatttcttctagaagtt
gcttgactttttttagcattttctttagctgtttttaatttttcttctcttatagctgcat
aaaatttttgttgttttaaaataccatttgtggcaactgccgccattttattcgggatta
aaaaatttcttgcataaccagaagctactttgataacgtcattactcttgccaagttttt
gaatattctcttttagcacaacatttataactttttttactcatctt-------aattcta

aaaa----atctgtattatcctattatacctgttgtatacagatttt----aataggaag
tatttatttgttgatataatattcttgtaaaa--aaaggtaatttttttatttttt-ttctg
atataatcactttcacaataattc-----attaggagaggtggccgagtggttgaaggcg
cagcattggaaatgctgtttaggagcaatcttaacgagggttcgaatccctctctctccg
tacaa--aaaatataaaaagttttatagagtccaatctaaatctccaataatattcttcc
aagcatttaatccaccccgaactctaacaatatttagtttttgagctattaaaaactcag
aagcaaatattgatctagaatctaagctacagtaaacaaaacaaattttatcttgtaaat
tgatatgagagtaattcattcctttattttttgtaaaggtagatttaatgaatatatta
aatgacttttatgatattcttcatgatttcttacatctattaaaatatattgtggattcc
tactaattaaaaacttttgtaattgaataacactaatttctcgtacaaatgtattagatt
tactgccataatatttgttccaatgctttttgt-------ggataatacaaatttagta
tttataatcttaaatttgttaaatgatgaagttaatgagttgtacgttaatatagttcca
cttaatatagacttataacccagaacgattttgacagcttcagttgcttgaagtgtacca
acaatacccggtaagagacctaaaactccggaattactacaagtatctcttgcgctctct
ttattttcagtttcactgtagaagtctcgataaacaggtccaccttgataattaaataca
ctaacttgtccttcaaattgaaaaatggcaccataaatatgtattttatttaactctagg
caagtatcattcaagagatatcgagtatcgaagttatcagatccatctataataatatca
tattgtctaataatgtcgattgaattctcatagcttagtcttgttttgaacacagttact
atacactgtggatttaaatctaata-tttttttttagctatttctacttttgaatatcct
atatcatgcatcgtgtataaaatttgcctctgtaaattagagagatcaattatgtcatcg
tctataattccaatgctgccaattcctgcagcagcaaggtaaatgattcctggagatcct
agcccaccagcaccaatgaataaaacttttgcttcttttaatctttcttgtccttctaat
tgaatttgtggcaatactaaatgcttagaatatcttgtatattcttctagtgagtatttt
gtattttctgttttaaaatttagcataaaaatttatagtttatttaatagcactta----
--aaagctagctgataagtttattaaaatgacttttgtaaacttggttcttctctatcta
aaatagctccttgtgtaggacagacctgaacacaaatactacaatcaatacaggcagaaa
agtcaatccaataccaattagtgttattattgtttttcccctttccttgatgaatacaag
ctacggggcaagcttccacgcattcagcaactccaatacatttttctgttacaattgtat
gagacatattatttaattaat-aaaagataagctatttacatctagtagttttgttgtat
actaaatttacatatgctatccaggccaatatttaaggtagtctatttctacagtttact
aatttgatatgcgcctttagttcagttggtagaacgcaggtctccaaaacctgatgtcga
gggttcaagtccttcagggcgcgtttag-ttttatcgtaattcaataatatcattttatc
tttttgcaaacatcaaaatgatatgatataaaagatcatattttatacattgaatattct
atgtactagtactg-ttttttaatatgttattattgataagttaattatactaattctat
ttttgtaataagt-aaaaacagttctaattaagtatgcacgtatttaatagtc-ttttgt
atatat--aacttgaaaaaatttaaactatggctaaaaaagttactggaattgttaaact

agcattaaatgcaggtaaagctactcctgcgccacctgtcggacctgctttaggacagca
cggcgtgaatattgttatgttctgcaaagagtacaatgcacgtactgctgacaagtcagg
actagtaatccctgtagagatctcaatatatgaagatagaagttttacttttatacttaa
aactcctcctgcttctgtactaatcgctaaagctgctggactaaataaaggttcgggtga
accaaataccaaaaaagtaggcagcataacaaataaacagttagaatctattgcagaaac
aaaacttcccgatttgaacactaacaatatacctcaagctatgaaaatagttggaggtac
ggctaaaaatatgggaattctaattaaagatt-aaaagtttcaattatatttacta-tt-
-tttatgaaaaaattttcacgtcgacttacaacattaaaatcaaaagttgagcctaaact
ttacactattaatgaagcagtatctatattaaaagcaacgtcaaatgctaaatttaagga
aactgcagaagctcatattgctctaggtttaaatcctaaatatgcagaccagcaactcag
agcaacagttatattaccgaaaggtacaggtaaattgataaaagtagcagttattgctaa
gggagaaaaattaacagaagcaattagtgcgggagctgatgttagtggctccgaagaact
aattgatgaaatactgaaaggtagattagattttgataagctgatagcaacaccagatgt
tatgcctttaatagcaaagctgggacgagtattaggccctcgagggttaatgccttctcc
taaagcaggcacggtaacattggacgtagcaaaagccgtgaatgaatttaaaggggggtaa
agttgaatacagagttgatagaacaggtataattcatgtaccttttggaaaatctagttt
ttcacaagaagatctagttttgaacctccaaacaattaaagaatcgattgatagaaacaa
gccttctggagcaaaagggaagtactggaaaactttttcttatctagcaccatggggcc
atctattcaaattgacatcactagccttctatgaaaaatttttgtataattatagaactt
gaataatctattatttcaaatta-taatttgctagcattattaacgtaacaacttgctta
ctatatcttatctgcattaaaaacttt-ttatatatcttattaattatgagtacaaaggt
tgaaaatatcttggaagagctaaaatctttaaaccttctagaggctgctgaactagttaa
acaaatagaagaaacgtttgatgttgatgcatctgcggcttcaggaggtatgatgatggc
cgcaccaacttcagcaccagcgtctgctgaggttgaagagaagacagaatttgatgttgt
cctagaggaagtcccagcacctaaaaagatttctgtattaaaagctgttcgctctctgac
tggtttgggtttaaaagaagcaaaagatcttgttgaatctacgccaaaagtcttaaaaga
aggtgcttctaaagatgatgcagaaactatgaaaaaacagctagaagatgctggcgcaac
agttattgttaagtaatga-----agaagcgcttctgtttaagtataaacagaagtgcct
ctttatttattataagcaattatactagaaaagtccaagtgtaattgcattgttgatagg
catagttgcaccaatacctagccaaatacttacaacggtaccaattaagaaaacagtggt
tgcaattggtcttctaaatggattttgaaatttgttaacattttcaatgaatggaacagt
aagtaaaccagctggtacagcagccatacttagaacacctaatagtttattaggaatcac
tcttaatagattaaatgttggaaagaagtaccattctggtaaaatctctaatggagtcgc
aaatggattagacttttcacctatagaagatggttctaaaattgcgagtccaatactgca
ggcaaaagtgccaataattacaactggaaagacgtataatagatcatttggccaagctgg
ctctccgtaataattatggcccattccttttgctaatttagctctcagcttcggatctgt

taaatctggtttttttaagaattgacatattg-tttttgtgtttaatataaagattagtct
aattactgttttaaagtgtaattttcaactattataatggtccagaaattccttgtttac
gtatcattaaaaaatgcataagcatgaaaacggcagttagtagcggaagtacaaaagtgt
gtaaactatagaacctagttaaagttccttggcctacactgactcctcctcttaacaatt
ctactatactcgctccgacaactggaacagcatcagggacacctgtaacaatttttacag
cccagtatccgatttgatcccatggcaaagaataacctgttaccccaaaagaaacagtaa
gcactcctaaaattacacctgttacccatgtcaattctctaggttttttgaaacctcctg
ttaaataaacacggaatacatgtagaatcatcatcaagaccatcatgctcgctgaccatc
tatgaattgatctaataagccatccaaagtttacatcagtcataatgtactctactgatg
taaaagcttctgcaactgtaggtctataatagaatgtcattgcaaatccagttgcaactt
gaattagaaaagatacaaatacaataccccctaagcaataaaaaatattaacatgaggtg
gtacatacttactagaaatatcgtcagcaattgcttgaatttctaatctctcttcaaacc
agtcataaattttactcataaaatagcttc-aaaagctcttctaatata--ttttgcgtt
taagctactattaattcaatag------ttattgaacttatatt-tttttataatcaata
cttattaactattataacatctttttattgattt--------------------aaaatt
ctctgacttagtaaaacagggctatgtactataattcttttttttgtatagagattaat
ttcgtttttagtaaattagctaaaattcttgttacactgactctattactacctataatc
tgtgctaaaattttatgtgtaatagtaaagttaagcataataccattattaacttgagtt
ccattttgctcagctaataataataataagctcgccagtctactggttatacttttatgt
gaaatgatttctataaaatgataagcttttgccgagcaaaatactaaatgatttacaaaa
aataaattgaatggagcgcagttactacacgcttttataatagtagtatagttaatacag
gcaacttctgttttatctattgcttctgcttcgtagtaaaaattatcgtcgaccaattct
atttgtccaaatgtatctcctgtagttaataagttaagagttactttatgtgtatttctc
aggacttttgtaataattaaagatcctatcaatataatgtataatctagagctgctatta
aacaaaagagtatcatttttcttaagtgaaaaaatttgataattaatttattacgatta
aaaaatagtagccatggattgcaagaaaaaaaatcacctgaactattttcagagaatatt
ggaatacaattttcttgatttaaacaatttttagtcataattactactttataa------
---aaaaatattctaataat-taagaactgaaattaaatgtcatataaattgatgttagt
tgagaatgacattgtattatcaaaggccattcaagaatacttaatagatcaaggcttcaa
tgtttatattgccaataatggattagaagcactgaatctagcttatcaatataatttcga
cttgataatttctgatataatgatgccgctagttaatggctatgaactattagcaaaact
taaaaaaaacaaagcattatccaaaatacctgttatctttttgacagctaaaggaatgac
taaagacagaataaaaggctacgacatggggtgctatggttatttatctaaaccatttga
tcctaatgagttgctttcaattattaacaacttaatcgctagagatgtcttaaaagaagc
ttctttacaaaacttggcaacatcaaaccagcaattaaatcataaaatacgtttgacacc
tagagaaaaaagtattttagatcttgttgttgatggacttactaataaagaaatatctac

tatactgaatactagtgttagaaatgtagaaaaatatgttagtcgactcttacacaaaac
taatatgaagaataggactttgctagtcaagtattctataaataataatctattaaataa
tgagatcaatgaaagggcgaatgacgggactcgaacccgcgaatgatggagccacaaccc
attgccttaaccccttggccacactcgccatattacacataccactatagcttgtttttt
gatatatcgtctagttttcgagttatatatttatctaaaacaatatttactttgaatttt
-tatgatacatagcaatattagtattcaaattaatggggaaccatttaattgctcaaagc
ccatctcattgcaatttttattaaattatcttgattttaattccgagcgtgtagcagtcg
agctaaatgacattctgttaccagagactctgtttcactcaacttatttgaatgatcaag
ataagctagaggttattaccattgtaggtggtggataatctatttgtacatac-------
-acagttctctatctcaaatgtttcatagatagagataatcttccttgtttttatctac
atgaataataacagctttaatagtgtcacctattttaaattgtgatgatatctgctctaa
ttctttgatattaatctcagaaatatggacaagacctttgagatttcctacctttataaa
taatccgtaaggtgtaatctgatttattatgccttcaataatattaccaacaattaaatt
agatgatgcttgagatattagagctcttctatgactcagtattaaattattagatttctc
ttcgacattgagtaattttagcttaataaatttattattaaattgctcactttttttgaaa
attaccgagatgagaattaggtacaaatccagatataccttcaaggttaattatcattcc
tcccttattgaatccttttatcattacattaagtaaagagtcttcagctaatagttgcct
aattcttttccatgctcttatatattcaaggcgacgaattgataatattaattgtttcga
ctgaatattataatctaataaaaaaaattctcttgtgtcattaatgtttaaagaagtgaa
attatttaaatcttgattactagatacttcttgaataggcaagtatgcagatattggtgt
tcctatatctactaatacccccatttaattcaaagctaaatatagttccagctacaatatc
tccaagattcaaatcatacttgtatttttgtaaaacagctgcaaaatttctatgagtaaa
tccttcattattttttgtcatattgaatagtatgaattaaagagtaactttgtacaatat
attaaggtaaataaatgtttgtcgaattgtatgaaaaaattttaatttgttcggttgtaa
ttaagtatataaacatattaaaatatctaggagacatgaaaagtagacgtagatagctgc
agatttaa-tactatataatctgaggaaagtccgggctccacaaatacaatttatgctgg
agaaatcccagtgtaggtaactgcgaggatagtaccacagaaaaaaaccgccagaataag
t----tatattagctggtaagggtgcaaaggcaagttaaaagcttaccaaaagtactgca
aagtatttgttaggtaaaccccctaaaatggagcaaagctactaaacaaatatttgtgtat
attttatctagtttagttgaaaatactgcatgaagttattagtaacaataactcgagagg
aatagctatcctttacaatatatc-tcgattatattgaaagaacagaacccggcttatgt
agtacttttatgtcttgtattattatgtctattgtcgaatctttaaatttaactgtttg
acaattttttttttgtagctgatgttgtttataatctatttctgaattggttaaagttttt
tcattagatcgatatgtaaatcttagacctatactttttttctcttttccaattgattca
tctatatattgatcaaataatgttgtactttctaaatctttatcataaaattgatccaat
aattttaataagtatttaatttccatattcttaggtattattaaagagagatctcttatg

atcgatggatacttagagtatggttgaatctgataacttaaataattgaattcattatga
caatcttctaaaatgtttaagttaacttcaagaaaaaaaagttttgtgttgagtcctaat
tcactatatgttgcttgattaagttcactgaatatgccaataataatattgttatagatt
agtgtggcaaattttcctttttgaatgaactttattctaccatcgactatatcttttttt
gtccactcaatttgtctattaatcttttgaaaaaaattttctacgatccctttagcttca
taccaatttaaagaatgcgcagggtgtgaccatgtagatcggatatctaaattaccaccc
aatatgatagctagattattagtttctacaattttgttttgatcattatgaaatactgtt
cctatctcaaagctatctatagtttggttgctttgtttaatattataaagatttgattca
attaatccttccagcaaactgccgcgtaaattagaataatcttgaataagaggattgttt
aaagctatatttcctttggactttattaaagaataatgaactaattcagttaatcctaga
tttcttaaaatacttctacttttatctatgaattgtctctttgtggataactttttgttg
aattgaatatttggtactgagctcctgaatttatgataaccataaattctagcaatttct
tcaataacatctatttctcgaaaaacatcttctcgtctataatttggaacagtaacttct
atattttccttattttgataaataagatcaaaatttaaagagtgaagaatattttgaatt
tcttcaaagaataaaaaacgtgtttgaccattatacataattggtcctaaaatgtcatga
actttttaattgataaattaatattaagagcatgatcatttatttttctcgacagaaa
gtctctctaatatttccgcctgttagatctgtgattaaagcaagagcttcaaaatgtgca
tttttccaattatctacatttagcccacgttcttgtctaattgaactttctgttcgaata
ttaagaactcgagaagattttctaactacagattgtttaaatatagcagactctacaagt
atcgattttgtattataatctgtatcaaaatcagagttgcttccaataccagctatacta
gttacatttgtatttacttgagtaactaaaatatttttgtttaactcaatattttcattg
tttaacttaatctgatcattacttccaagaggaaaattgcttcgaattgttagactattt
ttatgattcatattattaattttattcaaatcaacaatattaataggctgtccccatttc
aacataatataattgcttatatctgttaacaaatttctgtgtgtaaacccagaagagagc
aaccgatttttagccaatctggagaatctttaactcttatttcgtctataatggcagca
aaataattgtcacaatttaataaatcgtgattactgattatagt-ctccttttagagaa
atatctattattgggttgatgtatagctgataacattgaggaccctgttaaagccgagac
ttctcttgatagaccaatcatgcttaacacgtctgacctgttagctgttgatgatatatc
taatatatgatcaatctcacttccaattataatagattcaatagcttcaacttcaaaacc
tgcttgtgttagttgattagttaaattgtctatatctattgttttgatattaccaagttc
tttcaaccaatttaaagaaactttcataattaattgtcatctatcttatagaagtattta
ttttacttatgattaaaaatcaatttaaaaaattcatcaaattaatataagttatttgat
gaatttgcttatacaatatattattgtgatatcttacttttatatggcagttattaagaa
ctagcttttgtaaaagttagcccattatcttgggcatatctttccataaatctcatgaat
ctatcccaatcatcaggacttttaataacatgtacagcttctattgcttgtggtttacca
ttaataaacttggcattaacatctcttgtaataatttgtccctctgtgtccatcaggtac

atacctgtgatttcacctttgtctgccatactagcatctaaaattttgggatttgtaaac
cgaaaagttgctgttcctgtactaccatctcgagaccttgttaaacgtacatctggtaca
acttcttcattgataccttgaataaattgaattgttgccatatcaatcttctttatttgt
atataatttatattggataaaactgatttctagttcttgaatgcttgttgagaaactgtt
ttaaaagcttatagtagcagtagatcaaattttatgtattatataaaactgtaacttttc
-ttttttg--

----------------------atctgttcaaagaactaacttattataagctaatcata
cgaatattagccatataatgttcgtaagt-gaaattaaggggttgtagctcagaaggata
gagcaagcgcctcctaagcgctaggtcagcggttcaagtccgctcaaccctgttaacgaa
ttaagtagtattatttaacttgttctgcaaatcagtattgatctaagttaaagtacttga
cagctaaaacaaaagaatctgagaaatattttcatttaaagaacgaacaattatcttaac
tctaaatatctgataattattaa-aaaattct----------------------------
----ctttttaaaacacaagatgatg-----aagatatttaaactaataattgaaattaa
gtttttgaagttatagaagccaat--ataatactttctattaacaga-----------tg
cttgcttctattactatctgtttacaaga--aatt----atgagtatcaaaata------
--attttagctcta-------------------------------------ctgac----

-----------------------------tactaataaata-------------------
------------------tttattttagtctagacaag------atgaatcttcatggta
taacag--

--------------agctattctttgtgtcatacttatataaaattaggat-------ac
tgtt--

---------------ttcattattataactggggtgggaggattcgaacctgcgaatggc

ggagtcaaagtccgctgccttaccacttggctacaccccaacatgtgaacattataatag
cagtcgtataccctgccatgtcaatagactattgtctttatttaataattttgctgctc
ttgtagaataaaatcttttttttcaaataagatacgctatgtttaagttgcacgactgtt
atgttttcttgcttttgactgaacagccacttgatagtaattattccttctgcaatttcg
ttacttcctataataatacaagctacagctctttttttatttgcttgcttaatctgctta
ctaaaattacttgagctaacatcgatttctattttaaaaaactgctgatgtaaaaaacgc
attatttgcataccagtttcgttagcttttgtaccttgtgtagcaatataaaaatcgata
gattgattaggcaagtctatattatcttttgcaatcagtaataggcgttctaatcctatt
gcacatccaacagctggtgttgctttgccacctaattggtgaactaagctgtcatatctt
ccaccaccacatatagtatcttgcccttttgatgtcaatgtcttaatttcgaaagcagta
tcattataataatctaatcctcgaactaacttattatttatattgtagggaatattaagt
aattttagataattgcaaacgtcaccaaagtgtttttgtgattccaaacttaaaaaatca
gaaattttggagcctctgtcagtatttttgtgtattagaatcttttgaatctaaaatt
ctaattggattactagttaatctttttgtgagtcagtgtctaaatcatcatggtacttt
gtaagataatctcgtaatttaacttgataaatactacgatcctctacttttccaattgaa
ttcagatcaagtttcaagttgtgcagattgagattgttaaatatgctcatagctaaatgt
ataacttcactatctgctcttgcatctaagctaccaataaactcaatgccaagttggtga
aattgtcgttgtcgtccactttgtggtctttcgtacctgaacattggaccgctataccat
aatctttgtaaactatggtggtaactcattttattttcaataaaagatctcacaataccg
gcagtaccttctggccttaaggtaatatctctattgctgcgatcatgaaaccgatacatt
tctttatttacaatatcagtatcttcaccaatacccctatcatataattcactattttca
aaaataggtgttctaatttcttggtaatttgcacattctaataatttggaaattttatta
tgtataaactgccaatattgaatttcatcgggaagaatatcttttgtccctctaatagcc
tgaattttgccatgataatttttgattaccttcttaatatgtctgcattaaatagcag
ttgtagactgttattcagtcatgtattatacattag--aacatactgatg-taatttata
aagtacgggcaaggagggattcgaacccccgacaccatggttcgtagccatgtgctctaa
tccactgagctacaagcccacttaataagtaccttaactatatcattgttcactaatgta
tacaactatattcttttt------atcttacatgtgaaatttttgtaaaagtaaaaaatt
at----gtttgtttttacaattggagctattcatatataatgcttaatttattataact
ttgtaaa--aatatctataagtttaattgtatgattctattatattggtaatcttgttat
aattttgcgctcttatttttcaattgg------ctagcatttaattaacttatatttcaa
ataaaaaaatcttatctaaaatgagtaaacaaattctatatcaggatgatgccagaaaag
cattagaaaaaggcatggatattttaacagaagctgtttctgtgactttaggaccaaaag
gaagaaatgttgtcttagaaaaaaaattcggtgcccctcaaattattaatgatggtgtaa
cgattgcaaaggaaattagtttagaaaatcatattgaaaataccggagtcgcactgatta
gacaagcagcatctaaaacaaatgatgtagctggtgatggtacaacaacagctacagtac

tggcttcagcaattgttaagcaaggaatgagaaatgttgcagcaggttcgaatccgatgg
ctattaaaaaaggtattgaaaaagcaacaaattttgttgttagtaaaatagctgagtatg
ctaagccagtagaagatacaaaagctattatacaagttgcttctttgtcatcaggaaatg
atatagaggtaggtaaaatgatagcgaatgctatagaaaaagttggcagagaaggagtta
tttctttagaagaagggaaatcaactaatacgattcttgagatcacagaaggaatgcagt
ttgaaaaaggctttatttctccttatttcgttacagatacagaacgaatggaagttcttc
aagaaaatccttttattttatttacagacaaaaaaattactttggtgcagcaagaacttg
tgccattgcttgagcaaattgcaaaaacatctcggcctttattaataatagctgaagaca
tcgagaaagaagcattagccacaattgtagttaataaattaagagggattttgaatgttg
tagcggttagagctcctggattcggtgatagaagaaaatctttacttgaagatatgagta
tcttaacgaacggacaagtaattactgaagatgcaggtttatcacttgatacagttcaat
tagatatgttaggaaaagcccgaagagttattgttactaaagactcgacaacaataattg
cagatggtcatgaaattaaagttaaatcaagatgtgagcaaattaagcggcaaatagaga
cgagtgactctttatatgaaagagaaaaattgcaagaacgattagcaaagctttctggag
gagttgccgtcattaaagttggtgcagctacagaaacagagatgaaagataaaaaactaa
gactagaagatgcaattaatgcaacaaaagcagcaattgaagaaggtattgtaccaggag
gaggagctactaatgtccatatctctagtgaattatttacatgggctaaaaacaatttag
ttgaagatgaattaattggtgctttaatagttgaacgagctgtgacctatccgttaagac
gaatagcttttaatgcaggtgataatggagcagtaatagtggaaaaagttaaaagtcacg
attttcatataggctatgatgcagcaaacggtaatattgtaaatatgtacgatagaggta
ttatagatccagcgaaagtagctagatcagctttgcaaaacgcagcttctattgcagcaa
tggttttaactactgaatgtattgtagttgataaagtcgatgattgagctata------a
gataatagataatctcaaacaaagaataatttctaattatagaaattattctttgtttca
attaatctaattaattgatcaaatgtataattttataataaagccaaagtttaaacacg
tcttctttttcaactattaagtagagaagatataagcccgtaatacctaaattatcaatt
aaaatattattttcatatatagattttctaaagatctaaaaaatgacattttgaaaaa
taataccgaaatcttctcttatagttatttgctaaatcattactgtctaaagattgattt
ccaattgaattcagaacctttaacagaatacttttaaatctttatctgactttatttga
gaatcaataataatttgattcaattgtatgtcaaaactaagattaaaaatattacttgtt
aaagatatttgattttgaatatctagagattttatagccaagaagattaaacttttattg
ttcattgtaaaatataagacaattgatgatgtattctttaaatacta-aatttatcttct
tgtgcttctatcatttttgt-aaaataattaatgttttaatttttagttttagttact
acccgaaaaaataaattctggaaatctagcttgagctttagctagagagtgtttttccc
ttcttcttgccagtaattgataacttctgttgttttgtttaataactcaattctgtctct
ttcagcaatccacggcttggattctagttctgcttttaattcttcccaagcatcattcct
tggccagaaaaaataagaagtaagtggactagtgccatttccaacaatctgatcaatagc

aatagcaatattattttctaaccaaagtacttttagtgtaaattttgacaagtttttatc
tccattattaatattaagtattgg----tacagtagaatactgtactagttatttttttc
agcaacaatttgagctttgtttaaaatgtttttaccgttttagtagcttgtgcaccttg
agataatcgttgcaaaattttgaaatgttaacacgagtttcattatttataggattata
aaatcctaattcttctatagctttaccatcgcgtttatttctgctatccattacaacaat
tctatagcttggttgtttttttcttccgtatcgctttaaccttaacttgaccatattagt
cacctcgcttattttt--atatctttattaaattatatcttacgacattatgcaataat
tcaaacaattaagctgattcaagtctaacattattaaatattaccattaaacattgtaga
aaaataatacctaacataggtgacatgtccattccaaacatgggaggaatgcttcctctg
aatagttttaagtatggatcagtaattctatttaatgaacagaatggttcattgtaccaa
tttacagttggaaaccatgccaatgataatttaagtaaaattaaaattaaataaatctca
gaaaaattagctatagatccaagcaataaatttaatgtaccaggaagagtgttcatacgt
attttataattattatatacttcgttatactaatagtttgattatatattataatcgatg
taaataaat-cagctaaacaaaaaacttatccttaaaatcatcttctcttgagcattttt
ttttatctacattagtatttacttttgtcgtataaatttccagtttagaccaaatatgtc
ctaactgatttaattgagatgttccacattctaaacaggctatttttatattgctcaaaa
cagcacctttttttataaaatcgttcaataatggcgtaatagttgcttcatccaaaaata
aatctaagacaagtattttttgtaaatgaatttaattgatctgggacattgttaataatat
cattataattaactaaaactatattagctgtaggaagtaaagatcgtgctccttccgcta
agataaagccataaggcataataattacaataatatatttataatgactgctaataagac
ttatagtggtattagcttttaattcgattgtttctgttactagccactctctcattattt
cgtaagtaatccatttgcctagttctgagcaagcagttcttaaaatcgtacctggattat
tgtcgttttcgagaatccctgaccaatgttgtattaatggatgggatgctatattaattt
ggagttgcatgttttgttaaatatatttaacatttaatttgttgattgaatcttcaattc
ctatttgttgagtattattaaaatagcat----atatattaaagaaaataattat-aaaa
aatgaaaaaaagtctttggctgtggggttttacagatagtgctgaaacttggaatggtag
atttgcaatgattggttttatatcagttatttttatcgaagtagttacaggacaaggatt
attatatttaataggtatgatgtcataataaaaatccaaaaaagttcttgtaattttta
taagaactttttttgg-atttttataaatctaatctagcggtctcatagatagaacaggtt
cattctctctattgataccttttcaaatccagcagcagcagctcttgctctgcctgcat
gccaaagatgtccaataaataggaagaatcctaggaagaaatgagatgttgttaaccaag
atctaggagatacatagttaactgaattaatttctgtagctacaccaccaacggaattca
aagaacctagtggtgcgtgagtcatatattctgcggcacgcctttcctgccacggttgaa
tatcgttcttgatttatttaaatcaagaccatttggtcctctaagaggctcaacccaag
gagctcgcaagtcccaaaatctcatggtttcaccaccaaaaataatttctccactaggag
atctcattaggtatttacctaatccagtaggtccttgagaagatgcaacattcgcaccta

gtctttgatctctaactaagaaagtaaaagcttgagcttgtgaagcttcaggaccagttg
gtccatagaattcactaggataagctgtgttgttataccaaacaaaattagaagccgtaa
gccccatgattgataaagcacctagactataagataagtaagcctcaccagaccatacaa
aagctcttctagcccaagcaaaaggttttgttagtatatgccagattccgccagcaatac
agataatgccaatccagacatgaccacctacaagatcttccatgttatttacacttacga
tccatccatcaccgccaaacggagactttaagacataaccaaagataactaggggattaa
gtgtaggattgctaacaaatctaacatctccaccacctggagcccaagtgtcgtataccc
caccaataaataaagctttaataactaacagaaaagcgccaatgcctaataacactaagt
gtataccaagtattgttgtcattttgtttttatcccgccaatcgtatccgaagaaaggaa
atgattcttcaagagtatcagggccaatcagagaatgataaagacctccaaagccaagaa
cagctgaagaaattaaatgcactacgcctactacaaagtatgggtatgtattgaaaatct
cgccgcctggaccaacaccccagcctagtgtagctaagtgcggaattagaataaaccctt
gttcgtacaaaggcttctcgggaacaaagtgtgctacctcaaaaagagtcatggcaccag
tccaaaagaccattatacctgcatgagcaacatgagcaccaagcaatttgccagaaacgt
taattaagcgtgcattgccagaccaccaggcaaacccggtagactcaatgtctctaccgc
caacaccaacagttgtattaaagggcgtttccacgtggtaaaacctcctcagggaatata
aagttttcatgaggttgatcttgagcagccatccaagagcgaataccttcgttcaatagg
atatttttagtataaaaagtttcaaactcaggatcttccgcagctctcaactcttgagaa
acaaaatcgtaagctcttaaatttagagctagtccaacaattccaaatgcgcttgtccat
aatccagttactggtacaaataacatgaaaaaatgtagccaacgtttattagagaaagca
acaccaaaaatctgtgaccagaatctattagctgttaccatcgaataagtttcttcagat
tgtgttggagtaaatgcacgaaaagtatctgcagcatcaccatcttcgaataaggtattc
tgtacagttgcaccgtgaatagcacatagtaaagcaccacctaagattcctgcaacgccc
atcatatggaacggattcagagtccagttatgaaatccttgcaaaaatagcaaaaatcta
aaaattgcagcaactccaaggcttggggcaaagaaccagctcgcttggcctagagggtac
attagaaagacggaaacgaatactgcaattggtccagaaaatgcaatagcgttgtatggt
ctaagaccaactagcctagcaatttcaaattgtcttaaacagaatccaattagtccaaat
gatccatgtaaagctataaatgcccacagaccaccaatttggcaccagcgagtaaaatct
ccttgagcttcaggtccccaaaggaaaagaagtgaatgtcccatgctgtttgctggggta
gaaacagccgcagttaaaaaattgcatccttctagatatgaactggctagcccatgagta
taccaagaagtaacaaaagtagtcccagttagccagccacctacagcaaggtaagcgcaa
ggaaacagaagtagtccagaccaacctacgaatacaaatcggtctctttttagccaatcg
tctacaagatcaaaaccaccacgagtttttttcttgtccaattgctatggtcataattta
atctccagagcaaatttaataagtaatttgtttaaataaaca---ctttagatatactct
aaaattttacttttctttacggtcaatataattataagtcaaaattaaccaaaattcacg
cttttaataaca-tttttttagctcattaagttaa-ttttttaatatataaacagtaata

gagttattgttt-aacttacaatactcataatttattcatttctataatcctttgaaaaa
gatatcctgttcctcgtgctgttaaaatcaagtcgggattacttggatcatcttctagtt
tagctctcaatcgtgaaatatgaacatcaacaactctcgtgtcaacatgccgttcgggag
tatagccccaaacttcttgcaagatagaagctctagaaaaaggttctccagccttactaa
ctaaaagctcgagaagactgaattccatacctgttaatcgaactctttcattatttttat
aaacttgtcttttgttagtatctattttagaaatccaatactaataatcccagagtttg
ggactccaagactagtggtgattttatcagctcttcgaagaacagaacgaatgcgagctt
ctagctccttaggagaaaatggtttaacgacatagtcatcagcacctatttctaatccgg
taattctatcgcagacttcacctaacgctgttagcattatgataggaacatctgattctt
ttcgaagttcttgacaaacaccgtaaccatctagtttaggcatcataacatctaaaacaa
caaggctaggatactcttttctaaacacgagtaaagcttcttctccatttgaagcagtga
taacttcataacctattattgttagtcttgtttctaaaattcttcttatgctggcctcat
cgtcaacaacgagaatcttttctttctggttctccaa-tttcttatttactcctataact
caccgatcatgattactataaccttaaatttttagcgctatgagtatctttattcacttt
agaa-----tttagttattataaattttgcaagtctagtttacattaattagtaccagt-
-caatatttttgt-----------tttaagttatgac---------tattttaatccata
agttgactctagtttgcagaattcaatatatacata-ttttctagccaagtaaattgtat
tctattttctaaataaaaaagggggttgacaaaccagttgatagaaagtattctacattac
tataag----ttaattccaacaaagaagttttaa-----atcttctttcggagtaatgaa
tactttaat
>SRR9587921
tatatgata---aattaagacgtctgttatgtgagaatttctaaaatatacaaaactttt
tatttaatatagaaaaagtaaattaaaataagttgaacatactttgctctaatctaaacc
tcttaattgag------attctaattagagacaaaagtatttacttattttgtatctata
ggaataaaaatgaaacttgcagtttatggtaaaggtggtataggtaaatctacaactagt
tgcaatatttctgtagcactttcaaaaagaggtaaaaaagttctacaaattggctgcgac
cctaaacatgatagtacatttacgctaacaggttttttaatcccaacaattatcgatact
cttcaatccaaggattaccactatgaggacgtttggcctgaagatgtaatctataaggga
tatggcggtgtagactgtgttgaagctggtggaccgccggctggcgctggttgcggaggc
tacgttgtaggtgaaacagttaaactttaaaagaattaaatgcttttgatgaatacgat
attatcttatttgatgttttaggtgatgttgtatgtggaggttttgcagctccattaaat
tatgcagactactgcttaatcattacagacaatggatttgatgctttatttgcagctaat
agaatagcagcttcagtacgagaaaaagctagaacgcactctctgagattagctggactt
gttggtaatagaacagataaaagagatctaattgataaatatatagattgtgttccaatg
ccagtattagaagtcttgcccttgattgaagatattagagtgtccagagtaaaaggtaaa
actttatttgaaatggcagaaattgataaggatttagcatatgtatgcgattactatttg

aatattgcagatcagttgattacaaggccagaaggtgtagttcctaaagaatctccggat
agagaattatttagtcttttatctgatttttacttaaatcctaaatcaaaggtaggacaa
gaaaaagtagatcaagaagaattagatttgatgatagtgtaaataatattttcagcataa
tataacaaggaataagataatgtctacagctcaatcagatgctcttacttttgaatgtga
aacaggtaattatcatacttttttgtcccatcagttgcgtttcttggttatatcaaaaaat
tgaagacagtttttcttagttatagggactaaaacttgtggttatttcttgcaaaatgc
aatgggagtaatgatttttgctgaaccgagatatgccatggcagaattagaggaaggaga
tatttcagctaagctaaatgattatggtgaactccgtagattatgcttacaaataaaaaa
agatagaaatcctagtgttatattttggattggaacatgtacaaccgaaattataaaaat
ggatctggaaggaattgctccaaaattggaagcagaaattcgtgttcctatagtagttgc
tagagctaatggtttagattatgcctttacccaaggtgaagatacagttctagcagctat
ggctcaaaggtgtccattaaatttaaagaatcaatcagataacacgagtcttaaaacttc
tcctcatattcctttagttttatttggatctcttccggatccagttgtaacccagttaac
tatggaattaaaaaaacaaggtatttttgtttctggttggttgccatcaaagaggtatac
tgaattaccggttataaaagaaggatattatgttgcgggagttaatccatttcttagtcg
cacagctactacactaatgagacgccgaaaaacaaaactaattggtgcaccatttccaat
aggtccagatggtactcgagcctggattgaaaaaatctgttcagtaatgaatgtaaagcc
tattggattagaagatagagaaaaagcaatttgggcttctttagaagactatatttcttt
aatccgaggtaaatcagttttttttatgggtgataatttactagaagtatctcttgcaag
atttttaactagatgtggaatgactgtatatgaaattggtattccttatatggataaacg
ctatcaagcagcagagctagctttattaaaagctacatgtgataaaatgaacgttatgat
gccaacaattgtagaaaaaccagataattataatcaagtagaccgaatccgtgatctgaa
acccgatttagttatcactggtatggctcatgcaaacccctttagaagccagaggtattaa
tacaaaatggtcagtagaattcacatttgcacaaattcatggctttaccaatgcaagaga
tattcttgaactcgtcacaagaccattacggcgaaatcttagtttgtcagaattaggctg
ggatgtttatagcaagcaaagctagtattcctc----tttatgacttacatggtataaaa
gccttgtaagtct--tttattttatgttaaagaagcatttcttactctgccatcaccagc
ccaagcttgtaaactttctacttgttctttgtctgtaaatgctaaaggcacaaactgttt
gatagctattttaatatcttcagtactaaattctctctcttcactaaacgccgtatgcat
actttcaataattgcttgctcaatttcagcgccagaaaatttattacataataaactcaa
ttcatgaatatcatattcttgccacgatcttggtctaacttttgataaatgtatcttaaa
aattgactctctctcttgacgattaggtaaatctaagaaaaaaatttcatcgaacctgcc
tttccttaacatttcggaaggtaaactttgaattttattagctgttgcgacaacaaatac
tggagctttttttctctgatagccaggtaataaatgtaccaaatactctggcactagtacc
actatcaccttgactatgtaaaccagaaaaagctttatctatttcatcaatccataatat
gcaaggcgataatccttcagagatatttatcatttctctcatttttgattccgattcacc

aactagtccaccaaacagttttcccatgtcaagacgtagcagaggcagcatccaatcgct
tgcaatagctttcgcggttaaggatttacccgtcccctgtataccaactagcaataaacc
tttgggcgaaggaataccgtaatttaaactttgcttcgaaaatgagcgagaccttttttg
tagccattgttttaatacatctaagccaccaatatccctatttactttactataaggata
aaattctaatagatgcgtttgattaataatttgtctttttttcttcaataataataggtaa
gctccgagagtctatctgattatattgtgcaataatttagtaatcactttgcggattct
atctatcgataatccttggcaggattttgttatattattaacgagctctgaattcaaatt
aagattcaaagctttactcaatcttatgatctctttttgatttctagaagactaggtaa
gggcaaatctattaccgttatgatatcattcagagcaaaaggtatattgattttgcaaga
aataataataatgtgcttagattgcgttttgattatttagctaaattgcgaagtttcct
aattaatactatttcgtttaggaaagagtcaaaatcttttaaaagaaaaagatttaaaga
ttcattattcaagttttcaataaattctaatgctaataaaggatttctttttgcataacc
attatcgctaggattgttagtgtatccatctacaaagtcccaggaatatacttgttgact
gtttgaacagtttaagctgtgttttataatatactctagtctatcttcttctctagtatt
tattacaataataggatatcgagattttaaaagtaatcgtaaatcttgagtgaaattcat
gtcaatatttgtttccatcagacttttgtacttcaccaattgaagaaagactaaataaga
ttatctaatattccttggatatattcaaaagtc------atctctctatttctaaatta
ttgctattactaatttaacttaccatgatttttttcttccttttcttcgtcgactattt
aatatagagcgtccacatggtgttttcattcttgctctgaaaccagaaactctaattttt
tttcttttagatccttgtagtgttcttttagtcatgatgtttcctctgttgtaaaagatt
aaatactattttatcatcttttatatatctttatgatatagatatgtattaatatcaaag
tt-aacagcaataatttatattaatcaagaattatttatataatattaaacattaaatat
----------taactttatgatattggctttgcccatttttttatttaagtattttgacca
tctttttgctaatcttaaactggttaattttttcagcagctaaaaacgattttgttattag
aatctcaatttaagtattttgttgataaaagccaaaatcgtaaattagaacccgaagaaa
gttttgcttttgcaaaagtatgtgtagctaaaaaatatttttctaaagctattattgaag
gtcaattagctttaaaaaactatcgagacctgaatatcttagataataatatagtaattg
ctaatttgtataatatgctaggttttatttattttgaagcaggccaaacaagttttgcaa
agaacttttatgagcaagccttacaaataaaccctaattatgttgtagctttaaataacc
tggcaaaaaatttacgaagaggtaaaagatttaaaaaaagccgaatctctatacgataaag
tgttaactcttaacttaaataataaaactgctaatagaaggaaagattttatagcaaaaa
cta---------aaaacatctaatactatttgtaatcgggatagcaggatttgaacctgc
gacatcctgctcccaaagcaggcgcgctaccaaactgcgctatatcccgtaagacataat
tattactatataccttttctctaattatgtctactctttctgtattttatctaggatacc
aaaacatgcctttaactccatctggatctttgataaatcctaatttttttataaaaactaa
caacatcaggttctgcgaatagtgtaatagtgctaatttctgcctgtctcaactgctgta

ttaattgatgtatgattacttttcctaaacctaaaccttggaaatctggatgaataacta
cgtcccagatagttgcattaaatccgttgtctgatgttgctctagcaaatcctacaagtt
tactatttgcatctttttttgtattaaagaaataataatagaactatttttaatgcga
tctttactttttttagaggtcttttaacccatccgactgaatcacaaagctgctccaatt
cgtataaattaatattcttattgctactcagatagatatctttaaattcgattttatcgc
aagttttatctaaaacaataagtttttgaaatttttttcagagctagcattgatatcgg
agttttgaaaaaaatttttccagaagatcataatgattatgatataatta--aataaata
tatatattacaatattgctttaacaaacaatatattaatatatgctcaaatttttacatg
aatctattaagatagaagattgttaaaatatagagaaataaaaattttgtaaaatcttt
caataaaatgatataaagagtgcttttaagactaacatctattattataaataaagtct
cgaaattgagatgaaataacacattattctatgtctaatgccttttaaaggagttatttg
tgaaaaaatctatgtgtctaacctgtttacttgctttactaattatgagcaacccaataa
tagctaacgcagaagtagctggattggtgccctgcaaagattctgcggcattcaataagc
gtatggtaaatagtgtgaaaaaacttcaagccagattagctaaatatgatgcagacacgc
caccagcgttagctttaaataagcaaatagagaaaactaaaactagatttgcaacttatg
gtcgagcaggtttgctgtgtggtactgatggattgccacatttaatttctgatggtcgat
ggagtagagccggggactttgtttttccaggactattattcttgtatattacaggatgga
ttggctgggtaggtagaggatatcttttatctgttgctaagactagtaagccaacagaaa
aggaaatcattttagatgtgccattagctatcaaatttatgtcatccggatttgcatggc
cgctagcggcttggcaagaatttagtagtggacaattaattgcttccaatgacgatatta
ctgtttcaccccgttagtaaaaaatttatatgaataataattttaccaaatacttatcaa
cagcacctgtaattggtgtattgtggatgacgtttacagccggatttattatagaattga
accgctttttcccagatgtcttatacttttactt----ataaataa--gatcgtataaa-
--aaactacataaatatagagaaatactattatttgtgtagtttta---atatataaca
taattattta-tttttaaaaatgtttatcttttcgattattttaatattagatcttacat
aataaaata-atatcaatattatgagcttagtaacccaaattatagttaatgccgatgat
gaattaagatatcctacaattggagaattgcagtcaattcaagactacttaactacagga
agcaatagaattagaattgctactattattagagataaagaaaaggagattatacagaaa
gctagtaagcaaatttttcagttacatccagaatatatagctccaggtggtaatgcagca
ggttctaggaaaagatcgttatgcttacgtgattatggttggtatctcagactaattaca
tacggagtattagctggggataaagattctatagaaacaattggtattataggagtaaga
gaaatgtacaattctttaggtgtacctataattggaatgttagatgcaatccagtgttta
aaggaagcatctttagaaatgcttggtcaagatgatattagaatcatttctccttatttt
gattatataattcgtggaatgtcataaatcatagcttcaattaattagttgaataaaagc
ttttataatgttataattacaataagcccgaaagcatataattgtaaaaactaaatcttg
tcagaaccggaaggtagcagcaataatgtttcaatacaaaagttatga-ttttcgggtgt

ttattgtttttatctaatagtctcgacttttaattagtatgctatgtataaatctgtata
tgacatgtaatccatcaaaccttccagcaaaaataatttaataatttgatattaatacgt
aaaacacctatac-tcttttataaaatttctttatatctcatctaaacaatggaaactta
acttaatgggtgttcatattttatcaacaggttcgtctgtcccaaatttttctgtagaga
atcaacaatttgaagatatgatcgaaacttccgaccattggatttcaacaagaacaggaa
taaaaaaaagacatctagctccatcttctacttctttaactaaattagcggcagaagctg
caaacaaagccttatatgcagctaacttaaaacctactgagatcagtttaattattttag
ctacgtctacgcctgatgatttatttggtagtgctagccagttgcaagcagaaataggtg
caacaacatcggtagcttttgatattacggctgcctgctccggttttattgttgctttag
taacagcagctcagttcattcaaactggttcctatgacaatattttagttgttggagcag
acacaatgtctagatggattaattggtcagatagaactacctgtattttatttggtgatg
gtgctggagcagtagtattggggcaaagcctcaaaaatagtattttaggctttaagttat
gtacagatggtcagctaaacagtcatttacaattaatgaataaacctgtaaataatcaaa
aatttggtgttacagaaattcctcatggaaactataattccataacaatgaatggcaagg
aagtgtacaagttcgctgtatttcaagttccaacagtaattagacaatgtttgaataatt
taaacatttcaatagatgaagttgattggtttatattgcatcaagcaaacactagaatca
tagaagcaattgcgagcagattatcagtaccttttttctaaaatgattacgaacttagagc
attatggaaatacatctgcagcgtcaatccctttagcgttagatgaagctattcaatcca
ataaaattcaaccaggccaaattattgttttatctggttttggagcaggcttgacttggg
gagcaattgtcttgaagtggtgat---ttatatattgcggatgacgagactcgaactcgt
aaagctttcgctacacacccctcaagcgtgcgtgtataccaatttcaccacatccgcatt
ttttaccatgaata-ttaaaaatttataatatttatctaaatattcatataa--tgtgta
tatatatatacattcttgtcagaaactctcttatattttattttaagtgataaaattgag
t----ttagtatagatagtaaaatttttatttaaaatcataa----aaataaatatataa
ggaaaataaaatatgacaccatctttatcaagtttttttgaatagtcttattcttggggca
gtaattgtagtcgttcccataactttggctcttttatttgttagtcaaaaagacaggaca
atccggtcctaaaaataacttagaaagatatggagaagcaatttaaaatgaataataaaa
tatttttcatactaataagctctccatatattgctatact--ttagaagaatttttattt
ttttattttgtaacatcctgtaaagtttattccgttctttctttttccc-atttactctc
ttataattaatgatacaaatgtcagg-ttaataatag--tttttttattctgtatctttc
---------------attttatatacgtattattatgttcaattaataaagaaaagctca
tgtctttgtctaattggcctctcaaaaaagaaaattctgaagcatataatattaagaatt
caaaacaaatcacaattcctgatggtttatggataaaatgcttcgactgtggtctattaa
tgtattctaaagtactgaagagaaatttaaaagtttgccctcaatgtagttatcattttc
aagcttctagtaacgaaagaattgatcaattgatagaccaaggtagttggcaaccaatgg
atgttcacttgatctctacagatccattaggctttaaagaccaaaagctttatagtcaaa

ggttaaaagatactgctttcaagactggcctgcaagacgcagttcaaacaggtactggga
ctatgcaaggtaaaaaagtatgcttaggtattatggattttagattcatggggggaagca
tgggatctgttgtaggcgaaaaactaacaagactgctagaaaaagcaactcaagaaaagt
tgcctgcaattatactttgtgcatcaggcggagctagaatgcaagaaggtatgttgagct
taatgcaaatggcaaaaatttcttctgctctagaaatgcataaaaaagaaaatctactat
atatatctgttttaacctctcccacaacaggggggtgtaacagctagttttgctatgcttg
gagacttaattattgcagagccaaaagctcttattgcatttgctggtagacgagttatag
aacaaacaatcaaagaagacttgccagataattttcaaagttcagaatatttatttgaac
atggtttcctagatttaattgtatccagaactcagcttagatcaaagttaatacaaattt
tgtccttacataatcatagtaagtaatgaattgaatattgactataatatcaaaatgcta
gtaaaaattaagatattaagaaattttcactaaagatatttgtagactaactagaattac
aattactattgtagaaaaatttgtcttaatgcca-tttttcagtcttttaacaaattg-
tttttatgactttaaataattgttagtttaatacaaatttataaagagaata-----cat
actaagctaaatatagtaatattatgttttcctataatactgacctatcaaatttcgagg
aaatttatgcttaaaagatcttcttggcttgcggctttattgggactattaacagtagtt
tctacaagtacgcatacatatgccatagagttagacgaggcaacaagaactgttccatta
gaatcttctggcagaactgtaattcttacaccagaacaagttaaaagaggcaagcgatta
tttaataattcttgtgctatttgccataatggtggtatcacgaaaacaaatccgaatatt
ggacttgacccagaatctttaggattagctacgccacaaagagataccattgaaggacta
gttgactatatgaaagatccgactagttatgatggtgcagagtcaatcgcagaattgcat
ccaagtattaaaagtgctgaaatttttcctaaaatgcgcaatctaaccgatgaagaccta
tttacaatcgcaggtcatatcttacttcaacctaaaattgtttctgaaaagtggggcgga
ggaaaaatttactattagaacttcaaagacctaaaccttgtgaatactagtttatgta-t
ttgttgcttaagtgtgattattttgttagatcatgatatatattaaagatggagtttgtt
gacagcaagttacttttacccgaacttgtttacactattcgtctagttattaaaaagga
gacgttaaattgaagaagaagctttcagttcttttcactgtttttagttttttgtaata
ggtttcgcacaaattgcttttgctgcagatctagataatggagaaaaagtttttttctgct
aattgtgcagcatgtcatgctggcggtaataacgccattatgccagataaaaccttaaaa
aaagatgtacttgaagctaatagtatgaatactattgatgctattacttatcaagtacaa
aatggtaaaaatgccatgcctgctttcggaggtagactggttgatgaagatattgaagat
gcagcaaattatgtattatctcaatctgaaaaaggttggtaattatacttgattttatcc
tgtattaaagaatagacaatctatttagttgttcattagattgtctattctttgtttatg
---ctattatataaagatatttacacaatatt----ttatgatgaaaagaatacccgcaa
ttcttgtactagaagacggtgcgtattataaaggatggtcattccagcaagataaacaag
agattactattggtgaagtagtttttaatactggaatgacaggatatcaagaaataatca
cagatcctagttacttccatcaaattgtcgcttttacctacccggaaattgggaatacag

gtattaataatcaagatattgaatctcacagtattagtattaaaggacttattgcaaaaa
atatttgtaaaatttcaagcagctggagagagcagcaatctttagttacgtatttaagta
gtaataatattccttttattttcggaatagatacaaggtctttaacccaatacttgcgtc
aatttggtacaatgaacggttgtatctctactgataatttaaatcatagttacttaaaac
agaaaatttgtgagattccaagtatgcaaggtttagatttaatcccgcatgtaactacaa
gaaatgtttaccccctgggatgaaaaaagttttccaaattggtatttgacagataacatta
gagtgcaccgagttattcagttgaaagttattgttatagattttggagttaaactaaata
tactcagaagactagctacacttggatgtcagataacggttgtgcctgcccacactcctt
taaaagatattttggcttaccagcctgatggtatattactctctaatggtccaggagatc
catcagcagtacattacggcatccagacagttacaaaattactagattacaatgtgccta
tatttgggatttgtatggggcatcaaattttaaatttagctcttaaagctaaaactttca
aacttaaatttggtcatagaggtattaaccatccatccggattgaaccagcaagttgaaa
taactagtcaaaatcatggctttgcagttgaattgacttcggtttttgaatctcctgtaa
gagtgactcatttaatctaaatgacactactattgcaggaactggacataatcaaagtc
cttatttttctgtgcaatatcatccagaatcgagcccaggccctcatgatgctgattatc
tattcgaaaattttatagaaataatgacaaagtccaagaataaagttag-----------
ttagtaattttcccatgctttatgagtgaataaagcggatagtacttgtactcctcgtaa
ttgattaaagagaggcaaatgtcctctggggccatgagaattccattgaaattcctcagg
atatctgcaaggaattctatcaacttcccagccgatttttttgccagaattttccccaatc
ttttccaacactaagccatatttgtctttgaacaaatagcccaaatttaccttttgagtg
tgtatgccataacttatcaatagtttgtaaatcttgagcaggtattttttttatatctgt
aaaatatagccaattacgagtttgtgcatttacaccagctagttgaataagcttttgttg
agttagctgatctgctttgagcaaatcccgatgagtcagtaacatttgtaaatctttgta
attcatctgttgagcagagcgcaatggaacaatgccatcgggacataagttagaagcaaa
tttcactatttcttgattttttactgttaagtaacttttcataaattaaaccgtcaacaca
attacttttatagttaggcccagtaatcctctcaaaaaatagatctgctaaatcttttaa
ctcgatagagtctttgctatttatattctcaataatttcaagttgttgcttga---catt
attagacttagtattcttattcaattctaaaagctgggctcggatttgatttggcatctg
atttgtttaattagttttaatagtc----ataaggtcttagagagaattagattgttttt
tgaaattagaagagcgccgcagtatttctaaagaagttcgcataatttgattcaataagt
tttgtcctgcataaattataatttgaccaattaaaaaaattgtgctttttattttaggaa
caaaaaaatcttgtacctcaagcagaaaagtaattattatttgcattggtgacaacatct
gtaattcattcgatctgcaagcatatatatactgacagtttaagccttcttgataaaatc
cccatactttgtaacgactttcgtaaattgctcgtggtcgctcaagatagtttttaatta
tagtattccaaattaggttattctttaatttttctaattttctatcagatagaaatttta
cattacagagataacttgcatttgtctcatttacagtattaaaattttgaactaacatat

gagcaataatgttgcttagctgaattaaataattttcaagaaaaatttcgacctgcttaa
ctggcatattgtaagtaaagagattaaaagttttattcacagtttgagatgacccaaaaa
gtagctgaataattaaaatttgtagtagcatcttatagtcgttcagtagatagttcaaat
catgaatatcttggtaaatgtcaatacagtccagttcatataacttacaaaatcttttga
tacatctatggaataaatcaattaaaatattttcggtcaagtttgtaatatcatttacat
ctaaatttgaattataaatttctaaaagtatcttttccagttcagctaatatgattttta
ataagtttctttttacttcagtgcgaaaaacatctaaaattaacacttcctgagagcaat
tagttaatctcttattaatctttatagaagttctaactaatagctctgctacctcttgat
tgagtgttggtccctgagagcttggccaataattattcacgttataccatagtaagaaat
attgaatttaatgttatacgataacttttttaatatactgaaaagcaagttaattactcta
tagcaactag-------tttttacagatcttaatagttatttatcaaataaagttatatg
atagtagcgattgagctaattttattaaaattta----taataatgacaacttattactt
cgcccttgcgagtcaaaattttctattagtacaagaaccactagaagaagtttttagaga
gagagttaactactatcagtcaaataataaagcaattgatttttggctaataccaaaccc
ttcttttctagagaaaccagaaatgatttcatttaaaaaccttgtacctaaagacgctgt
agctataatctctactaatccaatatttattaattggttaaagctaagaatcggctatat
ctgtattgggcaatttgaagataacctacaactttctgaagaatcgttaaatattactgt
tttaacagacaaaatttaacaatgcttttattatgactagtgttaaattattttggttaa
gttataattatagtcttttagtattta-gactcttatagtttatcacttagtattttatg
ctgagatggagtgacaaatagtgtaagtatttcttggctaaacgtttcagttgccttaga
tttatatctatttggattaactataatggatagcattcgttttatagtaacattttcaat
ttgagcccaatgtacaattccaagttccaattccttagcaatagctgaaacggaaacaaa
ggcagctcctagccctgattgcacagcgtttttgatagcttctatagaattcaattccat
ctctatttaaagcgactgctgtcaataccatgttgactgagtactttatctatgacttt
tctaattgttgattgagtatctaacgcaataaatctaagcctgtataagtcttctttttg
aatgtctcctagtttagaaaaaggatgtgatttgggtaatataagtgctaattcgtcttc
cgcataagaagtaacctgcaaaacatcttgcaattcagtaggcacttctcctccaataat
tgctaaatcaacttgaccattagctacgctccatgaaataagtctagttgaatgtacttg
caattgaacagccacttgtggatatctttgcctaaatagtccgattaatcttggcatcaa
atatgtcccagttgtctggctagctccaataattaatgtgccaccttgtaagttttgtaa
gtcgtcaagagctcgacaagtttcttcgcagagagctaaaattctgcccccatatcgtaa
aagaagacttcctgcctcagttaaagttgccttcttattacctctttcaaatagggaaac
attcaattggcgctctaaattttgaatttgcaaactaatagctggctgagaaacatacaa
gctattagctgctttttttaaaactgccctctttggcaattgcttttaatattcttaactg
atccaatgtaaatggaaggtctgtcattaaagaattgtagtataatg---------tata
ttatttagtattgatcaatgtatgcatttgccaactaaaatatttgtcattacgcttcat

tattttcaaaataataaaaagtataatgttaaataagctgtattaaaatttagtatctgt
ttgatattagatttatacaagagatatcgtgaatttaattctaactataaaatataggag
acaat---atggactctagacttttagttgtactaataccagttttagcagcagcatctt
gggcagtttacaatattggtagagttgcactacagcaatttagaaaaatgacatcttagt
tttgcttagaatata-atttttagactttatgagatagaggactttaaaatccctatctc
tattgtatatcttaataaaaaaag-aaagc--ccccttgtacttactgttattacacatt
aatattattggt-ggtaccta----gataataagtttaagctaacttatttttttatttta
taattaattaaattaataacacaattatggctgttccaaagaaaagaacatctaaagcta
aaaaaaatgcacgcaaagcaaattggaaaaatcaagcaaaaactgaagctcaaagagctt
tgtctttagcaaaatcagtattaactagaaaatccaatggatttatttataatctaactg
aagcatcagatacttttagcgattagaagtgttt-ttactacaagtctgaaaaagtatta
cgcaacaagttctcagtaatttttatctag----taatcatttgaaaatcattctatttg
ataataaaataaatagcagattaaataaaatcacattaacaaattatgcagcaaagctag
atcaaactagtgaaatttggttgttcaattgtattgaaaatattcagcatatttttttta
aaagtcaattaaaatcaagtcatattactaaaatttttatttctggtactagctttgaat
ataccgcaggtctaccaggattattatccagcttaacactaagtggtagactacatccta
ttagtatatatagtccccagtctctcaaaaagtatcttgaagcatgtaccaaatattccc
aaactaatttttcttttcctattaatttcataatttacaatacggaggacaagttgtta
accaattttatacagtaatttgtttaccgttgagcaaaaagagcctgctgtatggattta
ttattctaaaaaaagaaaagcagggagtatttaatttagcacaagctaaaaccttgaata
ttcttcaaggacctatatatggaaaactcaaagaaaaggataatttttttaagtccagatg
gttactatctatccggccaagacttctcttctaatacaataatgggacataaaatatcgc
ttccgttattagtcagatattctagaattatttctgagatgcattggttttgctcttatc
ctattagattaaatacttattcgcatcagcaaggagcaaagtgtttaccacataatgttt
taactgatattatgaaatcccaaatatatcaagataacagttttgttgaataatacttat
tcttgatatataatggtctcggtaatctaaataaaatactagaaataatttcttaaattt
atttgtctatattaaatttacaatcatttatgacatacgcaattattgaagcaagtggca
cgcagctttggatagaagaaggccgctactatgatttaaatcatatacctgttgatccag
gtcagtcgattatattaggaaaagtcttattattgaataaaaaatggggaggttactttag
gccgcccttgtatagaaggggttacgataaaggctacagtaatgaggcacttgcgaggaa
agaagataactgttttcaaaatgaaaccaaagaagaaaatgagattaaaaaaaggtcatc
gacaagaattgactcgtttaatgatcgattctataacatcttaga------taaaatcta
gactcatattttt--aa-aaactttttaatatttaactaaaatagatataatggcacata
aaaaaggtagtggtagtacaagaaatggcagagactctaattccaagcgtttaggtgtta
aaaaatatggtggagagcaagtaacagcaggtaatattttaatcagacaacggggaacta
aagttaagcctggccaaaatgttggaaaaggaaaagatgatacattgttttctctaattg

atggtttcgtgctgtttgaaaagtcaaatcaaaagcaaaaaacaattagtgtttattctg
ctaagaaatagttaaaacaattggtgcaagataacttgagt----gtatttatttcactt
acagtggcaccatatatttgatttattaagaaatggagttccttaatgaaccaatgaatt
ttattaaagatttgctgtgaaatttaatgacaaacactattgtaatttcctgtctacaca
atatggctgctattctatattgtggtcagatacagaaactagtagtagcaaatgctcatt
atcaagtaagtgatatctacctaggttgtgttgataaaatcttctcaggaataaatgcgg
catttattaacctaggaaagaatgagtacagtggttttatacatatcagtgataccggtc
cgcttaaaaagaaatattatgtcaataatattactaacattttaacaatacggcaaaaaa
ttttagtacaaattattaaagagccaactttaaataaaggtccaaggctcactgccaata
ttacattatcaggtcgatatattgtattaatgccttttagtcaatcaatctgtatatctc
gaaaaatatatgatgaagatgagcgtcattatttgaagtctttagctattttaattaaac
cggcaacaatgggcttgctatttagaccttctgctgtaggtgtcgatgaggaaataatat
taagcgaattaaaaaatctaaaagaacaatggaactttgttcaaaaatctgcaattaata
gttattcacctgttcttctatataaagatgaagatattgttaaaaaggtaatccgagatt
tttataataataatacaaacaatatagtaattgattcaaacctgggattaaaacaattaa
attattatatccacacttggcactgtaataactctagcacagttcctaagattaagcttt
atagtaataatcaatgtatactagatgcttttggtatcaatcaggcaatttccagagctc
tcattccaaaagttgatcttatacttggtggctatatgtttattgaaactttagaagctt
ttactattattgacgttaattctggatcttttaataattccactagtgcacgagaaacag
ttttaaaaaccaactgttctgcagcaacagaaatagcttatcagttacaaattagaaata
ttactggtgtaattataatcgacttcattgatatggaatcacaaagagatcaattgcaat
tattagaacactttaataaagagctatcacttgatgatgctaaaccacaaattgtacagt
tatctgaattaggtttagttgaattgactagaagaagacagggcaaaagtttgtacgagt
taattagtagtgattctaattactttatttttcacacaatcagagagatctcagtctc
ttaagagattcgatgatagacagcagaaacaacagattttttaataaatcttggctatctg
cagagattaatactattaacaaggtctttttcaaaagtcaaatttgtgcagacctgcta
acttttacctaattcgtaatctctatatagttaaaagtagtattacttataaacaaaatt
atttattaactcacagatcaaaacttatttattctaaagaatacagcaaagtattaccaa
gca------gttattatctagctagtctcaataagaatagtaatcaagagttcctacctt
aagattgttatatcaactgtt---------------------------------------
-------------------gaaaaaaagaagctctctcttatataagagagagcttc-tt
ttgtagcttactaacta-tctagttagttaattaaaaattaaccgttaacagctggagct
gttagagctactggtaaagattcaccagaagctagatctagagggaagttgtgagcgtta
cgttcgtgcattacttccatacctaggttagcacggttgatgatatcagcccatgtgtta
attacacgaccttggctatcaacaacagattggttaaagttaaaaccattcaagttgaat
gccattgtgcttacagataaagctgttagccagataccaactacaggccatagacctaag

aagaaatgtagagaacgagagttgttgaaactagcatattggaagattaaacgaccgaag
tagccatgagctgcaacgatgttataagtttcttcttcttgtccgaatttgtaaccatag
ttagcagattcgttttcgcttgtttcacgaattaagctagatgtaactagggatccgtgc
atagcactgaacagagaaccaccaaacacaccagcaacacctagttggtggaatgggtgc
attaaaatgttgtgctcagcttggaatacaagcatgaagttaaatgttccggagatacct
agaggcattccatcagagaaactaccttggccaattgggtatactaggaatactgctgct
gctgctgctactggagcagtaaaagcaacggaaatccatggacgcatacctaggcggtag
cttagttcccactcacgaccaatgtagcaagctacgccagttaggaaatgaagaacaact
aattggtaaggaccaccgttgtatagccattcgtctaaagaagcagcttcccagattggg
tagaagtgaataccgatagctgcagaacttggaataacagcaccagaaatgatgttgttt
ccgtatagaagggaaccagcaactggctcacgaattccatcaatgtctactggaggtgca
gctacgaatgcaatgatgaatacagatgtggcagttaatagagttggaatcattaataca
ccaaaccaaccaatgtataggcggttttcagtactagtaatccaagagcagaaacgttcc
cacaagctagcgctttcgcgtctttgtaaagtagcagtcataatttttatcaattttta
aggatttaacaaagatgcttcccaaacaataagttgtctgttatggaaattattacttaa
ggccttttcaaatgagaacattttaaaaaaaaca-tagcaaaatctaatctcagtaact
aa-aattt-tacttgttcatatattgcttaataattcaa-----------taataaattt
tt-gct-------------ttttgcaaaattaacactattttagggat---ataataata
atgcatattaatgctgcttattttgattttaatagtttatataattttgccctacttaaa
tacgcatgtcacactttacaaaaattcagacgaccatacaagatttaaatctgttaaagc
atgctttaacagatttgggtttgatttggcaaatgaattctagtcatataaaagttggtg
aaaataatcagcacaaggtagatattttaattaaacaagataatttgtcacatattggct
ttacttggaatgataatagatatcacttagttgccgacttacaactttgggaacaaccct
ggtctctagaagtattttggataagttatctcagaagtatgcttactactctatcatcg
aagaaacaaagaagcaaggctttgaaaaaatgcagcagatttcacaaaaagatggatcaa
ttaagttaattgtgcaacgctggaattattaactaacatacaaatgcgggcggagagact
tgaactctcacgagattatctcactagaacctaaatctagcgcgtctaccaattccacca
cgcccgcactgttgagac--aatattactctatcataatctatactaaaacacaaactaa
-tagtataaactagcttccaagcttaaaagcgtctacaaatagaccgtatgttataccaa
ttttgaagttgttgaataaattaatttttatatttaattgcttgtggctagagtagacta
ttttactagtcaactcagttgctgcaactattagactggctgcaataataccccaatctc
ctgtttgtccaggaatcgtagacaaacctgttgaaataaaaaatcctagcaataaactaa
ttaagccagttgttaactcgctcaaagaataataaagcttattatttaagtttttatta
agtaagcaaaaaaagttgaaagtctagttttaatcatagtatttagtaacggagttttaa
aaggatgacaagtattaacag-tcattaatattgtcgtatccttttaaaactcgttaata
caaacagctgttcttgt---------ttttaaaggtcttgttcgcttaaagcattaataa

```
gatgctgaaacggctcttgtataatatctgcgcgtttaattgattgcaaatctaattctt
cttctatgatttcttgtaataactgtatgcttcttactgttggagcaacaggtacagata
gtgaattgtaagtatcttttagcccatttaatacacgctgatcgagaatatttgtactac
cagaaaccaaggcataactagcataccgtaggtaatattctatatcccttagacaagctg
catatcttcttgttgtataagaatttcctccaggccttaataattcaggctgttcttcgt
acaactgagctgcggcctcttttaaaatattggtagcttgatcattaataatttcagcta
tttttattcgatctagtccactcgaaaagaaagattctagttgtccgacagctgtcttat
ctagataacgaccagtaagatcgtaacgatttaatattgctgttatagcatcttgcataa
attaattctcctac-ttgctatcagagcggtattataaatgactaatattgtaatataat
cattagtgtttatgaaatttatttaaaacttattctttattgcttaatgtgcaattaaaa
actcgcatagattttacacatatagagtatacgtagtttttaatta-tttttttataaaa
accaagaaataaagctttttt-taagaggttcgtgaatttgttaatataacatgagcttt
cctcctaaataagttttgtaaagtac--aaagtaaaaaaacgattagtcaataatagtaa
------------------------------------------------------------
------------------------------------------------------------
------------------------------------------------------------
------------------------------------------------------------
------------------------------------------------------------
------------------------------------------------------------
------------------------------------------------------------
------------------------------------------------------------
------------------------------------------------------------
------------------------------------------------------------
--------------------------acaatattaatatgtatcaa--------------
------------------------------------------------------------
------------------------------------------------------------
------------------------------------------------------------
------------------------------------------------------------
------------------------------------------------------------
------------------------------------------------------------
------------------------------------------------------------
------------------------------------------------------------
------------------------------------------------------------
------------------------------------------------------------
------------------------------------------------------------
------------------------------------------------------------
```


-----------------agtataaataagctttactccagcatcaaaaaaaatatttga
gatatgttgatacaactgattattcaagtgaaaacttagatttagaccaatttagctatt
ttatcattacaattaataaaactgataaatgtattcttatagagcaattttgttataatg
ctgaaggccatatttactcaatattcttcaaaggtccaacagctaaacatctttcttcta
taatttgtcattttcaacaattaaatacaataatatctacagctcatgccatatatttag
gacgagagctaatgaagtcagaattagctctcgtgctagatcaacaatatattcaagatt
aaa--ttttgtaagtattaattatatttatgctagtataatattagtaagggcatgtaac
tcagtggatagagtatcagattccgattctgatggccgtgggttcgaatcccgccctgcc
c---gagtattcagagtact-atactaaaacatct-------taatagctagtttgataa
tagtatgggcattggggctgcaaggtttctacattatgaaaagaagaaaatatg-aaaaa
aaacaagctctccaaaagagcttttagtacaatcaataaatgcagaaaacaatattgttt
ctttttctcggaaattagctcttgcttaaacactgttaattttggcattcgacatgtta
aactcttattcatggcgaatattctgtcagagtcgctcttagtttaagaaaagtcagaaa
aaatatttatctttacgtttt-tacctattttatttaaataaaggttagta-ttttcaa
cttttatagtggacgtgggttcgagtcccaccagctccatcaatttgatt---------t
gtataattagctagtttaaccttttaataaccagatatataattggaggaaatatgggt
ttcataacaattactaagcccgctttaaagcaaattgcaatcttgaaaaatgatcatgaa
aatgatgtacatctaagaataggtgttagacaaggcggatgttcaggtatgtcatactca
atgaattttgaacatgttgacaaattaaaagatacagatgaacggcttcgtctagataat
ttttctgttgtctgcgaccctaaaagtcttctttatctttatggattatcattagatttt
agctcggaattaataggaggaggatttcaattttctaacccgaatgccagccaaacttgt
ggctgcggtaaatctttttcaggctgacc-ctctaacacgtttttttatatattttgtatt
atatatattattttagcaagttg-tttattagagacaagcaatgctctgttaaaaac---
----------------------------taatttcaagagttttattgtattatt-----
----ttatatatttattatcaactacttgaaaataaattagctaagcaatctaatttgtt
atcagtaccaaactttaaatgattaacttttattgatatatgctaatcttt-atcagcc

taaacatatctgtacattacaatcttttgacataaaaagagtcttaacatacctctcaa-
----------------------aaataaattcaa----------tattttataaatactc
aacatgaatctgtcacaacttatcgttaattcttctatgatgaaaattcctaataacttt
tattgcaatataaaagctatttcttctgagaatttaagtcacttacaattatcaaaaaca
aatttattatctcctacaaaaataattgcttttggactacctaaatttgattttatttct
acacagattacaagttctattagagaaacagttgttgtgtctactattgatgataataat
ctttttgctgccccaactaatgattggataaaaaaacacaaggataaaaatcatcattct
ttgttttataaaaaagtttttaaagctttgctaaaccataaaattagagttgttgctgag
ccatt------tcataactccgaaataaataaagaaaagagcataatggcaagtcaaaga
tacatctggggtaaaagctggaagccttcaattattttatcttgcttaaaaaacaaaaaa
aatagcaaaatttctgatgcatcgaaaaaatttttaactgaacagttatctgcatcacca
gtatttgtagtaaaaaatggttttaatgaaattattttaggtcacccattatctcgtgtt
aaaagaggaggagttaataatctgatgcatgcgttttctaatttattaaatcagtctaat
gcaacttatcctatatctactggtctattctttttttcatccagatgatgcatttgaattt
aaagactttataatatcggtcaatccactggcggccaaacatatggagataagcgtagag
cctgttggtctacactttgcttataaaatgaatagaaatatatcgtcagatactcaattt
tcgtttattccagattttaaagaagtaggagatttattattcaaatatagaaaaggtaat
catttagtttttcataagaatcagcattatggcaaagatttttttcaggggcaaccaata
tatatgattcagccgatcacttttaaagatcgagccggaaagctaaatattatcaaattt
acggggctaaatgacacaagagaaattatttttactaatcttgaagctgctaatagatca
tgggcacattttataaagaggaattcacaattaaaatcaattaaaaatcctactctatta
gtctataatttagaaagtttttttaaaagatcaagaacgattggataatcaagatttaaat
aagtttgtggtgatcactaataaagaatcttatcttgccacaaaagagttaatagcttta
cccgattctaatagcttttctaagcacttaaaattaaatataaagcctaaactcttttt
gtgaaactatgggtaagacggttattttccaccttaacttatgaat-aaatgctatt-gc
ctttattttctcgaataatattctataactagtaattcattgagttgtaaagcaacccat
tccctatcaatgactccattgactttccctgataaattggatttattcaattctaagtgg
ctaggaatattagctagtccagggaaagctaagtaattttctactagttttcgagatgct
tcttgatttttttacactaattgattctcctggtttacattgataactacagatagatact
acttgtccattaatacaaatatgaccatggttcactagttgtctagcggcaggaattgta
ggagccataccaagtctaaaaacagtattgtctagcctcatctctagtagctgtaacaaa
atttgacctgtcgacccttgtagtttttttagctgcttttacgtacttaaataactgtttc
tcacttaatccataattaaatcgcaacttctgtttttcttctaatcttacagcatactca
gaaggttttctggacttttgtccatgttctccaggtggataagatcttttaatcgctttt
ctactgagtccgggtaaatcacctaatctacgggaaatgcgtactcgtggccctctgtat
ctagacatattatttaaattctcctaatgtaaat-gattaag----------taatgagt

agctatttttaacacaaaaagagcattaaaacaaagc-ttttgctaaaaaatatttaca
aaaaatatagatttggacaaaa---tacattactatgc-tttttaagcgggtagcgggaa
tcgaacccgcatcattagcttggaaggctaaggttttaccactaaactatacccgcaata
ttcaacagattgtcactaactcaatataacatagttatgttaagttaagataatcttaaa
tttaatagaatt-ttaaattatttatgctccttctaaaaccacatcaagaaaacgagcaa
tttcggcagcttgttcttcaacttctgataataataatggttgaccgaccctagtcaatg
gaattactcttttatcttttgtacataaataaatttcacgtcttggatttagcccctctt
taatatctatttttattgatttgatttcttttatattaaactgaagacagattttctat
tctttccagggaaacctagccgaaaaatttttacgatacctttatctttgttaaattcat
tatatccagcccctatattccaaataatagttagccacagaaacaaactcagaaagactc
ctatactgccatagaatgtcattacaataccttgtggaataaataccaaatcggttgaat
ttgtaaaaggtaacaaatcaacttgaaaataacttgatagtccagcaagaagaaagccca
aagccccaataaatattatagtagcccaccagtaattactcagtcttcgtgaacctaaaa
ttaaatcttttctaactgagtgtatagataatgtttttttcataatttgttcttttaaaa
cagta-tactaatgcaaaatggaaaaattttgacaattttt--atgcgcgctttagattt
tttaaataagcttgattgcttttagtcctaaaaacagtcctgttgctaacccaaaaaaag
cagcaagaaatattatatatcctagaaaaactgacatattattattatttattattattg
ttacaatagtgtttacaaaaatagtgaaaatctattactacggtattatattacgttaaa
tgccttgctgtctacaggaaaaaactttttttcagtttttagcattgtgtttatattagaa
ataatattctagtaaatttattaaacttctggagaacagaatcttatgtcagaatttatc
aagccttataacgatgatccttttgtaggcaatttgtccacgccagttagtacgtcaagt
tttagtaaaggacttctaggaaatctaccagcttaccgtcgaggtttatctccgcttctt
agaggattagaaataggaatggcacatggatactttttaattggaccttttgataaattg
ggccctttacgaggtacagatgtagcgttactagcgggatttctatcttcggtcggcctc
attattattctcactacatgtttatccatgtatggtaatgtatcttttactagagcagat
tcgaaagatccactacaaacttctgaaggctggggacaattcactgcaggatttctagtt
ggagcagtaggtggttcaggatttgcttatttattgctagctaatatacctgtattacag
actgcaggtcttagtttattctcttaagctagtaaggggacttgaacccgtaacctactg
attacaaatcagttgctctaccaattgagctatactagcattcagttcacaataacacat
aaagatatttgttgcaatctgctattttcagatgtaacaaatatctttttttatttaaaca
gaactgttatgttaacac-------gtatattaattagaatctgaattatctgactctgc
agaagattcctgatcacagtttagataataacaaattgtttgatctagacaagttgcaga
ccaaccaacaggtgtttctcctgataattcttgcatatctacagaattataaaaattatc
atttgccttattactgtttgcttgcataaatatcactcccaaaacactctgaacttaatg
tgaagtaatttaacttctcaataatatattaacacaaaatattaaatatggtgattattc
tttaattgtaaagatgctgctaactttataaagtttgagtgagagtcttgatagcttttg

ttgaaataagcattttaacccatttattttgccctctcgaccatactttttttgtgttgta
aattaactttttgtagtttttttgttcgtttatgagaatgagatacggcatatccattat
tagctacttttcccgtaagctgacactttttttgacataatttt-------ctaacttata
tatttatttagagtacttgctaaagttgacttgggcacagctccaatcactgtgtctact
ctttctcctgctttaaagatcattaaagtaggtatacttctaatgccatattcagctgca
atagtaggattatcgtctgtgtttatttttactaccttaatagatgattcatattcttcc
gcaatttcatcaactacaggagaaaccattctacaaggaccacaccatggcgcccaaaaa
tctacgagtacaggtaagttattgttaataacttcttgtttgaaagaggcatctgtaact
tgagatactgacatattctttaacctttaatatatgttccttgctaagcaaatcttatca
caaatttcaagaaaaatcttctatagtgaattactattagtgttgaattatttttttaaa
aattagacattaaaaaaacttattacctatataaataaagtggtggccgagtgatatctt
gatagtgataatttttatattaaggttgaacactagaaataacttttcaagcaaactctt
gtttaaaaa--atttctctgtgaccataaaaacttaattatattacaatatattttatta
tttgtaacttagttctgataatggtataaacaacgcaaaagatactgccttataatcaag
gaggaatacatgtctcaatccgtagaatcacggactaggattaaaagcgaacgttacgaa
tctggagtaatcccctacgctaaaatgggctactgggatgctgactatgtgattaaagaa
acagatattctagctcttttcagaatcactcctcaaccaggtgttgacccgattgaagca
tctgctgcaattgcaggtgaatcttcaacagctacttggacagttgtatggactgattta
ttaacagcttgtgacttatacagagcaaaagcatatcgagtagatccagttccaaacgtg
gcagatcaatattttgcttacatagcttatgatattgatttgtttgaagaaggttccatt
gcgaacttaactgcttcaattattggtaacgtttttgggtttaaagctgttaaagctctt
cgcttggaagatatgcgtatgccagtagcttatctaaaaacgttccaaggtcctgcaact
ggattgattgtagaacgtgagcgtatggataagttcggtagacctttcttaggtgctaca
gttaaacctaaactaggtttatctggcaaaaactacggaagagttgtatacgaaggcctg
aaaggcggtcttgatttccttaaagatgatgagaatattaactcacaaccatttatgcgt
tggagagaaagatttttatattctatggaaggtgtaaataaagcatcggcttctgctggc
gaaattaaaggtcattaccttaacgtaacagccgcgacaatggaagatatgtatgagaga
gccgaattctctaaagaggttggtagtatcatttgtatgattgaccttgtgattggttat
actgcgattcaaagtatggcaatttgggctcgtaaacatgacatgattttacatttacat
agagctggtaactcaacttactctcgtcaaaaaaatcatggtatgaacttccgagttatt
tgcaaatggatgcgtatggctggtgttgaccatattcacgcaggtacagttgtaggtaag
cttgaaggagatcctttaatgattaaaggcttctacaatactctacttgaaagcgacaca
gatatcaacctacctcaaggtctgttctttgctcaaaattgggcttccctacgtaaagtt
gtaccagtagcatctggtggtattcatgctggtcaaatgcaccaacttcttgattactta
ggtgatgatgtagttcttcagtttggtggtggtacaattggacatcctgatggtatccaa
gcaggtgcaactgctaacagagtagcactagagtccatggttatggcaagaaatgaaggc

cgtaactatgtagcagaaggtccacaaatcttgagggacgctgctaaaacttgtgggcct
ctacaaacagctttagatttatggaaagatattagtttcaactacacttccacagataca
gctgatttcgttgagactccaacagcaaacatctagtttaatgactacttactgatactt
taaatagtcaattgtaagtggaattaacttataacaataaggagcatagaatagtgagac
taacacaagggacttttccttccttccagatttaactgatgagcaaattaataaacagc
ttgcttatatcgtttctaaaggcttatcagcaaacgttgagtatactgacgatcctcatc
caagaaactcctattgggaactgtggggtttacctttatttgatgtaaaagatgcttctg
ctgttatgtacgaaattagctcatgcagaaaagcaaaacctaattattatgttaaagtta
acgcttttgataatactagaggtattgaaagttgtgtaatgtctttcattgtaaatagac
ctgctaatgaaccaggattcttattacaacgccaagacttcgaaggtagaactatgaagt
atagtcttcatagctatgctactgaaaagcctgaaggagctaggtattaatattaattaa
gaattaatattggctaatta--ttaccctcttaaaaatcataattaatttagtaattgtg
atttttaagagggatagcttcccaagtaaattttgactaaa-tagattgaataattaaaa
atacacagaaataatgcaatcacaggatataatttccaacgatactcttgttaatttaca
agaagaatatgatagaacacaaatccaagaagttttaaatgagttaaatcaagaacttat
aggattagtgcctgtaaagaccagaattcgcgaaattgctgcgctattattgattgacag
attacgcagaaaactagaactagtttctggtaatccaggattacacatgtcatttacagg
tagtccaggaactggtaaaactacagttgctatgaaaatggctgatattttgcacagact
tggatatataaaaaaagggcatttgttgacagttacaagagatgatcttgtaggtcaata
tattggacatactgcccctaaaactaaggaagttcttaaacaagcaatgggaggagtttt
atttattgacgaagcttactatctatataaagcagataatgaaagagactatggctctga
ggcaattgaaattttattacaagtaatggaaaaccaaagaaatgacttagttgttatctt
tgctggatataaagatagaatggaaaaattctacgaatccaacccaggactctcttctag
agtagctaatcatgtagacttcccagattatacttcagatgaattattacaaatagctaa
aatgatgatagaagaacagcagtactgttttacagaagaagcagataaaactcttttaga
gtataccgagcgaagaatgaaacagccttatttgctaatgcaagaagtattcgcaatgc
tattgacagggctagaatgagacaagccaataggatttttgccagtggagaaaaagtatt
aacaaaagctgatttggtaacgattgaagcagaagatatcttgaaaagtagattattttc
attacctaatgcttaatatacacgtgatttcatgaaactatatattattaaaaag-tttc
ataatcagttgcaaatttcttgaaaaacctttattattatcttaatgcaggtg-tggcgg
catagccaagtggtaaggcagaggattgcaaatccttcatcccccagttcaaatctgggt
gccgcctagtactaaaaaagggggggtgtggtggaatggtagacacaacagacttaaaatc
tgttgatttttagtaatcgtgagggttcaagtccctccaccccccatatattgatttataa
ataaaaagagatgtgtatttgtattaattgcaaccatattaccaaatgtaatacctatca
cttaatagaatctcaacataaacagcctcatcttactagaagtccattgtttatacctaa
atatcctgtagttcatgttaatatatctaacaactgtacctataatcaaattgattggga

tttagtggagtgtttatcatttgtagaaaaaccgaatagttggaatttagacgctaatta
gttacaagaaaaattatgtaccataagcaacaactctatttttagatactcggtattaa
ttttggatgttctaaccagtaagattttacccgttcttgctatctctataatgccaaatt
tagttagtaattgttcaatagcaacaatctttcccggatctccagtaacctctataatta
aaagatcttctgcaatgtctactatcttagctctaaaaattcttacaatttctaaagctt
ctgttctagtctgagaattgatctgaatcttaattaacattagttctctttcaactgaag
gaatatttgttacatcttgaacgttaaggatatttactaacttatataattgtttcgtaa
gttgttcaatagttctattgtctccttgaactaccattgtgattctagagaccccaattt
gctctgctggtccaactgctaaacttgcgatattaaaccctctccgggcaaatagaccag
atattcttgacagtactccggcttcatcttgaactaaaactgataaggtgtgtttcatga
aaaattaaatttaatctatttgatctgttaagtatattctaagaaaattacagaaaaaat
actagttagtctataactaaattatccaaactaaacaaaatcctactatcagataatata
tctaccttgtaatgctatactattaacttagtatttaatgatattctaattgcaaataga
atgct-aatttttaaaatttgttaggaacttgtgctagaaaaatacaaaaacagtaaaa
aactctctcgagatttacctcaaatcaatgatcgcattagatttccaaaagtccgagtaa
ttgatgacgaaggtgaacaactaggtatttttgtgcctgaagaagctatacaattagctg
tccaacaaggtttagacttagttgttgttagtgataaatcggacccgccagtatgccgaa
tattagactatggtaaatataagtttacacaagaaaaaagagctagagaagctaagaaaa
agcaacataacagtagtattaaagaagtaaaaatgcgatataagatagaagagcatgatt
ataaagttagaataaaccaggcatccaaatttattcaagcaggagataaagtaaaagcaa
ctatcacatttcgggggcgtgaaatccagcactctaatttagctatagatttattgaata
aaatggcaagcgatctaaatgcaatagctgaaattcagcaagctccatcaagagatggca
gaaatgtcataatgctcttatctcccaaaaaagttagctaaactaatttttatttaaatg
catctggctggattcgaaccagcgacgtctctttcgaaatggcggattatgagtccgctg
ccttcggcccctcggccacagatgcatcacttaaagtttatacctcataaagcatataaa
aatcaatgtttagctatttttaataaagtctataagagtctattattcattcatattgtg
atatgtcgcaactgctgaaggggaaacttgatttaagtatcgaaatatccagtatttaaa
aattgtgtcgagtataactggaaaagttgaaataaataaaaaaataaaatctcgactttc
cggaagtcctaaatgtcttaaaattacttcaataataacttcccatccatgaggagaatg
aaaacccacaaacatatcagtaaaaagaatgattaaaaaagctttagctgtatcactaag
accataaataatttcatttaaaaaagatttaacgactgcaatttgtctttgtccagttat
cattagtaatataaagacaagtattgatacaagatcggataaaatatttttaacagcatt
tgcactttcatttgcataatattctcccaactctctagccttcagctgcactctttttc
aatgatttcgtaagatatatcttccgaaggattcagaagaacttcaaagtgaattttctc
ttcaaatctttgtaattcagcaaaagctctttcctcttgagaggaatttaaaaaaatttt
aggctgttcttggttccataaataatcaatacaaggtccaaatacaaaaaattttgaagc

ctgattaactagtactggagagatgaataataataaaatatattttacggaagtaatagt
ttggtgtctagatattctaaactcctctatagcttcagattcgccattaggatctaactc
tttacgaaacttttcaaaagtattagtaattgacctaggtattggcccaactttttcaaa
ggcgaattgattatttcgttttaaattccaatatttcatttttattgctgctaaaatct
ctattaatcaataattgatcacgtattcactataaatattaattctagaactaaacatat
aaaacaatatttatgaaaaataaaatacaattaaaaattaattatgcattagttctatct
gcagctacagcagtttatggatataaatcttatcagccaactataaatacactcttaatc
aaagatgaagaaaactggcctttttctctaaaaggcagactgcctttattcctataatt
gtattaaaaaaaaatcatcaaaatccctgaactcagaacctagaaatagtaagttatatc
ttaatggaattggaagtagaaaatttatcgacagaattgtcactctacttggatatttag
aaaaacaggcaaactatactgaatacttgaaccaaataaaaacaagtggttatttttctt
tagtctacctagacagtgatataatgtataagaaaaatattagtaacatttttttactac
ctaatgatattctaaaaagaatctatatttgcaataagaaaaaaaagcttataccgcatt
cattcttaataaatttatttcagaaacagataggctatccaaaaagttttgctaatttaa
attgggccttctccaaaattataaaatggtattatgatagaggataccaatggtctctag
ttgaagttaaacaagcgtctgatgcatcttctattgtaattgatattcatgaaggggtag
tgaagacaattataacagaatactacactctatcttataaaagagtctcaggtatcttat
gcgtagagtcaatagagcagtaccttggagtaagagtgggagctccactaaatataattg
atttacaaaaaaaaattacttacttaaaagataatcaactagttggcgacattatttata
gtattgaacgatcaaataatagctcaatgagtttggatatcaaatttcaaatacaagaat
taaaagataaagagataatagtgcttgcaaaaagttcttcta------------ttatct
cgcatgcatgtaatttacttaatc---aatataga---------aataggctagtagctt
ctaatatagtttcactatcaactaataaattacacgcatgctattttaattataaacttg
actatcaatataagtatataaacacgtctaatcttataaaattgttagcttattctattt
cttgtaggaagacactacttattcattattttaatttacaaactttaataagctatacaa
aaaaaaatactattggatttcagctatatttacgaaatctaagttttgggaaagctttct
gtgtgcttagtatgaaatttatcaaaaatggacttaatataaaaatttttatatataaatc
cttcattgatagttgatcagaattttgtatttcaatttgctatacaaatcattaagcaat
atcatactgcta------aacctccagctt------------------------------
------tatttttaacgaatctagatcttgaacaatatgttgctgaaagtttattaatgt
accattttacatcttgcttctcaatatctgaaaaaatattattatctcgaattatgcata
cagattctttatttttcaactctgaaa------attttcattttgatgatagaactaacg
cgatcaacagttatgatacttttaaacaaaatacaaaaatattttatcaagagtttcttt
ctttattattaagtttacgttatcaaaatttttaattatttaggttggcctttaaaaggcc
attttttgaaataaaatctttatacttggctccatttcaaaagagtg------------
---atttttctgatagtcgtaagaccctattcttccacaaaatgtcgttaaagcaagtat

ctaattttaatttaccaatatcttttaaaagccaccttaaccatattttagttagtacta
ttaaatgtcagtcaaatttgaatatgagaactgtatctttgctattaattgattcacctg
ctgaatatatgctatacaaatctattcttaatttctctattaaagttaggatgcagtatt
tcatacctatgagcaacaatataagattatctctattttataattatttagattgttttt
taataagatcatctcagagtcgcattcatatatggcaagatctaagaactcttacaccaa
ttcaaaattttggttaaaaaaattttcatatggagctggaattcagcttaaattaccaa
ttaagcagatgccacctctttcaattgaatatactgtaactagtagtcgctacttttgta
tttaccttcgtacttattatcaacgataactattactaaaatgaaaaatttaatttcttt
ttttgatcgcagcaaagggaaatggatttcacaaagaacaacttatgaattatctaataa
aaacatgagttcgatacagtctcagatgacaatgaaaataggcaactcattgtcaggatc
tataatacttgcatcgttaaattgggggacatttatagacaagttgcccattacgctaa
aaatcacagccggagtgaatatgataataagtttaacttgcaattcagtaatcaactaaa
taatcataaattattaacgctgtgcatagtgacagatcctagtctaattagttttaaaac
tcgctatggaagtactaccatagatgaaacatattggttcgcaacaaataatttacgtct
aagtactagtattgttaaacgattcaacacttgtgtggcagtatctttttgttcagaaat
taaagtttaa-actctatatagaagcaactaatagcctaaa-ggctattagttgcttcta
tatatctaaattttgtattaaaaattat----aatactatattattctaagtctttacga
ttgggattcctggaaggatcattagagaggaacccaaagacaaacaaagaaataaagaaa
ataacggtagtatatacaaagattttcaaagtaaacatattttcatccttaaaagctatt
agtgcg----atatttacatatctccacataattttatatcatgatataagttccagcac
tattttataaataaatcaatatctttattttttagtaatgatttatatttaaatatgcgt
gatgtactaaatactaaatcttttccgacttgttttgtcttgtttgtaaatattttgtta
tttctatggagtttaccttcaattatcacatagtctaacttctttagctgtttaaaagta
tatgaagatttttttgttccaaatagttagattgattataactaattgttttctttttaac
agtcttgcttttaacttaattatttggcttttattttcactaattctgatactcttacaa
ctaagaatttggactagaagagtacagctattcataatttataacaa------tttttta
aaaaatctactaaaatagatgattttacagaactaaaatgatatttcattatcttgttga
ccttgctcataagtaatatctttcaatacttttaatatttttccaaaatactcttgcagg
tatttctctaaagatattacttccaacctgtcaattccaagaatactgtaaacttcatcc
atcggagctgtaaattgctctccgctacttaaaatttcggcaaatgctaaacgatcagaa
atattccatgtccattgaagagttttagtaattcttcttaaggcttttaataatccaatc
ggaatttgtgagatctgtgtttttttgcccagataatttttcacataatttaataatttca
gctgatgtccaagcggtattaccaactaacggcagggtcctattttctgtggaaggtact
ccaagacttttttataactaactttgctgcatcttgagtatcaatgtatgcaatgggtgta
gattctcctgtaacccagactgatttttttgtctaaaataggaatggcatattggttgatt
aaaccttgaaaaaatcctcctaaagaaaacacagtatatttcacattggacttctgaagg

aaatctactacctgagactttaagttcatcaatggaacatctgggtatttctctgaattt
aatattgaaaagaaaataaatctttcaactttagccgctttagctgcttcaattaatgca
attttcccatctaaatctatttttttctgcattgtaaggatcagtaggacgagatgtagaa
gcatctatgattgctgttaccccacaaaaagattgcaaaatactttcgggtaattttaaa
tcaccatatataagttctgctccccactctttgagaaaggcagactttcttaaatttctt
accatgcattttacattgtagccttcatctaaagctcgtcttacaatttgacgtcctaaa
gttccggttgctccaattactaaaagagtcatattagttaaaaataagtatgtataaaga
cattgatttggttgacggcagagagtaaattaggtagagagggaatcgaaccctcatgac
cgaagtcgtcacattttgagtgtgatgcgtataccaatttcgccatctaccctattatag
accaactactacaagtaatttagtctttagcgtaaaaataatactaagtttcaatggata
tgtcaacttttaaatttatattaataaaaaaacggagtctcttatggccacttttgaatt
aattccttatagattgtatttgagtcaatctaacacatggatccatagaataaaagcaga
aattaagatatatatagtaacgcttttatggatttcaatttttattttttcttactttaa
actatgtattattgctttaagcttaattgccataagttttactataagaagtaaacagaa
tattatccaaaaacatttgttacaaactttattaataacgttcttaactactgttttgtc
ttttagtgtggctattagttataaacaatatgcagaacaagaacaatcgcagtatttatc
tgattccaagaaatataaaaattccagcagctactattatatacaaatagctaacgatca
aaggatcaaaagacaatacttaattactactttaaagccttccttgtactttttttattac
tatatattctattaaactagttatgataacaacttctccagaagttttagtaattactat
ttatagatctaggataataaataaaatatttaaaaatgaattgctatttatcttccttct
ttcgtcacatattgttactagtattattaatagaatcgataaagtgattcaagtaactag
cttaagaggaagcttgaatttatataattcattgacaaggcctttaatgttttctttgtt
aatatttcaagtcttctttttggagattattcgggagtcaaaagaaatagctcaagctct
ttatactagaaatctcaatcaagaaaacaataatttttttgaaaatatatacagtaaaatc
taactttagtgatcggctcaatataattattagcactttgtactttattattttagctct
agcgtaatagaatccacttattcttctgactctagtaatacgaagagtaataaaattagt
tagtagtttaaatagatagaat-taagatattatataaataattaaatgtttcattatgg
ttaatactcaaaatcaaatttcacaaacttcagaacttgattatatagttaatcaacctt
ataaatatggctttaaaacttctgttgaatctgagcaatttcctaggggaataagtgaag
atattgtccgcttgatctccaagaaaaaagatgagcctgaatatctattgaattttaggc
ttaaagcatataaaaaatggaaaaagatgagtagtccgtcatgggctcatattaagcatc
cgaatatagactttaatacgattatttattacgctgttcctaaattaaaaaaagaattga
aaagtctagatgaggttgatccagaaattcttgacacttttaacaagttaggtatatctt
taaatgagcaaaagcgaatttccaatgtagccgttgatgctgtttttgatagtgtatcta
ttgcaactacttttaagaaagaactgtctgaagctggtgttatattctgttcaatttctg
aagctataagggattatccagaattaataaaaaaatacttaggtactgttgtaccagctg

gtgataattattttgctgcattgaactctgcggtatttagtgatggctctttttgctata
tccctcctaatacagtttgccctttagaattatcaacttactttcgtattaacaacgaag
aatctgggcaatttgaaaggacactaattattgctgatcgtggcagtaaagtaagttatc
ttgaaggttgtactgctcctcaatttgacacaaatcaattacatgcagcgattgtagaat
tagtagctcttgaaggagccgaaattaaatattctacagtacagaattggtatgctggta
acaaagaaggtaaaggtggtatatacaattttgttactaaacgaggcttatgctcgggta
acaattcaaaaatttcatggactcaagtagaaactggatctgcaattacctggaaatatc
caagctgtattttagctggtgaaaattctcagggagaattttactctgtagctttaacaa
ataactatcaagaggctgatacagggactaaaatgatccatattggtaacaatacaaaaa
gtagaattatttctaaaggtatttctgcgggtagatcgaaaaacagttatcgaggcctag
taaaagttggccctcagtcatttaattctcgtaattattctcaatgcgattctttattaa
ttggtcaatcatctcaagctaatacatttccctatattcaagtacagaatccaacatcaa
aagtagaacatgaagcatccacttcaaaaattagcgaggaccaaatttttttatttttac
aaagagggatcaatttagaagaatctattgctcttatgatcagtggtttttgtaaagatg
tctttaatgaattgcctatggaatttgctactgaagctgatcgtttactgagtttaaaat
tagagggaactgtaggatgagccagactattttagaaatcaaagatttatatgcttctgt
tggtgaaacaacaattttaaaaggggtaaacttatctattcgagccggcgaaatacatgc
gattatggggcctaacggttcaggtaaaagtacattatcaaaagtaattgcaggacatcc
agcgtattcactaataagcggagacattttatttttggacaaagtattcttgaaatgga
gccagacgagagggcaaaagcaggtatttttttagcttttcagtatcctgttgaaattcc
tggagtcagtaattctgatttcttaagaattgcattaaatgctcgcagaaagtttcaagg
attatcggagtttagccccttagaatttttcaactaataacagaaaaaatagatcttgt
tggcatgcaagaaagtttttttgacaagaaatgttaatgaaggattttctggtggagaaaa
aaaacgtaacgaaattcttcaaatggctttactagatagcaaaatatctattttagatga
aacagattctggactagatattgatgcattacgagtagttgcaaaaggaattaacacttt
agctaaatcaacaaattcaattattttgattactcactatcaaagattattagattatat
tattccagattttgttcatattatgagtaacggacaaattgtaaaaactggtagtgttac
tcttgcccaagatctagaaaagcacggatatgactggattacgcagacataactatttac
tac-------aatattgagactaaagaatattctttagtctcaatatt-tttgcaaatgc
aaagtt-aattatttaagctttatcaaaaccttgcttaacatcttcaattgctcttttta
gcaattcttcactaactggttctaatttttttagtactacgaacattttcaccaaattctg
gtttagaattctttaaatcttctcgaagttctctaataaattcagctacttttgaaactt
caatatcatctagatagccattaatgccagtgtaaataatagcagtctgttcttcaactg
gaataggagaattttgagcttgtttcaaaatttctcgaagacgttgacctcttgctagtt
gattttgggttgctttatctagatcagatgcaaactgagaaaaagcttctaattcagcaa
attgtgctaactctagttttaacttgcctgccacttgtttcatggcttttatttgcgcag

cagagcctactctagagactgagatcccaacattaatagctggtctaatccctgagttaa
ataagtcaccagatagaaatatttgtccatctgtaattgaaattacatttgttggtatat
atgcagatacgtccccagcttgagtttcaataatagggagagctgtcatactacctccac
ctaattcagcattcaatttagctgctctctctagcaatctagagtgtaaatagaatacat
ccccaggataggcttctcgtcccggaggtcttctaagcaaaagagacatctgacgataag
cttgagcttgcttagttaaatcatcataaataactaatgttgctttacctttgtacataa
aatattcagctaaagccgcacctgtataaggagcaatatattgtaaagtcgcaggactat
ctgcattagctgcaactataatagtataatcaagagctcctttatcctgtagtgaagata
ctacttgggctactgaagatgccttttgtcctatcgcaacatatacacaaacaacatctt
ggccttttgattaataattgtgtccaatgcaacagctgttttacctgtttgacggtcac
caataattaattctcgctgacctctaccaattgggatcatagagtcaatagcagttatcc
ctgtttgcataggttcacaaactgattgccttccaattataccaggggccattgactcaa
ttagccttgttccattacttgcaggttcacctttatcatctattggccgagctaatggat
caacaactctacctaaaaaagcatctccgaccggaatttgagcaattcgaccagtccctt
ttacggaactgccctctaaaatatccctaccatctcccatgagtactacaccaacgttat
cactttctaaatttaaagcaaccccaatagttttatcttcaaattcaagtagttcaccag
ccatgacttcatcaagtccatatactcgagcgataccatcaccaacttgtaaaactgttc
ctatattggctacttctacatcctgatcatacttttcaatttgttgacgaataatactac
ttatttcatcaggtctaatatttaccatatttttaatattatttttattaattggaaatt
ctaaagcggtaattagtcagactaatgctatctgtctttttttactttattttaagttact
acaacatcaaggtgtgaagccatttgtcttagctgtcctctaatactagtatcaatgact
tttgatccaatctgaatagtaaagccaccaattagctcgggctcgactgaaataattagt
ttaacttctttagccttagtcataaccttgatttttttctgttaatagagtttcctgatca
gggcttaaagcaatagaagtattaatatttgcaattgttaaagattccatttggtaagcc
agttccaaatacttactagctatagcatctaacattccaattcgcttgcgatcaactaaa
atcattagaaatgataatgtattttcgttaatttgatcgccaacagttgcaataattact
tctttttttgcctctacagtttttaatggattagct---aaaaaaattttttagcttgtca
gattgcgctaaaatattttgtattgactgaatatcttggctcaccttttcagtaactttt
ttagttttagctaaatcaagaagagctactgcataaggctgagctatttttataacaaca
ttgttactgctcatagttgatctcctaacttagcaatattattatcaattatacgcagtt
gcatttcggagctcatttgattttccagctgcaaagtaactcttttaagagcaagaaatg
ttatttgttgctggatttgccttcggatctgtttttcagctgtttctatatttgatttac
ctgttatggcaagtctttcaatatctaattggccttgagctaatatggaacttctaactt
ttccagctgttagctgagcttctttcttaatttgatctataatgatttgagtttgagcaa
gttgtttttctgattctgacaatctagcacttgcttgctctagtctttcttcggattctt
gtatagctgctaaaacttttatctgtcttgcatttaaacttgatccgagaaattgcttca

aaacataaataagaccaaataaaagcaacaatatattaataacatttgcttcaaaaatat
cagaattaaagccgaatttgtgttcactactatgttctgataaaatagtaattatttgtg
gtatttttactatattattcatttaaaattct----------tactattttaatatttta
ctctggaatcatgattttcaagattgactacttaataatttaactttaatttgatcgctc
aaagtgtcaacctgattttctaaagttttaagagcttcttcttttttgaacatttaactgt
ttcgatgcttcagcaatcagtttctctgcattcaattgagcttttttttatatcttctgag
actatattttgagcttctttttgagaagaggctatttttaattgtgcattgcggcgagct
tcagataaatcttcttcatattttgcagctagctcatctgctttaacaagcatagacgat
gcagtagtcaatgttgttcgaatatattcatctcgttcgtctagtactttagttacaggt
ttataaaaaatagtatttaataaaaccattaaagtaagaaactgtaatgccattaaaggc
aaagtaccattgaagtcaaataagccaccttcaatttcttcggctaatagaaatggtaaa
tcaatcattttactaattatgtttatagttaataattttgtttcaattaagaaaaataac
tttatagtgcgcttagtctcatcagactaagcgcaaatgattattttaaccaacgtatgg
attagcgaacaataaagacaacgcaacaactaggccgtaaattgttaaagattccataaa
tgctaaacttagaagaagtgtaccccgaatctttccttctacttctggctgtctagcaat
accttcgacagcattagctgctgcactaccttgaccaatgcctgggcctattgcagcaag
acctacagcaagaccggcagcgataacggatgcggctgaaacgattgaatccataatgaa
ttttaatttttttgttatatgagtagaaaattgacaaatttcgtataatacttaaaatcta
tataattaagtataatagattctaatat-----tgtaattatagtatattactcttctcc
atgaccttccattgcttctccaatataagctgcggacaaagtagaaaagattaatgcctg
tattgaactcgcaaataatccgagtatcataactggtaatggtattaaaattggaattag
caaagtaaatacagaaacaactaattcatctgctaagacgtttccaaataatcgaaaact
taaagatagtggtttcgtaaaatcttctagaatattgattgggagtaatactggagttgg
ctgaatatatctagcaaagtagcctaacccttttttacttaaacctgcataaaaataagc
taaagatgtaagtaaagacagggccacagttgtattaatgtcatttgttggggcagctag
ctcaccttcaggcaagtgaattaacttccaaggaattaatgcacctgcccagttacaacc
tagaataaatagaaataaagttgcaatgtaaggcacccaagggcgatattcatgctcacc
aatctgatttttttgcaatatcttgcagaaattctaatataaattccataaaattctggaa
tttttcaggaattctttgtagatttctagtacctagaaatgataaagttaaaagtgtagc
aattactaaccaagagacaatgaaaacttgcccatgcaattgtaagctacctattttcca
atatagatgttttccaacttctactgctgataaactattatatgggttaaaatctatgag
actgttttgatacataataaatattt-ttttaaatagtgcaagaatcaaaaaaataag-tt
tttgaagaattaatatgcaattaatttattcttaaaattagactattaataaattaaaag
caaaaataaattctaatagagatagctatttttagtagtgattattttttgtaaaaaaca
attattgctacctataatgcttttacgtttaattatatacccatatattaattatttaat
atgattataggtattttgctacaaactttattgttttatccattct----aaagattgtt

atagtttatttttatttaaaatatctgcgacttcatctgcaaaattaactttcaaattt
tcttctcccccctcccagaagaaatcttacaaatcttctaattttaatattttcacctaga
acagcaatattttggtttattaaatcttcaatactaatatcttgattgcggataaacatt
tgatctaataatgagagctccttaagtctcttttcattcgtccttcaatgattgtttcg
attctatcaactggtttgtttttcaagtcatctttaccagcttcaactctttgttccaga
ttaataatttcactaggtatatcctgtgttgatacgtattccacattaggcgatgccgct
atttgcatagcaatatctttagctaacttttggaactctggacgtcgtgctacaaaatct
gtttcacagttgacttcaactaatacacctattcttccgccagtatgaatataactctct
agtaaaccttcaatagcggtacgattagatttcttgttagctgaagccagccctttttgt
cttaatgattctaaagcttttcttcattgccattattagcttgtaaagctttcttgcag
tccatcatacctgctccagtcttatcacgtaaggcttttacgacttgagcagaaatttgt
agtgtcatatgctttcaaaaattttaagtatttaaatt----------aattattataa
ttcactttgagtaggatgagttatttcttctaaatctgtttgcccgtatttaccatcata
aattgcatctgctattttacctacaattagtttaattgacctaatggcatcatcattagc
tgggattggaatattaataatttctggactacaattagtatcaagtatacaaatagttgg
aatacctaattttaaacactcttgtatagctgttgtttctctcttttgatctacaactac
aacaatatcaggtaaacgagtcatattttaataccatttagatgtttacgaagtttatc
taattctcttcgtaaaacagcagcttctttttgggtaactgatcaatcataccactttt
atcttgttcttctagctgtctcagacgattaactcgagacttaatcgttacccaattagt
taacattcctccaagccatctctggttaacataataagaatcacagcgttgtgcttcctg
tgccactataccagcggcttgtctttttgttcctagaaataaaactttttttaccatccga
cgaagcctgttttataaattcacaagcttctgttaataattgtgcagtttggactagatc
aataatatgtatgccatttctttctgtataaatatatggaaacattttaggattccatct
acgcgcttgatgtccaaaatgaacacctgcttctagtaattctgctaatgtaacaatagc
cataagattattaatatttaaataatgtaattattcgggaaatccc-tttgtttagaaac
tgat-----tcaactaacttatttgaattttagtaaactaataataaattaacacacttt
taaagcagatcaacaaaatagatcctgaagaattcat----tctacacttttattgctaa
aataattacgcgcggttctgtcgtctaaaatgatatcatctaaatcatctcgtacagatg
aaatagtactttcgtcattagtatttgcagttaaattctttttctctaacgatgagccat
tacagttatcatacatattgaagccggtaccagccggtataagccgacctataataacat
tttctttcagacctcttaaccaatctaatttaccagatatagcagcttcagttaatactt
ttgtagtttcttgaaaacttgctgcagaaataaagctttctgtatttaaggaagcttgag
taatacctaatagcacaggcctatatgatgcattaatcttatttctcaaggttatggctt
tattagtctgttcaatcttctgtaactctacaagctctccaggtaaatagcctgtttctt
caccattttctattttcactttagaagtcatctgccgtactattacttctacatgcttat
cagaaatattcactccttgagattgatataccaattgtacttctttaacaagtagtaact

gaatctcttgaaagcttaatcgagcagcttcatataaggctaatccacattcaatataca
aatcaaaacttgcatcgaggatatcatgaggatttaacaatacgtctgttttctttcttg
cttcaagaatttcttcaattcttggaagcccttgtataatgtcacctgtttttgctctat
caaaaaccaatatagctaatgtttctccccttctaattaatgcattgttatcaacatgta
aaatagcaccattagaaaccaagtaaggtctagcaattcttaatgtaactgatctagaag
aaatttcaataatttgtccagagtctaaagaagtcatattttcagtaataaaatcaccac
aacgaatccaatcacccacacttactttaactattgattttcctatattaaaggtctttt
tatcagaagatgtcgcaatcaaaatgcgcctgctagtattcttttcagaataaatctcct
caacagtaccttcactcattgcaataatctcagtactagcaacaactgtaaatggcttga
tatattcaccatttttacaataattctagtttgactttgttgcttctctgatttattta
catcaatacttttaattgataatgtttcaaaagttgacagccttaagtcaaaataattat
tgccactttctttagcagaaaactcaattgaggatattaaattatcatctcgatgctcaa
tttcagccacaagattagttgttactaaatgaactccttctatagatttcactctttctc
cgtcccgaaaaggagttcttttaactagctttagtttagtttgacttggtttttgtgaaa
ctagattttcaattagactggattttgtttctggtattgaataaacaataacaggtctaa
taagaatatagggcatttgctcgttttctatatactcccaataaactaacttatctgtag
aaatattattatgaagtctttctccaggtcttaaaaaacctctacttttatcattattac
tatacacctcactaagtttatagatagatccaggcttaataataatttctcgaactattc
catccttttcgattatttcaacaatacctgagcttttagaaaaaatatttttaactagct
ctgttccactttctataacatctccattatgcactagtagtagtgatgaatctttattaa
tttcgtgagtttcttctgaaatccataagatatatcccggacttaaaatttcgtaggcat
ctttatctaagcctgttttctttttgatacatttaagtctaaatatttaataataccac
cacttgtcgttttatacgtatcagatattagttctgcgactgtgtaaccatcttgaa---
ttttttggtttggaatagctttgaaaagaaattttttgtttttttttctgtttctaaaatat
aagattctttttgtggattacatctgtataaatataacaacctggaattacaatagatt
cagtaataacttgtatattggtaatactgctattagtattctggtgtatccttacttctc
cagcataatgatttattaattctgtctgtgcaagtatcgtaccagctttagttttatctt
ctttatgaacaataacattagcagaatcagatatactataaacttcacctgagagcaccc
agatcaagcctccagttttagtaattcttgtagtatattgattattatctgtttcttcaa
ctgttaagttggagaaacagattttgccagagagatctgagactacatgtttttgagctc
tttctgtcatgagtcgatttctgcggggtgattcagcaatcacttgatccttcgatacta
attcaccatcacatattaaaagagtagtacctttacttaagttaattattgacttctgat
ttttcttagatctgattgtaacttgggttggctttttttgttattaatgcttgctcaccat
gtctggtacgaacatctgtatacgattcaatatctagatctatcaattgaccatctatag
gtgcgtagatttgttctgctaattcaccggtaaatactcctccagtatgaaaggtcctca
ttgtcaattgagtgccaggctcaccaatagattgagccgcaataataccaacagcctctc

ctaaatcaactaagcggccatgtgcaagattccatccgtaacaatactgacaaacagaac
ttctagagttacatgtaacaggagatcttactaaaactttttt---gataccagctttag
ttatctcttttgctaacttaggactgacatcttggttcgtatgagcaattaaacaatttg
tctctggatggaatacattttctgctaatactcggccagataaagcttgttctagattaa
ttaatattttctgcgtatctactaaatcttctagtatgatgcctttattggtcttacagt
ctacttctctaattattacatcttgagaaacatcgacaagtcgacgcgtcaaatagccgg
aatctgctgttcttaaagcagtatcaactaatccttttcttgctccgtatgatgatataa
aataatcagtaacagttagtccttctctaaaattactcgatattggaagatcaataattt
gtccctgtggatcagccataagccctctcatgcccactaactgtcttacttgagaaatat
tacctctagcacctgagaacgccatcatataaacagcatttagaggatctgtctctttaa
agtatttaataacttcttgtttcaaggattcactcgcattattccacgtatcaataactt
tttgaaatcgttcaactgctgtaatctcacctcggcgatatttgttttcagtggccttaa
tatcttcaatcgtgcctaataaaagactttgcttactaggaggaattcttaaatcttcta
aacttaaagaaattcctgcctgggtcgcataatgaaatccaagatctttaagcttgtcgg
ccatattggctgctcgggctataccataattacgaaaagcccagacaattaaattttta
gttcgtttttatcaataaccttatttgaaaaacttggctgtgcaagacttcgtctattat
ccactaaatcttctccatattaaatgttcgtt--attaactatgctactaaagactcttg
aataatttattaaagattatacggccagctgttgttctaatatattgcacaattctctg
attttcagcgtcttcttttatgatatggttattaaaaaatttgttgtactattgtcaga
atgtttttcaatcttaatcggatcatgaggttgatcaccatctactaatccatcaaaacg
agcccatatataagaatgtaaatcaattttcttctgttcataagccataactacatcttc
taagcttgcaaaatactgattagatccctgctgctgagatggattattagctgttaaata
atagcaacctaaaaccatgtcttggcttggcataataattggttgacctgttgctggaga
gagaaaattatgaggggctaacattagcaatctagcttcggcttgagcctctaaagacaa
aggaacatgaacagccatttgatctccgtcaaaatcagcgttaaacgctgggcatactaa
tggatgaagcttaatagctcttccttctacaagaataggttcaaatgcttgaatacctaa
tctgtgtagtgtgggagctctgttcaatagtactgggtgaccttgaataacttcatttaa
gacattccaaatagaagattcatttttttgaatcatctttttggcagctttaatattgtt
aactaatccctgcaaaattagtctgtgaataacaaaaggctgaaataactccaaagccat
ttctcgtggcaatccacattgatgtagttttaaatgaggacctacaacaattacagatct
accggaataatctactcttttacctagcaagttttgtctaaaacgaccttgttttccttc
tataatatctgacaaagattttaatggtctattatttgcacctacaacagttctacctct
acgaccattatccatgagagagtcaacggcctcttgcagcattctttttttcatttctgat
aataatctccggagctaaaattgactttagacgagataatctattatttctattaataat
tcgacgataaaactcattcaaatctgccgtcgcaaatctacctccatctaattggaccat
aggccgtaagtctggaggtatcacaggaatgactgtgaataccatccaagaaggatctgc

acctgttgctatgaaattttcaatcaatcttaagcgtttcatttttttattgaattttaa
tgaaggtgtcttaaaactttttggtggatttgtagcttcagaccttaaggtttcagctat
gtgctctaaatctaaatctttcaacagtttctgaatagcttctgcacctattcctacttc
tacttggttattctcatcttgattttggtagatttcctcttcaagactttccattcgta
accttcgagaagttgcttgtattttaaattaatatctgtattagattgcgtaactacata
agaatgaaaataaacaatttttctacttctttgacttttaagtctaatgctaaagcaat
atagcttgtacttccttttaagtaccaaacatgagttactggtgaggcaagttcaatata
agccatccgatgtcttctcacgcgtgattctgtaacttctacgccacatcgttcacaaac
tatacctttgtagcgaaaacgtttatatttaccacagtgacattcccaatccttaactgg
gccaaaaattttctcacagaataaaccatccatttctggctttaaagttctgtaattaat
tgtttcaggctttgtaatttcaccaacaatttgtccgttgggcaaacttctttcgcccca
ctgtcgaatttttcgggagaagctaaactaattttacgtagtcaaaatattgctcaaa
ctttgtcataaatttgaataccttaaaatcaaataattagccaatttatttacatgcttc
atattgaaaaatgaatatcttaataaaggaattgttcaaaatcgtctactggaggagtat
cataattagaacgatctactcggttatctttagaatccgacatcaagtcaacttcaactg
tccgtctttggccatcttcaaatagtttcaatttatgtactgcaatgtctaaacctaatg
actgcagctctcgcattaagactttaaaagattctggagttccaggcttcggtataggct
tccctttaactattgcatttaatgcttcatttctagcttgcatatcatccgactttacag
ttaataattcttgcaaagtatatgctgcaccaaaagcttctaaagcccacacttccattt
cacctaatctttgacctccatgctgtgctctacctcctaatggctgctgcgtaactagag
agtaaggaccagtggatcttgcatggattttatcatcaacaagatgaacaagcttaagca
tataagctctaccaactgtaacaggattatcaaaaggctctcctgttctaccatcaaaaa
cttgcattttacctggatgctgatcattaaaaagccatttattgctagtaatcaatgatg
cttcttttaatttctatttacaagtgctcttgatgcttctgctccatacatttcatcaa
aaggtattattttaaatctctttcctaaatagccacctgctaagcctagtaggcactcaa
atacttgaccaacattcattctagaaggtacacctaaaggatttaaaacaatgtccactg
gtgttccatctgataaataaggcatatcttgtttaggtaagatcctagaaatgatacctt
tattgccgtgacgaccagccattttatcacccacttggatttttctcttctgcgcaacat
agacgcgaatcatagcatttgttccgggaggcagctcatctcctttctgcctagtaaaaa
ccctaaccttaactactctacctttttgcagcattaggaagccgtaaagatgtatctctca
catctcgagccttttctccaaaaatagctcgcaacaatttaccttctggtagttgatcag
cttcacctttaggagtaatttttcctactaaaatatctccagcttcaacccaagaaccac
cgacaacaattccatttctatctaaatccttcagagagttatcgctgacgttaggaatct
ctctagtaatttcttctggtcctaatttagtttgacggcattctacttcatacttttcga
tatggatagaagtatacaagtcatcataaactaatctttcgctaattaaaaatgcatctt
cgtagttataaccttcccaaggcatataagcaactagaatatttctccctaaagcgattt

ccccctccgtctgtggatgcgccatcagctaaagtttggccaacaactatttttctccta
cccaaacaattgggcgctgattaatacaagtatcttggttagaccgataatatttttta
agcgataatgaactgttctaccactattatcctgaatgcctattttattagcagaaacat
aatttacatgaccagatgttctgctaataacaaccataccagagtctcgagctattttag
tctcaagaccagtaccaattattggcttttctggatataataatggaacagcttgccttt
gcatattagatcccattagagctctattcgcatcatcatgttccaaaaaaggtattaatg
atgttgcagctgaaattacttgaataggtgaaattgcaatataatctacttgggttgggg
ttgttgtaataaactcttgacgataacgtacaggaataatatctccttcaatgtaatgtt
gtttactaactttaacatctcctggagctactcggaaatcatcttcctcatctgctgtta
gatagacaggactattgtgataaatcacttgaccttggtttaccggataaaatggggttt
ctatgaaaccaaaaacattgacccgcgcacaggttgctagtgaaccaattaaacctgcgt
tcggaccttccggagtttcaattgggcaaattctaccatagtgactaggatgtaaatcac
gaactgcaaaacctgccctgtctttattaaaacctcctggacctaaggcactaattcttc
tcttgtgagttagttctgcaactggattagtttggtccataaattgagaaagctgactag
atccaaagaattctctcactgacgcaattaatggcttgggattaattagatttgataaac
tcaatgaatctatatcacatatcatcattctctctctaataatgcgttctaaacgattga
gacctactctgaattggttctgcagcaattctccaactgaacgaactcttctattaccaa
gatgatctatatcatcaagattacctgagtttttatccttaatattaattagataatcaa
ttgaggataagatgtcttgaggagacaatactcggaacgttttaggtatattgagaccta
gtttttattaattttatatctaccaacttcacccaagtcatatcttttaggatcaaaaa
aacgagagtaaagcatctgcttagcaacagcaactgttgctggttcattaggtctaagct
tagaataaactatgagcagggcttcctcatcagttacttcttcaatattatttttccaa
tttctttagctaattctttaactgaatatatttgggatgcagaaataagaaaagcatatt
tactaagtccttttcaatttcatctttattgagtccaatagctcgcagaaaaatataag
catttactttgtgggtcttatctattcttatccaaatttctcccttaggatcaatttcaa
attttaaccaagaacctctattagaaattaggcttgcactataaatctgcttaccatttt
tgtctatctcttgtttataatagattcctggactacggattatttggttaataataactc
tttccgtcccagatacaataaaagttcctctatttgtcattattggcaagtctccaataa
aaattaatctctttttgtatttttttatttttatctgcttttcagcactgagctgtagat
gggttgatgataaatctaatgatttaatattttttcctggtttttagaaggtagatcaa
tatcttttcttgttaactttgcaggaacatatatttgagcactataagttttatccctat
tttttgcctgtctgacactataacgaggaaattttatcttgtactcattaccaaataatt
gtaattcgagtcgactagtagggtctgatatcttcggaaaaatttcaagaacttccgtca
aaccttctaataaaaaccatttaaaactggccctctgaatttcaactaaatctggaagaa
gtttatttttttaagcttatacgttgaaccatagatctcctttataacaaattttgaaatc
gaagagtttaa-tactattaatagagtactggtagataatgcataagcatagtttgctac

attatttcttctctagacataactagaacatcatattttattcaa--aaaaagattttta
aaatttttcttcac--acacactatctataagtactttgctgcta--aaagatagttcaa
gctattaaa-------aaaaacgaaattaatacttgtctgtctgtcaattaattgacatc
aagatagacaagtattatttatttaatcaattctg-tttttctgagcaaaagacaatgct
ctcgcgagtctagctttttttctagcgcctgtattagggtgaaaggccccttttttaca
gctttatcaatcttactatatacttgagaaatacttgactgcacatcattgaaattatca
ctttctaaattatcaatatttagaagacatcttttagttaaagttttgacaaccgattta
tacttacggttaatgagacgatttctttccgaagttttaatgcgtttaatcgcagaaagg
ttcttagccacagtaaatccaataaaactttataaaatatttttagaaacgaaaataca
caaatctgggatctattcaaaaagggaatatgcggcgtattataacattaaagaaaaaca
ataggcaatagttcaccaagagctcttattattacacaatagagattgtcattgcgaaaa
actttgtaagatagtatttaattaaaataaactaactagacatataaatatatatgaaaa
aaattgaagctattattagacctttcaagcttaatgaagtaaaacttgctctagtcaagg
aaggtattggaggaatgacggttatcaaagtttctggatttgggagacagaagggtcaaa
ctgaaagatataaaggatccgaatactctattgatatcattgataaaataaaaattgaaa
ttattattagcgatgataaagtagagaaaattgtagagacgattattaaggcttctaaaa
caggggaaattggagatggaaaaatatttattagtagtattgaaagagtaataagaatca
ggactaatgacttaaattttgaagctttatagtatttttacttt-tttagacttgatact
tcttcactaaacatgagataataagcgtttattgtatacttataactaaggtgtaata--
ctaatggctaaaagcaaaggtgcacgaatcgtaataactttagagtgctctgataaagct
ggagagtttgctcaaaaaaggaaacctggcgtttttcgatatacaactactaaaaataga
cgaaatacaccaagtagaattgaattaaacaagttttgtcctaattgtaatcagcattgt
atcttcaaagaaattaaatagttatttatcttattaaaat--aatcatattatggctata
tacagaaaaagaatatctccaattaagccaacagatgctgttgactacaaagatattgac
ttgctaagaaaatttattacagaacaaggcaaaatactgcctaaaaggtcaactggattg
acttcgaagcagcaaaaaaaacttactaaagcgattaaacaagctagaatcctttcttta
ttaccttttttaaataaagattaa--atatatagcctaacttcatattttccatctaaa
tattaaaaatttctatagatttgtttattaaaatgatctaacatactgaat---------
----------tactaagaaga-tctactcattacaagttttgttcatttataattaata
atagcatcataattta---ttaagctagattcagacaagacataaag-gtaacctctttt
aattgattatattatatagtctatttaaaagaggaaatttttttgcttc----------t
agcaattcaagattctttaagctttaacaaggtatttaaaacaaagatttttgtttaggt
atcagatcaaatgaatgaatcttatctcccgactgccataattgaaattcagagatgaaa
attccacattcgttgccagcttgaatttcttcgacatcttctctaacgcgctttaaagat
tcaatttttccttgataaataactttttcttctcgaatcactttaatccaagagtttttt
aatagcttattattgatcactcgacatcctgctatttttctatttgctaacgagaatact

gtacttacttctgcttctcccaccggtacttctgaatattcaggatcaagtaagtcttcc
atccttcttttaatgtcttcaattaaggcataaatgatttgatagttttctattaatata
ttcgactttgccgatgcttgctttgttcctggagcaaagttggtattgaacccaatgagg
gtagaatttgtggttgaagctagttcaacatctgtagcagtaatttctccaggcatgatt
gagacaacattgagttgaactttactctgaggaaattgagataaagaatctaggattgct
tctgtagagccttgattatctgttttaataattaaagaaatttgcttactaatatcttta
gaattagtgtttttttaaagtatctaatgtaattcgactattaagtgctctttgtttctgt
ataattgatgagtctttagaggtattttcaatcgctttaagtttggcttctttatcactt
ttaactgctaaagctatttctcctgtagctggtacagatgacaatccccaaatttcaaca
acagatgatggtattgctaaattaattttctctttagcgttgttaattataactcgaatc
tttgcgtaagctgatccaataaccaaattatcactaatattcaatgtaccattttgtatt
aatagagttgctactggaccatgcgatttatctaaatgggcttctattataataccttgt
gcaggctgtgttggatcagctttcaaatcctctaattcagctaacaaggtaatagtttct
aataatttatcaacattttgaccagtaagagaactaataggaattataggcacttggcca
ccaagtttctccgacatgacattgtattttaataaatcttgctcaataatatctgtatta
gaaccagctttatctatcttagatattgctatgacaaaagggacattcgctttttgaata
tgattaatagcctctatggtttgaggtttaactccgtcatcagcagctataataataata
gcgacatctgttaaattagctcccctagacctcatacttgtaaaagcttcatggcctgga
gtatccaagaaaacaatttttttgtttattatctttcttaatatactcaacttcataagct
gcaattgcttgagtaattcctccaatttctttgttagcattattagattttcgaatataa
tctaatagcgttgttttgccatgatctacatgtcccatcactgtgacaattggaggtctt
ttaatataattgccagtttcataaagagcattagagtgatctaaattactagaaagtcca
ttattatttcctttacatttgattctactgctataccaaaattatcagccactgatgat
attattgaggcgtctatggtttgattcatagtaactgatattcttttaagaaataaatat
tttataatatctgtttcttgaacacaaattagtttagataattcttgaatagttagagga
ttagtaatactaatagactctggcggagaattagattgaatgcttgctgaattttgacta
ggagttactgctactttttttctgtcttatagatttttttattactagtcagtttttttaca
acttccacttttggcttaggtggacgcattaaagagatagctaaatctcctgcagtctgc
gaaacattagaattagagtctctaaagttgtca---tcatcatcgtcaatatgtatttta
gtttttattttt-ttgcgctgtctattctta--ttttttttactatctactaagtcgtga
attttattaaaatttttactttttttatctagtttaggtggagaacttaattctagatgc
tgttcaccacc---tgtatgtgattctgatttatcaaggtctaaatttaacagattatca
ttattgactgactctaatctaattttatagataatctgaggatttttcaaatctacaatt
gtttcagaagaattgatatttgagctagatctagaggatctattttcaaagttttgatta
tttaaaaacataaatattggttttattatctaatagtaactttctaattttaatacagag
cttaatataaacaagaatcgaatatctgtataaataatacaaatgt---agagaattaat

ctatgttgtattagatttaatataacactaaagtcctttgttcgttatacaagtttaaaa
ctttt-tatgatgataaacactagattctagtttattggtatacaatctatttatctaaa
taatactgtttttgtcagggcttttaaa----ttatctaatgttatgttcattatgaaat
tctattaaaatttaaaaa-ctcaacaattaagtaagaatcgttaatcacctccacgacac
ttatgccacgatctcaaaaaaatgataattttattgacaaaacttttacagtattagctg
atattgtattaaaaatacttcctacaagtaaagaggaaaaagaagctttctcttactatc
gagatggaatgtctgcacaatctgaaggtgagtatgcggaagctttagaaaattattatg
aggctttaaaactagaggaagatccatatgatagaagctatattttatataatatagggc
ttatttatgctagtaatggtgagtatgtaaaagctttagaatactaccatcaaggattag
aactaaattttaaattacctcaggcccttaataatatagccgtgatataccattaccaag
gagttcaagcaattgaggataaagatacagagctatctaaattaatgtttgataaggcgg
ctcaatattggcagcaggctattaagttagctcctgataattatattgaagctcaaaatt
ggctgaaaacgacaggacgaatgaggaatatacaaggatattaatatatttaagatataa
taaagatatcaattaataaattaaaattagttaaagaaactggtactatatagagtactt
-tactatatattataatattatactagttga------tctctaagacttagctgaataaa
cgaaattaaatctattgactatattcatgaatacaaataagcataatggttagtacaaca
aaaagtaccggatctgttactcaaattattggaccagttttagatattgcatttcctaac
ggacagcttccgaaagtattcaacgcactcaaagtacaaagctcagaaggaactattact
tgtgaagtacaacaacttttgggtgacaacaaggtacgagctgtttctatgagttccact
gaaggactacaaagaggggtagaagttattgatactggatcccctatatctgttcctgta
ggtacagatactcttggacgtatttttaatgttttaggtgaacctgtagataatttgggt
cccgttgattctgagagtactttacctatccatcgaccagcacctgcttttactaagtta
gagacaaaaccaaatatttttgaaacaggtattaaagtcgtcgatttacttgctccttat
agaagaggtgggaaaattggtttattcggaggtgctggagtaggtaaaactgtattaatt
atggaactaattaataacatcgctaaagctcatggtggagtatctgtatttggaggcgtt
ggtgaaagaacaagagaaggaaacgacctatatatggaaatgaaagagtctaaagtaatt
gatgcagataatctgaaagaatctaaagtagcattagtatatggtcaaatgaatgaacct
cctggagcacgtatgcgcgttggcttaactgcattgacaatggcagaatactttagagat
attaacaaacaagacgttctattgtttattgataatattttttcggtttgtacaagctgga
tcagaagtatcagctctactaggccgtatgccatctgctgtgggttaccaaccaactcta
gcaactgaaatgggagcacttcaagaaagaattacttcaacaacagaaggatcaattaca
tctattcaagctgtatatgtgccggctgatgatttaacagacccagctccggccactaca
tttgcacatttagatgcaacaacagtactttcgaggaacttggcggcaaaaggaatttac
cctgcagtggatccactggattcaacatcaacaatgctacagcctggaattgttggaact
gagcattattctactgctcaagaggtaaaatcaactttacaaagatataaagaactgcaa
gatattattgctattcttggtcttgatgaactttcagaggaagacagacaaactgtatca

agagcaagaaaaattgaaagatttttatctcaaccttttttcgtggcagaagtgtttact
ggatcacctgggaaatatgtatctttggaagatgcaattaaagggtttcaaatgatctta
aaaggcaatttagacgacttgcctgagcaggcattttatctagtaggtgatatagatgaa
gctatacaaaaagctgacagcatgaaagattaatataattaatgatgactttaaatataa
gaattattgctcctgatcggactgtttgggatgcagaagcacaagaaattattttaccaa
gtagtacagggcaacttggtattttaacaggccatgcacctttgcttacagctttagata
ttggagttatgagagtaagagtagataaagaatggatgccgattgttttgctgggcggtt
ttgcagaaatagagaacaatcaattaactattctggttaatggcgcagaagaagctagtc
aaattgatttatcagaagcagaaaaaaatttagacactgcaactcaactcttaagtgatg
cctcgtctaataaagaaaaaatagaagcaacacaaaaaatacgaaaagctcgagctcgag
tacaagctgcaacagcagcaacttcgtaa-aagattcc------tcttaaaacataaaac
tttagtaatgattactaaa-------gttttatgttttatttgtatggttataggcagct
taaataaacttagcttagacctgaacaaatataatcaaaatataatcccatctctttacc
tgcatctggtccaactaaactaatagtaacttctttcatagctaaaatagcttgaatcgt
tgcaccaataggaacacctagagaattgtatgtttcttttagaccatttaatactctttc
ttcaaggatagatggatctccagctagcattccataagtagcatagcgtaagtagtagtc
taaatcacggatgcatgcagcataacgtcgagttgtatacatattaccacctgggcgtgt
aatatccgaataaagtagagattttgctacggattctttaataattgttgctgcattagc
tgcaatagtagcagcagctctgactcgtagctcacctgtttgaaaatagcctcttaattt
ttcaacagagctatcatctaaatatttaccttggacatcagctgcattaataacagaagt
aattgcgtcttgcataactttcacacattccttaattttaaatatctaatacta--gggg
tatataacaaaattactagatcttttagcaatatcagtattactgcatagcacccaaagt
ataatcaaagtaaaaaccagcttctgctgaatcttcacctgcaagcagtgagcaagctac
acttttcatgcattttacaccttcagcaactcctgaaattggtgttcctaaagaattata
catttctttaacacccactaatccaatttcttcaataggagttacatcgccagcaactat
tccgtaagttactaagcgaagataatagtccagatctcgtagacaagtagcagtcatctc
ttcgccataagcatttccaccaggagaaactacatcaggtctttttttgaaacagttgttg
gccgccttgctttacaatacgctcacgattatctgttaaaatttgagctattcttaaacg
acgttgtccagataaaacaaaactttttaattctatctaattccccaggacttaaatatct
tgcttctgcatctgcatttacaattgactttgtaacaatactcatggataaaactcctgt
aatgcttt-aaaaaaaagcttaatactaattatttatgatataactcagaaagtaatgta
ctttttgag-aattttataaccgaaacacgttcatgatatatgaagcaggatagatactg
aggtaagtactaaaacgagaaagttatatgtaggaacttctgctattgatttcctagtac
ttttttaaaacttggtaccacaattgatatatcctgcttagtcaaacgattatagagtgt
ttctgtattaggaaagttagctgctggtaaagtcggaaatcttctgtatggaacagtatc
tttgccatataccgcattgtattcaacactttcaactaaattacttataaatgcagataa

gccttttgaagctaaaatttgattaaagaatctaatttctgcctgattattaggagctct
tcctaagatatgcttagtacctaattctataacttttgtatttggatatggctgataaaa
ttctttaccatatagttcagatagcgccagtttttctactagttctttgacacagatttg
tctattcaagaaagctgacttaatatccaagaattcaccgccgacactaaaagagttaag
atctctttcgaaaatttgacgatatgctgctcttaaagcttgttctaacatttctttgtt
actatcactattgacttcaaagacaatagattgatctcttaaagaagttactccctgtga
aatacgagactgaatatcattactactcctcatttctttgacagtgcctaattcaacaaa
cctagcagatttactagcaataactttttgaatctctggtcaataattcctggtcttaa
gcttcttaaagcaacaccagctggagtactatatctttcataaggaacagtattatctcc
aaaagtttctgtgtactccgagctatcaattattgtgtctattacttggtaatagccttg
cttatatgcaatatcaaaatatttattaatttcttgccgaccatatgtagggcgacccag
taagcggttatgaatatattcaatagctttacaaatatataatggctcccaataaagaga
tctaaatatacttgattttgctaactgtctaacaaactcgcgaactgtaatttgattatc
ttttagctgactttctattggcttgagaattagtttttcttcttgatacacttctctgcc
aaaaactcttaaataagcaacttttgtgacaacttctactgaattttcaaaattttggga
tgaatctgactttataagagcgttggacaatttaaagattttggtcccagggatcctgc
tgacttaggtcttacctgtggattactaatctgattgtagattcctggcccacgtctaac
taatatccttcttgtatctttaccaaaaagagcttgtctttttcttggatctttattttc
tttagggaaaatagctccaaattgaattgataatggatcattacctattccatatggatg
ttgatcaggtaatgattgcttataatcactaaataaagttataaattgaggaaccttacg
aaaaggcgcactataggtgaaaagatctatttgaggcccccaattacggcactcttgagg
ctcttcacctaaatttctaaagtaaggcacagtctcttcaccgaaataatctgtatactc
agaagaatttaaaagtgcattcactaaaccacttaaaccagtagatgataaaatagcaaa
atattttgaaactcttctaaagagctaggacctctacccaagaaatgtctaaatgctaa
ttctaatgctcgactattgacaaaaggttcataaaattgctttctataaatacttgaagt
tcctaacgaacgaatgaattccttgattgaaatttggccatttttacttgcgactccaa
attcgataaagagaggtcatatgctttagcaatatctctttcaaaaatttgtctataaca
tgctttcacaacaatatttttctcatcagcagataaactaggcttcataacaaaacgagg
tgttgacactcctgcctgtacataagtttgaggaagacgcaaaccttgtaaatctccaga
tattctttttcttaatttatcagtcaaagatggagcttcaaattcagaaatcacaacatt
aaaatactctttaactaaatcttgaccttttatatcttcctcaaagattaataatgctgt
ccgtctcatctctctaagagccacaattgctgctgcacttgagcaagcgttatcaattaa
ctctctcaatcctcgtatattaacagataaaatattagggtcgcctgatacaattgcata
tgtcaaataccgtaaaaaccagtctaaatctcgaagagattttctcattcttgtagtacc
gtatctcaagacgtttataggtttaaaccctggcggagtagctcccccagcattaaataa
agatcggaaactttggccaaaatctccttggatatttcctgataactcattaattttatc

ttgagaactttgatctcctgcaataataacagctgcttgcggacgttctaaataagaaat
tgcagacccacctacgaatattttatctgctgctcgtgctaccagaatatttgcattttt
agttaaaatatctgcaacttccagtctttttttgtccagaatttaaaaaagaaactaattg
atttaattcaccaagctgtaaaaatcggtcttgttgctctgcctgagtaatagttaaaat
tgaggcagtccgataaagctgggggcgtgctaatgggcttccgccacttgctttgatact
cattactttatttatctccttaactgatattgcttgctagcgaatacaaatatagcttga
tatttctaaatatttaaagatactatgcaagcaca-ctgttttctatattattaggtact
agtacttatagtacagagagtcttataactaacttctataaatacaaattttgtaatact
tctattaacatttgtcaaaaaaatatttaagtgctcactcttttatattaaaattcaatt
agtgagagattgatgtgaaataaagtatactaagtaagtttatagataggaaaattaaa
agatttattctacaaacattaatca------------cttgaatgtttagcaattgtaca
attgttattgcttttaattggtttagactagttttatatttttctaacataaacaatat
taatgagcaatttgtatgaatttaaagccacaaaacaacctattaactaacgaaaatata
aatcatgtagatatacccgaaaatgatataccaatgtcaattacggaacatttagaagaa
ttaagacaacggactttatttgtgtttttattctttttgtttgctacaactataagtttc
acacaaattaagatcattgttgcaatactgcaagctcctgctgttggtattaagtttta
caactggcaccaggagaatatttttttcatctattaaggttgcaatatattgcgggatt
gttgcaacaactccgtttgccgtttatcaagttatattatatatactcccaggactaact
ggaaaagaaagaaaaattattttgcctctattaattagttctgtattgctttttattaca
ggtggtatatttgcttactttgttctcgcaccagcagctttaacattttaattagctat
gggtctgatattgtagaaccattatggtctttcgaacaatactttgactttattctatta
cttttacttagtacaggattagcatttgagataccaatcatacaattattgctcggtgtt
tcagggacattttcttctagtcaaatgatacgagcttggagatatattattattatagca
acaattgctggagctattctgaccccttcgactgatcccgttacacaattaataatgtct
tcagctgttttattactttattttggtggaattgttatattattagtcttaaaaaagtag
agtaaat---------attttcta-ttaatccaatatatttgttatc-----tgggcaaa
taatctataaaaacaattgct------------------agatttaagtatcaggcttct
aaatatttcaa---------gtctcttttatcatttaaatacaatc------------

--gatacattgg
cttgtttttcatcaattatttgtgttgcacac-tttttagcactctctttattgttctt

agaattatttctttaatctatttattaaat----agatatgaagagatttaagtattgat
tatggtaatctatatcaacgtaaaaagtattttattgtgagttttatttatataaaggct
tattcattatatagttataacagtaagtctaattaatag-ttttcatgcttaattaaagt
aataactcttaattcttttggcagaaaaatctgtcttaatattttatc--aaaaaaacag
-aaaaatgaagctaaatagtctaattaacttaattcaaaagtctatatattcttgtacac
ttttactaattattttaaatattatttgtgtcgcacctaattctagtaatgcatttccaa
tttatgcgcaacaagcttatgaaagtccaagagaagcaactggtaggatagtatgtgcca
attgtcatcttgctcaaaagcctgtggagatagaagcgcctcaagcagtactacctaata
ctgttttcgagactgttgtgaagattccatatgacagcaatgctaaacagattttaggta
atggcagtaaaggaggcttaaatgttggagccgttgtaatattacctgaaggatttaagt
tagctcctgttaatagattatctacagagttaaaagaaaagactagaaatctttacattc
agccgtacagtgctaaacaagacaacatttagtaattggacctatttctggtgataaaa
atagagaaatagttttcccaatactatctcccgatcctgcaaaagataaaaaagctcatt
ttttcaagtatccaatatatgttggcggaaatagaggacgaggccagatttatccaacgg
gtgacaaaagcaacaataatattgtctctgctttaagcagtggtaaaattaataaaattg
aattactagacaaaggtggatttataatacatgtgactaacagtagcaatgtagagtcaa
cacagaagatttcacctggtctcgaacttagagtaaaagaagggatacaattcagcttg
atcaagctttgaatagtgatccaaatgtaggtggttttggtcagaacgaaacagaaatag
ttttacagagtccaaatagaattaagggcatgattgttttctttttcgctagtgttctag
ctcaaatttcttcgtattaaagaaaaaacaatttgaaaaagttcaagcagctgaaatga
atttttaaagcaatataaaactggcacttactataaagaaagta-taaacactttattca
caggatgtaccagtttttatgttatgtaataatcgctttgacggaagttatgatactttt
aacagaatctacaatctgactaggctgaattactgtagcctgctctaagcttccattata
tggtgtgggtatatcctgcgaagatagcctcacgacaggagcatctaattcatcaaaaag
gtactcattaatttgtgcaattagctctgctccaattccggctgttttcatacactcttc
tacaattaaaactttgtgagttttctttactgagatagatatagagtctatatccagagg
ttttaaagatataagatctataacttctggatcataaccttcttttaataaggccggtaa
tgcttggataacatgatgcctcattctagaataggttaaaatcgtaatatcttttccttt
tcgtacaaattcaactttattgagaggtaagaaatattcttcttgaggaatctcttcttg
taaattataaagtagaacgtgctcaaagaatacaactggattattgtctcgaattgcaga
tttcagtaatccttttgcattataaggagtagaacaagcaactatttttaagccggggat
ggcttggaaataagcttccagtctctgagaatgctctgcacctaactgcctaccaactcc
tcccggtcctctaataactaaaggcaatgtaaaattacctccagaagtataacgtaacat
tcctgcattattagaaatttgattgaatgctaataataaaaaactcatgttcataccttc
aacaattggtcttagccctgttatagctgcaccaattgccataccagtaaagctattttc
tgctattggtgtatcaagaactcttaaatccccatatttgctatgcaaatctttagttac

cttataagatccaccatagtgaccaacatcttctcctattacgcagacagttggatcttt
tgccatttcctcatctgttgccgctcgtaaagcgtcaaacataaagatttactcatttt
aatatttgatttttatgatttgaccactgttttaaagtaacagttaagacaccatattct
atttagttatctgcaaaaagatatcgtttcaattctgacatgttaggttctggactggaa
atagcaaatttcacagcttgttccaattcggttttaacagcattctgaatctcattaagt
tcacctatattagcaatttcattatctagaatatattttttgagtttttttgataggatct
cttgccacccaagcctctttttcttgtcttgatcttagttcatcaggatctgcgagagag
tgaccacgaaatctatatgttaatgcttctattaaggttggaccatcaccttggcgagct
ctttgaactgcttgttttgcagcttgtcttacagctagcacatccattccatcaacttca
atcccaggaagcccaaaagcttctgctttttttatgtatttcaggtattgaagaagaccgg
tgatgtgccatacctatagcccactgattattttcaacaacaaatataataggtagtttc
cagagaactgccatattcagacattcaaaaaattgcccattattggtagtcccatcacca
aaaaagcaagccgtgactcttaaatcctctgtttctttaagtacttgctggcggtagata
ctttgaaaggctgcccctgttgcaaccggtataccttccgcaataaaagcaaagccacct
aaaaaattgtgaggcgcagaaaaaatatgcatcgaacctcctctgcctttactacaacca
gtctcttttccaaataactcagccatcacattttttagatgggacgcctttacttaaagca
tgtacatggtctcgataggtactgcaaacataatcagttggattcagaagtttaattaca
cctgtagaaacagcttcttgaccattataaagatgaacaaaaccaaacatctttccttta
taatacatctgagcacacatatcttcaaaatttctgcctaacaacatgtcttcatataaa
actaataaattactcttattaagattaagacctgttgaattatagttagtcagcggcaat
tgaactttcttaggataactcataatttgtaaagaaacctct----------tattgtaa
ataggaattttgaagaaacagctatcaatagaaaacacacagataaaaacttataaatta
taatatgtttttttactaaagactttaataattatttgaaactagttcagtatatcaaatt
aattttgcttatactacggatgtctaaaacttatataaaaaaagtagt-a-aaaaagttta
ttaaaagaatagttaaaaacagatataaatagaagtaaacagaattatt-gaaaaaaaat
gattattattaattaa----tacttaatatttctaaatattttacaattaatggctatgt
caccatctttattttatcctgttgaacaagaactatgtagtcttgaaaaaaaatctgaaag
ctgttgctgggactcgtcatccaattttatatgccgcagcaaagcatctattcgatgctg
gaggaaaacgagttagaccagctcttgtattttttagtggctaaagcaacctctgagaagc
aagatataaatactggacaaaaaaggctagcagaaattactgaaattatacatactgcta
gtttggtacatgatgatattattgacgagtgcacaacacgtagaggagtcaaaactgtac
ataatttattcaatactaagattgctgtgctagcaggagatttttttatttgcacagtctt
cttggtatttagctaatattgaaaatttagccgtagtcaaagctatttctaaagtcatca
ccgactttgcagaaggagaaattaggcaaggcttagttcatttcgatcccagtatttcaa
tagatgcttacattgagaaatcattttacaagactgcttcactcattgctgctagttgtc
ggggtgcagctatgcttaatggttccaatcatcaaataaataatgatctttatctttacg

gtaaacatatgggattagcatttcaaattatggacgatgttctagatataactggttcta
ctaagagcttaggaaaacctgctggcgctgatctaataaatggaaatttgacctctcctc
tcctttttcacttactcaagaagcaagtttaaatgatcttattgatagggagttctgta
atagtacagatatagcctcaacattatttcttataaaaagaagcgggggaattacaaaag
ctaaagatttagctaaagaacaggtgcaggcggcactttcctgccttcagtttttaccac
aatctacacctgtatctagtttaaaagaattaacacatttcataatcacaagattgtcat
aaaggacttgctaaaattaaaatgttattaactaatttttttactgtttctaaaacagtct
gaaaagtttgaatatcattactagctagctgcgctagcattttacgatttaacgcaatat
tctctttttttaatgcactaataaaagtactataattcatgccttgattgtgagcagcag
catttattctagttatccaaagacgacgaaaatctctcttttttccttttttcgaccaacat
aagaatatcgaagagctttaaggacttgctgcttagctgttcgaaataaacatttatgtg
cacctttaaagcctttagctagcttaaagatttagctcgtcttttttttgcaacgttac
ctcttttaactctactcataaaatattac-ttaaattttaattattaccagcttactaaa
ttcttatactctttg----attataaataaggtaatttcattgcaatgtttttaatatct
tttaagtcaaccgaacaagtagaagaaagatgtcttctttgttttgatgacttttttttgt
aataaatgacttttagaagctttatgtcgaagaaattttccggatgaagagactttaaat
ctttttgctattgcttttgatgtttttaacttaggcatattataaacttttaatgattaa
caaaagcatgaaaagtctgaggatatcaatcttagacctttcaatacttagaataatctt
aacacaatttattttacttactacataggttaactgaaacaatttatatatataatacta
atcagttaatcaaccatagctaaagtggatttatattttgctttgaacttttgtatagta
ttagcaattagcattgttataattttaagactgcttgaattcaattcttcgaacattttc
atatttaatgtaaatgaaatattggcttctgagacaatattttgaatttgaacatcagac
aagggaatcatatctaatgcagctctatattgatctttaaataatttatcgtcttttatt
tggtcaaaatcgtaaaatttcgttcctcctgaatctgataaattcatagcccctctagct
atttttttttaaaatttggccaccagaaaggtcacccaaatagcgcgtataagcatgagca
actagtaattcaggttgtttatgtcctatagtgtgaattctatcaacataaatttttgta
gcaggagatggttcaataaaatctaaccaatctgatccataataatagtttaaatcttca
gatagactcgctttcctattaagctctgtaaaatatataggtttaatagctggatgattt
ttattagaaaataactcttcctctattgcacaatagacaaagtatagatttgcaaccaat
ttacgatatgacttcttatctacaactcctcccaaaaaaagatttaacaaaactaacattt
tctgccatactatgggacttagtagtaccttctcttagttcattcgctaaagtattaacc
ataatatcaatattccattttt-aaagataacaaattaaca---ataaatagttatccgt
atatttataattgagttgcgctaactaggctgctgcaaaataatttttttgattttattgg
atctggattcattgttttgtcaccaggcttccaattagctggacaaacttcatctggatg
agattgtacatattgaattgcttgtaaaaccctcaaagtttcttctacactcctgccaaa
ctctagattattgattgtagagtactgaataattcctttcgggtctataataaatagtcc

tcttagagctacaccatcactattcaaaacattataagctgcactgatctctttttttaa
gtctgatactaatggatatgaaagatctcctaatccacctgattctctgtcagtctgtaa
ccaagcaagatgagaatactcactatcaaccgagactccaagaacttctgtattaagttc
cgaaaaagcattatacttatcgctaaatgctgtaatttctgtagggcacacaaatgtaaa
atctaaaggataaaagaataagacaatatacttattcttgagatcagataattttaacgt
tttaaattcttggtcataaacagctgtagctgaaaaatcaggagctagctgacctactcg
aagacaatttggtccagaaatcattaatttttcccagaagaattgatataattttgttat
ttaataaataattatataacaaaatttagtatatattaaaatactaccagtctttttttaa
attac--------taaatgatataatcaatactatctttatagcggggaatggatttgaa
ccattgaccttcgggttatgagcccgacgagctaccagactgctctaccccgcggataac
taaaatcatattagtctacatatatatataaaagcaaatgcataatagattttagaagta
aaaaaatagaagaactggtccactgatcattaaacttagaaacgacaaagtatttttat
cttatttaaaagaggaaagacttcatataaaccaataaatctatcttttaaaagaatttc
ggacggctttacccaaatttgaccgtcataccagcctgattcttcataaaatactgtagc
actcattaatcgttttactacgtatgaccagcctaaatataatctgattaaaataaatcc
tgtcattaggctggaagtaataaattctgaaaagaaaaatttcaaaggtagtttagtgat
tggaaaaattgataataatataggactaactaataaacagttaagtagtagtgcaatagt
tattttttgttataagatctatggcttaaagttggccagcaaaagaaccaagaattttt
caaagaagtatattcatgaacaggttgctgctcctttggcacaggacattgagtattata
taaattcattgacattaaaaagtattataataaagac--tttgtttatattatctaacat
aggtacgacattaaatattaactaactgcaattatagtagtgagtagaagaaataaaact
aaaaatagccaagtaattttatttaaagtattctcagtactacgagtattactaaagaac
tgattctgagcacctacacttcctaaaccttcggatttaggattgtgtattagaatagtg
aagattaatattattgtagatgagtaccaaaaaaattttaaaatttgttccattatgtaa
aagttaat-aaaaatagcacaaagacaatcgtctaaaagatattgattgtcttatcatat
taaaaagtatttattcaccctgtacttttaaaagtaaaaatcctattgccagccctacca
aaactaggactgaacttaatacagcactttctacaatttctccgaccatt-ttatttttc
cttattattttattattg---tcattcttttgacaagattatattgagctaa-ctaaaat
ccgtttctaccccaaactaccattgcaattgaaaatgtaaacattgccattagagctgcc
caccccaaacttaaaatatccataatttataatattc--ctaaaaataagatctctttta
cttaaactataatattcttaatattaaaaatatcattatataataagctaattataacag
agtactacttaattcgtatttttagtgtatttaaagaagattgaa-ttttttcgaggtac
tggataagtatttcatcctaagaaccatattcaac----ttttgatttattataatgctt
aaatctaaatata--tatcactcaagaatcattagtaattatgatgtttttaattatacg
att---aacatatttcaaaatagaagaattcatagcgactaagttaa-----tatgctaa
tataatttatcaat--aaaaaggaagttagtatttcaattatgcaaacaactataaataa

tgggcaaacgtctagtaaagaaactttactaacacctagattctacacgactgactttga
agagatggctaatatggatatttccggcaatcaagaagatttttggctatcctcgaaga
atttcgagctgactataatagtgaacattttattagagatgaagagtttaatcaatcttg
gtctaatttagaacataaaactaaatccttatttattgagtttttagaaagatcttgtac
cgcagaattttcaggtttttactatataaggaattatccagaaaattaaaagacagaaa
tccggttatagctgagtgctttttattaatgtctagagatgaagctaggcatgccggttt
tttaaataaagctatcggggacttcaatttatctttagatttaggattttaacgaagag
tcgcaagtatactttttctcacctaaatttattttttatgcaacctatctttctgaaaa
aattggatactggagatacataactatttaccgccatcttgaacaacatccagaacaccg
tatttatccaatctttagatttttgaaaattggtgtcaggatgaaaatcgtcacggaga
tttttttgctgctttgctcaaatcccaacctcatttttaaatgactggaaagcaaaaat
gtggtgcagatttttctattaagtgtatttgcaacaatgtacttaaacgactttcaaag
aattgattttataatgccataggcctagactctagacagtatgatatgcaagtaatacg
aaaaactaacgaaagtgccgctagagttttcccggttgctttagacgtggacaatccaaa
atttttcaaatatttagatacttgtgcatgtgataatagagctctgatcgatattgataa
taacaattctccattattcattaaatctatcgtaaagatacctctatattttccttatt
tgcaaatttactaaagatatatttgattaagccaatagactcaaaaacagtatggaatac
agttcgatagttaaatacaaggaatgctttttcttataaaaagctatctagtgattaa
tataaaat------aaaagcttccttct-----cgatggaaggagcttttattttgtcaa
ga---atataatgggattatatagatc-tagaactaagaacttacttttcttttgcttc
atacataacctctaaagtaattagatttttagaacaatcacgggcaaatttctctgtatt
tctttttactttacctctaacaaacccaggaattttatttaattctttctgtgcttcttc
tgaccaattaatactatcaacagcagaaattcctatacttaatgcttctgttgtatcatg
accaccaaatatttctagcaaatgatcttccataccaagtgtaaacgaattatatactaa
atcagcaatttgatttgttccctcataaccaagaaatggacggtaacttaaaggaaagtt
ttgtatatgcactggtgaagaaataacgccacaaggaatatttagacgcttaccaatatg
tctttccatctgtgttccaaaaattgctgcaggttcagttttagcaattaaatctccaat
taaaccatgatcatctgaaacgattacttcatcgcagaattcttgaacttgatctttaaa
ccactcttcatcatatttgcaataagttccacaccaggcgacatgtatacccatttcctg
gtgtaaaattcgagttattgctgcagcatgagttgcatctccaaatacaatagccttttt
acctgttaaattctgacaatcaattgatcttgaaaaccatgcagattgtgaaataaatcg
ggtttgctcgtctatgtatttttcgtagtctacagcagctcccaatgcatttactaattg
ctgtattgatcggatgcatgcagctgtttgaacaatacccataggtgtaatgtcaacata
aggcatattaaattctttctctaaatatcgagctgtcattagtccagtttctctatatgg
aataaaattaaaccatgcagagggtaatttttttaaatcttgaactgatgcattttctgg
aataatttggtttatttggatatctagatcttgaaataaccttttttaattctgctatatc

atgttgattatgaaatcctaagcttactgctccaataatattaacagatggagttttagt
cttttgagttaaaacttgattattagatttagctttttccatatagaaagtgacaatttg
ttctaacgttctatcacctgcttgcagttcatttactctataatgattaacatcagctaa
taaaacatctgcttctgtttctatagatgctctgctaacaaagttttgcaaatcttcttg
caaaatactcgaagtacaagttggtgttagaataactaaatcagggctttcttctctgtc
ttttctagtaatattttctacaactttttcttgagaaccacgggctaacacatgtctatc
tacaacacttgctgtaacaggagtaaaatctctgtctctttcaagcattgaacgcattac
attaaaataatcgtctccaagaggcgcatgcataattgcatgaactttttaaaagaact
cgcaattcttaaagttccaatatgagcagggcctgcatacatccaataagctaatttcat
attaaattttcctatgatttatttagacatagtaatacttttaaagctaactaacaaaca
ttagccaaacatctaatttaactccttgacaattttgtaatatttttttaaatgaagctta
tcattataaatatttaaaaaatataattcagaataga----aataactgttagatcaagc
ttgaaagtaaagatttcttgcagttatagtttttctttaagtaaacatgtaaaaattgta
ctatatataaatgtactatatataaactttttttactgaatattggtaatgccagataca
attaacttaaatatgccttccccaacgtttggtggaagcactggcggttggttaagagcc
gcagaagtagaagaaaagtatgcgataacatggacaggcaaaaatgaaagtaagtttgaa
atgccaactggtggtaccgcaactatgagaaatggagaaaacttactctatttagctaag
aaagaacagtgtttagctttaggtactcaactaaaaagtaaatttaaaatatctgactat
aagatttacagagtttttcctaacggagaagtgcaatatttgcatcctaaagatggagtc
tttccagaaaaagttaatactgggagagctagcgtcaacagtgttgatcattctattgga
caaaatattaatcctgtagatgtcaaattcatgaataaagcaacttacgattagtctctc
-aaaaacctagacataaaataacttttatatagtctaggtttttgataatgtatactgat
agtatggaagggtggccgagtggttgaaggcgtctgatttgaaatcagttgaacta--tc
tggttccgtgggttcgaatcccaccctttccgttataaaact------------atagtt
ggcttctcaaggcaagctagctatacactgatagtattaagtttagacttaataaagtct
acaacttgtgacaggttagaaatttttcggcatcctcatctggtatctctatactaaat
ttttcttctatggccattaccagctcaactgtatctagagaatcggctcccaggtcactt
gaaaaatttgcttccctagtcactatttttttttcaattcctagctgttctgctacaata
tcttgaactttttcaaagatttcattatcttgcataatatattatttcctctaaggtgtt
atcaaaaatttttcggcttatattattatccaactctaaaaaattagtaatttttagtcg
agattaaaatatttatacctatatgatatactattattctggataaatacgaagtttacg
atatggctcttctccaaaactacgagctctcaactggtagttgtcaactagattatgctg
catttttcgtatataggctgatcgaggtgtaagctgaactattgaactttcattcaagat
aatagtttcaatagccaatttagcttcttgaagagcctggatctcatcaaaatttttatt
tttacagagttctacccaattaagaccagatgaagtatgaatgtttaatatttttctgag
tgcacgagtaatttgtggaaccgtactattctgaattgtatatataataatttgttttga

ttttgcaatttgtctaagttttgtattttgctttacttgattacgtagcgctagaatagc
atcagacttttctatctccttggttaaaataattggtagatctagtgaagaaataactga
tgtaatatgctgccaactcaaagaataagcatataagtactgatgaggtatttccaattg
caaagattgtttattaatattaataggggcattgaaagtagttgacaataaagatgtatc
acgattttgtgatttattcaaatctaaggacttatttcttaattctctatattgtaaaac
cggaggcgcctgctgtgcgttaattgatttacgagcattagagatgtttacaggtaagac
ttctatagattgcgatggataacatttaattaaaattctaccattagcttgaatttgtct
tttttgcacaaaaggttgatggccttgcaaaatttgatctattgtctctttaactttatt
atgaactatccaaacatttcgctcgtgtatttcgattgcaatctgaaaagcaggctcagc
ttttctttctagtatactcttttgcgtacctctacgtttagcttcatcgtcacctaatgt
tacatattgaattccaccaattaggtccgccaatgttggatttttaattaaactttctaa
atgatttccatgcgctgtacctactaattgaactcctctttctgctatagttcgagcagc
taacgcttctaattccgtgccaatttcatctataataataacctcgggcatgtgattttc
aacagcttctatcattacttgatgttgcaaatcgggccttgcgacttgcattcttctagc
tctaccaatagctggatgaggaatatctccgtcaccagctatttcatttgaagtatctat
aataactactctttttttccatttcgtcagctaaaacacgggccatttctcgaactgcggt
tgttttaccaactcctggttttcctaagagtaaaattgaatcaccttgctgtagtaaatc
tcgaataatactaattgtaccaaagactgctctgccgacacgacaggttaagccaataac
actaccttctctattacgcagtgaactaatacgatgcaaggtttttttcgataccagctcg
attatcgccgctaaaattaccaactttttttacacaataatctaagtcttgccaactaat
agatctttgagataaatattctgggttatctggaaatctagcttctggacgacgacctaa
atccataacgacttctattaaattatttctattaggatgctgttgtaaaggttctttgac
aaaatttggcaaaatttctagcaacttatctaagtcatctgcaataagcatgattgagta
tataataattaattaatcaaatagaagattcaagattctaaagattacaatacatattaa
agaatattatgattggaatcaattttatacttaaattgaacaggaagtta-tagatgtaa
aagaactgattaaactttacgcaataaatcgattggtcaaaattaatagtcgtgttaaca
tgtttattggacaaaaagttcgtattaaatatagcaaacaaaaagttgctagtgatatag
cagataaagttggtgaattaggagttattaagggaataaagtttattaatagtcaatgtg
ttactattattgttgagtttgacaatcacactaggctttggatgtttagagaggaattaa
tctgtctaaatgaat------taaaaacatgaaacataatttattttatttattatacaa
aatttcttatgagtcgttaaaataataaattttaaaggagtacttttaattgtgcaaatt
actattaaaaaattacaagacttactttcgtctgtacaaagaaaaaagatccagacatta
aaactaaaacaaggtaagtttgaacttctattaaataagacctataaaaaagtcaatcaa
gaaattataccttcgcagaaatctgctgtattacaaaatagtccatctacgataattaaa
tcaataaataatacaaaaaaatcttctgttgttaatgaagaccgtacagaatatgccact
attgtttctccaatggttggaacgttttatcattcacctgctcctggtgaaaaaatttt

gtacaagttggcgatgaggtcaaattcaatcaaacagtctgtattattgaagcaatgaag
ttaatgaacgaaatcgaggcagaaattgaaggcaagattatagaaattcttgttaaagat
ggtgatatagtagattgcgggcaagccttaatgaaggttgaaacataatgctaatttttc
cattttacatattgttaacagaaattatttagatagaaaaattagcttatactatagtta
tgaaaacttatttcgcaaaggcac-taaaaactaatatataaaattatttactttatctg
ccaaaaatagaaataagcgttttgattccttgtctcaagagaggagaatctcaatggcaa
ttagctcaaaagagcaagagacaaagaaggtaaaaatctcggttgataaaaatcccgtag
atacttctttcgaaaagtgggcccaaccaggccatttttctcgtacactagcaaaaggac
caaaaactactacttggatttggaatcttcatgctgatgctcacgacttcgatagtcaaa
ccagttctttagaagaagtttcacgtaagattttcagtgcacattttgggcagctgtctg
taatatttttatggcttagtggaatgtattttcacggagcccgcttctctaactatgttg
cttggttaagtaatccaacaggtattaagccaagtgcgcaggttgtttggcctatagttg
ggcaagaaattttaaatggcgatgtaggtggtggctttcaaggagtacaagttacatctg
gatggttccaactgtggagagcatcaggaattactacagaatttcagctttactgtactg
ctattggcggattagctatggctgctttaatgctgtttgcaggatggtttcattatcata
aagctgctccaaagttagaatggtttcaaaatgttgaatcaatgatgaatcaccatttag
ctgggcttttaggcttaggctgtttaggctgggcaggccatcaaatccatttgtctttgc
ctattaataagctactagattctggcgtgtctccgcaagaaattccactacctcatgagt
ttttaattaatagagagcttatggctcagctgtatccaagttttagtaaaggattagttc
cattctttactttaaattgggctgaatattccgactttttaacttttaaaggaggtttaa
accctgttactggaggtttatggctaagtgatactgctcatcatcatctagcattagctg
ttctatttcttgctgcaggtcatatgtatagaaccaattggggtattggacatagcatga
aagaaattctagaagctcacaaaggaccttttaccggcaacggtcacgaaggtctatatg
aaattcttacaacttcttggcatgcacagcttgcaattaatttagctatgatgggatctt
taagcatcattgtagcacatcacatgtatgcaatgcctccttatccatatattgctactg
attacccgactcagttatcgctcttcactcatcatatgtggattggaggattttgtattg
ttggagcaggagcgcatgcttctatatttatggtaagggattataatcctgcagaaaatt
ataacaatcttttagatagagtcattaggcatcgagatgctattgtttctcatctaaatt
gggtatgtatatttcttggattccattcatttggtttatacattcacaatgatactatgc
gggcacttggaagatctcaagatatgttctctgatacagctatacagttacaacctattt
ttgctcaatgggtacaaagtatacacactttagctcctggaaatacagctccaaatgcat
tagcaacagctagttatgcatttggaggagatattgtttctgttggtaacaaagttgcaa
tgatgcctatttctttaggtactgcagatttttagttcaccatatacatgcatttacta
ttcatgtaactgttttaattttagttaaagggttccttttctcaagaaactctagactaa
ttcctgacaaggccaatcttggcttcaggtttccatgtgatggacctggtagaggtggta
cttgccaagtgtctggctgggatcatgtttttcttggcttattctggatgtacaattctc

tgtctgtagcaatttttcactttagttggaaaatgcaatcagatgtttggggtagtgtat
ctccgtctggaaatgtttctcatattactggcggtaattttgcacagagtgcaattacaa
tcaatggatggctgagagatttcctttgggctcaagcatctcaagttattcaatcatacg
gttctgcgctatctgcatatggattaatttcttagcagcacattttgtatgggcattca
gtttgatgttcttatttagtggtagaggttattggcaagagcttatagaatcaatcgtat
gggcgcataataagataaaagttgctcctgcaattcaaccaagagctttaagtattactc
aaggtagagcagtcggtgttgcacactacttattaggtggaattggtacaacttgggcat
tctttttagcgagaattatttcagtaggctaatagtgaaaataggataaaaaaaacaatt
atggcaacaaaatttcctaagtttagccaagctttatcacaagatcctacaactagaagg
atttggtatggtattgctacggcacatgactttgaaagtcatgatggaatgacagaagaa
aatttatatcaaaagatattcgcttcgcactttggacatctagcaattatcttcttatgg
acatctggtaatttattccatgtagcttggcaaggcaactttgaacagtgggtattaaat
cctttgaaagttaaaccaattgctcatgcaatttgggatccgcattttggacaacctgct
ttgaaagcttttagtaaaggtggctcagcttatccagtaaatatagcatattctggcgta
tatcactggtggtatactattggtatgagaagcaatcaagacctgtattctggggcttta
ttcttactagttttatcagctttactcttatttggagggtggctacatctacaaccaaaa
ttcaagcctggtttatcatggtttaaaaataacgaatcaagattaaatcatcatttatct
ggattatttggggttagttctttagcctggacaggtcatttagtacatgttgctatacct
gaggcaagaggacaacatgtaggatgggataattttacaaccgtattacctcatccagcc
ggtttacagccgtttttcagtggtaattggagtgtatatgctcaaaatccagatacagct
caacatttattcggaactaatgaaggtgcgggtacagcaattctgacatttctaggagga
tttcatcctcaaagtcagtctttgtggctaactgatatggctcatcaccatttggctatt
gcagtagtattcattgttgctggacatatgtataggactaattggggaattgggcacaat
ctaaaagatatttagatgctcatagaccacctagtggtagattaggagctggacataaa
gggctatttgatactattactaattctttacacatacagttgggattagcattggcttcc
ctaggtgtaattacttcgttggtagctcaacatatgtatgctatgcctccatatgctttc
atggctaaagatttcacaactcaagcatctttgtacacacatcatcaatatattgctggg
tttctaatggttggagcttttgctcatggggcaatattctttgttcgagactatgaccct
gaacagaataaagataatgttttagctcgtatgctagaacataaagaagctatcatttct
catttaagttgggtaactctatttttagggtttcatacattaggcctttatgttcacaat
gacacaatgattgcttttggaactcctgaaaaacaaattctaattgagccggtatttgct
caatggattcaagcctcttcagggaaagcactttatgggtttgatgtgttactatcatcc
tctactaatatcgcaacacaagctggtagcaatatttggctgccaggctggttagaagcg
attaatagcggaaaaaattcattgtttttaacaattggtcctggtgacttcttagttcat
catgcaattgcattgggattacatactactacgttaattttagttaaaggtgctttagat
gcaagaggctctaaacttatgccggacaaaaaagactttggatatagttttccttgcgat

ggacctggcagaggcggcacctgtgatatatctgcatgggatgcgttctatttagctgta
ttttggatgctaaatacaataggttgggtaacattttattggcattggaaacatattaca
atatggcaaggcaatgcaactcaattcaatgagtcttcaacttatctaatgggatggttt
agagattacttatggctaaattcttctccattaattaatggttataatccatatggcatg
aataatttatcagtatggtcatggatgttcctatttggacatttagtatgggcaacagga
tttatgttcttgatctcctggcgtggctattggcaagagttaattgaaactctagcatgg
gcgcatgaacgtacgcctttagcaaacttgattcgttggaaagataaacctgttgcatta
tcaattgtacaggcaagattagtaggtttagcacatt-tttctgtaggatatgtattaac
ttacgcagcttttgtattagcttcaacagcaggcaaatttggttagactaaa-----ttc
gaattagct-a-----aaaaaagtcagtcataata-gttttatgactgactttttagat
ttaaaacaaaaacttactaaagtttcacctcaatatctacaccagaaggcaaatttaact
tcattaatgcatctatcgtttgagaagaaggttgatgaatatcaataattcttctgtgag
atcttatttcaaagtgttctcttgagtctttatctacatgtggagaacgtaaaacacaat
aaattcttctttttgtcggtaaaggaattggccctactgcaacggcattagttctagatg
cggtatctagtattttgtcacacgacgtattaagtataatagaattatatgctttcagtt
taattctgattttgtctgctgagtaattgtcatgttaaaaagatcttattatttaagaa
ttttagagacaacacctgcgcctacggtacggccaccttctctaatagcgaaacgcatac
cttgctcaattgcaattgcattaattaattcagcagtcatcttaattctgtcaccaggca
taaccatttctgcatcagtaccatcatcagcagtaaactgattaatagtaccagttacat
cagttgttctaacataaaactgaggtctatatcctggaaaaaatggagtatgtcttccgc
cctcctctttagttaaaatataaacttctgcttcaaattgagtatgaggtgtaattgtac
caggtttagctaataccatacctctttcaatatctttttttctgcacacctcttaaaagaa
ttccaatattatcacccgctagaccttcttctaacgtttttttgaaacatttctaatccag
taatagtcgttgtacgagtttctcgtaaacctacaatttcaattgtgtcaccaactttaa
taatgcctctttcaattctaccagtggcaacagttccacgtcctgtaatagaaaaaacat
cttctacagccattaagaaagttttatcgacatctctctccggcgttggaatatatgtgt
caactgcttccataagtgaaaaaatcttgtcaacccatttatcttcaccttgcttagtag
ctgggtttttcgtcacagcttctaatgccaataaggcagaacctgcaacaaaaggaatat
catctccaggaaagtcgtattgacttaataattcccttccttctaattctactaattcta
gtagctcttcgtcatctacttgatcttccttatttaggaatactactaatgtaggaacac
ctacttgttttgctaataaaatatgttcacgagtttgtggcattggaccgtctgccgcag
atacaactaaaatagctccatccatctgagcagcacccgtaatcatatttttttacgtagt
cggcatggcctggacaatctacgtgagcataatgacgattatctgtttcgtattcaacat
gagcagtattaatagtaatacctctagctttttcttctggagcggcatcaatttcatcaa
attttttcgctgcagtagaccctaaagttgataaagtcgcggagatcgctgctgttaaag
ttgtcttaccatgatcaacatgaccaattgtgccaatattgacatgaggtttttttacgtt

caaatttagatcgagccatgctttttgttttccttatagtaaaagttgctttatagttac
ttttaacgataaatcttgtgaattgaaagatttattatttaatacagacaaga-----ta
caatataactaatctaatatcgatagtgagcaaacgctttatttgcttctgccattctat
gtgtatcctctctctttctaatagaatttcctgtttcattagctgcatccataatttcat
tagctaatttcatagacatacttttaccagatctgtctctagagaatttagtaatccatc
ttagtgctaaattcgtacctctataagctcgtacttcaataggaacttggtaagtagaac
caccaactcttcttgcttttacttctactagaggagtaatatttcggatggctttttcta
aaatatttaaaggatctgattctgttctctctttaacgatatctaaagcctgatatataa
ttctttgagataaagtttttttaccacttttcaaaatacgaacagttaacatacttacga
gtctgcttttatataaaggatcaggtgatgcaaaccttttttttagctgtattacgacgag
acatagtatttaaaattggttattgtaataa-tgttc-tattagcaggtatttaagattt
aggctttttttgtcccatatttagatcggcttttacgacgatcttttactcctgcagcatc
taaagtaccacgtactacatgatatcggactccaggcaaatctttaattcggccgcccct
aatcagtaccacagaatgctcttgaatattatgacctacgccaggaatataagctgtaac
ttcaaatccagatgttaatctaacgcgcgctacttttcttaaagcagagttcggtttttt
aggagttgtagtatatactcttgtacaaacacctcttctttgagggcaactttgaagagc
tggagattttgtttttttatgtatttttcgtctttcggatctaacaagttgttgaattgt
tggcataataaaattgatttaaagtctttaatagatactatttgaacctgctttacatga
attggctagtttttatttatcttatacttacgcataaatctttctactctaccttctgta
tcaataattctctgtgaaccagtaaagaatggatggtttcctgaccagatatctacgtgt
agttctggctttgtggacccaatcgtcataattaactggccgtcacagtaaacttttgct
tctggataccaatttggatgtatattatcttttgccattgtttttataattatataaatg
tatcaataataactattaacgcttagaaaattgaggagcctttcttgctttctttaaacc
atatttttttctctcttttactcttggatctcttgttaaatagccttcagacttgagtgt
cgttctattttctggattaattgagcacaatgctcttgcaacacctaaacgaattgcatc
agcttgccctgtcaaacctcctcctctagcatttacatggatatcatactggtttagtag
tcctaaaacttgtaatggtgcatatgaaactcttaagtaattaggactaaattgaagata
agactctcctggtataccgtttataattaaattacctgaccctgggactagtcttacttg
tgcaacagaacacttccggcgacctgttccagaatagattgcgcgagttttaattaattc
tgtcgacataacattccttgataactaaataaatatacattctttatactatatactctt
gtggcttttgcgcgacatgtgggtgaattgggccagaatacactttaagcttcgtaaata
gttttctgcctaatggacctttaggaagcatacctttaaccgatttttcaataattctgt
taggtaatcttgtctgaagctgatcaaatgtttcaacttttaatccgccaggctgtccag
aatgtcttctgtatagtttttgattcgttttatttccacttacagatacgtgagcagaat
taataacgattacataatctccagtatctagatagggtgtataagaaggcttgtttttac
ctcttaagatattagaaatatgagtagatattctaccaagtgtctgatttttagcgtcta

taacataccaatgagaattagtatttaatgagggtgattgcgttttattcatgaaaagat
taatacttttggttgacaataagagaatatgtaaatggtttacattcaatttctatacaa
gtagtattt-aaaaatatta-------------ttaacaaagagaacttttctaacagtt
atttaaattgtttgtcagcaagaaaatttctagttatcaataacttatactatttttaat
aatataaaatatattaatcactttttcttttggcaaacttatacctagttttttttgc-
aaggcatatataacttcttcagctgatttctggccaaaattttaatttctagcagttct
tcttgagagtaatctagtaaatctgcaatagaatggatttgagctcgctttaagcagtta
taagccctaacagataactgcagttcttctattaaaacttgactaatttttttatcttct
ttgctacgataattatctgctgatttaaagtctaaatttcttagtgaacaaaaaagatta
gttaaaactgtagctccttgacttattgcttcttggggagatatgctcccatttgtccag
atttgtataatcaatctatcttttatgctgttgctaccaatacggacttcttctacttta
taattaactttattaactggcataaaaacagagtctacttgcaaaaaatccacagataat
tcatctacagctttttcagctaagcgatacccgcaatttttttcaattttaaactccatt
tcaaatattgtgttattgcaaatagttgcaatatactgtctaggatctactacctctata
tcagaagataattcaaataggccggctgtaactatagctggcccttgaactctaattcga
ccaatttgagattctttgttataacttttaaatactacttcttttagattaagtaatatt
tctaacacatcttctcttaccccaggaattgtggaaaactcatggttcactccagcaatc
cgtacagcaactatagcagtaccttcaagatctgacaatattgatcttctcaaagcatta
cctaatgtaataccttgtccttgatttaatggttctattacaaaactaccgtactgccca
cgcgccccatctgttcttgactctatgcattcaatttgaaattgagccacctaagaaagc
tcc-tttaaaataatcagtgattagtaagtctctctaaaacaagaaggaatttatactcg
gcgtttcttaggagggcgacatccattatgaggtacaggagtaatatcttttattagagt
aatctctaatcctgcagcctgcaaagctctaattgctgtttctcgacccgctcctggacc
atttactagaacttcagtttggcgcataccttgatccatagcttgtctagctgcttttc
agctgctgtttgagctgcaaaaggtgttcctttttagctcccttaaatccacttgcacc
agaggatgaccatgataatgtttctcctttaaattagtaatagtaacaattgtattatt
gaatgtagatttaatatgtgtaataccgttaactgcattacgtttagtttttcttgctcc
ggatttttttatttgtctagccatcgtgtctcgtctattatatagttaattaaaagatta
ttttcttggagctttttcttacctgctactgtttttttacctcctctgcgtgttctagc
attagttctagttctttgtcctctcaaaggaagaccaagacggtgtcttctacctctata
agtactaatttccataagtctcttaatgctcatagactcaaaacgtttgagatccccttc
aatctgataattagactcaagaatttctcttatactgacaacttgttgatcatttaaatc
ttgacacttaatatcagcgtctatgtttgtttttctaatatttctttcgagcgagatag
tccaataccataaatatatgttaaagctatctctattcttttgtttcttggaagatctac
tccagcaattctggccactttgtcttgtctccaataaataatatgtat-ttttataacgg
ttatttata-agcttagccttgtctctgcttatgtttaggattagtgcaaattaccatca

cctttctgtgacgccgaattattctacatttttcacacatttttcgaacagaaggacgaa
ctttcatattaa----ctctatatactaggaatattactatattaactctattaattatc
gacattaaaaatgtcaacaatatattgtattactttaactgttgtagattttacttcgtc
atactatcatatttttttgatatgacgtatgtctgaatttgcttagctgtgtctattgcg
actccaactaaaataagtaaagacgtagccccgagacctcttaagttttggatctgagta
accttttctattataaacggaatcagcgctactgtaaataaaaacgaggctcctaggaat
gtcagtctatttaatatgacttgtaaataatcaatagtcgcttgacctggacgaatatta
ggaatgcttgcacccatttttttaaatttatagcaatatcttctggattcattactatt
gatgtataaaaatagctaaagaaaaggatcaaagcacaataaagaagaaggtatagtgaa
ccattaggacaaaacaggtataggatttggagtaatgttttattttgaataatttgggtt
agataagatgggagggccatagatgcagatgcaaaaacaataggcataactccaccttga
tttaatttcaagggcaagtaactatttggatccaaaattgaagattttcctagctgtctt
gctgaaataatttaattcttcttgttccttcttgtacacaaattgtaattattatcatt
aataaaaagatcgctataaataatccgaacttaagacttgcattactataactagcatca
aaaaatgactgtgtaaaattctttggtagtcctgacacaatgttttgaaaaataagtaaa
gaggctccattaccaattccctttccgtaattaactctgataaccacataataatcata
gagcctgctgttaaagctaaaacagactcgcagacaaatgcaaaattccaattaaaaaca
tatggttttacccatatagagattgctccagattgtaaagtcgcccaacctagagctaag
taccttgtaatttgagttattttttggcggcccaattcaccttcttccttctgtaatttt
tccagattggggacaattttcgtaagtagctgcattacaattgaagagttaatataagga
acaatacctaacgcaaaaatccctattgttgaaaaacctcctccagaaaaaatattcaga
aaatttactaaagtatttttttctacacttgcataaaaggcgtcatgatctatacctgga
acaggtataaatattcctaaacgtgctaaaactaatagaaagagagtaaagataatacga
tttcttagatcactttttggctcataaataaattttaaatgaaaagagatacttt--tt
tatgtaaaaaccctattccaagacttgtataagtttaaaatagggaagtaa---aatatt
atagtaatattaata-taatattctatgtagaatacaggctttctattggaactcctcga
tctttagctgcttcactaaaagttcttaattgagttaagccattgagaacagctcgcgcg
ttatttaatgtattattagaacctaattgcttagctaatatattctgtacacccgacaat
tctaggactgtccttacagaacctccggcaattacacctgagccaggtgcagagggcctt
aatataactttcgcggcaccagaaattccgttaataggatgaggtatagaattcgatttt
gtcagtggaactgtaactagatgtttttttagcatctgttactcctttttttactgcacca
attacatcgcttgcttttcccacgccgacaccaacttggccttgctcattaccaataaca
agaataactcgaaagcttagtttttttacctcctttttacaactttagtaactcttttaact
tgtacgactctttcttcccagccactatctttatcttttcctttgctctgtttttttacga
ttggccattttaagaattattcctaattagttaatta-cgattctagaaacccatgcctg
cttccttagcagcttcagccaaggccttaactcttccatgatataactttcctcctctat

cgaagacgacatttttaatgccttctttcatagactgttctgctaactgtttaccaacta
cgcgagaagtatcacagtttggtcttatgttatcagattctttattatttaaattaacag
atgatgtagctactaatgtaataccttgtgtgtcatcaattatttgtgcgtatatatgtt
tattagatctaaatacacacagacgaggcctacttgaggttccttgaactttttccgaa
ctcttttatgcttatgaattctagtttgtttagtgtttagtttcattattatttaccttt
tccagctttcccagcttttcttctaacaaattcaccttgatatctaattccttttccttt
ataaggctcaggaggcctaatagaacgaatagttgaagcaacctgaccgacaacttcttt
atctatgccagagacagtaatatttgtgttgttttcaactttaatttcaatattggcagg
aggtttaattttcactacatgactatagccaacacttaagattaaatcttggttatcaat
ttgagaacgatagcctacaccttgtatttgcagtttttaaaaaatccattagaaacacc
ttcaatcatattactaataagagttctggatagcccatgtaactgactagccatttttgt
ttgtccactcgtcttgacagcaattgtgtcgttaagtatttctaaattaataccagctgg
taaagttctggatagggtacctttaggtcctgtcacggtgatagtctgaccatcgaactg
agtactaagatttgtcggcaataaaattatttttttccaatacgagacat-attccacc
tataaaatagttaccaaatatagcataatatttcaccaccaagaccatcatgacgagctt
gtctatctgtcataacgcctctagaagtagaaataagagcgatgcctaaacctccaagaa
ctctgggcaactctttatgatttgcgtagactcttagtccgggtttactaattcttttca
gagcagtaataactggttgacgattttaccattatacttaagagaaatcattaaatgag
tctctataccttcacccatttgttcgaaattttgaacaaatccttcttcttttagtactg
ttgccatattgcatgtcattttcgttgctggaacttgcacaatttgatgtcttgctaagt
ttgcgttacgaatacgtgtcagcatatcggcgatcgtatcgttgaccaccttgatcctcc
ttgcga-----tgaattatttgaaatattttcaagattccctgaaaggcatccctagctt
ttttaacaaagctagaccttcttgatctgttttagctgtagtgacaattgatatatctag
accacgaatttgatcaatattatcatagtctatttctggaaagatcaactgttcacgtaa
acctaaattgtaattacctttgccatcaaaacttctaggactaattcctctaaagtctct
aattcttggtaatgttaaattaattaatttctctaaaaaagaatacatcttgtcttttct
tagatgcacaacaattccgataggaacctcttctcgaattttaaatcctgcaatagattt
tttagcttttgtgacaattggtttttgtccagttattaatgttaattcttgaatactact
ttcaagagccttagcattctgagaagcttcacctaagccacggttaatagtaattttagt
aaacctaggaacttcatgtacatttttgtactgaaattcatcttttaaagattgagtaac
agttgttttatatttttcttttaatcctattgccattatattttcatcaattatttaata
agttcgccagtctttttcagttttcgaa-ttttttggcctttatcattaattattactga
ggatcgactagcaatattattttgttcactaaataacataacgttagaggtatgtattgg
agcttcaaatttgataatttctcccgtttctccttcttgctgaggtttttttatgcttcac
tttgagattaattcctttaacaatcaccttatttgttttataaatgattgcaataatttc
acctgtttttgttttatcacttccagaaataacttgaactaagtcccctttttttaattt

aatttttgtattatttttggttgttttagaaggacctttcattatactacctccggtgca
agagaaactatttagaaaaatttttatctcttaactctcgggctataggtccgaagact
cttgtgccacgtggattattatcttgattaatgataactgccgcattatctccaaatcta
atgctcataccgtcagttctccgtaaagctttacgagttcttactacaacagctctgaca
acatcagatcgtttaactggcatattgggagacgcatctttgactactccaataataaca
tctccaatagatgcataagaaggattactagtgcctaatactctaatacacattattttt
ctagcaccactattatctgcaacattaagatagctttgagtctgtatcatacttttattc
tgtataagattaattatcaaaagacttagataagatattaaccattgtccaacactttgt
gcgacttaaaggtcgtgtctcttgtattgtaacaatatcgccgattgtgcattcattatt
ttcatcatgcgctttatatttctttgtccggatcatagtttttgcgtactttctatgaga
gattctattttctacagctactactatagttttattcattttatcgcttactactttacc
tgttgtttcttttaaaggcatagtctttatattttttatatat---------aattaaat
tttaagattattacatggttgacttagtacgagacttttcaacagttaaaagttgggcta
atctatgcttagaatgtttaaatagatgaggctggaaatcttgccttgtggctcttttta
gccttaaatcaaaaagctctctttttattacaaggatttcttcagctaaagaagaagagt
ccaggtttgtaacatctgatattttagggaaagtcatattttaggattctgttgtattcc
gaactataaatttagtcttgattggtaatttataagaagctaatttcatagcttgttgag
cagtttttgtggtacacctgtaatttcaaatagaatatgtccaggcttaataactgcaa
cccaatattctggagctcctttacctgaccccatgcgagtttcagctggacgggcagtga
ctggtttatctggaaatactctaatccatagtttaccacctcttctcacatatctagtaa
tagttcttcgagtagcttctatttgtctagaagttaaccaaactggctctgtcgcttgta
atgcataatcaccgaacgcaattgtgttacctttgctagcagaacctttcattctacctc
tatgttgttttctaaattttgttttcttggggcttagcataaaaattaaaaaataataat
aattaagaagctgttgaatcaggagtttcctgattataagcagattctagacttttcgat
tctggcaaaatttctcctttaaaaagccaaacttttactccgagcacaccatacgtagtg
tgagcttggcgatgacaataatcaatatcggctcttaaagtttgtaaaggtacacgacct
tctctaacccattcacttctagctatctctgcaccattaagccgaccagatacttgaatt
ttaacaccctgtgtatttgctctttgagctctttgaactgcttgtctgacagctctacga
aaggccactctttttttctagctgttgagtaataaattctgctactaaggttgcttcagaa
tctggatctgcaatctctacaacatttactctgagttgtttgctagggtctaggattaag
gacagcgattttcttaaagactcaattcccgctccagattttccaagtacgattcctggt
cgagctgttgctatgagaatttctacttgatcaactttacgattaatttcaattttagca
atactagcattactaagttttgaatgtataaatgagcgaattttatgatcttcttgtaaa
agaactgggtagtctttagagttagcaaaccatgaagaacgatgtttttgggtaatgcct
atgcgaaagcccaaaggatgaatttttttgacccacagtatccttgattataagttaaaag
ctataatgactaattgacgttaaa--------agactacagttctgaaacacctaatgta

atatgacaagttggtttatgtattggaaaggctcttccttgtgctctaggctgaaatctt
tttaaagtcgggcctttgtctgcaaaggctttactaacaaacaattggttttatttaat
ccatcattatgctcagcattcgctgcagcagattccaggatttgctttatatgagaacaa
actcggtatggcataaattccagaataattaatgcttcttggtattttctacctctaatt
tggtctaatacacgacggactttatgcggagaaagacgaatgtatttccctactgcttta
gtttcttttacgtttttgtaatactcatagttatagtttaacgacgggcttttctgtca
ccttttacgtgagtgcggaaagttcttgttggaacaaactctcctaatttatgtcctacc
atttggtctgacacaaagacaggaaaatgttgtttaccattataaacagctattgtatgc
cctaccatatcgggaataatagtagatgctctcgaccaagtttttaggacttcttttttt
cctgaaatatttaatgcttctatacgtttaagaagactaacatcaataaaagggccttta
tgtatagatcttgacataataataaatcattagattcttaataaaataaattctatttac
ggcgacgtaagacgtacgggttactatatttatttggatttcgtgttttaacacccaatg
caggtttaccccaaggtgttacaggacgagctcgtccaattggagatttgccttcaccac
caccatgtggatggtctacaggattcatgacaacacctctaacagtaggtcttttaccta
accaacgatttctaccagctttaccaagagtaatattactagcgtcaatattaccaactt
gtccaatagtagcgtagcattcttttcgaatcattcgaacttcgcttgaaggcaatttta
cagtaacaaaagtaccttcttttgctactatttgagcataagttcctgctgcacgaacaa
tctgtcctccgcacgaaggtcttaattctatattatgtactgctgttcctaaaggaatac
tagataaaggcaaagcatttccaacttcgataggggcagtaggaccagaaagaaccatag
atcctacactgagagatcgagggtgtagaatatatcttttctcaccatcaagatagtgta
ataatgcaattcttgcatttctattcggatcatattcaatagaagcaactttagcaacta
tattatgtctatttcttttaaagtcaattaatctgtactgctgcttatgtccaccacctt
tatgacgacaggtaatgacacctctattatttcgacctttgcagaaatggtgtttaacta
ttaatgatttttctggcttatcagtagtaatctctgaaaaggtagaaaccgttctatttc
ttgtccctggtgtgtaggcacgatataaacgaattgccatataagaagatttgagaagag
taaatgttgaa------ctatgtgatatatttaagtttctggaaacaaattaatagaatc
ttctgaggcaagtgttacaattgctttcttgtaatgtggtcgcttccctacgaatctacc
gatgctccttttttttcttaggaggatgacaagtgttaacacctgtaacttgcacgttgaa
aatatactgtatagcagccttaatattaatttttgttgctttgggatcaacggcaaaaca
atactgattttcttctaataatttagttgtcttatcagtaatgatgggatatttaaccag
atctaataaacctcttgaatcaatgctatccattatatacctcttgtattttagataaag
catcaactgtaataataattttatgtgcagctaacaaagccataatatttagtgtatcag
ctgaaataagttctacgttgtgtagattgcgaatagaaagataaacattcggatcttttt
tgtctacgatgactaaaactttttatttaaatcaagattccatcgatgtatagcttcca
taaatagtttagtcttgggctgatgaaaataactgttaaaattttctacaatcagtgtat
taactgatttgttatttaatgctgtccttaatgctaattgtctttctttttttattcattt

ttttggtaaaactacgaggttttggcccaaatattacaccaccacctctccatagaggtg
aacggatagaacctgctcttgctcgaccagtcccttttgacgccatggtttacgacctc
cacctcgaacttcacttcttgttttagtattagcagaaccctggcgcttttcattacttt
gctttactaaagctctatggactaagtacatcccagaatcttgactgaccttaagattta
aatcagcgttgccacttacttgaccttcccaattatagacttgataatttaattgcgtgt
taactgtcatgaaatatatcgtatgaaagaatattgctacttactaattttactaaagc
gccaggtttaccaggaacagctcccttaacgatcaacaaatcattctctgagttaatact
tacaatttgcaaatttttattgtaactttttattaccaagttggccagccatgtttt
acctggataaactcttccaggcgttgtaccggctccaatagagccaggttgcctatgatt
tttcgaaccatgtgacattgggcctctactaaaatgatgcctcttttggtaaccggagaa
acctttacctacacttctagaagagacattaatcttttggcctacttgaaatagatctgt
agatagtatttggctaacttcgaaatcatcagtagatttcatttcgtattcacgtaaata
ttttagtgggggagcctgtgatttttttaagtgtcccaacaatggtttatttaattttg
ttcagcaacttgcttgtaaccgacttgaatagcattgtagccatcagtagatacagcttt
aatttgagtaataacacatggcccaacttgaattacagtaactggaattgataaaccagc
ttcatcaaagaattgggtcatacctactttagtaccgagtataccaacagacactagatt
tctcctttttgagagatttatactatcattgtacataaaaattctacttcatagttactg
aag--taaattaaactaacagatgcatgatattattcatgacaacataaaggttaaaaac
tttacctatttaatctaaaatagaaacagctttttgtctagttacagcgtatataaatta
gaagtatgcttattaaatttttataaactact-tagatttaactgatattgatatccgt
aacttacttgcagtaatatc----aatcaacatgtataagtttaattgcctaaactagaa
ccagctattattagaaataattcaagtatagataaaagcgtatttgaacttttatatatt
gacaaatataatagaatatattgaggttcggtaactacaccttttatttttacatttat
tgtgca--gtatatgtacatctaatgcagtaaacataagaatataac----tgtatagca
aatgtaattacacaaattagagaggtattaatgggcaaagtcgttggaattgatcttgga
acaacgaattctgtaattgctgttatggaaggaggtaaacctaccgtcataccgaatgca
gaaggttttagaactacagcttctgttgttgcatatactaaaagtggagataaacttgta
ggacaaattgccaggaggcaagctgttattaacccagaaaacactttctactctgtcaaa
agattcataggacgtaaacagaatgaaatttcgcaagagattcggcaaacatcatataat
gttaaaactagtggatcaagcataaaaattgcttgccctgcactgaataaagattttgct
ccagaagaaatttcagctcaagtactgagaaaacttgttgaagatgctagtacgtactta
ggtgagactgttacacaagcagtaataactgtaccggcttactttaacgattcacaaaga
caagctactaaagacgcaggtaaaatagcaggcttagatgtattgagaattattaatgag
cctaccgcagcttctttgtcttatggactagacaaacaaaataatgaaacaatactagta
tttgaccttggtgggggcacatttgatgtatctgtattagaagttggagatggagtattt
gaagtactctcaacttctggagatacacatttaggcggagatgactttgatcagcagatt

gtagaatggctaatcaaagattttaaacagaatgaaggtattgatcttggtaaagataga
caagcacttcagagattgaccgaggctgcagaaaaagcaaagatagaactgtcaaattta
actcaaacagagatcaatcttccttttattactgccacacaagatggtccaaaacattta
gaaaaaactgtaactagaggaaagtttgaagaactttgttcaaatttaatagataaatgt
agtatccctgtaaataatgctctgaaagatgcaaaactagaagcttccagtattgatgaa
gttgttttagttggtggatctacaaggattccagccatacagcaaatggttaaaagatta
attggtaaagatccaaaccaaagtgtcaatccagatgaagttgttgctattggtgcagcc
gtacaagctggagttttagcaggcgaagtcaaagatattctattactagatgtgacgccg
ttatctttaggagtggaaactttgggtggcgtgatgacaaagattataccaagaaatact
actattcctacaaaaaaatcagaagtattttctacagctgtagataatcaaccaaatgtg
gaaattcaagtacttcaaggcgaaagagaactaacaaaagacaataaaagcttagggacg
ttccgattagatggcattatgcctgcacctagaggagttcctcaaattgaagttaccttt
gatattgacgctaacggaattttatctgtaaaagccaaagaaaaggctactggtaaagag
caatcaattactatatctggagcatcaactttacctaaagatgatgttgaaagaatggta
aaagaagctgaagaaaatttcgatacagaccaaaaaagaagaaagaatattgacacaaag
aatcaagcagagtccctatgttaccaagctgaaaagcaagttaaagagtttgaagacaaa
attagccaagatttaaaaataaaaatagaggagctaattacagagcttagatctagtcta
gagaaagaagaatatgacaatattgaatccatttctcagcaattacaaaatgctctgatg
gacattggaaaaaatgctgctcagactgaaagtaaagatacaaaagcgaaggatgacgac
actgtgattgacactgatttctctgaagctaagt-aaaagta-agcgggtaacgcgattc
gaacgcgcgacatcaaccttggcaaggttgcgctctaccactgagctatacccgcataga
ttgtattattacaaaaattaagatatttgtcaatctgtta------atctatac---atc
tagactattaattttggaattacatattagctaactgttgtaaatataacaattgctata
attttttaa-------aagtacttatatcat-tatataagtacttttatatt-------
-caaaaatacaaataagtattatag-------ctaaactactaaggagttaactaagcta
gtaacaattactaatcctgtccataagccggctcctgtatatattaatccttttgattgc
tcccattggccaggggatgctaaaacaacaggtacgcctactactaacacagtcgataat
gcaattaataatagcactaataattggatcgcaataatcatttaatatgttctcctgaaa
aattcaaatgttaataatactgtatataatataatgaagcttttgaccttcagcaattta
tctatgaagaaataatttaaaattattgttttctaaataaaccttaaattaagtattaaa
atctttattgtaaacaataatactatattttttta--gtagataaaaaatttaaagaaag
tctttttaaaaaaaaattaagataaggtgatatgctgtataaaaagctaagtattatta
attattagagaaatttaaa-ataagaggtaaaacatatgaactcagcccttttttttagca
aaactaccagaagcttatgccattttaaaccgatcattgatatcttacccgtaattcct
gtattttttcttcttttagcttttgtttggcaagcagcaattggttttagataacattta
ggttataaaaagaataacaaatatacagagtgccaatttttgagctctaatcttatatct

tacacaataataactattatggttgaacccttactatcaggaattgttcttggacttatt
cctattactttattcggattattagtagcagcttatttacaatatcaacggggcaaccaa
ttaggactgtaaagcctaaattatagatatttataaaaactacaataattatagtttta
taaatatctacaaataagctaattttaaatacagaaataatactattgaaactagagtga
taacaaaaaaaatttatgtaa---taa---------------------------------

---------------------------caagctaattgtttaccagctcgacttagtaac
tcctggtaaaagacattcatgagacatttctcgaaaaacatggcgagataatccaaaatc
tcgataataacctctacttctacctgttaaccaacatctatttctccctctaacagcggc
gctgtttctaggcatttcttggagtttttgtcttaaatccattttgtcagaaaagctgcc
agcttctttcagttgatctttaatagccagtctttttaaataatatttttttgcaagttt
cgatctcttaatctctctttggatcatattctttttagccatgctgtaa-aactctcttt
taaaattatttattcaatatattagctaaaaaagctcctgcctgcaactttatatattt
aaaaataaatagcataaagtagtgaaatgcaattgatgtttaatcaacgatacc---act
ttatgctattatcaaaaatttatttagtaaagttcttgttcaacatgagttgaaatagtg
caatcagatgaaggatatgcaatacaggttaaaacatagccagctaaaagttgatcatca
tctaagaatgattgatcagcttgatctacagttccttctgtaactttacctgcacaagtt
gaacaagctccagctctacaagagtaaggaagttcaattccttcttcttctgctgcatct
agaatatatgtatcctctgcacaattaaatgtgacatcaattccttcttcttcacatagt

aaatgaattttataatcagccatttcttcttactccataataaagatacacttgaaactt
aataaagcaaaat-aatttaaagaacttaccaaaaaattatatactagtttcattatgca
tgatagactggaaaaaaagaaaatagtatactattctgaaaaatcaagctacatttccta
ctaaatagaactatagtaggtccaaagtatatacataaattcatatagttaaaattactt
ttatcatttaaatagctgaacttgatagta---------tagattgatcatc------aa
gctagtcttctgc---caataaataattaattatgatttcaatgcttaatcaacaagtag
aacttaagcctattcttaaatttcagataacacaaatatattcgcattctcttattcagg
aaattaaagccttaataactagacttgttttgcaagtttggagaaggccagctacattga
tggcaggtattatccaacctttattatggttaattctatttggtgggcttttctacaatg
ctcctataaatttgttcactattaatacaagctataattgtttttttgagctctgggatta
taattttcacctcttttactggagctttgaattcaggtcttccattaatgtttgatagag
aatttggattttaaatagattattaacggctcccttagtatcgaggacttctatcattt
tatcttctgctacttttatgacttgtattagtttaatacaagttgtatttatagttacag
cttctcttttatgggaaacccacctctaaatagcgatagtactatgatttttggactta
tgattctattagtgactgtaggagttacaatgcttagtttagctttatctttcactctgc
caggtcatattgagctattagcatttatttagtagttaacttgcccttttatttcta
gtacggctttagctcctttatatttcatgccgccatggcttcagttaattgcaagtctca
atccattgagttatgcaatagaaggtacaagatacttatattcaagcgtaaactggaatt
ttacagagtgtgtgattaagattagctggggagatatttgtttagggcaaattattattt
tattaatcgctttagatataatggcagcttatcttgtgtctaatatattaaaagctaaac
ttaatta-----------------aaattttaataaaaaatttattaaatttattttaca
aagattttactatataatactaatagtagtatggataattgaaatagaaatagtttttc
aaaaccaaagctattatcaacaatgataaacatttgtaagaaagtcaacaaagtatgttc
ttattcataggaggcatgtagtcaatgggactaccatggtaccgtgtacacacggttgtt
ttaaatgatcctggacggttaattgcagtccacctaatgcatactgcacttgtagcgggt
tgggcaggatctatggcattatacgaactagctgtatttgatccttcagatccagtatta
aatccaatgtggcgacaaggcatgtttgttatgccatttatggctagactgggcgtaaca
gattcatggggaggatggagtataacaggagagagcgtatccaatcctggactgtggagt
tttgaaggtgtagccttaactcatatagtcctttctggcatgctttttctagctgctatt
tggcattgggtttattgggatttggaattatttagagatccacgaactggcgagccagcc
ttagatttaccgaaaatttttggaattcatttattgctatcaagtctactttgtttcgga
tttggagctttccatgtaactggacttttggaccaggaatgtgggtatcagatgggtac
ggagtaaccggaaaggtattaccagtagctccagcatggggaccagaaggattcaacccg
tttaatcctggaggagttgcatctcaccacattgccgcaggtactgtaggtatattagct
ggtgttttccatttaactgttagaccaccacaaagactgtatcgtgctctaagaatgggt
aatattgaaactgtattatcaagtagtatctctgctgtttttttctcagcttttgtgact

tgtggtacgatgtggtacggctctgcaactacgcctattgaattatttggtccaactaga
tatcagtgggatagtggatattttcagcaagaaattgagaaacgagtagaaaatgctatt
gctgatggtgctgcacctagcgaagcatggtcaagaattcctgacaagttggcattctat
gactatattggtaataatccagcaaaaggaggattattcagagcgggccctatgaataaa
ggcgacggagttgctgaagcatggcttggacatcctgtattccaagataaagaaggaaga
gagcttagtgttcgcagaatgcctgctttttcgaaactttcctgtaattttagttgat
aaagatggtattatacgagctgacattccatttagaagagcagagtctaagtatagtatt
gaacaagtaggtgtaacagctagtttttatggtggcaaattaaatggccaagttttcaac
gatgctcctagcgttaaaaaatatgcaaggaaagctcaattaggtgaggtatttgaattt
gatcgaactacattagaatcagatggagtgtttagaagtagtcctagaggctggtttaca
ttcggtcatgctaatttcgctttaattttcttctttggacatctttggcatggttcaaga
actatcttccgggatgtgtttgcgggaatcggagctgaagttactgaacaagttgaattt
ggagctttccaaaaattaggagatagaagtagtaaaaaacaaggagctgtataagaccaa
agtcaaagatagtctttatc---aaatatttctaatattaaaatagttaagacaaagttt
attatctaatagttactaatcaggagaatttatggaagccttagtctatgtatttttact
aacaggaacgctaatggttatattctttgcgatctttttagagaacctccaagaatagc
aaagtaacttgattccgtttaaacttataataatagaaccactttccaacttattagtgg
aaagtggttttattgtagattacattgtattattcttcatgctcttcaaaaggatctcgg
agatcttttgaagcaggaccaaatgctgtgtatatcgagtaacctgtaatacctaaaagc
agactcgaaataaaaatactaagaactgttgcagtttccataatttaagcagtattaaga
tgaactttttctataatgatattatcaatattaaaacatttaattattaaaattatcaaa
atatactatggcacttagaactagacttggagaaattttaagacctttaaattcagagta
tggaaaagttgctccaggttggggcacaactcctattatgggaattttcatgttactatt
tttcttgttttattaatcattttacaaatctataattcttcattagtattagaaaatgt
agatgtagattgggctactttaggtag----ctaactagagaagttctttga--------
ttacacaatattaaagacaac----------------aagcgatcgctattagattatag
tagcgatcgctttaaaagtattttactatatatccagagaaaattatttacctaaattaa
ttaactcggtatctgcaaaactatttgtgttaacattattatagttaactttttcaaacc
ttactaatactggatatttgataccacttttatcaattgctgcaacagtaccaatttcct
gataccagtacgattcttttctcaatatttttacttttgagcctctttccatatttttag
tcctcagatagattattaaagattattatattaaaaaatt-taaaaaactaaacaaaata
tataaaaatactaccgacaattgtaatatataactaataaaaggagagagagggattcga
accctcgataacaaaagttatgacaattttcgaaattgttgcaataaaccactctgccat
ctctcctgaattaaaatactgttttaaaaaattagacaagaataacatagaactaaggta
gttgcctagaaaattaaaactatgttaaatataaattataatctttctatagatatattt
aaaaaatatgtacatagaatctatatcagcttatttcaagatttttaatcttatacaat

ttttactaaataaataaattctctcataatatgaaactatcttggaaaacacttcttctt
tggtctttaccaatatttgtaattggtttctttttctggcaaggtttttaggtccaact
actacagatgttggcagtaatatcgcaagttctagaatgacatatggacgatttttagaa
tatttagatatgggttgggtgaaacgggttgacctctatgaaaataatcatacagcaatt
gtagaagctgttgggccagaattaggaaatagggttcaacgaattcgagttgaactgcca
gcaagtgcgccagaattaattacaaaattacgcaaagccaatgttgatctagatgctcac
ccccctaaaagtacaagtgcagtatggggactattaggcaatttactatttcctttacta
ttagttggcgggttagctttcttatttagaagatctaataatgctagtggtggacctggt
caagcaatgtcatttggcaaatcgaaagctttatttcaaatggaggctaaaacgggagta
gtatttaatgatgtagctggagttgaagaggcaaaggaagaatttcaagaagtggtaaca
tttttaaaacagcctgaatcatttactgctgttggtgcaaaaataccaaaaggcgtttta
ttagttggacctcctggcacaggcaaaacattactagcaaaagctattgctggcgaggct
agtgttcctttttttagtatctcaggctcagaatttgtagaaatgtttgttggtgttggc
gcttctcgtgtgagagacctattcaagaaagcaaaagacaatgcgccttgtatcgttttt
attgatgaaattgatgctgttggtagacaacgaggaacaggtgttggaggtggtaatgat
gaaagggaacaaacattaaatcaactattgactgaaatggatggctttgaaggaaatact
ggtgttattgtaattgccgctactaacagagctgatattttagattctgcattattaaga
cctggaagatttgatagacaagtttctgtagatgtaccagattttaaaggcaggttagca
attcttgaagttcatgctaaaaataagaaaatggaacctaaagtatctttagaaaccatt
gccagaagaactcccggcttttcaggagctgatttagctaacttactaaatgaggctgct
atcttaacggctcgacgaagaaaaaaatgcaatgactatgtctgaaattgatacatcaatt
gatcgagtagtagccgggatggaaggcactcctttaattgacagtaaaagcaaaagatta
attgcgtatcacgaagtgggtcacgcaataataggcagtttattagagcatcatgatcct
gtgcaaaaagttacattaataccaagagggcaagcaagaggcttaacttggtttactcct
agtgatgatcaaagtctaatatcaagatctcaaatactagcccgtatcgtaggtgctctt
ggtggcagagctgcagaagaaatcattttcggtgacgcagaagttactactggtgcaagt
aatgatttacagcaagtaacatcaatggcaagacaaatggtcactcgatttggaatgtct
aaaattggacctttatctcttgaaagccaaggaggagacccatttttaggtagaggcatg
ggaggaggctcagaatattcagatgaagttgcaactaatattgataagcaagtaagggaa
attgtcagtgaatgctatgcacaagctaaacacattattatagataatcgagtagtgata
gatagattagttgatttactaattgaaaaagaaacaattgaaggcaatgaatttagagac
atcgttaaggaatacactgcaattcctgaaaaaaattactacatatcacaattttaa---
---------------attaaacgggactgacgggattcgaacccgcaacttccgccgtga
cagggcggtgctctaaccagttgaactacagtccc-aaaaagatgaatacccagagataa
tctcatagttagcttaaacttgtcaaattagaatattaaatatgaagagta---------
--------tttgtaataatactcttcatatttaata-tagtagctaaaatatttcttta

gaaggagagagagggattcgaaccctcggtacgaagttaatcgtacagcagattagcaat
ctgccgctttcgaccactcagccacctctccatga---tatatattatatatatatcaaa
tatacttatattttaataggtgtgcataaatatttatggagctaagcggattcgaaccgc
tgaccctctcaatgccattgagatgctctaccaactgagctataaccc----ctttggta
agatattaagaaatggctcaagcgggatttgaacctgcgaccttgggcttatgaatcccc
tgctctaaccactgagctactgagccatagtataaaatattattaagtattataccataa
attatggtcaaatataaatcaataaaagaataattactatca-tttactacttttg----
-----------------tgcaagttgtaaatattcgaataaaatttcaaaattaaaaatg
tctttaactttttgttaga-------atta-ttgattacgaaa-----------gtattg
atatttagaattaac---
--------------------aataaatttg------------------------------
---tctttattt
g---

-------tagaca---
-------------------------------caaaaatattggaaa--------------
------------------------ttttcatcaat-----------------taattagt

------------------taaactataaccgacatac-----------------------

---aagaattattgaacta---

---cgttaaat---

-------------------------------------tgtgatgcatt------------
-----gggcttgatcaaaacttctctaaaatcaatagaatatagt---------------
---aattcaa
gtagatcaaatactaacaaatgtgacaaacgcttaaaaaaataata--------------
caattagaatcgtaaaaatttggtttttga-ctgtgttaaataaatttgattatcataga
tttaatatttaaaagtgttctattacttgta-----ttgagtaattgt-aatatttaata

tcttctttttagataacaatcataatataagctaaaaaatagtttt---tattaaacaa
ctagcatagagccaataccttgatcagttaatatttctagcaagagagcatgatctattc
taccatctaaaatatgcgctgaagctacaccctgagccaaagaacgaatacaacaattca
ctttaggaatcatgccaccagaaattactgctgtttgagttaaatctctagcttcctgta
tattcagatgactaattaatgttgaaggatctgaagaattacgtaaaatgccgggcgtat
ctgtgagtagaattagtttttcggcattaagagcggctgctatttctcctgcgacagtat
cagcattaatattatacgattggccttccttgtctgctgcaacactagctataacaggta
tataattattattaattaatatcttcagtaatttagtatcaatattttgtacttctccaa
caaaaccaaggtttgcttttccatttggtctcggagtaataagtaatccatcttttcccg
acaaacctacacttttaccaccttgtttattaatagttgcgacaagatctttattgactc
ttccaactaaaaccatttccactatatccatggtaggttgatctgttactctaacgccat
catcgaattttggtaatattttgaggcgatctaaccaaaaattaatttctggtcctccac
catgaactaaaatgggacgtagtcctataaaagacagaaaaacaagatcactaatcactt
gatcttttagtttctgatttttcatagccgctcccccatatttataacaataattctgg
aagaaaattgctgaatataaggcagagcttcacttaagacttttactctttctgaattag
tcaacataaataaagtaggattgtgatatataaaattatatacacagcttgtgttaattt
aaaagataccaatgggt---------acttgaaaaaatagaaatattactaacag-----
----ttaaaactatagggattataattatgaataaattttgggataatgtattaagattt
cctcgatttttagtcagcgtcattttaggattaattttgataattattagtcctttttc
gtgttattaaaaaaaccactgacaagtttttttttcatcatatcattggccggattaatc
acagttttggcaataataa-tacaaaaaatgataaatatcgaatgttgttgagagaaata
aataaatttaattaaaatttttgaacatatataatattatattatcattagatatgataa
ttt-atttccttttctat---aaaacccataaatattaaatattatttgataaatattat
gccactaaaacaaagagttagctctgaaaagaccggtgccttcgcacttttggatagtat
tgttaggcacggagttaaacatatatttggttatccaggtggagctattcttcctattta
tgatgagctttatgcttgggaagaagcctctctaattaaacatatccttgttcgtcatga
gcaaggcgctgctcatgctgcagattcttattctagatcaacaggagaggttggagtatg
cttcgctacttctggcccaggagcaactaatcttgtctcaggtatagctacagcacatat
tgattctgtacctatattagctataacaggtcaagttgggagagcttttattggtacaga
tgctttccaagaagtagatattttgggattacacttcctattgtaaaacattcatatgt
agttcgtgaccctagagacatgtctagaattgttgcggaagcatttttatttgtaaaca
cggtagaccaggtccagtattaattgatgttcctaaagatgtaggattagagaagtttaa
ttatttttctgttgagcccggaaaagttaatattcctggctgtaggccaattaccagcct
aaagtcaagacaaatccttatggcagctaaaatgatacagcaatctagccagccattgtt
gtatattggtggaggagccataatctctgattcacatcaaattattaaagaacttgttga
tttttataaaatacctgttactactactttgatggggaaggggattttaatgaggatag

cgattattgtctagggatgttaggtatgcatggtactgcgtatgctaattttgcagttag
tgagtgcgatcttttaattgctttaggagctagatttgatgatagagttactggaaaatt
agatgaatttgcttgtaatgcccaagtgattcacgtagacattgatcctgctgaagtagg
aaaaaataggattcctcaagttgctattgtcggtgacgtagcagaagttgttagtgaaat
attgaatttattaaagacttctttccccccttatccagagcagattatatcttggcaaga
aagaattaatcgttggcgtcaacagtatcctttactggttcctagaaaatcaacaagcat
ttcacctcaagagattcttgttgcaactaataaattagcccaaaatgcttattttactac
agatgttggccagcatcaaatgtggtcagctcaatttctgaaagtaaaagctaagcattg
gctttcaagtgctggattaggcacgatgggttatggtttacctgcagcaattggcgctca
agtagcacatccaaatgacgtagtcatttgtattagtggtgattctagttttcaaatgaa
tatgcaagagttaggaactatcgcgcaataccagttaccagttaaaattattattattaa
taatcgatggcaagggatggttagacagtggcaacaagctttctatggtgaaaggtattc
acactcaagaatgacagaaggagcacctgattttcaaaagcttgcagaagcttttggtat
taaagcttttactattaataataggcaaaatatgcaatctgctttacaagttgctattga
ttatcctggtccagttttattagattgccaagttacagaaaatgaaaactgttatccaat
ggttgctcctggaaaaagtaatgcacaaatgataggtatcgctaaaccgcagagaggtac
tgcttccaactatattaataatagtgtttgaagtaa------------------------

---------------atagttagtatttttta-aacaaagaata-aagtaca--------
-taaatgtattttttttatactatta-aaaactatgtaagaacagcctttgacgagaatt
gaactcgtgaccttccccttaccaaggggatgctctacctctgagccacaaaggc-tttt
tactatattgggccgggttggatttgaaccaacgtaggcgaagccagcggatttacagtc
cgcccccattaaccactcgggcaccgaccctaattaaaccttattcaagatatgacctat
aagatatcataactatgttgctaaaaacaagtactttgataaataaaactgaataagtaa
tggacatagctggacttgaaccagcgactttcacgatgtcgacgtgacactctaaccaac
tgagttacatgtccaaaactttaattatcattataacactgtttagtgattttataagta
tctatttctaattgaagatttgttttaatcgtccggctttaccgagcagatctcttagat
aatacagtttagctctacgtattttagctcttctaatcactttgacaaaagttattcgcg
gtgaattgagaaaaaatactctttcaactccaataccttgaaatgagcatcttaaagtta
aactagtatttaagcttttttttcttcttcttgctaatactactccttcacataattgtt
cccgagttttactaccttccttaaccattagtccaagttgtattgtatcgccaactttaa
tttcaggtacttctgttttgataaaaggtatctcaacacttttcattagctgacttagct
ttgtactatttgctttcatatataggattagtcttgtgaca-tgttttgaaatattatac
attaaaaaaacaaatatgaaaaattttagcgaataagcatattagtatttataaaac-tc

tttttatct---
--ccattata--------
----------------cagatccatt------------atattcaact------------

----------agattctttcatagtttc--------------------------------

--------tttgataatcagtac-------------------------------------

---------------------------atcta----------------------------

----gatgatcaaatattattttcaaacatggcttatgtatgaaaaagttctata---aa
aacctaatgac--------taaaattagggcttaagtatttcttagttttgtgttataat
tagttactatcaaaggtagtggcataacacgaagcatcaa-aatgttcgaacgctttact
gaaaaagctataaaagtcataatgctagcacaggaagaagctagacgcttaggacataat
tttgtcggaactgagcagatactattaggattagttggtgaaggtactggaatcgcagct
caagttttgaaatcgatgaatgtgaatctaaaagatgcaagagttgaagtcgaaaaaatt
ataggaagagggtctggttttgtagcggttgaaattcctttcactcctcgagcaaaaaga
gtactagaattatctttagaggaagcacgtcaactaggccataactatattggcacagaa
cacttgctaatgggcttagtccgagaaggagaaggagttgcggcaagagttttagaaaat

ttggcagtcgatgtttcttcaattagagctgaagttatacaaatgctcggagaaaatgcg
gaagccaatgtaagtggaagcaatactacgcaagctagaagtaaaacaccaacattagaa
gagttcggatctaacttaactcaaatggctatggaaggtggtttagatcctgtagtcgga
agacaaaaggaaatagaacgagttattcaaatcttaggtagaagaactaaaaataatcct
gtcttaattggggagcctggtgtaggtaagacagcgattgcggaaggattagctcaaaga
attgctaatagagatgtaccttctatttagaagataaattagttattactcttgatgtc
ggtctattagtagccggaactaaatatagaggtgaatttgaagaaagactaaaacgtatt
atggatgagattaaatcagctgataatgtaatattagtgattgatgaagttcatacattg
attggggctggtgctgcagaaggagcaatagatgcagctaatctgcttaagccagcttta
gcaaggggagaattgcaatgtataggtgcaacaactttagaagaatatagaaaacatata
gaaaaagatccagcattagaaagaagatttcaaccagttgtagttggagagccaagtgtt
gaagaaacaattgaaattttgtttggtcttagagaccgttatgaaaagcaccatcaatta
acaatgtcagatggagctttggctgcagctgctaaatacgctaatcagtatatttctgac
cgatttttgccagataaagcaattgatttaattgatgaagctggttctagagtccgttta
ctaaattctcaattacctcctgctgccagagaattagataaagagttaagagctgtatta
aaaacaaaagatgaagctattagagctcaaaaatatgaaacagcagagcagtatagagca
agagaaatggaaattaaagctcaaattgcagcaattgctcaaagtaaaaagaatgagcct
gatttaaatttagaagatcctgttgttacagaagatgatattgctgaaattgttgctgca
tggactggtataccagtaactaagcttactaaaagtgagtcagaaaaattaatgcacatg
gaagaaactttgcatggacgtattattggtcaagacgaagcggttgtagctgtctctaga
gcgataagacgcgcaagagtaggtctaaaaaatcctaacagaccaattgcaagctttatt
ttttccggaccgacgggtgtaggaaaaacagaattaacaaaagctttggcttcttatttc
tttggttcagaagcttctatgatacggctagatatgtcagaatacatggaaagacacact
gtatctaaactaattggttctcctccaggatatgtgggttatagtgaaggtggttatcta
acagaagcggtaagaaaaaaaccatatactgtcatcttatttgacgaaattgaaaaagct
catccggatattttaatctacttcttcaaattttagaagatggccgactaacagatgca
aaaggcagaactattgattttaagaatactcttttaattatgacttctaatattggatct
aaagttattgaaaaaggaggaggtagtttaggctttgaattatcagaagatcaaacagaa
tcccaatatactagagtacgatctttagtaaatgaagaactgaaacaatactttagacca
gagtttctaaatcgattagatgaaattattgtatttcgtcagcttactaaagatgaagtt
agagaaattgcagaattaatgcttaatgaagtctttgcgagaattaagcagcaagatatt
caattaaatgtaacagaacgatttaagcaacgattagtagaagaaggatataatccaagc
tatggagctagaccacttagacgagcagtaatgaggcttttagaagatagtctggctgaa
gaagttttatctggtaaaattaaagctggtgatagtgcagtagtagatgttactaatgaa
ggagaagttacagttttattaggtgaaaaattagaactgttaacataaa------aaaca
atttattaat--taaagcattgaagaggttat--attcaatgctat---gaaaaaaatta

ataagatgggttgctataattgacaacctctgatattctttatttgtatata---aagcc
gggatagctcagttggtagagcagtggattgaaaatcctcgtgtcaccagttcaaatctg
gttcttggcat-tttaagcatcagaaaattttgctatctgagcattaaattttaattgaa
atgttcctgtgggaccattcctgtgttttgctattataacttctgtgaagtctctacttc
tagtttcttgtgtatagtaactttctctatatagcattattactaaatctgcatcttgtt
ctatagaattatgaacaataaagttatttgaaataaaattgcataaaggtttacattcta
aatcaaacactatttgtaaacttgtaacgtttatttttttgtatactttcaaaagtcaaag
atgaaa------aagaattaaaaacatttttttgttcttcaaagttttt------tattt
gaatagcaatcatgtcattttgattaattttatcacatcttttccatccttgtgttgtga
gtagtttatggttacttgttaattgtatatatttcccagcttcggtaataatttatata
cagtttttttccctgttttcgaaatactacatttaattgctagaaataactgtcgttttt
gcgcactaaagttaatgacttca-atctttttataatagcaattaaataatatttg--ag
gttgattatataatgatgtctgaagataattgaatttggatatacacccactttctctta
aatcggataacaaaggccttttgtcactccgagtttcaaggtttctatttagttgagata
atgccaagataggcaagcttaaatcttttgctaatattttaagtgatcgagtaattaatg
atagttcttgagatctattgtcagattgtctactctcctgcagtaattgaagataatcta
taataattaattctatatttttttccttgtaatttaaagaattttacttttgtctttatag
tgtcagtagaaatcttagcgctatcatcaatataaagattaaggttagcaagagttttac
tttttttgaataacatattgccattcatcattattaagctgaccagattgaattttttggc
cgtttaaattacactcttgagctaaaattcttcttagtagttgctctgtagacatctcta
agctaaataaaataacataagatcttttctgattaattacatatctagttatattaatag
caaaagcagttttccccattgaaggtcttcctgcaagaataattaaatctgacttttgaa
acccttgtgttatagagtcgagttctgtaaaaccagaaagtatactactatttatactaa
tctttttccttgtatccaaatggactaatagtcttgcaagaattgtagctaatgtttgcg
tatctttttcgtcaagtatttcataagcttttgttaattgactaacaatagaagttatgt
ttttttgttcaataagttgttttgagtagctaattaaacataatgaatctccacatgcta
aaagtaatcttttaatgtagttatctataacgactgctgaatattcgcttatagtatcag
ataggggagcttgttctattagactaaaaacttcatttagctcattcaagtcttgaatca
tttttcatttttaagttaatgaaaaaatttcttacgcttttttgtctttgcatggttaa
cattttcgagtgctgctctataaagtaatgaagttgatgtaaaataaaaaaaaatcaggag
agatttttttctactgattttagtaatgatatcgattgctgagttaatattatactaatta
atattttttcagctaaaatattatgaggaggtagatatttataa------atacttatta
cttcctcc-ttgtaacattactcttgtg--------gtattagttaactattgatttaat
ttgattctggaagaacttgcaatttaatatttgcagttacttgatgtaatagcttaattt
ctacatcgtaaatgccaattgttttcacgtctggtaaagagatactttgtttttcaatgt
ctatgttggtagtgtttttaataatttgtgaaatttctttttctgtgacactaccaaaaa

tattgtggccatctcctgtttttttactaacgctaaacctttgtatttcttctagaagtt
gcttgactttttttagcattttctttagctgtttttaatttttcttctcttatagctgcat
aaaatttttgttgttttaaaataccatttgtggcaactgccgccattttattcgggatta
aaaaatttcttgcataaccagaagctactttgataacgtcattactcttgccaagttttt
gaatattctcttttagcacaacatttataactttttttactcatctt-------aattcta
aaaa----atctgtattatcctattatacctgttgtatacagatttt----aataggaag
tatttatttgttgatataatattcttgtaaaa--aaaggtaatttttttattttt-ttctg
atataatcactttcacaataattc-----attaggagaggtggccgagtggttgaaggcg
cagcattggaaatgctgtttaggagcaatcttaacgagggttcgaatccctctctctccg
tacaa--aaaatataaaaagttttatagagtccaatctaaatctccaataatattcttcc
aagcatttaatccaccccgaactctaacaatatttagtttttgagctattaaaaactcag
aagcaaatattgatctagaatctaagctacagtaaacaaaacaaatttatcttgtaaat
tgatatgagagtaattcattcctttttatttttttgtaaaggtagatttaatgaatatatta
aatgacttttatgatattcttcatgatttcttacatctattaaaatatattgtggattcc
tactaattaaaaacttttgtaattgaataacactaatttctcgtacaaatgtattagatt
tactgccataatatttgttccaatgctttttttgt-------ggataatacaaatttagta
tttataatcttaaatttgttaaatgatgaagttaatgagttgtacgttaatatagttcca
cttaatatagacttataacccagaacgattttgacagcttcagttgcttgaagtgtacca
acaatacccggtaagagacctaaaactccggaattactacaagtatctcttgcgctctct
ttattttcagtttcactgtagaagtctcgataaacaggtccaccttgataattaaataca
ctaacttgtccttcaaattgaaaaatggcaccataaatatgtattttatttaactctagg
caagtatcattcaagagatatcgagtatcgaagttatcagatccatctataataatatca
tattgtctaataatgtcgattgaattctcatagcttagtcttgttttgaacacagttact
atacactgtggatttaaatctaata-tttttttttagctatttctactttttgaatatcct
atatcatgcatcgtgtataaaatttgcctctgtaaattagagagatcaattatgtcatcg
tctataattccaatgctgccaattcctgcagcagcaaggtaaatgattcctggagatcct
agcccaccagcaccaatgaataaaacttttgcttcttttaatctttcttgtccttctaat
tgaatttgtggcaatactaaatgcttagaatatcttgtatattcttctagtgagtatttt
gtattttctgttttaaaatttagcataaaaatttatagtttatttaatagcactta----
--aaagctagctgataagtttattaaaatgacttttgtaaacttggttcttctctatcta
aaatagctccttgtgtaggacagacctgaacacaaatactacaatcaatacaggcagaaa
agtcaatccaataccaattagtgttattattgtttttcccctttccttgatgaatacaag
ctacggggcaagcttccacgcattcagcaactccaatacatttttctgttacaattgtat
gagacatattatttaattaat-aaaagataagctatttacatctagtagttttgttgtat
actaaatttacatatgctatccaggccaatatttaaggtagtctatttctacagtttact
aatttgatatgcgcctttagttcagttggtagaacgcaggtctccaaaacctgatgtcga

gggttcaagtccttcagggcgcgtttag-ttttatcgtaattcaataatatcattttatc
tttttgcaaacatcaaaatgatatgatataaaagatcatatttatacattgaatattct
atgtactagtactg-tttttaatatgttattattgataagttaattatactaattctat
ttttgtaataagt-aaaaacagttctaattaagtatgcacgtatttaatagtc-ttttgt
atatat--aacttgaaaaaatttaaactatggctaaaaaagttactggaattgttaaact
agcattaaatgcaggtaaagctactcctgcgccacctgtcggacctgctttaggacagca
cggcgtgaatattgttatgttctgcaaagagtacaatgcacgtactgctgacaagtcagg
actagtaatccctgtagagatctcaatatatgaagatagaagttttacttttatacttaa
aactcctcctgcttctgtactaatcgctaaagctgctggactaaataaaggttcgggtga
accaaataccaaaaaagtaggcagcataacaaataaacagttagaatctattgcagaaac
aaaacttcccgatttgaacactaacaatatacctcaagctatgaaaatagttggaggtac
ggctaaaaatatgggaattctaattaaagatt-aaaagtttcaattatatttacta-tt-
-tttatgaaaaaattttcacgtcgacttacaacattaaaatcaaaagttgagcctaaact
ttacactattaatgaagcagtatctatattaaaagcaacgtcaaatgctaaatttaagga
aactgcagaagctcatattgctctaggtttaaatcctaaatatgcagaccagcaactcag
agcaacagttatattaccgaaaggtacaggtaaattgataaaagtagcagttattgctaa
gggagaaaaattaacagaagcaattagtgcgggagctgatgttagtggctccgaagaact
aattgatgaaatactgaaaggtagattagattttgataagctgatagcaacaccagatgt
tatgcctttaatagcaaagctgggacgagtattaggccctcgagggttaatgccttctcc
taaagcaggcacggtaacattggacgtagcaaaagccgtgaatgaatttaaaggggggtaa
agttgaatacagagttgatagaacaggtataattcatgtaccttttggaaaatctagttt
ttcacaagaagatctagttttgaacctccaaacaattaaagaatcgattgatagaaacaa
gccttctggagcaaaagggaagtactggaaaactttttcttatctagcaccatggggcc
atctattcaaattgacatcactagccttctatgaaaaattttgtataattatagaactt
gaataatctattatttcaaatta-taatttgctagcattattaacgtaacaacttgctta
ctatatcttatctgcattaaaaacttt-ttatatatcttattaattatgagtacaaaggt
tgaaaatatcttggaagagctaaaatctttaaaccttctagaggctgctgaactagttaa
acaaatagaagaaacgtttgatgttgatgcatctgcggcttcaggaggtatgatgatggc
cgcaccaacttcagcaccagcgtctgctgaggttgaagagaagacagaatttgatgttgt
cctagaggaagtcccagcacctaaaaagatttctgtattaaaagctgttcgctctctgac
tggtttgggtttaaaagaagcaaaagatcttgttgaatctacgccaaaagtcttaaaaga
aggtgcttctaaagatgatgcagaaactatgaaaaaacagctagaagatgctggcgcaac
agttattgttaagtaatga-----agaagcgcttctgtttaagtataaacagaagtgcct
ctttatttattataagcaattatactagaaaagtccaagtgtaattgcattgttgatagg
catagttgcaccaatacctagccaaatacttacaacggtaccaattaagaaaacagtggt
tgcaattggtcttctaaatggattttgaaatttgttaacattttcaatgaatggaacagt

aagtaaaccagctggtacagcagccatacttagaacacctaatagtttattaggaatcac
tcttaatagattaaatgttggaaagaagtaccattctggtaaaatctctaatggagtcgc
aaatggattagacttttcacctatagaagatggttctaaaattgcgagtccaatactgca
ggcaaaagtgccaataattacaactggaaagacgtataatagatcatttggccaagctgg
ctctccgtaataattatggcccattccttttgctaatttagctctcagcttcggatctgt
taaatctggttttttaagaattgacatattg-tttttgtgtttaatataaagattagtct
aattactgttttaaagtgtaattttcaactattataatggtccagaaattccttgtttac
gtatcattaaaaaatgcataagcatgaaaacggcagttagtagcggaagtacaaaagtgt
gtaaactatagaacctagttaaagttccttggcctacactgactcctcctcttaacaatt
ctactatactcgctccgacaactggaacagcatcagggacacctgtaacaatttttacag
cccagtatccgatttgatcccatggcaaagaataacctgttaccccaaaagaaacagtaa
gcactcctaaaattacacctgttacccatgtcaattctctaggtttttttgaaacctcctg
ttaaataaacacggaatacatgtagaatcatcatcaagaccatcatgctcgctgaccatc
tatgaattgatctaataagccatccaaagtttacatcagtcataatgtactctactgatg
taaaagcttctgcaactgtaggtctataatagaatgtcattgcaaatccagttgcaactt
gaattagaaaagatacaaatacaataccccctaagcaataaaaaatattaacatgaggtg
gtacatacttactagaaatatcgtcagcaattgcttgaatttctaatctctcttcaaacc
agtcataaattttactcataaaatagcttc-aaaagctcttctaatata--ttttgcgtt
taagctactattaattcaatag------ttattgaacttatatt-tttttataatcaata
cttattaactattataacatcttttattgattt---------------------aaaatt
ctctgacttagtaaaacagggctatgtactataattcttttttttttgtatagagattaat
ttcgtttttagtaaattagctaaaattcttgttacactgactctattactacctataatc
tgtgctaaaattttatgtgtaatagtaaagttaagcataataccattattaacttgagtt
ccattttgctcagctaataataataataagctcgccagtctactggttatacttttatgt
gaaatgatttctataaaatgataagcttttgccgagcaaaatactaaatgatttacaaaa
aataaattgaatggagcgcagttactacacgcttttataatagtagtatagttaatacag
gcaacttctgttttatctattgcttctgcttcgtagtaaaaattatcgtcgaccaattct
atttgtccaaatgtatctcctgtagttaataagttaagagttactttatgtgtatttctc
aggacttttgtaataattaaagatcctatcaatataatgtataatctagagctgctatta
aacaaaagagtatcatttttcttaagtgaaaaaatttgataattaattttattacgatta
aaaaatagtagccatggattgcaagaaaaaaaatcacctgaactattttcagagaatatt
ggaatacaatttttcttgatttaaacaatttttagtcataattactactttataa------
---aaaaatattctaataat-taagaactgaaattaaatgtcatataaattgatgttagt
tgagaatgacattgtattatcaaaggccattcaagaatacttaatagatcaaggcttcaa
tgtttatattgccaataatggattagaagcactgaatctagcttatcaatataatttcga
cttgataatttctgatataatgatgccgctagttaatggctatgaactattagcaaaact

taaaaaaaacaaagcattatccaaaatacctgttatctttttgacagctaaaggaatgac
taaagacagaataaaaggctacgacatggggtgctatggttatttatctaaaccatttga
tcctaatgagttgctttcaattattaacaacttaatcgctagagatgtcttaaaagaagc
ttctttacaaaacttggcaacatcaaaccagcaattaaatcataaaatacgtttgacacc
tagagaaaaaagtattttagatcttgttgttgatggacttactaataaagaaatatctac
tatactgaatactagtgttagaaatgtagaaaaatatgttagtcgactcttacacaaaac
taatatgaagaataggactttgctagtcaagtattctataaataataatctattaaataa
tgagatcaatgaaagggcgaatgacgggactcgaacccgcgaatgatggagccacaaccc
attgccttaaccccttggccacactcgccatattacacataccactatagcttgtttttt
gatatatcgtctagttttcgagttatatatttatctaaaacaatatttactttgaatttt
-tatgatacatagcaatattagtattcaaattaatggggaaccatttaattgctcaaagc
ccatctcattgcaattttttattaaattatcttgattttaattccgagcgtgtagcagtcg
agctaaatgacattctgttaccagagactctgtttcactcaacttatttgaatgatcaag
ataagctagaggttattaccattgtaggtggtggataatctatttgtacatac-------
-acagttctctatctcaaatgtttcatagatagagataatcttccttgtttttttatctac
atgaataataacagctttaatagtgtcacctattttaaattgtgatgatatctgctctaa
ttctttgatattaatctcagaaatatggacaagacctttgagatttcctacctttataaa
taatccgtaaggtgtaatctgatttattatgccttcaataatattaccaacaattaaatt
agatgatgcttgagatattagagctcttctatgactcagtattaaattattagatttctc
ttcgacattgagtaattttagcttaataaatttattattaaattgctcactttttttgaaa
attaccgagatgagaattaggtacaaatccagatataccttcaaggttaattatcattcc
tcccttattgaatccttttatcattacattaagtaaagagtcttcagctaatagttgcct
aattcttttccatgctcttatatattcaaggcgacgaattgataatattaattgtttcga
ctgaatattataatctaataaaaaaaattctcttgtgtcattaatgtttaaagaagtgaa
attatttaaatcttgattactagatacttcttgaataggcaagtatgcagatattggtgt
tcctatatctactaataccccatttaattcaaagctaaatatagttccagctacaatatc
tccaagattcaaatcatacttgtatttttgtaaaacagctgcaaaatttctatgagtaaa
tccttcattattttttgtcatattgaatagtatgaattaaagagtaactttgtacaatat
attaaggtaaataaatgtttgtcgaattgtatgaaaaaattttaatttgttcggttgtaa
ttaagtatataaacatattaaaatatctaggagacatgaaaagtagacgtagatagctgc
agatttaa-tactatataatctgaggaaagtccgggctccacaaatacaatttatgctgg
agaaatcccagtgtaggtaactgcgaggatagtaccacagaaaaaaaccgccagaataag
t----tatattagctggtaagggtgcaaaggcaagttaaaagcttaccaaaagtactgca
aagtatttgttaggtaaaccccctaaaatggagcaaagctactaaacaaatatttgtgtat
attttatctagtttagttgaaaatactgcatgaagttattagtaacaataactcgagagg
aatagctatcctttacaatatatc-tcgattatattgaaagaacagaacccggcttatgt

agtactttttatgtcttgtattattatgtctattgtcgaatctttaaatttaactgtttg
acaatttttttttgtagctgatgttgtttataatctatttctgaattggttaaagttttt
tcattagatcgatatgtaaatcttagacctatactttttttctcttttccaattgattca
tctatatattgatcaaataatgttgtactttctaaatctttatcataaaattgatccaat
aatttaataagtatttaatttccatattcttaggtattattaaagagagatctcttatg
atcgatggatacttagagtatggttgaatctgataacttaaataattgaattcattatga
caatcttctaaaatgtttaagttaacttcaagaaaaaaaagttttgtgttgagtcctaat
tcactatatgttgcttgattaagttcactgaatatgccaataataatattgttatagatt
agtgtggcaaattttcctttttgaatgaactttattctaccatcgactatatctttttt
gtccactcaatttgtctattaatcttttgaaaaaaatttctacgatccctttagcttca
taccaatttaaagaatgcgcagggtgtgaccatgtagatcggatatctaaattaccaccc
aatatgatagctagattagtagtttctacaattttgttttgatcattatgaaatactgtt
cctatctcaaagctatctatagtttggttgctttgtttaatattataaagatttgattca
attaatccttccagcaaactgccgcgtaaattagaataatcttgaataagaggattgttt
aaagctatatttcctttggactttattaaagaataatgaactaattcagttaatcctaga
tttcttaaaatacttctacttttatctatgaattgtctctttgtggataactttttgttg
aattgaatatttggtactgagctcctgaatttatgataaccataaattctagcaatttct
tcaataacatctatttctcgaaaaacatcttctcgtctataatttggaacagtaacttct
atattttccttattttgataaataagatcaaaatttaaagagtgaagaatattttgaatt
tcttcaaagaataaaaaacgtgtttgaccattatacataattggtcctaaaatgtcatga
actttttaattgataaattaatattaagagcatgatcatttattttttctcgacagaaa
gtctctctaatatttccgcctgttagatctgtgattaaagcaagagcttcaaaatgtgca
tttttccaattatctacatttagcccacgttcttgtctaattgaactttctgttcgaata
ttaagaactcgagaagattttctaactacagattgtttaaatatagcagactctacaagt
atcgattttgtattatgatctgtatcaaaatcagagttgcttccaataccagctatacta
gttacatttgtatttacttgagtaactaaaatatttttgtttaactcaatattttcattg
tttaacttaatctgatcattacttccaagaggaaaattgcttcgaattgttagactattt
ttatgattcatattattaattttattcaaatcaacaatattaataggctgtccccatttc
aacataatataattgcttatatctgttaacaaatttctgtgtgtaaacccagaagagagc
aaccgatttttttagccaatctggagaatctttaactcttatttcgtctataatggcagca
aaataattgtcacaatttaataaatcgtgattactgattatagt-ctccttttttagagaa
atatctattattgggttgatgtatagctgataacattgaggaccctgttaaagccgagac
ttctcttgatagaccaatcatgcttaacacgtctgacctgttagctgttgatgatatatc
taatatatgatcaatctcacttccaattataatagattcaatagcttcaacttcaaaacc
tgcttgtgttagttgattagttaaattgtctatatctattgttttgatattaccaagttc
tttcaaccaatttaaagaaactttcataattaattgtcatctatcttatagaagtattta

ttttacttatgattaaaaatcaatttaaaaaattcatcaaattaatataagttatttgat
gaatttgcttatacaatatattattgtgatatcttacttttatatggcagttattaagaa
ctagcttttgtaaaagttagcccattatcttgggcatatctttccataaatctcatgaat
ctatcccaatcatcaggacttttaataacatgtacagcttctattgcttgtggtttacca
ttaataaacttggcattaacatctcttgtaataatttgtccctctgtgtccatcaggtac
atacctgtgatttcacctttgtctgccatactagcatctaaaattttgggatttgtaaac
cgaaaagttgctgttcctgtactaccatctcgagaccttgttaaacgtacatctggtaca
acttcttcattgataccttgaataaattgaattgttgccatatcaatcttctttatttgt
atataatttatattggataaaactgatttctagttcttgaatgcttgttgagaaactgtt
ttaaaagcttatagtagcagtagatcaaattttatgtattatataaaactgtaacttttc
-ttttttg---

----------------------atctgttcaaagaactaacttattataagctaatcata
cgaatattagccatataatgttcgtaagt-gaaattaaggggttgtagctcagaaggata
gagcaagcgcctcctaagcgctaggtcagcggttcaagtccgctcaaccctgttaacgaa
ttaagtagtattatttaacttgttctgcaaatcagtattgatctaagttaaagtacttga
cagctaaaacaaaagaatctgagaaatattttcatttaaagaacgaacaattatcttaac
tctaaatatctgataattattaa-aaaattct----------------------------
----cttttaaaacacaagatgatg-----aagatatttaaactaataattgaaattaa
gtttttgaagttatagaagccaat--ataatactttctattaacaga-----------tg
cttgcttctattactatctgtttacaaga--aatt----atgagtatcaaaata------
--attttagctcta-------------------------------------ctgac----

-----------------------------tactaataaata-------------------
------------------tttattttagtctagacaag------atgaatcttcatggta
taacag--

--------------agctattctttgtgtcatacttatataaaattaggat-------ac
tgtt--

---------------ttcattattataactggggtgggaggattcgaacctgcgaatggc
ggagtcaaagtccgctgccttaccacttggctacaccccaacatgtgaacattataatag
cagtcgtataccctgccatgtcaatagactattgtctttatttaataattttgctgctc
ttgtagaataaaatctttttttcaaataagatacgctatgtttaagttgcacgactgtt
atgttttcttgcttttgactgaacagccacttgatagtaattattccttctgcaatttcg
ttacttcctataataatacaagctacagctctttttttatttgcttgcttaatctgctta
ctaaaattacttgagctaacatcgatttctattttaaaaaactgctgatgtaaaaaacgc
attatttgcataccagtttcgttagcttttgtaccttgtgtagcaatataaaaatcgata
gattgattaggcaagtctatattatcttttgcaatcagtaataggcgttctaatcctatt
gcacatccaacagctggtgttgctttgccacctaattggtgaactaagctgtcatatctt
ccaccaccacatatagtatcttgcccttttgatgtcaatgtcttaatttcgaaagcagta
tcattataataatctaatcctcgaactaacttattatttatattgtagggaatattaagt
aattttagataattgcaaacgtcaccaaagtgtttttgtgattccaaacttaaaaaatca
gaaatttttggagcctctgtcagtatttttttgtgtattagaatcttttgaatctaaaatt
ctaattggattactagttaatctttttttgtgagtcagtgtctaaatcatcatggtacttt
gtaagataatctcgtaatttaacttgataaatactacgatcctctacttttccaattgaa
ttcagatcaagtttcaagttgtgcagattgagattgttaaatatgctcatagctaaatgt
ataacttcactatctgctcttgcatctaagctaccaataaactcaatgccaagttggtga
aattgtcgttgtcgtccactttgtggtctttcgtacctgaacattggaccgctataccat
aatctttgtaaactatggtggtaactcattttattttcaataaaagatctcacaataccg
gcagtaccttctggccttaaggtaatatctctattgctgcgatcatgaaaccgatacatt
tctttatttacaatatcagtatcttcaccaatacccctatcatataattcactattttca
aaaataggtgttctaatttcttggtaatttgcacattctaataatttggaaattttatta
tgtataaactgccaatattgaatttcatcgggaagaatatcttttgtccctctaatagcc
tgaattttgccatgataatttttttgattaccttcttaatatgtctgcattaaatagcag
ttgtagactgttattcagtcatgtattatacattag--aacatactgatg-taatttata
aagtacgggcaaggagggattcgaacccccgacaccatggttcgtagccatgtgctctaa
tccactgagctacaagcccacttaataagtaccttaactatatcattgttcactaatgta
tacaactatattctttt------atcttacatgtgaaattttgtaaaagtaaaaaatt
at----gtttgtttttacaattggagctattcatatataatgcttaatttattataact
ttgtaaa--aatatctataagtttaattgtatgattctattatattggtaatcttgttat
aattttgcgctcttatttttcaattgg------ctagcatttaattaacttatatttcaa

ataaaaaaatcttatctaaaatgagtaaacaaattctatatcaggatgatgccagaaaag
cattagaaaaaggcatggatattttaacagaagctgtttctgtgactttaggaccaaaag
gaagaaatgttgtcttagaaaaaaaattcggtgcccctcaaattattaatgatggtgtaa
cgattgcaaaggaaattagtttagaaaatcatattgaaaataccggagtcgcactgatta
gacaagcagcatctaaaacaaatgatgtagctggtgatggtacaacaacagctacagtac
tggcttcagcaattgttaagcaaggaatgagaaatgttgcagcaggttcgaatccgatgg
ctattaaaaaaggtattgaaaaagcaacaaattttgttgttagtaaaatagctgagtatg
ctaagccagtagaagatacaaaagctattatacaagttgcttctttgtcatcaggaaatg
atatagaggtaggtaaaatgatagcgaatgctatagaaaaagttggcagagaaggagtta
tttctttagaagaagggaaatcaactaatacgattcttgagatcacagaaggaatgcagt
ttgaaaaaggctttatttctccttatttcgttacagatacagaacgaatggaagttcttc
aagaaaatcctttttatttatttacagacaaaaaaattactttggtgcagcaagaacttg
tgccattgcttgagcaaattgcaaaaacatctcggcctttattaataatagctgaagaca
tcgagaaagaagcattagccacaattgtagttaataaattaagagggatttgaatgttg
tagcggttagagctcctggattcggtgatagaagaaaatctttacttgaagatatgagta
tcttaacgaacggacaagtaattactgaagatgcaggtttatcacttgatacagttcaat
tagatatgttaggaaaagcccgaagagttattgttactaaagactcgacaacaataattg
cagatggtcatgaaattaaagttaaatcaagatgtgagcaaattaagcggcaaatagaga
cgagtgactctttatatgaaagagaaaaattgcaagaacgattagcaaagctttctggag
gagttgccgtcattaaagttggtgcagctacagaaacagagatgaaagataaaaaactaa
gactagaagatgcaattaatgcaacaaaagcagcaattgaagaaggtattgtaccaggag
gaggagctactaatgtccatatctctagtgaattatttacatgggctaaaaacaatttag
ttgaagatgaattaattggtgctttaatagttgaacgagctgtgacctatccgttaagac
gaatagcttttaatgcaggtgataatggagcagtaatagtggaaaaagttaaaagtcacg
attttcatataggctatgatgcagcaaacggtaatattgtaaatatgtacgatagaggta
ttatagatccagcgaaagtagctagatcagctttgcaaaacgcagcttctattgcagcaa
tggttttaactactgaatgtattgtagttgataaagtcgatgattgagctata------a
gataatagataatctcaaacaaagaataatttctaattatagaaattattctttgtttca
attaatctaattaattgatcaaatgtataatttttataataaagccaaagtttaaacacg
tcttctttttcaactattaagtagagaagatataagcccgtaatacctaaattatcaatt
aaaatattattttcatatatagatttttctaaagatctaaaaaatgacatttttgaaaaa
taataccgaaatcttctcttatagttatttgctaaatcattactgtctaaagattgattt
ccaattgaattcagaacctttaacagaatactttttaaatctttatctgactttatttga
gaatcaataataatttgattcaattgtatgtcaaaactaagattaaaaatattacttgtt
aaagatatttgattttgaatatctagagattttatagccaagaagattaaacttttattg
ttcattgtaaaatataagacaattgatgatgtattctttaaatacta-aatttatcttct

tgtgcttctatcgtttttttgt-aaaataattaatgttttaattttttagttttagttact
acccgaaaaaaataaattctggaaatctagcttgagctttagctagagagtgtttttttccc
ttcttcttgccagtaattgataacttctgttgttttgtttaataactcaattctgtctct
ttcagcaatccacggcttggattctagttctgcttttaattcttcccaagcatcattcct
tggccagaaaaaataagaagtaagtggactagtgccatttccaacaatctgatcaatagc
aatagcaatattattttctaaccaaagtactttagtgtaaattttgacaagtttttatc
tccattattaatattaagtattgg----tacagtagaatactgtactagttattttttc
agcaacaatttgagctttgtttaaaatgtttttaccgttttagtagcttgtgcaccttg
agataatcgttgcaaaatttttgaaatgttaacacgagtttcattatttataggattata
aaatcctaattcttctatagctttaccatcgcgtttatttctgctatccattacaacaat
tctatagcttggttgtttttttcttccgtatcgctttaaccttaacttgaccatattagt
cacctcgcttatttttt--atatctttattaaattatatcttacgacattatgcaataat
tcaaacaattaagctgattcaagtctaacattattaaatattaccattaaacattgtaga
aaaataatacctaacataggtgacatgtccattccaaacatgggaggaatgcttcctctg
aatagttttaagtatggatcagtaattctatttaatgaacagaatggttcattgtaccaa
tttacagttggaaaccatgccaatgataatttaagtaaaattaaaattaaataaatctca
gaaaaattagctatagatccaagcaataaatttaatgtaccaggaagagtgttcatacgt
atttataattattatatacttcgttatactaatagtttgattatatattataatcgatg
taaataaat-cagctaaacaaaaaacttatccttaaaatcatcttctcttgagcattttt
ttttatctacattagtatttacttttgtcgtataaatttccagtttagaccaaatatgtc
ctaactgatttaattgagatgttccacattctaaacaggctatttttatattgctcaaaa
cagcacctttttttataaaatcgttcaataatggcgtaatagttgcttcatccaaaaata
aatctaagacaagtatttttgtaaatgaatttaattgatctgggacattgttaataatat
cattataattaactaaaactatattagctgtaggaagtaaagatcgtgctccttccgcta
agataaagccataaggcataataattacaataatatatttataatgactgctaataagac
ttatagtggtattagcttttaattcgattgtttctgttactagccactctctcattattt
cgtaagtaatccatttgcctagttctgagcaagcagttcttaaaatcgtacctggattat
tgtcgttttcgagaatccctgaccaatgttgtattaatggatgggatgctatattaattt
ggagttgcatgttttgttaaatatatttaacatttaatttgttgattgaatcttcaattc
ctatttgttgagtattattaaaatagcat----atatattaaagaaaataattat-aaaa
aatgaaaaaaagtctttggctgtggggttttacagatagtgctgaaacttggaatggtag
atttgcaatgattggttttatatcagttatttttatcgaagtagttacaggacaaggatt
attatatttaataggtatgatgtcataataaaaatccaaaaaagttcttgtaattttta
taagaactttttttgg-atttttataaatctaatctagcggtctcatagatagaacaggtt
cattctctctattgatacctttttcaaatccagcagcagcagctcttgctctgcctgcat
gccaaagatgtccaataaataggaagaatcctaggaagaaatgagatgttgttaaccaag

atctaggagatacatagttaactgaattaatttctgtagctacaccaccaacggaattca
aagaacctagtggtgcgtgagtcatatattctgcggcacgcctttcctgccacggttgaa
tatcgttcttgatttatttaaatcaagaccatttggtcctctaagaggctcaacccaag
gagctcgcaagtcccaaaatctcatggtttcaccaccaaaaataatttctccactaggag
atctcattaggtatttacctaatccagtaggtccttgagaagatgcaacattcgcaccta
gtctttgatctctaactaagaaagtaaaagcttgagcttgtgaagcttcaggaccagttg
gtccatagaattcactaggataagctgtgttgttataccaaacaaaattagaagccgtaa
gccccatgattgataaagcacctagactataagataagtaagcctcaccagaccatacaa
aagctcttctagcccaagcaaaaggttttgttagtatatgccagattccgccagcaatac
aaataatgccaatccagacatgaccacctacaagatcttccatgttatttacacttacga
tccatccatcaccgccaaacggagactttaagacataaccaaagataactaggggattaa
gtgtaggattgctaacaaatctaacatctccaccacctggagcccaagtgtcgtataccc
caccaataaataaagctttaataactaacagaaaagcgccaatgcctaataacactaagt
gtataccaagtattgttgtcattttgtttttatcccgccaatcgtatccgaagaaaggaa
atgattcttcaagagtatcagggccaatcagagaatgataaagacctccaaagccaagaa
cagctgaagaaattaaatgcactacgcctactacaaagtatgggtatgtattgaaaatct
cgccgcctggaccaacaccccagcctagtgtagctaagtgcggaattagaataaaccctt
gttcgtacaaaggcttctcgggaacaaagtgtgctacctcaaaaagagtcatggcaccag
tccaaaagaccattatacctgcatgagcaacatgagcaccaagcaatttgccagaaacgt
taattaagcgtgcattgccagaccaccaggcaaacccggtagactcaatgtctctaccgc
caacaccaacagttgtattaaagggcgtttccacgtggtaaaacctcctcagggaatata
aagttttcatgaggttgatcttgagcagccatccaagagcgaataccttcgttcaatagg
atatttttagtataaaaagtttcaaactcaggatcttccgcagctctcaactcttgagaa
acaaaatcgtaagctcttaaatttagagctagtccaacaattccaaatgcgcttgtccat
aatccagttactggtacaaataacatgaaaaaatgtagccaacgtttattagagaaagca
acaccaaaaatctgtgaccagaatctattagctgttaccatcgaataagtttcttcagat
tgtgttggagtaaatgcacgaaaagtatctgcagcatcaccatcttcgaataaggtattc
tgtacagttgcaccgtgaatagcacatagtaaagcaccacctaagattcctgcaacgccc
atcatatggaacggattcagagtccagttatgaaatccttgcaaaaatagcaaaaatcta
aaaattgcagcaactccaaggcttggggcaaagaaccagctcgcttggcctagagggtac
attagaaagacggaaacgaatactgcaattggtccagaaaatgcaatagcgttgtatggt
ctaagaccaactagcctagcaatttcaaattgtcttaaacagaatccaattagtccaaat
gatccatgtaaagctataaatgcccacagaccaccaatttggcaccagcgagtaaaatct
ccttgagcttcaggtccccaaaggaaaagaagtgaatgtcccatgctgtttgctggggta
gaaacagccgcagttaaaaaattgcatccttctagatatgaactggctagcccatgagta
taccaagaagtaacaaaagtagtcccagttagccagccacctacagcaaggtaagcgcaa

ggaaacagaagtagtccagaccaacctacgaatacaaatcggtctctttttagccaatcg
tctacaagatcaaaaccaccacgagtttttcttgtccaattgctatggtcataatttta
atctccagagcaaatttaataagtaatttgtttaaataaaca---ctttagatatactct
aaaatttactttctttacggtcaatataattataagtcaaaattaaccaaaattcacg
cttttaataaca-tttttttagctcattaagttaa-tttttaatatataaacagtaata
gagttattgttt-aacttacaatactcataatttattcatttctataatcctttgaaaaa
gatatcctgttcctcgtgctgttaaaatcaagtcgggattacttggatcatcttctagtt
tagctctcaatcgtgaaatatgaacatcaacaactctcgtgtcaacatgccgttcgggag
tatagccccaaacttcttgcaagatagaagctctagaaaaaggttctccagccttactaa
ctaaaagctcgagaagactgaattccatacctgttaatcgaactctttcattatttttat
aaacttgtcttttgttagtatctatttttagaaatccaatactaataatcccagagtttg
ggactccaagactagtggtgattttatcagctcttcgaagaacagaacgaatgcgagctt
ctagctccttaggagaaaatggtttaacgacatagtcatcagcacctatttctaatccgg
taattctatcgcagacttcacctaacgctgttagcattatgataggaacatctgattctt
ttcgaagttcttgacaaacaccgtaaccatctagtttaggcatcataacatctaaaacaa
caaggctaggatactcttttctaaacacgagtaaagcttcttctccatttgaagcagtga
taacttcataacctattattgttagtcttgtttctaaaattcttcttatgctggcctcat
cgtcaacaacgagaatcttttctttctggttctccaa-tttcttatttactcctataact
caccgatcatgattactataaccttaaatttttagcgctatgagtatctttattcacttt
agaa-----tttagttattataaattttgcaagtctagtttacattaattagtaccagt-
-caatatttttgt-----------tttaagttatgac---------tattttaatccata
agttgactctagtttgcagaattcaatatatacata-ttttctagccaagtaaattgtat
tctattttctaaataaaaaaggggttgacaaaccagttgatagaaagtattctacattac
tataag----ttaattccaacaaagaagttttaa-----atcttctttcggagtaatgaa
tactttaat
>SRR9587922
tatatgata---aattaagacgtctgttatgtgagaatttctaaaatatacaaaactttt
tatttaatatagaaaaagtaaattaaaataagttgaacatactttgctctaatctaaacc
tcttaattgag------attctaattagagacaaaagtatttacttattttgtatctata
ggaataaaaatgaaacttgcagtttatggtaaaggtggtataggtaaatctacaactagt
tgcaatatttctgtagcactttcaaaaagaggtaaaaaagttctacaaattggctgcgac
cctaaacatgatagtacatttacgctaacaggtttttaatcccaacaattatcgatact
cttcaatccaaggattaccactatgaggacgtttggcctgaagatgtaatctataaggga
tatggcggtgtagactgtgttgaagctggtggaccgccggctggcgctggttgcggaggc
tacgttgtaggtgaaacagttaaacttttaaaagaattaaatgcttttgatgaatacgat
attatcttatttgatgttttaggtgatgttgtatgtggaggttttgcagctccattaaat

tatgcagactactgcttaatcattacagacaatggatttgatgctttatttgcagctaat
agaatagcagcttcagtacgagaaaaagctagaacgcactctctgagattagctggactt
gttggtaatagaacagataaaagagatctaattgataaatatatagattgtgttccaatg
ccagtattagaagtcttgcccttgattgaagatattagagtgtccagagtaaaaggtaaa
actttatttgaaatggcagaaattgataaggatttagcatatgtatgcgattactatttg
aatattgcagatcagttgattacaaggccagaaggtgtagttcctaaagaatctccggat
agagaattatttagtcttttatctgatttttacttaaatcctaaatcaaaggtaggacaa
gaaaaagtagatcaagaagaattagatttgatgatagtgtaaataatattttcagcataa
tataacaaggaataagataatgtctacagctcaatcagatgctcttacttttgaatgtga
aacaggtaattatcatacttttttgtcccatcagttgcgtttcttggttatatcaaaaaat
tgaagacagtttttttcttagttatagggactaaaacttgtggttatttcttgcaaaatgc
aatgggagtaatgattttttgctgaaccgagatatgccatggcagaattagaggaaggaga
tatttcagctaagctaaatgattatggtgaactccgtagattatgcttacaaataaaaaa
agatagaaatcctagtgttatattttggattggaacatgtacaaccgaaattataaaaat
ggatctggaaggaattgctccaaaattggaagcagaaattcgtgttcctatagtagttgc
tagagctaatggtttagattatgcctttacccaaggtgaagatacagttctagcagctat
ggctcaaaggtgtccattaaatttaaagaatcaatcagataacacgagtcttaaaacttc
tcctcatattcctttagttttatttggatctcttccggatccagttgtaacccagttaac
tatggaattaaaaaaacaaggtatttttgtttctggttggttgccatcaaagaggtatac
tgaattaccggttataaaagaaggatattatgttgcgggagttaatccatttcttagtcg
cacagctactacactaatgagacgccgaaaaacaaaactaattggtgcaccatttccaat
aggtccagatggtactcgagcctggattgaaaaaatctgttcagtaatgaatgtaaagcc
tattggattagaagatagagaaaaagcaatttgggcttctttagaagactatatttcttt
aatccgaggtaaatcagtttttttttatgggtgataatttactagaagtatctcttgcaag
atttttaactagatgtggaatgactgtatatgaaattggtattccttatatggataaacg
ctatcaagcagcagagctagctttattaaaagctacatgtgataaaatgaacgttatgat
gccaacaattgtagaaaaaccagataattataatcaagtagaccgaatccgtgatctgaa
acccgatttagttatcactggtatggctcatgcaaaccctttagaagccagaggtattaa
tacaaaatggtcagtagaattcacatttgcacaaattcatggctttaccaatgcaagaga
tattcttgaactcgtcacaagaccattacggcgaaatcttagtttgtcagaattaggctg
ggatatttatagcaagcaaagctagtattcctc----tttatgacttacatggtataaaa
gccttgtaagtct--tttattttatgttaaagaagcatttcttactctgccatcaccagc
ccaagcttgtaaactttctacttgttctttgtctgtaaatgctaaaggcacaaactgttt
gatagctattttaatatcttcagtactaaattctctctcttcactaaacgccgtatgcat
actttcaataattgcttgctcaatttcagcgccagaaaatttattacataataaactcaa
ttcatgaatatcatattcttgccacgatcttggtctaacttttgataaatgtatcttaaa

aattgactctctctcttgacgattaggtaaatctaagaaaaaaatttcatcgaacctgcc
tttccttaacatttcggaaggtaaactttgaattttattagctgttgcgacaacaaatac
tggagctttttttctctgatagccaggtaataaatgtaccaaatactctggcactagtacc
actatcaccttgactatgtaaaccagaaaaagctttatctatttcatcaatccataatat
gcaaggcgataatccttcagagatatttatcatttctctcatttttgattccgattcacc
aactagtccaccaaacagttttcccatgtcaagacgtagcagaggcagcatccaatcgct
tgcaatagctttcgcggttaaggatttacccgtcccctgtataccaactagcaataaacc
tttgggcgaaggaataccgtaatttaaactttgcttcgaaaatgagcgagaccttttttg
tagccattgttttaatacatctaagccaccaatatccctatttactttactataaggata
aaattctaatagatgcgtttgattaataatttgtctttttttcttcaataataataggtaa
gctccgagagtctatctgattatattgtgcaataattttagtaatcactttgcggattct
atctatcgataatccttggcaggattttgttatattattaacgagctctgaattcaaatt
aagattcaaagctttactcaatcttatgatctcttttttgatttctagaagactaggtaa
gggcaaatctattaccgttatgatatcattcagagcaaaaggtatattgattttgcaaga
aataataataatgtgcttagattgcgttttgattattttagctaaattgcgaagtttcct
aattaatactatttcgtttaggaaagagtcaaaatcttttaaaagaaaaagatttaaaga
ttcattattcaagttttcaataaattctaatgctaataaaggatttctttttgcataacc
attatcgctaggattgttagtgtatccatctacaaagtcccaggaatatacttgttgact
gtttgaacagtttaagctgtgttttataatatactctagtctatcttcttctctagtatt
tattacaataataggatatcgagattttaaaagtaatcgtaaatcttgagtgaaattcat
gtcaatatttgtttccatcagacttttgtacttcaccaattgaagaaagactaaataaga
ttatctaatattccttggatatattcaaaagtc------atctctctatttctaaatta
ttgctattactaatttaacttaccatgattttttttcttccttttcttcgtcgactattt
aatatagagcgtccacatggtgttttcattcttgctctgaaaccagaaactctaatttttt
tttcttttagatccttgtagtgttcttttagtcatgatgtttcctctgttgtaaaagatt
aaatactattttatcatcttttatatatctttatgatatagatatgtattaatatcaaag
tt-aacagcaataatttatattaatcaagaattatttatataatattaaacattaaatat
----------taactttatgatattggctttgcccatttttttatttaagtattttgacca
tctttttgctaatcttaaactggttaatttttcagcagctaaaaacgatttttgttattag
aatctcaatttaagtattttgttgataaaagccaaaatcgtaaattagaacccgaagaaa
gttttgcttttgcaaaagtatgtgtagctaaaaaatatttttctaaagctattattgaag
gtcaattagctttaaaaaactatcgagacctgaatatcttagataataatatagtaattg
ctaatttgtataatatgctaggttttatttattttgaagcaggccaaacaagttttgcaa
agaacttttatgagcaagccttacaaataaaccctaattatgttgtagctttaaataacc
tggcaaaaatttacgaagaggtaaaagatttaaaaaaagccgaatctctatacgataaag
tgttaactcttaacttaaataataaaactgctaatagaaggaaagattttatagcaaaaa

cta---------aaaacatctaatactatttgtaatcgggatagcaggatttgaacctgc
gacatcctgctcccaaagcaggcgcgctaccaaactgcgctatatcccgtaagacataat
tgttactatataccttttctctaattatgtctactctttctgtattttatctaggatacc
aaaacatgcctttaactccatctggatctttgataaatcctaatttttataaaaactaa
caacatcaggttctgcgaatagtgtaatagtgctaatttctgcctgtctcaactgctgta
ttaattgatgtatgattacttttcctaaacctaaaccttggaaatctggatgaataacta
cgtcccagatagttgcattaaatccgttgtctgatgttgctctagcaaatcctacaagtt
tactatttgcatctttttttgtattaaagaaataataatagaactatttttaatgcga
tctttactttttttagaggtcttttaacccatccgactgaatcacaaagctgctccaatt
cgtataaattaatattcttattgctactcagatagatatctttaaattcgattttatcgc
aagttttatctaaaacaataagtttttgaaatttttttcagagctagcattgatatcgg
agttttgaaaaaaattttccagaagatcataatgattatgatataatta--aataaata
tatatattacaatattgctttaacaaacaatatattaatatatgctcaaatttttacatg
aatctattaagatagaagattgttaaaatatagagaaataaaaatttttgtaaaatcttt
caataaaatgatataaagagtgctttttaagactaacatctattattataaataaagtct
cgaaattgagatgaaataacacattattctatgtctaatgccttttaaaggagttatttg
tgaaaaaatctatgtgtctaacctgtttacttgctttactaattatgagcaacccaataa
tagctaacgcagaagtagctggattggtgccctgcaaagattctgcggcattcaataagc
gtatggtaaatagtgtgaaaaaacttcaagccagattagctaaatatgatgcagacacgc
caccagcgttagctttaaataagcaaatagagaaaactaaaactagatttgcaacttatg
gtcgagcaggtttgctgtgtggtactgatggattgccacatttaatttctgatggtcgat
ggagtagagccggggactttgtttttccaggactattattcttgtatattacaggatgga
ttggctgggtaggtagaggatatcttttatctgttgctaagactagtaagccaacagaaa
aggaaatcatttttagatgtgccattagctatcaaatttatgtcatccggatttgcatggc
cgctagcggcttggcaagaatttagtagtggacaattaattgcttccaatgacgatatta
ctgtttcaccccgttagtaaaaaatttatatgaataataatttaccaaatacttatcaa
cagcacctgtaattggtgtattgtggatgacgtttacagccggatttattatagaattga
accgctttttcccagatgtcttatacttttactt----ataaataa--gatcgtataaa-
--aaactacataaatatagagaaatactattatttgtgtagtttttta---atatataaca
taattattta-tttttaaaaatgtttatcttttcgattattttaatattagatcttacat
aataaaata-atatcaatattatgagcttagtaacccaaattatagttaatgccgatgat
gaattaagatatcctacaattggagaattgcagtcaattcaagactacttaactacagga
agcaatagaattagaattgctactattattagagataaagaaaaggagattatacagaaa
gctagtaagcaaatttttcagttacatccagaatatatagctccaggtggtaatgcagca
ggttctaggaaaagatcgttatgcttacgtgattatggttggtatctcagactaattaca
tacggagtattagctggggataaagattctatagaaacaattggtattataggagtaaga

gaaatgtacaattctttaggtgtacctataattggaatgttagatgcaatccagtgttta
aaggaagcatctttagaaatgcttggtcaagatgatattagaatcatttctccttatttt
gattatataattcgtggaatgtcataaatcatagcttcaattaattagttgaataaaagc
ttttataatgttataattacaataagcccgaaagcatataattgtaaaaactaaatcttg
tcagaaccggaaggtagcagcaataatgtttcaatacaaaagttatga-ttttcgggtgt
ttattgtttttatctaatagtctcgacttttaattagtatgctatgtataaatctgtata
tgacatgtaatccatcaaaccttccagcaaaaataatttaataatttgatattaatacgt
aaaacacctatac-tcttttataaaatttctttatatctcatctaaacaatggaaactta
acttaatgggtgttcatattttatcaacaggttcgtctgtcccaaatttttctgtagaga
atcaacaatttgaagatatgatcgaaacttccgaccattggatttcaacaagaacaggaa
taaaaaaaagacatctagctccatcttctacttctttaactaaattagcggcagaagctg
caaacaaagccttatatgcagctaacttaaaacctactgagatcagtttaattattttag
ctacgtctacgcctgatgatttatttggtagtgctagccagttgcaagcagaaataggtg
caacaacatcggtagcttttgatattacggctgcctgctccggttttattgttgctttag
taacagcagctcagttcattcaaactggttcctatgacaatattttagttgttggagcag
acacaatgtctagatggattaattggtcagatagaactacctgtattttatttggtgatg
gtgctggagcagtagtattggggcaaagcctcaaaaatagtattttaggctttaagttat
gtacagatggtcagctaaacagtcatttacaattaatgaataaacctgtaaataatcaaa
aatttggtgttacagaaattcctcatggaaactataattccataacaatgaatggcaagg
aagtgtacaagttcgctgtatttcaagttccaacagtaattagacaatgtttgaataatt
taaacatttcaatagatgaagttgattggtttatattgcatcaagcaaacactagaatca
tagaagcaattgcgagcagattatcagtaccttttctaaaatgattacgaacttagagc
attatggaaatacatctgcagcgtcaatccctttagcgttagatgaagctattcaatcca
ataaaattcaaccaggccaaattattgttttatctggttttggagcaggcttgacttggg
gagcaattgtcttgaagtggtgat---ttatatattgcggatgacgagactcgaactcgt
aaagctttcgctacacacccctcaagcgtgcgtgtataccaatttcaccacatccgcatt
ttttaccatgaata-ttaaaaatttataatatttatctaaatattcatataa--tgtgta
tatatatatacattcttgtcagaaactctcttatattttattttaagtgataaaattgag
t----ttagtatagatagtaaaattttattttaaaatcataa----aaataaatatataa
ggaaaataaaatatgacaccatctttatcaagtttttgaatagtcttattcttggggca
gtaattgtagtcgttcccataactttggctcttttatttgttagtcaaaaagacaggaca
atccggtcctaaaaataacttagaaagatatggagaagcaatttaaaatgaataataaaa
tatttttcatactaataagctctccatatattgctatact--ttagaagaatttttattt
ttttattttgtaacatcctgtaaagtttattccgttctttctttttccc-atttactctc
ttataattaatgatacaaatgtcagg-ttaataatag--tttttttattctgtatctttc
---------------atttatatacgtattattatgttcaattaataaagaaaagctca

tgtctttgtctaattggcctctcaaaaaagaaaattctgaagcatataatattaagaatt
caaaacaaatcacaattcctgatggtttatggataaaatgcttcgactgtggtctattaa
tgtattctaaagtactgaagagaaatttaaaagtttgccctcaatgtagttatcattttc
aagcttctagtaacgaaagaattgatcaattgatagaccaaggtagttggcaaccaatgg
atgttcacttgatctctacagatccattaggctttaaagaccaaaagctttatagtcaaa
ggttaaaagatactgctttcaagactggcctgcaagacgcagttcaaacaggtactggga
ctatgcaaggtaaaaaagtatgcttaggtattatggattttagattcatgggggaagca
tgggatctgttgtaggcgaaaaactaacaagactgctagaaaaagcaactcaagaaaagt
tgcctgcaattatactttgtgcatcaggcggagctagaatgcaagaaggtatgttgagct
taatgcaaatggcaaaaatttcttctgctctagaaatgcataaaaaagaaaatctactat
atatatctgttttaacctctcccacaacaggggggtgtaacagctagttttgctatgcttg
gagacttaattattgcagagccaaaagctcttattgcatttgctggtagacgagttatag
aacaaacaatcaaagaagacttgccagataattttcaaagttcagaatatttatttgaac
atggtttcctagatttaattgtatccagaactcagcttagatcaaagttaatacaaattt
tgtccttacataatcatagtaagtaatgaattgaatattgactataatatcaaaatgcta
gtaaaaattaagatattaagaaattttcactaaagatatttgtagactaactagaattac
aattactattgtagaaaaatttgtcttaatgcca-ttttttcagtcttttaacaaattg-
tttttatgactttaaataattgttagtttaatacaaatttataaagagaata-----cat
actaagctaaatatagtaatattatgttttcctataatactgacctatcaaatttcgagg
aaatttatgcttaaaagatcttcttggcttgcggctttattgggactattaacagtagtt
tctacaagtacgcatacatatgccatagagttagacgaggcaacaagaactgttccatta
gaatcttctggcagaactgtaattcttacaccagaacaagttaaaagaggcaagcgatta
tttaataattcttgtgctatttgccataatggtggtatcacgaaaacaaatccgaatatt
ggacttgacccagaatctttaggattagctacgccacaaagagataccattgaaggacta
gttgactatatgaaagatccgactagttatgatggtgcagagtcaatcgcagaattgcat
ccaagtattaaaagtgctgaaatttttcctaaaatgcgcaatctaaccgatgaagaccta
tttacaatcgcaggtcatatcttacttcaacctaaaattgtttctgaaaagtggggcgga
ggaaaaatttactattagaacttcaaagacctaaaccttgtgaatactagtttatgta-t
ttgttgcttaagtgtgattattttgttagatcatgatatatattaaagatggagtttgtt
gacagcaagttacttttacccgaacttgtttacactattcgtctagttattaaaaagga
gacgttaaattgaagaagaagctttcagttcttttcactgtttttagttttttgtaata
ggtttcgcacaaattgcttttgctgcagatctagataatggagaaaaagtttttttctgct
aattgtgcagcatgtcatgctggcggtaataacgccattatgccagataaaaccttaaaa
aaagatgtacttgaagctaatagtatgaatactattgatgctattacttatcaagtacaa
aatggtaaaaatgccatgcctgctttcggaggtagactggttgatgaagatattgaagat
gcagcaaattatgtattatctcaatctgaaaaaggttggtaattatacttgattttatcc

tgtattaaagaatagacaatctatttagttgttcattagattgtctattctttgtttatg
---ctattatataaagatatttacacaatatt----ttatgatgaaaagaatacccgcaa
ttcttgtactagaagacggtgcgtattataaaggatggtcattccagcaagataaacaag
agattactattggtgaagtagttttaatactggaatgacaggatatcaagaaataatca
cagatcctagttacttccatcaaattgtcgctttacctacccggaaattgggaatacag
gtattaataatcaagatattgaatctcacagtattagtattaaaggacttattgcaaaaa
atatttgtaaaatttcaagcagctggagagagcagcaatctttagttacgtatttaagta
gtaataatattcctttatttcggaatagatacaaggtctttaacccaatacttgcgtc
aatttggtacaatgaacggttgtatctctactgataatttaaatcatagttacttaaaac
agaaaatttgtgagattccaagtatgcaaggtttagatttaatcccgcatgtaactacaa
gaaatgtttacccctgggatgaaaaaagttttccaaattggtatttgacagataacatta
gagtgcaccgagttattcagttgaaagttattgttatagattttggagttaaactaaata
tactcagaagactagctacacttggatgtcagataacggttgtgcctgcccacactcctt
taaaagatattttggcttaccagcctgatggtatattactctctaatggtccaggagatc
catcagcagtacattacggcatccagacagttacaaaattactagattacaatgtgccta
tatttgggatttgtatggggcatcaaattttaaatttagctcttaaagctaaaactttca
aacttaaatttggtcatagaggtattaaccatccatccggattgaaccagcaagttgaaa
taactagtcaaaatcatggctttgcagttgaattgacttcggtttttgaatctcctgtaa
gagtgactcattttaatctaaatgacactactattgcaggaactggacataatcaaagtc
cttatttttctgtgcaatatcatccagaatcgagcccaggccctcatgatgctgattatc
tattcgaaaattttatagaaataatgacaaagtccaagaataaagttag-----------
ttagtaattttcccatgctttatgagtgaataaagcggatagtacttgtactcctcgtaa
ttgattaaagagaggcaaatgtcctctggggccatgagaattccattgaaattcctcagg
atatctgcaaggaattctatcaacttcccagccgatttttgccagaattttccccaatc
ttttccaacactaagccatatttgtctttgaacaaatagcccaaatttacctttgagtg
tgtatgccataacttatcaatagtttgtaaatcttgagcaggtattttttttatatctgt
aaaatatagccaattacgagtttgtgcatttacaccagctagttgaataagcttttgttg
agttagctgatctgctttgagcaaatcccgatgagtcagtaacatttgtaaatctttgta
attcatctgttgagcagagcgcaatggaacaatgccatcgggacataagttagaagcaaa
tttcactatttcttgattttactgttaagtaacttttcataaattaaaccgtcaacaca
attacttttatagttaggcccagtaatcctctcaaaaaatagatctgctaaatcttttaa
ctcgatagagtctttgctatttatattctcaataatttcaagttgttgcttga---catt
attagacttagtattcttattcaattctaaaagctgggctcggatttgatttggcatctg
atttgtttaattagttttaatagtc----ataaggtcttagagagaattagattgttttt
tgaaattagaagagcgccgcagtatttctaaagaagttcgcataatttgattcaataagt
tttgtcctgcataaattataatttgaccaattaaaaaaattgtgcttttattttaggaa

caaaaaaatcttgtacctcaagcagaaaagtaattattatttgcattggtgacaacatct
gtaattcattcgatctgcaagcatatatatactgacagtttaagccttcttgataaaatc
cccatactttgtaacgactttcgtaaattgctcgtggtcgctcaagatagtttttaatta
tagtattccaaattaggttattctttaatttttctaatttctatcagatagaaatttta
cattacagagataacttgcatttgtctcatttacagtattaaaattttgaactaacatat
gagcaataatgttgcttagctgaattaaataattttcaagaaaaatttcgacctgcttaa
ctggcatattgtaagtaaagagattaaaagttttattcacagtttgagatgacccaaaaa
gtagctgaataattaaaatttgtagtagcatcttatagtcgttcagtagatagttcaaat
catgaatatcttggtaaatgtcaatacagtccagttcatataacttacaaaatcttttga
tacatctatggaataaatcaattaaaatattttcggtcaagtttgtaatatcatttacat
ctaaatttgaattataaatttctaaaagtatcttttccagttcagctaatatgattttta
ataagtttctttttacttcagtgcgaaaaacatctaaaattaacacttcctgagagcaat
tagttaatctcttattaatctttatagaagttctaactaatagctctgctacctcttgat
tgagtgttggtccctgagagcttggccaataattattcacgttataccatagtaagaaat
attgaatttaatgttatacgataacttttttaatatactgaaaagcaagttaattactcta
tagcaactag-------tttttacagatcttaatagttatttatcaaataaagttatatg
atagtagcgattgagctaattttattaaaattta----taataatgacaacttattactt
cgcccttgcgagtcaaaattttctattagtacaagaaccactagaagaagtttttagaga
gagagttaactactatcagtcaaataataaagcaattgatttttggctaataccaaaccc
ttcttttctagagaaaccagaaatgatttcatttaaaaaccttgtacctaaagacgctgt
agctataatctctactaatccaatatttattaattggttaaagctaagaatcggctatat
ctgtatcgggcaatttgaagataacctacaactttctgaagaatcgttaaatattactgt
tttaacagacaaaatttaacaatgcttttattatgactagtgttaaattattttggttaa
gttataattatagtcttttagtattta-gactcttatagtttatcacttagtattttatg
ctgagatggagtgacaaatagtgtaagtatttcttggctaaacgtttcagttgccttaga
tttatatctatttggattaactataatggatagcattcgttttatagtaacattttcaat
ttgagcccaatgtacaattccaagttccaattccttagcaatagctgaaacggaaacaaa
ggcagctcctagccctgattgcacagcgtttttgatagcttctatagaattcaattccat
ctctattttaaagcgactgctgtcaataccatgttgactgagtactttatctatgacttt
tctaattgttgattgagtatctaacgcaataaatctaagcctgtataagtcttcttttttg
aatgtctcctagtttagaaaaaggatgtgatttgggtaatataagtgctaattcgtcttc
cgcataagaagtaacctgcaaaacatcttgcaattcagtaggcacttctcctccaataat
tgctaaatcaacttgaccattagctacgctccatgaaataagtctagttgaatgtacttg
caattgaacagccacttgtggatatctttgcctaaatagtccgattaatcttggcatcaa
atatgtcccagttgtctggctagctccaataattaatgtgccaccttgtaagttttgtaa
gtcgtcaagagctcgacaagtttcttcgcagagagctaaaattctgcccccatatcgtaa

aagaagacttcctgcctcagttaaagttgccttcttattacctctttcaaatagggaaac
attcaattggcgctctaaattttgaatttgcaaactaatagctggctgagaaacatacaa
gctattagctgctttttaaaactgccctctttggcaattgcttttaatattcttaactg
atccaatgtaaatggaaggtctgtcattaaagaattgtagtataatg---------tata
ttatttagtattgatcaatgtatgcatttgccaactaaaatatttgtcattacgcttcat
tattttcaaaataataaaaagtataatgttaaataagctgtattaaaatttagtatctgt
ttgatattagatttatacaagagatatcgtgaatttaattctaactataaaatataggag
acaat---atggactctagacttttagttgtactaataccagttttagcagcagcatctt
gggcagtttacaatattggtagagttgcactacagcaatttagaaaaatgacatcttagt
tttgcttagaatata-atttttagactttatgagatagaggactttaaaatccctatctc
tattgtatatcttaataaaaaaag-aaagc--ccccttgtacttactgttattacacatt
aatattattggt-ggtaccta----gataataagtttaagctaacttatttttttattta
taattaattaaattaataacacaattatggctgttccaaagaaaagaacatctaaagcta
aaaaaaatgcacgcaaagcaaattggaaaaatcaagcaaaaactgaagctcaaagagctt
tgtctttagcaaaatcagtattaactagaaaatccaatggatttatttataatctaactg
aagcatcagatacttttagcgattagaagtgttt-ttactacaagtctgaaaaagtatta
cgcaacaagttctcagtaatttttatctag----taatcatttgaaaatcattctatttg
ataataaaataaatagcagattaaataaaatcacattaacaaattatgcagcaaagctag
atcaaactagtgaaatttggttgttcaattgtattgaaaatattcagcatatttttttta
aaagtcaattaaaatcaagtcatattactaaaatttttatttctggtactagctttgaat
ataccgcaggtctaccaggattattatccagcttaacactaagtggtagactacatccta
ttagtatatatagtccccagtctctcaaaaagtatcttgaagcatgtaccaaatattccc
aaactaatttttcttttcctattaattttcataatttacaatacggaggacaagttgtta
accaatttatacagtaatttgtttaccgttgagcaaaaagagcctgctgtatggattta
ttattctaaaaaaagaaaagcagggagtatttaatttagcacaagctaaaaccttgaata
ttcttcaaggacctatatatggaaaactcaaagaaaaggataatttttaagtccagatg
gttactatctatccggccaagacttctcttctaatacaataatgggacataaaatatcgc
ttccgttattagtcagatattctagaattatttctgagatgcattggttttgctcttatc
ctattagattaaatacttattcgcatcagcaaggagcaaagtgtttaccacataatgttt
taactgatattatgaaatcccaaatatatcaagataacagttttgttgaataatacttat
tcttgatatataatggtctcggtaatctaaataaaatactagaaataatttcttaaattt
atttgtctatattaaatttacaatcatttatgacatacgcaattattgaagcaagtggca
cgcagctttggatagaagaaggccgctactatgatttaaatcatatacctgttgatccag
gtcagtcgattatattaggaaaagtcttattattgaataaaaatggggaggttactttag
gccgcccttgtatagaaggggttacgataaaggctacagtaatgaggcacttgcgaggaa
agaagataactgttttcaaaatgaaaccaaagaagaaaatgagattaaaaaaaggtcatc

gacaagaattgactcgtttaatgatcgattctataacatcttaga------taaaatcta
gactcatattttt--aa-aaacttttaatatttaactaaaatagatataatggcacata
aaaaaggtagtggtagtacaagaaatggcagagactctaattccaagcgtttaggtgtta
aaaaatatggtggagagcaagtaacagcaggtaatatttaatcagacaacggggaacta
aagttaagcctggccaaaatgttggaaaaggaaaagatgatacattgttttctctaattg
atggtttcgtgctgtttgaaaagtcaaatcaaaagcaaaaaacaattagtgtttattctg
ctaagaaatagttaaaacaattggtgcaagataacttgagt----gtatttatttcactt
acagtggcaccatatatttgatttattaagaaatggagttccttaatgaaccaatgaatt
ttattaaagatttgctgtgaaatttaatgacaaacactattgtaatttcctgtctacaca
atatggctgctattctatattgtggtcagatacagaaactagtagtagcaaatgctcatt
atcaagtaagtgatatctacctaggttgtgttgataaaatcttctcaggaataaatgcgg
catttattaacctaggaaagaatgagtacagtggttttatacatatcagtgataccggtc
cgcttaaaaagaaatattatgtcaataatattactaacattttaacaatacggcaaaaaa
ttttagtacaaattattaaagagccaactttaaataaaggtccaaggctcactgccaata
ttacattatcaggtcgatatattgtattaatgccttttagtcaatcaatctgtatatctc
gaaaaatatatgatgaagatgagcgtcattatttgaagtctttagctattttaattaaac
cggcaacaatgggcttgctatttagaccttctgctgtaggtgtcgatgaggaaataatat
taagcgaattaaaaaatctaaaagaacaatggaactttgttcaaaaatctgcaattaata
gttattcacctgttcttctatataaagatgaagatattgttaaaaaggtaatccgagatt
tttataataataatacaaacaatatagtaattgattcaaacctgggattaaaacaattaa
attattatatccacacttggcactgtaataactctagcacagttcctaagattaagcttt
atagtaataatcaatgtatactagatgcttttggtatcaatcaggcaatttccagagctc
tcattccaaaagttgatcttatacttggtggctatatgtttattgaaactttagaagctt
ttactattattgacgttaattctggatcttttaataattccactagtgcacgagaaacag
ttttaaaaaccaactgttctgcagcaacagaaatagcttatcagttacaaattagaaata
ttactggtgtaattataatcgacttcattgatatggaatcacaaagagatcaattgcaat
tattagaacactttaataaagagctatcacttgatgatgctaaaccacaaattgtacagt
tatctgaattaggtttagttgaattgactagaagaagacagggcaaaagtttgtacgagt
taattagtagtgattctaattactttatttttcacacaatcagagagatctcagtctc
ttaagagattcgatgatagacagcagaaacaacagattttaataaatcttggctatctg
cagagattaatactattaacaaggtctttttcaaaagtcaaatttgtgcagacctgcta
acttttacctaattcgtaatctctatatagttaaaagtagtattacttataaacaaaatt
atttattaactcacagatcaaaacttatttattctaaagaatacagcaaagtattaccaa
gca------gttattatctagctagtctcaataagaatagtaatcaagagttcctacctt
aagattgttatatcaactgtt---------------------------------------
-------------------gaaaaaaagaagctctctcttatataagagagagcttc-tt

ttgtagcttactaacta-tctagttagttaattaaaaattaaccgttaacagctggagct
gttagagctactggtaaagattcaccagaagctagatctagagggaagttgtgagcgtta
cgttcgtgcattacttccatacctaggttagcacggttgatgatatcagcccatgtgtta
attacacgaccttggctatcaacaacagattggttaaagttaaaaccattcaagttgaat
gccattgtgcttacagataaagctgttagccagataccaactacaggccatagacctaag
aagaaatgtagagaacgagagttgttgaaactagcatattggaagattaaacgaccgaag
tagccatgagctgcaacgatgttataagtttcttcttcttgtccgaatttgtaaccatag
ttagcagattcgttttcgcttgtttcacgaattaagctagatgtaactagggatccgtgc
atagcactgaacagagaaccaccaaacacaccagcaacacctagttggtggaatgggtgc
attaaaatgttgtgctcagcttggaatacaagcatgaagttaaatgttccggagatacct
agaggcattccatcagagaaactaccttggccaattgggtatactaggaatactgctgct
gctgctgctactggagcagtaaaagcaacggaaatccatggacgcatacctaggcggtag
cttagttcccactcacgaccaatgtagcaagctacgccagttaggaaatgaagaacaact
aattggtaaggaccaccgttgtatagccattcgtctaaagaagcagcttcccagattggg
tagaagtgaataccgatagctgcagaacttggaataacagcaccagaaatgatgttgttt
ccgtatagaagggaaccagcaactggctcacgaattccatcaatgtctactggaggtgca
gctacgaatgcaatgatgaatacagatgtggcagttaatagagttggaatcattaataca
ccaaaccaaccaatgtataggcggttttcagtactagtaatccaagagcagaaacgttcc
cacaagctagcgctttcgcgtctttgtaaagtagcagtcataatttttatcaattttta
aggatttaacaaagatgcttcccaaacaataagttgtctgttatggaaattattacttaa
ggccttttcaaatgagaacatttttaaaaaaaaca-tagcaaaatctaatctcagtaact
aa-aattt-tacttgttcatatattgcttaataattcaa-----------taataaattt
tt-gct-------------ttttgcaaaattaacactattttagggat---ataataata
atgcatattaatgctgcttattttgattttaatagtttatataattttgccctacttaaa
tacgcatgtcacactttacaaaaattcagacgaccatacaagatttaaatctgttaaagc
atgctttaacagatttgggtttgatttggcaaatgaattctagtcatataaaagttggtg
aaaataatcagcacaaggtagatattttaattaaacaagataatttgtcacatattggct
ttacttggaatgataatagatatcacttagttgccgacttacaactttgggaacaaccct
ggtctctagaagtatttttggataagttatctcagaagtatgcttactactctatcatcg
aagaaacaaagaagcaaggctttgaaaaaaatgcagcagatttcacaaaaagatggatcaa
ttaagttaattgtgcaacgctggaattattaactaacatacaaatgcgggcggagagact
tgaactctcacgagattatctcactagaacctaaatctagcgcgtctaccaattccacca
cgcccgcactgttgagac--aatattactctatcataatctatactaaaacacaaactaa
-tagtataaactagcttccaagcttaaaagcgtctacaaatagaccgtatgttataccaa
ttttgaagttgttgaataaattaatttttatatttaattgcttgtggctagagtagacta
ttttactagtcaactcagttgctgcaactattagactggctgcaataataccccaatctc

ctgtttgtccaggaatcgtagacaaacctgttgaaataaaaaatcctagcaataaactaa

ttaagccagttgttaactcgctcaaagaataataaagcttattatttaagtttttatta

agtaagcaaaaaaagttgaaagtctagttttaatcatagtatttagtaacggagttttaa

aaggatgacaagtattaacag-tcattaatattgtcgtatccttttaaaactcgttaata

caaacagctgttcttgt---------ttttaaaggtcttgttcgcttaaagcattaataa

gatgctgaaacggctcttgtataatatctgtgcgtttaattgattgcaaatctaattctt

cttctatgatttcttgtaataactgtatgcttcttactgttggagcaacaggtacagata

gtgaattgtaagtatcttttagcccatttaatacacgctgatcgagaatatttgtactac

cagaaaccaaggcataactagcataccgtaggtaatattctatatcccttagacaagctg

catatcttcttgttgtataagaatttcctccaggccttaataattcaggctgttcttcgt

acaactgagctgcggcctcttttaaaatattggtagcttgatcattaataatttcagcta

tttttattcgatctagtccactcgaaaagaaagattctagttgtccgacagctgtcttat

ctagataacgaccagtaagatcgtaacgatttaatattgctgttatagcatcttgcataa

attaattctcctac-ttgctatcagagcggtattataaatgactaatattgtaatataat

cattagtgtttatgaaatttatttaaaacttattctttattgcttaatgtgcaattaaaa

actcgcatagattttacacatatagagtatacgtagtttttaatta-tttttttataaaa

accaagaaataaagctttttt-taagaggttcgtgaatttgttaatataacatgagcttt

cctcctaaataagttttgtaaagtac--aaagtaaaaaaacgattagtcaataatagtaa

--------------------------acaatattaatatgtatcaa--------------

-----------------agtataaataagcttttactccagcatcaaaaaaaatatttga
gatatgttgatacaactgattattcaagtgaaaacttagatttagaccaatttagctatt
ttatcattacaattaataaaactgataaatgtattcttatagagcaattttgttataatg
ctgaaggccatatttactcaatattcttcaaaggtccaacagctaaacatctttcttcta
taatttgtcattttcaacaattaaatacaataatatctacagctcatgccatatatttag
gacgagagctaatgaagtcagaattagctctcgtgctagatcaacaatatattcaagatt
aaa--ttttgtaagtattaattatatttatgctagtataatattagtaagggcatgtaac
tcagtggatagagtatcagattccgattctgatggccgtgggttcgaatcccgccctgcc
c---gagtattcagagtact-atactaaaacatct-------taatagctagtttgataa
tagtatgggcattggggctgcaaggtttctacattatgaaaagaagaaaatatg-aaaaa
aaacaagctctccaaaagagcttttagtacaatcaataaatgcagaaaacaatattgttt
ctttttctcgaaaattagctcttgcttaaacactgttaatttttggcattcgacatgtta
aactcttattcatggcgaatattctgtcagagtcgctcttagtttaagaaaagtcagaaa
aaatatttatctttacgtttt-tacctatttttatttaaataaaggttagta-ttttcaa
cttttatagtggacgtgggttcgagtcccaccagctccatcaatttgatt---------t
gtataattagctagtttaacctttttaataaccagatatataattggaggaaatatgggt
ttcataacaattactaagcccgctttaaagcaaattgcaatcttgaaaaatgatcatgaa
aatgatgtacatctaagaataggtgttagacaaggcggatgttcaggtatgtcatactca
atgaattttgaacatgttgacaaattaaaagatacagatgaacggcttcgtctagataat
ttttctgttgtctgcgaccctaaaagtcttctttatctttatggattatcattagatttt
agctcggaattaataggaggaggatttcaattttctaacccgaatgccagccaaacttgt

ggctgcggtaaatctttttcaggctgacc-ctctaacacgtttttatatatttgtatt
atatatattatttagcaagttg-tttattagagacaagcaatgctctgttaaaaac---
---------------------------taatttcaagagtttttattgtattatt-----
----ttatatatttattatcaactacttgaaaataaattagctaagcaatctaatttgtt
[truncated: 632,603 more chars]
