# Supplementary material for: Genomic diversity of 39 samples of Pyropia species grown in Japan
Source: PLoS One. 2021 Jun 9;16(6):e0252207. doi: 10.1371/journal.pone.0252207 (PMC8189503; doi:10.1371/journal.pone.0252207)
Supplement: Attachment — Submitted filename: Point-by-point_response _to_the_reviewers.docx [file pone.0252207.s015.docx]

Point-by-point response to the reviewers

Reviewer #1: This manuscript was already published in “bioRxiv – the preprint server for biology” under the title of “Genomic diversity of 39 Pyropia species grown in Japan (doi: https://doi.org/10.1101/2020.05.15.099044). This case is the redundant publication and I wonder the guideline of “PLOS ONE” can allow this case. I think that the nature of “bioRxiv” is not the process of the pre-review system and it is just one of the publication. Therefore, I cannot accept this manuscript for the publication

The following website states that "PLOS encourages authors to post preprints as a way to accelerate the dissemination of research.”

https://journals.plos.org/plosone/s/preprints

Therefore, this opinion is contrary to one of editorial policies.

Reviewer #2: This manuscript analyzed the phylogenetic relations of 39 accessions of Pyropia species grown in Japan by chloroplast, mitochondrial and nuclear genome using high-throughput sequencing. In addition, the author also analyzed the genetic background of hybrid between P. yezoensis and P. tenera. The results of this study will be useful for breeding and the conservation of Pyropia species in Japan.

However, there are some problems existing in the structure and contents of the whole manuscript. The author attempted to elucidate the phylogenetic relationship of the samples from Japan. This part is the focus of the whole manuscript. In addition, the author also confirmed the hybrid relationship between between P. yezoensis and P. tenera, which is not consistent with the first part and can be deleted.

Phylogenetics is not the only subject of this paper; analysing the population structure and admixture is also the subject of this paper. Therefore, we thank the reviewer for pointing out that our description was not appropriate. To show the importance of this subject, we have added the following sentences or phrase to the Introduction.

Line numbers 40-42: “In addition, many of these previous studies analysed the genetic differences between species by phylogenetic analysis. In other words, few have analysed population structure or admixture within species.”

Line numbers 53-54: “Besides, whole-genome sequencing of the nuclear genome is suitable for analysing the population structure and admixture within a species.”

Line numbers 56-57: “To analyse the population structure and admixture,”

Because the population structure and admixture including the hybridisation history are also the subject of this paper, we have kept the description of the hybrid relationship between *P. yezoensis* and *P. tenera*.

About the phylogenetic analysis, the author should make model test using different softwares, such as PAUP、PYLIP and Bayesian. Otherwise, the author referred that the topological structure of phylogenetic trees based on chloroplast, mitrochondrial and nuclear genome sequences are not scientific.

Thank you for pointing out an important point. In the revised manuscript, we used ModelTest-NG to select the model for each phylogenetic analysis of chloroplasts and mitochondria. According to the selected models, we performed phylogenetic analysis of chloroplast and mitochondrial genomes. We have modified the “Materials and Methods” and “Figure Legends” to reflect these changes.

For the nuclear genome, traditional phylogenetic methods are not suitable because hybridisation has occurred. Therefore, we constructed a phylogenetic tree using the program SVDQuartet (Fig. 7), because this program assumes that intragenic recombination exists. We also carried out MDS (Fig. 5 and 8) and admixture (Fig 6) analyses to analyse the populations where hybridisation has occurred.

Furthermore, the conclusion about the evoltionary history of chloroplast and mitrochondrial genome is not identical is also not scientific. So the author should analyze the evolutionary speed of chloroplast and mitrochondrial genome.

Thank you for pointing out an important point. We agree that differences in the rate of chloroplast and mitochondrial evolution have caused differences in the topological structures of the phylogenetic trees. Therefore, we have created the S4 Fig to show the differences in the rate of evolution. We have also added the following paragraphs to show this.

Line numbers 134-140: “There were differences in topological structures between the chloroplast phylogenetic trees (Fig 2, S3 Fig) and the mitochondrial phylogenetic tree (Fig 3); in the chloroplast phylogenetic trees, the Japanese and Chinese *P. yezoensis* were clearly separated, but in the mitochondrial phylogenetic tree, the two were not separated. This may be due to differences in the rate of evolution between the chloroplast and mitochondrial genomes. Comparisons of the phylogenetic trees showed that the rate of evolution of the mitochondrial genome was about 10 times faster than that of the chloroplast genome (S4 Fig).”

To reflect this change, we deleted the following sentence from the Abstract and Results, “Thus, the chloroplast and mitochondrial genomes of P. yezoensis have different evolutionary histories” and “Thus, the chloroplast and mitochondrial genomes had different evolutionary histories.”

In addition, we deleted the following paragraph from the Discussion, “A comparison of Japanese and Chinese accessions of P. yezoensis revealed that the chloroplast and mitochondrial genomes had different evolutionary histories (Figs 2, 3 and S3 Fig). Incomplete lineage sorting [38] may cause this difference. However, we also need to consider the possibility that chloroplasts and mitochondria may have different inheritance mechanisms, which has not been studied in P. yezoensis. Therefore, hybridisation may have contributed to the occurrence of different evolutionary histories. However, further study is required to elucidate these two possibilities.”

The author should show the detailed information about the sequences from chloroplast and mitrochondrial genome used in constructing phylogenetic tree in the method section.

Thank you for the useful suggestion. We added the FASTA data for the phylogenetic analyses (S7 Fig, S8 Fig, S9 Fig, S10 Fig, and S11 Fig). In addition, we added the following sentences in the Materials and Methods.

Line numbers 374-378: “S7 Fig is the multi-FASTA file of the large single copy sections of chloroplast genomes used to create the phylogenetic tree in Fig 2. S8 Fig is the multi-FASTA file of the large single copy sections of chloroplast genomes used to create the phylogenetic tree in S4 Fig. S9 Fig is the multi-FASTA file of the assembled complete sequences of mitochondrial genomes used to create the phylogenetic tree in S4 Fig.”

Line numbers 407-409: “S10 Fig is the multi-FASTA file of variables sites in the chloroplast genomes used to create the phylogenetic tree in S3 Fig. S11 Fig is the multi-FASTA file of variables sites in mitochondrial genomes used to create the phylogenetic tree in Fig 3.”

The author should explain the reasons why China cultivar MK695880 clustered with Japan samples in Fig2.

Thank you for the useful suggestion. We added the following sentence.

Line numbers 95-97: “This suggests that this Chinese cultivar was introduced from Japan, where the cultivation of *P. yezoensis* was established.”

The taxonomy of some samples were confused by the author previously. They were corrected based this manuscript. Yet this is the mistake by the author in the previous research and is not similar with the wild sample identification using phylogenetic analysis. So these related analysis should be re-writen.

We did not understand the question. However, there was no difference between the taxonomy in the previous study and that in this study. We have also noted this in the text (line numbers 108-114).

In the result section, I am not sure whether the author removed the sequences from the contaminant from bacterial nuclear genome. Or the author should clearly state the genome sequences used for constructing the phylogenetic tree were indeed mapped to the reference genome.

Thank you for the valuable suggestion.

To make S5 Fig, bacterial contamination may have slightly influenced the results. Therefore, we added the following sentence.

Line numbers 160-163: “Observations of the mapping results suggests that there were very few regions that have been incorrectly mapped with bacterial sequences, but we cannot exclude the possibility that bacterial contamination may have influenced the results shown in S5 Fig.”

To make Table2, the calculation method that we used removes the contamination. Therefore, we added the following sentence.

Line numbers 179-180: “In principle, this calculation method effectively excludes the regions that have been mapped incorrectly with bacterial sequences.”

In addition, to make Fig 5, 6, 7, and 8, we effectively removed the regions that have been mapped incorrectly with bacterial sequences because we removed loci containing potentially heterozygous loci. Therefore, we added the following sentence.

Line numbers 196-197: “The removal of such loci can remove loci that have been mapped incorrectly with bacterial sequences.”
