# Supplementary material for: Genomic diversity of 39 samples of Pyropia species grown in Japan
Source: PLoS One. 2021 Jun 9;16(6):e0252207. doi: 10.1371/journal.pone.0252207 (PMC8189503; doi:10.1371/journal.pone.0252207)
Supplement: Attachment — Submitted filename: Reviwers comments#.docx.doc [file pone.0252207.s016.doc]

**Reviewer # comments**

**Manuscript number: PONE-D-20-27527R1**

This research paper is already available in public database/repository, so in the current scenario this unethical to publish. Paper doesn’t have authenticity and novelty. It may resubmit to the journal only after withdrawal from bioRxiv preprint server (Cold spring harbor laboratory) <https://www.biorxiv.org/content/10.1101/2020.05.15.099044v1.full>.

Nevertheless, the manuscript submitted to the Journal entitled “*Genomic diversity of 39 accessions of Pyropia species grown in Japan*" is an informative piece of work and seems useful in view of harnessing the potential of these important marine algae in breeding and genetics. Although, authors have included limited (39) number of accessions in the study, work may help to breeders and researcher to generate the information for identification of best planting material.

Based on my observations I recommend the manuscripts for major revision. To improve the manuscript quality, the following comments and suggestions are required to be addressed;

- The economic importance and medicinal values of the *Pyropia* species is lacking in the introduction section of the manuscript.
- Why the assessment of the genetic diversity is important within the the *Pyropia* species. Explain in introduction in brief.
- What is Ariake sound, mention clearly in M&M of MS.
- In material and methods part of the MS, the construction of DNA library is missing, include some lines about that.
- How organellar (mitochondrial and chloroplast) DNA was extracted..?
- What type of data was used to construct a phylogenetic tree, need to clearly mention in materials and methods.
- Discuss the results that mitochondrial DNA sequences couldn’t revealed genetic variability among the of 39 accessions of Pyropia species; What may be the probable cause of that.
- And also explain how about the chloroplast based genetic variations showed in gbPyropia species.
- Nuclear DNA based genetic analysis was significant in compared to that of organellar genetic diversity, what may be the possible reasons for this distinctive nature of between organellar and nuclear genetics.
- Line 403 is the repeat of 402; these can be clubbed in a single sentence.
- In results section, lines 73 -75 should be part of materials methods.
- There are discussions parts mingled with results which enlarged the size of results, check thoroughly and trim the results section.
