# Supplementary material for: Genomic diversity of 39 samples of Pyropia species grown in Japan
Source: PLoS One. 2021 Jun 9;16(6):e0252207. doi: 10.1371/journal.pone.0252207 (PMC8189503; doi:10.1371/journal.pone.0252207)
Supplement: Attachment — Submitted filename: Response to Reviewers.docx [file pone.0252207.s017.docx]

Journal requirements:

When submitting your revision, we need you to address these additional requirements.

1. Please ensure that your manuscript meets PLOS ONE's style requirements, including those for file naming. The PLOS ONE style templates can be found at

https://journals.plos.org/plosone/s/file?id=wjVg/PLOSOne_formatting_sample_main_body.pdf and

https://journals.plos.org/plosone/s/file?id=ba62

/PLOSOne_formatting_sample_title_authors_affiliations.pdf

We greatly appreciate your helpful suggestions.

We have revised our manuscript to meet the style requirements of PLOS ONE. We apologize for not meeting the style requirements.

2. In your Methods section, please provide additional details regarding the blades used in your study and ensure you have described the source. For more information regarding PLOS' policy on materials sharing and reporting, see https://journals.plos.org/plosone/s/materials-and-software-sharing#loc-sharing-materials.

In lines 81-89, we created a paragraph explaining the details of the samples as follows: “The collection sites were represented on a map generated with SimpleMappr [38] (Fig 1) and shown in Table 1. When the collection was performed, a single blade (a mixture of four types of haploid cell) was isolated. Next, carpospores (diploid spores) were collected from this blade and cultured. The culture of carpospores derived from single blade was defined as ‘culture’ in this study. Sometimes, one of the cultures was selected for aquaculture, which was defined as ‘strain’ in this study. A culture was also created from a strain, which is indicated in Table 1. Each culture was used as a sample in this study. In Japan, various cultures/strains are preserved in various institutions. Therefore, the acquisition of culture was not necessarily performed by the authors, so some cultures were provided by other researchers. From these cultures, this study focused on those grown in the Ariake sound.”

3. We note that you are reporting an analysis of a microarray, next-generation sequencing, or deep sequencing data set. PLOS requires that authors comply with field-specific standards for preparation, recording, and deposition of data in repositories appropriate to their field. Please upload these data to a stable, public repository (such as ArrayExpress, Gene Expression Omnibus (GEO), DNA Data Bank of Japan (DDBJ), NCBI GenBank, NCBI Sequence Read Archive, or EMBL Nucleotide Sequence Database (ENA)). In your revised cover letter, please provide the relevant accession numbers that may be used to access these data. For a full list of recommended repositories, see http://journals.plos.org/plosone/s/data-availability#loc-omics or http://journals.plos.org/plosone/s/data-availability#loc-sequencing.

In the "Data availability" section of the PLOS ONE submission system, we included the following statement: “Sequences are available at the DNA Data Bank of Japan Sequence Read Archive (https://www.ddbj.nig.ac.jp/dra/index-e.html; Accession no. DRA010178).” In addition, we provided the accession number in our revised cover letter.

4. We note that Figure 1 in your submission contain map images which may be copyrighted. All PLOS content is published under the Creative Commons Attribution License (CC BY 4.0), which means that the manuscript, images, and Supporting Information files will be freely available online, and any third party is permitted to access, download, copy, distribute, and use these materials in any way, even commercially, with proper attribution. For these reasons, we cannot publish previously copyrighted maps or satellite images created using proprietary data, such as Google software (Google Maps, Street View, and Earth). For more information, see our copyright guidelines: http://journals.plos.org/plosone/s/licenses-and-copyright.

We require you to either (1) present written permission from the copyright holder to publish these figures specifically under the CC BY 4.0 license, or (2) remove the figures from your submission:

As shown in the legend of Figure 1, we created this map using SimpleMappr (https://www.simplemappr.net/), which is a website to create free maps or publications and presentation. On this page (https://www.simplemappr.net/#tabs=6), the author states that "All versions of SimpleMappr map data found on this website are in the Public Domain. You may use the maps in any manner, including modifying the content and design, electronic dissemination, and offset printing. The primary author, David P. Shorthouse has waived all copyright, related or neighboring rights, and financial claim to the maps and invites you to use them for personal, educational, and commercial purposes. No permission is needed to use SimpleMappr. Crediting the author is unnecessary. However, if you wish to cite the application, simply use the following.”

In addition, to clarify, we added the sentence "The collection sites were represented on a map generated with SimpleMappr [38] (Fig 1) and shown in Table 1." to the Materials and methods (lines 81-82).

Additional Editor Comments (if provided):

1 - The authors are right in saying that submitting a pre-print to bioRxiv is actually encouraged by PLOS ONE. The best way forward (or the prefered and suggested one) from there, however, is to upload a revised version to bioRxiv and use the "direct transfer to journal" possibility there. In this way, the various versions of the manuscript stay tightly connected to each other. bioRxiv points later readers to the journal article, if the material gets published. So a bioRxiv item is not an independent publication on its own, and that's why PLOS ONE encourages it.

We apologize for uploading a different version to bioRxiv as a preprint. We had been remiss in fixing the old version with the new one. We are so sorry. Because preprint is a system that has only recently been actively used, we did not yet fully understand how it works. We greatly appreciate your guidance.

The current manuscript in bioRxiv is the same as this revised manuscript. Since we did not know how to transfer the revised manuscript to PLOS ONE directly, we submitted it to PLOS ONE independently.

2 - The style of the manuscript is not according to PLOS ONE criteria in that Results and Discussion come before Material and Methods here. So this must be corrected by the authors.

We have revised our manuscript to meet the style requirements of PLOS ONE. We apologize for not meeting the style requirements.

Now, Results and Discussion come after Material and Methods.

3 - I found the abstract not as informative about what was actually done in this study, as opposed to the last paragraph of the introduction, which gives a far better overview of the study. So I would like to encourage the authors to re-write their abstract accordingly, so that it gives a clear story of what was done in this study.

We have revised the abstract substantially (lines 13-29).

4 - It would help for the understanding of the sampling strategy to categorize/summarize the samples as, e.g. numbers of varieties, hybrids, related species, et cetera, and to also summarize the geographical sampling sites. Readers not familiar with the geography of Japan currently have bit of a hard time following the text, even when refering to the geographical map. This should be addressed and the text improved, in my opinion.

To improve the description, we have made the following changes.

1) We added the details of the sample processing to Materials and Methods (82-90 lines).
2) We described the details of the samples to Results (190-200 lines).
3) We added the information of Ariake sound to Introduction (66-68 lines).
4) Fig 1 was improved to understand the geographical information. In new figure, the Ariake sound is shown in red, and geographical names were added.

5 - Moreover, for the international and diverse readership of PLOS ONE, it would help I think if the authors briefly give a definition of these terms - varieties, hybrids, pure accessions et cetera - for non-seaweed specialists (as myself). This is important because of the implications for genomic data, i.e., whether samples are homozygous or heterozygous - what is the expectation in the various categories (varieties, hybrids, wild accessions, ...)? I found this a bit confusing when the 'allodiploid' nature of the hybrid is mentioned and discussed. My understanding after reading the text is that normally, these species are diploid; while the hybrid must then be allotretraploid, and by sampling an originally haploid cell line (which, in this case, must be allodiploid, correct?) which then undergoes diploidization (correct?), in this case we arrive at an allotetraploid? So when they write "allodiploid" here, they really mean an F1 hybrid cell lineage?

Please clarify all this; maybe my assumptions are wrong, or different terminology is used in the seaweed world; but a clarification would greatly improve the manuscript.

We apologize for writing descriptions that only some people will understand.

For clarity, we decided to use only the words ‘culture’, ‘strain’, and ‘sample’. These words were properly defined in Materials and Methods (lines 82-90). We stopped using the words ‘variety’ and ‘accession’.

We stopped using ‘pure’. Instead, we used the expression “Culture containing homozygous diploid cells derived from a single haploid cell was prepared.” (line 93). The wording has been changed to be similar in other places.

We defined ‘hybrid’ when the word first appeared as follows: “the offspring resulting from cross-breeding between *P. yezoensis* and *P. tenera*)” (lines 196-199).

Thank you for pointing out our mistake regarding allodiploid. We wrote about this issue clearly, as follows: “In other words, if the haploid genome of *P. yezoensis* and *P. tenera* is A and B, respectively, the blade cell of the hybrid has the allodiploid genome of AB, and the conchocelis cell of the hybrid has the allotetraploid genome of AABB.” (lines 288-290). In our study analyzing conchocelis, we observed allotetraploid rather than allodiploid, so we used the term ‘allotetraploid’ in many places in our manuscript.

6 - further, I have the following minor editorial remarks:

L 109 - ... addition, they can be crossed WITH each other[32]

We corrected it (line 391).

L 122 - Thus, mitochondrial sequences are determinant for clustering in the previous report[26] - ? I do not understand the meaning of this sentence.

We corrected it as follows: “Thus, mitochondrial sequences, rather than chloroplast sequences, are responsible for clustering in the previous report [32].” (lines 239-240).

L 147 - The mean coverage of Pyr_35 (P. dentata), Pyr_44 (P. tenuipedalis), and Pyr_45 (P. haitanensis) was 2.7, 8.8, and 4.2, respectively. - Use the X sign to indicate coverage, which is a convention that is useful I think: 2.7 X et cetera.

We corrected them and the other related sentences (lines 266-267 and lines 270-273).

L 173 - The Pyr_27 is also a pure accession,... - define what "pure accession" means. See remark 5 just above.

We stopped using “pure” as described above.

L 194 - Of 34 accessions of P. yezoensis, 31 accessions were pure,so they should be homozygous. - define "pure" as opposed to (or identical to ?) "homozygous". Again, see remark 5 above.

We corrected this sentence as follows: “Of 34 samples of *P. yezoensis*, 31 samples were derived from a single haploid cell, so they should be homozygous diploid.” (lines 314-315).

L 300 - In contrast, there were two types of rRNA genes in the chloroplast genome. - Have the auhors considered the possibility of nuclear and/or mitochondrial copies of chloroplast rRNA genes? There are many publications on this phenomenon in the plant world. Or is this another peculiarity in the seaweed world that chloroplasts are "added" when species hybridize? Or is it just simply unknown (as they also point out that the mode of inheritance of organelles is not known in this group of seaweeds, at another location in the manuscript)?

Pyropia has two types of rRNA genes, the sequences of which are slightly different. Because two types of rRNA genes has similar sequence, they are from chloroplast. Because there are some publications on this phenomenon in the *Pyropia* world, we added the following sentence: ”The presence of two non-identical direct repeats carrying two different rRNA operons are general features of *Pyropia* chloroplast genomes [32,65,66].” (lines 214-216). We don't think this is a unique phenomenon. Indeed, we also encountered this phenomenon when we analyzed the *Vibrio* genome last year (unpublished result).

L 367 - GetOrganelle - which reference genomes or 'baits' (if any?) were used to extract and map (assemble) the chloropalsts and mitochondria to?

"target genome(s) or sequence(s) as the seed" are used in GetOrganelle, when I look at the description of this programme.

We added the following sentences: “As the seed sequences for chloroplast genome assembly, other_pt.fasta included in this program was used. As the seed sequences for mitochondrial genome assembly, the complete genome sequence of Pyropia yezoensis (NCBI accession number: NC_017837) was used.” (lines 119-122).

Reviewer #3: This research paper is already available in public database/repository, so in the current scenario this unethical to publish. Paper doesn’t have authenticity and novelty. It may resubmit to the journal only after withdrawal from bioRxiv preprint server (Cold spring harbor laboratory) https://www.biorxiv.org/content/10.1101/2020.05.15.099044v1.full.

We greatly appreciate your helpful suggestions.

We handled this matter according to the editor's instructions.

The editor wrote: “The authors are right in saying that submitting a pre-print to bioRxiv is actually encouraged by PLOS ONE. The best way forward (or the prefered and suggested one) from there, however, is to upload a revised version to bioRxiv and use the "direct transfer to journal" possibility there. In this way, the various versions of the manuscript stay tightly connected to each other. bioRxiv points later readers to the journal article, if the material gets published. So a bioRxiv item is not an independent publication on its own, and that's why PLOS ONE encourages it.”

Therefore, we revised the current manuscript in bioRxiv. The current manuscript in bioRxiv is the same as this revised manuscript.

By the way, because preprint is a system that has only recently been actively used, many scientists, including us, did not yet fully understand how it works. The journal I (corresponding author) edit decided to allow preprints a few years ago, but I didn't learn enough. We are so sorry. Information about preprint can be obtained from the following sites.

https://www.biorxiv.org/about/FAQ

https://en.wikipedia.org/wiki/Preprint

https://en.wikipedia.org/wiki/List_of_academic_publishers_by_preprint_policy

Nevertheless, the manuscript submitted to the Journal entitled “Genomic diversity of 39 accessions of Pyropia species grown in Japan" is an informative piece of work and seems useful in view of harnessing the potential of these important marine algae in breeding and genetics. Although, authors have included limited (39) number of accessions in the study, work may help to breeders and researcher to generate the information for identification of best planting material.

Based on my observations I recommend the manuscripts for major revision. To improve the manuscript quality, the following comments and suggestions are required to be addressed;

The economic importance and medicinal values of the Pyropia species is lacking in the introduction section of the manuscript.

We edited the first paragraph of the Introduction as follows: “The genus *Pyropia* is a marine red alga belonging to the family Bangiaceae. Within the genus *Pyropia*, *P. yezoensis* Ueda (Susabi-nori in Japanese) and *P. haitanensis* Chang et Zheng (tan-zicai in Chinese) are economically important marine crops that are consumed in many countries [1]. These are farmed in Japan, China and Korea. In recent years, they have been eaten as ingredients of sushi and snacks around the world, and their consumption is increasing. Its production in 2018 was 2 million tonnes [2]. Other species are cultivated or wild, and some are occasionally used as edibles. For example, *P. tenera* Kjellman (Asakusa-nori in Japanese) was once cultivated in Japan but has now been replaced by *P. yezoensis* in many places. *P. yezoensis* has also been shown to have pharmacological and nutritional properties [reviewed in 3,4]. For example, a peptide derived from this seaweed induces apoptosis in cancer cells [5,6]. The eicosapentaenoic acid-rich lipid has also been shown to be beneficial to health by alleviating hepatic steatosis [7].” (lines 31-41).

Why the assessment of the genetic diversity is important within the Pyropia species. Explain in introduction in brief.

We edited the previous sentences as follows: “Inter- and intraspecific genetic diversity of *Pyropia* has not been well studied genomically. Studying the gemomic diversity of *Pyropia* species is necessary for planning the breeding and conservation of these seaweeds. This information is also important for the use of species that have not been utilized previously. Especially, utilization of underutilized species and breeding are important to address climate change.” (lines 42-46).

What is Ariake sound, mention clearly in M&M of MS.

The editor also pointed out this problem. Therefore, we made the following changes.

1) We added the information of Ariake sound to Introduction (lines 66-68).
2) Fig 1 was improved to understand the geographical information. In new figure, the Ariake sound is shown in red.

In material and methods part of the MS, the construction of DNA library is missing, include some lines about that.

We added the following sentence: “Sequencing libraries of total DNAwere generated using the NEBNext Ultra DNA library prep kit for Illumina (NEB, USA) by Novogene (Beijing, China).” (109-110 lines).

How organellar (mitochondrial and chloroplast) DNA was extracted..?

We did not extract organellar (mitochondrial and chloroplast) DNA. We used the short reads obtained from whole-genome resequencing to assemble organellar DNA. For the sake of clarity, we added the following sentence: “The short reads obtained from whole-genome resequencing can be appropriate in another application, the *de novo* assembly of the organellar genome [37].” (61-62 lines).

What type of data was used to construct a phylogenetic tree, need to clearly mention in materials and methods.

We used the FASTA files.

We described the process of creating a FASTA file properly as follows:

“Multiple alignments of the assembled chloroplast genome were performed by MAFFT [45] (version 7.455) to create FASTA files for phylogenetic analyses.” (lines 125-127).

“The vcf files were converted to the FASTA format file using the VCF-kit [58] (version 0.1.6).” (line 58).

“For the phylogenetic analysis of the nuclear genome, vcf files were converted to the FASTA format file using the VCF-kit [58] (version 0.1.6) and then were converted to NEXUS format file using MEGA X [59] (version 10.0.5).” (lines 176-178).

Discuss the results that mitochondrial DNA sequences couldn’t revealed genetic variability among the of 39 accessions of Pyropia species; What may be the probable cause of that.

We add the following sentences in the Discussion: “The chloroplast and mitochondrial sequences did not clearly discriminate each sample of Japanese *P. yezoensis*, probably because the size of them is smaller than that of nuclear genome. Indeed, in this study, we were able to distinguish the Japanese samples by analyzing the nuclear genomes” (lines 385-388). Thus, the reason is simple.

And also explain how about the chloroplast based genetic variations showed in gbPyropia species.

Same as above. We discussed mitochondria and chloroplasts problems in the same description.

Nuclear DNA based genetic analysis was significant in compared to that of organellar genetic diversity, what may be the possible reasons for this distinctive nature of between organellar and nuclear genetics.

Same as above. We discussed this problem in the same description.

Line 403 is the repeat of 402; these can be clubbed in a single sentence.

We changed the sentences as follows: “For the phylogenetic analysis of the chloroplast, the rRNA regions were removed from the vcf file. The vcf files were converted to the FASTA format file using the VCF-kit [58] (version 0.1.6)” (lines 168-169).

In results section, lines 73 -75 should be part of materials methods.

We deleted these sentences.

There are discussions parts mingled with results which enlarged the size of results, check thoroughly and trim the results section.

We moved two long descriptions to the Discussion (lines 389-396 and lines 428-442). However, we left a brief discussion in the Results, which should be associated with the results.

Reviewer #4: Dear authors,

The research work presented in the manuscript detailed about genetic diversity of 39 accessions of Pyropia species grown in Japan.The study seems important.

We greatly appreciate your helpful suggestions.

In my view the work will be of more significance if the authors could make out correlation of phenotypic differences with their genotypes.

As shown in lines 371-377 and lines 466-468, we examined those that clearly differed in phenotype.

We would like to perform GWAS analysis in the future. However, the present study has two problems: the number of samples is too small to do so, and the phenotypic differences between the samples used in this study are small because they are related to farmed seaweed.

The results are organized into headings which does not go well, the results of sequencing are not mentioned in the results section.

We added the “Sequencing” section in lines 202-206.

In the M&M section the authors wrote that DNA and RNA was isolated but in the heading they have mentioned DNA sequencing only. No clarity about what was sequenced. Details need to be incorporated.

We added the “DNA purification” section in lines 101-106 and, as mentioned above, sample details were described in Materials and Methods (lines 82-90) and Results (lines 190-200).
