# Supplementary material for: Genomic diversity of 39 samples of Pyropia species grown in Japan
Source: PLoS One. 2021 Jun 9;16(6):e0252207. doi: 10.1371/journal.pone.0252207 (PMC8189503; doi:10.1371/journal.pone.0252207)
Supplement: Attachment — Submitted filename: Response_to_Reviewers.docx [file pone.0252207.s018.docx]

Reviewer #4: Authors should merge the DNA purification and DNA sequencing sub-sections and I recommend to rewrite the DNA purification step in a proper way. When DNA is isolated RNAase treatment is done to get rid of RNA contamination, but that need not be written as DNA and RNA is isolated, when your aim is to isolate or purify only DNA.

We appreciated your helpful comments.

We have merged the two sections and changed the title to "DNA purification and sequencing".

In addition, we have changed the sentence about DNA purification as follows: “DNA was extracted from the conchocelis of each sample using the DNAs-ici!-F (Rizo, Tsukuba, Japan) according to the instructions of the manufacturer, followed by RNase A (NIPPON GENE, Tokyo, Japan) treatment.”

Reviewer #5: Review of the manuscript PONE-D-20-27527 entitled: “Genomic diversity of 39 samples of Pyropia species grown in Japan”

The submitted manuscript is an article that presents a phylogenetic analysis of 39 samples of Pyropia yezoensis, an important marine crop that is used as ingredients of sushi and snacks around the world. The Authors using HTS methods were able to perform a deep study of molecular variability of P. yezoensis isolated from different localities.

In my opinion, the revised paper after several corrections is well written and introduces the reader to the subject matter presented here. The methodology has been selected and presented correctly. The description of the results is presented clearly. The discussion section is revised according to reviewers' comments.

The main complaint relating to an earlier publication of the text in the bioRxiv repository has already been explained.

We are encouraged by your comments as we have made repeated revisions (*^-^*)

Additional Changes

In addition to the changes mentioned above, we have made the following changes. We apologize for these corrections.

1. We made some minor changes, such as spelling errors.
2. We have moved the legend in Figure 3 to the appropriate position.
3. The internal title of the PDF file of S9 Fig was S2 Fig, so we changed it to S9 Fig.
4. There was a mistake in Figure 8 that we failed to fix in the previous revision. In order to fit the figure to the manuscript, we changed the strain name from "S-18" to "Shin Saga 4 gou" in Figure 8.
